# Supplementary material for: Spatial Variation in Stream-Water Bacterial Communities Across Sites Differing in Wild Chinese Giant Salamander Detection Frequency
Source: Biology (Basel). 2026 Sep 18;15(18):1647. doi: 10.3390/biology15181647 (PMC13604818; doi:10.3390/biology15181647)
Supplement: Supplementary file 1 [file biology-15-01647-s001.zip › Data Related to “Spatial Variation in Stream-Water Bacterial Communities across Sites Differing in Wild Chinese Giant Salamander Detection Frequency”/Microbial Data/result/02.FeatureAnalysis/featureSeqs_qzv/index.html]

q2\_feature\_table : tabulate\_seqs


### Sequence Length Statistics

Download sequence-length statistics as a TSV

| Sequence Count | Min Length | Max Length | Mean Length | Range | Standard Deviation |
| --- | --- | --- | --- | --- | --- |
| 5738 | 358 | 509 | 418.0 | 151 | 12.22 |

### Seven-Number Summary of Sequence Lengths

Download seven-number summary as a TSV

| Percentile: | 2% | 9% | 25% | 50% | 75% | 91% | 98% |
| --- | --- | --- | --- | --- | --- | --- | --- |
| Length\* (nts): | 403 | 404 | 404 | 424 | 429 | 429 | 430 |

\*Values rounded down to nearest whole number.

### Sequence Table

To BLAST a sequence against the NCBI nt database, click the
sequence and then click the *View report*
button on the resulting page.

Download your sequences as a raw FASTA file

*Click on a Column header to sort the table.*

| Feature ID | Sequence Length | Sequence |
| --- | --- | --- |
| ASV1413 | 429 | `TGGGGAATATTGGACAATGGGCGAAAGCCTGATCCAGCCATGCCGCGTGTGTGAAGAAGGTCTTCGGATTGTAAAGCACTTTAAGTTGGGAGGAAGGGCATTAACCTAATACGTTAGTGTTTTGACGTTACCGACAGAATAAGCACCGGCTAACTCTGTGCCAGCAGCCGCGGTAATACAGAGGGTGCAAGCGTTAATCGGAATTACTGGGCGTAAAGCGCGCGTAGGTGGTTTGTTAAGTTGAATGTGAAATCCCCGGGCTCAACCTGGGAACTGCATCCAAAACTGGCAAGCTAGAGTATGGTAGAGGGTAGTGGAATTTCCTGTGTAGCGGTGAAATGCGTAGATATAGGAAGGAACACCAGTGGCGAAGGCGACTACCTGGACTGATACTGACACTGAGGTGCGAAAGCGTGGGGAGCAAACAGG` |
| ASV5149 | 424 | `TGGGGAATTTTGGACAATGGGGGAAACCCTGATCCAGCAACGCCGCGTGAAGGATGAAGGTCTTCGGATTGTAAACTTCTTTTGTAGGGGACGAATCGTTTTGTGAATAACAAAAAATGACGGTACCCTGCGAATAAGTCACGGCTAACTTCGTGCCAGCAGCCGCGGTAATACGAAGGTGGCAAGCGTTACTCGGAATTACTAGGCGTAAAGGGTAGGTAGGTGGTTAGGTAAGTCTGTAATGAAATCTCCCGGCTTAACCGGGAGGGGTTTATGGATACTGCTTGACTCGAGTATGGAAGAGGGTGCTGGAATTCCAGGTGTAGCGGTGAAATGCGTAGATATCTGGAGGAACACCAATGGCGAAGGCAGGCACCTGGTCCATTACTGACACTGAGCTACGAAAGCTAGGGGAGCAAACAGG` |
| ASV4643 | 424 | `TGAGGAATATTGGACAATGGGCGCAAGCCTGATCCAGCCATGCCGCGTGCAGGAATAAGGTCCTATGGATTGTAAACTGCTTTTATACAGGAAGAAACACTCGGACGTGTCCGAACTTGACGGTACTGTATGAATAAGGACCGGCTAACTCCGTGCCAGCAGCCGCGGTAATACGGAGGGTCCAAGCGTTATCCGGAATCATTGGGTTTAAAGGGTCCGTAGGCGGTTAAATAAGTCAGTGGTGAAATCTGGTCGCTCAACGATCAAACGGCCATTGATACTGTTTAACTTGAATTATTAGGAAGTAACTAGAATATGTAGTGTAGCGGTGAAATGCTTAGAGATTACATGGAATACCAATTGCGAAGGCAGGTTACTACTAATATATTGACGCTGATGGACGAAAGCGTGGGGAGCGAACAGG` |
| ASV1678 | 429 | `TGGGGAATTTTGGACAATGGGGGGAACCCTGATCCAGCAACGCCGCGTGAATGAAGAAGGCCTTAGGGTTGTAAAGTTCTTTTGTCAGGGAGCAAAGTCTAGCAGCTAATATCTGTTGGAGCTGAGAGTACCTGAAGAATAAGCACCGGCTAACTACGTGCCAGCAGCCGCGGTAATACGTAGGGTGCGAGCGTTAATCGGAATTACTGGGCGTAAAGCGTGCGCAGGCGGATATGTAAGTTAGGAGTGAAATCCCCGGGCTCAACCTGGGAACTGCTTTTAAGACTGCATATCTAGAGTTTGTCAGAGGGGGGTGGAATTCCAAGTGTAGCAGTGAAATGCGTAGAGATTTGGAGGAACACCGATGGCGAAGGCAGCCCCCTGGGATAGAACTGACGCTCATGTACGAAAGCGTGGGTAGCAAACAGG` |
| ASV3409 | 404 | `TGGGGAATATTGGACAATGGGCGCAAGCCTGATCCAGCAATACCGCGTGAGTGATGAAGGCCTTCGGGTTGTAAAGCTCTTTTAGTGAGAAAGATAATGACGGTACTCACAGAAAAAGCACCGGCAAACTTCGTGCCAGCAGCCGCGGTAATACGAAGGGTGCTAGCGTTGTTCGGAATTATTGGGCGTAAAGCGTATGTAGGCGGTTTTGTAAGTCGGATGTGAAAGCCCTGGGCTCAACCTAGGAATTGCATTCGAAACTGCATTACTCGAGTCTGGTAGGGGATAGTGGAATTCCTAGTGTAGGGGTGAAATCCGTAGATATTAGGAGGAATACCAATGGCGAAGGCAACTATCTGGGCCAGTACTGACGCTGAGATACGAAAGCGTGGGGAGCAAACAGG` |
| ASV4725 | 422 | `TAAGGAATATTGGACAATGGTGGCAACACTGATCCAGCCATGCCGCGTGAAGGATGAAGGCCCTATGGGTTGTAAACTTCTTTTATATGATAAAAAACCACTTTACGTGTAAAGTGCTGATTGTCGTAAGAATAAGGGTCGGCTAACTCCGTGCCAGCAGCCGCGGTAATACGGAGGACCCAAGCGTTATTCGGATTTATTGGGTTTAAAGGGTGCGTAGGCGGATTGGTAAGTCAGAGGTGAAATCCCACAGCTTAACTGTGGAACTGCCTTTGATACTGCCAACCTTGAGTACAGATGATGTGGGCGGAATATGACATGTAGCGGTGAAATGCTTAGATATGTCATAGAACACCGATTGCGAAGGCAGCTCACAAAACTGTAACTGACGCTGAGGCACGAAAGCATGGGGATCGAACAGG` |
| ASV2902 | 430 | `TGGGGAATCTTGCACAATGGGCGAAAGCCTGATGCAGCAACGCCGCGTGGGTGAAGACGGCCTTTGGGTTGTAAAGCCCTTTCAGTAGGAAAGAAGGGCATTACGGTAAATAATCGTAGTGCTTGACGGTACCTAAAGAAGAAGCACCGGCTAATCTCGTGCCAGCAGCCGCGGTAATACGAGAGGTGCAAGCGTTGTTCGGAATGACTGGGCGTAAAGCGCACGTAGGCGGATTCGCAAGTAGGATGTGAAAGCCCCAGGCTCAACCTGGGAATGGCACCCTAAACTACGGATCTTGAATGCTTAAGAGGTTGGTGGAATTCCTGGTGTAGGAGTGAAATCCGTAGATATCAGGAGGAATATCGGAGGCGAAGGCGGCCAACTGGTGATGCATTGACGCTAAGGTGCGAAAGCGTGGGGAGCAAACAGG` |
| ASV5705 | 408 | `TGGGGAATATTGGGCAATGGAGGCAACTCTGACCCAGCAACGCCGCGTGAGTGATTGAAGGTCTTCGGATTGTAAAGCTCTGTGATATGGGACGAAGAAAGTGACGGTACCATAACAGCAAGCCACGGCTAACTACGTGCCAGCAGCCGCGGTAATACGTAGGTGGCAAGCGTTGTCCGGAATGACTGGGCGTAAAGGGTGCGTAGGTGGCTATATAAGTTAGTAGTGAAATCCCGAGGCTCAACCTCGGTGCTACTACTAAAACTATATAGCTTGAGTGCAGGAGAGGTAAGTGGAATTCCTAGTGTAGCGGTGGAATGCGTAGATATTAGGAGGAACACCAGTGGCGAAGGCGACTTACTGGACTGTAACTGACACTGAGGCACGAAAGCGTGGGGAGCAAACAGG` |
| ASV2081 | 404 | `TGGGGAATATTGGACAATGGGCGCAAGCCTGATCCAGCCATGCCGCGTGAGTGATGAAGGCCCTAGGGTTGTAAAGCTCTTTTGTGCGGGAAGATAATGACTGTACCGCAAGAATAAGCCCCGGCTAACTTCGTGCCAGCAGCCGCGGTAATACGAAGGGGGCTAGCGTTGCTCGGAATCACTGGGCGTAAAGCGCACGTAGGCGGACTCTTAAGTCGGTGGTGAAATCCTGGAGCTCAACTCCAGAACTGCCTTCGATACTGGGAGTCTCGAGTTCGGGAGAGGTGAGTGGAACTGCGAGTGTAGAGGTGAAATTCGTAGATATTCGCAAGAACACCAGTGGCGAAGGCGGCTCACTGGCCCGATACTGACGCTGAGGTGCGAAAGCGTGGGGAGCAAACAGG` |
| ASV2317 | 403 | `TGGGGAATATTGCACAATGGGCGAAAGCCTGATGCAGCAACGCCGCGTGAGTGATGAAGGCTTTCGGGTCGTAAAACTCTGTCCTCAAGGAAGATAATGACGGTACTTGAGGAGGAAGCCCCGGCTAACTACGTGCCAGCAGCCGCGGTAATACGTAGGGGGCTAGCGTTATCCGGATTTACTGGGCGTAAAGGGTGCGTAGGTGGTTTTTTAAGTCAGGAGTGAAAGGCTACGGCTCAACCGTAGTAAGCTCTTGAAACTGAAAAACTTGAGTGCAGGAGAGGAAAGTGGAATTCCTAGTGTAGCGGTGAAATGCGTAGATATTAGGAGGAACACCAGTAGCGAAGGCGGCTTTCTGGACTGTAACTGACACTGAGGCACGAAAGCGTGGGGAGCAAACAGG` |
| ASV113 | 429 | `TGGGGAATTTTGGACAATGGGCGCAAGCCTGATCCAGCAATGCCGCGTGCAGGATGAAGGCCTTCGGGTTGTAAACTGCTTTTGTACGGAACGAAACGGTCCTTTTTAATACAGGGGGCTAATGACGGTACCGTAAGAATAAGCACCGGCTAACTACGTGCCAGCAGCCGCGGTAATACGTAGGGTGCGAGCGTTAATCGGAATTACTGGGCGTAAAGCGTGCGCAGGCGGTTATATAAGACAGATGTGAAATCCCCGGGCTCAACCTGGGACCTGCATTTGTGACTGTATAGCTAGAGTACGGTAGAGGGGGATGGAATTCCGCGTGTAGCAGTGAAATGCGTAGATATGCGGAGGAACACCGATGGCGAAGGCAATCCCCTGGACCTGTACTGACGCTCATGCACGAAAGCGTGGGGAGCAAACAGG` |
| ASV2927 | 408 | `TGAGGAATTTTCCGCAATGGGCGAAAGCCTGACGGAGCAATACCGCGTGGAGGATTGACAGCTTATGGGCCGTAAACTCCTTTTCTCAGAAAAGAAAAAATTGACGGTATCTGAGGAATAAGCATCGGCTAATTCTGTGCCAGCAGCCGCGGTAAGACAGGAGATGCAAGTGTTATCCGGAATGATTGGGCGTAAAGGGTCTGTAGGTGGTTTGAAAAGTCTGTTGTTAAATACTAGGGCTTAACCCTGGTCAGGCAATAGAAACTATTAGACTAGAGTACGGTAGAGGCAGAGGGAATTTTCGGTGGAGTGGTGAAATGCGTAGATCTCGAAAAGAACACCAATGGCGAAGGCACTCTGCTGGGCCGATACTGACATTGAGAGACGAAAGCTAGGGGAGCAAAAGGG` |
| ASV5340 | 429 | `TGGGGAATATTGGACAATGGGGGCAACCCTGATCCAGCGACGCCGCGTGGAGGAAGAAGGTTTTCGGATTGTAAACTCCTGTCGTCAGGGGCGAAGATCTAGCTATGAATAATGGTTAGGTTTGACAGTACCTGAGGAGGAAGCACCGGCTAACTCCGTGCCAGCAGCCGCGGTAATACGGAGGGTGCAAGCGTTGTTCGGAATTACTGGGCGTAAAGCGTGTCTCGGTGGATAGGTAAGTCAGATGTTAAAGATCCGGGCTCAACCCGGAGAATGCGTTTGATACTGCTTGTCTTGAGTACGGGAGGGATGAGCGGAATTCCTGGTGTAGCAGTGAAATGCGTAGAGATCAGGAGGAACACCTGTGGCGAAAGCGGCTCATTGGTCCGTTACTGACACTCAAGCACGAAAGCGTGGGTAGCAAACAGG` |
| ASV4741 | 423 | `TAGGGAATATTGGTCAATGGGTGGAAGCCTGAACCAGCCATGCCGCGTGCAGGAAGAAGGCCTTCTGGGTTGTAAACTGCTTTTATCGGGGGATAAAAAGACTGTGCGCAGTTACTTGAAGGTACCCGGTGAATAAGCCACGGCTAACTACGTGCCAGCAGCCGCGGTAATACGTAGGTGGCAAGCGTTGTCCGGATTTATTGGGTTTAAAGGGTGCGTAGGCGGCCCTGTAAGTCAGTGGTGAAATACTCTAGCTTAACTAGAGGGGTGCCAATGATACTGCAGGGCTTGAGTACAGATGAGGTAGGCGGAATTGACGGTGTAGCGGTGAAATGCATAGATATCGTCAAGAACTCCTATAGCGAAGGCAGCTTACTGGGCTGTAACTGACGCTGATGCACGAAAGTGTGGGGATCAAACAGG` |
| ASV2462 | 429 | `TGGGGAATTTTGGACAATGGGCGCAAGCCTGATCCAGCCATTCCGCGTGCAGGATGAAGGCCTTCGGGTTGTAAACTGCTTTTGTACGGAACGAAAAGGCTCTGGTTAATACCTGGGGCTCATGACGGTACCGTAAGAATAAGCACCGGCTAACTACGTGCCAGCAGCCGCGGTAATACGTAGGGTGCAAGCGTTAATCGGAATTACTGGGCGTAAAGCGTGCGCAGGCGGTTTTGTAAGACAGAGGTGAAATCCCCGGGCTCAACCTGGGAACTGCCTTTGTGACTGCAAGGCTGGAGTACGGCAGAGGGGGATGGAATTCCGCGTGTAGCAGTGAAATGCGTAGATATGCGGAGGAACACCGATGGCGAAGGCAATCCCCTGGGCCTGTACTGACGCTCATGCACGAAAGCGTGGGGAGCAAACAGG` |
| ASV2432 | 404 | `TGGGGAATATTGGACAATGGGGGCAACCCTGATCCAGCAATGCCGCGTGAGTGAAGAAGGCCTTAGGGTTGTAAAGCTCTTTCGGCGGGGAAGATGATGACGGTACCCGCAGAAGAAGCCCCGGCTAACTTCGTGCCAGCAGCCGCGGTAATACGAAGGGGGCTAGCGTTGTTCGGAGTTACTGGGCGTAAAGGGCGCGTAGGCGGCTTGACAAGTCAGGCGTGAAAGCCCCGGGCTTAACCCGGGAATTGCGCTTGATACTGTCTGGCTAGAGTCCGGGAGAGGATGGCGGAATTCCCAGTGTAGAGGTGAAATTCGTAGATATTGGGAAGAACACCGGTGGCGAAGGCGGCCATCTGGACCGGTACTGACGCTGAGGCGCGAAAGCGTGGGGAGCAAACAGG` |
| ASV3352 | 404 | `TGGGGAATATTGGACAATGGGGGCAACCCTGATCCAGCGATGCCGCGTGAGTGATGAAGGCCTTCGGGTTGTAAAGCTCTTTTGTTAGGGACGATGATGACGGTACCTAACGAATAAGCCCCGGCTAACTTCGTGCCAGCAGCCGCGGTAATACGAAGGGGGCTAGCGTTGTTCGGAATGACTGGGCGTAAAGGGCGCGTAGGCGGTTTGATAAGTTTGGTGTGAAAGTCCTGGGCTTAACCTAGGGATTGCACTGGATACTGTTAGACTAGAGTCCGAGAGAGGAAAATGGAATTGCGAGTGTAGAGGTGAAATTCGTAGATATTCGCAAGAACACCGGTGGCGAAGGCGGTTTTCTGGCTCGGGACTGACGCTAAGGCGCGAAAGCGTGGGGAGCAAACAGG` |
| ASV46 | 429 | `TGGGGAATTTTGGACAATGGACGCAAGTCTGATCCAGCCATGCCGCGTGCGGGAAGAAGGCCTTCGGGTTGTAAACCGCTTTTGTCAGGGAAGAAATCTTCTAGGCTAATACCTTGGGAGGATGACGGTACCTGAAGAATAAGCACCGGCTAACTACGTGCCAGCAGCCGCGGTAATACGTAGGGTGCAAGCGTTAATCGGAATTACTGGGCGTAAAGCGTGCGCAGGCGGTTATGCAAGACAGGTGTGAAATCCCCGAGCTCAACTTGGGAACTGCACTTGTGACTGCATAGCTAGAGTACGGTAGAGGGGAGTGGAATTCCGCGTGTAGCAGTGAAATGCGTAGATATGCGGAGGAACACCGATGGCGAAGGCAGCTCCCTGGACCTGTACTGACGCTCATGCACGAAAGCGTGGGGAGCAAACAGG` |
| ASV4068 | 404 | `TGGGGAATATTGGACAATGGGGGAAACCCTGATCCAGCCATGCCGCGTGAGTGATGAAGGCCTTAGGGTTGTAAAGCTCTTTTATCAGGGACGATGATGACGGTACCTGAAGAATAAGACACGGCTAACTTCGTGCCAGCAGCCGCGGTAATACGAAGGTGTCTAGCGTTGTTCGGAATTACTGGGCGTAAAGCGCGCGTAGGCGGCTTTTCAAGTCAGAGGTGAAAGCCTGGAGCTTAACTCCAGAACTGCCTTTGAAACTGTTGAGCTGGAGTATTGGAGAGGAGTGGGGAATACCTAATGTAGCGGTGAAATGCGTAGATATTAGGTGGAACACCGATGGCGAAGGCACCACTCTGGACAATAACTGACGCTGAGGCGCGAAAGCGTGGGGATCAAACAGG` |
| ASV2456 | 430 | `TGGGGAATCTTGCGCAATGGACGAAAGTCTGACGCAGCAACGCCGCGTGAGTGAAGAAGGCCTTCGGGTTGTAAAGCTCTGTCGTACGGGACGAATATCTTTGCGGTTAACAGCCGCAGAGACTGACGGTACCGTAGAAGGAAGCACCGGCTAACTTCGTGCCAGCAGCCGCGGTAATACGAGGGGTGCAAGCGTTGCTCGGAATTATTGGGCGTAAAGGGTGTGTAGGTTGTCTCGTTTGTCTGAGGTGAAATCCCCGAGCTTAACTTGGGAAGTGCCTTAGAAACGGCGAGACTAGAGTTCTATAGAGGGTAGCGGAATTCCCGGTGTAGCGGTGAAATGCGTAGAGATCGGGAGGAACATCAGAGGCGAAGGCGGCTACCTGGAGAGATACTGACACTAAAGCACGAAAGCGTGGGGAGCAAACAGG` |
| ASV2550 | 429 | `TGGGGAATATTGGACAATGGGCGCAAGCCTGATCCAGCAATGCCGCGTGTGTGAAGAAGGCCTTCGGGTTGTAAAGCACTTTTATCAGGAACGAAAAGCGTGGGGTTAATAACTCTACGTGCTGACGGTACCTGAGGAATAAGCACCGGCTAACTTCGTGCCAGCAGCCGCGGTAATACGAAGGGTGCAAGCGTTAATCGGAATTACTGGGCGTAAAGCGTGCGTAGATGGTTTGTTAAGTCGAATGTGAAAGCCCCGGGCTCAACCTGGGAACTGCATCCGATACTGGCAGACTAGAGTGTGGTAGAGGATGGCGGAATTCCCGGTGTAGCGGTGAAATGCGTAGAGATCGGGAGGAACATCCGTGGCGAAGGCGGCCATCTGGACCAACACTGACGTTGAGGCACGAAAGCGTGGGGAGCAAACAGG` |
| ASV1683 | 429 | `TGGGGAATATTGGACAATGGGCGAAAGCCTGATCCAGCCATGCCGCGTGTGTGAAGAAGGTCTTCGGATTGTAAAGCACTTTAAGTTGGGAGGAAGGGCAGTAAGTTAATACCTTGCTGTTTTGACGTTACCGACAGAATAAGCACCGGCTAACTCTGTGCCAGCAGCCGCGGTAATACAGAGGGTGCAAGCGTTAATCGGAATTACTGGGCGTAAAGCGCGCGTAGGTGGTTCGTTAAGTTGGATGTGAAATCCCCGGGCTCAACCTGGGAACTGCATCCAAAACTGGCGAGCTAGAGTAGGGCAGAGGGTGGTGGAATTTCCTGTGTAGCGGTGAAATGCGTAGATATAGGAAGGAACACCAGTGGCGAAGGCGACCACCTGGGCTCATACTGACACTGAGGTGCGAAAGCGTGGGGAGCAAACAGG` |
| ASV4261 | 407 | `TCGGGAATATTGGACAATGGAGGGAACTCTGATCCAGTGACGCCGCGTGAAGGATGAAGGTCTTCGGATTGTAAACTTATTTTATCAGGGAAGAAAAAAATGACTGTACCTGAAGAAAAAGCACCGGCTAACTACGTGCCAGCAGCCGCGGTAATACGTAGGGTGCAAGCGTTATCCGAATTTACTGGGTGTAAAGGGCGAGTAGACGGGAAATTAAGTTGAATGTGAAAACTCAGGGCTCAACCTTGAGACTGCATCCAAAACTGATTTTCTTGAGTGTTGGAGAGGTAAGTGGAATTCCTAGTGTAGCGGTGAAATGTGTAGATATTAGGAGGAACACCAGTGGCGAAGGCGACTTACTGGACAATAACTGACGTTGAGGCGCGAAAGTGTGGGGAGCAAACAGG` |
| ASV1611 | 424 | `TGAGGAATATTGGTCAATGGACGCAAGTCTGAACCAGCCATGCCGCGTGCAGGATGACGGTCCTATGGATTGTAAACTGCTTTTGTACGAGAAGAAACATCCCGACGTGTCGGGACTTGACGGTATCGTAAGAATAAGGATCGGCTAACTCCGTGCCAGCAGCCGCGGTAATACGGAGGATCCAAGCGTTATCCGGAATCATTGGGTTTAAAGGGTCCGTAGGCGGTTTAGTAAGTCAGTGGTGAAAGCCCATCGCTCAACGGTGGAACGGCCATTGATACTGCTGAACTTGAATTATTAGGAAGTAACTAGAATATGTAGTGTAGCGGTGAAATGCTTAGATATTACATGGAATACCAATTGCGAAGGCAGGTTACTACTAATTTATTGACGCTGATGGACGAAAGCGTGGGGAGCGAACAGG` |
| ASV3650 | 429 | `TGGGGAATTTTGGACAATGGGGGCAACCCTGATCCAGCCATTCCGCGTGAGTGAAGAAGGCCTTCGGGTTGTAAAGCTCTTTCGGCAGGAACGAAACGGTTCATCCTAATACGGTGGGCTAATGACGGTACCTGAAGAAGAAGCACCGGCTAACTACGTGCCAGCAGCCGCGGTAATACGTAGGGTGCGAGCGTTAATCGGAATTATTGGGCGTAAAGCGTGCGCAGGCGGTTTTATAAGACAGATGTGAAATCCCCGGGCTTAACCTGGGAACTGCGTTTGTGACTGTAAGACTTGAGTGTGGCAGAGGGGGGTGGAATTCCACGTGTAGCAGTGAAATGCGTAGATATGTGGAGGAACATCGATGGCGAAGGCAGCCCCCTGGGTCAACACTGACGCTCATGCACGAAAGCGTGGGGAGCAAACAGG` |
| ASV1964 | 429 | `TGGGGAATTTTGGACAATGGGCGCAAGCCTGATCCAGCAATGCCGCGTGCAGGATGAAGGCCTTCGGGTTGTAAACTGCTTTTGTACGGAGCGAAAAGGCTTCCTCTAATACAGGGAGTTCATGACGGTACCGTAAGAATAAGCACCGGCTAACTACGTGCCAGCAGCCGCGGTAATACGTAGGGTGCAAGCGTTAATCGGAATTACTGGGCGTAAAGCGTGCGCAGGCGGTTATGTAAGACAGACGTGAAATCCCCGGGCTCAACCTGGGAACTGCGTTTGTGACTGCATAGCTAGAGTACGGTAGAGGGGGGTGGAATTCCGCGTGTAGCAGTGAAATGCGTAGATATGCGGAGGAACACCGATGGCGAAGGCAGCCCCCTGGACCTGTACTGACGCTCATGCACGAAAGCATGGGGAGCAAACAGG` |
| ASV5450 | 406 | `TTGGGAATCTTGCACAATGGGGGAAACCCTGATGCAGCGACGCCGCGTGGAGGATGAAGGATCTAGGTCTGTAAACTCCTTTTACCGAGAAAGACTTAGGACGGTACTCGGTGAATAAGCCCCGGCTAACTACGTGCCAGCAGCCGCGGTAAGACGTAGGGGGCGAGCGTTGTCCGGATTTACTGGGCGTAAAGGGCGCGTAGGCGGTCTGTTAAGTGAGAAGTGAAATCTCCAGAGCTCAACTCGGAAACTGCTTTTCATACTGATAGACTGGAGGAACGCAGAGGTAACTGGAATTCCTGGTGTAACGGTGAAATGTGTTGATATCAGGAGGAACTCCGATGGCGAAGGCAGGTTACTGGGCGTTATCTGACGCTGAGGCGCGAAAGCGTGGGTAGCAAACAGG` |
| ASV3001 | 407 | `TGGGGAATATTGGGCAATGGGCGAAAGCCTGACCCAGCAACGCCGCGTGAAGGAAGAAGGTTTTCGGATTGTAAACTTCTGTCCTTGGTGAAGAAAAAAATGACGGTAGCCAAGGAGGAAGCCCCGGCTAACTACGTGCCAGCAGCCGCGGTAATACGTAGGGGGCAAGCGTTGTCCGGAATGACTGGGCGTAAAGGGCGCGTAGGCGGCTTGATAAGTCTGGAGTGAAAGTCCTGCTTTTAAGGTGGGAATTGCTTTGGAGACTGTCGGGCTTGAGTGCAGAAGAGGTAAGTGGAATTCCCAGTGTAGCGGTGAAATGCGTAGAGATTGGGAAGAACACCAGTGGCGAAGGCGACTTACTGGGCTGTAACTGACGCTGAGGCGCGAAAGCGTGGGGAGCGAACAGG` |
| ASV3622 | 429 | `TGGGGAATTTTGGACAATGGGCGAAAGCCTGATCCAGCAATGCCGCGTGCAGGAAGAAGGCCTTCGGGTTGTAAACTGCTTTTGTACGGAACGAAAAAGTCCTTTCTAATAAAGAGGGCTCATGACGGTACCGTAAGAATAAGCACCGGCTAACTACGTGCCAGCAGCCGCGGTAATACGTAGGGTGCGAGCGTTAATCGGAATTACTGGGCGTAAAGCGTGCGCAGGCGGTTCTATAAGACAGATGTGAAATCCCCGGGCTCAACCTGGGAATGGCATTTGTGACTGTAGAGCTAGAGTACGGTAGAGGGGGATGGAATTCCGCGTGTAGCAGTGAAATGCGTAGATATGCGGAGGAACACCGATGGCGAAGGCAATCCCCTGGACCTGTACTGACGCTCATGCACGAAAGCGTGGGGAGCAAACAGG` |
| ASV5045 | 404 | `TGGGGAATATTGGACAATGGGGGAAACCCTGATCCAGCGATGCCGCGTGAGTGAAGAAGGCCTTCGGGTTGTAAAGCTCTTTCAGAGGGAATGATGATGACGGTACCCTCAGAAGAAGCACCGGCTAACTTCGTGCCAGCAGCCGCGGTAATACGAAGGGTGCAAGCGTTGTTCGGAATTACTGGGCGTAAAGCGCGCGTAGGCGGCTTATCAAGTCAGGCGTGAAAGCCCTGGGCTCAACCCGGGAATTGCGCTTGAAACTGGTAAGCTTGAGTGTCAGAGGGGTGAGTGGAATTCCTAGTGTAGAGGTGAAATTCGTAGATATTAGGAGGAACACCAGTGGCGAAGGCGACTCACTGGCTGAATACTGACGCTAAGGCGCGAAAGCGTGGGGAGCAAACAGG` |
| ASV28 | 424 | `TGAGGAATATTGGACAATGGGCGCAAGCCTGATCCAGCCATGCCGCGTGCAGGAAGACGGTCCTATGGATTGTAAACTGCTTTTGTACAGGAAGAAACCCTCCCACGTGTGGGAGATTGACGGTACTGTAAGAATAAGGATCGGCTAACTCCGTGCCAGCAGCCGCGGTAATACGGAGGATCCAAGCGTTATCCGGAATCATTGGGTTTAAAGGGTCCGTAGGCGGTTTAGTAAGTCAGTGGTGAAAGCCCATCGCTCAACGGTGGAACGGCCATTGATACTGCTAGACTTGAATTACAAGGAAGTAACTAGAATATGTAGTGTAGCGGTGAAATGCTTAGAGATTACATGGAATACCAATTGCGAAGGCAGGTTACTACTTGTGGATTGACGCTGATGGACGAAAGCGTGGGTAGCGAACAGG` |
| ASV5514 | 424 | `TAGGGAATATTGGGCAATGGAGGCAACTCTGACCCAGCCATCCCGCGTGCAGGATGAAGGATCTATGGTTCGTAAACTGCTTTTGTTAGGGAAGAAAAAGACGCATTCATGCGTAACTGACGGTACCTAACGAATAAGCACCGGCTAACTCCGTGCCAGCAGCCGCGGTAATACGGAGGGTGCAAGCGTTATCCGGATTCACTGGGTTTAAAGGGTGCGTAGGCGGCTATATAAGTCAGTGGTGAAAGCCCGCAGCTCAACTGTGGAACTGCCATTGAAACTGCATAGCTTGAATCTGGTTGAAGTGGGCGGAATACATCATGTAGCGGTGAAATGCTTAGATATGATGTAGAACACCGATTGCGAAGGCAGCTCACTAAGCCAACATTGACGCTGAGGCACGAAAGCGTGGGTAGCGAACAGG` |
| ASV2778 | 409 | `TGGGGAATATTGCACAATGGGCGAAAGCCTGATGCAGCGACGCCGCGTGAGGGATGACGGCCTTCGGGTTGTAAACCTCTTTCAGCAGGGACGAAGCGAAAGTGACGGTACCTGCAGAAGAAGCACCGGCCAACTACGTGCCAGCAGCCGCGGTAATACGTAGGGTGCGAGCGTTGTCCGGAATTACTGGGCGTAAAGAGCTCGTAGGCGGTTTGTCGCGTCGTCTGTGAAAACCAGCAGCTCAACTGCTGGCTTGCAGGCGATACGGGCAGACTTGAGTACTGCAGGGGAGACTGGAATTCCTGGTGTAGCGGTGAAATGCGCAGATATCAGGAGGAACACCGGTGGCGAAGGCGGGTCTCTGGGCAGTAACTGACGCTGAGGAGCGAAAGCGTGGGTAGCGAACAGG` |
| ASV1887 | 405 | `TGGGGAATATTGGTCAATGGGCGCAAGCCTGAACCAGCGATTCTGCGTGAAGGATGAAGCCTCGCGGGGTGTAAACTTCTTTTTTGGGGGAAGACGAATGACGGTACCCCACGAATAAGGGACGGCTAACTACGTGCCAGCAGCCGCGGTAATACGTAGGTCCCAAGCGTTATCCGGATTTACTGGGCGTAAAGAGTCTGTAGCTGGTTATATAAGTCTATTTTCAAAGCCCTGGGCTCAACCCAGGAAAGGGAATGGATACTGTATATCTGGAGGTTTTTAGGGGTTAGTAGAATTCCCGGTGGAGGGGTGAAATCCGTTGATATTGGGAGGAATGCCGAAAGCGAAAGCAGCTAACTATAATTATCCTGACAGTGAGAGACGAAAGCATGGGTAGCAAACGGG` |
| ASV4594 | 430 | `TGGGGAATCTTGCGCAATGGGCGGAAGCCTGACGCAGCCACGCCGCGTGAGTGAAGAAGGCCTTAGGGTTGTAAAGCTCTGTCGGAGGGGACGAAAAGCATTTGGGTTAATAGCCCAAGTGCCTGACGGTACCCTTAAAGGAAGCACCGGCTAACTTCGTGCCAGCAGCCGCGGTAATACGAGGGGTGCAAGCGTTGCTCGGAATCATTGGGCGTAAAGGGCAGGTAGGTGGTCATATTTGTCTGAGGTGAAATCCCTGGGCTTAACCCAGGAAGTGCCTCAGAAACGGTATGACTAGAGTTCTAGAGGGGTTTGTGGAATTCCCGGTGTAGCGGTGAAATGCGTAGAGATCGGGAGGAACACCAGAGGCGAAGGCGGCAAACTGGATAGACACTGACACTCAACTGCGAAAGCGTGGGGAGCAAACAGG` |
| ASV3376 | 404 | `TGGGGAATATTGGACAATGGGCGCAAGCCTGATCCAGCCATGCCGCGTGAGTGATGAAGGCCTTAGGGTTGTAAAACTCTTTTACCCGGGAAGATAATGACTGTACCGGGAGAATAAGCTCCGGCTAACTTCGTGCCAGCAGCCGCGGTAATACGAAGGGAGCTAGCGTTGTTCGGATTTACTGGGCGTAAAGCGCACGTAGGCGGACGATTAAGTCAGAGGTGAAAGCCTGGGGCTCAACCCCAGAATTGCCTTTGATACTGGTCGTCTTGAGTATGATAGAGGTTGGTGGAATTCCTAGTGTAGAGGTGAAATTCGTAGATATTAGGAAGAACACCGGTGGCGAAGGCGGCCAACTGGATCATAACTGACGCTGAGGTGCGAAAGCGTGGGGAGCAAACAGG` |
| ASV3511 | 429 | `TGGGGAATTTTGGACAATGGGCGCAAGCCTGATCCAGCCATGCCGCGTGAGTGAAGAAGGCCTTCGGGTTGTAAAGCTCTTTCGGCGGGGACGAAACGGTTCGTGCCAACACCACGGACTAATGACGGTACCCGAAGAAGAAGCACCGGCTAACTACGTGCCAGCAGCCGCGGTAATACGTAGGGTGCGGGCGTTAATCGGAATTACTGGGCGTAAAGCGTGCGCAGGCGGTTTGTTAAGACAGGTGTGAAATCCCCGGGCTTAACCTGGGAATTGCGCTTGTTACTGGCAGACTCGAGTGTGGCAGAGGGGGGTGGAATTCCAGGTGTAGCAGTGAAATGCGTAGAGATCTGGAGGAACACCGATGGCGAAGGCAGCCCCCTGGGTTAACACTGACGCTCATGCACGAAAGCGTGGGGAGCAAACAGG` |
| ASV3450 | 430 | `TAGGGAATATTGCACAATGGAGGAAACTCTGATGCAGCAACGCCGCGTGAGTGAAGAAGGCCCTTGGGTCGTAAAGCTCTTTAGCCTGGAAAGAAGTGTTTAGGAGTAAATAATTCCTAGATGTGACGGTACCAGGAGAATAAGGACCGGCTAATTTCGTGCCAGCAGCCGCGGTAATACGAAAGGTCCAAGCGTTACTCGGAATTACTGGGCGTAAAGCGCGCGTAGGCGGCTTTGTATGTCGATTGTGAAAGCCCCGGGCTCAACCTGGGAACTGCAGTCGAAACTGCAAAGCTTGAATGTTGGAGAGGTTGGGGGAATACCTAGTGGAGAAGTGAAATTCGTAGAGATTAGGTGGAACACCAGAGGCGAAGGCGCCTGACTGGCCAAACATTGACGCTAAGGCGCGAAAGCGTGGGTAGCAAACAGG` |
| ASV4828 | 429 | `TGGGGAATATTGGACAATGGGGGCAACCCTGATCCAGCAATACCGCGTGTGTGAAGAAGGCCTTCGGGTTGTAAAGCACTTTTAGTTGGGAAGAAGTGTTCTATTCGAATAGAGTAGTGCATTGACGTTACCGACAGAAAAAGCACCGGCTAACTCTGTGCCAGCAGCCGCGGTAATACAGAGGGTGCAAGCGTTAATCGGAATTACTGGGCGTAAAGGGCGCGTAGGTTGCTATGTAAGTTGGATGTGAAATGTTCGGGCTTAACCTGAAACGGCCATCCAATACTGCATAGCTAGAGTACAGTAGAGGGAAGGGGAATTTCCGGTGTAGCGGTGAAATGCGTAGATATCGGAAGGAACATCAGTGGCGAAGGCGCCTTCCTGGACTGATACTGACACTGAGGCGCGACAGCGTGGGGAGCGAACAGG` |
| ASV24 | 423 | `TAGGGAATATTGGTCAATGGGCGCAAGCCTGAACCAGCCATGCCGCGTGCAGGAAGAATGCCCTCTGGGTTGTAAACTGCTTTTGACAGGGAAGAAATCACTTATGCGTAAGTGTCTGACGGTACCTGTAGAATAAGCACCGGCTAACTCCGTGCCAGCAGCCGCGGTAATACGGAGGGTGCAAGCGTTATCCGGATTTATTGGGTTTAAAGGGTGCGTAGGCGGTAGAGTAAGTCAGTGGTGAAAGCCTGTTGCCTAACAACAGAACTGCCATTGATACTGCTTTGCTTGAGTATAGATGAGGTAGGCGGAATTAATAGTGTAGCGGTGAAATGCTTAGATATTATTAAGAACTCCGATAGCGAAGGCAGCTTACTAAACTATAACTGACGCTGAGGCACGAAAGTGTGGGGATCAAACAGG` |
| ASV1313 | 429 | `TGGGGAATTTTGGACAATGGGCGCAAGCCTGATCCAGCCATGCCGCGTGCGGGAAGAAGGCCTTCGGGTTGTAAACCGCTTTTGTCAGGGAAGAAATCTTCTGGGTTAATACCTCGGGAGGATGACGGTACCTGAAGAATAAGCACCGGCTAACTACGTGCCAGCAGCCGCGGTAATACGTAGGGTGCAAGCGTTAATCGGAATTACTGGGCGTAAAGCGTGCGCAGGCGGTCTTGCAAGACAGGTGTGAAATCCCCGGGCTTAACCTGGGAACTGCACTTGTGACTGCAAGGCTGGAGTACGGCAGAGGGGGATGGAATTCCGCGTGTAGCAGTGAAATGCGTAGATATGCGGAGGAACACCGATGGCGAAGGCAATCCCCTGGGCCTGTACTGACGCTCATGCACGAAAGCGTGGGGAGCAAACAGG` |
| ASV233 | 421 | `TGGGGAATATTGCGCAATGGACGAAAGTCTGACGCAGCGACGCCGCGTGTAGGATGACGCCTTTCGGGGTGTAAACTACTGTTGCCCGGGACGAAACTCCTTTTTCGAGAGGACTGACGGTACCGGGTGAGGAAGCACCGGCTAACTCTGTGCCAGCAGCCGCGGTAATACAGAGGGTGCGAGCGTTGTCCGGAATCACTGGGCGTAAAGGGTGCGTAGGTGGCTTGGTAAGCGTGTGGTGAAAGTCCGGGGCTCAACCCCGGATCTGCCGTGCGAACTGCCGAGCTCGAGCAATGTAGAGGCAGGTGGAATTTCGGGTGTAGCGGTGGAATGCGTAGAGATCCGAAAGAACACCAGTGGCGAAGGCGGCCTGCTGGGCATTAGCTGACACTGAGGCACGACAGCGTGGGGAGCAAACAGG` |
| ASV4365 | 424 | `TGAGGAATATTGGACAATGGGCGCAAGCCTGATCCAGCCATGCCGCGTGCAGGAAGAAGCATCTATGGTGTGTAAACTGCTTTTGTACGGGAAGAAAAAAAGCTACGCGTAGTTTACTGACGGTACCGTAAGAATAAGGATCGGCTAACTCCGTGCCAGCAGCCGCGGTAATACGGAGGATCCAAGCGTTATCCGGAATCATTGGGTTTAAAGGGTCCGTAGGCGGCCTAATAAGTCAGCGGTGAAAGTTTTTGGCTTAACCAAAAAATTGCCATTGATACTGTTAGGCTTGAATATTTGTGAAGTAACTAGAATATGTAGTGTAGCGGTGAAATGCTTAGATATTACATGGAATACCAATTGCGAAGGCAGGTTACTAACAAATGATTGACGCTGATGGACGAAAGCGTGGGTAGCGAACAGG` |
| ASV1860 | 424 | `TGAGGAATATTGGACAATGGGCGCAAGCCTGATCCAGCCATGCCGCGTGCAGGAAGAAGGTCCTATGGATTGTAAACTGCTTTTATACAGGAAGAAACACCTCTACGTGTAGAGACTTGACGGTACTGTATGAATAAGCATCGGCTAACTCCGTGCCAGCAGCCGCGGTAATACGGAGGATGCAAGCGTTATCCGGAATCATTGGGTTTAAAGGGTCCGTAGGCGGTTAGATAAGTCAGTGGTGAAAGCCCATCGCTCAACGATGGAACGGCCATTGATACTGTTTAACTTGAATTATTAGGAAGTAACTAGAATATGTAGTGTAGCGGTGAAATGCTTAGAGATTACATGGAATACCAATTGCGAAGGCAGGTTACTACTAATATATTGACGCTGATGGACGAAAGCGTGGGGAGCGAACAGG` |
| ASV1745 | 421 | `TAAGGAATATTGCGCAATGGGCGAAAGCCTGACGCAGCGACGCCGCGTGGGGGATGAAGGTCTTCGGATTGTAAACCCCTTTCGGGAGGGAAGATGGAGTGGGGTAACCCATTCGGACGGTACCTCCAGAAGCAGCCACGGCTAACTTCGTGCCAGCAGCCGCGGTAATACGAAGGTGGCAAGCGTTATTCGGATTTACTGGGCGTACAGGGAGCGTAGGCGGTTCGGTAAGCCCTCTGTGAAATCTTCAGGCTTAACCTGGAACAGTCGGAGGGGACTGCTGGGCTAGAGGACGGGAGAGGAGCGCGGAATTCCCGGTGTAGCGGTGAAATGCGTAGAGATCGGGAGGAAGGCCGGTGGCGAAGGCGGCGCTCTGGAACGTTTCTGACGCTGAGGCTCGAAAGCGTGGGGAGCAAACAGG` |
| ASV3035 | 404 | `TGGGGAATATTGGGCAATGGGCGAAAGCCTGACCCAGCCATGCCGCGTGAATGATGAAGGCCTTAGGGTTGTAAAGTTCTTTCGCAGGTGAAGATAATGACGGTAACCTGATAAGAAGCCCCGGCTAACTTCGTGCCAGCAGCCGCGGTAATACGAAGGGGGCTAGCGTTGTTCGGAATTACTGGGCGTAAAGCGTATGTAGGCGGATATCTAAGCTAGAGGTGAAAGCCCAAGGCTCAACCTTGGAACTGCCTTTAGAACTGGATATCTGGAGTTTTGGAGGGGAGTGCGGAATTTCTAATGTAGAGGTGAAATTCGTAGATATTAGAAGGAACACCGGCGGCGAAGGCGGCACTCTGGACAAATACTGACGCTGAGATACGAAAGCGTGGGGAGCAAACAGG` |
| ASV3764 | 429 | `TGGGGAATTTTGGACAATGGGGGAAACCCTGATCCAGCCATGCCGCGTGAGTGAAGAAGGCCTTCGGGTTGTAAAGCTCTTTCGGCTGGGAAGAAATCGCACAGGCTAATACTCTGTGTGGATGACGGTACCAGCACAAGAAGCACCGGCTAACTACGTGCCAGCAGCCGCGGTAATACGTAGGGTGCGAGCGTTAATCGGAATTACTGGGCGTAAAGCGTGCGCAGGCGGTTGTGCAAGACAGATGTGAAATCCCCGGGCTCAACCTGGGAACTGCATTTGTGACTGCACGACTAGAGTACGGCAGAGGGGGGTAGAATTCCACGTGTAGCAGTGAAATGCGTAGAGATGTGGAGGAATACCGATGGCGAAGGCAGCCCCCTGGGCCGATACTGACGCTCATGCACGAAAGCGTGGGGAGCAAACAGG` |
| ASV4113 | 421 | `TGGGGAATATTGCACAATGGACGAAAGTCTGATGCAGCGACGCCGCGTGAGGGATGAAGGCCTTCGGGTTGTAAACCTTTTTCGCTCTTGACCAAGGCATCATTTTGGTGGTGTTGAGGGTAGGGGGTAAAGAAGCTCCGGCTAACTACGTGCCAGCAGCCGCGGTAATACGTAGGGAGCGAGCGTTGTCCGGATTTATTGGGCGTAAAGGGCTTGTAGGTGGCGTGTCGCGTCTGTCGTGAAAGGCTCCGGCTTAACTGGGGTAGTGCGGTGGGTACGGGCATGCTTGAGTGTGGTAGGGGAGATTGGAATTCCTGGTGTAGCGGTGGAATGCGCAGATATCAGGAGGAACACCGGTGGCGAAGGCGGATCTCTGGGCCATTACTGACGCTGAGGAGCGAAAGCGTGGGGAGCGAACAGG` |
| ASV5574 | 429 | `TGAGGAATCTTGCGCAATGGGCGAAAGCCTGACGCAGCAACGCCGCGTGGAGGATGAAGGTCTTCGGATTGTAAACTCCTGTCAGATGGGGCGAAGGTCCACTGCCTAATATGCGGTGGGCTTGACGGTACCATCAGAGGAAGCCACGGCTAACTCTGTGCCAGCAGCCGCGGTAATACAGAGGTGGCGAGCGTTATTCGGAATTATTGGGCGTAAAGGGCGCGTAGGCGGCCAGATAAGTCCCGGGTGAAATCCCTCGGCTCAACCGAGGAACTGCCTTGGAAACTGTCTGGCTCGAGGCCGGGAGAGGGTAGTGGAATTCCCAGTGTAGCGGTGAAATGCGTAGATACTGGGAGGAACACCGGTGGCGAAGGCGGCTACCTGGACCGGTTCTGACGCTGATGCGCGAAAGCGTGGGGAGCAAACAGG` |
| ASV4310 | 407 | `TAGGGAATATTGCACAATGGACGAAAGTCTGATGCAGCGACGCCGCGTGAGTGATGAAGGCCTTCGGGTCGTAAAGCTCTGTCGTATGGGACTAACACAATGAAGGTACCATGCAAGAAAGGATCGGCTAACTTCGTGCTAGCAGTTGCGGTAAGACGAGGGATCCTAGCGTTGTTCGGAATCATTGGGCGTAAAGCGGGTGTAGGCGGCTTTATAAGTCAGGTGTGAAAGCCCGGGGCTCAACCCCGGAAGTGCACTTGATACTGTTTAGCTTGAGTGTGGTAGAGGCTACTGGAATTTCTGGTGTAGTGGTGAAATACGTAGATATCAGAAGGAATACCGGAGGCGAAGGCGGGTAGCTGGGCCAACACTGACGCTGAGACCCGAAAGCGTGGGGATCAAACAGG` |
| ASV4270 | 409 | `TAGGGAATATTGCGCAATGGGCGAAAGCCTGACGCAGCGACGCCGCGTGGAGGATGAAGGCCTTCGGGTTGTAAACTCCTTTTCTCTGGGACGAATTTCTGACGGTACCAGAGGAATAAGAGGTTGCTGACTCTGTGCCAGCAGCAGCGGTAATACAGAGACCTCAAGCGTTATCCGGAATCACTGGGCGTAAAGCGTTCCGACAGGCGGCTCAAAAAGTTCGGGGTAAAATCCAGAGGCTCAACTTCTGGAACACTCTGAAGACTTTTGAGCTAGAGGGGCAAAGAGGTGCTTGGAACGTACGGTGTAGCAGTGAAATGCGTTGATATCGTACGGAACACCGATAGCGAAGGCAGAGCACTGGGTGCCACCTGACGCTGCTAGGACGAAAGCGTGGGGAGCGAATGGG` |
| ASV3118 | 403 | `TGGGGAATATTGCGCAATGGGCGAAAGCCTGACGCAGCGACGCCGCGTGAGTGACGAAGTCTTTCGGGATGTAAAACTCTGTCAAGAGGGACGATTATGACGGTACCTCTAAAGGAAGCCCCGGCTAACTACGTGCCAGCAGCCGCGGTAATACGTAGGGGGCGAGCGTTATCCGGATTTACTGGGCATAAAGAGCTTGTAGGCGGCGATCTAAGTCGGATGTTAAATTCTTCAGCTCAACTGAAGCATGCATTGGATACTGGATTGCTAGAGATCAGGAGAGGAAAGCGGAACTCACGGTGTAGCGGTGGAATGCGTAGATATCGTGAGGAACACCAGTTGCGAAGGCGGCTTTCTGGACTGAATCTGACGCTCAAGAGCGAAAGCGTGGGGAGCGAACGGG` |
| ASV4943 | 441 | `TGGGGAATCTTGCGCAATGGTCGAAAGACTGACGCAGCGACGCCGCGTGCGGGAGGAAGCTCTTCGGGGTGTAAACCGCTGTTGCCCGGAACGAAACTCCAGATTCGTCTGGATTGACGGTACCGGGTGAGGAAGCGCCGGCTAACTCTGTGCCAGCAGCCGCGGTAATACAGAGGGCGCGAGCGTTGTCCGGAATCACTGGGCGTAAAGGGCGCGTAGGTGGCCACCATAAGGCCGCGGTGAAAGTTCGGGGCTCAACCCTGAATCTGCCGCGGCGACTGTGGGGCTTGGGCACCGTAGAGGCAGATGGAATTCCGGGTGTAGCGGTGGAATGCGTAGAGATCCGGAAGAACACCGGTGGCGAAGGCGGTCTGCTGGGCGGTGGACTCGTAAGGGTCCAATAGCCGACACTGAGGCGCGACAGCGTGGGGAGCAAACAGG` |
| ASV1234 | 404 | `TGGGGAATATTGGACAATGGGCGAAAGCCTGATCCAGCCATGCCGCGTGAGTGATGAAGGCCTTAGGGTTGTAAAGCTCTTTCACCGATGAAGATAATGACGGTAGTCGGAGAAGAAGCCCCGGCTAACTTCGTGCCAGCAGCCGCGGTAATACGAAGGGGGCTAGCGTTGTTCGGAATCACTGGGCGTAAAGCGCACGTAGGCGGATTGTTAAGTGAGGGGTGAAATCCCGGGGCTCAACCCCGGAACTGCCTTTCATACTGGCAGTCTTGAGTTCGAGAGAGGTAAGTGGAATTCCGAGTGTAGAGGTGAAATTCGTAGATATTCGGAGGAACACCAGTGGCGAAGGCGGCTTACTGGATCGATACTGACGCTGAGGTGCGAAAGCGTGGGGAGCAAACAGG` |
| ASV3411 | 429 | `TGGGGAATCTTCCGCAATGGACGAAAGTCTGACGGAGCAACGCCGCGTGAGTGATGAAGGTTTTCGGATCGTAAAACTCTGTTGTTAAGGACGAATGTATAGATTGTGAATAATGGTTTATAATGACGGTACTTAACGAGGAAGCCACGGCTAACTACGTGCCAGCAGCCGCGGTAATACGTAGGTGGCAAGCGTTGTCCGGAATTATTGGGCGTAAAGGGAGCGCAGGTGGGAATGTAAGTCAATCTTAAAAGTGCGGGGCTCAACCCCGTGATGGGATAGAAACTACATTTCTTGAGTGCAGGAGAGGAAAGTGGAATTCCTAGTGTAGCGGTGAAATGCGTAGATATTAGGAGGAACACCAGTGGCGAAGGCGACTTTCTGGACTGTTACTGACACTGAGGCTCGAAAGCTAGGGGAGCGAACGGG` |
| ASV4559 | 429 | `TGGGGAATTTTGGACAATGGGGGCAACCCTGATCCAGCCATTCCGCGTGAGTGAAGAAGGCCTTCGGGTTGTAAAGCTCTTTTGTCCGGAGCGAAACGGTACGGGTGAATATCCCGTGCTACTGACGGTACCGGAAGAATAAGCACCGGCTAACTACGTGCCAGCAGCCGCGGTAATACGTAGGGTGCGAGCGTTAATCGGAATTACTGGGCGTAAAGCGTGCGCAGGCGGTTGTGTAAGCCAGACGTGAAATCCCCGGGCTTAAGCTGGGAATGGCGTTTGGGACTGCACGGCTTGAGTGTGGCAGAGGGGACTAGAATTCCTGGTGTAGCAGTGAAATGCGTAGATATCAGGAGGAATACCGATGGCGAAGGCAGGTCCCTGGGTTAACACTGACGCTCATGCACGAAAGCGTGGGGAGCAAACAGG` |
| ASV5722 | 404 | `TGGGGAATATTGGACAATGGGCGAAAGCCTGATCCAGCGACGCCGCGTGGGTGATGAAGGCCTTCGGGTTGTAAAGCCCTTTCGTCAGGGAAGATAATGACGGTACCTGAAGAAGAAGCTCCGGCTAACTTCGTGCCAGCAGCCGCGGTAATACGAAGGGAGCTAGCGTTACTCGGAATCATTGGGCGTACAGCGTGCGTAGGCGGCTTTGTAAGTTAGGAGTGAAAGCCTGGGGCTCAACCCCAGAATTGCTTTTAATACTGCAAAGCTAGAGTGTCAGAGGGGATAGCGGAATTCCTAGTGTAGAAGTGAAATTCGTAGATATTAGGAGGAACACCGGTGGCGAAGGCGGCTATCTGGCTGAATACTGACGCTGTTGCACGAAAGCGTGGGGAGCAAACAGG` |
| ASV2921 | 429 | `TGGGGAATCTTGGACAATGGGGGCAACCCTGATCCAGCGATGCCGCGTGAGTGAAGAAGGCCTTCGGGTCGTAAAGCTCTTTAGGTTGGGAGGAAGGCTAGTAGTTTAAGAGATAACTAGTTTGACTGTACCGACAGAATAAGCACCGGCAAACTCTGTGCCAGCAGCCGCGGTAATACAGAGGGTGCGAGCGTTAATCGGATTTACTGGGCGTAAAGGGCGCGTAGGCGGTCGTATGAGTGTGATGTGAAAGCCCCGGGCTTAACCTGGGAAGTGCATCGCAAACGATACGACTGGAGTAGGTGAGAGGGTGGCGGAATTTCCGGTGTAGCGGTGAAATGCGTAGAGATCGGAAGGAACGTCAATGGCGAAGGCAGCCACCTGGTACCATACTGACGCTGAGGCGCGAAAGCGTGGGGATCGAACAGG` |
| ASV4049 | 428 | `TGGGGAATTTTGCACAATGGGCGCAAGCCTGATGCAGCAACGCCGCGTGTGTGATGAAGGTCTTCGGATCGTAAAGCACTTTCGACCGGGACGAAACAGCGACACCCAACACGTGTCGCCTTGACGGTACCGGGAGAAGAAGCACCGGCTAACTCTGTGCCAGCAGCCGCGGTAATACAGAGGGTGCAAGCGTTGTTCGGAATTATTGGGCGTAAAGCGCGTGTAGGCGGCTTTGCAAGTCGGGTGTGAAAGCCCTCAGCTCAACTGAGGAAGTGCGCCCGAAACTGCAGAGCTTGAGTACCGGAGAGGATGGCGGAATTCCCCAAGTAGAGGTGAAATTCGTAGATATGGGGAGGAACACCGGTGGCGAAGGCGGCCATCTGGACGGTCACTGACGCTGAGACGCGAAAGCGTGGGTAGCAAACAGG` |
| ASV201 | 429 | `TGGGGAATCTTGGACAATGGGCGAAAGCCTGATCCAGCAATGCCGCGTGCGTGAAGAAGGCCTTCGGGTTGTAAAGCGCTTTTGTCGGGGAGGAAATCCTAACGGTTAATAACTGTTGGGGATGACAGTACCCGAAGAATAAGGACCGGCTAACTACGTGCCAGCAGCCGCGGTAATACGTAGGGTCCAAGCGTTAATCGGAATTACTGGGCGTAAAGCGTCCGCAGGTGGCTTGATAAGATAGACGTGAAATCCCTGGGCTCAACCTAGGAATTGCGTTTATGACTGTCTCGCTAGAGTATGGGAGAGGGGGGTGGAATTCCGCGTGTAGCAGTGAAATGCGTAGAGATGCGGAGGAACACCGATGGCGAAGGCAACCCCCTGGCCTAATACTGACACTCATGGACGAAAGCGTGGGGAGCAAACAGG` |
| ASV3944 | 430 | `TAGGGAATATTGCGCAATGGACGAAAGTCTGACGCAGCCACGCCGCGTGAGTGAGGAAGGCCTTCGGGTTGTAAAGCTCTGTCGGCTGGGAAAAACGGCTATCCGGGGAAAGTTCGGGTAGCGTGATGGTACCAGCAAAGGAAGCACCGGCAAACTCTGTGCCAGCAGCCGCGGTAATACAGAGGGTGCAAGCGTTGTTCGGAATTACTGGGCGTAAAGCGTGCGTAGTCGGTATTGAGAGTCGCGGGTGAAATCCCAGGGCTTAACCCTGGAACTGCCTGCGAGACCTCAGTACTAGAGTGTGAGAGGGGATAGTGGAATACCCAGTGTAGCGGTGAAATGCGTAGAGATTGGGTGGAACACCGGTGGCGAAGGCGGCTATCTGGCTCACAACTGACGATCAGGCACGAAAGCGTGGGGAGCAAACAGG` |
| ASV5651 | 408 | `TCGAGAATTTTCCACAATGGACGAAAGTCTGATGGAGCGACGCCGCGTGGTTGATGAAGTACTTAGGTATGTAAAAACCTTTTATGAGGGAAAAAGTTTATTGATTGTACCTCATGAATAAGGGGTTGCTAAACTCGTGCCAGCAGCAGCGGTAATACGAGTGCCCCAAGCGTTATCCGGAATTATTGGGCGTAAAGGGTGAGTAGGTGGTTGTATTAGTCTTGTGTTAAAGACTCCGGCTCAACCGGAGAAGTGCATAGGAAACGGTACAACTAGAGTATGTGAGAGGTGTGTAGAACTCATGGAGTAGGGGTGAAATCCGTTGATATCATGGGGAATACCAAAAGCGTAGGCAGCACACTGGCACATTACTGACACTGAATCACGAAAGCGTGGGTAGCGAATGGG` |
| ASV1017 | 429 | `TGGGGAATTTTGGACAATGGGCGCAAGCCTGATCCAGCAATGCCGCGTGCAGGATGAAGGCCTTCGGGTTGTAAACTGCTTTTGTACGGAGCGAAACGGTCTACCCTAATACGGTGGGCTAATGACGGTACCGTAAGAATAAGCACCGGCTAACTACGTGCCAGCAGCCGCGGTAATACGTAGGGTGCGAGCGTTAATCGGAATTACTGGGCGTAAAGCGTGCGCAGGCGGTTATATAAGACAGATGTGAAATCCCCGGGCTCAACCTGGGACCTGCATTTGTGACTGTATAGCTAGAGTACGGTAGAGGGGGATGGAATTCCGCGTGTAGCAGTGAAATGCGTAGATATGCGGAGGAACACCGATGGCGAAGGCAATCCCCTGGACCTGTACTGACGCTCATGCACGAAAGCGTGGGGAGCAAACAGG` |
| ASV2476 | 404 | `TGGGGAATATTGGACAATGGGCGCAAGCCTGATCCAGCCATGCCGCGTGTGTGATGAAGGCCTTAGGGTTGTAAAGCACTTTCACCGGTGAAGATAATGACGGTAACCGGAGAAGAAGCCCCGGCTAACTTCGTGCCAGCAGCCGCGGTAATACGAAGGGGGCTAGCGTTGTTCGGAATTACTGGGCGTAAAGCGCACGTAGGCGGATATTTAAGTCAGGGGTGAAATCCCAGAGCTCAACTCTGGAACTGCCTTTGATACTGGGTATCTTGAGTATGGAAGAGGTGAGTGGAATTCCGAGTGTAGAGGTGAAATTCGTAGATATTCGGAGGAACACCAGTGGCGAAGGCGGCTCACTGGTCCATAACTGACGCTGAGGTGCGAAAGCGTGGGGAGCAAACAGG` |
| ASV2955 | 424 | `TGAGGAATATTGCGCAATGGAGGAAACTCTGACGCAGCCATGCCGCGTGCAGGAAGAAGGCGCTACGCGTTGTAAACTGCTTTTAACCGGGAAGAAACCCCTTTACGTGTAAAGGGTTGACGGTACCGGAGGAATAAGCACCGGCTAACTTCGTGCCAGCAGCCGCGGTAATACGAAGGGTGCAAGCGTTATCCGGATTCATTGGGTTTAAAGGGTGCGTAGGCGGGGCGTTAAGTCAGTGGTGAAATCCTGCAGCTCAACTGTAGACTTGCCATTGATACTGACGCTCTTGAGTGCGCTTGAAGTAGGCGGAATGTGCCGTGTAGCGGTGAAATGCTTAGATATGGCACAGAACACCAATTGCGAAGGCAGCTTACTAAGGCGATACTGACGCTGAGGCACGAAAGCGTGGGGATCGAACAGG` |
| ASV3340 | 404 | `TGGGGAATCTTAGACAATGGGCGCAAGCCTGATCTAGCCATGCCGCGTGAGTGATGAAGGCCTTAGGGTTGTAAAGCTCTTTCAGTGGGGAAGATAATGACGGTACCCACAGAAGAAGCCCCGGCTAACTCCGTGCCAGCAGCCGCGGTAATACGGAGGGGGCTAGCGTTGTTCGGAATTACTGGGCGTAAAGCGCATGTAGGCGGGACGGCAAGTCAGAGGTGAAATCCCGGGGCTCAACCCCGGAACTGCCTTTGAAACTTTCGTTCTTGAGATCGAGAGAGGTGAGTGGAATTCCGAGTGTAGAGGTGAAATTCGTAGATATTCGGAGGAACACCAGTGGCGAAGGCGGCTCACTGGCTCGATACTGACGCTGAGATGCGAAAGCGTGGGGAGCAAACAGG` |
| ASV676 | 422 | `TGAGGAATATTGGTCAATGGGCGCAAGCCTGAACCAGCAATGCCGCGTGTAGGACGAAGGGGCTTCGCCCTGTAAACTACTGTCAGATGGGTTGAAAAGGAAGCTTAGCTTCTGGAGACGGTACCATCAGAGGAAGGGACGGCTAACTACGTGCCAGCAGCCGCGGTAATACGTAGGTCCCGAGCGTTGTCCGGATTTACTGGGTATAAAGGGTGCGTAGGCGGTTTTGTGCGTCAGAGGTGAAAGGTCAGTGCTTAACATTGATACTGCCTTTGAAACGGCGAAACTTGAGTATGAGAGAGGTTTGTAGAATTCCTGGTGTAGCGGTGAAATGCGTAGATATCAGGAGGAATACCGGTGGCGAAGGCGGCAAACTGGCTCATAACTGACGCTGAGGCACGAAAGCGTGGGGATCAAACAGG` |
| ASV1491 | 424 | `TGAGGAATATTGGACAATGGGCGCAAGCCTGATCCAGCCATGCCGCGTGCAGGATGACGGTCCTATGGATTGTAAACTGCTTTTATACGAGAAGAAACACTACTTCGTGAAGTAGCTTGACGGTATCGTAAGAATAAGGATCGGCTAACTCCGTGCCAGCAGCCGCGGTAATACGGAGGATCCAAGCGTTATCCGGAATCATTGGGTTTAAAGGGTCCGTAGGCGGTTTAGTAAGTCAGTGGTGAAAGCCCATCGCTCAACGGTGGAACGGCCATTGATACTGCTGAACTTGAATTATTAGGAAGTAACTAGAATATGTAGTGTAGCGGTGAAATGCTTAGAGATTACATGGAATACCAATTGCGAAGGCAGGTTACTACTAATTGATTGACGCTGATGGACGAAAGCGTGGGTAGCGAACAGG` |
| ASV2866 | 424 | `TAAGGAATATTGGTCAATGGAGGCAACTCTGAACCAGCCATGCCGCGTGCAGGAAGACTGCCCTATGGGTTGTAAACTGCTTTTATCCGGGAATAAACCTACTTACGTGTAAGTAGCTGAATGTACCGGAAGAATAAGGATCGGCTAACTCCGTGCCAGCAGCCGCGGTAATACGGAGGATCCAAGCGTTATCCGGATTTATTGGGTTTAAAGGGTGCGTAGGCGGCCTATTAAGTCAGGGGTGAAAGACGGTAGCTCAACTATCGCAGTGCCTTTGATACTGATGGGCTTGAATGAACTAGAGGTAGGCGGAATGAGACAAGTAGCGGTGAAATGCATAGATATGTCTCAGAACACCGATTGCGAAGGCAGCTTACTATGGTTTAATTGACGCTGAGGCACGAAAGCGTGGGGATCAAACAGG` |
| ASV5183 | 414 | `TGGGGAATTTTGGACAATGGGGGCAACCCTGATCCAGCAACTCTGCGTGAGGGACGAAGTCCTTCGGGATGTAAACCTCTTTTGTCGGGGACGAACGCCGCAAGGCTTGACGGTACCCGGCGAATAAGCCACGGCTAACTATGTGCCAGCAGCCGCGGTAAGACATAGGTGGCGAGCGTTATTCGGAATTACTAGGCGTAAAGCGAGTGTAGGCGGGCGCTTAAGTCCGTTGTGAAATCTCCCGGCTCAACTGGGAGGGGTCAACGGATACTGGGTGTCTTGAGTGGGGTAGGGGGCGCTGGAATTCCCGGTGTAGCGGTGAAATGCGTAGATATCGGGAGGAACACCAATGGCGAAAGCAGGCGCCTGGGCCTCTACTGACGCTAAGACTCGAAAGCTAGGGGAGCAAACAGG` |
| ASV3084 | 407 | `TGGGGAATTTTCCGCAATGGGCGAAAGCCTGACGGAGCAAGACCGCGTGAGGGAAGAAGGTCTGTGGATTGTAAACCTCTTTTGATAGGGAAGAAGACAGTGACGGTACCTATCGAATCAGCCTCGGCTAACTCCGTGCCAGCAGCCGCGGTAATACGGAGGAGGCAAGCGTTATCCGGAATTATTGGGCGTAAAGCGTCCGTAGGTGGGTTATCAAGTCAGTTGTTAAATAGTGAAGCTTAACTTCATAGAGGCAATTGAAACTGATAGTCTAGAGTGTGGTAGGGGTAGAGGGAATTTCCAGTGTAGCGGTGAAATGCGTAGATATTGGAAAGAACACCGGTGGCGAAAGCGCTCTACTGGGCCTGCACTGACACTGAGGGACGAAAGCTAGGGGAGCGAAAGGG` |
| ASV4300 | 428 | `TGGGGAATCTTGCGCAATGGGCGAAAGCCTGACGCAGCGACGCCGCGTGAGGGAAGAAGGCCTTCGGGTTGTAAACCTCTTTCAGCAGGGACGAAGCTACTCGGGTTAATAGCCCAGAGGGTGACGGTACCTGCAGAAGAAGCCCCGGCTAACTACGTGCCAGCAGCCGCGGTAATACGTAGGGGGCAAGCGTTGTCCGGATTTACTGGGCGTAAAGAGCGTGTAGGCGGCCAAGCAGGTCTGTTGTGAAAACTGGAGGCTCAACCTCCAGACGTCGACAGAAACCGCTCGGCTAGAGTCCGGAAGAGGAGAGTGGAATTCCTGGTGTAGCGGTGAAATGCGCAGATATCAGGAAGAACACCAATGGCGAAGGCAGCTCTCTGGGACGGTACTGACGCTGAGACGCGAAAGCGTGGGGAGCGAACAGG` |
| ASV3941 | 430 | `TGGGGAATATTGGACAATGGGGGGAACCCTGATCCAGCGATGCCGCGTGTGTGAAGAAGGCCTGCGGGTTGTAAAGCACTTTTGTTGGGGAAGAGGGTTATGTGCGTGAATAGCGTATGTAATTGACGGTACCCAAAGAATAAGCACCGGCTAACTCTGTGCCAGCAGCCGCGGTAATACAGAGGGTGCGAGCGTTAATCGGAATTACTGGGCGTAAAGGGCGCGTAGGTGGAAGAGTGAGTCAGATGTGAAAGTCCTGGGCTTAACCTAGGGATTGCATCTGATACTGCTTTTCTTGAGTGAAGTAGAGGGTGGCGGAATTTCCGGTGTAGCGGTGAAATGCGTAGATATCGGAAAGAACACCAGTGGCGAAGGCGGCCACCTGGACTTTAACTGACACTGAGGCGCGAAAGCATGGGGAGCGAACAGG` |
| ASV957 | 429 | `TGGGGAATTTTGGACAATGGGGGCAACCCTGATCCAGCAATGCCGCGTGAGTGAAGAAGGCCTTCGGGTTGTAAAGCTCTTTTGTTAGGGAAGAAAAGGTCTCGGATAATACCCGAGGCTGATGACGGTACCTAAAGAATAAGCACCGGCTAACTACGTGCCAGCAGCCGCGGTAATACGTAGGGTGCAAGCGTTAATCGGAATTACTGGGCGTAAAGCGTGCGCAGGCGGCCTCGCAAGTCAGATGTGAAATCCCCGAGCTCAACTTGGGAACTGCATTTGAAACTACGAGGCTAGAGTGTGTCAGAGGGAGGTAGAATTCCACGTGTAGCAGTGAAATGCGTAGATATGTGGAGGAATACCAGTGGCGAAGGCGGCCTCCTGGGATAACACTGACGCTCATGTACGAAAGCGTGGGGAGCAAACAGG` |
| ASV5034 | 406 | `TGGGGAATATTGGGCAATGGGCGAAAGCCTGACCCAGCAACGCCGCGTGAGGGAAGAAGGCTTTCGGGTCGTAAACCTCTGTCCCATGGGACGAGAAGAAGACGGTACCATGGGAGGAAGCCCCGGCTAACTACGTGCCAGCAGCCGCGGTAATACGTAGGGGGCGAGCGTTGTCCGGAATGACTGGGCGTAAAGGGCGCGTAGGCGGCCTTGTAAGTCTGGAGTGAAAGTCCTACTTTTAAGGTGGGAATTGCTTTGGAAACTGTGAGGCTTGAGTATGGGAGAGGATAGCGGAATTCCCGGTGTAGCGGTGAAATGCGTAGAGATCGGGAGGAACACCAGTGGCGAAGGCGGCTATCTGGACCAAAACTGACGCTGAGGCGCGAAAGCGTGGGTAGCAAACAGG` |
| ASV5109 | 424 | `TGAGGAATATTGGACAATGGGCGCAAGCCTGATCCAGCCATGCCGCGTGCAGGATGACGGTCCTATGGATTGTAAACTGCTTTTATACGAGAAGAAACACCCCGACGTGTCGGGACTTGACGGTATCGTAAGAATAAGGATCGGCTAACTCCGTGCCAGCAGCCGCGGTAATACGGAGGATCCGAGCGTTATCCGGAATCATTGGGTTTAAAGGGTCCGTAGGCTGTTTAATAAGTCAGTGGTGAAATCTGGTCGCTCAACGATCAAATTGCCATTGATACTGTTAGACTTGAATTATTTGGAAGTAACTAGAATATGTAGTGTAGCGGTGAAATGCTTAGATATTACATGGAATACCAATTGCGAAAGCAGGTTACTACAAATACATTGACGCTGATGGACGAAAGCGTGGGTAGCGAACAGG` |
| ASV5666 | 429 | `TGGGGAATCTTCCGCAATGGACGAAAGTCTGACGGAGCAACGCCGCGTGAGTGAAGAAGGTTTTCGGATCGTAAAGCTCTGTCGTTCGGGACGAATGTTACTTTTGTAAATAATGGGAGTAAATGACGGTACCGAGCAAGAAAGCCACGGCTAACTACGTGCCAGCAGCCGCGGTAATACGTAGGTGGCAAGCGTTGTCCGGAATTATTGGGCGTAAAGGGAGCGCAGGTGGGAATATCAGTCTGTTTTAAAAGATCGGGGCTCAACCCCGTTAGGGAACAGAAACTGTATTTCTTGAGTGCAGGAGAGGTAAGTGGAATTCCTAGTGTAGCGGTGAAATGCGTAGATATTAGGAGGAACACCAGTGGCGAAGGCGACTTACTGGACTGTAACTGACACTGAGGCTCGAAAGCCAGGGGAGCGAACGGG` |
| ASV3203 | 404 | `TGGGGAATATTGGACAATGGGCGAAAGCCTGATCCAGCAATACCGCGTGAATGATGAAGGTCTTAGGGTTGTAAAGTTCTTTTAGTTGGAAAGATAATGACGGTACCAACAAAAAAAGCCCCGGCTAACTCCGTGCCAGCAGCCGCGGTAAGACGGAGGGGGCTAGCGTTGTTCGGAATTACTGGGCGTAAAGAGCGCGTAGGTGGATTAGTAAGTTGGGAGTGAAAGCCCGGGGCTTAACCTCGGAACTGCTTTCAAAACTGCTAGTCTTGAGTGAAGTAGGGGATGATGGAATTCCTAGTGTAGAGGTGAAATTCTTAGATATTAGGAGGAACACCGGTGGCGAAGGCGGTCATCTGGACTTCAACTGACGCTGAGGCGCGAAAGCGTGGGGAGCAAACAGG` |
| ASV2336 | 429 | `TGGGGAATTTTGGACAATGGGGGGAACCCTGATCCAGCAACGCCGCGTGAATGAAGAAGGCCTTCGGGTTGTAAAGTTCTTTTGTCAGAGAGCAAAGGATGGTGGCTAATACCCGCTATCGCTGAGAGTATCTGAAGAATAAGCACCGGCTAACTACGTGCCAGCAGCCGCGGTAATACGTAGGGTGCGAGCGTTAATCGGAATTACTGGGCGTAAAGGGTGCGCAGGCGGATAATTAAGTTAGGAGTGAAATCCCCGAGCTCAACTTGGGAATTGCTTTTAAGACTGGTTATCTAGAGTTTGTCAGAGGGGGGTAGAATTCCAGGTGTAGCAGTGAAATGCGTAGAGATCTGGAGGAATACCAATGGCGAAGGCAGCCCCCTGGGATAAAACTGACGCTCATGCACGAAAGCGTGGGTAGCAAACAGG` |
| ASV5699 | 423 | `TAGGGAATATTGGTCAATGGAGGGAACTCTGAACCAGCCATGCCGCGTGCAGGAAGAAGGCCCTCTGGGTTGTAAACTGCTTTTGACGGGGAACAAATGTGCCATGCGTGGCAAGTTGAGTGTACCCGTAGAATAAGCCACGGCTAACTACGTGCCAGCAGCCGCGGTAATACGTAGGTGGCGAGCGTTGTCCGGATTTATTGGGTTTAAAGGGTGCGTAGGCGGCCCGTTAAGTCAGTTGTGAAATACCCCGGCTCAACCGGGGGGGTGCGATTGATACTGTCGGGCTTGAGTGGCAGCGAGGTTGGCGGAATTGACGGTGTAGCGGTGAAATGCATAGATATCGTCAGGAACACCGATAGCGAAGGCAGCCAACTAGGGGCCCACTGACGCTGAGGCACGAGAGTGCGGGGATCAAACAGG` |
| ASV2876 | 429 | `TGGGGAATCTTGGACAATGGGCGAAAGCCTGATCCAGCAATGCCGCGTGTGTGAAGAAGGCCTTCGGGTTGTAAAGCACTTTTGTCAAGAACGAAAAGTTACTTATTAATACTAGGTAATGATGACGGTACTTGAAGAATAAGCACCGGCTAACTACGTGCCAGCAGCCGCGGTAATACGTAGGGTGCAAGCGTTAATCGGAATTACTGGGCGTAAAGCGTGCGCAGGCGGTAACCCAAGTCAGATGTGAAATCCCCGGGCTCAACCTGGGAACTGCATTTGAAACTAGGTCACTAGAGTGTGTCAGAGGGGGGTAGAATTCCACGTGTAGCAGTGAAATGCGTAGATATGTGGAGGAATACCGATGGCGAAGGCAGCCCCCTGGGATAACACTGACGCTCATGCACGAAAGCGTGGGGAGCAAACAGG` |
| ASV3183 | 407 | `TAGGGAATATTGCGCAATGGACGAAAGTCTGACGCAGCGACGCCGCGTGAGTGATGAAGGCCTTAGGGTCGTAAAGCTCTGTTGAATGGGAAGAAAAAAATGACGGTACCATTCGAGGAAGGACCGGCTAACTTCGTGCCAGCAGCCGCGGTAATACGAGGGGTCCTAGCGTTGTTCGGAATCATTGGGCGTAAAGGGTGTGTAGGTGGCTATGTAAGTCAGGTGTGAAAGCCCCGGGCTCAACCCGGGAAGTGCATTTGATACTGCGTAGCTTGAGTACCGAAGAGGATAGTGGAATTCCAGGTGTAGTGGTGAAATACGTAGATATCTGGAGGAACACCGGTGGCGAAGGCGGCTATCTGGTCGAGTACTGACACTGAGACACGAAAGCATGGGGATCAAACAGG` |
| ASV1059 | 387 | `GCGAGAAAACTTTGCAATGTGCGAAAGCACGACAAGGTTAATCCGAGTGGTTTCTGCTAAAGAAACCTTTTGTCAGTCCTAGAAACACTGATGAATAAGGGGTGGGCAAGTTCTGGTGTCAGCCGCCGCGGTAAAACCAGCACCTCAAGTGGTCAGGATGATTATTGGGCCTAAAGCATCCGTAGCTCGTTTTGTAAGTTTTCGGTTAAATCCATGCGCTTAACGTATGGGCTGCCGGGAATACTGCATAACTAGGAAGTGGGAGAGGTAGACGGTACTCGGTAGGAAGGGGTAAAATCCTTTGATCTATTGATGACCACCTGTGGCGAAGGCGGTCTACCAGAACACGTTCGACGGTGAGGGATGAAAGCTGGGGGAGCAAACCGG` |
| ASV5452 | 423 | `TAGGGAATATTGGTCAATGGGTGAGAGCCTGAACCAGCCATGCCGCGTGCAGGAAGAAGGCCTTCTGGGTTGTAAACTGCTTTTGCCAGGGGATAAAACGGTCGTGCGCGACTAATTGAAGGTACCTGGTGAATAAGCCACGGCTAACTACGTGCCAGCAGCCGCGGTAATACGTAGGTGGCAAGCGTTGTCCGGATTTATTGGGTTTAAAGGGTGCGTAGGCGGCCCTGTAAGTCAGTGCTGAAATCCCCCGGCTTAACCGGGGTGGTGGCATTGATACTGCAGGGCTTGAGTACAGATGAGGTAGGCGGAATTGACGGTGTAGCGGTGAAATGCATAGATATCGTCAAGAACACCGATAGCGAAGGCAGCTTACTGGGCTGTAACTGACGCTGATGCACGAAAGTGTGGGGATCAAACAGG` |
| ASV40 | 429 | `TGGGGAATTTTGGACAATGGGCGCAAGCCTGATCCAGCAATGCCGCGTGCAGGATGAAGGCCTTCGGGTTGTAAACTGCTTTTGTACGGAGCGAAAAGGTCTCTTCTAATACAGGAGGCCCATGACGGTACCGTAAGAATAAGCACCGGCTAACTACGTGCCAGCAGCCGCGGTAATACGTAGGGTGCAAGCGTTAATCGGAATTACTGGGCGTAAAGCGTGCGCAGGCGGTGTTGTAAGACAGAGGTGAAATCCCCGGGCTCAACCTGGGAACTGCCTTTGTGACTGCAACGCTAGAGTACGGCAGAGGGGGATGGAATTCCGCGTGTAGCAGTGAAATGCGTAGATATGCGGAGGAACACCGATGGCGAAGGCAATCCCCTGGGCCTGTACTGACGCTCATGCACGAAAGCGTGGGGAGCAAACAGG` |
| ASV4331 | 429 | `TGGGGAATTTTGGACAATGGGGGCAACCCTGATCCAGCCATGCCGCGTGTGTGAAGAAGGCCTTCGGGTTGTAAAGCACTTTCGGACGGAACGAAATCGCGCGGGTGAATATCCCGCGTGGATGACGGTACCGTAAGAAGAAGCACCGGCTAACTACGTGCCAGCAGCCGCGGTAATACGTAGGGTGCGAGCGTTAATCGGAATTACTGGGCGTAAAGTGTGCGCAGGCGGCCCCGCAAGTCGAGTGTGAAATCCCCGGGCTTAACTTGGGAATGGCGCTCGAAACTACGGGGCTTGAGTGTGGCAGAGGGGGGTGGAATTCCACGTGTAGCGGTGAAATGCGTAGAGATGTGGAGGAACACCAATGGCGAAGGCAGCCCCCTGGGCCAACACTGACGCTCATGCACGAAAGAGTGGGGAGCAAACAGG` |
| ASV1212 | 373 | `TAGTCCTACGCGTGAGGGAAGACTGCCTATGGGTTGTAAACCTCTTTTTTCAGGGAGGAACAAAATGACGTGTACCTGAAGAATAAGCATCGGCTAACTCCGTGCCAGCAGCCGCGGTAAGACGGAGGATGCAAGTGTTATCCGGAATCACTGGGCGTAAAGCGTCTGTAGGTGGTTTAATAAGTCAACTGTTAAATCTTGAGGCTCAACTTCAAAATCGCAGTTGAAACTATTAGACTAGAGTATAGTAGAGGTAAAGGGAATTTCCAGTGGAGCGGTGAAATGCGTAGATATTGGAAAGAACACCGATGGCGAAAGCACTTTACTGGGCTATTACTAACACTCAGAGACGAAAGCTAGGGTAGCAAATGGG` |
| ASV2688 | 429 | `TGAGGAATATTGCACAATGGGCGAAAGCCTGATGCAGCGACGCCGCGTGGAGGATGAAGGTTTTCGGATTGTAAACTCCTGTTAAGTGTGAAAAAAGAGCTATCTTTAATAGAGATAGAAGATGATTGTAGCACTAAAGAAAGCACCGGCTAACTTCGTGCCAGCAGCCGCGGTAATACGAGGGGTGCAAACGTTATTCGGAATCACTGGGCGTAAAGGGTGCGTAGACGGCAAGATAAGTCAATTATTAAATCTCCTGGCTTAACCAGGAACCTGTAATTGAAACTGACTTGCTTGAGGATGGAAGAGAGAAGCGGAATTCTCGGAGTAGCGGTAAAATGCGTAGATCTCGAGAGGAACACCGATGGCGAAGGCAGCTTCTTGGTCCATTTCTGACGTTGAGGCACGAAAGCGTGGGGAGCAAACAGG` |
| ASV5241 | 405 | `TAGGGAATCTTCCGCAATGGACGAAAGTCTGACGGAGCAATACCGCGTGGTCGATGAAGCCCCTTGGGGTGTAAAGACCTTTTCTGAGAGAAGAATTATGACGGTATCTTAGGAATAAGCACCGGCTAACTCTGTGCCAGCAGCCGCGGTAATACAGAGGGTGCGAGCGTTATCCGGAATTATTGGGCGTAAAGCGTCCGCAGGTGGTCTACTAAGTAGGATATTAAAGCCAACGGCTCAACCGTTGTAAAGTGTCCTAAACTGGTAAACTAGAGACTCATAGAGGTACGTAGAATTCTGCATGTAGGGGTAAAATCCGTAGATATGCAGAGGAATACCAAAAGCGAAAGCAGCGTACTGGATGAGTTCTGACACTCAGGGACGAAAGCGTGGGGAGCAAACAGG` |
| ASV287 | 429 | `TGGGGAATTTTGGACAATGGGCGCAAGCCTGATCCAGCCATGCCGCGTGAGTGAAGAAGGCCTTCGGGTTGTAAAGCTCTTTTGTCCGGGAGCAAATCCTGATTCCTAATACGGATTGGGGCTGAGAGTACCGGAAGAATAAGGACCGGCTAACTACGTGCCAGCAGCCGCGGTAATACGTAGGGTCCAAGCGTTAATCGGAATTACTGGGCGTAAAGGGTGCGCAGGTGGTTGTGCAAGCCAGATGTGAAATCCCCGGGCTTAACCTGGGAACTGCATTTGGGACTGCACGGCTAGAGTGTGGCAGAGGGGGGTGGAATTCCGCGTGTAGCAGTGAAATGCGTAGAGATGCGGAGGAACACCAATGGCGAAGGCAGCCCCCTGGGTTGACACTGACACTCATGCACGAAAGCGTGGGGAGCAAACAGG` |
| ASV2293 | 423 | `TAGGGAATATTGGTCAATGGGCGCAAGCCTGAACCAGCCATGCCGCGTGCAGGATGAAGGCCCTCAGGGTCGTAAACTGCTTTTGTACGGGAACAAAAAGCTCCTGCGGGAGCCATTGAGGGTACCGTAAGAATAAGCACCGGCTAACTCCGTGCCAGCAGCCGCGGTAATACGGAGGGTGCAAGCGTTATCCGGATTTACTGGGTTTAAAGGGTGCGTAGGCGGCTTTTTAAGTCAGTGGTGAAAGCCTGGCGCTTAACGCCAGAATTGCCATTGATACTGGGAAGCTTGAGTCGAGAAGAGGTAAGCAGAATTCATGGTGTAGCGGTGAAATGCATAGATACCATGAGGAATTCCAATAGCGAAGGCAGCTTACTGGTCTCTGACTGACGCTGAGGCACGAAAGCGTGGGGAGCAAACAGG` |
| ASV4889 | 424 | `TGAGGAATATTGGTCAATGGCCGCAAGGCTGAACCAGCCAAGTCGCGTGAGGGAATAAGGCCCTACGGGTCGTAAACCTCTTTTGCCGGGGAGCAAGGATTTCCACGCGTGGAGATTCGGAGAGTACCCGGAGAAAAAGCATCGGCTAACTCCGTGCCAGCAGCCGCGGTAATACGGAGGATGCGAGCGTTATCCGGATTTATTGGGTTTAAAGGGTGCGTAGGCGGACTGACAAGTCAGCGGTCAAAACCCGGGGCTCAACCCCGGCCTGCCGTTGAAACTGTCAGCCTTGAGTGAGCGAGAAGTAAGCGGAATGCGTGGTGTAGCGGTGAAATGCATAGATATCACGCAGAACCCCGATTGCGAAGGCAGCTTACCGGCGCTCAACTGACGCTGAGGCACGAAAGTGCGGGGATCAAACAGG` |
| ASV846 | 429 | `TGGGGAATTTTGGACAATGGACGCAAGTCTGATCCAGCCATTCCGCGTGCAGGACGAAGGCCTTCGGGTTGTAAACTGCTTTTGTACAGAACGAAAAGGTCTCTATTAATACTAGGGGCTCATGACGGTACTGTAAGAATAAGCACCGGCTAACTACGTGCCAGCAGCCGCGGTAATACGTAGGGTGCAAGCGTTAATCGGAATTACTGGGCGTAAAGCGTGCGCAGGCGGTTATATAAGACAGATGTGAAATCCCCGGGCTCAACCTGGGAACTGCATTTGTGACTGTATAGCTGGAGTGCGGCAGAGGGGGATGGAATTCCGCGTGTAGCAGTGAAATGCGTAGATATGCGGAGGAACACCGATGGCGAAGGCAATCCCCTGGGCCTGCACTGACGCTCATGCACGAAAGCGTGGGGAGCAAACAGG` |
| ASV2397 | 429 | `TAGGGAATCTTCCACAATGGACGAAAGTCTGATGGAGCAACGCCGCGTGAGTGAAGAAGGGTTTCGGCTCGTAAAACTCTGTTATTGGAGAAGAAGGATAAGGTAAGTAACTGTACTTTATTTGACGGTACCCAACCAGAAAGTCACGGCTAACTACGTGCCAGCAGCCGCGGTAATACGTAGGTGGCAAGCGTTATCCGGATTTATTGGGCGTAAAGCGAGTGCAGGCGGTTTTATAAGTCTGATGTGAAAGCCCTCGGCTCAACCGAGGAACTGCATCAGAAACTGTGAGACTTGAGTGCAGAAGAGGAGAGTGGAACTCCATGTGTAGCGGTGGAATGCGTAGATATATGGAAGAACACCAGTGGCGAAGGCGGCTCTCTGGTCTGTAACTGACGCTGAGGCTCGAAAGCATGGGTAGCGAACAGG` |
| ASV3032 | 404 | `TGGGGAATATTGGACAATGGGGGAAACCCTGATCCAGCCATGCCGCGTGAGTGATGAAGGCCTTAGGGTTGTAAAGCTCTTTTGTCAGGGAAGATGATGACGGTACCTGAAGAATAAGCACCGGCTAACTTCGTGCCAGCAGCCGCGGTAATACGAAGGGTGCTAGCGTTGTTCGGAATCACTGGGCGTAAAGCGCGCGTAGGCGGCTTGTCAAGTCAGGGGTGAAATCCCACAGCTTAACTGTGGAACTGCCTTTGAAACTGCCAAGCTGGAGTGTCGGAGGGGATAGCGGAATTGCTAATGTAGAGGTGAAATTCGTAGATATTAGCAGGAACACCGGTGGCGAAGGCGGCTATCTGGACGACAACTGACGCTGAGGCGCGAAAGCGCGGGGATCAAACAGG` |
| ASV2417 | 429 | `TCGAGAATCTTTCACAATGGGGGAAACCCTGATGGAGCGACGCCGCGTGGAGGATGACGGTCTTCGGATTGTAAACTCCTGTCATGAAAGAACAAGCGGAGCGGGTGAACAGCCCGCCGAGTTGATAGTATTTCAAGAGGAAGGGACGGCTAACTTCGTGCCAGCAGCCGCGGTAATACGAAGGTCCCAAGCGTTGTTCGGAATCACTGGGCGTAAAGAGTGCGTAGGCGGCGCGGAAAGTCAGATGTGAAATCCCGGGGCTCAACCTCGGAACGGCATCCGATACTCCCGCGCTTGAGGACTGGAGGGGAGTCTGGAATTCACGGTGTAGCAGTGAAATGCGTAGATATCGTGAGGAACACTAGTGGCGAAGGCGAGACTCTGGACAGTTCCTGACGCTGAGGCACGAAGGCCAGGGGAGCGAAGGGG` |
| ASV5157 | 411 | `TCGAGGATCTTCGGCAATGGACGCAAGTCTGACCGAGCAACGCCGCGTGTGCGATGAAGGCCTTCGGGTTGTAAAGCACTGTCGAGAGGGAACAAGCCGCAAGGTTGAGTGACCTTTGGAGGAAGGACGGGCTAAGTTCGTGCCAGCAGCCGCGGTAAGACGAACCGTCCAAACGTTATTCGGAATCACTGGGCTTAAAGGGCGCGTAGGCGGGCGATCAAGTCAGGGGTGAAATCCTCCAGCTCAACTGGAGAAGTGCCTTTGATACTGGTTGTCTAGAGTGAGGTAGGGGCATGTGGAACTTCCGGTGGAGCGGTGAAATGCGTAGATATCGGAAGGAACGCCAGTGGCGAAAGCGATGTGCTGGATCTCAACTGACGCTGAGGCGCGAAAGCTAGGGGATCAAACGGG` |
| ASV2750 | 407 | `TAGGGAATATTGCGCAATGGGGGAAACCCTGACGCAGCAATTCCGCGTGGAGGATGACGGATTTCGGTCTGTAAACTCCTGTCAGCAGGGAAGTGAATAAGGACGGTACCTGCAGAGGAAGCACCGGCTAACTACGTGCCAGCAGCCGCGGTAATACGTAGGGTGCAAGCGTTGTCCGGAATTATTGGGCGTAAAGCGTCCGTAGGCGGTTAATCAAGTCATACGTTAAAGGTACGGGCTCAACCCGTGTATGGTGTATGAAACTGTTTGACTCGAATTAGGTAGAGGTTGCTGGAATTCCTGGTGTAGCGGTGAAATGCGTAGATATCAGGAGGAACACCTGTGGCGAAAGCGTGCAACTGGGCCTTAATTGACGCTAATGGACGAAGGCTAGGGTAGCGAAAGGG` |
| ASV2193 | 429 | `TGGGGAATTTTGGACAATGGACGCAAGTCTGATCCAGCAATGCCGCGTGCAGGACGAAGGCCTTCGGGTTGTAAACTGCTTTTGTACGGAACGAAACGGTGAGCTCTAATACAGTTTGCTAATGACGGTACCGTAAGAATAAGCACCGGCTAACTACGTGCCAGCAGCCGCGGTAATACGTAGGGTGCGAGCGTTAATCGGAATTACTGGGCGTAAAGCGTGCGCAGGCGGTTATATAAGACAGATGTGAAATCCCCGGGCTCAACCTGGGACCTGCATTTGTGACTGTATAGCTAGAGTACGGTAGAGGGGGATGGAATTCCGCGTGTAGCAGTGAAATGCGTAGATATGCGGAGGAACACCGATGGCGAAGGCAATCCCCTGGACCTGTACTGACGCTCATGCACGAAAGCGTGGGGAGCAAACAGG` |
| ASV5634 | 407 | `TGAGGAATTTTCCGCAATGGGCGAAAGCCTGACGGAGCAATGCCGCGTGGAGGACGAAGGCCCGTGGGTTGTAAACTCCTTTTCTCAGAGAAGAAGACATGACGGTATCTGAGGAATAAGCTCCGGCTAACTCTGTGCCAGCAGCCGCGGTAATACAGAGGGAGCAAGCGTTATCCGGAATGATTGGGCGTAAAGCGTCTGTAGGTGGTTCATTAAGTCTACTGTCAAATACCAGGGCTTAACCTTGGGCAGGCAGTGGAGAACTAATGAGCTAGAGTATGGTAGAGGCAGAGGGAATTCCATGTGGAGCGGTGAAATGCGTAGAGATATGGAAGAACACCAGTGGCGAAGGCGCTCTGCTGGGCCAAAACTGACACTGAGAGACGAAAGCCAGGGGAGCAAATAGG` |
| ASV591 | 429 | `TGGGGAATTTTGGACAATGGGCGCAAGCCTGATCCAGCAATGCCGCGTGAGTGAAGAAGGCCTTCGGGTTGTAAAGCTCTTTTGTCAGGGAAGAAACGGGAGTGGCTAATATCTGCTTCTAATGACGGTACCTGAAGAATAAGCACCGGCTAACTACGTGCCAGCAGCCGCGGTAATACGTAGGGTGCAAGCGTTAATCGGAATTACTGGGCGTAAAGCGTGCGCAGGCGGTTTTGTAAGTCTGTCGTGAAATCCCCGGGCTCAACCTGGGAATTGCGATGGAGACTGCAAGGCTAGAATCTGGCAGAGGGGGGTAGAATTCCACGTGTAGCAGTGAAATGCGTAGAGATGTGGAGGAACACCGATGGCGAAGGCAGCCCCCTGGGTCAAGATTGACGCTCATGCACGAAAGCGTGGGGAGCAAACAGG` |
| ASV5341 | 424 | `TGAGGAATATTGGTCAATGGCCGGAAGGCTGAACCAGCCAAGTCGCGTGAGGGAATAAGGCCCTACGGGTCGTAAACCTCTTTTGTCCGGGAGCAAGGACATCCACGTGTGGATGTTCGGAGAGTACCGGAAGAAAAAGCATCGGCTAACTCCGTGCCAGCAGCCGCGGTAATACGGAGGATGCGAGCGTTATCCGGATTTATTGGGTTTAAAGGGTGCGTAGGCGGCATGAAAAGTCAGCGGTAAAATAGCGGGGCTCAACCCCGTCGAGCCGTTGAAACTTTCAGGCTTGAGTGAGCGAGAAGTATGCGGAATGCGTGGTGTAGCGGTGAAATGCATAGATATCACGCAGAACTCCGATTGCGAAGGCAGCATACCGGCGCTCAACTGACGCTGAGGCACGAAAGTGCGGGGATCAAACAGG` |
| ASV2216 | 407 | `TGGGGAATTTTCCGCAATGGGGGCAACCCTGACGGAGCAATACCGCGTGAGGGACGAAGGCTTTTGGGTCGTAAACCTCTTTTGTTTGGGAAGAAAAACCTGACGGTACCAAACGAATAAGCATCGGCTAACTCCGTGCCAGCAGCCGCGGTAAGACGGAGGATGCAAGCGTTATCCGGAATTATTGGGCGTAAAGCGTCCGCAGGTGGCCATTCAAGTCTGTTGTCAAAGAGCAAGGCTCAACCTTGTAAAGGCAATGGAAACTGAATAGCTAGAGTGCGGTAGGGGTAGAGGGAATTCCCGGTGTAGCGGTGAAATGCGTAGATATCGGGAAGAACACCAGTGGCGAAAGCGCTCTACTGGGCCTGCACTGACACTGAGGGACGAAAGCTAGGGGAGCGAAAGGG` |
| ASV1480 | 429 | `TGGGGAATTTTGGACAATGGGGGCAACCCTGATCCAGCCATTCCGCGTGAGTGAAGAAGGCCTTCGGGTTGTAAAGCTCTTTTGTCGGGGACGAAACGGTTAAGGCTAATAACCTTGACTACTGACGGTACCCGAAGAATAAGCACCGGCTAACTACGTGCCAGCAGCCGCGGTAATACGTAGGGTGCAAGCGTTAATCGGAATTACTGGGCGTAAAGCGTGCGCAGGCGGTTTTGTAAGCCAGATGTGAAATCCCCGGGCTCAACCTGGGAATGGCATTTGGGACTGCAAGGCTAGAGTATGGCAGAGGAGACTAGAATTCCTGGTGTAGCAGTGAAATGCGTAGATATCAGGAGGAATACCGATGGCGAAGGCAGGTCTCTGGGTCAATACTGACGCTCATGCACGAAAGCGTGGGGAGCAAACAGG` |
| ASV5420 | 429 | `TGGGGAATCTTGGACAATGGGCGCAAGCCTGATCCAGCAATGCCGCGTGCGTGAAGAAGGCCTTCGGGTTGTAAAGCGCTTTTGTCCGGGAGCAAATCCTTGTGGCTAATACCCACTGGGGATGAGAGTACTGGAAGAATAAGGACCGGCTAACTACGTGCCAGCAGCCGCGGTAATACGTAGGGTCCAAGCGTTAATCGGAATTACTGGGCGTAAAGCGTGCGCAGGTGGCCCGTTAAGAGTGATGTGAAATCCCCGAGCTTAACTTGGGAATTGCATTGCTAACTGGCGGGCTAGAGTACGGGAGAGGGGGGTGGAATTCCACGTGTAGCAGTGAAATGCGTAGAGATGTGGAGGAACACCGATGGCGAAGGCAGCCCCCTGGCCTGCTACTGACACTCATGCACGAAAGCGTGGGGAGCAAACAGG` |
| ASV5075 | 430 | `TGGGGAATATTGGACAATGGGGGGAACCCTGATCCAGCAATGCCGCGTGTGTGAAGAAGGCCTGAGGGTTGTAAAGCACTTTCGGTGGTGAGGAAGGGGCATAGGTTAAGAGCTAATGCCACTTGACGGTAGCCACATAAGAAGCACCGGCTAACTCTGTGCCAGCAGCCGCGGTAATACAGAGGGTGCGAGCGTTAATCGGAATTACTGGGCGTAAAGCGCACGTAGGTGGTTAGATAAGTTAGATGTGAAATACCAGGGCTTAACCTTGGAGGGTCATTTAATACTGTTTAGCTAGAGTACAGTAGAGGGAAGTGGAATTTCCGGTGTAGCGGTGAAATGCGTAGATATCGGAAGGAACATCAGTGGCGAAGGCGGCTTTCTGGACTGATACTGACACTGAGGTGCGAAAGCGTGGGGAGCAAACAGG` |
| ASV624 | 404 | `TGGGGAATATTGGACAATGGGGGAAACCCTGATCCAGCAATGCCGCGTGTGTGATGAAGGCCTTCGGGTTGTAAAGCACTTTTGTCAGGGACGATGATGACGGTACCTGACGAATAAGCTCCGGCTAACTTCGTGCCAGCAGCCGCGGTAATACGAGGGGAGCTAGCGTTGTTCGGAATTACTGGGCGTAAAGGGCGTGTAGGCGGTACCATAAGTCAGATGTGAAAGCCCTAGGCTTAACCTAGGAGGTGCATTTGATACTGTGGAACTAGAGATCGAGAGAGGTAAGTGGAATTACGAGTGTAGAGGTGAAATTCGTAGATATTCGTAAGAACACCAGTGGCGAAGGCGACTTACTGGCTCGATACTGACGCTAAGGCGCGAAAGCGTGGGGAGCAAACAGG` |
| ASV1440 | 429 | `TGGGGAATTTTGGACAATGGGCGAAAGCCTGATCCAGCAATGCCGCGTGAGTGAAGAAGGCCTTCGGGTTGTAAAGCTCTTTTGTCAGGGAAGAAACGGTGAAGGCTAATATCCTTTGCTAATGACGGTACCTGAAGAATAAGCACCGGCTAACTACGTGCCAGCAGCCGCGGTAATACGTAGGGTGCAAGCGTTAATCGGAATTACTGGGCGTAAAGCGTGCGCAGGCGGTTTTGTAAGTCTGATGTGAAATCCCCGGGCTCAACCTGGGAATTGCATTGGAGACTGCAAGGCTAGAATCTGGCAGAGGGGGGTAGAATTCCACGTGTAGCAGTGAAATGCGTAGATATGTGGAGGAACACCGATGGCGAAGGCAGCCCCCTGGGTCAAGATTGACGCTCATGCACGAAAGCGTGGGGAGCAAACAGG` |
| ASV3922 | 404 | `TGAGGAATATTGGACAATGGGCGCAAGCCTGATCCAGCCATGCCGCGTGAGTGATGAAGGCCTTCGGGTTGTAAAACTCTTTTAGATGTGACGATGATGACGGTAACATCAGAAAAAGCCCCGGCTAACTTCGTGCCAGCAGCCGCGGTAATACGAAGGGGGCGAGCGTTGTTCGGAATCACTGGGCTTAAAGCGTGCGTAGGTGGGCAGGATAGTCAGAAGTGAAATCCCGGGGCTTAACCCCGGAACTGCTTTTGAAACTTCCTGTCTGGAATTCGGTAGAGGCTGATGGAATACCCAGTGTAGAGGTGAAATTCGCAGATATTGGGTGGAACACCAGTGGCGAAGGCGATCAGCTGGGCCGATATTGACACTGAGGCACGAAAGCGTGGGTAGCAAACAGG` |
| ASV1501 | 404 | `TGGGGAATATTGGACAATGGGGGAAACCCTGATCCAGCAATGCCGCGTGAATGAAGAAGGCCTTAGGGTTGTAAAATTCTTTTGTCCGTGACGATAATGACGGTAGCGGACGAATAAGCCCTGGCTAACTCTGTGCCAGCAGCCGCGGTAATACAGAGAGGGCAAGCGTTGTCCGGAATGACTGGGCGTAAAGCGCGCGCAGGCGGTTTCTTAAGTTGGATGTGAAATCCCCGGGCTTAACCTGGGAACTGCATTCGATACTGGGGAACTAGAGGCCGAGAGAGGAAAGCGGAATGACGAGTGTAGAGGTGAAATTCGTAGATATTCGTCGGAACACCAGAGGCGAAGGCGGCTTTCTGGCTCGGACCTGACGCTCAGGCGCGAAAGCGTGGGGAGCAAACAGG` |
| ASV1746 | 429 | `TGGGGAATTTTGGACAATGGACGCAAGTCTGATCCAGCAATGCCGCGTGCAGGATGAAGGCCTTCGGGTTGTAAACTGCTTTTGTACGGAGCGAAAAGGTCTGTCCTAATACGGCGGGCCCATGACGGTACCGTAAGAATAAGCACCGGCTAACTACGTGCCAGCAGCCGCGGTAATACGTAGGGTGCGAGCGTTAATCGGAATTACTGGGCGTAAAGCGTGCGCAGGCGGTTATATAAGACAGATGTGAAATCCCCGGGCTCAACCTGGGACCTGCATTTGTGACTGTATAGCTAGAGTACGGTAGAGGGGGATGGAATTCCGCGTGTAGCAGTGAAATGCGTAGATATGCGGAGGAACACCGATGGCGAAGGCAATCCCCTGGACCTGTACTGACGCTCATGCACGAAAGCGTGGGGAGCAAACAGG` |
| ASV1258 | 404 | `TGGGGAATATTGGACAATGGGGGCAACCCTGATCCAGCCATGCCGCGTGCGTGAAGAAGGCCTTAGGGTTGTAAAGCGCTTTTGTCAGGGAAGATAATGACTGTACCTGAAGAATAAGCCCCGGCTAACTCCGTGCCAGCAGCCGCGGTAAGACGGAGGGGGCTAGCGTTGTTCGGAATTACTGGGCGTAAAGGGCGCGTAGGCTGTTATGTGTGTCAGGTGTGAAATCCCAAGGCTTAACCTTGGAACTGCATCTGAAACTGCATGACTAGAGTGAGTAAGAGGCGGACGGAATTCCGAGTGTAGCAGTGAAATGCGCAGATATTCGGAGGAACATCGGTGGCGAAGGCGGTTCGCTGGTACTCTACTGACGCTGAGGCGCGAAAGCGTGGGGAGCAAACAGG` |
| ASV3938 | 413 | `TCGAGAATATTGGACAATGGACGCAAGTCTGATCCAGCGACGCCGCGTGCGGGATGACAGCCTTCGGGTTGTAAACCGCTTTTATTGGGGAAGAACCCGCAAGGGTGACGGTACCTGATGAATAAGGGGGTGCTAATTCTGTGCCAGCAGCCGCGGTAAAACAGAACCCCCAAGCGTTACCCGAATTTATTGGGCGTAAAGTGTCTGTAGCCGGTTTGGTAAGTTTTGGGTTAAATACCGAAAGCTCAACTTTTGGGATGCTCAAGAAACTGCCAGACTAGAGGGTGTTAGGGGCTGATAGAACTCTTGGTGGAGGGGTGAAATCCGTTGATATCAAGAGGAATACCAAAAGCGAAGGCATTCAGCTGGGACAACCCTGACGGTGAGAGACGAAAGCGTGGGGAGCAAAAAGG` |
| ASV1844 | 430 | `TAGGGAATATTGCGCAATGGGGGAAACCCTGACGCAGCAACGCCGCGTGAGTGAGGAAGGTCTTCGGATTGTAAAACTCTTTTGCTAGGGAAAAATGACCAGATGGCTAATATCCATTTGGAGTGATGGTACCTAAAGAATAAGGACCGGCTAACTTCGTGCCAGCAGCCGCGGTAATACGAAGGGTCCAAGCGTTGTTCGGAATCATTGGGCGTAAAGCGAGCGCAGGCGGATCAGCAAGTCAGATGTGAAATCTCGAAGCTCAACTTCGAAACTGCGTCTGAAACTGCTAGTCTAGAATGTCGGAGGGGATAGGGGAATTTCACGTGTAGGGGTAAAATCCGTAGAGATGTGAAGGAACACCGGAGGCGAAGGCGCCTATCTGGACGACTATTGACGCTGAGGCTCGAAAGCGTGGGGAGCAAACAGG` |
| ASV3939 | 407 | `TTAGGAATATTGGGCAATGGAGGAAACTCTGACCCAGCAACGCCGCGTGAAGGAAGAAGGTTTTCGGATTGTAAACTTCTGTATTATGGGACAAAGAAAGTGATGGTACCATAATAGAAAGCTACGGCAAACTACGTGCCAGCAGCCGCGGTAATACGTAGGTAGCAAGCGTTGTCCGGAATAACTGGGCGTAAAGGGAGCGTAGGCGGTTTCTTAAGTTCAATGTGAAAGACTGTGGCTTAACCACAGAAGTGCATTGGATACTGGGAGACTGGAGTACGGAAGAGGTAAACGGAATTACTAGTGTAGCGGTGGAATGCGTAGATATTAGTAGGAACACCAGAGGCGAAGGCGGTTTACTGGGACGATACTGACGCTGAGGCTCGAAAGCGTGGGGAGCAAACAGG` |
| ASV3239 | 404 | `TGGGGAATATTGGACAATGGGCGCAAGCCTGATCCAGCAATACCGCGTGAGTGATGAAGGTCTTCGGATTGTAAAGCTCTTTTAGTGGAAAAGATAATGACGGTATCCACAGAAAAAGCACCGGCAAACTTCGTGCCAGCAGCCGCGGTAATACGAAGGGTGCTAGCGTTGTTCGGAATCATTGGGCGTAAAGCGTACGTAGGCGGCATAGTAAGTCGATTGTGAAAGCCCCAAGCTCAACTTGGGAATTGCAATCGATACTGCTTTGCTAGAGTTTGGTGGGGGATAGTGGAATTCCTAGTGTAGGGGTGAAATCCGTAGATATTAGGAGGAACACCAGTGGCGAAGGCGACTATCTACGCCAATACTGACGCTAAGGTACGAAAGCGTGGGGAGCAAACAGG` |
| ASV2497 | 404 | `TGGGGAATTTTCCGCAATGGGCGAAAGCCTGACGGAGCAATACCGCGTGAGGGAGGAAGGCTCTTGGGTCGTAAACCTCTTTTCTCAAGGAAGATAATGACGGTACTTGAGGAATAAGCATCGGCTAACTCCGTGCCAGCAGCCGCGGTAATACGGAGGATGCAAGCGTTATCCGGAATGATTGGGCGTAAAGAGTTCGTAGGTGGTTTTTCAAGTCGTTCGTTAAAGAGTGTAGCTCAACTACATAAAGGCGGAGGAAACTGGAAGACTAGAGTAGTGTAGGGGTAGAGGGAATTCCCAGTGTAGCGGTGAAATGCGTAGAGATTGGGAAGAACACCGGTGGCGAAAGCGCTCTACTGGACTAATACTGACACTGAGGAACGAAAGCTAGGGGAGCGAAAGGG` |
| ASV763 | 404 | `TAAGGAATATTGGACAATGGAGGCAACTCTGATCCAGCCATGCCGCGTGAGTGAAGAAGGCCTTAGGGTTGTAAAGCTCTTTCAATCGTGAAGATAATGACGGTAGCGATAGAAGAAGCCCCGGCTAAATTCGTGCCAGCAGCCGCGGTAATACGAATGGGGCAAGCGTTGTTCGGAATCACTGGGCTTAAAGCGTGCGTAGGCGGTCTTGCAAGTCAGAAGTGAAAGCCTGGGGCTCAACCCCAGAATTGCTTTTGAAACTGCAAGACTAGAATACCGGAGAGGTTGGGAGAATTCCTAGTGTAGAGGTGAAATTCGCAGATATTAGGAGGAATACCAGTGGCGTAGGCGCCCAACTGGACGGTTATTGACGCTCAGGCACGAAAGCGTGGGGAGCAAACAGG` |
| ASV3299 | 404 | `TGGGGAATATTGGACAATGGGCGCAAGCCTGATCCAGCAATACCGCGTGAGTGATGAAGGTCTTCGGATTGTAAAGCTCTTTTAGTAGGGAAGATAATGACGGTACCTACAGAAAAAGCACCGGCAAACTTCGTGCCAGCAGCCGCGGTAATACGAAGGGTGCTAGCGTTGTTCGGAATCATTGGGCGTAAAGCGTACGTAGGCGGTTTAGTAAGTCGATTGTGAAAGCCCTAAGCTCAACTTAGGAACTGCAATCGATACTGCTTTGCTTGAGTTTGGTGGGGGATGGTGGAATTCCTAGTGTAGGGGTGAAATCCGTAGATATTAGGAGGAATATCGGTGGCGAAAGCGACCATCTACGCCAATACTGACGCTAAGGTACGAAAGCGTGGGGAGCAAACAGG` |
| ASV4793 | 400 | `GTGGAGCGGGGTCGGTTGTAATTAGTGGAAACACCGTAACACGCACAAATCCAATAGGTGGAGAGGTACGAGATATTGCAGGTATCGCAGTTTTCCGTAGAAGTGTACTTTCTGGTAATGTTGATGTTCCCTACGGAACTGTCGTAAGTGGAAATACCGTTTCGGGTTATACTCAACCAAGCAATAGCGATGGTTTTGGTATCGTGATGGAAGGTATCAACCATACAGTATCTGGAAATATAGTATCTGGAAATGATGTTGGTATTCAGCGTCAAGCGGGCTACACTGGCTACCCCAGCGATGGAAGTGATGCAAACTTGGTAGATACTTATTTTGGTCGAGGCAACTCGCCTGTTAGTTGTGGTATAAATCTGAGTGGCAATACGCTTTCATCAAATAC` |
| ASV1616 | 410 | `TGAGGAATCTTGGGCAATGAGCGAAAGCTTGACCCAGTGAGAATTTGTGTGTGATGAAGGCATTTAGTTGTAAAGCACTTTTAGGTAAGAAGATAATGACTTGTATTACATCAAGAAAAAGTACTGGCTAAGTCTGTGCCAGCAGCCGCGGTAATACAGACAGTGCGAGCGTTATTCGGATTGAATGGGCGTAAAAGGTATGTAGGCAGTTCTAGGAATTAGATCAGAAATTCTCGAGTCATAACTTGAGGTGTTGTTCTAAAGATTAATGGACTTGGAGTTTGTATTGAGTTGGAGGAATTTCTTGTGTCAACGGTTGAATGTTATTATATAAGAAGGACCATCAAAGGTGAAGACATCTTTCTAATACAAACTGACGCTGAGGTACGGAGGCGTGGGGATCAAACAGG` |
| ASV4496 | 424 | `TAAGGAATATTGGTCAATGGACGAAAGTCTGAACCAGCCATGCCGCGTGGAGGATGAAGGCCCTCTGGGTTGTAAACTTCTTTTATATGGGACGAAAAAGGGTCTTTCTAGATCAACTGACGGTACCATTTGAATAAGCACCGGCTAACTCCGTGCCAGCAGCCGCGGTAATACGGAGGGTGCAAGCGTTATCCGGATTCACTGGGTTTAAAGGGTGCGTAGGTGGGTTGGTAAGTCAGTGGTGAAATCTCCAAGCTTAACTTGGAAACTGCCATTGATACTATCAGTCTTGAATATTGTGGAGGTTAGCGGAATATGTCATGTAGCGGTGAAATGCTTAGATATGACATAGAACACCAATTGCGAAGGCAGCTGGCTACACATATATTGACACTGAGGCACGAAAGCGTGGGGATCAAACAGG` |
| ASV5143 | 410 | `TGGGGAATATTGCACAATGGGCGCAAGCCTGATGCAGCGACGCCGCGTGGGGGATGACGGCCTTCGGGTTGTAAACTCCTTTCGCAACCGACGAAGCTTTTGTGACGGTAGGTTGAGAAGAAGCACCGGCTAACTACGTGCCAGCAGCCGCGGTAATACGTAGGGTGCGAGCGTTGTCCGGAATTACTGGGCGTAAAGAGCTCGTAGGTGGTTTGTCGCGTCGTTTGTGGAATACCGCAGCTTAACTGCGGGGTTGCAGGCGATACGGGCATAACTTGAGTGCTGTAGGGGAGACTGGAATTCCTGGTGTAGCGGTGGAATGCGCAGATATCAGGAGGAACACCGATGGCGAAGGCAGGTCTCTGGGCAGTAACTGACGCTGAGGAGCGAAAGCATGGGGAGCGAACAGG` |
| ASV2504 | 430 | `TGGGGAATATTGGACAATGGGCGGAAGCCTGATCCAGCAATACCGCGTGTGTGAAGAAGGCCTTCGGGTTGTAAAGCACTTTTGTTAGGGAGGATGATTGCTTAAGCTAATACCTTAAGTAGTTGACGTTACCTAAAGAATAAGCACCGGCTAACTCCGTGCCAGCAGCCGCGGTAATACGGAGGGTGCAAGCGTTAATCGGATTTACTGGGCGTAAAGAGTACGTAGGTGGTTTTTTAAGTCAGATGTGAAAGCCCCGGGCTCAACCTGGGAATTGCATTTGAAACTGGAAGACTAGAATGTGGTAGAGGGTAGCGGAATTCCTGGTGTAGCGGTGAAATGCGTAGATATCAGGAGGAACATCAATGGCGAAGGCAGCTACCTGGGCCAACATTGACGCTGAGGTACGAAAGCGTGGGGAGCAAACAGG` |
| ASV1747 | 404 | `TGGGGAATCTTGGACAATGGGCGCAAGCCTGATCCAGCCATGCCGCGTGAGTGAAGAAGGTCTTCGGATTGTAAAGCTCTTTTACCAGGGACGATAATGACGGTACCTGGAGAATAAGCCCCGGCAAACTTCGTGCCAGCAGCCGCGGTAATACGAAGGGGGCTAGCGTTGTTCGGAATTACTGGGCGTAAAGCGCACGTAGGCGGGTCGTTAAGTCAGGGGTGAAATCCCGGAGCTCAACTCCGGAACTGCCTTTGATACTGGCGATCTAGAGATCGGAAGAGGTTAGTGGAATTCCCAGTGTAGAGGTGAAATTCGTAGATATTGGGAAGAACACCAGTGGCGAAGGCGGCTAACTGGTCCGATACTGACGCTGAGGTGCGAAAGCGTGGGGAGCAAACAGG` |
| ASV4411 | 423 | `TAGGGAATATTGGTCAATGGAGGCAACTCTGAACCAGCCATGCCGCGTGTAGGAAGAAGGCGTTATGCGTTGTAAACTACTTTTGAACGGGAATAAAAAGCTCTTGCGAGAGAAATTGAATGTACCGTTAGAATAAGCCACGGCTAACTACGTGCCAGCAGCCGCGGTAATACGTAGGTGGCAAGCGTTATCCGGATTTATTGGGTTTAAAGGGAGCGTAGGCGGTTTTGTAAGTCAGTGGTGAAAGTAGGCAGCTTAACTGTCGGATTGCCATTGATACTGCAAAGCTAGAGTATAGTAGAGGTAGGCGGAATTTATGATGTAGCGGTGAAATGCATAGATATCATAAAGAACACCGATTGCGAAGGCAGCTTACTGGACTATAACTGACGCTGAGGCTCGAAAGTGCGGGTATCAAACAGG` |
| ASV3194 | 403 | `TGGGGAATATTGCACAATGGGCGAAAGCCTGATGCAGCAACGCCGCGTGAGCGATGAAGGCCTTCGGGTCGTAAAGCTCTGTCCTCAAGGAAGATAATGACGGTACTTGAGGAGGAAGCCCCGGCTAACTACGTGCCAGCAGCCGCGGTAATACGTAGGGGGCTAGCGTTATCCGGAATTACTGGGCGTAAAGGGTGCGTAGGTGGTTTTTTAAGTCAGAAGTGAAAGGCTACGGCTCAACCGTAGTAAGCTTTTGAAACTGAAAAACTTGAGTGCAGGAGAGGAGAGTAGAATTCCTAGTGTAGCGGTGAAATGCGTAGATATTAGGAGGAATACCAGTAGCGAAGGCGGCTCTCTGGACTGTAACTGACACTGAGGCACGAAAGCGTGGGGAGCAAACAGG` |
| ASV4215 | 430 | `TAGGGAATTTTCCACAATGGGCGCAAGCCTGATGGAGCAACGCCGCGTGAGGGATGAAGCATTTCGGTGTGTAAACCTCTTTGGAGCGGGAAGAATGGGCTCTGGGTGAATAATCCGGGGCAGTGACGGTACCGCTAGAACAAGACACGGCTAACTTCGTGCCAGCAGCCGCGGTAATACGAAGGTGTCAAACGTTGTCCGGAATTATTGGGCGTAAAGGGCGCGTAGGCGGCCGGGTAAGTCAGATGTGAAAGCTCTGGGCTCAACCCAGGAATTGCATTTGATACTGCTCGGCTTGAGATTGGTAGAGGAGGGCGGAATTCCAGAAGTAGCGGTGAAATGCGTAGATATCTGGAGGAACACCCGAGGCGAAGGCGGCTCTCTGGACCAATTCTGACGCTGAGGCGCGAAAGGTAGGGGAGCAAACAGG` |
| ASV1587 | 430 | `TGGGGAATATTGGACAATGGGGGCAACCCTGATCCAGCCATGCCGCGTGTGTGAAGAAGGCCTTTTGGTTGTAAAGCACTTTAAGCGAGGAGGAGGCTACTTGGATTAATACTCTGGGATAGTGGACGTTACTCGCAGAATAAGCACCGGCTAACTCTGTGCCAGCAGCCGCGGTAATACAGAGGGTGCAAGCGTTAATCGGATTTACTGGGCGTAAAGCGTGCGTAGGTGGCCAATTAAGTCAAATGTGAAATCCCCGAGCTTAACTTGGGAATTGCATTCGATACTGGTTGGCTAGAGTATGGGAGAGGATGGTAGAATTCCAGGTGTAGCGGTGAAATGCGTAGAGATCTGGAGGAATACCGATGGCGAAGGCAGCCATCTGGCCTAATACTGACACTGAGGTACGAAAGCATGGGGAGCAAACAGG` |
| ASV884 | 404 | `TGGGGAATATTGGACAATGGGCGAAAGCCTGATCCAGCAATGCCGCGTGAGTGATGAAGGCCTTAGGGTTGTAAAGCTCTTTTACCCGGGATGATAATGACAGTACCGGGAGAATAAGCCCCGGCTAACTCCGTGCCAGCAGCCGCGGTAATACGGAGGGGGCTAGCGTTGTTCGGAATTACTGGGCGTAAAGCGCGCGTAGGCGGCTTTGTAAGTTAGAGGTGAAAGCCCGGAGCTCAACTCCGGAATTGCCTTTAAGACTGCATCGCTAGAATCATGGAGAGGTGAGTGGAATTCCGAGTGTAGAGGTGAAATTCGTAGATATTCGGAAGAACACCAGTGGCGAAGGCGACTCACTGGACATGTATTGACGCTGAGGTGCGAAAGCGTGGGGAGCAAACAGG` |
| ASV5128 | 429 | `TGGGGAATATTGCGCAATGGGCGAAAGCCTGACGCAGCCATGCCGCGTGTGTGAAGAAGGCCTTCGGGTTGTAAAGCACTTTCAGTGGGGAGGAAAGACTCAAGGCTAATACCCTTGGGGGTTGACGTTACCCACAGAAGAAGCACCGGCTAATTCCGTGCCAGCAGCCGCGGTAATACGGAAGGTGCGAGCGTTAATCGGAATTACTGGGCGTAAAGCGCGCGTAGGCGGCTTGGTAAGTCGGATGTGAAAGCCCCGGGCTCAACCTGGGAACTGCATTCGATACTGCTGAGCTGGAGTACGAGAGAGGGGGGTGGAATTCCAGGTGTAGCGGTGAAATGCGTAGAGATCTGGAGGAACACCGGTGGCGAAGGCGGCCCCCTGGTTCGATACTGACGCTGAGGTGCGAAAGCGTGGGGAGCAAACAGG` |
| ASV3819 | 404 | `TGAGGAATATTGGACAATGGGGGAAACCCTGATCCAGCAATGCCGCGTGAATGAAGAAGGCCTTAGGGTTGTAAAGTTCTTTCGCCTGTGAAGATAATGACGGTAGCAGGATAAGAAGTCCCGGCTAACTCTGTGCCAGCAGCCGCGGTAATACAGAGGGGGCAAGCGTTATTCGGAATGACTGGGCGTAAAGGGTGCGTAGGCGGATCTTTAAGTAAGGTGTTAAATCCCTGGGCTTAACCCAGGAAGTGCGCTTTAAACTGGAGATCTGGAGTATGGAAGAGGAGAGTGGAATTCCTAGTGTAGAGGTGAAATTCGTAGATATTAGGAGGAACATCAGAGGCGAAGGCGGCTCTCTGGTCCATTACTGACGCTGAGGCACGAAAGCGTGGGGAGCAAACAGG` |
| ASV3581 | 404 | `TGGGGAATCTTAGACAATGGGGGAAACCCTGATCTAGCCATGCCGCGTGATCGATGAAGGCCTTAGGGTTGTAAAGATCTTTCAGTGGGGAAGATAATGACGGTACCCACAGAAGAAGCCCCGGCTAACTCCGTGCCAGCAGCCGCGGTAATACGGAGGGGGCTAGCGTTGTTCGGAATTACTGGGCGTAAAGCGCACGTAGGCGGGCAGAGAAGTCAGAGGTGAAATCCCAGGGCTCAACCTTGGAACTGCCTTTGAAACTTTCTGTCTTGAGGTCGAGAGAGGTGAGTGGAATTCCGAGTGTAGAGGTGAAATTCGTAGATATTCGGAGGAACACCAGTGGCGAAGGCGGCTCACTGGCTCGATACTGACGCTGAGGTGCGAAAGCGTGGGGAGCAAACAGG` |
| ASV2057 | 404 | `TGGGGAATATTGGACAATGGGCGCAAGCCTGATCCAGCCATGCCGCGTGTGTGATGAAGGCCTTAGGGTTGTAAAGCACTTTCGCCGGTGAAGATAATGACGGTAACCGGAGAAGAAGCCCCGGCTAACTTCGTGCCAGCAGCCGCGGTAATACGAAGGGGGCAAGCGTTGCTCGGAATCACTGGGCGTAAAGCGTACGTAGGCGGGTCGTTAAGTCAGAGGTGAAATCCTGGAGCTCAACTCCAGAACTGCCTTTGATACTGGCGACCTTGAGTTCGAGAGAGGTTGGTGGAACTGCGAGTGTAGAGGTGAAATTCGTAGATATTCGCAAGAACACCAGTGGCGAAGGCGGCCAACTGGCTCGATACTGACGCTGAGGTACGAAAGCGTGGGGAGCAAACAGG` |
| ASV5250 | 422 | `TAGGGAATATTGGTCAATGGAGGCAACTCTGAACCAGCCATGCCGCGTGCAGGAAGAAGGCGTTCTGCGTTGTAAACTGCTTTTTTATGGGAAGAAAAACTTACGTGTAAGCATATGACGGTACCATAAGAATAAGCACCGGCTAACTCCGTGCCAGCAGCCGCGGTAATACGGAGGGCGCAAGCGTTGTCCGGATTTATTGGGTTTAAAGGGTGCGTAGGCGGTTTTTTAAGTCAGAGGTGAAAGCCCACAGCTCAACTGTGGAACTGCCTTTGATACTGGAAGACTTGAGTACACTAGAGGTTGGCGGAATTTATGATGTAGCGGTGAAATGCATAGATATCATAAAGAACACCGATAGCGCAGGCAGCTAACTGGAGTGTAACTGACGCTGAGGCACGAAAGCGTGGGTAGCAAACAGG` |
| ASV5560 | 404 | `TGAGGAATATTGAACAATGGGCGAAAGCCTGATTCAGCAATGCCGCGTGAGTGATGAAGGCCCATTGGGTTGTAAAGCTCTTTTGGTGGGGAAGATTGTGACGGTACCCACAGAATAAGGACCGACTAACTACGTGCCAGCAGTCGCGGTAATACGTAGGGTCCTAGCGTTGCTCGGAATTACTGGGCGTAAAGGGTGCGTAGGCCGATCTCCAAGTCAGGGGGTAAATCCCGAGGCTCAACCTCGGACTTCTTCTGAAACTAGAGATCTTGAGTATGGTAGAAGGCAGTGGAATTCCTAGTGTAGGGGTAAAATCCGTAGATATTAGGAGGAACACCAATGGCGAAGGCAGCTGCCTGGGCCATTACTGACGCTGAGGCACGAAAGTGTGGGGAGCAAACAGG` |
| ASV4027 | 408 | `TCGAGAATCTTCCGCAATGGGCGAAAGCCTGACGGAGCGACGCCGCGTGGTGGATGAAGCCCTTTGGGGCGTAAACACCTTTTATGGAGGAGAAAGTTTATTGATGTTACTCCATGAATAAGGGGCTCCCAACTCTGTGCCAGCAGGAGCGGTAATACAGAGGCCCCAAGCATTATCCGGATTTACTGGGCGTAAAGGGTGCGTAGGCGGTGTTATTAGTCGGGTGTTAAATCCTGGGGCTCAACCTCAGAATCGCATTCGAAACGGTAACACTAGAAGAAGTCAGAGGTGAACAGAACTCTCGGTGTAGGGGTGAAATCCGTTGATATCGAGGGGAATACCAAATGCGAAGGCAGTTCACTGGGGCTTTCTTGACGCTGAGGCACGAAAGCGTGGGTAGCGAATCGG` |
| ASV3638 | 404 | `TGGGGAATATTGGACAATGGGCGCAAGCCTGATCCAGCCATGCCGCGTGAGTGATGAAGGCCTTAGGGTTGTAAAGCTCTTTTGCCAGGGAAGATAATGACTGTACCTGGAGAATAAGCCCCGGCTAACTTCGTGCCAGCAGCCGCGGTAATACGAAGGGGGCTAGCGTTGTTCGGAATCACTGGGCGTAAAGCGCACGTAGGCGGACTCTTAAGTCAGGGGTGAAAGCCTGAGGCTCAACCTCAGAACTGCCTTTGATACTGAGAGTCTTGAGTGTGGTAGAGGTTGGTGGAACTGCGAGTGTAGAGGTGAAATTCGTAGATATTCGCAAGAACACCAGTGGCGAAGGCGGCCAACTGGACCATTACTGACGCTGAGGTGCGAAAGCGTGGGGAGCAAACAGG` |
| ASV3059 | 404 | `TGGGGAATATTGGACAATGGGCGAAAGCCTGATCCAGCAATGCCGCGTGGGTGAAGAAGGTCTTCGGATTGTAAAGCCCTTTCGGCGGGGACGATGATGACGGTACCCGCAGAAGAAGCCCCGGCTAACTTCGTGCCAGCAGCCGCGGTAATACGAAGGGGGCTAGCGTTGCTCGGAATTACTGGGCGTAAAGGGCGCGTAGGCGGCACCATCAGTCAGGCGTGAAATTCCCGGGCTTAACCTGGGGACTGCGCTTGATACAGTGGAGCTAGAGGACGGAAGAGGCTCGTGGAATTCCCAGTGTAGAGGTGAAATTCGTAGATATTGGGAAGAACACCGGTGGCGAAGGCGGCGAGCTGGTCCGTTACTGACGCTGAGGCGCGACAGCGTGGGGAGCAAACAGG` |
| ASV5189 | 407 | `TGGGGAATTTTCCGCAATGGGCGAAAGCCTGACGGAGCAACGCCGCGTGGGTGATGAAGTTTTTCGGAACGTAAAACCCTTTCGCCAGGGACGAAGCGAGTGACGGTACCTGGAGAAGAAGCATCGGCTAACTACGTGCCAGCAGCCGCGGTAATACGTAGGATGCGAGCGTTGTCCGGAATTATTGGGCGTAAAGCGTACGTAGGTGGTTTGTTAAGTTCGGTCTTAAAGACCAAGGCTCAACCTTGGGAGTGGACTGAAGACTGGCAGACTAGAGGACGTCAGAGGAAAATGGAATTCCGAGTGTAGCGGTGAAATGCGTAGATATTCGGAGGAACACCAGAGGCGAAAGCGGTTTTCTGGGGCGGTCCTGACACTGAGGTACGAAAGCTTGGGGAGCAAACAGG` |
| ASV1529 | 404 | `TGGGGAATATTGGACAATGGGCGAAAGCCTGATCCAGCAATGCCGCGTGAGTGAAGAAGGCCTTCGGGTTGTAAAGCTCTTTTGGCGGGGACGATAATGACGGTACCCGCAGAATAAGCTCCGGCTAACTTCGTGCCAGCAGCCGCGGTAATACGAAGGGAGCTAGCGTTGTTCGGAATTACTGGGCGTAAAGCGCACGTAGGCGGCTTATCAAGTTAGAGGTGAAAGCCTGGGGCTCAACCCCAGAACTGCCTTTAAGACTGGTAAGCTTGAATCCGGGAGAGGTGAGTGGAATTCCGAGTGTAGAGGTGAAATTCGTAGATATTCGGAAGAACACCAGTGGCGAAGGCGGCTCACTGGACCGGCATTGACGCTGAGGTGCGAAAGCGTGGGGAGCAAACAGG` |
| ASV5667 | 406 | `TGGGGAATTTTCCGCAATGGGCGAAAGCCTGACGGAGCAATGCCGCGTGGAGGTAGAAGGCCCACGGGTCGTGAACTTCTTTTCCCGGAGAAGAAGCAATGACGGTATCTGGGGAATAAGCATCGGCTAACTCTGTGCCAGCAGCCGCGGTAAGACAGAGGATGCAAGCGTTATCCGGAATGATTGGGCGTAAAGCGTCTGTAGGTGGCTTTTTAAGTCCGCCGTCAAATCCCAGGGCTCAACCCTGGACAGGCGGTGGAAACTACCAAGCTGGAGTACGGTAGGGGCAGAGGGAATTTCCGGTGGAGCGGTGAAATGCGTAGAGATCGGAAAGAACACCAACGGCGAAAGCACTCTGCTGGGCCGACACTGACACTGAGAGACGAAAGCTAGGGGAGCGAATGGG` |
| ASV2751 | 423 | `TAGGGAATATTGGGCAATGGAGGCAACTCTGACCCAGCCATGCCGCGTGCAGGACGAAGGCGTTATGCGTCGTAAACTGCTTTTATACGGGAAGAAAAAGGCTATGCGTAGCAAATTGACGGTACCGTATGAATAAGCACCGGCTAACTCCGTGCCAGCAGCCGCGGTAATACGGAGGGTGCAAGCGTTGTCCGGATTTATTGGGTTTAAAGGGTGCGTAGGCGGCTTTTTAAGTCAGCGGTGAAAGCCGATTGCTCAACAATCGAACTGCCGTTGATACTGGAGAGCTTGAGTTTGATTGAGGTAGCTGGAATGGATAGTGTAGCGGTGAAATGCTTAGATATTATCCAGAACGCCAATTGCGTAGGCAGGTTACTAAGTCATTACTGACGCTGAGGCACGAAAGTGCGGGTATCAAACAGG` |
| ASV3435 | 404 | `TGGGGAATCTTAGACAATGGGGGAAACCCTGATCTAGCCATGCCGCGTGAGCGATGAAGGCCTTAGGGTTGTAAAGCTCTTTCGTGGGGGAAGATAATGACTGTACCCCAAGAAGAAGCCCCGGCTAACTCCGTGCCAGCAGCCGCGGTAATACGGAGGGGGCTAGCGTTGTTCGGAATTACTGGGCGTAAAGCGCACGTAGGCGGACGAGAAAGTCAGGGGTGAAATCCCGGGGCTCAACCCCGGAACTGCCTTTGAAACTCCTTGTCTTGAGGTCGTGAGAGGTGGGTGGAATTCCGAGTGTAGAGGTGAAATTCGTAGATATTCGGAGGAACACCAGTGGCGAAGGCGGCCCACTGGCACGATACTGACGCTGAGGTGCGAAAGCGTGGGGAGCAAACAGG` |
| ASV2164 | 419 | `TAAGGAATATTGCGCAATGGACGAAAGTCTGACGCAGCGACGCCGCGTGGGGGATGAAGGTCTTCGGATTGTAAACCCCTTTCGGCAGGGAAGATGGAACGGGTAACCGTTCGGACGGTACCTGCAGAAGCAGCCACGGCTAACTTCGTGCCAGCAGCCGCGGTAATACGAAGGTGGCAAGCGTTGTTCGGATTCACTGGGCGTACAGGGAGCGTAGGCGGTTGAGTAAGCCCTCCGTGAAATCTCCGGGCCTAACCCGGAAAGTGCAGAGGGGACTGCTCGGCTAGAGGATGGGAGAGGAGCGCGGAATTCCCGGTGTAGCGGTGAAATGCGTAGAGATCGGGAGGAAGGCCGGTGGCGAAGGCGGCGCTCTGGAACATTTCTGACGCTGAGGCTCGAAAGCGTGGGGAGCAAACAGG` |
| ASV3007 | 404 | `TGGGGAATCTTGGACAATGGGCGCAAGCCTGATCCAGCCATGCCGCGTGAGTGACGAAGGCCTTCGGGTTGTAAAGCTCTTTTGCCGGGGACGATAATGACGGTACCCGGAGAATAAGTCCCGGCTAACTTCGTGCCAGCAGCCGCGGTAATACGAAGGGGACTAGCGTTGTTCGGAATTACTGGGCGTAAAGCGCACGTAGGCGGATGTGTCAGTCAGAGGTGAAATCCCGGAGCTCAACTTCGGAACTGCCTTTGATACAGCATATCTTGAATCCGATAGAGGTGGGTGGAATTCCTAGTGTAGAGGTGAAATTCGTAGATATTAGGAAGAACACCGGTGGCGAAGGCGGCCCACTGGATCGGTATTGACGCTGAGGTGCGAAAGCGTGGGGAGCAAACAGG` |
| ASV3488 | 404 | `TGGGGAATATTGGACAATGGGCGAAAGCCTGATCCAGCAATACCGCGTGAATGATGAAGGCCTTAGGGTTGTAAAGTTCTTTTAATAGGGAAGATAATGACGGTACCTATAGAAAAAGCCCCGGCTAACTCCGTGCCAGCAGCCGCGGTAAGACGGAGGGGGCTAGCGTTATTCGGAATTACTGGGCGTAAAGGGCGCGTAGGCGGATTAGTAAGTTGGGAGTGAAATCCCGAGGCTTAACCTCGGAATTGCTCTCAAAACTGCTAGTCTTGAGTGAAGTAGAGGATGATGGAATTCCTAGTGTAGAGGTGAAATTCTTAGATATTAGGAGGAACACCGGTGGCGAAAGCGGTCATCTGGACTTCAACTGACGCTGAGGCGCGAAAGCGTGGGGAGCAAACAGG` |
| ASV5594 | 387 | `GCGCGAAAACTTTCCAATGCACGCAAGTGTGAGAAGGGAACCCAGAGTGCTTAGCTCTAAGCTAGGCTTTTGCCAAGCGTAAAAAGCTTGGCGAATAAGTGGTGGGCAAGACTGGTGCCAGCCGCCGCGGTAACCCCAGCGCCACAAGTGGTGGTCCGCATTATTTGGCCTAAAGCGTCCGTAGCTGGTTTAGTAAATCTCTTGTGAAATCGTTCTGCTTAACAGGGCGGCGTGCAGGAGACACTGCTTGACTAGGGATTGGGAGGAGTTAGGGGTATGCTGTAGGGAGCGGTAAAATGCTATAATCTACAGTAGACCACCTGTGGCGAAGGCGCCTAACTAGAACAAGTCCGACAGTGAGGGACGAAAGCCAGGGGAGCAAACCGG` |
| ASV3562 | 430 | `TGGGGAATTTTCCGCAATGGACGAAAGTCTGACGGAGCAACGCCGCGTGAATGAAGAAGGCCTTCGGGTTGTAAAGTTCTGTCATTAGGGAAGAATGTTCTATATGTAAATAATGTATAGGAGTGACGGTACCTAAGGAGGAAGCCACGGCTAACTACGTGCCAGCAGCCGCGGTAATACGTAGGTGGCGAGCGTTGTCCGGAATTACTGGGCGTAAAGGGCGCGTAGGCGGATATTTAAGTCAGATGTGAAAGCCCCGGGCTTAACTTGGGGATTGCATATGATACTGGATATCTTGAGGACAGGAGAGGAAAGCGGAATTCCTAGTGTAGCGGTGAAATGCGTAGATATTAGGAGGAACACCAGTGGCGAAGGCGGCTTTCTGGACTGTAACTGACGCTGAGGCGCGAAAGCGTGGGGAGCGAACGGG` |
| ASV1554 | 424 | `TAAGGAATATTGGTCAATGGAGGCAACTCTGAACCAGCCATGCCGCGTGCAGGAAGACAGCCCTCTGGGTCGTAAACTGCTTTTATTCGGGAATAAACTTCCGCTCGTGAGCGGAACTGAATGTACCGAAGGAATAAGGATCGGCTAACTCCGTGCCAGCAGCCGCGGTAATACGGAGGATCCAAGCGTTATCCGGATTTATTGGGTTTAAAGGGTGCGTAGGCGGCCTGTTAAGTCAGGGGTGAAAGACGGTAGCTCAACTATCGCAGTGCCCTTGATACTGATGGGCTTGAATACACTAGAGGTAGGCGGAATGTGACAAGTAGCGGTGAAATGCATAGATATGTCACAGAACACCGATTGCGAAGGCAGCTTACTATGGTGTCATTGACGCTGAGGCACGAAAGCGTGGGGATCAAACAGG` |
| ASV1389 | 404 | `TGGGGAATATTGGACAATGGGCGCAAGCCTGATCCAGCAATACCGCGTGAGTGATGAAGGCCTTCGGGTTGTAAAACTCTTTTAGTAGGGAAGATAATGACGGTACCTACAGAAAAAGCACCGGCTAACTTCGTGCCAGCAGCCGCGGTAATACGAAGGGTGCTAGCGTTGTTCGGAATTACTGGGCGTAAAGCGTGCGTAGGCGGTTTAGCAAGTCGATTGTGAAAGCCCAGGGCTCAACCCTGGAATTGCAATCGAAACTACTTTGCTAGAGTGTGGTGGGGGATAGTAGAATTCCTAGTGTAGGGGTGAAATCCGTAGATATTAGGAGGAATACCAGTGGCGAAGGCGACTATCTACACCATTACTGACGCTGAGGCACGAAAGCGTGGGGAGCAAACAGG` |
| ASV3876 | 405 | `TGGGGAATCTTGCGCAATGGACGAAAGTCTGACGCAGCAACGCCGCGTGGAGGACGAAGGCTCTCGGGTTGTAAACTCCTTTCAGTGGGAACGATAATGACGGTACCCACAGAAGAAGCACCGGCTAACTACGTGCCAGCAGCCGCGGTAATACGTAGGGTGCAAGCGTTGTCCGGATTTATTGGGCGTAAAGAGCTCGTAGGCGGTTCAGTAAGTCAGATGTGAAAGCTCAGGGCTCAACCCAGAGACTGCATTTGATACTGCTGTGACTAGAGGTTGGTAGGGGATCACGGAATTCCTGGTGTAGCGGTGGAATGCGCAGATATCAGGAGGAACACCGATGGCGAAGGCAGTGATCTGGGCCAATTCTGACGCTGAGGAGCGAAAGCGTGGGTAGCAAACAGG` |
| ASV5349 | 408 | `TCGAGAATATTCGACAATGGGCGAAAGCCTGATCGAGCGACGCCGCGTGCAGGATGAAGGTCTTAGGATTGTAAACTGCTTTTCCACAGGAAAAACTTTTGTGATTGTACTGTGGGAATAAGAAGCTGCTAACTTCGTGCCAGCAGCCGCGGTAATACGAAGGCTTCAAGCGTTATCCGGATTTATTGGGCGTAAAGCGTGTGTAGGAGGATATGTAAGTTTGATGTTAAATCTCAGGGCTTAACTTTGAGGCAGCATCGAATACTGCGTATCTAGAGTGCGTGAGAGGTTCATAGAACGCATGGTGTAGGGGTGACATCCGTTGATATCATGCGGAATACCAAAGGCGAAGGCATTGAACTGGCGCGTTACTGACTCTAAGACACGAAAGCGTGGGGAGCAAAAAGG` |
| ASV1464 | 429 | `TGGGGAATTTTGGACAATGGGGGGAACCCTGATCCAGCAACGCCGCGTGAATGAAGAAGGCCTTAGGGTTGTAAAGTTCTTTTGTCAGGGAAGAAAGTTTCTGTGCTAATACCATAGAACGATGACGGTACCTGAAGAATAAGCACCGGCTAACTACGTGCCAGCAGCCGCGGTAATACGTAGGGTGCAAGCGTTAATCGGAATTACTGGGCGTAAAGCGTGCGCAGGCGGATATGTAAGTTAGAAGTGAAATCCCCGGGCTCAACCTGGGAACTGCTTTTAAGACTGCGTATCTAGAGTTTGTCAGAGGGGGGTGGAATTCCAAGTGTAGCAGTGAAATGCGTAGAGATTTGGAGGAACACCGATGGCGAAGGCAGCCCCCTGGGATAAAACTGACGCTCATGCACGAAAGCGTGGGTAGCAAACAGG` |
| ASV4038 | 430 | `TGGGGAATTTTCCGCAATGGGCGAAAGCCTGACGGAGTGACGCCGCGTGGGGGACGAAGGCCTTCGGGTCGTAAACCCCTGTCAGAGGGAAAGAAGGGTCGGCGCGTGAATATCGCGTCGATCTGACGGTACCTTCAGAGGAAGCCCCGGCCAACTCTGTGCCAGCAGCCGCGGTAAGACAGAGGGGGCAAGCGTTGCTCGGAATCACTGGGCGTAAAGGGCGCGTAGGCGGGATGGCAAGTCGGTCGTGAAATCCCTGGGCTCAACTCAGGGGGGTCGGCCGATACTGCCGTTCTTGAGGGATGCAGAGGAGACTGGAATTCCCGGTGTAGCGGTGAAATGTGTAGAGATCGGGAGGAACACCGGTGGCGAAGGCGGGTCTCTGGGCATTCCCTGACGCTGAGGCGCGAAAGCGTGGGGAGCAAACGGG` |
| ASV565 | 429 | `TGGGGAATTTTGGACAATGGGGGCAACCCTGATCCAGCCATGCCGCGTGAGTGAAGAAGGCCTTCGGGTTGTAAAGCTCTTTCGGCCGGGAAGAAATCGTATGGGCTAATACCCTGTGCGGATGACGGTACCGGCATAAGAAGCACCGGCTAACTACGTGCCAGCAGCCGCGGTAATACGTAGGGTGCGAGCGTTAATCGGAATTACTGGGCGTAAAGCGTGCGCAGGCGGTTTTGTAAGACAGGCGTGAAATCCCCGGGCTCAACCTGGGAACTGCGTTTGTGACTGCAAGGCTAGAGTACGGCAGAGGGGGGTGGAATTCCACGTGTAGCAGTGAAATGCGTAGAGATGTGGAGGAACACCAATGGCGAAGGCAGCCCCCTGGGTCGATACTGACGCTCATGCACGAAAGCGTGGGTAGCAAACAGG` |
| ASV5313 | 430 | `TGGGGAATCTTGCGCAATGGGCGGAAGCCTGACGCAGCCACGCCGCGTGAGTGAAGAAGGCCTTAGGGTTGTAAAGCTCTGTCGGAGGGGACGAAAAGCATTTGGGTTAATAGCCCAGATGCCTGACGGTACCCTTAAAGGAAGCACCGGCTAACTTCGTGCCAGCAGCCGCGGTAATACGAGGGGTGCAAGCGTTGCTCGGAATCATTGGGCGTAAAGGGCAGGTAGGTGGTCTTATTTGTCTGAGGTGAAATCCCTGGGCTTAACCCAGGAAGTGCCTCAGAAACGGTAGGACTAGAGTTCTAGAGGGGTTTGTGGAATTCCCGGTGTAGCGGTGAAATGCGTAGAGATCGGGAGGAACACCAGAGGCGAAGGCGGCAAACTGGATAGACACTGACACTCAACTGCGAAAGCGTGGGGAGCAAACAGG` |
| ASV1240 | 429 | `TGGGGAATTTTGGACAATGGGGGCAACCCTGATCCAGCAATGCCGCGTGAGTGAAGAAGGCCTTCGGGTTGTAAAGCTCTTTTGTTAGGGAAGAAAAGGTCATTGATAATACCATTGGCTGATGACGGTACCTAAAGAATAAGCACCGGCTAACTACGTGCCAGCAGCCGCGGTAATACGTAGGGTGCAAGCGTTAATCGGAATTACTGGGCGTAAAGCGTGCGCAGGCGGCCTCGCAAGTCAGATGTGAAATCCCCGAGCTTAACTTGGGAACTGCATTTGAAACTACGGGGCTAGAGTGTGTCAGAGGGAGGTAGAATTCCACGTGTAGCAGTGAAATGCGTAGAGATGTGGAGGAATACCAGTGGCGAAGGCGGCCTCCTGGGATAACACTGACGCTCATGTACGAAAGCGTGGGGAGCAAACAGG` |
| ASV1020 | 409 | `TGGGGAATATTGCACAATGGGCGCAAGCCTGATGCAGCAACGCCGCGTGAGGGACGACGGCCTTCGGGTTGTAAACCTCTTTTAGCAGGGAAGAAGCGAAAGTGACGGTACCTGCAGAAAAAGCACCGGCTAACTACGTGCCAGCAGCCGCGGTAATACGTAGGGTGCAAGCGTTGTCCGGAATTATTGGGCGTAAAGAGCTCGTAGGCGGTTTGTCGCGTCTGCTGTGAAAACGCGAGGCTCAACCTCGCGCCTGCAGTGGGTACGGGCAAACTAGAGTGCGGTAGGGGAGATTGGAATTCCTGGTGTAGCGGTGGAATGCGCAGATATCAGGAGGAACACCGATGGCGAAGGCAGATCTCTGGGCCGTAACTGACGCTGAGGAGCGAAAGCATGGGGAGCGAACAGG` |
| ASV2634 | 429 | `TGGGGAATTTTGGACAATGGGGGCAACCCTGATCCAGCCATTCCGCGTGAGTGAAGAAGGCCTTCGGGTTGTAAAGCTCTTTCGCAAGGGAAGAAACGATACCGGTGAATATCCGGTGTTAATGACGGTACCTTGATAAGAAGCACCGGCTAACTACGTGCCAGCAGCCGCGGTAATACGTAGGGTGCGAGCGTTAATCGGAATTACTGGGCGTAAAGCGTGCGCAGGCTGTTTGGCAAGTCAGATGTGAAATCCCCGAGCTCAACTTGGGAACTGCGTTTGAAACTGCCAGACTAGAATAGGTCAGAGGGGGGTAGAATTCCACGTGTAGCAGTGAAATGCGTAGAGATGTGGAGGAATATCAATGGCGAAGGCAGCCCCCTGGGATCATATTGACGCTCATGCACGAAAGCGTGGGGAGCAAACAGG` |
| ASV5292 | 429 | `TGGGGAATTTTGGACAATGGGGGCAACCCTGATCCAGCCATGCCGCGTGAGTGAAGAAGGCCTTCGGGTTGTAAAGCTCTTTCGGTTGGGAAGAAATTGCACGGGTTAATACCCTGTGTAGATGACGGTACCAGCATAAGAAGCACCGGCTAACTACGTGCCAGCAGCCGCGGTAATACGTAGGGTGCGAGCGTTAATCGGAATTACTGGGCGTAAAGCGTGCGCAGGCGGTTGTATAAGTCAGATGTGAAATCCCCGGGCTCAACCTGGGAACTGCATTTGGGACTGTACGGCTAGAGTGTGGCAGAGGGGGGTAGAATTCCACGTGTAGCAGTGAAATGCGTAGAGATGTGGAGGAATACCGATGGCGAAGGCAGCCCCCTGGGCCAATACTGACGCTCATGCACGAAAGCGTGGGGAGCAAACAGG` |
| ASV3687 | 429 | `TAGGGAATCTTCCGCAATGGACGAAAGTCTGACGGAGCAACGCCGCGTGAGTGAAGAAGGTTTTCGGATCGTAAAACTCTGTTGTCAGAGAAGAACAAGTTGGAGAGTAACTGCTCCAGCCTTGACGGTATCTGACCAGAAAGCCACGGCTAACTACGTGCCAGCAGCCGCGGTAATACGTAGGTGGCAAGCGTTGTCCGGATTTATTGGGCGTAAAGCGAGCGCAGGCGGTTCCTTAAGTCTGATGTGAAAGCCCACGGCTCAACCGTGGAAGGTCATTGGAAACTGGGGAACTTGAGTGCAGAAGAGGAGAGTGGAATTCCATGTGTAGCGGTGAAATGCGTAGATATATGGAGGAACACCAGTGGCGAAGGCGACTCTCTGGTCTGTAACTGACGCTGAGGCTCGAAAGCGTGGGGAGCAAACAGG` |
| ASV5387 | 406 | `TTGGGAATCTTGCACAATGGGGGAAACCCTGATGCAGCGACGCCGCGTGGAGGATGACGGATTTAGGTCTGTAAACTCCTTTTTCAGGGAAAGACTTAGGACGGTACCCTGAGAATAAGCACCGGCTAACTACGTGCCAGCAGCCGCGGTAAGACGTAGGGTGCAAGCGTTATCCGGATTTACTGGGCGTAAAGCGCGCGTAGGCGGCTTGTTAAGTGTGAAGTGAAATCTCCAGGGCTCAACCCGGAAACTGCTTCGCATACTGGCAAGCTTGAGGAAGGCAGAGGTTAGTAGAATTCCTGGTGTAGCGGTGAAATGCATAGATATCAGGAGGAATACCAACGGCGAAGGCAGCTAACTGGGCCTTATCTGACGCTGAGGCGCGAAAGCGTGGGTAGCAAACAGG` |
| ASV5074 | 429 | `TGGGGAATATTGGACAATGGGCGCAAGCCTGATCCAGCAATGCCGCGTGTGTGAAGAAGGCCTGCGGGTTGTAAAGCACTTTCGAGAGGGAAGAAAGGCATATGGTGAAAAGCCATATGAGTTGACGTTACCTCTATAAGAAGCACCGGCTAACTCTGTGCCAGCAGCCGCGGTAATACAGAGGGTGCAAGCGTTAATCGGAATTACTGGGCGTAAAGGGTACGTAGGTGGAAGACTAAGTTTTGTGTGAAATGTCTGGGCTTAACCTGGAACGTGCATAGGATACTGGTCGTCTAGAGTATGGTAGAGGGAAGTGGAATTTCCGGTGTAGCGGTGAAATGCGTAGATATCGGAAGGAACACCAGTGGCGAAGGCGGCTTCCTGGACTAATACTGACACTGAAGTACGAAAGCGTGGGTAGCGAACAGG` |
| ASV2714 | 404 | `TGGGGAATCTTGCGCAATGGGCGAAAGCCTGACGCAGCCATGCCGCGTGAATGATGAAGGTCTTAGGATTGTAAAATTCTTTTAGCAGGGACGATAATGACGGTACCTGCAGAAAAAGCCCCGGCTAACTTCGTGCCAGCAGCCGCGGTAATACGAAGGGGGCTAGCGTTGCTCGGAATTACTGGGCGTAAAGGGAGCGTAGGCGGGTTATCAAGTTGGAGGTGAAAGCCCAGGGCTCAACCTTGGAATTGCCTTCAAAACTGATAACCTAGAGGATGATAGAGGTAAGTGGAACTCCGAGTGTAGAGGTGAAATTCGTAGATATTCGGAAGAACACCAGTGGCGAAGGCGACTTACTGGATCATTACTGACGCTGAGGCTCGAAAGCGTGGGGAGCAAACAGG` |
| ASV4768 | 404 | `TGGGGAATATTGGACAATGGGCGAAAGCCTGATCCAGCCATGCCGCGTGAGTGATGAAGGCCCTAGGGTTGTAAAGCTCTTTCGCCAGGGACGATAATGACGGTACCTGGATAAGAAGCCCCGGCTAACTTCGTGCCAGCAGCCGCGGTAATACGAAGGGGGCTAGCGTTGTTCGGATTTACTGGGCGTAAAGCGCACGTAGGCGGATTTTTAAGTCAGGGGTGAAATCCCAAGGCTCAACCTTGGAACTGCCTTTGATACTGGAAGTCTCGAGTCCGGGAGAGGTGAGTGGAACTGCGAGTGTAGAGGTGAAATTCGTAGATATTCGCAAGAACACCAGTGGCGAAGGCGGCTCACTGGCCCGGAACTGACGCTGAGGTGTGAAAGCGTGGGGAGCAAACAGG` |
| ASV1813 | 407 | `TGGGGAATTTTCCGCAATGGGCGAAAGCCTGACGGAGCAATACCGCGTGAGGGATGAAGGCCTTTGGGTTGTAAACCTCTTTTATCAAGGAGGAAGTTCTGACGTGTACTTGATGAATAAGCATCGGCTAACTCCGTGCCAGCAGCCGCGGTAAGACGGAGGATGCAAGTGTTATCCGGAATCACTGGGCGTAAAGCGTCTGTAGGTGGTTTAAAAAGTCCACTGTTAAAGATTGAAGCTTAACTTCAAAAATGCCGTGGAAACTTTTAAACTTGAGTATGATAGGGGTAGAGGGAATTTCCAGTGGAGCGGTGAAATGCGTAGATATTGGAAAGACCACCAATGGCGAAGGCACTCTACTGGGCCATTACTGACACTGAGAGACGAAAGCTAGGGGAGCAAATGGG` |
| ASV2127 | 429 | `TGGGGAATATTGGACAATGGGCGGAAGCCTGATCCAGCAATGCCGCGTGTGTGAAGAAGGCCTTAGGGTTGTAAAGCACTTTCAGTGGGGAGGAAGGTTGAGACGCTAATATCGTTTTGATTTGACGTTACCCACAGAAGAAGCACCGGCTAACTCTGTGCCAGCAGCCGCGGTAATACAGAGGGTGCGAGCGTTAATCGGAATTACTGGGCGTAAAGGGTGTGTAGGTGGATAGGTAAGTTAGATGTGAAATCCCCGGGCTTAACCTGGGAACTGCGTTTAAAACTGCCGATCTAGAGTAATGTAGAGGATAGTGGAATTTCCAGTGTAGCGGTGAAATGCGTAGATATTGGAAGGAACACCAGTGGCGAAGGCGACTATCTGGACAGATACTGACACTGAGACACGAAAGCGTGGGGAGCAAACAGG` |
| ASV2701 | 408 | `TCGAGAATTTTCCGCAATGGACGAAAGTCTGACGGAGCGACGCCGCGTGGTTGATGAAGTACTTAGGTATGTAAAAACCTTTTATGAGGGAAAAAGTTTATTGATTGTACCTCATGAATAAGGGGTTGCTAAACTCGTGCCAGCAGCAGCGGTAATACGAGTGCCCCAAGCGTTATCCGGAATTATTGGGCGTAAAGGGTGAGTAGGTGGTTATATTAGTCTTGTGTTAAAGACCCCGGCTCAACCGGGGAAGTGCATAGGAAACGGTATAACTAGAGTATGTGAGAGGTGTGTAGAACTCATGGAGTAGGGGTGAAATCCGTTGATATCATGGGGAATACCAAAAGCGTAGGCAGCACACTGGCACATTACTGACACTGAATCACGAAAGCGTGGGTAGCGAATGGG` |
| ASV3889 | 404 | `CAAGGAATTTTCGTCAATGGGCGCAAGCCTGAACGAGCAACGCCGCGTGGAGGATGACGGCCTTCGGGTTGTAAACTCCTTTTTCGGGGGACGATAGTGACGGTACCCCGGGAATAAGGCTCGGCTAACTCTGTGCCAGCAGCCGCGGTAATACAGAGGAGCCAAGCGTTGTCCGGATTGACTGGGCGTAAAGCGCACGCAGGCGGCTGCGCGCGTGGGGTGTGAAATCTGGCCGCTTAACGGCCAGGCGCCATCCCATACGGCGCGGCTTGAGCCTTGCAGAGGGCGGTGGAATTGCCGGTGTAGTGGTGAAATGCGTAGAGATCGGCAGGAACACCCAGGGGGAAACCAGCCGCCTGGGCAAGCGCTGACGCTGAGGTGCGAGAGCGTGGGGAGCGAACCGG` |
| ASV874 | 429 | `TGGGGAATTTTGGACAATGGGCGCAAGCCTGATCCAGCCATGCCGCGTGCGGGAAGAAGGCCTTCGGGTTGTAAACCGCTTTTGTCAGGAACGAAACGGTATTGGTTAATAACCGATGCTAATGACGGTACCTGAAGAATAAGCACCGGCTAACTACGTGCCAGCAGCCGCGGTAATACGTAGGGTGCAAGCGTTAATCGGAATTACTGGGCGTAAAGCGTGCGCAGGCGGTTATGTAAGACAGGTGTGAAATCCCCGGGCTCAACCTGGGAACTGCATTTGTGACTGCATAGCTAGAGTTTGTCAGAGGGGGGTGGAATTCCACGTGTAGCAGTGAAATGCGTAGATATGTGGAAGAACACCGATGGCGAAGGCAGCCCCCTGGGATAAGACTGACGCTCATGCACGAAAGCGTGGGGAGCAAACAGG` |
| ASV3538 | 429 | `TGGGGAATATTGCGCAATGGGGGCAACCCTGACGCAGCCATGCCGCGTGTGTGAAGAAGGCCTTCGGGTTGTAAAGCACTTTCAATGGGGAAGAAAAGCGGGTCGTTAATAGCGGCTCGCCGTGACATTACCTATAGAAGAAGCACCGGCTAACTCCGTGCCAGCAGCCGCGGTAATACGGAGGGTGCGAGCGTTAATCGGAATTACTGGGCGTAAAGCGCGCGTAGGCGGTTTGTTAAGTCAGCTGTGAAAGCCCCGGGCTCAACCTGGGAACTGCAGTTGATACTGGCCGACTAGAGTACGAGAGAGGGAGGTAGAATTCCATGTGTAGCGGTGAAATGCGTAGATATATGGAGGAATACCAGTGGCGAAGGCGGCCTCCTGGCTCGATACTGACGCTGAGGTGCGAAAGCGTGGGGATCAAACAGG` |
| ASV1305 | 430 | `TGGGGAATATTGCGCAATGGGGGAAACCCTGACGCAGCAACGCCGCGTGAGTGAGGAAGGACTTCGGTCTGTAAAGCTCTGTTAATGTGGAAAAATGGTAGCTGGTCTAATAGGCCGGCTATTTGATGGTACACATAGAGGAAGCACCGGCTAACTTCGTGCCAGCAGCCGCGGTAATACGAAGGGTGCAAGCGTTGTTCGGATTTATTGGGCGTAAAGCGCGCGTAGGCGGACCTGTAAGTCAGATGTGAAATCTCGGGGCTCAACCTCGAAACTGCGTCTGAAACTGCAGGTCTAGAATCTCGGAGGGGGAAGGGGAATTTCGCATGTAGGGGTAAAATCCGTAGATATGCGAAGGAACACCAGAGGCGAAGGCGCCTTCCTGGACGAGTATTGACGCTGAGGCGCGAAAGCGTGGGGATCAAACAGG` |
| ASV3509 | 424 | `TGAGGAATATTGGACAATGGGCGCAAGCCTGATCCAGCCATGCCGCGTGCAGGAAGACGCATCTATGGTGTGTAAACTGCTTTTGTACGGGAAGAAACACTCTCTCGTGAGAGAGCTTGACGGTACCGTAAGAATAAGGATCGGCTAACTCCGTGCCAGCAGCCGCGGTAATACGGAGGATCCAAGCGTTATCCGGAATCATTGGGTTTAAAGGGTCCGTAGGCGGTTTAGTAAGTCAGTGGTGAAAGCCCATCGCTCAACGATGGAACGGCCATTGATACTGCTAGACTTGAATTACTAGGAAGTAACTAGAATATGTAGTGTAGCGGTGAAATGCTTAGATATTACATGGAATACCAATTGCGAAGGCAGGTTACTACTAGTGGATTGACGCTGATGGACGAAAGCGTGGGGAGCGAACAGG` |
| ASV380 | 404 | `TGGGGAATATTGGACAATGGGCGAAAGCCTGATCCAGCAATGCCGCGTGAGTGATGAAGGCCTTAGGGTTGTAAAGCTCTTTTACCAGGGATGATAATGACAGTACCTGGAGAATAAGCTCCGGCTAACTCCGTGCCAGCAGCCGCGGTAATACGGAGGGAGCTAGCGTTGTTCGGAATTACTGGGCGTAAAGCGTGCGTAGGCGGCTATTCAAGTCAGAGGTGAAAGCCTGGAGCTCAACTCCAGAACTGCCTTTGAAACTAGATAGCTAGAATCTTGGAGAGGTTAGTGGAATTCCGAGTGTAGAGGTGAAATTCGTAGATATTCGGAAGAACACCAGTGGCGAAGGCGACTAACTGGACAAGTATTGACGCTGAGGTACGAAAGCGTGGGGAGCAAACAGG` |
| ASV2260 | 404 | `TGGGGAATATTGGACAATGGGCGCAAGCCTGATCCAGCCATGCCGCGTGAGTGATGAAGGCCTTAGGGTTGTAAAGCTCTTTTACCAGGGACGATAATGACGGTACCTGGAGAATAAGCCCCGGCTAACTTCGTGCCAGCAGCCGCGGTAATACGAAGGGGGCTAGCGTTGTTCGGAATCACTGGGCGTAAAGCGCACGTAGGCGGATCTTTAAGTCAGGGGTGAAAGCCCGGAGCTCAACTCCGGAATTGCCTTTGATACTGGGGATCTTGAGTATGGTAGAGGTTGGTGGAATTGCGAGTGTAGAGGTGAAATTCGTAGATATTCGCAAGAACACCAGTGGCGAAGGCGGCCAACTGGACCATAACTGACGCTGAGGTGCGAAAGCGTGGGGAGCAAACAGG` |
| ASV3451 | 405 | `TAGGGAATTTTCCACAATGGACGAAAGTCTGATGGAGCAACGCCGCGTGCAGGATGAAGGCTTTCGGGTCGTAAACTGCTTTTATGAGTGATGATTATGACAGTAGCTCAAGAATAAGGGTCGGCTAACTACGTGCCAGCAGCCGCGGTCATACGTAGGACCCAAGCGTTATCCGGAATTACTGGGCGTAAAGAGTTGCGTAGGCGGCTTGTTAAGCAAGATGTGAAATCGTGAGGCTCAACCTCATACACATATTTTGAACTGGCAAGCTAGAGTATGGTAGAGGTCACTGGAATTCCAAGTGTAGGAGTGAAATCCGTAGATATTTGGAGGAACACCGATGGCGTAGGCAGGTGACTGGACCATTACTGACGCTAAGGCACGAAAGCGTGGGGAGCAAACGGG` |
| ASV898 | 407 | `TGGGGAATTTTCCGCAATGGGCGCAAGCCTGACGGAGCAAGACCGCGTGAGGGAAGAAGGTCTGTGGATTGTAAACCTCTTTTGATGGGGAAGAAGACAGTGACGGTACCCATCGAATCAGCCTCGGCTAACTCCGTGCCAGCAGCCGCGGTAATACGGAGGAGGCAAGCGTTATCCGGAATTATTGGGCGTAAAGCGTCCGTAGGTGGGTTATCAAGTCAGTTGTTAAATAGTGAAGCTTAACTTCATAGGGGCAATTGAAACTGATAGTCTAGAGTGTGGTAGGGGTAGAGGGAATTTCCAGTGTAGCGGTGAAATGCGTAGATATTGGAAAGAACACCGGTGGCGAAAGCGCTCTACTGGGCCTGCACTGACACTGAGGGACGAAAGCTAGGGGAGCGAAAGGG` |
| ASV3377 | 429 | `TGGGGAATTTTGGACAATGGGGGAAACCCTGATCCAGCCATGCCGCGTGTGTGAAGAAGGCCTTCGGGTTGTAAAGCACTTTCGGACGGAACGAAATCGCGTGAGCTAATAGTTCGCGTGGATGACGGTACCGTAAGAAGAAGCACCGGCTAACTACGTGCCAGCAGCCGCGGTAATACGTAGGGTGCGAGCGTTAATCGGAATTACTGGGCGTAAAGTGTGCGCAGGCGGCCGCGCAAGTCGAGTGTGAAATCCCCGAGCTTAACTTGGGAATTGCGCTCGAAACTACGTGGCTGGAGTATGGCAGAGGAAGGTGGAATTCCACGTGTAGCGGTGAAATGCGTAGAGATGTGGAGGAACACCGATGGCGAAGGCAGCCTTCTGGGCCAATACTGACGCTCATGCACGAAAGCGTGGGGAGCAAACAGG` |
| ASV5364 | 429 | `TGGGGAATATTGGACAATGGGCGCAAGCCTGATCCAGCCATACCGCGTGAGTGAAGAAGGCCCTCGGGTTGTAAAGCTCTTTTGTTGGGAAAGAAATCCTGCCGGTTAATACCTGGCAGGGATGACGGTACCCAAAGAATAAGCACCGGCTAACTTCGTGCCAGCAGCCGCGGTAATACGAAGGGTGCAAGCGTTACTCGGAATTACTGGGCGTAAAGCGTGCGTAGGTGGTTGTTTAAGTCTGTCGTGAAAGCCCTGGGCTCAACCTGGGAATTGCGATGGATACTGGGCAACTAGAGTGTGGTAGAGGGTGGCGGAATTCCCGGTGTAGCAGTGAAATGCGTAGAGATCGGGAGGAACATCCGTGGCGAAGGCGGCCACCTGGGCCAACACTGACACTGAGGCACGAAAGCGTGGGGAGCAAACAGG` |
| ASV5679 | 389 | `GCGCGAAACCTCCACAATGCGCGAAAGCGTGATGGGGGGATCCCGAGTGCTTATGCTTTTCGCATAGGCTTTTGCCATGAGTAAAAATCATGGCGAATAAGTGGTGGGTAAGACTGGTGCCAGCCGCCGCGGTAACACCAGCGCCACAAGTGGGGACCACGTTTATTGGGCCTAAAGCGTTCGTAGCCGGTTTTCTAAATGCCCTGTGAAATCGTTACGCCTAACGTAACTACGTGCAGGGTAGACTGGAGAACTTGGGACCGGGAGGGGTCAGGAGTATTTCTGGGGGAGCGGTAAAATGTTATAATCCCAGAAGGACTACCTGTGGCGAAGGCGCCTGACCAGAACGGATCCGACGGTGAGGAACGAAAGCTAGGGGAGCAAAACGG` |
| ASV3014 | 430 | `TAGGGAATCTTGCGCAATGGGGGAAACCCTGACGCAGCAATACCGCGTGAGTGAGGAAGGTCTTCGGATTGTAAAGCTCTGTCGTTGGGGAAAAATGGTTATTGGTCTAATAGGCCATTAATTTGATGGTACCCAAGAAGGAAACACCGGCTAACTTCGTGCCAGCAGCCGCGGTAATACGAAGGGTGTAAGCGTTGTTCGGATTTATTGGGCGTAAAGCGCGCGCAGGTGGATTTTTAAGTCAGATGTGAAATCTCGGGGCTCAACCCCGAACGTGCGTCTGAAACTGGAGATCTAGAATATTACAGGGGGTAGAGGAATTTCACATGTAGGGGTAAAATCCGTAGAGATGTGAAGGAACACCAGAGGCGAAGGCGTCTACCTGGGTAAGTATTGACACTGAGGCGCGAAAGCGTGGGGAGCAAACAGG` |
| ASV5319 | 404 | `TGGGGAATATTGGACAATGGGCGAAAGCCTGATCCAGCCATGCCGCGTGTGTGATGAAGGCCTTAGGGTTGTAAAGCACTTTCAATGGTGAAGATAATGACGGTAACCATAGAAGAAGCCCCGGCTAACTTCGTGCCAGCAGCCGCGGTAATACGAAGGGGGCTAGCGTTGTTCGGAATTACTGGGCGTAAAGCGCGCGTAGGCGGCTTGTCAAGCCAGAGGTGAAATCCCAGAGCTCAACTCTGGAACTGCCTTTGGAACTGTCAAGCTAGAGTATCGGAGGGGACAGTGGAATTCCTAGTGTAGAGGTGAAATTCGTAGATATTAGGAGGAACACCAGTGGCGAAGGCGACTGTCTGGACGATAACTGACGCTGAGGCGCGAAAGCGTGGGGAGCAAACAGG` |
| ASV1038 | 424 | `TGAGGAATATTGGTCAATGGGCGAGAGCCTGAACCAGCCATGCCGCGTGCAGGAAGACGGTCCTATGGATTGTAAACTGCTTTTATTTGGGAATAAACCTATTTACGTGTAGATAGCTGAAGGTACCAAATGAATAAGCACCGGCTAACTCCGTGCCAGCAGCCGCGGTAATACGGAGGGTGCAAGCGTTATCCGGAATCATTGGGTTTAAAGGGTCCGCAGGCGGAAGGATAAGTCAGTGGTGAAATCTCTCGGCTCAACCGAGAAACTGCCATTGATACTGTCTTTCTTGAATTCGATCGAAGTGGGCGGAATGTGACATGTAGCGGTGAAATGCTTAGATATGTCACAGAACACCGATAGCGAAGGCAGCTCACTAGGTCTGGATTGACGCTCAGGGACGAAAGCGTGGGGAGCAAACAGG` |
| ASV3592 | 408 | `TCGAGAATCTTCCGCAATGGACGAAAGTCTGACGGAGCGACGCCGCGTGGTGGATGAAGTCCTTAGGGACGTAAACACCTTTTATGGAGGAGGAAGTTTATTGACGTTACTCCATGAATAAGGGGCTCCCAACTCTGTGCCAGCAGGAGCGGTAATACAGAGGCCCCAAGCATTATCCGGATTTACTGGGCGTAAAGGGTACGTAGGCGGTTATATTAGTCAGGTGTTAAATCCTGGGGCTCAACCCCAGAATCGCATTTGAAACGGTATAACTAGAAGAAGTCAGAGGCAAGCAGAACTCACGGTGTAGGGGTGAAATCCGTTGATATCGTGGGGAATACCAAATGCGAAGGCAGCTTGCTGGGACTCTCTTGACGCTGAGGTACGAAAGCGTGGGTAGCGAAGCGG` |
| ASV4575 | 430 | `TGGGGAATTGTTCGCAATGGGCGCAAGCCTGACGACGCAACGCCGCGTGGAGGATGAAGGTCTTCGGATTGTAAACTCCTGTTGATCGGGACGAACAACCATCGGGATAACACCCCGGAGGTCTGACGGTACCGATTGAGGAAGCCCCGGCTAACTCTGTGCCAGCAGCCGCGGTAATACAGAGGGGGCAAGCGTTGTTCGGAATTACTGGGCGTAAAGGGCGCGTAGGCGGCCGCCTAAGTCAGACGTGAAATCCCTCGGCTCAACCGGGGAACTGCGTCTGATACTGGGTGGCTTGAATTCGGGAGAGGGATGCGGAATTCCAGGTGTAGCGGTGAAATGCGTAGATATCTGGAGGAACACCGGTGGCGAAGGCGGCATCCTGGACCGAGATTGACGCTGAGGCGCGAAAGCCAGGGGAGCAAACGGG` |
| ASV123 | 404 | `TGGGGAATCTTAGACAATGGGCGCAAGCCTGATCTAGCCATGCCGCGTGATCGATGAAGGCCTTAGGGTTGTAAAGATCTTTCAGTGGGGAAGATAATGACTGTACCCACAGAAGAAGCCCCGGCTAACTCCGTGCCAGCAGCCGCGGTAATACGGAGGGGGCTAGCGTTGTTCGGAATTACTGGGCGTAAAGCGCACGTAGGCGGACAGAGAAGTCAGAGGTGAAATCCCAGGGCTCAACCTTGGAACTGCCTTTGAAACTTTTTGTCTTGAGGTCGAGAGAGGTGAGTGGAATTCCGAGTGTAGAGGTGAAATTCGTAGATATTCGGAGGAACACCAGAGGCGAAGGCGGCTCACTGGCTCGATACTGACGCTGAGGTGCGAAAGCGTGGGGAGCAAACAGG` |
| ASV420 | 404 | `TGGGGAATCTTAGACAATGGGCGCAAGCCTGATCTAGCCATGCCGCGTGATCGATGAAGGCCTTAGGGTTGTAAAGATCTTTCAGTGGGGAAGATAATGACGGTACCCACAGAAGAAGCCCCGGCTAACTCCGTGCCAGCAGCCGCGGTAATACGGAGGGGGCTAGCGTTATTCGGAATTACTGGGCGTAAAGCGCACGTAGGCGGATTAACAAGTCAGAGGTGAAATCCCAGGGCTCAACCTTGGAACTGCCTTTGAAACTGTTAGTCTTGAGGTCGTGAGAGGTGAGTGGAATTCCGAGTGTAGAGGTGAAATTCGTAGATATTCGGAGGAACACCAGTGGCGAAGGCGGCTCACTGGCACGATACTGACGCTGAGGTGCGAAAGCGTGGGGAGCAAACAGG` |
| ASV3070 | 405 | `TGGGGAATCTTGCACAATGGGCGAAAGCCTGATGCAGCAACGCCGCGTGCGGGAAGAAGGCCCTAGGGTTGTAAACCGCTTTCAGTAGGGAAGAAAATGACGGTACCTACAGAAGAAGGTGCGGCCAACTACGTGCCAGCAGCCGCGGTGACACGTAGGCACCAAGCGTTATCCGGATTTATTGGGCGTAAAGAGCTCGTAGGCGGTTTGGTAAGTCGGGTGTGAAAACTCTGGGCTCAACCCAGAGAGGCCACTCGATACTGCTATGACTAGAGTACGGTAGGGGAGCGGGGAATTCATGGTGTAGCGGTGAAATGCGCAGATATCATGAGGAACACCAGCGGCGAAGGCGCCGCTCTGGGCCGTAACTGACGCTGAGGAGCGAAAGCATGGGTAGCAAACAGG` |
| ASV4745 | 429 | `TGGGGAATATTGGACAATGGGCGAAAGCCTGATCCAGCAATGCCGCGTGTGTGAAGAAGGCCTGCGGGTTGTAAAGCACTTTCAGTGGGGAAGAAAAAGTCAAGACTAATATTCTTGAAAATTGACGTTACCCAAAGAAGAAGCACCGGCTAACTCTGTGCCAGCAGCCGCGGTAATACAGAGGGTGCGAGCGTTAATCGGAATTACTGGGCGTAAAGGGCGCGTAGGTGGTTTGGTAAGTTAAATGTGAAAGCCCCGGGCTTAACCTGGGAACTGCATTTAATACTGCCAAGCTAGAGTAAAGTAGAGGGAAGTGGAATTTCCGGTGTAGCGGTGAAATGCGTAGATATCGGAAGGAACACCAGTGGCGAAGGCGGCTTCCTGGACTTATACTGACACTGAGGCGCGAAAGCGTGGGGAGCAAACAGG` |
| ASV585 | 424 | `TGAGGAATATTGGTCAATGGACGCAAGTCTGAACCAGCCATGCCGCGTGCAGGAAGACGGCTCTATGAGTTGTAAACTGCTTTTGTACGAGGGTAAACTCATCTACGAGTAGGTGATTGAAAGTATCGTACGAATAAGGATCGGCTAACTCCGTGCCAGCAGCCGCGGTAATACGGAGGATCCAAGCGTTATCCGGATTTATTGGGTTTAAAGGGTGCGTAGGCGGTTTGATAAGTTAGAGGTGAAATATTCGGGCTCAACCCGGAAACTGCCTCTAATACTGTCAGACTAGAGAATAGTTGCGGTAGGCGGAATGTATGGTGTAGCGGTGAAATGCTTAGAGATCATACAGAACACCGATTGCGAAGGCAGCTTACCAAACTATATCTGACGTTGAGGCACGAAAGCGTGGGTAGCGAACAGG` |
| ASV4170 | 404 | `TGGGGAATATTGGACAATGGGGGAAACCCTGATCCAGCAATGCCGCGTGTGTGATGAAGGCCTTCGGGTTGTAAAGCACTTTTGTTAGGGAAGATAATGACGGTACCTAAAGAATAAGCCCCGGCTAACTTCGTGCCAGCAGCCGCGGTAATACGAAGGGGGCTAGCGTTGTTCGGAATGACTGGGCGTAAAGGGCGCGTAGGCGGTTTTATAAGTCAGATGTGAAAGACCTGGGCTCAACCTGGGAAGTGCATTTGAAACTGTAGGACTGGAGGACGAGAGAGGAACGTGGAATTGCGAGTGTAGAGGTGAAATTCGTAGATATTCGCAAGAACACCGATGGCGAAGGCAGCGTTCTGGCTCGTATCTGACGCTAAGGCGCGAAAGCGTGGGGAGCAAACAGG` |
| ASV4610 | 429 | `TGGGGAATTTTGGACAATGGGGGCAACCCTGATCCAGCAATGCCGCGTGTGCGAAGAAGGCCTTCGGGTTGTAAAGCACTTTTGTCAGGGAAGAAAAGGTTTGTGCTAATACCATAAGCCGATGACGGTACCTGAAGAATAAGCACCGGCTAACTACGTGCCAGCAGCCGCGGTAATACGTAGGGTGCAAGCGTTAATCGGAATTACTGGGCGTAAAGCGTGCGCAGGCGGTTGTGTAAGCCAGATGTGAAATCCCCGGGCTTAACCTGGGAATGGCATTTGTGACTGCACGACTAGAGTGTGTCAGAGGGGGGTGGAATTCCACGTGTAGCAGTGAAATGCGTAGAGATGTGGAGGAACACCAATGGCGAAGGCAGCCCCCTGGGATAACACTGACGCTCATGCACGAAAGCGTGGGGAGCAAACAGG` |
| ASV5661 | 430 | `TGGGGAATATTGCGCAATGGGCGAAAGCCTGACGCAGCGACGCCGTGTGGGTGATGAAGGCCTTCGGGTCGTAAAGCCCTGTCAGGGGGGACGAACCCTCGTCGACCTAATACGTCGGCGACCTGACGGTACCCCCAAAGGAAGCACCGGCTAACTCCGTGCCAGCAGCCGCGGTAATACGGAGGGTGCGAGCGTTGTTCGGAATTACTGGGCGTAAAGCGCGTGTAGGCGGCTTCTTAAGTCTGGTGTGAAAGCCCGGGGCTCACCCCCGGAAGGGCATTGGATACTGGGAAGCTAGAGTACCGGAGAGGAAGGTGGAATTCCTGGTGTAGCGGTGAAATGCGTAGATATCAGGAGGAACACCGGTGGCGAAGGCGACCTTCTGGACGGATACTGACGCTGAGACGCGAAAGCGTGGGGAGCAAACAGG` |
| ASV3412 | 404 | `TCGGGAATATTGGACAATGGGCGAAAGCCTGATCCAGCAATGCCGCGTGAGTGATGAAGGCCTTAGGGTTGTAAAGCTCTTTTGACAGGGAAGATAATGACGGTACCTGTAGAATAAGCACCGGCTAATTTCGTGCCAGCAGCCGCGGTAATACGAAAGGTGCTAGCGTTACTCGGATTTATTGGGCGTAAAGAGCGTGTAGGTGGATTTGTAAGTTGGTTGTTAAATCTCGGAGCTTAACTCCGTTGGTGCAATCAAAACTGCTTATCTTGAGTGTGATAGGGGGCAATGGAATTCCTGGTGTAGGGGTAAAATCCGTAGATATCAGGAGGAACACCAAAGGCGAAGGCAGTTGCCTGGGTCACTACTGACACTGATATGCGAAAGCGTGGGGAGCAAAAAGG` |
| ASV2943 | 407 | `TGGGGAATTTTCCGCAATGGGCGAAAGCCTGACGGAGCAATACCGCGTGAGGGAGGAAGGCTCTTGGGTCGTAAACCTCTTTTGTCAGGGAAGAACACAATGACGGTACCTGACGAATCAGCATCGGCTAACTCCGTGCCAGCAGCCGCGGTAATACGGGGGATGCAAGCGTTATCCGGAATTATTGGGCGTAAAGCGTCCGCAGGTGGTTATCCAAGTCTGCTGTTAAAGCATGGGGCTTAACTCCATATAGGCAGTGGAAACTGGATGACTAGAGTACGGTAGGGGTCGCGGGAATTCCCAGTGTAGCGGTGAAATGCGTAGATATTGGGAAGAACACCAGTGGCGAAAGCGCGCGGCTGGACCTGTACTGACACTGAGGGACGAAAGCTAGGGGAGCGAAAGGG` |
| ASV4348 | 429 | `TGGGGAATATTGCACAATGGGCGAAAGCCTGATGCAGCCATGCCGCGTGTGTGAAGAAGGCCTTCGGGTTGTAAAGCACTTTAGGCGAGGAGGAAAAGCGTACGGTTAATAACCGTGCGCCGTGACGTTACTCGCAGAATAAGCACCGGCTAACTCCGTGCCAGCAGCCGCGGTAATACGGAGGGTGCAAGCGTTAATCGGAATTACTGGGCGTAAAGCGCGCGTAGGCGGTTTGATAAGCGGAGTGTGAAATCCCCGGGCTCAACCTGGGAACTGCATTCCGAACTGTCGAGCTAGAGTATGAGAGAGGGTGGCGGAATTCCGAGTGTAGCGGTGAAATGCGTAGATATTCGGAGGAACACCAGTGGCGAAGGCGGCCACCTGGATCAATACTGACGCTGAGGCGCGAAAGCGTGGGTAGCAAACAGG` |
| ASV1098 | 404 | `TGGGGAATATTGGACAATGGGCGAAAGCCTGATCCAGCAATGCCGCGTGAGTGATGAAGGCCTTAGGGTTGTAAAGCTCTTTTACCAGGGATGATAATGACAGTACCTGGAGAATAAGCTCCGGCTAACTCCGTGCCAGCAGCCGCGGTAATACGGAGGGAGCTAGCGTTGTTCGGAATTACTGGGCGTAAAGCGTACGTAGGCGGTTATTCAAGTTAGAGGTGAAAGCCTGGTGCTCAACATCAGAACTGCCTTTAAAACTAGATAGCTAGAATCACGGAGAGGTGAGTGGAATTCCGAGTGTAGAGGTGAAATTCGTAGATATTCGGAAGAACACCAGTGGCGAAGGCGGCTCACTGGACGTGTATTGACGCTGAGGTACGAAAGCGTGGGGAGCAAACAGG` |
| ASV4253 | 429 | `TGGGGAATTTTGGACAATGGGGGGAACCCTGATCCAGCAATGCCGCGTGAGTGAAGAAGGCCCTCGGGTTGTAAAGCTCTTTTGTCGGGGAAGAAAAGGTCGTTTTAAATAGAGATGACTACTGACGGTACCCGAAGAATAAGCACCGGCTAACTACGTGCCAGCAGCCGCGGTAATACGTAGGGTGCAAGCGTTAATCGGAATTACTGGGCGTAAAGGGTGCGCAGGCGGTTTAGTAAGTCAGCGGTGAAATCCCTGGGCTCAACCTAGGAATGGCCGTTGAGACTGCTAGGCTAGAGTGTGTCAGAGGGGGGTAGAATTCCACGTGTAGCAGTGAAATGCGTAGAGATGTGGAGGAATACCGATGGCGAAGGCAGCCCCCTGGGATAACACTGACGCTCAGGCACGAAAGCGTGGGGAGCAAACAGG` |
| ASV1175 | 429 | `TGGGGAATATTGGACAATGGGCGAAAGCCTGATCCAGCCATGCCGCGTGTGTGAAGAAGGTCTTCGGATTGTAAAGCACTTTAAGTTGGGAGGAAGGGCAGTAAGTTAATACCTTGCTGTTTTGACGTTACCAACAGAATAAGCACCGGCTAACTTCGTGCCAGCAGCCGCGGTAATACGAAGGGTGCAAGCGTTAATCGGAATTACTGGGCGTAAAGCGCGCGTAGGTGGTTCAGCAAGTTGGATGTGAAATCCCCGGGCTCAACCTGGGAACTGCATCCAAAACTACTGAGCTAGAGTACGGTAGAGGGTGGTGGAATTTCCTGTGTAGCGGTGAAATGCGTAGATATAGGAAGGAACACCAGTGGCGAAGGCGACCACCTGGACTGATACTGACACTGAGGTGCGAAAGCGTGGGGAGCAAACAGG` |
| ASV1643 | 430 | `TGGGGAATATTGCGCAATGGGGGAAACCCTGACGCAGCAACGCCGCGTGAGTGAGGAAGGACTTCGGTCTGTAAAGCTCTGTTAATGTGGAAAAATGGTAGTTGGTCTAATAGGCCGATTATTTGATGGTACACATAGAGGAAGCACCGGCTAACTTCGTGCCAGCAGCCGCGGTAATACGAAGGGTGCAAGCGTTGTTCGGATTTATTGGGCGTAAAGCGCGCGTAGGCGGGCCTGCAAGTCAGATGTGAAATCTCGAGGCTCAACCTCGAAACTGCGTCTGAAACTACAGGTCTAGAATCTCGGAGGGGGAAGGGGAATTTCGCATGTAGGGGTAAAATCCGTAGATATGCGAAGGAACACCAGAGGCGAAGGCGCCTTCCTGGACGAGTATTGACGCTGAGGCGCGAAAGCGTGGGGATCAAACAGG` |
| ASV563 | 404 | `TGGGGAATCTTAGACAATGGGGGGAACCCTGATCTAGCCATGCCGCGTGATCGATGAAGGCCTTAGGGTTGTAAAGATCTTTCAGATGGGAAGATAATGACGGTACCATCAGAAGAAGCCCCGGCTAACTCCGTGCCAGCAGCCGCGGTAATACGGAGGGGGCTAGCGTTATTCGGAATTACTGGGCGTAAAGCGCACGTAGGCGGACTGGAAAGTCAGAGGTGAAATCCCAGGGCTCAACCTTGGAACGGCCTTTGAAACTCCCCGTCTTGAGATCGTGAGAGGTGAGTGGAATTCCGAGTGTAGAGGTGAAATTCGTAGATATTCGGAGGAACACCAGTGGCGAAGGCGGCTCACTGGCACGATACTGACGCTGAGGTGCGAAAGCGTGGGGAGCAAACAGG` |
| ASV4152 | 416 | `TGGGGAATTTTGGACAATGGGGGCAACCCTGATCCAGCAACTCTGCGTGAGGGACGAAGGTCTTCGGATCGTAAACCTCTTTTTTACGGGACGAAAACCCGCAAGGGTCTGACGGTACCGTGAGAATAAGCCACGGCTAACTATGTGCCAGCAGCCGCGGTAAGACATAGGTGGCGAGCGTTATTCGGAATTACTAGGCGTAAAGCGAGTGTAGGCGGACGCTTAAGTCTGACGTTAAATCTCCGGGCTTAACCCGGAGCTGTCGTCGGATACTGAGCGCCTCGAATGGGGTAGGGGGCAGGGGAATTCCCGGTGTAGCGGTGAAATGCGTAGATATCGGGAGGAACACCTATGGCGAAAGCACCTGCCTGGGCCTCTATTGACGCTAAGACTCGAAAGCTGGGGGAGCAAACAGG` |
| ASV5181 | 404 | `TGGGGAATTTTGGACAATGGACGAAAGTCTGATCCAGCAATTCTGCGTGCACGATGAAGGCCTGCGGGTTGTAAAGTGCTTTTTTGAGGGAAAATGACTGATGGTACCTCAAGAATAAGGGACGGCTAACTCTGTGCCAGCAGCCGCGGTAATACAGAGGTCCCGAGCGTTATCCGGATTTACTGGGCGTAAAGAGTCCGTAGCTGGTTTTGTAAGTCTTAATTCAAATACTCTGACTCACTCAGAGAAAGGGTTAGGATACTGCATTACTAGAGATCATAGGGGGTTAGTGGAATTTCCGGTGGAGGGGTGAAATCCGTTGATATCGGAAGGAATGCCAAAAGCGAAAGCAGCTAACTACTATATTCTGACAGTCAAGGACGAAAGCTTGGGGATCGAACGGG` |
| ASV4593 | 430 | `TGGGGAATCTTGCGCAATGGACGAAAGTCTGACGCAGCCACGCCGCGTGAGTGAAGAAGGCCTTCGGGTTGTAAAGCTCTGTCGGAGGGGACAAAAAATAGTAAGGTTAATAGCCTTATTACTTGATGGTACCCTTAAAGGAAGCACCGGCTAACTTCGTGCCAGCAGCCGCGGTAATACGAAGGGTGCAAGCGTTGCTCGGAATTATTGGGCGTAAAGGGTAGGTAGGTGGTTGCGTATGTCTGGGGTGAAATCCCTGAGCTTAACTCAGGAAGTGCCTTGGAAACGGCGTAACTAGAGTGCTAGAGAGGTTCGTGGAATTCCCAGTGTAGCGGTGAAATGCGTAGAGATTGGGAGGAACACCAGAGGCGAAGGCGGCGAACTGGATAGCAACTGACACTGAACTACGAAAGCGTGGGTAGCAAACAGG` |
| ASV973 | 424 | `TGAGGAATATTGGTCAATGGACGCAAGTCTGAACCAGCCATGCCGCGTGCAGGATGACGGCTCTATGAGTTGTAAACTGCTTTTGTACGAGGGTAAACTTGGATACGTGTATTCAATTGAAAGTATCGTACGAATAAGGATCGGCTAACTCCGTGCCAGCAGCCGCGGTAATACGGAGGATCCGAGCGTTATCCGGATTTATTGGGTTTAAAGGGTGCGTAGGCGGCCTGATAAGTTAGAGGTGAAATCCACGGGCTTAACCCGTGAACTGCCTCTGATACTGTTGGGCTAGAGATTAGTTGCAGATGGCGGAATGTGTAGTGTAGCGGTGAAATGCTTAGATATTACACAGAACACCGATTGCGAAGGCAGCTGTCTAAGCTATATCTGACGCTGAGGCACGAAAGCGTGGGGAGCAAACAGG` |
| ASV984 | 429 | `TGGGGAATTTTGGACAATGGGGGCAACCCTGATCCAGCAATGCCGCGTGAGTGAAGAAGGCCTTCGGGTTGTAAAGCTCTTTTGTCAGGGAAGAAACGGTGTTTCCTAATACGGAATGCTAATGACGGTACCTGAAGAATAAGCACCGGCTAACTACGTGCCAGCAGCCGCGGTAATACGTAGGGTGCAAGCGTTAATCGGAATTACTGGGCGTAAAGCGTGCGCAGGCGGTTTTATAAGTCTGATGTGAAATCCCCGGGCTCAACCTGGGAACTGCATTGGAGACTGTAAGGCTAGAGTGTGTCAGAGGGGGGTAGAATTCCACGTGTAGCAGTGAAATGCGTAGATATGTGGAGGAATACCGATGGCGAAGGCAGCCCCCTGGGATAACACTGACGCTCATGCACGAAAGCGTGGGGAGCAAACAGG` |
| ASV1146 | 403 | `TGAGGAATCTTCCACAATGGGCGAAAGCCTGATGGAGCGACACCGCGTGAAGGATGAAGCCTTTGTTGGTGTAAACTTCTTTTTTCTGGGAAGATAATGACGGTACCAGAAGAATAAGGGGCGGCAAACTTCGTGCCAGCAGCCGCGGTAATACGAAGGCCCCAAGCGTTATCCGGAATTACTGGGTGTAAAGCGTCTGTAGGTGGTTTTTTAAGTTTCGTGTGAAACTTCGGGGCTCAACCCCGAATTGCACGGAAAACTGGAGAACTTGAGTGTGGGAGAGGGAAGCAGAACAGTAAGAGTAGGGGTGCAATCCGTTGATACTTACTAGAATACCAAAAGCGAAGGCAGCTTCCTGGAACACTACTGACACTGAGAGACGAAAGCGTGGGGAGCAAAAGGG` |
| ASV5289 | 404 | `TGGGGAGTATTGGACAATGGGGGCAACCCTGATCCAGCAATGCCGCGTGGGTGATGAAGGCCTTCGGGTTGTAAAGCCCTTTCGGCGGGGACGATGATGACGGTACCCGCAGAAGAAGCCCCGGCTAACTTCGTGCCAGCAGCCGCGGTAATACGAAGGGGGCTAGCGTTGTTCGGAATGACTGGGCGTAAAGGGCGCGTAGGCGGCCTATTTAGTCAGGCGTGAAAGCCCCGGGCTTAACCTGGGAATTGCGCTTGATACTGATGGGCTTGAGTGCGGGAGAGGATGGTGGAATTCCCAGTGTAGAGGTGAAATTCGTAGATATTGGGAAGAACACCGGTGGCGAAGGCGGCCATCTGGACCGTAACTGACGCTGAGGCGCGAAAGCGTGGGGAGCAAACAGG` |
| ASV2469 | 424 | `TAAGGAATATTGGTCAATGGAGGCAACTCTGAACCAGCCATGCCGCGTGCAGGAAGAAGGCCCTATGGGTCGTAAACTGCTTTTGTACCAGAGAAAACCCGAGTACGTGTACTCGGTTGATAGTATGGTAAGAATAAGCATCGGCTAACTTCGTGCCAGCAGCCGCGGTAAGACGAAGGATGCAAGCGTTATCCGGATTCATTGGGTTTAAAGGGTGCGTAGGCGGACTTGTAAGTCAGTGGTGAAATCTCTCAGCTTAACTGAGAAACTGCCATTGATACTGCAGGTCTAGAGTATAGATGACGTTGGCGGAATATGACATGTAGTGGTGAAATACTTAGATATGTCATAGAACACCGATTGCGAAGGCAGCTAACGAAACTATAACTGACGCTGAGGCACGAAAGTGCGGGGATCAAACAGG` |
| ASV5171 | 410 | `TTTGGAATTTTGGACAATGGGCGAAAGCCTGATCCAGCGACGCCGCGTGCACGAAGAAGGTCTTCGGATTGTAAAGTGCTTTTGACGCCGACTAACTTATGAATGTAGGCGTAGAATAAGAGGTTGCTAACTCTGTGCCAGCAGCAGCGGTAATACAGAGACCTCAAGCGTTATCCGGAATCATTGGGCGTAAAGCGTACCGACAGGTGGTTTAGATAGTTTTGGGTAAAAGCCAGAAGCTTAACTTCTGCGTTTGCTCAAAATACTTCTAGACTAGAGGGGCAAAGAGGTGCTTGGAACGAACGGTGTAGTAGTGAAATGCGTTGATATCGTTCGGAACACCGAAAGCGAAGGCAGAGCACTGGGTGCCACCTGACACTGCTAGGACGAAAGCGTGGGGAGCGAATGGG` |
| ASV3504 | 408 | `TGAGGAATTTTCCGCAATGGGCGAAAGCCTGACGGAGCAATACCGCGTGGAGGATTGACAGCTTATGGGCCGTAAACTCCTTTTCTCAGAAAAGAAAAAATTGACGGTATCTGAGGAATAAGCATCGGCTAATTCTGTGCCAGCAGCCGCGGTAAGACAGGAGATGCAAGTGTTATCCGGAATGATTGGGCGTAAAGGGTCTGTAGGTGGTTTGAAAAGTCTGTTGTTAAATACTAGGGCTTAACCCTGGTTAGGCAATAGAAACTATTAGACTAGAGTACGGTAGAGGCAGAGGGAATTTTCGGTGGAGTGGTGAAATGCGCAGATCTCGAAAAGAACACCAATGGCGAAGGCACTCTGCTGGGCCGATACTGACATTGAGAGACGAAAGCTAGGGGAGCAAAAGGG` |
| ASV4356 | 425 | `TAAGGAATATTGGACAATGGAGGAAACTCTGATCCAGCCATGCCGCGTGAAGGATGAAGGCGCTCAGCGTTGTAAACTTCTTTTGAGTGGGAACAAAAAACGGGACTTGTCCCGGCCTGAGGGTACCACTAGAATAAGCACCGGCTAACTCCGTGCCAGCAGCCGCGGTAATACGGAGGGTGCAAGCGTTATCCGGAATCACTGGGTTTAAAGGGTGCGTAGGCGGTCAGATAAGTCAGTCGTGAAATGCCCCGGCTTAACCGGCGGAACTGCGATTGATACTGTTTGACTTGAATCAAGTTTAGGCAGGCGGAATGTGACATGTAGCGGTGAAATGCTTAGATATGTCATAGAACACCGATTGCGAAGGCAGCTTGCTGGGCTTAGATTGACGCTGAGGCACGAAAGCGTGGGGAGCAAACAGG` |
| ASV1006 | 429 | `TAGGGAATATTGCGCAATGGGGGAAACCCTGACGCAGCAACGCCGCGTGAGTGAGGAAGGTCTTCGGATTGTAAAGCTCTGTTCTTAGGGAAAAATGGTTAAGGAGCTAATACCCCTTGATTTGATGGTACCTAAGTAGAAAGGACCGGCTAACTTCGTGCCAGCAGCCGCGGTAATACGAAGGGTCCAAGCGTTGTTCGGATTTATTGGGCGTAAAGCGCGCGCAGGCGGATCGGCAAGTCAGATGTGAAATCTCGGGGCTCAACCCCGAAACTGCGTCTGAAACTGCCAGTCTAGAGTATCGGAGGGGGTAGGGGAATTTCACGTGTAGGGGTAAAATCCGTAGAGATGTGAAGGAACACCGGAGGCGAAGGCGCCTACCTGGACGATTACTGACGCTGAGGCGCGAAAGCGTGGGGAGCAAACAGG` |
| ASV3398 | 404 | `TGGGGAATATTGGACAATGGGCGAAAGCCTGATCCAGCCATGCCGCGTGAGTGATGAAGGCTTTAGGGTTGTAAAGCTCTTTCGCCGATGAAGATAATGACGGTAGTCGGAGAAGAAGCCCCGGCTAACTTCGTGCCAGCAGCCGCGGTAATACGAAGGGGGCTAGCGTTGTTCGGAATTACTGGGCGTAAAGCGAGCGTAGGTTGTGTCGCAAGTCAGTGGTGAAATCCCGGAGCTTAACTTCGGAACTGCCATTGAAACTGCGATGCTAGAATCTCGGAGGGGGTAGCGGAATTCCAAATGTAGGGGTGAAATCCGTAGATATTTGGAGGAACACCGGTGGCGAAGGCGGCTACCTGGACGAGTATTGACACTGAGGCTCGAAAGCGTGGGGATCAAACAGG` |
| ASV4616 | 429 | `TGGGGAATATTGGACAATGGGGGAAACCCTGATCCAGCAATGCCGCGTGTGTGAAGAAGGCCTGCGGGTTGTAAAGCACTTTCAGTGGGGAGGAAAGGGTGTAGGTTAAGAGCTAACACCTGTGACGTTACCCACAGAAGAAGCACCGGCTAACTCTGTGCCAGCAGCCGCGGTAATACAGAGGGTGCAAGCGTTAATCGGACTTACTGGGCGTAAAGGGTGCGTAGGTGGTTTATTAAGTTGATTGTGAAAACTCCGGGCTTAACCTGGGACTTGCAGTCGATACTGGTAGACTAGAGTACAGCAGAGGGAAGTGGAATTTCCGGTGTAGCGGTGAAATGCGTAGATATCGGAAGGAACACCAGTGGCGAAGGCGGCTTCCTGGGCATGTACTGACACTGAGGCACGAAAGCGTGGGGAGCAAACAGG` |
| ASV4237 | 430 | `TAGGGAATATTGCGCAATGGAGGAAACTCTGACGCAGCGACGCCGCGTGGGTGACGAAGGTCTTCGGATCGTAAAGCCCTGTCGGATGGGAAGAAAAACGCTATGGCTAATATCCATAGCGCTTGACGGTACCATAAGAGGAAGCCCCGGCTAACTTCGTGCCAGCAGCCGCGGTAATACGAAGGGGGCAAGCGTTGTTCGGAATCATTGGGCGTAAAGCGCGTGTAGGCTGTCTATTAAGTCGGGTGTGAAATCCCTGGGCTCAACCGAGGAAGTGCATTCGAAACTGATAGACTAGAAGATGGTAGAGGAAGGTGGAATTCCAAGTGTAGAGGTGAAATTCGTAGATATTTGGAGGAATACCGGTGGCGAAGGCGGCCTTCTGGGCCATTCTTGACGCTGAGACGCGAAAGCGTGGGGAGCAAACAGG` |
| ASV1189 | 429 | `TGGGGAATTTTGGACAATGGGCGCAAGCCTGATCCAGCAATGCCGCGTGCAGGATGAAGGCCTTCGGGTTGTAAACTGCTTTTGTACGGAGCGAAACGGTCCTTTCTAATACAAAGGGCTAATGACGGTACCGTAAGAATAAGCACCGGCTAACTACGTGCCAGCAGCCGCGGTAATACGTAGGGTGCGAGCGTTAATCGGAATTACTGGGCGTAAAGCGTGCGCAGGCGGTTATATAAGACAGATGTGAAATCCCCGGGCTCAACCTGGGACCTGCATTTGTGACTGTATAGCTAGAGTACGGTAGAGGGGGATGGAATTCCGCGTGTAGCAGTGAAATGCGTAGATATGCGGAGGAACACCGATGGCGAAGGCAATCCCCTGGACCTGTACTGACGCTCATGCACGAAAGCGTGGGGAGCAAACAGG` |
| ASV3924 | 430 | `TCGGGAATTTTGGGCAATGGGCGAAAGCCTGACCCAGCGACGCCGCGTGAGGGATGAAGGCCTTCGGGTCGTAAACCTCTGTCGGAAGGGGCGAAAGGTTCTTGGGCGAATAGGCCAAGGGCTTGACGGTACCTTCAAAGGAAGCGCCGGCTAACTCCGTGCCAGCAGCCGCGGTAATACGGAGGGCGCAAGCGTTGTTCGGAATTACTGGGCGTAAAGCGCATGTAGGCGGCCTGAAAAGTCGGGTGTGAAATCCCGGAGCTCAACTCCGGAACTGCATCCGATACTGTCAGGCTCGAGTCTCGGAGAGGATGGCGGAATTTCCGGTGTAGAGGTGAAATTCGTAGATATCGGAAGGAACACCAGTGGCGAAGGCGGCCGTCTGGACGATGACTGACGCTGAGATGCGAAAGCGTGGGGAGCAAACAGG` |
| ASV5256 | 405 | `TAGGGAATATTGGACAATGGGCGAAAGCCTGATCCAGCAATACCGCGTGAGTGATGAATGCCCTATGGGTTGTAAAGCTCTTTTATCAGGGAAGATTATGACGGTACCTGAAGAATAAGGACTGACTAACTACGTGCCAGCAGTCGCGGTAATACGTAGAGTCCAAGCGTTACTCGGAATCACTGGGCGTAAAGAATGCGTAGGCGGCTTAGTAAGTCATCAATGAAATACCGGGGCTCAACTTCGGTGCTGTTGATGATACTGCTTCGCTAGAGTTTGGAAGAGGGAAGTGGAATTCCTAGTGTAGGGGTAAAATCCTTAGAGATTAGGAGGAACACCAGAGGCGAAAGCGGCTTCCTGGGACAATACTGACGCTGAGGCATGAAAGTGTGGGGAGCAAACAGG` |
| ASV4739 | 427 | `TCGAGAATTTTTCTCAATGGGGGAAACCCTGAAGGAGCGACGCCGCGTGGAGGATGAAGGTCTTCGGATTGTAAACTCCTGTCATTAGGGAACAAATCTTCCAAGTAACTACTGGAAGTCTGATAGTACCTGAAGAGGAAGGGACGGCTAACTCTGTGCCAGCAGCCGCGGTAATACAGAGGTCCCAACCGTTGTTCGGATTCATTGGGCGTAAAGGGTGCGTAGGCGGCGGAGTAAGTCAGGTGTGAAATCTCGGGGCTCAACCCCGAAACTGCACTTGATACTGCTTCGCTAGAGTACTGGAGAGGAGACTGGAATTTACGGTGTAGCAGTGAAATGCGTAGATATCGTAAGGAAGACCAGTGGCGAAGGCGAGTCTCTGGACAGTTACTGACGCTGAGGCACGAAGGCCAGGGGAGCAAACGGG` |
| ASV4144 | 424 | `TGGGGAATATTGGTCAATGGGCGAAAGCCTGAACCAGCAATGCCGCGTGGGTGATGAAGGCCTTCGGGTTGTAAAGCCCTGTCAGTGGGAAAGAAAAAGACGGTTAACACCCGTACTTGACGGTACCCACAAAGGAAGCACCGGCTAACTCTGTGCCAGCAGCCGCGGTAATACAGAGGGTGCAAGCGTTGTTCGGAATTACTGGGCGTAAAGCGCGTGTAGGCGGCTTATTAAGTCGGACGTGAAATCCCTTGGCTCAACCAAGGAACTGCGTCCGATACTGTTAAGCTTGAGTATCGGAGGGGGTGGCGGAATTCCTGGTGTAGAGGTGAAATTCGTAGATATCAGGAGGAACACCTGTGGCGAAGGCGGCCACCTGGAAGATAACTGACGCTGAGACGCGAAAGCGTGGGGAGCAAACAGG` |
| ASV1939 | 405 | `TGGGGGATATTGCACAATGGAGGAAACTCTGATGCAGCGACGCCGCGTGTAGGAAGACGGTCCTATGGATTGTAAACTACTGTCTTTGGGGACGATAATGACGGTACCCAAAGAGGAAGCTCCGGCTAACTACGTGCCAGCAGCCGCGGTAATACGTAGGGAGCGAGCGTTGTCCGGAATTACTGGGTGTAAAGGGAGCGTAGGCGGGAAGGTAAGTTGGATGTGAAAACTATCGGCTCAACCGGTAGATTGCATTCAAAACTGCTTTTCTTGAGTGAAGTAGAGGCAGGCGGAATTCCTAGTGTAGCGGTGAAATGCGTAGATATTAGGAGGAACACCAGTGGCGAAGGCGGCCTGCTGGGCTTTTACTGACGCTGAGGCTCGAAAGTGTGGGTAGCAAACAGG` |
| ASV4981 | 429 | `TGGGGAATTTTGGACAATGGGGGCAACCCTGATCCAGCAATGCCGCGTGAGTGAAGAAGGCCTTCGGGTTGTAAAGCTCTTTTGTCAGGGAAGAAACGGTCTTGGTTAATACCTGGGGCTAATGACGGTACCTGAAGAATAAGCACCGGCTAACTACGTGCCAGCAGCCGCGGTAATACGTAGGGTGCAAGCGTTAATCGGAATTACTGGGCGTAAAGCGTGCGCAGGCGGTTTTGTAAGTCTGTCGTGAAATCCCCGGGCTCAACCTGGGAATTGCGATGGAGACTGCAAGGCTAGAATCTGGCAGAGGGGGGTAGAATTCCACGTGTAGCAGTGAAATGCGTAGAGATGTGGAGGAACACCGATGGCGAAGGCAGCCCCCTGGGTCAAGATTGACGCTCATGCACGAAAGCGTGGGGAGCAAACAGG` |
| ASV3653 | 429 | `TGGGGAATATTGGACAATGGGGGCAACCCTGATCCAGCGATGCCGCGTGGGTGAAGAAGGCCTTCGGGTTGTAAAGCCCTTTAGGTTGGGAAGAAGTTATGAGGGTGAATATCCCTTGTAATTGACGGTACCGACAGAATAAGCACCGGCAAACTCTGTGCCAGCAGCCGCGGTAATACAGAGGGTGCGAGCGTTAATCGGAGTTACTGGGCGTAAAGGGCGCGTAGGTGGTTATGTGAGTGTGATGTGAAAGCCCTGGGCTTAACCTAGGAAGTGCATCGCAAACGACATAACTGGAGTATAAGAGAGGGTGGTGGAATTTCCGGTGTAGCGGTGAAATGCGTAGAGATCGGAAGGAACGTCAATGGCGAAGGCAGCCACCTGGCTTAATACTGACACTGAGGCGCGAAAGCGTGGGGATCAAACAGG` |
| ASV1680 | 430 | `TGGGGAATCTTCCGCAATGGGCGAAAGCCTGACGGAGCAACGCCGCGTGAATGAAGAAGGCCTTCGGGTCGTAAAGTTCTGTCTTTAGCGAGGAAGTCTCCTTGTGTAAATAATGCGAGGAAATGACGGTAGCTAATGAGGAAGCTCCGGCTAACTACGTGCCAGCAGCCGCGGTAATACGTAGGGAGCAAGCGTTGTCCGGAATTACTGGGCGTAAAGGGCGTGTAGGCGGGTGTTTAAGTCAGATGTGAAAACTCCGGGCTCAACTTGGAGACTGCATTTGAAACTAGATATCTTGAGGGCAGGAGAGGAAAGTGGAATTCCTAGTGTAGCGGTGAAATGCGTAGATATTAGGAGGAACACCAGTGGCGAAGGCGACTTTCTGGACTGTACCTGACGCTGAGGCGCGAAAGCGTGGGGAGCGAACGGG` |
| ASV2248 | 404 | `TGGGGAATCTTGGACAATGGGCGCAAGCCTGATCCAGCCATGCCGCGTGAGTGAAGAAGGCCTTCGGGTTGTAAAGCTCTTTTGTCAGGGACGATAATGACGGTACCTGAAGAATAAGCCCCGGCAAACTTCGTGCCAGCAGCCGCGGTAATACGAAGGGGGCTAGCGTTGTTCGGAATTACTGGGCGTAAAGCGCACGTAGGCTGATTGTTAAGTGAGGGGTGAAATCCCAAGGCTCAACCTTGGAACTGCCTTTCATACTGGCAATCTCGAGTCCGGAAGAGGTTAGTGGAATTCCTAGTGTAGAGGTGAAATTCGTAGATATTAGGAAGAACACCAGTGGCGAAGGCGGCTAACTGGTCCGGTACTGACGCTGAGGTGCGAAAGCGTGGGGAGCAAACAGG` |
| ASV4124 | 429 | `TGGGGAATATTGCGCAATGGGGGAAACCCTGACGCAGCAACGCCGCGTGAGTGATGAAGGCCTTCGGGTCGTAAAGCTCTGTCGGAAGGGACGAAGGTTACGGGGTAAATAATCCCGTGACTTGACGGTACCTTTAGAGGAAGCACCGGCTAACTCCGTGCCAGCAGCCGCGGTAATACGGAGGGTGCAAGCGTTGTTCGGAATTACTGGGCGTAAAGCGCGCGTAGGTGGTCTTTTAAGTCAGGTGTGAAAGACCTCGGCTCAACCGAGGAATTGCGCTTGAAACTAAAAGTCTTGAGTGTCGGAGAGGAAAGCAGAATTCCTGGTGTAGAGGTGAAATTCGTAGATATCAGGAGGAATACCGGAGGCGAAAGCGGCTTTCCAATCGACTACTGACACTGAGGCGCGAAAGCGTGGGGAGCAAACAGG` |
| ASV2653 | 404 | `TGAGGAATATTGGACAATGGAGGAAACTCTGATCCAGCCATGCCGCGTGAGTGACGAAGGCCTTCGGGTTGTAAAACTCTTTAAGTGGGGAAGATAATGACGGTACCCACAGAATAAGCACCGGCTAACTTCGTGCCAGCAGCCGCGGTAATACGAAGGGTGCTAGCGTTACTCGGAATCACTGGGCGTAAAGCGTGCGTAGGCGGAAATATAAGTCAGAGGTGAAATCCCAGGGCTCAACCCTGGAATTGCCTTTGAAACTGTATTTCTTGAGTATGGTAGAGGATGGTGGAATTCCTAGTGTAGAGGTGAAATTCGTAGATATTAGGAGGAACACCGGTGGCGAAGGCGGCCATCTGGGCCATTACTGACGCTGAGGCACGAAAGCGTGGGTAGCAAACAGG` |
| ASV51 | 423 | `TAGGGAATATTGGGCAATGGACGGAAGTCTGACCCAGCCATGCCGCGTGCAGGATGAAGGCCCTCTGGGTTGTAAACTGCTTTTGACAGGGAAGAAATACATCATGCGTGATGTGCTGACGGTACCTGTAGAATAAGCACCGGCTAACTCCGTGCCAGCAGCCGCGGTAATACGGAGGGTGCAAGCGTTGTCCGGATTTATTGGGTTTAAAGGGTGCGTAGGCGGTTCTGTAAGTCAGTGGTGAAAGCTAGCAGCTTAACTGTTAAACTGCCATTGATACTGCAGAGCTTGAGTTCAGATGAGGTAGGCGGAATTTATAGTGTAGCGGTGAAATGCTTAGATACTATAAAGAACTCCGATAGCGTAGGCAGCTTACTAAACTGATACTGACGCTGAGGCACGAAAGTGTGGGGATCAAACAGG` |
| ASV5557 | 423 | `TAGGGAATATTGGGCAATGGGCGCAAGCCTGACCCAGCCATGCCGCGTGCAGGAAGAAGGCGTTATGCGTTGTAAACTGCTTTTATATGGGAAGAAAAAGGCCATGCGTGGCAAACTGACGGTACCATATGAATAAGCACCGGCTAACTCCGTGCCAGCAGCCGCGGTAATACGGAGGGTGCAAGCGTTGTCCGGATTTATTGGGTTTAAAGGGTGCGTAGGCGGCTTTTTAAGTCAGCGGTGAAAGCCGGTCGCTCAACGACCGAACTGCCGTTGATACTGATTAGCTTGAGTTTGATTGAGGCAGCCGGAATGGATAGTGTAGCGGTGAAATGCATAGATATTATCCAGAACGCCAATAGCGTAGGCAGGTTGCTAAGTCATAACTGACGCTGAGGCACGAGAGTGCGGGGATCAAACAGG` |
| ASV3865 | 429 | `TGGGGAATATTGGACAATGGGGGCAACCCTGATCCAGCCATGCCGCGTGTGTGAAGAAGGCCTTCGGGTTGTAAAGCACTTTCAGCGGGGAGGAAGGCATCATGACTAATATTCATGGTGATTGACGTTACCCGCAGAAGAAGCACCGGCTAACTCCGTGCCAGCAGCCGCGGTAATACGGAGGGTGCGAGCGTTTATCGGAATTACTGGGCGTAAAGCGCGCGTAGGCGGCTTGTTAAGTCGGATGTGAAATCCCCGGGCTCAACCTGGGAATTGCATCCGATACTGGCGAGCTGGAGTGCGATAGAGGGGGGTAGAATTCCTAGTGTAGCGGTGAAATGCGTAGATATTAGGAGGAATACCGGTGGCGAAGGCGGCCCCCTGGATCGACACTGACGCTGAGGTGCGAAAGCGTGGGTAGCAAACAGG` |
| ASV611 | 430 | `TAGGGAATATTGCGCAATGGGGGAAACCCTGACGCAGCAACGCCGCGTGAGTGAGGAAGGTCTTCGGATTGTAAAACTCTTTTGCTAGGGAAAAATGACTTAACGGTTAATACCCGTTAGGAGTGATGGTACCTAAAGAATAAGGACCGGCTAACTTCGTGCCAGCAGCCGCGGTAATACGAAGGGTCCAAGCGTTGTTCGGAATCATTGGGCGTAAAGCGCGCGCAGGCGGATCAGCAAGTCAGATGTGAAATCTCGAAGCTCAACTTCGAAACTGCGTCTGAAACTGCTAGTCTAGAGTGTCGGAGGGGGAAGGGGAATTTCACGTGTAGGGGTAAAATCCGTAGAGATGTGAAGGAACACCGGGGGCGAAGGCGCCTTCCTGGACGACTACTGACGCTGAGGCGCGAAAGCGTGGGGAGCAAACAGG` |
| ASV2888 | 429 | `TGGGGAATATTGCACAATGGACGAAAGTCTGATGCAGCAACGCCGCGTGAGTGATGAAGGCCCTTGGGTTGTAAAGCTCTTTCGTCTGGGAAGAAGGACGTACGTCGAATAGACGTTGCGTTTGACGGTACCAGAGGAAGAAGTACCGGCTAATTCCGTGCCAGCAGCCGCGGTAATACGGAAGGTGCAAGCGTTGTTCGGAATGACTGGGCGTAAAGCGCACGTAGGCGGGTTTTTAAGTCGATTGTGAAAGCCCCGGGCTCAACCTGGGATTGGCAGTCGAAACTGAAGATCTTGAATGCCTGAGAGGTTAGTGGAATTTGTGGTGTAGGAGTGAAATCCGTAGAGATCACAAGGAACACCAGAGGCGAAGGCGACTAACTGGTGGTGCATTGACGCTGATGTGCGAAAGCGTGGGTAGCAAACAGG` |
| ASV2409 | 424 | `TGAGGAATATTGGACAATGGGCGCAAGCCTGATCCAGCCATGCCGCGTGCAGGATGAAGGTCCTATGGATTGTAAACTGCTTTTGCACAGGAAGAAACACCCCCTCGTGAGGGGGCTTGACGGTACTGTGAGAATAAGGATCGGCTAACTCCGTGCCAGCAGCCGCGGTAATACGGAGGATCCAAGCGTTATCCGGAATCATTGGGTTTAAAGGGTCCGTAGGCGGTCTTATAAGTCAGTGGTGAAATCTCCCCGCTCAACGGGGAAACGGCCATTGATACTGTAGGACTTGAATTATTAGGAAGTAACTAGAATATGTAGTGTAGCGGTGAAATGCTTAGAGATTACATGGAATACCAATTGCGAAGGCAGGTTACTACTAATGGATTGACGCTGATGGACGAAAGCGTGGGTAGCGAACAGG` |
| ASV5229 | 429 | `TAGGGAATCTTGCGCAATGGGGTCACCCCTGACGCAGCAATACCGCGTGAGTGAGGAAGGCCTTCGGGTTGTAAAGCTCTTTTCTTGGGGAAAAAGGAATAAAGGTGAATAATCTTTGTTATTGATGGTACCCAAGGAATAAGCACCGGCTAACTTCGTGCCAGCAGCCGCGGTAATACGAAGGGTGCAAGCGTTGTTCGGATTTATTGGGCGTAAAGGGTGCGTAGGCGGATCAGCAAGTCAGGTGTGAAATCTCGGGGCTTAACCCCGAAACTGCGCCTGAAACTACTAATCTAGAATACTGGAGAGGAAAGGGGAATTTCGCATGTAGGGGTAAAATCCGTAGATATGCGAAGGAACACCAGAGGCGAAGGCGCCTTTCTGGCCAGTTATTGACGCTGAGGCACGAAAGCGTGGGGAGCAAACAGG` |
| ASV1242 | 424 | `TGAGGAATATTGGACAATGGGCGCAAGCCTGATCCAGCCATGCCGCGTGCAGGAAGACGGTCCTATGGATTGTAAACTGCTTTTATACAGGAAGAAACACCTCTACGTGTAGAGACTTGACGGTACTGTATGAATAAGCATCGGCTAACTCCGTGCCAGCAGCCGCGGTAATACGGAGGATGCAAGCGTTATCCGGAATCATTGGGTTTAAAGGGTCCGTAGGCGGCCAAATAAGTCAGTGGTGAAAGCCCATCGCTCAACGATGGAACGGCCATTGATACTGTTTGGCTTGAATTATTAGGAAGTAACTAGAATATGTAGTGTAGCGGTGAAATGCTTAGAGATTACATGGAATACCAATTGCGAAGGCAGGTTACTACTAATATATTGACGCTGATGGACGAAAGCGTGGGGAGCGAACAGG` |
| ASV4867 | 404 | `TGAGGAATCTTCCACAATGGGCGAAAGCCTGATGGAGCGACACCGCGTGAAGGATGAAGCCTTTGTTGGTGTAAACTTCTTTTCTCTCCGAAGATAATGACGGTAGGAGAGGAATAAGGGGCGGCAAACTTCGTGCCAGCAGCCGCGGTAATACGAAGGCCCCAAGCGTTATCCGGAATTACTGGGTGTAAAGCGTCTGTAGGTGGTTCGGAAAGTTTCAAGTGAAAGGTCAGGGCTCAACCCTGTACTTGCTTGGAAAACTACCTGACTTGAGTATGGGAGAGGCAAGCAGAATAATAAGAGTAGGGGTGCAATCCGTTGATACTTATTAGAATACCAAAAGCGAAGGCAGCTTGCTGGAACATTACTGACACTGAGAGACGAAAGCGTGGGGAGCAAAAGGG` |
| ASV34 | 412 | `TCGAGAATCATTCACAATGGGGGAAACCCTGATGGTGCGACGCCGCGTGGGGGAAGAAGGTCCTCGGATCGTAAACCCCTGTCATGTGGGAGCAAGCAGCGATGTAGATAGTACCACAAGAGGAAGAGACGGCTAACTCTGTGCCAGCAGCCGCGGTAATACAGAGGTCTCAAGCGTTGTTCGGAATCACTGGGCGTAAAGAGTACGTAGGCTGTCAAGTAAGTCTGGCGTGAAAGGCAAGGGCTCAACCCTTGGACTGCGCCGGATACTGCGTGACTAGAGTAATGGAGGGGGAACCGGAATTCTCGGTGTAGCAGTGAAATGCGTAGATATCGAGAGGAACACTCGTGGCGAAGGCGGGTTCCTGGACATTAACTGACGCTGAGGTACGAAGGCTAGGGAAGCGAAAGGG` |
| ASV3123 | 429 | `TGGGGAATATTGGACAATGGGGGCAACCCTGATCCAGCCATGCCGCGTGTGTGAAGAAGGCCCTTGGGTTGTAAAGCACTTTCAGTGGGGAGAAAAGTCTGGCACCTAATACGTGTTGGAATTGATGTTACCCACAGAAGAAGCACCGGCTAACTCCGTGCCAGCAGCCGCGGTAATACGGAGGGTGCAAGCGTTAATCGGAATTACTGGGCGTAAAGCGCACGTAGGTGGTTTTTTAAGTCAGCTGTGAAATCCCCGGGCTCAACCTGGGCACTGCAGTTGAAACTGAGAAGCTAGAGTAGGGTAGAGGGGTGTGGAATTCCAGGTGTAGCGGTGAAATGCGTAGATATCTGGAGGAACATCAGTGGCGAAGGCGACACCCTGGACTCATACTGACACTGAGGTGCGAAAGCGTGGGGAGCAAACAGG` |
| ASV840 | 429 | `TGGGGAATTTTGGACAATGGGCGCAAGCCTGATCCAGCAATGCCGCGTGAGTGAAGAAGGCCTTCGGGTTGTAAAGCTCTTTTGTCAGGGAAGAAACGGTTGTGGCTAATATCTGCAGCTAATGACGGTACCTGAAGAATAAGCACCGGCTAACTACGTGCCAGCAGCCGCGGTAATACGTAGGGTGCAAGCGTTAATCGGAATTACTGGGCGTAAAGCGTGCGCAGGCGGTTTTGTAAGTCTGTCGTGAAATCCCCGGGCTCAACCTGGGAATGGCGATGGAGACTGCAAGGCTAGAATCTGGCAGAGGGGGGTAGAATTCCACGTGTAGCAGTGAAATGCGTAGAGATGTGGAGGAACACCGATGGCGAAGGCAGCCCCCTGGGTCAAGATTGACGCTCATGCACGAAAGCGTGGGGAGCAAACAGG` |
| ASV5217 | 430 | `TGGGGAATATTGCACAATGGGGGAAACCCTGATGCAGCAACGCCGCGTGAGTGAAGAAGGCCTTAGGGTTGTAAAGCTCTTTCGGCAGGGAAGAGGGGCTTTGTTCCTAATACGAACAAAGTCTGACGGTACCTGAAGAAGAAGCACCGGCTAACTCCGTGCCAGCAGCCGCGGTAAGACGGAGGGTGCAAGCGTTGTTCGGAATTATTGGGCGTAAAGCGCGCGCAGGCGGGCTGATAAGTTGGTTGTGAAATCCCCGAGCTTAACTTGGGAACTGCAGCCAAAACTGTCAGTCTTGAATACCTGAGAGGGTGGGAGAATTCCTGGTGTAGAAGTGAAATTCGTAGATATCAGGAGGAATACCGGAGGCGAAGGCGCCTGCCTGGCAGAGTATTGACGCTGAGGCGCGAAAGCGTGGGGAGCAAACAGG` |
| ASV3563 | 421 | `TGAGGAATATTGCGCAATGGACGGAAGTCTGACGCAGCAATGCCGCGTGCGGGATGAAGGCGCTATGCGTCGTAAACCGCTGTAGCGTGGGGCAAAAAGCACCCTCGGGGTGTTAGATGGTACCACGAAAGTAAGCATCGGCTAACTACGTGCCAGCAGCCGCGGTAATACGTAGGATGCAAGCGTTGTCCGGATTTACTGGGTGTAAAGGGTGCGCAGGCGGGCTTGTAAGTCAGAGGTGAAAGCCCGGAGCTTAACTCCGGAAATGCCTTTGATACTGCGAGTCTTGAATGTGAGAGAGGTTCATGGAATATGTGGTGTAGCGGTGAAATGCGTAGATATCACATAGAACACCAGTGGCGAAGGCGGTGAACTGGCTCATTATTGACGCTCAGGCACGAAAGTGTGGGTAGCAAACAGG` |
| ASV218 | 429 | `TGGGGAATTTTGGACAATGGGCGCAAGCCTGATCCAGCAATGCCGCGTGCAGGAAGAAGGCCTTCGGGTTGTAAACTGCTTTTGTACGGAGCGAAAAGGCTCTCTCTAATACAGGGGGTTCATGACGGTACCGTAAGAATAAGCACCGGCTAACTACGTGCCAGCAGCCGCGGTAATACGTAGGGTGCGAGCGTTAATCGGAATTACTGGGCGTAAAGCGTGCGCAGGCGGTTTTGTAAGACAGGCGTGAAATCCCCGGGCTCAACCTGGGAATGGCGCTTGTGACTGCAAAGCTGGAGTGCGGCAGAGGGGGATGGAATTCCGCGTGTAGCAGTGAAATGCGTAGATATGCGGAGGAACACCGATGGCGAAGGCAATCCCCTGGGCCTGCACTGACGCTCATGCACGAAAGCGTGGGGAGCAAACAGG` |
| ASV700 | 429 | `TGGGGAATTTTGGACAATGGGCGCAAGCCTGATCCAGCAATGCCGCGTGCAGGATGAAGGCCTTCGGGTTGTAAACTGCTTTTGTACGGAGCGAAACGGTCTCTTCTAATACAGGGGGCTAATGACGGTACCGTAAGAATAAGCACCGGCTAACTACGTGCCAGCAGCCGCGGTAATACGTAGGGTGCAAGCGTTAATCGGAATTACTGGGCGTAAAGCGTGCGCAGGCGGTTATATAAGACAGATGTGAAATCCCCGGGCTCAACCTGGGACCTGCATTTGTGACTGTATAGCTAGAGTACGGTAGAGGGGGATGGAATTCCGCGTGTAGCAGTGAAATGCGTAGATATGCGGAGGAACACCGATGGCGAAGGCAATCCCCTGGACCTGTACTGACGCTCATGCACGAAAGCGTGGGGAGCAAACAGG` |
| ASV3597 | 429 | `TGGGGAATTTTGGACAATGGGGGCAACCCTGATCCAGCCATTCCGCGTGAGTGAAGAAGGCCTTCGGGTTGTAAAGCTCTTTTGTCCGGAGCGAAACGGTTAGCGTGAATATCGCTGACTACTGACGGTACCGGAAGAATAAGCACCGGCTAACTACGTGCCAGCAGCCGCGGTAATACGTAGGGTGCGAGCGTTAATCGGAATTACTGGGCGTAAAGCGTGCGCAGGCGGGTTTTTAAGCCAGACGTGAAATCCCCGGGCTTAACCTGGGAATGGCGTTTGGGACTGGAAGTCTGGAGTGCGGCAGAGGAGACTGGAATTCCTGGTGTAGCAGTGAAATGCGTAGATATCAGGAGGAACACCGATGGCGAAGGCAGGTCTCTGGGCTGACACTGACGCTCATGCACGAAAGCGTGGGGAGCAAACAGG` |
| ASV5606 | 429 | `TGGGGAATATTGGACAATGGGGGCAACCCTGATCCAGCAATGCCGCGTGTGTGAAGAAGGCCTGCGGGTTGTAAAGCACTTTCAGTGGGGAGGAAGGCCTCGGTGGTAATATCGCCGAGGATTGACGTTACCCAAAGAAGAAGCACCGGCTAACTCTGTGCCAGCAGCCGCGGTAATACAGAGGGTGCAAGCGTTAATCGGAATTACTGGGCGTAAAGGGCGCGTAGGTGGTTAGTTAAGTTAGATGTGAAATCCCTGGGCTTAACCTAGGAATTGCATTTAATACTGATTAGCTAGAGTGTGGTAGAGGGAAGTGGAATTTCCAGTGTAGCGGTGAAATGCGTAGATATTGGAAGGAACACCAGTGGCGAAGGCGACTTCCTGGACCACTACTGACACTGAGGCGCGAAAGCGTGGGGAGCGAACAGG` |
| ASV659 | 408 | `TGGGGAATTTTCCGCAATGGGCGAAAGCCTGACGGAGCAATACCGCGTGAGGGAAGACTGCCTATGGGTTGTAAACCTCTTTTTTCAGGGAGGAATAAAATGACGTGTACCTGAAGAATAAGCATCGGCTAACTCCGTGCCAGCAGCCGCGGTAAGACGGAGGATGCAAGTGTTATCCGGAATCACTGGGCGTAAAGCGTCTGTAGGTGGTTTAATAAGTCAACTGTTAAATCTTGAGGCTCAACTTCAAAATCGCAGTCGAAACTATTAGACTAGAGTATAGTAGAGGTAAAGGGAATTTCCAGTGGAGCGGTGAAATGCGTAGATATTGGAAAGAACACCGATGGCGAAAGCACTTTACTGGGCTATTACTAACACTCAGAGACGAAAGCTAGGGTAGCAAATGGG` |
| ASV3658 | 404 | `TGGGGAATATTGGACAATGGGCGAAAGCCTGATCCAGCAATGCCGCGTGAGTGATGAAGGCTTTCGAGTTGTAAAGCTCTTTCAGCGAGGAAGATAATGACGGTACTCGCAGAAGAAGCCCCGGCTAACTCCGTGCCAGCAGCCGCGGTAATACGGAGGGGGCTAGCGTTGTTCGGAATTACTGGGCGTAAAGCGCGCGTAGGCGGTCTCGCAAGTTGGAAGTGAAATCCCGGAGCTCAACTTCGGAATTGCTTTCAAAACTGCATGACTTGAGAATGGTAGAGGATAGTGGAATTCCCAGTGTAGAGGTGAAATTCGTAGATATTGGGAGGAACACCAGTGGCGAAGGCGACTATCTGGGCCATTTCTGACGCTAAGGCGCGAAAGCGTGGGGAGCAAACAGG` |
| ASV3082 | 429 | `TGGGGAATATTGGACAATGGGGGCAACCCTGATCCAGCGATGCCGCGTGGGTGAAGAAGGCCTTCGGGTTGTAAAGCCCTTTAGGTTGGGAAGAAGTGTACGGGGCGAATAACCTCGTATGTTGACGGTACCGACAAAATAAGCACCGGCAAACTCTGTGCCAGCAGCCGCGGTAATACAGAGGGTGCGAGCGTTAATCGGAGTTACTGGGCGTAAAGGGCGCGTAGGCGGTTGTGTGAGTGTGATGTGAAAGCCCTGGGCTTAACCTGGGAAGTGCATCGCAAACGACATAACTGGAGTATAAGAGAGGGTGGCGGAATTTCCGGTGTAGCGGTGAAATGCGTAGAGATCGGAAGGAACGTCAATGGCGAAGGCAGCCACCTGGCTTAATACTGACGCTGAGGCGCGAAAGCGTGGGTAGCGAACAGG` |
| ASV96 | 429 | `TGGGGAATATTGGACAATGGGCGCAAGCCTGATCCAGCCATGCCGCGTGTGTGAAGAAGGCCTTCGGGTTGTAAAGCACTTTTGTTGGGGAAGAAAAGACCTTGGTTAATAACCGAGGTTCATGACGGTACCCAAAGAATAAGCACCGGCTAACTTCGTGCCAGCAGCCGCGGTAATACGAAGGGTGCAAGCGTTACTCGGAATTACTGGGCGTAAAGCGTGCGTAGGTGGTTCGTTAAGTCTGCCGTGAAAGCCCCGGGCTCAACCTGGGAATGGCGGTGGATACTGGCGGACTAGAGTGCGGTAGAGGGTGGTGGAATTCCCGGTGTAGCAGTGAAATGCGTAGAGATCGGGAGGAACATCTGTGGCGAAGGCGGCCACCTGGACCAGCACTGACACTGAGGCACGAAAGCGTGGGGAGCAAACAGG` |
| ASV2729 | 405 | `TGGGGAATTTTCCGCAATGGGCGAAAGCCTGACGGAGCAATACCGCGTGAGGGAGGAAGGCTCTTGGGTTGTAAACCTCAAAACTTAGGGAAGAAGAAAGTGACGGTACCTAATATAAGCATCGGCTAACTCCGTGCCAGCAGCCGCGGTAATACGGAGGATGCAAGCGTTATCCGGAATCATTGGGCGTAAAGAGTCTGTAGGTGGCATTTAAAGTCTGCTGTCAAAGACCGAAGCTCAACTTCGGGCAGGCGGTGGAAACTAAAAAGCTAGAGTATAGTAGGGGTAGAGGGAATTCCTAGTGTAGCGGTGAAATGCGTAGAGATTAGGAAGAACACCAGTGGCGAAGGCGCTCTACTGGGCATATACTGACACTGAGAGACGAAAGCTAGGGTAGCGAAAGGG` |
| ASV2591 | 405 | `TGGGGAATCTTGCGCAATGGGCGAAAGCCTGACGCAGCCATGCCGCGTGAATGATGAAGGTCTTAGGATTGTAAAGTTCTTTCGCCCGTGACGATGATGACGGTAACGGGAGAAGAAGCCCCGGCTAACTTCGTGCCAGCAGCCGCGGTAATACGAAGGGGGCTAGCGTTGTTCGGAATTACTGGGCGTAAAGCGCGCGTAGGCGGCTTTGCAAGCCAGAGGTGAAATGCCTGAGCTCAACTTAGGAATTGCCTTTGGGACTGCGAGGCTTCGAGGACTGGAGAGGTGAGTGGAATTCCCAGTGTAGAGGTGAAATTCGTAGATATTGGGAAGAACACCGGTGGCGAAGGCGGCTCACTGGCCAGTTTCTGACGCTGTAGCGCGAAAGCGTGGGGATCAAACAGG` |
| ASV743 | 429 | `TGGGGAATATTGGACAATGGGGGGAACCCTGATCCAGCAATGCCGCGTGTGTGAAGAAGGCCTGAGGGTTGTAAAGCACTTTCAGTGGGGAGGAGGGTTGAATGGTTAAGAGCTATTTAATTGGACGTTACCCACAGAAGAAGCACCGGCTAACTCCGTGCCAGCAGCCGCGGTAATACGGGGGGTGCAAGCGTTAATCGGAATTACTGGGCGTAAAGGGTGTGTAGGTGGTTTGATAAGATATCTGTGAAATTCCTGGGCTTAACCTGGGCTTGTCAGATAAGACTGTTAGACTAGAGTATGGGAGAGGGTAGTGGAATTTCCGGTGTAGCGGTGAAATGCGTAGAGATCGGAAGGAACACCAGTGGCGAAGGCGGCTACCTGGCCTAATACTGACACTGAGGCACGAAAGCGTGGGGAGCAAACAGG` |
| ASV5110 | 405 | `TAGGGAATTTTCCACAATGGGCGAAAGCCTGATGGAGCAACGCCGCGTGCAGGATGAAGGCTTTCGGGTCGTAAACTGCTTTTATATGTGAAGATTATGACGGTAGCATATGAATAAGGGTCGGCTAACTACGTGCCAGCAGCCGCGGTCATACGTAGGACCCAAGCGTTATCCGGAATTACTGGGCGTAAAGAGTTGCGTAGGTGGCATAGTAAGCGGGTAGTGAAATCGTGTGGCTCAACCATACACACATTACTTGAACTGCTAAGCTAGAGGATGAGAGAGGTCACTGGAATTCCAAGTGTAGGAGTGAAATCCGTAGATATTTGGAGGAACACCGATGGCGTAGGCAGGTGACTGGCTCATTCCTGACACTAAGGCACGAAAGCGTGGGTAGCAAACGGG` |
| ASV4188 | 429 | `TGGGGAATATTGGACAATGGGGGCAACCCTGATCCAGCGATGCCGCGTGGGTGAAGAAGGCCTTCGGGTTGTAAAGCCCTTTAGGTTGGGAAGAAGTGTAGTGGTTTAAGAGATAGCTACATTGACGGTACCGGCAGAATAAGCACCGGCAAACTCTGTGCCAGCAGCCGCGGTAATACAGAGGGTGCGAGCGTTAATCGGATTTACTGGGCGTAAAGGGCGCGTAGGCGGATAGGTGAGTGTGATGTGAAAGCCCTGGGCTTAACCTAGGAAGTGCATTGCAAACGACCTAACTGGAGTAGATGAGAGGGTGGCGGAATTTCCGGTGTAGCGGTGAAATGCGTAGAGATCGGAAGGAACGTCAATGGCGAAGGCAGCCACCTGGCATCATACTGACGCTGAGGCGCGAAAGCGTGGGTAGCGAACAGG` |
| ASV386 | 404 | `TGGGGAATATTGGACAATGGGCGAAAGCCTGATCCAGCAATGCCGCGTGAGTGAAGAAGGCCTTCGGGTTGTAAAGCTCTTTTACCCGGGAAGATAATGACTGTACCGGGAGAATAAGCCCCGGCTAACTCCGTGCCAGCAGCCGCGGTAATACGGAGGGGGCTAGCGTTGTTCGGAATTACTGGGCGTAAAGCGTACGTAGGCGGCCATTCAAGTCAGAGGTGAAAGCCCGGGGCTCAACTCCGGAACTGCCTTTGAAACTAGGTGGCTTGAACACGGGAGAGGTGAGTGGAATTCCGAGTGTAGAGGTGAAATTCGTAGATATTCGGAAGAACACCAGTGGCGAAGGCGGCTCACTGGACCGTTGTTGACGCTGAGGTACGAAAGCGTGGGGAGCAAACAGG` |
| ASV5500 | 407 | `TCGAGAATATTCGACAATGGGCGAAAGCCTGATCGAGCGACGCCGCGTGCAGGATGAAGGTCTTAGGATTGTAAACTGCTTTTCCACGGGAAAAAGTTATTGATGGTACCGTGGGAATAAGAAGCTGCTAACTTCGTGCCAGCAGCCGCGGTAATACGAAGGCTTCAAGCGTTATCCGGATTTATTGGGCGTAAAGCGTGTGTAGGAGGATGTGTAAGTTTGATGTTAAATCTCCGGGCTTAACCTGGAACCAGCATTGAATACTGCATATCTAGAGTGTGTGAGAGGTTCATAGAACGCATGGTGTAGGGGTGACATCCGTTGATATCATGCGGAATACCAAAGGCGAAGGCATTGAACTGGCGCATTACTGACTCTAAGACACGAAAGCGTGGGGAGCAAAAAGG` |
| ASV265 | 425 | `TAGGGAATATTGGGCAATGGGCGGAAGCCTGACCCAGCCACGCCGCGTGCAGGAAGACGGCCCTCTGGGTTGTAAACTGCTTTTAACTGGGAAGAAAGCAGCTCATGCGTGAGAAATTGACGGTACCAGTTGAATAAGCCACGGCTAACTACGTGCCAGCAGCCGCGGTAATACGTAGGTGGCGAGCGTTATCCGGATTTATTGGGTTTAAAGGGAGCGTAGGCGGCTGATTAAGTCAGCGGTGAAAGGTAGCAGCTTAACTGTTTTACATGCCGTTGATACTGGTTAGCTTGAGTTGACAGAAGGCAGATAGAATTCCTGATGTAGCGGTGAAATGCTTAGATATCAGGAGGAATACCGATTGCGAAGGCAGTCTGCTGCAGTCACACTGACGCTGATGCTCGAAGGTGCGGGGATCAAACAGG` |
| ASV3242 | 404 | `TGGGGAATATTGGACAATGGGGGCAACCCTGATCCAGCCATGCCGCGTGAGTGATGAAGGCCTTCGGGTTGTAAAGCTCTTTTGGCGGGGACGATGATGACGGTACCCGCAGAATAAGCCCCGGCTAACTTCGTGCCAGCAGCCGCGGTAAGACGAAGGGGGCTAGCGTTGTTCGGAATTACTGGGCGTAAAGCGCGTGTAGGCGGTCGTCCAAGTTGGATGTGAAAGCCTTGAGCTTAACTCAAGAAATGCATTCAGGACTGGATGACTAGAGGACCGGAGAGGATAGTGGAATTCCCAGTGTAGTGGTGAAATACGTAGAGATTGGGAAGAACACCAGTGGCGAAGGCGGCTATCTGGACGGTTTCTGACGCTAAGACGCGAAAGCGTGGGGAGCAAACAGG` |
| ASV4842 | 407 | `TAGGGAATATTGCGCAATGGGGGAAACCCTGACGCAGCGACGCCGCGTGAGTGATGAAGGACTTCGGTTCGTAAAGCTCTGTTCTCAGGGAATAAGAAAGTGAATGTACCTGAGAAGAAAGGATCGGCTAACTTCGTGCCAGCAGCCGCGGTAAGACGAGGGATCCTAGCGTTGCTCGGAATCATTGGGCGTAAAGCGGACGTAGGTGGCTTTGTAAGTCAGGCGTGAAAGCCCTGGGCTCAACCCAGGAAGTGCGCTTGATACTGCTTAGCTTGAATGTAGGAGAGGTAAGTGGAATACCTGGTGTAGTGGTGAAATACGTAGATATCAGGTGGAACACCGGTGGCGAAGGCGGCTTACTGGCCTAACATTGACGCTGAGGTCCGAAAGCGTGGGGATCAAACAGG` |
| ASV2341 | 429 | `TGGGGAATTTTGGACAATGGGCGAAAGCCTGATCCAGCAATGCCGCGTGTGCGAAGAAGGCCTTCGGGTTGTAAAGCACTTTTGTCAGAGAAAAAAAGACTTATGCTAATATCATAGGTTTATGATGGTATCTGAAGAATAAGCACCGGCTAACTACGTGCCAGCAGCCGCGGTAATACGTAGGGTGCAAGCGTTAATCGGAATTATTGGGCGTAAAGCGTGCGCAGGCGGTTGTATAAGATAGATGTGAAATCCTTGGGCTCAACCTGAGAACTGCATTTATGACTGTACGACTAGAGTGTGTCAGAGGGGGGTAGAATTCCACGTGTAGCAGTGAAATGCGTAGAGATGTGGAGGAATACCGATGGCGAAGGCAGCCCCCTGGGATAACACTGACGCTCATGCACGAAAGCGTGGGGAGCAAACAGG` |
| ASV5447 | 404 | `TGGGGAATCTTGCGCAATGGGGGCAACCCTGACGCAGCCATACCGCGTGCGCGATGACGGCCCTCGGGTTGTAAAGCGCTTTTGGCGGGGACGACGATGACGGTACCCGCAGAATAAGTCACGGCTAACTTCGTGCCAGCAGCCGCGGTAATACGAAGGTGGCGAATGTTGCTCGGATTCACTGGGCGTAAAGCGTCTGTAGGTGGTTTTGTAAGTTAGAGGTGAAAGCCCCGCGCTCAACGTGGGAATTGCCTTTAAAACTGCACAACTAGAAGATATGAGAGGATGGCGGAATGACTGGTGTAGAGGTGAAATTCGTAGATATCAGTCGGAACACCCATGGCGAAGGCAGCTATCTGGCATATTCTTGACACTGAGGGACGAAAGCGTGGGGAGCAAACAGG` |
| ASV49 | 429 | `TGGGGAATTTTGGACAATGGGCGCAAGCCTGATCCAGCAATGCCGCGTGCAGGATGAAGGCCTTCGGGTTGTAAACTGCTTTTGTACGGAGCGAAAAGGCTTCTTCTAATACAGGAGGCTCATGACGGTACCGTAAGAATAAGCACCGGCTAACTACGTGCCAGCAGCCGCGGTAATACGTAGGGTGCAAGCGTTAATCGGAATTACTGGGCGTAAAGCGTGCGCAGGCGGTTATGTAAGACAGTCGTGAAATCCCCGGGCTCAACCTGGGAACTGCGATTGTGACTGCATAGCTAGAGTACGGTAGAGGGGGGTGGAATTCCGCGTGTAGCAGTGAAATGCGTAGATATGCGGAGGAACACCGATGGCGAAGGCAGCCCCCTGGACCTGTACTGACGCTCATGCACGAAAGCATGGGGAGCAAACAGG` |
| ASV2035 | 429 | `TGGGGAATATTGGACAATGGGCGGAAGCCTGATCCAGCAATGCCGCGTGTGTGAAGAAGGCCTTAGGGTTGTAAAGCACTTTCAGTTGGGAGGAAGGGGCTATGGATAATACCCGTAGTCATTGACGCTACCGACAGAAGAAGCACCGGCTAACTCTGTGCCAGCAGCCGCGGTAATACAGAGGGTGCAAGCGTTAATCGGAATTACTGGGCGTAAAGGGTGCGTAGGCGGTTAAATAAGTTAGATGTGAAATCCCCAGGCTTAACCTGGGAACTGCGTTTAATACTGTATAACTAGAGTACTGAAGAGGTTAGTGGAATTTCCAGTGTAGCGGTGAAATGCGTAGATATTGGAAGGAACACCAGTGGCGAAGGCGACTAACTGGTCAGATACTGACGCTGAGGCACGAAAGCGTGGGGAGCAAACAGG` |
| ASV4371 | 408 | `TCAAGAACATTCGACAATGGGCGAAAGCCTGATCGAGCGACGCCGCGTGGAGGATGAAGGCCTTCGGGTCGTAAACTCCTTTTCTATGGGAAAAACTTTTGTGATGGTACCGTAGGAATAAGGGGCTGCAAACTTCGTGCCAGCAGCCGCGGTAATACGAAGGCCCCAAGCGTTATCCGGATTTATTGGGCGTAAAGGGCGCGTAGGAGGTTTGGTGTGTTTCCGGTTAAAGACCAAAGCTTAACTTTGGGAGTGCCGGGAAAACTGCCAGACTAGAGGACGTTAGAGGTGAGTGGAACTCATGGTGTAGGGGTGAAATCCGTTGATATCATGGGGAACACCAAAGGCGAAGGCATCTCACTGGGACGTTCCTGACTCTGAGGCGCGAAAGCGTGGGGAGCAAAAAGG` |
| ASV3909 | 425 | `TAAGGAATATTGGACAATGGTCGGAAGACTGATCCAGCCATGCCGCGTGAAGGACGAAGGCGCTCTGCGTTGTAAACTTCTTTTGGACGGGAACAAAACCAGGCCTGCGGGCCTGTCTGAGGGTACCGTCAGAATAAGCACCGGCTAACTCCGTGCCAGCAGCCGCGGTAATACGGAGGGTGCAAGCGTTATCCGGAATCACTGGGTTTAAAGGGTGCGTAGGCGGTCCGCTAAGTCAGTTGTGAAATTCCCTCGCTTAACGAGTGGGACTGCGATTGATACTGGCAGACTTGAATCAGGTTGAGGTAGGCGGAATGTGACATGTAGCGGTGAAATGCATAGATATGTCATAGAACACCGATTGCGAAGGCAGCTTGCTGGACCTGAATTGACGCTGAGGCACGAAAGCGTGGGGAGCAAACAGG` |
| ASV3999 | 404 | `TGAGGAATATTGGACAATGGAGGAAACTCTGATCCAGCCATGCCGCGTGAGTGACGAAGGCCTTCGGGTTGTAAAACTCTTTAAGTGGGGAAGATAATGACGGTACCCACAGAATAAGCACCGGCTAACTTCGTGCCAGCAGCCGCGGTAATACGAAGGGTGCTAGCGTTACTCGGAATCACTGGGCGTAAAGCGTGCGTAGGCGGAAATACAAGTCAGAGGTGAAATCCCGGAGCTTAACTTCGGAATTGCCTTTGAAACTGTATTTCTTGAGTATGGTAGAGGATGGTGGAATTCCTAGTGTAGAGGTGAAATTCGTAGATATTAGGAGGAACACCGGTGGCGAAGGCGGCCATCTGGGCCATCACTGACGCTGAGGCACGAAAGCGTGGGTAGCAAACAGG` |
| ASV410 | 424 | `TGAGGAATATTGGACAATGGGCGCAAGCCTGATCCAGCCATGCCGCGTGCAGGATGACGGTCCTATGGATTGTAAACTGCTTTTGCACAGGAAGAAACACTCCCTCGTGAGGGAGCTTGACGGTACTGTGAGAATAAGGATCGGCTAACTCCGTGCCAGCAGCCGCGGTAATACGGAGGATCCAAGCGTTATCCGGAATCATTGGGTTTAAAGGGTCCGTAGGCGGTCTTATAAGTCAGTGGTGAAATCTCCCCGCTCAACGGGGAAACGGCCATTGATACTGTAGGACTTGAATTATTAGGAAGTAACTAGAATATGTAGTGTAGCGGTGAAATGCTTAGAGATTACATGGAATACCAATTGCGAAGGCAGGTTACTACTAATGGATTGACGCTGATGGACGAAAGCGTGGGTAGCGAACAGG` |
| ASV1606 | 407 | `TGAGGAATTTTCCGCAATGGGCGAAAGCCTGACGGAGCAATACCGCGTGAGGGATGAAGGCCTTTGGGTTGTAAACCTCTTTTATCAAGGAGGAAGTTCTGACGTGTACTTGATGAATAAGCATCGGCTAACTCCGTGCCAGCAGCCGCGGTAAGACGGAGGATGCAAGTGTTATCCGGAATCACTGGGCGTAAAGCGTCTGTAGGTGGTTTAAAAAGTCTGCTGTTAAAAGTTGAAGCTTAACTTCAAAGATGCCGTAGAAACTCTTAAACTCGAGTATGGTAGGGGTAGAGGGAATTTCCAGTGGAGCGGTGAAATGCGTAGATCTTGGAAAGACCACCAATGGCGAAGGCACTCTACTGGGCCATTACTGACACTGAGAGACGAAAGCTAGGGGAGCAAATGGG` |
| ASV4187 | 429 | `TGGGGAATATTGGACAATGGGCGCAAGCCTGATCCAGCCATGCCGCGTGTGTGAAGAAGGCCTTCGGGTTGTAAAGCACTTTCAGTTGGGAGGAAGGGTGTTGTGTTAATAGTACAGCACTTTGACGTTACCAACAGAAGAAGCACCGGCTAACTCTGTGCCAGCAGCCGCGGTAATACAGAGGGTGCAAGCGTTAATCGGAATTACTGGGCGTAAAGCGCACGTAGGCGGTTTTTTAAGTCAGATGTGAAAGCCCCGGGCTCAACCTGGGAATTGCATTTGAAACTGGGGAACTAGAGTGTGTGAGAGGGGGGTAGAATTCCAAGTGTAGCGGTGAAATGCGTAGAGATTTGGAGGAATACCAGTGGCGAAGGCGGCCCCCTGGCACAACACTGACGCTCAGGTGCGAAAGCGTGGGGAGCAAACAGG` |
| ASV132 | 409 | `TGGGGAATATTGGGCAATGGAGGAAACTCTGACCCAGCGACGCCGCGTGCGGGATGAAGGCCTTCGGGTTGTAAACCGCTTTCAGCAGGGAAGAAGCGAAAGTGACGGTACCTGCAGAAGAAGCACCGGCTAACTATGTGCCAGCAGCCGCGGTAATACATAGGGTGCAAGCGTTGTCCGGAATTATTGGGCGTAAAGAGCTCGTAGGTGGTTCGTCACGTCGGATGTGAAACTCTGGGGCTTAACCCCAGACCTGCATTCGATACGGGCGAGCTTGAGTATGGTAGGGGAGTCTGGAATTCCTGGTGTAGCGGTGGAATGCGCAGATATCAGGAGGAACACCAATGGCGAAGGCAGGACTCTGGGCCATTACTGACACTGAGGAGCGAAAGCGTGGGGAGCGAACAGG` |
| ASV2674 | 430 | `TGGGGAATATTGCGCAATGGACGAAAGTCTGACGCAGCGACGCCGCGTGAGGGATGAAGGCCTTCGGGTTGTAAACCTCTGTCAGGAGGGAAGAACCGCCATGGTAGTAACTGCCCATGGTCTGACGGTACCTCCAAAGGAAGCACCGGCTAACTCCGTGCCAGCAGCCGCGGTAATACGGAGGGTGCAAGCGTTAATCGGAATCACTGGGCGTAAAGCGCGCGTAGGCGGCTTTGTAAGTCAGAGGTGAAAGCCCACGGCTCAACCGTGGAACTGCCTTTGATACTGCAAGGCTACGAGTTTGTGAGAGGATAGTGGAATTCCTGGTGTAGGAGTGAAATCCGTAGAGATCAGGAGGAACATCAGTGGCGAAGGCGACTATCTGGCACAACACTGACGCTGAGGTGCGAAAGCGTGGGTATCAAACAGG` |
| ASV3957 | 429 | `TGGGGAATTTTGGACAATGGGCGCAAGCCTGATCCAGCCATGCCGCGTGCGGGAAGAAGGCCTTCGGGTTGTAAACCGCTTTTGTTCGGGAAGAAATCGACCGGGTTAATACCCTGGTTGGATGACGGTACCGGAAGAATAAGCACCGGCTAACTACGTGCCAGCAGCCGCGGTAATACGTAGGGTGCAAGCGTTAATCGGAATTACTGGGCGTAAAGCGTGCGCAGGCGGTTTCGCAAGTCAGATGTGAAAGCCCCGGGCTTAACCTGGGAACTGCATTTGAAACTACGAGGCTAGAGTGTGTCAGAGGGGGGTAGAATTCCACGTGTAGCAGTGAAATGCGTAGAGATGTGGAGGAATACCGATGGCGAAGGCAGCCCCCTGGGATAACACTGACGCTCATGCACGAAAGCGTGGGGAGCAAACAGG` |
| ASV5380 | 429 | `TGGGGAATATTGGACAATGGGGGCAACCCTGATCCAGCAATGCCGCGTGTGTGAAGACGGCCTTCGGGTTGTAAAGCACTTTAGTTGGGGAAGAAGTTGAGCTGTTTAAGAGATGGCTTAGTTGACGGTACCCAAAGAATAAGCACCGGCTAACTCTGTGCCAGCAGCCGCGGTAATACAGAGGGTGCGAGCGTTAATCGGAATTACTGGGCGTAAAGGGCGCGTAGGCGGATGATTAAGTTGGATGTGAAATCCCTGGGCTTAACCTAGGAACTGCATACAATACTGGTCGACTAGAGTGCGGTAGAGGGTAGTGGAATTTCCGGTGTAGCGGTGAAATGCGTAGATATCGGAAGGAACACCAGTGGCGAAGGCGGCTACCTGGACTGACACTGACGCTGAGGCGCGAAAGCGTGGGGAGCAAACAGG` |
| ASV3098 | 409 | `TGAGGAATCTTCCGCAATGGACGAAAGTCTGACGGAGCGACGCCGCGTGGTGGATGAAGGCCTTAACTGGTCGTAAACACCTGTTCTGAGGGACGAAATTTTTGACGGTACCTCAGGAGAAAGTATCGGCAAACCCTGTGCCAGCAGCCGCGGTAATACAGGGGATACAAGCGTTACTCGGAATCACTGGGCGTAAAGCGTCTGTAGGTTCTTTAGTAAGTCTGCGGTAAAAGTCCGGGGCTCAACCCCGGGAGCGTCGTGGAAACTACTAAAGTTGAGCAGTAGAGAGGCATCTGGAATGCCGCATGTAGGAGTAAAATCCGTAGATATGCGGTAGAACGCCAAAAGCGAAGGCAGGATGCTAGCTACTTGCTGACACTGAGAGACGAAAGCGTGGGGAGCGAAGGGG` |
| ASV5338 | 386 | `TCGCGAAACCTTCGCAATGCGCGAAAGCGTGACGAGGGTAGTCTGAGTGCCATCCGAAAGGATGGCTTTTAGTCAGCTTGAATTGCTGGCTGAATAAGTGGCGGGTAATTGCTGTGCCAGCCGCCGCGGTAACACAGCTGCCACGAGTGTTGTCCACGAATATTGAGTCTAAAGCGCGTGTAGCGGGCTTGGCAAATTCCCTGTGAAATCTCTCGGCTCAACCGAGAGGCGTGCAGGGAACACTACCAGGCTTGGGAGTGGGGGAGGTCAAGGGTACTTTGGGGGGAGGGGTAAAATCCTTTAATCCTCAAAGGACCATCGGTGGCGAAAGCGCTTGACCAAAACACGTCCGACCGTGAGACGCGAAGGCCAGGGGAGCAAATCGG` |
| ASV5234 | 429 | `TGGGGAATATTGGACAATGGGCGAAAGCCTGATCCAGCAATGCCGCGTGTGTGAAGAAGGCCTTCGGGTTGTAAAGCACTTTCAGTAGAGAAGAAGAATATTGGATTAATACCCTAATATTTTGACGTTATTTACAGAAGAAGCACCGGCTAACTCTGTGCCAGCAGCCGCGGTAATACAGAGGGTGCGAGCGTTAATCGGAATTATTGGGCGTAAAGGGCGTGTAGGCGGATGTTTAAGTTAGGTGTGAAATACTCGGGCTCAACCTGAGGACTGCACCTAAAACTGGACACCTAGAGTATAGTAGAGGGAAGTGGAATTTCCGGAGTAGCGGTGGAATGCATAGATACCGGAAGGAACATCAGTGGCGAAGGCGGCTTCCTGGACTAATACTGACGCTGAGGCGCGAAAGCATGGGTAGCAAACAGG` |
| ASV2820 | 424 | `TGAGGAATATTGGACAATGGGCGCAAGCCTGATCCAGCCATGCCGCGTGCAGGAAGACGGTCCTATGGATTGTAAACTGCTTTTGTACAGGAAGAAACATTCTCACGTGTGAGAGCTTGACGGTACTGTAAGAATAAGGATCGGCTAACTCCGTGCCAGCAGCCGCGGTAATACGGAGGATCCAAGCGTTATCCGGAATCATTGGGTTTAAAGGGTCCGTAGGCGGTTTAGTAAGTCAGTGGTGAAAGCCCATCGCTCAACGGTGGAACGGCCATTGATACTGCTAGACTTGAATTATTTGGAAGTAACTAGAATATGTAGTGTAGCGGTGAAATGCTTAGAGATTACATGGAATACCAATTGCGAAGGCAGGTTACTACAAATGGATTGACGCTGATGGACGAAAGCGTGGGTAGCGAACAGG` |
| ASV3791 | 425 | `TAAGGAATATTGGACAATGGTCGGAAGACTGATCCAGCCATGCCGCGTGAAGGAAGAAGGCGCTCTGCGTTGTAAACTTCTTTTGTACAGGAACAAAATCAGGTCTGCGGATTTGACTGAGGGTACTGTAAGAATAAGCACCGGCTAACTCCGTGCCAGCAGCCGCGGTAATACGGAGGGTGCGAGCGTTATCCGGAATCACTGGGTTTAAAGGGTGCGTAGGCGGTCTGATAAGTCAGTCGTGAAATCACGTGGCTTAACCATAGTAACTGCGATTGATACTGTCAGACTTGAATCAAGTTGAGGTAGGCGGAATGAGACATGTAGCGGTGAAATGCATAGATATGTCTTAGAACACCGATTGCGAAGGCAGCTTGCTGGACTTAGATTGACGCTGAGGCACGAAAGCGTGGGGAGCAAACAGG` |
| ASV4166 | 389 | `GCGCGAAACCTCTACAATGTACGAAAGTGCGATAGGGGAATCCCAAGTGCTTACACTTTTAGTGTAAGCTTTTGCTAAGCGTAAATAACTTGGCGAATAAGTGGTGGGAAAGACCGGTGCCAGCCGCCGCGGTAATCCCGGCGCCACAAGTGGCAATCGCCTTTATTGGGCCTAAAGCGTCCGTAGCAGGTTTTGTAAATCTTTTGTGAAAGCGTTGCACTCAATGTAACGAACTGCAAGAGACACTGCAAAACTAGGAACCGGGAGGAGTTAGGGGTATTCTTGGGGGAGCGGTAAAATGTTATAATCCCAAGAGGACCACCTGTGGCGAAGGCGCCTAACTAGAACGGATTCGACTGTGAGGGACGAAAGCTAGGGGAGCAATCCGG` |
| ASV2501 | 405 | `TGGGGAATCTTGCGCAATGGGCGAAAGCCTGACGCAGCAACGCCGCGTGGGGGACGAAGGCTTTCGGGTTGTAAACCCCTTTCAGCAGGAACGAAATTGACGGTACCTGCAGAAGAAGCCCCGGCTAACTACGTGCCAGCAGCCGCGGTAATACGTAGGGGGCAAGCGTTGTCCGGATTTATTGGGCGTAAAGAGCTCGTAGGCGGCTTCGTAAGTCGGGTGTGAAAGCCCCAGGCTCAACCTGGGGATGCCACTCGATACTGCGATAGCTAGAGTTTGGTAGGGGATCACGGAATTCGTGGTGTAGCGGTGAAATGCGCAGATATCACGAGGAACACCAGTGGCGAAGGCGGTGGTCTGGGCCAATACTGACGCTGAGGAGCGAAAGCGTGGGGAGCAAACAGG` |
| ASV19 | 429 | `TGGGGAATATTGGACAATGGGCGCAAGCCTGATCCAGCCATGCCGCGTGTGTGAAGAAGGCCTTAGGGTTGTAAAGCACTTTCAGTGGGGAGAAAAGTCATTCACCTAATACGTGAGTGAATTGATGTTACCCACAGAAGAAGCACCGGCTAACTCCGTGCCAGCAGCCGCGGTAATACGGAGGGTGCAAGCGTTAATCGGAATTACTGGGCGTAAAGCGTACGTAGGTGGTTTGTTAAGCCAGCTGTGAAATCCCCGGGCTCAACCTGGGCACTGCAGTTGGAACTGGCAAGCTAGAGTAGGGTAGAGGGGTGTGGAATTCCAGGTGTAGCGGTGAAATGCGTAGATATCTGGAGGAACATCAGTGGCGAAGGCGACACCCTGGACTCATACTGACACTGAGGTACGAAAGCGTGGGGAGCAAACAGG` |
| ASV3453 | 404 | `TGGGGAATATTGCACAATGGACGAAAGTCTGATGCAGCCATGCCGCGTGCGTGATGAAGGCCTTCGGGTTGTAAAGCGCTTTTGGCGGGGACGATAATGACGGTACCCGCAGAATAAGCCCCGGCTAACTTCGTGCCAGCAGCCGCGGTAATACGAAGGGGGCGAGCATTGTTCGGAATTACTGGGCGTAAAGGGTGCGTAGGCGGATTGGATAGTCAGATGTGAAAGCCCAAGGCTTAACCTTGGAATTGCATTTGATACTGCCAGTCTGGAGTATTGGAGAGGAGCGTGGAATTCTCAGTGTAGAGGTGAAATTCGTAGATATTGAGAGGAACACCAGTGGCGTAGGCGACGCTCTGGCCAAATACTGACGCTGAGGCACGAAAGTGTGGGGAGCAAACAGG` |
| ASV5558 | 389 | `TCGGGAGAATTTTGCAATACACGAAAGTGTGACAGAGCAAGCCAGAGTGCTGTTCTATATTTGAACAGCTTTTGCGGGACGTAAAAAGTCCCGTGAATAAGGACTGGGCAAGACTGGTGCCAGCCGCCGCGGTAATCCCAGCAGTCCAAGTCGCAGCCACATTTGTTGGGTCTAAAATATCCGTAGCTTGCCTTGCAAGTCTTTTGTGAAATCGGGACTCTTAAGGTTCCGGCGGGCAAAAGATACTGCTTGGCTAGAGACCGGGAGACGTAAGAAGTACGATAGGGGTAACGGTAAAATGTGTTGATCCCTTTTGGACTAACAATAGCGAAGGCATCTTACGAGAACGGTTCTGACAGTGAGGGATGAAGGCTAGGGGCGCAAAGTGG` |
| ASV127 | 404 | `TGGGGAATATTGGACAATGGGCGAAAGCCTGATCCAGCAATGCCGCGTGAGTGAAGAAGGCCTTCGGGTTGTAAAGCTCTTTTACCCGGGAAGATAATGACTGTACCGGGAGAATAAGCTCCGGCTAACTCCGTGCCAGCAGCCGCGGTAATACGGAGGGAGCTAGCGTTGTTCGGAATTACTGGGCGTAAAGCGTACGTAGGCGGCTTTGTAAGTCAGAGGTGAAAGCCCACGACTCAATCGTGGAACTGCCTTTGAGACTGCTTCGCTTGAAGATGGGAGAGGTTAGTGGAATTCCGAGTGTAGAGGTGAAATTCGTAGATATTCGGAAGAACACCAGTGGCGAAGGCGGCTAACTGGACCATTCTTGACGCTGAGGTACGAAAGCGTGGGGAGCAAACAGG` |
| ASV1055 | 424 | `TGAGGAATATTGCGCAATGGAGGCAACTCTGACGCAGCCATGCCGCGTGCAGGAAGAAGGCGCTACGCGTTGTAAACTGCTTTTAACCGGGAAGAAACCCCAGGACGTGTCCTGGGTTGACGGTACCGGAGGAATAAGCACCGGCTAACTTCGTGCCAGCAGCCGCGGTAATACGAAGGGTGCAAGCGTTATCCGGATTCATTGGGTTTAAAGGGTGCGTAGGCGGAGCGTTAAGTCAGTGGTGAAATCCTGCAGCTTAACTGTAGACTTGCCATTGATACTGGCGCTCTTGAGTGCGCTTGAAGTAGGCGGAATGTGCCGTGTAGCGGTGAAATGCTTAGATATGGCACAGAACACCAATTGCGAAGGCAGCTTACTAAGGCGATACTGACGCTGAGGCACGAAAGCGTGGGGAGCGAACAGG` |
| ASV4155 | 429 | `TGGGGAATTTTGGACAATGGGGGAAACCCTGATCCAGCCATGCCGCGTGAGTGAAGAAGGCCTTCGGGTTGTAAAGCTCTTTCGGACAGAAAGAAATCGCACGGGTAAATAATCTGTGCGGATGACGGTACTGTAAGAAGAAGCACCGGCTAACTACGTGCCAGCAGCCGCGGTAATACGTAGGGTGCGAGCGTTAATCGGAATTACTGGGCGTAAAGCGTGCGCAGGCGGTTGCATAAGACAGATGTGAAATCCCCGGGCTTAACCTGGGAACTGCGTTTGTGACTGTGTAACTGGAGTACGGCAGAGGGGGGTGGAATTCCTGGTGTAGCAGTGAAATGCGTAGATATCAGGAGGAACACCGATGGCGAAGGCAGCCCCCTGGGCCTGTACTGACGCTCATGCACGAAAGCGTGGGGAGCAAACAGG` |
| ASV4266 | 428 | `TCGAGAATTTTTCTCAATGGGCGAAAGCCTGAAGGAGCGACGCCGCGTGGGGGATGAATGGCTTCGGCCCGTAAACCCCTGTCATTTGCGAACAAACCCTGCCATTTAAAAGATGTCAGGTTGATTGTAGCGAAAGAGGAAGGGACGGCTAACTCTGTGCCAGCAGCCGCGGTAATACAGAGGTCCCAAGCGTTGTTCGGATTCACTGGGCGTAAAGGGTGCGTAGGTGGTTGGGTAAGTCTAATGTGAAATCTCCGAGCCTAACTCGGAAACTGCATCGGATACTATCTAGCTAGAGGATTGGAGGGGGGACTGGAATACTTGGTGTAGCAGTGAAATGCGTAGATATCAAGTGGAACACCAGTGGCGAAGGCGAGTCCCTGGACAACTCCTGACACTGAGGCACGAAAGCTAGGGGAGCAAACAGG` |
| ASV2690 | 428 | `TGGGGAATCTTGCGCAATGGGCGAAAGCCTGACGCAGCGACGCCGCGTGAGCGATGAAGGCCTTCGGGTTGTAAAGCTCTGTGGGGAGGAAAGAATAAGGCTTGGCTAATATCCAAGTTGATGACGGTACCTCCTTAGCAAGCACCGGCTAACTCTGTGCCAGCAGCCGCGGTAAGACAGAGGGTGCAAACGTTGTTCGGAATTACTGGGCGTAAAGCGCGTGTAGGCGGCCCTGCAAGTCGGATGTGAAATCCCACGGCTCAACCGTGGAAGTGCACTCGAAACTGCAGGGCTAGAGTCCTGGAGAGGAAGGTGGAATGCTTGGTGTAGAGGTGAAATTCGTAGATATCAAGCGGAACACCGGTGGCGAAGGCGGCCTTCTGGACAGTGACTGACGCTGAGACGCGAAAGCGTGGGGAGCAAACAGG` |
| ASV4766 | 404 | `TGAGGAATATTGGACAATGGGCGCAAGCCTGATCCAGCCATGCCGCGTGAGTGAAGAAGGCCTTCGGGTTGTAAAGCTCTTTTAGATGTGACGATAATGACGGTAGCATCAGAAAAAGCCCCGGCTAAATTCGTGCCAGCAGCCGCGGTAATACGAATGGGGCAAGCGTTGTTCGGAATCACTGGGCTTAAAGCGTGCGCAGGCGGACTAGCAAGTCAGGAGTGAAATCCCCGGGCTCAACCCGGGAATTGCTTTTGAAACTGCAAGTCTAGAATCCCGGAGAGGTTAGCAGAATTCCGAGTGTAGAGGTGAAATTCGCAGATATTCGGAGGAATACCAATGGCGAAGGCAGCTAACTGGACGGGCATTGACGCTCAGGCACGAAAGCGTGGGTAGCAAACAGG` |
| ASV2535 | 409 | `TGGGGAATATTGCACAATGGGCGCAAGCCTGATGCAGCAACGCCGCGTGAGGGACGACGGCCTTCGGGTTGTAAACCTCTTTTAGTAGGGAAGAAGCGAAAGTGACGGTACCTGCAGAAAAAGCACCGGCTAACTACGTGCCAGCAGCCGCGGTAATACGTAGGGTGCAAGCGTTATCCGGAATTATTGGGCGTAAAGAGCTCGTAGGCGGTTTGTCGCGTCTGCTGTGAAAACTGGGGGCTCAACCCCCAGCCTGCAGTGGGTACGGGCAGACTAGAGTGCGGTAGGGGAGATTGGAATTCCTGGTGTAGCGGTGGAATGCGCAGATATCAGGAGGAACACCGATGGCGAAGGCAGATCTCTGGGCCGTAACTGACGCTGAGGAGCGAAAGCATGGGGAGCGAACAGG` |
| ASV213 | 404 | `TGGGGAATATTGGACAATGGGCGAAAGCCTGATCCAGCAATGCCGCGTGAGTGATGAAGGCCTTAGGGTTGTAAAGCTCTTTTACCCGGGATGATAATGACAGTACCGGGAGAATAAGCTCCGGCTAACTCCGTGCCAGCAGCCGCGGTAATACGGAGGGAGCTAGCGTTGTTCGGAATTACTGGGCGTAAAGAGTACGTAGGCGGTGATTCAAGTCAGAGGTGAAAGCCTGGAGCTCAACTCCAGAACTGCCTTTGAAACTAGATCGCTAGAATCATGGAGAGGTGAGTGGAATTCCGAGTGTAGAGGTGAAATTCGTAGATATTCGGAAGAACACCAGTGGCGAAGGCGACTCACTGGACATGTATTGACGCTGAGGTACGAAAGCGTGGGGAGCAAACAGG` |
| ASV5160 | 405 | `TAGGGAATTTTCCACAATGGACGAAAGTCTGATGGAGCAACGCCGCGTGCAGGATGAAGGCCCTTGGGTCGTAAACTGCTTTTATGTGCGAAGATTATGACAGTAACACATGAATAAGGGTCGGCTAACTACGTGCCAGCAGCCGCGGTCATACGTAGGACCCAAGCGTTATCCGGAATTACTGGGCGTAAAGAGTTGCGTAGGTGGCATAGTAAGTAAGAAGTGAAAGCGTTCGGCTCAACCGAATATACATTTCTTAAACTGCTAAGCTAGAGAACGAGAGAGGTAGATGGAATTCCCAGTGTAGGAGTGAAATCCGTAGATATTGGGAGGAACACCGATGGCGTAGGCAGTCTACTGGCTCGTTTCTGACACTAAGGCACGAAAGCGTGGGGAGCAAACAGG` |
| ASV664 | 429 | `TGGGGAATTTTGGACAATGGGCGCAAGCCTGATCCAGCCATTCCGCGTGCAGGATGAAGGCCTTCGGGTTGTAAACTGCTTTTGTACGGAACGAAACGGTCTGCCCTAATACGGCGGGCTAATGACGGTACCGTAAGAATAAGCACCGGCTAACTACGTGCCAGCAGCCGCGGTAATACGTAGGGTGCAAGCGTTAATCGGAATTACTGGGCGTAAAGCGTGCGCAGGCGGTTATATAAGACAGATGTGAAATCCCCGGGCTCAACCTGGGAACTGCATTTGTGACTGTATAGCTAGAGTACGGCAGAGGGGGATGGAATTCCGCGTGTAGCAGTGAAATGCGTAGATATGCGGAGGAACACCGATGGCGAAGGCAATCCCCTGGGCCTGTACTGACGCTCATGCACGAAAGCGTGGGGAGCAAACAGG` |
| ASV1882 | 404 | `TGAGGAATCTTCCGCAATGGGCGAAAGCCTGACGGAGCGACACCGCGTGGAGGATGACGCGTTTACTCGTGTAAACTCCTTTTCTGTGGGAAGATAATGACGGTACCACAGGAATAAGGGGCGGCAAACTACGTGCCAGCAGCCGCGGTAATACGTAGGCCCCAAGCGTTATCCGGAATTACTGGGTGTAAAGCGTCTGTAGGTGGGGATATAAGTCTCTCATGAAAGACCGGAGCTCAACTCCGTGTTTGTGAGAGATACTGTATTTCTAGAATCGGGGAGAGGGAAGCAGAATTGTATGAGTAGGGGTGCAATCCGTTGATACATACAAGAATACCAAAAGCGAAGGCAGCTTCCTGGAACCGTATTGACACTGAGAGACGAAAGCGTGGGGAGCGAAAAGG` |
| ASV309 | 429 | `TGGGGAATCTTGGACAATGGGCGAAAGCCTGATCCAGCAATGCCGCGTGCGTGAAGAAGGCCTTCGGGTTGTAAAGCGCTTTTGTCGGGGAGGAAATCCTAGTGGCTAATAACTACTGGGGATGACAGTACCCGAAGAATAAGGACCGGCTAACTACGTGCCAGCAGCCGCGGTAATACGTAGGGTCCAAGCGTTAATCGGAATTACTGGGCGTAAAGCGTCCGCAGGTGGCTTGATAAGATAGACGTGAAATCCCTGGGCTCAACCTAGGAATTGCGTTTATGACTGTCTCGCTAGAGTATGGGAGAGGGGGGTGGAATTCCGCGTGTAGCAGTGAAATGCGTAGAGATGCGGAGGAACACCGATGGCGAAGGCAACCCCCTGGCCTAATACTGACACTCATGGACGAAAGCGTGGGGAGCAAACAGG` |
| ASV1886 | 404 | `TGGGGAATATTGGACAATGGGGGCAACCCTGATCCAGCAATACCGCGTGAGTGATGAAGGCCTTCGGGTTGTAAAGCTCTTTTGTTAGGGACGATAATGACGGTACCTAACGAATAAGCTCCGGCTAACTTCGTGCCAGCAGCCGCGGTAATACGAAGGGAGCTAGCGTTGTTCGGAATTACTGGGCGTAAAGGGCGCGTAGGCGGTAATCTAAGTCAGATGTGAAAGCCTTGGGCTTAACCTGAGAATTGCATTTGATACTGGATAACTAGAGATCGAGAGAGGTAAGTGGAATTACGAGTGTAGAGGTGAAATTCGTAGATATTCGTAAGAACACCAGTGGCGAAGGCGGCTTACTGGCTCGATACTGACGCTAAGGCGCGAAAGCGTGGGGAGCAAACAGG` |
| ASV3912 | 409 | `TGGGGAATATTGGACAATGGGCGGAAGCCTGATCCAGCAACGCCGCGTGAGGGATGACGGCCTTCGGGTTGTAAACCTCTTTCACCTCGGACGAAGCGCAAGTGACGGTACGAGGGGAAGAAGCACCGGCCAACTACGTGCCAGCAGCCGCGGTAATACGTAGGGTGCGAGCGTTGTCCGGAATTATTGGGCGTAAAGGGCTCGTAGGCGGTTTGTCGCGTCGGGAGTGAAAACCTCGGGCTTAACTCGGGGCTTGCTTTCGATACGGGCAGACTGGAGGTATGCAGGGGAGAACGGAATTCCTGGTGTAGCGGTGAAATGCGCAGATATCAGGAGGAACACCGGTGGCGAAGGCGGTTCTCTGGGCATTACCTGACGCTGAGGAGCGAAAGTGTGGGGAGCGAACAGG` |
| ASV4463 | 404 | `TGGGGAGTATTGGACAATGGGGGAAACCCTGATCCAGCCATGCCGCGTGAGTGAAGAAGGCCTTAGGGTTGTAAAGCTCTTTCGCACGCGACGATGATGACGGTAGCGTGAGAAGAAGCCCCGGCTAACTTCGTGCCAGCAGCCGCGGTAATACGAAGGGGGCAAGCGTTGTTCGGATTTACTGGGCGTAAAGGGCGCGTAGGCGGAACGACAAGTCAGAGGTGAAAGCCCTGGGCTCAACCCGGGAATTGCCTTTGGGACTGCCGTTCTAGAGTTCGGAAGAGGATGGCGGAATACCCAGTGTAGAGGTGAAATTCGTAGATATTGGGTAGAACACCGGTGGCGAAGGCGGCCATCTGGTCCGACACTGACGCTGAGGCGCGAAAGCGTGGGGAGCAAACAGG` |
| ASV5631 | 389 | `TCGGGAAAATTTTGCAATGCGCGAAAGCGTGACAGAGCAAGCCAGAGTGATTTTCCATATTGGAAAATCTTTTGTGCGATCTAAACAGTCGCACGAATAAGGACTGGGAGAGACTGGTGCCAGCCGCCGCGGTAATCCCAGCAGTCCGAGTCGCAGCCACATTTGTTGGGTCTAAAACATCCGTAGCTTGCTTTTCAAGTCTCATGTGAAATCTGGCCTCTTAAGGGTCGGGCGAGCATGAGATACTGTTAAGCTAGAGACCGGGAGACGTAAGAAGTACGAACGGGGTAGCGGTAAAATGTGTTGATCCCATTTGGACTAACAACAGCGAAGGCATCTTACGAGAACGGATCTGACAGTGAGGGATGAAGGCTAGGGGCGCAAAGTGG` |
| ASV4122 | 404 | `TGAGGAATATTGGACAATGGGGGAAACCCTGATCCAGCCATGCCGCGTGAATGAAGAAGGCCTTAGGGTTGTAAAGTTCTTTTAGCTGTGAAGATAATGACGGTAACAGCAGAAAAAGCTCCGGCTAACTCCGTGCCAGCAGCCGCGGTAATACGGAGGGAGCGAGCGTTGTTCGGAATCACTGGGCGTAAAGCGTGCGCAGGCGGCGTGACAAGTCGGAAGTGAAAGCCCGGGGCTTAACCTCGGAACTGCTTTCGAAACTGTCATGCTAGAGTCCCGGAGAGGTAAGCGGAATTCCGAGTGTAGAGGTGAAATTCGTAGATATTCGGAGGAACACCAGTGGCGTAAGCGGCTTACTGGACGGGTACTGACGCTCAGGCACGAAAGCGTGGGGAGCAAACAGG` |
| ASV1751 | 404 | `TGGGGAATATTGGACAATGGGCGAAAGCCTGATCCAGCAATGCCGCGTGAGTGATGAAGGCCTTAGGGTTGTAAAGCTCTTTTACCCGGGATGATAATGACAGTACCGGGAGAATAAGCTCCGGCTAACTCCGTGCCAGCAGCCGCGGTAATACGGAGGGAGCTAGCGTTGTTCGGAATTACTGGGCGTAAAGCGCACGTAGGCGGTCTTTTAAGTCAGAGGTGAAAGCCCGGGGCTCAACCCCGGAATAGCCTTTGAAACTGGAAAACTTGAATCATGGAGAGGTCAGTGGAATTCCGAGTGTAGAGGTGAAATTCGTAGATATTCGGAAGAACACCAGTGGCGAAGGCGACTGACTGGACATGTATTGACGCTGAGGTGCGAAAGCGTGGGGAGCAAACAGG` |
| ASV2342 | 424 | `TGAGGAATATTGGACAATGGGCGCAAGCCTGATCCAGCCATGCCGCGTGCAGGATGACGCATCTATGGTGTGTAAACTGCTTTTGTACGAGAAGAAACACTCCGACGTGTCGGAGCTTGACGGTATCGTAAGAATAAGGATCGGCTAACTCCGTGCCAGCAGCCGCGGTAATACGGAGGATCCAAGCGTTATCCGGAATCATTGGGTTTAAAGGGTCCGTAGGCGGTCTTATAAGTCAGTGGTGAAATCTCTCCGCTCAACGGGGAAACGGCCATTGATACTGTAGGACTTGAATTATTAGGAAGTAACTAGAATATGTAGTGTAGCGGTGAAATGCTTAGAGATTACATGGAATACCAATTGCGAAGGCAGGTTACTACTAATGGATTGACGCTGATGGACGAAAGCGTGGGTAGCGAACAGG` |
| ASV165 | 404 | `TGGGGAATATTGGACAATGGGCGAAAGCCTGATCCAGCAATGCCGCGTGAGTGATGAAGGCCTTAGGGTTGTAAAGCTCTTTTACCCGGGAAGATAATGACTGTACCGGGAGAATAAGCCCCGGCTAACTCCGTGCCAGCAGCCGCGGTAATACGGAGGGGGCTAGCGTTATTCGGAATTACTGGGCGTAAAGCGCGCGTAGGCGGTTTTTCAAGTCAGAGGTGAAAGCCCGGAGCTCAACTCCGGAATTGCCTTTGAAACTGGATCACTAGAATCATGGAGAGGTCAGTGGAATTCCGAGTGTAGAGGTGAAATTCGTAGATATTCGGAAGAACACCAGTGGCGAAGGCGACTGACTGGACATGTATTGACGCTGAGGTGCGAAAGCGTGGGGAGCAAACAGG` |
| ASV5719 | 404 | `TGGGGAATATTGGACAATGGGGGCAACCCTGATCCAGCCATGCCGCGTGAGTGATGAAGGCCTTAGGGTTGTAAAGCTCTTTCGCACGCGACGATGATGACGGTAGCGTGAGAAGAAGCCCCGGCTAACTTCGTGCCAGCAGCCGCGGTAATACGAAGGGGGCAAGCGTTGTTCGGAATTACTGGGCGTAAAGCGCACGCAGGCGGCGTGACGCGTCAGAAGTGAAATCCCCGGGCTCAACCTGGGAACTGCTTTTGAAACGGTCATGCTTGAATCATGGAGAGGGTAGCGGAATTCCGAGTGTAGAGGTGAAATTCGTAGATATTCGGAAGAACACCGGTGGCGAAGGCGGCTACCTGGCCATGTATTGACGCTCAGGTGCGAAAGCGTGGGGAGCAAACAGG` |
| ASV3844 | 412 | `TTAGGAATCTTCCACAATGGGCGAAAGCCTGATGGAGCAACGCCGCGTGAAGGATGAAGGTTTTCGGATCGTAAACTTCTGAACTAGGGACGAAAGATAACCAAAATGACGGTACCTAGGTAATAGCACCGGCTAACTCCGTGCCAGCAGCCGCGGTAATACGGAGGGTGCAAGCGTTACCCGGAATCACTGGGCGTAAAGGGCGTGTAGGCGGTTCATTAAGTCTGACTTTAAAGACTGGGGCTCAACCCCAGGAATGGGCTGGATACTGATGAACTAGACGACTGGAGAGGCGATTGGAATTTCTGGTGTAGCGGTGGAATGCGTAGATACCAGAAGGAACATCAATGGCGAAGGCAGGTCGCTGGACAGATCGTGACGCTGAGGCGCGAAAGTGTGGGGAGCAAACCGG` |
| ASV4599 | 424 | `TGAGGAATATTGGACAATGGGTGAAAGCCTGATCCAGCCATCCCGCGTGAAGGATGACGGTCCTATGGATTGTAAACTTCTTTTGTATAGGGATAAACCTTTCTACGTGTAGAAAGCTGAAGGTACTATACGAATAAGCACCGGCTAACTCCGTGCCAGCAGCCGCGGTAATACGGAGGGTGCAAGCGTTATCCGGATTTATTGGGTTTAAAGGGTCCGTAGGCGGACCGATAAGTCAGTGGTGAAATCTCATAGCTTAACTATGAAACTGCCATTGATACTGTCGGTCTTGAGTAAATTTGAAGTGGCTGGAATAAGTAGTGTAGCGGTGAAATGCATAGATATTACTTAGAACACCAATTGCGAAGGCAGGTCACTAAGATTTAACTGACGCTGATGGACGAAAGCGTGGGTAGCGAACAGG` |
| ASV1318 | 404 | `TGAGGAATATTGGACAATGGGCGCAAGCCTGATCCAGCCATGCCGCGTGAATGATGAAGGCCTTAGGGTTGTAAAGTTCTTTTAGATGGGAAGATAATGACGGTACCATCAGAAAAAGCCCCGGCTAACTCCGTGCCAGCAGCCGCGGTAATACGGAGGGGGCGAGCGTTGTTCGGATTTACTGGGCGTAAAGCGTGCGTAGGCGGTTTCCCAAGTTAGAAGTGAAAGCCCAGGGCTTAACCCTGGAACGGCTTTTAAAACTGGGTCACTAGAATCTAGGAGAGGTAAATGGAATTCCGAGTGTAGAGGTGAAATTCGTAGATATTCGGAGGAACACCAATGGCGAAAGCAGTTTACTGGACTAGTATTGACGCTGAGGCACGAAAGCGTGGGGAGCAAACAGG` |
| ASV4192 | 429 | `TGGGGAATTTTGGACAATGGGCGAAAGCCTGATCCAGCCATGCCGCGTGCAGGATGAAGGCCTTCGGGTTGTAAACTGCTTTTGTACGGAACGAAAAGGTTCTTTCTAATAAAGAGGACTGATGACGGTACCGTAAGAATAAGCACCGGCTAACTACGTGCCAGCAGCCGCGGTAATACGTAGGGTGCAAGCGTTAATCGGAATTACTGGGCGTAAAGCGTGCGCAGGCGGTTATGTAAGACAGATGTGAAATCCCCGGGCTCAACCTGGGAACTGCATTTGTGACTGCATAGCTAGAGTACGGCAGAGGGGGATGGAATTCCGCGTGTAGCAGTGAAATGCGTAGATATGCGGAGGAACACCGATGGCGAAGGCAATCCCCTGGGCCTGTACTGACGCTCATGCACGAAAGCGTGGGGAGCAAACAGG` |
| ASV3821 | 430 | `TGGGGAATATTGGACAATGGGCGAAAGCCTGATCCAGCGATGCCGCGTGTGTGATGAAGGCCTGCGGGTTGTAAAGCACTTTCAGTAGGGAAAAAGAACGCTGTAAGAATATGACGGCGTTTTGATATTATCTACAGAAGAAGCACCGGCTAACTCTGTGCCAGCAGCCGCGGTAATACAGAGGGTGCAAGCGTTAATCGGAATTATTGGGCGTAAAGGGCGCGTAGGTGGTTAGGTAAGTTCAATGTGAAATCCTCTGGGCTCAACCCAGGAACTGCATTGAATACTGCTTATCTAGAGTAGGGTAGAGGGAAGTGGAATTTCCGGTGTAGCGGTGAAATGCGTAGATATCGGAAAGAACACCAGTGGCGAAGGCGACTTCCTGGATTCATACTGACACTGAGGCGCGAAAGCATAGGGAGCAAACAGG` |
| ASV5499 | 404 | `TTCAGAATCTTGCACAATGGGCGAAAGCCTGATGCAGCGATGCCGCGTGGGTGAAGAAGGCCCTCGGGTCGTAAAGCCCTGTCAGAGGGAAAGATAATGACGGTACCCTCTAAGGAAGCCACGGCTAATTACGTGCCAGCAGCCGCGGTAATACGTAAGTGGCGAGCGTTATCCGGAATCACTGGGCGTAAAGCGTGCGTAGGCGGTTTGGCAAGTCAGATTTGAAAGCCCGGGGCTCAACTCCGGACCCGGATCTGAAACTGCCAGACTCGAGCGGATCAGAGGACAGCGGAATTCCCGGTGTAGCGGTAATATGCGTAGATATCGGGAGGAACACCAGTGGCGAAGGCGGCTGTCTGGGGTCTTGCTGACGCTGAGGCACGAAAGCCAGGGGAGCGAACGGG` |
| ASV896 | 424 | `TAAGGAATATTGGTCAATGCTCGGAAGAGTGAACCAGCCACGCCGCGTGGAGGATGAAGGCCCTCTGGGTTGTAAACTTCTTTAGAATGGGAAGAAAGTGCGAGATTTATCTTGTTTTGACGGTACCATTAGAATAAGCACCGGCTAACTCCGTGCCAGCAGCCGCGGTAATACGGAGGGTGCGAGCGTTATCCGGAATCACTGGGTTTAAAGGGTGCGTAGGCTGTTAGATAAGTCAGTGGTGAAATATTTTCGCATAACGAAGAAACTGCCATTGATACTGTTTAGCTAGAATTGGGATGAGGTAACTGGAATGTGACATGTAGCGGTGAAATGCATAGAGATGTCATAGAATACCGATTGCGAAGGCAGGTTACTGGCCCTTGATTGACGCTGAGGCACGAAAGCGTGGGTAGCGAACAGG` |
| ASV1694 | 424 | `TGAGGAATATTGGACAATGGGCGCAAGCCTGATCCAGCCATGCCGCGTGCAGGATGACGGTCCTATGGATTGTAAACTGCTTTTATACGAGAAGAAACACTCCTTCGTGAAGGAGCTTGACGGTATCGTAAGAATAAGGATCGGCTAACTCCGTGCCAGCAGCCGCGGTAATACGGAGGATCCAAGCGTTATCCGGAATCATTGGGTTTAAAGGGTCTGTAGGCGGTTTAGTAAGTCAGTGGTGAAAGCCCATCGCTCAACGGTGGAACGGCCATTGATACTGCTAAACTTGAATTATAAGGAAGTAACTAGAATATGTAGTGTAGCGGTGAAATGCTTAGAGATTACATGGAATACCAATTGCGAAGGCAGGTTACTACTTATGGATTGACGCTGATGGACGAAAGCGTGGGTAGCGAACAGG` |
| ASV4153 | 429 | `TGGGGAATATTGGACAATGGGCGCAAGCCTGATCCAGCCATGCCGCGTGTGTGAAGAAGGCCTTCGGGTTGTAAAGCACTTTCAGTGATGAGGAAGTTAGGTTGTTTAAGAGATAGCTTAATTGACGTTAGTCACAGAAGAAGCACCGGCTAACTCCGTGCCAGCAGCCGCGGTAATACGGAGGGTGCGAGCGTTAATCGGAATTACTGGGCGTAAAGGGCATGTAGGCGGTTAATTAAGTTGGATGTGAAATCCCCGAGCTCAACTTGGGAACTGCATTCAAGACTGGTTAGCTAGAGTATCGTAGAGGGGGGTAGAATTCCAGGTGTAGCGGTGAAATGCGTAGAGATCTGGAGGAATACCGGTGGCGAAGGCGGCCCCCTGGACGAATACTGACGCTGAGGTGCGAAAGCGTGGGGATCAAACAGG` |
| ASV1062 | 429 | `TGGGGAATTTTGGACAATGGGCGCAAGCCTGATCCAGCAATGCCGCGTGCAGGAAGAAGGCCTTCGGGTTGTAAACTGCTTTTGTCAGGGAAGAAATCCTCTGGGTTAATACCTCGGGGGGATGACGGTACCTGAAGAATAAGCACCGGCTAACTACGTGCCAGCAGCCGCGGTAATACGTAGGGTGCGAGCGTTAATCGGAATTACTGGGCGTAAAGCGTGCGCAGGCGGTTGTGCAAGACAGGTGTGAAATCCCCGGGCTTAACCTGGGAACTGCACTTGTGACTGCACGGCTAGAGTACGGTAGAGGGGGATGGAATTCCGCGTGTAGCAGTGAAATGCGTAGATATGCGGAGGAACACCAATGGCGAAGGCAATCCCCTGGACCTGTACTGACGCTCATGCACGAAAGCGTGGGGAGCAAACAGG` |
| ASV4932 | 404 | `TCGGGAATTTTGGGCAATGGGCGAAAGCCTGACCCAGCAACATCGCGTGGAGGAAGAAGTATCTCGGTATGTAAACTCCGAAAGTATGAAAAGAATCAATGACGGTATCATATATAAGCTCCGGCTAACTTCGTGCCAGCAGCCGCGGTAATACGAGGGGAGCAAGCGTTGTTCGGATTTACTGGGCGTAAAGGGCGCGTAGGCGGTTAATTAAGTTACTTGTTAAATCTCCGAGCTTAACTCGGAACTGTCAAGTAATACTGATAAACTAGAGTGCAGAAGAGGCAACCGGAATTCTTGGTGTAGCGGTGAAATGCGTAGATATCAAGAGGAACACCTGTGGCGAAGGCGGGTTGCTGGGCTGACACTGACGCTGAGGCGCGAAAGCTAGGGTAGCAAACGGG` |
| ASV3601 | 408 | `TGGGGAATATTGGGCAATGGGCGAAAGCCTGACCCAGCAATGCCGCGTGAGTGATGAAGGTATTTTTATTGTAAAGCTCTTTTCTTTGGAACGAACAAAATGACGGTACCAAAGGAATAAGCCCCGGCTAACTACGTGCCAGCAGCCGCGGTAATACGTAGGGGGCGAGCGTTGTCCGGAATGACTGGGTGTAAAGGGTGTGTAGGTGGTTTGACAAGTTAGAAGTGTAATACCCAGGGCTTAACTCGGGTGCTGCTTCTAAAACTGTCAGACTTGAGTGCAGGAGAGGATAGTGGAATTCCTAGTGTAGCGGTGGAATGCATAGATATTAGGAGGAACATCAGTGGCGAAGGCGACTATCTGGACTGTAACTGACACTGAGGCACGAAAGCGTGGGGAGCAAACAGG` |
| ASV1945 | 423 | `TAGGGAATATTGGGCAATGGAGGCAACTCTGACCCAGCCATGCCGCGTGCAGGATGAAGGCGTTATGCGTTGTAAACTGCTTTTATATAGGAAGAAAAAGACCTTGCGAGGTAGATTGACGGTACTATATGAATAAGCACCGGCTAACTCCGTGCCAGCAGCCGCGGTAATACGGAGGGTGCAAGCGTTGTCCGGATTTATTGGGTTTAAAGGGTGCGTAGGCGGTTTATTAAGTCAGTGGTGAAAGACGGTCGCTCAACGATTGCAGTGCCATTGATACTGGTAGACTTGAGTGAATTTGAGGTAGCTGGAATGGATAGTGTAGCGGTGAAATGCATAGATATTATCCAGAACACCAATTGCGAAGGCAAGTTACTAAGATTCAACTGACGCTGATGCACGAAAGTGTGGGTATCAAACAGG` |
| ASV1245 | 404 | `TGGGGAATATTGCACAATGGGGGAAACCCTGATGCAGCAACGCCGCGTGAGTGATGACGGTCTTCGGATTGTAAAGCTCTGTCTTTAGGGACGATAATGACGGTACCTAAGGAGGAAGCCACGGCTAACTACGTGCCAGCAGCCGCGGTAATACGTAGGTGGCAAGCGTTGTCCGGATTTACTGGGCGTAAAGGGAGCGTAGGTGGATATTTAAGTGGGATGTGAAATACCCGGGCTTAACCTGGGTGCTGCATTCCAAACTGGATATCTAGAGTGCAGGAGAGGAAAGGAGAATTCCTAGTGTAGCGGTGAAATGCGTAGAGATTAGGAAGAATACCAGTGGCGAAGGCGCCTTTCTGGACTGTAACTGACACTGAGGCTCGAAAGCGTGGGGAGCAAACAGG` |
| ASV1409 | 429 | `TGGGGAATTTTGGACAATGGGCGCAAGCCTGATCCAGCAATGCCGCGTGCAGGATGAAGGCCTTCGGGTTGTAAACTGCTTTTGTACGGAACGAAACGGTGAGCTCTAATAAAGCTTGCTAATGACGGTACCGTAAGAATAAGCACCGGCTAACTACGTGCCAGCAGCCGCGGTAATACGTAGGGTGCGAGCGTTAATCGGAATTACTGGGCGTAAAGCGTGCGCAGGCGGTTATATAAGACAGATGTGAAATCCCCGGGCTCAACCTGGGACCTGCATTTGTGACTGTATAGCTAGAGTACGGTAGAGGGGGATGGAATTCCGCGTGTAGCAGTGAAATGCGTAGATATGCGGAGGAACACCGATGGCGAAGGCAATCCCCTGGACCTGTACTGACGCTCATGCACGAAAGCGTGGGGAGCAAACAGG` |
| ASV3318 | 404 | `TGGGGAATATTGGACAATGGGGGAAACCCTGATCCAGCAATGCCGCGTGAATGATGAAGGCCTTCGGGTTGTAAAGTTCTTTTACCTGAGAAGATAATGACGGTATCAGGGGAAAAAGCACCGGCTAACTTCGTGCCAGCAGCCGCGGTAATACGAAGGGTGCAAGCGTTGTTCGGAATGACTGGGCGTAAAGGGCGCGTAGGCGGTTTGGTACGTTTGATGTGAAAGTCCTGGGCTTAACCTGGGGACGGCATTGGATACGGCTAGACTAGAGAGCGAGAGAGGGAGACGGAATAGTGCGTGTAGAGGTGAAATTCGTAGATATGCACTAGAACACCGGAGGCGAAGGCGGTTTCCTGGCTCGCAACTGACGCTAAGGCGCGAAAGCGTGGGGAGCAAACAGG` |
| ASV5185 | 404 | `TAGGGAATTTTGCGCAATGGGCGAAAGCCTGACGCAGCAATACCGCGTGGAGGATGAAGTCTTTTGGGACGTAAACTCCTGTCGGCAGGGACGATTATGACGGTACCTGCAAAGGAAGCATCGGCTAACTACGTGCCAGCAGCCGCGGTAATACGTAGGATGCAAGCGTTGTCCGGAATTATTGGGCGTAAAGCGTTCGTAGGCGGTTTGTCAAGTCTGGTGTTAAATTCCGGGGCTCAACCCCGGCAAAGCACTGGAAACTGACAGACTAGAGTACGGTAGAGGTAACGGGAATTCCCGGTGTAGCGGTGAAATGCGTAGATATCGGGAGGAACACCGATGGCGAAAGCACGTTACTGGGCCAATACTGACGCTAAGGAACGAAAGCCAGGGGAGCGAAAGGG` |
| ASV4967 | 407 | `TGGGGAATTTTCCGCAATGGGCGAAAGCCTGACGGAGCAAGACCGCGTGAGGGAGGAAGGCTCTTGGGTTGTAAACCTCTTTTCTTGGGGAAGAACAAAATGACGGTACCCAAGGAATCAGCATCGGCTAACTCCGTGCCAGCAGCCGCGGTAATACGGAGGATGCAAGCGTTATCCGGAATTATTGGGCGTAAAGCGTCCGCAGGTGGCATTTCAAGTCTGCTGTCAAAGACCGGGGCTTAACCCTGGAAAGGCAGTGGAAACTGAAGAGCTAGAGTGTCGTAGGGGTAGAGGGAATTCCTGGTGTAGCGGTGAAATGCGTAGAGATCAGGAAGAACATCGGTGGCGAAAGCGCTCTACTGGACGACAACTGACACTCAGGGACGAAAGCTAGGGGAGCGAATGGG` |
| ASV987 | 429 | `TGGGGAATTTTGGACAATGGACGAAAGTCTGATCCAGCAATGCCGCGTGCAGGACGAAGGCCTTCGGGTTGTAAACTGCTTTTGTACGGAGCGAAAAGGTTTTCTTTAATAAAGAGGGCTCATGACGGTACCGTAAGAATAAGCACCGGCTAACTACGTGCCAGCAGCCGCGGTAATACGTAGGGTGCAAGCGTTAATCGGAATTACTGGGCGTAAAGCGTCCGCAGGCGGTTGTGTAAGACAGATGTGAAATCCCCGGGCTCAACCTGGGAACTGCATTTGTGACTGCACGGCTAGAGTACGGCAGAGGGGGATGGAATTCCGCGTGTAGCAGTGAAATGCGTAGATATGCGGAGGAACACCGATGGCGAAGGCAATCCCCTGGGCCTGTACTGACGCTCATGGACGAAAGCGTGGGGAGCAAACAGG` |
| ASV5426 | 404 | `TGGGGAATCTTGGACAATGGGGGCAACCCTGATCCAGCCATGCCGCGTGAATGATGAAGGCCTTAGGGTTGTAAAGTTCTTTCGTCGGGGACGATAATGACGGTACCCGAAGAAGAAGCCCCGGCTAACTTCGTGCCAGCAGCCGCGGTAATACGAAGGGGGCTAGCGTTGCTCGGAATTACTGGGCGTAAAGCGCGTGTAGGCGGATTGTTAAGTCGGAGGTGAAAGCCCAGAGCTCAACTCTGGAATTGCCTTCGATACTGGCAGTCTGGAGAGTAGGAGAGGCAAGGGGAATTCCGAGTGTAGAGGTGAAATTCGTAGATATTCGGAGGAACACCAGTGGCGAAGGCGCCTTGCTGGACTACATCTGACGCTGAGACGCGAAAGCGTGGGGAGCAAACAGG` |
| ASV3611 | 404 | `TGGGGAGTATTGGACAATGGGGGAAACCCTGATCCAGCAATGCCGCGTGGGTGATGAAGGTCTTCGGATTGTAAAGCCCTTTTGGCAGGGACGATGATGACGGTACCTGCAGAATAAGCTCCGGCTAACTTCGTGCCAGCAGCCGCGGTAATACGAAGGGAGCAAGCGTTGTTCGGATTTACTGGGCGTAAAGGATGCGTAGGTGGTCCGTTAAGTTGGAAGTGAAAGCCCTGGGCTTAACCCAGGAACTGCTTTCAAGACTGGCGGACTAGAGTGTGGAAGAGGTTGGTGGAATTCCCAGTGTAGAGGTGAAATTCGTAGATATTGGGAAGAACATCCGTGGCGAAGGCGGCCAACTGGTCCACAACTGACACTGAGGCATGACAGCGTGGGGAGCAAACAGG` |
| ASV1774 | 429 | `TGGGGAATTTTGGACAATGGGCGCAAGCCTGATCCAGCCATGCCGCGTGCGGGAAGAAGGCCTTCGGGTTGTAAACCGCTTTTGTCAGGGAAGAAACGCTCCGAACTAATACTTTGGGGTAATGACGGTACCTGAAGAATAAGCACCGGCTAACTACGTGCCAGCAGCCGCGGTAATACGTAGGGTGCAAGCGTTAATCGGAATTACTGGGCGTAAAGCGTGCGCAGGCGGTCTTGTAAGACAGAGGTGAAATCCCCGGGCTCAACCTGGGAACTGCCTTTGTGACTGCAAGGCTTGAGTACGGTAGAGGGGGATGGAATTCCGCGTGTAGCAGTGAAATGCGTAGATATGCGGAGGAACACCGATGGCGAAGGCAATCCCCTGGACCTGTACTGACGCTCATGCACGAAAGCGTGGGGAGCAAACAGG` |
| ASV5716 | 404 | `TGGGGAATATTGGACAATGGGCGCAAGCCTGATCCAGCAATACCGCGTGAGTGATGAAGGCCTTCGGGTTGTAAAACTCTTTTAGTAGGGAAGATAATGACGGTACCTACAGAAAAAGCACCGGCTAACTTCGTGCCAGCAGCCGCGGTAATACGAAGGGTGCTAGCGTTGTTCGGAATTACTGGGCGTAAAGCGTGCGTAGGCGGTTTAGCAAGTCGATTGTGAAAGCCCAGGGCTCAACCCTGGAATTGCAATCGAAACTACTTTGCTAGAGTGTGGTGGGGGATAGTAGAATTCCTAGTGTAGGGGTGAAATCCGTAGATATTAGGAGGAATACCAGTGGCGAAGGCGACTATCTACACCAATACTGACGCTGAGGCACGAAAGCGTGGGGAGCAAACAGG` |
| ASV16 | 404 | `TGGGGAATCTTGCGCAATGGGCGAAAGCCTGACGCAGCCATGCCGCGTGAATGATGAAGGTCTTAGGATTGTAAAATTCTTTCACCGGGGACGATAATGACGGTACCCGGAGAAGAAGCCCCGGCTAACTTCGTGCCAGCAGCCGCGGTAATACGAAGGGGGCTAGCGTTGCTCGGAATTACTGGGCGTAAAGGGAGCGTAGGCGGACATTTAAGTCAGGGGTGAAATCCCAGAGCTCAACTCTGGAACTGCCTTTGATACTGGATGTCTTGAGTGTGAGAGAGGTATGTGGAACTCCGAGTGTAGAGGTGAAATTCGTAGATATTCGGAAGAACACCAGTGGCGAAGGCGACATACTGGCTCATTACTGACGCTGAGGCTCGAAAGCGTGGGGAGCAAACAGG` |
| ASV289 | 404 | `TGGGGAATATTGGACAATGGGCGAAAGCCTGATCCAGCGACGCCGCGTGAGTGAAGAAGGCCTTAGGGTTGTAAAGCTCTTTCAGTAGGGAAGATAATGACGGTACCTACAGAAGAAGCTCCGGCTAACTTCGTGCCAGCAGCCGCGGTAATACGAAGGGAGCTAGCGTTACTCGGAATTATTGGGCGTAAAGCGTGCGTAGGCTGTTTTGTAAGTCAGAAGTGAAAGCCTGAGGCTCAACCTCAGAATAGCTTTTGAAACTGCAAGGCTAGAATACCAGAGAGGATGACGGAATTCCTAGTGTAGAGGTGAAATTCGTAGATATTAGGAGGAACACCGGTGGCGAAGGCGGTCATCTGGCTGGTTATTGACGCTGTTGCACGAAAGCGTGGGGAGCAAACAGG` |
| ASV5174 | 374 | `CGGCAAGCCAATGATACCATGACCCTGGTTACGCACGTTAGCCCTGCCAACGATATGATGCTGTTTGTGGGTAAGCTGAAAGGAGCCGCTGCTCACCTGACCCCTGAAAAAGCCCTTGAAAAACTGCTGGCCGACTTTGGGGTGAAAAGCAACCGTATATTCCCAACCAAATATAACGGCATCTCATTTCTTGAAACAACTGGTTCTGGCGTGATAAACTACCATCCGGTGCAGTATGATGCCATGGCAGCCATACACCGGGGTAACGCTATTTTGGTCTATGTATCGGCCAGCCCCGAAGCCTATGAGCAGCATAAGTCGATGATGACCGAAATTATTCACAGCATGTCGCCCTACCGAGGAAAGTAGCCCTG` |
| ASV3998 | 429 | `TAGGGAATCTTCCACAATGGACGCAAGTCTGATGGAGCAACGCCGCGTGAGTGAAGAAGGTCTTCGGATCGTAAAACTCTGTTGTTAGAGAAGAACACGAGTGAGAGTAACTGTTCATTCGATGACGGTATCTAACCAGCAAGTCACGGCTAACTACGTGCCAGCAGCCGCGGTAATACGTAGGTGGCAAGCGTTGTCCGGATTTATTGGGCGTAAAGGGAACGCAGGCGGTCTTTTAAGTCTGATGTGAAAGCCTTCGGCTTAACCGGAGTAGTGCATTGGAAACTGGAAGACTTGAGTGCAGAAGAGGAGAGTGGAACTCCATGTGTAGCGGTGAAATGCGTAGATATATGGAAGAACACCAGTGGCGAAAGCGGCTCTCTGGTCTGTAACTGACGCTGAGGTTCGAAAGCGTGGGTAGCAAACAGG` |
| ASV442 | 409 | `TGGGGAATATTGCACAATGGGCGCAAGCCTGATGCAGCGACGCCGCGTGAGGGATGAAGGCCTTCGGGTTGTAAACCTCTTTCAGCAGGGAAGAAGCGCAAGTGACGGTACCTGCAGAAGAAGCGCCGGCTAACTACGTGCCAGCAGCCGCGGTAATACGTAGGGCGCAAGCGTTGTCCGGAATTATTGGGCGTAAAGAGCTCGTAGGCGGTTTGTCGCGTCTGGTGTGAAAACTCAAGGCTCAACCTTGAGCTTGCATCGGGTACGGGCAGACTAGAGTGCGGTAGGGGTGACTGGAATTCCTGGTGTAGCGGTGGAATGCGCAGATATCAGGAGGAACACCGATGGCGAAGGCAGGTCACTGGGCCGCAACTGACGCTGAGGAGCGAAAGCATGGGGAGCGAACAGG` |
| ASV1227 | 429 | `TGGGGAATTTTGGACAATGGGCGCAAGCCTGATCCAGCCATGCCGCGTGCGGGAAGAAGGCCTTCGGGTTGTAAACCGCTTTTGTCAGGGAAGAAACGCGCCGAGCTAATATCTTGGCGTAATGACGGTACCTGAAGAATAAGCACCGGCTAACTACGTGCCAGCAGCCGCGGTAATACGTAGGGTGCAAGCGTTAATCGGAATTACTGGGCGTAAAGCGTGCGCAGGCGGTCCTACAAGACAGATGTGAAATCCCCGGGCTCAACCTGGGAACTGCATTTGTGACTGTAGGGCTGGAGTGCGGCAGAGGGGGATGGAATTCCGCGTGTAGCAGTGAAATGCGTAGATATGCGGAGGAACACCGATGGCGAAGGCAATCCCCTGGGCCTGCACTGACGCTCATGCACGAAAGCGTGGGGAGCAAACAGG` |
| ASV5567 | 407 | `TGGGGAATATTGCACAATGGGGGAAACCCTGATGCAGCAACGCCGCGTGAAGGACGAAGGGGTTCGCCTCGTAAACTTCTTTAGGCAGGGACGAAGAAAGTGACGGTACCTGCAGAATAAGCCCCGGCTAACTACGTGCCAGCAGCCGCGGTAATACGTAGGGGGCAAGCGTTATCCGGAATTACTGGGTGTAAAGGGTGAGTAGGCGGTTCGATAAGTCATATGTGAAATTCCCGAGCTTAACTTGGGCGCTGCATAAGAAACTGTCGGGCTAGAGTACAGGAGAGGTAAGCGGAATTCCTAGTGTAGCGGTGAAATGCGTAGATATTAGGAAGAACACCAGTGGCGAAGGCGGCTTACTGGACTGAAACTGACGCTGAGTCACGAAAGCGTGGGGAGCGAACAGG` |
| ASV2203 | 423 | `TAGGGAATATTGGACAATGGGTGAGAGCCTGATCCAGCCATGCCGCGTGCAGGAAGACGGCCTTCTGGGTTGTAAACTGCTTTTATCTGGGGATAAAATTCCCTTGGGAGGGAGACTGAAGGTACCAGATGAATAAGCCACGGCTAACTACGTGCCAGCAGCCGCGGTAATACGTAGGTGGCAAGCGTTGTCCGGATTTATTGGGTTTAAAGGGTGCGTAGGCGGTCTATTAAGTCAGCGGTGAAAGACGACAGCTCAACTGTCGAGGTGCCATTGATACTGATGGACTTGAGTGCACACGAGGTAAGCGGAATGGACGGTGTAGCGGTGAAATGCATAGATATCGTCCAGAACACCGATAGTGAAGACAGCTTACCAGAGTGCAACTGACGCTGAGGCACGAAAGTGTGGGGATCAAACAGG` |
| ASV2331 | 404 | `TGGGGAATCTTAGACAATGGGGGAAACCCTGATCTAGCCATGCCGCGTGAGCGATGAAGGCCTTAGGGTTGTAAAGCTCTTTCGTGGGGGAAGATAATGACTGTACCCCAAGAAGAAGCCCCGGCTAACTCCGTGCCAGCAGCCGCGGTAATACGGAGGGGGCTAGCGTTGTTCGGAATTACTGGGCGTAAAGCGCACGTAGGCGGATCGGATAGTCAGAGGTGAAATCCCGGGGCTCAACCCCGGAACGGCCTTTGAAACTCCCGGTCTTGAGGTCGTGAGAGGTGGGTGGAATTCCGAGTGTAGAGGTGAAATTCGTAGATATTCGGAGGAACACCAGTGGCGAAGGCGGCCCACTGGCACGATACTGACGCTGAGGTGCGAAAGCGTGGGGAGCAAACAGG` |
| ASV5733 | 428 | `TCGAGAATTTTTCACAATGGGCGAAAGCCTGATGGAGCGACGCCGCGTGGGGGATGAATGGCTTCGGCCCGTAAACCCCTGTCATTTGCGAACAAATTGGTTCATTTAATACCTGAACCATTGATAGTAGCGGAAGAGGAAGGGACGGCTAACTCTGTGCCAGCAGCCGCGGTAATACAGAGGTCCCAAGCGTTGTTCGGATTCACTGGGCGTAAAGGGTGCGTAGGCGGTCGGGTAAGTCTGATGTGAAATCTCTGGGCTCAACCCAGAAATGGCATTGGATACTATTCGGCTTGAGGTCTGGAGGGGGGACTGGAATTCTCGGTGTAGCAGTGAAATGCGTAGATATCGAGAGGAACACCAGTGGCGAAGGCGAGTCCCTGGACAGGTCCTGACGCTGAGGCACGAAAGCTAGGGGAGCAAACAGG` |
| ASV2170 | 424 | `TGAGGAATATTGGTCAATGGGCGCAAGCCTGAACCAGCCATGCCGCGTGCAGGATGACGGTCCTATGGATTGTAAACTGCTTTTGTACAGGAAGAAACACTCGGACGTGTCCGAGCTTGACGGTACTGTAAGAATAAGGATCGGCTAACTCCGTGCCAGCAGCCGCGGTAATACGGAGGATCCAAGCGTTATCCGGAATCATTGGGTTTAAAGGGTTCGTAGGCGGTCTTATAAGTCAGTGGTGAAAGCCCATCGCTCAACGATGGAACGGCCATTGATACTGTAAGACTTGAATTATTTGGAAGTAACTAGAATATGTAGTGTAGCGGTGAAATGCTTAGAGATTACATGGAATACCAATTGCGAAGGCAGGTTACTACAAATGGATTGACGCTGATGGACGAAAGCGTGGGTAGCGAACAGG` |
| ASV5427 | 430 | `TGGGGAATATTGCGCAATGGGCGAAAGCCTGACGCAGCAACGCCGCGTGAGTGATGAAGGCCTTCGGGTTGTAAAGCTCTTTCGGCCGGGAAGAAGGGGTATGGTGTTAATAGCGCTGTACTTTGATGGTACCGGAAGAAGAAGCACCGGCTAACTTCGTGCCAGCAGCCGCGGTAATACGAAGGGTGCGAGCGTTGTTCGGAATTACTGGGCGTAAAGGGTTCGTAGGCTGGAATTTAAGTCAAGTGTGAAATCCCCAAGCTCAACTTGGGACGTGCACTTGAAACTGAGTTTCTTGAGTATCGGAGAGGGTGGTGGAATTGTTGGTGTAGGAGTGACATCCGTAGAGATCAGCAGGAACATCAGATGCGAAAGCGACCGCCTGGCCGATTACTGACGCTGAGGAACGAAAGCGTGGGGAGCAAACAGG` |
| ASV5633 | 405 | `TGGGGAATCTTGCGCAATGGGGGAAACCCTGACGCAGCGACGCCGCGTGCGGGATGAAGGCCTTCGGGTTGTAAACCGCTTTCAGCAGGGACGAAATTGACGGTACCTGCAGAAGAAGGCCCGGCCAACTACGTGCCAGCAGCCGCGGTAATACGTAGGGGCCTAGCGTTGTCCGGATTTATTGGGCGTAAAGAGCTCGTAGGCGGTTCGGCAAGTCGGATGTGAAATCTCCACGCTTAACGTGGAGGGGCCATTCGAAACTGCCGTGACTCGAGTCCGGTAGGGGAGTGTGGAATTCCTGGTGTAGCGGTGAAATGCGCAGATATCAGGAGGAACACCAGTAGCGAAGGCGGCACTCTGGGCCGGTACTGACGCTGAGGAGCGAAAGCGTGGGGAGCGAACAGG` |
| ASV3386 | 429 | `TGGGGAATATTGGACAATGGGGGAAACCCTGATCCAGCGACGCCGCGTGGGTGAAGAAGGCCTGCGGGTTGTAAAGCCCTTTCGGTAGGGACGAAAGCTCTTGCCCTAACAAGGCAGGAGGTTGACTTAACCTACATAAGAAGCACCGGCTAACTCTGTGCCAGCAGCCGCGGTAATACAGAGGGTGCGAGCGTTAATCGGAATTACTGGGCGTAAAGCGCACGTAGTCGGCTTGTCAAGTCGGGTGTGAAATCCCCGGGCTCAACCTGGGAACTGCATCCGAGACTGACTTGCTAGAGTATGGGAGAGGGAAGCGGAATTTCTGGTGTAGCAGTGAAATGCGTAGATATCAGAAGGAACATCAGTGGCGAAGGCGGCTTCCTGGACCAATACTGACGATCAGGTGCGAAAGCGTGGGGAGCAAACAGG` |
| ASV2545 | 430 | `TGGGGAATCTTGCACAATGGGCGAAAGCCTGATGCAGCAACGCCGCGTGAGTGAAGAAGGTCTTTGGATTGTAAAGCTCTTTCGGCCAGAAAGAAGGGCTTTAAGGTAAATAATCTTAGAGTCTGACGGTACTGGAAGAAGAAGCACCGGCTAACTCCGTGCCAGCAGCCGCGGTAATACGGAGGGTGCAAGCGTTGTTCGGATTGATTGGGCGTAAAGCGCTCGTAGGCGGGTTCATAAGTCGAATGTGAAAGCCCTGGGCTTAACCTGGGAATTGCATCCGAAACTATGTATCTTGAATGCTCGAGAGGTTGGTGGAATTCTTGGTGTAGGAGTGAAATCCGTAGATATCAAGAGGAATATCGGAGGCGAAGGCGGCCGACTGGCGATGCATTGACGCTGAGGAGCGAAAGCGTGGGGAGCAAACAGG` |
| ASV2933 | 430 | `TAGGGAATATTGCGCAATGGGGGAAACCCTGACGCAGCAACGCCGCGTGAGTGAGGAAGGTCTTCGGATTGTAAAACTCTTTTATTTGGGAAAAATGTCCATATGGCTAATATCCATGTGGAGTGATGGTACCAAAGGAATAAGGACCGGCTAACTTCGTGCCAGCAGCCGCGGTAATACGAAGGGTCCAAGCGTTGTTCGGAATCATTGGGCGTAAAGCGCGCGCAGGCGGATCAGCAAGTCAGATGTGAAATCTCGGAGCTCAACTTCGAAACTGCGTCTGAAACTGCTAGTCTAGAATGTCGGAAGGGGTAGGGGAATTTCACGTGTAGGGGTAAAATCCGTAGAGATGTGAAGGAACACCGGAGGCGAAGGCGCCTGCCTGGACGACTATTGACGCTGAGGCGCGAAAGCGTGGGGAGCAAACAGG` |
| ASV4598 | 404 | `TGGGGAATATTGGACAATGGGGGAAACCCTGATCCAGCAATGCCGCGTGGGTGATGAAGGCCTTCGGGTTGTAAAGCCCTTTTGGCGGGGACGATGATGACGGTACCCGCAGAAGAAGCCCCGGCTAACTTCGTGCCAGCAGCCGCGGTAATACGAAGGGGGCTAGCGTTGTTCGGAATTACTGGGCGTAAAGGGCGCGTAGGCGGCGTCGATAGTCGGGCGTGAAATACCTGGGCTCAACCCGGGAATGGCGCTCGATACTTCGATGCTTGAGTGTGGGAGAGGATAGTGGAATTCCCAGTGTAGAGGTGAAATTCGTAGATATTGGGAAGAACACCAGTGGCGAAGGCGGCTATCTGGACCACAACTGACGCTGAGGCGCGAAAGCGTGGGGAGCAAACAGG` |
| ASV3067 | 404 | `TGGGGAATATTGGGCAATGGGCGCAAGCCTGACCCAGCCATGCCGCGTGAGTGATGAAGGCCTTAGGGTTGTAAAGCTCTTTTAGTGGTGAAGATGATGACTGTAGCCACAGAAAAAGCCCCGGCTAACTTCGTGCCAGCAGCCGCGGTAATACGAAGGGGGCTAGCGTTGTTCGGAATTACTGGGCGTAAAGCGCACGCAGGCGGCTTGACAAGTCAGAGGTGAAATCCCGAAGCTTAACTTCGGAACTGCCTTTGAAACTGTCTGGCTAGAGATCGAGAGAGGTGAGTGGAATTCCTAGTGTAGCAGTGAAATGCGTAGATATTAGGAGGAACACCAGTGGCGAAGGCGGCTCACTGGCTCGATACTGACGCTCATGTGCGAAAGCGTGGGGAGCAAACAGG` |
| ASV1862 | 404 | `TGGGGAATATTGGACAATGGGGGAAACCCTGATCCAGCCATGCCGCGTGAGTGATGACGGCCTTAGGGTTGTAAAGCTCTTTTACTCGGGAAGATAATGACTGTACCGAGAGAATAAGCCCCGGCAAACTTCGTGCCAGCAGCCGCGGTGATACGAAGGGGGCTAGCGTTGTTCGGATTTACTGGGCGTAAAGCGCACGTAGGCGGACTGTTAAGTCGGAGGTGAAATCCCAGGGCTCAACCCTGGAATTGCCTTCGATACTGGCAGTCTTGAGTATGATAGAGGTTAGTGGAATTCCTAGTGTAGAGGTGAAATTCGTAGATATTAGGAAGAACACCAGTGGCGAAGGCGGCTAACTGGATCATAACTGACGCTGAGGTGCGAAAGCGTGGGGATCAAACAGG` |
| ASV2852 | 429 | `TGGGGAATTTTGGACAATGGGCGAAAGCCTGATCCAGCAATGCCGCGTGTGCGAAGAAGGCCTTCGGGTTGTAAAGCACTTTTATCAGAGAAAAAAAGACGATTGCTAATATCAATTGTTTATGATGGTATCTGAAGAATAAGCACCGGCTAACTACGTGCCAGCAGCCGCGGTAATACGTAGGGTGCGAGCGTTAATCGGAATTATTGGGCGTAAAGCGTGCGCAGGCGGTTGTATAAGATAGATGTGAAATCCTTGGGCTCAACCTGAGAACTGCATTTATGACTGTACGACTAGAGTGTGTCAGAGGGGGGTAGAATTCCACGTGTAGCAGTGAAATGCGTAGAGATGTGGAGGAATACCGATGGCGAAGGCAGCCCCCTGGGATAACACTGACGCTCATGCACGAAAGCGTGGGGAGCAAACAGG` |
| ASV4184 | 428 | `TCGAGAATTTTTCTCAATGGGGGAAACCCTGAAGGAGCGACGCCGCGTGGGGGATGAAGGACTTCGGTTCGTAAACCCCTGTCACTTGGGAACAAAAGCTGCAACCTAACACGTTGCAGCCTGATAGTACCAGGAGAGGAAGGCACGGCTAACTCTGTGCCAGCAGCCGCGGTAATACAGAGGTGCCAAGCGTTGTTCGGATTTATTGGGCGTAAAGGGTGCGTAGGCGGTCATGCAAGTCGGGTGTGAAATACCGGAGCCTAACTCCGGAACGGCATTCGAAACTGCACGGCTAGAGATCTGGAGAGGTGATTGGAATTCTCGGTGTAGCAGTGAAATGCGTAGATATCGAGAGGAACACCAGAGGCGAAAGCGAATCACTGGACAGAATCTGACGCTGAGGCACGAAAGCCAGGGGAGCAAACGGG` |
| ASV2593 | 404 | `TGGGGAATATTGGACAATGGGCGAAAGCCTGATCCAGCAATGCCGCGTGAGTGAAGAAGGCCTTCGGGTTGTAAAGCTCTTTTACCCGGGAAGATAATGACTGTACCGGGAGAATAAGCCCCGGCTAACTCCGTGCCAGCAGCCGCGGTAATACGGAGGGGGCTAGCGTTGTTCGGAATTACTGGGCGTAAAGCGTACGTAGGCGGCTTGGTAAGTCAGAGGTGAAAGCCCACGACTCAATCGTGGAACTGCCTTTGAGACTGCTTGGCTTGAACACGGGAGAGGTGAGTGGAATTCCGAGTGTAGAGGTGAAATTCGTAGATATTCGGAAGAACACCAGTGGCGAAGGCGGCTCACTGGACCGTTGTTGACGCTGAGGTACGAAAGCGTGGGGAGCAAACAGG` |
| ASV2760 | 429 | `TGGGGAATTTTGGACAATGGGCGCAAGCCTGATCCAGCCATGCCGCGTGAGTGAAGAAGGCCTTCGGGTTGTAAAGCTCTTTTGTCCGGGAGCAAATCCTAGTCGTTAATACCGGCTGGGGATGAGAGTACTGGAAGAATAAGCACCGGCTAACTACGTGCCAGCAGCCGCGGTAATACGTAGGGTGCGAGCGTTAATCGGAATTACTGGGCGTAAAGCGTGCGCAGGTGGTTTTGTAAGCCAGATGTGAAATCCCCGGGCTTAACCTGGGAATGGCATTTGGGACTGCAAGGCTAGAGTGTGGCAGAGGGGGGTGGAATTCCGCGTGTAGCAGTGAAATGCGTAGAGATGCGGAGGAACACCGATGGCGAAGGCAGCCCCCTGGGCTAACACTGACACTCATGCACGAAAGCGTGGGGAGCAAACAGG` |
| ASV4066 | 404 | `TGGGGAATCTTGGACAATGGGCGAAAGCCTGATCCAGCCATGCCGCGTGAGTGAAGAAGGCCTTAGGGTTGTAAAGCTCTTTTACCCAGGAAGATAATGACTGTACTGGGAGAATAAGCCCCGGCTAACTTCGTGCCAGCAGCCGCGGTAATACGAAGGGGGCTAGCGTTGCTCGGAATTACTGGGCGTAAAGCGCACGTAGGCGGGTTATTAAGTCAGAGGTGAAAGCCTGGAGCTCAACTCCAGAACTGCCTTTGATACTGATAGTCTTGAGTTTGTGAGAGGTAAGTGGAATACCGAGTGTAGAGGTGAAATTCGTAGATATTCGGTGGAACACCAGTGGCGAAGGCGACTTACTGGCACAATACTGACGCTGAGGTGCGAAAGCGTGGGGATCAAACAGG` |
| ASV1948 | 429 | `TGGGGAATTTTGGACAATGGGCGCAAGCCTGATCCAGCAATGCCGCGTGCAGGATGAAGGCCTTCGGGTTGTAAACTGCTTTTGTACGGAGCGAAACAGCCTCTTCTAATACAGGGGGTGAATGACGGTACCGTAAGAATAAGCACCGGCTAACTACGTGCCAGCAGCCGCGGTAATACGTAGGGTGCAAGCGTTAATCGGAATTACTGGGCGTAAAGCGTGCGCAGGCGGTCTTGTAAGACAGATGTGAAATCCCCGGGCTTAACCTGGGACCTGCATTTGTGACTGCAAGGCTAGAGTACGGCAGAGGGGGATGGAATTCCGCGTGTAGCAGTGAAATGCGTAGATATGCGGAGGAACACCGATGGCGAAGGCAGTCCCCTGGGCCTGTACTGACGCTCATGCACGAAAGCGTGGGGAGCAAACAGG` |
| ASV2677 | 429 | `TGGGGAATATTGGACAATGGGCGCAAGCCTGATCCAGCCATACCGCGTGGGTGAAGAAGGCCTTCGGGTTGTAAAGCCCTTTTGTTGGGAAAGAAAAGCAGCGGGCTAATACCTTGCTGTTCTGACGGTACCCAAAGAATAAGCACCGGCTAACTTCGTGCCAGCAGCCGCGGTAATACGAAGGGTGCAAGCGTTACTCGGAATTACTGGGCGTAAAGCGTGCGTAGGTGGTCGTTTAAGTCTGTTGTGAAAGCCCTGGGCTCAACCTGGGAACTGCAGTGGAAACTGGACGACTAGAGTGTGGTAGAGGGTAGCGGAATTCCCGGTGTAGCAGTGAAATGCGTAGAGATCGGGAGGAACATCCATGGCGAAGGCAGCTACCTGGACCAACACTGACACTGAGGCACGAAAGCGTGGGGAGCAAACAGG` |
| ASV409 | 424 | `TGAGGAATATTGGACAATGGGCGCAAGCCTGATCCAGCCATGCCGCGTGCAGGAAGACGGTCCTATGGATTGTAAACTGCTTTTATACAGGAAGAAACACTCCCTCGTGAGGGAACTTGACGGTACTGTATGAATAAGCATCGGCTAACTCCGTGCCAGCAGCCGCGGTAATACGGAGGATGCAAGCGTTATCCGGAATCATTGGGTTTAAAGGGTCCGTAGGCGGTTTAATAAGTCAGTGGTGAAAGCCCATCGCTCAACGATGGAACGGCCATTGATACTGTTAAACTTGAATTATTAGGAAGTAACTAGAATATGTAGTGTAGCGGTGAAATGCTTAGAGATTACATGGAATACCAATTGCGAAGGCAGGTTACTACTAATATATTGACGCTGATGGACGAAAGCGTGGGGAGCGAACAGG` |
| ASV1064 | 404 | `TGGGGAATATTGGACAATGGGCGCAAGCCTGATCCAGCCATGCCGCGTGGATGATGAAGGCCCTAGGGTTGTAAAATCCTTTCGCCGGAGAAGATAATGACGGTATCCGGTAAAGAAGCCCCGGCTAACTTCGTGCCAGCAGCCGCGGTAATACGAAGGGGGCTAGCGTTGCTCGGAATTACTGGGCGTAAAGCGCACGTAGGCGGGTCATTAAGTCAGAGGTGAAAGCCCACAGCTCAACTGTGGAACTGCCTTTGATACTGGTGACCTAGACCAAAGTAGAGGCAGGTGGAATTCCGAGTGTAGAGGTGGAATTCGTAGATATTCGGAGGAACACCAGTGGCGAAGGCGGCCTGCTGGACTTTTGGTGACGCTGAGGTGCGAAAGCGTGGGGAGCAAACAGG` |
| ASV4923 | 406 | `TGGGGAATTTTCCGCAATGGGCGAAAGCCTGACGGAGCAATACCGCGTGAGGGAGGAAGGCTCTTGGGTTGTAAACCTCTTTTCTCAGGGAAGAATAAATGACGGTACCTGAGGAATAAGCATCGGCTAACTCCGTGCCAGCAGCCGCGGTAATACGGAGGATGCAAGCGTTATCCGGAATGATTGGGCGTAAAGCGTCCGCAGGTGGTTTTGTATGTCTGCTGTTAAAGCGTGCGGCTTAACCGCATAAGGGCGGTGGAAACTACAAGACTAGAGTACGTTCGGGGCAGAGGGAATTCCCAGTGTAGCGGTGAAATGCGTAGAGATTGGGAAGAACATCGGTGGCGAAGGCGCTCTGCTAGGCCTGTACTGACACTGAGGGACGAAAGCTAGGGGAGCGAATGGG` |
| ASV3461 | 404 | `TGGGGAATATTGGACAATGGGGGAAACCCTGATCCAGCAATGCCGCGTGAGTGATGAAGGCCTTAGGGTTGTAAAGCTCTTTTACCTGCGATGATAATGACAGTAGCAGGGGAATAAGCTCCGGCTAACTTCGTGCCAGCAGCCGCGGTAATACGAAGGGAGCTAGCGTTGTTCGGAATTACTGGGCGTAAAGGGCGCGTAGGCGGCGCTGTAAGTGAGATGTGAAAGACCTGGGCTTAACCTGGGAAGTGCATTTCAGACTGCAGTGCTAGAGTGCGAGAGAGGAAAGTGGAATGACGCATGTAGAGGTGAAATTCGTAGATATGCGTTGGAACACCAATGGCGAAGGCAACTTTCTGGCTCGCAACTGACGCTAAGGCGCGAAAGCGTGGGGAGCAAACAGG` |
| ASV117 | 429 | `TGGGGAATATTGGACAATGGGCGAAAGCCTGATCCAGCCATGCCGCGTGTGTGAAGAAGGTCTTCGGATTGTAAAGCACTTTAAGTTGGGAGGAAGGGTAGTAACTTAATACGTTGCTACTTTGACGTTACCGACAGAATAAGCACCGGCTAACTTCGTGCCAGCAGCCGCGGTAATACGAAGGGTGCAAGCGTTAATCGGAATTACTGGGCGTAAAGCGCGCGTAGGTGGTTCAGTAAGTTGGAAGTGAAATCCCCGGGCTCAACCTGGGAACTGCTTTCAAAACTGCTGAGCTAGAGTACGGTAGAGGGTGGTGGAATTTCCTGTGTAGCGGTGAAATGCGTAGATATAGGAAGGAACACCAGTGGCGAAGGCGACCACCTGGACTGATACTGACACTGAGGTGCGAAAGCGTGGGGAGCAAACAGG` |
| ASV4317 | 389 | `CAGGGAATTTTTTGCAATGCGCGAAAGCGTGACAGAGCGAGCCAGAGTGCTTTCTCTTTTTGAGAAAGCTTTTATGAGATGTAAAAAGTCTCGTGAATAAGGACCGGGAGAGACTGGTGCCAGCCGCCGCGGTAATCCCAGCGGTCCAAGTCGTAACCACATTTATTGGGTCTAAAACATCCGTAGCTTGCTTTGTAAGTCTCTTGTGAAATCTGACATCTCAAGTGTCGGGCGAGCAAGAGATACTGTAGAGCTAGAGACCGGGAGACGTAAGAAGTACAGTTAGAGTAGCGGTTAAATGCGTTGATCCTTACTGGACTAACAATAGCGAAGGCATCTTACGAGAACGGATCTGACAGTGAGGGATGAAGGCTAGGGGCGCAAATTGG` |
| ASV67 | 424 | `TGAGGAATATTGGTCAATGGACGCAAGTCTGAACCAGCCATGCCGCGTGCAGGATGACGGCTCTATGAGTTGTAAACTGCTTTTGTACTAGGGTAAACTTATCTACGAGTAGATAATTGAAAGTATAGTACGAATAAGGATCGGCTAACTCCGTGCCAGCAGCCGCGGTAATACGGAGGATCCAAGCGTTATCCGGATTTATTGGGTTTAAAGGGTGCGTAGGCGGTTTGATAAGTTAGAGGTGAAATATTGGGGCTCAACCCCGAAACTGCCTCTAATACTGTCAGGCTAGAGAATAGTTGCGGTAGGCGGAATGTATGGTGTAGCGGTGAAATGCTTAGAGATCATACAGAACACCGATTGCGAAGGCAGCTTACCAAACTATATCTGACGTTGAGGCACGAAAGCGTGGGGAGCAAACAGG` |
| ASV4652 | 403 | `TGAGGAATCTTCCACAATGGACGAAAGTCTGATGGAGCGACACCGCGTGAAGGATGAAGCCTTTGTTGGTGTAAACTTCTTTTTTCTGGGAAGATAATGACGGTACCAGAAGAATAAGGGGCGGCAAACTTCGTGCCAGCAGCCGCGGTAATACGAAGGCCCCAAGCGTTATCCGGAATTACTGGGCGTAAAGAGTCTGTAGGTGGTCTGATAAGTCTCGTGTGAAAGTCCAGGGCTCAACCCTGGGTTGCACGAGAAACTGCCAGACTCGAGTACGGGAGAGGTGAGCGGAATGGTATGAGTAGGGGTGCAATCCGTTGATACATACCAGAACACCAAAAGCGAAGGCAGCTCACTGGAACGTTACTGACACTGAGAGACGAAAGCGTGGGGAGCGAAAGGG` |
| ASV1924 | 429 | `TGGGGAATATTGGACAATGGGGGCAACCCTGATCCAGCAATGCCATGTGTGCGATGAAGGCCTTCGGGTTGTAAAGCACTTTAGTGGGGGAGGAAGGGTGACGTGTTAATACCACGTTATTCAGACAGTACCCCAAGAATAAGCACCGGCAAACTCTGTGCCAGCAGCCGCGGTAATACAGAGGGTGCAAGCGTTAATCGGAATTATTGGGCGTAAAGGGCTTGTAGGTGGTTGTTTAAGTTAGCTGTGAAAACCCTGGGCTTAACCTGGGACGTGCAGTTAATACTGGATAGCTAGAGTATGGTAGAGGAGAGGGGAATTCCTGGTGTAGCAGTGAAATGCGTAGAGATCAGGAGGAACATCAGTGGCGAAGGCGCCTCTCTGGACTAATACTGACACTGAAAAGCGAAGGCGTGGGGATCAAACAGG` |
| ASV2629 | 429 | `TGGGGAATTTTGGACAATGGGCGCAAGCCTGATCCAGCCATGCCGCGTGTCTGAAGAAGGCCTTCGGGTTGTAAAGGACTTTTGTCTGGGAGCAAATACCCGGTGTTAATAGCACCGGGGGCTGAGAGTACCAGAAGAATAAGCACCGGCTAACTACGTGCCAGCAGCCGCGGTAATACGTAGGGTGCAAGCGTTAATCGGAATTACTGGGCGTAAAGCGTGCGCAGGCGGTTTTGTAAGCCAGATGTGAAAGCCCCGGGCTCAACCTGGGAACGGCGTTTGGAACTACAAGACTAGAGTGCGTCAGAGGGGGGTGGAATGCCGCGTGTAGCAGTGAAATGCGTAGAGATGCGGCGGAACACCGATGGCGAAGGCAGCCCCCTGGGATGACACTGACGCTCATGCACGAAAGCGTGGGGAGCAAACAGG` |
| ASV2283 | 429 | `TGGGGAATATTGGACAATGGGGGCAACCCTGATCCAGCAATGCCGCGTGTGTGAAGAAGGCCTGAGGGTTGTAAAGCACTTTCAGTGGGGAGGAGGATGCCTAGGTTAAGAGCTAGGGTATTGGACGTTACCCACAGAAGAAGCACCGGCTAACTCCGTGCCAGCAGCCGCGGTAATACGGAGGGTGCGAGCGTTAATCGGAATTACTGGGCGTAAAGGGTGCGTAGGTGGTTTGGTAAGTTATCTGTGAAATCCCTGGGCTCAACCTGGGCAGGTCAGATAAAACTGCTGGACTCGAGTATGGGAGAGGGTAGTGGAATTTCCGGTGTAGCGGTGAAATGCGTAGAGATCGGAAGGAACACCAGTGGCGAAGGCGGCTACCTGGCCTAATACTGACACTGAGGCACGAAAGCGTGGGGAGCAAACAGG` |
| ASV5102 | 404 | `TAAGGAATATTGGACAATGGGGGAAACCCTGATCCAGCCATGCCGCGTGAGTGATGAAGGCCTTCGGGTTGTAAAGCTCTTTTAGATGTGACGATGATGACGGTAACATCAGAAAAAGCTCCGGCTAACTTCGTGCCAGCAGCCGCGGTAATACGAAGGGAGCGAGCGTTGTTCGGAATTACTGGGCTTAAAGCGTGCGTAGGCGGACTGAAAAGTCAGAAGTGAAATCCCAGGGCTTAACCCTGGAATTGCTTTTGAAACTCTCGGTCTGGAATTCGGAAGAGGTTGGCGGAATACCCAGTGTAGAGGTGAAATTCGCAGATATTGGGTAGAACACCGGTGGCGAAGGCGGCCAACTGGTCCGACATTGACGCTGAGGCACGAAAGCGTGGGGAGCAAACAGG` |
| ASV5212 | 425 | `TGAGGAATATTGGTCAATGGACGAGAGTCTGAACCAGCCAAGTAGCGTGAAGGATGACTGCCCTATGGGTTGTAAACTTCTTTTATAGGGGAATAAAATGCACCACGCGCTGGTGTTTTGTATGTACCCTACGAATAAGGATCGGCTAACTCCGTGCCAGCAGCCGCGGTAATACGGAGGATCCGAGCGTTATCCGGATTTATTGGGTTTAAAGGGTGCGCAGGCGGACTGATAAGTCAGCTGTGAAAGTTTACGGCTCAACCGTGAAATTGCAGTTGAAACTGTCAGTCTTGAGTACACATGAGGTGAGCGGAATTTGTGGTGTAGCGGTGAAATGCTTAGATATCACAAGGAACTCCAATTGCGAAGGCAGCTCACTGGGGTGTAACTGACGCTCATGCACGAAGGTGTGGGTATCAAACAGG` |
| ASV3871 | 429 | `TAGGGAATTTTCCACAATGGGCGAAAGCCTGATGGAGCAACGCCGCGTGTGCGAGGAAGGTCTTCGGATTGTAAAGCACTGTTGTAAGGGAAGAATAATGTTAAGAGGAAATGATTAACAGATGACGGTACCTTACCAGAAAGTCACGGCTAACTACGTGCCAGCAGCCGCGGTAATACGTAGGTGGCAAGCGTTGTCCGGAATTATTGGGTGTAAAGGGCGTGTAGGCGGCCCGATAAGTCTGATGTGAAATCTCACGGCTCAACCGTGAAACTGCATTGGAAACTGTCAGGCTAGAGTGTCGGAGAGGAAAGCGGAATTCCATGTGTAGCGGTGGAATGCGTAGATATATGGAGGAACACCAGTGGCGAAGGCGGCTTTCTGGACGACAACTGACGCTGAGGCGCGAAAGCGTGGGGAGCAAACAGG` |
| ASV4758 | 405 | `TCGAGAATATTCGACAATGGACGAAAGTCTGATCGAGCGACGCCGCGTGGAGGAAGAAGGTCTTCGGATTGTAAACTCCTTTTCCATTCTAGAAAGTTATTGATCAGAGTGGGAATAAGTAGCGGCAAACTTCGTGCCAGCAGCAGCGGTAATACGAAGGCTACAAGCGTTATCCGGATTTATTGGGCGTAAAGAGCGAGTAGGTGGTTTGGTGCGTCTGTTGTTAAATGTCACCGCTTAACGGTGTCACCGCAATGGAAACGGCCGAACTAGAGAGTGCTAGAGGCAAATAGAACGCACGGTGTAGGGGTGAAATCCGTTGATATCGTGCGGAATACCAAAGGCGAAGGCATTTTGCTGGGGCATTTCTGACACTCAATCGCGAAAGCGTGGGGAGCAAAAAGG` |
| ASV3491 | 404 | `TGGGGAATATTGGACAATGGGCGAAAGCCTGATCCAGCCATGCCGCGTGTGTGATGAAGGCCTTAGGGTTGTAAAGCACTTTCACTGGGGAAGATAATGACGGTACCCAGAGAAGAAGCCCCGGCTAACTTCGTGCCAGCAGCCGCGGTAATACGAAGGGGGCTAGCGTTGTTCGGAATCACTGGGCGTAAAGCGCACGTAGGCGGACACTTAAGTCAGAGGTGAAATCCCAAGGCTCAACCTTGGAACTGCCTTTGATACTGGGTGTCTTGAGTTCGGGAGAGGTGAGTGGAACTGCGAGTGTAGAGGTGAAATTCGTAGATATTCGCAAGAACACCAGTGGCGAAGGCGGCTCACTGGCCCGATACTGACGCTGAGGTGCGAAAGCGTGGGGAGCAAACAGG` |
| ASV4194 | 428 | `TGGGGAATCTTGCGCAATGGGCGAAAGCCTGACGCAGCGACGCCGCGTGAGTGATGAAGGCCTTCGGGTTGTAAAGCTCTGTGGGGAGAGAAGAATAAGCCATGGCTAATATCCATGGTGATGACGGTATCTCCTTAGCAAGCACCGGCTAACTCTGTGCCAGCAGCCGCGGTAAGACAGAGGGTGCAAACGTTGTTCGGAATTACTGGGCGTAAAGCGTGTGTAGGCGGTGAGGTAAGTCAGGTGTGAAAGCCCGGAGCTCAACTTCGGAAGTGCACTTGATACTGCTTCGCTTGAGTACTGGAGAGGTTGGTGGAATTCTCGGTGTAGAGGTGAAATTCGTAGATATCGAGAGGAACATCGGTGGCGAAGGCGGCCAACTGGACAGATACTGACGCTGAGACACGAAAGCGCGGGGAGCAAACAGG` |
| ASV2719 | 424 | `TGAGGAATATTGGACAATGGGCGCAAGCCTGATCCAGCCATGCCGCGTGCAGGAAGACGGTCCTATGGATTGTAAACTGCTTTTGTACGGGAAGAAACACTCCTACGTGTAGGAGCTTGACGGTACCGTAAGAATAAGGATCGGCTAACTCCGTGCCAGCAGCCGCGGTAATACGGAGGATCCAAGCGTTATCCGGAATCATTGGGTTTAAAGGGTCCGTAGGCGGTTTAGTAAGTCAGTGGTGAAAGCCCATCGCTCAACGATGGAACGGCCATTGATACTGCTAGACTTGAATTACAAGGAAGTAACTAGAATATGTAGTGTAGCGGTGAAATGCTTAGATATTACATGGAATACCAATTGCGAAGGCAGGTTACTACTTGTGGATTGACGCTGATGGACGAAAGCGTGGGGAGCGAACAGG` |
| ASV2758 | 424 | `TGAGGAATATTGGACAATGGGCGCAAGCCTGATCCAGCCATGCCGCGTGCAGGATGACGGTCCTATGGATTGTAAACTGCTTTTATACAGGAAGAAACCCTGGTTCGTGAACCAGCTTGACGGTACTGTAAGAATAAGGATCGGCTAACTCCGTGCCAGCAGCCGCGGTAATACGGAGGATCCAAGCGTTATCCGGAATCATTGGGTTTAAAGGGTCCGTAGGCGGTTTAGTAAGTCAGTGGTGAAAGCCCATCGCTCAACGGTGGAACGGCCATTGATACTGCTAAACTTGAATTATTAGGAAGTAACTAGAATATGTAGTGTAGCGGTGAAATGCTTAGAGATTACATGGAATACCAATTGCGAAGGCAGGTTACTACTAATTGATTGACGCTGATGGACGAAAGCGTGGGTAGCGAACAGG` |
| ASV3434 | 407 | `TAGGGAATATTGCGCAATGGGCGAAAGCCTGACGCAGCGACGCCGCGTGAGTGATGAAGGCCTTAGGGTCGTAAAGCTCTGTTGAATGGGAAGAAAAAAATGACGGTACCATTCGAGGAAGGACCGGCTAACTTCGTGCCAGCAGCCGCGGTAATACGAGGGGTCCTAGCGTTGTTCGGAATCATTGGGCGTAAAGGGTGTGTAGGTGGCCTTATAAGTCAGGTGTGAAAGCCCCGGGCTCAACCCGGGAAGTGCATCTGATACTGTTTGGCTTGAGTACCGAAGAGGATAGTGGAATTCCAGGTGTAGTGGTGAAATACGTAGATATCTGGAGGAACACCGGTGGCGAAGGCGGCTATCTGGTCGAGTACTGACACTGAGACACGAAAGCATGGGGATCAAACAGG` |
| ASV3996 | 429 | `TGGGGAATATTGGACAATGGGGGAAACCCTGATCCAGCAATGCCGCGTGTGTGAAGAAGGCCTGCGGGTTGTAAAGCACTTTCGGTCGGGAGGAAAGGTTGAATGTTAATAGCATTCAGCTGTGACGTTACCGGCATAAGAAGCACCGGCTAACTCTGTGCCAGCAGCCGCGGTAATACAGAGGGTGCGAGCGTTAATCGGAATTACTGGGCGTAAAGGGTGCGTAGGCGGTTTAGTAAGTCAGATGTGAAATCCCCGGGCTTAACCTGGGAACGGCATTTGATACTGCTAGGCTAGAGTATGGTAGAGGAAAGCGGAATTTCCGGTGTAGCGGTGAAATGCGTAGATATCGGAAGGAACACCAGTGGCGAAGGCGGCTTTCTGGACTAATACTGACGCTGAGGCACGAAAGCATGGGGAGCAAACAGG` |
| ASV2547 | 430 | `TGGGGAATATTGGACAATGGGCGAAAGCCTGATCCAGCAATACCGCGTGTGTGAAGAAGGCCTTCGGGTTGTAAAGCACTTTAGTCAGGGAGGATGGTGATTTGGGCTAATACCCCAGATTGCTGACGTTACCTGAAGAATAAGCACCGGCTAACTCCGTGCCAGCAGCCGCGGTAATACGGGGGGTGCAAGCGTTAATCGGAATTACTGGGCGTAAAGGGCGCGTAGGCGGCCTCTTAAGTCAGATGTGAAATCCCTGGGCTCAACCTGGGAATGGCATTTGATACTGTGAGGCTAGAATGTGGTAGAGGATAGCGGAATTTCTGGTGTAGCGGTGAAATGCGTAGATATCAGAAGGAACATCAATGGCGAAGGCAGCTATCTGGGCCAACATTGACGCTGAGGCGCGAAAGCGTGGGGAGCAAACAGG` |
| ASV5693 | 424 | `TAAGGAATATTGGTCAATGGAGGGAACTCTGAACCAGCCATGCCGCGTGCAGGACGACTGCCCTATGGGTTGTAAACTGCTTTTGTACAGGAATAACCGTATCTACGAGTAGATACCTGAATGTACTGTAAGAATAAGGATCGGCTAACTCCGTGCCAGCAGCCGCGGTAATACGGAGGATCCGAGCGTTATCCGGATTTATTGGGTTTAAAGGGTGCGTAGGCGGCTTTATAAGTCAGGGGTGAAATACGGCAGCTCAACTGTCGCAGTGCCTTTGATACTGTAGAGCTTGAATGTGGTTGAAGATGGCGGAATGAGACAAGTAGCGGTGAAATGCATAGATATGTCTCAGAACACCGATTGCGAAGGCAGCTGTCTAAGCCATAATTGACGCTGATGCACGAAAGCGTGGGGATCAAACAGG` |
| ASV253 | 430 | `TAGGGAATATTGCACAATGGAGGAAACTCTGATGCAGCAACGCCGCGTGAGTGAAGAAGGCCCTTGGGTCGTAAAGCTCTTTAGCGAGGGAAGAAGCTGTGTACGGTGAATAATCGTGCATGGTGACGGTACCTCGAGAATAAGCACCGGCTAATTTCGTGCCAGCAGCCGCGGTAATACGAAAGGTGCAAGCGTTACTCGGAATCACTGGGCGTAAAGCGCACGTAGGCGGACTCGTAGGTCAGTTGTGAAATACCTGAGCTCAACTCAGGAATTGCAGCTGAAACCGCTTGTCTTGAATACCTGAGAGGGTGGGGGAATACCTGGTGGAGAAGTGAAATTCGTAGAGATCAGGTGGAACACCGGAGGCGAAGGCGCCTGCCTGGCAGTGTATTGACGCTGAGGTGCGAAAGTGCGGGGAGCAAACAGG` |
| ASV198 | 429 | `TCGAGAATAATTCACAATGGGGGCAACCCTGATGGTGCAACGCCGCGTGGAGGATGACGGTCTTCGGATTGTAAACTCCTGTCATCTGGGAGTAAGGCATGGTCGTTAATAGCGAACATGATTGATAGTACCAGAAGAGGAAGGGACGGCTAACTTCGTGCCAGCAGCCGCGGTAATACGAAGGTCCCAAGCGTTGTTCGGAATCACTGGGCGTAAAGGGAGCGTAGGCGGCGTGGTAAGTCAGATGTGAAATCCCGGGGCTCAACCCCGGAACTGCATCCGATACTGCCATGCTAGAGGATTGGAGAGGTAGCTGGAATTCTTGGTGTAGCAGTGAAATGCGTAGAGATCAAGAGGAACACTCGTGGCGAAAGCGAGCTACTGGACAATTCCTGACGCTGAGGCTCGAAGGCTAGGGTAGCGAAAGGG` |
| ASV2018 | 404 | `TGGGGAATTTTCCGCAATGGGCGAAAGCCTGACGGAGCAACGCCGCGTGAGGGAGGAAGGTCTTTGGATTGTAAACCTCTTTTCTCTAGGAAGATGATGACGGTACTAGAGGAATCAGCATCGGCTAACTCCGTGCCAGCAGCCGCGGTAATACGGGGGATGCGAGCGTTATCCGGAATTATTGGGCGTAAAGCGTCCGTAGGCGGCCGAGCAAGTTTGTTGTTAAAGCGTGCAGCTTAACTGCATAAGGGCAATGAAAACTGTTTGGCTAGAGTGTGGTAGGGGTAGAGGGAATTCCCGGTGTAGCGGTGAAATGCGTAGATATCGGGAAGAACACCAGTGGCGAAAGCGCTCTGCTGGGCCACAACTGACGCTGATGGACGAAAGCTAGGGGAGCGAAAGGG` |
| ASV3603 | 430 | `TGGGGAATCTTCCGCAATGGGCGAAAGCCTGACGGAGCAACGCCGCGTGAGTGAAGAAGGCCTTCGGGTCGTAAAGCTCTGTCTTTAGGGAAGAAACACGTATATGCTAATATCATATACGCTTGACGGTACCTGAGGAGGAAGCTCCGGCTAACTACGTGCCAGCAGCCGCGGTAATACGTAGGGAGCAAGCGTTGTCCGGAATTACTGGGCGTAAAGGGCGTGTAGGCGGGAATTTAAGTAAGGTGTGAAAACTCCAGGCTCAACCTGGAGACTGCACTTTAAACTGAGTTTCTTGAGGGCAGGAGAGGAAAGTGGAATTCCTAGTGTAGCGGTGAAATGCGTAGATATTAGGAGGAACACCAGTGGCGAAGGCGACTTTCTGGACTGTACCTGACGCTGAGGCGCGAAAGCGTGGGGAGCGAACAGG` |
| ASV1373 | 404 | `TGGGGAATATTGGACAATGGAGGAAACTCTGATCCAGCCATGCCGCGTGAGTGAAGAAGGCCTTCGGGTTGTAAAGCTCTTTTGTCCCTGACGATAATGACGGTAGGGGAAGAAAAAGCCACGGCTAACTTCGTGCCAGCAGCCGCGGTAATACGAAGGTGGCAAGCGTTGTTCGGATTTACTGGGCGTAAAGCGTGCGTAGGCGGTTTAACAAGTCAGGAGTGAAAGCCCAAGGCTCAACCCTGGAATTGCTTTTGAAACTGTTAGACTCGAATCCGGTAGAGGTTGGCGGAATTCTCAGTGTAGAGGTGAAATTCGCAGATATTGAGAGGAACACCAGTGGCGTAAGCGGCCAACTGGACCGGTATTGACGCTGAGGCACGAAAGCATGGGGAGCAAACAGG` |
| ASV4125 | 407 | `TAGGGAATATTGCGCAATGGGGGAAACCCTGACGCAGCGACGCCGCGTGAGTGATGAAGGCCTTCGGGTTGTAAAGCTCTGTCGTAAGGGAATAAAAAAATGAAGGTACCTTACAAGAAAGGACCGGCTAACTTCGTGCCAGCAGCCGCGGTAATACGAGGGGTCCTAGCGTTGCTCGGAATCATTGGGCGTAAAGCGGGTGTAGGCGGACAACCAAGTCAGATGTGAAAGCCCCGGGCTCAACCCGGGAAGTGCATTTGAAACTGGTTATCTTGAGTGCAGTAGAGGAAGGTGGAATTCCAAGTGTAGTGGTGAAATACGTAGATATTTGGAGGAACATCGGTGGCGAAGGCGACCTTCTGGACTGTTACTGACGCTGAGACCCGAAAGCGTGGGGATCAAACAGG` |
| ASV5592 | 406 | `TGAGGAATCTTCCGCAATGGGCGAAAGCCTGACGGAGCGACACCGCGTGGAGGACGAAGGCCTTTTGGTCGTAAACTCCTTTTCTGAGGGAATAAATGATGAAGGTACCTCAGGAATAAGCACCGGCTAACTACGTGCCAGCAGCCGCGGTAATACGTAGGGTGCAAGCGTTATCCGGAATTATTGGGCGTAAAGCGTTCGTAGGCGGTTTATTAAGTTTGTGGTTAAATCTGGTGGCTCAACCATCAAATCGCCGCAAAAACTGATAAACTTGAGAATGACAGAGGCAACTGGAATTCCACATGTAGGGGTAAAATCCGTTGATATGTGGAGGAACACCAAAAGCGAAGGCAGGTTGCTGGGTCATTTCTGACGCTGAGGAACGAAAGCGTGGGGAGCAAACAGG` |
| ASV1973 | 404 | `TGGGGAATCTTGGACAATGGGGGAAACCCTGATCCAGCCATGCCGCGTGAATGAAGACGGCCTTCGGGTTGTAAAGTTCTTTCAGTAGGGAAGATAATGACTGTACCTACAGAAGAAGCCCCGGCTAACTCCGTGCCAGCAGCCGCGGTAAGACGGAGGGGGCTAGCGTTGTTCGGAATTACTGGGCGTAAAGGGTGCGTAGGCGGTTATGTAAGTCAGAGGTGAAATTCCCGAGCTCAACTTGGGCGCTGCCTTTGATACTGCATGACTAGAGATTGGAAGAGGTAGGTAGAATTCCTAGTGTAGAGGTGAAATTCGTAGATATTAGGAGGAATACCAGTGGCGAAGGCGGCCTACTGGTCCACATCTGACGCTGAGGCACGAAAGCGTGGGGATCAAACAGG` |
| ASV1537 | 429 | `TGGGGAATATTGCACAATGGGCGAAAGCCTGATGCAGCCATGCCGCGTGTGTGAAGAAGGCCTTCGGGTTGTAAAGCACTTTAGGCGAGGAGGAAAAGCGTACGGTTAATAACCGTGCGCCGTGACGTTACTCGCAGAATAAGCACCGGCTAACTCCGTGCCAGCAGCCGCGGTAATACGGAGGGTGCAAGCGTTAATCGGAATTACTGGGCGTAAAGCGCGCGTAGGCGGTTTGATAAGCGGAGTGTGAAATCCCCGGGCTTAACCTGGGAACTGCATTCCGAACTGTCGAGCTAGAGTATGAGAGAGGGTGGCGGAATTCCGAGTGTAGCGGTGAAATGCGTAGATATTCGGAGGAACACCAGTGGCGAAGGCGGCCACCTGGATCAATACTGACGCTGAGGCGCGAAAGCGTGGGTAGCAAACAGG` |
| ASV2374 | 424 | `TAAGGAATATTGGACAATGGTGGCAACACTGATCCAGCCATGCCGCGTGAAGGATGAAGGCCCTATGGGTTGTAAACTTCTTTTGTACGGGAAAAAACCTCTTTACGTGTAAAGAGCTGATTGTACTGTAAGAATAAGGATCGGCTAACTCCGTGCCAGCAGCCGCGGTAATACGGAGGATCCAAGCGTTATCCGGATTCATTGGGTTTAAAGGGTGCGTAGGCGGAATGATAAGTCAGTGGTGAAATCCTTCAGCTTAACTGGAGAATTGCCATTGATACTGTCAATCTTGAGTACATTTGATGTGGGCGGAATGTGTCATGTAGCGGTGAAATGCTTAGATATGACACAGAACACCGATTGCGAAGGCAGCTCACAAAACTGTAACTGACGCTGAGGCACGAAAGCGTGGGTATCAAACAGG` |
| ASV1198 | 429 | `TGGGGAATTTTGGACAATGGGGGAAACCCTGATCCAGCCATTCCGCGTGAGTGAAGAAGGCCTTCGGGTTGTAAAGCTCTTTCAGCAGGAACGAAACGGTAGGCTTTAATAAAGTTTGCTAATGACGGTACCTGAAGAAGAAGCACCGGCTAACTACGTGCCAGCAGCCGCGGTAATACGTAGGGTGCGAGCGTTAATCGGAATTACTGGGCGTAAAGCGTGCGCAGGCGGTTTTGTAAGACAGATGTGAAATCCCCGGGCTTAACCTGGGAACTGCGTTTGTGACTGCAAGACTAGAGTGTGGCAGAGGGGGGTAGAATTCCACGTGTAGCAGTGAAATGCGTAGAGATGTGGAGGAATACCGATGGCGAAGGCAGCCCCCTGGGTCAGCACTGACGCTCATGCACGAAAGCGTGGGGAGCAAACAGG` |
| ASV4512 | 406 | `TGGGGAATATTGCGCAATGGGGGAAACCCTGACGCAGCAACGCCGCGTGAAGGATGAAGGTTTTCGGATTGTAAACTTCTTTGATGGGGGACGAATAAATGACGGTACCCCAAAAACAAGCCACGGCTAACTACGTGCCAGCAGCCGCGGTAATACGTAGGTGGCGAGCGTTGTCCGGAATTACTGGGTGTAAAGGGCGTGTAGGCGGGATTGTAAGTCAGATGTGAAATTCCAGAGCTTAACTCTGGAGCTGCATCTGAAACTGTGATTCTTGAGTGCTGGAGAGGATAGTGGAATTCCTAGTGTAGCGGTGAAATGCGTAGATATTAGGAGGAACACCAGTGGCGAAGGCGGCTATCTGGACAGTAACTGACGCTGAGGCGCGAAAGCGTGGGGAGCAAACAGG` |
| ASV1511 | 404 | `TGGGGAATATTGGACAATGGACGAAAGTCTGATCCAGCAATACCGCGTGAGTGATGAAGGCCTTAGGGTTGTAAAGCTCTTTTAGCAGGGAAGATAATGACGGTACCTGCAGAAAAAGCCCCGGCTAACTCCGTGCCAGCAGCCGCGGTAAGACGGAGGGGGCTAGCGTTGTTCGGAATTACTGGGCGTAAAGAGCGCGTAGGCGGTTTGGTAAGTTGGAAGTGAAAGCCCGGGGCTTAACCTCGGAACTGCTTTCAAAACTGCCAAGCTAGAGTATGGTAGGGGGTGATGGAATTCCTAGTGTAGAGGTGAAATTCTTAGATATTAGGAGGAACACCGGTGGCGAAGGCGGTCACCTGGGCCATTACTGACGCTGAGGCGCGAAAGCGTGGGGAGCAAACAGG` |
| ASV1211 | 404 | `TGGGGAATATTGGACAATGGGCGAAAGCCTGATCCAGCAATGCCGCGTGAGTGATGAAGGCCTTAGGGTTGTAAAGCTCTTTTACCCGGGATGATAATGACAGTACCGGGAGAATAAGCCCCGGCTAACTCCGTGCCAGCAGCCGCGGTAATACGGAGGGGGCTAGCGTTGTTCGGAATTACTGGGCGTAAAGCGCACGTAGGCGGCTTTGTAAGTTAGAGGTGAAAGCCCGGGGCTCAACTCCGGAACTGCCTTTAAGACTGCATCGCTAGAATTGTGGAGAGGTAAGTGGAATTCCGAGTGTAGAGGTGAAATTCGTAGATATTCGGAAGAACACCAGTGGCGAAGGCGACTTACTGGACACATATTGACGCTGAGGTGCGAAAGCGTGGGGAGCAAACAGG` |
| ASV5038 | 426 | `TTAAGAATCTTGCGCAATGGGGGAAACCCTGACGCAGCCACGCCGCGTGATTGATGAAGGCCTTCGGGTTGTAAAAATCGGTGAGACGGGAAGAATTAGTCAAGTCGAACAGGCTTGATGATGACGGTACCGTCCTAAAGCCCCGGCTAATTACGTGCCAGCAGCCGCGGTAATACGTATGGGGCAAGCGTTGTTCGGAATTATTGGGCGTAAAGGGCACGCAGGCGGACTATTAAGTTGGATGTATAATCTTCAGGCTCAACCTGATTCTTGCATCCAAAACTGGTGGTCTTGAGTTTTGCAGAGGAAAGCGGAATTCTCGGTGTAGCGGTGGAATGCGTAGATATCGAGAGGAACACCAATGGCGAAGGCAGCTTTCTGGGCAGAAACTGACGCTCATGTGCGAAAGTGTGGGGAGCAAACAGG` |
| ASV3052 | 425 | `TGGGGAATATTGGTCAATGGGCGAAAGCCTGAACCAGCAATGCCGCGTGAGTGATGAAGGCCTTCGGGTTGTAAAGCTCTGTCGGGAGGGACGAAAAAGGGCGGGTAACGCCGCAACTTGACGGTACCTCCAAAGGAAGCACCGGCTAACTCTGTGCCAGCAGCCGCGGTAATACAGAGGGTGCAAGCGTTGTTCGGAATTACTGGGCGTAAAGCGCGTGTAGGCTGCAATGTAAGTCGGATGTGAAAGCCCTTGGCTCAACCAAGGAAGTGCATCCGATACTGCGTCGCTTGAGTGTCGGAGGGGTCGGCGGAATTCCCGGTGTAGAGGTGAAATTCGTAGATATCGGGAGGAACACCTGTGGCGAAGGCGGCCGACTGGACGATAACTGACGCTGAGACGCGAAAGCGTGGGTAGCAAACAGG` |
| ASV4377 | 407 | `TTGGGAATTTTGCACAATGGACGAAAGTCTGATGCAGCAACGCCGCGTGCGGGATGAAGAATTTCGGTTTGTAAACCGCTTTTATAGGGGAGTAACTGTTTGAAAGTACCCTATGAATAAGTACCGGCTAACTCCGTGCCAGCAGCCGCGGTAATACGGAGGGTACAAACGTTGTCCGGAATCACTGGGCGTAAAGCGCATGTAGGTGGCCTTATACGTGGACGATTAAAGACCGTCGCTTAACGACGGGACTGTTGTCCACACGGTAAGGCTCGAGGAATGCAGGGGTAAGCGGAACTTTGAGTGTAGGAGTGATATCCGTAGATATTCAAAGGAACACCAATGGCGAAGGCAGCTTACTGGGCATTTACTGACACTCAGATGCGAAAGCGTGGGGAGCAAACGGG` |
| ASV712 | 421 | `TGGGGAATCTTGCGCAATGGCCGAGAGGCTGACGCAGCGACGCCGCGTGGGGGATGAAGCATTTCGGTGTGTAAACCCCTGTTGCTCGGGACGAACGATCCGTTTCGACGGATGTGACGGTACCGAGTGAGGAAGCACCGGCTAACTCCGTGCCAGCAGCCGCGGTAATACGGAGGGTGCGAGCGTTGTCCGGAATCACTGGGCGTAAAGGGCGCGTAGGTGGTTGGATGCGCCGGTGGTGAAAGCGCAGGGCTCAACCCTGCGTCGGCCATCGGGACGGTCCGACTGGAGCACTGTAGAGGCAGGTGGAATTCCGGGTGTAGCGGTGGAATGCGTAGAGATCCGGAAGAACACCGGTGGCGAAGGCGGCCTGCTGGGCAGTTGCTGACACTGAGGCGCGACAGCGTGGGGAGCAAACAGG` |
| ASV4694 | 404 | `TGGGGAATATTGGACAATGGGGGAAACCCTGATCCAGCCATGCCGCGTGAGTGATGAAGGCCTTAGGGTTGTAAAGCTCTTTTGTCAGGGAAGATGATGACGGTACCTGAAGAATAAGCACCGGCTAACTTCGTGCCAGCAGCCGCGGTAATACGAAGGGTGCTAGCGTTGTTCGGAATCACTGGGCGTAAAGCGCGCGTAGGCGGCTATTCAAGTCAGGGGTGAAATCCCGGAGCTTAACTCCGGAATTGCCTTTGAAACTGTATAGCTGGAGTGTCGGAGGGGATAGCGGAATTCCTAATGTAGAGGTGAAATTCGTAGATATTAGGAGGAACACCGGTGGCGAAGGCGGCTATCTGGACGACAACTGACGCTGAGGCGCGAAAGCGTGGGTATCAAACAGG` |
| ASV5345 | 404 | `TTAGGAATTTTGGGCAATGGGCGAAAGCCTGACCCAGCAACGCCGCGTGGGTGATGAAGGCCTTCGGGTCGTAAAGCCCTTTTTCAGGGGAAGATGATGACGGTACCCTGAGAATAAGCCCCGGCTAACTACGTGCCAGCAGCCGCGGTAATACGTAGGGGGCAAGCGTTGTCCGGATTTATTGGGCGTAAAGCGCGCGTAGGCGGCGCGGTATGTCGTGCGTGAAATCTCCCGGCTTAACTGGGAGGGGCCGTGCGAAACTGCCGGGCTTGAGGTCGGAAGGGGAAAGTGGAATTCCGGGTGTAGTGGTGGAATACGTAGATATCCGGAGGAACACCAGCGGCGAAGGCGGCTTTCTGGTCCGATACTGACGCTGAGGTGCGAAAGCGTGGGGAGCGATCCGG` |
| ASV5129 | 404 | `TGAGGAATCTTGGACAATGGGCGCAAGCCTGATCCAGCCATGCCGCGTGAGTGATGAAGGCCTTAGGGTTGTAAAGCTCTTTTGCCGGGGATGATAATGACCGTACCCGGAGAATAAGCCACGGCTAAATTCGTGCCAGCAGCCGCGGTAATACGAATGTGGCGAGCGTTGTTCGGAATCACTGGGCGTAAAGAGTACGTAGGCGGTTTAGCAAGTCAGGAGTGAAATCCCCAGGCTCAACCTGGGAACTGCTTTTGAAACTGCTAGACTAGAAGCTCGGAGAGGTTGGCGGAATTCTCAGTGTAGAGGTGAAATTCGCAGATATTGAGAGGAACACCAGTGGCGTAAGCGGCCAACTGGACGAGTATTGACGCTGAGGTACGAAAGCGTGGGGATCAAACAGG` |
| ASV1046 | 429 | `TAGGGAATATTGGACAATGGGCGCAAGCCTGATCCAGCCATGCCGCGTGTGTGAAGAAGGCCTTCGGGTTGTAAAGCACTTTTATCCGGAAAGAAAAGCGCTGGGTTAATACCCTGGTGTCCTGACGGTACCGGAAGAATAAGCACCGGCTAACTTCGTGCCAGCAGCCGCGGTAATACGAAGGGTGCAAGCGTTACTCGGAATTACTGGGCGTAAAGCGTGCGTAGGTGGTTTGTTAAGTCTGATGTGAAAGCCCTGGGCTCAACCTGGGAATTGCATTGGATACTGGCAGGCTAGAGTGCGGTAGAGGGTAGTGGAATTCCCGGTGTAGCAGTGAAATGCGTAGAGATCGGGAGGAACATCTGTGGCGAAGGCGACTACCTGGACCAGCACTGACACTGAGGCACGAAAGCGTGGGGAGCAAACAGG` |
| ASV1845 | 404 | `TGGGGAATATTGGACAATGGGGGCAACCCTGATCCAGCCATGCCGCGTGCGTGAAGAAGGCCTTAGGGTTGTAAAGCGCTTTTGTCAGGGAAGATAATGACTGTACCTGAAGAATAAGCCCCGGCTAACTCCGTGCCAGCAGCCGCGGTAAGACGGAGGGGGCTAGCGTTGTTCGGAATTACTGGGCGTAAAGGGCGCGTAGGCTGTTATGTGTGTCAGATGTGAAATCCCAAGGCTTAACCTTGGAACTGCATTTGAAACTGCATGACTAGAGTGAGTAAGAGGCGGACGGAATTCCGAGTGTAGCAGTGAAATGCGCAGATATTCGGAGGAACATCGGTGGCGAAGGCGGTTCGCTGGTACTCTACTGACGCTGAGGCGCGAAAGCGTGGGGAGCAAACAGG` |
| ASV2430 | 423 | `TAGGGAATATTGGGCAATGGAGGCAACTCTGACCCAGCCATGCCGCGTGCAGGAAGAAGGCGTTATGCGTTGTAAACTGCTTTTATATAGGAAGAAATAGTCCTTGCGAGGAAAGTTGACGGTACTATATGAATAAGCACCGGCTAACTCCGTGCCAGCAGCCGCGGTAATACGGAGGGTGCAAGCGTTGTCCGGATTTATTGGGTTTAAAGGGTGCGTAGGCGGTTTATTAAGTCAGTGGTGAAAGACGGTCGCTCAACGATTGCAGTGCCATTGATACTGGTAGACTTGAGTGGGATTGAGGTAGCTGGAATGGATAGTGTAGCGGTGAAATGCATAGATATTATCCAGAACACCAATTGCGTAGGCAAGTTACTAAGTCTCAACTGACGCTGAGGCACGAAAGTGTGGGTATCAAACAGG` |
| ASV934 | 430 | `TGGGGAATATTGGACAATGGGGGGAACCCTGATCCAGCCATGCCGCGTGTGTGAAGAAGGCCTTTTGGTTGTAAAGCACTTTAAGCGAGGAGGAGGCTACCGAGATTAATACTCTTGGATAGTGGACGTTACTCGCAGAATAAGCACCGGCTAACTCTGTGCCAGCAGCCGCGGTAATACAGAGGGTGCAAGCGTTAATCGGATTTACTGGGCGTAAAGCGCGCGTAGGTGGCCAATTAAGTCAAATGTGAAATCCCCGAGCTTAACTTGGGAATTGCATTCGATACTGGTTGGCTAGAGTATGGGAGAGGATGGTAGAATTCCAGGTGTAGCGGTGAAATGCGTAGAGATCTGGAGGAATACCGATGGCGAAGGCAGCCATCTGGCCTAATACTGACACTGAGGTGCGAAAGCATGGGGAGCAAACAGG` |
| ASV2704 | 404 | `TGGGGAATATTGGACAATGGGCGAAAGCCTGATCCAGCCATGCCGCGTGTGTGATGAAGGCCTTAGGGTTGTAAAGCACTTTTGGCGGGGAAGATAATGACGGTACCCGCAGAATAAGCCCCGGCTAACTTCGTGCCAGCAGCCGCGGTAATACGAAGGGGGCTAGCGTTGTTCGGAATCACTGGGCGTAAAGAGCGCGTAGGCGGTGATGTAAGTCAGAGGTGAAATCCCAGGGCTCAACCCTGGAATTGCCTTTGAAACTACATCGCTGGAGTCTTGAAGGGGGTGGTGGAATTTCTAGTGTAGGGGTGAAATCCGTAGATATTAGAAGGAACACCAGTGGCGAAGGCGGCCACCTGGTCAAGTACTGACGCTGAGGCGCGAAAGCGTGGGGATCAAACAGG` |
| ASV2167 | 407 | `TTAGGAATTTTGCGCAATGGGGGAAACCCTGACGCAGCAACGCCGCGTGGAGGATGACGGCCTTCGGGTTGTAAACTCCTTTTAGCAGGGAAGAAACAAATGACGGTATCTGCAGAAAAAGCACCGGCTAACTCTGTGCCAGCAGCCGCGGTAATACAGAGGGTGCAAGCGTTATTCGGAATCACTGGGCGTAAAGCGCTCGTAGGCTGTTAATTAAGTCAGATGTGAAATCCCTGGGCTCAATCCAGGACGTGCATCTGATACTGATTGACTAGAGTGCAGGAGAGGAAAGCGGAATTCCAGGTGTAGAGGTGAAATTCGTAGATATCTGGAGGAACAACAGAGGCGAAGGCGGCTTTCTGGACTGTCACTGACGCTGAGGAGCGAAAGCGTGGGGAGCAAACAGG` |
| ASV1004 | 429 | `TGGGGAATATTGCGCAATGGAGGAAACTCTGACGCAGCGACGCCGCGTGGGTGATGAAGGCCTTCGGGTTGTAAAGCCCTGTCGAATGGGAAAAAGGTAGTCGTGCTAATACCACGATTGCTTGATGGTACCATTAAAGGAAGCACCGGCTAACTCTGTGCCAGCAGCCGCGGTAATACAGAGGGTGCAAGCGTTGTTCGGAATTACTGGGCGTAAAGGGCGCGTAGGCGGTTTGGTAAGTCAGATGTGAAAGCCCGGGGCTCAACCCCGGAAGTGCATTTGAAACTACCAGGCTAGAGTGCAGTAGAGAATGGCGGAATTCCCAGTGGAGAGGTGAAATTCGTAGATATTGGGAGGAACACCGGTGGCGAAGGCGGCCATTTGGGCTGTAACTGACGCTGAGGCGCGAGAGCGTGGGTAGCAAACAGG` |
| ASV4165 | 431 | `TCGAGGATATTTGGCAATGGACGAAAGTCTGACCATGCGACGCCGCGTGCGGGATGAAGGCCTTCGGGTCGTAAACCGCTGTTACGAGGGACGAAGATCTCATCTGCGAACAGTGGGTGAGGTTTGACGGTACCTCGAAAGAAAGCCACGGCTAACTCCGTGCCAGCAGCCGCGGTAATACGGAGGTGGCAAGCGTTACTCGGATTTACTGGGTGTAAAGGGCACGTAGGCGGTCCTATAAGCCCTGCCTAAAAGGCTCTGGCTTAACCAGAGTATGTGGTGGGGGACTGCAGGACTTGAGGGTGGGAGAGGCGAGCGGAATTCCCGGTGTAAGGGTGAAATCTGTAGATATCGGGAGGAACACCAGTGGCGAAAGCGGCTCGCTGGCCCATTTCTGACGCTAAGGTGCGAAAGCTAGGGGAGCAAACAGG` |
| ASV5237 | 430 | `TAGGGAATATTGCTCAATGGGGGAAACCCTGAAGCAGCAACGCCGCGTGAGTGATGAAGGCCTTCGGGTTGTAAAGCTCTGTTGTACGAGACGAATGAGCGGGTAGATAATAACTATCCGCAGTGACGGTATCGTATAAGAAAGGACCGGCTAACTTCGTGCCAGCAGCCGCGGTAAGACGGGGGGTCCAAGCGTTGTTCGGAATCATTGGGCGTAAAGCGTGTGTAGGTGGCCTTGTAAGTTAGATGTGAAAGCCTTGGGCTTAACCCAAGAAGTGCGTTTAAAACTGCAAGACTTGAGTAAGGGAGAGGATCGTGGAATTCCGGGTGTAGTGGTGAAATACGTAGATATCCGGAGGAACACCAGCGGCGAAGGCGGCGATCTGGCCCTATACTGACACTGATACACGAAAGCGTGGGTAGCAAACAGG` |
| ASV2454 | 405 | `TAGGGAATATTGGACAATGGGCGAAAGCCTGATCCAGCAATACCGCGTGGATGATGAAGGCGTTATGCGTTGTAAAATCCTTTTGTCTGGGAAGATAATGACGGTACCAGAAGAATAAGCACCGACTAACTATGTGCCAGCAGTCGCGGTAATACATAGGGTGCTAGCGTTGCTCGGAATTACTGGGCGTAAAGCATGTGTAGGAGGATTTGTAAGTCAAAAGTGTAATCCCTAGGCTCAACCTAGGAACTGCTTTTGATACTACAAATCTAGAGTGAGATAGAGGATAGCGGAATTCTTAGTGTAGGGGTAAAATCCGTAGATATTAAGAGGAACACCGAAAGCGAAGGCAGCTATCTGGGTCTTTACTGACTCTGAGACATGACAGCGTGGGGAGCAAACAGG` |
| ASV2723 | 422 | `TGAGGAATATTGGTCAATGGGAGCAATCCTGAACCAGCAATGCCGCGTGTGGGATGACAGGGCTTCGCCTTGTAAACCACTGTCGGACGGGACGAAAGGAGGCCTCGGGCCTCTGGGACGGTACCGTCAAAGGAAGGGTCGGCTAACTACGTGCCAGCAGCCGCGGTAATACGTAGGACCCGAGCGTTGTCCGGATTTACTGGGTATAAAGGGTGCGTAGGCGGCCTTGTGCGTCAGAGGTGAAATATCCGGGCTTAACCCGGAGGGTGCCTTTGATACGGCAGGGCTTGAGTGCGAGAGAGGATGATGGAATTCCTGGTGTAGCGGTGAAATGCGTAGATATCAGGAGGAACACCGGTGGCGAAGGCGGTCATCTGGCTCGTAACTGACGCTGAGGCACGAAAGCGCGGGGATCAAACAGG` |
| ASV2917 | 404 | `TGGGGAATATTGGACAATGGGCGAAAGCCTGATCCAGCAATGCCGCGTGAATGATGAAGGCCTTAGGGTTGTAAAGTTCTTTTAGCAGGGAAGATAATGACGGTACCTGCAGAAAAAGCCCCGGCTAACTCCGTGCCAGCAGCCGCGGTAAGACGGAGGGGGCTAGCGTTGTTCGGAATTACTGGGCGTAAAGAGCGCGTAGGCGGTTTAGTAAGTTGGGAGTGAAAGCCCGGGGCTTAACCTCGGAATTGCTTTCAAAACTGCTAATCTAGAGTGTAGTAGGGGATGATGGAATTCCTAGTGTAGAGGTGAAATTCTTAGATATTAGGAGGAACACCGGTGGCGAAGGCGGTCATCTGGGCTACAACTGACGCTGATGCGCGAAAGCGTGGGGAGCAAACAGG` |
| ASV2946 | 428 | `TCGAGAATCTTTCACAATGGGGGCAACCCTGATGGAGCGACGCCGCGTGGAGGATAAGGTCTTCGGATTGTAAACTCCTGTCATGTGTGAACAAGGTTGTCTCGTTAATAGCGGGGCAAATTGATGGTAACACAAGAGGAAGAGACGGCTAACTCTGTGCCAGCAGCCGCGGTAATACAGAGGTCTCAAGCGTTGTTCGGAATCACTGGGCGTAAAGGGTGCGTAGGTGGCGTGGAAAGTCAGATGTGAAAGCCCGGGGCTCAACCTCGGAATAGCATCCGATACTACCATGCTAGAGTACTGAAGAGGTGACTAGAATTCTCGGTGTAGCAGTGAAATGCGTAGATATCGAGAGGAATACCAACGGCGAAGGCAGGTCACTGGGCAGTTACTGACACTGAGGCACGAAGGCCAGGGGAGCAAACGGG` |
| ASV4743 | 407 | `TGGGGAATTTTGGACAATGGGCGCAAGCCTGATCCAGCAACGCCGCGTGAGGGATGAAGGTCTTCGGATTGTAAACCTCTTCTATAGGGGACGAATAAATTGACGGTACCCTATGAATAAGTCACGGCTAACTTCGTGCCAGCAGCCGCGGTAATACGAAGGTGGCAAGCGTTACTCGGATTGACTAGGTGTAAAGCGCAGGTAGGTGGTGAAGTAAGTCCGCAATGGAATCTCCCGGCTTAACCGGGAGGGGTTTGTGGAAACTGCTTTGCTTGAGTGTGGGAGAGGATGCTGGAATTCCAGGTGTAGCGGTGAAATGCGTAGATATCTGGAGGAACACCAATGGCGAAAGCAGGCATCTGGCCCAATACTGACACTGAGCTGCGAAAGCTAGGGGAGCAAACAGG` |
| ASV2484 | 429 | `TGGGGAATTTTGGACAATGGGCGCAAGCCTGATCCAGCCATGCCGCGTGCGGGAAGAAGGCCTTCGGGTTGTAAACCGCTTTTGTCAGGGAAGAAATCCTTTGAGCTAATACCTCGGGGGGATGACGGTACCTGAAGAATAAGCACCGGCTAACTACGTGCCAGCAGCCGCGGTAATACGTAGGGTGCAAGCGTTAATCGGAATTACTGGGCGTAAAGCGTGCGCAGGCGGTTATGCAAGACAGATGTGAAATCCCCGGGCTCAACCTGGGAACTGCATTTGTGACTGCATAGCTGGAGTGCGGCAGAGGGGGATGGAATTCCGCGTGTAGCAGTGAAATGCGTAGATATGCGGAGGAACACCGATGGCGAAGGCAATCCCCTGGGCCTGCACTGACGCTCATGCACGAAAGCGTGGGGAGCAAACAGG` |
| ASV2878 | 430 | `TCGGGAATTTTGGGCAATGGGCGAAAGCCTGACCCAGCGACGCCGCGTGAGGGATGAAGGCCTTCGGGTCGTAAACCTCTGTCGGAAGGGGCGAAAGGTTCTTGGGCGAATAGGCCAAGGGCTTGACGGTACCTTCAAAGGAAGCTCCGGCTAACTCCGTGCCAGCAGCCGCGGTAATACGGAGGGAGCAAGCGTTGTTCGGAATTACTGGGCGTAAAGCGCATGTAGGCGGCCTGGCAAGTCGGGTGTGAAATCCCGGAGCTCAACTCCGGAACTGCATCCGATACTGCCAGGCTCGAGTCTCGGAGAGGATGGCGGAATTTCCGGTGTAGAGGTGAAATTCGTAGATATCGGAAGGAACACCAGTGGCGAAGGCGGCCGTCTGGACGATGACTGACGCTGAGATGCGAAAGCGTGGGGAGCAAACAGG` |
| ASV597 | 407 | `TGGGGAATTTTCCGCAATGGGCGAAAGCCTGACGGAGCAATACCGCGTGAGGGAAGAAGGCTCTTGGGTTGTAAACCTCTTTTCTTAGGGAAGAAAAAAATGACGGTACCTAAGGAATCAGCATCGGCTAACTCCGTGCCAGCAGCCGCGGTAATACGGAGGATGCAAGCGTTATCCGGAATGATTGGGCGTAAAGCGTCCGCAGGTGGCATTTCAAGTCTGCGGTTAAAGGTTCTGGCTCAACCAGAGACAAGCCGTGGAAACTGAAAAGCTAGAGTATGGTAGGGGCAGAGGGAATTCCCAGTGTAGCGGTGAAATGCGTAGAGATTGGGAAGAACACCGGTGGCGAAAGCGCTCTGCTAGGCCAAAACTGACACTGAGGGACGAAAGCTAGGGGAGCGAATGGG` |
| ASV2092 | 424 | `TAGGGAATATTGGACAATGGGCGCAAGCCTGATCCAGCCATGCCGCGTGCAGGATGAAGGCCCTATGGGTCGTAAACTGCTTTTGTACGGGAAGAAACCTCACTACGTGTAGTGAGTTGACGGTACCGTGAGAATAAGGATCGGCTAACTTCGTGCCAGCAGCCGCGGTAATACGAAGGATCCAAGCGTTGTCCGGATTTACTGGGTTTAAAGGGTGCGTAGGCGGACTTTTAAGTCAGTGGTGAAAGCCTGTAGCTTAACTATAGAATTGCCATTGAAACTGAAAGTCTCGAGTGTGGTTGAGGTAACTGGAATATATCATGTAGCGGTGAAATGCTTAGATATGATATAGAACACCAATTGCGAAGGCAGGTTACTGAGCCATAACTGACGCTGAGGCACGAAAGCGTGGGGATCAAACAGG` |
| ASV1985 | 429 | `TGGGGAATTTTGGACAATGGGCGCAAGCCTGATCCAGCAATGCCGCGTGCAGGATGAAGGCCTTCGGGTTGTAAACTGCTTTTGTACGGAACGAAAAGCCTTGGGCTAATACCCCGAGGCCATGACGGTACCGTAAGAATAAGCACCGGCTAACTACGTGCCAGCAGCCGCGGTAATACGTAGGGTGCAAGCGTTAATCGGAATTACTGGGCGTAAAGCGTCCGCAGGCGGTAATGTAAGACAGAGGTGAAATCCCCGAGCTCAACTTGGGAACGGCCTTTGTGACTGCATCACTAGAGTACGGCAGAGGGGGATGGAATTCCGCGTGTAGCAGTGAAATGCGTAGATATGCGGAGGAACACCGATGGCGAAGGCAATCCCCTGGGCCTGTACTGACGCTCATGGACGAAAGCGTGGGGAGCAAACAGG` |
| ASV1565 | 424 | `TGAGGAATATTGGACAATGGGCGCAAGCCTGATCCAGCCATGCCGCGTGCAGGAAGACGGTCCTATGGATTGTAAACTGCTTTTGTACAGGAAGAAACACTCCGACGTGTCGGAGCTTGACGGTACTGTAAGAATAAGGATCGGCTAACTCCGTGCCAGCAGCCGCGGTAATACGGAGGATCCAAGCGTTATCCGGAATCATTGGGTTTAAAGGGTCCGTAGGCGGTTTAATAAGTCAGTGGTGAAATCTGGTCGCTCAACGATCAAACGGCCATTGATACTGTTAAACTTGAATTATTAGGAAGTAACTAGAATATGTAGTGTAGCGGTGAAATGCTTAGATATTACATGGAATACCAATTGCGAAGGCAGGTTACTACTAATGGATTGACGCTGATGGACGAAAGCGTGGGGAGCGAACAGG` |
| ASV200 | 404 | `TGGGGAATATTGGACAATGGGCGAAAGCCTGATCCAGCAATGCCGCGTGAGTGATGAAGGCCTTAGGGTTGTAAAGCTCTTTTACCCGGGATGATAATGACAGTACCGGGAGAATAAGCCCCGGCTAACTCCGTGCCAGCAGCCGCGGTAATACGGAGGGGGCTAGCGTTGTTCGGAATCACTGGGCGTAAAGCGTGCGTAGGCGGTGACTCAAGTCAGAGGTGAAAGCCTGGAGCTCAACTCCAGAACTGCCTTTGAAACTAGGTCGCTAGAATGTCGGAGAGGTGAGTGGAATTCCGAGTGTAGAGGTGAAATTCGTAGATATTCGGAAGAACACCAGTGGCGAAGGCGGCTCACTGGACGACTATTGACGCTGAGGCACGAAAGCGTGGGGAGCAAACAGG` |
| ASV153 | 404 | `TGGGGAATATTGGACAATGGGCGAAAGCCTGATCCAGCAATGCCGCGTGAGTGATGAAGGCCTTAGGGTTGTAAAGCTCTTTTACCAGGGATGATAATGACAGTACCTGGAGAATAAGCTCCGGCTAACTCCGTGCCAGCAGCCGCGGTAATACGGAGGGAGCTAGCGTTGTTCGGAATTACTGGGCGTAAAGAGTACGTAGGCGGTGATTCAAGTCAGAGGTGAAAGCCTGGAGCTCAACTCCAGAACTGCCTTTGAAACTAGATCGCTAGAATTATGGAGAGGTTAGTGGAATTCCGAGTGTAGAGGTGAAATTCGTAGATATTCGGAAGAACACCAGTGGCGAAGGCGACTAACTGGACATATATTGACGCTGAGGTACGAAAGCGTGGGGAGCAAACAGG` |
| ASV1663 | 429 | `TGGGGAATTTTGGACAATGGGCGCAAGCCTGATCCAGCCATGCCGCGTGCGGGAAGAAGGCCTTCGGGTTGTAAACCGCTTTTGTCCGGGAAGAAATTGACTGGGTTAATACCCCGGTTAGATGACGGTACCGGAAGAATAAGGACCGGCTAACTACGTGCCAGCAGCCGCGGTAATACGTAGGGTCCAAGCGTTAATCGGAATTACTGGGCGTAAAGCGTGCGCAGGCGGTTTCGCAAGTCAGATGTGAAAGCCCCGGGCTTAACCTGGGAACTGCATTTGAAACTACGAGGCTAGAGTGTGTCAGAGGGGGGTAGAATTCCACGTGTAGCAGTGAAATGCGTAGAGATGTGGAGGAATACCGATGGCGAAGGCAGCCCCCTGGGATGACACTGACGCTCATGCACGAAAGCGTGGGGAGCAAACAGG` |
| ASV421 | 424 | `TAGGGAATATTGGTCAATGGGGGCAACCCTGAACCAGCCATGCCGCGTGCAGGAATAAGGCCCTATGGGTCGTAAACTGCTTTTGAACGGGAAGAAATCTATTTACGTGTAGATAGTTGACGGTACCGTTAGAATAAGGATCGGCTAACTTCGTGCCAGCAGCCGCGGTAATACGAAGGATCCAAGCGTTGTCCGGATTTACTGGGTTTAAAGGGTGCGTAGGCGGACTCATAAGTCAGTGGTGAAAGCCTTCAGCTTAACTGAAGAACTGCCATTGAAACTGTGAGTCTCGAGTTCGGTTGAGGTCATTGGAATGTAACATGTAGCGGTGAAATGCATAGATATGTTACAGAACACCGATTGCGAAGGCAGATGACTAAACCGAAACTGACGCTGAGGCACGAAAGCGTGGGGATCAAACAGG` |
| ASV4773 | 429 | `TGGGGAATATTGGACAATGGGGGCAACCCTGATCCAGCCATGCCGCGTGTGTGAAGAAGGCCTTCGGGTTGTAAAGCACTTTTGTCCGGAACGAAAAGCGTTCGGTTAATAACCGGGCGTCATGACGGTACCGGAAGAATAAGCACCGGCTAACTTCGTGCCAGCAGCCGCGGTAATACGAAGGGTGCAAGCGTTACTCGGAATTACTGGGCGTAAAGCGTGCGTAGGTGGTGCGTTAAGTCTGTCGTGAAAGCCCCGGGCTCAACCTGGGAATGGCGATGGATACTGGCGCGCTAGAGTGCGGTAGAGGGTGGTGGAATTCCCGGTGTAGCAGTGAAATGCGTAGAGATCGGGAGGAACATTCGTGGCGAAGGCGGCCACCTGGACCAGCACTGACACTGAGGCACGAAAGCGTGGGGAGCAAACAGG` |
| ASV1181 | 405 | `TGGGGAATCTTGCGCAATGGGCGAAAGCCTGACGCAGCCATGCCGCGTGAATGATGAAGGTCTTAGGATTGTAAAGTTCTTTCGCTCGTGACGATGATGACGGTAACGAGAGAAGAAGCCCCGGCTAACTTCGTGCCAGCAGCCGCGGTAATACGAAGGGGGCTAGCGTTGTTCGGAATTACTGGGCGTAAAGCGCGCGTAGGCGGTTTTGCAAGCCAGAGGTGAAATGCCTGAGCTCAACTTAGGAATTGCCTTTGGGACTGCGAGGCTTTGAGGACTGGAGAGGTGAGTGGAATTCCCAGTGTAGAGGTGAAATTCGTAGATATTGGGAAGAACACCAGTGGCGAAGGCGGCTCACTGGCCAGTTTCTGACGCTGCAGCGCGAAAGCGTGGGGATCAAACAGG` |
| ASV340 | 404 | `TGGGGAATATTGGACAATGGGCGCAAGCCTGATCCAGCAATTCCGCGTGGGTGAAGAAGGTCTTCGGATTGTAAAGCCCTTTCGTTGGGGACGATGATGACGGTACCCAAAGAAGAAGCCCCGGCTAACTTCGTGCCAGCAGCCGCGGTAATACGAAGGGGGCTAGCGTTGCTCGGAATGACTGGGCGTAAAGGGCGCGTAGGCGGCACAACTCGTCAGGCGTGAAATTCCCGGGCTTAACCTGGGGACTGCGTTTGATACGGTTGAGCTAGAGGATGGAAGAGGCTCGTGGAATTCCCAGTGTAGAGGTGAAATTCGTAGATATTGGGAAGAACACCGGTGGCGAAGGCGGCGAGCTGGTCCATTACTGACGCTGAGGCGCGAAAGCGTGGGGAGCAAACAGG` |
| ASV1174 | 424 | `TGAGGAATATTGGACAATGGGCGCAAGCCTGATCCAGCCATGCCGCGTGCAGGATGACGGTCCTATGGATTGTAAACTGCTTTTGCACAGGAAGAAACAACTCTACGTGTAGAGTCTTGACGGTACTGTGAGAATAAGGATCGGCTAACTCCGTGCCAGCAGCCGCGGTAATACGGAGGATCCAAGCGTTATCCGGAATCATTGGGTTTAAAGGGTCCGTAGGCGGTTTAGTAAGTCAGTGGTGAAAGCCCATCGCTCAACGGTGGAACGGCCATTGATACTGCTAGACTTGAATTATTAGGAAGTAACTAGAATATGTAGTGTAGCGGTGAAATGCTTAGAGATTACATGGAATACCAATTGCGAAGGCAGGTTACTACTAATGGATTGACGCTGATGGACGAAAGCGTGGGTAGCGAACAGG` |
| ASV3540 | 429 | `TGGGGAATATTGGACAATGGGGGCAACCCTGATCCAGCGATGCCGCGTGAGTGAAGAAGGCCTTCGGGTCGTAAAGCTCTTTAGGTTGGGAAGAAGTGCATTCGGTGAATAGCCGAGTGTGTTGACGGTACCGACAGAATAAGCACCGGCAAACTCTGTGCCAGCAGCCGCGGTAATACAGAGGGTGCGAGCGTTAATCGGAGTTACTGGGCGTAAAGGGCGCGTAGGCGGTAAGATGTGTGTGATGTGAAAGCCCCGGGCTTAACCTGGGAAGTGCATCGCAAACTATCTAACTGGAGTAGATGAGAGGGTGGCGGAATTTCCGGTGTAGCGGTGAAATGCGTAGAGATCGGAAGGAACGTCAATGGCGAAGGCAGCCACCTGGCATCATACTGACGCTGAGGCGCGAAAGCGTGGGGAGCGAACAGG` |
| ASV2517 | 424 | `TAAGGAATATTGGTCAATGGACGAAAGTCTGAACCAGCCATGCCGCGTGGAGGATGAAGGTCCTCTGGATTGTAAACTTCTTTTATATGGGACGAAAAAAGGCTTTTCTAAGTCGTCTGACGGTACCATATGAATAAGCACCGGCTAACTCCGTGCCAGCAGCCGCGGTAATACGGAGGGTGCAAGCGTTATCCGGATTCACTGGGTTTAAAGGGTGCGTAGGTGGGTTGGTAAGTCAGTGGTGAAATCCCCGAGCTTAACTCGGGAACTGCCATTGATACTATTAGTCTTGAATACCGTGGAGGTCAGCGGAATATGTCATGTAGCGGTGAAATGCTTAGATATGACATAGAACACCAATTGCGAAGGCAGCTGGCTACTCGAGTATTGACACTGAGGCACGAAAGCGTGGGGATCAAACAGG` |
| ASV4759 | 429 | `TGGGGAATTTTGGACAATGGGGGCAACCCTGATCCAGCAATGCCGCGTGTGTGAAGAAGGCCTTCGGGTTGTAAAGCACTTTTGTTCGGAAAGAAATCGCCCTGGTTAATACCTGGGGTGGATGACGGTACCGGAAGAATAAGCACCGGCTAACTACGTGCCAGCAGCCGCGGTAATACGTAGGGTGCGAGCGTTAATCGGAATTACTGGGCGTAAAGCGTGCGCAGGCGGTTGTGCAAGACAGATGTGAAATCCCCGGGCTTAACCTGGGAACTGCATTTGTGACTGCACGGCTAGAGTGTGTCAGAGGGGGGTAGAATTCCACGTGTAGCAGTGAAATGCGTAGATATGTGGAGGAATACCGATGGCGAAGGCAGCCCCCTGGGATAACACTGACGCTCATGCACGAAAGCGTGGGGAGCAAACAGG` |
| ASV4189 | 429 | `TGGGGAATATTGGACAATGGGCGCAAGCCTGATCCAGCCATACCGCGTGGGTGAAGAAGGCCTTCGGGTTGTAAAGCCCTTTTGTTGGGAAAGAAATCCAGCCGGCTAATACCTGGTTGGGATGACGGTACCCAAAGAATAAGCACCGGCTAACTTCGTGCCAGCAGCCGCGGTAATACGAAGGGTGCAAGCGTTACTCGGAATTACTGGGCGTAAAGCGTGCGTAGGTGGTTGTTTAAGTCTGTTGTGAAAGCCCTGGGCTCAACCTGGGAACTGCAGTGGAAACTGGACAACTAGAGTGTGGTAGAGGGTAGCGGAATTCCCGGTGTAGCAGTGAAATGCGTAGAGATCGGGAGGAACATCCATGGCGAAGGCAGCTACCTGGACCAACACTGACACTGAGGCACGAAAGCGTGGGGAGCAAACAGG` |
| ASV3582 | 429 | `TGGGGAATATTGGACAATGGGCGAAAGCCTGATCCAGCCATGCCGCGTGTGTGAAGAAGGCCCTAGGGTTGTAAAGCACTTTCAGTGGGGAGAAAGGTTTGCCACCTAATACGTGGTGAAATTGATGTTACCCACAGAAGAAGCACCGGCTAACTCCGTGCCAGCAGCCGCGGTAATACGGAGGGTGCAAGCGTTAATCGGAATTACTGGGCGTAAAGCGCATGTAGGTGGTTTGCTAAGCCAGCTGTGAAATCCCCGGGCTCAACCTGGGACTTGCAGTTGGAACTGGTAAGCTAGAGTAGGGTAGAGGGGTGTGGAATTCCAGGTGTAGCGGTGAAATGCGTAGATATCTGGAGGAACATCAGTGGCGAAGGCGACACCCTGGACTCATACTGACACTGAGATGCGAAAGCGTGGGGAGCAAACAGG` |
| ASV4285 | 405 | `TGAGGAATATTGCACAATGGGCGAAAGCCTGATGCAGCGACGCCGCGTGGAGGATGAAGGATTTCGGTCTGTAAACTCCTGTCAGTAGGGAAGAGAATGGACGGTACCTACTGAGGAAGCTTCGGCTAACTACGTGCCAGCAGCCGCGGTAAGACGTAGGAAGCAAGCGTTGTCCGGAATTACTGGGCGTAAAGAGCTCGTAGGCGGGATCGTAAGTCTTAGAGTAAATCTCACGGCTCAACCGTGGGGCCTTCTGAGATACTGCGATTCTTGAGGATGTCAGAGGAAAGTGGAATTCCCGGTGTAGCGGTGAAATGCGTAGATATCGGGAGGAACACCAGTGGCGAAGGCGACTTTCTGGGACATTCCTGACGCTGAGGAGCGAAAGCGTGGGGAGCAAACGGG` |
| ASV2784 | 429 | `TGTGGAATATTGGACAATGGGCGAAAGCCTGATCCAGCAACGCTGCGTGAACGATGAAGGCCTTCGGGTCGTAAAGTTCAAAACTCCGAGAAGAATAACGTGTCGTGAATAACTACACATATGACGGTATCGGAGGTTAAGCACCGGCTAACTACGTGCCAGCAGCTGCGGTAATACGTAGGGTGCAAGCATTAATCGGAATTATTGGGCGTAAAGGGCGCGTAGGCGGATAAACAAGTCAGATGTGAAATTCCAAAGCTCAACTTTGGAGCTGCGTCTGAAACTATTTGTCTCAAGAGGTTTGGAGGAGAAAGCGGAATTCCACAAGTAGCGGTGAAATGCGTAGATATGTGGAAGAACACCTGTGGCGAAAGCGGCTTTCTATCCTTTACCTGACGCTGAGGCGCGAGAGCGTGGGGAGCAAACAGG` |
| ASV6 | 424 | `TGAGGAATATTGGACAATGGGCGCAAGCCTGATCCAGCCATGCCGCGTGCAGGATGACGGTCCTATGGATTGTAAACTGCTTTTGTACAGGAAGAAACACTCCCTCGTGAGGGAGCTTGACGGTACTGTAAGAATAAGGATCGGCTAACTCCGTGCCAGCAGCCGCGGTAATACGGAGGATCCAAGCGTTATCCGGAATCATTGGGTTTAAAGGGTCCGTAGGCGGTTTAGTAAGTCAGTGGTGAAAGCCCATCGCTCAACGGTGGAACGGCCATTGATACTGCTAAACTTGAATTATTAGGAAGTAACTAGAATATGTAGTGTAGCGGTGAAATGCTTAGAGATTACATGGAATACCAATTGCGAAGGCAGGTTACTACTAATGGATTGACGCTGATGGACGAAAGCGTGGGTAGCGAACAGG` |
| ASV1157 | 429 | `TGGGGAATTTTGGACAATGGGCGCAAGCCTGATCCAGCAATGCCGCGTGCAGGATGAAGGCCTTCGGGTTGTAAACTGCTTTTGTACGGAGCGAAACGGCGGGTTCTAATAAAACCCGCTAATGACGGTACCGTAAGAATAAGCACCGGCTAACTACGTGCCAGCAGCCGCGGTAATACGTAGGGTGCAAGCGTTAATCGGAATTACTGGGCGTAAAGCGTGCGCAGGCGGTTATATAAGTCAGATGTGAAATCCCCGGGCTCAACCTGGGACCTGCATTTGAGACTGTATAGCTAGAGTACGGTAGAGGGGGATGGAATTCCGCGTGTAGCAGTGAAATGCGTAGATATGCGGAGGAACACCGATGGCGAAGGCAATCCCCTGGACCTGTACTGACGCTCATGCACGAAAGCGTGGGGAGCAAACAGG` |
| ASV1818 | 407 | `TAGGGAATATTGCACAATGGACGAAAGTCTGATGCAGCGACGCCGCGTGAGTGATGAAGGCCTTCGGGTCGTAAAGCTCTGTCGCATGGGACTAACACAATGAAGGTACCATGCAAGAAAGGATCGGCTAACTTCGTGCCAGCAGCCGCGGTAAGACGAGGGATCCTAGCGTTGTTCGGAATTATTGGGCGTAAAGCGGGTGTAGGCGGCTCTATAAGTCAGGTGTGAAAGCCCGAGGCTCAACCTCGGAAGTGCACTTGATACTGTAGAGCTTGAATGTGGTAGAGGTTACTGGAATTGCTGGTGTAGTGGTGAAATACGTAGATATCAGCAGGAATACCGGAGGCGAAGGCGGGTAACTGGGCCAACATTGACGCTGAGACCCGAAAGCGTGGGGATCAAACAGG` |
| ASV1352 | 429 | `TGGGGAATATTGGACAATGGGCGCAAGCCTGATCCAGCAATGCCGCGTGTGTGAAGAAGGCCTTCGGGTTGTAAAGCACTTTCGGGGGGGAGAAAGAGGCCATGCCTAATAGGGATGGAAGAAGATGTTACCCCCAGAAGAAGCACCGGCTAACTCTGTGCCAGCAGCCGCGGTAATACAGAGGGTGCGAGCGTTAATCGGAATTACTGGGCGTAAAGGGTGCGTAGGTGGTTATGTAAGTTAGGTGTGAAAGTCCCGGGCTTAACCTGGGGATTGCGCCTAATACTGTGTAGCTAGAGTCCTGTAGAGGAAAGTGGAATTTCCGGTGTAGCGGTGAAATGCGTAGATATCGGAAGGAACATCAGTGGCGAAGGCGACTTTCTGGATAGAGACTGACACTGAGGCACGAAGGCATGGGGAGCAAACAGG` |
| ASV4245 | 404 | `TGGGGAATATTGGACAATGGGCGCAAGCCTGATCCAGCAATGCCGCGTGAGTGAAGAAGGCCTTCGGGTTGTAAAGCTCTTTCGATGGGGACGATGATGACGGTACCCATAGAAGAAGCCCCGGCAAACTTCGTGCCAGCAGCCGCGGTAATACGAAGGGGGCAAGCGTTGTCCGGAATTACTGGGCGTAAAGGGCGCGCAGGCGGGTGGTCAAGTTAGATGTGAAAGCCCCGGGCTCAACCTGGGAACAGCATTTAAGACTGGTCATCTTGAATACGAGAGAGGGTTGTGGAATTCCCAGTGTAGAGGTGAAATTCGTAGATATTGGGAAGAACACCAGTGGCGAAGGCGGCAACCTGGCTCGATATTGACGCTGAGGCGCGAAAGCGTGGGGAGCAAACAGG` |
| ASV4299 | 429 | `TGGGGAATTTTGGACAATGGGCGAAAGCCTGATCCAGCCATTCCGCGTGAGTGAAGAAGGCCTTCGGGTTGTAAAGCTCTTTCGCAAGGGAAGAACACTTAATCTCTAACATAGATTGAGGTTGACGGTACCTTGATAAGAAGCACCGGCTAACTACGTGCCAGCAGCCGCGGTAATACGTAGGGTGCGAGCGTTAATCGGAATTACTGGGCGTAAAGCGTGCGCAGGCTGTTTTGTAAGTCAGATGTGAAATCCCTGGGCTCAACCTAGGAACTGCGTTTGAAACTACAAGACTAGAATATGTCAGAGGGGGGTAGAATTCCACGTGTAGCAGTGAAATGCGTAGAGATGTGGAGGAATACCAATGGCGAAGGCAGCCCCCTGGGATAATATTGACGCTCATGCACGAAAGCGTGGGGAGCAAACAGG` |
| ASV2267 | 429 | `TGGGGAATTTTGGACAATGGGCGCAAGCCTGATCCAGCAATGCCGCGTGCAGGATGAAGGCCTTCGGGTTGTAAACTGCTTTTGTACGGAGCGAAACGGCCTACCCTAATACGGTGGGCTAATGACGGTACCGTAAGAATAAGCACCGGCTAACTACGTGCCAGCAGCCGCGGTAATACGTAGGGTGCGAGCGTTAATCGGAATTACTGGGCGTAAAGCGTGCGCAGGCGGTTATGTAAGACAGATGTGAAATCCCCGGGCTCAACCTGGGACCTGCATTTGTGACTGCATAGCTAGAGTACGGTAGAGGGGGATGGAATTCCGCGTGTAGCAGTGAAATGCGTAGATATGCGGAGGAACACCGATGGCGAAGGCAATCCCCTGGACCTGTACTGACGCTCATGCACGAAAGCGTGGGGAGCAAACAGG` |
| ASV4206 | 422 | `TAGGGAATTTTGCGCAATGGGGGAAACCCTGACGCAGCAACGCCATGTGTGGGATGAAGCATTTCGGTGTGTAAACCACTGTCGGCAGGGAATAAAGCCCGCGTTTACGCGGGGGTGAATGTACCTGCAGAGGAAGCCCCGGCTAACTTCGTGCCAGCAGCCGCGGTAATACGAGGGGGGCAAGTGTTGTTCGGAATCACTGGGCGTAAAGGGAGCGTAGGCGGAAGCTTAAGTTGGGTGTTTAAAACCGGGGCCCAACCCCGGGGCGGCACTCAAAACTGGGCTTCTTGAATGGGACAGAGGCTAGTGGAATTGGGGGTGTAGCGGTGGAATGCGTAGAGATCCCCAAGAACACCGATTGCGAAGGCAGCTAGCTGGGTCCACATTGACGCTGAGGCTCGAAAGCATGGGGAGCAAACAGG` |
| ASV5438 | 404 | `TGGGGAATATTGGACAATGGGGGCAACCCTGATCCAGCCATGCCGCGTGAGTGATGAAGGCCTTAGGGTTGTAAAGCTCTTTTGTCCGGGAAGATAATGACTGTACCGGAAGAATAAGCCCCGGCTAACTTCGTGCCAGCAGCCGCGGTAATACGAAGGGGGCTAGCGTTGTTCGGAATCACTGGGCGTAAAGCGCACGTAGGCGGATTGTTAAGTCAGAGGTGAAATCCCGGAGCTCAACTTCGGAACTGCCTTTGATACTGGCAATCTCGAGTTTGGAAGAGGTTGGTGGAACTGCGAGTGTAGAGGTGAAATTCGTAGATATTCGCAAGAACACCAGTGGCGAAGGCGGCCAACTGGTCCAATACTGACGCTGAGGTGCGAAAGCGTGGGGAGCAAACAGG` |
| ASV69 | 424 | `TAGGGAATATTGGGCAATGGGCGCAAGCCTGACCCAGCCATGCCGCGTGCAGGAAGAAGGCGCTCAGCGTCGTAAACTGCTTTTATCTGGGAAGAATGCTGGTTGTGCGCAACCATTTGACGGTACCAGAGGAATAAGCACCGGCTAACTCCGTGCCAGCAGCCGCGGTAATACGGAGGGTGCAAGCGTTGTCCGGATTTATTGGGTTTAAAGGGTGCGTAGGTGGGTGTTTAAGTCTGGTTTGAAAGCAAGCGGCTCAACCGTTTGATGTGGCTGGAAACTGGGCATCTTGAATGGGTTAGCGGTAGCCGGAATGGGTCATGTAGCGGTGAAATGCATAGATATGACCCGGAACACCGATTGCGAAGGCAGGCTACTGGGACTTGATTGACACTGAGGCACGAGAGCATGGGTAGCGAACAGG` |
| ASV1857 | 429 | `TGGGGAATTTTGGACAATGGGGGCAACCCTGATCCAGCCATGCCGCGTGAGTGAAGAAGGCCTTCGGGTTGTAAAGCTCTTTCGGCCGGGAAAAAATCGCATGGGTTAATACCCTGTGTGGATGATGGTACCGGAATAAGAAGCACCGGCTAACTACGTGCCAGCAGCCGCGGTAATACGTAGGGTGCGAGCGTTAATCGGAATTACTGGGCGTAAAGCGTGCGCAGGCGGTTTTGTAAGACAGGCGTGAAATCCCCGGGCTCAACCTGGGAACTGCGCTTGTGACTGCAAGGCTAGAGTACGGCAGAGGGGGGTGGAATTCCACGTGTAGCAGTGAAATGCGTAGAGATGTGGAGGAACACCAATGGCGAAGGCAGCCCCCTGGGTCGATACTGACGCTCATGCACGAAAGCGTGGGTAGCAAACAGG` |
| ASV3002 | 401 | `TGGGGAATATTGGACAATGGGCGAAAGCTTGATCCAGCAATATCATGTGAGTGATGAAGACAATGTTTGTAAAGCTCTAAAAACAAGGAGGATAATGACGTTACTTGTAGAAAAGCCCTGGCTAACTTCGTGCCAGCAGCCGCGGTAAAACGGAGGGGGCAAGTGTTACTCATGATGACTGGGCGTAAAGGGTACGTAGATGGTTTTTTAAGTTAAAAATAAAAGAATAGTGTAAGACATTATAAATATTTTTAATACTGAAAATCTAGAGTGCGAGAGAGGAAAATGGTATTCTTATTGTAGAGGTGAAATTCTGAGATAATAGGAGGACGATCGAAGGCGAAGGCAATTTTCTGGTTCGCTACTGACATTGAGGTACGAAAGCATGGGGAGCAAACAGG` |
| ASV2699 | 404 | `TGGGGAATATTGGACAATGGGCGAAAGCCTGATCCAGCAATACCACGTGAGTGATGAAGGCCTTAGGGTTGTAAAACTCTTTTAGTAGGGAAGATAATGACTGTACCTACAGAAAAAGCACCGGCTAACTTCGTGCCAGCAGCCGCGGTAATACGAAGGGTGCTAGCGTTGTTCGGAATCATTGGGCGTAAAGCGTGCGTAGGCGGCTTGGTAAGTCAGCTGTGAAAGCCCAGGGCTCAACCCTGGAATTGCAGTTGAAACTGCCTGGCTAGAGTATGAAGAGGGATAGTGGAATTCTTAGTGTAGGGGTGAAATCCGTAGATATTAAGAGGAACACCGGTGGCGAAAGCGACTATCTATGTCATCACTGACGCTGTTGCACGAAAGCGTGGGGAGCAAACAGG` |
| ASV1387 | 424 | `TAGGGAATATTGCGCAATGGACGAAAGTCTGACGCAGCAACTCCACGTGTGGGATGACGCATTTCGGTGTGTAAACCACTGTCGGATGGGAATAACGGCGCGATGTTGAATCGCGTATGAATGTACCATCAAAGGAAGCATCGGCTAACTTCGTGCCAGCAGCCGCGGTAATACGAGGGATGCAAGCGTTGTTCGGAATCACTGGGCGTAAAGAGAGCGTAGGCGGGAAAATAAGTTTGGTGTTTAAAGCAAGGGCCTAACCCTTGTGTGGCACTGAATACTGTTTTTCTTGAGTAGGTTAGGGGTTATCGGAATTTCTGGTGTAGCGGTGGAATGCGTAGATATCAGAAAGAACACCAATGGCGAAGGCAGATAACTGGGACCATACTGACGCTGAGGCTCGAAAGTGCGGGGAGCAAACAGG` |
| ASV4148 | 429 | `TGGGGAATATTGGACAATGGGGGCAACCCTGATCCAGCAATGCCGCGTGTGTGAAGAAGGCCTGCGGGTTGTAAAGCACTTTTGGCAGGTAGGAAGGGTGCCGGATTAACACTCCGGTGCATTGACTTAACCTGCAGAATAAGCACCGGCTAACTCTGTGCCAGCAGCCGCGGTAATACAGAGGGTGCAAGCGTTAATCGGAATTACTGGGCGTAAAGCGCGCGTAGGCCGTTGAGTAAGTCGGATGTGAAATCCCCGGGCTTAACCTGGGAATTGCATTCGATACTGCTTGACTAGAGTGTGGAAGAGGGAAGCGGAATTTCCGGTGTAGCGGTGAAATGCGTAGATATCGGAAGGAACACCAGTGGCGAAGGCGGCTTCCTGGTCCAACACTGACGCTGAGGCGCGAAAGCGTGGGGAGCGAACAGG` |
| ASV3128 | 424 | `TAAGGAATATTGGTCAATGGACGCAAGTCTGAACCAGCCATGCCGCGTGGAGGATGAAGGTCCTCTGGATTGTAAACTTCTTTTCTCAATAAAGAAAACTCTTGTTTTTACGAGATTTGACGGTAATTGAGGAATAAGCACCGGCTAACTCCGTGCCAGCAGCCGCGGTAATACGGAGGGTGCAAGCGTTATCCGGATTCACTGGGTTTAAAGGGAGCGTAGGTGGGTTTGTAAGTCAGTGGTGAAATACCCGAGCTTAACTTGGGAACTGCCATTGATACTGCAGATCTTGAATATCGTAGAGGTAAGCGGAATATGTCATGTAGCGGTGAAATGCTTAGATATGACATAGAACACCGATTGCGAAGGCAGCTTACTATGCGTATATTGACACTAATGCTCGAAAGCGTGGGGATCAAACAGG` |
| ASV1668 | 429 | `TGGGGAATATTGGACAATGGGGGCAACCCTGATCCAGCCATGCCGCGTGTGTGAAGAAGGCCTTCGGGTTGTAAAGCACTTTCAGTGGGGAGAAAGGCGATTTACCTAACACGTAAGTTGATTGATGCTACCCACAGAAGAAGCACCGGCTAACTCCGTGCCAGCAGCCGCGGTAATACGGAGGGTGCAAGCGTTAATCGGAATTACTGGGCGTAAAGCGCACGTAGGTGGTTTGTTAAGCTAGCTGTGAAATCCCCGGGCTCAACCTGGGCACTGCAGTTAGAACTGGCAAGCTAGAGTAGGGTAGAGGGGTGTGGAATTCCAGGTGTAGCGGTGAAATGCGTAGATATCTGGAGGAACATCAGTGGCGAAGGCGACACCCTGGACTCATACTGACACTGAGGTGCGAAAGCGTGGGGAGCAAACAGG` |
| ASV318 | 424 | `TGAGGAATATTGGTCAATGGGCGATAGCCTGAACCAGCCAAGTAGCGTGAAGGATGACTGCCCTATGGGTTGTAAACTTCTTTTATAGGGGAATAAAGTCCGATACGTGTATCGGTTTGTATGTACCCTACGAATAAGGATCGGCTAACTCCGTGCCAGCAGCCGCGGTAATACGGAGGATTCGAGCGTTATCCGGATTTATTGGGTTTAAAGGGTGCGTAGGTGGAAGATTAAGTCAGTTGTGAAAGTTTGCGGCTCAACCGTAAAATTGCAGTTGATACTGGTTTTCTTGAGTACACATAAGGTAGGCGGAATTCGTGGTGTAGCGGTGAAATGCTTAGATATCACGAAGAACTCCAATTGCGAAGGCAGCTTACCGGGGTGTAACTGACACTGAGGCACGAAAGTGTGGGTATCAAACAGG` |
| ASV5480 | 404 | `TGGGGAATCTTGCGCAATGGAGGCAACTCTGACGCAGCCATGCCGCGTGGGTGAAGAAGGCCTTAGGGTTGTAAAACCCTTTTAGTGGTGAAGATGATGACGGTAGCCACAGAAAAAGCTCCGGCTAACTTCGTGCCAGCAGCCGCGGTAATACGAAGGGAGCAAGCGTTGTTCGGAATTACTGGGCGTAAAGGGCGTGTAGGCGGATGTATCAGTCAGAGGTGAAATCCCTGGGCTTAACCTAGGAACTGCCTTTGAGACGTTACATCTAGAATTCGGGAGAGGATAGTGGAATACCCAGTGTAGAGGTGAAATTCGTAGATATTGGGTGGAACACCAGTGGCGAAGGCGACTATCTGGACCGACATTGACGCTGAGGCGCGAAAGCGTGGGGAGCAAACAGG` |
| ASV3076 | 429 | `TGGGGAATTTTGGACAATGGGGGCAACCCTGATCCAGCAATGCCGCGTGAGTGAAGAAGGCCTTCGGGTTGTAAAGCTCTTTTGTCAGGGAAGAAATCCTTAATGATAATACCATTAGGGGATGACGGTACCTGAAGAATAAGCACCGGCTAACTACGTGCCAGCAGCCGCGGTAATACGTAGGGTGCAAGCGTTAATCGGAATTACTGGGCGTAAAGCGTGCGCAGGCGGCTTCGCAAGTCAGATGTGAAATCCCCGAGCTCAACTTGGGAACTGCATTTGAAACTACGAAGCTAGAGTGTGTCAGAGGGAGGTAGAATTCCACGTGTAGCAGTGAAATGCGTAGAGATGTGGAGGAATACCAGTGGCGAAGGCGGCCTCCTGGGATAACACTGACGCTCATGTACGAAAGCGTGGGGAGCAAACAGG` |
| ASV4978 | 404 | `TCGGGAATTTTGGGCAATGGGCGAAAGCCTGACCCAGCAACGCCGCGTGAAGGATGAAGTATTTCGGTATGTAAACTTCGAAAGAATAGGAAGAATAAATGACGGTACTATTTATAAGGTCCGGCTAACTACGTGCCAGCAGCCGCGGTAATACGTAGGGACCAAGCGTTGTTCGGATTTACTGGGCGTAAAGGGCGCGTAGGCGGCTCTGTAAGTCACTTGTGAAATCTCTGAGCTTAACTCAGAACGGCCAAGTGATACTGCAGAGCTAGAGTGTAGAAGGGGCAATCGGAATTCTTGGTGTAGCGGTGAAATGCGTAGATATCAAGAGGAACACCTGAGGTGAAGACGGGTTGCTGGTCTAACACTGACGCTGAGGCGCGAAAGCCAGGGGAGCAAACGGG` |
| ASV5445 | 407 | `TAGGGAATATTGCACAATGGAGGAAACTCTGATGCAGCGACGCCGCGTGAGTGATGAAGGCCTTCGGGTTGTAAAGCTCTGTTCTCAGGGAATAATAAATTGAAGGTACCTGAGAAGAAAGGACCGGCTAACTTCGTGCCAGCAGCCGCGGTAAGACGGGGGGTCCAAGCGTTGTTCGGAATTATTGGGCGTAAAGCGGGTGTAGGTGGCTTTGTAAGTCAGAAGTGAAAGCCCTGGGCTCAACCCAGGAAGTGCTTTTGATACTGCGAAGCTTGAGTGTGGGAGAGGATAGTAGAATTCCTAGTGTAGTGGTGAAATACGTAGATATTAGGAGGAATACCGGTGGCGAAGGCGGCTATCTGGCCCAACACTGACACTCAGACCCGAAAGCGTGGGGATCAAACAGG` |
| ASV3077 | 424 | `TGAGGAATATTGGTCAATGGACGCAAGTCTGAACCAGCCATGCCGCGTGCAGGATGACGGTCCTATGGATTGTAAACTGCTTTTGTACAGGAAGAAACACTCCCTCGTGAGGGAACTTGACGGTACTGTAAGAATAAGGATCGGCTAACTCCGTGCCAGCAGCCGCGGTAATACGGAGGATCCAAGCGTTATCCGGAATCATTGGGTTTAAAGGGTCCGTAGGCGGTTTAGTAAGTCAGTGGTGAAAGCCCATCGCTCAACGGTGGAACGGCCATTGATACTGCTAGACTTGAATTACTAGGAAGTAACTAGAATATGTAGTGTAGCGGTGAAATGCTTAGAGATTACATGGAATACCAATTGCGAAGGCAGGTTACTACTAGTTTATTGACGCTGATGGACGAAAGCGTGGGTAGCGAACAGG` |
| ASV249 | 429 | `TGGGGAATATTGGACAATGGGCGCAAGCCTGATCCAGCCATGCCGCGTGTGTGAAGAAGGCCTTCGGGTTGTAAAGCACTTTTGTTGGGAAAGAAATCCTGTCGATTAATACTCGGCGGGGATGACGGTACCCAAAGAATAAGCACCGGCTAACTTCGTGCCAGCAGCCGCGGTAATACGAAGGGTGCAAGCGTTACTCGGAATTACTGGGCGTAAAGCGTGCGTAGGTGGTTCGTTAAGTCAGATGTGAAAGCCCCGGGCTCAACCTGGGAATTGCATTTGATACTGGCGGACTAGAGTGCGGTAGAGGAGAGTGGAATTCCCGGTGTAGCAGTGAAATGCGTAGAGATCGGGAGGAACATCAGTTGCGAAGGCGGCTCTCTGGACCAGCACTGACACTGAGGCACGAAAGCGTGGGGAGCAAACAGG` |
| ASV894 | 429 | `TGGGGAATATTGCACAATGGGGGAAACCCTGATGCAGCCATGCCGCGTGTGTGAAGAAGGCCTTCGGGTTGTAAAGCACTTTCAGCGAGGAGGAAAGGTTGGCAGCTAATATCTGTCAGCTGTGACGTTACTCGCAGAAGAAGCACCGGCTAACTCCGTGCCAGCAGCCGCGGTAATACGGAGGGTGCAAGCGTTAATCGGAATTACTGGGCGTAAAGCGCACGCAGGCGGTTGGATAAGTTAGATGTGAAAGCCCCGGGCTCAACCTGGGAATTGCATTTAAAACTATTCAGCTAGAGTCTTGTAGAGGGGGGTAGAATTCCAGGTGTAGCGGTGAAATGCGTAGAGATCTGGAGGAATACCGGTGGCGAAGGCGGCCCCCTGGACAAAGACTGACGCTCAGGTGCGAAAGCGTGGGGAGCAAACAGG` |
| ASV3217 | 429 | `TGGGGAATTTTGGACAATGGGCGCAAGCCTGATCCAGCCATGCCGCGTGAGTGAAGAAGGCCTTCGGGTTGTAAAGCTCTTTCGGCGGGGACGAAACGGTTCGTGCCAACACCACGGACTAATGACGGTACCCGAAGAAGAAGCACCGGCTAACTACGTGCCAGCAGCCGCGGTAATACGTAGGGTGCAGGCGTTAATCGGAATTACTGGGCGTAAAGCGTGCGCAGGCGGTTTGTTAAGACAGGTGTGAAATCCCCGGGCTTAACCTGGGAACTGCGCTTGTTACTGGCAGACTCGAGTGTGGCAGAGGGGGGTGGAATTCCAGGTGTAGCAGTGAAATGCGTAGAGATCTGGAGGAACACCGATGGCGAAGGCAGCCCCCTGGGCTAACACTGACGCTCATGCACGAAAGCGTGGGGAGCAAACAGG` |
| ASV1596 | 404 | `TGGGGAATATTGGGCAATGGACGAAAGTCTGACCCAGCCATGCCGCGTGAATGATGAAGGCCTTAGGGTTGTAAAGTTCTTTCGCCGATGAAGATAATGACGGTAGTCGGAGAAGAAGCCCCGGCTAACTTCGTGCCAGCAGCCGCGGTAATACGAAGGGGGCTAGCGTTGTTCGGAATTACTGGGCGTAAAGCGAGCGTAGGTTGCTCGACAAGTCAGTGGTGAAAGCCCAGAGCTCAACTCTGGAACTGCCATTGAAACTGTCGAGCTGGAATGTCAGAGGGGATAGCGGAATTCCAAATGTAGGGGTGAAATCCGTAGATATTTGGAGGAACACCGGTGGCGAAGGCGGCTATCTGGATGACTATTGACACTGAGGCTCGAAAGCGTGGGGATCAAACAGG` |
| ASV2566 | 429 | `TGGGGAATATTGGACAATGGGGGCAACCCTGATCCAGCCATGCCGCGTGTGTGAAGAAGGCCTTCGGGTTGTAAAGCACTTTAAGCGGGGAGGAAGGCTCTGTGGCTAATATCCACGGGGATTGACGTTACCCGCAGAATAAGCACCGGCAAACTTCGTGCCAGCAGCCGCGGTAATACGAAGGGTGCGAGCGTTAATCGGAATTACTGGGCGTAAAGCGCGCGTAGGTGGTTGAGTAAGCGATCTGTGAAAGCCCTGGGCTTAACCTGGGAACTGCAGGTCGAACTGCTTGGCTAGAGTATGGTAGAGGGGTGTGGAATTTCCTGTGTAGCGGTGAAATGCGTAGATATAGGAAGGAACATCAGTGGCGAAGGCGACACCCTGGACCGATACTGACACTGAGGCGCGAAAGCGTGGGTAGCGAACAGG` |
| ASV5014 | 424 | `TGAGGAATATTGGTCAATGGGCGCAAGCCTGAACCAGCCATGCCGCGTGCAGGATGAAGCATCTATGGTGTGTAAACTGCTTTTATACGGGAAGAAACCCTGGCTCGTGAGCCAGATTGACGGTACCGTAAGAATAAGGACCGGCTAACTCCGTGCCAGCAGCCGCGGTAATACGGAGGGTCCAAGCGTTATCCGGAATCATTGGGTTTAAAGGGTCCGTAGGCGGTCAGGTAAGTCAGTGGTGAAAGCCCATCGCTCAACGGTGGAACGGCCATTGATACTGCCTGACTCGAATTACTGGGAAGTAACTAGAATATGTAGTGTAGCGGTGAAATGCTTAGATATTACATGGAATACCAATTGCGAAGGCAGGTTACTACCAGTGGATTGACGCTGATGGACGAAAGCGTGGGGAGCGAACAGG` |
| ASV2702 | 404 | `TGAGGAATCTTCCACAATGGGCGAAAGCCTGATGGAGCGACACCGCGTGAAGGATGAAGCCTTTGTTGGTGTAAACTTCTTTTTTCTGGGAAGATAATGACGGTACCAGAAGAATAAGGGGCGGCAAACTTCGTGCCAGCAGCCGCGGTAATACGAAGGCCCCAAGCGTTATCCGGAATTACTGGGTGTAAAGGGTATGTAGGTGGTTCATTAAGTCTATCTGTTAAAATTCGGGGCTCAACCCCGAATCGCAGTAGAAACTGGTGAACTTGAGTGTGGGAGAGGCAAGCAGAACAGTAAGAGTAGGGGTGCAATCCGTTGATACTTACTAGAATACCAAAAGCGAAGGCAGCTTGCTGGAACACTACTGACACTGAGATACGAAAGCGTGGGGAGCGAAAGGG` |
| ASV280 | 429 | `TGGGGAATTTTGGACAATGGGGGCAACCCTGATCCAGCAATGCCGCGTGAGTGAAGAAGGCCTTCGGGTTGTAAAGCTCTTTTGTCAGGGAAGAAAAGGTATCCTCTAATACAGGGTGCTCATGACGGTACCTGAAGAATAAGCACCGGCTAACTACGTGCCAGCAGCCGCGGTAATACGTAGGGTGCAAGCGTTAATCGGAATTACTGGGCGTAAAGCGTGCGCAGGCGGTTATGTAAGTCAGATGTGAAATCCCCGGGCTCAACCTGGGAACTGCATTTGAGACTGCATGGCTAGAGTGTGTCAGAGGGGGGTAGAATTCCACGTGTAGCAGTGAAATGCGTAGATATGTGGAGGAATACCGATGGCGAAGGCAGCCCCCTGGGATAACACTGACGCTCATGCACGAAAGCGTGGGGAGCAAACAGG` |
| ASV3803 | 429 | `TGGGGAATATTGCACAATGGGGGAAACCCTGATGCAGCCATGCCGCGTGTGTGAAGAAGGCCTTCGGGTTGTAAAGCACTTTAGGCGAGGAGGAAAAGCTAACGATTAATAATCGTTAGCCGTGACGTTACTCGCAGAATAAGCACCGGCTAACTCCGTGCCAGCAGCCGCGGTAATACGGAGGGTGCAAGCGTTAATCGGAATTACTGGGCGTAAAGAGTGCGTAGGCGGTTTGATAAGCGGAGTGTGAAATCCCCGGGCTTAACCTGGGAACTGCATTCCGAACTGTCAAGCTAGAGTATGGTAGAGGGTGGCGGAATTCCGAGTGTAGCGGTGAAATGCGTAGATATTCGGAGGAACACCAGTGGCGAAGGCGGCCACCTGGACTAATACTGACGCTGAGGCACGAAAGCGTGGGGAGCAAACAGG` |
| ASV210 | 429 | `TCGAGAATAATTCACAATGGGGGCAACCCTGATGGTGCAACGCCGCGTGGAGGATGAAGGTCTTCGGATTGTAAACTCCTGTCATCCGGGAGCAAGACCTGGCTGTGAATAGCAGACAGGGTTGATAGTACCGGAAGAGGAAGGGACGGCTAACTTCGTGCCAGCAGCCGCGGTAATACGAAGGTCCCGAGCGTTGTTCGGAATCACTGGGCGTAAAGGGAGCGTAGGCGGCGTGGTAAGTCAGATGTGAAATCCCGGGGCTCAACCCCGGAACTGCATCCGATACTGCCGTGCTTGAGTGATGGAGAGGTAGCTGGAATTTACGGTGTAGCAGTGAAATGCGTGGATATCGTAAGGAACACTCGTGGCGAAAGCGAGCTACTGGACATTTACTGACGCTGAGGCTCGAAGGCCAGGGTAGCGAAAGGG` |
| ASV3475 | 429 | `TGGGGAATATTGCGCAATGGAGGAAACTCTGACGCAGCGACGCCGCGTGGGTGATGAAGGCCTTCGGGTTGTAAAGCCCTGTCGGCTGGGAAAAAGGTAGATGCACTAATACTGCAACTGCTTGATGGTACCAGCAAAGGAAGCACCGGCTAACCCTGTGCCAGCAGCCGCGGTAATACAGGGGGTGCTAGCGTTGTTCGGAATTACTGGGCGTAAAGGGCGCGTAGGCGGTTTAGCAAGTCAGATGTGAAATCCCGGGGCTTAACTCCGGAAGTGCATTTGAAACTGCTAGGCTAGAGTGCAGTAGAGGTTATTGGAATTCCCAGTGTAGAGGTGAAATTCGTAGATATTGGGAGGAACACCAGTGGCGAAGGCGGATAACTGGGCTGTAACTGACGCTGAGGCGCGAAAGCGTGGGGATCAAACAGG` |
| ASV717 | 424 | `TAGGGAATATTGGGCAATGGTCGAGAGACTGACCCAGCCATGCCGCGTGCAGGAAGACGGCCTTATGGGTTGTAAACTGCTTTTATACAGGAATAATACTTGATACGTGTATTGAGATGAATGTACTGTAAGAATAAGGATCGGCTAACTCCGTGCCAGCAGCCGCGGTAATACGGAGGATCCAAGCGTTATCCGGATTTATTGGGTTTAAAGGGTGCGCAGGCGGATTGATAAGTCAGCGGTGAAATATTTCGGCTTAACCGAGACACTGCCGTTGATACTGTTAGTCTAGAGTTTAGTTGATGTGGGCGGAATATGTCATGTAGCGGTGAAATGCGTAGATATGACATAGAACACCGATCGGGAAGCCAGCTCACAAAACTATAACTGACGCTCATGCACGAAAGCGTGGGGATCAAACAGG` |
| ASV426 | 404 | `TGGGGAATATTGGACAATGGGGGCAACCCTGATCCAGCCATGCCGCGTGAGTGATGAAGGCCTTCGGGTTGTAAAGCTCTTTTGGCGGGGACGATGATGACGGTACCCGCAGAATAAGCCCCGGCTAACTTCGTGCCAGCAGCCGCGGTAAGACGAAGGGGGCTAGCGTTGTTCGGAATTACTGGGCGTAAAGCGCGTGTAGGCGGTTTTCCAAGTTGGGTGTGAAAGCCTTGGGCTTAACCCAAGAAATGCACTCAGTACTGGAAGACTAGAGGACCGGAGAGGATAGTGGAATTCCCAGTGTAGTGGTGAAATACGTAGAGATTGGGAAGAACACCAGTGGCGAAGGCGGCTATCTGGACGGTTTCTGACGCTAAGACGCGAAAGCGTGGGGAGCAAACAGG` |
| ASV532 | 422 | `TGAGGAATATTGGTCAATGGGCGCAAGCCTGAACCAGCAATGCCGCGTGTAGGACGAAGGGGCTTCGCCCTGTAAACTACTGTCAGATGGGTTGAAAAGGAAGCATGGCTTCTGGAGACGGTACCATCAGAGGAAGGGACGGCTAACTACGTGCCAGCAGCCGCGGTAATACGTAGGTCCCGAGCGTTGTCCGGATTTACTGGGTATAAAGGGTGCGTAGGCGGTTTTGTGCGTCAGAGGTGAAAGGTCAGTGCTTAACATTGATACTGCCTTTGAAACGGCGGAACTTGAGTATGAGAGAGGTTTGTAGAATTCCTGGTGTAGCGGTGAAATGCGTAGATATCAGGAGGAATACCGGTGGCGAAGGCGGCAAACTGGCTCATAACTGACGCTGAGGCACGAAAGCGTGGGGATCAAACAGG` |
| ASV3742 | 407 | `TGGGGAATTTTCCGCAATGGGCGCAAGCCTGACGGAGCAATACCGCGTGAGGGAGGAAGGCTCTTGGGTCGTAAACCTCTTTTATCAGGGAAGAAGAAGATGACGGTACCTGATGAATCAGCATCGGCTAACTCCGTGCCAGCAGCCGCGGTAATACGGAGGATGCAAGCGTTATCCGGAATTATTGGGCGTAAAGCGTCCGCAGGTGGTTTAGGAAGTCTGCTGTCAAAGCGCGAGGCTTAACTTCGTACAGGCAGTGGAAACTTTTAGACTAGAGTTTGGTAGGGGTCACGGGAATTCCCAGTGTAGCGGTGAAATGCGTAGAGATTGGGAAGAACACCAGCGGCGAAAGCGCGTGACTGGGCCTGAACTGACACTCATGGACGAAAGCTAGGGGAGCGAAAGGG` |
| ASV5166 | 421 | `TGGGGAATATTGCACAATGGACGAAAGTTTGATGCAGCGACGCCGCGTGGAGGGTGTAGGCCTTCGGGTTGTGAACTCCTTTTTCTCGCGAAAAAGGCACAGTTTTGGCTGTGTTGATGGTAGTGGGGGAAGAAGCGCCGGCTAACTACGTGCCAGCAGCCGCGGTAATACGTAGGGCGCGAGCGTTGTCCGGATTTATTGGGCGTAAAGAGCTCGTAGGTGGTTCGTCGCGTCTGTCGTGAAAGCCAGCAGCTTAACTGTTGGTCTGCGGTGGGTACGGGCGGGCTTGAGTGCGGTAGGGGTGACTGGAATTCCTGGTGTAGCGGTGGAATGCGCAGATATCAGGAGGAACACCGATGGCGAAGGCAGGTCACTGGGCCGTTACTGACACTGAGGAGCGAAAGCGTGGGGAGCGAACAGG` |
| ASV4016 | 430 | `TGGGGAATTTTGCGCAATGGACGAAAGTCTGACGCAGCGACGCCGCGTGAGTGAAGAAGGCCTTCGGGTTGTAAAGCTCTGTCGGAGGGGACGAACATCGTCTTGGTTAATAGCCTTGGCGACTGACGGTACCCTCAAAGGAAGCACCGGCTAACTCTGTGCCAGCAGCCGCGGTAATACAGAGGGTGCAAGCGTTGCTCGGAATTATTGGGCGTAAAGCGTGGGTAGGTGGTCTTGTTTGTTAGAGGTGAAAGCCTGGGGCTTAACCCCAGAAGTGCCTCTAAAACGGCAAGACTAGAGTACCGAAGAGGTTCGTGGAATTCCCGGTGTAGCGGTGAAATGCGTAGAGATCGGGAGGAACACCAGAGGCGAAGGCGACGAACTGGTAGGTAACTGACACTCAACCACGAAAGCACGGGTAGCAAACAGG` |
| ASV5681 | 390 | `TTAGGGATCTTTTGCAATGCGCGCAAGCGTGACAAAGCGAGCCAGAGTGTTTTTCTAATAATTGAAAAACTTTTGCCAGTTGTAAAAAGACTGGCGAATAAGGACTGGGCAAGACCGGTGCCAGCCGCCGCGGTAATCCCGGCAGTCCAAGTCGCAGCCACTATTACTGGGTCTAAGACATCCGTAGCTTGCATAGTAAGTCTCTTGTGAAATCGGGCATCTTAAGTGTCCGTCGTGCAGGAGATACTGCTTTGCTAGAGACTGGGGGACGTGAGGAGTACGATGGAGGTAGTGGTTAAATACGTTAATCTCAATCGGACTAACAACAGCGAAGGCACCTCACGATTACAGTTCTGACAGTAAGGGATGAAGGCTAGGGGCGCAAAATGG` |
| ASV2012 | 404 | `TCGGGAATTTTGGGCAATGGGCGAAAGCCTGACCCAGCAACGCCGCGTGAAGGATGAAGTTTTTCGGAATGTAAACTTCGTAAGAATGAGAAGAATAAATGACGGTATCATTTGTAAGGTCCGGCTAACTACGTGCCAGCAGCCGCGGTAATACGTAGGGACCAAGCGTTGTTCGGATTTACTGGGCGTAAAGGGCGCGTAGGCGGCAATTCAAGTCATCTGTGAAATCTCCGGGCTTAACTCGGAACGGTCAGATGATACTGCTTTGCTAGAGTGCAGAAGGGGCAATCGGAATTCTTGGTGTAGCGGTGAAATGCGTAGATATCAAGAGGAACACCTGAGGTGAAGACGGGTTGCTGGGCTGACACTGACGCTGAGGCGCGAAAGCTAGGGTAGCAAACGGG` |
| ASV3337 | 429 | `TGGGGAATTTTGGACAATGGGGGCAACCCTGATCCAGCAACGCCGCGTGAATGAAGAAGGCCTTAGGGTTGTAAAGTTCTTTTATCTGTGAGCAAAGCGCTGATGTTAACAGCATGAGTGTATGAGAGTAACAGAAGAATAAGCACCGGCTAACTACGTGCCAGCAGCCGCGGTAATACGTAGGGTGCGAGCGTTAATCGGAATTACTGGGCGTAAAGGGTGCGCAGGCGGATATGTAAGTTAGATGTGAAATCCCCGAGCTTAACTTGGGAATGGCATTTAAGACTGCATGTCTAGAGTTTGTCAGAGGGGGGTAGAATTCCAAGTGTAGCAGTGAAATGCGTAGAGATTTGGAGGAATACCGGTGGCGAAGGCGACCCCCTGGGATAAAACTGACGCTCAGGCACGAAAGCGTGGGTAGCAAACAGG` |
| ASV483 | 424 | `TAAGGAATATTGGTCAATGGAGGCAACTCTGAACCAGCCATGCCGCGTGCAGGAAGACTGCCCTATGGGTTGTAAACTGCTTTTATACGGGAATAAACCTTAATTCGTGAATTAAGCTGAATGTACCGTAAGAATAAGGATCGGCTAACTCCGTGCCAGCAGCCGCGGTAATACGGAGGATCCAAGCGTTATCCGGATTTATTGGGTTTAAAGGGAGCGTAGGCGGCTCATTAAGTCAGAGGTGAAAACTTGCAGCTCAACTGTAAGCTTGCCTTTGATACTGATGAGCTTGAATGTAGTTGAGGTGGGCGGAATGTGACAAGTAGCGGTGAAATGCATAGATATGTCACAGAACACCAATTGCGAAGGCAGCTCACTAAGCTACGATTGACGCTGAGGCTCGAAAGCGTGGGGATCAAACAGG` |
| ASV3971 | 404 | `TGGGGAATATTGGACAATGGGCGCAAGCCTGATCCAGCAATGCCGCGTGGATGAAGAAGGTCTTCGGATTGTAAAGTCCTTTCGGCGGGGACGATGATGACGGTACCCGCAGAAGAAGCCCCGGCTAACTTCGTGCCAGCAGCCGCGGTAATACGAAGGGGGCTAGCGTTGCTCGGAATGACTGGGCGTAAAGGGCGCGTAGGCGGTTCGGTTAGTTAGGCGTGAAATTCCTGGGCTTAACCTGGGGGCTGCGCTTAATACAGCCGGGCTTGAGTGTGGAAGAGGGTCGTGGAATTCCCAGTGTAGAGGTGAAATTCGTAGATATTGGGAAGAACACCGGTGGCGAAGGCGGCGACCTGGTCCATTACTGACGCTGAGGCGCGAAAGCGTGGGGAGCAAACAGG` |
| ASV2287 | 404 | `TGGGGAATATTGGACAATGGGGGCAACCCTGATCCAGCCATGCCGCGTGAGTGAAGAAGGCCTTAGGGTTGTAAAGCTCTTTTGTCAGGGATGATAATGACCGTACCTGAAGAATAAGCCCCGGCTAACTCCGTGCCAGCAGCCGCGGTAAGACGGAGGGGGCTAGCGTTGTTCGGATTTACTGGGCGTAAAGGGCGCGTAGGCTGCCATGTGTGTCAGGGGTGAAATCCCAAGGCTTAACCTTGGAACTGCTTCTGAAACTGCATGGCTAGAGGAATAGAGAGGCGAGTGGAATTCCGAGTGTAGCAGTGAAATGCGCAGATATTCGGAGGAACATCAGTGGCGAAGGCGACTCGCTGGCTATTATCTGACGCTGAGGCGCGAAAGCGTGGGGAGCAAACAGG` |
| ASV941 | 424 | `TAGGGAATATTGGACAATGGGGGCAACCCTGATCCAGCCATGCCGCGTGCAGGAAGAAGGCCCTATGGGTCGTAAACTGCTTTTGTACGGGAAGAAACCTCACTACGTGTAGTGAGTTGACGGTACCGTGAGAATAAGGATCGGCTAACTTCGTGCCAGCAGCCGCGGTAATACGAAGGATCCAAGCGTTGTCCGGATTTACTGGGTTTAAAGGGTGCGTAGGCGGACTTTTAAGTCAGTGGTGAAAGCCTGTAGCTTAACTATAGAATTGCCATTGAAACTGAAAGTCTCGAGTGTGGTTGAGGTAACTGGAATATATCATGTAGCGGTGAAATGCTTAGATATGATATAGAACACCAATTGCGAAGGCAGGTTACTGAGCCATTACTGACGCTGAGGCACGAAAGCGTGGGGATCAAACAGG` |
| ASV4707 | 429 | `TGGGGAATATTGGACAATGGGGGCAACCCTGATCCAGCCATGCCGCGTGTGTGAAGAAGGCCTTCGGGTTGTAAAGCACTTTCAGTGGGGAGAAAGGCTGCTTACCTAATACGTAAGCGGATTGATGTTACCCACAGAAGAAGCACCGGCTAACTCCGTGCCAGCAGCCGCGGTAATACGGAGGGTGCAAGCGTTAATCGGAATTACTGGGCGTAAAGCGCATGTAGGTGGTTTTTTAAGCCAGCTGTGAAATCCCCGGGCTCAACCTGGGCACTGCAGTTGGTACTGGAAAGCTAGAGTAGGGTAGAGGGGTGTGGAATTCCAGGTGTAGCGGTGAAATGCGTAGATATCTGGAGGAACATCAGTGGCGAAGGCGACACCCTGGACTCATACTGACACTGAGATGCGAAAGCGTGGGGAGCAAACAGG` |
| ASV3840 | 428 | `TCGAGAATTTTTCTCAATGGGCGAAAGCCTGAAGGAGCGACGCCGCGTGGGGGATGAATGGCTTCGGCCCGTAAACCCCTGTCATTTGCGAACAAACCTTTTCATTTAACACCTGAAAAGCTGATTGTAGCGAAAGAGGAAGGGACGGCTAACTCTGTGCCAGCAGCCGCGGTAATACAGAGGTCCCAAGCGTTGTTCGGATTCACTGGGCGTAAAGGGTGCGTAGGTGGTTGGGTAAGTCTGATGTGAAATCTCTGAGCTCAACTCAGAAACTGCATTGGATACTATCCGGCTAGAGAATCGGAGGGGGGACTGGAATACTTGGTGTAGCAGTGAAATGCGTAGATATCAAGTGGAACACCAGTGGCGAAGGCGAGTCCCTGGACGATTTCTGACACTGAGGCACGAAAGCTAGGGGAGCAAACAGG` |
| ASV4146 | 411 | `TTTGGAATTTTGGACAATGGGCGAAAGCCTGATCCAGCGACGCCGCGTGGACGATGAAGGCCTTCGGGTTGTAAAGTCCTTTTGCTCGGGAACAATATTTTGAGCGTACCGAGAGAATAAGAGGTTGCTAACTCTGTGCCAGCAGCAGCGGTAATACAGAGACCTCAAGCGTTATCCGGAATTATTGGGCGTAAAGCGTACCGACAGGTGGTTTAACAAGTCAGAAGTGAAATCTCCAAGCTTAACTTGGAATCTGTCTTTTGAAACTGTTAAACTAGAAGGGCAAAGAGGAAGCTGGAACGAACGGTGTAGTAGTGAAATGCGTTGATATCGTTCGGAACACCAATAGCGTAGGCAGGCTTCTGGGTGCCACTTGACACTGCTAGGACGAAAGCGTGGGTAGCGAATGGG` |
| ASV4127 | 404 | `TGGGGAATATTGGACAATGGGCGCAAGCCTGATCCAGCAATACCGCGTGAGTGATGAAGGTCTTCGGATTGTAAAGCTCTTTTAGTAGGGAAGATAATGACGGTACCTACAGAAAAAGCACCGGCAAACTTCGTGCCAGCAGCCGCGGTAATACGAAGGGTGCTAGCGTTGTTCGGAATTATTGGGCGTAAAGCGTGTGTAGGCGGCTTAGTAAGTCAGTTGTGAAATCCCCGAGCTCAACTTGGGAATTGCGACCGAAACTGCTTCGCTTGAGTTTGGTGGGGGATGGTGGAATTCCTAGTGTAGGAGTGAAATCCGTAGAGATTAGGAGGAATATCGGTGGCGAAGGCGACCATCTACGCCAATACTGACGCTAAGACACGAAAGCGTGGGGAGCAAACAGG` |
| ASV4256 | 404 | `TGGGGAGTATTGGACAATGGGGGCAACCCTGATCCAGCAATGCCGCGTGGGTGATGAAGGCCTTCGGGTTGTAAAGCCCTTTCGGCGGGGACGATGATGACGGTACCCGCAGAAGAAGCCCCGGCTAACTTCGTGCCAGCAGCCGCGGTAATACGAAGGGGGCTAGCGTTGTTCGGAATGACTGGGCGTAAAGGGCGCGTAGGCGGCGATTTTAGTCAGGCGTGAAAGCCCTGGGCTCAACCTAGGAACTGCGTTTGATACTGGATCGCTTGAGTGTGGGAGAGGATGGTGGAATTCCCAGTGTAGAGGTGAAATTCGTAGATATTGGGAAGAACACCGGTGGCGAAGGCGGCCATCTGGACCACTACTGACGCTGAGGCGCGAAAGCGTGGGGAGCAAACAGG` |
| ASV3617 | 404 | `TGAGGAATCTTCCACAATGGGCGAAAGCCTGATGGAGCGACACCGCGTGAAGGATGAAGCCTTTGTTGGTGTAAACTTCTTTTCTCTGGGAAGATAATGACGGTACCAGAGGAATAAGGGGCGGCAAACTTCGTGCCAGCAGCCGCGGTAATACGAAGGCCCCAAGCGTTATCCGGAATTACTGGGCGTAAAGCGTCTGTAGGTGGTTCGGAAAGTCTCAAGTGAAAGGTCAGGGCTCAACCCTGTACTTGCTTGGGAAACTATCGAACTTGAGTGTGGGAGAGGCAAGCAGAACTGTATGAGTAGGGGTGCAATCCGTTGATACATACAAGAATACCAAAAGCGAAGGCAGCTTGCTGGAACACTACTGACACTGAGAGACGAAAGCGTGGGGAGCAAAAGGG` |
| ASV3420 | 407 | `TCGGGAATATTGGGCAATGGAGGAAACTCTTACCCAGCAATGCCGCGTGAATGAAGAAGGTCTTCGGATTGTAAAGTTCTTTAGTCAGGGACGAAAAAAATGACGGTACCTGAAAAATAAGCTCCGGCTAACTACGTGCCAGCAGCCGCGGTAATACGTAGGGAGCAAGCGTTGTCCGGAATGACTGGGCGTAAAGGGTGTGTAGGTGGTTTGGCAAGTTGGGAGTGTAATACCTGGGCTTAACTTAGGTGCTGCTTTCAAAACTGCTAGACTTGAGTGCGGAAGAGGTTAATGGAATTCCTAGTGTAGCGGTGGAATGCGTAGATATTAGGAGGAACACCAGAGGCGAAGGCGATTAACTGGTCCGTAACTGACACTGAAACACGAAAGTGTGGGGAGCAAACAGG` |
| ASV303 | 429 | `TGGGGAATTTTGGACAATGGGCGAAAGCCTGATCCAGCAATGCCGCGTGAGTGAAGAAGGCCTTCGGGTTGTAAAGCTCTTTTGTCAGGGAAGAAACGGTGAGGGCTAATATCCCTTGCTAATGACGGTACCTGAAGAATAAGCACCGGCTAACTACGTGCCAGCAGCCGCGGTAATACGTAGGGTGCAAGCGTTAATCGGAATTACTGGGCGTAAAGCGTGCGCAGGCGGTTTTGTAAGTCTGTCGTGAAATCCCCGGGCTCAACCTGGGAATTGCGATGGAGACTGCAAGGCTAGAATCTGGCAGAGGGGGGTAGAATTCCACGTGTAGCAGTGAAATGCGTAGAGATGTGGAGGAACACCGATGGCGAAGGCAGCCCCCTGGGTCAAGATTGACGCTCATGCACGAAAGCGTGGGGAGCAAACAGG` |
| ASV3915 | 424 | `TGGGGAATATTGGTCAATGGGCGAAAGCCTGAACCAGCAATGCCGCGTGAGTGATGAAGGCCTTAGGGTTGTAAAGCTCTGTCAGTGGGAACGAATAAGCCGGTTAATACCCGGTGGTGACGGTACCCACAAAGGAAGCACCGGCTAACTCTGTGCCAGCAGCCGCGGTAATACAGAGGGTGCAAGCGTTGTTCGGAATTACTGGGCGTAAAGCGCGTGTAGGCGGTTTTGTAAGTCGGTCGTGAAAGCCCTGGGCTTAACCCAGGAACTGCGATCGAAACTGTGAGACTAGAGTATCGGAGGGGGTGGCGGAATTCCCGGTGTAGAGGTGAAATTCGTAGATATCGGGAGGAACACCTGTGGCGAAGGCGGCCACCTGGAAGATTACTGACGCTGAGACGCGAAAGCGTGGGGAGCAAACAGG` |
| ASV2717 | 404 | `TGGGGAATCTTGGACAATGGGCGCAAGCCTGATCCAGCCATGCCGCGTGAGTGAAGAAGGCCTTCGGGTTGTAAAGCTCTTTTGTCAGGGACGATAATGACGGTACCTGAAGAATAAGCCCCGGCTAACTTCGTGCCAGCAGCCGCGGTAATACGAAGGGGGCTAGCGTTGTTCGGAATTACTGGGCGTAAAGCGCACGTAGGCTGATACTTAAGTGAGGGGTGAAATCCCGAGGCTCAACCTCGGAACTGCCTTTCATACTGGGTATCTCGAGTCCGGAAGAGGTTAGTGGAATTCCCAGTGTAGAGGTGAAATTCGTAGATATTGGGAAGAACACCAGTGGCGAAGGCGGCTAACTGGTCCGGTACTGACGCTGAGGTGCGAAAGCGTGGGGAGCAAACAGG` |
| ASV2156 | 404 | `TGGGGAATATTGGACAATGGGCGAAAGCCTGATCCAGCAATGCCGCGTGAGTGATGAAGGCCTTAGGGTTGTAAAGCTCTTTTACCCGGGATGATAATGACAGTACCGGGAGAATAAGCCCCGGCTAACTCCGTGCCAGCAGCCGCGGTAATACGGAGGGGGCTAGCGTTGTTCGGAATTACTGGGCGTAAAGCGCGCGTAGGCGGCTTTGTAAGTCAGAGGTGAAATCCCGGGGCTCAACTCCGGAACTGCCTTTGAAACTGCATCGCTTGAATCCGGGAGAGGTGAGTGGAATTCCGAGTGTAGAGGTGAAATTCGTAGATATTCGGAAGAACACCAGTGGCGAAGGCGGCTCACTGGACCGGCATTGACGCTGAGGTGCGAAAGCGTGGGGAGCAAACAGG` |
| ASV2488 | 429 | `TGGGGAATTTTGGACAATGGGCGAAAGCCTGATCCAGCAATGCCGCGTGCAGGACGAAGGCCCTCGGGTTGTAAACTGCTTTTGTACGGAAAGAAACGGCAGGCTCTAATACAGCTTGTGACTGACGGTACCGTAAGAATAAGCACCGGCTAACTACGTGCCAGCAGCCGCGGTAATACGTAGGGTGCAAGCGTTAATCGGAATTACTGGGCGTAAAGCGTGCGCAGGCGGTTTTGTAAGTCAGATGTGAAATCCCCGGGCTCAACCTGGGACCTGCATTTGAGACTGCAAAGCTAGAGTGCGGCAGAGGGGGATGGAATTCCGCGTGTAGCAGTGAAATGCGTAGATATGCGGAGGAACACCGATGGCGAAGGCAATCCCCTGGGCCTGCACTGACGCTCATGCACGAAAGCGTGGGGAGCAAACAGG` |
| ASV3078 | 386 | `GCGCGAAACCTTCGCAATAGGCGAAAGCCTGACGAGGTTAATCCGAGTGTTTTCTGCTAAAGGAATCTTTTGTCAGCTCTAAATAGGCTGATGAATAAGGGGTGGGCAAGCCTGGTGTCAGCCGCCGCGGTAATACCAGCACCCCGAGTGGTCGGGATGATTATTGGGCCTAAAGCATCCGTAGCCGGTCTTGTAAGTCTTCCGTTAAATCCACCTGCTCAACAGTTGGGCTGCGGGAGATACTGCAGGACTAGGAGGCGGGAGAGGCGAGGGGTATTCCTTGGGTAGGGGTAAAATCCTCTGATCCATGGAGGACCACCAGTGGCGAAGGCGTCTCGCCAGAACGCGCTCGACGGTGAGGGATGAAAGCTGGGGGAGCAAACCGG` |
| ASV3873 | 429 | `TGGGGAATATTGCGCAATGGGCGAAAGCCTGACGCAGCGACGCCGCGTGAGGGATGAAGGTTCTCGGATCGTAAACCTCTGTCAGAAGGGAAGAAAAACTAGGGGAGTAACTGCCCTTAGCCTGACGGTACCTTCAAAGGAAGCACCGGCTAACTCCGTGCCAGCAGCCGCGGTAATACGGAGGGTGCAAGCGTTAATCGGAATCACTGGGCGTAAAGCGCACGTAGGCGGCTTTGTAAGTCAGCGGTGCAAGCCCTTCGCTTAACGAAGGAATTGCCGTTGATACTGCTTAGCTTGAGTTTGTGAGAGGATAGTGGAATTCCAGGTGTAGGAGTGAAATCCGTAGAGATCTGGAGGAACATCAGTGGCGAAGGCGACTATCTGGCACAATACTGACGCTGAGGTGCGAAAGCGTGGGGATCAAACAGG` |
| ASV4528 | 426 | `TCGAGAATCTTCGGCAATGGGCGAAAGCCTGACCGAGCGACGCCGCGTGCGGGATGAAGGTCTTCGGATTGTAAACCGCTGTCAGAGGGGATGAAGTCATAGAGGGTTCTCCCTTTATGTTGACAGAGCCTCAGAGGAAGCACGGGCTAAGTATGTGCCAGCAGCCGCGGTAATACATACTGTGCGAACGTTATTCGGATTCACTGGGCTTAAAGGGTGCGTAGGCGGCTTGTTAAGTAGGGTGTGAAAGCCCCCAGCTCAACTGGGGAACTGCACTCTATACTGGCGAGCTGGAGTGAGATAGGGGTGTGTGGAACTGCCAGTGGAGCGGTGAAATGTGTTGATATTGGCAGGAACGCCGGTGGCGAAAGCGACACACTGGGTCTCAACTGACGCTGAGGCACGAAAGCCAGGGGAGCGAACGGG` |
| ASV1457 | 429 | `TGGGGAATTTTGGACAATGGGCGCAAGCCTGATCCAGCCATGCCGCGTGCGGGAAGAAGGCCTTCGGGTTGTAAACCGCTTTTGTCAGGGAAGAAACGGCTCTTACTAATATTAGGAGCTAATGACGGTACCTGAAGAATAAGCACCGGCTAACTACGTGCCAGCAGCCGCGGTAATACGTAGGGTGCAAGCGTTAATCGGAATTACTGGGCGTAAAGCGTGCGCAGGCGGCTATGCAAGACAGATGTGAAATCCCCGGGCTCAACCTGGGAACTGCATTTGTGACTGCATGGCTAGAGTACGGTAGAGGGGGATGGAATTCCGCGTGTAGCAGTGAAATGCGTAGATATGCGGAGGAACACCGATGGCGAAGGCAATCCCCTGGACCTGTACTGACGCTCATGCACGAAAGCGTGGGGAGCAAACAGG` |
| ASV3478 | 404 | `TGGGGAATCTTGGACAATGGGCGCAAGCCTGATCCAGCCATGCCGCGTGAGTGACGAAGGCCTTAGGGTTGTAAAGCTCTTTTGCCGGGGACGATAATGACGGTACCCGGAGAATAAGTCCCGGCTAACTTCGTGCCAGCAGCCGCGGTAATACGAAGGGGACTAGCGTTGTTCGGAATTACTGGGCGTAAAGCGCACGTAGGCGGATAGTTAAGTCAGGGGTGAAATCCCGGGGCTCAACCTCGGAACTGCCTTTGATACTGGCTATCTAGAGTCCGGAAGAGGTGGGTGGAATTCCTAGTGTAGAGGTGAAATTCGTAGATATTAGGAAGAACACCGGTGGCGAAGGCGGCCCACTGGTCCGGTACTGACGCTGAGGTGCGAAAGCGTGGGGAGCAAACAGG` |
| ASV2102 | 406 | `TGGGGAATTTTCCGCAATGGGCGAGAGCCTGACGGAGCAACGCCGCGTGAGGGAGGAAGGCCTGTGGGTTGTAAACCTCTTTTCTCAGGGAAGAAGTTCTGACGGTACCTGAGGAATCAGCATCGGCTAACTCCGTGCCAGCAGCCGCGGTAAGACGGAGGATGCAAGCGTTATCCGGAATTATTGGGCGTAAAGCGTCCGTAGGCGGTTTCAAAAGTCTGTTGTTAAAGCTCACAGCTCAACTGTGAATCGGCAATGGAAACTGTGAGACTAGAGAGTGGTAGGGGTAGAGGGAATTCCTAGTGTAGCGGTGAAATGCGTAGATATTAGGAAGAACACCAGTGGCGAAGGCGCTCTACTGGGCCATTACTGACGCTGATGGACGAAAGCTAGGGGAGCGAAAGGG` |
| ASV3124 | 426 | `TTTCGAATTATTTGCAATGGGGGCAACCCTGACAGTGCAACGCCGCGTGAGGGATGAAGGTCTTCGGATCGTAAACCTCTGTCACCGGACATTAATGTGCGCATGTTAATAGCATGTGTATTGAAAATCCGGAGAGGAAGCAGTGGCTAACTCCGTGCCAGCAGCCGCGGTAATACGGAGACTGCAAGCGTTACTCGGAATCACTGGGCGTAAAGGGTGCGTAGGTTGTTGGGTGTGACTGACGTGAAATCCCAGGGCTTAACCCTGGAACTGCGTTGGTTCCTACCCATCTAGAGTATCAGAGAGGCAGGCGGAATTCCCGGAGTAGCGGTGAAATGCGTAGATATCGGGAAGAACACCGAAAGCGAAGGCAGCCTGCTGGATGATAACTGACACTGAGGCACGAAAGCGCGGGTAGCAAAAGGG` |
| ASV1304 | 407 | `TGGGGAATTTTCCGCAATGGGCGAAAGCCTGACGGAGCAATACCGCGTGCGGGACGAAGGCTTTTGGGTCGTAAACCGCTTTTGTTAGGGAAGAACACAATGACGGTACCTAACGAATCAGCATCGGCTAACTCCGTGCCAGCAGCCGCGGTAATACGGAGGATGCAAGCGTTATCCGGAATTATTGGGCGTAAAGCGTCCGCAGGTGGTCTATCAAGTCTGCTGTTAAATCGTAGGGCTTAACCCTATACAGGCAGTGGAAACTGAGAGACTAGAGTACGGTAGGGGTAGCGGGAATTCCCAGTGTAGCGGTGAAATGCGTAGATATTGGGAAGAACACCAGCGGCGAAGGCGCGCTACTGGGCCGAAACTGACACTGATGGACGAAAGCTAGGGGAGCGAAAGGG` |
| ASV2523 | 406 | `TGAGGAATATTCCACAATGGGCGAAAGCCTGATGGAGCAACGCCGCGTGCAGGATGAAGGCCTTCGGGTCGTAAACTGCTTTTATAAGTGAATAACATGAATGTATCTTATGAATAAGCACCGGCTAACTACGTGCCAGCAGCCGCGGTCATACGTAGGGTGCAAGCATTATCCGGAGTGACTGGGCGTAAAGAGTTGCGTAGGTGGTTTATCAAGTGAATAGTGAAATCTGGTGGCTCAACCACATAGACTATTATTCAAACTGGTAAACTCGAGAGTAGCAGAGGTAACTGGAATTTCTAGTGTAGGAGTGAAATCCGTAGATATTAGAAGGAACACCAATGGCATAGGCAGGTTACTGGGCTATTTCTGACACTAAGGCACGAAAGCGTGGGGAGCGAACCGG` |
| ASV5247 | 409 | `TGGGGAATATTGCACAATGGGCGAAAGCCTGATGCAGCGACGCCGCGTGAGGGATGACGGCCTTCGGGTTGTAAACCTCTTTCAGCAGGGACGAAGCGCAAGTGACGGTACCTGCAGAAGAAGCACCGGCTAACTACGTGCCAGCAGCCGCGGTAATACGTAGGGTGCAAGCGTTGTCCGGAATTACTGGGCGTAAAGAGTTCGTAGGCGGTTTGTCGCGTCGTTTGTGAAAACCAGCAGCTCAACTGCTGGCTTGCAGGCGATACGGGCAGACTTGAGTACTGCAGGGGAGACTGGAATTCCTGGTGTAGCGGTGAAATGCGCAGATATCAGGAGGAACACCGGTGGCGAAGGCGGGTCTCTGGGCAGTAACTGACGCTGAGGAACGAAAGCGTGGGTAGCGAACAGG` |
| ASV1697 | 424 | `TGAGGAATATTGCGCAATGGAGGCAACTCTGACGCAGCCATGCCGCGTGCAGGAAGAAGGCGCTACGCGTTGTAAACTGCTTTTAACCGGGAAGAAACCCCAGGACGTGTCCTGGGTTGACGGTACCGGAGGAATAAGCACCGGCTAACTTCGTGCCAGCAGCCGCGGTAATACGAAGGGTGCAAGCGTTATCCGGATTCATTGGGTTTAAAGGGTGCGTAGGCGGGGCGTTAAGTCAGTGGTGAAATCCTACAGCTCAACTGTAGACTTGCCATTGATACTGACGCTCTTGAGTGCGCTTGAAGTAGGCGGAATGTGCCGTGTAGCGGTGAAATGCTTAGATATGGCACAGAACACCGATTGCGAAGGCAGCTTACTAAGGCGATACTGACGCTGAGGCACGAAAGCGTGGGGATCGAACAGG` |
| ASV2263 | 424 | `TGAGGAATATTGGACAATGGGCGCAAGCCTGATCCAGCCATGCCGCGTGCAGGAAGAAGCATCTATGGTGTGTAAACTGCTTTTGTACGGGAAGAAACACTCCTTCGTGAAGGAGCTTGACGGTACCGTAAGAATAAGGATCGGCTAACTCCGTGCCAGCAGCCGCGGTAATACGGAGGATCCAAGCGTTATCCGGAATCATTGGGTTTAAAGGGTCCGTAGGCGGTTTGATAAGTCAGTGGTGAAAGCCCATCGCTCAACGATGGAACGGCCATTGATACTGTCAGACTTGAATTACTAGGAAGTAACTAGAATATGTAGTGTAGCGGTGAAATGCTTAGATATTACATGGAATACCAATTGCGAAGGCAGGTTACTACTAGTTTATTGACGCTGATGGACGAAAGCGTGGGGAGCGAACAGG` |
| ASV1342 | 404 | `TGGGGAATATTGGACAATGGGCGCAAGCCTGATCCAGCAATACCGCGTGAATGATGAAGGTCTTCGGATTGTAAAGTTCTTTTAGTGAGGAAGATAATGACGGTACTCACAGAAAAAGCACCGGCTAACTTCGTGCCAGCAGCCGCGGTAATACGAAGGGTGCTAGCGTTGTTCGGAATTATTGGGCGTAAAGCGTATGTAGGCGGTTTGGTAAGTCAGTTGTGAAAGCCCCGAGCTCAACTCGGGAATTGCAGCTGAAACTGCCTTGCTTGAATTTGGTGGGGGATAGTGGAATTCTTAGTGTAGGGGTGAAATCCGTAGATATTAAGAGGAACACCGGCGGCGAAAGCGACTATCTACACCAACATTGACGCTGAGATACGAAAGCGTGGGGAGCAAACAGG` |
| ASV3197 | 407 | `TGGGGAATATTGGACAATGGGCCACGAGCCTGATCCAGCAATTCTGTGTGCACGATGAAGGTCTTCGGATTGTAAAGTGCTTTCAGTTGGGAAGAAGAAAGTGACGGTACCAACAGAAGAAGCGACGGCTAAATATGTGCCAGCAGCCGCGGTAATACATATGTCGCAAGCGTTATCCGGATTTATTGGGCGTAAAGCGCGTCTAGGCGGTTTACTAAGTCTGATGTGAAAATGCGGGGCTCAACTCCGTATTGCGTTGGAAACTGGTAGACTAGAATACTGGAGAGGTGGGCGGAACTACAAGAGTAGAGGTGGAATTCGTAGATACTTGTAGGAATGCCGATGGAGAAGTCAGCTCACTGGACAGATATTGACGCTAAAGCGCGAAAGCGTGGGGAGCAAACAGG` |
| ASV4701 | 404 | `TTAGGAATATTGGACAATGGGCGAAAGCCTGATCCAGCCATGCCGCGTGAACGATGAAGGCCCTAGGGTTGTAAAGTTCTTTCGTGGGCGAAGATAATGACGGTAACCCAAGAAGAAGCTCCGGCTAACTCCGTGCCAGCAGCCGCGGTAATACGGAGGGAGCGAGCGTTGTTCGGAATCACTGGGCGTAAAGGGTGCGTAGGCGGATTAACAAGTCAGGAGTGAAATCCCCAGGCTTAACCTGGGAACTGCTTTTGATACTGTTATTCTAGAGTTTGGTAGAGGTTGGCGGAATTCCTAGTGTAGAGGTAAAATTCGTAGATATTAGGAGGAACACCAGTGGCGTAGGCGGCCAACTGGACCATTACTGACGCTGAGGCACGAAAGCGTGGGGAGCAAACAGG` |
| ASV942 | 404 | `TGGGGAATATTGGACAATGGGCGAAAGCCTGATCCAGCCATGCCGCGTGAGTGATGAAGGCCTTAGGGTTGTAAAGCTCTTTTGTCAGGGAAGATAATGACGGTACCTGAAGAATAAGCACCGGCTAACTTCGTGCCAGCAGCCGCGGTAATACGAAGGGTGCTAGCGTTGTTCGGAATCACTGGGCGTAAAGCGCGCGTAGGCGGCTTAACAAGTCAGAGGTGAAAGCCTGGAGCTCAACTCCAGAATTGCCTTTGAAACTGTTAAGCTAGAGTGTCGGAGGGGATAGCGGAATTGCTAATGTAGAGGTGAAATTCGTAGATATTAGCAGGAACACCGGTGGCGAAGGCGGCTATCTGGACGACAACTGACGCTGAGGCGCGAAAGCGTGGGGATCAAACAGG` |
| ASV1742 | 429 | `TGGGGAATATTGGACAATGGGCGAAAGCCTGATCCAGCCATGCCGCGTGTGTGAAGAAGGTCTTAGGATCGTAAAGCACTTTAAGTTGGGAGGAAGGGCAGTAACCTAATACGTTATTGTTTTGACGTTACCGACAGAATAAGCACCGGCTAACTCTGTGCCAGCAGCCGCGGTAATACAGAGGGTGCAAGCGTTAATCGGAATTACTGGGCGTAAAGCGCGCGTAGGTGGTTCGTTAAGTTGGATGTGAAATCCCCGGGCTCAACCTGGGAACTGCATTCAAAACTGTCGAGCTAGAGTATGGTAGAGGGTGGTGGAATTTCCTGTGTAGCGGTGAAATGCGTAGATATAGGAAGGAACACCAGTGGCGAAGGCGACCACCTGGACTGATACTGACACTGAGGTGCGAAAGCGTGGGGAGCAAACAGG` |
| ASV2560 | 407 | `TGGGGAATATTGGGCAATGGGCGAAAGCCTGACCCAGCGACGCCGCGTGAGGGAAGAAGGTCTTCGGATTGTAAACCTTTGTAGACAGGGACGAAAAAAATGACGGTACCTGTAAAGTAAGCCACGGCTAACTACGTGCCAGCAGCCGCGGTAATACGTAGGTGGCGAGCGTTATCCGGATTTATTGGGTGTAAAGGGTGAGTAGGCGGGTTTGCAAGTCAGATGTGAAATACTGTGGCTTAACCACAGAATAGCATTTGAAACTGTGAATCTTGAGAGTCGGAGAGGTAATCGGAATTCCCGGTGTAGCGGTGAAATGCGTAGAGATCGGGAGGAACACCAGTGGCGAAGGCGGATTACTGGACGACAACTGACGCTGAGACACGAAAGCGTGGGGAGCAAACAGG` |
| ASV3773 | 404 | `TGGGGAATATTGGACAATGGGGGAAACCCTGATCCAGCCATGCCGCGTGAGTGATGACGGCCTTAGGGTTGTAAAGCTCTTTTACCAGGGAAGATAATGACTGTACCTGGAGAATAAGTTCCGGCTAACTTCGTGCCAGCAGCCGCGGTGATACGAAGGGAACTAGCGTTGTTCGGATTTACTGGGCGTAAAGAGTTCGTAGGCGGACCATTAAGTCGGAGGTGAAATCCCAGGGCTCAACCCTGGAATTGCCTTCGATACTGGTGGTCTTGAGTATGATAGAGGTTGGTGGAATTCCTAGTGTAGAGGTGAAATTCGTAGATATTAGGAAGAACACCGGTGGCGAAGGCGGCCAACTGGATCATAACTGACGCTGAGGAACGAAAGCGTGGGGAGCAAACAGG` |
| ASV864 | 429 | `TGGGGAATTTTGGACAATGGGGGCAACCCTGATCCAGCCATTCCGCGTGAGTGAAGAAGGCCTTCGGGTTGTAAAGCTCTTTTGTCGGGGACGAAACGGTTAAGGTTAATAACCTTGACTACTGACGGTACCCGAAGAATAAGCACCGGCTAACTACGTGCCAGCAGCCGCGGTAATACGTAGGGTGCGAGCGTTAATCGGAATTACTGGGCGTAAAGCGTGCGCAGGCGGTTTTGTAAGCCAGATGTGAAATCCCCGGGCTCAACCTGGGAATGGCATTTGGGACTGCAAGGCTAGAGTATGGCAGAGGAGACTAGAATTCCTGGTGTAGCAGTGAAATGCGTAGATATCAGGAGGAATACCGATGGCGAAGGCAGGTCTCTGGGTCAATACTGACGCTCATGCACGAAAGCGTGGGGAGCAAACAGG` |
| ASV1334 | 404 | `TGGGGAATATTGGACAATGGGCGCAAGCCTGATCCAGCCATGCCGCGTGAGTGATGAAGGCCTTAGGGTTGTAAAGCTCTTTTACCAGGGATGATAATGACAGTACCTGGAGAATAAGCTCCGGCTAACTCCGTGCCAGCAGCCGCGGTAATACGGAGGGAGCTAGCGTTGTTCGGAATTACTGGGCGTAAAGAGTACGTAGGCGGTTTTTTAAGTCAGAGGTGAAATCCCGGAGCTCAACTCCGGAACTGCCTTTGAAACTGGAAAGCTAGAATCATGGAGAGGTTAGTGGAATTCCGAGTGTAGAGGTGAAATTCGTAGATATTCGGAAGAACACCAGTGGCGAAGGCGACTGACTGGACATGTATTGACGCTGAGGTACGAAAGCGTGGGGAGCAAACAGG` |
| ASV4438 | 416 | `TTAGGAATATTGTGCAATGGAGGGAACTCTGACACAGCGACGCCGCGTGAGGGATGAAGGTTTTAGGATCGTAAACCTCTGAATTAGGGACGAAAGACGCGTAAGCGGGATGACGGTACCTAAGTAATAGCACCGGCTAACTCCGTGCCAGCAGCCGCGGTAATACGGAGGGTGCAAGCGTTACCCGGAATCACTGGGCGTAAAGGGCGTGTAGGCGGTGAATTAAGTCTGGATTTAAAGACTGAAGCTCAACTTCAGGAATGGTTCGGATACTGGCTCACTAGACAAATGGAGAGGTAATTGGAATTCTTGGTGTAGCGGTGAAATGCGTAGATACCAAGAGGAACACCAATGGCGAAGGCAGGTTACTGGACATTTTGTGACGCTGAGGCGCGAAAGTGTGGGGAGCAAACCGG` |
| ASV774 | 429 | `TGGGGAATATTGGACAATGGGGGCAACCCTGATCCAGCAATGCCGCGTGTGTGAAGAAGGCCTTAGGGTTGTAAAGCACTTTTGGCAGGGAGGAAGGTATAGAGTTTAAGAGACACTATATTTGACTGTACCTGCAGAATAAGCACCGGCTAACTCTGTGCCAGCAGCCGCGGTAATACAGAGGGTGCAAGCGTTAATCGGAGTTACTGGGCGTAAAGGGCGCGTAGGCGGTTATATAAGTGAGATGTGAAATCCCTGGGCTTAACCTAGGAACTGCATCTCATACTGTATAGCTAGAGTATTGTAGAGGGTAGTGGAATTTCCGGTGTAGCGGTGAAATGCGTAGAGATCGGAAGGAACACCAGTGGCGAAGGCGGCTACCTGGACAAATACTGACGCTGAGGCGCGAAAGCGTGGGGAGCAAACAGG` |
| ASV1050 | 429 | `TGGGGAATTTTGGACAATGGGCGAAAGCCTGATCCAGCAATGCCGCGTGCAGGAAGAAGGCCTTCGGGTTGTAAACTGCTTTTGTACGGAACGAAACGGCTCGCTTTAATAAAGTGGGCTAATGACGGTACCGTAAGAATAAGCACCGGCTAACTACGTGCCAGCAGCCGCGGTAATACGTAGGGTGCAAGCGTTAATCGGAATTACTGGGCGTAAAGCGTGCGCAGGCGGTTATATAAGACAGTTGTGAAATCCCCGGGCTCAACCTGGGAATTGCATCTGTGACTGTATGGCTAGAGTACGGTAGAGGGGGATGGAATTCCGCGTGTAGCAGTGAAATGCGTAGATATGCGGAGGAACACCGATGGCGAAGGCAATCCCCTGGACCTGTACTGACGCTCATGCACGAAAGCGTGGGGAGCAAACAGG` |
| ASV1539 | 404 | `TGGGGAATCTTGCGCAATGGGCGAAAGCCTGACGCAGCCATGCCGCGTGAATGATGAAGGTCTTAGGATTGTAAAATTCTTTCACCGGGGACGATAATGACGGTACCCGGAGAAGAAGCCCCGGCTAACTTCGTGCCAGCAGCCGCGGTAATACGAAGGGGGCTAGCGTTGCTCGGAATTACTGGGCGTAAAGGGAGCGTAGGCGGACTGTTAAGTTAGAGGTGAAAGCCCAGGGCTCAACCTTGGAATTGCCTTTGATACTGGCAGTCTTGAGTACGGAAGAGGTATGTGGAACTCCGAGTGTAGAGGTGAAATTCGTAGATATTCGGAAGAACACCAGTGGCGAAGGCGACATACTGGTCCGTTACTGACGCTGAGGCTCGAAAGCGTGGGGAGCAAACAGG` |
| ASV2075 | 404 | `TGGGGAATTTTCCGCAATGGGCGAAAGCCTGACGGAGCAATACCGCGTGAGGGACGAAGGCTCTTGGGTCGTAAACCTCTTTTCTCAAGGAAGATTAAGACGGTACTTGAGGAATAAGCATCGGCTAACTCTGTGCCAGCAGCCGCGGTAATACAGAGGATGCAAGCGTTATCCGGAATGATTGGGCGTAAAGCGTCCGCAGGTGGTTTAATAAGTCTGCTGTTAAAGCGTTAGGCTCAACCTAATAAGGGCAGTGGAAACTATTCGACTAGAGTATGTTAGGGGTAGAGGGAATTCCCAGTGTAGCGGTGAAATGCGTAGAGATTGGGAAGAACACCGGTGGCGAAAGCGCTCTGCTAGGACAATACTGACACTGAGGGACGAAAGCTAGGGGAGCGAATGGG` |
| ASV989 | 424 | `TGAGGAATATTGGACAATGGGCGCAAGCTTGATCCAGCCATGCCGCGTGCAGGAAGACGGTCCTATGGATTGTAAACTGCTTTTGTACAGGAAGAAACCCTCTCACGTGTGAGAGATTGACGGTACTGTAAGAATAAGGATCGGCTAACTCCGTGCCAGCAGCCGCGGTAATACGGAGGATCCAAGCGTTATCCGGAATCATTGGGTTTAAAGGGTCCGTAGGCGGTTTGTTAAGTCAGTGGTGAAAGCCCATCGCTCAACGGTGGAACGGCCATTGATACTGACAGACTTGAATTATAAAGAAGTAACTAGAATATGTAGTGTAGCGGTGAAATGCTTAGAGATTACATGGAATACCAATTGCGAAGGCAGGTTACTATTTATGGATTGACGCTGATGGACGAAAGCGTGGGTAGCGAACAGG` |
| ASV5626 | 408 | `TGAGGAATCTTCCGCAATGGGCGAAAGCCTGACGGAGCGACACCGCGTGAAGGATGAAACTCGTTTCGGGTGTAAACTTCTGTTCTGAGGGACGAAATTTTTGACGGTACCTCAGGAGCAAGCACCGGCTAATCCTGTGCCAGCAGCCGCGGTAAGACAGGAGGTGCAAGCGTTACTCGGAATTACTGGGCGTAAAGCGTCTGCAGGCTTCCTCCCACGTCTGGCGTGAAATGTCGACGCTCAACGTCGTCCCCACGCTGGAAACGAGGAGGATCGAGGCACTCAGAGGCATCTGGAATGTCGTGTGTAGGGGTAAAATCCGTTGATACACGATGGAACGCCGAAAGCGAAGGCAGGATGCTGGGAGTCGCCTGACGCTCAGAGACGAAAGCGTGGGGAGCAAAGGGG` |
| ASV2479 | 405 | `TGGGGAATCTTGCACAATGGGCGAAAGCCTGATGCAGCAACGCCGCGTGCGGGAAGAAGGCCCTAGGGTTGTAAACCGCTTTCAGTAGGGAAGAAAATGACGGTACCTACAGAAGAAGGTGCGGCCAACTACGTGCCAGCAGCCGCGGTAACACGTAGGCACCAAGCGTTATCCGGATTTATTGGGCGTAAAGAGCTCGTAGGCGGTTCAGTCAGTCGGGTGTGAAAACTTGAGGCTTAACCTCAAGAGGTCACCCGATACTACTGTGACTAGAGTGCGGTAGGGGAGCGGGGAATTCCTGGTGTAGCGGTGAAATGCGCAGATATCAGGAGGAACACCAGTGGCGAAGGCGCCGCTCTGGGCCGTAACTGACGCTGAGGAGCGAAAGCATGGGTAGCAAACAGG` |
| ASV2783 | 424 | `TGAGGAATATTGGTCAATGGACGCAAGTCTGAACCAGCCATGCCGCGTGCAGGATGACGGTGCTATGCATTGTAAACTGCTTTTGTACGAGGGTAAACTTGGGTACGTGTACCCAACTGAAAGTATTGTACGAATAAGGATCGGCTAACTCCGTGCCAGCAGCCGCGGTAATACGGAGGATCCGAGCGTTATCCGGATTTATTGGGTTTAAAGGGTGCGTAGGCGGTTTAATAAGTTAGAGGTGAAAGCTCGATGCTCAACATCGAAATTGCCTCTGATACTGTTAGACTAGAGTGTAGTTGCGGAAGGCGGAATGTGTGGTGTAGCGGTGAAATGCTTAGATATCACACAGAACACCGATTGCGAAGGCAGCTTTCCAAGCTACCACTGACGCTGAGGCACGAAAGCGTGGGGAGCGAACAGG` |
| ASV2107 | 429 | `TGGGGAATATTGGACAATGGGGGCAACCCTGATCCAGCAATGCCGCGTGTGTGAAGAAGGCCTGCGGGTTGTAAAGCACTTTGGTTGGGGAAGAAAAGCGTGGTTTTAATAGGACCATGAATTGACGTTACCCAAAGAACAAGCACCGGCTAACTCTGTGCCAGCAGCCGCGGTAATACAGAGGGTGCGAGCGTTAATCGGAATTACTGGGCGTAAAGGGCGCGTAGGCGGATAGATAAGTTAGGTGTGAAATGTCTGGGCTTAACCTAGAAATAGCATTTAATACTGTTTATCTAGAGTAGAGTAGAGGGAAGTGGAATTTCCGGTGTAGCGGTGAAATGCGTAGATATCGGAAAGAACACCAGTGGCGAAGGCGACTTCCTGGACTCAAACTGACGCTGAGGCGCGAAAGCATGGGTAGCAAACAGG` |
| ASV1716 | 404 | `TGGGGAATCTTGCGCAATGGGGGCAACCCTGACGCAGCCATACCGCGTGTGTGATGAAGGTCTTCGGATTGTAAAGCACTTTCAGTGGGGACGATGATGACGGTACCCACAGAAGAAGTCACGGCTAACTTCGTGCCAGCAGCCGCGGTAATACGAAGGTGGCGAATGTTGCTCGGATTCACTGGGCGTAAAGCGTCGGTAGGTGGTTTGTTAAGTCAGAGGTGAAAGCCCCGCGCTCAACGTGGGAATTGCCTTTGAAACTGATGAACTTGAAGATATGAGAGGATGGCGGAATGACTGGTGTAGAGGTAAAATTCATAGATATCAGTCGGAACACCCAAGGCGAAGGCAGCTATCTGGCATATTCTTGACACTGAGCGACGACAGCGTGGGGAGCAAACAGG` |
| ASV4783 | 404 | `TGGGGAATATTGGGCAATGGGCGAAAGCCTGACCCAGCAACGCCGCGTGAAGGATGAAGGCCTTAGGGTTGTAAACTTCTTTTACCAGGGAAGATAATGACTGTACCTGGAGAATAAGCATCGGCTAACTTCGTGCCAGCAGCCGCGGTAATACGAAGGATGCTAGCGTTACTCGGAATTATTGGGCGTAAAGAATGCGTAGGCGGACTTGTAAGTTGATGGTGAAATTCCTGGGCTCAACCCGGGCGCTGCTGTCAAAACTGCATGTCTTGAATATATCAGGGAGTGAGGGAATTTCTAGTGTAGGGGTAAAATCCGTAGATATTAGAAGGAACACCAGAGGCGAAAGCGCTTACTTGGGATATTATTGACGCTGAGGCATGAAAGCGTGGGGAGCAAACAGG` |
| ASV3414 | 430 | `TGGGGAATATTGCGCAATGGGCGAAAGCCTGACGCAGCGACGCCGCGTGGGTGATGAAGGCCTTCGGGTCGTAAAGCCCTGTCAAGGGGGACGAAACCTCGCCGACCTAATATGTCGGCGACTTGACGGTACCTCTGAAGGAAGCACCGGCTAACTCCGTGCCAGCAGCCGCGGTAATACGGAGGGTGCGAGCGTTGTTCGGAATCACTGGGCGTAAAGCGCGTGTAGGCGGCCTCTTCAGTCTGGTGTGAAAGCCCGGGGCTCACCCCCGGAAGTGCATTGGATACTGGGAGGCTGGAGTACCGGAGAGGAGGGTGGAATTCCTGGTGTAGCGGTGAAATGCGTAGATATCAGGAGGAACACCGGTGGCGAAGGCGGCCCTCTGGACGGATACTGACGCTGAGACGCGAAAGCGTGGGGAGCAAACAGG` |
| ASV1996 | 404 | `TGGGGAATATTGGGCAATGGACGAAAGTCTGACCCAGCCATGCCGCGTGAATGATGAAGGCCTTAGGGTTGTAAAGTTCTTTCGCCGATGAAGATAATGACGGTAGTCGGAGAAGAAGCCCCGGCTAACTTCGTGCCAGCAGCCGCGGTAATACGAAGGGGGCTAGCGTTGTTCGGAATTACTGGGCGTAAAGCGAGCGTAGGTTGCTCGACAAGTCAGTGGTGAAAGCCCAAGGCTCAACCTTGGAACTGCCATTGAAACTGTCGAGCTGGAATCTCAGAGGGGGTAGCGGAATTCCAAATGTAGGGGTGAAATCCGTAGATATTTGGAGGAACACCGGTGGCGAAGGCGGCTACCTGGATGAGTATTGACACTGAGGCTCGAAAGCGTGGGGATCAAACAGG` |
| ASV4810 | 423 | `TAGGGAATATTGGACAATGGGGGGAACCCTGATCCAGCCATGCCGCGTGAGGGAAGAAGGTCCTCTGGATTGTAAACCTCTTTTGGCGGGGGACAAACCTGCGCTACACGCGCAGCTGAGGGTACCCGCAGAATAAGCACCGGCTAACTCCGTGCCAGCAGCCGCGGTAATACGGAGGGTGCAAGCGTTGTCCGGATTCATTGGGTTTAAAGGGTGCGTAGGCGGTCTTTTAAGTCAGCGGTGAAATCCCCCGGCTCAACTGGGGAACTGCCGTTGATACTGAGAGACTTGAGTTCGTCCGAGGTCGCTGGAATGCGTGGTGTAGCGGTGAAATGCATAGATATCACGCAGAACACCGATTGCGAAGGCAGGTGACTGGGACGCGACTGACGCTGAGGCACGAAAGTGTGGGGAGCAAACAGG` |
| ASV4876 | 404 | `TGGGGAATATTGGACAATGGGCGAAAGCCTGATCCAGCTATGTCACGTGAACGATGAAGGTCTTCGGATTGTAAAGTTCTTTTAGTGGGGATGATAATGACAGTACCCACAAAAAAAGCCCCGGCTAACTTCGTGCCAGCAGCCGCGGTAATACGAAGGGGGCTAGCGTTATTCGGAATGATTGGGCGTAAAGGACACGTAGGCGGTTTTGTAAGTTATGGGTGAAATCCCTGGGCTCAACCCAGGAACTGCTCATAATACTGCATGACTTGAGTATGGGGGAGGACAGTGGAATCTTTGGTGTAGAGGTGAAATTCTTAGATATCAGAGAGAACGTCGGTGGCGAAAGCGACTGTCTAAAACATTACTGACGCTGAGGTGTGAAGGCGTGGGGAGCAAATCGG` |
| ASV135 | 404 | `TGGGGAATATTGGACAATGGGCGAAAGCCTGATCCAGCAATGCCGCGTGAGTGAAGAAGGCCTTCGGGTTGTAAAGCTCTTTTACCCGGGAAGATAATGACTGTACCGGGAGAATAAGCCCCGGCTAACTCCGTGCCAGCAGCCGCGGTAATACGGAGGGGGCTAGCGTTGTTCGGAATTACTGGGCGTAAAGCGTACGTAGGCGGCTATTCAAGTCAGGGGTGAAAGCCTGGGGCTCAACCTCAGAATTGCCCTTGAAACTAGATAGCTTGAACACGGGAGAGGTGAGTGGAATTCCGAGTGTAGAGGTGAAATTCGTAGATATTCGGAAGAACACCAGTGGCGAAGGCGGCTCACTGGACCGTTGTTGACGCTGAGGTACGAAAGCGTGGGGAGCAAACAGG` |
| ASV276 | 429 | `TGGGGAATATTGGACAATGGGCGCAAGCCTGATCCAGCCATGCCGCGTGTGTGAAGAAGGCCTTCGGGTTGTAAAGCACTTTTGTTGGGAAAGAAATCCTGTCGGTTAATACCCGGCGGGGCTGACGGTACCCAAAGAATAAGCACCGGCTAACTTCGTGCCAGCAGCCGCGGTAATACGAAGGGTGCAAGCGTTACTCGGAATTACTGGGCGTAAAGCGTGCGTAGGTGGTTTGTTAAGTCAGATGTGAAAGCCCCGGGCTCAACCTGGGAATTGCATTTGATACTGGCAGACTAGAGTGCGGTAGAGGAGAGTGGAATTCCCGGTGTAGCAGTGAAATGCGTAGAGATCGGGAGGAACATCAGTTGCGAAGGCGGCTCTCTGGACCAGCACTGACACTGAGGCACGAAAGCGTGGGGAGCAAACAGG` |
| ASV1664 | 423 | `TAGGGAATATTGGTCAATGGGCGAAAGCCTGAACCAGCCATGCCGCGTGCAGGAAGAAGGCCCTCTGGGTTGTAAACTGCTTTTGACAGGGAACAAGAAGTCCATGCGTGGATTAGTGAGTGTACCTGTAGAATAAGCCACGGCTAACTACGTGCCAGCAGCCGCGGTAATACGTAGGTGGCAAGCGTTGTCCGGATTTATTGGGTTTAAAGGGTGCGTAGGCGGCCGCCTAAGTCAGTTGTGAAATACCCCGGCTCAACCGGGGGGGTGCGATTGATACTGGGCGGCTCGAGTAACAGCGAGGTTGGCGGAATTGACGGTGTAGCGGTGAAATGCATAGATATCGTCAAGAACACCGATAGCGAAGGCAGCCAACTAGGGGTTAACTGACGCTGAGGCACGAAAGTGCGGGGATCAAACAGG` |
| ASV1011 | 429 | `TGGGGAATTTTGGACAATGGGCGCAAGCCTGATCCAGCAATGCCGCGTGAGTGAAGAAGGCCTTCGGGTTGTAAAGCTCTTTTGTCAGGGAAGAAACGGTCTAGGTTAATACCTTGGGCTAATGACGGTACCTGAAGAATAAGCACCGGCTAACTACGTGCCAGCAGCCGCGGTAATACGTAGGGTGCAAGCGTTAATCGGAATTACTGGGCGTAAAGCGTGCGCAGGCGGTTTTGTAAGTCTGTCGTGAAAGCCCCGGGCTCAACCTGGGAATTGCGATGGAGACTGCAAGGCTTGAATCTGGCAGAGGGGGGTAGAATTCCACGTGTAGCAGTGAAATGCGTAGAGATGTGGAGGAACACCGATGGCGAAGGCAGCCCCCTGGGTCAAGATTGACGCTCATGCACGAAAGCGTGGGGAGCAAACAGG` |
| ASV2965 | 404 | `TGGGGAATATTGGACAATGGGGGCAACCCTGATCCAGCCATGCCGCGTGAATGATGAAGGCCTTCGGGTTGTAAAGTTCTTTTAGCGGGGAAAATAATGATGGTACCCGCAGAAAAAGCACCGGCTAACTTCGTGCCAGCAGCCGCGGTAATACGAAGGGTGCTAGCGTTGCTCGGAATTACTGGGCGTAAAGGGTTTGTAGGCGGTCTTTTAAGCTAGTGGTGAAAGCCCAGGGCTCAACCCTGGAATTGCCATTAGAACTGGAAGGCTAGAGAGTGTTAGAGGTAAGTGGAATTCCTAGTGTAGGAGTGAAATCCGTAGAGATTAGGAGGAATATCGATGGCGAAGGCAGCTTACTGGGACATTTCTGACGCTGAAAAACGAAAGCGTGGGTAGCAAACAGG` |
| ASV4297 | 407 | `TCGAGAATTTTTCACAATGGGGGAAACCCTGATGGAGCGACGCCGCGTGGAGGACGAAGGCCTTCGGGTCGTAAACTCCTGTCATGCGGGAACAAGAAAGTGATAGTACCGCAAGAGGAAGGGACGGCTAACTCTGTGCCAGCAGCCGCGGTAATACAGAGGTCCCAAGCGTTGTTCGGATTCATTGGGCGTAAAGGGTGCGTAGGTGGCGATGTAAGTCTGGTGTGAAATCTCAGGGCTCAACCTTGAAATTGCATTGGATACTGCGTTGCTTGAGGATTGTAGAGGAGAGTGGAATTCACGGTGTAGCAGTGAAATGCGTAGATATCGTGAGGAAGACCAGTTGCGAAGGCGACTCTCTGGGCAATTCCTGACACTGAGGCACGAAGGCCAGGGGAGCAAACGGG` |
| ASV1471 | 429 | `TGGGGAATTTTGGACAATGGGCGCAAGCCTGATCCAGCAATGCCGCGTGCAGGAAGAAGGCCTTCGGGTTGTAAACTGCTTTTGTACGGAACGAAAAGGCTCTGGTTAATACCTGGGGCTCATGACGGTACCGTAAGAATAAGCACCGGCTAACTACGTGCCAGCAGCCGCGGTAATACGTAGGGTGCAAGCGTTAATCGGAATTACTGGGCGTAAAGCGTGCGCAGGCGGTTTTGTAAGACAGTCGTGAAATCCCCGGGCTCAACCTGGGAATGGCGATTGTGACTGCAAAGCTGGAGTGCGGCAGAGGGGGATGGAATTCCGCGTGTAGCAGTGAAATGCGTAGATATGCGGAGGAACACCGATGGCGAAGGCAATCCCCTGGACCTGTACTGACGCTCATGCACGAAAGCGTGGGGAGCAAACAGG` |
| ASV2031 | 429 | `TGGGGAATTTTGGACAATGGGGGCAACCCTGATCCAGCAATGCCGCGTGAGTGAAGAAGGCCTTCGGGTTGTAAAGCTCTTTTGTTAGGGAAGAAAAGGTCTTCGATAATACCGAGGACTGATGACGGTACCTAAAGAATAAGCACCGGCTAACTACGTGCCAGCAGCCGCGGTAATACGTAGGGTGCAAGCGTTAATCGGAATTACTGGGCGTAAAGCGTGCGCAGGCGGCCTCGCAAGTCAGATGTGAAATCCCCGAGCTCAACTTGGGAACTGCATTTGAAACTACGAGGCTAGAGTGTGTCAGAGGGAGGTAGAATTCCACGTGTAGCAGTGAAATGCGTAGATATGTGGAGGAATACCAGTGGCGAAGGCGGCCTCCTGGGATAACACTGACGCTCATGTACGAAAGCGTGGGGAGCAAACAGG` |
| ASV175 | 424 | `TGAGGAATATTGGACAATGGGCGCAAGCCTGATCCAGCCATGCCGCGTGCAGGATGACGGTCCTATGGATTGTAAACTGCTTTTGCACAGGAAGAAACACTACTTCGTGAAGTAGCTTGACGGTACTGTGAGAATAAGGATCGGCTAACTCCGTGCCAGCAGCCGCGGTAATACGGAGGATCCAAGCGTTATCCGGAATCATTGGGTTTAAAGGGTCCGTAGGCGGTTTAGTAAGTCAGTGGTGAAAGCCCATCGCTCAACGGTGGAACGGCCATTGATACTGCTAGACTTGAATTATTAGGAAGTAACTAGAATATGTAGTGTAGCGGTGAAATGCTTAGAGATTACATGGAATACCAATTGCGAAGGCAGGTTACTACTAATGGATTGACGCTGATGGACGAAAGCGTGGGTAGCGAACAGG` |
| ASV464 | 404 | `TGGGGAATCTTAGACAATGGGCGCAAGCCTGATCTAGCCATGCCGCGTGAGCGATGAAGGCCTTAGGGTTGTAAAGCTCTTTCAGGGGGGAAGATAATGACTGTACCCCCAGAAGAAGCCCCGGCTAACTCCGTGCCAGCAGCCGCGGTAATACGGAGGGGGCTAGCGTTGTTCGGAATTACTGGGCGTAAAGCGCACGTAGGCGGACTGGAAAGTCAGAGGTGAAATCCCAGGGCTCAACCTTGGAACTGCCTTTGAAACTCCCGGTCTTGAGGTCGAGAGAGGTGAGTGGAATTCCGAGTGTAGAGGTGAAATTCGTAGATATTCGGAGGAACACCAGTGGCGAAGGCGGCTCACTGGCTCGATACTGACGCTGAGGTGCGAAAGCGTGGGGAGCAAACAGG` |
| ASV1869 | 425 | `TGGGGAATATTGGACAATGGGCGCAAGCCTGATCCAGCAATGCCGCGTGAGTGATGAAGGCCTTCGGGTTGTAAAGCTCTGTCGGGAGGGACGAAAAAGTCAGGGTAACGCCTGATATTGACGGTACCTCCAAAGGAAGCACCGGCTAACTCTGTGCCAGCAGCCGCGGTAATACAGAGGGTGCAAGCGTTGTTCGGAATTACTGGGCGTAAAGCGCGTGTAGGCGGCTTCGTTAGTCGGATGTGAAAGCCCTCGGCTCAACCGAGGAAGTGCATCCGATACTGCGATGCTTGAGTGTTGGAGGGGGTGGCGGAATTCCCGGTGTAGAGGTGAAATTCGTAGATATCGGGAGGAACACCTGTGGCGAAGGCGGCCACCTGGACAACAACTGACGCTGAGACGCGAAAGCGTGGGTAGCAAACAGG` |
| ASV2718 | 404 | `TGGGGAATATTGGACAATGGGCGAAAGCCTGATCCAGCAATGCCGCGTGAGTGATGAAGGCCTTAGGGTTGTAAAGCTCTTTTACCCGGGAAGATAATGACTGTACCGGGAGAATAAGCCCCGGCTAACTCCGTGCCAGCAGCCGCGGTAATACGGAGGGGGCTAGCGTTGTTCGGAATTACTGGGCGTAAAGCGCACGTAGGCGGCTTTGTAAGTTAGAGGTGAAAGCCCGGGGCTCAACTCCGGAACTGCCTTTAAGACTGCATCGCTAGAATTGTGGAGAGGTGAGTGGAATTCCGAGTGTAGAGGTGAAATTCGTAGATATTCGGAAGAACACCAGTGGCGAAGGCGACTCACTGGACACATATTGACGCTGAGGTGCGAAAGCGTGGGGAGCAAACAGG` |
| ASV1913 | 405 | `TAGGGAATCTTGCTCAATGGGCGAAAGCCTGAAGCAGCGACGCCGCGTGCGGGACGAAGGCCCTCGGGTCGTAAACCGCTTTCAGCAGGAACGAAATTGACGGTACCTGCAGAAGAAGCCCCGGCCAACTACGTGCCAGCAGCCGCGGTGATACGTAGGGGGCAAGCGTTGTCCGGATTTATTGGGCGTAAAGAGCTCGTAGGTGGTTCGGTAAGTCGGGTGTGAAATATCAGGGCTCAACCCTGAGAGGCCACTCGATACTGCTGTGACTAGAGTCCGGTAGAGGAGTGTGGAATTCCTGGTGTAGCGGTGAAATGCGCAGATATCAGGAGGAACACCCATGGCGAAGGCAGCACTCTGGGCCGGTACTGACACTGAGGAGCGAAAGCGTGGGGAGCGAACAGG` |
| ASV2256 | 429 | `TGGGGAATTTTGGACAATGGGCGCAAGCCTGATCCAGCCATGCCGCGTGCGGGAAGAAGGCCTTCGGGTTGTAAACCGCTTTTGTCAGGGAAGAAACGCTCCGGAATAATACTCTGGGGTAATGACGGTACCTGAAGAATAAGCACCGGCTAACTACGTGCCAGCAGCCGCGGTAATACGTAGGGTGCAAGCGTTAATCGGAATTACTGGGCGTAAAGCGTGCGCAGGCGGTTATGCAAGACAGATGTGAAATCCCCGGGCTCAACCTGGGAACTGCATTTGTGACTGCATAGCTAGAGTACGGTAGAGGGGGATGGAATTCCGCGTGTAGCAGTGAAATGCGTAGATATGCGGAGGAACACCGATGGCGAAGGCAATCCCCTGGACCTGTACTGACGCTCATGCACGAAAGCGTGGGGAGCAAACAGG` |
| ASV4581 | 430 | `TGGGGAATCTTGCACAATGGGCGAAAGCCTGATGCAGCAACGCCGCGTGAGTGAAGAAGGTTCTTGGATTGTAAAGCTCTTTCGGCTAGAAAGAAGGGCGACACTGCGAATAGTTTTGTCGTTTGACGGTACTAGAAGAAGAAGCACCGGCTAACTCCGTGCCAGCAGCCGCGGTAATACGGAGGGTGCAAGCGTTGTTCGGATTGATTGGGCGTAAAGCGCACGTAGGCGGGTTCACAAGTCGGATGTGAAAGCCCCGGGCTTAACCTGGGAATTGCATCTGAAACTATGAGTCTTGAATGCTTGAGGGGTTGGTGGAATTCTTGGTGTAGGAGTGAAATCCGTAGATATCAAGAGGAATACCGGAGGCGAAGGCGGCCGACTGGCAATGCATTGACGCTGAGGTGCGAAAGTGCGGGGAGCAAACAGG` |
| ASV3114 | 424 | `TAAGGAATATTGGTCAATGGACGCAAGTCTGAACCAGCCATGCCGCGTGCAGGATGACTGCCCTATGGGTTGTAAACTGCTTTTGTACGGGAAGAAACCTCTCTACGTGTAGAGAGTTGACGGTACCGTAAGAATAAGGATCGGCTAACTCCGTGCCAGCAGCCGCGGTAATACGGAGGATCCAAGCGTTATCCGGATTTATTGGGTTTAAAGGGTGCGTAGGCGGCCTATTAAGTCAGTGGTGAAAGCCTGGAGCTTAACTCCAGAACTGCCATTGATACTGATTGGCTTGAGTACAGTTGAGGTAGGCGGAATGTGTCATGTAGCGGTGAAATGCTTAGATATGACACAGAACACCGATTGCGAAGGCAGCTTGCTAAACTGTAACTGACGCTGAGGCACGAAAGCGTGGGTAGCAAACAGG` |
| ASV1295 | 404 | `TGGGGAATCTTGCACAATGGAGGAAACTCTGATGCAGCGACGCCGCGTGGACGATGAAGCCCTTCGGGGTGTAAAGTCCTTTCGACCGGAACGATAATGACGGTACCGGTGGAAGAAGCTGCGGCTAACTACGTGCCAGCAGCCGCGGTAATACGTAGGCAGCAAGCGTTGTTCGGAATTACTGGGCGTAAAGAGTTCGTAGGCGGTGCGGCAAGTTTGGTGTGAAATCTCCCGGCTCAACTGGGAGGGTGCGCCGAATACTGCCGTGCTCGAGTATAGGAGAGGTAAGCGGAATTCCTGGTGTAGCGGTGAAATGCGTAGATATCAGGAGGAACACCTGTGGTGTAGACGGCTTACTGGACTATAACTGACGCTGAGGAACGAAAGCGTGGGTAGCAAACAGG` |
| ASV4649 | 407 | `TTAGGAATTTTGCGCAATGGGAGAAATCCTGACGCAGCAACGCCGCGTGAGTGATGAAGGCCTTCGGGTTGTAAAGCTCTTTTAGTAGGAACGAAAAAAATGACGGTACCTACAGAATAAGCACCGGCTAACTCTGTGCCAGCAGCCGCGGTAATACAGAGGGTGCAAGCGTTATTCGGAATTATTGGGCGTAAAGGGCGCGTAGGTGGTTTCGTAAGTCTGGTGTGAAATCCCTGGGCTTAATCCAGGAAGTGCATTGGATACTGCGAGACTAGAGTACAGGAGAGGAGAACGGAATTCCTGGTGTAGAGGTGAAATTCGTAGATATCAGGAGGAACAACAGTGGCGAAGGCGGTTCTCTGGACTGTAACTGACACTGAGGCGCGACAGCGTGGGGAGCAAACAGG` |
| ASV4858 | 407 | `TGGGGAATTTTCCGCAATGGGCGAAAGCCTGACGGAGCAACGCCGCGTGGGTGATGAAGATTTTCGGATCGTAAAGCCCTTTCAGCAGGGACGAAGCGAGTGACGGTACCTGCAGAAGAAGCATCGGCTAACTACGTGCCAGCAGCCGCGGTAATACGTAGGATGCAAGCGTTGTCCGGATTTATTGGGCGTAAAGCGAACGTAGGCGGTTTCTTAAGTTTGGTCTTAAAGACTGGGGCTCAACCTCAGGAGTGGACTGAATACTGGGAGACTAGAGGATGTTAGAGGAAAGTGGAATTCCAAGTGTAGCGGTGAAATGCGTAGATATTTGGAGGAACACCAGTGGCGAAAGCGGCTTTCTGGGACATTCCTGACGCTGAGGTTCGAAAGCTCGGGGAGCAAACAGG` |
| ASV3044 | 429 | `TGGGGAATTTTGGACAATGGGCGAAAGCCTGATCCAGCAATGCCGCGTGTGCGAAGAAGGCCTTCGGGTTGTAAAGCACTTTTGTCAGAGAAAAAAAGACTTTTGCTAATATCAGAAGTTTATGATGGTATCTGAAGAATAAGCACCGGCTAACTACGTGCCAGCAGCCGCGGTAATACGTAGGGTGCAAGCGTTAATCGGAATTATTGGGCGTAAAGCGTGCGCAGGCGGTTGTATAAGATAGATGTGAAATCCTTGGGCTCAACCTGAGAACTGCATTTATGACTGTACGACTAGAGTGTGTCAGAGGGGGGTAGAATTCCACGTGTAGCAGTGAAATGCGTAGAGATGTGGAGGAATACCGATGGCGAAGGCAGCCCCCTGGGATAACACTGACGCTCATGCACGAAAGCGTGGGGAGCAAACAGG` |
| ASV3385 | 404 | `TGGGGAATATTGGACAATGGGGGCAACCCTGATCCAGCCATGCCGCGTGAGTGATGAAGGCCTTAGGGTTGTAAAGCTCTTTTACTCGGGAAGATAATGACTGTACCGAGAGAATAAGCCCCGGCTAACTTCGTGCCAGCAGCCGCGGTAATACGAAGGGGGCTAGCGTTGTTCGGAATTACTGGGCGTAAAGCGCATGTAGGCGGCGATTTAAGTCGGGGGTGAAATCCCAGAGCTCAACTCTGGAACTGCCTTCGATACTATTTTGCTAGAGTATGGAAGAGGTTGGTGGAACTGCAAGTGTAGAGGTGAAATTCGTAGATATTTGCAAGAACACCGGTGGCGAAGGCGGCCAACTGGTCCATCACTGACGCTGAGATGCGAAAGCGTGGGGAGCAAACAGG` |
| ASV3041 | 430 | `TAGGGAATATTGCGCAATGGGGGAAACCCTGACGCAGCAACGCCGCGTGAGTGAGGAAGGTCTTCGGATTGTAAAACTCTTTTATCAGGGAAAAATGATTTAGTGGTTAATACCCATTAGAAGTGATGGTACCTGAGGAATAAGCACCGGCTAACTTCGTGCCAGCAGCCGCGGTAATACGAAGGGTGCAAGCGTTGTTCGGAATCATTGGGCGTAAAGCGCGCGCAGGCGGATCAGCAAGTCAGATGTGAAATCTCGGAGCTCAACTCCGAAACTGCGTCTGAAACTGCTAGTCTAGAATGTCAGAGGGGGTAGGGGAATTTCACGTGTAGGGGTAAAATCCGTAGAGATGTGAAGGAACACCGGAGGCGAAGGCGCCTGCCTGGATGACTATTGACGCTGAGGCGCGAAAGCGTGGGGAGCAAACAGG` |
| ASV130 | 429 | `TGGGGAATTTTGGACAATGGACGCAAGTCTGATCCAGCCATGCCGCGTGCGGGAAGAAGGCCTTCGGGTTGTAAACCGCTTTTGTCAGGGAAGAAACGCTCTGGGCTAATACCCTGGGGTAATGACGGTACCTGAAGAATAAGCACCGGCTAACTACGTGCCAGCAGCCGCGGTAATACGTAGGGTGCAAGCGTTAATCGGAATTACTGGGCGTAAAGCGTGCGCAGGCGGTTATGCAAGACAGATGTGAAATCCCCGGGCTCAACCTGGGAACTGCATTTGTGACTGCATGGCTAGAGTACGGTAGAGGGGGATGGAATTCCGCGTGTAGCAGTGAAATGCGTAGATATGCGGAGGAACACCGATGGCGAAGGCAATCCCCTGGACCTGTACTGACGCTCATGCACGAAAGCGTGGGGAGCAAACAGG` |
| ASV4907 | 429 | `TGGGGAATATTGCACAATGGAGGAAACTCTGATGCAGCGACGCCGCGTGGGTGATGAAGGCCTTCGGGTTGTAAAGCCCTGTCGGCTGGGAACAAGTTAGACGTGCTCATACCACGTTTGATTGAGTGTACCAGCAAAGGAAGCACCGGCTAACCCTGTGCCAGCAGCCGCGGTAATACAGGGGGTGCTAGCGTTGTTCGGAATTACTGGGCGTAAAGGGCGCGTAGGCGGTGCCGCAAGTCAGGGGTGAAATCCCGGAGCTTAACTCCGGAATTGCTTTTGATACTGCGGTGCTAGAGTGTGTTAGAGGATGGCGGAATTCCTAGTGTAGAAGTGAAATTCGTAGATATTAGGAAGAACACCGGTGGCGAAGGCGGCCATCTGGGACACAACTGACGCTGAGGCGCGAAAGCGTGGGGATCAAACAGG` |
| ASV3559 | 430 | `TGGGGAATCTTGCACAATGGGCGAAAGCCTGATGCAGCAACGCCGCGTGGGTGAAGACGGCCTTCGGGTTGTAAAGCCCTTTCGGTAGGAAAGAAGGGTGCTACGGTAAATAATCGTGGCATTTGACGGTACCTAAAGAAGAAGCACCGGCTAATTTCGTGCCAGCAGCCGCGGTAATACGAAAGGTGCAAGCGTTGTTCGGAATGACTGGGCGTAAAGCGCACGTAGGCGGATTCGCAAGTAGGATGTGAAAGCCCCAGGCTCAACCTGGGAATGGCACCCTAAACTACGGATCTTGAATGCTTAAGAGGTTGGTGGAATTCCTGGTGTAGGAGTGAAATCCGTAGATATCAGGAGGAATATCGGAGGCGAAGGCGGCCAACTGGTGATGCATTGACGCTGAGGTGCGAAAGCGTGGGGAGCAAACAGG` |
| ASV4312 | 405 | `TGGGGAATTTTCCGCAATGGGCGAAAGCCTGACGGAGCAAGACCGCGTGAGGGAGGAAGGCCTATTGGTTGTAAACCTCTTTTCTCAGGGAAGATAATGACGGTACCTGAGGAATCAGCATCGGCTAACTCCGTGCCAGCAGCCGCGGTAAGACGGAGGATGCAAGCGTTATCCGGAATGATTGGGCGTAAAGCGTCCGTAGGTGGTTAAATAAGTTTGCTGTCAAAGAGCGGAGCTTAACTCCGTCAAGGCGGTGGATACTGTTTGACTAGAGTGCGGTAGGGGTAGAGGGAATTCCAGGTGTAGCGGTGAAATGCGTAGATATCTGGAAGAACACCAGTGGCGAAAGCGCTCTACTGGACCTGACACTGACACTGAGGGACGAAAGCTAGGGGAGCGAATGGG` |
| ASV2231 | 429 | `TGGGGAATTTTGGACAATGGACGAAAGTCTGATCCAGCCATTCCGCGTGCAGGATGAAGGCCCTCGGGTTGTAAACTGCTTTTGTACGGAACGAAAAGGTCTCTCCTAATACGAGAGGCTCATGACGGTACCGTAAGAATAAGCACCGGCTAACTACGTGCCAGCAGCCGCGGTAATACGTAGGGTGCAAGCGTTAATCGGAATTACTGGGCGTAAAGCGTGCGCAGGCGGTTATATAAGACAGATGTGAAATCCCCGGGCTCAACCTGGGAACTGCATTTGTGACTGTATAGCTAGAGTACGGTAGAGGGGGATGGAATTCCGCGTGTAGCAGTGAAATGCGTAGATATGCGGAGGAACACCGATGGCGAAGGCAATCCCCTGGACCTGTACTGACGCTCATGCACGAAAGCGTGGGGAGCAAACAGG` |
| ASV2601 | 404 | `TGGGGAATATTGGACAATGGGCGAAAGCCTGATCCAGCAATGCCGCGTGAGTGATGAAGGCCTTAGGGTTGTAAAGCTCTTTTACCCGGGATGATAATGACAGTACCGGGAGAATAAGCCCCGGCTAACTCCGTGCCAGCAGCCGCGGTAATACGGAGGGGGCTAGCGTTGTTCGGAATTACTGGGCGTAAAGCGCACGTAGGCGGCTTTGTAAGTTAGAGGTGAAAGCCCGGGGCTCAACTCCGGAATTGCCTTTAAGACTGCATCGCTAGAACGTCGGAGAGGTGAGTGGAATTCCGAGTGTAGAGGTGAAATTCGTAGATATTCGGAAGAACACCAGTGGCGAAGGCGACTCACTGGACGACTGTTGACGCTGAGGTGCGAAAGCGTGGGGAGCAAACAGG` |
| ASV778 | 387 | `GCGAGAAACCTTTGCAATGTGCGCAAGCACGACAAGGTTAATCCGAGTGTTTCCTGCTAAAGGAATCTTTTGTTAGTTCTAAAACCACTGATGAATAAGAGGTGGGCAAGTTCTGGTGTCAGCCGCCGCGGTAAAACCAGCACCTCAAGTGGTCAGGAGGATTATTGGGCCTAAAGCATCCGTAGCCGGTTCTGTAAGTTTTCGGTTAAATCCACTAGCTTAACTAATGGGCTGCCGAAAATACTGCAGAACTAGGGAGTGGGAGAGGTAGACGGTACTCGGTAGGAAGGGGTAAAATCCTTTGATCTATTGATGACCACCTGTGGCGAAGGCGGTCTACCAGAACACGTTCGACGGTGAGGGATGAAAGCTGGGGGAGCAAACCGG` |
| ASV4792 | 429 | `CTAAGAATCTTCCGCAATGGGCGAAAGCCTGACGGAGCGATGCCGCGTGATCGATGAAGGTCGAGAGATTGTAAAGATCTTTTCCAACTGAGGAATAAGTGGACCAGGGAATGGGTTCATGATGACGTTAAGTTGGGAATAAGCCCCGGCTAATTACGTGCCAGCAGCCGCGGTAACACGTAAGGGGCGAGCGTTGTTCGGAATTATTGGGCGTAAAGGGCACGCAGGCGGTTTTACAAGTCCGATGTTAAAGACCTGGGCTTAACTCAGGGAATGCGTTGGAAACTGTAAGACTAGAATCTTGGAGGGGGAGTCGGAATTCCTAGTGTAGGGGTGAAATCTGTTGATATTAGGAAGAACACCGGTGGCGAAGGCGGACTCCTGGCCGAAGATTGACGCTGAGGTGCGAAAGCATAGGGAGCAAACAGG` |
| ASV484 | 429 | `TGGGGAATATTGGACAATGGGCGCAAGCCTGATCCAGCCATGCCGCGTGTGTGAAGAAGGCCTTAGGGTTGTAAAGCACTTTCAGTGGGGAGAAAGGTCATTCACCTAATACGTGAGTGAATTGATGTTACCCACAGAAGAAGCACCGGCTAACTCCGTGCCAGCAGCCGCGGTAATACGGAGGGTGCAAGCGTTAATCGGAATTACTGGGCGTAAAGCGTACGTAGGTGGTTTGTTAAGCCAGCTGTGAAATCCCCGGGCTCAACCTGGGCACTGCAGTTGGAACTGGCAAGCTAGAGTAGGGTAGAGGGGTGTGGAATTCCAGGTGTAGCGGTGAAATGCGTAGATATCTGGAGGAACATCAGTGGCGAAGGCGACACCCTGGACTCATACTGACACTGAGGTACGAAAGCGTGGGGAGCAAACAGG` |
| ASV188 | 423 | `TGAGGAATATTGGTCAATGGGCGCGAGCCTGAACCAGCCAAGTCGCGTGAGGGAAGACGGTTCTATGGATTGTAAACCTCTTTTGTCAGGGAGCAACGGGTGCTACGCGTAGCGCCATGAGAGTACCTGAAGAAAAAGCATCGGCTAACTCCGTGCCAGCAGCCGCGGTAATACGGAGGATGCGAGCGTTATCCGGATTTATTGGGTTTAAAGGGTGCGTAGGCGGGCTATCAAGTCAGCGGTAAAATTTCGGGGCTCAACCCCGACTCGCCGTTGAAACTGACAGTCTAGAGTGAGAGTGAGGTATTCGGAATGTGTAGTGTAGCGGTGAAATGCATAGATATTACGCAGAACTCCAATTGCGAAGGCAGAGTACCAACTCTTAACAGACGCTGAGGCACGAAAGTGTGGGTATCGAACAGG` |
| ASV1582 | 429 | `TGGGGAATTTTGGACAATGGGCGCAAGCCTGATCCAGCCATGCCGCGTGTGTGAAGAAGGCCTTCGGGTTGTAAAGCACTTTCGGCCGGAACGAAATCGCCTTTGTTAACAGCAAGGGTGGATGACGGTACCGGAAGAAGAAGCACCGGCTAACTACGTGCCAGCAGCCGCGGTAATACGTAGGGTGCGAGCGTTAATCGGAATTACTGGGCGTAAAGGGTGCGCAGGCGGTTGCGCAAGTCAGGCGTGAAATCCCCGGGCTTAACCTGGGAATGGCGCTTGAAACTACGTGACTCGAGTATGGCAGAGGGAGGTGGAATTCCACGTGTAGCGGTGAAATGCGTAGAGATGTGGAGGAACACCGATGGCGAAGGCAGCCTCCTGGGCCAATACTGACGCTCATGCACGAAAGCGTGGGGAGCAAACAGG` |
| ASV10 | 423 | `TAGGGAATATTGGGCAATGGAGGCAACTCTGACCCAGCCATGCCGCGTGCAGGATGAAGGCGTTATGCGTTGTAAACTGCTTTTATATAGGAAGAAATAGACCTTGCGAGGTAAGTTGACGGTACTATATGAATAAGCACCGGCTAACTCCGTGCCAGCAGCCGCGGTAATACGGAGGGTGCAAGCGTTGTCCGGATTTATTGGGTTTAAAGGGTGCGTAGGCGGTTTATTAAGTCAGTGGTGAAAGACGGTCGCTCAACGATTGCAGTGCCATTGATACTGATAGACTTGAGTGGGATTGAGGTAGCTGGAATGGATAGTGTAGCGGTGAAATGCATAGATATTATCCAGAACACCAATTGCGTAGGCAAGTTACTAAGTCTCAACTGACGCTGAGGCACGAAAGTGTGGGTATCAAACAGG` |
| ASV4217 | 428 | `TCGAGAATTTTTCTCAATGGGGGAAACCCTGAAGGAGCGACGCCGCGTGGGGGATGAAGGACTTCGGTTCGTAAACCCCTGTCACTTGGGAACAAAAGTTTCAACCTAACACGTTGAGGCCTGATAGTACCAGGAGAGGAAGGCACGGCTAACTCTGTGCCAGCAGCCGCGGTAATACAGAGGTGCCAAGCGTTGTTCGGATTTATTGGGCGTAAAGGGTGCGTAGGCGGTCATGCAAGTCGGGTGTGAAATACCGGAGCCTAACTCCGGAACGGCATTCGAAACTGCATGGCTCGAGAACTGGAGAGGTGACTGGAATTCTCGGTGTAGCAGTGAAATGCGTAGATATCGAGAGGAACACCAGAGGCGAAAGCGAGTCACTGGACAGATTCTGACGCTGAGGCACGAAAGCCAGGGGAGCAAACGGG` |
| ASV234 | 429 | `TGGGGAATTTTGGACAATGGACGAAAGTCTGATCCAGCCATTCCGCGTGCAGGATGAAGGCCCTCGGGTTGTAAACTGCTTTTGTACGGAACGAAAAGGTTTCTATTAATACTAGGAGCTCATGACGGTACCGTAAGAATAAGCACCGGCTAACTACGTGCCAGCAGCCGCGGTAATACGTAGGGTGCAAGCGTTAATCGGAATTACTGGGCGTAAAGCGTGCGCAGGCGGTTATATAAGACAGATGTGAAATCCCCGGGCTCAACCTGGGAACTGCATTTGTGACTGTATAGCTAGAGTACGGTAGAGGGGGATGGAATTCCGCGTGTAGCAGTGAAATGCGTAGATATGCGGAGGAACACCGATGGCGAAGGCAATCCCCTGGACCTGTACTGACGCTCATGCACGAAAGCGTGGGGAGCAAACAGG` |
| ASV1110 | 424 | `TAGGGAATATTGGGCAATGGATGCAAGTCTGACCCAGCCATGCCGCGTGCAGGAAGAAGGTCCTCTGGATTGTAAACTGCTTTTATCGGGGAAGAATAGTTTTCTTGCGAGGGAATGTGACGGTACCCGATGAATAAGCCACGGCTAACTACGTGCCAGCAGCCGCGGTAATACGTAGGTGGCAAGCGTTGTCCGGATTTATTGGGTTTAAAGGGTGCGTAGGTGGTTTTATAAGTCAGTGGTGAAAGCTGGTTGCTCAACAATCAAGTTGCCATTGATACTGTTAGACTTGAGAGAAGTGGAGGTTCATGGAATGGATGGTGTAGCGGTGAAATGCATAGATATCATCCAGAACGTCGATTGCGAAGGCAGTGGGCTGTACTTTTTCTGACACTGAGGCACGAAAGCGTGGGGATCAAACAGG` |
| ASV4029 | 387 | `GCGCGAAAACTCCACACTGCACGCAAGTGTGATGGGGGGATCCTAAGTGCGTTACTCTTAGTAACGCTTTTGCCACGAGTACAAATCTTGGCGAATAAGTGGTGGGTAAGACATGTGCCAGCCGCCGCGGTAACACCTGCGCCACAAGTGGCAACCGCTATTATTGGGCCTAAAGCGTTCGTAGCCGGTTTTGTAAATTTTCTGTGAAATCGTTTCACTTAATGGAACGACGTGCAGGGAACACTGCAGAACTAGGGACCGGGAGGCGTCAGAGGTATTCTCAAGGGAGCGGTAAAATGCTATAATCTTGAGAGGACCCCCTGTGGCGAAGGCGTCTGACGAGAACGGATCCGACGGTGAGGAACGAAAGCCAGGGGAGCAAACCGG` |
| ASV2844 | 404 | `TGGGGAATATTGGACAATGGGGGAAACCCTGATCCAGCAATGCCGCGTGAGTGAAGAAGGCCTTAGGGTTGTAAAGCTCTTTCAATAGGGAAGATAATGACGGTACCTATAGAAGAAGCCCCGGCTAAATCCGTGCCAGCAGCCGCGGTAAGACGGATGGGGCTAGCGTTATTCGGAATTACTGGGCGTAAAGGGCGCGTAGGCGGTGAATTAAGTTAGGCGTGAAAGCCCTGGGCTCAACCCAGGAACTGCGTTTAATACTGGTTCGCTAGAGTTTGGTAGAGGAAAGCGGAATTCCTAGTGTAGAGGTGAAATTCGTAGATATTAGGAAGAACACCAGAGGCGAAGGCGGCTTTCTGGACCAATACTGACGCTGAGGCGCGAAAGCGTGGGGAGCAAACAGG` |
| ASV3383 | 424 | `TAGGGAATATTGGTCAATGGGGGCAACCCTGAACCAGCCATGCCGCGTGCAGGATGAAGGCCCTATGGGTCGTAAACTGCTTTTGTACGGGAAGAAATCACATTACGTGTAATGTGTTGACGGTACCGTGAGAATAAGGATCGGCTAACTTCGTGCCAGCAGCCGCGGTAATACGAAGGATCCAAGCGTTGTCCGGATTTACTGGGTTTAAAGGGTGCGTAGGCGGGCTATTAAGTCAGTGGTGAAAGCTTGCAGCTTAACTGTAAAATTGCCATTGAAACTGATAGCCTCGAATATGGTTGAGGTGGTTGGAATGTATCATGTAGCGGTGAAATGCTTAGATATGATACAGAACACCGATTGCGAAGGCAGATCACTAAACCATTATTGACGCTGAGGCACGAAAGCGTGGGGATCAAACAGG` |
| ASV2106 | 429 | `TGGGGAATTTTGGACAATGGGCGCAAGCCTGATCCAGCAATGCCGCGTGCAGGATGAAGGCCTTCGGGTTGTAAACTGCTTTTGTACGGAACGAAAAGGTTCCCTCTAATACAGGGAGCCCATGACGGTACCGTAAGAATAAGCACCGGCTAACTACGTGCCAGCAGCCGCGGTAATACGTAGGGTGCAAGCGTTAATCGGAATTACTGGGCGTAAAGCGTGCGCAGGCGGTTATGTAAGTCAGATGTGAAATCCCCGGGCTCAACCTGGGACCTGCATTTGAGACTGCATAGCTGGAGTGCGGCAGAGGGGGATGGAATTCCGCGTGTAGCAGTGAAATGCGTAGATATGCGGAGGAACACCGATGGCGAAGGCAATCCCCTGGGCCTGCACTGACGCTCATGCACGAAAGCGTGGGGAGCAAACAGG` |
| ASV4823 | 424 | `TGAGGAATATTGGTCAATGGGCGCGAGCCTGAACCAGCCATCCCGCGTGCAGGAATAAGGCGTTATGCGTTGTAAACTGCTTTTGTATAGGGATAAACCTACTTACGTGTAAGTAGCTGAAGGTACTATACGAATAAGCACCGGCTAACTCCGTGCCAGCAGCCGCGGTAATACGGAGGGTGCAAGCGTTATCCGGATTTATTGGGTTTAAAGGGTCCGTAGGCGGCTTAGTAAGTCAGTGGTGAAATCCTATCGCTTAACGATAGAACTGCCATTGAAACTACTAGGCTTGAATTTGAGTGCAGTGGTTGGAATGTGTAGTGTAGCGGTGAAATGCTTAGATATTACACAGAACACCGATTGCGAAGGCAGATCACTAACTCATGATTGACGCTGAGGGACGAAAGCGTGGGGAGCGAACAGG` |
| ASV1715 | 429 | `TGGGGAATTTTGGACAATGGGGGCAACCCTGATCCAGCCATTCCGCGTGAGTGAAGAAGGCCTTCGGGTTGTAAAGCTCTTTCGGCAGGAACGAAACGGTGGGCTCTAACATAGCCTGCTAATGACGGTACCTGAAGAAGAAGCACCGGCTAACTACGTGCCAGCAGCCGCGGTAATACGTAGGGTGCGAGCGTTAATCGGAATTACTGGGCGTAAAGCGTGCGCAGGCGGTCCTATAAGACAGATGTGAAATCCCCGGGCTTAACCTGGGAACTGCGTTTGTGACTGTAGAACTAGAGTGTGGCAGAGGGGGGTGGAATTCCACGTGTAGCAGTGAAATGCGTAGAGATGTGGAGGAACACCGATGGCGAAGGCAGCCCCCTGGGTCAACACTGACGCTCATGCACGAAAGCGTGGGGAGCAAACAGG` |
| ASV5527 | 429 | `TGGGGAATATTGGACAATGGGCGCAAGCCTGATCCAGCAATGCCGCGTGTGTGAAGAAGGCCTGCGGGTTGTAAAGCACTTTCAGTGGGGAGGAAGGCTCTATGTTTAATAGACATAGGGATTGACGTTACCCACAGAAGAAGCACCGGCTAACTCTGTGCCAGCAGCCGCGGTAATACAGAGGGTGCAAGCGTTAATCGGAATTACTGGGCGTAAAGGGTGTGTAGGTGGAAACTTAAGTCAAGTGTGAAAGCCCCAGGCTTAACCTGGGCAGTGCGCATGATACTGAGTTTCTTGAGTACAGTAGAGGGTAGCGGAATTTCCGGTGTAGCGGTGAAATGCGTAGATATCGGAAGGAACACCAGTGGCGAAGGCGGCTATCTGGACTGATACTGACACTGAGACACGAAAGCGTGGGGAGCAAACAGG` |
| ASV1647 | 409 | `TGGGGAATATTGCACAATGGGCGAAAGCCTGATGCAGCGACGCCGCGTGAGGGATGACGGCCTTCGGGTTGTAAACCTCTTTCAGTAGGGAAGAAGCGAAAGTGACGGTACCTGCAGAAGAAGCGCCGGCTAACTACGTGCCAGCAGCCGCGGTAATACGTAGGGCGCAAGCGTTATCCGGAATTATTGGGCGTAAAGAGCTCGTAGGCGGTTTGTCGCGTCTGCCGTGAAAGTCCGGGGCTCAACTCCGGATCTGCGGTGGGTACGGGCAGACTAGAGTGATGTAGGGGAGACTGGAATTCCTGGTGTAGCGGTGAAATGCGCAGATATCAGGAGGAACACCGATGGCGAAGGCAGGTCTCTGGGCATTAACTGACGCTGAGGAGCGAAAGCATGGGGAGCGAACAGG` |
| ASV2716 | 404 | `TGAGGAATTTTGCGCAATGGGGGAAACCCTGACGCAGCGACGCCGCGTGGAGGATGAAGTATTTCGGTATGTAAACTCCTGTCAGTGGGGATGATGATGACAGTACCCACAGAGGAAGCACCGGCTAACTCCGTGCCAGCAGCCGCGGTAAGACGGAGGGTGCAAGCGTTGTTCGGAATTACTGGGCGTAAAGCGTACGTAGGCGGCGGATTAAGTCGAATGTTAAAGCCTGAGGCTCAACCTCAGAGATGCATTCGATACTGGTCTGCTAGAGGGTGATAGAGGAAAGTGGAATTCCCAGTGTAGCGGTGAAATGCGTAGATATTGGGAGGAACATCAGTAGCGAAGGCGACTTTCTGGGTCACACCTGACGCTGAGGTACGAAAGCGTGGGGAGCAAACGGG` |
| ASV1512 | 407 | `TCGAGAATCTTCCACAATGGACGAAAGTCTGATGGAGCGACGCCGCGTGATGGATGAAGTCCCTCGGGATGTAAACATCTTTTATGGATGAAGAAATTATTGACATTAGTCCATGAATAAGAGGTTGCTAAACTCGTGCCAGCAGCAGCGGTAATACGAGTGCCTCAAGCGTTATCCGGAATTATTGGGTGTAAAGGGTGTGTAGGCGGCGTTATTAGTCTCTTGTTAAATATGGCAGCTCAACTGTCAAAATGCAGGGGAAACGGTAATGCTCGAGGGTATGAGGGGTGAACAGAACTCTATGTGTAGGGGTGAAATCCGTTGATATATAGGGGAATACCAAAAGCGAAGGCAGTTCACTGGCATATTTCTGACGCTGAAACACGAAAGCGTGGGTAGCGAATGGG` |
| ASV264 | 430 | `TGGGGAATATTGGACAATGGGCGGAAGCCTGATCCAGCCATGCCGCGTGTGTGAAGAAGGCCTTTTGGTTGTAAAGCACTTTAAGCGAGGAGGAGGCTACTTAGATTAATACTCTAGGATAGTGGACGTTACTCGCAGAATAAGCACCGGCTAACTCTGTGCCAGCAGCCGCGGTAATACAGAGGGTGCGAGCGTTAATCGGATTTACTGGGCGTAAAGCGTGCGTAGGCGGCTGATTAAGTCGGATGTGAAATCCCTGAGCTTAACTTAGGAATTGCATTCGATACTGGTCAGCTAGAGTATGGGAGAGGATGGTAGAATTCCAGGTGTAGCGGTGAAATGCGTAGAGATCTGGAGGAATACCGATGGCGAAGGCAGCCATCTGGCCTAATACTGACGCTGAGGTACGAAAGCATGGGGAGCAAACAGG` |
| ASV4505 | 408 | `TGAGGAATCTTCCGCAATGGGCGAAAGCCTGACGGAGCGACACCGCGTGGAGGATGAAGCCCTTTCCGGGCGTAAACTCCTGTTCTGAGGGACGAAATTTTTGACGGTACCTCAGGAGAAAGCATCGGCTAACCCTGTGCCAGCAGCCGCGGTAAGACAGGGGATGCAAGCGTTACTCGGAATTACTGGGCGTAAAGCGTCTGCAGGTTCCATGGTAAGTCTGTCGTAAAAGCACGGGGCTCAACCCCGTGTACGTGACGGAAACTACCGTGGTCGAGCCACTCAGAGGCATCTGGAATGTCGTGTGTAGGGGTAAAATCCGTAGATCCACGATGGAACGCCAAAAGCGAAGGCAGGATGCTGGGAGTGCGCTGACACTCAGAGACGAAAGCGTGGGGAGCAAAGGGG` |
| ASV1652 | 429 | `TGGGGAATTTTGGACAATGGGCGAAAGCCTGATCCAGCAATGCCGCGTGAGTGAAGAAGGCCTTCGGGTTGTAAAGCTCTTTTGTCAGGGAAGAAACGGTGAGGGCTAATATCCCTCGCTAATGACGGTACCTGAAGAATAAGCACCGGCTAACTACGTGCCAGCAGCCGCGGTAATACGTAGGGTGCAAGCGTTAATCGGAATTACTGGGCGTAAAGCGTGCGCAGGCGGTTTTGTAAGTCTGACGTGAAATCCCCGGGCTCAACCTGGGAATTGCGTTGGAGACTGCAAGGCTAGAATCTGGCAGAGGGGGGTAGAATTCCACGTGTAGCAGTGAAATGCGTAGAGATGTGGAGGAACACCGATGGCGAAGGCAGCCCCCTGGGTCAAGATTGACGCTCATGCACGAAAGCGTGGGGAGCAAACAGG` |
| ASV4410 | 407 | `TGGGGAATTTTCCGCAATGGGGGCAACCCTGACGGAGCAAGACCGCGTGGGGGAGGAAGGTCTGTGGATTGTAAACCCCTTTTGACTGGGAAGAACACAATGACGGTACCAGTCGAATCAGCCTCGGCTAACTCCGTGCCAGCAGCCGCGGTAATACGGAGGAGGCAAGCGTTATCCGGATTTATTGGGCGTAAAGCGTCCGCAGGTGGTTTATCAAGTCTGCTGTCAAAGCGCGAAGCTTAACTTCGTAAAGGCAGTGGAAACTGATGGACTTGAGGATGATAGGGGCAGAGGGAATTCCTAGTGTAGCGGTGAAATGCGTAGAGATTAGGAAGAACACCGGTGGCGAAAGCGCTCTGCTGGGTCATTACTGACACTGAGGGACGAAAGCTAGGGGAGCGAAAGGG` |
| ASV612 | 429 | `TGGGGAATTTTGGACAATGGGCGCAAGCCTGATCCAGCAATGCCGCGTGAGTGAAGAAGGCCTTCGGGTTGTAAAGCTCTTTTGTCAGGGAAGAAAAGGGTCTGGCTAATATCTGGGCCTCATGACGGTACCTGAAGAATAAGCACCGGCTAACTACGTGCCAGCAGCCGCGGTAATACGTAGGGTGCAAGCGTTAATCGGAATTACTGGGCGTAAAGCGTGCGCAGGCGGTTTTGTAAGACTGTCGTGAAATCCCCGGGCTCAACCTGGGAATGGCGATGGTGACTGCAAGGCTAGAGTTTGGCAGAGGGGGGTAGAATTCCACGTGTAGCAGTGAAATGCGTAGATATGTGGAGGAACACCGATGGCGAAGGCAGCCCCCTGGGTCAAAACTGACGCTCATGCACGAAAGCGTGGGGAGCAAACAGG` |
| ASV1263 | 430 | `TGGGGAATATTGGACAATGGGCGAAAGCCTGATCCAGCAATGCCGCGTGTGTGAAGAAGGCCTGCGGGTTGTAAAGCACTTTCAGGGGGGAAAAAGGAGACGGTGTTAATAGCACTGCGCTTTTGATATTACCCCCAGAAGAAGCACCGGCTAACTCTGTGCCAGCAGCCGCGGTAATACAGAGGGTGCGAGCGTTAATCGGAATAACTGGGCGTAAAGGGTGCGTAGGCGGAACGCTAAGTCAGATGTGAAAGCCCCGGGCTCAACCTGGGAATGGCATTTGAAACTGGCGATCTAGAGTAGAGAAGAGGAGAGTGGAATTTCAGGTGTAGCGGTGAAATGCGTAGATATCTGAAGGAACACCAGTGGCGAAGGCGGCTCTCTGGTCTCATACTGACGCTGAGGCACGACAGCGTGGGGAGCAAACAGG` |
| ASV1689 | 423 | `TAGGGAATATTGGGCAATGGGCGCAAGCCTGACCCAGCCATGCCGCGTGCAGGAAGAAGGCGTTATGCGTTGTAAACTGCTTTTATACAGGAAGAAAAAGGCCATGCGTGGCAAACTGACGGTACTGTATGAATAAGCACCGGCTAACTCCGTGCCAGCAGCCGCGGTAATACGGAGGGTGCAAGCGTTGTCCGGATTTATTGGGTTTAAAGGGTGCGTAGGCGGCTTTTTAAGTCAGCGGTGAAAGACTGTCGCTCAACGACGGAACTGCCGTTGATACTGGGAAACTTGAGTATGATTGAGGTAGCCGGAATGGATAGTGTAGCGGTGAAATGCATAGATATTATCCAGAACGCCAATAGCGTAGGCAGGTTACTAAGTCATTACTGACGCTGAGGCACGAAAGTGCGGGGATCAAACAGG` |
| ASV2546 | 429 | `TGGGGAATTTTGGACAATGGGCGCAAGCCTGATCCAGCCATGCCGCGTGAGTGAAGAAGGCCTTCGGGTTGTAAAGCTCTTTTGTCAGGAAGGAAATCCTAGTGGTTAATACCTGCTGGGGATGACAGTACCTGAAGAATAAGCACCGGCTAACTACGTGCCAGCAGCCGCGGTAATACGTAGGGTGCGAGCGTTAATCGGAATTACTGGGCGTAAAGCGTGCGCAGGTGGTTGTGTAAGCCAGATGTGAAATCCCCGGGCTCAACCTGGGAATTGCATTTGGGACTGCACGGCTAGAGTGTGGCAGAGGGGGGTGGAATTCCGCGTGTAGCAGTGAAATGCGTAGAGATGCGGAGGAACACCGATGGCGAAGGCAGCCCCCTGGGCTAACACTGACACTCATGCACGAAAGCGTGGGGAGCAAACAGG` |
| ASV715 | 423 | `TAGGGAATATTGGGCAATGGGCGCAAGCCTGACCCAGCCATGCCGCGTGCAGGAAGAAGGCCCTCTGGGTTGTAAACTGCTTTTGGGAGGGGACAAAAAGCCAATGCGTTGGTAATTGAGTGTACCTCCAGAATAAGCACCGGCTAACTCCGTGCCAGCAGCCGCGGTAATACGGAGGGTGCAAGCGTTGTCCGGATTTATTGGGTTTAAAGGGTGCGTAGGCGGCCCGATAAGTCGGGGGTGAAACCCACAGGCTTAACCTGTGGAGTGCCCTCGATACTGTCGGGCTTGGGTTATCTCTAGGCGGGCGGAATGGAAGGTGTAGCGGTGAAATGCATAGATATCTACCAGAACGCCGATAGCGAAGGCAGCTCGCTGGGGATATACCGACGCTGAGGCACGATAGCGTGGGGATCAAACAGG` |
| ASV3443 | 404 | `TGGGGAATCTTGGACAATGGGCGCAAGCCTGATCCAGCCATGCCGCGTGAGTGAAGAAGGCCTTCGGGTTGTAAAGCTCTTTTACCAGGGACGATAATGACGGTACCTGGAGAATAAGCCCCGGCAAACTTCGTGCCAGCAGCCGCGGTAATACGAAGGGGGCTAGCGTTGTTCGGAATTACTGGGCGTAAAGCGCTCGTAGGCTGACATTTAAGTCAGGGGTGAAATCCCGAGGCTCAACCTCGGAACTGCCTTTGATACTGGGTGTCTCGAGTCCGGAAGAGGTGAGTGGAATTCCTAGTGTAGAGGTGAAATTCGTAGATATTAGGAAGAACACCAGTGGCGAAGGCGGCTCACTGGTCCGGTACTGACGCTGAGGAGCGAAAGCGTGGGGAGCAAACAGG` |
| ASV5098 | 429 | `TGGGGAATATTGCGCAATGGGCGAAAGCCTGACGCAGCAACGCCGCGTGAAGGATGAAGGCCTTCGGGTCGTAAACTTCTGTCAGTTGGGAAGAAATCTCGCCGGTAAATAGCCGGTGAGTTTGACGGTACCAGCAGAGGAAGCCCCGGCTAACTCCGTGCCAGCAGCCGCGGTAATACGGAGGGGGCAAGCGTTATTCGGAATTACTGGGCGTAAAGGGCGCGTAGGCGGCCTGACAGGTCGCAGGTGAAATCCCTCGGCTCAACCGAGGAACTGCCTGCGAAACCGTTGGGCTAGAGGCCGGGAGAGGGTGGCGGAATTCCCAGTGTAGCGGTGAAATGCGTAGATATTGGGAGGAACACCAGTGGCGAAGGCGGCCACCTGGACCGGTTCTGACGCTGAGGCGCGAAAGCGTGGGTAGCAAACAGG` |
| ASV5687 | 403 | `TGGGGAATATTGCACAATGGAGGGAACTCTGATGCAGCGACGCCGCGTGAACGAAGAAGGCTTTCGAGTCGTAAAGTTCTTTTATATGGGAAGATAATGACGGTACCATAAGAAAAAGCCCCGGCTAACTACGTGCCAGCAGCCGCGGTAATACGTAGGGGGCTAGCGTTGTCCGGAATCACTGGGCGTAAAGGGTTCGCAGGCGGAAATGCAAGTCAGGTGTAAAAGGCAGTAGCTTAACTACTGTAAGCATTTGAAACTGCATATCTTGAGAAGAGTAGAGGTAAGTGGAATTTTTAGTGTAGCGGTGAAATGCGTAGATATTAAAAAGAATACCGGTGGCGAAGGCGACTTACTGGGCTCATTCTGACGCTGAGGAACGAAAGCGTGGGTAGCAAACAGG` |
| ASV5523 | 429 | `TGGGGAATATTGGACAATGGGGGGAACCCTGATCCAGCAATGCCGCGTGTGTGAAGAAGGCCTGAGGGTTGTAAAGCACTTTTAGTGGTGAGGAGAGATGTCATTTTAAGAGATAGACATTGAGACGTTAACCAAAGAAAAAGCACCGGCTAACTCCGTGCCAGCAGCCGCGGTAATACGGAGGGTGCAAGCGTTAATCGGAATGATTGGGCGTAAAGGGCGCGTAGGTGGTTTACTGAGTGAGTTGTGAAATCCATGGGCTTAACCTGTGGTAGTCAACTCAGACGGGTAGACTAGAGTACAGTAGAGGGTTGTGGAATTTCCGGTGTAGCGGTGAAATGCGTAGAGATCGGAAGGAACACCAGTGGCGAAGGCGACAACCTGGGCTGAAACTGACACTGAGGCGCGAAAGCGTGGGGAGCAAACAGG` |
| ASV878 | 404 | `TGGGGAATATTGGACAATGGGCGAAAGCCTGATCCAGCAATGCCGCGTGAGTGATGAAGGCCTTAGGGTTGTAAAGCTCTTTTACCAGGGATGATAATGACAGTACCTGGAGAATAAGCTCCGGCTAACTCCGTGCCAGCAGCCGCGGTAATACGGAGGGAGCTAGCGTTGTTCGGAATTACTGGGCGTAAAGCGTGCGTAGGCGGTTATTTAAGTCAGGGGTGAAATCCCGGAGCTCAACTCCGGAACTGCCCTTGAAACTAGATGACTAGAATCATGGAGAGGTGAGTGGAATTCCGAGTGTAGAGGTGAAATTCGTAGATATTCGGAAGAACACCAGTGGCGAAGGCGACTCACTGGACATGTATTGACGCTGAGGTACGAAAGCGTGGGGAGCAAACAGG` |
| ASV1759 | 429 | `TGGGGAATTTTGGACAATGGGCGAAAGCCTGATCCAGCCATGCCGCGTGAGTGAAGAAGGCCTTCGGGTTGTAAAGCTCTTTCGGACAGAAAGAAATCGCACGAACTAATACTTTGTGCGGATGACGGTACTGTAAGAAGAAGCACCGGCTAACTACGTGCCAGCAGCCGCGGTAATACGTAGGGTGCGAGCGTTAATCGGAATTACTGGGCGTAAAGGGTGCGCAGGCGGTTATATAAGACAGATGTGAAATCCCCGGGCTCAACCTGGGAACTGCGTTTGTGACTGTATAACTAGAGTACGGTAGAGAGGGGTGGAATTCCTGGTGTAGCAGTGAAATGCGTAGATATCAGGAGGAACATCGATGGCGAAGGCAGCCCCTTGGACCTGTACTGACGCTCATGCACGAAAGCGTGGGGAGCAAACAGG` |
| ASV1013 | 429 | `TGGGGAATTTTGGACAATGGGCGCAAGCCTGATCCAGCAATGCCGCGTGCAGGAAGAAGGCCTTCGGGTTGTAAACTGCTTTTGTACGGAGCGAAAAGGCTCTCTCTAATACAGGGGGCTCATGACGGTACCGTAAGAATAAGCACCGGCTAACTACGTGCCAGCAGCCGCGGTAATACGTAGGGTGCAAGCGTTAATCGGAATTACTGGGCGTAAAGCGTGCGCAGGCGGTTTTGTAAGACAGGCGTGAAATCCCCGGGCTCAACCTGGGAATGGCGCTTGTGACTGCAAAGCTGGAGTGCGGCAGAGGGGGATGGAATTCCGCGTGTAGCAGTGAAATGCGTAGATATGCGGAGGAACACCGATGGCGAAGGCAATCCCCTGGGCCTGCACTGACGCTCATGCACGAAAGCGTGGGGAGCAAACAGG` |
| ASV2362 | 407 | `TGGGGAATATTGGGCAATGGGCGCAAGCCTGACCCAGCAACGCCGCGTGAAGGAAGAAGGCTTTCGGGTTGTAAACTTCTTTTAAGAGGGACGAAACAAATGACGGTACCTCTTGAATAAGCCACGGCTAACTACGTGCCAGCAGCCGCGGTAATACGTAGGTGGCGAGCGTTATCCGGATTTACTGGGTGTAAAGGGCGTGTAGGCGGGACTGTAAGTCAGGCGTGAAAACTGGTGGCTCAACCACCAGCCTGCGTTTGAAACTGCGGTTCTTGAGTGTCGGAGAGGCAATCGGAATTCCGTGTGTAGCGGTGAAATGCGTAGATATACGGAGGAACACCAGTGGCGAAGGCGGATTGCTGGACGATAACTGACGCTGAGGCGCGAAAGCGTGGGGAGCAAACAGG` |
| ASV1152 | 423 | `TAGGGAATATTGGGCAATGGAAGAAATTCTGACCCAGCCATGCCGCGTGCAGGATGAAGGCGCTATGCGTTGTAAACTGCTTTTGTCAGGGAAGAAAAAGGCTATGCGTAGCAAATTGACGGTACCTGAAGAATAAGCATCGGCTAACTCCGTGCCAGCAGCCGCGGTAATACGGAGGATGCAAGCGTTGTCCGGATTTATTGGGTTTAAAGGGTGCGTAGGCGGTCTGATAAGTCAGTGGTTAAATAACCTTGCTTAACGAGGGAGATGCCATTGATACTGTCAGACTTGAATACTGTTTGGGTAGGCGGAATTGGTAGTGTAGCGGTGAAATGCTTAGATATTACCAGGAACACCGATAGCGAAGGCAGCTTACTGAAGAGATATTGACGCTGAGGCACGAAAGTGCGGGTATCAAACAGG` |
| ASV1642 | 429 | `TGGGGAATTTTGGACAATGGGCGAAAGCCTGATCCAGCCATGCCGCGTGCAGGATGAAGGCCTTCGGGTTGTAAACTGCTTTTGTACGGAACGAAAAGACCTCTTCTAATAAAGGGGGTCCATGACGGTACCGTAAGAATAAGCACCGGCTAACTACGTGCCAGCAGCCGCGGTAATACGTAGGGTGCAAGCGTTAATCGGAATTACTGGGCGTAAAGCGTGCGCAGGCGGTTATGTAAGACAGATGTGAAATCCCCGGGCTCAACCTGGGAACTGCATTTGTGACTGCATAGCTAGAGTACGGTAGAGGGGGATGGAATTCCGCGTGTAGCAGTGAAATGCGTAGATATGCGGAGGAACACCGATGGCGAAGGCAATCCCCTGGACCTGTACTGACGCTCATGCACGAAAGCGTGGGGAGCAAACAGG` |
| ASV729 | 404 | `TGGGGAATATTGGACAATGGGCGAAAGCCTGATCCAGCAATACCGCGTGAATGATAAAGGCCTTAGGGTTGTAAAGTTCTTTTAGTAGGGAAGATAATGACGGTACCTACAGAAAAAGCCCCGGCTAACTCCGTGCCAGCAGCCGCGGTAAGACGGAGGGGGCTAGCGTTGTTCGGAATTACTGGGCGTAAAGAGCGCGTAGGCGGATTAGTAAGTTGGGAGTGAAAGCCCGAGGCTTAACCTCGGAATTGCTCTCAAAACTATTAGTCTTGAGTGTAGTAGGGGATGATGGAATTCCTAGTGTAGAGGTGAAATTCTTAGATATTAGGAGGAACACCGGTGGCGAAGGCGGTCATCTGGGCTACAACTGACGCTGAGGCGCGAAAGCGTGGGGAGCAAACAGG` |
| ASV4167 | 404 | `TGGGGAATATTGGACAATGGGGGAAACCCTGATCCAGCGATGCCGCGTGTATGATGAAGGTCTTCGGATTGTAAAGTACTTTTATTGGTGACGATGATGACGGTAACCAATGAATAAGCTCCGGCTAACTTCGTGCCAGCAGCCGCGGTAATACGAAGGGAGCTAGCGTTGTTCGGAATAACTGGGCGTAAAGGGCGCGTAGGTGGATGTGTGCGTTAGATGTGAAAGCCCCGGGCTCAACCTGGGAATGGCATTTAATACGGCACGTCTAGAGGACGAGAGGGGAAAGCGGAATGGCGAGTGGAGCAGTGAAATGCATTGATATTCGCTGGAACACCAGAGGCGAAGGCGGCTTTCTGGCTCGTTACTGACACTAAAGCGCGAAAGCGTGGGGAGCAAACAGG` |
| ASV3657 | 430 | `TGGGGAATTTTCCGCAATGGGCGAAAGCCTGACGGAGTGACGCCGCGTGGGGGACGAAGGCCTTCGGGTTGTAAACCCCTGTCAGAGGGAAAGAAGGGTGTCGGCGCGATAACCGCCGGCACTTGACGGTACCTTCAGAGGAAGCCCCGGCCAACTCTGTGCCAGCAGCCGCGGTAAGACAGAGGGGGCAAGCGTTGCTCGGAATCACTGGGCGTAAAGGGCGCGTAGGCGGGATGGCAAGTCGGTCGTGAAATCCCTCGGCTCAACCGAGGAACGTCGGCCGATACTGCCGTTCTTGAGGGATGCAGAGGAGACTGGAATTCCCGGTGTAGCGGTGAAATGTGTAGATATCGGGAGGAACACCGGTGGCGAAGGCGGGTCTCTGGGCATTTCCTGACGCTGAGGCGCGAAAGCGTGGGGAGCAAACGGG` |
| ASV3482 | 424 | `TAAGGAATATTGGTCAATGGACGAAAGTCTGAACCAGCCATGCCGCGTGGAGGATGAAGGCCCTCTGGGTTGTAAACTTCTTTTATCTGGGACGAAAAAAGGCTTTTCTAAGTCGTCTGACGGTACCAGGTGAATAAGCACCGGCTAACTCCGTGCCAGCAGCCGCGGTAATACGGAGGGTGCAAGCGTTATCCGGATTCACTGGGTTTAAGGGGTGCGTAGGTGGGTTGGTAAGTCAGTGGTGAAATCCCCGAGCTTAACTTGGGAACTGCCATTGATACTATTAATCTTGAATACCGTGGAGGTTAGCGGAATATGTCATGTAGCGGTGAAATGCTTAGATATGACATAGAACACCAATTGCGAAGGCAGCTAGCTACACGAATATTGACACTGAGGCACGAAAGCGTGGGGATCAAACAGG` |
| ASV4412 | 424 | `TGAGGAATATTGGTCAATGGGCGAGAGCCTGAACCAGCCAAGTAGCGTGCAGGACGACGGCCCTATGGGTTGTAAACTGCTTTTATGCGGGGATAAAGTGAGGGACGTGTCCCTTTTTGTAGGTACCGCATGAATAAGGACCGGCTAATTCCGTGCCAGCAGCCGCGGTAATACGGAAGGTCCGGGCGTTATCCGGATTTATTGGGTTTAAAGGGAGCGTAGGCCGGGGATTAAGCGTGTTGTGAAATGTAGTCGCTCAACGTCTGCACTGCAGCGCGAACTGGTTCCCTTGAGTACGCCGGAAGTCGGCGGAATTCGTGGTGTAGCGGTGAAATGCTTAGATATCACGAAGAACTCCGATTGCGAAGGCAGCTGACTGTAGCGTAACTGACGCTGAAGCTCGAAAGTGTGGGTATCGAACAGG` |
| ASV3695 | 405 | `TGGGGAATCTTGCGCAATGGGCGAAAGCCTGACGCAGCAACGCCGCGTGCGGGATGAAGGCCTTCGGGCTGTAAACCGCTTTCAGCAGGAACGAAAATGACGGTACCTGCAGAAGAAGGAGCGGCCAACTACGTGCCAGCAGCCGCGGTGACACGTAGGCTCCAAGCGTTGTCCGGATTTATTGGGCGTAAAGAGCTCGTAGGCGGTTGAGTAAGTCGGGTGTGAAAACTCTGGGCTTAACCCGGAGACGCCATCCGATACTGCTCTGACTAGAGTTCAGGAGGGGAGTGGGGAATTCCTAGTGTAGCGGTGAAATGCGCAGATATTAGGAGGAACACCGGTGGCGAAGGCGCCACTCTGGACTGAAACTGACGCTGAGGAGCGAAAGCATGGGTAGCAAACAGG` |
| ASV58 | 404 | `TGGGGAATCTTGGACAATGGGCGCAAGCCTGATCCAGCCATGCCGCGTGGATGATGAAGGCCTTCGGGTTGTAAAATCCTTTCAACGGGGACGATAATGACGGTACCCGTAGAAGAAGCCCCGGCTAACTTCGTGCCAGCAGCCGCGGTAATACGAAGGGGGCTAGCGTTGCTCGGAATTACTGGGCGTAAAGCGCACGTAGGCGGATATTTAAGTCGGAGGTGAAAGCCCAGGGCTCAACCCTGGAACTGCCCTCGATACTGGGTATCTGGACCAAAGTAGAGGTTAGTGGAATTCCGAGTGTAGAGGTGAAATTCGTAGATATTCGGAGGAACACCAGTGGCGAAGGCGACTAACTGGACTTTTGGTGACGCTGAGGTGCGAAAGCGTGGGGAGCAAACAGG` |
| ASV1449 | 429 | `TGGGGAATTTTGGACAATGGGGGAAACCCTGATCCAGCAATGCCGCGTGTCTGAAGAAGGCCTTCGGGTTGTAAAGGACTTTTGTTGGGGAGCAAGGTGCACAGGTTAAGAGCTAGTGTACTTGAGAGTACCCAAAGAATAAGGACCGGCTAACTACGTGCCAGCAGCCGCGGTAATACGTAGGGTCCAAGCGTTAATCGGAATTACTGGGCGTAAAGAGTGCGCAGGCGGCTTTGTAAGCTTGATGTGAAATCCCCGGGCTCAACCTGGGAACTGCATTGAGGACTGCAAGGCTAGAGTGTAGCAGAGGGGGGTAGAATTCCACGTGTAGCAGTGAAATGCGTAGAGATGTGGAGGAATACCGATGGCGAAGGCAGCCCCCTGGGTTAACACTGACGCTCATGCACGAAAGCGTGGGGAGCAAACAGG` |
| ASV5717 | 404 | `TGGGGAATATTGGACAATGGGCGCAAGCCTGATCCAGCCATGCCGCGTGAGTGACGAAGGTCTTCGGATTGTAAAGCTCTTTTGGCGGGGACGATAATGACGGTACCCGCAGAATAAGCCCCGGCTAACTTCGTGCCAGCAGCCGCGGTAATACGAAGGGGGCTAGCGTTGTTCGGAATCACTGGGCGTAAAGCGCACGTAGGCGGATGTGTCAGTCAGGGGTGAAATCCCGGAGCTCAACTCCGGAACTGCCTCTGATACAGCACGTCTTGAGTCCGATAGAGGTGGGTGGAATTCCTAGTGTAGAGGTGAAATTCGTAGATATTAGGAAGAACACCGGTGGCGAAGGCGGCCCACTGGATCGGTACTGACGCTGAGGTGCGAAAGCGTGGGGAGCAAACAGG` |
| ASV5640 | 404 | `TGGGGGATATTGCACAATGGAGGAAACTCTGATGCAGCAACGCCGCGTGAAGGAAGAAGGTTTTCGGATTGTAAACTTCTATCAGCAGGGAAGAAAATGACTGTACCTGACTAAGAAGCCCCGGCTAACTACGTGCCAGCAGCCGCGGTAATACGTAGGGGGCAAGCGTTATCCGGAATTACTGGGTGTAAAGGGTACGTAGGCGGTTCATTAAGTCAGATGTGAAAGCCCGAGGCTCAACCTCGGGACTGCATTTGAAACTGGTGAACTAGAGTACAGGAGAGGTAAGTGGAATTCCTAGTGTAGCGGTGAAATGCGTAGATATTAGGAAGAACACCAGTGGCGAAGGCGGCTTACTGGACTGTAACTGACGCTGAGGTACGAAAGCGTGGGGAGCGAACAGG` |
| ASV677 | 429 | `TGGGGAATTTTGGACAATGGGCGCAAGCCTGATCCAGCCATGCCGCGTGTGGGAAGAAGGCCTTCGGGTTGTAAACCACTTTTGTCAGGGAAGAAACGGCTCTTACTAATATTAGGGGCTAATGACGGTACCTGAAGAATAAGCACCGGCTAACTACGTGCCAGCAGCCGCGGTAATACGTAGGGTGCGAGCGTTAATCGGAATTACTGGGCGTAAAGCGTGCGCAGGCGGCTTTGCAAGACAGATGTGAAATCCCCGGGCTCAACCTGGGAACTGCATTTGTGACTGCAAGGCTAGAGTACGGTAGAGGGGGATGGAATTCCGCGTGTAGCAGTGAAATGCGTAGATATGCGGAGGAACACCGATGGCGAAGGCAATCCCCTGGACCTGTACTGACGCTCATGCACGAAAGCGTGGGGAGCAAACAGG` |
| ASV4954 | 429 | `TGGGGAATTTTGGACAATGGGGGAAACCCTGATCCAGCAACGCCGCGTGAATGAAGAAGGCCTTCGGGTTGTAAAGTTCTTTTGTCAGAGAGCAAAGGATAGTGGCTAATACCCGCTATCGCTGAGAGTATCTGAAGAATAAGCACCGGCTAACTACGTGCCAGCAGCCGCGGTAATACGTAGGGTGCGAGCGTTAATCGGAATTACTGGGCGTAAAGGGTGCGCAGGCGGATAATTAAGTTAGGAGTGAAATCCCCGAGCTCAACTTGGGAATTGCTTTTAAGACTGGTTATCTAGAGTTTGTCAGAGGGGGGTAGAATTCCAGGTGTAGCAGTGAAATGCGTAGAGATCTGGAGGAATACCAATGGCGAAGGCAGCCCCCTGGGATAAAACTGACGCTCATGCACGAAAGCGTGGGTAGCAAACAGG` |
| ASV5325 | 429 | `TGGGGAATTTTGGACAATGGGGGCAACCCTGATCCAGCCATGCCGCGTGAGTGAAGAAGGCCTTCGGGTTGTAAAGCTCTTTCGGCGGGAACGAAAGCGCACTTATTAATACTAGGTGTGTATGACGGTACTCGCAGAAGAAGCACCGGCTAACTACGTGCCAGCAGCCGCGGTAATACGTAGGGTGCGAGCGTTAATCGGAATTACTGGGCGTAAAGCGTGCGCAGGCGGTTATTTAAGACAGGCGTGAAAGCCCCGGGCTTAACCTGGGAGGTGCGCTTGTGACTGGATAGCTAGAGTACGGCAGAGGGGGGTGGAATTCCACGTGTAGCAGTGAAATGCGTAGAGATGTGGAGGAACACCGATGGCGAAGGCAGCCCCCTGGGCTAGTACTGACGCTCAGGCACGAAAGCGTGGGGAGCAAACAGG` |
| ASV4244 | 424 | `TAAGGAATATTGGTCAATGGGCGAAAGCCTGAACCAGCCACGCCGCGTGAAGGAAGACGGCCCTACGGGTTGTAAACTTCTTTTCTACGGGAATAAACCTCAGGACGTGTCCTGAGCTGAAGGTACCGTAGGAATAAGGATCGGCTAACTCCGTGCCAGCAGCCGCGGTAATACGGAGGATCCAAGCGTTATCCGGATTTATTGGGTTTAAAGGGTGCGTAGGTGGAATTATAAGTCAGAGGTGAAAGCTAGCAGCTTAACTGTTAAATTGCCTTTGATACTGTTTTTCTTGAGTGTAGTTGGCGTTGGCGGAATGTGACATGTAGCGGTGAAATGCGTAGATATGTCACAGAACACCGATCGCGAAGGCAGCTAACGAAGCTATCACTGACACTGATGCACGAAAGCGTGGGGAGCAAACAGG` |
| ASV2773 | 404 | `TGGGGAATCTTAGACAATGGGCGCAAGCCTGATCTAGCCATGCCGCGTGAGCGATGAAGGCCTTAGGGTTGTAAAGCTCTTTCGTGGGGGAAGATAATGACTGTACCCCAAGAAGAAGCCCCGGCTAACTCCGTGCCAGCAGCCGCGGTAATACGGAGGGGGCTAGCGTTGTTCGGAATTACTGGGCGTAAAGCGCACGTAGGCGGACGGACAAGTCAGAGGTGAAATCCCAGGGCTCAACCCTGGAACTGCCTTTGAAACTTTTCGTCTTGAGGTCGAGAGAGGTGAGTGGAATTCCGAGTGTAGAGGTGAAATTCGTAGATATTCGGAGGAACACCAGTGGCGAAGGCGGCTCACTGGCTCGATACTGACGCTGAGGTGCGAAAGCGTGGGGAGCAAACAGG` |
| ASV5710 | 429 | `TGGGGAATATTGGACAATGGGCGCAAGCCTGATCCAGCCATGCCGCGTGTGTGAAGAAGGCCTTTGGGTTGTAAAGCACTTTAAGCGGGGAGGAAGGCTCGAGCGTTAATAGCGTTCGGGATTGACGTTACCCGCAGAATAAGCACCGGCAAACTTCGTGCCAGCAGCCGCGGTAATACGAAGGGTGCGAGCGTTAATCGGAATTACTGGGCGTAAAGCGCGCGTAGGTGGTTTAGTAAGCGATATGTGAAAGCCCTGGGCTTAACCTAGGAACTGCATGTCGAACTGCTAGGCTAGAGTATGGTAGAGGGGTGTGGAATTTCCGGTGTAGCGGTGAAATGCGTAGATATCGGAAGGAACATCAGTGGCGAAGGCGACACCCTGGCCCAATACTGACACTGAGGCGCGAAAGCGTGGGTAGCGAACAGG` |
| ASV2785 | 406 | `TGGGGAATATTGGGCAATGGGGGGAACCCTGACCCAGCGACGCCGCGTGGGTGAAGAAATCCTTCGGGATGTAAAGCCCTGTTGTATGGGAAGAGCAGAAGACGGTACCATACGAGGAAGCCCCGGCAAACTACGTGCCAGCAGCCGCGGTAATACGTAGGGGGCAAGCGTTGTCCGGAATTACTGGGCGTAAAGCGCACGCAGGCTGAATTATAAGTCGGTAGTCAAAGGCGGAGGCTCAACTTCCGTTTATCTATCGAAACTGTAAATCTAGAGTATGTGAGAGGGAAGTGGAATTCCCGGTGTAGCGGTGAAATGCGTAGATATCGGGAGGAACACCAGTGGCGAAGGCGGCTTCCTGGCACAATACTGACGCTCATGTGCGAAAGCTAGGGTAGCGAACGGG` |
| ASV956 | 424 | `TGAGGAATATTGGTCAATGGGCGCAAGCCTGAACCAGCCATGCCGCGTGCAGGAAGACGGTCCTATGGATTGTAAACTGCTTTTATTAGGGAATAAACCTCTTTACGTGTAGAGAGCTGAAGGTACCTAATGAATAAGCACCGGCTAACTCCGTGCCAGCAGCCGCGGTAATACGGAGGGTGCAAGCGTTATCCGGAATCATTGGGTTTAAAGGGTCCGCAGGCGGGAAGATAAGTCAGTGGTGAAATCTCACAGCTCAACTGTGAAACTGCCATTGATACTGTCTTTCTTGAATTCGATCGAAGTGGGCGGAATATGACATGTAGCGGTGAAATGCTTAGATATGTCATAGAACACCGATAGCGAAGGCAGCTCACTAGGTCTGGATTGACGCTCAGGGACGAAAGCGTGGGGAGCAAACAGG` |
| ASV3427 | 429 | `TGGGGAATATTGCACAATGGGCGAAAGCCTGATGCAGCCATGCCGCGTGTGTGAAGAAGGCCTTCGGGTTGTAAAGCACTTTCAGCGGGGAGGAAGTTGGTAGGGTTAATAGCTTTATTAATTGACGTTACCCGCAGAAGAAGCACCGGCTAACTCCGTGCCAGCAGCCGCGGTAATACGGAGGGTGCGAGCGTTAATCGGAATTACTGGGCGTAAAGGGCTTGTAGGCGGTTGATTAAGTTAGATGTGAAATCCCTGGGCTTAACCTAGGAATGGCATTTAAGACTGGTGAGCTAGAGTACTGTAGAGGGGGGTAGAATTCCAGGTGTAGCGGTGAAATGCGTAGAGATCTGGAGGAATACCGGTGGCGAAGGCGGCCCCCTGGACAGTGACTGACGCTGAGGAGCGAAAGCGTGGGGAGCAAACAGG` |
| ASV1166 | 429 | `TGGGGAATTTTGGACAATGGGCGCAAGCCTGATCCAGCAATGCCGCGTGAGTGAAGAAGGCCTTCGGGTTGTAAAGCTCTTTTGTCAGGGAAGAAACGGGTCTTTCTAATACAGAGATCTAATGACGGTACCTGAAGAATAAGCACCGGCTAACTACGTGCCAGCAGCCGCGGTAATACGTAGGGTGCAAGCGTTAATCGGAATTACTGGGCGTAAAGCGTGCGCAGGCGGTTTTGTAAGACTGTCGTGAAATCCCCGGGCTCAACCTGGGAATGGCGATGGTGACTGCAAGGCTAGAGTTTGGCAGAGGGGGGTAGAATTCCACGTGTAGCAGTGAAATGCGTAGATATGTGGAGGAACACCGATGGCGAAGGCAGCCCCCTGGGTCAAAACTGACGCTCATGCACGAAAGCGTGGGGAGCAAACAGG` |
| ASV1865 | 429 | `TGGGGAATTTTGGACAATGGGCGCAAGCCTGATCCAGCAATGCCGCGTGAGTGAAGAAGGCCTTCGGGTTGTAAAGCTCTTTTGTCAGGGAAGAAACGGGATTGGCTAATATCCGACCTTAATGACGGTACCTGAAGAATAAGCACCGGCTAACTACGTGCCAGCAGCCGCGGTAATACGTAGGGTGCAAGCGTTAATCGGAATTACTGGGCGTAAAGCGTGCGCAGGCGGTTTTGTAAGTCTGACGTGAAATCCCCGGGCTCAACCTGGGAATTGCGTTGGAGACTGCAAGGCTGGAGTCTGGCAGAGGGGGGTAGAATTCCACGTGTAGCAGTGAAATGCGTAGAGATGTGGAGGAACACCGATGGCGAAGGCAGCCCCCTGGGTCAAGACTGACGCTCATGCACGAAAGCGTGGGGAGCAAACAGG` |
| ASV2949 | 429 | `TGGGGAATTTTGGACAATGGGCGCAAGCCTGATCCAGCAATGCCGCGTGCAGGATGAAGGCCTTCGGGTTGTAAACTGCTTTTGTACGGAACGAAAAGCCTGGGGCTAATATCCCTGGGTCATGACGGTACCGTAAGAATAAGCACCGGCTAACTACGTGCCAGCAGCCGCGGTAATACGTAGGGTGCAAGCGTTAATCGGAATTACTGGGCGTAAAGCGTGCGCAGGCGGTTATGTAAGACAGAGGTGAAATCCCCGGGCTCAACCTGGGAACTGCCTTTGTGACTGCATGGCTAGAGTACGGTAGAGGGGGATGGAATTCCGCGTGTAGCAGTGAAATGCGTAGATATGCGGAGGAACACCGATGGCGAAGGCAATCCCCTGGACCTGTACTGACGCTCATGCACGAAAGCGTGGGGAGCAAACAGG` |
| ASV810 | 424 | `TAAGGAATATTGGTCAATGGGGGCAACCCTGAACCAGCCATGCCGCGTGCAGGAAGACGGCCCTCTGGGTTGTAAACTGCTTTTATTCGGGAATAAACCTACTTACGTGTAAGTAGCTGAATGTACCGAAGGAATAAGGATCGGCTAACTCCGTGCCAGCAGCCGCGGTAATACGGAGGATCCAAGCGTTATCCGGATTTATTGGGTTTAAAGGGTGCGTAGGCGGCCTGTTAAGTCAGGGGTGAAAGACGGTAGCTCAACTATCGCAGTGCCCTTGATACTGATGGGCTTGAATGGACTAGAGGTAGGCGGAATGTGACAAGTAGCGGTGAAATGCATAGATATGTCACAGAACACCGATTGCGAAGGCAGCTTACTATGGTCTTATTGACGCTGAGGCACGAAAGCGTGGGGATCAAACAGG` |
| ASV2709 | 404 | `TGGGGAATATTGGACAATGGGGGCAACCCTGATCCAGCGATGCCGCGTGAGTGAAGAAGGCCTTCGGGTTGTAAAGCTCTTTTGTCGGGGACGATAATGACGGTACCCGACGAATAAGTCCCGGCTAACTTCGTGCCAGCAGCCGCGGTAATACGAAGGGGGCTAGCGTTGTTCGGAATGACTGGGCGTAAAGGGCGCGTAGGCGGTTGAACAAGTTTGGTGTGAAAGTCCTGGGCTTAACCTGGGGATTGCATTGAAAACTGTTCATCTAGAGTTCGAGAGAGGAAAATGGAATTGCGAGTGTAGAGGTGAAATTCGTAGATATTCGCAAGAACACCGGTGGCGAAGGCGGTTTTCTGGCTCGGGACTGACGCTAAGGCGCGAAAGCGTGGGGAGCAAACAGG` |
| ASV3680 | 404 | `TGGGGAATATTGGACAATGGGCGCAAGCCTGATCCAGCAATGCCGCGTGTGTGAAGAAGGTCTTCGGATTGTAAAGCACTTTCGGCGGGGACGATGATGACGGTACCCGCAGAAGAAGCCCCGGCTAACTTCGTGCCAGCAGCCGCGGTAATACGAAGGGGGCTAGCGTTGCTCGGAATTACTGGGCGTAAAGGGCGCGTAGGCGGCTCTGTCAGTCGGGCGTGAAATTCCTGGGCTCAACCTGGGGGCTGCGTTCGATACGGCGGGGCTAGAGGACGGAAGAGGCTCGTGGAATTCCCAGTGTAGAGGTGAAATTCGTAGATATTGGGAAGAACACCGGTGGCGAAGGCGGCGAGCTGGTCCGTAACTGACGCTGAGGCGCGACAGCGTGGGGAGCAAACAGG` |
| ASV3440 | 424 | `TAGGGAATATTGGTCAATGGATGGAAGTCTGAACCAGCCATGCCGCGTGCAGGACGAAGGCCCTCTGGGTTGTAAACTGCTTTTGACGAGGGAGAAAAGGTAGAGTGCGCTCTATTATGACGGTACTCGTTGAATAAGCACCGGCTAACTACGTGCCAGCAGCCGCGGTAATACGTAGGGTGCAAGCGTTGTCCGGATTTATTGGGTTTAAAGGGTGCGTAGGTGGTTTGATAAGTCAGTGGTGAAATATGGTTGCTCAACAATCAATGTGCCATTGATACTGTCGAACTTGAGTAAAGTGGAGGCTGGAGGAATGGATGGTGTAGCGGTGAAATGCATAGATATCATCCAGAACGTCAATTGCGAAGGCATCTGGCTGTACTTTTACTGACACTGAGGCACGAAAGCGTGGGGATCAAACAGG` |
| ASV2984 | 429 | `TCGAGAATAATTCACAATGGGGGCAACCCTGATGGTGCAACGCCGCGTGGAGGATGACGGTCTTCGGATTGTAAACTCCTGTCATCCGGGAGTAAGACCTGGGAGTTAATAGCTCACAGGGTTGATAGTACCGGAAGAGGAAGGGACGGCTAACTTCGTGCCAGCAGCCGCGGTAATACGAAGGTCCCAAGCGTTGTTCGGAATCACTGGGCGTAAAGGGAGCGTAGGCGGCGTGGTAAGTCAGATGTGAAATCCCGGGGCTCAACCCCGGAACTGCATCCGATACTGCCATGCTAGAGGATTGGAGAGGTAGCTGGAATTCTTGGTGTAGCAGTGAAATGCGTGGATATCAAGAGGAACACTCGTGGCGAAAGCGAGCTACTGGACAATTCCTGACGCTGAGGCTCGAAGGCCAGGGTAGCGAAAGGG` |
| ASV3852 | 408 | `TCGAGAATCTTCCGCAATGGACGAAAGTCTGACGGAGCGACGCCGCGTGGTTGATGAAGTGCTTAGGCATGTAAAAACCTTTTATGAGGGAAAAAGTTTATTGATTGTACCTCATGAATAAGGGGTTGCTAAACTCGTGCCAGCAGCAGCGGTAATACGAGTGCCCCAAGCGTTATCCGGAATTATTGGGCGTAAAGGGTGCGTAGGTGGTGGTATTAGTCTAATGTTAAAGCCCCCGGCTTAACCGGGGAATCGCATTAGAAACGGTACCACTAGAGGATGTGAGAGGTCTGTAGAACTCATGGAGTAGGGGTGAAATCCGTTGATATCATGGGGAATACCAAAAGCGTAGGCAGCAGACTGGCACATTTCTGACACTGAAGCACGAAAGCGTGGGTAGCGAATGGG` |
| ASV435 | 404 | `TGGGGAATATTGGACAATGGGCGAAAGCCTGATCCAGCAATGCCGCGTGAGTGATGAAGGCCTTAGGGTTGTAAAGCTCTTTTACCCGGGAAGATAATGACTGTACCGGGAGAATAAGCCCCGGCTAACTCCGTGCCAGCAGCCGCGGTAATACGGAGGGGGCTAGCGTTATTCGGAATTACTGGGCGTAAAGCGCGCGTAGGCGGCTATTCAAGTCAGAGGTGAAAGCCCGGAGCTCAACTCCGGAATTGCCTTTGAAACTAGATAGCTTGAATCATGGAGAGGTCAGTGGAATTCCGAGTGTAGAGGTGAAATTCGTAGATATTCGGAAGAACACCAGTGGCGAAGGCGACTGACTGGACATGTATTGACGCTGAGGTGCGAAAGCGTGGGGAGCAAACAGG` |
| ASV5321 | 429 | `TGGGGAATATTGCGCAATGGACGAAAGTCTGACGCAGCGACGCCGCGTGAGGGATGAAGGCCTTAGGGTCGTAAACCTCTGTCAGGAGGGAAGAACCGCCAGGGGAGGAAATGCCCTTGGTCTGACGGTACCTCCAAAGGAAGCACCGGCTAACTCCGTGCCAGCAGCCGCGGTAATACGGAGGGTGCAAGCGTTAATCGGAATCACTGGGCGTAAAGCGCGCGTAGGCGGCTTGGTCAGTCAGGGGTGAAATCCCACAGCTTAACTGTGGAACTGCCTTTGATACTGCCTTGCTTGAGTTTGTGAGAGGATAGTGGAATTCCAGGTGTAGGAGTGAAATCCGTAGAGATCTGGAGGAACATCAGTGGCGAAGGCGACTATCTGGCACAACACTGACGCTGAGGTGCGAAAGCGTGGGTATCAAACAGG` |
| ASV272 | 429 | `TGGGGAATATTGCGCAATGGGGGCAACCCTGACGCAGCCATGCCGCGTGTGTGAAGAAGGCCTTCGGGTTGTAAAGCACTTTAAGTGGGGAGAAAAGGGTAGGCGTTAATAGCGTTTACCTGTGATGTTACCCACAGAATAAGCACCGGCTAACTCCGTGCCAGCAGCCGCGGTAATACGGAGGGTGCGAGCGTTAATCGGAATTACTGGGCGTAAAGCGCGCGTAGGCGGTTTGTTAAGTCAGCTGTGAAAGCCCCGGGCTCAACCTGGGAACTGCAGTTGATACTGGCCGACTAGAGTACGAGAGAGGGAGGTAGAATTCCATGTGTAGCGGTGAAATGCGTAGATATATGGAGGAATACCGGTGGCGAAGGCGGCCTCCTGGCTCGATACTGACGCTGAGGTGCGAAAGCGTGGGGATCAAACAGG` |
| ASV1988 | 404 | `TGGGGAATATTGGACAATGGGCGAAAGCCTGATCCAGCGACGCCGCGTGGGTGATTAAGGCCTTCGGGTTGTAAAGCCCTTTCAGTAGGAAAGATGATGACGGTACCTACAGAAGAAGCTCCGGCTAACTTCGTGCCAGCAGCCGCGGTAATACGAAGGGAGCTAGCGTTACTCGGAATTATTGGGCGTAAAGCGTGCGTAGGCTGCTTTGCAAGTTGGAAGTGAAAGCCCAGGGCTCAACCCTGGAATTGCTTTCAAAACTACAGAGCTAGAATATCAGAGAGGATAGCGGAATTACTAGTGTAGAGGTGAAATTCGTAGATATTAGTAGGAACACCGGAGGCGAAAGCGGCTATCTGGCTGATTATTGACGCTGTTGCACGAAAGCGTGGGGAGCAAACAGG` |
| ASV788 | 430 | `TGGGGAATCTTGCGCAATGGACGAAAGTCTGACGCAGCCACGCCGCGTGAGTGAAGAAGGCCTTCGGGTTGTAAAGCTCTGTCGTGTGGGACGAAAAACATCTTGGTTAATAGCCTTGGTGTCTGACGGTACCATGAAAGGAAGCACCGGCTAACTCCGTGCCAGCAGCCGCGGTAATACGGAGGGTGCAAGCGTTGCTCGGAATTATTGGGCGTAAAGGGCAGGTAGGTGGTCCTATTTGTCTGGGGTGAAATCCCTGAGCTTAACTCAGGAAGTGCCTCAGAAACGGTAGGACTAGAGTCTTAGAGAGGTTCGTGGAATTCCCGGTGTAGCGGTGAAATGCGTAGAGATCGGGAGGAACACCAGAGGCGAAGGCGGCGAACTGGATAAGTACTGACACTCAACTGCGAAAGCGTGGGGAGCAAACAGG` |
| ASV610 | 404 | `TGGGGAATATTGGACAATGGGGGAAACCCTGATCCAGCCATGCCGCGTGAGTGATGAAGGCCTTAGGGTTGTAAAGCTCTTTTGCCAGGGAAGATAATGACTGTACCTGGAGAATAAGCCCCGGCTAACTTCGTGCCAGCAGCCGCGGTAATACGAAGGGGGCTAGCGTTGTTCGGAATCACTGGGCGTAAAGCGCACGTAGGCGGGCCGATAAGTCGGAGGTGAAAGCCTGGGGCTCAACCCCAGAATTGCCTTCGATACTGTCAGTCTAGAATGCAGGAGAGGTTGGTGGAACTCCGAGTGTAGAGGTGAAATTCGTAGATATTCGGAAGAACACCAGTGGCGAAGGCGGCCAACTGGCCTGTGATTGACGCTGAGGTGCGAAAGCGTGGGGAGCAAACAGG` |
| ASV3960 | 424 | `TAAGGAATATTGGTCAATGGACGCAAGTCTGAACCAGCCATGCCGCGTGGAGGATGAAGGTCCTCTGGATTGTAAACTTCTTTTATCTGTGAAGAAATGCACGGTTTCTACTGTGTTTGACGGTAACAGATGAATAAGCACCGGCTAACTCCGTGCCAGCAGCCGCGGTAATACGGAGGGTGCAAGCGTTATCCGGATTCACTGGGTTTAAAGGGAGCGTAGGTGGGCAGGTAAGTCAGTGGTGAAATCCCCGAGCTTAACTTGGGAACTGCCATTGATACTATCTGTCTTGAATACCTTGGAGGTAAGCGGAATATGTCATGTAGCGGTGAAATGCTTAGATATGACATAGAACACCAATTGCGAAGGCAGCTTACTACACGGTTATTGACACTGAGGCTCGAAAGCGTGGGGATCAAACAGG` |
| ASV2175 | 429 | `TGGGGAATTTTGGACAATGGGCGAAAGCCTGATCCAGCCATTCCGCGTGCAGGATGAAGGCCTTCGGGTTGTAAACTGCTTTTGTACGGAACGAAACGGTCCTTTCTAATACAGGGGGCTAATGACGGTACCGTAAGAATAAGCACCGGCTAACTACGTGCCAGCAGCCGCGGTAATACGTAGGGTGCAAGCGTTAATCGGAATTACTGGGCGTAAAGCGTGCGCAGGCGGTTATGTAAGACAGATGTGAAATCCCCGGGCTCAACCTGGGAACTGCATTTGTGACTGTATAGCTAGAGTACGGCAGAGGGGGATGGAATTCCGCGTGTAGCAGTGAAATGCGTAGATATGCGGAGGAACACCGATGGCGAAGGCAATCCCCTGGGCCTGTACTGACGCTCATGCACGAAAGCGTGGGGAGCAAACAGG` |
| ASV205 | 424 | `TAGGGAATATTGGACAATGGGCGCAAGCCTGATCCAGCCATGCCGCGTGCAGGATGAAGGCCCTATGGGTCGTAAACTGCTTTTGTACGGGAAGAAACCTTACTACGTGTAGTAAGCTGACGGTACCGTGAGAATAAGGATCGGCTAACTTCGTGCCAGCAGCCGCGGTAATACGAAGGATCCAAGCGTTGTCCGGATTTACTGGGTTTAAAGGGTGCGTAGGCGGACTTTTAAGTCAGTGGTGAAAGCCTGTAGCTTAACTATAGAATTGCCATTGAAACTGAAAGTCTCGAGTGTGGTTGAGGTAACTGGAATATATCATGTAGCGGTGAAATGCTTAGATATGATATAGAACACCAATTGCGAAGGCAGGTTACTGAGCCACAACTGACGCTGAGGCACGAAAGCGTGGGGATCAAACAGG` |
| ASV5065 | 404 | `TGGGGAATATTGGACAATGGGGGAGACCCTGATCCAGCCATGTCGCGTGAGTGATGAAGGCCTTCGGGTTGTAAAGCTCTTTTACATGGGAAGATAATGACGGTACCATGAGAATAAGCACCGGCTAACTTCGTGCCAGCAGCCGCGGTAATACGAAGGGTGCAAGCGTTGTTCGGAATTACTGGGTGTAAAGGGCGTGTAGGCGGACGATCGAGTTAGTGGTGAAAGGCTTGGGCTTAACCTGAGACGTGCCATTGATACTGATTGTCTGGAGTATCAGAGAGGATGACGGAATATCCAGTGTAGAGGTGAAATTCGTAGATATTGGATAGAACACCGGTGGCGAAGGCGGTTATCTGGCTGATTACTGACGCTGAGGCGCGAGAGCGTGGGGAGCAAACAGG` |
| ASV8 | 429 | `TGGGGAATTTTGGACAATGGGCGCAAGCCTGATCCAGCAATGCCGCGTGCAGGATGAAGGCCTTCGGGTTGTAAACTGCTTTTGTACGGAACGAAACGGTGAGCTCTAATACAGTTTGCTAATGACGGTACCGTAAGAATAAGCACCGGCTAACTACGTGCCAGCAGCCGCGGTAATACGTAGGGTGCGAGCGTTAATCGGAATTACTGGGCGTAAAGCGTGCGCAGGCGGTGATGTAAGACAGATGTGAAATGCCCGGGCTCAACCTGGGACCTGCATTTGTGACTGCATCGCTAGAGTACGGTAGAGGGGGATGGAATTCCGCGTGTAGCAGTGAAATGCGTAGATATGCGGAGGAACACCGATGGCGAAGGCAATCCCCTGGACCTGTACTGACGCTCATGCACGAAAGCGTGGGGAGCAAACAGG` |
| ASV2572 | 424 | `TGAGGAATATTGGACAATGGGCGCAAGCCTGATCCAGCCATGCCGCGTGCAGGATGACGGTCCTATGGATTGTAAACTGCTTTTGTACGGGAAGAAACCACTCTACGAGTAGAGTATTGACGGTACCGTAAGAATAAGGATCGGCTAACTCCGTGCCAGCAGCCGCGGTAATACGGAGGATCCAAGCGTTATCCGGAATCATTGGGTTTAAAGGGTCCGTAGGCGGCCCGATAAGTCAGCGGTGAAATCTCCCGGCTCAACCGGGAAATGGCCATTGATACTGTCGGGCTTGAATTACTAGGAAGTAACTAGAATATGTAGTGTAGCGGTGAAATGCTTAGATATTACATGGAATACCAATTGCGAAGGCAGGTTACTACTAGTTGATTGACGCTGATGGACGAAAGCGTGGGTAGCGAACAGG` |
| ASV4784 | 424 | `TAGGGAATATTGGGCAATGGGAGCAATCCTGACCCAGCCATGCCGCGTGCCGGATGAAGGCTTTCTGAGTCGTAAACGGCTTTTGCCAGGGAAGAATAAGGGGGATGCGTCCTCCGACGACGGTACCTGGTGAATAAGCACCGGCTAACTCCGTGCCAGCAGCCGCGGTAATACGGAGGGTGCAAGCGTTGTCCGGATTTATTGGGTTTAAAGGGTGCGTAGGCGGCTTCTTAAGTCTGGGGTGAAAGCCCGTTGCTCAACAACGGAACTGCCCTGGAAACTGGGAGGCTTGAGTACAGACGAGGTTGGCGGAATGGATGCTGTAGCGGTGAAATGCATAGATAGCATCCAGAACCCCGATTGCGAAGGCAGCTGACTAGGCTGTAACTGACGCTGAGGCACGAAAGCGTGGGGAGCGAACAGG` |
| ASV4847 | 428 | `TGGGGAATCTTGCGCAATGGGCGAAAGCCTGACGCAGCGACGCCGCGTGAGCGATGAAGGCCTTCGGGTTGTAAAGCTCTGTGGGGAGAGAAGAATAAGGGTACGCTAACACCGTAGTCGATGACGGTATCTCCTTAGCAAGCACCGGCTAACTCTGTGCCAGCAGCCGCGGTAAGACAGAGGGTGCAAACGTTGTTCGGAATTACTGGGCGTAAAGCGCGTGTAGGCGGCTAAGTGTGTCGGATGTGAAATCCCGCGGCTCAACCGTGGAAGTGCATTCGAAACTGCTTAGCTTGAGTTTCGGAGAGGAAGGCGGAATGCTTGGTGTAGAGGTGAAATTCGTAGATATCAAGCAGAACACCTGCGGCGAAGGCGGCCTTCTGGACGATGACTGACGCTGAGACGCGAAAGCGTGGGGAGCAAACAGG` |
| ASV5328 | 429 | `TGGGGAATCTTGGACAATGGGGGCAACCCTGATCCAGCGATGCCGCGTGGGTGAAGAAGGCCTTCGGGTTGTAAAGCCCTTTAGGCAGGGAAGAAGGGTATGGGTTGAATACGCCTGTACTTTGACGGTACCTGCAGAATAAGCACCGGCAAACTCTGTGCCAGCAGCCGCGGTAATACAGAGGGTGCAAGCGTTAATCGGATTTACTGGGCGTAAAGGGCGCGTAGGCGGTTGTGTGAGTGTGATGTGAAAGCCCCGGGCTCAACCTGGGAAGTGCATCGCAAACGACACGACTGGAGTAGATGAGAGGGTGGCGGAATTTCCAGTGTAGCGGTGAAATGCGTAGAGATTGGAAGGAACGTCAATGGCGAAGGCAGCCACCTGGCATCATACTGACGCTGAGGCGCGAAAGCGTGGGGAGCGAACAGG` |
| ASV983 | 429 | `TGGGGAATATTGGACAATGGGCGCAAGCCTGATCCAGCCATGCCGCGTGTGTGAAGAAGGCCTTCGGGTTGTAAAGCACTTTCAGCGAGGAGGAAGAGTTGTGTGTTAATAGTACACAGCTTTGACGTTACTCGCAGAAGAAGCACCGGCTAACTCTGTGCCAGCAGCCGCGGTAATACAGAGGGTGCAAGCGTTAATCGGAATTACTGGGCGTAAAGCGCACGTAGGCGGTTTTTTAAGTCAGATGTGAAAGCCCCGGGCTCAACCTGGGAATTGCATTTGAAACTGGAAAACTAGAGTGTGTGAGAGGGGGGTAGAATTCCAAGTGTAGCGGTGAAATGCGTAGAGATTTGGAGGAATACCAGTGGCGAAGGCGGCCCCCTGGCACAACACTGACGCTCAGGTGCGAAAGCGTGGGGAGCAAACAGG` |
| ASV5540 | 418 | `TGGGGAATATTGGACAATGGGGGAAACCCTGATCCAGCAATACCGCGTGTGTGAAGAAGGCCTGAGGGTTGTAAAGCACTTTAGTTGGGGAAGAAGACTACTTCGGTAGTTTGACGTTACCCAAAGAATAAGCACCGGCTAACTCTGTGCCAGCAGCCGCGGTAATACAGAGGGTGCGAGCGTTAATCGGAATGACTGGGCGTAAAGGGCACGTAGGTGGTTTTTTTAGCTAGATGTGAAAGCCCTTGGCTTAACCAAGGAACTGCATTTAGAACTGAATGACTAGAGAGAAGTAGAGGTAAGTGGAATTTCCAGAGTAGCGGTGAAATGCGTAGATACTGGAAGGAACACCAGTGGCGAAGGCGACTTACTGGACTTCTTCTGACACTGAGGTGCGACAGCGTGGGTAGCGAACAGG` |
| ASV3542 | 404 | `TGGGGAATATTGGACAATGGGCGAAAGCCTGATCCAGCCATGCCGCGTGTGTGATGAAGGCCTTAGGGTTGTAAAGCACTTTTGTCAGGGAAGATAATGACGGTACCTGAAGAATAAGCACCGGCTAACTTCGTGCCAGCAGCCGCGGTAATACGAAGGGTGCTAGCGTTGTTCGGAATCACTGGGCGTAAAGCGCGCGTAGGCGGCGATACAAGTCAGAGGTGAAAGCCCAAGGCTCAACCTTGGAATTGCCTTTGAAACTGTATTGCTTGAGTATTGGAGGGGATAGCGGAATTGCTAATGTAGAGGTGAAATTCGTAGATATTAGCAGGAACACCGGTGGCGAAGGCGGCTATCTGGACAATTACTGACGCTGAGGCGCGAAAGCGTGGGGATCAAACAGG` |
| ASV5309 | 404 | `TGGGGAATATTGGACAATGGGCGCAAGCCTGATCCAGCAATGCCGCGTGAGTGATGAAGGCCTTCGGGTTGTAAAGCTCTTTTACCGGGGACGATAATGACGGTACCCGGAGAATAAGCACCGGCTAACTTCGTGCCAGCAGCCGCGGTAATACGAAGGGTGCTAGCGTTGTTCGGAATTACTGGGCGTAAAGCGTACGTAGGCGGTCTACCAAGTCAGAGGTGAAAGCCCAGGGCTCAACCCTGGAACTGCCTTTGAGACTGGTAGACTAGAGTATGGGAGAGGTTGGTGGAATTCCAAGTGGAGAGGTGAAATTCGTAGATATTTGGAAGAACACCGGTGGCGAAGGCGGCCAACTGGAACATTACTGACGCTGAGGTACGAAAGCGTGGGGAGCAAACAGG` |
| ASV2322 | 404 | `TGGGGAATCTTGCACAATGGAGGAAACTCTGATGCAGCGACGCCGCGTGGACGATGAAGCCCTTCGGGGTGTAAAGTCCTTTCGACCGGAACGATAATGACGGTACCGGTGGAAGAAGCTGCGGCTAACTACGTGCCAGCAGCCGCGGTAATACGTAGGCAGCAAGCGTTGTTCGGAATTACTGGGCGTAAAGAGTTCGTAGGCGGTGCGGCAAGTTTGGTGTGAAATCTCCCGGCTCAACTGGGAGGGTGCGCCGAATACTGCCGTGCTAGAGTATAGGAGAGGTAAGCGGAATTCCTGGTGTAGCGGTGAAATGCGTAGATATCAGGAGGAACACCTGTGGTGTAGACGGCTTACTGGACTATAACTGACGCTGAGGAACGAAAGCGTGGGTAGCAAACAGG` |
| ASV1350 | 424 | `TGAGGAATATTGGTCAATGGGCGCAAGCCTGAACCAGCCATGCCGCGTGCAGGAAGACGGTCCTATGGATTGTAAACTGCTTTTATTTGGGAATAAACCTCTTTACGTGTAGAGAGCTGAAGGTACCAAATGAATAAGCACCGGCTAACTCCGTGCCAGCAGCCGCGGTAATACGGAGGGTGCAAGCGTTATCCGGAATCATTGGGTTTAAAGGGTCCGCAGGCGGTCTTATAAGTCAGTGGTGAAAGCCTACAGCTCAACTGTAGAACTGCCATTGATACTGTAAGACTTGAATTCGATCGAAGTGGGCGGAATATGACATGTAGCGGTGAAATGCTTAGATATGTCATAGAACACCGATAGCGAAGGCAGCTCACTAGGTCTGGATTGACGCTCAGGGACGAAAGCGTGGGGAGCAAACAGG` |
| ASV506 | 429 | `TGGGGAATCTTGGACAATGGGCGAAAGCCTGATCCAGCAATGCCGCGTGCGTGAAGAAGGCCTTCGGGTTGTAAAGCGCTTTTGTCGGGGAGGAAATCCTAACAGTTAATACCTGTTGGGGATGACAGTACCCGAAGAATAAGCACCGGCTAACTACGTGCCAGCAGCCGCGGTAATACGTAGGGTGCAAGCGTTAATCGGAATTACTGGGCGTAAAGCGTCCGCAGGTGGCTTGATAAGATAGACGTGAAATCCCTGGGCTCAACCTAGGAATTGCGTATATGACTGTCTCGCTAGAGTATGGGAGAGGGGGGTGGAATTCCGCGTGTAGCAGTGAAATGCGTAGAGATGCGGAGGAACACCGATGGCGAAGGCAACCCCCTGGCCTAATACTGACACTCATGGACGAAAGCGTGGGGAGCAAACAGG` |
| ASV3023 | 429 | `TGGGGAATTTTGGACAATGGGCGCAAGCCTGATCCAGCCATGCCGCGTGAGTGAAGAAGGCCTTCGGGTTGTAAAGCTCTTTCAGCCGGGAAGAAAACGCATGGGTTAATACCCTGTGTGGATGACGGTACCGGAATAAGAAGCACCGGCTAACTACGTGCCAGCAGCCGCGGTAATACGTAGGGTGCGAGCGTTAATCGGAATTACTGGGCGTAAAGCGTGCGCAGGCGGTTTTGTAAGACAGGCGTGAAATCCCCGGGCTCAACCTGGGAACTGCGTTTGTGACTGCAAGGCTAGAGTATGGCAGAGGGGGGTGGAATTCCACGTGTAGCAGTGAAATGCGTAGAGATGTGGAGGAACACCGATGGCGAAGGCAGCCCCCTGGGCCAATACTGACGCTCATGCACGAAAGCGTGGGTAGCAAACAGG` |
| ASV4087 | 425 | `TAGGGAATATTGGGCAATGGAGGCAACTCTGACCCAGCCATGCCGCGTGCAGGAAGACGGTCCTCTGGATTGTAAACTGCTTTTGAATGGGAAGAATGTGCGGTTTGCGAACTGTAATGACGGTACCATTAGAATAAGCCACGGCTAACTACGTGCCAGCAGCCGCGGTAATACGTAGGTGGCGAGCGTTATCCGGATTTATTGGGTTTAAAGGGTGCGTAGGCGGCTTAATAAGTCGGTGGTAAAAGGTAGCAGCTTAACTGTTTTACAAGCCATCGATACTGCTAAGCTTGAGTTATTTGGAGGCAGCTAGAATTTCCGGTGTAGCGGTGAAATGCATAGATACCGGAAGGAATACCGATTGCGAAGGCAGGTTGCTACGAATATACTGACGCTGATGCACGAAAGCGTGGGGATCAAACAGG` |
| ASV4421 | 404 | `TGGGGAATATTGGACAATGGGCGAAAGCCTGATCCAGCAATGCCGCGTGAGTGAAGAAGGCCTTCGGGTTGTAAAGCTCTTTTACCCGGGAAGATAATGACTGTACCGGGAGAATAAGCCCCGGCTAACTCCGTGCCAGCAGCCGCGGTAATACGGAGGGGGCTAGCGTTGTTCGGAATTACTGGGCGTAAAGCGCACGTAGGCGGCTTTACAAGTCAGGGGTGAAAGCCCGCGGCTCAACCGCGGAATTGCCCTTGAGACTGTATCGCTTGAACACGGGAGAGGTGAGTGGAATTCCGAGTGTAGAGGTGAAATTCGTAGATATTCGGAAGAACACCAGTGGCGAAGGCGGCTCACTGGACCGTAGTTGACGCTGAGGTGCGAAAGCGTGGGGAGCAAACAGG` |
| ASV4488 | 406 | `CGAGGAATCTTGGGCAATGGGCGAAAGCCTGACCCAGCGACGCCGCGTGGAGGATGAAGGCCTTCGGGTTGTAAACTCCTTTTGCCGGGGAAGAAGTTCTGACGGTACCCGGCGAATAAGCCACGGCTAACTACGTGCCAGCAGCCGCGGTAATACGTAGGTGGCAAGCGTTGTCCGGATTTACTGGGCGTAAAGGGCTTGTAGGCGGTTCGTTAAGTCCGGTGTGAAATCTCCCGGCTCAACTGGGAGCGGCCATCGGATACTGGCGAGCTAGAGGTGGGCAGAGGAAGGCGGAATTCCCGGTGTAGCGGTGAAATGCGTAGATATCGGGAGGAACACCAGTGGCGAAGGCGGCCTTCTGGGCCTACCCTGACGCTGAGGAGCGAAAGCGTGGGGAGCAAACCGG` |
| ASV2962 | 404 | `TTAGGAATATTGGACAATGGGCGCAAGCCTGATCCAGCCATGCCGCGTGAGTGATGAAGGCCTTCGGGTTGTAAAGCTCTTTAGTCCACGATTATAATGACGGTAGTGGAAGAATAAGCCCCGGCTAACTTCGTGCCAGCAGCCGCGGTAATACGAAGGGGGCGAGCGTTATTCGGAATCACTGGGCGTAAAGCGTGCGTAGGCTGTCTTAAAAGTTAGGAGTGAAAGCCCCAGGCTCAACCTGGGAATTGCTTTTAATACTGTAAGACTCGAATTCGGGAGAGGATAGCGGAATTCTCAGTGTAGCAGTGAAATGCGTAGATATTGAGAGGAACACCAGTGGCGTAAGCGGCTATCTGGCCCGACATTGACGCTGAGGCACGAAAGCGTGGGGATCAAACAGG` |
| ASV2922 | 429 | `TGGGGAATATTGGACAATGGGCGAAAGCCTGATCCAGCCATGCCGCGTGTGTGAAGAAGGTCTTCGGATTGTAAAGCACTTTAAGTTGGGAGGAAGGGCAGTTACCTAATACGTAATTGTTTTGACGTTACCGACAGAATAAGCACCGGCTAACTCTGTGCCAGCAGCCGCGGTAATACAGAGGGTGCAAGCGTTAATCGGAATTACTGGGCGTAAAGCGCGCGTAGGTGGTTTGTTAAGTTGAATGTGAAATCCCCGGGCTCAACCTGGGAACTGCATCCAAAACTGGCAAGCTAGAGTATGGTAGAGGGTGGTGGAATTTCCTGTGTAGCGGTGAAATGCGTAGATATAGGAAGGAACACCAGTGGCGAAGGCGACCACCTGGACTGATACTGACACTGAGGTGCGAAAGCGTGGGGAGCAAACAGG` |
| ASV1735 | 429 | `TGGGGAATATTGGACAATGGGGGCAACCCTGATCCAGCAATACCGCGTGTGTGAAGAAGGCCTGAGGGTTGTAAAGCACTTTCAGTGGGGAGGAGGTTTGCTAGGTTAAGAGCTAAGTAGATAGACGTTACCCACAGAAGAAGCACCGGCTAACTCCGTGCCAGCAGCCGCGGTAATACGGAGGGTGCAAGCGTTAATCGGAATTACTGGGCGTAAAGGGTGCGTAGGTGGTTTGGTAAGTTATCTGTGAAATCCCCGGGCTCAACCTGGGCAGGTCAGATAATACTGCTGAACTCGAGTATGGGAGAGGGTAGTGGAATTTCCGGTGTAGCGGTGAAATGCGTAGAGATCGGAAGGAACACCAGTGGCGAAGGCGGCTACCTGGCCCAATACTGACACTGAGGCACGAAAGCGTGGGGAGCAAACAGG` |
| ASV4313 | 405 | `TGGGGAATTTTCCGCAATGGGCGCAAGCCTGACGGAGCAATACCGCGTGAGGGAAGAAGGCTCTTGGGTCGTAAACCTCTTTTGTTTGGGAAGAAACCTGACGGTACCAAACGAATAAGCCTCGGCTAACTCCGTGCCAGCAGCCGCGGTAAGACGGAGGAGGCAAGCGTTATCCGGAATTATTGGGCGTAAAGCGTCCGCAGGTGGTTTTACAAGTCTGTTGTCAAAGCGCGAGGCTCAACCTTGTATAGGCAATGGAAACTGTGGGACTAGAGTGCGGTAGGGGTAGAGGGAATTCCCGGTGTAGCGGTGAAATGCGTAGATATCGGGAAGAACACCAGTGGCGAAGGCGCTCTACTGGGCCTGCACTGACACTGAGGGACGAAAGCTAGGGGAGCGAAAGGG` |
| ASV5620 | 404 | `TGGGGAATATTGGACAATGGGCGAAAGCCTGATCCAGCAATACCGCGTGAGTGATGAAGGCCTTAGGGTTGTAAAACTCTTTCAGTAGGGAAGATAATGACGGTACCTACAGAAGAAGCACCGGCTAACTCCGTGCCAGCAGCCGCGGTAATACGGAGGGTGCAAGCGTTGTTCGGAATTACTGGGCGTAAAGAGTGCGTAGGCGGCTTGGCAAGTTGGGAGTGAAATCCCGGGGCTCAACCTCGGAATTGCTCTCAAAACTGCTGAGCTAGAGTATGGTAGGGGATAGTGGAATTCCTAGTGTAGGGGTGAAATCCGTAGATATTAGGAGGAACACCAGAGGCGAAGGCGACTATCTGGGCCATTACTGACGCTAAGGTACGAAAGCGTGGGGAGCAAACAGG` |
| ASV1549 | 404 | `TGGGGAATCTTGGACAATGGGCGCAAGCCTGATCCAGCCATGCCGCGTGAGTGAAGAAGGCCTTCGGGTTGTAAAGCTCTTTTGTCAGGGACGATAATGACGGTACCTGAAGAATAAGCCCCGGCAAACTTCGTGCCAGCAGCCGCGGTAATACGAAGGGGGCTAGCGTTGTTCGGAATTACTGGGCGTAAAGCGCACGTAGGCTGATTGTTAAGTCAGGGGTGAAATCCCGGAGCTCAACTCCGGAACTGCCTTTGATACTGGCAATCTCGAGTCCGGAAGAGGTTAGTGGAATTCTCAGTGTAGAGGTGAAATTCGTAGATATTGAGAAGAACACCAGTGGCGAAGGCGGCTAACTGGTCCGGTACTGACGCTGAGGTGCGAAAGCGTGGGGAGCAAACAGG` |
| ASV3720 | 429 | `TGGGGAATATTGGACAATGGGCGCAAGCCTGATCCAGCAATGCCGCGTGTGTGAAGAAGGCCTTCGGGTTGTAAAGCACTTTTGTTCGGGAAGAAATCCGTGAGGCTAATATCCTTACGGGATGACGGTACCGAAAGAATAAGCACCGGCTAACTTCGTGCCAGCAGCCGCGGTAATACGAAGGGTGCAAGCGTTAATCGGAATTACTGGGCGTAAAGCGTGCGTAGGCGGTTTGTTAAGTCAGATGTGAAAGCCCCGGGCTCAACCTGGGAACTGCATTTGATACTGGCAAGCTAGAGTGTGGTAGAGGGTGGTGGAATTCCCGGTGTAGCGGTGAAATGCGTAGAGATCGGGAGGAACATCCGTGGCGAAGGCGGCCACCTGGACCAACACTGACGCTGAGGCACGAAAGCGTGGGGAGCAAACAGG` |
| ASV5103 | 429 | `TGGGGAATATTGGACAATGGGGGAAACCCTGATCCAGCCATGCCGCGTGTGTGAAGAAGGCCTTTTGGTTGTAAAGCACTTTAAGCAGTGAAGAAGACTCTTCGGTTAATACCCGGAGACGATGACATTAGCTGCAGAATAAGCACCGGCTAACTCTGTGCCAGCAGCCGCGGTAATACAGAGGGTGCAAGCGTTAATCGGAATTACTGGGCGTAAAGCGAGCGTAGGTGGCTTGATAAGTCAGATGTGAAATCCCCGGGCTTAACCTGGGAACTGCATCTGAAACTGTTAGGCTAGAGTAGGTGAGAGGGAGGTAGAATTTCAGGTGTAGCGGTGAAATGCGTAGAGATCTGAAGGAATACCGATGGCGAAGGCAGCCTCCTGGCATCATACTGACACTGAGGCTCGAAAGCGTGGGTAGCAAACAGG` |
| ASV1358 | 429 | `TGGGGAATTTTGGACAATGGGCGAAAGCCTGATCCAGCAATGCCGCGTGCAGGATGAAGGCCTTCGGGTTGTAAACTGCTTTTGTACGGAGCGAAAAGGCTTCCATTAATACTGGGAGTTCATGACGGTACCGTAAGAATAAGCACCGGCTAACTACGTGCCAGCAGCCGCGGTAATACGTAGGGTGCAAGCGTTAATCGGAATTACTGGGCGTAAAGCGTGCGCAGGCGGTTATGTAAGACAGACGTGAAATCCCCGGGCTCAACCTGGGAACTGCGTTTGTGACTGCATAGCTAGAGTACGGTAGAGGGGGGTGGAATTCCGCGTGTAGCAGTGAAATGCGTAGATATGCGGAGGAACACCGATGGCGAAGGCAGCCCCCTGGACCTGTACTGACGCTCATGCACGAAAGCATGGGGAGCAAACAGG` |
| ASV4515 | 428 | `TGGGGAATATTGGACAATGGGCGAAAGCCTGATCCAGCAATGCCGCGTGTGTGAAGAAGGCCTTCGGGTTGTAAAGCACTTTAGGCAGGGAAGAAAAAATCGGCGATAACATCGCCGATCTTGACGGTACCTGAGGAATAAGCACCGGCTAACTCTGTGCCAGCAGCCGCGGTAATACAGAGGGTGCAAGCGTTAATCGGATTTACTGGGCGTAAAGCGTGTGTAGGTGGTTGGGAAAGTCAATTGTGAAATCCCTGGGCTCAACCCGGGAACTGCTTTTGATACTGTCTGACTAGAATTCGGTAGAGGGAGGCGGAACTCCAGGTGTAGCGGTGAAATGCGTAGATATCTGGAAGAACACCAATGGCGAAGGCAACCTCCTGGGCCTGAATTGACACTGAGACACGAAAGCGTGGGGATCAAACAGG` |
| ASV805 | 424 | `TGAGGAATATTGGACAATGGGCGCAAGCCTGATCCAGCCATGCCGCGTGCAGGAAGAAGCATCTATGGTGTGTAAACTGCTTTTGTACAGGAAGAAACACCCCTACGTGTAGGGACTTGACGGTACTGTAAGAATAAGGATCGGCTAACTCCGTGCCAGCAGCCGCGGTAATACGGAGGATCCAAGCGTTATCCGGAATCATTGGGTTTAAAGGGTCCGTAGGCGGTTTAATAAGTCAGTGGTGAAATCTGGTCGCTCAACGATCAAACGGCCATTGATACTGTTAGACTTGAATTATTGTGAAGTAACTAGAATATGTAGTGTAGCGGTGAAATGCTTAGATATTACATGGAATACCAATTGCGAAGGCAGGTTACTAACAATATATTGACGCTGATGGACGAAAGCGTGGGGAGCGAACAGG` |
| ASV5253 | 429 | `TGGGGAATATTGGACAATGGGGGCAACCCTGATCCAGCAATGCCGCGTGTGTGAAGAAGGCCTTAGGGTTGTAAAGCACTTTAGGCAGGGAAGAAGGTGAGTAGTTTAAGAGATGACTCATTTGACGGTACCTGCAGAATAAGCACCGGCTAACTCTGTGCCAGCAGCCGCGGTAATACAGAGGGTGCAAGCGTTAATCGGAATTACTGGGCGTAAAGGGCGCGTAGGTGGTTATGTAAGTTAGATGTGAAATCCCTGGGCTTAACCTAGGAACTGCATCTAAGACTGTGTGACTAGAGTACTGTAGAGGGTAGTGGAATTTCCGGTGTAGCGGTGAAATGCGTAGAGATCGGAAGGAACACCAGTGGCGAAGGCGGCTACCTGGACAGATACTGACACTGAGGCGCGAGAGCGTGGGGAGCAAACAGG` |
| ASV5409 | 430 | `TAGGGAATTTTGCACAATGGGCGCAAGCCTGATGCAGCAACGCCGCGTGAGTGAAGAAGGCCTTTGGGTTGTAAAGCTCTTTCGGCTGGGAAGATGGGTCTGTTTGCGAATAGCACTCAGATTTGACGGTACCAGAAGAAGAAGCACCTGCTAACTACGTGCCAGCAGCAGCGGTAATACGTAGGGTGCAAGCGTTGTTCGGAATTATTGGGCGTAAAGCGCGCGTAGGCGGGTTTGTAAGTCGATTGTGAAAGCCCTGGGCTTAACCTGGGAATGGCAGTCGAAACTGCAAATCTTGAATACCTAAGAGGATGGCGGAATTCCCGGTGTAGTAGTGAAATACGTAGATATCGGGAGGAACACCTGAGGCGAAGGCGGCTGTCTGGTGGTGTATTGACGCTGAGGCGCGAAAGCGTGGGGAGCAAACAGG` |
| ASV4118 | 429 | `TCGAGAATAATTCGCAATGGACGAAAGTCTGACGATGCGACGCCGTGTGAATGATGAAGGCCTTAGGGTTGTAAAGTTCTGTCATTTGCGAACAAAGATTTTGTACGAATACTACAGAATATTGAGCGTAGCAGAAGAGGAAGCACCGGCTAACTCCGTGCCAGCAGCTGCGGTAATACGGAGGGTGCAAGCGTTAATCGGAATTACTGGGCGTAAAGAGCGCGTAGGCGGAGATGTAAGTCAGTTGTGAAATTTCGCAGCTCAACTGCGAAACAGCACCTGAAACTGTATTTCTAGAGGGATGATGGAGGAAACGGAATTCCACGTGTAGCGGTGAAATGCGTAGATATGTGGAAGAACACCCGTGGCGAAGGCGGTTTCCTAATCTACCCCTGACGCTGAGGCGCGAAAGCAAGGGGAGCAAACAGG` |
| ASV4864 | 404 | `TGGGGAATATTGGACAATGGGGGAAACCCTGATCCAGCAATGCCGCGTGAGTGATGAAGGCCTTCGGGTTGTAAAGCTCTTTTGGCGGGGACGATGATGACGGTACCCGCAGAATAAGCTCCGGCTAACTTCGTGCCAGCAGCCGCGGTAATACGAGGGGAGCAAGCGTTGTTCGGAATTACTGGGCGTAAAGAGCGCGTAGGCGGCTTGATAAGTTGGGCGTGAAAGCCCTGGGCTCAACCCAGGAACTGCGCTCAAAACTATCTGGCTTGAGAGTGGTAGAGGATAGTGGAATACCCAGTGTAGAGGTGAAATTCGTAGATATTGGGTAGAACACCGGTGGCGAAGGCGGCTATCTGGACCATTTCTGACGCTGAGGCGCGAAAGCGTGGGGAGCAAACAGG` |
| ASV2612 | 406 | `TGGGGAATTTTCCGCAATGGGCGAAAGCCTGACGGAGCAATGCCGCGTGGAGGTAGAAGGCCCACGGGTCGTGAACTTCTTTTCCCGGAGAAGAAGCAATGACGGTATCCGGGGAATAAGCATCGGCTAACTCTGTGCCAGCAGCCGCGGTAAGACAGAGGATGCAAGCGTTATCCGGAATGATTGGGCGTAAAGCGTCTGTAGGTGGCTTTTTAAGTCCGCCGTCAAATCCCAGGGCTCAACCCTGGACAGGCGGTGGAAACTACCAAGCTGGAGTACGGTAGGGGCAGAGGGAATTTCCGGTGGAGCGGTGAAATGCGTAGAGATCGGAAAGAACACCAACGGCGAAAGCACTCTGCTGGGCCGACACTGACACTGAGAGACGAAAGCTAGGGGAGCGAATGGG` |
| ASV4393 | 424 | `TAAGGAATATTGGTCAATGGAGGCAACTCTGAACCAGCCATGCCGCGTGCAGGAAGACGGACCTATGGTTTGTAAACTGCTTTTGTACGAGAGAAAACCTCTTTACGTGTAGAGAGTTGATAGTATCGTAAGAATAAGGATCGGCTAACTTCGTGCCAGCAGCCGCGGTAATACGAAGGATCCAAGCGTTATCCGGATTCATTGGGTTTAAAGGGTGCGTAGGCGGGCTGATAAGTCAGCGGTGAAATCTCTCGGCTTAACCGAGAAACTGCCGTTGATACTGTTGGTCTTGAGTATAGATGATGTTGGCGGAATATGACATGTAGCGGTGAAATGCTTAGATATGTCATAGAACACCGATTGCGAAGGCAGCTGACAAAACTATAACTGACGCTGAGGCACGAAAGTGCGGGGATCAAACAGG` |
| ASV4353 | 428 | `CTAAGAATCTTCCACAATGGACGGAAGTCTGATGGAGCGACGCCGCGTGGACGATGAAGGCCGTGAGGTTGTAAAGTCCTTTTCGGGAGGGGGAATAACCTGGGGAGGGAATGCCCCGGGGATGACGTGAATCCCGGAATAAGCCCCGGCTAACTACGTGCCAGCAGCCGCGGTAATACGTAGGGGGCGAGCGTTGTTCGGAATCATTGGGCGTAAAGGGTGCGCAGGTGGCCTTTCAAGTCTGGCGTTAAAGTTCGGGGCTCAACTCCGTGATGCGCTGGAAACTGGAGGGCTAGAGTACAGCAGGGGGCATCGGAATTCCAGGTGTAGGGGTGAAATCTGTAGATATCTGGAAGAACACCGGTGGCGAAGGCGGATGCCTGGGCATGTACTGACACTGAGGCACGAAGGTGCGGGGAGCAAACAGG` |
| ASV2112 | 429 | `TGGGGAATTTTGGACAATGGGCGCAAGCCTGATCCAGCCATTCCGCGTGCAGGATGAAGGCCCTCGGGTTGTAAACTGCTTTTGTACGGAACGAAAAGGCCTCTTCTAATACAAGGGGCTCATGACGGTACCGTAAGAATAAGCACCGGCTAACTACGTGCCAGCAGCCGCGGTAATACGTAGGGTGCAAGCGTTAATCGGAATTACTGGGCGTAAAGCGTGCGCAGGCGGTGATGTAAGACAGTTGTGAAATCCCCGGGCTCAACCTGGGAACTGCATCTGTGACTGCATCGCTGGAGTGCGGCAGAGGGGGATGGAATTCCGCGTGTAGCAGTGAAATGCGTAGATATGCGGAGGAACACCGATGGCGAAGGCAATCCCCTGGGCCTGCACTGACGCTCATGCACGAAAGCGTGGGGAGCAAACAGG` |
| ASV4375 | 389 | `TCGGGAAACTTTTGCAATATGCGCAAGCGTGACAAAGCAAGCCAGAGTGCTGTTCTATTTTTGAACAGCTTTTGCGGGATGTAAAAAGTCTCGAGAATAAGGACTGGGCAAGACTGGTGCCAGCCGCCGCGGTAATCCCAGCAGTCCGAGTCGCAGCCACATTTGTTGGGTCTAAAACATTCGTAGCTCGTCTTGTAAGTCCTTTGTGAAATCGGGCCTCTTAAGGGTCCGGCGGGCAAAGGATACTACTTGGCTAGAGACCGGGAGGCGCAGGAAGTACGCACAGGGTAGCGGTAAAATGTGTTGATCCTGTGCGGACTCACAGTCGCGAAGGCGTCCTGCGAGAACGGTTCTGACAGTGAGGGATGAAGGCTAGGGGCGCAAAGTGG` |
| ASV5201 | 424 | `TGAGGAATATTGGTCAATGGACGAGAGTCTGAACCAGCCAAGTAGCGTGAAGGATGAAGGTCCTACGGATTGTAAACTTCTTTTATAAGGGAATAAAACCTCCCACGTGTGGGAGCTTGTATGTACCTTATGAATAAGCATCGGCTAACTCCGTGCCAGCAGCCGCGGTAATACGGAGGATGCGAGCGTTATCCGGATTTATTGGGTTTAAAGGGAGCGCAGACGGGTCGTTAAGTCAGCTGTGAAAGTTTGGGGCTCAACCTTAAAATTGCAGTTGATACTGGCGTCCTTGAGTGCGGTTGAGGTGTGCGGAATTCGTGGTGTAGCGGTGAAATGCTTAGATATCACGAAGAACTCCGATTGCGAAGGCAGCACACTAAGCCGTAACTGACGTTCATGCTCGAAAGTGTGGGTATCAAACAGG` |
| ASV3975 | 423 | `TAGGGAATATTGGACAATGGGTGAGAGCCTGATCCAGCCATGCCGCGTGCAGGAAGACGGCCTTCTGGGTTGTAAACTGCTTTTATCTGGGGATAAAATACCCATGCGTGGGTAACTGAAGGTACCAGATGAATAAGCCACGGCTAACTACGTGCCAGCAGCCGCGGTAATACGTAGGTGGCAAGCGTTGTCCGGATTTATTGGGTTTAAAGGGTGCGTAGGCGGTTTATTAAGTCAGTGGTGAAAGACGGTAGCTTAACTATCGAGGTGCCATTGATACTGATGAACTTGAGTGCAGTCGAAGTAGGCGGAATGGACGGTGTAGCGGTGAAATGCATAGATATCGTCCAGAACACCGATAGTGAAGACAGTTTACTAGGCTGTAACTGACGCTGAGGCACGAAAGTGTGGGGATCAAACAGG` |
| ASV5111 | 424 | `TGGGGAATATTGGTCAATGGGCGAAAGCCTGAACCAGCAATGCCGCGTGAGTGATGAAGGCCTTCGGGTTGTAAAGCTCTGTCAGTGGGAACGAATAAGCCGTTTAATACACGGTGGTGACGGTACCCACAAAGGAAGCACCGGCTAACTCTGTGCCAGCAGCCGCGGTAATACAGAGGGTGCAAGCGTTGTTCGGAATTACTGGGCGTAAAGCGCGTGTAGGCGGTTTGGTAAGTCGGATGTGAAATCCCTTGGCTCAACCAAGGAACTGCATTCGAAACTGCCAGACTTGAGTGTCGGAGGGGATGGCGGAATTCCCGGTGTAGAGGTGAAATTCGTAGATATCGGGAGGAACACCTGTGGCGAAGGCGGCCATCTGGAAGACAACTGACGCTGAGACGCGAAAGCGTGGGGAGCAAACAGG` |
| ASV947 | 429 | `TGGGGAATTTTGGACAATGGGCGCAAGCCTGATCCAGCAATGCCGCGTGCAGGAAGAAGGCCTTCGGGTTGTAAACTGCTTTTGTACGGAACGAAACGGTCTGGATTAATACCCTGGGCTAATGACGGTACCGTAAGAATAAGCACCGGCTAACTACGTGCCAGCAGCCGCGGTAATACGTAGGGTGCAAGCGTTAATCGGAATTACTGGGCGTAAAGCGTGCGCAGGCGGTTTTGTAAGACAGTCGTGAAATCCCCGGGCTCAACCTGGGAATTGCGATTGTGACTGCAAAGCTGGAGTGCGGCAGAGGGGGATGGAATTCCGCGTGTAGCAGTGAAATGCGTAGATATGCGGAGGAACATCGATGGCGAAGGCAATCCCCTGGGCCTGCACTGACGCTCATGCACGAAAGCGTGGGGAGCAAACAGG` |
| ASV2738 | 429 | `TGGGGAATTTTGGACAATGGGCGAAAGCCTGATCCAGCCATTCCGCGTGAGTGAAGAAGGCCTTCGGGTTGTAAAGCTCTTTCGGCAGGGAAGAAACGGTCTAGGTTAATACCTTGGGCTAATGACGGTACCTGAAGAAGAAGCACCGGCTAACTACGTGCCAGCAGCCGCGGTAATACGTAGGGTGCGAGCGTTAATCGGAATTACTGGGCGTAAAGCGTGCGCAGGCGGTTTTGTAAGACAGGTGTGAAATCCCCGGGCTTAACCTGGGAACTGCGCTTGTGACTGCAAGACTCGAGTACGGCAGAGGGGGGTAGAATTCCACGTGTAGCAGTGAAATGCGTAGAGATGTGGAGGAATACCGATGGCGAAGGCAGCCCCCTGGGTCGATACTGACGCTCATGCACGAAAGCGTGGGGAGCAAACAGG` |
| ASV1916 | 404 | `TTAGGAATATTGGACAATGGGGGAAACCCTGATCCAGCCATGCCGCGTGTGTGAAGAAGGCCTTAGGGTTGTAAAGCACTTTCGGCTGTGAAGATAATGACAGTAGCAGAAGAAGAAGCTCCGGCTAAATTCGTGCCAGCAGCCGCGGTAATACGAATGGAGCGAGCGTTGTTCGGAATCACTGGGCGTAAAGCGTGCGCAGGCGGTTATGAAAGTCAGATGTGAAAGCCCAGGGCTTAACCCTGGAATTGCATTTGAAACTCCATGACTAGAGTACTGGAGGGGTTGGCGGAATTTCGAGTGTAGCAGTGAAATGCGTAGATATTCGAAGGAACACCGATGGCGTAGGCAGCCAACTGGACAGTAACTGACGCTCATGCACGAAAGCGTGGGGATCAAACAGG` |
| ASV826 | 424 | `TGAGGAATATTGGTCAATGGACGAGAGTCTGAACCAGCCAAGTAGCGTGAAGGATGAAGGCCCTATGGGTCGTAAACTTCTTTTATAGGGGAATAAAGTGCGGAACGTGTTCCGCTTTGTATGTACCCTACGAATAAGGATCGGCTAACTCCGTGCCAGCAGCCGCGGTAATACGGAGGATCCGAGCGTTATCCGGATTTATTGGGTTTAAAGGGAGCGTAGGTGGACGTATAAGTCAGTTGTGAAAGTTTGCGGCTCAACCGTAAAATTGCAGTTGATACTGTATGTCTTGAGTGTACAAGAGGTGGGCGGAATTCGTGGTGTAGCGGTGAAATGCTTAGATATCACGAAGAACTCCGATTGCGAAGGCAGCTCACTAGAGTACAACTGACACTGAGGCTCGAAAGTGTGGGTATCAAACAGG` |
| ASV3503 | 429 | `TGGGGAATTTTGGACAATGGGCGCAAGCCTGATCCAGCCATGCCGCGTGCGGGAAGAAGGCCTTCGGGTTGTAAACCGCTTTTGTCAGGGAAGAAATCCTTTGAGCCAATACCTCGAGGGGATGACGGTACCTGAAGAATAAGCACCGGCTAACTACGTGCCAGCAGCCGCGGTAATACGTAGGGTGCAAGCGTTAATCGGAATTACTGGGCGTAAAGCGTGCGCAGGCGGTTTTGTAAGACAGAGGTGAAATCCCCGGGCTTAACCTGGGAACTGCCTTTGTGACTGCAAAGCTTGAGTGCGGCAGAGGGGGATGGAATTCCGCGTGTAGCAGTGAAATGCGTAGATATGCGGAGGAACACCGATGGCGAAGGCAATCCCCTGGGCCTGCACTGACGCTCATGCACGAAAGCGTGGGGAGCAAACAGG` |
| ASV3639 | 429 | `TGGGGAATTTTGGACAATGGGCGCAAGCCTGATCCAGCAATGCCGCGTGCAGGATGAAGGCCTTCGGGTTGTAAACTGCTTTTGTACGGAACGAAAAGCCTTGGGCTAATACCCCGAGGCCATGACGGTACCGTAAGAATAAGCACCGGCTAACTACGTGCCAGCAGCCGCGGTAATACGTAGGGTGCAAGCGTTAATCGGAATTACTGGGCGTAAAGCGTCCGCAGGCGGTAATGTAAGACAGAGGTGAAATCCCCGAGCTCAACTTGGGAACAGCCTTTGTGACTGCATCACTAGAGTACGGCAGAGGGGGATGGAATTCCGCGTGTAGCAGTGAAATGCGTAGATATGCGGAGGAACACCGATGGCGAAGGCAATCCCCTGGGCCTGTACTGACGCTCATGGACGAAAGCGTGGGGAGCAAACAGG` |
| ASV3092 | 429 | `TGGGGAATTTTGGACAATGGGGGAAACCCTGATCCAGCCATTCCGCGTGAGTGAAGAAGGCCTTCGGGTTGTAAAGCTCTTTCGGCAGGGAAGAAACGCTCTGGATTAATACTCCGGGGTAATGACGGTACCTGAAGAAGAAGCACCGGCTAACTACGTGCCAGCAGCCGCGGTAATACGTAGGGTGCGAGCGTTAATCGGAATTACTGGGCGTAAAGCGTGCGCAGGCGGTTTTGTAAGACAGGTGTGAAATCCCCGGGCTTAACCTGGGAACTGCGCTTGTGACTGCAAGACTTGAGTATGGCAGAGGGGGGTAGAATTCCACGTGTAGCAGTGAAATGCGTAGAGATGTGGAGGAATACCGATGGCGAAGGCAGCCCCCTGGGTCAATACTGACGCTCATGCACGAAAGCGTGGGGAGCAAACAGG` |
| ASV727 | 404 | `TGGGGAATCTTAGACAATGGGCGCAAGCCTGATCTAGCCATGCCGCGTGAGCGATGAAGGCCTTAGGGTTGTAAAGCTCTTTCGGGGGGGAAGATAATGACTGTACCCCCAGAAGAAGCCCCGGCTAACTCCGTGCCAGCAGCCGCGGTAATACGGAGGGGGCTAGCGTTGTTCGGAATTACTGGGCGTAAAGCGCACGTAGGCGGACGGAGAAGTCAGAGGTGAAATCCCAGGGCTCAACCTTGGAACTGCCTTTGAAACTTTCTGTCTTGAGGTCGAGAGAGGTGAGTGGAATTCCGAGTGTAGAGGTGAAATTCGTAGATATTCGGAGGAACACCAGTGGCGAAGGCGGCTCACTGGCTCGATACTGACGCTGAGGTGCGAAAGCGTGGGGAGCAAACAGG` |
| ASV1681 | 428 | `TGGGGAATATTGGACAATGGGGGCAACCCTGATCCAGCAATGCCGCGTGTGTGAAGAAGGCCTGCGGGTTGTAAAGCACTTTCAGAGGGGAAGAAAAAATCGGATCTAACACATCCGAGCTTGACGTTACCCTTAGAAGAAGCACCGGCTAACTCCGTGCCAGCAGCCGCGGTAATACGGAGGGTGCAAGCGTTAATCGGAATTACTGGGCGTAAAGCGCACGCAGGTGGCTGAGAAAGTCGATTGTGAAAGCCCTGGGCTTAACCTGGGAATTGCAGTCGAAACTACTCGGCTAGAGTATGGGAGAGGGTAGTGGAATTCCCGGTGTAGCGGTGAAATGCGTAGATATCGGGAGGAACATCAGTGGCGAAGGCGGCTACCTGGCCCAATACTGACACTCAGGTGCGACAGCGTGGGGAGCAAACAGG` |
| ASV3142 | 404 | `TGGGGAATCTTGGACAATGGGCGCAAGCCTGATCCAGCCATGCCGCGTGAGTGAAGAAGGCCTTAGGGTTGTAAAGCTCTTTTGCCAGGGACGATAATGACGGTACCTGGAGAATAAGCCCCGGCAAACTTCGTGCCAGCAGCCGCGGTAATACGAAGGGGGCTAGCGTTGTTCGGATTTACTGGGCGTAAAGCGCACGTAGGCGGGCCGTTAAGTCAGGGGTGAAATCCCGGAGCTCAACTCCGGAACTGCCTTTGATACTGGCGGCCTCGAGGCCGGAAGAGGTGAGTGGAATTCCCAGTGTAGAGGTGAAATTCGTAGATATTGGGAAGAACACCAGTGGCGAAGGCGGCTCACTGGTCCGGTACTGACGCTGAGGTGCGAAAGCGTGGGGATCAAACAGG` |
| ASV1126 | 404 | `TGGGGAATATTGGACAATGGGCGAAAGCCTGATCCAGCAATGCCGCGTGAGTGAAGAAGGCCTTCGGGTTGTAAAGCTCTTTTACCCGGGAAGATAATGACTGTACCGGGAGAATAAGCCCCGGCTAACTCCGTGCCAGCAGCCGCGGTAATACGGAGGGGGCTAGCGTTGTTCGGAATTACTGGGCGTAAAGCGCACGTAGGCGGCTTTTTAAGTCAGAGGTGAAAGCCCGGGGCTCAACTCCGGAATTGCCTTTGAAACTGGAAAGCTTGAACATGGGAGAGGTGAGTGGAATTCCGAGTGTAGAGGTGAAATTCGTAGATATTCGGAGGAACACCAGTGGCGAAGGCGGCTCACTGGACCATTGTTGACGCTGAGGTGCGAAAGCGTGGGGAGCAAACAGG` |
| ASV1124 | 429 | `TGGGGAATATTGGACAATGGGCGAAAGCCTGATCCAGCCATGCCGCGTGTGTGAAGAAGGTCTTCGGATTGTAAAGCACTTTAAGTTGGGAGGAAGGGTAGTAACCTAATACGTTGCTACTTTGACGTTACCGACAGAATAAGCACCGGCTAACTTCGTGCCAGCAGCCGCGGTAATACGAAGGGTGCAAGCGTTAATCGGAATTACTGGGCGTAAAGCGCGCGTAGGTGGTTCAGCAAGTTGGATGTGAAAGCCCTGGGCTCAACCTGGGAACTGCATCCAAAACTACTGAGCTAGAGTACGGTAGAGGGTAGTGGAATTTCCTGTGTAGCGGTGAAATGCGTAGATATAGGAAGGAACACCAGTGGCGAAGGCGACTACCTGGACTGATACTGACACTGAGGTGCGAAAGCGTGGGGAGCAAACAGG` |
| ASV3176 | 422 | `TAGGGAATATTGGGCAATGGAGGCAACTCTGACCCAGCCACGCCGCGTGCAGGATGAAGGCCCTCTGGGTTGTAAACTGCTTTTGTATGGGAAGAAAATGCTTCTGCGGAAGTGACTGACGGTACCATACGAATAAGCATCGGCTAACTCCGTGCCAGCAGCCGCGGTAATACGGAGGATGCGAGCGTTGTCCGGATTTACTGGGTTTAAAGGGTGCGTAGGCGGGTTAGTAAGTGAGAGTTGAAAGGCAGTTGCTCAACAATTGTAAGGCTCTCATACTGCTAATCTTGAGTGTACTGGAGGCAGATAGAATGGGTGGAGTAGCGGTGAAATGCATAGATACCATCCAGAATACCGATTGCGAAGGCATTCTGCTACATTACAACTGACGCTGAGGCACGAAAGTGTGGGGATCAAACAGG` |
| ASV3710 | 424 | `TAAGGAATATTGGTCAATGGAGGCAACTCTGAACCAGCCATGCCGCGTGCAGGAAGACGGCCCTATGGGTTGTAAACTGCTTTTGTACCAGAGAAAACCCTTCTACGTGTAGGAGGTTGATAGTATGGTAAGAATAAGCATCGGCTAACTTCGTGCCAGCAGCCGCGGTAAGACGAAGGATGCAAGCGTTATCCGGATTCATTGGGTTTAAAGGGTGCGTAGGCGGACCTGTAAGTCAGTGGTGAAATCTCTCAGCTTAACTGAGAAACTGCCATTGATACTGCAAGTCTAGAGTATAGATGACGTTGGCGGAATATGACATGTAGTGGTGAAATACTTAGATATGTCATAGAACACCGATTGCGAAGGCAGCTAACGAAACTATAACTGACGCTGAGGCACGAAAGTGCGGGGATCAAACAGG` |
| ASV4020 | 430 | `TGGGGAATCTTGCGCAATGGAGGAAACTCTGACGCAGTGATACCGCGTGAGTGAGGAAGGCCTTCGGGTTGTAAAGCTCTGTCAAAATGGAAAAACGATGGTATGTCGAACAGGCATATCAAGTGATGGTACATTGAGAGGAATCACCGGCTAACTTCGTGCCAGCAGCCGCGGTAATACGAAGGGTGAGAGCGTTGTTCGGATTTATTGGGCGTAAAGCGCGCGCAGGCGGGTAATCAAGTCAGATGTGAAATCTCGGGGCTCAACTCCGAAACAGCGTCTGAAACTAGTTATCTAGAGTCTTGGCAGGGGAGGGGGAATTTCACATGTAGGGGTAAAATCCGTAGAGATGTGAAGGAACACCAGAGGCGAAGGCGCCCTCCTGGACAAGTACTGACGCTGAGGCGCGAAAGCGTGGGGAGCAAACAGG` |
| ASV2584 | 423 | `TAGGGAATATTGGGCAATGGAGGCAACTCTGACCCAGCCATGCCGCGTGCAGGATGAAGGCGTTATGCGTTGTAAACTGCTTTTATATAGGAAGAAAAAGACCTTGCGAGGTAGATTGACGGTACTATATGAATAAGCACCGGCTAACTCCGTGCCAGCAGCCGCGGTAATACGGAGGGTGCAAGCGTTGTCCGGATTTATTGGGTTTAAAGGGTGCGTAGGCGGTTTATTAAGTCAGTGGTGAAAGACGGTCGCTCAACGATTGCAGTGCCATTGATACTGGTAGACTTGAGTGAGATTGAGGTAGCTGGAATGGATAGTGTAGCGGTGAAATGCATAGATATTATCCAGAACACCAATTGCGAAGGCAAGTTACTAAGTCTCAACTGACGCTGATGCACGAAAGTGTGGGTATCAAACAGG` |
| ASV5407 | 428 | `TCGAGAATTTTTCACAATGGGCGAAAGCCTGATGGAGCGACGCCGCGTGGGGGATGAATGGCTTCGGCCCGTAAACCCCTGTCATTTGCGATCAAACCGCGTAGTTTAAGAGATGACGCGCTGATAGTAGCGGAAGAGGAAGGGACGGCTAACTCTGTGCCAGCAGCCGCGGTAATACAGAGGTCCCAAGCGTTGTTCGGATTCACTGGGCGTAAAGGGTGCGTAGGCGGTCGCGCAAGTTTGACGTGAAATCTCCGGGCTCAACCCGGAAATTGCGTTGGATACTGCGCGGCTTGAGGATTGGAAGGGAGACTGGAATGCTCGGTGTAGCAGTGAAATGCGTAGATATCGAGCGGAACACCAGTGGCGAAGGCGAGTCTCTGGACAATTCCTGACGCTGAGGCACGAAAGCCAGGGGAGCAAACGGG` |
| ASV3949 | 424 | `TGAGGAATATTGGTCAATGGACGCAAGTCTGAACCAGCCAAGTAGCGTGCAGGATGACGGCCCTATGGGTTGTAAACTGCTTTTATGCGGGGATAAAGTGTGCCATGTATGGCATTTTGTAGGTACCGCATGAATAAGGACCGGCTAATTCCGTGCCAGCAGCCGCGGTAATACGGAAGGTCCGGGCGTTATCCGGATTTATTGGGTTTAAAGGGAGCGTAGGCCGTCGTTTAAGCGTGTTGTGAAATGCCGCGGCTCAACCGTGGCACTGCAGCGCGAACTGGATGACTTGAGTTCGCGGGAGGTAGGCGGAATTCGTGGTGTAGCGGTGAAATGCTTAGATATCACGAAGAACTCCGATTGCGAAGGCAGCTTACCGTAGCGTTACTGACGCTGAAGCTCGAAAGTGCGGGTATCGAACAGG` |
| ASV4653 | 429 | `TAGGGAATTTTCCACAATGGACGAAAGTCTGATGGAGCAACGCCGCGTGTGCGAAGAAGGCCTTCGGGTTGTAAAGCACTGTTGTAAGGGAAGAACAGGTGTGAGAGGAAATGCTCATGCTGTGACGGTACCTTACCAGAAAGCCACGGCTAACTACGTGCCAGCAGCCGCGGTAATACGTAGGTGGCGAGCGTTATCCGGAATTATTGGGCGTAAAGGGCGTGTAGGCGGTACTTTAAGTCTGACGTGAAAGCCAACGGCTCAACCGTTGAAATGCGTTGGAAACTGAAGTACTTGAGTGTCGGAGAGGAAAGCGGAATTCCTAGTGTAGCGGTGAAATGCGTAGATATTAGGAGGAACACCAGTGGCGAAGGCGGCTTTCTGGACGACAACTGACGCTGAGGCGCGAAAGCGTGGGGAGCAAACAGG` |
| ASV5360 | 429 | `TGGGGAATTTTGGACAATGGGCGAAAGCCTGATCCAGCCATGCCGCGTGAGTGAAGAAGGCCTTCGGGTTGTAAAGCTCTTTCAGACGGAAAGAAATTGCTTCGGTTAATAACTGGAGTAGATGACGGTACCGTAAGAAGAAGCACCGGCTAACTACGTGCCAGCAGCCGCGGTAATACGTAGGGTGCGAGCGTTAATCGGAATTACTGGGCGTAAAGCGTGCGCAGGCGGTTATATAAGACAGATGTGAAATCCCCGGGCTTAACCTGGGAACTGCGTTTGTGACTGTATAACTAGAGTACGGTAGAGGGGGGTGGAATTCCTGGTGTAGCAGTGAAATGCGTAGATATCAGGAGGAACATCGATGGCGAAGGCAGCCCCCTGGACCTGTACTGACGCTCATGCACGAAAGCGTGGGGAGCAAACAGG` |
| ASV2481 | 407 | `TAGGGAATATTGCGCAATGGAGGAAACTCTGACGCAGCGACGCCGCGTGAGCGATGAAGGCCTTCGGGTTGTAAAGCTCTGTCGCAGGGGAATAAGACAGTGAATGTACTCTGCAAGAAAGGATCGGCTAACTTCGTGCCAGCAGCCGCGGTAAGACGAGGGATCCTAGCGTTGTTCGGAATTATTGGGCGTAAAGCGGGTGTAGGTGGCTTGGTAAGTCAGGTGTGAAAGCCCAGGGCTTAACCCTGGAAGTGCATTTGATACTGCCGAGCTTGAGTGTAGGAGAGGTTATTAGAATTCTTGGTGTAGTGGTGAAATACGTAGATATCAAGAGGAATACCGGAGGCGAAGGCGGATAACTGGCCTAACACTGACACTCAGACCCGAAAGCGTGGGGATCAAACAGG` |
| ASV5304 | 404 | `TGGGGAATATTGGACAATGGGCGCAAGCCTGATCCAGCCATGCCGCGTGAGTGATGAAGGCCCTAGGGTTGTAAAGCTCTTTCGCCAGGGACGATAATGACGGTACCTGGATAAGAAGCCCCGGCTAACTTCGTGCCAGCAGCCGCGGTAATACGAAGGGGGCTAGCGTTGTTCGGAATCACTGGGCGTAAAGCGCACGTAGGCGGGCTGTTAAGTCGGAGGTGAAATCCTGGGGCTCAACCCCAGAACTGCCTTCGATACTGGCGGTCTTGAGTGTGGAAGAGGTTGGTGGAACTCCGAGTGTAGAGGTGAAATTCGTAGATATTCGGAAGAACACCAGTGGCGAAGGCGGCCAACTGGTCCACTACTGACGCTGAGGTGCGAAAGCGTGGGGAGCAAACAGG` |
| ASV4892 | 430 | `TGGGGAATCTTCCGCAATGGGCGAAAGCCTGACGGAGTGACGCCGCGTGGGGGATGAAGGCCTTCGGGTCGTAAACCCCTGTCAGAGGGAAAGAAGGGCGTCGGCGCGACAACCGCCGGCGTTTGACGGTACCCTCAGAGGAAGCCCCGGCCAACTCTGTGCCAGCAGCCGCGGTAAGACAGAGGGGGCAAGCGTTGCTCGGAATTACTGGGCGTAAAGAGCGCGTAGGCGGGGCGGTAAGTCGGTCGTAAAAGCCTCCGGCTCAACTGGAGAATGTCGATCGATACTGCTGTCCTTGAGGGTTGCAGGGGAGACTGGAATTCCCGGTGTAGCGGTGAAATGTGTAGAGATCGGGAGGAACACCGGTGGCGAAGGCGGGTCTCTGGGCAACTCCTGACGCTGAGGCGCGAAAGCGTGGGGAGCAAACGGG` |
| ASV488 | 429 | `TCGAGAATAATTCACAATGGGGGCAACCCTGATGGTGCAACGCCGCGTGGAGGATGACGGTCTTCGGATTGTAAACTCCTGTCATCTGGGAGTAAGGCATGGTCGTTAATAGCGGACATGATTGATAGTACCAGAAGAGGAAGGGACGGCTAACTTCGTGCCAGCAGCCGCGGTAATACGAAGGTCCCAAGCGTTGTTCGGAATCACTGGGCGTAAAGGGAGCGTAGGCGGCGTGGTAAGTCAGATGTGAAATCCCGGGGCTCAACCCCGGAACTGCATCCGATACTGCCATGCTAGAGGATTGGAGAGGTAGCTGGAATTCTTGGTGTAGCAGTGAAATGCGTAGAGATCAAGAGGAACACTCGTGGCGAAAGCGAGCTACTGGACAATTCCTGACGCTGAGGCTCGAAGGCTAGGGTAGCGAAAGGG` |
| ASV548 | 429 | `TGGGGAATATTGGACAATGGGGGAAACCCTGATCCAGCCATGCCGCGTGTGTGAAGAAGGCCTTCGGGTTGTAAAGCACTTTCGGTCAGAAAGAAGGGAGCGTAGTTAATAACTGCGTTCTTTGACGGTACTGACATAAGAAGCACCGGCTAACTCCGTGCCAGCAGCCGCGGTAATACGGAGGGTGCAAGCGTTAATCGGAATTACTGGGCGTAAAGCGCGCGTAGGCGGTTTGTTAAGTCGGATGTGAAAGCCCTGGGCTCAACCTGGGAACTGCATACGATACTGGCAAGCTAGAGTGCAATAGAGGGGGGTAGAATTCCAGGTGTAGCGGTGAAATGCGTAGATATCTGGAGGAATACCGGTGGCGAAGGCGGCCCCCTGGATTGACACTGACGCTGAGGTGCGAAAGCGTGGGTAGCAAACAGG` |
| ASV4534 | 426 | `TTAAGAATCTTGCGCAATGGACGAAAGTCTGACGCAGCGACGCCGCGTGAAGGATGAAGGCTTTCGGGTTGTAAACTTCGGTAGACTGGGAAGAAAAAGTATGTTCGTTGAGGGCATACATTGACGGTACCAGTCTGAAGCCCCGGCTAATTACGTGCCAGCAGCCGCGGTAATACGTATGGGGCAAGCGTTGTTCGGAATTATTGGGCGTAAAGGGCTCGCAGGTGGTTTGTTAAGTTGGTGGTTTAATCTCTGGGCTCAACCCAGAGTCAGCCTCCAAAACTGGCGAACTTGAGTACGATAGGGGATAGCGGAATTCTCGGTGTAGCGGTGGAATGCGTAGATATCGAGAGGAACACCAATGGCGAAGGCAGCTATCTGGATCGTAACTGACACTCATGAGCGAAAGTGCGGGGAGCAAACAGG` |
| ASV2375 | 429 | `TGGGGAATTTTGGACAATGGGGGCAACCCTGATCCAGCCATGCCGCGTGCGGGAAGAAGGCCTTCGGGTTGTAAACCGCTTTTGTCAGGGAAGAAACGCTCTAAGATAATACCTTGGGGTAATGACGGTACCTGAAGAATAAGCACCGGCTAACTACGTGCCAGCAGCCGCGGTAATACGTAGGGTGCAAGCGTTAATCGGAATTACTGGGCGTAAAGCGTGCGCAGGCGGTTATGCAAGACAGATGTGAAATCCCCGGGCTCAACCTGGGAACTGCATTTGTGACTGCATAGCTGGAGTGCGGCAGAGGGGGATGGAATTCCGCGTGTAGCAGTGAAATGCGTAGATATGCGGAGGAACACCAATGGCGAAGGCAATCCCCTGGGCCTGCACTGACGCTCATGCACGAAAGCGTGGGGAGCAAACAGG` |
| ASV1563 | 428 | `TGGGGAATTGTTCGCAATGGGCGCAAGCCTGACGACGCAACGCCGCGTGGAGGATGAAGGTCTTCGGATTGTAAACTCCTGTTGCCCGGGACGAAACGCTCCAACCTAACACGTCGGAGTCTGACGGTACCGGGTGAGGAAGCCCCGGCTAACTCTGTGCCAGCAGCCGCGGTAATACAGAGGGGGCGAGCGTTGTTCGGAATTACTGGGCGTAAAGGGCGCGTAGGCGGCCTGTTAAGTCGAACGTGAAATCCCCGGGCTCAACCCGGGAACTGCGTCCGAGACTGATAGGCTCGAATCCGGGAGAGGGATGTGGAATTCCAGGTGTAGCGGTGAAATGCGTAGATATCTGGAGGAACACCGGTGGCGAAGGCGGCATCCTGGACCGGCATTGACGCTGAGGCGCGAAAGCCAGGGGAGCAAACGGG` |
| ASV4474 | 406 | `TGGGGAATTTTGGACAATGGGCGCAAGCCTGATCCAGCGACGCCGCGTGGAGGATGAAGGCCTTCGGGTTGTAAACTCCTTTTGGAGGGGAAGAATAAATTGACGGTACCCTCAGAAAAAGCCACGGCTAACTACGTGCCAGCAGCCGCGGTAATACGTAGGTGGCGAGCGTTACTCGGAATTACTAGGCGTAAAGCGTATGTAGGCTGTCGTATAAGTCTATGGTGAAATTTCCTGGCTCAACTGGGAAGGGACATAGATACTGTATGGCTTGAGTGTTGGAGGGGGAGACGGAATTCCTGGTGTAGCGGTGAAATGCGTAGATATCAGGAGGAACACCGATGGCGAAAGCAGTCTCCTGGCCAAATACTGACGCTGAGGTACGAAAGCTAGGGTAGCAAACAGG` |
| ASV5146 | 409 | `TGGGGAATATTGCACAATGGGCGCAAGCCTGATGCAGCGACGCCGCGTGAGGGATGACGGCCTTCGGGTTGTAAACCTCTTTCACCAGGGACGAAGCGGAAGTGACGGTACCTGGAGAAGAAGCACCGGCCAACTACGTGCCAGCAGCCGCGGTAATACGTAGGGTGCGAGCGTTGTCCGGAATTACTGGGCGTAAAGAGCTCGTAGGCGGTTTGTCGCGTCGTCTGTGAAATTCTGCAACTCAATTGCAGGCGTGCAGGCGATACGGGCAGACTTGAGTACTACAGGGGAGACTGGAATTCCTGGTGTAGCGGTGAAATGCGCAGATATCAGGAGGAACACCGGTGGCGAAGGCGGGTCTCTGGGTAGTAACTGACGCTGAGGAGCGAAAGCGTGGGTAGCGAACAGG` |
| ASV1863 | 404 | `TGGGGAATATTGGACAATGGGCGCAAGCCTGATCCAGCCATGCCGCGTGAGTGATGAAGGCCTTAGGGTTGTAAAGCTCTTTTGCCAGGGAAGATAATGACTGTACCTGGAGAATAAGCCCCGGCTAACTTCGTGCCAGCAGCCGCGGTAATACGAAGGGGGCTAGCGTTGTTCGGAATCACTGGGCGTAAAGCGCACGTAGGCGGACTTTTAAGTCAGGGGTGAAAGCCTGAGGCTCAACCTCAGAATTGCCCTTGATACTGGGAGTCTTGAGTGTGGTAGAGGTAGGTGGAACAGCAAGTGTAGAGGTGAAATTCGTAGATATTTGCTAGAACACCAGTGGCGAAGGCGGCCTACTGGACCATAACTGACGCTGAGGTGCGAAAGCGTGGGGAGCAAACAGG` |
| ASV1748 | 404 | `TGGGGAATCTTGGACAATGGGCGCAAGCCTGATCCAGCCATGCCGCGTGAGTGACGAAGGCCTTAGGGTTGTAAAGCTCTTTTGCCGGGGACGATAATGACGGTACCCGGAGAATAAGTCCCGGCTAACTTCGTGCCAGCAGCCGCGGTAATACGAAGGGGACTAGCGTTGTTCGGAATTACTGGGCGTAAAGCGCACGTAGGCGGATTTGTCAGTCAGGGGTGAAATCCCGGGGCTCAACCTCGGAACTGCCTTTGATACAGCAAGTCTAGAATCCGGTAGAGGTGGGTGGAATTCCTAGTGTAGAGGTGAAATTCGTAGATATTAGGAAGAACACCGGTGGCGAAGGCGGCCCACTGGACCGGTATTGACGCTGAGGTGCGAAAGCGTGGGGAGCAAACAGG` |
| ASV4490 | 424 | `TGAGGAATATTGGACAATGGGCGCAAGCCTGATCCAGCCATGCCGCGTGCAGGATGAAGCATCTATGGTGTGTAAACTGCTTTTATACGGGAAGAAGCCCCGCCTCGTGAGGCGGACTGACGGTACCGTAAGAATAAGGACCGGCTAACTCCGTGCCAGCAGCCGCGGTAATACGGAGGGTCCAAGCGTTATCCGGAATCATTGGGTTTAAAGGGTCCGTAGGCGGTCTGGTAAGTCAGTGGTGAAAGCCCATCGCTCAACGGTGGAACGGCCATTGATACTGCCAGACTTGAATTACTAGGAAGCAACTAGAATATGTAGTGTAGCGGTGAAATGCTTAGATATTACATGGAATACCAATTGCGAAGGCAGGTTGCTACTAGTTGATTGACGCTGATGGACGAAAGCGTGGGTAGCGAACAGG` |
| ASV3518 | 404 | `TAGGGAATATTGGGCAATGGACGAAAGTCTGACCCAGCCATGCCGCGTGTGTGATGAAGGCCTTAGGGTTGTAAAGCACTTTCGCCGATGAAGATAATGACGGTAGTCGGATAAGAAGCCCCGGCTAACTTCGTGCCAGCAGCCGCGGTAATACGAAGGGGGCAAGCGTTGTTCGGAATTACTGGGCGTAAAGCGCATGTAGGCGGTCCTGCAAGCCAGGGGTGAAATCCCAGAGCTCAACTCTGGAATTGCCTTTGGAACTGTAGGACTGGAGTCTCGGAGAGGATGGTGGAATTCCAAGTGTAGAGGTGAAATTCGTAGATATTTGGAGGAACACCGGTGGCGAAGGCGGCCATCTGGACGAGTACTGACGCTGAGATGCGAAAGCGTGGGGAGCAAACAGG` |
| ASV4639 | 409 | `TGAGGAATCTTCCGCAATGGGCGCAAGCCTGACGGAGCGACGCCGCGTGAAGGATGAAGGCCCTTACGGGTCGTAAACTTCTGTTCTGAGGGACGAAATTTTTGACGGTACCTCAGGAGAAAGTATCGGCAAACCCTGTGCCAGCAGCCGCGGTAAGACAGGGGATACAAGCGTTACTCGGAATCACTGGGCGTAAAGCGTCTGTAGGTGCTTTGGTAAGTCTGCGGTAAAAGCTCGGGGCTTAACCCTGAGAGCGTCGCGGAAACTACCGAAGTTGAGCAGTAGAGAGGCATCTGGAATGCCGCATGTAGGAGTAAAATCCGTAGATATGCGGTAGAACGCCAAAAGCGTAGGCAGGATGCTAGCTACTTGCTGACACTGAGAGACGAAAGCGTGGGGAGCGAAGCGG` |
| ASV764 | 429 | `TGGGGAATTTTGGACAATGGGCGCAAGCCTGATCCAGCAATGCCGCGTGCAGGATGAAGGCCTTCGGGTTGTAAACTGCTTTTGTACGGAACGAAACGGCTCTTTCTAATAAAGAGAGTTAATGACGGTACCGTAAGAATAAGCACCGGCTAACTACGTGCCAGCAGCCGCGGTAATACGTAGGGTGCAAGCGTTAATCGGAATTACTGGGCGTAAAGCGTGCGCAGGCGGTGATGTAAGACAGATGTGAAATCCCCGGGCTCAACCTGGGACCTGCATTTGTGACTGCATCGCTAGAGTACGGTAGAGGGGGATGGAATTCCGCGTGTAGCAGTGAAATGCGTAGATATGCGGAGGAACACCGATGGCGAAGGCAATCCCCTGGACCTGTACTGACGCTCATGCACGAAAGCGTGGGGAGCAAACAGG` |
| ASV5290 | 404 | `TGAGGAATATTGGACAATGGGGGCAACCCTGATCCAGCCATGCCGCGTGAGTGATGAAGGCCTTCGGGTTGTAAAGCTCTTTCAGATGCGAAGATAATGACGGTAACATCAGAAGAAGCCCCGGCTAAATTCGTGCCAGCAGCCGCGGTAATACGAATGGGGCAAGCGTTGTTCGGAATCACTGGGCTTAAAGCGTGCGCAGGCGGCCAAGCAAGTCAGAAGTGAAATCCCAGGGCTTAACCCTGGAATTGCTTTTGAAACTGCATGGCTAGAATATGGGAGAGGTTAGCAGAATTCCGAGTGTAGAGGTGAAATTCGCAGATATTCGGAGGAATACCAGTGGCGAAGGCGGCTAACTGGACCATTATTGACGCTCAGGCACGAAAGCGTGGGGATCAAACAGG` |
| ASV609 | 429 | `TGGGGAATTTTGGACAATGGGCGCAAGCCTGATCCAGCCATGCCGCGTGAGTGAAGAAGGCCTTCGGGTTGTAAAGCTCTTTCGGCGGGGACGAAACGGTCCGTGACAACACCACGGACTAATGACGGTACTCGCAGAAGAAGCACCGGCTAACTACGTGCCAGCAGCCGCGGTAATACGTAGGGTGCGGGCGTTAATCGGAATTACTGGGCGTAAAGCGTGCGCAGGCGGCTCCTTAAGTCAGATGTGAAATCCCCGGGCTTAACCTGGGAACTGCGTTTGAAACTGAGGGGCTTGAGTGTGGAAGAGGGGGGTGGAATTCCAGGTGTAGCAGTGAAATGCGTAGAGATCTGGAGGAACACCGATGGCGAAGGCAGCCCCCTGGGCTAACACTGACGCTCATGCACGAAAGCGTGGGGAGCAAACAGG` |
| ASV5417 | 424 | `TGGGGAATATTGGTCAATGGGCGAAAGCCTGAACCAGCAATGCCGCGTGAGTGATGAAGGCCTTCGGGTTGTAAAGCTCTGTCGGAAGGAAAGAAAAAGCCAGCTAACAACTGGTGTTGACGGTACCTTCAAAGGAAGCACCGGCTAACTCTGTGCCAGCAGCCGCGGTAATACAGAGGGTGCAAGCGTTGTTCGGAATTATTGGGCGTAAAGCGCGTGTAGGCGGTTTAGTAAGTCAGACGTGAAAGCCCTCGGCTTAACCGAGGAAGTGCATCTGATACTGCTTTACTTGAGTGTCGGAGGGGATGGTAGAATTCCCGGTGTAGAGGTGAAATTCGTAGATATCGGGAGGAATACCTGTGGCGAAGGCGACCATCTGGAAGACAACTGACGCTGAGACGCGAAAGCGTGGGGAGCAAACAGG` |
| ASV4263 | 429 | `TGGGGAATATTGGACAATGGGGGCAACCCTGATCCAGCGATGCCGCGTGTGTGAAGAAGGCCTGCGGGTTGTAAAGCACTTTCAGTGGGGAGGAGAACTTAGAGGTTAAGAGCTACTAAGTTGGACGTTACCCACAGAAGAAGCACCGGCTAACTCCGTGCCAGCAGCCGCGGTAATACGGAGGGTGCAAGCGTTAATCGGAATTACTGGGCGTAAAGAGTGCGTAGATGGTTTTATAAGTTAAGTGTGAAAGCCCCGGGCTTAACCTGGGATAGTCACTTAAGACTGTAGGACTAGAGTATGGGAGAGGGTAGTGGAATTTCCGGTGTAGCGGTGAAATGCGTAGAGATCGGAAGGAACACCAGTGGCGAAGGCGGCTACCTGGCCCAATACTGACGTTGAGGCACGAAAGCGTGGGGAGCAAACAGG` |
| ASV2289 | 404 | `TGGGGAATATTGGACAATGGGGGAAACCCTGATCCAGCAATGCCGCGTGAATGACGAAGGCCTTAGGGTTGTAAAGTTCTTTCACCTGTGAAGATAATGACGGTAGCAGGAGAAGAAGCCCCGGCTAACTCCGTGCCAGCAGCCGCGGTAAGACGGAGGGGGCTAGCGTTGTTCGGAATGACTGGGCGTAAAGGGCGCGTAGGCGGTTTTTTAAGTGAGGCGTGAAAGCCCTGGGCTTAACCCAGGAGGTGCGTTTCATACTGGAAGACTTGAGTACGAGAGAGGAAAGCGGAATTCCTAGTGTAGAGGTGAAATTCGTAGATATTAGGAAGAACACCAGTGGCGAAGGCGGCTTTCTGGCTCGTAACTGACGCTGAGGCGCGAAAGCGTGGGGAGCAAACAGG` |
| ASV4141 | 406 | `GCGAGAATATTCGACAATGGACGAAAGTCTGATCGAGCGACGCCGCGTGTACGATGAAGTTTTTCGGAATGTAAAGTACTTTTATGAGGGAAGAACGTTGACGGTACCTCATGAATAAGTGGTTGCTAACTCTGTGCCAGCAGCAGCGGTAATACAGAGACCACAAGCGTTATCCGGAATTATTGGGCGTAAAGGGTGAGTAGGTGGTATTGTTAGTCGGGTTTGAAAGATCGTGGGCTCAACCCATGATATGGACCCGAAACGGCAAAACTAGAATATGTAAGAGGTTAGTGGAACTATATGTGTAGGGGTAAAATCCGTTGATATATATGGGAACACCTAAAGCGAAGGCAGCTAACTGGTACATTATTGACACTGAATCACGAAAGCGTGGGTAGCGATACGG` |
| ASV5645 | 429 | `TGGGGAATATTGGACAATGGGGGCAACCCTGATCCAGCGATGCCGCGTGTGCGAAGAAGGCCTGCGGGTTGTAAAGCACTTTGGTTGGGGAAGAAGGTTGGGCGTTGAATAGATGCCTGAATTGACGTTACCCAAAGAACAAGCACCGGCTAACTCTGTGCCAGCAGCCGCGGTAATACAGAGGGTGCGAGCGTTAATCGGAATTACTGGGCGTAAAGGGTGCGTAGGCGGTTAAATAAGTTAGATGTGAAATCCCCGGGCTTAACCTGGGAATTGCATTTAATACTGTATGACTAGAGTAGAGTAGAGGGAAGTGGAATTTCCGGTGTAGCGGTGAAATGCGTAGATATCGGAAAGAACATCAGTGGCGAAGGCGGCTTCCTGGACTCATACTGACGCTGAGGCACGAAAGCATGGGGAGCAAACAGG` |
| ASV5068 | 429 | `TGGGGAATATTGGACAATGGGGGCAACCCTGATCCAGCAATGCCGCGTGAGTGAAGAAGGCCTTAGGGTTGTAAAGCTCTTTTGTAGGGGAGGAAAGGGCTTGTGTGAATAACACGAGCCTATGACGTTACCCTAAGAATAAGCACCGGCTAACTCTGTGCCAGCAGCCGCGGTAATACAGAGGGTGCGAGCGTTAATCGGAATGACTGGGCGTAAAGGGTGCGTAGGTGGTTTGTTAAGTTAGCTGTGAAATCCCTGGGCTTAACCTGGGAATTGCAGCTAAAACTGATGGACTCGAGTAGGTGAGAGGAGTGTGGAATTTCCGGTGTAGCGGTGAAATGCGTAGAGATCGGAAAGAACACCGGTGGCGAAGGCGGCACTCTGGTACCATACTGACACTGAGGCACGAAGGCGTGGGGAGCAAACAGG` |
| ASV3874 | 409 | `TCGAGAATCTTCCACAATGGACGAAAGTCTGATGGAGCGACGCCGCGTGATTGATGAAGTCCCTCTGGGACGTAAAGATCTTTTATGAGGGAAGAAGTTTATTGACTGTACCTCATGAATAAGAGGCTCCTAATCTCGTGCCAGCAGGAGCGGTAATACGAGAGCCTCGAGCGTTATCCGGAATTATTGGGCGTAAAGGGTGCGTAGGTTGTTTTGTTAGTCTTTTGTCAAAGCCCCGAGCTTAACTTGGGATCCGCAAAAGAAACGGCAGAACTTGAAAGTGCGAGAGGTATACGGAACTTATGGTGTAGGGGTAAAATCCGTTGATATCATAGGGAACACCAAATGCGAAAGCAGTATACTGGCGCATATTTGACACTGAAGCACGAAAGCGTGGGTAGCGAATGGG` |
| ASV4992 | 428 | `CCAGGAATCTTGGGCAATGGGCGAAAGCCTGACCCAGCAACACCGTGTGGGCGATGAAGGCTCTAGGGTCGTAAAGCCCTGTTGATAGGGACGAAGGGCGAAGGGTTAATAGCCCCTAGCTTGACGGTACCTTTCGAGGAAGCCCCGGCTAACTACGTGCCAGCAGCCGCGGTAATACGTAGGGGGCGAGCGTTGTCCGGAATTATTGGGCGTAAAGAGCGTGTAGGCGGTTCGGTAAGTCTGCTGTGAAATCTTGGGGCTCAACCCTGAGCGTGCAGCGGATACTGCCGGGCTAGAGGGTGGTAGAGGCGAGTGGAATTCCGAGTGTAGCGGTGAAATGCGCAGATATTCGGAGGAACACCAGTAGCGAAGGCGGCTCGCTGGGCCACACCTGACGCTGAGACGCGAAAGCGTGGGGAGCGAACAGG` |
| ASV3379 | 423 | `TAGGGAATATTGGGCAATGGAGGCAACTCTGACCCAGCCATGCCGCGTGCAGGATGAAGGCGTTATGCGTTGTAAACTGCTTTTATATAGGAAGAAAGAGTCTTTGCGAAGAAAATTGACGGTACTATATGAATAAGCACCGGCTAACTCCGTGCCAGCAGCCGCGGTAATACGGAGGGTGCAAGCGTTGTCCGGATTTATTGGGTTTAAAGGGTGCGTAGGCGGTTTATTAAGTCAGTGGTGAAAGACGGTCGCTCAACGATTGCAGTGCCATTGATACTGGTAGACTTGAGTGAGATTGAGGTAGCTGGAATGGATAGTGTAGCGGTGAAATGCATAGATATTATCCAGAACACCAATTGCGAAGGCAAGTTACTAAGTCTCAACTGACGCTGATGCACGAAAGTGTGGGTATCAAACAGG` |
| ASV4360 | 430 | `TGGGGAATTTTGCGCAATGGACGAAAGTCTGACGCAGCCACGCCGCGTGAGTGAAGAAGGCCTTCGGGTTGTAAAGCTCTGTCAATGGGGACAAAAACCCTCAAGGTTAATAGCCTCGAGGCTTGATGGTACCCATGGAGGAAGCACCGGCTAACTTCGTGCCAGCAGCCGCGGTAATACGAAGGGTGCAAGCGTTGCTCGGAATTATTGGGCGTAAAGGGTAGGTAGGTGGTCTTACAAGTCTGGGGTGAAATCCCTGAGCTCAACTCAGGAAGTGCCTTGGAAACTGTAAGACTGGAGTATCGGAGAGGGTCGTGGAATTCCCGGTGTAGCGGTGAAATGCGTAGAGATCGGGAGGAACATCAGAGGCGAAGGCGACGACCTGGACGATTACTGACACTAAACTACGAAAGCGTGGGGAGCAAACAGG` |
| ASV2877 | 404 | `TGAGGAATATTGGACAATGGGGGCAACCCTGATCCAGCAATGCCGCGTGAGTGATGAAGGCCTTCGGGTTGTAAAACTCTTTTGTCAGGGACGATAATGACGGTACCTGACGAATAAGCTCCGGCTAACTTCGTGCCAGCAGCCGCGGTAATACGAAGGGAGCTAGCGTTGTTCGGAATTACTGGGCGTAAAGGGCGCGTAGGCGGTCTGATAAGTTTGATGTGAAAGCCCTGGGCTTAACCTAGGAATTGCATTGGATACTGTTAGACTAGAGACCGAGAGAGGAAAATGGAATTGCGAGTGTAGAGGTGAAATTCTTAGATATTCGCAAGAACACCAGTGGCGAAGGCGATTTTCTGGCTCGGTACTGACGCTAAGGCGCGAAAGCGTGGGGAGCAAACAGG` |
| ASV926 | 429 | `TGGGGAATTTTGGACAATGGGGGAAACCCTGATCCAGCCATTCCGCGTGAGTGAAGAAGGCCTTCGGGTTGTAAAGCTCTTTCAGCAGGAACGAAACGGTAGGCTTTAATAAAGTTTGCTAATGACGGTACCTGAAGAAGAAGCACCGGCTAACTACGTGCCAGCAGCCGCGGTAATACGTAGGGTGCGAGCGTTAATCGGAATTACTGGGCGTAAAGCGTGCGCAGGCGGTTTTGTAAGACAGATGTGAAATCCCCGGGCTTAACCTGGGAACTGCGTTTGTGACTGCAAGACTAGAGTGTGGCAGAGGGGGGTAGAATTCCACGTGTAGCAGTGAAATGCGTAGAGATGTGGAGGAATACCGATGGCGAAGGCAGCCCCCTGGGTCAACACTGACGCTCATGCACGAAAGCGTGGGGAGCAAACAGG` |
| ASV3651 | 429 | `TGGGGAATTGTTCACAATGGGCGCAAGCCTGATGACGCAACGCCGCGTGGGGGATGAAGGTCTTCGGATTGTAAACCCCTGTCGAACGGGACGAAAGACTCGGGAGCTAATCATCTCGAGAGTGACGGTACCGTCAAAGGAAGCCACGGCTAACTCTGTGCCAGCAGCCGCGGTAATACAGAGGTGGCAAGCGTTGTTCGGAATTACTGGGCGTAAAGGGCGCGTAGGCGGCCTTCTAAGTCAGACGTGAAATCCCCCGGCTTAACCTGGGAACTGCGTCTGATACTGGAGGGCTCGAGTTTGGGAGAGGGATGTAGAATTCCAGGTGTAGCGGTGAAATGCGTAGATATCTGGAGGAATACCGGTGGCGAAGGCGGCATCCTGGACCAACACTGACGCTGAGGCGCGAAAGCTAGGGGAGCAAACGGG` |
| ASV795 | 424 | `TAAGGAATATTGGACAATGGTCGGAAGACTGATCCAGCCATGCCGCGTGGAGGATGAAGGCCCTCTGGGTTGTAAACTTCTTTTATTGGGGGCGAAAAAAGGGAATTCTTTCTCACTTGACGGTACCCAATGAATAAGCACCGGCTAACTCCGTGCCAGCAGCCGCGGTAATACGGAGGGTGCAAGCGTTATCCGGATTCACTGGGTTTAAAGGGTGCGTAGGCGGGTATGTAAGTCAGTGGTGAAATCTCCGAGCTTAACTTGGAAACTGCCATTGATACTATATATCTTGAATGCTCTGGAGGTTTGCGGAATATGTCATGTAGCGGTGAAATGCATAGATATGACATAGAACACCTATTGCGAAGGCAGCAGGCTACAGAGATATTGACGCTGAGGCACGAAAGCGTGGGGATCAAACAGG` |
| ASV1506 | 406 | `TAGGGAATATTGCGCAATGGGGGAAACCCTGACGCAGCGACGCCGCGTGAGTGATGAAGGCCTTCGGGTCGTAAAGCTCTGTTATATGGGAATAAGATCTGAATGTACCATGTGAGAAAGGATCGGCTAACTTCGTGCCAGCAGCCGCGGTAAGACGAGGGATCCTAGCGTTGCTCGGAATCATTGGGCGTAAAGCGGGTGTAGGTGGCTTTATAAGTCAGGTGTGAAAGCCCAGGGCTCAACCCTGGAAGTGCATTTGATACTGTTTAGCTTGAGTGTGGGAGAGGTTACTGGAATACCTGGTGTAGTGGTGAAATACGTAGATATCAGGTGGAACACCGGAGGCGAAGGCGGGTAACTGGCCCAACACTGACACTCAGACCCGAAAGCGTGGGGATCAAACAGG` |
| ASV651 | 407 | `TGGGGAATTTTCCGCAATGGGCGAAAGCCTGACGGAGCAATACCGCGTGAGGGAAGAAGGCTCTTGGGTTGTAAACCTCTTTTCTTGGGGAAGAAAAAAATGACGGTACCCAAGGAATCAGCATCGGCTAACTCCGTGCCAGCAGCCGCGGTAATACGGAGGATGCAAGCGTTATCCGGAATGATTGGGCGTAAAGCGTCCGCAGGTGGCATTTCAAGTCTGCGGTTAAAGGTTCTGGCTCAACCAGAGACAAGCCGTGGAAACTGAAAAGCTAGAGTATGGTAGGGGCAGAGGGAATTCCCAGTGTAGCGGTGAAATGCGTAGAGATTGGGAAGAACACCGGTGGCGAAAGCGCTCTGCTAGGCCAAAACTGACACTGAGGGACGAAAGCTAGGGGAGCGAATGGG` |
| ASV1719 | 424 | `TAAGGAATATTGGTCAATGGACGAAAGTCTGAACCAGCTATGCCGCGTGGAGGATGAAGGCCCTCTGGGTTGTAAACTTCTTTTATATGGGACGAAAAAGGGCCTTTCTAGGTCAACTGACGGTACCATGTGAATAAGCACCGGCTAACTCCGTGCCAGCAGCCGCGGTAATACGGAGGGTGCAAGCGTTATCCGGATTCACTGGGTTTAAAGGGTGCGTAGGTGGGTTGGTAAGTCAGTGGTGAAATCTCCGAGCTTAACTTGGAAACTGCCATTGATACTATCAATCTTGAATATTGTGGAGGTTAGCGGAATATGTCATGTAGCGGTGAAATGCTTAGATATGACATAGAACACCAATTGCGAAGGCAGCTGGCTACACATATATTGACACTGAGGCACGAAAGCGTGGGGATCAAACAGG` |
| ASV306 | 429 | `TGGGGAATATTGGACAATGGGGGAAACCCTGATCCAGCGATGCCGCGTGTGTGAAGAAGGCCTGAGGGTTGTAAAGCACTTTTGTTGGGGAGGAGATTTGTTTGGTTAAGAGCTGAATGAAGAGACGTTACCCAAAGAATAAGCACCGGCTAACTCCGTGCCAGCAGCCGCGGTAATACGGGGGGTGCAAGCGTTAATCGGAATGACTGGGCGTAAAGGGTGCGTAGGTTGTTTTGTAAGTTACATGTAAAAGCCCTGGGCTTAACCTGGGAAGGCCATGTAATACTGCGAGACTAGAGTATAGGAGAGGGTAGTGGAATTTCCGGTGTAGCGGTGAAATGCGTAGAGATCGGAAGGAACACTAGTGGCGAAGGCGGCTACCTGGCCTAATACTGACACTGAGGCACGAAAGCATGGGGAGCAAACAGG` |
| ASV5674 | 405 | `TGAGGAATATTCGGCAATGGGGGGAACCCTGACCGAGCAACGCCGCGTGGAGGAAGACGGTTTTCGGATTGTAAACTCCTTTTCTGCGGGAAGAGCAAGGACGGTACCGCAGGAATCAGTCACGGCTAACTACGTGCCAGCAGCCGCGGTAATACGTAGGTGGCAAGCGTTATCCGGAATTATTGGGCGTAAAGCGCACGCAGGTGGCTGCTTAAGTCTGACGTGAAAGCTCCTGGCTTAACTGGGAGAGGTCGTTGGGTACTGGGCAGCTTGAGGCAAGGAGAGGGGCGTAGAATTCCCGGTGTAGTGGTGGAATGCGTAGATATCGGGAGGAATACCAGAGGCGCAAGCGGCGCCCTGGCCTTGGCCTGACGCTCAGGTGCGAAAGCGTGGGGAGCGAACGGG` |
| ASV831 | 430 | `TGGGGAATATTGGACAATGGGCGGAAGCCTGATCCAGCAATACCGCGTGTGTGAAGAAGGCCTTCGGGTTGTAAAGCACTTTCAGTTGGGAAGAAGTTGTGCATGGGAATACTGTGCATGATTGACGTTACCGACAGAAGAAGCACCGGCTAACTCTGTGCCAGCAGCCGCGGTAATACAGAGGGTGCAAGCGTTAATCGGAATTACTGGGCGTAAAGGGTGCGTAGGCGGAACAACAAGATGGGCGTGAAATCCCTGGGCTCAACCTGGGAACTGCGTTCAAGACTGTTGATCTAAGAGTGCAGTAGAGGAAAGTGGAATTTCCGGTGTAGCGGTGAAATGCGTAGATATCGGAAGGAACATCAGTGGCGAAGGCGACTTTCTGGACTGACACTGACGCTGAGGCACGAAAGCGTGGGGAGCAAACAGG` |
| ASV1627 | 424 | `TAAGGAATATTGGTCAATGGAGGCAACTCTGAACCAGCCATGCCGCGTGCAGGAAGACGGCCCTATGGGTTGTAAACTGCTTTTGAATGGGAGAAAACCCCGGTACGTGTACTGGGTTGATAGTACCATTAGAATAAGCATCGGCTAACTTCGTGCCAGCAGCCGCGGTAAGACGAAGGATGCAAGCGTTATCCGGATTCATTGGGTTTAAAGGGTGCGTAGGCGGACTTGTAAGTCAGTGGTGAAATCTCTTCGCTTAACGAAGAAACTGCCATTGATACTGCAGGTCTAGAGTATAGATGACGTTGGCGGAATATGACATGTAGTGGTGAAATACTTAGATATGTCATAGAACACCGATTGCGAAGGCAGCTAACGAAACTATAACTGACGCTGAGGCACGAAAGTGCGGGGATCAAACAGG` |
| ASV1328 | 429 | `TGGGGAATTTTGGACAATGGGCGCAAGCCTGATCCAGCAATGCCGCGTGCAGGATGAAGGCCTTCGGGTTGTAAACTGCTTTTGTACGGAGCGAAAAGGCTCTCTCTAATAAAGGGGGCTCATGACGGTACCGTAAGAATAAGCACCGGCTAACTACGTGCCAGCAGCCGCGGTAATACGTAGGGTGCAAGCGTTAATCGGAATTACTGGGTGTAAAGCGTGCGCAGGCGGTTATGTAAGACAGATGTGAAATCCCCGGGCTCAACCTGGGACCTGCATTTGTGACTGCATAGCTAGAGTACGGCAGAGGGGGATGGAATTCCGCGTGTAGCAGTGAAATGCGTAGATATGCGGAGGAACACCGATGGCGAAGGCAATCCCCTGGGCCTGTACTGACGCTCATGCACGAAAGCGTGGGGAGCAAACAGG` |
| ASV282 | 424 | `TGAGGAATATTGGACAATGGGCGCAAGCCTGATCCAGCCATGCCGCGTGCAGGAAGACGCATCTATGGTGTGTAAACTGCTTTTGTACGGGAAGAAACACTCCGACGTGTCGGAGCTTGACGGTACCGTAAGAATAAGGATCGGCTAACTCCGTGCCAGCAGCCGCGGTAATACGGAGGATCCAAGCGTTATCCGGAATCATTGGGTTTAAAGGGTCCGTAGGCGGTTTAATAAGTCAGTGGTGAAATCTGGTCGCTCAACGATCAAACGGCCATTGATACTGTTAGACTTGAATTATAAGGAAGTAACTAGAATATGTAGTGTAGCGGTGAAATGCTTAGATATTACATGGAATACCAATTGCGAAGGCAGGTTACTACTTATGGATTGACGCTGATGGACGAAAGCGTGGGGAGCGAACAGG` |
| ASV3233 | 429 | `TGGGGAATATTGGACAATGGGGGAAACCCTGATCCAGCGACGCCGCGTGTGTGAAGAAGGCCTGCGGGTTGTAAAGCACTTTTAGTGGGGAAGAAATGCTCGGCGCTAACACCGTCGGGAACTGACCTAACCCAAAGAAAAAGCACCGGCTAACTCTGTGCCAGCAGCCGCGGTAATACAGAGGGTGCAAGCGTTAATCGGAATTACTGGGCGTAAAGCGTGCGTAGACGGTTGTTTAAGTCGAGTGTGAAATCCCCGGGCTCAACCTGGGAATGGCATTCGAAACTGAATAGCTAGGGTGCGGAAGAGGGAAGCGGAATTTCCGGTGTAGCGGTGAAATGCGTAGATATCGGAAGGAACATCAGTGGCGAAAGCGGCTTCCTGGTCCAGCACCGACGTTCAGGCACGAAAGCGTGGGGAGCAAACAGG` |
| ASV4940 | 428 | `TCGAGAATTTTTCTCAATGGGCGAAAGCCTGAAGGAGCGACGCCGCGTGGGGGATGAAGGGCTTCGGTCCGTAAACCCCTGTCATTTGTGAACAAACCTTACCGGTAAACAGCCGGTGAGCTGATTGTAATGGAAGAGGAAGGGACGGCTAACTCTGTGCCAGCAGCCGCGGTAATACAGAGGTCCCAAGCGTTGTTCGGATTCACTGGGCGTAAAGGGTGCGTAGGCGGTTTGATAAGTTTGGCGTGAAAGCCCCGGGCCTAACCCGGGAAATGCGTTGAAAACTGTTGAACTAGAGGATTGGAGGGGAGACTGGAATTCTTGGTGTAGCAGTGAAATGCGTAGATATCAAGAGGAACATCAGTGGCGAAGGCGGGTCTCTGGACAAATCCTGACGCTGAGGCACGAAAGCTAGGGGAGCAAACAGG` |
| ASV3171 | 404 | `TGGGGAATATTGGACAATGGGCGCAAGCCTGATCCAGCCATGCCGCGTGAGTGATGAAGGCTTTAGGGTTGTAAAGCTCTTTCGCCGATGAAGATAATGACGGTAGTCGGAGAAGAAGCCCCGGCTAACTTCGTGCCAGCAGCCGCGGTAATACGAAGGGGGCTAGCGTTGTTCGGAATTACTGGGCGTAAAGCGTGCGTAGGCTGTGCGACAAGTCAGTGGTGAAATCCCGGAGCTTAACTTCGGAACTGCCATTGAAACTGTCGTGCTAGAATCTTAGAGGGGGTAGCGGAATTCCAAATGTAGGGGTGAAATCCGTAGATATTTGGAGGAACACCGGTGGCGAAGGCGGCTACCTGGATGAGTATTGACGCTGAGGCACGAAAGCGTGGGGATCAAACAGG` |
| ASV4898 | 430 | `TGAGGAATCTTGCGCAATGGGGGAAACCCTGACGCAGCGACGCCGCGTGGAGGACGAAGGCCTTCGGGTCGTAAACTCCTGTCGAGCGGGACGAATGCCGCGGGGATGAATGAGCCCCGCGGTTGACGGTACCGCTGGAGGAAGCCCCGGCTAACTCCGTGCCAGCAGCCGCGGTAATACGGAGGGGGCCAGCGTTGTTCGGAATCATTGGGCGTAAAGGGCGCGTAGGCGGCTCGGCAAGTCAGACGTGAAATCCCGCGGCTCAACCGCGGAACTGCGTTTGAAACTACCGGGCTTGAGTCCCGGAGAGGGTAGTGGAATTCCCAGTGTAGCGGTGAAATGCGTAGATACTGGGAGGAACACCGGTGGCGAAGGCGGCTACCTGGACGGGTACTGACGCTGAGGCGCGAAAGCGTGGGGAGCAAACAGG` |
| ASV3881 | 425 | `TGGGGAATATTGGTCAATGGGCGAAAGCCTGAACCAGCAATGCCGCGTGAGTGATGAAGGCCTTCGGGTTGTAAAGCTCTGTCGGGAGGGACGAAAAAAGCCGGGTCATTCCGGAACTTGACGGTACCTCCAAAGGAAGCACCGGCTAACTCTGTGCCAGCAGCCGCGGTAATACAGAGGGTGCAAGCGTTGTTCGGAATTACTGGGCGTAAAGCGCGTGTAGGCTGCGAGATAAGTCGGACGTGAAATCCCTTGGCTTAACCAAGGAAGTGCGTCCGATACTGCTTCGCTTGAGTGTCGGAGGGGGTGGCGGAATTCCCGGTGTAGAGGTGAAATTCGTAGATATCGGGAGGAACACCTGTGGCGAAGGCGGCCACCTGGACGACAACTGACGCTGAGACGCGAAAGCGTGGGTAGCAAACAGG` |
| ASV5193 | 390 | `CCGGGTATATTCTGCAATGTACGAAAGTATGACAGAGCGAGCCAGAGTGATTCACATTATTTTTGTGAATCTTTTGTGACATGTAGATAGTGTTACGAATAAGGACCGGGCAAGACTGGTGCCAGTCGCCGCGGTAATACCAGCGGTCCAAGTCGCAACCACAATTATTGGGTCTAAAACATCCGTAGCTTGCTTAGTAAGTCTCCTGTGAAATCTGACCTCTTAAGGGTCAAAGAGCAGGAGATACTGCAAAGCTAGAGACCGGGAGAGGTAGGAAGTACGGTCGGGGTACCGGTAAAATGGGCTGATCCCGTCTGGACTAACAACAGCGAAGGCATCCTACCAGCACGGTTCTGACAGTGAGGGATGAAGGCTAGGGGCGCAAAAGGG` |
| ASV5159 | 429 | `TGGGGAATATTGGACAATGGGGGCAACCCTGATCCAGCGATGCCGCGTGTGTGAAGAAGGCCTGCGGGTTGTAAAGCACTTTAGTTATGGAGGAAGTTGCTGCGTTTAAGAGATGTGGTAATTGACGTTACATAAAGAATAAGCACCGGCTAACTCTGTGCCAGCAGCCGCGGTAATACAGAGGGTGCGAGCGTTAATCGGAATTACTGGGCGTAAAGGGTGCGTAGGCGGAAATTTAAGTCAGGCGTGAAATACTCGAGCTTAACTTGGGTACTGCGTCTGATACTGAATATCTAGAGTACAGTAGAGGGAAGTGGAATTTCCAGTGTAGCGGTGAAATGCGTAGATATTGGAAGGAACACCAGTGGCGAAGGCGACTTTCTGGGCTGATACTGACGCTGAGGCACGAAAGCGTGGGGAGCAAACAGG` |
| ASV2696 | 387 | `GCGAGAAACCTTTGCAATGTGCGAAAGCACGACAAGGTTAATCCGAGTGTCTCCTGCTAAAGGAGTCTTTTGCCGGTCATAGAACCACCGGTGAATAAGGGGTGGGCAAGTTCTGGTGTCAGCCGCCGCGGTAAAACCAGCACCTCGAGTGGTCAGGAGGTTTATTGGGCCTAAAGCATCCGTAGCCGGCTGCTCAAGTTTTCGGTTAAATCTATGCGCTTAACGTATAGGCCGCCGAAAATACTGGGCAGCTAGGGAGTGGGAGAGGTACACGGTACTCGATAGGTAGGGGTAAAATCCTTTGATCTATTGATGACCACCTGTGGCGAAGGCGGTCTACCAGAACACGTTCGACGGTGAGGGATGAAAGCTGGGGGAGCAAACCGG` |
| ASV1000 | 404 | `TGGGGAATCTTAGACAATGGGCGCAAGCCTGATCTAGCCATGCCGCGTGAGCGATGAAGGCCTTAGGGTTGTAAAGCTCTTTCAGGGGGGAAGATAATGACTGTACCCCCAGAAGAAGCCCCGGCTAACTCCGTGCCAGCAGCCGCGGTAATACGGAGGGGGCTAGCGTTGTTCGGAATTACTGGGCGTAAAGCGCACGTAGGCGGACGGAGAAGTCAGAGGTGAAATCCCAGGGCTCAACCTTGGAACTGCCTTTGAAACTTTCTGTCTTGAGGTCGTGAGAGGTGAGTGGAATTCCGAGTGTAGAGGTGAAATTCGTAGATATTCGGAGGAACACCAGTGGCGAAGGCGGCTCACTGGCACGATACTGACGCTGAGGTGCGAAAGCGTGGGGAGCAAACAGG` |
| ASV3702 | 429 | `TGGGGAATATTGGACAATGGGGGCAACCCTGATCCAGCAATGCCGCGTGTGTGAAGAAGGCCTGCGGGTTGTAAAGCACTTTGATGGGGGAAGAAGGTCTTTTGGCTAATAACCAAGAGAATTGACACTACCCCAAGAACAAGCACCGGCTAACTCTGTGCCAGCAGCCGCGGTAATACAGAGGGTGCAAGCGTTAATCGGAATGACTGGGCGTAAAGGGCGCGTAGGTGGTTGCTTAAGTTAGATGTGAAAGCCCCGGGCTTAACCTGGGAACTGCATCTAATACTGAGCAGCTTGAGTAGAATAGAGGGGAGTGGAATTTCCGGTGTAGCGGTGGAATGCGTAGATATCGGAAGGAACACCAGTGGCGAAGGCGGCTCCCTGGATTCATACTGACACTGAGGCGCGAAAGCGTGGGGAGCAAACAGG` |
| ASV3008 | 404 | `TGGGGAATATTGGACAATGGGCGCAAGCCTGATCCAGCCATGCCGCGTGAGTGATGAAGGCCTTAGGGTTGTAAAGCTCTTTTGTCCGGGAAGATAATGACGGTACCGGAAGAATAAGCCCCGGCTAACTTCGTGCCAGCAGCCGCGGTAATACGAAGGGGGCTAGCGTTGTTCGGAATCACTGGGCGTAAAGCGCTGGTAGGCGGACTTCTAAGTCAGGGGTGAAATCCCGGGGCTCAACCTCGGAACTGCCTTTGATACTGGAAGTCTTGAGTATGGAAGAGGTTGGTGGAACTCCGAGTGTAGAGGTGAAATTCGTAGATATTCGGAAGAACACCAGTGGCGAAGGCGGCCAACTGGTCCATTACTGACGCTGAGCAGCGAAAGCGTGGGGAGCAAACAGG` |
| ASV1044 | 429 | `TGGGGAATTTTGGACAATGGGCGAAAGCCTGATCCAGCAATGCCGCGTGAGTGAAGAAGGCCTTCGGGTTGTAAAGCTCTTTTGTCAGGGAAGAAACGGTGAGAGCTAATATCTTTTGCTAATGACGGTACCTGAAGAATAAGCACCGGCTAACTACGTGCCAGCAGCCGCGGTAATACGTAGGGTGCAAGCGTTAATCGGAATTACTGGGCGTAAAGCGTGCGCAGGCGGTTTTGTAAGTCTGATGTGAAATCCCCGGGCTCAACCTGGGAATTGCATTGGAGACTGCAAGGCTAGAATCTGGCAGAGGGGGGTAGAATTCCACGTGTAGCAGTGAAATGCGTAGATATGTGGAGGAACACCGATGGCGAAGGCAGCCCCCTGGGTCAAGATTGACGCTCATGCACGAAAGCGTGGGGAGCAAACAGG` |
| ASV408 | 404 | `TGGGGAATATTGGACAATGGGCGAAAGCCTGATCCAGCAATGCCGCGTGAGTGATGAAGGCCTTAGGGTTGTAAAGCTCTTTTACCAGGGATGATAATGACAGTACCTGGAGAATAAGCTCCGGCTAACTCCGTGCCAGCAGCCGCGGTAATACGGAGGGAGCTAGCGTTGTTCGGAATTACTGGGCGTAAAGCGCACGTAGGCGGCTTTTTAAGTCAGGGGTGAAATCCCGGAGCTCAACTCCGGAACTGCCCTTGAAACTGGGAAGCTAGAATCTTGGAGAGGCGAGTGGAATTCCGAGTGTAGAGGTGAAATTCGTAGATATTCGGAAGAACACCAGTGGCGAAGGCGACTCGCTGGACAAGTATTGACGCTGAGGTGCGAAAGCGTGGGGAGCAAACAGG` |
| ASV920 | 429 | `TGGGGAATTTTGGACAATGGACGCAAGTCTGATCCAGCCATGCCGCGTGCGGGAAGAAGGCCTTCGGGTTGTAAACCGCTTTTGTCAGGGAAGAAAAGGTTCTGGTTAATACCTGGGACTCATGACGGTACCTGAAGAATAAGCACCGGCTAACTACGTGCCAGCAGCCGCGGTAATACGTAGGGTGCAAGCGTTAATCGGAATTACTGGGCGTAAAGCGTGCGCAGGCGGTTATGCAAGACAGAGGTGAAATCCCCGGGCTCAACCTGGGAACTGCCTTTGTGACTGCATAGCTAGAGTACGGTAGAGGGGGATGGAATTCCGCGTGTAGCAGTGAAATGCGTAGATATGCGGAGGAACACCGATGGCGAAGGCAATCCCCTGGACCTGTACTGACGCTCATGCACGAAAGCGTGGGGAGCAAACAGG` |
| ASV3445 | 407 | `TGGGGAATTTTCCGCAATGGGCGCAAGCCTGACGGAGCAAGACCGCGTGAGGGAAGAAGGTCTGTGGATTGTAAACCTCTTTTGATAGGGAAGAAGACAGTGACGGTACCTATCGAATCAGCCTCGGCTAACTCCGTGCCAGCAGCCGCGGTAATACGGAGGAGGCAAGCGTTATCCGGAATTATTGGGCGTAAAGCGTCCGTAGGTGGGTTATCAAGTCAGTTGTTAAATAGTGAAGCTTAACTTCATAGAGGCAAATGAAACTGATAGTCTAGAGTGTGGTAGGGGTAGAGGGAATTTCCAGTGTAGCGGTGAAATGCGTAGATATTGGAAAGAACACCGGTGGCGAAAGCGCTCTACTGGGCCTGCACTGACACTGAGGGACGAAAGCTAGGGGAGCGAAAGGG` |
| ASV2739 | 429 | `TGGGGAATTTTGGACAATGGGCGCAAGCCTGATCCAGCCATTCCGCGTGAGTGAAGAAGGCCTTCGGGTTGTAAAGCTCTTTCGCAAGGGAAGAAAACTTAACCTCTAACATAGGTTGAGGCTGACGGTACCTTGATAAGAAGCACCGGCTAACTACGTGCCAGCAGCCGCGGTAATACGTAGGGTGCGAGCGTTAATCGGAATTACTGGGCGTAAAGCGTGCGCAGGCGGCATGATAAGTCAGATGTGAAATCCCCGAGCTCAACTTGGGAACTGCGTTTGAAACTATCAAGCTAGAATATGTCAGAGGGGGGTAGAATTCCACGTGTAGCAGTGAAATGCGTAGAGATGTGGAGGAATACCAATGGCGAAGGCAGCCCCCTGGGATAATATTGACGCTCATGCACGAAAGCGTGGGGAGCAAACAGG` |
| ASV2046 | 430 | `TGGGGAATATTGGACAATGGGGGGAACCCTGATCCAGCCATGCCGCGTGTGTGAAGAAGGCCTTTTGGTTGTAAAGCACTTTAAGCGAGGAGGAGGCTACTCTAGTTAATACCTAGGGATAGTGGACGTTACTCGCAGAATAAGCACCGGCTAACTCTGTGCCAGCAGCCGCGGTAATACAGAGGGTGCGAGCGTTAATCGGATTTACTGGGCGTAAAGCGTGCGTAGGTGGCCAATTAAGTCAAATGTGAAATCCCCGAGCTTAACTTGGGAATTGCATTCGATACTGGTTGGCTAGAGTATGGGAGAGGATGGTAGAATTCCAGGTGTAGCGGTGAAATGCGTAGAGATCTGGAGGAATACCGATGGCGAAGGCAGCCATCTGGCCTAATACTGACACTGAGGTACGAAAGCATGGGGAGCAAACAGG` |
| ASV2666 | 404 | `TGGGGAATATTGGACAATGGGCGAAAGCCTGATCCAGCCATGCCGCGTGAGTGATGAAGGCCTTAGGGTTGTAAAGCTCTTTTGTCAGGGAAGATAATGACGGTACCTGAAGAATAAGCACCGGCTAACTTCGTGCCAGCAGCCGCGGTAATACGAAGGGTGCTAGCGTTGTTCGGAATCACTGGGCGTAAAGCGCGCGTAGGCGGCGTCGTAAGTCAGAGGTGAAATCCCAAGGCTCAACCTTGGAATTGCCTTTGAAACTACGATGCTAGAGTGTCGGAGGGGATGGCGGAATTGCTAATGTAGAGGTGAAATTCGTAGATATTAGCAGGAACACCGGTGGCGAAGGCGGCCATCTGGACGACAACTGACGCTGAGGCGCGAAAGCGTGGGGATCAAACAGG` |
| ASV2830 | 404 | `TGGGGAATCTTGGACAATGGGCGCAAGCCTGATCCAGCCATGCCGCGTGAGTGACGAAGGCCTTAGGGTTGTAAAGCTCTTTTGGCGGGGACGATAATGACGGTACCCGCAGAATAAGTCCCGGCTAACTTCGTGCCAGCAGCCGCGGTAATACGAAGGGGACTAGCGTTGTTCGGAATCACTGGGCGTAAAGCGCACGTAGGCGGATATGTCAGTCAGGGGTGAAATCCCGGAGCTCAACTTCGGAACTGCCTTTGATACAGCATATCTTGAATCCGGTAGAGGTGAGTGGAATTCCTAGTGTAGAGGTGAAATTCGTAGATATTAGGAAGAACACCAGTGGCGAAGGCGGCTCACTGGACCGGTATTGACGCTGAGGTGCGAAAGCGTGGGGAGCAAACAGG` |
| ASV1651 | 429 | `TGGGGAATTTTGGACAATGGGCGCAAGCCTGATCCAGCAATGCCGCGTGAGTGAAGAAGGCCTTCGGGTTGTAAAGCTCTTTTGTCAGGGAAGAAACGGTGCGGACTAATATTCTGCGCTAATGACGGTACCTGAAGAATAAGCACCGGCTAACTACGTGCCAGCAGCCGCGGTAATACGTAGGGTGCAAGCGTTAATCGGAATTACTGGGCGTAAAGCGTGCGCAGGCGGTTTTGTAAGTCTGTCGTGAAAGCCCCGGGCTCAACCTGGGAATTGCGATGGAGACTGCAAGGCTTGAATCTGGCAGAGGGGGGTAGAATTCCACGTGTAGCAGTGAAATGCGTAGAGATGTGGAGGAACACCGATGGCGAAGGCAGCCCCCTGGGTCAAGATTGACGCTCATGCACGAAAGCGTGGGGAGCAAACAGG` |
| ASV5475 | 429 | `TGGGGAATATTGGACAATGGGGGAAACCCTGATCCAGCAATACCGCGTGGAGGAAGAAGGCCTTTGGGTTGTAAACTCCTGTCGTCGGGGACAAAGACAATTCGGTGAATAATCGGGTTGTTTGATGGTACCCGAGGAGGAAGCACCGGCTAACTCCGTGCCAGCAGCCGCGGTAATACGGAGGGTGCAAGCGTTGTTCGGAATCACTGGGCGTAAAGCGCGTCTAGGCTGTTTGCTAAGTTTGTTGTGAAAGCCCCTGGCTCAACCAGGGATGTGCAATGGATACTGGCAGACTTGAATAATGGAGAGGTGGGCGGAATTCCGAGTGTAGCAGTGAAATGCGTAGATATTCGGAGGAACACCAGAGGCGAAGGCGGCTCACTGGCCATTTATTGACGCTGAAGCGCGAAAGCATGGGGATCAAACAGG` |
| ASV2236 | 404 | `TGGGGAATCTTGGACAATGGGGGGAACCCTGATCCAGCCATGCCGCGTGAGCGATGAAGGCCTTCGGGTTGTAAAGCTCTTTCGTGGGGGAAGATAATGACTGTACCCCAAGAAGAAGCCCCGGCTAACTCCGTGCCAGCAGCCGCGGTAATACGGAGGGGGCTAGCGTTGTTCGGAATTACTGGGCGTAAAGCGCACGTAGGCGGGACGGAAAGTCAGAGGTGAAATCCCAGGGCTCAACTCTGGAACTGCCTTTGAAACTCCCGTTCTTGAGGTCGAGAGAGGTGGGTGGAATTCCGAGTGTAGAGGTGAAATTCGTAGATATTCGGAGGAACACCAGTGGCGAAGGCGGCCCACTGGCTCGATACTGACGCTGAGGTGCGAAAGCGTGGGGAGCAAACAGG` |
| ASV1602 | 424 | `TGAGGAATATTGGTCAATGGGCGCAAGCCTGAACCAGCCATGCCGCGTGCAGGATGACGCATCTATGGTGTGTAAACTGCTTTTGCACAGGAAGAAACACTCCCTCGTGAGGGAGCTTGACGGTACTGTGAGAATAAGGATCGGCTAACTCCGTGCCAGCAGCCGCGGTAATACGGAGGATCCAAGCGTTATCCGGAATCATTGGGTTTAAAGGGTCCGTAGGCGGTTTATTAAGTCAGTGGTGAAAGCCCATCGCTCAACGGTGGAACGGCCATTGATACTGCTAGACTTGAATTATTAGGAAGTAACTAGAATATGTAGTGTAGCGGTGAAATGCTTAGATATTACATGGAATACCAATTGCGAAGGCAGGTTACTATCAACATATTGACGCTGATGGACGAAAGCGTGGGTAGCGAACAGG` |
| ASV438 | 424 | `TAAGGAATATTGGTCAATGGACGCAAGTCTGAACCAGCCATGCCGCGTGAAGGATTAAGGTCCTCTGGATTGTAAACTTCTTTTATATGGGACGAAAAAAGGTCTTTCTAGACCCCTTGACGGTACCATATGAATAAGCACCGGCTAACTCCGTGCCAGCAGCCGCGGTAATACGGAGGGTGCAAGCGTTATCCGGATTCACTGGGTTTAAAGGGTGCGTAGGCGGGTAGGTAAGTCAGTGGTGAAATCCTGGAGCTCAACTCCAGAACTGCCATTGATACTATCTATCTTGAATATTGTGGAGGTAAGCGGAATATGTCATGTAGCGGTGAAATGCTTAGATATGACATAGAACACCCATTGCGAAGGCAGCTTACTACGCATATATTGACGCTGAGGCACGAAAGCGTGGGGATCAAACAGG` |
| ASV997 | 430 | `TGGGGAATATTGCGCAATGGGGGAAACCCTGACGCAGCAACGCCGCGTGAGTGAGGAAGGACTTCGGTCTGTAAAGCTCTGTTAATGAGGAAAAATGGTAGTTGGTCTAATAGGCCGATTATTTGATGGTACTCATAGAGGAAGCACCGGCTAACTTCGTGCCAGCAGCCGCGGTAATACGAAGGGTGCAAGCGTTGTTCGGATTTATTGGGCGTAAAGCGCGCGTAGGCGGACCTGCAAGTCAGATGTGAAATCTCGAGGCTCAACCTCGAAACTGCGTCTGAAACTACAGGTCTAGAATCTCGGAGGGGGAAGGGGAATATCGCATGTAGGGGTAAAATCCGTAGATATGCGATGGAACACCAGAGGCGAAGGCGCCTTCCTGGACGAGTATTGACGCTGAGGCGCGAAAGCGTGGGGATCAAACAGG` |
| ASV1353 | 423 | `TAGGGAATATTGGTCAATGGGCGGAAGCCTGAACCAGCCATGCCGCGTGCAGGAAGAATGCCCTCTGGGTTGTAAACTGCTTTTGACAGGGAACAAGTAGCCCATGCGTGGGTTGGTGAGTGTACCTGTAGAATAAGCCACGGCTAACTACGTGCCAGCAGCCGCGGTAATACGTAGGTGGCAAGCGTTGTCCGGATTTATTGGGTTTAAAGGGTGCGTAGGCGGCCATGTAAGTCAGTTGTGAAATACCCCGGCTTAACCGGGGGGGTGCGATTGATACTGCGTGGCTTGAGTGGCAGCGAGGTTGGCGGAATTGACGGTGTAGCGGTGAAATGCATAGATATCGTCAAGAACACCGATAGCGAAGGCAGCCAACTAGGAGCTAACTGACGCTGAGGCACGAAAGTGCGGGGATCAAACAGG` |
| ASV4949 | 428 | `TGGGGAATCTTGCGCAATGGGCGAAAGCCTGACGCAGCGACGCCGCGTGAGTGATGAAGGCCTTCGGGTTGTAAAGCTCTGTGGGGAGAGACGAATAAGCTGTGGCTAATATCCACGGTGATGACGGTATCTCCTTAGCAAGCACCGGCTAACTCTGTGCCAGCAGCCGCGGTAAGACAGAGGGTGCAAACGTTGTTCGGAATTACTGGGCGTAAAGCGCATGTAGGCGGTCTAGCAGGTCGGATGTGAAAGCCCTCGGCTCAACCGAGGAAGTGCATTCGAAACCGCAAGACTCGAGTCCCGGAGAGGGTAGTGGAATTCTCGGTGTAGAGGTGAAATTCGTAGATATCGAGAGGAACACCGGTGGCGAAAGCGGCTACCTGGACGAGGACTGACGCTGAGATGCGAAAGCGTGGGGAGCAAACAGG` |
| ASV1979 | 429 | `TCGAGAATTTTTCACAATGGGGGAAACCCTGATGGAGCGACGCCGCGTGGAGGATGAAGGTTTTCGGATTGTAAACTCCTGTCACCACAGAACAAGCAATGTAGTTTAATAAATTACAAAGTTGATAGTATGTGGGGAGGAAGGGACGGCTAACTCTGTGCCAGCAGCCGCGGTAATACAGAGGTCCCAAGCGTTGTTCGGATTCACTGGGCGTAAAGGGTGTGTAGGAGGTTTGATAAGTCTGATGTGAAATCCCGTCGCTCAACGACGAAACTGCATTGGATACTGTCAGGCTAGAGGGTCTGAGGGGGAAGCGGAATTCTTGGTGTAGCGGTGAAATGCGTAGATATCAAGAGGAACACCCGTGGCTAACGCGGCTTCCTGGAAGACTCCTGACTCTGAAACACGAAAGCCAGGGTAGCGAATGGG` |
| ASV199 | 408 | `TGGGGAATTTTCCGCAATGGGCGAAAGCCTGACGGAGCAATACCGCGTGAGGGATGAAGGCCTATGGGTTGTAAACCTCTTTTTTCAGGGAGGAACAAAATGACGTGTACCTGAAGAATAAGCATCGGCTAACTCCGTGCCAGCAGCCGCGGTAAGACGGGGGATGCAAGTGTTATCCGGAATCACTGGGCGTAAAGCGTCTGTAGGTGGTTAAGTAAGTCAACTGTTAAATCTTGAGGCTTAACCTCAAAATCGCAGTCGAAACTGTTTGACTAGAGTATAGTAGGGGTAAAGGGAATTTCCAGTGGAGCGGTGAAATGCGTAGATATTGGAAAGAACACCGATGGCGAAGGCACTTTACTGGGCTATTACTAACACTGAGAGACGAAAGCTAGGGTAGCAAATGGG` |
| ASV4950 | 424 | `TGAGGAATATTGGTCAATGGAGGCAACTCTGAACCAGCCATGCCGCGTGTAGGACGAATGCCCTATGGGTTGTAAACTACTTTTATTGGGGAAGAAACCTTTCTACGTGTAGAGAGCTGACGGTACCCAAGGAATAAGGACCGGCTAACTCCGTGCCAGCAGCCGCGGTAATACGGAGGGTCCAAGCGTTATCCGGAATCATTGGGTTTAAAGGGTGCGCAGGCGGTAAAGTAAGTCAGTGGTGAAATCTCTCGGCTCAACCGAGAAACTGCCATTGATACTGCTATACTTGAATATAGTCGAAGTGGGCGGAATGTGACATGTAGCGGTGAAATGCTTAGATATGTCACAGAACACCGATAGCGAAGGCAGCTCACTAGACTAACATTGACGCTCATGCACGAAAGCGTGGGGAGCGAACAGG` |
| ASV301 | 404 | `TGGGGAATCTTAGACAATGGGGGAAACCCTGATCTAGCCATGCCGCGTGATCGATGAAGGCCTTAGGGTTGTAAAGATCTTTCAGGTGGGAAGATAATGACTGTACCACCAGAAGAAGCCCCGGCTAACTCCGTGCCAGCAGCCGCGGTAATACGGAGGGGGCTAGCGTTATTCGGAATTACTGGGCGTAAAGCGCACGTAGGCGGACTGGAAAGTCAGAGGTGAAATCCCAGGGCTCAACCTTGGAACTGCCTTTGAAACTCTCAGTCTTGAGGTCGAGAGAGGTGAGTGGAATTCCGAGTGTAGAGGTGAAATTCGTAGATATTCGGAGGAACACCAGTGGCGAAGGCGGCTCACTGGCTCGATACTGACGCTGAGGTGCGAAAGCGTGGGGAGCAAACAGG` |
| ASV1271 | 430 | `TGGGGAATATTGGACAATGGGCGAAAGCCTGATCCAGCCATGCCGCGTGTGTGAAGAAGGCCTTTTGGTTGTAAAGCACTTTAAGCGAGGAGGAGGCTACCGAGATTAATACTCTTGGATAGTGGACGTTACTCGCAGAATAAGCACCGGCTAACTCTGTGCCAGCAGCCGCGGTAATACAGAGGGTGCGAGCGTTAATCGGATTTACTGGGCGTAAAGCGTGCGTAGGCGGCTTTTTAAGTCGGATGTGAAATCCCTGAGCTTAACTTAGGAATTGCATTCGATACTGGGAAGCTAGAGTATGGGAGAGGATGGTAGAATTCCAGGTGTAGCGGTGAAATGCGTAGAGATCTGGAGGAATACCGATGGCGAAGGCAGCCATCTGGCCTAATACTGACGCTGAGGTACGAAAGCATGGGGAGCAAACAGG` |
| ASV2264 | 404 | `TGGGGAATATTGGACAATGGGGGCAACCCTGATCCAGCAATGCCGCGTGTGTGAAGAAGGTCTTCGGATTGTAAAGCACTTTCGGCGGGGACGATGATGACGGTACCCGCAGAAGAAGCCCCGGCTAACTTCGTGCCAGCAGCCGCGGTAATACGAAGGGGGCTAGCGTTGCTCGGAATGACTGGGCGTAAAGGGCGCGTAGGCGGCGGCATAAGTCAGGCGTGAAATTCCTGGGCTCAACCTGGGGACTGCGCTTGAGACTGTGTTGCTAGAGGATGGAAGAGGCTCGTGGAATTCCCAGTGTAGAGGTGAAATTCGTAGATATTGGGAAGAACACCGGTGGCGAAGGCGGCGAGCTGGTCCATTACTGACGCTGAGGCGCGATAGCGTGGGGAGCAAACAGG` |
| ASV621 | 429 | `TCGAGAATAATTCACAATGGGGGCAACCCTGATGGTGCAACGCCGCGTGGAGGATGACGGTCTTCGGATTGTAAACTCCTGTCATCTGGGAGAAAGACCTGGCCGTTAATAGCGGACAGGGTTGATAGTACCAGAAGAGGAAGGGACGGCTAACTTCGTGCCAGCAGCCGCGGTAATACGAAGGTCCCAAGCGTTGTTCGGAATCACTGGGCGTAAAGGGAGCGTAGGCGGCGCGGTAAGTCAGATGTGAAATCCCGGGGCTCAACCCCGGAACTGCATCCGATACTGCCGTGCTTGAGCAATGGAGAGGTAGCTGGAATTCTCGGTGTAGCAGTGAAATGCGTAGATATCGAGAGGAACACTCGTGGCGAAAGCGAGCTACTGGACATTTGCTGACGCTGAGGCTCGAAGGCTAGGGGAGCGAAAGGG` |
| ASV4207 | 407 | `TAGGGAATATTGCACAATGGGGGAAACCCTGATGCAGCGACGCCGCGTGAGTGATGAAGGACTTCGGTTTGTAAAGCTCTGTTGTCTGGGAATAAAAAAATGAATGTACCAGACGAGAAAGGGACGGCTAACTTCGTGCCAGCAGCCGCGGTAATACGAAGGTCCCAAGCGTTGCTCGGAATCATTGGGCGTAAAGCGGGCGCGGGTGGCTTTGTAAGTCAGGAGTGAAAGCCCAGGGCTCAACCCTGGAAGTGCTTTTGATACTGCAAAGCTTGAGTGTGGGAGAGGGTGCTAGAATACCTAGTGTAGTGGTGAAATACGTAGATATTAGGTGGAACTCCGGTGGCGAAGGCGGGTGCCTGGCCCAACACTGACACTTAGGCCCGAAAGCGTGGGGATCAAACAGG` |
| ASV1586 | 404 | `TGGGGAATCTTAGACAATGGGGGGAACCCTGATCTAGCCATGCCGCGTGATCGATGAAGGCCTTAGGGTTGTAAAGATCTTTCAGTGGGGAAGATAATGACGGTACCCACAGAAGAAGCCCCGGCTAACTCCGTGCCAGCAGCCGCGGTAATACGGAGGGGGCTAGCGTTATTCGGAATTACTGGGCGTAAAGCGCACGTAGGCGGATAAGAAAGTCAGAGGTGAAATCCCAGGGCTCAACCTTGGAACTGCCTTTGAAACTCCTTGTCTTGAGGTCGTGAGAGGTGAGTGGAATTCCGAGTGTAGAGGTGAAATTCGTAGATATTCGGAGGAACACCAGTGGCGAAGGCGGCTCACTGGCACGATACTGACGCTGAGGTGCGAAAGCGTGGGGAGCAAACAGG` |
| ASV5489 | 387 | `GCGCGAAAACTTTGCAATGCGGGAAACCGCGACAGGGGGACTCCGAGTGTCCGTACTTAGTACGGACTTTTCAGGTGCCTAAAAAGCACCCGGAATAAGGGCTGGGTAAGACGGGTGCCAGCCGCCGCGGTAATACCCGCAGCCCGAGTGGTGGTCGCTATTATTGGGTCTAAAGGGTCCGTAGTCGGCCCGGGAAGTCCCTGGGGAAATCCGGCCGCTCAACGGTCGGGAGGCTCAGGGATACTCCCAGGCTTGGGATCGGGTGAGGGCAGGGGTACTCCGGGGGTAGCGGTGAAATGCTGTAATCCTCGGGGGACCACCAGTGGCGAAGGCGCCTGCCCAGAACGACTCCGACGATGAGGGACGAAAGCCAGGGGCGCGAACGGG` |
| ASV1819 | 422 | `TAGGGAATTTTGCGCAATGGGGGAAACCCTGACGCAGCAACGCCATGTGTGGGATGAAGCATTTCGGTGTGTAAACCACTGTCAGCAGGGAATAAACGGCGGCTTCGGGCCGCTTTGAATGTACCTGCAAAGGAAGCCCCGGCTAACTTCGTGCCAGCAGCCGCGGTAATACGAGGGGGGCAAATGTTGTTCGGAATCACTGGGCGTAAAGGGAGCGTAGGCGGGTTTTTAAGTCAGGTGTTTAATCCCGGGGCCCAACCCCGGACCGGCACTTGAAACTGGAAATCTTGAATAGTGGAGAGGTCAGTGGAATTGGGGGTGTAGCGGTGGAATGCGTAGAGATCCCCAAGAACACCAGTTGCGAAGGCGGCTGACTGGACACATATTGACGCTGAGGCTCGAAAGCATGGGGAGCAAACAGG` |
| ASV283 | 429 | `TGAGGAATATTGCACAATGGGCGAAAGCCTGATGCAGCGACGCCGCGTGAAGGATGAAGGTCTTCGGATTGTAAACTTCTGTTAAATGGGAAAAAAGAGTTCTTCTGAATAGGAAGAAAAGATGATGGTACCATTAGAGAAAGCACCGGCTAACTTCGTGCCAGCAGCCGCGGTAATACGAGGGGTGCAAGCGTTATTCGGATTTATTGGGCGTAAAGGGTTTGTAGGCGGTCTGTTAAGTCATTTATTAAATATCCTAGCTTAACTAGGAAAATGTGAGTGAAACTGGGAGACTAGAGGATGGAAGAGAGAAGTGGAATTCTCGGAGTAGCGGTAAAATGCGTAGATCTCGAGAGGAACACCGATGGCGAAGGCAGCTTCTTGGTCCATATCTGACGCTGAAAAACGAAAGCGTGGGGAGCAAACAGG` |
| ASV2097 | 429 | `TGGGGAATTTTGGACAATGGGCGCAAGCCTGATCCAGCAATGCCGCGTGAGTGAAGAAGGCCTTCGGGTTGTAAAGCTCTTTTGTCAGGGAAGAAACGGCTGCGGCTAATATCTGCGGCTAATGACGGTACCTGAAGAATAAGCACCGGCTAACTACGTGCCAGCAGCCGCGGTAATACGTAGGGTGCAAGCGTTAATCGGAATTACTGGGCGTAAAGCGTGCGCAGGCGGTTTTGTAAGTCTGTCGTGAAAGCCCCGGGCTCAACCTGGGAATTGCGATGGAGACTGCAAGGCTTGAATCTGGCAGAGGGGGGTAGAATTCCACGTGTAGCAGTGAAATGCGTAGAGATGTGGAGGAACACCGATGGCGAAGGCAGCCCCCTGGGTCAAGATTGACGCTCATGCACGAAAGCGTGGGGAGCAAACAGG` |
| ASV4062 | 410 | `TGGGGAATATTGCGCAATGGGCGGAAGCCTGACGCAGCGACGCCGCGTGGGGGATGACGGCCTTCGGGTTGTAAACCTCTTTCGCCAGGGACGAAGCTTTTGTGACGGTACCTGGATAAGAAGCACCGGCCAACTACGTGCCAGCAGCCGCGGTAACACGTAGGGTGCGAGCGTTGTCCGGAATTATTGGGCGTAAAGAGCTCGTAGGCGGTGTGTCGCGTCGGCCGTGAAAACTTGGGGCTTAACTCTGAGCGTGCGGTCGATACGGGCATCACTTGAGTTCGGCAGGGGAGACTGGAATTCCTGGTGTAGCGGTGAAATGCGCAGATATCAGGAGGAACACCGGTGGCGAAGGCGGGTCTCTGGGCCGATACTGACGCTGAGGAGCGAAAGCGTGGGGAGCGAACAGG` |
| ASV3604 | 404 | `TGGGGAATATTGGACAATGGGCGCAAGCCTGATCCAGCCATGCCGCGTGAGTGATGAAGGCCTTAGGGTTGTAAAGCTCTTTCAGTGGGGAAGATAATGACGGTACCCACAGAAGAAGCCCCGGCTAACTTCGTGCCAGCAGCCGCGGTAATACGAAGGGGGCTAGCGTTGTTCGGATTTACTGGGCGTAAAGCGCACGTAGGCGGATTGGTCAGTTAGGGGTGAAATCCCAGAGCTCAACTCTGGAACTGCCTCTAATACTGCCAATCTCGAGTCCGGAAGAGGTGAGTGGAACTCCTAGTGTAGAGGTGGAATTCGTAGATATTAGGAAGAACACCAGTGGCGAAGGCGGCTCACTGGTCCGGTACTGACGCTGAGGTGCGAAAGCGTGGGGAGCAAACAGG` |
| ASV4305 | 404 | `TGGGGAATATTGGACAATGGGCGCAAGCCTGATCCAGCCATGCCGCGTGAGTGATGACGGCCTTAGGGTTGTAAAGCTCTTTCGCACGCGACGATAATGACGGTAGCGTGAGAAGAAGCCCCGGCTAACTTCGTGCCAGCAGCCGCGGTAATACGAAGGGGGCTAGCGTTGTTCGGAATCACTGGGCGTAAAGGGTGCGCAGGCGGGTTCATAAGTCAGGGGTGAAATCCCGAGGCTCAACCTCGGAACTGCCTTTGATACTGTGGATCTTGAGTCCGGAAGAGGTGAGTGGAACTGCGAGTGTAGAGGTGAAATTCGTAGATATTCGCAAGAACACCGGTGGCGAAGGCGGCTCACTGGTCCGGAACTGACGCTGAGGCACGACAGCGTGGGGAGCAAACAGG` |
| ASV4231 | 429 | `TGGGGAATTTTGGACAATGGGCGCAAGCCTGATCCAGCCATGCCGCGTGAGTGAAGAAGGCCTTCGGGTTGTAAAGCTCTTTCGGCGGGAACGAAATGGTTCGTGCTAATACCACGGATTGATGACGGTACCTGAAGAAGAAGCACCGGCTAACTACGTGCCAGCAGCCGCGGTAATACGTAGGGTGCGGGCGTTAATCGGAATTACTGGGCGTAAAGCGTGCGCAGGCGGTTTCTTAAGTCAGATGTGAAAGCCCCGGGCTTAACCTGGGAACTGCGTTTGAAACTGGGAGACTCGAGTGTGACAGAGGGGGGTGGAATTCCACGTGTAGCAGTGAAATGCGTAGATATGTGGAGGAACACCGATGGCGAAGGCAGCCCCCTGGGTCAACACTGACGCTCATGCACGAAAGCGTGGGGAGCAAACAGG` |
| ASV5501 | 362 | `GACGATCTACCAAACCAACGCCATGACCAGTTATTCAGCAGGACGTTTGGCACAGCTGCAGGATGGCGCATTCGAATTCTGGATCTACAGGCACAACGACTCGGTGCAGAACCCACGCCCTGAGCACAAGAAGCTGGACGGCCTGACCCTGCCAGCCAAACATCCGTTCTGGAATCGCTATTACCCGCCCAATGGCTGGGGTTGCCAGTGCTACGTGGTGGGTGCGCGTTCTGCAGCTGGTGCACGTCGCCTCGGTGGCGACCCGGACAAGGCCATCCTGGAAGACTGGGACGCCGACGAAGCCCCAGGCATCGACGAAGGCTGGGACTATCAACCAGGTGCCCGAGTGGCCAAGGCTGTTT` |
| ASV4327 | 407 | `TGGGGAATATTGGACAATGGGCCACAAGCCTGATCCAGCAATTCTGTGTGCACGATGAAGGTCTTAGGATTGTAAAGTGCTTTCAGTGGGGAAGAAGTCAGTGACGGTACCCACAGAAGAAGCGACGGCTAAATATGTGCCAGCAGCCGCGGTAATACATATGTCGCAAGCGTTATCCGGATTTATTGGGCGTAAAGCGCGTCTAGGCGGTTTACTAAGTCTGATGTGAAAATGCAGGGCTCAACTCTGTATTGCGTTGGAAACTGGTAGACTAGAGTATTGGAGAGGTGGGCGGAACTACAAGTGTAGAGGTGGAATTCGTAGATATTTGTAGGAATGCCGATGGAGAAGTCAGCTCACTGGACATATACTGACGCTAAAGCGCGAAAGCGTGGGGAGCAAACAGG` |
| ASV3129 | 407 | `TAGGGAATATTGCACAATGGAGGAAACTCTGATGCAGCGACGCCGCGTGAGTGATGAAGGCCTTCGGGTCGTAAAGCTCTGTCGCATGGGAATAATAAAGTGAATGTACCATGCAAGAAAGGATCGGCTAACTTCGTGCCAGCAGCCGCGGTAAGACGAGGGATCCTAGCGTTGTTCGGAATTATTGGGCGTAAAGCGGGTGTAGGTGGCTTTATAAGTCAGGTGTGAAAGCCCGGGGCTCAACCCCGGAAGTGCATTTGATACTGTAAAGCTTGAGTGTGGGAGAGGCTATTGGAATTCCAGGTGTAGTGGTGAAATACGTAGATATCTGGAGGAACACCGGAGGCGAAAGCGGATAGCTGGCCCAACACTGACACTCAGACCCGAAAGCGTGGGGATCAAACAGG` |
| ASV620 | 429 | `TGGGGAATTTTGGACAATGGGCGCAAGCCTGATCCAGCAATGCCGCGTGCAGGAAGAAGGCCTTCGGGTTGTAAACTGCTTTTGTACGGAACGAAAAGGCTCTGGTTAATACCTGGGGCACATGACGGTACCGTAAGAATAAGCACCGGCTAACTACGTGCCAGCAGCCGCGGTAATACGTAGGGTGCAAGCGTTAATCGGAATTACTGGGCGTAAAGCGTGCGCAGGCGGTTTTGTAAGACAGGCGTGAAATCCCCGGGCTTAACCTGGGAATTGCGTTTGTGACTGCAAGGCTGGAGTGCGGCAGAGGGGGATGGAATTCCGCGTGTAGCAGTGAAATGCGTAGATATGCGGAGGAACACCGATGGCGAAGGCAATCCCCTGGGCCTGCACTGACGCTCATGCACGAAAGCGTGGGGAGCAAACAGG` |
| ASV5587 | 406 | `CTGGGAATTTTGCACAATGGACGAAAGTCTGATGCAGTGACACTGCGTGTGGGATGAAGGCCCTCGGGTCGTAAACCACTGTGATAGGGGAAAACTAACGATGGTACCCTATTAGAAAGCACCTGCTAACCACGTGCCAGAAGCCTCGGTAATACGTGGGGTGCAAGCGTTGTCCGGATTTATTGGGCGTAAAGAGTATCGTAGGCGGTTTTTTAAGTATCGGGTTAAAGCCCCCGGCTCAACTGGGGAGATGCTCGATAAACTGAAAAACTTGAGGGTGTTTGGGGTTGCTAGAATTCTTGGTGGAGGAGTGAAATCCGTTGATATCAAGAAGAATACCAAAAGCGAAGGCAGGCAACCAGAACACTCCTGACGCTGATGAACGAAAGCTAGGGGAGCGAAAGGG` |
| ASV3761 | 429 | `TGGGGAATATTGGACAATGGGGGCAACCCTGATCCAGCGATGCCGCGTGGGTGAAGAAGGCCTTCGGGTTGTAAAGCCCTTTAGGCCGGGACGAAGTGCAGTAGTTTAAGAGATGATTGCATTGACGGTACCGGCAGAATAAGCACCGGCAAACTCTGTGCCAGCAGCCGCGGTAATACAGAGGGTGCGAGCGTTAATCGGAGTTACTGGGCGTAAAGGGCGCGTAGGCGGTTGTGTGAGTGTGATGTGAAAGCCCTGGGCTTAACCTAGGAAGTGCATCGCAAACGACATAACTGGAGTATATGAGAGGGTGGTGGAATTTCCGGTGTAGCGGTGAAATGCGTAGAGATCGGAAGGAACGTCAATGGCGAAGGCAGCCACCTGGCATAATACTGACGCTGAGGCGCGAAAGCGTGGGGATCAAACAGG` |
| ASV3919 | 404 | `TGAGGAATATTGGACAATGGAGGAAACTCTGATCCAGCCATGCCGCGTGAGTGAAGAAGGCCTTCGGGTTGTAAAGCTCTTTTACCCCTGACGATAATGACGGTAAGGGGAGAAAAAGCCACGGCTAACTTCGTGCCAGCAGCCGCGGTAATACGAAGGTGGCAAGCGTTGTTCGGATTTACTGGGCGTAAAGAGTGCGTAGGCGGTTTTACAAGTCAGAAGTGAAAGCCCAAGGCTCAACCTTGGAATTGCTTTTGAAACTGTTTAACTTGAATCCGGTAGAGGTTGGCGGAATTCTCAGTGTAGAGGTGAAATTCGCAGATATTGAGAGGAACACCAGTGGCGTAAGCGGCCAACTGGACCGGTTTTGACGCTGAGGCACGAAAGCATGGGGAGCAAACAGG` |
| ASV2710 | 404 | `TGGGGAATATTGGACAATGGGCGCAAGCCTGATCCAGCCATGCCGCGTGAGTGATGAAGGCCTTAGGGTTGTAAAGCTCTTTCACCGGTGAAGATAATGACGGTAACCGGAGAAGAAGCCCCGGCTAACTTCGTGCCAGCAGCCGCGGTAATACGAAGGGGGCTAGCGTTGTTCGGAATTACTGGGCGTAAAGCGCACGTAGGCGGATATTTAAGTCAGGGGTGAAATCCCAGAGCTCAACTCTGGAACTGCCTTTGATACTGGGTATCTTGAGTATGGAAGAGGTAAGTGGAATTGCGAGTGTAGAGGTGAAATTCGTAGATATTCGCAGGAACACCAGTGGCGAAGGCGGCTTACTGGTCCATTACTGACGCTGAGGTGCGAAAGCGTGGGGAGCAAACAGG` |
| ASV5072 | 424 | `TAAGGAATATTGGACAATGGGCGGAAGCCTGATCCAGCCATCCCGCGTGCAGGACGACGGCCCTCTGGGTTGTAAACTGCTTTTGTTAGGGAAGAAACCCCTGGATTCATCCGGGGCTGACGGTACCTAAAGAATAAGCACCGGCTAACTCCGTGCCAGCAGCCGCGGTAATACGGAGGGTGCAAGCGTTATCCGGAATCACTGGGTTTAAAGGGTGCGTAGGCGGCGTTATAAGTCAGAGGTGAAAGGCCACCGCTTAACGGTGGGACTGCCTTTGATACTGTAGTGCTTGAATAAGGTTGAGGTTAGCGGAATGTGACATGTAGCGGTGAAATGCATAGATATGTCATAGAACACCAATTGCGAAGGCAGCTGGCTAGACCTATATTGACGCTGAGGCACGAAAGCGTGGGGAGCGAACAGG` |
| ASV4624 | 429 | `TGGGGAATCTTGGACAATGGGCGAAAGCCTGATCCAGCAATGCCGCGTGTGTGAAGAAGGCCTTCGGGTTGTAAAGCACTTTTGTCAAGAACGAAAAGTTACTTACTAATATTAGGTAATGATGACGGTACTTGAAGAATAAGCACCGGCTAACTACGTGCCAGCAGCCGCGGTAATACGTAGGGTGCAAGCGTTAATCGGAATTACTGGGCGTAAAGCGTGCGCAGGCGGTGGCTCAAGTCAGATGTGAAATCCCCGAGCTTAACTTGGGAACTGCATTTGAAACTAGGTCACTAGAGTGTGTCAGAGGGGGGTAGAATTCCACGTGTAGCAGTGAAATGCGTAGATATGTGGAGGAATACCGATGGCGAAGGCAGCCCCCTGGGATAACACTGACGCTCATGCACGAAAGCGTGGGGAGCAAACAGG` |
| ASV2964 | 405 | `TGGGGAATCTTGCGCAATGGGCGAAAGCCTGACGCAGCAACGCCGCGTGGGGGATGAAGGCTTTCGGGTTGTAAACCCCTTTCAGCAGGAACGAAATTGACGGTACCTGCAGAAGAAGCCCCGGCTAACTACGTGCCAGCAGCCGCGGTAATACGTAGGGGGCGAGCGTTGTCCGGATTTATTGGGCGTAAAGAGCTCGTAGGCGGCTTCGTAAGTCGGGTGTGAAAGCCCCAGGCTCAACCTGGGGATGCCACTCGATACTGCGATAGCTAGAGTCTGGTAGGGGATCACGGAATTCCTGGTGTAGCGGTGAAATGCGCAGATATCAGGAGGAACACCGGTGGCGAAGGCGGTGATCTGGGCCAGTACTGACGCTGAGGAGCGAAAGCGTGGGGAGCAAACAGG` |
| ASV4803 | 404 | `TCGGGAATTTTCCACAATGGACGAAAGTCTGATGGAGCAACGCCGCGTGCAGGATGACGGCCTTCGGGTTGTAAACTGCTTTTATATGGGAAGATTATGACGGTACCATATGAATAAGCACCGGCTAACTACGTGCCAGCAGCCGCGGTAATACGTAGGGTGCAAGCGTTATCCGGAATCACTGGGCGTAAAGCGTGCGTAGGCGGAAATTTAAGTGTGGTGTTAAATCGTGTGGCTCAACCATATCGATGCATCGCATACTGGGTTTCTAGAGGATGGCAGAGGCAAGTGGAATTGGCAGTGTAGCAGTGAAATGCGTAGAGATTGCCAGGAACACCAATGGCGAAGGCAGCTTGCTGGGCCATTTCTGACGCTGAGGCACGAAAGCGTGGGGAGCGAACAGG` |
| ASV5130 | 416 | `TGGGGAATTTTGGACAATGGGGGAAACCCTGATCCAGCAACTCTGCGTGAGGGACGAAGGTCTTCGGATTGTAAACCTCTTTTGCTAGGGACGAAATCCCGCAAGGGTGTGACGGTACCTAGCGAATAAGCCACGGCTAACTATGTGCCAGCAGCCGCGGTAAGACATAGGTGGCGAGCGTTATTCGGAATTACTAGGCGTAAAGCGAGTGTAGGCGGACGCTTAAGTCTGACGTTAAATCTCCAGGCTTAACTTGGAGCTGTCGTCGGATACTGAGCGCCTCGAATGGGGTAGGGGGCAGGGGAATTCCCGGTGTAGCGGTGAAATGCGTAGATATCGGGAGGAACACCTATGGCGAAAGCACCTGCCTGGGCCTCTATTGACGCTGAGACTCGAAAGCCGGGGGAGCAAACAGG` |
| ASV1301 | 429 | `TGGGGAATTTTGGACAATGGGGGAAACCCTGATCCAGCCATGCCGCGTGCGGGAAGAAGGCCTTCGGGTTGTAAACCGCTTTTAGTCGGGAAGAAAAGGCGGCCCCTAATACGGGTCGTTTTTGACGGTACCGGCGGAATAAGCACCGGCTAACTACGTGCCAGCAGCCGCGGTAATACGTAGGGTGCGAGCGTTAATCGGAATTACTGGGCGTAAAGCGTGCGCAGGCGGTTCGAAAAGTCCGATGTGAAAGCCCCGGGCTCAACCTGGGAATGGCATTGGAAACTGGCGGACTAGAGTGTGGCAGAGGGGGGTGGAATTCCGCGTGTAGCAGTGAAATGCGTAGAGATGCGGAGGAACACCGATGGCGAAGGCAGCCCCCTGGGCTAACACTGACGCTCAGGCACGAAAGCGTGGGGAGCAAACAGG` |
| ASV3579 | 429 | `TGGGGAATTTTGGACAATGGGCGCAAGCCTGATCCAGCAATGCCGCGTGCAGGATGAAGGCCTTCGGGTTGTAAACTGCTTTTGTACGGGGCGAAAAGGTTCTCTCTAATACAGGGGGCTAATGACGGTACCGTAAGAATAAGCACCGGCTAACTACGTGCCAGCAGCCGCGGTAATACGTAGGGTGCAAGCGTTAATCGGAATTACTGGGCGTAAAGCGTGCGCAGGCGGTTTTGTAAGACAGGCGTGAAATCCCCGGGCTCAACCTGGGAACTGCGTTTGTGACTGCAAGGCTGGAGTGCGGCAGAGGGGGATGGAATTCCGCGTGTAGCAGTGAAATGCGTAGATATGCGGAGGAACACCGATGGCGAAGGCAATCCCCTGGGCCTGCACTGACGCTCATGCACGAAAGCGTGGGGAGCAAACAGG` |
| ASV4861 | 406 | `TTAGGAATCTTGCACAATGGGGGAAACCCTGATGCAGCGACGCCGCGTGAAGGACGAAGGTTCTCGGATCGTAAACTTCTTTTAAGTGGGAAGAAATTTGACGGTACCACTTGAATAAGCCCCGGCTAACTACGTGCCAGCAGCCGCGGTAATACGTAGGGGGCGAGCGTTGTCCGAAGTTACTGGGCGTAAAGCGCGCGTAGGCGGTTTCTTAAGTCTGGGGTGAAAGGTTCAACGCTCAACGTGAACAGTGCCTTGGATACTGGGAGACTTGAGTTAGGGAGAGGGTAGTAGAATTCCTGGTGTAGCGGTGAAATGCGTAGATATCAGGAGGAATACCAATGGCGAAGGCAGCTACCTGGCCTATAACTGACGCTGAGGTGCGAAAGCGTGGGGAGCAAACGGG` |
| ASV690 | 404 | `TGGGGAATCTTAGACAATGGGGGCAACCCTGATCTAGCCATGCCGCGTGATCGATGAAGGCCTTAGGGTTGTAAAGATCTTTCAGATGGGAAGATAATGACGGTACCATCAGAAGAAGCCCCGGCTAACTCCGTGCCAGCAGCCGCGGTAATACGGAGGGGGCTAGCGTTATTCGGAATTACTGGGCGTAAAGCGCACGTAGGCGGACTGGAAAGTCAGAGGTGAAATCCCAGGGCTCAACCTTGGAACGGCCTTTGAAACTCCCCGTCTTGAGATCGTGAGAGGTGAGTGGAATTCCGAGTGTAGAGGTGAAATTCGTAGATATTCGGAGGAACACCAGTGGCGAAGGCGGCTCACTGGCACGATACTGACGCTGAGGTGCGAAAGCGTGGGGAGCAAACAGG` |
| ASV5236 | 429 | `TGGGGAATTTTTCGCAATGGGGGCAACCCTGACGAAGCAACGCCGCGTGGGGGATGAAGGTCTTCGGATCGTAAACCCCTGTCGACCGGGACGAAAGCACCCGACCTAACACGTCGAAGTGTTGACTGTACCGGTGGAGGAAGCCACGGCTAACTCTGTGCCAGCAGCCGCGGTAATACAGAGGTGGCAAGCGTTGTTCGGAATTACTGGGCGTAAAGGGCGCGTAGGCGGTCTCATAAGTCCCGTGTGAAAGCCCCCGGCTCAACTGGGGAATGGCACGGGAAACTGCGAGGCTTGAGTTCGGGAGAGGGAAGCGGAATTCCGGGTGTAGCGGTGAAATGCGTAGATATCCGGAGGAACACCGGTGGCGAAGGCGGCTTCCTGGACCGATACTGACGCTGAGGCGCGAAAGCTAGGGGAGCAAACGGG` |
| ASV3262 | 424 | `TGAGGAATATTGGTCAATGGGCGCAAGCCTGAACCAGCCATGCCGCGTGCAGGATGACGCATCTATGGTGTGTAAACTGCTTTTGTACAGGAAGAAACAGTTCTTTGCGAAGAACCTTGACGGTACTGTAAGAATAAGGATCGGCTAACTCCGTGCCAGCAGCCGCGGTAATACGGAGGATCCAAGCGTTATCCGGAATCATTGGGTTTAAAGGGTCCGTAGGCGGCCTTATAAGTCAGCGGTGAAAGTTTTTGGCTTAACCAAAAAATTGCCATTGATACTGTAGGGCTTGAATGTTTGTGAAGTAACTAGAATATGTAGTGTAGCGGTGAAATGCTTAGATATTACATGGAATACCAATTGCGAAGGCAGGTTACTAACAAATGATTGACGCTGATGGACGAAAGCGTGGGTAGCGAACAGG` |
| ASV4142 | 418 | `TGGGGAATATTGGACAATGGGCGAAAGCCTGATCCAGCAATGCCGCGTGTGTGAAGAAGGCCTTCGGGTTGTAAAGCACTTTAGTGGGGGAAGAAAGCTACGCAAGTAGATTGACGGTACCCCAAGAATAAGCACCGGCTAACTCTGTGCCAGCAGCCGCGGTAATACAGAGGGTGCGAGCGTTAATCGGAATGACTGGGCGTAAAGAGCGCGTAGGTGGATGTGTGTGTTGGATGTGAAAGCCCCGGGCTTAACCTGGGAATTGCATACAAAACTGCAGATCTAGAGTAGAGCAGAGGTGAGTGGAATTTCCGGAGTAGCGGTGAAATGCGTAGATACCGGAAGGAACACCAGTGGCGAAGGCGACTCACTGGGCTCAAACTGACACTGAGGCGCGAAAGCGTGGGGAGCAAACAGG` |
| ASV3575 | 429 | `TGGGGAATTTTGGACAATGGGCGCAAGCCTGATCCAGCCATGCCGCGTGTGTGAAGAAGGCCTTCGGGTTGTAAAGCACTTTCGGACGGAACGAAATCGCGCGGTTGAATATTCCGCGTGGATGACGGTACCGTAAGAAGAAGCACCGGCTAACTACGTGCCAGCAGCCGCGGTAATACGTAGGGTGCGAGCGTTAATCGGAATTACTGGGCGTAAAGGGTGCGCAGGCGGCTCCGCGTGTCAGACGTGAAATCCCCGGGCTCAACTTGGGAATGGCGTTTGAAACTACGGAGCTGGAGTGTGGCAGAGGGAGGTGGAATTCCACGTGTAGCGGTGAAATGCGTAGAGATGTGGAGGAACACCGATGGCGAAGGCAGCCTCCTGGGCCAACACTGACGCTCATGCACGAAAGCGTGGGGAGCAAACAGG` |
| ASV765 | 429 | `TGGGGAATTTTGGACAATGGGCGCAAGCCTGATCCAGCAATGCCGCGTGCAGGATGAAGGCCTTCGGGTTGTAAACTGCTTTTGTACGGGGCGAAACGGCTCACTTTAATACAGTGGGCTAATGACGGTACCGTAAGAATAAGCACCGGCTAACTACGTGCCAGCAGCCGCGGTAATACGTAGGGTGCGAGCGTTAATCGGAATTACTGGGCGTAAAGCGTGCGCAGGCGGTGATGTAAGACAGATGTGAAATCCCCGGGCTCAACCTGGGACCTGCATTTGTGACTGCATCGCTAGAGTACGGTAGAGGGGGATGGAATTCCGCGTGTAGCAGTGAAATGCGTAGATATGCGGAGGAACACCGATGGCGAAGGCAATCCCCTGGACCTGTACTGACGCTCATGCACGAAAGCGTGGGGAGCAAACAGG` |
| ASV257 | 429 | `TGGGGAATTTTGGACAATGGGCGCAAGCCTGATCCAGCAATGCCGCGTGCAGGATGAAGGCCTTCGGGTTGTAAACTGCTTTTGTACGGAACGAAACGGTCTCTCCTAATACGAGGGGCTAATGACGGTACCGTAAGAATAAGCACCGGCTAACTACGTGCCAGCAGCCGCGGTAATACGTAGGGTGCGAGCGTTAATCGGAATTACTGGGCGTAAAGCGTGCGCAGGCGGTTATATAAGACAGATGTGAAATCCCCGGGCTCAACCTGGGACCTGCATTTGTGACTGTATAGCTAGAGTACGGTAGAGGGGGATGGAATTCCGCGTGTAGCAGTGAAATGCGTAGATATGCGGAGGAACACCGATGGCGAAGGCAATCCCCTGGACCTGTACTGACGCTCATGCACGAAAGCGTGGGGAGCAAACAGG` |
| ASV4405 | 424 | `TAAGGAATATTGGACAATGCCCGCAAGGGTGATCCAGCCATGCCGCGTGCAGGAAGACGGCCCTATGGGTTGTAAACTGCTTTTATACAAGAGAAAACCCATCTACGTGTAGATGGCTGATAGTATTGTAGGAATAAGCATCGGCTAACTTCGTGCCAGCAGCCGCGGTAAGACGAAGGATGCAAGCGTTATCCGGATTCATTGGGTTTAAAGGGAGCGTAGGCGGTCTTATAAGTCAGTGGTGAAAGCTCCTCGCTTAACGAGGAAATTGCCATTGATACTGTAGGACTTGAGTACAGTTGCCGTTGGCGGAATATGACATGTAGTGGTGAAATACATAGAGATGTCATAGAACACCGATTGCGAAGGCAGCTAACGAAACTGTAACTGACGCTGAGGCTCGAAAGTGCGGGGATCAAACAGG` |
| ASV5280 | 404 | `TTAGGAATCTTGGGCAATGGGGGCAACCCTGACCCAGCCATGCCGCGTGGATGATGAAGGTCTTAGGATTGTAAAATCCTTTCACCGGGGATGATAATGACAGTACCCGGAGAAGAAGCCCCGGCTAACTTCGTGCCAGCAGCCGCGGTAATACGAAGGGGGCTAGCGTTGCTCGGAATTACTGGGCGTAAAGGGCGCGTAGGCGGATCAGCAAGTTGGGGGTGAAATCCCGGGGCTTAACCTCGGAACTGCCTCCAAAACTGCTTATCTTGAGTACGGGAGAGGTGAGTGGAACTCCGAGTGTAGAGGTGAAATTCGTAGATATTCGGAAGAACACCAGTGGCGAAGGCGACTCACTGGCCCGTTACTGACGCTGAGGCGCGAAAGCGTGGGGAGCAAACAGG` |
| ASV2328 | 407 | `TAGGGAATATTGGGCAATGGAGGCAACTCTGACCCAGCAACACCGCGTGAATGATGAAGGTCTTCGGATTGTAAAGTTCTGTGACGGGGGACGAAGAAAGTGACGGTACCCCGTAAGCAAGCCACGGCTAACTACGTGCCAGCAGCCGCGGTAATACGTAGGTGGCAAGCGTTGTCCGGAATGACTGGGCGTAAAGGGTGCGTAGGTGGTTATTCAAGTTAGTAGTGAAATCCCGGGGCTCAACTCCGGTGCTACTACTAAAACTGTTTAACTTGAGTGCAGGAGGGGTAAGTGGAATTCCTAGTGTAGCGGTGGAATGCGTAGATATTAGGAGGAACACCAGTGGCGAAGGCGACTTACTGGACTGTAACTGACACTGAGGCACGAAAGCGTGGGGAGCAAACAGG` |
| ASV4469 | 404 | `TGGGGAATATTGGGCAATGGACGAAAGTCTGACCCAGCCATGCCGCGTGAGTGATGAAGGCTTTAGGGTTGTAAAGCTCTTTCGCCGATGAAGATAATGACGGTAGTCGGAGAAGAAGCCCCGGCTAACTTCGTGCCAGCAGCCGCGGTAATACGAAGGGGGCTAGCGTTGTTCGGAATTACTGGGCGTAAAGCGAGCGTAGGTTGCTCGATGTGTCAGTGGTGAAAGCCCGGGGCTCAACCCCGGAACTGCCATTGAAACTGTCGAGCTGGAATCTCAGAGGGGGTAGCGGAATTCCAAATGTAGGGGTGAAATCCGTAGATATTTGGAGGAACACCGGTGGCGAAGGCGGCTACCTGGATGAGTATTGACACTGAGGCTCGAAAGCGTGGGGATCAAACAGG` |
| ASV4311 | 409 | `TGGGGAATATTGCACAATGGGCGCAAGCCTGATGCAGCGACGCCGCGTGGGGGATGACGGCCTTCGGGTTGTAAACCTCTTTCAGTACCGACGAAGCGTAAGTGACGGTAGGTACAGAAGAAGCACCGGCCAACTACGTGCCAGCAGCCGCGGTAATACGTAGGGTGCGAGCGTTGTCCGGAATTACTGGGCGTAAAGAGCTCGTAGGTGGTTTGTCGCGTTGTCCGTGAAAACTCACAGCTTAACTGTGGGCGTGCGGGCGATACGGGCAGACTAGAGTACTGCAGGGGAGACTGGAATTCCTGGTGTAGCGGTGGAATGCGCAGATATCAGGAGGAACACCGGTGGCGAAGGCGGGTCTCTGGGCAGTAACTGACGCTGAGGAGCGAAAGCGTGGGGAGCGAACAGG` |
| ASV4754 | 429 | `TGGGGAATATTGGACAATGGGGGAAACCCTGATCCAGCAATGCCGCGTGTGTGAAGAAGGCCTGCGGGTTGTAAAGCACTTTCGGAGGGGACAAAAAGCGTCGGGCTAACACCCAGGCGTCTTGATTTAACCCTCATAAGAAGCACCGGCTAACTCTGTGCCAGCAGCCGCGGTAATACAGAGGGTGCAAGCGTTAATCGGAATTACTGGGCGTAAAGCGCACGCAGGCGGCTGCATAAGCCGGTCGTGAAATCCCCGGGCTTAACCTGGGAATTGCGATCGGGACTGTGCGGCTAGAGTGTTGGAGAGGACAGCGGAATTCCCGGTGTAGCAGTGAAATGCGTAGATATCGGGAGGAACATCAGTGGCGAAGGCGGCTGTCTGGCCAAACACTGACGCTCAGGTGCGAAAGCGTGGGGAGCAAACAGG` |
| ASV5471 | 422 | `TCGAGAATCTTCCGCAATGGGCGCAAGCCTGACGGAGCGACGCCGCGTGTGGGATGAAGGCCTTCGGGTTGTAAACCACTGTCGTAGAGGAGGAAGGACCGGTGCAGAGCCGGTTTTGACCTATTCTAGGAGGAAGGGTGGGCTAAGTTCGTGCCAGCAGCCGCGGTAAGACGAACCACCCAAACGTTATTCGGAATTACTGGGCTTAAAGGGTGCGTAGGCGGCGCGGAAAGTCAGGTGTGAAATCCCTCGGCTCAACCGAGGAACTGCGCTTGAAACTACCGTGCTCGAGTGAGACAGAGGTAAGCGGAACTTCGGGTGGAGCGGTGAAATGCGTTGATATCCGAAGGAACACCGGTGGCGAAAGCGGCTTACTGGGTCTTTTCTGACGCTGATGCACGAAAGCTAGGGGAGCAAACGGG` |
| ASV1904 | 424 | `TGAGGAATATTGGACAATGGGCGCAAGCCTGATCCAGCCATGCCGCGTGCAGGATGACGGTCCTATGGATTGTAAACTGCTTTTATACAGGAAGAAACCCTGGTTCGTGAACCAGCTTGACGGTACTGTAAGAATAAGGATCGGCTAACTCCGTGCCAGCAGCCGCGGTAATACGGAGGATCCAAGCGTTATCCGGAATCATTGGGTTTAAAGGGTCCGTAGGCGGTTTAATAAGTCAGTGGTGAAAGCCCATCGCTCAACGGTGGAACGGCCATTGATACTGTTAAACTTGAATTATTAGGAAGTAACTAGAATATGTAGTGTAGCGGTGAAATGCTTAGAGATTACATGGAATACCAATTGCGAAGGCAGGTTACTACTAATATATTGACGCTGATGGACGAAAGCGTGGGTAGCGAACAGG` |
| ASV32 | 428 | `TAGGGAATTTTCGTCAATGGGGGAAACCCTGAACGAGCAATGCCGCGTGAGTGAAGAAGGTTTTCGGATCGTAAAGCTCTGTTGTAAGTGAAGAACGACCAGTATAGGAAATGATATTGGAGTGACGGTAGCTTACCAGAAAGCCACGGCTAACTACGTGCCAGCAGCCGCGGTAATACGTAGGTGGCAAGCGTTATCCGGAATTATTGGGCGTAAAGGGTGCGTAGGTGGTGCATTAAGTCTGAAGTAAAAGCCAGTGGCTCAACCACTGTAAGCTTTGGAAACTGGTGCACTAGAGTACAGAAGAGGATGATGGAATTCCATGTGTAGCGGTAAAATGCGTAGATATATGGAGGAACACCAGTGGCGAAGGCGGTCATCTGGTCTGTAACTGACACTGAGGCACGAAAGCGTGGGGAGCAAATAGG` |
| ASV342 | 404 | `TGGGGAATCTTAGACAATGGGGGAAACCCTGATCTAGCCATGCCGCGTGATCGATGAAGGCCTTAGGGTTGTAAAGATCTTTCAGATGGGAAGATAATGACGGTACCATCAGAAGAAGCCCCGGCTAACTCCGTGCCAGCAGCCGCGGTAATACGGAGGGGGCTAGCGTTATTCGGAATTACTGGGCGTAAAGCGCACGTAGGCGGATCAGAAAGTCAGAGGTGAAATCCCAGGGCTCAACCTTGGAACTGCCTTTGAAACTCCTGGTCTTGAGGTCGTGAGAGGTGAGTGGAATTCCGAGTGTAGAGGTGAAATTCGTAGATATTCGGAGGAACACCAGTGGCGAAGGCGGCTCACTGGCACGATACTGACGCTGAGGTGCGAAAGCGTGGGGAGCAAACAGG` |
| ASV964 | 429 | `TGGGGAATTTTGGACAATGGGCGCAAGCCTGATCCAGCAATGCCGCGTGAGTGAAGAAGGCCTTCGGGTTGTAAAGCTCTTTTGTCAGGGAAGAAAAGGTGCGCTCTAACATAGCGTACTGATGACGGTACCTGAAGAATAAGCACCGGCTAACTACGTGCCAGCAGCCGCGGTAATACGTAGGGTGCAAGCGTTAATCGGAATTACTGGGCGTAAAGCGTGCGCAGGCGGTTTTATAAGTCTGATGTGAAATCCCCGGGCTCAACCTGGGAACTGCATTGGAGACTGTAAGGCTAGAGTGTGTCAGAGGGGGGTAGAATTCCACGTGTAGCAGTGAAATGCGTAGATATGTGGAGGAATACCGATGGCGAAGGCAGCCCCCTGGGATAACACTGACGCTCATGCACGAAAGCGTGGGGAGCAAACAGG` |
| ASV2467 | 429 | `TGGGGAATATTGGACAATGGGGGCAACCCTGATCCAGCCATGCCGCGTGTGTGAAGAAGGCCTTAGGGTTGTAAAGCACTTTCAGTGGGGAGAAAGGTGATTCACCTAATACGTGAGTCAATTGATGCTACCCACAGAAGAAGCACCGGCTAACTCCGTGCCAGCAGCCGCGGTAATACGGAGGGTGCAAGCGTTAATCGGAATTACTGGGCGTAAAGCGCACGTAGGTGGTTTGTTAAGCCAGCTGTGAAATCCCCGGGCTCAACCTGGGCACTGCAGTTGGAACTGGCAAGCTAGAGTAGGGTAGAGGGGTGTGGAATTCCAGGTGTAGCGGTGAAATGCGTAGATATCTGGAGGAACATCAGTGGCGAAGGCGACACCCTGGACTCATACTGACACTGAGGTGCGAAAGCGTGGGGAGCAAACAGG` |
| ASV2509 | 429 | `TGGGGAATCTTGGACAATGGGCGAAAGCCTGATCCAGCAATGCCGCGTGGGTGAAGAAGGCCTTCGGGTTGTAAAGCCCTTTTGTCAGGGAGGAAATCCTGAGTGTTAATAGCATTCGGGGATGACAGTACCTGAAGAATAAGGACCGGCTAACTACGTGCCAGCAGCCGCGGTAATACGTAGGGTCCAAGCGTTAATCGGAATTACTGGGCGTAAAGCGTCCGCAGGTGGCTTGATAAGATAGACGTGAAATCCCTGGGCTCAACCTAGGAATTGCGTTTATGACTGTCTCGCTAGAGTATGGGAGAGGGGGGTGGAATTCCACGTGTAGCAGTGAAATGCGTAGAGATGTGGAGGAACACCGATGGCGAAGGCAACCCCCTGGCCTAATACTGACACTCATGGACGAAAGCGTGGGGAGCAAACAGG` |
| ASV4108 | 430 | `TAGGGAATATTGCGCAATGGGGGAAACCCTGACGCAGCCACGCCGTGTGAGTGAGGAAGGCCTTCGGGTTGTAAAGCTCTGTCATCGGGGAAGAAACTCTATCTGGGGAAAGTTCAGGTAGATTGACGGTACCCGAGGAGGAAGCACCGGCTAACTCTGTGCCAGCAGCCGCGGTAATACAGAGGGTGCGAGCGTTGTTCGGAATTACTGGGCGTAAAGCGTTTGTAGGCGGTGCTGAGAGTCCTAGGTGAAATCCCCGAGCTCAACTCGGGAACTGCCTAGGAGACCTCAGTACTAGAGTACTGAAGGGGTTACTGGAATTCCATGTGTAGCGGTGAAATGCGTAGATATATGGAGGAACACCTGTGGCGAAGGCGGGTAACTGGTCAGAGACTGACGCTGAGAAACGAAAGCGTGGGGAGCAAACAGG` |
| ASV66 | 404 | `TGGGGAATATTGGACAATGGGCGAAAGCCTGATCCAGCAATGCCGCGTGAGTGATGAAGGCCTTAGGGTTGTAAAGCTCTTTTACCAGGGATGATAATGACAGTACCTGGAGAATAAGCTCCGGCTAACTCCGTGCCAGCAGCCGCGGTAATACGGAGGGAGCTAGCGTTGTTCGGAATTACTGGGCGTAAAGCGTACGTAGGCGGTGATTCAAGTCAGAGGTGAAAGCCTGGAGCTCAACTCCAGAACTGCCTTTGAAACTAGATCGCTAGAATTGCGGAGAGGTGAGTGGAATTCCGAGTGTAGAGGTGAAATTCGTAGATATTCGGAAGAACACCAGTGGCGAAGGCGGCTCACTGGACGCATATTGACGCTGAGGTACGAAAGCGTGGGGAGCAAACAGG` |
| ASV4910 | 429 | `TGGGGAATTTTGGACAATGGGCGAAAGCCTGATCCAGCAATGCCGCGTGAGTGAAGAAGGCCTTCGGGTTGTAAAGCTCTTTTGTCAGGGAAGAAACGGTAGAGGCTAATATCCTTTGCTAATGACGGTACCTGAAGAATAAGCACCGGCTAACTACGTGCCAGCAGCCGCGGTAATACGTAGGGTGCAAGCGTTAATCGGAATTACTGGGCGTAAAGCGTGCGCAGGCGGTTGTGCAAGACAGATGTGAAATCCCCGGGCTCAACCTGGGAATTGCATTTGTGACTGCACGGCTAGAGTGTGTCAGAGGGGGGTAGAATTCCACGTGTAGCAGTGAAATGCGTAGAGATGTGGAGGAATACCGATGGCGAAGGCAGCCCCCTGGGATAACACTGACGCTCATGCACGAAAGCGTGGGGAGCAAACAGG` |
| ASV4959 | 424 | `TAGGGAATATTGGGCAATGGGCGGGAGCCTGACCCAGCCATGCCGCGTGCAGGAAGACGGCCTTATTGGTTGTAAACTGCTTTTGGCGGGGAAGAAACCGGGGGATGCGTCCCCTTCTGACGGTACCCGCCGAATAAGCGCCGGCTAACCCCGTGCCAGCAGCCGCGGTAATACGGAGGGCGCGAGCGTTGTCCGGAATCATTGGGTTTAAAGGGTGCGCAGGCGGCCCGATAAGCCGGGGGTGAAATACCGGGGCTCAACCCCGTTGCCGCCCCCGGAACTGCCGGGCTTGAGTACGGTTAAGGCAGGCGGAATTTGTGGTGTAGCGGTGAAATGCATAGATACCACAAGGAACACCGAAAGCGAAGGCAGCCTGCCGGGCCGGCACTGACGCTCATGCACGAAAGCGCGGGTAGCGAACAGG` |
| ASV836 | 404 | `TGGGGAATATTGGACAATGGGGGAAACCCTGATCCAGCGATGCCGCGTGAATGAAGAAGGCCTTAGGGTTGTAAAGTTCTTTCACCTGTGACGATGATGACGGTAACAGGAGAAGAAGCCCCGGCTAACTCCGTGCCAGCAGCCGCGGTAAGACGGAGGGGGCTAGCGTTGTTCGGAATGACTGGGCGTAAAGGGCGCGTAGGCGGCTTATCAAGTCAGGTGTGAAAGCCTTGGGCTCAACCCAAGAACTGCGCTTGATACTGATAAGCTTGAGGATGATAGAGGAAAGCGGAATTCCTAGTGTAGAGGTGAAATTCGTAGATATTAGGAAGAACACCAGAGGCGAAGGCGGCTTTCTGGATCATATCTGACGCTGAGGCGCGAAAGCGTGGGGAGCAAACAGG` |
| ASV3073 | 426 | `TCGAGAATCTTCGGCAATGGACGAAAGTCTGACCGAGCGACGCCGCGTGCGGGATGAAGGCCTTCGGGTTGTAAACCGCTGTCGTAGGGAATCAAATGCATAGGGGTACTCCCCTTTGTTTGAGAGATCCTAGGAGGAAGGAGAGGCTAAGCTCGTGCCAGCAGCCGCGGTAAGACGAGCTCTCCAAACGTTATTCGGTATTACTGGGCTTAAAGAGTTCGTAGGCGGCTCTGTAGGTGAGATGTGAAAGCCCTCGGCTTAACCGAGGAACTGCGTTTCAAACCACAGGGCTTGAGGGAGATAGGGGTAAGCGGAACAGATGGTGGAGCGGTGAAATGCATTGATATCATCTGGAACACCGGTGGCGAAGGCGGCTTACTGGGTCTTTTCTGACGCTGAGGAACGAAAGCTAGGGTAGCGAACGGG` |
| ASV3256 | 429 | `TGGGGAATTTTGGACAATGGGGGCAACCCTGATCCAGCCATGCCGCGTGAGTGAAGAAGGCCTTCGGGTTGTAAAGCTCTTTCGGCCGGGAAGAAATCGTGCAGTCTAATACACTGTATGGATGACGGTACCGGAATAAGAAGCACCGGCTAACTACGTGCCAGCAGCCGCGGTAATACGTAGGGTGCGAGCGTTAATCGGAATTACTGGGCGTAAAGCGTGCGCAGGCGGTTTTGTAAGACAGGCGTGAAATCCCCGGGCTCAACCTGGGCATGGCGCTTGTGACTGCAAGGCTAGAGTACGGCAGAGGGGGGTAGAATTCCACGTGTAGCAGTGAAATGCGTAGAGATGTGGAGGAATACCGATGGCGAAGGCAGCCCCCTGGGTCGATACTGACGCTCATGCACGAAAGCGTGGGGAGCAAACAGG` |
| ASV5175 | 430 | `TAGGGAATCTTGCACAATGGGCGAAAGCCTGATGCAGCGATGCCGCGTGAGTGAAGAAGGCCTTTGGGTTGTAAAGCTCTTTCGGCCGGGAAGAAGGGTCTGTTTGTGAATAATTAGCAGATTTGACTGTACCGGAAGAAGAAGTACCTGCCAACTACGTGCCAGCAGCAGCGGTAATACGTAGGGTACAAGCGTTACTCGGAATCACTGGGCGTAAAGCGCATGTAGGCGGGTTTGTAAGTCGATTGTGAAAGCCCTGGGCTCAACCTGGGAATGGCAGTCGATACTGCAGATCTTGAATGTTCGAGAGGATGGCAGAATTCCAGGTGTAGAAGTGAAATTCGTAGATATCTGGAGGAATACCGGTGGTGAAGACGGCTGTCTGGCGAAACATTGACGCTGAGGTGCGAAAGTGCGGGGATCAAACAGG` |
| ASV463 | 406 | `TGGGGAATATTGGACAATGGGCGAAAGCCTGATCCAGCAATACCGAGTGGGTGATGAAGGCCTTAGGGTTGTAAAGCCCTTTTCAGCAGGGAAGATAATGACGGTACCTGACCAAGAAAGCCCCGGCTAACTCCGTGCCAGCAGCCGCGGTAAGACGGAGGGGGCTAGCGTTGTTCGGAATTACTGGGCGTAAAGAGTGCGTAGGCGGTTTAGTAAGTTGGAAGTGAAAGCCCGAGGCTTAACCTCGGAACTGCTTTCAAAACTATTAATCTAGAGTGTAGTAGGGGATGATGGAATTCCTAGTGTAGAGGTGAAATTCTTAGATATTAGGAGGAACACCGGTGGCGAAGGCGGTCATCTAGGCTACAACTGACGCTGATGCACGAAAGCGTGGGGAGCAAACAGG` |
| ASV3837 | 407 | `TTGGGAATTTTGGACAATGGGCGCAAGCCTGATCCAGCCACGCCGCGTGAATGATGAAGGCCTTCGGGTCGTAAAGTTCTTTTAGTTGGGAAAAACACAATGATGGTACCAACAGAAAAAGCACCGGCTAACTCCGTGCCAGCAGCCGCGGTAATACGGAGGGTGCAAGCGTTGCTCGGAATTATTGGGCGTAAAGCGTATGTAGGCGGTTTTCTAAGTCTGATGTGAAATCCCGGGGCTCAACTCCGGAACTGCATTGGATACTGGAAAGCTAGAGGACGATAGAGGATGGTGGAATTCCAGGTGTAGGGGTGAAATCCGTAGATATCTGGAGGAACATCAGTGGCGAAGGCGGCCATCTGGATCGTACCTGACGCTGAGATACGAAAGCGTGGGGAGCAAACAGG` |
| ASV2775 | 404 | `TAGGGAATATTGGACAATGGGGGCAACCCTGATCCAGCAATGCCGCGTGAGTGATGAAGGCCTTAGGGTTGTAAAGCTCTTTTACCCGAGATGATAATGACAGTATCGGGAGAATAAGCTCCGGCTAACTCCGTGCCAGCAGCCGCGGTAATACGGAGGGAGCTAGCGTTGTTCGGAATTACTGGGCGTAAAGCGCACGTAGGCGGCGATTTAAGTCAGAGGTGAAAGCCCGGGGCTCAACCCCGGAACTGCATTTGAGACTGGATTGCTTGAATCCTGGAGAGGTGAGTGGAATTCCGAGTGTAGAGGTGAAATTCGTAGATATTCGGAAGAACACCAGTGGCGAAGGCGGCTCACTGGACAGGTATTGACGCTGAGGTGCGAAAGCGTGGGGAGCAAACAGG` |
| ASV4111 | 404 | `TGGGGAATATTGGACAATGGGCGAAAGCCTGATCCAGCTATGTCGCGTGAACGATGAAGGTCTTCGGATTGTAAAGTTCTTTTAGTAGGGATGATAATGACAGTACCTACAGAAAAAGCCCCGGCTAACTTCGTGCCAGCAGCCGCGGTAATACGAAGGGGGCTAGCGTTATTCGGAATGATTGGGCGTAAAGGACACGTAGGCGGTTTTGTAAGTTTTGTGTGAAATCCCTGGGCTTAACCCAGGAATTGCGCAAAATACTGCATGACTTGAGTATGGGGGAGGATAGTGGAATCTTTGGTGTAGAGGTGAAATTCTTAGATATCAGAGAGAACGTCGGAGGCGAAAGCGACTATCTAAAACATTACTGACGCTGAGGTGTGAAGGCGTGGGGAGCAAATCGG` |
| ASV713 | 429 | `TAGGGAATCTTCCACAATGGACGAAAGTCTGATGGAGCAACGCCGCGTGAGTGATGAAGGTTTTCGGATCGTAAAACTCTGTTGTAAGGGAAGAACAGGTATGAGAGGTAATGCTCATGCTATGACGGTACCTTACGAGAAAGCCACGGCTAACTACGTGCCAGCAGCCGCGGTAATACGTAGGTGGCAAGCGTTGTCCGGAATTATTGGGCGTAAAGCGCGCGCAGGCGGCCTTTTAAGTCTGATGTGAAAGCCCCCGGCTCAACCGGGGAGGGTCATTGGAAACTGGAAGGCTTGAGTACAGAAGAGAAGAGTGGAATTCCATGTGTAGCGGTGAAATGCGTAGAGATGTGGAGGAACACCAGTGGCGAAGGCGACTCTTTGGTCTGTAACTGACGCTGAGGCGCGAAAGCGTGGGGAGCAAACAGG` |
| ASV5692 | 407 | `TGGGGAATATTGCGCAATGGGGGAAACCCTGACGCAGCAACGCCGCGTGAAGGATGAAGGTTTTCGGATTGTAAACTTCTTTGATGGGGGACGAAAAAAATGACGGTACCCCAAAAACAAGCCACGGCTAACTACGTGCCAGCAGCCGCGGTAATACGTAGGTGGCGAGCGTTGTCCGGAATTACTGGGTGTAAAGGGCGTGTAGGCGGGATTGCAAGTCAGATGTGAAATTCTTGAGCTTAACTCAAGAGCTGCATCTGAAACTGTGATTCTTGAGTACTGGAGAGGATAGTGGAATTCCTAGTGTAGCGGTGAAATGCGTAGATATTAGGAGGAACACCAGTGGCGAAGGCGGCTATCTGGACAGTAACTGACGCTGAGGCGCGAAAGCGTGGGGAGCAAACAGG` |
| ASV1702 | 429 | `TGGGGAATTTTGGACAATGGGCGCAAGCCTGATCCAGCAATGCCGCGTGCAGGATGAAGGCCTTCGGGTTGTAAACTGCTTTTGTACGGAGCGAAACGGCTTTTTCTAATAAAGAGAGCTAATGACGGTACCGTAAGAATAAGCACCGGCTAACTACGTGCCAGCAGCCGCGGTAATACGTAGGGTGCAAGCGTTAATCGGAATTACTGGGCGTAAAGCGTGCGCAGGCGGTTGTGTAAGACAGATGTGAAATCCCCGGGCTTAACCTGGGACCTGCATTTGTGACTGCATAGCTAGAGTACGGTAGAGGGGGATGGAATTCCGCGTGTAGCAGTGAAATGCGTAGATATGCGGAGGAACACCGATGGCGAAGGCAATCCCCTGGACCTGTACTGACGCTCATGCACGAAAGCGTGGGGAGCAAACAGG` |
| ASV520 | 404 | `TGGGGAATATTGGACAATGGGGGAAACCCTGATCCAGCAATGCCGCGTGAATGAAGAAGGCCTTAGGGTTGTAAAGTTCTTTCACCTGTGAAGATAATGACGGTAGCAGGAGAAGAAGCCCCGGCTAACTCCGTGCCAGCAGCCGCGGTAAGACGGAGGGGGCTAGCGTTGTTCGGAATGACTGGGCGTAAAGGGCGCGTAGGCGGTTTTTTAAGTGAGGCGTGAAAGCCCTGGGCTTAACCCAGGAGGTGCGTTTCAGACTGGAAGACTTGAGTGCGAGAGAGGAAAGTAGAATTTCTAGTGTAGAGGTGAAATTCGTAGATATTAGAAAGAACACCCGTGGCGAAGGCGGCTTTCTGGCTCGCAACTGACGCTGAGGCGCGAAAGCGTGGGGAGCAAACAGG` |
| ASV3447 | 430 | `TGGGGAATATTGCGCAATGGGGGAAACCCTGACGCAGCGACGCCGCGTGGGTGATGAAGGCCTTCGGGTCGTAAAGCCCTGTCGTAGGGAAAGAACTGGTTGCAGGCTAATATCCTGCAGCCTTGACGGTACCCTACAAGAAAGCACCGGCTAACTCCGTGCCAGCAGCCGCGGTAATACGGAGGGTGCAAGCGTTGTTCGGAATTACTGGGCGTAAAGAGCGTGTAGGCGGCTGATCAAGTCCGGTGTGAAAGCCCGGGGCTTACCCCCGGAAGTGCGCTGGAAACTGATCAGCTAGAGTATGGGAGAGGGAAGTGGAATTCCTGGTGTAGCGGTGAAATGCGTAGATATCAGGAGGAACACCGGCGGCGAAGGCGGCTTCCTGGACCAATACTGACGCTGAGACGCGAAAGCGTGGGGAGCAAACAGG` |
| ASV1251 | 429 | `TGGGGAATATTGGACAATGGGCGCAAGCCTGATCCAGCCATACCGCGTGGGTGAAGAAGGCCTTCGGGTTGTAAAGCCCTTTTGTTGGGAAAGAAAACTTGCAGGTTAATACCCAGCAAGTCTGACGGTACCCAAAGAATAAGCACCGGCTAACTTCGTGCCAGCAGCCGCGGTAATACGAAGGGTGCAAGCGTTACTCGGAATTACTGGGCGTAAAGCGTGCGTAGGTGGTTGTTTAAGTCTGTCGTGAAAGCCCTGGGCTCAACCTGGGAATTGCGATGGATACTGGGCGACTAGAGTGTGGTAGAGGACAGTGGAATTTCCGGTGTAGCAGTGAAATGCGTAGAGATCGGAAGGAACATCTGTGGCGAAGGCGACTGTCTGGGCCAACACTGACACTGAGGCACGAAAGCGTGGGGAGCAAACAGG` |
| ASV1727 | 429 | `TGGGGAATTTTGGACAATGGGCGCAAGCCTGATCCAGCCATGCCGCGTGTGTGAAGAAGGCCTTCGGGTTGTAAAGCACTTTCGGCCGGAACGAAATCGCCTTTGTTAATAGCAAGGGTGGATGACGGTACCGGAAGAAGAAGCACCGGCTAACTACGTGCCAGCAGCCGCGGTAATACGTAGGGTGCGAGCGTTAATCGGAATTACTGGGCGTAAAGGGTGCGCAGGCGGTTGCGCAAGTCAGGCGTGAAATCCCCGGGCTTAACCTGGGAATGGCGCTTGAAACTACGTGACTGGAGTATGGCAGAGGGAGGTGGAATTCCACGTGTAGCGGTGAAATGCGTAGAGATGTGGAGGAACACCGATGGCGAAGGCAGCCTCCTGGGCCAATACTGACGCTCATGCACGAAAGCGTGGGGAGCAAACAGG` |
| ASV5305 | 424 | `TAAGGAATATTGGGCAATGGAGGCAACTCTGACCCAGCCATCCCGCGTGAAGGAATAAGGCCCTATGGGTTGTAAACTTCTTTTATCTGGGAAGAAATCTGGACATTCATGTGCAGTTGACGGTACCAGAGGAATAAGCACCGGCTAACTCCGTGCCAGCAGCCGCGGTAATACGGAGGGTGCAAGCGTTATCCGGAATCACTGGGTTTAAAGGGTGCGTAGGCGGTCCGATAAGTCAGAGGTGAAAGCTGGCAGCTTAACTGTCAAATTGCCTTTGATACTGTTGGACTTGAATCAGGTTGAGGTATGCAGAATGTGGCATGTAGCGGTGAAATGCATAGATATGCCATAGAATACCAATTGCGAAGGCAGCATGCTGGGCCTGAATTGACGCTGAGGCACGAAAGCGTGGGGATCAAACAGG` |
| ASV3311 | 429 | `TGGGGAATTTTGGACAATGGGCGAAAGCCTGATCCAGCCATTCCGCGTGCAGGATGAAGGCCCTCGGGTTGTAAACTGCTTTTGTACGGAACGAAACGGTCTTGGTTAATACCTGAGGCTAATGACGGTACCGTAAGAATAAGCACCGGCTAACTACGTGCCAGCAGCCGCGGTAATACGTAGGGTGCGAGCGTTAATCGGAATTACTGGGCGTAAAGCGTGCGCAGGCGGTGATGTAAGACAGATGTGAAATCCCCGGGCTTAACCTGGGAACTGCATTTGTGACTGCATCGCTAGAGTGCGGCAGAGGGGGATGGAATTCCGCGTGTAGCAGTGAAATGCGTAGATATGCGGAGGAACACCGATGGCGAAGGCAATCCCCTGGGCCTGCACTGACGCTCATGCACGAAAGCGTGGGGAGCAAACAGG` |
| ASV2025 | 404 | `TGGGGAATATTGGACAATGGGCGAAAGCCTGATCCAGCAATACCGCGTGAACGATGAAGGCCTTAGGGTTGTAAAGTTCTTTTAGTAGGGAAGATAATGACGTTACCTACAGAAAAAGCCCCGGCTAACTCCGTGCCAGCAGCCGCGGTAAGACGGAGGGGGCTAGCGTTATTCGGAATTACTGGGCGTAAAGGGCGCGTAGGCGGATTAGTAAGTTGGGAGTGAAATCCCGGGGCTCAACCTCGGAATTGCTTTCAAAACTGCTAGTCTAGAGTGAAGTAGGGGGTGATGGAATTCCTAGTGTAGAGGTGAAATTCTTAGATATTAGGAGGAACACCGGTGGCGAAGGCGGTCACCTGGACTTCAACTGACGCTGAGGCGCGAAAGCGTGGGGAGCAAACAGG` |
| ASV5161 | 424 | `TAAGGAATATTGGACAATGGGCGCAAGCCTGATCCAGCCATCCCGCGTGCAGGATGAAAGCCCTATGGGTTGTAAACTGCTTTTCTTTGGGAAGAAAACCTTGATTTCTATCGAGGCTGACGGTACCAGAGGAATAAGCACCGGCTAACTCCGTGCCAGCAGCCGCGGTAATACGGAGGGTGCAAGCGTTATCCGGATTTACTGGGTTTAAAGGGTGCGTAGGCGGGCTATTAAGTCAGTGGTGAAAGCTCCCGGCTCAACCGGGAAATTGCCATTGATACTGGTAGCCTTGAGTGCGGTTGAGGTGGACGGAATACAGCATGTAGCGGTGAAATGCTTAGATATGCTGTAGAACACCAATTGCGAAGGCAGTTCACCAAACCGTTACTGACGCTGAGGCACGAAAGCGTGGGGATCGAACAGG` |
| ASV647 | 424 | `TAAGGAATATTGGACAATGGAGGAAACTCTGATCCAGCCATGCCGCGTGAAGGATGAAGGCGCTCAGCGTTGTAAACTTCTTTTGGACGGGAACAAAAGCACTTATTTATAAGTGTCTGAGGGTACCGTCAGAATAAGCACCGGCTAACTCCGTGCCAGCAGCCGCGGTAATACGGAGGGTGCAAGCGTTATCCGGAATCACTGGGTTTAAAGGGTGCGTAGGCGGTCCGATAAGTCAGTCGTGAAATCGTGTGGCTTAACCATGCAACTGCGATTGATACTGTTGGACTTGAATCAGGTTGAGGTAGGCGGAATGTGACATGTAGCGGTGAAATGCATAGATATGTCATAGAACACCGATTGCGAAGGCAGCTTACTGGGCCTGAATTGACGCTGAGGCACGAAAGCGTGGGGATCAAACAGG` |
| ASV3302 | 404 | `TGGGGAATCTTGCGCAATGGGCGAAAGCCTGACGCAGCCATGCCGCGTGAATGATGAAGGTCTTAGGATTGTAAAATTCTTTCACCGGGGACGATAATGACGGTACCCGGAGAAGAAGCCCCGGCTAACTTCGTGCCAGCAGCCGCGGTAATACGAAGGGGGCTAGCGTTGCTCGGAATTACTGGGCGTAAAGGGAGCGTAGGCGGACAGTTAAGTCAGAGGTGAAAGCCCAGGGCTCAACCTTGGAATTGCCTTTGATACTGATTGTCTTGAGTATGGGAGAGGTAAGCGGAACTCCGAGTGTAGAGGTGAAATTCGTAGATATTCGGAAGAACACCAGTGGCGAAGGCGGCTTACTGGCCCATTACTGACGCTGAGGCTCGAAAGCGTGGGGAGCAAACAGG` |
| ASV1028 | 429 | `TGGGGAATTTTGGACAATGGGCGCAAGCCTGATCCAGCAATGCCGCGTGCAGGATGAAGGCCTTCGGGTTGTAAACTGCTTTTGTACGGAGCGAAACGGCTTCCACTAATACTGGGGGCTAATGACGGTACCGTAAGAATAAGCACCGGCTAACTACGTGCCAGCAGCCGCGGTAATACGTAGGGTGCGAGCGTTAATCGGAATTACTGGGCGTAAAGCGTGCGCAGGCGGTTATGTAAGACAGATGTGAAATCCCCGGGCTTAACCTGGGAACTGCATTTGTGACTGCATAGCTAGAGTACGGTAGAGGGGGATGGAATTCCGCGTGTAGCAGTGAAATGCGTAGATATGCGGAGGAACACCGATGGCGAAGGCAATCCCCTGGACCTGTACTGACGCTCATGCACGAAAGCGTGGGGAGCAAACAGG` |
| ASV3431 | 424 | `TAGGGAATATTGGACAATGGAGGGAACTCTGATCCAGCCATGCCGCGTGAACGATGAAGGCGCTCTGCGTTGTAAAGTTCTTTTAGATGGGGCGAAAAAGGGGGACTTGTCCTCAACTGACGGTACCATCGGAATAAGCACCGGCTAACTCCGTGCCAGCAGCCGCGGTAATACGGAGGGTGCAAGCGTTATCCGGAATCACTGGGTTTAAAGGGTGAGTAGGCGGGTAGATAAGTCAGAGGTGAAATGTGATTGCTTAACGATTAAATTGCCTTTGAAACTGTTTATCTTGAATCGAGATGAGGTATGCGGAATGAGACATGTAGCGGTGAAATGCATAGAGATGTCTTAGAACACCAATTGCGAAGGCAGCATACTGGGTTTGTATTGACGCTGAGGCACGAAAGCGTGGGGATCAAACAGG` |
| ASV2460 | 405 | `TGGGGAATATTGGACAATGGGCGAAAGCCTGATCCAGCGATTCTGCGTGAAGGATGAAGCCTCGTGGGGTGTAAACTTCTTTTCTGGGAGAAGACGAATGACGGTATCTCAGGAATAAGAGACGGCTAACTACGTGCCAGCAGCCGCGGTAATACGTAGGTCTCAAGCGTTATCCGGAATTACTGGGCGTAAAGCGTCTGTAGCCGGATGTATAAGTCTGTCTTTAAAGACTCTGATTCAATCGGAGGAAAGGGATAGATACTGTATATCTAGAGAATTTTGGGGGTTAGTGGAATTTCCGGTGGAGCGGTGAAATGCGTTGATATCGGAAGGAATGCCAAAAGCGAAAGCAGCTAACTACGAAACTTCTGACGGTCAGAGACGACAGCTTGGGGAGCAAACGGG` |
| ASV1630 | 416 | `TTAGGAATCTTCCCCAATGGGCGAAAGCCTGAGGGAGCGACGCCGCTTGAAGGATGAAGGTTTTCGGATCGTAAACTTCTGAAACAAGGACGAAATCCCTGCAAAGGGTCTGACGGTACTTGTGTAATAGCACCGGCTAACTCCGTGCCAGCAGCCGCGGTAATACGGAGGGTGCAAGCGTTACCCGGAATCACTGGGCGTAAAGGGCGTGTAGGCGGCTTGGTAAGTCTGGATTTAAAGACCGAAGCTCAACTTCGGGAATGGTCTGGATACTGCCTTGCTTGACGACTGGAGGGGAGAGTGGAATTCTCGGTGTAGCGGTGGAATGCGTAGATACCGAGAGGAACACCAGTGGCGAAGGCGGCTCTCTGGACAGTGCGTGACGCTGAGGCGCGAAAGTGTGGGGAGCGAACCGG` |
| ASV1990 | 428 | `TCGAGAATCATTCACAATGGGCGAAAGCCTGATGGTGTGACGCCGCGTGGAGGATGAAGGTCTTCGGATTGTAAACTCCTGTCATGTGAGAGTAAAGCCGGGCGTTAACTGCGTGCCGGTTTGAGAGTATCACAAGAGGAAGAGACGGCTAACTCTGTGCCAGCAGCCGCGGTAATACAGAGGTCTCAAGCGTTGTTCGGAATCACTGGGCGTAAAGGGTGCGTAGGCTGCACGGTAAGTCAGAGGTGAAATCCCGGAGCTCAACTTCGGAATTGCCTCCGATACTGCCGTGCTTGAGGACTGGAGAGGAGATTGGAATTCACAGTGGAGCAGTGAAATGCGTAGATATTGTGAGGAACACTAGTGGCGAAGGCGAATCTCTGGACAGTTCCTGACGCTGAGGCACGAAGGCCAGGGGAGCAAACGGG` |
| ASV5055 | 404 | `TGGGGAATTTTGGACAATGGGCGCAAGCCTGATCCAGCGACGCCGCGTGGACGATGAAGGCCTTCGGGTTGTAAAGTCCTTTCGGTAGGAAAGATTATGACGGTACCTACAGAAGAAGCTCCGGCTAACTTCGTGCCAGCAGCCGCGGTAATACGAAGGGAGCTAGCGTTACTCGGAATTATTGGGCGTAAAGCGTGCGTAGGCGGCTAAGAAAGTTGGAAGTGAAAGCCCAGGGCTCAACCCTGGAATTGCTTCCAAAACTCCTTAGCTTGAGTATCAGAGGGGATAGTGGAATTCCTAGTGTAGAGGTGAAATTCGTAGATATTAGGAGGAACACCGGTGGCGAAAGCGACTATCTGGCTGATTACTGACGCTGAGGTGCGAAAGCGTGGGGAGCAAACAGG` |
| ASV4953 | 404 | `TGGGGAATATTGGACAATGGGCGAAAGCCTGATCCAGCAATGCCGCGTGAATGATGAAGGCCTTAGGGTTGTAAAGTTCTTTCGACGGGGAAGATAATGACGGTACCCGTAGAAGAAGCCCCGGCTAACTTCGTGCCAGCAGCCGCGGTAATACGAAGGGGGCTAGCGTTGTTCGGAATTACTGGGCGTAAAGGGCGCGTAGGCGGTTTGGTAAGTTAGGAGTGAAATGCCGGGGCTTAACCTCGGAACTGCTCTTAAGACTGCCTGACTAGAGTTCGGGAGAGGATGGCGGAATTCCTAGTGTAGAGGTGAAATTCGTAGATATTAGGAGGAACACCGGTGGCGAAGGCGGCCATCTGGACCGATACTGACGCTGAGGCGCGAAAGCGTGGGGAGCAAACAGG` |
| ASV3732 | 424 | `TTTCGAATCATTCACAATGGGGGCAACCCTGATGGTGCGACGCCGCGTGGAGGATGAAGGTCTTCGGATTGTAAACTCCTGTCAAGGGGGACTAACCACATGGCTCATACCCATGTCTGCATTAACCCCTAGAGGAAGCAGTGGCTAACTCTGTGCCAGCAGCCGCGGTAATACAGAGACTGCAAGCGTTACTCGGATTCACTGGGCGTAAAGGGAGCGCAGGCGGATTGGTGTGTTGGATGTGAAATCCCGGGGCTCAACTCCGGGGCTGCGTCCAAAACTACCGATCTAGAGATTCGGAGGGGCTAGTGGAATTCCTGGTGGAGCAGTGAAATGCGTAGATATCAGGAGGAACACCAACGGCGAAGGCAGCTAGCTGGACGAAATCTGACGCTCAGGCTCGAAAGTATGGGGAGCAAAAAGG` |
| ASV5675 | 407 | `CCGAGAATATTGGACAATGGGCGAAAGCCTGATCCAGCGACATTGCGTGGAGGATGAAGACGTTCGCGCCGTAAACTCCTTTTATAGGGAAAGAAGTAATTGACGGTACCCTATGAATAAGCGGGGGCTAATTCTGTGCCAGCAGCAGCGGTAATACAGAACCCGCAAGCGTTATCCGGATTTATTGGGCGTAAAGTGTTCGTAGGCGGTTCGGAAAGTCTGATATCAAATATCCGGGCTCAACCCGGGACGCGTATCGGAAACTTCCAAACTTGAAGGTGCTAGGGGCTATCGGAACGCACGGTGTAGGGGTGAAATCCGTTGATATCGTGCGGAACACCAAAAGCGAAGGCAGATAGCTGGGGCATTCTTGACGCTGAGGAACGAAAGCGTGGGGAGCAAAAAGG` |
| ASV4 | 407 | `TGGGGAATTTTCCGCAATGGGCGAAAGCCTGACGGAGCAATCTCGCGTGAGGGATGACTGCCTATGGGTTGTAAACCTCTTTTTTCAGGGAGGAATAAATGACGTGTACCTGAAGAATAAGCATCGGCTAACTCCGTGCCAGCAGCCGCGGTAAGACGGAGGATGCAAGTGTTATCCGGAATCACTGGGCGTAAAGCGTCTGTAGGTGGTTTGATAAGTCAACTGTTAAATCTTGAAGCTCAACTTCAAAATCGCAGTCGAAACTATCAGACTAGAGTATAGTAGGGGTAAAGGGAATTTCCAGTGGAGCGGTGAAATGCGTAGAGATTGGAAAGAACACCGATGGCGAAGGCACTTTACTGGGCTATTACTGACACTCAGAGACGAAAGCTAGGGTAGCAAATGGG` |
| ASV904 | 429 | `TGGGGAATTTTGGACAATGGACGCAAGTCTGATCCAGCCATGCCGCGTGCGGGAAGAAGGCCTTCGGGTTGTAAACCGCTTTTGTCAGGGAAGAAAAGGGTGTTTCTAATAAAGATGCCTCATGACGGTACCTGAAGAATAAGCACCGGCTAACTACGTGCCAGCAGCCGCGGTAATACGTAGGGTGCAAGCGTTAATCGGAATTACTGGGCGTAAAGCGTGCGCAGGCGGTTATGCAAGACAGATGTGAAATCCCCGGGCTCAACCTGGGAACTGCATTTGTGACTGCATGGCTAGAGTACGGTAGAGGGGGATGGAATTCCGCGTGTAGCAGTGAAATGCGTAGATATGCGGAGGAACACCGATGGCGAAGGCAATCCCCTGGACCTGTACTGACGCTCATGCACGAAAGCGTGGGGAGCAAACAGG` |
| ASV2678 | 406 | `TGAGGAATATTCTGCAATGGGCGAAAGCCTGACAGAGCAATACCGCGTGAAGGATGAAAGCCTATTGGTTGTAAACTTCTTTTCTCAAAGACGAAGATCTGACATTATTTGAGGAATAAGCATCGGCTAACCCTGTGCCAGCAGCCGCGGTAATACAGGGGATGCAAGTGTTATCCGGAATCATTGGGCGTAAAGCGTTTGTAGGTGGATATTTAAGTCTTATGTTAAATCTCTAAGCTTAACTTAGAACAGGCAAAAGATACTGAATATCTTGAGTATGGTAGAGGTAAAGGGAATTTTCAGTGGAGCGGTGAAATGCGTAGATATTGAAAAGAACACCAATAGCGAAAGCACTTTACTGGGCCATCACTGACACTCAGAAACGAAAGCTGGGGGAGCAAACAGG` |
| ASV5474 | 430 | `TGGGGAATATTGCGCAATGGCCGAAAGGCTGACGCAGCGACGCCGCGTGTGGGAGGAAGCCCTTCGGGGTGTAAACCACTGTCAGGGGGGACGAATCGCGAGCGGGCAAATAGCCCGAGCGCTTGACGGTACCTCCAAAGGAAGCACCGGCTAACTCCGTGCCAGCAGCCGCGGTAAGACGGAGGGTGCAAGCGTTGTTCGGAATTACTGGGCGTAAAGCGCGGGCAGGTGGTCCTTTAAGTCCTTTGTGAAATCCCGGGGCTCAACTCCGGATGTGCATAGGATACTGGAGGACTGGAGACTGGTAGAGGCGAGCGGAATTCCTGGTGTAGCGGTGGAATGCGTAGATATCAGGAAGAACACCGGTGGCGAAGGCGGCTCGCTGGGCCAGTTCTGACACTCATCCGCGAAAGCGTGGGGAGCAAACAGG` |
| ASV3234 | 407 | `TGGGGAATTTTCCGCAATGGGCGCAAGCCTGACGGAGCAAGACCGCGTGAGGGAAGAAGGTCTGTGGATTGTAAACCTCTTTTGATGGGGAAGAAGAAAGTGACGGTACCCATCGAATCAGCCTCGGCTAACTCCGTGCCAGCAGCCGCGGTAATACGGAGGAGGCAAGCGTTATCCGGAATTATTGGGCGTAAAGAGTCCGTAGGTGGGTTATCAAGTCAGCTGTTAAATAGTGAAGCTTAACTTCATAAAGGCGGCTGAAACTGATAGTCTAGAGTGTGGTAGGGGTAGAGGGAATTTCCAGTGTAGCGGTGAAATGCGTAGATATTGGAAAGAACACCGGTGGCGAAAGCGCTCTACTGGGCCTGCACTGACACTGAGGGACGAAAGCTAGGGGAGCGAAAGGG` |
| ASV4471 | 406 | `TGAGGAATCTTCCGCAATGGGCGAAAGCCTGACGGAGCAACACCGCGTGAAGGATGAAGTGGTTCGCCATGTAAACTTCTTTTCTGAGGGAAGAATTCTGACGGTACCTTAGGAATAAGCACCGGCTAACTCTGTGCCAGCAGCCGCGGTAAGACAGAGGGTGCGAGCGTTATCCGGATTTATTGGGCGTAAAGCGTGTGTAGGCGGATTGTTAAGTCGGGGATTAAATTTCCAAGCCCAACTTGGAAAGTGTTCCCGAAACTGGCAGTCTTGGAAGAATGCAGAGGCGCGTGGAATTCTGAGTGTAGGGGTAAAATCCGTAGATACTCGGAGGAACACCAAAAGCGAAAGCAGCGCGCTGGGCATTTCTTGACGCTGAGACACGAAAGCGTGGGTAGCAAACAGG` |
| ASV950 | 429 | `TGGGGAATATTGCACAATGGGCGCAAGCCTGATGCAGCCATGCCGCGTGTGTGAAGAAGGCCTTCGGGTTGTAAAGCACTTTCAGCGAGGAGGAAGGGTTCAGTGTTAATAGCACTGTTCATTGACGTTACTCGCAGAAGAAGCACCGGCTAACTCCGTGCCAGCAGCCGCGGTAATACGGAGGGTGCAAGCGTTAATCGGAATTACTGGGCGTAAAGCGCACGCAGGCGGTTTGTTAAGTCAGATGTGAAATCCCCGAGCTTAACTTGGGAACTGCATTTGAAACTGGCAAGCTAGAGTCTTGTAGAGGGGGGTAGAATTCCAGGTGTAGCGGTGAAATGCGTAGAGATCTGGAGGAATACCGGTGGCGAAGGCGGCCCCCTGGACAAAGACTGACGCTCAGGTGCGAAAGCGTGGGGAGCAAACAGG` |
| ASV2913 | 429 | `TGGGGAATTTTGGACAATGGGCGCAAGCCTGATCCAGCAATGCCGCGTGTGTGAAGAAGGCCTTCGGGTTGTAAAGCACTTTTGGCGGGAACGAAACGGTTGGGGCTAATATCCCTGGCTAATGACGGTACCCGCAGAATAAGCACCGGCTAACTACGTGCCAGCAGCCGCGGTAATACGTAGGGTGCAAGCGTTAATCGGAATTACTGGGCGTAAAGCGTGCGCAGGCGGTTATGCAAGACCGATGTGAAATCCCCGGGCTCAACCTGGGAATTGCATTAGTGACTGCATGGCTAGAGTGTGTCAGAGGGGGGTAGAATTCCACGTGTAGCAGTGAAATGCGTAGAGATGTGGAGGAATACCGATGGCGAAGGCAGCCCCCTGGGATAACACTGACGCTCATGCACGAAAGCGTGGGGAGCAAACAGG` |
| ASV975 | 424 | `TGAGGAATATTGGACAATGGGCGCAAGCCTGATCCAGCCATGCCGCGTGCAGGATGACGGTCCTATGGATTGTAAACTGCTTTTGTACAGGAAGAAACCTCCCGACGTGTCGGGAATTGACGGTACTGTAAGAATAAGGATCGGCTAACTCCGTGCCAGCAGCCGCGGTAATACGGAGGATCCAAGCGTTATCCGGAATCATTGGGTTTAAAGGGTCCGTAGGCGGTTTAATAAGTCAGTGGTGAAAGCCCATCGCTCAACGGTGGAACGGCCATTGATACTGTTAGACTTGAATTATAAGGAAGTAACTAGAATATGTAGTGTAGCGGTGAAATGCTTAGATATTACATGGAATACCAATTGCGAAGGCAGGTTACTACTTATTGATTGACGCTGATGGACGAAAGCGTGGGTAGCGAACAGG` |
| ASV5580 | 390 | `TCGGGAAAACTTCGCAATGCGCGAAAGCGTGACGAGGGGATTCAGAGTGCTCAGGAATTAATTCCTGGGCTTTTGTCAAGGGCAAATACCTTGGCGAATAAGTGGTGGGCAAGACTGGTGCCAGCCGCCGCGGTAACCCCAGCGCCACAAGTGGGAACCACTTTTATTGGGTCTAAAGCATTCGTAGCCGGTTCGGCAAATGCCTTGTGAAATGGTTGCGCTCAACGTAACCACGTGCAAGGTAGACTACCGAACTAGGGACCAGAAGATGTTAGCAGTATTCCTGGGGGAACGGTAAAATGTTATAATCCCAGGAGGACTACCGATGGCGAAGGCAGCTAACAAGTATGGATCCGACGGTGAGGAATGAAAGCTAGGGGAGCGATCCGG` |
| ASV3619 | 404 | `TGGGGAATATTGGACAATGGGGGCAACCCTGATCCAGCCATGCCGCGTGGGTGATGAAGGCCCTAGGGTTGTAAAGCCCTTTCGGCGGGGAAGATAATGACGGTACCCGCAGAAGAAGCCCCGGCTAACTTCGTGCCAGCAGCCGCGGTAATACGAAGGGGGCTAGCGTTGCTCGGAATCACTGGGCGTAAAGCGCACGTAGGCGGATTCCTAAGTCGGGGGTGAAATCCTGGAGCTCAACTCCAGAACTGCCTTCGATACTGGGAATCTTGAGTCCGGGAGAGGTGAGTGGAACTGCGAGTGTAGAGGTGAAATTCGTAGATATTCGCAAGAACACCAGTGGCGAAGGCGGCTCACTGGCCCGGTACTGACGCTGAGGTGCGAAAGCGTGGGGAGCAAACAGG` |
| ASV2278 | 424 | `TGAGGAATATTGGTCAATGGGCGCAAGCCTGAACCAGCCATGCCGCGTGCAGGATGACGGTCCTATGGATTGTAAACTGCTTTTGTACGGGAAGAAACACTGATTCGTGAATCAGCTTGACGGTACCGTAAGAATAAGGATCGGCTAACTCCGTGCCAGCAGCCGCGGTAATACGGAGGATCCAAGCGTTATCCGGAATCATTGGGTTTAAAGGGTCCGTAGGCGGTTTGGTAAGTCAGTGGTGAAAGCCCATCGCTCAACGGTGGAACGGCCATTGATACTGCTAAACTTGAATTATTGGGAAGTAACTAGAATATGTAGTGTAGCGGTGAAATGCTTAGAGATTACATGGAATACCAATTGCGAAGGCAGGTTACTACCAATTGATTGACGCTGATGGACGAAAGCGTGGGTAGCGAACAGG` |
| ASV3417 | 428 | `TGGGGAATATTGGACAATGGGCGCAAGCCTGATCCAGCCACGCCGCGTGAGTGATGAAGGCCTTCGGGTCGTAAAGCTCTGTGGGGAGGGACGAACCGCTGCACGTTCATAGCGTGCAGCATGACGGTACCTCCTTAGCAAGCACCGGCTAACTTCGTGCCAGCAGCCGCGGTAATACGAAGGGTGCAAACGTTGCTCGGAATTATTGGGCGTAAAGCGCACGTAGGCGGCCCGACAAGTCGGATGTGAAATCCCTCGGCTCAACCAAGGAAGTGCATCCGAAACTGTCAGGCTTGAGTACGAAAGAGGATCGCGGAATTCCCGGTGTAGAGGTGAAATTCGTAGATATCGGGAGGAACACCAGTGGCGAAGGCGGCGATCTGGGTCGATACTGACGCTGAGGTGCGAAAGCGTGGGGAGCAAACAGG` |
| ASV3640 | 404 | `TGGGGAATATTGGGCAATGGACGAAAGTCTGACCCAGCCATGCCGCGTGAATGATGAAGGCTTTAGGGTTGTAAAGTTCTTTCACCGATGAAGATAATGACGGTAGTCGGAGAAGAAGCCCCGGCTAACTTCGTGCCAGCAGCCGCGGTAATACGAAGGGGGCTAGCGTTGTTCGGAATTACTGGGCGTAAAGCGAGCGTAGGCTGCTCGGAAAGTCAGTGGTGAAATCCCAGAGCTTAACTCTGGAACTGCCATTGAAACTTCCGAGCTGGAATCTCAGAGGGGGTAGCGGAATTCCAAATGTAGGGGTGAAATCCGTAGATATTTGGAGGAACACCGGTGGCGAAGGCGGCTACCTGGATGAGTATTGACGCTGAGGCTCGAAAGCGTGGGGATCAAACAGG` |
| ASV4982 | 424 | `TAAGGAATATTGGACAATGGTCGCAAGACTGATCCAGCCATGCCGCGTGAAGGATGACGGCCCTATGGGTTGTAAACTTCTTTTGAACGGGAGAAAACCCCTTTACGTGTAAAGGGCTGATAGTACCGTTAGAATAAGGATCGGCTAACTCCGTGCCAGCAGCCGCGGTAATACGGAGGATCCAAGCGTTATCCGGATTCATTGGGTTTAAAGGGTGCGTAGGCGGAATGATAAGTCAGTGGTGAAATCTCTTCGCTTAACGAAGAAACTGCCATTGATACTGTCAATCTTGAGTACATTTGATGTGGGCGGAATGTGTCATGTAGCGGTGAAATGCTTAGATATGACACAGAACACCGATTGCGAAGGCAGCTCACAAAACTGTAACTGACGCTGAGGCACGAAAGCGTGGGTATCAAACAGG` |
| ASV3214 | 424 | `TAAGGAATATTGGTCAATGGACGAAAGTCTGAACCAGCCATCCCGCGTGCAGGATGACTGCCCTATGGGTTGTAAACTGCTTTTAGTAGAGAAGAAACGCAGTCATTTATGATTGCCTGACGGTACCTACAGAATAAGCACCGGCTAACTCCGTGCCAGCAGCCGCGGTAATACGGAGGGTGCGAGCGTTATCCGGAATCACTGGGTTTAAAGGGTGCGTAGGCGGGGAATTAAGTCAGTGGTGAAATCTTGTCGCTTAACGATAAAATTGCCATTGATACTGATTTTCTTGAATTGGGTTGAGGTTAGCGGAATGTGACATGTAGCGGTGAAATGCATAGATATGTCATGGAACACCAATTGCGAAGGCAGCTAGCTGGGCCTTGATTGACGCTGAGGCACGAAAGCGTGGGTAGCGAACAGG` |
| ASV3349 | 407 | `TAGGGAATATTGCGCAATGGGGGAAACCCTGACGCAGCGACGCCGCGTGAGTGACGAAGGCCTTAGGGTCGTAAAGCTCTGTTGAATGGGAAGAAACAAATGACGGTACCATTCGAGAAAGGACCGGCTAACTTCGTGCCAGCAGCCGCGGTAAGACGAGGGGTCCTAGCGTTGTTCGGAATCATTGGGCGTAAAGCGTGTGTAGGCTGCCATTCAAGTCAGTTGTGAAATCCCCGGGCTTAACCCGGGAAGTGCTTCTGATACTGTTTGGCTTGAGTACTGGAGAGGATAGTAGAATTCCTGGTGTAGTGGTGAAATACGTAGATATCAGGAGGAATACCGGTGGCGAAGGCGGCTATCTGGCCATGTACTGACGCTGAGACACGAAAGCGTGGGGAGCAAACAGG` |
| ASV1937 | 429 | `TGGGGAATTTTGGACAATGGACGCAAGTCTGATCCAGCAATGCCGCGTGCAGGACGAAGGCCTTCGGGTTGTAAACTGCTTTTGTACGGAACGAAACGGTGAGCTTTAATACAGTTTGCTAATGACGGTACCGTAAGAATAAGCACCGGCTAACTACGTGCCAGCAGCCGCGGTAATACGTAGGGTGCGAGCGTTAATCGGAATTACTGGGCGTAAAGCGTGCGCAGGCGGTTATATAAGACAGATGTGAAATCCCCGGGCTCAACCTGGGACCTGCATCTGTGACTGTATAGCTAGAGTACGGTAGAGGGGGATGGAATTCCGCGTGTAGCAGTGAAATGCGTAGATATGCGGAGGAACACCGATGGCGAAGGCAATCCCCTGGACCTGTACTGACGCTCATGCACGAAAGCGTGGGGAGCAAACAGG` |
| ASV5120 | 405 | `TTGGGAATCTTGCGCAATGGGGGAAACCCTGACGCAGCGACGTCGCGTGGACGATGAAGGCCTTAGGGTTGTAAAGTCCTTTTTTCAGGAAAGACTTAGGACGGTACCTGAAGAATAAGCTCCGGCTAACTACGTGCCAGCAGCCGCGGTAAGACGTAGGGAGCAAGCGTTGTCCGGATTTACTGGGCGTAAAGAGCGCGTAGGCGGTCTGTTAAGTGAGAAGTGAAATCTCCCTGCTCAACAGGGAAACTGCTTTTCATACTGGCAGACTAGAGGAATGGAGAGGTAACTGGAATTCCTGGTGTAGCGGTGAAATGCGTTGATATCAGGAGGAACACCCATGGCGAAGGCAGGTTACTGGCCATTATCTGACGCTGAGGCGCGAAAGCGTGGGTAGCAAACAGG` |
| ASV4185 | 424 | `TGAGGAATATTGGTCAATGGAGGCAACTCTGAACCAGCCATGCCGCGTGCAGGATGACGGTCCTATGGATTGTAAACTGCTTTTGTACAGGAAGAAAAAGTACCACGTGTGGTATATTGACGGTACTGTAAGAATAAGGATCGGCTAACTCCGTGCCAGCAGCCGCGGTAATACGGAGGATCCAAGCGTTATCCGGAATCATTGGGTTTAAAGGGTCCGTAGGCGGCTTTATAAGTCAGTGGTGAAAGTTTGCAGCTTAACTGTAAAATTGCCATTGATACTGTAGAGCTTGAATTTTTGTGAAGTAACTAGAATATGTAGTGTAGCGGTGAAATGCTTAGATATTACATGGAATACCAATTGCGAAGGCAGGTTACTAACAAACAATTGACGCTGATGGACGAAAGCGTGGGTAGCGAACAGG` |
| ASV3627 | 424 | `TAAGGAATATTGGTCAATGGAGGCAACTCTGAACCAGCCATGCCGCGTGCAGGAAGACAGCCCTATGGGTCGTAAACTGCTTTTATACGTGAATAAACCTTGATTCGTGAATCAAGCTGAATGTACCGTAAGAATAAGGATCGGCTAACTCCGTGCCAGCAGCCGCGGTAATACGGAGGATCCAAGCGTTATCCGGATTTATTGGGTTTAAAGGGAGCGTAGGCGGCCTATTAAGTCAGGGGTGAAAGACGGTGGCTCAACCATCGCAGTGCCTTTGATACTGATGGGCTTGAATATAGTTGAGGTAGGCGGAATGTGACAAGTAGCGGTGAAATGCATAGATATGTCACAGAACACCGATTGCGAAGGCAGCTTACTAAGCTATTATTGACGCTGAGGCTCGAAAGCGTGGGGATCAAACAGG` |
| ASV5512 | 429 | `TGGGGAATATTGGACAATGGGGGAAACCCTGATCCAGCAATACCATGTGTGTGAAGAAGGCCTTAGGGTTGTAAAGCACTTTAGTTGGGGAGGAAAGCAATTAGGTTAAGAGCTAAGTTGTGTGACGTTACCCAAAGAATAAGCACCGGCTAACTCTGTGCCAGCAGCCGCGGTAATACAGAGGGTGCAAGCGTTAATCGGAATTACTGGGCGTAAAGGGTCTGTAGGTGGTTAAATAAGTCAGATGTGAAATCCCCGAGCTTAACTTAGGAATGGCATTTGATACTGTTTGGCTAGAGTTTGGTAGAGGATTGGGGAATTTCTGGTGTAGCGGTGAAATGCGTAGATATCAGAAGGAACATCAATGGCGAAGGCAACAATCTGGACCTGAACTGACACTGAGGGACGAAAGCATGGGGAGCAAACAGG` |
| ASV5079 | 430 | `TGGGGAATCTTCCGCAATGGACGAAAGTCTGACGGAGCAACGCCGCGTGAGTGAAGAAGGTTTTCGGATCGTAAAACTCTGTTTTCAGGGACGAATGTGCGATGTGTAAATAATGCATTGTAATGACGGTACCTGAGTAGAAAGCCACGGCTAACTACGTGCCAGCAGCCGCGGTAATACGTAGGTGGCAAGCGTTGTCCGGAATTATTGGGCGTAAAGCGCGCGCAGGTGGGATATTAAGTCTGGTGTTAAAGTTCGGGGCTCAACCCCGTGAAGCATTTGGAAACTGATATTCTTGAGTGCAGGAGAGGAAAGTGGAATTCCTAGTGTAGCGGTGAAATGCGTAGATATTAGGAGGAACACCAGTGGCGAAGGCGACTTTCTGGACTGTGTCTGACACTGAGGCGCGAAAGCCAGGGTAGTGAACGGG` |
| ASV2655 | 429 | `TCGAGAATAATTCACAATGGGGGCAACCCTGATGGTGCAACGCCGCGTGGAGGATGACGGTCTTCGGATTGTAAACTCCTGTCATCTGGGAGTAAGACCTGGCTGTGAATAGCAGACAGGGTTGATAGTACCAGAAGAGGAAGGGACGGCTAACTTCGTGCCAGCAGCCGCGGTAATACGAAGGTCCCGAGCGTTGTTCGGAATCACTGGGCGTAAAGGGAGCGTAGGCGGCGCGGTAAGTCAGATGTGAAATCCCGGGGCTCAACCCCGGAACTGCATCCGATACTGCCGTGCTCGAGCATTGGAGAGGTAGCTGGAATTCTTGGTGTAGCAGTGAAATGCGTAGAGATCAAGAGGAACACTCGTGGCGAAAGCGAGCTACTGGACAATTGCTGACGCTGAGGCTCGAAGGCCAGGGTAGCGAAAGGG` |
| ASV2185 | 404 | `TGAGGAATCTTCCGCAATGGGCGAAAGCCTGACGGAGCGACACCGCGTGAAGGATGAAGCGTTTACTCGTGTAAACTTCTTTTCTGTGGGAAGATAATGACGGTACCACAGGAATAAGGGGCGGCAAACTACGTGCCAGCAGCCGCGGTAATACGTAGGCCCCAAGCGTTATCCGGAATTACTGGGTGTAAAGGGTTTGTAGGCGGGACGGAAAGTCAGATGTGAAAGGTATGGGCTCAACCCATTACTTGCATCCGAAACTCCCGTTCTTGAATTAGGGAGAGGTAAGCAGAATCGTATGAGTAGGGGTGCAATCCGTTGATACATACGAGAATACCAAAAGCGAAGGCAGCTTACTGGAACTATATTGACGCTGAGGAACGAAAGCGTGGGGAGCGAAAAGG` |
| ASV3408 | 429 | `TGGGGAATTTTGGACAATGGGGGCAACCCTGATCCAGCCATGCCGCGTGAGTGAAGAAGGCCTTCGGGTTGTAAAGCTCTTTCAGACGGAAAGAAAACGGATAAGCTAATACCCTATCTGGATGACGGTACCGTCAGAAGAAGCACCGGCTAACTACGTGCCAGCAGCCGCGGTAATACGTAGGGTGCGAGCGTTAATCGGAATTACTGGGCGTAAAGCGTGCGCAGGCGGTTGTGTAAGACAGATGTGAAAGCCCCGGGCTTAACCTGGGAACTGCGTTTGTGACTGCACAGCTAGAGTACGGCAGAGGGGGGTGGAATTCCACGTGTAGCAGTGAAATGCGTAGATATGTGGAGGAACACCGATGGCGAAGGCAGCCCCCTGGGCCTGTACTGACGCTCATGCACGAAAGCGTGGGGAGCAAACAGG` |
| ASV4750 | 430 | `TGGGGAGTATTGCACAATGGGGGAAACCCTGATGCAGCAACGCCGCGTGAGTGAAGAAGGCCCTTGGGTTGTAAAGCTCTTTCGGCAGGGAAGAAGGGCGGCTAGGTAAACAATCTAGTTGTTTGACGGTACCTGAAGAAGAAGCATCGGCTAACTTCGTGCCAGCAGCCGCGGTAATACGAAGGATGCAAGCGTTGTTCGGAATTATTGGGCGTAAAGCGCGCGTAGGCGGGTTCGTAAGTCGATTGTGAAATCCCTGGGCTTAACCTGGGAATTGCAGTCGAAACTACTTATCTTGAATGCTAGAGAGGGTGGCGGAATTCCTGGTGTAGAAGTGAAATTCGTAGATATCAGGAGGAACACCGGCGGCGAAGGCGGCTACCTGGCTATGCATTGACGCTGAGGCGCGAAAGCGTGGGGAGCAAACAGG` |
| ASV5515 | 429 | `TGGGGAATTTTGGACAATGGGGGCAACCCTGATCCAGCAACGCCGCGTGTCTGATGAAGGCCTTAGGGTTGTAAAAGACTTTTGTTGGGGAGCAAAACTTGCCTAATAACACTGGGTGAGGATGAGAGTACCCAAAGAATAAGCACCGGCTAACTACGTGCCAGCAGCCGCGGTAATACGTAGGGTGCAAGCGTTAATCGGAATGACTGGGCGTAAAGGGTGCGCAGGCGGTTGTACAAGTTTGGTGTGAAATCCTCGGGCTTAACCTGAGAACTGCATTGAAGACTGTACGGCTAGAATGTGTCAGAGGGGGGTAGAATTCCACGTGTAGCAGTGAAATGCATAGAGATGTGGAGGAATACCAATGGCGAAGGCAGCCCCCTGGGATAACATTGACGCTCATGCACGAAAGCGTGGGGAGCAAACAGG` |
| ASV1219 | 429 | `TGGGGAATATTGGACAATGGGGGGAACCCTGATCCAGCAATGCCGCGTGTGTGAAGAAGGCCTGCGGGTTGTAAAGCACTTTCAGTGGGGAGGAGGGATGATTGGTTAAGAGCTGATTATTTGGACGTTACCCACAGAAGAAGCACCGGCTAACTCCGTGCCAGCAGCCGCGGTAATACGGAGGGTGCGAGCGTTAATCGGAATTACTGGGCGTAAAGGGTGCGTAGGTGGTTTAATAAGTTAAGTGTGAAAGCCCTGGGCTCAACCTAGGAAGGCCATTTAAGACTGTTAGACTTGAGTGTAGGAGAGGGTAGTGGAATTTCCGGTGTAGCGGTGAAATGCGTAGAGATCGGAAGGAACACCAGTGGCGAAGGCGGCTACCTGGCCGGACACTGACACTGAAGCACGAAAGCGTGGGGAGCAAACAGG` |
| ASV3469 | 430 | `TGGGGAATCTTGCGCAATGGGCGAAAGCCTGACGCAGCGACGCCGTGTGGGGGACGAAGGCCTTCGGGTTGTAAACCCCTGTCGGGTGGAACGAATCCCCCGAGGACGAATACTCCTCGGGGTTGACGGTACCGCCGGAGGAAGCCCCGGCTAACTACGTGCCAGCAGCCGCGGTAATACGTAGGGGGCGAGCGTTGCTCGGATTTACTGGGCGTAAAGAGCGCGTAGGCGGCCAGGGAAGTCTGGTGTGAAATCCCTCGGCTCAACCGAGGACCCGCATTGGAGACTCCCTGGCTTGAGTCCAGGAGGGGAGGGTGGAATTCCCGGTGTAGCGGTGAAATGCGTAGAGATCGGGAGGAACACCGGTGGCGAAGGCGGCCCTCTGGAATGGTACTGACGCTGAGGCGCGAAAGCTGGGGGAGCAAACGGG` |
| ASV3686 | 424 | `TGAGGAATATTGGTCAATGGACGAGAGTCTGAACCAGCCAAGTCGCGTGAAGGATGACTGCCCTACGGGTTGTAAACTTCTTTTGTATGGGAGTAAAAAAGTCTACGAGTAGACTATTGCAAGTACCATACGAATAAGCATCGGCTAACTCCGTGCCAGCAGCCGCGGTAATACGGAGGATGCAAGCGTTATCCGGATTTATTGGGTTTAAAGGGTGCGTAGGCGGGACAATAAGTCAGCGGTGAAAGTTTGAGGCTCAACCTTAAAATTGCCGTTGAAACTGTTATTCTTGAGTATATTTGAAGTAAGCGGAATTCGTGGTGTAGCGGTGAAATGCATAGATATCACGAAGAACTCCAATTGCGAAGGCAGCTTACTAAACTATAACTGACGCTGAAGCACGAAAGCGTGGGTATCAAACAGG` |
| ASV5395 | 430 | `TGGGGAATCTTGCGCAATGGACGAAAGTCTGACGCAGCCACGCCGCGTGAGTGAAGAAGGCCTTCGGGTTGTAAAGCTCTGTCGCCCGGGACGAAAATGCATTGGGTTAACAGCCCAGTGAACTGACGGTACCGGGAGAGGAAGCACCGGCTAACTTCGTGCCAGCAGCCGCGGTAATACGAGGGGTGCAAGCGTTGCTCGGAATTATTGGGCGTAAAGGGTAGGTAGGTGGTCTTATTTGTCTGGGGTGAAAGCCTTGAGCTTAACTCAAGAAGTGCCCCAGAAACGGTAAGACTGGAGTCCTGGAGAGGCTAGCGGAATTCCCGGTGTAGCGGTGAAATGCGTAGAGATCGGGAGGAACACCAGAGGCGAAGGCGGCTAGCTGGACAGGCACTGACACTCAACTACGAAAGCGTGGGGAGCAAACAGG` |
| ASV628 | 404 | `TGGGGAATATTGGACAATGGGGGAAACCCTGATCCAGCAATGCCGCGTGAGTGATGAAGGCCTTCGGGTTGTAAAGCTCTTTTGTCAGGGACGATGATGACGGTACCTGACGAATAAGCTCCGGCTAACTTCGTGCCAGCAGCCGCGGTAATACGAGGGGAGCTAGCGTTGTTCGGAATTACTGGGCGTAAAGGGCGCGTAGGCGGTACTGTAAGTCAGATGTGAAAGCCCCGGGCTTAACCTGGGAGGTGCATTTGAAACTGCAGAACTTGAGATCGAGAGAGGAAAGTGGAATTACGAGTGTAGAGGTGAAATTCGTAGATATTCGTAAGAACACCAGTGGCGAAGGCGACTTTCTGGCTCGATACTGACGCTAAGGCGCGAAAGCGTGGGGAGCAAACAGG` |
| ASV1158 | 404 | `TGGGGAATATTGGACAATGGGGGCAACCCTGATCCAGCCATGCCGCGTGAGTGATGAAGGCCTTCGGGTTGTAAAGCTCTTTTGGCGGGGACGATGATGACGGTACCCGCAGAATAAGCCCCGGCTAACTTCGTGCCAGCAGCCGCGGTAAGACGAAGGGGGCTAGCGTTGTTCGGAATTACTGGGCGTAAAGCGAGTGTAGGTGGTTGTCCAAGTTGGATGTGAAAGCCTTGAGCTCAACTCAAGAAATGCATTCAGGACTGGATGACTAGAGGACCGGAGAGGATAGTGGAATTCCCAGTGTAGTGGTGAAATACGTAGAGATTGGGAAGAACACCAGTGGCGAAGGCGGCTATCTGGACGGTTTCTGACACTAAGACTCGAAAGCGTGGGGAGCAAACAGG` |
| ASV4778 | 429 | `TGGGGAATATTGGACAATGGGCGAAAGCCTGATCCAGCAATGCCGCGTGTGTGAAGAAGGCCTGCGGGTTGTAAAGCACTTTCAGTGGGGAAGAAAAGCTTGGTGTGAATAGCGCCAAGTTTTGACGTTACCCACAGAAGAAGCACCGGCTAACTCCGTGCCAGCAGCCGCGGTAATACGGAGGGTGCGAGCGTTAATCGGAATCACTGGGCGTAAAGCGCGCGTAGGCGGCTAAGTAAGTCGGGTGTGAAAGCCCTGGGCTTAACCCAGGAACTGCACTCGATACTGCTTAGCTAGAGTATGGTAGAGGGAAGCGGAATTCCGGGTGTAGCGGTGAAATGCGTAGAGATCCGGAGGAACACCAGTGGCGAAGGCGGCTTCCTGGACCAATACTGACGCTGAGGTGCGAAAGCGTGGGGAGCAAACAGG` |
| ASV3704 | 404 | `TGGGGAATATTGGACAATGGGCGAAAGCCTGATCCAGCCATGCCGCGTGTGTGATGAAGGCCTTAGGGTTGTAAAGCACTTTTGTCAGGGAAGATAATGACGGTACCTGAAGAATAAGCACCGGCTAACTTCGTGCCAGCAGCCGCGGTAATACGAAGGGTGCTAGCGTTGTTCGGAATCACTGGGCGTAAAGCGCGCGTAGGCGGCGATACAAGTCAGAGGTGAAAGCCCAAGGCTCAACCTTGGAATTGCCTTTGAAACTGTATTGCTTGAGTATTGGAGGGGATAGCGGAATTGCTAATGTAGAGGTGAAATTCGTAGATATTAGCGGGAACACCGGTGGCGAAGGCGGCTATCTGGACAATTACTGACGCTGAGGCGCGAAAGCGTGGGGATCAAACAGG` |
| ASV529 | 424 | `TGAGGAATATTGGTCAATGGGCGCAAGCCTGAACCAGCCATGCCGCGTGCAGGATGACGGTCCTATGGATTGTAAACTGCTTTTGTACAGGAAGAAACACTCCCTCGTGAGGGAGCTTGACGGTACTGTAAGAATAAGGATCGGCTAACTCCGTGCCAGCAGCCGCGGTAATACGGAGGATCCAAGCGTTATCCGGAATCATTGGGTTTAAAGGGTCCGTAGGCGGTTTTATAAGTCAGTGGTGAAATCTGGTCGCTCAACGATCAAACGGCCATTGATACTGTAAGACTTGAATTACTTGGAAGTAACTAGAATATGTAGTGTAGCGGTGAAATGCTTAGAGATTACATGGAATACCAATTGCGAAGGCAGGTTACTACGAGTATATTGACGCTGATGGACGAAAGCGTGGGGAGCGAACAGG` |
| ASV1571 | 404 | `TGAGGAATTTTGCGCAATGGGGGAAACCCTGACGCAGCGACGCCGCGTGGAGGATGAAGTATCTCGGTATGTAAACTCCTGTCAGTGGGGATGATGATGACAGTACCCACAGAGGAAGCACCGGCTAACTCCGTGCCAGCAGCCGCGGTAAGACGGAGGGTGCAAGCGTTGTTCGGAATTACTGGGCGTAAAGCGTACGTAGGCGGTAGATTAAGTCGAATGTTAAAGCCTGAGGCTCAACCTCAGAGATGCATTCGATACTGGTTTACTAGAGGGTGATAGAGGAAAGCGGAATTCCGAGTGTAGCGGTGAAATGCGTAGATATTCGGAGGAACATCAGTAGCGAAGGCGGCTTTCTGGGTCACACCTGACGCTGAGGTACGAAAGCGTGGGGAGCAAACGGG` |
| ASV3154 | 404 | `TGGGGAATATTGGACAATGGGCGAAAGCCTGATCCAGCCATGCCGCGTGAGTGATGAAGGCCCTAGGGTTGTAAAGCTCTTTTACCTGGGAAGATAATGACTGTACCAGGAGAATAAGCCCCGGCTAACTTCGTGCCAGCAGCCGCGGTAATACGAAGGGGGCTAGCGTTGTTCGGAATCACTGGGCGTAAAGCGCACGTAGGCGGATTTTTAAGTCAGGGGTGAAATCCCGGGGCTCAACCTCGGAACTGCCTTTGATACTGGAGATCTTGAGTCCGGAAGAGGTGAGTGGAACTGCGAGTGTAGAGGTGAAATTCGTAGATATTCGCAAGAACACCAGTGGCGAAGGCGGCTCACTGGTCCGGTACTGACGCTGAGGTGCGAAAGCGTGGGGAGCAAACAGG` |
| ASV5089 | 428 | `TCGAGGATCTTTAGCAATGCCCGAAAGGGTGACTATGCGACGCCGCGTGCAGGATGAAGGCCTTCGGGTTGTAAACTGCTGTCAGGGGGGAAGAAACCTTACGTGTTAACAGCGCGTAAGCTGACATCACCCCCAAAGGAAGCCACGGCTAACTCTGTGCCAGCAGCCGCGGTAAGACAGAGGTGGCAAGCGTTGTTCGGATTCATTGGGTGTAAAGGGCAGGTAGGTGGTTGCGTAAGTCCGGTGTGAAATCCCAGGGCTCAACTCTGGAACGGCATTGGAAACTGCGTGACTTGAGTACCGGAGAGGTGAGGGGAATTCTCGGTGTAAGGGTGAAATCTGTAGATATCGAGAGGAACACCAGTGGCGAAGGCGCCTCACTGGCCGGTTACTGACACTGAGCTGCGAAAGCAAGGGGAGCAAACAGG` |
| ASV5016 | 406 | `TGAGGAATCTTCCGCAATGGGCGAAAGCCTGACGGAGCGACACCGCGTGGAGGATGACGGCCTTTTGGTTGTAAACTCCTTTTCTGGGGGAATAATTGATGAAGGTACCCCAGGAATAAGCACCGGCTAACTACGTGCCAGCAGCCGCGGTAATACGTAGGGTGCAAGCGTTATCCGGAATCATTGGGCGTAAAGAGTTCGTAGGCGTCCCTATAAGTCTTCTGTTAAATCCATCAGCTCAACTGATGAACCGCAGAGGAAACTATTGGGATTGAGATCAGCAGGGGCAACTGGAATGCCGCATGTAGGGGTAAAATCCGTTGATATGCGGTGGAACACCAAAAGCGAAGGCAGGTTGCTGGGCTGTTTCTGACGCTGAGGAACGAAAGCGTGGGGAGCAAACAGG` |
| ASV1632 | 422 | `TGAGGAATTTTGCACAATGGAGGTAACTCTGATGCAGCGACGCCGTGTGTAGGATGAAGGGGCTCTGCCCTGTAAACTACTGTCGGATAGGAATAAAAGTGGGGTTGCCCTACTGGGAATGTACTATCAAAGGAAGGATCGGCTAACTACGTGCCAGCAGCCGCGGTAATACGTAGGATCCGAGCGTTGTCCGGATTTACTGGGTATAAAGGGTGCGTAGGCGGGTTTGTGCTTCAGAGGTTAAAGACCGCAGCTTAACTGCGGGAGTGCCTCTGATAAGGCAAATCTTGGATATGAGAGGGGTAGGTAGAATTCCTGGTGTAGCGGTGAAATGCGTAGATATCAGGAGGAATACCAGTGGCGAAGGCGGCCTACTGGCTCATAATCGACGCTGAGGCACGAAAGCGTGGGGATCAAACAGG` |
| ASV4902 | 404 | `TGGGGAATCTTGCGCAATGGGCGAAAGCCTGACGCAGCAACGCCGCGTGCGGGATGAAGGCCTTCGGGTTGTAAACCGCTTTCAGCAGGGACGAAAATGACGGTACCTGCAGAAGAAGCCCCGGCTAACTACGTGCCAGCAGCCGCGGTAATACGTAGGGGGCAAGCGTTGTCCGGATTTATTGGGCGTAAAGAGCTCGTAGGCGGTTCCGTAAGTCAGGTGTGAAAAATCTGGGCTCAACCCAGTGGAGCACCTGATACTGCGGTGACTTGAGTCCGGTAGGGGAGTGTGGAATTCCTGGTGTAGCGGTGAAATGCGCAGATATCAGGAGGAACACCAACAGCGAAGGCAGCACTCTGGGCCGGAACTGACGCTGAGGAGCGAAAGCGTGGGGAGCGAACAGG` |
| ASV1382 | 403 | `TGGGGAATATTGCACAATGGAGGAAACTCTGATGCAGCGACGCCGCGTGAGGGAAGAAGGTTTTCGGATTGTAAACCTCTGTCTTCGGGGACGATAATGACGGTACCCGAGGAGGAAGCCACGGCTAACTACGTGCCAGCAGCCGCGGTAATACGTAGGTGGCAAGCGTTGTCCGGATTTACTGGGTGTAAAGGGAGCGCAGGTGGAAAAGTAAGTAGGATGTTTAATCTACGGGCTCAACCCGTATCAGCATTCTAAACTATTTTTCTTGAGTGGAGTAGAGGCAGGCGGAATTCCAGGTGTAGCGGTGGAATGCGTAGATATCTGGAGGAACACCAGTGGCGAAGGCGGCCTGCTGGGCTCTAACTGACACTGAGGCTCGAAAGCGTGGGTAGCAAACAGG` |
| ASV3672 | 404 | `TGGGGAATATTGCGCAATGGGCGAAAGCCTGACGCAGCAACGCCGCGTGGAGGAAGAAGGTATTCGTATTGTAAACTCCTGTCTAGTGGGACGATAATGACGGTACCACTGGAGGAAGCCACGGCTAACTACGTGCCAGCAGCCGCGGTAATACGTAGGTGGCAAGCGTTGTCCGGAATTACTGGGCGTAAAGCGCGTGTAGGCGGCACTTTAAGTTAGATGTGAAAAGCCGAGGCTTAACCTCGGACCTGCATTTAAAACTGAAGAGCTTGAGTGTCGGAGAGGAAAGCGGAATTCCTAGTGTAGCGGTGAAATGCGTAGATATTAGGAGGAACACCAGTGGCGAAGGCGGCTTTCTGGACGACAACTGACGCTGAGATGCGAAAGCGTGGGGAGCAAACAGG` |
| ASV3997 | 404 | `TGGGGAATATTGGACAATGGGGGCAACCCTGATCCAGCAATGCCGCGTGAATGATGAAGGCCTTAGGGTTGTAAAGTTCTTTCGCACACGACGATGATGACGGTAGTGTGAGAAGAAGCCCCGGCAAACTTCGTGCCAGCAGCCGCGGTAATACGAAGGGGGCTAGCGTTGTTCGGAATTACTGGGCGTAAAGGGCATGTAGGCGGCCCGATAAATTAGAAGTGAAATGCCTGGGCTTAACCCGGGAACTGCTTTTAAGACTGTCGGGCTTGAATCCGTGAGAGGACAGTGGAATTCCCAGTGTAGAGGTGAAATTCGTAGATATTGGGAGGAACACCAGTAGCGAAGGCGACTGTCTGGTGCGGTATTGACGCTGAGATGCGAAAGCGTGGGGAGCAAACAGG` |
| ASV3227 | 429 | `TGGGGAATATTGGACAATGGGGGAAACCCTGATCCAGCGATGCCGCGTGGGTGAAGAAGGCCTTCGGGTTGTAAAGCCCTTTAGGTTGGGAAGAAGGGTAGATGTGGATAATACATTTACTTTGACGGTACCGACAGAATAAGCACCGGCAAACTCTGTGCCAGCAGCCGCGGTAATACAGAGGGTGCGAGCGTTAATCGGATTTACTGGGCGTAAAGGGCACGTAGGCGGTAAGATGTGTGTGATGTGAAAGCCCCGGGCTCAACCTGGGAAGTGCATCGCAAACTATCTAACTAGAGTATATGAGAGGGTGGCGGAATTTCCGGTGTAGCGGTGAAATGCGTAGAGATCGGAAGGAACGTCAATGGCGAAGGCAGCCACCTGGCATAATACTGACGCTGAGGTGCGAAAGCGTGGGGATCGAACAGG` |
| ASV2010 | 406 | `TGAGGAATTTTCCGCAATGGGCGAAAGCCTGACGGAGCAATGCCGCGTGGAGGATGAAAGCCTATGGGTTGTAAACTCCTTTTCTCAGAGAAGAAATTTTGACGGTATCTGAGGAATAAGCATCGGCTAACTCCGTGCCAGCAGCCGCGGTAATACGGGGGATGCAAGTGTTATCCGGAATAATTGGGCGTAAAGCGTCTGTAGGTGGTTGACCAAGTCTGTTGTTAAAAATCAGGGCTTAACCCTGATCCGGCAACAGAAACTAGTAAACTTGAGTACGGTAGGGGCAGAGGGAATTCTCGGTGTAGTGGTGAAATACGTAGATATCGAGAAGAACACCAATAGCGAAAGCACTCTGCTGGGCCGAAACTGACATTGAGAGACGAAAGCTAGGGGAGCAAAAGGG` |
| ASV3735 | 404 | `TAAGGAATATTGGACAATGGGGGAAACCCTGATCCAGCCATGCCGCGTGAGTGAAGAAGGCCTTCGGGTTGTAAAGCTCTTTTAGATGTGACGATGATGACGGTAGCATCAGAAAAAGCTCCGGCTAACTTCGTGCCAGCAGCCGCGGTAATACGAAGGGAGCGAGCGTTGTTCGGAATCACTGGGCTTAAAGCGTACGTAGGCGGATTTACAAGTCAGGAGTGAAATCCCAGGGCTTAACCCTGGAATTGCTTTTGAAACTGTAAGTCTGGAATCCGGTAGAGGTTGGCGGAACTCCGAGTGTAGAGGTGAAATTCGCAGATATTCGGAAGAACACCAGTGGCGAAGGCGGCCAACTGGACCGGTATTGACGCTGAGGTACGAAAGCGTGGGTAGCAAACAGG` |
| ASV84 | 404 | `TGGGGAATCTTGCGCAATGGGCGAAAGCCTGACGCAGCCATGCCGCGTGTATGATGAAGGTCTTAGGATTGTAAAATACTTTCACCGGTGAAGATAATGACTGTAGCCGGAGAAGAAGCCCCGGCTAACTTCGTGCCAGCAGCCGCGGTAATACGAAGGGGGCTAGCGTTGCTCGGAATTACTGGGCGTAAAGGGAGCGTAGGCGGACATTTAAGTCAGAGGTGAAATCCCGGAGCTTAACTTCGGAACTGCCTTTGATACTGGGTGTCTTGAGTGTGAGAGAGGTATGTGGAACTCCGAGTGTAGAGGTGAAATTCGTAGATATTCGGAAGAACACCAGTGGCGAAGGCGACATACTGGCTCATTACTGACGCTGAGGCTCGAAAGCGTGGGGAGCAAACAGG` |
| ASV4276 | 416 | `TCGAGAATTTTTCACAATGGGGGAAACCCTGATGGAGCGACGCCGCGTGGAGGATGAAGGTTTTCGGATTGTAAACTCCTGTCATGAGGGAACAAAGCCAGCAATGGCCTGATAGTACCTCAAGAGGAAGGGACGGCTAACTCTGTGCCAGCAGCCGCGGTAATACAGAGGTCCCAAGCGTTGTTCGGATTCACTGGGCGTAAAGGGTGCGTAGGCGGCGAGGTAAGTTGAGTGTTAAATTTCGGGGCTTAACCCCGAAACCGCTCTCAATACTGCTTTGCTAGAGGAATGTAGAGGAGACTGGAATTCATGGTGTAGCAGTGAAATGCGTAGATATCATGAGGAAGGCCAGTGGCGAAAGCGGGTCTCTGGGCATTTCCTGACGCTGAGGCACGAAGGCTGGGGGAGCGAACGGG` |
| ASV251 | 387 | `TCGCGAAAACTTCACAATGGGGGCAACCCCGATGAGGGAATTCCTAGTGCTAGCACATTAGTGTTAGCTTTTCTTTAGCGTAGATAACTAGAGGAATAAGGGCTGGGTAAGACGGGTGCCAGCCGCCGCGGTAATACCCGCAGCCCAAGTGGTGGTCGATTTTATTGAGTCTAAAACGTTCGTAGCCGGTTTGATAAATCTTTGGGTAAATCGGAAAGCTTAACTTTCCGACTTCCGAGGAGACTGTCAAACTTGGGACCGGGAGAGGCAAGAGGTACTTGGGGGGTAGGGGTAAAATCCTGTAATCCTTCAAGGACCACCGGTGGCGAAGGCGTCTTGCTAGAACGGATCCGACGGTGAGGGACGAAGCCCTGGGTCGCAAACGGG` |
| ASV5208 | 429 | `TGGGGAATATTGGACAATGGGCGCAAGCCTGATCCAGCGATGCCGCGTGTGTGAAGAAGGCCTGCGGGTTGTAAAGCACTTTCAGTGGGGAGAAAGATTCCTGGGTTAATAACCTAGGGCGAAGATGTTACCCACAGAAGAAGCACCGGCTAACTCTGTGCCAGCAGCCGCGGTAATACAGAGGGTGCAAGCGTTAATCGAAATTACTGGGCGTAAAGGGTGCGTAGGTGGTTTGATAAGTTAGATGTGAAAGTCCTGGGCTCAACCTGGGGATTGCGTCTGATACTGTTGAGCTAGAGTTCTGTAGAGGGAGGTGGAATTTCCGGAGTAGCGGTGAAATGCGTAGATATCGGAAGGAACATCGGTGGCGAAGGCGGCCTCCTGGACAGGAACTGACACTGAGGCACGAAAGCGTGGGGAGCAAACAGG` |
| ASV3854 | 427 | `TGGGGAATATTGCACAATGGGCGCAAGCCTGATGCAGCGACGCCGCGTGGGGGATGACGGCCTTCGGGTTGTAAACTCCTTTCGTTAGGGACGAAGCCACACCTTTTTTTGGGTGTGGTGACGGTACCTTTGTTAAGAAGCACCGGCTAACTACGTGCCAGCAGCCGCGGTAATACGTAGGGTGCGAGCGTTGTCCGGAATTACTGGGCGTAAAGAGCTCGTAGGTGGTTTGTTGCGTCGTCTGTGAAATTCCGGGGCTTAACTTCGGGGTGGCAGGCGATACGGGCATAACTAGAGTGCTGTAGGGGAGACTGGAATTCCTGGTGTAGCGGTGGAATGCGCAGATATCAGGAGGAACACCGATGGCGAAGGCAGGTCTCTGGGCAGTAACTGACGCTGAGGAGCGAAAGCATGGGGAGCGAACAGG` |
| ASV5264 | 404 | `TGGGGAATATTGGACAATGGGGGCAACCCTGATCCAGCAATGCCGCGTGAGTGATGAAGGCCTTAGGGTTGTAAAGCTCTTTTACCAGGGACGATGATGACGGTACCTGGAGAATAAGCCCCGGCTAACTTCGTGCCAGCAGCCGCGGTAATACGAAGGGGGCTAGCGTTGTTCGGAATTACTGGGCGTAAAGGGCGCGTAGGCGGCCGATCAAGTCAGATGTGAAAGTCCTGGGCTTAACCTGGGGACTGCATTTGATACTGATTGGCTTGAATTCGGGAGAGGATAGCGGAATTCCCAGTGTAGAGGTGAAATTCGTAGATATTGGGAAGAACACCGGTGGCGAAGGCGGCTATCTGGACCGACATTGACGCTGAGGCGCGAAAGCGTGGGGAGCAAACAGG` |
| ASV4249 | 409 | `TGAGGAATCTTCCGCAATGGACGAAAGTCTGACGGAGCGACGCCGCGTGGTGGATGAAGGCCTTAACTGGTCGTAAACACCTGTTCTGAGGGACGAAATTTTTGACGGTACCTCAGGAGAAAGTATCGGCAAACCCTGTGCCAGCAGCCGCGGTAATACAGGGGATACAAGCGTTACTCGGAATTACTGGGCGTAAAGCGTCTGTAGGTTCCTATGAAAGTCTGGGGTAAAAACTCAGTGCTTAACACTGAGATCGTCCCGGAAACTCCATAGGTTGAGCAGTGGAGAGGCATCTGGAATGCCGCATGTAGGAGTAAAATCCGTAGATATGCGGTAGAACGCCAAAAGCGAAGGCAGGATGCTAGCCACTTGCTGACACTGAGAGACGAAAGCGTGGGGAGCAAAGGGG` |
| ASV445 | 404 | `TGGGGAATATTGGACAATGGGCGAAAGCCTGATCCAGCCATGCCGCGTGAGTGATGAAGGCCTTCGGGTTGTAAAGCTCTTTTATCCGGGAAGATAATGACTGTACCGGAAGAATAAGCCCCGGCTAACTTCGTGCCAGCAGCCGCGGTAATACGAAGGGGGCTAGCGTTGCTCGGAATCACTGGGCGTAAAGGGTGCGTAGGCGGATTTTTAAGTCGGAGGTGAAAGCCTGGGGCTCAACCCCAGAATTGCCTTCGATACTGAGAGTCTTGAGTTCGGAAGAGGTTGGTGGAACTGCGAGTGTAGAGGTGAAATTCGTAGATATTCGCAAGAACACCGGTGGCGAAGGCGGCCAACTGGTCCGATACTGACGCTGAGGCACGAAAGCGTGGGGAGCAAACAGG` |
| ASV5552 | 430 | `TGGGGAATATTGCGCAATGGGGGCAACCCTGACGCAGCAACGCCGCGTGAGTGAAGAAGGCCTTTGGGTTGTAAAGCTCTTTCGGCTGGGGAGAAGGTGGTAAGTGTTAATAGTACTTATTATTGATGGTACCAGAAGAAGAAGCACCGGCAAACTTCGTGCCAGCAGCCGCGGTAATACGAAGGGTGCAAGCGTTGTTCGGAATTATTGGGCGTAAAGGGTTCGTAGGCGGGGATATAAGTAGGTTGTGAAAACCCTGGGCTCAACCTGGGACGTGCGACTTAGACTGTATTTCTTGAGTTATGGAGAGGGTAATGGAATTGCTGGTGTAGGAGTGACATCCGTAGAGATCAGCAGGAACGCCGGAGGCGAAAGCGGTTACCTGGCCAATGACTGACGCTGAGGAACGAAAGCGTGGGGAGCAAACAGG` |
| ASV2034 | 424 | `TGAGGAATATTGGACAATGGGCGCAAGCCTGATCCAGCCATGCCGCGTGCAGGAAGACGGTCCTATGGATTGTAAACTGCTTTTATACAGGAAGAAACACCTCTACGTGTAGAGACTTGACGGTACTGTATGAATAAGCATCGGCTAACTCCGTGCCAGCAGCCGCGGTAATACGGAGGATGCAAGCGTTATCCGGAATCATTGGGTTTAAAGGGTCCGTAGGCGGTTTAATAAGTCAGTGGTGAAAGCCCATCGCTCAACGATGGAACGGCCATTGATACTGTTAGACTTGAATTATTAGGAAGTAACTAGAATATGTAGTGTAGCGGTGAAATGCTTAGATATTACATGGAATACCAATTGCGAAGGCAGGTTACTACTAATGGATTGACGCTGATGGACGAAAGCGTGGGGAGCGAACAGG` |
| ASV2706 | 407 | `CCGAGAATATTCGACAATGGGCGAAAGCCTGATCGAGCGATACCGCGTGGTGGATGAAGCGCTTCGGCGCGTAAACATCTTTTATGAGGGAGGAAGTTATTGACGTTACCTCATGAATAAGGGGCTCCTAACTCTGTGCCAGCAGGAGCGGTAATACAGAGGCCCCAAGCATTACCCGGAATCACTGGGCGTAAAGGGTGCGTAGGTGGTCGTATTAGTCGTTCGTTAAAACCCGGGGCTTAACCCCGGAATCGCGAGCGAAACGGTACGACTCGAGAGAGTGAGAGGTGAATGGAACTCATGGTGTAGGGGTGAAATCCGTTGATATCATGGGGAACACCGAAAGCGAAGGCAATTCACTGGCACTTTTCTGACACTCAAGCACGAAAGCGTGGGTAGCGAACTGG` |
| ASV2345 | 424 | `TGAGGAATATTGGTCAATGGGCGCAAGCCTGAACCAGCCATGCCGCGTGCAGGAAGAATGCCCTATGGGTTGTAAACTGCTTTTATTTGGGAATAAACCTCTTTACGTGTAGAGAGCTGAAGGTACCAAATGAATAAGCACCGGCTAACTCCGTGCCAGCAGCCGCGGTAATACGGAGGGTGCAAGCGTTATCCGGAATCATTGGGTTTAAAGGGTCCGCAGGCGGATTTATAAGTCAGTGGTGAAATCCTATCGCTTAACGATAGAACTGCCATTGATACTGTAAGTCTTGAATTCGGTCGGAGTGGGCGGAATGTGTAGTGTAGCGGTGAAATGCATAGATATTACACAGAACACCGATAGCGAAGGCAGCTCACTAGGCCTGAATTGACGCTCAGGGACGAAAGCGTGGGGATCAAACAGG` |
| ASV2713 | 409 | `TGGGGAATATTGCGCAATGGGCGAAAGCCTGACGCAGCGACGCCGCGTGAGGGATGAAGGCCTTCGGGTTGTAAACCTCTTTCAGCAGGGACGAAGCGAAAGTGACTGTACCTGCAGAAGAAGCACCGGCTAACTACGTGCCAGCAGCCGCGGTAATACGTAGGGTGCGAGCGTTGTCCGGATTTATTGGGCGTAAAGAGCTCGTAGGCGGCTCGTCGCGTCGGATGTGAAAACTCGGGGCTTAACCCCGAGCCTGCATTCGATACGGGCGAGCTAGAATTCGGTAGGGGAGACTGGAATTCCTGGTGTAGCGGTGAAATGCGCAGATATCAGGAGGAACACCGATGGCGAAGGCAGGTCTCTGGGCCGATATTGACGCTGAGGAGCGAAAGCGTGGGGAGCGAACAGG` |
| ASV328 | 429 | `TGGGGAATATTGGACAATGGGCGCAAGCCTGATCCAGCCATGCCGCGTGGGTGAAGAAGGCCTTCGGGTTGTAAAGCCCTTTTGTCCGGAAAGAAAAGCACGGGATTAATACCCTCGTGTGATGACGGTACCGGAAGAATAAGCACCGGCTAACTTCGTGCCAGCAGCCGCGGTAATACGAAGGGTGCAAGCGTTACTCGGAATTACTGGGCGTAAAGCGTGCGTAGGTGGTTTGTTAAGTCTGTCGTGAAATCCCCGGGCTCAACCTGGGAATGGCGATGGATACTGGCAAGCTAGAGTGAGGTAGAGGAGAGTGGAATTCCCGGTGTAGCAGTGAAATGCGTAGAGATCGGGAGGAACATCAGTTGCGAAGGCGGCTCTCTGGACCATCACTGACACTGAGGCACGAAAGCGTGGGGAGCAAACAGG` |
| ASV150 | 429 | `TGGGGAATTTTGGACAATGGGCGCAAGCCTGATCCAGCAATGCCGCGTGCAGGAAGAAGGCCTTCGGGTTGTAAACTGCTTTTGTACGGAACGAAAAGGCTCTGGTTAATACCTGGGGTTCATGACGGTACCGTAAGAATAAGCACCGGCTAACTACGTGCCAGCAGCCGCGGTAATACGTAGGGTGCAAGCGTTAATCGGAATTACTGGGCGTAAAGCGTGCGCAGGCGGTTTTGTAAGACAGTCGTGAAATCCCCGGGCTCAACCTGGGAATGGCGATTGTGACTGCAAAGCTGGAGTGCGGCAGAGGGGGATGGAATTCCGCGTGTAGCAGTGAAATGCGTAGATATGCGGAGGAACACCGATGGCGAAGGCAATCCCCTGGGCCTGCACTGACGCTCATGCACGAAAGCGTGGGGAGCAAACAGG` |
| ASV4178 | 404 | `TGGGGAATATTGGACAATGGGCGCAAGCCTGATCCAGCAACGCCGCGTGAATGATGAAGGCCTTCGGGTCGTAAAGTTCTTTTGTAAGGGAAGAAAATGACGGTACCTTAAGAATAAGCTCCGGCTAACTTCGTGCCAGCAGCCGCGGTAATACGAAGGGGGCTAGCGTTGTTCGGAATTACTGGGCGTAAAGGGTGCGTAGGTTGTTTAACAAGTTAGTGGTGAAAGCCCAGGGCTCAACTCTGGAACTGCCATTAAAACTGTTAAGCTAGAGAGTGTTAGAGGTGAGTGGAATTTCTAGTGTAGGAGTGAAATCCGTAGATATTAGAAGGAACATCAATGGCGAAGGCAGCTCACTGGGTCACATCTGACACTGAGGCACGAAAGCGTGGGTAGCAAACAGG` |
| ASV1799 | 430 | `TGGGGAATTGTTCACAATGGGCGCAAGCCTGATGACGCAACGCCGCGTGGGGGATGAAGGTCTTCGGATCGTAAACCCCTGTCGAATGGGACGAATAGCCTTCGGATTAATACCCCGGAGGAATGACGGTACCGTTAAAGGAAGCCACGGCTAACTCTGTGCCAGCAGCCGCGGTAATACAGAGGTGGCAAGCGTTGTTCGGAATTACTGGGCGTAAAGGGCGCGTAGGCGGCCATCTAAGTCAGACGTGAAATCCCCCGGCTTAACCTGGGAACTGCGTCTGATACTGGAAGGCTTGAGTTTGGGAGAGGGATGTAGAATTCCAGGTGTAGCGGTGAAATGCGTAGATATCTGGAGGAATACCGGTGGCGAAGGCGGCATCCTGGACCAATACTGACGCTGAGGCGCGAAAGCTAGGGGAGCAAACGGG` |
| ASV5064 | 429 | `TGGGGAATATTGGACAATGGGGGCAACCCTGATCCAGCAATGCCGCGTGTGTGAAGAAGGCCTGCGGGTTGTAAAGCACTTTCGGCGGGGAGGAGGAATTTTTCGTTAATAGCGGAGAGTTCGGACGTTACCCGCATAAGAAGCACCGGCTAACTCTGTGCCAGCAGCCGCGGTAATACAGAGGGTGCAAGCGTTAATCGGAATTACTGGGCGTAAAGCGCGCGTAGGTGGATAATTAAGTTAGCTGTGAAAGCCCCGGGCTCAACCTGGGAAAGCCAGCTAAAACTGGTTGTCTAGAGTATGGTAGAGGGAAGTGGAATTTCCGGTGTAGCGGTGAAATGCGTAGATATCGGAAGGAACATCAGTGGCGAAGGCGACTTCCTGGACCAATACTGACACTGAGGCGCGAAAGCGTGGGTAGCAAACAGG` |
| ASV4548 | 405 | `TAGGGAATCTTCCACAATGGGCGAAAGCCTGATGGAGCAACGCCGCGTGCAGGATGAAGGCTTTCGGGTCGTAAACTGCTTTTATATGGGAAGATTATGACGGTACCATATGAATAAGGATTGGCTAACTCCGTGCCAGCAGCCGCGGTCATACGGAGGATCCAAGCGTTACCCGGATTTATTGGGCGTAAAGCGTTGCGTAGGTGGTTTAGTAAGTGAGGTATGAAATGTTAGGGCTCAACCCTGAAGGTGTATCTCAAACTACTAAACTAGAGCATATTAGAGGCAAGTGGAATTCCTGGTGTAGGAGTGAAATCCGTAGATATCAGGAGGAACACCAATGGCGAAGGCAGCTTGCTGGGATATTGCTGACACTGAGGCACGAAAGCGTGGGGAGCGAACCGG` |
| ASV2073 | 405 | `TGGGGGATATTGCACAATGGAGGAAACTCTGATGCAGCGACGCCGCGTGTAGGAAGACGGTCTTATGGATTGTAAACTACTGTCTTTGGGGACGATAATGACGGTACCCAAAGAGGAAGCTCCGGCTAACTACGTGCCAGCAGCCGCGGTAATACGTAGGGAGCGAGCGTTGTCCGGAATTACTGGGTGTAAAGGGAGCGTAGGCGGGGAGGCAAGTTGGATGTGAAAACTATCGGCTCAACCGATAGATTGCATTCAAAACTGTTTCTCTTGAGTGAAGTAGAGGCAGGCGGAATTCCTAGTGTAGCGGTGAAATGCGTAGATATTAGGAGGAACACCAGTGGCGAAGGCGGCCTGCTGGGCTTTTACTGACGCTGAGGCTCGAAAGTGTGGGTAGCAAACAGG` |
| ASV13 | 404 | `TGGGGAATCTTAGACAATGGGGGAAACCCTGATCTAGCCATGCCGCGTGAGCGATGAAGGCCTTAGGGTTGTAAAGCTCTTTCAGTGGGGAAGATAATGACTGTACCCACAGAAGAAGCCCCGGCTAACTCCGTGCCAGCAGCCGCGGTAATACGGAGGGGGCTAGCGTTGTTCGGAATTACTGGGCGTAAAGCGCACGTAGGCGGACCGGAAAGTCAGAGGTGAAATCCCAGGGCTCAACCTTGGAACTGCCTTTGAAACTCCTGGTCTTGAGGTCGTGAGAGGTGAGTGGAATTCCGAGTGTAGAGGTGAAATTCGTAGATATTCGGAGGAACACCAGTGGCGAAGGCGGCTCACTGGCACGATACTGACGCTGAGGTGCGAAAGCGTGGGGAGCAAACAGG` |
| ASV877 | 423 | `TAGGGAATATTGGGCAATGGGCGCAAGCCTGACCCAGCCATGCCGCGTGCAGGAAGAAGGCGTTATGCGTTGTAAACTGCTTTTATACAGGAAGAAACGACCCTTGCGAGGGGCATTGACGGTACTGTATGAATAAGCACCGGCTAACTCCGTGCCAGCAGCCGCGGTAATACGGAGGGTGCAAGCGTTGTCCGGATTTATTGGGTTTAAAGGGTGCGCAGGTGGTTTATTAAGTCAGTGGTGAAAGACGGTCGCTCAACGATTGCAGTGCCATTGATACTGGTAGACTTGAGTAAAGTAGAGGTGGGCGGAATTGATAGTGTAGCGGTGAAATGCATAGATATTATCAAGAACTCCAATTGCGTAGGCAGCTCACTTGGCTTTTACTGACACTCATGCACGAAAGTGTGGGTATCAAACAGG` |
| ASV5384 | 429 | `TGGGGAATCTTGGACAATGGGGGCAACCCTGATCCAGCGATGCCGCGTGGGTGAAGAAGGCCTTCGGGTTGTAAAGCCCTTTAGGCTGGGACGAAGGGTAGTGGGTGAATAGCCCATTACCTTGACGGTACCGGCAAAATAAGCACCGGCAAACTCTGTGCCAGCAGCCGCGGTAATACAGAGGGTGCGAGCGTTAATCGGAATTACTGGGCGTAAAGGGCGCGTAGGCGGATGCAATAGTGTGATGTGAAAGCCCCGGGCTTAACCTGGGAAGCGCATCGCAAACGTTGCGACTAGAGTACAGGAGAGGGTGGCGGAATTTCCGGTGTAGCGGTGAAATGCGTAGAGATCGGAAGGAACGTCGATGGCGAAGGCAGCCACCTGGCCTGATACTGACGCTGAGGCGCGAAAGCGTGGGGATCGAACAGG` |
| ASV5028 | 429 | `TGGGGAATTTTGGACAATGGGCGAAAGCCTGATCCAGCCATGCCGCGTGTGGGAAGAAGGCCTTCGGGTTGTAAACCACTTTTGTCGGGAACGAAACGGTCTGTGATAACACCATGGGCTAATGACGGTACCCGAAGAATAAGCACCGGCTAACTACGTGCCAGCAGCCGCGGTAATACGTAGGGTGCAAGCGTTAATCGGAATTACTGGGCGTAAAGCGTGCGCAGGCGGTTATGTAAGACAGATGTGAAATCCCCGGGCTTAACCTGGGAACTGCATTTGTGACTGCATAGCTTGAGTACGGTAGAGGGGGGTAGAATTCCACGTGTAGCAGTGAAATGCGTAGAGATGTGGAGGAATACCGATGGCGAAGGCAGCCCCCTGGACCGTAACTGACGCTCATGCACGAAAGCGTGGGGAGCAAACAGG` |
| ASV1784 | 429 | `TGGGGAATTTTGGACAATGGGCGCAAGCCTGATCCAGCCATGCCGCGTGAGTGAAGAAGGCCTTCGGGTTGTAAAGCTCTTTCGGTGAAGACGAAACGGTTAGCGCTAATAACGCTGACTAATGACGGTACTCACAGAAGAAGCACCGGCTAACTACGTGCCAGCAGCCGCGGTAATACGTAGGGTGCAGGCGTTAATCGGAATTACTGGGCGTAAAGCGTGCGCAGGCGGTTTGTTAAGACAGGTGTGAAATCCCCGGGCTTAACCTGGGAATTGCGCTTGTAACTGGCAAGCTAGAGTGTGGCAGAGGGGGGTGGAATTCCACGTGTAGCAGTGAAATGCGTAGATATGTGGAGGAACACCGATGGCGAAGGCAGCCCCCTGGGTTAACACTGACGCTCAGGCACGAAAGCGTGGGGAGCAAACAGG` |
| ASV314 | 424 | `TGAGGAATATTGGACAATGGGCGCAAGCCTGATCCAGCCATGCCGCGTGCAGGATGACGGTCCTATGGATTGTAAACTGCTTTTGCACAGGAAGAAACACTCGGACGTGTCCGAGCTTGACGGTACTGTGAGAATAAGGATCGGCTAACTCCGTGCCAGCAGCCGCGGTAATACGGAGGATCCAAGCGTTATCCGGAATCATTGGGTTTAAAGGGTCCGTAGGCGGTTTAGTAAGTCAGTGGTGAAAGCCCATCGCTCAACGGTGGAACGGCCATTGATACTGCTAGACTTGAATTATTAGGAAGTAACTAGAATATGTAGTGTAGCGGTGAAATGCTTAGAGATTACATGGAATACCAATTGCGAAGGCAGGTTACTACTAATGGATTGACGCTGATGGACGAAAGCGTGGGTAGCGAACAGG` |
| ASV1968 | 429 | `TGGGGAATTTTGGACAATGGGCGCAAGCCTGATCCAGCCATGCCGCGTGAGTGAAGAAGGCCTTCGGGTTGTAAAGCTCTTTCGGCGGGGACGAAATGGTTCGTGCTAATACCACGGACTGATGACGGTACCCGAAGAAGAAGCACCGGCTAACTACGTGCCAGCAGCCGCGGTAATACGTAGGGTGCAGGCGTTAATCGGAATTACTGGGCGTAAAGCGTGCGCAGGCGGTTTGCTAAGACAGGTGTGAAATCCCCGGGCTTAACCTGGGAACTGCGCTTGTAACTGGCAAGCTAGAGTGTGGCAGAGGGGGGTGGAATTCCAAGTGTAGCAGTGAAATGCGTAGATATTTGGAGGAACACCGATGGCGAAGGCAGCCCCCTGGGCTAACACTGACGCTCATGCACGAAAGCGTGGGGAGCAAACAGG` |
| ASV1920 | 408 | `TGGGGAATTTTCCGCAATGGGCGAAAGCCTGACGGAGCAATACCGCGTGAGAGATGAAGGCCTATGGGTTGTAAACCTCTTTTTTCAGGGAGGAATCAAATGACGTGTACCTGAAGAATAAGCATCGGCTAACTCCGTGCCAGCAGCCGCGGTAAGACGGGGGATGCAAGTGTTATCCGGAATCACTGGGCGTAAAGCGTCTGTAGGTGGTCCAATAAGTCAACTGTTAAATCTTGGGGCTCAACCTCAAAATCGCAGTCGAAACTATTAGACTAGAGTATAGTAGGGGTAAAGGGAATTTCCAGTGGAGCGGTGAAATGCGTAGATATTGGAAAGAACACCGATGGCGAAGGCACTTTACTGGGCTATTACTAACACTCAGAGACGAAAGCTAGGGTAGCAAATGGG` |
| ASV2529 | 430 | `TGGGGAATCTTGCGCAATGGACGAAAGTCTGACGCAGCCACGCCGCGTGAGTGAAGAAGGCCTTCGGGTTGTAAAGCTCTGTCGTGTGGGACGAAAAACACTAAGTTTAAGAGATGTAGTGTCTGACGGTACCATGAAAGGAAGCACCGGCTAACTCTGTGCCAGCAGCCGCGGTAATACAGAGGGTGCAAGCGTTGCTCGGAATTATTGGGCGTAAAGGGCAGGTAGGTGGTCCTATTTGTCTGGGGTGAAATCCCTGAGCTTAACTCAGGAAGTGCCCCAGAAACGGTAGGACTAGAGTTTTGGAGAGGTTCGTGGAATTCCCAGTGTAGCGGTGAAATGCGTAGAGATTGGGAGGAACACCAGCGGCGAAGGCGGCGAACTGGACAAACACTGACACTCAACTGCGAAAGCGTGGGTAGCAAACAGG` |
| ASV3786 | 407 | `CTAGGAATTATGCACAATGGACGAAAGTCTGATGCTGCGACATCGCGTGGAGGATGAAGGTCTTAGGATCGTAAACTCCTTTTATGGGTGAACAAGTTCTGAGTGTAACCCATGAATAAGTGTCTGCCAACCACGTGCCAGAAGCATCGGTAATACGTGGGACACAAGCGTTATCCGGATTTACTGGGCGTAAAGAGATGCGTAGGCGACTTCTTAAGTTTTCTGTTAAATCTTTCGATTCAATCGAAAAACCGCAGAAGATACTGAGTTGATTGAGTAAGGTAGAGAAGAGGGGAATTCCTGGTGGAGAGGTTAAATTCGTTGATATCAGGAGGAACACCAATGGCGAAAGCACCTCTTTGGACCTTTACTGACGCTAAGGCTCGAAAGCGTGGGGAGCAAAACGG` |
| ASV1319 | 429 | `TGGGGAATATTGGACAATGGGCGAAAGCCTGATCCAGCGACGCCGCGTGTGTGAAGAAGGCCTGCGGGTTGTAAAGCACTTTCGATGGGAACGAAATGTGCCGGGTTAATACCCCGGTAAGCTGACGTTACTCAATGAAGAAGCACCGGCTAACTCTGTGCCAGCAGCCGCGGTAATACAGAGGGTGCGAGCGTTAATCGGAATTACTGGGCGTAAAGCGCACGTAGGTGGCGACGTAAGTCGGGTGTGAAAGCCCCGGGCTCAACCTGGGAATTGCATTCGAGACTGCATTGCTGGAGTACGGAAGAGGAGGGCGGAATTCCCGGTGTAGCGGTGAAATGCGTAGATATCGGGAGGAACATCAGTGGCGAAGGCGGCCCTCTGGTCCAGTACTGACACTCAAGTGCGAAAGCGTGGGGAGCAAACAGG` |
| ASV3029 | 404 | `TGGGGAATATTGGACAATGGGGGAAACCCTGATCCAGCGATGCCGCGTGAGTGAAGAAGGCCTTCGGGTTGTAAAACTCTTTCACCTGTGATGATGATGACAGTAGCAGGAGAAGAAGCACCGGCTAACTCCGTGCCAGCAGCCGCGGTAAGACGGAGGGTGCTAGCGTTGTTCGGATTGACTGGGCGTAAAGGGCGCGTAGGCGGTCTTATTAGTCGGGCGTGAAAGCCCCGGGCTTAACCTGGGAACTGCGCTCGATACTGTAAGACTTGAGAATGGAAGAGGAAAGTGGAATTCCTAGTGTAGAGGTGAAATTCGTAGATATTAGGAGGAACACCAGAGGCGAAGGCGGCTTTCTGGTCCATTACTGACGCTGAGGCGCGAAAGCGTGGGGAGCAAACAGG` |
| ASV2648 | 404 | `TGGGGAATCTTGGACAATGGGGGCAACCCTGATCCAGCCATGCCGCGTGGGTGATGAAGGCCTTAGGGTCGTAAAGCCCTTTCGCTGGGGAAGACAATGACGGTACCCAGAGAAGAAGCCCCGGCTAACTCCGTGCCAGCAGCCGCGGTAATACGGAGGGGGCTAGCGTTGTTCGGAATGACTGGGCGTAAAGCGCGCGTAGGCGGATCATCAAGTCGGGGGTGAAATCCCGGGGCTCAACCCCGGAACGGCCTTCGAGACTGGTGGTCTTGAGTTCGAGAGAGGTGGGTGGAATTCCGAGTGTAGAGGTGAAATTCGTAGATATTCGGAGGAACACCAGTGGCGAAGGCGGCCCACTGGCTCGACACTGACGCTGAGGCGCGAAAGCGTGGGGAGCAAACAGG` |
| ASV549 | 424 | `TAGGGAATATTGGGCAATGGATGAAAGTCTGACCCAGCCATGCCGCGTGCAGGAAGAAAGTCCTCTGGACTGTAAACTGCTTTTTTCAGGGAAGAATAGGTGGGATGCGTCCTACAGTGACGGTACTTGAAGAATAAGCACCGGCTAACTACGTGCCAGCAGCCGCGGTAATACGTAGGGTGCGAGCGTTGTCCGGATTTATTGGGTTTAAAGGGTGCGTAGGTGGATTTTTAAGTCAGCGGTGAAATATGGTTGCTCAACAATCAGATTGCCATTGATACTGGAAGTCTTGAGAGAAGTGGAGGCTGTTGGAATGGATGGTGTAGCGGTGAAATGCATAGATATCATCCAGAACGTCAATTGCGAAGGCAGATGGCTGTACTTTTTCTGACACTGAGGCACGAAAGCGTGGGGATCAAACAGG` |
| ASV4443 | 424 | `TAGGGAATATTGGGCAATGGACTAGCGTCTGACCCAGCCATGCCGCGTGCAGGATGAAGGCCCTCTGGGTTGTAAACTGCTTTTACTAAGGAAGAAGAGCGCTCTTGCGAGAGTGTGTGACGGTACTTAGGGAATCAGCACCGGCTAACTCCGTGCCAGCAGCCGCGGTAATACGGAGGGTGCAAGCGTTGTCCGGATTTATTGGGTTTAAAGGGTGCGTAGGTGGCCTGATAAGTCAGCTTTGAAAGTGGCTTGCTTAACAAGACAGGGTGGGTTGATACTGTCAGGCTTGAATGGTGGTGAGGTTTCTGGAACGGGTCGTGTAGCGGTGAAATGCATAGATATGACCCAGAACTCCAATTGCGAAGGCAGGAGACTAATCACCGATTGACACTGAGGCACGAGAGCATGGGGAGCAAACAGG` |
| ASV4090 | 424 | `TAAGGAATATTGGTCAATGGACGAAAGTCTGAACCAGCCATGCCGCGTGAAGGATGAAGGCCCTCTGGGTTGTAAACTTCTTTTATCTGGGACGAAAAAAGGGTATTCTTACTCACTTGACGGTACCAGAGGAATAAGCACCGGCTAACTCCGTGCCAGCAGCCGCGGTAATACGGAGGGTGCAAGCGTTATCCGGATTCACTGGGTTTAAAGGGAGCGTAGGTGGGCAGGTAAGTCAGTGGTGAAATCTTTGGGCTTAACCCGAAAACTGCCATTGATACTATCTGTCTTGAATTATCTGGAGGTAAGCGGAATATGTCATGTAGCGGTGAAATGCTTAGATATGACATAGAACACCCATTGCGAAGGCAGCTTACTACGGATATATTGACACTGAGGCTCGAAAGCGTGGGGATCAAACAGG` |
| ASV3812 | 429 | `TGGGGAATTTTGGACAATGGGGGCAACCCTGATCCAGCCATTCCGCGTGAGTGAAGAAGGCCTTCGGGTTGTAAAGCTCTTTCGGCAGGAACGAAACGGTCCTAGTTAATACCTGGGGCTAATGACGGTACCTGAAGAAGAAGCACCGGCTAACTACGTGCCAGCAGCCGCGGTAATACGTAGGGTGCGAGCGTTAATCGGAATTACTGGGCGTAAAGCGTGCGCAGGCGGTTCTATAAGACAGATGTGAAATCCCCGGGCTTAACCTGGGAACTGCGTTTGTGACTGTAGGACTCGAGTGTGGCAGAGGGGGGTGGAATTCCACGTGTAGCAGTGAAATGCGTAGAGATGTGGAGGAACACCGATGGCGAAGGCAGCCCCCTGGGTCAACACTGACGCTCATGCACGAAAGCGTGGGTAGCAAACAGG` |
| ASV618 | 429 | `TGGGGAATCTTGGACAATGGGCGAAAGCCTGATCCAGCAATGCCGCGTGCGTGAAGAAGGCCTTCGGGTTGTAAAGCGCTTTTGTCAGGGAGGAAATCCTAACTTTTAATAAAGGTTGGGGATGACAGTACCTGAAGAATAAGGACCGGCTAACTACGTGCCAGCAGCCGCGGTAATACGTAGGGTCCAAGCGTTAATCGGAATTACTGGGCGTAAAGCGTCCGCAGGTGGCTTGATAAGATAGACGTGAAATCCCTGGGCTCAACCTAGGAATTGCGTTTATGACTGTCTCGCTAGAGTATGGGAGAGGGGGGTGGAATTCCACGTGTAGCAGTGAAATGCGTAGAGATGTGGAGGAACACCGATGGCGAAGGCAACCCCCTGGCCTAATACTGACACTCATGGACGAAAGCGTGGGGAGCAAACAGG` |
| ASV3341 | 429 | `TGGGGAATTTTGGACAATGGGGGCAACCCTGATCCAGCCATGCCGCGTGAGTGAAGAAGGCCTTCGGGTTGTAAAGCTCTTTCGGACGGAACGAAACGGTTAGCGTGAATAACGCTGACTAATGACGGTACCGTAAGAAGAAGCACCGGCTAACTACGTGCCAGCAGCCGCGGTAATACGTAGGGTGCGAGCGTTAATCGGAATTACTGGGCGTAAAGCGTGCGCAGGCGGTTCGCCAAGTCAGGTGTGAAATCCCCGGGCTTAACCTGGGAATTGCGCTTGAAACTAGCGGGCTTGAGTGCGGCAGAGGGGGGTGGAATTCCACGTGTAGCAGTGAAATGCGTAGAGATGTGGAGGAACACCGATGGCGAAGGCAGCCCCCTGGGTCGACACTGACGCTCATGCACGAAAGCGTGGGGAGCAAACAGG` |
| ASV2884 | 429 | `TGGGGAATATTGGACAATGGGCGCAAGCCTGATCCAGCAATGCCGCGTGTGTGAAGAAGGCCTGCGGGTTGTAAAGCACTTTTATCGGGAAAGAAAAGCTTAGGGCTAATACCTCTAAGCCTTGACGGTACCCGATGAATAAGCACCGGCTAACTCCGTGCCAGCAGCCGCGGTAATACGGAGGGTGCAAGCGTTAATCGGAATTACTGGGCGTAAAGCGAGTGTAGGTGGTCGAGTAAGTCGGATGTGAAAGCCCTGGGCTTAACCTGGGAATTGCATTCGATACTGCTTGGCTAGAGTCTGATAGAGGGAAGTGGAATTCCAGGTGTAGCGGTGAAATGCGTAGATATCTGGAGGAACATCAGTGGCGAAGGCGGCTTCCTGGATCAAGACTGACACTGAGGCTCGAAAGCGTGGGGAGCAAACAGG` |
| ASV4173 | 405 | `TGGGGAATATTGGACAATGGGCGAAAGCCTGATCCAGCGATTCTGCGTGAAGGATGAAGCCTCGTGGGGTGTAAACTTCTTTTCTGGGGGAAGACGAATGACGGTACCTCAGGAATAAGAGACGGCTAACTACGTGCCAGCAGCCGCGGTAATACGTAGGTCTCGAGCGTTATCCGGAATTACTGGGCGTAAAGCGTCCGCAGCCGGATATATAAGTCTGTCTTTAAATATCCCGATTCAATCGGGAGAAAGGGATAGATACTGTATATCTAGAGTACTTTAGGGGTTAGTGGAATTTCCGGTGGAGCGGTGAAATGCGTTGATATCGGAAGGAATGCCAAAAGCGAAAGCAGCTATCTATAAAGTGTCTGACGGTCAGGGACGACAGCTTGGGGAGCAAACGGG` |
| ASV4609 | 429 | `TGGGGAATATTGGACAATGGGGGAAACCCTGATCCAGCAATGCCGCGTGTGTGAAGAAGGCCTTCGGGTTGTAAAGCACTTTTAGTTGGGAGGAAAAGTTTACGGTTAATAACCGTAAACCCTGACGTTACCAACAGAAAAAGCACCGGCTAACTCTGTGCCAGCAGCCGCGGTAATACAGAGGGTGCAAGCGTTAATCGGAATTACTGGGCGTAAAGCGCACGCAGGTGGATTGTTAAGTTGGATGTGAAAGCCCTGGGCTCAACCTGGGAACGGCATTCAAAACTGGCAATCTAGAGTATGGGAGAGGGAAGTGGAATTTCCGGTGTAGCAGTGAAATGCGTAGATATCGGAAGGAACATCAGTGGCGAAGGCGACTTCCTGGCCTAATACTGACACTCAGGTGCGAAAGCGTGGGGAGCAAACAGG` |
| ASV2895 | 404 | `TGGGGAATATTGGACAATGGGGGCAACCCTGATCCAGCCATGCCGCGTGCGTGAAGAAGGCCTTAGGGTTGTAAAGCGCTTTTGTCAGGGAAGATAATGACTGTACCTGAAGAATAAGCCCCGGCTAACTCCGTGCCAGCAGCCGCGGTAAGACGGAGGGGGCTAGCGTTGTTCGGAATTACTGGGCGTAAAGGGCGCGTAGGCTGTCATGTGTGTCAGGTGTGAAATCCCAGGGCTCAACCTTGGAATGGCATCTGAAACTGCATGACTAGAGTGAGTAAGAGGCGGACGGAATTCCGAGTGTAGCAGTGAAATGCGCAGATATTCGGAGGAACATCGGTGGCGAAGGCGGTTCGCTGGTACTCTACTGACGCTGAGGCGCGAAAGCGTGGGGAGCAAACAGG` |
| ASV3607 | 404 | `TGGGGAATATTGGACAATGGGCGAAAGCCTGATCCAGCCATGCCGCGTGAGTGACGAAGGCCTTAGGGTTGTAAAGCTCTTTTGTCAGGGAAGATAATGACGGTACCTGAAGAATAAGACACGGCTAACTTCGTGCCAGCAGCCGCGGTAATACGAAGGTGTCTAGCGTTGTTCGGAATCACTGGGCGTAAAGCGCGCGTAGGCGGCTTTTCAAGTCAGAGGTGAAAGCCTGGAGCTTAACTCCAGAATTGCCTTTGAAACTGTAGAGCTAGAGTATTGGAGAGGAGTGCGGAATACCTAATGTAGCGGTGAAATGCGTAGATATTAGGTGGAACACCGATGGCGAAGGCAGCACTCTGGACAATAACTGACGCTGAGGTGCGAAAGCGTGGGGATCAAACAGG` |
| ASV1482 | 407 | `TGGGGAATTTTCCGCAATGGGCGAAAGCCTGACGGAGCAATACCGCGTGAGGGAGGAAGGCTCTTGGGTTGTAAACCTCTTTTCTGAAGGAAGAAAAAAATGACGGTACTTCAGGAATCAGCATCGGCTAACTCCGTGCCAGCAGCCGCGGTAATACGGAGGATGCAAGCGTTATCCGGAATGATTGGGCGTAAAGCGTCCGCAGGTGGCATTTCAAGTCTGCGGTTAAAGGTCGAGGCTCAACCTCGAACAGGCCGTGGAAACTGAGAAGCTAGAGTGTGGTAGGGGCAGAGGGAATTCCCAGTGTAGCGGTGAAATGCGTAGAGATTGGGAAGAACACCGGTGGCGAAAGCGCTCTGCTAGGCCAAGACTGACACTGAGGGACGAAAGCTAGGGGAGCGAATGGG` |
| ASV998 | 404 | `TGGGGAATCTTAGACAATGGGGGAAACCCTGATCTAGCCATGCCGCGTGAGCGATGAAGGCCTTAGGGTTGTAAAGCTCTTTCAGTGGGGAAGATAATGACTGTACCCACAGAAGAAGCCCCGGCTAACTCCGTGCCAGCAGCCGCGGTAATACGGAGGGGGCTAGCGTTGTTCGGAATTACTGGGCGTAAAGCGCACGTAGGCGGACGGAGAAGTCAGAGGTGAAATCCCAGGGCTCAACCTTGGAACTGCCTTTGAAACTTTCTGTCTTGAGGTCGTGAGAGGTGAGTGGAATTCCGAGTGTAGAGGTGAAATTCGTAGATATTCGGAGGAACACCAGTGGCGAAGGCGGCTCACTGGCACGATACTGACGCTGAGGTGCGAAAGCGTGGGGAGCAAACAGG` |
| ASV5066 | 425 | `TAAGGAATATTGGACAATGGCCGAAAGGCTGATCCAGCCATGCCGCGTGAAGGATGAAGGCGCTCTGCGTTGTAAACTTCTTTTGTGCAGGAACAAAACCATCCATGCGTGGATGTCTGAGGGTACTGCAAGAATAAGCACCGGCTAACTCCGTGCCAGCAGCCGCGGTAATACGGAGGGTGCAAGCGTTATCCGGAATCACTGGGTTTAAAGGGTGCGTAGGCGGTTCGATAAGTCAGTCGTGAAATACCTCCGCTTAACGGAAGGAACTGCGATTGATACTGTTGAACTTGAATCAAGTTGAGGTCGGCGGAATGAAACATGTAGCGGTGAAATGCATAGATATGTTTTAGAACACCAATTGCGAAGGCAGCTGGCTGGGCTTTGATTGACGCTGAGGCACGAAAGCGTGGGGAGCAAACAGG` |
| ASV4129 | 404 | `TGGGGAATATTGGACAATGGGGGCAACCCTGATCCAGCCATGCCGCGTGAGTGATGAAGGCCTTCGGGTTGTAAAGCTCTTTCGCCTGTGACGATGATGACGGTAACAGGAGAAGAAGCACCGGCTAACTTCGTGCCAGCAGCCGCGGTAATACGAAGGGTGCAAGCGTTGTTCGGAATTACTGGGCGTAAAGGGAGCGTAGGCCGTTTGCCAAGTCAGGCGTGAAATCCCCGGGCTCAACCTGGGAACTGCGTTTGATACTGGTAGACTAGAGTATGGGAGAGGATGGTAGAATTCCGAGTGTAGAGGTGAAATTCGTAGATATTCGGAGGAATACCCGTGGCGAAGGCGGCCATCTGGACCATTACTGACGCTGAGGCTCGAAAGCGTGGGGAGCAAACAGG` |
| ASV1756 | 404 | `TGGGGAATATTGGGCAATGGGCGAAAGCCTGACCCAGCAATACCGCGTGAGTGATGAAGGTCGAAAGATTGTAAAACTCTTTTATCAGGGAAGATTATGACGGTACCTGAAGAATAAGGACTGACTAACTTCGTGCCAGCAGTCGCGGTAATACGAAGAGTCCAAGCGTTACTCGGAATCACTGGGCGTAAAGAATGCGCAGGCTGTTTGGTAAGTTAATAGTGAAATACCGGAGCACAACTTCGGTGCTGCTATTAATACTGCCTTACTAGAGTTTGGTAGAGGATGATGGAATTCTTGGTGTAGGGGTTAAATCCTTTGATATCAAGAGGAACGCCGGAGGCGAAAGCGATCATCTGGGCCAATACTGACGCTCATGCATGACAGCGTGGGGAGCAAACAGG` |
| ASV4603 | 424 | `TAAGGAATATTGGTCAATGGACGCAAGTCTGAACCAGCCATGCCGCGTGGAGGATGAAGGTCCTCTGGATTGTAAACTTCTTTTATATGGGACGAAAAAAGGCTTTTCTAAGTCGTCTGACGGTACCATATGAATAAGCACCGGCTAACTCCGTGCCAGCAGCCGCGGTAATACGGAGGGTGCAAGCGTTATCCGGATTCACTGGGTTTAAAGGGAGCGTAGGTGGGTTGGTAAGTCAGTGGTGAAATCCCCGAGCTTAACTTGGGAACTGCCATTGATACTATCAGTCTTGAATACCGTGGAGGTCAGCGGAATATGTCATGTAGCGGTGAAATGCTTAGATATGACATAGAACACCAATTGCGAAGGCAGCTGGCTACACGAATATTGACACTGAGGCTCGAAAGCGTGGGGATCAAACAGG` |
| ASV996 | 404 | `TGGGGAATATTGGACAATGGGCGAAAGCCTGATCCAGCAATGCCGCGTGAGTGATGAAGGCCTTAGGGTTGTAAAGCTCTTTTACCAGGGATGATAATGACAGTACCTGGAGAATAAGCTCCGGCTAACTCCGTGCCAGCAGCCGCGGTAATACGGAGGGAGCTAGCGTTGTTCGGAATTACTGGGCGTAAAGCGTGCGTAGGCGGTTATTCAAGTCAGAGGTGAAAGCCTGGAGCTCAACTCCAGAACTGCCTTTGAAACTAGATAACTAGAATCTTGGAGAGGTTAGTGGAATTCCGAGTGTAGAGGTGAAATTCGTAGATATTCGGAAGAACACCAGTGGCGAAGGCGACTAACTGGACAAGCATTGACGCTGAGGTACGAAAGCGTGGGGAGCAAACAGG` |
| ASV4323 | 429 | `TGGGGAATATTGGACAATGGGGGCAACCCTGATCCAGCCATGCCGCGTGTGTGAAGAAGGCCTTCGGGTTGTAAAGCACTTTTGTTCGGGAAGAAAAGCTGCCGGTTAATACCTGGTGGTGATGACGGTACCGAAAGAATAAGCACCGGCTAACTTCGTGCCAGCAGCCGCGGTAATACGAAGGGTGCAAGCGTTACTCGGAATTACTGGGCGTAAAGCGTGCGTAGGCGGTTGGTCAAGTCTGTCGTGAAAGCCCCGGGCTCAACCTGGGAATGGCGATGGAAACTGGCGAGCTAGAGTACGGTAGAGGGCAGTGGAATTCCCGGTGTAGCAGTGAAATGCGTAGAGATCGGGAGGAACATCAGTTGCGAAGGCGGCTGCCTGGACCAGTACTGACGCTGAGGCACGAAAGCGTGGGGAGCAAACAGG` |
| ASV4268 | 404 | `TGGGGAATATTGGACAATGGGGGAAACCCTGATCCAGCGATGCCGCGTGAATGAAGAAGGCCTTAGGGTTGTAAAGTTCTTTCACCCACGACGATGATGACGGTAGTGGGAGAAGAAGCCCCGGCTAACTCCGTGCCAGCAGCCGCGGTAATACGGAGGGGGCTAGCGTTGTTCGGAATGACTGGGCGTAAAGGGCGCGTAGGCGGTTTATCAAGTGAGGCGTGAAAGCCCCGGGCTTAACCTGGGAATTGCGTTTCATACTAGTAGACTTGAGGACGAGAGAGGAAAGTGGAATTCCTAGTGTAGAGGTGAAATTCGTAGATATTAGGAAGAACACCAGTGGCGAAGGCGACTTTCTGGCTCGTATCTGACGCTGAGGCGCGAAAGCGTGGGGAGCAAACAGG` |
| ASV3009 | 429 | `TGGGGAATTTTGGACAATGGGGGCAACCCTGATCCAGCCATGCCGCGTGTGTGAAGAAGGCCTTCGGGTTGTAAAGCACTTTCGGCCGGAACGAAATCGCCTTCGTTAATAGCGGGGGTGGATGACGGTACCGGAAGAAGAAGCACCGGCTAACTACGTGCCAGCAGCCGCGGTAATACGTAGGGTGCGAGCGTTAATCGGAATTACTGGGCGTAAAGGGTGCGCAGGCGGCGTCGCAAGTCAGGCGTGAAATCCCCGGGCTTAACCTGGGAATGGCACTTGAAACTACGATGCTGGAGTATGGCAGAGGGAGGTGGAATTCCACGTGTAGCGGTGAAATGCGTAGAGATGTGGAGGAACACCGATGGCGAAGGCAGCCTCCTGGGCCAATACTGACGCTCATGCACGAAAGCGTGGGGAGCAAACAGG` |
| ASV5562 | 430 | `TGGGGAATATTGCGCAATGGGCGAAAGCCTGACGCAGCGACGCCGCGTGGGTGATGAAGGCCTTCGGGTCGTAAAGCCCTGTCTGGTGGAACGAATGGCTTGGCGGCAAATAACCGCTGGGCGTGACGGTACCACCGAAGGAAGCCCCGGCTAACTACGTGCCAGCAGCCGCGGTAAGACGTAGGGGGCGAGCGTTGTTCGGATTTACTGGGCGTAAAGCGCGCGTAGGCGGCTAGATAAGTCGGGTGTGAAATCCCTCGGCTTAACCGAGGACCCGCATCCGATACTGTCTGGCTCGAGTCTAGGAGAGGAAAGCGGAATTCCCAGTGTAGCGGTGAAATGCGTAGATATTGGGAGGAACACCTGTGGCGAAGGCGGCTTTCCGGACTAGCACTGACGCTGAGGCGCGAAAGCTAGGGGAGCGAACGGG` |
| ASV3848 | 406 | `TGGGGAATTTTCCGCAATGGGCGAAAGCCTGACGGAGCAACGCCGCGTGAGGGATGAAGGCCTCTGGGCTGTAAACCTCTTTTCTCAAGGAAGAAGACATGACGGTACTTGAGGAATAAGCCACGGCTAATTCCGTGCCAGCAGCCGCGGTAATACGGGAGTGGCAAGCGTTATCCGGAATTATTGGGCGTAAAGCGTCCGCAGGCGGCCTTGTAAGTCTGTCGTTAAAGCGTGGAGCTTAACTCCATTTCAGCGATGGAAACTACAAGGCTTGAGTGTGGTAGGGGCAGAGGGAATTCCCGGTGTAGCGGTGAAATGCGTAGATATCGGGAAGAACACCAGTGGCGAAGGCGCTCTGCTGGGCCATAACTGACGCTCATGGACGAAAGCCAGGGGAGCGAAAGGG` |
| ASV2628 | 423 | `TAGGGAATATTGGTCAATGGGCGCAAGCCTGAACCAGCCATGCCGCGTGCAGGATGAAGGCCCTCAGGGTCGTAAACTGCTTTTGTACGGGAACAAAAAGCCCCTGCGGGGGCCATTGAGGGTACCGTAAGAATAAGCACCGGCTAACTCCGTGCCAGCAGCCGCGGTAATACGGAGGGTGCAAGCGTTATCCGGATTTACTGGGTTTAAAGGGTGCGTAGGCGGCTTTTTAAGTCAGTGGCGAAAGCCTGGCGCTTAACGCCAGAATTGCCATTGATACTGGAGAGCTTGAGTCGAGAAGAGGTAAGCAGAATTCATGGTGTAGCGGTGAAATGCATAGATACCATGAGGAATTCCAACAGCGAAGGCAGCTTACTGGTCTCTGACTGACGCTGAGGCACGAAAGCGTGGGGAGCAAACAGG` |
| ASV2232 | 429 | `TGGGGAATTTTGGACAATGGACGCAAGTCTGATCCAGCCATGCCGCGTGCGGGAAGAAGGCCTTCGGGTTGTAAACCGCTTTTGTCAGGGAAGAAAAGGGCGTTTCTAATAAAGATGCCTCATGACGGTACCTGAAGAATAAGCACCGGCTAACTACGTGCCAGCAGCCGCGGTAATACGTAGGGTGCAAGCGTTAATCGGAATTACTGGGCGTAAAGCGTGCGCAGGCGGTTATGCAAGACAGATGTGAAATCCCCGGGCTCAACCTGGGAACTGCATTTGTGACTGCATGGCTAGAGTACGGTAGAGGGGGATGGAATTCCGCGTGTAGCAGTGAAATGCGTAGATATGCGGAGGAACACCGATGGCGAAGGCAATCCCCTGGACCTGTACTGACGCTCATGCACGAAAGCGTGGGGAGCAAACAGG` |
| ASV3882 | 404 | `TGAGGAATATTGGACAATGGGCGCAAGCCTGATCCAGCCATGCCGCGTGAGTGAAGAAGGCCTTCGGGTTGTAAAGCTCTTTTAGATGTGACGATAATGACGGTAGCATCAGAAAAAGCCCCGGCTAAATTCGTGCCAGCAGCCGCGGTAATACGAATGGGGCAAGCGTTGTTCGGAATCACTGGGCTTAAAGCGTGCGCAGGCGGACTAGCAAGTCAGGAGTGAAATCCCAGGGCTCAACCCTGGAATTGCTTTTGAAACTGCAAGTCTAGAATCCCGGAGAGGTTAGCAGAATTCCGAGTGTAGAGGTGAAATTCGCAGATATTCGGAGGAATACCAATGGCGAAGGCAGCTAACTGGACGGGCATTGACGCTCAGGCACGAAAGCGTGGGTAGCAAACAGG` |
| ASV2668 | 429 | `TGGGGAATTTTGGACAATGGGCGAAAGCCTGATCCAGCCATGCCGCGTGCAGGAAGAAGGCCTTCGGGTTGTAAACTGCTTTTGTCAGGGAAGAAATCTTCTGGGTTAATACCTCGGGAGGATGACGGTACCTGAAGAATAAGCACCGGCTAACTACGTGCCAGCAGCCGCGGTAATACGTAGGGTGCAAGCGTTAATCGGAATTACTGGGCGTAAAGCGTGCGCAGGCGGTTGTATAAGACAGATGTGAAATCCCCGGGCTCAACCTGGGAACTGCATTTGTGACTGTACAGCTGGAGTGCGGCAGAGGGGGATGGAATTCCGCGTGTAGCAGTGAAATGCGTAGATATGCGGAGGAACACCGATGGCGAAGGCAATCCCCTGGGCCTGCACTGACGCTCATGCACGAAAGCGTGGGGAGCAAACAGG` |
| ASV570 | 404 | `TGGGGAATATTGCACAATGGACGAAAGTCTGATGCAGCAACGCCGCGTGGAGGATGACACATTTCGGTGCGTAAACTCCTTTTATATGGGAAGATAATGACGGTACCATATGAATAAGCACCGGCTAACTCCGTGCCAGCAGCCGCGGTAATACGGAGGGTGCAAGCGTTACTCGGAATCACTGGGCGTAAAGAGCGTGTAGGCGGATATATAAGTCAGAAGTGAAATCCAATAGCTTAACTATTGAACTGCTTTTGAAACTGTATATCTAGAATGTGGGAGAGGTAGATGGAATTTCTGGTGTAGGGGTAAAATCCGTAGAGATCAGAAGGAATACCGATTGCGAAGGCGATCTACTGGAACATTATTGACGCTGAGACGCGAAAGCGTGGGGAGCAAACAGG` |
| ASV744 | 424 | `TGAGGAATATTGGACAATGGGCGCAAGCCTGATCCAGCCATGCCGCGTGCAGGATGACGGTCCTATGGATTGTAAACTGCTTTTGCACAGGAAGAAACAACATTACGTGTAATGTCTTGACGGTACTGTGAGAATAAGGATCGGCTAACTCCGTGCCAGCAGCCGCGGTAATACGGAGGATCCAAGCGTTATCCGGAATCATTGGGTTTAAAGGGTCCGTAGGCGGTTTAGTAAGTCAGTGGTGAAAGCCCATCGCTCAACGGTGGAACGGCCATTGATACTGCTAGACTTGAATTATTAGGAAGTAACTAGAATATGTAGTGTAGCGGTGAAATGCTTAGAGATTACATGGAATACCAATTGCGAAGGCAGGTTACTACTAATGGATTGACGCTGATGGACGAAAGCGTGGGTAGCGAACAGG` |
| ASV793 | 429 | `TGGGGAATTTTGGACAATGGGCGCAAGCCTGATCCAGCAATGCCGCGTGCAGGATGAAGGCCTTCGGGTTGTAAACTGCTTTTGTACGGAACGAAACGGCTCTTTCTAATAAAGAGGGCTAATGACGGTACCGTAAGAATAAGCACCGGCTAACTACGTGCCAGCAGCCGCGGTAATACGTAGGGTGCGAGCGTTAATCGGAATTACTGGGCGTAAAGCGTGCGCAGGCGGTTATGTAAGACAGATGTGAAATCCCCGGGCTCAACCTGGGACCTGCATTTGTGACTGCATAGCTAGAGTACGGTAGAGGGGGATGGAATTCCGCGTGTAGCAGTGAAATGCGTAGATATGCGGAGGAACACCGATGGCGAAGGCAATCCCCTGGACCTGTACTGACGCTCATGCACGAAAGCGTGGGGAGCAAACAGG` |
| ASV4103 | 405 | `TAGGGAATATTGCACAATGGGGGAAACCCTGATGCAGCCATGCCGCGTGTGTGAAGAAGGCCCTAGGGTTGTAAAGCACTTTCAGTGGGGAGGAAATTTGACGTTACCCACAGAAGAAGCACCGGCTAACTCCGTGCCAGCAGCCGCGGTAATACGGAGGGTGCAAACGTTAATCGGAATAACTGGGCGTAAAGGGCATGTAGGCGGAGAGGCAAGTAAGATGTGAAAGACTTGGGCTCAACCTGAGCTGGTCATTTTAAACTACCTTTCTAGAGTATCGCAGAGGAAGATGGAATTTCAGGTGTAGCAGTGGAATGCGTAGAGATCTGAAAGAACACCAGTGGCGAAGGCGGTCTTCTGGGCGAATACTGACGCTGAGGTGCGAAAGCGTGGGGAGCAAACAGG` |
| ASV4402 | 404 | `TTAGGAATTTTGGGCAATGGGGGGAACCCTGACCCAGCAACGCCGCGTGGGTGATGACGGCCTTCGGGTTGTAAAGCCCTTTTCCCAGGGAAGATGGTGACGGTACCTGGGGAAGAAGCCCCGGCTAACTACGTGCCAGCAGCCGCGGTAATACGTAGGGGGCGAGCGTTGTCCGGATTTATTGGGCGTAAAGGGCCCGCAGGTGGTCTTGTGCGTCGCGCGTGAAATACCCCGGCTCAACTGGGGGGTGTCGTGCGATACGGCGAGACTGGAGGCCAGTAGGGGGATGCGGAATTCCGGGTGTAGTGGTGGAATGCGTAGAGATCCGGAGGAACACCAGCGGCGAAGGCGGCATCCTGGACTGGTCCTGACACTCAGGGGCGAAAGCGTGGGGAGCGATCCGG` |
| ASV5050 | 406 | `TGGGGAATTTTCCGCAATGGGCGCAAGCCTGACGGAGCAAGACCGCGTGGGGGACGAAGGTTTGTGGATTGTAAACCCCTTTTGATTGGGAAGAAGTTCTGACGGTACCAATCGAATCAGCATCGGCTAACTCCGTGCCAGCAGCCGCGGTAATACGGAGGATGCAAGCGTTATCCGGAATTATTGGGCGTAAAGCGTCCGTAGGTGGCTATTCAAGTCAGTTGTTAAAGCGTGGGGCTTAACTCCATAAAGGCAAATGAAACTGATTGGCTAGAGTGCGATAGGGGCAAGGGGAATTTCCAGTGTAGCGGTGAAATGCGTAGATATTGGAAAGAACACCGGTGGCGAAAGCGCCTTGCTGGGTCTGCACTGACACTGAGGGACGAAAGCTAGGGGAGCGAAAGGG` |
| ASV1082 | 404 | `TGGGGAATATTGGACAATGGGCGCAAGCCTGATCCAGCCATGCCGCGTGAGTGATGAAGGCCTTAGGGTTGTAAAGCTCTTTCACCGGTGAAGATAATGACGGTAACCGGAGAAGAAGCCCCGGCTAACTTCGTGCCAGCAGCCGCGGTAATACGAAGGGGGCTAGCGTTGTTCGGAATTACTGGGCGTAAAGCGCACGTAGGCGGATATTTAAGTCAGGGGTGAAATCCCGCAGCTCAACTGCGGAACTGCCTTTGATACTGGGTATCTTGAGTATGGAAGAGGTAAGTGGAATTGCGAGTGTAGAGGTGAAATTCGTAGATATTCGCAGGAACACCAGTGGCGAAGGCGGCTTACTGGTCCATTACTGACGCTGAGGTGCGAAAGCGTGGGGAGCAAACAGG` |
| ASV1908 | 424 | `TGAGGAATATTGGTCAATGGAGGCAACTCTGAACCAGCCATGCCGCGTGAAGGATGAAGGTCCTCTGGATTGTAAACTTCTTTTATAGAAGAAGAAACCCCAGTATTCTTACTGGGTTGACGGTACTCTATGAATAAGCACCGGCTAACTCCGTGCCAGCAGCCGCGGTAATACGGAGGGTGCAAGCGTTATCCGGATTCACTGGGTTTAAAGGGAGCGTAGGTGGGTCGGTAAGTCAGTGGTGAAATCCCCGAGCTTAACTTGGGAACTGCCATTGATACTATCGATCTTGAATTCTGTGGAGGTAAGCGGAATATATCATGTAGCGGTGAAATGCTTAGATATGATATAGAACACCGATAGCGAAGGCAGCTTACTACGCAGATATTGACACTGAGGCTCGAAAGCGTGGGGATCAAACAGG` |
| ASV4753 | 429 | `TGGGGAATATTGGACAATGGGGGCAACCCTGATCCAGCAATGCCGCGTGTGTGAAGAAGGCCTGAGGGTTGTAAAGCACTTTCAGTGGGGAAGAAGGCGTTAGTGTGAATAATACTAACGATTGACGTTACCCAAAGAAGAAGCACCGGCTAACTCTGTGCCAGCAGCCGCGGTAATACAGAGGGTGCAAGCGTTAATCGGAATCACTGGGCGTAAAGAGCGCGTAGGTTGATAATTAAGTTGGATGTGAAAGCCCTGGGCTCAACCCGGGATGGTCATTCAAGACTGGTTATCTAGAGTATAGTAGAGGGAAGTGGAATTTCCGGTGTAGCGGTGAAATGCGTAGATATCGGAAGGAACACCAGTGGCGAAGGCGGCTTCCTGGACTAATACTGACACTGAGGCGCGAAAGCGTGGGTAGCAAACAGG` |
| ASV5619 | 424 | `TAAGGAATATTGGTCAATGGAGGCAACTCTGAACCAGCCATGCCGCGTGGAGGATGAAGGTCCTCTGGATTGTAAACTTCTTTTATATGGGACGAAAAAACGGTTTTTCAACTGGACTGACGGTACCATATGAATAAGCACCGGCTAACTCCGTGCCAGCAGCCGCGGTAATACGGAGGGTGCAAGCGTTATCCGGATTCACTGGGTTTAAAGGGAGCGTAGGTGGGCTAGTAAGTCCGTGGTGAAATCTCCGAGCTTAACTCGGAAACTGCCGTGGATACTATTAGTCTTGAATATTGTGGAGGTTAGCGGAATATGTCATGTAGCGGTGAAATGCTTAGATATGACATAGAACACCAATTGCGAAGGCAGCTGGCTACACATATATTGACACTGATGCTCGAAAGCGTGGGGATCAAACAGG` |
| ASV345 | 404 | `TGGGGAATCTTAGACAATGGGGGAAACCCTGATCTAGCCATGCCGCGTGAGCGATGAAGGCCTTAGGGTTGTAAAGCTCTTTCAGTGGGGAAGATAATGACGGTACCCACAGAAGAAGCCCCGGCTAACTCCGTGCCAGCAGCCGCGGTAATACGGAGGGGGCTAGCGTTGTTCGGAATTACTGGGCGTAAAGCGCACGTAGGCGGACTTGAAAGTCAGAGGTGAAATCCCAGGGCTCAACCTTGGAACTGCCTTTGAAACTCCTGGTCTTGAGGTCGTGAGAGGTGAGTGGAATTCCGAGTGTAGAGGTGAAATTCGTAGATATTCGGAGGAACACCAGTGGCGAAGGCGGCTCACTGGCACGATACTGACGCTGAGGTGCGAAAGCGTGGGGAGCAAACAGG` |
| ASV176 | 428 | `TGGGGAATTTTGGACAATGGGGGAAACCCTGATCCAGCAATGCCGCGTGAGTGAAGAAGGCCTTCGGGTTGTAAAGCTCTTTTGTCAGGGAAGAAACACCGGCTCTAACACAGTCCGGGAATGACGGTACCTGAAGAATAAGCACCGGCTAACTACGTGCCAGCAGCCGCGGTAATACGTAGGGTGCGAGCGTTAATCGGAATTACTGGGCGTAAAGCGTGCGCAGGCGGTTATACAAGACAGGCGTGAAATCCCCGGGCTTAACCTGGGAATGGCGCCTGTGACTGTATAGCTAGAGTGTGTCAGAGGGGGGTAGAATTCCACGTGTAGCAGTGAAATGCGTAGATATGTGGAGGAATACCAATGGCGAAGGCAGCCCCCTGGGATAACACTGACGCTCATGCACGAAAGCGTGGGGAGCAAACAGG` |
| ASV1376 | 404 | `TGGGGAATATTGGACAATGGGCGAAAGCCTGATCCAGCCATGCCGCGTGAGTGATGAAGGCCTTAGGGTTGTAAAGCTCTTTCACCGATGAAGATAATGACGGTAGTCGGAGAAGAAGCCCCGGCTAACTTCGTGCCAGCAGCCGCGGTAATACGAAGGGGGCTAGCGTTGTTCGGAATCACTGGGCGTAAAGCGCACGTAGGCGGATTGTTAAGTGAGGGGTGAAATCCCAGGGCTCAACCCTGGAACTGCCTCTCATACTGGCAATCTCGAGTTCGAGAGAGGTAAGTGGAATTCCGAGTGTAGAGGTGAAATTCGTAGATATTCGGAGGAACACCAGTGGCGAAGGCGGCTTACTGGATCGATACTGACGCTGAGGTGCGAAAGCGTGGGGAGCAAACAGG` |
| ASV5267 | 403 | `TGGGGGATATTGCACAATGGAGGAAACTCTGATGCAGCGACGCCGCGTGAGGGAAGAAGGTTTTCGGATTGTAAACCTCTGTCTTCAAGGACGATAATGACGGTACTTGAAGAGGAAGCCACGGCTAACTACGTGCCAGCAGCCGCGGTAATACGTAGGTGGCAAGCGTTGTCCGGAATTACTGGGTGTAAAGGGAGCGTAGGCGGTTCTGCAAGTTGAATGTTTAAACTATCGGCTCAACCGGTAGTCGCGTTCAAAACTGTGGGACTTGAGTGAAGTAGAGGCAGGCGGAATTCCCGGTGTAGCGGTGAAATGCGTAGATATCGGGAGGAACACCAGTGGCGAAGGCGGCCTGCTGGGCTTTTACTGACGCTGAGGCTCGAAAGTGTGGGGAGCAAACAGG` |
| ASV5429 | 407 | `TAGGGAATATTGCGCAATGGGGGAAACCCTGACGCAGCGACGCCGCGTGAGTGATGAAGGCCTTCGGGTCGTAAAGCTCTGTTGCAAGGGAATAACAAAATGAATGTACCTTGTGAGGAAGGATCGGCTAACTTCGTGCCAGCAGCCGCGGTAAGACGAGGGATCCTAGCGTTGCTCGGAATCATTGGGCGTAAAGCGGGTGTAGGGGGCTTTGTAAGTCAGGTGTGAAAGCCCAGGGCTCAACCCTGGAAGTGCATTTGATACTGCGAAGCTTGAGTGTTGGAGAGGCTACTGGAATACCTGGTGTAGTGGTGAAATACGTAGATATCAGGTGGAACACCGGAGGCGAAGGCGGGTAGCTGGCCAAACACTGACCCTGAGACCCAAAAGCGTGGGGATCAAACAGG` |
| ASV2848 | 430 | `TGGGGAATCTTGCGCAATGGGGGAAACCCTGACGCAGCGATGCCGCGTGAGTGAGGAAGGCCTTCGGGTTGTAAAGCTCTGTCGTATGGGAAAAAGGGCAAACGGTCTAATAGGCCGTATGTTTGATGGTACCATAAAAGGAAGCACCGGCTAACTTCGTGCCAGCAGCCGCGGTAATACGAAGGGTGCAAGCGTTGTTCGGATTTATTGGGCGTAAAGCGCGCGCAGGCGGACCTGCAAGTCAGATGTGAAATCTCGGGGCTCAACTCCGAACGTGCGTCTGAAACTGCGGGACTAGAATATTGGAGAGGGAAGGGGAATTTCTCATGTAGGGGTAAAATCCGTAGAGATGAGAAGGAACACCAGAGGCGAAAGCGCCTTCCTGGCCAAAGATTGACGCTGAGGCGCGAAAGCGTGGGGAGCAAACAGG` |
| ASV5113 | 416 | `TTAGGAATCTTCCCCAATGGGCGCAAGCCTGAGGGAGCGACGCCGCGTGCGGGATGAAGGCCTTCGGGTCGTAAACCGCTGAAACGAGGACGAAAACCCCGCAAGGGATCTGACGGTACTCGTGTAATAGCACCGGCTAACTCCGTGCCAGCAGCCGCGGTAATACGGAGGGTGCAAGCGTTACCCGGAATCACTGGGCGTAAAGGGCATGTAGGCGGTCATTTAAGTCCGAATTTAAAGACCGAGGCTCAACCTCGGCAGTGGTTTGGATACTGGATGACTGGACGACTGGAGGGGAGAGTGGAATTCTCGGTGTAGCGGTGGAATGCGTAGATACCGAGAGGAACACCAGTGGCGAAGGCGGCTCTCTGGACAGTGCGTGACGCTGAGGTGCGAAAGTGTGGGGAGCAAACCGG` |
| ASV5737 | 404 | `TGGGGAATATTGCACAATGGGCGAAAGCCTGATGCAGCGACGCCGCGTGAGCGAAGAAGTATTTCGGTATGTAAAGCTCTATCAGCAGGGAAGAAAATGACGGTACCTGACTAAGAAGCACCGGCTAAATACGTGCCAGCAGCCGCGGTAATACGTATGGTGCAAGCGTTATCCGGATTTACTGGGTGTAAAGGGAGCGTAGGCGGCTTTGTAAGTCTGATGTGAAAACCCGGGGCTCAACCCCGGGACTGCATTGGAAACTACGAAGCTAGAGTGTCGGAGAGGTAAGTGGAATTCCTAGTGTAGCGGTGAAATGCGTAGATATTAGGAGGAACACCAGTGGCGAAGGCGACTTACTGGACGATAACTGACGCTGAGGCTCGAAAGCGTGGGGAGCAAACAGG` |
| ASV23 | 429 | `TGGGGAATTTTGGACAATGGGCGCAAGCCTGATCCAGCAATGCCGCGTGCAGGATGAAGGCCTTCGGGTTGTAAACTGCTTTTGTACGGAACGAAACGGCGAGCTCTAATACAGCTTGCTAATGACGGTACCGTAAGAATAAGCACCGGCTAACTACGTGCCAGCAGCCGCGGTAATACGTAGGGTGCGAGCGTTAATCGGAATTACTGGGCGTAAAGCGTGCGCAGGCGGTGATGTAAGACAGATGTGAAATCCCCGGGCTCAACCTGGGACCTGCATTTGTGACTGCATCGCTAGAGTACGGTAGAGGGGGATGGAATTCCGCGTGTAGCAGTGAAATGCGTAGATATGCGGAGGAACACCGATGGCGAAGGCAATCCCCTGGACCTGTACTGACGCTCATGCACGAAAGCGTGGGGAGCAAACAGG` |
| ASV2228 | 405 | `TGGGGAATATTGGACAATGGGGGAAACCCTGATCCAGCGATGCCGCGTGAGTGATGAAGGCCTTCGGGTTGTAAAGCTCTTTTGTCCGGGACGATAATGACGGTACCGGAAGAATAAGCTCCGGCTAACTTCGTGCCAGCAGCCGCGGTAATACGAAGGGAGCTAGCGTTGTTCGGAATTACTGGGCGTAAAGGGCGCGTAGGTGGTTCAATAAGTGAGATGTGAAAGCCCCGGGCTTAACCTGGGAAATGCATTTCATACTGTTGGACTAAGTGTGTAAGAGGGGAAAGCGGAATGACGAGTGGAGCAGTGAAATGCGTTGATATTCGTTGGAACACCAGAGGCGAAGGCGGCTTTCTAGCTTACAACAGACACTAAGGCGCTAAAGCGTGGGGAGCAAACAGG` |
| ASV4003 | 405 | `TAGGGAATTTTCCACAATGGGCGAAAGCCTGATGGAGCAACGCCGCGTGCAGGATGAAGGCCTTCGGGTCGTAAACTGCTTTTATGAGTGATGATTATGACAGTAGCTCAAGAATAAGGGTCGGCTAACTACGTGCCAGCAGCCGCGGTCATACGTAGGACCCAAGCGTTATCCGGAATTACTGGGCGTAAAGAGTTGCGTAGGCGGCTTATTAAGCAAGATGTGAAAGCACACGGCTCAACCGTGTAAACATATTTTGAACTGGTAAGCTAGAGTATGGGAGAGGTTATTGGAATTGCTAGTGTAGGAGTGAAATCCGTAGATATTAGCAGGAACACCGATGGCGTAGGCAGATAACTGGCCCATTACTGACGCTCAGGCACGAAAGCGTGGGGAGCAAACGGG` |
| ASV1018 | 429 | `TGGGGAATTTTGGACAATGGGCGCAAGCCTGATCCAGCAATGCCGCGTGCAGGAAGAAGGCCTTCGGGTTGTAAACTGCTTTTGTACGGAACGAAACGGTCTGGGTTAATACCCTGGGCTAATGACGGTACCGTAAGAATAAGCACCGGCTAACTACGTGCCAGCAGCCGCGGTAATACGTAGGGTGCAAGCGTTAATCGGAATTACTGGGCGTAAAGCGTGCGCAGGCGGTTTTGTAAGACAGTCGTGAAATCCCCGGGCTCAACCTGGGAATTGCGATTGTGACTGCAAAGCTGGAGTGCGGCAGAGGGGGATGGAATTCCGCGTGTAGCAGTGAAATGCGTAGATATGCGGAGGAACACCGATGGCGAAGGCAATCCCCTGGGCCTGCACTGACGCTCATGCACGAAAGCGTGGGGAGCAAACAGG` |
| ASV5233 | 429 | `TGGGGAATATTGGACAATGGGGGCAACCCTGATCCAGCAATGCCACGTGTGTGAAGAAGGCCTGAGGGTTGTAAAGCACTTTTGTCAATGAGGAAGCTTGTTAGGTTAAGAGCTAGACAAGTTGACGTTAGTTGAAGAAAAAGCACCGGCAAACTCTGTGCCAGCAGCCGCGGTAATACAGAGGGTGCAAGCGTTAATCGGAATGACTGGGCGTAAAGCGCACGTAGGTGGATATTTAAGTCGGTTGTGAAACCCCTGGGCTCAACCTAGGAATGGCAATCGATACTGGATATCTGGAGTACGATAGAGGGTAGAGGAATTTCTGGTGTAGCGGTGAAATGCGTAGATATCAGAAGGAACATCGGTGGCGAAGGCGTCTACCTGGATTGATACTGACACTGAGGAGCGAAAGCATGGGTAGCAAACAGG` |
| ASV4364 | 409 | `TGGGGAATATTGGACAATGGGCGAAAGCCTGATCCAGCAACGCCGCGTGAGGGATGACGGCCTTCGGGTTGTAAACCTCTTTCAGCAGGGACGAAGCGCAAGTGACGGTACCTGCAGAAGAAGCACCGGCCAACTACGTGCCAGCAGCCGCGGTAATACGTAGGGTGCGAGCGTTGTCCGGAATTATTGGGCGTAAAGGGCTCGTAGGCGGTTTGTCGCGTCGGGAGTGAAAACTCAGGGCTTAACCCTGAGCCTGCTTCCGATACGGGCAGACTAGAGGTATGCAGGGGAGAACGGAATTCCTGGTGTAGCGGTGAAATGCGCAGATATCAGGAGGAACACCGGTGGCGAAGGCGGTTCTCTGGGCATTACCTGACGCTGAGGAGCGAAAGTGTGGGGAGCGAACAGG` |
| ASV3829 | 430 | `TGGGGAATTTTGGACAATGGGGGAAACCCTGATCCAGCCATGCCGCGTGCAGGAAGAAGGCCTTCGGGTTGTAAACTGCTTTAGTCAGGGGAAAATGTATTGACCTTTAATACGGGTTGATGCTGATGGTACCTGAAGAATAAGCACCGGCTAACTACGTGCCAGCAGCCGCGGTAATACGTAGGGTGCAAGCGTTAATCGGAATTACTGGGCGTAAAGCGTGCGCAGGCGGTTTCATAAGACAGATGTGAAAGCCCCGGGCTTAACCTGGGAACTGCATTTGTGACTGTGAGGCTAGAGTACAGCAGAGGGGGGTAGAATTCCACGTGTAGCAGTGAAATGCGTAGAGATGTGGAGGAATACCAATGGCGAAGGCAACCCCCTGGGCTGATACTGACGCTCATGTACGAAAGCGTGGGGAGCAAACAGG` |
| ASV185 | 429 | `TGGGGAATTTTGGACAATGGGCGCAAGCCTGATCCAGCCATGCCGCGTGCGGGAAGAAGGCCTTCGGGTTGTAAACCGCTTTTGTCAGGGAAGAAACGCTCCGGGCTAATATCCTGGGGTAATGACGGTACCTGAAGAATAAGCACCGGCTAACTACGTGCCAGCAGCCGCGGTAATACGTAGGGTGCAAGCGTTAATCGGAATTACTGGGCGTAAAGCGTGCGCAGGCGGTCCTACAAGACAGATGTGAAATCCCCGGGCTCAACCTGGGAACTGCATTTGTGACTGTAGGGCTGGAGTGCGGCAGAGGGGGATGGAATTCCGCGTGTAGCAGTGAAATGCGTAGATATGCGGAGGAACACCGATGGCGAAGGCAATCCCCTGGGCCTGCACTGACGCTCATGCACGAAAGCGTGGGGAGCAAACAGG` |
| ASV1564 | 429 | `TAGGGAATATTGCGCAATGGGGGAAACCCTGACGCAGCAACGCCGCGTGAGTGAGGAAGGTCTTCGGATTGTAAAACTCTTTTATTTGGGAAAAATGGTCATGGAGCTAATACCCCATGATTTGATGGTACCAAAAGAATAAGCACCGGCTAACTTCGTGCCAGCAGCCGCGGTAATACGAAGGGTGCAAGCGTTGTTCGGAATCATTGGGCGTAAAGCGCGCGCAGGCGGGCCAGCAAGTCAGATGTGAAATCTCGGAGCTCAACTCCGAAACTGCGTCTGAAACTGCTAGTCTAGAGTGTCAGAGGGGGTAGGGGAATTTCACGTGTAGGGGTAAAATCCGTAGAGATGTGAAGGAACACCGGAGGCGAAGGCGCCTACCTGGATGACTACTGACGCTGAGGCGCGAAAGCGTGGGGAGCAAACAGG` |
| ASV5040 | 427 | `TGGGGAATCTTCCGCAATGGGCGAAAGCCTGACGGAGCAACGCCGCGTGAATGAAGAAGGCCTTCGGGTCGTAAAGTTCTGTCATTAGGGACGAATAAGCCTTGTAAATAATGAGGTGTATGACGGTACCTAAGGAGGAAGCTCCGGCTAACTACGTGCCAGCAGCCGCGGTAATACGTAGGGAGCAAGCGTTGTCCGGAATTACTGGGCGTAAAGGGCGCGTAGGCGGATATTTATGTCAGATGTGAAAACTCCAGGCTCAACCTGGAGACTGCATTTGAAACTGGGTATCTTGAGGGCAGGAGAGGAAAGTGGAATTCCTAGTGTAGCGGTGAAATGCGTAGATATTAGGAGGAACACCAGTGGCGAAGGCGACTTTCTGGACTGTACCTGACGCTGAGGCGCGAAAGCGTGGGGAGCAAACGGG` |
| ASV2753 | 404 | `TGGGGAATTTTCCGCAATGGGCGAAAGCCTGACGGAGCAATACCGCGTGAGGGACGAAGGCCTGTGGGTTGTAAACCTCTTTTGTTAGGGAAGATAATGACGGTACCTAACGAATAAGCATCGGCTAACTCCGTGCCAGCAGCCGCGGTAAGACGGAGGATGCAAGCGTTATCCGGAATTATTGGGCGTAAAGCGTACGTAGGCTGTTTGATAAGTCTGTTGTCAAAGCGCGAGGCTCAACCTTGTAAAGGCAATGGAAACTGTGAGACTAGAGAGAGATAGGGGCAGGAGGAATTCCAGGTGTAGCGGTGAAATGCGTAGATATCTGGAAGAACACCAGTGGCGAAAGCGTCCTGCTGGATCTCAACTGACGCTGAAGTACGAAAGCTAGGGGAGCGAATGGG` |
| ASV3327 | 404 | `TGAGGAATATTGGACAATGGAGGAAACTCTGATCCAGCCATGCCGCGTGAGTGATGAAGGCCTTCGGGTTGTAAAGCTCTTTCAGGTGTGAAAATGATGATGGTAACACCAGAAGAAGCCCCGGCTAACTCCGTGCCAGCAGCCGCGGTAATACGGAGGGGGCGAGCGTTGTTCGGAATCACTGGGCGTAAAGAGTGCGTAGGCTGTTATACAAGTCAGGAGTGAAATCCCAGGGCTTAACCCTGGAATTGCTTTTGAGACTGTATGACTAGAGATCTAGAGAGGTTGGGGGAATTCCGAGTGTAGAGGTGAAATTCGCAGATATTCGGAGGAACACCAGTGGCGTAGGCGCCCAACTGGCTAGATACTGACGCTGAGGCACGAAAGCGTGGGGAGCAAACAGG` |
| ASV270 | 430 | `TGGGGAATATTGCGCAATGGGCGAAAGCCTGACGCAGCAACGCCGCGTGAGTGAAGAAGGCCTTTGGGTTGTAAAGCTCTTTCGGCTGGGAAGAAGGGTTTTTGTGTTAATAGCGCAAGAACTTGATGGTACCAGAATAAGAAGCACCGGCTAACTTCGTGCCAGCAGCCGCGGTAATACGAAGGGTGCGAGCGTTGTTCGGAATTACTGGGCGTAAAGGGTTTGTAGGCTGAAATGTAAGTCAAGTGTGAAATCCCTGAGCTTAACTTGGGACGTGCATTTGAAACTGTGTTTCTTGAGTATCGTAGAGGATGGTGGAATTGTAGGTGTAGGAGTGACATCCGTAGAGATCTGCAGGAACATCAGAGGCGAAAGCGACCATCTGGGCGATTACTGACGCTGAGGAACGAAAGCGTGGGGAGCAAACAGG` |
| ASV2827 | 429 | `TGGGGAATTTTGGACAATGGGGGCAACCCTGATCCAGCAATGCCGCGTGAGTGAAGAAGGCCTTCGGGTTGTAAAGCTCTTTTGTTAGGGAAGAAAAGGTGAGCAATAATACTGTTTACTGATGACGGTACCTAAAGAATAAGCACCGGCTAACTACGTGCCAGCAGCCGCGGTAATACGTAGGGTGCAAGCGTTAATCGGAATTACTGGGCGTAAAGCGTGCGCAGGCGGCCTCGCAAGTCAGATGTGAAATCCCCGAGCTCAACTTGGGAACTGCATTTGAAACTACGAGGCTAGAGTGTGTCAGAGGGAGGTAGAATTCCACGTGTAGCAGTGAAATGCGTAGATATGTGGAGGAATACCAGTGGCGAAGGCGGCCTCCTGGGATAACACTGACGCTCATGTACGAAAGCGTGGGGAGCAAACAGG` |
| ASV2611 | 429 | `TGGGGAATATTGCGCAATGGGGGAAACCCTGACGCAGCGACGCCGCGTGAGTGACGAAGGCCTTCGGGTTGTAAAGCTCTGTCAGCTGGGAAGAAATTCCAGGTGCTAATATCACCTGGCGATGACGGTACCAGCAGAGGAAGCACCGGCTAACCCTGTGCCAGCAGCCGCGGTAATACAGGGGGTGCGAGCGTTGTTCGGAATTACTGGGCGTAAAGCGCGTGTAGGCGGTTTGATAAGTCAGATGTGAAAGCCTGGGGCTTAACTCCAGAAGTGCATTTGAAACTGTCAAGCTTGAGTGATGTAGAGGATGGCGGAATACCCAGTGTAGAGGTGAAATTCGTAGAGATTGGGTAGAACACCGGTGGCGAAGGCGGCCATCTGGGCATTAACTGACGCTGAGACGCGAAAGCATGGGGAGCAAACAGG` |
| ASV1335 | 425 | `TAAGGAATATTGGGCAATGGTCGGAAGACTGACCCAGCCATGCCGCGTGCAGGAAGAAGGCCCTCTGGGTTGTAAACTGCTTTTATACGGGAAGAAAAAGCCCTTGCGGGGGTGTGTTTGACGGTACCGTATGAATAAGCACCGGCTAACTACGTGCCAGCAGCCGCGGTAATACGTAGGGTGCAAGCGTTGTCCGGATTTATTGGGTTTAAAGGGTGCGTAGGCGGTTAATTAAGTTAGGGGTGAAAGTTTTCGGCTTAACCGGAAAATTGCCTTTAATACTGGTTAGCTTGGATACGGTTGAGGTAAGCGGAATTGGTGGTGTAGCGGTGAAATGCTTAGATACCATCAGGAACACCGATTGCGAAGGCAGCTTACTAAGCCGTTATTGACGCTGAGGCACGAAAGCGTGGGGATCAAACAGG` |
| ASV2654 | 404 | `TGGGGAATATTGGACAATGGGCGAAAGCCTGATCCAGCAATACCGCGTGAATGATGAAGGCCTTCGGGTTGTAAAATTCTTTTAGTGGGGAAGATAATGACGGTACCCACAGAAAAAGCACCGGCTAACTTCGTGCCAGCAGCCGCGGTAATACGAAGGGTGCTAGCGTTGTTCGGAATTACTGGGCGTAAAGCGTGCGTAGGCGGCTTGGTAAGTCGATTGTGAAAGCCCAGGGCTCAACTCTGGAACTGCAATCGATACTGCCTTGCTTGAGTATGGTTGGGGATAGTAGAATTCCTAGTGTAGGGGTGAAATCCGTAGATATTAGGAGGAATACCAGTGGCGAAAGCGGCTATCTGGACCATTACTGACGCTGAGGTACGAAAGCGTGGGGAGCAAACAGG` |
| ASV2130 | 404 | `TGGGGAATATTGGACAATGGGCGCAAGCCTGATCCAGCCATGCCGCGTGAGTGATGAAGGCCTTAGGGTTGTAAAGCTCTTTTGCCAGGGAAGATAATGACTGTACCTGGAGAATAAGCCCCGGCTAACTTCGTGCCAGCAGCCGCGGTAATACGAAGGGGGCTAGCGTTGTTCGGAATCACTGGGCGTAAAGCGCACGTAGGCGGACTTTTAAGTCAGGGGTGAAAGCCTGAGGCTCAACCTCAGAATTGCCCTTGATACTGGGAGTCTTGAGTGTGGTAGAGGTTGGTGGAACAGCAAGTGTAGAGGTGAAATTCGTAGATATTTGCTAGAACACCAGTGGCGAAGGCGGCCAACTGGACCATAACTGACGCTGAGGTGCGAAAGCGTGGGGAGCAAACAGG` |
| ASV1095 | 430 | `TGGGGAATATTGGACAATGGGCGCAAGCCTGATCCAGCCATGCCGCGTGTGTGAAGAAGGCCTTTTGGTTGTAAAGCACTTTAAGCGAGGAGGAGGGTGTCGTGGTTAATACCCATGAACATCGGACGTTACTCGCAGAATAAGCACCGGCTAACTCTGTGCCAGCAGCCGCGGTAATACAGAGGGTGCGAGCGTTAATCGGAATTACTGGGCGTAAAGCGCGCGTAGGCGGTTATGTAAGTTGGATGTGAAATCCCCGGGCTTAACCTGGGCACTGCATTCAAAACTGCATAGCTAGAGTATGGGAGAGGAAGGTAGAATTCCAGGTGTAGCGGTGAAATGCGTAGAGATCTGGAGGAATACCGATGGCGAAGGCAGCCTTCTGGCCTAATACTGACGCTGAGGTGCGAAAGCATGGGGAGCAAACAGG` |
| ASV4763 | 429 | `TGGGGAATATTGGACAATGGGGGCAACCCTGATCCAGCAATACCGCGTGTGTGAAGAAGGCCTTCGGGTTGTAAAGCACTTTGGGCGGGGAAGAAGTTATTCTGTTTAAGAGATGGGGTAATTGACGGTACCCGCAGAACAAGCACCGGCTAACTCTGTGCCAGCAGCCGCGGTAATACAGAGGGTGCGAGCGTTAATCGGAATTACTGGGCGTAAAGGGCGCGTAGGCGGTTTATCAAGTGAGATGTGAAATCCCCGGGCTTAACCTGGGAACTGCATCTTAAACTGATGAGCTGGAGTACTGTAGAGGATAGTGGAATTTCCGGTGTAGCGGTGAAATGCGTAGAGATCGGAAGGAACATCAGTGGCGAAGGCGACTGTCTGGACAGATACTGACGCTGAGGCGCGAAAGCGTGGGGAGCAAACAGG` |
| ASV191 | 429 | `TGGGGAATTTTGGACAATGGGCGAAAGCCTGATCCAGCCATTCCGCGTGCAGGATGAAGGCCCTCGGGTTGTAAACTGCTTTTGTACAGAACGAAAAGGTTTCTACTAATACTAGGAGCTCATGACGGTACTGTAAGAATAAGCACCGGCTAACTACGTGCCAGCAGCCGCGGTAATACGTAGGGTGCAAGCGTTAATCGGAATTACTGGGCGTAAAGCGTGCGCAGGCGGTTATATAAGTCAGATGTGAAATCCCCGGGCTCAACCTGGGAACTGCATTTGAGACTGTATAGCTAGAGTACGGTAGAGGGGGATGGAATTCCGCGTGTAGCAGTGAAATGCGTAGATATGCGGAGGAACACCGATGGCGAAGGCAATCCCCTGGACCTGTACTGACGCTCATGCACGAAAGCGTGGGGAGCAAACAGG` |
| ASV244 | 404 | `TGGGGAATCTTAGACAATGGGGGAAACCCTGATCTAGCCATGCCGCGTGATCGATGAAGGCCTTAGGGTTGTAAAGATCTTTCAGTGGGGAAGATAATGACGGTACCCACAGAAGAAGCCCCGGCTAACTCCGTGCCAGCAGCCGCGGTAATACGGAGGGGGCTAGCGTTGTTCGGAATTACTGGGCGTAAAGCGCACGTAGGCGGACCGGAAAGTCAGAGGTGAAATCCCAGGGCTCAACCTTGGAACTGCCTTTGAAACTCCTGGTCTTGAGGTCGTGAGAGGTGAGTGGAATTCCGAGTGTAGAGGTGAAATTCGTAGATATTCGGAGGAACACCAGAGGCGAAGGCGGCTCACTGGCACGATACTGACGCTGAGGTGCGAAAGCGTGGGGAGCAAACAGG` |
| ASV2389 | 404 | `TGGGGAATATTGGACAATGGGGGCAACCCTGATCCAGCCATGCCGCGTGAGTGATGAAGGCCCTAGGGTTGTAAAGCTCTTTTGTGCGGGAAGATAATGACGGTACCGCAAGAATAAGCCCCGGCTAACTTCGTGCCAGCAGCCGCGGTAATACGAAGGGGGCTAGCGTTGCTCGGAATCACTGGGCGTAAAGGGTGCGTAGGCGGGTCTTTAAGTCAGGGGTGAAATCCTGGAGCTCAACTCCAGAACTGCCTTTGATACTGAGGATCTTGAGTCCGGGAGAGGTGAGTGGAACTGCGAGTGTAGAGGTGAAATTCGTAGATATTCGCAAGAACACCAGTGGCGAAGGCGGCTCACTGGCCCGGTACTGACGCTGAGGCACGAAAGCGTGGGGAGCAAACAGG` |
| ASV573 | 404 | `TGGGGAATCTTAGACAATGGGCGCAAGCCTGATCTAGCCATGCCGCGTGAGTGATGAAGGCCTTAGGGTCGTAAAGCTCTTTCGCTGGGGAAGATAATGACGGTACCCAGTAAAGAAACCCCGGCTAACTCCGTGCCAGCAGCCGCGGTAATACGGAGGGGGTTAGCGTTGTTCGGAATTACTGGGCGTAAAGCGCGCGTAGGCGGATCAGAAAGTTGGGGGTGAAAGCCCAGGGCTCAACCCTGGAATTGCCTTCAAAACTCCTGGTCTTGAGTTCGAGAGAGGTGAGTGGAATTCCGAGTGTAGAGGTGAAATTCGTAGATATTCGGAGGAACACCAGTGGCGAAGGCGGCTCACTGGCTCGATACTGACGCTGAGGTGCGAAAGCGTGGGGAGCAAACAGG` |
| ASV239 | 425 | `TGAGGAATATTGGACAATGGGTGAAAGCCTGATCCAGCCATCCCGCGTGAAGGAATAAGGTTCTCTGGATCGTAAACTTCTTTTATACTAGGATAAACCTACCCACGAGCTGGGTAGCTGAAGGTATAGTATGAATAAGCACCGGCTAACTCCGTGCCAGCAGCCGCGGTAATACGGAGGGTGCAAGCGTTATCCGGATTTATTGGGTTTAAAGGGTCCGTAGGCGGACTTATAAGTCAGTGGTGAAATCTCGATGCTTAACATCGAAACTGCCATTGATACTGTAAGTCTTGAGTAAGGTAGAGGTAGCTGGAATGAGTAGTGTAGCGGTGAAATGCATAGATATTACTCGGAACACCGATTGCGAAGGCAGGTTACCATGTCTTAACTGACGCTGATGGACGAAAGCGTGGGGAGCGAACAGG` |
| ASV1225 | 429 | `TGGGGAATTTTGGACAATGGGGGCAACCCTGATCCAGCAATGCCGCGTGAGTGAAGAAGGCCTTCGGGTTGTAAAGCTCTTTTGTTAGGGAAGAAAAGGTATCCAATAATACTGGATGCTGATGACGGTACCTAAAGAATAAGCACCGGCTAACTACGTGCCAGCAGCCGCGGTAATACGTAGGGTGCAAGCGTTAATCGGAATTACTGGGCGTAAAGCGTGCGCAGGCGGCCTATTAAGTCAGATGTGAAATCCCCGAGCTCAACTTGGGAACTGCATTTGAAACTGATAGGCTAGAGTGTGTCAGAGGGAGGTAGAATTCCACGTGTAGCAGTGAAATGCGTAGAGATGTGGAGGAATACCAGTGGCGAAGGCGGCCTCCTGGGATAACACTGACGCTCATGTACGAAAGCGTGGGGAGCAAACAGG` |
| ASV5355 | 421 | `TGGGGAATATTGCGCAATGGACGAAAGTCTGACGCAGCGACGCCGCGTGTGGGATGAAGCTCTTCGGGGTGTAAACCACTGTTGCCCGGGAAGAACCTCCCCTTTCGAGGGGACTGACGGTACCGGGTGAGGAAGCACCGGCTAACTCTGTGCCAGCAGCCGCGGTAATACAGAGGGTGCGAGCGTTGTCCGGAATCACTGGGCGTAAAGGGCGCGTAGGTGGCTTGGTAAGTGTGCGGTGAAAGCCCGGGGCTCAACCCCGGGTCTGCCGTGCAGACTGCCTGGCTCGAGCATAGTAGAGGCAGGTGGAATTCCGGGTGTAGCGGTGGAATGCGTAGAGATCCGGAAGAACACCGGTGGCGAAGGCGGCCTGCTGGGCTATTGCTGACACTGAGGCGCGACAGCGTGGGGAGCAAACAGG` |
| ASV4682 | 429 | `TGGGGAATTTTGGACAATGGGGGCAACCCTGATCCAGCAATGCCGCGTGAGTGAAGAAGGCCTTCGGGTTGTAAAGCTCTTTTGTCAGGGAAGAAACGGCGCTTACTAATACTAGGCGCTAATGACGGTACCTGAAGAATAAGCACCGGCTAACTACGTGCCAGCAGCCGCGGTAATACGTAGGGTGCGAGCGTTAATCGGAATTACTGGGCGTAAAGCGTGCGCAGGCGGTTGTATAAGACAGGTGTGAAATCCCCGGGCTTAACCTGGGAACTGCATCTGTGACTGTACGGCTAGAGTGTGTCAGAGGGGGGTGGAATTCCACGTGTAGCAGTGAAATGCGTAGAGATGTGGAGGAACACCGATGGCGAAGGCAGCCCCCTGGGATAACACTGACGCTCATGCACGAAAGCGTGGGGAGCAAACAGG` |
| ASV2589 | 406 | `TGGGGAATTTTCCGCAATGGGCGAAAGCCTGACGGAGCAATGCCGCGTGGAGGTAGAAGGCCTACGGGTCGTGAACTTCTTTTCCCGGAGAAGAAGCAATGACGGTATCTGGGGAATAAGCATCGGCTAACTCTGTGCCAGCAGCCGCGGTAATACAGAGGATGCAAGCGTTATCCGGAATGATTGGGCGTAAAGCGTCTGTAGGTGGCTTTTTAAGTCCGCCGTCAAATCCCAGGGCTCAACCCTGGACAGGCGGTGGAAACTACCAAGCTGGAGTACGGTAGGGGCAGAGGGAATTTCCGGTGGAGCGGTGAAATGCGTAGAGATCGGAAAGAACACCAACGGCGAAAGCACTCTGCTGGGCCGACACTGACACTGAGAGACGAAAGCTAGGGGAGCGAATGGG` |
| ASV1322 | 424 | `TGGGGAATATTGGTCAATGGGCGAAAGCCTGAACCAGCAATGCCGCGTGAGTGATGAAGGCCTTAGGGTTGTAAAGCTCTGTCAGTGGGAACGAATAAGCCGGTTAATACCCGGTGGTGACGGTACCCACAAAGGAAGCACCGGCTAACTCTGTGCCAGCAGCCGCGGTAATACAGAGGGTGCAAGCGTTGTTCGGAATTACTGGGCGTAAAGCGCGTGTAGGCGGTTTCGTAAGTCGGTCGTGAAAGCCCTGGGCTTAACCCAGGAACTGCGATCGAAACTGTGAGACTAGAGTATCGGAGGGGGTGGCGGAATTCCCGGTGTAGAGGTGAAATTCGTAGATATCGGGAGGAACACCTGTGGCGAAGGCGGCCACCTAGAAGATTACTGACGCTGAGACGCGAAAGCGTGGGGAGCAAACAGG` |
| ASV401 | 404 | `TGGGGAATATTGGACAATGGGCGAAAGCCTGATCCAGCAATGCCGCGTGAGTGATGAAGGCCTTCGGGTTGTAAAGCTCTTTTGGTAGGGAAAATAATGATGGTACCTACAGAATAAGCTCCGGCTAACTTCGTGCCAGCAGCCGCGGTAATACGAAGGGAGCTAGCGTTGTTCGGAATTACTGGGCGTAAAGAGTACGTAGGCTGTTTTTTAAGTTAGGCGTGAAAGCCCCGGGCTCAACCCGGGAATTGCGCTTAAAACTGGAAAACTTGAGATTGGGAGAGGAAAGTGGAATAACTAGTGTAGAGGTGAAATTCGTAGATATTAGTTAGAACACCAGTGGCGAAGGCGACTTTCTGGACCAATACTGACGCTGAGGTACGAAAGCGTGGGGAGCAAACAGG` |
| ASV1701 | 404 | `TGGGGAATTTTCCGCAATGGGCGAAAGCCTGACGGAGCAATACCGCGTGAGGGACGAAGGCCTGTGGGTTGTAAACCTCTTTTGTTAGGGAAGATAATGACGGTACCTAACGAATAAGCATCGGCTAACTCCGTGCCAGCAGCCGCGGTAAGACGGAGGATGCAAGCGTTATCCGGAATTATTGGGCGTAAAGCGTACGTAGGCTGTTTAATAAGTCTGTTGTCAAAGCCCGAGGCTTAACCTTGGATCGGCAATGGAAACTGTAAGACTAGAGAGAGATAGGGGCAGGAGGAATTCCAGGTGTAGCGGTGAAATGCGTAGATATCTGGAAGAACACCAGTGGCGAAAGCGTCCTGCTGGATCTCAACTGACGCTGAAGTACGAAAGCTAGGGGAGCGAATGGG` |
| ASV2421 | 430 | `TGGGGAATATTGCGCAATGGGGGAAACCCTGACGCAGCAACGCCGCGTGAGTGAGGAAGGACTTCGGTCTGTAAAGCTCTGTTAATGAGGAAAAATGGCATCTGGTCTAATAGGCCGGATGTTTGATGGTACTCATAGAGGAAGCACCGGCTAACTTCGTGCCAGCAGCCGCGGTAATACGAAGGGTGCAAGCGTTGTTCGGATTTATTGGGCGTAAAGCGCGCGTAGGTGGACCTGCAAGTCAGATGTGAAATCTCGGGGCTCAACCTCGAAACTGCGTCTGAAACTACAGGTCTAGAGTCTCGGAGGGGGAAGGGGAATTTCGCATGTAGGGGTAAAATCCGTAGATATGCGAAGGAACACCAGAGGCGAAGGCGCCTTCCTGGACGAGTACTGACACTGAGGCGCGAAAGCGTGGGGATCAAACAGG` |
| ASV711 | 387 | `GCGAGAAACCTTTGCAATGTGCGAAAGCACGACAAGGTTAATCCGAGTGATTCCTGCTAAAGGAATCTTTTGTTAGTCATAAAACCACTGATGAATAAGGGGTGGGCAAGTTCTGGTGTCAGCCGCCGCGGTAATACCAGCACCTCGAGTGGTCAGGAGGTTTATTGGGCCTAAAGCATCCGTAGCCGGCTGCTCAAGTTTCCGGTTAAATCCACACGCTTAACGTATGGGCTGCCGGAAATACTGTTCAGCTAGGGAGTGGGAGAGGTAGACGGTACTCGATAGGAAGGGGTAAAATCCTTTGATCTATTGATGACCACCTGTGGCGAAGGCGGTCTACCAGAACACGTTCGACGGTGAGGGATGAAAGCTGGGGGAGCAAACCGG` |
| ASV1947 | 424 | `TGAGGAATATTGGACAATGGGCGCAAGCCTGATCCAGCCATGCCGCGTGCAGGAAGACGGTCCTATGGATTGTAAACTGCTTTTATACAGGAAGAAACACACCCTCGTGAGGGTACTTGACGGTACTGTATGAATAAGCATCGGCTAACTCCGTGCCAGCAGCCGCGGTAATACGGAGGATGCAAGCGTTATCCGGAATCATTGGGTTTAAAGGGTCCGTAGGCGGTTTAATAAGTCAGTGGTGAAAGCCCATCGCTCAACGATGGAACGGCCATTGATACTGTTAAACTTGAATAATTAGGAAGTAACTAGAATATGTAGTGTAGCGGTGAAATGCTTAGATATTACATGGAATACCAATTGCGAAGGCAGGTTACTACTAATTTATTGACGCTGATGGACGAAAGCGTGGGTAGCGAACAGG` |
| ASV830 | 406 | `TAGGGAATATTGGACAATGGGCGAAAGCCTGATCCAGCAATGCCGCGTGAGTGATGAAGGCCTTAATCGGTCGTAAAGCTCTTTTGCCAGGGAAGATTATGACTGTACCTGGAGAATAAGGACCGACTAACTATGTGCCAGCAGTCGCGGTAATACATAGGGTCCTAGCGTTACTCGGAATTACTGGGCGTAAAGGGCGCGTAGGCGGATTTGTAAGTTATGTGTGAAATCTTCGGGCTCAACCTGAAATCTGCTCATAATACTGCAAGTCTAGAGTACAATCGAAGATGATAGAATTTCTGGTGTAGGGGTAAAATCCGTAGATATCAGAAGGAATACCGGAAGCGAAAGCGATCATCTAGGTTGGCACTGACGCTGAGGCGCGACAGCGTGGGGAGCAAACAGG` |
| ASV5365 | 405 | `TGAGGAATTTTGGTCAATGGGCGAAAGCCTGAACCAGCAACGCCGCGTGGGGGATGAAGTCTTTCGGGATGTAAACCCCTTTTCACCTTGAAAAACACTGATTGTAAAGGTGGAATAAGCAATGGCTAACTACGTGCCAGCAGCCGCGGTAATACGTAGGTTGCAAGCGTTACTCGGATTCACTAGGCGTAAAGCGGGTGTAGGTGGGCTTTTAAGTTTCCTGTGTAAGCCCCAGGCTTAACCTGGGGAGGTCAGGGAAAACTAATTGTCTTGAGTAAGGTAGGGGAGGTTGGAATTCCCGGTGTAGCGGTGAAATGCGTAGATATCGGGAGGAACACCAATGGCGAAGGCAGGCCTCTGGGCCTTTACTGACACTGAGACCCTAAAGCTAGGGGAGCAAACAGG` |
| ASV5205 | 428 | `TGGGGAATTTTGCGCAATGGGCGAAAGCCTGACGCAGCCACGCCGCGTGAGTGAAGAAGGCCTTCGGGTCGTAAAGCTCTGTCATTAGGGACGAAAATGTATGGGTGAACAGCCCATACACTGACGGTACCTAAGGAGGAAGCACCGGCTAACTCCGTGCCAGCAGCCGCGGTAATACGGAGGGTGCAAGCGTTGTTCGGATTCATTGGGCGTAAAGGGTAGGTAGGTGGTCTCGTGTGTCTGAGGTGAAATCCCTGGGCTTAACCCAGGAACTGCCTCGGAAACTGCGAGACTGGAGTCTCGGAGAGGTTCGTGGAATTCCCGGTGTAGAGGTGAAATTCGTAGAGATCGGGAGGAACACCAGAGGCGAAAGCGGCGAACTGGACGAGTACTGACACTCAACTACGAAAGCGTGGGGATCAAACAGG` |
| ASV3684 | 429 | `TGGGGAATTTTGGACAATGGGCGCAAGCCTGATCCAGCAATGCCGCGTGTGTGAAGAAGGCCTTCGGGTTGTAAAGCACTTTTGTTCGGGAAGAAAAGATTCGTGCTAATATCATGGATTGATGACGGTACCGGAAGAATAAGCACCGGCTAACTACGTGCCAGCAGCCGCGGTAATACGTAGGGTGCAAGCGTTAATCGGAATTACTGGGCGTAAAGCGTGCGCAGGCGGCTATGTAAGACAGATGTGAAATCCCCGGGCTCAACCTGGGAACTGCATTTGTGACTGCATGGCTAGAGTGTGTCAGAGGGGGGTAGAATTCCACGTGTAGCAGTGAAATGCGTAGATATGTGGAGGAATACCGATGGCGAAGGCAGCCCCCTGGGATAACACTGACGCTCATGCACGAAAGCGTGGGGAGCAAACAGG` |
| ASV2972 | 429 | `TGGGGAATATTGGACAATGGGCGCAAGCCTGATCCAGCAATGCCGCGTGTGTGAAGAAGGCCTTAGGGTTGTAAAGCACTTTCAGTTGGGAGGAATGGCTTATGGCTAATATCCATGAGTCTTGACGCTACCGACAGAAGAAGCACCGGCTAACTCTGTGCCAGCAGCCGCGGTAATACAGAGGGTGCAAGCGTTAATCGGAATTACTGGGCGTAAAGGGTGCGTAGGCGGTTATGTAAGTTAGATGTGAAATCCCCAGGCTTAACCTGGGAACTGCGTTTAATACTGTGTAGCTAGAGTACTGAAGAGGTTAGTGGAATTTCCAGTGTAGCGGTGAAATGCGTAGATATTGGAAGGAACACCAGTGGCGAAGGCGACTAACTGGTCAGATACTGACGCTGAGGCACGAAAGCGTGGGGAGCAAACAGG` |
| ASV3348 | 428 | `TGGGGAATTGTTCGCAATGGGCGCAAGCCTGACGACGCAACGCCGCGTGGAGGATGAAGGTCTTCGGATCGTAAACTCCTGTTGATCGGGACGAAAAGCTTCGACCTAACACGTCGAAGACTGACGGTACCGGTTGAGGAAGCCACGGCTAACTCTGTGCCAGCAGCCGCGGTAATACAGAGGTGGCAAGCGTTGTTCGGAATTACTGGGCGTAAAGGGCGCGTAGGCGGCCTTCTAAGTCGAACGTGAAAGCCCCAGGCTTAACCTGGGAACTGCGTTTGATACTGGGAGGCTTGGATTCGGGAGAGGGATGTGGAATTCCAGGTGTAGCGGTGAAATGCGTAGATATCTGGAGGAACACCGGTGGCGAAGGCGGCATCCTGGACCGAGATCGACGCTGAGGCGCGAAAGCTAGGGGAGCAAACGGG` |
| ASV1365 | 429 | `TGGGGAATATTGCGCAATGGGGGCAACCCTGACGCAGCCATGCCGCGTGTGTGAAGAAGGCCTTCGGGTTGTAAAGCACTTTCAATGGGGAAGAAAAGCGTAGCGTTAATAGCGTTGCGCCGTGACGTTACCCATAGAAGAAGCACCGGCTAACTCCGTGCCAGCAGCCGCGGTAATACGGAGGGTGCGAGCGTTAATCGGAATTACTGGGCGTAAAGCGCGCGTAGGCGGTTTGTTAAGTCAGCTGTGAAAGCCCCGGGCTCAACCTGGGAACTGCAGTTGATACTGGCCGACTAGAGTACGAGAGAGGGAGGTAGAATTCCATGTGTAGCGGTGAAATGCGTAGATATATGGAGGAATACCGGTGGCGAAGGCGGCCTCCTGGCTCGATACTGACGCTGAGGTGCGAAAGCGTGGGGATCAAACAGG` |
| ASV4573 | 429 | `TGGGGAATATTGGACAATGGGGGCAACCCTGATCCAGCGATGCCGCGTCGAGGAAGAAGGCCTTTGGGTTGTAAACTCGTGTCGTTAGGGAAAAAGGTTATGCAGTGAATAGCTGTATAGCTTGATGGTACCTAAGGAGGAAGCACCGGCTAACCTCGTGCCAGCAGCCGCGGTAATACGAGGGGTGCAAGCGTTGTTCGGAATCACTGGGCGTAAAGCGTGTCTAGGTGGATTGTTAAGTTCATTGTGAAAGCCCCAGGCTTAACCTGGGATGTGCAGTGGATACTGGCAGTCTTGAGTAATGGAGAGATGGGCGGAATTCCGGGTGTAGCAGTGAAATGCGTAGAGATCCGGAGGAACATCAGAGGCGAAGGCGGCTCATTGGCCATTTACTGACACTAAAGCACGAAAGCGTGGGTAGCAAACAGG` |
| ASV5311 | 428 | `TGGGGAATATTGGACAATGGGGGAAACCCTGATCCAGCAATGCCGCGTGTGTGAAGAAGGCCTTAGGGTTGTAAAGCACTTTCAGTTGGGAGAAATATCCTGCCGCTAATAACAGCAGGTTTGATGCTACCGACAGAAGAAGCACCGGCTAACTCTGTGCCAGCAGCCGCGGTAATACAGAGGGTGCAAGCGTTAATCGGAATTACTGGGCGTAAAGCGCGCGTAGGTGGATAAGTAAGTTGGATGTGAAAGCCCCGGGCTCAACCTGGGAACTGCATTCAAAACTGCTTTTCTAGAGTATGGTAGAGGAGAGTGGAATTTCCGGTGTAGCGGTGAAATGCGTAGATATCGGAAGGAACACCAGTGGCGAAGGCGACTCTCTGGACCAATACTGACACTGAGGTGCGAAAGCGTGGGGAGCAAACAGG` |
| ASV367 | 404 | `TGGGGAATCTTAGACAATGGGCGCAAGCCTGATCTAGCCATGCCGCGTGAGCGATGAAGGCCTTAGGGTTGTAAAGCTCTTTCAGTGGGGAAGATAATGACGGTACCCACAGAAGAAGCCCCGGCTAACTCCGTGCCAGCAGCCGCGGTAATACGGAGGGGGCTAGCGTTGTTCGGAATTACTGGGCGTAAAGCGCACGTAGGCGGACTGGAAAGTCAGGGGTGAAATCCCGGGGCTCAACCCCGGAACTGCCTTTGAAACTCCCAGTCTTGAGGTCGAGAGAGGTGAGTGGAATTCCGAGTGTAGAGGTGAAATTCGTAGATATTCGGAGGAACACCAGTGGCGAAGGCGGCTCACTGGCTCGATACTGACGCTGAGGTGCGAAAGCGTGGGGAGCAAACAGG` |
| ASV893 | 404 | `TGGGGAATATTGGACAATGGGGGAAACCCTGATCCAGCAATGCCGCGTGAGTGATGAAGGCCTTAGGGTCGTAAAGCTCTTTTACCAGGGATGATAATGACAGTACCTGGAGAATAAGCTCCGGCTAACTCCGTGCCAGCAGCCGCGGTAATACGGAGGGAGCTAGCGTTGTTCGGAATTACTGGGCGTAAAGCGCACGTAGGCGGTTATTCAAGTCAGAGGTGAAAGCCCGGGGCTCAACCCCGGAACTGCCTTTGAAACTAGATGACTGGAATCTTGGAGAGGTCAGTGGAATTCCGAGTGTAGAGGTGAAATTCGTAGATATTCGGAAGAACACCAGTGGCGAAGGCGACTGACTGGACAAGTATTGACGCTGAGGTGCGAAAGCGTGGGGAGCAAACAGG` |
| ASV178 | 424 | `TGAGGAATATTGGACAATGGGCGCAAGCCTGATCCAGCCATGCCGCGTGCAGGATGACGGTCCTATGGATTGTAAACTGCTTTTGTACAGGAAGAAACACTGGTTCGTGAACCAGCTTGACGGTACTGTAAGAATAAGGATCGGCTAACTCCGTGCCAGCAGCCGCGGTAATACGGAGGATCCAAGCGTTATCCGGAATCATTGGGTTTAAAGGGTCCGTAGGCGGTTTAGTAAGTCAGTGGTGAAAGCCCATCGCTCAACGGTGGAACGGCCATTGATACTGCTAGACTTGAATTATTAGGAAGTAACTAGAATATGTAGTGTAGCGGTGAAATGCTTAGAGATTACATGGAATACCAATTGCGAAGGCAGGTTACTACTAATGGATTGACGCTGATGGACGAAAGCGTGGGTAGCGAACAGG` |
| ASV3517 | 429 | `TGGGGAATATTGGACAATGGGGGAAACCCTGATCCAGCAATGCCGCGTGTGTGAAGAAGGCCTGAGGGTTGTAAAGCACTTTCAGTGGGGAGGAGGGTATATTGATTAAGAGTTGATATACTGGACGTTACCCACAGAAGAAGCACCGGCTAACTCCGTGCCAGCAGCCGCGGTAATACGGAGGGTGCGAGCGTTAATCGGAATTACTGGGCGTAAAGCGTGCGTAGGTGGTTTTTTAAGTTATCTGTAAAATCCCCGGGCTCAACCTGGGAAGGCCAGATAAAACTGAGGGACTCGAGTATTGGAGAGGGTAGTGGAATTTCCGGTGTAGCGGTGAAATGCGTAGAGATCGGAAGGAACACCAGTGGCGAAGGCGGCTACCTGGCCAAATACTGACACTGAGGCACGAAAGCGTGGGGAGCAAACAGG` |
| ASV4086 | 405 | `TTAGGAATCTTGCGCAATGGGCGAAAGCCTGACGCAGCGACGCCTCGTGGGGGATGAAGGCTCTCGGGTTGTAAACCCCTTTCGGCAGGAACGAAATTGACGGTACCTGAAGAAGAAGCACCGGCCAACTACGTGCCAGCAGCCGCGGTAATACGTAGGGTGCAAGCGTTGTCCGGATTTATTGGGCGTAAAGAGCTCGTAGGCGGCTTCGTAAGTCGGGTGTGAAACCTCCAGGCTTAACCTGGAGACGCCACCCGATACTGCGATAGCTAGAGTCTGGTAGGGGATCACGGAATTCCTGGTGTAGCGGTGAAATGCGCAGATATCAGGAGGAACACCGATGGCGAAGGCAGTGATCTGGGCCAGTACTGACGCTGAGGAGCGAAAGCGTGGGGAGCAAACAGG` |
| ASV4727 | 404 | `TAGGGAATATTGGACAATGGGGGAAACCCTGATCCAGCCATGCCGCGTGAGTGATGAAGGCCTTAGGGTTGTAAAGCTCTTTTGCCGGGGAAGATAATGACGGTACCCGGAGAATAAGTTCCGGCTAACTTCGTGCCAGCAGCCGCGGTAATACGAAGGGAACTAGCGTTGTTCGGAATCACTGGGCGTAAAGCGCGCGCAGGCGGGCATTCAAGTCAGGGGTGAAATCCTGAGGCTTAACCTCAGAATTGCCTTTGAGACTGTATGCCTGGAGTGTGTGAGGGGGTAGCGGAATTCCAAATGTAGAGGTGAAATTCGTAGATATTTGGAGGAACACCGGTGGCGAAGGCGGCTACCTGGCACACAACTGACGCTGAGGCGCGAAAGCGTGGGGATCAAACAGG` |
| ASV3746 | 424 | `TGAGGAATATTGGTCAATGGGCGTTAGCCTGAACCAGCCAAGTAGCGTGAAGGATGACTGCCCTATGGGTTGTAAACTTCTTTTATAAGGGAATAAAACGGGGAACGTGTTCCCCTTTGCATGTACCTTACGAATAAGGATCGGCTAACTCCGTGCCAGCAGCCGCGGTAATACGGAGGATCCGAGCGTTATCCGGATTTATTGGGTTTAAAGGGTGCGTAGGCGGAATGATCAGTCAGTTGTGAAAGTTTGAGGCTCAACCTTAAAATTGCAGTTGATACTGTCATTCTTGAGTGTACAAGAGGTGGGCGGAATTCGTGGTGTAGCGGTGAAATGCTTAGATATCACGAAGAACTCCGATTGCGAAGGCAGCTCACTGGGGTACAACTGACGCTGAGGCACGAAAGTGTGGGTATCAAACAGG` |
| ASV5108 | 405 | `TGGGGAATTTTCCGCAATGGGCGAAAGCCTGACGGAGCAATACCGCGTGAGGGAGGAAGGCTCTTGGGTTGTAAACCTCTTTTCTCAGGGAAGAATAATGACGGTACCTGAGGAATCAGCATCGGCTAACTCCGTGCCAGCAGCCGCGGTAATACGGAGGATGCAAGCGTTATCCGGAATTATTGGGCGTAAAGCGTCTGTAGGTGGATGTTCAAGTCTGCTGTCAAAGACCGGGGCTTAACCCTGGAAAGGCAGTGGAAACTGAAAATCTAGAGTGCGGTAGGGGCAGAGGGAATTCCCAGTGTAGCGGTGAAATGCGTAGAGATTGGGAAGAACACCGGTGGCGAAAGCGCTCTGCTAGGCCAAAACTGACACTAAGGGACGAAAGCTAGGGGAGCGAATGGG` |
| ASV4749 | 407 | `TGGGGAATATTGGGCAATGGAGGAAACTCTGACCCAGCAACGCCGCGTGAAGGATGAAGGTCTTCGGATTGTAAACTTCTGTTCTAAGGGAAGAAACAAATGACGGTACCTTAGAAGAAAGCCCCGGCAAACTACGTGCCAGCAGCCGCGGTGATACGTAGGGGGCGAGCGTTGTCCGGAATGACTGGGCGTAAAGGGAGTGTAGGCGGTTTTTTAAGTTAAATGTGAAAGCCCGGGGCTTAACTCCGGAACTGCATTTAATACTGGAAGACTTGAGTGTGGGAGAGGTAAGTGGAATTCCTAGTGTAGCGGTGGAATGCGTAGATATTAGGAGGAACATCAGTGGCGAAGGCGACTTACTGGACCATTACTGACGCTGAGGCTCGAAAGCGTGGGGAGCAAACAGG` |
| ASV1398 | 404 | `TGGGGAATCTTAGACAATGGGCGCAAGCCTGATCTAGCCATGCCGCGTGAGCGATGAAGGCCTTAGGGTTGTAAAGCTCTTTCGTGGGGGAAGATAATGACTGTACCCCAAGAAGAAGCCCCGGCTAACTCCGTGCCAGCAGCCGCGGTAATACGGAGGGGGCTAGCGTTGTTCGGAATTACTGGGCGTAAAGCGCACGTAGGCGGACTGGAAAGTCAGAGGTGAAATCCCAGGGCTCAACCTTGGAACTGCCTTTGAAACTCCCAGTCTTGAGGTCGAGAGAGGTGAGTGGAATTCCGAGTGTAGAGGTGAAATTCGTAGATATTCGGAGGAACACCAGTGGCGAAGGCGGCTCACTGGCTCGATACTGACGCTGAGGTGCGAAAGCGTGGGGAGCAAACAGG` |
| ASV4398 | 429 | `TCGAGAATAATTCACAATGGGGGCAACCCTGATGGTGCAACGCCGCGTGGAGGATGACGGTCTTCGGATTGTAAACTCCTGTCATCTGGGAGTAAGGCATGGCTGTGAATAGCGGACATGATTGATAGTACCAGAAGAGGAAGGGACGGCTAACTTCGTGCCAGCAGCCGCGGTAATACGAAGGTCCCAAGCGTTGTTCGGAATCACTGGGCGTAAAGGGAGCGTAGGCGGCGTGGTAAGTCAGATGTGAAATCCCGGGGCTCAACCCCGGAACTGCATCCGATACTGCCATGCTAGAGTGATGGAGAGGTAGCTGGAATTCTTGGTGTAGCAGTGAAATGCGTGGATATCAAGAGGAACACTCGTGGCGAAAGCGAGCTACTGGACATTTACTGACGCTGAGGCTCGAAGGCCAGGGTAGCGAAAGGG` |
| ASV528 | 424 | `TAAGGAATATTGGTCAATGGACGCAAGTCTGAACCAGCCATCCCGCGTGCAGGATTAAGGTCCTATGGATTGTAAACTGCTTTTAGTAGGGACGAAACGCTTCCATTTATGGGAGTCTGACGGTACCTACAGAATAAGCACCGGCTAACTCCGTGCCAGCAGCCGCGGTAATACGGAGGGTGCAAGCGTTATCCGGAATCACTGGGTTTAAAGGGTGCGTAGGCGGGTTATTAAGTCAGTGGTGAAATACTGTCGCTTAACGATAGAACTGCCATTGATACTGATAGTCTTGAATTGGGTTGAGGTTAGCGGAATGTGACATGTAGCGGTGAAATGCATAGATATGTCATGGAACACCAATTGCGAAGGCAGCTAACTGGGCCTTGATTGACGCTGAGGCACGAAAGCGTGGGTAGCGAACAGG` |
| ASV4935 | 424 | `TGAGGAATATTGGTCAATGGGCGGTAGCCTGAACCAGCCAAGTCGCGTGAGGGAAGACGGCCCTAAGGGTTGTAAACCTCTTTTGCCGGGGAGCAAGGCGCGCCACGTGTGGCGCGACGGAGAGTACCCGGAGAAAAAGCATCGGCTAACTCCGTGCCAGCAGCCGCGGTAATACGGAGGATGCGAGCGTTATCCGGATTTATTGGGTTTAAAGGGTGCGCAGGCGGAGAGTCAAGCCGGCGGTAAAATTGCGGGGCCCAACCCCGTACCGCCGTCGGAACTGGCTCCCTTGAGTGGACGAGAAGTAAGCGGAATGCGTGGTGTAGCGGTGAAATGCATAGATATCACGCAGAACGCCGATTGCGAAGGCAGCTTACCGGTGTCCAACTGACGCTCAGGCACGAAAGCGTGGGGATCGAACAGG` |
| ASV3324 | 429 | `TGGGGAATTTTGGACAATGGGCGCAAGCCTGATCCAGCAACGCCGCGTGCAGGATGAAGGCCTTCGGGTTGTAAACTGCTTTTGTACAGAGCGAAAAGGCTTCCATTAATACTGGGGGCTCATGACGGTACTGTAAGAATAAGCACCGGCTAACTACGTGCCAGCAGCCGCGGTAATACGTAGGGTGCAAGCGTTAATCGGAATTACTGGGCGTAAAGCGTGCGCAGGCGGTTGTGTAAGACAGTTGTGAAATCCCCGGGCTCAACCTGGGAACTGCAATTGTGACTGCACGGCTAGAGTACGGTAGAGGGGGGTGGAATTCCGCGTGTAGCAGTGAAATGCGTAGATATGCGGAGGAACACCGATGGCGAAGGCAGCCCCCTGGACCTGTACTGACGCTCATGCACGAAAGCGTGGGGAGCAAACAGG` |
| ASV2037 | 424 | `TAGGGAATATTGGGCAATGGGTGGAAGCCTGACCCAGCCATGCCGCGTGCAGGAAGAAGGTCCTCTGGATTGTAAACTGCTTTTATCGGGGGAGAAGAGACAGGATGCGTCCTGTTGTGACGGTACCCGATGAATAAGCCACGGCTAACTACGTGCCAGCAGCCGCGGTAATACGTAGGTGGCAAGCGTTGTCCGGATTTATTGGGTTTAAAGGGTGCGTAGGTGGTTTAATAAGTCAGTGGTGAAAGATGGTTGCTCAACAATCAAATTGCCATTGATACTGTTAGACTTGAGTGAAGTGGAGGTTGTTGGAATGGATGGTGTAGCGGTGAAATGCATAGATATCATCCAGAACGTCAATTGCGAAGGCAGATGGCTGTACTTTTACTGACACTGAGGCACGAAAGCGTGGGGATCAAACAGG` |
| ASV3495 | 404 | `TGGGGAATCTTAGACAATGGGCGCAAGCCTGATCTAGCCATGCCGCGTGGGTGATGAAGGCCTTAGGGTCGTAAAGCCCTTTCGCCGGGGAAGATAATGACGGTACCCGGTAAAGAAACCCCGGCTAACTCCGTGCCAGCAGCCGCGGTAATACGGAGGGGGTTAGCGTTGTTCGGAATGACTGGGCGTAAAGCGCGCGTAGGCGGATCGGATAGTTGGGGGTGAAATCCCGGGGCTCAACCCCGGAATGGCCTTCAAAACTCCCGGTCTAGAGTTCGAGAGAGGTGAGTGGAATTCCGAGTGTAGAGGTGAAATTCGTAGATATTCGGAGGAACACCAGTGGCGAAGGCGGCTCACTGGCTCGATACTGACGCTGAGGTGCGAAAGCGTGGGGAGCAAACAGG` |
| ASV3914 | 430 | `TGGGGAATATTGCGCAATGGGGGAAACCCTGACGCAGCAACGCCGCGTGAGTGAGGAAGGTCTTCGGATTGTAAAGCTCTGTCACTGTAGAAAAATGTCTGTTGGTCTAATAGGCCAGCAGAGTGATGGTATACAGAGAGGAAGCACCGGCTAACTTCGTGCCAGCAGCCGCGGTAATACGAAGGGTGCAAGCGTTGTTCGGATTTATTGGGCGTAAAGCGCGCGCAGGCGGATAATTAAGCCAGGTGTGAAATCTCGGGGCTCAACCCCGAAACTGCGTCTGGAACTGATTATCTAGAGTACTGGAGGGGAAAGGGGAATTTCGCATGTAGGGGTAAAATCCGTAGAGATGCGAAGGAACACCAGCGGGGAAACCGCCTTTCTGGACAGCTACTGACGCTGAGGCGCGAAAGCGTGGGGATCAAACAGG` |
| ASV1253 | 407 | `TAGGGAATATTGCGCAATGGGGGAAACCCTGACGCAGCGACGCCGCGTGAGTGATGAAGGCCTTCGGGTCGTAAAGCTCTGTCGTATGGGAATAACACAATGAATGTACCATGCAAGAAAGGATCGGCTAACTTCGTGCCAGCAGCCGCGGTAAGACGAGGGATCCTAGCGTTGTTCGGAATCATTGGGCGTAAAGCGGGTGTAGGTGGGCTTCTAAGTCAGATGTGAAATCCCAGGGCTCAACCCTGGAAGTGCATTTGATACTGGAAGTCTTGAGTGTGGTAGAGGTTACTGGAATTGTTGGTGTAGTGGTGAAATACGTAGATATCAACAGGAATACCGGAGGCGAAGGCGAGTAACTGGGCCAACACTGACACTGAGACCCGAAAGCGTGGGGATCAAACAGG` |
| ASV2306 | 408 | `TGAGGAATCTTCCGCAATGGGCGAAAGCCTGACGGAGCGACACCGCGTGAAGGATGAAGCCGGTTTCCGGTGTAAACTTCTGTTCTGAGGGACGAAATTTTTGACGGTACCTCAGGAGAAAGCATCGGCTAATTCTGTGCCAGCAGCCGCGGTAAGACAGAAGATGCAAGCGTTACTCGGAATTACTGGGCGTAAAGGGTCCGCAGGTGACTGACCACGTCTGGGGTAAAAGCACGTCGCTCAACGGCGTGTATGTCCCGGAAACGAGTCGGATTGAGCTACTCAGAGGCATCTGGAATGTTGTGTGTAGGGGTAAAATCCGTTGATCCACAATGGAACGCCAAAAGCGAAAGCAGGATGCTGGGGGTATGCTGACACTCATGGACGAAAGCGTGGGGAGCAAACGGG` |
| ASV5625 | 430 | `TGGGGAATCTTGCGCAATGGACGAAAGTCTGACGCAGCAACGCCGCGTGAGTGAAGAAGGCCTTCGGGTTGTAAAGCTCTGTCGGGCGGGACGAAAATCTATACAGTTAAGAGCTGGGTAGACTGACGGTACCGCCAAAGGAAGCACCGGCTAACTCTGTGCCAGCAGCCGCGGTAATACAGAGGGTGCAAGCGTTGCTCGGAATTATTGGGCGTAAAGGGTAGGTAGGTGGTCTTGTTTGTCAGGGGTGAAATCCCTGGGCTCAACCCAGGAAGTGCCCCTGAAACGGCAAGACTAGAGTTCTGGAGAGGATCGTGGAATTCCCGGTGTAGCGGTGAAATGCGTAGAGATCGGGAGGAACACCTGAGGCGAAGGCGGCGATCTGGACAGATACTGACACTCAACTACGAAAGCGTGGGTAGCAAACAGG` |
| ASV952 | 424 | `TAGGGAATATTGGACAATGGAGGAAACTCTGATCCAGCCATGCCGCGTGATAGATGAAGGCCCTCTGGGTTGTAAATATCTTTTATCTGGGAAGAAACAATCTTATTCTTAAGATCTTGACGGTACCAGAGGAATAAGCACCGGCTAACTCCGTGCCAGCAGCCGCGGTAATACGGAGGGTGCAAGCGTTATCCGGATTCACTGGGTTTAAAGGGTGCGTAGGCGGGCTGATAAGTCAGTGGTGAAAGCTCCGAGCTTAACTTGGAAACTGCCATTGATACTGCCAGTCTTGAATGTCGTAGAGGTAAGCGGAATGTTGCATGTAGCGGTGAAATGCTTAGATATGTTACAGAACACCAATTGCGAAGGCAGCTTACTATGCGATGATTGACGCTGAGGCACGAAAGCGTGGGGAGCAAACAGG` |
| ASV708 | 405 | `TGGGGAATCTTGCGCAATGGGCGAAAGCCTGACGCAGCAACGCCGCGTGGGGGATGAAGGCTCTCGGGTTGTAAACCCCTTTCAGCAGGGACGAATCAAGACGGTACCTGCAGAAGAAGCCCCGGCTAACTACGTGCCAGCAGCCGCGGTAACACGTAGGGGGCAAGCGTTGTCCGGATTTATTGGGCGTAAAGAGCTCGTAGGTGGCTGAGTAAGTCGGATGTTAAATCCCCAGGCTCAACCTGGGACACCACCCGATACTGCTCTGGCTAGAGTCCGGTAGGGGAGCGTGGAATTCCTGGTGTAGCGGTGAAATGCGCAGATATCAGGAGGAACACCAGCGGCGAAGGCGGCGCTCTGGGCCGGTACTGACACTGAGGAGCGAAAGCGTGGGTAGCAAACAGG` |
| ASV2029 | 406 | `TGGGGGATATTGGTCAATGGGGGGAACCCTGAACCAGCGACGCCGCGTGAGGGAAGACGGTCTTCGGATTGTAAACCTCTGTCTTCAGGGACGAAAAAAATGACGGTACCTGAAGAGGAAGCTCCGGCTAACTACGTGCCAGCAGCCGCGGTAATACGTAGGGAGCGAGCGTTGTCCGGAATTACTGGGTGTAAAGGGAGCGTAGGCGGGGAATCAAGTTGAATGTTAAAACTATCGGCTCAACCGGTAGCTGCGTTCAAAACTGAATCTCTTGAGTGAAGTAGAGGCAGGCGGAATTCCTAGTGTAGCGGTGAAATGCGTAGATATTAGGAGGAACACCAGTGGCGAAGGCGGCCTGCTGGGCTTTAACTGACGCTGAGGCTCGAAAGTGTGGGGAGCAAACAGG` |
| ASV4808 | 429 | `TAGGGAATATTGCGCAATGGACGAAAGTCTGACGCAGCAACTCCGCGTGAAGGATGAAGGTTTTCGGATCGTAAACTTCTTTTATTTGGGAAGAAATATCATGGCAGGAAATGGTCATGAGTTGACGGTACCTTATGAATAAGCTCCGGCTAAATACGTGCCAGCAGCCGCGGTAATACGTATGGAGCGAGCGTTACTCGGAATCACTGGGCGTAAAGAGTTCGTAGGCGGATTCGTAAGTCAGAGGTGAAATTTTGGGGCTTAACCCCGAAACTGCCTTTGAAACTGCGTATCTTGAGTATGTGAGAGGAAGATGGAATTCCTGGTGTAGCGGTGGAATGCGTAGATATCAGGAAGAACACCGGTGGCGAAGGCGGTCTTCTGGCACAATACTGACGCTGAGGAACGAAAGCGTGGGGAGCAAACAGG` |
| ASV4692 | 429 | `TGGGGAATATTGGACAATGGGCGCAAGCCTGATCCAGCCATGCCGCGTGAGTGAAGAAGGCCTTCGGGTTGTAAAGCTCTTTTGTTGGGGAAGAAAAGCTTCCGGTTAATACCCGGGAGTCCTGACGGTACCCAAAGAATAAGCACCGGCTAACTTCGTGCCAGCAGCCGCGGTAATACGAAGGGTGCAAGCGTTACTCGGAATTACTGGGCGTAAAGCGTGCGTAGGTGGTTCGTTAAGTCTGATGTGAAAGCCCTGGGCTCAACCTGGGAACTGCATTGGATACTGGCGATCTGGAGTGCGGTAGAGGGTGGCGGAATTCCCGGTGTAGCAGTGAAATGCGTAGATATCGGGAGGAACATCCGTGGCGAAGGCGGCCACCTGGACCAGCACTGACACTGAGGCACGAAAGCGTGGGGAGCAAACAGG` |
| ASV4852 | 407 | `TAGGGAATATTGCGCAATGGGGGAAACCCTGACGCAGCGACGCCGCGTGAGTGATGAAGGCCTTCGGGTCGTAAAGCTCTGTTGCAAGGGAATAATAAACTGAATGTACCTTGCGAGGAAGGATCGGCTAACTTCGTGCCAGCAGCCGCGGTAAGACGAGGGATCCTAGCGTTGTTCGGAATTATTGGGCGTAAAGCGGGTGCAGGTGGCTTTGTAAGTCAGGTGTGAAATCCCAGAGCTTAACTCTGGAAGTGCATTTGATACTGCGAAGCTTGAGTGTGGGAGAGATGAGTAGAATTCCAGGTGTAGTGGTGAAATACGTAGATATCTGGAGGAATACCGGAGGCGAAGGCGGCTCATTGGCCCAACACTGACACTCAGACCCGAAAGCGTGGGGATCAAACAGG` |
| ASV4782 | 430 | `TGGGGAATATTGCACAATGGGGGAAACCCTGATGCAGCAACGCCGCGTGAGTGAAGAAGGCCCTTGGGTCGTAAAGCTCTTTAGGTTGGGAAGAAGGTTGTGGCGGTTAATACCCGCGAGAACTGACGGTACCAGCAGAATAAGCACTGGCTAATTCCGTGCCAGCAGCCGCGGTAATACGGAAGGTGCAAGCGTTGTTCGGATTGACTGGGCGTAAAGAGCACGTAGGCGGGCTCGTGTGTCGATTGTGAAATCCCTGGGCTTAACCTGGGAACTGCAGTCGAAACTGCGAGTCTTGAATATCTGAGGGGTTGGTGGAATTCCAGGTGTAGGAGTGAAATCCGTAGATATCTGGAGGAACATCGGAGGCGAAGGCGGCCAACTGGCAGAATATTGACGCTGAGGTGCGAAAGCGTGGGGAGCAAACAGG` |
| ASV1572 | 404 | `TAAGGAATATTGGACAATGGGGGAAACCCTGATCCAGCCATGCCGCGTGAATGAAGAAGGCCTTAGGGTTGTAAAGTTCTTTTAGAGGTGAAGATAATGACGGTAGCCTCAGAAAAAGCTCCGGCTAACTCCGTGCCAGCAGCCGCGGTAATACGGAGGGAGCGAGCGTTGTTCGGAATTACTGGGCGTAAAGCGTGCGCAGGCGGCTTTGTAAGTTGGAAGTGAAATCCCTGGGCTCAACCCAGGAATTGCTTTCAAAACTACTAAGCTAGAGTATCGGAGAGGTTGGCGGAATTCCCAGTGTAGAGGTGAAATTCGTAGATATTGGGAGGAACACCAGTGGCGTAAGCGGCCAACTAGACGATAACTGACGCTCAGGCACGAAAGTATGGGGATCAAACAGG` |
| ASV552 | 424 | `TGAGGAATATTGGTCAATGGACGCAAGTCTGAACCAGCCATGCCGCGTGCAGGATGACGGCTCTATGAGTTGTAAACTGCTTTTATACGAGGGTAAATTCATCTACGTGTAGGTGATTGAAAGTATCGTACGAATAAGGATCGGCTAACTCCGTGCCAGCAGCCGCGGTAATACGGAGGATCCAAGCGTTATCCGGATTTATTGGGTTTAAAGGGTGCGTAGGCGGTTTAATAAGTTAGAGGTGAAATATTAGGGCTTAACCCTGAAACTGCCTCTAATACTGTTAGACTAGAGAATAGTTGCGGTAGGCGGAATGTATGGTGTAGCGGTGAAATGCTTAGAGATCATACAGAACACCGATTGCGAAGGCAGCTTACCAAACTATATCTGACGTTGAGGCACGAAAGCGTGGGGAGCAAACAGG` |
| ASV5080 | 388 | `TCGGGAAACTTCTGCAATGCGCGAAAGCGTGACAGAGCAAGCCAGAGTGATTTTCCATTAGGAAAATCTTTTGCGGGATGTAAAAAGTCTTGCGAATAAGGACTGGGCAAGACTGGTGCCAGCCGCCGCGGTAATCCCAGCGGTCCAAGTCGCAGCCACTTTTATTGGGTCTAAAACATCCGTAGCTTGCTTCGTAAGTCTCTTGTGAAATCCGGCCTCTTAAGGGTCGGGCGGGCAAGAGATACTGCGTTGCTAGGGACCGGGAGACGTAAGGAGTATGCACTGGGTAGCGGTAAAATGCGTTGATCCAATGCAGACTAACGACAGCGAAGGCACCTTACGAGAACGGATCCGACAGTGAGGGATGAAGGCTAGGGGCGCAAATTGG` |
| ASV2883 | 423 | `TAGGGAATATTGGTCAATGGATGGAAGTCTGAACCAGCCATGCCGCGTGCAGGAAGAAGGCCTTCTGGGTTGTAAACTGCTTTTGCCAGGGGATAAAAATTCTTTGCGAAGAAAATTGAAGGTACCTGGTGAATAAGCACCGGCTAACTACGTGCCAGCAGCCGCGGTAATACGTAGGGTGCAAGCGTTGTCCGGATTTATTGGGTTTAAAGGGTGCGTAGGCGGCCCTGTAAGTCAGTGGTGAAATACTCCAGCTTAACTGGAGGGGTGCCATTGATACTGCAGGGCTTGAGTTAAGTTGAAGTTGGCGGAATTGACGGTGTAGCGGTGAAATGCTTAGATATCGTCAAGAACACCGATAGTGAAGACAGCTAACTAAACTTTTACTGACGCTGAGGCACGAAAGTGTGGGGATCAAACAGG` |
| ASV1058 | 429 | `TGGGGAATTTTGGACAATGGGCGCAAGCCTGATCCAGCAATGCCGCGTGAGTGAAGAAGGCCTTCGGGTTGTAAAGCTCTTTTGTCAGGGAAGAAAAGGTTACGGCTAATATCCGTGGCTCATGACGGTACCTGAAGAATAAGCACCGGCTAACTACGTGCCAGCAGCCGCGGTAATACGTAGGGTGCAAGCGTTAATCGGAATTACTGGGCGTAAAGCGTGCGCAGGCGGTTTTGTAAGTCTGTTGTGAAATCCCCGGGCTCAACCTGGGAATGGCAATGGAGACTGCAAGGCTAGAGTTTGGCAGAGGGGGGTAGAATTCCACGTGTAGCAGTGAAATGCGTAGATATGTGGAGGAACACCGATGGCGAAGGCAGCCCCCTGGGTCAAAACTGACGCTCATGCACGAAAGCGTGGGGAGCAAACAGG` |
| ASV658 | 404 | `TGAGGAATTTTCCGCAATGGGCTCAAGCCTGACGGAGCAATGCCGCGTGAAGGATGAAGGCCTGCGGGTTGTAAACTTCTTTTCTTAGAGAAGATAATGACGGTATCTGAGGAATAAGCATCGGCTAACCCTGTGCCAGCAGCCGCGGTAATACAGGGGATGCAAGCGTTATCCGGAATAATTGGGCGTAAAGCGTCTGTAGGTGGTTTTGAAAGTCGATTGTCAAAAACCAAGGCTTAACCTTGGGCCGGCAATGGAAACTTCAAGACTAGAGTATGGTAGAGGTAGAGGGAATTACCGGTGTAACGGTGAAATGTGCAGATATCGGTAAGAACACCAAAGGCGAAAGCACTCTACTGGGCCAAAACTGACACTCAGAGACGAAAGCTAGGGGAGCGAATAGG` |
| ASV3003 | 406 | `TGGGGAATTTTCCGCAATGGGCGAAAGCCTGACGGAGCAATACCGCGTGAGGGACGACGGCTTTTGGGTTGTAAACCTCTTTTCCCTGGGAAGAAGATCTGACGGTACCAGGGGAATCAGCATCGGCTAACTTCGTGCCAGCAGCCGCGGTAATACGAGGGATGCAAGCGTTATCCGGAATTATTGGGCGTAAAGCGTCCGCAGGTGGCTCTGTAAGTCTGTCGTTAAAGCGTGGAGCTTAACTCCATATCGGCGATGGAAACTACAAGGCTAGAGTTTGGTAGGGGTCACGGGAATTCTTAGTGTAGCGGTGAAATGCGTAGATATTAAGAAGAACACCAGCGGCGAAAGCGCGTGACTGGGCCAATACTGACACTCATGGACGAAAGCTAGGGGAGCGAAAGGG` |
| ASV3321 | 404 | `TGAGGAATCTTCCACAATGGGCGAAAGCCTGATGGAGCGACACCGCGTGAAGGATGAAGCCTTTATTGGTGTAAACTTCTTTTTTGAGGGAAGATAATGACGGTACCTCAAGAATAAGGGGCGGCAAACTATGTGCCAGCAGCCGCGGTAATACATAGGCCCCAAGCGTTATCCGGAATTACTGGGCGTAAAGAGTTCGTAGGCGGAGGAATAAGTCAGAAGTGAAAGGTCCGGGCTCAACCCGGTACTTGCTTTTGAAACTATTCCCCTTGAATGAGGGAGAGGGAGGCAGAATGGTACGAGTAGGGGTGCAATCCGTTGATACGTACCGGAATACCAAAAGCGAAGGCAGCCTCCTGGAACTCTATTGACGCTGAGGAACGAAAGCGTGGGGAGCGAAAAGG` |
| ASV2519 | 404 | `TGGGGAATATTGGACAATGGGGGAAACCCTGATCCAGCAATGCCGCGTGAATGATGAAGGCCTTCGGGTTGTAAAGTTCTTTTACCTGAGAAGATAATGACGGTATCAGGGGAAAAAGCACCGGCTAACTTCGTGCCAGCAGCCGCGGTAATACGAAGGGTGCAAGCGTTGTTCGGAATGACTGGGCGTAAAGGGCGCGTAGGCGGTTTTGTACGTTTGATGTGAAAGTCCTGGGCTTAACCTGGGGACGGCATTGGATACGGCAGAGCTAGAGAGCGAGAGAGGGAGACGGAATAGTGCGTGTAGAGGTGAAATTCGTAGATATGCACTAGAACACCGGAGGCGAAGGCGGTTTCCTGGCTCGCAACTGACGCTAAGGCGCGAAAGCGTGGGGAGCAAACAGG` |
| ASV4879 | 388 | `TCGGGAAATTTCTGCAATGCGCGCAAGCGTGACAGAGCAAGCCAGAGTGCATTCTCGTTTTGAGAACGCTTTTGTGTGATGTAAAAAGTCGCACGAATAAGGACTGGGCAAGACTGGTGCCAGCCGCCGCGGTAATCCCAGCAGTCCAAGTCGCAATCACATTTATTGGGTCTAAAACATCCGTAGCTTGTTCTGTAAGTCTCTTGTGAAATCCTGCCTCTTAAGGGTAGACGTGCAAGAGATACTGCAGAGCTCGAGACCGGTAGACGCGAGAAGTACAGTTAGGGTAGCGGTAAAATGCGTTGATCCTTACTGGACTAACAACAGCGAAGGCATCTCGCGAGAACGGATCTGACAGTGAGGGATGAAGGCTAGGGGCGCAAAACGG` |
| ASV1641 | 404 | `TGGGGAATATTGGACAATGGGCGAAAGCCTGATCCAGCAATGCCGCGTGAGTGAAGAAGGCCTTCGGGTTGTAAAGCTCTTTTACCCGGGAAGATAATGACTGTACCGGGAGAATAAGCCCCGGCTAACTCCGTGCCAGCAGCCGCGGTAATACGGAGGGGGCTAGCGTTGTTCGGAATCACTGGGCGTAAAGCGTACGTAGGCGGCTATTCAAGTCAGAGGTGAAAGCCTGGAGCTCAACTCCAGAACTGCCTTTGAAACTAGGTAGCTTGAACATGGGAGAGGTGAGTGGAATTCCGAGTGTAGAGGTGAAATTCGTAGATATTCGGAAGAACACCAGTGGCGAAGGCGGCTCACTGGACCATTGTTGACGCTGAGGTACGAAAGCGTGGGGAGCAAACAGG` |
| ASV4732 | 405 | `TAGGGAATTTTCCACAATGGACGAAAGTCTGATGGAGCAACGCCGCGTGCAGGATGAAGGCTTTCGGGTCGTAAACTGCTTTTATATGTGACGATTATGACGGTAGCATATGAATAAGGATCGGCTAACTCCGTGCCAGCAGCCGCGGTCATACGGAGGATCCAAGCGTTATCCGGAATTACTGGGCGTAAAGAGTTGCGTAGGTGGCAGAGTAAGTAGGTAGTGAAAGCGTTCGGCTCAACCGAATAAACATTATCTAAACTGCTCAGCTAGAGGATGAGAGAGGTTATTGGAATTCCTAGTGTAGGAGTGAAATCCGTAGATATTAGGAGGAACACCGATGGCGTAGGCAGATAACTGGCTCATTTCTGACACTAAGGCACGAAAGCGTGGGTAGCAAACGGG` |
| ASV2767 | 430 | `TGGGGAATCTTGCACAATGGGCGAAAGCCTGATGCAGCAACGCCGCGTGAGTGAAGAAGGTCTTTGGATTGTAAAGCTCTTTCGGCTAGGAAGAAGGGCCTTTAGGTAAATAATCTTGAGGTCTGACGGTACTAGAAGAAGAAGCACCGGCTAACTCCGTGCCAGCAGCCGCGGTAATACGGAGGGTGCAAGCGTTGTTCGGATTGATTGGGCGTAAAGCGCTCGTAGGCGGGTTCATAAGTCGAATGTGAAAGCCCCGGGCTTAACCTGGGAATTGCATCCGAAACTATGTATCTTGAATACTCGAGAGGTTGGTGGAATTCTTGGTGTAGGAGTGAAATCCGTAGATATCAAGAGGAATATCGGAGGCGAAGGCGGCCGGCTGGCGATGTATTGACGCTGAGGAGCGAAAGCGTGGGGAGCAAACAGG` |
| ASV3636 | 419 | `TGAGGAATATTGCGCAATGGGCGAAAGCCTGACGCAGCGACGCCGCGTGGGGGATGAAGGTCTTCGGATTGTAAACCCCTTTCGGGAGGGAAGATGGGACGGGCAACCGTTCGGACGGTACCTCCAGAAGCAGCCACGGCTAACTTCGTGCCAGCAGCCGCGGTAATACGAAGGTGGCAAGCGTTGTTCGGATTTACTGGGCGTACAGGGAGCGTAGGCGGTTGGGTAAGCCCTTCGGGAAATCTCCCGGCTTAACCGGGAAAGGTCGGGGGGGACTGCCTGGCTAGAGGACGGGAGAGGAGCGCGGAATTCCCGGTGTAGCGGTGAAATGCGTAGAGATCGGGAGGAAGGCCGGTGGCGAAGGCGGCGCTCTGGAACGTTCCTGACGCTGAGGCTCGAAAGCGTGGGGAGCAAACAGG` |
| ASV5402 | 428 | `TGGGGAATATTGGACAATGGGCGCAAGCCTGATCCAGCCACGCCGCGTGAGTGATGAAGGCCTTCGGGTCGTAAAGCTCTGTGGGGAGGGACGAACCGCCAACCGTTCATAGCGGGTGGCATGACGGTACCTCCTTAGCAAGCACCGGCTAACTTCGTGCCAGCAGCCGCGGTAATACGAAGGGTGCAAACGTTGCTCGGAATCATTGGGCGTAAAGCGCACGTAGGCGGCCCTAAAAGTCGGATGTGAAATCCCTCGGCTCAACCAAGGAAGTGCATCTGATACTGTAGGGCTTGAGTACCAAAGAGGATCGCGGAATTCCCGGTGTAGAGGTGAAATTCGTAGATATCGGGAGGAACACCAGTGGCGAAGGCGGCGATCTGGGTGGTTACTGACGCTGAGGTGCGAAAGCGTGGGGAGCAAACAGG` |
| ASV643 | 404 | `TGGGGAATATTGGACAATGGGCGAAAGCCTGATCCAGCCATGCCGCGTGTGTGATGAAGGCCTTAGGGTTGTAAAGCACTTTTGTCCGGGAAGATAATGACTGTACCGGAAGAATAAGCCCCGGCTAACTTCGTGCCAGCAGCCGCGGTAATACGAAGGGGGCTAGCGTTGCTCGGAATCACTGGGCGTAAAGCGCACGTAGGCGGATTATTAAGTCGGAGGTGAAAGCCTGGGGCTCAACCCCAGAATTGCCTTCGATACTGATAGTCTCGAGTTTGGAAGAGGTTGGTGGAACTCCGAGTGTAGAGGTGAAATTCGTAGATATTCGGAAGAACACCAGTGGCGAAGGCGGCCAACTGGTCCAATACTGACGCTGAGGTGCGAAAGCGTGGGGAGCAAACAGG` |
| ASV5254 | 429 | `TGGGGAATATTGGACAATGGGGGAAACCCTGATCCAGCAATACCGCGTGTGTGAAGAAGGCCTATGGGTTGTAAAGCACTTTCAGTGGGGAGGAAAGTATTATGGTTAATACCCATAATAATTGACGCTACCCACAGAAGAAGCACCGGCTAACTCCGTGCCAGCAGCCGCGGTAATACGGAGGGTGCAAGCGTTAATCGGAATTACTGGGCGTAAAGAGTGCGTAGGTGGTTTAGTGTGTTAGATGTGAAATCCATGGGCTTAACCTGTGAATTGCATTTAAAACTGCTAGGCTAGAGTGTTGTAGAGGGCGGCGGAATTTCCAGTGTAGCGGTGAAATGCGTAGATATTGGAAGGAACACCAGTGGCGAAGGCGGCCGCCTGGACAAACACTGACACTGAGGCACGAAAGCGTGGGTAGCGAACAGG` |
| ASV1706 | 424 | `TGAGGAATATTGGTCAATGGGCGAGAGCCTGAACCAGCCAAGTAGCGTGAAGGATGAAGGCCCTATGGGTCGTAAACTTCTTTTATAGGGGAATAAAGTGCGGAACGTGTTCCGCTTTGTATGTACCCTACGAATAAGGATCGGCTAACTCCGTGCCAGCAGCCGCGGTAATACGGAGGATCCGAGCGTTATCCGGATTTATTGGGTTTAAAGGGAGCGTAGGTGGATGTATAAGTCAGTTGTGAAAGTTTGCGGCTCAACCGTAAAATTGCAGTTGATACTGTATGTCTTGAGTGTACAAGAGGTGGGCGGAATTCGTGGTGTAGCGGTGAAATGCTTAGATATCACGAAGAACTCCGATTGCGAAGGCAGCTCACTAGGGTACAACTGACACTGAGGCTCGAAAGTGTGGGTATCAAACAGG` |
| ASV2640 | 424 | `TGAGGAATATTGGACAATGGGCGCAAGCCTGATCCAGCCATGCCGCGTGCAGGAAGACGGTCCTATGGATTGTAAACTGCTTTTGTACGGGAAGAAACACCTCTTCGTGAAGAGGCTTGACGGTACCGTAAGAATAAGGATCGGCTAACTCCGTGCCAGCAGCCGCGGTAATACGGAGGATCCAAGCGTTATCCGGAATCATTGGGTTTAAAGGGTCCGTAGGCGGTTTAGTAAGTCAGTGGTGAAAGCCCATCGCTCAACGATGGAACGGCCATTGATACTGCTAGACTTGAATTACAAGGAAGTAACTAGAATATGTAGTGTAGCGGTGAAATGCTTAGATATTACATGGAATACCAATTGCGAAGGCAGGTTACTACTTGTGGATTGACGCTGATGGACGAAAGCGTGGGGAGCGAACAGG` |
| ASV3654 | 429 | `TGGGGAATATTGCGCAATGGAGGAAACTCTGACGCAGCGACGCCGCGTGGGTGATGAAGGCCTTCGGGTTGTAAAGCCCTGTCGGCAGGGAAAAAGGTAGCTTACCTAATACGTAAGTTGCTTGATGGTACCTGCAAAGGAAGCACCGGCTAACTCTGTGCCAGCAGCCGCGGTAATACAGAGGGTGCAAGCGTTGTTCGGAATTACTGGGCGTAAAGGGCGCGTAGGCGGCGATACAAGTCAGATGTGAAAACCCATGGCTCAACTATGGACGTGCATTTGAAACTGTATCGCTAGAGTGCAGTAGAGGTTGGTGGAATTCCCAGTGTAGAGGTGAAATTCGTAGATATTGGGAGGAACACCGGAGGCGAAGGCGGCCAACTGGGCTGTAACTGACGCTGAGGCGCAAAAGCGTGGGGATCAAACAGG` |
| ASV4746 | 428 | `TGGGGAATATTGGGCAATGGGCGCAAGCCTGACCCAGCAATACCGCGTGTGTGAAGAAGGCCTGAGGGTTGTAAAGCACTTTCAATGGGGAGGAAAAAGCCAGGGCAAATACCCCTGGCCTTGACATTACCCATACAAGAAGCACCGGCTAACTCCGTGCCAGCAGCCGCGGTAATACGGAGGGTGCGAGCGTTAATCGGAATTACTGGGCGTAAAGCGTGCGTAGGCGGCCTGCCAAGCCAGGTGTGAAAGCCCCGGGCTCAACCTGGGAACGGCACTTGGAACTGGCAGGCTCGAGTTGGGTAGAGGCAGGCGGAATTCCGGGTGTAGCGGTGAAATGCGTAGATATCCGGAGGAACACCAGTGGCGAAGGCGGCCTGCTGGCCCCAAACTGACGCTGAGGCACGAAAGCGTGGGTAGCAAACAGG` |
| ASV4883 | 403 | `TGGGGAATCTTGCACAATGGGGGCAACCCTGATGCAGCGACGCCGCGTGAGCGATGAAGGTTTTCGAATCGTAAAGCTCTGTCCTATGGGAAGATAATGACGGTACCATAGGAGGAAGCCCCGGCTAACTACGTGCCAGCAGCCGCGGTAATACGTAGGGGGCGAGCGTTGTCCGGAATTACTGGGCGTAAAGGGTTCGCAGGCGGCATGGCAAGTCCGATGTAAAAGGCGAAGGCTCAACCTTCGTAAGCATCGGAAACTGTCAAGCTTGAGTGAAGGAGAGGCAAGTGGAATTCCTAGTGTAGCGGTGGAATGCGTAGATATTAGGAGGAATACCGGTGGCGAAGGCGACTTGCTGGACTTCAACTGACGCTGAGGAACGAAAGCGTGGGTAGCAAACAGG` |
| ASV1347 | 404 | `TGGGGAATATTGGACAATGGGCGAAAGCCTGATCCAGCAATACCGCGTGAATGATGAAGGCCTTAGGGTTGTAAAGTTCTTTTAGCAGGGAAGATAATGACGGTACCTGCAGAAAAAGCCCCGGCTAACTCCGTGCCAGCAGCCGCGGTAAGACGGAGGGGGCTAGCGTTGTTCGGAATTACTGGGCGTAAAGAGCGCGTAGGCGGATTAGTAAGTTGGGAGTGAAAGCCCAGGGCTTAACCCTGGAATTGCTTTCAAAACTGCTAGTCTTGAGTGAAGTAGGGGGTGATGGAATTCCTAGTGTAGAGGTGAAATTCTTAGATATTAGGAGGAACACCGGTGGCGAAGGCGGTCACCTGGGCTTCAACTGACGCTGAGGCGCGAAAGCGTGGGGAGCAAACAGG` |
| ASV3065 | 404 | `TGGGGAATATTGGACAATGGGCGCAAGCCTGATCCAGCCATGCCGCGTGAGTGATGAAGGCCTTAGGGTTGTAAAGCTCTTTTATCCGGGAAGATAATGACTGTACCGGAAGAATAAGCCCCGGCTAACTTCGTGCCAGCAGCCGCGGTAATACGAAGGGGGCTAGCGTTGCTCGGAATCACTGGGCGTAAAGGGCGCGTAGGCGGCTTTTTAAGTCGGAGGTGAAAGCCTGGGGCTCAACCCCAGAATTGCCTTCGATACTAGATGGCTTGAGAGTGAGAGAGGTAAGTGGAACTGTGAGTGTAGAGGTGAAATTCGTAGATATTCACAAGAACACCGGTGGCGAAGGCGGCTTACTGGCTCATTTCTGACGCTGAGGCGCGAAAGCGTGGGGAGCAAACAGG` |
| ASV4893 | 415 | `TGGGGAATTTTGGACAATGGGCGAAAGCCTGATCCAGCAACTCTGCGTGAGGGACGAAGGTCTTCGGATCGTAAACCTCTTTTACATGGGAAGAATACCCGCAAGGGTTCGACGGTACCATGTGAATAAGCCACGGCTAACTATGTGCCAGCAGCCGCGGTAAGACATAGGTGGCAAGCGTTATTCGGAATTACTAGGCGTAAAGCGAGTGTAGGCGGACGATTAAGTCCGATGTTAAATCTCCAGGCTTAACTTGGAGTCGCATCGGATACTGGTCGCCTCGAGTGGGGTAGGGGGCAGCGGAATTCCCGGTGTAGCGGTGAAATGCGTAGATATCGGGAGGAACACCTATGGCGAAAGCAGCTGCCTGGGCCTTTACTGACGCTAAGACTCGAAAGCTGGGGGAGCAAACAGG` |
| ASV1047 | 429 | `TGGGGAATTTTGGACAATGGGCGCAAGCCTGATCCAGCCATTCCGCGTGCAGGATGAAGGCCTTCGGGTTGTAAACTGCTTTTGTACGGAACGAAACGGCTCTCTCTAATACAGGGGGCTAATGACGGTACCGTAAGAATAAGCACCGGCTAACTACGTGCCAGCAGCCGCGGTAATACGTAGGGTGCAAGCGTTAATCGGAATTACTGGGCGTAAAGCGTGCGCAGGCGGTTATGTAAGACAGTTGTGAAATCCCCGGGCTCAACCTGGGAACTGCATCTGTGACTGCATAGCTAGAGTACGGCAGAGGGGGATGGAATTCCGCGTGTAGCAGTGAAATGCGTAGATATGCGGAGGAACACCGATGGCGAAGGCAATCCCCTGGGCCTGTACTGACGCTCATGCACGAAAGCGTGGGGAGCAAACAGG` |
| ASV1134 | 429 | `TGGGGAATATTGGACAATGGGCGCAAGCCTGATCCAGCCATACCGCGTGGGTGAAGAAGGCCTTCGGGTTGTAAAGCCCTTTTGTTGGGAAAGAAAACTTGCAGGTTAATACCCAGCAAGTCTGACGGTACCCAAAGAATAAGCACCGGCTAACTTCGTGCCAGCAGCCGCGGTAATACGAAGGGTGCAAGCGTTACTCGGAATTACTGGGCGTAAAGCGTGCGTAGGTGGTTGTTTAAGTCTGTCGTGAAAGCCCTGGGCTCAACCTGGGAATTGCGATGGATACTGGGCGACTAGAGTGTGGTAGAGGATAGTGGAATTTCCGGTGTAGCAGTGAAATGCGTAGAGATCGGAAGGAACATCTGTGGCGAAGGCGACTATCTGGGCCAACACTGACACTGAGGCACGAAAGCGTGGGGAGCAAACAGG` |
| ASV5013 | 409 | `TGAGGAATCTTCCGCAATGGGCGAAAGCCTGACGGAGCGACGCCGCGTGGTGGATGAAGGCCCTAACGGGTCGTAAACACCTGTTCTGAGGGACGAAATTTTTGACGGTACCTCAGGAGAAAGTATCGGCAAACCCTGTGCCAGCAGCCGCGGTAAGACAGGGGATACAAGCGTTACTCGGAATCACTGGGCGTAAAGCGTCTGTAGGTGCCTAGTCAAGTCTGCGGTAAAAGCTCGGGGCTTAACCCCGAGAGCGTCGCGGAAACTAATTAGGTTGAGCAGTGGAGAGGCATCTGGAATGTCGCATGTAGGAGTAAAATCCGTAGAGATGCGATGGAACGCCAAAAGCGAAGGCAGGATGCTAGCCACTTGCTGACACTGAGAGACGAAAGCGTGGGGAGCGAAGGGG` |
| ASV4908 | 430 | `TCGAGAATCATTCGCAATGGGCGAAAGCCTGACGGTGCGACGCCGCGTGGAGGATGAAGGCCCTAGGGTCGTAAACTCCTGTCATCAGGGAGTAATGGCTCGCGGAGTAACTGCCGCGGGTCTTGATAGTACCTGAAGAGGAAGGGACGGCAAACTTCGTGCCAGCAGCCGCGGTAATACGAAGGTCCCAAGCGTTGTTCGGATTTACTGGGCGTAAAGAGTCTGTAGGCGGCGGGGTAAGTCAGATGTGAAATCCCGGGGCTCAACCCCGGAACTGCATCCGATACTGCCTTGCTTGAGTATTGGAGGGGAGTCTGGAATTCTCGGTGTAGCAGTGAAATGCGTAGATATCGAGAGGAACACTAGTGGCGAAGGCGAGACTCTGGACAATTACTGACGCTGAGAGACGAAGGTCAGGGGAGCGAAAAGG` |
| ASV2603 | 404 | `TGGGGAATATTGGACAATGGGCGCAAGCCTGATCCAGCAATTCCGCGTGGGTGAAGAAGGTCTTCGGATTGTAAAGCCCTTTCGGCGGGGACGATGATGACGGTACCCGCAGAAGAAGCCCCGGCTAACTTCGTGCCAGCAGCCGCGGTAATACGAAGGGGGCTAGCGTTGCTCGGAATGACTGGGCGTAAAGGGCGCGTAGGCGGCACGGACAGTCAGGCGTGAAATTCCTGGGCTTAACCTGGGGGCTGCGTTTGATACGTCCGAGCTAGAGGATGGAAGAGGCTCGTGGAATTCCCAGTGTAGAGGTGAAATTCGTAGATATTGGGAAGAACACCGGTGGCGAAGGCGGCGAGCTGGTCCATTACTGACGCTGAGGCGCGAAAGCGTGGGGAGCAAACAGG` |
| ASV833 | 429 | `TGGGGAATATTGGACAATGGGCGCAAGCCTGATCCAGCCATGCCGCGTGTGTGAAGAAGGCCTTCGGGTTGTAAAGCACTTTCACTTGGGAAGAAAAGTTGGTCGTTAATAGCGGCCAGCCCTGACATTACCTTGAGAAGAAGCACCGGCTAACTCCGTGCCAGCAGCCGCGGTAATACGGAGGGTGCGAGCGTTAATCGGAATTACTGGGCGTAAAGCGCGCGTAGGCGGTTTGTTAAGTCAGCTGTGAAAGCCCCGGGCTCAACCTGGGAACTGCAGTTGATACTGGCCGACTAGAGTACGAGAGAGGGAGGTAGAATTCCATGTGTAGCGGTGAAATGCGTAGATATATGGAGGAATACCGGTGGCGAAGGCGGCCTCCTGGCTCGATACTGACGCTGAGGTGCGAAAGCGTGGGGATCAAACAGG` |
| ASV5708 | 430 | `TGGGGAATATTGCGCAATGGACGAAAGTCTGACGCAGCAACGCCGCGTGAGTGAAGAAGGCCTTTGGGTTGTAAAGCTCTTTCGGCCGGGAAGAAGGGTTGGGGTGTTAATAGTATCCCAATTTGATGGTACCGGAAGAAGAAGCACCGGCTAACTTCGTGCCAGCAGCCGCGGTAATACGAAGGGTGCGAGCGTTGTTCGGAATTACTGGGCGTAAAGGGTTTGTAGGCTGAAATGTAAGTCAAGTGTGAAATCCCCGAGCTAAACTTGGGACGTGCATTTGAAACTGTGTTTCTTGAGTATCGTAGAGGGTGGTGGAATTGTAGGTGTAGGAGTGACATCCGTAGAGATCTGCAGGAACATCAGAGGCGAAGGCGACCACCTGGCCGATTACTGACGCTGAGGAACGAAAGCGTGGGGAGCAAACAGG` |
| ASV1535 | 428 | `TCGGGAATCTTCCGCAATGGGCGAAAGCCTGACGGAGCGACGCCGCGTGAGTGATGAAGGCCTTCGGGTTGTAAAGCTCTGTGGGGAGAGAAGAATAAGGGTGGGCGAATATCCCATCTGATGACGGTATCTCCTTAGCAAGCACCGGCTAACTCTGTGCCAGCAGCCGCGGTAAGACAGAGGGTGCAAACGTTGTTCGGATTTACTGGGCGTAAAGCGCGTGTAGGCGGCTTGGCAAGTTGGATGTGAAAGCCCACGGCTCAACCGTGGAAGCGCATCCAAAACTGCTTCGCTCGAGTCTCGGAGAGGAAGGTGGAATGCTTGGTGTAGAGGTGAAATTCGTAGATATCAAGCAGAACACCTGCGGCGAAGGCGGCCTTCTGGACGATGACTGACGCTGAGACGCGAAAGCGTGGGGAGCAAACAGG` |
| ASV2682 | 430 | `TAGGGAATATTGCACAATGGACGCAAGTCTGATGCAGCAACGCCGCGTGAGTGAAGAAGGCCCTTGGGTCGTAAAGCTCTTTAGCCTGGAAAGAAGTGCCATCCCGTAAACAATAGGATGGTGTGACGGTACCAGGAGAATAAGGACCGGCTAATTTCGTGCCAGCAGCCGCGGTAATACGAAAGGTCCAAGCGTTACTCGGAATTACTGGGCGTAAAGCGCGCGTAGGCGGCTTTGTATGTCGATTGTGAAAGCCCCAGGCTCAACCTGGGAACTGCAGTCGAAACTGCAAAGCTTGAATGTTGGAGAGGTTGGGGGAATACCTAGTGGAGAAGTGAAATTCGTAGAGATTAGGTGGAACACCAGAGGCGAAGGCGCCTGACTGGCCAAACATTGACGCTAAGGTGCGAAAGCGTGGGTAGCAAACAGG` |
| ASV3646 | 430 | `TGGGGAATCTTGCGCAATGGACGAAAGTCTGACGCAGCCACGCCGCGTGAGTGAAGAAGGCCTTCGGGTTGTAAAGCTCTGTCGAGCGGGACGAAAAGTTTTTAGGTAAATAGCCTAAGAATCTGACGGTACCGCTAAAGGAAGCACCGGCTAACTCCGTGCCAGCAGCCGCGGTAATACGGAGGGTGCAAGCGTTGCTCGGAATTATTGGGCGTAAAGGGTAGGTAGGTGGTCTTATTAGTCTGGGGTGAAAGCCTTGAGCTTAACTCAAGAAGTGCCCCAGAAACGGTGAGACTAGAGTCCTGGAGAGGGTCGTGGAATTCCCGGTGTAGCGGTGAAATGCGTAGATATCGGGAGGAACACCTGAGGCGAAAGCGGCGACCTGGACAGGTACTGACACTCAACTACGAAAGCGTGGGGAGCAAACAGG` |
| ASV5652 | 407 | `TTAGGAATCTTGCGCAATGGGGGAAACCCTGACGCAGCAACGCCGCGTGAGTGATGAAGGCCTTCGGGTTGTAAAGCTCTTTTAGTAGGGAAGAAAAAAATGACGGTACCTACAGAAAAAGCACCGGCTAACTCTGTGCCAGCAGCCGCGGTAATACAGAGGGTGCAAGCGTTATTCGGATTCATTGGGCGTAAAGGGCGCGTAGGCGGTTTGGTAAGTCTGGTGTGAAAACCCTGGGCTCAATCCAGGAGGTGCATTGGATACTGCCGAACTAGAGTACAGGAGGGGAGAACGGAATTCCTGGTGTAGAGGTGAAATTCGTAGATATCAGGAGGAACAACAGTGGCGAAGGCGGTTCTCTGGACTGTAACTGACGCTGAGGCGCGAAAGCGTGGGGAGCAAACAGG` |
| ASV5275 | 424 | `TGGGGAATATTGGACAATGGGCGAAAGCCTGATCCAGCAATGCCGCGTGAGTGATGAAGGCCTTCGGGTTGTAAAGCTCTGTCGGGAGGGACGAAAAAGCCGGTTAAAACCCGGTATTGACGGTACCTCCAAAGGAAGCACCGGCTAACTCTGTGCCAGCAGCCGCGGTAATACAGAGGGTGCAAGCGTTGTTCGGAATTACTGGGCGTAAAGCGCGTGTAGGTTGTTTGATAAGTCGGATGTGAAAGCCCTCGGCTCAACCGAGGAAGTGCATCCGATACTGTCAGACTTGAGTGTCGGAGGGGATGGCGGAATTCTAGGTGTAGAGGTGAAATTCGTAGATATCTAGAAGAACACCTGTGGCGAAGGCGGCCATCTGGAAGACAACTGACACTGAGACGCGAAAGCGTGGGTAGCAAACAGG` |
| ASV1833 | 404 | `TGGGGAATCTTGGACAATGGGCGAAAGCCTGATCCAGCCATGCCGCGTGGATGATGAAGGCCCTAGGGTTGTAAAATCCTTTCGACGGGGAAGATAATGACGGTACCCGTAGAAGAAGCTCCGGCTAACTTCGTGCCAGCAGCCGCGGTAATACGAAGGGGGCTAGCGTTGTTCGGAATTACTGGGCGTAAAGCGCACGTAGGCGGATCGATTAGTCAGGGGTGAAAGCCCAGAGCTCAACTCTGGAACTGCCTTTGATACTGTCGATCTGGAGCAGAGTAGAGGTGAGTGGAATTCCGAGTGTAGAGGTGAAATTCGTAGATATTCGGAGGAACACCAGTGGCGAAGGCGGCTCACTGGACTCTTGCTGACGCTGAGGTGCGAAAGCGTGGGGAGCAAACAGG` |
| ASV3193 | 430 | `TAGGGAATATTGCGCAATGGACGAAAGTCTGACGCAGCGACGCCGCGTGAGTGAAGAAGGCCTTCGGGTTGTAAAGCTCTGTCGAGCGGGACGAAAAGCGGATGGGTTAATAGCCCAGTCGCCTGACGGTACCGCTAAAGGAAGCACCGGCTAACTCCGTGCCAGCAGCCGCGGTAATACGGAGGGTGCAAGCGTTGCTCGGAATTATTGGGCGTAAAGGGTAGGTAGGTGGTCTCGTTTGTCTTGGGTGAAATCCCTGGGCTTAACTCAGGAAGTGCCTTGGAAACGGCGAGACTAGAGTACTGGAGAGGGTCGTGGAATTCCCGGTGTAGCGGTGAAATGCGTAGAGATCGGGAGGAACACCAGAGGCGAAAGCGGCGACCTGGACAGTTACTGACACTCAACTACGAAAGCGTGGGGAGCAAACAGG` |
| ASV1900 | 408 | `TCGAGAATCTTCCGCAATGGGCGAAAGCCTGACGGAGCGACGCCGCGTGGTGGATGAAGTCCTTCGGGACGTAAACACCTTTTATGAGGGAGAAAGTTTATTGATGTTACCTCATGAATAAGGGGCTCCCAACTCTGTGCCAGCAGGAGCGGTAATACAGAGGCCCCAAGCATTATCCGGATTTACTGGGCGTAAAGGGTGCGTAGGCGGTTATATTAGTCAGGTGTTAAATCCTGGGGCTCAACCCCAGAACTGCATTTGAAACGGTATAACTAGAATAAGTCAGAGGCAAGCAGAACTCACGGTGTAGGGGTGAAATCCGTTGATATCGTGGGGAATACCAAATGCGAAGGCAGCTTGCTGGGACTTTATTGACGCTGAGGCACGAAAGCGTGGGTAGCGAATCGG` |
| ASV2541 | 404 | `TGGGGAATATTGGACAATGGGCGAAAGCCTGATCCAGCAATGCCGCGTGAGTGAAGAAGGCCTTCGGGTTGTAAAGCTCTTTTGCCCGGGAAGATAATGACTGTACCGGGAGAATAAGCCCCGGCTAACTCCGTGCCAGCAGCCGCGGTAATACGGAGGGGGCTAGCGTTGTTCGGAATTACTGGGCGTAAAGCGTGCGTAGGCGGCTTTGTAAGTCAGAGGTGAAAGCCCACGGCTCAACCGTGGAATTGCCTTTGAGACTGCATCGCTTGAACACGGGAGAGGTGAGTGGAATTCCGAGTGTAGAGGTGAAATTCGTAGATATTCGGAAGAACACCAGTGGCGAAGGCGGCTCACTGGACCGTTGTTGACGCTGAGGCACGAAAGCGTGGGGAGCAAACAGG` |
| ASV2023 | 429 | `TGGGGAATTTTGGACAATGGGGGCAACCCTGATCCAGCCATGCCGCGTGCAGGATGAAGGCCTTCGGGTTGTAAACTGCTTTTGTTAGGAACGAAAAGCCACTGAATAACACTCAGTGGTGCTGACGGTACCTAAAGAATAAGCACCGGCTAACTACGTGCCAGCAGCCGCGGTAATACGTAGGGTGCAAGCGTTAATCGGAATTACTGGGCGTAAAGCGTGCGCAGGCGGTTATATAAGTCAGATGTGAAATCCCTGGGCTCAACCTAGGAACTGCATTTGAGACTGTATGGCTAGAGTGTGTCAGAGGGGGGTAGAATTCCACGTGTAGCAGTGAAATGCGTAGAGATGTGGAGGAATACCAATGGCGAAGGCAACCCCCTGGGATAACACTGACGCTCATGTACGAAAGCGTGGGGAGCAAACAGG` |
| ASV97 | 429 | `TGGGGAATATTGCACAATGGGCGCAAGCCTGATGCAGCCATGCCGCGTGTATGAAGAAGGCCTTCGGGTTGTAAAGTACTTTCAGCGAGGAGGAAGGGGATGTGGTTAATAACCGCATTCATTGACGTTACTCGCAGAAGAAGCACCGGCTAACTCCGTGCCAGCAGCCGCGGTAATACGGAGGGTGCAAGCGTTAATCGGAATTACTGGGCGTAAAGCGCACGCAGGCGGTCTGTCAAGTCGGATGTGAAATCCCCGGGCTCAACCTGGGAACTGCATCCGAAACTGGCAGGCTAGAGTCTTGTAGAGGGGGGTAGAATTCCAGGTGTAGCGGTGAAATGCGTAGAGATCTGGAGGAATACCGGTGGCGAAGGCGGCCCCCTGGACAAAGACTGACGCTCAGGTGCGAAAGCGTGGGGAGCAAACAGG` |
| ASV1264 | 429 | `TGGGGAATATTGCACAATGGGCGCAAGCCTGATGCAGCCATGCCGCGTGTGTGAAGAAGGCCTTCGGGTTGTAAAGCACTTTCAGCGAGGAGGAAGGGTAATGTGTTAATAGCACACTACATTGACGTTACTCGCAGAAGAAGCACCGGCTAACTCCGTGCCAGCAGCCGCGGTAATACGGAGGGTGCAAGCGTTAATCGGAATTACTGGGCGTAAAGCGCACGCAGGCGGTTTGTTAAGTCAGATGTGAAATCCCCGCGCTTAACGTGGGAACTGCATTTGAAACTGGCAAGCTAGAGTCTTGTAGAGGGGGGTAGAATTCCAGGTGTAGCGGTGAAATGCGTAGAGATCTGGAGGAATACCGGTGGCGAAGGCGGCCCCCTGGACAAAGACTGACGCTCAGGTGCGAAAGCGTGGGGAGCAAACAGG` |
| ASV100 | 429 | `TGGGGAATCTTCCGCAATGGACGAAAGTCTGACGGAGCAACGCCGCGTGAGTGATGAAGGTTTTCGGACTGTAAAACTCTGTTGTTTAGGACGAAGGTGCTTAGTGCAAATAGTGCTAGGTATTGACGGTACTGAACGAGGAAGCCACGGCTAACTACGTGCCAGCAGCCGCGGTAATACGTAGGTGGCAAGCGTTGTCCGGAATTATTGGGCGTAAAGAGCATGTAGGTGGACTGATAAGTCTGTTGTGAAAATGCGGGGCTCAACCCCGTATGGCAATGGAAACTGTCAGCCTTGAGTGCAGGAGAGGAAAGGGGAATTCCCAGTGTAGCGGTGAAATGCGTAGATATTGGGAGGAACACCAGTGGCGAAGGCGCCTTTCTGGACTGTGTCTGACACTGAGATGCGAAAGCTAGGGTAGCGAACGGG` |
| ASV2809 | 385 | `CTACGAAAACTTTACAATGCACGAAAGTGTGATAAGGGAATTCCGAGTGCTCATGCATTGCATGGGCTTTTGCCAAAGGTAAACACTTTGGCGAATAAGTGGTGGGTAAGACTGGTGCCAGCAGCCGCGGTAACACCAGCGCCACAAGTGGGGGCCGTGTATTTGGTCTAAAGCGTCCGTAGCCGGTTCTGTAAATTTCCTGTGAAATCATTTCGCTTAACGAAATGGCGGGCAGGGAAAACTGCAGAACTAGAGACCGGGAGGAGTTCGAAGTATTCTGTGGGGAGCGGTAAAATGCATTAATCCACAGAGGACTACCGATGGCGAAGGCATCGAACCAGAACGGATCTGACGGTGAGGGACGAAAGCTAGGGGAGCGATCCGG` |
| ASV853 | 429 | `TCGAGAATAATTCACAATGGGGGCAACCCTGATGGTGCAACGCCGCGTGGAGGATGACGGTCTTCGGATTGTAAACTCCTGTCATCTGGGAGTAAGACCTGGTCGTTAATAGCGAACAGGGTTGATAGTACCAGAAGAGGAAGGGACGGCTAACTTCGTGCCAGCAGCCGCGGTAATACGAAGGTCCCGAGCGTTGTTCGGAATCACTGGGCGTAAAGGGAGCGTAGGCGGCGCGGTAAGTCAGATGTGAAATCCCGGGGCTCAACCCCGGAACTGCATCCGATACTGCCGTGCTCGAGCATTGGAGAGGTAGCTGGAATTCTTGGTGTAGCAGTGAAATGCGTAGAGATCAAGAGGAACACTCGTGGCGAAAGCGAGCTACTGGACAATTGCTGACGCTGAGGCTCGAAGGCTAGGGTAGCGAAAGGG` |
| ASV2441 | 428 | `TAGGGAATTTTCGGCAATGGGCGCAAGCCTGACCGAGCAACGCCGCGTGAGTGATTAAGGCCTTCGGGTTGTAAAGCTCTGTTGTAAGGGAAGAACATTCAGGATAGGAAATGATCTTGAACTGACGGTACCTTACCAGAAAGCCACGGCTAACTACGTGCCAGCAGCCGCGGTAATACGTAGGTGGCAAGCGTTATCCGGAATTATTGGGCGTAAAGGGTGCGTAGGCGGTTTATTAAGTCTATGGTGAAAGTGCGGGGCTCAACCCCGTATGGCCATAGAAACTAGTAGACTTGAGTGCAGGAGAGGGCAATGGAATTCCATGTGTAGCGGTAAAATGCGTAGATATATGGAGGAACACCAGTGGCGAAGGCGGTTGCCTGGCCTGTAACTGACGCTGAGGCACGAAAGCGTGGGGAGCAAATAGG` |
| ASV1677 | 429 | `TGGGGAATTTTGGACAATGGGGGCAACCCTGATCCAGCAATGCCGCGTGAGTGAAGAAGGCCTTCGGGTTGTAAAGCTCTTTTGTTGGGGAAGAAACGGCGGGTGCTAATATCACTCGTTAATGACGGTACCCAAAGAATAAGCACCGGCTAACTACGTGCCAGCAGCCGCGGTAATACGTAGGGTGCGAGCGTTAATCGGAATTACTGGGCGTAAAGGGTGCGCAGGCGGTATGTTAAGTCAGATGTGAAATCCCCGGGCTCAACCTGGGAATGGCATTTGAGACTGGCAAGCTAGAGTGTGTCAGAGGGGGGTAGAATTCCACGTGTAGCAGTGAAATGCGTAGAGATGTGGAGGAATACCGATGGCGAAGGCAGCCCCCTGGGATAACACTGACGCTCATGCACGAAAGCGTGGGGAGCAAACAGG` |
| ASV1085 | 404 | `TGGGGAATCTTGGACAATGGGCGCAAGCCTGATCCAGCCATGCCGCGTGAGTGAAGAAGGCCTTCGGGTTGTAAAGCTCTTTTGTCAGGGACGATAATGACGGTACCTGAAGAATAAGCCCCGGCAAACTTCGTGCCAGCAGCCGCGGTAATACGAAGGGGGCTAGCGTTGTTCGGAATTACTGGGCGTAAAGCGCACGTAGGCTGACACTTAAGTGAGGGGTGAAATCCCAAGGCTCAACCTTGGAACTGCCTTTCATACTGGGTGTCTCGAGTCCGGAAGAGGTTAGTGGAATTCCCAGTGTAGAGGTGAAATTCGTAGATATTGGGAAGAACACCAGTGGCGAAGGCGGCTAACTGGTCCGGTACTGACGCTGAGGTGCGAAAGCGTGGGGAGCAAACAGG` |
| ASV2925 | 428 | `TCGAGAATCTTTCACAATGGGGGCAACCCTGATGGAGCGACGCCGCGTGGAGGATAAGGTCTTCGGATTGTAAACTCCTGTCATGCGAGAACAAGGTAGCTTGGTTAATAACCTTGCTGCTTGATGGTACCGCAAGAGGAAGAGACGGCTAACTCTGTGCCAGCAGCCGCGGTAATACAGAGGTCTCAAGCGTTGTTCGGAATCACTGGGCGTAAAGGGTGCGTAGGCTGCGCCGTAAGTCAGATGTGAAAGCTCAGGGCTCAACCCTGAAATTGCATCCGATACTGCGGTGCTAGAGAAATGAAGGGGAGTCTAGAATTTTCGGTGTAGCAGTGAAATGCGTAGATATCGAAAGGAATACCAAAGGCGAAGGCAGGACTCTGGGCATTTTCTGACGCTCATGCACGAAGGCTAGGGTAGCGAACGGG` |
| ASV2633 | 424 | `TGAGGAATATTGGACAATGGGCGCAAGCCTGATCCAGCCATGCCGCGTGCAGGAATAAGGTCTTATGGATTGTAAACTGCTTTTGTACAGGAAGAAACCTCATTATGTATAATGAATTGACGGTACTGTAAGAATAAGGATCGGCTAACTCCGTGCCAGCAGCCGCGGTAATACGGAGGATCCGAGCGTTATCCGGAATCATTGGGTTTAAAGGGTCCGTAGGCGGTTTAATAAGTCAGTGGTGAAATTCCAACGCTCAACGTTGGGACTGCCATTGATACTGTTAGGCTTGAATTATTAGGAAGTAACTAGAATATGTAGTGTAGCGGTGAAATGCTTAGATATTACATGGAATACCGATTGCGAAAGCAGGTTACTACTAATTGATTGACGCTGATGGACGAAAGCGTGGGTAGCGAACAGG` |
| ASV4628 | 429 | `TGGGGAATATTGGACAATGGGCGAAAGCCTGATCCAGCAATGCCGCGTGTGTGAAGAAGGCCTTCGGGTTGTAAAGCACTTTCAGTGGGGAAGAAAAGTTTGAGGTTAATACCCTTAAATATTGACGGTACCTACAGAAGAAGCACCGGCTAACTCTGTGCCAGCAGCCGCGGTAATACAGAGGGTGCAAGCGTTAATCGGAATTACTGGGCGTAAAGCGCGCGTAGGCGGGCATCTAAGTTGGGTGTGAAAGCCCCGGGCTTAACCTGGGAATTGCACTCAAGACTGGATGCCTTGAGTATGGTAGAGGAAAGTGGAATTCCCGGTGTAGCGGTGAAATGCGTAGATATCGGGAGGAACACCAGTGGCGAAGGCGGCTTTCTGGACCAATACTGACGCTGAGGTGCGAAAGCGTGGGGAGCAAACAGG` |
| ASV1427 | 404 | `TGGGGAATATTGGACAATGGGCGCAAGCCTGATCCAGCCATGCCGCGTGTGTGATGAAGGCCTTAGGGTTGTAAAGCACTTTTGTCAGGGAAGATAATGACGGTACCTGAAGAATAAGCACCGGCTAACTTCGTGCCAGCAGCCGCGGTAATACGAAGGGTGCTAGCGTTGTTCGGAATCACTGGGCGTAAAGCGCGCGTAGGCGGCGTAACAAGTCAGAGGTGAAATCCCCAGGCTCAACCTGGGAATTGCCTTTGAAACTGTTATGCTTGAGTGTCGGAGGGGATAGCGGAATTGCTAATGTAGAGGTGAAATTCGTAGATATTAGCAGGAACACCGGTGGCGAAGGCGGCTATCTGGACGACTACTGACGCTGAGGCGCGAAAGCGTGGGGATCAAACAGG` |
| ASV3057 | 404 | `TCGGGAATTTTGCGCAATGGACGAAAGTCTGACGCAGCAACGCCGCGTGAGGGATGACGGCCTTCGGGTTGTAAACCTCTTTTGTCAGGGACGATGATGACGGTACCTGACGAATAAGCCACGGCTAACTACGTGCCAGCAGCCGCGGTAATACGTAGGTGGCAAGCGTTGTCCGGATTTACTGGGCGTAAAGAGCGCGCAGGCGGTCGTTCAAGTCGCGTGTGAAAGCCCCCGGCTCAACTGGGGAGGGTCACGCGATACTGATCGACTCGAAGGCAGGAGAGGGTAGTGGAATTCCCGGTGTAGTGGTGAAATGCGTAGATATCGGGAGGAACACCAGTGGCGAAGGCGACTACCTGGCCTGTTCTTGACGCTGAGGCGCGAAAGCTAGGGGAGCAAACGGG` |
| ASV1516 | 423 | `TAGGGAATATTGGGCAATGGGCGCGAGCCTGACCCAGCCATGCCGCGTGCCGGATGAAGGCCTTCTGGGTTGTAAACGGCTTTTCTCAGGGAAGAAAAAGGTGATGCGTCACAAACTGACGGTACCTGAGGAATAAGCACCGGCTAACTCCGTGCCAGCAGCCGCGGTAATACGGAGGGTGCAAGCGTTGTCCGGATTTATTGGGTTTAAAGGGTGCGTAGGTGGCCCGTTAAGTCCGGGGTGAAAGCCCACAGCTCAACTGTGGAACTGCCCTGGATACTGGCGGGCTTGAGTCCAGACGAGGTTGGCGGAATGGAAGATGTAGCGGTGAAATGCATAGATATCTTCCAGAACCCCGATTGCGTAGGCAGCTGACTAGGCTGGTACTGACACTGAGGCACGAAAGCGTGGGGAGCGAACAGG` |
| ASV5646 | 424 | `TAGGGAATATTGGGCAATGGAAGAGATTCTGACCCAGCCATGCCGCGTGCAGGATGAAGGCTTTCTGAGTCGTAAACTGCTTTTGCCAGGGAAGAAAAAAGGGGATGCGTCCCCTACTGACGGTACCTGGTGAATAAGCACCGGCTAACTCCGTGCCAGCAGCCGCGGTAATACGGAGGGTGCAAGCGTTGTCCGGATTTATTGGGTTTAAAGGGTGCGTAGGCGGTCCGTTAAGTCCGGGGTGAAAGCCCACTGCTCAACAGTGGAACTGCCCTGGAAACTGGCGGACTTGAGTACAGACGAGGGCGGCGGAATGGATGGTGTAGCGGTGAAATGCATAGATACCATCCAGAACCCCGATTGCGAAGGCAGCTGCCTAGACTGTAACTGACGCTGAGGCACGAAAGCGTGGGGAGCGAACAGG` |
| ASV4790 | 428 | `TAGGGAATCTTCCGCAATGGGCGAAAGCCTGACGGAGCGACGCCGCGTGAGTGATGAAGGCCTTCGGGTTGTAAAGCTCTGTGGGGAGGGAAGAATAAGTTCAGATCAATACTCTGAATGATGACGGTACCTCCTTAGCAAGCACCGGCTAACTCTGTGCCAGCAGCCGCGGTAAGACAGAGGGTGCGAACGTTGTTCGGAATTACTGGGCGTAAAGCGTGTGTAGGCGGCAAGGCAAGTCGGATGTGAAATCCCGTGGCTCAACCATGGACGTGCACTCGAAACTGCCTAGCTAGAGTCTCGGAGAGGAAGGTGGAATGCTTGGTGTAGAGGTGAAATTCGTAGATATCAAGCAGAACACCTGTGGCGAAGGCGGCCTTCTGGACGATGACTGACGCTGAGACACGAAAGCGTGGGGAGCAAACAGG` |
| ASV2744 | 423 | `TAGGGAATATTGGGCAATGGTCGGAAGACTGACCCAGCCATGCCGCGTGCAGGATGAAGGCGCTATGCGTTGTAAACTGCTTTTGTTAGGGAAGAAAATGGCTATGCGTAGCAACTTGACGGTACCTAAAGAATAAGCATCGGCTAACTCCGTGCCAGCAGCCGCGGTAATACGGAGGATGCAAGCGTTGTCCGGATTTATTGGGTTTAAAGGGTGCGTAGGCGGTCTTATAAGTCAGTGGTTAAATTACTTTGCTCAACAAGGGAACTGCCATTGATACTGTAAGACTTGAATACTGACTAGGTAGGCGGAATTGGTAGTGTAGCGGTGAAATGCTTAGATATTACCAGGAACACCGATAGCGAAAGCAGCTTACTGGATAGTAATTGACGCTGAGGCACGAAAGTGCGGGTATCAAACAGG` |
| ASV1598 | 429 | `TGGGGAATTTTGGACAATGGGCGCAAGCCTGATCCAGCAATGCCGCGTGCAGGATGAAGGCCTTCGGGTTGTAAACTGCTTTTGTACGGAACGAAAAGAGACTGCCTAATACGTGGTCTTACTGACGGTACCGTAAGAATAAGCACCGGCTAACTACGTGCCAGCAGCCGCGGTAATACGTAGGGTGCAAGCGTTAATCGGAATTACTGGGCGTAAAGCGTGCGCAGGCGGTCTTATAAGACAGATGTGAAATCCCCGGGCTCAACCTGGGAACTGCATTTGTGACTGTAAGGCTGGAGTGCGGCAGAGGGGGATGGAATTCCGCGTGTAGCAGTGAAATGCGTAGATATGCGGAGGAACACCGATGGCGAAGGCAATCCCCTGGGCCTGCACTGACGCTCATGCACGAAAGCGTGGGGAGCAAACAGG` |
| ASV2141 | 429 | `TGGGGAATTTTGGACAATGGGCGCAAGCCTGATCCAGCCATTCCGCGTGAGTGAAGAAGGCCTTCGGGTTGTAAAGCTCTTTCGGCAGGGAAGAAACGGTTTGGGCTAATACCCCAAGCTAATGACGGTACCTGAAGAAGAAGCACCGGCTAACTACGTGCCAGCAGCCGCGGTAATACGTAGGGTGCGAGCGTTAATCGGAATTACTGGGCGTAAAGCGTGCGCAGGCGGTTTTGTAAGACAGATGTGAAATCCCCGGGCTTAACCTGGGAACTGCGTTTGTGACTGCAAGACTCGAGTGCGGCAGAGGGGGGTGGAATTCCACGTGTAGCAGTGAAATGCGTAGAGATGTGGAGGAACACCGATGGCGAAGGCAGCCCCCTGGGTCGACACTGACGCTCATGCACGAAAGCGTGGGGAGCAAACAGG` |
| ASV5288 | 404 | `TGGGGAATATTGGACAATGGGGGCAACCCTGATCCAGCAATGCCGCGTGAGTGATGAAGGCCTTAGGGTTGTAAAGCTCTTTTGCCAGGGACGATGATGACGGTACCTGGAGAATAAGCCCCGGCTAACTTCGTGCCAGCAGCCGCGGTAATACGAAGGGGGCAAGCGTTGTTCGGAATTACTGGGCGTAAAGGGCGCGTAGGCGGCTTGACAAGTCAGGCGTGAAAGCCCCGGGCTTAACCTGGGAGGTGCGTTTGAGACTGTTGGGCTTGAGAGTGGGAGAGGATGGTGGAATTCCCAGTGTAGAGGTGAAATTCGTAGATATTGGGAAGAACACCGGTGGCGAAGGCGGCCATCTGGCCCACCACTGACGCTGAGGCGCGAAAGCGTGGGGAGCAAACAGG` |
| ASV576 | 424 | `TGAGGAATATTGGACAATGGGCGCAAGCCTGATCCAGCCATGCCGCGTGCAGGATGACGGTCCTATGGATTGTAAACTGCTTTTGTACAGGAAGAAACACCTCTTCGTGAAGAGACTTGACGGTACTGTAAGAATAAGGATCGGCTAACTCCGTGCCAGCAGCCGCGGTAATACGGAGGATCCAAGCGTTATCCGGAATCATTGGGTTTAAAGGGTCCGTAGGCGGTTTGATAAGTCAGTGGTGAAAGCCCATCGCTCAACGGTGGAACGGCCATTGATACTGTTAGACTTGAATTATTAGGAAGTAACTAGAATATGTAGTGTAGCGGTGAAATGCTTAGAGATTACATGGAATACCAATTGCGAAGGCAGGTTACTACTAATTGATTGACGCTGATGGACGAAAGCGTGGGTAGCGAACAGG` |
| ASV655 | 429 | `TCGAGAATAATTCACAATGGGGGCAACCCTGATGGTGCAACGCCGCGTGGAGGATGACGGTCTTCGGATTGTAAACTCCTGTCATCCGGGAGTAAGACCTGGCTGTTAATAGCAGACAGGGTTGATAGTACCGGAAGAGGAAGGGACGGCTAACTTCGTGCCAGCAGCCGCGGTAATACGAAGGTCCCGAGCGTTGTTCGGAATCACTGGGCGTAAAGGGAGCGTAGGCGGCGTGGTAAGTCAGATGTGAAATCCCGGGGCTCAACCCCGGAACTGCATCCGATACTGCCATGCTAGAGGATTGGAGAGGTAGCTGGAATTCTTGGTGTAGCAGTGAAATGCGTGGAGATCAAGAGGAACACTCGTGGCGAAAGCGAGCTACTGGACAATATCTGACGCTGAGGCTCGAAGGCCAGGGTAGCGAAAGGG` |
| ASV2536 | 404 | `TGGGGAATCTTGCGCAATGGACGAAAGTCTGACGCAGCCATGCCGCGTGAATGATGAAGGTCTTAGGATTGTAAAGTTCTTTCGACGGGGACGATGATGACGGTACCCGTAGAAGAAGCCCCGGCTAACTTCGTGCCAGCAGCCGCGGTGATACGAAGGGGGCTAGCGTTGTTCGGAATTACTGGGCGTAAAGCGTGCGCAGGCGGTTTTTTAAGCCAGAGGTGAAATCCCGGAGCTCAACTTCGGAATGGCCTTTGGAACTGGAAAGCTTGAGAACGGTAGAGGTGAGTGGAATTCCCAGTGTAGAGGTGAAATTCGTAGATATTGGGAAGAACACCGGTGGCGAAGGCGGCTCACTGGACCGTTTCTGACGCTCAGGCACGAAAGCGTGGGGAGCAAACAGG` |
| ASV1936 | 429 | `TAGGGAATCTTCCGCAATGGACGAAAGTCTGACGGAGCAATGCCGCGTGAGTGAAGAAGGTTTTCGGATCGTAAAACTCTGTTGTTAGAGAAGAACAAGGATGAGAGTAACTGCTCATCCCCTGACGGTATCTAACCAGAAAGCCACGGCTAACTACGTGCCAGCAGCCGCGGTAATACGTAGGTGGCAAGCGTTGTCCGGATTTATTGGGCGTAAAGCGAGCGCAGGCGGTTCTTTAAGTCTGATGTGAAAGCCCCCGGCTCAACCGGGGAGGGTCATTGGAAACTGGAGAACTTGAGTGCAGAAGAGGAGAGTGGAATTCCATGTGTAGCGGTGAAATGCGTAGATATATGGAGGAACACCAGTGGCGAAGGCGACTCTCTGGTCTGTAACTGACGCTGAGGCTCGAAAGCGTGGGGAGCAAACAGG` |
| ASV4433 | 430 | `TGGGGAATATTGCGCAATGGGCGAAAGCCTGACGCAGCAACGCCGCGTGAGTGATGAAGGCCTTAGGGTTGTAAAGCTCTTTCGGTTGGGAAGAAGGGTATTCTAGTTAAAAGCTGGAGTATTTGATGGTACCAAAAGAAGAAGCACCGGCTAACTTCGTGCCAGCAGCCGCGGTAATACGAAGGGTGCAAGCGTTGTTCGGATTTACTGGGCGTAAAGGGTTCGTAGGCGGGAAAACAAGTTGGGAGTGAAATCCCTGGGCTCAACCCAGGACGTGCTTTCAAAACTGTTTTTCTTGAGTACTGGAGAGGGTGGTGGAATTGCTGGTGTAGGAGTGACATCCGTAGAGATCAGCAGGAACATCAGAGGCGAAAGCGACCACCTGGCCTGAAACTGACGCTGAGGAACGAAAGCGTGGGGAGCAAACAGG` |
| ASV4214 | 406 | `TAAGGAATCTTCCGCAATGGGCGAAAGCCTGACGGAGCGACACCGCGTGAAGGATGAAGGCCTATGGGTTGTAAACTTCTTTTCTGTGGGAAGAAAATCTGACGGTACCACAGGAATAAGGACCGGCTAATTACGTGCCAGCAGCCGCGGTAATACGTAAGGTCCGAGCGTTACCCGGAATTATTGGGTGTAAAGCGTCCGCAGGTGGCGCAAAAAGTCAGTTATTAAATACCGGAGCTCAACTTCGGTGCTGTAATTGAAACTTTTGTGCTAGAGGATGGGAGAGGTATATGGAATTGTGCAAGTAGGGGTAAAATCCGTAGATATGCACAGGAACACCAAAAGCGAAGGCAGTATACTGGACCATTCCTGACACTCATGGACGAAAGCGTGGGGAGCGAAACGG` |
| ASV1659 | 424 | `TAGGGAATATTGGTCAATGGGGGCAACCCTGAACCAGCCATGCCGCGTGCAGGAAGACTGCCCTATGGGTTGTAAACTGCTTTTGTACGGGAAGAAATGTCACTATGCGTAGTGATTTGACGGTACCGTAAGAATAAGGATCGGCTAACTTCGTGCCAGCAGCCGCGGTAAGACGAAGGATCCAAGCGTTGTCCGGATTTACTGGGTTTAAAGGGAGCGTAGGCGGATACATAAGTCAGTGGTGAAAGCCTTCAGCTTAACTGAAGAACTGCCATTGAAACTGTGTGTCTCGAGTACGGCTGAGGTGACTGGAATGTAACATGTAGCGGTGAAATGCTTAGATATGTTACAGAACACCAATTGCGAAGGCAGGTCACTAAACCGTAACTGACGCTGAGGCTCGAAAGCGTGGGGAGCAAACAGG` |
| ASV4764 | 424 | `TGAGGAATATTGGTCAATGGGCGAGAGCCTGAACCAGCCATGCCGCGTGCAGGATGACGCATCTATGGTGTGTAAACTGCTTTTGTACAGGAAGAAACACCGCTACGTGTAGCGGCTTGACGGTACTGTAAGAATAAGGATCGGCTAACTCCGTGCCAGCAGCCGCGGTAATACGGAGGATCCAAGCGTTATCCGGAATCATTGGGTTTAAAGGGTCCGTAGGCGGTTTTATAAGTCAGTGGTGAAATCCGGCAGCTCAACTGTCGAACTGCCATTGATACTGTAGGACTTGAATTATTGTGAAGTAACTAGAATATGTAGTGTAGCGGTGAAATGCTTAGATATTACATGGAATACCGATTGCGAAGGCAGGTTACTAACAATACATTGACGCTGATGGACGAAAGCGTGGGGAGCGAACAGG` |
| ASV2494 | 405 | `TGGGGAATCTTGCGCAATGGGCGAAAGCCTGACGCAGCCATGCCGCGTGAATGATGAAGGTCTTAGGATTGTAAAGTTCTTTCGCTCGTGACGATGATGACGGTAACGAGAGAAGAAGCCCCGGCTAACTTCGTGCCAGCAGCCGCGGTAATACGAAGGGGGCTAGCGTTGTTCGGAATTACTGGGCGTAAAGCGCGCGTAGGCGGTTTTGCAAGCCAGAGGTGAAATGCCTGAGCTCAACTTAGGAATTGCCTTTGGGACTGCGAGGCTTTGAGGACGGAAGAGGTGAGTGGAATTCCCAGTGTAGAGGTGAAATTCGTAGATATTGGGAAGAACACCGGTGGCGAAGGCGGCTCACTGGTCCGTTTCTGACGCTGTAGCGCGAAAGCGTGGGGATCAAACAGG` |
| ASV3220 | 429 | `TGGGGAATTTTGGACAATGGGCGCAAGCCTGATCCAGCCATGCCGCGTGTGGGAAGAAGGCCTTCGGGTTGTAAACCACTTTTGTCGGGGAAGAAACGGTTTGGGCTAATACCCTGAGCTAATGACGGTACCCGAAGAATAAGCACCGGCTAACTACGTGCCAGCAGCCGCGGTAATACGTAGGGTGCAAGCGTTAATCGGAATTACTGGGCGTAAAGCGTGCGCAGGCGGCTTTGCAAGACAGATGTGAAATCCCCGGGCTCAACCTGGGAACTGCATTTGTGACTGCATGGCTAGAGTGCGGCAGAGGGGGATGGAATTCCGCGTGTAGCAGTGAAATGCGTAGATATGCGGAGGAACACCGATGGCGAAGGCAATCCCCTGGGCCTGCACTGACGCTCATGCACGAAAGCGTGGGGAGCAAACAGG` |
| ASV1925 | 404 | `TGGGGAATCTTGCGCAATGGGGGCAACCCTGACGCAGCCATACCGCGTGTGTGATGAAGGTCTTCGGATTGTAAAGCACTTTCAGTAGGGACGACGATGACGGTACCTACAGAAGAAGTCACGGCTAACTTCGTGCCAGCAGCCGCGGTAATACGAAGGTGGCGAATGTTGCTCGGATTCACTGGGCGTAAAGCGTCGGTAGGTGGTTATGTTAGTCAGAGGTGAAAGCCCCGCGCTCAACGTGGGAACTGCCTTTGAAACTGCATGACTTGAAGGTATGAGAGGATGGCGGAATGACTGGTGTAGAGGTAAAATTCATAGATATCAGTCGGAACACCCAAGGCGAAGGCAGCTATCTGGCATATTCTTGACACTGAGCGACGACAGCGTGGGGAGCAAACAGG` |
| ASV3436 | 404 | `TAGGGAATATTGCTCAATGGGGGAAACCCTGAAGCAGCAACGCCGCGTGGAGGATGAAGGTTTTCGGATTGTAAACTCCTTTTGTTAGAGAAGATTATGACGGTATCTAACGAATAAGCACCGGCTAACTCCGTGCCAGCAGCCGCGGTAATACGGAGGGTGCAAGCGTTACTCGGAATCACTGGGCGTAAAGAGCGCGTAGGCGGGATAGCAAGTCAGATGTGAAATCCTATGGCTTAACCATAGAACTGCATTTGAAACTGCTATTCTAGAGTATGGGAGAGGTAGGTGGAATTCTTGGTGTAGGGGTAAAATCCGTAGAGATCAAGAGGAATACTCATTGCGAAGGCGACCTGCTAGAACATAACTGACGCTGATGCGCGAAAGCGTGGGGAGCAAACAGG` |
| ASV385 | 404 | `TGGGGAATCTTAGACAATGGGGGAAACCCTGATCTAGCCATGCCGCGTGATCGATGAAGGCCTTAGGGTTGTAAAGATCTTTCAGGTGGGAAGATAATGACTGTACCACCAGAAGAAGCCCCGGCTAACTCCGTGCCAGCAGCCGCGGTAATACGGAGGGGGCTAGCGTTGTTCGGAATTACTGGGCGTAAAGCGCACGTAGGCGGACTGAAAAGTCAGAGGTGAAATCCCAGGGCTCAACCTTGGAACTGCCTTTGAAACTCTCAGTCTTGAGGTCGAGAGAGGTGAGTGGAATTCCGAGTGTAGAGGTGAAATTCGTAGATATTCGGAGGAACACCAGTGGCGAAGGCGGCTCACTGGCTCGATACTGACGCTGAGGTGCGAAAGCGTGGGGAGCAAACAGG` |
| ASV4958 | 404 | `TTAGGAATATTGGACAATGGGCGCAAGCCTGATCCAGCCATGCCGCGTGAGTGATGAAGGCCTTCGGGTTGTAAAGCTCTTTAGCCGACGATTATAATGACTGTAGTCGGAAAATAAGCCCCGGCTAACTTCGTGCCAGCAGCCGCGGTAATACGAAGGGGGCGAGCGTTATTCGGAATCACTGGGCGTAAAGCGTGCGTAGGCGGTTTTGTAAGTTGGAAGTGAAATCCCGGGGCTCAACCCCGGAATTGCTTTCAAAACTACAAAACTCGAATTCGGGAGAGGATAGCGGAATTCTCAGTGTAGCAGTGAAATGCGTAGATATTGAGAGGAACACCAATGGCGCAGGCAGCTATCTGGACCGATATTGACGCTGAGGCACGAAAGCGTGGGGATCAAACAGG` |
| ASV4219 | 421 | `TGGGGAATATTGGTCAATGGGCGAAAGCCTGAACCAGCAATGCCGCGTGAGTGATGAAGGCCTTCGGGTTGTAAAGCTCTGTCGGAGGGGACGAAAAAAGTGGGCAACCAAACTTGACGGTACCCTCAAAGGAAGCACCGGCTAACTCTGTGCCAGCAGCCGCGGTAATACAGAGGGTGCAAGCGTTGTTCGGAATTACTGGGCGTAAAGCGCGTGTAGGCGGCGATGTAAGTCAGGTGTGAAATCCCTCGGCTTAACCGAGGAAGTGCATCTGATACTGTATTGCTTGAGTGCCGGAGGGGATGGCGGAATTCCCGGTGTAGAGGTGAAATTCGTAGATATCGGGAGGAACACCTGTGGCGAAGGCGGCCATCTGGACGGTAACTGACGCTGAGACGCGAAAGCGTGGGTAGCAAACAGG` |
| ASV5070 | 404 | `TGAGGAATATTGGACAATGGGCGCAAGCCTGATCCAGCCATGCCGCGTGAGTGATGAAGGCCCTAGGGTTGTAAAACTCTTTTAGTGGGGAAAATAATGATGGTACCCACAGAAAAAGCTCCGGCTAACTTCGTGCCAGCAGCCGCGGTAATACGAAGGGAGCTAGCGTTACTCGGAATCACTGGGCGTAAAGCGCATGTAGGCGGAATTACAAGTCAGGGGTGAAATCCCAGAGCTTAACTCTGGAATTGCCTTTGAAACTGTAATTCTTGATTATGTCAGAGGATGGTGGAATTTCCAGTGTAGAGGTGAAATTCGTAGATATTGGAAAGAACACCGGTGGCGAAGGCGGCCATCTGGGACATTAATGACGCTGAGATGCGAAAGCGTGGGGAGCGAACGGG` |
| ASV2977 | 429 | `TGGGGAATATTGGACAATGGGCGCAAGCCTGATCCAGCCATGCCGCGTGTGTGAAGAAGGCCTTCGGGTTGTAAAGCACTTTTGTTCGGGAAGAAATCGTACGGATTAATACTTTGTACGGATGACGGTACCGAAAGAATAAGCACCGGCTAACTTCGTGCCAGCAGCCGCGGTAATACGAAGGGTGCAAGCGTTACTCGGAATCACTGGGCGTAAAGCGTGCGTAGGCGGTTCGTTAAGTCTGCTGTGAAAGCCCTGGGCTCAACCTGGGAACTGCAGTGGATACTGGCGGACTAGAGTGTGATAGAGGATGGTGGAATTCCCGGTGTAGCGGTGAAATGCGTAGAGATCGGGAGGAACACCAGTGGCGAAGGCGGCCATCTGGATCAACACTGACGCTGAGGCACGAAAGCGTGGGGAGCAAACAGG` |
| ASV2022 | 429 | `TGGGGAATTTTGGACAATGGGCGCAAGCCTGATCCAGCCATGCCGCGTGCGGGAAGAAGGCCTTCGGGTTGTAAACCGCTTTTGGCAGGAACGAAACGGCGCTCGCTAATACCGAGCGCTAATGACGGTACCTGCAGAATAAGCACCGGCTAACTACGTGCCAGCAGCCGCGGTAATACGTAGGGTGCGAGCGTTAATCGGATTTACTGGGCGTAAAGCGTACGCAGGCGGCTTGGTAAGACAGGTGTGAAATCCCCGGGCTTAACCTGGGAACTGCATTTGTGACTGCCTGGCTAGAGTCTGTCAGAGGGGGGTGGAATTCCACGTGTAGCAGTGAAATGCGTAGAGATGTGGAGGAACACCGATGGCGAAGGCAGCCCCCTGGGATGAGACTGACGCTCATGTACGAAAGCGTGGGGAGCAAACAGG` |
| ASV4979 | 429 | `TGGGGAATTTTGGACAATGGGCGCAAGCCTGATCCAGCAATGCCGCGTGAGTGAAGAAGGCCTTCGGGTTGTAAAGCTCTTTTGTCAGGGAAGAAACGGCCGGGGCTAATATCCTCGGCTAATGACGGTACCTGAAGAATAAGCACCGGCTAACTACGTGCCAGCAGCCGCGGTAATACGTAGGGTGCAAGCGTTAATCGGAATTACTGGGCGTAAAGCGTGCGCAGGCGGTTTTGTAAGTCTGACGTGAAATCCCCGGGCTCAACCTGGGAATTGCGTTGGAGACTGCAAGGCTGGAGTCTGGCAGAGGGGGGTAGAATTCCACGTGTAGCAGTGAAATGCGTAGAGATGTGGAGGAACACCGATGGCGAAGGCAGCCCCCTGGGTCAAGACTGACGCTCATGCACGAAAGCGTGGGGAGCAAACAGG` |
| ASV2580 | 407 | `TGGGGAATTTTCCGCAATGGGCGAAAGCCTGACGGAGCAATACCGCGTGAGGGAAGAAGGCTCTTGGGTTGTAAACCTCTTTTCTTGGGGAAGAAAAAAATGACGGTACCCAAGGAATCAGCATCGGCTAACTCCGTGCCAGCAGCCGCGGTAATACGGAGGATGCAAGCGTTATCCGGAATGATTGGGCGTAAAGCGTCCGCAGGTGGCAATTCAAGTTTGCGGTTAAAGGCTCTGGCTCAACCAGGGACAGGCCGTGAAAACTGAATAGCTAGAGTATGGTAGGGGCAGAGGGAATTCCCAGTGTAGCGGTGAAATGCGTAGAGATTGGGAAGAACACCGGTGGCGAAAGCGCTCTGCTAGGCCAAAACTGACACTGAGGGACGAAAGCTAGGGGAGCGAATGGG` |
| ASV2762 | 429 | `TGGGGAATTTTGGACAATGGGCGCAAGCCTGATCCAGCCATGCCGCGTGAGTGAAGAAGGCCTTCGGGTTGTAAAGCTCTTTCGGCCGGGAAGAAATCGCACAGGTTAATACCTTGTGTGGATGACGGTACCGGAATAAGAAGCACCGGCTAACTACGTGCCAGCAGCCGCGGTAATACGTAGGGTGCGAGCGTTAATCGGAATTACTGGGCGTAAAGCGTGCGCAGGCGGTTTCGTAAGACAGATGTGAAATCCCCGGGCTCAACCTGGGAACTGCATTTGTGACTGCGAGGCTAGAGTATGGCAGAGGGGGGTGGAATTCCACGTGTAGCAGTGAAATGCGTAGAGATGTGGAGGAACACCGATGGCGAAGGCAGCCCCCTGGGCCAATACTGACGCTCATGCACGAAAGCGTGGGGAGCAAACAGG` |
| ASV248 | 429 | `TGGGGAATTTTGGACAATGGACGCAAGTCTGATCCAGCCATTCCGCGTGCAGGATGAAGGCCCTCGGGTTGTAAACTGCTTTTGTACGGAACGAAAAGGTTTCTCTTAATACGAGGAGCTCATGACGGTACCGTAAGAATAAGCACCGGCTAACTACGTGCCAGCAGCCGCGGTAATACGTAGGGTGCAAGCGTTAATCGGAATTACTGGGCGTAAAGCGTGCGCAGGCGGTTATGTAAGACAGAGGTGAAATCCCCGGGCTCAACCTGGGAACTGCCTTTGTGACTGCATAGCTAGAGTACGGTAGAGGGGGATGGAATTCCGCGTGTAGCAGTGAAATGCGTAGATATGCGGAGGAACACCGATGGCGAAGGCAATCCCCTGGACCTGTACTGACGCTCATGCACGAAAGCGTGGGGAGCAAACAGG` |
| ASV568 | 429 | `TGGGGAATTTTGGACAATGGGCGCAAGCCTGATCCAGCAATGCCGCGTGCAGGACGAAGGCCTTCGGGTTGTAAACTGCTTTTGTACGGAACGAAACGGTCTCTTCTAATACAGGGGGCTAATGACGGTACCGTAAGAATAAGCACCGGCTAACTACGTGCCAGCAGCCGCGGTAATACGTAGGGTGCGAGCGTTAATCGGAATTACTGGGCGTAAAGCGTGCGCAGGCGGTGATGTAAGACAGATGTGAAATCCCCGGGCTCAACCTGGGACCTGCATTTGTGACTGCATCGCTAGAGTACGGTAGAGGGGGATGGAATTCCGCGTGTAGCAGTGAAATGCGTAGATATGCGGAGGAACACCGATGGCGAAGGCAATCCCCTGGACCTGTACTGACGCTCATGCACGAAAGCGTGGGGAGCAAACAGG` |
| ASV17 | 407 | `TGGGGAATTTTCCGCAATGGGCGAAAGCCTGACGGAGCAATACCGCGTGAGGGATGACGGCCTATGGGTTGTAAACCTCTTTTTTCAGGGAGGAATAAATGACGTGTACCTGAAGAATAAGCATCGGCTAACTCCGTGCCAGCAGCCGCGGTAAGACGGGGGATGCAAGTGTTATCCGGAATCACTGGGCGTAAAGCGTCTGTAGGTGGTTTAATAAGTCAACTGTTAAATCTTGAGGCTCAACCTCGAAATCGCAGTCGAAACTATTAGACTAGAGTATAGTAGGGGTAAAGGGAATTTCCAGTGGAGCGGTGAAATGCGTAGATATTGGAAAGAACACCGATGGCGAAGGCACTTTACTGGGCTATTACTAACACTGAGAGACGAAAGCTAGGGTAGCAAATGGG` |
| ASV2994 | 430 | `TGGGGAATATTGGACAATGGGGGCAACCCTGATCCAGCAATGCCGCGTGTGTGAAGAAGGCCTGCGGGTTGTAAAGCACTTTTGTTCGAGACGAAATCTTGTTGATTAATACCTGACAAGGATGACGGTATCGAAAGAATAAGCACCGGCTAACTTCGTGCCAGCAGCCGCGGTAATACGAAGGGTGCAAGCGTTAATCGGAATTACTGGGCGTAAAGCGTGCGTAGACGGCACATTAAGTCGACTGTGAAACCCCCGGGCTCAACCTGGGACCTGCAGTTGATACTGGTGTGCTTGGAGTTTGAGAGAGGGTGGTGGAATTCCTGGTGTAGCGGTGAAATGCGTAGAGATCAGGAGGAACACTAGTGGCGAAGGCGGCCACCTGGCTTAAAACTGACGTTGAGGCACGAAAGCGTGGGGAGCAAACAGG` |
| ASV1223 | 404 | `TGGGGAATATTGGACAATGGGGGAAACCCTGATCCAGCAATGCCGCGTGAGTGAAGAAGGCCTTCGGGTTGTAAAGCTCTTTTGTCTGTGAAGATAATGACGGTAACAGACGAATAAGCACCGGCTAACTTCGTGCCAGCAGCCGCGGTAATACGAAGGGTGCTAGCGTTGTTCGGAATAACTGGGCGTAAAGGGCGTGTAGGCGGTTTTATGCGTCAGATGTGAAAGTCCTGGGCTCAACCTGGGGACGGCATTTGATACGGTAGGACTAGAGGTTATGAGAGGAAGGCGGAATTGTGCGTGTAGGAGTGAAATCCGTAGAGATGCACAGGAACACCAGTTGCGAAGGCGGCCTTCTGGTATTAACCTGACGCTAAGGCGCGAAAGCGTGGGGAGCAAACAGG` |
| ASV1770 | 404 | `TGGGGAATCTTGGACAATGGGGGCAACCCTGATCCAGCCATGCCGCGTGAGTGATGAAGGCCTTAGGGTTGTAAAACTCTTTCGTCGGGGACGATAATGACGGTACCCGAAGAAGAAGCTCCGGCTAACTTCGTGCCAGCAGCCGCGGTAATACGAAGGGGGCTAGCGTTGTTCGGAATTACTGGGCGTAAAGCGCGTGTAGGCGGACCGTTAAGTCGGGGGTGAAAGCCCGGGGCTCAACCTCGGAATTGCCTTCGATACTGGCGGTCTGGAGCAGAGGAGAGGCGAGTGGAATACCGAGTGGAGAGGTGAAATTCGCAGATATTCGGTGGAACACCAGTGGCGAAGGCGGCTCGCTGGACTCTTGCTGACGCTGAGACGCGAAAGCGTGGGGAGCAAACAGG` |
| ASV33 | 424 | `TAGGGAATATTGGACAATGGGGGCAACCCTGATCCAGCCATGCCGCGTGCAGGAAGAAGGCCCTATGGGTCGTAAACTGCTTTTGTACGGGAAGAAACCTTACCATGCGTGGTAAGTTGACGGTACCGTGAGAATAAGGATCGGCTAACTTCGTGCCAGCAGCCGCGGTAATACGAAGGATCCAAGCGTTGTCCGGATTTACTGGGTTTAAAGGGTGCGTAGGCGGACTTTTAAGTCAGTGGTGAAAGCCTGTAGCTTAACTATAGAATTGCCATTGAAACTGAAAGTCTCGAGTGTGGTTGAGGTAACTGGAATATATCATGTAGCGGTGAAATGCTTAGATATGATATAGAACACCAATTGCGAAGGCAGGTTACTGAGCCATAACTGACGCTGAGGCACGAAAGCGTGGGGATCAAACAGG` |
| ASV1940 | 404 | `TTAGGAATATTGGACAATGGGCGCAAGCCTGATCCAGCCATGCCGCGTGAACGATGAAGGCCTTCGGGTTGTAAAGTTCTTTAGCCGATGATTATAATGACTGTAGTCGGAAAATAAGCCCCGGCTAACTTCGTGCCAGCAGCCGCGGTAATACGAAGGGGGCGAGCGTTATTCGGAATCACTGGGCGTAAAGCGTGCGTAGGCGGTCTCGTAAGTTAGGAGTGAAAGCCCAGGGCTCAACCCTGGAATTGCTCTTAATACTGCAAGGCTAGAATCTCGGAGAGGATAGCGGAATTTCCAGTGTAGCAGTGAAATGCGTAGATATTGGAAGGAACACCAGTGGCGTAAGCGGCTATCTGGACGAGTATTGACGCTGAGGCACGAAAGCGTGGGGATCAAACAGG` |
| ASV361 | 429 | `TGGGGAATATTGGACAATGGGCGCAAGCCTGATCCAGCCATGCCGCGTGTGTGAAGAAGGCCTTCGGGTTGTAAAGCACTTTCAGCGAGGAGGAAGGGGTAGGTGTTAATAGCATCTACTTTTGACGTTACTCGCAGAAGAAGCACCGGCTAACTCTGTGCCAGCAGCCGCGGTAATACAGAGGGTGCAAGCGTTAATCGGAATTACTGGGCGTAAAGCGCACGCAGGCGGTTGGTTAAGTCAGATGTGAAAGCCCCGGGCTCAACCTGGGAATTGCATTTGAAACTGGCCAACTAGAGTATGTGAGAGGGGGGTAGAATTCCAAGTGTAGCGGTGAAATGCGTAGAGATTTGGAGGAATACCAGTGGCGAAGGCGGCCCCCTGGCACAATACTGACGCTCAGGTGCGAAAGCGTGGGGAGCAAACAGG` |
| ASV736 | 405 | `TGGGGAATCTTGCACAATGGGCGAAAGCCTGATGCAGCAACGCCGCGTGCGGGATGAAGGCCCTAGGGTTGTAAACCGCTTTCAGTAGGGAAGAAAATGACGGTACCTACAGAAGAAGGTGCGGCCAACTACGTGCCAGCAGCCGCGGTGACACGTAGGCACCAAGCGTTATCCGGATTTATTGGGCGTAAAGAGCTCGTAGGCGGTTCGGTAAGTCGGGTGTGAAAACTCTGGGCTCAACCCAGAGACGCCACCCGATACTGCTGTGACTAGAGTGCGGTAGGGGAGCGGGGAATTCATGGTGTAGCGGTGAAATGCGCAGATATCATGAGGAACACCAGCGGCGAAGGCGCCGCTCTGGGCCGTAACTGACGCTGAGGAGCGAAAGCATGGGTAGCAAACAGG` |
| ASV5272 | 404 | `TGGGGAATATTGCACAATGGGGGGAACCCTGATGCAGCGACGCCGCGTGAGTGAAGAAGTACTTCGGTATGTAAAGCTCTATCAGCAGGGAAGAAAATGACGGTACCTGACTAAGAAGCCCCGGCTAACTACGTGCCAGCAGCCGCGGTAATACGTAGGGGGCAAGCGTTATCCGGAATTACTGGGTGTAAAGGGTGCGTAGGTGGCAGGGCAAGTCAGAAGTGAAAACCCGGGGCTTAACTCCGGGACTGCTTTTGAAACTGTCCAGCTGGAGTGCAGGAGAGGTAAGCGGAATTCCTAGTGTAGCGGTGAAATGCGTAGATATTAGGAGGAACATCAGTGGCGAAGGCGGCTTACTGGACTGAAACTGACACTGAGGCACGAAAGCGTGGGGAGCAAACAGG` |
| ASV1836 | 424 | `TGAGGAATATTGGTCAATGGACGCAAGTCTGAACCAGCCATGCCGCGTGCAGGATGACGGTCCTATGGATTGTAAACTGCTTTTGTACAGGAAGAAACAGTTCTACGTGTAGAACCTTGACGGTACTGTAAGAATAAGGATCGGCTAACTCCGTGCCAGCAGCCGCGGTAATACGGAGGATCCAAGCGTTATCCGGAATCATTGGGTTTAAAGGGTCCGTAGGCGGTTTAGTAAGTCAGTGGTGAAAGCCCATCGCTCAACGGTGGAACGGCCATTGATACTGCTAGACTTGAATTATTAGGAAGTAACTAGAATATGTAGTGTAGCGGTGAAATGCTTAGAGATTACATGGAATACCAATTGCGAAGGCAGGTTACTACTAATGGATTGACGCTGATGGACGAAAGCGTGGGTAGCGAACAGG` |
| ASV1504 | 429 | `TGGGGAATTTTGGACAATGGGCGCAAGCCTGATCCAGCCATGCCGCGTGAGTGAAGAAGGCCTTCGGGTTGTAAAGCTCTTTCGGTGAAGACGAAACGGTTAGCGTTAATAACGCTGACTAATGACGGTACTCACAGAAGAAGCACCGGCTAACTACGTGCCAGCAGCCGCGGTAATACGTAGGGTGCAGGCGTTAATCGGAATTACTGGGCGTAAAGCGTGCGCAGGCGGTTTGCTAAGACAGGTGTGAAATCCCCGGGCTTAACCTGGGAATTGCGCTTGTAACTGGCAAGCTAGAGTGTGGCAGAGGGGGGTGGAATTCCACGTGTAGCAGTGAAATGCGTAGATATGTGGAGGAACACCGATGGCGAAGGCAGCCCCCTGGGTTAACACTGACGCTCAGGCACGAAAGCGTGGGGAGCAAACAGG` |
| ASV5437 | 429 | `TGGGGAATATTGGACAATGGGGGCAACCCTGATCCAGCAATGCCGCGTGTGTGAAGAAGGCCTGAGGGTTGTAAAGCACTTTCAGTGGGGAGGAGGGCAATTAGGTTAAGAGCTAGATTGTTGGACGTTACCCACAGAAGAAGCACCGGCTAACTCCGTGCCAGCAGCCGCGGTAATACGGAGGGTGCGAGCGTTAATCGGAATTACTGGGCGTAAAGGGTGCGTAGGTGGTTTTTTAAGTTATCTGTGAAATTCCTGGGCTCAACCTGGGGTGGTCAGATAAGACTGGAAGACTTGAGTGTGGGAGAGGGTAGTGGAATTTCCGGTGTAGCGGTGAAATGCGTAGAGATCGGAAGGAACACCAGTGGCGAAGGCGGCTACCTGGCCCAACACTGACACTGAGGCACGAAAGCGTGGGGAGCAAACAGG` |
| ASV2316 | 406 | `TAGGGAATTTTGCGCAATGGGGGAAACCCTGACGCAGCAACGCCGCGTGAACGATTAAGCCCTTCGGGGTGTAAAGTTCTGTCAGTGGGGACGAAAACATGACTGTACCCACAGAGGAAGCACCGGCTAACTCCGTGCCAGCAGCCGCGGTAATACGGAGGGTGCAAGCGTTGTCCGGAATCATTGGGCGTAAAGAGTTCGTAGGTGGCCTGTTAAGTCAGGTGTTAAAGGCAGAAGCTCAACTTCTGTAAGGCACTTGATACTGGCAAGCTTGAATATGGTAGAGGTAAAGGGAATTCCTGGTGTAGCGGTGAAATGCGTAGATATCAGGAGGAACATCGGTGGCGTAAGCGCTTTACTGGGCCATTATTGACACTGAGGAACGAAAGCCGGGGTAGCAAATGGG` |
| ASV2116 | 429 | `TGGGGAATTTTGGACAATGGGGGCAACCCTGATCCAGCAATGCCGCGTGAGTGAAGAAGGCCTTCGGGTTGTAAAGCTCTTTTGTCAGGGAAGAAAAGGCTGTGGCTAATATCCACAGCTGCTGACGGTACCTGAAGAATAAGCACCGGCTAACTACGTGCCAGCAGCCGCGGTAATACGTAGGGTGCAAGCGTTAATCGGAATTACTGGGCGTAAAGCGTGCGCAGGCGGTTTCGTAAGTCTGTCGTGAAATCCCCGGGCTTAACCTGGGAATGGCGATGGAGACTGCGAGGCTAGAGTTTGGCAGAGGGGGGTAGAATTCCACGTGTAGCAGTGAAATGCGTAGAGATGTGGAGGAACACCGATGGCGAAGGCAGCCCCCTGGGTCAAAACTGACGCTCATGCACGAAAGCGTGGGGAGCAAACAGG` |
| ASV3832 | 404 | `TAGGGAATCTTGCGCAATGGGCGAAAGCCTGACGCAGCAACTCCGCGTGGAGGATGACGTATTTCGGTACGTAAACTCCTGTCAGTAGGGACGATAATGACTGTACCTACAGAGGAAGCACCGGCTAACTCCGTGCCAGCAGCCGCGGTAATACGGGGGGTGCAAGCGTTGTCCGGAATTATTGGGCGTAAAGAGTTCGTAGGCTGTTAATTAAGTCTCATGTTAAAGACAGAAGCTCAACTTCTGCGTGGCATTGGAAACTGGTTAACTTGAGTCCACTAGAGGAAGCGGGAATTCCAGGTGTAGCGGTGAAATGCGTAGATATCTGGAGGAACACCGATGGCGAAAGCACGCTTCTGGGGTGGTACTGACGCTGAGGAACGAAAGCCAGGGTAGCAAAAGGG` |
| ASV1989 | 423 | `TAAGGAATATTGGTCAATGGTGGTAACACTGAACCAGCCATGCCGCGTGCAGGAAGAAGGCGTTTTGCGTTGTAAACTGCTTTTATAAGGGAATAAAAGGCTCTTGCGAGAGAAATTGAAGGTACCTTGTGAATAAGCATCGGCTAACTACGTGCCAGCAGCCGCGGTAATACGTAGGATGCAAGCGTTGTCCGGATTTATTGGGTTTAAAGGGTGCGTAGGCGGTTTTATAAGTCTCGACTGAAAGCTTGTTGCTCAACAATAAGACTGGTTTAGATACTGTATAACTTGAATTACATTAAGGTAAATGGAATTTATGATGTAGCGGTGAAATGCTCAGATATCATAAGGAACGTCGATTGCGTAGGCATTTTACTAGGTGTATATTGACGCTGAGGCACGAAAGTCTGGGGATCAAACAGG` |
| ASV161 | 404 | `TGGGGAATATTGGACAATGGGCGAAAGCCTGATCCAGCAATGCCGCGTGAGTGATGAAGGCCTTAGGGTTGTAAAGCTCTTTTACCCGGGATGATAATGACAGTACCGGGAGAATAAGCTCCGGCTAACTCCGTGCCAGCAGCCGCGGTAATACGGAGGGAGCTAGCGTTGTTCGGAATTACTGGGCGTAAAGCGCACGTAGGCGGCTTTGTAAGTTAGAGGTGAAAGCCTGGAGCTCAACTCCAGAATTGCCTTTAAGACTGCATCGCTTGAATCCAGGAGAGGTGAGTGGAATTCCGAGTGTAGAGGTGAAATTCGTAGATATTCGGAAGAACACCAGTGGCGAAGGCGGCTCACTGGACTGGTATTGACGCTGAGGTGCGAAAGCGTGGGGAGCAAACAGG` |
| ASV1270 | 424 | `TGAGGAATATTGGACAATGGGCGCAAGCCTGATCCAGCCATGCCGCGTGCAGGATGACGGTCCTATGGATTGTAAACTGCTTTTGTACAGGAAGAAACACTGGTTCGTGAACCAGCTTGACGGTACTGTAAGAATAAGGATCGGCTAACTCCGTGCCAGCAGCCGCGGTAATACGGAGGATCCAAGCGTTATCCGGAATCATTGGGTTTAAAGGGTCCGTAGGCGGTTTGGTAAGTCAGTGGTGAAAGCCCATCGCTCAACGGTGGAACGGCCATTGATACTGCTAGACTTGAATTATTAGGAAGTAACTAGAATATGTAGTGTAGCGGTGAAATGCTTAGAGATTACATGGAATACCAATTGCGAAGGCAGGTTACTACTAATGGATTGACGCTGATGGACGAAAGCGTGGGTAGCGAACAGG` |
| ASV1834 | 429 | `TGGGGAATATTGGACAATGGGCGCAAGCCTGATCCAGCCATGCCGCGTGTGTGAAGAAGGCCCTTGGGTTGTAAAGCACTTTCAGTGGGGAGAAAGGTCTGTCACCTAATACGTGATGGAATTGATGTTACCCACAGAAGAAGCACCGGCTAACTCCGTGCCAGCAGCCGCGGTAATACGGAGGGTGCAAGCGTTAATCGGAATTACTGGGCGTAAAGCGCACGTAGGTGGTTTTCTAAGCCAGCTGTGAAATCCCCGGGCTCAACCTGGGCACTGCAGTTGGAACTGGAAAGCTAGAGTAGGGTAGAGGGGTGTGGAATTCCAGGTGTAGCGGTGAAATGCGTAGATATCTGGAGGAACATCAGTGGCGAAGGCGACACCCTGGACTCATACTGACACTGAGGTGCGAAAGCGTGGGGAGCAAACAGG` |
| ASV1043 | 404 | `TGGGGAATATTGGACAATGGGCGAAAGCCTGATCCAGCAATACCGCGTGAATGATGAAGGCCTTAGGGTTGTAAAGTTCTTTTAGCAGGGAAGATAATGACGGTACCTGCAGAAAAAGCCCCGGCTAACTCCGTGCCAGCAGCCGCGGTAAGACGGAGGGGGCTAGCGTTGTTCGGAATTACTGGGCGTAAAGAGCGCGTAGGCGGCTTGGTAAGTTGGAAGTGAAATCCCGGGGCTTAACCCCGGAACTGCTTTCAAAACTGCTAGGCTAGAGTATAGTAGGGGGTGATGGAATTCCTAGTGTAGAGGTGAAATTCTTAGATATTAGGAGGAACACCGGAAGCGAAAGCAATCACCTGGGCTATAACTGACGCTGATGCGCGAAAGCGTGGGGAGCAAACAGG` |
| ASV3605 | 429 | `TGGGGAATATTGCACAATGGGGGAAACCCTGATGCAGCCATGCCGCGTGTGTGAAGAAGGCCTTCGGGTTGTAAAGCACTTTCAGTAGGGAGGAAAGGGTGTAGTTTAATACGCTATATCTGTGACGTTACCTACAGAAGAAGGACCGGCTAACTCCGTGCCAGCAGCCGCGGTAATACGGAGGGTCCGAGCGTTAATCGGAATTACTGGGCGTAAAGCGTGCGCAGGCGGTTTGTTAAGCGAGATGTGAAAGCCCTGGGCTCAACCTAGGAATAGCATTTCGAACTGGCGAACTAGAGTCTTGTAGAGGGGGGTAGAATTCCAGGTGTAGCGGTGAAATGCGTAGAGATCTGGAGGAATACCGGTGGCGAAGGCGGCCCCCTGGACAAAGACTGACGCTCATGCACGAAAGCGTGGGGAGCAAACAGG` |
| ASV4477 | 429 | `TAGGGAATCTTCGGCAATGGGGGCAACCCTGACCGAGCAACGCCGCGTGAGTGAAGAAGGTTTTCGGATCGTAAAGCTCTGTTGTAAGAGAAGAACGTGTGTGAGAGTGGAAAGTTCACACAGTGACGGTAACTTACCAGAAAGGGACGGCTAACTACGTGCCAGCAGCCGCGGTAATACGTAGGTCCCGAGCGTTGTCCGGATTTATTGGGCGTAAAGCGAGCGCAGGCGGTTTAATAAGTCTGAAGTTAAAGGCAGTGGCTTAACCATTGTTCGCTTTGGAAACTGTTAAACTTGAGTGCAGAAGGGGAGAGTGGAATTCCATGTGTAGCGGTGAAATGCGTAGATATATGGAGGAACACCGGTGGCGAAAGCGGCTCTCTGGTCTGTAACTGACGCTGAGGCTCGAAAGCGTGGGGAGCAAACAGG` |
| ASV1893 | 407 | `TGAGGAATATTGGACAATGGACGAAAGTCTGATCCAGCGATGCCGCGTGGGTGAAGAAGGTCTTCGGATTGTAAAGCCCTTTTAGCAGGGAAGAAAAAAATGACGGTACCTGCAGAAAAAGCTCCGGCTAACTCCGTGCCAGCAGCCGCGGTAATACGGAGGGAGCGAGCGTTGTTCGGATTTACTGGGCGTAAAGGGCGCGTAGGTTGTTAGATAAGTCAGAAGTGAAATCCCAAAGCTTAACTTTGGAACTGCTTTTGAAACTGTCTTTCTAGAGTTTGGTAGGGGGTGATGGAATTCCTAGTGTAGCGGTGAAATGCTTAGATATTAGGAGGAACACCGATGGCGAAAGCGATCACCTGGGCCACAACTGACACTGAGGCGCGAAAGCATAGGGAGCAAACAGG` |
| ASV2042 | 429 | `TGGGGAATTTTGGACAATGGGCGCAAGCCTGATCCAGCAATGCCGCGTGAGTGAAGAAGGCCTTCGGGTTGTAAAGCTCTTTTGTCAGGGAAGAAACGGTGTGGGCTAATATCCCATGCTAATGACGGTACCTGAAGAATAAGCACCGGCTAACTACGTGCCAGCAGCCGCGGTAATACGTAGGGTGCAAGCGTTAATCGGAATTACTGGGCGTAAAGCGTGCGCAGGCGGTTTTGTAAGTCTGTCGTGAAATCCCCGGGCTCAACCTGGGAATGGCGATGGAGACTGCAAGGCTAGAGTTTGGCAGAGGGGGGTAGAATTCCACGTGTAGCAGTGAAATGCGTAGATATGTGGAGGAACACCGATGGCGAAGGCAGCCCCCTGGGTCAAAACTGACGCTCATGCACGAAAGCGTGGGGAGCAAACAGG` |
| ASV2094 | 404 | `TGGGGAATATTGGACAATGGGCGCAAGCCTGATCCAGCCATGCCGCGTGGGTGATGAAGGCCCTAGGGTTGTAAAGCCCTTTCGGCGGGGAAGATAATGACGGTACCCGCAGAAGAAGCCCCGGCTAACTTCGTGCCAGCAGCCGCGGTAATACGAAGGGGGCTAGCGTTGCTCGGAATCACTGGGCGTAAAGCGCACGTAGGCGGATCTTTAAGTCGGTGGTGAAATCCTGGAGCTCAACTCCAGAACTGCCTTCGATACTGAGGGTCTCGAGTCCGGGAGAGGTGAGTGGAACTGCGAGTGTAGAGGTGAAATTCGTAGATATTCGCAAGAACACCAGTGGCGAAGGCGGCTCACTGGCCCGGTACTGACGCTGAGGTGCGAAAGCGTGGGGAGCAAACAGG` |
| ASV1927 | 429 | `TGGGGAATTTTGGACAATGGGCGCAAGCCTGATCCAGCAATGCCGCGTGCAGGAAGAAGGCCTTCGGGTTGTAAACTGCTTTTGTACGGAGCGAAAAAGCTCTCCTTAATACGGGGGGTTCATGACGGTACCGTAAGAATAAGCACCGGCTAACTACGTGCCAGCAGCCGCGGTAATACGTAGGGTGCGAGCGTTAATCGGAATTACTGGGCGTAAAGCGTGCGCAGGCGGTTTTGTAAGATAGGCGTGAAATCCCCGGGCTCAACCTGGGAATGGCGCTTGTGACTGCAAGGCTAGAGTGCGGCAGAGGGGGATGGAATTCCGCGTGTAGCAGTGAAATGCGTAGATATGCGGAGGAACACCGATGGCGAAGGCAATCCCCTGGGCCTGCACTGACGCTCATGCACGAAAGCGTGGGGAGCAAACAGG` |
| ASV5162 | 408 | `TCGAGAATCTTCCACAATGGACGAAAGTCTGATGGAGCGACGCCGCGTGATTGATGAAGTCCTTAGGGACGTAAAGATCTTTTATGAGGGAAGAAGTTTATTGACGGTACCTCAGGAATAAGGGGTTGCTAAACTCGTGCCAGCAGCAGCGGTAATACGAGTGCCCCGAGCGTTATCCGGAATTATTGGGCGTAAAGGATGTGTAGGTGGTGTTGTTAGTCTCTTGTTAAAACCCCGAGCTTAACTCGGGGACTGCGAGGGAAACGGCAACGCTAGAGGGTGCGAGGGGTGAATGGAACTCATGGTGTAGGGGTGAAATCCGTTGATATCATGGGGAACACCAAAAGCGAAGGCAATTCACTGGCGCATTTCTGACACTGAAACATGAAAGCGTGGGTAGCGAATGGG` |
| ASV3515 | 429 | `TGGGGAATTTTGGACAATGGGCGCAAGCCTGATCCAGCCATTCCGCGTGAGTGAAGAAGGCCTTCGGGTTGTAAAGCTCTTTAGTCAGGGAGGAAAGCTTAGTGGTTAATACCTACTGAGTATGACAGTACCTGAAGAATAAGCACCGGCTAACTACGTGCCAGCAGCCGCGGTAATACGTAGGGTGCAAGCGTTAATCGGAATTACTGGGCGTAAAGCGTGCGCAGGCGGATAATTAAGTCAGACGTGAAAGCCCCGAGCTTAACTTGGGAACGGCGTTTGAAACTGGTTGTCTAGAATACGTCAGAGGGGGGTGGAATTCCACGTGTAGCAGTGAAATGCGTAGAGATGTGGAGGAACACCGATGGCGAAGGCAGCCCCCTGGGATGATATTGACGCTCATGCACGAAAGCGTGGGGAGCAAACAGG` |
| ASV1149 | 424 | `TGAGGAATATTGGACAATGGGTGAGAGCCTGATCCAGCCATCCCGCGTGAAGGACGACGGCCCTATGGGTTGTAAACTTCTTTTGTATAGGGATAAACCTACTCTCGTGAGAGTAGCTGAAGGTACTATACGAATAAGCACCGGCTAACTCCGTGCCAGCAGCCGCGGTAATACGGAGGGTGCAAGCGTTATCCGGATTTATTGGGTTTAAAGGGTCCGTAGGCGGACTCGTAAGTCAGTGGTGAAATCTCATAGCTTAACTATGAAACTGCCATTGATACTGCGGGTCTTGAGTAAGGTAGAGGTAGCTGGAATAAGTAGTGTAGCGGTGAAATGCATAGATATTACTTAGAACACCAATTGCGAAGGCAGGTTACCATGTCTTAACTGACGCTGATGGACGAAAGCGTGGGGAGCGAACAGG` |
| ASV2879 | 429 | `TGGGGAATATTGGACAATGGGGGCAACCCTGATCCAGCGATGCCGCGTGTGTGAAGAAGGCCTGAGGGTTGTAAAGCACTTTCGCAGGGGAGGAAAGTAATGATTTTAATAGAGTCATTGCGTGACGTTACCCTGATAAGAAGCACCGGCTAACTCTGTGCCAGCAGCCGCGGTAATACAGAGGGTGCAAGCGTTAATCGGAATGACTGGGCGTAAAGGGCGCGTAGGCGGAAAGATGTGTCATAGGTGAAATCCCCGGGCTTAACCTGGGAACTGCCTGTGAAACTGTCTATCTAGGATATAGGAGAGGGAAGTGGAATTTCCGGTGTAGCGGTGAAATGCGTAGATATCGGAAAGAACACCAGTGGCGAAGGCGACTTCCTGGCCTAATATTGACGCTGAGGCGCGAAAGCGTGGGGAGCAAACGGG` |
| ASV3500 | 429 | `TGGGGAATATTGGACAATGGGCGCAAGCCTGATCCAGCAATGCCGCGTGTGTGAAGAAGGCCTTAGGGTTGTAAAGCACTTTCAGTTGGGAGGAAGGGCTTATGGTTAATACCCATAAGTCTTGACGCTACCAACAGAAGAAGCACCGGCTAACTCTGTGCCAGCAGCCGCGGTAATACAGAGGGTGCGAGCGTTAATCGGAATTACTGGGCGTAAAGAGTGCGTAGGCGGTTGTATAAGTTAGATGTGAAATCCCCAGGCTTAACCTGGGAACTGCGTTTAATACTGTATAGCTAGAGTACTGAAGAGGTTAGTGGAATTTCCAGTGTAGCGGTGAAATGCGTAGATATTGGAAGGAACACCAGTGGCGAAGGCGACTAATTGGTCAGATACTGACGCTGAGGCACGAAAGCGTGGGGAGCAAACAGG` |
| ASV4708 | 404 | `TGGGGAATATTGGACAATGGGGGCAACCCTGATCCAGCCATACCGCGTGAGTGATGACGGCCTTCGGGTTGTAAAGCTCTTTTACCCCTGACGATAATGACGGTAAGGGGAGAAAAAGCCACGGCTAACTTCGTGCCAGCAGCCGCGGTAATACGAAGGTGGCAAGCGTTGTTCGGATTTACTGGGCGTAAAGCGTGCGTAGGCGGTTTAACAAGTCAGAAGTGAAAGCCCCGGGCTCAACCTGGGAATTGCTTTTGAAACTGTTTTACTTGAATCCGGTAGAGGTTGGCGGAATTCCCAGTGTAGAGGTGAAATTCGCAGATATTGGGAGGAACACCAGTGGCGTAAGCGGCCAACTGGACCGGTTTTGACGCTGAGGCACGAAAGCGTGGGTAGCAAACAGG` |
| ASV3102 | 429 | `TCGAGAATAATTCACAATGGGGGCAACCCTGATGGTGCAACGCCGCGTGGAGGATGACGGTCTTCGGATTGTAAACTCCTGTCATCTGGGAGTAAGGCATGGTTGTGAAAAGCAGACATGATTGATAGTACCAGAAGAGGAAGGGACGGCTAACTTCGTGCCAGCAGCCGCGGTAATACGAAGGTCCCAAGCGTTGTTCGGAATCACTGGGCGTAAAGGGAGCGTAGGCGGCGTGGTAAGTCAGATGTGAAATCCCGGGGCTCAACCCCGGAACTGCATCCGATACTGCCATGCTAGAGGATTGGAGAGGTAGCTGGAATTCTTGGTGTAGCAGTGAAATGCGTAGAGATCAAGAGGAACACTCGTGGCGAAAGCGAGCTACTGGACAATATCTGACGCTGAGGCTCGAAGGCTAGGGTAGCGAAAGGG` |
| ASV4840 | 404 | `TGGGGAATATTGGGCAATGGGCGAAAGCCTGACCCAGCTATGCCGCGTGAGTGATGAAGGCCTTCGGGTTGTAAAGCTCTTTTAGGTGGGACGATGATGACGGTACCACCAGAAAAAGCCCCGGCTAACTTCGTGCCAGCAGCCGCGGTAATACGAAGGGGGCAAGCGTTGCTCGGATTTATTGGGCGTAAAGGGTGTGTAGGTGGTCCTACAAGTTAGATGTGAAAGCCCCGGGCTTAACCCGGGAACTGCATTTAAAACTGTAGGACTTGAAGATCACAGAGGTAAGCAGAATTTCCAGTGTAGAGGTGAAATTCGTAGATATTGGAAAGAATACCAACGGCGTAGGCAGCTTACTGGGTGACTCTTGACACTGAGGCACGAAAGCGTGGGGAGCAAACAGG` |
| ASV5628 | 428 | `TCGAGAATCTTTCACAATGGGGGCAACCCTGATGGAGCGACGCCGCGTGGAGGATAAGGTCTTCGGATTGTAAACTCCTGTCATGCGAGAGCAATGCTCACTGGTTAAGAGCTGGTGAGAGGGATAGTATCGCAAGAGGAAGAGACGGCTAACTCTGTGCCAGCAGCCGCGGTAATACAGAGGTCTCAAGCGTTGTTCGGAATTACTGGGCGTAAAGGGTGTGTAGGCTGCGAATTAAGTCAGATGTGAAAATCCGGGGCTCAACCTCGGACATGCATCCGATACTGGTTTGCTAGAGTGACTTAGGGGGACATGGAATTCACAGTGTAGCAGTGAAATGCGTAGATATTGTGAAGAACACCAACGGCGAAGGCAATGTCCTGGGGGTTAACTGACGCTCAAACACGAAGGCCAAGGGAGCGAACGGG` |
| ASV1261 | 430 | `TGGGGAATATTGCGCAATGGACGAAAGTCTGACGCAGCGACGCCGCGTGAGGGATGAAGGTCTTCGGATCGTAAACCTCTGTCAGGGGGGAAGAAACACCGGTGTGTGAATAATGCATCGGCTTGACGGTACCCCCAAAGGAAGCACCGGCTAACTCCGTGCCAGCAGCCGCGGTAATACGGAGGGTGCGAGCGTTAATCGGAATCACTGGGCGTAAAGCGCATGTAGGCGGCTTGTTAAGTCAGGGGTGAAATCCCACAGCTCAACTGTGGAACTGCCCTTGATACTGGCAGGCTTGAGTACCGGAGAGGGTGGCGGAATTCCAGGTGTAGGAGTGAAATCCGTAGATATCTGGAGGAACACCGGTGGCGAAGGCGGCCACCTGGACGGTAACTGACGCTGAGATGCGAAAGCGTGGGTAGCAAACAGG` |
| ASV4315 | 429 | `TGGGGAATTTTGGACAATGGGCGAAAGCCTGATCCAGCCATGCCGCGTGAGTGAAGAAGGCCTTCGGGTTGTAAAGCTCTTTTGTCAGGGAAGAAATCGCTTAGGATAATACCTTAGGTGGATGACGGTACCTGAAGAATAAGCACCGGCTAACTACGTGCCAGCAGCCGCGGTAATACGTAGGGTGCAAGCGTTAATCGGAATTACTGGGCGTAAAGCGTGCGCAGGCGGTTTTGCAAGTCAGATGTGAAAGCCCCGGGCTCAACCTGGGAATGGCATTTGAAACTACAAGGCTAGAGTGTGTCAGAGGGGGGTAGAATTCCACGTGTAGCAGTGAAATGCGTAGAGATGTGGAGGAATACCGATGGCGAAGGCAGCCCCCTGGGATAACACTGACGCTCATGCACGAAAGCGTGGGGAGCAAACAGG` |
| ASV5610 | 428 | `TCGAGAATTTTTCTCAATGGGGGAAACCCTGAAGGAGCGACGCCGCGTGGGGGATGAATGGCTTCGGCCCGTAAACCCCTGTCATTCGGGATCAAACGTCATTCATGAACAATGGGTGGCCTGATTGTACCGGAAGAGGAAGGGACGGCTAACTCTGTGCCAGCAGCCGCGGTAATACAGAGGTCCCAAGCGTTGTTCGGATTTACTGGGCGTAAAGGGTGCGTAGGCGGTGGGGTAAGTTTGAGGTGAAAGCTCCGGGCTCAACCCGGAAATGGCCTTGAAGACTATCTCGCTAGAGGGTTGGAGGGGAGACTGGAATTCTCGGTGTAGCAGTGAAATGCGTAGATCTCGAGAGGAACACCAGTGGCGAAGGCGGGTCTCTGGACAACACCTGACGCTGAGGCACGAAAGCCAGGGGAGCAAACGGG` |
| ASV1056 | 430 | `TAGGGAATATTGCGCAATGGGGGAAACCCTGACGCAGCAACGCCGCGTGAGTGAGGAAGGTCTTAGGATTGTAAAACTCTTTTCTTAGGGAAAAATGTCCTTATGGTTAATACCCATGAGGAGTGATGGTACCTAAGGAATAAGCACCGGCTAACTTCGTGCCAGCAGCCGCGGTAATACGAAGGGTGCAAGCGTTATTCGGAATCATTGGGCGTAAAGCGCGCGCAGGCGGATTAATAAGTCAGGTGTGAAATCTCGAGGCTCAACCTCGAAACTGCGCCTGAAACTATTAGTCTAGAGTGTTGGAGGGGGTAGGGGAATTTCACGTGTAGGGGTAAAATCCGTAGAGATGTGAAGGAACACCGGAGGCGAAGGCGCCTACCTGGACAACTACTGACGCTGAGGCGCGAAAGCGTGGGGAGCAAACAGG` |
| ASV290 | 429 | `TGGGGAATATTGGACAATGGGCGCAAGCCTGATCCAGCCATGCCGCGTGTGTGAAGAAGGCCTTCGGGTTGTAAAGCACTTTTGTCCGGAAAGAAATCTTCCAACTTAATACGTTGGGAGGATGACGGTACCGGAAGAATAAGCACCGGCTAACTTCGTGCCAGCAGCCGCGGTAATACGAAGGGTGCAAGCGTTACTCGGAATTACTGGGCGTAAAGCGTGCGTAGGCGGTTCGTTAAGTCTGACGTGAAATCCCCGGGCTCAACCTGGGACGTGCGTTGGAAACTGGCGATCTAGAGTACGGTAGAGGGTGGTGGAATTCCCGGTGTAGCAGTGAAATGCGTAGAGATCGGGAGGAACACTCGTGGCGAAGGCGGCCACCTGGACCAGTACTGACGCTGAGGCACGAAAGCGTGGGGAGCAAACAGG` |
| ASV5359 | 404 | `TGGGGAATTTTCCGCAATGGGCGAAAGCCTGACGGAGCAACGCCGCGTGAGGGAGGAAGGTCTTTGGATTGTAAACCTCTTTTCTCAAGGAAGATAATGACGGTACTTGAGGAATCAGCATCGGCTAACTCCGTGCCAGCAGCCGCGGTAATACGGGGGATGCAAGCGTTATCCGGAATTATTGGGCGTAAAGCGTCCGTAGGCGGCGATACAAGTCTGCTGTTAAAGAGTGCAGCTTAACTGCATAGCGGCGGTGGAAACTGTATAGCTAGAGTGTGGTAGGGGTAGAGGGAATTCCCGGTGTAGCGGTGAAATGCGTAGATATCGGGAAGAACACCAGTGGCGAAGGCGCTCTACTGGGCCACAACTGACGCTGATGGACGAAAGCTAGGGGAGCGAAAGGG` |
| ASV4267 | 406 | `TGAGGAATTTTCCGCAATGGGCGCACGCCTGACGGAGCAATGCCGCGTGGAGGATGACAGCCTATGGGTCGTAAACTCCTTTTCTTGGAGAAGAAATTTTGACGGTATCTAAGGAATAAGCATCGGCTAACTCCGTGCCAGCAGCCGCGGTAATACGGAGGATGCAAGCGTTGTCCGGAATCATTGGGCGTAAAGGGTCCTGAGGTGGTTTGCTTAGTCCAGTGTGAAAGTTCAGGGCTTACCCTTGAAAATGCGGTGGAAACTAGCAGGCTAGAGTACGGTGGGGGCAGAGGGAATTCCCGATGTAGCGGTGAAATGCATAGATATCGGGAAGAACACCGATGGCGAAGGCACTCTGCTGGGCCGAAACTGACACTGAAAGACGAAAGCCAGGGGAGCGAATGGG` |
| ASV2177 | 408 | `TCGAGAATCTTCCGCAATGGACGCAAGTCTGACGGAGCGACACCGCGTGATCGATGAAGTGCTTCGGTATGTAAAGATCTTTTATGAGGGAAGAAGTTTATTGACGGTACCTCATGAATAAGGGCCTGCAAAACTCGTGCCAGCAGCAGCGGTAATACGAGTGGCCCAAGCGTTATCCGGAATTATTGGGCGTAAAGGATGTGTAGGTGGTCATATTAGTCTTTGGTCTAACCTCTGTGCTCAACATGGAGACAGCCAAGGAAACGGTACGACTAGAGTAGAGTAGGGGTAAGCGGAACTCATAGTGTAGGGGTGAAATCCGTTGATATTATGGGGAACACCAAAAGCGAAGGCAGCTTACTGGACTCTTACTGACACTGAAACATGAAAGCGTGGGTAGCGAATGGG` |
| ASV858 | 424 | `TAAGGAATATTGGACAATGCCCGCAAGGGTGATCCAGCCATGCCGCGTGCAGGAAGACGGCCCTATGGGTTGTAAACTGCTTTTACACCGGAGAAAACCTCCCCTCGTGAGGGGAGCTGATAGTATGGTGAGAATAAGCATCGGCTAACTTCGTGCCAGCAGCCGCGGTAAGACGAAGGATGCAAGCGTTATCCGGATTCATTGGGTTTAAAGGGAGCGTAGGTGGACTGATAAGTCAGTGGTGAAATCTTCGAGCTTAACTCGGAAATTGCCATTGATACTGTTGGTCTTGAGTACAGTTGCTGTGGGCGGAATATGACATGTAGTGGTGAAATACATAGAGATGTCATAGAACACCGATTGCGAAGGCAGCTCACAAAGCTGTAACTGACACTGAGGCTCGAAAGTGCGGGGATCAAACAGG` |
| ASV1096 | 429 | `TGGGGAATTTTGGACAATGGGCGCAAGCCTGATCCAGCAATGCCGCGTGCAGGATGAAGGCCTTCGGGTTGTAAACTGCTTTTGTATGGAACGAAACGGTCTGCCTTAATACGGTGGGCTAATGACGGTACCGTAAGAATAAGCACCGGCTAACTACGTGCCAGCAGCCGCGGTAATACGTAGGGTGCGAGCGTTAATCGGAATTACTGGGCGTAAAGCGTGCGCAGGCGGTTATATAAGACAGATGTGAAATCCCCGGGCTCAACCTGGGACCTGCATTTGTGACTGTATAGCTAGAGTACGGTAGAGGGGGATGGAATTCCGCGTGTAGCAGTGAAATGCGTAGATATGCGGAGGAACACCGATGGCGAAGGCAATCCCCTGGACCTGTACTGACGCTCATGCACGAAAGCGTGGGGAGCAAACAGG` |
| ASV2095 | 404 | `TGGGGAATTTTCCGCAATGGGCGCAAGCCTGACGGAGCAATACCGCGTGCGGGAGGAAGCTTTTTGGAGTGTAAACCGCTTTTCTCAGGGAAGATGATGACGGTACCTGAGGAATCAGCATCGGCTAACTTCGTGCCAGCAGCCGCGGTAATACGAGGGATGCAAGCGTTATCCGGAATTATTGGGCGTAAAGCGTTCGTAGGTGGCTAAGTTAGTCTGTCGTTAAAGCGTGGAGCTTAACTCCATACCGGCGATGGAAACTGCTTGGCTAGAGTATGGTAGGGGTAAGAGGAATTCCCAGTGTAGCGGTGAAATGCGTAGATATTGGGAAGAACACCAGCGGCGAAAGCGTCTTACTGGGCCATTACTGACACTGAGGGACGAAAGCTAGGGGAGCGAAAGGG` |
| ASV2064 | 423 | `TAGGGAATATTGGTCAATGGAGGCAACTCTGAACCAGCCATGCCGCGTGCAGGAAGAAGGCCCTATGGGTTGTAAACTGCTTTTTTACGGGAATAAAAAGCTCATGCGTGAGAAATTGAAGGTACCGTGAGAATAAGCCTCGGCTAACTACGTGCCAGCAGCCGCGGTAATACGTAGGAGGCAAGCGTTGTCCGGATTTATTGGGTTTAAAGGGTGCGTAGGCGGCCCTATAAGTCAGTGGTGAAAGCCGACAGCTTAACTGTCGAACTGCCATTGATACTGTAGGGCTTGAGTACAAATGAGGTAGGCGGAATTGATGGTGTAGCGGTGAAATGCTTAGATACCATCAAGAACACCGATTGCGAAGGCAGCTTACTAAGATGTAACTGACGCTGAGGCACGAAAGTGCGGGGATCAAACAGG` |
| ASV5173 | 407 | `TCGAGAATATTCGACAATGGGCGAAAGCCTGATCGAGCGACACCGCGTGGAGGATGAAGTCCTTCGGGACGTAAACTTCTTTTAGCAGGGAAGAATTCAATGACGGTACCTGCAGAATAAGGGCTTGCAAATCTCGTGCCAGCAGCCGCGGTAATACGAGAGGCCCGAGCGTTATCCGGAGTTACTGGGCGTAAAGGGTGTGTAGGAGGTTGTGTTAGTCATTGTTTAAATCTTTGGGCCCAACCCAAAAACTGGCAGTGAAACGGCACAACTGGAGGGCGTGAGAGGCAAGTGGAACTCATGGTGGAGGGGTGAAATCCGTTGATATCATGGGGAACACCAAAGGCGAAGGCAACTTGCTGGCGCGTACCTGACTCTGAAACACGAAAGCGTGGGGATCAAAAAGG` |
| ASV692 | 429 | `TGGGGAATTTTGGACAATGGGCGCAAGCCTGATCCAGCAATGCCGCGTGCAGGAAGAAGGCCTTCGGGTTGTAAACTGCTTTTGTACGGAGCGAAAAGGTCTTCTCTAATACAGAAGGCTGATGACGGTACCGTAAGAATAAGCACCGGCTAACTACGTGCCAGCAGCCGCGGTAATACGTAGGGTGCGAGCGTTAATCGGAATTACTGGGCGTAAAGCGTGCGCAGGCGGTTTTGTAAGACAGGCGTGAAATCCCCGGGCTCAACCTGGGAATGGCGCTTGTGACTGCAAAGCTGGAGTGCGGCAGAGGGGGATGGAATTCCGCGTGTAGCAGTGAAATGCGTAGATATGCGGAGGAACACCGATGGCGAAGGCAATCCCCTGGGCCTGCACTGACGCTCATGCACGAAAGCGTGGGGAGCAAACAGG` |
| ASV3817 | 428 | `TCGAGAATCATTCACAATGGGCGAAAGCCTGATGGTGTGACGCCGCGTGGAGGATGAAGGTCTTCGGATTGTAAACTCCTGTCAAGTGAGAGTAAAGCCGGGCGTTAACTGCGTCCCGGTCTGAGAGTATCACTAGAGGAAGAGACGGCTAACTCTGTGCCAGCAGCCGCGGTAATACAGAGGTCTCAAGCGTTGTTCGGAATCACTGGGCGTAAAGGGTGCGTAGGCCGCAATGTAAGTCAGATGTGAAAGCCCCGGGCTCAACCCGGGAATTGCATCCGATACTGCATTGCTTGAGGACTGGAGAGGAGTCTGGAATTCATGGTGGAGCAGTGAAATGCGTAGATATCATGAGGAACACTAGTGGCGAAGGCGAGACTCTGGACAGTTCCTGACGCTGAGGCACGAAGGCCAGGGGAGCAAACGGG` |
| ASV1217 | 426 | `TGGGGAATATTGGACAATGGGCGCAAGCCTGATCCAGCAATACCGCGTGTGTGAAGAAGGCCTTAGGGTTGTAAAGCACTTTCAGTTGGGAGGAAACGCACAAGGTGAACCTTGTGAACTGACGTTACCAACAGAAGAAGCACCGGCTAACTCCGTGCCAGCAGCCGCGGTAATACGGAGGGTGCGAGCGTTAATCGGAATTACTGGGCGTAAAGCGCGTGTAGGCGGATAAGTAAGTCAGATGTGAAATCCCTGGGCTCAACCTGGGAACGGCATCTGAAACTGCTAATCTAGAGTGTAGTAGAGGGAAGTGGAATTTCCGGTGTAGCGGTGAAATGCGTAGATATCGGAAGGAACATCAGTGGCGAAGGCGGCTTCCTGGACAAACACTGACGCTGAGATGCGAAAGCGTGGGTAGCGAACGGG` |
| ASV4308 | 430 | `TGGGGAATCTTGCACAATGGGCGAAAGCCTGATGCAGCAACGCCGCGTGGGTGAAGAAGGTTCTTGGATTGTAAAGCCCTTTCGGCTGGAAAGAAGGGCTCATCGGTTAATACCCGGTGGGTCTGACGGTACCAGAAGAAGAAGCACCGGCTAATTTCGTGCCAGCAGCCGCGGTAATACGAAAGGTGCAAGCGTTGTTCGGAATGACTGGGCGTAAAGCGCGCGTAGGCGGGTTCGCAAGTAGGATGTGAAAGCCCAAGGCTCAACCTTGGAATGGCACCCTAAACTACGGACCTTGAATGCTTGAGGGGTTGGTGGAATTCCTGGTGTAGGAGTGAAATCCGTAGATATCAGGAGGAATTTCGGCGGCGAAGGCGGCCAACTGGTGATGCATTGACGCTGAGGCGCGAAAGCGTGGGGAGCAAACAGG` |
| ASV5555 | 428 | `TCGAGAATCTTTCACAATGGGGGCAACCCTGATGGAGCGACGCCGCGTGGAGGATAAGGTCTTCGGATTGTAAACTCCTGTCATGCGAGAGCAATGCTCATTGGTTAAGAGCTGGTGAGAGGGATAGTATCGCAAGAGGAAGAGACGGCTAACTCTGTGCCAGCAGCCGCGGTAATACAGAGGTCTCAAGCGTTGTTCGGAATTACTGGGCGTAAAGGGTGTGTAGGCTGCGAATTAAGTCAGATGTGAAAATCCGGGGCTCAACCTCGGACATGCATCCGATACTGGTTTGCTAGAGTGACTTAGGGGGACATGGAATTCACAGTGTAGCAGTGAAATGCGTAGATATTGTGAAGAACACCAACGGCGAAGGCAATGTCCTGGGGGTTTACTGACGCTGAAACACGAAGGCCAAGGGAGCGAACGGG` |
| ASV1 | 429 | `TGGGGAATTTTGGACAATGGACGCAAGTCTGATCCAGCAATGCCGCGTGCAGGACGAAGGCCTTCGGGTTGTAAACTGCTTTTGTACGGAACGAAACGGTCTGCTTTAATACAGTGGGCTAATGACGGTACCGTAAGAATAAGCACCGGCTAACTACGTGCCAGCAGCCGCGGTAATACGTAGGGTGCGAGCGTTAATCGGAATTACTGGGCGTAAAGCGTGCGCAGGCGGTTATATAAGTCAGATGTGAAATCCCCGGGCTCAACCTGGGACCTGCATTTGAGACTGTATAGCTAGAGTACGGTAGAGGGGGATGGAATTCCGCGTGTAGCAGTGAAATGCGTAGATATGCGGAGGAACACCGATGGCGAAGGCAATCCCCTGGACCTGTACTGACGCTCATGCACGAAAGCGTGGGGAGCAAACAGG` |
| ASV1938 | 408 | `TGGGGAATTTTCCGCAATGGGCGAAAGCCTGACGGAGCAATACCGCGTGAGGGATGAAGGCCTATGGGTTGTAAACCTCTTTTTTCAGGGAGGAACAAAATGACGTGTACCTGAAGAATAAGCATCGGCTAACTCCGTGCCAGCAGCCGCGGTAAGACGGGGGATGCAAGTGTTATCCGGAATCACTGGGCGTAAAGCGTCTGTAGGTGGTTAAGTAAGTCAACTGTTAAATCTTGAGGCTTAACCTCAAAATCGCAGTCGAAACTGCTTGACTAGAGTATAGTAGGGGTAAAGGGAATTTCCAGTGGAGCGGTGAAATGCGTAGATATTGGAAAGAACACCGATGGCGAAGGCACTTTACTGGGCTATTACTAACACTGAGAGACGAAAGCTAGGGTAGCAAATGGG` |
| ASV2553 | 430 | `TAGGGAATATTGCACAATGGGGGAAACCCTGATGCAGCAACGCCGCGTGAGTGAAGAAGGCCCTTGGGTCGTAAAGCTCTTTAGCGAGGGAAGAGGGTTGATACGGTGAATAATCGTGTCAGCTGACGGTACCTCGAGAATAAGCACCGGCTAATTTCGTGCCAGCAGCCGCGGTAATACGAAAGGTGCAAGCGTTACTCGGAATCACTGGGCGTAAAGCGCACGTAGGCGGACTCATAGGTCAGTTGTGAAATACCTGGGCTCAACCCGGGAACTGCAGCTGAAACCATGAGCCTTGAATATCTGAGAGGGTGGGGGAATACCTGGTGGAGAAGTGAAATTCGTAGAGATCAGGTGGAACACCGGAGGCGAAGGCGCCTGCCTGGCAGTATATTGACGCTGAGGTGCGAAAGTGCGGGGAGCAAACAGG` |
| ASV4344 | 404 | `TGGGGAATTTTCCGCAATGGGCGAAAGCCTGACGGAGCAATACCGCGTGAGGGACGAAGGCCTGTGGGTTGTAAACCTCTTTTGTTAGGGAAGATAATGACGGTACCTAACGAATAAGCATCGGCTAACTCCGTGCCAGCAGCCGCGGTAAGACGGAGGATGCAAGCGTTATCCGGAATTATTGGGCGTAAAGCGTACGTAGGCTGTTTTATAAGTCTGTTGTCAAAGCCCGAGGCTCAACCTTGGATCGGCAATGGAAACTGTGAGACTAGAGAGAGATAGGGGCAGGAGGAATTCCAGGTGTAGCGGTGAAATGCGTAGATATCTGGAAGAACACCAGTGGCGAAAGCGTCCTGCTGGATCTCAACTGACGCTGAAGTACGAAAGCTAGGGGAGCGAATGGG` |
| ASV4536 | 429 | `TGGGGAATATTGGACAATGGGGGCAACCCTGATCCAGCGACGCCGCGTGGGTGAAGAAGGCCTGCGGGTTGTAAAGCCCTTTCGATGGGGACGAAAGCTGTCCACCTAATACGTGGATAGTCTGACTTAACCCATATAAGAAGCACCGGCTAACTCTGTGCCAGCAGCCGCGGTAATACAGAGGGTGCGAGCGTTAATCGGAATTACTGGGCGTAAAGCGCACGTAGGCGGCTTGGTATGTCGGGTGTGAAATCCCCGGGCTCAACCTGGGAACTGCATCCGAAACTGCCTTGCTAGAGTATGGGAGAGGGAAGTGGAATTTCCGGTGTAGCAGTGAAATGCGTAGATATCGGAAGGAACACCAGTGGCGAAGGCGACTTCCTGGACCAATACTGACGCTCAGGTGCGAAAGCGTGGGGAGCAAACAGG` |
| ASV2954 | 429 | `TGGGGAATATTGGACAATGGGGGCAACCCTGATCCAGCAATGCCGCGTGTGTGAAGAAGGCCTAAGGGTTGTAAAGCACTTTCAGTGGGGAGGAGGATTGTTAGGTTAAGAGCTAGATAATTGGACGTTACCCACAGAAGAAGCACCGGCTAACTCCGTGCCAGCAGCCGCGGTAATACGGGGGGTGCAAGCGTTAATCGGAATTACTGGGCGTAAAGGGTGCGTAGGTGGTTGATTAAGTTATCTGTAAAATCCCTGGGCTTAACCTGGGCATGTCAGATAAGACTGGTCGACTCGAGTATGGGAGAGGGTAGTGGAATTTCCGGTGTAGCGGTGAAATGCGTAGAGATCGGAAGGAACACCAGTGGCGAAGGCGGCTACCTGGCCTAATACTGACACTGAGGCACGAAAGCGTGGGGAGCAAACAGG` |
| ASV1810 | 428 | `TGGGGAATTGTTCGCAATGGGCGCAAGCCTGACGACGCAACGCCGCGTGGAGGATGAAGGTCTTCGGATTGTAAACTCCTGTTGCCCGGGACGAAACGCTCCGACCTAACACGTCGGAGCCTGACGGTACCGGGTGAGGAAGCCCCGGCTAACTCTGTGCCAGCAGCCGCGGTAATACAGAGGGGGCGAGCGTTGTTCGGAATTACTGGGCGTAAAGGGCGCGTAGGCGGCCTGTTAAGTCGAACGTGAAATCCCCGGGCTCAACCCGGGAACTGCGTCCGAGACTGACAGGCTCGAATCCGGGAGAGGGATGTGGAATTCCAGGTGTAGCGGTGAAATGCGTAGATATCTGGAGGAACACCGGTGGCGAAGGCGGCATCCTGGACCGGCATTGACGCTGAGGCGCGAAAGCCAGGGGAGCAAACGGG` |
| ASV560 | 404 | `TGGGGAATATTGGACAATGGGCGAAAGCCTGATCCAGCAATGCCGCGTGAGTGATGAAGGCCTTAGGGTTGTAAAGCTCTTTTACCAGGGATGATAATGACAGTACCTGGAGAATAAGCTCCGGCTAACTCCGTGCCAGCAGCCGCGGTAATACGGAGGGAGCTAGCGTTGTTCGGAATTACTGGGCGTAAAGCGTACGTAGGCGGTGACTCAAGTCAGAGGTGAAAGCCTGGAGCTCAACTCCAGAACTGCCTTTGAAACTAGGTCGCTAGAATCTTGGAGAGGTGAGTGGAATTCCGAGTGTAGAGGTGAAATTCGTAGATATTCGGAAGAACACCAGTGGCGAAGGCGACTCACTGGACAAGTATTGACGCTGAGGTACGAAAGCGTGGGGAGCAAACAGG` |
| ASV626 | 424 | `TGAGGAATATTGGTCAATGGGCGCAAGCCTGAACCAGCCATGCCGCGTGCAGGAAGACGGTCCTATGGATTGTAAACTGCTTTTATACAGGAAGAAACCCTCCCACGTGTGGGAGATTGACGGTACTGTAGGAATAAGGATCGGCTAACTCCGTGCCAGCAGCCGCGGTAATACGGAGGATCCAAGCGTTATCCGGAATCATTGGGTTTAAAGGGTCCGTAGGCGGTCTTATAAGTCAGTGGTGAAATCCCATCGCTCAACGATGGAACTGCCATTGATACTGTAAGACTTGAATACTTAGGAAGTAACTAGAATATGTAGTGTAGCGGTGAAATGCTTAGATATTACATGGAATACCAATTGCGAAGGCAGGTTACTACTAAGTGATTGACGCTGATGGACGAAAGCGTGGGGAGCGAACAGG` |
| ASV2724 | 423 | `TAAGGAATATTGGACAATGGACGGAAGTCTGATCCAGCCATCCCGCGTGGAGGAATAAGGCCCTATGGGTTGTAAACTCCTTTAGCAGGGGAATAAAAAGGTCATTTATGATCAATTGAAGGTACCCTGAGAATAAGCACCGGCTAACTCCGTGCCAGCAGCCGCGGTAATACGGAGGGTGCAAGCGTTATCCGGATTTACTGGGTTTAAAGGGTGCGTAGGCGGACTATTAAGTCAAATGTGAAAGCTTGCAGCTTAACTGTAAGATTGCGTTTGATACTGGTAGTCTTGAATATGGTAGAAGTTGGCGGAATAAATCATGTAGCGGTGAAATGCTTAGATATGATTTAGAACACCGATTGCGAAGGCAGCTAGCTATGTCATAATTGACGCTGAGGCACGAAAGCGTGGGGATCAAACAGG` |
| ASV4047 | 429 | `TGGGGAATTTTGGACAATGGGCGAAAGCCTGATCCAGCAATGCCGCGTGTGGGAAGAAGGCCTTCGGGTTGTAAACCACTTTTGTCGGGAACGAAACGGTCTGCACTAATAATGCGGACTAATGACTGTACCCGAAGAATAAGCACCGGCTAACTACGTGCCAGCAGCCGCGGTAATACGTAGGGTGCAAGCGTTAATCGGAATTACTGGGCGTAAAGCGTGCGCAGGCGGTTATGTAAGTCAGATGTGAAATCCCCGGGCTTAACCTGGGAACTGCATTTGAAACTGCATGGCTTGAGTGCGGTAGAGGGGGGTAGAATTCCACGTGTAGCAGTGAAATGCGTAGAGATGTGGAGGAATACCGATGGCGAAGGCAGCCCCCTGGACCGTAACTGACGCTCATGCACGAAAGCGTGGGGAGCAAACAGG` |
| ASV1207 | 404 | `TTAGGAATATTGGACAATGGGGGAAACCCTGATCCAGCCATGCCGCGTGTGTGATGAAGGCCTTAGGGTTGTAAAGCACTTTCGTTGGGGATGATAATGACCGTACCCAAAGAAGAAGCACCGGCTAACTTCGTGCCAGCAGCCGCGGTAATACGAAGGGTGCAAGCGTTGTTCGGAATCACTGGGCGTAAAGAATGCGTAGGCGGCTTGATAAGTCGGGAGTGAAATCCCAGGGCTCAACCCTGGAACTGCTTTCGAAACTATCTTGCTAGAGTCCCGGAGGGGTTAGCGGAATTCCGAGTGTAGAGGTGAAATTCGCAGATATTCGGAGGAACACCAGTGGCGAAGGCGGCTAACTGGACGGGAACTGACGCTGAGGCATGAAAGTGTGGGGATCAAACAGG` |
| ASV2048 | 407 | `TAGGGAATATTGCGCAATGGACGAAAGTCTGACGCAGCGACGCCGCGTGAGTGATGAAGGCCTTAGGGTCGTAAAGCTCTGTTGAATGGGAAGAAAAAAATGACGGTACCATTCGAGGAAGGACCGGCTAACTTCGTGCCAGCAGCCGCGGTAATACGAGGGGTCCTAGCGTTGTTCGGAATCATTGGGCGTAAAGGGTGTGTAGGTGGCTATGTAAGTCAGGTGTGAAAGCCCCGGGCTCAACCCGGGAAGTGCATCTGATACTGCGTAGCTTGAGTACCGAAGAGGATAGTGGAATTCCAGGTGTAGTGGTGAAATACGTAGATATCTGGAGGAACACCGGTGGCGAAGGCGGCTATCTGGTCGAGTACTGACACTGAGACACGAAAGCATGGGGATCAAACAGG` |
| ASV5468 | 429 | `TAGGGAATCTTCGGCAATGGGGGCAACCCTGACCGAGCAACGCCGCGTGAGTGAAGAAGGTTTTCGGATCGTAAAGCTCTGTTGTAAGTCAAGAACGAGTGTGAGAGTGGAAAGTTCACACTGTGACGGTAGCTTACCAGAAAGGGACGGCTAACTACGTGCCAGCAGCCGCGGTAATACGTAGGTCCCGAGCGTTGTCCGGATTTATTGGGCGTAAAGCGAGCGCAGGCGGTTTGATAAGTCTGAAGTTAAAGGCTGTGGCTCAACCATAGTTCGCTTTGGAAACTGTCAAACTTGAGTGCAGAAGGGGAGAGTGGAATTCCATGTGTAGCGGTGAAATGCGTAGATATATGGAGGAACACCGGTGGCGAAAGCGGCTCTCTGGTCTGTAACTGACGCTGAGGCTCGAAAGCGTGGGGAGCGAACAGG` |
| ASV3167 | 429 | `TGGGGAATTTTGGACAATGGGGGCAACCCTGATCCAGCCATGCCGCGTGAGTGAAGAAGGCCTTCGGGTTGTAAAGCTCTTTCGGCGGGGAAGAAATGGCAACGGCTAATATCCGTTGTTGATGACGGTACCCGCATAAGAAGCACCGGCTAACTACGTGCCAGCAGCCGCGGTAATACGTAGGGTGCGAGCGTTAATCGGAATTACTGGGCGTAAAGCGTGCGCAGGCGGTTTCGTAAGACAGACGTGAAATCCCCGGGCTCAACCTGGGAACTGCGTTTGTGACTGCGAGGCTAGAGTACGGCAGAGGGGGGTAGAATTCCACGTGTAGCAGTGAAATGCGTAGAGATGTGGAGGAATACCGATGGCGAAGGCAGCCCCCTGGGTTAGTACTGACGCTCATGCACGAAAGCGTGGGGAGCAAACAGG` |
| ASV1142 | 429 | `TGGGGAATATTGGACAATGGGCGGAAGCCTGATCCAGCAATGCCGCGTGTGTGAAGAAGGCCTGAGGGTTGTAAAGCACTTTCAGTGGGGAGAAAGAGGCTATCGCTAATATCGATAGTGGATGATGTTACCCACAGAAGAAGCACCGGCTAACTCTGTGCCAGCAGCCGCGGTAATACAGAGGGTGCGAGCGTTAATCGGAATTACTGGGCGTAAAGGGTGCGTAGGTGGTTTTGTAAGTTAGATGTGAAATTCCTGGGCTTAACCTGGGGACTGCGTCTGATACTGCGAAGCTAGAGTACTGTAGAGGGAAGTGGAATTTCCGGTGTAGCAGTGAAATGCGTAGATATCGGAAGGAACATCAGTGGCGAAGGCGACTTCCTGGACAGATACTGACACTGAGGCACGAAAGCGTGGGGAGCAAACAGG` |
| ASV1946 | 407 | `TGGGGAATTTTCCGCAATGGGCGCAAGCCTGACGGAGCAAGACCGCGTGAGGGAAGAAGGTCTGTGGATTGTAAACCTCTTTTGATGGGGAAGAAGACAGTGACGGTACCCATCGAATCAGCCTCGGCTAACTCCGTGCCAGCAGCCGCGGTAATACGGAGGAGGCAAGCGTTATCCGGAATTATTGGGCGTAAAGCGTCCGTAGGTGGGTTATCAAGTCAGTTGTTAAATAGTGAAGCTTAACTTCATAGAGGCAATTGAAACTGATAGTCTAGAGTGTGGTAGGGGTAGAGGGAATTTCCAGTGTAGCGGTGAAATGCGTAGATATTGGAAAGAACACCGGTGGCGAAGGCGCTCTACTGGGCCATCACTGACACTGAGGGACGAAAGCTAGGGGAGCGAAAGGG` |
| ASV2781 | 404 | `TGGGGAATATTGGACAATGGGCGCAAGCCTGATCCAGCCATGCCGCGTGGATGATGAAGGCCCTAGGGTTGTAAAGTCCTTTTAACGGGGAAGATAATGACGGTACCCGTAGAATAAGCCCCGGCTAACTTCGTGCCAGCAGCCGCGGTAATACGAAGGGGGCTAGCGTTGCTCGGAATCACTGGGCGTAAAGCGCACGTAGGCGGATTCTTAAGTCGGTGGTGAAAGCCTGGAGCTCAACTCCAGAACTGCCTTCGATACTGAGAGTCTTGAGTTCGGGAGAGGTGAGTGGAACTGCGAGTGTAGAGGTGAAATTCGTAGATATTCGCAAGAACACCAGTGGCGAAGGCGGCTCACTGGCCCGATACTGACGCTGAGGTGCGAAAGCGTGGGGAGCAAACAGG` |
| ASV4709 | 429 | `TGGGGAATTTTGGACAATGGGGGCAACCCTGATCCAGCCATGCCGCGTGCGGGAAGAAGGCCTTCGGGTTGTAAACCGCTTTTGGTCGGGAAGAAAAGGTTATCCCTAATAAGGATGACTCTTGACGGTACCGGCAGAATAAGCACCGGCTAACTACGTGCCAGCAGCCGCGGTAATACGTAGGGTGCGAGCGTTAATCGGAATTACTGGGCGTAAAGCGTGCGCAGGCGGTTTGTTAAGTCTGATGTGAAAGCCCCGGGCTCAACCTGGGAATGGCATTGGAAACTGGCAAGCTTGAGTATGGCAGAGGGGGGTGGAATTCCGCGTGTAGCAGTGAAATGCGTAGAGATGCGGAGGAACACCGATGGCGAAGGCAGCCCCCTGGGCCAATACTGACGCTCAGGCACGAAAGCGTGGGGAGCAAACAGG` |
| ASV3304 | 424 | `TGAGGAATATTGGTCAATGGGCGCAAGCCTGAACCAGCCATGCCGCGTGCAGGAAGACGGTCCTATGGATTGTAAACTGCTTTTGTACAGGAAGAAACCCTTGCTCGTGAGCAAGATTGACGGTACTGTAAGAATAAGGATCGGCTAACTCCGTGCCAGCAGCCGCGGTAATACGGAGGATCCAAGCGTTATCCGGAATCATTGGGTTTAAAGGGTCCGTAGGCGGTTTAATAAGTCAGTGGTGAAATCCCATCGCTCAACGGTGGAACGGCCATTGATACTGTTAGACTTGAATTATTAGGAAGTAACTAGAATATGTAGTGTAGCGGTGAAATGCTTAGAGATTACATGGAATACCAATTGCGAAGGCAGGTTACTACTAATTGATTGACGCTGATGGACGAAAGCGTGGGTAGCGAACAGG` |
| ASV3053 | 404 | `TGGGGAATCTTAGACAATGGGCGCAAGCCTGATCTAGCCATGCCGCGTGATCGATGAAGGCCTTAGGGTTGTAAAGATCTTTCGTGGGGGAAGATAATGACGGTACCCCAAGAAGAAGCCCCGGCTAACTCCGTGCCAGCAGCCGCGGTAATACGGAGGGGGCTAGCGTTATTCGGAATTACTGGGCGTAAAGCGCACGTAGGCGGATTGGAAAGTCAGAGGTGAAATCCCAGAGCTCAACTTTGGAACTGCCTTTGAAACTCCCAGTCTTGAGGTCGAGAGAGGTGAGTGGAATTCCGAGTGTAGAGGTGAAATTCGTAGATATTCGGAGGAACACCAGTGGCGAAGGCGGCTCACTGGCTCGATACTGACGCTGAGGTGCGAAAGCGTGGGGAGCAAACAGG` |
| ASV4514 | 423 | `TAGGGAATATTGGACAATGGGTGCGAGCCTGATCCAGCCATGCCGCGTGCAGGAAGAAGGCCTTCTGGGTTGTAAACTGCTTTTGCCAGGGGATAAAAAGCCCTTGCGAGGGAAATTGAAGGTACCTGGTGAATAAGCCACGGCTAACTACGTGCCAGCAGCCGCGGTAATACGTAGGTGGCAAGCGTTGTCCGGATTTATTGGGTTTAAAGGGTGCGTAGGCGGTCTTATAAGTCAGCGGTGAAATCCGGCAGCTCAACTGTCGATGTGCCATTGATACTGTAGGACTTGAGTACTGATGAGGTAGGCGGAATTGACGGTGTAGCGGTGAAATGCTTAGATATCGTCAAGAACACCGATAGCGAAGGCAGCTTACTAAGGAGTAACTGACGCTGATGCACGAAAGTGTGGGGATCAAACAGG` |
| ASV2769 | 429 | `TGGGGAATTTTGGACAATGGGCGCAAGCCTGATCCAGCCATGCCGCGTGCGGGAAGAAGGCCTTCGGGTTGTAAACCGCTTTTGTCAGGGAAGAAACGGCCTTTTCTAATACAGGAGGCTAATGACGGTACCTGAAGAATAAGCACCGGCTAACTACGTGCCAGCAGCCGCGGTAATACGTAGGGTGCAAGCGTTAATCGGAATTACTGGGCGTAAAGCGTGCGCAGGCGGCTTTGCAAGACAGATGTGAAATCCCCGGGCTCAACCTGGGAACTGCATTTGTGACTGCAAGGCTAGAGTACGGTAGAGGGGGATGGAATTCCGCGTGTAGCAGTGAAATGCGTAGATATGCGGAGGAACACCGATGGCGAAGGCAATCCCCTGGACCTGTACTGACGCTCATGCACGAAAGCGTGGGGAGCAAACAGG` |
| ASV3361 | 404 | `TGGGGAATATTGGACAATGGGCGCAAGCCTGATCCAGCAATACCGCGTGGGTGATGAAGGTCTTCGGATTGTAAAGCCCTTTTAGTGGAGAAGATAATGACGGTATCCACAGAAAAAGCACCGGCAAACTTCGTGCCAGCAGCCGCGGTAATACGAAGGGTGCTAGCGTTGTTCGGAATCATTGGGCGTAAAGCGTACGTAGGCGGCTTAGTAAGTCGATTGTGAAAGCCCGGGGCTTAACCCTGGAACTGCAACCGAAACTGCTTCGCTTGAGTTTGGTGGGGGATGATAGAATTCCTAGTGTAGGGGTGAAATCCGTAGATATTAGGAGGAATACCGGTGGCGAAAGCGATCATCTACGCCAATACTGACGCTAAGGTACGAAAGCGTGGGGAGCAAACAGG` |
| ASV4520 | 429 | `TAGGGAATATTGCGCAATGGGGGAAACCCTGACGCAGCAACGCCGCGTGAGTGAGGAAGGTCTTCGGATTGTAAAACTCTTTTGTTAGGGAAAAATGGTCACGTAACTAATAGCGCGTGATTTGATGGTACCTAAAGAATAAGCACCGGCTAACTTCGTGCCAGCAGCCGCGGTAATACGAAGGGTGCAAGCGTTATTCGGAATCATTGGGCGTAAAGCGCGCGCAGGCGGATCAGCAAGTCAGATGTGAAATCTCGGAGCTCAACTCCGAAACTGCGTCTGAAACTGCTAGTCTAGAGTGTCGGAGGGGGTAGGGGAATTTCACGTGTAGGGGTAAAATCCGTAGAGATGTGAAGGAACACCGGGGGCGAAGGCGCCTACCTGGACGACTACTGACGCTGAGGCGCGAAAGCGTGGGGAGCAAACAGG` |
| ASV1113 | 429 | `TGGGGAATTTTGGACAATGGGCGCAAGCCTGATCCAGCCATTCCGCGTGAGTGAAGAAGGCCTTCGGGTTGTAAAGCTCTTTCGCAAGGGAAGAAACGATTTAGGTAAATAATCTAAGTTAATGACGGTACCTTGATAAGAAGCACCGGCTAACTACGTGCCAGCAGCCGCGGTAATACGTAGGGTGCGAGCGTTAATCGGAATTACTGGGCGTAAAGCGTGCGCAGGCTGTTTTGTAAGTCAGATGTGAAATCCCTGGGCTCAACCTAGGAACTGCGTTTGAAACTACAAGACTAGAATATGTCAGAGGGGGGTAGAATTCCACGTGTAGCAGTGAAATGCGTAGAGATGTGGAGGAATATCAATGGCGAAGGCAGCCCCCTGGGATAATATTGACGCTCATGCACGAAAGCGTGGGGAGCAAACAGG` |
| ASV3671 | 405 | `TCGGGAATATTGCGCAATGGAGGAAACTCTGACGCAGTGACGCCGCGTGCAGGAAGAAGGTTTTCGGATTGTAAACTGCTTTAGACAGGGAAGAAGAAAGACAGTACCTGTAGAATAAGCTCCGGCTAACTACGTGCCAGCAGCCGCGGTAATACGTAGGGAGCAAGCGTTATCCGGATTTATTGGGTGTAAAGGGTGCGTAGACGGGAAGTCAAGTTAGTTGTGAAATCCCTCGGCTCAACTGAGGAACTGCAACTAAAACTAACTTTCTTGAGTGCTGGAGAGGAAAGTGGAATTCCTAGTGTAGCGGTGAAATGCGTAGATATTAGGAGGAACACCAGTGGCGAAGGCGACTTTCTGGACAGTAACTGACGTTGAGGCACGAAAGTGTGGGGAGCAAACAGG` |
| ASV3158 | 404 | `TGGGGAATATTGGACAATGGGGGAAACCCTGATCCAGCAATGCCGCGTGGGTGATGAAGGCCTTCGGGTTGTAAAGCCCTTTTGGCGGGGACGATGATGACGGTACCCGCAGAAGAAGCCCCGGCTAACTTCGTGCCAGCAGCCGCGGTAATACGAAGGGGGCTAGCGTTGTTCGGAATTACTGGGCGTAAAGGGCGCGTAGGCGGCCTTGATAGTTGGGCGTGAAAGGCCTGGGCTTAACCCAGGAACTGCGTTCAATACTGCGAGGCTAGAGTGTGGGAGAGGTTGGTGGAATTCCCAGTGTAGAGGTGAAATTCGTAGATATTGGGAAGAACACCGGTGGCGAAGGCGGCCAACTGGACCACAACTGACGCTGAGGCGCGAAAGCGTGGGGAGCAAACAGG` |
| ASV1814 | 429 | `TAGGGAATTTTCCACAATGGACGGAAGTCTGATGGAGCAACGCCGCGTGTGCGAAGAAGGTTTTCGGATTGTAAAGCACTGTTGTAAGGGAAGAATAAGGTAAACAGGAAATGGTTTATCGATGACGGTACCTTACCAGAAAGCCACGGCTAACTACGTGCCAGCAGCCGCGGTAATACGTAGGTGGCAAGCGTTGTCCGGAATTATTGGGCGTAAAGGGCGTGTAGGCGGTAAAATAAGTCTGATGTGAAATCTCATAGCTCAACTATGAAACTGCATTGGAAACTGTTTAACTAGAGTGTCGGAGAGGAAAGCGGAATTCCATGTGTAGCGGTGAAATGCGTAGATATATGGAGGAACACCAGTGGCGAAGGCGGCTTTCTGGACGACTACTGACGCTGAGGCGCGAAAGCGTGGGGAGCAAACAGG` |
| ASV341 | 429 | `TGGGGAATTTTGGACAATGGACGCAAGTCTGATCCAGCCATGCCGCGTGCGGGAAGAAGGCCTTCGGGTTGTAAACCGCTTTTGTCAGGGAAGAAAAGGTTCTGGTTAATACCTGGGGCTGATGACGGTACCTGAAGAATAAGCACCGGCTAACTACGTGCCAGCAGCCGCGGTAATACGTAGGGTGCAAGCGTTAATCGGAATTACTGGGCGTAAAGCGTGCGCAGGCGGTTATGCAAGACAGAGGTGAAATCCCCGGGCTCAACCTGGGAACTGCCTTTGTGACTGCATAGCTAGAGTACGGTAGAGGGGGATGGAATTCCGCGTGTAGCAGTGAAATGCGTAGATATGCGGAGGAACACCGATGGCGAAGGCAATCCCCTGGACCTGTACTGACGCTCATGCACGAAAGCGTGGGGAGCAAACAGG` |
| ASV4994 | 404 | `TGGGGAATCTTGGACAATGGGGGCAACCCTGATCCAGCCATGCCGCGTGAGTGATGAAGGCCTTCGGGTTGTAAAACTCTTTCGGCGGGGAAAATAATGATGGTACCCGCAGAAGAAGCCCCGGCTAACTTCGTGCCAGCAGCCGCGGTAATACGAAGGGGGCTAGCGTTGTTCGGAATTACTGGGCGTAAAGCGCGCGTAGGCGGTTCGACAAGTCGGACGTGAAATCCCCGGGCTCAACCCGGGAATTGCATTCGAAACTGTCGAGCTAGAGGCCTGGAGAGGAGAGTGGAATTCCCAGTGTAGAGGTGAAATTCGTAGATATTGGGAAGAACACCAGTGGCGAAGGCGGCTCTCTGGCCAGGTTCTGACGCTGAGGCGCGAAAGTGTGGGGAGCAAACAGG` |
| ASV157 | 429 | `TGGGGAATATTGGACAATGGGCGCAAGCCTGATCCAGCCATGCCGCGTGTGTGAAGAAGGCCTTCGGGTTGTAAAGCACTTTCAGCGAGGAGGAAGGGAGTTGTGTTAATAGCACAGCTTCTTGACGTTACTCGCAGAAGAAGCACCGGCTAACTCTGTGCCAGCAGCCGCGGTAATACAGAGGGTGCAAGCGTTAATCGGAATTACTGGGCGTAAAGCGCACGTAGGCGGTTTTTTAAGTCAGATGTGAAAGCCCCGGGCTCAACCTGGGAATTGCATTTGAAACTGGAAAACTAGAGTGTGTGAGAGGGGGGTAGAATTCCAAGTGTAGCGGTGAAATGCGTAGAGATTTGGAGGAATACCAGTGGCGAAGGCGGCCCCCTGGCACAACACTGACGCTCAGGTGCGAAAGCGTGGGGAGCAAACAGG` |
| ASV5145 | 404 | `TGGGGAATATTGGACAATGGGGGAAACCCTGATCCAGCAATGCCGCGTGAGTGAAGAAGGCCTTCGGGTTGTAAAGCTCTTTTATTGGGGAAGATAATGACGGTACCCAAAGAATAAGCTCCGGCTAACTTCGTGCCAGCAGCCGCGGTAATACGAAGGGAGCTAGCGTTGTTCGGAATTACTGGGCGTAAAGGGCGCGTAGGCGGCCGTTTAAGTTAGATGTGAAAGCCCCGGGCTTAACCTGGGAATTGCATTTAATACTGAATGGCTTGAGATCGAGAGAGGAAAGTGGAATTGTGAGTGTAGAGGTGAAATTCGTAGATATTCACAAGAACACCAGTGGCGAAGGCGGCTTTCTGGCTCGATACTGACGCTAAGGCGCGAAAGCGTGGGGAGCAAACAGG` |
| ASV3048 | 404 | `TGGGGAATCTTAGACAATGGGCGCAAGCCTGATCTAGCCATGCCGCGTGATCGATGAAGGCCTTAGGGTTGTAAAGATCTTTCAGGTGGGAAGATAATGACTGTACCACCAGAAGAAGCCCCGGCTAACTCCGTGCCAGCAGCCGCGGTAATACGGAGGGGGCTAGCGTTATTCGGAATTACTGGGCGTAAAGCGCACGTAGGCGGATTGGAAAGTCAGAGGTGAAATCCCAGGGCTCAACCTTGGAACGGCCTTTGAAACTCCCAGTCTTGAGATCGAGAGAGGTGAGTGGAATTCCGAGTGTAGAGGTGAAATTCGTAGATATTCGGAGGAACACCAGTGGCGAAGGCGGCTCACTGGCTCGATACTGACGCTGAGGTGCGAAAGCGTGGGGAGCAAACAGG` |
| ASV2082 | 429 | `TGGGGAATATTGGACAATGGGGGCAACCCTGATCCAGCCATGCCGCGTGTGTGAAGAAGGCCTTCGGGTTGTAAAGCACTTTAAGGGGGGAGGAAGGTTCGCTGGCTAATATCCAGCGGAATTGACGTTACCCCCAGAATAAGCACCGGCTAACTCCGTGCCAGCAGCCGCGGTAATACGGAGGGTGCAAGCGTTAATCGGAATTACTGGGCGTAAAGCGCGCGTAGGCGGCTTGATAAGTCGGATGTGAAATCCCTGGGCTCAACCTGGGAACTGCATTCGATACTGTCTGGCTAGAGTGTGGGAGAGGGTGGTGGAATTCCCGGTGTAGCGGTGAAATGCGTAGATATCGGGAGGAACATCAGTGGCGAAGGCGACCACCTGGCCCAACACTGACGCTGAGGTGCGAAAGCGTGGGTAGCAAACAGG` |
| ASV1390 | 429 | `TGGGGAATTTTGGACAATGGGCGAAAGCCTGATCCAGCCATTCCGCGTGAGTGAAGAAGGCCTTCGGGTTGTAAAGCTCTTTCGGCAGGGAAGAAACGGTTTTGGCTAATACCCAGAGCTAATGACGGTACCTGAAGAAGAAGCACCGGCTAACTACGTGCCAGCAGCCGCGGTAATACGTAGGGTGCGAGCGTTAATCGGAATTACTGGGCGTAAAGCGTGCGCAGGCGGTTTTGTAAGACAGATGTGAAATCCCCGGGCTCAACCTGGGAACTGCGTTTGTGACTGCAAGACTTGAGTATGGCAGAGGGGGGTAGAATTCCACGTGTAGCAGTGAAATGCGTAGAGATGTGGAGGAATACCGATGGCGAAGGCAGCCCCCTGGGTCAATACTGACGCTCATGCACGAAAGCGTGGGGAGCAAACAGG` |
| ASV137 | 429 | `TGGGGAATTTTGGACAATGGGCGCAAGCCTGATCCAGCCATGCCGCGTGCAGGATGAAGGCCTTCGGGTTGTAAACTGCTTTTGTACGGAACGAAAAGACTCTGGTTAATACCTGGGGTCCATGACGGTACCGTAAGAATAAGCACCGGCTAACTACGTGCCAGCAGCCGCGGTAATACGTAGGGTGCAAGCGTTAATCGGAATTACTGGGCGTAAAGCGTGCGCAGGCGGTTATGTAAGACAGATGTGAAATCCCCGGGCTCAACCTGGGAACTGCATTTGTGACTGCATAGCTAGAGTACGGTAGAGGGGGATGGAATTCCGCGTGTAGCAGTGAAATGCGTAGATATGCGGAGGAACACCGATGGCGAAGGCAATCCCCTGGACCTGTACTGACGCTCATGCACGAAAGCGTGGGGAGCAAACAGG` |
| ASV479 | 429 | `TGGGGAATATTGGACAATGGGGGCAACCCTGATCCAGCAATGCCGCGTGTGTGAAGAAGGCCTGCGGGTTGTAAAGCACTTTTGTTTGGAACGAAAAGCTTGAGGTTAATACGTTCAAGCGCTGACGGTACCAAAAGAATAAGCACCGGCTAACTTCGTGCCAGCAGCCGCGGTAATACGAAGGGTGCAAGCGTTAATCGGAATTACTGGGCGTAAAGCGTGCGTAGGTGGCCTGTTAAGTTTGCTGTGAAAGCCCCGGGCTTAACCTGGGAACGGCAGCGAAAACTGGTGGGCTAGAGTCGGTTAGAGGGTAGTGGAATTCCCGGTGTAGCGGTGAAATGCGTAGAGATCGGGAGGAACATCAGTTGCGAAGGCGGCTACCTGGAACCAGACTGACACTGAGGCACGAAAGCGTGGGGAGCAAACAGG` |
| ASV531 | 404 | `TGGGGAATCTTGGACAATGGGCGCAAGCCTGATCCAGCCATGCCGCGTGGATGATGAAGGCCTTAGGGTTGTAAAATCCTTTCAACGGGGACGATAATGACGGTACCCGTAGAAGAAGCCCCGGCTAACTTCGTGCCAGCAGCCGCGGTAATACGAAGGGGGCTAGCGTTGCTCGGAATTACTGGGCGTAAAGCGCACGTAGGCGGATATTTAAGTCGGAGGTGAAATCCCAGGGCTCAACCCTGGAACTGCCCTCGATACTGGGTATCTGGACCAAAGTAGAGGTAAGTGGAATTCCGAGTGTAGAGGTGAAATTCGTAGATATTCGGAGGAACACCAGTGGCGAAGGCGACTTACTGGACTTTTGGTGACGCTGAGGTGCGAAAGCGTGGGGAGCAAACAGG` |
| ASV316 | 429 | `TGGGGAATTTTGGACAATGGGCGCAAGCCTGATCCAGCCATGCCGCGTGCGGGAAGAAGGCCTTCGGGTTGTAAACCGCTTTTGTCAGGGAAGAAACGGCTTTTTCTAATACAGAAGGCTAATGACGGTACCTGAAGAATAAGCACCGGCTAACTACGTGCCAGCAGCCGCGGTAATACGTAGGGTGCAAGCGTTAATCGGAATTACTGGGCGTAAAGCGTGCGCAGGCGGTTATGCAAGACAGAGGTGAAATCCCCGGGCTCAACCTGGGAACTGCCTTTGTGACTGCATAGCTAGAGTACGGTAGAGGGGGATGGAATTCCGCGTGTAGCAGTGAAATGCGTAGATATGCGGAGGAACACCGATGGCGAAGGCAATCCCCTGGACCTGTACTGACGCTCATGCACGAAAGCGTGGGGAGCAAACAGG` |
| ASV2077 | 428 | `TTTCGAATCATTCACAATGGGCGAAAGCCTGATGGTGCGACGCCGCGTGAGGGATGAAGGCCTTCGGGTCGTAAACCTCTGTCACCGGGGAAGAAACGCTTCAAGTTAATAGCTTGAAGTCTGACTTAACCCGGAGAGGAAGCAGTGGCTAACTCTGTGCCAGCAGCCGCGGTAATACAGAGACTGCAAGCGTTATTCGGATTCACTGGGCGTAAAGGGTGCGCAGGCGGCCAAATGTGTGAGGTGTGAAAGCCCGGGGCTTAACCCCGGAATTGCACCTCAAACTATTTGGCTAGAGCATTGGAGAGGGTAGCAGAATTCACGGTGTAGCAGTGAAATGCGTAGATATCGTGAGGAATACCAGAGGCGAAGGCGGCTACCTGGACAATTGCTGACGCTCAGGCACGAAAGCGTGGGGAGCAAAAGGG` |
| ASV3664 | 404 | `TGGGGAATATTGGACAATGGGGGAAACCCTGATCCAGCAATGCCGCGTGAGTGAAGAAGGCCTTCGGGTTGTAAAGCTCTTTTGTGGGGAAAGATAATGACGGTCCCTCACGAATAAGCCCCGGCTAACTTCGTGCCAGCAGCCGCGGTAATACGAAGGGGGCTAGCGTTGTTCGGAATGACTGGGCGTAAAGGGCGCGTAGGCGGTTGATTAAGTTTGTTGTGAAAGGCCTGGGCTCAACCTAGGATTGGCAATGAAGACTGGTCAACTAGAGTTTGGAAGGGGAGAATGGAATTGCGTATGGAGAGGTGAAATTCGTAGATATACGCAGGAACACCGGTGGTGAAAACGATTCTCTGGTACAAGACTGACGCTGAGGCGCGAAAGCGTGGGGAGCAAACAGG` |
| ASV3423 | 405 | `TGAGGAATATTGCACAATGGGCGCAAGCCTGATGCAGCGACGCCGCGTGGGTGAAGAAGGTTTTCGGATCGTAAAACCCTTTTCTGTGTGACGAGCAAGGACGGTAGCACAGGAATAAGTCTCGGCTAACTACGTGCCAGCAGCCGCGGTAAAACGTAGGAGACAAACGTTATCCGGAGTTACTGGGCGTAAAGGGCGTGCAGGCGGCAGTCTAAGTCTGGCGTGAAATATCCCGGCTCAACCGGGGAAGGTCGTCGGATACTGGGCAGCTCGAGGACGGTAGAGGCGTGTGGAATTCCGGGTGTAGTGGTGAAATGCGTAGAGATCCGGAGGAACACCAGTGGCGAAGGCGACACGCTGGGCCGTACCTGACGCTAAGACGCGACAGCATGGGGAGCAAACAGG` |
| ASV131 | 404 | `TGGGGAATCTTAGACAATGGGGGAAACCCTGATCTAGCCATGCCGCGTGATCGATGAAGGCCTTAGGGTTGTAAAGATCTTTCAGATGGGAAGATAATGACGGTACCATCAGAAGAAGCCCCGGCTAACTCCGTGCCAGCAGCCGCGGTAATACGGAGGGGGCTAGCGTTATTCGGAATTACTGGGCGTAAAGCGCACGTAGGCGGACTGGAAAGTCAGAGGTGAAATCCCAGGGCTCAACCTTGGAACGGCCTTTGAAACTCCCAGTCTTGAGATCGTGAGAGGTGAGTGGAATTCCGAGTGTAGAGGTGAAATTCGTAGATATTCGGAGGAACACCAGTGGCGAAGGCGGCTCACTGGCACGATACTGACGCTGAGGTGCGAAAGCGTGGGGAGCAAACAGG` |
| ASV2473 | 407 | `TGGGGAATATTGCACAATGGGCGAAAGCCTGATGCAGCAACGCCGCGTGAAGGAAGACGGTTTTCGGATTGTAAACTTCTGTTCTTAGTGAAGAAAAAAATGACGGTAACTAAGCAGCAAGCTCCGGCTAACTACGTGCCAGCAGCCGCGGTAATACGTAGGGAGCGAGCGTTATCCGGATTTACTGGGTGTAAAGGGAGCGCAGGCGGGACTGCAAGTTGGATGTGAAATACCGGGGCTCAACCCCGGAGCTGCATCCAAAACTGTAGTTCTTGACTGAAGTAGAGGCAAGTGGAATTCCGAGTGTAGCGGTGAAATGCGTAGATATTCGGAGGAACACCAGTGGCGAAGGCGACTTGCTGGGATTTAACTGACGCTGAGGCTCGAAAGTGTGGGGAGCAAACAGG` |
| ASV3132 | 404 | `TGAGGAATATTGGGCAATGGGGGAAACCCTGACCCAGCCATGCCGCGTGAATGATGAAGGCCTTAGGGTTGTAAAGTTCTTTCGCCGATGAAGATAATGACGGTAGTCGGAGAAGAAGCCCCGGCTAACTTCGTGCCAGCAGCCGCGGTAATACGAAGGGGGCTAGCGTTGTTCGGAATCACTGGGCGTAAAGCGCGCGTAGGCGGAATGGTAAGTCAGATGTGAAATCCCGGGGCCTAACCTCGGAACTGCATTTGAAACTATCATTCTAGAGACTCGGAGGGGGTGGCGGAATTCCAAATGTAGGGGTGAAATCCGTAGATATTTGGAGGAACACCGGTGGCGAAGGCGGCCACCTGGACGAGTACTGACGCTGAGGCGCGAAAGCGTGGGGAGCAAACAGG` |
| ASV1986 | 424 | `TGAGGAATATTGGACAATGGGCGCAAGCCTGATCCAGCCATGCCGCGTGCAGGATGACGGTCCTATGGATTGTAAACTGCTTTTGTACAGGAAGAAACAGTTCTACGTGTAGAACCTTGACGGTACTGTAAGAATAAGGATCGGCTAACTCCGTGCCAGCAGCCGCGGTAATACGGAGGATCCAAGCGTTATCCGGAATCATTGGGTTTAAAGGGTCCGTAGGCGGTTTAGTAAGTCAGTGGTGAAAGCCCATCGCTCAACGGTGGAACGGCCATTGATACTGCTAGACTTGAATTATTAGGAAGTAACTAGAATATGTAGTGTAGCGGTGAAATGCTTAGAGATTACATGGAATACCAATTGCGAAGGCAGGTTACTACTAATGGATTGACGCTGATGGACGAAAGCGTGGGTAGCGAACAGG` |
| ASV5589 | 446 | `GCATCTTGAGTTGTGTGTGCGGCGGACTTTGTAGCTGCTCCTTCCTCTCCGATGGGTTGGGCCACGGCTTGCTGATATGAGTGATTTACAAACACAATAAGTTATATTGGCCTTACACTCGACAGGATCTGGGCGCCTTCCACTGACTCAGGACTTTGAAGAAGGTTGGAGCTAGCGCCTACAACTCACGCAATTCGCGATGGAGGCGTGGGGTCGATCCTCGTCGCCAATTGTAGAACCGCCCTCTACGCTCCTTTGTCGACGCATTAAACAGACTCCCGTTCTCACCTCCTCCTCGCAGTGCGACTCAAAGGGAACTGGTCTTTATTTACATCAGATGTGACAGCTGAGTTTTGACCTCGTGGTACCATACATGATCACAGTTACATATGCGTATACAACAATGTGCTTTGTATAATAACGACTGTATAACAAAGACAGTTGTC` |
| ASV686 | 429 | `TGGGGAATTTTGGACAATGGGCGAAAGCCTGATCCAGCAATGCCGCGTGCAGGAAGAAGGCCTTCGGGTTGTAAACTGCTTTTGTACGGAACGAAACGGTCCGCTTTAATACAGTGGGCTAATGACGGTACCGTAAGAATAAGCACCGGCTAACTACGTGCCAGCAGCCGCGGTAATACGTAGGGTGCGAGCGTTAATCGGAATTACTGGGCGTAAAGCGTGCGCAGGCGGTTGTATAAGACAGTTGTGAAATCCCCGGGCTCAACCTGGGAATTGCATCTGTGACTGTATAGCTAGAGTACGGTAGAGGGGGATGGAATTCCGCGTGTAGCAGTGAAATGCGTAGATATGCGGAGGAACACCGATGGCGAAGGCAATCCCCTGGACCTGTACTGACGCTCATGCACGAAAGCGTGGGGAGCAAACAGG` |
| ASV4798 | 430 | `TGGGGAATTTTGCACAATGGACGAAAGTCTGATGCAGCAACGCCGCGTGAGTGAAGAAGGCCCTTGGGTTGTAAAGCTCTTTCGGTTGGGAAGAAGGGTGTCGTAGTTAATACCTGCGGCATTTGACGGTACCAGATTAAGAAGCACTGGCTAATTCCGTGCCAGCAGCCGCGGTAATACGGAAAGTGCAAGCGTTGTTCGGATTGATTGGGCGTAAAGGGCGCGTAGGCGGGTTTGTCAGTCGATTGTGAAATCCCCAGGCTTAACCTGGGAACTGCAGTCGATACTGCAAATCTTGAATGTTCGAGAGGTTGGTGGAATTCCAGGTGTAGAAGTGAAATTCGTAGATATCTGGAGGAACACCGGAGGCGAAGGCGGCCAACTGGCGAAACATTGACGCTGAGGCGCGAAAGCGTGGGGAGCAAACAGG` |
| ASV3131 | 429 | `TGGGGAATCTTGGACAATGGGGGAAACCCTGATCCAGCAATGCCGCGTGTGTGAAGAAGGCCTGCGGGTTGTAAAGCACTTTCAGTGGGGAAGAAAAGCTTCGTGTTAACACCACGGAGTTTTGACGTTACCCAAAGAAGAAGCACCGGCTAACTCTGTGCCAGCAGCCGCGGTAATACAGAGGGTGCGAGCGTTAATCGGAATTACTGGGCGTAAAGCGCACGTAGGTGGCCTGTTAAGTCGGATGTGAAAGCCCTGGGCTTAACCTGGGAATTGCATTCGATACTGGCAGGCTAGAGTGTGGTAGAGGGAAGTGGAATTCCCGGTGTAGCGGTGAAATGCGTAGATATCGGGAGGAACACCAGTGGCGAAGGCGGCTTCCTGGACCAACACTGACACTGAGGTGCGAAAGCGTGGGGAGCAAACAGG` |
| ASV2220 | 405 | `TGGGGAATTTTCCGCAATGGGCGCAAGCCTGACGGAGCAATACCGCGTGAGGGATGAAGGCTCTTGGGTCGTAAACCTCTTTTCTCAGGGAAGAAACCTGACGGTACCTGAGGAATAAGCATCGGCTAACTCCGTGCCAGCAGCCGCGGTAAGACGGAGGATGCAAGCGTTATCCGGAATTATTGGGCGTAAAGCGTCCGCAGGTGGTTTTTCAAGTCTGTTTTCAAAGAGCGAGGCTCAACCTTGTAAGGGGGATGGAAACTGAAAGACTAGAGTTCGGTAGGGGTAGAGGGAATTCCAGGTGTAGCGGTGAAATGCGTAGATATCTGGAAGAACACCAGTGGCGAAAGCGCTCTACTGGGCCTGAACTGACACTGAGGGACGAAAGCTAGGGGAGCGAAAGGG` |
| ASV1620 | 404 | `TGGGGAATATTGGACAATGGGCGCAAGCCTGATCCAGCCATGCCGCGTGAGTGATGAAGGTCTTAGGATTGTAAAGCTCTTTCACCGATGAAGATAATGACGGTAGTCGGAGAAGAAGCCCCGGCTAACTTCGTGCCAGCAGCCGCGGTAATACGAAGGGGGCTAGCGTTGTTCGGAATTACTGGGCGTAAAGCGCACGTAGGCGGGTATTTAAGTCAGGGGTGAAATCCCGCAGCTCAACTGCGGAACTGCCTTTGATACTGGGTATCTTGAGTATGGAAGAGGTAAGTGGAATTGCGAGTGTAGAGGTGAAATTCGTAGATATTCGCAGGAACACCAGTGGCGAAGGCGGCTTACTGGTCCATTACTGACGCTGAGGTGCGAAAGCGTGGGGAGCAAACAGG` |
| ASV3195 | 429 | `TGGGGAATATTGGACAATGGGGGCAACCCTGATCCAGCGACGCCGCGTGTGTGAAGAAGGCCTGCGGGTTGTAAAGCACTTTTAGTGGGGATGAAAAGCACAGCGTTAACACCGTTGTGTATTGACCTAACCCAAAGAAAAAGCACCGGCTAACTCTGTGCCAGCAGCCGCGGTAATACAGAGGGTGCAAGCGTTAATCGGAATTACTGGGCGTAAAGCGTGCGTAGACGGTTATTTAAGTCGGGTGTGAAAGCCCCGGGCTCAACCTGGGAATTGCATTCGAGACTGAATAGCTAGGGTACGGTAGAGGGAAGTGGAATTTCCGGTGTAGCGGTGAAATGCGTAGATATCGGAAGGAACATCAGTGGCGAAAGCGACTTCCTGGACCAGTACCGACGTTCAGGCACGAAAGCGTGGGGAGCAAACAGG` |
| ASV1431 | 429 | `TGGGGAATTTTGGACAATGGGGGCAACCCTGATCCAGCAATGCCGCGTGAGTGAAGAAGGCCTTCGGGTTGTAAAGCTCTTTTGTCAGGGAAGAAACGGTGAGTTCTAATACAGCTTGCTAATGACGGTACCTGAAGAATAAGCACCGGCTAACTACGTGCCAGCAGCCGCGGTAATACGTAGGGTGCAAGCGTTAATCGGAATTACTGGGCGTAAAGCGTGCGCAGGCGGTTATATAAGACAGAGGTGAAATCCCCGGGCTCAACCTGGGAACTGCCTTTGTGACTGTATGGCTAGAGTGTGTCAGAGGGGGGTAGAATTCCACGTGTAGCAGTGAAATGCGTAGAGATGTGGAGGAATACCGATGGCGAAGGCAGCCCCCTGGGATAACACTGACGCTCATGCACGAAAGCGTGGGGAGCAAACAGG` |
| ASV1962 | 424 | `TGAGGAATATTGGACAATGGGCGCAAGCCTGATCCAGCCATGCCGCGTGCAGGATGACGGTCCTATGGATTGTAAACTGCTTTTGCACAGGAAGAAACACTCCCTCGTGAGGGAGCTTGACGGTACTGTGAGAATAAGGATCGGCTAACTCCGTGCCAGCAGCCGCGGTAATACGGAGGATCCAAGCGTTATCCGGAATCATTGGGTTTAAAGGGTCCGTAGGCGGTTTAGTAAGTCAGTGGTGAAAGCCCATCGCTCAACGGTGGAACGGCCATTGATACTGCTAGACTTGAATTACAAAGAAGTAACTAGAATATGTAGTGTAGCGGTGAAATGCTTAGAGATTACATGGAATACCAATTGCGAAGGCAGGTTACTATTTGTGGATTGACGCTGATGGACGAAAGCGTGGGTAGCGAACAGG` |
| ASV2163 | 428 | `TCGAGAATCATTCACAATGGGCGAAAGCCTGATGGTGTGACGCCGCGTGGAGGATGAAGGTCTTCGGATTGTAAACTCCTGTCAAGTGAGAGTAAAGCCGGGCGTTAACTGCGAGCCGGTGTGAGAGTATCACTAGAGGAAGAGACGGCTAACTCTGTGCCAGCAGCCGCGGTAATACAGAGGTCTCAAGCGTTGTTCGGAATCACTGGGCGTAAAGGGTGCGTAGGCTGCACGGTAAGTCAGAGGTGAAATCCCGGGGCTCAACCCCGGAATTGCCTCCGATACTGCCGTGCTTGAGGACTGGAGAGGAGTCTGGAATTCACAGTGGAGCAGTGAAATGCGTAGATATTGTGAGGAACACTAGTGGCGAAGGCGAGACTCTGGACAGTTCCTGACGCTGAGGCACGAAGGCCAGGGGAGCAAACGGG` |
| ASV59 | 404 | `TGGGGAATCTTGCGCAATGGGCGAAAGCCTGACGCAGCCATGCCGCGTGTATGATGAAGGTCTTAGGATTGTAAAATACTTTCACCGGTGAAGATAATGACTGTAGCCGGAGAAGAAGCCCCGGCTAACTTCGTGCCAGCAGCCGCGGTAATACGAAGGGGGCTAGCGTTGCTCGGAATTACTGGGCGTAAAGGGAGCGTAGGCGGACATTTAAGTCAGGGGTGAAATCCCAGAGCTCAACTCTGGAACTGCCTTTGATACTGGGTGTCTTGAGTGTGAGAGAGGTATGTGGAACTCCGAGTGTAGAGGTGAAATTCGTAGATATTCGGAAGAACACCAGTGGCGAAGGCGACATACTGGCTCATTACTGACGCTGAGGCTCGAAAGCGTGGGGAGCAAACAGG` |
| ASV3130 | 405 | `TGGGGAATATTGGACAATGGGCGCAAGCCTGATCCAGCCATGCCGCGTGAGTGATGAAGGCCTTAGGGTTGTAAAGCTCTTTTAGCAGGGACGATAATGACGGTACCTGCAGAAAAAGCCCCGGCCAACTTCGTGCCAGCAGCCGCGGTAATACGAAGGGGGCTAGCGTTGCTCGGAATCACTGGGCGTAAAGCGCACGTAGGTGGGTCGTTAAGTCAGGGGTGAAATCCCGGAGCTCAACTCCGGAACTGCCTTTGATACTGGCGACCTTCGAGTTCGGGAGAGGTGAGTGGAACTGCGAGTGTAGAGGTGAAATTCGTAGATATTCGCAAGAACACCAGTGGCGAAGGCGGCTCACTGGCCCGATACTGACACTGAGGTGCGAAAGCGTGGGGAGCAAACAGG` |
| ASV4880 | 404 | `TGGGGAATATTGGACAATGGGGGAAACCCTGATCCAGCAATGCCGCGTGTGTGATGAAGGCCTTCGGGTTGTAAAGCACTTTCGTCCACGACGATGATGACAGTAGTGGAAGAAGAAGCCCCGGCTAACTTCGTGCCAGCAGCCGCGGTAATACGAAGGGGGCTAGCGTTGCTCGGAATTACTGGGCGTAAAGGGCGCGTAGGCGGCTTAACAAATCAGGTGTGAAAGCCCTGGGCTCAACCCGGGAATTGCATTTGAGACTGTTTCGCTTGGGTATGGGAGAGGAGAGTGGAATTCCTAGTGTAGAGGTGAAATTCGTAGATATTAGGAGGAACACCAGTGGCGAAGGCGACTCTCTGGACCATTACCGACGCTGAGGCGCGAAAGCATGGGGAGCAAACAGG` |
| ASV3645 | 430 | `TGAGGAATTTTGCGCAATGGGCGAAAGCCTGACGCAGCAACGCCGCGTGGGGGATGAAGGCTTTCGGGTTGTAAACCCCTTTCGATCGGAAAGAAACTTTCAATGGTGAATAATCATTGAAACTGACGGTACCGAGAGAAGAAGCCACGGCTAACTCTGTGCCAGCAGCCGCGGTAAGACAGAGGTGGCAAGCGTTGTTCGGATTTACTGGGCGTAAAGCGCGTGTAGGCGGTTTAGTAAGTCAGGCGTGAAATCTTCCGACTTAATCGGGAAACTGCGCTTGATACTGCTTTACTTGAGTGTGGGAGAGGAGAGTGGAATTCCCGGTGTAGCGGTGAAATGCGTAGATATCGGGAGGAACACCAGTGGCGAAGGCGGCTCTCTGGACCACTACTGACGCTGAGACGCGAAAGCGTGGGGAGCAAACAGG` |
| ASV3005 | 429 | `TGGGGAATATTGGACAATGGGGGGAACCCTGATCCAGCAATGCCGCGTGTGTGAAGAAGGCCTGAGGGTTGTAAAGCACTTTCAGTGGGGAGGAGGGATGAGAGATTAAGAGTCACTTATTTGGACGTTACCCACAGAAGAAGCACCGGCTAACTCCGTGCCAGCAGCCGCGGTAATACGGAGGGTGCGAGCGTTAATCGGAATTACTGGGCGTAAAGAGTGCGTAGGTGGTTTTTTAAGTTATCTGTAAAATCCCCGGGCTCAACCTGGGAAGGCCAGATAATACTGGAGAACTCGAGTATTGGAGAGGGTAGTGGAATTTCCGGTGTAGCGGTGAAATGCGTAGAGATCGGAAGGAACACCAGTGGCGAAGGCGGCTACCTGGCCAAATACTGACACTGAGGCACGAAAGCGTGGGGAGCAAACAGG` |
| ASV3322 | 424 | `TGAGGAATATTGGTCAATGGGCGCAAGCCTGAACCAGCCATGCCGCGTGCAGGATGAAGCATCTATGGTGTGTAAACTGCTTTTATACAGGAAGAAACACTCCCTCGTGAGGGAGCTTGACGGTACTGTAGGAATAAGGATCGGCTAACTCCGTGCCAGCAGCCGCGGTAATACGGAGGATCCAAGCGTTATCCGGAATCATTGGGTTTAAAGGGTCCGTAGGCGGCCCTGTAAGTCAGTGGTGAAATCTCCCCGCTCAACGGGGAAACGGCCATTGATACTGCAGGGCTTGAATTATTGGGAAGTAACTAGAATATGTAGTGTAGCGGTGAAATGCTTAGATATTACATGGAATACCAATTGCGAAGGCAGGTTACTATCAATACATTGACGCTGATGGACGAAAGCGTGGGTAGCGAACAGG` |
| ASV5720 | 428 | `TTTCGAATCATTCACAATGGGCGAAAGCCTGATGGTGCGACGCCGCGTGAGGGATGAAGGTCTTCGGATTGTAAACCTCTGTCACCGGGGACGAAACTTTTTGGTTTAACAGACTTAAAATTGACTTAACCCGGAGAGGAAGCAGTGGCTAACTCTGTGCCAGCAGCCGCGGTAATACAGAGACTGCAAGCGTTATTCGGATTTACTGGGCGTAAAGGGTGCGCAGGCGGTTTTGTGTGTGATATGTGAAATCCCAAGGCTCAACCTTGGAATTGCATCTCAAACTACAAGACTAGAGCATTGGAAAGGATAGCAGAATTCATGGTGTAGCAGTGAAATGCGTAGATATCATGAGGAATACCAGAGGCGAAGGCGGCTATCTAGACAATTGCTGACGCTCAGGCACGAAAGCGTGGGGAGCAAAAGGG` |
| ASV4502 | 429 | `TGGGGAATTTTGGACAATGGGCGCAAGCCTGATCCAGCAATACCGCGTGCAGGAAGAAGGCCTTCGGGTTGTAAACTGCTTTTGTACGGAGCGAAAAGGTCTTTCCTAATACGAGAGGCTGATGACGGTACCGTAAGAATAAGCACCGGCTAACTACGTGCCAGCAGCCGCGGTAATACGTAGGGTGCAAGCGTTAATCGGAATTACTGGGCGTAAAGCGTGCGCAGGCGGTTTTGTAAGACAGGCGTGAAATCCCCGGGCTCAACCTGGGAATGGCGCTTGTGACTGCAAAGCTGGAGTGCGGCAGAGGGGGATGGAATTTTGCGTGTAGCAGTGAAATGCGTAGATATGCAAAGGAACACCGATGGCGAAGGCAATCCCCTGGGCCTGCACTGACGCTCATGCACGAAAGCGTGGGGAGCAAACAGG` |
| ASV4404 | 429 | `TGGGGAATCTTGGGCAATGGGCGAAAGCCTGACCCAGCCATGCCGCGTGGGTGAAGAAGGCCTTCGGGTTGTAAAGCCCTTTCGGGAGGGACAAAGGGGTGGGCGTGAATAACGCTTATTTTTGATGGTACCTCCAAAAGCAGCACCGGCTAACTCTGTGCCAGCAGCCGCGGTAATACAGAGGGTGCAAGCGTTAATCGGAATTACTGGGCGTAAAGCGTGCGTAGGTGGTTGAAGAAGTGGACTGTGAAAGCCCTGGGCTTAACCTGGGAATGGCAGTTCAAACTGCTGAACTCGAGTCTGAAAGAGGAGAGTGGAATTTCCGGTGTAGCGGTGAAATGCGTAGAGATCGGAAAGAACATCCGTGGCGAAGGCGGCTCTCTGGTTCAAGACTGACACTGAGGTACGAAAGCGTGGGGAGCAAACAGG` |
| ASV1190 | 429 | `TGGGGAATATTGCGCAATGGACGAAAGTCTGACGCAGCGACGCCGCGTGAGGGATGAAGGTCTTCGGATCGTAAACCTCTGTCAGAGGGGAAGAAACTACTTGGTGCTAATCAGCCAGGTACTGACGGTACCCTCAAAGGAAGCACCGGCTAACTCCGTGCCAGCAGCCGCGGTAATACGGAGGGTGCAAGCGTTAATCGGAATCACTGGGCGTAAAGCGCTCGTAGGCTGTTTGTTAAGTCAGAGGTGAAATCCCACGGCTCAACCGTGGAACTGCCTTTGATACTGGCAGACTAGAATCCGGGAGAGGGTGGTGGAATTCCAGGTGTAGGAGTGAAATCCGTAGATATCTGGAGGAACATCAGTGGCGAAGGCGACCACCTGGATCGGTATTGACGCTGAGGAGCGAAAGCGTGGGGAGCAAACAGG` |
| ASV411 | 404 | `TGGGGAATATTGGACAATGGGCGAAAGCCTGATCCAGCAATGCCGCGTGAGTGATGAAGGCCTTAGGGTTGTAAAGCTCTTTTACCAGGGATGATAATGACAGTACCTGGAGAATAAGCTCCGGCTAACTCCGTGCCAGCAGCCGCGGTAATACGGAGGGAGCTAGCGTTGTTCGGAATTACTGGGCGTAAAGCGCACGTAGGCGGCTATTCAAGTCAGAGGTGAAAGCCCGGGGCTCAACCCCGGAACTGCCTTTGAAACTAGATAGCTAGAATCTTGGAGAGGTCAGTGGAATTCCGAGTGTAGAGGTGAAATTCGTAGATATTCGGAAGAACACCAGTGGCGAAGGCGACTGACTGGACAAGTATTGACGCTGAGGTGCGAAAGCGTGGGGAGCAAACAGG` |
| ASV982 | 429 | `TGGGGAATATTGGACAATGGGCGAAAGCCTGATCCAGCCATGCCGCGTGTGTGAAGAAGGTCTTCGGATTGTAAAGCACTTTAAGTTGGGAGGAAGGGCAGTTACCTAATACGTGATTGTTTTGACGTTACCGACAGAATAAGCACCGGCTAACTCTGTGCCAGCAGCCGCGGTAATACAGAGGGTGCAAGCGTTAATCGGAATTACTGGGCGTAAAGCGCGCGTAGGTGGTTTGTTAAGTTGGATGTGAAATCCCCGGGCTCAACCTGGGAACTGCATTCAAAACTGACTGACTAGAGTATGGTAGAGGGTGGTGGAATTTCCTGTGTAGCGGTGAAATGCGTAGATATAGGAAGGAACACCAGTGGCGAAGGCGACCACCTGGACTAATACTGACACTGAGGTGCGAAAGCGTGGGGAGCAAACAGG` |
| ASV4151 | 418 | `TAGGGAATTTTGCGCAATGGGGGAAACCCTGACGCAGCAACGCCATGTGAGGGATGAAGCATTTCGGTGTGTAAACCTCTGTCAGTAGGGAATAAATGGTCGCAAGACAGTTGAATGTACCTACAAAGGAAGCCCCGGCTAACTTCGTGCCAGCAGCCGCGGTAATACGAGGGGGGCAAGTGTTGTTCGGAATCACTGGGCGTAAAGGGAGCGTAGGCGGGAATTTAAGTTGGATGTTTAAACCTGGGGCCCAACCCCAGCTCGGCATCCAAAACTGGATTTCTTGAATAGTGGAGAGGTAGATGGAATTTCCGGTGTAGCGGTGGAATGCGTAGATATCGGAAAGAACACCAGTTGCGAAGGCGATCTACTGGACACATATTGACGCTGAGGCTCGAAAGCATGGGGAGCAAACAGG` |
| ASV3153 | 422 | `TGGGGAATATTGGGCAATGGGCGGAAGCCTGACCCAGCCATGCCGCGTGCAGGAAGAAGGTCCACTGGATTGTAAACTGCTTTTCTTTGGGGATAAGGCGTCATTAGATGGCGATTGAAGGTACCAAGGGAATAAGCACCGGCAAACTACGTGCCAGCAGCCGCGGTAATACGTAGGGTGCAAGCGTTGTCCGGATTTATTGGGTTTAAAGGGTGCGTAGGCGGTTTAGTAAGTCGTAGATGAAATGTTTGGGCTCAACCTAGGCACTGTTTACGATACTGCTAGACTTGAATAAGCTTGAGGTGGCTAGAATGAGTGGTGTAGCGGTGAAATGCGTAGAGATCACTCAGAATACCAATTGCGAAGGCAGGTCACTGGAGCTATATTGACGCTGAGGCACGAAAGCGTGGGGAGCAAACAGG` |
| ASV978 | 423 | `TAGGGAATATTGGTCAATGGGGGCAACCCTGAACCAGCCATGCCGCGTGCAGGAAGAAGGCCTTCTGGGTTGTAAACTGCTTTTGACAGGGAACAAATGCCATTTGCGAATGGAGCTGAGTGTACCTGTAGAATAAGCCACGGCTAACTACGTGCCAGCAGCCGCGGTAATACGTAGGTGGCAAGCGTTGTCCGGATTTATTGGGTTTAAAGGGTGCGTAGGCGGCCTCTTAAGTCAGCGGTGAAATCCTTCAGCTTAACTGAAGACGTGCCATTGATACTGGGAGGCTTGAATATAGTTGAGGTGGGCGGAATTGACGGTGTAGCGGTGAAATGCATAGATATCGTCAAGAACACCTATTGCGAAGGCAGCTCACTAAGCTATAATTGACGCTGAGGCACGAAAGTGTGGGGATCAAACAGG` |
| ASV600 | 430 | `TCGAGGATCATTCGCAATGGACGAAAGTCTGACGATGCGACGCTGCGTGAAGGATGAAGGTCTTCGGATTGTAAACTTCTGTCATGTGGGAAAAGGCAATCATCAATAACATTGGTGTGCGTTGATGGTACCACAAGAGGAAGCCACGGCTAACTCTGTGCCAGCAGCCGCGGTAATACAGAGGTGGCAAGCGTTGTTCGGATTTACTGGGCGTAAAGGGCGCGTAGGCGGTTTCGTATGTCGGGTGTGAAAGCCCCAGGCTCAACCTGGGAAGTGCGCCCGAAACTGCGAAGCTTTGAGTACGGAAGGGGGATGCGGAATGTAGGGTGTAGCGGTGAAATGCGTTGATATCCTACAGAACACCGGAGGCGAAGGCGGCATCCTGGGACGTAACTGACGCTGAGGCGCGAAAGCAGGGGGAGCAAACAGG` |
| ASV792 | 423 | `TGAGGAATTTTGCGCAATGGACGAAAGTCTGACGCAGCGACGCCGCGTGTGGGATGAAGGGGTTATCGCCTTGTAAACCACTGTCGGATGGGAATAAAAGTTTGCTTTGCGGACTGGGAATGTACCATCAAAGGAAGGACCGGCTAACTACGTGCCAGCAGCCGCGGTAATACGTAGGGTCCGAGCGTTATCCGGAATTACTGGGTGTAAAGGGTGCGTAGGCGGGCTTTTGCGTCGAGGGTTAAAGACCACAGCTCAACTGTGGGAGTGCCCTCGAAACGAGAAGTCTTGAATGTGAGAGAGGTAGGTAGAATTCCTGGTGTAGCAGTGAAATGCGTAGATATCAGGAGGAATACCAGTGGCGAAGGCGGCCTACTGGCTCATTATTGACGCTGAGGCACGAAAGTGTGGGTAGCGAACAGG` |
| ASV5044 | 429 | `TGGGGAATATTGGACAATGGGCGCAAGCCTGATCCAGCAATGCCGCGTGTGTGAAGAAGGCCTGCGGGTTGTAAAGCACTTTCAGTGGGGAGGAAGGTCTTGGTATTAATACTGCCAAGAATTGACGTTACCCAAAGAAGAAGCACCGGCTAACTCTGTGCCAGCAGCCGCGGTAATACAGAGGGTGCAAGCGTTAATCGGAATTACTGGGCGTAAAGGGCGCGTAGGTGGTTGACTAAGTCAGATGTGAAATCCCTGGGCTTAACCTAGGAATTGCGTTTGATACTGGTTGACTAGAGTGTGGGAGAGGGAAGTGGAATTTCCAGTGTAGCGGTGAAATGCGTAGATATTGGAAGGAACACCAGTGGCGAAGGCGACTTCCTGGACCACTACTGACACTGAGGCGCGAAAGCGTGGGGAGCGAACAGG` |
| ASV2113 | 429 | `TGGGGAATATTGGACAATGGGGGCAACCCTGATCCAGCAATGCCGCGTGTGTGAAGAAGGCCTGCGGGTTGTAAAGCACTTTCAGTAGGGAAGAAAAGCCTGGGACTAATACTCTCGGGTCTTGACGTTACCTAAAGAAGAAGCACCGGCTAACTCCGTGCCAGCAGCCGCGGTAATACGGAGGGTGCGAGCGTTAATCGGAATTACTGGGCGTAAAGCGCACGTAGGCGGTTAGATAAGTCGGATGTGAAAGCCCTGGGCTTAACCTGGGAACTGCATTCGATACTGTCTGGCTAGAGTATGGGAGAGGGAAGCAGAATTTCCGGTGTAGCGGTGAAATGCGTAGATATCGGAAGGAATACCAATGGCGAAGGCAGCTTCCTGGCCCAATACTGACGCTCAGGTGCGAAAGCGTGGGGATCAAACAGG` |
| ASV2821 | 424 | `TAGGGAATATTGGGCAATGGGCGAGAGCCTGACCCAGCCATGCCGCGTGCAGGACGAAGGCCCTCTGGGTTGTAAACTGCTTTTTTGGGGGAAGAATAGCATTCCTGCGGGGGTGTATGACGGTACCCTAAGAATAAGCACCGGCTAACTCCGTGCCAGCAGCCGCGGTAATACGGAGGGTGCAAGCGTTGTCCGGATTTATTGGGTCTAAAGGGTGCGTAGGTGGGTGAGTAAGTCTGGTTTGAAAGTTGGTCGCTTAACGATCAAGGGTGGCTGGATACTGTTCATCTTGAATATTTTGGAGGTAGCTGGAATGGGTTGTGTAGCGGTGAAATGCATAGAGATGACCCGGAACTCCAATTGCGTAGGCAGGCTACTACGAAATGATTGACACTGAGGCACGAGAGCATGGGTAGCAAACAGG` |
| ASV3372 | 429 | `TGGGGAATATTGCGCAATGGAGGAAACTCTGACGCAGCGACGCCGCGTGGGTGATGAAGGCCTTCGGGTTGTAAAGCCCTGTCGGCTGGGAAAAAGGTAGTTGCACTAATACTGCAATTGCTTGATGGTACCAGCAAAGGAAGCACCGGCTAACCCTGTGCCAGCAGCCGCGGTAATACAGGGGGTGCTAGCGTTGTTCGGAATTACTGGGCGTAAAGGGCGCGTAGGCGGTTTAGCAAGTCAGATGTGAAAGCCCGGGGCTTAACTCCGGAAGTGCATTTGAAACTGCTAGGCTAGAGTACAGTAGAGGTTATTGGAATTCCCAGTGTAGAGGTGAAATTCGTAGATATTGGGAGGAACACCAGTGGCGAAGGCGGATAACTGGGCTGTAACTGACGCTGAGGCGCGAAAGCGTGGGGATCAAACAGG` |
| ASV4002 | 429 | `TGGGGAATTTTGGACAATGGGGGCAACCCTGATCCAGCCATGCCGCGTGCGGGAAGAAGGCCTTCGGGTTGTAAACCGCTTTTGTCAGGGAAGAAAAGACTCCTACTAATACTGGGGGTTCATGACGGTACCTGAAGAATAAGCACCGGCTAACTACGTGCCAGCAGCCGCGGTAATACGTAGGGTGCAAGCGTTAATCGGAATTACTGGGCGTAAAGCGTGCGCAGGCGGTTATGCAAGACAGATGTGAAATCCCCGGGCTCAACCTGGGAACTGCATTTGTGACTGCATGGCTAGAGTACGGTAGAGGGGGATGGAATTCCGCGTGTAGCAGTGAAATGCGTAGATATGCGGAGGAACACCGATGGCGAAGGCAATCCCCTGGACCTGTACTGACGCTCATGCACGAAAGCGTGGGGAGCAAACAGG` |
| ASV967 | 424 | `TAGGGAATATTGGGCAATGGATGCAAGTCTGACCCAGCCATGCCGCGTGCAGGAAGAAGGTCCTCTGGATTGTAAACTGCTTTTATCGGGGAAGAATAGGTTTCTTGCGAGGGACTGTGACGGTACCCGATGAATAAGCCACGGCTAACTACGTGCCAGCAGCCGCGGTAATACGTAGGTGGCAAGCGTTGTCCGGATTTATTGGGTTTAAAGGGTGCGTAGGTGGTTTTATAAGTCAGTGGTGAAAGCTGGTTGCTCAACAATCAAGTTGCCATTGATACTGTTAGACTTGAGAGAAGTGGAGGTTCATGGAATGGATGGTGTAGCGGTGAAATGCATAGATATCATCCAGAACGTCGATTGCGAAGGCAGTGGGCTGTACTTTTTCTGACACTGAGGCACGAAAGCGTGGGGATCAAACAGG` |
| ASV2802 | 429 | `TGGGGAATTTTGGACAATGGGGGCAACCCTGATCCAGCCATGCCGCGTGAGTGAAGAAGGCCTTCGGGTTGTAAAGCTCTTTCGGACGGAACGAAATGGTTACGGTGAATATCCGTGACTGATGACGGTACTGTAAGAAGAAGCACCGGCTAACTACGTGCCAGCAGCCGCGGTAATACGTAGGGTGCGAGCGTTAATCGGAATTACTGGGCGTAAAGCGTGCGCAGGCGGCCGCGCAAGTCAGGTGTGAAATCCCCGGGCTTAACCTGGGAATTGCGCTTGAAACTATGCGGCTTGAGTGTGGCAGAGGGGGGTGGAATTCCACGTGTAGCAGTGAAATGCGTAGAGATGTGGAGGAACACCGATGGCGAAGGCAGCCCCCTGGGCCAACACTGACGCTCAGGCACGAAAGCGTGGGGAGCAAACAGG` |
| ASV1684 | 406 | `TGGGGAATCTTGCGCAATGGGCGAAAGCCTGACGCAGCAACGCCGCGTGCGGGATGACGGCCTTCGTGGTTGTAAACCGCTTTCAGCAGGGACGAAAATGACGGTACCTGCAGAAGAAGCCCCGGCTAACTACGTGCCAGCAGCCGCGGTAATACGTAGGGGGCAAGCGTTGTCCGGAATCATTGGGCGTAAAGAGCTCGTAGGCGGTTCAGTAAGTCGGGTGTGAAAGCTCCGAGCTCAACTCGGAGAGGCCACTCGATACTGCTGTGACTAGAATCCAGTAGGGGAGTGTGGAACTCCTGGTGTAGCGGTGAAATGCGCAGATATCAGGAAGAACACCAGCGGCGAAGGCGGCACTCTGGGCTGGTATTGACGCTGAGGAGCGAAAGTGTGGGTAGCAAACAGG` |
| ASV495 | 404 | `TGGGGAATATTGGACAATGGGCGAAAGCCTGATCCAGCAATGCCGCGTGAGTGATGAAGGCCTTCGGGTCGTAAAGCTCTTTTGGTGGGGAAAATAATGATGGTACCCACAGAATAAGCTCCGGCTAACTTCGTGCCAGCAGCCGCGGTAATACGAAGGGAGCTAGCGTTGTTCGGAATTACTGGGCGTAAAGAGTACGTAGGCGGTTTTTTAAGTTAGGTGTGAAAGCCCCGGGCTCAACCCGGGAATTGCACTTAAAACTGAAAAGCTTGAGATTGGGAGAGGAAAGTGGAATTACTAGTGTAGAGGTGAAATTCGTAGATATTAGTAAGAACACCAGTGGCGAAGGCGACTTTCTGGACCAATACTGACGCTGAGGTACGAAAGCGTGGGGAGCAAACAGG` |
| ASV5432 | 429 | `TGGGGAATTTTGGACAATGGGCGCAAGCCTGATCCAGCCATGCCGCGTGAGTGAAGAAGGCCTTCGGGTTGTAAAGCTCTTTCGGTGGGGAAGAAATGGTTTCGGTTAATACCCGGAACTGATGACGGTACCCGCATAAGAAGCACCGGCTAACTACGTGCCAGCAGCCGCGGTAATACGTAGGGTGCGAGCGTTAATCGGAATTACTGGGCGTAAAGCGTGCGCAGGCGGTTGTATAAGTCAGATGTGAAATCCCCGGGCTCAACCTGGGAACTGCATTTGAGACTGTACGGCTAGAGTGTGGCAGAGGGGGGTAGAATTCCACGTGTAGCAGTGAAATGCGTAGAGATGTGGAGGAATACCGATGGCGAAGGCAGCCCCCTGGGCCAATACTGACGCTCATGCACGAAAGCGTGGGGAGCAAACAGG` |
| ASV4076 | 426 | `TTAAGAATCTTGCGCAATGGGGGAAACCCTGACGCAGCGACGCCGCGTGAAGGATGAAGGCTTTCGGGTTGTAAACTTCGGTAGACTGGGAAGAAGAAATACGTTCGTTGAGGGCGTATAGTGACGGTACCAGTCTGAAGCCCCGGCTAATTACGTGCCAGCAGCCGCGGTAATACGTATGGGGCAAGCGTTGTTCGGAATTATTGGGCGTAAAGGGCTCGCAGGTGGTTTGTTAAGTTGGTGGTTTAATCTCTGGGCTCAACCCAGAGTCAGCCATCAAAACTGGCGAACTTGAGTACGATAGGGGATAGCGGAATTCTCGGTGTAGCGGTGGAATGCGTAGATATCGAGAGGAACACCAATGGCGAAGGCAGCTATCTGGATCGTAACTGACACTCATGAGCGAAAGTGCGGGGAGCAAACAGG` |
| ASV1092 | 406 | `TAGGGAATATTGCACAATGGAGGAAACTCTGATGCAGCGACGCCGCGTGAGTGATGAAGGACTTCGGTTCGTAAAGCTCTGTTGCAAGGGAAAAAGTTCTGATTGTACCTTGTGAGGAAGGGTCGGCTAACTTCGTGCCAGCAGCCGCGGTAAGACGAGGGACCCTAGCGTTGTTCGGAATCATTGGGCGTAAAGAGGGCGTAGGTGGCTTTGTAAGTCAGGTGTGAAATCCCAGGGCTCAACCCTGGACGTGCATTTGATACTGCGAAGCTTGAGTGTTGGAGAGGTTACTGGAATACCTGGTGTAGTGGTGAAATACGTAGATATCAGGTGGAACACCAGAGGCGAAGGCGGGTAACTGGCCAAACACTGACGCTGAGGCCCGAAAGCGTGGGGATCAAACAGG` |
| ASV4751 | 404 | `TGGGGAATCTTGGACAATGGGCGCAAGCCTGATCCAGCCATGCCGCGTGAATGATGAAGGCCTTAGGGTTGTAAAATTCTTTCGCCGGGGACGATAATGACGGTACCCGGAGAAGAAGCCCCGGCTAACTTCGTGCCAGCAGCCGCGGTAATACGAAGGGGGCTAGCGTTGTTCGGAATTACTGGGCGTAAAGCGCGTGTAGGCGGATTTTTAAGTCGGGGGTGAAATCCCAGAGCTCAACTCTGGAACTGCCTTCGATACTGGAAGTCTGGAGCAAGATAGAGGCGGGTGGAATTGCGAGTGTAGAGGTGAAATTCGTAGATATTCGCAGGAACACCAGTGGCGAAGGCGACTCGCTGGATCTTTGCTGACGCTGAGACGCGAAAGCGTGGGTAGCAAACAGG` |
| ASV5100 | 430 | `TGGGGAATCTTGCGCAATGGACGAAAGTCTGACGCAGCAACGCCGCGTGAGTGAAGAAGGCCTTTGGGTTGTAAAGCTCTTTCGACTGGAAAGAAGGGTTTCGTTGCGAATAGTTACGGAACTTGACGGTACCAGGAGAAGAAGTCCCGGCTAACTTCGTGCCAGCAGCCGCGGTAATACGAAGGGGACAAGCGTTGTTCGGAATCATTGGGCGTAAAGGGCGCGTAGGCGGACTTGTAAGTCGATTGTGAAATCCCTGGGCTTAACCCGGGAATTGCAGTCGAAACTGCAAGTCTTGAGTACTGGAGAGGATGGTGGAATTCCTGGAGTAGTAGTGAAATACGTAGATATCAGGAGGAACACCGGAGGCGAAGGCGGCCATCTGGCCAGTCACTGACGCTGAGGCGCGAAAGCATGGGGAGCAAACAGG` |
| ASV3545 | 407 | `TGGGGAATTTTCCGCAATGGGCGCAAGCCTGACGGAGCAATACCGCGTGAGGGAGGAAGGCTCTTGGGTTGTAAACCTCTTTTCTCTGGGAAGAACACAATGACGGTACCAGAGGAATCAGCATCGGCTAACTCCGTGCCAGCAGCCGCGGTAATACGGAGGATGCAAGCGTTATCCGGAATTATTGGGCGTAAAGCGTCCGCAGATGGTTCTACAAGTCTATTGTCAAAGCGCGGGGCTTAACCTCGTACAGGCGGTGGAAACTGTAAGACTAGAGTTCGGTAGGGGTCAGGGGAATTCCCAGTGTAGCGGTGAAATGCGTAGATATTGGGAAGAACACCAGCGGCGAAAGCGCCTGACTGGGCCTGAACTGACATTCATGGACGAAAGCTAGGGGAGCGAAAGGG` |
| ASV102 | 404 | `TGGGGAATCTTAGACAATGGGCGCAAGCCTGATCTAGCCATGCCGCGTGAGCGATGAAGGCCTTAGGGTTGTAAAGCTCTTTCGTGGGGGAAGATAATGACTGTACCCCAAGAAGAAGCCCCGGCTAACTCCGTGCCAGCAGCCGCGGTAATACGGAGGGGGCTAGCGTTGTTCGGAATTACTGGGCGTAAAGCGCACGTAGGCGGACCGGAAAGTCAGAGGTGAAATCCCAGGGCTCAACCTTGGAACTGCCTTTGAAACTCCTGGTCTTGAGGTCGTGAGAGGTGAGTGGAATTCCGAGTGTAGAGGTGAAATTCGTAGATATTCGGAGGAACACCAGTGGCGAAGGCGGCTCACTGGCACGATACTGACGCTGAGGTGCGAAAGCGTGGGGAGCAAACAGG` |
| ASV4965 | 388 | `GCGCGAAAACTTTACAATGCTGGCAACAGCGATAAGGGAACCTCGAGTGCCAGGTTACAAATCTGGCTGTCGAGATGCCTAAAAAGCATTTCATAGCAAGGGCCGGGCAAGACCGGTGCCAGCCGCCGCGGTAACACCGGCGGCTCGAGTGGTAACCGTTATTATTGGGTCTAAAGGGTCTGTAGCCGGCCGGATAAGTCTCTTGAGAAATCTGGCAGCTTAACTGTCAGGCTTTCAGGAGATACTGTCTGGCTCGAGGCCGGGAGAGGTGAGAGGTACTTCAGGGGTAGGGGTGAAATCTTGTAATCCTTGAAGGACCACCAGTGGCGAAGGCGTCTCACCAGAACGGACCTGACGGCAAGGGACGAAAGCTAGGGGCACGAACCGG` |
| ASV42 | 424 | `TGAGGAATATTGGTCAATGGGCGCAAGCCTGAACCAGCCATGCCGCGTGCAGGAAGACGGCTCTATGAGTTGTAAACTGCTTTTATACGAGGGTAAACTCGGGTACGTGTACCCGATTGAAAGTATCGTAAGAATAAGGATCGGCTAACTCCGTGCCAGCAGCCGCGGTAATACGGAGGATTCGAGCGTTATCCGGATTTATTGGGTTTAAAGGGTGCGTAGGTGGTTTTATAAGTTAGTCGTTAAATCGGGAGGCTCAACCTTTCAGTGGCGATTAATACTGTAGGACTAGAGAACAGATGCTGTGGGCGGAATGTGTAGTGTAGCGGTGAAATGCTTAGATATTACACAGAACACCAATTGCGAAGGCAGCTCACAAAACTGTTTCTGACACTGAGGCACGAAAGCGTGGGGATCAAACAGG` |
| ASV2686 | 405 | `TAGGGAATTTTGCGCAATGGGCGAAAGCCTGACGCAGCAACGCCGCGTGAATGATGAAGCCCTTCGGGGTGTAAAATTCTGTCAGTGGGGACGAATTTTGACGGTACCCACAGAGGAAGCACCGGCTAACTCCGTGCCAGCAGCCGCGGTAATACGGAGGGTGCAAGCGTTGTCCGGAATCATTGGGCGTAAAGAGTTCGTAGGCGGTATGTAAAGTCCGGTGTTAAATCCCGAAGCTCAACTTCGGCATGGCACTGGATACTTGCAAACTAGAATGCGGTAGAGGTAAAGGGAATTCCTGGTGTAGCGGTGAAATGCGTAGATATCAGGAGGAACATCGGTGGCGAAAGCGCTTTACCGGGCCGTTATTGACGCTGAGGAACGAAAGCCGGGGTAGCAAATGGG` |
| ASV4685 | 429 | `TAGGGAATCTTCCACAATGGACGAAAGTCTGATGGAGCAACGCCGCGTGAGCGATGAAGGCCTTCGGGTCGTAAAGCTCTGTTGTTAGGGAAGAACAAGTACCGGAGTAACTGCCGGTACCTTGACGGTACCTAACCAGAAAGCCACGGCTAACTACGTGCCAGCAGCCGCGGTAATACGTAGGTGGCAAGCGTTGTCCGGAATTATTGGGCGTAAAGCGCGCGCAGGCGGTCCTTTAAGTCTGATGTGAAAGCCCACGGCTCAACCGTGGAGGGTCATTGGAAACTGGGGGACTTGAGTGCAGAAGAGGAAAGCGGAATTCCACGTGTAGCGGTGAAATGCGTAGAGATGTGGAGGAACACCAGTGGCGAAGGCGGCTTTCTGGTCTGTAACTGACGCTGAGGCGCGAAAGCGTGGGGAGCAAACAGG` |
| ASV4903 | 430 | `TGGGGAATATTGCGCAATGGGCGAAAGCCTGACGCAGCAACGCCGCGTGAGCGATGAAGGCCTTCGGGTTGTAAAGCTCTTTCGGTTGGGAAGAAGGGTGGAGTGGTGAATAACCATTTCATTTGATGGTACCAAAAGAAGAAGCACCGGCTAACTTCGTGCCAGCAGCCGCGGTAATACGAAGGGTGCAAGCGTTGTTCGGAATTACTGGGCGTAAAGGGTTCGTAGGCGGGAATCTAAGTCAAGTGTGAAATCCCCAGGCTCAACCTGGGACGTGCACCTGAAACTGGATTTCTTGAGTACTAGAGAGGGTGGTGGAATTGCTGGTGTAGGAGTGACATCCGTAGAGATCAGCAGGAACACCGGAGGCGAAGGCGACCACCTGGCTAGACACTGACGCTGAGGAACGAAAGCGTGGGGAGCAAACAGG` |
| ASV1349 | 429 | `TGGGGAATTTTGGACAATGGGCGCAAGCCTGATCCAGCCATGCCGCGTGCGGGAAGAAGGCCTTCGGGTTGTAAACCGCTTTTGTCAGGGAAGAAACGGCTCTGGATAATACCTGGAGCTAATGACGGTACCTGAAGAATAAGCACCGGCTAACTACGTGCCAGCAGCCGCGGTAATACGTAGGGTGCAAGCGTTAATCGGAATTACTGGGCGTAAAGCGTGCGCAGGCGGTCTTGCAAGACAGATGTGAAATCCCCGGGCTCAACCTGGGAACTGCATTTGTGACTGCAAGGCTAGAGTACGGTAGAGGGGGATGGAATTCCGCGTGTAGCAGTGAAATGCGTAGATATGCGGAGGAACACCGATGGCGAAGGCAATCCCCTGGACCTGTACTGACGCTCATGCACGAAAGCGTGGGGAGCAAACAGG` |
| ASV4159 | 428 | `TCGAGAATTTTTCTCAATGGACGAAAGTCTGAAGGAGCGACGCCGCGTGGGGGATGAAGGGCTTCGGTCCGTAAACCCCTGTCATTTGCGAACAAACCTCGTTATTGAATATTTGACGAGCTGATTGTAGCGAAAGAGGAAGGGACGGCTAACTCTGTGCCAGCAGCCGCGGTAATACAGAGGTCCCAAGCGTTGTTCGGATTCACTGGGCGTAAAGGGTGCGTAGGCGGTGAGGTAAGTCTGGTGTGAAAGCTCGGAGCTCAACTCCGAAATGTCATCGGAAACTACTTCACTAGAGGTTTGGAGGGGGGACTGGAATTCTCGGTGTAGCAGTGAAATGCGTAGATATCGAGAGGAACACCAGTGGCGAAGGCGAGTCCCTGGACAAATCCTGACGCTGAGGCACGAAAGCTAGGGGAGCAAACAGG` |
| ASV986 | 429 | `TGGGGAATATTGGACAATGGGCGCAAGCCTGATCCAGCAATGCCGCGTGTGTGAAGAAGGCCTTAGGGTTGTAAAGCACTTTCAGAGGGGAGGAAAAGCTCATACTTAATAAGTGTGAGAAAAGACGTTACCTTCAGAAGAAGCACCGGCTAACTCTGTGCCAGCAGCCGCGGTAATACAGAGGGTGCAAGCGTTAATCGGAATTATTGGGCGTAAAGCGTGTGTAGGTGGTATAACAAGTCAGTCGTGAAAACCCCGGGCTCAACCTGGGAACTGCGATTGAAACTGTTGAACTAGAGTGTGGAAGAGGGAAGTGGAATTTCCGGTGTAGCGGTGAAATGCGTAGATATCGGAAGGAACATCAGTGGCGAAGGCGACTTCCTGGTCCAACACTGACACTCAGACACGAAAGCGTGGGGAGCAAACAGG` |
| ASV1975 | 430 | `TAGGGAATATTGCGCAATGGGGGAAACCCTGACGCAGCAACGCCGCGTGAGTGAGGAAGGTCTTCGGATTGTAAAACTCTTTTGCTAGGGAAAAATGATCTAGTGGCTAATATCCATTAGAAGTGATGGTACCTAAAGAATAAGGACCGGCTAACTTCGTGCCAGCAGCCGCGGTAATACGAAGGGTCCAAGCGTTGTTCGGAATCATTGGGCGTAAAGCGCGCGCAGGCGGGTCAGCAAGTCAGATGTGAAATCTCGAAGCTCAACTTCGAAACTGCGTCTGAAACTGCTAGTCTAGAGTGTCGGAGGGGGTAGGGGAATTTCACGTGTAGGGGTAAAATCCGTAGAGATGTGAAGGAACACCGGGGGCGAAGGCGCCTACCTGGACGACTACTGACGCTGAGGCGCGAAAGCGTGGGGAGCAAACAGG` |
| ASV2089 | 404 | `TGGGGAATATTGGACAATGGGCGCAAGCCTGATCCAGCCATGCCGCGTGAGTGATGAAGGCCTTCGGGTTGTAAAGCTCTTTTGTCCGGGAAGATAATGACTGTACCGGAAGAATAAGCCCCGGCAAACTTCGTGCCAGCAGCCGCGGTAATACGAAGGGGGCTAGCGTTGTTCGGAATCACTGGGCGTAAAGCGCACGTAGGCGGCTTGTTAAGTCAGAGGTGAAATCCTGGAGCTCAACTCCAGAACTGCCTTTGATACTGGCGAGCTCGAGTATGACAGAGGTTGGTGGAACTCCGAGTGTAGAGGTGAAATTCGTAGATATTCGGAAGAACACCAGTGGCGAAGGCGGCCAACTGGGTCATTACTGACGCTGAGGTGCGAAAGCGTGGGGAGCAAACAGG` |
| ASV3046 | 404 | `TGGGGAATATTGGACAATGGGCGCAAGCCTGATCCAGCAATGCCGCGTGAGTGATGAAGGCCTTCGGGTTGTAAAGCTCTTTCGCACGCGACGATGATGACGGTAGCGTGAGAAGAAGCCCCGGCTAACTTCGTGCCAGCAGCCGCGGTAATACGAAGGGGGCAAGCGTTGTTCGGAATTACTGGGCGTAAAGGGCGCGTAGGCGGCTCTCTAAGTCAGGCGTGAAAGCCCCGGGCTCAACCTGGGAACTGCGCTTGAAACTGGCGGGCTTGAGTTCGGGAGAGGTGAGTGGAATTCCCAGTGTAGAGGTGAAATTCGTAGATATTGGGAAGAACACCAGTGGCGAAGGCGGCTCACTGGACCGACACTGACGCTGAGGCGCGAAAGCGTGGGGAGCAAACAGG` |
| ASV4944 | 429 | `TGGGGAATCTTGCACAATGGGCGAAAGCCTGATGCAGCCATGCCGCGTGTGTGAAGAAGGCCTTCGGGTTGTAAAGCACTTTAAGCGGGGAGAAAAGGGTAGTTGCTAACAACAGCTATCTATGATGTTACCCGCAGAATAAGCACCGGCAAACTTCGTGCCAGCAGCCGCGGTAATACGAAGGGTGCGAGCGTTAATCGGAATTACTGGGCGTAAAGAGCACGTAGGCGGTTTGATAAGCGGAATGTGAAAGCCCCGGGCTTAACCTGGGAACTGCATCCCGAACTGTCAGGCTAGAGTATGATAGAGGTGAGCGGAATTTCCTGTGTAGCGGTGAAATGCGTAGATATAGGAAGGAACATCAGTTGCGAAGGCGGCTCACTGGATCAATACTGACGCTGAGGTGCGAAAGCGTGGGTAGCGAACAGG` |
| ASV2930 | 386 | `GCGCGAAACCTTCGCAATAGGCGAAAGCCTGACGAGGTTAATCCGAGTGTTCCCTGCTAAAGGGATCTTTTGTCAGCTCTAAAAATGCTGGTGAATAAGGGGTGGGCAAGCCTGGTGTCAGCCGCCGCGGTAATACCAGCACCCCGAGTGGTCGGGATGATTATTTGGCCTAAAGCATCCGTAGCCGGTGCAGTAAGTCTTCCGTTAAATCCACCTGCTCAACAGATGGGCTGCGGGAGATACTGCTGCACTAGGAGGCGGGAGAGGCGAGGGGTATTCCATGGGTAGGGGTAAAATCCTCTGATCCATGGAGGACCACCAGTGGCGAAGGCGTCTCGCCAGAACGCGCTCGACGGTGAGGGATGAAAGCTGGGGGAGCAAACCGG` |
| ASV4680 | 405 | `TAAGGAATCTTTCGCAATGGGGGAAACCCTGACGAAGCAACACCGCGTGGAGGATGAAGGCGTTTTCGTTGTAAACTCCTTTTTTGAGGGAATAACCTTGAAGGTACCTCAAGAATAAGCATCGGCTAACTACGTGCCAGCAGCCGCGGTAAGACGTAGGATGCAAGCGTTATCCGGAATTATTGGGCGTAAAGCGTTCGTAGGTGGTTTGTTAAGTAGGGCGTTAAATCTTCGGGCTTAACCCGAAATTGGCGTCCTAAACTGGCTCACTAGAGATAGGTAGAGGTAAGCGGAATTCTGTATGTAGGGGTAATATCCGTAGATATGCAGAGGAACACCAAAAGCGAAAGCAGCTTACTGGACCTGTTCTGACACTCAAGAACGAAAGCGTGGGGAGCAAACGGG` |
| ASV5686 | 405 | `TGAGGAATCTTGCGCAATGGGCGAAAGCCTGACGCAGCAATGCCGCGTGAGTGAAGAAGGTCTTCGGATCGTAAAGCTCTTTATCTCTGAGAAGATGATGACGGTATCAGAGGAAGAAGCACCGGCTAACTACGTGCCAGCAGCCGCGGTAATACGTAGGGTGCGAGCGTTGTTCGGAATTACTGGGCGTAAAGGGTGTGTAGGCGGATAGGTCAGCTAGAGGTGAAATCCCGGGGCTCAACCCCGGAACCGCTTTTAGAACTGCCTAACTAGAGAGGGACAGAGGCTAGCGGAATTCCTGGTGTAGAGGTGAAATTCGTAGATATCAGGAGGAACACCGGCGGCGTAAGCGGCTGGCTGGGTCCACTCTGACGCTGAGGCACGAAAGCGTGGGTAGCAAACGGG` |
| ASV120 | 429 | `TGGGGAATTTTGGACAATGGACGAAAGTCTGATCCAGCCATGCCGCGTGCGGGAAGAAGGCCTTCGGGTTGTAAACCGCTTTTGTCAGGGAAGAAACGGGTTTCTCTAATACAGAGACCTAATGACGGTACCTGAAGAATAAGCACCGGCTAACTACGTGCCAGCAGCCGCGGTAATACGTAGGGTGCAAGCGTTAATCGGAATTACTGGGCGTAAAGCGTGCGCAGGCGGTGATGCAAGACAGATGTGAAATCCCCGGGCTCAACCTGGGAACTGCATTTGTGACTGCATCGCTAGAGTACGGTAGAGGGGGATGGAATTCCGCGTGTAGCAGTGAAATGCGTAGATATGCGGAGGAACACCGATGGCGAAGGCAATCCCCTGGACCTGTACTGACGCTCATGCACGAAAGCGTGGGGAGCAAACAGG` |
| ASV3804 | 409 | `TGGGGAATATTGGGCAATGGAGGAAACTCTGACCCAGCGACGCCGCGTGCGGGATGAAGGCCTTCGGGTTGTAAACCGCTTTCAGCAGGGAAGAAGCGAAAGTGACGGTACCTGCAGAAGAAGCACCGGCTAACTATGTGCCAGCAGCCGCGGTAATACATAGGGTGCAAGCGTTGTCCGGAATTATTGGGCGTAAAGAGCTCGTAGGTGGTTCGTCACGTCGGATGTGAAACTCTGGGGCTTAACCCCAGACCTGCATTCGATACGGGCGAGCTAGAGTGTGGTAGGGGAGACTGGAATTCCTGGTGTAGCGGTGGAATGCGCAGATATCAGGAGGAACACCAATGGCGAAGGCAGGTCTCTGGGCCATTACTGACACTGAGGAGCGAAAGCGTGGGGAGCGAACAGG` |
| ASV4626 | 404 | `TGAGGAATATTGGACAATGGGCGAAAGCCTGATCCAGCAATACCACGTGAATGATGAAGGCCTTAGGGTTGTAAAGTTCTTTTAATAGGGAAGATAATGACGGTACCTATAGAAAAAGCCCCGGCTAACTCCGTGCCAGCAGCCGCGGTAAGACGGAGGGGGCTAGCGTTGTTCGGAATTACTGGGCGTAAAGAGCGCGTAGGCGGATTAGTAAGTTGGAAGTGAAAGCCCAGGGCTTAACCTTGGAAATGCTTTCAAAACTGTTAATCTTGAGTGTAGTAGGGGATGATGGAATTCCTAGTGTAGAGGTGGAATTCTTAGATATTAGGAGGAACACCGGTGGCGAAGGCGGTCATCTGGGCTACAACTGACGCTGAGGCGCGAAAGCGTGGGGAGCAAACAGG` |
| ASV748 | 431 | `TGGGGAATATTGGACAATGGGCGCAAGCCTGATCCAGCAATACCGCGTGTGTGAAGAAGGCCTTCGGGTTGTAAAGCACTTTTGTTAGGGAGGATGGTGATTTTGGCGAATACCCAAAGTCATTGACGTTACCTAAAGAATCAGCACCGGCTAACTCCGTGCCAGCAGCCGCGGTAATACGGAGGGTGCAAGCGTTAATCGGATTTACTGGGCGTAAAGGGTGCGTAGGCGGTTACTTAAGTCAGATGTGAAATCCCCGGGCTCAACCTGGGAATTGCATTTGATACTGGGTGACTTAGAATGTGGTAGAGGGTAGCGGAATTTCTGGTGTAGCGGTGAAATGCGTAGATATCAGAAGGAACATCAATGGCGAAGGCAGCTACCTGGGCCAACATTGACGCTGAGGCACGAAAGCGTGGGGAGCAAACAGG` |
| ASV2761 | 404 | `TGGGGAATATTGGGCAATGGGCGCAAGCCTGATCCAGCAATACCACGTGAGTGATGAAGGCCTTAGGGTTGTAAAACTCTTTTAGTGGGGAAGATAATGACTGTACCCACAGAAAAAGCACCGGCTAACTTCGTGCCAGCAGCCGCGGTAATACGAAGGGTGCTAGCGTTGTTCGGAATCATTGGGCGTAAAGCGTGCGTAGGCGGCTTGACAAGTCAGCTGTGAAAGCCCAGGGCTCAACCTTGGAATTGCAGTTGAAACTGTTTCGCTAGAGTCTGAAGAGGGATAGTGGAATTCTTAGTGTAGGGGTGAAATCCGTAGATATTAAGAGGAACACCGGTGGCGAAAGCGACTATCTATGTCAGTACTGACGCTGTTGCACGAAAGCGTGGGGAGCAAACAGG` |
| ASV3655 | 428 | `TGGGGAATCTTCCGCAATGGACGAAAGTCTGACGGAGCGACGCCGCGTGAGTGATGAAGGCCCTTGGGTCGTAAAGCTCTGTCATTGGGGACGAACGACCGCCTCAGGAAATGGGGGCGGAGTGACGGTACCCAAGAAGGAAGCCACGGCTAACTACGTGCCAGCAGCCGCGGTAATACGTAGGTGGCAAGCGTTGTCCGGAATTATTGGGCGTAAAGCGCGCGCAGGTGGGATCTTAAGTCCATTTTGAAAGTTCGGAGCTCAACTCCGTGATGGGATGGAAACTGGGATTCTTGAGTATCGGAGAGGAAAGCGGAATTCCCCGTGTAGCGGTGAAATGCGTAGATATGGGGAGGAACACCAGTGGCGAAGGCGGCTTTCTGGACGATGTCTGACACTGAGGCGCGAAAGCCAGGGGAGCGAACGGG` |
| ASV3040 | 406 | `TGGGGAATATTGCACAATGGGCGAAAGCCTGATGCAGCAACGCCGCGTGAACGAAGAAGGCCTTTGGGTCGTAAAGTTCTGTTCTTAGGGAAGAAAACAATGACGGTACCTAAGGAGAAAGCCCCGGCTAACTACGTGCCAGCAGCCGCGGTAATACGTAGGGGGCAAGCGTTATCCGGAATTATTGGGCGTAAAGAGTACGTAGGCGGTTTTATAAGCGTAAGGTGAAAGGCAGTGGCTCAACCATTGTAAGCCTTATGAACTGTAAAACTTGAGTGCAGGAGAGGAGAGTGGAATTCCTAGTGTAGCGGTGAAATGCGTAGATATTAGGAGGAACACCAGTGGCGAAGGCGACTCTCTGGACTGAAACTGACGCTGAGGTACGAAAGCGTGGGGAGCAAACAGG` |
| ASV4419 | 405 | `TGGGGAATCTTGCGCAATGGGCGAAAGCCTGACGCAGCAACGCCGCGTGCGGGATGAAGGCCCTCGGGTTGTAAACCGCTTTCAGCAGGGACGAAGATGACGGTACCTGCAGAAGAAGCCCCGGCCAACTACGTGCCAGCAGCCGCGGTAACACGTAGGGGGCGAGCGTTGTCCGGATTTATTGGGCGTAAAGAGCTCGTAGGCGGTTCCGTCAGTCGGGTGTGAAAACTCTGGGCTCAACCCAGAGACGCCACCCGATACTGCGGTGACTAGAGTCCGGTAGGGGAGTGCGGAATTCCTGGTGTAGCGGTGAAATGCGCAGATATCAGGAGGAACACCAACGGCGAAGGCAGCACTCTGGGCCGGTACTGACGCTGAGGAGCGAAAGCGTGGGGAGCGAACAGG` |
| ASV5490 | 429 | `TGGGGAATCTTGGACAATGGGGGCAACCCTGATCCAGCGATGCCGCGTGGGTGAAGAAGGCCTTCGGGTTGTAAAGCCCTTTAGGCCGGGAGGAAGGTGAATAGCTGAATAAGTTATTTGCTTGACTGTACCGGCAGAATAAGCACCGGCTAACTCTGTGCCAGCAGCCGCGGTAATACAGAGGGTGCGAGCGTTAATCGGAATTACTGGGCGTAAAGGGCGCGTAGGCGGTAGCGTGTGAGTGATGTGAAAGCCCCGGGCTTAACCTGGGAAGAGCATCGCTAACTGCGCAACTAGAGTACAGCAGAGGGTGGCGGAATTTCCGGTGTAGCGGTGAAATGCGTAGAGATCGGAAGGAACGTCGGTGGCGAAGGCGGCCACCTGGGCTGATACTGACGCTGAGGCGCGAGAGCGTGGGGAGCGAACAGG` |
| ASV1029 | 404 | `TGGGGAATATTGGGCAATGGGCGAAAGCCTGACCCAGCCATGCCGCGTGAATGATGAAGGCCTTAGGGTTGTAAAGTTCTTTCGCAGGTGAAGATAATGACGGTAACCTGATAAGAAGCCCCGGCTAACTTCGTGCCAGCAGCCGCGGTAATACGAAGGGGGCTAGCGTTGTTCGGAATTACTGGGCGTAAAGCGTATGTAGGCGGATACATAAGCTAGAGGTGAAATCCCAAGGCTCAACCTTGGAACTGCCTTTAGAACTGTGTATCTGGAGTTTTGGAGGGGAGTGCGGAATTCCTAATGTAGAGGTGAAATTCGTAGATATTAGGAGGAACACCGGCGGCGAAGGCGGCACTCTGGACAAATACTGACGCTGAGATACGAAAGCGTGGGGAGCAAACAGG` |
| ASV796 | 429 | `TGGGGAATATTGGACAATGGGGGCAACCCTGATCCAGCCATGCCGCGTGTGTGAAGAAGGCCTTAGGGTTGTAAAGCACTTTCAGTGGGGAGAAAGGTTATTCAGCTAATATCTGAGTAAATTGATGCTACCCACAGAAGAAGCACCGGCTAACTCCGTGCCAGCAGCCGCGGTAATACGGAGGGTGCAAGCGTTAATCGGAATTACTGGGCGTAAAGCGCACGTAGGTGGTTTTTTAAGTCAGCTGTGAAATCCCCGGGCTCAACCTGGGCACTGCAGTTGAAACTGAAAAGCTAGAGTAGGGTAGAGGGGTGTGGAATTCCAGGTGTAGCGGTGAAATGCGTAGATATCTGGAGGAACATCAGTGGCGAAGGCGACACCCTGGACTCATACTGACACTGAGGTGCGAAAGCGTGGGGAGCAAACAGG` |
| ASV5223 | 404 | `TCGGGAATATTGGACAATGGGCGAAAGCCTGATCCAGCAATGCCGCGTGAATGATGAAGGTCTTAGGATTGTAAAGTTCTTTTGACGAGGAAGATAATGACGGTACTCGTAGAATAAGCACCGGCTAATTTCGTGCCAGCAGCCGCGGTAATACGAAAGGTGCTAGCGTTACTCGGATTTACTGGGCGTAAAGAGCGTGTAGGCGGGCTTGTAAGTTAGTTGTTAAAACCCGAAGCTTAACTTCGGATCTGCGACTAAAACTACAGGCCTTGAATATGGTAGGGGACAGTGGAATTTCTTGTGTAGGGGTAAAATCCGTAGATATAAGAAGGAACACCAAAGGCTAACGCAACTGTCTGGGCCATTATTGACGCTGAGACGCGAAAGCGTGGGGAGCAAACAGG` |
| ASV2692 | 429 | `TGGGGAATATTGGACAATGGGCGAAAGCCTGATCCAGCCATGCCGCGTGTGTGAAGAAGGCCCTAGGGTTGTAAAGCACTTTCAGTGGGGAGAAAGGTCGTTCACTTAATACGTGAATGAATTGATGTTACCCACAGAAGAAGCACCGGCTAACTTCGTGCCAGCAGCCGCGGTAATACGAGGGGTGCAAGCGTTAATCGGAATTACTGGGCGTAAAGCGTACGTAGGTGGTTTGTTAAGCCAGCTGTGAAATCCCCGGGCTCAACCTGGGCACTGCAGTTGGAACTGGCGAGCTAGAGTAGGGTAGAGGGGTGTGGAATTCCAGGTGTAGCGGTGAAATGCGTAGATATCTGGAGGAACATCAGTGGCGAAGGCGACACCCTGGACTCATACTGACACTGAGGTACGAAAGCGTGGGGAGCAAACAGG` |
| ASV4711 | 429 | `TGGGGAATATTGGACAATGGGGGCAACCCTGATCCAGCAATACCGCGTGTGTGAAGAAGGCCTTCGGGTTGTAAAGCACTTTGGGCGGGGAAGAAGGTGTGGAGTTTAAGAGATGCCACATTTGACGGTACCCGCAGAACAAGCACCGGCTAACTCTGTGCCAGCAGCCGCGGTAATACAGAGGGTGCGAGCGTTAATCGGAATTACTGGGCGTAAAGGGCGCGTAGGCGGTTCTACAAGTGAGATGTGAAATCCCCGGGCTTAACCTGGGAACTGCATATTAGACTGTAGGACTGGAGTACTGTAGAGGACAGTGGAATTTCCGGTGTAGCGGTGAAATGCGTAGAGATCGGAAGGAACACCAGTGGCGAAGGCGACTGTCTGGACAGATACTGACGCTGAGGCGCGAAAGCGTGGGGAGCAAACAGG` |
| ASV1337 | 429 | `TGGGGAATTTTGGACAATGGGGGCAACCCTGATCCAGCCATGCCGCGTGAGTGAAGAAGGCCTTCGGGTTGTAAAGCTCTTTCGGCCGGGAAGAAATCGTGCAGGCTAATACTCTGTATGGATGACGGTACCGGAATAAGAAGCACCGGCTAACTACGTGCCAGCAGCCGCGGTAATACGTAGGGTGCGAGCGTTAATCGGAATTACTGGGCGTAAAGCGTGCGCAGGCGGTTTTGTAAGACAGGCGTGAAATCCCCGGGCTCAACCTGGGAATGGCGCTTGTGACTGCAAGGCTAGAGTACGGCAGAGGGGGGTAGAATTCCACGTGTAGCAGTGAAATGCGTAGAGATGTGGAGGAATACCGATGGCGAAGGCAGCCCCCTGGGTCGATACTGACGCTCATGCACGAAAGCGTGGGGAGCAAACAGG` |
| ASV1978 | 404 | `TGGGGAATCTTAGACAATGGGCGCAAGCCTGATCTAGCCATGCCGCGTGAGCGATGAAGGCCTTAGGGTTGTAAAGCTCTTTCGTGGGGGAAGATAATGACGGTACCCCAAGAAGAAGCCCCGGCTAACTCCGTGCCAGCAGCCGCGGTAATACGGAGGGGGCTAGCGTTGTTCGGAATTACTGGGCGTAAAGCGCACGTAGGCGGATCGGATAGTCAGAGGTGAAATCCCAGGGCTCAACCTTGGAACTGCCTTTGAAACTCCCGGTCTTGAGGTCGAGAGAGGTGAGTGGAATTCCGAGTGTAGAGGTGAAATTCGTAGATATTCGGAGGAACACCAGTGGCGAAGGCGGCTCACTGGCTCGATACTGACGCTGAGGTGCGAAAGCGTGGGGAGCAAACAGG` |
| ASV4664 | 502 | `GCGCGAAACCTTTGCAAGGAACTTTTCAGAAAAGTTCCATCAAAAGCTGGAAAGTTCGCAAATTGGGTGAAAGCCTGACAAGGGAATCCTGAGTGGCAGCGCTTAGCGCTGTCTTTTCTTCACTGTAAAAAGGTGGAGGAATAAGCGGTGGGCAAGACGGTTGCCAGCCGCCGCGGTAATAACCGCGCCGCAAGGGGTAGTCACGTTTATTTGGCCTAAAGCATCCGTAGCCGGTTCGATGTGTCTTTGGTGAAATTCGAGAATAGCGAAGCTATTCTGAATTTACTGTTCTGTAATTCAGATATGCTGTTCTCAGAACACTAAATCTTATAGAAAACTATAAGGCTGGCTGGAGATACTATCGGACTTGGGACCGGGAGAGGCTGAGGGTACTCTACGGGTAGGGGTAAAATCCTTTAATCCGTAGGGGACCACCTGTGGCGAAGGCGCTCAGCTGGAACGGGTCCGACGGTGAGGGATGAAAGCGTGGGGAGCGATCGGG` |
| ASV1634 | 424 | `TGAGGAATATTGGACAATGGGCGCAAGCCTGATCCAGCCATGCCGCGTGCAGGAAGAAGCATCTATGGTGTGTAAACTGCTTTTGTACGGGAAGAAACACTCCTACGTGTAGGAGCTTGACGGTACCGTAAGAATAAGGATCGGCTAACTCCGTGCCAGCAGCCGCGGTAATACGGAGGATCCAAGCGTTATCCGGAATCATTGGGTTTAAAGGGTCCGTAGGCGGTTTAATAAGTCAGTGGTGAAAGCCCATCGCTCAACGATGGAACGGCCATTGATACTGTTAGACTTGAATTACAAGGAAGTAACTAGAATATGTAGTGTAGCGGTGAAATGCTTAGATATTACATGGAATACCAATTGCGAAGGCAGGTTACTACTTGTGGATTGACGCTGATGGACGAAAGCGTGGGGAGCGAACAGG` |
| ASV1816 | 423 | `TAGGGAATATTGGGCAATGGGCGAGAGCCTGACCCAGCCATGCCGCGTGCCGGATGAAGGCCTTCTGGGTTGTAAACGGCTTTTCTCAGGGAAGAAAAAGACCATGCGTGGTACACTGACGGTACCTGAGGAATAAGCACCGGCTAACTCCGTGCCAGCAGCCGCGGTAATACGGAGGGTGCAAGCGTTGTCCGGATTTATTGGGTTTAAAGGGTGCGTAGGCGGCCCGTTAAGTCCGGGGTGAAAGCCCACAGCTCAACTGTGGAACTGCCCTGGATACTGGCGGGCTTGAGTCCAGACGAGGTTGGCGGAATGGAAGGTGTAGCGGTGAAATGCATAGATACCTTCCAGAACCCCGATTGCGTAGGCAGCTGACTAGGCTGGTACTGACGCTGAGGCACGAAAGCGTGGGGAGCGAACAGG` |
| ASV4201 | 387 | `TCGGGAAAATTCTGCAATGTACGAAAGTATGACAGAGCAAGCCAAAGTGCTCAACTTCGTGTTGAGCTTTTGTGCGCTGTAAAAAGGCGCACGAATAAGGACTGGGCAAGACTGGTGCCAGCCGCCGCGGTAATCCCAGCAGTCCAAGTCGCAGCCACATTTGTTGGGTCTAAAACATTCGTAGCTTGCTATTCAAGTCTTTTGTGAAATCGGGACTCTTAAGGTTCCGGCGGGCAAAAGATACTGTTTAGCTAGAGACCGGTAGACGCATCGAGTACGGATAGGGTAGTGGTAAAATACGTTGATCCTTTCCGGACTAACAATAGCGAAGGCACGATGCGAGCACGGTTCTGACAGTGAGGGATGAAGGCTAGGGGCGCAAAGTGG` |
| ASV2173 | 429 | `TGGGGAATATTGGACAATGGGGGCAACCCTGATCCAGCCATGCCGCGTGTGTGAAGAAGGCCTTCGGGTTGTAAAGCACTTTAAGCGGGGAGGAAGGCTCTATGGCTAATATCCATGGGGATTGACGTTACCCGCAGAATAAGCACCGGCAAACTTCGTGCCAGCAGCCGCGGTAATACGAAGGGTGCGAGCGTTAATCGGAATTACTGGGCGTAAAGCGCGCGTAGGTGGTTTGGTAAGCGATATGTGAAATCCCCGGGCTTAACCTGGGAACTGCATGTCGAACTGCCAGGCTAGAGTATGGTAGAGGGGTGTGGAATTTCCTGTGTAGCGGTGAAATGCGTAGATATAGGAAGGAACATCAGTGGCGAAGGCGACACCCTGGACCGATACTGACACTGAGGCGCGAAAGCGTGGGTAGCGAACAGG` |
| ASV1024 | 406 | `TCGAGAATCTTCCGCAATGGGCGAAAGCCTGACGGAGCGACGCCGCGTGGTGGATGAAGTCCCTCGGGACGTAAACACCTTTTATGAGGGAGGAAATTTTGACGTTACCTCATGAATAAGGGGCTCCTAACTCTGTGCCAGCAGGAGCGGTAATACAGAGGCCCCAAGCATTATCCGGATTTACTGGGCGTAAAGGGTGCGTAGGCGGTTATATTAGTCAGGTGTTAAATCCTGGGGCTCAACCCCAGAATCGCATTTGAAACGGTATAACTAGAAGAAGTCAGAGGCAAGCAGAACTCATGGTGTAGGGGTGAAATCCGTTGATATCATGGGGAATACCAAATGCGAAGGCAGCTTGCTGGGACTTTCTTGACGCTGAGGCACGAAAGCGTGGGTAGCGAATCGG` |
| ASV3049 | 424 | `TAAGGAATATTGGTCAATGGAGGGAACTCTGAACCAGCCACGCCGCGTGGACGATGAAGGCCCTATGGGTTGTAAAGTTCTTTAGTCTGGGACGAAAAAGGGTGATTTATCATCAACTGACGGTACCAGAAGAATAAGCACCGGCTAACTCCGTGCCAGCAGCCGCGGTAATACGGAGGGTGCAAGCGTTATCCGGAATCACTGGGTTTAAAGGGTGCGTAGGCTGTTTTATAAGTCAGCGGTGAAAGGTGTCGGCTCAACCGACGAACTGCCGTTGATACTGTTTAACTAGAATTGGGATGAGGTAACTAGAATGTAACATGTAGCGGTGAAATGCTTAGATATGTTATAGAACACCAATTGCGAAGGCAGGTTACTGGACCTTGATTGACGCTGAGGCACGAAAGCGTGGGTAGCGAACAGG` |
| ASV5617 | 404 | `TGAGGAATATTGGACAATGGGCGCAAGCCTGATCCAGCCATGCCGCGTGAGTGATGAAGGCCTTCGGGTTGTAAAACTCTTTGAGTAGGGAAGATAATGACGGTACCTACAGAACAAGCCACGGCAAACTTCGTGCCAGCAGCCGCGGTAATACGAAGGTGGCAAGCGTTACTCGGAATTACTGGGCGTAAAGCGCATGTAGGCGGATAAGTAAGTCAGAGGTGAAATCCCGGGGCTCAACCTCGGAATTGCCTTTGAAACTACTTATCTAGAGTGTATCAGGGGATGGTGGAATTTCCAGTGTAGAGGTGAAATTCGTAGATATTGGAAGGAACATCGGTGGCGAAGGCGACCATCTGGGATACAACTGACGCTGAGATGCGAAAGCGTGGGGAGCAAACGGG` |
| ASV2607 | 424 | `TAGGGAATATTGGGCAATGGAGGCAACTCTGACCCAGCCATGCCGCGTGCAGGACGAAGGCTCTTCGAGTCGTAAACTGCTTTTATCGGGGAAGAACGGCTTTCTTGCGAGGGAGTGTGACGGTATCCGATGAATAAGCACCGGCTAACTCCGTGCCAGCAGCCGCGGTAATACGGAGGGTGCAAGCGTTGTCCGGATTTATTGGGTTTAAAGGGTGCGTAGGCGGGTGTTTAAGTCTGGTTTAAAATCAGGTCGCTCAACGATCTAACGTGGCTGGATACTGGACATCTTGAAATTGTTGGCCGCAGCCGGAATGGGTCATGTAGCGGTGAAATGCATAGATATGCCCCGGAACCCCGATTGCGAAGGCAGGCTGCGACGACTGATTTGACGCTGAGGCACGAGAGCATGGGTAGCAAACAGG` |
| ASV707 | 404 | `TGGGGAATATTGGACAATGGGCGCAAGCCTGATCCAGCAATGCCGCGTGAGTGATGAAGGCCTTAGGGTTGTAAAGCTCTTTCACCGGGGAAGATAATGACGGTACCCGGAGAAGAAGCCCCGGCTAACTCCGTGCCAGCAGCCGCGGTAATACGGAGGGGGCTAGCGTTGTTCGGAATTACTGGGCGTAAAGCGCGCGTAGGCGGCCTGACAAGTTGGAGGTGAAAGCCCGGAGCTCAACTTCGGAATTGCCTTCAAGACTGTCTCGCTCGAGGCAGGGAGAGGAGAGCGGAATACCCAGTGTAGAGGTGAAATTCGTAGATATTGGGTGGAACACCGGTGGCGAAGGCGGCTCTCTGGCCCTGATCTGACGCTAAGGCGCGAAAGCGTGGGGAGCAAACAGG` |
| ASV1876 | 405 | `TGGGGAATCTTGGTCAATGGGCGAAAGCCTGAACCAGCGATTCTGCGTGAAGGATGAAGCCTCGCGGGGTGTAAACTTCTTTTATGGGGGAAGACGAATGACGGTACCCCACGAATAAGGGACGGCTAACTACGTGCCAGCAGCCGCGGTAATACGTAGGTCCCAAGCGTTATCCGGATTTACTGGGTGTAAAGAGTCTGTAGCTGGTTATATAAGTCTATTTTTAAAGACCTTGGCTCAACTAAGGGAAGGGAATGGATACTATATATCTGGAGGTTTTTAGGGGTTAGTAGAATTCCCGGTGGAGGGGTGAAATCCGTTGATATTGGGAGGAATGCCGAAAGCGAAAGCAGCTAACTATAATTATCCTGACAGTGAGAGACGAAAGCATGGGTAGCAAACGGG` |
| ASV4890 | 389 | `TCGGGAAACTTCTGCAATGTACGCAAGTATGACAGAGCAAGCCAGAGTGCGCTTCTATAACTGAAGCGCTTTTGCCAGATGTAAAAAGTCTGGCGAATAAGGACTGGGCAAGACTGGTGCCAGCCGCCGCGGTAATACCAGCGGTCCAAGTGGCAGCCGCGTTTATTGGGTCTAAAACATCCGTAGCTCGCTTTGTAAGTCTCTTGTGAAATCTGGCCTCTCAAGGGTCAGGCGTGCAAGAGATACTGCTTAGCTAGAGACCGGGAGGCGTAAGGAGTACAGTTAGGGTAGCGGTTAAATGCGTTGATCCTTACTGGACTAACGATAGCGAAGGCACCTTACGAGAACGGATCTGACAGTGAGGGATGAAGGCTAGGGGCGCAAAACGG` |
| ASV4136 | 429 | `TGGGGAATATTGGACAATGGGGGGAACCCTGATCCAGCAACGCCGCGTGAATGAAGAAGGCCTTCGGGTTGTAAAGTTCTTTTGTCTGGGAGCAAATCTGTGCTGTGAATAGCGGAATAGGATGAGAGTACCAGAAGAATAAGCACCGGCTAACTACGTGCCAGCAGCCGCGGTAATACGTAGGGTGCGAGCGTTAATCGGAATTACTGGGCGTAAAGGGTGCGCAGGCTGGAATACAAGTTAGATGTGAAATACCCGAGCTTAACTTGGGAGGTGCATTTAAGACTGTATACCTAGAGTTAGTCAGAGGATGGGGGAATTCCGAGTGTAGCAGTGAAATGCGTAGATATTCGGAGGAACATCGGTGGCGAAGGCGCCCATCTGGGATATAACTGACGCTCATGCACGAAAGCGTGGGGAGCAAACAGG` |
| ASV4196 | 404 | `TGGGGAATATTGGACAATGGAGGAAACTCTGATCCAGCCATGCCGCGTGAGTGAAGAAGGCCTTCGGGTTGTAAAGCTCTTTTGTCCCTGACGATAATGACGGTAGGGGAAGAAAAAGCCACGGCTAACTTCGTGCCAGCAGCCGCGGTAATACGAAGGTGGCAAGCGTTGTTCGGATTTACTGGGCGTAAAGAGTGCGTAGGCGGTTTAACAAGTCAGGAGTGAAAGCCCAAGGCTCAACCCTGGAATTGCTTTTGAAACTGTTAGACTCGAATCCGGTAGAGGTTGGCGGAATTCTCAGTGTAGAGGTGAAATTCGCAGATATTGAGAGGAACACCAGTGGCGTAAGCGGCCAACTGGACCGGTATTGACGCTGAGGCACGAAAGCATGGGGAGCAAACAGG` |
| ASV5539 | 412 | `TCGAGAATCATTCACAATGGGCGCAAGCCTGATGGTGCGACGCCGCGTGGGGGAGGAAGGTCTTCGGATTGTAAACCCCTGTCATGCAGGAGCAAGGCGCAAGCTTGATAGTACTGCAAGAGGAAGAGACGGCAAACTCTGTGCCAGCAGCCGCGGTAATACAGAGGTCTCAAGCGTTGTTCGGAATCACTGGGCGTAAAGGGTACGTAGGCTGCACCATAAGTCGCGCGTGAAAGGCAGGGGCTCAACCTCTGGAGTGCGCATGATACTGTGGTGCTGGAGTAATGGAGGGGGAACCGGAATTCTTGGTGTAGCAGTGAAATGCGTAGATATCAAGAAGAAGACCCGAGGCGAAGGCGGGTTCCTGGACATTAACTGACGCTAAGGTACGAAGGCCGGGGTAGCGAAAGGG` |
| ASV266 | 429 | `TGGGGAATTTTGGACAATGGGCGCAAGCCTGATCCAGCAATGCCGCGTGCAGGATGAAGGCCTTCGGGTTGTAAACTGCTTTTGTACGGAACGAAACGGCGAGCTCTAATAAAGCTCGCTAATGACGGTACCGTAAGAATAAGCACCGGCTAACTACGTGCCAGCAGCCGCGGTAATACGTAGGGTGCAAGCGTTAATCGGAATTACTGGGCGTAAAGCGTGCGCAGGCGGTGATGTAAGACAGATGTGAAATCCCCGGGCTCAACCTGGGACCTGCATTTGTGACTGCATCGCTAGAGTACGGTAGAGGGGGATGGAATTCCGCGTGTAGCAGTGAAATGCGTAGATATGCGGAGGAACACCGATGGCGAAGGCAATCCCCTGGACCTGTACTGACGCTCATGCACGAAAGCGTGGGGAGCAAACAGG` |
| ASV2020 | 429 | `TGGGGAATATTGGACAATGGGCGCAAGCCTGATCCAGCCATGCCGCGTGTGTGAAGAAGGCCTTCGGGTTGTAAAGCACTTTCAGCGAGGAGGAAGGGAGTTGTGTTAATAGCACGGCTTTTTGACGTTACTCGCAGAAGAAGCACCGGCTAACTCTGTGCCAGCAGCCGCGGTAATACAGAGGGTGCAAGCGTTAATCGGAATTACTGGGCGTAAAGCGCACGCAGGCGGTTGGTTAAGTCAGATGTGAAAGCCCCGGGCTCAACCTGGGAATTGCATTTGAAACTGGCCAACTAGAGTATGTGAGAGGGGGGTAGAATTCCAAGTGTAGCGGTGAAATGCGTAGAGATTTGGAGGAATACCAGTGGCGAAGGCGGCCCCCTGGCACAATACTGACGCTCAGGTGCGAAAGCGTGGGGAGCAAACAGG` |
| ASV4060 | 407 | `TGGGGAATATTGGGCAATGGGCGAAAGCCTGACCCAGCAACGCCGCGTGAGGGAAGAAGGTTTTCGGATTGTAAACCTCTGTCCTCAGGGAAGAAAAAAATGACGGTACCTGAGGAGGAAGCCCCGGCTAACTACGTGCCAGCAGCCGCGGTAATACGTAGGGGGCAAGCGTTGTCCGGAATGACTGGGCGTAAAGGGCGCGTAGGCGGCCAATTAAGTCTGATGTGAAATACCCGTTTTTAAGGCGGGTCGTGCATTGGAAACTGAATGGCTTGAGTGCAGAAGAGGTAAGTGGAATTCCCAGTGTAGCGGTGAAATGCGTAGATATTGGGAGGAACACCAGTGGCGAAGGCGACTTACTGGGCTGCAACTGACGCTGAGGCGCGAAAGCGTGGGGAGCAAACAGG` |
| ASV4518 | 428 | `TGGGGAATCTTGCGCAATGGGCGAAAGCCTGACGCAGCGACGCCGCGTGAGCGATGAAGGCCCTCGGGTTGTAAAGCTCTGTGGAGAGGGAAGAATAAGGGTACGTTAATAGCGTGCTCGATGACGGTACCTCTTTAGCAAGCACCGGCTAACTCTGTGCCAGCAGCCGCGGTAATACAGAGGGTGCAAACGTTGTTCGGAATTACTGGGCGTAAAGCGCGTGTAGGCGGCAATGTAAGTCGGATGTGAAAGCCCATGGCTCAACCATGGAAGTGCACTCGAAACTGCGTTGCTAGAGTCTCGGAGAGGAAGGTGGAATGCTTGGTGTAGAGGTGAAATTCGTAGATATCAAGCGGAACACCGGTGGCGAAGGCGGCCTTCTGGACGAGTACTGACGCTGAGACGCGAAAGCGTGGGTAGCAAACAGG` |
| ASV434 | 430 | `TGGGGAATATTGGACAATGGGGGCAACCCTGATCCAGCAATGCCGCGTGTGTGAAGAAGGCCTGCGGGTTGTAAAGCACTTTTGTTCGAGAAGAAATCGTGTGTATGAATAGTGCATATGGATGACGGTATCGAAAGAATAAGCACCGGCTAACTTCGTGCCAGCAGCCGCGGTAATACGAAGGGTGCAAGCGTTAATCGGAATTACTGGGCGTAAAGCGTGCGTAGATGGCTTTTTAAGTCGACTGTGAAACCCCTGGGCTCAACCTGGGACGTGCAGTCGATACTGAGGAGCTTGGAGTCTGAGAGAGGGTGGTGGAATTCCTGGTGTAGCGGTGAAATGCGTAGAGATCAGGAGGAACACTAGTGGCGAAGGCGGCCACCTGGCTTAAGACTGACGTTGAGGCACGAAAGCGTGGGGAGCAAACAGG` |
| ASV7 | 429 | `TGGGGAATTTTGGACAATGGGCGCAAGCCTGATCCAGCAATGCCGCGTGCAGGATGAAGGCCTTCGGGTTGTAAACTGCTTTTGTACGGAACGAAACGGTCTGCCCTAATACGGCGGGCTAATGACGGTACCGTAAGAATAAGCACCGGCTAACTACGTGCCAGCAGCCGCGGTAATACGTAGGGTGCGAGCGTTAATCGGAATTACTGGGCGTAAAGCGTGCGCAGGCGGTTATATAAGACAGATGTGAAATCCCCGGGCTCAACCTGGGACCTGCATTTGTGACTGTATAGCTAGAGTACGGTAGAGGGGGATGGAATTCCGCGTGTAGCAGTGAAATGCGTAGATATGCGGAGGAACACCGATGGCGAAGGCAATCCCCTGGACCTGTACTGACGCTCATGCACGAAAGCGTGGGGAGCAAACAGG` |
| ASV427 | 429 | `TGGGGAATTTTGGACAATGGGCGAAAGCCTGATCCAGCAATGCCGCGTGCAGGAAGAAGGCCTTCGGGTTGTAAACTGCTTTTGTACGGAGCGAAAAGGCTCTCTCTAATACAGGGGGCTCATGACGGTACCGTAAGAATAAGCACCGGCTAACTACGTGCCAGCAGCCGCGGTAATACGTAGGGTGCAAGCGTTAATCGGAATTACTGGGCGTAAAGCGTGCGCAGGCGGTTTTGTAAGACAGGCGTGAAATCCCCGGGCTCAACCTGGGAATGGCGCTTGTGACTGCAAAGCTGGAGTGCGGCAGAGGGGGATGGAATTCCGCGTGTAGCAGTGAAATGCGTAGATATGCGGAGGAACACCGATGGCGAAGGCAATCCCCTGGGCCTGCACTGACGCTCATGCACGAAAGCGTGGGGAGCAAACAGG` |
| ASV5393 | 429 | `TGGGGAATTTTGGACAATGGGCGAAAGCCTGATCCAGCCATGCCGCGTGTCTGAAGAAGGCCTTCGGGTTGTAAAGGACTTTTGTCCGGGAGCAAAGCCCAGTAGCTAATACCTACTGGGGCTGAGAGTACCGGAAGAATAAGCACCGGCTAACTACGTGCCAGCAGCCGCGGTAATACGTAGGGTGCAAGCGTTAATCGGAATTACTGGGCGTAAAGCGTGCGCAGGCGGCTGTGCAAGTCTGATGTGAAAGCCCTAGGCTTAACCTGGGAACGGCATTGGAGACTGCATAGCTAGAGTGCGTCAGAGGGGGGTAGAATTCCGCGTGTAGCAGTGAAATGCGTAGATATGCGGAAGAATACCAATGGCGAAGGCAGCCCCCTGGGATGACACTGACGCTCATGTACGAAAGCGTGGGGAGCAAACAGG` |
| ASV2314 | 404 | `TGAGGAATATTGGACAATGGGCGCAAGCCTGATCCAGCCATGCCGCGTGAGTGATGAAGGCCTTCGGGTTGTAAAGCTCTTTCAATGGTGAAGATAATGACGGTAGCCATATAAGAAGCCCCGGCTAAATTCGTGCCAGCAGCCGCGGTAATACGAATGGGGCAAGCGTTGTTCGGAATCACTGGGCTTAAAGCGTGCGCAGGCGGCCTGTAAAGTCAGGAGTGAAATCCCGGGGCTTAACCCCGGAATTGCTTCTGAAACTCACAGGCTAGAATCCGGGAGAGGTTGGGAGAATTCCTAGTGTAGAGGTGAAATTCGCAGATATTAGGAGGAATACCAGTGGCGTAGGCGCCCAACTGGACCGGTATTGACGCTCAGGTACGAAAGCGTGGGTAGCAAACAGG` |
| ASV734 | 429 | `TGGGGAATTTTGGACAATGGGGGCAACCCTGATCCAGCAATGCCGCGTGTGTGAAGAAGGCCTTCGGGTTGTAAAGCACTTTTGGCGGGAACGAAACGGTGGTCTCTAATACAGACTGCTAATGACGGTACCCGCAGAATAAGCACCGGCTAACTACGTGCCAGCAGCCGCGGTAATACGTAGGGTGCAAGCGTTAATCGGAATTACTGGGCGTAAAGCGTGCGCAGGCGGTTTTGTAAGTCTGTCGTGAAATCCCCGGGCTCAACCTGGGAATGGCGATGGAGACTGCAAGGCTGGAATCTGGCAGAGGGGGGTAGAATTCCACGTGTAGCAGTGAAATGCGTAGAGATGTGGAGGAACACCGATGGCGAAGGCAGCCCCCTGGGCTAAGATTGACGCTCATGCACGAAAGCGTGGGGAGCAAACAGG` |
| ASV5151 | 425 | `TAGGGAATATTGGGCAATGGGCGAAAGCCTGACCCAGCCATGCCGCGTGCAGGAAGAATGCCCTCTGGGTTGTAAACTGCTTTTTTGGGGAAAGAAAAAGGGCTTTGCGAAGCCCCTTGACGGTACCCTAAGAATAAGCCACGGCTAACTACGTGCCAGCAGCCGCGGTAATACGTAGGTGGCGAGCGTTATCCGGATTTATTGGGTTTAAAGGGTGCGTAGGCGGGTATTTAAGTCGGTGGTGAAAGGTAGCAGCTCAACTGTTTTACATGCTGCCGATACTGAATATCTTGAATTATGAGAAGGCAGATAGAATTTCTGGTGTAGCGGTGAAATGCTTAGATACCAGAAGGAATACCGATTGCGAAGGCAGTCTGCTGCACATACATTGACGCTGAGGCACGAAAGTATGGGGATCAAACAGG` |
| ASV2101 | 428 | `TAGGGAATCTTCCGCAATGGACGAAAGTCTGACGGAGCAACGCCGCGTGAGTGATGAAGGTTTTCGGATCGTAAAGCTCTGTTGCCAGGGAAGAACGTCCGATAGAGTAACTGCTATCGGAGTGACGGTACCTGAGAAGAAAGCCCCGGCTAACTACGTGCCAGCAGCCGCGGTAATACGTAGGGGGCAAGCGTTGTCCGGAATTATTGGGCGTAAAGCGCGCGCAGGCGGCTATTTAAGTCTGGTGTTTAAACCTTGGGCTCAACCTAAGGTCGCACTGGAAACTGGGTGGCTTGAGTACAGAAGAGGAAAGTGGAATTCCACGTGTAGCGGTGAAATGCGTAGATATGTGGAGGAACACCAGTGGCGAAGGCGACTTTCTGGGCTGTAACTGACGCTGAGGCGCGAAAGCGTGGGGAGCAAACAGG` |
| ASV4641 | 428 | `CTTGGAATATTTCTCAATGGGGGCAACCCTGAAGGAGCGACGCCGCGTGGGGGATGAAGGACTTCGGTTCGTAAACCCCTGTCACCTGGGAACAAAAGTTTCAACCTAATACGTTGAAATCTGATAGTACCAGGAGAGGAAGGCACGGCTAACTCTGTGCCAGCAGCCGCGGTAATACAGAGGTGCCAAGCGTTGTTCGGATTTATTGGGCGTAAAGGGTGCGTAGGCGGTTATACAAGTCGGGTGTGAAATCCCGGAGCTCAACTCCGGAATGGCATTCGAAACTGTATAGCTAGAGGTCTGGAGAGGTGACTGGAATTCTCGGTGTAGCAGTGAAATGCGTAGATATCGAGAGGAACACCAGAGGCGAAGGCGAGTCACTGGACAGAACCTGACGCTGAGGCACGAAAGCTAGGGGAGCAAACGGG` |
| ASV3525 | 424 | `TAAGGAATATTGGTCAATGGACGAAAGTCTGAACCAGCCATGCCGCGTGCAGGAAGACGGTCCTCTGGATTGTAAACTGCTTTTGTTAGGGAAGAAACCCCTGGATGCGTCCGGGGCTGACGGTACCTAAAGAATAAGGATCGGCTAACTCCGTGCCAGCAGCCGCGGTAATACGGAGGATCCAAGCGTTATCCGGATTTATTGGGTTTAAAGGGTGCGTAGGCGGCGTTGTAAGTCAGTGGTGAAAGTTTGCAGCTTAACTGTAAAATTGCCATTGATACTGCAGTGCTTGAGTACAGATGAGGTGGGCGGAATGTGTCATGTAGCGGTGAAATGCATAGATATGACACAGAACACCGATTGCGAAGGCAGCTCACTAAACTGTAACTGACGCTGAGGCACGAAAGCGTGGGGATCAAACAGG` |
| ASV136 | 423 | `TAGGGAATATTGGGCAATGGACGGAAGTCTGACCCAGCCATGCCGCGTGCAGGATGAAGGCCCTCTGGGTTGTAAACTGCTTTTGACAGGGAAGAAATAGACCATGCGTGGTTTGCTGACGGTACCTGTAGAATAAGCACCGGCTAACTCCGTGCCAGCAGCCGCGGTAATACGGAGGGTGCAAGCGTTGTCCGGATTTATTGGGTTTAAAGGGTGCGTAGGCGGTTCTGTAAGTCAGTGGTGAAAGCTAGCAGCTTAACTGTTAAACTGCCATTGATACTGCAGAGCTTGAGTTCAGATGAGGTAGGCGGAATTTATAGTGTAGCGGTGAAATGCTTAGATACTATAAAGAACTCCGATAGCGTAGGCAGCTTACTAAACTGATACTGACGCTGAGGCACGAAAGTGTGGGGATCAAACAGG` |
| ASV3681 | 409 | `TGGGGAATATTGCGCAATGGGCGAAAGCCTGACGCAGCGACGCCGCGTGAGGGATGAAGGCCTTCGGGTTGTAAACCTCTTTCAGCAGGGAAGAAGCGAAAGTGACGGTACCTGCAGAAGAAGCACCGGCTAACTACGTGCCAGCAGCCGCGGTAATACGTAGGGTGCAAGCGTTGTCCGGAATTATTGGGCGTAAAGAGCTCGTAGGCGGTTTGTCGCGTCTGCTGTGAAAACGCGGGGCTCAACTCCGCGCCTGCAGTGGGTACGGGCAGACTAGAGTGCAGTAGGGGAGACTGGAATTCCTGGTGTAGCGGTGGAATGCGCAGATATCAGGAGGAACACCGGTGGCGAAGGCGGGTCTCTGGGCTGTAACTGACGCTGAGGAGCGAAAGCGTGGGGAGCGAACAGG` |
| ASV1445 | 429 | `TGGGGAATTTTGGACAATGGACGCAAGTCTGATCCAGCCATGCCGCGTGCGGGAAGAAGGCCTTCGGGTTGTAAACCGCTTTTGTCAGGGAAGAAAAGGTTCTGGTTAATACCTGGGACTCATGACGGTACCTGAAGAATAAGCACCGGCTAACTACGTGCCAGCAGCCGCGGTAATACGTAGGGTGCAAGCGTTAATCGGAATTACTGGGCGTAAAGCGTGCGCAGGCGGTTATGCAAGACAGATGTGAAATCCCCGGGCTCAACCTGGGAACTGCATTTGTGACTGCATAGCTAGAGTACGGTAGAGGGGGATGGAATTCCGCGTGTAGCAGTGAAATGCGTAGATATGCGGAGGAACACCGATGGCGAAGGCAATCCCCTGGACCTGTACTGACGCTCATGCACGAAAGCGTGGGGAGCAAACAGG` |
| ASV397 | 429 | `TGGGGAATATTGGACAATGGGCGCAAGCCTGATCCAGCCATGCCGCGTGTGTGAAGAAGGCCTTCGGGTTGTAAAGCACTTTTGTTGGGAAAGAAATCCTGTCGGCTAATACCCGGCGGGGATGACGGTACCCAAAGAATAAGCACCGGCTAACTTCGTGCCAGCAGCCGCGGTAATACGAAGGGTGCAAGCGTTACTCGGAATTACTGGGCGTAAAGCGTGCGTAGGTGGTTCGTTAAGTCAGATGTGAAAGCCCCGGGCTCAACCTGGGAATTGCATTTGATACTGGCGGACTAGAGTGCGGTAGAGGAGAGTGGAATTCCCGGTGTAGCAGTGAAATGCGTAGAGATCGGGAGGAACATCAGTTGCGAAGGCGGCTCTCTGGACCAGCACTGACACTGAGGCACGAAAGCGTGGGGAGCAAACAGG` |
| ASV2660 | 421 | `TGGGGAATATTGCGCAATGGACGAAAGTCTGACGCAGCGACGCCGCGTGTGGGATGAAGCTCTTCGGGGTGTAAACCACTGTTGCCAGGGAAGAATCTCCCGATTCATCGGGATTGACGGTACCTGGTGAGGAAGCACCGGCTAACTCTGTGCCAGCAGCCGCGGTAATACAGAGGGTGCGAGCGTTGTCCGGAATCACTGGGCGTAAAGGGCGCGTAGGTGGCTGGGTAAGTGTGCGGTGAAAGCCCGGGGCTCAACCCCGGGTCGGCCGTGCAGACTGCTTGGCTTGAGCATAGTAGAGGCAGGTGGAATTCCGGGTGTAGCGGTGGAATGCGTAGAGATCCGGAAGAACACCGGTGGCGAAGGCGGCCTGCTGGGCTATTGCTGACACTGAGGCGCGACAGCGTGGGGAGCAAACAGG` |
| ASV1755 | 430 | `TGGGGAATATTGCGCAATGGACGAAAGTCTGACGCAGCAACGCCGCGTGAGTGAAGAAGGCCTTTGGGTTGTAAAGCTCTTTCGGCTGGGAAGAAGTGTTTGAGTGTTAATAGCATTTGAATGTGATGGTACCAGAAGAAGAAGCACCGGCTAACTTCGTGCCAGCAGCCGCGGTAATACGAAGGGTGCGAGCGTTGTTCGGAATTACTGGGCGTAAAGGGTTTGTAGGCTGAAAGATAAGTCAAGTGTGAAATCCCCGAGCTAAACTTGGGACGTGCACCTGAAACTGTTTTTCTTGAGTATCGTAGAGGGTGGTGGAATTGTAGGTGTAGGAGTGACATCCGTAGAGATCTGCAGGAACATCAGAGGCGAAGGCGACCACCTGGCCGATTACTGACGCTGAGGAACGAAAGCGTGGGGAGCAAACAGG` |
| ASV1736 | 404 | `TGGGGAATATTGGACAATGGGCGCAAGCCTGATCCAGCCATGCCGCGTGAGTGATGAAGGCCTTCGGGTTGTAAAGCTCTTTTGTCCGGGAAGATAATGACTGTACCGGAAGAATAAGCCCCGGCAAACTTCGTGCCAGCAGCCGCGGTAATACGAAGGGGGCTAGCGTTGTTCGGATTTACTGGGCGTAAAGCGCACGTAGGCGGCTTGTTAAGTCAGGGGTGAAATCCTGGAGCTCAACTCCAGAACTGCCCTTGATACTGGCGAGCTCGAGTGTGGAAGAGGTTGGTGGAACTGCGAGTGTAGAGGTGAAATTCGTAGATATTCGCAAGAACACCGGTGGCGAAGGCGGCCAACTGGTCCATTACTGACGCTGAGGTGCGAAAGCGTGGGGAGCAAACAGG` |
| ASV1010 | 404 | `TGGGGAATATTGGACAATGGGCGCAAGCCTGATCCAGCAATGCCGCGTGAGTGATGAAGGCCTTAGGGTTGTAAAGCTCTTTCGCACGTGACGATTATGACGGTAACGTGAGAAGAAGCCCCGGCTAACTTCGTGCCAGCAGCCGCGGTAATACGAAGGGGGCTAGCGTTGTTCGGAATTACTGGGCGTAAAGGGCGCGTAGGCGGCCCGATTAGTCAGAAGTGAAAGCCCCGGGCTCAACCTGGGAACTGCTTTTGATACTGTCGGGCTTGAGTTTCGGAGAGGATGGTGGAATTCCCAGTGTAGAGGTGAAATTCGTAGATATTGGGAAGAACACCAGTGGCGAAGGCGGCCATCTGGACGAACACTGACGCTGAGGCGCGAAAGCGTGGGGAGCAAACAGG` |
| ASV2422 | 429 | `TGGGGAATATTGCGCAATGGAGGAAACTCTGACGCAGCGACGCCGCGTGGGTGATGAAGGCCTTCGGGTTGTAAAGCCCTGTCGAATGGGAAAAAGGTAGTCGTGCTAATACCACGATTGCTTGATGGTACCATTAAAGGAAGCACCGGCTAACTCTGTGCCAGCAGCCGCGGTAATACAGAGGGTGCAAGCGTTGTTCGGAATTACTGGGCGTAAAGGGCGCGTAGGCGGTTTGGTAAGTCAGATGTGAAAGCCCGGGGCTCAACCCCGGAAGTGCATTTGAAACTACCAAGCTAGAGTGCAGTAGAGAATGGCGGAATTCCCAGTGGAGAGGTGAAATTCGTAGATATTGGGAGGAACACCGGTGGCGAAGGCGGCCATTTGGGCTGTAACTGACGCTGAGGCGCGAGAGCGTGGGTAGCAAACAGG` |
| ASV809 | 429 | `TGGGGAATTTTGGACAATGGGGGAAACCCTGATCCAGCCATGCCGCGTGCGGGAAGAAGGCCTTCGGGTTGTAAACCGCTTTTAGTCGGGAAGAAAAGGCGGCCCCTAATACGGGTCGTTTTTGACGGTACCGGCGGAATAAGCACCGGCTAACTACGTGCCAGCAGCCGCGGTAATACGTAGGGTGCGAGCGTTAATCGGAATTACTGGGCGTAAAGCGTGCGCAGGCGGTTCGAAAAGTCCGATGTGAAAGCCCCGGGCTCAACCTGGGAATGGCATTGGAAACTGGCGGGCTAGAGTGTGGCAGAGGGGGGTGGAATTCCGCGTGTAGCAGTGAAATGCGTAGAGATGCGGAGGAACACCGATGGCGAAGGCAGCCCCCTGGGCTAACACTGACGCTCAGGCACGAAAGCGTGGGGAGCAAACAGG` |
| ASV2093 | 404 | `TGGGGAATATTGGACAATGGGCGAAAGCCTGATCCAGCCATGCCGCGTGAGTGATGAAGGCCTTAGGGTTGTAAAGCTCTTTTACCAGGGAAGATAATGACTGTACCTGGAGAATAAGCCCCGGCTAACTTCGTGCCAGCAGCCGCGGTAATACGAAGGGGGCTAGCGTTGTTCGGAATCACTGGGCGTAAAGCGCACGTAGGCGGATTTTTAAGTCAGGGGTGAAATCCTGAGGCTCAACCTCAGAACTGCCTTTGATACTGGAAATCTTGAGTATGGTAGAGGTCGGTGGAACAGCAAGTGTAGAGGTGAAATTCGTAGATATTTGCTAGAACACCAGTGGCGAAGGCGGCCGACTGGACCATAACTGACGCTGAGGTGCGAAAGCGTGGGGAGCAAACAGG` |
| ASV4078 | 404 | `TGGGGAATCTTGCGCAATGGGCGAAAGCCTGACGCAGCCATGCCGCGTGAATGATGAAGGTCTTAGGATTGTAAAATTCTTTCACCGGGGAAGATAATGACGGTACCCGGAGAAGAAGCCCCGGCTAACTTCGTGCCAGCAGCCGCGGTAATACGAAGGGGGCTAGCGTTGCTCGGATTTACTGGGCGTAAAGGGCGCGTAGGCGGACAGTTTAGTCAGAGGTGAAAGCCCGGGGCTCAACCCCGGAATAGCCTTTGATACTGGTTGTCTTGAGTTTGGGAGAGGTATGCGGAACTCCGAGTGTAGAGGTGAAATTCGTAGATATTCGGAAGAACACCAGTGGCGAAGGCGGCATACTGGCCCAATACTGACGCTGAGGCGCGAAAGCGTGGGGAGCAAACAGG` |
| ASV2935 | 409 | `TGGGGAATATTGGACAATGGGCGGAAGCCTGATCCAGCAACGCCGCGTGAGGGATGACGGCCTTCGGGTTGTAAACCTCTTTCAGCACAGACGAAGCGAAAGTGACGGTATGTGCAGAAGAAGCACCGGCCAACTACGTGCCAGCAGCCGCGGTAATACGTAGGGTGCGAGCGTTGTCCGGAATTATTGGGCGTAAAGGGCTCGTAGGCGGTTTGTCGCGTCGGGAGTGAAAACACACAGCTTAACTGTGTGCTTGCTTCCGATACGGGCAGACTAGAGGTATGCAGGGGAGAACGGAATTCCTGGTGTAGCGGTGAAATGCGCAGATATCAGGAGGAACACCGGTGGCGAAGGCGGTTCTCTGGGCATTACCTGACGCTGAGGAGCGAAAGTGTGGGGAGCGAACAGG` |
| ASV156 | 404 | `TGGGGAATATTGGACAATGGGCGCAAGCCTGATCCAGCAATGCCGCGTGAGTGATGAAGGCCTTCGGGTTGTAAAGCTCTTTTACCTACGACGATGATGACGGTAGTAGGAGAATAAGCCCCGGCTAACTTCGTGCCAGCAGCCGCGGTAATACGAAGGGGGCTAGCGTTGTTCGGAATTACTGGGCGTAAAGGGCGCGTAGGCGGCCCTGTCAGTCAAGCGTGAAAGCCCCGGGCTCAACCTGGGAATTGCGCCTGATACTGCAGGGCTTGAGGGTCGGAGAGGATGGTGGAATTCCCAGTGTAGAGGTGAAATTCGTAGATATTGGGAAGAACACCGGTGGCGAAGGCGGCCATCTGGACGACATCTGACGCTGAGGCGCGAAAGCGTGGGGAGCAAACAGG` |
| ASV4930 | 389 | `TCGGGAAACTTTTGCAATACACGAAAGTGTGACAAAGCAAGCCAGAGTGCTTTTCTATTTTTGAAAAGCTTTTGTGAGATGTAAAAAGTCTTACGAATAAGGACTGGGCAAGACTGGTGCCAGCCGCCGCGGTAATCCCAGCAGTCCAAGTCGCAGCCACATTTGTTGGGTCTAAAATATCCGTAGCTTGCTTTTCAAGTCTCTTGTGAAATCGGGACTCTTAAGGTTCCGGCGTGCAAGAGATACTGTTTAGCTAGAGACCGGGAGGCGTAAGAAGTACGGATGGGGTAACGGTAAAATGTGTTGATCCCATCTGGACTAACAGTCGCGAAGGCGTCTTACGAGAACGGTTCTGACAGTGAGGGATGAAGGCTAGGGGCGCAAAGTGG` |
| ASV2956 | 406 | `TGGGGAATTTTCCGCAATGGGCGCAAGCCTGACGGAGCAATACCGCGTGAGGGAGGAAGGCTCTTGGGTCGTAAACCTCTTTTCTCTGGGAAGAAGAACTGACGGTACCAGAGGAATCAGCATCGGCTAACTCCGTGCCAGCAGCCGCGGTAATACGGGGGATGCAAGCGTTATCCGGAATTATTGGGCGTAAAGCGTCCGCAGGTGGCTGATCAAGTCTGTCGTCAAAGCGCGGAGCTCAACTCCGTACAGGCGATGGAAACTGATAGGCTAGAGTTAGGTAGGGGTAAGAGGAATTCCCAGTGTAGCGGTGAAATGCGTAGATATTGGGAAGAACACCGGCGGCGAAAGCGTCTTACTGGGCCTGTACTGACACTGAGGGACGAAAGCTAGGGGAGCGAAAGGG` |
| ASV388 | 430 | `TAGGGAATATTGCACAATGGGGGAAACCCTGATGCAGCAACGCCGCGTGAGTGAAGAAGGCCCTTGGGTCGTAAAGCTCTTTAGCGAGGGAAGAGGGTTGTTATGGTGAATAATCATGGCAGCTGACGGTACCTCGAGAATAAGCACCGGCTAATTTCGTGCCAGCAGCCGCGGTAATACGAAAGGTGCAAGCGTTACTCGGAATCACTGGGCGTAAAGCGCACGTAGGCGGACTCGTAGGTCAGTTGTGAAATACCTGGGCTCAACCCAGGAACTGCAGCTGAAACCGCTTGTCTTGAATACCTGAGAGGGTGGGGGAATACCTGGTGGAGAAGTGAAATTCGTAGAGATCAGGTGGAACACCGGAGGCGAAGGCGCCTGCCTGGCAGTGTATTGACGCTGAGGTGCGAAAGTGCGGGGAGCAAACAGG` |
| ASV4439 | 429 | `TGGGGAATTTTGGACAATGGGGGAAACCCTGATCCAGCAATGCCGCGTGAGTGATGAAGGCCTTCGGGTTGTAAAGCTCTTTTGCGAGGAAAGAAAAGACTTGAGCTAATACCTCAGGTTCATGACGTTACCTCGAGAATAAGCACCGGCTAACTACGTGCCAGCAGCCGCGGTAATACGTAGGGTGCAAGCGTTAATCGGAATGACTGGGCGTAAAGCGTGCGTAGGTGGTTAATCAAGTCAAGCGTGAAAGCCCCGAGCTCAACTCGGGAACTGCGTTTGATACTGTTTAGCTAGAGTGCGGCAGAGGGAAGCGGAATTCCGCGTGTAGCAGTGAAATGCGTAGATATGCGGAGGAACACCGGTGGCGAAGGCAGCTTCCTGGGCCAAAACTGACACTGAGGCACGAAAGCGTGGGGAGCAAACAGG` |
| ASV1795 | 429 | `TGGGGAATTTTGGACAATGGGCGAAAGCCTGATCCAGCCATTCCGCGTGAGTGAAGAAGGCCTTCGGGTTGTAAAGCTCTTTAGTCAGGGAGCAAATCCTACGTATAAACAATGCGTGGGGATGAGAGTACCTGAAGAATAAGCACCGGCTAACTACGTGCCAGCAGCCGCGGTAATACGTAGGGTGCAAGCGTTAATCGGAATTACTGGGCGTAAAGCGTGCGCAGGCGGCCTTAAAAGTCAGATGTGAAATCCCTGGGCTCAACCTAGGAACTGCGTTTGAAACTCTAAGGCTAGAGTATGTGAGAGGGGGGTAGAATTCCACGTGTAGCAGTGAAATGCGTAGAGATGTGGAGGAATACCGATGGCGAAGGCAGCCCCCTGGCATAATACTGACGCTCATGCACGAAAGCGTGGGGAGCAAACAGG` |
| ASV4673 | 404 | `TAGGGAATATTGCGCAATGGGCGAAAGCCTGACGCAGCAATTCCGCGTGGAGGATGACATATCTCGGTATGTAAACTCCTGTCGGCGGGGACGATTATGACGGTACCCGCAAAGGAAGCACCGGCTAACTACGTGCCAGCAGCCGCGGTAATACGTAGGGTGCAAGCGTTGTCCGGATTTATTGGGCGTAAAGCGTCCGTAGGCGGTTTTGAAAGTCTGGTGTTAAAGGCTGGAGCTCAACTCCAGTAGGGCATTGGAAACTACTTAACTAGAGTGCAATAGGGGAAGCTGGAATTTCCGGTGTAGCGGTGAAATGCGTAGATATCGGAAGGAACACCAGTGGCGAAAGCGGGCTTCTGGATTGCAACTGACGCTGATGGACGAAAGCTAGGGGAGCGAAAGGG` |
| ASV879 | 408 | `TCGAGAATCTTCCGCAATGGACGAAAGTCTGACGGAGCGACGCCGCGTGGTGGATGAAGTCCTTAGGGACGTAAACACCTTTTATGGAGGAGAAAGTTTATTGATGTTACTCCATGAATAAGGGGCTCCCAACTCTGTGCCAGCAGGAGCGGTAATACAGAGGCCCCAAGCATTATCCGGATTTACTGGGCGTAAAGGGTGCGTAGGCGGTTATATTAGTCAGGTGTTAAATCTTTGGGCTCAACCCAAAAATCGCATTTGAAACGGTATAACTAGAAGAAGTCAGAGGCAAGCAGAACTCACGGTGTAGGGGTGAAATCCGTTGATATCGTGGGGAATACCAAATGCGAAGGCAGCTTGCTGGGACTCTCTTGACGCTGAGGCACGAAAGCGTGGGTAGCGAAGCGG` |
| ASV1147 | 404 | `TGGGGAATATTGGACAATGGGCGAAAGCCTGATCCAGCAATGCCGCGTGAGTGAAGAAGGCCTTCGGGTTGTAAAGCTCTTTTACCCGGGATGATAATGACAGTACCGGGAGAATAAGCCCCGGCTAACTCCGTGCCAGCAGCCGCGGTAATACGGAGGGGGCTAGCGTTGTTCGGAATTACTGGGCGTAAAGCGTACGTAGGCGGCTTGGTAAGTCAGAGGTGAAAGCCCACGACTCAATCGTGGAACTGCCTTTGAGACTGCTTGGCTTGAAGATGGGAGAGGTTAGTGGAATTCCGAGTGTAGAGGTGAAATTCGTAGATATTCGGAAGAACACCAGTGGCGAAGGCGGCTAACTGGACCATTCTTGACGCTGAGGTACGAAAGCGTGGGGAGCAAACAGG` |
| ASV1462 | 429 | `TGGGGAATTTTGGACAATGGGCGCAAGCCTGATCCAGCAATGCCGCGTGCAGGATGAAGGCCTTCGGGTTGTAAACTGCTTTTGTACGGAGCGAAAAGGTTTTCTCTAATACAGGAGGCCCATGACGGTACCGTAAGAATAAGCACCGGCTAACTACGTGCCAGCAGCCGCGGTAATACGTAGGGTGCGAGCGTTAATCGGAATTACTGGGCGTAAAGCGTGCGCAGGCGGTTTTGTAAGACAGATGTGAAATCCCCGGGCTTAACCTGGGACCTGCATTAGTGACTGCAAAGCTAGAGTACGGTAGAGGGGGATGGAATTCCGCGTGTAGCAGTGAAATGCGTAGATATGCGGAGGAACACCGATGGCGAAGGCAATCCCCTGGACCTGTACTGACGCTCATGCACGAAAGCGTGGGGAGCAAACAGG` |
| ASV1623 | 404 | `TGAGGAATCTTCCACAATGGACGAAAGTCTGATGGAGCGACACCGCGTGAAGGATGAAGGCCTAACGGTTGTAAACTTCTTTTCTGAGGGAGCATAATGAGAGTACTTCAGGAATAAGGAGCGGCAAATCACGTGCCAGCAGCCGCGGTAATACGTGAGCTCCAAGCGTTACCCGGAATCATTGGGTGTAAAGGATGCGTAGGTGGATTTTTAAGTCAGGTATGAAAGACCGGAGCTCAACTCCGCGTTTGTGCTTGAAACTGAAAATCTGGAATCAGGGAGAGGTTAGCGGAATTCTAAGTGTAGGGGTGCAATCCGTAGATACTTAGAAGAACACCAAAAGCGAAGGCAGCTAACTGGAACTGTATTGACACTGAGGCATGAAAGCGTGGGGAGCAAAAAGG` |
| ASV4385 | 388 | `GCGCGAAAACTTTACAATGCGCGAAAGCGTGATAAGGGAATTCCCAGTGCTTTGCCTTCGGGTAAAGCTTTTGTCGAGTCTAAGAATCTCGGCGAATAAGGGGTGGGTAAGACGGGTGCCAGCCGCCGCGGTAACACCCGCGCCCCAAGTGGAGCTCACATTTATTGGGTCTAAAGCGTTCGTAGCCGGGCGGGCAAATCTCTTGTGAAATCGTTGCGCTTAACGTAACGTCGTGCAGGAGAGACTGCTCGTCTTGAGACCGGGAAGAGGGCGGGGTACGCGAGGGGAAGCGGTAAAATGTGATAATCCTTCGCGGACCACCAGTGGTGGAGACGCCGCTCTAGAACGGGTCTGACGGTGAGGGACGAAGGCCAGGGGAGCAAAACGG` |
| ASV3673 | 423 | `TAGGGAATATTGGGCAATGGAAGGAATTCTGACCCAGCCATGCCGCGTGCAGGATGAAGGCGCTATGCGTTGTAAACTGCTTTTGTCAGGGAAGAAAAAGGCTATGCGTAGCAAATTGACGGTACCTGAAGAATAAGCATCGGCTAACTCCGTGCCAGCAGCCGCGGTAATACGGAGGATGCAAGCGTTGTCCGGATTTATTGGGTTTAAAGGGTGCGTAGGCGGTCTGATAAGTCAGTGGTTAAATAACCTTGCTTAACGAGGGAGATGCCATTGATACTGTTAGACTTGAATACTGTTTGGGTAGGCGGAATTGGTAGTGTAGCGGTGAAATGCTTAGATATTACCAGGAACACCGATAGCGAAGGCAGCTTACTGAAGAGATATTGACGCTGAGGCACGAAAGTGCGGGTATCAAACAGG` |
| ASV5467 | 429 | `TGGGGAATATTGCGCAATGGGGGAAACCCTGACGCAGCGACGCCGCGTGGGTGATGAAGGCCTTCGGGTTGTAAAGCTCTGTCGGCTGGGAAAAAGGTGTTCGTGCTAATACCACGAACGCTTGATGGTACCAGCAAAGGAAGCACCGGCTAACTCTGTGCCAGCAGCCGCGGTAATACAGAGGGTGCAAGCGTTGTTCGGAATTACTGGGCGTAAAGGGCGTGTAGGCGGCTTAGCAAGTCAGATGTGAAATCCCGGGGCTTAACTTCGGAAGTGCATTTGAAACTGCTAGGCTAGAGTGAAGTAGAGGTTGGTGGAATTCCCAGTGTAGAGGTGAAATTCGTAGATATTGGGAGGAACACCGGTGGCGAAGGCGGCCAACTGGGCTTTAACTGACGCTGAGGCGCGAAAGCGTGGGGAGCAAACAGG` |
| ASV2135 | 424 | `TAAGGAATATTGGGCAATGGACGCAAGTCTGACCCAGCCATGCCGCGTGAAGGATGAAGGTCCTCTGGATTGTAAACTTCTTTTATCTGGGGCGAAAACAACTCTTTCTAGAGTTCTTGACAGTACCAGAGGAATAAGCACCGGCTAACTCCGTGCCAGCAGCCGCGGTAATACGGAGGGTGCAAGCGTTATCCGGATTCACTGGGTTTAAAGGGTGCGTAGGCGGGTTAGTAAGTCAGTGGTGAAATCCCCGAGCTTAACTTGGGAACTGCCGTTGATACTATTAGTCTTGAATATCGTGGAGGTAAGCGGAATATGTCATGTAGCGGTGAAATGCTTAGATATGACATAGAACACCAATTGCGAAGGCAGCTTGCTACACGATTATTGACGCTGAGGCACGAAAGCGTGGGGATCAAACAGG` |
| ASV2266 | 404 | `TGGGGAATATTGGACAATGGGCGCAAGCCTGATCCAGCAATTCCGCGTGGGTGAAGAAGGTCTTCGGATTGTAAAGCCCTTTCGTTGGGGACGATGATGACGGTACCCAAAGAAGAAGCCCCGGCTAACTTCGTGCCAGCAGCCGCGGTAATACGAAGGGGGCTAGCGTTGCTCGGAATGACTGGGCGTAAAGGGCGCGTAGGCGGCATAACTCGTCAGACGTGAAATTCCTGGGCTTAACCTGGGGGCTGCGTTTGATACGGTTGGGCTAGAGGATGGAAGAGGCTCGTGGAATTCCCAGTGTAGAGGTGAAATTCGTAGATATTGGGAAGAACACCGGTGGCGAAGGCGGCGAGCTGGTCCATTACTGACGCTGAGGCGCGAAAGCGTGGGGAGCAAACAGG` |
| ASV366 | 429 | `TGGGGAATTTTGGACAATGGGCGAAAGCCTGATCCAGCAATGCCGCGTGAGTGAAGAAGGCCTTCGGGTTGTAAAGCTCTTTTGTCAGGGAAGAAACGGTTTGGGCTAATATCCTGAGCTAATGACGGTACCTGAAGAATAAGCACCGGCTAACTACGTGCCAGCAGCCGCGGTAATACGTAGGGTGCAAGCGTTAATCGGAATTACTGGGCGTAAAGCGTGCGCAGGCGGTTTTGTAAGTCTGACGTGAAATCCCCGGGCTCAACCTGGGAATTGCGTTGGAGACTGCAAGGCTAGAATCTGGCAGAGGGGGGTAGAATTCCACGTGTAGCAGTGAAATGCGTAGAGATGTGGAGGAACACCGATGGCGAAGGCAGCCCCCTGGGTCAAGATTGACGCTCATGCACGAAAGCGTGGGGAGCAAACAGG` |
| ASV1451 | 428 | `TGGGGAATATTGGACAATGGGGGAAACCCTGATCCAGCGATGCCGCGTGAGTGAAGAAGGCCTTCGGGTTGTAAAGCTCTTTAGTCAGGAAAGAAATGTTGTTTCGGAAATGTGACAACTATGACGTTACCTGAAGAATAAGCACCGGCTAACTCTGTGCCAGCAGCCGCGGTAATACAGAGGGTGCAAGCGTTAATCGGATTTACTGGGCGTAAAGCGCGCGTAGGTGGTTTGATAGGTTGGATGTGAAATCCTTGGGCTTAACCCAAGAATGGCATTCAAAACGGTCAAGCTAGAGTGTTGGAGAGGGAAGTAGAATTTCTGGTGTAGCGGTGAAATGCGTAGATATCAGAAAGAATGACAGTGGCGAAGGCGGCTTCCTGGCCAAATACTGACACTGAGGCGCGAAAGCGTGGGTAGCGAACGGG` |
| ASV991 | 429 | `TGGGGAATATTGCACAATGGGCGCAAGCCTGATGCAGCCATGCCGCGTGTGTGAAGAAGGCCTTCGGGTTGTAAAGCACTTTCAGCGAGGAGGAAGGGTAGTGTGTTAATAGCACACTATATTGACGTTACTCGCAGAAGAAGCACCGGCTAACTCCGTGCCAGCAGCCGCGGTAATACGGAGGGTGCAAGCGTTAATCGGAATTACTGGGCGTAAAGCGCACGCAGGCGGTTTGTTAAGTCAGATGTGAAATCCCCGCGCTTAACGTGGGAACTGCATTTGAAACTGGCAAGCTAGAGTCTTGTAGAGGGGGGTAGAATTCCAGGTGTAGCGGTGAAATGCGTAGAGATCTGGAGGAATACCGGTGGCGAAGGCGGCCCCCTGGACAAAGACTGACGCTCAGGTGCGAAAGCGTGGGGAGCAAACAGG` |
| ASV278 | 404 | `TGGGGAATCTTAGACAATGGGCGCAAGCCTGATCTAGCCATGCCGCGTGAGCGATGAAGGCCTTAGGGTTGTAAAGCTCTTTCGTGGGGGAAGATAATGACTGTACCCCAAGAAGAAGCCCCGGCTAACTCCGTGCCAGCAGCCGCGGTAATACGGAGGGGGCTAGCGTTGTTCGGAATTACTGGGCGTAAAGCGCACGTAGGCGGACCAGAAAGTCAGAGGTGAAATCCCAGGGCTCAACCTTGGAACTGCCTTTGAAACTCCTGGTCTTGAGGTCGAGAGAGGTGAGTGGAATTCCGAGTGTAGAGGTGAAATTCGTAGATATTCGGAGGAACACCAGTGGCGAAGGCGGCTCACTGGCTCGATACTGACGCTGAGGTGCGAAAGCGTGGGGAGCAAACAGG` |
| ASV804 | 429 | `TGGGGAATATTGGACAATGGGGGAAACCCTGATCCAGCAATGCCGCGTGTGTGAAGAAGGCCTGAGGGTTGTAAAGCACTTTCAGTGGGGAGGAGTCTTATTAGGTTAAGAGCTAAATAAGAGGACGTTACCCACATAAGAAGCACCGGCTAACTCCGTGCCAGCAGCCGCGGTAATACGGAGGGTGCGAGCGTTAATCGGAATTACTGGGCGTAAAGGGTGCGTAGGTGGTTTGATAAGTTATCTGTGAAAGCCCTGGGCTCAACCTAGGAAGGCCAGATAAGACTGTTAGACTCGAGTATGGGAGAGGGTAGTGGAATTTCCGGTGTAGCGGTGAAATGCGTAGAGATCGGAAGGAACACCAGTGGCGAAGGCGGCTACCTGGCCTAATACTGACACTGAAGCACGAAAGCGTGGGGAGCAAACAGG` |
| ASV2184 | 423 | `TAGGGAATATTGGGCAATGGGCGCAAGCCTGACCCAGCCATGCCGCGTGCAGGAAGAAGGCGTTATGCGTTGTAAACTGCTTTTATATAGGAAGAAAAAGACTTTGCGAAGTAAATTGACGGTACTATATGAATAAGCACCGGCTAACTCCGTGCCAGCAGCCGCGGTAATACGGAGGGTGCAAGCGTTGTCCGGATTTATTGGGTTTAAAGGGTGCGTAGGCGGCCTATTAAGTCAGTGGTGAAAGACCGTCGCTCAACGACGGAACTGCCATTGATACTGATTGGCTTGAGTTTGATTGAGGCAGCCGGAATGGATAGTGTAGCGGTGAAATGCATAGATATTATCCAGAACGCCAATAGCGTAGGCAGGTTGCTAAGTCATAACTGACGCTGAGGCACGAGAGTGCGGGGATCAAACAGG` |
| ASV3231 | 405 | `TGAGGAATCTTGCACAATGGGCGAAAGCCTGATGCAGCGACGCCGCGTGAGGGACGAAGGCCTTCGGGTTGTAAACCTCTGTCAGTAGGGAAGAAGATTGACGGTACCTACTGAGGAAGCTTCGGCTAACTACGTGCCAGCAGCCGCGGTAATACGTAGGAAGCAAGCGTTGCCCGGAATTACTGGGCGTAAAGAGCTCGTAGGCGGGGGCGTGCGTCCGAGAAGAAATCTCAAGGCTCAACCTTGGGGCTATCTCGGATACGGCGCTTCTTGAGGGTGAGAGAGGAAAGCGGAATTCCAGGTGTAGCGGTGAAATGCGTAGATATCTGGAGGAACACCAGCGGCGAAGGCGGCTTTCTGGCTCATTCCTGACGCTGAGGAGCGAAAGCGTGGGTAGCGAACGGG` |
| ASV9 | 429 | `TGGGGAATATTGGACAATGGGCGCAAGCCTGATCCAGCCATGCCGCGTGTGTGAAGAAGGCCTTCGGGTTGTAAAGCACTTTTGTTGGGAAAGAAATCCTGCCGGTTAATACCCGGCGGGGCTGACGGTACCCAAAGAATAAGCACCGGCTAACTTCGTGCCAGCAGCCGCGGTAATACGAAGGGTGCAAGCGTTACTCGGAATTACTGGGCGTAAAGCGTGCGTAGGTGGTTTGTTAAGTCAGATGTGAAAGCCCCGGGCTCAACCTGGGAATTGCATTTGATACTGGCAGACTAGAGTGCGGTAGAGGAGAGTGGAATTCCCGGTGTAGCAGTGAAATGCGTAGAGATCGGGAGGAACATCAGTTGCGAAGGCGGCTCTCTGGACCAGCACTGACACTGAGGCACGAAAGCGTGGGGAGCAAACAGG` |
| ASV5669 | 429 | `TGGGGAATATTGGACAATGGGGGCAACCCTGATCCAGCGATGCCGCGTGTGCGAAGAAGGCCTGCGGGTTGTAAAGCACTTTGATGGGGGAAGAAGGTTTTGTATCGAATAGATACGAGAATTGACGTTACCCCAAGAACAAGCACCGGCTAACTCTGTGCCAGCAGCCGCGGTAATACAGAGGGTGCGAGCGTTAATCGGAATTACTGGGCGTAAAGGGCGCGTAGGCGGCTATGTAAGTTAGATGTGAAATCCTTGGGCTTAACCTGAGCATTGCATTTAATACTGTATGGCTAGAGTAAAGTAGAGGGAAGTGGAATTTCCGGTGTAGCGGTGAAATGCGTAGATATCGGAAAGAACATCAGTGGCGAAGGCGGCTTTCTGGACTTATACTGACGCTGAGGCGCGAAAGCATGGGTAGCAAACAGG` |
| ASV2771 | 429 | `TGGGGAATTTTGGACAATGGGCGCAAGCCTGATCCAGCAATGCCGCGTGCAGGAAGAAGGCCTTCGGGTTGTAAACTGCTTTTGTACGGAACGAAACGGTCTGGGTTAATACCCTGGGCTAATGACGGTACCGTAAGAATAAGCACCGGCTAACTACGTGCCAGCAGCCGCGGTAATACGTAGGGTGCAAGCGTTAATCGGAATTACTGGGCGTAAAGCGTGCGCAGGCGGTGATGTAAGACAGTCGTGAAATCCCCGGGCTCAACCTGGGAATTGCGATTGTGACTGCATCGCTGGAGTGCGGCAGAGGGGGATGGAATTCCGCGTGTAGCAGTGAAATGCGTAGATATGCGGAGGAACACCGATGGCGAAGGCAATCCCCTGGGCCTGCACTGACGCTCATGCACGAAAGCGTGGGGAGCAAACAGG` |
| ASV1670 | 430 | `TGGGGAATATTGGACAATGGGCGAAAGCCTGATCCAGCAATGCCGCGTGTGTGAAGAAGGCCTGCGGGTTGTAAAGCACTTTCAGGGGGGAAAAAGAAGACTGTGTGAATAGCACAGCGCTTTTGATACTACCCCCAGAAGAAGCACCGGCTAACTCTGTGCCAGCAGCCGCGGTAATACAGAGGGTGCGAGCGTTAATCGGAATAACTGGGCGTAAAGGGTGCGTAGGTGGCACGCTAAGTCAGATGTGAAATCCCCGGGCTTAACCTGGGAACTGCATTTGAAACTGGCGAGCTAGAGTAGAGTAGAGGAAAGTGGAATTTCAGGTGTAGCGGTGAAATGCGTAGATATCTGAAGGAACACCAGTGGCGAAGGCGGCTTTCTGGACTCATACTGACACTGAGGCACGACAGCGTGGGGAGCAAACAGG` |
| ASV5227 | 430 | `TGGGGAATCTTGCGCAATGGGCGAAAGCCTGACGCAGCAACGCCGCGTGAGTGAAGAAGGCCTTTGGGTTGTAAAGCTCTTTCGACTGGGAAGAAGGGTGTCGTCGCGAATAGTAACGACATTTGACGGTACCAGGAGAAGAAGCCCCGGCTAACTTCGTGCCAGCAGCCGCGGTAATACGAAGGGGGCAAGCGTTGTTCGGAATCATTGGGCGTAAAGGGCGCGTAGGCGGGCCTGTAAGTCGATTGTGAAATCCCTGGGCTTAACCCGGGAATTGCAGTCGAAACTGCAGGTCTTGAGTTCTCAAGAGGATGGTGGAATTCCTGGAGTAGTAGTGAAATACGTAGATATCAGGAGGAACACCGGCGGCGAAGGCGGCCATCTGGTGAGACACTGACGCTGAGGCGCGAAAGCATGGGGAGCAAACAGG` |
| ASV2673 | 406 | `GCGAGAATATTCGACAATGGACGAAAGTCTGATCGAGCGACGCCGCGTGTACGATGAAGTTTTTCGGAATGTAAAGTACTTTTATTAGGGAAGAACGTTGACGGTACCTAATGAATAAGGGGTTGCTAACTCTGTGCCAGCAGCAGCGGTAATACAGAGACCCCAAGCGTTATCCGGAATCATTGGGCGTAAAGGGTGAGTAGGTGGTATTGTTAGTCGGGTTTTAAAGATTATGGGCTCAACCCATGATATGGACCCGAAACGGCAAAACTAGAATATGTAAGAGGTTAGTGGAACTATATGTGTAGGGGTAAAATCCGTTGATATATATGGGAACACCTAAAGCGAAGGCAGCTAACTGGGACATTATTGACACTGAATCACGAAAGCGTGGGTAGCGATACGG` |
| ASV550 | 424 | `TGAGGAATATTGGTCAATGGGCGGGAGCCTGAACCAGCCAAGTAGCGTGAAGGACGAAGGCCCTATGGGTCGTAAACTTCTTTTATAGGGGAATAAAGTGGAGAACGTGTTCTCCTTTGTATGTACCCTTTGAATAAGGATCGGCTAACTCCGTGCCAGCAGCCGCGGTAATACGGAGGATCCGAGCGTTATCCGGATTTATTGGGTTTAAAGGGAGCGTAGGTGGATATATAAGTCAGTTGTGAAAGTTTACGGCTCAACCGTGAAATTGCAGTTGATACTGTGTATCTTGAGTGTACAAGAGGTGGGCGGAATTCGTGGTGTAGCGGTGAAATGCTTAGATATCACGAAGAACTCCGATTGCGAAGGCAGCTCACTGGGGTACAACTGACACTGAGGCTCGAAAGTGTGGGTATCAAACAGG` |
| ASV4314 | 416 | `TTAGGAATCTTCCACAATGGGCGCAAGCCTGATGGAGCGACGCCGCGTGAGGGATGAAGGTTTTCGGATCGTAAACCTCTGAATGGGGGACGAAAGGGCCGTAAGGCAGATGACGGTACCCCAGTAATAGCACCGGCTAACTCCGTGCCAGCAGCCGCGGTAATACGGAGGGTGCAAGCGTTACCCGGAATCACTGGGCGTAAAGGGCGTGTAGGCGGTTCACTAAGTCTGGTTTTAAAGACCGACGCTCAACGTCGGGCATGGACTGGCGACTGGTGGACTAGACCTCTGGAGAGGCAATCGGAATTCCTGGTGTAGCGGTGGAATGCGTAGATACCAGGAGGAACACCAACGGCGAAGGCAGATTGCTGGACAGAAGGTGACGCTGAGGCGCGAAAGTGTGGGGAGCGAACCGG` |
| ASV1942 | 429 | `TGGGGAATTTTGGACAATGGGGGCAACCCTGATCCAGCCATTCCGCGTGAGTGAAGAAGGCCTTCGGGTTGTAAAGCTCTTTTGTCGGGGACGAAACGGTTTTGGCTAATAACCAAGACTACTGACGGTACCCGAAGAATAAGCACCGGCTAACTACGTGCCAGCAGCCGCGGTAATACGTAGGGTGCGAGCGTTAATCGGAATTACTGGGCGTAAAGCGTGCGCAGGCGGTTTTGTAAGCCAGATGTGAAATCCCCGGGCTTAACCTGGGAATGGCATTTGGGACTGCAAGGCTAGAGTATGGCAGAGGAGACTAGAATTCCTGGTGTAGCAGTGAAATGCGTAGATATCAGGAGGAATACCGATGGCGAAGGCAGGTCTCTGGGTCAATACTGACGCTCATGCACGAAAGCGTGGGGAGCAAACAGG` |
| ASV1267 | 429 | `TGGGGAATTTTGGACAATGGGGGAAACCCTGATCCAGCAACGCCGCGTGAATGAAGAAGGCCTTAGGGTTGTAAAGTTCTTTTGTCAGGGAAGAAAGTTTCTGTGCTAATACCATAGAACGATGACGGTACCTGAAGAATAAGCACCGGCTAACTACGTGCCAGCAGCCGCGGTAATACGTAGGGTGCAAGCGTTAATCGGAATTACTGGGCGTAAAGCGTGCGCAGGCGGATATGTAAGTTAGAAGTGAAATCCCCGGGCTCAACCTGGGAACTGCTTTTAAGACTGCGTATCTAGAGTTTGTCAGAGGGGGGTGGAATTCCAAGTGTAGCAGTGAAATGCGTAGAGATTTGGAGGAACACCGATGGCGAAGGCAGCCCCCTGGGATAAAACTGACGCTCATGCACGAAAGCGTGGGTAGCAAACAGG` |
| ASV18 | 404 | `TGGGGAATCTTAGACAATGGGGGAAACCCTGATCTAGCCATGCCGCGTGAGCGATGAAGGCCTTAGGGTTGTAAAGCTCTTTCGTGGGGGAAGATAATGACTGTACCCCAAGAAGAAGCCCCGGCTAACTCCGTGCCAGCAGCCGCGGTAATACGGAGGGGGCTAGCGTTGTTCGGAATTACTGGGCGTAAAGCGCACGTAGGCGGACCGGAAAGTCAGAGGTGAAATCCCAGGGCTCAACCTTGGAACTGCCTTTGAAACTCCTGGTCTTGAGGTCGAGAGAGGTGAGTGGAATTCCGAGTGTAGAGGTGAAATTCGTAGATATTCGGAGGAACACCAGTGGCGAAGGCGGCTCACTGGCTCGATACTGACGCTGAGGTGCGAAAGCGTGGGGAGCAAACAGG` |
| ASV4818 | 407 | `TAGGGAATATTGCACAATGGGGGAAACCCTGATGCAGCGACGCCGCGTGAGTGATGAAGGCCTTCGGGTCGTAAAGCTCTGTTGCATGGGAAAAAACAAATGATGGTACCATGCGAGAAAGGACCGGCTAACTTCGTGCCAGCAGCCGCGGTAAGACGAAGGGTCCTAGCGTTGCTCGGAATCATTGGGCGTAAAGCGTGCGTAGGCTGCCTTGTAAGTCAGATGTGAAAGCCTTGGGCTCAACCCAAGAAGTGCATTTGATACTGCGAGGCTTGAGTACAGGAGAGGGTAGTAGAATTCCTGGTGTAGTGGTGAAATACGTAGATATCAGGAGGAATACCGGTGGCGAAGGCGGCTACCTGGCCTAGTACTGACGCTGAGGCACGAAAGCGTGGGGATCAAACAGG` |
| ASV4971 | 424 | `TGAGGAATATTGGGCAATGGAGGCAACTCTGACCCAGCCATGCCGCGTGCAGGAAGACGGCCCTCTGGGTTGTAAACTGCTTTTGTTTGGGAATAAACCCTCCTACGTGTAGGAGGCTGAAGGTACCTTACGAATAAGCATCGGCTAACTCCGTGCCAGCAGCCGCGGTAATACGGAGGATGCAAGCGTTATCCGGATTTATTGGGTTTAAAGGGTCCGTAGGCGGGTCTTTAAGTCAGTGGTGAAAGCCCACAGCTCAACTGTGGAACTGCCATTGATACTGGAGATCTTGAGTGTAGTAGAAGTAGGCGGAATAGGGCATGTAGCGGTGAAATGCATAGATATGCCCTAGAACACCGATTGCGAAGGCAGCTTACTATGTTACAACTGACGCTGAGGGACGAAAGCGTGGGGAGCAAACAGG` |
| ASV3458 | 428 | `TGGGGAATTTTCCACAATGGGCGAAAGCCTGATGGAGCAACGCCGCGTGTGTGATGAAGGTTTTCGGATCGTAAAGCACTTTCGACCGGGACGAAAAACCTCTGGCTAACATCCAGGGGCCTGACGGTACCGGGAGAAGAAGCACCGGCTAACTCTGTGCCAGCAGCCGCGGTAATACAGAGGGTGCAAGCGTTGTTCGGAATTATTGGGCGTAAAGCGCGTGTAGGCGGCTTGGCAAGTCAGATGTGAAAGCCCTCGGCTTAACCGAGGAAGTGCGTCTGAAACTGCTGAGCTTGAGTGTCGGAGAGGATGGCGGAATTCCGCAAGTAGAGGTGAAATTCGTAGATATGCGGAGGAACACCGGTGGCGAAGGCGGCCATCTGGACGACTACTGACGCTGAGACGCGAAAGCGTGGGGAGCAAACAGG` |
| ASV3264 | 424 | `TGAGGAATATTGGTCAATGGGCGCGAGCCTGAACCAGCCAAGTAGCGTGAAGGATGAAGGCTCTATGGGTCGTAAACTTCTTTTATATGGGAATAAAGTGAGCCACGTGTGGCTTTTTGTATGTACCATATGAATAAGGACCGGCTAACTCCGTGCCAGCAGCCGCGGTAATACGGAGGGTCCGAGCGTTATCCGGATTTATTGGGTTTAAAGGGAGCGTAGGCGGATTGATAAGTCAGTTGTGAAAGTTTGCGGCTCAACCGTAAAATTGCAGTTGATACTGTCAGTCTTGAGTACAGTAGAGGTAAGCGGAATTCGTGGTGTAGCGGTGAAATGCTTAGATATCACGAAGAACTCCAATTGCGAAGGCAGCTTGCTGGACTGTCACTGACGCTGAGGCTCGAAAGTGTGGGTATCAAACAGG` |
| ASV2419 | 424 | `TGAGGAATATTGGACAATGGGCGCAAGCCTGATCCAGCCATGCCGCGTGCAGGATGAAGCATCTATGGTGTGTAAACTGCTTTTGTACGAGAAGAAACACTCCTTCGTGAAGGGGCTTGACGGTATCGTAAGAATAAGGATCGGCTAACTCCGTGCCAGCAGCCGCGGTAATACGGAGGATCCAAGCGTTATCCGGAATCATTGGGTTTAAAGGGTCCGTAGGCGGGCTAGTAAGTCAGTGGTGAAATCTCTCGGCTCAACCGGGAAATGGCCATTGATACTGCTAGTCTTGAATTATTAGGAAGTAACTAGAATATGTAGTGTAGCGGTGAAATGCTTAGAGATTACATGGAATACCAATTGCGAAGGCAGGTTACTACTAATGGATTGACGCTGATGGACGAAAGCGTGGGTAGCGAACAGG` |
| ASV3940 | 429 | `TGGGGAATATTGGACAATGGGGGCAACCCTGATCCAGCGATGCCGCGTGTGTGAAGAAGGCCTGCGGGTTGTAAAGCACTTTAGTTGGGGAGGAGAGTATGTGTATGAATAATGCATGTATGAGACGTTACCCAAAGAATAAGCACCGGCAAACTCTGTGCCAGCAGCCGCGGTAATACAGAGGGTGCAAGCGTTAATCGGAGTTACTGGGCGTAAAGGGCACGTAGGCGGTCGAATAAGTCAGTTGTGAAATCCCCGGGCTTAACCTGGGAACTGCAAATGATACTGTGCGACTAGAGTCGAAGAGAGGGAAGTGGAATTTCCGGTGTAGCGGTGAAATGCGTAGATATCGGAAGGAACATCAGTGGCGAAGGCGACTTCCTGGCTTCAGACTGACGCTGAGGTGCGAAAGCGTGGGTAGCGAACAGG` |
| ASV5597 | 389 | `GCGCGCAAACTCTCCAATGTGCGAAAGCGTGAGAGGGGAACTCCTAGTGCTACAGGTTTAACCTGTGGCTTTTGCCAAGTGTAAATAGCTTGGCGAATAAGTGGTGGGCAAGACTGGTGCCAGCCGCCGCGGTAACACCAGCGCCACAAGTGGCCATCACGATTATTGGGCCTAAAGCGTCCGTAGTCGGTTGTATACATCCTCTGTGAAATCGCAGCGCTTAACGTTGCGGCATGCGGAGGAAACGGTACAGCTCGAGAGCGGGAGGCGTCGGGGGTACTCCAAGGGGACCGGTAAAATGGTATAATCCTTGGAAGACCACCTGTGGCGAAGGCGCCCGACGAGAACGCATCTGACGATGAGGGACGAAAGCCAGGGGAGCAAACCGG` |
| ASV1359 | 404 | `TGGGGAATCTTGCACAATGGACGAAAGTCTGATGCAGCGACGCCTCGTGAGTGATGAAGGTCTTCGGATCGTAAAGCTCTTTCGACTGGGAAGATAATGACGGTACCAGGAGAAGAAGCTGCGGCTAACTACGTGCCAGCAGCCGCGGTAATACGTAGGCAGCAAGCGTTGTTCGGAATTACTGGGCGTAAAGAGTGTGTAGGCGGTGTCGTAAGTTTGGCGTGAAATCTCCCGGCTTAACTGGGAGGGTGCGCCGAAGACTGCGATGCTTGAGGGTTGGAGAGGAAAGTGGAATTTCTGGTGTAGCGGTGAAATGCGTAGATATCAGAAGGAACACCTGTGGTGTAGACGGCTTTCTGGACAACTCCTGACGCTGAGACACGAAAGCGTGGGTAGCAAACAGG` |
| ASV1877 | 429 | `TGGGGAATTTTGGACAATGGGGGCAACCCTGATCCAGCAATGCCGCGTGCAGGAAGAAGGCCTTCGGGTTGTAAACTGCTTTTGTCAGGGAAGAAATCTTCTGGGCTAATACCTCGGGAGGATGACGGTACCTGAAGAATAAGCACCGGCTAACTACGTGCCAGCAGCCGCGGTAATACGTAGGGTGCGAGCGTTAATCGGAATTACTGGGCGTAAAGCGTGCGCAGGCGGCTTTGCAAGACAGATGTGAAATCCCCGGGCTTAACCTGGGAACTGCATTTGTGACTGTAAGGCTAGAGTACGGTAGAGGGGGATGGAATTCCGCGTGTAGCAGTGAAATGCGTAGATATGCGGAGGAACACCGATGGCGAAGGCAATCCCCTGGACCTGTACTGACGCTCATGCACGAAAGCGTGGGGAGCAAACAGG` |
| ASV555 | 424 | `TGAGGAATATTGGTCAATGGACGCAAGTCTGAACCAGCCATGCCGCGTGCAGGAAGACGGTCCTATGGATTGTAAACTGCTTTTATACGGGAAGAAACACTTCGACGTGTCGAAGCTTGACGGTACCGTAAGAATAAGGATCGGCTAACTCCGTGCCAGCAGCCGCGGTAATACGGAGGATCCAAGCGTTATCCGGAATCATTGGGTTTAAAGGGTCCGTAGGCGGTTAGTTAAGTCAGTGGTGAAATCCCGTCGCTCAACGATGGAACTGCCATTGATACTGATTAACTTGAATAACTGGGAAGTAACTAGAATATGTAGTGTAGCGGTGAAATGCTTAGATATTACATGGAATACCAATTGCGAAGGCAGGTTACTATCAGTTTATTGACGCTGATGGACGAAAGCGTGGGGAGCGAACAGG` |
| ASV141 | 404 | `TGGGGAATATTGGACAATGGGCGAAAGCCTGATCCAGCAATGCCGCGTGAGTGAAGAAGGCCTTCGGGTTGTAAAGCTCTTTTACCCGGGAAGATAATGACTGTACCGGGAGAATAAGCCCCGGCTAACTCCGTGCCAGCAGCCGCGGTAATACGGAGGGGGCTAGCGTTGTTCGGAATTACTGGGCGTAAAGCGTACGTAGGCGGCTTTGTAAGTCAGAGGTGAAAGCCCACGACTCAATCGTGGAACTGCCTTTGAGACTGCTTCGCTTGAAGATGGGAGAGGTTAGTGGAATTCCGAGTGTAGAGGTGAAATTCGTAGATATTCGGAAGAACACCAGTGGCGAAGGCGGCTAACTGGACCATTCTTGACGCTGAGGTACGAAAGCGTGGGGAGCAAACAGG` |
| ASV4359 | 429 | `TGGGGAATTTTGGACAATGGGCGCAAGCCTGATCCAGCCATGCCGCGTGAGTGAAGAAGGCCTTCGGGTTGTAAAGCTCTTTCGGTGAAGACGAAACGGTTCGCGCTAATAACGCGGACTAATGACGGTACTCACAGAAGAAGCACCGGCTAACTACGTGCCAGCAGCCGCGGTAATACGTAGGGTGCAGGCGTTAATCGGAATTACTGGGCGTAAAGCGTGCGCAGGCGGTTTGCTAAGACAGGTGTGAAATCCCCGAGCTTAACTTGGGAATTGCGCTTGTAACTGGCAAGCTAGAGTGTGGCAGAGGGGGGTGGAATTCCACGTGTAGCAGTGAAATGCGTAGATATGTGGAGGAACACCGATGGCGAAGGCAGCCCCCTGGGTTAACACTGACGCTCATGCACGAAAGCGTGGGGAGCAAACAGG` |
| ASV2078 | 404 | `TTAGGAATATTGGACAATGGGCGCAAGCCTGATCCAGCCATGCCGCGTGAGTGATGAAGGCCTTCGGGTTGTAAAGCTCTTTAGCCGATGATTATAATGACTGTAGTCGGAAAATAAGCCCCGGCTAACTTCGTGCCAGCAGCCGCGGTAATACGAAGGGGGCGAGCGTTATTCGGAATCACTGGGCGTAAAGCGTGCGTAGGCGGTTTTGTAAGTTGGAAGTGAAAGCCCAGGGCTCAACCCTGGAATTGCTTTCAAAACTACAAGACTCGGATTCGGGAGAGGATAGCGGAATTGTCAGTGTAGCAGTGAAATGCGTAGATATTGACAGGAACACCAGTGGCGCAAGCGGCTATCTGGACCGACATCGACGCTGAGGCACGAAAGCGTGGGGATCAAACAGG` |
| ASV1357 | 404 | `TGGGGAATCTTGGACAATGGGCGCAAGCCTGATCCAGCCATGCCGCGTGAGTGAAGAAGGCCTTCGGGTTGTAAAGCTCTTTTACCAGGGACGATAATGACGGTACCTGGAGAATAAGCCCCGGCAAACTTCGTGCCAGCAGCCGCGGTAATACGAAGGGGGCTAGCGTTGTTCGGAATTACTGGGCGTAAAGCGCACGTAGGCGGATTGTTAAGTCAGAGGTGAAATCCCGGAGCTCAACTTCGGAACTGCCTTTGATACTGGCAATCTAGAGTCCGGAAGAGGTTAGTGGAATTCCCAGTGTAGAGGTGAAATTCGTAGATATTGGGAAGAACACCAGTGGCGAAGGCGGCTAACTGGTCCGGTACTGACGCTGAGGTGCGAAAGCGTGGGGAGCAAACAGG` |
| ASV3222 | 404 | `TGGGGAATCTTGGACAATGGGGGCAACCCTGATCCAGCGACGCCGCGTGGGTGACGAAGGCCTTCGGGTTGTAAAGCCCTTTTGTCGGGGACGATGATGACGGTACCCGGCGAATCAGCACCGGCTAACTCCGTGCCAGCAGCCGCGGTAAGACGGAGGGTGCTAGCGTTGTTCGGATTTACTGGGCGTAAAGGGCGCGTAGGCGGCTTGGTCAGTCGGGTGTGAAAGCCCGGGGCTCAACCCCGGAACGGCACCCGAGACGGCCAGGCTGGAGACCGGGAGAGGAGGGTGGAATAGCCAGTGTAGAGGTGAAATTCGTAGATATTGGCTGGAACACCGGTGGCGAAAGCGGCTCTCTGGCCCGGTTCTGACGCTGAGGCGCGAAAGCGTGGGGAGCAAACAGG` |
| ASV5022 | 419 | `TAAGGAATATTGCGCAATGGGCGAAAGCCTGACGCAGCGACGCCGCGTGGGGGATGAAGGTCTTCGGATTGTAAACCCCTTTTGGGAGGGAAGATGGGACGAGCAATCGTCCGGACGGTACCTCCAGAAAAAGCCACGGCTAACTTCGTGCCAGCAGCCGCGGTAATACGAAGGTGGCAAGCGTTGTTCGGATTTACTGGGCGTACAGGGAGCGTAGGCGGTTTGGTAAGCCCTTCGTGAAATCTTCGGGCTCAACCCGGAAAGTTCGGAGGGGACTGCCAGGCTAGAGGACGGGAGAGGAGCGCGGAATTCCCGGTGTAGCGGTGAAATGCGTAGAGATCGGGAGGAAGGCCGGTGGCGAAGGCGGCGCTCTGGAACGTTTCTGACGCTGAGGCTCGAAAGCGTGGGGAGCAAACAGG` |
| ASV2493 | 429 | `TGGGGAATTTTGGACAATGGGGGAAACCCTGATCCAGCAATGCCGCGTGTGCGAAGAAGGCCTTCGGGTTGTAAAGCACTTTTGTCAGGGAAGAAAACCTATGGACTAATAATCTATTGGGATGACGGTACCTGAAGAATAAGCACCGGCTAACTACGTGCCAGCAGCCGCGGTAATACGTAGGGTGCAAGCGTTAATCGGAATTACTGGGCGTAAAGCGTGCGCAGGCGGTTCTATAAGACAGGTGTGAAATCCCCGGGCTTAACCTGGGAATGGCATTTGTGACTGTAGGACTAGAGTGTGTCAGAGGGGGGTGGAATTCCACGTGTAGCAGTGAAATGCGTAGATATGTGGAGGAACACCAATGGCGAAGGCAGCCCCCTGGGATAACACTGACGCTCATGCACGAAAGCGTGGGGAGCAAACAGG` |
| ASV1628 | 424 | `TAAGGAATATTGGTCAATGGACGAAAGTCTGAACCAGCCATCCCGCGTGCAGGATGACGGCCCTATGGGTTGTAAACTGCTTTTAGCAGAGACGAAACGCTGTCATTTATGATGGTCTGACGGTATCTGCAGAATAAGCACCGGCTAACTCCGTGCCAGCAGCCGCGGTAATACGGAGGGTGCAAGCGTTATCCGGAATCACTGGGTTTAAAGGGTGCGTAGGCGGAAAATTAAGTCAGTGGTGAAAGCCTGTCGCTTAACGATAGAACTGCCATTGATACTGATTTTCTTGAATTGGGTTGAGGTTAGCGGAATGTGACATGTAGCGGTGAAATGCATAGATATGTCATGGAACACCAATTGCGAAGGCAGCTAGCTGGGCCTTGATTGACGCTGAGGCACGAAAGCGTGGGTAGCGAACAGG` |
| ASV4837 | 424 | `TGAGGAATATTGGTCAATGGACGTAAGTCTGAACCAGCCAAGTAGCGTGCAGGATGACGGCCCTATGGGTTGTAAACTGCTTTTATGCGGGGATAAAGTTTGGGATGTATCCCTTTTTGCAGGTACCGCATGAATAAGGACCGGCTAATTCCGTGCCAGCAGCCGCGGTAATACGGAAGGTCCGGGCGTTATCCGGATTTATTGGGTTTAAAGGGAGCGTAGGCCGTCGTTTAAGCGTGTTGTGAAATGCCGCGGCTCAACCGTGGCACTGCAGCGCGAACTGGATGACTTGAGTTCGCGGGAGGTAGGCGGAATTCGTGGTGTAGCGGTGAAATGCTTAGATATCACGAAGAACTCCGATTGCGAAGGCAGCTTACCGTAGCGTTACTGACGCTGAAGCTCGAAAGTGCGGGTATCGAACAGG` |
| ASV3018 | 429 | `TGGGGAATTTTGGACAATGGGCGCAAGCCTGATCCAGCCATGCCGCGTGCGGGAAGAAGGCCTTCGGGTTGTAAACCGCTTTTGTCAGGGAAGAAATACCTTGGGTTAATACCCTGGGGTGATGACGGTACCTGAAGAATAAGCACCGGCTAACTACGTGCCAGCAGCCGCGGTAATACGTAGGGTGCGAGCGTTAATCGGAATTACTGGGCGTAAAGCGTGCGCAGGCGGTTGTGTAAGACAGATGTGAAATCCCCGGGCTCAACCTGGGAACTGCATTTGTGACTGCACGGCTTGAGTGCGGCAGAGGGGGATGGAATTCCGCGTGTAGCAGTGAAATGCGTAGATATGCGGAGGAACACCGATGGCGAAGGCAATCCCCTGGGCCTGCACTGACGCTCATGCACGAAAGCGTGGGGAGCAAACAGG` |
| ASV678 | 424 | `TGAGGAATATTGGACAATGGGCGCAAGCCTGATCCAGCCATGCCGCGTGCAGGAAGACGCATCTATGGTGTGTAAACTGCTTTTGTACGGGAAGAAACACTCCTTCGTGAAGGAGCTTGACGGTACCGTAAGAATAAGGATCGGCTAACTCCGTGCCAGCAGCCGCGGTAATACGGAGGATCCAAGCGTTATCCGGAATCATTGGGTTTAAAGGGTCCGTAGGCGGTTTAGTAAGTCAGTGGTGAAAGCCCATCGCTCAACGATGGAACGGCCATTGATACTGCTAGACTTGAATTACAAGGAAGTAACTAGAATATGTAGTGTAGCGGTGAAATGCTTAGATATTACATGGAATACCAATTGCGAAGGCAGGTTACTACTTGTGGATTGACGCTGATGGACGAAAGCGTGGGGAGCGAACAGG` |
| ASV767 | 424 | `TAGGGAATATTGGACAATGGGCGCAAGCCTGATCCAGCCATGCCGCGTGCAGGATGAAGGCCCTCTGGGTCGTAAACTGCTTTTGTACGGGAAGAAACCTCATTACGTGTAGTGAGTTGACGGTACCGTGAGAATAAGGATCGGCTAACTTCGTGCCAGCAGCCGCGGTAATACGAAGGATCCAAGCGTTGTCCGGATTTACTGGGTTTAAAGGGTGCGTAGGCGGACTTTTAAGTCAGTGGTGAAAGCCTGTAGCTTAACTATAGAATTGCCATTGAAACTGAAAGTCTCGAGTGTGGTTGAGGTAACTGGAATATATCATGTAGCGGTGAAATGCTTAGATATGATATAGAACACCAATTGCGAAGGCAGGTTACTGAGCCATAACTGACGCTGAGGCACGAAAGCGTGGGGATCAAACAGG` |
| ASV3333 | 406 | `TAGGGAATTTTGCGCAATGGACGAAAGTCTGACGCAGCAACACCGCGTGTAGGATGAAGCCTTTTGGGGTGTAAACTACTGTCAGCAGGGAAGAAACAATGACTGTACCTGCAGAGGAAGCATCGGCTAACTACGTGCCAGCAGCCGCGGTAATACGTAGGATGCAAGCGTTGTCCGGAATTATTGGGCGTAAAGAGTTCGTAGGCGGTTACCCCAGTCTGGTGTTAAATCCCAAGGCTTAACCTTGGCATGGCATTGGAAACTAGGTAGCTAGAGTGTGGTAGAGGTAAAGGGAATTCCAGGTGTAGCGGTGAAATGCGTAGATATCTGGAGGAACACCGGCGGCGAAAGCGCTTTACTGGGCCATAACTGACGCTGAGGAACGAAAGCCAGGGTAGCAAAAGGG` |
| ASV5067 | 404 | `TGGGGAATATTGGACAATGGGGGCAACCCTGATCCAGCCATGCCGCGTGAATGATGAAGGCCTCCGGGTTGTAAAGTTCTTTTGGCGGGGACGATGATGACGGTACCCGCAGAATAAGCTCCGGCTAACTTCGTGCCAGCAGCCGCGGTAATACGAAGGGAGCTAGCGTTGTTCGGAATTACTGGGCGTAAAGGGCGCGTAGGCGGTTAAACAAGTCAGGCGTGAAAGCCCCGGGCTCAACCCGGGAACAGCGCTTGAGACTGTTTACCTAGAGTACGGTAGAGGTAAGTGGAATTCCCAGTGTAGCAGTGAAATGCGTAGATATTGGGAGGAACACCAGTGGCGAAGGCGGCTTACTGGACCGTAACTGACGCTGAGGCGCGAAAGCGTGGGGAGCAAACAGG` |
| ASV1635 | 404 | `TGGGGAATATTGGACAATGGGCGCAAGCCTGATCCAGCAATGCCGCGTGTGTGAAGAAGGTCTTCGGATTGTAAAGCACTTTCGGCGGGGACGATGATGACGGTACCCGCAGAAGAAGCCCCGGCTAACTTCGTGCCAGCAGCCGCGGTAATACGAAGGGGGCTAGCGTTGCTCGGAATTACTGGGCGTAAAGGGCGCGTAGGCGGCGCTGCTAGTCAGGCGTGAAATTCCTGGGCTTAACCTGGGGACTGCGCTTGATACGGCAGTGCTAGAGGGCGGAAGAGGGTCGTGGAATTCCCAGTGTAGAGGTGAAATTCGTAGATATTGGGAAGAACACCGGTGGCGAAGGCGGCGACCTGGTCCGTTACTGACGCTGAGGCGCGACAGCGTGGGGAGCAAACAGG` |
| ASV4511 | 425 | `TAGGGAATATTGGACAATGGGCGGAAGCCTGATCCAGCCATGCCGCGTGGAGGAAGACGGCCCTCTGGGTTGTAAACTCCTTTTGAAAGGGAAGAAACCTCTCTATTTATGGATGAGCTGACGGTACCTTTCGAATAAGCACCGGCTAACTCCGTGCCAGCAGCCGCGGTAATACGGAGGGTGCAAGCGTTATCCGGATTTACTGGGTTTAAAGGGTGCGTAGGCGGTTTTTTAAGTCAGTGGTGAAATACCCGTGCTCAACATGGGAATTGCCATTGATACTGAGGAACTTGAATATGAGTGAGGCAGGCGGAATGATGCATGTAGCGGTGAAATGCTTAGATATGTTTCAGAACACCTATTGCGAAGGCAGCTTGCTAATTCATGATTGACGCTGAGGCACGAAAGCGTGGGGAGCAAACAGG` |
| ASV814 | 429 | `TGGGGAATTTTGGACAATGGGCGAAAGCCTGATCCAGCCATGCCGCGTGCGGGAAGAAGGCCTTCGGGTTGTAAACCGCTTTTGTCAGGGAAGAAATATTCCGGGCTAATACCCCGGGAGGATGACGGTACCTGAAGAATAAGCACCGGCTAACTACGTGCCAGCAGCCGCGGTAATACGTAGGGTGCGAGCGTTAATCGGAATTACTGGGCGTAAAGCGTGCGCAGGTGGTCATGTAAGACAGATGTGAAATCCCCGGGCTTAACCTGGGAATTGCATTTGTGACTGCATGGCTAGAGTGTGGCAGAGGGGGATGGAATTCCGCGTGTAGCAGTGAAATGCGTAGATATGCGGAGGAACACCGATGGCGAAGGCAGTCCCCTGGGCCTGCACTGACACTCATGCACGAAAGCGTGGGGAGCAAACAGG` |
| ASV1933 | 421 | `TAAGGAATATTGCGCAATGGGCGAAAGCCTGACGCAGCGACGCCGCGTGGGGGATGAAGGTCTTCGGATTGTAAACCCCTTTCGGGAGGGAAGATGGAATGGGGCAACCCATTCGGACGGTACCTCCAGAAGCAGCCACGGCTAACTTCGTGCCAGCAGCCGCGGTAATACGAAGGTGGCAAGCGTTGTTCGGATTTACTGGGCGTACAGGGAGCGTAGGCGGTTCGGTAAGCCCTTTGTGAAATCTCCAGGCTTAACCTGGAACAGTCGGAGGGGACTGCCGGGCTAGAGGACGGGAGAGGAGCGCGGAATTCCCGGTGTAGCGGTGAAATGCGTAGAGATCGGGAGGAAGGCCGGTGGCGAAGGCGGCGCTCTGGAACGTTTCTGACGCTGAGGCTCGAAAGCGTGGGGAGCAAACAGG` |
| ASV163 | 404 | `TGGGGAATCTTAGACAATGGGCGCAAGCCTGATCTAGCCATGCCGCGTGATCGATGAAGGCCTTAGGGTTGTAAAGATCTTTCAGTGGGGAAGATAATGACTGTACCCACAGAAGAAGCCCCGGCTAACTCCGTGCCAGCAGCCGCGGTAATACGGAGGGGGCTAGCGTTGTTCGGAATTACTGGGCGTAAAGCGCACGTAGGCGGACGGAGAAGTCAGAGGTGAAATCCCAGGGCTCAACCTTGGAACTGCCTTTGAAACTTTCTGTCTTGAGGTCGAGAGAGGTGAGTGGAATTCCGAGTGTAGAGGTGAAATTCGTAGATATTCGGAGGAACACCAGTGGCGAAGGCGGCTCACTGGCTCGATACTGACGCTGAGGTGCGAAAGCGTGGGGAGCAAACAGG` |
| ASV625 | 407 | `TAGGGAATATTGCACAATGGGGGAAACCCTGATGCAGCGACGCCGCGTGAGTGATGAAGGCCTTCGGGTCGTAAAGCTCTGTCGTATGGGACTAACAAAATGAAGGTACCATGCAAGAAAGGATCGGCTAACTTCGTGCCAGCAGCCGCGGTAAGACGAGGGATCCTAGCGTTGTTCGGAATCATTGGGCGTAAAGCGGGTGTAGGCGGATTTATAAGTCAGGTGTGAAATCCCAGAGCTCAACTCTGGAAGTGCACTTGATACTGTAAATCTTGAATGTGGTAGAGGTTACTGGAATTCCTGGTGTAGTGGTGAAATACGTAGATATCAGGAGGAATACCGGAGGCGAAGGCGAGTAACTGGGCCAACATTGACGCTGAGACCCGAAAGCGTGGGGATCAAACAGG` |
| ASV2033 | 404 | `TGGGGAATATTGGACAATGGGCGGAAGCCTGATCCAGCAATACCGCGTGAATGATGAAGGCCTTAGGGTTGTAAAGTTCTTTTAGTTGGGAAGATAATGACGGTACCAACAGAAAAAGCCCCGGCTAACTCCGTGCCAGCAGCCGCGGTAAGACGGAGGGGGCTAGCGTTGTTCGGAATTACTGGGCGTAAAGGGCGCGTAGGCGGATTAGTAAGTTGGGAGTGAAAGCCCGGGGCTTAACCTCGGAACTGCTTTCAAAACTGCTAGTCTTGAGTGAAGTAGGGGATGATGGAATTCCTAGTGTAGAGGTGAAATTCTTAGATATTAGGAGGAACACCGGTGGCGAAGGCGGTCATCTGGACTTCAACTGACGCTGAGGCGCGAAAGCGTGGGGAGCAAACAGG` |
| ASV3037 | 409 | `TGGGGAATATTGCACAATGGGCGCAAGCCTGATGCAGCAACGCCGCGTGAGGGATGAAGGCCTTCGGGTTGTAAACCTCTTTTAGCAGGGAAGAAGCGAAAGTGACGGTACCTGCAGAAAAAGCACCGGCTAACTACGTGCCAGCAGCCGCGGTAATACGTAGGGTGCAAGCGTTGTCCGGAATTATTGGGCGTAAAGAGCTCGTAGGCGGTTTGTCGCGTCTGCTGTGAAAACCCGAGGCTCAACCTCGGACCTGCAGTGGGTACGGGCAAGCTAGAGTGCGGTAGGGGAGATGGGAATTCCTGGTGTAGCGGTGGAATGCGCAGATATCAGGAGGAACACCGATGGCGAAGGCACATCTCTGGGCCGTAACTGACGCTGAGGAGCGAAAGCGTGGGGAGCGAACAGG` |
| ASV1026 | 404 | `TGGGGAATATTGGACAATGGGCGCAAGCCTGATCCAGCAATTCCGCGTGGGTGAAGAAGGTCTTCGGATTGTAAAGCCCTTTCGTTGGGGACGATGATGACGGTACCCAAAGAAGAAGCCCCGGCTAACTTCGTGCCAGCAGCCGCGGTAATACGAAGGGGGCTAGCGTTGCTCGGAATGACTGGGCGTAAAGGGCGCGTAGGCGGCACAACTAGTCAGGCGTGAAATTCCTGGGCTTAACCTGGGGGCTGCGTTTGATACGGTTGGGCTAGAGGATGGAAGAGGCTCGTGGAATTCCCAGTGTAGAGGTGAAATTCGTAGATATTGGGAAGAACACCGGTGGCGAAGGCGGCGAGCTGGTCCATTACTGACGCTGAGGCGCGAAAGCGTGGGGAGCAAACAGG` |
| ASV109 | 424 | `TAGGGAATATTGGGCAATGGATGCAAGTCTGACCCAGCCATGCCGCGTGCAGGAAGAAGGTCCTCTGGACTGTAAACTGCTTTTTTCAGGGAAGAATAGGTGGGATGCGTCCTACAGTGACGGTACTTGAAGAATAAGCACCGGCTAACTACGTGCCAGCAGCCGCGGTAATACGTAGGGTGCGAGCGTTGTCCGGATTTATTGGGTTTAAAGGGTGCGTAGGTGGATTTTTAAGTCAGCGGTGAAATATGGTTGCTCAACAATCAGATTGCCATTGATACTGGAAGTCTTGAGAGAAGTGGAGGCTGTTGGAATGGATGGTGTAGCGGTGAAATGCATAGATATCATCCAGAACGTCAATTGCGAAGGCAGATGGCTGTACTTTTTCTGACACTGAGGCACGAAAGCGTGGGGATCAAACAGG` |
| ASV2918 | 407 | `TAGGGAATATTGCACAATGGGGGAAACCCTGATGCAGCGACGCCGCGTGAGTGATGAAGGCCTTCGGGTCGTAAAGCTCTGTCGTATGGGACTAACACAATGAAGGTACCATGCAAGAAAGGATCGGCTAACTTCGTGCCAGCAGCCGCGGTAAGACGAGGGATCCTAGCGTTGTTCGGAATTATTGGGCGTAAAGCGGGTGTAGGCGGATTTGTAAGTCAGGTGTGAAATCCCAGGGCTCAACCCTGGAAGTGCACTTGATACTGCTAATCTTGAGTGTGGTAGAGGTTACTGGAATTCCTGGTGTAGTGGTAAAATACGTAGATATCAGGAGGAATACCGGAGGCGAAGGCGAGTAACTGGGCCAACACTGACGCTGAGACCCGAAAGCGTGGGGATCAAACAGG` |
| ASV63 | 429 | `TGGGGAATATTGCACAATGGGGGAAACCCTGATGCAGCCATGCCGCGTGTGTGAAGAAGGCCTTCGGGTTGTAAAGCACTTTCAGCGAGGAGGAAAGGTTGGCAGCTAATATCTGTCAGCTGTGACGTTACTCGCAGAAGAAGCACCGGCTAACTCCGTGCCAGCAGCCGCGGTAATACGGAGGGTGCAAGCGTTAATCGGAATTACTGGGCGTAAAGCGCACGCAGGCGGTTGGATAAGTTAGATGTGAAAGCCCCGGGCTCAACCTGGGAATTGCATTTAAAACTGTTCAGCTAGAGTCTTGTAGAGGGGGGTAGAATTCCAGGTGTAGCGGTGAAATGCGTAGAGATCTGGAGGAATACCGGTGGCGAAGGCGGCCCCCTGGACAAAGACTGACGCTCAGGTGCGAAAGCGTGGGGAGCAAACAGG` |
| ASV5015 | 428 | `TGGGGAATATTGGACAATGGGCGCAAGCCTGATCCAGCCATGCCGCGTGAGTGATGAAGGCCTTCGGGTCGTAAAGCTCTGTGGGGAGGGACGAACCGCCAAGGGTGAATAGCCAGTGGCATGACGGTACCTCCTTAGCAAGCACCGGCTAACTTCGTGCCAGCAGCCGCGGTAATACGAAGGGTGCAAACGTTGCTCGGAATCATTGGGCGTAAAGCGCACGTAGGCGGTCTGAAAAGTCGGATGTGAAAGCCCTCGGCTCAACCAAGGAAGTGCATCCGAAACTGACAGACTTGAGTATCATAGAGGATCGCGGAATTCCCGGTGTAGAGGTGAAATTCGTAGAGATCGGGAGGAACATCAGTGGCGAAGGCGGCGATCTGGGTGATTACTGACGCTGAGGTGCGAAAGCGTGGGGAGCAAACAGG` |
| ASV5488 | 431 | `TAGGGAATATTGCGCAATGGGCGAAAGCCTGACGCAGCGATGCCGCGTGAGTGAGGAAGGCCTTCGGGTTGTAAAGCTCTGTCCTAAGGGAAAAACGGCCAGTATGCTAACAGTATATTGGAGTGATGGTACCTTGGAAGAAAGCACCGGCCAACTTCGTGCCAGCAGCCGCGGTAATACGAAGGGTGCGAGCGTTGTTCGGATTTACTGGGCGTAAAGAGCATGTAGGCGGGTTATGAAAGTCAGGTGTGAAATCTCGGAGCTTAACTCCGAAAGTGCGCCTGAAACTTCTAATCTAGAGTATCGGAGAGGAAAGGGGAATTTCTCATGTAGGGGTAAAATCCGTAGAGATGAGAAGGAACATCAGAGGCGAAGGCGCCTTTCTGGCCGATAACTGACGCTAAGATGCGAAAGCGTGGGGAGCAAACAGG` |
| ASV5097 | 429 | `TGGGGAATTTTGCGCAATGGGCTAACGCCTGACGCAGCAACGCCGCGTGGAGGATGAAGGTCTTTGGATTGTAAACTCCTGTCAGCAGGGAAAAAGGGACTGGGTCTAATACGCCCGGGACTTGATTGTACCTGCAGAGGAAGCCCCGGCTAACTCTGTGCCAGCAGCCGCGGTAATACAGAGGGGGCAAGCGTTATTCGGAATTATTGGGCGTAAAGGGCGCGTAGGCGGCCATTCAAGTGACGGGTGAAAGCCCTCGGCTTAACCGAGGAACTGCCTGTCAGACTGGATGGCTTGAGACCGGGAGAGGTGAGTGGAATTCCCAGTGTAGCGGTGAAATGCGTAGATATTGGGAGGAACACCAGTGGCGAAGGCGGCTCACTGGACCGGAACTGACGCTGAGGCGCGAAAGCGTGGGGAGCAAACAGG` |
| ASV5300 | 404 | `TGGGGAATATTGGACAATGGGCGCAAGCCTGATCCAGCCATGCCGCGTGAGTGACGAAGGCCTTAGGGTTGTAAAACTCTTTCGCCGGGGAAGATAATGACGGTACCCGGAGAAGAAGCTCCGGCTAACTTCGTGCCAGCAGCCGCGGTAATACGAAGGGAGCTAGCGTTGTTCGGAATTACTGGGCGTAAAGCGCACGTAGGCGGGCTTGTCAGTCAGGGGTGAAATCCCAAGGCTCAACCTTGGAACTGCCTCCGATACAGCAAGCCTTGAGATCGGGAGAGGTGAGTGGAATTCCTAGTGTAGAGGTGAAATTCGTAGATATTAGGAAGAACACCAGTGGCGAAGGCGGCTCACTGGCCCGATACTGACGCTGAGGTGCGAAAGCGTGGGGAGCAAACAGG` |
| ASV4642 | 429 | `TGGGGAATATTGCACAATGGGCGCAAGCCTGATGCAGCCATGCCGCGTGTGTGAAGAAGGCCTTCGGGTTGTAAAGCACTTTCAGTGATGAGGAAGGGGTGTGCGTTAATAGCGTACACTTTTGACGTTATTCACAGAAGAAGCACCGGCTAACTCCGTGCCAGCAGCCGCGGTAATACGGAGGGTGCGAGCGTTAATCGGAATTACTGGGCGTAAAGGGTGTGCAGGCGGTTGCTTAAGTTAGATGTGAAATCCCCGAGCTTAACTTGGGAATTGCATTTAAGACTGGGCGGCTAGAGTCTTGTAGAGGGGGGTAGAATTCCACGTGTAGCGGTGAAATGCGTAGAGATGTGGAGGAATACCGGTGGCGAAGGCGGCCCCCTGGACAAAGACTGACGCTCAAACACGAAAGCGTGGGGAGCAAACAGG` |
| ASV1540 | 407 | `TCGAGAATTTTTCACAATGGGGGAAACCCTGATGGAGCGACGCCGCGTGGAGGATGAAGGCGTTTTCGTCGTAAACTCCTGTCAAACGGGAACAAGAAAGTGATAGTACCGTTAGAGGAAGGGACGGCTAACTCTGTGCCAGCAGCCGCGGTAATACAGAGGTCCCAAGCGTTGTTCGGATTCATTGGGCGTAAAGGGTGCGTAGGCGGCATGGTAAGTTGAAGGTGAAATCTCGGAGCTCAACTCCGAAACTGCCTTCAATACTGCTGTGCTTGAGGACTGTAGAGGAGAATGGAATTCACGGTGTAGCAGTGAAATGCGTAGATATCGTGAGGAAGACCAGTGGCGAAGGCGATTCTCTGGGCAGTTCCTGACGCTGAGGCACGAAGGCTAGGGGAGCAAACGGG` |
| ASV3297 | 429 | `TGGGGAATATTGGACAATGGGGGCAACCCTGATCCAGCCATGCCGCGTGTGTGAAGAAGGCCTTCGGGTTGTAAAGCACTTTAAGTGGGGAGAAAGGTTCGCCGGTTAATAGTCGGCGGAATTGATGTTACCCACAGAATAAGCACCGGCTAACTCTGTGCCAGCAGCCGCGGTAATACAGAGGGTGCAAGCGTTAATCGGAATTACTGGGCGTAAAGCGCGCGTAGGCGGCTGTCTAAGTCGGGTGTGAAAGCCCCGGGCTCAACCTGGGAACTGCATTCGATACTGGACGGCTAGAGTATGAGAGAGGGAGGTAGAATTCCACGTGTAGCGGTGAAATGCGTAGATATGTGGAGGAATACCGGTGGCGAAGGCGGCCTCCTGGTTCAATACTGACGCTGAGGTGCGAAAGCGTGGGGAGCAAACAGG` |
| ASV186 | 404 | `TGGGGAATCTTAGACAATGGGCGCAAGCCTGATCTAGCCATGCCGCGTGAGTGATGAAGGCCTTAGGGTCGTAAAGCTCTTTCGCTGGGGAAGATAATGACGGTACCCAGTAAAGAAACCCCGGCTAACTCCGTGCCAGCAGCCGCGGTAATACGGAGGGGGTTAGCGTTGTTCGGAATTACTGGGCGTAAAGCGCGCGTAGGCGGATCGGAAAGTTGGGGGTGAAATCCCAGGGCTCAACCCTGGAATTGCCTTCAAAACTCCTGGTCTTGAGTTCGAGAGAGGTGAGTGGAATTCCGAGTGTAGAGGTGAAATTCGTAGATATTCGGAGGAACACCAGTGGCGAAGGCGGCTCACTGGCTCGATACTGACGCTGAGGTGCGAAAGCGTGGGGAGCAAACAGG` |
| ASV310 | 404 | `TGGGGAATATTGGACAATGGGCGCAAGCCTGATCCAGCCATGCCGCGTGAGTGATGAAGGCCTTCGGGTTGTAAAGCTCTTTTGTCCGGGAAGATAATGACTGTACCGGAAGAATAAGCCCCGGCAAACTTCGTGCCAGCAGCCGCGGTAATACGAAGGGGGCTAGCGTTGTTCGGATTTACTGGGCGTAAAGCGCACGTAGGCGGCTTGTTAAGTCAGAGGTGAAAGCCTGGAGCTCAACTCCAGAACTGCCTTTGATACTGGCAAGCTCGAGTATAGGAGAGGTTGGTGGAACTGCGAGTGTAGAGGTGAAATTCGTAGATATTCGCAAGAACACCAGTGGCGAAGGCGGCCAACTGGCCTATAACTGACGCTGAGGTGCGAAAGCGTGGGGAGCAAACAGG` |
| ASV3360 | 404 | `TGGGGAATATTGGACAATGGGGGCAACCCTGATCCAGCCATGCCGCGTGAGTGATGAAGGCCTTCGGGTTGTAAAGCTCTTTTGGCGGGGACGATGATGACGGTACCCGCAGAATAAGCTCCGGCTAACTTCGTGCCAGCAGCCGCGGTAATACGAAGGGAGCTAGCGTTGTTCGGAATTACTGGGCGTAAAGGGCGCGTAGGCGGCCCTGTCAGTCAGGCGTGAAAGCCCCGGGCTTAACCTGGGAAATGCGTTTGATACTGCGGGGCTTGAGTGTGGGAGAGGTTGGTGGAATTCCCAGTGTAGAGGTGAAATTCGTAGATATTGGGAAGAACACCGGTGGCGAAGGCGGCCAACTGGACCACAACTGACGCTGAGGCGCGAAAGCGTGGGGAGCAAACAGG` |
| ASV4811 | 404 | `TGGGGAATATTGGACAATGGGCGAAAGCCTGATCCAGCCATGCCGCGTGAGTGAAGAAGGTCTTCGGATTGTAAAGCTCTTTTGTCCGGGAAGATGATGACTGTACCGGAAGAATAAGCCCCGGCTAACTTCGTGCCAGCAGCCGCGGTAATACGAAGGGGGCTAGCGTTGTTCGGAATCACTGGGCGTAAAGCGCGCGCAGGTGGTTTGCTAAGTTAGAAGTGAAAGCCCAGGGCTCAACCTTGGAACGGCTTTTAAGACTGGTAAACTAGAATCTGGTAGGGGAATGCGGAATACCGAGTGTAGAGGTGAAATTCGTAGATATTCGGTGGAACACCAATGGCGAAGGCAGCATTCTGGAACATGATTGACACTGAGGCGCGAAAGCGTGGGGAGCAAACAGG` |
| ASV2554 | 405 | `TGGGGAATTTTCCGCAATGGGCGAAAGCCTGACGGAGCAATACCGCGTGAGGGAGGAAGGCTCTTGGGTTGTAAACCTCTTTTCTCAGGGAAGAATAATGACGGTACCTGAGGAATAAGCATCGGCTAACTCCGTGCCAGCAGCCGCGGTAATACGGAGGATGCAAGCGTTATCCGGAATGATTGGGCGTAAAGCGTCCGCAGGTGGTTTTGTATGTCTGCTGTTAAAGCGTGCGGCTTAACCGCATAAGGGCGGTAGAAACTACAAGACTCGAGTACGTTCGGGGCAGAGGGAATTCCCAGTGTAGCGGTGAAATGCGTAGAGATTGGGAAGAACATCGGTGGCGAAAGCGCTCTGCTAGGCCTGTACTGACACTGAGGGACGAAAGCTAGGGGAGCGAATGGG` |
| ASV4241 | 429 | `TGAGGAATCTTGCGCAATGGGCGAAAGCCTGACGCAGCGACGCCGCGTGGAGGATGAAGGTCTTCGGATTGTAAACTTCTGTTAAGTGGGAAGAAATAGTCTGGCCTAATACGTCAGATGCATGACGGTACCACTAGAGAAAGCACCGGCTAACTTCGTGCCAGCAGCCGCGGTAATACGAAGGGTGCAAGCGTTATTCGGATTGATTGGGTGTAAAGGGTGTGTAGGTGGTGTGCTAAGTCAACTGTTAAATTTCCTGGCCTAACCAGGAGTCGGCAGTAGATACTGGCGCGCTAGAGGATGGAAGAGATAAGTGGAATTCTCGGAGTAGCGGTAAAATGCGTAGATCTCGAGAAGAACACCGATGGCGAAGGCAGCTTATTGGTCCATTCCTGACACTGAGGCACGAAAGCGTGGGGAGCAAACAGG` |
| ASV3365 | 404 | `TTAGGAATATTGGACAATGGGCGCAAGCCTGATCCAGCCATGCCGCGTGAGTGATGAAGGCCTTCGGGTTGTAAAGCTCTTTAGCCGACGATTATAATGACTGTAGTCGGAAAATAAGCCCCGGCTAACTTCGTGCCAGCAGCCGCGGTAATACGAAGGGGGCGAGCGTTATTCGGAATCACTGGGCGTAAAGCGTGCGTAGGCCGTTTGGCAAGTTGGAAGTGAAAGCCCAGGGCTCAACCTTGGAATTGCTTTCAAAACTGTCAGACTAGAGTCCGGGAGAGGATAGCGGAATTTCCAGTGTAGTAGTGAAATACGTAGATATTGGAAGGAACACCAGTGGCGTAAGCGGCTATCTGGACCGGTACTGACGCTGAGGCACGAAAGCGTGGGGATCAAACAGG` |
| ASV5235 | 424 | `TGGGGAATTTTGGACAATGGGGGCAACCCTGATCCAGCGACGCCGCGTGAAGGATGAAGGTCTTCGGATTGTAAACTTCTTTTGAAGGGGATGAATGTCTCGGTGAATAGCCGAGAGTGACAGTACTCTTCGAATAAGCCACGGCTAACTACGTGCCAGCAGCCGCGGTAATACGTAGGTGGCAAGCGTTACTCGGAATCACTAGGCGTAAAGCGCAGGTAGGCGGTTGGATAAGTCCGTTGTGAAAGCTCCCGGCTCAACTGGGAGAGGCCAACGGAAACTATCCGGCTAGAGTGTGGTAGAGGTAGCTGGAATTCCCGGTGTAGCGGTGAAATGCGTAGATATCGGGAGGAATACCTATGGCGAAAGCAGGCTACTGGACCATCACTGACGCTGATCTGCGAAAGCTAGGGGAGCAAACAGG` |
| ASV5282 | 429 | `TGGGGAATATTGGACAATGGGGGCAACCCTGATCCAGCGATGCCGCGTGGGTGAAGAAGGCCTTCGGGTTGTAAAGCCCTTTAGGCCGGGAAGAAGTGTGTGGGGGGAATATCCCTGCATATTGACGGTACCGGCAGAATAAGCACCGGCAAACTCTGTGCCAGCAGCCGCGGTAATACAGAGGGTGCGAGCGTTAATCGGAGTTACTGGGCGTAAAGGGCGCGTAGGCGGTGAGATGTGTGTGATGTGAAAGCCCCGGGCTTAACCTGGGAAGTGCATCGCAAACTATCTGACTAGAGTATATGAGAGGGTGGTGGAATTTCCGGTGTAGCGGTGAAATGCGTAGAGATCGGAAGGAACGTCAATGGCGAAGGCAGCCACCTGGCATAATACTGACGCTGAGGCGCGAAAGCGTGGGGAGCGAACAGG` |
| ASV638 | 429 | `TGGGGAATATTGCACAATGGGGGAAACCCTGATGCAGCCATGCCGCGTGTGTGAAGAAGGCCTTCGGGTTGTAAAGCACTTTCAGCGAGGAGGAAAGGTTGATGCCTAATACGCATCAGCTGTGACGTTACTCGCAGAAGAAGCACCGGCTAACTCCGTGCCAGCAGCCGCGGTAATACGGAGGGTGCAAGCGTTAATCGGAATTACTGGGCGTAAAGCGCACGCAGGCGGTTGGATAAGTTAGATGTGAAAGCCCCGGGCTCAACCTGGGAATTGCATTTAAAACTGTCCAGCTAGAGTCTTGTAGAGGGGGGTAGAATTCCAGGTGTAGCGGTGAAATGCGTAGAGATCTGGAGGAATACCGGTGGCGAAGGCGGCCCCCTGGACAAAGACTGACGCTCAGGTGCGAAAGCGTGGGGAGCAAACAGG` |
| ASV1019 | 429 | `TGGGGAATTTTGGACAATGGGCGCAAGCCTGATCCAGCCATGCCGCGTGCGGGAAGAAGGCCTTCGGGTTGTAAACCGCTTTTGTCAGGGAAGAAACGCTCTGGGCTAATACCCCGGGGTAATGACGGTACCTGAAGAATAAGCACCGGCTAACTACGTGCCAGCAGCCGCGGTAATACGTAGGGTGCAAGCGTTAATCGGAATTACTGGGCGTAAAGCGTGCGCAGGCGGTCTTGCAAGACAGATGTGAAATCCCCGGGCTCAACCTGGGAACTGCATTTGTGACTGCAAGGCTAGAGTACGGCAGAGGGGGATGGAATTCCGCGTGTAGCAGTGAAATGCGTAGATATGCGGAGGAACACCGATGGCGAAGGCAATCCCCTGGGCCTGTACTGACGCTCATGCACGAAAGCGTGGGGAGCAAACAGG` |
| ASV1625 | 404 | `TGGGGAATATTGGGCAATGGGCGCAAGCCTGACCCAGCAATGCCGCGTGGGTGAAGAAGGTCTTCGGATTGTAAAGCCCTTTCGGCGGGGACGATGATGACGGTACCCGCAGAAGAAGCCCCGGCTAACTTCGTGCCAGCAGCCGCGGTAATACGAAGGGGGCTAGCGTTGCTCGGAATTACTGGGCGTAAAGGGCGCGTAGGCGGTTCTGTTAGTCAGGCGTGAAATTCCTGGGCTCAACCTGGGGACTGCGCTTGAAACGGCGGGACTTGAGGACGGAAGAGGGTCGTGGAATTCCCAGTGTAGAGGTGAAATTCGTAGATATTGGGAAGAACACCGGTGGCGAAGGCGGCGACCTGGTCCGTTACTGACGCTGAGGCGCGACAGCGTGGGGAGCAAACAGG` |
| ASV1102 | 425 | `TGGGGAATATTGGACAATGGGCGCAAGCCTGATCCAGCAATGCCGCGTGAGTGATGAAGGCCTTCGGGTTGTAAAGCTCTGTCGGGAGGGACGAAAAAGCGGGGGTAATGCCCCGTATTGACGGTACCTCCAAAGGAAGCACCGGCTAACTCTGTGCCAGCAGCCGCGGTAATACAGAGGGTGCAAGCGTTGTTCGGAATTACTGGGCGTAAAGCGCGTGTAGGCGGCTTCGTTAGTCGGATGTGAAAGCCCTCGGCTCAACCGAGGAAGTGCATCCGATACTGCGATGCTTGAGTGTTGGAGGGGGTGGCGGAATTCCCGGTGTAGAGGTGAAATTCGTAGATATCGGGAGGAACACCTGTGGCGAAGGCGGCCACCTGGACAACAACTGACGCTGAGACGCGAAAGCGTGGGTAGCAAACAGG` |
| ASV4791 | 422 | `TAGGGAATATTGGGCAATGGGCGGAAGCCTGACCCAGCCATGCCGCGTGTGCGATGAAGGCTTTCGGGTCGTAAAGCACTTTTATTCGGGAAGACACGCACTGTGCGCAGTGCTATGACGGTACCGAAGGAATAAGCACCGGCTAACTCCGTGCCAGCAGCCGCGGTAATACGGAGGGTGCAAGCGTTGTCCGGATTCATTGGGTTTAAAGGGTGCGTAGGCGGCCTGATAAGTCAGGGGTGAAATACCGCGGCTCAACCGCGGGGCTGCCCCTGATACTGCCAGGCTTGGCTGCCGGAAGGGCATGCGGAATTTGCGGTGTAGCGGTGAAATGCATAGATACCGCAAGGAACGCCAGAGGCGAAGGCGGCGTGCTAGCCGGCCAGCGACGCTGATGCACGAAAGCGCGGGTAGCGAACAGG` |
| ASV195 | 424 | `TAAGGAATATTGGTCAATGGAGGCAACTCTGAACCAGCCATGCCGCGTGCAGGAAGACAGCCCTCTGGGTCGTAAACTGCTTTTATTCGGGAATAAACCTATCTACGTGTAGATAGCTGAATGTACCGAAGGAATAAGGATCGGCTAACTCCGTGCCAGCAGCCGCGGTAATACGGAGGATCCAAGCGTTATCCGGATTTATTGGGTTTAAAGGGTGCGTAGGCGGCCTATTAAGTCAGGGGTGAAAGACGGTAGCTCAACTATCGCAGTGCCCTTGATACTGATGGGCTTGAATACACTAGAGGTAGGCGGAATGTGACAAGTAGCGGTGAAATGCATAGATATGTCACAGAACACCGATTGCGAAGGCAGCTTACTATGGTGTTATTGACGCTGAGGCACGAAAGCGTGGGGATCAAACAGG` |
| ASV2124 | 424 | `TGAGGAATATTGGACAATGGGCGCAAGCCTGATCCAGCCATGCCGCGTGCAGGATGACGGTCCTATGGATTGTAAACTGCTTTTGTACAGGAAGAAACACCTCTACGTGTAGAGACTTGACGGTACTGTAAGAATAAGGATCGGCTAACTCCGTGCCAGCAGCCGCGGTAATACGGAGGATCCAAGCGTTATCCGGAATCATTGGGTTTAAAGGGTCCGTAGGCGGTCTTATAAGTCAGTGGTGAAATCTCCCCGCTCAACGGGGAAACGGCCATTGATACTGTAAGACTTGAATTATTAGGAAGTAACTAGAATATGTAGTGTAGCGGTGAAATGCTTAGAGATTACATGGAATACCAATTGCGAAGGCAGGTTACTACTAATGGATTGACGCTGATGGACGAAAGCGTGGGTAGCGAACAGG` |
| ASV4255 | 404 | `TGGGGAATATTGGGCAATGGGCGAAAGCCTGACCCAGCAATACCGCGTGAGTGATGAAGGTCGAAAGATTGTAAAACTCTTTTACCAGGGAAGATTATGACGGTACCTGGAGAATAAGGACTGACTAACTTCGTGCCAGCAGTCGCGGTAATACGAAGAGTCCAAGCGTTACTCGGAATCACTGGGCGTAAAGAATGCGCAGGCTGTTTGGTAAGTTAATAGTGAAATATCGGGGCATAACTTCGGTGCTGCTATTAATACTGCCTTACTAGAGTTTGGTAGAGGATGATGGAATTCTTGGTGTAGGGGTTAAATCCTTTGATATCAAGAGGAACGCCGGAGGCGAAAGCGATCATCTGGGCCAATACTGACGCTCATGCATGACAGCGTGGGGAGCAAACAGG` |
| ASV4105 | 405 | `TGGGGAATATTGGACAATGGGCGAAAGCCTGATCCAGCAATGCCGCGTGAGTGATGAAGGCCCTATGGGTTGTAAAGCTCTTTTATTAGGGAAGATAATGACGGTACCTAATGAATAAGCACCGACTAACTACGTGCCAGCAGTCGCGGTAATACGTAGGGTGCTAGCGTTACTCGGATTTACTGGGCGTAAAGCACACGTAGGTGGACTTGTAAGACAGAAGTGCAATCCCTAAGCTCAACTTAGGAACCGCTTTTGTTACTGCTAGTCTGGAGTGCGATAGGGGATAATGGAATTTCTAGTGGAGGGGTAAAATCCGTAGATATTAGAAGGAACACCAAAAGCGAAAGCAATTATCTGGGTCGCTACTGACACTGAGGTGTGAAAGCGTGGGGAGCAAACAGG` |
| ASV4662 | 431 | `TAGGGAATCTTCCGCAATGGGCGAAAGCCTGACGGAGCAATGCCGCGTGAACGATGAAGGCCTTCGGGTCGTAAAGTTCTGTTGTGGGGGAAGAAAACCCAATTAGATAACACCTAGTTGGGCTTGACGGTACTCCACGAGGAAGCCCCGGCTAATTACGTGCCAGCAGCCGCGGTAATACGTAAGGGGCAAGCGTTGTCCGGAATCACTGGGCGTAAAGCGCACGTAGGCGGTTTGTCAAGTTGGGGGTGTAAGCTCTAGGCCTAACCTAGAAATTGCCTCCAAAACTAGCATACTTGAGTATTAGAGGGGAAGCTGGAATTCACGGTGTAGCGGTGAAATGCGTAGATATCGTGGAGAACACCCGTGGCGAAGGCGGGCTTCTGGCTAATTACTGACGCTGAGGTGCGAAAGCTAGGGGAGCGAACGGG` |
| ASV1187 | 424 | `TGAGGAATATTGGTCAATGGACGAGAGTCTGAACCAGCCAAGTAGCGTGAAGGATGAAGGCCCTATGGGTCGTAAACTTCTTTTATAGGGGAATAAAGTGCGGAACGTGTTCCGCTTTGTATGTACCCTACGAATAAGGATCGGCTAACTCCGTGCCAGCAGCCGCGGTAATACGGAGGATCCGAGCGTTATCCGGATTTATTGGGTTTAAAGGGAGCGTAGGTGGACGTATAAGTCAGTTGTGAAAGTTTGCGGCTCAACCGTAAAATTGCAGTTGATACTGTATGTCTTGAGTGTACAAGAGGTGGGCGGAATTCGTGGTGTAGCGGTGAAATGCTTAGATATCACGAAGAACTCCGATTGCGAAGGCAGCTCACTAGCGTACAACTGACACTGAGGCTCGAAAGTGTGGGTATCAAACAGG` |
| ASV3312 | 424 | `TAAGGAATATTGGGCAATGGACGCAAGTCTGACCCAGCCATGCCGCGTGGAGGATGAAGGCCCTCTGGGTTGTAAACTTCTTTTCTCAATAAAGAAAACTCTGGTTTCTACTAGATTTGACGGTAATTGAGGAATAAGCACCGGCTAACTCCGTGCCAGCAGCCGCGGTAATACGGAGGGTGCAAGCGTTATCCGGATTCACTGGGTTTAAAGGGAGCGTAGGTGGGCTGATAAGTCAGTGGTGAAATACCCGAGCTTAACTCGGGAACTGCCATTGATACTGTTGGTCTTGAATATCGTAGAGGTAAGCGGAATATGTCATGTAGCGGTGAAATGCTTAGATATGACATAGAACACCGATTGCGAAGGCAGCTTACTATGCGAATATTGACACTGATGCTCGAAAGCGTGGGGATCAAACAGG` |
| ASV4017 | 429 | `TGGGGAATATTGGACAATGGGCGAAAGCCTGATCCAGCGACGCCGCGTGGGTGAAGAAGGCCTGCGGGTTGTAAAGCCCTTTCGGTTGGGAAGAAAAGTATTGGCCTAATACGCTAGAACCTTGACGTAACCAACAAAAGAAGCACCGGCTAACTCTGTGCCAGCAGCCGCGGTAATACAGAGGGTGCAAGCGTTAATCGGAATTACTGGGCGTAAAGCGCGCGTAGGCGGCTTGATAAGTCGAGTGTGAAATCCCCGGGCTTAACCTGGGACCTGCATTCGAAACTGTCTTGCTAGAGTATGGGAGAGGGAAGTGGAATTTCAGGTGTAGCGGTGAAATGCGTAGATATCTGAAGGAACATCAGTGGCGAAAGCGACTTCCTGGACCAATACTGACGCTCAGGTGCGAAAGCGTGGGGAGCAAACAGG` |
| ASV4461 | 404 | `TGGGGAATATTGGACAATGGGGGAAACCCTGATCCAGCGATGCCGCGTGAGTGAAGAAGGCCTTCGGGTTGTAAAGCTCTTTCAGAGGGGATGATGATGACGGTACCCTCAGAAGAAGCACCGGCTAACTTCGTGCCAGCAGCCGCGGTAATACGAAGGGTGCAAGCGTTGTTCGGAATTACTGGGCGTAAAGGGCGCGTAGGCGGCTTATCAAGTCAGGCGTGAAATCCCTGGGCTCAACCCGGGAATTGCGTTTGAAACTGGTAAGCTTGAGTGTCAGAGGGGTGAGTGGAATTCCTAGTGTAGAGGTGAAATTCGTAGATATTAGGAGGAACACCAGTGGCGAAGGCGACTCACTGGCTGAATACTGACGCTAAGGCGCGAAAGCGTGGGGAGCAAACAGG` |
| ASV3925 | 424 | `TGAGGAATATTGGTCAATGGACGCAAGTCTGAACCAGCCATGCCGCGTGCAGGATTAAGGCTCTATGAGTTGTAAACTGCTTTTGTATGCGGGTAAAGTGAGTTACGTGTAGCTCATTGAAAGTAGTATACGAATAAGGATCGGCTAACTCCGTGCCAGCAGCCGCGGTAATACGGAGGATCCGAGCGTTATCCGGATTTATTGGGTTTAAAGGGTGCGTAGGCGGTGGATTAAGTTAGTCGTTAAATCGGGAGGCTCAACCTTCTCATGGCGATTAATACTGGTTCACTAGAGATTAGATGCAGAGGGCGGAATGTGTAGTGTAGCGGTGAAATGCTTAGATATTACACAGAACACCGATTGCGAAGGCAGCTCTCTAATCTACATCTGACGCTCAGGCACGAAAGCGTGGGGATCAAACAGG` |
| ASV2072 | 423 | `TAGGGAATATTGGGCAATGGCCGAGAGGCTGACCCAGCCATGCCGCGTGCCGGATGACGGCCTTCTGGGTTGTAAACGGCTTTTCTCGGGGAAGAAAAAGGGTCTGCGGACCACACTGACGGTACCCGAGGAAGAAGCACCGGCTAACTCCGTGCCAGCAGCCGCGGTAATACGGAGGGTGCAAGCGTTGTCCGGATTTATTGGGTTTAAAGGGTGCGTAGGCGGCCCGTTAAGTCCGGGGTGAAAGCCCACGGCTCAACCGTGGAACTGCCCTGGATACTGGCGGGCTTGAGTCCAGGCGAGGTTGGCGGAATGGACGCTGTAGCGGTGAAATGCATAGATAGCGTCCAGAACCCCGATTGCGTAGGCAGCTGACTAGGCTGGTACTGACGCTGAGGCACGAAAGCGTGGGGAGCGAACAGG` |
| ASV861 | 424 | `TGAGGAATATTGGACAATGGGCGCAAGCCTGATCCAGCCATGCCGCGTGCAGGACGACGCATCTATGGTGTGTAAACTGCTTTTGTACAGGAAGAAACACTCCCTCGTGAGGGAGCTTGACGGTACTGTAAGAATAAGGATCGGCTAACTCCGTGCCAGCAGCCGCGGTAATACGGAGGATCCAAGCGTTATCCGGAATCATTGGGTTTAAAGGGTCCGTAGGCGGTTTGATAAGTCAGTGGTGAAATCCCATCGCTCAACGATGGAACGGCCATTGATACTGTCAGACTTGAATTACTAGGAAGTAACTAGAATATGTAGTGTAGCGGTGAAATGCTTAGATATTACATGGAATACCAATTGCGAAGGCAGGTTACTACTAATGGATTGACGCTGATGGACGAAAGCGTGGGTAGCGAACAGG` |
| ASV4912 | 388 | `TCGGGAAACTTTTGCAATACGCGCAAGCGTGACAAAGCAAGCCAAAGTGCTGCTCATCTTTGAGCAGCTTTTGTGGGATGTAAAAAGTCCTGCGAATAAGGACTGGGCAAGACTGGTGCCAGCCGCCGCGGTAATCCCAGCAGTCCAAGTCGCAGCCACATTTGTTGGGTCTAAAACATCCGTAGCTTGCCTAGTAAGTCCTTGGTGAAATCGGGCCTCTTCAGGGTCCGGCGTGCCTAGGATACTGCTTGGCTAGAGACCGGGAGGCGCAGAAGGTACGTACAGGGTAGCGGTAAAATGTGTTGATCCTGTATGGACCTACAGTCGCGAAGGCGTCCTGCGAGAACGGATCTGACAGTGAGGGATGAAGGCTAGGGGCGCAAAGTGG` |
| ASV985 | 407 | `TAGGGAATATTGCACAATGGGGGAAACCCTGATGCAGCGACGCCGCGTGAGTGATGAAGGCCTTCGGGTCGTAAAGCTCTGTCGTATGGGACTAACACAATGAAGGTACCATGCAAGAAAGGATCGGCTAACTTCGTGCCAGCAGCCGCGGTAAGACGAGGGATCCTAGCGTTGTTCGGAATTATTGGGCGTAAAGCGGGTGTAGGCGGATTTATAAGTCAGGTGTGAAATCCCAGGGCTCAACCCTGGAAGTGCACTTGATACTGTAGATCTTGAGTGTGGTAGAGGTTACTGGAATTCCTGGTGTAGTGGTAAAATACGTAGATATCAGGAGGAATACCGGAGGCGAAGGCGAGTAACTGGGCCAACACTGACGCTGAGACCCGAAAGCGTGGGGATCAAACAGG` |
| ASV5330 | 405 | `TGGGGAATATTGCGCAATGGGCGAAAGCCTGACGCAGCAACGCCGCGTGAGGGATGAATGCCTTCGGGTTGTAAACCTCTTTCAGCAGGGACGAAAAAAGACTGTACCTGCAGAAGAAGCACCGGCTAACTACGTGCCAGCAGCCGCGGTAATACGTAGGGTGCAAGCGTTGTCCGGATTTATTGGGCGTAAAGAGCTCGTAGGCGGTCTGTTGCGTCGGATGTGAAAACCCGGAACTCAACTCCGGGCCTGCATTCGATACGGGCAGACTTGAGTATGGCAGGGGAGACTGGAATTCCTGGTGTAGCGGTGAAATGCGCAGATATCAGGAGGAACACCGGTGGCGAAGGCGGGTCTCTGGGCCAATACTGACGCTGAGGAGCGAAAGCGTGGGGAGCAAACAGG` |
| ASV910 | 424 | `TGAGGAATATTGGTCAATGGACGCAAGTCTGAACCAGCCATGCCGCGTGCAGGATGACGGTCCTATGGATTGTAAACTGCTTTTGTACGAGAAGAAACACTCCTTCGTGAAGGAGCTTGACGGTATCGTAAGAATAAGGATCGGCTAACTCCGTGCCAGCAGCCGCGGTAATACGGAGGATCCAAGCGTTATCCGGAATCATTGGGTTTAAAGGGTCCGTAGGCGGTTTAGTAAGTCAGTGGTGAAAGCCCATCGCTCAACGGTGGAACGGCCATTGATACTGCTAAACTTGAATTATTAGGAAGTAACTAGAATATGTAGTGTAGCGGTGAAATGCTTAGAGATTACATGGAATACCAATTGCGAAGGCAGGTTACTACTAATGGATTGACGCTGATGGACGAAAGCGTGGGTAGCGAACAGG` |
| ASV5396 | 405 | `TGGGGAATCTTGCGCAATGGGCGAAAGCCTGACGCAGCAACGCCGCGTGCGGGATGAAGGCCTTCGGGTTGTAAACCGCTTTTACCAGGAACGAAAATGACGGTACCTGGAGAATAAGGTGCGGCCAACTACGTGCCAGCAGCCGCGGTGACACGTAGGCACCAAGCGTTGTCCGGATTTATTGGGCGTAAAGAGCTCGTAGGCGGTTCAGTAAGTCGGGTGTGAAAACTCTGGGCTTAACCCAGAGACGCCACTCGATACTACTGTGACTAGAGTTCGGTAGGGGAGCAGGGAATTCCTGGTGTAGCGGTGAAATGCGCAGATATCAGGAGGAACACCGATGGCGAAGGCACTGCTCTGGGCCGAAACTGACGCTGAGGAGCGAAAGCGTGGGTAGCAAACAGG` |
| ASV4921 | 429 | `TGGGGAATATTGGACAATGGGGGCAACCCTGATCCAGCAATGCCGCGTGTGTGAAGAAGGCCTGAGGGTTGTAAAGCACTTTCAGAGGGGAGGAAGGCGTTTTGTCGAATAGGCGAAATGATTGACGTTACCTTCAGAAGAAGCACCGGCTAACTCTGTGCCAGCAGCCGCGGTAATACAGAGGGTGCAAGCGTTAATCGGAATTACTGGGCGTAAAGGGTGCGTAGGTGGATAAATAAGTTGGACGTGAAAGCCCCGGGCTCAACCTGGGATGGTCGTCCAATACTGTTTATCTAGAGTATGGTAGAGGGTGGTAGAATTTCCGGTGTAGCGGTGAAATGCGTAGATATCGGAAGGAATATCAATGGCGAAGGCAGCCACCTGGGCCAATACTGACACTGAGGCACGAAAGCATGGGGAGCAAACAGG` |
| ASV360 | 429 | `TCGAGAATCATTCACAATGGGCGAAAGCCTGATGGTGTGACGCCGCGTGGAGGATGAAGGTCTTCGGATTGTAAACTCCTGTCATGTGAGAGTAAGGTTGGGGTAGTAACTGACTGCCAACTTGAGAGTATCACAAGAGGAAGAGGCGGCTAACTCTGTGCCAGCAGCCGCGGTAATACAGAGGCCTCAAGCGTTGTTCGGAATCACTGGGCGTAAAGGGTGCGTAGGCGGCGCGGAAAGTCAGGGGTGAAATCCCGGAGCTCAACTCCGGAACTGCCTTTGATACTCCCGCGCTTGAGAACTGGAGGGGACATCGGAATTCACGGTGGAGCAGTGAAATGCGTGGATATCGTGAGGAACACTAGTGGCGAAGGCGGATGTCTGGACAGTATCTGACGCTGAGGCACGAAGGCCAGGGGAGCGAACGGG` |
| ASV4288 | 426 | `TTAAGAATCTTGCGCAATGGGGGCAACCCTGACGCAGCGACGCCGCGTGAAGGATGAAGGCTTTCGGGTTGTAAACTTCGGTAGACTGGGAAGAAGAAATACGTTCGTTGAGGGCGTATAGTGACGGTACCAGTCTGAAGCCCCGGCTAATTACGTGCCAGCAGCCGCGGTAATACGTATGGGGCAAGCGTTGTTCGGAATTATTGGGCGTAAAGGGCTCGCAGGTGGTTTGTTAAGTTGGTGGTTTAATCTCTGGGCTCAACCCAGAGTCAGCCTCCAAAACTGGCGAACTTGAGTACGATAGGGGATAGCGGAATTCTCGGTGTAGCGGTGGAATGCGTAGATATCGAGAGGAACACCAATGGCGAAGGCAGCTATCTGGATCGTAACTGACACTCATGAGCGAAAGTGCGGGGAGCAAACAGG` |
| ASV1881 | 404 | `TGGGGAATATTGGACAATGGGCGCAAGCCTGATCCAGCCATGCCGCGTGAGTGACGAAGGCCTTCGGGTTGTAAAACTCTTTTGGCGGGGAAGATAATGACGGTACCCGCAGAATAAGCTCCGGCTAACTTCGTGCCAGCAGCCGCGGTAATACGAAGGGAGCTAGCGTTGTTCGGAATCACTGGGCGTAAAGCGCACGTAGGCGGATTTGTCAGTCAGGGGTGAAATCCCGGGGCTCAACCTCGGAACTGCCTTTGATACAGCAAGTCTCGAGTCCGATAGAGGTGGGTGGAATTCCTAGTGTAGAGGTGAAATTCGCAGATATTAGGAAGAACACCGGTGGCGAAGGCGGCCCACTGGATCGGTACTGACGCTGAGGTGCGAAAGCGTGGGGAGCAAACAGG` |
| ASV514 | 428 | `TCGAGAATCATTCACAATGGGCGAAAGCCTGATGGTGTGACGCCGCGTGGAGGATTAAGGTCTTCGGATTGTAAACTCCTGTCAAGTGAGAGTAAATTTGCGCGTTAACTGCGTCGCAAACTGAGAGTATCACTAGAGGAAGAGACGGCTAACTCTGTGCCAGCAGCCGCGGTAATACAGAGGTCTCAAGCGTTGTTCGGAATCACTGGGCGTAAAGGGTGCGTAGGCTGTGTTGTAAGTCAGAGGTGAAATCCCGGGGCTCAACCTCGGAACTGCCTCCGATACTGCAAGACTTGAGGACTGGAGAGGAGATTGGAATTCATGGTGGAGCAGTGAAATGCGTAGATATCATGAGGAACGCTAGTGGCGTAGGCGAATCTCTGGACAGTTCCTGACGCTGAGGCACGAAGGCTAGGGGAGCAAACGGG` |
| ASV4988 | 430 | `TCGAGGATATTTTCCAATGGGCGCAAGCCTGAGAATGCGACGCCGCGTGCGGGATGAAGGCCTTCGGGTCGTAAACCGCTGTCGGACCGGAAGAATACCTGTCCGTCGAATAGACGGATCGGTTGACGGTACGGTCGAAGGAAGCCACGGCCAACTCCGTGCCAGCAGCCGCGGTAATACGGAGGTGGCGAGCGTTGCTCGGATTTATTGGGTGTAAAGGGTCGGTAGGCGGTCCAGTAAGTGCCCGGTGAAATCCTGGGGCTTAACCCCAGGGCGCCCGGGTAGACTGCCGGACTTGAGGGCTGGAGAGGCAAGCGGAATTCCCGGTGTAAGAGTGAAATCTGTAGATATCGGGAGGAACACCAGTGGCGAAGGCGGCTTGCTGGACAGCACCTGACGCTGAGCGACGAAAGCGTGGGGAGCAAACAGG` |
| ASV2780 | 404 | `TGGGGAATATTGGACAATGGGCGAAAGCCTGATCCAGCCATGCCGCGTGAGTGACGAAGGCCTTAGGGTTGTAAAGCTCTTTTATCAGGGACGATGATGACGGTACCTGAAGAATAAGACACGGCTAACTTCGTGCCAGCAGCCGCGGTAATACGAAGGTGTCTAGCGTTGTTCGGAATCACTGGGCGTAAAGCGCGCGTAGGCGGCTTTGCAAGTCAGAGGTGAAATCCTGAGGCTTAACCTCAGCATTGCCTTTGAAACTGCAAAGCTAGAGTATTGGAGAGGAGTGGGGAATTCCTAATGTAGCAGTGAAATGCGTAGATATTAGGAGGAACACCGATGGCGAAGGCACCACTCTGGACAATTACTGACGCTGAGGTGCGAAAGCGTGGGGATCAAACAGG` |
| ASV3683 | 424 | `TGGGGAATATTGGTCAATGGGCGAAAGCCTGAACCAGCAATGCCGCGTGAGTGATGAAGGCCTTCGGGTTGTAAAGCTCTGTCAGGGGGAACGAAAAAGCCGATTAATAACCGGCCTTGACGGTACCCCCAAAGGAAGCACCGGCTAACTCTGTGCCAGCAGCCGCGGTAATACAGAGGGTGCAAGCGTTGTTCGGAATTACTGGGCGTAAAGCGCGTGTAGGCGGCCTATTAAGTCAGATGTGAAATCCCTTGGCTCAACCAAGGAACTGCATCTGATACTGGTAGGCTTGAGTGTCGGAGGGGATGGTAGAATTCCTGGTGTAGAGGTGAAATTCGTAGATATCAGGAGGAATACCTGTGGCGAAGGCGACCATCTGGAAGACAACTGACGCTGAGACGCGAAAGCGTGGGTAGCAAACAGG` |
| ASV2578 | 404 | `TGGGGAATATTGGACAATGGGCGAAAGCCTGATCCAGCAATGCCGCGTGAGTGATGAAGGCCTTAGGGTTGTAAAGCTCTTTTACCAGGGATGATAATGACAGTACCTGGAGAATAAGCTCCGGCTAACTCCGTGCCAGCAGCCGCGGTAATACGGAGGGAGCTAGCGTTGTTCGGAATTACTGGGCGTAAAGCGTGCGTAGGCGGCGATTCAAGTCAGAGGTGAAAGCCTGGAGCTCAACTCCAGAACTGCCTTTGAAACTAGATCGCTAGAATCTTGGAGAGGTTAGTGGAATTCCGAGTGTAGAGGTGAAATTCGTAGATATTCGGAAGAACACCAGTGGCGAAGGCGACTAACTGGACAAGCATTGACGCTGAGGTACGAAAGCGTGGGGAGCAAACAGG` |
| ASV3134 | 429 | `TGGGGAATTTTGGACAATGGGCGAAAGCCTGATCCAGCCATTCCGCGTGAGTGAAGAAGGCCTTCGGGTTGTAAAGCTCTTTCGTGAGGGAAGAAACGTGTACTGCTAATACCAGTGCAAACTGACGGTACCTTAAGAAGAAGCACCGGCTAACTACGTGCCAGCAGCCGCGGTAATACGTAGGGTGCAAGCGTTAATCGGAATTACTGGGCGTAAAGCGTGCGCAGGCGGTTTTTTAAGTCAGATGTGAAAGCCCCGGGCTCAACCTGGGAACTGCGTTTGAAACTGAAGAACTAGAGTCTGTCAGAGGGGGGTAGAATTCCACGTGTAGCAGTGAAATGCGTAGAGATGTGGAGGAACACCGATGGCGAAGGCAGCCCCCTGGGATAAGACTGACGCTCATGCACGAAAGCGTGGGGAGCAAACAGG` |
| ASV323 | 404 | `TGGGGAATCTTAGACAATGGGCGCAAGCCTGATCTAGCCATGCCGCGTGAGCGATGAAGGCCTTAGGGTTGTAAAGCTCTTTCAGTGGGGAAGATAATGACTGTACCCACAGAAGAAGCCCCGGCTAACTCCGTGCCAGCAGCCGCGGTAATACGGAGGGGGCTAGCGTTGTTCGGAATTACTGGGCGTAAAGCGCACGTAGGCGGACGGAGAAGTCAGAGGTGAAATCCCAGGGCTCAACCTTGGAACTGCCTTTGAAACTTTCTGTCTTGAGGTCGAGAGAGGTGAGTGGAATTCCGAGTGTAGAGGTGAAATTCGTAGATATTCGGAGGAACACCAGTGGCGAAGGCGGCTCACTGGCTCGATACTGACGCTGAGGTGCGAAAGCGTGGGGAGCAAACAGG` |
| ASV5658 | 410 | `TGGGGGATCTTGCGCTAATGCGCGAAAGCGTGACGCAGCGACGCCGCGTGGGGGATGAAGGCCCTCGGGTTGTAAACCCCTTTCGGCAGGGACGAAGCGAAAGTGACGGTACCTGCAGAAGAAGTCCCGGCTAACTACGTGCCAGCAGCCGCGGTAACACGTAGGGGACGAGCGTTGTCCGGAATCATTGGGCGTAAAGCGCGCGTAGGCGGCCTGGAAAGTCGGTTGTGAAAGCCCGGGGCTCAACCCCGGGACTGCATCCGATACTTCCAGGCTGGAGGTAGGTAGGGGAGATCGGAATTCCTGGTGTAGCGGTGAAATGCGCAGATATCAGGAGGAACACCGGTGGCGAAGGCGGATCTCTGGGCCTTACCTGACGCTGAGGCGCGAAAGCTAGGGGAGCGAACAGG` |
| ASV671 | 404 | `TGGGGAATATTGGACAATGGACGCAAGTCTGATCCAGCAATACCACGTGAGTGATGAAGGCCTTCGGGTTGTAAAACTCTTTTAGTAGGGAAGATAATGACGGTACCTACAGAAAAAGCACCGGCTAACTTCGTGCCAGCAGCCGCGGTAATACGAAGGGTGCTAGCGTTGTTCGGAATCATTGGGCGTAAAGCGTGCGTAGGCGGTTTGATAAGTCGGCTGTGAAAGCCCAGGGCTTAACCTTGGAATTGCAGTCGAAACTATCAAGCTAGAGTATGGCCGGGGATAGTGGAATTCCTAGTGTAGGGGTGAAATCCGTAGATATTAGGAGGAACACCGGTGGCGAAAGCGACTATCTAGACCATTACTGACGCTAAGGCACGAAAGCGTGGGGAGCAAACAGG` |
| ASV2326 | 429 | `TGGGGAATTTTGGACAATGGGGGCAACCCTGATCCAGCAATGCCGCGTGAGTGAAGAAGGCCTTCGGGTTGTAAAGCTCTTTTGTCAGGGAAGAAACGGTTGTTCCTAATACGGACGACTAATGACGGTACCTGAAGAATAAGCACCGGCTAACTACGTGCCAGCAGCCGCGGTAATACGTAGGGTGCAAGCGTTAATCGGAATTACTGGGCGTAAAGCGTGCGCAGGCGGTTTTGTAAGACAGATGTGAAATGCCCGGGCTTAACCTGGGAACTGCATTTGTGACTGCAAGGCTAGAGTGTGTCAGAGGGGGGTAGAATTCCACGTGTAGCAGTGAAATGCGTAGATATGTGGAGGAACACCGATGGCGAAGGCAGCCCCCTGGGATAACACTGACGCTCATGCACGAAAGCGTGGGGAGCAAACAGG` |
| ASV2115 | 430 | `TGGGGAATCTTGCACAATGGGCGAAAGCCTGATGCAGCAACGCCGCGTGAGTGAAGAAGGCCTTTGGGTTGTAAAGCTCTTTCGGCTGGGGAGAAGGGAATATGTGCTAATACCACATATTTTTGACGGTACCAGCATAAGAAGCACCGGCTAATTCCGTGCCAGCAGCCGCGGTAATACGGGAGGTGCAAGCGTTGTTCGGAGTGACTGGGCGTAAAGCGCACGTAGGTGGGCTTGTAAGTCGGATGTGAAATCCCTGGGCTTAACCTAGGAACTGCATCCGAAACTACAAGTCTTGAATGCTTCAGAGGTTGGTAGAATTCCTGGTGTAGGAGTGAAATCCGTAGATATCAGGAGGAATACCGGAGGCGAAGGCGGCCAACTGGGAGTGCATTGACACTGAGGTGCGAAAGCGTGGGGAGCAAACAGG` |
| ASV5126 | 407 | `TGGGGAATATTGCGCAATGGGGGAAACCCTGACGCAGCAACGCCGCGTGATTGAAGAAGGCCTTCGGGTTGTAAAGATCTTTAATTGGGGACGAAAAAAATGACGGTACCCAAAGAATAAGCTCCGGCTAACTACGTGCCAGCAGCCGCGGTAATACGTAGGGAGCAAGCGTTATCCGGATTTACTGGGTGTAAAGGGCGAGTAGGCGGGCTGGCAAGTTGGGAGTGAAATCCGGGGGCTCAACCCCCGAACTGCTTTCAAAACTGCTGGTCTTGAGTGATGGAGAGGCAGGCGGAATTCCCAGTGTAGCGGTGAAATGCGTAGATATTGGGAGGAACACCAGTGGCGAAGGCGGCCTGCTGGACATTAACTGACGCTGAGGCGCGAAAGCGTGGGGAGCAAACAGG` |
| ASV3833 | 426 | `TTTCGAATTATTTGCAATGGGGGCAACCCTGACAGTGCAACGCCGCGTGAGGGATGAAGGTCTTCGGATCGTAAACCTCTGTCACCGGACATTAATGTGCGTTAGTTAATAGCTGACGTATTGAAAATCCGGAGAGGAAGCAGTGGCTAACTCCGTGCCAGCAGCCGCGGTAATACGGAGACTGCAAGCGTTACTCGGAATCACTGGGCGTAAAGGGTGCGTAGGTTGTTGGGTGTGACTGACGTGAAATCCCAGGGCTTAACCCTGGAACTGCGTTGGTTCCTACCCATCTAGAGTATCAGAGAGGCAGGCGGAATTCCCGGAGTAGCGGTGAAATGCGTAGATATCGGGAAGAACACCGAAAGCGAAGGCAGCCTGCTGGATGATAACTGACACTGAGGCACGAAAGCGCGGGTAGCAAAAGGG` |
| ASV121 | 429 | `TCGAGAATAATTCACAATGGGGGCAACCCTGATGGTGCAACGCCGCGTGGAGGATGACGGTCTTCGGATTGTAAACTCCTGTCATCTGGGAGTAAGGCATGGTTGTTAATAGCAAACATGATTGATAGTACCAGAAGAGGAAGGGACGGCTAACTTCGTGCCAGCAGCCGCGGTAATACGAAGGTCCCAAGCGTTGTTCGGAATCACTGGGCGTAAAGGGAGCGTAGGCGGCGTGGTAAGTCAGATGTGAAATCCCGGGGCTCAACCCCGGAACTGCATCCGATACTGCCATGCTAGAGGATTGGAGAGGTAGCTGGAATTCTTGGTGTAGCAGTGAAATGCGTAGAGATCAAGAGGAACACTCGTGGCGAAAGCGAGCTACTGGACAATTCCTGACGCTGAGGCTCGAAGGCTAGGGTAGCGAAAGGG` |
| ASV825 | 429 | `TGGGGAATATTGCGCAATGGGGGCAACCCTGACGCAGCCATGCCGCGTGTGTGAAGAAGGCCTTCGGGTTGTAAAGCACTTTCAATGGGGAAGAAAAGCGTAGCGTTAATAGCGTTGCGCCGTGACGTTACCCATAGAAGAAGCACCGGCTAACTCCGTGCCAGCAGCCGCGGTAATACGGAGGGTGCGAGCGTTAATCGGAATTACTGGGCGTAAAGCGCGCGTAGGCGGTTTGTTAAGTCAGCTGTGAAAGCCCTGGGCTCAACCTGGGAACTGCAGTTGATACTGGCCGACTAGAGTACGAGAGAGGGAGGTAGAATTCCATGTGTAGCGGTGAAATGCGTAGATATATGGAGGAATACCGGTGGCGAAGGCGGCCTCCTGGCTCGATACTGACGCTGAGGTGCGAAAGCGTGGGGATCAAACAGG` |
| ASV1030 | 429 | `TGGGGAATATTGGACAATGGGCGCAAGCCTGATCCAGCCATGCCGCGTGTGTGAAGAAGGCCTTCGGGTTGTAAAGCACTTTTGTTGGGGAAGAAAAGCCTTCGGTTAATACCCGGGGGTTCTGACGGTACCCAAAGAATAAGCACCGGCTAACTTCGTGCCAGCAGCCGCGGTAATACGAAGGGTGCAAGCGTTACTCGGAATTACTGGGCGTAAAGCGTGCGTAGGTGGTGAGTTAAGTCTGTCGTGAAAGCCCCGGGCTCAACCTGGGAATGGCGATGGATACTGGCTCGCTAGAGTGCGGTAGAGGAGAGTGGAATTCCCGGTGTAGCAGTGAAATGCGTAGAGATCGGGAGGAACATCAGTTGCGAAGGCGGCTCTCTGGACCAGCACTGACACTGAGGCACGAAAGCGTGGGGAGCAAACAGG` |
| ASV5622 | 404 | `TGGGGAGTATTGGACAATGGGGGCAACCCTGATCCAGCAATGCCGCGTGGGTGATGAAGGTCTTCGGATTGTAAAGCCCTTTCGGCGGGGACGATGATGACGGTACCCGCAGAAGAAGCCCCGGCTAACTTCGTGCCAGCAGCCGCGGTAATACGAAGGGGGCTAGCGTTGTTCGGAATCATTGGGCGTAAAGGGCGCGTAGGCGGTTCCGCAAGTCGGGCGTGAAAGCCCTGGGCTTAACCCAGGAATTGCGTTCGAAACTGCGGGGCTAGAGGCCGGGAGAGGAGAGTGGAATTCCCAGTGTAGAGGTGAAATTCGTAGATATTGGGAAGAACACCGGTGGCGAAGGCGGCTCTCTGGACCGGTTCTGACGCTGAGGCGCGAAAGCGTGGGGAGCAAACAGG` |
| ASV4424 | 423 | `TAGGGAATATTGGTCAATGGGTGCAAGCCTGAACCAGCCATGCCGCGTGCAGGAAGAAGGCCTTCTGGGTTGTAAACTGCTTTTGCCAGGGGATAAAATGACCATGCGTGGTTAATTGAAGGTACCTGGTGAATAAGCCACGGCTAACTACGTGCCAGCAGCCGCGGTAATACGTAGGTGGCAAGCGTTGTCCGGATTTATTGGGTTTAAAGGGTGCGTAGGCGGTCTATTAAGTCAGTGGTGAAATATCGCAGCTCAACTGTGAGGGTGCCATTGATACTGATGGACTTGAGTGAAGTCGAAGTAGGCGGAATTGACGGTGTAGCGGTGAAATGCTTAGATATCGTCAAGAACACCGATAGTGAAGACAGCTTACTAGGCTTATACTGACGCTGAGGCACGAAAGTGTGGGGATCAAACAGG` |
| ASV3942 | 411 | `TCGAGGATCTTCGGCAATGGACGAAAGTCTGACCGAGCGACGCCGCGTGTGCGATGAAGGCCTTCGGGTTGTAAAGCACTGTCGAGAGGGAGAAAAGCGAAAGTTTGATCTATCTCTGGAGGAAGGACGGGCTAAGTTCGTGCCAGCAGCCGCGGTAAGACGAACCGTCCAAACGTTATTCGGAATCACTGGGCTTAAAGGGCGCGTAGGCGGGCTATCAAGTCCGGGGTGAAATCCTCTAGCTTAACTAGAGAACGGCCTCGGATACTGATGGTCTCGAGGAAGATAGGGGCATGCGGAACTGGTGGTGGAGCGGTGAAATGCGTTGATATCACTAGGAACTCCGGTGGCGAAGGCGGCATGCTGGATCTTTTCTGACGCTGAGGCGCGAAAGCCAGGGGAGCGAACGGG` |
| ASV3752 | 404 | `TGGGGAATATTGGACAATGGGCGCAAGCCTGATCCAGCCATGCCGCGTGAGTGATGACGGCCTTAGGGTTGTAAAGCTCTTTCACCCACGACGATAATGACGGTAGTGGGAGAAGAAGCCCCGGCTAACTTCGTGCCAGCAGCCGCGGTAATACGAAGGGGGCTAGCGTTGTTCGGAATCACTGGGCGTAAAGCGTACGTAGGCGGATCGTTAAGTCAGAGGTGAAATCCCGGAGCTCAACTTCGGAATTGCCTTTGATACTGGCGGTCTTGAGTTCGGGAGAGGTGAGTGGAACTGCTAGTGTAGAGGTGAAATTCGTAGATATTAGCAAGAACACCAGTGGCGAAGGCGGCTCACTGGCCCGATACTGACGCTGAGGTACGAAAGCGTGGGGAGCAAACAGG` |
| ASV1773 | 422 | `TAGGGAATTTTGCGCAATGGAGGAAACTCTGACGCAGCAACGCCATGTGTGGGATGAAGCATTTCGGTGTGTAAACCACTGTCAGCGGGGAATAAATGGTCTCTTCGGAGGCAGTTGAATGTACCCGCAAAGGAAGCCCCGGCTAACTTCGTGCCAGCAGCCGCGGTAATACGAGGGGGGCAAATGTTGTTCGGAATCACTGGGCGTAAAGGGAGCGTAGGCGGGCTTTTAAGTTAGGTGTTTAATCCCGGGGCCCAACCCCGGACAGGCACCTAAAACTGGGAGTCTTGAATAGTGGAGAGGACGGCGGAATTGGGGGTGTAGCGGTGGAATGCGTAGAGATCCCCAAGAACACCAGTTGCGAAGGCGGCCGTCTGGACACATATTGACGCTGAGGCTCGAAAGCATGGGGAGCAAACAGG` |
| ASV350 | 404 | `TGGGGAATATTGGACAATGGGCGAAAGCCTGATCCAGCAATGCCGCGTGAGTGATGAAGGCCTTAGGGTTGTAAAGCTCTTTTACCCGGGATGATAATGACAGTACCGGGAGAATAAGCTCCGGCTAACTTCGTGCCAGCAGCCGCGGTAATACGAGGGGAGCTAGCGTTGTTCGGAATTACTGGGCGTAAAGCGCACGTAGGCGGTTTTTTAAGTCAGAGGTGAAAGCCCGGGGCTCAACCCCGGAATAGCCTTTGAAACTGGAAAACTTGAATCATGGAGAGGTCAGTGGAATTCCGAGTGTAGAGGTGAAATTCGTAGATATTCGGAAGAACACCAGTGGCGAAGGCGACTGACTGGACATGTATTGACGCTGAGGTGCGAAAGCGTGGGGAGCAAACAGG` |
| ASV5484 | 429 | `TGGGGAATATTGGACAATGGGGGCAACCCTGATCCAGCAATGCCGCGTGTGTGAAGAAGGCCTTAGGGTTGTAAAGCACTTTCAGTTAGGAAGAGGGATACTAGGTTAAGAGCTAGGTATTCGGACATTACTGACAGAAGAAGCACCGGCTAACTCTGTGCCAGCAGCCGCGGTAATACAGAGGGTGCGAGCGTTAATCGGAATTACTGGGCGTAAAGAGTGCGCAGGTGGTGTGTTAAGTGTTGTGTGAAATGCCTGGGCTTAACCCAGGAATTGCATAATAGACTGTCACACTAGAGTCTTGTAGAGGATAGCGGAATTCCCGGTGTAGCGGTGAAATGCGTAGATATCGGGAGGAACACCAGTTGCGAAGGCGGCTATCTGGACATAGACTGACACTGAGGCACGAAAGCGTGGGTAGCGAACAGG` |
| ASV4906 | 406 | `TTGGGAATCTTGCACAATGGGGGAAACCCTGATGCAGCGACGCCGCGTGGAGGACGAAGGATCTAGGTCTGTAAACTCCTTTTTCACGGAAAGACTTAGGACGGTACCGTGAGAATAAGCTCCGGCTAACTACGTGCCAGCAGCCGCGGTAAGACGTAGGGAGCGAGCGTTGTCCGGATTTACTGGGCGTAAAGAGCGCGTAGGCGGCTCGTTAAGTATGAAGTGAAATCTCCAGGGCTCAACCCGGAAACTGCTTCGTAGACTGGCGAGCTTGAGGAATGCAGAGGTAAGTAGAATTCCTGGTGTAGCGGTGAAATGCGTAGATATCAGGAGGAATACCGACGGCGAAGGCAGCTTACTGGGCATTATCTGACGCTGAGGCGCGAAAGCGTGGGTAGCAAACAGG` |
| ASV207 | 404 | `TGGGGAATATTGGACAATGGGCGAAAGCCTGATCCAGCCATGCCGCGTGAGTGATGAAGGCCTTAGGGTTGTAAAGCTCTTTTACCAGGGAAGATAATGACTGTACCTGGAGAATAAGCCCCGGCTAACTTCGTGCCAGCAGCCGCGGTAATACGAAGGGGGCTAGCGTTGTTCGGAATCACTGGGCGTAAAGCGCACGTAGGCGGATTTTTAAGTCAGGGGTGAAATCCCGAGGCTCAACCTCGGAACTGCCTTTGATACTGGGAATCTTGAGTATGGTAGAGGTCGGTGGAACAGCAAGTGTAGAGGTGAAATTCGTAGATATTTGCTAGAACACCAGTGGCGAAGGCGGCCGACTGGACCATAACTGACGCTGAGGTGCGAAAGCGTGGGGAGCAAACAGG` |
| ASV5600 | 405 | `TGAGGAATATTCGGCAATGGGGGGAACCCTGACCGAGCAACGCCGCGTGGAGGAAGACGGTTTTCGGATTGTAAACTCCTTTTCTGCGGGAAGAGCGAGGACGGTACCGCAGGAATCAGTCACGGCTAACTACGTGCCAGCAGCCGCGGTAATACGTAGGTGGCAAGCGTTATCCGGAATTATTGGGCGTAAAGCGCACGCAGGTGGCGGCTTAAGTCTGACGTGAAAGCTCCTGGCTTAACTGGGAGAGGTCGTTGGGTACTGGGCGGCTTGAGGCAAGGAGAGGGGCGTAGAATTCCCGGTGTAGTGGTGGAATGCGTAGATATCGGGAGGAATACCAGAGGCGCAAGCGGCGCCCTGGCCTTGGCCTGACGCTCAGGTGCGAAAGCGTGGGGAGCGAACGGG` |
| ASV2148 | 429 | `TGGGGAATTTTGGACAATGGGCGAAAGCCTGATCCAGCCATGCCGCGTGAGTGAAGAAGGCCTTCGGGTTGTAAAGCTCTTTCAGCCGGGAAGAAAATGTACGGGCGAATACCCCGTGCAGATGACGGTACCGGAAGAAGAAGCACCGGCTAACTACGTGCCAGCAGCCGCGGTAATACGTAGGGTGCGAGCGTTAATCGGAATTACTGGGCGTAAAGCGTGCGCAGGCGGTTGTGTAAGACAGATGTGAAATCCCCGGGCTCAACCTGGGAACTGCGTTTGTGACTGCACGACTAGAATACGGCAGAGGGGGGTGGAATTCCACGTGTAGCAGTGAAATGCGTAGATATGTGGAGGAACACCGATGGCGAAGGCAGCCCCCTGGGCCTGTATTGACGCTCATGCACGAAAGCGTGGGGAGCAAACAGG` |
| ASV3148 | 404 | `TAGGGAATATTGCACAATGGACGAAAGTCTGATGCAGCGACGCCGCGTGAGTGATGAAGGCCTTCGGGTCGTAAAACTCTGTGATAAGGGAAGATGATGACGGTACCTTATCAGAAAAGACCGGCTAACTTCGTGCCAGCAGCCGCGGTAATACGAGGGGTCTAAGCGTTGTTCGGAATCACTGGGCGTAAAGCGGGTGTAGGTGGCTATGCAAGTTGGATGTGAAATCCCCGGGCTCAACCCGGGAAGTGCATCCAAAACTACGTAGCTTGAGTGCGGGAGAGAAAGGCAGAATTCCTGGTGTAGTGGTGAAATACGTAGATATCAGGAGGAACTCCGGTGGCGAAGGCGGCCTTTTGGCCCAGCACTGACACTCAGACCCGAAAGCGTGGGTAGCAAACAGG` |
| ASV2114 | 424 | `TAAGGAATATTGGACAATGGTCGCAAGACTGATCCAGCCATGCCGCGTGCAGGATGACTGCCCTATGGGTTGTAAACTGCTTTTGTACGAGAAAAAACCTCTTTACGTGTAAAGAGCTGATTGTATCGTAAGAATAAGGATCGGCTAACTCCGTGCCAGCAGCCGCGGTAATACGGAGGATCCAAGCGTTATCCGGATTTATTGGGTTTAAAGGGTGCGTAGGCGGAATGATAAGTCAGAGGTGAAAACCTGCAGCTCAACTGCAGAACTGCCTTTGATACTGTCAATCTTGAGTATATTTGATGTGGGCGGAATGTGTCATGTAGCGGTGAAATGCTTAGATATGACACAGAACACCGATTGCGAAGGCAGCTCACAAAACTATAACTGACGCTGAGGCACGAAAGCGTGGGGATCAAACAGG` |
| ASV4449 | 430 | `TGGGGAATATTGCGCAATGGGCGAAAGCCTGACGCAGCGACGCCGCGTGAGCGATGAAGGTCTTCGGATCGTAAAGCTCTGTCGGCAGGGAAGATGGGCTTCATGGCTAACATCTATGAAGTTTGACTGTACCTGAAAAGGAAGCACCGGCTAACTCTGTGCCAGCAGCCGCGGTAAGACAGAGGGTGCAAGCGTTGTTCGGATTTACTGGGCGTAAAGCGCGCGTAGGCTGTCTCAAAAGTCGAATGTGAAATCTCATGGCTCAACCGTGAAAGTGCGTTCGATACTATGAGACTTGAGTTTCAGAGGGGACAGTGGAATTCCCAGTGTAGAGGTGAAATTCGTAGATATTGGGAGGAACATCGGTGGCGAAGGCGACTGTCTGGATGAACACTGACGCTGAGGCGCGAAAGCGTGGGTAGCAAACAGG` |
| ASV3456 | 429 | `TGGGGAATATTGCACAATGGGCGCAAGCCTGATGCAGCCATGCCGCGTGTATGAAGAAGGCCTTCGGGTTGTAAAGTACTTTCAGTAGGGAGGAAGGTGTGAACGTTAATAGCGCTCACAATTGACGTTACCTACAGAAGAAGCACCGGCTAACTCCGTGCCAGCAGCCGCGGTAATACGGAGGGTGCAAGCGTTAATCGGAATTACTGGGCGTAAAGCGCACGCAGGCGGTTTGTTAAGTTGGATGTGAAATCCCCGGGCTTAACCTGGGAACTGCATCCAAGACTGGCAAGCTAGAGTCTCGTAGAGGGAGGTAGAATTCCAGGTGTAGCGGTGAAATGCGTAGAGATCTGGAGGAATACCGGTGGCGAAGGCGGCCTCCTGGACGAAGACTGACGCTCAGGTGCGAAAGCGTGGGGAGCAAACAGG` |
| ASV440 | 429 | `TGGGGAATTTTGGACAATGGGCGAAAGCCTGATCCAGCAATGCCGCGTGAGTGAAGAAGGCCTTCGGGTTGTAAAGCTCTTTTGTCAGGGAAGAAACGGTGAGGGCTAATATCCTTTGCTAATGACGGTACCTGAAGAATAAGCACCGGCTAACTACGTGCCAGCAGCCGCGGTAATACGTAGGGTGCAAGCGTTAATCGGAATTACTGGGCGTAAAGCGTGCGCAGGCGGTTTTGTAAGTCTGTCGTGAAATCCCCGGGCTCAACCTGGGAATTGCGATGGAGACTGCAAGGCTAGAATCTGGCAGAGGGGGGTAGAATTCCACGTGTAGCAGTGAAATGCGTAGAGATGTGGAGGAACACCGATGGCGAAGGCAGCCCCCTGGGTCAAGATTGACGCTCATGCACGAAAGCGTGGGGAGCAAACAGG` |
| ASV1966 | 429 | `TCGAGAATAATTCACAATGGGGGCAACCCTGATGGTGCAACGCCGCGTGGAGGATGAAGGTCTTCGGATTGTAAACTCCTGTCATCTGGGAGTAAGGCATGGTTGTTAATAGCAAACATGATTGATAGTACCAGAAGAGGAAGGGACGGCTAACTTCGTGCCAGCAGCCGCGGTAATACGAAGGTCCCGAGCGTTGTTCGGAATCACTGGGCGTAAAGGGAGCGTAGGCGGTGTGGTAAGTCAGATGTGAAATCCCGGGGCTCAACCCCGGAACTGCATCCGATACTGCCATACTAGAGGATTGGAGAGGTAGCTGGAATTCTTGGTGTAGCAGTGAAATGCGTAGAGATCAAGAGGAACACTCGTGGCGAAAGCGAGCTACTGGACAATTCCTGACGCTGAGGCTCGAAGGCTAGGGTAGCGAAAGGG` |
| ASV2211 | 404 | `TAGGGAATATTGGGCAATGGGCGAAAGCCTGACCCAGCCATGCCGCGTGAATGATGAAGGCCTTAGGGTTGTAAAGTTCTTTCGCAGGTGAAGATAATGACGGTAACCTGATAAGAAGCCCCGGCTAACTTCGTGCCAGCAGCCGCGGTAATACGAAGGGGGCTAGCGTTGTTCGGAATTACTGGGCGTAAAGCGCATGTAGGCGGTCCTACAAGCCAGAGGTGAAATCCCGGAGCTCAACTTCGGAATTGCCTTTGGAACTGTAGGACTGGAGTCTCGGAGGGGATGGTGGAATTCCAAGTGTAGAGGTGAAATTCGTAGATATTTGGAGGAACACCGGTGGCGAAGGCGGCCATCTGGACGAGTACTGACGCTGAGATGCGAAAGCGTGGGGAGCAAACAGG` |
| ASV3842 | 425 | `TAAGGAATATTGGACAATGGACGGAAGTCTGATCCAGCCATGCCGCGTGCAGGATGAAGGCGCTCAGCGTTGTAAACTGCTTTTGGACGGGGACAAACCACTCGATGCGTCGGGTGCTGAGGGTACCGTCAGAATAAGCACCGGCTAACTCCGTGCCAGCAGCCGCGGTAATACGGAGGGTGCAAGCGTTATCCGGAATCACTGGGTTTAAAGGGTGCGTAGGCGGTTTGTTAAGTCAGTTGTGAAATTTCCTCGCTTAACGAGAGAGACTGCGATTGATACTGACAGACTTGAATGAGGTTGAGGTAGGCGGAATGTGGCATGTAGCGGTGAAATGCATAGATATGCCATAGAACACCGATTGCGAAGGCAGCTTGCTGGACCTTCATTGACGCTGAGGCACGAAAGCGTGGGGAGCAAACAGG` |
| ASV4082 | 404 | `TGGGGAATCTTGGACAATGGGCGCAAGCCTGATCCAGCCATGCCGCGTGAGTGACGAAGGCCTTAGGGTTGTAAAGCTCTTTTACTGGGGACGATAATGACGGTACCCAGAGAATAAGTCCCGGCTAACTTCGTGCCAGCAGCCGCGGTAATACGAAGGGGACTAGCGTTGTTCGGAATTACTGGGCGTAAAGCGCACGTAGGCGGATATGCCAGTCAGAGGTGAAATCCCGGAGCTCAACTTCGGAACTGCCTTTGATACAGCATATCTGGAGTCCGATAGAGGTGAGTGGAATTCCTAGTGTAGAGGTGAAATTCGTAGATATTAGGAAGAACACCGGTGGCGAAGGCGGCTCACTGGATCGGTACTGACGCTGAGGTGCGAAAGCGTGGGGAGCAAACAGG` |
| ASV5590 | 405 | `TGGGGAATCTTGCACAATGGACGAAAGTCTGATGCAGCAATTTCGCGTGAAGGATGAAGCATTACGGTGTGTAAACTTCTTTTTTGGCAGAAGACGAATGACGGTATGTCAAGAATAAGAGACGGCTAACTACGTGCCAGCAGCCGTGGTAATACGTAGGTCTCAAGCGTTGTCCGGATTTACTGGGCGTAAAGTGTCCGTAGTCTGAATTGTAAGTCTGTTTTCAAATCCTACGACTCAATCGTAGAAAGGGAGTGGATACTGCAGTTCTGGAAGTATCTGGGGGTTAGTGGAATTTCCGGTGGAGCGGTGAAATGCGTTGATATCGGAAGGAACGCCGAAAGCGAAAGCAGCTAACTACAGAATACTTGACGATGAGGGACGACAGTTCGGGTAGCAAACAGG` |
| ASV2721 | 424 | `TAAGGAATATTGGGCAATGGACGAAAGTCTGACCCAGCCACGTCGCGTGCAGGATGAATGCCTTATGGGTTGTAAACTGCTTTTTTACAGGAAGAAATCTATGGATGCGTTCATAGTTGACGGTACTGTGAGAATAAGGATCGGCTAACTCCGTGCCAGCAGCCGCGGTAATACGGAGGATCCAAGCGTTATCCGGATTTACTGGGTTTAAAGGGTGCGTAGGTGGACTTTTAAGTCAGTGGTGAAATCTGATAGCTTAACTATCAAAGTGCCATTGATACTGATTGTCTTGAGTATGGTTGGAGTGAATGGAATGTGTTGTGTAGCGGTGAAATGCTTAGATATAACACAGAACACCAATCGCGAAGGCAGTTCACTAAGCCTTTACTGACACTGATGCACGAAAGCTTGGGGAGCAAACAGG` |
| ASV1788 | 429 | `TGGGGAATATTGGACAATGGGGGCAACCCTGATCCAGCAATGCCGCGTGTGTGAAGAAGGCCTGCGGGTTGTAAAGCACTTTTGTTCGGGGCGAAATCGTGTGGATTAATACTTCATGCGGATGACGGTACCGAAAGAATAAGCACCGGCTAACTTCGTGCCAGCAGCCGCGGTAATACGAAGGGTGCGAGCGTTAATCGGAATTACTGGGCGTAAAGGGTGCGTAGATGGTTTTTTAAGTCAGCTGTGAAATTCCCGGGCTTAACCTGGGCGCTGCAGCTGAAACTGGAAAGCTAGAGTTTGAGAGAGGACGGTGGAATTCCTGGTGTAGCGGTGAAATGCGTAGAGATCAGGAGGAACACTAGTGGCGAAGGCGGCCGTCTGGCTTAAAACTGACGTTGAGGCACGAAAGCGTGGGGAGCAAACAGG` |
| ASV2366 | 403 | `TGGGGGATATTGCACAATGGAGGAAACTCTGATGCAGCGACGCCGCGTGAGGGAAGACGGTCTTCGGATTGTAAACCTCTGTCTTTGGGGAAGAAAATGACGGTACCCAAAGAGGAAGCTCCGGCTAACTACGTGCCAGCAGCCGCGGTAATACGTAGGGAGCAAGCGTTGTCCGGAATTACTGGGTGTAAAGGGAGCGTAGGCGGGACAGTAAGTTGGATGTTTAATCGATCGGCTCAACCGATCGCTGCATCCAAAACTACGGTTCTTGAGTGAAGTAGAGGCAGGCGGAATTCCTAGTGTAGCGGTGAAATGCGTAGATATTAGGAGGAACACCAGTGGCGAAGGCGGCCTGCTGGGCTTTAACTGACGCTGAGGCTCGAAAGCGTGGGGAGCAAACAGG` |
| ASV4456 | 404 | `CAAGGAATCTTCCGCAATGGGCGCAAGCCTGACGGAGCAACGCCGCGTGGAGGACGACGGCCTTCGGGTTGTAAACTCCTTTTCTCAGGGACGAAACGGACGGTACCTGGGGAATCAGCCCCGGCTAACTCTGTGCCAGCAGCCGCGGTAAGACAGAGGGGGCAAGCGTTGTCCGGATTTACTGGGCGTACAGCGCGCGCAGGCGGTCGTGTAGGTACCTGGTGACAGTCTCGGGCTCAACTCGAGGGGGTCTGGGTAGACCGCGCGACTGGAGGCAGGGAGAGGGATGTGGAATACCCGGTGGAGCGGTGAAATGCGTAGAGATCGGGTGGAACACCAGTGGCGAAGGCGGCATCCTGGCCCTGACCTGACGCTGAGGCGCGAGAGCGTGGGGAGCAAACGGG` |
| ASV3701 | 429 | `TGGGGAATATTGGACAATGGGGGAAACCCTGATCCAGCAATGCCGCGTGTGTGAAGAAGGCCTGCGGGTTGTAAAGCACTTTCGGAGGGGACAAAAAGCGTCGGGCTAACACCCAGGCGTCTTGATTTAACCCTTATAAGAAGCACCGGCTAACTCTGTGCCAGCAGCCGCGGTAATACAGAGGGTGCAAGCGTTAATCGGAATTACTGGGCGTAAAGCGCACGCAGGCGGCTGCATAAGCCGGTCGTGAAATCCCCGGGCTTAACCTGGGAATTGCGATCGGGACTGTGCGGCTAGAGTGTTGGAGAGGACAGCGGAATTCCCGGTGTAGCAGTGAAATGCGTAGATATCGGGAGGAACATCAGTGGCGAAGGCGGCTGTCTGGCCAAACACTGACGCTCAGGTGCGAAAGCGTGGGGAGCAAACAGG` |
| ASV3950 | 404 | `TGGGGAATATTGGACAATGGGCGAAAGCCTGATCCAGCGACGCCACGTGAATGATGAAGGCCTTAGGGTTGTAAAGTTCTTTTAGTGGAGAAGATAATGACGGTATCCACAGAAAAAGTCCCGGCTAACTTCGTGCCAGCAGCCGCGGTAATACGAAGGGGGCAAGCGTTACTCGGAATTATTGGGCGTAAAGCGTGCGCAGGCGGTTTCATACGTTGAAAGTGAAAGCCTTTGGCTCAACCAAAGAATTGCTTACAAAACGGTGGAACTAGAGTATTAGAGAGGATAGAGGAATTCCTGATGTAGGGGTGAAATCCGTAGATATCAGGAAGAATATCGAAGGCGAAGGCATCTATCTGGCTAAATACTGACGCTGTTGCACGAAAGCGTGGGGAGCAAACAGG` |
| ASV3527 | 428 | `TGGGGAATCTTGGTCAATGGGCGCAAGCCTGAACCAGCGACGCCGCGTGAGTGATGAAGGCCTTCGGGTTGTAAAGCTCTGTGGGGAGAGACGAAAGAATCGGTTCTAACAGGACCGAAGTTGACGGTATCTCCTTAGCAAGCACCGGCTAACTCTGTGCCAGCAGCCGCGGTAAGACAGAGGGTGCAAACGTTGCTCGGAATCACTGGGCGTAAAGGGCGTGTAGGCGGGAATGTAAGTCGGGCGTGAAATCCCTAGGCTCAACCTAGGAACTGCACTCGAAACTGCGTTTCTTGAGTACTGGAGAGGAAAGCGGAATACCTGGTGTAGAGGTGAAATTCGTAGATATCAGGTGGAACACCAGCGGCGAAGGCGGCTTTCTGGACAGTAACTGACGCTGAGACGCGAAAGCGTGGGGAGCAAACAGG` |
| ASV4181 | 404 | `TGGGGAATATTGGACAATGGGCGAAAGCCTGATCCAGCGACGCCGCGTGGATGATGAAGGCCTTCGGGTTGTAAAGTCCTTTCGGTAGGGAAGATAATGACGGTACCTACAAAAGAAGCTCCGGCTAACTTCGTGCCAGCAGCCGCGGTAATACGAAGGGAGCTAGCGTTACTCGGAATCATTGGGCGTAAAGCGTGCGTAGGCGGTCTTGTAAGTTAGGAGTGAAATCCTGGGGCTCAACCCCAGAACTGCTCTTAATACTGCTCGACTAGAATAACAGAGGGGACAGTAGAATTCCTAGTGTAGGGGTGAAATCCGTAGATATTAGGAGGAATACCAGTGGCGAAAGCGACTGTCTGGCTGATTATTGACGCTGTTGCACGAAAGCGTGGGGAGCAAACAGG` |
| ASV2510 | 424 | `TGAGGAATATTGGACAATGGGCGCAAGCCTGATCCAGCCATGCCGCGTGCAGGATGACGGTCCTATGGATTGTAAACTGCTTTTATACGAGAAGAAACACTCCTTCGTGAAGGAATTTGACGGTATCGTAAGAATAAGGATCGGCTAACTCCGTGCCAGCAGCCGCGGTAATACGGAGGATCCAAGCGTTATCCGGAATCATTGGGTTTAAAGGGTCCGTAGGCGGTCTTGTAAGTCAGTGGTGAAAGCCCATCGCTCAACGGTGGAACGGCCATTGATACTGCTGGACTTGAATTATTAGGAAGTAACTAGAATATGTAGTGTAGCGGTGAAATGCTTAGAGATTACATGGAATACCAATTGCGAAGGCAGGTTACTACTAATTGATTGACGCTGATGGACGAAAGCGTGGGTAGCGAACAGG` |
| ASV2161 | 429 | `TGGGGAATATTGGACAATGGGGGCAACCCTGATCCAGCCATGCCGCGTGTGTGAAGAAGGCCTTAGGGTTGTAAAGCACTTTAAGTTGTGAGGAAGTTGACGCTGTGAATAATGGTGTTGATTGACGTTAGCAACAGAATAAGCACCGGCTAACTCCGTGCCAGCAGCCGCGGTAATACGGAGGGTGCGAGCGTTAATCGGAATTACTGGGCGTAAAGAGCACGTAGGCGGTGCGTTAAGTTGTGTGTGAAATCCCTGGGCTTAACCTGGGAACTGCATGCAAAACTGGCGGACTAGAGTAAGTCAGAGGAGAGCGGAATTTCCGGTGTAGCGGTGAAATGCACAGATATCGGAAGGAACATCAGTGGCGAAGGCGGCTCTCTGGGATATTACTGACGCTGAGGTGCGAAAGCGTGGGGATCAAACAGG` |
| ASV2838 | 429 | `TGGGGAATATTGGACAATGGGGGAAACCCTGATCCAGCAATGCCGCGTGTGTGAAGAAGGCCTGAGGGTTGTAAAGCACTTTCAGTGGGGAGGAGGGATGAGAGATTAAGAGTCACTTATTTGGACGTTACCCACAGAAGAAGCACCGGCTAACTCCGTGCCAGCAGCCGCGGTAATACGGAGGGTGCGAGCGTTAATCGGAATTACTGGGCGTAAAGAGTGCGTAGGTGGTTTTTTAAGTTATCTGTAAAATCCCCGGGCTCAACCTGGGAAGGCCAGATAATACTGGAGAACTCGAGTATTGGAGAGGGTAGTGGAATTTCCGGTGTAGCGGTGAAATGCGTAGAGATCGGAAGGAACACCAGTGGCGAAGGCGGCTGCCTGGCCAAATACTGACACTGAGGCACGAAAGCGTGGGGAGCAAACAGG` |
| ASV2812 | 424 | `TAGGGAATATTGGGCAATGGAGGCAACTCTGACCCAGCCATGCCGCGTGAACGAAGAAGGCGCTATGCGTCGTAAAGTTCTTTTAGAGGGGGCGAAAACACTTGATTTATCAAGTACTGACGGTACCTTCGGAATAAGCACCGGCTAACTCTGTGCCAGCAGCCGCGGTAATACAGAGGGTGCAAGCGTTATCCGGAATCACTGGGTTTAAAGGGTGCGTAGGCGGGATTATAAGTCAGAGGTGAAATCTCGTCGCTTAACGACGGAATTGCCTTTGATACTGTTTTTCTTGAAATGTGTTGAGGTTGGCGGAATGAGACATGTAGCGGTGAAATGCTTAGATATGTCTTAGAACACCGATTGCGAAGGCAGCTGACTGGACACTATTTGACGCTGAGGCACGAAAGCGTGGGGATCAAACAGG` |
| ASV305 | 429 | `TGGGGAATATTGGACAATGGGCGCAAGCCTGATCCAGCCATGCCGCGTGTGTGAAGAAGGCCTTCGGGTTGTAAAGCACTTTTGTTGGGGAAGAAAAGACCGCGGTTAATAACCGTGGTTCATGACGGTACCCAAAGAATAAGCACCGGCTAACTTCGTGCCAGCAGCCGCGGTAATACGAAGGGTGCAAGCGTTACTCGGAATTACTGGGCGTAAAGCGTGCGTAGGCGGTTTGTTAAGTCTGTCGTGAAAGCCCCGGGCTCAACCTGGGAATGGCGGTGGATACTGGCAGGCTAGAGTACGGTAGAGGATGGTGGAATTCCCGGTGTAGCAGTGAAATGCGTAGAGATCGGGAGGAACATCTATGGCGAAGGCAGCCATCTGGACCAGTACTGACGCTGAGGCACGAAAGCGTGGGGAGCAAACAGG` |
| ASV2853 | 424 | `TGAGGAATATTGGACAATGGGCGCAAGCCTGATCCAGCCATGCCGCGTGCAGGAAGAAGCATCTATGGTGTGTAAACTGCTTTTGTACGGGAAGAAACACCACTACGTGTAGTGACTTGACGGTACCGTAAGAATAAGGATCGGCTAACTCCGTGCCAGCAGCCGCGGTAATACGGAGGATCCAAGCGTTATCCGGAATCATTGGGTTTAAAGGGTCCGTAGGCGGTTTAGTAAGTCAGTGGTGAAAGCCTATCGCTCAACGATAGAACGGCCATTGATACTGCTAGACTTGAATTACAAGGAAGTAACTAGAATATGTAGTGTAGCGGTGAAATGCTTAGATATTACATGGAATACCAATTGCGAAGGCAGGTTACTACTTGTGGATTGACGCTGATGGACGAAAGCGTGGGGAGCGAACAGG` |
| ASV3983 | 424 | `TAAGGAATATTGGACAATGGTCGGAAGACTGATCCAGCCATGCCGCGTGGAGGATGAAGGCCCTCTGGGTTGTAAACTTCTTTTATTGGGGGCGAAAAAAGGGAATTCTTTCTCACTTGACGGTACCCAATGAATAAGCACCGGCTAACTCCGTGCCAGCAGCCGCGGTAATACGGAGGGTGCAAGCGTTATCCGGATTCACTGGGTTTAAAGGGTGCGTAGGCGGGTATGTAAGTCAGTGGTGAAATCTCCGAGCTTAACTTGGAAACTGCCATTGATACTATATATCTTGAATGCTCTGGAGGTTTGCGGAATATGTCATGTAGCGGTGAAATGCATAGATATGACATAGAACACCTATTGCGAAGGCAGCAGGCTACAGAGAAATTGACGCTGAGGCACGAAAGCGTGGGGATCAAACAGG` |
| ASV2622 | 405 | `TGGGGGATATTGCACAATGGAGGAAACTCTGATGCAGCGACGCCGCGTGTAGGAAGACGGTCCTCTGGATTGTAAACTACTGTCTTTGGGGACGATAATGACGGTACCCAAAGAGGAAGCTCCGGCTAACTACGTGCCAGCAGCCGCGGTAATACGTAGGGAGCGAGCGTTGTCCGGAATTACTGGGTGTAAAGGGAGCGTAGGCGGGAAGGCAAGTTGGATGTGAAAACTATCGGCTCAACCGATAGACTGCATTCAAAACTGTTTTTCTTGAGTGAAGTAGAGGCAGGCGGAATTCCTAGTGTAGCGGTGAAATGCGTAGATATTAGGAGGAACACCAGTGGCGAAGGCGGCCTGCTGGGCTTTTACTGACGCTGAGGCTCGAAAGTGTGGGTAGCAAACAGG` |
| ASV1502 | 430 | `TGGGGAATATTGGACAATGGGCGGAAGCCTGATCCAGCAATACCGCGTGTGTGAAGAAGGCCTTCGGGTTGTAAAGCACTTTTGTTAGGGAGGATGGTTATTTAGGCGAATACCCTAAGTAATTGACGTTACCTAAAGAATCAGCACCGGCTAACTCCGTGCCAGCAGCCGCGGTAATACGGAGGGTGCAAGCGTTAATCGGATTTACTGGGCGTAAAGGGTGCGTAGGCGGCTCGTTAAGTCAGATGTGAAATCCCCGGGCTCAACCTGGGAATTGCATTTGATACTGGCGAGCTAGAATGTGGTAGAGGGTAGCGGAATTTCTGGTGTAGCGGTGAAATGCGTAGATATCAGAAGGAACATCAATGGCGAAGGCAGCTACCTGGGCCAACATTGACGCTGAGGCACGAAAGCGTGGGGAGCAAACAGG` |
| ASV3967 | 428 | `TGGGGAATTTTCCACAATGGGCGAAAGCCTGATGGAGCAACGCCGCGTGTGTGATGAAGGTTTTCGGATCGTAAAGCACTTTCGACCGGGACGAAAAACCTCTGGCTAACATCCAGGGGCCTGACGGTACCGGGAGAAGAAGCACCGGCTAACTCTGTGCCAGCAGCCGCGGTAATACAGAGGGTGCAAGCGTTGTTCGGAATTATTGGGCGTAAAGCGCGTGTAGGCGGCTTTGCAAGTCAGATGTGAAAGCCCTCGGCTTAACCGAGGAAGTGCGTCTGAAACTGCAGAGCTTGAGTGTCGGAGAGGATGGTGGAATTCCGCAAGTAGAGGTGAAATTCGTAGATATGCGGAGGAACACCGGTGGCGAAGGCGACCATCTGGACGACGACTGACGCTGAGACGCGAAAGCGTGGGGAGCAAACAGG` |
| ASV3180 | 404 | `TGGGGAATATTGGACAATGGGGGAAACCCTGATCCAGCAATGCCGCGTGAGTGAAGAAGGCCTTCGGGTTGTAAAGCTCTTTTAGTAGGGACGATAATGACGGTACCTATTGAATAAGCACCGGCTAACTCTGTGCCAGCAGCCGCGGTAATACAGAGGGTGCTAGCGTTGTTCGGAATTACTGGGCGTAAAGGGCACGTAGGTTGCCTATTAAGTTAGACGTGAAAGGCCTGGGCTCAACCTAGGAACGGCGTTTAATACTGGTAGGCTAGAAGACGGGAGAGGGAAGTGGAATTCCTAGTGTAGAGGTGAAATTCGTAGATATTAGGAGGAACACCGGAGGCGAAGGCGGCTTCCTGGACCGATCTTGACACTGAGGTGCGAAAGCGTGGGGAGCAAACAGG` |
| ASV3958 | 404 | `TGGGGAATATTGGACAATGGGCGCAAGCCTGATCCAGCCATGCCGCGTGAGTGATGAAGGCCTTAGGGTTGTAAAGCTCTTTTATCCGGGAAGATAATGACTGTACCGGAAGAATAAGCCCCGGCTAACTTCGTGCCAGCAGCCGCGGTAATACGAAGGGGGCTAGCGTTGCTCGGAATCACTGGGCGTAAAGGGCGCGTAGGCGGATGATTAAGTCGGAGGTGAAAGCCCAGGGCTCAACCCTGGAATTGCCTTCGATACTGATCATCTCGAGAGTGGTAGAGGTAAGTGGAACTCCGAGTGTAGAGGTGAAATTCGTAGATATTCGGAAGAACACCAGTGGCGAAGGCGACTTACTGGACCATTTCTGACGCTGAGGCGCGAAAGCGTGGGGATCAAACAGG` |
| ASV3467 | 387 | `GCGCGAAAACTTTACACTACGCGCAAGCGTGATAAGGGGATCCAGAGTGCTCATGCACTGCATGGGCTTTTGCCAACGGTAAATACGTTGGCGAATAAGTGGTGGACAAGACTGGTGCCAGCCGCCGCGGTAACCCCAGCACCACGAGTGGGGATCGTACTTATTTGGCCTAAAGCGCTCGTAGCCGGTCATGTAAATCTCTTGTGAAATCGTTGCGCTTAACGTAACGGCGTGCAGGAGAAACTGCAAGACTAGGGACTGGGAGGCGTCGAGGGTATTCTTTGGGGAGCAGTAAAATGCTATAATCCTAAGAGGACCACCTGTGGCGAAGGCGCTCGACGAGAACAGGTTCGACGGTGAGGGGCGAAGGCTAGGGGAGCGATCCGG` |
| ASV2626 | 424 | `TGAGGAATATTGGACAATGGCCGAGAGGCTGATCCAGCCATGCCGCGTGCGGGAGGACGGCCCTATGGGTTGTAAACCGCTTTTGTTGGGGAGCAATAAGGTCTACGTGTAGACTGATGAGAGTACCCAGCGAATAAGCATCGGCTAACTCCGTGCCAGCAGCCGCGGTAATACGGAGGATGCGAGCGTTATCCGGATTTATTGGGTTTAAAGGGTGCGTAGGCGGGTGCTTAAGCGTGAGGTGAAAGCTCGGAGCTCAACTCCGGAACTGCCTCGCGAACTGGGTGCCTTGAATGTCCGTGCCGTGGGAGGAATGTGTGGTGTAGCGGTGAAATGCATAGAGATCACACAGAACACCGATTGCGAAGGCATCTCACGAACGGATGATTGACGCTGAGGCACGAAAGCGTGGGGATCAAACAGG` |
| ASV2213 | 421 | `TGAGGAATATTGCGCAATGGACGGAAGTCTGACGCAGCAATGCCGCGTGCGGGAAGAAGGAGCTATGCTTCGTAAACCGCTGTAGCGTGGGACAAAAAGCACCTTATGGGTGTGAGATGGTACCACGAAAGTAAGCATCGGCTAACTACGTGCCAGCAGCCGCGGTAATACGTAGGATGCAAGCGTTGTCCGGATTTACTGGGTGTAAAGGGTGCGCAGGCGGCTTGGTAAGTCAGAGGTGAAAGCCCGCAGCTCAACTGCGGAAATGCCTTTGATACTGCCTAGCTTGAATATGATAGAGGCTCATGGAATATGTGGTGTAGCGGTGAAATGCGTAGATATCACATAGAACACCAGTGGCGAAGGCGGTGAGCTGGGTCATTATTGACGCTCATGCACGAAAGTGTGGGTAGCAAACAGG` |
| ASV4254 | 429 | `TGGGGAATATTGGACAATGGGGGCAACCCTGATCCAGCAATGCCATGTGTGCGAAGAAGGCCTTCGGGTTGTAAAGCACTTTAGTGAGGGAGGAAGGTATAGAGGTTAATACCCATTATATTTGACAGTACCTCAAGAATAAGCACCGGCAAACTCTGTGCCAGCAGCCGCGGTAATACAGAGGGTGCAAGCGTTAATCGGAATTATTGGGCGTAAAGGGCTTGTAGGCGGTTATATAAGTTAGATGTGAAAACCCTGGGCTTAACCTGGGACGTGCATATAATACTGTATAGCTAGAGTATGGTAGAGGAGTAGGGAATTCCTGGTGTAGCAGTGAAATGCGTAGAGATCAGGAGGAACATCAGTGGCGAAGGCGCTACTCTGGACTAATACTGACGCTGAGGAGCGAAGGCGTGGGGATCAAACAGG` |
| ASV2897 | 429 | `TGGGGAATTTTGGACAATGGGCGAAAGCCTGATCCAGCAATGCCGCGTGAGTGAAGAAGGCCTTCGGGTTGTAAAGCTCTTTCGGACAGAACGAAATCTCTTGCTCTAACATAGCGAGAGTCTGACGGTACTGTAATAAGAAGCACCGGCTAACTACGTGCCAGCAGCCGCGGTAATACGTAGGGTGCGAGCGTTAATCGGAATTACTGGGCGTAAAGCGTGCGCAGGCGGTTATATAAGCCAGATGTGAAATCCCCGAGCTTAACTTGGGAATGGCGTTTGGAACTGTATAACTAGAATTCAGCAGAGGGGGATGGAATTCCACGTGTAGCAGTGAAATGCGTAGAGATGTGGAGGAACACCAATGGCGAAGGCAGTCCCCTGGGCTAGAATTGACGCTCATGCACGAAAGCGTGGGGAGCAAACAGG` |
| ASV2834 | 429 | `TGGGGAATTTTGGACAATGGGCGCAAGCCTGATCCAGCAATGCCGCGTGCAGGATGAAGGCCTTCGGGTTGTAAACTGCTTTTGTACGGAACGAAACGGCAGGCTCTAATAAAGTTTGCTAATGACGGTACCGTAAGAATAAGCACCGGCTAACTACGTGCCAGCAGCCGCGGTAATACGTAGGGTGCGAGCGTTAATCGGAATTACTGGGCGTAAAGCGTGCGCAGGCGGTTATATAAGACAGATGTGAAATCCCCGGGCTCAACCTGGGACCTGCATTTGTGACTGTATAGCTAGAGTACGGTAGAGGGGGATGGAATTCCGCGTGTAGCAGTGAAATGCGTAGATATGCGGAGGAACACCGATGGCGAAGGCAATCCCCTGGACCTGTACTGACGCTCATGCACGAAAGCGTGGGGAGCAAACAGG` |
| ASV1965 | 404 | `TGGGGAATATTGGGCAATGGACGAAAGTCTGACCCAGCCATGCCGCGTGTGTGATGAAGGCCTTAGGGTTGTAAAGCACTTTCGTTGGTGACGATAATGACGGTAACCAAAGAAGAAGCCCCGGCTAACTTCGTGCCAGCAGCCGCGGTAATACGAAGGGGGCTAGCGTTGTTCGGAATAACTGGGCGTAAAGAGTTCGTAGGCTGCACAGCAAGTCAGTGGTGAAATCCCGGAGCTTAACTTCGGAACTGCCATTGAAACTGTTGTGCTGGAATCTCAGAGGGGATAGCGGAATTCCAAATGTAGGGGTGAAATCCGTAGATATTTGGAGGAACACCGGTGGCGAAGGCGGCTATCTGGATGAGTATTGACGCTGAGGAACGAAAGCGTGGGGATCAAACAGG` |
| ASV4400 | 409 | `CCGAGAATATTCGACAATGGGCGAAAGCCTGATCGAGCGACGCCGCGTGCAGGAAGAAGTTCTTCGGGACGTAAACTGCTTTTATAGGGGAGGAACGTAAGTGACTGTACCCTATGAATAAGGACTTGCTAATCTCGTGCCAGCAGCCGCGGTAATACGAGAGGTCCAAGCGTTATCCGGATTTACTGGGCGTAAAGGGTGCGTAGGAGGTTGTGTTAGTCTTCTGTTAAAGCCTGATGCTTAACATCAGAACTGCGGAGGAAACGGCATAACTTGGAGGATGCCAGAGGCAAGCGGAACTCAAGGTGTAGGGGTGAAATCCGTTGATATCTTGGGGAACACCAAAGGCGAAGGCAGCTTGCTGGGGCAGTCCTGACTCTGAGGCACGAAAGCGTGGGGATCAAAAAGG` |
| ASV679 | 429 | `TGGGGAATTTTGGACAATGGGCGCAAGCCTGATCCAGCCATGCCGCGTGTCTGAAGAAGGCCTTCGGGTTGTAAAGGACTTTTGTCTGGGAGCAAATACCCGGTGTTAATAGCGCCGGGGGCTGAGAGTACCAGAAGAATAAGCACCGGCTAACTACGTGCCAGCAGCCGCGGTAATACGTAGGGTGCAAGCGTTAATCGGAATTACTGGGCGTAAAGCGTGCGCAGGCGGTTTTGTAAGCCAGATGTGAAAGCCCCGGGCTCAACCTGGGAACGGCGTTTGGAACTACAAGACTAGAGTGCGTCAGAGGGGGGTGGAATGCCGCGTGTAGCAGTGAAATGCGTAGAGATGCGGCGGAACACCGATGGCGAAGGCAGCCCCCTGGGATGACACTGACGCTCATGCACGAAAGCGTGGGGAGCAAACAGG` |
| ASV1513 | 429 | `TGGGGAATTTTGGACAATGGGGGCAACCCTGATCCAGCAACGCCGCGTGAATGAAGAAGGCCTTAGGGTTGTAAAGTTCTTTTATTTGGGAGCAAAGTTCATGTGTTAATAGCGTATGAATCTGAGAGTACCAAAGGAATAAGCACCGGCTAACTACGTGCCAGCAGCCGCGGTAATACGTAGGGTGCGAGCGTTAATCGGAATTACTGGGCGTAAAGGGTGCGTAGGCGGAAATATAAGTTAGATGTGAAAAGCCTGGGCTCAACCTTGGTCGTGCATTTAAGACTGTATATCTAGAGTTTGTAAGAGGGGCGTAGAATTCCAAGTGTAGCAGTGAAATGCGTAGATATTTGGAGGAATACCGATGGCGAAGGCAGCGCCCTGGTATAGAACTGACGCTGAGGCACGAAAGCGTGGGTAGCAAACAGG` |
| ASV4535 | 387 | `CCGGGAATCATCTGCAATACGCGCAAGCGTGACAGTTTGAACTGGAGTGCTTTCCAAATGGAAAGCTTTTGTGAAATGTAAAAAGTTTTACGAATAAGGACTGGGTAAGACGAGTGCCAGCCGCCGCGGTAATACTCGCGGTCCAAGTCGCAGCCATTTTTATTGGGTCTAAAACATCCGTAGCTTGCTTTGCAAGTTCTCTGTGAAATCCCGTTTCTCAAGGGCGGGGCGAGCAAAGAATACTACAATAGCTAGAGATTGGAAGACGTAAGAAGTATGTTCGAAGTAGCGGTAAAATGTGTTAATCTCGAACAGACTAACAAGAGCGAAGGCATCTTACGAGGACAGTTCTGACAGTAAGGGATGAAGGCCAGGGGCGCGAAAGGG` |
| ASV5498 | 421 | `ACAGAAATACCGATGCGGGGCCCTCTGGGCCGCCGCAATTGGAATGAGCACAATGTACACGCGTTAGCGAGTAGCAACAAGAGGGCAAGCCTGGTGCCAGCAGCCGCGGTAATTCCAGCTCTTGTAGCGTATATTAACGTTGCTGCGTTTGAAAAGCCCGTAGTCAAAGGAGTGTTACCTTGAACAAATCGGAGTGCTCAGCGCAGACGCGGACGTCTGCACGATCAAGCATGGGATAACGGAAGAGGCTGCGGCCTGCGTTGGTTTACGCGGCGCGGAATGATCAAGAGGGACAGTTGGGGGCATTAGTATTCTGCAGTCAGAGGTGAAATTCTTGGATTTGCAGAAGACTCGCTGGTGCGAAAGCGTCTGCCAAGGATGTTTTCATTGATCAAGGACGAAAGTTGGGGGATCGAAGACG` |
| ASV2274 | 430 | `TGGGGAATCTTGCGCAATGGACGAAAGTCTGACGCAGCCACGCCGCGTGAGTGAAGAAGGCCTTCGGGTTGTAAAGCTCTGTCGAACGGGACGAAAAGTCGTTAGGTGAATAGCCTAGCGATGTGACGGTACCGTTAAAGGAAGCACCGGCTAACTCCGTGCCAGCAGCCGCGGTAATACGGAGGGTGCAAGCGTTGCTCGGAATTATTGGGCGTAAAGGGTAGGTAGGTGGTCTCATTTGTCTGGGGTGAAAGCCTTGAGCTTAACTCAAGAAGTGCCCCAGAAACGGTGAGACTAGAGTCCTGGAGAGGGTCGTGGAATTCCCGGTGTAGCGGTGAAATGCGTAGAGATCGGGAGGAACACCAGAGGCGAAGGCGGCGACCTGGACAGGTACTGACACTCAACTACGAAAGCGTGGGGAGCAAACAGG` |
| ASV2663 | 404 | `TGGGGAATATTGGACAATGGGCGCAAGCCTGATCCAGCCATGCCGCGTGAGTGACGAAGGCCTTAGGGTTGTAAAGCTCTTTTAACGGGGAAGATAATGACGGTACCCGTAGAATAAGCTCCGGCTAACTTCGTGCCAGCAGCCGCGGTAATACGAAGGGGGCTAGCGTTGTTCGGAATTACTGGGCGTAAAGAGTTCGTAGGCGGTCTGCTAAGCTAGATGTGAAATCCCAGGGCTTAACCTTGGAACTGCATTTAGAACTGGTAGGCTAGAGAGTGTTAGAGGTTGGTAGAATTCCTAGTGTAGGGGTGAAATCCGTAGATATTAGGAGGAATACCGATGGCGAAGGCAGCCAACTGGGACATTTCTGACGCTGAGGAACGAAAGCGTGGGGAGCAAACAGG` |
| ASV737 | 429 | `TGGGGAATTTTGGACAATGGGCGCAAGCCTGATCCAGCCATTCCGCGTGCAGGATGAAGGCCTTCGGGTTGTAAACTGCTTTTGGACGGAACGAAAAGCCACTTTCTAATACAAGGTGGTCATGACGGTACCGTCAGAATAAGCACCGGCTAACTACGTGCCAGCAGCCGCGGTAATACGTAGGGTGCAAGCGTTAATCGGAATTACTGGGCGTAAAGCGTGCGCAGGCGGTTATGTAAGACAGTTGTGAAATCCCCGGGCTCAACCTGGGAACTGCATCTGTGACTGCATAGCTAGAGTACGGTAGAGGGGGATGGAATTCCGCGTGTAGCAGTGAAATGCGTAGATATGCGGAGGAACACCGATGGCGAAGGCAATCCCCTGGACCTGTACTGACGCTCATGCACGAAAGCGTGGGGAGCAAACAGG` |
| ASV969 | 429 | `TGGGGAATATTGGACAATGGGCGAAAGCCTGATCCAGCCATGCCGCGTGTGTGAAGAAGGTCTTCGGATTGTAAAGCACTTTAAGTTGGGAGGAAGGGCAGTTACCTAATACGTGACTGTCTTGACGTTACCGACAGAATAAGCACCGGCTAACTCTGTGCCAGCAGCCGCGGTAATACAGAGGGTGCAAGCGTTAATCGGAATTACTGGGCGTAAAGCGCGCGTAGGTGGTTTGTTAAGTTGAATGTGAAATCCCCGGGCTCAACCTGGGAACTGCATCCAAAACTGGCAAGCTAGAGTATGGTAGAGGGTAGTGGAATTTCCTGTGTAGCGGTGAAATGCGTAGATATAGGAAGGAACACCAGTGGCGAAGGCGACTACCTGGACTGATACTGACACTGAGGTGCGAAAGCGTGGGGAGCAAACAGG` |
| ASV2385 | 404 | `TGGGGAATCTTGCGCAATGGTCGAAAGACTGACGCAGCCATGCCGCGTGAATGATGAAGGTCTTAGGATTGTAAAATTCTTTTACCAGGGACGATAATGACGGTACCTGGAGAAAAAGCCCCGGCTAACTTCGTGCCAGCAGCCGCGGTAATACGAAGGGGGCTAGCGTTGCTCGGAATTACTGGGCGTAAAGGGAGCGTAGGCGGGTCGACAAGTTGGAGGTGAAAGCCCAGGGCTCAACCTTGGAATTGCCTTCAAAACTGTCGATCTTGAGTATGGGAGAGGTAAGCGGAACTCCGAGTGTAGAGGTGAAATTCGTAGATATTCGGAAGAACACCAGTGGCGAAGGCGGCTTACTGGCCCATTACTGACGCTGAGGCTCGAAAGCGTGGGGAGCAAACAGG` |
| ASV1739 | 429 | `TCGAGAATAATTCACAATGGGGGCAACCCTGATGGTGCAACGCCGCGTGGAGGATGAAGGTCTTCGGATCGTAAACTCCTGTCATCTGGGAGCAAGACCTGGCGGTAAATAGCCGACAGGGTTGATAGTACCAGAAGAGGAAGGGACGGCTAACTTCGTGCCAGCAGCCGCGGTAATACGAAGGTCCCAAGCGTTGTTCGGATTCACTGGGCGTAAAGGGAGCGTAGGCTGCGTGGTAAGTCAGATGTGAAATCCCAGGGCTCAACCCTGGAACTGCATCCGATACTGCCATGCTAGAGGTTTGGAGAGGTAACTGGAATTCTCGGTGTAGCAGTGAAATGCGTAGATATCGAGAGGAACACTCGTTGCGAAAGCGAGTTACTGGACAACACCTGACGCTGAGGCTCGAAGGCTAGGGGAGCGAAAGGG` |
| ASV5177 | 405 | `TGGGGAATCTTGGACAATGAGTGAAAACTTGATCCAGCGATATCACATGAGTGAAGAAGGCTATTTGGTCGTAAAACTCTTTCGTCAACGAAGATTATGACATTAGTTGAAGTAAGAAGTCCTGGCAAATCCTGTGCCAGCAGCCGCGGTAATACAGGAGGGGCAAGCGTTATTCGGATTTAATGGGCGTAAAGGGTGCGTAGGCTATAAATTAGATTAAATATGAAATCCCAAAGCTTAACTTTGGAACTGTATTTAAAATCGATTTATTTGAGTTAGATAGGGGTTAGTGGAATTCTTAAAGTAAAGGTAATATTTGTCGATATTAGGAGGAATACCCGAAGCGAAGGCGGCTTTCTGGATCTATACTGACGCTGAGGCACGAAAGCGTGGGGAGCAAACAGG` |
| ASV1854 | 403 | `TGGGGAATTTTGCGCAATGGGGGAAACCCTGACGCAGCAACGCCGCGTGCGGGATGACGGCCTTCGGGTTGTAAACCGCTTTCAGCAGGGAAGATAGTGACGGTACCTGCAGAAGAAGCCCCGGCTAACTACGTGCCAGCAGCCGCGGTAATACGTAGGGGGCGAGCGTTATCCGGATTCATTGGGCGTAAAGAGCGCGTAGGCGGTCTGTTAAGCGGAATCTCTAATCCGAGGGCTCAACCCCCGGCCGGATTCCGAACTGGCGGACTCGAGTTTGGTAGAGGAAGATGGAATTCCCGGTGTAGCGGTGGAATGCGCAGATATCGGGAAGAACACCGATGGCGAAGGCAGTCTTCTGGGCCATAACTGACGCTGAGGCGCGAAAGCTAGGGGAGCGAACAGG` |
| ASV1201 | 429 | `TGGGGAATATTGCACAATGGGCGCAAGCCTGATGCAGCCATGCCGCGTGTGTGAAGAAGGCCTTCGGGTTGTAAAGCACTTTCAGCGAGGAGGAAGGGTGGTGTGTTAATAGCGCACCACATTGACGTTACTCGCAGAAGAAGCACCGGCTAACTCCGTGCCAGCAGCCGCGGTAATACGGAGGGTGCAAGCGTTAATCGGAATTACTGGGCGTAAAGCGCACGCAGGCGGTTTGTTAAGTCAGATGTGAAATCCCCGCGCTTAACGTGGGAACTGCATTTGAAACTGGCAAGCTAGAGTCTTGTAGAGGGGGGTAGAATTCCAGGTGTAGCGGTGAAATGCGTAGAGATCTGGAGGAATACCGGTGGCGAAGGCGGCCCCCTGGACAAAGACTGACGCTCAGGTGCGAAAGCGTGGGGAGCAAACAGG` |
| ASV4531 | 408 | `TGGGGAATTTTCCACAATGGGCGAAAGCCTGATGGAGCAATACCGCGTGAGGGATGAAGGACCGTGGTTCGTAAACCTCTTTTCTTAAGGAAGAACACAATGACGGTACTTAAGGAATAAGCACCGGCTAACTCCGTGCCAGCAGCCGCGGTAATACGGGGGGTGCAAGCGTTGTCCGGAATGATTGGGCGTAAAGCATCTGTAGGCGGTTTCGAAAGTCGACTGTTAAAGCCCAGGGCTTAACTTTGGAAAAGCAGTCGAGGACTTCGGAACTGGAGTTTGGTAGGGGTAAAGGGAATTCCTAGTGGAGCGGTGAAATGCACAGAGATTAGGAAGAACACCTATGGCGAAAGCACTTTACTGGGCCAAAACTGACGCTGAGAGATGAAAGTTGGGGGAGCAAATAGG` |
| ASV126 | 404 | `TGGGGAATATTGGACAATGGGGGAAACCCTGATCCAGCAATGCCGCGTGAGTGATGAAGGCCTTCGGGTTGTAAAGCTCTTTTACCTGCGACGATGATGACGGTAGCAGGAGAATAAGCCCCGGCAAACTTCGTGCCAGCAGCCGCGGTAATACGAAGGGGGCTAGCGTTGTTCGGAGTTACTGGGCGTAAAGGGCGCGTAGGCGGCGGGTACAGTTTGGCGTGAAAGCCCGGGGCTCAACCTCGGAATTGCGCTGAATACTAGCTCGCTTGAGTACCGGAGAGGATAGTGGAATTCCCAGTGTAGAGGTGAAATTCGTAGATATTGGGAAGAACACCAGTGGCGAAGGCGGCTATCTGGACGGTAACTGACGCTGAGGCGCGAAAGCGTGGGGAGCAAACAGG` |
| ASV4055 | 429 | `TGGGGAATATTGGACAATGGGGGCAACCCTGATCCAGCAATGCCGCGTGTGTGAAGAAGGCCTGAGGGTTGTAAAGCACTTTCAGTGGGGAGGAGGGGTGATTGGTTAAGAGCTGGTTATCTGGACGTTACCCACAGAAGAAGCACCGGCTAACTCCGTGCCAGCAGCCGCGGTAATACGGAGGGTGCGAGCGTTAATCGGAATTACTGGGCGTAAAGGGTGCGTAGGTGGTTTGGTAAGTTATCTGTAAAATCCCTGGGCTTAACCTGGGCAGGTCAGATAATACTGCTGAACTCGAGTATGGGAGAGGGTAGTGGAATTTCCGGTGTAGCGGTGAAATGCGTAGATATCGGAAGGAACACCGGTGGCGAAGGCGGCTACCTGGCCTAATACTGACACTGAGGCACGAAAGCGTGGGGAGCAAACAGG` |
| ASV4260 | 404 | `TGGGGAATATTGGACAATGGGCGCAAGCCTGATCCAGCAACGCCGCGTGAATGATGAAGGCCTTCGGGTCGTAAAGTTCTTTTAGCAGGGAAGAAAATGACGGTACCTGCAGAATAAGCACCGGCTAACTTCGTGCCAGCAGCCGCGGTAATACGAAGGGTGCTAGCGTTGTTCGGAATTACTGGGCGTAAAGGGTGCGTAGGTTGTTTATCAAGCTAGTGGTGAAAGCCCGGGGCTTAACCCCGGAATTGCCATTAGAACTGATAGGCTAGAGAGTGTTAGAGGTGAGTGGAATTTCTAGTGTAGGAGTGAAATCCGTAGATATTAGAAGGAACATCAATGGCGAAGGCAGCTCACTGGGTCACATCTGACACTGAGGCACGAAAGCGTGGGTAGCAAACAGG` |
| ASV1117 | 429 | `TGGGGAATTTTGGACAATGGGCGAAAGCCTGATCCAGCAATGCCGCGTGAGTGAAGAAGGCCTTCGGGTTGTAAAGCTCTTTTGTCAGGGAAGAAACGGTTTGGGCTAATATCCTGGGCTAATGACGGTACCTGAAGAATAAGCACCGGCTAACTACGTGCCAGCAGCCGCGGTAATACGTAGGGTGCAAGCGTTAATCGGAATTACTGGGCGTAAAGCGTGCGCAGGCGGTTTTGTAAGTCTGACGTGAAATCCCCGGGCTCAACCTGGGAATTGCGTTGGAGACTGCAAGGCTAGAATCTGGCAGAGGGGGGTAGAATTCCACGTGTAGCAGTGAAATGCGTAGAGATGTGGAGGAACACCGATGGCGAAGGCAGCCCCCTGGGTCAAGATTGACGCTCATGCACGAAAGCGTGGGGAGCAAACAGG` |
| ASV2604 | 424 | `TAAGGAATATTGGTCAATGGACGGAAGTCTGAACCAGCCATGCCGCGTGCAGGATGACGGCCCTCTGGGTTGTAAACTGCTTTTATAGGGGACGAAAAAAGGGAATTCTTTCCCACTTGACGGTACCCTAGGAATAAGCACCGGCTAACTCCGTGCCAGCAGCCGCGGTAATACGGAGGGTGCAAGCGTTATCCGGATTTACTGGGTTTAAAGGGTGCGTAGGTGGGTCGGTAAGTCAGCGGTGAAATCTTCGAGCTTAACTCGGAAACTGCCATTGATACTATCGATCTTGAATCATCTAGAGGTAAGCGGAATATGTCATGTAGCGGTGAAATGCTTAGATATGACATAGAACACCAATTGCGAAGGCAGCTTACTATGGATGTATTGACACTGAGGCACGAAAGCGTGGGGATCAAACAGG` |
| ASV3257 | 408 | `TCGAGAATCTTCCGCAATGGACGAAAGTCTGACGGAGCGACGCCGCGTGGTGGATGAAGTCCCTCGGGACGTAAACACCTTTTATGAGGGAGGAAGTTTATTGACGTTACCTCATGAATAAGGGGCTCCCAACTCTGTGCCAGCAGGAGCGGTAATACAGAGGCCCCAAGCATTATCCGGATTTACTGGGCGTAAAGGGTGCGTAGGCGGTTATATTAGTCAGGTGTTAAATCCCGGGGCCCAACCCCGGAATCGCATTTGAAACGGTATAACTAGAATAAGTCAGAGGCAAGCAGAACTCTCGGTGTAGGGGTGAAATCCGTTGATATCGAGGGGAATACCAAATGCGAAGGCAGCTTGCTGGGACTTTATTGACGCTGAGGCACGAAAGCGTGGGTAGCGAAGCGG` |
| ASV1257 | 429 | `TGGGGAATATTGGACAATGGGGGAAACCCTGATCCAGCCATGCCGCGTGTGTGAAGAAGGCCCTAGGGTTGTAAAGCACTTTAAGCAGGGAGGAAAGGGTGATGGCTAATATCCATCATCATTGACGCTACCTGCAGAATAAGCACCGGCTAACTTCGTGCCAGCAGCCGCGGTAATACGAAGGGTGCAAGCGTTAATCGGAATTACTGGGCGTAAAGCGCGCGTAGGCGGTTTGGTAAGTGTGATGTGAAATCCCCGGGCTCAACCTGGGAACTGCATCACATACTGCCAGGCTAGAGTACGGTAGAGGGGGGTAGAATTCCACGTGTAGCGGTGAAATGCGTAGAGATGTGGAGGAATACCAGTGGCGAAGGCGGCCCCCTGGATCGATACTGACGCTGAGGTGCGAAAGCGTGGGGAGCAAACAGG` |
| ASV3472 | 407 | `TAGGGAATATTGCACAATGGAGGAAACTCTGATGCAGCGACGCCGCGTGAGTGACGAAGGACTTCGGTTCGTAAAGCTCTGTTGCACGGGAATAAGAAAGTGAATGTACCGTGTGAGGAAGGGTCGGCTAACTTCGTGCCAGCAGCCGCGGTAAGACGAGGGACCCTAGCGTTGTTCGGAATCATTGGGCGTAAAGCGGAGGTAGGTGGCTTGGTAAGTCGAGTGTGAAAGCCCAGGGCTCAACCCTGGAAGTGCATTCGAAACTGCTGAGCTTGAGTGCTAGAGAGGTTACTAGAATGCCTGGTGTAGTGGTGAAATACGTAGATATCAGGCGGAATACCGGAGGCGAAGGCGGGTAACTGGCTAGACACTGACACTCAATTCCGAAAGCGTGGGGATCAAACAGG` |
| ASV4558 | 404 | `TGGGGAATTTTGGACAATGGGGGAAACCCTGATCCAGCGATGCCGCGTGAGTGATGAAGGCCTTAGGGTTGTAAAGCTCTTTCGCCCGTGACGATGATGACGGTAACGGGATAAGAAGCCCCGGCTAACTCCGTGCCAGCAGCCGCGGTAATACGGAGGGGGCTAGCGTTGTTCGGAATTACTGGGCGTAAAGCGCGCGTAGGCGGCTCGCCAAGTCGGACGTGAAAGGCCCGGGCTTAACCTGGGACCGGCGTTCGAGACTGGCGGGCTAGGGTCCGGAAGAGGGCAGTGGAATTCCCAGTGTAGAGGTGAAATTCGTAGAGATTGGGAAGAACACCGGTGGCGAAGGCGGCTGCCTGGTCCGTGTCCGACGCTGAGGCGCGAAAGCGTGGGGAGCAAACAGG` |
| ASV370 | 429 | `TGGGGAATTTTGGACAATGGGCGCAAGCCTGATCCAGCAATGCCGCGTGAGTGAAGAAGGCCTTCGGGTTGTAAAGCTCTTTTGTCAGGGAAGAAACGGTAGTCTCTAATACAGATTGCTAATGACGGTACCTGAAGAATAAGCACCGGCTAACTACGTGCCAGCAGCCGCGGTAATACGTAGGGTGCAAGCGTTAATCGGAATTACTGGGCGTAAAGCGTGCGCAGGCGGTTATATAAGTCAGATGTGAAATCCCCGGGCTCAACCTGGGAACTGCATTTGAGACTGTATGGCTAGAGTGTGTCAGAGGGGGGTAGAATTCCACGTGTAGCAGTGAAATGCGTAGATATGTGGAGGAATACCGATGGCGAAGGCAGCCCCCTGGGATAACACTGACGCTCATGCACGAAAGCGTGGGGAGCAAACAGG` |
| ASV4627 | 424 | `TGGGGAATATTGGACAATGGGCGAAAGCCTGATCCAGCAATGCCGCGTGAGTGATGAAGGCCTTCGGGTTGTAAAGCTCTGTCGGGAGGGACGAAAAAGCCGGTTAACACCCGGACTTGACGGTACCTCCAAAGGAAGCACCGGCTAACTCTGTGCCAGCAGCCGCGGTAATACAGAGGGTGCAAGCGTTGTTCGGAATTACTGGGCGTAAAGCGCGTGTAGGTGGCGGCGTAAGTCGGGTGTGAAATCCCTCAGCTCAACTGAGGAAGTGCACTCGATACTGCGTCGCTTGAGTACCGGAGGGGATGGCGGAATTCCCGGTGTAGAGGTGAAATTCGTAGATATCGGGAGGAACACCTGTGGCGAAGGCGGCCATCTGGACGGTCACTGACACTGAGACGCGAAAGCGTGGGTAGCAAACAGG` |
| ASV5219 | 405 | `TAAGGAATATTGCGCAATGGGCGAAAGCCTGACGCAGCAACGCCGTGTGGAGGATGAAGGTCTTTGGATTGTAAACTCCTGTCAGTGGGGACGAAATTTGACGGTACCCACAGAGGAAGCACCGGCTAACTACGTGCCAGCAGCCGCGGTAAGACGTAGGGTGCAAGCGTTGTCCGGATTTATTGGGCGTAAAGCGTCCGTAGGCGGTGAAGAAAGTTTGGTGTTAAAGATCAGAGCTCAACTCTGTCCAGGCACTGAAAACTACTTTACTAGAGTGCGGTAGAGGTAAAGGGAATTCCTGGTGTAGCGGTGAAATGCGTAGATATCAGGAGGAACGCCAGTGGCGAAAGCGCTTTACTGGGCCGTTACTGACGCTAAGGGACGAAAGCCAGGGTAGCGAAAGGG` |
| ASV4543 | 404 | `TGAGGAATCTTCCACAATGGGCGCAAGCCTGATGGAGCGACACCGCGTGAAGGATGAAGATCTAACGATTGTAAACTTCTTTTCTGAGGGAGCATAATGAGAGTACTTCAGGAATAAGCACCGGCTAAACTCGTGCCAGCAGCCGCGGTAATACGAGTGGTGCAAGCATTGCCCGGAATCACTGGGTGTAAAGGGTTTGTAGGCGGATAAGCAAGTCAAGTATGAAAGACCGAAGCTCAACTTCGTGTTTGTGCTTGAAACTATTTATCTAGAATGAGGAAGGGGTAAGCGGAATTCTAAGTGTAGGGGTGCAATCCGTAGATACTTAGAGGAACACCGAAAGCGAAGGCAGCTTACTGGGACTCTATTGACGCTGAAAAACGAAAGCGTGGGGAGCGAAAAGG` |
| ASV2338 | 429 | `TGGGGAATATTGGACAATGGGGGCAACCCTGATCCAGCAATGCCGCGTGTGTGAAGAAGGCCTGAGGGTTGTAAAGCACTTTCAGTGGGGAGGAGGGTTAGTAGGTTAAGAGCTGACTAATTGGACGTTACCCACAGAAGAAGCACCGGCTAACTCCGTGCCAGCAGCCGCGGTAATACGGAGGGTGCGAGCGTTAATCGGAATTACTGGGCGTAAAGGGTGCGTAGGTGGTTTGATAAGTTACGTGTAAAATTCCCGGGCTTAACCTGGGGCTGTCATGTAAGACTGTTAGACTAGAGTATGGGAGAGGGTAGTGGAATTTCCGGTGTAGCGGTGAAATGCGTAGATATCGGAAGGAACATCGGTGGCGAAGGCGGCTACCTGGCCTAATACTGACACTGAGGCACGAAAGCGTGGGGAGCAAACAGG` |
| ASV1345 | 404 | `TGGGGAATATTGGACAATGGGCGCAAGCCTGATCCAGCCATGCCGCGTGAGTGACGAAGGCCTTAGGGTTGTAAAGCTCTTTTAACGGGGAAGATAATGACGGTACCCGTAGAATAAGCTCCGGCTAACTTCGTGCCAGCAGCCGCGGTAATACGAAGGGGGCTAGCGTTGTTCGGAATTACTGGGCGTAAAGAGTTCGTAGGCGGTTTATTAAGCTAGATGTGAAATCCCAAGGCTCAACCTTGGAACTGCATTTAGAACTGGTAGGCTAGAGAGTGTTAGAGGTTGGTAGAATTCCTAGTGTAGGGGTGAAATCCGTAGATATTAGGAGGAATACCGATGGCGAAGGCAGCCAACTGGGACATTTCTGACGCTGAGGAACGAAAGCGTGGGTAGCAAACAGG` |
| ASV2557 | 407 | `TGGGGAATATTGGGCAATGGAGGAAACTCTTACCCAGCAATGCCGCGTGACTGAAGAAGGTCTTCGGATTGTAAAGGTCTTTGATTGGGGACGAATAAATGACGGTACCCAAGGAACAAGCCCCGGCTAACTATGTGCCAGCAGCCGCGGTAATACATAGGGGGCAAGCGTTGTCCGGAATGACTGGGCGTAAAGGGTGTGTAGGTGGTCTGGCAAGTTGGAAGTGTAATACCTAGGGCTTAACCCAGGTGCTGCTTCCAAAACTGTTGGACTTGAGTATCGGAGAGGAAAGTGGAATTCCTAGTGTAGCGGTGGAATGCGTAGATATTAGGAGGAACACCAGAGGCGAAAGCGACTTTCTGGACGATAACTGACACTGAGGCACGAAAGCGTGGGGAGCAAACAGG` |
| ASV3838 | 405 | `TAGGGAATCTTGCGCAATGGGCGAAAGCCTGACGCAGCGACGCCGCGTGGGGGATGAAGGCCTTCGGGTTGTAAACCCCTTTCATGAGGGAAGAACACTGACCTAACCTCAGGAAGAAGCACCGGCTAACTCCGTGCCAGCAGCTGCGGTAATACGGAGAGTGCGAACGTTGTTCGGATTCACTGGGCGTAAAGGGCACGTAGGCGGCTCGGCAAATCAGTCGTGGAATCCCGTCGCTTACCGGCGGAACGGCGGCTGAAAATACCGGGCTCGAGGGCGTGAGGGGTCGCTGGAACTCCTGGTGAAGCGGTGAAATGCGTAGATATCAGGAGGAACGCCGGAGGCGAAAGCGAGCGACTGGCGCGTTCCTGACGCTGAGGTGCGAAAGCTGGGGGAGCAAACAGG` |
| ASV2728 | 424 | `TGAGGAATATTGGTCAATGGGCGCAAGCCTGAACCAGCCATGCCGCGTGAAGGATGAAGGTTCTATGGATTGTAAACTTCTTTTATTTGGGAATAAACCTATTTACGTGTAGATAGCTGAAGGTACCAAATGAATAAGCACCGGCTAACTCCGTGCCAGCAGCCGCGGTAATACGGAGGGTGCAAGCGTTATCCGGAATCATTGGGTTTAAAGGGTCCGCAGGCGGATTTATAAGTCAGTGGTGAAAGCCTACAGCTTAACTGTAGAACTGCCATTGATACTGTAAGTCTTGAATTTGGTCGAAGTGGGCGGAATATGACATGTAGCGGTGAAATGCATAGATATGTCATAGAACACCGATAGCGAAGGCAGCTCACTAGGCCAACATTGACGCTCATGGACGAAAGCGTGGGGAGCAAACAGG` |
| ASV5069 | 404 | `TGGGGGATATTGCACAATGGAGGAAACTCTGATGCAGCGACGCCGCGTGAGTGAAGACGGCCTTCGGGTTGTAAAGCTCTGTAGACGGGGAAGATAATGACGGTACCCGTAAAGTAAGCCACGGCTAACTACGTGCCAGCAGCCGCGGTAATACGTAGGTGGCAAGCGTTGTCCGGAATTACTGGGTGTAAAGGGAGCGTAGGCGGGAGCCCAAGTTGGATGTGAAATACCGCAGCTTAACTGCGGGGCTGCATCCAAAACTGGGTTTCTTGAGTGAAGTAGAGGTAGGCGGAATTCCTAGTGTAGCGGTGAAATGCGTAGATATTAGGAGGAACACCAGTGGCGAAGGCGGCCTACTGGGCTTTAACTGACGCTGAGGCTCGAAAGCGTGGGGAGCAAACAGG` |
| ASV2003 | 404 | `TGGGGAATATTGGACAATGGGCGAAAGCCTGATCCAGCAATGCCGCGTGAGTGATGAAGGCCTTAGGGTTGTAAAGCTCTTTTACCCGGGATGATAATGACAGTACCGGGAGAATAAGCCCCGGCTAACTCCGTGCCAGCAGCCGCGGTAATACGGAGGGGGCTAGCGTTGTTCGGAATTACTGGGCGTAAAGCGCACGTAGGCGGCTGTTCAAGTCAGAGGTGAAAGCCTGGAGCTCAACTCCAGAACTGCCTTTGAAACTAGACAGCTAGAATCATGGAGAGGTGGGTGGAATTCCGAGTGTAGAGGTGAAATTCGTAGATATTCGGAAGAACACCAGTGGCGAAGGCGGCCCACTGGACATGTATTGACGCTGAGGTGCGAAAGCGTGGGGAGCAAACAGG` |
| ASV2426 | 429 | `TGGGGAATTTTGGACAATGGGCGCAAGCCTGATCCAGCCATGCCGCGTGCGGGAAGAAGGCCTTCGGGTTGTAAACCGCTTTTGTCAGGGAAGAAACGCTCCGGGCTAATACCCTGGGGTAATGACGGTACCTGAAGAATAAGCACCGGCTAACTACGTGCCAGCAGCCGCGGTAATACGTAGGGTGCAAGCGTTAATCGGAATTACTGGGCGTAAAGCGTGCGCAGGCGGTCTTGCAAGACAGATGTGAAATCCCCGGGCTTAACCTGGGAACTGCATTTGTGACTGCAAGGCTGGAGTACGGCAGAGGGGGATGGAATTCCGCGTGTAGCAGTGAAATGCGTAGATATGCGGAGGAACACCGATGGCGAAGGCAATCCCCTGGGCCTGTACTGACGCTCATGCACGAAAGCGTGGGGAGCAAACAGG` |
| ASV1281 | 429 | `TGGGGAATTTTGGACAATGGGCGAAAGCCTGATCCAGCCATTCCGCGTGAGTGAAGAAGGCCTTCGGGTTGTAAAGCTCTTTCGGCAGGGAAGAAACGGTCCTGGATAATACCTTGGGCTAATGACGGTACCTGAAGAAGAAGCACCGGCTAACTACGTGCCAGCAGCCGCGGTAATACGTAGGGTGCGAGCGTTAATCGGAATTACTGGGCGTAAAGCGTGCGCAGGCGGTTTTGTAAGACAGGTGTGAAATCCCCGGGCTTAACCTGGGAACTGCGCTTGTGACTGCAAGACTTGAGTACGGCAGAGGGGGGTAGAATTCCACGTGTAGCAGTGAAATGCGTAGAGATGTGGAGGAATACCGATGGCGAAGGCAGCCCCCTGGGTCGATACTGACGCTCATGCACGAAAGCGTGGGGAGCAAACAGG` |
| ASV3268 | 430 | `TGGGGAATATTGCGCAATGGGCGAAAGCCTGACGCAGCAACGCCGCGTGAGTGATGAAGGCCTTCGGGTTGTAAAGCTCTTTCGGCTGGGAAGAAGGGCATCAAGGTGAATAGCTTTGGTGTTTGATGGTACCAGAAGAAGAAGCACCGGCTAACTTCGTGCCAGCAGCCGCGGTAATACGAAGGGTGCGAGCGTTGTTCGGAATTACTGGGCGTAAAGGGTTCGTAGGCGGGAAGATAAGTTGAGTGTGAAAACCCCGGGCTTAACCTGGGACGTGCATTCAAAACTGTTTTTCTTGAGTATCGGAGAGGGTTGTGGAATTGCTGGTGTAGGAGTGAAATCCGTAGAGATCAGCAGGAACACCTGAGGCGAAAGCGACAACCTGGTCGAATACTGACGCTGAGGAACGAAAGCGTGGGGAGCAAACAGG` |
| ASV3554 | 404 | `TGGGGAATATTGGACAATGGGGGCAACCCTGATCCAGCAATGCCGCGTGAGTGAAGAAGGCCTTCGGGTTGTAAAGCTCTTTTGTCTGTGATGATAATGACAGTAGCAGACGAATAAGCCCCGGCTAACTTCGTGCCAGCAGCCGCGGTAATACGAAGGGGGCTAGCGTTGTTCGGAATAACTGGGCGTAAAGGGCGCGTAGGCGGTTAGATACGTCTGATGTGAAAGTCCTGGGCTTAACCTAGGGACTGCATTGGATACGGTTTAGCTTGAGTGCGAGAGGGGAAAATGGAATTGCGCGTGTAGAGGTGAAATTCGTAGAGATGCGCAAGAACACCTGTGGCGAAGGCGATTTTCTGGCTCGCAACTGACGCTGAGGCGCGAAAGCGTGGGGAGCAAACAGG` |
| ASV526 | 424 | `TAGGGAATATTGGGCAATGGATGAAAGTCTGACCCAGCCATGCCGCGTGCAGGAAGAAGGTCCTCTGGACTGTAAACTGCTTTTTTCAGGGAAGAATAGGTGGGATGCGTCCTACAGTGACGGTACTTGAAGAATAAGCACCGGCTAACTACGTGCCAGCAGCCGCGGTAATACGTAGGGTGCGAGCGTTGTCCGGATTTATTGGGTTTAAAGGGTGCGTAGGTGGATTTTTAAGTCAGCGGTGAAATATGGTTGCTCAACAATCAGATTGCCATTGATACTGGAAGTCTTGAGAGAAGTGGAGGCTGTTGGAATGGATGGTGTAGCGGTGAAATGCATAGATATCATCCAGAACGTCAATTGCGAAGGCAGATGGCTGTACTTTTTCTGACACTGAGGCACGAAAGCGTGGGGATCAAACAGG` |
| ASV2394 | 429 | `TGGGGAATTTTGGACAATGGGCGAAAGCCTGATCCAGCAATGCCGCGTGAGTGAAGAAGGCCTTCGGGTTGTAAAGCTCTTTTGTCAGGGAAGAAACGGTTTGGGCTAATACCCTGAGCTAATGACGGTACCTGAAGAATAAGCACCGGCTAACTACGTGCCAGCAGCCGCGGTAATACGTAGGGTGCAAGCGTTAATCGGAATTACTGGGCGTAAAGCGTGCGCAGGCGGTTTTGTAAGTCTGACGTGAAATCCCCGGGCTCAACCTGGGAATTGCGTTGGAGACTGCAAGGCTAGAATCTGGCAGAGGGGGGTAGAATTCCACGTGTAGCAGTGAAATGCGTAGAGATGTGGAGGAACACCGATGGCGAAGGCAGCCCCCTGGGTCAAGATTGACGCTCATGCACGAAAGCGTGGGGAGCAAACAGG` |
| ASV4430 | 429 | `TCAGGAATATTGGGCAATGGGCGAAAGCCTGACCCAGCAACGCCGCGTGAAGGATGAAGGCCTTCGGGTCGTAAACTTCTGTCGAGAGGGAAGAATGTGTACGGGTGAATAATCCGTGCATTTGACGGTACCTCCAAAGGAAGCACCGGCTAACTCCGTGCCAGCAGCCGCGGTAATACGGAGGGTGCAAGCGTTGTTCGGATTCATTGGGCGTAAAGCGAGTGTAGGCGGTTTGACAAGTCGGATGTGAAATCCCGGGGCCCAACCCCGGAACTGCATCCGATACTGTCTGGCTAGAGTCCCGGAGAGGGTGGCGGAATTCCCGGTGTAGGGGTGAAATCCGTAGATATCGGGAGGAACACCAGTGGCGAAGGCGGCCACCTGGACGGTGACTGACGCTGAGACTCGAAAGCGTGGGTAGCAAACAGG` |
| ASV5615 | 404 | `TGGGGAATATTGGACAATGGGGGAAACCCTGATCCAGCCATGCCGCGTGAATGATGAAGGCCTTAGGGTTGTAAAGTTCTTTTACCAGGGAAGATGATGACGGTACCTGGAGAATAAGCACCGGCTAACTTCGTGCCAGCAGCCGCGGTAATACGAAGGGTGCTAGCGTTGTTCGGAATTACTGGGCGTAAAGCGCGCGTAGGCGGCCAATCAAGTCAGAGGTGAAAGCCTGAAGCTTAACTTCAGAATTGCCTTTGAAACTGTTTGGCTGGAATCTCGGAGGGGAGTGTGGAATTCCTAAAGTAGCGGTGAAATGCGTAGATATTAGGAGGAACACCGGTGGCGAAGGCGACACTCTGGACGAGCATTGACGCTGAGGCGCGAAAGCGTGGGGATCAAACAGG` |
| ASV1967 | 404 | `TGAGGAATCTTCCACAATGGACGAAAGTCTGATGGAGCGACACCGCGTGAAGGATGAAGGTTTTGTTATTGTAAACTTCTTTTCTCAGGGACGATAATGACGGTACCTGAGGAATAAGGGACGGCAAACTATGTGCCAGCAGCCGCGGTAATACATAGGTCCCAAGCGTTATCCGGAATTACTGGGCGTAAAGCGTCTGCAGGTGGAAAAGTGTGTGAGATGTGAAAGACCGGGGCTCAACCCCGTGTTTGTGTCTCAAACTGCTTTTCTCGAGCGAGCAAGAGGTATGCGGAATTTATGGAGTAGGAGTGCAATCCGTTGACACCATAAAGAACATCAAAAGCGAAGGCAGCATACTGGGGCTCCGCTGACACTCAGAGACGAAAGCGTGGGGAGCGAAAGGG` |
| ASV630 | 423 | `TAGGGAATATTGGGCAATGGGCGGAAGCCTGACCCAGCCATGCCGCGTGCAGGAAGAAGGCGTTCTGCGTTGTAAACTGCTTTTTTACAGGAAGAAAAAGTCCATGCGTGGAAAATTGACGGTACTGTAAGAATAAGCACCGGCTAACTCCGTGCCAGCAGCCGCGGTAATACGGAGGGTGCAAGCGTTGTCCGGATTTATTGGGTTTAAAGGGTGCGTAGGTGGTTTATTAAGTCAGTGGTGAAAGACGGTCGCTCAACGATTGCAGTGCCATTGAAACTGATAGACTTGAGTTTCGTTAAGGTAGGTGGAATTGATAGTGTAGCGGTGAAATGCATAGATATTATCAAGAACTCCAATTGCGTAGGCAACTTACTAGTCGAAAACTGACACTGATGCACGAAAGTGTGGGTATCAAACAGG` |
| ASV1500 | 409 | `TGGGGAATATTGCACAATGGGCGAAAGCCTGATGCAGCAACGCCGCGTGAGGGATGAAGGCCTTCGGGTTGTAAACCTCTTTTAGCAGGGAAGAAGCGAAAGTGACGGTACCTGCAGAAAAAGCACCGGCTAACTACGTGCCAGCAGCCGCGGTAATACGTAGGGTGCAAGCGTTGTCCGGAATTATTGGGCGTAAAGAGCTCGTAGGCGGTTTGTCGCGTCTGCTGTGAAAATCCGAGGCTCAACCTCGGACCTGCAGTGGGTACGGGCAAACTAGAGTGCGGTAGGGGAGATGGGAATTCCTGGTGTAGCGGTGGAATGCGCAGATATCAGGAGGAACACCGATGGCGAAGGCACATCTCTGGGCCGTAACTGACGCTGAGGAGCGAAAGCGTGGGGAGCGAACAGG` |
| ASV2392 | 404 | `TGGGGAATCTTGGACAATGGGCGCAAGCCTGATCCAGCCATGCCGCGTGAGTGAAGAAGGCCTTCGGGTTGTAAAGCTCTTTTAGCGGGGAAGATAATGACGGTACCCGCAGAAAAAGCCCCGGCTAACTCCGTGCCAGCAGCCGCGGTAATACGGAGGGGGCTAGCGTTGTTCGGAATCACTGGGCGTAAAGCGCGCGTAGGCGGCTTGATAAGTTAGAAGTGAAAGCCCTGGGCTTAACCTAGGAATTGCTTTTAAAACTGTCGAGCTAGAGAATGGTAGAGGAGAGTGGAATTCCTAGTGTAGAGGTGAAATTCGTAGATATTAGGAGGAACACCAGAGGCGAAGGCGGCTCTCTGGGCCATTTCTGACGCTAAGGCGCGAAAGCGTGGGGAGCAAACAGG` |
| ASV5623 | 404 | `TGGGGAATCTTAGACAATGGGGGCAACCCTGATCTAGCCATGCCGCGTGAGTGAAGAAGGCCTTCGGGTTGTAAAACTCTTTCACTTGTGAGGATGATGACAGTAGCAAGAGAAGAAGCACCGGCTAACTCCGTGCCAGCAGCCGCGGTAAGACGGAGGGTGCTAGCGTTGTTCGGAATGACTGGGCGTAAAGGGTGCGTAGGCGGTTTTATAAGTCAGATGTGAAATCCCCGGGCTTAACCCTGGAATGGCATATGATACTGTGAGACTAGAGACTGGAAGAGGAAAGCGGAATTCCTAGTGTAGAGGTGAAATTCGTAGATATTAGGAGGAACACCAGAGGCGAAGGCGGCTTTCTGGTCCAGATCTGACGCTGAGGCACGAAAGCGTGGGGAGCAAACAGG` |
| ASV4399 | 428 | `TCGCGAATTTTTCACAATGGACGCAAGTCTGATGAAGCCACGCCGTGTGCGGGATGAAGGCTCTTTGAGTTGTAAACCGCTGTCAAGTGGAGGGAAACGTACGGTGTTAACAGCGCCGTGCTTGACTGTCCGCTAAAGGAAGCCCCGGCAAAACATGTGCCAGCAGCCGCGGTAATACATGTGGGGCAAGCGTTGTTCGGAATTACTGGGCATAAAGGGTGCGTAGGCGGCATGTTAAGTCAGGGGTGAAATCCCACGGCCCAACCGTGGAACTGCCTTTGATACTGGCGTGCTTGAGTGCGTGAGGGGTAACCGGAACGAGTAGTGTAGCGGTGAAATGCGTAGATATTACTCGGAAGGCCAAAGGCGAAGGCAGGTTACTGGTACGCAACTGACGCTGAGGCACGAAAGCGTGGGGAGCAAACAGG` |
| ASV5379 | 429 | `TGGGGAATATTGGACAATGGGGGAAACCCTGATCCAGCGACGCCGCGTGGGTGAAGAAGGCCTGCGGGTTGTAAAGCCCTTTCGGTGAGGAAGAAAAGTTGCGACCTAACACGTCGCGACCTTGACGTAACTCATTAAAGAAGCACCGGCTAACTCTGTGCCAGCAGCCGCGGTAATACAGAGGGTGCGAGCGTTAATCGGAATTACTGGGCGTAAAGCGCGCGTAGGCGGCTTTGCAAGTCGGGTGTGAAATCCCCAGGCCTAACCTGGGAACTGCACTCGAGACTGCATGGCTAGAGTATCGGAGAGGGAAGTGGAATTTCAGGTGTAGCGGTGAAATGCGTAGATATCTGAAGGAACATCAGTGGCGAAAGCGACTTCCTGGACGAATACTGACGCTCATGTGCGAAAGCGTGGGGAGCAAACAGG` |
| ASV2268 | 404 | `TCGGGAATTTTGGGCAATGGGCGAAAGCCTGACCCAGCAACGCCGCGTGAAGGATGAAGTATTTCGGTATGTAAACTTCGAAAGAATAGGAAGAATCAATGACGGTACTATTTATAAGGTCCGGCTAACTACGTGCCAGCAGCCGCGGTAATACGTAGGGACCAAGCGTTGTTCGGATTTACTGGGCGTAAAGGGCGCGTAGGCGGCGCGGTAAGTCACTTGTGAAATCTCTGAGCTTAACTCAGAACGGCCAAGTGATACTGCAGTGCTAGAGTGCGGAAGGGGCAATCGGAATTCTTGGTGTAGCGGTGAAATGCGTAGATATCAAGAGGAACACCTGAGGTGAAGACGGGTTGCTGGGCCGACACTGACGCTGAGGCGCGAAAGCCAGGGGAGCAAACGGG` |
| ASV4226 | 404 | `TGGGGAATCTTGCGCAATGGGCGAAAGCCTGACGCAGCCATGCCGCGTGAATGATGAAGGTCTTAGGATTGTAAAATTCTTTCAGTAGGGACGATAATGACGGTACCTACAGAAGAAGCCCCGGCTAACTTCGTGCCAGCAGCCGCGGTAATACGAAGGGGGCTAGCGTTGCTCGGAATTACTGGGCGTAAAGGGAGCGTAGGCGGACATTTAAGTCAGGGGTGAAATCCCGGGGCTCAACCTCGGAATTGCCTTTGATACTGGGTGTCTTGAGTATGAGAGAGGTATGTGGAACTCCGAGTGTAGAGGTGAAATTCGTAGATATTCGGAAGAACACCAGTGGCGAAGGCGACATACTGGCTCATTACTGACGCTGAGGCTCGAAAGCGTGGGGAGCAAACAGG` |
| ASV5476 | 417 | `TTAGGAATCTTCCACAATGGGCGAAAGCCTGATGGAGCGACGCCGCGTGAGGGATGAAGGTTTTAGGATTGTAAACCTCTGAATTAGGGACGAAAGACGCTTTATGCGGGATGACGGTACCTAAGTAATAGCACCGGCTAACTCCGTGCCAGCAGCCGCGGTAATACGGAGGGTGCAAGCGTTACCCGGAATCACTGGGCGTAAAGGGCGTGTAGGCGGAACGTTAAGTCTGGTTTTAAAGACCGTCGCTCAACGACGGGCATGGACTGGATACTGACGATCTTGACCTCTGGAGAGGTAACTGGAATTTCTGGTGTAGCGGTGGAATGCGTAGATACCAGAAGGAACACCAATGGCGAAGGCAAGTTACTGGACAGAAGGTGACGCTGAGGCGCGAAAGTGTGGGGAGCGAACCGG` |
| ASV1103 | 404 | `TGGGGAATCTTGGACAATGGGCGCAAGCCTGATCCAGCCATGCCGCGTGAGTGAAGAAGGCCTTCGGGTTGTAAAGCTCTTTTACCAGGGACGATAATGACGGTACCTGGAGAATAAGCCCCGGCAAACTTCGTGCCAGCAGCCGCGGTAATACGAAGGGGGCTAGCGTTGTTCGGAATTACTGGGCGTAAAGCGCACGTAGGCTGATTGTTAAGTCAGGGGTGAAATCCCAGAGCTCAACTCTGGAACTGCCCTTGATACTGGCAATCTCGAGTCCGGAAGAGGTTAGTGGAATTCCTAGTGTAGAGGTGAAATTCGTAGATATTAGGAAGAACACCAGTGGCGAAGGCGGCTAACTGGTCCGGTACTGACGCTGAGGTGCGAAAGCGTGGGGAGCAAACAGG` |
| ASV623 | 429 | `TGGGGAATTTTGGACAATGGGCGAAAGCCTGATCCAGCAATGCCGCGTGCAGGATGAAGGCCTTAGGGTTGTAAACTGCTTTTGTACGGAACGAAACGGCCCCTTCTAATACAAGGGGCTAATGACGGTACCGTAAGAATAAGCACCGGCTAACTACGTGCCAGCAGCCGCGGTAATACGTAGGGTGCGAGCGTTAATCGGAATTACTGGGCGTAAAGCGTGCGCAGGCGGTTATATAAGACAGATGTGAAATCCCCGGGCTCAACCTGGGACCTGCATTTGTGACTGTATAGCTAGAGTACGGTAGAGGGGGATGGAATTCCGCGTGTAGCAGTGAAATGCGTAGATATGCGGAGGAACACCGATGGCGAAGGCAATCCCCTGGACCTGTACTGACGCTCATGCACGAAAGCGTGGGGAGCAAACAGG` |
| ASV543 | 387 | `GCGAGAAAACTTTGCAATGTGCGAAAGCACGACAAGGTTAATCCGAGTGGTTTCTGCTAAAGAAACCTTTTGTCAGTCCTAGAAACACTAACGAATAAGGGGTGGGCAAGTTCTGGTGTCAGCCGCCGCGGTAAAACCAGCACCTCAAGTGGTCAGGATGATTATTGGGCCTAAAGCATCCGTAGCTCGTTTTGTAAGTTTTCGGTTAAATCCATGCGCTTAACGTATGGGCTGCCGGGAATACTGCATAACTAGGAAGTGGGAGAGGTAGACGGTACTCGGTAGGAAGGGGTAAAATCCTTTGATCTATTGATGACCACCTGTGGCGAAGGCGGTCTACCAGAACACGTTCGACGGTGAGGGATGAAAGCTGGGGGAGCAAACCGG` |
| ASV3621 | 404 | `TAGGGAATATTGCACAATGGGGGAAACCCTGATGCAGCGACGCCGCGTGAGCGAAGAAGTATCTCGGTATGTAAAGCTCTATCAGCAGGGAAGAAGATGACGGTACCTGACTAAGAAGCACCGGCTAAATACGTGCCAGCAGCCGCGGTAATACGTATGGTGCAAGCGTTATCCGGATTTACTGGGTGTAAAGGGAGCGTAGACGGAGAAGCAAGTCTGGAGTGAAAGCCCGGGGCTCAACCCCGGGACTGCTTTGGAAACTGTTATTCTTGAGTGCCGGAGAGGTAAGCGGAATTCCTAGTGTAGCGGTGAAATGCGTAGATATTAGGAGGAACACCAGTGGCGAAGGCGGCTTACTGGACGGTAACTGACGTTGAGGCTCGAAAGCGTGGGGAGCAAACAGG` |
| ASV1320 | 429 | `TGGGGAATATTGGACAATGGGGGCAACCCTGATCCAGCAATGCCGCGTGTGTGAAGAAGGCCTGCGGGTTGTAAAGCACTTTTGGCAGGGATGAAAAGCACTGGATTAATAACCCAGTGTGTTGACCTAACCTGCAGAAGAAGCACCGGCTAACTCTGTGCCAGCAGCCGCGGTAATACAGAGGGTGCAAGCGTTAATCGGAATTACTGGGCGTAAAGCGCGCGTAGGCCGTCGAGTAAGTCGGATGTGAAATCCCCGGGCTTAACCTGGGAATTGCATTCGATACTGCTTGGCTAGAGTGTGGAAGAGGGAAGCGGAATTTCCGGTGTAGCGGTGAAATGCGTAGATATCGGAAGGAACACCAGTGGCGAAGGCGGCTTCCTGGTCCAACACTGACGCTGAGGCGCGAAAGCGTGGGGAGCGAACAGG` |
| ASV5607 | 431 | `TGGGGAATATTGCGCAATGGGCGAAAGCCTGACGCAGCGACGCCGCGTGGAGGATGAAGGTCTTAGGATTGTAAACTCCTGTTAGATAGGAAGAAAAGTCTTGTTTATAATACAGACAAGAAAATGACTGTACTGTCAGAGAAAGCACCGGCTAACTTCGTGCCAGCAGCCGCGGTAATACGAGGGGTGCTAGCGTTATTCGGAATTACTGGGCGTAAAGGGTGCGTAGACGGCGTGTTAAGTCCGTTGTTAAATTCTTCGGCCTAACCGAAGATCAGCGATGGAAACTGGCATGCTAGAGGATGAGAGAGAGAAGTGGAATTCCCGGAGTAGCGGTAATATGCGTAGATCTCGGGAGGAACACCAATGGCGAAGGCAGCTTCTTGGCTCATTTCTGACGTTGAGGCACGAAAGTGTGGGGAGCAAACAGG` |
| ASV3038 | 404 | `TAGGGAATATTGCACAATGGACGAAAGTCTGATGCAGCGACGCCGCGTGAGTGATGAAGGCCTTCGGGTCGTAAAGCTCTGTGATAAGGGAAGATAATGACGGTACCTTATCAGAAAAGACCGGCTAACTTCGTGCCAGCAGCCGCGGTAATACGAGGGGTCTAAGCGTTGTTCGGAATCACTGGGCGTAAAGCGGGTGTAGGTGGCTATGCAAGTTGGATGTGAAATCCCCGGGCTCAACCCGGGAAGTGCATCCAAAACTACGTAGCTTGAGTGCGGGAGAGAAAAGCAGAATTCCTGGTGTAGTGGTGAAATACGTAGATATCAGGAGGAACTCCGGTGGCGAAGGCGGCTTTTTGGCCCAGCACTGACACTCAGACCCGAAAGCGTGGGTAGCAAACAGG` |
| ASV5671 | 418 | `TGGGGAATTTTGCGCAATGGGGGAAACCCTGACGCAGCAACGCCGCGTGGACGATGAAGCCCCTTGGGGTGTAAAGTCCTTTCGGCAGGGACGAAGTCCGCGCAAGCGGATTGACGGTACCTGAAGAAGAAGCCCCGGCTAACTCCGTGCCAGCAGCCGCGGTAATACGGGGGGGGCAAGCGTTGTTCGGAATTACTGGGCGTAAAGGGAGCGTAGGCGGTGCGGCAAGTCCGGTGTGAAATCTCCGAGCTTAACTCGGAGCGGTCATTGGAAACTGCCGTGCTAGAGTGTGGGAGAGGAGAGTGGAATTCCCGGTGTAGCGGTGAAATGCGTAGATATCGGGAGGAACACCAGTGGCGAAGGCGGCTCTCTGGACCACAACTGACGCTGAGGCTCGAAAGCTAGGGGAGCAAACAGG` |
| ASV1316 | 404 | `TGGGGAATTTTCCGCAATGGGCGAAAGCCTGACGGAGCAATACCGCGTGAGGGAGGAAGGCTCTTGGGTCGTAAACCTCTTTTCTCTGGGAAGATAATGACGGTACCAGAGGAATAAGCATCGGCTAACTCCGTGCCAGCAGCCGCGGTAATACGGAGGATGCAAGCGTTATCCGGAATGATTGGGCGTAAAGAGTCCGTAGGTGGTTGTGAAAGTCTGTGGTTAAAGCGCGAAGCTTAACTTCGTAAAGGCCATGGAAACTATGCGACTAGAGTGAGATAGGGGTAGAGGGAATTCTTGGTGTAGCGGTGAAATGCGTAGATATCAAGAAGAACACCGGTGGCGAAAGCGCTCTACTGGATCTCAACTGACACTGAGGGACGAAAGCTAGGGGAGCGAAAGGG` |
| ASV5259 | 404 | `TGGGGAATATTGGACAATGGGCGCAAGCCTGATCCAGCAATGCCGCGTGTGTGAAGAAGGTCTTCGGATTGTAAAGCACTTTCGACAGGGACGATGATGACGGTACCTGTAGAAGAAGCCCCGGCTAACTTCGTGCCAGCAGCCGCGGTAATACGAAGGGGGCTAGCGTTGCTCGGAATTACTGGGCGTAAAGGGCGCGTAGGCGGCGAGATCAGTCAGGCGTGAAATTCCTGGGCTTAACCTGGGGGCTGCGCTTGATACGGTCATGCTAGAGGGCAGAAGAGGGTCGTGGAATTCCCAGTGTAGAGGTGAAATTCGTAGATATTGGGAAGAACACCGGTGGCGAAGGCGGCGACCTGGTCTGTTACTGACGCTGAGGCGCGAAAGCGTGGGGAGCAAACAGG` |
| ASV1707 | 404 | `TGGGGAATATTGGACAATGGGCGCAAGCCTGATCCAGCCATGCCGCGTGGATGATGAAGGCCCTAGGGTTGTAAAGTCCTTTCGGCGGGGAAGATAATGACGGTACCCGCAGAAGAAGCCCCGGCTAACTTCGTGCCAGCAGCCGCGGTAATACGAAGGGGGCTAGCGTTGCTCGGAATCACTGGGCGTAAAGCGCACGTAGGCGGACTCTTAAGTCGGTGGTGAAATCCTGGAGCTCAACTCCAGAACTGCCTTCGATACTGAGAGTCTCGAGTCCGGGAGAGGTGAGTGGAACTGCGAGTGTAGAGGTGAAATTCGTAGATATTCGCAAGAACACCAGTGGCGAAGGCGGCTCACTGGCCCGGTACTGACGCTGAGGTGCGAAAGCGTGGGGAGCAAACAGG` |
| ASV4781 | 388 | `TCGGGTATTTTCTGCAATGTACGCAAGTATGACAGGGCGAGCCAGAGTGATTTTCTATATTTGAAAATCTTTTGTGACATGTAAAAAGTGTTACGAATAAGGACCGGGCAAGACTGGTGCCAGTCGCCGCGGTAATCCCAGCGGTCCAAGTCGCAACCACAATTATTGGGTCTAAAACATCCGTAGCTTGCTTGATAAGTCTCTTGTGAAATCCGGCCTCTTAAGGGTCGAAGAGCGAGAGATACTGTCGGGCTAGAGACCGGGAGAGGTAAGAAGTACGGTCGGGGTACCGGTAAAATGGGTTGATCCCGTCTGGACTAACAACAGCGAAGGCATCTTACCAGCACGGATCTGACAGTAAGGGATGAAGGCTAGGGGCGCAAAAGGG` |
| ASV883 | 430 | `TGGGGAATATTGGACAATGGGCGGAAGCCTGATCCAGCAATACCGCGTGTGTGAAGAAGGCCTTCGGGTTGTAAAGCACTTTTGTTAGGGAGGATGATTGTTTAAGCTAATATCTTAAGCAGTTGACGTTACCTAAAGAATAAGCACCGGCTAACTCCGTGCCAGCAGCCGCGGTAATACGGAGGGTGCAAGCGTTAATCGGATTTACTGGGCGTAAAGGGTGCGTAGGCGGCCTATTAAGTCAGATGTGAAAGCCCTGGGCTCAACCTGGGAATTGCATTTGAAACTGGTAGGCTAGAATGTGGTAGAGGGTAGCGGAATTTCTGGTGTAGCGGTGAAATGCGTAGATATCAGAAGGAACATCAATGGCGAAGGCAGCTACCTGGGCCAACATTGACGCTGAGGCACGAAAGCGTGGGGAGCAAACAGG` |
| ASV2768 | 424 | `TAAGGAATATTGGTCAATGGACGCAAGTCTGAACCAGCCATGCCGCGTGGAGGATGAAGGTCCTCTGGATTGTAAACTTCTTTTATATGGGACGAAAAAAGGCTTTTCTAAGTCGTCTGACGGTACCATATGAATAAGCACCGGCTAACTCCGTGCCAGCAGCCGCGGTAATACGGAGGGTGCAAGCGTTATCCGGATTCACTGGGTTTAAAGGGTGCGTAGGTGGGTTGGTAAGTCAGTGGTGAAATCCCCGAGCTTAACTTGGGAACTGCCATTGATACTATCAGTCTTGAATACCGTGGAGGTCAGCGGAATATGTCATGTAGCGGTGAAATGCTTAGATATGACATAGAACACCAATTGCGAAGGCAGCTGGCTACTCGGATATTGACACTGAGGCACGAAAGCGTGGGGATCAAACAGG` |
| ASV1828 | 429 | `TGGGGAATTTTGGACAATGGGGGCAACCCTGATCCAGCCATTCCGCGTGAGTGAAGAAGGCCTTCGGGTTGTAAAGCTCTTTCGCAAGGGAAGAAACGTTACGAGCGAATAACTCGTGATAATGACGGTACCTTGATAAGAAGCACCGGCTAACTACGTGCCAGCAGCCGCGGTAATACGTAGGGTGCAAGCGTTAATCGGAATTACTGGGCGTAAAGCGTGCGCAGGCGGTTTTGTAAGTCAGATGTGAAATCCCCGAGCTCAACTTGGGAACTGCGTTTGAAACTACAAGGCTAGAATATGTCAGAGGGGGGTAGAATTCCACGTGTAGCAGTGAAATGCGTAGAGATGTGGAGGAATACCAATGGCGAAGGCAGCCCCCTGGGATAATATTGACGCTCATGCACGAAAGCGTGGGGAGCAAACAGG` |
| ASV1843 | 429 | `TGGGGAATTTTGGACAATGGGCGCAAGCCTGATCCAGCCATGCCGCGTGAGTGAAGAAGGCCTTCGGGTTGTAAAGCTCTTTCGGTTGGGAAGAAATTGCCTGGGCTAATACCCTAGGTAGATGACGGTACCAGCATAAGAAGCACCGGCTAACTACGTGCCAGCAGCCGCGGTAATACGTAGGGTGCGAGCGTTAATCGGAATTACTGGGCGTAAAGCGTGCGCAGGCGGTTGTGTAAGTCAGATGTGAAATCCCCGGGCTCAACCTGGGAACTGCATTTGAGACTGCACGGCTAGAGTGTGGCAGAGGGGGGTAGAATTCCACGTGTAGCAGTGAAATGCGTAGAGATGTGGAGGAATACCGATGGCGAAGGCAGCCCCCTGGGCCAATACTGACGCTCATGCACGAAAGCGTGGGGAGCAAACAGG` |
| ASV2831 | 404 | `TGGGGAATATTGGACAATGGGCGAAAGCCTGATCCAGCCATGCCGCGTGAATGATGAAGGCCTTAGGGTTGTAAAGTTCTTTTGTCAGGGAAGATGATGACGGTACCTGAAGAATAAGCACCGGCTAACTTCGTGCCAGCAGCCGCGGTAATACGAAGGGTGCTAGCGTTGTTCGGAATCACTGGGCGTAAAGCGCGCGTAGGCGGCTTGACAAGTCAGAGGTGAAATCCCAGAGCTTAACTCTGGAACTGCCTTTGAAACTGTCAAGCTGGAGTGTCGGAGGGGATGGCGGAATTGCTAATGTAGAGGTGAAATTCGTAGATATTAGCAGGAACACCGGTGGCGAAGGCGGCCATCTGGACGACAACTGACGCTGAGGCGCGAAAGCGTGGGGATCAAACAGG` |
| ASV3801 | 423 | `TAGGGAATATTGGACAATGGGTGAGAGCCTGATCCAGCCATGCCGCGTGCAGGAAGACGGCCTTCTGGGTTGTAAACTGCTTTTATCTGGGGATAAAATTCCCTTGCGAGGGAGACTGAAGGTACCAGATGAATAAGCCACGGCTAACTACGTGCCAGCAGCCGCGGTAATACGTAGGTGGCAAGCGTTGTCCGGATTTATTGGGTTTAAAGGGTGCGTAGGCGGTCTATTAAGTCAGCGGTGAAAGACGACAGCTCAACTGTCGAGGTGCCATTGATACTGATGGACTTGAGTGCACACGAGGTAAGCGGAATGGACGGTGTAGCGGTGAAATGCATAGATATCGTCCAGAACACCGATAGTGAAGGCAGCTTACTAAGATGTAACTGACGCTGAGGCACGAAAGTGCGGGGATCAAACAGG` |
| ASV880 | 429 | `TGGGGAATTTTGGACAATGGGCGCAAGCCTGATCCAGCAATGCCGCGTGAGTGAAGAAGGCCTTCGGGTTGTAAAGCTCTTTTGTCAGGGAAGAAACGGGTCTTTCTAATACAGAGGCCTAATGACGGTACCTGAAGAATAAGCACCGGCTAACTACGTGCCAGCAGCCGCGGTAATACGTAGGGTGCAAGCGTTAATCGGAATTACTGGGCGTAAAGCGTGCGCAGGCGGTTTTGTAAGTCTGTTGTGAAATCCCCGGGCTCAACCTGGGAATGGCAATGGAGACTGCAAGGCTAGAGTTTGGCAGAGGGGGGTAGAATTCCACGTGTAGCAGTGAAATGCGTAGATATGTGGAGGAACACCGATGGCGAAGGCAGCCCCCTGGGTCAAAACTGACGCTCATGCACGAAAGCGTGGGGAGCAAACAGG` |
| ASV4556 | 429 | `TGGGGAATATTGGACAATGGGCGCAAGCCTGATCCAGCCATGCCGCGTGTGTGAAGAAGGCCTTCGGGTTGTAAAGCACTTTTGTCCGGAAAGAAATCCTTTTGATTAATACTCGGAAGGGATGACGGTACCGGAAGAATAAGCACCGGCTAACTTCGTGCCAGCAGCCGCGGTAATACGAAGGGTGCAAGCGTTACTCGGAATTACTGGGCGTAAAGCGTGCGTAGGTGGTTTGTTAAGTCTGATGTGAAAGCCCTGGGCTCAACCTGGGAACTGCATTGGATACTGGCTTACTAGAGTGCGGTAGAGGGTAGTGGAATTCCCGGTGTAGCAGTGAAATGCGTAGATATCGGGAGGAACATCTGTGGCGAAGGCGACTACCTGGACCAGCACTGACACTGAGGCACGAAAGCGTGGGGAGCAAACAGG` |
| ASV5073 | 424 | `TGGGGAATATTGGACAATGGGCGCAAGCCTGATCCAGCAATTCCGCGTGTGTGAAGAAGGCCTTCGGGTTGTAAAGCACTTTAAGCAGGGAAGAAAAAGCTTGGCCACACCAAGCCTTGACGGTACCTGAAGAATAAGCACCGGCTAACTCTGTGCCAGCAGCCGCGGTAATACAGAGGGTGCGAGCGTTAATCGGATTTACTGGGCGTAAAGGGTGCGTAGGTGGTTAGATAAGTCGGATGTGAAATCCTCGGGCTCAACCTGAGAACTGCATTCGATACTGTTTGACTAGAGATCGGTAGAGGGTGGCGGAATTCCTGGTGTAGCAGTGAAATGCGTAGATATCAGGAGGAACATCAATGGCGAAGGCAGCCACCTGGGCCTGATCTGACACTGAGGCACGAAAGCGTGGGTAGCAAACAGG` |
| ASV1983 | 429 | `TGGGGAATATTGGACAATGGGGGAAACCCTGATCCAGCAATGCCGCGTGTGTGAAGAAGGCCTGAGGGTTGTAAAGCACTTTCAGTGGGGAGGAGGATTGACAGGTTAAGAGCTAGTTAATTGGACGTTACCCACAGAAGAAGCACCGGCTAACTCCGTGCCAGCAGCCGCGGTAATACGGAGGGTGCGAGCGTTAATCGGAATTACTGGGCGTAAAGGGTGCGTAGGTGGTTGGATAAGTTATCTGTGAAATCCCCGGGCTCAACCTGGGAAGGTCAGATAAGACTGTTCGACTCGAGTATAGGAGAGGGTAGTGGAATTTCCGGTGTAGCGGTGAAATGCGTAGAGATCGGAAGGAACACCAGTGGCGAAGGCGGCTACCTGGCCTAATACTGACACTGAGGCACGAAAGCGTGGGGAGCAAACAGG` |
| ASV240 | 404 | `TGGGGAATATTGGACAATGGGCGCAAGCCTGATCCAGCCATGCCGCGTGAGTGATGAAGGCCTTAGGGTTGTAAAGCTCTTTTATCCGGGAAGATAATGACTGTACCGGAAGAATAAGCCCCGGCTAACTTCGTGCCAGCAGCCGCGGTAATACGAAGGGGGCTAGCGTTGCTCGGAATCACTGGGCGTAAAGGGCGCGTAGGCGGATGATTAAGTCGGAGGTGAAAGCCCAGGGCTCAACCCTGGAATTGCCTTCGATACTGATCGTCTCGAGAATGGAAGAGGTTAGTGGAACTCCGAGTGTAGAGGTGAAATTCGTAGATATTCGGAAGAACACCAGTGGCGAAGGCGACTGACTGGTCCATTTCTGACGCTGAGGCGCGAAAGCGTGGGGATCAAACAGG` |
| ASV4396 | 430 | `TAGGGAATTTTGCACAATGGGCGCAAGCCTGATGCAGCAACGCCGCGTGAGTGAAGAAGGCTCTTGGGTTGTAAAGCTCTTTCGGCTGGGAAGAAGGGGTTGCCCTCGAACAGGTGGCAATTCTGACGGTACCAGAAGAAGAAGCACCTGCTAACTACGTGCCAGCAGCAGCGGTAATACGTAGGGTGCAAGCGTTGTTCGGAATTACTGGGCGTAAAGCGCGCGTAGGCGGGCTTGTATGTCGATTGTGAAAGCCCTGGGCTTAACCCGGGAACTGCAGTCGAAACTGCAAGTCTTGAGTGCCTGAGAGGATGGCGGAATTCCCGGTGTAGAAGTGAAATTCGTAGATATCGGGAAGAACACCTGAGGCGAAGGCGGCTATCTGGCGGTGCACTGACGCTAAGGCGCGAAAGCGTGGGGAGCAAACAGG` |
| ASV5677 | 404 | `CGACGAATCTTCTGCAATGGGCGAAAGCCTGACAGAGCAATGCCGCGTGTGGGATGAAGCGTCTATGACGTGTAAACCACTGTCAGGGGGCGGAACCAATGATTGCCCCCAGAGGAAGAGCCGGCTAACCCTGTGCCAGCAGCCGCGGTAATACAGGGGGCTCGAGCGTTACGCGGAATCACTGGGCTTAAAGGGTGCGTAGGTGGCTTGCAAAGCATTACGTGAAAGCCCCGGGCTCAACCCGGGAATTGCGCGATGTACTGGCAAGCTTGAGGTAAGTAGGGGTTGACGGAACAGTGGGTGGAGCGGTGAAATGCGTAGAGATCCACTGGAACGCCGATGGTGAAGACGGTCAACTGGGCTTATCCTGACACTGAGGCACGAAAGCGTGGGGAGCAAACAGG` |
| ASV1129 | 429 | `TGGGGAATATTGCACAATGGGCGCAAGCCTGATGCAGCCATGCCGCGTGTGTGAAGAAGGCCTTAGGGTTGTAAAGCACTTTCAGTGAGGAGGAAGGGTATAACTTTAATAGAGTTGTGCTTTGACGTTACTCACAGAAGAAGCACCGGCTAACTCCGTGCCAGCAGCCGCGGTAATACGGAGGGTGCGAGCGTTAATCGGAATTACTGGGCGTAAAGGGCACGCAGGCGGTTTGTTAAGTCAGATGTGAAATCCCCGCGCTTAACGTGGGAACTGCATTTGAAACTGGCAAGCTAGAGTCTTGTAGAGGGGGGTAGAATTCCATGTGTAGCGGTGAAATGCGTAGAGATGTGGAGGAATACCGGTGGCGAAGGCGGCCCCCTGGACAAAGACTGACGCTCATGTGCGAAAGCGTGGGGAGCAAACAGG` |
| ASV4693 | 429 | `TGGGGAATATTGGACAATGGGGGCAACCCTGATCCAGCCATGCCGCGTGTGTGAAGAAGGCCCTTGGGTTGTAAAGCACTTTCAGTGGGGAAAAAGGTCTGGAACCTAATACGTTTTGGAATTGATGTTACCCACAGAAGAAGCACCGGCTAACTCCGTGCCAGCAGCCGCGGTAATACGGAGGGTGCAAGCGTTAATCGGAATTACTGGGCGTAAAGCGCACGTAGGTGGTTTACTAAGCCAGCTGTGAAATCCCCGGGCTCAACCTGGGCACTGCAGTTGGAACTGGTAAGCTAGAGTATGGTAGAGGGGTGTGGAATTCCAGGTGTAGCGGTGAAATGCGTAGATATCTGGAGGAACATCAGTGGCGAAGGCGACACCCTGGACTAATACTGACACTGAGGTGCGAAAGCGTGGGGAGCAAACAGG` |
| ASV5033 | 404 | `TGGGGAATATTGCACAATGGAGGAAACTCTGATGCAGCAACGCCGCGTGAGTGACGAAGGCCTTCGGGTTGTAAAACTCTGTCTTAGCTGACGATAATGACGGTAAGCTAGGAGGAAGCCACGGCTAACTACGTGCCAGCAGCCGCGGTAATACGTAGGTGGCGAGCGTTGTCCGGAATTACTGGGCGTAAAGGGAGCGTAGGCGGTTTTTTAAGTGGGATGTGAAATCCCCGGGCTTAACCTGGGAATGGCATTCCAAACTGGAAGACTAGAGTGCAGGAGAGGAAAGTGGAATTCCAAGTGTAGCGGTGAAATGCGTAGAGATTTGGAGGAACACCAGTGGCGAAGGCGACTTTCTGGACTGATACTGACGCTGAGGCTCGAGAGCGTGGGGAGCAAACAGG` |
| ASV3728 | 424 | `TAAGGAATATTGGACAATGGTCGCAAGACTGATCCAGCCATGCCGCGTGCAGGATGACTGCCCTATGGGTTGTAAACTGCTTTTGTACGAGAAAAAACCTCTTTACGTGTAAAGAGCTGATTGTATCGTAAGAATAAGGATCGGCTAACTCCGTGCCAGCAGCCGCGGTAATACGGAGGATCCAAGCGTTATCCGGATTTATTGGGTTTAAAGGGTGCGTAGGCGGAATGATAAGTCAGTGGTGAAAGCCTGCAGCTTAACTGTAGAATTGCCGTTGATACTGTCGTTCTTGAGTATATTTGATGTGGGCGGAATGTGTCATGTAGCGGTGAAATGCTTAGATATGACACAGAACACCGATTGCGAAGGCAGCTCACAAAACTATAACTGACGCTGAGGCACGAAAGCGTGGGGATCAAACAGG` |
| ASV1172 | 429 | `TCGAGAATAATTCACAATGGGGGCAACCCTGATGGTGCAACGCCGCGTGGAGGATGAAGGTCTTCGGATTGTAAACTCCTGTCATCTGGGAGTAAGGCATGGCTGTGAATAGCAGACATGATTGATAGTACCAGAAGAGGAAGGGACGGCTAACTTCGTGCCAGCAGCCGCGGTAATACGAAGGTCCCAAGCGTTGTTCGGAATCACTGGGCGTAAAGGGAGCGTAGGCTGCGTGGTAAGTCAGATGTGAAATCCCGGGGCTCAACCCCGGAACTGCATCCGATACTGCCATGCTAGAGGATTGGAGAGGTAGCTGGAATTCTTGGTGTAGCAGTGAAATGCGTAGAGATCAAGAGGAACACTCGTGGCGAAAGCGAGCTACTGGACAATAACTGACGCTGAGGCTCGAAGGCTAGGGTAGCGAAAGGG` |
| ASV4444 | 416 | `TCGAGAATTTTTCACAATGGGGGAAACCCTGATGGAGCGACGCCGCGTGGAGGATGAAGGTTTTCGGATTGTAAACTCCTGTCATGAGAGAACAAAACCCGCAAGGGCTTGATAGTATCTCAAGAGGAAGGGACGGCTAACTCTGTGCCAGCAGCCGCGGTAATACAGAGGTCCCAAGCGTTGTTCGGATTCACTGGGCGTAAAGGGTGCGTAGGTGGCGGGGTAAGTTGCGTGTTAAATCTCAAGGCTCAACCTTGAAACCGCTCGCAATACTGCTCTGCTCGAGGACTGTAGAGGAGAATGGAATTCACGGTGTAGCAGTGAAATGCGTAGATATCGTGAGGAAGACCAGTGGCGAAGGCGATTCTCTGGGCAGTTCCTGACACTGAGGCACGAAGGCCAGGGGAGCAAACGGG` |
| ASV369 | 424 | `TGAGGAATATTGGACAATGGGCGCAAGCCTGATCCAGCCATGCCGCGTGCAGGAAGAAGCATCTATGGTGTGTAAACTGCTTTTGTACGGGAAGAAACATCACTACGTGTAGTGACTTGACGGTACCGTAAGAATAAGGATCGGCTAACTCCGTGCCAGCAGCCGCGGTAATACGGAGGATCCAAGCGTTATCCGGAATCATTGGGTTTAAAGGGTCCGTAGGCGGTTTAATAAGTCAGTGGTGAAATCTGGTCGCTCAACGATCAAACGGCCATTGATACTGTTAAACTTGAATTATTGTGAAGTAACTAGAATATGTAGTGTAGCGGTGAAATGCTTAGATATTACATGGAATACCAATTGCGAAGGCAGGTTACTAACAATATATTGACGCTGATGGACGAAAGCGTGGGGAGCGAACAGG` |
| ASV1206 | 404 | `TGGGGAATATTGGACAATGGGGGAAACCCTGATCCAGCAATGCCGCGTGAGTGAAGAAGGCCTTCGGGTTGTAAAGCTCTTTTAGATGAGACGATGATGACGGTATCATCAGAAAAAGCACCGGCTAACTCTGTGCCAGCAGCCGCGGTAATACAGAGGGTGCTAGCGTTGTTCGGAATTACTGGGCGTAAAGAGCGCGTAGGCGGCCTGTTGAGTTAAGTGTGAAAGCCCAAGGCTTAACCTTGGAACTGCATTTAACACTGGCAGGCTAGAGGTCGCAAGAGGACAGTGGAATTCCTAGTGTAGAGGTGAAATTCGTAGATATTAGGAAGAACATCAATGGCGAAGGCAGCTGTCTGGGGCGATACTGACGCTAAGGCGCGAAAGCGTGGGGAGCAAACAGG` |
| ASV4820 | 404 | `TGGGGAATATTGGACAATGGGGGAAACCCTGATCCAGCCATGCCGCGTGAGTGATGAAGGCCTTCGGGTTGTAAAGCTCTTTTGGGTGGGACGATAATGACGGTACCACCAGAATAAGATCCGGCAAACTTCGTGCCAGCAGCCGCGGTAATACGAAGGGATCTAGCGTTGTTCGGATTTACTGGGCGTAAAGGGCGCGTAGGCGGTTAGATAAGTAAGGCGTGAAAGCCCTGGGCTTAACCCAGGAACAGCGCTTTAAACTGTCTAGCTTGAGTATGGGAGAGGTAAGTAGAATTTCCAGTGTAGAGGTGAAATTCGTAGATATTGGAAAGAATACCAGTGGCGAAGGCGACTTACTGGACCATAACTGACGCTGAGGCGCGAAAGCGTGGGGAGCAAACAGG` |
| ASV5214 | 430 | `TAGGGAATTTTGCACAATGGGCGCAAGCCTGATGCAGCAACGCCGCGTGAGTGAAGAAGGCTCTTGGGTTGTAAAGCTCTTTCGGCCAAGAAGAAGGGATCACTTGCGAACAGTCTGTGATTCTGACGGTATTGGAAGAAGAAGCACCTGCTAACTTCGTGCCAGCAGCAGCGGTAATACGAAGGGTGCAAGCGTTGTTCGGAATTACTGGGCGTAAAGCGCGCGTAGGCGGGTCTGTAAGTTGATTGTGAAATCCCTGGGCTTAACCTGGGACGTGCAGTCAAAACTGCAGATCTTGAATGTCTGAGAGGATGGCGGAATAACCGGTGTAGTAGTGAAATACGTAGATATCGGTTAGAACACCTGAGGCGAAGGCGGCTATCTGGCGGCACATTGACGCTAAGGCGCGAAAGCGTGGGGAGCAAACAGG` |
| ASV5114 | 424 | `TGAGGAATATTGGTCAATGGGCGCAAGCCTGAACCAGCCATGCCGCGTGCAGGATGACGCATCTATGGTGTGTAAACTGCTTTTGTACAGGAAGAAACATCCCGACGAGTCGGGACTTGACGGTACTGTAAGAATAAGGATCGGCTAACTCCGTGCCAGCAGCCGCGGTAATACGGAGGATCCAAGCGTTATCCGGAATCATTGGGTTTAAAGGGTCCGTAGGCGGCTTAATAAGTCAGCGGTGAAAGTTTTTGGCTTAACCAAAAAATTGCCATTGATACTGTTAGGCTTGAATTTTTGTGAAGTAACTAGAATATGTAGTGTAGCGGTGAAATGCTTAGATATTACATGGAATACCAATTGCGAAGGCAGGTTACTAACAAACGATTGACGCTGATGGACGAAAGCGTGGGTAGCGAACAGG` |
| ASV3216 | 428 | `TTTCGAATCATTCACAATGGGCGAAAGCCTGATGGTGCGACGCCGCGTGAGGGATGAAGGCCTTCGGGTCGTAAACCTCTGTCACCGGGGAAGAAACGCTGCAATTTAACAGATTGCAGCCTGACTTAACCCGGAGAGGAAGCAGTGGCTAACTCTGTGCCAGCAGCCGCGGTAATACAGAGACTGCAAGCGTTATTCGGATTCACTGGGCGTAAAGGGTGCGCAGGCGGCCATGTGTGTCAGGTGTGAAAGCCCGAGGCTCAACCTCGGAATTGCGCCTGAAACTACATGGCTAGAGTACTGGAGAGGGTAGCGGAATTCATGGTGTAGCAGTGAAATGCGTAGATATCATGAGGAACACCAGAGGCGAAGGCGGCTACCTGGACAGTTACTGACGCTCAGGCACGAAAGCGTGGGGAGCAAAAGGG` |
| ASV3066 | 405 | `TGGGGAATATTGGACAATGGGCGCAAGCCTGATCCAGCCATGCCGCGTGAGTGATGAAGGCCTTAGGGTTGTAAAGCTCTTTTAGCGGAGACGATAATGACGGTATCCGCAGAAAAAGCCCCGGCCAACTTCGTGCCAGCAGCCGCGGTAATACGAAGGGGGCTAGCGTTGCTCGGAATCACTGGGCGTAAAGCGCACGTAGGCGGGTCGTTAAGTCGGGGGTGAAATCCCAGAGCTCAACTCTGGAACTGCCTTCGATACTGGCGACCTTCGAGTCCGGGAGAGGTGAGTGGAACTGCGAGTGTAGAGGTGAAATTCGTAGATATTCGCAAGAACACCAGTGGCGAAGGCGGCTCACTGGCCCGGTACTGACGCTGAGGTGCGAAAGCGTGGGGAGCAAACAGG` |
| ASV4829 | 424 | `TGGGGAATATTGGACAATGGGCGAAAGCCTGATCCAGCAATTCCGCGTGTGTGAAGAAGGCCTTCGGGTTGTAAAGCACTTTAAGCAGGGAAGAAAAAGCTTGGCCACACCAAGCCTTGACGGTACCTGAAGAATAAGCACCGGCTAACTCTGTGCCAGCAGCCGCGGTAATACAGAGGGTGCAAGCGTTAATCGGATTTACTGGGCGTAAAGGGTGCGTAGGTGGTTTGATAAGTCGGATGTGAAATCCTCGGGCTCAACCTGAGAACTGCATTCGATACTGTCAGACTCGAGATCGGTAGAGGGCGGCGGAATTTCCGGTGTAGCAGTGAAATGCGTAGATATCGGAAGGAACATCAATGGCGAAGGCAGCCGCCTGGGCCTGATCTGACACTGAGGCACGAAAGCGTGGGTAGCAAACAGG` |
| ASV2222 | 404 | `TGGGGAATATTGGACAATGGGCGCAAGCCTGATCCAGCAATATCACGTGAATGATGAAGGCCTTAGGGTTGTAAAATTCTTTTAGTAGGGAAGATAATGACGGTACCTACAGAAAAAGCACCGGCTAACTTCGTGCCAGCAGCCGCGGTAATACGAAGGGTGCTAGCGTTGTTCGGAATTATTGGGCGTAAAGCGTGCGTAGGCGGCATAGTAAGTCAGCTGTGAAAGCCCAGGGCTCAACCTTGGAATTGCGGTTGAAACTATTAAGCTAGAGTGTGGCGGGGGATAGTGGAATTCTTAGTGTAGGGGTGAAATCCGTAGATATTAAGAGGAACACCAGTGGCGAAAGCGACTATCTACACCATTACTGACGCTGAGGTACGAAAGCGTGGGGAGCAAACAGG` |
| ASV2088 | 429 | `TGGGGAATTTTGGACAATGGGGGCAACCCTGATCCAGCCATGCCGCGTGCGGGAAGAAGGCCTTCGGGTTGTAAACCGCTTTTGTCAGGGAAGAAACGGCTTGGGTTAATACCTCGAGCTAATGACGGTACCTGAAGAATAAGCACCGGCTAACTACGTGCCAGCAGCCGCGGTAATACGTAGGGTGCAAGCGTTAATCGGAATTACTGGGCGTAAAGCGTGCGCAGGCGGCTTTGCAAGACAGATGTGAAATCCCCGGGCTCAACCTGGGAACTGCATTTGTGACTGCAAAGCTAGAGTGCGGCAGAGGGGGATGGAATTCCGCGTGTAGCAGTGAAATGCGTAGATATGCGGAGGAACACCGATGGCGAAGGCAATCCCCTGGGCCTGCACTGACGCTCATGCACGAAAGCGTGGGGAGCAAACAGG` |
| ASV2555 | 428 | `TGGGGAATATTGGACAATGGGCGAAAGCCTGATCCAGCAATACCGCGTGTGTGAAGAAGGCCTTAGGGTTGTAAAGCACTTTCAATTGGGAGGAAAACCTATCGGTTAATACCCGGTAGCTTGACATTACCTTTAGAAGAAGCACCGGCTAACTCCGTGCCAGCAGCCGCGGTAATACGGAGGGTGCAAGCGTTAATCGGAATTACTGGGCGTAAAGCGTGCGTAGGCGGTTCGTTAAGTCAGATGTGAAAGCCCTGGGCTCAACCTGGGAACTGCATTTGAAACTGGTGGACTAGAGTTTAGTAGAGGGGAGTGGAATTTCAGGTGTAGCGGTGAAATGCGTAGATATCTGAAGGAACACCAGTGGCGAAGGCGACTCCCTGGACTAGAACTGACGCTGAGGTACGAAAGCGTGGGTAGCAAACAGG` |
| ASV4457 | 423 | `TAAGGAATATTGGTCAATGGGCGGAAGCCTGAACCAGCCATGCCGCGTGAAGGAAGAAGGTGCTCAGCATCGTAAACTTCTTTTGTCAGGGAAGAAAACGCTCCTGCGGGAGAACTTGCCGGTACCTGATGAATAAGCATCGGCTAACTACGTGCCAGCAGCCGCGGTAATACGTAGGATGCGAGCGTTATCCGGATTTATTGGGTTTAAAGGGTGCGTAGGCGGTTTTGTAAGTCATGGGTGAAAGTCAGATGCTTAACGTCTGGATTGCCTGTGATACTGCAAGACTTGGATTCGGTGGAGGTAATTGGAATGACTGATGTAGCGGTGAAATGCATAGATATCAGTTGGAACGCCAATTGCGTAGGCAGGTTACTGTACCGAGATTGACGCTGAGGCACGAAAGCGTGGGGAGCAAACAGG` |
| ASV1292 | 429 | `TGGGGAATATTGGACAATGGGGGGAACCCTGATCCAGCAATGCCGCGTGTGTGAAGAAGGCCTGAGGGTTGTAAAGCACTTTCAGTGGGGAGGAGTTTTCATAGGTTAAGAGCTGATGAGGAGGACGTTACCCACAGAAGAAGCACCGGCTAACTCCGTGCCAGCAGCCGCGGTAATACGGAGGGTGCAAGCGTTAATCGGAATTACTGGGCGTAAAGGGTGCGTAGGTGGTTTGGTAAGTTATCTGTGAAATCCCTGGGCTCAACCTGGGCAGGTCAGATAAAACTGCTGGACTCGAGTATGGGAGAGGGTAGTGGAATTTCCGGTGTAGCGGTGAAATGCGTAGAGATCGGAAGGAACACCAGTGGCGAAGGCGGCTACCTGGCCTAATACTGACACTGAGGCACGAAAGCGTGGGGAGCAAACAGG` |
| ASV1065 | 429 | `TGGGGAATTTTGGACAATGGGGGCAACCCTGATCCAGCCATGCCGCGTGAGTGAAGAAGGCCTTCGGGTTGTAAAGCTCTTTTGTCCGGAAAGAAATCGCCTAGGTTAATACCTTGGGTGGATGACGGTACCGGAAGAATAAGCACCGGCTAACTACGTGCCAGCAGCCGCGGTAATACGTAGGGTGCAAGCGTTAATCGGAATTACTGGGCGTAAAGCGTGCGCAGGCGGTTTTGCAAGTCAGATGTGAAAGCCCCGGGCTCAACCTGGGAATGGCATTTGAAACTACAAGGCTAGAGTGTGTCAGAGGGGGGTGGAATTCCACGTGTAGCAGTGAAATGCGTAGATATGTGGAGGAACACCGATGGCGAAGGCAGCCCCCTGGGATAACACTGACGCTCATGCACGAAAGCGTGGGGAGCAAACAGG` |
| ASV1273 | 429 | `TGGGGAATTTTGGACAATGGGCGCAAGCCTGATCCAGCCATGCCGCGTGTCTGAAGAAGGCCTTCGGGTTGTAAAGGACTTTTGTCTGGGAGCAAATACCCGGTGTTAATAGCACCGGGGGCTGAGAGTACCAGAAGAATAAGCACCGGCTAACTACGTGCCAGCAGCCGCGGTAATACGTAGGGTGCAAGCGTTAATCGGAATTACTGGGCGTAAAGCGTGCGCAGGCGGTTTTGTAAGCCAGATGTGAAAGCCCCGGGCTCAACCTGGGAACGGCGTTTGGAACTACAAGACTAGAGTGTGTCAGAGGGGGGTGGAATTCCGCGTGTAGCAGTGAAATGCGTAGAGATGCGGAGGAACACCGATGGCGAAGGCAGCCCCCTGGGATAACACTGACGCTCATGCACGAAAGCGTGGGGAGCAAACAGG` |
| ASV5172 | 430 | `TGGGGAATATTGCGCAATGGGCGAAAGCCTGACGCAGCGACGCCGTGTGGGTGATGAAGGCCTTCGGGTCGTAAAGCCCTGTCGGGTGGAACGAATCTCCCGGTGGCAAATAACCACGGGGATTGACGGTACCACCGAAGGAAGCTCCGGCTAACTACGTGCCAGCAGCCGCGGTAATACGTAGGGAGCGAGCGTTGTTCGGATTTACTGGGCGTAAAGCGCGCGTAGGCGGCCTGATAAGTCGGGTGTGAAAGCCCTCGGCTCAACCGAGGATCCGCGCCCGAGACTGCCAGGCTCGAGTCTAGGAGAGGAGAGCGGAATTCCCAGTGTAGCGGTGAAATGCGTAGATATTGGGAGGAACACCAGTGGCGAAGGCGGCTCTCTGGACTAGTACTGACGCTGAGGCGCGAAAGCTAGGGGAGCGAACGGG` |
| ASV232 | 428 | `TAGGGAATTTTCGTCAATGGGGGGAACCCTGAACGAGCAATGCCGCGTGAGTGATGAAGGCCTTCGGGTCGTAAAGCTCTGTTGTAAGTGAAGAACGACCATGAGAGGGAATGCTCATGGAGTGACGGTAGCTTACCAGAAAGCCACGGCTAACTACGTGCCAGCAGCCGCGGTAATACGTAGGTGGCAAGCGTTATCCGGAATGATTGGGCGTAAAGGGTGCGTAGGTGGTCCGATAAGTCTGAGGTAAAAGCTCGCAGCTCAACTGCGATAAGCCTTGGAAACTGTTGGACTAGAGTGTAGGAGAGGCAAATGGAATTCCATGTGTAGCGGTAAAATGCGTAGATATATGGAGGAACACCAGTGGCGAAGGCGGTTTGCTGGCCTACAACTGACACTGAGGCACGAAAGCGTGGGGAGCAAATAGG` |
| ASV971 | 429 | `TGGGGAATTTTGGACAATGGGCGAAAGCCTGATCCAGCCATTCCGCGTGAGTGAAGAAGGCCTTCGGGTTGTAAAGCTCTTTCGCAAGGGAAGAAAGATTACTCTCTAACATAGAGTAATTATGACGGTACCTTGATAAGAAGCACCGGCTAACTACGTGCCAGCAGCCGCGGTAATACGTAGGGTGCGAGCGTTAATCGGAATTACTGGGCGTAAAGCGTGCGCAGGCGGTTTTGTAAGTCAGATGTGAAATCCCCGAGCTCAACTTGGGAACTGCGTTTGAAACTACAAGACTAGAATATGTCAGAGGGGGGTAGAATTCCACGTGTAGCAGTGAAATGCGTAGAGATGTGGAGGAATACCAATGGCGAAGGCAGCCCCCTGGGATAATATTGACGCTCATGCACGAAAGCGTGGGGAGCAAACAGG` |
| ASV915 | 404 | `TGGGGAATCTTAGACAATGGGGGAAACCCTGATCTAGCCATGCCGCGTGAGTGACGAAGGCCTTAGGGTCGTAAAGCTCTTTCGCTGGGGAAGATAATGACGGTACCCAGTAAAGAAACCCCGGCTAACTCCGTGCCAGCAGCCGCGGTAATACGGAGGGGGTTAGCGTTGTTCGGAATTACTGGGCGTAAAGCGCACGTAGGCGGGCTGTTAAGTCGGGGGTGAAATCCCGGGGCTCAACCCCGGAACTGCCTTCGATACTGGCAGCCTTGAGTTCGAGAGAGGTGAGTGGAATTCCGAGTGTAGAGGTGAAATTCGTAGATATTCGGAGGAACACCAGTGGCGAAGGCGGCTCACTGGCTCGATACTGACGCTGAGGTGCGAAAGCGTGGGGAGCAAACAGG` |
| ASV2675 | 429 | `TGGGGAATATTGGACAATGGGGGAAACCCTGATCCAGCAATGCCGCGTGTGTGAAGAAGGCCTGAGGGTTGTAAAGCACTTTCGGCGGGGAGGAGGGTTGATTGGTTAAGAGCTGATTAACTGGACGTTACCCGCATAAGAAGCACCGGCTAACTCCGTGCCAGCAGCCGCGGTAATACGGAGGGTGCGAGCGTTAATCGGAATTACTGGGCGTAAAGGGTGCGTAGGTGGTTTGATAAGTTATCTGTGAAAGCCCTGGGCTCAACCTGGGAATGCCAGATAGTACTGTTGAACTCGAGTATTGGAGAGGGTAGTGGAATTTCCGGTGTAGCGGTGAAATGCGTAGAGATCGGAAGGAACACCAGTGGCGAAGGCGGCTACCTGGCCAGATACTGACACTGAGGCACGAAAGCGTGGGGAGCAAACAGG` |
| ASV3875 | 423 | `TAAGGAATATTGGACAATGGACGCAAGTCTGATCCAGCCATCCCGCGTGGAGGAATAAGGCCCTATGGGTTGTAAACTCCTTTAGCAGGGGAATAAAAAGGTGATTTATCATCAATTGAAGGTACCCTGAGAATAAGCACCGGCTAACTCCGTGCCAGCAGCCGCGGTAATACGGAGGGTGCAAGCGTTATCCGGATTTACTGGGTTTAAAGGGTGCGTAGGCGGACTTTTAAGTTAAATGTGAAAGCTTGCAGCTTAACTGTAAGATTGCGTTTGATACTGAAAGTCTTGAATTTAGTAGAAGTGGGCGGAATAAATCATGTAGCGGTGAAATGCATAGATATGATTTAGAACACCGATTGCGAAGGCAGCTCGCTATGCTAATATTGACGCTGAGGCACGAAAGCGTGGGGATCAAACAGG` |
| ASV4905 | 429 | `TGGGGAATATTGGACAATGGGCGCAAGCCTGATCCAGCAATACCGCGTGTGTGAAGAAGGCCTGCGGGTTGTAAAGCACTTTCAGTGGGGAGGAAGGCTTAATGGTTAATAACCATTAGGATTGACGTTACCCACAGAAGAAGCACCGGCTAACTCTGTGCCAGCAGCCGCGGTAATACAGAGGGTGCGAGCGTTAATCGGAATTACTGGGCGTAAAGGGTGCGTAGGTGGTTTGATAAGTCAGATGTGAAAGCCCCGGGCTTAACCTGGGAACTGCATTTGAGACTATTAGGCTAGAGTATGGTAGAGGGAAGTGGAATTTCCGGTGTAGCGGTGAAATGCGTAGATATCGGAAGGAACATCAGTGGCGAAGGCGACTTCCTGGACCAACACTGACACTGAGGCACGAAAGCGTGGGGAGCAAACAGG` |
| ASV897 | 429 | `TGGGGAATATTGGACAATGGGCGCAAGCCTGATCCAGCCATGCCGCGTGTGTGAAGAAGGCCTTCGGGTTGTAAAGCACTTTTGTCCGGAAAGAAAAGCTTCGGGTTAATACCCCGAGGTCCTGACGGTACCGGAAGAATAAGCACCGGCTAACTTCGTGCCAGCAGCCGCGGTAATACGAAGGGTGCAAGCGTTACTCGGAATTACTGGGCGTAAAGCGTGCGTAGGTGGTTTGTTAAGTCTGATGTGAAAGCCCTGGGCTCAACCTGGGAATTGCATTGGATACTGGCAAGCTAGAGTGCGGTAGAGGATGGCGGAATTCCCGGTGTAGCAGTGAAATGCGTAGAGATCGGGAGGAACATCTGTGGCGAAGGCGGCCATCTGGACCAGCACTGACACTGAGGCACGAAAGCGTGGGGAGCAAACAGG` |
| ASV3178 | 405 | `TGGGGAATATTGGACAATGGGCGCAAGCCTGATCCAGCCATGCCGCGTGAGTGATGAAGGCCTTAGGGTTGTAAAGCTCTTTTAGCAGGGACGATAATGACGGTACCTGCAGAAAAAGCCCCGGCCAACTTCGTGCCAGCAGCCGCGGTAATACGAAGGGGGCTAGCGTTGCTCGGAATCACTGGGCGTAAAGCGCACGTAGGTGGGTCGTTAAGTCGAGGGTGAAATCCCAGAGCTCAACTCTGGAACTGCCTTCGATACTGGCGACCTTCGAGTCCGGGAGAGGTGAGTGGAACTGCGAGTGTAGAGGTGAAATTCGTAGATATTCGCAAGAACACCAGTGGCGAAGGCGGCTCACTGGCCCGGTACTGACACTGAGGTGCGAAAGCGTGGGGAGCAAACAGG` |
| ASV3834 | 387 | `GCGCGAAAACTTTCCAATGCACGCAAGTGTGAGAAGGGAATCCCGAGTGCTTATACTAAGTATAGGCTTTTGCCATGGGTAAATACCATGGAGAATAAGTGGGGGGCAATACCTGTGCCAGCCGCCGCGGTAACCCAGGACCCACAAGTGGCAACCGCTTTTATTGGGCCTAAAGCGTCCGTAGCCGGTTTTGTAAATCTTCTGTGAAATTCTTCGGCTTAACCGAAGAGCGCGCAGGAGACACTGCATCACTAGGGACCGGGAGGCGTGAGGGGTATTTCGGGGGGAGCGGTAAAATGTTATAATCCTCGAAGGACCACCTGTGGCGAAGGCGCCTCACGAGAACGGATCCGACGGTGAGTGACGAAAGCTAGGGGAGCAAAACGG` |
| ASV3783 | 405 | `TGGGGAATATTGGACAATGGGGGAAACCCTGATCCAGCCATGCCGCGTGAGTGATGAAGGCCTTCGGGTTGTAAAGCTCTTTTGTCAGGGAAGATAATGACTGTACCTGAAGAATAAGCCCCGGCTAACTTCGTGCCAGCAGCCGCGGTAATACGAAGGGGGCTAGCGTTGTTCGGAATTACTGGGCGTAAAGGGCGCGTAGGCGGTTTACCAAGTCAGAAGTGAAAGCCCTGGGCTCAACCCGGGAATTGCTTTTGAAACTGGTAAGCTTTGAGTTCGAGAGAGGTAAGTGGAATATCCTGTGTAGAGGTAAAATTCGTAGATATAGGATGGAACACCAGTGGCGAAGGCGGCTTACTGGCTCGATACTGACGCTGAGGCGCGAAAGCGTGGGGAGCAAACAGG` |
| ASV1608 | 404 | `TGGGGAATTTTGGACAATGGGGGCAACCCTGATCCAGCAATGCCGCGTGAGTGAAGAAGGCCTTCGGGTTGTAAAGCTCTTTTACTGATGAAGATGATGACGGTAATCAGAGAATAAGTTCCGGCAAACTTCGTGCCAGCAGCCGCGGTAATACGAAGGGAACTAGCGTTGTTCGGAATCACTGGGCGTAAAGCGTGAGTAGGCGGCTGTATAAGTCAGATGTGAAATCCTTGATCTCAAGTCAAGAACTGCATTTGAAACTGTACAGCTTGAGAGTAGTAGGGGGTAATGGAATTCCAAGTGTAGAGGTGAAATTCGTAGATATTTGGAGGAACACCAGTAGCGAAGGCGTTTACCTGGGCTACAACTGACGCTGAGTCACGAAAGTGTGGGGAGCAAACAGG` |
| ASV88 | 429 | `TGGGGAATTTTGGACAATGGGCGCAAGCCTGATCCAGCCATGCCGCGTGCGGGAAGAAGGCCTTCGGGTTGTAAACCGCTTTTGTCAGGGAAGAAACGCTCCGAAATAATACTTTGGGGTAATGACGGTACCTGAAGAATAAGCACCGGCTAACTACGTGCCAGCAGCCGCGGTAATACGTAGGGTGCAAGCGTTAATCGGAATTACTGGGCGTAAAGCGTGCGCAGGCGGTCTTGTAAGACAGAGGTGAAATCCCCGGGCTCAACCTGGGAACTGCCTTTGTGACTGCAAGGCTTGAGTACGGTAGAGGGGGATGGAATTCCGCGTGTAGCAGTGAAATGCGTAGATATGCGGAGGAACACCGATGGCGAAGGCAATCCCCTGGACCTGTACTGACGCTCATGCACGAAAGCGTGGGGAGCAAACAGG` |
| ASV3567 | 404 | `TGGGGAATATTGGGCAATGGGCGAAAGCCTGACCCAGCTATGCCGCGTGAATGATGAAGGCCTTAGGGTTGTAAAGTTCTTTTAGATGGGAAGATGATGACGGTACCATCAGAAAAAGCCCCGGCAAACTTCGTGCCAGCAGCCGCGGTAATACGAAGGGGGCAAGCGTTGCTCGGATTTATTGGGCGTAAAGGGTGTGTAGGCGGTTTGGTAAGTTAGATGTGAAAGCCCTGGGCTCAACCCGGGAATTGCATTTAAGACTGCTAGACTTGAAGATCGAAGAGGTAAGCAGAATTCCCAGTGTAGAGGTGAAATTCGTAGATATTGGGAAGAATACCAATGGCGTAGGCAGCTTACTGGTCGATACTTGACGCTGAGGCACGAAAGCGTGGGGAGCAAACAGG` |
| ASV2330 | 429 | `TGGGGAATTTTGGACAATGGGCGCAAGCCTGATCCAGCCATTCCGCGTGCAGGACGAAGGCCTTCGGGTTGTAAACTGCTTTTGTACGGAACGAAAAGGCTCTGGTTAATACCCGGGGCTCATGACGGTACCGTAAGAATAAGCACCGGCTAACTACGTGCCAGCAGCCGCGGTAATACGTAGGGTGCAAGCGTTAATCGGAATTACTGGGCGTAAAGCGTGCGCAGGCGGTGATGTAAGACAGATGTGAAATCCCCGGGCTCAACCTGGGAACTGCATTTGTGACTGCATCGCTGGAGTGCGGCAGAGGGGGATGGAATTCCGCGTGTAGCAGTGAAATGCGTAGATATGCGGAGGAACACCGATGGCGAAGGCAATCCCCTGGGCCTGCACTGACGCTCATGCACGAAAGCGTGGGGAGCAAACAGG` |
| ASV5335 | 509 | `GCGCGAAAATTCCGCAATGCGCGAAAGCGTGACGGGGGAACTCCGAGTGCTGGTCTTACGACCGGCTTTTTGCCAGCTTGAATAGCTGACAGAATAAGTAGTGGGTAAATCCTGTGCCAGCCGCCGCGGTAATACAGGTAGTACAAGTGGTGCCCGCGAATATTGAGTCTAAAGCGCCCGTAGCCGGCTTGGCAAGTCCTCTGTGAAATTTACAGGCTCAACCTGTAAGCGTGCAGTGGATACTACCAGGCTTGGGAGTGGGGGAGGTGAAGAGTACTCTAGGGGGAGGAGTAACTGACTGAGCCTACCTTTTCTTTCTTCCAAGAAAGAAAAGTCAGGCACCCCAAAAGAAAGAAGGAAAAAATTTTTTCTTTTGGAATCAGCTTTCGTTTTTATATGAAAGCACAGCCAACTTAGAATCCTGTAATCCTTAGAGGACTTCCAGTAGCGAAGGCGCTTCACCAAAACACGTCCGACGGTGAGGGGCGAAGGCTAGGGGAGCAAATCGG` |
| ASV2782 | 404 | `TGGGGAATATTGGACAATGGACGAAAGTCTGATCCAGCAATGCCGCGTGAATGAAGAAGGCCTTCGGGTCGTAAAGTTCTTTCAGTAGGGAAGATAATGACGGTACCTACAGAAGAAGCACCGGCTAACTTCGTGCCAGCAGCCGCGGTAATACGAAGGGTGCTAGCGTTGTTCGGAATTATTGGGCGTAAAGAGCGCGTAGGCGGCTTAGTTAGTTAGGGGTGAAATCCCGGGGCTTAACCCCGGAACTGCTTCTAAAACTGCTAGGCTAGAGTTTGATAGGGGGTGACGGAATTCCTAGTGTAGAGGTGAAATTCGTAGATATTAGGAGGAACACCGGTGGCGAAGGCGGTCACCTAGGTCAAAACTGACGCTGTGGCGCGAAAGCGTGGGGAGCAAACAGG` |
| ASV3737 | 429 | `TCGAGAATCTTTCACAATGGGGGAAACCCTGATGGAGCGACGCCGCGTGGAGGATGAAGGTCTTCGGATTGTAAACTCCTGTCATGAAAGAACAAGTGATGCGGGTGAACAGCCCGCAAAGTTGATAGTATTTCAAGAGGAAGGGACGGCTAACTTCGTGCCAGCAGCCGCGGTAATACGAAGGTCCCAAGCGTTGTTCGGAATCACTGGGCGTAAAGAGTGCGTAGGCGGCGCGGAAAGTCAGATGTGAAATCCCGGGGCTCAACCTCGGAACGGCATCCGATACTCCCGCGCTTGAGGACTGGAGGGGAGTCTGGAATTCACGGTGTAGCAGTGAAATGCGTAGATATCGTGAGGAACACTAGTGGCGAAGGCGAGACTCTGGACAGTTCCTGACGCTGAGGCACGAAGGCCAGGGGAGCGAAGGGG` |
| ASV5481 | 420 | `TCGAGAATCATTCGCAATGGGGGAAACCCTGACGGTGCGACGCCGCGTGGAGGAAGAAGGCCCTCGGGTTGTAAACTCCTGTCAAGTGAGAGTAAGGTTGTAGCAATACAGCTTGAGAGTATCACTAGAGGAAGGGGCGGCTAACTCCGTGCCAGCAGCCGCGGTAATACGGAGGCCCCAAGCGTTGTTCGGATTTATTGGGCGTAAAGGGTGCGTAGGCGGCGTGGTAAGTCTGGTGTGAAAGGCGGGGGCTCAACCCTCGGACTGCATTGGATACTGCCATGCTAGAGGACTGGAGAGGAGAATGGAATTCACGGTGGAGCAGTGAAATGCGTAGATATCGTGAGGAAGGCCAGTGGCGAAGGCGATTCTCTGGACAGTAACTGACGCTGAGGCACGAAGGCTGGGGGAGCGAACAGG` |
| ASV5458 | 404 | `TGGGGAATATTGGACAATGGGCGAAAGCCTGATCCAGCCATGCCGCGTGAGTGATGAAGGCCTTAGGGTTGTAAAGCTCTTTTATGAGGGAAGATGATGACGGTACCTCAAGAATAAGCACCGGCTAACTTCGTGCCAGCAGCCGCGGTAATACGAAGGGTGCTAGCGTTGTTCGGAATCACTGGGCGTAAAGAGTACGTAGGTGGCTTGGCCAGTCAGGGGTGAAATCCCACAGCTCAACTGTGGAACTGCCTCTGAAACTGCCAAGCTAGAGTGTCGGAGGGGATAGCGGAATTGCTAATGTAGAGGTGAAATTCGTAGATATTAGCAGGAACACCGGTGGCGAAGGCGGCTATCTGGACGACAACTGACACTGAGGTACGAAAGTGTGGGGAGCAAACAGG` |
| ASV2528 | 404 | `TAAGGAATATTGGACAATGGGCGAAAGCCTGATCCAGCCATGCCGCGTGAATGATGAAGGCCTTAGGGTTGTAAAGTTCTTTCGCAGGTGAAGATAATGACGGTAACCTGATAAGAAGCCCCGGCTAACTTCGTGCCAGCAGCCGCGGTAATACGAAGGGGGCTAGCGTTGTTCGGAATTACTGGGCGTAAAGCGCGCGCAGGCGGGCTGACAAGCCAGAGGTGAAATCCCCAAGCTTAACTTGGGAACTGCCTTTGGAACTGTCAGTCTAGAGTCTTGGAGGGGGTGGTGGAATTCCTAGTGTAGAGGTGAAATTCGTAGATATTAGGAGGAACACCGGTGGCGAAAGCGACCACCTGGACAAGTACTGACGCTGAGGCGCGAAAGCGTGGGGAGCAAACAGG` |
| ASV2187 | 404 | `TGGGGAATATTGCACAATGGGGGAAACCCTGATGCAGCAACGCCGCGTGAGTGATGAAGGTTTTCGGATCGTAAAACTCTGTCTTTGGGGACGATAATGACGGTACCCAAGGAGGAAGCCACGGCTAACTACGTGCCAGCAGCCGCGGTAATACGTAGGTGGCAAGCGTTGTCCGGATTTACTGGGCGTAAAGGGAGCGTAGGTGGACTTTTAAGTGAGATGTGAAATACTCGGGCTCAACCTGAGAACTGCATTTCAAACTGGAAGTCTAGAGTGCAGGAGAGGAAAGTGGAATTCCTAGTGTAGCGGTGAAATGCGTAGATATTAGGAAGAACACCAGTGGCGAAGGCGACTTTCTGGACTGTAACTGACACTGAGGCTCGAAAGCGTGGGGAGCAAACAGG` |
| ASV3953 | 423 | `TAGGGAATATTGGACAATGGGCGCAAGCCTGATCCAGCCATGCCGCGTGAAGGATGAAGGCCCTCTGGGTTGTAAACTTCTTTTAGCAGGGGATAAACGATCACTATACGTGGTCCTGAAGGTACCTGCAGAATAAGCACCGGCTAACTCCGTGCCAGCAGCCGCGGTAATACGGAGGGTGCAAGCGTTGTCCGGATTTATTGGGTTTAAAGGGTGCGTAGGCGGACCTGTAAGTCAGCGGTGAAATCCTGAGGCTTAACCTTAGAATTGCCATTGATACTGCAGGTCTTGAATCAGGTCGAGGTAGTTGGAATACGTAGTGTAGCGGTGAAATGCTTAGATATTACGTAGAACACCTATTGCGAAGGCAGATTACTAGGCCTGAATTGACGCTGAGGCACGAAAGTGTGGGGAGCAAACAGG` |
| ASV3249 | 423 | `TAAGGAATATTGGTCAATGGACGCAAGTCTGAACCAGCCATGCCGCGTGAAGGATGAAGGTCCTCTGGATTGTAAACTTCTTTTATATGGGACGAAACCTCTTTTTCTAAGGAGACTGACGGTACCATAAGAATAAGCACCGGCTAACTCCGTGCCAGCAGCCGCGGTAATACGGAGGGTGCAAGCGTTATCCGGATTCACTGGGTTTAAAGGGTGCGTAGGAGGGCAGGTAAGTCAGTGGTGAAATCTTCGAGCTTAACTCGGAAACTGCCGTTGATACTATCTGTCTTGAATATTGTGGAGGTGAGCGGAATATGTCATGTAGCGGTGAAATGCTTAGATATGACATAGAACACCAATTGCGAAGGCAGCTCGCTACACATATATTGACTCTGAGGCACGAAAGCGTGGGGATCAAACAGG` |
| ASV4403 | 430 | `TGGGGAATATTGGACAATGGGCGAAAGCCTGATCCAGCCATGCCGCGTGTGTGAAGAAGGCCTTTTGGTTGTAAAGCACTTTAAGCGGGGAGGAGGGCACCACGGTTAATACCCGTGAATGTCGGACGTTACCCGCAGAATAAGCACCGGCTAACTCTGTGCCAGCAGCCGCGGTAATACAGAGGGTGCGAGCGTTAATCGGAATTACTGGGCGTAAAGCGCGCGTAGGCGGCTTGTTAAGTCAAATGTGAAATCCCCGAGCTTAACTTGGGCATTGCATTCGATACTGGCAAGCTAGAGTATGGGAGAGGAAGGTAGAATTCCAGGTGTAGCGGTGAAATGCGTAGAGATCTGGAGGAATACCGATGGCGAAGGCAGCCTTCTGGCCTAATACTGACGCTGAGGTGCGAAAGCATGGGGAGCAAACAGG` |
| ASV2867 | 404 | `TAGGGAATATTGCACAATGGAGGAAACTCTGATGCAGCAACGCCGCGTGTGTGATGAAGGCTCTAGGGTCGTAAAGCACTGTCGTATGGGAAGATAATGACGGTACCATACAAGAAAGGACCGGCTAACTTCGTGCCAGCAGCCGCGGTAATACGAGGGGTCCTAGCGTTGTTCGGAATCATTGGGCGTAAAGCGGGTGTAGGTGGCTTTGTAAGTCAGGTGTGAAATCCCAGGGCTCAACCCTGGACGTGCACTTGATACTGCTTAGCTTGAGTGTTGGAGAGGTTCGTGGAATTCCTGGTGTAGTGGTGAAATACGTAGATATCAGGAGGAACATCGGTGGGGAAGCCGGCGAACTGGCCAAACACTGACACTCAGACCCGAAAGCGTGGGGATCAAACAGG` |
| ASV459 | 429 | `TGGGGAATTTTGGACAATGGGGGCAACCCTGATCCAGCCATTCCGCGTGAGTGAAGAAGGCCTTCGGGTTGTAAAGCTCTTTCGCAAGGGAAGAAACGATTTAGGTAAATAATCTAAGTTAATGACGGTACCTTGATAAGAAGCACCGGCTAACTACGTGCCAGCAGCCGCGGTAATACGTAGGGTGCGAGCGTTAATCGGAATTACTGGGCGTAAAGCGTGCGCAGGCTGTTTTGTAAGTCAGATGTGAAATCCCTGGGCTCAACCTAGGAACTGCGTTTGAAACTACAAGACTAGAATATGTCAGAGGGGGGTAGAATTCCACGTGTAGCAGTGAAATGCGTAGAGATGTGGAGGAATATCAATGGCGAAGGCAGCCCCCTGGGATAATATTGACGCTCATGCACGAAAGCGTGGGGAGCAAACAGG` |
| ASV537 | 404 | `TGGGGAATATTGGACAATGGGCGAAAGCCTGATCCAGCAATGCCGCGTGAGTGAAGAAGGCCTTCGGGTTGTAAAGCTCTTTTACCCGGGATGATAATGACCGTACCGGGAGAATAAGCCCCGGCTAACTCCGTGCCAGCAGCCGCGGTAATACGGAGGGGGCTAGCGTTGTTCGGAATCACTGGGCGTAAAGCGTACGTAGGCGGCTATTCAAGTCAGAGGTGAAAGCCTGGAGCTCAACTCCAGAACTGCCTTTGAAACTAGGTAGCTTGAACACGGGAGAGGTGAGTGGAATTCCGAGTGTAGAGGTGAAATTCGTAGATATTCGGAAGAACACCAGTGGCGAAGGCGGCTCACTGGACCGTTGTTGACGCTGAGGTACGAAAGCGTGGGGAGCAAACAGG` |
| ASV4428 | 409 | `TGAGGAATCTTCCGCAATGGGCGAAAGCCTGACGGAGCGACACCGCGTGAAGGATGAAGCCCTTTCCGGGCGTAAACTTCTGTTTTGAGGGACGAAATTCTTGACGGTACCTCAAGAGAAAGCACCGGCTAATCCTGTGCCAGCAGCCGCGGTAAGACAGGAGGTGCAAGCGTTACTCGGAATTACTGGGCGTAAAGCGTCTGCAGGCGTCTTTGAAAGTCTGGTGTGAAATGGCGATGCTCAACATCGTCTCCGCATCGGAAACTTCAGAGATCGAGGCATTTTAGAGGCATCTGGAATGTCGTGTGTAGGGGTAAAATCCGTTGATACACGATGGAACGCCAAAAGCGAAAGCAGGATGCTGGGAATGTCCTGACGCTCAGAGACGAAAGCGTGGGGAGCAAAGGGG` |
| ASV1932 | 429 | `TGGGGAATATTGCGCAATGGGCGAAAGCCTGACGCAGCGACGCCGCGTGAGGGATGAAGGTTCTCGGATCGTAAACCTCTGTCAGAAGGGAAGAATAGCATTAGTAGTAACTGACTAGTGTATGACGGTACCTTCAAAGGAAGCACCGGCTAACTCCGTGCCAGCAGCCGCGGTAATACGGAGGGTGCGAGCGTTAATCGGAATTACTGGGCGTAAAGCGCACGTAGGCGGCTTTATAAGTCAGCGGTGAAATCCCAGGGCTCAACCTTGGAACTGCCGTTGAAACTGTATGGCTTGAGTTCGTAAGAGGGTGGCAGAATTCCAGGTGTAGGAGTGAAATCCGTAGAGATCTGGAGGAATATCAGTGGCGAAGGCGGCCACCTGGTACGACACTGACGCTGAGGTGCGAAAGCGTGGGGAGCAAACAGG` |
| ASV3697 | 429 | `TGGGGAATCTTGGGCAATGGGCGAAAGCCTGACCCAGCCATGCCGCGTGTGTGAAGAAGGCCTTCGGGTTGTAAAGCACTTTCAGTCGTAAAGAAACGCTTCAGGCCAATACCTTGAAGCTTTGACGTTAGCGACAGAAGAAGCACCGGCTAACTCCGTGCCAGCAGCCGCGGTAATACGGAGGGTGCAAACGTTAATCGGAATTACTGGGCGTAAAGCGCGCGTAGGCGGTTTGGTAAGTGGGATGTGAAAGCCCCGGGCTTAACCTGGGAACTGCATTCCAAACTGCCAAGCTAGAGTTTGGTAGAGGGAGGTGGAATTTCCTGTGTAGCGGTGAAATGCGTAGATATAGGAAGGAACACCAGTGGCGAAGGCGGCCTCCTGGACCAAAACTGACGCTGAGGTGCGAAAGCGTGGGGAGCAAACAGG` |
| ASV482 | 429 | `TGGGGAATATTGGACAATGGGCGAAAGCCTGATCCAGCCATGCCGCGTGTGTGAAGAAGGTCTTCGGATTGTAAAGCACTTTAAGTTGGGAGGAAGGGCATTAACCTAATACGTTAGTGTTTTGACGTTACCGACAGAATAAGCACCGGCTAACTCTGTGCCAGCAGCCGCGGTAATACAGAGGGTGCAAGCGTTAATCGGAATTACTGGGCGTAAAGCGCGCGTAGGTGGTTTGTTAAGTTGGATGTGAAATCCCCGGGCTCAACCTGGGAACTGCATTCAAAACTGACAAGCTAGAGTATGGTAGAGGGTGGTGGAATTTCCTGTGTAGCGGTGAAATGCGTAGATATAGGAAGGAACACCAGTGGCGAAGGCGACCACCTGGACTGATACTGACACTGAGGTGCGAAAGCGTGGGGAGCAAACAGG` |
| ASV2725 | 430 | `TGGGGAATATTGGACAATGGGGGCAACCCTGATCCAGCAATACCGCGTGTGTGAAGAAGGCCTTCGGGTTGTAAAGCACTTTTGTTAGGGAGGATGGTTATTTGTATTAATACTGCAAGTAGCTGACGTTACCTAAAGAATAAGCACCGGCTAACTCCGTGCCAGCAGCCGCGGTAATACGGAGGGTGCGAGCGTTAATCGGAATTACTGGGCGTAAAGGGTGCGTAGGCGGTTAATTAAGTCAGATGTGAAATACCTGGGCTCAACCTGGGAACTGCATTTGAAACTGTTTGACTAGAGTATGGTAGAGGGTAGCGGAATTTCTGGTGTAGCGGTGAAATGCGTAGATATCAGAAGGAACATCAATGGCGAAGGCAGCTACCTGGGCCAATACTGACGCTGAGGCACGAAAGCGTGGGGAGCAAACAGG` |
| ASV2756 | 429 | `TGGGGAATATTGGACAATGGGGGAAACCCTGATCCAGCAATGCCGCGTGTGTGAAGAAGGCCTTCGGGTTGTAAAGCACTTTCAGTGAAGAAGAAGTAAAATGGTTTAAGAGATCATTTTGTTGACGTTACTCACAGAAGCAGCACCGGCAAACTCTGTGCCAGCAGCCGCGGTAATACAGAGGGTGCAAGCGTTAATCGGAATGACTGGGCGTAAAGGGCGCGTAGGCGGGATAGTAAGTGCGTTGTGAAAGACTTGGGCTCAACCTGAGGAAGTCATCGTAGACTGTTATTCTAGAGTGAGAGAGAGGTCATAGGAATTTCCGGAGGAGCGGTGAAATGCGTAGATTTCGGAAGGAACATCAGTGGCGAAGGCGAGTGACTGGCTCTTAACTGACGCTGAGGCGCGAAGGCGTGGGTAGCAAACAGG` |
| ASV3590 | 424 | `TGAGGAATATTGGTCAATGGGCGAGAGCCTGAACCAGCCAAGTAGCGTGAAGGATGAAGGCTCTATGGGTCGTAAACTTCTTTTATATGGGAATAAAGTTACCTACGTGTAGGTATTTGTATGTACCATACGAATAAGGATCGGCTAACTCCGTGCCAGCAGCCGCGGTAATACGGAGGATCCGAGCGTTATCCGGATTTATTGGGTTTAAAGGGTGCGTAGGTGGAAAAATAAGTCAGTTGTGAAAGTTTGCGGCTCAACCGTAAAATTGCAGTTGAAACTGTTTTTCTTGAGTATAGATGAGGTAGGCGGAATTCGTGGTGTAGCGGTGAAATGCTTAGATATCACGAAGAACTCCGATTGCGTAGGCAGCTTACTAAACTATAACTGACACTGAGGCACGAAAGTGTGGGTATCAAACAGG` |
| ASV1866 | 404 | `TAAGGAATATTGGACAATGGGGGAAACCCTGATCCAGCCATGCCGCGTGAGTGACGAAGGCCTTAGGGTTGTAAAGCTCTTTTGGTGGAGAAGATAATGACTGTATCCACAGAATAAGCCCCGGCTAAATTCGTGCCAGCAGCCGCGGTAATACGAATGGGGCAAGCGTTGTTCGGAATCACTGGGCGTAAAGCGTGCGTAGGCGGTTGTGCAAGTCAGAAGTGAAAGCCCAGGGCTCAACCCTGGAATTGCTTTTGAAACTGCACTACTTGAATCCCGGAGAGGTTAGTAGAATTCCGAGTGTAGAGGTGAAATTCGCAGATATTCGGAGGAATACCAGTGGCGTAAGCGGCTAACTGGACGGGCATTGACGCTGAGGTACGAAAGCGTGGGGATCAAACAGG` |
| ASV1385 | 425 | `TAAGGAATATTGGACAATGGCCGCAAGGCTGATCCAGCCATCCAGCGTGCAGGAAGAAGGCCCTATGGGTTGTAAACTGCTTTTATCAGGGAATAAACCTCTGGATTCACTCCGGAGCTGAAGGTACCTGAAGAATAAGCACCGGCTAACTCCGTGCCAGCAGCCGCGGTAATACGGAGGGTGCGAGCGTTATCCGGAATTACTGGGTTTAAAGGGTGCGTAGGCGGCTTTGTAAGTCAGGAGTGAAAGTTTGCGGCTCAACCGTAAAATTGCTTCTGATACTGCATGGCTAGAATTAGGATGAGGTCAGCGGAATGTGGCATGTAGCGGTGAAATGCATAGATATGCCATAGAACACCAATTGCGAAGGCAGCTGGCTAGACCTGGATTGACGCTGAGGCACGAAAGCGTGGGGAGCGAACAGG` |
| ASV3976 | 429 | `TGGGGAATTTTGGACAATGGGCGCAAGCCTGATCCAGCAATGCCGCGTGCAGGATGAAGGCCTTCGGGTTGTAAACTGCTTTTGTACGGAGCGAAAAGCCTCTTTCTAATAAAGAGGGGTCATGACGGTACCGTAAGAATAAGCACCGGCTAACTACGTGCCAGCAGCCGCGGTAATACGTAGGGTGCGAGCGTTAATCGGAATTACTGGGCGTAAAGCGTGCGCAGGCGGTTATGTAAGACAGTTGTGAAATCCCCGGGCTCAACCTGGGAACTGCATCTGTGACTGCATAGCTGGAGTGCGGCAGAGGGGGATGGAATTCCGCGTGTAGCAGTGAAATGCGTAGATATGCGGAGGAACACCGATGGCGAAGGCAATCCCCTGGGCCTGCACTGACGCTCATGCACGAAAGCGTGGGGAGCAAACAGG` |
| ASV5648 | 409 | `TGGGGAATATTGCACAATGGGCGAAAGCCTGATGCAGCGACGCCGCGTGAGGGATGAAGGCCTTCGGGTTGTAAACCTCTTTCAGCAGGGAAGAAGCGAAAGTGACGGTACCTGCAGAAGAAGCACCGGCTAACTACGTGCCAGCAGCCGCGGTAATACGTAGGGTGCAAGCGTTGTCCGGAATTATTGGGCGTAAAGAGCTCGTAGGCGGTCTGTCGCGTCTGCTGTGAAAACTCGGGGCTCAACCCCGGGCCTGCAGTGGGTACGGGCAGACTAGAGTGCGGTAGGGGAGACTGGAATTCCTGGTGTAGCGGTGAAATGCGCAGATATCAGGAGGAACACCGGTGGCGAAGGCGGGTCTCTGGGCCGTAACTGACGCTGAGGAGCGAAAGCATGGGTAGCGAACAGG` |
| ASV4812 | 418 | `TGGGGAATATTGGACAATGGGCGCAAGCCTGATCCAGCAATGCCACGTGTGTGAAGAAGGCCTGCGGGTTGTAAAGCACTTTCAGAAGGGAAGAAGGACGCGCAAGCGTATTGACATTACCTTCAGAAGAAGCACCGGCTAACTCTGTGCCAGCAGCCGCGGTAATACAGAGGGTGCGAGCGTTAATCGGAATTACTGGGCGTAAAGCGCGCGTAGGCGGTTATTTAAGTTGGACGTGAAATCCCCGAGCTTAACTTGGGAATTGCGTCCAAAACTGGATGACTGGAGTGGAGTAGAGGTGAGTGGAATTTCCGGAGTAGCGGTGAAATGCGTAGATACCGGAAGGAACACCAGTGGCGAAGGCGACTCACTGGACTCTAACTGACGCTGAGGTGCGAAAGCGTGGGGAGCAAACAGG` |
| ASV3137 | 429 | `TGGGGAATTTTGGACAATGGGCGCAAGCCTGATCCAGCAATGCCGCGTGAGTGAAGAAGGCCTTCGGGTTGTAAAGCTCTTTTGTCAGGGAAGAAACGGTGTTTCCTAATACGGAATGCTAATGACGGTACCTGAAGAATAAGCACCGGCTAACTACGTGCCAGCAGCCGCGGTAATACGTAGGGTGCAAGCGTTAATCGGAATTACTGGGCGTAAAGCGTGCGCAGGCGGTTATATAAGTCAGATGTGAAATCCCCGGGCTCAACCTGGGAACTGCATTTGAGACTGTATGGCTAGAGTGTGTCAGAGGGGGGTAGAATTCCACGTGTAGCAGTGAAATGCGTAGATATGTGGAGGAATACCGATGGCGAAGGCAGCCCCCTGGGATAACACTGACGCTCATGCACGAAAGCGTGGGGAGCAAACAGG` |
| ASV1143 | 423 | `TAGGGAATATTGGTCAATGGACGGAAGTCTGAACCAGCCATGCCGCGTGCAGGAAGAAGGCGCTCTGCGTTGTAAACTGCTTTAGACTAGGAACAAAAGGCCTCTGCGGAGGATATTGAGTGTACTAGTAGAATAAGCCACGGCTAACTACGTGCCAGCAGCCGCGGTAATACGTAGGTGGCAAGCGTTGTCCGGATTTATTGGGTTTAAAGGGTGCGTAGGCGGCTAAATAAGTCAGTGGTGAAATACTCGAGCTTAACTTGAGAACGGCCAATGATACTGTTTAGCTTGAGTACAAACGAAGTGGGCGGAATTGAAAGTGTAGCGGTGAAATGCATAGATATTTTCAAGAACACCGATAGCGAAGGCAGCTCACTAGGATGTAACTGACGCTGAAGCACGAAAGTGTGGGGATCAAACAGG` |
| ASV1467 | 407 | `TAGGGAATATTGGACAATGGGGGAAACCCTGATCCAGCGACGCCGCGTGAGTGACGAAGGCCTTCGGGTCGTAAAGCTCTGTTGCAGGGGAAAAACAAAATGATGGTACCCTGCTAGAAAGGATCGGCTAACTTCGTGCCAGCAGCCGCGGTAAGACGAGGGATCCTAGCGTTGTTCGGAATTATTGGGCGTAAAGCGGGTGCAGGTGGCTTGGTAAGTCAGATGTGAAAGCCTAGGGCTCAACCCTAGAAGTGCATTTGATACTGCTGAGCTTGAGTGCTGGAGAGGCTATTAGAATTCCTGGTGTAGTGGTGAAATACGTAGATATCAGGAGGAATACCGGAGGCGAAGGCGGATAGCTGGCCAGACACTGACACTCAGACCCGAAAGCGTGGGGATCAAACAGG` |
| ASV2191 | 430 | `TGGGGAATTTTGGACAATGGGGGCAACCCTGATCCAGCCATGCCGCGTGCGGGAAGAAGGCCTTCGGGTTGTAAACCGCTTTAGTCCGGGAAAAATGTTCCTATAGTTAATACCTATGGGAGTTGATGGTACTGGAAGAATAAGCACCGGCTAACTACGTGCCAGCAGCCGCGGTAATACGTAGGGTGCAAGCGTTAATCGGAATTACTGGGCGTAAAGCGTGCGCAGGCGGTTTCATAAGACAGATGTGAAAGCCCCGGGCTTAACCTGGGAATGGCATTTGTGACTGTGAGGCTAGAGTGTGTCAGAGGGGGGTGGAATTCCGCGTGTAGCAGTGAAATGCGTAGATATGCGGAGGAACACCGATGGCGAAGGCAGCCCCCTGGGATAACACTGACGCTCATGCACGAAAGCGTGGGGAGCAAACAGG` |
| ASV4917 | 406 | `TGAGGAATTTTCCGCAATGGGCGAAAGCCTGACGGAGCAATGCCGCGTGGAGGAAGACAGCCTGTGGGTCGTAAACTCCTTTTCTCAGAGAAGAAGTTCTGACGGTATCTGAGGAAAAAGCATCGGCTAACTCCGTGCCAGCAGCCGCGGTAATACGGAGGATGCAAGCGTTATCCGGAATGATTGGGCGTAAAGCGTCTGTAGGTGGTTTCTGAAGTCTGCTGTCAAATATCAAGGCTTAACCTTGAGCAGGCAGTCGAAACTCAGAAGCTTGAGTCCGGTAGGGGCAGAGGGAATTCCCGGTGTAGCGGTGAAATGCGTAGAGATCGGGAAGAACACCAAGAGCGAAAGCACTCTGCTGGGCCGAAACTGACACTAAGAGACGAAAGCTAGGGGAGCAAATGGG` |
| ASV2873 | 423 | `TAGGGAATATTGGGCAATGGGCGCAAGCCTGACCCAGCCATGCCGCGTGCAGGAAGAATGCCCTCTGGGTTGTAAACTGCTTTTGGGAGGGGACAAAAAGCCAATGCGTTGGTAATTGAGTGTACCTCCAGAATAAGCACCGGCTAACTCCGTGCCAGCAGCCGCGGTAATACGGAGGGTGCAAGCGTTGTCCGGATTTATTGGGTTTAAAGGGTGCGTAGGCGGCCTAATAAGTCGGAGGTGAAACCCACAGGCTTAACCTGTGGAGTGCCTTCGATACTGTTAGGCTTGGGTTATCTCTAGGCGGGCGGAATGGAAGGTGTAGCGGTGAAATGCATAGATATCTACCAGAACGCCGATAGCGAAGGCAGCTCGCTGGGGATATACCGACGCTGAGGCACGATAGCGTGGGGATCAAACAGG` |
| ASV108 | 404 | `TGGGGAATATTGGACAATGGGCGAAAGCCTGATCCAGCAATGCCGCGTGAGTGATGAAGGCCTTAGGGTTGTAAAGCTCTTTTACCAGGGATGATAATGACAGTACCTGGAGAATAAGCTCCGGCTAACTCCGTGCCAGCAGCCGCGGTAATACGGAGGGAGCTAGCGTTGTTCGGAATTACTGGGCGTAAAGCGTACGTAGGCGGTTATTCAAGTCAGGGGTGAAAGCCTGGAGCTCAACTCCAGAACTGCCCTTGAAACTAGATAACTAGAATCTTGGAGAGGTGAGTGGAATTCCGAGTGTAGAGGTGAAATTCGTAGATATTCGGAAGAACACCAGTGGCGAAGGCGACTCACTGGACAAGTATTGACGCTGAGGTACGAAAGCGTGGGGAGCAAACAGG` |
| ASV3393 | 389 | `GCGCGAAAACTCTCCAATGTGCGAAAGCGTGAGAGGGGAACTCCTAGTGCTTACGGTTCATCCGTAGGCTTTTGCCATGTGTAAATAGCATGGCGAATAAGTGGTGGGTAAGACTGGTGCCAGCCGCCGCGGTAACACCAGCGCCACGAGTGGCCACCGCGATTATTGGGCCTAAAGCGTCCGTAGTCGGTTCTGTGCATCTTCTGTGAAATCGCTGCGCTTAACGTAGCGGCATGCGGAAGACACGGCAGAACTTGAGACCGGGAGGCGTGAAGGGTACTCCGGGGGGACCGGTAAAATGGTATAATCCCCGGAAGACCACCTGTGGTGAAGACGCTTCACGAGAACGGATCTGACGATGAGGGACGAAAGCCAGGGGAGCAAACCGG` |
| ASV5632 | 429 | `TGGGGAATATTGGACAATGGGGGAAACCCTGATCCAGCAATGCCGCGTGTGTGAAGAAGGCCTGAGGGTTGTAAAGCACTTTTAGTGGTGAGGAGAGACGTGAGTTTAAGAGATACACGTTGAGACGTTAACCAAAGAAAAAGCACCGGCTAACTCCGTGCCAGCAGCCGCGGTAATACGGAGGGTGCAAGCGTTAATCGGATTGATTGGGCGTAAAGGGCGCGTAGGTGGTCTACTGAGTGAGTTGTGAAAGACCTGGGCTTAACCTGGGGGTGTCAACTCAGACGGGTAGACTGGAGTACAGTAGAGGGTTGTGGAATTTCCGGTGTAGCGGTGAAATGCGTAGAGATCGGAAGGAACACCAGTGGCGAAGGCGACAACCTGGGCTGAAACTGACACTGAGGCGCGAAAGCGTGGGGAGCAAACAGG` |
| ASV5478 | 430 | `TGGGGAATTTTGCGCAATGGACGAAAGTCTGACGCAGCAACGCCGTGTGAGTGAGGAAGGATTTCGGTCTGTAAAGCTCTGTCAGGTTGGAAAAATACCCAAGGGTCTAATAGGCCCATGGATTGATGGTACAACCAAAGGAAGCACCGGCAAACTTCGTGCCAGCAGCCGCGGTAATACGAAGGGTGCAAGCGTTGTTCGGATTTATTGGGCGTAAAGCGCGCGCAGGCGGGCTTGCAAGTCAGATGTGAAATCTCGGAGCTCAACTCCGAAACGGCGTCTGAAACTACAAGTCTAGAATACTGGAGAGGGTAACGGAATATCGCATGTAGGGGTAAAATCCGTAGAGATGCGATGGAACATCAGAGGCGAAGGCGGTTACCTGGCCGGTTATTGACGCTGAGGCGCGAAAGCGTGGGGAGCAAACAGG` |
| ASV3240 | 429 | `TGGGGAATATTGGACAATGGGGGCAACCCTGATCCAGCAATGCCGCGTGTGTGAAGAAGGCCTTCGGGTTGTAAAGCACTTTTATCAGGAAAGAAATCCGGTGGATTAATACTTTGCCGGGATGACGGTACCTGAGGAATAAGCACCGGCTAACTTCGTGCCAGCAGCCGCGGTAATACGAAGGGTGCAAGCGTTAATCGGAATTACTGGGCGTAAAGGGTGCGTAGGCGGCCCGTTAAGTTCGATGTGAAAGCCCCGGGCTCAACCTGGGAATGGCATCGAAGACTGGCAGGCTGGAGTGCGGTAGAGGGTGGTGGAATTCCCGGTGTAGCGGTGAAATGCGTAGAGATCGGGAGGAACATCTGTGGCGAAGGCGGCCACCTGGACCAGCACTGACGCTGAGGCACGAAAGCGTGGGGAGCAAACAGG` |
| ASV2938 | 429 | `TGGGGAATATTGGACAATGGGGGCAACCCTGATCCAGCCATGCCGCGTGTGTGAAGAAGGCCTTCGGGTTGTAAAGCACTTTAAGTGGGGAGAAAGGCTCCAAGGTTAATACCTTTGGGGATTGATGCTACCCACAGAATAAGCACCGGCTAACTCTGTGCCAGCAGCCGCGGTAATACAGAGGGTGCAAGCGTTAATCGGAATTACTGGGCGTAAAGCGCGCGTAGGCGGCTGATTAAGTCGGATGTGAAATCCCTGGGCTTAACCTAGGAACTGCATTCGATACTGATCGGCTAGAGTATGAGAGAGGGAGGTAGAATTCCACGTGTAGCGGTGAAATGCGTAGATATGTGGAGGAATACCGGTGGCGAAGGCGGCCTCCTGGCTTAATACTGACGCTGAGGTGCGAAAGCGTGGGGAGCAAACAGG` |
| ASV2932 | 430 | `TGGGGAATATTGGACAATGGGCGAAAGCCTGATCCAGCGACGCCGCGTGAGTGATGAAGGCCTTCGGGTTGTAAAGCTCTGTTGAGGGAGAAGAAAACAGTTGGAGCGAATAGGTCTAACTCTTGACGGTATCCCTTTAGAAAGCACCGGCTAACTCTGTGCCAGCAGCCGCGGTAAGACAGAGGGTGCAAACGTTGCTCGGAATTACTGGGCGTAAAGCGCGTGTAGGCGGACCTGTAAGTTGGACGTGAAAGCCCTGGGCTCAACCCAGGAATTGCATCCAAAACTACAGGTCTAGAGTGATGGAGAGGAAAGCAGAATTCCTGGTGTAGAGGTGAAATTCGTAGATATCAGGAGGAACACCGGTGGCGAAGGCGGCTTTCTGGACATCAACTGACGCTGAGACGCGAAAGCGTGGGGAGCAAACAGG` |
| ASV1007 | 429 | `TGGGGAATTTTGGACAATGGGCGAAAGCCTGATCCAGCAATGCCGCGTGAGTGAAGAAGGCCTTCGGGTTGTAAAGCTCTTTTGTCAGGGAAGAAACGGTTCGGGCTAATATCCTGAGCTAATGACGGTACCTGAAGAATAAGCACCGGCTAACTACGTGCCAGCAGCCGCGGTAATACGTAGGGTGCAAGCGTTAATCGGAATTACTGGGCGTAAAGCGTGCGCAGGCGGTTTTGTAAGTCTGACGTGAAATCCCCGGGCTCAACCTGGGAATTGCGTTGGAGACTGCAAGGCTAGAATCTGGCAGAGGGGGGTAGAATTCCACGTGTAGCAGTGAAATGCGTAGAGATGTGGAGGAACACCGATGGCGAAGGCAGCCCCCTGGGTCAAGATTGACGCTCATGCACGAAAGCGTGGGGAGCAAACAGG` |
| ASV5566 | 423 | `TAGGGAATATTGGGCAATGGTCGGCAGACTGACCCAGCCATGCCGCGTGCAGGATGACGGCGCTATGCGTTGTAAACTGCTTTTATTAGGGAAGAAAATGGCTATGCGTAGCAACTTGACGGTACCTAAAGAATAAGCATCGGCTAACTCCGTGCCAGCAGCCGCGGTAATACGGAGGATGCAAGCGTTGTCCGGATTTATTGGGTTTAAAGGGTGCGTAGGCTGTTTTATAAGTCAGTGGTTAAATTATTTTGCTTAACAAGAGACCTGCCATTGATACTGTAAAACTTGAATACTGACTAGGTAGGCGGAATTGGTAGTGTAGCGGTGAAATGCTTAGATATTACCAGGAACACCGATAGCGAAAGCAGCTTACTGGAGAGTAATTGACGCTGAGGCACGAAAGTGCGGGTATCAAACAGG` |
| ASV4274 | 404 | `TGGGGAATATTGGACAATGGGGGAAACCCTGATCCAGCGATGCCGCGTGAGTGAAGAAGGCCTTCGGGTTGTAAAGCTCTTTTGCCTGTGACGATGATGACAGTAACAGGAGAATAAGCCCCGGCTAACTCCGTGCCAGCAGCCGCGGTAAGACGGAGGGGGCTAGCGTTGTTCGGAATGACTGGGCGTAAAGGGCGCGTAGGCGGCAAATCAAGTCAGGTGTGAAAGCCTTGGGCTCAACCCAAGAATAGCGCTTGATACTGGTTAGCTAGAGGATGGAAGAGAAAAGCGGAATTCCTAGTGTAGAGGTGAAATTCGTAGATATTAGGAAGAACACCAGAGGCGAAGGCGGCTTTTTGGTCCATATCTGACGCTGAGGCGCGAAAGCGTGGGGAGCAAACAGG` |
| ASV4458 | 404 | `TAGGGAATTTTGCGCAATGGGCGAAAGCCTGACGCAGCAATTCCGCGTGGAGGATGAAGTTTCTCGGAACGTAAACTCCTTTCGGTAGTGACGATTATGACGGTAACTACAAAAGAAGCACCGGCTAACTACGTGCCAGCAGCCGCGGTAATACGTAGGGTGCAAGCGTTGTCCGGAATTATTGGGCGTAAAGAGTTCGTAGGCGGTTTACCAAGTCCGTTGTTAAAGGTCGAGGCTCAACCTCGGTATGGCATCGGAAACTGGTAGGCTAGAGTGCAGTAGAGGCAAGGGGAATTCCCGGTGTAGCGGTGAAATGCGTAGATATCGGGAGGAACACCAGTGGCGAAAGCGCCTTGCTGGGCTGTAACTGACGCTGAGGAACGAAAGCTAGGGTAGCGAAAGGG` |
| ASV1811 | 429 | `TGGGGAATTTTGGACAATGGACGCAAGTCTGATCCAGCCATGCCGCGTGCGGGAAGAAGGCCTTCGGGTTGTAAACCGCTTTTGTCAGGGAAGAAACGGCCTGGGATAATACCTTGGGCTAATGACGGTACCTGAAGAATAAGCACCGGCTAACTACGTGCCAGCAGCCGCGGTAATACGTAGGGTGCAAGCGTTAATCGGAATTACTGGGCGTAAAGCGTGCGCAGGCGGTTATGCAAGACAGATGTGAAATCCCCGGGCTCAACCTGGGAACTGCATTTGTGACTGCATAGCTAGAGTACGGTAGAGGGGGATGGAATTCCGCGTGTAGCAGTGAAATGCGTAGATATGCGGAGGAACACCGATGGCGAAGGCAATCCCCTGGACCTGTACTGACGCTCATGCACGAAAGCGTGGGGAGCAAACAGG` |
| ASV5516 | 429 | `TGGGGAATTTTGGACAATGGGGGAAACCCTGATCCAGCGATGCCGCGTGGGTGAAGAAGGCCTGCGGGTTGTAAAGCCCTTTTAGTGAGGACGAAATGCGCTGACCTAATACGTCGGCGAGTTGACTTAACTCACAGAAAAAGCACCGGCTAACTCCGTGCCAGCAGCCGCGGTAATACGGAGGGTGCGAGCGTTAATCGGAATTACTGGGCGTAAAGCGCACGTAGGCGGTCAAGTAAGTCAGTTGTGAAAGCCCCGGGCTTAACCTGGGAATTGCAGCTGATACTGCTCGACTAGAGTGTGGTAGAGGAAAGCGGAATTCCCGGTGTAGCGGTGAAATGCGTAGATATCGGGAGGAACACCAGTGGCGAAGGCGGCTTTCTGGACCAACACTGACGCTCAGGTGCGAAAGCGTGGGGAGCAAACAGG` |
| ASV2900 | 428 | `TCGAGAATTTTTCACAATGGGCGAAAGCCTGATGGAGCGACGCCGCGTGGGGGATGAAGGGCTTCGGCTCGTAAACCCCTGTCATTCGCGAACAAACCCTGCGGCTTAACAGGCTGCAGGCTGATAGTAGCGGAAGAGGAAGGGACGGCTAACTCTGTGCCAGCAGCCGCGGTAATACAGAGGTCCCAAGCGTTGTTCGGATTCACTGGGCGTAAAGGGTGCGTAGGCGGCGAGGAAAGTTCTAGGTGAAATCTCCAGGCTCAACTTGGAAAATGCCTAGAAAACTTCCTCGCTAGAGGGTTGCAGAGGAGATTGGAATTCTCGGTGTAGCAGTGAAATGCGTAGATATCGAGAGGAACATCAGTGGCGAAAGCGAATCTCTGGGCAACATCTGACGCTGAGGCACGAAAGCCAGGGGAGCAAACGGG` |
| ASV2667 | 404 | `TGAGGAATATTGGACAATGGGGGAAACCCTGATCCAGCCATGCCGCGTGAGTGAAGAAGGCCTTAGGGTTGTAAAGCTCTTTCAATAGTGAAGATAATGACGGTAGCTATAGAAGAAGCCCCGGCTAAATTCGTGCCAGCAGCCGCGGTAATACGAATGGGGCAAGCGTTGTTCGGAATCACTGGGCTTAAAGCGTGCGCAGGCGGATTTGCAAGTCAGGAGTGAAATCCTTGGGCTCAACCCAAGAATTGCTTCTGAAACTGCATATCTAGAATCCGGTAGAGGTCAGGAGAATTCCTAGTGTAGAGGTAAAATTCGCAGATATTAGGAGGAATACCAGTGGCGAAGGCGCCTGACTGGGCCGGTATTGACGCTCATGTACGAAAGCGTGGGGAGCAAACAGG` |
| ASV1087 | 424 | `TGAGGAATATTGGACAATGGGCGCAAGCCTGATCCAGCCATGCCGCGTGCAGGATGACGGTCCTATGGATTGTAAACTGCTTTTGCACAGGAAGAAACACTCCCTCGTGAGGGAGCTTGACGGTACTGTGAGAATAAGGATCGGCTAACTCCGTGCCAGCAGCCGCGGTAATACGGAGGATCCAAGCGTTATCCGGAATCATTGGGTTTAAAGGGTCCGTAGGCGGTTTAGTAAGTCAGTGGTGAAAGCCCATCGCTCAACGGTGGAACGGCCATTGATACTGCTAAACTTGAATTATTAGGAAGTAACTAGAATATGTAGTGTAGCGGTGAAATGCTTAGAGATTACATGGAATACCAATTGCGAAGGCAGGTTACTACTAATGGATTGACGCTGATGGACGAAAGCGTGGGTAGCGAACAGG` |
| ASV817 | 404 | `TGGGGAATCTTGGACAATGGGCGCAAGCCTGATCCAGCCATGCCGCGTGGATGATGAAGGCCTTCGGGTTGTAAAATCCTTTCAACGGGGACGATAATGACGGTACCCGTAGAAGAAGCCCCGGCTAACTTCGTGCCAGCAGCCGCGGTAATACGAAGGGGGCTAGCGTTGCTCGGAATTACTGGGCGTAAAGCGCACGTAGGCGGATATTTAAGTCGGAGGTGAAATCCCAGGGCTCAACCCTGGAACTGCCCTCGATACTGGGTATCTGGACCAAAGTAGAGGTTAGTGGAATTCCGAGTGTAGAGGTGAAATTCGTAGATATTCGGAGGAACACCAGTGGCGAAGGCGACTAACTGGACTTTTGGTGACGCTGAGGTGCGAAAGCGTGGGGAGCAAACAGG` |
| ASV3317 | 404 | `TGGGGAATATTGGACAATGGGCGAAAGCCTGATCCAGCCATGCCGCGTGAGTGATGAAGGCCTTAGGGTTGTAAAGCTCTTTTGTGGGGGAAGATGATGACGGTACCCCAAGAATAAGCACCGGCTAACTTCGTGCCAGCAGCCGCGGTAATACGAAGGGTGCTAGCGTTGTTCGGAATCACTGGGCGTAAAGCGCGCGTAGGCGGCTTTACAAGTCAGAGGTGAAATCCCCAAGCTTAACTTGGGAACTGCCTTTGAAACTGTAAAGCTGGAGTGTCGGAGGGGATAGCGGAATTGCTAATGTAGCGGTGAAATGCGTAGATATTAGCAGGAACACCGGTGGCGAAGGCGGCTATCTGGACGACAACTGACGCTGAGGCGCGAAAGCGTGGGGATCAAACAGG` |
| ASV2854 | 430 | `TGGGGAATCTTGCACAATGGGCGAAAGCCTGATGCAGCAACGCCGCGTGAGTGAAGAAGGTCTTTGGATTGTAAAGCTCTTTCGGCTAGAAAGAAGGGTACGTTAGTTAATACCTAATGTACTTGACGGTACTAGAAGAAGAAGCACCGGCTAATTTCGTGCCAGCAGCCGCGGTAATACGAAAGGTGCAAGCGTTGTTCGGAATGACTGGGCGTAAAGCGCACGTAGGTGGGAGGATAAGTCGGATGTGAAAGCCCTGGGCTCAACCTGGGAATGGCATTCGAAACTATCTTTCTTGAATACTCGAGAGGTTGGTAGAATTCCTGGTGTAGGAGTGAAATCCGTAGATATCAGGAGGAAGATCGGTGGCGAAGGCGGCCGACTGGCGATGTATTGACACTGAGGTGCGAAAGCGTGGGGAGCAAACAGG` |
| ASV2818 | 430 | `TCGGGAATTTTGCACAATGGAGGCAACTCTGATGCAGCAATGCCGCGTGAGTGAAGAAGGCCCTTGGGTCGTAAAGCTCTTTCGGCCGGGAAGAAGGGTTTCCAAGCGAATAGGTTGGGAATTTGACGGTACCGGAAGAAGAAGCACCTGCTAACTTCGTGCCAGCAGCAGCGGTAATACGAAGGGTGCAAGCGTTGTTCGGAATTATTGGGCGTAAAGGGTGCGTAGGCGGAAACATATGTCGATTGTGAAATCCCTGGGCTCAACCTGGGAATTGCAGTCGAAACTGTGTTTCTTGAATGCCTGAGAGGATGGCGGAATAACCGGTGTAGTAGTGAAATACGTAGATATCGGTTAGAACACCTGAGGCGAAGGCGGCTATCTGGCAGTGCATTGACGCTGAGGCACGAAAGCGTGGGGAGCAAACAGG` |
| ASV566 | 404 | `TGGGGAATATTGGACAATGGGCGAAAGCCTGATCCAGCAATGCCGCGTGAGTGATGAAGGCCTTAGGGTTGTAAAGCTCTTTTACCAGGGATGATAATGACAGTACCTGGAGAATAAGCTCCGGCTAACTCCGTGCCAGCAGCCGCGGTAATACGGAGGGAGCTAGCGTTGTTCGGAATTACTGGGCGTAAAGCGTACGTAGGCGGTTATTCAAGTCAGAGGTGAAAGCCTGGAGCTCAACTCCAGAACTGCCTTTGAAACTAGATAACTAGAATCACGGAGAGGTTAGTGGAATTCCGAGTGTAGAGGTGAAATTCGTAGATATTCGGAAGAACACCAGTGGCGAAGGCGACTAACTGGACGTGTATTGACGCTGAGGTACGAAAGCGTGGGGAGCAAACAGG` |
| ASV3555 | 418 | `TAGGGAATTTTGCGCAATGGGGGCAACCCTGACGCAGCAACGCCATGTGAGGGATGAAGCATTTCGGTGTGTAAACCTCTGTCAGCAGGGACTAAACGGCCATTCGGTCTTTGAATGTACCTGCAAAGGAAGCCCCGGCTAACTTCGTGCCAGCAGCCGCGGTAATACGAGGGGGGCTAGTGTTGTTCGGAATCACTGGGCGTAAAGGGAGCGTAGGCGGGAGCCTAAGTTGGGTGTTTAATTCCGGGGCCCAACCCCGGGCGTGCACTCAAAACTGGGTTTCTTGAATAGGGGAGAGGTCGGTGGAATTGGAGGTGTAGCGGTGGAATGCGTAGAGATCTCCAAGAACACCCGTTGCGAAGGCGGCCGACTGGACCCATATTGACGCTGAGGCTCGAAAGCATGGGGAGCAAACAGG` |
| ASV4247 | 422 | `TAGGGAATATTGGGCAATGGAGGCAACTCTGACCCAGCCATGCCGCGTGCAGGAAGAAGGCCCTCTGGGTTGTAAACTGCTTTTGAACGGGAAGAAAAGCCCATGCGTGGGTCTATGACGGTACCGTTAGAATAAGCCACGGCTAACTACGTGCCAGCAGCCGCGGTAATACGTAGGTGGCAAGCGTTGTCCGGATTTATTGGGTTTAAAGGGTGCGTAGGCGGCCCCGTAAGTCAGTGGTGAAATACCGCAGCTCAACTGTGGGGGTGCCATTGATACTGCGGGGCTTGAGTGGGGTCGGGGTGGCCGGAATTTACGGTGTAGCGGTGAAATGCATAGATACCGTAAAGAACACCGATAGCGAAGGCAGGCCACTGGGCCCCAACTGACGCTGAGGCACGAAAGCGTGGGGATCAAACAGG` |
| ASV2066 | 429 | `TGGGGAATTTTGGACAATGGGCGCAAGCCTGATCCAGCAATGCCGCGTGCAGGATGAAGGCCTTCGGGTTGTAAACTGCTTTTGTACGGAGCGAAAAGGCTTCCACTAATACTGGGGGCTCATGACGGTACCGTAAGAATAAGCACCGGCTAACTACGTGCCAGCAGCCGCGGTAATACGTAGGGTGCAAGCGTTAATCGGAATTACTGGGCGTAAAGCGTGCGCAGGCGGTTATGTAAGACAGATGTGAAATCCCCGGGCTTAACCTGGGAACTGCATTTGTGACTGCATGGCTAGAGTACGGTAGAGGGGGATGGAATTCCGCGTGTAGCAGTGAAATGCGTAGATATGCGGAGGAACACCGATGGCGAAGGCAATCCCCTGGACCTGTACTGACGCTCATGCACGAAAGCGTGGGGAGCAAACAGG` |
| ASV3522 | 404 | `TAGGGAATATTGGACAATGGGGGCAACCCTGATCCAGCAATGCCGCGTGAGTGATGAAGGCCTTAGGGTTGTAAAGCTCTTTTGGCAGGGACGATGATGACGGTACCTGCAGAATAAGCCCCGGCTAACTTCGTGCCAGCAGCCGCGGTAATACGAAGGGGGCTAGCGTTGTTCGGAGTTACTGGGCGTAAAGGGCGCGTAGGCGGCCTTCCAAGTCGGGCGTGAAATCCCTGGGCTCAACCCGGGAATGGCGCACGAGACTGGCGGGCTTGAGTGCGGGAGAGGATGGTGGAATTCCCAGTGTAGAGGTGAAATTCGTAGATATTGGGAAGAACACCGGTGGCGAAGGCGGCCATCTGGTCCGCAACTGACGCTGAGGCGCGAAAGCGTGGGGAGCAAACAGG` |
| ASV4024 | 404 | `TGAGGAATATTGGACAATGGGCGAAAGCCTGATCCAGCGACGCCGCGTGAGTGATGAAGGCCTTAGGGTTGTAAAGCTCTTTTAGTGGGGAAGATAATGACGGTACCCACAGAAAAAGCCCCGGCTAACTTCGTGCCAGCAGCCGCGGTAATACGAAGGGGGCTAGCGTTACTCGGAATTATTGGGCGTAAAGCGTGCGTAGGCGGTTTAGTAAGTCAGAAGTGAAAGCCTTGAGCTCAACTTGAGAATTGCTTTTGAAACTACTAAACTTGAGTATTGGAGAGGATAGCGGAATTCCTAGTGTAGGGGTGAAATCCGTAGATATTAGGAGGAACACCGGTGGCGAAAGCGGCTATCTGGACAATAACTGACGCTGTTGCACGAAAGCGTGGGGAGCAAACAGG` |
| ASV3371 | 429 | `TGGGGAATTTTGGACAATGGGCGCAAGCCTGATCCAGCCATTCCGCGTGCAGGATGAAGGCCTTCGGGTTGTAAACTGCTTTTGTACGGAGCGAAAAGGCTCTCTCTAATACAGGGGGCTCATGACGGTACCGTAAGAATAAGCACCGGCTAACTACGTGCCAGCAGCCGCGGTAATACGTAGGGTGCAAGCGTTAATCGGAATTACTGGGCGTAAAGCGTGCGCAGGCGGTTATGTAAGACAGAGGTGAAATCCCCGGGCTCAACCTGGGAACTGCCTTTGTGACTGCATAGCTAGAGTACGGCAGAGGGGGATGGAATTCCGCGTGTAGCAGTGAAATGCGTAGATATGCGGAGGAACACCGATGGCGAAGGCAATCCCCTGGGCCTGTACTGACGCTCATGCACGAAAGCGTGGGGAGCAAACAGG` |
| ASV5381 | 430 | `TGGGGGATATTGCGCAATGGGCGAAAGCCTGACGCAGCGACGCCGCGTGGGTGATGAAGGCCTTCGGGTCGTAAAGCCCTGTCGAGGGGAACGAAACGGTCACGACCTAACACGTCGTGGCCCTGACGGTACCCCTAAAGGAAGCACCGGCTAACTCCGTGCCAGCAGCCGCGGTAATACGGAGGGTGCGAGCGTTGTTCGGAATCACTGGGCGTAAAGGGCGTGTAGGCGGTCTGCTAAGTCTGATGTGAAAGCCCGGGGCTCACCCCCGGAAGGGCATTGGAAACTGGCGGACTCGAGTCCCGGAGAGGAGAGTGGAATTCCTGGTGTAGCGGTGAAATGCGTAGATATCAGGAGGAACACCGGTGGCGAAGGCGGCTCTCTGGACGGTGACTGACGCTGAGACGCGAAAGCGTGGGGAGCAAACAGG` |
| ASV4552 | 406 | `TGGGGAATTTTCCGCAATGGGCGAAAGCCTGACGGAGCAATACCGCGTGAGGGACGACGGCTTTTGGGTTGTAAACCTCTTTTCCCTGGGAAGAAGATCTGACGGTACCAGGGGAATCAGCATCGGCTAACTTCGTGCCAGCAGCCGCGGTAATACGAGGGATGCAAGCGTTATCCGGAATTATTGGGCGTAAAGCGTCCGCAGGTGGCTCTGTAAGTCTGTCGTTAAAGAATGGGGCTCAACCCCATACAGGCGATGGAAACTACAAGGCTAGAGTTTGGTAGGGGTCACGGGAATTCTTAGTGTAGCGGTGAAATGCGTAGATATTAAGAAGAACACCAGCGGCGAAAGCGCGTGACTGGGCCAGTACTGACACTCATGGACGAAAGCTAGGGGAGCGAAAGGG` |
| ASV486 | 429 | `TCGAGAATCATTCACAATGGGGGCAACCCTGATGGTGCAACGCCGCGTGGAGGATGAAGGTCTTCGGATTGTAAACTCCTGTCATCTAGGAGCAAGGCATGGTGGTGAATAGCCAACATGATTGATAGTACTAGAAGAGGAAGGGACGGCTAACTTCGTGCCAGCAGCCGCGGTAATACGAAGGTCCCGAGCGTTATTCGGAATCACTGGGCGTAAAGGGAGCGTAGGCGGCGTGGAAAGTCAGATGTGAAATCCCGGGGCTCAACCCCGGAACTGCATCCGATACTACCATGCTAGAGTATTGGAGGGGTAACTGGAATTCTCGGTGTAGCAGTGAAATGCGTGGATATCGAGAGGAACACTCGTGGCGAAAGCGAGTTACTGGACAATTACTGACGCTGAGGCTCGAAGGCTAGGGGAGCAAAAGGG` |
| ASV3994 | 428 | `TCGAGAATCTTTCACAATGGGGGCAACCCTGATGGAGCGACGCCGCGTGGAGGATAAGGTCTTCGGATTGTAAACTCCTGTCATGCGAGAACAAGGTAGTCAGGTTAATAACCTGGCTGCTTGATGGTACCGCAAGAGGAAGAGACGGCTAACTCTGTGCCAGCAGCCGCGGTAATACAGAGGTCTCAAGCGTTGTTCGGAATCACTGGGCGTAAAGGGTGCGTAGGCTGCGCCGTAAGTCAGATGTGAAAGCTCAGGGCTCAACCCTGAAATTGCATCCGATACTGCGGTGCTAGAGAAATGAAGGGGAGTCTAGAATTTTCGGTGTAGCAGTGAAATGCGTAGATATCGAAAGGAATACCAAAGGCGAAGGCAGGACTCTGGGCATTTTCTGACGCTCATGCACGAAGGCTAGGGTAGCGAACGGG` |
| ASV2858 | 404 | `TGGGGAATATTGGACAATGGGCGCAAGCCTGATCCAGCCATGCCGCGTGAGTGATGAAGGCCTTAGGGTTGTAAAGCTCTTTCGTCCGGGACGATAATGACGGTACCGGAAGAAGAAGCCCCGGCTAACTTCGTGCCAGCAGCCGCGGTAATACGAAGGGGGCTAGCGTTGTTCGGAATCACTGGGCGTAAAGGGCGCGTAGGCGGCTTTATAAGTCGGGGGTGAAAGCCTGTGGCTCAACCACAGAATTGCCTTCGATACTGTAGAGCTTGAGACCGGAAGAGGTAAGTGGAACTGCGAGTGTAGAGGTGAAATTCGTAGATATTCGCAAGAACACCAGTGGCGAAGGCGGCTTACTGGTCCGGTTCTGACGCTGAGGCGCGAAAGCGTGGGGAGCAAACAGG` |
| ASV3981 | 410 | `TTTGGAATTTTGGACAATGGACGAAAGTCTGATCCAGCGACGCCGCGTGCACGAAGAAGGCCTTCGGGTTGTAAAGTGCTTTTGCTTGGGAAAAATTTTTGATTGTACCAAGAGAATAAGAGGTTGCTAACTCTGTGCCAGCAGCAGCGGTAATACAGAGACCTCAAGCGTTATCCGGAATCATTGGGCGTAAAGCGTACCGATAGGTGGTTTTGAAAGTTTTGGGTAAAAGCCAGAAGCTCAACTTCTGAGTTTGCTCAAAATACTGCAAGACTAGAGGGGCAAAGAGGTGCTTGGAACGAACGGTGTAGTAGTGAAATGCGTTGATATCGTTCGGAACACCGAAAGCGAAGGCAGAGCACTGGGTGCCACCTGACACTGCTAGGACGAGAGCGTGGGGAGCGAATGGG` |
| ASV546 | 404 | `TGGGGAATATTGGACAATGGGCGAAAGCCTGATCCAGCAATGCCGCGTGAGTGATGAAGGCCTTCGGGTCGTAAAGCTCTTTTACCAGGGATGATAATGACAGTACCTGGAGAATAAGCTCCGGCTAACTCCGTGCCAGCAGCCGCGGTAATACGGAGGGAGCTAGCGTTGTTCGGAATTACTGGGCGTAAAGAGTACGTAGGCGGTTATTCAAGTCAGAGGTGAAAGCCCGGGGCTCAACCCCGGAACTGCCTTTGAAACTAGATAGCTAGAGTCTTGGAGAGGTTAGTGGAATTCCGAGTGTAGAGGTGAAATTCGTAGATATTCGGAAGAACACCAGTGGCGAAGGCGACTAACTGGACAAGTACTGACGCTGAGGTACGAAAGCGTGGGGAGCAAACAGG` |
| ASV2480 | 404 | `TGGGGAATATTGGACAATGGGGGAAACCCTGATCCAGCAATGCCGCGTGAATGAAAAAGGCCTTCGGGTTGTAAAGTTCTTTCGCCTGTGACGATAATGACGGTAGCAGGAAAAGAAGTCCCGGCTAACTCCGTGCCAGCAGCCGCGGTAATACGGAGGGGACTAGCGTTGCTCGGAATGACTGGGCGTAAAGGGTGCGTAGGCGGTTCTTTAAGTAAGGCGTGAAAGCCCTGGGCTTAACCCAGGAAGTGCGTTTTAAACTGAAGAGCTAGAGTGTGAGAGAGGAAAGTGGAATTCCTAGTGTAGAGGTGAAATTCGCAGATATTAGGAAGAACGCCAGAGGCGAAAGCGGCTTTCTGGCTCACAACTGACGCTGAGGCACGAAAGCGTGGGGAGCAAACAGG` |
| ASV3210 | 424 | `TAGGGAATATTGGGCAATGGATGCAAGTCTGACCCAGCCATGCCGCGTGCAGGAAGAAGGTCCTCTGGATTGTAAACTGCTTTTTTCGGGGAAGAAGAGTTCTCTTGCGAGGGAATGTGACGGTACCCGATGAATAAGCCACGGCTAACTACGTGCCAGCAGCCGCGGTAATACGTAGGTGGCAAGCGTTGTCCGGATTTATTGGGTTTAAAGGGTGCGTAGGTGGTTTGATAAGTCAGTGGTGAAAGCTGGTTGCTCAACAATCAAGTTGCCATTGATACTGTCGGACTTGAGAGAAGTGGAGGTTCATGGAATGGATGGTGTAGCGGTGAAATGCATAGATATCATCCAGAACGTCGATTGCGAAGGCAGTGGGCTGTACTTTTTCTGACACTGAGGCACGAAAGCGTGGGGATCAAACAGG` |
| ASV2357 | 424 | `TAAGGAATATTGGACAATGGAGGAAACTCTGATCCAGCCATGCCGCGTGAAGGATGAAGGCGCTCAGCGTTGTAAACTTCTTTTGGACGGGAACAAAAGCAATCATTTATGATTGTCTGAGGGTACCGTCAGAATAAGCACCGGCTAACTCCGTGCCAGCAGCCGCGGTAATACGGAGGGTGCAAGCGTTATCCGGAATCACTGGGTTTAAAGGGTGCGTAGGCGGTCCGATAAGTCAGTCGTGAAATCGTGTGGCTTAACCATGCAACTGCGATTGATACTGTTGGACTTGAATCAGGTTGAGGTAGGCGGAATGTGACATGTAGCGGTGAAATGCATAGATATGTCATAGAACACCGATTGCGAAGGCAGCTTACTGGGCCTGAATTGACGCTGAGGCACGAAAGCGTGGGGATCAAACAGG` |
| ASV1368 | 424 | `TAAGGAATATTGGACAATGCCCGCAAGGGTGATCCAGCCATGCCGCGTGCAGGAAGACAGCCCTATGGGTCGTAAACTGCTTTTACACCGGAGAAAACCCCCCTACGTGTAGGGGGCTGATAGTATGGTGAGAATAAGCATCGGCTAACTTCGTGCCAGCAGCCGCGGTAAGACGAAGGATGCAAGCGTTATCCGGATTCATTGGGTTTAAAGGGAGCGTAGGCGGCCCAATAAGTCAGTGGTGAAATCTCCTGGCTTAACTAGGAAATTGCCATTGATACTGTTGGGCTTGAGTACAGTTGCCGTTGGCGGAATATGACATGTAGTGGTGAAATACATAGAGATGTCATAGAACACCGATTGCGAAGGCAGCTAACGAAACTGTAACTGACGCTGAGGCTCGAAAGTGCGGGGATCAAACAGG` |
| ASV4683 | 429 | `TGGGGAATTTTGGACAATGGGGGCAACCCTGATCCAGCCATCCCGCGTGTGCGATGAAGGCCTTCGGGTTGTAAAGCACTTTTGTTAGGGAAGAAAAGGCGCTGGTTAATACCTGGCGCTGCTGACGGTACCTGACGAATAAGCACCGGCTAACTACGTGCCAGCAGCCGCGGTAATACGTAGGGTGCAAGCGTTAATCGGAATTACTGGGCGTAAAGCGTGCGCAGGCGGTTCGGAAAGAAGGATGTGAAATCCCAGGGCTTAACCTTGGAATGGCATTTTTAACTACCGGGCTAGAGTGTGTCAGAGGGAGGTGGAATTCCGCGTGTAGCAGTGAAATGCGTAGATATGCGGAGGAACATCGATGGCGAAGGCAGCCTCCTGGGATAACACTGACGCTCATGCACGAAAGCGTGGGGAGCAAACAGG` |
| ASV5318 | 404 | `TGGGGAATATTGGACAATGGGGGAAACCCTGATCCAGCAATGCCGCGTGAGTGATGAAGGCCTTCGGGTTGTAAAGCTCTTTTGCCGATGAAGATAATGACTGTAGTCGGAGAATAAGCCCCGGCAAACTTCGTGCCAGCAGCCGCGGTAATACGAAGGGGGCTAGCGTTGTTCGGAATTACTGGGCGTAAAGGGCGCGTAGGCGGTTATGTAAGTCAGAAGTGAAAGCCCCGGGCTCAACCTGGGAATTGCTTTTGAGACTGCATAGCTTGAGTACAGGAGAGGTGAGTGGAATTCCAAGTGTAGAGGTGAAATTCGTAGATATTTGGAAGAACACCAGTGGCGAAGGCGACTCACTGGACTGTAACTGACGCTGAGGCGCGAAAGCGTGGGGAGCAAACAGG` |
| ASV1148 | 429 | `TGGGGAATTTTGGACAATGGGCGCAAGCCTGATCCAGCCATGCCGCGTGTGGGAAGAAGGCCTTCGGGTTGTAAACCACTTTTGTCAGGGAAGAAACGGTCTGATCTAATAAATCGGACTAATGACGGTACCTGAAGAATAAGCACCGGCTAACTACGTGCCAGCAGCCGCGGTAATACGTAGGGTGCAAGCGTTAATCGGAATTACTGGGCGTAAAGCGTGCGCAGGCGGCTATGCAAGACAGATGTGAAATCCCCGGGCTCAACCTGGGAACTGCATTTGTGACTGCATGGCTAGAGTACGGCAGAGGGGGATGGAATTCCGCGTGTAGCAGTGAAATGCGTAGATATGCGGAGGAACACCGATGGCGAAGGCAATCCCCTGGGCCTGTACTGACGCTCATGCACGAAAGCGTGGGGAGCAAACAGG` |
| ASV4562 | 424 | `TAAGGAATATTGGACAATGGACGCAAGTCTGATCCAGCCATGCCGCGTGAAGGATTAAGGTCCTCTGGATTGTAAACTTCTTTTATTTGGGAAGAAAAAAGGGAATTCTTTCTCTCTTGACGGTACCAAACGAATAAGCACCGGCTAACTCCGTGCCAGCAGCCGCGGTAATACGGAGGGTGCAAGCGTTATCCGGATTTACTGGGTTTAAAGGGTGCGTAGGCGGGCAATTAAGTCAGTGGTGAAATCCTTGAGCTTAACTCGAGAACTGCCATTGATACTATTTGTCTTGAATATTGTGGAGGTAAGCGGAATATGTCATGTAGCGGTGAAATGCTTAGATATGACATAGAACACCTATTGCGAAGGCAGCTTACTACGCACATATTGACGCTGAGGCACGAAAGCGTGGGGATCAAACAGG` |
| ASV449 | 404 | `TGGGGAATATTGGACAATGGGCGAAAGCCTGATCCAGCAATACCGCGTGAATGATGAAGGCCTTAGGGTTGTAAAGTTCTTTTAGTTGGGAAGATAATGACGGTACCAACAAAAAAAGCCCCGGCTAACTCCGTGCCAGCAGCCGCGGTAAGACGGAGGGGGCTAGCGTTGTTCGGAATTACTGGGCGTAAAGGGCGCGTAGGCGGATTAGTAAGTTGGGAGTGAAAGCCCGGGGCTTAACCTCGGAACTGCTTTCAAAACTGCTAGTCTTGAGTGAAGTAGGGGATGATGGAATTCCTAGTGTAGAGGTGAAATTCTTAGATATTAGGAGGAACACCGGTGGCGAAGGCGGTCATCTGGACTTCAACTGACGCTGAGGCGCGAAAGCGTGGGGAGCAAACAGG` |
| ASV3572 | 404 | `TGGGGAATATTGGACAATGGGGGAAACCCTGATCCAGCAATGCCGCGTGAGTGAAGAAGGCCTTAGGGTTGTAAAGCTCTTTTGGTAGGGAAGATAATGACGGTACCTATCGAATAAGCACCGGCTAACTCTGTGCCAGCAGCCGCGGTAATACAGAGGGTGCTAGCGTTGTTCGGAATTACTGGGCGTAAAGGGCGCGTAGGTGGCTCTTTAAGTCAGACGTGAAAGGCCTGGGCTTAACCTAGGAACTGCGTTTGATACTGAAGAGCTTGAAGACGGGAGAGGGAAGTGGAATTCCTAGTGGAGAGGTGAAATTCGTAGATATTAGGAGGAACACCGGAGGCGAAGGCGGCTTCCTGGACCGATCTTGACACTGAGGCGCGAAAGCGTGGGGAGCAAACAGG` |
| ASV2118 | 421 | `TGGGGAATATTGCGCAATGGACGAAAGTCTGACGCAGCGACGCCGCGTGTGGGATGAAGCTCTTCGGGGTGTAAACCACTGTTGCCCGGGAAGAATCTCCTGATTCATCAGGATTGACGGTACCGGGTGAGGAAGCACCGGCTAACTCTGTGCCAGCAGCCGCGGTAATACAGAGGGTGCGAGCGTTGTCCGGAATCACTGGGCGTAAAGGGCGCGTAGGTGGCTGGGTAAGTGTGCGGTGAAAGCCCGGGGCTCAACCCCGGGTCGGCCGTGCAGACTGCTTGGCTCGAGCATAGTAGAGGCACGTGGAATTCCGGGTGTAGCGGTGGAATGCGTAGAGATCCGGAAGAACACCGGTGGCGAAGGCGGCGTGCTGGGCTATTGCTGACACTGATGCGCGACAGCGTGGGGAGCAAACAGG` |
| ASV2337 | 429 | `TGGGGAATTTTGGACAATGGGGGCAACCCTGATCCAGCAATGCCGCGTGAGTGAAGAAGGCCTTCGGGTTGTAAAGCTCTTTTGTCAGGGAAGAAACGGTGTGAGCTAATATCTCATGCTAATGACGGTACCTGAAGAATAAGCACCGGCTAACTACGTGCCAGCAGCCGCGGTAATACGTAGGGTGCAAGCGTTAATCGGAATTACTGGGCGTAAAGCGTGCGCAGGCGGTTTTGTAAGACTGTCGTGAAATCCCCGGGCTCAACCTGGGAATGGCGATGGTGACTGCAAGGCTAGAGTTTGGCAGAGGGGGGTAGAATTCCACGTGTAGCAGTGAAATGCGTAGATATGTGGAGGAACACCGATGGCGAAGGCAGCCCCCTGGGTCAAAACTGACGCTCATGCACGAAAGCGTGGGGAGCAAACAGG` |
| ASV5077 | 408 | `TGAGGAATCTTCCGCAATGGGCGAAAGCCTGACGGAGCGACACCGCGTGGAGGATGAAGCCCTTTCCGGGTGTAAACTCCTGTTCTGAGGGACGAAATTTTTGACGGTACCTCAGGAGAAAGCATCGGCTAATTCTGTGCCAGCAGCCGCGGTAAGACAGAAGATGCAAGCGTTACTCGGAATTACTGGGCGTAAAGGGTCCGCAGGTTCTTTGCCACGTCTGGGGTAAAAGCACGTCGCTCAACGGCGTGTACGTCCCGGAAACGAGCAGAGTTGAGCCATTCAGAGGCATCTGGAATGCCGTGTGTAGGGGTAAAATCCGTTGATCCACGGTGGAACGCCGAAAGCGAAAGCAGGATGCTGGGAATGTGCTGACACTCATGGACGAAAGCGTGGGGAGCAAACGGG` |
| ASV3992 | 404 | `TGGGGAATATTGGACAATGGGGGAAACCCTGATCCAGCAATGCCGCGTGAGTGATGAAGGCCTTCGGGTTGTAAAGCTCTTTTATCAGGGACGATAATGACGGTACCTGATGAATAAGCACCGGCTAACTTCGTGCCAGCAGCCGCGGTAATACGAAGGGTGCTAGCGTTGTTCGGAATGACTGGGCGTAAAGGGAGCGTAGGCGGTTGAGTACGTTTGATGTGAAAGTCCTGGGCTTAACCTGGGGACTGCATTGAATACGGCTTGACTTGAGGTATAGAGGGGAAGATGGAATTGCGTGTGTAGAGGTGAAATTCGTAGATATACGCAAGAACACCAGTGGCGAAGGCGATCTTCTGGCTATAACCTGACGCTAAGGCTCGAAAGCGTGGGGAGCAAACAGG` |
| ASV4478 | 423 | `TAGGGAATATTGGACAATGGAGGCAACTCTGATCCAGCCATGCCGCGTGCAGGAAGAAGGCGTTATGCGTTGTAAACTGCTTTTGACAGGGAATAAAAAGACCATGCGTGGTAAATTGAATGTACCTGTAGAATAAGCCACGGCTAACTACGTGCCAGCAGCCGCGGTAATACGTAGGTGGCGAGCGTTATCCGGATTTATTGGGTTTAAAGGGAGCGTAGGCGGTTCTGTAAGTCAGTGGTGAAAGTCGGCCGCTTAACGGTTGGATTGCCATTGATACTGCAGAGCTAGAGTATAGTAGAGGTAGGGAGAATTGACGGTGTAGCGGTGAAATGCATAGATATCGTCAAGAATACCGATTGCGAAGGCACCTTACTGGACTATAACTGACGCTGAGGCTCGAAAGTATGGGAATCAAACAGG` |
| ASV2576 | 404 | `TGGGGAATCTTGGACAATGGGGGAAACCCTGATCCAGCCATGCCGCGTGGACGATGAAGGCCTTAGGGTTGTAAAGTCCTTTCGCCAGGGAAGATAATGACGGTACCTGGTAAAGAAGCCCCGGCTAACTCCGTGCCAGCAGCCGCGGTAATACGGAGGGGGCTAGCGTTGTTCGGAATTACTGGGCGTAAAGCGCACGTAGGCGGATCAGTCAGTCAGGGGTGAAATCCCGGGGCTCAACCCCGGAACTGCCTTTGATACTGCTGGTCTGGAGGTCGAGAGAGGTGAGTGGAATTCCGAGTGTAGAGGTGAAATTCGTAGATATTCGGAGGAACACCAGTGGCGAAGGCGGCTCACTGGCTCGATACTGACGCTGAGGTGCGAAAGCGTGGGGAGCAAACAGG` |
| ASV2727 | 408 | `TAGGGAATATTGGGCAATGGAGGAAACTCTTACCCAGCAATGCCGCGTGAATGAAGAAGGTCTTCGGATTGTAAAGTTCTTTAATTGGGGACGAAGAAAGTGACGGTACCCAAAGAATAAGCTCCGGCTAACTACGTGCCAGCAGCCGCGGTAATACGTAGGGAGCGAGCGTTGTCCGGAATGACTGGGCGTAAAGGGTGTGTAGGCGGTCGATCAAGTTAGAAGTGTAATACCTCGGGCTTAACCCAGGTGCTGCTTCTAAAACTGATAGACTTGAGTGCAGGAGAGGAAAGTGGAATTCCTAGTGTAGCGGTGGAATGCGTAGATATTAGGAGGAACATCAGTGGCGAAGGCGACTTTCTGGACTGTAACTGACGCTGAGGCACGAAAGTGTGGGGAGCAAACAGG` |
| ASV3514 | 424 | `TAAGGAATATTGGGCAATGGAGGCAACTCTGACCCAGCCATGCCGCGTGCAGGATGACGGCCCTATGGGTTGTAAACTGCTTTTGTATAGGAATAAACCCTCGCTCGTGAGCGAGGCTGAATGTACTATAAGAATAAGCATCGGCTAACTCCGTGCCAGCAGCCGCGGTAATACGGAGGATGCAAGCGTTATCCGGATTTATTGGGTTTAAAGGGTGCGTAGGCGGGATAATAAGTCAGTGGTGAAAGCCTACAGCTCAACTGTGGAACTGCCATTGATACTGTTAACCTAGAGTACATTTGATGTGGGCGGAATGTGTCATGTAGCGGTGAAATGCTTAGATATGACACAGAACACCGATTGCGAAGGCAGCTCACAAAACTGTAACTGACGCTGAGGCACGAAAGCGTGGGGAGCAAACAGG` |
| ASV95 | 429 | `TGGGGAATTTTGGACAATGGGGGCAACCCTGATCCAGCAATGCCGCGTGAGTGAAGAAGGCCTTCGGGTTGTAAAGCTCTTTTGTCAGGGAAGAAACGGTGAGTTCTAATACAACTTGCTAATGACGGTACCTGAAGAATAAGCACCGGCTAACTACGTGCCAGCAGCCGCGGTAATACGTAGGGTGCAAGCGTTAATCGGAATTACTGGGCGTAAAGCGTGCGCAGGCGGTTATATAAGTCAGATGTGAAATCCCCGGGCTCAACCTGGGAACTGCATTTGAGACTGTATGGCTAGAGTGTGTCAGAGGGGGGTAGAATTCCACGTGTAGCAGTGAAATGCGTAGAGATGTGGAGGAATACCGATGGCGAAGGCAGCCCCCTGGGATAACACTGACGCTCATGCACGAAAGCGTGGGGAGCAAACAGG` |
| ASV3530 | 396 | `GCTCGAGCGTCCGGGCGCGCCCGGCCTCGACGGCGTCGGCCAGCGCGCTCGGGTAGCCGGTCCACGGCTCGACGATCACGTGATACTCGCCGCGCCATCCGCCGTACGAGGCCCACAGCCAGACCACGGGGAAGATGGTGCGGTCGAACGTGAGCCCGAAGCCGCGCCGGGATTCGGTGTCGGTGCACGCCAGCCAGCCGTCGGAGAGTTCCGTCAGGTAGTGCAGCGTGAAATCGACGGCCTCCGGCGCGAGGACCCGGCGCATGTCGCGGTCCCCGAGCATCGGCCAGGCGTACGTGTCACCGGTGCGCCCCAGGACCCCGCCGCCGTCTTCATCGACCTCCGCCAGCGCGGCAGGGACGTCGATCCGATGCGTGGGTGCGATCCCCTGCACCG` |
| ASV3738 | 429 | `TGGGGAATATTGCGCAATGGGCGAAAGCCTGACGCAGCCATGCCGCGTGTGTGAAGAAGGCCTTCGGGTTGTAAAGCACTTTCAGTGGGGAGGAAAGGGGGATGGCTAATATCCATCACCGGTGACGCTACCCACAGAAGAAGCACCGGCTAATTCCGTGCCAGCAGCCGCGGTAATACGGAAGGTGCAAGCGTTAATCGGAATTACTGGGCGTAAAGCGCGCGTAGGCGGCTTGGTAAGTCGGGTGTGAAAGCCCCGGGCTCAACCTGGGAACGGCACTCGATACTGCTGGGCTGGAGTACGAGAGAGGGGGGTGGAATTCCAGGTGTAGCGGTGAAATGCGTAGAGATCTGGAGGAACACCGGTGGCGAAGGCGGCCCCCTGGCTCGATACTGACGCTGAGGTGCGAAAGCGTGGGGAGCAAACAGG` |
| ASV2384 | 429 | `TGGGGAATTTTGGACAATGGGCGCAAGCCTGATCCAGCAATGCCGCGTGCAGGATGAAGGCCTTCGGGTTGTAAACTGCTTTTGTACGGAACGAAAAGGCTCTGGATAATACCTGGGGCCCATGACGGTACCGTAAGAATAAGCACCGGCTAACTACGTGCCAGCAGCCGCGGTAATACGTAGGGTGCGAGCGTTAATCGGAATTACTGGGCGTAAAGCGTGCGCAGGCGGTAATGCAAGACAGATGTGAAATCCCCGGGCTTAACCTGGGAACTGCATTTGTGACTGCATTGCTGGAGTGCGGCAGAGGGGGATGGAATTCCGCGTGTAGCAGTGAAATGCGTAGATATGCGGAGGAACACCGATGGCGAAGGCAATCCCCTGGGCCTGCACTGACGCTCATGCACGAAAGCGTGGGGAGCAAACAGG` |
| ASV2198 | 424 | `TAAGGAATATTGGTCAATGGAGGCAACTCTGAACCAGCCATGCCGCGTGCAGGAAGACGGCCCTCTGGGTTGTAAACTGCTTTTATTCGGGAATAAACCTACTTACGTGTAAGTAGCTGAATGTACCGAAGGAATAAGGATCGGCTAACTCCGTGCCAGCAGCCGCGGTAATACGGAGGATCCAAGCGTTATCCGGATTTATTGGGTTTAAAGGGTGCGTAGGCGGCCTGTTAAGTCAGGGGTGAAAGACGGTAGCTCAACTATCGCAGTGCCCTTGATACTGATGGGCTTGAATACACTAGAGGTAGGCGGAATGTGACAAGTAGCGGTGAAATGCATAGATATGTCACAGAACACCGATTGCGAAGGCAGCTTACTATGGTGTTATTGACGCTGAGGCACGAAAGCGTGGGGATCAAACAGG` |
| ASV202 | 429 | `TGGGGAATTTTGGACAATGGGCGAAAGCCTGATCCAGCAATGCCGCGTGCAGGATGAAGGCCTTCGGGTTGTAAACTGCTTTTGTACGGAGCGAAAAGGTCTTCTCTAATACAGGGGACTGATGACGGTACCGTAAGAATAAGCACCGGCTAACTACGTGCCAGCAGCCGCGGTAATACGTAGGGTGCAAGCGTTAATCGGAATTACTGGGCGTAAAGCGTGCGCAGGCGGTTTTGTAAGACAGGCGTGAAATCCCCGGGCTCAACCTGGGAACTGCGTTTGTGACTGCAAGGCTGGAGTGCGGCAGAGGGGGATGGAATTCCGCGTGTAGCAGTGAAATGCGTAGATATGCGGAGGAACACCGATGGCGAAGGCAATCCCCTGGGCCTGCACTGACGCTCATGCACGAAAGCGTGGGGAGCAAACAGG` |
| ASV1607 | 429 | `TGGGGAATTTTGGACAATGGGGGCAACCCTGATCCAGCCATTCCGCGTGAGTGAAGAAGGCCTTCGGGTTGTAAAGCTCTTTCGGCAGGAACGAAACGGTGTCCCCTAATACGGGATGCTAATGACGGTACCTGAAGAAGAAGCACCGGCTAACTACGTGCCAGCAGCCGCGGTAATACGTAGGGTGCGAGCGTTAATCGGAATTACTGGGCGTAAAGCGTGCGCAGGCGGTTCTATAAGACAGATGTGAAATCCCCGGGCTCAACCTGGGAACTGCGTTTGTGACTGTAGGACTAGAGTGTGGCAGAGGGGGGTGGAATTCCACGTGTAGCAGTGAAATGCGTAGAGATGTGGAGGAACACCGATGGCGAAGGCAGCCCCCTGGGTTAACACTGACGCTCATGCACGAAAGCGTGGGGAGCAAACAGG` |
| ASV4010 | 406 | `TGGGGAATATTGCACAATGGGCGAAAGCCTGATGCAGCAACGCCGCGTGAGCGAAGAAGGCCTTTGGGTCGTAAAGCTCTGTCGGGAGGGAAGAAACAAATGACGGTACCTTCCGAGGAAGCCCCGGCTAACTACGTGCCAGCAGCCGCGGTAATACGTAGGGGGCAAGCGTTATCCGGAATTATTGGGCGTAAAGAGTGCGTAGGTGGTCTTGTAAGCGTGGGGTGAAAGGCAATAGCTTAACTATTGTAAGCCCTGCGAACTGTGAGACTTGAGTGCAGGAGAGGAAAGTGGAATTCCTAGTGTAGCGGTGAAATGCGTAGATATTAGGAGGAACACCAGTGGCGAAGGCGACTTTCTGGACTGTAACTGACACTGAGGCACGAAAGCGTGGGGAGCAAACAGG` |
| ASV4252 | 406 | `TGGGGAATTTTCCGCAATGGGCGAAAGCCTGACGGAGCAATACCGCGTGAGGGAGGAAGGCTCTTGGGTTGTAAACCTCTTTTCTCAGGGAAGAATAAATGACGGTACCTGAGGAATAAGCATCGGCTAACTCCGTGCCAGCAGCCGCGGTAATACGGAGGATGCAAGCGTTATCCGGAATGATTGGGCGTAAAGAGTCCGTAGGTGGCAATTCAAGTCTGCTGTTAAAGACCGGGGCTCAACCCTGGAAAGGCGGTGGAAACTGAATAGCTAGAGGCTGGTAGGGGTAGAGGGAATTCCCAGTGTAGCGGTGAAATGCGTAGAGATTGGGAAGAACACCGGTGGCGAAAGCGCTCTACTGGACCAGGACTGACACTGAGGGACGAAAGCTAGGGGAGCGAATGGG` |
| ASV3966 | 405 | `TCGAGAATATTCGACAATGGGCGAAAGCCTGATCGAGCGACGCCGCGTGCAGGAAGAAGGGCTTCGGTCTGTAAACTGCTTTTAATGGATAATAAGTTATTGAATATCCATGGAATAAGGGGCTGCAAACCTCGTGCCAGCAGCCGCGGTAATACGAGGGCCCCAAGCGTTATCCGGATTTATTGGGCGTAAAGAGCGAGTAGGTGGTTTTGCGCGTCTGTTGTTAAATGTCTCCGCTTAACGGGGTCACCGCAATGGATACGGCAAAACTAGAGGGTACTAGAGGCAAATAGAACGCACGGTGTAGGGGTGAAATCCGTTGATATCGTGCGGAATACCAAAGGCGAAGGCATTTTGCTGGAGTACTCCTGACACTCAATCGCGAAAGCGTGGGGAGCAAAAAGG` |
| ASV2671 | 404 | `TGGGGAATATTGCACAATGGGCGAAAGCCTGATGCAGCGACGCCGCGTGAGTGAAGAAGTATTTCGGTATGTAAAGCTCTATCAGCAGGGAAGATAATGACGGTACCTGACTAAGAAGCACCGGCTAAATACGTGCCAGCAGCCGCGGTAATACGTATGGTGCAAGCGTTATCCGGATTTACTGGGTGTAAAGGGAGCGTAGGCGGCGCGATAAGTCTGGTGTGAAAGCCCGGGGCTCAACCCCGGGACTGCATTGGAAACTGTCGTGCTTGAGTGTCGGAGAGGTAAGTGGAATTCCTAGTGTAGCGGTGAAATGCGTAGATATTAGGAGGAACACCAGTGGCGAAGGCGACTTACTGGACGATAACTGACGCTGAGGCTCGAAAGCGTGGGGAGCAAACAGG` |
| ASV12 | 429 | `TGGGGAATATTGGACAATGGGCGCAAGCCTGATCCAGCCATGCCGCGTGTGTGAAGAAGGCCTTCGGGTTGTAAAGCACTTTTGTCCGGAAAGAAAAGCTTCGGGTTAATACCCCGGGGTCCTGACGGTACCGGAAGAATAAGCACCGGCTAACTTCGTGCCAGCAGCCGCGGTAATACGAAGGGTGCAAGCGTTACTCGGAATTACTGGGCGTAAAGCGTGCGTAGGTGGTTTGTTAAGTCTGATGTGAAAGCCCTGGGCTCAACCTGGGAATTGCATTGGATACTGGCAAGCTAGAGTGCGGTAGAGGATGGCGGAATTCCCGGTGTAGCAGTGAAATGCGTAGAGATCGGGAGGAACATCTGTGGCGAAGGCGGCCATCTGGACCAGCACTGACACTGAGGCACGAAAGCGTGGGGAGCAAACAGG` |
| ASV1753 | 429 | `TGGGGAATTTTGGACAATGGACGCAAGTCTGATCCAGCCATGCCGCGTGCGGGAAGAAGGCCTTCGGGTTGTAAACCGCTTTTGTCAGGGAAGAAACGGCTTTCTCTAATACAGAGGGCTAATGACGGTACCTGAAGAATAAGCACCGGCTAACTACGTGCCAGCAGCCGCGGTAATACGTAGGGTGCAAGCGTTAATCGGAATTACTGGGCGTAAAGCGTGCGCAGGCGGTTATGCAAGACAGAGGTGAAATCCCCGGGCTCAACCTGGGAACTGCCTTTGTGACTGCATGGCTAGAGTACGGTAGAGGGGGATGGAATTCCGCGTGTAGCAGTGAAATGCGTAGATATGCGGAGGAACACCGATGGCGAAGGCAATCCCCTGGACCTGTACTGACGCTCATGCACGAAAGCGTGGGGAGCAAACAGG` |
| ASV3705 | 404 | `TGGGGAATCTTAGACAATGGGCGCAAGCCTGATCTAGCCATGCCGCGTGATCGATGAAGGCCTTAGGGTTGTAAAGATCTTTCAGGTGGGAAGATAATGACTGTACCACCAGAAGAAGCCCCGGCTAACTCCGTGCCAGCAGCCGCGGTAATACGGAGGGGGCTAGCGTTATTCGGAATTACTGGGCGTAAAGCGCACGTAGGCGGATTGGAAAGTTGGAGGTGAAATCCCAGGGCTCAACCTTGGAACGGCCTTCAAAACTCCCAGTCTTGAGATCGAGAGAGGTGAGTGGAATTCCGAGTGTAGAGGTGAAATTCGTAGATATTCGGAGGAACACCAGTGGCGAAGGCGGCTCACTGGCTCGATACTGACGCTGAGGTGCGAAAGCGTGGGGAGCAAACAGG` |
| ASV1696 | 429 | `TGGGGAATATTGCACAATGGGCGCAAGCCTGATGCAGCCATGCCGCGTGTATGAAGAAGGCCTTCGGGTTGTAAAGTACTTTCAGCGAGGAGGAAGGCATTAAGGTTAATAACCTTAGTGATTGACGTTACTCGCAGAAGAAGCACCGGCTAACTCCGTGCCAGCAGCCGCGGTAATACGGAGGGTGCAAGCGTTAATCGGAATTACTGGGCGTAAAGCGCACGCAGGCGGTCTGTCAAGTCGGATGTGAAATCCCCGGGCTCAACCTGGGAACTGCATTCGAAACTGGCAGGCTAGAGTCTTGTAGAGGGGGGTAGAATTCCAGGTGTAGCGGTGAAATGCGTAGAGATCTGGAGGAATACCGGTGGCGAAGGCGGCCCCCTGGACAAAGACTGACGCTCAGGTGCGAAAGCGTGGGGAGCAAACAGG` |
| ASV4401 | 406 | `TGAGGGATATTGGGCAATGGGCGCAAGCCTGACCCAGCGACGCCGCGTGGAGGAGAAGGGCTTCGGCTCGTAAACTCCTTTTCCCGAGGACGAGAAGCAGGACGGTACTCGGGGAATAAGTCTCGGCTAACTACGTGCCAGCAGCCGCGGTAATACGTAGGAGGCAAGCGTTATCCGGAATTACTGGGCGTAAAGGGCGTCTAGGTGGGCTGCCAAGCGGGGTGTGAAAGCGCCTGGCTTAACTGGGCGAGTGCGCTCTGAACTGGCAACCTAGAGGGCAGGCGACGGAGGTGGAATACCCGGTGGAGTGGTGAAATGCGTAGAGATCGGGTGGAACACCAGTGGCGAAGGCGGCCTTCGAGCCTGTTGCTGACACTGAAGCGCGAGAGCGTGGGGAGCAAACGGG` |
| ASV3796 | 428 | `TGGGGAATATTGGACAATGGGCGCAAGCCTGATCCAGCCACGCCGCGTGAGTGATGAAGGCCTTCGGGTCGTAAAGCTCTGTGGGGAGGGACGAACCGCTGGTGGTGCATAGCCATCAGCATGACGGTACCTCCTTAGCAAGCACCGGCTAACTTCGTGCCAGCAGCCGCGGTAATACGAAGGGTGCAAACGTTGCTCGGAATTATTGGGCGTAAAGCGCACGTAGGCGGCTCCGCAAGTCGGATGTGAAATCCCTCGGCTCAACCAAGGAAGTGCATCCGAAACTGCGAAGCTTGAGTACTTAAGAGGATCGCGGAATTCCCGGTGTAGAGGTGAAATTCGTAGATATCGGGAGGAACACCAGTGGCGAAGGCGGCGATCTGGGAAGATACTGACGCTGAGGTGCGAAAGCGTGGGGAGCAAACAGG` |
| ASV5018 | 423 | `TAGGGAATATTGGACAATGGGTGAGAGCCTGATCCAGCCATGCCGCGTGCAGGAAGAAGGCCTTCTGGGTTGTAAACTGCTTTTATTTGGGGATAAAAGACCCGTGCGCGGGAGACTGAAGGTACCAGATGAATAAGCCACGGCTAACTACGTGCCAGCAGCCGCGGTAATACGTAGGTGGCAAGCGTTGTCCGGATTTATTGGGTTTAAAGGGTGCGTAGGCGGTCTATTAAGTCAGTGGTGAAATACGATAGCTTAACTATCGATGTGCCATTGATACTGATGGACTTGAGTGAACGCGAGGTAGGCGGAATGGACGGTGTAGCGGTGAAATGCATAGATATCGTCCAGAACACCGATAGTGAAGACAGCTTACCAGAGTTCAACTGACGCTGAGGCACGAAAGTGTGGGGATCAAACAGG` |
| ASV425 | 424 | `TGAGGAATATTGGACAATGGGCGCAAGCCTGATCCAGCCATGCCGCGTGCAGGATGACGGTCCTATGGATTGTAAACTGCTTTTGCACAGGAAGAAACCTCCCGACGTGTCGGGACTTGACGGTACTGTGAGAATAAGGATCGGCTAACTCCGTGCCAGCAGCCGCGGTAATACGGAGGATCCAAGCGTTATCCGGAATCATTGGGTTTAAAGGGTCCGTAGGCGGTTTAGTAAGTCAGTGGTGAAAGCCCATCGCTCAACGGTGGAACGGCCATTGATACTGCTAGACTTGAATTATTAGGAAGTAACTAGAATATGTAGTGTAGCGGTGAAATGCTTAGAGATTACATGGAATACCAATTGCGAAGGCAGGTTACTACTAATGGATTGACGCTGATGGACGAAAGCGTGGGTAGCGAACAGG` |
| ASV400 | 429 | `TGGGGAATATTGGACAATGGGCGAAAGCCTGATCCAGCCATGCCGCGTGTGTGAAGAAGGTCTTAGGATCGTAAAGCACTTTAAGTTGGGAGGAAGGGCAGTAACTTAATACGTTGCTGTTTTGACGTTACCGACAGAATAAGCACCGGCTAACTCTGTGCCAGCAGCCGCGGTAATACAGAGGGTGCAAGCGTTAATCGGAATTACTGGGCGTAAAGCGCGCGTAGGTGGTTCGTTAAGTTGGATGTGAAATCCCCGGGCTCAACCTGGGAACTGCATTCAAAACTGTCGAGCTAGAGTATGGTAGAGGGTGGTGGAATTTCCTGTGTAGCGGTGAAATGCGTAGATATAGGAAGGAACACCAGTGGCGAAGGCGACCACCTGGACTGATACTGACACTGAGGTGCGAAAGCGTGGGGAGCAAACAGG` |
| ASV2496 | 404 | `TGGGGAATATTGGACAATGGGCGAAAGCCTGATCCAGCAATGCCGCGTGAGTGATGAAGGCCTTAGGGTTGTAAAGCTCTTTTACTGGGGATGATAATGACAGTACCCAGAGAATAAGCTCCGGCTAACTCCGTGCCAGCAGCCGCGGTAATACGGAGGGAGCTAGCGTTGTTCGGAATTACTGGGCGTAAAGCGCACGTAGGCGGCTCTTTAAGTCAGGGGTGAAATCCCGGGGCTCAACCCCGGAACTGCCCTTGAAACTGGAAAGCTAGAATCCTGGAGAGGTGAGTGGAATTCCGAGTGTAGAGGTGAAATTCGTAGATATTCGGAAGAACACCAGTGGCGAAGGCGACTCACTGGACAGGTATTGACGCTGAGGTGCGAAAGCGTGGGGAGCAAACAGG` |
| ASV3952 | 429 | `TGGGGAATATTGGACAATGGGCGCAAGCCTGATCCAGCCATGCCGCGTGAGTGAAGAAGGCCTTCGGGTTGTAAAGCTCTTTGAGCGGGGAGGAAGGTTCGTTGATTAATAATCAGCGGAATTGACGTTACCTGCAGAACAAGCACCGGCTAACTCTGTGCCAGCAGCCGCGGTAATACAGAGGGTGCGAGCGTTAATCGGAATTACTGGGCGTAAAGCGTGCGTAGGCGGTTTGATAAGCTGGATGTGAAATCCCCGGGCTTAACCTGGGAACTGCATTCAGGACTGTTTGACTAGAGTCTGGGAGAGGGTGGTGGAATTTCCTGTGTAGCGGTGAAATGCGTAGATATAGGAAGGAACATCAGTGGCGAAGGCGACTGCCTGGCCCAAGACTGACGCTGAGGCACGAAAGCGTGGGTAGCAAACAGG` |
| ASV613 | 429 | `TGGGGAATTTTGGACAATGGGCGAAAGCCTGATCCAGCAATGCCGCGTGCAGGATGAAGGCCTTCGGGTTGTAAACTGCTTTTGTACGGAGCGAAAAGGTCTTCTCTAATACAGGGGACTGATGACGGTACCGTAAGAATAAGCACCGGCTAACTACGTGCCAGCAGCCGCGGTAATACGTAGGGTGCAAGCGTTAATCGGAATTACTGGGCGTAAAGCGTGCGCAGGCGGTTTTGTAAGACAGGCGTGAAATCCCCGGGCTTAACCTGGGAACTGCGTTTGTGACTGCAAGGCTGGAGTGCGGCAGAGGGGGATGGAATTCCGCGTGTAGCAGTGAAATGCGTAGATATGCGGAGGAACACCGATGGCGAAGGCAATCCCCTGGGCCTGCACTGACGCTCATGCACGAAAGCGTGGGGAGCAAACAGG` |
| ASV2190 | 424 | `TAAGGAATATTGGTCAATGGGCGCAAGCCTGAACCAGCCATGCCGCGTGCAGGAAGACGGACCTATGGTTTGTAAACTGCTTTTGTACGAGAGAAAACCCTTCTACGTGTAGGAGGTTGATAGTATCGTAAGAATAAGGATCGGCTAACTTCGTGCCAGCAGCCGCGGTAATACGAAGGATCCAAGCGTTATCCGGATTCATTGGGTTTAAAGGGTGCGTAGGCGGGCTGATAAGTCAGCGGTGAAATCTCTCGGCTTAACCGAGAACCTGCCGTTGATACTGTCAGTCTTGAGTATAGATGATGTTGGCGGAATATGACATGTAGCGGTGAAATGCTTAGATATGTCATAGAACACCGATTGCGAAGGCAGCTGACAAAACTATAACTGACGCTGAGGCACGAAAGTGCGGGGATCAAACAGG` |
| ASV821 | 417 | `TTAGGAATCTTCCACAATGGGCGAAAGCCTGATGGAGCGACGCCGCGTGAGGGATGAAGGTTTTCGGATCGTAAACCTCTGAATCAGGGACGAAAGACGCTTTATGCGGGATGACGGTACCTGAGTAATAGCACCGGCTAACTCCGTGCCAGCAGCCGCGGTAATACGGAGGGTGCAAGCGTTACCCGGAATCACTGGGCGTAAAGGGCGTGTAGGCGGGATGTTAAGTCTGGTTTTAAAGACTGCGGCTCAACCGCAGGGATGGACTGGATACTGGCATTCTTGACCTCTGGAGAGAGAACTGGAATTCCTGGTGTAGCGGTGGAATGCGTAGATACCAGGAGGAACACCAATGGCGAAGGCAGGTTCTTGGACAGAAGGTGACGCTGAGGCGCGAAAGTGTGGGGAGCGAACCGG` |
| ASV3698 | 429 | `TGGGGAATATTGGACAATGGGGGAAACCCTGATCCAGCCATGCCGCGTGTGTGAAGAAGGCTCTAGGGTTGTAAAGCACTTTAAGCTGTGAGGAAGGCTCAATGGTTAATAGTCATTGGGATTGACGTTAGCAGCGGAATAAGCACCGGCTAACTCCGTGCCAGCAGCCGCGGTAATACGGAGGGTGCGAGCGTTAATCGGAATTACTGGGCGTAAAGGGCGCGTAGGTTGTTGCTTAAGTGAGATGTGAAAGCCCTGGGCTTAACCTGGGAACTGCATTTCAAACTGGACAACTAGAGTGCGGTAGAGGGAGGTGGAATTTCCGGTGTAGCGGTGAAATGCGTAGAGATCGGAAGGAACACCAGTGGCGAAGGCGACCTCCTGGACCGACACTGACACTGAGGCGCGAAAGCGTGGGTAGCAAACAGG` |
| ASV2990 | 429 | `TGGGGAATTTTGGACAATGGGCGAAAGCCTGATCCAGCAATGCCGCGTGCAGGACGAAGGCCCTCGGGTTGTAAACTGCTTTTGTACGGAAAGAAACGGCAGGCTCTAATACAGCCTGTGACTGACGGTACCGTAAGAATAAGCACCGGCTAACTACGTGCCAGCAGCCGCGGTAATACGTAGGGTGCAAGCGTTAATCGGAATTACTGGGCGTAAAGCGTGCGCAGGCGGTTTTGTAAGTCAGATGTGAAATCCCCGGGCTCAACCTGGGACCTGCATTTGAGACTGCAAAGCTAGAGTGCGGCAGAGGGGGATGGAATTCCGCGTGTAGCAGTGAAATGCGTAGATATGCGGAGGAACACCGATGGCGAAGGCAATCCCCTGGGCCTGCACTGACGCTCATGCACGAAAGCGTGGGGAGCAAACAGG` |
| ASV4043 | 429 | `TGGGGAATATTGGACAATGGGGGCAACCCTGATCCAGCAATGCCGCGTGTGTGAAGAAGGCCTTCGGGTTGTAAAGCACTTTAGGCTGGGAAGAAGGTAAGTAGTTTAAGAGATGACTTATTTGACTGTACCGGCAGAATAAGCACCGGCTAACTCTGTGCCAGCAGCCGCGGTAATACAGAGGGTGCGAGCGTTAATCGGAATTACTGGGCGTAAAGGGCGCGTAGGTGGTTAAATAAGATTGTTGTGAAATCCCTGGGCTTAACCTAGGAACTGCAACGATGACTGTATAGCTTGAGTATTGTAGAGGGTAGTGGAATTTCCGGTGTAGCGGTGAAATGCGTAGAGATCGGAAGGAACACCAGTGGCGAAGGCGGCTACCTGGACAAATACTGACACTGAGGCGCGAGAGCGTGGGGAGCAAACAGG` |
| ASV4806 | 421 | `TCGAGAATATTCCACAATGGGCGAAAGCCTGATGGAGCGACGCCGCGTGCAGGATGACACCCTTCGGGGCGTAAACTGCTTTTCTATGGGAGGAAGTCCGTCTTATGGGCGGATTGACAGTACCGTAGGAATAAGAGGTTGCAAACTTCGTGCCAGCAGCCGCGGTAATACGAAGGCCTCAAGCGTTATCCGGATTTATTGGGCGTAAAGGGCGCGTAGGGGGTTTGGTGTGTTTTTGGTTAAATCCTGGAGCTTAACTTCGGGTCTGCCAAGAAAACTACCAAACTTGAGAGCGTTAGAGGTAAGCGGAACGCACGGTGTAGGGGTGAAATCCGTTGATATCGTGCGGAACACCAAAGGCGAAGGCAGCTTACTGGGACGTTTCTGACCCTGAGGCGCGAAAGCGTGGGGAGCAAAAAGG` |
| ASV4015 | 404 | `TGGGGAATATTGGACAATGGGCGAAAGCCTGATCCAGCAATACCGCGTGAACGATGAAGGCCTTAGGGTTGTAAAGTTCTTTTAGCAGGGAAGATAATGACGGTACCTGCAGAAAAAGCCCCGGCTAACTCCGTGCCAGCAGCCGCGGTAAGACGGAGGGGGCTAGCGTTGTTCGGAATTACTGGGCGTAAAGGGCGCGTAGGCGGCTTAGTAAGTTGGAAGTGAAATCCCGAGGCTTAACCTCGGAACTGCTTTCAAAACTGCTAGGCTAGAGTATAGTAGAGGGTGATGGAATTCCTAGTGTAGAGGTGAAATTCTTAGATATTAGGAGGAACACCAGAAGCGAAAGCGATCACCTGGGCTATAACTGACGCTGATGCGCGAAAGCGTGGGGAGCAAACAGG` |
| ASV4857 | 426 | `TGGGGAATATTGGACAATGGGCGCAAGCCTGATCCAGCAATACCGCGTGTGTGAAGAAGGCCTTAGGGTTGTAAAGCACTTTCAGTTGGGAGGAAACGCACAAGGTGAACCTTGTGAACTGACGTTACCAACAGAAGAAGCACCGGCTAACTCCGTGCCAGCAGCCGCGGTAATACGGAGGGTGCAAGCGTTAATCGGAATTACTGGGCGTAAAGCGCGTGTAGGCGGATAAGTAAGTCAGATGTGAAATCCCTGGGCTCAACCTGGGAACGGCATCTGAAACTGCTAATCTAGAGTGTAGTAGAGGGAAGTGGAATTTCCGGTGTAGCGGTGAAATGCGTAGATATCGGAAGGAACATCAGTGGCGAAGGCGGCTTCCTGGACAAACACTGACGCTGAGATGCGAAAGCGTGGGTAGCGAACGGG` |
| ASV3969 | 405 | `TGGGGAATTTTCCGCAATGGGCGCAAGCCTGACGGAGCAATACCGCGTGAGGGAAGAAGGCTCTTGGGTCGTAAACCTCTTTTGTTTGGGAAGAAACCTGACGGTACCAAACGAATAAGCCTCGGCTAACTCCGTGCCAGCAGCCGCGGTAAGACGGAGGAGGCAAGCGTTATCCGGAATTATTGGGCGTAAAGCGTCCGCAGGTGGTTTTTCAAGTCTGTTGTTAAAGCGCGAGGCTCAACCTTGTATAGGCAATGGAAACTGAAAGACTAGAGTGCGGTAGGGGTAGAGGGAATTCCCGGTGTAGCGGTGAAATGCGTAGATATCGGGAAGAACACCAGTGGCGAAGGCGCTCTACTGGGCCTGCACTGACACTGAGGGACGAAAGCTAGGGGAGCGAAAGGG` |
| ASV1454 | 404 | `TGGGGAATATTGCACAATGGAGGAAACTCTGATGCAGCGACGCCGCGTGAGGGAAGAAGGTTTTCGGATTGTAAACCTCTGTCTTTGGGGACGATAATGACGGTACCCAAAGAGGAAGCACCGGCTAACTACGTGCCAGCAGCCGCGGTAAAACGTAGGGTGCAAGCGTTGTCCGGAATTACTGGGTGTAAAGGGAGCGCAGGCGGAAAGGTAAGTTGGGAGTGAAAACTATGGGCTCAACCCATAAATTGCTTTCAAAACTATTTTTCTTGAGTGGTGCAGAGGTAGGCGGAATTCCCGGTGTAGCGGTGGAATGCGTAGATATCGGGAGGAACACCAGTGGCGAAGGCGGCCTACTGGGCACTAACTGACGCTGAGGCTCGAAAGCATGGGTAGCAAACAGG` |
| ASV1036 | 429 | `TGGGGAATTTTGGACAATGGGCGCAAGCCTGATCCAGCCATGCCGCGTGCGGGAAGAAGGCCTTCGGGTTGTAAACCGCTTTTGTCAGGGAAGAAACGCTCTGGGTTAATACCTCGGGGTAATGACGGTACCTGAAGAATAAGCACCGGCTAACTACGTGCCAGCAGCCGCGGTAATACGTAGGGTGCAAGCGTTAATCGGAATTACTGGGCGTAAAGCGTGCGCAGGCGGTCTTGCAAGACAGATGTGAAATCCCCGGGCTCAACCTGGGAACTGCATTTGTGACTGCAAGGCTGGAGTGCGGCAGAGGGGGATGGAATTCCGCGTGTAGCAGTGAAATGCGTAGATATGCGGAGGAACACCGATGGCGAAGGCAATCCCCTGGGCCTGCACTGACGCTCATGCACGAAAGCGTGGGGAGCAAACAGG` |
| ASV3775 | 430 | `TGGGGAATATTGCGCAATGGGCGAAAGCCTGACGCAGCAACGCCGCGTGAGTGATGAAGGCCTTCGGGTTGTAAAGCTCTTTCGGTTGGGAAGAAGGGTGTGCGGGCGAACAGGTCGTACATTTGATGGTACCAAAAGAAGAAGCACCGGCTAACTTCGTGCCAGCAGCCGCGGTAATACGAAGGGTGCGAGCGTTGTTCGGAATTACTGGGCGTAAAGGGTTCGTAGGCGGGAACTTAAGTCAAGTGTGAAATCCCCAGGCTCAACCTGGGACGTGCACCTGAAACTGAGTTTCTTGAGTGATGGAGAGGGCGATGGAATTGCTGGTGTAGGAGTGACATCCGTAGAGATCAGCAGGAACACCGGAGGCGAAGGCGATCGCCTGGCCATTTACTGACGCTGAGGAACGAAAGCGTGGGGAGCAAACAGG` |
| ASV5449 | 404 | `TGGGGAATATTGGACAATGGGCGCAAGCCTGATCCAGCCATGCCGCGTGAGTGATGAAGGCCTTCGGGTTGTAAAGCTCTTTCGCTAGGGACGATAATGACGGTACCTAGACAAGAAGCCCCGGCTAACTTCGTGCCAGCAGCCGCGGTAATACGAAGGGGGCTAGCGTTGTTCGGAATTACTGGGCGTAAAGCGCACGTAGGCGGATCTTTAAGTCAGAGGTGAAAGCCTGGAGCTCAACTCCAGAACTGCCTTTGATACTGGGGATCTCGAGTTTGGAAGAGGTTGGTGGAACTCCTAGTGTAGAGGTGAAATTCGTAGATATTAGGAAGAACACCAGTGGCGAAGGCGGCCAACTGGTCCAATACTGACGCTGAGGTGCGAAAGCGTGGGGAGCAAACAGG` |
| ASV899 | 404 | `TTAGGAATATTGGACAATGGGCGAAAGCCTGATCCAGCCATGCCGCGTGTGTGATGAAGGCCTTAGGGTTGTAAAGCACTTTCGTTGGGGATGATAATGACCGTACCCAAAGAAGAAGCACCGGCTAACTTCGTGCCAGCAGCCGCGGTAATACGAAGGGTGCAAGCGTTGTTCGGAATCACTGGGCGTAAAGAATGCGTAGGCGGCTTAATAAGTCGGAAGTGAAATCCCAGGGCTCAACCCTGGAACTGCTTTCGAAACTATTTTGCTAGAGTCCCGGAGGGGTTAGCGGAATTCCGAGTGTAGAGGTGAAATTCGCAGATATTCGGAGGAACACCAGTGGCGAAGGCGGCTAACTGGACGGGAACTGACGCTGAGGCATGAAAGTGTGGGGATCAAACAGG` |
| ASV2226 | 425 | `TAAGGAATATTGGACAATGGAGGGAACTCTGATCCAGCCATGCCGCGTGAAGGAAGAAGGCCCTCTGGGTTGTAAACTTCTTTTATATGGGAACAAACCTGTGGATTGATCCGCAGCTGAGGGTACCATAAGAATAAGCACCGGCTAACTCCGTGCCAGCAGCCGCGGTAATACGGAGGGTGCAAGCGTTATCCGGAATCACTGGGTTTAAAGGGTGCGTAGGCGGTCTTATAAGTCAGTCGTGAAATCGCTGGGCTCAACCCATGCAATTGCGATTGATACTGTTTGACTTGAATCAGGATGAGGTAGGCGGAATGTGGCATGTAGCGGTGAAATGCATAGATATGCCATAGAACACCGATTGCGAAGGCAGCTTGCTGGACCTGAATTGACGCTGAGGCACGAAAGCGTGGGGAGCAAACAGG` |
| ASV398 | 404 | `TGGGGAATCTTAGACAATGGGGGAAACCCTGATCTAGCCATGCCGCGTGAGCGATGAAGGCCTTAGGGTTGTAAAGCTCTTTCAGATGGGAAGATAATGACGGTACCATCAGAAGAAGCCCCGGCTAACTCCGTGCCAGCAGCCGCGGTAATACGGAGGGGGCTAGCGTTGTTCGGAATTACTGGGCGTAAAGCGCGCGTAGGCGGATTGGAAAGTTGGGGGTGAAATCCCGGGGCTCAACCTCGGAACTGCCTTCAAAACTCCCAGTCTTGAGTTCGAGAGAGGTGAGTGGAATTCCGAGTGTAGAGGTGAAATTCGTAGATATTCGGAGGAACACCAGTGGCGAAGGCGGCTCACTGGCTCGATACTGACGCTGAGGTGCGAAAGCGTGGGGAGCAAACAGG` |
| ASV1123 | 424 | `TAAGGAATATTGGTCAATGGACGAAAGTCTGAACCAGCCATGCCGCGTGAAGGATTAAGGCCCTCTGGGTTGTAAACTTCTTTTATATGGGACGAAAAAAGGGAATTCTTTCTCACTTGACGGTACCATAGGAATAAGCACCGGCTAACTCCGTGCCAGCAGCCGCGGTAATACGGAGGGTGCAAGCGTTATCCGGATTTACTGGGTTTAAAGGGAGCGTAGGTGGGCAGGTAAGTCAGTGGTGAAATCTCTGGGCTTAACCCGGAAACTGCCATTGATACTATTTGTCTTGAATTATCTGGAGGTAAGCGGAATATGTCATGTAGCGGTGAAATGCTTAGATATGACATAGAACACCCATTGCGAAGGCAGCTTACTACGGATATATTGACACTGAGGCTCGAAAGCGTGGGGATCAAACAGG` |
| ASV4030 | 404 | `TGGGGAATATTGGACAATGGGCGCAAGCCTGATCCAGCGACGTCGCGTGGGTGATTAAGGCCTTCGGGTTGTAAAGCCCTTTCGGCAGGGAAGATTATGACGGTACCTGCATAAGAAGCTCCGGCTAACTTCGTGCCAGCAGCCGCGGTAATACGAAGGGAGCTAGCGTTACTCGGAATTATTGGGCGTAAAGCGTGCGTAGGCGGTTTTATAAGTCAGAAGTGAAAGCCTGGGGCTCAACCCTAGAATTGCTTTTGAAACTGTGAAACTTGAGTATCAGAGAGGATAGTGGAATTCCTAGTGTAGAAGTGAAATTCGTAGATATTAGGAGGAACATCGGTGGCGAAGGCGGCTATCTGGCTGAATACTGACGCTGTTGCACGAAAGCGTGGGGAGCAAACAGG` |
| ASV5591 | 406 | `TGAGGAATTTTGGACAATGGGCGGAAGCTTGATCCAGCAATACTATGTGAGTGAAGAAGGCTAAAAGGTTGTAAAGCTCTTTCAATAAGGAGGAATAAAGACAGTACTTATAAAAGAAGTTCCGGCCAACTCCGTGCCAGCAGCCGCGGTAATACGGGGGGAGCGAGCATTATTCATAATGATTAGGCGTAAAGGGTTTGTAGGTGGTTTTTTAAGTTGAAAAAAAAAGCCTAAAGCTCAACTTTAGTTCTTTTTTCAATACTGAAAAACTTGAGTATATATAGAGGGTAATGGAATTTCTATTGAAAGGATAAAATCTGTTGATAATAGTAAGAAGACCAAAAGCGAAGGCAATTACCTGGGTTATTACTGACACTGAGAAACGAAAGCTTGGGTAGCGAACGGG` |
| ASV3079 | 404 | `TGGGGAATCATAGACAATGGGCGAAAGCCTGATCTTGCCATGCCGCGTGGACGATGAAGGCCTTAGGGTTGTAAAGTCCTTTCACCGGGGAAGATAATGACGGTACCCGGAGAAGAAGCCCCGGCTAACTCCGTGCCAGCAGCCGCGGTAATACGGAGGGGGCTAGCGTTGTTCGGAATTACTGGGCGTAAAGCGCACGTAGGCGGACTGTTAAGTCGGGGGTGAAATCCCGGGGCTCAACCCCGGAACTGCCTTCGATACTGGCAGTCTAGAGGACGGTAGAGGCTAGTGGAATGGCGAGTGTAGAGGTGAAATTCGTAGATATTCGCCGGAACACCAGTGGCGAAGGCGACTGGCTGGACCGTTACTGACGCTGAGGTGCGAAAGCGTGGGGAGCAAACAGG` |
| ASV5127 | 423 | `TAGGGAATATTGGGCAATGGGCGCAAGCCTGACCCAGCCATGCCGCGTGCAGGAAGAAGGCGTTATGCGTTGTAAACTGCTTTTATATAGGAAGAAAACGGCCATGCGTGGTCAACTGACGGTACTATATGAATAAGCACCGGCTAACTCCGTGCCAGCAGCCGCGGTAATACGGAGGGTGCAAGCGTTGTCCGGATTTATTGGGTTTAAAGGGTGCGTAGGCGGTTGATTAAGTCAGCGGTGAAAGACGGTTGCTCAACAATCGCATTGCCGTTGATACTGATTTACTTGAGTTTGATTGAGGCAGCCGGAATGGATAGTGTAGCGGTGAAATGCATAGATATTATCCAGAACGCCAATAGCGTAGGCAGGTTGCTAAGTCATTACTGACGCTGAGGCACGAGAGTGCGGGGATCAAACAGG` |
| ASV4227 | 406 | `CCGAGAATATTCGACAATGGGCGAAAGCCTGATCGAGCGATACCGCGTGATCGATGAAGTGCTTCGGCACGTAAAGATCTTTTATGGATGAGAAATATATGATGTTAGTCCATGAATAAGGGGCTCCTAACTCTGTGCCAGCAGGAGCGGTAATACAGAGGCCCCAAGCGTTACCCGGAATCACTGGGCGTAAAGGGTGTGTAGGTGGTTGTGTTAGTCGTGCTTTAAAACCTGGGGCTTAACCTCAGACATGGGTACGAAACGGCACAACTCGAGTGCGACAGAGGTCTGTGGAATTCTACGTGTAGGGGTAAAATCCGTTGATATGTAGGAGAACACCAAATGCGAAGGCAGCAGACTGGGTCGTAACTGACACTCAAACACGAAAGCGTGGGTAGCGAACCGG` |
| ASV3767 | 404 | `TGGGGAATCTTGGACAATGGGGGAAACCCTGATCCAGCCATGCCGCGTGAGTGATGAAGGCCCTAGGGTTGTAAAACTCTTTCGGCGGGGACGATAATGACGGTACCCGCAGAAGAAGCTCCGGCTAACTTCGTGCCAGCAGCCGCGGTAATACGAAGGGGGCTAGCGTTGTTCGGAATTACTGGGCGTAAAGCGCGCGCAGGCGGTCCTTTAAGTCAGGGGTGAAAGCCCAGAGCTCAACTCTGGAATTGCCCTTGAAACTAGAGGACTTGGGTACGGGAGAGGTGAGTGGAATTCCGAGTGTAGAGGTGAAATTCGTAGATATTCGGAAGAACACCAGTGGCGAAGGCGGCTCACTGGCCCGTTACCGACGCTCAGGCGCGACAGCGTGGGGAGCAAACAGG` |
| ASV1176 | 429 | `TGGGGAATTTTGGACAATGGACGCAAGTCTGATCCAGCAATGCCGCGTGCAGGATGAAGGCCTTCGGGTTGTAAACTGCTTTTGTACGGAGCGAAAAGGTTTTTCCTAATACGAGAGGCCCATGACGGTACCGTAAGAATAAGCACCGGCTAACTACGTGCCAGCAGCCGCGGTAATACGTAGGGTGCGAGCGTTAATCGGAATTACTGGGCGTAAAGCGTGCGCAGGCGGTTATATAAGACAGATGTGAAATCCCCGGGCTCAACCTGGGACCTGCATTTGTGACTGTATAGCTAGAGTACGGTAGAGGGGGATGGAATTCCGCGTGTAGCAGTGAAATGCGTAGATATGCGGAGGAACACCGATGGCGAAGGCAATCCCCTGGACCTGTACTGACGCTCATGCACGAAAGCGTGGGGAGCAAACAGG` |
| ASV4058 | 430 | `TAGGGAATATTGCACAATGGGGGAAACCCTGATGCAGCAACGCCGCGTGAGTGAAGAAGGCCCTTGGGTTGTAAAGCTCTTTAGCGAGGGAAGAGGGTAGGCATGGTGAATAATCATGTCTGCTGACGGTACCTCGAAAATAAGCACCGGCTAATTTCGTGCCAGCAGCCGCGGTAATACGAAAGGTGCAAGCGTTACTCGGAATCACTGGGCGTAAAGCGCACGTAGGCGGACTTACAGGTCAATTGTGAAATACCTGGGCTCAACCCAGGAACTGCAGTTGAAACCGTTTGTCTTGAATACTTGAGAGGGTGGGGGAATACCTGGTGGAGAAGTGAAATTCGTAGAGATCAGGTGGAACACCGGAGGCGAAGGCGCCTGCCTGGCAATGTATTGACGCTGAGGTGCGAAAGTGCGGGGAGCAAACAGG` |
| ASV5209 | 424 | `TAAGGAATATTGGTCAATGGACGGAAGTCTGAACCAGCCATGCCGCGTGAAGGATGAAGGTCCTCTGGATTGTAAACTTCTTTTATGGGGGACGAAAAAAGACCATTTTTGGTCACTTGACGGTACCCTAGGAATAAGCACCGGCTAACTCCGTGCCAGCAGCCGCGGTAATACGGAGGGTGCGAGCGTTATCCGGATTCACTGGGTTTAAAGGGTGCGTAGGCGGGTTTGTAAGTCAGTGGTGAAAGCCTTCAGCTTAACTGAAGAACTGCCATTGATACTGCGAATCTTGAATACTGTGGAGGTATGCGGAATAGGTCATGTAGCGGTGAAATGCATAGATATGACCTAGAACACCTATTGCGAAGGCAGCATACTACGCAGCGATTGACGCTGAGGCACGAAAGCGTGGGGAGCAAACAGG` |
| ASV4848 | 429 | `TGGGGAATATTGCGCAATGGGCGAAAGCCTGACGCAGCGACGCCGCGTGGAGGATGAAGGTCTTCGGATTGTAAACTTCTGTTAAACGGGAAGAAAGACTCACGACGAATAATCGTGGGGGATGACGGTACCGTTAGAGAAAGCCCCGGCTAACTTCGTGCCAGCAGCCGCGGTAATACGAAGGGGGCGAGCATTATTCGGATTTATTGGGCGTAAAGGGTGTGTAGGTGGTTTGTTAAGTTGGTTGTTAAATCTCATGGCCTAACCATGAGGCTGCGATCAAAACTGACAGACTAGAGGATGAAAGAGAAGAGTGGAATTCTCGGAGTAGCGGTTAAATGCGTGGATCTCGAGAGGAACATCATTGGCGAAGGCAGCTCTTTGGTTCATTTCTGACACTAAGACACGAAGGCGTGGGGAGCAAACAGG` |
| ASV2367 | 424 | `TGAGGAATATTGGTCAATGGGCGCAAGCCTGAACCAGCCAAGTCGCGTGAAGGAAGAAGGCTCTATGGGTCGTAAACTTCTTTTAACTGGGAATAAAACGGGGGATGTATCCCCCCTTGCATGTACCAGCAGAATAAGCATCGGCTAACTCCGTGCCAGCAGCCGCGGTAATACGGAGGATGCAAGCGTTATCCGGATTTATTGGGTTTAAAGGGTGCGTAGGCGGACAGATAAGTCAGCGGTGAAATCCTGAGGCTCAACCTCAGCACTGCCGTTGAAACTGTCTATCTTGAGTGTAAATGAAGTAAGCGGAATGCGTGGTGTAGCGGTGAAATGCATAGATATCACGCAGAACTCCAATTGCGAAGGCAGCTTACTAAGCTACAACTGACGCTGAAGCACGAAAGCGTGGGTATCGAACAGG` |
| ASV501 | 421 | `TGGGGAATATTGCGCAATGGCCGCAAGGCTGACGCAGCGACGCCGCGTGGGGGATGAAGCATTTCGGTGTGTAAACCCCTGTTGCTCGGGACGAACAAGCCTTTTCGAGGGCTCTGACGGTACCGAGTGAGGAAGCACCGGCTAACTCCGTGCCAGCAGCCGCGGTAATACGGAGGGTGCGAGCGTTGTCCGGAATCACTGGGCGTAAAGGGCGCGTAGGTGGTCTGCTAAGCGTGCGGTGAAAGCCTGGGGCTCAACCCCAGGTCTGCCGTGCGAACTGGTGGACTGGAGCACTGTAGAGGCAGGTGGAATTCCGGGTGTAGCGGTGGAATGCGTAGAGATCCGGAAGAACACCGGTGGCGAAGGCGGCCTGCTGGGCAGTTGCTGACACTGAGGCGCGACAGCGTGGGGAGCAAACAGG` |
| ASV1807 | 429 | `TGGGGAATTTTGGACAATGGGCGCAAGCCTGATCCAGCCATGCCGCGTGTGGGAAGAAGGCCTTCGGGTTGTAAACCACTTTTGTCAGGGAAGAAACGGCCTTTACTAATATTAGAGGCTAATGACGGTACCTGAAGAATAAGCACCGGCTAACTACGTGCCAGCAGCCGCGGTAATACGTAGGGTGCAAGCGTTAATCGGAATTACTGGGCGTAAAGCGTGCGCAGGCGGCTATGCAAGACAGATGTGAAATCCCCGGGCTTAACCTGGGAACTGCATTTGTGACTGCATGGCTAGAGTACGGTAGAGGGGGATGGAATTCCGCGTGTAGCAGTGAAATGCGTAGATATGCGGAGGAACACCGATGGCGAAGGCAATCCCCTGGACCTGTACTGACGCTCATGCACGAAAGCGTGGGGAGCAAACAGG` |
| ASV2596 | 404 | `TGAGGAATATTGGACAATGGAGGAAACTCTGATCCAGCCATGCCGCGTGAGTGAAGAAGGCCTTCGGGTTGTAAAGCTCTTTTACCCCTGACGATAATGACGGTAAGGGGAGAAAAAGCCACGGCTAACTTCGTGCCAGCAGCCGCGGTAATACGAAGGTGGCAAGCGTTGTTCGGATTTACTGGGCGTAAAGAGTGCGTAGGCGGTTTAACAAGTCAGGAGTGAAAGCCCAAGGCTCAACCTTGGAATTGCTTTTGAAACTGTTTTACTTGAATCCGGTAGAGGTTGGCGGAATTCCCAGTGTAGAGGTGAAATTCGCAGATATTGGGAGGAACACCAGTGGCGTAAGCGGCCAACTGGACCGGTTTTGACGCTGAGGCACGAAAGCATGGGTAGCAAACAGG` |
| ASV4494 | 404 | `TGGGGAATATTGGACAATGGGCGCAAGCCTGATCCAGCAATGCCGCGTGAGTGATGAAGGCCTTAGGGTTGTAAAGCTCTTTCGCACGTGACGATAATGACGGTAACGTGAGAAGAAGCCCCGGCTAACTTCGTGCCAGCAGCCGCGGTAATACGAAGGGGGCTAGCGTTGTTCGGAATTACTGGGCGTAAAGGGCGCGTAGGCGGCCCGATCAGTCAGAAGTGAAAGCCCCGGGCTCAACCTGGGAACCGCTTTTGATACTGTCGAGCTTGAGTTCCGGAGAGGATGGTGGAATTCCCAGTGTAGAGGTGAAATTCGTAGATATTGGGAAGAACACCGGTGGCGAAGGCGGCCATCTGGACGGACACTGACGCTGAGGCGCGAAAGCGTGGGGAGCAAACAGG` |
| ASV1224 | 429 | `TGGGGAATATTGCGCAATGGGCGAAAGCCTGACGCAGCCATGCCGCGTGTGTGAAGAAGGCCTTCGGGTTGTAAAGCACTTTCAATTGGGAAGAAAGGTTGCGTGTTAATAGCACGTAGCTGTGACGTTACCTTTAGAAGAAGCACCGGCTAACTCCGTGCCAGCAGCCGCGGTAATACGGAGGGTGCGAGCGTTAATCGGAATTACTGGGCGTAAAGCGCGCGTAGGCGGTTTGCTAAGTCGGATGTGAAATCCCCGGGCTCAACCTGGGAATGGCATTCGATACTGGCCGACTAGAGTACGAGAGAGGGAGGTAGAATTCCACGTGTAGCGGTGAAATGCGTAGATATGTGGAGGAATACCGGTGGCGAAGGCGGCCTCCTGGCTCGATACTGACGCTCAGGTGCGAAAGCGTGGGTAGCAAACAGG` |
| ASV1726 | 429 | `TGGGGAATTTTGGACAATGGGCGAAAGCCTGATCCAGCAATGCCGCGTGTGTGAAGAAGGCCTTCGGGTTGTAAAGCACTTTTGGCGGGAACGAAACGGTATTGGCTAATATCCGATACTAATGACGGTACCCGCAGAATAAGCACCGGCTAACTACGTGCCAGCAGCCGCGGTAATACGTAGGGTGCAAGCGTTAATCGGAATTACTGGGCGTAAAGCGTGCGCAGGCGGTTATGTAAGTCTGATGTGAAATCCCCGGGCTCAACCTGGGAATTGCATTGGAGACTGCATAGCTAGAATCTGGCAGAGGGGGGTAGAATTCCACGTGTAGCAGTGAAATGCGTAGAGATGTGGAGGAACACCGATGGCGAAGGCAGCCCCCTGGGCTAAGATTGACGCTCATGCACGAAAGCGTGGGGAGCAAACAGG` |
| ASV1185 | 429 | `TGGGGAATTTTGGACAATGGGCGCAAGCCTGATCCAGCCATGCCGCGTGCGGGAAGAAGGCCTTCGGGTTGTAAACCGCTTTTGTCAGGGAAGAAAAGCTTTGGGCTAATACCCCGAGGTGATGACGGTACCTGAAGAATAAGCACCGGCTAACTACGTGCCAGCAGCCGCGGTAATACGTAGGGTGCAAGCGTTAATCGGAATTACTGGGCGTAAAGCGTGCGCAGGCGGTTGTGCAAGACAGATGTGAAATCCCCGGGCTCAACCTGGGAACTGCATTTGTGACTGCACAGCTAGAGTACGGTAGAGGGGGATGGAATTCCGCGTGTAGCAGTGAAATGCGTAGATATGCGGAGGAACACCGATGGCGAAGGCAATCCCCTGGACCTGTACTGACGCTCATGCACGAAAGCGTGGGGAGCAAACAGG` |
| ASV2399 | 404 | `TTAGGAATATTGGACAATGGGCGCAAGCCTGATCCAGCCATGCCGCGTGAGTGATGAAGGCCTTCGGGTTGTAAAGCTCTTTAGCCGACGAATATAATGACTGTAGTCGGAGAATAAGCCCCGGCTAACTTCGTGCCAGCAGCCGCGGTAATACGAAGGGGGCGAGCGTTATTCGGAATCACTGGGCGTAAAGCGTGCGTAGGCTGTTTTGTAAGTTGGGAGTGAAAGCCCAGGGCTCAACCTTGGAATTGCTCTCAAAACTACAAGACTTGAATCTCGGAGAGGATAGCGGAATTGTCAGTGTAGCAGTGAAATGCGTAGATATTGACAGGAACACCAGTGGCGTAAGCGGCTATCTGGACGAGTATTGACGCTGAGGCACGAAAGCGTGGGGATCAAACAGG` |
| ASV2804 | 429 | `TGGGGAATTTTGGACAATGGGCGAAAGCCTGATCCAGCAATGCCGCGTGCAGGATGAAGGCCCTCGGGTTGTAAACTGCTTTTGTACGGAGCGAAAAGGTTCTCTCTAATACAGGGGGCTGATGACGGTACCGTAAGAATAAGCACCGGCTAACTACGTGCCAGCAGCCGCGGTAATACGTAGGGTGCAAGCGTTAATCGGAATTACTGGGCGTAAAGCGTGCGCAGGCGGTTTTGTAAGACAGGCGTGAAATCCCCGGGCTCAACCTGGGAACTGCGTTTGTGACTGCAAGGCTGGAGTGCGGCAGAGGGGGATGGAATTCCGCGTGTAGCAGTGAAATGCGTAGATATGCGGAGGAACACCGATGGCGAAGGCAATCCCCTGGGCCTGCACTGACGCTCATGCACGAAAGCGTGGGGAGCAAACAGG` |
| ASV2975 | 408 | `TGAGGAATTTTCCGCAATGGGCGAAAGCCTGACGGAGCAATACCGCGTGAAGGATGAAGGTCCGTGGATCGTAAACTTCTTTTCTCGGGGAAGAAATTAATGACGGTACCCGAGGAATAAGCACCGGCTAACTCTGTGCCAGCAGCCGCGGTAATACAGAGGGTGCAAGCGTTATCCGGAATGATTGGGCGTAAAGCGTTTGTAGGTTGTTTGAGAAGTCTACTGTCAAATCCCAGAGCTCAACTTTGGACAGGCAGTGGAGCACTCTTAGACTAGAGTACGGTAGGGGTAGAGGGAATTCCCGGTGGAGCGGTGAAATGCACAGAGATCGGGAAGAACACCAGTGGCGAAAGCGCTCTACTAGGCCGTGACTGACACTGAGAGACGATAGCTAGGGGAGCGAATGGG` |
| ASV1617 | 429 | `TGGGGAATATTGGACAATGGGGGCAACCCTGATCCAGCAATGCCACGTGTGTGAAGAAGGCCTAAGGGTTGTAAAGCACTTTTGGGAGTGAGGAAGGTTATGTGGTTAATACCTACATAATTTGACGTTAGCTCCAGAATAAGCACCGGCTAACTCTGTGCCAGCAGCCGCGGTAATACAGAGGGTGCAAGCGTTAATCGGAGTGACTGGGCGTAAAGGGTGCGTAGGTGGTTAAGTAGGTAACTTGTGAAAAGCTTGGGCTCAACCCAAGTCGTGCGAGTTAAACCGCATAACTAGAGTGAAGTAGAGGATAGTGGAATTCCCGGAGTAGTAGTGAAATGCGTAGATATCGGGAGGAACATCAGTGGCGAAGGCGGCTATCTGGACTTTAACTGACACTGAGGCACGAAAGCGTGGGTAGCAAACAGG` |
| ASV4290 | 407 | `TGGGGAATTTTGGACAATGGGCGAAAGCCTGATCCAGCAACGATGCGTGGAGGATGAAGGTTCTCGGATTGTAAACTCCTTTTGAGAGGGAAGAAAAAAATGACGGTACCTCTCGAATAAGCCACGGCTAACTACGTGCCAGCAGCCGCGGTAAGACGTAGGTGGCAAGCGTTACTCGGAATTACTAGGCGTAAAGCGCGCGTAGGCGGAATGTTAAGTCTGTTGTGTAATCTCTGGGCTCAACCCAGAAACTGCAACAGAAACTGGCGTTCTTGAGTGAGGCAGAGGAAATCGGAATTCCTAGTGTAGCAGTGAAATGCGTAGATATTAGGAGGAACACCGGTGGCGAAGGCGGATTTCTGGGCCTTTACTGACGCTAAAGTGCGAAAGCCAGGGGAGCAAACGGG` |
| ASV2348 | 429 | `TGGGGAATTTTGGACAATGGGCGCAAGCCTGATCCAGCCATTCCGCGTGAGTGAAGAAGGCCTTCGGGTTGTAAAGCTCTTTCGCAAGGGAAGAAACGATTTAGGTGAATAATCTAAGTTAATGACGGTACCTTGATAAGAAGCACCGGCTAACTACGTGCCAGCAGCCGCGGTAATACGTAGGGTGCGAGCGTTAATCGGAATTACTGGGCGTAAAGCGTGCGCAGGCTGTTTGGCAAGTCAGATGTGAAATCCCCGAGCTCAACTTGGGAACTGCGTTTGAAACTGCCAGACTAGAATAGGTCAGAGGGGGGTAGAATTCCACGTGTAGCAGTGAAATGCGTAGAGATGTGGAGGAATATCAATGGCGAAGGCAGCCCCCTGGGATCATATTGACGCTCATGCACGAAAGCGTGGGGAGCAAACAGG` |
| ASV3802 | 424 | `TGAGGAATATTGGACAATGGGTGAAAGCCTGATCCAGCCATCCCGCGTGAAGGATGACGGTCCTATGGATTGTAAACTTCTTTTGTATAGGAATAAACCTTTCTACGTGTAGAAAGCTGAAGGTACTATACGAATAAGCACCGGCTAACTCCGTGCCAGCAGCCGCGGTAATACGGAGGGTGCAAGCGTTATCCGGATTTATTGGGTTTAAAGGGTCCGTAGGCGGACCGATAAGTCAGTGGTGAAATCTCATAGCTTAACTATGAAACTGCCATTGATACTGTCGGTCTTGAGTAAATTTGAAGTGGCTGGAATAAGTAGTGTAGCGGTGAAATGCATAGATATTACTTAGAACACCAATTGCGAAGGCAGGTCACTAAGATTTAACTGACGCTGATGGACGAAAGCGTGGGTAGCGAACAGG` |
| ASV5333 | 409 | `TCGAGAATATTCGACAATGGGCGAAAGCCTGATCGAGCGACGCCGCGTGCAGGATGAAGGGCTTCGGCCTGTAAACTGCTTTTAGCAGATAGAAAGCGCAAGTTGATCATCTGTTGAATAAGAGGCGGCAAACTTCGTGCCAGCAGCAGCGGTAATACGAAGGCCTCAAGCGTTATCCGGATTTATTGGGCGTAAAGAGCGCGTAGGTGGTCCCGTAAGTTTCGCGTTAAATGCCAGAGCTTAACTTTGGCGCTGCGCGGAATACTGCGGAACTAGAGGGTGTGAGAGGCACATGGAACTCACGGTGTAGGGGTGAAATCCGTTGATATCGTGGGGAACACCGAAGGCGAAGGCAATGTGCTGGTACACTCCTGACACTCAGTGCGCGAAAGCGTGGGGAGCAAAAAGG` |
| ASV3978 | 430 | `TGGGGAATATTGGACAATGGGGGCAACCCTGATCCAGCAATGCCGCGTGTGTGAAGAAGGCCTGCGGGTTGTAAAGCACTTTTGTTCGAGAAGAAATCCTGTTTATGAATAGTATGCGGGGATGACGGTATCGAAAGAATAAGCACCGGCTAACTTCGTGCCAGCAGCCGCGGTAATACGAAGGGTGCAAGCGTTAATCGGAATTACTGGGCGTAAAGCGTGCGTAGACGGCTTTTTAAGTCGACTGTGAAACCCCCGGGCTCAACCTGGGACCTGCAGTCGATACTGGAAAGCTTGGAGTTTGAGAGAGGGTGGTGGAATTCCTGGTGTAGCGGTGAAATGCGTAGAGATCAGGAGGAACACTAGTGGCGAAGGCGGCCACCTGGCTTAAAACTGACGTTGAGGCACGAAAGCGTGGGGAGCAAACAGG` |
| ASV4389 | 404 | `TGGGGAATATTGGACAATGGGGGCAACCCTGATCCAGCAATGCCGCGTGTGTGAAGAAGGTCTTCGGATTGTAAAGCACTTTCGGCGGGGACGATGATGACGGTACCCGCAGAAGAAGCCCCGGCTAACTTCGTGCCAGCAGCCGCGGTAATACGAAGGGGGCTAGCGTTGCTCGGAATGACTGGGCGTAAAGGGCGCGTAGGCGGCTGATCAAGTCAGACGTGAAATTCCTGGGCTCAACCTGGGGACTGCGTTTGAGACTGATTGGCTAGAGGATGGAAGAGGCTCGTGGAATTCCCAGTGTAGAGGTGAAATTCGTAGATATTGGGAAGAACACCGGTGGCGAAGGCGGCGAGCTGGTCCATGACTGACGCTGAGGCGCGATAGCGTGGGGAGCAAACAGG` |
| ASV4678 | 405 | `TAGGGAATTTTCCACAATGGGCGAAAGCCTGATGGAGCAACGCCGCGTGCAGGATGAATGCCTTCGGGTTGTAAACTGCTTTTATATGTGACGATTTTGACGGTAGCATATGAATAAGGATCGGCTAACTCCGTGCCAGCAGCCGCGGTCATACGGGGGATCCAAGCGTTATCCGGAATTACTGGGCGTAAAGAGTTGCGTAGGTGGCAGAGTAAGTAGATAGTGAAAGCGTTCGGCTCAACCGAATATCCATTATCTAAACTGCTCAGCTAGAGAATGAGAGAGGTAGCTAGAATTCCTTGTGTAGGAGTGAAATCCGTAGATATAAGGAGGAATACCGATGGCGTAGGCAGGCTACTGGCTCATTTCTGACACTAAGGCACGAAAGCGTGGGGAGCGACCGGG` |
| ASV5198 | 424 | `TAAGGAATATTGGACAATGGAGGCAACTCTGATCCAGCCATGCCGCGTGAAGGATGAAGGCCCTATGGGTCGTAAACTTCTTTTGTACGAGAGAAAACCATTTCACGTGTGAGATGTTGATAGTATCGTAAGAATAAGGATCGGCTAACTTCGTGCCAGCAGCCGCGGTAAGACGAAGGATCCAAGCGTTATCCGGATTCATTGGGTTTAAAGGGTGCGTAGGCGGATTTGTAAGTCAGTGGTGAAAGCCGATAGCTCAACTATCGAACTGCCATTGATACTGCAGATCTAGAATATAGATGATGTTGGCGGAATATGACATGTAGCGGTGAAATGCATAGATATGTCATAGAACACCGATTGCGAAGGCAGCTGACAAAACTATTATTGACGCTGATGCACGAAAGTGCGGGGATCAAACAGG` |
| ASV2563 | 424 | `TAGGGAATATTGGTCAATGGGGGCAACCCTGAACCAGCCATGCCGCGTGCAGGAATAAGGCCCTATGGGTCGTAAACTGCTTTTGAACGGGAAGAAATCTATTTACGTGTAAATAGTTGACGGTACCGTTAGAATAAGGATCGGCTAACTTCGTGCCAGCAGCCGCGGTAATACGAAGGATCCAAGCGTTGTCCGGATTTACTGGGTTTAAAGGGTGCGTAGGCGGACTCATAAGTCAGTGGTGAAAGCCTTCAGCTTAACTGAAGAACTGCCATTGAAACTGTGAGTCTCGAGTTCGGTTGAGGTCATTGGAATGTAACATGTAGCGGTGAAATGCATAGATATGTTACAGAACACCGATTGCGAAGGCAGATGACTAAACCGAAACTGACGCTGAGGCACGAAAGCGTGGGGATCAAACAGG` |
| ASV1407 | 404 | `TGGGGAATATTGGACAATGGGCGAAAGCCTGATCCAGCAATACCGCGTGAATGATGAAGGCCTTAGGGTTGTAAAGTTCTTTTAATAGGGAAGATAATGACGGTACCTATAGAAAAAGCCCCGGCTAACTCCGTGCCAGCAGCCGCGGTAAGACGGAGGGGGCTAGCGTTATTCGGAATTACTGGGCGTAAAGGGCGCGTAGGCGGATTAGTAAGTTGGGAGTGAAATCCCGAGGCTTAACCTCGGAATTGCTCTCAAAACTGCTAGTCTTGAGTGAAGTAGAGGATGATGGAATTCCTAGTGTAGAGGTGAAATTCTTAGATATTAGGAGGAACACCGGTGGCGAAGGCGGTCATCTGGACTTCAACTGACGCTGAGGCGCGAAAGCGTGGGGAGCAAACAGG` |
| ASV3097 | 404 | `TGGGGAATATTGGGCAATGGGCGAAAGCCTGACCCAGCCATGCCGCGTGAGTGATGAAGGCCTTAGGGTTGTAAAGCTCTTTTAGTGGGGAAGATGATGACGGTACCCACAGAATAAGCTCCGGCTAACTTCGTGCCAGCAGCCGCGGTAATACGAAGGGAGCAAGCGTTACTCGGATTTACTGGGCGTAAAGGGCGTGTAGGCGGCTTGGTCAGTCAGATGTGAAAGCCCTGGGCTTAACCCGGGAACTGCATTTGATACTGCCAAGCTTGAGTATAAGAGAGGTAAGCGGAATTTCCAGTGTAGCAGTGAAATGCGTAGATATTGGAAGGAACACCGATGGCGAAGGCAGCTTACTGGCTTATAACTGACGCTGAGGCGCGAAAGCGTGGGGAGCAAACAGG` |
| ASV1121 | 421 | `TGGGGAATATTGCGCAATGGCCGCAAGGCTGACGCAGCGACGCCGCGTGGGGGATGAAGCATTTCGGTGTGTAAACCCCTGTTGCTCGGGACGAACAAGCCTTTTCGAGGGTTCTGACGGTACCGAGTGAGGAAGCACCGGCTAACTCCGTGCCAGCAGCCGCGGTAATACGGAGGGTGCGAGCGTTGTCCGGAATCACTGGGCGTAAAGGGCGCGTAGGTGGTCTGCTAAGCGTGCGGTGAAAGCCTGGGGCTCAACCCCAGGTCTGCCGTGCGAACTGGTGGACTTGAGCACTGTAGAGGCAGGTGGAATTCCGGGTGTAGCGGTGGAATGCGTAGAGATCCGGAAGAACACCGGTGGCGAAGGCGGCCTGCTGGGCAGTAGCTGACACTGAGGCGCGACAGCGTGGGGAGCAAACAGG` |
| ASV2471 | 424 | `TGAGGAATATTGGACAATGGGCGCAAGCCTGATCCAGCCATGCCGCGTGCAGGATGACGGTCCTATGGATTGTAAACTGCTTTTATACGAGAAGAAACACTCCTTCGTGAAGGAATTTGACGGTATCGTAAGAATAAGGATCGGCTAACTCCGTGCCAGCAGCCGCGGTAATACGGAGGATCCAAGCGTTATCCGTAATCATTGGGTTTAAAGGGTCCGTAGGCGGTCTTGTAAGTCAGTGGTGAAAGCCCATCGCTCAACGGTGGAACGGCCATTGATACTGCAGGACTTGAATTACTAGGAAGTAACTAGAATATGTAGTGTAGCGGTGAAATGCTTAGAGATTACATGGAATACCAATTGCGAAGGCAGGTTACTACTAGTGGATTGACGCTGATGGACGAAAGCGTGGGTAGCGAACAGG` |
| ASV2408 | 430 | `TAGGGAATATTGCGCAATGGACGAAAGTCTGACGCAGCCACGCCGCGTGAGTGAGGAAGGCCTTCGGGTTGTAAAGCTCTGTCGTCCGGGAAAAACGGCTATTCGGGGAAAGTTCGAGTAGCGTGATGGTACCGGAGAAGGAAGCACCGGCAAACTCTGTGCCAGCAGCCGCGGTAATACAGAGGGTGCAAGCGTTGTTCGGAATTACTGGGCGTAAAGCGTGCGTAGTCGGTATTGAGAGTCGCGGGTGAAATCCCAGGGCTTAACCCTGGAACTGCCTGCGAGACCTCAGTACTAGAGTGTGAGAGGGGATAGTGGAATACCAAGTGTAGCGGTGAAATGCGTAGAGATTTGGTAGAACACCGGTGGCGAAGGCGGCTATCTGGCTCACAACTGACGATCAGGCACGAAAGCGTGGGTAGCAAACAGG` |
| ASV2885 | 404 | `TGAGGAATATTGGACAATGGAGGAAACTCTGATCCAGCCATGCCGCGTGAGTGATGAAGGCCTTCGGGTTGTAAAGCTCTTTCGGGTGTGAAAATGATGATGGTAACACCAGAAGAAGCCCCGGCTAACTCCGTGCCAGCAGCCGCGGTAATACGGAGGGGGCGAGCGTTGTTCGGAATCACTGGGCGTAAAGAGTGCGTAGGCGGTTACACAAGTCAGAAGTGAAATCCCAGGGCTTAACCCTGGAATTGCTTTTGAGACTGTGTGACTAGAGATCTAGAGAGGTTGGGGGAATTCCGAGTGTAGAGGTGAAATTCGCAGATATTCGGAGGAACACCAGTGGCGTAGGCGCCCAACTGGCTAGATACTGACGCTGAGGCACGAAAGCGTGGGGAGCAAACAGG` |
| ASV1995 | 407 | `TGGGGAATTTTCCGCAATGGGCGAAAGCCTGACGGAGCAATACCGCGTGAGGGATGAAGGCCTTTGGGTTGTAAACCTCTTTTATCAAGGAGGAAGTTCTGACGTGTACTTGATGAATAAGCATCGGCTAACTCCGTGCCAGCAGCCGCGGTAAGACGGAGGATGCAAGTGTTATCCGGAATCACTGGGCGTAAAGCGTCTGTAGGTGGTTTAAAAAGTCCACTGTTAAAAATTGAAGCTTAACTTCAAAGATGCCGTGGAAACTTTTAAACTTGAGTATGGTAGGGGTAGAGGGAATTTCCAGTGGAGCGGTGAAATGCGTAGATATTGGAAAGACCACCAATGGCGAAGGCACTCTACTGGGCCATTACTGACACTGAGAGACGAAAGCTAGGGGAGCAAATGGG` |
| ASV2759 | 404 | `TGGGGAATATTGGACAATGGGCGAAAGCCTGATCCAGCAATGCCGCGTGAGTGATGAAGGCCTTAGGGTTGTAAAGCTCTTTTACCCGGGATGATAATGACAGTACCGGGAGAATAAGCCCCGGCTAACTCCGTGCCAGCAGCCGCGGTAATACGGAGGGGGCTAGCGTTATTCGGAATTACTGGGCGTAAAGCGCACGTAGGCGGCTATTCAAGTCAGAGGTGAAAGCCCGGAGCTCAACTCCGGAACTGCCTTTGAAACTAGATAGCTAGAATCACGGAGAGGTAAGTGGAATTCCGAGTGTAGAGGTGAAATTCGTAGATATTCGGAAGAACACCAGTGGCGAAGGCGGCTTACTGGACGTGTATTGACGCTGAGGTGCGAAAGCGTGGGGAGCAAACAGG` |
| ASV3853 | 429 | `TGGGGAATATTGGACAATGGGGGAAACCCTGATCCAGCGACGCCGCGTGGGTGAAGAAGGCCTGCGGGTTGTAAAGCCCTTTCGGTAGGGACGAAAGCTTCCAGTCTAACACACCGGGAGGTTGACTTAACCTACAAAAGAAGCACCGGCTAACTCTGTGCCAGCAGCCGCGGTAATACAGAGGGTGCGAGCGTTAATCGGAATTACTGGGCGTAAAGCGCACGTAGGCGGCTTGGTATGTCGGGTGTGAAATCCCCGGGCTCAACCTGGGAACTGCATCCGAAACTGCCTTGCTCGAGTATGGGAGAGGGAAGTGGAATTTCCGGTGTAGCGGTGAAATGCGTAGATATCGGAAGGAACATCAGTGGCGAAGGCGACTTCCTGGACCAATACTGACGCTCAGGTGCGAAAGCGTGGGGAGCAAACAGG` |
| ASV2835 | 429 | `TGGGGAATTTTGGACAATGGGGGCAACCCTGATCCAGCCATGCCGCGTGTGTGAAGAAGGCCTTCGGGTTGTAAAGCACTTTCGGCCGGAACGAAATCGCGCGGGTGAATATCCCGCGTGGATGACGGTACCGGAAGAAGAAGCACCGGCTAACTACGTGCCAGCAGCCGCGGTAATACGTAGGGTGCGAGCGTTAATCGGAATTACTGGGCGTAAAGTGTGCGCAGGTGGCCGCGCAAGTCGAGTGTGAAAGCCCCGAGCTTAACTTGGGAATTGCGCTCGAAACTACGTGGCTAGAGTGTGGCAGAGGAAGGTGGAATTCCACGTGTAGCGGTGAAATGCGTAGAGATGTGGAGGAACACCGATGGCGAAGGCAGCCTTCTGGGCCAACACTGACGCTCATGCACGAAAGCGTGGGGAGCAAACAGG` |
| ASV4264 | 408 | `TCGAGAATCTTCCGCAATGGACGAAAGTCTGACGGAGCGACGCCGCGTGGTGGATGAAGCCCTTTGGGGCGTAAACACCTTTTATGAGGGAGGAAGTTTATTGACGTTACCTCATGAATAAGGGGCTCCCAACTCTGTGCCAGCAGGAGCGGTAATACAGAGGCCCCAAGCATTATCCGGATTTACTGGGCGTAAAGGGTGCGTAGGCGGTTATATTAGTCAGGTGTTAAATCCTGGGGCTCAACCCCAGAATCGCATTTGAAACGGTATAACTAGAATCAGTTAGAGGCAAGCAGAACTCACGGTGTAGGGGTGAAATCCGTTGATATCGTGGGGAATACCAAATGCGAAGGTAGCTTGCTGGGACTTGATTGACGCTGAGGCACGAAAGCGTGGGTAGCGAATCGG` |
| ASV5265 | 429 | `TGGGGAATATTGGACAATGGGCGCAAGCCTGATCCAGCAATGCCGCGTGTGTGAAGAAGGCCTTCGGGTTGTAAAGCACTTTAAGCGGGGAGGAAGCGCAGTCAGCTAATACCTGGTTGCGTTGACGTTACCCGCAGAATAAGCACCGGCTAACTCTGTGCCAGCAGCCGCGGTAATACAGAGGGTGCAAGCGTTAATCGGAATTACTGGGCGTAAAGCGCGCGTAGGCGGTTTGTTCAGTCCGTTGTGAAATCCCTGGGCTCAACCTGGGAATGGCGATGGATACTGGCAAGCTAGAGTATGAGAGAGGTGGGTAGAATTCCAGGTGTAGCGGTGAAATGCGTAGATATCTGGAGGAATACCGGTGGCGAAGGCGGCCCACTGGCTTAATACTGACGCTGAGGTGCGAAAGCGTGGGGAGCAAACAGG` |
| ASV593 | 429 | `TGGGGAATTTTGGACAATGGGCGAAAGCCTGATCCAGCAATGCCGCGTGCAGGATGAAGGCCTTCGGGTTGTAAACTGCTTTTGTACGGAACGAAAAGTCTCGGGATAATACCCTGGGACCATGACGGTACCGTAAGAATAAGCACCGGCTAACTACGTGCCAGCAGCCGCGGTAATACGTAGGGTGCAAGCGTTAATCGGAATTACTGGGCGTAAAGCGTGCGCAGGCGGTTATATAAGACAGATGTGAAATCCCCGGGCTCAACCTGGGAACTGCATTTGTGACTGTATAGCTAGAGTACGGTAGAGGGGGATGGAATTCCGCGTGTAGCAGTGAAATGCGTAGATATGCGGAGGAACACCGATGGCGAAGGCAATCCCCTGGACCTGTACTGACGCTCATGCACGAAAGCGTGGGGAGCAAACAGG` |
| ASV1850 | 424 | `TAGGGAATATTGGTCAATGGGGGCAACCCTGAACCAGCCATGCCGCGTGCAGGAATAAGGCCCTATGGGTCGTAAACTGCTTTTGAACGGGAAGAAATCTATTTACGTGTAGATAGTTGACGGTACCGTTAGAATAAGGATCGGCTAACTTCGTGCCAGCAGCCGCGGTAATACGAAGGATCCAAGCGTTGTCCGGATTTACTGGGTTTAAAGGGTGCGTAGGCGGACTTATAAGTCAGTGGTGAAAGCCTTCAGCTTAACTGAAGAACTGCCATTGAAACTGTAAGTCTCGAGTTCGGTTGAGGTCATTGGAATGTAACATGTAGCGGTGAAATGCATAGATATGTTACAGAACACCGATTGCGAAGGCAGATGACTAAACCGAAACTGACGCTGAGGCACGAAAGCGTGGGGATCAAACAGG` |
| ASV3462 | 429 | `TGGGGAATATTGCGCAATGGGCGAAAGCCTGACGCAGCCATGCCGCGTGTGTGAAGAAGGCCTTAGGGTTGTAAAGCACTTTCAGTGGGGAGGAAGGGGTGTAGGCGAATACCCTGCATCATTGACGTTACCCACAGAAGAAGCACCGGCTAACTCCGTGCCAGCAGCCGCGGTAATACGGAGGGTGCAAGCGTTAATCGGAATTACTGGGCGTAAAGCGCGCGTAGGCGGCTAGGTCAGTCGGATGTGAAAGCCCCGGGCTCAACCTGGGAACGGCATTCGATACTGCCTGGCTAGAGTACGAGAGAGGGGGGTGGAATTCCTAGTGTAGCGGTGAAATGCGTAGATATTAGGAGGAACACCAGTGGCGAAGGCGGCCCCCTGGCTCGATACTGACGCTGAGGTGCGAAAGCGTGGGTAGCAAACAGG` |
| ASV5206 | 404 | `TGGGGAATATTGCACAATGGGGGAAACCCTGATGCAGCGACGCCGCGTGGAGGAAGAAGGTCTTCGGATTGTAAACTCCTGTTGTTGGGGAAAAGAAGGATGGTACCCAACAAGGAAGTGACGGCTAACTACGTGCCAGCAGCCGCGGTAAAACGTAGGTCACAAGCGTTGTCCGGAATTACTGGGTGTAAAGGGAGCGCAGGCGGGTTTTCAAGTTGGGAGTGAAATACATGGGCTCAACCCATGAACTGCTCTCAAAACTGGAGATCTTGAGTAGTGCAGAGGTAGGCGGAATTCCCGGTGTAGCGGTGGAATGCGTAGAGATCGGGAGGAACACCAGTGGCGAAGGCGGCCTACTGGGCACCAACTGACGCTGAGGCTCGAAAGTGTGGGTAGCAAACAGG` |
| ASV1009 | 430 | `TGGGGAATATTGGACAATGGGGGGAACCCTGATCCAGCCATGCCGCGTGTGTGAAGAAGGCCTTTTGGTTGTAAAGCACTTTAAGCGAGGAGGAGGCTCTTCTAGTTAATACCTAGGATGAGTGGACGTTACTCGCAGAATAAGCACCGGCTAACTCTGTGCCAGCAGCCGCGGTAATACAGAGGGTGCAAGCGTTAATCGGATTTACTGGGCGTAAAGCGTGCGTAGGTGGTCTTTTAAGTCGGATGTGAAATCCCTGAGCTTAACTTAGGAATTGCATTCGATACTGGGAGACTAGAGTATGGGAGAGGATGGTAGAATTCCAGGTGTAGCGGTGAAATGCGTAGAGATCTGGAGGAATACCGATGGCGAAGGCAGCCATCTGGCCTAATACTGACACTGAGGTACGAAAGCATGGGGAGCAAACAGG` |
| ASV4658 | 427 | `TGGGGAATCTTGCGCAATGGGCGAAAGCCTGACGCAGCGACGCCGCGTGAGCGATGAAGGCCTTCGGGTTGTAAAGCTCTGTGGGGAGAGAAGAATAAGGCGGTGCTAATCAGCCGCTGATGACGGTATCTCCTTAGCAAGCACCGGCTAACTCTGTGCCAGCAGCCGCGGTAAGACAGAGGGTGCAAACGTTGTTCGGAATTACTGGGCGTAAAGCGCGTGTAGGTGGCTTCGCAAGTCGAATGTGAAAACCCCAGGCTCACCCTGGGAAGAGCATCCGAAACTGCGTCGCTAGAGTCCCGGAGAGGAAGGTGGAATGCTTGGTGTAGAGGTGAAATTCGTAGATATCAAGCGGAACACCAGTGGCGAAGGCGGCCTTCTGGACGGTGACTGACACTGAGACGCGAAAGCGTGGGGAGCAAACAGG` |
| ASV2431 | 429 | `TGGGGAATATTGGACAATGGGCGCAAGCCTGATCCAGCCATGCCGCGTGGGTGAAGAAGGCCTTCGGGTTGTAAAGCCCTTTTGTCCGGAAAGAAAAGCATCGGGCTAATACCTCGATGTTCTGACGGTACCGGAAGAATAAGCACCGGCTAACTTCGTGCCAGCAGCCGCGGTAATACGAAGGGTGCAAGCGTTACTCGGAATTACTGGGCGTAAAGCGTGCGTAGGTGGTTCGTTAAGTCTGATGTGAAAGCCCTGGGCTCAACCTGGGAATTGCATTGGATACTGGCGAGCTAGAGTGCGGTAGAGGATGGCGGAATTCCCGGTGTAGCAGTGAAATGCGTAGAGATCGGGAGGAACATCTGTGGCGAAGGCGGCCATCTGGACCAGCACTGACACTGAGGCACGAAAGCGTGGGGAGCAAACAGG` |
| ASV1420 | 429 | `TGGGGAATTTTGGACAATGGGCGCAAGCCTGATCCAGCAATGCCGCGTGTCTGAAGAAGGCCTTAGGGTTGTAAAGGACTTTTGTTAGTGAGCAAGTACGCTAGGTTAAGAGCTAGGTGTATTGAGAGTAGCTAAAGAATAAGGACCGGCTAACTACGTGCCAGCAGCCGCGGTAATACGTAGGGTCCAAGCGTTAATCGGAATTACTGGGCGTAAAGGGTGAGTAGGCGGTTATATAAGATTGATGTGAAAGCCCCGGGCTTAACCTGGGATGTGCATTGAAGACTGTATAGCTAGAGTGAAGCAGAGGGGGGTAGAATTCCACGTGTAGCAGTGAAATGCGTAGAGATGTGGAGGAATACCGATGGCGAAGGCAGCCCCCTGGGTTTACACTGACGCTGAGTCACGAAAGCGTGGGGAGCAAACAGG` |
| ASV749 | 424 | `TGAGGAATATTGGACAATGGGCGCAAGCCTGATCCAGCCATGCCGCGTGCAGGATGACGCATCTATGGTGTGTAAACTGCTTTTGCACAGGAAGAAACACTCCCTCGTGAGGGAGCTTGACGGTACTGTGAGAATAAGGATCGGCTAACTCCGTGCCAGCAGCCGCGGTAATACGGAGGATCCAAGCGTTATCCGGAATCATTGGGTTTAAAGGGTCCGTAGGCGGTTTAATAAGTCAGTGGTGAAAGCCCATCGCTCAACGGTGGAACGGCCATTGATACTGTTAGACTTGAATTATAAGGAAGTAACTAGAATATGTAGTGTAGCGGTGAAATGCTTAGAGATTACATGGAATACCAATTGCGAAGGCAGGTTACTACTTATGGATTGACGCTGATGGACGAAAGCGTGGGTAGCGAACAGG` |
| ASV2100 | 429 | `TGGGGAATTTTGGACAATGGGGGCAACCCTGATCCAGCAATACCGCGTGAGTGAAGAAGGCCTTCGGGTTGTAAAGCTCTTTTGTTAGGGAAGAAAAGGTTGGGGCTAATATCTCTGACTGATGACGGTACCTAAAGAATAAGCACCGGCTAACTACGTGCCAGCAGCCGCGGTAATACGTAGGGTGCAAGCGTTAATCGGAATTACTGGGCGTAAAGCGTGCGCAGGCGGTTTGGTAAGACAGGTGTGAAATCCCCGAGCTCAACTTGGGAACTGCACTTGTGACTGCCAGACTAGAGTGTGTCAGAGGGGGGTAGAATTCCACGTGTAGCAGTGAAATGCGTAGAGATGTGGAGGAATACCGATGGCGAAGGCAGCCCCCTGGGATAACACTGACGCTCATGTACGAAAGCGTGGGGAGCAAACAGG` |
| ASV1902 | 407 | `TGGGGAATTTTCCGCAATGGGCGAAAGCCTGACGGAGCAATACCGCGTGAGGGAGGAAGGTTTTTGGATTGTAAACCTCTTTTTTAAAGGAAGAAGAAAGTGACGGTACTTTAAGAATCAGCATCGGCTAACTCCGTGCCAGCAGCCGCGGTAATACGGAGGATGCAAGCGTTATCCGGAATCATTGGGCGTAAAGCGTCCGCAGGTGGCGATTCAAGTCTGCTGTTAAAGACAGAAGCTCAACTTCTGATAGGCAGTGGAAACTGGATAGCTAGAGTATGGTAGGGGTTGAGGGAATTCCCAGTGTAGCGGTGAAATGCGTAGATATTGGGAAGAACACCAGTGGCGAAGGCGCTCAACTGGGCCATAACTGACACTCAGGGACGAAAGCTAGGGGAGCGAAAGGG` |
| ASV4730 | 429 | `TGGGGAATATTGCGCAATGGGCGAAAGCCTGACGCAGCGACGCCGCGTGAGGGATGAAGGTCTTCGGATCGTAAACCTCTGTCAGAAGGGAAGAAGTGTAGTGAGAGTAACTGCTCGCTATTTGACGGTACCTTCAAAGGAAGCACCGGCTAACTCCGTGCCAGCAGCCGCGGTAATACGGAGGGTGCAAGCGTTAATCGGAATTACTGGGCGTAAAGCACATGTAGGCTGTTGTATAAGTCAGGGGTGAAATCCCACGGCTCACCCGTGGAACTGCCCTTGATACTGTATGACTAGAGTCTGTGAGAGGGTAGTGGAATTCCAGGTGTAGGAGTGAAATCCGTAGAGATCTGGAGGAACATCAGTGGCGAAGGCGACTACCTGGTGCAGTACTGACGCTGAGATGCGAAAGCGTGGGGATCAAACAGG` |
| ASV2752 | 428 | `TGGGGAATATTGGACAATGGGCGCAAGCCTGATCCAGCCACGCCGCGTGAGTGATGAAGGCCTTCGGGTCGTAAAGCTCTGTGGGGAGGGACGAACCGCTGGTGGTTCATAGCCACCAGCATGACGGTACCTCCTTAGCAAGCACCGGCTAACTTCGTGCCAGCAGCCGCGGTAATACGAAGGGTGCAAACGTTGCTCGGAATTATTGGGCGTAAAGCGCACGTAGGCGGCCTATCAAGTCGGATGTGAAATCCCTCGGCTCAACCAAGGAAGTGCATCCGAAACTGGCAGGCTTGAGTACTTAAGAGGATCGCGGAATTCCCGGTGTAGAGGTGAAATTCGTAGATATCGGGAGGAACACCAGTGGCGAAGGCGGCGATCTGGGAAGATACTGACGCTGAGGTGCGAAAGCGTGGGGAGCAAACAGG` |
| ASV3296 | 429 | `TGGGGAATTTTGGACAATGGGGGCAACCCTGATCCAGCCATGCCGCGTGTGCGAAGAAGGCCTTCGGGTTGTAAAGCACTTTCGGCCGGAACGAAATCGCGCGGGTGAATATCCCGCGTGGATGACGGTACCGGAAGAAGAAGCACCGGCTAACTACGTGCCAGCAGCCGCGGTAATACGTAGGGTGCGAGCGTTAATCGGAATTACTGGGCGTAAAGTGTGCGCAGGCGGCGTCGCAAGTCGAGTGTGAAATCCCCGAGCTTAACTTGGGAATTGCGCTCGAAACTACGATGCTGGAGTATGGCAGAGGAAGGTGGAATTCCACGTGTAGCGGTGAAATGCGTAGAGATGTGGAGGAACACCGATGGCGAAGGCAGCCTTCTGGGCCAATACTGACGCTCATGCACGAAAGCGTGGGGAGCAAACAGG` |
| ASV383 | 429 | `TGGGGAATATTGCACAATGGGGGAAACCCTGATGCAGCCATGCCGCGTGTGTGAAGAAGGCCTTCGGGTTGTAAAGCACTTTCAGCGAGGAGGAAAGGTTGGTAGCTAATAACTGCCAGCTGTGACGTTACTCGCAGAAGAAGCACCGGCTAACTCCGTGCCAGCAGCCGCGGTAATACGGAGGGTGCAAGCGTTAATCGGAATTACTGGGCGTAAAGCGCACGCAGGCGGTTGGATAAGTTAGATGTGAAAGCCCCGGGCTCAACCTGGGAATTGCATTTAAAACTGTCCGGCTAGAGTCTTGTAGAGGGGGGTAGAATTCCAGGTGTAGCGGTGAAATGCGTAGAGATCTGGAGGAATACCGGTGGCGAAGGCGGCCCCCTGGACAAAGACTGACGCTCAGGTGCGAAAGCGTGGGGAGCAAACAGG` |
| ASV407 | 429 | `TGGGGAATTTTGGACAATGGGGGCAACCCTGATCCAGCCATTCCGCGTGAGTGAAGAAGGCCTTCGGGTTGTAAAGCTCTTTCGCAAGGGAAGAAAACTTATCCTCTAACATAGGATGAGGCTGACGGTACCTTGATAAGAAGCACCGGCTAACTACGTGCCAGCAGCCGCGGTAATACGTAGGGTGCGAGCGTTAATCGGAATTACTGGGCGTAAAGCGTGCGCAGGCTGTTTGGCAAGTCAGATGTGAAATCCCCGGGCTCAACCTGGGAACTGCGTTTGAAACTGCCAGACTAGAATAGGTCAGAGGGGGGTAGAATTCCACGTGTAGCAGTGAAATGCGTAGAGATGTGGAGGAATATCAATGGCGAAGGCAGCCCCCTGGGATCATATTGACGCTCATGCACGAAAGCGTGGGGAGCAAACAGG` |
| ASV2277 | 404 | `TGGGGAATCTTAGACAATGGGCGCAAGCCTGATCTAGCCATGCCGCGTGAGCGATGAAGGCCTTAGGGTTGTAAAGCTCTTTCGTGGGGGAAGATAATGACTGTACCCCAAGAAGAAGCCCCGGCTAACTCCGTGCCAGCAGCCGCGGTAATACGGAGGGGGCTAGCGTTGTTCGGAATTACTGGGCGTAAAGCGCACGTAGGCGGACGAGAAAGTCAGGGGTGAAATCCCGGGGCTCAACCCCGGAACTGCCTTTGAAACTCCTTGTCTTGAGGTCGTGAGAGGTGGGTGGAATTCCGAGTGTAGAGGTGAAATTCGTAGATATTCGGAGGAACACCAGTGGCGAAGGCGGCCCACTGGCACGATACTGACGCTGAGGTGCGAAAGCGTGGGGAGCAAACAGG` |
| ASV5224 | 407 | `TGGGGAATATTGCACAATGGGCGGAAGCCTGATGCAGCAACGCCGCGTGAAGGATGAAGGGTTTCGGCTCGTAAACTTCTTTAGGCGGGGACGAAAAAAATGACGGTACCCGCAGAATAAGCCCCGGCTAACTACGTGCCAGCAGCCGCGGTAATACGTAGGGGGCAAGCGTTATCCGGAATTACTGGGTGTAAAGGGTGAGTAGGCGGCCAAATAAGTCACATGTGAAAGCCCCGGGCTTAACCCGGGGACTGCATGAGAAACTGTTTGGCTCGAGTACAGGAGAGGTAAGCGGAATTCCTAGTGTAGCGGTGAAATGCGTAGATATTAGGAAGAACACCAGTGGCGAAGGCGGCTTACTGGACTGAAACTGACGCTGAGTCACGAAAGCGTGGGGAGCGAACAGG` |
| ASV2199 | 424 | `TGAGGAATATTGGTCAATGGGCGAGAGCCTGAACCAGCCATGCCGCGTGCAGGAAGACGGTCCTATGGATTGTAAACTGCTTTTATTTGGGAATAAACTCCTCTACGTGTAGAGGCCTGAAGGTACCAAATGAATAAGCACCGGCTAACTCCGTGCCAGCAGCCGCGGTAATACGGAGGGTGCAAGCGTTATCCGGAATCATTGGGTTTAAAGGGTCCGCAGGCGGATGTATAAGTCAGTGGTGAAAGCCTACAGCTCAACTGTAGAACTGCCATTGATACTGTACGTCTTGAATTCGATCGAAGTGGGCGGAATATGACATGTAGCGGTGAAATGCTTAGATATGTCATAGAACACCGATAGCGAAGGCAGCTCACTAGGTCTGGATTGACGCTCAGGGACGAAAGCGTGGGGAGCAAACAGG` |
| ASV1476 | 404 | `TAAGGAATATTGGACAATGGGGGAAACCCTGATCCAGCCATGCCGCGTGAGTGAAGAAGGCCTTAGGGTTGTAAAGCTCTTTCAATGGTGAAGATAATGACGGTAGCCATAGAAGAAGCCCCGGCTAAATTCGTGCCAGCAGCCGCGGTAATACGAATGGGGCAAGCGTTGTTCGGAATCACTGGGCTTAAAGCGTGCGCAGGCGGATATGAAAGTCAGGAGTGAAATCCCGGGGCTCAACCCCGGAATTGCTTTTGAAACTTCATATCTTGAATCCGGTAGAGGTTGGGAGAATTCCTAGTGTAGAGGTGAAATTCGCAGATATTAGGAGGAATACCAGTGGCGTAGGCGCCCAACTGGACCGGTATTGACGCTCAGGCACGAAAGCGTGGGGAGCAAACAGG` |
| ASV5019 | 405 | `TGGGGAATCTTGCGCAATGGGCGAAAGCCTGACGCAGCAACGCCGCGTGCGGGATGACGGCCTTCGGGTTGTAAACCGCTTTCAGCAGGGACGAAATTGACGGTACCTGCAGAAGAAGCCCCGGCCAACTACGTGCCAGCAGCCGCGGTAACACGTAGGGGGCGAGCGTTGTCCGGATTTATTGGGCGTAAAGAGCTCGTAGGCGGCTCGGTAAGTCGGGTGTGAAACCTCCAGGCTCAACCTGGAGACGCCACCTGATACTGCTGTGGCTAGAGTCCGGTAGGGGAGCGTGGAACTCCTGGTGTAGCGGTGAAATGCGCAGATATCAGGAAGAACACCAGCGGCGAAGGCGGCGCTCTGGGCCGGAACTGACGCTGAGGAGCGAAAGCGTGGGTAGCAAACAGG` |
| ASV1838 | 429 | `TGGGGAATTTTGGACAATGGGCGAAAGCCTGATCCAGCCATGCCGCGTGCGGGAAGAAGGCCTTCGGGTTGTAAACCGCTTTTGTCAGGGAAGAAACGCTCCTGTCTAATACACGGGGGTAATGACGGTACCTGAAGAATAAGCACCGGCTAACTACGTGCCAGCAGCCGCGGTAATACGTAGGGTGCAAGCGTTAATCGGAATTACTGGGCGTAAAGCGTGCGCAGGCGGCCATGCAAGACAGATGTGAAATCCCCGGGCTCAACCTGGGAACTGCATTTGTGACTGCATGGCTGGAGTGCGGCAGAGGGGGATGGAATTCCGCGTGTAGCAGTGAAATGCGTAGATATGCGGAGGAACACCGATGGCGAAGGCAATCCCCTGGGCCTGCACTGACGCTCATGCACGAAAGCGTGGGGAGCAAACAGG` |
| ASV1787 | 430 | `TGGGGAATATTGCGCAATGGGCGAAAGCCTGACGCAGCGACGCCGCGTGGAGGAAGACGGTCTTCGGATTGTAAACTCCTGTTAAGTGGGAAGAAAGACCAAGTGCTAATACCACTTGGGGATGACGGTACCACTAGAGAAAGCCCCGGCTAACTTCGTGCCAGCAGCCGCGGTAATACGAAGGGGGCGAGCGTTATTCGGAATTACTGGGCGTAAAGGACGCGTAGGCGGTTTATTAAGTCAGCTGTTAAATTTCAGGGCCTAACCCTGAGTATGCAGATGATACTGATGGAACTAGAGTATGGAAGAGAGGGGTGGAATTCTCGGAGTAGCGGTAAAATGCGTAGATCTCGAGAGGAACACCGATGGCGAAGGCAGCCCCTTGGTCCAATACTGACGCTGAGGCGTGAAAGCGTGGGGAGCAAACAGG` |
| ASV4897 | 404 | `TGGGGAATATTGGACAATGGGGGGAACCCTGATCCAGCAATGCCGCGTGAGTGAAGAAGGCCTTCGGGTTGTAAAGCTCTTTTATGAGGGAAGATGATGACGGTACCTCAAGAAAAAGCTCCGGCTAACTTCGTGCCAGCAGCCGCGGTAATACGAAGGGAGCAAGCGTTGTTCGGAATAACTGGGCGTAAAGGGCGCGTAGGCGGCGATATAAGTTAGTTGTGAAAGACCTGGGCTTAACCGAGGAACAGCAATTGATACTGTATGGCTAGAGTCTGGAAGGGGAGAATGGAATTGTGTGTGGAGAGGTGAAATTCGTAGAGATGCACAGGAACACCGGTGGTGAAAACGATTCTCTGGGACAAGACTGACGCTGAGGCGCGAGAGCGTGGGGAGCAAACAGG` |
| ASV745 | 422 | `TGAGGAATTTTGCACAATGGAGGAAACTCTGATGCAGCGACGCCGTGTGTAGGATGAAGGGGCTCTGCCTTGTAAACTACTGTCGGATAGGAATAAAAGTATGCTTTGCGTACTGGGAATGTACTATCAAAGGAAGGACCGGCTAACTACGTGCCAGCAGCCGCGGTAATACGTAGGGTCCGAGCGTTGTCCGGATTTACTGGGTATAAAGGGTGCGTAGGCGGATTTGTGCTTCAGAGGTTAAAGACCGCAGCTCAACTGCGGGAGTGCCTCTGATAAGGCAGATCTTGGATATGAGAGGGGTAGGTAGAATTCCTGGTGTAGCGGTGAAATGCGTAGATATCAGGAGGAATACCAGTGGCGAAGGCGGCCTACTGGCTCATAATCGACGCTGAGGCACGAAAGCGTGGGGATCAAACAGG` |
| ASV2435 | 419 | `TGGGGAATATTGCACAATGGGCGCAAGCCTGATGCAGCGACGCCGCGTGAGGGATGAAGGCCTTCGGGTTGTAAACCTCTTTCAGTAGGGAAGAAGCCGGCCCTTGGGTTGGTGACGGTACCTGCAGAAGAAGCGCCGGCTAACTACGTGCCAGCAGCCGCGGTAATACGTAGGGCGCAAGCGTTATCCGGAATTATTGGGCGTAAAGAGCTCGTAGGCGGTTTGTCGCGTCTGCCGTGAAAGTCCGGGGCTTAACTCCGGATCTGCGGTGGGTACGGGCAGACTAGAGTGCAGTAGGGGAGACTGGAATTCCTGGTGTAGCGGTGAAATGCGCAGATATCAGGAGGAACACCGATGGCGAAGGCAGGTCTCTGGGCTGTAACTGACGCTGAGGAGCGAAAGCATGGGGAGCGAACAGG` |
| ASV4687 | 429 | `TGGGGAATTTTGGACAATGGGGGCAACCCTGATCCAGCAACGCCGCGTGAATGAAGAAGGCCTTCGGGTTGTAAAGTTCTTTTGTCAGGGAGCAAAGTCTGATGCCTAATAAGTATTGGAGCTGAGAGTACCTGAAGAATAAGCACCGGCTAACTACGTGCCAGCAGCCGCGGTAATACGTAGGGTGCGAGCGTTAATCGGAATTACTGGGCGTAAAGCGTGCGCAGGCGGATATTTAAGTTAGGAGTGAAATCCCCGGGCTCAACCTGGGAACTGCTCTTAAGACTGGGTATCTAGAGTTTGTCAGAGGGGGGTGGAATTTCAAGTGTAGCAGTGAAATGCGTAGAGATTTGAAGGAACACCGATGGCGAAGGCAGCCCCCTGGGATAAAACTGACGCTCATGTACGAAAGCGTGGGTAGCAAACAGG` |
| ASV2457 | 494 | `CAGGAACAGCAGGGCCAGCACATAGGCAAACCAGTTTTCGATGCCGCCACCGGCCAGGTCGAAGGTCTTGATCAGCACCGGGCCAAGGAATACCTCCGACACTTTTTCGCCCACCCCAATGATCAGGCCGCCGATGATGGCGCCGGGAATCGACGTGAAGCCCCCCAGGATCACCACCGGCAAGGCCTTGAGCGCCAGTTGCGCCAGCGAAAACTGCACGCCCAGCTTGGCGCCCCACACCATGCCAGCCACCAGCGCAACGAAGCCGGCCACCGCCCAGACGATCACCCAGATGCGGTTCAGGGGAATGCCGATCGACTGCGCGGCCTGGTGGTCGTCAGCCACCGCACGCAGCGCGCGGCCGGTTGCCGTCTTCTGGAAGAAGATGGCCAACAGGATCACGAGGATCGTCGCGATCACGGTCGCCACCAGATCTTCCTTGCTGACCAGAATGCCGCCCTGGAACATGGAATCGAGAATGAAGGTTGGATCCT` |
| ASV3270 | 429 | `TGGGGAATTTTGGACAATGGGGGCAACCCTGATCCAGCAATGCCGCGTGAGTGAAGAAGGCCTTCGGGTTGTAAAGCTCTTTTGTCAGGGAAGAAACGGTTGTTCCTAATACGGATGACTAATGACGGTACCTGAAGAATAAGCACCGGCTAACTACGTGCCAGCAGCCGCGGTAATACGTAGGGTGCAAGCGTTAATCGGAATTACTGGGCGTAAAGCGTGCGCAGGCGGTTTTGTAAGACAGATGTGAAATGCCCGGGCTTAACCTGGGAACTGCATTTGTGACTGCAAGGCTAGAGTGTGTCAGAGGGGGGTAGAATTCCACGTGTAGCAGTGAAATGCGTAGATATGTGGAGGAACACCGATGGCGAAGGCAGCCCCCTGGGATAACACTGACGCTCATGCACGAAAGCGTGGGGAGCAAACAGG` |
| ASV50 | 404 | `TGGGGAATCTTAGACAATGGGCGCAAGCCTGATCTAGCCATGCCGCGTGATCGATGAAGGCCTTAGGGTTGTAAAGATCTTTCAGTGGGGAAGATAATGACGGTACCCACAGAAGAAGCCCCGGCTAACTCCGTGCCAGCAGCCGCGGTAATACGGAGGGGGCTAGCGTTATTCGGAATTACTGGGCGTAAAGCGCACGTAGGCGGATTAGAAAGTCAGAGGTGAAATCCCAGGGCTCAACCTTGGAACTGCCTTTGAAACTTCTAGTCTTGAGGTCGTGAGAGGTGAGTGGAATTCCGAGTGTAGAGGTGAAATTCGTAGATATTCGGAGGAACACCAGTGGCGAAGGCGGCTCACTGGCACGATACTGACGCTGAGGTGCGAAAGCGTGGGGAGCAAACAGG` |
| ASV3618 | 429 | `TGGGGAATATTGGACAATGGGGGCAACCCTGATCCAGCAATGCCGCGTGTGTGAAGAAGGCCTTCGGGTTGTAAAGCACTTTAGGTTAAGAAGAAGGTATGTAGTTTAAGAGATGACATACTTGACGGTATTAACAGAATAAGCACCGGCTAACTCTGTGCCAGCAGCCGCGGTAATACAGAGGGTGCAAGCGTTAATCGGAGTTACTGGGCGTAAAGGGCGCGTAGGCGGTTATTTAAGTGAGATGTGAAATCCCTGGGCTTAACCTAGGAACTGCATCTCATACTGGATAGCTAGAGTATTGTAGAGGGTAGTGGAATTTCCGGTGTAGCGGTGAAATGCGTAGAGATCGGAAGGAACACCAGTGGCGAAGGCGACTACCTGGACAAATACTGACGCTGAGGCGCGAGAGCGTGGGGAGCAAACAGG` |
| ASV1437 | 403 | `TGAGGAATCTTCCACAATGGGCGAAAGCCTGATGGAGCGACACCGCGTGAAGGATGAAGCCTTTGTTGGTGTAAACTTCTTTTCTCTGGGAAGATAATGACGGTACCAGAGGAATAAGGGGCGGCAAACTTCGTGCCAGCAGCCGCGGTAATACGAGGGCCCCAAGCGTTATCCGGAATTACTGGGCGTAAAGCGTCTGTAGGTGGTTTGGAAAGTCTCAAGTGAAAGATCAGGGCTCAACCCTGATTCGCTTGGGAAACTATCAAACTTGAGTGCGGGAGAGGCAAGCAGAACTGTATGAGTAGGGGTGCAATCCGTTGATACATACAAGAATACCAAAAGCGAAGGCAGCTTGCTGGAACGCCACTGACACTGAGAGACGAAAGCGTGGGGAGCGAAAGGG` |
| ASV3659 | 385 | `GCGCGAAAACTTTACAATGCACGAAAGTGTGATAGGGGAACTCAGCGTGCTCATGGAAACCATGGGCTTTTCTACAGGGTAAATACCTGTGGGAATAAAGGATGGGCAAGACCGGTGCCAGCAGCCGCGGTAACACCGGCGTCCTTAGTGGGAATCACTTATTAGGTCTAAAGCGCTCGTAGTCGGCTTTGTAAATTTCTTGTGAAATCGTGTCGCTTAACGGCACGGCGTGCAGGAAACACTGCAGAGCTTGGGACCGGGAGGCGTCAGAAGTATTCCTTAGGGAGCGGTAAAATGTGATAATCTAAGGAGGACTACCTGTGGCGAAGGCGTCTGACGAGAACGGGTTCGACGATCAGGAGCGAAGGCTAGGGGAGCAAACCGG` |
| ASV801 | 430 | `TGGGGAATATTGCGCAATGGGGGAAACCCTGACGCAGCAACGCCGCGTGAGTGAGGAAGGACTTCGGTCTGTAAAGCTCTGTTAATGTGGAAAAATGGTAGTTGGTCTAATAGGCCGATTATTTGATGGTACACATAGAGGAAGCACCGGCTAACTTCGTGCCAGCAGCCGCGGTAATACGAAGGGTGCAAGCGTTGTTCGGATTTATTGGGCGTAAAGCGCGCGTAGGCGGACCTGCAAGTAAGATGTGAAATCTCGAGGCTCAACCTCGAAACTGCGTCTTAAACTATAGGTCTAGAATCTCGGAGGGGGAAGGGGAATTTCGCATGTAGGGGTAAAATCCGTAGATATGCGAAGGAACACCAGAGGCGAAGGCGCCTTCCTGGACGAGTATTGACGCTGAGGCGCGAAAGCGTGGGGATCAAACAGG` |
| ASV2631 | 404 | `TGGGGAATCTTGGACAATGGACGAAAGTCTGATCCAGCAATTCTGCGTGCGGGATGAAGGTCTATGGATTGTAAACCGCTTTTCTGAAGGAAGACGAATGACGGTACTTCAGGAATAAGCGACGGCTAACTTCGTGCCAGCAGCCGCGGTAATACGAAGGTCGCAAGCGTTATCCGGAGTTACTGGGCGTAAAGAGTGCGTAGATGGTTTAGTAAGTCTGATTTTAAAGACTCTGTCTCAAGCAGAGGAAGGGGTCAGATACTGCTATTCTGGAGATAACTGGGGGTTAGTGGAATTACCGGTGGAGGGGTGAAATCCGTTGATATCGGTAGGAACGCCGAAAGCGAAAGCAACTAACTATGGTTTTCTGACATTCAAGCACGAAAGCTCGGGGATCAAACGGG` |
| ASV1982 | 429 | `TAGGGAATCTTCGGCAATGGACGGAAGTCTGACCGAGCAACGCCGCGTGAGTGAAGAAGGTTTTCGGATCGTAAAGCTCTGTTGTAAGAGAAGAACGAGTGTGAGAGTGGAAAGTTCACACTGTGACGGTATCTTACCAGAAAGGGACGGCTAACTACGTGCCAGCAGCCGCGGTAATACGTAGGTCCCGAGCGTTGTCCGGATTTATTGGGCGTAAAGCGAGCGCAGGCGGTTAGATAAGTCTGAAGTTAAAGGCTGTGGCTTAACCATAGTACGCTTTGGAAACTGTTTAACTTGAGTGCAAGAGGGGAGAGTGGAATTCCATGTGTAGCGGTGAAATGCGTAGATATATGGAGGAACACCGGTGGCGAAAGCGGCTCTCTGGCTTGTAACTGACGCTGAGGCTCGAAAGCGTGGGGAGCAAACAGG` |
| ASV1786 | 408 | `TCGAGAATCTTCCGCAATGGACGCAAGTCTGACGGAGCGATGTCGCGTGATTGATGAAGTCTTTCGGGATGTAAAGATCTTTTCTCAAGGAGGAAGTTTATTGACGTTACTTGAGGAATAAGGGGTTGCTAAACTCGTGCCAGCAGCAGCGGTAATACGAGTGCCCCAAGCGTTATCCGGAATTATTGGGCGTAAAGGGTCCGCAGGCGGTTTTATTAGTCTTGTGTGAAATCTCCCGGCTTAACTGGGAAAACGCATAGGAAACGGTAAGACTAGAGGATGTGAGAGGTATGTGGAACTCATGGTGTAGGGGTGAAATCCGTTGATATCATGGGGAACACCAAATGCGAAGGCAACATACTGGCACATTCCTGACGCTCAGGGACGAAAGCACGGGTAGCGAATGGG` |
| ASV4991 | 425 | `TAAGGAATATTGGACAATGGTCGGAAGACTGATCCAGCCATGCCGCGTGAAGGATGAAGGCGCTCAGCGTTGTAAACTTCTTTTGGACGGGAACAAAATGCGGGATTTATCCTGTACTGAGGGTACCGTCAGAATAAGCACCGGCTAACTCCGTGCCAGCAGCCGCGGTAATACGGAGGGTGCAAGCGTTATCCGGAATCACTGGGTTTAAAGGGTGCGTAGGCGGTCTGATAAGTCAGTCGTGAAATCGCTGGGCTCAACCCATGCAACTGCGATTGATACTGTCAGACTTGAATCAGGTTGAGGTAGGCGGAATGTGGCATGTAGCGGTGAAATGCTTAGATATGCCATAGAACACCGATTGCGAAGGCAGCTTGCTGGGCCTGAATTGACGCTGAGGCACGAAAGCGTGGGGAGCAAACAGG` |
| ASV3649 | 404 | `TGGGGAATATTGGACAATGGGGGCAACCCTGATCCAGCAATGCCGCGTGAGTGATGAAGGCCTTAGGGTTGTAAAGCTCTTTTACCGGGGACGATGATGACGGTACCCGGAGAATAAGCCCCGGCTAACTTCGTGCCAGCAGCCGCGGTAATACGAAGGGGGCGAGCGTTGTTCGGAGTTACTGGGCGTAAAGGGCGCGTAGGCGGCGATCCAAGTTAGGCGTGAAAGCCCCGGGCTCAACCTGGGAATTGCGCTTAAGACTGGATTGCTCGAGTGCGGAAGAGGTGAGTGGAATTCCCAGTGTAGAGGTGAAATTCGTAGATATTGGGAAGAACACCAGTGGCGAAGGCGGCTCACTGGTCCGCAACTGACGCTGAGGCGCGAAAGCGTGGGGAGCAAACAGG` |
| ASV5711 | 424 | `TAGGGAATATTGGACAATGGAGGCAACTCTGATCCAGCCATGCCGCGTGCAGGAAGACTGCCCTATGGGTTGTAAACTGCTTTTGTACGGGAAGAAACCCACCTACGAGTAGGAGGTTGACGGTACCGTAAGAATAAGGATCGGCTAACTTCGTGCCAGCAGCCGCGGTAATACGAAGGATCCAAGCGTTGTCCGGATTTACTGGGTTTAAAGGGAGCGTAGGCGGTTTTATAAGTCAGTGGTGAAAGCCTTCAGCTTAACTGAAGAATAGCCATTGAAACTGTAGGACTCGAGTATGGTTGAGGTCACTGGAATGTAACATGTAGCGGTGAAATGCTTAGATATGTTACAGAACACCAATTGCGAAGGCAGGTGACTAAGCCATAACTGACGCTGAGGCTCGAAAGCGTGGGGAGCAAACAGG` |
| ASV4429 | 429 | `TGGGGAATATTGCACAATGGGGGAAACCCTGATGCAGCCATGCCGCGTGTGTGAAGAAGGCCTTCGGGTTGTAAAGCACTTTCAGTGGTGAGGAAAGGCAGGAACCTAATACGTTTTTGCTGTGACGTTAACCACAGAAGAAGCACCGGCTAACTCCGTGCCAGCAGCCGCGGTAATACGGAGGGTGCAAGCGTTAATCGGAATAACTGGGCGTAAAGCGCACGCAGGCGGTTTGTTAAGCCAGATGTGAAAGCCCCGAGCTCAACTCGGGAACTGCATTTGGAACTGGCAAACTAGAGTCTTGTAGAGGGGGGTAGAATTTCCAGTGTAGCGGTGAAATGCGTAGAGATTGGAAGGAATACCAGTGGCGAAGGCGGCCCCCTGGACAAAGACTGACGCTCATGTGCGAAAGCGTGGGGAGCAAACAGG` |
| ASV4815 | 428 | `TGGGGAATATTGGACAATGGGCGAAAGCCTGATCCAGCAATACCGCGTGTGTGAAGAAGGCCTTCGGGTTGTAAAGCACTTTAAGTTGGGAAGAAAAAGCTTCACCTAATACGTGGAGCCTTGACGGTACCGACAGAATAAGCACCGGCTAACTCTGTGCCAGCAGCCGCGGTAATACAGAGGGTGCTAGCGTTAATCGGATTTACTGGGCGTAAAGCGTGCGTAGGTGGTTTGATAAGTCGGTTGTGAAAGCCCCGGGCTCAACCTGGGAATTGCGGCCGATACTGTCAGACTCGAAAACGGTAGAGGGAGGTGGAACTCCAGGTGTAGCGGTGAAATGCGTAGATATCTGGAAGAACACCGATGGCGAAGGCAGCCTCCTGGGCCTGTTTTGACACTGAGGCACGAAAGCGTGGGTAGCAAACAGG` |
| ASV4493 | 404 | `TGGGGAATATTGGACAATGGGCGAAAGCCTGATCCAGCAATGCCGCGTGAGTGATGAAGGCCTTAGGGTTGTAAAGCTCTTTCAGCAGGGAAGATAATGACGGTACCTGCAGAAGAAGCTCCGGCTAACTCCGTGCCAGCAGCCGCGGTAATACGGAGGGAGCTAGCGTTGTTCGGAATTACTGGGCGTAAAGAGCGCGTAGGCGGTTTGTAAAGTTGGAAGTGAAAGCCCAAGGCTTAACCTTGGAAATGCTTTCAAAACTCACAGACTAGAGATTGGTAGAGGATGGTGGAATTCCTAGTGTAGAGGTGAAATTCGTAGATATTAGGAGGAACACCGGTGGCGAAGGCGGCCATCTGGGCCAAAACTGACGCTGAGGCGCGAAAGCGTGGGGAGCAAACAGG` |
| ASV3104 | 429 | `TGGGGAATATTGGACAATGGGCGCAAGCCTGATCCAGCCATGCCGCGTGTGTGAAGAAGGCCTTCGGGTTGTAAAGCACTTTCAGCGAGGAGGAAGGGTGGTGTGTTAATAGCACATCATTTTGACGTTACTCGCAGAAGAAGCACCGGCTAACTCTGTGCCAGCAGCCGCGGTAATACAGAGGGTGCAAGCGTTAATCGGAATTACTGGGCGTAAAGCGCACGTAGGCGGTTGGTTAAGTTGGATGTGAAAGCCCCGGGCTCAACCTGGGAATTGCATTCAAAACTGGCCAACTAGAATACGGGAGAGGGGGGTAGAATTCCAAGTGTAGCGGTGAAATGCGTAGAGATTTGGAGGAATACCAGTGGCGAAGGCGGCCCCCTGGCCCGATATTGACGCTGAGGTGCGAAAGCGTGGGGAGCAAACAGG` |
| ASV4970 | 430 | `TGGGGAATTTTGGACAATGGGGGCAACCCTGATCCAGCCATGCCGCGTGCGGGAAGAAGGCCTTCGGGTTGTAAACCGCTTTAGTCTAGGAAAAAGGAATGCAGGATTAATACCCTTGTGTTTTGATGGTACTGGAAGAATAAGCACCGGCTAACTACGTGCCAGCAGCCGCGGTAATACGTAGGGTGCGAGCGTTAATCGGAATTACTGGGCGTAAAGCGTGCGCAGGCGGTCTGTTAAGACAGGCGTGAAATCCCCGGGCTCAACCTGGGAATGGCGCTTGTGACTGGCAGGCTAGAGTGTGTCAGAGGGGGGTGGAATTCCACGTGTAGCAGTGAAATGCGTAGAGATGTGGAGGAACACCGATGGCGAAGGCAGCCCCCTGGGATAACACTGACGCTCATGCACGAAAGCGTGGGGAGCAAACAGG` |
| ASV3145 | 407 | `TCGAGAATCTTCCGCAATGCACGAAAGTGTGACGGAGCGACGCCGCGTGATTGATGACGCTCTTCGGAGTGTAAAAATCTTTTATAAGGGAGGAAATTTTTGACGTTACCTTATGAATAAGGGGCTCCTAATCTCGTGCCAGCAGGAGCGGTAATACGAGAGCCTCAAGCGTTATCCGGAATTATTGGGCGTAAAGGGTGCGTAGGTGGTTATATTAGTCGAATGTTAAAGACTCGGGCTTAACCCGAGGAATGCATTCGAAACGGTATGACTAGAGAATGTGAGAGGTGTACGGAACTAACGGTGTAGGGGTGAAATCCGTTGATATCGTTGGGAACACCAAAAGCGAAGGCAGTACACTGGCACATTTCTGACACTGAGGCACGAAACCGTGGGTAGCGAATGGG` |
| ASV5434 | 429 | `TGGGGAATATTGGACAATGGGCGAAAGCCTGATCCAGCAATGCCGCGTGTGTGAAGAAGGCCTGCGGGTTGTAAAGCACTTTTGTCGGGAAAGAAAATCACAGGGTTAATACCCCTGTGCCTTGACGGTACCCGAAGAATAAGCACCGGCTAACTCCGTGCCAGCAGCCGCGGTAATACGGAGGGTGCAAGCGTTAATCGGAATTACTGGGCGTAAAGCGAGTGTAGGTGGCTATGTAAGTCGGATGTGAAATCCCCGGGCTCAACCTGGGAACTGCATTCGATACTGCATGGCTAGAGTCTGATAGAGGGAAGTGGAATTCCAGGTGTAGCGGTGAAATGCGTAGATATCTGGAAGAACACCAGTGGCGAAGGCGGCTTCCTGGATCAAGACTGACACTGAGGCTCGAAAGCGTGGGGAGCAAACAGG` |
| ASV3278 | 430 | `TGGGGAATATTGCGCAATGGGGGAAACCCTGACGCAGCAACGCCGCGTGAGTGATGAAGGCCTTCGGGTTGTAAAGCTCTTTCGGTTGGGAAGAAGGATATTACGGCGAACAGTCGTGGTATTTGATGGTACCAAAAGAAGAAGCACCGGCTAACTTCGTGCCAGCAGCCGCGGTAATACGAAGGGTGCAAGCGTTGTTCGGATTTATTGGGCGTAAAGGGTTCGTAGGCTGGATTATAAGTTACATGTGAAACCCCAGGGCTCAACCTTGGACGTGCATGTAATACTGTAGTTCTTGAATGTCGGAGAGGGTAGCGGAATTGCTGGTGTAGGAGTGACATCCGTAGAGATCAGCAGGAACACCGGAGGCGAAGGCGGCTACCTGGTCGAACATTGACGCTGAGGAACGAAAGCGTGGGGAGCAAACAGG` |
| ASV2464 | 405 | `TGGGGAATCTTGCGCAATGGGCGAAAGCCTGACGCAGCAACGCCGCGTGCGGGAAGAAGGCCTTCGGGTTGTAAACCGCTTTCAGAAGGAACGAAAATGACGGTACTTTCAGAAGAAGGTGCGGCCAACTACGTGCCAGCAGCCGCGGTGACACGTAGGCACCAAGCGTTGTCCGGATTTATTGGGCGTAAAGAGCTCGTAGGCGGTTCGGTAAGTCGGGTGTGAAAACTTTGGGCTTAACCCAAAGCCTGCATCCGATACTGCTGTGACTAGAGTTCGGTAGGGGAGCGGGGAATTCCTAGTGTAGCGGTGAAATGCGCAGATATTAGGAGGAACACCGGTGGCGAAGGCGCCGCTCTGGGCCGATACTGACGCTGAGGAGCGAAAGCGTGGGTAGCAAACAGG` |
| ASV3107 | 404 | `TGGGGAATCTTGGACAATGGGGGCAACCCTGATCCAGCCATGCCGCGTGGACGATGAAGGCCTTAGGGTTGTAAAGTCCTTTCGCCGGGGAAGATAATGACGGTACCCGGTAAAGAAGCCCCGGCTAACTCCGTGCCAGCAGCCGCGGTAATACGGAGGGGGCTAGCGTTGTTCGGAATTACTGGGCGTAAAGCGCACGTAGGCGGATCAGTCAGTCAGGGGTGAAATCCCGGGGCTCAACCCCGGAACTGCCTTTGATACTGCTGATCTGGAGGTCGAGAGAGGCGAGTGGAATTCCGAGTGTAGAGGTGAAATTCGTAGATATTCGGAGGAACACCAGTGGCGAAGGCGGCTCGCTGGCTCGATACTGACGCTGAGGTGCGAAAGCGTGGGGAGCAAACAGG` |
| ASV503 | 429 | `TGGGGAATTTTGGACAATGGGCGCAAGCCTGATCCAGCCATGCCGCGTGCGGGAAGAAGGCCTTCGGGTTGTAAACCGCTTTTGTCAGGGAAGAAACGGCTTTTGCTAATACCGAGAGCTAATGACGGTACCTGAAGAATAAGCACCGGCTAACTACGTGCCAGCAGCCGCGGTAATACGTAGGGTGCAAGCGTTAATCGGAATTACTGGGCGTAAAGCGTGCGCAGGCGGTCTTGCAAGACAGGTGTGAAATCCCCGGGCTTAACCTGGGAGCTGCATTTGTGACTGCAAGGCTAGAGTACGGTAGAGGAGGATGGAATTCCGCGTGTAGCAGTGAAATGCGTAGATATGCGGAGGAACACCGATGGCGAAGGCAATCCCCTGGACCTGTACTGACGCTCATGCACGAAAGCGTGGGGAGCAAACAGG` |
| ASV4437 | 429 | `TAGGGAATCTTCCACAATGGACGAAAGTCTGATGGAGCAACGCCGCGTGAGTGAAGAAGGTTTTCGGATCGTAAAACTCTGTTATTGGAGAAGAACGTATTTGGTAGTAACTGGCCAGATAGTGACGGTATCCAATCAGAAAGCCACGGCTAACTACGTGCCAGCAGCCGCGGTAATACGTAGGTGGCAAGCGTTGTCCGGATTTATTGGGCGTAAAGCGAGTGCAGGCGGTTTTTTAAGTCTGATGTGAAAGCCTTCGGCTTAACCGAAGAAATGCATTGGAAACTGGGAAACTTGAGTGCAGAAGAGGAGAGTGGAACTCCATGTGTAGCGGTGAAATGCGTAGATATATGGAAGAACACCAGTGGCGAAGGCGGCTCTCTGGTCTGTAACTGACGCTGAGGCTCGAAAGCGTGGGTAGCAAACAGG` |
| ASV1163 | 424 | `TGAGGAATATTGGTCAATGGGCGCAAGCCTGAACCAGCCATGCCGCGTGCAGGATGACGGTCCTATGGATTGTAAACTGCTTTTGTACAGGAAGAAACACTCCCTCGTGAGGGAGCTTGACGGTACTGTAAGAATAAGGATCGGCTAACTCCGTGCCAGCAGCCGCGGTAATACGGAGGATCCAAGCGTTATCCGGAATCATTGGGTTTAAAGGGTCCGTAGGCGGTTAAATAAGTCAGTGGTGAAATCTGGTCGCTCAACGATCAAACGGCCATTGATACTGTTTAACTTGAATTACTTGGAAGTAACTAGAATATGTAGTGTAGCGGTGAAATGCTTAGAGATTACATGGAATACCAATTGCGAAGGCAGGTTACTACAAGTATATTGACGCTGATGGACGAAAGCGTGGGGAGCGAACAGG` |
| ASV3916 | 429 | `TGGGGAATATTGGACAATGGGCGAAAGCCTGATCCAGCGATGCCGCGTGTGTGAAGAAGGCCTGCGGGTTGTAAAGCACTTTCAGTGGGGAGAATGAACCTGTTCCTAATATGAATAGGGAAAGATGTTACCCACAGAAGAAGCACCGGCTAACTCTGTGCCAGCAGCCGCGGTAATACAGAGGGTGCGAGCGTTAATCGGAATTACTGGGCGTAAAGCGTGCGTAGGTGGTTGCATAAGTTAGGTGTGAAATTCCTGGGCTTAACCTGGGAACTGCGCCTAATACTGTATAGCTAGAGTATTGTAGAGGAAAGTGGAATTTCCGGTGTAGCAGTGAAATGCGTAGATATCGGAAGGAACATCAGTGGCGAAGGCGACTTTCTGGACAAATACTGACACTGAGGCACGAAAGCATGGGGAGCAAACAGG` |
| ASV1578 | 430 | `TGGGGAATTTTGGACAATGGGCGCAAGCCTGATCCAGCCATGCCGCGTGCAGGATGAAGGCCTTCGGGTTGTAAACTGCTTTAGTTCGGGAAAAATGTCCTGTGCGCTAATAACGCATGGGGTTGATGGTACCGGAAGAATAAGCACCGGCTAACTACGTGCCAGCAGCCGCGGTAATACGTAGGGTGCAAGCGTTAATCGGAATTACTGGGCGTAAAGCGTGCGCAGGCGGTTCGCTAAGACAGACGTGAAATCCCCGGGCTTAACCTGGGAACTGCGTTTGTGACTGGCGGGCTAGAGTACGGCAGAGGGGGGTGGAATTCCACGTGTAGCAGTGAAATGCGTAGATATGTGGAGGAACACCGATGGCGAAGGCAGCCCCCTGGGTCGATACTGACGCTCATGCACGAAAGCGTGGGGAGCAAACAGG` |
| ASV1793 | 424 | `TGAGGAATATTGGACAATGGGTGAAAGCCTGATCCAGCCATCCCGCGTGAAGGACGACGGCCCTACGGGTTGTAAACTTCTTTTATATAGGAATAAACCCAGATACGTGTATTTGGCTGAAGGTACTATATGAATAAGCACCGGCTAACTCCGTGCCAGCAGCCGCGGTAATACGGAGGGTGCAAGCGTTATCCGGATTTATTGGGTTTAAAGGGTCCGTAGGCGGATCTGTAAGTCAGTGGTGAAATCTCATAGCTTAACTATGAAACTGCCATTGATACTGCAGGTCTTGAGTGAATTTGAGGTAGCTGGAATAAGTAGTGTAGCGGTGAAATGCATAGATATTACTTAGAACACCAATTGCGAAGGCAGGTTACCAAGATTCAACTGACGCTGAGGGACGAAAGCGTGGGGAGCGAACAGG` |
| ASV3867 | 404 | `TGGGGAATTTTCCGCAATGGGCGAAAGCCTGACGGAGCAATACCGCGTGAGGGAAGAAGGTCTTTGGATTGTCAACCTCTTTTCTCAGGGAAGATAATGACGGTACCTGAGGAATAAGCATCGGCTAACTCCGTGCCAGCAGCCGCGGTAATACGGAGGATGCAAGCGTTATCCGGAATCATTGGGCGTAAAGCGTCCGTAGGTGGTTAGTCAAGTCCATTGTCAAAGAAAGCAGCTTAACTGCAGGCCGGCAGTGGAAACTGAAAAACTAGAGCCAAATAGGGGTAGAGGGAATTCCCGGTGTAGCGGTGAAATGCGTAGAGATCGGGAAGAACACCAGTGGCGAAAGCGCTCTACTGGATTAAGGCTGACACTGAGGGACGAAAGCTAGGGGAGCGAAAGGG` |
| ASV1994 | 403 | `TGGGGGATATTGCACAATGGGGGAAACCCTGATGCAGCGACGCCGCGTGTGGGAAGACGGTCTTCGGATTGTAAACCACTGTCTTCGGGGACGATAATGACGGTACCCGAGGAGGAAGCTCCGGCTAACTACGTGCCAGCAGCCGCGGTAATACGTAGGGAGCGAGCGTTGTCCGGAATTACTGGGTGTAAAGGGAGCGTAGGCGGGAAGACAAGTTGGGTGTGAAATCTATCGGCTCAACCGGTAGTTGCATTCAAAACTGTTTTTCTTGAGTGAAGTAGAGGTTGGCGGAATTCCTAGTGTAGCGGTGAAATGCGTAGATATTAGGAGGAACACCAGTGGCGAAGGCGGCCAACTGGGCTTTTACTGACGCTGAGGCTCGAAAGCGTGGGGAGCAAACAGG` |
| ASV619 | 429 | `TGGGGAATTTTGGACAATGGGCGCAAGCCTGATCCAGCAATGCCGCGTGAGTGAAGAAGGCCTTCGGGTTGTAAAGCTCTTTTGTCAGGGAAGAAACGGTATCTCCTAATACGGGGTGCTAATGACGGTACCTGAAGAATAAGCACCGGCTAACTACGTGCCAGCAGCCGCGGTAATACGTAGGGTGCAAGCGTTAATCGGAATTACTGGGCGTAAAGCGTGCGCAGGCGGTTATATAAGTCAGATGTGAAATCCCCGGGCTCAACCTGGGAACTGCATTTGAGACTGTATGGCTAGAGTGTGTCAGAGGGGGGTAGAATTCCACGTGTAGCAGTGAAATGCGTAGATATGTGGAGGAATACCGATGGCGAAGGCAGCCCCCTGGGATAACACTGACGCTCATGCACGAAAGCGTGGGGAGCAAACAGG` |
| ASV321 | 429 | `TGGGGAATTTTGGACAATGGGCGCAAGCCTGATCCAGCAATGCCGCGTGCAGGATGAAGGCCTTCGGGTTGTAAACTGCTTTTGTACGGAACGAAAAGGTCTGCCTTAATACGGTGGGCCCATGACGGTACCGTAAGAATAAGCACCGGCTAACTACGTGCCAGCAGCCGCGGTAATACGTAGGGTGCGAGCGTTAATCGGAATTACTGGGCGTAAAGCGTGCGCAGGCGGTTATATAAGACAGATGTGAAATCCCCGGGCTCAACCTGGGACCTGCATTTGTGACTGTATAGCTAGAGTACGGTAGAGGGGGATGGAATTCCGCGTGTAGCAGTGAAATGCGTAGATATGCGGAGGAACACCGATGGCGAAGGCAATCCCCTGGACCTGTACTGACGCTCATGCACGAAAGCGTGGGGAGCAAACAGG` |
| ASV4460 | 429 | `TGAGGAATTTTGCGCAATGGGGGAAACCCTGACGCAGCAACGCCGCGTGAGTGATGAAGGCCTTTTGGTTGTAAAGCTCTGTCGCCTGGAAAGAAACTCTATGGTAGGAAATGACTATAGATTGACGGTACCAGGAAAGGAAGCACCGGCTAACTCCGTGCCAGCAGCCGCGGTAATACGGGGGGTGCAAGCGTTATTCGGAATCATTGGGTGTAAAGCGCATGTAGGCGGTCAGGTATGTCAGATGTGAAAGCCCGGGGCTTAACCCCGGAAGTGCATTTGAAACTGCCTGGCTTGAGTCTCGGAGAGGAAAGTGGAATTCCCAGTGTAGAGGTGGAATTCGTAGATATTGGGAGGAACACCGGTGGCGAAAGCGACTTTCTGGACGATGACTGACGCTCAGATGCGAAAGCGTGGGTAGCAAACAGG` |
| ASV782 | 429 | `TGGGGAATCTTGGACAATGGGGGAAACCCTGATCCAGCAATGCCGCGTGCAGGAAGAAGGCCTTCGGGTTGTAAACTGCTTTTGTTCGGAACGAAACGGTTCTTACTAATACTAGGAGCTAATGACGGTACCGGAAGAATAAGCACCGGCTAACTACGTGCCAGCAGCCGCGGTAATACGTAGGGTGCGAGCGTTAATCGGAATTACTGGGCGTAAAGCGTGCGCAGGCGGTTTTGTAAGATAGATGTGAAATCCCCGGGCTCAACCTGGGAATTGCATTTATGACTGCAAGGCTAGAGTACGGTAGAGGGGGGTAGAATTCCGCGTGTAGCAGTGAAATGCGTAGATATGCGGAGGAACACCGATGGCGAAGGCAGCCCCCTGGACCTGTACTGACGCTCATGCACGAAAGCGTGGGGAGCAAACAGG` |
| ASV5119 | 389 | `TCGGGAAAACTTCGCAATGCGCGAAAGCGTGACGAGGGGATTCAGAGTGCTCAGGAATTATTCCTGGGCTTTTGCCAAGGGCAAATACCTTGGCGAATAAGTGGTGGGCAAGACTGGTGCCAGCCGCCGCGGTAACCCCAGCGCCACAAGTGGGAACCACTTTTATTGGGTCTAAAGCATCCGTAGCCGGTCCGGCAAATGCCTTGTGAAATGGTTGCGCTCAACGTAACCACGTGCAAGGTAGACTACCAGACTAGGGACCAGAGGAGGTTGGCGGTATTCTTGGGGGAGCGGTAAAATGTTATAATCCCAAGAGGACCACCGATGGCGAAGGCAGCCAACCAATATGGATCCGACGGTGAGGGATGAAAGCTAGGGGAGCGATCCGG` |
| ASV275 | 404 | `TGGGGAATATTGGACAATGGGCGAAAGCCTGATCCAGCCATGCCGCGTGAATGATGAAGGCCTTAGGGTTGTAAAGTTCTTTCAACGGTGAAGATAATGACGGTAACCGTAGAAGAAGCCCCGGCTAACTTCGTGCCAGCAGCCGCGGTAATACGAAGGGGGCTAGCGTTGTTCGGAATTACTGGGCGTAAAGCGCACGTAGGCGGACTTTTAAGTCAGAGGTGAAATCCCGGGGCTCAACCCCGGAACTGCCTTTGATACTGGGAGTCTAGAGTATGGTAGAGGTGAGTGGAATACCGAGTGTAGAGGTGGAATTCGTAGATATTCGGTAGAACACCAGTGGCGAAGGCGGCTCACTGGACCATTACTGACGCTGAGGTGCGAAAGCGTGGGGAGCAAACAGG` |
| ASV1714 | 404 | `TGGGGAATATTGGACAATGGGGGCAACCCTGATCCAGCCATGCCGCGTGAGTGATGAAGGCCCTAGGGTTGTAAAGCTCTTTCACCGGAGAAGATAATGACGGTATCCGGAGAAGAAGCCCCGGCTAACTTCGTGCCAGCAGCCGCGGTAATACGAAGGGGGCTAGCGTTGTTCGGAATTACTGGGCGTAAAGCGCACGTAGGCTGATATTTAAGTCAGGGGTGAAATCCCAGAGCTCAACTCTGGAACTGCCTTTGATACTGGGTATCTTGAGTATGGAAGAGGTAAGTGGAATTCCGAGTGTAGAGGTGAAATTCGTAGATATTCGGAGGAACACCAGTGGCGAAGGCGGCTTACTGGTCCATTACTGACGCTGAGGTGCGAAAGCGTGGGGAGCAAACAGG` |
| ASV1216 | 429 | `TGGGGAATCTTGGACAATGGGCGAAAGCCTGATCCAGCAATGCCGCGTGCGTGAAGAAGGCCTTCGGGTTGTAAAGCGCTTTTGTCGGGGAGGAAATCCTGGTCTCTAATAAAGGCCGGGGATGACAGTACCCGAAGAATAAGGACCGGCTAACTACGTGCCAGCAGCCGCGGTAATACGTAGGGTCCAAGCGTTAATCGGAATTACTGGGCGTAAAGCGTCCGCAGGTGGCTTGATAAGATAGACGTGAAATCCCTGGGCTCAACCTAGGAATTGCGTTTATGACTGTCTCGCTAGAGTATGGGAGAGGGGGGTGGAATTCCACGTGTAGCAGTGAAATGCGTAGAGATGTGGAGGAACACCGATGGCGAAGGCAACCCCCTGGCCTAATACTGACACTCATGGACGAAAGCGTGGGGAGCAAACAGG` |
| ASV2511 | 428 | `TAGGGAATCTTGCGCAATGGGCGAAAGCCTGACGCAGCGACGCCGCGTGAGCGATGAAGGCCTTCGGGTTGTAAAGCTCTGTGGGGAGGGAAGAATAAGGATTGGCTAATACCCAGTCTGATGACGGTACCTCCTTAGCAAGCACCGGCTAACTCTGTGCCAGCAGCCGCGGTAAGACAGAGGGTGCAAACGTTGTTCGGAATTACTGGGCGTAAAGCGCGTGTAGGCGGCTATGTAAGTCGGGCGTGAAATCCCACGGCTCAACCGTGGAAGTGCACCCGAAACTGCGTAGCTAGAGTCCTGGAGAGGAAGGTGGAATGCTTGGTGTAGAGGTGAAATTCGTAGATATCAAGCGGAACACCGGTGGCGAAGGCGGCCTTCTGGACAGTGACTGACGCTGAGACGCGAAAGCGTGGGGAGCAAACAGG` |
| ASV2015 | 429 | `TGGGGAATCTTGTGCAATGGGCGAAAGCCTGACACAGCCATGCCGCGTGTGTGAAGAAGGCCTTCGGGTTGTAAAGCACTTTCAGTCGTAAAGAAAAGCTTCGGGCTAATACTTCGAGGTCTTGACGTTAGCGACAGAAGAAGCACCGGCTAACTCCGTGCCAGCAGCCGCGGTAATACGGAGGGTGCAAACGTTAATCGGAATTACTGGGCGTAAAGCGCGCGTAGGCGGTTTGTTAAGTGGGATGTGAAAGCCCCGGGCTTAACCTGGGAACTGCATTCCAAACTGACAGGCTAGAGTTTGGTAGAGGGAGGTGGAATTTCCTGTGTAGCGGTGAAATGCGTAGATATAGGAAGGAACACCAGTGGCGAAGGCGGCCTCCTGGACCAAAACTGACGCTGAGGTGCGAAAGCGTGGGGAGCAAACAGG` |
| ASV5063 | 429 | `TGGGGAATATTGGACAATGGGGGAAACCCTGATCCAGCAATGCCGCGTGTGTGAAGAAGGCCTTAGGGTTGTAAAGCACTTTCGGTTAGAAGAAAAAGCTTGTGACTAATAATCATGAGTCTTGATGTTATCAACAGAAGAAGCACCGGCTAACTCTGTGCCAGCAGCCGCGGTAATACAGAGGGTGCAAGCGTTAATCGGAATTATTGGGCGTAAAGCGCACGTAGGCGGACTAGAAAGTTGGGTGTGAAATCCCTAGGCTCAACCTGGGAACTGCATTCAAAACTACTAATCTAGAGTATTGGAGAGGAGAGTGGAATTTCCGGTGTAGCAGTGAAATGCGTAGATATCGGAAGGAACATCAGTGGCGAAGGCGACTCTCTGGCCAAATACTGACGCTGAGGTGCGAAAGCGTGGGGAGCAAACAGG` |
| ASV1776 | 429 | `TGGGGAATATTGGACAATGGGGGAAACCCTGATCCAGCCATGCCGCGTGTGTGAAGAAGGTCTTCGGATTGTAAAGCACTTTAAGTTGGGAGGAAGGGTAGTAACTTAATACGTTGCTACTTTGACGTTACCGACAGAATAAGCACCGGCTAACTCTGTGCCAGCAGCCGCGGTAATACAGAGGGTGCAAGCGTTAATCGGAATTACTGGGCGTAAAGCGCGCGTAGGTGGTTTGTTAAGTTGGATGTGAAAGCCCCGGGCTCAACCTGGGAACTGCATCCAAAACTGGCAAGCTAGAGTACGGTAGAGGGTGGTGGAATTTCCTGTGTAGCGGTGAAATGCGTAGATATAGGAAGGAACACCAGTGGCGAAGGCGACCACCTGGACTGATACTGACACTGAGGTGCGAAAGCGTGGGGAGCAAACAGG` |
| ASV147 | 404 | `TGGGGAATCTTAGACAATGGGGGAAACCCTGATCTAGCCATGCCGCGTGATCGATGAAGGCCTTAGGGTTGTAAAGATCTTTCAGGTGGGAAGATAATGACTGTACCACCAGAAGAAGCCCCGGCTAACTCCGTGCCAGCAGCCGCGGTAATACGGAGGGGGCTAGCGTTATTCGGAATTACTGGGCGTAAAGCGCACGTAGGCGGACTGAAAAGTCAGAGGTGAAATCCCAGGGCTCAACCTTGGAACTGCCTTTGAAACTCTCAGTCTTGAGGTCGAGAGAGGTGAGTGGAATTCCGAGTGTAGAGGTGAAATTCGTAGATATTCGGAGGAACACCAGTGGCGAAGGCGGCTCACTGGCTCGATACTGACGCTGAGGTGCGAAAGCGTGGGGAGCAAACAGG` |
| ASV4859 | 404 | `TCGGGAATTTTGCGCAATGGGCGAAAGCCTGACGCAGCAACGCCGCGTGAGGGATGAAGGCCTTCGGGTTGTAAACCTCTTTTCTCAGGGACGATGATGACGGTACCTGAGGAATAAGTCACGGCTAACTACGTGCCAGCAGCCGCGGTAATACGTAGGTGACAAGCGTTGTCCGGATTTACTGGGCGTAAAGAGCGCGCAGGCGGTCGTTCAAGTCGAGTGTGAAAGCCCCCGGCTCAACTGGGGAGGGTCACTCGATACTGATCGACTCGAAGGCGGGAGAGGGAAGCGGAATTCCCGGTGTAGTGGTGAAATGCGTAGATATCGGGAGGAACACCAGTGGCGAAGGCGGCTTCCTGGCCCGTTCTTGACGCTGAGGCGCGAAAGCTAGGGGAGCAAACGGG` |
| ASV4222 | 406 | `TGAGGAATTTTCCGCAATGGGCGAAAGCCTGACGGAGCAATGCCGCGTGAAGGAAGAAGGCTCACGGGTCGTAAACTTCTTTTCTCGGAGAAGAATAAATGACGGTATCTGAGGAATAAGCATCGGCTAACTCTGTGCCAGCAGCCGCGGTAAAACAGAGGATGCAAGCGTTATCCGGAATTATTGGGCGTAAAGTGTCTGTAGGTGGCTTTTCAAGTCCGTCGTCAAATCCCAGGGCTCAACCCTGGACAGGCGGTGGAAACTATCAAGCTAGAGTACGGTAGAGGCAGAGGGAATTTCCGGTGGAGCGGTGAAATGCGTTGAGATCGGGAGGAACACCAAGGGCGAAAGCACTCTGCTGGGCCGTTACTGACACTCAGAGACGAAAGCTAGGGGAGCAAATGGG` |
| ASV3624 | 429 | `TGGGGAATATTGGACAATGGGGGCAACCCTGATCCAGCCATGCCGCGTGTGTGAAGAAGGCCTTCGGGTTGTAAAGCACTTTAAGTGGGGAGAAAAGCTCCAAAGCTAATATCTTTGGGGATTGATGCTACCCACAGAATAAGCACCGGCTAACTCTGTGCCAGCAGCCGCGGTAATACAGAGGGTGCAAGCGTTAATCGGAATTACTGGGCGTAAAGCGCGCGTAGGCGGCTGCTTAAGTCGGATGTGAAATCCCCGGGCTCAACCTGGGAACTGCATTCGATACTGAGCGGCTAGAGTATGAGAGAGGGAGGTAGAATTCCACGTGTAGCGGTGAAATGCGTAGATATGTGGAGGAATACCGGTGGCGAAGGCGGCCTCCTGGCTTAATACTGACGCTGAGGTGCGAAAGCGTGGGGAGCAAACAGG` |
| ASV2937 | 408 | `TGGGGAATTTTCCGCAATGGGCGAAAGCCTGACGGAGCAATCTCGCGTGAGGGATGACGGCCTATGGGTTGTAAACCTCTTTTTTCAGGGATGAATAAAATGACGTGTACCTGAAGAATAAGCATCGGCTAACTCCGTGCCAGCAGCCGCGGTAAGACGGAGGATGCAAGTGTTATCCGGAATCACTGGGCGTAAAGCGTCTGTAGGTGGTTTAGTAAGTCAACTGTTAAATCTTGAAGCTTAACTTCAAAATCGCAGTCGAAACTACTAGACTAGAGTATAGTAGGGGTAAAGGGAATTTCCAGTGGAGCGGTGAAATGCGTAGAGATTGGAAAGAACACCGATGGCGAAGGCACTTTACTGGGCTATTACTGACACTCAGAGACGAAAGCTAGGGTAGCAAATGGG` |
| ASV5093 | 428 | `TCGAGAATTTTTCTCAATGGGGGAAACCCTGAAGGAGCGACGCCGCGTGGGGGATGAAGGACTTCGGTTCGTAAACCCCTGTCACTTGGGAACAAACGCTCCGACCTAATACGTCGGAGCCTGATAGTACCAGGAGAGGAAGGCACGGCTAACTCTGTGCCAGCAGCCGCGGTAATACAGAGGTGCCAAGCGTTGTTCGGATTTACTGGGCGTAAAGGGTGCGTAGGCGGTTTTGCAAGTCGGATGTGAAATACCGGAGCCTAACTCCGGAACGGCATTCGAAACTGCAAGGCTAGAGGCTTGGAGAGGTGACTGGAATTCTTGGTGTAGCAGTGAAATGCGTAGATATCAAGAGGAACACCAGAGGCGAAAGCGAGTCACTAGACAGAGCCTGACGCTGAGGCACGAAAGCCAGGGGAGCAAACGGG` |
| ASV3816 | 429 | `TGGGGAATTTTGGACAATGGGCGAAAGCCTGATCCAGCAATGCCGCGTGAGTGAAGAAGGCCTTCGGGTTGTAAAGCTCTTTTGTCAGGGAAGAAATCCTAATGAATAATACTTGTTGGGGATGACGGTACCTGAAGAATAAGCACCGGCTAACTACGTGCCAGCAGCCGCGGTAATACGTAGGGTGCAAGCGTTAATCGGAATTACTGGGCGTAAAGCGTGCGCAGGCGGTTTGGTAAGACAGGTGTGAAATCCCCGAGCTCAACTTGGGAACTGCACTTGTGACTGCCAGACTAGAGTGTGTCAGAGGGAGGTAGAATTCCACGTGTAGCAGTGAAATGCGTAGATATGTGGAGGAATACCGGTGGCGAAGGCGGCCTCCTGGGATAACACTGACGCTCATGTACGAAAGCGTGGGGAGCAAACAGG` |
| ASV4309 | 404 | `TAGGGAATATTGGACAATGGACGAAAGTCTGATCCAGCAATGCCGCGTGAATGATGAAGGCCTTAGGGTTGTAAAGTTCTTTTAGTGGGGAAGATAATGACGGTACCCACAGAAAAAGCACCGGCTAACTCCGTGCCAGCAGCCGCGGTAATACGGAGGGTGCAAGCGTTGTTCGGAATTACTGGGCGTAAAGCACGCGTAGGCGGCTTGGTAAGTTGGGAGTGAAATCCCGGGGCTTAACCCCGGAACTGCTTTCAAAACTATCAAGCTAGAGTATGGCAGGGGACAGTGGAATTCCTAGTGTAGAGGTGAAATTCGTAGATATTAGGAGGAACACCAGTGGCGAAAGCGACTGTCTGGACCATTACTGACGCTGAGGCGTGAAAGCGTGGGGAGCAAACAGG` |
| ASV2583 | 423 | `TAGGGAATATTGGGCAATGGAAGAAATTCTGACCCAGCCATGCCGCGTGCAGGATGAAGGCGCTATGCGTTGTAAACTGCTTTTGTCAGGGAAGAAAAAGGCTATGCGTAGCAAATTGACGGTACCTGAAGAATAAGCATCGGCTAACTCCGTGCCAGCAGCCGCGGTAATACGGAGGATGCAAGCGTTGTCCGGATTTATTGGGTTTAAAGGGTGCGTAGGCGGTCTAATAAGTCAGTGGTTAAATTACCTTGCTTAACGAGAGAGATGCCATTGATACTGTTAGACTTGAATACTGTTTGGGTAGGCGGAATTGGTAGTGTAGCGGTGAAATGCTTAGATATTACCAGGAACACCGATAGCGAAGGCAGCTTACTGAAGAGATATTGACGCTGAGGCACGAAAGTGCGGGTATCAAACAGG` |
| ASV3096 | 404 | `TGGGGAATATTGGACAATGGGCGAAAGCCTGATCCAGCCATGCCGCGTGAGTGATGAAGGCCCTAGGGTTGTAAAGCTCTTTCACCGGTGAAGATAATGACGGTAACCGGAGAAGAAGCCCCGGCTAACTTCGTGCCAGCAGCCGCGGTAATACGAAGGGGGCTAGCGTTGTTCGGAATTACTGGGCGTAAAGCGCACGTAGGCGGATATTTAAGTCAGGGGTGAAATCCCGGGGCTCAACCCCGGAACTGCCTTTGATACTGGGTATCTCGAGTCCGGAAGAGGTGAGTGGAATTCCGAGTGTAGAGGTGAAATTCGTAGATATTCGGAGGAACACCAGTGGCGAAGGCGGCTCACTGGTCCGGTACTGACGCTGAGGTGCGAAAGCGTGGGGAGCAAACAGG` |
| ASV3446 | 429 | `TGGGGAATATTGGACAATGGGGGAAACCCTGATCCAGCGACGCCGCGTGGGTGAAGAAGGCCTGCGGGTTGTAAAGCCCTTTCGGTAGGGACGAAAGCTCTTGCCTTAACACGGCAGGAGGTTGACTTAACCTACATAAGAAGCACCGGCTAACTCTGTGCCAGCAGCCGCGGTAATACAGAGGGTGCGAGCGTTAATCGGAATTACTGGGCGTAAAGCGCACGTAGTCGGCCTTGCAAGTCGGGTGTGAAATCCCCGGGCTCAACCTGGGAACTGCATCCGAGACTGCATTGCTAGAGTATGGGAGAGGGAAGCGGAATTTCTGGTGTAGCAGTGAAATGCGTAGATATCAGAAGGAACATCAGTGGCGAAGGCGGCTTCCTGGACCAATACTGACGATCAGGTGCGAAAGCGTGGGGAGCAAACAGG` |
| ASV1048 | 429 | `TGGGGAATTTTGGACAATGGGCGAAAGCCTGATCCAGCCATGCCGCGTGAGTGAAGAAGGCCTTCGGGTTGTAAAGCTCTTTCAGACGGAAAGAAATCTTTCGGGTGAATATCCCGGGAGGATGACGGTACCGTAAGAAGAAGCACCGGCTAACTACGTGCCAGCAGCCGCGGTAATACGTAGGGTGCGAGCGTTAATCGGAATTACTGGGCGTAAAGCGTGCGCAGGCGGTTGCATAAGACAGATGTGAAATCCCCGGGCTCAACCTGGGAACTGCGTTTGTGACTGTGCGACTGGAGTACGGTAGAGGGGGGTGGAATTCCTGGTGTAGCAGTGAAATGCGTAGATATCAGGAGGAACATCGATGGCGAAGGCAGCCCCCTGGACCTGTACTGACGCTCATGCACGAAAGCGTGGGGAGCAAACAGG` |
| ASV994 | 429 | `TGGGGAATATTGGACAATGGGCGCAAGCCTGATCCAGCCATGCCGCGTGTGTGAAGAAGGCCTTCGGGTTGTAAAGCACTTTTGTTGGGGAAGAAAAGACCGTGGCTAATACCCGTGGTTCATGACGGTACCCAAAGAATAAGCACCGGCTAACTTCGTGCCAGCAGCCGCGGTAATACGAAGGGTGCAAGCGTTACTCGGAATTACTGGGCGTAAAGCGTGCGTAGGTGGTTCGTTAAGTCTGCCGTGAAAGCCCCGGGCTCAACCTGGGAATGGCGGTGGATACTGGCGGACTAGAGTGCGGTAGAGGGTGGTGGAATTCCCGGTGTAGCAGTGAAATGCGTAGAGATCGGGAGGAACATCTGTGGCGAAGGCGGCCACCTGGACCAGCACTGACACTGAGGCACGAAAGCGTGGGGAGCAAACAGG` |
| ASV3339 | 428 | `TGGGGAATATTGGACAATGGGCGCAAGCCTGATCCAGCCACGCCGCGTGAGTGATGAAGGCCTTCGGGTCGTAAAGCTCTGTGGGGAGGGACGAACCGCTGCACGTGCATAGCGTGCAGCATGACGGTACCTCCTTAGCAAGCACCGGCTAACTTCGTGCCAGCAGCCGCGGTAATACGAAGGGTGCAAACGTTGCTCGGAATTATTGGGCGTAAAGCGCACGTAGGCGGCTTAGCAAGTCGGATGTGAAATCCCTCGGCTCAACCAAGGAAGTGCATCCGAAACTGCTGAGCTTGAGTACGAAAGAGGATCGCGGAATTCCCGGTGTAGAGGTGAAATTCGTAGATATCGGGAGGAACACCAGTGGCGAAGGCGGCGATCTGGGTCGATACTGACGCTGAGGTGCGAAAGCGTGGGGAGCAAACAGG` |
| ASV308 | 429 | `TGGGGAATATTGGACAATGGGCGCAAGCCTGATCCAGCCATGCCGCGTGTGTGAAGAAGGTCTTCGGATTGTAAAGCACTTTAAGTTGGGAGGAAGGTTAGTAACCTAATACGTTGCTAATTTGACGTTACCGACAGAATAAGCACCGGCTAACTCTGTGCCAGCAGCCGCGGTAATACAGAGGGTGCAAGCGTTAATCGGAATTACTGGGCGTAAAGCGCGCGTAGGTGGTTTTGTAAGTTGGAGGTGAAATCCCCGGGCTCAACCTGGGAACTGCCTCCAAAACTGCAAAGCTAGAGTACGGTAGAGGGTGGTGGAATTTCCTGTGTAGCGGTGAAATGCGTAGATATAGGAAGGAACACCAGTGGCGAAGGCGACCACCTGGACTGATACTGACACTGAGGTGCGAAAGCGTGGGGAGCAAACAGG` |
| ASV1492 | 425 | `TAAGGAATATTGGTCAATGGGCGCAAGCCTGAACCAGCCATGCCGCGTGAAGGACGAAGGCGCTCTGCGTTGTAAACTTCTTTTGTATGGGAAGAATAGCCGAGATTCATCTTGGAAGCGACGGTACCATACGAATAAGCACCGGCTAACTCCGTGCCAGCAGCCGCGGTAATACGGAGGGTGCAAGCGTTATCCGGAATTACTGGGTTTAAAGGGTACGTAGGCGGTTAGTTAAGTCAATAGTGAAAGCCCGTTGCTCAACAACGGATGTGCTGTTGATACTGGCTGACTTGAAACAAGTTAGAGTCGGCGGAATGGGTCATGTAGCGGTGAAATGCTTAGATATGACTTGGAACACCGAAAGCGAAGGCAGCTGACTGAGCTTAGTTTGACGCTGAGGTACGAAAGTGTGGGTAGCGAACAGG` |
| ASV2079 | 404 | `TGGGGAATCTTAGACAATGGGGGAAACCCTGATCTAGCCATGCCGCGTGATCGATGAAGGCCTTAGGGTTGTAAAGATCTTTCAGTTGGGAAGATAATGACGGTACCAACAGAAGAAGCCCCGGCTAACTCCGTGCCAGCAGCCGCGGTAATACGGAGGGGGCTAGCGTTATTCGGAATTACTGGGCGTAAAGCGCACGTAGGCGGACTGGAAAGTCAGAGGTGAAATCCCAGGGCTCAACCTTGGAACTGCCTTTGAAACTCCCAGTCTTGAGGTCGAGAGAGGTGAGTGGAATTCCGAGTGTAGAGGTGAAATTCGTAGATATTCGGAGGAACACCAGTGGCGAAGGCGGCTCACTGGCTCGATACTGACGCTGAGGTGCGAAAGCGTGGGGAGCAAACAGG` |
| ASV2196 | 429 | `TGGGGAATTTTGGACAATGGGGGAAACCCTGATCCAGCAATGCCGCGTGTGTGAAGAAGGCCTTCGGGTTGTAAAGCACTTTTGTCAGGAACGAAACGGTGAGGGCTAATATCCCTTGCTAATGACGGTACCTGAAGAATAAGCACCGGCTAACTACGTGCCAGCAGCCGCGGTAATACGTAGGGTGCAAGCGTTAATCGGAATTACTGGGCGTAAAGCGTGCGCAGGCGGTTGTGCAAGACAGATGTGAAATCCCCGGGCTTAACCTGGGAATGGCATTTGTGACTGCACGGCTAGAGTGTGTCAGAGGGGGGTAGAATTCCACGTGTAGCAGTGAAATGCGTAGATATGTGGAGGAATACCGATGGCGAAGGCAGCCCCCTGGGATAACACTGACGCTCATGCACGAAAGCGTGGGGAGCAAACAGG` |
| ASV5105 | 404 | `TGGGGAATATTGGACAATGGGCGAAAGCCTGATCCAGCCATGCCGCGTGAGTGATGACGGCCTTAGGGTTGTAAAGCTCTTTTATCCGGGAAGATAATGACTGTACCGGAAGAATAAGCCCCGGCTAACTTCGTGCCAGCAGCCGCGGTAATACGAAGGGGGCTAGCGTTGCTCGGAATCACTGGGCGTAAAGGGCGCGTAGGCGGCTATGTAAGTCGGAGGTGAAAGCCTGGGGCTCAACCCCAGAATTGCCTTCGATACTATATGGCTTGAGAGCGAGAGAGGTAAGTGGAACTGCGAGTGTAGAGGTGAAATTCGTAGATATTCGCAAGAACACCAGTGGCGAAGGCGGCTTACTGGCTCGTTTCTGACGCTGAGGCGCGAAAGCGTGGGGAGCAAACAGG` |
| ASV4545 | 404 | `TGGGGAATCTTGGACAATGGGCGCAAGCCTGATCCAGCCATGCCGCGTGGGCGATGAAGGTCTTCGGATTGTAAAGCCCTTTTGGCGGGGACGATAATGACGGTACCCGCAGAATAAGCTCCGGCTAACTTCGTGCCAGCAGCCGCGGTAATACGAAGGGGGCTAGCGTTGTTCGGAATTACTGGGCGTAAAGCGTGCGCAGGCGGTTTTTCAAGTCAGGGGTGAAAGCCCTGGGCTCAACCCAGGAATGGCCTTTGAGACTGGAAAGCTAGAGTGTGAGAGAGGTGAGTGGAATTCCCAGTGTAGAGGTGAAATTCGTAGATATTGGGAAGAACACCCGTGGCGAAGGCGGCTCACTGGCTCGCAACTGACGCTCAGGCACGACAGCGTGGGGAGCAAACAGG` |
| ASV666 | 429 | `TCGAGAATCATTCACAATGGGCGAAAGCCTGATGGTGTGACGCCGCGTGGAGGATGAAGGTCTTCGGATTGTAAACTCCTGTCATGTGAGAGTAAGAACCAAGAAGTAACTGCCTGTGGTTTTGAGAGTATCACAAGAGGAAGAGGCGGCTAACTCTGTGCCAGCAGCCGCGGTAATACAGAGGCCTCAAGCGTTGTTCGGAATCACTGGGCGTAAAGGGTGCGTAGGCGGCGCGGAAAGTCAGGGGTGAAATCCCGGAGCTCAACTCCGGAACTGCCTTTGATACTCCCGCGCTTGAGAACTGGAGGGGACATCGGAATTCACGGTGGAGCAGTGAAATGCGTGGATATCGTGAGGAACACTAGTGGCGAAGGCGGATGTCTGGACAGTATCTGACGCTGAGGCACGAAGGCCAGGGGAGCGAACGGG` |
| ASV2168 | 429 | `TGGGGAATTTTGGACAATGGGCGCAAGCCTGATCCAGCAATGCCGCGTGCAGGATGAAGGCCTTCGGGTTGTAAACTGCTTTTGTACGGAGCGAAAAGGTCTGCCCTAATACGGTGGGCTCATGACGGTACCGTAAGAATAAGCACCGGCTAACTACGTGCCAGCAGCCGCGGTAATACGTAGGGTGCAAGCGTTAATCGGAATTACTGGGCGTAAAGCGTGCGCAGGCGGTTATATAAGACAGATGTGAAATCCCCGGGCTTAACCTGGGACCTGCATTTGTGACTGTATAGCTAGAGTACGGTAGAGGGGGATGGAATTCCGCGTGTAGCAGTGAAATGCGTAGATATGCGGAGGAACACCGATGGCGAAGGCAATCCCCTGGACCTGTACTGACGCTCATGCACGAAAGCGTGGGGAGCAAACAGG` |
| ASV1360 | 429 | `TGGGGAATTTTGGACAATGGGCGCAAGCCTGATCCAGCAATGCCGCGTGAGTGAAGAAGGCCTTCGGGTTGTAAAGCTCTTTTGTCAGGGAAGAAACGGTGTTTTCTAATACAGAACGCTAATGACGGTACCTGAAGAATAAGCACCGGCTAACTACGTGCCAGCAGCCGCGGTAATACGTAGGGTGCAAGCGTTAATCGGAATTACTGGGCGTAAAGCGTGCGCAGGCGGTTTTGTAAGACTGTCGTGAAATCCCCGGGCTCAACCTGGGAATGGCGATGGTGACTGCAAGGCTAGAGTTTGGCAGAGGGGGGTAGAATTCCACGTGTAGCAGTGAAATGCGTAGATATGTGGAGGAACACCGATGGCGAAGGCAGCCCCCTGGGTCAAAACTGACGCTCATGCACGAAAGCGTGGGGAGCAAACAGG` |
| ASV4579 | 404 | `TGGGGAATATTGGACAATGGGGGAAACCCTGATCCAGCAATGCCGCGTGGGTGATGAAGGCCTTCGGGTTGTAAAGCCCTTTTGGCGGGGACGATGATGACGGTACCCGCAGAAGAAGCCCCGGCTAACTTCGTGCCAGCAGCCGCGGTAATACGAAGGGGGCTAGCGTTGTTCGGAATTACTGGGCGTAAAGGGCGCGCAGGCGGCTTTGCAAGTTAGGCGTGAAAGGCCTGGGCTTAACCTGGGAATGGCGCTTAAGACTGCGGAGCTCGAGGGTGGGAGGGGATGGTGGAATTCCCAGTGTAGAGGTGAAATTCGTAGATATTGGGAAGAACACCAATGGCGAAGGCGGCCATCTGGACCACATCTGACGCTGAGGCGCGAAAGCGTGGGGAGCAAACAGG` |
| ASV5726 | 405 | `TGGGGAATATTGGACAATGGGCGAAAGCCTGATCCAGCAATGCCGCGTGAGTGATGAAGGCCTATTAGGTTGTAAAGCTCTTTTAGCAGGGAAGATAATGACGGTACCTGCAGAAAAAGCCCCGGCTAACTACGTGCCAGCAGCCGCGGTAATACGTAGGGGGCTAGCGTTGTTCGGATTTACTGGGCGTAAAGAGCGCGTAGGTGGTTATACAAGTTAGGGGTGAAATCCCGGAGCTTAACTTCGGAACTGCTTCTAAAACTGTATAGCTTGAGTGTGGTAGGGGGTAATAGAATTCCTAGTGTAGAGGTGAAATTCTTAGATATTAGGAGGAATACCGATGGCGAAGGCGATTACCTGGGCCACTACTGACACTGAGGCGCGAAAGCGTGGGGAGCAAACAGG` |
| ASV2406 | 429 | `TGGGGAATTTTGGACAATGGGCGCAAGCCTGATCCAGCCATGCCGCGTGCGGGAAGAAGGCCTTCGGGTTGTAAACCGCTTTTGTCAGGGAAGAAAAGCTTCGGGCCAATACCCCGGGGTAATGACGGTACCTGAAGAATAAGCACCGGCTAACTACGTGCCAGCAGCCGCGGTAATACGTAGGGTGCAAGCGTTAATCGGAATTACTGGGCGTAAAGCGTGCGCAGGCGGTTGTGCAAGACAGATGTGAAATCCCCGGGCTCAACCTGGGAACTGCATTTGTGACTGCACAGCTAGAGTACGGTAGAGGGGGATGGAATTCCGCGTGTAGCAGTGAAATGCGTAGATATGCGGAGGAACACCGATGGCGAAGGCAATCCCCTGGACCTGTACTGACGCTCATGCACGAAAGCGTGGGGAGCAAACAGG` |
| ASV5308 | 424 | `TAGGGAATATTGGGCAATGGTCGAGAGACTGACCCAGCCATGCCGCGTGCAGGATGAAGGCTTTCTGAGTCGTAAACTGCTTTTGCCAGGGAAGAATAAGCCCCATGCGTGGGGTGACGACGGTACCTGGTGAATAAGCACCGGCTAACTCCGTGCCAGCAGCCGCGGTAATACGGAGGGTGCAAGCGTTGTCCGGATTTATTGGGTTTAAAGGGTGCGTAGGCGGTTCATTAAGTCTGGGGTGAAAGCCCGTTGCTCAACAACGGAACTGCCCTGGAAACTGGCGAACTTGAGTACAGACGAGGGTGGCGGAATAGATGGTGTAGCGGTGAAATGCATAGATACCATCTAGAACCCCGATTGCGAAGGCAGCTGCCTAGACTGTAACTGACGCTGAGGCACGAAAGCGTGGGGAGCGAACAGG` |
| ASV1864 | 429 | `TGGGGAATTTTGGACAATGGGCGCAAGCCTGATCCAGCAATGCCGCGTGCAGGATGAAGGCCTTCGGGTTGTAAACTGCTTTTGTACGGAACGAAACGGCTCTGGTTAATACCTGGGGCTAATGACGGTACCGTAAGAATAAGCACCGGCTAACTACGTGCCAGCAGCCGCGGTAATACGTAGGGTGCAAGCGTTAATCGGAATTACTGGGCGTAAAGCGTGCGCAGGCGGTTATGTAAGACAGTCGTGAAATCCCCGGGCTCAACCTGGGAACTGCGATTGTGACTGCATAGCTAGAGTACGGTAGAGGGGGGTGGAATTCCGCGTGTAGCAGTGAAATGCGTAGATATGCGGAGGAACACCGATGGCGAAGGCAGCCCCCTGGACCTGTACTGACGCTCATGCACGAAAGCATGGGGAGCAAACAGG` |
| ASV3213 | 404 | `TGGGGAATTTTGGACAATGGGCGAAAGCCTGATCCAGCGATGCCGCGTGAGTGATGAAGGCCTTCGGGTTGTAAAACTCTTTCGGTAGGGAAGATAATGACGGTACCTACAGAAGAAGCTCCGGCTAACTTCGTGCCAGCAGCCGCGGTAATACGAAGGGAGCTAGCGTTACTCGGAATTACTGGGCGTAAAGCGTGCGCAGGCGGTCTGTTAAGTTGGAAGTGAAAGCCCAGGGCCTAACCCTGGAACTGCTTTCAAAACTAGCGGACTTGAATATTGGAGGAGATGGCGGAATTCCTAGTGTAGAGGTGAAATTCGTAGATACTAGGAGGAACACCGGTGGCGAAAGCGGCCATCTGGCCAAATATTGACGCTGTTGCACGAAAGCGTGGGGAGCAAACAGG` |
| ASV2412 | 410 | `TGGGGAATATTGCACAATGGGCGCAAGCCTGATGCAGCAACGCCGCGTGCGGGATGACGGCCTTCGGGTTGTAAACCGCTTTCAGCAGGGACGAAGCTTTTTGTGACGGTACCTGCAGAAGAAGCACCGGCTAACTACGTGCCAGCAGCCGCGGTGATACGTAGGGTGCGAGCGTTGTCCGGATTTATTGGGCGTAAAGGGCTCGTAGGCGGTTGATCGCGTCGGAAGTGGAAACTTGATGCTTAACGTTGAGCGTGCTTTCGATACGGGTTGACTTGAGGAAGGTAGGGGAGAATGGAATTCCTGGTGGAGCGGTGGAATGCGCAGATATCAGGAGGAACACCAGTGGCGAAGGCGGTTCTCTGGACCTTTCCTGACGCTGAGGAGCGAAAGCGTGGGGAGCGAACAGG` |
| ASV2060 | 429 | `TGGGGAATATTGGACAATGGGCGCAAGCCTGATCCAGCCATGCCGCGTGTGTGAAGAAGGCCTTCGGGTTGTAAAGCACTTTCACTTGGGAAGAAAAGCTGACCGTTAATAGCGGTTAGCCGTGACATTACCTTGAGAAGAAGCACCGGCTAACTCCGTGCCAGCAGCCGCGGTAATACGGAGGGTGCGAGCGTTAATCGGAATTACTGGGCGTAAAGCGCGCGTAGGCGGTTTGTTAAGTCAGCTGTGAAAGCCCCGGGCTCAACCTGGGAACTGCAGTTGATACTGGCCGACTAGAGTACGAGAGAGGGAGGTAGAATTCCATGTGTAGCGGTGAAATGCGTAGATATATGGAGGAATACCGGTGGCGAAGGCGGCCTCCTGGCTCGATACTGACGCTGAGGTGCGAAAGCGTGGGGATCAAACAGG` |
| ASV1976 | 429 | `TGGGGAATTTTGGACAATGGACGCAAGTCTGATCCAGCCATGCCGCGTGCGGGAAGAAGGCCTTCGGGTTGTAAACCGCTTTTGTCAGGGAAGAAAAGGGCGACTCTAATACAGTTGCCTCATGACGGTACCTGAAGAATAAGCACCGGCTAACTACGTGCCAGCAGCCGCGGTAATACGTAGGGTGCAAGCGTTAATCGGAATTACTGGGCGTAAAGCGTGCGCAGGCGGTTATGCAAGACAGAGGTGAAATCCCCGGGCTCAACCTGGGAACTGCCTTTGTGACTGCATAGCTAGAGTACGGTAGAGGGGGATGGAATTCCGCGTGTAGCAGTGAAATGCGTAGATATGCGGAGGAACACCGATGGCGAAGGCAATCCCCTGGACCTGTACTGACGCTCATGCACGAAAGCGTGGGGAGCAAACAGG` |
| ASV124 | 429 | `TGGGGAATTTTGGACAATGGGCGAAAGCCTGATCCAGCCATTCCGCGTGCAGGATGAAGGCCCTCGGGTTGTAAACTGCTTTTGTACAGAACGAAAAGGTCTCTATTAATACTAGGGGCTCATGACGGTACTGTAAGAATAAGCACCGGCTAACTACGTGCCAGCAGCCGCGGTAATACGTAGGGTGCAAGCGTTAATCGGAATTACTGGGCGTAAAGCGTGCGCAGGCGGTTATATAAGTCAGATGTGAAATCCCCGGGCTCAACCTGGGAACTGCATTTGAGACTGTATAGCTAGAGTACGGTAGAGGGGGATGGAATTCCGCGTGTAGCAGTGAAATGCGTAGATATGCGGAGGAACACCGATGGCGAAGGCAATCCCCTGGACCTGTACTGACGCTCATGCACGAAAGCGTGGGGAGCAAACAGG` |
| ASV2074 | 408 | `TCGAGAATCTTCCACAATGGGCGCAAGCCTGATGGAGCGATGTCGCGTGATTGATGAAGTGTTTCGGCATGTAAAGATCTTTTCTAGAGGAGGAAGTTTATTGACGTTACTCTAGGAATAAGGGGTTGCTAAACTCGTGCCAGCAGCAGCGGTAATACGAGTGCCCCAAGCGTTATCCGGAATTATTGGGCGTAAAGGGTCCGCAGGCGGTCTTATTAGTCTTGTGTGAAATCTCCCGGCTTAACTGGGAAAACGCATAGGAAACGGTAAGACTAGAGGATGTGAGAGGTATGTGGAACTCATGGTGTAGGGGTGAAATCCGTTGATATCATGGGGAACACCAAATGCGAAGGCAACATACTGGCACATTCCTGACGCTCAGGGACGAAAGCATGGGTAGCGAATGGG` |
| ASV1546 | 421 | `TGGGGAATATTGCGCAATGGCCGTAAGGCTGACGCAGCGACGCCGCGTGGGGGATGAAGCATTTCGGTGTGTAAACCCCTGTTGCTTGGGACGAACAAGCCTTTTCGAGGGCTCTGACGGTACCGAGTGAGGAAGCACCGGCTAACTCCGTGCCAGCAGCCGCGGTAATACGGAGGGTGCGAGCGTTGTCCGGAATCACTGGGCGTAAAGGGCGCGTAGGCGGTCTGCTAAGCGTGTGGTGAAAGCTCGGGGCTCAACCCCGCGTCGGCCATGCGAACTGGTGGACTAGAGCACTGTAGAGGCAGGTGGAATTCCGGGTGTAGCGGTGGAATGCGTAGAGATCCGGAAGAACACCGGTGGCGAAGGCGGCCTGCTGGGCAGTAGCTGACGCTGAGGCGCGATAGCGTGGGGAGCAAACAGG` |
| ASV2726 | 417 | `TTAGGAATCTTCCACAATGGGCGCAAGCCTGATGGAGCGACGCCGCGTGAGGGATGACGGTTTTCGGATTGTAAACCTCTGAATCTGGGACGAAAGACGCGACGAGCGGGATGACGGTACCAGAGTAATAGCACCGGCTAACTCCGTGCCAGCAGCCGCGGTAATACGGAGGGTGCAAGCGTTACCCGGAATCACTGGGCGTAAAGGGCGTGTAGGCGGATCACTCAGTCTGGTTTTAAAGACCGGGGCTCAACCCCGGGGATGGACTGGATACTGGTGACCTTGACCTCTGGAGAGAGAACTGGAATTCCTGGTGTAGCGGTGGAATGCGTAGATACCAGGAGGAACACCAATGGCGAAGGCAGGTTCTTGGACAGAAGGTGACGCTGAGGCGCGAAAGTGTGGGGAGCGAACCGG` |
| ASV493 | 429 | `TGGGGAATATTGGACAATGGGCGCAAGCCTGATCCAGCCATGCCGCGTGTGTGAAGAAGGCCTTCGGGTTGTAAAGCACTTTCACTTGGGAAGAAAAGTTGGTCGTTAATAGCGGCCAGCCGTGACATTACCTTGAGAAGAAGCACCGGCTAACTCCGTGCCAGCAGCCGCGGTAATACGGAGGGTGCGAGCGTTAATCGGAATTACTGGGCGTAAAGCGCGCGTAGGCGGTTTGTTAAGTCAGCTGTGAAAGCCCCGGGCTCAACCTGGGAACTGCAGTTGATACTGGCCGACTAGAGTACGAGAGAGGGAGGTAGAATTCCATGTGTAGCGGTGAAATGCGTAGATATATGGAGGAATACCGGTGGCGAAGGCGGCCTCCTGGCTCGATACTGACGCTGAGGTGCGAAAGCGTGGGGATCAAACAGG` |
| ASV2770 | 430 | `TGGGGAATTTTGGACAATGGGCGCAAGCCTGATCCAGCCATGCCGCGTGCAGGAAGAAGGCCTTCGGGTTGTAAACTGCTTTAGTCTAGGAAAAAGGGATGCAGGGTTAATACCTCTGTGTTTTGATGGTACTGGAAGAATAAGCACCGGCTAACTACGTGCCAGCAGCCGCGGTAATACGTAGGGTGCGAGCGTTAATCGGAATTACTGGGCGTAAAGCGTGCGCAGGCGGTCTTGTAAGACAGTCGTGAAAGCCCCGGGCTTAACCTGGGAACTGCGATTGTGACTGCAAGGCTAGAGTGTGTCAGAGGGGGGTGGAATTCCACGTGTAGCAGTGAAATGCGTAGAGATGTGGAGGAACACCGATGGCGAAGGCAGCCCCCTGGGATAACACTGACGCTCATGCACGAAAGCGTGGGGAGCAAACAGG` |
| ASV2695 | 423 | `TAGGGAATATTGGTCAATGGGTGAGAGCCTGAACCAGCCATGCCGCGTGCAGGAAGAAGGCCTTCTGGGTTGTAAACTGCTTTTGCCGGGGGATAAAATAGCTTTGCGAAGCAAATTGAAGGTACCCGGTGAATAAGCCACGGCTAACTACGTGCCAGCAGCCGCGGTAATACGTAGGTGGCAAGCGTTGTCCGGATTTATTGGGTTTAAAGGGTGCGTAGGCGGTTCTATAAGTCAGTGGTGAAATACAGCCGCTTAACGGTTGAGGTGCCATTGATACTGCAGAACTTGAGTACAGACGAGGTAGGCGGAATTGACGGTGTAGCGGTGAAATGCTTAGATATCGTCAAGAACACCGATAGCGAAGGCAGCTTACTAGACTGTAACTGACGCTGAGGCACGAAAGTGTGGGGATCAAACAGG` |
| ASV3219 | 404 | `TGGGGAATCTTGGACAATGGGCGCAAGCCTGATCCAGCCATGCCGCGTGAGTGAAGAAGGCCTTCGGGTTGTAAAGCTCTTTTGTCAGGGACGATAATGACGGTACCTGAAGAATAAGCCCCGGCAAACTTCGTGCCAGCAGCCGCGGTAATACGAAGGGGGCTAGCGTTGTTCGGAATTACTGGGCGTAAAGCGCTCGTAGGCTGATTGTTAAGTGAGGGGTGAAATCCCAAGGCTCAACCTTGGAACTGCCTTTCATACTGGCAATCTCGAGTCCGGAAGAGGTGAGTGGAATTCCTAGTGTAGAGGTGAAATTCGTAGATATTAGGAAGAACACCAGTGGCGAAGGCGGCTCACTGGTCCGGTACTGACGCTGAGGAGCGAAAGCGTGGGGAGCAAACAGG` |
| ASV1959 | 424 | `TAAGGAATATTGGTCAATGGACGCAAGTCTGAACCAGCCATGCCGCGTGAAGGATGAAGGCCCTCTGGGTTGTAAACTTCTTTTATCTGGGACGAAAAAAGGGGATTCTTCCTCACTTGACGGTACCAGAGGAATAAGCACCGGCTAACTCCGTGCCAGCAGCCGCGGTAATACGGAGGGTGCAAGCGTTATCCGGATTTACTGGGTTTAAAGGGTGCGTAGGTGGGTCTGTAAGTCAGTGGTGAAATCTCCGAGCTTAACTTGGAAACTGCCATTGATACTATAGGTCTTGAATTGTCTGGAGGTAAGCGGAATATGTCATGTAGCGGTGAAATGCTTAGATATGACATAGAACACCCATTGCGAAGGCAGCTTACTACGGATTGATTGACACTGAGGCACGAAAGCGTGGGGATCAAACAGG` |
| ASV3357 | 429 | `TGGGGAATATTGGACAATGGGCGCAAGCCTGATCCAGCCATGCCGCGTGAGTGAAGAAGGCCTTCGGGTTGTAAAGCTCTTTGAGCGGGGAGGAAGGGCATGCGGTTAATAACCGTGTGCATTGACGTTACCTGCAGAACAAGCACCGGCTAACTCTGTGCCAGCAGCCGCGGTAATACAGAGGGTGCGAGCGTTAATCGGAATTACTGGGCGTAAAGCGTGCGTAGGCGGTTACATAAGTTGGGTGTGAAAGCCCCGGGCTTAACCTGGGAACTGCACTCAAGACTGTGTCACTAGAGTCTGGGAGAGGGTGGTGGAATTTCCTGTGTAGCGGTGAAATGCGTAGATATAGGAAGGAACATCAGTGGCGAAGGCGACTGCCTGGCCCAAGACTGACGCTGAGGTACGAAAGCGTGGGGAGCAAACAGG` |
| ASV662 | 423 | `TAGGGAATATTGGGCAATGGGCGCAAGCCTGACCCAGCCATGCCGCGTGCAGGAAGAAGGTCCTCTGGATTGTAAACTGCTTTTGTCGAGGACGAAAAAGGTGCTGCGGCACAAATTGACGGTATTCGACGAATAAGCCACGGCTAACTACGTGCCAGCAGCCGCGGTAATACGTAGGTGGCAAGCGTTGTCCGGATTTATTGGGTTTAAAGGGTGCGTAGGTGGCTTGGTAAGTCAGCGGTGAAAGACGGTCGCTTAACGATTGCAGTGCCGTTGATACTGCCGAGCTTGAGTAAAGATGAGGTAGGCGGAACGGATAGTGTAGCGGTGAAATGCATAGATATTATCCAGAACACCGATTGCGAAGGCAGCTTACTGGACTTTAACTGACACTGAGGCACGAGAGCGTGGGGAGCAAACAGG` |
| ASV519 | 429 | `TGGGGAATTTTGGACAATGGGCGAAAGCCTGATCCAGCAATGCCGCGTGCAGGAAGAAGGCCTTCGGGTTGTAAACTGCTTTTGTACGGAACGAAAAGGCCAGCCCTAATACGGCAGGCCCATGACGGTACCGTAAGAATAAGCACCGGCTAACTACGTGCCAGCAGCCGCGGTAATACGTAGGGTGCGAGCGTTAATCGGAATTACTGGGCGTAAAGCGTGCGCAGGCGGTTGTGTAAGACAGTTGTGAAATCCCCGGGCTCAACCTGGGAATTGCATCTGTGACTGCACAGCTAGAGTACGGTAGAGGGGGATGGAATTCCGCGTGTAGCAGTGAAATGCGTAGATATGCGGAGGAACACCGATGGCGAAGGCAATCCCCTGGACCTGTACTGACGCTCATGCACGAAAGCGTGGGGAGCAAACAGG` |
| ASV3062 | 429 | `TGGGGAATTTTGGACAATGGGGGCAACCCTGATCCAGCAATGCCGCGTGAGTGAAGAAGGCCTTCGGGTTGTAAAGCTCTTTTGTTAGGGAAGAAAAGGACTTCAATAATACTGGGGTCTGATGACGGTACCTAAAGAATAAGCACCGGCTAACTACGTGCCAGCAGCCGCGGTAATACGTAGGGTGCAAGCGTTAATCGGAATTACTGGGCGTAAAGCGTGCGCAGGCGGCCTATTAAGTCAGATGTGAAATCCCCGAGCTCAACTTGGGAACTGCATTTGAAACTGATAGGCTAGAGTGTGTCAGAGGGAGGTAGAATTCCACGTGTAGCAGTGAAATGCGTAGAGATGTGGAGGAATACCAGTGGCGAAGGCGGCCTCCTGGGATAACACTGACGCTCATGTACGAAAGCGTGGGGAGCAAACAGG` |
| ASV1544 | 424 | `TAAGGAATATTGGTCAATGGACGCAAGTCTGAACCAGCCATGCCGCGTGAAGGATGACGGCCCTCTGGGTTGTAAACTTCTTTTATCTGGGACGAAAAAAGGTCATTCTTGATCACTTGACGGTACCAGAGGAATAAGCACCGGCTAACTCCGTGCCAGCAGCCGCGGTAATACGGAGGGTGCAAGCGTTATCCGGATTTACTGGGTTTAAAGGGTGCGTAGGTGGGTCTGTAAGTCAGTGGTGAAATCTCCGAGCTTAACTCGGAAACTGCCATTGATACTATAGGTCTTGAATTATCTGGAGGTGAGCGGAATATGTCATGTAGCGGTGAAATGCTTAGATATGACATAGAACACCAATTGCGAAGGCAGCTCACTACGGATGTATTGACACTGAGGCACGAAAGCGTGGGGATCAAACAGG` |
| ASV4532 | 426 | `TGAGGAATATTGCGCAATGGGCGAAAGCCTGACGCAGCGACGCCGCGTGAAGGACGAAGGTCTTCGGATTGTAAACTTCTGTTAAGCGGGAAGAAAACCTGGCATAACCTGCCGGGCATGACTGTACCGCTAGAGAAAGCACTGGCTAACCTCGTGCCAGCAGCCGCGGTAATACGAGGGGTGCAAACGTTATTCGGATTAACTGGGCGTAAAGGGTGTGTAGACGGCTCTTTAAGTTCGTTGTCAAATCTCCCGGCCTAACTGGGAATTCGCGATGAAAACTAAATGAGCTTGAGGGTGAAAGAGAGAAGTGGAATTCTCGGAGTAGCGGTGAAATGCGTAGATATCGAGAGGAACACCGATGGCGAAGGCAGCTTCTTGGTTCACTCCTGACGTTGAGACACGAAAGCGTGGGTAGCAAACAGG` |
| ASV4702 | 404 | `TGGGGAATATTGGACAATGGGGGAAACCCTGATCCAGCCATGCCGCGTGAGTGATGACGGCCTTAGGGTTGTAAAGCTCTTTTACTCGGGAAGATAATGACTGTACCGAGAGAATAAGCCCCGGCAAACTTCGTGCCAGCAGCCGCGGTGATACGAAGGGGGCTAGCGTTGTTCGGATTTACTGGGCGTAAAGCGCACGTAGGCGGACTGTTAAGTCGGAGGTGAAAGCCCAGGGCTCAACCCTGGAATTGCCTTCGATACTGGCAGTCTTGAGTGTGATAGAGGTTAGTGGAATTCCTAGTGTAGAGGTGAAATTCGTAGATATTAGGAAGAACACCAGTGGCGAAGGCGGCTAACTGGATCACAACTGACGCTGAGGTGCGAAAGCGTGGGGATCAAACAGG` |
| ASV5263 | 429 | `TGGGGAATATTGCGCAATGGAGGAAACTCTGACGCAGCGACGCCGCGTGGGTGATGAAGGCCTTCGGGTTGTAAAGCCCTGTCGGCTGGGAAAAAGGTTGTCGTGCTAATACCACGATAATTTGATGGTACCAGCAAAGGAAGCACCGGCTAACTCTGTGCCAGCAGCCGCGGTAATACAGAGGGTGCAAGCGTTGTTCGGAATTACTGGGCGTAAAGGGCGCGTAGGCGGCCTTGTAAGTCAGATGTGAAAGCCCGAGGCTTAACTTCGGAAGTGCATTTGAAACTACAAGGCTAGAGTTCAGTAGAGGTTGGCGGAATACCCAGTGGAGAGGTGAAATTCGTAGATATTGGGTGGAACACCGGTGGCGAAGGCGGCCAACTGGGCTGACACTGACGCTGAGGCGCGAGAGCATGGGTAGCAAACAGG` |
| ASV709 | 404 | `TGGGGAATATTGGACAATGGGCGCAAGCCTGATCCAGCCATGCCGCGTGAGTGATGAAGGCCTTAGGGTTGTAAAGCTCTTTCAGTAGGGACGATAATGACGGTACCTACAGAAGAAGCCCCGGCTAACTTCGTGCCAGCAGCCGCGGTAATACGAAGGGGGCTAGCGTTGTTCGGATTTACTGGGCGTAAAGCGCACGTAGGCGGATTGTTAAGTGAGGGGTGAAATCCCAGGGCTCAACCCTGGAACTGCCTTTCATACTGGCAATCTAGAGTCCGGAAGAGGTGAGTGGAACTCCTAGTGTAGAGGTGGAATTCGTAGATATTAGGAAGAACACCAGTGGCGAAGGCGGCTCACTGGTCCGGTACTGACGCTGAGGTGCGAAAGCGTGGGGAGCAAACAGG` |
| ASV639 | 429 | `TGGGGAATTTTGGACAATGGGGGCAACCCTGATCCAGCAATGCCGCGTGTGTGAAGAAGGCCTTCGGGTTGTAAAGCACTTTTGTCAGGAACGAAACGGTCACTGCTAATATCGGTGGCTAATGACGGTACCTGAAGAATAAGCACCGGCTAACTACGTGCCAGCAGCCGCGGTAATACGTAGGGTGCAAGCGTTAATCGGAATTACTGGGCGTAAAGCGTGCGCAGGCGGTTGTGTAAGTCAGATGTGAAATCCCCGGGCTTAACCTGGGAATGGCATTTGAGACTGCACGGCTAGAGTGTGTCAGAGGGGGGTAGAATTCCACGTGTAGCAGTGAAATGCGTAGAGATGTGGAGGAACACCGATGGCGAAGGCAGCCCCCTGGGATAACACTGACGCTCATGCACGAAAGCGTGGGGAGCAAACAGG` |
| ASV4931 | 430 | `TGGGGAATATTGGACAATGGGGGGAACCCTGATCCAGCAATACCGCGTGTGTGAAGAAGGCCTTCGGGTTGTAAAGCACTTTTGTTAGGGAGGATGGTAATTTAGGTTAATACCCTGAGTTATTGACGTTACCTAAAGAATAAGCACCGGCTAACTCCGTGCCAGCAGCCGCGGTAATACGGAGGGTGCAAGCGTTAATCGGAATTACTGGGCGTAAAGGGCGCGTAGGTGGTCTTTTAAGTCAGATGTGAAAGCCCTGGGCTCAACCTGGGAATTGCATTTGATACTGGGAGACTAGAATGTGGTAGAGGGTAGCGGAATTTCTGGTGTAGCGGTGAAATGCGTAGATATCAGAAGGAACATCAATGGCGAAGGCAGCTACCTGGGCCAACATTGACGCTGAGGCGCGAAAGCGTGGGGAGCAAACAGG` |
| ASV4220 | 406 | `TTGGGAATCTTGCACAATGGGGGAAACCCTGATGCAGCGACGCCGCGTGGAGGATGACGGATCTAGGTCTGTAAACTCCTTTTTCAGGGAAAGACTTAGGACGGTACCCTGAGAATAAGCTCCGGCTAACTACGTGCCAGCAGCCGCGGTAAGACGTAGGGAGCAAGCGTTGTCCGGATTTACTGGGCGTAAAGGGCGCGTAGGCGGTCTGTTAAGTGAGTACTAAAATCTCCAGGGCTCAACCCGGAAACCGGTTCTCATACTGACAGACTGGAGGAAGGCAGAGGTAACTGGAATTCCTGGTGTAGCGGTGAAATGCGTTGATATCAGGAGGAACTCCAATGGCGAAGGCAGGTTACTGGGCCTTATCTGACGCTGAGGCGCGAAAGCGTGGGTAGCAAACAGG` |
| ASV4604 | 429 | `TGGGGAATATTGGACAATGGGGGCAACCCTGATCCAGCAATGCCGCGTGTGTGAAGAAGGCCTGAGGGTTGTAAAGCACTTTCAGTGGGGAAGAAGGCGTTAGTGTGAATAATACTAACGATTGACGTTACCCAAAGAAGAAGCACCGGCTAACTCTGTGCCAGCAGCCGCGGTAATACAGAGGGTGCAAGCGTTAATCGGAATCACTGGGCGTAAAGAGCGCGTAGGTTGATAATTAAGTTGGATGTGAAAGCCCTGGGCTCAACCCGGGATGGTCATTCAAGACTGGTTGTCTAGAGTATAGTAGAGGGAAGTGGAATTTCCGGTGTAGCGGTGAAATGCGTAGATATCGGAAGGAACACCAGTGGCGAAGGCGGCTTCCTGGACTAATACTGACACTGAGGCGCGAAAGCGTGGGTAGCAAACAGG` |
| ASV2941 | 404 | `TGGGGAATATTGCACAATGGAGGAAACTCTGATGCAGCAACGCCGCGTGGAGGATGACGCATTTCGGTGTGTAAACTCCTTTTATAAGGGAAGATAATGACGGTACCTTATGAATAAGCACCGGCTAACTCCGTGCCAGCAGCCGCGGTAATACGGAGGGTGCAAGCGTTACTCGGAATCACTGGGCGTAAAGGATGCGTAGGCTGGAAATCAAGTCGAGAGTGAAATCCAACGGCTCAACCGTTGAACTGCTCTCGAAACTGGTTACCTAGAATATGGGAGAGGTAGATGGAATTGGTGGTGTAGGGGTAAAATCCGTAGATATCACCAGGAATACCGATTGCGAAGGCGATCTACTGGAACATTATTGACGCTGAGGCATGAAAGCGTGGGGAGCAAACAGG` |
| ASV2620 | 429 | `TGGGGAATTTTGGACAATGGGCGCAAGCCTGATCCAGCAATGCCGCGTGAGTGAAGAAGGCCTTCGGGTTGTAAAGCTCTTTTGTCAGGGAAGAAAAGGTTGCGAATAATACTCGCAGCTCATGACGGTACCTGAAGAATAAGCACCGGCTAACTACGTGCCAGCAGCCGCGGTAATACGTAGGGTGCAAGCGTTAATCGGAATTACTGGGCGTAAAGCGTGCGCAGGCGGTTTTGTAAGTCTGTTGTGAAATCCCCGGGCTCAACCTGGGAATGGCAATGGAGACTGCAAGGCTAGAGTTTGGCAGAGGGGGGTAGAATTCCACGTGTAGCAGTGAAATGCGTAGATATGTGGAGGAACACCGATGGCGAAGGCAGCCCCCTGGGTCAAAACTGACGCTCATGCACGAAAGCGTGGGGAGCAAACAGG` |
| ASV3709 | 429 | `TGGGGAATTTTGGACAATGGGCGCAAGCCTGATCCAGCAATGCCGCGTGCAGGAAGAAGGCCTTCGGGTTGTAAACTGCTTTTGTACGGAACGAAACGGACTGGGATAATACCCTGGTCTAATGACGGTACCGTAAGAATAAGCACCGGCTAACTACGTGCCAGCAGCCGCGGTAATACGTAGGGTGCAAGCGTTAATCGGAATTACTGGGCGTAAAGCGTGCGCAGGCGGTTTTGTAAGACAGGCGTGAAATCCCCGGGCTCAACCTGGGAATGGCGCTTGTGACTGCAAAGCTGGAGTGCGGCAGAGGGGGATGGAATTCCGCGTGTAGCAGTGAAATGCGTAGATATGCGGAGGAACACCGATGGCGAAGGCAATCCCCTGGGCCTGCACTGACGCTCATGCACGAAAGCGTGGGGAGCAAACAGG` |
| ASV3437 | 406 | `TGGGGAATTTTCCGCAATGGGCGAAAGCCTGACGGAGCAATGCCGCGTGGAGGTAGAAGGCCTACGGGTCGTGAACTTCTTTTCCCGGAGAAGAAGCAATGACGGTATCTGGGGAATAAGCATCGGCTAACTCTGTGCCAGCAGCCGCGGTAATACAGAGGATGCAAGCGTTATCCGGAATGATTGGGCGTAAAGCGTCTGTAGGTGGCTTTTTAAGTCCGCCGTCAAATCCCAGGGCTCAACCCCGGACAGGCGGTGGAAACTACCAAGCTGGAGTACGGTAGGGGCAGAGGGAATTTCCGGTGGAGCGGTGAAATGCGTAGAGATCGGAAAGAACACCAACGGCGAAAGCACTCTGCTGGGCCGACACTGACACTGAGAGACGAAAGCTAGGGGAGCGAATGGG` |
| ASV5164 | 388 | `TCGGGAAAATTTTGCAATGCGCGCAAGCGTGACAGAGCAAGCCAGAGTGCGTTTCATTTTTGAAACGCTTTTGTGAGATGTAAACAGTCTTACGAATAAGGACTGGGAGAGACTGGTGCCAGCCGCCGCGGTAATCCCAGCAGTCCAAGTCGCAGCCACATTTGTTGGGTCTAAAACATCCGTAGCTTGTCTTGCAAGTCCTATGTGAAATCGGGCATCTTAAGTGTCCGGCGTGCATAAGATACTGCTTGGCTAGAGACCGGGAGGCGCAGAAAGTACGCATAGAGTAGCGGTTAAATGTGTTGATCCTATGCGGACTAACAGTCGCGAAGGCGTTCTGCGAGAACGGTTCTGACAGTGAGGGATGAAGGCTAGGGGCGCAAAGTGG` |
| ASV4955 | 424 | `TGAGGAATATTGGTCAATGGGCGCAAGCCTGAACCAGCCATGTCGCGTGCAGGATGACTGCCCTATGGGTTGTAAACTGCTTTTGTATGGGAAGAAAGTGAGATACGTGTATCTCTTTGCCGGTACCATACGAATAAGGATCGGCTAACTCCGTGCCAGCAGCCGCGGTAATACGGAGGATCCAAGCGTTATCCGGATTTATTGGGTTTAAAGGGTGCGTAGGCGGTTTAATAAGTCAGTGGTGAAATCTTGCAGCTTAACTGTAAAATTGCCATTGATACTGCTAGACTTGAGTATAGTTGAGGTGGGCGGAATGTGTTGTGTAGCGGTGAAATGCATAGATATAACACAGAACACCAATTGCGAAGGCAGCTCACTAAGCTATAACTGACGCTGAGGCACGAAAGCGTGGGTAGCGAACAGG` |
| ASV5519 | 404 | `TTAGGAATATTGGACAATGGGCGAAAGCCTGATCCAGCCATGCCGCGTGAACGATGAAGGCCTTAGGGTTGTAAAGTTCTTTCGTGGGCGAAGATAATGACGGTAACCCAAGAAGAAGCTCCGGCTAACTCCGTGCCAGCAGCCGCGGTAATACGGAGGGAGCGAGCGTTGTTCGGAATCACTGGGCGTAAAGGGTGCGTAGGCGGATTAGCAAGTCAGGAGTGAAATCCCCGGGCTTAACCCGGGAACTGCTTTTGATACTGCTATTCTAGAGTTTGGCAGAGGTTGGCGGAATTCCTAGTGTAGAGGTAAAATTCGTAGATATTAGGAGGAACACCAGTGGCGTAGGCGGCCAACTGGGCCATTACTGACGCTGAGGCACGAAAGCGTGGGGAGCAAACAGG` |
| ASV3898 | 404 | `TCGGGAATTTTGGGCAATGGGCGAAAGCCTGACCCAGCAACGCCGCGTGAAGGATGAAGTATTTCGGTATGTAAACTTCGAAAGAATAGGAAGAATAAATGACGGTACTATTTATAAGGTCCGGCTAACTACGTGCCAGCAGCCGCGGTAATACGTAGGGACCAAGCGTTGTTCGGATTTACTGGGCGTAAAGGGCGCGTAGGCGGCAATTTAAGTCATCTGTGAAATCTCCGGGCTTAACTCGGAACGGTCAGATGATACTGCTTTGCTAGAGTGCAGAAGGGGCAATCGGAATTCTTGGTGTAGCGGTGAAATGCGTAGATATCAAGAGGAACACCTGAGGTGAAGACGGGTTGCTGGGCTGACACTGACGCTGAGGCGCGAAAGCTAGGGGAGCAAACGGG` |
| ASV4065 | 404 | `TGGGGAATCTTGGACAATGGGCGCAAGCCTGATCCAGCCATGCCGCGTGAGTGAAGAAGGCCTTCGGGTTGTAAAGCTCTTTTGTCAGGGACGATAATGACGGTACCTGAAGAATAAGCCCCGGCAAACTTCGTGCCAGCAGCCGCGGTAATACGAAGGGGGCTAGCGTTGTTCGGATTTACTGGGCGTAAAGCGTACGTAGGCGGATTGTTAAGTGAGAGGTGAAATCCCAAGGCTCAACCTTGGAACTGCCTTTCATACTGGCAATCTTGAGTTCGGAAGAGGTGAGTGGAATTCCTAGTGTAGCAGTGAAATGCGTAGATATTAGGAAGAACACCGGTGGCGAAGGCGGCTCACTGGTCCGATACTGACGCTGAGGTACGAAAGCGTGGGGAGCAAACAGG` |
| ASV5479 | 405 | `TAAGGAATCTTCCACAATGGGGGCAACCCTGATGGAGCAACGCCGCGTGCAGGATGAAGGCTTTCGAGTCGTAAACTGCTTTTATTAGTGAGGAATATGACGGTAACTAATGAATAAGGGTCTGCTAACTACGTGCCAGCAGCCGCGGTCATACGTGGGACCCAAGCGTTATCCGGATTGACTGGGCGTAAAGCGTTGCGTAGGTGGTCTGTTAAGCGAATAGTGAAATCGAGTGGCTCAACCATTCGGCTATTATTCGAACTGGCAGACTAGAGAACAGTAGAGGTACCTGGAATTTCTAGTGTAGGGGTAATATCCGTAGATACTAGAAGGAACACCAATGGCGTAGGCAGGGTACTGGGCTGTTTCTGACACTGAGGCACGAAAGCGTGGGGAGCGAACGGG` |
| ASV379 | 429 | `TGGGGAATATTGCACAATGGGGGAAACCCTGATGCAGCCATGCCGCGTGTGTGAAGAAGGCCTTCGGGTTGTAAAGCACTTTCAGCGAGGAGGAAAGGTTGTTGGCTAATATCCAGCAACTGTGACGTTACTCGCAGAAGAAGCACCGGCTAACTCCGTGCCAGCAGCCGCGGTAATACGGAGGGTGCAAGCGTTAATCGGAATTACTGGGCGTAAAGCGCACGCAGGCGGTTGGATAAGTTAGATGTGAAAGCCCCGGGCTCAACCTGGGAATTGCATTTAAAACTGTCCAGCTAGAGTCTTGTAGAGGGGGGTAGAATTCCAGGTGTAGCGGTGAAATGCGTAGAGATCTGGAGGAATACCGGTGGCGAAGGCGGCCCCCTGGACAAAGACTGACGCTCAGGTGCGAAAGCGTGGGGAGCAAACAGG` |
| ASV1139 | 423 | `TAGGGAATATTGGGCAATGGGCGGGAGCCTGACCCAGCCATGCCGCGTGCAGGAAGAAGGCGTTATGCGTTGTAAACTGCTTTTATACGGGAAGAAAAGGCCCATGCGTGGGACATTGCCGGTACCGTATGAATAAGCACCGGCTAACTCCGTGCCAGCAGCCGCGGTAATACGGAGGGTGCAAGCGTTGTCCGGATTTATTGGGTTTAAAGGGTGCGTAGGCGGCCGATTAAGTCAGCGGTGAAAGTCTGTGGCTCAACCATAGAAGCGCCGTTGATACTGGTTGGCTTGAGTGCCGTATGGGTACATGGAATTGATGGTGTAGCGGTGAAATGCATAGATACCATCAGGAACACCGATAGCGTAGGCATTGTACTGTGCGGCAACTGACGCTGAGGCACGAAAGCATGGGTAGCGAACAGG` |
| ASV2136 | 424 | `TGAGGAATATTGGTCAATGGGCGCAAGCCTGAACCAGCCATGCCGCGTGCAGGAAGACGGTCCTATGGATTGTAAACTGCTTTTATACAGGAAGAAACAACTCTACGTGTAGAGTCTTGACGGTACTGTAGGAATAAGGATCGGCTAACTCCGTGCCAGCAGCCGCGGTAATACGGAGGATCCAAGCGTTATCCGGAATCATTGGGTTTAAAGGGTCCGTAGGCGGTCTTATAAGTCAGTGGTGAAATCTCCTAGCTCAACTAGGAAACTGCCATTGATACTGTAAGACTTGAATTTTTGTGAAGTAACTAGAATATGTAGTGTAGCGGTGAAATGCTTAGATATTACATGGAATACCAATTGCGAAGGCAGGTTACTAACAAACGATTGACGCTGATGGACGAAAGCGTGGGGAGCGAACAGG` |
| ASV992 | 429 | `TGGGGAATTTTGGACAATGGGCGAAAGCCTGATCCAGCCATGCCGCGTGAGTGAAGAAGGCCTTCGGGTTGTAAAGCTCTTTCGGACAGAAAGAAATCGCATGGACTAATACTCTGTGCGGATGACGGTACTGTAAGAAGAAGCACCGGCTAACTACGTGCCAGCAGCCGCGGTAATACGTAGGGTGCGAGCGTTAATCGGAATTACTGGGCGTAAAGGGTGCGCAGGCGGTTATATAAGACAGATGTGAAATCCCCGGGCTCAACCTGGGAACTGCGTTTGTGACTGTATGACTAGAGTACGGTAGAGAGGGGTGGAATTCCTGGTGTAGCAGTGAAATGCGTAGATATCAGGAGGAACATCGATGGCGAAGGCAGCCCCTTGGACCTGTACTGACGCTCATGCACGAAAGCGTGGGGAGCAAACAGG` |
| ASV2340 | 409 | `TGGGGAATATTGCACAATGGGCGAAAGCCTGATGCAGCAACGCCGCGTGAGGGATGACGGCCTTCGGGTTGTAAACCTCTTTTAGTAGGGAAGAAGCGAAAGTGACGGTACCTGCAGAAAAAGCACCGGCTAACTACGTGCCAGCAGCCGCGGTAATACGTAGGGTGCAAGCGTTGTCCGGAATTATTGGGCGTAAAGAGCTCGTAGGCGGTTTGTCGCGTCTGCTGTGAAATCTGGAGGCTCAACCTCCAGCCTGCAGTGGGTACGGGCAGACTAGAGTGCGGTAGGGGAGATTGGAATTCCTGGTGTAGCGGTGGAATGCGCAGATATCAGGAGGAACACCGATGGCGAAGGCAGATCTCTGGGCCGTAACTGACGCTGAGGAGCGAAAGCATGGGGAGCGAACAGG` |
| ASV36 | 404 | `TGGGGAATCTTGCGCAATGGGCGAAAGCCTGACGCAGCCATGCCGCGTGAATGATGAAGGTCTTAGGATTGTAAAATTCTTTCACCGGGGACGATAATGACGGTACCCGGAGAAGAAGCCCCGGCTAACTTCGTGCCAGCAGCCGCGGTAATACGAAGGGGGCTAGCGTTGCTCGGAATTACTGGGCGTAAAGGGAGCGTAGGCGGATAGTTTAGTCAGAGGTGAAAGCCCAGGGCTCAACCTTGGAATTGCCTTTGATACTGGCTATCTTGAGTACGGAAGAGGTATGTGGAACTCCGAGTGTAGAGGTGAAATTCGTAGATATTCGGAAGAACACCAGTGGCGAAGGCGACATACTGGTCCGTTACTGACGCTGAGGCTCGAAAGCGTGGGGAGCAAACAGG` |
| ASV4427 | 403 | `TTAGGAATCTTCCCCAATGGGCGCAAGCCTGAGGGAGCGACACTGCATGAAGGATGAAGGTTTTCGGATCGTAAACTTCTGAACTAGGGACGAATAATGACGGTACCTAGGTAATAGCACCGGCTAACTCTGTGCCAGCAGCCGCGGTAATACAGAGGGTGCGAGCGTTATCCGGAATTACTGGGCGTAAAGGGCGTGTAGGTGGTTTGTTAAGTTCGATGTTAAAGCGTGAGGCTCAACCTCATAATGGCGTTGAATACTGGCAAGCTAGACGACTGGAGAGGAAAGCAGAATTACCAGTGTAGCGGTGAAATGCGTAGATACTGGTAGGAATACCCATTGCGAAGGCAGCTTTCTGGACAGTTTGTGACACTCAGACGCGAAAGTGTGGGGAGCAAACCGG` |
| ASV15 | 429 | `TGGGGAATTTTGGACAATGGGCGCAAGCCTGATCCAGCAATGCCGCGTGCAGGAAGAAGGCCTTCGGGTTGTAAACTGCTTTTGTACGGAACGAAACGGTCCTGGTTAATACCTGGGGCTAATGACGGTACCGTAAGAATAAGCACCGGCTAACTACGTGCCAGCAGCCGCGGTAATACGTAGGGTGCAAGCGTTAATCGGAATTACTGGGCGTAAAGCGTGCGCAGGCGGTTTTGTAAGACAGGCGTGAAATCCCCGGGCTTAACCTGGGAATGGCGCTTGTGACTGCAAAGCTGGAGTGCGGCAGAGGGGGATGGAATTCCGCGTGTAGCAGTGAAATGCGTAGATATGCGGAGGAACACCGATGGCGAAGGCAATCCCCTGGGCCTGCACTGACGCTCATGCACGAAAGCGTGGGGAGCAAACAGG` |
| ASV3058 | 424 | `TAAGGAATATTGGTCAATGGAGGAAACTCTGAACCAGCCATCCCGCGTGCAGGATTAAGGTCCTATGGATTGTAAACTGCTTTTGGTAGGGAAGAAACGCCTCCATTTATGGGGGTCTGACGGTACCTACAGAATAAGCACCGGCTAACTCCGTGCCAGCAGCCGCGGTAATACGGAGGGTGCAAGCGTTATCCGGAATCACTGGGTTTAAAGGGTGCGTAGGCGGGTTATTAAGTCAGTGGTGAAAGCCGGTCGCTCAACGATCGAACTGCCATTGATACTGATAGTCTTGAATTGGGTTGAGGTTAGCGGAATGTGACATGTAGCGGTGAAATGCGTAGATATGTCATGGAACACCAATTGCGAAGGCAGCTAGCTAGGCCTTGATTGACGCTGAGGCACGAAAGCGTGGGTAGCGAACAGG` |
| ASV372 | 424 | `TGAGGAATATTGGACAATGGGCGCAAGCCTGATCCAGCCATGCCGCGTGCAGGATGACGGTCCTATGGATTGTAAACTGCTTTTGTACGAGAAGAAACACTCCTACGTGTAGGAGCTTGACGGTATCGTAAGAATAAGGATCGGCTAACTCCGTGCCAGCAGCCGCGGTAATACGGAGGATCCAAGCGTTATCCGGAATCATTGGGTTTAAAGGGTCCGTAGGCGGTTTAGTAAGTCAGTGGTGAAAGCCCATCGCTCAACGGTGGAACGGCCATTGATACTGCTAAACTTGAATTATTAGGAAGTAACTAGAATATGTAGTGTAGCGGTGAAATGCTTAGAGATTACATGGAATACCAATTGCGAAGGCAGGTTACTACTAATGGATTGACGCTGATGGACGAAAGCGTGGGTAGCGAACAGG` |
| ASV1383 | 429 | `TGGGGAATATTGGACAATGGGGGCAACCCTGATCCAGCCATGCCGCGTGTGTGAAGAAGGCCTTCGGGTTGTAAAGCACTTTAAGCGAGGAGGAAGGTCACCATACTAATATTATGGTGAATTGACGTTACTCGCAGAATAAGCACCGGCTAACTCTGTGCCAGCAGCCGCGGTAATACAGAGGGTGCAAGCGTTAATCGGAATTACTGGGCGTAAAGCGCGCGTAGGCGGTTTGTTAAGTCGGATGTGAAATCCCTGGGCTCAACCTAGGCACTGCATCCGATACTGGCTAACTAGAGTGTGGGAGAGGAAGGTAGAATTCCAGGTGTAGCGGTGAAATGCGTAGAGATCTGGAGGAATACCGATGGCGAAGGCAGCCTTCTGGCCTAACACTGACGCTGAGGTGCGAAAGCATGGGGAGCAAACAGG` |
| ASV288 | 424 | `TGAGGAATATTGGTCAATGGACGCAAGTCTGAACCAGCCATGCCGCGTGCAGGATGACGGTCCTATGGATTGTAAACTGCTTTTGTACGAGAAGAAACACTCCTACGTGTAGGAGCTTGACGGTATCGTAAGAATAAGGATCGGCTAACTCCGTGCCAGCAGCCGCGGTAATACGGAGGATCCAAGCGTTATCCGGAATCATTGGGTTTAAAGGGTCCGTAGGCGGTTTAGTAAGTCAGTGGTGAAAGCCCATCGCTCAACGGTGGAACGGCCATTGATACTGCTAAACTTGAATTATTAGGAAGTAACTAGAATATGTAGTGTAGCGGTGAAATGCTTAGAGATTACATGGAATACCAATTGCGAAGGCAGGTTACTACTAATGGATTGACGCTGATGGACGAAAGCGTGGGTAGCGAACAGG` |
| ASV3081 | 430 | `TGGGGAATATTGCGCAATGGGCGAAAGCCTGACGCAGCAACGCCGCGTGAGTGAAGAAGGCCTTTGGGTTGTAAAGCTCTTTCGGCCGGGAAGAAGGGGGATGGTGTTAATAGCATTATTCTTTGATGGTACCGGAAGAAGAAGCACCGGCTAACTTCGTGCCAGCAGCCGCGGTAATACGAAGGGTGCGAGCGTTGTTCGGAATTATTGGGCGTAAAGGGTTTGTAGGCTGAAATGTAAGTCAAGTGTGAAATCCCCGAGCTAAACTTGGGACGTGCACTTGAAACTGTGTTTCTTGAGTATCGTAGAGGGTGGTGGAATTGTAGGTGTAGGAGTGACATCCGTAGAGATCTGCAGGAACATCAGAGGCGAAGGCGACCACCTGGCCGATTACTGACGCTGAGGAACGAAAGCGTGGGGAGCAAACAGG` |
| ASV970 | 429 | `TGGGGAATTTTGGACAATGGACGAAAGTCTGATCCAGCCATGCCGCGTGCGGGAAGAAGGCCTTCGGGTTGTAAACCGCTTTTGTCAGGGAAGAAAAGGTTCTGGTTAATACCTGGGACTCATGACGGTACCTGAAGAATAAGCACCGGCTAACTACGTGCCAGCAGCCGCGGTAATACGTAGGGTGCAAGCGTTAATCGGAATTACTGGGCGTAAAGCGTGCGCAGGCGGTTATGCAAGACAGAGGTGAAATCCCCGGGCTCAACCTGGGAACTGCCTTTGTGACTGCATAGCTAGAGTACGGTAGAGGGGGATGGAATTCCGCGTGTAGCAGTGAAATGCGTAGATATGCGGAGGAACACCGATGGCGAAGGCAATCCCCTGGACCTGTACTGACGCTCATGCACGAAAGCGTGGGGAGCAAACAGG` |
| ASV524 | 424 | `TGAGGAATATTGGACAATGGGCGCAAGCCTGATCCAGCCATGCCGCGTGCAGGAAGACGGTCCTATGGATTGTAAACTGCTTTTATACAGGAAGAAACACTCGGACGTGTCCGAACTTGACGGTACTGTATGAATAAGCATCGGCTAACTCCGTGCCAGCAGCCGCGGTAATACGGAGGATGCAAGCGTTATCCGGAATCATTGGGTTTAAAGGGTCCGTAGGCGGTTTAATAAGTCAGTGGTGAAAGCCCATCGCTCAACGATGGAACGGCCATTGATACTGTTAAACTTGAATTATTAGGAAGTAACTAGAATATGTAGTGTAGCGGTGAAATGCTTAGAGATTACATGGAATACCAATTGCGAAGGCAGGTTACTACTAATATATTGACGCTGATGGACGAAAGCGTGGGGAGCGAACAGG` |
| ASV716 | 423 | `TAGGGAATATTGGGCAATGGAAGGAATTCTGACCCAGCCATGCCGCGTGCAGGATGAAGGCGCTATGCGTTGTAAACTGCTTTTGTCAGGGAAGAAAATGGCTATGCGTAGCAACTTGACGGTACCTGAAGAATAAGCATCGGCTAACTCCGTGCCAGCAGCCGCGGTAATACGGAGGATGCAAGCGTTGTCCGGATTTATTGGGTTTAAAGGGTGCGTAGGCGGTCTTATAAGTCAGTGGTTAAATTACCTTGCTTAACGAGGGAGATGCCATTGATACTGTTGGACTTGAATACTGTTTGGGTAGGCGGAATTGGTAGTGTAGCGGTGAAATGCTTAGATATTACCAGGAACACCGATAGCGAAGGCAGCTTACTGAAGAGATATTGACGCTGAGGCACGAAAGTGCGGGTATCAAACAGG` |
| ASV1088 | 429 | `TGGGGAATTTTGGACAATGGGGGCAACCCTGATCCAGCAATGCCGCGTGAGTGAAGAAGGCCTTCGGGTTGTAAAGCTCTTTTGTCAGGGAAGAAAAGGTATCCTCTAATACAGGATGCTCATGACGGTACCTGAAGAATAAGCACCGGCTAACTACGTGCCAGCAGCCGCGGTAATACGTAGGGTGCAAGCGTTAATCGGAATTACTGGGCGTAAAGCGTGCGCAGGCGGTTATGTAAGTCAGATGTGAAATCCCCGGGCTCAACCTGGGAACTGCATTTGAGACTGCATGGCTAGAGTGTGTCAGAGGGGGGTAGAATTCCACGTGTAGCAGTGAAATGCGTAGATATGTGGAGGAATACCGATGGCGAAGGCAGCCCCCTGGGATAACACTGACGCTCATGCACGAAAGCGTGGGGAGCAAACAGG` |
| ASV1675 | 429 | `TGGGGAATTTTGGACAATGGGCGCAAGCCTGATCCAGCCATGCCGCGTGCGGGAAGAAGGCCTTCGGGTTGTAAACCGCTTTTGTTCGGGAAGAAATTGACTGGGCTAATACCCCGGTTAGATGACGGTACCGGAAGAATAAGCACCGGCTAACTACGTGCCAGCAGCCGCGGTAATACGTAGGGTGCGAGCGTTAATCGGAATTACTGGGCGTAAAGCGTGCGCAGGCGGTTTCGCAAGTCAGATGTGAAAGCCCCGGGCTCAACCTGGGAACTGCATTTGAAACTACGAAGCTAGAGTGTGTCAGAGGGGGGTAGAATTCCACGTGTAGCAGTGAAATGCGTAGAGATGTGGAGGAATACCAATGGCGAAGGCAGCCCCCTGGGATAACACTGACGCTCATGCACGAAAGCGTGGGGAGCAAACAGG` |
| ASV3534 | 429 | `TGGGGAATTTTGGACAATGGGGGAAACCCTGATCCAGCCATGCCGCGTGCAGGACGAAGGCCCTCGGGTTGTAAACTGCTTTTGTCAGGGAAGAAATCCTGCTTGATAATACCAGGCGGGGATGACGGTACCTGAAGAATAAGTACCGGCTAACTACGTGCCAGCAGCCGCGGTAATACGTAGGGTGCAAGCGTTAATCGGAATTACTGGGCGTAAAGCGTGCGCAGGCGGCCTTACAAGACCGATGTGAAATCCCCGGGCTTAACCTGGGAACTGCATTGGTGACTGTAAAGCTAGAGTGCGGTAGAGGGGGGTGGAATTTTGCGTGTAGCAGTGAAATGCGTAGATATGCAAAGGAACACCAATGGCGAAGGCAGCCCCCTGGACCTGCACTGACGCTCATGCACGAAAGCGTGGGGAGCAAACAGG` |
| ASV4053 | 429 | `TGGGGAATATTGGACAATGGGCGAAAGCCTGATCCAGCCATGCCGCGTGTGTGAAGAAGGCCCTAGGGTTGTAAAGCACTTTCAGTGGGGAGAAAGGTTTACCACCTAATACGTGGTGAAATTGATGTTACCCACAGAAGAAGCACCGGCTAACTCCGTGCCAGCAGCCGCGGTAATACGGAGGGTGCAAGCGTTAATCGGAATTACTGGGCGTAAAGCGCATGTAGGTGGTTTACTAAGCCAGCTGTGAAATCCCCGGGCTCAACCTGGGACTTGCAGTTGGAACTGGTAAGCTAGAGTAGGGTAGAGGGGTGTGGAATTCCAGGTGTAGCGGTGAAATGCGTAGATATCTGGAGGAACATCAGTGGCGAAGGCGACACCCTGGACTCATACTGACACTGAGATGCGAAAGCGTGGGGAGCAAACAGG` |
| ASV3155 | 423 | `TAGGAAATATTGGGCAATGGGCGAGAGCCTGACCCAGCCATGCCGCGTGCCGGATGAAGGCCTTCTGGGTTGTAAACGGCTTTTCTCAGGGAAGAAAAAGAGGATGCGTCCTACACTGACGGTACCTGAGGAATAAGCACCGGCTAACTCCGTGCCAGCAGCCGCGGTAATACGGAGGGTGCAAGCGTTGTCCGGATTTATTGGGTTTAAAGGGTGCGTAGGCGGCCCGTTAAGTCCGGGGTGAAAGCCCACAGCTCAACTGTGGAACTGCCCTGGATACTGGCGGGCTTGAGTCCAGACGAGGTTGGCGGAATGGAAGGTGTAGCGGTGAAATGCATAGATACCTTCCAGAACCCCGATTGCGTAGGCAGCTGACTAGGCTGGTACTGACGCTGAGGCACGAAAGCGTGGGGAGCGAACAGG` |
| ASV667 | 407 | `TGGGGAATTTTCCGCAATGGGCGAAAGCCTGACGGAGCAATACCGCGTGAGGGATGACGGCCTATGGGTTGTAAACCTCTTTTTTCAGGGAGGAATCAATGACGTGTACCTGAGGAATAAGCATCGGCTAACTCCGTGCCAGCAGCCGCGGTAAGACGGAGGATGCAAGTGTTATCCGGAATCACTGGGCGTAAAGCGTCTGTAGGTGGTCAAATAAGTCAACTGTTAAATCTTGAGGCTCAACCTCAAAATCGCAGTCGAAACTATTCGACTAGAGTATAGTAGGGGTAAAGGGAATTTCCAGTGGAGCGGTGAAATGCGTAGAGATTGGAAAGAACACCGATGGCGAAGGCACTTTACTGGGCTATTACTAACACTGAGAGACGAAAGCTAGGGTAGCAAATGGG` |
| ASV4240 | 430 | `TGGGGAATCTTGCACAATGGGCGAAAGCCTGATGCAGCAACGCCGCGTGAGTGATGAAGGTCTTTGGATTGTAAAGCTCTTTCGATCGGGAAGAAGGGATAGGGTATTAATACTGCCTTATTTTGACGGTACCGATATAAGAAGCACTGGCTAATTCCGTGCCAGCAGCCGCGGTAATACGGAAGGTGCAAGCGTTGTTCGGAGTGACTGGGCGTAAAGGGCGCGTAGGCTGGAAGATAAGACGATTGTGAAAACCCCGGGCTTAACCTGGGACGTGCAGTCGTGACTGTTTTTCTTGAATGTCTAAGAGGTTAGTGGAATTCCAGGTGTAGGAGTGAAATCCGTAGATATCTGGAGGAACACCGGAGGCGAAGGCGGCTAACTGGTGGAGCATTGACGCTGAGGCGCGAAAGCGCGGGGAGCAAACAGG` |
| ASV4293 | 408 | `TGAGGAATCTTCCGCAATGGGCGAAAGCCTGACGGAGCGACACCGCGTGGAGGATGAAGCCCTTTCCGGGCGTAAACTCCTGTTCTGAGGGACGAAATTTTTGACGGTACCTCAGGAGAAAGCATCGGCTAACCCTGTGCCAGCAGCCGCGGTAAGACAGGGGATGCAAGCGTTACTCGGAATTACTGGGCGTAAAGCGTCTGCAGGTGCCCCGTAAAGTCTGGTGTGAAATGGCGATGCTTAACATCGTCACTGTGCCGGAAACTTACGGGGTTGAGTCACTCAAAGGCATCTGGAATGTCGTGTGTAGGGGTAAAATCCGTAGATCCACGATGGAACGCCGAAAGCGAAAGCAGGATGCTGGGAGTGCACTGACACTCAGAGACGAAAGCGTGGGGAGCAAAGGGG` |
| ASV5002 | 423 | `TAGGGAATATTGGTCAATGGAGGAAACTCTGAACCAGCCATGCCGCGTGCAGGAAGAAGGCCCTCTGGGTTGTAAACTGCTTTTGACAGGGAACAAGAAGGCCATGCGTGGTTTAGTGAGTGTACCTGTAGAATAAGCCACGGCTAACTACGTGCCAGCAGCCGCGGTAATACGTAGGTGGCAAGCGTTGTCCGGATTTATTGGGTTTAAAGGGTGCGTAGGCGGCTTTTTAAGTCAGTTGTGAAATATCTCGGCTTAACCGAGAGGGTGCGATTGATACTGGGAAGCTTGAGTTACAGCGAGGTCAGCGGAATTGATGGTGTAGCGGTGAAATGCATAGATATCATCAAGAACACCGATAGCGAAGGCAGCTGACTAGGAGTACACTGACGCTGAGGCACGAAAGTGCGGGGATCAAACAGG` |
| ASV547 | 424 | `TGAGGAATATTGGACAATGGGCGCAAGCCTGATCCAGCCATGCCGCGTGCAGGATGACGGTCCTATGGATTGTAAACTGCTTTTGTACAGGAAGAAACACTCCCTCGTGAGGGAGCTTGACGGTACTGTAAGAATAAGGATCGGCTAACTCCGTGCCAGCAGCCGCGGTAATACGGAGGATCCAAGCGTTATCCGGAATCATTGGGTTTAAAGGGTCCGTAGGCGGTTTAGTAAGTCAGTGGTGAAAGCCCATCGCTCAACGGTGGAACGGCCATTGATACTGCTAAACTTGAATTATTAGGAAGTAACTAGAATATGTAGTGTAGCGGTGAAATGCTTAGAGATTACATGGAATACCAATTGCGAAGGCAGGTTACTACTAATTGATTGACGCTGATGGACGAAAGCGTGGGTAGCGAACAGG` |
| ASV806 | 429 | `TGGGGAATATTGCACAATGGGGGAAACCCTGATGCAGCCATGCCGCGTGTGTGAAGAAGGCCTTCGGGTTGTAAAGCACTTTCAGCGAGGAGGAAAGGTTGTTGGCTAATACCCAGCAACTGTGACGTTACTCGCAGAAGAAGCACCGGCTAACTCCGTGCCAGCAGCCGCGGTAATACGGAGGGTGCAAGCGTTAATCGGAATTACTGGGCGTAAAGCGCACGCAGGCGGTTGGATAAGTTAGATGTGAAAGCCCCGGGCTCAACCTGGGAATTGCATTTAAAACTGTCCAGCTAGAGTCTTGTAGAGGGGGGTAGAATTCCAGGTGTAGCGGTGAAATGCGTAGAGATCTGGAGGAATACCGGTGGCGAAGGCGGCCCCCTGGACAAAGACTGACGCTCAGGTGCGAAAGCGTGGGGAGCAAACAGG` |
| ASV1849 | 429 | `TGGGGAATTTTGGACAATGGGGGAAACCCTGATCCAGCCATGCCGCGTGTCTGAAGAAGGCCTTCGGGTTGTAAAGGACTTTTGTCCGGGAGGAAACCCTGATCGTTAATAGCGGTCGGGACTGACAGTACCGGAAGAATAAGCACCGGCTAACTACGTGCCAGCAGCCGCGGTAATACGTAGGGTGCAAGCGTTAATCGGAATTACTGGGCGTAAAGCGTGCGCAGGCGGTGAGATAAGTCAGCTGTGAAAGCCCCGGGCTCAACCTGGGAACTGCGGCCGAAACTGTCTCGCTAGAGTACGTCAGAGGGGGGTGGAATTCCACGTGTAGCAGTGAAATGCGTAGAGATGTGGAGGAACACCAATGGCGAAGGCAGCCCCCTGGGATGATACTGACGCTCATGCACGAAAGCGTGGGGAGCAAACAGG` |
| ASV4040 | 429 | `TGGGGAATTTTGGACAATGGGCGCAAGCCTGATCCAGCAATGCCGCGTGAGTGAAGAAGGCCTTCGGGTTGTAAAGCTCTTTTGTCAGGGAAGAAACGGTGGTGGTTAATACCTGCTGCTAATGACGGTACCTGAAGAATAAGCACCGGCTAACTACGTGCCAGCAGCCGCGGTAATACGTAGGGTGCAAGCGTTAATCGGAATTACTGGGCGTAAAGCGTGCGCAGGCGGTTATATAAGTCAGATGTGAAATCCCCGGGCTCAACCTGGGAACTGCATTTGAGACTGTATGGCTAGAGTGTGTCAGAGGGGGGTAGAATTCCACGTGTAGCAGTGAAATGCGTAGATATGTGGAGGAATACCGATGGCGAAGGCAGCCCCCTGGGATAACACTGACGCTCATGCACGAAAGCGTGGGGAGCAAACAGG` |
| ASV3629 | 429 | `TGGGGAATTTTGGACAATGGGGGCAACCCTGATCCAGCAATGCCGCGTGAGTGAAGAAGGCCTTCGGGTTGTAAAGCTCTTTTGTCAGGGAAGAAACGGGTCCAGCTAATATCTGGGCCTAATGACGGTACCTGAAGAATAAGCACCGGCTAACTACGTGCCAGCAGCCGCGGTAATACGTAGGGTGCAAGCGTTAATCGGAATTACTGGGCGTAAAGCGTGCGCAGGCGGTTTCGTAAGTCTGTCGTGAAATCCCCGGGCTTAACCTGGGAATGGCGATGGAGACTGCGAGGCTAGAGTTTGGCAGAGGGGGGTAGAATTCCACGTGTAGCAGTGAAATGCGTAGAGATGTGGAGGAACACCGATGGCGAAGGCAGCCCCCTGGGTCAAAACTGACGCTCATGCACGAAAGCGTGGGGAGCAAACAGG` |
| ASV3232 | 429 | `TGGGGAATATTGGACAATGGGCGCAAGCCTGATCCAGCAATACCGCGTGTGTGAAGAAGGCCTTCGGGTTGTAAAGCACTTTCAGTAGCTAAGAAATACTCAAAACTAATACTTTTGAGAGTTGACGTAAACTACAGAAGAAGCACCGGCTAACTCTGTGCCAGCAGCCGCGGTAATACAGAGGGTGCAAGCGTTAATCGGAATTACTGGGCGTAAAGCGCACGTAGGTGGGTGATTAAGTCGAATGTGAAACCCCCGAGCTTAACTTGGGAACTGCATGCGATACTGAACACCTAGAGTATAGTAGAGGGAAGTGGAATTTCCGGAGTAGCGGTGAAATGCGTAGATATCGGAAGGAACACCAGTGGCGAAGGCGGCTTCCTGGGCTAATACTGACACTGAGGTGCGAAAGCGTGGGGAGCAAACAGG` |
| ASV2401 | 407 | `TAGGGAATATTGCACAATGGAGGAAACTCTGATGCAGCGACGCCGCGTGAGTGATGAAGGCCTTCGGGTCGTAAAGCTCTGTCGTAAGGGAATAAAAAAATGAAGGTACCTTACAAGAAAGGACCGGCTAACTTCGTGCCAGCAGCCGCGGTAATACGAGGGGTCCTAGCGTTGCTCGGAATCATTGGGCGTAAAGCGGGTGTAGGTGGATAACTAAGTCAGATGTGAAAGCCCCAGGCTCAACCTGGGAAGTGCATTTGAAACTGGATATCTTGAGTGCAGTAGAGGAAGGTGGAATTCCAAGTGTAGTGGTGAAATACGTAGATATTTGGAGGAACATCGGTGGCGAAGGCGACCTTCTGGACTGTTACTGACACTGAGACCCGAAAGCGTGGGGATCAAACAGG` |
| ASV2227 | 429 | `TCGAGAATCATTCACAATGGGGGCAACCCTGATGGTGCAACGCCGCGTGGAGGATGAAGGTCTTCGGATTGTAAACTCCTGTCATCTCGGAGCAAGGCATGGTGGTGAATAGCCAACATGATTGATAGTACGAGAAGAGGAAGGGACGGCTAACTTCGTGCCAGCAGCCGCGGTAATACGAAGGTCCCGAGCGTTATTCGGAATCACTGGGCGTAAAGGGAGCGTAGGCGGCGTGGAAAGTCAGATGTGAAATCCCGGGGCTCAACCCCGGAACTGCATCCGATACTACCATGCTTGAGTATTGGAGGGGTAACTGGAATTCTCGGTGTAGCAGTGAAATGCGTGGATATCGAGAGGAACACCTGTGGCGAAAGCGAGTTACTGGACAATTACTGACGCTGAGGCTCGAAGGCTAGGGGAGCAAAAGGG` |
| ASV5650 | 406 | `TGGGGAATATTGCGCAATGGGGGAAACCCTGACGCAGCCACGCCGCGTGAGTGATGAAGGCCTTCGGGTTGTAAAACTCTGTCGGGAGGGAAGAAGTTCTGACGGTACCTCCCAAGAAAGCGCCGGCTAACCCTGTGCCAGCAGCCGCGGTAATACAGGGGGCGCGAGCGTTGCTCGGAATTACTGGGCGTAAAGCGTGCGCAGGCGGACTGATAAGTCGGGCGTGAAATCCCAGGGCTCAACCCTGGACCTGCGTTCGAGACTGTCAGCCTTGAGTACGGGAGAGGTGGGTGGAATTCCTAGTGTAGCGGTGAAATGCGTAGATATTAGGAGGAACACCGGTGGCGAAAGCGGCCCACTGGCCCGATACTGACGCTCATGTACGAAAGCGCGGGGAGCAAACAGG` |
| ASV3083 | 423 | `TAGGGAATATTGGTCAATGGACGCAAGTCTGAACCAGCCATGCCGCGTGCAGGATGAAGGCCCTCTGGGTTGTAAACTGCTTTTGATAGGGAGCAAATGCCACTTGCGAGTGGAGCTGAGAGTACCTATAGAATAAGCACCGGCTAACTCCGTGCCAGCAGCCGCGGTAATACGGAGGGTGCAAGCGTTGTCCGGATTTATTGGGTTTAAAGGGTGCGTAGGCGGCTCAATAAGTCAGTGGTGAAAGCCTCCAGCTTAACTGGAGAACTGCCATTGATACTGTTGAGCTTGAGTATAGTTGAGGTAGGCGGAATTGACAGTGTAGCGGTGAAATGCTTAGATATTGTCAAGAACACCGATTGCGAAGGCAGCTTACTAAGCTATAACTGACGCTGAGGCACGAAAGTGTGGGTATCAAACAGG` |
| ASV4116 | 404 | `TGGGGAATATTGGACAATGGGGGCAACCCTGATCCAGCCATGCCGCGTGAATGATGAAGGCCTCCGGGTTGTAAAGTTCTTTTGGCGGGGACGATGATGACGGTACCCGCAGAATAAGCTCCGGCTAACTTCGTGCCAGCAGCCGCGGTAATACGAAGGGAGCTAGCGTTGTTCGGAATTACTGGGCGTAAAGGGCGCGTAGGCGGTTAAACAAGTCAGGCGTGAAAGCCCCGGGCTCAACCCGGGAACTGCGCTTGAGACTGTTTACCTAGAGTACGGTAGAGGTAAGTGGAATTCCCAGTGTAGCAGTGAAATGCGTAGATATTGGGAGGAACACCAGTGGCGAAGGCGGCTTACTGGACCGTAACTGACGCTGAGGCGCGAAAGCGTGGGGAGCAAACAGG` |
| ASV3519 | 429 | `TGGGGAATATTGGACAATGGGGGCAACCCTGATCCAGCAATGCCGCGTGTGTGAAGAAGGCCTGCGGGTTGTAAAGCACTTTTGGCAGGGATGAAAAGCACTGGATTAATAACCTAGTGTGTTGACCTAACCTGCAGAAGAAGCACCGGCTAACTCTGTGCCAGCAGCCGCGGTAATACAGGGGGTGCGAGCGTTAATCGGAATTACTGGGCGTAAAGGGCGCGTAGGCGGTCGAGTAAGTCGGATGTGAAATCCCCGGGCTTAACCTGGGAATTGCATTCGATACTGCTTGGCTAGAGTGTGGAAGAGGGAAGCGGAATTTCCGGTGTAGCGGTGAAATGCGTAGATATCGGAAGGAACACCAGTGGCGAAGGCGGCTTCCTGGTCCAACACTGACGCTGAGGCGCGAAAGCGTGGGGAGCAAACAGG` |
| ASV4333 | 408 | `TGGGGAATATTGGGCAATGGAGGAAACTCTTACCCAGCAATGCCGCGTGAGTGAATGAAGGTCTTCGGATTGTAAAGCTCTTTGATTGGGGACGAATAAATGACGGTACCCAAGGAACAAGCCCCGGCTAACTATGTGCCAGCAGCCGCGGTAATACATAGGGGGCAAGCGTTGTCCGGAATGACTGGGCGTAAAGGGTGCGTAGGCGGTTTAACAAGTTGGAAGTGTAATACCTAGAGCTTAACTCAGGTGCTGCTTCCAAAACTGTTAGACTTGAGTGATGGAGAGGAAAATGGAATTCCTAGTGTAGCGGTGGAATGCGTAGATATTAGGAGGAACACCAGAGGCGAAGGCGATTTTCTGGACATTAACTGACGCTGAGGCACGAAAGCGTGGGTAGCAAACAGG` |
| ASV665 | 429 | `TGGGGAATATTGGACAATGGGGGCAACCCTGATCCAGCAATGCCGCGTGTGTGAAGAAGGCCTGAGGGTTGTAAAGCACTTTCAGTGGGGAGGAGGGTTGATTGGTTAAGAGCTGATTGATTGGACGTTACCCACAGAAGAAGCACCGGCTAACTCTGTGCCAGCAGCCGCGGTAATACAGAGGGTGCAAGCGTTAATCGGAATTACTGGGCGTAAAGGGTGCGTAGGTGGTTAGATAAGTTATCTGTAAAATCCCTGGGCTTAACCTGGGCAGGTCAGATAAGACTGTTTGACTCGAGTATGGGAGAGGGTAGTGGAATTTCCGGTGTAGCGGTGAAATGCGTAGAGATCGGAAGGAACACCAGTGGCGAAGGCGGCTACCTGGCCTAATACTGACACTGAGGCACGAAAGCGTGGGGAGCAAACAGG` |
| ASV2592 | 429 | `TGAGGAATATTGCACAATGGGCGAAAGCCTGATGCAGCGACGCCGCGTGGAGGATGAAGGTCTTAGGATTGTAAACTCCTGTTAAGTGAGAAGAAAGTACTACTTTTAATAGAAGTGGGAGATGACGTTATCACTAGAGAAAGCACCGGCTAACTTCGTGCCAGCAGCCGCGGTAATACGAGGGGTGCAAGCGTTATTCGGAATGACTGGGCGTAAAGGGTGCGTAGACGGCACGTTAAGTCTTTTGTTAAATACCTCAGCCTAACTGAGGATCAGCAGGGGAAACTGGCGAGCTTGAGATTGGAAGAGAGAAGTGGAATTCTCGGAGTAGCGGTAAAATGCGTAGATCTCGAGAGGAACACCAATGGCGAAGGCAGCTTCTTGGTCCAATTCTGACGTTGAGGCACGAAAGCGTGGGGAGCAAACAGG` |
| ASV4001 | 405 | `TAGGGAATTTTCCACAATGGGCGAAAGCCTGATGGAGCAACGCCGCGTGCAGGATGAAGGTTTTCGGATCGTAAACTGCTTTTATAAGTGAAGAATATGACGGTAACTTATGAATAAGGGTCGGCTAACTACGTGCCAGCAGCCGCGGTCATACGTAGGACCCAAGCGTTATCCGGAATTACTGGGCGTAAAGAGTTGCGTAGGTGGCATAGTAAGTGGGTAGTGAAATCGTGTGGCTCAACCATACACACATTATCTAAACTGCTAAGCTTGAGTGAGATAGAGGTAACTGGAATTCCAAGTGTAGGAGTGAAATCCGTAGATATTTGGAGGAACACCGATGGCGTAGGCAGGTTACTGGATCTCAACTGACACTGAGGCACGAAAGCATGGGGAGCAAACGGG` |
| ASV2391 | 429 | `TGGGGAATTTTGGACAATGGGGGCAACCCTGATCCAGCAATGCCGCGTGAGTGAAGAAGGCCTTCGGGTTGTAAAGCTCTTTTGTCAGGGAAGAAACGGTCTGGGCTAATATCCTGGGCTAATGACGGTACCTGAAGAATAAGCACCGGCTAACTACGTGCCAGCAGCCGCGGTAATACGTAGGGTGCAAGCGTTAATCGGAATTACTGGGCGTAAAGCGTGCGCAGGCGGTTATGTAAGACAGGTGTGAAATCCCCGGGCTTAACCTGGGAATTGCATTTGTGACTGCATGGCTAGAGTGTGTCAGAGGGGGGTAGAATTCCACGTGTAGCAGTGAAATGCGTAGAGATGTGGAGGAATACCGATGGCGAAGGCAGCCCCCTGGGATAACACTGACGCTCATGCACGAAAGCGTGGGGAGCAAACAGG` |
| ASV273 | 429 | `TGGGGAATTTTGGACAATGGACGCAAGTCTGATCCAGCCATGCCGCGTGCGGGAAGAAGGCCTTCGGGTTGTAAACCGCTTTTGTCAGGGAAGAAACGCTCTGGGTTAATACCTCGGGGTAATGACGGTACCTGAAGAATAAGCACCGGCTAACTACGTGCCAGCAGCCGCGGTAATACGTAGGGTGCAAGCGTTAATCGGAATTACTGGGCGTAAAGCGTGCGCAGGCGGTTATGCAAGACAGATGTGAAATCCCCGGGCTCAACCTGGGAACTGCATTTGTGACTGCATGGCTAGAGTACGGTAGAGGGGGATGGAATTCCGCGTGTAGCAGTGAAATGCGTAGATATGCGGAGGAACACCGATGGCGAAGGCAATCCCCTGGACCTGTACTGACGCTCATGCACGAAAGCGTGGGGAGCAAACAGG` |
| ASV2 | 429 | `TGGGGAATTTTGGACAATGGGCGCAAGCCTGATCCAGCAATGCCGCGTGCAGGATGAAGGCCTTCGGGTTGTAAACTGCTTTTGTACGGAACGAAACGGCGAGCTCTAATACAGTTTGCTAATGACGGTACCGTAAGAATAAGCACCGGCTAACTACGTGCCAGCAGCCGCGGTAATACGTAGGGTGCGAGCGTTAATCGGAATTACTGGGCGTAAAGCGTGCGCAGGCGGTGATGTAAGACAGATGTGAAATCCCCGGGCTCAACCTGGGACCTGCATTTGTGACTGCATCGCTAGAGTACGGTAGAGGGGGATGGAATTCCGCGTGTAGCAGTGAAATGCGTAGATATGCGGAGGAACACCGATGGCGAAGGCAATCCCCTGGACCTGTACTGACGCTCATGCACGAAAGCGTGGGGAGCAAACAGG` |
| ASV3027 | 404 | `TGGGGAATATTGGACAATGGGGGCAACCCTGATCCAGCGATGCCGCGTGAGTGATGAAGGCCTTCGGGTTGTAAAGCTCTTTTGTGGGGGACGATGATGACGGTACCCCACGAATAAGCCCCGGCTAACTTCGTGCCAGCAGCCGCGGTAATACGAAGGGGGCTAGCGTTGTTCGGAATGACTGGGCGTAAAGGGTGCGTAGGCGGTTTAATAAGTTTGGTGTGAAAGTCCTGGGCTCAACCTGGGGATTGCACTGGAAACTATTAGACTAGAGATCGAGAAAGGAAAATGGAATTGCGAGTGTAGAGGTGAAATTCGTAGATATTCGCAAGAACACCGGTGGCGAAGGCGGTTTTCTGGCTCGATACTGACGCTAAGGCACGAAAGCGTGGGGAGCAAACAGG` |
| ASV1371 | 424 | `TAAGGAATATTGGTCAATGGACGCAAGTCTGAACCAGCCATGCCGCGTGGAGGATGAAGGTCCTCTGGATTGTAAACTTCTTTTCTCTGGGACGAAACCCCCGATTTCTATCGGGCCTGACGGTACCAGAGGAATAAGCACCGGCTAACTCCGTGCCAGCAGCCGCGGTAATACGGAGGGTGCAAGCGTTATCCGGATTCACTGGGTTTAAAGGGTGCGTAGGCGGGTTTGTAAGTCAGTGGTGAAATCCCCGAGCTTAACTTGGGAACTGCCATTGATACTGCAGATCTTGAATTTTCTGGAGGTAAGCGGAATATGTCATGTAGCGGTGAAATGCTTAGATATGACATAGAACACCCATTGCGAAGGCAGCTTACTACGGGAATATTGACGCTGAGGCACGAAAGCGTGGGGATCAAACAGG` |
| ASV4145 | 407 | `TGGGGAATTTTCCGCAATGGGCGAAAGCCTGACGGAGCAATACCGCGTGAGGGAGGAAGGCTCTTGGGTTGTAAACCTCTTTTCTTGGGGAAGAAAAAAATGACGGTACCCAAGGAATCAGCATCGGCTAACTCCGTGCCAGCAGCCGCGGTAATACGGAGGATGCAAGCGTTATCCGGAATGATTGGGCGTAAAGCGTCCGCAGGTGGCTCATCAAGTCGATTGTCAAAGATTGGGGCTTAACTCCGAATAGGCAGTGGAAACTGAAGAGCTAGAGTGTGATAGGGGTAGAGGGAATTCCCGGTGTAGCGGTGAAATGCGTAGAGATCGGGAAGAACACCAGTGGCGAAAGCGCTCTACTAGGTCACAACTGACACTCAGGGACGAAAGCTAGGGGAGCGAATGGG` |
| ASV4546 | 404 | `TGGGGAATATTGGACAATGGGGGCAACCCTGATCCAGCTATGCCGCGTGAGTGATGAAGGCCTTCGGGTTGTAAAGCTCTTTAGCTAGGGAAAATAATGATTGTACCTAGAGAATAAGCCCCGGCAAACTCTGTGCCAGCAGCCGCGGTAATACAGAGGGGGCTAGCGTTGTTCGGAATTACTGGGCGTAAAGGGTGCGTAGGCGGCTTACCAAGTCAGGTGTGAAATCCCGGGGCTCAACCTCGGAATTGCACTTGAAACTAGTAAGCTTGAGGACGAGAGAGGATAGTGGAATTCCTAGTGTAGGAGTGAAATCCGTAGATATTAGGAGGAACACCAGTGGCGAAGGCGGCTATCTGGCTCGTATCTGACGCTGAGGCACGAAAGCGTGGGGATCAAACAGG` |
| ASV3119 | 429 | `TGGGGAATATTGGACAATGGGGGCAACCCTGATCCAGCCATGCCGCGTGTGTGAAGAAGGCCTTCGGGTTGTAAAGCACTTTCAGCGGGGAGGAAAGGTTGACGGTTAATAACCGTGAGCTGTGACGTTACCCGCAGAAGAAGCACCGGCTAATTCAGTGCCAGCAGCCGCGGTAATACTGAAGGTGCAAGCGTTAATCGGAATTACTGGGCGTAAAGCGCGCGTAGGCGGCCAAGTCAGTCAGATGTGAAAGCCCTGGGCTCAACCTGGGAACTGCATTTGATACTGCTTGGCTAGAGTATGGGAGAGGGCTGTGGAATTCCACGTGTAGCGGTGAAATGCGTAGATATGTGGAGGAACATCAGTGGCGAAGGCGGCAGCCTGGCCCAATACTGACGCTGAGGTGCGAAAGCGTGGGGAGCAAACAGG` |
| ASV4724 | 405 | `TGGGGAATATTGGTCAATGGGCGAAAGCCTGAACCAGCGATTCTGCGTGAAGGATGAAGCATTACGGTGTGTAAACTTCTTTTTTGGGGGAAGACGAATGACGGTACCCCACGAATAAGGGACGGCTAACTACGTGCCAGCAGCCGCGGTAATACGTAGGTCCCAAGCGTTATCCGGAATTACTGGGCGTAAAGAGTCTGTAGCTGGTCTCGCAAGTCTACTTTCAAATACCTTGGCTTAACCAAGGAAAGGGGGTGGATACTGCGAGTCTGGAAGTTTTCAGGGGTAAGTAGAATTCCCGGTGGAGAGGTGAAATTCGTTGATATCGGGAGGAATGCCAAAAGCGAAAGCAGCTTACTATGAATATCTTGACAGTGAGAGACGAAAGCTTGGGTAGCAAACGGG` |
| ASV3287 | 424 | `TGAGGAATATTGGTCAATGGACGCAAGTCTGAACCAGCCATGCCGCGTGCAGGATGACGGTGCTATGCATTGTAAACTGCTTTTTTACGAGGGTAAACCTGGGTACGTGTACCCAGCTGAAAGTATCGTAAGAATAAGGGTCGGCTAACTCCGTGCCAGCAGCCGCGGTAATACGGAGGACCCGAGCGTTATCCGGATTTATTGGGTTTAAAGGGTGCGTAGGCGGATTAATAAGTTAGAGGTGAAAGCTCGACGCTCAACGTCGAAATTGCCTCTGATACTGTTAGTCTAGAGTATAGTTGCGGAAGGCGGAATGTGTGGTGTAGCGGTGAAATGCTTAGATATCACACAGAACACCGATTGCGAAGGCAGCTTTCCAAGCTATCACTGACGCTGAGGCACGAAAGCGTGGGGAGCGAACAGG` |
| ASV4729 | 421 | `TGAGGAATATTGGTCAATGGGAGCAATCCTGAACCAGCGACGCCGCGTGTGGGATGAAGGGGCTTAGCCTCGTAAACCACTGTCGGATGGGACGAAAGAAGATATGAATCTTTGGGACGGTACCATTAGAGGAAGGGTCGGCTAACTACGTGCCAGCAGCCGCGGTAATACGTAGGACCCAAGCGTTGTCCGGAATTACTGGGTGTAAAGGGTGCGTAGGCGGACGGGTGCGTCAGAGGTGAAATCTCCGGGCTTAACTTGGAGGGTGCCTTTGATACGGCTCGTCTTGAGTACGAGAGAGGATGATAGAATTCCTGGTGTAGCAGTGAAATGCGTAGATATCAGGAGGAATACCGGTGGCGAAGGCGGTCATCTGGCTCGTAACTGACGCTGAGGCACGAAAGCGTGGGGATCAAACAGG` |
| ASV294 | 429 | `TGGGGAATATTGGACAATGGGCGCAAGCCTGATCCAGCCATGCCGCGTGTGTGAAGAAGGCCTTCGGGTTGTAAAGCACTTTTGTCCGGAAAGAAATCTTCCAACTTAATACGTTGGGAGGATGACGGTACCGGAAGAATAAGCACCGGCTAACTTCGTGCCAGCAGCCGCGGTAATACGAAGGGTGCAAGCGTTACTCGGAATTACTGGGCGTAAAGCGTGCGTAGGCGGTTTGTTAAGTCTGATGTGAAATCCCCGGGCTCAACCTGGGACGTGCATTGGAAACTGGCGATCTAGAGTACGGTAGAGGGTGGTGGAATTCCCGGTGTAGCAGTGAAATGCGTAGAGATCGGGAGGAACACTCGTGGCGAAGGCGGCCACCTGGACCAGTACTGACGCTGAGGCACGAAAGCGTGGGGAGCAAACAGG` |
| ASV1765 | 430 | `TGGGGAATCTTGCGCAATGGGCGAAAGCCTGACGCAGCGACACCGCGTGAGTGAGGAAGGTCTTCGGATTGTAAAGCTCTGTCGGCAGGAAAGATGGGACAGGTGGCTAATACCCACTTGTCTTGACGGTACCTGCAAAGGAAGCACCGGCTAACTCTGTGCCAGCAGCCGCGGTAATACAGAGGGTGCAAGCGTTGTTCGGATTTACTGGGCGTAAAGCGCGCGTAGGCGGTATATCAAGTCGGATGTGAAAACCCAAGGCTTAACCATGGAAGTGCATTCGAAACTGGTACACTGGAGTCTCGGAGAGGGTGGTGGAATTCCTGGTGTAGAGGTGAAATTCGTAGATATCAGGAAGAACATCAGTGGCGAAGGCGACCACCTGGACGATGACTGACGCTGAGGCGCGAAAGCGTGGGTAGCAAACAGG` |
| ASV935 | 423 | `TAGGGAATATTGGGCAATGGAGGCAACTCTGACCCAGCCATGCCGCGTGCAGGAAGAAGGCGTTATGCGTTGTAAACTGCTTTTATATAGGAAGAAATAGACCTTGCGAGGTAAGTTGACGGTACTATATGAATAAGCACCGGCTAACTCCGTGCCAGCAGCCGCGGTAATACGGAGGGTGCAAGCGTTGTCCGGATTTATTGGGTTTAAAGGGTGCGTAGGCGGTTTATTAAGTCAGTGGTGAAAGACGGTCGCTCAACGATTGCAGTGCCATTGATACTGGTAGACTTGAGTGGGTTTGAGGTAGCTGGAATGGATAGTGTAGCGGTGAAATGCATAGATATTATCCAGAACACCAATTGCGTAGGCAAGTTACTAAGACTCAACTGACGCTGAGGCACGAAAGTGTGGGTATCAAACAGG` |
| ASV2279 | 404 | `TAGGGAATATTGGACAATGGACGAAAGTCTGATCCAGCAATGCCGCGTGAATGATGAAGGCCTTAGGGTTGTAAAGTTCTTTTAGTGGGGAAGATAATGACGGTACCCACAGAAAAAGCACCGGCTAACTCCGTGCCAGCAGCCGCGGTAATACGGAGGGTGCAAGCGTTGTTCGGAATTACTGGGCGTAAAGCACGCGTAGGCGGCTTGGTAAGTTGGGAGTGAAATCCCGAGGCTTAACCTCGGAATTGCTTTCAAAACTATCAAGCTAGAGTATGGCAGGGGACAGTGGAATTCCTAGTGTAGAGGTGAAATTCGTAGATATTAGGAGGAACACCAGTGGCGAAAGCGACTGTCTGGACCATTACTGACGCTGAGGCGTGAAAGCGTGGGGAGCAAACAGG` |
| ASV2684 | 429 | `TGGGGAATATTGGACAATGGGGGCAACCCTGATCCAGCAATACCGCGTGTGTGAAGAAGGCCTGAGGGTTGTAAAGCACTTTCAGTGGGGAGGAGGGTACTTAGGTTAAGAGCTAGAGTACTGGACGTTACCCACAGAAGAAGCACCGGCTAACTCCGTGCCAGCAGCCGCGGTAATACGGAGGGTGCAAGCGTTAATCGGAATTACTGGGCGTAAAGGGTGCGTAGGTGGTTTGGTAAGTTATCTGTGAAATCCCCGGGCTCAACCTGGGCAGGTCAGATAATACTGCTAGACTCGAGTATGGGAGAGGGTAGTGGAATTTCCGGTGTAGCGGTGAAATGCGTAGAGATCGGAAGGAACACCAGTGGCGAAGGCGGCTACCTGGCCCAATACTGACACTGAGGCACGAAAGCGTGGGGAGCAAACAGG` |
| ASV2302 | 424 | `TGAGGAATATTGCGCAATGGAGGCAACTCTGACGCAGCCATGCCGCGTGCAGGAAGAAGGCGCTACGCGTTGTAAACTGCTTTTAACCGGGAAGAAACCCCAGGACGTGTCCTGGGTTGACGGTACCGGAGGAATAAGCACCGGCTAACTTCGTGCCAGCAGCCGCGGTAATACGAAGGGTGCAAGCGTTATCCGGATTCATTGGGTTTAAAGGGTGCGTAGGTGGGGCAATAAGTCAGTGGTGAAATCTTGCGGCTCAACCGTAAAATTGCCATTGATACTGTTGCTCTTGAGTACGCTTGACGTGGGCGGAATGTGCCGTGTAGCGGTGAAATGCTTAGATATGGCACAGAACACCGATAGTGAAGACAGCTCACGAAGGCGAAACTGACACTGAGGCACGAAAGCGTGGGGATCGAACAGG` |
| ASV3272 | 424 | `TGAGGAATATTGGTCAATGGACGCAAGTCTGAACCAGCCAAGTCGCGTGAAGGATGACTGCCCTACGGGTTGTAAACTTCTTTTATAAGAGAGTAAAAAACAGTACGTGTACTGTCTTGCAAGTACCTTATGAATAAGCATCGGCTAACTCCGTGCCAGCAGCCGCGGTAATACGGAGGATGCAAGCGTTATCCGGATTTATTGGGTTTAAAGGGTGCGTAGGCGGTTTTTTAAGTCAGCGGTGAAAGTTTTAGGCTCAACCTAAAAATTGCCGTTGAAACTGGAAGACTTGAGTGTATATGAAGTGGGCGGAATTTGTGGTGTAGCGGTGAAATGCATAGATATCACGAGGAACTCCAATTGCGCAGGCAGCTCACTAAAATACAACTGACGCTGAGGCACGAAAGCGTGGGTATCCAACAGG` |
| ASV3763 | 406 | `TGGGGAATTTTCCGCAATGGGCGAAAGCCTGACGGAGCAATACCGCGTGTGGGAGGAAGGCTCTTGGGTTGTAAACCACTTTTCTCAGGGAAGAATAGATGACGGTACCTGAGGAATAAGCATCGGCTAACTCCGTGCCAGCAGCCGCGGTAATACGGAGGATGCAAGCGTTATCCGGAATCATTGGGCGTAAAGCGTCCGCAGGTGGCCTATCAAGTCTGCTGTCAAAGCGCAGAGCTCAACTCTGTAAAGGCAGTGGAAACTGATAAGCTAGAGTTCGGTAGGGGTAGCGGGAATTCCCAGTGTAGCGGTGAAATGCGTAGATATTGGGAAGAACATCGGTGGCGAAGGCGCGCTACTGGGCCGAAACTGACACTGAGGGACGAAAGCTAGGGGAGCGAAAGGG` |
| ASV924 | 404 | `TGGGGAATCTTGGACAATGGGCGCAAGCCTGATCCAGCCATGCCGCGTGGATGATGAAGGCCTTAGGGTTGTAAAATCCTTTCGTTGGGGACGATAATGACGGTACCCAAAGAAGAAGCTCCGGCTAACTTCGTGCCAGCAGCCGCGGTAATACGAAGGGGGCTAGCGTTGTTCGGATTTACTGGGCGTAAAGCGCGTGTAGGCGGACTGGAAAGTTGGGGGTGAAATCCCGAGGCTCAACCTCGGAACTGCCTCCAAAACTTCCAGTCTGGACTCAAGTAGAGGCAAGTGGAATTGCGAGTGTAGAGGTGAAATTCGTAGATATTCGCAGGAACACCAGTGGCGAAGGCGACTTGCTGGACTTGTAGTGACGCTGAGACGCGAAAGCGTGGGGAGCAAACAGG` |
| ASV3980 | 421 | `TGAGGAATATTGCGCAATGGACGGAAGTCTGACGCAGCAATGCCGCGTGCGGGATGAAGGCGCTATGCGTCGTAAACCGCTGTAGCGTGGGGCAAAAAGCACCCTTGGGGTGTTAGATGGTACCACGAAAGTAAGCATCGGCTAACTACGTGCCAGCAGCCGCGGTAATACGTAGGATGCAAGCGTTGTCCGGATTTACTGGGTGTAAAGGGTGCGCAGGCGGGCTTGTAAGTCAGAGGTGAAAGCCCGGAGCTTAACTCCGGAAATGCCTTTGATACTGCAAGTCTTGAATCTGATAGAGGCTCATGGAATATGTGGTGTAGCGGTGAAATGCGTAGATATCACATAGAACACCAGTGGCGAAGGCGGTGAGCTGGGTCAGTATTGACGCTCATGCACGAAAGTGTGGGTAGCAAACAGG` |
| ASV4526 | 406 | `TGGGGAATTTTCCGCAATGGGCGAAAGCCTGACGGAGCAAGACCGCGTGAGGGAAGAAGGCTCTTGGGTTGTAAACCTCTTTTATCAGGGAAGAAGAACTGACGGTACCTGGTGAATAAGCATCGGCTAACTCCGTGCCAGCAGCCGCGGTAAGACGGAGGATGCAAGCGTTATCCGGAATTATTGGGCGTAAAGCGTCCGCAGGTGGTTTTTCAAGTCTGTCGTTAAAGGACGGGGCTCAACCCCGGGAAGGCGGTGGAAACTGGAAGACTAGAGTACGGCAGGGGTAGAGGGAATTCTCAGTGTAGCGGTGAAATGCGTAGAGATTGAGAAGAACACCAGTGGCGAAAGCGCTCTACTGGACCTGTACTGACACTCAGGGACGAAAGCTAGGGGAGCAAAAGGG` |
| ASV354 | 429 | `TGGGGAATTTTGGACAATGGGCGAAAGCCTGATCCAGCAATGCCGCGTGCAGGATGAAGGCCTTCGGGTTGTAAACTGCTTTTGTACGGAGCGAAAAGGCTTCTTCTAATACAGGAGGCTCATGACGGTACCGTAAGAATAAGCACCGGCTAACTACGTGCCAGCAGCCGCGGTAATACGTAGGGTGCAAGCGTTAATCGGAATTACTGGGCGTAAAGCGTGCGCAGGCGGTGATGTAAGACAGTGGTGAAATCCCCGGGCTCAACCTGGGAACTGCCATTGTGACTGCATCGCTAGAGTACGGTAGAGGGGGGTGGAATTCCGCGTGTAGCAGTGAAATGCGTAGATATGCGGAGGAACACCGATGGCGAAGGCAGCCCCCTGGACCTGTACTGACGCTCATGCACGAAAGCATGGGGAGCAAACAGG` |
| ASV1577 | 429 | `TGGGGAATTTTGCGCAATGGAGGAAACTCTGACGCAGCGACGCCGCGTGGGTGACGAAGGCCTTCGGGTTGTAAAGCCCTGTCGGCTGGGAACAAGATAGTCGTGCTAATACCACGATTGTTTGAGGGTACCAGCAAAGGAAGCACCGGCTAACTCTGTGCCAGCAGCCGCGGTAATACAGAGGGTGCTAGCGTTGTTCGGAATTACTGGGCGTAAAGCGCGCGTAGGCGGCTTACCAAGTCAGGTGTGAAATCTCGGGGCTCAACTCCGAACGTGCATTTGAAACTGGTAGGCTAGAGTACATTAGAGGTTGGTGGAATACACAGTGTAGAGGTGAAATTCGTAGATATTGTGTGGAACACCGGTGGCGAAGGCGGCCAACTGGGATGTAACTGACGCTGAGGTGCGAAAGCGTGGGGATCAAACAGG` |
| ASV2253 | 405 | `TGGGGAATCTTGCGCAATGGGCGAAAGCCTGACGCAGCAACGCCGCGTGCGGGAAGAAGGCCTTCGGGTTGTAAACCGCTTTCAGAAGGAACGAAACTGACGGTACTTTCAGAAGAAGGTGCGGCCAACTACGTGCCAGCAGCCGCGGTGACACGTAGGCACCAAGCGTTGTCCGGATTTATTGGGCGTAAAGAGCTCGTAGGCGGTTCGGTAAGTCGGGTGTGAAAACTTTGGGCTCAACCCAAAGCGTGCACTCGATACTGCCGTGACTAGAGTTCGGTAGGGGAGTGGGGAATTCCTAGTGTAGCGGTGAAATGCGCAGATATTAGGAGGAACACCGGTGGCGAAGGCGCCACTCTGGGCCGAAACTGACGCTGAGGAGCGAAAGCGTGGGTAGCAAACAGG` |
| ASV1559 | 424 | `TAAGGAATATTGGTCAATGGACGGAAGTCTGAACCAGCCATCCCGCGTGCAGGATTAAGGTCCTATGGATTGTAAACTGCTTTTAGTAGGGAAGAAACGCCTTCATTTATGAGGGTCTGACGGTACCTACAGAATAAGCACCGGCTAACTCCGTGCCAGCAGCCGCGGTAATACGGAGGGTGCAAGCGTTATCCGGAATCACTGGGTTTAAAGGGTGCGTAGGCGGGTTATTAAGTCAGTGGTGAAAGCTGGTCGCTTAACGATCAAACTGCCATTGATACTGATAGTCTTGAATTGGGTTGAGGTTAGCGGAATGTGACATGTAGCGGTGAAATGCGTAGATATGTCATGGAACACCAATTGCGAAGGCAGCTAGCTAGGCCTTGATTGACGCTGAGGCACGAAAGCGTGGGTAGCGAACAGG` |
| ASV1783 | 429 | `TGGGGAATTTTGGACAATGGGCGCAAGCCTGATCCAGCCATGCCGCGTGCGGGAAGAAGGCCTTCGGGTTGTAAACCGCTTTTGTCAGGGAAGAAACGCTCTGGATTAATACTCTGGGGTAATGACGGTACCTGAAGAATAAGCACCGGCTAACTACGTGCCAGCAGCCGCGGTAATACGTAGGGTGCAAGCGTTAATCGGAATTACTGGGCGTAAAGCGTGCGCAGGCGGCTGTGCAAGACAGATGTGAAATCCCCGGGCTCAACCTGGGAACTGCATTTGTGACTGCATAGCTAGAGTACGGTAGAGGGGGATGGAATTCCGCGTGTAGCAGTGAAATGCGTAGATATGCGGAGGAACACCGATGGCGAAGGCAATCCCCTGGACCTGTACTGACGCTCATGCACGAAAGCGTGGGGAGCAAACAGG` |
| ASV3993 | 406 | `TGGGGAATTTTCCGCAATGGGCGCAAGCCTGACGGAGCAACGCCGCGTGAGGGAGGAAGGCCTTAGGGTTGTAAACCTCTTTTCTCTGGGAAGAATAACTGACGGTACCAGAGGAATAAGCCTCGGCTAACTCCGTGCCAGCAGCCGCGGTAAGACGGAGGAGGCAAGCGTTATCCGGAATTATTGGGCGTAAAGCGTCCGCAGGCGGTTAATTAAGTCTGTTGTCAAAGGTCACAGCTCAACTGTGGATCGGCAATGGAAACTGGTTGACTAGAGTGTGGTAGGGGTAGAGGGAATTCCCGGTGTAGCGGTGAAATGCGTAGATATCGGGAAGAACACCAGTGGCGAAGGCGCTCTACTGGGCCACAACTGACGCTGAGGGACGAAAGCTAGGGGAGCGAAAGGG` |
| ASV4995 | 404 | `TGGGGAATATTGGACAATGGGCGCAAGCCTGATCCAGCCATGCCGCGTGAGTGACGAAGGTCTTCGGATTGTAAAGCTCTTTTGGCGGGGACGATAATGACGGTACCCGCAGAATAAGCCCCGGCTAACTTCGTGCCAGCAGCCGCGGTAATACGAAGGGGGCTAGCGTTGTTCGGAATTACTGGGCGTAAAGCGCACGTAGGCGGACTCTTAAGTCAGGGGTGAAATCCCGGGGCTCAACCTCGGAACTGCCCTTGATACTGTGAGTCTCGAGACCGGAAGAGGTGAGTGGAATTCCTAGTGTAGAGGTGAAATTCGTAGATATTAGGAAGAACACCAGTGGCGAAGGCGGCTCACTGGTCCGGTTCTGACGCTGAGGTGCGAAAGCGTGGGGAGCAAACAGG` |
| ASV958 | 429 | `TGGGGAATTTTGGACAATGGGCGCAAGCCTGATCCAGCAATGCCGCGTGCAGGATGAAGGCCTTCGGGTTGTAAACTGCTTTTGTACGGAGCGAAACGGCTTTTTCTAATAAAGAGAGCTAATGACGGTACCGTAAGAATAAGCACCGGCTAACTACGTGCCAGCAGCCGCGGTAATACGTAGGGTGCAAGCGTTAATCGGAATTACTGGGCGTAAAGCGTGCGCAGGCGGTTATGTAAGACAGATGTGAAATCCCCGGGCTTAACCTGGGACCTGCATTTGTGACTGCATAGCTAGAGTACGGTAGAGGGGGATGGAATTCCGCGTGTAGCAGTGAAATGCGTAGATATGCGGAGGAACACCGATGGCGAAGGCAATCCCCTGGACCTGTACTGACGCTCATGCACGAAAGCGTGGGGAGCAAACAGG` |
| ASV965 | 407 | `TGGGGAATATTGGACAATGGGCCACAAGCCTGATCCAGCAATTCTGTGTGCACGATGAAGGTTTTAGGATCGTAAAGTGCTTTCAGTTGGGAAGAAAAAAATGACGGTACCAACAGAAGAAGCGACGGCTAAATACGTGCCAGCAGCCGCGGTAATACGTATGTCGCAAGCGTTATCCGGATTTATTGGGCGTAAAGCGCGTCTAGGCGGATTAGTAAGTCTGATGTTAAAATGCGGGGCTCAACTCCGTATTGCGTTGGAAACTGCTAGTCTAGAGTACTGGAGAGGTGGGCGGAACTACAAGTGTAGAGGTGAAATTCGTAGATATTTGTAGGAATGCCGATAGAGAAGTCAGCTCACTGGACAGATACTGACGCTGAAGCGCGAAAGCGTGGGGAGCAAACAGG` |
| ASV465 | 404 | `TGGGGAATATTGGACAATGGGCGCAAGCCTGATCCAGCAATTCCGCGTGGGTGAAGAAGGTCTTCGGATTGTAAAGCCCTTTCGTTGGGGACGATGATGACGGTACCCAAAGAAGAAGCCCCGGCTAACTTCGTGCCAGCAGCCGCGGTAATACGAAGGGGGCTAGCGTTGCTCGGAATGACTGGGCGTAAAGGGCGCGTAGGCGGCACAACTCGTCAGACGTGAAATTCCTGGGCTTAACCTGGGGGCTGCGTTTGATACGGTTGAGCTAGAGGATGGAAGAGGCTCGTGGAATTCCCAGTGTAGAGGTGAAATTCGTAGATATTGGGAAGAACACCGGTGGCGAAGGCGGCGAGCTGGTCCATTACTGACGCTGAGGCGCGAAAGCGTGGGGAGCAAACAGG` |
| ASV1892 | 429 | `TGGGGAATATTGGACAATGGGGGAAACCCTGATCCAGCAATGCCGCGTGTGTGAAGAAGGCCTTAGGGTCGTAAAGCACTTTTGTTAGGGAGGAAGGAGTATGCGCTAATAACGCATATGACTGACGTTACCTAAAGAATAAGCACCGGCTAACTCTGTGCCAGCAGCCGCGGTAATACAGAGGGTGCAAGCGTTAATCGGAATTACTGGGCGTAAAGGGTGCGTAGGTGGACGCATAAGTCGGATGTGAAATCCCTGGGCTTAACCTAGGAACTGCATTCGATACTGTGAGTCTAGAGTATGGTAGAGGTAAGTGGAATTTCCGGTGTAGCGGTGAAATGCGTAGATATCGGAAGGAACATCAGTGGCGAAGGCGACTTACTGGACTGATACTGACACTGAGGCACGAAAGCGTGGGGAGCAAACAGG` |
| ASV5727 | 429 | `TGGGGAATATTGCGCAATGGGCGAAAGCCTGACGCAGCCATACCGCGTGTGTGAAGAAGGCCTTCGGGTTGTAAAGCACTTTCAGTGGGGAGGAAAAGCTGGCGGCTAATATCCGTCGGCCGTGACGCTACCCACAGAAGAAGCACCGGCTAATTCCGTGCCAGCAGCCGCGGTAATACGGAAGGTGCGAGCGTTAATCGGAATTACTGGGCGTAAAGCGCACGTAGGTGGCTTGGTAAGTCGGATGTGAAAGCCCCGGGCTCAACCTGGGAACGGCATTCGATACTGCTGAGCTAGAGTACGAAAGAGGGGGGTGGAATTCCAGGTGTAGCGGTGAAATGCGTAGAGATCTGGAGGAACACCGGTGGCGAAGGCGGCCCCCTGGTTCGATACTGACGCTGAGGTGCGAAAGCGTGGGGAGCAAACAGG` |
| ASV4441 | 406 | `TGGGGAATTTTCCGCAATGGGCGAAAGCCTGACGGAGCAATACCGCGTGAGGGATGAAAGCCTGTGGGTTGTAAACCTCTTTTCTCAAGGAAGAAATTTTGACGGTACTTGAGGAATAAGCATCGGCTAACTCTGTGCCAGCAGCCGCGGTAATACAGAGGATGCAAGCGTTATCCGGAATCACTGGGCATAAAGCGTCTGTAGGTTGCATATTAAGTCTGTTGTTAAAGACTAGGGCCTAACCCTGGGAAAGCAATGGAAACTAGTACGCTTGAGTGTGGTAGAGGTAGAGGGAATTCCTGGTGTAGCGGTGAAATGCGTAGATATTAGGAAGAACACCAATGGCGAAAGCACTCTACTGGGCCATAACTGACACTGAGAGACGACAGCTAGGGGAGCAAATGGG` |
| ASV845 | 404 | `TGAGGAATATTGGACAATGGGCGAAAGCCTGATCCAGCAATACCGCGTGAGTGAAGAAGGCCTTCGGGTTGTAAAGCTCTTTCAGCAGGGAAAATGATGATGGTACCTGCAAAAGAAGTTCCGGCTAACTCCGTGCCAGCAGCCGCGGTAATACGGAGGGAACTAGCGTTGTTCGGATTTACTGGGCGTAAAGAGCGCGTAGGCGGTTTATTAAGTTAGAAGTGAAAGCCCAGGGCTTAACCCTGGAACTGCTTCTAAAACTGATAAACTAGAGTACGGGAGAGGATAGTGGAATTCCTAGTGTAGAGGTGAAATTCTTAGATATTAGGAGGAACACCGGTGGCGAAAGCGACTATCTGGAACGTTACTGACGCTGAGGCGCGAAAGCGTGGGGAGCAAACAGG` |
| ASV2371 | 421 | `TGGGGAATATTGCGCAATGGACGAAAGTCTGACGCAGCGACGCCGCGTGTGGGATGAAGCTCTTCGGGGTGTAAACCACTGTTGCCAGGGAAGAATCTCCTGATTTTTCAGGATTGACGGTACCTGGTGAGGAAGCACCGGCTAACTCTGTGCCAGCAGCCGCGGTAATACAGAGGGTGCGAGCGTTGTCCGGAATCACTGGGCGTAAAGGGCGCGTAGGTGGCTGGGTAAGTGTGCGGTGAAAGCCCGGGGCTCAACCCCGGGTCTGCCGTGCAGACTGCTTGGCTCGAGCATAGTAGAGGCACGTGGAATTCCGGGTGTAGCGGTGGAATGCGTAGAGATCCGGAAGAACACCGGTGGCGAAGGCGGCGTGCTGGGCTATTGCTGACACTGAGGCGCGACAGCGTGGGGAGCAAACAGG` |
| ASV5644 | 428 | `TCGCGAATTTTTCTCAATGGGCGCAAGCCTGAAGAAGCCACGCCGCGTGCGGGATGAAGGCCCTCTGGGTTGTAAACCGCTGTCAGGTGGGGGGAACCGCTGCAAGTGAACAGCTTGCAGTCTGACTGCCCGCCAAAGGAAGCCCCGGCAAAACATGTGCCAGCAGCCGCGGTAATACATGTGGGGCAAGCGTTGTTCGGAATCACTGGGCATAAAGGGTGCGTAGGTGGCGTCATAAGTCAGTGGTGAAATCCGGTGGCCCAACCATCGAACTGCCATTGATACTGTGATGCTTGAGTGCGTGAGGGGTGACTGGAACGCGTGGTGTAGCGGTGAAATGCGTAGATATCACGCGGAAGGCCAACGGCGAAGGCAGGTCACTGGTACGCAACTGACACTGAGGCACGAAAGCGTGGGGAGCAAACAGG` |
| ASV2787 | 429 | `TGGGGAATATTGGACAATGGGCGCAAGCCTGATCCAGCCATACCGCGTGGGTGAAGAAGGCCTTCGGGTTGTAAAGCCCTTTTGTTGGGAAAGAAAAGCAGCCGGTTAATACCTGGTTGTTCTGACGGTACCCAAAGAATAAGCACCGGCTAACTTCGTGCCAGCAGCCGCGGTAATACGAAGGGTGCAAGCGTTACTCGGAATTACTGGGCGTAAAGCGTGCGTAGGTGGTTGTTTAAGTCTGTCGTGAAAGCCCTGGGCTCAACCTGGGAATTGCGATGGAAACTGGGCGACTAGAGTGTGGCAGAGGATAGTGGAATTCCTGGTGTAGCAGTGAAATGCGTAGAGATCAGGAGGAACATCCGTGGCGAAGGCGACTGTCTGGGCCAACACTGACACTGAGGCACGAAAGCGTGGGGAGCAAACAGG` |
| ASV4659 | 404 | `TGGGGAATATTGGACAATGGGCGAAAGCCTGATCCAGCAATGCCGCGTGAGTGAAGAAGGCCTTAGGGTTGTAAAGCTCTTTTAGTAGGGAAGATAATGACGGTACCTACAGAAAAAGCACCGGCTAACTCCGTGCCAGCAGCCGCGGTAATACGGAGGGTGCTAGCGTTGTTCGGAATTACTGGGCGTAAAGCGTACGTAGGCGGTTTTATAAGTTGGAAGTGAAATCCCAAGGCTTAACCTTGGAATGGCTTTCAAAACTATAAAACTAGAGAATGGTAGGGGAAAGTGGAATTCCTAGTGTAGAGGTGAAATTCGTAGATATTAGGAGGAACACCAGAGGCGAAAGCGGCTTTCTGGACCATTTCTGACGCTAAGGTACGAAAGCGTGGGTAGCAAACAGG` |
| ASV3429 | 408 | `TCGAGAATCTTCCGCAATGGGCGAAAGCCTGACGGAGCGACACCGCGTGATGGATGAAGTGCTTCGGTATGTAAACATCTTTTATAGGGGAAAAAGTTTATTGATGGTACCCTATGAATAAGGACCTGCCAAACTCGTGCCAGCAGCAGCGGTAATACGAGTGGTCCAAGCGTTATCCGGAATTATTGGGCGTAAAGGGTGCGAAGATGGAATTGTTAGTCGGGTGTTTAACCCACAGGCTCAACCTGTGGATTGCATTCGAAACGTCAATTCTTGAGGTTGTTAGAGGTTAGCAGAACTAATAGTGTAGGGGTGAAATCCGTTGATATTATTGGGAATACCAAAAGCGAAGGCAGCTAACTAGAACACACCTGACATTATAGCACGAAAGCGTGGGTAGCGAATGGG` |
| ASV5112 | 404 | `TGGGGAATCTTGGACAATGGGGGAAACCCTGATCCAGCCATGCCGCGTGAATGAAGAAGGCCTTCGGGTTGTAAAGTTCTTTCAGTAGGGATGATAATGACCGTACCTACAGAAGAAGCCCCGGCTAACTCCGTGCCAGCAGCCGCGGTAAGACGGAGGGGGCTAGCGTTGTTCGGAATTACTGGGCGTAAAGGGTGCGTAGGCGGTTACATAAGTCAGAGGTGAAATTCCCGAGCTTAACTTGGGCGCTGCCTTTGATACTGTGTGACTAGAGATTGGGAGAGGTAGGTAGAATTCCTGGTGTAGAGGTGAAATTCGTAGATATCAGGAGGAATACCGGTGGCGAAGGCGGCCTACTGGCCCATATCTGACGCTGAGGCACGAAAGCGTGGGGATCAAACAGG` |
| ASV1594 | 424 | `TGAGGAATATTGGACAATGGGCGCAAGCCTGATCCAGCCATGCCGCGTGCAGGATGACGGTCCTATGGATTGTAAACTGCTTTTGTACGAGAAGAAACACTCCTTCGTGAAGGAGCTTGACGGTATCGTAAGAATAAGGATCGGCTAACTCCGTGCCAGCAGCCGCGGTAATACGGAGGATCCAAGCGTTATCCGGAATCATTGGGTTTAAAGGGTCCGTAGGCGGTTTAGTAAGTCAGTGGTGAAAGCCCATCGCTCAACGGTGGAACGGCCATTGATACTGCTAAACTTGAATTATTAGGAAGTAACTAGAATATGTAGTGTAGCGGTGAAATGCTTAGAGATTACATGGAATACCAATTGCGAAGGCAGGTTACTACTAATGGATTGACGCTGATGGACGAAAGCGTGGGTAGCGAACAGG` |
| ASV1801 | 404 | `TGGGGAATATTGGACAATGGGCGAAAGCCTGATCCAGCCATGCCGCGTGAATGATGAAGGCCTTAGGGTTGTAAAGTTCTTTCAACGGTGAAGATAATGACGGTAACCGTAGAAGAAGCCCCGGCTAACTTCGTGCCAGCAGCCGCGGTAATACGAAGGGGGCTAGCGTTGTTCGGAATTACTGGGCGTAAAGCGCGCGTAGGCGGATATTTAAGTCAGAGGTGAAATCCCGGGGCTCAACCCCGGAACTGCCTTTGATACTGGGTATCTAGAGTATGGTAGAGGTGAGTGGAATACCGAGTGTAGAGGTGGAATTCGTAGATATTCGGTAGAACACCAGTGGCGAAGGCGGCTCACTGGACCATTACTGACGCTGAGGTGCGAAAGCGTGGGGAGCAAACAGG` |
| ASV5718 | 424 | `TGAGGAATATTGGTCAATGGGCGCAAGCCTGAACCAGCCATGCCGCGTGCAGGAAGAATGCCCTATGGGTTGTAAACTGCTTTTATTTGGGAATAAACCTTCTTACGAGTAAGAAGCTGAAGGTACCAAAAGAATAAGCACCGGCTAACTCCGTGCCAGCAGCCGCGGTAATACGGAGGGTGCAAGCGTTATCCGGAATCATTGGGTTTAAAGGGTGCGCAGGCGGATCAATAAGTCAGTGGTGAAATCCCGTTGCTCAACAACGGAACTGCCATTGAAACTGTAGATCTTGAATTTAGTCGAAGTGGGCGGAATGTGACATGTAGCGGTGAAATGCTTAGATATGTCACAGAACACCGATAGCGAAGGCAGCTCACTAGGCTTGAATTGACGCTCATGCACGAAAGCGTGGGGAGCAAACAGG` |
| ASV1300 | 404 | `TGGGGAATATTGGACAATGGGCGAAAGCCTGATCCAGCGACGCCGCGTGGGTGATTAAGGCCTTCGGGTTGTAAAGCCCTTTCAGTAGGAAAGATGATGACGGTACCTACAGAAGAAGCTCCGGCTAACTTCGTGCCAGCAGCCGCGGTAATACGAAGGGAGCTAGCGTTACTCGGAATTATTGGGCGTAAAGCGTGCGTAGGCTGCTTTGCAAGTTGGAAGTGAAAGCCCAGGGCTCAACCTTGGAATTGCTTTCAAAACTACAGAGCTAGAATATCAGAGAGGATAGCGGAATTACTAGTGTAGAGGTGAAATTCGTAGATATTAGTAGGAACACCGGAGGCGAAAGCGGCTATCTGGCTGATTATTGACGCTGTTGCACGAAAGCGTGGGGAGCAAACAGG` |
| ASV1486 | 429 | `TGGGGAATTTTGGACAATGGGCGAAAGCCTGATCCAGCCATTCCGCGTGCAGGATGAAGGCCTTCGGGTTGTAAACTGCTTTTGTACGGAACGAAACGGCCTTTTCTAATACAAGAGGCTAATGACGGTACCGTAAGAATAAGCACCGGCTAACTACGTGCCAGCAGCCGCGGTAATACGTAGGGTGCAAGCGTTAATCGGAATTACTGGGCGTAAAGCGTGCGCAGGCGGTTATGTAAGACAGATGTGAAATCCCCGGGCTCAACCTGGGAACTGCATTTGTGACTGCATAGCTAGAGTACGGCAGAGGGGGATGGAATTCCGCGTGTAGCAGTGAAATGCGTAGATATGCGGAGGAACACCGATGGCGAAGGCAATCCCCTGGGCCTGTACTGACGCTCATGCACGAAAGCGTGGGGAGCAAACAGG` |
| ASV5504 | 358 | `GTGGTAGGCCGCGTTGATCGGCAGGCTGAGGCTGAAAGGCATCACCAGCCGGCCGGATTCGAGATCCGCCGAGGCCACCACCGGGATGCCCAGCACCACGCCGCGGCCGTCGGCGGCGGCTTCCATCGCCATCGCTACCTGGTTGAAGGTGGGGCCGCGCCGGTCGTTCACGCCGTGCACGCCGGCCGCGCGCAGCCAGATCGTCCAGTTGGGGCGCTCGTCGTCGGTCTCGTCCATCCCGGTTTCGTGGTGCAGCAGGGTGTGGTGCCGCAGATCCTGCGGCAGCCGCAGCGGGTAGGGGCCTTCGAGCAGGCGCGGGCTGCACATGGGAATGGCGTAGGCGGGAAACAGCGCATCC` |
| ASV3274 | 430 | `TGGGGAATATTGGACAATGGGCGGAAGCCTGATCCAGCAATGCCGCGTGTGTGAAGAAGGCCTTAGGGTTGTAAAGCACTTTCAGAGGTGAGGAAGCGATTATGGTTAATAGTCATGATTGTTGACGTTATCCTCAGAAGAAGCACCGGCTAACTCTGTGCCAGCAGCCGCGGTAATACAGAGGGTGCAAGCGTTAATCGGAATTACTGGGCGTAAAGGGTGTGTAGGTTGTTGAGGCAAGTCAGGTGTAAAAACCCCGGGCTTAACCTGGGAACGTCGCTTGAGACTGCCTAGCTAGAGTACTATAGAGGATAGTGGAATTTCCAGTGTAGCGGTGAAATGCGTAGATATTGGAAGGAACACCAGTGGCGAAGGCGACTATCTGGATAGAGACTGACACTGAAACACGAAAGCATGGGTAGCAAACAGG` |
| ASV3591 | 384 | `GCGCGCAAACTTTACAATGCGAGCAATCGTGATAAGGAAACCCTGAGTGCCTGTTGATGCAGGCTGTTCATATATCTAAATCGTATGTGAAGAAAGGGCAGGGCAAGACCGGTGCCAGCCGCCGCGGTAATACCGGCTGCTCGAGTGATGGCCACTATTACTGGGTTTAAAGCGTCCGTAGCTTGATTGTTAGGTCTCTTGGGAAATCTTCTGGCTTAACCAAAAGGCGTCTAAGAGATACCGGCAATCTAGGAACTGGGAGAGGTAAGCCGTACTTCGGGGGTAGGAGTGAAATCTTGTAATCCTCGAGGGACGACCTATGGCGAAGGCAGCTTACCAGAACAGCTTCGACAGTGAGGGACGAAAGCTGGGGGAGCAAACGGG` |
| ASV4779 | 407 | `TCGAGAATTTTTCACAATGGGGGAAACCCTGATGGAGCGACGCCGCGTGGAGGATGAAGGCCCTTGGGTCGTAAACTCCTGTCATAAGAGAACAAGAAAGTGATAGTATCTTAAGAGGAAGAGACGGCTAACTCTGTGCCAGCAGCCGCGGTAATACAGAGGTCTCAAGCGTTGTTCGGATTCATTGGGCGTAAAGGGTGCGCAGGCTGCGTGGTAAGTCGGGTGTGAAATTTAGGGGCTTAACCCCTAAACTGCATCCGATACTGCCATGCTAGAGGACTGTAGAGGAGATTGGAATTCACGGTGTAGCAGTGAAATGCGTAGATATCGTGAGGAAGACCAGTGGCGAAGGCGAATCTCTGGGCAGGTCCTGACGCTCATGCACGAAGGCTAGGGGAGCAAACGGG` |
| ASV1338 | 405 | `TAGGGAATTTTCCACAATGGACGAAAGTCTGATGGAGCAACGCCGCGTGCAGGATGACAGCCTTCGGGTCGTAAACTGCTTTTATGAGTGAAGATTATGACAGTAACTCAAGAATAAGGGACGGCTAACTACGTGCCAGCAGCCGCGGTCATACGTAGGTCCCAAGCATTATCCGGAATTACTGGGCGTAAAGAGTTGCGTAGGTGGTTTACTAAGCAGAACATGAAATCGTACGGCTCAACCGTATACACGTTTTCTGAACTGGTAAACTAGAGGATGAGAGAGGTTATTGGAATTTCTTGTGTAGGAGTGAAATCCGTAGATATAAGAAGGAACACCGATGGCGTAGGCAGATAACTGGCTCATACCTGACACTAAGGCACGAAAGCGTGGGTAGCAAACGGG` |
| ASV4878 | 424 | `TAGGGAATATTGGGCAATGGGCGCAAGCCTGACCCAGCCATGCCGCGTGGAGGATGAAGGCGCTCAGCGTTGTAAACTTCTTTTATCAACGAAGAACGGTGGGGGTGCGCCCCCATGTGACGGTAGTTGAGGAATAAGCACCGGCTAACTCCGTGCCAGCAGCCGCGGTAATACGGAGGGTGCAAGCGTTGTCCGGATTTATTGGGTTTAAAGGGTGCGTAGGTGGGTGACTAAGTCTGGTTTGAAAGCTGGGGGCTCAACCTTCCGATGTGGCTGGAAACTGGTTATCTTGAATGGGTTGGCGGTAGCCGGAATGGGTCATGTAGCGGTGAAATGCATAGATATGACCCGGAACACCGATTGCGAAGGCAGGCTACTACGACCTGATTGACACTGAGGCACGAGAGCATGGGTAGCGAACAGG` |
| ASV3330 | 403 | `TGGGGAATATTGCGCAATGGACGAAAGTCTGACGCAGCGACGCCGCGTGAGTGAAGAAGTCTTTAGGGATGTAAAGCTCTTTCGGGTGGGAAGATAATGACGGTACCACCAGAAGAAGCCCCGGCTAATTACGTGCCAGCAGCCGCGGTAATACGTAAGGGGCGAGCGTTATCCGGATTTACTGGGCATAAAGGGCGCGTAGGTGGTTTTTTAAGTCAGGTGTTAAAAGCAACAGCTCAACTGTTGTTCGCACTTGAAACTGGGAAACTAGAGATCAGGAGAGGAAAGCGGAACTCACAGTGTAGCGGTGAAATGCGTAGATATTGTGAGGAACACCAGTTGCGAAAGCGGCTTTCTGGACTGAATCTGACACTGAGGCGCGAAAGCGTGGGGAGCAAACAGG` |
| ASV2273 | 407 | `TGGGGAATTTTCCGCAATGGGCGCAAGCCTGACGGAGCAATACCGCGTGAGGGAGGAAGGCTCTTGGGTCGTAAACCTCTTTTCTCAGGGAAGAACAAAATGACGGTACCTGAGGAATAAGCATCGGCTAACTCTGTGCCAGCAGCCGCGGTAATACAGAGGATGCAAGCGTTATCCGGAATGATTGGGCGTAAAGCGTCCGTAGGTGGTTTTGTAAGTCTGTGGTTAAAGCGTGGAGCTTAACTCCATAACGGCCATGGAAACTACAAGACTTGAGTGAAGTAGGGGTAGAGGGAATTCCCAGTGTAGCGGTGAAATGCGTAGAGATTGGGAAGAACACCGGTGGCGAAAGCGCTCTACTGGACTTATACTGACACTGAGGGACGAAAGCTAGGGGAGCGAAAGGG` |
| ASV3691 | 404 | `TAAGGAATATTGGACAATGGGGGCAACCCTGATCCAGCCATGCCGCGTGTGTGATGAAGGCCTTAGGGTTGTAAAGCACTTTCAATGGTGAAGATAATGACGGTAGCCATAGAAGAAGCTCCGGCTAAATTCGTGCCAGCAGCCGCGGTAATACGAATGGAGCAAGCGTTGTTCGGAATCACTGGGCTTAAAGCGTGCGTAGGCGGTCTCGCAAGTCAGAAGTGAAAGCCTGGGGCTCAACCCCAGAATTGCTTTTGAAACTGCAAGACTAGAATATCGGAGAGGTTGGGAGAATTCCTAGTGTAGAGGTGAAATTCGCAGATATTAGGAGGAATACCAGTGGCGTAGGCGCCCAACTGGACGATTATTGACGCTGAGGCACGAAAGCGTGGGGAGCAAACAGG` |
| ASV4802 | 408 | `TTAGGAATCTTCCGCAATGGGCGAAAGCCTGACGGAGCGACACCGCGTGAAGGATGAAGCCCTTTTTGGGTGTAAACTTCTGTTCTGAGGGACGAAATTTTTGACGGTACCTCAGGAGAAAGCACCGGCTAATTCTGTGCCAGCAGCCGCGGTAAGACAGAAGGTGCAAGCGTTACTCGGAATTACTGGGCGTAAAGCGTCTGCAGGCGTCCTATTAAGTCTGGCGTGAAATGTCGGGGCTCAACCCCGTCTCCGTGCCGGAAACTGGCAGGATCGAGTCACTCAGAGGCATCTGGAATGTCGTGTGTAGGGGTAAAATCCGTAGATACACGATGGAACGCCGAAAGCGAAGGCAGGATGCTAGGAGTGTACTGACGCTCAGAGACGAAAGCGTGGGGAGCAAAGGGG` |
| ASV4021 | 424 | `TGAGGAATATTGGTCAATGGGCGGAAGCCTGAACCAGCCATGCCGCGTGCAGGAAGACGGTCCTATGGATTGTAAACTGCTTTTATACAGGAAGAAACACCCGCTCGTGAGCGGGCTTGACGGTACTGTAAGAATAAGGATCGGCTAACTCCGTGCCAGCAGCCGCGGTAATACGGAGGATCCGAGCGTTATCCGGAATCATTGGGTTTAAAGGGTCCGTAGGCGGTTAGATAAGTCAGTGGTGAAATCCTATCGCTTAACGATAGAACTGCCATTGATACTGTTTAACTTGAATAATTGGGAAGTAACTAGAATATGTAGTGTAGCGGTGAAATGCTTAGATATTACATGGAATACCAATTGCGAAGGCAGGTTACTATCAATTTATTGACGCTGATGGACGAAAGCGTGGGGAGCGAACAGG` |
| ASV5729 | 429 | `TAGGGAATCTTCCGCAATGGACGAAAGTCTGACGGAGCAACGCCGCGTGAGTGAAGAAGGTTTTCGGATCGTAAAACTCTGTTGTCAGAGAAGAACACGTTGGAGAGTAACTGCTCCAGCCTTGACGGTATCTGACCAGAAAGCCACGGCTAACTACGTGCCAGCAGCCGCGGTAATACGTAGGTGGCAAGCGTTGTCCGGATTTATTGGGCGTAAAGCGAGCGCAGGCGGTTCCTTAAGTCTGATGTGAAAGCCCACGGCTCAACCGTGGAAGGTCATTGGAAACTGGGGAACTTGAGTGCAGAAGAGGAGAGTGGAATTCCATGTGTAGCGGTGAAATGCGTAGATATATGGAGGAACACCAGTGGCGAAGGCGACTCTCTGGTCTGTAACTGACGCTGAGGCTCGAAAGCGTGGGGAGCAAACAGG` |
| ASV177 | 429 | `TCGAGAATAATTCACAATGGGGGCAACCCTGATGGTGCAACGCCGCGTGGAGGATGACGGTCTTCGGATTGTAAACTCCTGTCATCTGGGAGTAAGGCATGGTTGTTAATAGCAAACATGATTGATAGTACCAGAAGAGGAAGGGACGGCTAACTTCGTGCCAGCAGCCGCGGTAATACGAAGGTCCCGAGCGTTGTTCGGAATCACTGGGCGTAAAGGGAGCGTAGGCGGCGTGGTAAGTCAGATGTGAAATCCCGGGGCTCAACCCCGGAACTGCATCCGATACTGCCATGCTAGAGGATTGGAGAGGTAGCTGGAATTCTTGGTGTAGCAGTGAAATGCGTAGAGATCAAGAGGAACACTCGTGGCGAAAGCGAGCTACTGGACAATTCCTGACGCTGAGGCTCGAAGGCTAGGGTAGCGAAAGGG` |
| ASV3995 | 404 | `TGGGGAATATTGGACAATGGGCGAAAGCCTGATCCAGCAATACCGCGTGAGTGATGAAGGTCTTCGGATTGTAAAGCTCTTTTAGTAGGGAAGATAATGACGGTACCTACAGAAAAAGCACCGGCAAACTTCGTGCCAGCAGCCGCGGTAATACGAAGGGTGCTAGCGTTGTTCGGAATTATTGGGCGTAAAGCGTACGTAGGCGGTTAAGTAAGTCGATTGTGAAAGCCCCGAGCTCAACTTGGGAATTGCAACCGAAACTGCTTAGCTTGAGTTTGGTGGGGGATGATGGAATTCCTAGTGTAGGAGTGAAATCCGTAGAGATTAGGAGGAACACCGGTGGCGAAAGCGATCATCTACGCCAATACTGACGCTAAGGTACGAAAGCGTGGGGAGCAAACAGG` |
| ASV1766 | 404 | `TGAGGAATCTTCCACAATGGACGAAAGTCTGATGGAGCGACACCGCGTGAAGGATGAAGGTTTTGTTATTGTAAACTTCTTTTCTCAGGGACGATAATGACGGTACCTGAGGAATAAGGGACGGCAAACTATGTGCCAGCAGCCGCGGTAATACATAGGTCCCAAGCGTTATCCGGAATTACTGGGCGTAAAGCGTCTGCAGGTGGAAAAGTGTGTGAGATGTGAAAGACCGGGGCTTAACCCCGTGTTTGTGTCTCAAACTGCTTTTCTGGAGTGAGCAAGAGGTATGCGGAATTTATGGAGTAGGAGTGCAATCCGTTGACACCATAAAGAACACCAAAAGCGAAGGCAGCATACTGGGGCTCTACTGACACTCAGAGACGAAAGCGTGGGGAGCGAAAGGG` |
| ASV3334 | 404 | `TGGGGAATATTGGACAATGGGGGAAACCCTGATCCAGCAATGCCGCGTGAGTGATGAAGGCCTTCGGGTTGTAAAGCTCTTTCACCCACGACGATGATGACGGTAGTGGGAGAAGAAGCCCCGGCTAACTTCGTGCCAGCAGCCGCGGTAATACGAAGGGGGCTAGCGTTGTTCGGAGTTACTGGGCGTAAAGGGCGCGTAGGCGGCGCAATCAGTCTGGCGTGAAAGCCCGGGGCTCAACCCCGGAACGGCGCTGGAGACGGTTGTGCTAGGGTTCGGGAGAGGATAGTGGAATTCCCAGTGTAGAGGTGAAATTCGTAGATATTGGGAAGAACACCGGTGGCGAAGGCGGCTATCTGGACCGATACCGACGCTGAGGCGCGAAAGCGTGGGGAGCAAACAGG` |
| ASV2575 | 423 | `TGAGGAATATTGGTCAATGGGCGCGAGCCTGAACCAGCCAAGTCGCGTGAGGGAAGACGGTTCTATGGATTGTAAACCTCTTTTGTCAGGGAGCAACGGGTGTTACGAGTAGCGCCATGAGAGTACCTGAAGAAAAAGCATCGGCTAACTCCGTGCCAGCAGCCGCGGTAATACGGAGGATGCGAGCGTTATCCGGATTTATTGGGTTTAAAGGGTGCGTAGGCGGGCTATCAAGTCAGCGGTAAAATTTCGGGGCTCAACCCCGACTCGCCGTTGAAACTGACAGTCTAGAGTGAGAGTGAGGTATTCGGAATGTGTAGTGTAGCGGTGAAATGCATAGATATTACGCAGAACTCCAATTGCGAAGGCAGAGTACCAACTCTTAACAGACGCTGAGGCACGAAAGTGTGGGTATCGAACAGG` |
| ASV553 | 404 | `TGGGGAATATTGCACAATGGACGAAAGTCTGATGCAGCAACGCCGCGTGGAGGATGACACATTTCGGTGCGTAAACTCCTTTTATATAGGAAGATAATGACGGTACTATATGAATAAGCACCGGCTAACTCCGTGCCAGCAGCCGCGGTAATACGGAGGGTGCAAGCGTTACTCGGAATCACTGGGCGTAAAGAGCGTGTAGGCGGATATATAAGTCAGAAGTGAAATCCAATAGCTTAACTATTGAACTGCTTTTGAAACTGTATATCTAGAATGTGGGAGAGGTAGATGGAATTTCTGGTGTAGGGGTAAAATCCGTAGAGATCAGAAGGAATACCGATTGCGAAGGCGATCTACTGGAACATTATTGACGCTGAGACGCGAAAGCGTGGGGAGCAAACAGG` |
| ASV1662 | 429 | `TGGGGAATATTGGACAATGGGGGCAACCCTGATCCAGCCATGCCGCGTGTGTGAAGAAGGCCTTCGGGTTGTAAAGCACTTTAAGTGGGGAGAAAGGTTCGGCAATTAATACCTGCCGGAAGTGATGTTACCCACAGAATAAGCACCGGCTAACTCTGTGCCAGCAGCCGCGGTAATACAGAGGGTGCAAGCGTTAATCGGAATTACTGGGCGTAAAGCGCGCGTAGGCGGCTGTCTAAGTCGGGTGTGAAAGCCCCGGGCTCAACCTGGGAACTGCATTCGATACTGGACGGCTAGAGTATGAGAGAGGGAGGTAGAATTCCACGTGTAGCGGTGAAATGCGTAGATATGTGGAGGAATACCGGTGGCGAAGGCGGCCTCCTGGTTCAATACTGACGCTGAGGTGCGAAAGCGTGGGGAGCAAACAGG` |
| ASV4993 | 424 | `TGGGGAATATTGGTCAATGGGCGAAAGCCTGAACCAGCAATGCCGCGTGAGTGATGAAGGCCTTAGGGTTGTAAAGCTCTGTCAGTGGGAACGAATAAGCCGGTTAATACCCGGTGGTGACGGTACCCACAAAGGAAGCACCGGCTAACTCTGTGCCAGCAGCCGCGGTAATACAGAGGGTGCAAGCGTTGTTCGGAATTACTGGGCGTAAAGCGCGTGTAGGCGGTTTCGTAAGTCGGTCGTGAAAGCCCTGGGCTTAACCCAGGAACTGCGATCGAAACTGTGAGACTAGAGTATCGGAGGGGGTGGCGGAATTCCCGGTGTAGAGGTGAAATTCGTAGATATCGGGAGGAACACCTGTGGCGAAGGCGGCCACCTGGAAGATTACTGACGCTGAGACGCGAAAGCGTGGGGAGCAAACAGG` |
| ASV3363 | 423 | `TAGGGAATATTGGTCAATGGACGCAAGTCTGAACCAGCCATGCCGCGTGCAGGAAGACGGCCCTCTGGGTTGTAAACTGCTTTTGATAGGGAACAAATGCCACTTGCGAGTGGAGCTGAGTGTACCTATAGAATAAGCACCGGCTAACTCCGTGCCAGCAGCCGCGGTAATACGGAGGGTGCAAGCGTTGTCCGGATTTATTGGGTTTAAAGGGTGCGTAGGCGGCCTAATAAGTCAGTGGTGAAAGCCTCCCGCTTAACGGGAGAACTGCCATTGATACTGTTGGGCTTGAGTATACTTGAGGTAGGCGGAATTGACAGTGTAGCGGTGAAATGCATAGATATTGTCAAGAACACCAATTGCGAAGGCAGCTTACTAAATTATAACTGACGCTGAGGCACGAAAGTGTGGGTATCAAACAGG` |
| ASV3758 | 408 | `TGAGGAATCTTCCGCAATGGGCGAAAGCCTGACGGAGCGACACCGCGTGGAGGATGAAGACCTTTTTGGTCGTAAACTCCTGTTCTGAGGGACGAAATTTTTGACGGTACCTCAGGAGAAAGTATCGGCAAACCCTGTGCCAGCAGCCGCGGTAAGACAGGGGATACAAGCATTACTCGGAATTACTGGGCGTAAAGCGTCTGCAGGTGTCGTAGAAAGTCTGGTGTAAAAGCACGGAGCCTAACTCCGTGTACGTATCGGAAACTTCTACGATTGAGTCACTCAGAGGTACCTGGAATGCCGTGTGTAGGGGTAAAATCCGTAGATCCACGGTGGAACGCCAAAAGTGAAGACAGGGTACTGGGGGTGTACTGACACTCAGAGACGAAAGCGTGGGGAGCGAAGGGG` |
| ASV4414 | 405 | `TAGGGAATTTTCCACAATGGGGGCAACCCTGATGGAGCAACGCCGCGTGCAGGATGAAGGCCCTCGGGTCGTAAACTGCTTTTTTCTGTGATGAATATGACAGTAACAGACGAATAAGGGTCGGCTAACTCCGTGCCAGCAGCCGCGGTCATACGGAGGACCCAAGCGTTATCCGGATTTACTGGGCGTAAAGAGTTGCGTAGGTGGTTTGTTAAGCAAAGTATGAAATCGTGTGGCTCAACCATACGGCTATATTTTGAACTGGCAGACTTGAGAATGAGAGAGGTTATTGGAATTCCCAGTGTAGGAGTGAAATCCGTAGATATTGGGAGGAACACCGATGGCGTAGGCAGATAACTGGCTCATTTCTGACACTAAGGCACGAAAGCGTGGGTAGCAAACGGG` |
| ASV4006 | 429 | `TGGGGAATTTTGGACAATGGGCGCAAGCCTGATCCAGCCATGCCGCGTGAGTGAAGAAGGCCCTAGGGTTGTAAAGCTCTTTTATCCGGAAAGAAACCCTGCTAATGAACAATAAACAGGACTGACAGTACCGGAAGAATAAGCACCGGCTAACTACGTGCCAGCAGCCGCGGTAATACGTAGGGTGCAAGCGTTAATCGGAATTACTGGGCGTAAAGGGTGCGCAGGCGGTATAGCAAGTCAGATGTGAAATCCCTAGGCTCAACCTAGGAACGGCGTTTGAAACTACAATACTAGAGTATGTCAGAGGGGGGTGGAATTCCACGTGTAGCAGTGAAATGCGTAGAGATGTGGAGGAACACCAATGGCGAAGGCAGCCCCCTGGGATAATACTGACGCTCAAGCACGAAAGCGTGGGGAGCAAACAGG` |
| ASV2924 | 409 | `TGGGGAATATTGCACAATGGGCGAAAGCCTGATGCAGCAACGCCGCGTGAGGGATGACGGCCTTCGGGTTGTAAACCTCTTTTAGTAGGGAAGAAGCGAAAGTGACGGTACCTGCAGAAAAAGCACCGGCTAACTACGTGCCAGCAGCCGCGGTAATACGTAGGGTGCAAGCGTTATCCGGAATTATTGGGCGTAAAGAGCTCGTAGGCGGTTTGTCGCGTCTGCTGTGAAAACTGGAGGCTCAACCTCCAGCCTGCAGTGGGTACGGGCAGACTAGAGTGCGGTAGGGGAGATTGGAATTCCTGGTGTAGCGGTGGAATGCGCAGATATCAGGAGGAACACCAATGGCGAAGGCAGATCTCTGGGCCGTAACTGACGCTGAGGAGCGAAAGCATGGGGAGCGAACAGG` |
| ASV5721 | 424 | `TGAGGAATATTGGTCAATGGGCGCAAGCCTGAACCAGCTATGCCGCGTGCAGGAAGACGGCGCTATGCGTTGTAAACTGCTTTTGTACGAGGGTAACTATAGGCACGTGTGCTTATTTGAAAGTATCGTACGAATAAGGATCGGCTAACTCCGTGCCAGCAGCCGCGGTAATACGGAGGATTCGAGCGTTATCCGGATTTATTGGGTTTAAAGGGTGCGTAGGCGGTTTGATAAGTTAGTCGTTAAATATATGGGCTTAACCCGTAGCAGGCGATTGATACTGTTGGACTAGAGATAGGACGCTGTGGGCGGAATGTGTAGTGTAGCGGTGAAATGCTTAGATATTACACAGAACACCGATTGCGAAGGCAGCTCACAAGGCCTTATCTGACGCTGAGGCACGAAAGCGTGGGGATCAAACAGG` |
| ASV3106 | 405 | `TGGGGAATCTTGCGCAATGGGCGAAAGCCTGACGCAGCAACGCCGCGTGCGGGATGAAGGCTCTCGGGTTGTAAACCGCTTTCAGCAGGAACGAAAATGACGGTACTTGCAGAAGAAGGTGCGGCCAACTATGTGCCAGCAGCCGCGGTGATACATAGGCACCAAGCGTTGTCCGGATTTATTGGGCGTAAAGAGCTCGTAGGCGGTTCGGTAAGTCGGGTGTGAAAACTTTGGGCTCAACCCAAAGCCTGCATCCGATACTGCTGTGACTAGAGTTCGGTAGGGGAGCGGGGAATTCCTAGTGTAGCGGTGAAATGCGCAGATATTAGGAGGAACACCGGTGGCGAAGGCGCCGCTCTGGGCCGATACTGACGCTGAGGAGCGAAAGCGTGGGTAGCAAACAGG` |
| ASV3444 | 419 | `TGAGGAATATTGCGCAATGGGCGAAAGCCTGACGCAGCGACGCCGCGTGGGGGATGAAGGTCTTCGGATTGTAAACCCCTTTCGGGAGGGAAGATGGGACGGGCAACCGTTCGGACGGTACCTCCAGAAGCAGCCACGGCTAACTTCGTGCCAGCAGCCGCGGTAATACGAAGGTGGCAAGCGTTGTTCGGATTTACTGGGCGTACAGGGAGCGTAGGTGGTTTGGTAAGCCCTCCGGGAAATCTCCCGGCTTAACCGGGAAAGGTCGGAGGGGACTGCCTGACTAGAGGGCGGGAGAGGAGCGCGGAATTCCCGGTGTAGCGGTGAAATGCGTAGAGATCGGGAGGAAGGCCGGTGGCGAAGGCGGCGCTCTGGAACGATCCTGACACTGAGGCTCGAAAGCGTGGGGAGCAAACAGG` |
| ASV4492 | 429 | `TGGGGAATTTTGGACAATGGGGGCAACCCTGATCCAGCAATGCCGCGTGTGTGAAGAAGGCCTTCGGGTTGTAAAGCACTTTTGGCGGGAGCGAAACGGCTTGGGCTAATACCTCGAGCTAATGACGGTACCCGCAGAATAAGCACCGGCTAACTACGTGCCAGCAGCCGCGGTAATACGTAGGGTGCAAGCGTTAATCGGAATTACTGGGCGTAAAGCGAGCGCAGGCGGTTGTGTAAGACAGATGTGAAATCCCCGGGCTCAACCTGGGAACTGCATTTGTGACTGCACGGCTAGAGTGCGTCAGAGGGGGGTGGAACTCCACGTGTAGCAGTGAAATGCGTAGAGATGTGGAAGAACACCGATGGCGAAGGCAGCCCCCTGGGACGATACTGACGCTCATGCTCGAAAGCGTGGGGAGCAAACAGG` |
| ASV5087 | 375 | `GTGTCGACACATCGATGATGGGACAAGTAGCGCAGATGGACATGACCAACTTGATCAATGTTGGTCAACTCAAATTGCTGCTCGAAACCGATCGATTCACGATTGTGCTGTCCAACTTCACAATTCAATCCGAAACGAACGTGACCGCGAGTGTGTGGCCGTTGCCCGTGAGTGAAGAAGTGGTCGTTGTCGACATGCATGTGCACGAAGCGCGTGTGTCCATCGTGCCCATTGACAATCACACACTATCCATGGTTTCGTGTCATCTCGATAAGGCTAAAGTGGTCGGCGGCACACGCAACTACTGGTTATACGGCCAAACCGTGCGCCTTGGCGCATATGTGCTCGGTTCGAATCTCGAATCGTACGTGTGTC` |
| ASV5616 | 404 | `TGGGGAATATTGGACAATGGGGGCAACCCTGATCCAGCAATGCCGCGTGAGTGATGAAGGCCTTCGGGTTGTAAAGCTCTTTTGCCAGGGACGATGATGACGGTACCTGGAGAATAAGCCCCGGCTAACTTCGTGCCAGCAGCCGCGGTAATACGAAGGGGGCGAGCGTTGTTCGGAATTACTGGGCGTAAAGGGCGCGTAGGCGGTTTAGCGAGTCAGGCGTGAAATTCCCGGGCTTAACCTGGGGGCTGCGCTTGATACTGCTAGGCTTGAATTCGGGAGAGGATGGCGGAATTCCCAGTGTAGAGGTGAAATTCGTAGATATTGGGAAGAACACCGGTGGCGAAGGCGGCCATCTGGCCCGGGATTGACGCTGAGGCGCGAAAGCGTGGGGAGCAAACAGG` |
| ASV762 | 424 | `TGAGGAATATTGGACAATGGGCGCAAGCCTGATCCAGCCATGCCGCGTGCAGGATGACGGTCCTATGGATTGTAAACTGCTTTTGCACAGGAAGAAACACTCCCTCGAGAGGGAGCTTGACGGTACTGTGAGAATAAGGATCGGCTAACTCCGTGCCAGCAGCCGCGGTAATACGGAGGATCCAAGCGTTATCCGGAATCATTGGGTTTAAAGGGTCCGTAGGCGGTCTTATAAGTCAGTGGTGAAATCTCCCCGCTCAACGGGGAAACGGCCATTGATACTGTGGGACTTGAATTATTAGGAAGTAACTAGAATATGTAGTGTAGCGGTGAAATGCTTAGAGATTACATGGAATACCAATTGCGAAGGCAGGTTACTACTAATGGATTGACGCTGATGGACGAAAGCGTGGGTAGCGAACAGG` |
| ASV772 | 404 | `TGGGGAATATTGGACAATGGGCGAAAGCCTGATCCAGCAATGCCGCGTGAGTGAAGAAGGCCTTCGGGTTGTAAAGCTCTTTTACCCGGGAAGATAATGACTGTACCGGGAGAATAAGCCCCGGCTAACTCCGTGCCAGCAGCCGCGGTAATACGGAGGGGGCTAGCGTTGTTCGGAATTACTGGGCGTAAAGCGCACGTAGGCGGTCTTTTAAGTCAGAGGTGAAAGCCCGGGGCTCAACTCCGGAATTGCCTTTGAAACTGGAAGACTTGAACACGGGAGAGGTGAGTGGAATTCCGAGTGTAGAGGTGAAATTCGTAGATATTCGGAGGAACACCAGTGGCGAAGGCGGCTCACTGGACCGTTGTTGACGCTGAGGTGCGAAAGCGTGGGGAGCAAACAGG` |
| ASV4677 | 407 | `TGGGGAATATTGCACAATGGAGGAAACTCTGATGCAGCAACGCCGCGTGAAGGAAGAAGGCTTTCGGGTCGTAAACTTTTGTCTTTGGGGAAGAAAAAAATGACGGTACCCAAGGAGGAAGCCACGGCTAACTACGTGCCAGCAGCCGCGGTAATACGTAGGTGGCGAGCGTTGTCCGGAATTACTGGGTGTAAAGGGTGCGTAGGCGGGAAATAAAGTCAAGTGTGAAATGCCGTAGCTTAACTACGGAGCTGCACTTGAAACTTATTTTCTTGAGTGAAGTAGAGGCAAGTGGAATTCCCAGTGTAGCGGTGAAATGCGTAGATATTGGGAGGAACACCAGTGGCGAAGGCGACTTGCTGGGCTTTAACTGACGCTGAGGCACGAAAGCATGGGGAGCAAACAGG` |
| ASV847 | 429 | `TGGGGAATTTTGGACAATGGGGGCAACCCTGATCCAGCAATGCCGCGTGTGCGACGAAGGCCTTCGGGTTGTAAAGCACTTTTGGCGAGAACGAAACGGTTTGGATTAATACTCCGAACTAATGACGGTACTCGCAGAATAAGCACCGGCTAACTACGTGCCAGCAGCCGCGGTAATACGTAGGGTGCAAGCGTTAATCGGAATTACTGGGCGTAAAGCGAGCGCAGGCGGCTATATAAGACAGATGTGAAATCCCCGGGCTTAACCTGGGAACTGCATTTGTGACTGTATGGCTAGAGTGTGTCAGAGGGAGGTAGAATTCCGCGTGTAGCAGTGAAATGCGTAGATATGCGGAGGAATACCGATGGCGAAGGCAGCCTCCTGGGATAACACTGACGCTCATGCTCGAAAGCGTGGGGAGCAAACAGG` |
| ASV5368 | 429 | `TGGGGAATATTGCACAATGGGCGAAAGCCTGATGCAGCGATGCCGCGTGAAGGATGAAGGTTTTCGGATCGTAAACTTCTGTTAGGTGGGAAGAAAGACTCTTGGCGAATAACCAAGAGGGATGACGGTACCATCAGAGAAAGCCCCGGCTAACTTCGTGCCAGCAGCCGCGGTAATACGAGGGGGGCAAGCGTTATTCGGAATTACTGGGCGTAAAGAGCGTGGAGGTGGCCTCTTAAGTTATTTGTTAAATCCTACGGCTTAACCGTAGGCCCGCAAGTAAAACTGAGAGGCTAGAGGATGAAAGAGAGGGGTGGAACTCCTAGAGTAGCGGTAAAATGCGTGGATCCTAGGATGAACGTCGGTGGCGAAGGCGGCCCCTCAGGTCATATCTGACACTCGAGCGCGAAGGCGTGGGGAGCAAACAGG` |
| ASV3535 | 426 | `TCGAGAATCTTCGGCAATGGACGAAAGTCTGACCGAGCGACGCCGCGTGCGGGACGAAGGCCTTCGGGTTGTAAACCGCTGTCGTAGAGGATCAAAGGTACTTGGGTTCTCCCTTGTATTTGAGTGATTCTAGGAGGAAGGAGAGGCTAAGCTCGTGCCAGCAGCCGCGGTAAGACGAGCTCTCCAAACGTTATTCGGTATCACTGGGCTTAAAGAGTTCGTAGGCGGCTTTGTAGGTGGGGTGTGAAAGCCCTCGGCTTAACCGAGGAACTGCGCCCCAAACCACAAGGCTTGAGGGATATAGGGGTAAGCGGAACAGATGGTGGAGCGGTGAAATGCATTGATATCATCTGGAACACCGGTGGCGAAGGCGGCTTACTGGATATTTTCTGACGCTGAGGAACGAAAGCTAGGGTAGCGAACGGG` |
| ASV5635 | 430 | `TGGGGAATTGTTCGCAATGGGCGCAAGCCTGACGACGCAACGCCGCGTGGAGGATGAAGACCTTCGGGTCGTAAACTCCTTTCGATCGAGACGAATGGCTTTCGGGTGAACAGCCCGGGAGAATGACGGTACCGAGAGAAGAAGCCCCGGCTAACTCCGTGCCAGCAGCCGCGGTAATACGGGGGGGGCAAGCGTTGTTCGGAATTACTGGGCGTAAAGGGCTCGTAGGCGGCCAACTAAGTCATACGTGAAATCCCTCGGCTTAACCGGGGAACTGCGTGTGATACTGGATGGCTTGAGATTGGGAGAGGGATGCGGAATTCCAGGTGTAGCGGTGAAATGCGTAGATATCTGGAGGAACACCGGTGGCGAAGGCGGCATCCTAGACCAACACTGACGCTGAGGAGCGAAAGCCAGGGGAGCAAACGGG` |
| ASV2908 | 429 | `TGGGGAATTTTGGACAATGGACGCAAGTCTGATCCAGCAATGCCGCGTGCAGGACGAAGGCCTTCGGGTTGTAAACTGCTTTTGTACGGAACGAAAAGCCTTCTTCTAATACAGGAGGGTCATGACGGTACCGTAAGAATAAGCACCGGCTAACTACGTGCCAGCAGCCGCGGTAATACGTAGGGTGCAAGCGTTAATCGGAATTACTGGGCGTAAAGCGTGCGCAGGCGGTTGTGTAAGACAGGTGTGAAATCCCCGAGCTTAACTTGGGAATTGCACTTGTGACTGCACGGCTAGAGTGCGGCAGAGGGGGATGGAATTCCGCGTGTAGCAGTGAAATGCGTAGATATGCGGAGGAACACCGATGGCGAAGGCAATCCCCTGGGCCTGCACTGACGCTCATGCACGAAAGCGTGGGGAGCAAACAGG` |
| ASV4990 | 387 | `GCGCGAAAACTTTACACTGTACGAAAGTGCGATAAGGGAACTCCCAGTGCTCATGCAATGCATGGGCTTTTGTCAAGTGTAAATAACTTGGCGAATAAGTGGTGGGTAAGACGGGTGCCAGCCGCCGCGGTAACACCTGCGCCACAAGTGGCAATCGCATTTATTGGGCCTAAAGCGTTCGTAGCCGGATCAGACAGTCTTGAGTGAAATCGTTGCGCTTAACAAAACGACGTGCTTGAGATACTACTGATCTTGAGACCGGGAAGCGTTAGGAGTATTCCTGAGGGAGCGGTAAAATGCTATAATCTCAGGAGGACTACCTGTGGCGAAGGCGCCTAACGAGAACGGGTCTGACGGTGAGGAACGAAAGCTAGGGGAGCAAACCGG` |
| ASV3426 | 429 | `TGGGGAATCTTGGACAATGGGGGCAACCCTGATCCAGCGATGCCGCGTGTGTGAAGAAGGCCTTAGGGTTGTAAAGCACTTTAATGAGGGAGGAGTAGGGTTCGTTGAATAGGCGGATCTGAGGACGTTACCTCAAGAATAAGCACCGGCTAACTCTGTGCCAGCAGCCGCGGTAATACAGAGGGTGCGAGCGTTAATCGGAATTATTGGGCGTAAAGGGAGCGTAGGCGGATTGACAAGTGGTATGTGAAATACCCGGGCTTAACCTGGGGAGGTCATATCAGACTGTTAGTCTAGAGTATGGTAGAGGAAGGTGGAATTCCTGGTGTAGCGGTGAAATGCGTAGATATCAGGAAGAACATCGATGGCGAAGGCAGCCTTCTGGGCCATTACTGACGCTGAGGCTCGAAAGCGTGGGGAGCGAACAGG` |
| ASV545 | 424 | `TGAGGAATATTGGACAATGGGCGCAAGCCTGATCCAGCCATGCCGCGTGCAGGATGACGGTCCTATGGATTGTAAACTGCTTTTGCACAGGAAGAAACACTCCCTCGAGAGGGAGCTTGACGGTACTGTGAGAATAAGGATCGGCTAACTCCGTGCCAGCAGCCGCGGTAATACGGAGGATCCAAGCGTTATCCGGAATCATTGGGTTTAAAGGGTCCGTAGGCGGTCTTATAAGTCAGTGGTGAAATCTCCCCGCTCAACGGGGAAACGGCCATTGATACTGTAGGACTTGAATTATTAGGAAGTAACTAGAATATGTAGTGTAGCGGTGAAATGCTTAGAGATTACATGGAATACCAATTGCGAAGGCAGGTTACTACTAATGGATTGACGCTGATGGACGAAAGCGTGGGTAGCGAACAGG` |
| ASV3185 | 407 | `CCGAGAATATTCGACAATGGGCGAAAGCCTGATCGAGCGATACCGCGTGGTGGATGAAGTGCTTCGGCACGTAAACACCTTTTATGAGGGAGGAAGTTATTGACGTTACCTCATGAATAAGGGGCTCCTAACTCTGTGCCAGCAGGAGCGGTAATACAGAGGCCCCAAGCATTACCCGGAATCACTGGGCGTAAAGGGTGCGTAGGTGGTCGTATTAGTCGTTCGTTAAAACCCGGGGCTTAACCCCGGAATCGCGAGCGAAACGGTACGACTCGAGAGAGTGAGAGGTGAATGGAACTCATGGTGTAGGGGTGAAATCCGTTGATATCATGGGGAACACCGAAAGCGAAGGCAATTCACTGGCACTTTTCTGACACTCAAGCACGAAAGCGTGGGTAGCGAACTGG` |
| ASV5260 | 429 | `TGGGGAATATTGGACAATGGGGGAAACCCTGATCCAGCAATGCCGCGTGTGTGAAGAAGGCCTTCGGGTTGTAAAGCACTTTCAGTAGGGAAGAAAAATTTAGGGTTAATACCCCTGAATGTTGACGTTACCTACAGAAGAAGCACCGGCTAACTCTGTGCCAGCAGCCGCGGTAATACAGAGGGTGCGAGCGTTAATCGGAATTACTGGGCGTAAAGCGTGCGTAGGCGGATATTTAAGTCGGATGTGAAATCCCTGGGCTCAACCTGGGAACTGCATCCGATACTGGGTATCTGGAGTATGGTAGAGGAAAGTGGAATTCCCGGTGTAGCGGTGGAATGCGTAGATATCGGGAGGAACATCAGTGGCGAAGGCGACTTTCTGGATCAATACTGACGCTGAGGCACGAAAGCGTGGGGATCAAACAGG` |
| ASV5059 | 430 | `TGGGGAATTTTGCACAATGGACGCAAGTCTGATGCAGCGACGCCGCATGAGTGACGAAGGCCTTCGGGTTGTAAAGCTCTGTCGGTTGGGAAAAAAGGCTCTCTTATTAACATGAAGAGAGATTGATGGTACCAGCAAAGGAAGAGGTGGCTAACTCTGTGCCAGCAGCCGCGGTAATACAGAGACCTCAAGCGTTGTTCGGAATTACTGGGCGTAAAGGGTTCGTAGGTGGCGAAGCAAGTCAGATGTGAAAGCCCTAGGCCTAACTTAGGAAGTGCATTTGATACTGCTTTGCTAGAGTGCGGAAGAGGAAAATGGAATTCCAGGTGTAGGGGTGAAATCCGTAGATATCTGGAGGAATACCGATGGTGAAGACAGTTTTCTGGTCCGTTACTGACACTGAGGAACGAAAGCGTGGGGATCAAACAGG` |
| ASV5096 | 430 | `TAGGGAATATTGCGCAATGGAGGAAACTCTGACGCAGCGACGCCGCGTGGGTGATGAAGGTCTTCGGATCGTAAAGCCCTGTCGGATGGGAAGAAAAACACTGAGGGTAATATTCTCAGTGCTTGACGGTACCATTAAAGGAAGCCCCGGCTAACTTCGTGCCAGCAGCCGCGGTAATACGAAGGGGGCAAGCGTTGTTCGGAATCATTGGGCGTAAAGCGCGTGTAGGCGGCCTATTAAGTCGAGTGTGAAATCCCTGGGCTCAACCGAGGAAGTGCATTCGAAACTAATAGGCTAGAAGATGGTAGAGGATGGTGGAATTCCAGGTGTAGAGGTGAAATTCGTAGATATCTGGAGGAATACCGGTGGCGAAGGCGGCCATCTGGGCCATTCTTGACGCTGAGACGCGAAAGCGTGGGTAGCAAACAGG` |
| ASV73 | 429 | `TGGGGAATTTTGGACAATGGACGCAAGTCTGATCCAGCCATGCCGCGTGCGGGAAGAAGGCCTTCGGGTTGTAAACCGCTTTTGTCAGGGAAGAAAAGGTTCTGGTTAATACCTGGGACTGATGACGGTACCTGAAGAATAAGCACCGGCTAACTACGTGCCAGCAGCCGCGGTAATACGTAGGGTGCAAGCGTTAATCGGAATTACTGGGCGTAAAGCGTGCGCAGGCGGTTATGCAAGACAGAGGTGAAATCCCCGGGCTCAACCTGGGAACTGCCTTTGTGACTGCATAGCTAGAGTACGGTAGAGGGGGATGGAATTCCGCGTGTAGCAGTGAAATGCGTAGATATGCGGAGGAACACCGATGGCGAAGGCAATCCCCTGGACCTGTACTGACGCTCATGCACGAAAGCGTGGGGAGCAAACAGG` |
| ASV2909 | 430 | `TGGGGAATCTTGCGCAATGGGCGAAAGCCTGACGCAGCGACGCCGCGTGGGTGATGAAGGCCTTCGGGTCGTAAAGCCCTGTCAAGAGGGACGAAACCTCGTCGACCTAATACGTCGGCGACCTGACGGTACCTCTGAAGGAAGCACCGGCTAACTCCGTGCCAGCAGCCGCGGTAATACGGAGGGTGCGAGCGTTGTTCGGAATTACTGGGCGTAAAGCGCGTGTAGGCGGCCTCCTCAGTCTGGTGTGAAAGCCCGGGGCTCACCCCCGGAAGTGCATTGGATACTGGGAGGCTGGAGTACCGGAGAGGAGGGTGGAATTCCTGGTGTAGCGGTGAAATGCGTAGATATCAGGAGGAACACCGGTGGCGAAGGCGGCCCTCTGGACGGATACTGACGCTGAGACGCGAAAGCGTGGGGAGCAAACAGG` |
| ASV4004 | 429 | `TGGGGAATATTGGACAATGGGGGGAACCCTGATCCAGCAATGCCGCGTGTGTGAAGAAGGCCTGAGGGTTGTAAAGCACTTTCAGTGGGGAGGAGGATGCTTAGGTTAAGAGCTAGAGTATTGGACGTTACCCACAGAAGAAGCACCGGCTAACTCCGTGCCAGCAGCCGCGGTAATACGGAGGGTGCGAGCGTTAATCGGAATTACTGGGCGTAAAGAGTGCGTAGGTGGTTAATTAAGTTATCTGTGAAATTCCTGGGCTTAACCTGGGCAGGTCAGATAATACTGATTGACTCGAGTATGGGAGAGGGTAGTGGAATTTCCGGTGTAGCGGTGAAATGCGTAGAGATCGGAAGGAACACCAGTGGCGAAGGCGGCTACCTGGCCTGATACTGACACTGAGGCACGAAAGCGTGGGGAGCAAACAGG` |
| ASV735 | 429 | `TGGGGAATTTTGGACAATGGGCGAAAGCCTGATCCAGCAATGCCGCGTGCAGGAAGAAGGCCTTCGGGTTGTAAACTGCTTTTGTACGGAGCGAAAAGGTCTTCTTTAATACAGGAGGCTGATGACGGTACCGTAAGAATAAGCACCGGCTAACTACGTGCCAGCAGCCGCGGTAATACGTAGGGTGCGAGCGTTAATCGGAATTACTGGGCGTAAAGCGTGCGCAGGCGGTTTTGTAAGACAGGCGTGAAATCCCCGGGCTCAACCTGGGAATGGCGCTTGTGACTGCAAAGCTGGAGTGCGGCAGAGGGGGATGGAATTCCGCGTGTAGCAGTGAAATGCGTAGATATGCGGAGGAACACCGATGGCGAAGGCAATCCCCTGGGCCTGCACTGACGCTCATGCACGAAAGCGTGGGGAGCAAACAGG` |
| ASV1438 | 429 | `TGGGGAATTTTGGACAATGGGGGCAACCCTGATCCAGCCATGCCGCGTGAGTGAAGAAGGCCTTCGGGTTGTAAAGCTCTTTCGGCCGGGAAGAAATCGTATCAGCTAATACCTGGTGCGGATGACGGTACCGGCATAAGAAGCACCGGCTAACTACGTGCCAGCAGCCGCGGTAATACGTAGGGTGCGAGCGTTAATCGGAATTACTGGGCGTAAAGCGTGCGCAGGCGGTTTTGTAAGACAGGCGTGAAATCCCCGGGCTCAACCTGGGAACTGCGTTTGTGACTGCAAGGCTAGAGTACGGCAGAGGGGGGTGGAATTCCACGTGTAGCAGTGAAATGCGTAGAGATGTGGAGGAACACCAATGGCGAAGGCAGCCCCCTGGGTCGATACTGACGCTCATGCACGAAAGCGTGGGTAGCAAACAGG` |
| ASV1441 | 429 | `TGGGGAATTTTGGACAATGGGCGCAAGCCTGATCCAGCAATGCCGCGTGCAGGATGAAGGCCTTCGGGTTGTAAACTGCTTTTGTACGGAGCGAAACGATCTTTCCTAATACGAGAGGTTAATGACGGTACCGTAAGAATAAGCACCGGCTAACTACGTGCCAGCAGCCGCGGTAATACGTAGGGTGCAAGCGTTAATCGGAATTACTGGGCGTAAAGCGTGCGCAGGCGGTTATATAAGACAGATGTGAAATCCCCGGGCTCAACCTGGGACCTGCATTTGTGACTGTATAGCTAGAGTACGGTAGAGGGGGATGGAATTCCGCGTGTAGCAGTGAAATGCGTAGATATGCGGAGGAACACCGATGGCGAAGGCAATCCCCTGGACCTGTACTGACGCTCATGCACGAAAGCGTGGGGAGCAAACAGG` |
| ASV1074 | 424 | `TAAGGAATATTGGTCAATGGAGGCAACTCTGAACCAGCCATGCCGCGTGCAGGAAGACGGCCCTATGGGTTGTAAACTGCTTTTGTACCAGAGAAAACCCTTCTACGTGTAGGAGGTTGATAGTATGGTAAGAATAAGCATCGGCTAACTTCGTGCCAGCAGCCGCGGTAAGACGAAGGATGCAAGCGTTATCCGGATTCATTGGGTTTAAAGGGTGCGTAGGCGGACCTGTAAGTCAGTGGTGAAATCTCTCAGCTTAACTGAGAAACTGCCATTGATACTGCAGGTCTAGAGTATAGATGACGTTGGCGGAATATGACATGTAGTGGTGAAATACTTAGATATGTCATAGAACACCGATTGCGAAGGCAGCTAACGAAACTATAACTGACGCTGAGGCACGAAAGTGCGGGGATCAAACAGG` |
| ASV2829 | 424 | `TGAGGAATATTGGACAATGGGCGCAAGCCTGATCCAGCCATGCCGCGTGCAGGATGACGCATCTATGGTGTGTAAACTGCTTTTGCACAGGAAGAAACATCCCGACGTGTCGGGACTTGACGGTACTGTGAGAATAAGGATCGGCTAACTCCGTGCCAGCAGCCGCGGTAATACGGAGGATCCAAGCGTTATCCGGAATCATTGGGTTTAAAGGGTCCGTAGGCGGTTTATTAAGTCAGTGGTGAAAGCCCATCGCTCAACGGTGGAACGGCCATTGATACTGATAAACTTGAATTACTAGGAAGTAACTAGAATATGTAGTGTAGCGGTGAAATGCTTAGAGATTACATGGAATACCAATTGCGAAGGCAGGTTACTACTAGTGGATTGACGCTGATGGACGAAAGCGTGGGTAGCGAACAGG` |
| ASV3809 | 429 | `TGGGGAATATTGGACAATGGGCGCAAGCCTGATCCAGCCACGCCGCGTGAGTGATGAAGGCCTTCGGGTCGTAAAGCTCTGTGGGGAGAGACGAACCGCCAGGGGTTAATAGCCGCTTGGCATGACGGTATCTCCTTAGCAAGCACCGGCTAACTTCGTGCCAGCAGCCGCGGTAATACGAAGGGTGCAAACGTTGCTCGGAATTATTGGGCGTAAAGCGCACGTAGGCGGTTTTTCAAGTCGGATGTGAAAGCCCTCGGCTTAACCAAGGAAGTGCATCCGAAACTGAAAGACTTGAGTACTTAAGAGGATCGCGGAATTCCCGGTGTAGAGGTGAAATTCGTAGATATCGGGAGGAACACCAGTGGCGAAGGCGGCGATCTGGGAAGATACTGACGCTGAGGTGCGAAAGCGTGGGGAGCAAACAGG` |
| ASV5031 | 385 | `CTGACCAGCCCCGAGTCTGCCCTCCTCGCTACGCAGACGCTCTCTCTGCCTCCAGTCCGAAAGGATATTTTGGCAAAACCCGTCGCCTCGGATCCATATATGAGCGTCCTCTACAGTAGCGCCCTGATGGCGACAGGGTGGATGGATCCCGATCCGAATACCACAAAAGCGATATTCGGAGAGATGGTAGAAAGTATCGCTTCGGGGAGAGAGGAGATAGCAGGTGCTATCCGTACCGCGAGCGACAGAATGCAGCAGATCGTGCGGTAGGGAAGTATAAAATAGAAGAGCCCAAGATTTGCTTTCGCAAATCTTGGGCTAATTCTTTTAAATTTTACCGCATTCGAGGCGGTGGTTTTTCGTTTTGTTTTGGTGCGGGAGTAGG` |
| ASV319 | 404 | `TGGGGAATATTGGACAATGGGCGAAAGCCTGATCCAGCAATGCCGCGTGAGTGAAGAAGGCCTTCGGGTTGTAAAGCTCTTTTACCCGGGAAGATAATGACTGTACCGGGAGAATAAGCCCCGGCTAACTCCGTGCCAGCAGCCGCGGTAATACGGAGGGGGCTAGCGTTGTTCGGAATTACTGGGCGTAAAGCGTACGTAGGCGGCTTTTTAAGTCAGAGGTGAAAGCCCGGGGCTCAACTCCGGAATTGCCTTTGAAACTGAAGAGCTTGAACATGGGAGAGGTGAGTGGAATTCCGAGTGTAGAGGTGAAATTCGTAGATATTCGGAGGAACACCAGTGGCGAAGGCGGCTCACTGGACCATTGTTGACGCTGAGGTACGAAAGCGTGGGGAGCAAACAGG` |
| ASV5122 | 386 | `GCGCGAAAACTGCACAATGTGGGAAATCACGATGCGGGAAACCCAAGTGCCAGCACTATGTGTTGGCTGTCCTCAAGCGTTCGTTGCTTGGGAAGAAAGAGCTGGGTAAGACGGGTGCCAGCCGCCGCGGTAATACCCGCAGCTCAAGTGGTGGTCATTATTATTGGGCCTAAAGCGTCCGTAGCTGGTGTGTTAGATGCCTGGGTAAATCGAATCGCTCAACGGTTCGAATTCCGGGTAGACCGGCACACTCGAGACCGGGAGAGGCATGGGGTACTCGAGGGGTAGGGGTAAAATCCTGTAATCCTTCGGGGACGACCAGTGGCGAAGGCGCCATGCTAGAACGGCTCTGACAGTGAGGGACGAAGCCCTGGGGCGCAAACCGG` |
| ASV1603 | 424 | `TGAGGAATATTGGTCAATGGACAGAAGTCTGAACCAGCCAAGTCGCGTGAAGGAAGACTGCCCTATGGGTTGTAAACTTCTTTTAATTGGGAATAAAAGAGGGGATGTATCCCCTATTGCAAGTACCAAAAGAATAAGCATCGGCTAACTCCGTGCCAGCAGCCGCGGTAATACGGAGGATGCGAGCGTTATCCGGATTTATTGGGTTTAAAGGGTGCGTAGGCGGGCTACCAAGTCAGCGGTGAAATATTCGGGCTCAACCCGGAACGTGCCGTTGAAACTGGCAGCCTTGAGAGTAAAAGAAGTAGGCGGAATGCGTGGTGTAGCGGTGAAATGCATAGATATCACGCAGAACTCCGATTGCGAAGGCAGCTTACTATCCTACATCTGACGCTGAAGCACGAAAGCGTGGGTATCGAACAGG` |
| ASV1241 | 404 | `TGGGGAATTTTCCGCAATGGGCGCAAGCCTGACGGAGCAATACCGCGTGAGGGAGGAAGGCTCTTGGGTCGTAAACCTCTTTTCTCAAGGAAGATAATGACGGTACTTGAGGAATAAGCATCGGCTAACTCTGTGCCAGCAGCCGCGGTAATACAGAGGATGCAAGCGTTATCCGGAATGATTGGGCGTAAAGAGTCCGTAGGTGGTAATTCAAGTCTGTGGTTAAAGCGTGGAGCTCAACTCCATAAAGGCCATGGAAACTGAATAACTAGAGTGAGGTAGGGGTAGAGGGAATTCTCGGTGTAGCGGTGAAATGCGTAGATATCGGGAAGAACACCGGTGGCGAAAGCGCTCTACTGGACCTTAACTGACACTGAGGGACGAAAGCTAGGGGAGCGAAAGGG` |
| ASV789 | 424 | `TGAGGAATATTGGTCAATGGACGCAAGTCTGAACCAGCCATGCCGCGTGAAGGATGAAGGCCTTCTGGGTTGTAAACTTCTTTTATCTGGGAAGAAACCTCCGATTTCTATTGGAGTTGACGGTACCAGAGGAATAAGCACCGGCTAACTCCGTGCCAGCAGCCGCGGTAATACGGAGGGTGCAAGCGTTATCCGGATTTACTGGGTTTAAAGGGTGTGTAGGCGGGCTATTAAGTCAGTGGTGAAATCTCCGAGCTTAACTTGGAAACTGCCATTGATACTATTAGTCTTGAATTTTGTTGAGGTAGGCGGAATAAGTCATGTAGCGGTGAAATGCATAGATATGACTTAGAACACCAATTGCGAAGGCAGCTTGCTAAACAAATATTGACGCTGAGGCACGAAAGCGTGGGGATCAAACAGG` |
| ASV4209 | 430 | `TGGGGAATCTTGCGCAATGGACGAAAGTCTGACGCAGCCACGCCGCGTGAGTGAAGAAGGCCTTCGGGTTGTAAAGCTCTGTCACCTGGGACGAAAACCTTTGCGGTTAATAGCCGCAGAGCTTGACGGTACCAGGGGAGGAAGCACCGGCTAACTTCGTGCCAGCAGCCGCGGTAATACGAAGGGTGCAAGCGTTGCTCGGAATTATTGGGCGTAAAGGGTTGGTAGGTGGTCTTGTTTGTCTAAGGTGAAATCCCACGGCTTAACTGTGGAAGTGCCTTAGAAACGGCAAGACTAGAGTCCTAGAGGGGTTTGTGGAATTCCCGGTGTAGCGGTGAAATGCGTAGAGATCGGGAGGAACACCAGCGGCGAAGGCGGCAAACTGGATAGGTACTGACACTAAACAACGAAAGCGTGGGGATCAAACAGG` |
| ASV4737 | 404 | `TGGGGAATATTGGACAATGGGGGCAACCCTGATCCAGCAATGCCGCGTGAGTGATGAAGGCCTTCGGGTTGTAAAGCTCTTTTGTCTGTGATGATAATGACAGTAACAGACGAATAAGCCCCGGCTAACTTCGTGCCAGCAGCCGCGGTAATACGAAGGGGGCTAGCGTTGTTCGGAATAACTGGGCGTAAAGGGCGCGTAGGCGGTTAGATACGTCTGATGTGAAAGTCCTGGGCTTAACCTAGGGACCGCATTGGATACGGTTTAGCTTGAGTGCGAGAGGGGAAAATGGAATTGCGCGTGTAGAGGTGAAATTCGTAGAGATGCGCAAGAACACCAGTGGCGAAGGCGATTTTCTGGCTCGCAACTGACGCTGAGGCGCGAAAGCGTGGGGAGCAAACAGG` |
| ASV3292 | 430 | `TGGGGAATTTTGGACAATGGGCGAAAGCCTGATCCAGCCATGCCGCGTGCAGGAAGAAGGCCTTCGGGTTGTAAACTGCTTTAGTTCGGGAAAAAGGGATGCTTGGTTAATACCCGAGTGTTTTGATGGTACCGGAAGAATAAGCACCGGCTAACTACGTGCCAGCAGCCGCGGTAATACGTAGGGTGCGAGCGTTAATCGGAATTACTGGGCGTAAAGCGTGCGCAGGCGGTCTTGTAAGACAGTTGTGAAATCCCCGGGCTTAACCTGGGAACTGCAATTGTGACTGCAAGGCTAGAGTGTGTCAGAGGGGGGTGGAATTCCACGTGTAGCAGTGAAATGCGTAGAGATGTGGAGGAACACCGATGGCGAAGGCAGCCCCCTGGGATAACACTGACGCTCATGCACGAAAGCGTGGGGAGCAAACAGG` |
| ASV3755 | 404 | `TGAGGAATATTGGACAATGGGCGCAAGCCTGATCCAGCCATGCCGCGTGAGTGATGAAGGCCCTAGGGTTGTAAAACTCTTTTAGTGGGGAAAATAATGATGGTACCCACAGAAAAAGCTCCGGCTAACTTCGTGCCAGCAGCCGCGGTAATACGAAGGGAGCTAGCGTTACTCGGAATCACTGGGCGTAAAGCGCATGTAGGCGGAAATACAAGTCAGAGGTGAAAGCCCTGGGCTCAACCCAGGAATTGCCTTTGAAACTGTATTTCTTGAGTGTAACAGAGGATGGTGGAATTTTCAGTGTAGAGGTGAAATTCGTAGATATTGAAAAGAACACCGGTGGCGAAGGCGACCATCTGGGTTACAACTGACGCTGAGATGCGAAAGCGTGGGGAGCGAACGGG` |
| ASV1910 | 386 | `GCGCGAAAACTGCACAATGTGGGAAACCACGATGCGGGAAACCCAAGTGCCAGCACTAAGTGTTGGCTGTCCTCCAGTGTTCGTAACTGGGGAAGAAAGTGCTGGGTAAGACGGGTGCCAGCCGCCGCGGTAATACCCGCAGCACAAGTGGTGGTCATTATTATTGGGCCTAAAGCGTCCGTAGCAGGCTTGTTAGATGCCTGGGTAAATCGATGCGCTCAACGCATCGAACTCCGGGTAGACCGGCAAGCTCGAGACCGGGAGAGGTGTAGGCTACTCGCGGGGTAGGGGTAAAATCCTGTAATCCTGCGGGGAGGACCAGTGGCGAAGGCGCTACACTAGAACGGCTCTGACTGTGAGGGACGAAGCCCTGGGGCGCAAACCGG` |
| ASV3228 | 424 | `TAAGGAATATTGGGCAATGGACGAAAGTCTGACCCAGCCATGCCGCGTGGAGGATGAAGGCCCTCTGGGTTGTAAACTTCTTTTCTCTGTAAAGAAATGCACGGTTTCTACTGTGTTTGACGGTAACAGAGGAATAAGCACCGGCTAACTCCGTGCCAGCAGCCGCGGTAATACGGAGGGTGCAAGCGTTATCCGGATTCACTGGGTTTAAAGGGAGCGTAGGTGGGTAGATAAGTCAGCGGTGAAATACCTAAGCTTAACTTAGGAACTGCCGTTGATACTGTCTGTCTTGAATATCGTAGAGGTAAGCGGAATATGTCATGTAGCGGTGAAATGCTTAGATATGACATAGAACACCAATTGCGAAGGCAGCTTGCTATGCGAATATTGACACTAAGGCTCGAAAGCGTGGGGATCAAACAGG` |
| ASV2423 | 429 | `TGGGGAATTTTGGACAATGGGCGCAAGCCTGATCCAGCCATTCCGCGTGAGTGAAGAAGGCCTTCGGGTTGTAAAGCTCTTTCGGCAGGGAAGAAACGGCTACGGCTAATACTCGTGGCTAATGACGGTACCTGAAGAAGAAGCACCGGCTAACTACGTGCCAGCAGCCGCGGTAATACGTAGGGTGCGAGCGTTAATCGGAATTACTGGGCGTAAAGCGTGCGCAGGCGGTTTTGTAAGACAGGTGTGAAATCCCCGGGCTTAACCTGGGAACTGCGCTTGTGACTGCAAGACTCGAGTGCGGCAGAGGGGGGTGGAATTCCACGTGTAGCAGTGAAATGCGTAGAGATGTGGAGGAACACCGATGGCGAAGGCAGCCCCCTGGGTCGACACTGACGCTCATGCACGAAAGCGTGGGGAGCAAACAGG` |
| ASV5 | 429 | `TGGGGAATTTTGGACAATGGGCGCAAGCCTGATCCAGCAATGCCGCGTGCAGGATGAAGGCCTTCGGGTTGTAAACTGCTTTTGTACGGAACGAAACGGCTCTGGTTAATACCCGGGGCTAATGACGGTACCGTAAGAATAAGCACCGGCTAACTACGTGCCAGCAGCCGCGGTAATACGTAGGGTGCAAGCGTTAATCGGAATTACTGGGCGTAAAGCGTGCGCAGGCGGTTATGTAAGACAGTCGTGAAATCCCCGGGCTCAACCTGGGAACTGCGATTGTGACTGCATAGCTAGAGTACGGTAGAGGGGGGTGGAATTCCGCGTGTAGCAGTGAAATGCGTAGATATGCGGAGGAACACCAATGGCGAAGGCAGCCCCCTGGACCTGTACTGACGCTCATGCACGAAAGCATGGGGAGCAAACAGG` |
| ASV518 | 404 | `TTAGGAATATTGGACAATGGGGGAAACCCTGATCCAGCCATGCCGCGTGAGTGAAGAAGGCCTTAGGGTTGTAAAGCTCTTTCGGCTGTGAAGATAATGACAGTAGCAGAAGAAGAAGCCCCGGCTAAATTCGTGCCAGCAGCCGCGGTAATACGAATGGGGCGAGCGTTGTTCGGAATCACTGGGCGTAAAGCGTGCGCAGGCGGTCATGAAAGTCAGAAGTGAAAGCCCAGGGCTTAACCCTGGAATTGCTTTTGAAACTCCATGACTAGAGTACTGGAGAGGTTGGCGGAATTCCGAGTGTAGCAGTGAAATGCGTAGATATTCGGAGGAACACCGATGGCGTAGGCAGCCAACTGGACAGTAACTGACGCTCATGCACGAAAGCGTGGGGATCAAACAGG` |
| ASV4426 | 404 | `TGGGGAATATTGGACAATGGGCGCAAGCCTGATCCAGCCATGCCGCGTGAGTGATGAAGGCCTTAGGGTTGTAAAGCTCTTTAGCAGGGGAAGATAATGACGGTACCCTGAGAATAAGCACCGGCTAACTTCGTGCCAGCAGCCGCGGTAATACGAAGGGTGCTAGCGTTGTTCGGAATCACTGGGCGTAAAGCGTGCGTAGGCGGCTTGACAAGTCTGACGTGAAAGCCCGGGGCTTAACCCCGGATGTGCGTTGGAAACTGTCTTGCTGGAGGACGGAAGAGGAGAGTGGAATTCCTAGTGTAGAGGTGAAATTCGTAGATATTAGGAGGAACACCAGTGGCGAAGGCGACTCTCTGGTCCGTTTCTGACGCTGAGGCACGAAAGCGTGGGGATCAAACAGG` |
| ASV2282 | 429 | `TCGAGAATAATTCACAATGGGGGCAACCCTGATGGTGCAACGCCGCGTGGAGGATGACGGTCTTCGGATTGTAAACTCCTGTCATCTGGGAGTAAGACCTGGCTGTGAATAGCAGACAGGGTTGATAGTACCAGAAGAGGAAGGGACGGCTAACTTCGTGCCAGCAGCCGCGGTAATACGAAGGTCCCAAGCGTTGTTCGGAATCACTGGGCGTAAAGGGAGCGTAGGCGGCGTGGTAAGTCAGATGTGAAATCCCGGGGCTCAACCCCGGAACTGCATCCGATACTGCCATGCTAGAGGATTGGAGAGGTAGCTGGAATTCTTGGTGTAGCAGTGAAATGCGTAGAGATCAAGAGGAACACTCGTGGCGAAAGCGAGCTACTGGACAATACCTGACGCTGAGGCTCGAAGGCTAGGGGAGCGAAAGGG` |
| ASV146 | 404 | `TGGGGAATATTGGACAATGGGGGAAACCCTGATCCAGCGATGCCGCGTGAGTGAAGAAGGCCTTCGGGTTGTAAAACTCTTTCACCTGTGACGATGATGACGGTAGCAGGAGAAGAAGCACCGGCTAACTCCGTGCCAGCAGCCGCGGTAAGACGGAGGGTGCTAGCGTTGTTCGGATTGACTGGGCGTAAAGGGCGCGTAGGCGGTTTTATAAGTTGGGCGTGAAAGCCCCGGGCTCAACCCGGGAACTGCGTCCGATACTGTAAGACTAGAGGATGGAAGAGGAAAGTGGAATTCCTAGTGTAGAGGTGAAATTCGTAGATATTAGGAGGAACACCAGAGGCGAAGGCGGCTTTCTGGTCCATCACTGACGCTGAGGCGCGAAAGCGTGGGGAGCAAACAGG` |
| ASV3000 | 411 | `TTTGGAATTTTGGACAATGGGCGAAAGCCTGATCCAGCGACGCCGCGTGGACGATGACGGCCTTCGGGTTGTAAAGTCCTTTTGCTCGGGAACAATATTTTGAGCGTACCGAGAGAATAAGAGGTTGCTAACTCTGTGCCAGCAGCAGCGGTAATACAGAGACCTCAAGCGTTATCCGGAATCATTGGGCGTAAAGCGTACCGATCGGTGGCTTAACAAGCCAGAAGTGAAATCTCCGAGCTCAACTCGGAATCTGTCTTTTGGAACTGTTAGGCTTGAGGGGCAAAGAGGAAGCTGGAACGAACGGTGTAGTAGTGAAATGCGTTGATATCGTTCGGAACACCAATAGCGAAGGCGGGCTTCTGGGTGCCACCTGACACTGCTAGGACGAAAGCGTGGGGAGCGAATGGG` |
| ASV3325 | 429 | `TGGGGAATTTTGGACAATGGGCGCAAGCCTGATCCAGCAATGCCGCGTGAGTGAAGAAGGCCTTCGGGTTGTAAAGCTCTTTTGTCAGGGAAGAAACGGGGTTGGCTAATATCCGACCTTAATGACGGTACCTGAAGAATAAGCACCGGCTAACTACGTGCCAGCAGCCGCGGTAATACGTAGGGTGCAAGCGTTAATCGGAATTACTGGGCGTAAAGCGTGCGCAGGCGGTTTTGTAAGTCTGACGTGAAATCCCCGGGCTCAACCTGGGAATTGCGTTGGAGACTGCAAGGCTGGAGTCTGGCAGAGGGGGGTAGAATTCCACGTGTAGCAGTGAAATGCGTAGAGATGTGGAGGAACACCGATGGCGAAGGCAGCCCCCTGGGTCAAGACTGACGCTCATGCACGAAAGCGTGGGGAGCAAACAGG` |
| ASV1250 | 429 | `TGGGGAATTTTGGACAATGGGCGCAAGCCTGATCCAGCCATTCCGCGTGAGTGAAGAAGGCCTTCGGGTTGTAAAGCTCTTTCGCAAGGGAAGAAACGATTTAGGTAAATAATCTAAGTTAATGACGGTACCTTGATAAGAAGCACCGGCTAACTACGTGCCAGCAGCCGCGGTAATACGTAGGGTGCGAGCGTTAATCGGAATTACTGGGCGTAAAGCGTGCGCAGGCTGTTTTGTAAGTCAGATGTGAAATCCCCGAGCTCAACTTGGGAACTGCGTTTGAAACTACAAGACTAGAATATGTCAGAGGGGGGTAGAATTCCACGTGTAGCAGTGAAATGCGTAGAGATGTGGAGGAATATCAATGGCGAAGGCAGCCCCCTGGGATAATATTGACGCTCATGCACGAAAGCGTGGGGAGCAAACAGG` |
| ASV4602 | 405 | `TGGGGAATCTTGTGCAATGGGCGCAAGCCTGACACAGCAACGCCGCGTGCGGGAAGAAGGCCTTCGGGTCGTAAACCGCTTTCAGCAGGAACGAAAATGACGGTACCTGCAGAAGAAGATGCGGCCAACTACGTGCCAGCAGCCGCGGTGACACGTAGGCATCAAGCGTTGTCCGGATTTATTGGGCGTAAAGAGCTCGTAGGCGGTTCAGCAAGTCGGGTGTGAAAACTCTGGGCTTAACCCAGAGCCTGCATCCGAAACTGCTGTGACTAGAGTTCGGTAGGGGAGAGGGGAATTCCTAGTGTAGCGGTGAAATGCGCAGATATTAGGAGGAACACCGATGGCGAAGGCACCTCTCTGGGCCGATACTGACGCTGAGGAGCGAAAGCGTGGGTAGCAAACAGG` |
| ASV1499 | 429 | `TGGGGAATTTTGGACAATGGGCGAAAGCCTGATCCAGCCATGCCGCGTGAGTGAAGAAGGCCTTCGGGTTGTAAAGCTCTTTCGGACAGAAAGAAATCTTTCGGGTTAATACCTCGGAAGGATGACGGTACTGTAAGAAGAAGCACCGGCTAACTACGTGCCAGCAGCCGCGGTAATACGTAGGGTGCGAGCGTTAATCGGAATTACTGGGCGTAAAGGGTGCGCAGGCGGTTATATAAGACAGATGTGAAATCCCCGGGCTCAACCTGGGAACTGCGTTTGTGACTGTATGACTAGAGTACGGTAGAGAGGGGTGGAATTCCTGGTGTAGCAGTGAAATGCGTAGATATCAGGAGGAACATCGATGGCGAAGGCAGCCCCTTGGACCTGTACTGACGCTCATGCACGAAAGCGTGGGGAGCAAACAGG` |
| ASV2490 | 430 | `TGGGGAATCTTGCGCAATGGACGAAAGTCTGACGCAGCGACGCCGCGTGAGTGAAGAAGGCCTTCGGGTTGTAAAGCTCTGTCGGAGGGGACGAACATTCGATTAGTTAATAGCTAGTCGAACTGACGGTACCCTTAAAGGAAGCACCGGCTAACTCTGTGCCAGCAGCCGCGGTAATACAGAGGGTGCAAGCGTTGCTCGGAATTATTGGGCGTAAAGCGTGGGTAGGTTGTCTTGTTTGTCAGAGGTGAAAGCCTGGAGCTTAACTCCAGAAGTGCCTCTGAAACGGCAAGACTAGAGTGCTGAAGAGGTTCGTGGAATTCCCGGTGTAGCGGTGAAATGCGTAGAGATCGGGAGGAACATCAGAGGCGAAGGCGACGAACTGGGAAGTAACTGACACTCAACCACGAAAGCACGGGTAGCAAACAGG` |
| ASV2749 | 423 | `TAGGGAATATTGGTCAATGGGCGCAAGCCTGAACCAGCCATGCCGCGTGCAGGATGAAGGCCCTCAGGGTCGTAAACTGCTTTTGTACGGGAACAAAAAGCTCCTGCGGGGGCCATTGAGGGTACCGTAAGAATAAGCACCGGCTAACTCCGTGCCAGCAGCCGCGGTAATACGGAGGGTGCAAGCGTTATCCGGATTTACTGGGTTTAAAGGGTGCGTAGGCGGCTCTTTAAGTCAGTGGCGAAAGCCTGGCGCTTAACGTCAGAATTACCATTGATACTGGAGAGCTTGAGTCAAGAAGAGGTAAGCAGAATTCATGGTGTAGCGGTGAAATGCATAGATACCATGAGGAATTCCAACAGCGAAGGCAGCTTACTGGTCTTTGACTGACGCTGAGGCACGAAAGCGTGGGGAGCAAACAGG` |
| ASV2788 | 404 | `TGGGGGATATTGCACAATGGAGGAAACTCTGATGCAGCGACGCCGCGTGGAGGAAGACGGTTTTCGGATTGTAAACTCCTGTCTTCGGGGACGATAATGACGGTACCCGAGGAGGAAGCACCGGCTAACTACGTGCCAGCAGCCGCGGTAATACGTAGGGTGCAAGCGTTGTCCGGAATTACTGGGTGTAAAGGGAGCGTAGGCGGGATCGCAAGTTGGATGTGAAATATACTGGCTCAACCAGTAAACTGCATTCAAAACTGCGGTTCTTGAGTGAAGTAGAGGCAGGCGGAATTCCTAGTGTAGCGGTGAAATGCGTAGATATTAGGAGGAACACCAGTGGCGAAGGCGGCCTGCTGGGCTTTTACTGACGCTGAGGCTCGAAAGCATGGGGAGCAAACAGG` |
| ASV1621 | 430 | `TAGGGAATATTGCACAATGGGGGAAACCCTGATGCAGCAACGCCGCGTGAGTGAAGAAGGCCCTTGGGTTGTAAAGCTCTTTAGCGAGGGAAGAAGGTTGGTACGGTGAACAATCGGATCAGCTGACGGTACCTCGAGAATAAGCACCGGCTAATTTCGTGCCAGCAGCCGCGGTAATACGAAAGGTGCAAGCGTTACTCGGAATCACTGGGCGTAAAGCGCACGTAGGCGGACTTACAGGTCAATTGTGAAATACCTGGGCTCAACCCGGGAACTGCAGTTGAAACCGTTTGTCTTGAATACCTGAGAGGGTGGGGGAATACCTGGTGGAGAAGTGAAATTCGTAGAGATCAGGTGGAACACCGGAGGCGAAGGCGCCTGCCTGGCAGTGTATTGACGCTGAGGTGCGAAAGTGCGGGGAGCAAACAGG` |
| ASV4775 | 429 | `TGGGGAATATTGGACAATGGGGGGAACCCTGATCCAGCAATGCCGCGTGTGTGAAGAAGGCCTGAGGGTTGTAAAGCACTTTCAGTGGGGAGGAGGATTGTTTTGTTAATAGCAGGATGATTGGACGTTACCCACAGAAGAAGCACCGGCTAACTCCGTGCCAGCAGCCGCGGTAATACGGAGGGTGCAAGCGTTAATCGGAATTACTGGGCGTAAAGCGTGCGTAGGTTGTTGACTAAGTTATCTGTGAAATCCCTGGGCTCAACCTGGGGCGGTCAGATAAGACTGGTTGACTCGAGTATAGGAGAGGGTAGTGGAATTTCCGGTGTAGCGGTGAAATGCGTAGAGATCGGAAGGAACACCAGTGGCGAAGGCGGCTACCTGGCCAGATACTGACACTGAGGCACGAAAGCGTGGGGAGCAAACAGG` |
| ASV2746 | 404 | `TGGGGAATATTGGACAATGGGCGAAAGCCTGATCCAGCAATACCGCGTGAATGATGAAGGCCTTAGGGTTGTAAAGTTCTTTTAGTAGGGAAGATAATGACGGTACCTACAGAAAAAGCCCCGGCTAACTCCGTGCCAGCAGCCGCGGTAAGACGGAGGGGGCTAGCGTTGTTCGGAATTACTGGGCGTAAAGGGCGCGTAGGCGGATTAGTAAGTTGGAAGTGAAAGCCCGGGGCTTAACCTCGGAACTGCTTTCAAAACTGCTAGTCTAGAGTGAAGTAGAGGATGATGGAATTCCTAGTGTAGAGGTGAAATTCTTAGATATTAGGAGGAACACCGGTGGCGAAGGCGGTCATCTGGACTTCAACTGACGCTGAGGCGCGAAAGCGTGGGGAGCAAACAGG` |
| ASV87 | 429 | `TGGGGAATATTGGACAATGGGCGAAAGCCTGATCCAGCCATGCCGCGTGTGTGAAGAAGGTCTTAGGATCGTAAAGCACTTTAAGTTGGGAGGAAGGGTTGTAGATTAATACTCTGCAATTTTGACGTTACCGACAGAATAAGCACCGGCTAACTCTGTGCCAGCAGCCGCGGTAATACAGAGGGTGCAAGCGTTAATCGGAATTACTGGGCGTAAAGCGCGCGTAGGTGGTTCGTTAAGTTGGATGTGAAATCCCCGGGCTCAACCTGGGAACTGCATTCAAAACTGTCGAGCTAGAGTATGGTAGAGGGTGGTGGAATTTCCTGTGTAGCGGTGAAATGCGTAGATATAGGAAGGAACACCAGTGGCGAAGGCGACCACCTGGACTGATACTGACACTGAGGTGCGAAAGCGTGGGGAGCAAACAGG` |
| ASV2585 | 404 | `TGGGGAATATTGGGCAATGGGCGCAAGCCTGACCCAGCAATGCCGCGTGGGTGAAGAAGGTCTTCGGATTGTAAAGCCCTTTCGGCGGGGACGATGGTGACGGTACCCGCAGAAGAAGCCCCGGCCAACTTCGTGCCAGCAGCCGCGGTAATACGAAGGGGGCTAGCGTTGCTCGGAATCACTGGGCGTAAAGGGCGCGTAGGCGGCGCACCCAGTCGGGCGTGAAATCCCCGGGCTCAACCCGGGGGCTGCGCCCGAGACGGGCGTGCTGGAGGGCGGGAGAGGCTCGCGGAATTCCCAGTGTAGAGGTGAAATTCGTAGATATTGGGAAGAACACCGGTGGCGAAGGCGGCGAGCTGGCCCGTTTCTGACGCTGAGGCGCGACAGCGTGGGGAGCAAACAGG` |
| ASV1066 | 404 | `TGGGGAATATTGGGCAATGGACGAAAGTCTGACCCAGCCATGCCGCGTGAATGATGAAGGCTTTAGGGTTGTAAAGTTCTTTCACCGATGAAGATAATGACGGTAGTCGGAGAAGAAGCCCCGGCTAACTTCGTGCCAGCAGCCGCGGTAATACGAAGGGGGCTAGCGTTGTTCGGAATTACTGGGCGTAAAGCGAGCGTAGGTTGCTCGATAAGTCAGTGGTGAAAGCCCAGAGCTCAACTCTGGAACTGCCATTGAAACTGTCGAGCTGGAATCTCAGAGGGGGTAGCGGAATTCCAAATGTAGGGGTGAAATCCGTAGATATTTGGAGGAACACCGGTGGCGAAGGCGGCTACCTGGATGAGTATTGACACTGAGGCTCGAAAGCGTGGGGATCAAACAGG` |
| ASV2683 | 423 | `TAGGGAATATTGGTCAATGGGCGGAAGCCTGAACCAGCCATGCCGCGTGCAGGATGACGGCCTTCTGGGTTGTAAACTGCTTTTATCAGGGACGAAAACACCCATGCGTGGGGCATTGACGGTACCTGATGAATAAGCACCGGCTAACTCCGTGCCAGCAGCCGCGGTAATACGGAGGGTGCAAGCGTTGTCCGGATTTATTGGGTTTAAAGGGTGCGTAGGCGGCTTGTTAAGTCAGTGGTGAAAGCCGACAGCTCAACTGTCGAACTGCCATAGATACTGACAGGCTTGAGTGCAAACGAGGTATGCGGAATTTATGGTGTAGCGGTGAAATGCATAGATACCATAAAGAACACCGATAGCGAAGGCAGCATACTAGGTTGTAACTGACGCTGAGGCACGAAAGCATGGGTAGCAAACAGG` |
| ASV3281 | 408 | `TGAGGAATCTTCCGCAATGGGCGAAAGCCTGACGGAGCGACACCGCGTGAAGGATGAAGCCCTTTCCGGGTGTAAACTTCTGTTCTGAGGGACGAAATTTTTGACGGTACCTCAGGAGAAAGCATCGGCTAATTCTGTGCCAGCAGCCGCGGTAAGACAGAAGATGCAAGCGTTACTCGGAATTACTGGGCGTAAAGGGTCCGCAGGTGACTCACCACGTCTGGGGTAAAAGCACGTCGCTCAACGGCGTGTATGTCCCGGAAACGAGTGGGATTGAGCCATTCAGAGGCATCTGGAATGTCGTGTGTAGGGGTAAAATCCGTTGATCCACGATGGAACGCCAAAAGCGAAAGCAGGATGCTGGGGATGTGCTGACACTCATGGACGAAAGCGTGGGGAGCAAACGGG` |
| ASV2201 | 404 | `TGGGGAATATTGGACAATGGGGGCAACCCTGATCCAGCAATGCCGCGTGAGTGAAGAAGGCCTTCGGGTTGTAAAGCTCTTTTGTCCGTGACGATAATGACGGTAGCGGACGAATAAGCACCGGCTAACTTCGTGCCAGCAGCCGCGGTAATACGAAGGGTGCAAGCGTTGTTCGGAATGACTGGGCGTAAAGGGCGTGTAGGCGGCTTTGTGCGTCAGATGTGAAAGTCCTGGGCTTAACCTAGGGATTGCATTTGATACGGCAGGGCTAGAGGTAATGAGGGGAGAATGGAATTGCGCATGTAGAAGTGAAATTCGTAGAGATGCGCAGGAACACCAGTGGCGAAGGCGATTCTCTGGCACTTACCTGACGCTAAGGCGCGAAAGCGTGGGGAGCAAACAGG` |
| ASV3557 | 404 | `TGGGGAATATTGGACAATGGGCGCAAGCCTGATCCAGCCATGCCGCGTGTGTGATGAAGGCCTTAGGGTTGTAAAGCACTTTCACCGGAGAAGATAATGACGGTATCCGGAGAAGAAGCCCCGGCTAACTTCGTGCCAGCAGCCGCGGTAATACGAAGGGGGCTAGCGTTGTTCGGAATTACTGGGCGTAAAGCGCACGTAGGCGGATCGATCAGTCAGGGGTGAAATCCCGCAGCTCAACTGCGGAACTGCCTTTGATACTGTCGATCTAGAGTATGGAAGAGGTGAGTGGAATTCCGAGTGTAGAGGTGAAATTCGTAGATATTCGGAGGAACACCAGTGGCGAAGGCGGCTCACTGGTCCATTACTGACGCTGAGGTGCGAAAGCGTGGGGAGCAAACAGG` |
| ASV3858 | 429 | `TGGGGAATATTGGACAATGGGGGCAACCCTGATCCAGCCATGCCGCGTGTGTGAAGAAGGCCTTCGGGTTGTAAAGCACTTTCAGTGGGGAGGAAAGGTTGCAGGTTAATACTCTGTAGCTGTGACGTTACCCACAGAAGAAGCACCGGCTAACTCCGTGCCAGCAGCCGCGGTAATACGGGGGGTGCGAGCGTTAATCGGATTTACTGGGCGTAAAGCGCGCGTAGGCGGCTTGTTAAGTCGGATGTGAAAGCCCTGGGCTCAACCTAGGAACTGCATACGATACTGGCGAGCTAGAGTACGATAGAGGGTGGTAGAATTCCTAGTGTAGCGGTGAAATGCGTAGATATTAGGAGGAATACCGGTGGCGAAGGCGGCCACCTGGATCGATACTGACGCTGAAGTGCGAAAGCGTGGGGAGCAAACAGG` |
| ASV3965 | 404 | `TGGGGAATATTGGACAATGGGCGCAAGCCTGATCCAGCAATGCCGCGTGAATGATGAAGGCCTTAGGGTTGTAAAGTTCTTTCAGTAGGGAAGATAATGACGGTACCTACAGAAGAAGCTCCGGCTAACTCCGTGCCAGCAGCCGCGGTAATACGGAGGGAGCTAGCGTTGTTCGGAATTACTGGGCGTAAAGAGCGCGTAGGCGGTCTGTAAAGTTGGGAGTGAAAGCCCAAGGCTTAACCTTGGAACTGCTCTCAAAACTCACGGACTAGAGATTGGTAGAGGATGGCGGAATTCCTAGTGTAGAGGTGAAATTCGTAGATATTAGGAGGAACACCGGTGGCGAAGGCGGCCATCTGGGCCAATACTGACGCTGAGGCGCGAAAGCGTGGGGAGCAAACAGG` |
| ASV224 | 404 | `TGGGGAATATTGGACAATGGGCGAAAGCCTGATCCAGCCATGCCGCGTGAGTGATGAAGGCCTTAGGGTTGTAAAGCTCTTTTATCCGGGAAGATAATGACTGTACCGGAAGAATAAGCCCCGGCTAACTTCGTGCCAGCAGCCGCGGTAATACGAAGGGGGCTAGCGTTGCTCGGAATCACTGGGCGTAAAGGGCGCGTAGGCGGATGATTAAGTCGGAGGTGAAAGCCCAGGGCTCAACCCTGGAATTGCCTTCGATACTGATCGTCTCGAGAATGGAAGAGGTAAGTGGAATACCGAGTGTAGAGGTGAAATTCGTAGATATTCGGTGGAACACCAGTGGCGAAGGCGACTTACTGGTCCATTTCTGACGCTGAGGCGCGAAAGCGTGGGGATCAAACAGG` |
| ASV3641 | 429 | `TGGGGAATTTTGGACAATGGGGGCAACCCTGATCCAGCAATGCCGCGTGCAGGAAGAAGGCCTTCGGGTTGTAAACTGCTTTTGTCAGGGAAGAAATCCCTTGGGTTAATACCTCGGGGGGATGACGGTACCTGAAGAATAAGCACCGGCTAACTACGTGCCAGCAGCCGCGGTAATACGTAGGGTGCGAGCGTTAATCGGAATTACTGGGCGTAAAGCGTGCGCAGGCGGCTTTGCAAGACAGGTGTGAAATCCCCGGGCTTAACCTGGGAACTGCACTTGTGACTGCATCGCTAGAGTACGGTAGAGGGGGATGGAATTCCGCGTGTAGCAGTGAAATGCGTAGATATGCGGAGGAACACCGATGGCGAAGGCAATCCCCTGGACCTGTACTGACGCTCATGCACGAAAGCGTGGGGAGCAAACAGG` |
| ASV382 | 404 | `TGGGGAATATTGCACAATGGGCGAAAGCCTGATGCAGCGACGCCGCGTGAGTGAAGAAGTATTTCGGTATGTAAAGCTCTATCAGCAGGGAAGAAAATGACGGTACCTGACTAAGAAGCCCCGGCTAACTACGTGCCAGCAGCCGCGGTAATACGTAGGGGGCAAGCGTTATCCGGATTTACTGGGTGTAAAGGGAGCGTAGACGGTACCATAAGTCTGAAGTGAAAGCCCGCGGCTCAACCGCGGGACTGCTTTGGAAACTATGGAACTAGAGTGTTGGAGAGGTAAGTGGAATTCCTAGTGTAGCGGTGAAATGCGTAGATATTAGGAGGAACACCAGTGGCGAAGGCGACTTACTGGACAATAACTGACGTTGAGGCTCGAAAGCGTGGGGAGCAAACAGG` |
| ASV2007 | 404 | `TGGGGAATATTGGACAATGGGCGAAAGCCTGATCCAGCAATGCCGCGTGAGTGATGAAGGCCTTAGGGTTGTAAAGCTCTTTTACCAGGGATGATAATGACAGTACCTGGAGAATAAGCTCCGGCTAACTTCGTGCCAGCAGCCGCGGTAATACGAGGGGAGCTAGCGTTGTTCGGAATTACTGGGCGTAAAGCGTGCGTAGGCGGTTACTCAAGTCAGAGGTGAAAGCCCGGGGCTCAACCCCGGAACGGCCTTTGAAACTAGGTAACTAGAATCTTGGAGAGGTTAGTGGAATTCCGAGTGTAGAGGTGAAATTCGTAGATATTCGGAAGAACACCAGTGGCGAAGGCGACTAACTGGACAAGCATTGACGCTGAGGCACGAAAGCGTGGGGAGCAAACAGG` |
| ASV1732 | 430 | `TAGGGAATATTGCGCAATGGGGGAAACCCTGACGCAGCAACGCCGCGTGAGTGAGGAAGGTCTTCGGATTGTAAAACTCTTTTCTTAGAGAAAAATGTCCTTGTGGCTAATATCCACGAGGAGTGATGGTATCTAAGGAATAAGCACCGGCTAACTTCGTGCCAGCAGCCGCGGTAATACGAAGGGTGCAAGCGTTGTTCGGATTTATTGGGCGTAAAGCGCGCGCAGGCGGATCAGCAAGTCAGATGTGAAATCTCGGAGCTCAACTCCGAAACTGCGTCTGAAACTACTAGTCTAGAATGTCGGAGGGGGCAGGGGAATTTCACGTGTAGGGGTAAAATCCGTAGAGATGTGAAGGAACACCAGGGGCGAAGGCGCCTGCCTGGACGACTATTGACGCTGAGGCGCGAAAGCGTGGGGAGCAAACAGG` |
| ASV2238 | 420 | `TAGGGAATTTTGCGCAATGGGGGAAACCCTGACGCAGCAACGCCATGTGTGGGATGAAGCATTTCGGTGCGTAAACCACTGTCAGCAGGGAATAAAAGGTCCTCACGGACATTTGAATGTACCTGCAAAGGAAGCCCCGGCTAACTTCGTGCCAGCAGCCGCGGTAATACGAGGGGGGCAAGTGTTGTTCGGAATCACTGGGCGTAAAGGGAGCGTAGGCGGGCTTTTAAGTTAGGTGTTTAATCCCGGGGCCCAACCCCGGACCGGCACCTAAAACTGGGAGTCTTGAATAGTGGAGAGGTCAGTGGAATTGGGGGTGTAGCGGTGGAATGCGTAGAGATCCCCAAGAACACCAGTTGCGAAGGCGGCTGACTGGACACATATTGACGCTGAGGCTCGAAAGCATGGGGAGCAAACAGG` |
| ASV5306 | 430 | `TGGGGGATATTGCGCAATGGGCGAAAGCCTGACGCAGCGACGCCGCGTGGGTGATGAAGGCCTTCGGGTCGTAAAGCCCTGTCGAGGGAGACGAACCGGTCGAGGGCGAATAGGCCTCGGCCCTGACGGTACCCCTAAAGGAAGCACCGGCTAACTCCGTGCCAGCAGCCGCGGTAATACGGAGGGTGCGAGCGTTGTTCGGAATCACTGGGCGTAAAGCGCGTGTAGGCGGTCTCGTAAGTCTGGTGTGAAAGCCCGGGGCTCACCCCCGGAAGTGCACTGGAAACTGCAAGACTCGAGTCTCGGAGAGGAGGGTGGAATTCCTGGTGTAGCGGTGAAATGCGTAGATATCAGGAGGAACACCGGTGGCGAAGGCGGCCCTCTGGACGATGACTGACGCTGAGACGCGAAAGCGTGGGGAGCAAACAGG` |
| ASV4354 | 407 | `CAGGGAATCTTGCGCAATGGGCCGAAAGGCCTGACGCAGCGACACCGCGTGGGGGAAGAAGGCCTTCGGGTTGTAAACCCCTTTTCCCGGGGAAGAGAGAGGACGGTACCCGGGGAATAAGCCTCGGCTAACTACGTGCCAGCAGCCGCGGTAATACGTAGGAGGCGAGCGTTGTCCGGATTTACTGGGCGTAAAGGGCGAGTAGGCGGCCTGTTAAGTCCCGTGTGAAATCTCCCGGCCTAACTGGGAGGGGTCATGGGATACTGGCGGGCTTGAGGGCAGCAGAGGGAAGCGGAATTCCCGGAGTAGTGGTGAAATGCTTAGATACCGGGAGGAACACCAGCGGCGAAAGCGGCTTCCTGGGCTGACCCTGACGCTGAGGCGCGAAAGCATGGGGAGCAAACCGG` |
| ASV646 | 407 | `TGGGGAATTTTCCGCAATGGGCGCAAGCCTGACGGAGCAATACCGCGTGAGGGAGGAAGGCTCTTGGGTCGTAAACCTCTTTTCTCAGGGAAGAACAAAATGACGGTACCTGAGGAATCAGCATCGGCTAACTCCGTGCCAGCAGCCGCGGTAATACGGAGGATGCAAGCGTTATCCGGAATGATTGGGCGTAAAGCGTCCGTAGGTGGTTTTGTAAGTCCGTGGTTAAAGCGCGAAGCTTAACTTCGTAAGGGCCATGGAAACTACAAGACTTGAGTAAAGTAGGGGTAGAGGGAATTCCCAGTGTAGCGGTGAAATGCGTAGAGATTGGGAAGAACACCGGTGGCGAAAGCGCTCTACTGGACTTAAACTGACACTGAGGGACGAAAGCTAGGGGAGCGAAAGGG` |
| ASV1769 | 429 | `TGGGGAATTTTGGACAATGGGCGAAAGCCTGATCCAGCCATGCCGCGTGTCTGAAGAAGGCCTTCGGGTTGTAAAGGACTTTTGTCGGGGAGGAAATCCCCAGCGCTAATACCGCTGGGGGATGACAGTACCTGAAGAATAAGCACCGGCTAACTACGTGCCAGCAGCCGCGGTAATACGTAGGGTGCAAGCGTTAATCGGAATTACTGGGCGTAAAGCGTGCGCAGGCGGTTTGATAAGCCAGATGTGAAATCCCCGAGCTCAACTTGGGAACTGCGTTTGGAACTGTCAGACTAGAGTGCGTCAGAGGGGGGTGGAATTCCGCGTGTAGCAGTGAAATGCGTAGAGATGCGGAGGAACACCGATGGCGAAGGCAGCCCCCTGGGATGACACTGACGCTCATGCACGAAAGCGTGGGGAGCAAACAGG` |
| ASV1691 | 429 | `TGGGGAATATTGCACAATGGGCGCAAGCCTGATGCAGCCATGCCGCGTGTGTGAAGAAGGCCTTCGGGTTGTAAAGCACTTTCAGCGAGGAGGAAGGGTGGTGTGTTAATAGCGCATCACATTGACGTTACTCGCAGAAGAAGCACCGGCTAACTCCGTGCCAGCAGCCGCGGTAATACGGAGGGTGCAAGCGTTAATCGGAATTACTGGGCGTAAAGCGCACGCAGGCGGTTTGTTAAGTCAGATGTGAAATCCCCGCGCTTAACGTGGGAACTGCATTTGAAACTGGCAAGCTAGAGTCTTGTAGAGGGGGGTAGAATTCCAGGTGTAGCGGTGAAATGCGTAGAGATCTGGAGGAATACCGGTGGCGAAGGCGGCCCCCTGGACAAAGACTGACGCTCAGGTGCGAAAGCGTGGGGAGCAAACAGG` |
| ASV2618 | 404 | `TGGGGAATCTTGCACAATGGAGGAAACTCTGATGCAGCGACGCCGCGTGGACGACGAAGCCTTTCGGGGTGTAAAGTCCTTTCGGCAGGAACGATAATGACGGTACCTGCAGAAGAAGCTGCGGCTAACTACGTGCCAGCAGCCGCGGTAATACGTAGGCAGCAAGCGTTGTTCGGAATTACTGGGCGTAAAGAGTTCGTAGGCGGTTCGCTAAGTTTGGTGTGAAATCTCCCGGCTTAACCGGGAGGGTGCGCCGAAAACTGGCGGACTAGAGTGTCGGATAGGAAAGCGGAATTCCTGGTGTAGCGGTGAAATGCGTAGATATCAGGAGGAACACCTGTGGTGTAGACGGCTTTCTGGACGATAACTGACGCTGAGGAACGAAAGCGTGGGTAGCAAACAGG` |
| ASV3075 | 424 | `TTTCGAATTATTCACAATGGGCGAAAGCCTGATGATGCAACGCCGCGTGGGGGATGAAGGTCTTCGGATTGTAAACCCCTGTCATTAAGGAACAAAAAATAGGCTCATACCCTATATTGAGTTAACTTAAAAAGGAAGGAGTGGCAAACTCTGTGCCAGCAGCCGCGGTAATACAGAGACTCCAAGCGTTACTCGGATTCACTGGGCGTAAAGGGTGCGTAGGTGGCTAAATGTGTCAGGTGTGAAATCTCGGGGCTTAACTCCGAAACTGCGCCTGAAACTGTTTAGCTTGAGGCCTGGAGGGGTAAGCGGAATTCCTAGTGTAGCAGTGAAATGCGTAGATATTAGGAGGAACACCAAAGGCGAAGGCAGCTTACTGGACAGGTACTGACACTGAGGCACGAAAGCGTGGGGAGCAAAAAGG` |
| ASV1729 | 419 | `TAAGGAATATTGCGCAATGGGCGACAGCCTGACGCAGCGACGCCGCGTGGGGGATGAAGGTCTTCGGATTGTAAACCCCTTTCGGCAGGGAAGATGGAACGGGTAACCGTTCGGACGGTACCTGCAGAAGCAGCCACGGCTAACTTCGTGCCAGCAGCCGCGGTAATACGAAGGTGGCAAGCGTTGTTCGGATTTACTGGGCGTACAGGGAGCGTAGGCGGTTGGGTAAGCCCTTCGTGAAATCTCCGGGCCTAACCCGGAAAGTGCGGAGGGGACTGCTCGGCTAGAGGATGGGAGAGGAGCGCGGAATTCCCGGTGTAGCGGTGAAATGCGTAGAGATCGGGAGGAAGGCCGGTGGCGAAGGCGGCGCTCTGGAACATTTCTGACGCTGAGGCTCGAAAGCGTGGGGAGCAAACAGG` |
| ASV2056 | 430 | `TGGGGAATCTTGCACAATGGGCGAAAGCCTGATGCAGCAACGCCATGTGAGTGAAGAAGGCCCTTGGGTTGTAAAGCTCTTTCGGTTAGGAAGAAGGGTAGAGTAGTTAATACCTATTTTATCTGACGGTACTAGACTAAGAAGCACTGGCTAATTCCGTGCCAGCAGCCGCGGTAATACGGAAGGTGCAAGCATTGTTCGGATTGACTGGGCGTAAAGAGCGCGTAGGTGGGTTTGTATGTCGGATGTGAAATCCCTAGGCTTAACCTGGGAACTGCATCCGAAACGGCAGATCTTGAATGTTTGAGAGGTTGGCGGAATTCCAGGTGTAGGAGTGAAATCCGTAGATATCTGGAGGAACACCGGAGGCGAAGGCGGCTGACTAACAAAACATTGACACTGAGGTGCGAAAGCGTGGGTAGCAAACAGG` |
| ASV2011 | 430 | `TGGGGAATTGTTCACAATGGGCGCAAGCCTGATGACGCAACGCCGCGTGGGGGATGAAGGTCTTCGGATCGTAAACCCCTGTCGAATGGGACGAATAGCTTCCGGATTAATATCCCGGAGGAATGACGGTACCGTTAAAGGAAGCCACGGCTAACTCTGTGCCAGCAGCCGCGGTAATACAGAGGTGGCAAGCGTTGTTCGGAATTACTGGGCGTAAAGGGCGCGTAGGCGGCCATCTAAGTCAGACGTGAAATCCCCCGGCTCAACCTGGGAACTGCGTCTGATACTGGAGGGCTTGAGTTTGGGAGAGGGATGTAGAATTCCAGGTGTAGCGGTGAAATGCGTAGATATCTGGAGGAATACCGGTGGCGAAGGCGGCATCCTGGACCAATACTGACGCTGAGGCGCGAAAGCTAGGGGAGCAAACGGG` |
| ASV1794 | 404 | `TGGGGAATATTGGACAATGGGGGAAACCCTGATCCAGCAATGCCGCGTGAATGATGAAGGCCCTAGGGTTGTAAAGTTCTTTCGTTGGGGAAGATAATGACTGTACCCAAAGAAGAAGCCCCGGCTAACTTCGTGCCAGCAGCCGCGGTAATACGAAGGGGGCTAGCGTTGTTCGGAATCACTGGGCGTAAAGGGCGCGTAGGCGGCTATTTAAGTTAGGCGTGAAATCCCGGGGCTTAACCTCGGAATTGCGTTTAATACTGGATAGCTTGAGTTTGAGAGAGGATAGTGGAATTCCTAGTGTAGAGGTGAAATTCGTAGATATTAGGAAGAACACCAGTGGCGAAGGCGACTATCTGGCTCAATACTGACGCTGAGGCGCGAAAGCGTGGGGAGCAAACAGG` |
| ASV583 | 424 | `TGAGGAATATTGGACAATGGGCGCAAGCCTGATCCAGCCATGCCGCGTGCAGGATGACGGTCCTATGGATTGTAAACTGCTTTTGTACAGGAAGAAACACCTCTACGTGTAGAGATTTGACGGTACTGTAAGAATAAGGATCGGCTAACTCCGTGCCAGCAGCCGCGGTAATACGGAGGATCCAAGCGTTATCCGGAATCATTGGGTTTAAAGGGTCCGTAGGCGGTTTAGTAAGTCAGTGGTGAAAGCCCATCGCTCAACGGTGGAACGGCCATTGATACTGCTAGACTTGAATTATTAGGAAGTAACTAGAATATGTAGTGTAGCGGTGAAATGCTTAGAGATTACATGGAATACCAATTGCGAAGGCAGGTTACTACTAATTGATTGACGCTGATGGACGAAAGCGTGGGTAGCGAACAGG` |
| ASV79 | 404 | `TGGGGAATCTTAGACAATGGGGGAAACCCTGATCTAGCCATGCCGCGTGAGCGATGAAGGCCTTAGGGTTGTAAAGCTCTTTCAGTGGGGAAGATAATGACGGTACCCACAGAAGAAGCCCCGGCTAACTCCGTGCCAGCAGCCGCGGTAATACGGAGGGGGCTAGCGTTGTTCGGAATTACTGGGCGTAAAGCGCACGTAGGCGGACGAGAAAGTCAGAGGTGAAATCCCAGGGCTCAACCCTGGAACTGCCTTTGAAACTCCTTGTCTTGAGGTCGAGAGAGGTGAGTGGAATTCCGAGTGTAGAGGTGAAATTCGTAGATATTCGGAGGAACACCAGTGGCGAAGGCGGCTCACTGGCTCGATACTGACGCTGAGGTGCGAAAGCGTGGGGAGCAAACAGG` |
| ASV144 | 404 | `TTAGGAATATTGGACAATGGGCGAAAGCCTGATCCAGCCATGCCGCGTGTGTGATGAAGGCCTTAGGGTTGTAAAGCACTTTCGTTGGGGATGATAATGACCGTACCCAAAGAAGAAGCACCGGCTAACTTCGTGCCAGCAGCCGCGGTAATACGAAGGGTGCAAGCGTTGTTCGGAATCACTGGGCGTAAAGAATGCGTAGGCGGCTTAATAAGTCGGAAGTGAAATCCCAGGGCTTAACCCTGGAACTGCTTTCGAAACTATTTTGCTAGAGTCCCGGAGGGGTTAGCGGAATTCCGAGTGTAGAGGTGAAATTCGCAGATATTCGGAGGAACACCAGTGGCGAAGGCGGCTAACTGGACGGGAACTGACGCTGAGGCATGAAAGTGTGGGGATCAAACAGG` |
| ASV5728 | 404 | `TGAGGAATCTTCCACAATGGGCGAAAGCCTGATGGAGCGACACCGCGTGAAGGATGAAGCCTTTATTGGTGTAAACTTCTTTTCTGTGGGAAGATAATGACGGTACCACAGGAATAAGGGGCGGCAAACTATGTGCCAGCAGCCGCGGTAATACATAGGCCCCAAGCGTTATCCGGAATTACTGGGCGTAAAGAGTTCGTAGGCGGAGAAACAAGTCAGATGTGAAAGATACGGGCTCAACCCGTTATTAGCATCCGAAACTGTTTCCCTAGAATTAGGGAGAGGTAAGCAGAACTGTATGAGTAGGGGTGCAATCCGTTGATACATACAAGAATACCAAAAGCGAAGGCAGCTTACTGGAACTATATTGACGCTGAGGAACGAAAGCGTGGGGAGCGAAAAGG` |
| ASV5724 | 428 | `TGGGGAATCTTGCGCAATGGACGAAAGTCTGACGCAGCGACGCCGCGTGAGCGATGAAGGCCTTCGGGTTGTAAAGCTCTGTGGGGGGAGACGAATAAGGTGCAGCTAATACCTGCATCGATGACGGTATCCCCTTAGCAAGCACCGGCTAACTCTGTGCCAGCAGCCGCGGTAAGACAGAGGGTGCAAACGTTGTTCGGAATTACTGGGCGTAAAGCGCGTGTAGGCGGCGGCGTGTGTCGGGCGTGAAATCCCATGGCTCAACCATGGAAGTGCACCCGAAACTGCGTTGCTAGAGTCCTGGAGAGGAAGGTGGAATACTTGGTGTAGAGGTGAAATTCGTAGATATCAAGTGGAACACCGGTGGCGAAGGCGGCCTTCTGGACAGTGACTGACGCTGAGACGCGAAAGCGTGGGGAGCAAACAGG` |
| ASV5167 | 407 | `TGGGGAATATTGGGCAATGGGCGCAAGCCTGACCCAGCAACGCCGCGTGAGGGAAGAAGGCTTTCGGGTTGTAAACCTCTGTCCTTGGTGACGAAAAAAATGACGGTAGCCAAGGAGGAAGCCCCGGCTAACTACGTGCCAGCAGCCGCGGTAATACGTAGGGGGCGAGCGTTGTCCGGAATTACTGGGCGTAAAGGGTGCGTAGGTGGCGCCTTAAGTTGGATGTGAAATACCCGAGCTTAACTTGGGAACTGCATCCAAAACTGAGGTGCTAGAGTGCAGGAGAGGGAAGCGGAATTCCTAGTGTAGCGGTGAAATGCGTAGATATTAGGAGGAACACCAGTGGCGAAGGCGGCTTTCTGGACTGTAACTGACACTGAGGCACGAAAGCGTGGGGAGCAAACAGG` |
| ASV2000 | 424 | `TGAGGAATATTGGACAATGGGCGCAAGCCTGATCCAGCCATGCCGCGTGCAGGAAGAATGTCCTATGGATTGTAAACTGCTTTTGTACAGGAAGAAATACTCCGACGAGTCGGAATTTGACGGTACTGTAAGAATAAGGATCGGCTAACTCCGTGCCAGCAGCCGCGGTAATACGGAGGATCCAAGCGTTATCCGGAATCATTGGGTTTAAAGGGTCTGTAGGCGGTTTAATAAGTCAGTGGTGAAAGCCCATCGCTCAACGATGGAACGGCCATTGATACTGTTGAACTTGAATTATTAGGAAGTAACTAGAATATGTAGTGTAGCGGTGAAATGCTTAGATATTACATGGAATACCAATTGCGAAGGCAGGTTACTACTAATGGATTGACGCTGATGGACGAAAGCGTGGGGAGCGAACAGG` |
| ASV2252 | 421 | `TGGGGAATATTGCGCAATGGACGAAAGTCTGACGCAGCGACGCCGCGTGTGGGATGAAGCCTTTCGGGGCGTAAACCACTGTTGCCCGGGACGAACATCCTCTTTCGAGAGGACTGACGGTACCGGGTGAGGAAGCACCGGCTAACTCTGTGCCAGCAGCCGCGGTAATACAGAGGGTGCGAGCGTTGTCCGGAATCACTGGGCGTAAAGGGCGCGTAGGTGGCTTGGTAAGCGTGTGGTGAAAGTCCGGGGCTCAACCCCGGATCTGCCGTGCGAACTGCTGAGCTCGAGCAATGTAGAGGCAGGTGGAATTTCGGGTGTAGCGGTGGAATGCGTAGATATCCGAAAGAACACCAGTGGCGAAGGCGGCCTGCTGGGCATTAGCTGACACTGAGGCGCGACAGCGTGGGGAGCAAACAGG` |
| ASV3162 | 386 | `GCGAGAAACCTTAGCAATGTGCGAAAGCACGACTAGGTTAATCCGAGTGATTTCTGCTGAAGAAATCTTTTGTCAGTTCTAAAAATACCGATGAATAAGGGGTGGGCAAGTCTGGTGTCAGCCGCCGCGGTAATACCAGCACCTCGAGTGGTCAGGATGTTTATTGGGCCTAAAGCATCCGTAGCCGGCTTTACAAGTCTCCGGTTAAATCCAGCTGCCTAACAGATGGGCTGCCGGAGATACTACAAAGCTAGGGAGCGGGAGAGGCAGACGGTATTCGGTGGGTAGGGGTAAAATCCTCTGATCCATTGAGGACCACCAGTGGCGAAGGCGGTCTGCCAGAACGCGTTCGACGGTGAGGGATGAAAGCTGGGGGAGCAAACCGG` |
| ASV2833 | 424 | `TGAGGAATATTGGACAATGGGCGCAAGCCTGATCCAGCCATGCCGCGTGCAGGATGACGGTCCTATGGATTGTAAACTGCTTTTATACGAGAAGAAACACTCCTTCGTGAAGGAATTTGACGGTATCGTAAGAATAAGGATCGGCTAACTCCGTGCCAGCAGCCGCGGTAATACGGAGGATCCAAGCGTTATCCGGAATCATTGGGTTTAAAGGGTCCGTAGGCGGTTTAGTAAGTCAGTGGTGAAAGCCCATCGCTCAACGGTGGAACGGCCATTGATACTGCTAGACTTGAATTATTAGGAAGTAACTAGAATATGTAGTGTAGCGGTGAAATGCTTAGAGATTACATGGAATACCAATTGCGAAGGCAGGTTACTACTAATGGATTGACGCTGATGGACGAAAGCGTGGGTAGCGAACAGG` |
| ASV4862 | 407 | `TGAGGAATTTTCCGCAATGGGCGAAAGCCTGACGGAGCAATCCCGCGTGGAGGATGACACCTTTCGGGGCGTAAACTCCTTTTCTGAGGGAAGAAATTTTGACGGTACCTCAGGAATAAGCACCGGCTAACTACGTGCCAGCAGCCGCGGTAATACGTAGGGTGCGAGCGTTATCCGAAATTATTGGGCGTAAAGCGTTCGAGGGCGGATATACAGGTCTGGTGTTAAATACCTCGGCTCAACCGAGGTTCCGTACCGGAAACCGTATGTCTGGAGAAAGGCAGAGGCAGGTAGAATTTCCATTGTAGGGGTTAAATCCGTAGATAATGGAAGGAATACCAAAAGCGAAGGCAGCCTGCTGGGCCTTTTCTGACGCTGCACGAACGAAAGCGTGGGGAGCGAAAGGG` |
| ASV2308 | 407 | `TGGGGAATTTTCCGCAATGGGCGAAAGCCTGACGGAGCAATACCGCGTGAGGGAGGAAGGCTCTTGGGTCGTAAACCTCTTTTCTCAGGGAAGAACAAAATGACGGTACCTGAGGAATAAGCATCGGCTAACTCCGTGCCAGCAGCCGCGGTAATACGGAGGATGCAAGCGTTATCCGGAATGATTGGGCGTAAAGCGTCCGCAGGTGGCAATGTAAGTCTGCTGTCAAAGGTCAGGGCTTAACTCTGAACAGGCAGTGGAAACTACACAGCTAGAGTGCGGTGGGGGCAGAGGGAATTCCCAGTGTAGCGGTGAAATGCGTAGAGATTGGGAAGAACACCGGTGGCGAAAGCGCTCTGCTAGGCCGCAACTGACACTCATGGACGAAAGCTAGGGGAGCGAATGGG` |
| ASV1298 | 406 | `TAGGGAATATTGCGCAATGGGGGAAACCCTGACGCAGCGACGCCGCGTGAGTGATGAAGGCCTTCGGGTCGTAAAGCTCTGTTGCAAGGGAAAAATTTTTGATTGTACCTTGCGAGGAAGGATCGGCTAACTTCGTGCCAGCAGCCGCGGTAAGACGAGGGATCCTAGCGTTGTTCGGAATCATTGGGCGTAAAGCGGGTGCAGGTGGCTTTGTAAGTCAGGTGTGAAAGCCCAGGGCTCAACCCTGGAAGTGCATTTGATACTGCGAAGCTTGAGTGCTGGAGAGGCTACTAGAATACCTGGTGTAGTGGTGAAATACGTAGATATCAGGTGGAATACCGGAGGCGAAGGCGGGTAGCCAGCCAGACACTGACACTCAGACCCGAAAGCGTGGGGATCAAACAGG` |
| ASV4873 | 429 | `TGGGGAATTTTGCGCAATGGGCGAAAGCCTGACGCAGCGACGCCGCGTGTGCGATGAAGGCCTTTGGGTCGTAAAGCACTGTCGACAGGGAAGAAAGCTCGTCGGATAACACCCGGCGAGGTTGACGGTACCTGTAAAGGAAGCACCGGCTAACTCTGTGCCAGCAGCCGCGGTAATACAGAGGGTGCAAACGTTGCTCGGAATTACTGGGCGTAAAGCGCGTGTAGGCGGTTCGTTAAGTCGTCTGTGAAAGCCCATGGCTCAACCATGGAAGTGCAGCCGATACTGGCAAACTTGAGTCCTGGAGAGGGTGGCGGAATTCCCAGTGTAGAGGTGAAATTCGTAGATATTGGGAGGAACACCGGTGGCGAAGGCGGCCACCTGGACAGGTACTGACGCTGAGACGCGAAAGCGCGGGTAGCAAACAGG` |
| ASV636 | 424 | `TAAGGAATATTGGGCAATGGAGGCAACTCTGACCCAGCCATGCCGCGTGCAGGATGACTGCCCTATGGGTTGTAAACTGCTTTTGTACGGGAGAAAACCCTCGTTTGCGAACGAGGCTGATAGTACCGTAAGAATAAGCATCGGCTAACTCCGTGCCAGCAGCCGCGGTAATACGGAGGATGCAAGCGTTATCCGGATTCATTGGGTTTAAAGGGTGCGTAGGCGGTCTTATAAGTCAGTGGTGAAAGCCTGCAGCTTAACTGTAGAATTGCCATTGATACTGTAGGACTAGAGTACAGACGACGTTGGCGGAATATGACATGTAGCGGTGAAATGCTTAGATATGTCATAGAACACCGATTGCGAAGGCAGCTGACGAGACTGTAACTGACGCTGAGGCACGAAAGTGTGGGGATCAAACAGG` |
| ASV4097 | 424 | `TGAGGGATATTGGACAATGGAAGGAATTCTGATCCAGCCATGCCGCGTGCAGGACGACCGCCCTACGGGCGATGAACTGCTTTTGCCCCGGAGCAATAAGGGCCACGTGTGGCCCGACGAGAGTACGGGGCGAATAAGCATCGGCTAACTCCGTGCCAGCAGCCGCGGTAATACGGGGGATGCGAGCGTTATCCGGATTCATTGGGTTTAAAGGGTGCGTAGGCTGTTCCGCAAGCCGGTGGTGAAAAGCGCGGGCTCAACCCGTGGAGTGCCTCCGGGACTGCGGTTCTGGGATTCGGCCGCCGTGGGGGGAATGCGTGGTGTAGCGGTGAAATGCATAGATATCACGCAGAACGCCGATTGCGAAGGCACCTCACGAGGCCGCGATTGACGCTGATGCACGAAAGCGCGGGGATCGAACAGG` |
| ASV775 | 428 | `TTTCGAATCATTCACAATGGGCGAAAGCCTGATGGTGCGACGCCGCGTGAGGGATGAAGGTCTTCGGATTGTAAACCTCTGTCACCGGGGACGAAACTCTTTGAGTTAATAGTTCAAAGATTGACTTAACCCGGAGAGGAAGCAGTGGCTAACTCTGTGCCAGCAGCCGCGGTAATACAGAGACTGCAAGCGTTATTCGGATTTACTGGGCGTAAAGGGTGCGCAGGCGGTTATGTGTGTGATGTGTGAAATCCCATGGCTCAACCTTGGAATTGCACTTCAAACTACATAACTAGAGCATTGGAGAGGGTAGCAGAATTCATGGTGTAGCAGTGAAATGCGTAGATATCATGAGGAATACCAGAGGCGAAGGCGGCTACCTAGACAATTGCTGACGCTCAGGCACTAAAGCGTGGGGAGCAAAAGGG` |
| ASV3089 | 429 | `TGGGGAATTTTGGACAATGGGCGAAAGCCTGATCCAGCCATGCCGCGTGAGTGAAGAAGGCCTTCGGGTTGTAAAGCTCTTTCGGACAGAAAGAAATCGGTTGGGCTAATACCCCAACTGGATGACGGTACTGTAAGAAGAAGCACCGGCTAACTACGTGCCAGCAGCCGCGGTAATACGTAGGGTGCGAGCGTTAATCGGAATTACTGGGCGTAAAGCGTGCGCAGGCGGTTACATAAGACAGATGTGAAATCCCCGGGCTTAACCTGGGAACTGCGTTTGTGACTGTGTGACTAGAGTACGGCAGAGGGGGGTGGAATTCCTGGTGTAGCAGTGAAATGCGTAGATATCAGGAGGAACATCGATGGCGAAGGCAGCCCCCTGGGCCTGTACTGACGCTCATGCACGAAAGCGTGGGGAGCAAACAGG` |
| ASV4301 | 404 | `TGAGGAATATTGGACAATGGACGAAAGCCTGATCCAGCCATGCCGCGTGAATGATGAAGGCCTTAGGGTTGTAAAGTTCTTTTAGTAGTGAAGATGATGACGGTAACTACAGAAAAAGCTCCGGCTAACTCCGTGCCAGCAGCCGCGGTAATACGGAGGGAGCGAGCGTTGTTCGGATTTACTGGGCGTAAAGCGTGCGTAGGTGGTTTTTCAAGTCAGAAGTGAAAGCCCAGGGCTTAACCCTGGAACGGCTTTTGAAACTGGATCACTAGAATGTGGGAGAGGTAAGCGGAATTCCGAGTGTAGAGGTGAAATTCGTAGATATTCGGAGGAACACCAATGGCGTAAGCAGCTTACTGGAACACTATTGACACTGAGGCACGAAAGCGTGGGGAGCAAACAGG` |
| ASV3718 | 404 | `TGGGGAATATTGGACAATGGGCGAAAGCCTGATCCAGCCATGCCGCGTGAGTGATGAAGGCCTTAGGGTTGTAAAGCTCTTTTGTCAGGGAAGATAATGACGGTACCTGAAGAATAAGCACCGGCTAACTTCGTGCCAGCAGCCGCGGTAATACGAAGGGTGCTAGCGTTGTTCGGAATCACTGGGCGTAAAGGGCGCGTAGGCGGCTTAGCAAGTCAGAGGTGAAAGCCTGGAGCTCAACTCCAGAATTGCCTTTGAAACTGCAAAGCTTGAGTATTGGAGGGGATAGCGGAATTCCTAATGTAGAGGTGAAATTCGTAGATATTAGGAGGAACACCGGTGGCGAAGGCGGCTATCTGGACAATAACTGACGCTGAGGCGCGAAAGCGTGGGGATCAAACAGG` |
| ASV1648 | 430 | `TGGGGAATATTGGACAATGGGCGGAAGCCTGATCCAGCAATACCGCGTGTGTGAAGAAGGCCTTCGGGTTGTAAAGCACTTTTGTTAGGGAGGATGGTTATTTAGATTAATACCCTAAGTAGCTGACGTTACCTAAAGAATAAGCACCGGCTAACTCCGTGCCAGCAGCCGCGGTAATACGGAGGGTGCAAGCGTTAATCGGATTTACTGGGCGTAAAGGGTGCGTAGGCGGCCTATTAAGTCAGATGTGAAAGCCCTGGGCTCAACCTGGGAATTGCATTTGAAACTGGTAGGCTAGAATGTGGTAGAGGGTAGCGGAATTTCTGGTGTAGCGGTGAAATGCGTAGATATCAGAAGGAACATCAATGGCGAAGGCAGCTACCTGGGCCAACATTGACGCTGAGGCACGAAAGCGTGGGGAGCAAACAGG` |
| ASV2915 | 424 | `TAAGGAATATTGGTCAATGGACGCAAGTCTGAACCAGCCATGCCGCGTGAAGGATGAAGGCCCTCTGGGTTGTAAACTTCTTTTATCTGGGACGAAAAACGGGCTTTCTAGCTCACTTGACGGTACCAGAGGAATAAGCACCGGCTAACTCCGTGCCAGCAGCCGCGGTAATACGGAGGGTGCAAGCGTTATCCGGATTTACTGGGTTTAAAGGGTGCGTAGGTGGGTAGGTAAGTCAGTGGTGAAATCTTCAAGCTTAACTTGGAAACTGCCATTGATACTATTTATCTTGAATACTTTGGAGGTAAGCGGAATATGTCATGTAGCGGTGAAATGCTTAGATATGACATAGAACACCAATTGCGAAGGCAGCTTACTACACAGTTATTGACACTGAGGCACGAAAGCGTGGGGATCAAACAGG` |
| ASV2516 | 428 | `TGGGGAATATTGGACAATGGGGGCAACCCTGATCCAGCAATGCCGCGTGTGTGAAGAAGGCCTGCGGGTTGTAAAGCACTTTCAGAGGGGAAGAAAAAATCGGATCTAACACATCCGAGCTTGACGTTACCCTTAGAAGAAGCACCGGCTAACTCCGTGCCAGCAGCCGCGGTAATACGGAGGGTGCAAGCGTTAATCGGAATTACTGGGCGTAAAGCGCACGCAGGTGGCTGAGCGAGTCGATTGTGAAAGCCCTGGGCTTAACCTGGGAATTGCAGTCGATACTACTCAGCTAGAGTATGGGAGAGGGTAGTGGAATTCCCGGTGTAGCGGTGAAATGCGTAGATATCGGGAGGAACATCAGTGGCGAAGGCGGCTACCTGGCCCAATACTGACACTCAGGTGCGACAGCGTGGGGAGCAAACAGG` |
| ASV5581 | 422 | `TAGGGAATATTGGGCAATGGGTGGAAGCCTGACCCAGCCATACCGCGTGGTGGAAGAAGGTCTTCGGATTGTAAACACCTTTTATAGGAGAAAAAGTCGAGACTGCGGTCTTGGGTGATGGTATCCTGTGAATAAGCACCGGCTAACTACGTGCCAGCAGCCGCGGTAATACGTAGGGTGCAAGCGTTGTCCGGATTTATTGGGTTTAAAGGGTACGTAGGTGGTCTAATAAGTCAGTGGTGAAAGGTAGCCGCTTAACGGTTGTTATGCCATTGATACTGCTAGACTTGAGTGCGACAGGGGTTACTGGAATTTGTGGTGTAGCGGTGAAATGCATAGATACCACAAGGAACACCAAAAGCGAAGGCAGGTAACTGGGTCGCAACTGACACTGAGGTACGACAGTGCGGGTATCAAACAGG` |
| ASV2534 | 404 | `TGGGGAATATTGGACAATGGGCGAAAGCCTGATCCAGCAATGCCGCGTGAGTGATGAAGGCCTTAGGGTTGTAAAGCTCTTTTACTAGGGATGATAATGACAGTACCTAGAGAATAAGCTCCGGCTAACTCCGTGCCAGCAGCCGCGGTAATACGGAGGGAGCTAGCGTTGTTCGGAATTACTGGGCGTAAAGCGCACGTAGGCGGCCTAGCAAGTCAGGGGTGAAATCCCGGAGCTCAACTCCGGAACTGCCCTTGAAACTGCTAAGCTAGAATCTTGGAGAGGCGAGTGGAATTCCGAGTGTAGAGGTGAAATTCGTAGATATTCGGAAGAACACCAGTGGCGAAGGCGACTCGCTGGACAAGTATTGACGCTGAGGTGCGAAAGCGTGGGGAGCAAACAGG` |
| ASV1960 | 404 | `TGGGGAATATTGGACAATGGGCGAAAGCCTGATCCAGCCATGCCGCGTGAGTGATGAAGGCTTTAGGGTTGTAAAGCTCTTTCGCCGATGAAGATAATGACGGTAGTCGGAGAAGAAGCCCCGGCTAACTTCGTGCCAGCAGCCGCGGTAATACGAAGGGGGCTAGCGTTGTTCGGAATTACTGGGCGTAAAGCGAGCGTAGGTTGTGTCGCAAGTCAGTGGTGAAATCCCGGAGCTTAACTTCGGAACTGCCATTGAAACTGCGATGCTAGAATCTCAGAGGGGGTAGCGGAATTCCAAATGTAGGGGTGAAATCCGTAGATATTTGGAGGAACACCGGTGGCGAAGGCGGCTACCTGGATGAGTATTGACACTGAGGCTCGAAAGCGTGGGGATCAAACAGG` |
| ASV2087 | 429 | `TGGGGAATATTGGACAATGGGCGCAAGCCTGATCCAGCCATACCGCGTGGGTGAAGAAGGCCTTCGGGTTGTAAAGCCCTTTTGTTGGGAAAGAAATCCTGTTGATTAATACTCGATAGGGATGACGGTACCCAAAGAATAAGCACCGGCTAACTTCGTGCCAGCAGCCGCGGTAATACGAAGGGTGCAAGCGTTACTCGGAATTACTGGGCGTAAAGCGTGCGTAGGTGGTTGTTTAAGTCCGTTGTGAAAGCCCTGGGCTCAACCTGGGAATTGCAGTGGATACTGGGCAACTAGAGTGTGATAGAGGGTGGCGGAATTCCCGGTGTAGCAGTGAAATGCGTAGATATCGGGAGGAACATCCGTGGCGAAGGCGGCCACCTGGGTCAACACTGACACTGAGGCACGAAAGCGTGGGGAGCAAACAGG` |
| ASV3126 | 428 | `TGGGGAATTGTTCGCAATGGGCGCAAGCCTGACGACGCAACGCCGCGTGGAGGATGAAGGTCTTCGGATTGTAAACTCCTGTTGCCCGGGACGAAACGTTCCGACCTAACACGTCGGAGCCTGACGGTACCGGGTGAGGAAGCCCCGGCTAACTCTGTGCCAGCAGCCGCGGTAATACAGAGGGGGCGAGCGTTGTTCGGAATTACTGGGCGTAAAGGGCGCGTAGGCGGCCTGTTAAGTCGAACGTGAAATCCCCGGGCTCAACCCGGGAACTGCGTCCGAGACTGACAGGCTCGAATCCGGGAGAGGGATGTGGAATTCCAGGTGTAGCGGTGAAATGCGTAGATATCTGGAGGAACACCGGTGGCGAAGGCGGCATCCTGGACCGGTATTGACGCTGAGGCGCGAAAGCCAGGGGAGCAAACGGG` |
| ASV3015 | 429 | `TGGGGAATATTGGACAATGGGGGAAACCCTGATCCAGCAATGCCGCGTGTGTGAAGAAGGCCTTAGGGTTGTAAAGCACTTTTGTTGGGGAGGAAGGAATGTAGGCTAATAACCTACATGACAGACGTTACCCAAAGAATAAGCACCGGCTAACTCTGTGCCAGCAGCCGCGGTAATACAGAGGGTGCAAGCGTTAATCGGAATTACTGGGCGTAAAGGGTGCGTAGGTGGACGCATAAGTCGGATGTGAAATCCCTGGGCTTAACCTAGGAACTGCATTCGATACTGTGAGTCTAGAGTATGGTAGAGGTAAGTGGAATTTCCGGTGTAGCGGTGAAATGCGTAGATATCGGAAGGAACATCAGTGGCGAAGGCGACTTACTGGACTGATACTGACACTGAGGCACGAAAGCGTGGGGAGCAAACAGG` |
| ASV3315 | 428 | `TCGGGAATCTTCCGCAATGGGCGAAAGCCTGACGGAGCGACGCCGCGTGAGCGATGAAGGCCTTCGGGTTGTAAAGCTCTGTGGGGAGAGAAGAATAAGTTCAAGCGAATACCTTGAATGATGACGGTATCTCCTTAGCAAGCACCGGCTAACTCTGTGCCAGCAGCCGCGGTAAGACAGAGGGTGCAAACGTTGTTCGGATTTACTGGGCGTAAAGCGCGTGTAGGCGGCTTTGCAAGTCGGATGTGAAAGCCCACGGCTCAACCGTGGAAGTGCATCCGAAACTGCATGGCTGGAGTCTCGGAGAGGAAGGTGGAATGCTTGGTGTAGAGGTGAAATTCGTAGATATCAAGCAGAACACCTGCGGCGAAGGCGGCCTTCTGGACGATGACTGACGCTGAGACGCGAAAGCGTGGGGAGCAAACAGG` |
| ASV4999 | 404 | `TGGGGAATATTGCACAATGGGCGGAAGCCTGATGCAGCGACGCCGCGTGAGGGAAGACGGTCTTCGGATTGTAAACCTCTGTCTTCGGTGACGAAACAAGACGGTAGCCGAGGAGGAAGCCACGGCTAACTACGTGCCAGCAGCCGCGGTAATACGTAGGTGGCGAGCGTTGTCCGGAATTACTGGGTGTAAAGGGAGCGTAGGCGGGACCGCAAGTTAAATGTTAAAGGTATCGGCTTAACCGGTATTTGCGTTTAAAACTGCGGTTCTTGAGTGAAGTAGAGGCAGGCGGAATTCCTAGTGTAGCGGTGAAATGCGTAGATATTAGGAGGAACACCAGTGGCGAAGGCGGCCTGCTGGGCTTTAACTGACGCTGAGGCTCGAAAGCGTGGGGAGCAAACAGG` |
| ASV1461 | 429 | `TGGGGAATATTGGACAATGGGCGAAAGCCTGATCCAGCCATGCCGCGTGTGTGAAGAAGGTCTTCGGATTGTAAAGCACTTTAAGTTGGGAGGAAGGGCATTTACCTAATACGTAAGTGTTTTGACGTTACCGACAGAATAAGCACCGGCTAACTCTGTGCCAGCAGCCGCGGTAATACAGAGGGTGCAAGCGTTAATCGGAATTACTGGGCGTAAAGCGCGCGTAGGTGGTTTGTTAAGTTGAATGTGAAATCCCCGGGCTCAACCTGGGAACTGCATCCAAAACTGGCAAGCTAGAGTATGGTAGAGGGTGGTGGAATTTCCTGTGTAGCGGTGAAATGCGTAGATATAGGAAGGAACACCAGTGGCGAAGGCGACCACCTGGACTGATACTGACACTGAGGTGCGAAAGCGTGGGGAGCAAACAGG` |
| ASV3820 | 424 | `TGAGGAATATTGGTCAATGGGCGAGAGCCTGAACCAGCCAAGTCGCGTGAGGGAAGACGGTCCTACGGATTGTAAACCTCTTTTGTCGGGGAGCAATAAAGCGTACGTGTACGCGGATGCGAGTACCCGAAGAAAAAGCATCGGCTAACTCCGTGCCAGCAGCCGCGGTAATACGGAGGATGCGAGCGTTATCCGGATTTATTGGGTTTAAAGGGTGCGCAGGCGGAAAGTGCAAGTCAGCGGTCAAACGGCAGGGCTCAACCCTGTCTCGCCGTTGAAACTGCCTTTCTAGAGTGAGCGAGAAGTATGCGGAATGCGTGGTGTAGCGGTGAAATGCATAGATATCACGCAGAACTCCGATTGCGAAGGCAGCATACCGGCGCTCAACTGACGCTCATGCACGAAAGCGTGGGTATCGAACAGG` |
| ASV2503 | 429 | `TGGGGAATTTTGGACAATGGGGGCAACCCTGATCCAGCCATTCCGCGTGAGTGAAGAAGGCCTTCGGGTTGTAAAGCTCTTTTGTCCGGAGCGAAACGGGAGCGGTGAATATCCGCTCTCACTGACGGTACCGGAAGAATAAGCACCGGCTAACTACGTGCCAGCAGCCGCGGTAATACGTAGGGTGCGAGCGTTAATCGGAATTACTGGGCGTAAAGCGTGCGCAGGCGGTTTTGTAAGCCAGACGTGAAATCCCCGGGCTTAACCTGGGAATGGCGTTTGGGACTGCAAGGCTCGAGTATGGCAGAGGGGACTAGAATTCCTGGTGTAGCAGTGAAATGCGTAGATATCAGGAGGAATACCGATGGCGAAGGCAGGTCCCTGGGTTAATACTGACGCTCATGCACGAAAGCGTGGGGAGCAAACAGG` |
| ASV972 | 429 | `TGGGGAATATTGGACAATGGGCGCAAGCCTGATCCAGCCATGCCGCGTGTGTGAAGAAGGCCTTCGGGTTGTAAAGCACTTTTGTTAGGGAAGAAAAGCCGTCGGTTAATACCCGATGGTGATGACGGTACCTAAAGAATAAGCACCGGCTAACTTCGTGCCAGCAGCCGCGGTAATACGAAGGGTGCAAGCGTTACTCGGAATTACTGGGCGTAAAGCGTGCGTAGGTGGTTTGTTAAGTCAGATGTGAAAGCCCCGGGCTCAACCTGGGAATTGCATTTGATACTGGCGAACTAGAGTGAGGTAGAGGAGAGTGGAATTCCCGGTGTAGCAGTGAAATGCGTAGAGATCGGGAGGAACATCAGTTGCGAAGGCGGCTCTCTGGACCATCACTGACACTGAGGCACGAAAGCGTGGGGAGCAAACAGG` |
| ASV766 | 429 | `TGGGGAATTTTGGACAATGGGCGCAAGCCTGATCCAGCCATGCCGCGTGCGGGAAGAAGGCCTTCGGGTTGTAAACCGCTTTTGTCAGGGAAGAAATCCCTTGGGTTAATACCTCGGGGGGATGACGGTACCTGAAGAATAAGCACCGGCTAACTACGTGCCAGCAGCCGCGGTAATACGTAGGGTGCAAGCGTTAATCGGAATTACTGGGCGTAAAGCGTGCGCAGGCGGTTGTGCAAGACAGATGTGAAATCCCCGGGCTCAACCTGGGAACTGCATTTGTGACTGCACAGCTAGAGTACGGTAGAGGGGGATGGAATTCCGCGTGTAGCAGTGAAATGCGTAGATATGCGGAGGAACACCGATGGCGAAGGCAATCCCCTGGACCTGTACTGACGCTCATGCACGAAAGCGTGGGGAGCAAACAGG` |
| ASV2570 | 405 | `TGGGGAATATTGCACAATGGGCGAAAGCCTGATGCAGCAACGCCGCGTGCGGGAAGAAGGCCTTAGGGTTGTAAACCGCTTTCAGCAGGGAAGAAACTGACGGTACCTGCAGAAGAAGGTGCGGCCAACTACGTGCCAGCAGCCGCGGTGACACGTAGGCACCAAGCGTTGTCCGGATTTATTGGGCGTAAAGAGCTCGTAGGCGGTTCAGTAAGTCGGGTGTGAAAACTCTGGGCTCAACCCAGAGACGCCACCCGATACTGCTGTGACTTGAGTACGGTAGGGGAGTGGGGAATTCCTGGTGTAGCGGTGAAATGCGCAGATATCAGGAGGAACACCAGTGGCGAAGGCGCCACTCTGGGCCGTAACTGACGCTGAGGAGCGAAAGCGTGGGTAGCAAACAGG` |
| ASV5694 | 429 | `TGGGGAATATTGGACAATGGGGGGAACCCTGATCCAGCAATGCCGCGTGTGTGAAGAAGGCCTGAGGGTTGTAAAGCACTTTCAGTGGGGAAGAGGATTGATAGGTTAAGAGCTGATTAATTGGACTAAACCCACAGAAGAAGCACCGGCTAACTCCGTGCCAGCAGCCGCGGTAATACGGGGGGTGCAAGCGTTAATCGGAATTACTGGGCGTAAAGGGTGCGTAGGTAGTGTATTAAGTTATCTGTAAAATCCCTGGGCTTAACCTGGGCAGGTCAGATAAGACTGATAGACTCGAGTATGGGAGAGGGTAGTGGAATTTCCGGTGTAGCGGTGAAATGCGTAGAGATCGGAAGGAACACCAGTGGCGAAGGCGGCTACCTGGCCTAATACTGACACTGAGGCACGAAAGCGTGGGTAGCAAACAGG` |
| ASV5006 | 404 | `TGGGGAATATTGGACAATGGGGGCAACCCTGATCCAGCAATGCCGCGTGAGTGAAGAAGGCCTTCGGGTTGTAAAGCTCTTTTATTAGGGAAGATAATGACGGTACCTAATGAATAAGCTCCGGCTAACTTCGTGCCAGCAGCCGCGGTAATACGAAGGGAGCTAGCGTTGTTCGGAATTACTGGGCGTAAAGGGCGCGTAGGCGGCTCTGTAAGTCAGATGTGAAAGCCCCGGGCTTAACCTGGGAACTGCATTTGATACTGCAGAGCTAGAGAATGTGAGAGGTAAGTGGAATTATGAGTGTAGAGGTGAAATTCGTAGATATTCATAAGAACATCAGTGGCGAAGGCGGCTTACTGGCACATTACTGACGCTAAGGCGCGAAAGCGTGGGGAGCAAACAGG` |
| ASV3670 | 424 | `TGAGGAATATTGGTCAATGGGCGGAAGCCTGAACCAGCCATGCCGCGTGCAGGATGAAGGCTCTATGGGTCGTAAACTGCTTTTGTACGAGAGTAAAAGTAGCTATGCGTAGTTATTTGCAAGTATCGTACGAATAAGGATCGGCTAACTCCGTGCCAGCAGCCGCGGTAATACGGAGGATTCGAGCGTTATCCGGATTTATTGGGTTTAAAGGGTGCGTAGGTGGAATGTTAAGTTAGTCGTTAAATCGTGAGGCTCAACCTTACAGTGGCGATTGATACTGGCGTTCTAGAGAACAGTTGCGGTAGGCGGAATGAGTAGTGTAGCGGTGAAATGCTTAGATATTACTCAGAACACCGATTGCGAAGGCAGCTTACCAAGCTGTTTCTGACACTGAGGCACGAAAGCGTGGGGATCAAACAGG` |
| ASV4137 | 404 | `TGGGGAATCTTGGACAATGGGCGCAAGCCTGATCCAGCCATGCCGCGTGAGTGAAGAAGGCCTTCGGGTTGTAAAGCTCTTTTGCCAGGGACGATAATGACGGTACCTGGAGAATAAGCCCCGGCAAACTTCGTGCCAGCAGCCGCGGTAATACGAAGGGGGCTAGCGTTGTTCGGAATTACTGGGCGTAAAGCGCACGTAGGCGGATTGTTAAGTGAGGGGTGAAATCCCGAGGCTCAACCTCGGAACTGCCTTTCATACTGGCAATCTCGAGGCCGGAAGAGGTTAGTGGAATTCCCAGTGTAGAGGTGAAATTCGTAGATATTGGGAAGAACACCAGTGGCGAAGGCGGCTAACTGGTCCGGATCTGACGCTGAGGTGCGAAAGCGTGGGGAGCAAACAGG` |
| ASV1214 | 429 | `TGGGGAATATTGCGCAATGGGGGCAACCCTGACGCAGCCATGCCGCGTGTGTGAAGAAGGCCTTCGGGTTGTAAAGCACTTTCAATGGGGAAGAAAAGCGTAGCGTTAATAGCGCTGCGCCGTGACGTTACCCATAGAAGAAGCACCGGCTAACTCCGTGCCAGCAGCCGCGGTAATACGGAGGGTGCGAGCGTTAATCGGAATTACTGGGCGTAAAGCGCGCGTAGGCGGTTTGTTAAGTCAGCTGTGAAAGCCCCGGGCTCAACCTGGGAACTGCAGTTGATACTGGCCGACTAGAGTACGAGAGAGGGAGGTAGAATTCCATGTGTAGCGGTGAAATGCGTAGATATATGGAGGAATACCGGTGGCGAAGGCGGCCTCCTGGCTCGATACTGACGCTGAGGTGCGAAAGCGTGGGGATCAAACAGG` |
| ASV5268 | 429 | `TGGGGAATTTTGGACAATGGGGGCAACCCTGATCCAGCGATGCCGCGTGCAGGAAGAAGGCCTTCGGGTTGTAAACTGCTTTTGTCAGGGAAGAAATCCCAGGTGCTAAAACCACCTGGGGATGACGGTACCTGAAGAATCAGCACCGGCTAACTACGTGCCAGCAGCCGCGGTAATACGTAGGGTGCAAGCGTTAATCGGAATTACTGGGCGTAAAGGGTGCGCAGGCGGTCTTGCAAGACTGGTGTGAAATCCCCGGGCTTAACCTGGGAACTGCATTGGTAACTGCAAGGCTTGAGTGCGGTAGAGGGGGGTAGAATTTTGCGTGTAGCAGTGAAATGCGTAGATATGCAAAGGAATACCGATGGCGAAGGCAGCCCCCTGGACCTGCACTGACGCTCATGCACGAAAGCGTGGGGAGCAAACAGG` |
| ASV3019 | 424 | `TGAGGAATATTGGACAATGGGCGCAAGCCTGATCCAGCCATGCCGCGTGCAGGATGACGCATCTATGGTGTGTAAACTGCTTTTGTACGAGAAGAAACACTCCGACGTGTCGGAGCTTGACGGTATCGTAAGAATAAGGATCGGCTAACTCCGTGCCAGCAGCCGCGGTAATACGGAGGATCCAAGCGTTATCCGGAATCATTGGGTTTAAAGGGTCCGTAGGCGGCCTTATAAGTCAGTGGTGAAATCTCCCCGCTCAACGGGGAAACGGCCATTGATACTGTAGGGCTTGAATTATTAGGAAGTAACTAGAATATGTAGTGTAGCGGTGAAATGCTTAGAGATTACATGGAATACCAATTGCGAAGGCAGGTTACTACTAATTGATTGACGCTGATGGACGAAAGCGTGGGTAGCGAACAGG` |
| ASV4473 | 430 | `TAGGGAATATTGCGCAATGGAGGAAACTCTGACGCAGCGACGCCGCGTGGGCGATGAAGGCTTTCGGGTCGTAAAGCCCTGTCGGAAGAGAAGAAAAATATTGTGGCTAACATCCACAGTACTTGACGGTATCTTCAGAGGAAGCACCGGCTAACTACGTGCCAGCAGCCGCGGTAATACGTAGGGTGCAAGCGTTGTTCGGAATCATTGGGCGTAAAGCGCGTGTAGGTGGTCATCTAAGTCGAGTGTGAAAGCCCTGGGCTCAACCTAGGAAGTGCATTCGAAACTAGTTGACTAGAAGACAGTAGAGGAAGGTGGAATTCCAAGTGTAGAGGTGAAATTCGTAGATATTTGGAGGAATACCGGCGGCGAAGGCGGCCTTCTGGGCTGTTCTTGACACTGAGACGCGAAAGCGTGGGTAGCAAACAGG` |
| ASV2121 | 425 | `TAAGGAATATTGGACAATGGAGGGAACTCTGATCCAGCCATGCCGCGTGAAGGAAGAAGGCCCTCTGGGTTGTAAACTTCTTTTATATGGGAACAAACCTGTGGATTCATCCGCAGCCGAGGGTACCATAAGAATAAGCACCGGCTAACTCCGTGCCAGCAGCCGCGGTAATACGGAGGGTGCAAGCGTTATCCGGAATCACTGGGTTTAAAGGGTGCGTAGGCGGTCTTATAAGTCAGTCGTGAAATCGCTGGGCTCAACCCATGCAATTGCGATTGATACTGTTTGACTTGAATCAGGTTGAGGTAGGCGGAATGTGGCATGTAGCGGTGAAATGCATAGATATGCCATAGAACACCGATTGCGAAGGCAGCTTGCTGGACCTGAATTGACGCTGAGGCACGAAAGCGTGGGGAGCAAACAGG` |
| ASV3072 | 429 | `TGGGGAATTTTGGACAATGGGCGAAAGCCTGATCCAGCCATGCCGCGTGCAGGATGAAGGCCTTCGGGTTGTAAACTGCTTTTGTACGGAACGAAAAAGTTCTCTCTAATACAGGGGGCGAATGACGGTACCGTAAGAATAAGCACCGGCTAACTACGTGCCAGCAGCCGCGGTAATACGTAGGGTGCGAGCGTTAATCGGAATTACTGGGCGTAAAGCGTGCGCAGGCGGTTATATAAGACAGATGTGAAATCCCCGGGCTCAACCTGGGAACTGCATTAGTGACTGTATAGCTAGAGTGCGGCAGAGGGGGATGGAATTCCGCGTGTAGCAGTGAAATGCGTAGATATGCGGAGGAACACCGATGGCGAAGGCAATCCCCTGGGCCTGCACTGACGCTCATGCACGAAAGCGTGGGGAGCAAACAGG` |
| ASV4350 | 424 | `TAGGGAATATTGGGCAATGGGCGCAAGCCTGACCCAGCCACCCCGCGTGAAGGAAGAAGGTTCATAGAATTGTAAACTTCTTTTGTCTAGGGATAAAAGCCGTGTAACTCACGGAATTGAAGGTACTAGGCGAATAAGCGCCGGCTAACTACGTGCCAGCAGCCGCGGTAATACGTAGGGCGCAAGCGTTGTCCGGATTTACTGGGTTTAAAGGGTGCGTAGGCGGCTTAATAAGTCTGTGATGAAATGTCAGGGCTCAACCTTGGCGCCGTTACGGATACTGTTAGGCTTGAGTATAGATGAGGCTGTTAGAATGAGTGGTGTAGCGGTGAAATGCGTAGAGATCACTCAGAATACCGATTGCGAAGGCAGACAGCCGGGCTATAACTGACGCTGAGGCACGAAAGCGTGGGGATCAAACAGG` |
| ASV1721 | 428 | `TTTCGAATCATTCACAATGGGCGAAAGCCTGATGGTGCGACGCCGCGTGAGGGATGAAGGTCTTCGGATTGTAAACCTCTGTCACTGGGGAGAAAACGCTTCAATTTAATAGATTGAAGCCTGATTTAACCCGGAGAGGAAGCAGTGGCTAACTCTGTGCCAGCAGCCGCGGTAATACAGAGACTGCAAGCGTTATTCGGATTCACTGGGCGTAAAGGGTGCGCAGGCGGTTGGGTGTGTCAGATGTGAAATCCCGAGGCTCAACCTCGGAACTGCGTCTGAAACTACTCGACTAGAGTACTGGAGAGGGTAACGGAATTCACGGTGTAGCAGTGAAATGCGTAGATATCGTGAGGAACACCAGAGGCGAAGGCGGTTACCTGGACAGTTACTGACGCTCAGGCACGAAAGCATGGGGAGCAAAAGGG` |
| ASV4061 | 404 | `TGGGGAATATTGGACAATGGGCGAAAGCCTGATCCAGCTATGCCACGTGAGTGATGAAGGCCTTAGGGTTGTAAAGCTCTTTTGACAGGAAAGATAATGACGGTACCTGTAAAAAAAGTTCCGGCTAACTCCGTGCCAGCAGCCGCGGTAATACGGAGGGAACTAGCGTTGTTCGGAATTACTGGGCGTAAAGAGCGCGTAGGCGGTTTATTAAGTTGGGAGTGAAATCCCAGGGCTCAACCCTGGAACTGCTCTCAAAACTGGTAGACTAGAGTGTGATAGGGGGTGATGGAATTCCTAGTGTAGAGGTGAAATTCTTAGATATTAGGAGGAACATCGGTGGCGAAGGCGATCACCTGGATCATTACTGACGCTGAGGCGCGAAAGCGTGGGGAGCAAACAGG` |
| ASV3460 | 430 | `TAGGGAATATTGCGCAATGGAGGAAACTCTGACGCAGCCACGCCGTGTGAGTGAGGAAGGCCTTCGGGTTGTAAAGCTCTGTCATCGGGGAAGAAACTCTATCTGGGGAAAGTTCAGGTAGATTGACGGTACCCGAGAAGGAAGCACCGGCTAACTCTGTGCCAGCAGCCGCGGTAATACAGAGGGTGCAAGCGTTGTTCGGAATTACTGGGCGTAAAGCGTTTGTAGGCGGTGCTGAGAGTCCTAGGTGAAATCCCCGAGCTTAACTCGGGAACTGCCTGGGAGACCTCAGTACTAGAGTACTGAAGGGGTTGCTGGAATTCCATGTGTAGCGGTGAAATGCGTAGATATATGGAGGAACACCTGTGGCGAAGGCGGGCAACTGGTCAGAGACTGACGCTGAGAAACGAAAGCGTGGGGAGCAAACAGG` |
| ASV3283 | 430 | `TAGGGAATATTGCGCAATGGGGGAAACCCTGACGCAGCCACGCCGCGTGAGTGAGGAAGGCCTTCGGGTTGTAAAGCTCTGTCGGGAGGGAAAAATGGCAGCTCTAATAAAACGGGAGCTGATTGATGGTACCTCCGAAGGAAGCACCGGCTAACTTCGTGCCAGCAGCCGCGGTAATACGAAGGGTGCAAGCGTTGTTCGGATTTATTGGGCGTAAAGAGCTCGTAGGCGGGAATGAAAGTCAGGTGTGAAATCCCGGAGCTTAACTCCGGAACTGCGCCTGAAACTCCATTTCTAGAATGTCGGAGAGGGAAGTGGAATATCGCATGTAGGGGTAAAATCCGTAGAGATGCGATGGAACACCAGAGGCGAAGGCGACTTCCTGGACGACTATTGACGCTGAGGAGCGAAAGCGTGGGGAGCAAACAGG` |
| ASV2299 | 407 | `TGGGGAATATTGGGCAATGGGCGCAAGCCTGACCCAGCAACGCCGCGTGAAGGAAGAAGGCTTTCGGGTTGTAAACTTCTTTTATTCGGGACGAAACAAATGACGGTACCGAATGAATAAGCCACGGCTAACTACGTGCCAGCAGCCGCGGTAATACGTAGGTGGCAAGCGTTATCCGGATTTACTGGGTGTAAAGGGCGTGTAGGCGGGACTTCAAGTCAGATGTGAAAACTATGGGCTCAACCCATAGCCTGCATTTGAAACTGAGGTTCTTGAGTGATGGAGAGGCAGGCGGAATTCCCTGTGTAGCGGTGAAATGCGTAGATATAGGGAGGAACACCAGTGGCGAAGGCGGCCTGCTGGACATTAACTGACGCTGAGGCGCGAAAGCGTGGGGAGCAAACAGG` |
| ASV3373 | 429 | `TCGAGAATAATTCACAATGGGGGCAACCCTGATGGTGCAACGCCGCGTGGAGGATGAAGGTCTTCGGATTGTAAACTCCTGTCATCCGGGAGCAAGACCTGGTCGTGAATAGCGAACAGGGTTGATAGTACCGGAAGAGGAAGGGACGGCTAACTTCGTGCCAGCAGCCGCGGTAATACGAAGGTCCCGAGCGTTGTTCGGAATCACTGGGCGTAAAGGGAGCGTAGGCGGCGTGGTAAGTCAGATGTGAAATCCCGGGGCTCAACCCCGGAACTGCATCCGATACTGCCGTGCTAGAGTTTTGGAGAGGTAGCTGGAATTCACGGTGTAGCAGTGAAATGCGTGGATATCGTGAGGAACACTCGTGGCGAAAGCGAGCTACTGGACAACAACTGACGCTGAGGCTCGAAGGCCAGGGTAGCGAAAGGG` |
| ASV2951 | 404 | `TGGGGAATATTGCACAATGGACGAAAGTCTGATGCAGCAACGCCGCGTGGAGGATGACACATTTCGGTGCGTAAACTCCTTTTATATGAGAAGATAATGACGGTATTATATGAATAAGCACCGGCTAACTCCGTGCCAGCAGCCGCGGTAATACGGGGGGTGCAAGCGTTACTCGGAATCACTGGGCGTAAAGAGCATGTAGGCGGATTGATAAGTTTGAAGTGAAATCCTATAGCTTAACTATAGAACTGCTTTGAAAACTGTTAATCTAGAATGTGGGAGAGGTAGATGGAATTTCTGGTGTAGGGGTAAAATCCGTAGAGATCAGAAGGAATACCGATTGCGAAGGCGATCTACTGGAACATTATTGACGCTGAGATGCGAAAGCGTGGGGAGCAAACAGG` |
| ASV3677 | 404 | `TAAGGAATATTGGACAATGGGGGAAACCCTGATCCAGCCATGCCGCGTGAGTGATGAAGGCCTTCGGGTTGTAAAGCTCTTTTGTCCGGGACGATGATGACGGTACCGGAAGAATAAGCCCCGGCTAACTTCGTGCCAGCAGCCGCGGTAATACGAAGGGGGCAAGCGTTGTTCGGAATCACTGGGCGTAAAGCGTGCGTAGGCGGCTCGACAAGTCAGGAGTGAAATCCCCAGGCTCAACCTGGGAATTGCTTTTGAAACTGTCTTGCTTGAATCCGGGAGAGGTTGGCGGAATTCCAAGTGTAGAGGTGAAATTCGCAGATATTTGGAGGAACACCAGTGGCGTAAGCGGCCAACTGGACCGGTATTGACGCTGAGGCACGAAAGCGTGGGGATCAAACAGG` |
| ASV4123 | 424 | `TGAGGAATATTGGACAATGGGTGCAAGCCTGATCCAGCCATCCCGCGTGAAGGACGACGGCCCTACGGGTTGTAAACTTCTTTTATATGGGGATAAACCTACTTACGTGTAAGTAGCTGAAGGTACCATATGAATAAGCACCGGCTAACTCCGTGCCAGCAGCCGCGGTAATACGGAGGGTGCAAGCGTTATCCGGATTTATTGGGTTTAAAGGGTCCGTAGGCGGGCTAATAAGTCAGAGGTGAAATCCTACAGCTTAACTGTAGAACTGCCTTTGATACTGTTAGTCTTGAATTTTTGTGAAGTTGCTGGAATAAGTAGTGTAGCGGTGAAATGCATAGATATTACTTAGAACACCAATTGCGAAGGCAGGTAACTAACAAACGATTGACGCTGATGGACGAAAGCGTGGGGAGCGAACAGG` |
| ASV3714 | 404 | `TGGGGAATATTGGACAATGGGCGAAAGCCTGATCCAGCCATGCCGCGTGAGTGATGAAGGCCTTAGGGTTGTAAAGCTCTTTTGTCAGGGAAGATAATGACGGTACCTGAAGAATAAGCACCGGCTAACTTCGTGCCAGCAGCCGCGGTAATACGAAGGGTGCTAGCGTTGTTCGGAATCACTGGGCGTAAAGCGCGCGTAGGCGGCTATTCAAGTCAGAGGTGAAATCCTGGAGCTCAACTCCAGAATTGCCTTTGAAACTGGGTAGCTTGAGTGTCGGAGGGGATAGCGGAATTCCTAATGTAGAGGTGAAATTCGTAGATATTAGGAGGAACACCGGTGGCGAAGGCGGCTATCTGGACGACAACTGACGCTGAGGCGCGAAAGCGTGGGGATCAAACAGG` |
| ASV2486 | 430 | `TGGGGAATATTGGACAATGGGCGCAAGCCTGATCCAGCAATACCGCGTGTGTGAAGAAGGCCTTCGGGTTGTAAAGCACTTTTGTTAGGGAGGATGGTAATTTTGGCTAATACCCAAAGTTGCTGACGTTACCTAAAGAATAAGCACCGGCTAACTCCGTGCCAGCAGCCGCGGTAATACGGAGGGTGCAAGCGTTAATCGGATTTACTGGGCGTAAAGGGTGCGTAGGCGGCCTATTAAGTCAGATGTGAAAGCCCTGGGCTCAACCTGGGAATTGCATTTGAAACTGGTAGGCTAGAATGTGGTAGAGGGTAGCGGAATTTCTGGTGTAGCGGTGAAATGCGTAGATATCAGAAGGAACATCAATGGCGAAGGCAGCTACCTGGGCCAACATTGACGCTGAGGCACGAAAGCGTGGGGAGCAAACAGG` |
| ASV3872 | 428 | `TGGGGAATCTTGCGCAATGGGCGAAAGCCTGACGCAGCAACGCCGCGTGAGGGAAGACGGCTTTCGGGTTGTAAACCTCTTTCAGGGGGGACGAAGCCACCGGGGTTAATAGCCCACGGGGTGACGGTACCCCCAGAAGAAGCCCCGGCTAACTACGTGCCAGCAGCCGCGGTAATACGTAGGGGGCAAGCGTTGTCCGGAATCATTGGGCGTAAAGCGCGTGTAGGTGGCTTGGTAAGTCCGGTGTGAAACCTTGGGGCTCAACCCCAAGCCTGCATCGGATACTGCCTCGCTCGAGTTCGGGAGGGGAGAGCGGAATTCCTAGTGTAGCGGTGAAATGCGCAGATATTAGGAAGAACACCGGTGGCGAAGGCGGCTCTCTGGAACGATACTGACACTGAGACGCGAAAGCGTGGGTAGCGAACAGG` |
| ASV1685 | 430 | `TGGGGAATATTGCGCAATGGACGAAAGTCTGACGCAGCAACGCCGCGTGAGTGATGAAGGCCTTTGGGTTGTAAAGCTCTTTCGGCTGGGAAGAAGGGTATTTGTGTTAATAGCACAAGTATTTGATGGTACCAGAAGAAGAAGCACCGGCTAACTTCGTGCCAGCAGCCGCGGTAATACGAAGGGTGCGAGCGTTGTTCGGAATTACTGGGCGTAAAGGGTTTGTAGGCTGAAATGTAAGTCAAGTGTGAAATCCCCGAGCTAAACTTGGGACGTGCATTTGAAACTGTGTTTCTTGAGTATCGTAGAGGGTGGTGGAATTGTAGGTGTAGGAGTGACATCCGTAGAGATCTACAGGAACATCAGAGGCGAAGGCGACCACCTGGCCGATTACTGACGCTGAGGAACGAAAGCGTGGGGAGCAAACAGG` |
| ASV1957 | 404 | `TGGGGAATATTGGGCAATGGGCGAAAGCCTGACCCAGCCATGCCGCGTGAGTGATGAAGGCCTTAGGGTTGTAAAGCTCTTTCAGGGGGGAAGATAATGACGGTACCCCAAGAAGAAGCTCCGGCTAACTTCGTGCCAGCAGCCGCGGTAATACGAAGGGAGCGAGCGTTACTCGGATTTACTGGGCGTAAAGGGTGCGTAGGCGGTTTGATCAGTCAGATGTGAAAGCCCAGGGCTTAACCCTGGAACTGCATTTGATACTGTCAAACTTGAGTATAAGAGAGGTAAGTGGAATTCCCAGTGTAGCAGTGAAATGCGTAGATATTGGGAGGAACATCAGTGGCGAAGGCGACTTACTGGCTTATAACTGACGCTGAGGCACGAAAGCGTGGGGAGCAAACAGG` |
| ASV3347 | 404 | `TGGGGAATATTGGACAATGGGGGCAACCCTGATCCAGCAATGCCGCGTGAGTGATGAAGGCCTTAGGGTTGTAAAGCTCTTTTACCGGGGACGATGATGACGGTACCCGGAGAATAAGCCCCGGCTAACTTCGTGCCAGCAGCCGCGGTAATACGAAGGGGGCGAGCGTTGTTCGGAGTTACTGGGCGTAAAGGGCGCGTAGGCGGCGCTCCAAGTTAGGCGTGAAAGCCCCGGGCTCAACCTGGGAATTGCGCTTAAGACTGGAGTGCTCGAGTGCGGGAGAGGTGAGTGGAATTCCCAGTGTAGAGGTGAAATTCGTAGATATTGGGAAGAACACCAGTGGCGAAGGCGGCTCACTGGCCCGCAACTGACGCTGAGGCGCGAAAGCGTGGGGAGCAAACAGG` |
| ASV3668 | 404 | `TGGGGAATATTGGGCAATGGGCGAAAGCCTGACCCAGCAACGCCGCGTGAGGGAAGAAGGTCTTCGGATTGTAAACCTTTGTCTTATGGGACGATAATGACGGTACCATAGGAGGAAGCCACGACTAACTACGTGCCAGCAGTCGCGGTAATACGTAGGTGGCAAGCGTTATCCGGAATTATTGGGTGTAAAGGGTGCGTAGGCGGGATGTAAAGTCAGATGTGAAATCTAGGGGCTCAACCCTTAAACTGCATTTGAAACTTATGTTCTTGAGTAAAGTAGAGGCAAGCGGAATTCCCGGTGTAGCGGTGAAATGCGTAGATATCGGGAGGAACACCAGTGGCGAAGGCGGCTTGCTGGGCTTTTACTGACGCTGAGGCACGAAAGTGTGGGGAGCAAACAGG` |
| ASV2521 | 429 | `TGGGGAATTTTGGACAATGGACGAAAGTCTGATCCAGCCATTCCGCGTGCAGGATGAAGGCCTTCGGGTTGTAAACTGCTTTTGTACGGAACGAAAAGGCGACCTCTAATACGGGTTGTTGATGACGGTACCGTAAGAATAAGCACCGGCTAACTACGTGCCAGCAGCCGCGGTAATACGTAGGGTGCAAGCGTTAATCGGAATTACTGGGCGTAAAGCGTGCGCAGGCGGTTTTGTAAGACAGAGGTGAAATCCCCGGGCTCAACCTGGGAACTGCCTTTGTGACTGCAAAGCTAGAGTGCGGCAGAGGGGGATGGAATTCCGCGTGTAGCAGTGAAATGCGTAGATATGCGGAGGAACACCGATGGCGAAGGCAATCCCCTGGGCCTGCACTGACGCTCATGCACGAAAGCGTGGGGAGCAAACAGG` |
| ASV5536 | 430 | `TGGGGAATATTGGTCAATGGGCGCAAGCCTGAACCAGCGACGCCGCGTGAGTGATGAAGGCCTTCGGGTTGTAAAGCTCTGTGGGGAGGGAAGAAAACAGTCCGTTCGAATAGGTCGGGCTCTTGACGGTACCTCCTTAGCAAGCACCGGCTAACTCTGTGCCAGCAGCCGCGGTAAGACAGAGGGTGCAAACGTTGCTCGGAATTACTGGGCGTAAAGCGCGTGTAGGCTGGCCAACAAGTCAGATGTGAAAGCCCTGAGCTCAACTTAGGAACTGCATTTGAAACTGTTGGTCTTGAATAGTGGAGAGGGTCGTGGAATTCCTGGTGTAGAGGTGAAATTCGTAGATATCAGGAGGAACACCGGTGGCGAAGGCGGCGACCTGGACACTTATTGACGCTGAGACGCGAAAGCGTGGGGAGCAAACAGG` |
| ASV149 | 404 | `TGGGGAATATTGGACAATGGGCGAAAGCCTGATCCAGCGACGCCGCGTGGATGATGAAGGCCTTCGGGTTGTAAAGTCCTTTCGGTAGGGAAGATAATGACGGTACCTACAAAAGAAGCTCCGGCTAACTTCGTGCCAGCAGCCGCGGTAATACGAAGGGAGCTAGCGTTACTCGGAATCATTGGGCGTAAAGCGTGCGTAGGCGGTCTTGTAAGTTAGGAGTGAAATCCTGGGGCTCAACCCCAGAACTGCTCTTAATACTGCTCGACTAGAATACTTGAGGGGACAGTAGAATTCCTAGTGTAGGGGTGAAATCCGTAGATATTAGGAGGAATACCAGTGGCGAAAGCGACTGTCTGGCAAGTTATTGACGCTGTTGCACGAAAGCGTGGGGAGCAAACAGG` |
| ASV3835 | 414 | `TGGGGAATTTTTGGCAATGGGGGAAACCCTGACCAAGCAACGCCGCGTGGATGATGAAGTATCTTGGTACGTAAAATCCTTTCGACGGGGAAGAAAACCGCAAGGTTTGACGGTACCCGTAGAAGAAGCCCCGGCTAACTCCGTGCCAGCAGCCGCGGTAATACGGGGGGGGCAAGCGTTGTTCGGAATTACTGGGCGTAAAGGGCGCGTAGGCGGTCTGTTAAGTTGGATGTGAAAACTGTGGGCTCAACCTGCAGCCTGCATCCAATACTGACAGGCTAGAGTTCTGGAGGGGATAGCGGAATTCCTGGTGTAGCGGTGAAATGCGTAGATATCAGGAGGAACACCGGTGGCGAAGGCGGCTATCTGGACAGAGTCTGACGCTGAGGCGCGAAAGCTAGGGGAGCAAACAGG` |
| ASV3484 | 404 | `TAGGGAATTTTGCGCAATGGGCGAAAGCCTGACGCAGCAACGCCGCGTGAATGTTAAGCCCTTCGGGGTGTAAAGTTCTGTCAGTGGGGACGAAACTTGACGGTACCCACAGAGGAAGCACCGGCTAACTCCGTGCCAGCAGCCGCGGTAATACGGAGGGTGCAAGCGTTGTCCGGAATCATTGGGCGTAAAGCGTTCGTAGGCGGCACATCAAGTCTGGTGTTAAAGACCGGGGCTCAACTCCGGCATGGCATTGGATACTGATGAGCTTGAATGCGGCAGAGGTAAAGGGAATTCCTGGTGGAGCGGTGAAATGCTAAGATATCAGGAGGAACATCAGTGGCGAAAGCGCTTTACCGGACCGTGATTGACGCTGAGGAACGAAAGCCGGGGGAGCAAATGGG` |
| ASV4845 | 428 | `TCGAGAATTTTTCTCAATGGGCGAAAGCCTGAAGGAGCGACGCCGCGTGGGGGATGAAGGGCTTCGGCCCGTAAACCCCTGTCATTTGCGAACAAACCTTACCGGTTAACAACCGGTGAGCTGATTGTAGCGAAAGAGGAAGGGACGGCTAACTCTGTGCCAGCAGCCGCGGTAATACAGAGGTCCCAAGCGTTGTTCGGATTCACTGGGCGTAAAGGGTGCGTAGGTGGCGGGGTAAGTTTGATGTGAAATCTCCGGGCTTAACCCGGAAATGGCATTGAATACTATTCTGCTCGAGGGTTGGAAGGGAGACTGGAATTCTCGGTGTAGCAGTGAAATGCGTAGATATCGAGAGGAACACCAGTGGCGAAAGCGAGTCTCTGGACAACACCTGACACTGAGGCACGAAAGCCAGGGGAGCAAACGGG` |
| ASV5283 | 429 | `TGGGGAATCTTCCGCAATGGACGAAAGTCTGACGGAGCAACGCCGCGTGAGTGAAGAAGGTTTTCGGATCGTAAAGCTCTGTTGTTTGAGACGAATGTGTATATTAGTAATACGGATATACAATGACGGTATTAAACGAGAAAGCCACGGCTAACTACGTGCCAGCAGCCGCGGTAATACGTGGGTGGCAAGCGTTGTCCGGAATTATTGGGCGTAAAGGGAGCGCAGGTGGGAATGTAAGTCGGTCTTAAAAGTGCGGGGCTCAACCCCGTGAGGGGATCGAAACTACATTTCTTGAGTGCAGGAGAGGAAAGTGGAATTCCTAGTGTAGCGGTGAAATGCGTAGATATTAGGAGGAACACCAGTGGCGAAGGCGACTTTCTGGACTGTAACTGACACTGAGGCTCGAAAGCCAGGGGAGCGAACGGG` |
| ASV5339 | 387 | `GCGCGAAAACTTTACAATGCACGAAAGTGTGATAAGGGAATCCTAAGTGCTCGTACACAGTACGGGCTTTTGCCAAGGGTAAATACCTTGGAGAATAAGTGGTGGGTAAGACTGGTGCCAGCAGCCGCGGTAACCCCAGCGCCACAAGTGGGAGCCACAATTATTTGGCCTAAAGCGTCCGTAGCTGGCCTGGTAAATCTTTTGTGAAATCGGAAGGCTCAACCTTCCGGCGTGCAGAAGACACTGCCTGGCTTGAGACCGGGAGGGGTAAGGAGTATTCCTAGGGAACCGGTAAAATGGGATAATCCTAGGAAGACTACCTGTGGCGAAGGCGCCTTACCAGCACGGGTTTGACAGTGAGGGACGAAGGCTAGGGGAGCGAACCGG` |
| ASV3442 | 430 | `TGGGGAATCTTGCACAATGGGCGAAAGCCTGATGCAGCAACGCCGCGTGGGTGAAGAAGGTTCTTGGATTGTAAAGCCCTTTCGGCTAGAAAGAAGGGTTTCTAGATTAATACCCTAGAGATCTGACGGTACTAGAAGAAGAAGCACCGGCTAATTCCGTGCCAGCAGCCGCGGTAATACGGAAGGTGCGAGCGTTGTTCGGATTGATTGGGCGTAAAGCGCATGTAGGCGGGTTTGTAAGTCGGATGTGAAAGCCCTAGGCTCAACCTAGGAACTGCATCTGAAACTGCATATCTTGAATACTCGAGGGGTTGGTGGAATTCCAGGTGTAGGAGTGAAATCCGTAGATATCTGGAGGAACACCGGAGGCGAAGGCGGCCAACTGGCGATGTATTGACGCTGAGGTGCGAAAGCGTGGGGAGCAAACAGG` |
| ASV329 | 423 | `TAGGGAATATTGGTCAATGGGCGCAAGCCTGAACCAGCCATGCCGCGTGCAGGAAGAAGGCCCTCTGGGTTGTAAACTGCTTTTGACAGGGAACAAATGCCACTTGCGAGTGGAGCTGAGTGTACCTGTAGAATAAGCACCGGCTAACTCCGTGCCAGCAGCCGCGGTAATACGGAGGGTGCAAGCGTTGTCCGGATTTATTGGGTTTAAAGGGTGCGTAGGCGGCTCTTTAAGTCAGTGGTGAAAGCCTCCAGCTTAACTGGAGAACTGCCATTGATACTGAAGAGCTTGAGTATAGTTGAGGTAGGCGGAATTGACAGTGTAGCGGTGAAATGCTTAGATATTGTCAAGAACACCGATTGCGAAGGCAGCTTACTAAACTATAACTGACGCTGAGGCACGAAAGCGTGGGGATCAAACAGG` |
| ASV2747 | 424 | `TGAGGAATATTGGTCAATGGGCGCAAGCCTGAACCAGCCATGCCGCGTGCAGGAAGACTGCCCTATGGGTTGTAAACTGCTTTTATTTAGGAAGAATAGCATTTATGTATAGATGTATGACGGTACTAAAGGAATAAGCACCGGCTAACTCCGTGCCAGCAGCCGCGGTAATACGGAGGGTGCAAGCGTTATCCGGAATTATTGGGTTTAAAGGGTGCGTAGGTTGCTAATCAAGTCAGAGGTGAAATGCTGCAGCTTAACTGTAGGACTGCCTTTGATACTGTATAGCTTGAGATAGGTCGCCGTTGGCGGAATGAGTAGTGTAGCGGTGAAATGCATAGATATTACTCAGAACACCAATTGCGAAGGCAGCTAACGAGGCCTTATCTGACACTGAGGCACGAAAGCGTGGGGAGCAAACAGG` |
| ASV243 | 429 | `TGGGGAATTTTGGACAATGGGGGCAACCCTGATCCAGCAATGCCGCGTGCGGGATGAAGGCCTTCGGGTTGTAAACCGCTTTTGTCAGGAACGAAACGGCTGGGGCTAATACCTTTGGCTAATGACGGTACCTGAAGAATAAGCACCGGCTAACTACGTGCCAGCAGCCGCGGTAATACGTAGGGTGCGAGCGTTAATCGGAATTACTGGGCGTAAAGCGTGCGCAGGCTGCTGAGTAAGACAGATGTGAAAGCCCTGGGCTCAACCTGGGAACTGCATTTGTGACTGCTCAGCTGGAGTGCGGCAGAGGGGGATGGAATTCCGCGTGTAGCAGTGAAATGCGTAGATATGCGGAGGAACACCGATGGCGAAGGCAATCCCCTGGGCCTGCACTGACGCTCATGCACGAAAGCGTGGGGAGCAAACAGG` |
| ASV3202 | 424 | `TAAGGAATATTGGTCAATGGACGAAAGTCTGAACCAGCCATGCCGCGTGAAGGATGAAGGCCCTCTGGGTTGTAAACTTCTTTTATATGGGACGAAAAAAGGGAATTCTTTCTCACTTGACGGTACCATAGGAATAAGCACCGGCTAACTCCGTGCCAGCAGCCGCGGTAATACGGAGGGTGCAAGCGTTATCCGGATTTACTGGGTTTAAAGGGAGCGTAGGTGGGCAGTTAAGTCAGTGGTGAAATCTCTGGGCTTAACCCGGAAACTGCCATTGATACTATCTGTCTTGAATATTCTGGAGGTAAGCGGAATATGTCATGTAGCGGTGAAATGCTTAGATATGACATAGAACACCCATTGCGAAGGCAGCTTACTACGGAATTATTGACACTGAGGCTCGAAAGCGTGGGGATCAAACAGG` |
| ASV578 | 429 | `TGGGGAATTTTGGACAATGGGCGCAAGCCTGATCCAGCCATTCCGCGTGAGTGAAGAAGGCCTTCGGGTTGTAAAGCTCTTTCGCAAGGGAAGAAAAATTACGCTCTAACATAGCGTGATGATGACGGTACCTTGATAAGAAGCACCGGCTAACTACGTGCCAGCAGCCGCGGTAATACGTAGGGTGCGAGCGTTAATCGGAATTACTGGGCGTAAAGCGTGCGCAGGCTGTTTTGTAAGTCAGATGTGAAATCCCCGAGCTCAACTTGGGAACTGCGTTTGAAACTACAAGACTAGAATAGGTCAGAGGGGGGTAGAATTCCACGTGTAGCAGTGAAATGCGTAGAGATGTGGAGGAATATCAATGGCGAAGGCAGCCCCCTGGGATCATATTGACGCTCATGCACGAAAGCGTGGGGAGCAAACAGG` |
| ASV2772 | 429 | `TCGAGAATTTTTCACAATGGGGGAAACCCTGATGGAGCGACGCCGCGTGGAGGATGAAGGTTTTCGGATTGTAAACTCCTGTCACCACAGAACAAGCAATGTTATTTAACAAATGACAAAGTTGATAGTATGTGGGGAGGAAGGGACGGCTAACTCTGTGCCAGCAGCCGCGGTAATACAGAGGTCCCAAGCGTTGTTCGGATTCACTGGGCGTAAAGGGTGTGTAGGAGGTTTGATAAGTCTGATGTGAAATCCCGTCGCTCAACGACGGAACTGCATTGGATACTGTCAGGCTAGAGGGTCTGAGGGGGAAGCGGAATTCTTGGTGTAGCGGTGAAATGCGTAGATATCAAGAGGAACACCCGTGGCTAACGCGGCTTCCTGGAAGACTCCTGACTCTGAAACACGAAAGCCAGGGTAGCGAATGGG` |
| ASV2249 | 404 | `TGGGGAATCTTGGACAATGGGCGCAAGCCTGATCCAGCCATGCCGCGTGAGTGACGAAGGTCTTCGGATTGTAAAGCTCTTTTGGCGGGGACGATAATGACGGTACCCGCAGAATAAGCCCCGGCTAACTTCGTGCCAGCAGCCGCGGTAATACGAAGGGGGCTAGCGTTGTTCGGAATTACTGGGCGTAAAGCGCACGTAGGCGGATTTGTTAGTCAGGGGTGAAATCCCGGGGCTCAACCTCGGAACTGCCTTTGATACTGCAAGTCTCGAGTCCGGAAGAGGTGAGTGGAATTCCTAGTGTAGAGGTGAAATTCGTAGATATTAGGAAGAACACCAGTGGCGAAGGCGGCTCACTGGTCCGGTACTGACGCTGAGGTGCGAAAGCGTGGGGAGCAAACAGG` |
| ASV2656 | 424 | `TAAGGAATATTGGTCAATGGACGCAAGTCTGAACCAGCCATGCCGCGTGCAGGAAGACGGTCCTCTGGATTGTAAACTGCTTTTGTTAGGGAAGAAACCCCTGGATGCGTCCGGGGCTGACGGTACCTAAAGAATAAGGATCGGCTAACTCCGTGCCAGCAGCCGCGGTAATACGGAGGATCCAAGCGTTATCCGGATTTATTGGGTTTAAAGGGTGCGTAGGCGGCGTTGTAAGTCAGTGGTGAAAGTTTGCAGCTTAACTGTAAAATTGCCATTGATACTGCAGTGCTTGAGTATAGATGAGGTGGGCGGAATGTGTCATGTAGCGGTGAAATGCATAGATATGACACAGAACACCGATTGCGAAGGCAGCTCACTAAACTATAACTGACGCTGAGGCACGAAAGCGTGGGGATCAAACAGG` |
| ASV4896 | 404 | `TTAGGAATATTGGACAATGGGCGCAAGCCTGATCCAGCCATGCCGCGTGAACGATGAAGGCCTTCGGGTTGTAAAGTTCTTTAGCCGATGATTATAATGACTGTAGTCGGAAAATAAGCCCCGGCTAACTTCGTGCCAGCAGCCGCGGTAATACGAAGGGGGCGAGCGTTATTCGGAATCACTGGGCGTAAAGCGTGCGTAGGCGGTCTCGTAAGTTAGGAGTGAAAGCCCAGGGCTCAACCTTGGAATTGCTCTTAAAACTGCGAGGCTAGAATCTCGGAGAGGATAGCGGAATTTCCAGTGTAGCAGTGAAATGCGTAGATATTGGAAGGAACACCAGTGGCGTAAGCGGCTATCTGGACGAGTATTGACGCTGAGGCACGAAAGCGTGGGGATCAAACAGG` |
| ASV1749 | 429 | `TGGGGAATTTTGGACAATGGGCGAAAGCCTGATCCAGCCATTCCGCGTGCAGGATGAAGGCCTTCGGGTTGTAAACTGCTTTTGTACAGAACGAAAAGGTTTTCTTTAATAAAGAGGGCTCATGACGGTACTGTAAGAATAAGCACCGGCTAACTACGTGCCAGCAGCCGCGGTAATACGTAGGGTGCAAGCGTTAATCGGAATTACTGGGCGTAAAGCGTGCGCAGGCGGTTATATAAGACAGATGTGAAATCCCCGGGCTCAACCTGGGAACTGCATTTGTGACTGTATGGCTAGAGTACGGTAGAGGGGGATGGAATTCCGCGTGTAGCAGTGAAATGCGTAGATATGCGGAGGAACACCGATGGCGAAGGCAATCCCCTGGACCTGTACTGACGCTCATGCACGAAAGCGTGGGGAGCAAACAGG` |
| ASV5226 | 408 | `TGGGGAATATTGGGCAATGGGCGAAAGCCTTACCCAGCAATACCGCGTGAGGGAAGGAGGTCTTCGGATTGTAAACCTCTTTGATCAGGGACGAAGCAAGTGACGGTACCTGAAGAACAAGCCCCGGCTAACTACGTGCCAGCAGCCGCGGTAATACGTAGGGGGCGAGCGTTGTCCGGAATGACTGGGCGTAAAGGGTGCGTAGGCGGCTTGGCAAGTTAGAAGTGAAATACCTAGGGCTTAACTCAGGTGCTGCTTTTAAGACTGTCGGGCTGGAGTGCAGGAGAGGTTAATGGAATTCCTAGTGTAGCGGTGGAATGCGTAGATATTAGGAGGAACACCAGAGGCGAAGGCGATTAACTGGACTGTAACTGACGCTGAGGCACGAAAGTGTGGGGAGCAAACAGG` |
| ASV4195 | 428 | `TGGGGAATCTTGCGCAATGGACGAAAGTCTGACGCAGCGACGCCGCGTGGGTGATGAAGGCCTTCGGGTTGTAAAGCCCTGTGGGGAGGGACGAATAAGTGTTGGTTAATACCCAGCGCGATGACGGTACCTCCTTAGCAAGCACCGGCTAACTCTGTGCCAGCAGCCGCGGTAAGACAGAGGGTGCAAACGTTGTTCGGAATTACTGGGCGTAAAGCGCGTGTAGGCGGCTATGCAAGTCGGATGTGAAAGCCCTCGGCTCAACCGGGGAAGTGCACCCGAAACTGCAAAGCTAGAGTCTCGGAGAGGATCGTGGAATTCTCGGTGTAGAGGTGAAATTCGTAGATATCGAGAGGAACACCGGTGGCGAAGGCGGCGATCTGGACGATGACTGACGCTGAGACGCGAAAGCGTGGGGAGCAAACAGG` |
| ASV2376 | 430 | `TGGGGAATATTGGACAATGGGGGGAACCCTGATCCAGCCATGCCGCGTGTGTGAAGAAGGCCTTTTGGTTGTAAAGCACTTTAAGCGAGGAGGAGGATACTTGAGTTAATAGCTTAAGATATTGGACGTTACTCGCAGAATAAGCACCGGCTAACTCTGTGCCAGCAGCCGCGGTAATACAGAGGGTGCAAGCGTTAATCGGAATTACTGGGCGTAAAGCGCGCGTAGGCGGTTTTTTAAGTCGGATGTGAAATCCCTGAGCTTAACTTAGGAATTGCATTCGATACTGGGAAGCTAGAGTATGGGAGAGGATGGTAGAATTCCAGGTGTAGCGGTGAAATGTTCAGAGATCTGGAGGAATACCGATGGCGAAGGCAGCCATCTGGCCTAATACTGACGCTGAGGTGCGAAAGCATGGGGAGCAAACAGG` |
| ASV4964 | 429 | `TGGGGAATATTGCACAATGGGCGCAAGCCTGATGCAGCCATGCCGCGTGTATGAAGAAGGCCTTCGGGTTGTAAAGTACTTTCAGTCGTGAGGAAGGTTGTAGCGTTAATAGCGTTATGATTTGACGTTAGCGACAGAAGAAGCACCGGCTAACTCCGTGCCAGCAGCCGCGGTAATACGGAGGGTGCGAGCGTTAATCGGAATTACTGGGCGTAAAGCGCATGCAGGTGGTTTGTTAAGTCAGATGTGAAAGCCCTGGGCTCAACCTAGGAGTTGCATTTGAAACTGACAAGCTAGAGTACTGTAGAGGGGGGTAGAATTTCAGGTGTAGCGGTGAAATGCGTAGAGATCTGAAGGAATACCGGTGGCGAAGGCGGCCCCCTGGACAGATACTGACACTCAGATGCGAAAGCGTGGGGAGCAAACAGG` |
| ASV2837 | 429 | `TGGGGAATATTGGACAATGGGGGGAACCCTGATCCAGCGATGCCGCGTGTGTGAAGAAGGCCTGAGGGTTGTAAAGCACTTTAGTTGGGGAGGAAAGGCTCTCTTCTAACAGGAGAGAGAAAAGACGTTACCCAAAGAATAAGCACCGGCTAACTCTGTGCCAGCAGCCGCGGTAATACAGAGGGTGCAAGCGTTAATCGGAATGACTGGGCGTAAAGGGCACGTAGGCGGATAGATGTGTCATAGGTGAAATCCCCGGGCTTAACCTGGGAACTGCCTGTGAAACTGTGTATCTAGAATAGAGTAGAGGTAAGTGGAATTTCCGGTGTAGCGGTGAAATGCGTAGATATCGGAAAGAACACCAGTGGCGAAGGCGACTTACTGGACTCATATTGACGCTGAGGTGCGAAAGCGTGGGGAGCAAACGGG` |
| ASV704 | 429 | `TGGGGAATTTTGGACAATGGGGGAAACCCTGATCCAGCAACGCCGCGTGAATGAAGAAGGCCTTAGGGTTGTAAAGTTCTTTTGTCGCAGAGCAAAATGCTTGTACTAATACTATGAGTAGATGAGAGTATGCGAAGAATAAGCACCGGCTAACTACGTGCCAGCAGCCGCGGTAATACGTAGGGTGCAAGCGTTAATCGGAATTACTGGGCGTAAAGCGTGCGCAGGCGGATATATAAGTTAGAAGTGAAATCCCCGGGCTCAACCTGGGAACTGCTTTTAAGACTGTATATCTAGAGTTTGTCAGAGGGGGGTGGAATTCCAAGTGTAGCAGTGAAATGCGTAGAGATTTGGAGGAACACCGATGGCGAAGGCAGCCCCCTGGGATAAAACTGACGCTCAGGCACGAAAGCGTGGGTAGCAAACAGG` |
| ASV723 | 424 | `TAAGGAATATTGGTCAATGGGCGAAAGCCTGAACCAGCCATCCCGCGTGCAGGATTAAGGTCCTATGGATTGTAAACTGCTTTTATTAGGGAAGAAACGCCTTCATTTATGAGGGTCTGACGGTACCTAAGGAATAAGCACCGGCTAACTCCGTGCCAGCAGCCGCGGTAATACGGAGGGTGCAAGCGTTATCCGGAATCACTGGGTTTAAAGGGTGCGTAGGCGGGTTATTAAGTCAGTGGTGAAAGCTGGTCGCTCAACGATCAAATTGCCATTGATACTGATAGTCTTGAATTGAGTTGAGGTTAGCGGAATGTGACATGTAGCGGTGAAATGCGTAGATATGTCATGGAACACCAATTGCGAAGGCAGCTAACTAGGCTTATATTGACGCTGAGGCACGAAAGCGTGGGTAGCGAACAGG` |
| ASV5473 | 430 | `TGGGGAATATTGCACAATGGACGCAAGTCTGATGCAGCAATGCCGCGTGAGTGAAGAAGGCCCTTGGGTCGTAAAGCTCTTTCGATTGGAAAGAAGGGGCGCTTGGTAAATAGCCAAGTGTTCTGACGGTACCTAAAGAAGAAGCACCGGCTAATTTCGTGCCAGCAGCCGCGGTAATACGAAAGGTGCAAGCGTTGTTCGGAATCATTGGGCGTAAAGCGCACGTAGGCGGGGCTGTGTGTCGATTGTGAAAGCCCAGGGCTCAACCCTGGAATTGCAGTCGAAACTGCGGCTCTTGAATACTTGAGAGGGTGGGGGAATTCCTGGTGAAGCAGTGAAATGCGTAGATATCAGGAGGAACATCGGAGGCGAAGGCGCCTACCTGGCAAGATATTGACGCTGAGGTGCGAAAGCGTGGGGAGCAAACAGG` |
| ASV3713 | 429 | `TGGGGAATTTTGGACAATGGGGGCAACCCTGATCCAGCCATGCCGCGTGCGGGAAGAAGGCCTTCGGGTTGTAAACCGCTTTTAGTCGGGAAGAAAAGGCCAAGGCTAATACCCTTGGTTCTTGACGGTACCGGCGGAATAAGCACCGGCTAACTACGTGCCAGCAGCCGCGGTAATACGTAGGGTGCGAGCGTTAATCGGAATTACTGGGCGTAAAGCGTGCGCAGGCGGTTCGTTAAGTCTGATGTGAAAGCCCCGGGCTCAACCTGGGAATGGCATTGGAAACTGGCGAGCTGGAGTGCGGCAGAGGGGGGTGGAATTCCGCGTGTAGCAGTGAAATGCGTAGAGATGCGGAGGAACACCGATGGCGAAGGCAGCCCCCTGGGCCGACACTGACGCTCAGGCACGAAAGCGTGGGGAGCAAACAGG` |
| ASV654 | 404 | `TGGGGAATCTTGGACAATGGGCGCAAGCCTGATCCAGCCATGCCGCGTGAGTGAAGAAGGCCTTAGGGTTGTAAAACTCTTTCGCCGGAGAAGATAATGACGGTATCCGGAGAAGAAGCCCCGGCTAACTTCGTGCCAGCAGCCGCGGTAATACGAAGGGGGCTAGCGTTGTTCGGAATTACTGGGCGTAAAGCGCACGTAGGCGGGTTTGTAAGTAGAGGGTGAAATCCCAGGGCTCAACCCTGGAACTGCCTTCTAAACTACAAGCCTGGAGGTCAGGAGAGGCGAGTGGAATACCGAGTGTAGAGGTGAAATTCGTAGATATTCGGTGGAACACCAGTGGCGAAGGCGACTCGCTGGACTGATACTGACGCTGAGGTGCGAAAGCGTGGGGAGCAAACAGG` |
| ASV2119 | 423 | `TAGGGAATATTGGGCAATGGGCGCAAGCCTGACCCAGCCATGCCGCGTGCAGGAAGAAGGCGTTATGCGTTGTAAACTGCTTTTATACAGGAAGAAAATGACCATGCGTGGTAAACTGACGGTACTGTATGAATAAGCACCGGCTAACTCCGTGCCAGCAGCCGCGGTAATACGGAGGGTGCAAGCGTTGTCCGGATTTATTGGGTTTAAAGGGTGCGTAGGCGGCTTTTTAAGTCAGCGGTGAAAGACTGTCGCTCAACGACGGAACTGCCGTTGATACTGATTAGCTTGAGTATGATTGAGGCAGCCGGAATGGATAGTGTAGCGGTGAAATGCATAGATATTATCCAGAACGCCAATAGCGTAGGCAGGTTGCTAAGTCATTACTGACGCTGAGGCACGAGAGTGCGGGGATCAAACAGG` |
| ASV14 | 424 | `TGAGGAATATTGGACAATGGGCGCAAGCCTGATCCAGCCATGCCGCGTGCAGGATGACGGTCCTATGGATTGTAAACTGCTTTTGTACAGGAAGAAACACTCCCTCGTGAGGGAGCTTGACGGTACTGTAAGAATAAGGATCGGCTAACTCCGTGCCAGCAGCCGCGGTAATACGGAGGATCCAAGCGTTATCCGGAATCATTGGGTTTAAAGGGTCCGTAGGCGGTTTAATAAGTCAGTGGTGAAAGCCCATCGCTCAACGGTGGAACGGCCATTGATACTGTTAAACTTGAATTATTAGGAAGTAACTAGAATATGTAGTGTAGCGGTGAAATGCTTAGAGATTACATGGAATACCAATTGCGAAGGCAGGTTACTACTAATGGATTGACGCTGATGGACGAAAGCGTGGGTAGCGAACAGG` |
| ASV2542 | 405 | `TGGGGAATTTTACACAATGGGCGAAAGCCTGATGTAGCAACGCCGCGTGGAGGATGAAGGTTTTCGGATTGTAAACTCCTTTTATTAGGGAAGAAATTTGACGGTACCTAATGAATAAGCGCCGGCTAACTCCGTGCCAGCAGCCGCGGTAATACGGAGGGCGCAAGCGTTACTCGGAATCACTGGGCGTAAAGGGAGCGTAGGCTTTTTATCAAGTCTCGAGTGAAATGCTATAGCTTAACTATAGAACTGCTTGGGAAACTGATAGAATAGAGTATGGAAGAGGTAGGTGGAATTTCCAGTGTAGGGGTAAAATCCGTAGATATTGGAAGGAATACCAGAAGCGAAGGCGACCTACTGGGACATTACTGACGCTGAGGCTCGAAAGCGTGGGGAGCAAACAGG` |
| ASV5466 | 428 | `TGGGGAATATTGGACAATGGGCGCAAGCCTGATCCAGCAATGCCGCGTGTGTGAAGAAGGCCCGAGGGTTGTAAAGCACTTTCAGCGGGGAGGAAAAAGAGATTGTTAATACCGGTCTTATTGACATTACCCGCAGAAGAAGCACCGGCTAACTCCGTGCCAGCAGCCGCGGTAATACGGAGGGTGCGAGCGTTAATCGGAATTACTGGGCGTAAAGCGTGCGTAGGCGGCGCGTTAAGCCAGGTGTGAAAGCCCCGGGCTCAACCTGGGAACTGCACTTGGGACTGGCGCGCTAGAGTTGGGCAGAGGGGGGTGGAATTCCGGGTGTAGCGGTGAAATGCGTAGATATCCGGAGGAACACCAGTGGCGAAGGCGGCCCCCTGGGTCCAAACTGACGCTGAGGCACGAAAGCGTGGGTAGCAAACAGG` |
| ASV3505 | 429 | `TGGGGAATTTTGGACAATGGGGGCAACCCTGATCCAGCCATTCCGCGTGAGTGAAGAAGGCCTTCGGGTTGTAAAGCTCTTTCAGCAGGAACGAAACGGTGGCTACTAATACTAGCTACTAATGACGGTACCTGAAGAAGAAGCACCGGCTAACTACGTGCCAGCAGCCGCGGTAATACGTAGGGTGCGAGCGTTAATCGGAATTACTGGGCGTAAAGCGTGCGCAGGCGGTTATGTAAGACAGGTGTGAAATCCCCGGGCTTAACCTGGGAACTGCGCTTGTGACTGCATAACTCGAGTGTGGCAGAGGGGGGTGGAATTCCACGTGTAGCAGTGAAATGCGTAGAGATGTGGAGGAACACCGATGGCGAAGGCAGCCCCCTGGGTCAACACTGACGCTCATGCACGAAAGCGTGGGGAGCAAACAGG` |
| ASV4718 | 430 | `TGGGGAATATTGCGCAATGGGCGAAAGCCTGACGCAGCGACGCCGCGTGGGTGATGAAGGCCTTCGGGTCGTAAAGCCCTGTCGGGAGGGAAGAAATCTCGGTAGCCCAATACGCTGCCGACTTGACGGTACCTCCTAAGGAAGCACCGGCTAACTCCGTGCCAGCAGCCGCGGTAATACGGAGGGTGCGAGCGTTGTTCGGAATCACTGGGCGTAAAGCGCGTGTAGGCGGCCTCTTAAGTTTGGTGTGAAAGCCCGGGGCTCACCCCCGGAAGTGCACCGGATACTGGGCGGCTCGAGTACCGGAGAGGAGGGTGGAATTCCTGGTGTAGCGGTGAAATGCGTAGATATCAGGAGGAACACCGGCGGCGAAGGCGGCCCTCTGGACGGATACTGACGCTGAGACGCGAAAGCGTGGGGAGCAAACAGG` |
| ASV5691 | 404 | `TGGGGAATCTTGCGCAATGGGGGCAACCCTGACGCAGCGACGCCGCGTGCGGGACGAAGGCCTTCGGGTCGTAAACCGCTTTCAGCAGGGATGAGACAAGACGGTACCTGCAGAAGAAGCCCCGGCTAACTACGTGCCAGCAGCCGCGGTAATACGTAGGGGGCGAGCGTTATCCGGATTCATTGGGCGTAAAGCGCGCGTAGGCGGCCCGGCAGGCAGGGGGTCAAATGGCGGGGCTCAACCCCGTCCCGCCCCCTGAACCGCCGGGCTCGGGTCCGGTAGGGGAGGGTGGAACACCCGGTGTAGCGGTGGAATGCGCAGATATCGGGTGGAACACCGGTGGCGAAGGCGGCCCTCTGGGCCGAGACCGACGCTGAGGCGCGAAAGCTGGGGGAGCGAACAGG` |
| ASV4640 | 429 | `TGGGGAATATTGGACAATGGGGGCAACCCTGATCCAGCAATGCCGCGTGTGTGAAGAAGGCCTTCGGGTTGTAAAGCACTTTAGGCTGAGAGGAAGGTGGGGTGTTTAAGAGACGCGTCATTTGACTGTATCAGCAGAATAAGCACCGGCTAACTCTGTGCCAGCAGCCGCGGTAATACAGAGGGTGCAAGCGTTAATCGGAATTACTGGGCGTAAAGGGCGCGTAGGCGGTTAGTTAAGTGAGATGTGAAATCCCTGGGCTTAACCTAGGAACTGCATCTGAAACTGACTGGCTGGAGTACTGTAGAGGGTAGTGGAATTTCCGGTGTAGCGGTGAAATGCGTAGAGATCGGAAGGAACACCAGTGGCGAAGGCGGCTACCTGGACAGATACTGACGCTGAGGCGCGAGAGCGTGGGGAGCAAACAGG` |
| ASV4504 | 407 | `TGAGGAATCTTCCGCAATGGGCGAAAGCCTGACGGAGCGACACCGCGTGAAGGATGAAGTGTTTTTACACGTAAACTTCTGTTGTGAGGGACGAAATTTTTGACGGTACCTCACGAGAAAGCACCGGCTAATTCTGTGCCAGCAGCCGCGGTAAGACAGAAGGTGCAAGCGTTACTCGGAATTACTGGGCGTAAAGCGTCCGCAGCCGATCATGTCAGTCTGGTGTTAAAGCCCGGGGCCCAACCCCGGAATTGTGCCGGAAACTGCACGAATTGAGTCATACAGAGGCATCTGGAATATCGTGTGTAGGGGTAAAATCCGTTGATCCACGGTAGAACGCCAAAAGCGAAGGCAGGATGCTGGGTATGTACTGACGGTCAGGGACGAAAGCGTGGGGAGCAAACGGG` |
| ASV4509 | 429 | `TGGGGAATCTTGCGCAATGCGCGAAAGCGTGACGCAGCAACGCCGCGTGGGGGAAGAAGGCTCTCGGGTCGTAAACCCCTTTCAGTTGGGACGAAGCTTCGCCGGTGAATAGCCGGCCGGAGTGACGGTACCTTCACAAGAAGCCCCGGCTAACTACGTGCCAGCAGCCGCGGTAATACGTAGGGGGCAAGCGTTGTCCGGAATCATTGGGCGTAAAGCGCGTGTAGGCGGCTTGATAAGTCTGTTGTGAAAGTCCAGGGCTCAACCCTGGAATGCCGATGGATACTGTCAAGCTCGAGTCCGGAAGAGGCGAGTGGAATTCCTGGTGTAGCGGTGAAATGCGCAGATATCAGGAGGAACACCAATGGCGAAGGCAGCTCGCTGGGACGGTACTGACGCTGAGACGCGAAAGCGTGGGGAGCAAACAGG` |
| ASV5090 | 424 | `TGAGGAATATTGGTCAATGGGCGCGAGCCTGAACCAGCCAAGTAGCGTGCAGGAAGACGGCCCTATGGGTTGTAAACTGCTTTTGCGCGGGGATAACTTCCGCCACGTGTGGCGGCTTGCAGGTACCGCGTGAATAAGGACCGGCTAATTCCGTGCCAGCAGCCGCGGTAATACGGAAGGTCCGGGCGTTATCCGGATTCATTGGGTTTAAAGGGAGCGTAGGCCGCGCGTCAAGCGTGCCGTGAAATGCCGTGGCTCAACCATGGCACCGCGGCGCGAACTGTCGCGCTTGAGTGGGCTCGAGGCAGGCGGAATTCGTGGTGTAGCGGTGAAATGCTTAGATATCACGAGGAACCCCGATTGCGAAGGCAGCCTGCCAGCGCCGCACTGACGCTGAAGCTCGAAGGCGTGGGTATCGAACAGG` |
| ASV1638 | 429 | `TGGGGAATTTTGGACAATGGGGGCAACCCTGATCCAGCCATTCCGCGTGAGTGAAGAAGGCCTTCGGGTTGTAAAGCTCTTTCGCAAGGGAAGAAACGATTTAGGTAAATAATCTAAGTTAATGACGGTACCTTGATAAGAAGCACCGGCTAACTACGTGCCAGCAGCCGCGGTAATACGTAGGGTGCGAGCGTTAATCGGAATTACTGGGCGTAAAGCGTGCGCAGGCTGTTTTGTAAGTCAGATGTGAAATCCCCGAGCTCAACTTGGGAACTGCGTTTGAAACTACAAGACTAGAATATGTCAGAGGGGGGTAGAATTCCACGTGTAGCAGTGAAATGCGTAGAGATGTGGAGGAATATCAATGGCGAAGGCAGCCCCCTGGGATAATATTGACGCTCATGCACGAAAGCGTGGGGAGCAAACAGG` |
| ASV3136 | 430 | `TGGGGAATATTGCACAATGGGCGCAAGCCTGATGCAGCGACGTCGCGTGGAGGATGAAGGTTTTCGGATTGTAAACTCCTGTTAAACGGGAAGAAAGAACTGTGCCTAATAAGTACGGGAGATGACGTTACCGTTAAAGAAAGCACCGGCTAACTTCGTGCCAGCAGCCGCGGTAATACGAAGGGTGCGAGCGTTATTCGGAATAACTGGGCGTAAAGGGCGCGTAGGCGGTTTTCTAAGTTGACGGTTAAAAGCCATGGCTTAACCATGGAGATGCCGGCGATACTGGAATGACTAGAGGGTAGTAGAGAGGAGTGGAATTCTCGGAGTAGCGGTAAAATGCGTAGATCTCGAGAGGAACACCGATGGCGAAGGCAGCTCCTTGGGCTACATCTGACGCTAAGGCGCGAAAGCGTGGGGAGCAAACAGG` |
| ASV1108 | 407 | `TGGGGAATATTGGGCAATGGGCGAAAGCCTGACCCAGCAACGCCGCGTGAGGGAAGAAGGTCTTCGGATTGTAAACCTTTGTCCTATGGGACGAAACAAATGACGGTACCATAGGAGGAAGCTCCGGCTAACTACGTGCCAGCAGCCGCGGTAATACGTAGGGAGCAAGCGTTGTCCGGAATTACTGGGCGTAAAGGGTGCGTAGGTGGTTATGTAAGTCAGATGTGAAAGACCCGGGCTTAACCCGGGGATTGCATTTGAAACTGTGTAACTTGAGTACAGGAGAGGGAAGTGGAATTCCTAGTGTAGCGGTGAAATGCGTAGATATTAGGAGGAACACCGGTGGCGAAGGCGACTTTCTGGACTGTAACTGACACTGAGGCACGAAAGCGTGGGGAGCAAACAGG` |
| ASV3061 | 429 | `TGGGGAATATTGGACAATGGGGGCAACCCTGATCCAGCGACGCCGCGTGTGTGAAGAAGGCCTGCGGGTTGTAAAGCACTTTTAGTGGGGATGAAAAGCACATTACTAACACTGGTGTGTCTTGACCTAACCCAAAGAAAAAGCACCGGCTAACTCTGTGCCAGCAGCCGCGGTAATACAGAGGGTGCGAGCGTTAATCGGAATTACTGGGCGTAAAGCGTGCGTAGACGGTTATTTAAGTCGGGTGTGAAAGCCCCGGGCTCAACCTGGGAATTGCATTCGAGACTGAATAGCTAGGGTACGGTAGAGGGAAGTGGAATTTCCGGTGTAGCGGTGAAATGCGTAGATATCGGAAGGAACATCAGTGGCGAAAGCGACTTCCTGGACCAGTACCGACGTTCAGGCACGAAAGCGTGGGGAGCAAACAGG` |
| ASV5082 | 404 | `CGACGAATCTTCCGCAATGGGCGAAAGCCTGACGGAGCGACGCCGCGTGTGGGATGAAGCGGCTTTGCCGTGTAAACCACTGTCAGGGAATAGAAACATTGATCATTTCCAAAGGAAGGGGCGACTAACTCTGTGCCAGCAGTCGCGGTAATACAGAGGCCCCGAGCGTTAGTCGGAATTACTGGGCTTAAAGGGTGCGTAGGCGGACTTCTAAGTGTCGCGTGAAAGCCCACGGCTTAACCGTGGAACCGCGCGGCAAACTGGGAGTCTTGAGGAACGTATAGGCGGGTGGAACGATAGGTGGAGCGGTGGAATGCGTAGATATCTATCGGAACGCCGAAGGTGAAGACAGCCCGCTGGGCGTTTCCTGACGCTGAGGCACGAAAGCGTGGGTAGCAAACAGG` |
| ASV3474 | 424 | `TAGGGAATATTGGGCAATGGGCGAGAGCCTGACCCAGCCATGCCGCGTGCAGGACGAAGGCCCTCTGGGTTGTAAACTGCTTTTATCGGGGAAGAAGAGCAAGTATGCGTACTTGTGTGACGGTACCCGAGGAATCAGCACCGGCTAACTCCGTGCCAGCAGCCGCGGTAATACGGAGGGTGCAAGCGTTGTCCGGATTTATTGGGTTTAAAGGGTGCGTAGGTGGCCTGTTAAGTCCGCTTTGAAAGCTGGTCGCTCAACGATCAGGGGTGGGTGGATACTGGCGGGCTTGAATGATGTCGAGGTTACTGGAACGGGTCGTGTAGCGGTGAAATGCATAGATATGACCCAGAACTCCGATTGCGAAGGCAGGTGGCTAGGCATCGATTGACACTGAGGCACGAGAGCATGGGGAGCAAACAGG` |
| ASV3703 | 429 | `TGGGGAATATTGGACAATGGGCGCAAGCCTGATCCAGCAATGCCGCGTGTGTGAAGAAGGCCTTAGGGTTGTAAAGCACTTTCAGAAGGGAGGAAAAGCTCCTTCATAATACGAATGAGAAAAGACGTTACCTTCAGAAGAAGCACCGGCTAACTCTGTGCCAGCAGCCGCGGTAATACAGAGGGTGCAAGCGTTAATCGGAATTATTGGGCGTAAAGCGTGTGTAGGTGGTATGACAAGTCGGTTGTGAAAACCCCGGGCTCAACCTGGGAATTGCGATCGAAACTGTTGAACTAGAGTGTGGAAGAGGGAAGTGGAATTTCCGGTGTAGCGGTGAAATGCGTAGATATCGGAAGGAACATCAGTGGCGAAGGCGACTTCCTGGTCCAACACTGACACTGAGGCACGAAAGCGTGGGGAGCAAACAGG` |
| ASV4203 | 429 | `TGGGGAATATTGGACAATGGGCGAAAGCCTGATCCAGCAATGCCGCGTGTGTGAAGAAGGCCTTCGGGTTGTAAAGCACTTTCGTCGGGGAGGAAGGGTATATAGCAAATACCTATATACTTTGACGTTACCCGAATAAGAAGCACTGGCTAACTCTGTGCCAGCAGCCGCGGTAATACAGAGGGTGCGAGCGTTAATCGGAATTACTGGGCGTAAAGGGTGCGTAGGTTGCCAATCAAGTCAGATGTGAAAGCCCTGGGCTTAACCTAGGAATGGCATTTGATACTAGTTGGCTCGAGTGCAGTAGAGAGAGGCGGAATTTCCGGTGTAGCGGTGAAATGCGTAGATATCGGAAGGAACACCAGTGGCGAAGGCGGCCTCTTGGACTGTAACTGACACTGATGCACGAAAGCGTGGGGAGCAAACAGG` |
| ASV632 | 430 | `TGGGGAATATTGCGCAATGGGGGAAACCCTGACGCAGCAACGCCGCGTGAGTGAGGAAGGACTTCGGTCTGTAAAGCTCTGTTAATGTGGAAAAATGGTAGTTGGTCTAATAGGCCGATTATTTGATGGTACACATAGAGGAAGCACCGGCTAACTTCGTGCCAGCAGCCGCGGTAATACGAAGGGTGCAAGCGTTGTTCGGATTTATTGGGCGTAAAGCGCGCGTAGGCGGGCCTGCAAGTCAGATGTGAAATCTCGAGGCTCAACCTCGAAACTGCGTCTGAAACTACAGGTCTAGAGTCTCGGAGGGGGAAGGGGAATTTCGCATGTAGGGGTAAAATCCGTAGATATGCGAAGGAACACCAGAGGCGAAGGCGCCTTCCTGGACGAGTACTGACGCTGAGGCGCGAAAGCGTGGGGATCAAACAGG` |
| ASV3849 | 424 | `TGAGGAATATTGCGCAATGGAGGCAACTCTGACGCAGCAATACCGCGTGAACGAAGAAGGTCTATGGATTGTAAAGTTCTGTCGGGAGGGAAGAACGAGTGGGTTAGTAGCTCACTGTGACGGTACCTCCAAAGGAAGCACCGGCTAACTCCGTGCCAGCAGCCGCGGTAATACGGAGGGTGCAAGCGTTGTCCGGAATCATTGGGCGTAAAGGGTGCGTAGGTGGGAAGATACGTCTGAAGTGAAATCCTGCGGCTTAACCGTAGAACTGCTTTGGATACGGTCTTTCTTGAGTCATAGAGGGGGTTGCGGAATTTCAGGTGTAGCGGTGAAATGTGTAGATATCTGAAAGAACACCGGTGGCGAAGGCGGCAACCTGGCTATGTACTGACACTGAGGCACGAAAGTGCGGGGATCAAACAGG` |
| ASV4358 | 424 | `TAAGGAATATTGGTCAATGGGGGCAACCCTGAACCAGCCATGCCGCGTGCAGGAAGACGGCCCTATGGGTTGTAAACTGCTTTTGTACCGGAGAAAACCACCTTACGTGTAGGGTGTTGATAGTATGGTAAGAATAAGCATCGGCTAACTTCGTGCCAGCAGCCGCGGTAAGACGAAGGATGCAAGCGTTATCCGGATTCATTGGGTTTAAAGGGTGCGTAGGCGGACTGATAAGTCAGTGGTGAAATCTCTCAGCTTAACTGAGAAACTGCCATTGATACTGTTAGTCTAGAGTATAGATGACGTTGGCGGAATATGACATGTAGTGGTGAAATACTTAGATATGTCATAGAACACCGATTGCGAAGGCAGCTAACGAAACTATAACTGACGCTGAGGCACGAAAGTGCGGGGATCAAACAGG` |
| ASV1329 | 424 | `TGAGGAATATTGGTCAATGGGCGCAAGCCTGAACCAGCCATGCCGCGTGCAGGAAGACGGTCCTATGGATTGTAAACTGCTTTTGTACGAGAAGAAACACTCCTATGTATAGGAGCTTGACGGTATCGTAAGAATAAGGATCGGCTAACTCCGTGCCAGCAGCCGCGGTAATACGGAGGATCCAAGCGTTATCCGGAATCATTGGGTTTAAAGGGTCCGTAGGCGGTTTAGTAAGTCAGTGGTGAAAGCCCATCGCTCAACGGTGGAACGGCCATTGATACTGCTATACTTGAATTATTAGGAAGTAACTAGAATATGTAGTGTAGCGGTGAAATGCTTAGAGATTACATGGAATACCAATTGCGAAGGCAGGTTACTACTAATATATTGACGCTGATGGACGAAAGCGTGGGTAGCGAACAGG` |
| ASV2286 | 424 | `TAAGGAATATTGGACAATGGTCGCAAGACTGATCCAGCCATGCCGCGTGAAGGATGAAGGCCCTATGGGTTGTAAACTTCTTTTGTATGGGAAAAAACCCCTCTACGTGTAGAGGGCTGATTGTACCATAAGAATAAGGATCGGCTAACTCCGTGCCAGCAGCCGCGGTAATACGGAGGATCCAAGCGTTATCCGGATTCATTGGGTTTAAAGGGTGCGTAGGCGGAACGATAAGTCAGTGGTGAAAGCCCTCAGCTTAACTGAGGAACTGCCATTGATACTGTCGATCTTGAGTACATTTGATGTGGGCGGAATGTGTCATGTAGCGGTGAAATGCTTAGATATGACACAGAACACCGATTGCGAAGGCAGCTCACAAAACTGTAACTGACGCTGAGGCACGAAAGCGTGGGTATCAAACAGG` |
| ASV3543 | 430 | `TGGGGAATTGTTCGCAATGGGCGCAAGCCTGACGACGCAACGCCGCGTGGGGGATGAAGGTCTTCGGATTGTAAACCCTTGTCGAACGGGAAGAACGACTCGCGCATGAACATTGCGCGGGACTGACGGTACCGTTAAAGGAAGCCACGGCTAACTCTGTGCCAGCAGCCGCGGTAATACAGAGGTGGCAAGCGTTGTTCGGAATTACTGGGCGTAAAGGGCGCGTAGGCGGCCATCTAAGTCAGACGTGAAATCCCCCGGCTTAACCTGGGAACTGCGTCTGATACTGGAAGGCTCGAGTTTGGGAGAGGGATGTGGAATTCCAGGTGTAGCGGTGAAATGCGTAGATATCTGGAGGAACACCGGTGGCGAAGGCGGCATCCTGGACCAACACTGACGCTGAGGCGCGAAAGCTAGGGGAGCAAACGGG` |
| ASV2662 | 429 | `TGGGGAATTTTGGACAATGGGGGCAACCCTGATCCAGCCATGCCGCGTGAGTGAAGAAGGCCTTCGGGTTGTAAAGCTCTTTCGGCCGGGAAGAAATCGCACAGACTAATACTCTGTGTGGATGACGGTACCGGAATAAGAAGCACCGGCTAACTACGTGCCAGCAGCCGCGGTAATACGTAGGGTGCGAGCGTTAATCGGAATTACTGGGCGTAAAGCGTGCGCAGGCGGTTTTGTAAGACAGGCGTGAAATCCCCGGGCTCAACCTGGGCATTGCGCTAGTGACTGCAAGGCTAGAGTACGGCAGAGGGGGGTAGAATTCCACGTGTAGCAGTGAAATGCGTAGAGATGTGGAGGAATACCGATGGCGAAGGCAGCCCCCTGGGTCGATACTGACGCTCATGCACGAAAGCGTGGGGAGCAAACAGG` |
| ASV1495 | 404 | `TGGGGAATCTTAGACAATGGGGGCAACCCTGATCTAGCCATGCCGCGTGATCGATGAAGGCCTTAGGGTTGTAAAGATCTTTCAGTTGGGAAGATAATGACGGTACCAACAGAAGAAGCCCCGGCTAACTCCGTGCCAGCAGCCGCGGTAATACGGAGGGGGCTAGCGTTATTCGGAATTACTGGGCGTAAAGCGCACGTAGGCGGATCGGAAAGTTGGGGGTGAAAGCCCAGGGCTCAACCCTGGAACTGCCTTCAAAACTCCCGGTCTTGAGGTCGAGAGAGGTGAGTGGAATTCCGAGTGTAGAGGTGAAATTCGTAGATATTCGGAGGAACACCAGTGGCGAAGGCGGCTCACTGGCTCGATACTGACGCTGAGGTGCGAAAGCGTGGGGAGCAAACAGG` |
| ASV3271 | 429 | `TGGGGAATTTTGGACAATGGGCGCAAGCCTGATCCAGCAATGCCGCGTGCAGGAAGAAGGCCTTCGGGTTGTAAACTGCTTTTGTCAGGGAAGAAATCCCTTGGGTTAATACCTCGGGGGGATGACGGTACCTGAAGAATAAGCACCGGCTAACTACGTGCCAGCAGCCGCGGTAATACGTAGGGTGCGAGCGTTAATCGGAATTACTGGGCGTAAAGCGTGCGCAGGCGGCTTTGCAAGACAGATGTGAAATCCCCGGGCTCAACCTGGGAACTGCATTTGTGACTGCAAGGCTAGAGTACGGCAGAGGGGGATGGAATTCCGCGTGTAGCAGTGAAATGCGTAGATATGCGGAGGAACACCAATGGCGAAGGCAATCCCCTGGGCCTGTACTGACGCTCATGCACGAAAGCGTGGGGAGCAAACAGG` |
| ASV5388 | 404 | `TAAGGAATATTGGACAATGGAGGAAACTCTGATCCAGCCATGCCGCGTGAGTGATGAAGGCCTTCGGGTTGTAAAGCTCTTTCAGATGTGAAAATGATGATGGTAACATCAGAAGAAGCCCCGGCTAACTTCGTGCCAGCAGCCGCGGTAATACGAAGGGGGCGAGCGTTGTTCGGAATCACTGGGCTTAAAGCGTGCGTAGGCGGATTTATAAGTCAGAAGTGAAAGCCCTGGGCTCAACCCAGGAACTGCTTTTGAGACTGTAAGTCTAGAATTCGGTAGAGGTTAGTGGAATATCCAGTGTAGAGGTGAAATTCGCAGATATTGGATAGAACACCAGTGGCGAAGGCGACTAACTGGGCCGACATTGACGCTGAGGTACGAAAGCGTGGGTAGCAAACAGG` |
| ASV396 | 429 | `TGGGGAATTTTGGACAATGGGGGCAACCCTGATCCAGCAATGCCGCGTGAGTGAAGAAGGCCTTCGGGTTGTAAAGCTCTTTTGTTAGGGAAGAAAAGGTGAGCAATAATACTGCTCACTGATGACGGTACCTAAAGAATAAGCACCGGCTAACTACGTGCCAGCAGCCGCGGTAATACGTAGGGTGCAAGCGTTAATCGGAATTACTGGGCGTAAAGCGTGCGCAGGCGGCCTGTTAAGTCAGATGTGAAATCCCCGAGCTCAACTTGGGAACTGCATTTGAAACTGATAGGCTAGAGTGTGTCAGAGGGAGGTAGAATTCCACGTGTAGCAGTGAAATGCGTAGAGATGTGGAGGAATACCAGTGGCGAAGGCGGCCTCCTGGGATAACACTGACGCTCATGTACGAAAGCGTGGGGAGCAAACAGG` |
| ASV1466 | 424 | `TGAGGAATATTGGTCAATGGAGGCAACTCTGAACCAGCCATCCCGCGTGAAGGATGAATGCCCTAGTGGTTGTAAACTTCTTTTATATGGGAATAAACCTAGGTACGTGTGCCTAGCTGAAGGTACCATATGAATAAGCATCGGCTAACTCCGTGCCAGCAGCCGCGGTAATACGGAGGATGCAAGCGTTATCCGGATTTATTGGGTTTAAAGGGTCCGCAGGCGGACTATTAAGTCAGTGGTGAAAGCCTGCAGCTTAACTGTAGAACTGCCATTGATACTGATAGTCTTGAGTGTGGTTGATGTGGGCGGAATATGACATGTAGCGGTGAAATGCATAGAGATGTCATAGAACACCAATTGCGAAGGCAGCTCACAAAACCATTACTGACGCTCAGGGACGAAAGCGTGGGGAGCAAACAGG` |
| ASV4007 | 429 | `TGGGGAATATTGCGCAATGGGGGCAACCCTGACGCAGCCATGCCGCGTGTGTGAAGAAGGCCTTCGGGTTGTAAAGCACTTTCAGAGGGGAGGAAAGGCTCAGGGCTAATATCCCTGGGAGTTGACGCTACCCTCAGAAGAAGCACCGGCTAATTCCGTGCCAGCAGCCGCGGTAATACGGAAGGTGCAAGCGTTAATCGGAATTACTGGGCGTAAAGCGCGCGTAGGCGGCTTGGTAAGTCGGGTGTGAAAGCCCCGGGCTCAACCTGGGAACTGCATTCGATACTGCCGAGCTGGAGTACGAGAGAGGGGGGTGGAATTCCAGGTGTAGCGGTGAAATGCGTAGAGATCTGGAGGAACACCGGTGGCGAAGGCGGCCCCCTGGTTCGATACTGACGCTGAGGTGCGAAAGCGTGGGGAGCAAACAGG` |
| ASV5298 | 429 | `TGGGGAATATTGGACAATGGGGGAAACCCTGATCCAGCGATGCCGCGTGTGTGAAGAAGGCCTAAGGGTTGTAAAGCACTTTCGGTGGGGAGGAGGACATCTAGGTTAAGAGCTAGGATGTTGGACGTTACCCACATAAGAAGCACCGGCTAACTCCGTGCCAGCAGCCGCGGTAATACGGAGGGTGCAAGCGTTAATCGGAATTACTGGGCGTAAAGGGTGCGTAGGCGGTTTAATAAGTTAAGTGTGAAAGTCCCGGGCTTAACCTGGGGAAGTCACTTAAGACTGTTAGACTAGAGTATGGGAGAGGGTAGTGGAATTTCCGGTGTAGCGGTGAAATGCGTAGAGATCGGAAGGAACACCAGTGGCGAAGGCGGCTACCTGGCCCAATACTGACGCTGAGGCACGAAAGCGTGGGGAGCAAACAGG` |
| ASV4801 | 424 | `TAGGGAATATTGGGCAATGGAGGCAACTCTGACCCAGCCATGCCGCGTGAACGAAGAAGGCGCTATGCGTCGTAAAGTTCTTTTAGAGGGGGCGAAAACACTCGATTCATCGAGTACTGACGGTACCTTCGGAATAAGCACCGGCTAACTCTGTGCCAGCAGCCGCGGTAATACAGAGGGTGCAAGCGTTATCCGGAATCACTGGGTTTAAAGGGTGCGTAGGCGGGATTATAAGTCAGAGGTGAAATCTCGTCGCTTAACGACGGAATTGCCTTTGATACTGTTTTTCTTGAAATGTGTTGAGGTTGGCGGAATGAGACATGTAGCGGTGAAATGCTTAGATATGTCTTAGAACACCGATTGCGAAGGCAGCTGACTGGACACTATTTGACGCTGAGGCACGAAAGCGTGGGGATCAAACAGG` |
| ASV481 | 404 | `TGGGGAATATTGGACAATGGGCGAAAGCCTGATCCAGCAATGCCGCGTGAGTGATGAAGGCCTTAGGGTTGTAAAGCTCTTTTACCAGGGATGATAATGACAGTACCTGGAGAATAAGCTCCGGCTAACTCCGTGCCAGCAGCCGCGGTAATACGGAGGGAGCTAGCGTTGTTCGGAATTACTGGGCGTAAAGCGTACGTAGGCGGTTATTCAAGTCAGAGGTGAAAGCCTGGAGCTCAACTCCAGAACTGCCTTTGAAACTAGGTAGCTAGAATCACGGAGAGGTTAGTGGAATTCCGAGTGTAGAGGTGAAATTCGTAGATATTCGGAAGAACACCAGTGGCGAAGGCGACTAACTGGACGTGTATTGACGCTGAGGTACGAAAGCGTGGGGAGCAAACAGG` |
| ASV5573 | 388 | `TCGAGTATTTTCTGCAATGTACGAAAGTATGACAGAGCGAGCCAGAGTGGTTTTCTATAATTGAAAACCTTTTGTGACATGTAGATAGTGTTACGAATAAGGACCGGGCAAGACTGGTGCCAGTCGCCGCGGTAATCCCAGCGGTCCAAGTCGCAACCACAATTATTGGGTCTAAAACATCCGTAGCTTGCCTGGTAAGTCTCTTGTGAAATCCGGCCTCTTAAGGGTCGAAGAGCAAGAGATACTGCTGGGCTAGAGACCGGTAGAGGTAAGAAGTATGGTCGGGGTACCGGTAAAATGGGTTGATCCTGTCCAGACTAACAACAGCGAAGGCATCTTACCAGCACGGATCTGACAGTAAGGGATGAAGGCTAGGGGCGCAAAAGGG` |
| ASV2797 | 429 | `TGGGGAATTTTGGACAATGGGCGAAAGCCTGATCCAGCAATGCCGCGTGTGTGAAGAAGGCCTTCGGGTTGTAAAGCACTTTTGTCAGGAACGAAACGGTTGGGGCTAATATCCCTGGCTAATGACGGTACCTGAAGAATAAGCACCGGCTAACTACGTGCCAGCAGCCGCGGTAATACGTAGGGTGCAAGCGTTAATCGGAATTACTGGGCGTAAAGCGTGCGCAGGCGGTTATGTAAGACAGATGTGAAATGCCCGGGCTTAACCTGGGGACTGCATTTGTGACTGCATGGCTAGAATCTGGCAGAGGGGGGTAGAATTCCACGTGTAGCAGTGAAATGCGTAGAGATGTGGAGGAACACCGATGGCGAAGGCAGCCCCCTGGGCTAAGATTGACGCTCATGCACGAAAGCGTGGGGAGCAAACAGG` |
| ASV4479 | 430 | `TAGGGAATATTGCGCAATGGGCGAAAGCCTGACGCAGCAATACCGCGTGAGTGAGGAAGGTCTTCGGATTGTAAAACTCTGTCGGGATGGAAAAATGACGTATGGTCTAATAGGCCGTACGAGTGATGGTACATCCAAAGGAAGCACCGGCTAACTTCGTGCCAGCAGCCGCGGTAATACGAAGGGTGCAAGCGTTGTTCGGATTTATTGGGCGTAAAGCGCGCGCAGGCGGGTAATTAAGTCAGGTGTGAAATCTCAGGGCTCAACCCTGAAACTGCGCCTGAAACTGGTTATCTAGAATGTCGGAGAGGGAAGGGGAATTTCGCATGTAGGGGTAAAATCCGTAGATATGCGAAGGAATACCAGAGGCGAAGGCGCCTTCCTGGACGATGATTGACGCTGAGGCGCGAAAGCGTGGGGAGCAAACAGG` |
| ASV4734 | 428 | `TGGGGAATCTTGCGCAATGGGCGAAAGCCTGACGCAGCGACGCCGCGTGAGTGATGAAGGCCTTCGGGTTGTAAAGCTCTGTGGGGAGAGACGAATAAGTGTTGGCCAATATCCAGCATGATGACGGTATCTCTTTAGCAAGCACCGGCTAACTCTGTGCCAGCAGCCGCGGTAAGACAGAGGGTGCAAACGTTGTTCGGAATTACTGGGCGTAAAGCGTGTGTAGGCGGCCACGCAAGTCGGATGTGAAAGCCCCGGGCTCAACCCGGGAAGTGCACTCGATACTGCGGAGCTTGAGTGTCGGAGAGGTTGGTGGAATTCTCGGTGTAGAGGTGAAATTCGTAGATATCGAGAGGAACACCGGTGGCGAAGGCGGCCAACTGGACGAACACTGACGCTGAGACACGAAAGCGTGGGGAGCAAACAGG` |
| ASV3050 | 404 | `TGGGGAATATTGGACAATGGGCGCAAGCCTGATCCAGCAATACCGCGTGAGTGATGAAGGCCTTAGGGTTGTAAAGCTCTTTTAGCAGGGAAGATAATGACGGTACCTGCAGAAAAAGCCCCGGCTAACTCCGTGCCAGCAGCCGCGGTAAGACGGAGGGGGCTAGCGTTGTTCGGAATTACTGGGCGTAAAGAGCGCGTAGGCGGCTTGGCAAGTTGGAAGTGAAATCCCGGGGCTTAACCTCGGAACTGCTTTCAAAACTGCTGGGCTAGAGTATGGTAGGGGGTGATGGAATTCCTAGTGTAGAGGTGAAATTCTTAGATATTAGGAGGAACACCGGTGGCGAAGGCGGTCACCTGGGCCAATACTGACGCTGAGGCGCGAAAGCGTGGGGAGCAAACAGG` |
| ASV5215 | 430 | `TGGGGAATATTGGACAATGGGCGAAAGCCTGATCCAGCCATGCCGCGTGTGTGAAGAAGGTCTTCGGATTGTAAAGCACTTTAAGTCAGGAAGAAGGGCTCAGATCTAATACATCTTGAATCTTGACGCTACTGACAGAATAAGCACCGGCAAACTTCGTGCCAGCAGCCGCGGTAATACGAAGGGTGCAAGCGTTAATCGGAATCACTGGGCGTAAAGCGTGCGTAGGTGGTTTTACAGGTTGGATGTGAAAACCTTGGGCTTAACCTGAGGCCTGCATCCAAAACGGTAAGACTAGAGTAGAGCAGAGAGTAGTGGAACTTCCGGTGTAGCGGTGAAATGCGTAGAGATCGGAAAGAACATCAGTGGCGAAGGCGACTGCTTGGGCTCATACTGACACTGAGGTACGAAAGCGTGGGGAGCAAACAGG` |
| ASV2083 | 404 | `TGGGGAATATTGGACAATGGGCGCAAGCCTGATCCAGCAATGCCGCGTGAGTGATGAAGGCCTTCGGGTTGTAAAGCTCTTTTACCTGCGACGATGATGACGGTAGCAGGAGAATAAGCCCCGGCTAACTTCGTGCCAGCAGCCGCGGTAATACGAAGGGGGCTAGCGTTGTTCGGAATTACTGGGCGTAAAGGGCGCGTAGGCGGCCCTGTCAGTCAAGCGTGAAAGCCCCGGGCTCAACCTGGGAATTGCGCCTGATACTGCAGGGCTTGAGGGTCGGAGAGGATGGTGGAATTCCCAGTGTAGAGGTGAAATTCGTAGATATTGGGAAGAACACCGGTGGCGAAGGCGGCCATCTGGACGACATCTGACGCTGAGGCGCGAAAGCGTGGGGAGCAAACAGG` |
| ASV5246 | 424 | `TAAGGAATATTGGACAATGGACGAAAGTCTGATCCAGCCATGCCGCGTGAAGGATTAAGGTCCTCTGGATTGTAAACTTCTTTTATTTGGGACGAAAAAAGGGAATTCTTTCCCACTTGACGGTACCAGATGAATAAGCACCGGCTAACTCCGTGCCAGCAGCCGCGGTAATACGGAGGGTGCAAGCGTTATCCGGATTCACTGGGTTTAAAGGGTGCGTAGGTGGGCTTGTAAGTCAGTGGTGAAATCCCGAAGCTTAACTTCGGAACTGCCATTGATACTATTAGTCTTGAATATTGTGGAGGTAAACGGAATATGTCATGTAGCGGTGAAATGCTTAGATATGACATAGAACACCAATTGCGAAGGCAGTTTGCTACACATATATTGACACTGAGGCACGAAAGCGTGGGGATCAAACAGG` |
| ASV5376 | 408 | `TGAGGAATCTTCCGCAATGGGCGAAAGCCTGACGGAGCGACACCGCGTGAAGGATGAAGCCCTTTCTGGGTGTAAACTTCTGTTCTGAGGGACGAAATTTTTGACGGTACCTCAGGAGAAAGCATCGGCTAACCCTGTGCCAGCAGCCGCGGTAAGACAGGGGATGCAAGCGTTACTCGGAATTACTGGGCGTAAAGCGTCTGCAGGCGTCTTGCCACGTCTGGTGTAAAAGCACGGAGCTCAACTCCGTGTACGTGCCGGAAACGAGCGAGATCGAGTCACTCAGAGGCATCTGGAATGTCGTGTGTAGGGGTAAAATCCGTAGATCCACGATGGAACGCCGAAAGCGAAGGCAGGATGCTGGGAGTGTACTGACGCTCAGAGACGAAAGCGTGGGGAGCAAAGGGG` |
| ASV5423 | 404 | `TGGGGAATATTGGACAATGGGCGAAAGCCTGATCCAGCGACGCCGCGTGAGTGATGAAGGCCTTCGGGTTGTAAAGCTCTTTTAGTGGGAAAGATGATGACGGTACCTACAGAAAAAGCCCCGGCTAACTTCGTGCCAGCAGCCGCGGTAATACGAAGGGGGCTAGCGTTACTCGGAATTACTGGGCGTAAAGCGTGCGTAGGCGGTTAAGTAAGTTGGAAGTGAAAGCCTAGGGCTCAACCTTAGAATTGCTTTCAATACTGCTTGACTAGAGTATTAGAGGGGGTAGTGGAATTCCTAGTGTAGAGGTGAAATTCGTAGATATTAGGAGGAACATCGGAAGCGAAAGCGGCTACCTGGCTAGTAACTGACGCTGTTGCACGAAAGCGTGGGGAGCAAACAGG` |
| ASV2740 | 404 | `TGGGGAATATTGGACAATGGGCGCAAGCCTGATCCAGCAATACCGCGTGAGTGATGAAGGTCTTCGGATTGTAAAGCTCTTTTAGTAGGGAAGATAATGACGGTACCTACAGAAAAAGCACCGGCAAACTTCGTGCCAGCAGCCGCGGTAATACGAAGGGTGCTAGCGTTGTTCGGAATCATTGGGCGTAAAGCGTACGTAGGCGGTTAGGTAAGTCGATTGTGAAAGCCCTGGGCTTAACTCAGGAATTGCAACCGAAACTGCTTAGCTAGAGTATGGTAGGGGATAGTGGAATTCCTAGTGTAGGGGTGAAATCCGTAGATATTAGGAGGAATACCGGTGGCGAAAGCGACTATCTATGCCATTACTGACGCTAAGGTACGAAAGCGTGGGGAGCAAACAGG` |
| ASV5343 | 425 | `TGAGGAATTTTGGACAATGGGGGCAACCCTGATCCAGCGACGCCGCGTGAGGGACGAAGGTCTTCGGATTGTAAACCTCTTTTATTCGGGAATAAGAAACTTATGGAAACATAGGGAGTGAATGTACCGAATGAATAAGCCACGGCTAACTTCGTGCCAGCAGCCGCGGTAATACGAAGGTGGCAAGCGTTACTCGGAATTACTAGGCGTAAAGGGCAGGTAGGTGGTTTGATAAGTCTGTTGTGAAAGCTCCTGGCTTAACTGGGAGAGGTCAACAGAAACTATCAGGCTTGAGTATGGGAGAGGGTACTGGAATTCCCGGTGTAGCGGTGAAATGCGCAGAGATCGGGAGGAACACCTATGGCGAAAGCAGGTACCTGGACCATTACTGACACTCAGCTGCGAAAGCTAGGGGAGCAAACAGG` |
| ASV4014 | 425 | `TAGGGAATATTGGGCAATGGGCGAAAGCCTGACCCAGCCATGCCGCGTGCAGGAAGAATGCCCTCTGGGTTGTAAACTGCTTTTTTGGGGAAAGAAAAAGTTCTTTGCGAAGAACCTTGACGGTACCCTAAGAATAAGCCACGGCTAACTACGTGCCAGCAGCCGCGGTAATACGTAGGTGGCGAGCGTTATCCGGATTTATTGGGTTTAAAGGGTGCGTAGGCGGGTATTTAAGTCGGTGGTGAAAGGTAGCAGCTCAACTGTTTTACATGCTGCCGATACTGAATATCTTGAATTATGAGAAGGCAGATAGAATTTCTGGTGTAGCGGTGAAATGCTTAGATACCAGAAGGAATACCGATTGCGAAGGCAGTCTGCTGCACATATATTGACGCTGAGGCACGAAAGTATGGGGATCAAACAGG` |
| ASV5152 | 405 | `TGAGGAATCTTGCACAATGGGCGAAAGCCTGATGCAGCGACGCCGCGTGAGGGACGACGGCCTTCGGGTTGTAAACCTCTGTCAGTGGGGAAGAAGATTGACGGTACCCACTGAGGAAGCTTCGGCTAACTACGTGCCAGCAGCCGCGGTAATACGTAGGGAGCAAGCGTTGCCCGGAATTACTGGGCGTAAAGAGCTCGTAGGCGGGGGCGTGCGTCCGAGAAGAAATCTCATGGCTCAACCATGGGGCTATCTCGGATACGGCGCTTCTTGAGGGTGAGAGAGGAAAGCGGAATTCCAGGTGTAGCGGTGAAATGCGTAGATATCTGGAGGAACACCAGCGGCGAAGGCGGCTTTCTGGCTCATTCCTGACGCTGAGGAGCGAAAGCGTGGGTAGCGAACGGG` |
| ASV5528 | 406 | `TGGGGAATTTTCCGCAATGGGCGAAAGCCTGACGGAGCAATACCGCGTGAGGGAGGAAGGCCTTAGGGTTGTAAACCTCTTTTCTCTGGGAAGAAGATCTGACGGTACCAGAGGAATAAGCCTCGGCTAACTCCGTGCCAGCAGCCGCGGTAAGACGGAGGAGGCAAGCGTTATCCGGAATTATTGGGCGTAAAGCGTCCGTAGGCGGTTAATTAAGTCTGTTGTTAAAGCCCACAGCTCAACTGTGGATCGGCAATGGAAACTGGTTGACTAGAGTGTGGTAGGGGTAGAGGGAATTCCCGGTGTAGCGGTGAAATGCGTAGATATCGGGAAGAACACCAGTGGCGAAGGCGCTCTACTGGGCCACAACTGACGCTGATGGACGAAAGCTAGGGGAGCGAAAGGG` |
| ASV3161 | 430 | `TGGGGAATATTGGACAATGGGGGCAACCCTGATCCAGCAATGCCGCGTGTGTGAAGAAGGCCTGCGGGTTGTAAAGCACTTTTGTTCGAGACGAAATCTTGATGATTAATACTTATCAAGGATGACGGTATCGAAAGAATAAGCACCGGCTAACTTCGTGCCAGCAGCCGCGGTAATACGAAGGGTGCAAGCGTTAATCGGAATTACTGGGCGTAAAGCGTGCGTAGACGGCACATTAAGTCATTTGTGAAACCCCCGGGCTCAACCTGGGACCTGCAGATGATACTGATGTGCTTGGAGTTTGAGAGAGGGTGGTGGAATTCCTGGTGTAGCGGTGAAATGCGTAGAGATCAGGAGGAACACTAGTGGCGAAGGCGGCCACCTGGCTTAAAACTGACGTTGAGGCACGAAAGCGTGGGGAGCAAACAGG` |
| ASV5187 | 407 | `TTAGGAATTTTGCACAATGGGGGAAACCCTGATGCAGCGACGCCGTGTGGGTGATGAAGGCCTTCGGGTTGTAAAGCCCCTTTAGTCAAGACGAAAAAAATGACGGTATTGACAGAAAAAGCACCGGCTAACTCCGTGCCAGCAGCCGCGGTAATACGGAGGGTGCAAGCGTTACTCGGATTTACTGGGCGTAAAGCGCATGTAGGCGGTTAAATAAGTCTGTTGTGAAATCCCGGGGCTCAACCCCGGAAGTGCAGTGGATACTGTTTAACTAGAATTCAGGAGAGGAGAGTGGAATTCCTGGTGTAGAGGTGAAATTCGTAGATATCAGGAGGAACACCGGTGGCGAAGGCGGCTCTCTGGACTGACATTGACGCTGAGATGCGAAAGCGTGGGTAGCAAACAGG` |
| ASV1921 | 408 | `TCGAGAATCTTCCGCAATGGACGAAAGTCTGACGGAGCGACGCCGCGTGGTGGATGAAGCCCTTTGGGGCGTAAACACCTTTTATGGAGGAGAAAGTTTATTGATGTTACTCCATGAATAAGGGGCTCCCAACTCTGTGCCAGCAGGAGCGGTAATACAGAGGCCCCAAGCATTATCCGGATTTACTGGGCGTAAAGGGTGCGTAGGCGGTTATATTAGTCGGGTGTTAAATCCCGGGGCTCAACCCCGGAACTGCATCCGAAACGGTATGACTAGAATCAGTCAGAGGCAAGCAGAACTCACGGTGTAGGGGTGAAATCCGTTGATATCGTGGGGAATACCAAATGCGAAGGCAGCTTGCTGGGACTTGATTGACGCTGAGGCACGAAAGCGTGGGTAGCGAAGCGG` |
| ASV1545 | 429 | `TGGGGAATATTGGACAATGGGCGCAAGCCTGATCCAGCCATGCCGCGTGTGTGAAGAAGGCCTTCGGGTTGTAAAGCACTTTCAGTGGGGAGAAAGGTGATTCACCTAATACGTGAGTTAATTGATGTTACCCACAGAAGAAGCACCGGCTAACTCCGTGCCAGCAGCCGCGGTAATACGGAGGGTGCAAGCGTTAATCGGAATTACTGGGCGTAAAGCGCACGTAGGTGGTTTGTTAAGCCAGCTGTGAAATCCCCGGGCTCAACCTGGGCACTGCAGTTGGAACTGGCAAGCTAGAGTAGGGTAGAGGGGTGTGGAATTCCAGGTGTAGCGGTGAAATGCGTAGATATCTGGAGGAACATCAGTGGCGAAGGCGACACCCTGGACTCATACTGACACTGAGGTGCGAAAGCGTGGGGAGCAAACAGG` |
| ASV1306 | 424 | `TGAGGAATATTGGACAATGGGTGAGAGCCTGATCCAGCCATCCCGCGTGAAGGAAGACGGCCCTACGGGTTGTAAACTTCTTTTATATAGGAAGAAACCTACATACGTGTATGTAGCTGACGGTACTGTATGAATAAGCATCGGCTAACTCCGTGCCAGCAGCCGCGGTAATACGGAGGATGCGAGCGTTATCCGGATTTATTGGGTTTAAAGGGTCCGTAGGCGGTAGATTAAGTCAGTGGTGAAATCCTACAGCTCAACTGTAGAATTGCCATTGATACTGGTTTACTTGAGTAAGGTTGAAGTGGCTAGAATGTGTAGTGTAGCGGTGAAATGCATAGATATTACACGGAATACCGATAGCGAAGGCAGGTCACTAAGCCTTTACTGACGCTGAGGGACGAAAGCGTGGGGAGCAAACAGG` |
| ASV1919 | 407 | `TGGGGAATTTTCCGCAATGGGCGAAAGCCTGACGGAGCAATACCGCGTGAGGGAAGAAGGCTCTTGGGTTGTAAACCTCTTTTCTTGGGGAAGAAAAAAATGACGGTACCCAAGGAATCAGCATCGGCTAACTCCGTGCCAGCAGCCGCGGTAATACGGAGGATGCAAGCGTTATCCGGAATGATTGGGCGTAAAGCGTCCGCAGGTGGCAATTCAAGTTTGCGGTTAAAGGTTCTGGCTCAACCAGAGACAAGCCGTGAAAACTGAATAGCTAGAGTATGGTAGGGGCAGAGGGAATTCCCAGTGTAGCGGTGAAATGCGTAGAGATTGGGAAGAACACCGGTGGCGAAAGCGCTCTGCTAGGCCAAAACTGACACTGAGGGACGAAAGCTAGGGGAGCGAATGGG` |
| ASV473 | 429 | `TGGGGAATATTGGACAATGGGCGCAAGCCTGATCCAGCCATGCCGCGTGTGTGAAGAAGGCCTTCGGGTTGTAAAGCACTTTTGTTGGGGAAGAAAAGACCTGGGTTAATACCCTGGGTTCATGACGGTACCCAAAGAATAAGCACCGGCTAACTTCGTGCCAGCAGCCGCGGTAATACGAAGGGTGCAAGCGTTACTCGGAATTACTGGGCGTAAAGCGTGCGTAGGTGGTTCGTTAAGTCTGCCGTGAAAGCCCCGGGCTCAACCTGGGAATGGCGGTGGATACTGGCGGACTAGAGTGCGGTAGAGGGTGGTGGAATTCCCGGTGTAGCAGTGAAATGCGTAGAGATCGGGAGGAACATCTGTGGCGAAGGCGGCCACCTGGACCAGCACTGACACTGAGGCACGAAAGCGTGGGGAGCAAACAGG` |
| ASV1285 | 429 | `TGGGGAATTTTGGACAATGGGCGCAAGCCTGATCCAGCAATGCCGCGTGCAGGATGAAGGCCTTCGGGTTGTAAACTGCTTTTGTACGGAACGAAACGGTCCTTTCTAATACAGGGGGCTAATGACGGTACCGTAAGAATAAGCACCGGCTAACTACGTGCCAGCAGCCGCGGTAATACGTAGGGTGCAAGCGTTAATCGGAATTACTGGGCGTAAAGCGTGCGCAGGCGGTTATGTAAGACAGATGTGAAATCCCCGGGCTTAACCTGGGAACTGCATTTGTGACTGCATAGCTAGAGTACGGCAGAGGGGGATGGAATTCCGCGTGTAGCAGTGAAATGCGTAGATATGCGGAGGAACACCGATGGCGAAGGCAATCCCCTGGGCCTGTACTGACGCTCATGCACGAAAGCGTGGGGAGCAAACAGG` |
| ASV1808 | 409 | `TGGGGAATATTGCACAATGGGCGCAAGCCTGATGCAGCAACGCCGCGTGAGGGACGACGGCCTTCGGGTTGTAAACCTCTTTTAGCAGGGAAGAAGCGAAAGTGACGGTACCTGCAGAAAAAGCACCGGCTAACTACGTGCCAGCAGCCGCGGTAATACGTAGGGTGCAAGCGTTATCCGGAATTATTGGGCGTAAAGAGCTCGTAGGCGGTTTGTCGCGTCTGCTGTGAAAACTGGGGGCTCAACCCCCAGCCTGCAGTGGGTACGGGCAGACTAGAGTGCGGTAGGGGAGATTGGAATTCCTGGTGTAGCGGTGGAATGCGCAGATATCAGGAGGAACACCGATGGCGAAGGCAGATCTCTGGGCCGTAACTGACGCTGAGGAGCGAAAGGGTGGGGAGCAAACAGG` |
| ASV2564 | 387 | `GCGAGAAAACTTTGCAATGTGCGAAAGCACGACAAGGTTAATCCGAGTGGTTTCTGCTAAAGAAACCTTTTGTCAGTCCTAGAAACACTGGTGAATAAGGGGTGGGCAAGTTCTGGTGTCAGCCGCCGCGGTAAAACCAGCACCTCAAGTGGTCAGGATGATTATTGGGCCTAAAGCATCCGTAGCTCGTTTTGTAAGTTTTCGGTTAAATCCATGCGCTTAACGTATGGGCTGCCGGTAATACTGCATAACTAGGAAGTGGGAGAGGTAGACGGTACTCGGTAGGAAGGGGTAAAATCCTTTGATCTATTGATGACCACCTGTGGCGAAGGCGGTCTACCAGAACACGTTCGACGGTGAGGGATGAAAGCTGGGGGAGCAAACCGG` |
| ASV1820 | 429 | `TGGGGAATATTGCACAATGGGCGCAAGCCTGATGCAGCCATGCCGCGTGTGTGAAGAAGGCCTTCGGGTTGTAAAGCACTTTCAGCGAGGAGGAAGGGTTCAGTGTTAATAGCACTGTGCATTGACGTTACTCGCAGAAGAAGCACCGGCTAACTCCGTGCCAGCAGCCGCGGTAATACGGAGGGTGCAAGCGTTAATCGGAATTACTGGGCGTAAAGCGCACGCAGGCGGTTTGTTAAGTCAGATGTGAAATCCCCGAGCTTAACTTGGGAACTGCATTTGAAACTGGCAAGCTAGAGTCTTGTAGAGGGGGGTAGAATTCCAGGTGTAGCGGTGAAATGCGTAGAGATCTGGAGGAATACCGGTGGCGAAGGCGGCCCCCTGGACAAAGACTGACGCTCAGGTGCGAAAGCGTGGGGAGCAAACAGG` |
| ASV4697 | 404 | `TAGGGAATCTTGCGCAATGGGCGAAAGCCTGACGCAGCCATGCCGCGTGAATGATGAAGGTCTTAGGATTGTAAAATTCTTTCACCGGGGACGATAATGACGGTACCCGGAGAAGAAGCCCCGGCTAACTTCGTGCCAGCAGCCGCGGTAATACGAAGGGGGCTAGCGTTGCTCGGAATTACTGGGCGTAAAGGGCGCGTAGGCGGATGTTTAAGTTGGGGGTGAAAGCCCGGGGCTCAACCTCGGAATTGCCTTCAATACTGGACATCTTGAGTACGGGAGAGGTGAGTGGAACTCCGAGTGTAGAGGTGAAATTCGTAGATATTCGGAAGAACACCAGTGGCGAAGGCGACTCACTGGCCCGTTACTGACGCTGAGGCGCGAAAGCGTGGGGAGCAAACAGG` |
| ASV1419 | 408 | `TGGGGAATATTGGGCAATGGGCGAAAGCCTGACCCAGCAATGCCGCGTGAGTGATGAAGGTATTTTTATTGTAAAGCTCTTTTCTTTGGAACGAACAAAATGACGGTACCAAAGGAATAAGCCCCGGCTAACTACGTGCCAGCAGCCGCGGTAATACGTAGGGGGCGAGCGTTGTCCGGAATGACTGGGTGTAAAGGGTGTGTAGGTGGTTTGACAAGTTAGAAGTGTAATACCCAGGGCTTAACTCGGGTGCTGCTTCTAAAACTGTCAGACTTGAGTGCAGGAGAGGATAGTGGAATTCCTAGTGTAGCGGTGGAATGCGTAGATATTAGGAGGAACATCAGTGGCGAAGGCGACTATCTGGACTGTAACTGACACTGAGGCACGAAAGCGTGGGGAGCAAACAGG` |
| ASV3159 | 424 | `TAAGGAATATTGGTCAATGGACGAAAGTCTGAACCAGCCATGCCGCGTGAAGGATGAAGGCCCTCTGGGTTGTAAACTTCTTTTATCTGGGACGAAAAAAGGGTATTCTTACTCACTTGACGGTACCAGAGGAATAAGCACCGGCTAACTCCGTGCCAGCAGCCGCGGTAATACGGAGGGTGCAAGCGTTATCCGGATTCACTGGGTTTAAAGGGTGCGTAGGTGGGTTTGTAAGTCAGTGGTGAAATCTCCGGGCTTAACTCGGAAACTGCCATTGATACTATAGATCTTGAATCATCTGGAGGTAAGCGGAATATGTCATGTAGCGGTGAAATGCTTAGATATGACATAGAACACCAATTGCGAAGGCAGCTTACTACGGATATATTGACACTGAGGCACGAAAGCGTGGGGATCAAACAGG` |
| ASV3476 | 404 | `TGGGGAATATTGGGCAATGGGCGAAAGCCTGACCCAGCCATGCCGCGTGAGTGATGACGGCCTTAGGGTTGTAAAGCTCTTTCGCAGGTGAAGATAATGACGGTAACCTGATAAGAAGCCCCGGCTAACTTCGTGCCAGCAGCCGCGGTAATACGAAGGGGGCTAGCGTTGTTCGGAATTACTGGGCGTAAAGCGCATGTAGGCGGATATGCAAGCAAGAGGTGAAATCCCGGGGCTTAACCTCGGAATTGCCTTTTGAACTGCATATCTGGAGTTTTGGAGGGGAGTGCGGAATTTCTAATGTAGAGGTGAAATTCGTAGATATTAGAAGGAACACCGGTGGCGAAGGCGGCACTCTGGACAAATACTGACGCTGAGATGCGAAAGCGTGGGGAGCAAACAGG` |
| ASV99 | 429 | `TGGGGAATATTGGACAATGGGGGCAACCCTGATCCAGCCATGCCGCGTGTGTGAAGAAGGCCTTCGGGTTGTAAAGCACTTTTGTCCGGAAAGAAATCTTCCAGGTTAATAACCTGGGAGGATGACGGTACCGGAAGAATAAGCACCGGCTAACTTCGTGCCAGCAGCCGCGGTAATACGAAGGGTGCAAGCGTTACTCGGAATTACTGGGCGTAAAGCGTGCGTAGGCGGTTCGTTAAGTCTGACGTGAAATCCCCGGGCTCAACCTGGGACGTGCGTTGGAAACTGGCGATCTAGAGTACGGTAGAGGGTGGTGGAATTCCCGGTGTAGCAGTGAAATGCGTAGAGATCGGGAGGAACACTCGTGGCGAAGGCGGCCACCTGGACCAGTACTGACGCTGAGGCACGAAAGCGTGGGGAGCAAACAGG` |
| ASV5261 | 424 | `TGAGGAATATTGGTCAATGGGCGAGAGCCTGAACCGGCCAAGTCGCGTGAAGGATGAAGGTTCTATGGATTGTAAACTTCTTTTATTGGGGAATAAAGTGCTCTACGTGTAGAGTTTTGTATGTACCCATTGAATAAGCATCGGCTAACTCCGTGCCAGCAGCCGCGGTAATACGGAGGATGCGAGCGTTATCCGGATTTATTGGGTTTAAAGGGTGCGTAGGTGGTGACTTAAGTCAGCGGTGAAAGTTTGTCGCTCAACGATAAAATTGCCATTGATACTGAGTTTCTTGAGTATATTTGAGGTAGGCGGAATGCGTGGTGTAGCGGTGAAATGCTTAGATATCACGCAGAACTCCGATTGCGAAGGCAGCTTACTAAACTATAACTGACACTGAAGCACGAAAGCGTGGGTATCAAACAGG` |
| ASV5294 | 404 | `TGGGGAATATTGCACAATGGGCGAAAGCCTGATGCAGCAACGCCGCGTGAGTGATGAAGGTCTTCGGATTGTAAAGCTCTGTCTTTGGGGACGATAATGACGGTACCCAAGGAGGAAGCCACGGCTAACTACGTGCCAGCAGCCGCGGTAATACGTAGGTGGCAAGCGTTGTCCGGATTTATTGGGCGTAAAGGATGCGTAGGCGGATGTTTAAGTGAGATGTGAAATACCCGAGCTCAACTTGGGTACTGCATTTCAAACTGGATATCTAGAGTGCAGGAGAGGAGAATGGAATTCCTAGTGTAGCGGTGAAATGCGTAGAGATTAGGAAGAACACCAGTGGCGAAGGCGATTCTCTGGACTGTAACTGACGCTGAGGCATGAAAGCGTGGGTAGCAAACAGG` |
| ASV5522 | 404 | `TGGGGAATCTTGGACAATGGGGGCAACCCTGATCCAGCCATGCCGCGTGAGTGATGAAGGCCTTCGGGTTGTAAAGCTCTTTTATCAGGGAAGATAATGACTGTACCTGAAGAATAAGCCCCGGCTAACTTCGTGCCAGCAGCCGCGGTAATACGAAGGGGGCGAGCGTTGTTCGGAATTACTGGGCGTAAAGCGTACGTAGGCTGCTTAGCAAGTCAGAAGTGAAATCCCAGGGCTTAACCCTGGAATTGCTCTTGATACTGCTGAGCTCGAATTCGAGAGAGGATAGCGGAATACCCAGTGTAGAGGTGAAATTCGTAGATATTGGGTGGAACACCGGTGGCGAAGGCGGCTATCTGGCTCGACATTGACGCTGAGGTACGAAAGCGTGGGGAGCAAACAGG` |
| ASV4067 | 421 | `TGGGGAATATTGCGCAATGGTCGAAAGACTGACGCAGCGACGCCGCGTGGAGGATGAAGCCCCTCGGGGTGTAAACTCCTGTTGCTCGGGACGAACCTCTCCTTTCGAGGAGACTGACGGTACCGAGTGAGGAAGCACCGGCTAACTCTGTGCCAGCAGCCGCGGTAATACAGAGGGTGCGAGCGTTGTCCGGAATCACTGGGCGTAAAGGGCGCGTAGGCGGCTTGGTAAGCGTGCGGTGAAAGCTCAGGGCTCAACCCTGAGTCTGCCGTGCGAACTGCCGAGCTGGAGCACGGTAGAGGCACATGGAATTCCGGGTGTAGCGGTGGAATGCGTAGAGATCCGGAAGAACACCGGTGGCGAAGGCGGTGTGCTGGGCCGTTGCTGACGCTGAGGCGCGACAGCGTGGGGAGCAAACAGG` |
| ASV530 | 407 | `TGGGGAATTTTCCGCAATGGGCGAAAGCCTGACGGAGCAATACCGCGTGAGGGAGGAAGGTTTTTGGATTGTAAACCTCTTTTTTAAAGGAAGAAGAAAGTGACGGTACTTTAAGAATAAGCATCGGCTAACTCCGTGCCAGCAGCCGCGGTAATACGGAGGATGCAAGCGTTATCCGGAATCATTGGGCGTAAAGCGTCCGCAGGTGGTTCATCAAGTCTGCTGTTAAAGACAGAAGCTCAACTTCTGATAGGCGGTGGAAACTGGTGAACTAGAGTACGGTAGGGGTTGAGGGAATTCCCAGTGTAGCGGTGAAATGCGTAGATATTGGGAAGAACACCAGTGGCGAAGGCGCTCAACTGGGCCGTAACTGACACTCAGGGACGAAAGCTAGGGTAGCGAAAGGG` |
| ASV3546 | 429 | `TGGGGAATTTTGGACAATGGGGGCAACCCTGATCCAGCCATTCCGCGTGAGTGAAGAAGGCCTTCGGGTTGTAAAGCTCTTTTGTCCGGAGCGAAACGGGCGGTCCTAATACGATCGTCTACTGACGGTACCGGAAGAATAAGCACCGGCTAACTACGTGCCAGCAGCCGCGGTAATACGTAGGGTGCGAGCGTTAATCGGAATTACTGGGCGTAAAGCGTGCGCAGGCGGTTTTGTAAGCCAGATGTGAAATCCCCGGGCTCAACCTGGGAATGGCATTTGGGACTGCAAGGCTGGAGTGTGGCAGAGGGGACTAGAATTCCTGGTGTAGCAGTGAAATGCGTAGATATCAGGAGGAATACCGATGGCGAAGGCAGGTCCCTGGGCTAACACTGACGCTCATGCACGAAAGCGTGGGGAGCAAACAGG` |
| ASV1384 | 404 | `TAGGGAATATTGGACAATGGGCGAAAGCCTGATCCAGCCATGCCGCGTGAGTGATGAAGGCCTTAGGGTTGTAAAGCTCTTTCACTCGTGAAGATAATGACGGTAACGAGAGAAGAAGCCCCGGCTAACTTCGTGCCAGCAGCCGCGGTAATACGAAGGGGGCGAGCGTTGTTCGGATTTACTGGGCGTAAAGGGCGCGTAGGCGGCCTTTTAAGTTAGGTGTGAAAGCCCGAGGCTTAACCTCGGAACAGCACTTAATACTGGAAGGCTTGAGTATGGCAGAGGTTAGTGGAATTCCCAGTGGAGAGGTGAAATTCGTAGAGATTGGGAGGAACACCAGTAGCGAAGGCGGCTAACTGGGCCATAACTGACGCTGAGGCGCGAAAGCGTGGGTAGCAAACAGG` |
| ASV4161 | 404 | `TGGGGAATATTGGACAATGGGCGCAAGCCTGATCCAGCCATGCCGCGTGAGTGACGAAGGCCTTAGGGTTGTAAAGCTCTTTTGGCGGGGAAGATAATGACGGTACCCGCAGAATAAGCTCCGGCTAACTTCGTGCCAGCAGCCGCGGTAATACGAAGGGAGCTAGCGTTGTTCGGAATCACTGGGCGTAAAGCGCACGTAGGTGGATAGTTAAGTGAGGGGTGAAAGCCCGAGGCTCAACCTCGGAATTGCCTCTCATACTGGCTGTCTCGAGTCCGAGAGAGGTGGGTGGAATTCCTAGTGTAGAGGTGAAATTCGTAGATATTAGGAAGAACACCGGTGGCGAAGGCGGCCCACTGGCTCGGTACTGACACTGAGGTGCGAAAGCGTGGGGAGCAAACAGG` |
| ASV3814 | 404 | `TAAGGAATATTGGACAATGGGCGCAAGCCTGATCCAGCCATGCCGCGTGTGTGATGAAGGCCTTAGGGTTGTAAAGCACTTTCAAACGTGAAGATAATGACGGTAGCGTTAGAAGAAGCTCCGGCTAAATTCGTGCCAGCAGCCGCGGTAATACGAATGGAGCAAGCGTTGTTCGGAATCACTGGGCTTAAAGCGTGCGTAGGCGGCTTTGCAAGTCAGAAGTGAAAGCCTGGGGCTCAACCCCAGAACTGCTTTTGAAACTGCAAAGCTAGAATATCGGAGAGGTTGGGAGAATTCCTAGTGTAGAGGTGAAATTCGCAGATATTAGGAGGAATACCAGTGGCGTAGGCGCCCAACTGGACGATTATTGACGCTGAGGCACGAAAGCGTGGGGAGCAAACAGG` |
| ASV1879 | 429 | `TGGGGAATTTTGGACAATGGGCGCAAGCCTGATCCAGCCATGCCGCGTGCAGGATGAAGGCCTTCGGGTTGTAAACTGCTTTTGTACGGAACGAAACGGCTTTCTCTAATAAAGAAGGCTAATGACGGTACCGTAAGAATAAGCACCGGCTAACTACGTGCCAGCAGCCGCGGTAATACGTAGGGTGCAAGCGTTAATCGGAATTACTGGGCGTAAAGCGTGCGCAGGCGGTTATGTAAGACAGTTGTGAAATCCCCGGGCTCAACCTGGGAACTGCATCTGTGACTGCATAGCTAGAGTACGGTAGAGGGGGATGGAATTCCGCGTGTAGCAGTGAAATGCGTAGATATGCGGAGGAACACCGATGGCGAAGGCAATCCCCTGGACCTGTACTGACGCTCATGCACGAAAGCGTGGGGAGCAAACAGG` |
| ASV2398 | 423 | `TAGGGAATATTGGTCAATGGGCGAGAGCCTGAACCAGCCACGCCGCGTGCAGGATGAAGGCCTTCTGGGTTGTAAACTGCTTTTGACAGGGAACAAAGAATGCTTGCGAGCAGAGATGAGTGTACCTGTAGAATAAGCCACGGCTAACTACGTGCCAGCAGCCGCGGTAATACGTAGGTGGCAAGCGTTGTCCGGATTTATTGGGTTTAAAGGGAGCGTAGGCGGCTTAATAAGTCAGTGGTGAAAGAAGTGGGCTTAACCCACGAGCTGCCATTGATACTGTTTAGCTTGAGTACTGATGAGGTAGGCGGAATTGACAGTGTAGCGGTGAAATGCATAGATATTGTCAAGAACACCGATTGCGAAGGCAGCTTACTAAGGAGTTACTGACGCTGAGGCTCGAAAGCGTGGGGATCGAACAGG` |
| ASV320 | 405 | `TGGGGAATATTGGACAATGGGCGAAAGCCTGATCCAGCGATTCTGCGTGAAGGATGAAGCCTTGTGGGGTGTAAACTTCTTTTCTGGGAGAAGACGAATGACGGTATCTCAGGAATAAGAGACGGCTAACTACGTGCCAGCAGCCGCGGTAATACGTAGGTCTCGAGCGTTATCCGGAATTACTGGGCGTAAAGCGTCTGTAGCCGGATGTATAAGTCTGTCTTTAAATACTCTGATTCAATCGGAGAAAAGGGATAGATACTGTATATCTAGAGGATTTTAGAGGTTAGTGGAATTTCCGGTGGAGCGGTGAAATGCGTTGATATCGGAAGGAATGCCGAAAGCGAAAGCAGCTAACTATAAAATATCTGACGGTCAGAGACGACAGCTTGGGGAGCGAACGGG` |
| ASV1042 | 416 | `TTAGGAATCTTCCCCAATGGGGGCAACCCTGAGGGAGCGACGCCGCTTGCGGGATGAAGGTCTTCGGATCGTAAACCGCTGAAACAAGGACGAAATCCCTGCAAAGGGTCTGACGGTACTTGTGTAATAGCACCGGCTAACTCCGTGCCAGCAGCCGCGGTAATACGGAGGGTGCAAGCGTTACCCGGAATCACTGGGCGTAAAGGGCGTGTAGGTGGTTATTTAAGTCTGGATTTAAAGACCGAAGCTCAACTTCGGGAATGGTTTGGATACTGGATGACTTGACGACTGGAGGGGAGAGTGGAATTCTCGGTGTAGCGGTGGAATGCGTAGATACCGAGAGGAACACCAGTGGCGAAGGCGGCTCTCTGGACAGTGCGTGACACTGAGGCGCGAAAGTGTGGGGAGCGAACCGG` |
| ASV4833 | 405 | `TAGGGAATTTTCCACAATGGGGGCAACCCTGATGGAGCAACGCCGCGTGCAGGATGAATGTCTTCGGATTGTAAACTGCTTTTATCTGTGACGAATATGACGGTAGCAGATGAATAAGGATCGGCTAACTCCGTGCCAGCAGCCGCGGTCATACGGAGGATCCAAGCGTTATCCGGAATTACTGGGCGTAAAGAGTTGCGTAGGTGGCATTGTAAGTAGATAGTGAAATCGTGTGGCTCAACCATACTCACATTATCTAAACTGCAAAGCTAGAGTATGAGAGAGGTACCTGGAATTCCCTGTGTAGGAGTGAAATCCGTAGATATAGGGAGGAACACCGATGGCGTAGGCAGGGTACTGGCTCATTACTGACACTAAGGCACGAAAGCGTGGGTAGCAAACGGG` |
| ASV5220 | 407 | `TCGGGAATATTGGGCAATGGAGGAAACTCTGACCCAGCAATACCGCGTGAAGGATGAAGGTCTTCGGATTGTAAACTTCTGTTATAAAGGAAGAAATAAATGACGGTACTTTATAAGTAAGCCCCGGCAAACTACGTGCCAGCAGCCGCGGTGATACGTAGGGGGCAAGCGTTGTCCGGAATGACTGGGTGTAAAGGGTGTGTAGGTGGCTTATCAAGTTGGAAGTGAAAGCCTGGGGCTTAACTCCAGAACTGCTTTCAAAACTGGTTGGCTTGAGTGTGGGAGAGGTAAGTGGAACTCCTAGTGTAGCGGTGGAATGCGTAGATATTAGGAAGAACATCAGAGGCGAAGGCGACTTACTGGAACATTACTGACACTGAAACACGAAAGCGTGGGGAGCAAACAGG` |
| ASV890 | 429 | `TGGGGAATATTGGACAATGGGCGCAAGCCTGATCCAGCCATGCCGCGTGTGTGAAGAAGGTCTTCGGATTGTAAAGCACTTTAAGTTGGGAGGAAGGTCAGTAGCTTAATACGTTGCTGATTTGACGTTACCGACAGAATAAGCACCGGCTAACTCTGTGCCAGCAGCCGCGGTAATACAGAGGGTGCAAGCGTTAATCGGAATTACTGGGCGTAAAGCGCGCGTAGGTGGTTTTGTAAGTTGGAGGTGAAATCCCCGGGCTCAACCTGGGAACTGCCTCCAAAACTGCAAAGCTAGAGTACGGTAGAGGGTGGTGGAATTTCCTGTGTAGCGGTGAAATGCGTAGATATAGGAAGGAACACCAGTGGCGAAGGCGACCACCTGGACTGATACTGACACTGAGGTGCGAAAGCGTGGGGAGCAAACAGG` |
| ASV3856 | 423 | `TAGGGAATATTGGTCAATGGGCGAGAGCCTGAACCAGCCATGCCGCGTGCAGGAAGAAGGCCCTCTGGGTTGTAAACTGCTTTTGACAGGGGCCAAAATATCCATGCGTGGAGAATTGAGAGTACCTGTAGAATAAGCACCGGCTAACTCCGTGCCAGCAGCCGCGGTAATACGGAGGGTGCAAGCGTTGTCCGGATTTATTGGGTTTAAAGGGTGCGTAGGTGGCCTTATAAGTCAGTGGTGAAAGCCTGCAGCTTAACTGTAGGACTGCCATTGATACTGTAGGGCTTGAGTACTGATGAGGTAGGCGGAATTGATAGTGTAGCGGTGAAATGCATAGATATTATCAAGAACACCGATTGCGAAGGCAGCTTACTAAGGAGTTACTGACACTGAGGCACGAAAGCGTGGGGATCAAACAGG` |
| ASV657 | 429 | `TGGGGAATATTGGACAATGGGGGCAACCCTGATCCAGCCATGCCGCGTGTGTGAAGAAGGCCTTAGGGTTGTAAAGCACTTTCAGTGGGGAGAAAGGTGATTCACCTAATACGTGAGTCAATTGATGCTACCCACAGAAGAAGCACCGGCTAACTCCGTGCCAGCAGCCGCGGTAATACGGAGGGTGCAAGCGTTAATCGGAATTACTGGGCGTAAAGCGCACGTAGGTGGTTTGTTAAGCTAGCTGTGAAATCCCCGGGCTCAACCTGGGCACTGCAGTTAGAACTGGCAAGCTAGAGTAGGGTAGAGGGGTGTGGAATTCCAGGTGTAGCGGTGAAATGCGTAGATATCTGGAGGAACATCAGTGGCGAAGGCGACACCCTGGACTCATACTGACACTGAGGTGCGAAAGCGTGGGGAGCAAACAGG` |
| ASV2416 | 404 | `TGGGGAATATTGGACAATGGGGGAAACCCTGATCCAGCAATGCCGCGTGAATGAAGAAGGCCTTAGGGTTGTAAAATTCTTTTGTTCGTGAAGATAATGACGGTAGCGGACGAATAAGCCCTGGCTAACTCTGTGCCAGCAGCCGCGGTAATACAGAGAGGGCAAGCGTTGTCCGGAATGACTGGGCGTAAAGCGCGCGCAGGCGGTTTTTTAAGTTGGATGTGAAATCCCCGGGCTCAACCTGGGAACTGCATTCGATACTGGAAAACTAGAGGCCGAGAGAGGAAAGCGGAATGACGAGTGTAGAGGTGAAATTCGTAGATATTCGTCGGAACACCAGAGGCGAAGGCGGCTTTCTGGCTCGGACCTGACGCTCAGGCGCGAAAGCGTGGGGAGCAAACAGG` |
| ASV5269 | 405 | `TGGGGAATTTTGCGCAATGGGCGAAAGCCTGACGCAGCAATGCCGCGTGAGCGAAGAAGGCCTTCGGGTCGTAAAGCTCTTTATCTCTGGGAAGATGATGACGGTACCAGAGGAATAAGCACCGGCTAACTACGTGCCAGCAGCCGCGGTAATACGTAGGGTGCGAGCGTTGTTCGGAATTACTGGGCGTAAAGGGTGTGTAGGCGGACTGGTCAGCTAGAGGTGAAAGCCCGGGGCTCAACCCCGGAACTGCTTTTAGAACTGCCAGACTAGAAAAGGTCAGAGGTCAGCGGAATTCCTAGTGTAGAGGTGAAATTCGTAGATATTAGGAGGAACACCGGCGGCGCAAGCGGCTGGCTGGGGCCATTTTGACGCTGAGGCACGAAAGCGTGGGGAGCAAACGGG` |
| ASV2166 | 405 | `TAGGGAATATTGGACAATGGGCGAAAGCCTGATCCAGCAATACCGCGTGGATGATGAAGGCGTTATGCGTTGTAAAGTCCTTTTGTCTGGGAAGATAATGACGGTACCAGAAGAATAAGCACCGACTAACTATGTGCCAGCAGTCGCGGTAATACATAGGGTGCTAGCGTTGCTCGGAATTACTGGGCGTAAAGCATGTGTAGGAGGATTTGTAAGTCAAAAGTGCAATCCCTAGGCTCAACCTAGGAACTGCTTTTGATACTACAAATCTAGAGTGAGATAGAGGATAGCGGAATTCTTAGTGTAGGGGTAAAATCCGTAGATATTAAGAGGAACACCGAAAGCGAAGGCAGCTATCTGGGTCTTTACTGACTCTGAGACATGACAGCGTGGGGAGCAAACAGG` |
| ASV3010 | 429 | `TGGGGAATTTTGGACAATGGGGGCAACCCTGATCCAGCAATGCCGCGTGTGTGAAGAAGGCCTTCGGGTTGTAAAGCACTTTTGTCAGGAACGAAACGGTGGAGGCTAATACCTTCTGCTAATGACGGTACCTGAAGAATAAGCACCGGCTAACTACGTGCCAGCAGCCGCGGTAATACGTAGGGTGCGAGCGTTAATCGGAATTACTGGGCGTAAAGCGTGCGCAGGCGGTTGTGCAAGACAGATGTGAAATCCCCGGGCTCAACCTGGGAATTGCATTTGTGACTGCACGGCTAGAGTGTGTCAGAGGGGGGTAGAATTCCACGTGTAGCAGTGAAATGCGTAGATATGTGGAGGAATACCGATGGCGAAGGCAGCCCCCTGGGATAACACTGACGCTCATGCACGAAAGCGTGGGGAGCAAACAGG` |
| ASV2370 | 404 | `TGGGGAATATTGGACAATGGGCGAAAGCCTGATCCAGCAATGCCGCGTGAGTGATGAAGGCCTTAGGGTTGTAAAGCTCTTTTACCAGGGATGATAATGACAGTACCTGGAGAATAAGCTCCGGCTAACTCCGTGCCAGCAGCCGCGGTAATACGGAGGGAGCTAGCGTTGTTCGGAATTACTGGGCGTAAAGCGCACGTAGGCGGCTATTCAAGTCAGAGGTGAAAGCCCGGAGCTCAACTCCGGAACTGCCTTTGAAACTAGATGGCTCGAATCACAGAGAGGTGAGTGGAATTCCGAGTGTAGAGGTGAAATTCGTAGATATTCGGAAGAACACCAGTGGCGAAGGCGGCTCACTGGCTGTGTATTGACGCTGAGGTGCGAAAGCGTGGGGAGCAAACAGG` |
| ASV5614 | 404 | `TGGGGAATATTGGACAATGGGCGAAAGCCTGATCCAGCCATGCCGCGTGAGTGATGAAGGCCTTAGGGTTGTAAAGCTCTTTCGGCGCAGAATATAATGAACGTATGCGCAGAAGAAGCCCCGGCTAAATTCGTGCCAGCAGCCGCGGTAATACGAATGGGGCAAGCGTTGTTCGGAATCACTGGGCGTAAAGCGTGCGTAGGCGGTCTTACAAGTCAGAAGTGAAAGCCCTGGGCTTAACCTAGGAATTGCTTTTGAAACTGTAGGACTTGAGTCCCGGAGAGGTTGGCGGAATATCCAGTGTAGAGGTGAAATTCGCAGATATTGGATAGAACACCAGTGGCGTAAGCGGCCAACTGGACGGGTACTGACGCTGAGGCACGAAAGCGTGGGGATCAAACAGG` |
| ASV5463 | 429 | `TGGGGAATATTGGACAATGGGGGCAACCCTGATCCAGCAATGCCGCGTGTGTGAAGAAGGCCTTCGGGTTGTAAAGCACTTTAGGTTGAGAGGAAGGTGGCTATTTTAAGAGATAAGCTATTTGACTGTATCAGCAGAATAAGCACCGGCTAACTCTGTGCCAGCAGCCGCGGTAATACAGAGGGTGCAAGCGTTAATCGGAGTTACTGGGCGTAAAGGGCGCGTAGGCGGTTGATTAAGTTGGATGTGAAATCCCTGGGCTTAACCTAGGAACTGCATTCAAAACTAATTGACTAGAGTACTGTAGAGGGTAGTGGAATTTCCGGTGTAGCGGTGAAATGCGTAGAGATCGGAAGGAACACCAGTGGCGAAGGCGGCTACCTGGACAGATACTGACGCTGAGGCGCGAGAGCGTGGGGAGCAAACAGG` |
| ASV4936 | 428 | `TCGAGAATTTTTCACAATGGGCGAAAGCCTGATGGAGCGACGCCGCGTGGGGGATGAAGGGCTTCGGCTCGTAAACCCCTGTCATTTGCGAACAAAGGCGCCAACCTAACACGTTGTTGTCTGATAGTAGCAGAAGAGGAAGGGACGGCTAACTCTGTGCCAGCAGCCGCGGTAATACAGAGGTCCCAAGCGTTGTTCGGATTCACTGGGCGTAAAGGGTGCGTAGGTGGTGAATTAAGTCGGGTGTGAAATCTCCGGGCTCAACCCGGAGCGTGCGCCCGAAACTGATTTGCTAGAGGATAGGAGGGGAGACTGGAATTCTCGGTGTAGCAGTGAAATGCGTAGATATCGAGAGGAACACCGGTGGCGAAGGCGAGTCTCTGGACTATTCCTGACACTGAATCACGAAAGCCGGGGGAGCAAACAGG` |
| ASV1421 | 422 | `TAGGGAATATTGGGCAATGGAGGCAACTCTGACCCAGCCATGCCGCGTGAGGGAAGAAGGCTTTCTGAGTTGTAAACCTCTTTTATATAGGAAGAAAAGCTTACGTGTAAGTCTATGACGGTACTATATGAATAAGCACCGGCTAACTCCGTGCCAGCAGCCGCGGTAATACGGAGGGTGCAAGCGTTGTCCGGATTTATTGGGTTTAAAGGGTACGCAGGCGGTCCTTTAAGTCAGAGCTGAAATTCTACGGCTTAACCGTAGCACCGGCTTTGATACTGGAGGACTTGAATCCACTAAGGGTAGGCGGAATTGATTGTGTAGCGGTGAAATGCATAGATATGATCAAGAACACCGATAGCGAAGGCAGCTTACTGGAGTGGTATTGACGCTCATGTACGAAAGCGTGGGTAGCGAACAGG` |
| ASV5518 | 429 | `TGGGGAATATTGGACAATGGGCGAAAGCCTGATCCAGCGATGCCGCGTGTGTGAAGAAGGCCTGCGGGTTGTAAAGCACTTTCGGTAGGAAAGAAAAAGCGATCACTAATACTGATCGAAATTGACGTTACTTACAGAAGAAGCACCGGCTAACTCTGTGCCAGCAGCCGCGGTAATACAGAGGGTGCAAGCGTTAATCGGAATTACTGGGCGTAAAGGGCGCGTAGGTGGCTGTGTAAGTTGGATGTGAAATACCTGGGCTTAACCTGGGAATGGCATTCAAAACTGTACGGCTAGAGTAAGGTAGAGGGAAGTGGAATTTCCGGTGTAGCGGTGAAATGCGTAGATATCGGAAGGAACACCAGTGGCGAAGGCGGCTTCCTGGACCTATACTGACACTGAGACGCGAAAGCGTGGGGAGCAAACAGG` |
| ASV1993 | 404 | `TGGGGAATATTGGACAATGGGGGCAACCCTGATCCAGCAATGCCGCGTGAGTGATGAAGGCCTTCGGGTTGTAAAGCTCTTTCACCCACGACGATGATGACGGTAGTGGGAGAAGAAGCCCCGGCTAACTTCGTGCCAGCAGCCGCGGTAATACGAAGGGGGCTAGCGTTGTTCGGAGTTACTGGGCGTAAAGGGCGCGTAGGCGGCCTGATCAGTCTGGTGTGAAAGCCCGGGGCTCAACCCCGGAATTGCACTGGAGACGGTCAAGCTTGAGTGTGGGAGAGGATGGTGGAATTCCCAGTGTAGAGGTGAAATTCGTAGATATTGGGAAGAACACCGGTGGCGAAGGCGGCCATCTGGACCATTACTGACGCTGAGGCGCGAAAGCGTGGGGAGCAAACAGG` |
| ASV2415 | 430 | `TGGGGAATTTTGGACAATGGGCGAAAGCCTGATCCAGCCATGCCGCGTGCGGGAAGAAGGCCTTCGGGTTGTAAACCGCTTTAGTCTAGGAAAAAGGGGTGCGAGGTTAATACCCTCGTGCTTTGATGGTACTGGAAGAATAAGCACCGGCTAACTACGTGCCAGCAGCCGCGGTAATACGTAGGGTGCGAGCGTTAATCGGAATTACTGGGCGTAAAGCGTGCGCAGGCGGTCTTGTAAGACAGTCGTGAAAGCCCCGGGCTTAACCTGGGAACTGCGATTGTGACTGCAAGGCTAGAGTGTGTCAGAGGGGGGTGGAATTCCACGTGTAGCAGTGAAATGCGTAGAGATGTGGAGGAACACCGATGGCGAAGGCAGCCCCCTGGGATAACACTGACGCTCATGCACGAAAGCGTGGGGAGCAAACAGG` |
| ASV2381 | 429 | `TGGGGAATATTGGACAATGGGCGCAAGCCTGATCCAGCCATGCCGCGTGGGTGAAGAAGGCCTTCGGGTTGTAAAGCCCTTTTGTTGGGAAAGAAAAGCAGTCGGTTAATACCCGATTGTTCTGACGGTACCCAAAGAATAAGCACCGGCTAACTTCGTGCCAGCAGCCGCGGTAATACGAAGGGTGCAAGCGTTACTCGGAATTACTGGGCGTAAAGCGTGCGTAGGTGGTGGTTTAAGTCTGTTGTGAAAGCCCTGGGCTCAACCTGGGAATTGCAGTGGATACTGGGTCACTAGAGTGTGGTAGAGGGTAGCGGAATTCCCGGTGTAGCAGTGAAATGCGTAGAGATCGGGAGGAACATCCGTGGCGAAGGCGGCTACCTGGACCAACACTGACACTGAGGCACGAAAGCGTGGGGAGCAAACAGG` |
| ASV4841 | 429 | `TGGGGAATATTGGACAATGGGGGCAACCCTGATCCAGCAATGCCGCGTGTGTGAAGAAGGCCTTCGGGTTGTAAAGCACTTTAGGCCGGGAAGAAGATGAGTGGTTTAAGAGATGATTCATTTGACTGTACCGGCAGAATAAGCACCGGCTAACTCTGTGCCAGCAGCCGCGGTAATACAGAGGGTGCGAGCGTTAATCGGAATTACTGGGCGTAAAGGGCGCGTAGGTGGTTTGTTAAGATTGTTGTGAAATCCCTGGGCTTAACCTAGGAACTGCAACGATGACTGACAAGCTTGAGTATTGTAGAGGGTAGTGGAATTTCCGGTGTAGCGGTGAAATGCGTAGAGATCGGAAGGAACACCAGTGGCGAAGGCGGCTACCTGGACAAATACTGACACTGAGGCGCGAGAGCGTGGGGAGCAAACAGG` |
| ASV5621 | 404 | `TGGGGAATATTGGACAATGGGGGAAACCCTGATCCAGCGATGCCGCGTGAGTGAAGAAGGCCTTCGGGTTGTAAAACTCTTTCAGGGGGGAAGATAATGACGGTACCCCCAGAAGAAGCACCGGCTAACTCCGTGCCAGCAGCCGCGGTAAGACGGAGGGTGCAAGCGTTGTTCGGAATTACTGGGCGTAAAGGGCGTGTAGGCTGCTGTGTCAGTCAGGCGTGAAAGCCCCGGGCTTAACCTGGGAACCGCGTTTGATACTGCACAGCTAGAGTATTGGAGAGGAAAGTGGAATTCCTAGTGTAGAGGTGAAATTCGTAGATATTAGGAGGAACACCAGTGGCGAAAGCGGCTTTCTGGACAATTACTGACGCTGAGGCGCGAAAGCGTGGGGAGCAAACAGG` |
| ASV900 | 421 | `TGAGGAATATTGCGCAATGGACGAAAGTCTGACGCAGCAATGCCGCGTGCGGGAAGAAGGTGCTATGCATCGTAAACCGCTGTAGCGTGGGGCAAAAGGCGCCTTATGGGCGCGAGATGGTACCACGAAAGTAAGCATCGGCTAACTACGTGCCAGCAGCCGCGGTAATACGTAGGATGCAAGCGTTGTCCGGATTTACTGGGTGTAAAGGGTGCGCAGGCGGGACTGTAAGTCAGAGGTGAAAGCCCGGAGCTTAACTCCGGAAATGCCTTTGATACTGCGGTTCTTGAATATGATAGAGGCTCATGGAATATGTGGTGTAGCGGTGAAATGCGTAGATATCACATAGAACACCAGTGGCGAAGGCGGTGAGCTGGGTCATTATTGACGCTCATGCACGAAAGTGTGGGTAGCAAACAGG` |
| ASV4132 | 423 | `TAGGGAATATTGGACAATGGGTGCAAGCCTGATCCAGCCATGCCGCGTGCAGGAAGAAAGCCTTCTGGGTTGTAAACTGCTTTTACCAGGGGATAAAATAGTCTTGCGAGACGAATTGAAGGTACCTGGTGAATAAGCCACGGCTAACTACGTGCCAGCAGCCGCGGTAATACGTAGGTGGCAAGCGTTGTCCGGATTTATTGGGTTTAAAGGGTGCGTAGGCGGTAGCCTAAGTCAGCAGTGAAATACGGCAGCTTAACTGTCGAGGTGCTGTTGATACTGGGTTACTTGAGTACTGATGAGGTAGGCGGAATGGACGGTGTAGCGGTGAAATGCTTAGAGATCGTCCAGAACACCGATAGCGAAGGCAGCTTACTAAGGAGTAACTGACGCTGAGGCACGAAAGTGTGGGGATCAAACAGG` |
| ASV5133 | 430 | `TGGGGAATCTTGCGCAATGGGCGAAAGCCTGACGCAGCCACGCCGCGTGAGTGAAGAAGGCCTTCGGGTTGTAAAGCTCTGTCACCTGGGACGAAAATCCCGATAGTTAATAGCTATTGGGCCTGACGGTACCAGGGGAGGAAGCACCGGCTAACTTCGTGCCAGCAGCCGCGGTAATACGAAGGGTGCAAGCGTTGCTCGGAATTATTGGGCGTAAAGGGCAGGTAGGTGGTCTTATTTGTCTGAGGTGAAATCCCTGAGCTTAACTCAGGACGTGCCTTAGAAACGGTAGGACTAGAGTCTCAGAGAGGGTCGTAGAATTCCCAGTGTAGCGGTGAAATGCGTAGAGATTGGGAGGAATACCAGAGGCGAAGGCGGCGACCTGGATGAGAACTGACACTCAACTGCGAAAGCGTGGGGAGCAAACAGG` |
| ASV3303 | 424 | `TGAGGAATATTGGTCAATGGGCGCAAGCCTGAACCAGCCATGCCGCGTGCAGGAAGACGGCTCTATGAGTTGTAAACTGCTTTTATACGAGAGTAAACTTTGGTACGTGTACCGAATTGAAAGTATTGTATGAATAAGGATCGGCTAACTCCGTGCCAGCAGCCGCGGTAATACGGAGGATTCGAGCGTTATCCGGATTTATTGGGTTTAAAGGGTGCGTAGGTGGTTTAGTAAGTTAGTCGTTAAATCGGGAGGCTCAACCTTTCAGTGGCGATTAATACTGTTAGACTAGAGATCAGATGCTGTGGGCGGAATGTGTAGTGTAGCGGTGAAATGCTTAGATATTACACAGAACACCAATTGCGAAGGCAGCTCACAAAACTGTATCTGACACTGAGGCACGAAAGCGTGGGGATCAAACAGG` |
| ASV1581 | 422 | `TAGGGAATTTTGCGCAATGGAGGAAACTCTGACGCAGCAACGCCATGTGTGGGATGAAGCATTTCGGTGTGTAAACCACTGTCAGCAGGGAATAAATGGTCTCTTCGGAGGCAGTTGAATGTACCTGCAAAGGAAGCCCCGGCTAACTTCGTGCCAGCAGCCGCGGTAATACGAGGGGGGCAAATGTTGTTCGGAATCACTGGGCGTAAAGGGAGCGTAGGCGGGCTTTTAAGTTAGGTGTTTAATCCCGGGGCCCAACCCCGGACAGGCACCTAAAACTGGGAGTCTTGAATAGTGGAGAGGACGGCGGAATTGGGGGTGTAGCGGTGGAATGCGTAGAGATCCCCAAGAACACCAGTTGCGAAGGCGGCCGTCTGGACACATATTGACGCTGAGGCTCGAAAGCATGGGGAGCAAACAGG` |
| ASV1661 | 404 | `TAGGGAATATTGCGCAATGGGCGAAAGCCTGACGCAGCAATTCCGCGTGGAGGATGACGGATTTCGGTCTGTAAACTCCTGTCAGCAGGGAAGCAATGGACGGTACCTGCAGAGGAAGCACCGGCTAACTACGTGCCAGCAGCCGCGGTAATACGTAGGGTGCAAGCGTTGTCCGGAATTATTGGGCGTAAAGCGTCCGTAGGCGGTTTATTAAGTCTGACGTGAAAGGTCGTGGCTCAACCACGGTTATGTGTTGGAAACTAGTAGACTAGAATATGGTAGAGGTTGCTGGAATTCCTGGTGTAGCGGTGAAATGCGTAGATATCAGGAGGAACACCTGTGGCGAAAGCGTGCAACTGGGCCTATATTGACGCTAATGGACGAAAGCTAGGGTAGCGAAAGGG` |
| ASV4031 | 429 | `TGGGGAATATTGGACAATGGGGGCAACCCTGATCCAGCGATGCCGCGTGTGCGAAGAAGGCCTGCGGGTTGTAAAGCACTTTGATTGGGGAAGAAAAGCTTGTGTTTAAGAGATGCAAGATATGACGTTACCCAAAGAACAAGCACCGGCTAACTCTGTGCCAGCAGCCGCGGTAATACAGAGGGTGCGAGCGTTAATCGGAATTACTGGGCGTAAAGGGCGCGTAGGCGGTATATTAAGTTAGATGTGAAAATTCTGGGCTTAACCTAGGACGTGCATTTAAGACTGATAAGCTAGAGTATTGTAGAGGGAAGTGGAATTTCCGGTGTAGCGGTGAAATGCGTAGATATCGGAAAGAACATCAGTGGCGAAGGCGGCTTCCTGGACAAATACTGACGCTGAGGCGCGAAAGCATGGGTAGCAAACAGG` |
| ASV5550 | 404 | `TGGGGAATATTGGACAATGGGCGAAAGCCTGATCCAGCAATGCCGCGTGAGTGATGAAGGTCTTAGGATTGTAAAGCTCTTTTGCCGGAGAGGATAATGACAGTATCCGGAGAATAAGGACCGGCTAACTTCGTGCCAGCAGCCGCGGTAATACGAAGGGTCCTAGCGTTACTCGGATTTACTGGGCGTAAAGTGTATGTAGGCGGACTAACAAGTCAGAAGTCAAATCCCTGGGCTTAACCTAGGAATTGCTTTTGAAACTGTTAGTCTAGAGTATGGTAGGGAGAAATGGAATTCCTAGTGTAGGGGTAAAATCCGTAGATATTAGGAGGAACACCAGAGGCGAAGGCGGTTTCTTGGGCCATTACTGACGCTGAGATACGAAGGCGTGGGGAGCAAACAGG` |
| ASV4447 | 404 | `TGGGGAATATTGGGCAATGGACGCAAGTCTGACCCAGCCATGCCGCGTGAGTGATGAAGGCCTTAGGGTTGTAAAGCTCTTTCAGTGGTGAAGATGATGACTGTAACCACAAAAGAAGCCCCGGCTAACTTCGTGCCAGCAGCCGCGGTAATACGAAGGGGGCTAGCGTTGTTCGGATTTACTGGGCGTAAAGCGCACGCAGGCGGCCTAACAAGTCAGAGGTGAAATCCCAGGGCTCAACCTTGGAACTGCCTTTGAAACTGTTAGGCTAGAGATCGAGAGAGGTAAGTGGAATTCCTAGTGTAGAGGTGAAATTCGTAGATATTAGGAGGAACACCAGTGGCGAAGGCGGCTTACTGGCTCGATACTGACGCTCATGTGCGAAAGCGTGGGGAGCAAACAGG` |
| ASV3384 | 404 | `TGGGGAATCTTGGACAATGGGGGAAACCCTGATCCAGCCATGCCGCGTGAGTGATGAAGGCCTTAGGGTTGTAAAGCTCTTTCGCACGCGACGATGATGACGGTAGCGTGAGAAGAAGCCCCGGCTAACTTCGTGCCAGCAGCCGCGGTAATACGAAGGGGGCTAGCGTTGTTCGGAATTACTGGGCGTAAAGCGCACGTAGGCGGCTTGGTAAGTTGGGGGTGAAATCCCGGGGCTCAACCTCGGAATTGCCTTCAAAACTGCTTGGCTTGAGTTCGAGAGAGGTGAGTGGAATTCCCAGTGTAGAGGTGAAATTCGTAGAGATTGGGAAGAACACCAGTGGCGAAGGCGGCTCACTGGCTCGATACTGACGCTGAGGTGCGAAAGCGTGGGGAGCAAACAGG` |
| ASV3212 | 429 | `TGGGGAATTTTGGACAATGGGGGCAACCCTGATCCAGCAACGCCGCGTGAATGAAGAAGGCCTTAGGGTTGTAAAGTTCTTTTATCTGGGAGCAAATCCTGTTGGTGAATAGCTAATGGGGATGAGAGTACCAGAAGAATAAGCACCGGCTAACTACGTGCCAGCAGCCGCGGTAATACGTAGGGTGCGAGCGTTAATCGGAATTACTGGGCGTAAAGCGTGCGCAGGCGGATGTGTAAGTTAGATGTGAAATCCCCGAGCTTAACTTGGGAATAGCATTTAAGACTGCATGTCTAGAGTTTGTCAGAGGGGGGTAGAATTCCAAGTGTAGCAGTGAAATGCGTAGAGATTTGGAGGAATACCGGTGGCGAAGGCGGCCCCCTGGGATAGAACTGACGCTCATGCACGAAAGCGTGGGTAGCAAACAGG` |
| ASV268 | 407 | `TGGGGAATATTGGACAATGGGCCACAAGCCTGATCCAGCAATTCTGTGTGCACGATGAAGGTTTTCGGATCGTAAAGTGCTTTCAGTTGGGAAGAAGAAAGTGACGGTACCAACAGAAGAAGCGACGGCTAAATACGTGCCAGCAGCCGCGGTAATACGTATGTCGCAAGCGTTATCCGGATTTATTGGGCGTAAAGCGCGTCTAGGCGGAAAAGAAAGTCTGATGTTAAAATGCGGGGCTCAACTCCGTATTGCGTTGGAAACTGCTTTTCTAGAGTACTGGAGAGGTGGGCGGAACTACAAGTGTAGAGGTGAAATTCGTAGATATTTGTAGGAATGCCGATGGAGAAGTCAGCTCACTGGACAGATACTGACGCTAAAGCGCGAAAGCGTGGGGAGCAAACAGG` |
| ASV5453 | 429 | `TGGGGAATATTGGACAATGGGGGAAACCCTGATCCAGCCATACCGCGTGTGTGAAGAAGGCCTTTTGGTTGTAAAGCACTTTAAGCGAGGAGGAGGCTTTGTTGGTTAATAACTGACAAAGTGGACGTTACTCGCAGAATAAGCACCGGCTAACTCTGTGCCAGCAGCCGCGGTAATACAGAGGGTGCGAGCGTTAATCGGAATTACTGGGCGTAAAGCGCGCGTAGGCGGTTCGTCAAGTCGGATGTGAAATCCCTGAGCTCAACTTAGGAATTGCATTCGATACTGGCGAGCTAGAGTATGGGAGAGGATGGTAGAATTCCAGGTGTAGCGGTGAAATGCGTAGAGATCTGGAGGAATACCGATGGCGAAGGCAGCCATCTGGCCTAATACTGACGCTGAGGTGCGAAAGCATGGGGAGCAAACAGG` |
| ASV2065 | 429 | `TGGGGAATATTGGACAATGGGGGCAACCCTGATCCAGCAATGCCGCGTGTGTGAAGAAGGCCTGCGGGTTGTAAAGCACTTTTGGCAGGGATGAAAAGCACTGGATTAATAACCCAGTGTGTTGACCTAACCTGCAGAAGAAGCACCGGCTAACTCTGTGCCAGCAGCCGCGGTAATACAGAGGGTGCGAGCGTTAATCGGAATTACTGGGCGTAAAGGGCGCGTAGGCCGTTGAGTAAGTCGGATGTGAAATCCCCGGGCTTAACCTGGGAATTGCATTCGATACTGCTTGGCTAGAGTGTGGAAGAGGGAAGCGGAATTTCCGGTGTAGCGGTGAAATGCGTAGATATCGGAAGGAACACCAGTGGCGAAGGCGGCTTCCTGGTCCAACACTGACGCTGAGGCGCGAAAGCGTGGGGAGCAAACAGG` |
| ASV2643 | 404 | `TGGGGAATCTTGGACAATGGGCGCAAGCCTGATCCAGCCATGCCGCGTGAGTGAAGAAGGCCTTCGGGTTGTAAAGCTCTTTTGTCAGGGACGATAATGACGGTACCTGAAGAATAAGCCCCGGCAAACTTCGTGCCAGCAGCCGCGGTAATACGAAGGGGGCTAGCGTTGTTCGGAATTACTGGGCGTAAAGCGCACGTAGGCTGACATTTAAGTCAGGGGTGAAATCCCGAGGCTCAACCTCGGAACTGCCTTTGATACTGGATGTCTCGAGTCCGGAAGAGGTTAGTGGAATTCCCAGTGTAGAGGTGAAATTCGTAGATATTGGGAAGAACACCAGTGGCGAAGGCGGCTAACTGGTCCGGTACTGACGCTGAGGTGCGAAAGCGTGGGGAGCAAACAGG` |
| ASV2945 | 429 | `TGGGGAATTTTGGACAATGGGGGCAACCCTGATCCAGCCATGCCGCGTGAGTGAAGAAGGCCTTCGGGTTGTAAAGCTCTTTCGGCCGGGAAGAAATCGCACGGGTTAATACCCTGTGTGGATGACGGTACCGGAATAAGAAGCACCGGCTAACTACGTGCCAGCAGCCGCGGTAATACGTAGGGTGCGAGCGTTAATCGGAATTACTGGGCGTAAAGCGTGCGCAGGCGGTTTAGTAAGACAGGCGTGAAATCCCCGGGCTCAACCTGGGAACTGCGCTTGTGACTGCTAAGCTAGAGTACGGCAGAGGGGGGTGGAATTCCACGTGTAGCAGTGAAATGCGTAGAGATGTGGAGGAACACCAATGGCGAAGGCAGCCCCCTGGGTCGATACTGACGCTCATGCACGAAAGCGTGGGTAGCAAACAGG` |
| ASV3236 | 405 | `TAGGGAATTTTCCACAATGGACGAAAGTCTGATGGAGCAACGCCGCGTGCAGGATGAAGGCTTTCGGGTCGTAAACTGCTTTTATGAGTGATGATTATGACAGTAGCTCAAGAATAAGGGTCGGCTAACTACGTGCCAGCAGCCGCGGTCATACGTAGGACCCAAGCGTTATCCGGAATTACTGGGCGTAAAGAGTTGCGTAGGCGGCTTGTTAAGCAAGATGTGAAATCGTGAGGCTCAACCTCATAAACATATTTTGAACTGGCAAGCTAGAGTATGGTAGAGGTCACTGGAATTCCAAGTGTAGGAGTGAAATCCGTAGATATTTGGAGGAACACCGATGGCGTAGGCAGGTGACTGGACCATTACTGACGCTAAGGCACGAAAGCGTGGGGAGCAAACGGG` |
| ASV1943 | 429 | `TGGGGAATATTGCGCAATGGGGGCAACCCTGACGCAGCCATGCCGCGTGTGTGAAGAAGGCCTTCGGGTTGTAAAGCACTTTAAGTGGGGAGAAAAGGGTAGCGGTTAATAGCCGTTACCTGTGATGTTACCCACAGAATAAGCACCGGCTAACTCCGTGCCAGCAGCCGCGGTAATACGGAGGGTGCGAGCGTTAATCGGAATTACTGGGCGTAAAGCGCGCGTAGGCGGTTTGTTAAGTCAGCTGTGAAAGCCCCGGGCTCAACCTGGGAACTGCAGTTGATACTGGCCGACTAGAGTACGAGAGAGGGAGGTAGAATTCCATGTGTAGCGGTGAAATGCGTAGATATATGGAGGAATACCGGTGGCGAAGGCGGCCTCCTGGCTCGATACTGACGCTGAGGTGCGAAAGCGTGGGGATCAAACAGG` |
| ASV4046 | 429 | `TGGGGAATTTTGGACAATGGGCGCAAGCCTGATCCAGCCATGCCGCGTGAGTGAAGAAGGCCTTCGGGTTGTAAAGCTCTTTCGGTGGGGAAGAAATGGTTCGTGCTAATACCACGGACTGATGACGGTACCCGAAGAAGAAGCACCGGCTAACTACGTGCCAGCAGCCGCGGTAATACGTAGGGTGCAGGCGTTAATCGGAATTACTGGGCGTAAAGCGTACGCAGGCGGTTTGCTAAGACAGGTGTGAAATCCCCGGGCTTAACCTGGGAACTGCGCTTGTTACTGGCAAGCTAGAGTGTGGCAGAGGGGGGTAGAATTCCAGGTGTAGCAGTGAAATGCGTAGATATCTGGAGGAATACCGATGGCGAAGGCAGCCCCCTGGGTTAACACTGACGCTCATGTACGAAAGCGTGGGGAGCAAACAGG` |
| ASV1236 | 429 | `TGGGGAATCTTGGACAATGGGGGAAACCCTGATCCAGCAATGCCGCGTGCAGGAAGAAGGCCTTCGGGTTGTAAACTGCTTTTGTTCGGGACGAAACGGTTCTTACTAATACTAGGGGCTAATGACGGTACCGGAAGAATAAGCACCGGCTAACTACGTGCCAGCAGCCGCGGTAATACGTAGGGTGCGAGCGTTAATCGGAATTACTGGGCGTAAAGCGTGCGCAGGCGGTTTTGTAAGATAGATGTGAAATCCCCGGGCTCAACCTGGGAATTGCATTTATGACTGCAAGGCTAGAGTACGGTAGAGGGGGGTAGAATTCCGCGTGTAGCAGTGAAATGCGTAGATATGCGGAGGAACACCGATGGCGAAGGCAGCCCCCTGGACCTGTACTGACGCTCATGCACGAAAGCGTGGGGAGCAAACAGG` |
| ASV742 | 430 | `TGGGGAATTTTGGACAATGGGGGAAACCCTGATCCAGCCATGCCGCGTGCAGGAAGAAGGCCTTCGGGTTGTAAACTGCTTTAGTCTAGGAAAAAGGGGTGCACGATTAATACTCGTGTGCTTTGATGGTACTGGAAGAATAAGCACCGGCTAACTACGTGCCAGCAGCCGCGGTAATACGTAGGGTGCGAGCGTTAATCGGAATTACTGGGCGTAAAGCGTGCGCAGGCGGTCCGTTAAGACAGTCGTGAAAGCCCCGGGCTTAACCTGGGAACTGCGATTGTGACTGGCGGGCTAGAGTGTGTCAGAGGGGGGTGGAATTCCACGTGTAGCAGTGAAATGCGTAGAGATGTGGAGGAACACCGATGGCGAAGGCAGCCCCCTGGGATAACACTGACGCTCATGCACGAAAGCGTGGGGAGCAAACAGG` |
| ASV770 | 404 | `TGAGGAATCTTCCACAATGGGCGAAAGCCTGATGGAGCGACACCGCGTGAAGGATGAAGCCTTTGTTGGTGTAAACTTCTTTTCTCTCCGAAGATAATGACGGTAGGAGAGGAATAAGGGGCGGCAAACTTCGTGCCAGCAGCCGCGGTAATACGAAGGCCCCAAGCGTTATCCGGAATTACTGGGTGTAAAGCGTCTGTAGGTGGTTCGGAAAGTTTCAAGTGAAAGGTCAGGGCTCAACCCTGTACTTGCTTGGAAAACTATCGAACTTGAGTATGGGAGAGGCAAGCAGAATAATAAGAGTAGGGGTGCAATCCGTTGATACTTATTAGAATACCAAAAGCGAAGGCAGCTTGCTGGAACATTACTGACACTGAGAGACGAAAGCGTGGGGAGCAAAAGGG` |
| ASV3051 | 404 | `TGGGGAATATTGGACAATGGGGGCAACCCTGATCCAGCCATGCCGCGTGAGTGAAGAAGGCCTTCGGGTTGTAAAGCTCTTTCGGCGGGGACGATGATGACGGTACCCGCATAAGAAGCCCCGGCTAACTTCGTGCCAGCAGCCGCGGTAATACGAAGGGGGCTAGCGTTGTTCGGAATTACTGGGCGTAAAGCGCGCGTAGGCGGCGACCTTTGTCAGAGGTGAAATCCCGAGGCTCAACTTCGGAATTGCCTTTGAAACGGGGTTGCTTGAGTCCGAGAGAGGATGGCGGAATTCCTAGTGTAGAGGTGAAATTCGTAGATATTAGGAAGAACACCGGTGGCGAAGGCGGCCATCTGGCTCGGTACTGACGCTCAGGCGCGAAAGCGTGGGGATCAAACAGG` |
| ASV1109 | 404 | `TGGGGAATATTGGACAATGGGCGAAAGCCTGATCCAGCAATGCCGCGTGAGTGATGAAGGCCTTAGGGTTGTAAAGCTCTTTTACCCGGGAAGATAATGACTGTACCGGGAGAATAAGCCCCGGCTAACTCCGTGCCAGCAGCCGCGGTAATACGGAGGGGGCTAGCGTTATTCGGAATTACTGGGCGTAAAGCGCGCGTAGGCGGCTATTCAAGTCAGAGGTGAAAGCCCGGAGCTCAACTCCGGAATTGCCTTTGAAACTAGATAGCTTGAATCATGGAGAGGTCAGTGGAATTCCGAGTGTAGAGGTGAAATTCGTAGATATTCGGAAGAACACCAGTGGCGAAGGCGACTGACTGGACATGCATTGACGCTGAGGTGCGAAAGCGTGGGGAGCAAACAGG` |
| ASV913 | 429 | `TGGGGAATTTTGGACAATGGACGAAAGTCTGATCCAGCCATGCCGCGTGCGGGAAGAAGGCCTTCGGGTTGTAAACCGCTTTTGTCAGGGAAGAAACGGGTTTCTCTAATACAGAGGCCTAATGACGGTACCTGAAGAATAAGCACCGGCTAACTACGTGCCAGCAGCCGCGGTAATACGTAGGGTGCAAGCGTTAATCGGAATTACTGGGCGTAAAGCGTGCGCAGGCGGTTATGCAAGACAGAGGTGAAATCCCCGGGCTCAACCTGGGAACTGCCTTTGTGACTGCATAGCTAGAGTACGGTAGAGGGGGATGGAATTCCGCGTGTAGCAGTGAAATGCGTAGATATGCGGAGGAACACCGATGGCGAAGGCAATCCCCTGGACCTGTACTGACGCTCATGCACGAAAGCGTGGGGAGCAAACAGG` |
| ASV3250 | 430 | `TAGGGAATATTGCACAATGGAGGAAACTCTGATGCAGCAACGCCGCGTGAGTGAAGAAGGCCCTTGGGTCGTAAAGCTCTTTAGCGAGGGAAGAAGCTGATTATGGTGAATAATCATAGTTGGTGACGGTACCTCGAGAATAAGCACCGGCTAATTTCGTGCCAGCAGCCGCGGTAATACGAAAGGTGCAAGCGTTACTCGGAATCACTGGGCGTAAAGCGCACGTAGGCGGACTCGTAGGTCAGTTGTGAAATACCTGGGCTCAACCCAGGAATTGCAGCTGAAACCGCTTGTCTTGAATACCTGAGAGGGTGGGGGAATACCTGGTGGAGAAGTGAAATTCGTAGAGATCAGGTGGAACACCGGAGGCGAAGGCGCCTGCCTGGCAGTGTATTGACGCTGAGGTGCGAAAGTGCGGGGAGCAAACAGG` |
| ASV3903 | 430 | `TGGGGAATTTTGGACAATGGGGGCAACCCTGATCCAGCCATGCCGCGTGCGGGAAGAAGGCCTTCGGGTTGTAAACCGCTTTAGTCCGGGAAAAATGTTCTATCTCCTAATACGAGGTGGGATTGATGGTACTGGAAGAATAAGCACCGGCTAACTACGTGCCAGCAGCCGCGGTAATACGTAGGGTGCAAGCGTTAATCGGAATGACTGGGCGTAAAGCGAGCGCAGGCGGTTTGTTAAGACAGTCGTGAAATCCCCGGGCTTAACCTGGGAATGGCGATTGTGACTGGCAAGCTAGAGTGTGTCAGAGGGAGGTGGAATTCCGCGTGTAGCAGTGAAATGCGTAGAGATGCGGAGGAACACCGATGGCGAAGGCAGCCTCCTGGGATAACACTGACGCTCATGCTCGAAAGCGTGGGGAGCAAACAGG` |
| ASV5271 | 430 | `TGGGGAATCTTGCGCAATGGACGAAAGTCTGACGCAGCGACGCCGCGTGAGTGAAGAAGGCCTTCGGGTTGTAAAGCTCTGTCGTCTGGGACGAATAGGTTTGGGGTTAACAGCCTCAGATCCTGACGGTACCAGAAAAGGAAGCACCGGCTAACTCTGTGCCAGCAGCCGCGGTAATACAGAGGGTGCAAGCGTTGCTCGGAATCATTGGGCGTAAAGGGCAGGTAGGTGGTCTCATTTGTCTGAGGTGAAATCCTGGGGCTTAACCCCAGAAGTGCCTCAGAAACGGTGGGACTAGAGTGCTGGAGAGGGTCGTGGAATTCCCGGTGTAGCGGTGAAATGCGTAGAGATCGGGAGGAACACCAGAGGCGAAGGCGGCGACCTGGACAGTGACTGACACTCAACTGCGAAAGCGTGGGGAGCAAACAGG` |
| ASV4033 | 424 | `TGAGGAATATTGGACAATGGGTGAGAGCCTGATCCAGCCATCCCGCGTGAAGGACGACTGCCCTATGGGTTGTAAACTTCTTTTATACTGGGATAAACCTACTTACGTGTAAGTAGCTGAAGGTACAGTATGAATAAGCACCGGCTAACTCCGTGCCAGCAGCCGCGGTAATACGGAGGGTGCAAGCGTTATCCGGATTTATTGGGTTTAAAGGGTCCGTAGGCGGACTCGTAAGTCAGTGGTGAAATCTCATAGCTTAACTATGAAACTGCCATTGATACTGCGGGTCTTGAGTAAAGTAGAAGTGGCTGGAATAAGTAGTGTAGCGGTGAAATGCATAGATATTACTTAGAACACCAATTGCGAAGGCAGGTCACTATGTTTTAACTGACGCTGATGGACGAAAGCGTGGGGAGCGAACAGG` |
| ASV4663 | 430 | `TGGGGAATCTTGGACAATGGACGAAAGTCTGATCCAGCGACGCCGCGTGGGTGATGAAGGCCTTCGGGTCGTAAAGCCCTGTGGGCAGGGAAGAAAATGGTCACGGCGAACAGGCGTGGCCCTTGACGGTACCTGCTCAGAAAGCACCGGCTAACTCTGTGCCAGCAGCCGCGGTAAGACAGAGGGTGCGAACGTTGCTCGGAATTACTGGGCGTAAAGCGCGTGTAGGCTGACTCGTGTGTCGGGTGTGAAATCCCTGGGCTCAACCCAGGAACTGCATTCGAAACTGCGAGTCTAGAGTGGTGGAGAGGGTAGTGGAATTCCTGGTGTAGAGGTGAAATTCGTAGATATCAGGAGGAACACCGGTGGCGAAGGCGGCTACCTGGACACCAACTGACGCTGAGACGCGAAAGCGTGGGGAGCAAACAGG` |
| ASV939 | 429 | `TGGGGAATTTTGGACAATGGGCGCAAGCCTGATCCAGCCATTCCGCGTGAGTGAAGAAGGCCTTCGGGTTGTAAAGCTCTTTCGCAAGGGAAGAAAAATTACGCTCTAACATAGCGTGACGATGACGGTACCTTGATAAGAAGCACCGGCTAACTACGTGCCAGCAGCCGCGGTAATACGTAGGGTGCGAGCGTTAATCGGAATTACTGGGCGTAAAGCGTGCGCAGGCGGTTTTGTAAGTCAGATGTGAAATCCCCGAGCTCAACTTGGGAACTGCGTTTGAAACTACAAGACTAGAATAGGTCAGAGGGGGGTAGAATTCCACGTGTAGCAGTGAAATGCGTAGAGATGTGGAGGAATATCAATGGCGAAGGCAGCCCCCTGGGATCATATTGACGCTCATGCACGAAAGCGTGGGGAGCAAACAGG` |
| ASV4569 | 404 | `TGGGGAATATTGGACAATGGACGAAAGTCTGATCCAGCAACGCCGCGTGAAGGATGAAGGTCTTAGGATTGTAAACTTCTTTTGACAGGGAAGATAATGACTTTACCTGTAGAATAAGCACCGGCTAACTTCGTGCCAGCAGCCGCGGTAATACGAAGGGTGCTAGCGTTACTCGGAATTACTGGGCGTAAAGAATGCGTAGGCGGACTTGCAAGTTAGGAGTGAAATCCCTGGGCTCAACCTAGGAACTGCTTTTAAAACTGCTTGTCTTGAATGTTGGAGTGATTAGGGGAATTCCTGGTGTAGGGGTAATATCCGTAGATATCAGGAGGAAGACCGGAAGTGAAGACGCTTAATTAGCCAAATATTGACGCTGATGCATGAAAGCGTGGGGAGCAAACAGG` |
| ASV3464 | 404 | `TGAGGAATCTTCCACAATGGACGAAAGTCTGATGGAGCGACACCGCGTGAAGGATGAAGGCCTAACGGTTGTAAACTTCTTTTCTGAAGGAGCATAATGAGAGTACTTCAGGAATAAGGGACGGCTAAATACGTGCCAGCAGCCGCGGTAATACGTATGTCCCAAGCGTTACCCGGAATCACTGGGTGTAAAGGGTTCGTAGGTGGTTAATTAAGTTGGATATGAAAGACCGGAGCTCAACTTCGTGTTTGTGTTCAAAACTGATTAACTAGAATCAGGGAGAGGTAAGCGGAATTCTGTGTGTAGGGGTGCAATCCGTAGATACACAGAGGAACACCAAAAGCGAAGGCAGCTTACTGGAACTGTATTGACGCTCATGAACGAAAGCGTGGGGAGCGAAAAGG` |
| ASV3404 | 425 | `TAGGGAATATTGGGCAATGGAGGCAACTCTGACCCAGCCATGCCGCGTGCAGGAAGACGGTCCTCTGGATTGTAAACTGCTTTTGAATGGGAAGAACACTATCCTTGCGAGGGTAATTGACGGTACCATTAGAATAAGCCACGGCTAACTACGTGCCAGCAGCCGCGGTAATACGTAGGTGGCGAGCGTTATCCGGATTTATTGGGTTTAAAGGGTGCGTAGGCGGCTTAATAAGTCGGTGGTTAAAGGTAGCAGCTCAACTGTTTTACATGCCATCGATACTGTTTAGCTTGAGATATATGGAGGCAGCTAGAATTCCCGGTGTAGCGGTGAAATGCATAGATACCGGGAGGAATACCGATTGCGAAGGCATGTTGCTACGTATATTCTGACGCTGATGCACGAAAGCGTGGGGATCAAACAGG` |
| ASV4083 | 429 | `TGGGGAATTTTGGACAATGGGCGAAAGCCTGATCCAGCCATGCCGCGTGAGTGAAGAAGGCCTTCGGGTTGTAAAGCTCTTTCGGCTGGGAAGAAATCGCACAGGCTAATACTCTGTGTGGATGACGGTACCAGCACAAGAAGCACCGGCTAACTACGTGCCAGCAGCCGCGGTAATACGTAGGGTGCGAGCGTTAATCGGAATTACTGGGCGTAAAGCGTGCGCAGGCGGTTGTGCAAGACAGATGTGAAATCCCCGGGCTCAACCTGGGAACTGCATTTGTGACTGCACGACTAGAGTACGGCAGAGGGGGGTAGAATTCCACGTGTAGCAGTGAAATGCGTAGAGATGTGGAGGAATACCGATGGCGAAGGCAGCCCCCTGGGCCGATACTGACGCTCATGCACGAAAGCGTGGGGAGCAAACAGG` |
| ASV2543 | 428 | `TCGGGAATCTTCCGCAATGGGCGAAAGCCTGACGGAGCGACGCCGCGTGAGCGATGAAGGCCTTCGGGTTGTAAAGCTCTGTGAGGAGAGAAGAATAAGTTCAAGCGAATACCTTGAATGATGACGGTATCTCCTTAGCAAGCACCGGCTAACTCTGTGCCAGCAGCCGCGGTAAGACAGAGGGTGCAAACGTTGTTCGGATTTACTGGGCGTAAAGCGCGTGTAGGCGGCTTGGCAAGTCGGATGTGAAAGCCCACGGCTCAACCGTGGAAGCGCATCCGAAACGGCCTCGCTCGAGTCTCGGAGAGGAAGGTGGAATGCTTGGTGTAGAGGTGAAATTCGTAGATATCAAGCAGAACACCTGCGGCGAAGGCGGCCTTCTGGACGATGACTGACGCTGAGACGCGAAAGCGTGGGGAGCAAACAGG` |
| ASV685 | 429 | `TGGGGAATATTGGACAATGGGCGCAAGCCTGATCCAGCCATACCGCGTGGGTGAAGAAGGCCTTCGGGTTGTAAAGCCCTTTTGTTGGGAAAGAAATCCTGCCGGTTAATAACCAGCGGGGATGACGGTACCCAAAGAATAAGCACCGGCTAACTTCGTGCCAGCAGCCGCGGTAATACGAAGGGTGCAAGCGTTACTCGGAATTACTGGGCGTAAAGCGTGCGTAGGTGGTTGTTTAAGTCTGCTGTGAAAGCCCTGGGCTCAACCTGGGAATTGCAGTGGATACTGGGCGACTAGAGTGTGGTAGAGGACAGTGGAATTTCCGGTGTAGCAGTGAAATGCGTAGAGATCGGAAGGAACATCTGTGGCGAAGGCGACTGTCTGGGCCAACACTGACACTGAGGCACGAAAGCGTGGGGAGCAAACAGG` |
| ASV4434 | 429 | `TGGGGAATATTGGACAATGGGGGAAACCCTGATCCAGCAATGCCGCGTGTGTGAAGAAGGCCTGCGGGTTGTAAAGCACTTTCGGAGGGGACAAAAAGCGTCGGGTTAATAACCAGGCGTCTTGATTTAACCCTCATAAGAAGCACCGGCTAACTCTGTGCCAGCAGCCGCGGTAATACAGAGGGTGCAAGCGTTAATCGGAATTACTGGGCGTAAAGCGCACGCAGGCGGCTGCATAAGCCGGTCGTGAAATCCCCGGGCTTAACCTGGGAATTGCGATCGGGACTGTGCGGCTAGAGTGTTGGAGAGGACAGCGGAATTCCCGGTGTAGCAGTGAAATGCGTAGATATCGGGAGGAACATCAGTGGCGAAGGCGGCTGTCTGGCCAAACACTGACGCTCAGGTGCGAAAGCGTGGGGAGCAAACAGG` |
| ASV582 | 430 | `TGGGGAATATTGGACAATGGGGGGAACCCTGATCCAGCCATGCCGCGTGTGTGAAGAAGGCCTTTTGGTTGTAAAGCACTTTAAGCGAGGAGGAGGCTACTAGTACTAATACTACTGGATAGTGGACGTTACTCGCAGAATAAGCACCGGCTAACTCTGTGCCAGCAGCCGCGGTAATACAGAGGGTGCGAGCGTTAATCGGATTTACTGGGCGTAAAGCGTGCGTAGGCGGCCAATTAAGTCGGATGTGAAATCCCTGAGCTTAACTTAGGAATTGCATTCGATACTGTTTGGCTAGAGTATGGGAGAGGATGGTAGAATTCCAGGTGTAGCGGTGAAATGCGTAGAGATCTGGAGGAATACCGATGGCGAAGGCAGCCATCTGGCCTAATACTGACGCTGAGGTACGAAAGCATGGGGAGCAAACAGG` |
| ASV5577 | 424 | `TAAGGAATATTGGGCAATGGAGGCAACTCTGACCCAGCCATGCCGCGTGAAGGATGAAGGCCCTATGGGTTGTAAACTTCTTTTGTATGGGAATAAACGCAGTTACGTGTAACTGTTTGAATGTACCATAAGAATAAGCACCGGCTAACTCCGTGCCAGCAGCCGCGGTAATACGGAGGGTGCAAGCGTTATCCGGATTTATTGGGTTTAAAGGGAGCGTAGGCGGGATTGTAAGTCAGTGGTGAAAGCCCGTAGCTCAACTACGGAACTGCCATTGATACTGCAGACCTAGAGTACACTTGAAGTGGGCGGAATGTGTCATGTAGCGGTGAAATGCTTAGATATGACACAGAACACCAATTGCGAAGGCAGCTCACTAAATTGTAACTGACGCTGAGGCTCGATAGCGTGGGGATCAAACAGG` |
| ASV5613 | 404 | `TGGGGAATATTGGACAATGGGGGAAACCCTGATCCAGCCATGCCGCGTGAGTGATGAAGGCCTTAGGGTTGTAAAGCTCTTTTGTCAGGGAAGATAATGACGGTACCTGAAGAATAAGTTCCGGCTAACTTCGTGCCAGCAGCCGCGGTAATACGAAGGGAACTAGCGTTGTTCGGAATCACTGGGCGTAAAGCGCGCGTAGGCGGACTGTCAAGTCAGAGGTGAAATCCCAAGGCTCAACCTTGGAATTGCCTTTGAAACTGTCAGTCTGGAGTATGTGAGGGGATAGCGGAATTCCAAATGTAGCAGTGAAATGCGTAGATATTTGGAGGAACACCGGTGGCGAAGGCGGCTATCTGGCACATTACTGACGCTGAGGTGCGAAAGCGTGGGGAGCAAACAGG` |
| ASV2108 | 424 | `TGAGGAATATTGGTCAATGGACGCAAGTCTGAACCAGCCATGCCGCGTGCAGGATGACGGTCCTATGGATTGTAAACTGCTTTTGTACAGGAAGAAACCCTACTTCGTGAAGTAGATTGACGGTACTGTAAGAATAAGGATCGGCTAACTCCGTGCCAGCAGCCGCGGTAATACGGAGGATCCAAGCGTTATCCGGAATCATTGGGTTTAAAGGGTCCGTAGGCGGCCTAGCAAGTCAGTGGTGAAATCTCCCCGCTCAACGGGGAAACGGCCATTGATACTACTAGGCTTGAATTATTAGGAAGTAACTAGAATATGTAGTGTAGCGGTGAAATGCTTAGATATTACATGGAATACCAATTGCGAAGGCAGGTTACTACTAATGGATTGACGCTGATGGACGAAAGCGTGGGGAGCGAACAGG` |
| ASV2860 | 404 | `TGGGGAATCTTAGACAATGGGGGAAACCCTGATCTAGCCATGCCGCGTGAGCGATGAAGGCCTTAGGGTTGTAAAGCTCTTTCGGGGGGGAAGATAGTGACTGTACCCCCAGAAGAAGCCCCGGCTAACTCCGTGCCAGCAGCCGCGGTAATACGGAGGGGGCTAGCGTTGTTCGGAATTACTGGGCGTAAAGCGCACGTAGGCGGATCGGATAGTCAGAGGTGAAAGCCCGGGGCTCAACCCCGGAACGGCCTTTGAAACTCCCGGTCTTGAGGTCGAGAGAGGTGAGTGGAATTCCGAGTGTAGAGGTGAAATTCGTAGATATTCGGAGGAACACCAGTGGCGAAGGCGGCTCACTGGCTCGATACTGACGCTGAGGTGCGAAAGCGTGGGGAGCAAACAGG` |
| ASV2920 | 404 | `TGGGGAATATTGGACAATGGACGAAAGTCTGATCCAGCAACGCCGCGTGAAGGATGAATGTCTTAGGATTGTAAACTTCTTTTGACAGGGAAGATAATGACTGTACCTGTAGAATAAGCACCGGCTAACTTCGTGCCAGCAGCCGCGGTAATACGAAGGGTGCTAGCGTTACTCGGAATTATTGGGCGTAAAGAATGCGTAGGCGGACTTGTAAGTGAGGAGTGAAATCCCTGGGCTCAACCTAGGAACTGCTTTTCAAACTGCTTGTCTTGAATCTGATAGTGATTAGGGGAATTCCTAGTGTAGGGGTAATATCCGTAGATATTAGGAGGAAGACCGGAAGCGAAAGCGCTTAATTGGGTCAAGATTGACGCTGATGCATGAAAGCGTGGGGAGCAAACAGG` |
| ASV3028 | 403 | `TGGGGGATATTGCACAATGGGCGAAAGCCTGATGCAGCGACGCCGCGTGAGGGAAGACGGTCTTCGGATTGTAAACCTCTGTCTTCGGGGACGATAATGACGGTACCCGAGGAGGAAGCTCCGGCTAACTACGTGCCAGCAGCCGCGGTAATACGTAGGGAGCGAGCGTTGTCCGGAATTACTGGGTGTAAAGGGAGCGTAGGCGGGAAGACAAGTTGAATGTTAAATCCATCGGCTCAACCGGTGGCTGCGTTCAAAACTGTTTTTCTTGAGTGAAGTAGAGGTTGGCGGAATTCCTAGTGTAGCGGTGAAATGCGTAGATATTAGGAGGAACACCAGTGGCGAAGGCGGCCAACTGGGCTTTTACTGACGCTGAGGCTCGAAAGCGTGGGGAGCAAACAGG` |
| ASV627 | 407 | `TGGGGAATTTTCCGCAATGGGCGAAAGCCTGACGGAGCAATACCGCGTGAGGGAGGAAGGCTCTTGGGTTGTAAACCTCTTTTCTGAAGGAAGAAAAAAATGACGGTACTTCAGGAATCAGCATCGGCTAACTCCGTGCCAGCAGCCGCGGTAATACGGAGGATGCAAGCGTTATCCGGAATGATTGGGCGTAAAGCGTCCGCAGGTGGCATTTCAAGTCTGCGGTCAAAGGTCGAGGCTCAACCTCGAATCGGCCGTGGAAACTGAGAAGCTAGAGTGTGGTAGGGGCAGAGGGAATTCCCAGTGTAGCGGTGAAATGCGTAGAGATTGGGAAGAACACCGGTGGCGAAAGCGCTCTGCTAGGCCAAGACTGACACTGAGGGACGAAAGCTAGGGGAGCGAATGGG` |
| ASV3679 | 406 | `TAGGGAATTTTCCACAATGGACGAAAGTCTGATGGAGCAACGCCGCGTGCAGGATGAAGGCCTTCGGGTCGTAAACTGCTTTTATGAGTGATGACTTTAGACAGTAGCTCAAGAATAAGGGTCGGCTAACTACGTGCCAGCAGCCGCGGTCATACGTAGGACCCAAGCGTTATCCGGAATTACTGGGCGTAAAGAGTTGCGTAGGCGGCTTGTTAAGTTAGTAGTGAAATCGTGTGGCTCAACCACATAAACATTATTAAAACTGGCAAGCTAGAGTATAGAAGAGGTCACTGGAATTCCAAGTGTAGGAGTGAAATCCGTAGATATTTGGAGGAACACCGATGGCGTAGGCAGGTGACTGGTCTATTACTGACGCTAAGGCACGAAAGCGTGGGGAGCAAACGGG` |
| ASV3237 | 404 | `TGGGGAATCTTAGACAATGGGGGGAACCCTGATCTAGCCATGCCGCGTGATCGATGAAGGCCTTAGGGTTGTAAAGATCTTTCAGCTGGGAAGATAATGACTGTACCAGCAGAAGAAGCCCCGGCTAACTCCGTGCCAGCAGCCGCGGTAATACGGAGGGGGCTAGCGTTATTCGGAATTACTGGGCGTAAAGCGCACGTAGGCGGATAAGAAAGTCAGAGGTGAAATCCCAGGGCTCAACCTTGGAACTGCCTTTGAAACTCCTTGTCTTGAGGTCGTGAGAGGTGAGTGGAATTCCGAGTGTAGAGGTGAAATTCGTAGATATTCGGAGGAACACCAGTGGCGAAGGCGGCTCACTGGCACGATACTGACGCTGAGGTGCGAAAGCGTGGGGAGCAAACAGG` |
| ASV1424 | 404 | `TGGGGAATATTGGACAATGGGCGAAAGCCTGATCCAGCGACGCCGCGTGGATGAAGAAGGCCTTCGGGTTGTAAAGTCCTTTCAGTAGGGAAGATAATGACGGTACCTACAGAAGAAGCTCCGGCTAACTTCGTGCCAGCAGCCGCGGTAATACGAAGGGAGCTAGCGTTACTCGGAATTATTGGGCGTAAAGCGTGCGTAGGCTGTTTTGTCAGTCAGAAGTGAAATCCTGGGGCTCAACCTCAGAACCGCTTTTGATACTGCAAGACTAGAGTATGTGAGGGGATGGCGGAATTCCTAGTGTAGAAGTGAAATTCGTAGATATTAGGAGGAACACCGGTGGCGAAGGCGGCCATCTGGCACAAAACTGACGCTGTTGCACGAAAGCGTGGGGAGCAAACAGG` |
| ASV4387 | 429 | `TCGCGAATTTTTCTCAATGGGCGCAAGCCTGAAGAAGCCATGCCGCGTGCGGGACGAAGGCCCTCTGGGTTGTAAACCGCTGTCAGGGAAGGGGAAATGGTGTATGTGAATAGCGTGCATCTTGACTGCTTTCCAAAGGAAGCCCCGGCAAAACATGTGCCAGCAGCCGCGGTAATACATGTGGGGCAAGCGTTGTTCGGAATCACTGGGCATAAAGGGTGCGTAGGCGGCGCGACACGTCTTGGGTGAAATCCTGCGGCCTAACCGTGGGACTGCCTGGGATACGGTCGTGCTTGAGTGCGTGAGGGGTGACTGGAACGCGTGGTGTAGCGGTGAAATGCGTAGATATCACGCGGAAGGCCAACGGCGAAGGCAAGTCACTGGTACGCAACTGACGCTGAGTGCACGAAAGCGTGGGGAGCAAACAGG` |
| ASV5601 | 405 | `TAAAGAATCTTCGGCAATGGGCGAAAGCCTGACCGAGCAATACCGCGTGAACGATGAAGGTCGAAAGATTGTAAAGTTCTTTTCTGAGGGAAGAATTCTGACGGTACCTCAGGAATAAGCACCGGCTAACTCCGTGCCAGCAGCCGCGGTAATACGGAGGGTGCAAGCGTTACCCGGAATTATTGGGCGTAAAGCGTCCGCAGGCTGTTTGTTAAGTCAAATATTAAATCTTGGAGCCCAACTTCGAGGCTGTATTTGAAACTGGCAAACTAGAGATTTTCAGAGGCATGTGGAATTCTGTGTGTAGGGGTAAAATCCGTAGATATACAGAAGAACACCAAAAGCGAAGGCAGCATGCTGGGAAAATTCTGACGCTCATGGACGAAAGCGTGGGGAGCGAAGAGG` |
| ASV3463 | 430 | `TGGGGAATCTTGCACAATGGGCGAAAGCCTGATGCAGCAACGCCGCGTGGGTGAAGAAGGTCTTTGGATTGTAAAGCCCTTTCGGCTAGGAAGAAGGGATATTGCATTAATACTGCAGTATTCTGACGGTACTAGAAGAAGAAGCACCGGCTAATTCCGTGCCAGCAGCCGCGGTAATACGGAAGGTGCAAGCGTTATTCGAAATTACTGGGCGTAAAGAGCACGTAGGTGGGTATGTGTGTCGGATGTGAAATCCCTGGGCTCAACCTGGGAATGGCATTCGAAACTGCGTATCTTGAATACTTGAGGGGTTGGAGAAATTCCAGGTGTAGGAGTGAAATCCGTAGAGATCTGGAGGAATACCGGAGGCGAAGGCGTCCAACTGGCAATGTATTGACACTGAGGTGCGAAAGTGCGGGGAGCAAACAGG` |
| ASV3224 | 429 | `TGGGGAATTTTTCGCAATGGGCGCAAGCCTGACGAAGCAACGCCGCGTGGAGGATGAAGGTCTTCGGATTGTAAACTCCTGTCGACCGGGACGAATGCACTCGACCTAATACGTCGGAATGTTGACGGTACCGGTGGAGGAAGCCACGGCTAACTCTGTGCCAGCAGCCGCGGTAATACAGAGGTGGCAAGCGTTGTTCGGAATTACTGGGCGTAAAGGGCGCGTAGGCGGCCTTGTAAGTCTCGTGTGAAAGCCCTCGGCTTAACTGAGGATCTGCACGGGATACTGTAAGGCTTGAGTTCGGGAGAGGAAAGCGGAATTCCGGGTGTAGCGGTGAAATGCGTAGATATCCGGAGGAACACCAGTGGCGAAGGCGGCTTTCTGGACCGACACTGACGCTGAGGCGCGAAAGCTAGGGGAGCAAACGGG` |
| ASV71 | 404 | `TGGGGAATCTTAGACAATGGGGGAAACCCTGATCTAGCCATGCCGCGTGAGCGATGAAGGCCTTAGGGTTGTAAAGCTCTTTCAGTGGGGAAGATAATGACGGTACCCACAGAAGAAGCCCCGGCTAACTCCGTGCCAGCAGCCGCGGTAATACGGAGGGGGCTAGCGTTGTTCGGAATTACTGGGCGTAAAGCGCACGTAGGCGGATCGGAAAGTCAGAGGTGAAATCCCAGGGCTCAACCTTGGAACTGCCTTTGAAACTCCCGGTCTTGAGGTCGAGAGAGGTGAGTGGAATTCCGAGTGTAGAGGTGAAATTCGTAGATATTCGGAGGAACACCAGTGGCGAAGGCGGCTCACTGGCTCGATACTGACGCTGAGGTGCGAAAGCGTGGGGAGCAAACAGG` |
| ASV5627 | 429 | `TGGGGAATATTGGACAATGGGGGCAACCCTGATCCAGCGATGCCGCGTGTGTGAAGAAGGCCTGTGGGTTGTAAAGCACTTTAGGTGGGGAAGAAGGGTAAATACTTAATACGTATTTACATTGACGTTACCCACAGAATAAGCACCGGCTAACTCTGTGCCAGCAGCCGCGGTAATACAGAGGGTGCAAGCATTAATCGGAATTACTGGGTGTAAAGGGTGTGTAGGTGGTTAGTTAAGTGAGATGTGAAAGTCCCGGGCTTAACCTGGGGAGGTCATTTCATACTGGCTGACTAGAATAGGATAGAGGGAGGTGGAATTTCCGGTGTAGCGGTGAAATGCGTAGATATCGGAAGGAACACCAGTGGCGAAGGCGGCCTCCTGGATCCATATTGACACTAAGACACGAAAGCGTGGGGAGCAAACAGG` |
| ASV4814 | 413 | `TCGAGAATCATTCACAATGGGGGCAACCCTGATGGTGCGACGCCGCGTGGGGGAATGAAGGTCTTCGGATTGTAAACCCCTGTCAAGTGGGAGCAAGCAGAAATGTTGATAGTACCACTAGAGGAAGAGACGGCTAACTCTGTGCCAGCAGCCGCGGTAATACAGAGGTCTCAAGCGTTGTTCGGAATCACTGGGCGTAAAGAGTACGTAGGCGGCTAGGTAAGTCGTGTGTGAAAGGCAGGGGCTCAACCTCTGGACTGCACACGATACTGCTTGGCTTGAGAAATGGAGGGGAAGCTGGAATTCTCGGTGTAGCAGTGAAATGCGTAGATATCGAGAGGAACACTCGTGGCGAAGGCGAGCTTCTGGACATTATCTGACGCTGAGGTACGAAGGCTAGGGGAGCGAAAGGG` |
| ASV1192 | 424 | `TAAGGAATATTGGACAATGCCCGCAAGGGTGATCCAGCCATGCCGCGTGCAGGAAGACGGCCCTATGGGTTGTAAACTGCTTTTGAACTAGAGAAAACCCTCCTACGTGTAGGAGGCTGATAGTATAGTTAGAATAAGCATCGGCTAACTTCGTGCCAGCAGCCGCGGTAAGACGAAGGATGCAAGCGTTATCCGGATTCATTGGGTTTAAAGGGAGCGTAGGTGGACTGATAAGTCAGTGGTGAAAGCCCTCAGCTTAACTGAGGAATTGCCATTGATACTGTTGGTCTTGAGTATGGTTGCTGTGGGCGGAATATGACATGTAGAGGTGAAATTCATAGAGATGTCATAGAACACCGATTGCGAAGGCAGCTCACAAAGCCATTACTGACACTGAGGCTCGAAAGTGCGGGGATCAAACAGG` |
| ASV3536 | 404 | `TGGGGAATATTGGACAATGGGCGAAAGCCTGATCCAGCAATGCCGCGTGAGTGATGAAGGCCTTAGGGTTGTAAAGCTCTTTTACCCGGGAAGATAATGACTGTACCGGGAGAATAAGCCCCGGCTAACTCCGTGCCAGCAGCCGCGGTAATACGGAGGGGGCTAGCGTTGTTCGGAATTACTGGGCGTAAAGCGCACGTAGGCGGTTACTCAAGTCAGAGGTGAAAGCCCGGAGCTCAACTCCGGAACTGCCTTTGAAACTAGGTGACTTGAATCATGGAGAGGCGGGTGGAATTCCGAGTGTAGAGGTGAAATTCGTAGATATTCGGAAGAACACCAGTGGCGAAGGCGGCCCGCTGGACATGTATTGACGCTGAGGTGCGAAAGCGTGGGGAGCAAACAGG` |
| ASV2664 | 407 | `TAGGGAATATTGCACAATGGAGGAAACTCTGATGCAGCGACGCCGCGTGAGTGACGAAGGCCTTCGGGTTGTAAAGCTCTGTTCTCAGGGAAAAAGAAAGTGATTGTACCTGAGAAGAAAGGACCGGCTAACTTCGTGCCAGCAGCCGCGGTAAGACGAGGGGTCCAAGCGTTGTTCGGAATTATTGGGCGTAAAGCGGGTGTAGGTGGCTCTGTAAGTCAGGAGTGAAAGCCCCGAGCTTAACTTGGGAAGTGCTTTTGATACTGCAGAGCTTGAGTGTGGGAGAGGATCGTGGAATTCCAGGTGTAGTGGTGAAATACGTAGATATCTGGAGGAACACCGGTGGCGAAGGCGGCGATCTGGCCCAACACTGACACTGAGACCCGAAAGCGTGGGGATCAAACAGG` |
| ASV1562 | 424 | `TGAGGAATATTGGACAATGGGCGCAAGCCTGATCCAGCCATGCCGCGTGCAGGATGACGGTCCTATGGATTGTAAACTGCTTTTGCACAGGAAGAAACACTCCCTCGTGAGGGAGCTTGACGGTACTGTGAGAATAAGGATCGGCTAACTCCGTGCCAGCAGCCGCGGTAATACGGAGGATCCAAGCGTTATCCGGAATCATTGGGTTTAAAGGGTCCGTAGGCGGTTTAGTAAGTCAGTGGTGAAAGCCCATCGCTCAACGGTGGAACGGCCATTGATACTGCTAGACTTGAATTATTAGGAAGTAACTAGAATATGTAGTGTAGCGGTGAAATGCTTAGATATTACATGGAATACCAATTGCGAAGGCAGGTTACTACTAGTTTATTGACGCTGATGGACGAAAGCGTGGGGAGCGAACAGG` |
| ASV645 | 405 | `TGGGGAATTTTCCGCAATGGGCGAAAGCCTGACGGAGCAATACCGCGTGAGGGAGGAAGGCTCTTGGGTTGTAAACCTCAAAACTTAGGGAAGAAGAAAGTGACGGTACCTAATATAAGCATCGGCTAACTCCGTGCCAGCAGCCGCGGTAATACGGAGGATGCAAGCGTTATCCGGAATCATTGGGCGTAAAGAGTCCGTAGGTGGCAATTAAAGTCTGCTGTCAAAGACCGAAGCTCAACTTCGGGTAGGCGGTGGAAACTGAAGAGCTAGAGTATAGTAGGGGTAGAGGGAATTCCTAGTGTAGCGGTGAAATGCGTAGAGATTAGGAAGAACACCAGTGGCGAAGGCGCTCTACTGGGCATATACTGACACTGAGGGACGAAAGCTAAGGTAGCGAAAGGG` |
| ASV5470 | 404 | `CAAGGAATTTTCCCCAATGGGCGCAAGCCTGAGGGAGCAACGCCGCGTGGAGGATGACGGCTTTCGGGTTGTAAACTCCTTTTGGGTGGGAAGATGATGACGGTACCACCCGAATAAGCCCCGGCTAACTCCGTGCCAGCAGCCGCGGTAATACGGAGGGGGCAAGCGTTGTCCGGACTTACTGGGCGTAAAGCGCACGTAGGCGGTCTGTTGCGTCGGTGCTGAAAGCGCCAGGCTTAACCTGGCGAGGCGCGCCGATACGAGCAGACTGGAGGCCAGCAGAGGGTGGCGGAATTCCGGGTGTAGTGGTGAAATGCGTAGAGATCCGGAGGAACGCCTGTGGCGCAAGCGGCCACCTGGGCTGGACCTGACGCTGAGGTGCGAAAGCGTGGGGAGCGAACCGG` |
| ASV3351 | 404 | `TGGGGAATATTGGACAATGGGCGCAAGCCTGATCCAGCCATGCCGCGTGAATGATGAAGGCCTTAGGGTTGTAAAATTCTTTCACCGGGGACGATAATGACGGTACCCGGAGAAGAAGCCCCGGCTAACTTCGTGCCAGCAGCCGCGGTAATACGAAGGGGGCTAGCGTTGTTCGGAATTACTGGGCGTAAAGCGCACGTAGGCGGATCGATTAGTTGGTGGTGAAAGCCCAGGGCTCAACCTTGGAACTGCCATCAATACTGTCGATCTGGAGCATAGTAGAGGTTAGTGGAATTCCAAGTGTAGAGGTGAAATTCGTAGATATTTGGAGGAACACCAGTGGCGAAGGCGACTGACTGGGCTATTGCTGACGCTGAGGTGCGAAAGCGTGGGGAGCAAACAGG` |
| ASV2404 | 430 | `TAGGGAATTTTGCACAATGGGCGCAAGCCTGATGCAGCAACGCCGCGTGAGTGAAGAAGGCTCTTGGGTTGTAAAGCTCTTTCGGCTGGGAAGAAGGGTCTACGTGCAAATAGTTCGTAGGTCTGACGGTACCAGAATAAGAAGCACCTGCTAACTTCGTGCCAGCAGCAGCGGTAATACGAAGGGTGCAAGCGTTGTTCGGAATTATTGGGCGTAAAGCGCGCGTAGGCGGGCTGGAAAGTCGATTGTGAAATACCTGGGCTTAACCTGGGAACTGCAGTCGAAACTTCCTGTCTTGAGTGCCTGAGAGGATGGCGGAATTCCCGGTGTAGTAGTGAAATACGTAGATATCGGGAAGAACACCTGAGGCGAAGGCGGCTATCTGGCGGTGCACTGACGCTGAGGCGCGAAAGCGTGGGGAGCAAACAGG` |
| ASV181 | 429 | `TGGGGAATTTTGGACAATGGGCGCAAGCCTGATCCAGCCATGCCGCGTGCGGGAAGAAGGCCTTCGGGTTGTAAACCGCTTTTGTTAGGGAAGAAACGCTCTGGGCTAATACCCTGGGGTAATGACGGTACCTAAAGAATAAGCACCGGCTAACTACGTGCCAGCAGCCGCGGTAATACGTAGGGTGCGAGCGTTAATCGGAATTACTGGGCGTAAAGCGTGCGCAGGCGGTTTTGTAAGACAGATGTGAAATCCCCGGGCTCAACCTGGGAACTGCATTTGTGACTGCAAGGCTAGAGTACGGTAGAGGGGGGTGGAATTCCGCGTGTAGCAGTGAAATGCGTAGATATGCGGAGGAACACCGATGGCGAAGGCAGCCCCCTGGACCTGTACTGACGCTCATGCACGAAAGCGTGGGGAGCAAACAGG` |
| ASV1566 | 404 | `TAGGGAATATTGGACAATGGGCGAAAGCCTGATCCAGCAATGCCGCGTGAGCGATGAAGGCCTTAGGGTTGTAAAGCTCTTTTACCCGGGATGATAATGACAGTACCGGGAGAATAAGCCCCGGCTAACTCCGTGCCAGCAGCCGCGGTAATACGGAGGGGGCTAGCGTTGTTCGGAATTACTGGGCGTAAAGCGCACGTAGGCGGCTATTCAAGTCAGAGGTGAAAGCCCGGGGCTCAACCCCGGAATTGCCTTTGAAACTAGATAGCTCGAATCTGGGAGAGGTGAGTGGAATTCCGAGTGTAGAGGTGAAATTCGTAGATATTCGGAAGAACACCAGTGGCGAAGGCGGCTCACTGGACCAGTATTGACGCTGAGGTGCGAAAGCGTGGGGAGCAAACAGG` |
| ASV5701 | 407 | `TAGGGAATATTGCACAATGGAGGAAACTCTGATGCAGCGACGCCGCGTGAGTGATGAAGGACTTCGGTTCGTAAAGCTCTGTCGTATGGGAATAAGAAAGTGAATGTACCATGCAAGAAAGGATCGGCTAACTTCGTGCCAGCAGCCGCGGTAAGACGAGGGATCCTAGCGTTGTTCGGAATCATTGGGCGTAAAGCGGATGTAGGTGGCTTTGTAAGTCAGGTGTGAAAGCCCCGGGCTCAACCCGGGAAGTGCATTTGATACTGCGAAGCTTGAGTGCTAGAGAGGTTACTAGAATACCTGGTGTAGTGGTGAAATACGTAGATATCAGGTGGAATACCGGAGGCGAAGGCGGGTAACCAGCTAGACACTGACACTCAGATCCGAAAGTGTGGGGATCAAACAGG` |
| ASV412 | 404 | `TGGGGAATATTGGACAATGGGCGAAAGCCTGATCCAGCCATGCCGCGTGTGTGATGAAGGCCTTAGGGTTGTAAAGCACTTTTGTCAGGGAAGATAATGACGGTACCTGAAGAATAAGCACCGGCTAACTTCGTGCCAGCAGCCGCGGTAATACGAAGGGTGCTAGCGTTGTTCGGAATCACTGGGCGTAAAGCGCGCGTAGGCGGCGATACAAGTCAGAGGTGAAAGCCTGGAGCTCAACTCCAGAACTGCCTTTGAAACTGTATTGCTAGAGTGTTGGAGGGGATAGCGGAATTGCTAATGTAGAGGTGAAATTCGTAGATATTAGCAGGAACACCGGTGGCGAAGGCGGCTATCTGGACAACAACTGACGCTGAGGCGCGAAAGCGTGGGGATCAAACAGG` |
| ASV1197 | 423 | `TAGGGAATATTGGTCAATGGGCGCAAGCCTGAACCAGCCATGCCGCGTGCAGGAAGAATGCCCTCTGGGTTGTAAACTGCTTTTGACAGGGAAGAAATACGAGATGCGTCTCGGCCTGACGGTACCTGTAGAATAAGCACCGGCTAACTCCGTGCCAGCAGCCGCGGTAATACGGAGGGTGCAAGCGTTATCCGGATTTATTGGGTTTAAAGGGTGCGTAGGCGGTTTTGTAAGTCAGCGGTGAAAGCTCCCAGCCTAACTGGGAAACTGCCATTGATACTGCAAAGCTTGAGTATAGTTGAGGTAGGCGGAATTGATGGTGTAGCGGTGAAATGCATAGATATCATCAAGAACTCCAATAGCGAAGGCAGCTTACTAAACTATAACTGACGCTGAGGCACGAAAGTGTGGGGATCAAACAGG` |
| ASV3726 | 429 | `TGGGGAATATTGGACAATGGGGGCAACCCTGATCCAGCCATGCCGCGTGTGTGAAGAAGGCCTTAGGGTTGTAAAGCACTTTAAGTTGGGAGGAAAGGTGTTTGGCTAATATCCAAATGCTGTGACGTTACCAACAGAATAAGCACCGGCTAACTCCGTGCCAGCAGCCGCGGTAATACGGAGGGTGCAAGCGTTAATCGGAATTACTGGGCGTAAAGCGCGCGTAGGCGGCTTGGTAAGCTAGATGTGAAAGCCCTGGGCTCAACCTGGGAACTGCATATAGAACTGCTGAGCTAGAGTGCAGAAGAGGAGTGTGGAATTTCAGGTGTAGCGGTGAAATGCGTAGAGATCTGAAGGAACATCAGTGGCGAAGGCGACACTCTGGTCTGACACTGACGCTGAGGTGCGAAAGCGTGGGTAGCAAACAGG` |
| ASV3866 | 429 | `TAGGGAATATTGGACAATGGGGGCAACCCTGATCCAGCAATGCCACGTGTGTGAAGAAGGCCTGAGGGTTGTAAAGCACTTTTGTGGGGGAAGAAGTGGAGTAGGTTAAGAGCTAGCTCCATTGACATTACCCCAAGAAAAAGCACCGGCTAACTCTGTGCCAGCAGCCGCGGTAATACAGAGGGTGCAAGCGTTAATCGGACTTACTGGGCGTAAAGGGCGCGTAGGTGGATAGTTAAGTCAGGTGTGAAATCCCAGGGCTCAACCTTGGAATGGCACTTGATACTGGCTATCTAGAGTATGGTAGAGGGAAGGGGAATTTCCGGTGTAGCGGTGAAATGCGTAGATATCGGAAGGAACATCAGTGGCGAAGGCGCCTTCCTGGACCAATACTGACACTGAGGCGCGAAAGCGTGGGTAGCAAACAGG` |
| ASV1099 | 424 | `TAGGGAATATTGGACAATGGGGGCAACCCTGATCCAGCCATGCCGCGTGCAGGAAGACTGCCCTATGGGTTGTAAACTGCTTTTGTACGGGAAGAAACCCCTCTACGTGTAGAGGGTTGACGGTACCGTGAGAATAAGGATCGGCTAACTTCGTGCCAGCAGCCGCGGTAATACGAAGGATCCAAGCGTTGTCCGGATTTACTGGGTTTAAAGGGTGCGTAGGCGGGCTATTAAGTCAGTGGTGAAAGCCGATCGCTTAACGATCGAACTGCCATTGAAACTGATAGTCTTGAGTATGTTTGAGGTTACTGGAATATAACATGTAGCGGTGAAATGCTTAGATATGTTATAGAACACCAATTGCGAAGGCAGGTAACTAAGTCATAACTGACGCTGAGGCACGAAAGCGTGGGGAGCAAACAGG` |
| ASV1871 | 429 | `TGGGGAATTTTGGACAATGGGGGCAACCCTGATCCAGCCATGCCGCGTGCAGGATGAAGGCCTTCGGGTTGTAAACTGCTTTTGTTAGGAACGAAAAGCCACTGAATAACACTCAGTGGTGCTGACGGTACCTAAAGAATAAGCACCGGCTAACTACGTGCCAGCAGCCGCGGTAATACGTAGGGTGCAAGCGTTAATCGGAATTACTGGGCGTAAAGCGTGCGCAGGCGGTTATATAAGTCAGATGTGAAATCCCTGGGCTCAACCTAGGAACTGCATTTGAGACTGTATAGCTAGAGTGTGTCAGAGGGGGGTAGAATTCCACGTGTAGCAGTGAAATGCGTAGAGATGTGGAGGAATACCAATGGCGAAGGCAACCCCCTGGGATAACACTGACGCTCATGTACGAAAGCGTGGGGAGCAAACAGG` |
| ASV93 | 404 | `TGGGGAATCTTGCGCAATGGGCGAAAGCCTGACGCAGCCATGCCGCGTGAATGATGAAGGTCTTAGGATTGTAAAATTCTTTCACCGGGGACGATAATGACGGTACCCGGAGAAGAAGCCCCGGCTAACTTCGTGCCAGCAGCCGCGGTAATACGAAGGGGGCTAGCGTTGCTCGGAATTACTGGGCGTAAAGGGAGCGTAGGCGGACATTTAAGTCAGGGGTGAAATCCCGGGGCTCAACCTCGGAACTGCCTTTGATACTGGGTGTCTTGAGTGTGATAGAGGTATGTGGAACTCCGAGTGTAGAGGTGAAATTCGTAGATATTCGGAAGAACACCAGTGGCGAAGGCGACATACTGGATCATTACTGACGCTGAGGCTCGAAAGCGTGGGGAGCAAACAGG` |
| ASV1180 | 429 | `TGGGGAATATTGGACAATGGGCGCAAGCCTGATCCAGCAATGCCGCGTGTGTGAAGAAGGCCTTCGGGTTGTAAAGCACTTTTGTACGGGAAGAAAGCGAGCGGATTAATACTTCGTTCGGATGACGGTACCGTAAGAATAAGCACCGGCTAACTTCGTGCCAGCAGCCGCGGTAATACGAAGGGTGCAAGCGTTAATCGGAATTACTGGGCGTAAAGCGTGCGTAGGCGGTTTGTTAAGTCCGATGTGAAAGCCCTGGGCTCAACCTGGGAATGGCATTGGAAACTGGCGAGCTAGAGTCAGGTAGAGGAGTGCGGAATTCCTGGTGTAGCGGTGAAATGCGTAGAGATCAGGAGGAACATCCGTGGCGAAGGCGGCACTCTGGACCATGACTGACGCTGAGGCACGAAAGCGTGGGGAGCAAACAGG` |
| ASV1767 | 429 | `TGGGGAATTTTGGACAATGGGCGAAAGCCTGATCCAGCAATGCCGCGTGCAGGATGAAGGCCTTAGGGTTGTAAACTGCTTTTGTACGGAACGAAACGGCTCTCTTTAATACAGGGAGCTAATGACGGTACCGTAAGAATAAGCACCGGCTAACTACGTGCCAGCAGCCGCGGTAATACGTAGGGTGCGAGCGTTAATCGGAATTACTGGGCGTAAAGCGTGCGCAGGCGGTTATATAAGACAGATGTGAAATCCCCGGGCTCAACCTGGGACCTGCATTTGTGACTGTATAGCTAGAGTACGGTAGAGGGGGATGGAATTCCGCGTGTAGCAGTGAAATGCGTAGATATGCGGAGGAACACCGATGGCGAAGGCAATCCCCTGGACCTGTACTGACGCTCATGCACGAAAGCGTGGGGAGCAAACAGG` |
| ASV1842 | 429 | `TGGGGAATATTGCGCAATGGGGGCAACCCTGACGCAGCCATGCCGCGTGTGTGAAGAAGGCCTTCGGGTTGTAAAGCACTTTAAGTGGGGAGAAAAGGGTAGGCGTTAATAGCGTCTATCTGTGATGTTACCCACAGAATAAGCACCGGCTAACTCCGTGCCAGCAGCCGCGGTAATACGGAGGGTGCGAGCGTTAATCGGAATTACTGGGCGTAAAGCGCGCGTAGGTGGTTTGTTAAGTCAGCTGTGAAAGCCCCGGGCTCAACCTGGGAACTGCAGTTGATACTGGCCGACTAGAGTACGAGAGAGGGAGGTAGAATTCCATGTGTAGCGGTGAAATGCGTAGATATATGGAGGAATACCGGTGGCGAAGGCGGCCTCCTGGCTCGATACTGACGCTGAGGTGCGAAAGCGTGGGGATCAAACAGG` |
| ASV1744 | 429 | `TGGGGAATATTGGACAATGGGGGAAACCCTGATCCAGCGATGCCGCGTGTGTGAAGAAGGCCTTAGGGTTGTAAAGCACTTTAATGAGGGAGGATTAGGTTCTGTTGAATAGGCAGGGCTGAGGACGTTACCTCAAGAATAAGCACCGGCTAACTCTGTGCCAGCAGCCGCGGTAATACAGAGGGTGCGAGCGTTAATCGGAATTATTGGGCGTAAAGGGTGCGTAGGCGGATGTGCAAGTGATAGGTGAAATACCCGGGCTTAACCTGGGGAGGTCCTATCAGACTGCACGTCTAGAATGTGGTAGAGGAAGGTGGAATTCCTGGTGTAGCGGTGAAATGCGTAGATATCAGGAAGAACATCGATGGCGAAGGCAGCCTTCTGGGCCATTATTGACGCTGAGGCACGAAAGCGTGGGGAGCGAACAGG` |
| ASV3795 | 423 | `TAGGGAATATTGGGCAATGGTCGGAAGACTGACCCAGCCATGCCGCGTGCAGGATGAAGGCGCTATGCGTTGTAAACTGCTTTTGTTAGGGAAGAAAAAGGCTATGCGTAGCAACTTGACGGTACCTAAAGAATAAGCATCGGCTAACTCCGTGCCAGCAGCCGCGGTAATACGGAGGATGCAAGCGTTGTCCGGATTTATTGGGTTTAAAGGGTGCGTAGGCGGTCTTATAAGTCAGTGGTTAAATAACTTTGCTTAACAAGGGAGATGCCATTGATACTGTTAGACTTGAATACTGACTAGGTAGGCGGAATTGGTAGTGTAGCGGTGAAATGCTTAGATATTACCAGGAACACCGATAGCGAAGGCAGCTTACTGGACAGTTATTGACGCTGAGGCACGAAAGTGCGGGTATCAAACAGG` |
| ASV4126 | 429 | `TGAGGAATATTGCGCAATGGGCGAAAGCCTGACGCAGCGACGCCGCGTGAAGGATGAAGGTCTTAGGATTGTAAACTTCTGTTAAGTGGGAAGAAAGAATCACCAGTAATATGGGTGAGAGATGACGTTACCACTAGAGAAAGCACCGGCTAACTTCGTGCCAGCAGCCGCGGTAATACGAGGGGTGCAAGCGTTATTCGGAATTACTGGGCGTAAAGGGTGTGTAGACGGTTTAGTAAGTCTACTGTTAAAGACCCCGGCTTAACCGGGGAAAAGCAGTGGAAACTAGTAGACTTGAGGGTGGAAGAGAGAAGTGGAATTCTCGGAGTAGCGGTAAAATGCGTAGATCTCGAGAGGAACACCGATGGCGAAGGCAGCTTCTTGGTCCATTCCTGACGTTGAAACACGAAAGCGTGGGTAGCAAACAGG` |
| ASV4236 | 429 | `TGGGGAATTTTGGACAATGGGGGCAACCCTGATCCAGCCATTCCGCGTGTGTGAAGAAGGCCTTCGGGTTGTAAAGCACTTTTGTCAGGAAAGAAATAGCGCGGGTTAATACCTTGCGTTGATGACGGTACCTGAAGAATAAGCACCGGCTAACTACGTGCCAGCAGCCGCGGTAATACGTAGGGTGCAAGCGTTAATCGGAATTACTGGGCGTAAAGCGTGCGCAGGCGGTTCGGAAAGAAAGATGTGAAATCCCAGAGCTCAACTTTGGAACTGCATTTTTAACTACCGGGCTAGAGTATGTCAGAGGGGGGTAGAATTCCACGTGTAGCAGTGAAATGCGTAGATATGTGGAGGAATACCGATGGCGAAGGCAGCCCCCTGGGATAATACTGACGCTCATGCACGAAAGCGTGGGGAGCAAACAGG` |
| ASV1182 | 429 | `TGGGGAATTTTGGACAATGGGCGAAAGCCTGATCCAGCAATGCCGCGTGCAGGAAGAAGGCCTTCGGGTTGTAAACTGCTTTTGTACGGAACGAAACGGTCTTTTTTAATAAAGAGGGCTAATGACGGTACCGTAAGAATAAGCACCGGCTAACTACGTGCCAGCAGCCGCGGTAATACGTAGGGTGCGAGCGTTAATCGGAATTACTGGGCGTAAAGCGTGCGCAGGCGGTTATGCAAGACAGTTGTGAAATCCCCGGGCTCAACCTGGGAATTGCATCTGTGACTGCATAGCTAGAGTACGGTAGAGGGGGATGGAATTCCGCGTGTAGCAGTGAAATGCGTAGATATGCGGAGGAACACCGATGGCGAAGGCAATCCCCTGGACCTGTACTGACGCTCATGCACGAAAGCGTGGGGAGCAAACAGG` |
| ASV1829 | 425 | `TGAGGAATATTGGACAATGGGTGGAAGCCTGATCCAGCCATCCCGCGTGCAGGAAGAAGGTCCTATGGATTGTAAACTGCTTTTATACAGGAATAAACCTCTTTACGTGTAGAGAGCTGAAGGTACTGTATGAATAAGCACCGGCTAACTCCGTGCCAGCAGCCGCGGTAATACGGAGGGTGCAAGCGTTATCCGGATTTATTGGGTTTAAAGGGTCCGTAGGCGGCTAAATAAGTCAGTGGTGAAAGCCGGGCAGCTTAACTGTCGAACTGCCATTGATACTGTTTAGCTTGAATTGATTTGAAGTAGCTGGAATGTGTAGTGTAGCGGTGAAATGCATAGATATTACACAGAACATCGATAGCGAAGGCAGGTTACTAAGGTCATATTGACGCTGATGGACGAAAGCGTGGGGAGCGAACAGG` |
| ASV1481 | 404 | `TGGGGAATATTGGACAATGGGCGCAAGCCTGATCCAGCAATTCCGCGTGGGTGAAGAAGGTTTTCGGATTGTAAAGCCCTTTCGGCGGGGACGATGATGACGGTACCCGCAGAAGAAGCCCCGGCTAACTTCGTGCCAGCAGCCGCGGTAATACGAAGGGGGCTAGCGTTGCTCGGAATGACTGGGCGTAAAGGGCGCGTAGGCGGCGTGGTAAGTCAGGCGTGAAATTCCTGGGCTCAACCTGGGGGCTGCGCTTGAGACTGCTGTGCTTGAGGGTGAGAGAGGCTCGTGGAATTCCCAGTGTAGAGGTGAAATTCGTAGATATTGGGAAGAACACCGGTGGCGAAGGCGGCGAGCTGGCTCATTTCTGACGCTGAGGCGCGAAAGCGTGGGGAGCAAACAGG` |
| ASV3549 | 404 | `TGGGGAATATTGGACAATGGGGGCAACCCTGATCCAGCCATGCCGCGTGAGTGATGAAGGCCTTCGGGTTGTAAAGCTCTTTCGCCAGGGACGATAGTGACGGTACCTGGATAAGAAGCCCCGGCTAACTTCGTGCCAGCAGCCGCGGTGATACGAAGGGGGCTAGCGTTGTTCGGAATCACTGGGCGTAAAGCGCACGTAGGCGGGCCGTTAAGTCGGAGGCGAAAGCCTGGGGCTCAACCCCAGAACTGCCTTCGATACTGGCGGCCTAGAGTATGGGAGAGGTTGGTGGAACTCCGAGTGTAGAGGTGAAATTCGTAGATATTCGGAAGAACACCAGTGGCGAAGGCGGCCAACTGGCCCATTACTGACGCTGAGGTGCGAAAGCGTGGGGAGCAAACAGG` |
| ASV5713 | 423 | `TAGGGAATATTGGTCAATGGGTGCAAGCCTGAACCAGCCATGCCGCGTGCAGGAAGAAGGCCTTCTGGGTTGTAAACTGCTTTTGCCAGGGGATAAAATTTCCTTGCGAGGAAAATTGAAGGTACCTGGTGAATAAGCCACGGCTAACTACGTGCCAGCAGCCGCGGTAATACGTAGGTGGCAAGCGTTGTCCGGATTTATTGGGTTTAAAGGGTGCGTAGGCGGTTTTGTAAGTCAGTGGTGAAATATGTCAGCTTAACTGAGAGGGTGCCATTGATACTGCAGAACTTGAGTACAGATGAGGTAGGCGGAATTGACGGTGTAGCGGTGAAATGCTTAGATATCGTCAAGAACACCTATAGCGAAGGCAGCTTACTAGGCTGTAACTGACGCTGAGGCACGAAAGTGTGGGGATCAAACAGG` |
| ASV776 | 419 | `TAAGGAATATTGCGCAATGGACGAAAGTCTGACGCAGCGACGCCGCGTGGGGGATGAAGGTCTTCGGATTGTAAACCCCTTTCGGCAGGGAAGATGGAACGGGTAACCGTTCGGACGGTACCTGCAGAAGCAGCCACGGCTAACTTCGTGCCAGCAGCCGCGGTAATACGAAGGTGGCAAGCGTTGTTCGGATTCACTGGGCGTACAGGGAGCGTAGGCGGTTGGGTAAGCCCTCCGTGAAATCTCCGGGCCTAACCCGGAAAGTGCAGAGGGGACTGCTCGGCTAGAGGATGGGAGAGGAGCGCGGAATTCCCGGTGTAGCGGTGAAATGCGTAGAGATCGGGAGGAAGGCCGGTGGCGAAGGCGGCGCTCTGGAACATTTCTGACGCTGAGGCTCGAAAGCGTGGGGAGCAAACAGG` |
| ASV220 | 424 | `TAGGGAATATTGGGCAATGGGCGAGAGCCTGACCCAGCCATGCCGCGTGCAGGAAGACGGCCCTCTGGGTTGTAAACTGCTTTTATCGGGGAAGAACAGACGGGGTGCGCCCTGTTGTGACGGTACTCGATGAATAAGCACCGGCTAACTCCGTGCCAGCAGCCGCGGTAATACGGAGGGTGCAAGCGTTGTCCGGATTTATTGGGTTTAAAGGGTGCGTAGGTGGTTTTTTAAGTCTGGTTTGAAAGTTGGTCGCTTAACGATCAAGGGTGGCTGGATACTGGGGAACTTGAATATTGTGGAGGTAGCCGGAATGGGTTGTGTAGCGGTGAAATGCATAGATATGACCCGGAACACCAATTGCGAAGGCAGGCTACTACGCAATGATTGACACTGAGGCACGAAAGCATGGGGAGCAAACAGG` |
| ASV5317 | 424 | `TAAGGAATATTGGTCAATGGACGCAAGTCTGAACCAGCCATGCCGCGTGCAGGATGACTGCCCTATGGGTTGTAAACTGCTTTTGTACGGGAAGAAACCTCTCTACGTGTAGAGAGTTGACGGTACCGTAAGAATAAGGATCGGCTAACTCCGTGCCAGCAGCCGCGGTAATACGGAGGATCCAAGCGTTATCCGGATTTATTGGGTTTAAAGGGTGCGTAGGCGGCCTATTAAGTCAGTGGTGAAAGCCTGGAGCTTAACTCCAGAACTGCCATTGATACTGATGGGCTTGAGTACAGTTGAGGTAGGCGGAATGTGTCATGTAGCGGTGAAATGCTTAGATATGACACAGAACACCGATTGCGAAGGCAGCTTGCTAAACTGTAACTGACGCTGAGGCACGAAAGCGTGGGTAGCAAACAGG` |
| ASV5270 | 404 | `TGGGGAATATTGGACAATGGGCGCAAGCCTGATCCAGCCATGCCGCGTGAGTGATGAAGGCCTTAGGGTTGTAAAGCTCTTTTACCTGGGAAGATAATGACTGTACCAGGAGAATAAGCCCCGGCAAACTTCGTGCCAGCAGCCGCGGTAATACGAAGGGGGCTAGCGTTGCTCGGAATCACTGGGCGTAAAGGGCGCGTAGGTGGCGAATCAAGTCAGAGGTGAAAGCCTGAAGCTCAACTTCAGAACTGCCTTTGATACTGATTTGCTAGAGTTCGAGAGAGGTAAGTGGAATTCCGAGTGTAGAGGTGAAATTCGTAGATATTCGGAAGAACACCAGTGGCGAAGGCGGCTTACTGGCTCGATACTGACACTGAGGCGCGAAAGCGTGGGGAGCAAACAGG` |
| ASV4376 | 403 | `TGGGGAATATTGGGCAATGGGCGAAAGCCTGACCCAGCCATGCCGCGTGAGTGATAAGGCCTTAGGGTTGTAAAACTCTTTCACCGCTGAGGATAATGACGGTAAGCGGAGAAGAAGTCCCGGCTAACTTCGTGCCAGCAGCCGCGGTAATACGAAGGGGACTAGCGTTGTTCGGAATCACTGGGCGTAAAGCGCGCGTAGGCGGCTTGACAAGTCAGAGGTGAAAGCCTGAAGCTTAACTTCAGAACTGCCTTTGAAACTGTCAAGCTGGAATCTCGGAGAGGATAGTGGAATTTCTAGTGTAGAGGTGAAATTCGTAGATACTAGAAGGAACACCAGTGGCGAAGGCGGCTATCTGGACGAGTATTGACGCTGAGGTGCGAAAGCGTGGGTAGCAAACAGG` |
| ASV2111 | 424 | `TGAGGAATATTGGTCAATGGAGGCAACTCTGAACCAGCCATGCCGCGTGAAGGATGAAGGTCCTCTGGATTGTAAACTTCTTTTATAGAAGAAGAAACCCCATTTTTCTAAATGGGTTGACGGTACTCTATGAATAAGCACCGGCTAACTCCGTGCCAGCAGCCGCGGTAATACGGAGGGTGCAAGCGTTATCCGGATTCACTGGGTTTAAAGGGAGCGTAGGTGGGCTTGTAAGTCAGTGGTGAAATCTCCGAGCTTAACTTGGAAACTGCCATTGATACTACTGGTCTTGAATTCTGTGGAGGTAAGCGGAATATATCATGTAGCGGTGAAATGCTTAGATATGATATAGAACACCGATAGCGAAGGCAGCTTACTACGCAGATATTGACACTGAGGCTCGAAAGCGTGGGGATCAAACAGG` |
| ASV4204 | 430 | `TAGGGAATATTGCGCAATGGAGGAAACTCTGACGCAGCGACGCCGCGTGGGTGATGAAGGCCTTCGGGTCGTAAAGCCCTGTCGGAAGGGAAGAAAAACATTTTGGCTAACATCCAAAGTGCTTGACGGTACCTTCAAAGGAAGCACCGGCTAACTACGTGCCAGCAGCCGCGGTAATACGTAGGGTGCTAGCGTTGTTCGGAATCATTGGGCGTAAAGCGCGTGTAGGTGGTCAGCAAAGTTGGGTGTGAAATCCCTCGGCTCAACCGAGGAAGGGCATCCAAAACTAACTGGCTTGAGTATGGTAGAGGAAGGTGGAATTCCTAGTGTAGAGGTGAAATTCGTAGATATTAGGAGGAATACCGGAGGCGAAGGCGGCCTTCTGGACCATTACTGACACTGAGACGCGAAAGCGTGGGTAGCAAACAGG` |
| ASV3547 | 404 | `TGGGGAATATTGGACAATGGGCGCAAGCCTGATCCAGCCATGCCGCGTGAGTGATGAAGGCCTTCGGGTTGTAAAGCTCTTTTGGCGGGGACGATGATGACGGTACCCGCAGAATAAGCCCCGGCAAACTTCGTGCCAGCAGCCGCGGTAAGACGAAGGGGGCAAGCGTTGTTCGGAATTACTGGGCGTAAAGGGCGCGTAGGCGGCTCTTTAAGTCAGGCGTGAAAGCCCTGGGCTCAACCTGGGAGTGGCGCTTGATACTGAGGGGCTAGAGAGTGGGAGAGGTTGGTGGAATTCCCAGTGTAGAGGTGAAATTCGTAGATATTGGGAAGAACACCGGTGGCGAAGGCGGCCAACTGGACCATTTCTGACGCTGAGGCGCGAAAGCGTGGGGAGCAAACAGG` |
| ASV230 | 429 | `TGGGGAATATTGGACAATGGGCGAAAGCCTGATCCAGCCATGCCGCGTGTGTGAAGAAGGCCTTCGGGTTGTAAAGCACTTTAAGCGAGGAGGAATGGCTGAGGGTTAATAACCTTCAGCAGTGACGTTACTCGCAGAATAAGCACCGGCTAACTCTGTGCCAGCAGCCGCGGTAATACAGAGGGTGCAAGCGTTAATCGGAATTACTGGGCGTAAAGCGCGCGTAGGCGGTTACGTAAGTTGGATGTGAAATCCCCGGGCTCAACCTGGGAATTGCATTCAAAACTGCGTGGCTAGAGTGTGGGAGAGGAAGGTAGAATTCCAGGTGTAGCGGTGAAATGCGTAGATATCTGGAGGAATACCGATGGCGAAGGCAGCCTTCTGGCCTAACACTGACGCTGAGGTGCGAAAGCATGGGGAGCAAACAGG` |
| ASV60 | 404 | `TGGGGAATATTGGACAATGGGCGCAAGCCTGATCCAGCAATTCCGCGTGGGTGAAGAAGGTCTTCGGATTGTAAAGCCCTTTCGTTGGGGACGATGATGACGGTACCCAAAGAAGAAGCCCCGGCTAACTTCGTGCCAGCAGCCGCGGTAATACGAAGGGGGCTAGCGTTGCTCGGAATGACTGGGCGTAAAGGGCGCGTAGGCGGCACAACTCGTCAGGCGTGAAATTCCTGGGCTTAACCTGGGGGCTGCGTTTGATACGGTTGGGCTAGAGGATGGAAGAGGCTCGTGGAATTCCCAGTGTAGAGGTGAAATTCGTAGATATTGGGAAGAACACCGGTGGCGAAGGCGGCGAGCTGGTCCATTACTGACGCTGAGGCGCGAAAGCGTGGGGAGCAAACAGG` |
| ASV617 | 407 | `TGGGGAATTTTCCGCAATGGGCGAAAGCCTGACGGAGCAATACCGCGTGAGGGATGACGGCCTATGGGTTGTAAACCTCTTTTTTCAGGGAGGAATAAATGACGTGTACCTGAAGAATAAGCATCGGCTAACTCCGTGCCAGCAGCCGCGGTAAGACGGAGGATGCAAGTGTTATCCGGAATCACTGGGCGTAAAGCGTCTGTAGGTGGTTTAATAAGTCAACTGTTAAATCTTAAGGCTCAACCTTGAAATCGCAGTCGAAACTATTAGACTAGAGTATAGTAGGGGTAAAGGGAATTTCCAGTGGAGCGGTGAAATGCGTAGATATTGGAAAGAACACCGATGGCGAAGGCACTTTACTGGGCTATTACTAACACTCAGAGACGAAAGCTAGGGTAGCAAATGGG` |
| ASV669 | 424 | `TGAGGAATATTGGTCAATGGGCGCAAGCCTGAACCAGCCATGCCGCGTGCAGGATGACGGTCCTATGGATTGTAAACTGCTTTTGTGCAGGAAGAAACACTCCCTCGTGAGGGAGCTTGACGGTACTGTAAGAATAAGGATCGGCTAACTCCGTGCCAGCAGCCGCGGTAATACGGAGGATCCAAGCGTTATCCGGAATCATTGGGTTTAAAGGGTCCGTAGGCGGTTTTATAAGTCAGTGGTGAAATCTGGTCGCTCAACGATCAAACGGCCATTGATACTGTAAGACTTGAATTACTTGGAAGTAACTAGAATATGTAGTGTAGCGGTGAAATGCTTAGAGATTACATGGAATACCAATTGCGAAGGCAGGTTACTACAAGTGGATTGACGCTGATGGACGAAAGCGTGGGGAGCGAACAGG` |
| ASV4576 | 424 | `TGAGGAATATTGGTCAATGGACGCAAGTCTGAACCAGCCATGCCGCGTGCAGGATGACGGCTCTATGAGTTGTAAACTGCTTTTGTACGAGGGTAATTGTGATCACGTGTGGTCACTTGAAAGTATCGTACGAATAAGGATCGGCTAACTCCGTGCCAGCAGCCGCGGTAATACGGAGGATCCGAGCGTTATCCGGATTTATTGGGTTTAAAGGGTGCGTAGGTGGCGCTTTAAGTCAGCGGTTAAAGGTGAGAGCTCAACTCTCAATTTGCCGTTGAAACTGAAGCGCTAGAGAGTGGTTGCAGCAGGCGGAATGTGTAGTGTAGCGGTGAAATGCTTAGATATTACACAGAACACCGATTGCGAAGGCAGCTTGCTAAGCCATATCTGACACTGATGCACGAAAGCGTGGGGATCAAACAGG` |
| ASV1173 | 404 | `TGGGGAATATTGGACAATGGGCGAAAGCCTGATCCAGCCATGCCGCGTGTGTGATGAAGGCCTTAGGGTTGTAAAGCACTTTTGTCAGGGAAGATAATGACGGTACCTGAAGAATAAGCACCGGCTAACTTCGTGCCAGCAGCCGCGGTAATACGAAGGGTGCTAGCGTTGTTCGGAATCACTGGGCGTAAAGCGCGCGTAGGCGGCGTAACAAGTCAGAGGTGAAAGCCTGGAGCTCAACTCCAGAACTGCCTTTGAAACTGTTATGCTAGAGTGTTGGAGGGGATAGCGGAATTGCTAATGTAGAGGTGAAATTCGTAGATATTAGCAGGAACACCGGTGGCGAAGGCGGCTATCTGGACAACAACTGACGCTGAGGCGCGAAAGCGTGGGGATCAAACAGG` |
| ASV1584 | 424 | `TGAGGAATATTGGTCAATGGACGCAAGTCTGAACCAGCCATGCCGCGTGCAGGATGACGGTCCTATGGATTGTAAACTGCTTTTGTACAGGAAGAAACACTCCCTCGTGAGGGAGCTTGACGGTACTGTAAGAATAAGGATCGGCTAACTCCGTGCCAGCAGCCGCGGTAATACGGAGGATCCAAGCGTTATCCGGAATCATTGGGTTTAAAGGGTCCGTAGGCGGTTAAATAAGTCAGTGGTGAAATCTGGTCGCTCAACGATCAAACGGCCATTGATACTGTTTAACTTGAATTACTTGGAAGTAACTAGAATATGTAGTGTAGCGGTGAAATGCTTAGAGATTACATGGAATACCAATTGCGAAGGCAGGTTACTACAAGTATATTGACGCTGATGGACGAAAGCGTGGGGAGCGAACAGG` |
| ASV1256 | 404 | `TGGGGAATCTTGGACAATGGGCGCAAGCCTGATCCAGCCATGCCGCGTGGATGATGAAGGCCCTAGGGTTGTAAAATCCTTTCGTCAGGGACGATAATGACGGTACCTGAAGAAGAAGCCCCGGCTAACTTCGTGCCAGCAGCCGCGGTAATACGAAGGGGGCTAGCGTTGTTCGGAATTACTGGGCGTAAAGCGCATGTAGGCGGGTCGTTAAGTCGGGGGTGAAAGCCCAGAGCTCAACTCTGGAACTGCCTTCGATACTGGCGACCTGGACCCAAGGAGAGGCGAGTGGAATTGCGAGTGTAGAGGTGAAATTCGTAGATATTCGCAGGAACACCAGTGGCGAAGGCGACTCGCTGGACTTGTGGTGACGCTGAGATGCGAAAGCGTGGGGAGCAAACAGG` |
| ASV5310 | 404 | `TGGGGAATATTGGACAATGGACGCAAGTCTGATCCAGCCATGCCGCGTGAGTGATGAAGGCCTTCGGGTTGTAAAGCTCTTTGAGTGGGGACAATCATGATGGTACCCACAGAACAAGCCACGGCTAACTTCGTGCCAGCAGCCGCGGTAATACGAAGGTGGCTAGCGTTGTTCGGAATTACTGGGCGTAAAGCGCACGTAGGCGGTCGCTTAAGTCGAAAGTGAAATCCCGGGGCTTAACTCCGGAACTGCTTTCGAAACTGAGCGGCTTGAATGTGACAGAGGATGATGGAATTCCTAGTGTAGAGGTGAAATTCGTAGATATTAGGAGGAACACCAGTGGCGAAGGCGGTCATCTGGGTCAACATTGACGCTAAGGTGCGAAAGCGTGGGGAGCAAACAGG` |
| ASV660 | 424 | `TGAGGAATATTGGACAATGGGCGCAAGCCTGATCCAGCCATGCCGCGTGCAGGATGACGGTCCTATGGATTGTAAACTGCTTTTGCACAGGAAGAAACATCCCGACGTGTCGGGACTTGACGGTACTGTGAGAATAAGGATCGGCTAACTCCGTGCCAGCAGCCGCGGTAATACGGAGGATCCAAGCGTTATCCGGAATCATTGGGTTTAAAGGGTCCGTAGGCGGTTTAGTAAGTCAGTGGTGAAAGCCCATCGCTCAACGGTGGAACGGCCATTGATACTGCTAGACTTGAATTATTAGGAAGTAACTAGAATATGTAGTGTAGCGGTGAAATGCTTAGAGATTACATGGAATACCAATTGCGAAGGCAGGTTACTACTAATTGATTGACGCTGATGGACGAAAGCGTGGGTAGCGAACAGG` |
| ASV1131 | 424 | `TAGGGAATATTGGACAATGGAGGCAACTCTGATCCAGCCATGCCGCGTGCAGGAAGACTGCCCTATGGGTTGTAAACTGCTTTTGTACGGGAAGAAACCCATCTACGTGTAGGTGGTTGACGGTACCGTAAGAATAAGGATCGGCTAACTTCGTGCCAGCAGCCGCGGTAATACGAAGGATCCAAGCGTTGTCCGGATTTACTGGGTTTAAAGGGAGCGTAGGCGGTCTAATAAGTCAGTGGTGAAAGCCTTCAGCTTAACTGAAGAATTGCCATTGAAACTGCTAGACTCGAGTATGGTTGAGGTCACTGGAATGTAACATGTAGCGGTGAAATGCATAGATATGTTACAGAACACCAATTGCGAAGGCAGGTGACTAAGCCATAACTGACGCTGAGGCTCGAAAGCGTGGGGAGCAAACAGG` |
| ASV1370 | 429 | `TCGAGAATCTTTCACAATGGGGGCAACCCTGATGGAGCGACGCCGCGTGGAGGATAAGGTCTTCGGATTGTAAACTCCTGTCATGCGTGAACAAGATTGCAGGGTTAATAACCCGAGCAAGTTGATGGTAACGCAAGAGGAAGAGACGGCTAACTCTGTGCCAGCAGCCGCGGTAATACAGAGGTCTCAAGCGTTGTTCGGAATCACTGGGCGTAAAGGGTGCGTAGGTGGCGTGGAAAGTCAGATGTGAAAGCCCGGAGCTCAACTTCGGAATTGCATCCGATACTACCATGCTTGAGTACTGGAGAGGTGACTAGAATTCTAGGTGTAGCAGTGAAATGCGTAGATATCTAGAGGAATACCAAAGGCGTAGGCAGGTCACTGGACAGTTACTGACACTGAGGCACGAAGGCCAGGGGAGCGAACGGG` |
| ASV3024 | 429 | `TGGGGAATTTTGGACAATGGGGGAAACCCTGATCCAGCAATGCCGCGTGTGTGAAGAAGGCCTTCGGGTTGTAAAGCACTTTTGGCGGGAACGAAACGGATCTTTCTAATACAAGGATCTAATGACGGTACCCGCAGAATAAGCACCGGCTAACTACGTGCCAGCAGCCGCGGTAATACGTAGGGTGCAAGCGTTAATCGGAATTACTGGGCGTAAAGCGTGCGCAGGCGGTTATGCAAGACAGATGTGAAATCCCCGGGCTTAACCTGGGCACTGCATTTGTGACTGTATAGCTAGAATCTGGCAGAGGGGGGTAGAATTCCACGTGTAGCAGTGAAATGCGTAGAGATGTGGAGGAACACCGATGGCGAAGGCAGCCCCCTGGGCTAAGATTGACGCTCATGCACGAAAGCGTGGGGAGCAAACAGG` |
| ASV4633 | 429 | `TGGGGAATTTTGGACAATGGGGGCAACCCTGATCCAGCCATTCCGCGTGAGTGAAGAAGGCCTTCGGGTTGTAAAGCTCTTTTGTCCGGAGCGAAACGGATGCGGTGAACAGCCGCGTCTACTGACGGTACCGGAAGAATAAGCACCGGCTAACTACGTGCCAGCAGCCGCGGTAATACGTAGGGTGCGAGCGTTAATCGGAATTACTGGGCGTAAAGCGTGCGCAGGCGGTTTTGTAAGCCAGACGTGAAATCCCCGGGCTCAACCTGGGAATGGCGTTTGGGACTGCAAGGCTGGAGTATGGCAGAGGGGACTAGAATTCCTGGTGTAGCAGTGAAATGCGTAGATATCAGGAGGAATACCGATGGCGAAGGCAGGTCCCTGGGCTGATACTGACGCTCATGCACGAAAGCGTGGGGAGCAAACAGG` |
| ASV3754 | 405 | `TAGGGAATTTTCCACAATGGGCGAAAGCCTGATGGAGCAACGCCGCGTGCAGGATGAAGGCCTTCGGGTTGTAAACTGCTTTTTTCTGTGACGATTATGACGGTAGCAGACGAATAAGGACCGGCTAACTACGTGCCAGCAGCCGCGGTCATACGTAGGGTCCAAGCGTTATCCGGAATTACTGGGCGTAAAGAGTTGCGTAGGTGGCAGAGTAAGTTGGTAGTGAAAGCATGTGGCTCAACCATATATCCATTACTAAAACTGCTCAGCTAGAAGATGAGAGAGGTCGCTGGAATTCCTAGTGTAGGAGTGAAATCCGTAGATATTAGGAGGAACACCGATGGCGTAGGCAGGCGACTGGCTCATTCTTGACACTAAGGCACGAAAGCGTGGGGAGCGAACTGG` |
| ASV2861 | 404 | `TGGGGAATATTGGACAATGGGGGCAACCCTGATCCAGCCATGCCGCGTGAGTGAAGAAGGCCTTCGGGTTGTAAAGCTCTTTTGGCGGGGACGATGATGACGGTACCCGCAGAATAAGCCCCGGCTAACTTCGTGCCAGCAGCCGCGGTAAGACGAAGGGGGCTAGCGTTGTTCGGAATTACTGGGCGTAAAGGGCGCGTAGGCGGTTTGTTAAGTCAGGCGTGAAAGCCCTGGGCTCAACCTGGGATGTGCGTTTGATACTGGCAAGCTTGAGTGCAGGAGAGGATGGTGGAATTCCCAGTGTAGAGGTGAAATTCGTAGATATTGGGAAGAACACCGGTGGCGAAGGCGGCCATCTGGACTGCAACTGACGCTGAGGCGCGAAAGCGTGGGGAGCAAACAGG` |
| ASV4292 | 429 | `TGGGGAATATTGCGCAATGGGGGCAACCCTGACGCAGCCATGCCGCGTGAATGAAGAAGGCCTTCGGGTTGTAAAGTTCTTTCGGTAGCGAGGAAGGCATTTAGTTTAATAGACTAGGTGATTGACGTTAACTACAGAAGAAGCACCGGCTAACTCCGTGCCAGCAGCCGCGGTAATACGGAGGGTGCGAGCGTTAATCGGAATAACTGGGCGTAAAGGGCACGCAGGCGGTGACTTAAGTGAGGTGTGAAAGCCCCGGGCTTAACCTGGGAATTGCATTTCATACTGGGTCGCTAGAGTACTTTAGGGAGGGGTAGAATTCCACGTGTAGCGGTGAAATGCGTAGAGATGTGGAGGAATACCGAAGGCGAAGGCAGCCCCTTGGGAATGTACTGACGCTCATGTGCGAAAGCGTGGGGAGCAAACAGG` |
| ASV5465 | 404 | `TGGGGAATATTGGACAATGGGGGAAACCCTGATCCAGCAATGCCGCGTGAGTGACGAAGGCCTTAGGGTTGTAAAACTCTTTTCTAAGTGAAGATGATGACGGTAACTTAGGAATAAGCTCCGGCTAACTTCGTGCCAGCAGCCGCGGTAATACGAAGGGAGCGAGCGTTGTCCGGAATTACTGGGCGTAAAGAGCACGTAGGCGGCTATATTAGTTGGGTGTGAAATCCCAGGGCTTAACCCTGGAACTGCATCCAATACTGTATAGCTAGAGACCGAGAGAGGAAAATGGAATTACGAGTGTAGAGGTGAAATTCGTAGATATTCGTAGGAACACCAGAGGCGAAAGCGATTTTCTGGCTCGGTACTGACGCTCAGGTGCGAAAGCGTGGGGAGCAAACAGG` |
| ASV461 | 428 | `TGGGGAATCTTGCGCAATGGGCGAAAGCCTGACGCAGCGACGCCGCGTGAGTGATGAAGGCCTTCGGGTTGTAAAGCTCTGTGGAGGGGGAAGAATAAGCGCCGGGTAATATTCGGTGTGATGACGGTACCCCTATAGCAAGCACCGGCTAACTCTGTGCCAGCAGCCGCGGTAAGACAGAGGGTGCAAACGTTGTTCGGAATGACTGGGCGTAAAGCGTGTGTAGGCGGCCTTTTAGGTCTGATGTGAAAGCCCCGGGCTCAACCTGGGAAGTGCATTGGAAACCGGGAGGCTAGAGTGACGGAGAGGTTGGTGGAATTCTCGGTGTAGAGGTGAAATTCGTAGATATCGAGAGGAACATCGGTGGCGAAGGCGGCCAACTGGACGTAAACTGACGCTGAGACACGAAAGTGTGGGGAGCAAACAGG` |
| ASV197 | 404 | `TGGGGAATATTGCACAATGGGGGAAACCCTGATGCAGCGACGCCGCGTGAAGGATGAAGTATTTCGGTATGTAAACTTCTATCAGCAGGGAAGAAAATGACGGTACCTGACTAAGAAGCCCCGGCTAACTACGTGCCAGCAGCCGCGGTAATACGTAGGGGGCAAGCGTTATCCGGATTTACTGGGTGTAAAGGGAGCGTAGACGGTTGTGTAAGTCAGATGTGAAAGCCCGGGGCTCAACCCCGTGACTGCATTTGAAACTATGCAACTAGAGTGTCGGAGAGGTAAGTGGAATTCCTAGTGTAGCGGTGAAATGCGTAGATATTAGGAGGAACACCAGTGGCGAAGGCGGCTTACTGGACGATGACTGACGTTGAGGCTCGAAAGCGTGGGGAGCAAACAGG` |
| ASV3946 | 429 | `TGGGGAATTTTGGACAATGGGGGCAACCCTGATCCAGCCATGCCGCGTGAGGGAAGAAGGCCTTCGGGTTGTAAACCTCTTTCGGGCGGGACGAAACGGGGCGCGTGAATAACGCGCTTTACTGACGGTACCGCAAGAAGAAGCACCGGCTAACTACGTGCCAGCAGCCGCGGTAATACGTAGGGTGCGAGCGTTAATCGGAATTACTGGGCGTAAAGCGTGCGCAGGCGGTTTGTCAAGCCAGACGTGAAAGCCCCGGGCTCAACCTGGGAATGGCGTTTGGGACTGACAGACTGGAGTGCGGCAGAGGGAACTGGAATTCCTGGTGTAGCAGTGAAATGCGTAGATATCAGGAGGAACACCAATGGCGAAGGCAGGTTCCTGGGCCAGCACTGACGCTCATGCACGAAAGCGTGGGGAGCAAACAGG` |
| ASV1299 | 409 | `TGGGGAATATTGCGCAATGGGCGAAAGCCTGACGCAGCGACGCAGCGTGAGGGATGAAGGCCTTCGGGTTGTAAACCTCTTTCAGCGGGGACGAAGCGAAAGTGACGGTACCCGCAGAAGAAGCACCGGCTAACTACGTGCCAGCAGCCGCGGTAATACGTAGGGTGCAAGCATTGTCCGGATTTATTGGGCGTAAAGAGCTCGTAGGCGGCTTGTCACGTCGGATGTGAAAACTCGGGGCTCAACCCCGAGCCTGCATTCGATACGGGCTAGCTAGAGTGTTGCAGGGGAGACTGGAATTCCTGGTGTAGCGGTGAAATGCGCAGATATCAGGAGGAACACCGGTGGCGAAGGCGGGTCTCTGGGCAACTACTGACGCTGAGGAGCGAAAGCGTGGGGAGCGAACAGG` |
| ASV4382 | 406 | `TAGGGAATATTGGACAATGGGCGAAAGCCTGATCCAGCAATGCCGCGTGAGTGATGAAGGCCTTAATCGGTCGTAAAGCTCTTTTGCCAGGGAAGATTATGACTGTACCTGGAGAATAAGGACCGACTAACTATGTGCCAGCAGTCGCGGTAATACATAGGGTCCTAGCGTTACTCGGAATTACTGGGCGTAAAGGGCGCGTAGGCGGATTTATAAGTTATGTGTGAAATCTTCGGGCTCAACCTGAGGTCTGCTCATAATACTGTAAATCTAGAGTACAATCGAAGATGATAGAATTTCTGGTGTAGGGGTAAAATCCGTAGATATCAGAAGGAATACCAGAAGCGAAAGCGATCATCTAGGTTGGCACTGACGCTGAGGCGCGACAGCGTGGGGAGCAAACAGG` |
| ASV228 | 404 | `TGGGGAATATTGGACAATGGGCGAAAGCCTGATCCAGCCATGCCGCGTGTGTGATGAAGGCCTTAGGGTTGTAAAGCACTTTCACCGATGAAGATAATGACGGTAGTCGGAGAAGAAGCCCCGGCTAACTTCGTGCCAGCAGCCGCGGTAATACGAAGGGGGCTAGCGTTGTTCGGAATCACTGGGCGTAAAGCGCACGTAGGCGGATTGTTAAGTGAGGGGTGAAATCCCAGGGCTCAACCCTGGAACTGCCTTTCATACTGGCAATCTCGAGTTCGAGAGAGGTAAGTGGAATTCCGAGTGTAGAGGTGAAATTCGTAGATATTCGGAGGAACACCAGTGGCGAAGGCGGCTTACTGGATCGATACTGACGCTGAGGTGCGAAAGCGTGGGGAGCAAACAGG` |
| ASV4467 | 426 | `TGGGGAATATTGGACAATGGGGGGAACCCTGATCCAGCGATGCCGCGTGTGTGAAGAAGGCCTGCGGGTTGTAAAGCACTTTAGTTGGGGAGGAGAGTCTGATTCGAAGGGATGAGACGAGACGTTACCCAAAGAATAAGCACCGGCTAACTCTGTGCCAGCAGCCGCGGTAATACAGAGGGTGCGAGCGTTAATCGGAATTATTGGGCGTAAAGCGCACGTAGGCGGGCGATTAAGTTAGATGTAAAATACCTGGGCTCAACCTGGGTGGGTCATTTAAAACTGGTTGTCTAGAGTATAGGAGAGGAAGATAGAATTTCCGGTGTAGCGGTGAAATGCGTAGATATCGGAAGGAATACCCGTGGCGAAGGCGATCTTCTGGCCTAATACTGACGCTGAGGTGCGAAAGCGTGGGGAGCAAACAGG` |
| ASV3719 | 404 | `TGGGGAATATTGGACAATGGGCGCAAGCCTGATCCAGCCATGCCGCGTGAGTGATGACGGCCTTAGGGTTGTAAAGCTCTTTCGCACGCGACGATAATGACGGTAGCGTGAGAAGAAGCCCCGGCTAACTTCGTGCCAGCAGCCGCGGTAATACGAAGGGGGCTAGCGTTGTTCGGAATCACTGGGCGTAAAGGGTGCGTAGGCGGGATCTTAAGTCAGGGGTGAAATCCCGAGGCTCAACCTCGGAACTGCCTTTGATACTGGGGTCCTTGAGTCCGGAAGAGGTGAGTGGAACTGCGAGTGTAGAGGTGAAATTCGTAGATATTCGCAAGAACACCGGTGGCGAAGGCGGCTCACTGGTCCGGAACTGACGCTGAGGCACGACAGCGTGGGGAGCAAACAGG` |
| ASV2285 | 429 | `TGGGGAATTTTGGACAATGGGCGAAAGCCTGATCCAGCCATTCCGCGTGAGTGAAGAAGGCCTTCGGGTTGTAAAGCTCTTTCGCAAGAGAAGAAAACTTAGTTACTAATATTAACTGAGGTTGACGGTATCTTGATAAGAAGCACCGGCTAACTACGTGCCAGCAGCCGCGGTAATACGTAGGGTGCGAGCGTTAATCGGAATTACTGGGCGTAAAGCGTGCGCAGGCTGTTTTGTAAGTCAGATGTGAAATCCCCGAGCTCAACTTGGGAACTGCGTTTGAAACTACAAGACTAGAATATGTCAGAGGGGGGTAGAATTCCACGTGTAGCAGTGAAATGCGTAGAGATGTGGAGGAATATCAATGGCGAAGGCAGCCCCCTGGGATAATATTGACGCTCATGCACGAAAGCGTGGGGAGCAAACAGG` |
| ASV2210 | 429 | `TGGGGAATTTTGGACAATGGGCGCAAGCCTGATCCAGCCATGCCGCGTGCGGGAAGAAGGCCTTCGGGTTGTAAACCGCTTTTGTCAGGGAAGAAATCTTCTGGGTTAATACCCTGGGAGGATGACGGTACCTGAAGAATAAGCACCGGCTAACTACGTGCCAGCAGCCGCGGTAATACGTAGGGTGCAAGCGTTAATCGGAATTACTGGGCGTAAAGCGTGCGCAGGCGGTTGTGCAAGACAGGTGTGAAATCCCCGAGCTCAACTTGGGAACTGCACTTGTGACTGCACAGCTAGAGTACGGTAGAGGGGAGTGGAATTCCGCGTGTAGCAGTGAAATGCGTAGATATGCGGAGGAACACCGATGGCGAAGGCAGCTCCCTGGACCTGTACTGACGCTCATGCACGAAAGCGTGGGGAGCAAACAGG` |
| ASV5507 | 406 | `TGGGGAATATTGGGCAATGGGGGGAACCCTGACCCAGCGACGCCGCGTGGGTGAAGAAATCCTTCGGGATGTAAAGCCCTGTTGTATGGGAAGAGTAGAAGACGGTACCATACGAGGAAGCCCCGGCAAACTACGTGCCAGCAGCCGCGGTAATACGTAGGGGGCAAGCGTTGTCCGGAATTACTGGGCGTAAAGCGCACGCAGGCTGACTTATAAGTCGGTAGTCTAATGCGGAGGCTCAACTTCCGTTTATCTATCGATACTGTAAGACTAGAGTATGTGAGAGGGAAGTGGAATTCCCGGTGTAGCGGTGAAATGCGTAGATATCGGGAGGAACACCAGTGGCGAAGGCGACTTCCTGGCACAATACTGACGCTCATGTGCGAAAGCTAGGGTAGCGAACGGG` |
| ASV2582 | 429 | `TGGGGAATTTTGGACAATGGGCGCAAGCCTGATCCAGCAATGCCGCGTGCAGGAAGAAGGCCTTCGGGTTGTAAACTGCTTTTGTACGGAGCGAAAAGGCTTTCTCTAATAAAGGAGGCTGATGACGGTACCGTAAGAATAAGCACCGGCTAACTACGTGCCAGCAGCCGCGGTAATACGTAGGGTGCAAGCGTTAATCGGAATTACTGGGCGTAAAGCGTGCGCAGGCGGTTTTGTAAGACAGGCGTGAAATCCCCGGGCTCAACCTGGGAATGGCGCTTGTGACTGCAAGGCTGGAGTGCGGCAGAGGGGGATGGAATTCCGCGTGTAGCAGTGAAATGCGTAGATATGCGGAGGAACACCGATGGCGAAGGCAATCCCCTGGGCCTGCACTGACGCTCATGCACGAAAGCGTGGGGAGCAAACAGG` |
| ASV1025 | 421 | `TGGGGAATATTGCGCAATGGCCGAAAGGCTGACGCAGCGACGCCGCGTGGGGGATGAAGCATTTCGGTGTGTAAACCCCTGTTGCTCGGGACGAACAACTGGATTCTTCCAGTCTGACGGTACCGGGTGAGGAAGCACCGGCTAACTCCGTGCCAGCAGCCGCGGTAATACGGAGGGTGCGAGCGTTGTCCGGAATCACTGGGCGTAAAGGGCGCGTAGGCGGTCTGCTAAGCGTGCGGTGAAAGCCTGGGGCTCAACCCCAGGTCTGCCGTGCGAACTGGTGGACTAGAGCACTGTAGAGGCAGGTGGAATTCCGGGTGTAGCGGTGGAATGCGTAGAGATCCGGAAGAACACCGGTGGCGAAGGCGGCCTGCTGGGCAGTAGCTGACGCTGAGGCGCGACAGCGTGGGGAGCAAACAGG` |
| ASV2470 | 429 | `TCGAGAATAATTCACAATGGGGGCAACCCTGATGGTGCAACGCCGCGTGGAGGATGACGGTCTTCGGATTGTAAACTCCTGTCATCTGGGAGTAAGGCATGGCTGTTAATAGCAGACATGATTGATAGTACCAGAAGAGGAAGGGACGGCTAACTTCGTGCCAGCAGCCGCGGTAATACGAAGGTCCCGAGCGTTGTTCGGAATCACTGGGCGTAAAGGGAGCGTAGGCGGCGTGGTAAGTCAGATGTGAAATCCCGGGGCTCAACCCCGGAACTGCATCCGATACTGCCATGCTAGAGGATTGGAGAGGTAGCTGGAATTCTTGGTGTAGCAGTGAAATGCGTGGATATCAAGAGGAACACTCGTGGCGAAAGCGAGCTACTGGACAATTCCTGACGCTGAGGCTCGAAGGCTAGGGTAGCGAAAGGG` |
| ASV5144 | 405 | `TGGGGAATATTGGACAATGGGGACAACCCTGATCCAGCCATGCCGCGTGGATGATGAAGGCCTTCGGGTTGTAAAGTTCTTTTGCAGGGGACGATGATGACGGTACCCTGAGAATAAGCTCCGGCTAACTTCGTGCCAGCAGCCGCGGTAATACGAAGGGAGCTAGCGTTGTTCGGAATTACTGGGCGTAAAGGGCGCGTAGGCGGTTTAACAAGTCAGGCGTGAAAGCCCAAGGCTCAACCTTGGAATAGCGCTTGAGACTGTTTTTCTAAGAGTTTGGGAGAGGTAAGTGGAATTTCCAGTGTAGCAGTGAAATGCGTAGATATTGGAAAGAACACCAGTGGCGAAGGCGGCTTACTGGCCCAAAACTGACGCTGAGGCGCGAAAGCGTGGGGAGCAAACAGG` |
| ASV4644 | 407 | `TAGGGAATATTGCACAATGGGGGAAACCCTGATGCAGCAACGCCGCGTGAGTGATGAAGGCTCTAGGGTCGTAAAGCTCTGTCGTGTGGGAAGAAACAAATGACGGTACCACACAAGAAAGGACCGGCCAACTTCGTGCCAGCAGCCGCGGTAATACGGGGGGTCCAAGCGTTGTTCGGAATCACTGGGCGTAAAGCGCATGTAGGTGGCTTTTCAAGTCAACTGTGAAAGCCCCGGGCCTAACCCGGGAAGTGCAGTTGATACTGTTGAGCTTGAGTACGAGAGAGGATAGTGGAATTTCTGGTGTAGTGGTGAAATACGTAGATATCAGAAGGAACATCGGTGGCGAAGGCGGCTATCTGGCTCGATACTGACACTGAGATGCGAAAGCGTGGGGATCAAACAGG` |
| ASV1665 | 408 | `TGGGGAATTTTCCGCAATGGGCGAAAGCCTGACGGAGCAATACCGCGTGAGGGATGACGGCCTATGGGTTGTAAACCTCTTTTTTCAGGGAGGAATCAAATGACGTGTACCTGAAGAATAAGCATCGGCTAACTCCGTGCCAGCAGCCGCGGTAAGACGGAGGATGCAAGTGTTATCCGGAATCACTGGGCGTAAAGCGTCTGTAGGTGGTCCAATAAGTCAACTGTTAAATCTTGGGGCTCAACCTCAAAATCGCAGTCGAAACTATTAGACTAGAGTATAGTAGGGGTAAAGGGAATTTCCAGTGGAGCGGTGAAATGCGTAGATATTGGAAAGAACACCGATGGCGAAGGCACTTTACTGGGCTATTACTAACACTCAGAGACGAAAGCTAGGGTAGCAAATGGG` |
| ASV3025 | 404 | `TGGGGAATATTGGACAATGGGCGAAAGCCTGATCCAGCCATGCCGCGTGAATGATGAAGGCCTTAGGGTTGTAAAGTTCTTTTGTCAGGGAAGATGATGACGGTACCTGAAGAATAAGCACCGGCTAACTTCGTGCCAGCAGCCGCGGTAATACGAAGGGTGCTAGCGTTGTTCGGAATCACTGGGCGTAAAGCGCGCGTAGGCGGCGTTACAAGTCAGGGGTGAAATCCCAAGGCTCAACCTTGGAACTGCCTTTGAAACTGTAATGCTAGAGTGTCGGAGGGGATGGCGGAATTGCTAATGTAGAGGTGAAATTCGTAGATATTAGCAGGAACACCGGTGGCGAAGGCGGCCATCTGGACGACAACTGACGCTGAGGCGCGAAAGCGTGGGGATCAAACAGG` |
| ASV4555 | 430 | `TTGGGAATATTGCGCAATGGGGGAAACCCTGACGCAGCGACGCCGCGTGTGCGAAGAAGGTCTTCGGATTGTAAAGCACTGTCGAATGGGAAGAACGCATAGATAGTTAACTGCTATCAGTGTTGACTGTACCATTGGAGGAAGCACCGGCTAACTCTGTGCCAGCAGCCGCGGTAATACAGAGGGTGCAAGCGTTGTTCGGAATTACTGGGCGTAAAGAGTTCGTAGGCGGACTTGATAGTATGAGGTTAAATCCCAGGGCCTAACCCTGGACCTGCTTCATAAACGTCAAGTCTGGAGTATCGGAGAGGATAGCGGAATTCCTGGTGTAGAGGTGAAATTCGTAGATATCAGGAGGAACACCTGAGGCGAAGGCGGCTATCTGGACGAGAACTGACGCTAAAGAACGAAAGCATGGGGATCAAACAGG` |
| ASV134 | 424 | `TGAGGAATATTGGACAATGGGCGCAAGCCTGATCCAGCCATGCCGCGTGCAGGAAGAAGGTCCTATGGATTGTAAACTGCTTTTATACAGGAAGAAACACCTCTACGTGTAGAGACTTGACGGTACTGTATGAATAAGGACCGGCTAACTCCGTGCCAGCAGCCGCGGTAATACGGAGGGTCCAAGCGTTATCCGGAATCATTGGGTTTAAAGGGTCCGTAGGCGGCCAAATAAGTCAGTGGTGAAAGCCCATCGCTCAACGATGGAACGGCCATTGATACTGTTTGGCTTGAATTATTAGGAAGTAACTAGAATATGTAGTGTAGCGGTGAAATGCTTAGAGATTACATGGAATACCAATTGCGAAGGCAGGTTACTACTAATATATTGACGCTGATGGACGAAAGCGTGGGGAGCGAACAGG` |
| ASV4250 | 406 | `TGGGGAATTTTCCGCAATGGGCGAAAGCCTGACGGAGCAATACCGCGTGAGGGAGGAAGGCTCTTGGGTTGTAAACCTCTTTTCTCAAGGAAGAATAAATGACGGTACTTGAGGAATAAGCATCGGCTAACTCTGTGCCAGCAGCCGCGGTAATACAGAGGATGCAAGCGTTATCCGGAATGATTGGGCGTAAAGCGTCCGCAGGTGGTTTTGTATGTCTGCTGTTAAAGAGTTAGGCTTAACCTAATAAAGGCGGTGGAAACTACAAGACTAGAGTACGTTCGGGGCAGAGGGAATTCCCAGTGTAGCGGTGAAATGCGTAGAGATTGGGAAGAACACCGGTGGCGAAAGCGCTCTGCTAGGCCTGTACTGACACTGAGGGACGAAAGCTAGGGGAGCGAATGGG` |
| ASV1340 | 404 | `TAGGGAATATTGCACAATGGACGAAAGTCTGATGCAGCGACGCCGCGTGAGTGATGAAGGCCTTCGGGTTGTAAAACTCTGTGATAAGGGAAGATGATGACGGTACCTTATCAGAAAAGACCGGCTAACTTCGTGCCAGCAGCCGCGGTAATACGAGGGGTCTAAGCGTTGTTCGGAATCACTGGGCGTAAAGCGGGTGTAGGTGGCTCAGCAAGTTAGATGTGAAAGCCCCGGGCTCAACCCGGGAAGTGCATCTAAAACTACTGAGCTTGAGTACGGGAGAGGGAAGCAGAATTCCTGGTGTAGTGGTGAAATACGTAGATATCAGGAGGAACACCGGTGGCGAAGGCGGTTTCCTGGCCCAGTACTGACACTCAGACCCGAAAGCGTGGGTAGCAAACAGG` |
| ASV4797 | 424 | `TGAGGAATATTGGTCAATGGAGGCAACTCTGAACCAGCCATGCCGCGTGCAGGATGACGGTCCTATGGATTGTAAACTGCTTTTGTACGGGAAGAAATATAGTTACGTGTAATTATTTGACGGTACCGTAAGAATAAGGATCGGCTAACTCCGTGCCAGCAGCCGCGGTAATACGGAGGATCCGAGCGTTATCCGGAATTATTGGGTTTAAAGGGTTCGTAGGCGGTTTAGTAAGTCAGTGGTGAAATCTTGCAGCTTAACTGTAAAATTGCCGTTGATACTGCTAGACTTGAATAGTATGGAAGTAATTAGAATATGTAGTGTAGCGGTGAAATGCTTAGATATTACATGGAATACCAATTGCGAAGGCAGATTACTACGTACTTATTGACGCTGATGAACGAAAGCGTGGGTAGCGAACAGG` |
| ASV447 | 429 | `TAGGGAATCTTCCACAATGGACGAAAGTCTGATGGAGCAACGCCGCGTGAGTGATGAAGGTTTTCGGATCGTAAAACTCTGTTGTAAGGGAAGAACAGGTATGAGAGGTAATGCTCATGCTATGACGGTACCTTGCGAGAAAGCCACGGCTAACTACGTGCCAGCAGCCGCGGTAATACGTAGGTGGCAAGCGTTGTCCGGAATTATTGGGCGTAAAGCGCGCGCAGGCGGCCTTTTAAGTCTGATGTGAAAGCCCCCGGCTCAACCGGGGAGGGTCATTGGAAACTGGAAGGCTTGAGTACAGAAGAGAAGAGTGGAATTCCATGTGTAGCGGTGAAATGCGTAGAGATGTGGAGGAACACCAGTGGCGAAGGCGACTCTTTGGTCTGTAACTGACGCTGAGGCGCGAAAGCGTGGGGAGCAAACAGG` |
| ASV5503 | 406 | `TCAGGAATTGTGCGCAATGGACGAAAGTCTGACGCCGCGACGTCGCGTGGAGGATTAAGGCTCTAGGGTCGTAAACTCCTTTTGCCAGGGAACAAGATCTGAGTGTACCTGGAGAATAAGTGCCTGCTAACCACGTGCCAGAAGCATCGGTAATACGTGGGGCACAAGCGTTATCCGGATTTACTGGGCGTAAAGAGATGCGTAGGCGTCTGTTTAAGTCATTTGTGAAATCTTGAGGCTCACCCTCAAGCTGCAAGTGATACTGTACAGATTGAGAAAAGTAGGGGAAAGCGGAATTCCTGGTGGAGCGGTTAAATGCGTAGATATCAGGAGGAACACCGATGGCGAAGGCAGCTTTCTGGACTTTTTCTGACGCTAAGGCTCGAAAGCGTGGGGAGCAAAACGG` |
| ASV1276 | 424 | `TGAGGAATATTGGTCAATGGACGGAAGTCTGAACCAGCCATGCCGCGTGCAGGAAGACGGCTCTATGAGTTGTAAACTGCTTTTAAACGAGAGTAAACCCGCATACGTGTATGCGGTTGAAAGTATCGTAAGAATAAGGATCGGCTAACTCCGTGCCAGCAGCCGCGGTAATACGGAGGATCCAAGCGTTATCCGGATTTATTGGGTTTAAAGGGTGCGTAGGCGGTTTAATAAGTTAGAGGTGAAATCTCGGGGCTCAACCTCGAAATAGCCTCTAATACTGTTAAGCTAGAGAATAGTTGCGGTAGGCGGAATGTATGGTGTAGCGGTGAAATGCTTAGAGATCATACAGAACACCGATTGCGAAGGCAGCTTACCAAACTATATCTGACGTTGAGGCACGAAAGCGTGGGGAGCAAACAGG` |
| ASV2439 | 429 | `TGGGGAATTTTGGACAATGGGCGCAAGCCTGATCCAGCAATGCCGCGTGAGTGAAGAAGGCCTTCGGGTTGTAAAGCTCTTTTGTCAGGGAAGAAAAGGCTGCGGCTAATATCTGCGGCTCATGACGGTACCTGAAGAATAAGCACCGGCTAACTACGTGCCAGCAGCCGCGGTAATACGTAGGGTGCAAGCGTTAATCGGAATTACTGGGCGTAAAGCGTGCGCAGGCGGTTTTGTAAGACTGTCGTGAAATCCCCGGGCTCAACCTGGGAATGGCGATGGTGACTGCAAGGCTAGAGTTTGGCAGAGGGGGGTAGAATTCCACGTGTAGCAGTGAAATGCGTAGATATGTGGAGGAACACCGATGGCGAAGGCAGCCCCCTGGGTCAAAACTGACGCTCATGCACGAAAGCGTGGGGAGCAAACAGG` |
| ASV3986 | 429 | `TGGGGAATATTGGACAATGGGGGCAACCCTGATCCAGCAATGCCGCGTGTGTGAAGAAGGCCTTAGGGTCGTAAAGCACTTTTGTTGGGGAGGAAGGAGTATGTGCTAATAGTGCATATGACAGACGTTACCCAAAGAATAAGCACCGGCTAACTCTGTGCCAGCAGCCGCGGTAATACAGAGGGTGCAAGCGTTAATCGGAATTACTGGGCGTAAAGGGTGCGTAGGTGGACGCATAAGTCGGATGTGAAATCCCTGGGCTTAACCTAGGAACTGCATTCGATACTGTGAGTCTAGAGTATGGTAGAGGTAAGTGGAATTTCCGGTGTAGCGGTGAAATGCGTAGATATCGGAAGGAACATCAGTGGCGAAGGCGGCTTACTGGACTAATACTGACACTGAGGCACGAAAGCGTGGGGAGCAAACAGG` |
| ASV4436 | 406 | `CAAGGAATTTTGCGCAATGGACGAAAGTCTGACGCAGCAACGCCGCGTGGGGGATGAAGGCTCTCGGGTTGTAAACCCCTTTTGCGAGGGACGAAGATCTGACGGTACCTCGCGAATAAGCCACGGCTAACTACGTGCCAGCAGCCGCGGTAATACGTAGGTGGCGAGCGTTGTCCGGATTTACTGGGCGTAAAGCGCGCGCAGGCGGACTGATCAGTCCGGGGTGAAATCTCACGGCTCAACTGTGAGCGGTCCCCGGATACTGTCAGCCTTGAGATGTCTAGAGGAGAGCGGAATTCCCGGTGTAGTGGTGGAATGCGTAGATATCGGGAAGAACACCAGTGGCGAAAGCGGCTCTCTGGGGACCATCTGACGCTGAGGCGCGAAAGCGTGGGGAGCGAACCGG` |
| ASV3516 | 408 | `TCGAGAATCTTCCGCAATGGACGAAAGTCTGACGGAGCGACGCCGCGTGGTGGATGAAGTCCTTCGGGACGTAAACACCTTTTATGAGGGAGGAAGTTTATTGACGTTACCTCATGAATAAGGGGCTCCCAACTCTGTGCCAGCAGGAGCGGTAATACAGAGGCCCCAAGCATTATCCGGATTTACTGGGCGTAAAGGGTGCGTAGGCGGTTATATTAGTCGGGTGTTAAATCCCAGGGCCCAACCCTGGAATCGCATTCGAAACGGTATAACTAGAATAAGTCAGAGGCAAGCAGAACTCTCGGTGTAGGGGTGAAATCCGTTGATATCGAGGGGAATACCAAATGCGAAGGCAGCTTGCTGGGACTTTATTGACGCTGAGGCACGAAAGCGTGGGTAGCGAAGCGG` |
| ASV3424 | 404 | `TGGGGAATATTGGACAATGGGGGAAACCCTGATCCAGCGATGCCGCGTGAATGATGAAGGCCTTCGGGTTGTAAAGTTCTTTCATTTGTGATGATTATGACAGTAACAAAAGAAGAAGCTCCGGCTAACTTCGTGCCAGCAGCCGCGGTAATACGAGGGGAGCTAGCGTTGTTCGGAATTATTGGGCGTAAAGGGTGCGTAGGCGGCTCTGTAAGTATCATGTTAAATCCCCGGGCTCAACCTTGGGCTTGCATGATAAACTGCAGTGCTAGAATGTGATAGAGGGAAACGGAATTTCTAGTGTAGAGGTGAAATTCGTAGATATTAGAAGGAACGTCAGAGGCGAAGGCGGTTTCCTGGGTCACAATTGACGCTGAGGCACGAAAGCGTGGGGAGCAAACAGG` |
| ASV1035 | 424 | `TGAGGAATATTGGACAATGGGCGCAAGCCTGATCCAGCCATGCCGCGTGCAGGATGAAGCATCTATGGTGTGTAAACTGCTTTTGTACGAGAAGAAACACTCCTTCGTGAAGGAGCTTGACGGTATCGTAAGAATAAGGATCGGCTAACTCCGTGCCAGCAGCCGCGGTAATACGGAGGATCCAAGCGTTATCCGGAATCATTGGGTTTAAAGGGTCCGTAGGCGGGCTAGTAAGTCAGTGGTGAAATCTCTCGGCTCAACCGGGAAATGGCCATTGATACTGCTAGTCTTGAATTATTAGGAAGTAACTAGAATATGTAGTGTAGCGGTGAAATGCTTAGAGATTACATGGAATACCAATTGCGAAGGCAGGTTACTACTAATGGATTGACGCTGATGGACGAAAGCGTGGGTAGCGAACAGG` |
| ASV4296 | 404 | `TGAGGAATTTTCCGCAATGGGCGAAAGCCTGACGGAGCGACGCCGTGTGAGGGATGAAGGCCTTCGGGTTGTAAACCTCTGTCGATGGGGACGAAACTGACGGTACCCATAAAGGAAGCCCCGGCTAACTACGTGCCAGCAGCCGCGGTAATACGTAGGGGGCGAGCGTTGTCCGGAATCACTGGGCGTAAAGCGCGCGTAGGTGGCCCGATAAGTCCGGTGTGAAAGGGTGCGGCTCAACCGCACCATGTCGCCGGAAACTGCCGGGCTCGAGATCAGGAGAGGGAAGTGGAACAACCGGTGTAGCGGTGAAATGCGTAGATATCGGTTGGAACACCAGTGGCGAAGGCGGCTTCCTGGCCTGTTTCTGACACTGAGGCGCGAAAGCTCGGGGAGCAAACGGG` |
| ASV5094 | 406 | `TTGGGAATCTTGCACAATGGGGGAAACCCTGATGCAGCGACGCCGCGTGGACGATGAAGGCCTTAGGGTTGTAAAGTCCTTTTTTGGGGAAAGACTTAGGACGGTACCCCAAGAATAAGCTCCGGCTAACTACGTGCCAGCAGCCGCGGTAAGACGTAGGGAGCAAGCGTTGTCCGGATTTACTGGGCGTAAAGAGCTCGTAGGCGGCTCGTTAAGTGTGAAGTGAAATCTCCAGTGCTCAACACGGAAACTGCTTTACATACTGGCGAGCTTGAGGAATGCAGAGGTAACTGGAATTCCTGGTGTAGCGGTGAAATGCGTTGATATCAGGAGGAACACCAATGGCGAAGGCAGGTTACTGGGCATTATCTGACGCTGAGGAGCGAAAGCGTGGGTAGCAAACAGG` |
| ASV798 | 404 | `TGGGGAATATTGGACAATGGGCGAAAGCCTGATCCAGCGACGTCACGTGAATGATGAAGACCTTAGGGTTGTAAAATTCTTTTAGTGGAGAAGATAATGACGGTATCCACAGAAAAAGTCCCGGCTAACTTCGTGCCAGCAGCCGCGGTAATACGAAGGGGGCAAGCGTTACTCGGAATTATTGGGCGTAAAGCGTGCGTAGGCGGCTTTATAAGTTGGAAGTGAAAGCCTTTGGCTCAACCAAAGAATTGCTTACAAAACTGTAGAGCTAGAGTATTAGAGAGGATAGAAGAATTTCTGATGTAGGGGTGAAATCCGTAGATATCAGAAAGAATATCGAAGGCGAAAGCATCTGTCTGGCTAAATACTGACGCTGTTGCACGAAAGCGTGGGGAGCAAACAGG` |
| ASV4933 | 404 | `TAAGGAATATTGGACAATGGGGGAAACCCTGATCCAGCCATGCCGCGTGAGTGATGAAGGCCTTAGGGTTGTAAAGCTCTTTTAACGGTGAAGATAATGACGGTAACCGTAGAATAAGCCCCGGCTAACTTCGTGCCAGCAGCCGCGGTAATACGAAGGGGGCTAGCGTTGTTCGGAATTACTGGGCGTAAAGCGCGTGTAGGTTGACTATCAAGTCAGAGGTGAAATCCCGGAGCTTAACTTCGGAATTGCCTTTGAAACTGATAGTCTAGAGACTTGGAGGGGGAAGTGGAATTTCTAATGTAGGGGTGAAATCCGTAGAGATTAGAAGGAACACCGGTGGCGAAGGCGGCTTCCTGGACAAGTACTGACACTGAGACGCGAAAGCGTGGGGATCAAACAGG` |
| ASV169 | 429 | `TGGGGAATTTTGGACAATGGGCGAAAGCCTGATCCAGCAATGCCGCGTGAGTGAAGAAGGCCTTCGGGTTGTAAAGCTCTTTTGTCAGGGAAGAAACGGTGAGAGCTAATATCTCTTGCTAATGACGGTACCTGAAGAATAAGCACCGGCTAACTACGTGCCAGCAGCCGCGGTAATACGTAGGGTGCAAGCGTTAATCGGAATTACTGGGCGTAAAGCGTGCGCAGGCGGTTTTGTAAGTCTGATGTGAAATCCCCGGGCTCAACCTGGGAATTGCATTGGAGACTGCAAGGCTAGAATCTGGCAGAGGGGGGTAGAATTCCACGTGTAGCAGTGAAATGCGTAGATATGTGGAGGAACACCGATGGCGAAGGCAGCCCCCTGGGTCAAGATTGACGCTCATGCACGAAAGCGTGGGGAGCAAACAGG` |
| ASV5425 | 429 | `TCGAGAATCATTCACAATGGGGGGAACCCTGATGGTGCAACGCCGCGTGGAGGATGAAGGTCTTCGGATTGTAAACTCCTGTCATTTGAGAGCAAGGCACGGTGGTGAATAGCCAACGTGATTGATAGTATCAAAAGAGGAAGGGACGGCTAACTTCGTGCCAGCAGCCGCGGTAATACGAAGGTCCCGAGCGTTGTTCGGAATCACTGGGCGTAAAGGGAGCGTAGGCGGCGTGGTAAGTCAGATGTGAAATCCCGGAGCTTAACTCCGGAACTGCATCCGATACTGCCATGCTAGAGTATTGGAGGGGTAACTGGAATTTTCGGTGTAGCAGTGAAATGCGTGGATATCGAAAGGAACACCTGTGGCGAAAGCGAGTTACTGGACAATTACTGACGCTGAGGCTCGAAGGCTAGGGGAGCAAAAGGG` |
| ASV859 | 429 | `TAGGGAATATTGGACAATGGGCGCAAGCCTGATCCAGCCATGCCGCGTGAGTGAAGAAGGCCTTCGGGTTGTAAAGCTCTTTTGTCCGGAAAGAAAAGCATCCAGTTAATACCTGGGTGTCCTGACGGTACCGGAAGAATAAGCACCGGCTAACTTCGTGCCAGCAGCCGCGGTAATACGAAGGGTGCAAGCGTTACTCGGAATTACTGGGCGTAAAGCGTGCGTAGGTGGTTTGTTAAGTCTGATGTGAAAGCCCTGGGCTCAACCTGGGAATGGCATTGGATACTGGCAGGCTAGAGTGCGGTAGAGGGTAGTGGAATTCCCGGTGTAGCAGTGAAATGCGTAGAGATCGGGAGGAACATCTGTGGCGAAGGCGACTACCTGGACCAGCACTGACACTGAGGCACGAAAGCGTGGGGAGCAAACAGG` |
| ASV4417 | 404 | `TGGGGAATATTGGACAATGGGCGAAAGCCTGATCCAGCAATACCGCGTGAATGATGAAGGCCTTAGGGTTGTAAAGTTCTTTTAGTATGGAAGATAATGACTGTACATACAGAAAAAGCCCCGGCTAACTCCGTGCCAGCAGCCGCGGTAAGACGGAGGGGGCTAGCGTTGTTCGGAATTACTGGGCGTAAAGAGCGCGTAGGCGGATTAGTAAGTTGGAAGTGAAATCCCGGGGCTTAACCTCGGAACTGCTTTCAAAACTGCTAGTCTAGAGTGAAGTAGGGGGTGATGGAATTCCAAGTGTAGAGGTGAAATTCTTAGATATTTGGAGGAACACCGGTGGCGAAGGCGGTCACCTGGGCTTCAACTGACGCTGAGGCGCGAAAGCGTGGGGAGCAAACAGG` |
| ASV951 | 424 | `TAAGGAATATTGGTCAATGGACGCAAGTCTGAACCAGCCATGCCGCGTGAAGGATTAAGGTCCTCTGGATTGTAAACTTCTTTTATTTGGGACGAAAAAGGGTCTTTCTAGACCAACTGACGGTACCAGATGAATAAGCACCGGCTAACTCCGTGCCAGCAGCCGCGGTAATACGGAGGGTGCAAGCGTTATCCGGATTCACTGGGTTTAAAGGGTGCGTAGGCGGGTAGGTAAGTCAGTGGTGAAATCCTAGAGCTCAACTCTAGAACTGCCATTGATACTATTTATCTTGAATATTGTGGAGGTAAGCGGAATATGTCATGTAGCGGTGAAATGCTTAGATATGACATAGAACACCTATTGCGAAGGCAGCTTACTACGCATATATTGACGCTGAGGCACGAAAGCGTGGGGATCAAACAGG` |
| ASV5461 | 409 | `TGGGGAATATTGCACAATGGGCGAAAGCCTGATGCAGCGACGCCGCGTGAGGGATGACGGCCTTCGGGTTGTAAACCTCTTTCAGCAGGGAAGAAGCGCAAGTGACGGTACCTGCAGAAGAAGCACCGGCTAACTACGTGCCAGCAGCCGCGGTAATACGTAGGGTGCGAGCGTTGTCCGGAATTATTGGGCGTAAAGAGCTCGTAGGCGGCTTGTCGCGTCGGATGTGAAAGCCCGGGGCTTAACTCCGGGTCTGCATTCGATACGGGCAGGCTAGAGTTCGGTAGGGGAGATCGGAATTCCTGGTGTAGCGGTGAAATGCGCAGATATCAGGAGGAACACCGGTGGCGAAGGCGGATCTCTGGGCCGATACTGACGCTGAGGAGCGAAAGCGTGGGGAGCGAACAGG` |
| ASV3378 | 404 | `TGGGGAATATTGGACAATGGGCGCAAGCCTGATCCAGCCATGCCGCGTGAGTGATGAAGGCCTTAGGGTTGTAAAGCTCTTTTGCCGGGGAAGATAATGACGGTACCCGGAGAATAAGTCCCGGCTAACTTCGTGCCAGCAGCCGCGGTAATACGAAGGGGACTAGCGTTGTTCGGAATCACTGGGCGTAAAGCGCACGTAGGCGGATCATTTAGTCAGGGGTGAAATCCCGGGGCTCAACCTCGGAACTGCCTTTGATACTGATGGTCTCGAGTCCGATAGAGGTGGGTGGAATTCCTAGTGTAGAGGTGAAATTCGTAGATATTAGGAAGAACACCAGTGGCGAAGGCGGCCCACTGGATCGGTACTGACGCTGAGGTGCGAAAGCGTGGGGAGCAAACAGG` |
| ASV1953 | 429 | `TGGGGAATTTTGGACAATGGGGGCAACCCTGATCCAGCAACGCCGCGTGAGTGAAGAAGGCCTTCGGGTTGTAAAGCTCTTTTGGCAGGAAAGAAATCGCTTAGGTTAATACCTTGAGTGGATGACGGTACCTGCAGAATAAGCACCGGCTAACTACGTGCCAGCAGCCGCGGTAATACGTAGGGTGCAAGCGTTAATCGGAATTACTGGGCGTAAAGCGAGCGCAGGCGGTTCTGTAAGACAGATGTGAAAGCCCCGGGCTTAACCTGGGAATTGCATTTGTGACTGCAGAGCTAGAGTGTGTCAGAGGGAGGTAGAATTCCGCGTGTAGCAGTGAAATGCGTAGATATGCGGAGGAATACCGGTGGCGAAGGCGGCCTCCTGGGATAACACTGACGCTCATGCTCGAAAGCGTGGGGAGCAAACAGG` |
| ASV2735 | 429 | `TGGGGAATATTGGACAATGGGGGAAACCCTGATCCAGCGACGCCGCGTGGGTGAAGAAGGCCTGCGGGTTGTAAAGCCCTTTCGGTAGGGAAGAAATCTCTTGTTCTAACAAAACAAGGGTTTGACTTAACCTACATAAGAAGCACCGGCTAACTCTGTGCCAGCAGCCGCGGTAATACAGAGGGTGCGAGCGTTAATCGGAATTACTGGGCGTAAAGCGCACGTAGTCGGCTTGCCAAGTCGGGTGTGAAATCCCCGGGCTCAACCTGGGAACTGCATCCGAAACTGGCTTGCTAGAGTATGGGAGAGGGAGGCGGAATTTCTGGTGTAGCAGTGAAATGCGTAGATATCAGAAGGAACATCAGTGGCGAAGGCGGCCTCCTGGACCAATACTGACGATCAGGTGCGAAAGCGTGGGGAGCAAACAGG` |
| ASV4928 | 428 | `TGGGGAATCTTGCGCAATGGGCGAAAGCCTGACGCAGCGACGCCGCGTGAGTGATGAAGGCCTTCGGGTTGTAAAGCTCTGTGGGGAGAGACGAATAAGTCATGGCCAACATCCATGATGATGACGGTATCTCCTTAGCAAGCACCGGCTAACTCTGTGCCAGCAGCCGCGGTAATACAGAGGGTGCAAACGTTGTTCGGAATTACTGGGCGTAAAGCGCGTGTAGGCGGTCCTGTAGGTCAGGTGTGAAATCCCCCGGCTCAACCGAGGAAGTGCACTTGAAACCCCAGGACTTGAGTGTTGGAGAGGAAGGCGGAATTGTCGGTGTAGAGGTGAAATTCGTAGATATCGACAGGAACACCTGCGGCGAAAGCGGCCTTCTGGACAAACACTGACGCTGAGACGCGAAAGCGTGGGGAGCAAACAGG` |
| ASV1068 | 424 | `TGAGGAATATTGGTCAATGGGCGAGAGCCTGAACCAGCTATGCCGCGTGCAGGATGACGGCTCTATGAGTTGTAAACTGCTTTTGTAAAGGGGTAAAGGCGGGCACGAGTTGCTCGGATGAAAGTACTTTACGAATAAGGATCGGCTAACTCCGTGCCAGCAGCCGCGGTAATACGGAGGATTCGAGCGTTATCCGGATTTATTGGGTTTAAAGGGTGCGTAGGTTGTTTTGTAAGTTAGTGGTTAAACGGAGTGGCTCAACCACTTTTTGCCATTAAAACTGTGAAACTAGAGATTGGATGCAGATGGCGGAATGTGTAGTGTAGCGGTGAAATGCTTAGATATTACACAGAACACCGATTGCGAAGGCAGCTGTCTAATCCATATCTGACACTGAGGCACGAAAGCGTGGGTAGCGAACGGG` |
| ASV2372 | 404 | `TGAGGAATATTGGACAATGGGCGAAAGCCTGATCCAGCCATGCCGCGTGAGTGAAGAAGGCCTTCGGGTTGTAAAGCTCTTTCAGTTGTGAAGATAATGACGGTAGCAACAGAAGAAGCTCCGGCTAACTCCGTGCCAGCAGCCGCGGTAATACGGGGGGAGCGAGCGTTGTTCGGAATCACTGGGCGTAAAGCGTGCGCAGGCGGTTTGATAAGTCAGAAGTGAAATCCCAGGGCTCAACCCTGGAACTGCTTTTGAAACTATCTTACTAGAGTATGGTAGAGGTTGGGAGAATTCCTAGTGTAGAGGTGAAATTCGTAGATATTAGGAGGAATACCAGTGGCGTAGGCGCCCAACTGGACCATTACTGACGCTCAGGCACGAAAGCGTGGGGAGCAAACAGG` |
| ASV2047 | 428 | `TCGAGAATTTTTCTCAATGGGCGAAAGCCTGAAGGAGCGACGCCGCGTGGGGGATGAAGGGATTCGTCTTGTAAACCCCTGTCATTCGCGATCAAACCTCTTCACTTAACACGTGAGGAGCTGATAGTAGCGGAAGAGGAAGGGACGGCTAACTCTGTGCCAGCAGCCGCGGTAATACAGAGGTCCCAAGCGTTGTTCGGATTCACTGGGCGTAAAGGGTGCGTAGGTGGCGAGGTAAGTTTGATGTGAAAGCTCCGGGCTCAACCCGGAAATGGCATTGAATACTCTCTCGCTCGAGGGTTGGAGGGGAGTCTGGAATTCTCGGTGTAGCAGTGAAATGCGTAGATATCGAGAGGAACACCAGTGGCGAAGGCGAGACTCTGGACAATTCCTGACACTGAGGCACGAAAGCCAGGGGAGCAAACGGG` |
| ASV1791 | 429 | `TGGGGAATTTTGGACAATGGGCGCAAGCCTGATCCAGCAATGCCGCGTGCAGGACGAAGGCCTTCGGGTTGTAAACTGCTTTTGTACGGAACGAAACGGTCTTTTCTAATACAAGAGGCTAATGACGGTACCGTAAGAATAAGCACCGGCTAACTACGTGCCAGCAGCCGCGGTAATACGTAGGGTGCGAGCGTTAATCGGAATTACTGGGCGTAAAGCGTGCGCAGGCGGTTATATAAGACAGTTGTGAAATCCCCGGGCTCAACCTGGGAATTGCATCTGTGACTGTATAGCTAGAGTACGGTAGAGGGGGATGGAATTCCGCGTGTAGCAGTGAAATGCGTAGATATGCGGAGGAACACCGATGGCGAAGGCAATCCCCTGGACCTGTACTGACGCTCATGCACGAAAGCGTGGGGAGCAAACAGG` |
| ASV4794 | 405 | `CAAAGAATCTTGGGCAATGGGCGAAAGCCTGACCCAGCGACGCCGCGTGGAGGATGAAGGCCTTCGGGTTGTAAACTCCTTTTTCAGGGGAAGAGTAAGGACGGTACCCTGAGAATAAGCCCCGGCCAACTACGTGCCAGCAGCCGCGGTAATACGTAGGGGGCAAGCGTTACCCGGATTTACTGGGCGTAAAGAGCGCGTAGGCGGCCCAACAAGTCTCCTGTGAAATCTTCTGGCTCAACTGGAAGAGGTCAGGGGATACTGTTGGACTTGAGGGCAGCAGAGGAAGACGGAATTCCCGGTGTAGCGGTGAAATGCGTAGATATCGGGAGGAACACCAGTGGCAAAGGCGGTCTTCTGGGCTGTACCTGACGCTGAGGCGCGAAAGCGTGGGGAGCAAACCGG` |
| ASV3979 | 429 | `TGGGGAATTTTGGACAATGGGGGCAACCCTGATCCAGCCATGCCGCGTGTGTGAAGAAGGCCTTCGGGTTGTAAAGCACTTTCGGACGGAACGAAATCGCGCGGGTGAATATCCCGCGTGGATGACGGTACTGTAAGAAGAAGCACCGGCTAACTACGTGCCAGCAGCCGCGGTAATACGTAGGGTGCGAGCGTTAATCGGAATTACTGGGCGTAAAGCGTGCGCAGGCGGCTTCGCAAGTCAGACGTGAAATCCCCGGGCTTAACCTGGGAATGGCGTTTGAAACTACGGAGCTGGAGTGTGGCAGAGGGAGGTAGAATTCCACGTGTAGCGGTGAAATGCGTAGATATGTGGAGGAATACCGATGGCGAAGGCAGCCTCCTGGGCCAACACTGACGCTCATGCACGAAAGCGTGGGGAGCAAACAGG` |
| ASV3800 | 425 | `TGAGGAATATTGGTCAATGGTCGAAAGACTGAACCAGCCATGCCGCGTGCAGGACAGACGGCCCTAGAGGTTGTAAACTGCTTTTATATGGGAAGAATAAGGAGTACGCGTACTTTGATGACGGTACCATATGAATAAGCATCGGCTAACTCCGTGCCAGCAGCCGCGGTAATACGGAGGATGCGAGCGTTATTCGGAATCATTGGGTTTAAAGGGTCTGTAGGCGGGCTATTAAGTCAGAGGTGAAAGGTTTCAGCTTAACTGAGAAATTGCCTTTGATACTGGTAGTCTTGAATATCTGTGAAGTTCTTGGAATGTGTAGTGTAGCGGTGAAATGCTTAGATATTACACAGAACACCGATTGCGGAGGCAGGGGACTAACAGACAATTGACGCTGAGAGACGAAAGCGTGGGGAGCGAACAGG` |
| ASV4617 | 407 | `TGGGGAATATTGGGCAATGGGCGAAAGCCTGACCCAGCAACGCCGCGTGAAGGAAGAAGGCTCTCGGGTTGTAAACTTCTTTTAAGAGGGACGAAAAAAATGACGGTACCTCTTGAATAAGCCACGGCTAACTACGTGCCAGCAGCCGCGGTAATACGTAGGTGGCAAGCGTTATCCGGATTTACTGGGTGTAAAGGGCGTGTAGGCGGGACTGCAAGTCAGGCGTGAAAACTGGGGGCTCAACCTCCAGCCTGCGTTTGAAACTGTGGTTCTTGAGTGATGGAGAGGCAGGCGGAATTCCCTGTGTAGCGGTGAAATGCGTAGATATAGGGAGGAACACCAGTGGCGAAGGCGGCCTGCTGGACATTAACTGACGCTGAGGCGCGAAAGCGTGGGGAGCAAACAGG` |
| ASV2870 | 404 | `TGGGGAATATTGGACAATGGGCGAAAGCCTGATCCAGCGACGCCGCGTGGGTGATGAAGGCCTTCGGGTTGTAAAGCCCTTTCGGCAGGGAAGATAATGACGGTACCTGCAAAAGAAGCTCCGGCTAACTTCGTGCCAGCAGCCGCGGTAATACGAAGGGAGCTAGCGTTACTCGGAATCATTGGGTGTAAAGCGTGCGCAGGCGGTTTAATAAGTTAGAAGTGAAAGCCCAGAGCTTAACTTTGGAATGGCTTTTAAAACTGTTAAACTTGAATACCAGAGAGGATGGCGGAATTTCTAGTGTAGAAGTGAAATTCGTAGATATTAGAAGGAACACCGGAGGCGAAGGCGGCCATCTGGCTGGTTATTGACGCTGATGCACGAAAGCGTGGGGAGCAAACAGG` |
| ASV83 | 429 | `TGGGGAATTTTGGACAATGGGCGCAAGCCTGATCCAGCCATGCCGCGTGCGGGAAGAAGGCCTTCGGGTTGTAAACCGCTTTTGTCAGGGAAGAAACGCTCTGGGCTAATACCCTGGGGTAATGACGGTACCTGAAGAATAAGCACCGGCTAACTACGTGCCAGCAGCCGCGGTAATACGTAGGGTGCAAGCGTTAATCGGAATTACTGGGCGTAAAGCGTGCGCAGGCGGTCTTGCAAGACAGATGTGAAATCCCCGGGCTCAACCTGGGAACTGCATTTGTGACTGCAAGGCTAGAGTACGGCAGAGGGGGATGGAATTCCGCGTGTAGCAGTGAAATGCGTAGATATGCGGAGGAACACCGATGGCGAAGGCAATCCCCTGGGCCTGTACTGACGCTCATGCACGAAAGCGTGGGGAGCAAACAGG` |
| ASV5428 | 404 | `TGGGGAATATTGGACAATGGGGGAAACCCTGATCCAGCCATGCCGCGTGAGTGATGAAGGCCTTAGGGTTGTAAAGCTCTTTTACCAGGGAAGATGATGACGGTACCTGGAGAATAAGTTCCGGCTAACTTCGTGCCAGCAGCCGCGGTAATACGAAGGGAACTAGCGTTGTTCGGAATCACTGGGCGTAAAGCGCGCGTAGGCGGCTTTGCAAGTCAGGGGTGAAATGCCAAGGCTTAACCTTGGAACTGCCCTTGAAACTGCATCGCTGGAGTGTCGGAGGGGATAGCGGAATTGCTAATGTAGCGGTGAAATGCGTAGATATTAGCAGGAACACCGGTGGCGAAGGCGGCTATCTGGACGACAACTGACGCTGAGGCGCGAAAGCGTGGGGATCAAACAGG` |
| ASV3502 | 429 | `TGGGGAATATTGGACAATGGGCGCAAGCCTGATCCAGCAATGCCGCGTGTGTGAAGAAGGCCTTCGGGTTGTAAAGCACTTTCAGTGGGGAGGAAGGAGTGTGTGCTAATACCACATGCTATTGACGTTACCCACAGAAGAAGCACCGGCTAACTCTGTGCCAGCAGCCGCGGTAATACAGAGGGTGCAAGCGTTAATCGGAATTACTGGGCGTAAAGGGTGTGTAGGTGGTTATATAAGTTAGATGTGAAATCCCCGGGCTCAACCTGGGAACTGCGTTTAAGACTGTATAACTAGAGTACTGTAGAGGATAGTGGAATTTCCAGTGTAGCGGTGAAATGCGTAGATATTGGAAGGAACACCAGTGGCGAAGGCGACTATCTGGACAGATACTGACACTGAGACACGAAAGCGTGGGGAGCAAACAGG` |
| ASV3635 | 404 | `TGGGGAATCTTAGACAATGGGGGAAACCCTGATCTAGCCATGCCGCGTGAGCGATGAAGGCCTTAGGGTTGTAAAGCTCTTTCAGTGGGGAAGATAATGACGGTACCCACAGAAGAAGCCCCGGCTAACTCCGTGCCAGCAGCCGCGGTAATACGGAGGGGGCTAGCGTTGTTCGGAATTACTGGGCGTAAAGCGCACGTAGGCGGATAAGAAAGTCAGGGGTGAAATCCCGGGGCTCAACCCCGGAACTGCCTTTGAAACTCCTTGTCTTGAGGTCGTGAGAGGTGGGTGGAATTCCGAGTGTAGAGGTGAAATTCGTAGATATTCGGAGGAACACCAGTGGCGAAGGCGGCCCACTGGCACGATACTGACGCTGAGGTGCGAAAGCGTGGGGAGCAAACAGG` |
| ASV4537 | 405 | `TGGGGAATCTTGCTCAATGGGCGAAAGCCTGAAGCAGCAACGCCGCGTGAGGGACGAAGGCCTTCGGGTTGTAAACCTCTTTCAGCAGGGACGAAGATGACGGTACCTGCAGAAGAAGCTCCGGCCAACTACGTGCCAGCAGCCGCGGTAATACGTAGGGAGCGAGCGTTGTCCGGATTTATTGGGCGTAAAGAGCTCGTAGGCGGTTTGGTAAGTCGGGTGTGAAAGGCCGAGGCTCAACCTCGGGAAGCCACCCGATACTGCTATGACTAGAGTCCGGTAGAGGAGTGTGGAATTCCTGGTGTAGCGGTGAAATGCGCAGAGATCAGGAGGAACACCAATGGCGAAGGCAGCACTCTGGGCCGGTACTGACGCTGAGGAGCGAAAGCGTGGGTAGCAAACAGG` |
| ASV3329 | 429 | `TGGGGAATTTTGGACAATGGGGGCAACCCTGATCCAGCCATGCCGCGTGCGGGAAGAAGGCCTTCGGGTTGTAAACCGCTTTTGGTCGGGAAGAAAAGGTTATCCCTAATAAGGGTGACTCTTGACGGTACCGGCAGAATAAGCACCGGCTAACTACGTGCCAGCAGCCGCGGTAATACGTAGGGTGCGAGCGTTAATCGGAATTACTGGGCGTAAAGCGTGCGCAGGCGGTTTGTTAAGTCTGATGTGAAAGCCCCGGGCTCAACCTGGGAATGGCATTGGAAACTGGCAAGCTTGAGTATGGCAGAGGGGGGTGGAATTCCGCGTGTAGCAGTGAAATGCGTAGAGATGCGGAGGAACACCGATGGCGAAGGCAGCCCCCTGGGCCAATACTGACGCTCAGGCACGAAAGCGTGGGGAGCAAACAGG` |
| ASV4028 | 404 | `TGAGGAATCTTCCACAATGGGCGAAAGCCTGATGGAGCGACACCGCGTGAAGGATGAAGCCTTTGTTGGTGTAAACTTCTTTTCTCTGGGAAGATAATGACGGTACCAGAGGAATAAGGGGCGGCAAACTTCGTGCCAGCAGCCGCGGTAATACGAAGGCCCCAAGCGTTATCCGGAATTACTGGGCGTAAAGCGTCTGTAGGTGGTCTTTTAAGTCTTTCCGTGAAATTTCGGGGCTCAACCCTGAATTGCGGGAGAAACTGGAGGACTTGAGTGTGGGAGGGGGAAGCAGAACGGTAAGAGTAGGGGTGCAATCCGTTGATACTTACCAGAATACCAAAAGCGAAGGCAGCTTCCTGGAACACAACTGACACTGAGAGACGAAAGCGTGGGGAGCAAAAGGG` |
| ASV4332 | 429 | `TGGGGAATATTGGACAATGGGCGGAAGCCTGATCCAGCAATGCCGCGTGTGTGAAGAAGGCCTGCGGGTTGTAAAGCACTTTCAGTGGGGAGGAGAGGCCTGTGGCTAATATCCATAGGAAGAGACGTTACCCACAGAAGAAGCACCGGCTAACTCTGTGCCAGCAGCCGCGGTAATACAGAGGGTGCAAGCGTTAATCGGAATTACTGGGCGTAAAGGGTGCGTAGGTGGTTTGATAAGTTGGGTGTGAAATTCCTGGGCTTAACCTAGGAACTGCGCTCAATACTGTTGAGCTAGAGTACTGTAGAGGAAGGTGGAATTTCCAGTGTAGCGGTGAAATGCGTAGATATTGGAAGGAACATCAGTGGCGAAGGCGACTTTCTGGACAGATACTGACACTGAGGCACGAAAGCGTGGGGAGCAAACAGG` |
| ASV429 | 429 | `TGGGGAATTTTGGACAATGGGGGCAACCCTGATCCAGCCATCCCGCGTGTGCGATGAAGGCCTTCGGGTTGTAAAGCACTTTTGGCAGGAAAGAAACGGCGCGTGCTAATACCATGCGCAACTGACGGTACCTGCAGAATAAGCACCGGCTAACTACGTGCCAGCAGCCGCGGTAATACGTAGGGTGCAAGCGTTAATCGGAATTACTGGGCGTAAAGCGTGCGCAGGCGGTTCGGAAAGAAAGATGTGAAATCCCAGGGCTCAACCTTGGAACTGCATTTTTAACTACCGGACTAGAGTGTGTCAGAGGGAGGTGGAATTCCACGTGTAGCAGTGAAATGCGTAGATATGTGGAGGAACACCGATGGCGAAGGCAGCCTCCTGGGATAACACTGACGCTCATGCACGAAAGCGTGGGGAGCAAACAGG` |
| ASV81 | 429 | `TGGGGAATTTTGGACAATGGGCGCAAGCCTGATCCAGCAATGCCGCGTGCAGGAAGAAGGCCTTCGGGTTGTAAACTGCTTTTGTACGGAACGAAACGGTCCTGGTTAATACCTGGGGCTAATGACGGTACCGTAAGAATAAGCACCGGCTAACTACGTGCCAGCAGCCGCGGTAATACGTAGGGTGCAAGCGTTAATCGGAATTACTGGGCGTAAAGCGTGCGCAGGCGGTTTTGTAAGACAGGCGTGAAATCCCCGGGCTTAACCTGGGAATTGCGCTTGTGACTGCAAGGCTGGAGTGCGGCAGAGGGGGATGGAATTCCGCGTGTAGCAGTGAAATGCGTAGATATGCGGAGGAACACCGATGGCGAAGGCAATCCCCTGGGCCTGCACTGACGCTCATGCACGAAAGCGTGGGGAGCAAACAGG` |
| ASV2393 | 429 | `TGGGGAATTTTGGACAATGGGCGCAAGCCTGATCCAGCAATGCCGCGTGCAGGAAGAAGGCCTTCGGGTTGTAAACTGCTTTTGTACGGAACGAAACGGCTGGGGTTAATACCTCTGGCTAATGACGGTACCGTAAGAATAAGCACCGGCTAACTACGTGCCAGCAGCCGCGGTAATACGTAGGGTGCAAGCGTTAATCGGAATTACTGGGCGTAAAGCGTGCGCAGGCGGTGATGTAAGACAGGCGTGAAATCCCCGGGCTCAACCTGGGAATTGCGCTTGTGACTGCATCGCTGGAGTGCGGCAGAGGGGGATGGAATTCCGCGTGTAGCAGTGAAATGCGTAGATATGCGGAGGAACACCGATGGCGAAGGCAATCCCCTGGGCCTGCACTGACGCTCATGCACGAAAGCGTGGGGAGCAAACAGG` |
| ASV2936 | 429 | `TGGGGAATATTGGACAATGGGCGCAAGCCTGATCCAGCCATGCCGCGTGGGTGAAGAAGGCCTTCGGGTTGTAAAGCCCTTTTGTCCGGAAAGAAAAGCACTGGATTAATACTTCGGTGTTCTGACGGTACCGGAAGAATAAGCACCGGCTAACTTCGTGCCAGCAGCCGCGGTAATACGAAGGGTGCAAGCGTTACTCGGAATTACTGGGCGTAAAGCGTGCGTAGGTGGTTCGTTAAGTCTGATGTGAAAGCCCTGGGCTCAACCTGGGAATTGCATTGGATACTGGCGGGCTAGAGTGCGGTAGAGGATGGCGGAATTCCCGGTGTAGCAGTGAAATGCGTAGAGATCGGGAGGAACATCTGTGGCGAAGGCGGCCATCTGGACCAGCACTGACACTGAGGCACGAAAGCGTGGGGAGCAAACAGG` |
| ASV4422 | 406 | `TGAGGAATCTTCCACAATGGGCGAAAGCCTGATGGAGCAACGCCGCGTGCAGGATGAAGGCCTTCGGGTCGTAAACTGCTTTTATAAGTGAAGAATATGACGGTAACTTATGAATAAGCACCGGCTAACTACGTGCCAGCAGCCGCGGTCATACGTAGGGTGCAAGCATTATCCGGAGTGACTGGGCGTAAAGAGTTGCGTAGGTGGTTTGTTAAGTGAATGGTGAAATCTGGCGGCTCAACCGTACAGACTATTATTCAAACTGGCAAACTCGAGAATGGTAGAGGTCACTGGAATTTCTTGTGTAGGAGTGAAATCCGTAGATATAAGAAGGAACACCAATGGCGTAGGCAGGTGACTGGACCATTTCTGACACTGAGGCACGAAAGCGTGGGGAGCGAACCGG` |
| ASV2068 | 430 | `TGGGGAATATTGGACAATGGGGGGAACCCTGATCCAGCCATGCCGCGTGTGTGAAGAAGGTCGTAAGATTGTAAAGCACTTTAAGTGGGGAAGAGGGTGCCTTTGTTAATAGCAAGGGTTACTGGACATTACCCACAGAATAAGCACCGGCTAACTCTGTGCCAGCAGCCGCGGTAATACAGAGGGTGCGAGCGTTAATCGGAATTACTGGGCGTAAAGCGTGCGTAGGCGGGCTTATAAGTTGGATGTGAAATCCCCGAGCTTAACTTGGGAATTGCATTCAAGACTGTAAGTCTAGAGTATGGGAGAGGAAGGTAGAATTCCAGGTGTAGCGGTGAAATGCGTAGAGATCTGGAGGAATACCGATGGCGAAGGCAGCCTTCTGGCCTAATACTGACGCTGAGGCACGAAAGCATGGGGAGCAAACAGG` |
| ASV3663 | 429 | `TGGGGAATTGTTCGCAATGGGCGCAAGCCTGACGACGCAACGCCGCGTGGGGGATGAAGGTCTTCGGATTGTAAACCCCTGTCGAGCGGGACGAACGGCCTGAGAGGTAACTCTCTTGGGAGTGACGGTACCGCTAAAGGAAGCCACGGCTAACTCTGTGCCAGCAGCCGCGGTAATACAGAGGTGGCAAGCGTTGTTCGGAATTACTGGGCGTAAAGGGCGCGTAGGCGGCTCTTTTAGTCAGACGTGAAATCCCCCGGCTTAACCTGGGAACTGCGTCTGATACTGGAGAGCTTGAGTGCGGGAGAGGGATGTGGAATTCCAGGTGTAGCGGTGAAATGCGTAGATATCTGGAGGAACACCGGTGGCGAAGGCGGCATCCTGGACCGACACTGACGCTGAGGCGCGAAAGCTAGGGGAGCAAACGGG` |
| ASV247 | 407 | `TGGGGAATTTTCCGCAATGGGCGAAAGCCTGACGGAGCAATACCGCGTGAGGGATGACGGCCTATGGGTTGTAAACCTCTTTTTTCAGGGAGGAATCAATGACGTGTACCTGAAGAATAAGCATCGGCTAACTCCGTGCCAGCAGCCGCGGTAAGACGGAGGATGCAAGTGTTATCCGGAATCACTGGGCGTAAAGCGTCTGTAGGTGGCTAATTAAGTCAACTGTTAAATCTTGGGGCTTAACCTCAAAATCGCAGTCGAAACTGGTTGGCTAGAGTATAGTAGGGGTAAAGGGAATTTCCAGTGGAGCGGTGAAATGCGTAGATATTGGAAAGAACACCGATGGCGAAGGCACTTTACTGGGCTATTACTAACACTCAGAGACGAAAGCTAGGGTAGCAAATGGG` |
| ASV4938 | 429 | `TGGGGAATATTGGGCAATGGGCGCAAGCCTGACCCAGCCACGCCGCGTGAGCGATGAAGGCCTTCGGGTTGTAAAGCTCTGTGGGGAGGGGCGAAAGGCAGAAGTGCTAATAGTACTCTGTTTGACGGTACCTCCTTAGCAAGCACCGGCTAACTCTGTGCCAGCAGCCGCGGTAAGACAGAGGGTGCAAACGTTGCTCGGAATTACTGGGCGTAAAGCGCGTGTAGGCGGGAAGATAAGTCGGGTGTGAAATCCCTGGGCTCAACCTAGGAACTGCATCCGAAACTGCCTTTCTGGAGTGATGGAGAGGAAAGCGGAATTCCCGGTGTAGAGGTGAAATTCGTAGATATCGGGAGGAACACCAGTGGCGAAGGCGGCTTTCTGGCCATCAACTGACGCTGAGACGCGAAAGCGTGGGGAGCAAACAGG` |
| ASV699 | 404 | `TGGGGAATCTTAGACAATGGGCGCAAGCCTGATCTAGCCATGCCGCGTGAGCGATGAAGGCCTTAGGGTTGTAAAGCTCTTTCGGGGGGGAAGATAATGACGGTACCCCCAGAAGAAGCCCCGGCTAACTCCGTGCCAGCAGCCGCGGTAATACGGAGGGGGCTAGCGTTGTTCGGAATTACTGGGCGTAAAGCGCACGTAGGCGGATCGGAAAGTCAGGGGTGAAATCCCGGGGCTCAACCCCGGAACGGCCCTTGAAACTCCCGGTCTTGAGGTCGAGAGAGGTGGGTGGAATTCCGAGTGTAGAGGTGAAATTCGTAGATATTCGGAGGAACACCAGTGGCGAAGGCGGCCCACTGGCTCGATACTGACGCTGAGGTGCGAAAGCGTGGGGAGCAAACAGG` |
| ASV2040 | 429 | `TGGGGAATATTGGACAATGGGGGCAACCCTGATCCAGCCATGCCGCGTGTGTGAAGAAGGCCTTCGGGTTGTAAAGCACTTTTAATAGAGAAGAAGTGATATTGGCTAATACCTGATATTGTTGACGTTATTTATAGAAAAAGCACCGGCTAACTCCGTGCCAGCAGCCGCGGTAATACGGAGGGTGCTAGCGTTAATCGGAATTACTGGGCGTAAAGGGCATGTAGGCGGATGAGTAAGTTGGATGTGAAATCCCTGGGCTTAACCTAGGAACTGCATACAAAACTGCTTATCTAGAATGTAGTAGAGGATGATGGAATTTTCTGTGTAGTAGTAAAATGCTTAGAGATAGAAAGGAACACCAGAGGCGAAGGCGATCGTCTGGGCTACAATTGACGCTGAGGTGCGAAAGCGTGGGGAGCAAACAGG` |
| ASV1282 | 429 | `TGGGGAATATTGGACAATGGGGGCAACCCTGATCCAGCCATGCCGCGTGTGTGAAGAAGGCCTTCGGGTTGTAAAGCACTTTCAGCGGGGAGGAAGGCTCTATGGCTAATATCCATGGGGATTGACGTTACCCGCAGAAGAAGCACCGGCTAACTCCGTGCCAGCAGCCGCGGTAATACGGAGGGTGCGAGCGTTTATCGGAATTACTGGGCGTAAAGCGCGCGTAGGCGGCTTGTTAAGTCGGATGTGAAATCCCCGGGCTCAACCTGGGAATTGCATCCGATACTGGCGAGCTGGAGTGCGATAGAGGGGGGTAGAATTCCTGGTGTAGCGGTGAAATGCGTAGATATCAGGAGGAATACCGGTGGCGAAGGCGGCCCCCTGGATCGACACTGACGCTGAGGTGCGAAAGCGTGGGTAGCAAACAGG` |
| ASV5295 | 404 | `CTAGGAATCTTGGGCAATGGGCGAAAGCCTGACCCAGCAACGCCGCGTGGGTGACGAAGGCCTTCGGGTCGTAAAGCCCTTTTGCAGGGGAAGATAGTGACGGTACCCTGCGAATAAGCCACGGCTAACTACGTGCCAGCAGCCGCGGTAATACGTAGGTGGCGAGCGTTGTCCGGATTTACTGGGCGTAAAGCGCGTGCAGGCGGTTCGTTAAGTCTGGGGTGAAATCTCCCGGCTCAACTGGGAGGGGTTCTCAGATACTGGCGGACTTGAGGGAGGCAGAGGAGAGTAGAATTCTCGGTGTAGTGGTGATATGCGTAGATATCGAGAGGAATACCAGTGGCGAAGGCGGCTCTCTGGGCCTTACCTGACGCTAAGACGCGAAAGCGTGGGGAGCGAACCGG` |
| ASV4084 | 429 | `TGGGGAATTTTGGACAATGGGCGCAAGCCTGATCCAGCCATGCCGCGTGTGTGAAGAAGGCCTTCGGGTTGTAAAGCACTTTCGGACGGAACGAAATCGCGCGGGTGAATAATCCGCGTGGCTGACGGTACCGTAAGAAGAAGCACCGGCTAACTACGTGCCAGCAGCCGCGGTAATACGTAGGGTGCGAGCGTTAATCGGAATTACTGGGCGTAAAGGGTGCGCAGGCGGCTCCGCGTGTCAGGCGTGAAATCCCCGGGCTTAACTTGGGAATGGCGCTTGAAACTACGGGGCTGGAGTGTGGCAGAGGGAGGTGGAATTCCACGTGTAGCGGTGAAATGCGTAGAGATGTGGAGGAACACCGATGGCGAAGGCAGCCTCCTGGGCCAACACTGACGCTCATGCACGAAAGCGTGGGGAGCAAACAGG` |
| ASV4510 | 422 | `TAGGGAATTTTGCGCAATGGAGGCAACTCTGACGCAGCAACGCCATGTGTGGGATGAAGCATTTCGGTGTGTAAACCACTGTCAGCAGGGAATAAACGGTCGCTTCGGGCGACTTTGAATGTACCTGCAAAGGAAGCCCCGGCTAACTTCGTGCCAGCAGCCGCGGTAATACGAGGGGGGCAAATGTTGTTCGGAATCACTGGGCGTAAAGGGAGCGTAGGCGGGTTCTTAAGTAAGGTGTTTAATCCCGGGGCCCAACCCCGGACCGGCACTTTAAACTGGGAATCTTGAATAGTGGAGAGGTCAGTGGAATTGGGGGTGTAGCGGTGGAATGCGTAGAGATCCCCAAGAACACCAGTTGCGAAGGCGGCTGACTGGACACATATTGACGCTGAGGCTCGAAAGCATGGGGAGCAAACAGG` |
| ASV5137 | 404 | `TGGGGAATATTGGACAATGGGCGCAAGCCTGATCCAGCAATACCGCGTGAGTGATGAAGGTCTTCGGATTGTAAAGCTCTTTTAGTAGGGAAGATAATGACGGTACCTACAGAAAAAGCACCGGCAAACTTCGTGCCAGCAGCCGCGGTAATACGAAGGGTGCTAGCGTTGTTCGGAATCACTGGGCGTAAAGCGTACGTAGGTGGTCGAGTAAGTCGATTGTGAAAGCCCCAAGCTTAACTTGGGAATTGCAACCGAAACTGCTCAGCTAGAGTATGGTCGGGGATAGTGGAATTTCTAGTGTAGGGGTGAAATCCGTAGATATTAGAAGGAATACCGGTGGCGAAAGCGACTGTCTAGGCCATTACTGACACTGAGGTACGAAAGCGTGGGGAGCAAACAGG` |
| ASV5440 | 407 | `TAGGGAATATTGCGCAATGGGGGAAACCCTGACGCAGCGACGCCGCGTGAGTGATGAAGGCCTTAGGGTCGTAAAGCTCTGTTGAATGGGAATAAAAAAATGAAGGTACCATTCGAGAAAGGACCGGCTAACTTCGTGCCAGCAGCCGCGGTAATACGAGGGGTCCTAGCGTTGTTCGGAATCATTGGGCGTAAAGGGAGCGTAGGTGGCTATTCAAGTCAGTTGTGAAATCCCCGGGCTTAACCCGGGAAGTGCTTCTGATACTGTTTAGCTTGAGTACTGGAGAGGATAGTAGAATTCCTGGTGTAGTGGTGAAATACGTAGATATCAGGAGGAATACCGGTGGCGAAGGCGGCTATCTGGCCATGTACTGACACTGAAGCTCGAAAGCGTGGGGAGCAAACAGG` |
| ASV5656 | 429 | `TGGGGAATATTGGACAATGGGCGCAAGCCTGATCCAGCAACGCCGCGTGGAGGATGAAGGTCTTCGGATTGTAAACTCCTGTCAGATGGGAAGAATGACTGTCGGCAAATAGTCGATAGTCTTGACTGTACCATCAGAGGAAGCCCCGGCTAACTCTGTGCCAGCAGCCGCGGTAATACAGAGGGGGCAAGCGTTATTCGGAATTATTGGGCGTAAAGGGCGCGTAGGTGGCCTGATCAGTCAAAGGTGAAATCCCTCGGCTTAACCGAGGAACTGCCTTTGATACTGTCAGGCTTGAGGCCGGGAGAGGGTAGCGGAATTCCCAGTGTAGCGGTGAAATGCGTAGATATTGGGAGGAACACCGGTGGCGAAGGCGGCTACCTGGACCGGTTCTGACACTGAGGCGCGAAAGCGTGGGTAGCAAACAGG` |
| ASV3342 | 429 | `TGGGGAATTTTGGACAATGGGGGCAACCCTGATCCAGCCATGCCGCGTGAGTGAAGAAGGCCTTCGGGTTGTAAAGCTCTTTCAGCCGGGAAGAAATCGCATTCTCTAATATAGAGTGTGGATGACGGTACCGGAATAAGAAGCACCGGCTAACTACGTGCCAGCAGCCGCGGTAATACGTAGGGTGCGAGCGTTAATCGGAATTACTGGGCGTAAAGCGTGCGCAGGCGGTTTCGTAAGACAGAGGTGAAATCCCCGGGCTCAACCTGGGAACTGCCTTTGTGACTGCGAGGCTAGAGTATGGCAGAGGGGGGTGGAATTCCACGTGTAGCAGTGAAATGCGTAGAGATGTGGAGGAACACCGATGGCGAAGGCAGCCCCCTGGGCCAATACTGACGCTCATGCACGAAAGCGTGGGGAGCAAACAGG` |
| ASV5277 | 431 | `TGGGGAATCTTGCGCAATGGACGGAAGTCTGACGCAGCAACGCCGCGTGAGTGAAGAAGGCCTTCGGGTTGTAAAGCTCTGTCGTTGGGGACGAAAACTTTTAGGGTGAATAGCCTTAGGAGCTGACGGTACCTAGAGAGGAAGCACCGGCTAACTTCGTGCCAGCAGCCGCGGTAATACGAGGGGTGCAAGCGTTGCTCGGAATTATTGGGCGTAAAGGGTAGGTAGGTGGTCTCATTAGTCAGGGGTGAAAGCCTTGGGCTTAACTCAAGAAGTGCCTCTGAAACGGTGAGACTTGGAGTCCTGGAGGGGGTCGTGGAATTCCCGGTGTAGCGGTGAAATGCGTAGAGATCGGGAGGAACACCTGAGGCGAAGGCGGCGACCTGGACAGGAACTGACACTAAACTACGAAAGCGTGGGGAGCAAACAGG` |
| ASV862 | 424 | `TGAGGAATATTGGACAATGGGCGCAAGCCTGATCCAGCCATGCCGCGTGCAGGATGACGGTCCTATGGATTGTAAACTGCTTTTGTACAGGAAGAAACATTCCCTCGTGAGGGAGCTTGACGGTACTGTAAGAATAAGGATCGGCTAACTCCGTGCCAGCAGCCGCGGTAATACGGAGGATCCAAGCGTTATCCGGAATCATTGGGTTTAAAGGGTCCGTAGGCGGTTTAGTAAGTCAGTGGTGAAAGCCCATCGCTCAACGGTGGAACGGCCATTGATACTGCTAGACTTGAATTATTAGGAAGTAACTAGAATATGTAGTGTAGCGGTGAAATGCTTAGAGATTACATGGAATACCAATTGCGAAGGCAGGTTACTACTAATGGATTGACGCTGATGGACGAAAGCGTGGGTAGCGAACAGG` |
| ASV2970 | 430 | `TGGGGAATATTGCGCAATGGGGGAAACCCTGACGCAGCAACGCCGCGTGAGTGATGAAGGCCTTCGGGTTGTAAAGCTCTTTCGGTTGGGAAGAAGGGTTATTCGGTTAATAAGCGAGTAATTTGATGGTACCAAAAGAAGAAGCACCGGCAAACTTCGTGCCAGCAGCCGCGGTAATACGAAGGGTGCAAGCGTTGTTCGGAATTACTGGGCGTAAAGGGTTCGTAGGCGGGAATGCAAGTCAAGTGTGAAATCCCTAGGCTCAACCTAGGACGTGCATTTGAGACTGTGTATCTTGAGTTTCGGAGAGGGTGGTGGAATTGCTGGTGTAGGAGTGACATCCGTAGAGATCAGCAGGAACACCGGAGGCGAAGGCGACCACCTGGCCGAATACTGACGCTGAGGAACGAAAGCGTGGGGAGCAAACAGG` |
| ASV948 | 429 | `TGGGGAATTTTGGACAATGGGCGCAAGCCTGATCCAGCCATGCCGCGTGCAGGATGAAGGCCTTCGGGTTGTAAACTGCTTTTGTTAGGAACGAAAAGCCATCTAATAACACTAGGTGGTGCTGACGGTACCTAAAGAATAAGCACCGGCTAACTACGTGCCAGCAGCCGCGGTAATACGTAGGGTGCAAGCGTTAATCGGAATTACTGGGCGTAAAGCGTGCGCAGGCGGTTTTGTAAGACAGATGTGAAATCCCTGGGCTCAACCTAGGAACTGCATTTGTGACTGCAAAGCTAGAGTGTGTCAGAGGGGGGTAGAATTCCACGTGTAGCAGTGAAATGCGTAGAGATGTGGAGGAATACCAATGGCGAAGGCAACCCCCTGGGATAACACTGACGCTCATGTACGAAAGCGTGGGGAGCAAACAGG` |
| ASV3177 | 403 | `TGGGGAATATTGCACAATGGGGGAAACCCTGATGCAGCGACGCCGCGTGAGGGAAGAAGGTTTTCGGATTGTAAACCTCTGTCTTCGGGGACGATAATGACGGTACCCGAGGAGGAAGCACCGGCTAACTACGTGCCAGCAGCCGCGGTAAAACGTAGGGTGCAAGCGTTGTCCGGAATTACTGGGTGTAAAGGGAGCGCAGGTGGGATAGTAAGTTGGACGTTTAATCTATGGGCTCAACCCATAATCGCGTTCAAAACTATTATTCTTGAGTGGTGTAGAGGCAGATGGAATTCCCGGTGTAGCGGTGGAATGCGTAGATATCGGGAGGAACACCAGTGGCGAAGGCGGTCTGCTGGGCACTAACTGACACTGAGGCTCGAAAGCATGGGTAGCAAACAGG` |
| ASV4997 | 404 | `TTAGGAATATTGGACAATGGGGGCAACCCTGATCCAGCCATGCCGCGTGAGTGATGAAGGCCTTCGGGTTGTAAAGCTCTTTAGCCGACGATTATAATGACTGTAGTCGGAAAATAAGCCCCGGCTAACTTCGTGCCAGCAGCCGCGGTAATACGAAGGGGGCGAGCGTTGTTCGGAATCACTGGGCGTAAAGCGTGCGTAGGCGGTCATGACAGTCAGAAGTGAAAGCCCCAGGCTCAACTTGGGAATTGCTTTTGATACTACATGACTGGAATTCGGGAGAGGATAGCGGAATTGTCAGTGTAGCAGTGAAATGCGTAGATATTGACAGGAACACCAGTGGCGTAAGCGGCTATCTGGACCGACATTGACGCTGAGGCACGAAAGCGTGGGGATCAAACAGG` |
| ASV2992 | 404 | `TGGGGAATCTTGCGCAATGGGGGCAACCCTGACGCAGCCATACCGCGTGTGCGATGAAGGTCTTCGGATTGTAAAGCACTTTCAGTGGGGACGACGATGACGGTACCCACAGAAGAAGTCACGGCTAACTTCGTGCCAGCAGCCGCGGTAATACGAAGGTGGCGAATGTTGCTCGGATTCACTGGGCGTAAAGCGTCGGTAGGTGGTTTGTTAAGTCAGAGGTGAAAGCCCCGCGCTCAACGTGGGAATTGCCTTTGAAACTGACAGACTTGAAGGTATGAGAGGATGGCGGAATGACTGGTGTAGAGGTAAAATTCATAGAGATCAGTCGGAACACCCAAGGCGAAGGCAGCTATCTGGCATATTCTTGACACTGAGCGACGACAGCGTGGGGAGCAAACAGG` |
| ASV2396 | 429 | `TGGGGAATTTTGGACAATGGGCGAAAGCCTGATCCAGCCATGCCGCGTGTGGGAAGAAGGCCTTCGGGTTGTAAACCACTTTTGTCAGGGAAGAAACGGTTTTTACTAATACTAAAAACTAATGACGGTACCTGAAGAATAAGCACCGGCTAACTACGTGCCAGCAGCCGCGGTAATACGTAGGGTGCAAGCGTTAATCGGAATTACTGGGCGTAAAGCGTGCGCAGGCGGCTATGCAAGACAGATGTGAAATCCCCGGGCTTAACCTGGGAACTGCATTTGTGACTGCATAGCTAGAGTACGGTAGAGGGGGATGGAATTCCGCGTGTAGCAGTGAAATGCGTAGATATGCGGAGGAACACCGATGGCGAAGGCAATCCCCTGGACCTGTACTGACGCTCATGCACGAAAGCGTGGGGAGCAAACAGG` |
| ASV2514 | 407 | `TGGGGAATATTGGGCAATGGGCGCAAGCCTGACCCAGCAACGCCGCGTGAAGGAAGAAGGCTTTCGGGTTGTAAACTTCTTTAGACAGGGACGAAACAAATGACGGTACCTGTAAAATAAGCCACGGCTAACTACGTGCCAGCAGCCGCGGTAATACGTAGGTGGCGAGCGTTATCCGGATTTACTGGGTGTAAAGGGCGTGTAGGCGGTACAATAAGTCAGATGTGAAATCTCCCGGCTCAACTGGGAGGCTGCATCTGAAACTGTTGAACTTGAGTACTGGAGGGGAAAGCGGAATTCCTAGTGTAGCGGTAAAATGCGTAGATATTAGGAGGAACACCGGTGGCGAAGGCGGCTTTCTGGACAGTAACTGACGCTGAGGCGCGAAAGCGTGGGGAGCAAACAGG` |
| ASV3822 | 430 | `TAGGGAATATTGCGCAATGGACGCAAGTCTGACGCAGCGACGCCGCGTGGGTGACGAAGGCTTTCGGGTCGTAAAGCCCTGTCGAAAGGGACGAAAAACGGAATGGCTAATATCCATACCGCTTGACGGTACCTTTAGAGGAAGCACCGGCTAACTACGTGCCAGCAGCCGCGGTAATACGTAGGGTGCTAGCGTTGTTCGGAATCATTGGGCGTAAAGCGCGTGTAGGCGGTTCATTAAGTCGGGTGTGAAATCCCTGGGCTCAACCCAGGAAGGGCATCCGAAACTAGTGAGCTGGAAGATGGTAGAGGAAGGTGGAATTCCAAGTGTAGAGGTGAAATTCGTAGATATTTGGAGGAATACCGGTGGCGAAGGCGGCCTTCTGGACCATTCTTGACGCTGAGACGCGAAAGCGTGGGGAGCAAACAGG` |
| ASV3135 | 429 | `TAGGGAATATTGCGCAATGGGGGAAACCCTGACGCAGCAACGCCGCGTGAGTGAGGAAGGTCTTCGGATTGTAAAACTCTTTTCTTAGGGAAAAATGGTTATGGAGCTAATACCCCATGATTTGATGGTACCTAAGGAATAAGCACCGGCTAACTTCGTGCCAGCAGCCGCGGTAATACGAAGGGTGCAAGCGTTATTCGGAATCATTGGGCGTAAAGCGCGCGCAGGCGGATTGATAAGTCAGGTGTGAAATCTCGAGGCTCAACCTCGAAACTGCGCCTGAAACTATTAGTCTAGAATGTCGGAGGGGGCAGGGGAATTTCACGTGTAGGGGTAAAATCCGTAGAGATGTGAAGGAACACCGGAGGCGAAGGCGCCTGCCTGGACGACTATTGACGCTGAGGCGCGAAAGCGTGGGGAGCAAACAGG` |
| ASV5570 | 428 | `TGGGGAATATTGGACAATGGGGGCAACCCTGATCCAGCGATGCCGCGTGTGTGAAGAAGGCCTTCGGGTTGTAAAGCACTTTTGTCAGGAAAGAAAGGTTGTCTCGGAAATGGGATAACTGTGACGGTACCTGAAGAAAAAGCACCGGCTAACTCTGTGCCAGCAGCCGCGGTAATACAGAGGGTGCAAGCGTTAATCGGATTTACTGGGCGTAAAGAGCGCGTAGGTGGTTTGATAGGTTGAATGTGAAATCCTTGGGCTTAACCCAAGAGCGGCATTCAAAACGGTCAAGCTAGAGTGTTGGAGAGGGAAGTAGAATTTCTGGTGTAGCGGTGAAATGCGTAGATATCAGAAAGAATGACAGTGGCGAAGGCGGCTTCCTGGCCAAATACTGACACTGAGGCGCGAAAGCGTGGGTAGCGAACGGG` |
| ASV3577 | 404 | `TGGGGAATATTGGACAATGGGCGCAAGCCTGATCCAGCCATGCCGCGTGAGTGATGAAGGCCTTAGGGTTGTAAAGCTCTTTTGGCGGGGACGATAATGACGGTACCCGCAGAATAAGCCCCGGCTAACCTCGTGCCAGCAGCCGCGGTAATACGAGGGGGGCTAGCGTTGTTCGGAATTACTGGGCGTAAAGAGCACGTAGGCGGCTCGTTAAGTCAGGGGTGAAATCCCAGAGCTCAACTCTGGAACTGCCTTTGATACTGGCGAGCTCGAGTGTGGTAGAGGTTGGTGGAACTCCGAGTGTAGAGGTGAAATTCGTAGATATTCGGAAGAACACCAGTGGCGAAGGCGGCCAACTGGACCATTACTGACGCTGAGGTGCGAAAGCGTGGGGAGCAAACAGG` |
| ASV4623 | 424 | `TAGGGAATATTGGACAATGGAGGGAACTCTGATCCAGCCATGCCGCGTGAACGATGAAGGCGCTCTGCGTTGTAAAGTTCTTTTAGATGGGGCGAAAAAGGGTCATTTATGATCAACTGACGGTACCATCGGAATAAGCACCGGCTAACTCCGTGCCAGCAGCCGCGGTAATACGGAGGGTGCAAGCGTTATCCGGAATCACTGGGTTTAAAGGGTGAGTAGGCGGAAGGATAAGTCAGAGGTGAAATGTGATTGCTTAACGATTAAATTGCCTTTGAAACTGTTTTTCTTGAATCGAGATGAGGTATGCGGAATGAGACATGTAGCGGTGAAATGCATAGAGATGTCTTAGAACACCAATTGCGAAGGCAGCGTACTGGACTTGTATTGACGCTGAGGCACGAAAGCGTGGGGATCAAACAGG` |
| ASV2537 | 404 | `TGGGGAATATTGGACAATGGGCGAAAGCCTGATCCAGCAATGCCGCGTGAGTGATGAAGGCCTTAGGGTTGTAAAGCTCTTTTACCAGGGATGATAATGACAGTACCTGGAGAATAAGCTCCGGCTAACTCCGTGCCAGCAGCCGCGGTAATACGGAGGGAGCTAGCGTTGTTCGGAATTACTGGGCGTAAAGCGCGCGTAGGCGGTTTTTCAAGTCAGAGGTGAAAGCCCGGAGCTCAACTCCGGAACTGCCTTTGAAACTGGATTACTTGAATCTTGGAGAGGTCAGTGGAATTCCGAGTGTAGAGGTGAAATTCGTAGATATTCGGAAGAACACCAGTGGCGAAGGCGACTGACTGGACAAGTATTGACGCTGAGGTGCGAAAGCGTGGGGAGCAAACAGG` |
| ASV2652 | 429 | `TGGGGAATTTTGGACAATGGGGGCAACCCTGATCCAGCCATTCCGCGTGAGTGAAGAAGGCCTTCGGGTTGTAAAGCTCTTTCGGCAGGAACGAAACGGTCATTTCTAATAAAGATGGCTAATGACGGTACCTGAAGAAGAAGCACCGGCTAACTACGTGCCAGCAGCCGCGGTAATACGTAGGGTGCGAGCGTTAATCGGAATTATTGGGCGTAAAGCGTGCGCAGGCGGCTTCATAAGACAGATGTGAAATCCCCGGGCTTAACCTGGGAACTGCGTTTGTGACTGTGAGGCTAGAGTGTGGCAGAGGGGGGTGGAATTCCACGTGTAGCAGTGAAATGCGTAGATATGTGGAGGAACATCGATGGCGAAGGCAGCCCCCTGGGTTAACACTGACGCTCATGCACGAAAGCGTGGGGAGCAAACAGG` |
| ASV2849 | 404 | `TGGGGAATATTGGACAATGGGCGAAAGCCTGATCCAGCAATACCACGTGAGTGATGAAGGCCTTAGGGTTGTAAAACTCTTTTAGTGAGGAAGATAATGACTGTACTCACAGAAAAAGCACCGGCAAACTTCGTGCCAGCAGCCGCGGTAATACGAAGGGTGCTAGCGTTGTTCGGATTTATTGGGCGTAAAGCGTATGTAGGCTGTTTAGTAAGTCGATTGTGAAATCCCTGGGCTTAACCTAGGAACTGCATTCGAAACTGCTAAGCTAGATTATGGTGGGGGACAGTGGAATTCCTAGTGTAGGAGTGAAATCCGTAGATATTAGGAGGAACACCAGAGGCGAAGGCGACTGTCTACGCCATTAATGACGCTGAGATACGAAAGCGTGGGGAGCAAACAGG` |
| ASV592 | 429 | `TGGGGAATATTGGACAATGGGCGAAAGCCTGATCCAGCCATGCCGCGTGTGTGAAGAAGGTCTTAGGATCGTAAAGCACTTTAAGTTGGGAGGAAGGGCAGTAAATTAATACTTTGCTGTTTTGACGTTACCGACAGAATAAGCACCGGCTAACTCTGTGCCAGCAGCCGCGGTAATACAGAGGGTGCAAGCGTTAATCGGAATTACTGGGCGTAAAGCGCGCGTAGGTGGTTCGTTAAGTTGGATGTGAAATCCCCGGGCTCAACCTGGGAACTGCATTCAAAACTGTCGAGCTAGAGTATGGTAGAGGGTGGTGGAATTTCCTGTGTAGCGGTGAAATGCGTAGATATAGGAAGGAACACCAGTGGCGAAGGCGACCACCTGGACTGATACTGACACTGAGGTGCGAAAGCGTGGGGAGCAAACAGG` |
| ASV2255 | 424 | `TAGGGAATATTGGTCAATGGAGGCAACTCTGAACCAGCCATGCCGCGTGCAGGACTAAGGCCCTATGGGTCGTAAACTGCTTTTGAATGGGAAGAAATGTATCTACGTGTAGATATTTGACGGTACCATTAGAATAAGGATCGGCTAACTTCGTGCCAGCAGCCGCGGTAATACGAAGGATCCAAGCGTTGTCCGGATTTACTGGGTTTAAAGGGTGCGTAGGCGGACTCATAAGTCAGTGGTGAAAGCCTTCAGCTTAACTGAAGAACTGCCATTGAAACTGTAAGTCTCGAGTTCGGTTGAGGTCATTGGAATGTAACATGTAGCGGTGAAATGCATAGATATGTTACAGAACACCAATTGCGAAGGCAGATGACTAAACCGAAACTGACGCTGAGGCACGAAAGCGTGGGGATCAAACAGG` |
| ASV1327 | 404 | `TGGGGAATATTGGACAATGGGGGCAACCCTGATCCAGCAATGCCGCGTGGGTGAAGAAGGTCTTCGGATTGTAAAGCCCTTTCGACGGGGACGATGATGACGGTACCCGTAGAAGAAGCCCCGGCTAACTTCGTGCCAGCAGCCGCGGTAATACGAAGGGGGCTAGCGTTGTTCGGAATGACTGGGCGTAAAGGGCGCGTAGGCGGCTTTGTAAGTCAGGCGTGAAATTCCTGGGCTTAACCTGGGGGCTGCGTCTGAGACTGCGGAGCTAGAGGCCGAGAGAGGGTGGTGGAATTCCCAGTGTAGAGGTGAAATTCGTAGATATTGGGAAGAACACCGGTGGCGAAGGCGGCCACCTGGCTCGGAACTGACGCTGAGGCGCGACAGCGTGGGGAGCAAACAGG` |
| ASV267 | 404 | `TGGGGAATCTTGGACAATGGGGGCAACCCTGATCCAGCGATGCCGCGTGAATGAAGAAGGCCTTAGGGTTGTAAAGTTCTTTCACCCGTGACGATAATGACGGTAGCGGGAGAAGAAGCCCCGGCTAACTCCGTGCCAGCAGCCGCGGTAAGACGGAGGGGGCTAGCGTTGTTCGGAATGACTGGGCGTAAAGGGCGTGTAGGCTGTTGATCAAGTCAAGCGTGAAAGCCTCGGGCTTAACCCGAGAACTGCGTTTGATACTGGTTGACTTGAGGACGAGAGAGGAAAGTGGAATTCCGAGTGTAGAGGTGAAATTCGTAGATATTCGGAGGAACATCAGAGGCGAAGGCGGCTTTCTGGCTCGTATCTGACGCTGAGGCGCGAAAGCGTGGGGAGCAAACAGG` |
| ASV5533 | 404 | `TGGGGAATATTGGGCAATGGAGGCAACTCTGACCCAGCCATGCCGCGTGGGTGACGAAGGTCTTCGGATTGTAAAGCCCTTTCGGCGGGGACGATGATGACGGTACCCGCAGAAGAAGCCCCGGCTAACTTCGTGCCAGCAGCCGCGGTAATACGAAGGGGGCTAGCGTTGTTCGGAATTACTGGGCGTAAAGGGCGTGTAGGCGGCCCGCTAAGTCAGGTGTGAAAGGCCTGGGCTCAACCCAGGAAATGCGCCTGAGACTGACGGGCTTGAATCCGGGAGAGGATGGTGGAACTCCCAGTGTAGAGGTGAAATTCGTAGATATTGGGAAGAACACCCGTGGCGAAGGCGGCCATCTGGACCGGTATTGACGCTAAGGCGCGACAGCGTGGGGAGCAAACAGG` |
| ASV238 | 429 | `TGGGGAATTTTGGACAATGGGCGCAAGCCTGATCCAGCCATTCCGCGTGAGTGAAGAAGGCCTTCGGGTTGTAAAGCTCTTTCGCAAGGGAAGAAAACTTACTTTCTAATAAAGAGTGAGGTTGACGGTACCTTGATAAGAAGCACCGGCTAACTACGTGCCAGCAGCCGCGGTAATACGTAGGGTGCGAGCGTTAATCGGAATTACTGGGCGTAAAGCGTGCGCAGGCGGTTTTGTAAGTCAGATGTGAAATCCCCGAGCTCAACTTGGGAACTGCGTTTGAAACTACAAGACTAGAATATGTCAGAGGGGGGTAGAATTCCACGTGTAGCAGTGAAATGCGTAGAGATGTGGAGGAATACCAATGGCGAAGGCAGCCCCCTGGGATAATATTGACGCTCATGCACGAAAGCGTGGGGAGCAAACAGG` |
| ASV2446 | 428 | `TCGAGAATTTTTCACAATGGGCGAAAGCCTGATGGAGCGACGCCGCGTGGGGGATGAAGGGCTTCGGCTCGTAAACCCCTGTCATTCGAGAACAAACCCTACGATTGAATAGATCGTGGGCTGATAGTATCGGAAGAGGAAGGGACGGCTAACTCTGTGCCAGCAGCCGCGGTAATACAGAGGTCCCAAGCGTTGTTCGGATTTACTGGGCGTAAAGGGTGCGTAGGCGGCGAGGAAAGTTTGAGGTGAAATCTCCAGGCTCAACTTGGAAAATGCCTTGAAGACTTCCTTGCTCGAGGGTCGTAGAGGAGACTGGAATTCTCGGTGTAGCAGTGAAATGCGTAGATATCGAGAGGAACACCAGTGGCGAAAGCGGGTCTCTGGACGACACCTGACGCTGAGGCACGAAAGCCAGGGGAGCAAACGGG` |
| ASV263 | 424 | `TAAGGAATATTGGACAATGGACGCAAGTCTGATCCAGCCATGCCGCGTGAAGGATTAAGGTCCTCTGGATTGTAAACTTCTTTTATAAGGGACGAAAAAAGGTCTTTCTAGATCGTCTGACGGTACCTTATGAATAAGCACCGGCTAACTCCGTGCCAGCAGCCGCGGTAATACGGAGGGTGCAAGCGTTATCCGGATTCACTGGGTTTAAAGGGTGCGTAGGCGGGCAGGTAAGTCAGTGGTGAAATCCTAGAGCTTAACTCTAGAACTGCCATTGATACTATCTGTCTTGAATATTGTGGAGGTAAGCGGAATATGTCATGTAGCGGTGAAATGCTTAGATATGACATAGAACACCTATTGCGAAGGCAGCTTACTACGCATATATTGACGCTGAGGCACGAAAGCGTGGGGATCAAACAGG` |
| ASV2850 | 419 | `TGAGGAATATTGCGCAATGGGCGAAAGCCTGACGCAGCGACGCCGCGTGGGGGATGAAGGTCTTCGGATTGTAAACCCCTTTCGGGAGGGAAGATGGGACGGGCAACCGTTCGGACGGTACCTCCAGAAGCAGCCACGGCTAACTTCGTGCCAGCAGCCGCGGTAATACGAAGGTGGCAAGCGTTGTTCGGATTTACTGGGCGTACAGGGAGCGTAGGCGGTTAGGTAAGCCCTTCGGGAAATCTCCCGGCTTAACCGGGAAAGGTCGGAGGGGACTGCCTGGCTAGAGGACGGGAGAGGAGCGCGGAATTCCCGGTGTAGCGGTGAAATGCGTAGAGATCGGGAGGAAGGCCGGTGGCGAAGGCGGCGCTCTGGAACGTTCCTGACGCTGAGGCTCGAAAGCGTGGGGAGCAAACAGG` |
| ASV2206 | 429 | `TGGGGAATTTTGGACAATGGGCGAAAGCCTGATCCAGCAATGCCGCGTGAGTGAAGAAGGCCTTCGGGTTGTAAAGCTCTTTTGTCAGGGAAGAAATCCTAGTGGATAATACCTACTGGGGATGACGGTACCTGAAGAATAAGCACCGGCTAACTACGTGCCAGCAGCCGCGGTAATACGTAGGGTGCAAGCGTTAATCGGAATTACTGGGCGTAAAGCGTGCGCAGGCGGTTTGGTAAGACAGGTGTGAAATCCCCGAGCTCAACTTGGGAACTGCACTTGTGACTGCCAGACTAGAGTGTGTCAGAGGGAGGTAGAATTCCACGTGTAGCAGTGAAATGCGTAGATATGTGGAGGAATACCGGTGGCGAAGGCGGCCTCCTGGGATAACACTGACGCTCATGTACGAAAGCGTGGGGAGCAAACAGG` |
| ASV2540 | 404 | `TGGGGAATATTGGACAATGGGCGCAAGCCTGATCCAGCAATGCCGCGTGAGTGATGAAGGCCTTAGGGTTGTAAAGCTCTTTTACCCGGGATGATAATGACAGTACCGGGAGAATAAGCCCCGGCTAACTCCGTGCCAGCAGCCGCGGTAATACGGAGGGGGCTAGCGTTGTTCGGAATTACTGGGCGTAAAGCGCACGTAGGCGGCTTTGTAAGTCAGGGGTGAAAGCCTGGAGCTCAACTCCAGAACTGCCTTTGAGACTGCATCGCTTGAATCCGGGAGAGGTAAGTGGAATTCCGAGTGTAGAGGTGAAATTCGTAGATATTCGGAAGAACACCAGTGGCGAAGGCGGCTTACTGGACCGGAATTGACGCTGAGGTGCGAAAGCGTGGGGAGCAAACAGG` |
| ASV1468 | 429 | `TGGGGAATATTGGACAATGGGCGAAAGCCTGATCCAGCCATGCCGCGTGTGTGAAGAAGGTCTTCGGATTGTAAAGCACTTTAAGTTGGGAGGAAGGGTCGTTACCTAATACGTGACGATTTTGACGTTACCGACAGAATAAGCACCGGCTAACTCTGTGCCAGCAGCCGCGGTAATACAGAGGGTGCAAGCGTTAATCGGAATTACTGGGCGTAAAGCGCGCGTAGGTGGTTCGTTAAGTTGGATGTGAAATCCCCGGGCTCAACCTGGGAACTGCATCCAAAACTGGCGAGCTAGAGTAGGGCAGAGGGTGGTGGAATTTCCTGTGTAGCGGTGAAATGCGTAGATATAGGAAGGAACACCAGTGGCGAAGGCGACCACCTGGGCTCATACTGACACTGAGGTGCGAAAGCGTGGGGAGCAAACAGG` |
| ASV325 | 423 | `TAGGGAATATTGGTCAATGGGCGGAAGCCTGAACCAGCCATGCCGCGTGCAGGAAGACGGCCCTCTGGGTTGTAAACTGCTTTTGACTAGGAACAAACGAACCTTGCGAGGTGCATTGAGTGTACTAGTAGAATAAGCCACGGCTAACTACGTGCCAGCAGCCGCGGTAATACGTAGGTGGCAAGCGTTGTCCGGATTTATTGGGTTTAAAGGGTGCGTAGGCGGTTTATTAAGTCAGTCGTTAAATTACCCAGCTTAACTGGGGGCCTGCGAATGATACTGATAGACTTGAGTACAAATGAGGTTAGCGGAATTAATAGTGTAGCGGTGAAATGCATAGATATTATTAAGAACTCCGATAGCGAAGGCAGCTAACCAAGATGTAACTGACGCTGATGCACGAAAGTATGGGGATCAAACAGG` |
| ASV3054 | 405 | `TAGGGAATTTTCCACAATGGGCGAAAGCCTGATGGAGCAACGCCGCGTGCAGGATGAAGGCTTTCGGGTCGTAAACTGCTTTTATAGGTGAAGATTATGACGGTAACCTATGAATAAGGATCGGCTAATTACGTGCCAGCAGCCGCGGTCATACGTAAGATCCAAGCGTTATCCGGAATTACTGGGTGTAAAGAGTTGCGTAGGTGGCATAGTAAGTAGGGAGTGAAAGCGTTCGGCTCAACCGAATAAACATTTCTTAAACTGCTAAGCTAGAGAATGAGAGAGGTCACTGGAATTCCAAGTGTAGGAGTGAAATCCGTAGATATTTGGAGGAACACCGATGGCGTAGGCAGGTGACTGGCTCATTTCTGACACTAAGGCACGAAAGCGTGGGTAGCAAACAGG` |
| ASV3614 | 429 | `TGGGGAATATTGGACAATGGGCGCAAGCCTGATCCAGCCATGCCGCGTGTGTGAAGAAGGCCTTCGGGTTGTAAAGCACTTTAAGCGAGGAGGAAGGTCTTTGAACTAATATTTCAAAGAATTGACGTTACTCGCAGAATAAGCACCGGCTAACTCTGTGCCAGCAGCCGCGGTAATACAGAGGGTGCAAGCGTTAATCGGAATTACTGGGCGTAAAGCGCGCGTAGGCGGTATGTTAAGTCGGATGTGAAATCCCTGGGCTCAACCTAGGCATTGCATCCGATACTGGCACACTAGAGTGTGGGAGAGGAAGGTAGAATTCCAGGTGTAGCGGTGAAATGCGTAGAGATCTGGGGGAATACCGATGGCGAAGGCAGCCTTCTGGCCTGACACTGACGCTGAGGTGCGAAAGCATGGGGAGCAAACAGG` |
| ASV1027 | 429 | `TGGGGAATTTTGGACAATGGGCGCAAGCCTGATCCAGCCATGCCGCGTGCGGGAAGAAGGCCTTCGGGTTGTAAACCGCTTTTGTTAGGGAAGAAACGCTCTGGGTTAATACCCTGGGGTAATGACGGTACCTAAAGAATAAGCACCGGCTAACTACGTGCCAGCAGCCGCGGTAATACGTAGGGTGCGAGCGTTAATCGGAATTACTGGGCGTAAAGCGTGCGCAGGCGGTTTTGTAAGACAGATGTGAAATCCCCGGGCTCAACCTGGGAACTGCATTTGTGACTGCAAGGCTAGAGTACGGTAGAGGGGGGTGGAATTCCGCGTGTAGCAGTGAAATGCGTAGATATGCGGAGGAACACCGATGGCGAAGGCAGCCCCCTGGACCTGTACTGACGCTCATGCACGAAAGCGTGGGGAGCAAACAGG` |
| ASV5049 | 429 | `TGGGGAATTTTGGACAATGGGGGCAACCCTGATCCAGCAACGCCGCGTGAATGAAGAAGGCCTTAGGGTTGTAAAGTTCTTTTGTCAGGGAAGAAAAGTGTTAGGTTAATACCTTAGCATGCTGACGGTACCTGAAGAATAAGCACCGGCTAACTACGTGCCAGCAGCCGCGGTAATACGTAGGGTGCGAGCGTTAATCGGAATAACTGGGCGTAAAGAGTGCGTAGGCGGATATTTAAGTTAGATGTGAAATGCCCGAGCTTAACTTGGGACTGGCATTTAATACTGGGTATCTAGAGTTTGTCAGAGGGGGGTAGAATTCCACGTGTAGCAGTGAAATGCGTAGAGATGTGGAGGAATACCAATGGCGAAGGCAGCCCCCTGGGATAGAACTGACGCTGAGGCACGAAAGCGTGGGTAGCAAACAGG` |
| ASV101 | 429 | `TCGAGAATAATTCACAATGGGGGCAACCCTGATGGTGCAACGCCGCGTGGAGGATGAAGGTCTTCGGATTGTAAACTCCTGTCATCCGGGAGCAAGACCTGGCTGTGAATAGCAGACAGGGTTGATAGTACCGGAAGAGGAAGGGACGGCTAACTTCGTGCCAGCAGCCGCGGTAATACGAAGGTCCCGAGCGTTGTTCGGAATCACTGGGCGTAAAGGGAGCGTAGGCGGCGTGGTAAGTCAGATGTGAAATCCCGGGGCTCAACCCCGGAACTGCATCCGATACTGCCGTGCTTGAGTGATGGAGAGGTAGCTGGAATTCACGGTGTAGCAGTGAAATGCGTGGATATCGTGAGGAACACTCGTGGCGAAAGCGAGCTACTGGACATTTACTGACGCTGAGGCTCGAAGGCCAGGGTAGCGAAAGGG` |
| ASV362 | 429 | `TCGAGAATCATTCACAATGGGCGAAAGCCTGATGGTGTGACGCCGCGTGGAGGATGAAGGTCTTCGGATTGTAAACTCCTGTCATGTGAGAGTAAGGTTGGGGTAGTAACTGACTGCCAACTTGAGAGTATCACAAGAGGAAGAGGCGGCTAACTCTGTGCCAGCAGCCGCGGTAATACAGAGGCCTCAAGCGTTGTTCGGAATCACTGGGCGTAAAGGGTGCGTAGGCGGCGCGGAAAGTCAGGGGTGAAATCCCGGAGCTCAACTCCGGAACTGCCCTTGATACTCCCGCGCTTGAGAACTGGAGGGGACATCGGAATTCACGGTGGAGCAGTGAAATGCGTGGATATCGTGAGGAACACTAGTGGCGAAGGCGGATGTCTGGACAGTATCTGACGCTGAGGCACGAAGGCCAGGGGAGCGAACGGG` |
| ASV2225 | 429 | `TGGGGAATATTGGACAATGGGCGCAAGCCTGATCCAGCCATGCCGCGTGGGTGAAGAAGGCCTTCGGGTTGTAAAGCCCTTTTGTCCGGAAAGAAAAGCATCGGGCTAATACCTCGGTGTTCTGACGGTACCGGAAGAATAAGCACCGGCTAACTTCGTGCCAGCAGCCGCGGTAATACGAAGGGTGCAAGCGTTACTCGGAATTACTGGGCGTAAAGCGTGCGTAGGTGGTTCGTTAAGTCTGATGTGAAAGCCCTGGGCTCAACCTGGGAATTGCATTGGATACTGGCGGGCTAGAGTGCGGTAGAGGATGGCGGAATTCCCGGTGTAGCAGTGAAATGCGTAGAGATCGGGAGGAACATCTGTGGCGAAGGCGGCCATCTGGACCAGCACTGACACTGAGGCACGAAAGCGTGGGGAGCAAACAGG` |
| ASV4243 | 404 | `TGGGGAATATTGGACAATGGGGGCAACCCTGATCCAGCAATGCCGCGTGAGTGATGAAGGCCTTAGGGTTGTAAAGCTCTTTCGCCGACGACGATGATGACGGTAGTCGGAGAAGAAGCCCCGGCTAACTTCGTGCCAGCAGCCGCGGTAATACGAAGGGGGCTAGCGTTGCTCGGAATTACTGGGCGTAAAGGGCGCGTAGGCGGACCTGTCAGTCGGGCGTGAAAGCCCGGGGCTCAACCCCGGAATTGCGCTCGATACTTCGGGTCTTGAGTGCCGGAGAGGATGGTGGAATTCCCAGTGTAGAGGTGAAATTCGTAGATATTGGGAAGAACACCGGTGGCGAAGGCGGCCATCTGGACGGCAACTGACGCTGAGGCGCGAAAGCGTGGGGAGCAAACAGG` |
| ASV1825 | 428 | `TGGGGAATTTTGGACAATGGGCGCAAGCCTGATCCAGCCATGCCGCGTGAGTGAAGAAGGCCTTCGGGTTGTAAAGCTCTTTTGTCCGGGAGCAAACCTTTGCACCTAATACGTGTAGAGCTGAGAGTACCGGAAGAATAAGCACCGGCTAACTACGTGCCAGCAGCCGCGGTAATACGTAGGGTGCGAGCGTTAATCGGAATTACTGGGCGTAAAGCGTGCGCAGGTGGTTGTGTAAGCTTGATGTGAAATCCCCGGGCTTAACCTGGGAATGGCATTGAGGACTGCACGGCTAGAGTGTGGCAGAGGGGGGTGGAATTCCGCGTGTAGCAGTGAAATGCGTAGAGATGCGGAGGAACACCGATGGCGAAGGCAGCCCCCTGGGTTAACACTGACACTCATGCACGAAAGCGTGGGGAGCAAACAGG` |
| ASV5141 | 404 | `TGGGGAATATTGGACAATGGGCGAAAGCCTGATCCAGCCATGCCGCGTGAATGATGAAGGCCTTAGGGTTGTAAAGTTCTTTTGGTGGGGAAGATAATGACGGTACCCACAGAATAAGCCCCGGCTAACTTCGTGCCAGCAGCCGCGGTAATACGAAGGGGGCTAGCGTTGTTCGGAATTACTGGGCGTAAAGCGCGCGTAGGCGGTGCTGCAAGTCAGGGGTGAAATCCCAGAGCTCAACTCTGGAATTGCCTTTGAAACTGCAGCGCTGGAATCTCAGAGGGGGTAGTGGAATTTCTAGTGTAGGGGTGAAATCCGTAGATATTAGAAGGAACACCGGTGGCGAAGGCGGCTACCTGGCTGAGTATTGACGCTGAGGCGCGAAAGCGTGGGGATCAAACAGG` |
| ASV786 | 423 | `TAGGGAATATTGGTCAATGGATGCAAGTCTGAACCAGCCATGCCGCGTGCAGGAAGAAGGCCTTCTGGGTTGTAAACTGCTTTTACCAGGGGATAAAACGACCATGCGTGGTTAATTGAAGGTACCTGGTGAATAAGCCACGGCTAACTACGTGCCAGCAGCCGCGGTAATACGTAGGTGGCAAGCGTTGTCCGGATTTATTGGGTTTAAAGGGTGCGTAGGCGGTCTATTAAGTCAGTGCTGAAATACTCCAGCTTAACTGGAGGGGTGGCATTGATACTGATGGACTTGAGTACAGATGAGGTAGGCGGAATTGACGGTGTAGCGGTGAAATGCATAGATATCGTCAAGAACACCGATAGCGAAGGCAGCTTACTGGGCTGTAACTGACGCTGATGCACGAAAGTGTGGGGATCAAACAGG` |
| ASV4347 | 424 | `TGAGGAATATTGGTCAATGGGCGGTAGCCTGAACCAGCCAAGTCGCGTGAGGGAAGACGGCCCTAAGGGTTGTAAACCTCTTTTGCCGGGGAGCAAGGCGGATTACGAGTAATCCGACGGAGAGTACCCGGAGAAAAAGCATCGGCTAACTCCGTGCCAGCAGCCGCGGTAATACGGAGGATGCGAGCGTTATCCGGATTTATTGGGTTTAAAGGGTGCGCAGGCGGAGAGTCAAGCCGGCGGTAAAATTGCGGGGCCCAACCCCGTACCGCCGTCGGAACTGGCTCCCTTGAGTGGACGAGAAGTAAGCGGAATGCGTGGTGTAGCGGTGAAATGCATAGATATCACGCAGAACGCCGATTGCGAAGGCAGCTTACCGGTGTCCAACTGACGCTCAGGCACGAAAGCGTGGGGATCGAACAGG` |
| ASV5314 | 425 | `TAAGGAATATTGGACAATGGTCGGAAGACTGATCCAGCCATGCCGCGTGAAGGAAGAAGGCGCTCTGCGTTGTAAACTTCTTTTGTACAGGAACAAAATCAGGTTTGCGGACTTGACTGAGGGTACTGTAAGAATAAGCACCGGCTAACTCCGTGCCAGCAGCCGCGGTAATACGGAGGGTGCGAGCGTTATCCGGAATCACTGGGTTTAAAGGGTGCGTAGGCGGTCTGATAAGTCAGTCGTGAAATCACGTGGCTTAACCATAGTAACTGCGATTGATACTGTCAGACTTGAATCAAGTTGAGGTAGGCGGAATGAGACATGTAGCGGTGAAATGCATAGATATGTCTTAGAACACCGATTGCGAAGGCAGCTTGCTGGACTTAGATTGACGCTGAGGCACGAAAGCGTGGGGAGCAAACAGG` |
| ASV4464 | 404 | `TAGGGAATTTTGCGCAATGGGCGAAAGCCTGACGCAGCAATTCCGCGTGGAGGATGACGGCTCTCGGGTTGTAAACTCCTTTCGGTGGTGAAGATAATGACGGTAGCCACAAAAGAAGCACCGGCTAACTACGTGCCAGCAGCCGCGGTAATACGTAGGGTGCAAGCGTTGTCCGGATTTATTGGGCGTAAAGCGTCCGCAGGCTGTTTGATAAGTCTAATGTTAAATACCGGAGCTCAACTCCGGAAAAGCATTGGATACTGTCAGGCTAGAGTTTGGTAGAGGTAAGGGGAATTCCTGGTGTAGCGGTGAAATGCGTAGATATCAGGAGGAACACCGGTGGCGAAAGCGCCTTACTGGACCATAACTGACGCTCAGGGACGAAAGCTAGGGTAGCGAAAGGG` |
| ASV2013 | 404 | `TAAGGAATATTGGACAATGGGCGAAAGCCTGATCCAGCCATGCCGCGTGAGTGATGAAGGCCTTCGGGTTGTAAAGCTCTTTTGTCCGGGACGATGATGACGGTACCGGAAGAATAAGCCCCGGCTAACTTCGTGCCAGCAGCCGCGGTAATACGAAGGGGGCAAGCGTTGTTCGGAATCACTGGGCGTAAAGCGTGCGTAGGCGGCATTACAAGTCGGAAGTGAAATCCCTGGGCTTAACCCAGGAACTGCTTTCGAAACTGTTTTGCTTGAATCCGGGAGAGGTTGGCGGAATTCCAAGTGTAGAGGTGAAATTCGCAGATATTTGGAGGAACACCAGTGGCGTAAGCGGCCAACTGGACCGGTATTGACGCTGAGGCACGAAAGCGTGGGGATCAAACAGG` |
| ASV5258 | 429 | `TGGGGAATTTTGGACAATGGGGGCAACCCTGATCCAGCCATTCCGCGTGAGTGAAGAAGGCCTTCGGGTTGTAAAGCTCTTTCGCAAGGGAAGAAAACTTATGATCGAATAAATCATGAGGTTGACGGTACCTTGATAAGAAGCACCGGCTAACTACGTGCCAGCAGCCGCGGTAATACGTAGGGTGCGAGCGTTAATCGGAATTACTGGGCGTAAAGCGTGCGCAGGCGGTTTTGTAAGTCGGATGTGAAATCCCCGGGCTCAACCTGGGAACTGCGTTCGAAACTGCAAGGCTAGAGTGTGTCAGAGGGGGGTAGAATTCCACGTGTAGCAGTGAAATGCGTAGAGATGTGGAGGAATACCAATGGCGAAGGCAGCCCCCTGGGATAACACTGACGCTCATGCACGAAAGCGTGGGGAGCAAACAGG` |
| ASV1154 | 430 | `TGGGGAATATTGGACAATGGGCGAAAGCCTGATCCAGCCATGCCGCGTGTGTGAAGAAGGCCTTTTGGTTGTAAAGCACTTTAAGCGAGGAGGAGGGCACTTAGGTTAATACCCTGATGTGTCGGACGTTACTCGCAGAATAAGCACCGGCTAACTCTGTGCCAGCAGCCGCGGTAATACAGAGGGTGCGAGCGTTAATCGGAATTACTGGGCGTAAAGCGCGCGTAGGCGGTTATGTAAGTTGGATGTGAAATCCCCGGGCTTAACCTGGGCACTGCATTCAAAACTGCATAGCTAGAGTATGGGAGAGGAAGGTAGAATTCCAGGTGTAGCGGTGAAATGCGTAGAGATCTGGAGGAATACCGATGGCGAAGGCAGCCTTCTGGCCTAATACTGACGCTGAGGTGCGAAAGCATGGGGAGCAAACAGG` |
| ASV5195 | 429 | `TAGGGAATATTGCACAATGGGCGAAAGCCTGATGCAGCAACGCCGCGTGAGTGATGAAGGTTTTCGGATCGTAAAGCTCTGTCGTAAGGGAAAAAACAGGCGTAGGTTAATACCCTTGTCTTTGATTGTACCTTACAAGAAAGCACCGGCTAACTTCGTGCCAGCAGCCGCGGTAATACGAGGGGTGCAAGCGTTGTTCGGAATTACTGGGCGTAAAGCGTGTGTAGGTGGTCATGTAAGTCAGATGTGAAAGCCTGGGGCTCAACCCCAGAAGTGCATCTGAAACTATTTGACTTGAGTATGGGAGAGGGTAGCGGAATTCCTGGTGTAGTGGTAAAATACGTAGATATCAGGAGGAACATCGGTGGCGAAGGCGGCTACCTGGCCCAATACTGACACTGAGACACGAAAGCATGGGGATCAAACAGG` |
| ASV733 | 404 | `TGGGGAATATTGGACAATGGGGGCAACCCTGATCCAGCCATGCCGCGTGCGTGAAGAAGGCCTTAGGGTTGTAAAGCGCTTTTGTCAGGGAAGATAATGACTGTACCTGAAGAATAAGCCCCGGCTAACTCCGTGCCAGCAGCCGCGGTAAGACGGAGGGGGCTAGCGTTGTTCGGAATTACTGGGCGTAAAGGGCGCGTAGGCTGTTATGTGTGTCAGGTGTGAAATCCCAGGGCTTAACCTTGGAATGGCATCTGAAACTGCATGACTAGAGTGAGTAAGAGGCGGACGGAATTCCGAGTGTAGCAGTGAAATGCGCAGATATTCGGAGGAACATCGGTGGCGAAGGCGGTTCGCTGGTACTCTACTGACGCTGAGGCGCGAAAGCGTGGGGAGCAAACAGG` |
| ASV4081 | 429 | `TGGGGAATTTTGGACAATGGGCGAAAGCCTGATCCAGCCATGCCGCGTGCGGGATGAAGGCCTTCGGGTTGTAAACCGCTTTTGTTGGGGAAGAAATAGTCCGGGCTAATACCCTGGAAAGATGACGGTACCCAAAGAATAAGCACCGGCTAACTACGTGCCAGCAGCCGCGGTAATACGTAGGGTGCAAGCGTTAATCGGAATTACTGGGCGTAAAGCGTGCGCAGGCGGTCTTGCAAGACAGGTGTGAAATCCCAGGGCTTAACCTTGGAACTGCATTTGTGACTGCAAGGCTGGAGTACGGCAGAGGGGGATGGAATTCCGCGTGTAGCAGTGAAATGCGTAGAGATGCGGAGGAACACCGATGGCGAAGGCAATCCCCTGGGCCTGTACTGACGCTCATGCACGAAAGCGTGGGGAGCAAACAGG` |
| ASV980 | 429 | `TGGGGAATTTTGGACAATGGGGGCAACCCTGATCCAGCCATGCCGCGTGAGTGAAGAAGGCCTTCGGGTTGTAAAGCTCTTTCGGCCGGGAAAAAATCGCATAGGTTAATACCCTGTGTGGATGATGGTACCGGAATAAGAAGCACCGGCTAACTACGTGCCAGCAGCCGCGGTAATACGTAGGGTGCGAGCGTTAATCGGAATTACTGGGCGTAAAGCGTGCGCAGGCGGTTTTTTAAGACAGGCGTGAAATCCCCGGGCTCAACCTGGGAACTGCGCTTGTGACTGGAAGGCTAGAGTACGGCAGAGGGGGGTGGAATTCCACGTGTAGCAGTGAAATGCGTAGAGATGTGGAGGAACACCAATGGCGAAGGCAGCCCCCTGGGTCGATACTGACGCTCATGCACGAAAGCGTGGGTAGCAAACAGG` |
| ASV4306 | 429 | `TGGGGAATTTTGGACAATGGGGGCAACCCTGATCCAGCCATGCCGCGTGCGGGAAGAAGGCCTTCGGGTTGTAAACCGCTTTCGGCCGGAACGAAATCGCCGGGGAGAACATCTCCGGTGGATGACGGTACCGGAAGAAGAAGCACCGGCTAACTACGTGCCAGCAGCCGCGGTAATACGTAGGGTGCGAGCGTTAATCGGAATTACTGGGCGTAAAGCGTGCGCAGGTGGTTCGCTAAGACAGATGTGAAATCCCCGGGCTTAACCTGGGAACTGCGTTTGTGACTGGCGGGCTAGAGTGCGGAAGAGGGAGGTGGAATTCCACGTGTAGCAGTGAAATGCGTAGAGATGTGGAGGAACACCGATGGCGAAGGCAGCCTCCTGGTCTAGCACTGACGCTCATGCACGAAAGCGTGGGGAGCAAACAGG` |
| ASV4731 | 410 | `TGGGGAATATTGCACAATGGGCGCAAGCCTGATGCAGCGACGCCGCGTGAGGGATGACGGCCTTCGGGTTGTAAACCTCTGTTAGCAGGGAAGAAGAGAGATTGACGGTACCTGCAGAGAAAGCGCCGGCTAACTACGTGCCAGCAGCCGCGGTAATACGTAGGGCGCGAGCGTTGTCCGGAATTATTGGGCGTAAAGAGCTTGTAGGCGGTTTGTCGCGTCTGCTGTGAAAGGCCGGGGCTTAACCCCGTGTATTGCAGTGGGTACGGGCAGACTAGAGTGCAGTAGGGGAGACTGGAATTCCTGGTGTAGCGGTGGAATGCGCAGATATCAGGAGGAACACCGATGGCGAAGGCAGGTCTCTGGGCTGTAACTGACGCTGAGAAGCGAAAGCATGGGGAGCGAACAGG` |
| ASV3937 | 405 | `TTAAGAATCTTGCTCAATGGGGGAAACCCTGAAGCAGCGACGCCGCGTGAACGATGAAGGTCTTCGGATTGTAAAGTTCAATAAGGAGGGAAAAAAAAGATGATGGTACCTCCCTAAAGCACCGGCTAACTACGTGCCAGCAGCCGCGGTAATACGTATGGTGCAAGCGTTGTTCGGAATCATTGGGCGTAAAGGGTATGTAGGCGGAATGATAAGTCAGAAGTGAAAACTACGAGCTCAACTCGTAGCCTGCTTTTGATACTGTTATTCTAGAGTTTGGAAGGGGCAAGTGGAATTCCTGGTGTAGCGGTGAAATGCGTAGATATCAGGAGGAACACCAGTGGCGTAAGCGACTTGCTGGTCTGGAACTGACGCTGAGATACGAAAGTGTGGGGAGCAAACGGG` |
| ASV5700 | 430 | `TAGGGAATATTGCGCAATGGAGGAAACTCTGACGCAGCGACGCCGCGTGGGTGATGAAGGCCTTCGGGTCGTAAAGCCCTGTCGGTAGGGAAGAAAAATGATGTAGCTAACATCTACGTCACTTGACGGTACCTGCAAAGGAAGCACCGGCTAACTACGTGCCAGCAGCCGCGGTAATACGTAGGGTGCTAGCGTTGTTCGGAATCATTGGGCGTAAAGCGCGTGTAGGCGGCTATCCAAGTCGGGTGTGAAATCCCTGGGCTCAACCGAGGAAGTGCATCCGAAACTAGATAGCTGGAAGATGGTAGAGGAAGGTGGAATTCCTAGTGTAGAGGTGAAATTCGTAGATATTAGGAGGAATACCGGTGGCGAAGGCGGCCTTCTGGACCATTCTTGACGCTAAGACGCGAAAGCGTGGGGAGCAAACAGG` |
| ASV5703 | 429 | `TGGGGAATATTGGACAATGGGGGCAACCCTGATCCAGCAATGCCGCGTGTGTGAAGAAGGCCTGCGGGTTGTAAAGCACTTTCGGTGGGGAGGAGGACTGTTAGGTTAAGAGCTAGATAGTTGGACGTTACCCAAAGAAGAAGCACCGGCTAACTCCGTGCCAGCAGCCGCGGTAATACGGAGGGTGCGAGCGTTAATCGGAATTACTGGGCGTAAAGGGTGCGTAGGCGGTTTAATAAGTTAAGTGTGAAATCCCCGGGCTTAACCTGGGTTGGTCATTTAAGACTGTTAGACTAGAGTATGGGAGAGGGTAGTGGAATTTCCGGTGTAGCGGTGAAATGCGTAGAGATCGGAAGGAACACCAGTGGCGAAGGCGGCTACCTGGCCTAATACTGACGCTGAGGCACGAAAGCATGGGGAGCAAACAGG` |
| ASV2492 | 405 | `TGGGGAATTTTCCGCAATGGGGGCAACCCTGACGGAGCAATACCGCGTGAGGGACGAAGGCTTTTGGGTCGTAAACCTCTTTTGTTTGGGAAGAAACCTGACGGTACCAAACGAATAAGCATCGGCTAACTCCGTGCCAGCAGCCGCGGTAAGACGGAGGATGCAAGCGTTATCCGGAATTATTGGGCGTAAAGCGTCCGCAGGTGGTTTTACAAGTCTGCTGTCAAAGCGCGAGGCTCAACCTTGTATAGGCAGTGGAAACTGTAGGACTAGAGTTCGGTAGGGGTAGAGGGAATTCCCGGTGTAGCGGTGAAATGCGTAGATATCGGGAAGAACACCAGTGGCGAAGGCGCTCTACTGGGCCTGAACTGACACTGAGGGACGAAAGCTAGGGGAGCGAAAGGG` |
| ASV4600 | 429 | `TGGGGAATATTGCACAATGGGGGAAACCCTGATGCAGCCATGCCGCGTGTGTGAAGAAGGCCTTCGGGTTGTAAAGCACTTTCAGTAGGGAGGAAGGGTTGATGTCTAATACGCATCAGCATTGACGTTACCTACAGAAGAAGCACCGGCTAACTCCGTGCCAGCAGCCGCGGTAATACGGAGGGTGCAAGCGTTAATCGGAATAACTGGGCGTAAAGCGCACGCAGGCGGTTAGATAAGTCAGATGTGAAATCCCCGGGCTCAACCTGGGAACTGCATTTGAAACTGTCTGACTAGAGTCTTGTAGAGGGGGGTAGAATTCCAGGTGTAGCGGTGAAATGCGTAGAGATCTGGAGGAATACCGGTGGCGAAGGCGGCCCCCTGGACAAAGACTGACGCTCAGGTGCGAAAGCGTGGGGAGCAAACAGG` |
| ASV5538 | 404 | `TGAGGAATCTTCCGCAATGGACGAAAGTCTGACGGAGCGACGCCGCGTGGAGGATGACGCGTTTACTCGTGTAAACTCCTTTTCTGCGGGAAGATAATGACGGTACCGCAGGAATAAGGGGCGGCAAACTACGTGCCAGCAGCCGCGGTGATACGTAGGCCCCAAGCGTTATCCGGAATTACTGGGCGTAAAGCGTTCGTAGGCGGCATGGAAAGTCAGATGTGAAAGACCGGGGCTTAACCCCGTGTTTGCATCCGAAACTTCCAAGCTAGAATGAGGGAGAGGTAAGCGGAACGGTATGAGTAGGGGTGCAATCCGTTGATACATACCAGAACACCAAAAGCGAAGGCAGCTTACTGGAACTCTATTGACGCTGAGGAACGAAAGCGTGGGGAGCGAAAAGG` |
| ASV2899 | 404 | `TGAGGAATATTGGACAATGGACGCAAGTCTGATCCAGCCATGCCGCGTGAGTGATGAAGGCCTTCGGGTTGTAAAACTCTTTAAGTTGGGAAGATAATGACGGTACCAACAGAATAAGCCCCGGCTAACTTCGTGCCAGCAGCCGCGGTAATACGAAGGGGGCTAGCGTTACTCGGAATTACTGGGCGTAAAGCGCATGTAGGCGGACTTACAAGTCAGAGGTGAAATCCCGGGGCTCAACCTCGGAACTGCCTTTGAAACTGTAAGTCTTGAGTATGATAGAGGATAGTGGAATTCTTAGTGTAGAGGTGAAATTCGTAGATATTAAGAGGAACACCAGTGGCGAAGGCGACTATCTGGGTCATTACTGACGCTGAGATGCGAAAGCGTGGGGAGCAAACGGG` |
| ASV5303 | 404 | `TAGGGAATCTTGCGCAATGGGCGAAAGCCTGACGCAGCAACGCCGCGTGCGGGAAGAAGGCTCTCGGGTTGTAAACCGCTTTTGGCAGGAACGAAAATGACGGTACCTGCAGAATAAGGTGCGGCCAACTACGTGCCAGCAGCCGCGGTGACACGTAGGCACCAAGCGTTGTCCGGATTTATTGGGCGTAAAGAGCTCGTAGGCGGTTCAGTTAGTCGGGTGTGAAAACTCTGGGCTTAACCCAGAGACGCCACCCGATACTGCTGAACTTGAGTACGGTAGGGGAGTGGGGAATTCCTGGTGTAGCGGTGAAATGCGCAGATATCAGGAGGAACACCGGTGGCGAAGGCGCCACTCTGGGCCGAAACTGACGCTGAGGAGCGAAAGCGTGGGTAGCAAACAGG` |
| ASV3209 | 424 | `TGAGGAATATTGGTCAATGGACGCAAGTCTGAACCAGCCATGCCGCGTGCAGGATGACGGTCCTATGGATTGTAAACTGCTTTTGTACGAGAAGAAACACTGCTACGTGTGGCAGCTTGACGGTATCGTAAGAATAAGGATCGGCTAACTCCGTGCCAGCAGCCGCGGTAATACGGAGGATCCAAGCGTTATCCGGAATCATTGGGTTTAAAGGGTCCGTAGGCGGTTTAGTAAGTCAGTGGTGAAAGCCCATCGCTCAACGGTGGAACGGCCATTGATACTGCTAAACTTGAATTATTAGGAAGTAACTAGAATATGTAGTGTAGCGGTGAAATGCTTAGAGATTACATGGAATACCAATTGCGAAGGCAGGTTACTACTAATGGATTGACGCTGATGGACGAAAGCGTGGGTAGCGAACAGG` |
| ASV2036 | 404 | `TGGGGAATATTGGACAATGGGCGCAAGCCTGATCCAGCAATACCACGTGAGTGATGAAGGCCTTAGGGTTGTAAAACTCTTTTAGTGGGGAAGATAATGACTGTACCCACAGAAAAAGCACCGGCTAACTTCGTGCCAGCAGCCGCGGTAATACGAAGGGTGCTAGCGTTGTTCGGAATCATTGGGCGTAAAGCGTGCGTAGGCGGCTTGACAAGTCAGCTGTGAAAGCCCAGGGCTCAACCTTGGAATTGCAGTTGAAACTGTTTCGCTAGAGTCTGAAGAGGGATAGTGGAATTCTTAGTGTAGGGGTGAAATCCGTAGATATTAAGAGGAACACCGGTGGCGAAAGCGACTATCTATGTCAGTACTGACGCTGTTGCACGAAAGCGTGGGGAGCAAACAGG` |
| ASV663 | 429 | `TGGGGAATTTTGGACAATGGGCGCAAGCCTGATCCAGCAATGCCGCGTGCAGGATGAAGGCCTTCGGGTTGTAAACTGCTTTTGTATGGAACGAAACGGTGAGCTCTAATAAAGTTCGCTAATGACGGTACCATAAGAATAAGCACCGGCTAACTACGTGCCAGCAGCCGCGGTAATACGTAGGGTGCAAGCGTTAATCGGAATTACTGGGCGTAAAGCGTGCGCAGGCGGTGATGTAAGACAGATGTGAAATCCCCGGGCTCAACCTGGGACCTGCATTTGTGACTGCATCGCTAGAGTACGGTAGAGGGGGATGGAATTCCGCGTGTAGCAGTGAAATGCGTAGATATGCGGAGGAACACCGATGGCGAAGGCAATCCCCTGGACCTGTACTGACGCTCATGCACGAAAGCGTGGGGAGCAAACAGG` |
| ASV5563 | 402 | `TTAGGAATCTTCGTCAATGGGCGAAAGCCTGAACGAGCGATACCGCTTGAAGGATGAAGCCTTTCGGGGTGTAAACTTCTGAAACAGGGACGATAATGACGGTACCTGTGTAATAGCACCGGCTAACTCCGTGCCAGCAGCCGCGGTAATACGGAGGGTGCGAGCGTTACCCGGATTTACTGGGTGTAAAGGGCGTGTAGGCGGTTTCTCAAGTCCGGTGCTAAAGACTGGGGCTCAACCCCAGGAGTGCGCTGGATACTGTGAAGCTAGACGGTCGGAGAGGGTAGCGGAATTCTCGGAGTAGCGGTGAAATGCGCAGATACCGAGAGGAACGCCAATAGCGAAAGCAGCTACCTGGACGATTTGTGACGCTGAGGCGCGAAAGCGTGGGGAGCAAACCGG` |
| ASV4386 | 375 | `TCGGGAATCTTGCGCAATGGGCGAAAGCCTGACGCAGCGACACCGCGTGAAGGATGACGGCCTTCGGGTTGTAAACTTCTGTAAATAGCACCGGCTAACTACGTGCCAGCAGCAGCGGTCATACGTAGGGTGCGAGCATTATCCGGATTTATTGGGCGTAAAGAGTTCTGTAGGCGGTATTGTAAGTCAGGGGTTAAATCTTCCAGCTCAACTGGAAAACTGCTTCTGATACTGCAGTACTAGAGTCAGTTAGGGGATAGCAGAACCTATGGTGAAGCAGTGAAATGCGTTGATATCATAGGGAATACCAAGGGGGAAGCCAGCTATCTGGGACTGTACTGACGCTGAGAGACGAAAGCGTGGGGAGCGAAACGG` |
| ASV5170 | 429 | `TCGAGGATCTTTCGCAATGGGCGAAAGCCTGACGAAGCGACGCTGTGTGAGCGATGAAGGCCTTCGGGTTGTAAAGCTCTTTCGCATAAGAACAAGAGAGGTTGGTGAATAACCAGCCGATTTGAGTGTACTATGTAAAGAAGCACCGGCTAACTCCGTGCCAGCAGCTGCGGTAATACGGAGGGTGCAAGCATTAATCGGATTTATTGGGCGTAAAGGGCGCGTAGGCGGGTTTGTAAGTCAGATGTGAAATTCCGAAGCTCAACTTCGGAGCTGCATTTGAAACTACAAGTCTAGAGGAAAGGCGGAGAAAATGGAATTCCACGTGTAGCGGTGAAATGCGTAGATATGTGGAAGAACACCGGTGGCGAAAGCGGTTTTCTAGCTTATTCCTGACGCTGAGGCGCGAGAGCAAGGGGAGCAAACAGG` |
| ASV4574 | 429 | `TGGGGAATATTGGACAATGGGGGAAACCCTGATCCAGCAATACCGCGTGTGTGAAGAAGGCCTATGGGTTGTAAAGCACTTTCAGTGGGGAGGAAAGCGTTGAAGTTAATACCTTCGATGTATGACGCTACCCACAGAAGAAGCACCGGCTAACTCCGTGCCAGCAGCCGCGGTAATACGGAGGGTGCGAGCGTTAATCGGAATTACTGGGCGTAAAGAGTGCGTAGGTGGTTATGTGTGTTAGATGTGAAAGCCATGGGCTTAACCTGTGAATTGCATTTAAAACTGCATAGCTAGAGTGTTGTAGAGGGTGGCGGAATTTCCAGTGTAGCGGTGAAATGCGTAGATATTGGAAGGAACACCAGTGGCGAAGGCGGCCACCTGGACAAACACTGACACTGAGGCACGAAAGCGTGGGTAGCGAACAGG` |
| ASV3839 | 426 | `TGGGGAATTTTGCACAATGGAGGAAACTCTGATGCAGCAACGCCGCGTGAGTGAAGAAGGCCTTTGGGTCGTAAAGCTCTTTCGGCCGGGAAAAAGGGTATTGGGTAAACCCCAGTATTTGATGGTACCGGAGTAAGAAGCACCTGCTAACTACGTGCCAGCAGCAGCGGTAATACGTAGGGTGCAAGCGTTGTTCGGAATTACTGGGCGTAAAGGGCGCGTAGGCTGGTTTGTAAGTTGTTCGTGAAATTCCTGGGCTTAACCTGGGCGCTGCGGACAATACTGCAGATCTTGAGTGCTTGAGAGGATGGCGGAATTCCAGGTGTAGAAGTGAAATTCGTAGATATCTGGAAGAACACCTGAGGCGAAGGCGGCTGTCTGGCAAGACACTGACGCTGAGGCGCGAAAGCGTGGGGATCAAACAGG` |
| ASV3660 | 408 | `TGAGGAATCTTCCGCAATGGGCGAAAGCCTGACGGAGCGACACCGCGTGGAGGATGAAGCCCTTCTTGGGCGTAAACTCCTGTTCTGAGGGAACAAATTTTTGAGTGTACCTCAGGAGAAAGCATCGGCTAACCCTGTGCCAGCAGCCGCGGTAAGACAGGGGATGCAAGCGTTACTCGGAATTACTGGGCGTAAAGCGTCTGTAGGCGTCCCGTGAAGTCCGGCGTGAAAGTGCGAAGCTCAACTTCGTGAACGTGCTGGAAACTGGCGGGATCGAGATACTCAGAGGTGCCTGGAATGCTGTGTGTAGGGGTAAAATCCGTAGATACACAGTAGAACGCTCAAAGCGAAGGCAGGGCACTGGGGGTGTTCTGACGCTGAGAGACGAAAGCGTGGGGAGCAAACCGG` |
| ASV4882 | 429 | `TGGGGAATATTGGACAATGGGCGCAAGCCTGATCCAGCAATGCCGCGTGGGTGAAGAAGGCCTGCGGGTTGTAAAGCCCTTTAAGCGGGGAGGAAAGCCGGGGAGCGAATAGCGCCTCGGGGTGACGTTACCCGCAGAATAAGCACCGGCTAACTCCGTGCCAGCAGCCGCGGTAATACGGGGGGTGCGAGCGTTAATCGGAATGACTGGGCGTAAAGCGTGCGTAGGCGGTGTAATCAGTCAGCCGTGAAAGCCCCGGGCTTAACCTGGGAATGGCGGTTGAGACGGTTAAACTAGAGTGGGCTAGAGGACTGTGGAACTCCCGGTGTAGCGGTGAAATGCGTAGAGATCGGGAAGAACACCGATGGCGAAGGCAGCAGTCTGGGGCCACACTGACGCTGAGGCACGACGGCGTGGGGAGCAAACAGG` |
| ASV3226 | 421 | `TGAGGAATATTGCGCAATGGACGGAAGTCTGACGCAGCAATGCCGCGTGCGGGATGAAGGCGCTATGCGTCGTAAACCGCTGTAGCGTGGGGCAAAAAGCACCCTTGGGGTGTTAGATGGTACCACGAAAGTAAGCATCGGCTAACTACGTGCCAGCAGCCGCGGTAATACGTAGGATGCAAGCGTTGTCCGGATTTACTGGGTGTAAAGGGTGCGCAGGCGGGCTTGTAAGTCAGAGGTGAAAGCCCGGAGCTTAACTCCGGAAATGCCTTTGATACTGCGAGTCTTGAATATGAGAGAGGTTCATGGAATATGTGGTGTAGCGGTGAAATGCGTAGATATCACATAGAACACCAGTGGCGAAGGCGGTGAACTGGCTCATTATTGACGCTCAGGCACGAAAGTGTGGGTAGCAAACAGG` |
| ASV4225 | 418 | `TCGAGAATATTCGACAATGGGCGAAAGCCTGATCGAGCGACGCCGCGTGCAGGAAGAAGGCCTTCGGGTCGTAAACTGCTTTTCATAGGGAGGAACCCCGTTTTGCGGGATGACTGTACCTATGGAATAAGGAGCTGCAAACCATGTGCCAGCAGCCGCGGTAATACATGGGCTCCAAGCGTTATCCGGATTTATTGGGCGTAAAGGGTCTCCAGGCGGCCTGGTTAGTCGGGCGTTAAATCCCACTGCTTAACGGTGGGGCTGCGTTCGAAACGGCCAGGCTTGAGGGATGGAGAGGGAGTAGGAACTCACGGTGTAGGGGTGAAATCCGTTGATATCGTGGGGAACACCAAAGGCGAAGGCAAACTCCTGGCCATCTCCTGACGCTCATGAGACGAAAGCGTGGGGAGCGAATAGG` |
| ASV4336 | 424 | `TAAGGAATATTGGACAATGCCCGCAAGGGTGATCCAGCCATGCCGCGTGCAGGAAGACGGCCCTATGGGTTGTAAACTGCTTTTATACAAGAGAAAACCTCCCTACGTGTAGGGAGCTGATAGTATTGTAGGAATAAGCATCGGCTAACTTCGTGCCAGCAGCCGCGGTAAGACGAAGGATGCAAGCGTTATCCGGATTCATTGGGTTTAAAGGGAGCGTAGGCGGTCTTATAAGTCAGTGGTGAAAGCTCCTCGCTTAACGAGGAAATTGCCATTGATACTGTAGGACTTGAGTACAGTTGCTGTGGGCGGAATATGACATGTAGTGGTGAAATACATAGAGATGTCATAGAACACCGATTGCGAAGGCAGCTCACAAAACTGTAACTGACGCTGAGGCTCGAAAGTGCGGGGATCAAACAGG` |
| ASV4442 | 407 | `TTAGGAATTTTGCGCAATGGGGGAAACCCTGACGCAGCAACGCCGCGTGAGTGATGAAGGCCTTCGGGTTGTAAAGCTCTTTTAGCTGGAAAGAATAAAATGACGGTACCAGCAGAAAAAGCACCGGCTAACTCCGTGCCAGCAGCCGCGGTAATACGGAGGGTGCAAGCGTTACTCGGAATTATTGGGCGTAAAGCGCGTGTAGGCGGTTTGGTAAGTCTGATGTGAAATCCCTGGACTCAATTCAGGAAGTGCATTGGATACTGCTAGACTAGAGTTTTGGAGAGGTGAACGGAATTCCTGGTGTAGAGGTGAAATTCGTAGATATCAGGAGGAACAACAGAGGCGAAGGCGGTTCACTGGACAATAACTGACGCTCAGACGCGAAAGCGTGGGTAGCAAACAGG` |
| ASV2194 | 404 | `TGGGGAATATTGGACAATGGGCGAAAGCCTGATCCAGCCATGCCGCGTGAGTGATGAAGGCCTTAGGGTTGTAAAGCTCTTTTGTCAGGGAAGATAATGACGGTACCTGAAGAATAAGCACCGGCTAACTTCGTGCCAGCAGCCGCGGTAATACGAAGGGTGCTAGCGTTGTTCGGAATTACTGGGCGTAAAGCGCGCGTAGGCGGTCAATCAAGTCAGAGGTGAAAGCCCTGGGCTCAACCCAGGAATTGCCTTTGAAACTGGTTGGCTAGAGTATTGGAGGGGAGTGCGGAATTGCTAATGTAGAGGTGAAATTCGTAGATATTAGCAGGAACACCGGTGGCGAAGGCGGCACTCTGGACAATAACTGACGCTGAGGCGCGAAAGCGCGGGGATCAAACAGG` |
| ASV3694 | 424 | `TAAGGAATATTGGACAATGGAGGGAACTCTGATCCAGCCATGCCGCGTGAAGGATGAAGGCGCTCAGCGTTGTAAACTTCTTTTGGACGGGAACAAAAGCAATCATTTATGATTGTCTGAGGGTACCGTCAGAATAAGCACCGGCTAACTCCGTGCCAGCAGCCGCGGTAATACGGAGGGTGCAAGCGTTATCCGGAATCACTGGGTTTAAAGGGTGCGTAGGCGGTTCGATAAGTCAGTCGTGAAATCGTGTGGCTTAACCATGCAACTGCGATTGATACTGTTGGACTTGAATGAGGTTGAGGTAGGCGGAATGTGACATGTAGCGGTGAAATGCATAGATATGTCATAGAACACCGATTGCGAAGGCAGCTTACTGGGCCTGAATTGACGCTGAGGCACGAAAGCGTGGGGATCAAACAGG` |
| ASV4877 | 384 | `GTGGGAAACCTTTACAATACACGAAAGTGTGATAAGGGAACCCTAAGTGCCTACCAGTGGTGGGCTTTTATCAAGTGTAAATATCTTGATGAATAAGTGGTGGGTAAGACCGGTGCCAGCAGCCGCGGTAACCCCGGCGCCACAAGTGGTAATCACGTTTATTTGGCCTAAAGCGTCCGTAGTCGATGATGCAAGTCTTTTGTTAAATCCTTATGCTCAACATAGGGGCCGCAAGAGATACTACTTCATTAGAGATCGGGAGGAGACAAAGGTATTCCTTGGGTAGCGGTAAAATGCGATAATCCTCGGAAGACCACCAATGGCGAAGGCATTTGTCTAGAACGAATCTGACGATCAGGGACGAAAGCTAGGGGAGCAAAAGGG` |
| ASV2309 | 404 | `TGGGGAATATTGGACAATGGGCGAAAGCCTGATCCAGCAATACCGCGTGAATGATGAAGGCCTTAGGGTTGTAAAGTTCTTTTAGTAGGGAAGATAATGACGGTACCTACAGAAAAAGCCCCGGCTAACTCCGTGCCAGCAGCCGCGGTAAGACGGAGGGGGCTAGCGTTGTTCGGAATTACTGGGCGTAAAGAGCGCGTAGGCGGATTAGTAAGTTGGAAGTGAAAGCCCAAGGCTTAACCTTGGAAATGCTTCCAAAACTGCTAGTCTTGAGTATGGTAGGGGGTGATGGAATTCCTAGTGTAGAGGTGAAATTCTTAGATATTAGGAGGAACACCGGTGGCGAAGGCGGTCACCTGGGCCATAACTGACGCTGAGGCGCGAAAGCGTGGGGAGCAAACAGG` |
| ASV740 | 407 | `TGGGGAATTTTCCGCAATGGGCGCAAGCCTGACGGAGCAATACCGCGTGAGGGAGGAAGGCTCTTGGGTCGTAAACCTCTTTTCTCAGGGAAGAACACAATGACGGTACCTGAGGAATAAGCATCGGCTAACTCCGTGCCAGCAGCCGCGGTAATACGGAGGATGCAAGCGTTATCCGGAATGATTGGGCGTAAAGCGTCCGTAGGTGGTTTTGTAAGTCTGTGGTTAAAGCGTGTAGCTCAACTACATAACGGCCATGGAAACTACATAACTCGAGTCAAGTAGGGGTAGAGGGAATTCCCAGTGTAGCGGTGAAATGCGTAGAGATTGGGAAGAACACCGGTGGCGAAAGCGCTCTACTGGACTTGTACTGACACTGAGGGACGAAAGCTAGGGGAGCGAAAGGG` |
| ASV1081 | 429 | `TGGGGAATTTTGGACAATGGACGCAAGTCTGATCCAGCCATGCCGCGTGCGGGAAGAAGGCCTTCGGGTTGTAAACCGCTTTTGTCAGGGAAGAAACGGCTTTCTCTAATACAGAGAGCTAATGACGGTACCTGAAGAATAAGCACCGGCTAACTACGTGCCAGCAGCCGCGGTAATACGTAGGGTGCAAGCGTTAATCGGAATTACTGGGCGTAAAGCGTGCGCAGGCGGTTATGCAAGACAGATGTGAAATCCCCGGGCTCAACCTGGGAACTGCATTTGTGACTGCATGGCTAGAGTACGGTAGAGGGGGATGGAATTCCGCGTGTAGCAGTGAAATGCGTAGATATGCGGAGGAACACCGATGGCGAAGGCAATCCCCTGGACCTGTACTGACGCTCATGCACGAAAGCGTGGGGAGCAAACAGG` |
| ASV4945 | 428 | `TTTCGAATCATTCACAATGGGCGAAAGCCTGATGGTGCGACGCCGCGTGAGGGATGAAGGTCTTCGGATTGTAAACCTCTGTCACTGGGGAGGAAACTTCTTCACCTAACACGTGTAGAACTGACTTAACCCGGAGAGGAAGCAGTGGCTAACTCTGTGCCAGCAGCCGCGGTAATACAGAGACTGCAAGCGTTATTCGGATTCACTGGGCGTAAAGGGTGCGCAGGCGGCTTTGTGTGTGGGGCGTGAAAGCCCGAGGCTTAACCTCGGAATAGCGCCCCAAACTACAAGGCTAGAGCATTGGAGAGAGTGGCAGAATTCACGGTGTAGCAGTGAAATGCGTAGATATCGTGAGGAATACCAGAGGCGAAGGCGGCCACTTGGACAATTGCTGACGCTCAGGCACGAAAGCGTGGGGAGCAAAAGGG` |
| ASV2856 | 429 | `TGGGGAATTTTGGACAATGGGGGCAACCCTGATCCAGCCATTCCGCGTGAGTGAAGAAGGCCTTCGGGTTGTAAAGCTCTTTTGTCGGGGACGAAACGGTTTCAGTTAATACCTGAGACTACTGACGGTACCCGAAGAATAAGCACCGGCTAACTACGTGCCAGCAGCCGCGGTAATACGTAGGGTGCGAGCGTTAATCGGAATTACTGGGCGTAAAGCGTGCGCAGGCGGTTTTGTAAGCCAGATGTGAAATCCCCGGGCTTAACCTGGGAATGGCATTTGGGACTGCAAGGCTAGAGTATGGCAGAGGAGACTAGAATTCCTGGTGTAGCAGTGAAATGCGTAGATATCAGGAGGAATACCGATGGCGAAGGCAGGTCTCTGGGTCAATACTGACGCTCATGCACGAAAGCGTGGGGAGCAAACAGG` |
| ASV746 | 404 | `TGGGGAATCTTGGACAATGGGCGAAAGCCTGATCCAGCCATGCCGCGTGGATGATGAAGGCCTTAGGGTTGTAAAATCCTTTCAACGGGGACGATAATGACGGTACCCGTAGAAGAAGCCCCGGCTAACTTCGTGCCAGCAGCCGCGGTAATACGAAGGGGGCTAGCGTTGCTCGGAATTACTGGGCGTAAAGCGCACGTAGGCGGATATTTAAGTCGGGGGTGAAATCCCAGAGCTCAACTCTGGAACTGCCTTCGATACTGGGTATCTGGACCAAAGTAGAGGTAAGTGGAATTCCAAGTGTAGAGGTGAAATTCGTAGATATTTGGAGGAACACCAGTGGCGAAGGCGACTTACTGGACTTTTGGTGACGCTGAGGTGCGAAAGCGTGGGGAGCAAACAGG` |
| ASV1752 | 429 | `TGGGGAATTTTGGACAATGGGGGAAACCCTGATCCAGCAATGCCGCGTGTGCGAAGAAGGCCTTCGGGTTGTAAAGCACTTTTGTCAGGGAAGAAAACCTTTGGGCTAATACCCTAAGGGGATGACGGTACCTGAAGAATAAGCACCGGCTAACTACGTGCCAGCAGCCGCGGTAATACGTAGGGTGCGAGCGTTAATCGGAATTACTGGGCGTAAAGCGTGCGCAGGCGGTTCTATAAGACAGGTGTGAAATCCCCGGGCTTAACCTGGGAATTGCACTTGTGACTGTAGAGCTAGAGTGTGTCAGAGGGGGGTGGAATTCCACGTGTAGCAGTGAAATGCGTAGATATGTGGAGGAACACCAATGGCGAAGGCAGCCCCCTGGGATAACACTGACGCTCATGCACGAAAGCGTGGGGAGCAAACAGG` |
| ASV1553 | 429 | `TGGGGAATTTTGGACAATGGGGGCAACCCTGATCCAGCCATGCCGCGTGCGGGAAGAAGGCCTTCGGGTTGTAAACCGCTTTTGGTCGGGAAGAAAAGGTTACCCCTAATAAGGGTGACTGTTGACGGTACCGGCAGAATAAGCACCGGCTAACTACGTGCCAGCAGCCGCGGTAATACGTAGGGTGCGAGCGTTAATCGGAATTACTGGGCGTAAAGCGTGCGCAGGCGGTTTGTTAAGTCTGATGTGAAAGCCCCGGGCTCAACCTGGGAATGGCATTGGAAACTGGCAAGCTTGAGTATGGCAGAGGGGGGTGGAATTCCGCGTGTAGCAGTGAAATGCGTAGAGATGCGGAGGAACACCGATGGCGAAGGCAGCCCCCTGGGCCAATACTGACGCTCAGGCACGAAAGCGTGGGGAGCAAACAGG` |
| ASV1248 | 429 | `TGGGGAATATTGGACAATGGGGGCAACCCTGATCCAGCAATGCCGCGTGTGTGAAGAAGGCCTTAGGGTTGTAAAGCACTTTAGGTTGGGAAGAAGGTGTGTATTTTAAGAGATAGCACATTTGACGGTACCGACAGAATAAGCACCGGCTAACTCTGTGCCAGCAGCCGCGGTAATACAGAGGGTGCGAGCGTTAATCGGAGTTACTGGGCGTAAAGGGCGCGTAGGCGGTTATGTAAGTGAGATGTGAAATCCCTGGGCTTAACCTAGGAACTGCATCTTAAACTGTGTAGCTAGAGTACTGTAGAGGGTAGTGGAATTTCCGGTGTAGCGGTGAAATGCGTAGAGATCGGAAGGAACACCAGTGGCGAAGGCGGCTACCTGGACAGATACTGACGCTGAGGCGCGAGAGCGTGGGGAGCAAACAGG` |
| ASV1992 | 404 | `TGAGGAATCTTCCACAATGGGCGAAAGCCTGATGGAGCGACACCGCGTGAAGGATGAAGCCTTTATTGGTGTAAACTTCTTTTCTGTGGGAAGATAATGACGGTACCACAGGAATAAGGGGCGGCAAACTACGTGCCAGCAGCCGCGGTAATACGTAGGCCCCAAGCGTTATCCGGAATTACTGGGCGTAAAGAGTGCGTAGGCGGAGTGGAAAGTCAGATGTGAAAGGCCGGGGCTTAACCCCGTGTTTGCATCCGAAACTTCCACCCTAGAATCGGGGAGAGGTAAGCAGAATAGTATGAGTAGGGGTGCAATCCGTTGATACATACTAGAATACCAAAAGCGAAGGCAGCTTACTGGAACCGCATTGACGCTGAGGCACGAAAGCGTGGGGAGCGAAAAGG` |
| ASV3625 | 429 | `TGGGGAATTTTGGACAATGGGCGCAAGCCTGATCCAGCAATGCCGCGTGAGTGAAGAAGGCCTTCGGGTTGTAAAGCTCTTTTGTCAGGGAAGAAAAGGGATTGGCTAATATCTGATCCTCATGACGGTACCTGAAGAATAAGCACCGGCTAACTACGTGCCAGCAGCCGCGGTAATACGTAGGGTGCAAGCGTTAATCGGAATTACTGGGCGTAAAGCGTGCGCAGGCGGTTTTGTAAGACTGTCGTGAAATCCCCGGGCTCAACCTGGGAATGGCGATGGTGACTGCAAGGCTAGAGTTTGGCAGAGGGGGGTAGAATTCCACGTGTAGCAGTGAAATGCGTAGATATGTGGAGGAACACCGATGGCGAAGGCAGCCCCCTGGGTCAAAACTGACGCTCATGCACGAAAGCGTGGGGAGCAAACAGG` |
| ASV571 | 429 | `TGGGGAATTTTGGACAATGGGGGCAACCCTGATCCAGCAATGCCGCGTGAGTGAAGAAGGCCTTCGGGTTGTAAAGCTCTTTTGTTAGGGAAGAAAAGGTAGGCAATAATACTGCTTACTGATGACGGTACCTAAAGAATAAGCACCGGCTAACTACGTGCCAGCAGCCGCGGTAATACGTAGGGTGCAAGCGTTAATCGGAATTACTGGGCGTAAAGCGTGCGCAGGCGGCCTATTAAGTCAGATGTGAAATCCCCGAGCTCAACTTGGGAACTGCATTTGAAACTGATAGGCTAGAGTGTGTCAGAGGGAGGTAGAATTCCACGTGTAGCAGTGAAATGCGTAGATATGTGGAGGAATACCAGTGGCGAAGGCGGCCTCCTGGGATAACACTGACGCTCATGTACGAAAGCGTGGGGAGCAAACAGG` |
| ASV4163 | 425 | `TGGGGAATATTGCGCAATGGGCGAAAGCCTGACGCAGCCACGCCGCGTGAGCGATGAAGGATTTCGGTTCGTAAAGCTCTGTGGGGAGAGAAGAAGAAAGTTGGTGAAGAGCCAACCATGACGGTATCTCCTTAGCAAGCACCGGCTAACTCTGTGCCAGCAGCCGCGGTAATACAGAGGGTGCAAACGTTGCTCGGAATTATTGGGCGTAAAGCGCACGTAGGCGGTTTGTTAAGTTGAATGTGAAAACCCTGGGCTCAACCCTGGAAGTGCATCCAAAACTGACAAGCTAGAGTGTCGGAGGGGGTCGGAGAATTCCCGGTGTAGAGGTGAAATTCGTAGATATCGGGAGGAACATCTGTGGCGAAGGCGCCGACCTGGACGACAACTGACGCTGAGGTGCGAAAGCATGGGGAGCAAACAGG` |
| ASV3196 | 404 | `TAAGGAATATTGGACAATGGGGGCAACCCTGATCCAGCCATGCCGCGTGAGTGATGAAGGCCTTCGGGTTGTAAAGCTCTTTTAGATGTGACGATGATGACGGTAGCATCAGAAAAAGCTCCGGCTAACTTCGTGCCAGCAGCCGCGGTAATACGAAGGGAGCGAGCGTTGTTCGGAATCACTGGGCTTAAAGCGTACGTAGGCGGATTTACAAGTCAGGAGTGAAATCCCAGGGCTTAACCCTGGAATTGCTTTTGAAACTGTAAGTCTGGAATCCGGTAGAGGTTGGCGGAACTCCGAGTGTAGAGGTGAAATTCGCAGATATTCGGAAGAACACCAGTGGCGAAGGCGGCCAACTGGACCGGTATTGACGCTGAGGTACGAAAGCGTGGGTAGCAAACAGG` |
| ASV1286 | 404 | `TGGGGAATCTTGGACAATGGGCGAAAGCCTGATCCAGCCATGCCGCGTGGATGATGAAGGCCTTAGGGTTGTAAAATCCTTTCGTTGGGGACGATAATGACGGTACCCAAAGAAGAAGCTCCGGCTAACTTCGTGCCAGCAGCCGCGGTAATACGAAGGGGGCTAGCGTTGTTCGGATTTACTGGGCGTAAAGCGCGTGTAGGCGGACTGGAAAGTTGGGGGTGAAATCCCGAGGCTCAACCTCGGAACTGCCTCCAAAACTTCCAGTCTGGACTCAAGTAGAGGCAAGTGGAATTGCGAGTGTAGAGGTGAAATTCGTAGATATTCGCAGGAACACCAGTGGCGAAGGCGACTTGCTGGACTTGTAGTGACGCTGAGACGCGAAAGCGTGGGGAGCAAACAGG` |
| ASV3127 | 404 | `TGGGGAATCTTGGACAATGGGCGCAAGCCTGATCCAGCCATGCCGCGTGGATGATGAAGGCCCTAGGGTTGTAAAATCCTTTCGTTGGGGACGATAATGACGGTACCCAAAGAAGAAGCTCCGGCTAACTTCGTGCCAGCAGCCGCGGTAATACGAAGGGGGCTAGCGTTGTTCGGATTTACTGGGCGTAAAGCGCGTGTAGGCGGGTCGATAAGTTGGGGGTGAAATCCCGGAGCTCAACTCCGGAACTGCCTTCAAAACTGCCGACCTGGACCTAAGTAGAGGCAAGTGGAATTGCGAGTGTAGAGGTGAAATTCGTAGATATTCGCAGGAACACCAGTGGCGAAGGCGACTTGCTGGACTTATGGTGACGCTGAGACGCGAAAGCGTGGGGAGCAAACAGG` |
| ASV3471 | 424 | `TAAGGAATATTGGTCAATGGAGGCAACTCTGAACCAGCCATGCCGCGTGCAGGAAGACGGTCCTATGGATTGTAAACTGCTTTTGTACCAGAGAAAACCTACTCACGTGTGAGTAGCTGATAGTATGGTAAGAATAAGGATCGGCTAACTTCGTGCCAGCAGCCGCGGTAATACGAAGGATCCAAGCGTTATCCGGATTCATTGGGTTTAAAGGGTGCGTAGGCGGATTAGTAAGTCAGTGGTGAAATCTCCTCGCTTAACGAGGAAACTGCCATTGATACTGCAAATCTAGAGTACAGATGATGTTGGCGGAATGTGTCATGTAGCGGTGAAATGCTTAGATATGACACAGAACACCGATTGCGAAGGCAGCTGACAAAACTGTAACTGACGCTGATGCACGAAAGTGCGGGGATCAAACAGG` |
| ASV286 | 429 | `TGGGGAATTTTGGACAATGGGCGCAAGCCTGATCCAGCAATGCCGCGTGCAGGATGAAGGCCTTCGGGTTGTAAACTGCTTTTGTACGGAACGAAACGGCTCTGGTTAATACCCGGAGCTAATGACGGTACCGTAAGAATAAGCACCGGCTAACTACGTGCCAGCAGCCGCGGTAATACGTAGGGTGCAAGCGTTAATCGGAATTACTGGGCGTAAAGCGTGCGCAGGCGGTTATGTAAGACAGACGTGAAATCCCCGGGCTCAACCTGGGAACTGCGTTTGTGACTGCATAGCTAGAGTACGGTAGAGGGGGGTGGAATTCCGCGTGTAGCAGTGAAATGCGTAGATATGCGGAGGAACACCGATGGCGAAGGCAGCCCCCTGGACCTGTACTGACGCTCATGCACGAAAGCATGGGGAGCAAACAGG` |
| ASV1633 | 429 | `TGGGGAATATTGGACAATGGGGGGAACCCTGATCCAGCAATGCCGCGTGTGTGAAGAAGGCCTGAGGGTTGTAAAGCACTTTCAGTGGGGAGGAGGGTTGATTGGTTAAGAGCTGGTTAATTGGACGTTACCCACAGAAGAAGCACCGGCTAACTCCGTGCCAGCAGCCGCGGTAATACGGAGGGTGCGAGCGTTAATCGGAATTACTGGGCGTAAAGGGTGCGTAGGTGGTTGCATAAGTTATCTGTGAAATTCCTGGGCTTAACCTGGGGTGGTCAGATAAGACTGTGCGACTCGAGTATGGGAGAGGGTAGTGGAATTTCCGGTGTAGCGGTGAAATGCGTAGAGATCGGAAGGAACACCAGTGGCGAAGGCGGCTACCTGGCCTAATACTGACACTGAGGCACGAAAGCGTGGGGAGCAAACAGG` |
| ASV1713 | 404 | `TTGGGAATCTTGGACAATGGGCGAAAGCCTGATCCAGCCATGCCGCGTGAGTGATGAAGGCCTTAGGGTTGTAAAGCTCTTTTGTCAGGGAAGATAATGACTGTACCTGAAGAATAAGCTCCGGCTAACTTCGTGCCAGCAGCCGCGGTAATACGAAGGGGGCTAGCGTTGTTCGGAATTACTGGGCGTAAAGCGCGCGCAGGCGGCTTTTCAAGTCGAGGGTGAAAGCCCAGAGCTCAACTCTGGAATTGCCTTCGAAACTGGAAAGCTTGAGTACGAGAGAGGTGAGTGGAATTCCCAGTGTAGAGGTGAAATTCGTAGATATTGGGAAGAACACCGGTGGCGAAGGCGGCTCACTGGCTCGTTACTGACGCTCAGGCGCGATAGCGTGGGGATCAAACAGG` |
| ASV241 | 404 | `TGGGGAATATTGGACAATGGGCGCAAGCCTGATCCAGCCATGCCGCGTGTGTGATGAAGGCCTTAGGGTTGTAAAGCACTTTTGTCCGGGAAGATAATGACTGTACCGGAAGAATAAGCCCCGGCTAACTTCGTGCCAGCAGCCGCGGTAATACGAAGGGGGCTAGCGTTGCTCGGAATCACTGGGCGTAAAGGGCGCGTAGGCGGACTCTTAAGTCGGGGGTGAAAGCCCAGGGCTCAACCCTGGAATTGCCTTCGATACTGAGAGTCTTGAGTTCGGAAGAGGTTGGTGGAACTGCGAGTGTAGAGGTGAAATTCGTAGATATTCGCAAGAACACCAGTGGCGAAGGCGGCCAACTGGTCCGATACTGACGCTGAGGCGCGAAAGCGTGGGGAGCAAACAGG` |
| ASV4341 | 404 | `TAAGGAATATTGGACAATGGGGGAAACCCTGATCCAGCCATGCCGCGTGAGTGACGAAGGCCTTAGGGTTGTAAAGCTCTTTTGGTGGGGAAGATAATGACTGTACCCACAGAATAAGCCCCGGCTAAATTCGTGCCAGCAGCCGCGGTAATACGAATGGGGCAAGCGTTGTTCGGAATCACTGGGCGTAAAGCGTGCGTAGGCGGTTTTGCAAGTCAGAAGTGAAAGCCCAGGGCTCAACCCTGGAATTGCTTTTGAAACTGCATTACTTGAATCCCGGAGAGGTTAGTAGAATTCCGAGTGTAGAGGTGAAATTCGCAGATATTCGGAGGAATACCAGTGGCGTAAGCGGCTAACTGGACGGGCATTGACGCTGAGGTACGAAAGCGTGGGGATCAAACAGG` |
| ASV3859 | 428 | `TAGGGAATCTTGCGCAATGGGCGAAAGCCTGACGCAGCGACGCCGCGTGAGTGATGAAGGCCTTCGGGTTGTAAAGCTCTGTGGGGAGAGAAGAATAAGTGCAAGCTAATACTTTGCACGATGACGGTATCTCCTTAGCAAGCACCGGCTAACTCTGTGCCAGCAGCCGCGGTAAGACAGAGGGTGCAAACGTTGTTCGGAATCACTGGGCGTAAAGCGTGTGTAGGCGGCTTCTCAAGTCGGGTGTGAAATCCCAGGGCTCAACCCTGGAAGTGCATTCGAAACTGGGTAGCTAGAGTACTGGAGAGGAAGGTGGAATGCTTGGTGTAGAGGTGAAATTCGTAGATATCAAGCGGAACACCGGTGGCGAAGGCGGCCTTCTGGACAGATACTGACGCTGAGACACGAAAGCGTGGGGAGCAAACAGG` |
| ASV5336 | 391 | `GCGCGAAAACTTTCCAATGCACGAAAGTGTGAGAAGGGAATCCCAAGTGCTTCGTCATTTAATGGCGAAGCTTTTATCGAGTGTAAGACTCTCGGTGAATAAGTGGTGGGTAAGACGGGTGCCAGCCTCCGCGGTAATACCCGCGCCACAAGTGGTGATCGACTTTATTGGGTCTAAAGCGTCCGTAGCAGGTCATGTATGTCTCTTGTGAAATTGCTTCGCTCAACGAAACATCGGGCAAGGGAAACTGTATGACTTGAGACCGGGAGGAGATAGGAGTATTCCGTGGGGAGCGGTAAAATGTGATAATCCTCGGAGGACTACCTGTGGCGAAGGCGCCTATCTAGAACGGTTCTGACTGTGAGGGACGAAAGCTAGGGGATCAAAACGG` |
| ASV1140 | 424 | `TAAGGAATATTGGTCAATGGACGCAAGTCTGAACCAGCCATGCCGCGTGAAGGATTAAGGTCCTCTGGATTGTAAACTTCTTTTATTTGGGACGAAAAAGGGTCTTTCTAGACCAACTGACGGTACCAGATGAATAAGCACCGGCTAACTCCGTGCCAGCAGCCGCGGTAATACGGAGGGTGCAAGCGTTATCCGGATTCACTGGGTTTAAAGGGTGCGTAGGCGGGTAGGTAAGTCAGTGGTGAAATCCTAGAGCTTAACTCTAGAACTGCCATTGATACTATCTATCTTGAATATTGTGGAGGTAAGCGGAATATGTCATGTAGCGGTGAAATGCTTAGATATGACATAGAACACCTATTGCGAAGGCAGCTTACTACGCATATATTGACGCTGAGGCACGAAAGCGTGGGGATCAAACAGG` |
| ASV2777 | 404 | `TGGGGAATATTGGACAATGGAGGAAACTCTGATCCAGCCATGCCGCGTGAGTGATGAAGGCCTTAGGGTTGTAAAGCTCTTTTACCAGGGAAGATGATGACGGTACCTGGAGAATAAGCACCGGCTAACTTCGTGCCAGCAGCCGCGGTAATACGAAGGGTGCTAGCGTTGTTCGGAATCACTGGGCGTAAAGGGTGCGTAGGCGGCTAGACAAGTCAGAGGTGAAAGCCTGGAGCTTAACTCCAGAACTGCCTTTGAAACTGTCTGGCTAGAGTGTCGGAGGGGATAGCGGAATTTCTAATGTAGCGGTGAAATGCGTAGATATTAGAAGGAACACCGGTGGCGAAGGCGGCTATCTGGACGACAACTGACGCTGAGGCACGAAAGCGTGGGGATCAAACAGG` |
| ASV2353 | 422 | `TGGGGAATATTGGGCAATGGGCGCAAGCCTGACCCAGCCATGCCGCGTGAGGGAAGAAGGGGCATCGCCTTGTAAACCTCTTTTCTCTGGGGATAAGGCGCTATTAGATAGCGATTGAAGGTACCAGAGGAATAAGCACCGGCAAACTACGTGCCAGCAGCCGCGGTAATACGTAGGGTGCAAGCGTTGTCCGGATTTACTGGGTTTAAAGGGTGCGTAGGCGGGATAATAAGTCTGGGATGAAATGGCAGGGCTCAACCTTGTCGCTGTCTTGGATACTGTTATTCTGGAGTGGGAGTGAGGTAGCTGGAATGAGTGGTGTAGCGGTGAAATGCGTAGAGATCACTCAGAACACCGATTGCGAAGGCAGGCTACTGACTCTTAACTGACGCTGAGGCACGAAAGCGTGGGGATCAAACAGG` |
| ASV368 | 404 | `TGGGGAATCTTAGACAATGGGGGAAACCCTGATCTAGCCATGCCGCGTGAGCGATGAAGGCCTTAGGGTTGTAAAGCTCTTTCAGTGGGGAAGATAATGACGGTACCCACAGAAGAAGCCCCGGCTAACTCCGTGCCAGCAGCCGCGGTAATACGGAGGGGGCTAGCGTTGTTCGGAATTACTGGGCGTAAAGCGCACGTAGGCGGATCGGATAGTCAGAGGTGAAAGCCCGGGGCTCAACCCCGGAACGGCCTTTGAAACTCCCGGTCTTGAGGTCGAGAGAGGTGAGTGGAATTCCGAGTGTAGAGGTGAAATTCGTAGATATTCGGAGGAACACCAGTGGCGAAGGCGGCTCACTGGCTCGATACTGACGCTGAGGTGCGAAAGCGTGGGGAGCAAACAGG` |
| ASV3313 | 429 | `TGGGGAATTTTGGACAATGGACGAAAGTCTGATCCAGCAATGCCGCGTGCAGGAAGAAGGCCTTCGGGTTGTAAACTGCTTTTGTACGGAACGAAACGGTCTGAGTTAATACCTCGGGCTAATGACGGTACCGTAAGAATAAGCACCGGCTAACTACGTGCCAGCAGCCGCGGTAATACGTAGGGTGCAAGCGTTAATCGGAATTACTGGGCGTAAAGCGTGCGCAGGCGGTTTTGTAAGACAGGCGTGAAATCCCCGGGCTCAACCTGGGAATTGCGCTTGTGACTGCAAAGCTGGAGTGCGGCAGAGGGGGATGGAATTCCGCGTGTAGCAGTGAAATGCGTAGATATGCGGAGGAACACCGATGGCGAAGGCAATCCCCTGGGCCTGCACTGACGCTCATGCACGAAAGCGTGGGGAGCAAACAGG` |
| ASV5548 | 429 | `TGGGGAATCTTGCACAATGGGCGACAGCCTGATGCAGCAATGCCGCGTGAGGGAAGACGGCCTTCGGGTTGTAAACCTCTTTCAGTTGGGATGAAGGTTGGACGGTGAATAGCCGCGCCAACTGACCGTACCTTCAGAAGAAGCTCCGGCTAACTACGTGCCAGCAGCCGCGGTAATACGTAGGGAGCAAGCGTTGTCCGGAATCATTGGGCGTAAAGCGCGTGTAGGCGGTTTTGTAAGTCTGCTGTGAAAGTCGGAGGCTCAACCTTCGAATGCCAGTGGATACTGCAAGACTTGAGTACGGAAGAGGAGAGTGGAATTCCTGGTGTAGCGGTGAAATGCGCAGATATCAGGAGGAACACCAACGGCGAAGGCAGCTCTCTGGGACGTTACTGACGCTGAGACGCGAAAGCGTGGGGAGCAAACAGG` |
| ASV5578 | 429 | `TGGGGAATATTGCGCAATGGGCGAAAGCCTGACGCAGCGACGCCGCGTGGAGGATGAAGGTCTTAGGATTGTAAACTCCTGTCAGATGGAAAGAAAAGTTCATGTTTAATAGACGTGAAATATGACGGTACCATCAGAGGAAGCACCGGCTAACTTCGTGCCAGCAGCCGCGGTAATACGAAGGGTGCAAACGTTATTCGGAATCACTGGGCGTAAAGGGTGCGTAGACGGTATGTTAAGTTCGTTGTTAAAGGCTCCGGCCTAACTGGAGTACCGCAATGAAAACTGGCGTGCTAGAGGGTGAGAGAGAGAAGTGGAATTCCTGGAGTAGCGGTAAAATGCGTGGATCTCAGGAGGAACACCAATGGCGAAGGCAGCTTCTTGGCTCATTCCTGACGTTGAGGCACGAAAGCGTGGGGAGCAAACAGG` |
| ASV1014 | 404 | `TAGGGAATATTGCACAATGGACGAAAGTCTGATGCAGCGACGCCGCGTGAGTGATGAAGGCCTTCGGGTTGTAAAACTCTGTGATAAGGGAAGATGATGACGGTACCTTATCAGAAAAGACCGGCTAACTTCGTGCCAGCAGCCGCGGTAATACGAGGGGTCTAAGCGTTGTTCGGAATCACTGGGCGTAAAGCGGGTGTAGGTGGCTTAGTAAGTTAGGTGTGAAATCCCCGGGCTCAACCCGGGAAGTGCATCTAAAACTACTTAGCTTGAGTACGGGAGAGGGAAGCAGAATTCCTGGTGTAGTGGTGAAATACGTAGATATCAGGAGGAACACCGGTGGCGAAGGCGGTTTCCTGGCCCAGTACTGACACTCAGACCCGAAAGCGTGGGTAGCAAACAGG` |
| ASV1958 | 429 | `TGGGGAATTTTGGACAATGGGGGCAACCCTGATCCAGCCATGCCGCGTGCAGGATGAAGGCCTTCGGGTTGTAAACTGCTTTTGTTAGGAACGAAAAGCCACTGAATAACACTCAGTGGTGCTGACGGTACCTAAAGAATAAGCACCGGCTAACTACGTGCCAGCAGCCGCGGTAATACGTAGGGTGCAAGCGTTAATCGGAATTACTGGGCGTAAAGCGTGCGCAGGCGGTTCGTTAAGTCAGATGTGAAATCCCTGGGCTCAACCTAGGAACTGCATTTGAAACTGGCGAGCTAGAGTGTGTCAGAGGGGGGTAGAATTCCACGTGTAGCAGTGAAATGCGTAGAGATGTGGAGGAATACCAATGGCGAAGGCAACCCCCTGGGATAACACTGACGCTCATGTACGAAAGCGTGGGGAGCAAACAGG` |
| ASV5035 | 429 | `TAGGGAATCTTCGGCAATGGGCGAAAGCCTGACCGAGCAACGCCGCGTGAGCGATGAAGGCCTTCGGGTCGTAAAGCTCTGTTGTTAGAGAAGAACAAGTACGAGAGTAACTGCTCGTACCTTGACGGTACCTAACCAGAAAGCCACGGCTAACTACGTGCCAGCAGCCGCGGTAATACGTAGGTGGCAAGCGTTATCCGGAATTATTGGGCGTAAAGCGCGCGCAGGCGGTCTCTTAAGTCTGATGTGAAAGCCCACGGCTCAACCGTGGAGGGTCATTGGAAACTGGGAGACTTGAGTGCAGGAGAGAAAAGTGGAATTCCACGTGTAGCGGTGAAATGCGTAGAGATGTGGAGGAACACCAGTGGCGAAGGCGGCTTTTTGGCCTGTAACTGACGCTGAGGCGCGAAAGCGTGGGGAGCAAACAGG` |
| ASV661 | 430 | `TGGGGAATATTGGACAATGGGCGAAAGCCTGATCCAGCGACGCCGCGTGAGTGATGAAGGCCTTCGGGTTGTAAAGCTCTGTTGAGGGAGAAGAAAACAGTTAGGGCGAATAGGCCTAGCTCTTGACGGTATCCCTTTAGAAAGCACCGGCTAACTCTGTGCCAGCAGCCGCGGTAATACAGAGGGTGCAAACGTTGCTCGGAATTACTGGGCGTAAAGCGCGTGTAGGCGGACCTGTAAGTTGGACGTGAAAGCCCTGGGCTCAACCCAGGAATTGCGTACAAAACTACAGGCCTAGAGTGATGGAGAGGGCAGCAGAATTCCTGGTGTAGAGGTGAAATTCGTAGATATCAGGAGGAACACCGGTGGCGAAGGCGGCTGCCTGGACATCAACTGACGCTGAGACGCGAAAGCGTGGGGAGCAAACAGG` |
| ASV1790 | 404 | `TGGGGAATATTGGACAATGGGCGCAAGCCTGATCCAGCCATGCCGCGTGAGTGACGAAGGTCTTCGGATTGTAAAGCTCTTTTGGCGGGGACGATAATGACGGTACCCGCAGAATAAGCCCCGGCTAACTTCGTGCCAGCAGCCGCGGTAATACGAAGGGGGCTAGCGTTGTTCGGAATTACTGGGCGTAAAGCGCACGTAGGCGGATTTGTAAGTCAGGGGTGAAATCCCGGGGCTCAACCTCGGAACTGCCTTTGATACTGCAAGTCTCGAGACCGGAAGAGGTGAGTGGAATTCCTAGTGTAGAGGTGAAATTCGTAGATATTAGGAAGAACACCAGTGGCGAAGGCGGCTCACTGGTCCGGTTCTGACGCTGAGGTGCGAAAGCGTGGGGAGCAAACAGG` |
| ASV1650 | 425 | `TAAGGAATATTGGACAATGGAGGGAACTCTGATCCAGCCATGCCGCGTGAAGGAAGAAGGCCCTCTGGGTTGTAAACTTCTTTTATATGGGAACAAACCTGTGGATTCATCCGCAGCTGAGGGTACCATAAGAATAAGCACCGGCTAACTCCGTGCCAGCAGCCGCGGTAATACGGAGGGTGCAAGCGTTATCCGGAATCACTGGGTTTAAAGGGTGCGTAGGCGGTCTTATAAGTCAGTCGTGAAATCGCTGGGCTCAACCCATGCAATTGCGATTGATACTGTTTGACTTGAATCAGGATGAGGTAGGCGGAATGTGGCATGTAGCGGTGAAATGCATAGATATGCCATAGAACACCGATTGCGAAGGCAGCTTGCTGGACCTGAATTGACGCTGAGGCACGAAAGCGTGGGGAGCAAACAGG` |
| ASV1954 | 404 | `TGGGGAATATTGGACAATGGGGGCAACCCTGATCCAGCCATGCCGCGTGAGTGATGAAGGCCTTAGGGTTGTAAAGCTCTTTTGCCAGGGAAGATAATGACTGTACCTGGAGAATAAGCCCCGGCTAACTTCGTGCCAGCAGCCGCGGTAATACGAAGGGGGCTAGCGTTGTTCGGAATCACTGGGCGTAAAGCGCACGTAGGCGGATTTTTAAGTCAGGGGTGAAAGCCTGGGGCTCAACCTCAGAATTGCCTTTGATACTGGGAATCTTGAGTATGGTAGAGGTCGGTGGAATAGCAAGTGTAGAGGTGAAATTCGTAGATATTTGCTAGAACACCAGTGGCGAAGGCGGCCGACTGGACCATAACTGACGCTGAGGTGCGAAAGCGTGGGGAGCAAACAGG` |
| ASV1494 | 423 | `TAGGGAATATTGGGCAATGGACGAAAGTCTGACCCAGCCATGCCGCGTGAAGGAAGAAGGCGTTATGCGTCGTAAACTTCTTTTATAAGAGACGAAAGAGACGGTGCGCCGTTTGTTGACGGTATCTTATGAATAAGCATCGGCTAACTACGTGCCAGCAGCCGCGGTAATACGTAGGATGCAAGCGTTGTCCGGATTTACTGGGTTTAAAGGGTGCGTAGGCGGTTTTATAAGTCAGTGGTGAAATCTCATAGCTTAACTGTGAATGTGCCATTGATACTGTAAGACTTGAGTATTTGTGAGGTGGGCGGAATTGGTAGTGTAGCGGTGAAATGCATAGATATTACCAGGAACGTCGATAGCGAAGGCAGCCCACTAACAAATTACTGACGCTGAGGCACGAAAGTGCGGGGATCAAACAGG` |
| ASV1543 | 428 | `TCGAGAATCTTTCACAATGGGGGCAACCCTGATGGAGCGACGCCGCGTGGAGGATAAGGTCTTCGGATTGTAAACTCCTGTCATGCGCGAACAAGGTTGCAGGGATAATACCTTTGCAAATTGATGGTACCGCAAGAGGAAGAGACGGCTAACTCTGTGCCAGCAGCCGCGGTAATACAGAGGTCTCAAGCGTTGTTCGGAATCACTGGGCGTAAAGGGTGCGTAGGTGGCGTGGTAAGTCAGATGTGAAAGCCCGGGGCTCAACCTCGGAATTGCATCCGATACTACCATGCTTGAGTACTGAAGAGGAAACTAGAATTCTCGGTGTAGCAGTGAAATGCGTAGATATCGAGAGGAATACCAAAGGCGAAGGCAGGTTTCTTGGCAGTTACTGACACTGAGGCACGAAGGCCAGGGGAGCAAACGGG` |
| ASV2152 | 424 | `TGAGGAATATTGCGCAATGGACGAAAGTCTGACGCAGCCATGCCGCGTGCAGGATGACGGCCCTATGGGTTGTAAACTGCTTTTGTATGGGAATAAACCCTTCTACGTGTAGAAGGCTGAAGGTACCATAAGAATAAGCACCGGCTAACTCCGTGCCAGCAGCCGCGGTAATACGGAGGGTGCAAGCGTTATCCGGATTTATTGGGTTTAAAGGGTCCGTAGGCGGGCCGATAAGTCAGTGGTGAAAGCCCATAGCTCAACTATGGAACTGCCATTGATACTGTCGGTCTTGAGTATATGTGATGTTGGCGGAATGTGTAGTGTAGCGGTGAAATGCTTAGATATTACACAGAACACCGATTGCGAAGGCAGCTGACAAATATATAACTGACGCTGAGGGACGAAAGTGTGGGGATCAAACAGG` |
| ASV869 | 429 | `TGGGGAATATTGGACAATGGGGGCAACCCTGATCCAGCCATGCCGCGTGTGTGAAGAAGGCCTTAGGGTTGTAAAGCACTTTCAGTGGGGAGAAAGGTGATTCACCTAATACGTGAGTCAATTGATGCTACCCACAGAAGAAGCACCGGCTAACTCCGTGCCAGCAGCCGCGGTAATACGGAGGGTGCAAGCGTTAATCGGAATTACTGGGCGTAAAGCGTACGTAGGTGGTTTGTTAAGCCAGCTGTGAAATCCCCGGGCTCAACCTGGGCACTGCAGTTGGAACTGGCAAGCTAGAGTAGGGTAGAGGGGTGTGGAATTCCAGGTGTAGCGGTGAAATGCGTAGATATCTGGAGGAACATCAGTGGCGAAGGCGACACCCTGGACTCATACTGACACTGAGGTACGAAAGCGTGGGGAGCAAACAGG` |
| ASV1394 | 404 | `TGGGGAATATTGGACAATGGGGGAAACCCTGATCCAGCAATGCCGCGTGAGTGATGAAGGCCTTAGGGTTGTAAAGCTCTTTTCTTGGGGAAGATAATGACGGTACCCAAGGAATAAGCCCCGGCTAACTTCGTGCCAGCAGCCGCGGTAATACGAAGGGGGCAAGCGTTGTTCGGAATAACTGGGCGTAAAGGGCGCGTAGGCGGTGACACATGTCACATGTGAAAGACCTGGGCTTAACCTAGGAATAGCATGTGAAACGGTGTCGCTAGAGACCGAGAGGGGAAAAGGGAATTGTGCGTGTAGAGGTGAAATTCGTAGATATGCACAGGAACACCGGTGGCGAAGGCGCTTTTCTGGCTCGGATCTGACGCTAAGGCGCGAAAGCGTGGGGAGCAAACAGG` |
| ASV2587 | 404 | `TGGGGAATATTGGACAATGGGCGAAAGCCTGATCCAGCAATGCCGCGTGAGTGATGAAGGCCTTAGGGTTGTAAAGCTCTTTTACCCGGGATGATAATGACAGTACCGGGAGAATAAGCTCCGGCTAACTTCGTGCCAGCAGCCGCGGTAATACGAGGGGAGCTAGCGTTGTTCGGAATTACTGGGCGTAAAGCGCGCGTAGGCGGTTTTTTAAGTCAGAGGTGAAAGCCTGGTGCTCAACACCAGAACGGCCTTTGAAACTGGAAAACTTGAATCATGGAGAGGTCAGTGGAATTCCGAGTGTAGAGGTGAAATTCGTAGATATTCGGAAGAACACCAGTGGCGAAGGCGACTGACTGGACATGTATTGACGCTGAGGTGCGAAAGCGTGGGGAGCAAACAGG` |
| ASV492 | 424 | `TGAGGAATATTGGACAATGGGCGCAAGCCTGATCCAGCCATGCCGCGTGCAGGATGACGGTCCTATGGATTGTAAACTGCTTTTGCACAGGAAGAAACACTCCCTCGTGAGGGAGCTTGACGGTACTGTGAGAATAAGGATCGGCTAACTCCGTGCCAGCAGCCGCGGTAATACGGAGGATCCAAGCGTTATCCGGAATCATTGGGTTTAAAGGGTCCGTAGGCGGTTTGGTAAGTCAGTGGTGAAAGCCCATCGCTCAACGGTGGAACGGCCATTGATACTGCTAGACTTGAATTATTAGGAAGTAACTAGAATATGTAGTGTAGCGGTGAAATGCTTAGAGATTACATGGAATACCAATTGCGAAGGCAGGTTACTACTAATGGATTGACGCTGATGGACGAAAGCGTGGGTAGCGAACAGG` |
| ASV1354 | 429 | `TCGAGAATAATTCACAATGGGGGCAACCCTGATGGTGCAACGCCGCGTGGAGGATGACGGTCTTCGGATTGTAAACTCCTGTCATCTGGGAGTAAGACCTGGCTGTGAATAGCAGACAGGGTTGATAGTACCAGAAGAGGAAGGGACGGCTAACTTCGTGCCAGCAGCCGCGGTAATACGAAGGTCCCAAGCGTTATTCGGAATCACTGGGCGTAAAGGGAGCGTAGGCGGCGTGGTAAGTCAGATGTGAAATCCCGGGGCTCAACCCCGGAACTGCATCCGATACTGCCATGCTAGAGGATTGGAGAGGTAGCTGGAATTCTTGGTGTAGCAGTGAAATGCGTAGAGATCAAGAGGAACACTCGTGGCGAAAGCGAGCTACTGGACAATTCCTGACGCTGAGGCTCGAAGGCTAGGGTAGCGAAAGGG` |
| ASV4646 | 405 | `TAGGGAATTTTCCACAATGGGCGAAAGCCTGATGGAGCAACGCCGCGTGCAGGATGAAGGCCCTTGGGTCGTAAACTGCTTTTATCTGTGACGATTTTGACGGTAGCAGATGAATAAGGATCGGCTAACTCCGTGCCAGCAGCCGCGGTCATACGGAGGATCCAAGCGTTATCCGGAATCACTGGGCGTAAAGAGTTGCGTAGGTGGCATAGTAAGTTGGTGGTGAAAGCGTGTGGCTCAACCATATACACATCACCAAAACTACTAAGCTAGAGTATAAGAGAGGTAGTTGGAATTCCTAGTGTAGGAGTGAAATCCGTAGATATTAGGAGGAACACCAATGGCGTAGGCAGACTACTGGCTTATTACTGACACTAAGGCACGAAAGCGTGGGTAGCAAACGGG` |
| ASV261 | 424 | `TGAGGAATATTGGACAATGGGCGCAAGCCTGATCCAGCCATGCCGCGTGCAGGAAGACGGTCCTATGGATTGTAAACTGCTTTTGCACAGGAAGAAACAACATTACGTGTAATGTCTTGACGGTACTGTGAGAATAAGGATCGGCTAACTCCGTGCCAGCAGCCGCGGTAATACGGAGGATCCAAGCGTTATCCGGAATCATTGGGTTTAAAGGGTCCGTAGGCGGTTTAGTAAGTCAGTGGTGAAAGCCCATCGCTCAACGGTGGAACGGCCATTGATACTGCTAGACTTGAATTATTAGGAAGTAACTAGAATATGTAGTGTAGCGGTGAAATGCTTAGAGATTACATGGAATACCAATTGCGAAGGCAGGTTACTACTAATTGATTGACGCTGATGGACGAAAGCGTGGGTAGCGAACAGG` |
| ASV2303 | 407 | `TGGGGAATATTGGACAATGGACCAAAAGTCTGATCCAGCAATTCTGTGTGCACGATGAAGGTCTTAGGATTGTAAAGTGCTTTCAGTTGGGAAGAAGTCAGTGACGGTACCAACAGAAGAAGCGACGGCTAAATATGTGCCAGCAGCCGCGGTAATACATATGTCGCAAGCGTTATCCGGATTTATTGGGCGTAAAGCGCGTCTAGGCGGTTTACTAAGTCTGATGTGAAAATGCGGGGCTCAACCCCGTATTGCGTTGGAAACTGGTAGACTAGAGTACTGGAGAGGTGGGCGGAACTACAAGTGTAGAGGTGGAATTCGTAGATATTTGTAGGAATGCCGATAGAGAAGTCAGCTCACTGGACAGATACTGACGCTAAAGCGCGAAAGCGTGGGGAGCAAACAGG` |
| ASV3392 | 423 | `TAGGGAATATTGGTCAATGGGCGAGAGCCTGAACCAGCCATGCCGCGTGCAGGAAGAAGGCCTTCTGGGTTGTAAACTGCTTTTATCAGGGAAGAAAACGCTCTTGCGAGAGTAACTGACGGTACCTGAGGAATAAGCACCGGCTAACTCCGTGCCAGCAGCCGCGGTAATACGGAGGGTGCAAGCGTTGTCCGGATTTATTGGGTTTAAAGGGTGCGTAGGCGGGCTATTAAGTCAGTGGTGAAAGCCGGCAGCTCAACTGTCGAACTGCCATTGATACTGATAGCCTTGAGTACAGATGAGGTGGGCGGAATTTATGGTGTAGCGGTGAAATGCATAGATACCATAAAGAACACCGATAGCGAAGGCAGCTCACTAAACTGTAACTGACGCTGAGGCACGAAAGCATGGGGATCGAACAGG` |
| ASV2651 | 423 | `TAGGGAATATTGGGCAATGGGCGCAAGCCTGACCCAGCCATGCCGCGTGCAGGAAGAAGGCGTTATGCGTTGTAAACTGCTTTTATACAGGAAGAAAAAGGCCATGCGTGGCAAACTGACGGTACTGTATGAATAAGCACCGGCTAACTCCGTGCCAGCAGCCGCGGTAATACGGAGGGTGCAAGCGTTGTCCGGATTTATTGGGTTTAAAGGGTGCGTAGGCGGCTTTTTAAGTCAGCGGTGAAAGACTGTCGCTCAACGACGGAACTGCCGTTGATACTGGGAGGCTTGAGTATGATTGAGGTAGCCGGAATGGATAGTGTAGCGGTGAAATGCATAGATATTATCCAGAACGCCAATAGCGTAGGCAGGTTACTAAGTCACAACTGACGCTGAGGCACGAAAGTGCGGGGATCAAACAGG` |
| ASV3989 | 404 | `TGGGGAATATTGGACAATGGGCGAAAGCCTGATCCAGCAATACCGCGTGAGTGATGAAGGCCTTAGGGTTGTAAAGCTCTTTTAGTAGGGAAGATAATGACGGTACCTACAGAAAAAGCACCGGCTAACTCCGTGCCAGCAGCCGCGGTAAGACGGAGGGTGCTAGCGTTGTTCGGAATTACTGGGCGTAAAGAGCGCGTAGGCGGTTTGGTAAGTTGGAAGTGAAAGCCCGGGGCTTAACCTCGGAATTGCTTTCAAAACTGCTAGGCTAGAGTATGGTAGGGGGTGATAGAATTCCTAGTGTAGAGGTGAAATTCTTAGATATTAGGAGGAATACCGGTGGCGAAGGCGGTCACCTGGACCATTACTGACGCTGAGGCGCGAAAGCGTGGGGAGCAAACAGG` |
| ASV4888 | 387 | `GCGCGGAAACTTTGCAATGCACGAAAGTGTGACAAGGGAATCCCAAGTGCTTTGCTTCAAGCAAAGCTTTTGCCAAGAGCAAAAATCTTGGCGAATAAGTGGTGGGCAATACCTGTGCCAGCCGCCGCGGTAACCCAGGAGCCACAAGTGGCAGCCACTTTTATTGGGCCTAAAGCGTCCGTAGCCGGTTCGGTAAATTTCCTGTGAAATCGTTGGGCTTAACCTAACGGTGTGCAAGAAACACTGCCAAACTTGGGACCGGGAGGCGTCAGAGGTATTCCTGAGGGAGCGGTAAAATGCTATAATCTCAGGAGGACCCCCTGTGGCGAAGGCGTCTGACGAGAACGGATCCGACGGTGAGGGACGAAAGCTAGGGGAGCGAACCGG` |
| ASV307 | 429 | `TCGAGAATAATTCACAATGGGGGCAACCCTGATGGTGCAACGCCGCGTGGAGGATGACGGTCTTCGGATTGTAAACTCCTGTCATCTGGGAGTAAGGCATGGCTGTGAATAGCAGACATGATTGATAGTACCAGAAGAGGAAGGGACGGCTAACTTCGTGCCAGCAGCCGCGGTAATACGAAGGTCCCGAGCGTTGTTCGGAATCACTGGGCGTAAAGGGAGCGTAGGCGGCGTGGTAAGTCAGATGTGAAATCCCGGGGCTCAACCCCGGAACTGCATCCGATACTGCCATGCTAGAGGATTGGAGAGGTAGCTGGAATTCTTGGTGTAGCAGTGAAATGCGTAGAGATCAAGAGGAACACTCGTGGCGAAAGCGAGCTACTGGACAATTCCTGACGCTGAGGCTCGAAGGCTAGGGTAGCGAAAGGG` |
| ASV2548 | 406 | `TGGGGAATTTTCCGCAATGGGCGAAAGCCTGACGGAGCAATACCGCGTGCGGGACGAAGGCTTTTGGGTCGTAAACCGCTTTTGTTAGGGAAGAAGAACTGACGGTACCTAACGAATCAGCATCGGCTAACTCCGTGCCAGCAGCCGCGGTAATACGGAGGATGCAAGCGTTATCCGGAATTATTGGGCGTAAAGCGTCCGCAGGTGGTTTATCAAGTCTGCTGTTAAAGCATGGGGCTTAACCCCATATCGGCAGTGGAAACTGAGAGACTAGAGTACGGTAGGGGTAGCGGGAATTCCCAGTGTAGCGGTGAAATGCGTAGATATTGGGAAGAACACCAGCGGCGAAAGCGCGCTACTGGGCCGAAACTGACACTCATGGACGAAAGCTAGGGGAGCGAAAGGG` |
| ASV2155 | 429 | `TGGGGAATTTTGGACAATGGGCGCAAGCCTGATCCAGCCATGCCGCGTGTGTGAAGAAGGCCTTCGGGTTGTAAAGCACTTTCGGCCGGAACGAAATCGCCCGTGTTAACAGCGCGGGTGGATGACGGTACCGGAAGAAGAAGCACCGGCTAACTACGTGCCAGCAGCCGCGGTAATACGTAGGGTGCGAGCGTTAATCGGAATTACTGGGCGTAAAGGGTGCGCAGGCGGCTCCGCATGTCGGGCGTGAAATCCCCGGGCTTAACCTGGGAATGGCGTCCGAAACTATGGAGCTGGAGTGTGGCAGAGGGAGGTGGAATTCCACGTGTAGCGGTGAAATGCGTAGAGATGTGGAGGAACACCGATGGCGAAGGCAGCCTCCTGGGCCAACACTGACGCTCATGCACGAAAGCGTGGGGAGCAAACAGG` |
| ASV502 | 404 | `TGGGGAATCTTGGACAATGGGCGCAAGCCTGATCCAGCCATGCCGCGTGAGTGAAGAAGGCCTTAGGGTTGTAAAGCTCTTTTACCAGGGACGATAATGACGGTACCTGGAGAATAAGCCCCGGCAAACTTCGTGCCAGCAGCCGCGGTAATACGAAGGGGGCTAGCGTTGTTCGGAATTACTGGGCGTAAAGCGCACGTAGGCGGGTCGTTAAGTCAGGGGTGAAATCCCGGAGCTCAACTCCGGAACTGCCTTTGATACTGGCGATCTAGAGATCGAGAGAGGTGAGTGGAATTCCGAGTGTAGAGGTGAAATTCGTAGATATTCGGAAGAACACCAGTGGCGAAGGCGGCTCACTGGCTCGATACTGACGCTGAGGTGCGAAAGCGTGGGGAGCAAACAGG` |
| ASV1090 | 421 | `TGGGGAATATTGCGCAATGGCCGCAAGGCTGACGCAGCGACGCCGCGTGGGGGATGAAGCATTTCGGTGTGTAAACCCCTGTTGCTCGGGACGAACAAGCCTTTTCGAGGGCTCTGACGGTACCGAGTGAGGAAGCACCGGCTAACTCCGTGCCAGCAGCCGCGGTAATACGGAGGGTGCGAGCGTTGTCCGGAATCACTGGGCGTAAAGGGCGCGTAGGTGGTCTGCTAAGCGTGCGGTGAAAGCCTGGGGCTCAACCCCAGGTCTGCCGTGCGAACTGGTGGACTTGAGCACTGTAGAGGCAGGTGGAATTCCGGGTGTAGCGGTGGAATGCGTAGAGATCCGGAAGAACACCGGTGGCGAAGGCGGCCTGCTGGGCAGTAGCTGACACTGAGGCGCGACAGCGTGGGGAGCAAACAGG` |
| ASV1991 | 429 | `TGGGGAATATTGGACAATGGGCGCAAGCCTGATCCAGCAATACCGCGTGTGTGAAGAAGGCCTTCGGGTTGTAAAGCACTTTCAGTAGCTAAGAAATGCTCAAGGTTAATACCCTTGAGAGTTGACGTAAACTACAGAAGAAGCACCGGCTAACTCTGTGCCAGCAGCCGCGGTAATACAGAGGGTGCAAGCGTTAATCGGAATTACTGGGCGTAAAGCGCACGTAGGTGGGTGTTTAAGTCGAATGTGAAACCCTCGAGCTTAACTTGGGGACTGCATGCGATACTGAACACCTAGAGTATAGTAGAGGGAAGTGGAATTTCCGGAGTAGCGGTGAAATGCGTAGATATCGGAAGGAACACCAGTGGCGAAGGCGGCTTCCTGGGCTAATACTGACACTGAGGTGCGAAAGCGTGGGGAGCAAACAGG` |
| ASV5575 | 389 | `TCGGGAAAATTTTGCAATGCGCGAAAGCGTGACAGAGCAAGCCAGAGTGATTTTCCGTTTTGGAAAATCTTTTGTGGAATGTAGACAGTTCCACGAATAAGGACCGGGAGAGACTGGTGCCAGCCGCCGCGGTAATCCCAGCGGTCCAAGTCGCAGCCACATTTGTTGGGTCTAAAACATCCGTAGCTTGTCTTACAAGTTTCTTGTGAAATCGGGACTCTCAAGGTTCCGGCGGGCAAGAAATACTGTTTGACTCGAGACCGGGAGGTGCAGAGAGTACTTTGGGGGTAGCGGTAAAATGTGTTGATCCTCAAAGGACTAACAGTCGCGAAGGCGCTCTGCAAGAACGGATCTGACAGTGAGGGATGAAGGCTAGGGGCGCAAAGTGG` |
| ASV2307 | 424 | `TAGGGAATATTGGGCAATGGAGGCAACTCTGACCCAGCCATGCCGCGTGAACGAAGAAGGCGCTATGCGTCGTAAAGTTCTTTTAGAGGGGGCGAAAACACTCGATTTATCGAGTACTGACGGTACCTTCGGAATAAGCACCGGCTAACTCTGTGCCAGCAGCCGCGGTAATACAGAGGGTGCAAGCGTTATCCGGAATCACTGGGTTTAAAGGGTGCGTAGGCGGGATTATAAGTCAGAGGTGAAATCTCGTCGCTTAACGACGGAATTGCCTTTGATACTGTTTTTCTTGAAATGTGTTGAGGTTGGCGGAATGAGACATGTAGCGGTGAAATGCTTAGATATGTCTTAGAACACCGATTGCGAAGGCAGCTGACTGGACACTATTTGACGCTGAGGCACGAAAGCGTGGGGATCAAACAGG` |
| ASV1436 | 424 | `TGAGGAATATTGGACAATGGGCGCAAGCCTGATCCAGCCATGCCGCGTGCAGGATGACGGTCCTATGGATTGTAAACTGCTTTTGCACAGGAAGAAACACTCCCTCGTGAGGGAGCTTGACGGTACTGTGAGAATAAGGATCGGCTAACTCCGTGCCAGCAGCCGCGGTAATACGGAGGATCCAAGCGTTATCCGGAATCATTGGGTTTAAAGGGTCCGTAGGCGGTTTAATAAGTCAGTGGTGAAAGCCCATCGCTCAACGGTGGAACGGCCATTGATACTGTTAGACTTGAATTACTAGGAAGTAACTAGAATATGTAGTGTAGCGGTGAAATGCTTAGAGATTACATGGAATACCAATTGCGAAGGCAGGTTACTACTAATGGATTGACGCTGATGGACGAAAGCGTGGGTAGCGAACAGG` |
| ASV2857 | 407 | `TGGGGAATTTTCCGCAATGGGCGAAAGCCTGACGGAGCAATACCGCGTGAGGGATGACGGCCTATGGGTTGTAAACCTCTTTTTTCAGGGAGGAATCAATGACGTGTACCTGAAGAATAAGCATCGGCTAACTCCGTGCCAGCAGCCGCGGTAAGACGGGGGATGCAAGTGTTATCCGGAATCACTGGGCGTAAAGCGTCTGTAGGTGGTTTAATAAGTCAACTGTTAAATCTTGAGGCTCAACCTCGAAATCGCAGTCGAAACTATTAGACTAGAGTATAGTAGGGGTAAAGGGAATTTCCAGTGGAGCGGTGAAATGCGTAGATATTGGAAAGAACACCGATGGCGAAGGCACTTTACTGGGCTATTACTAACACTGAGAGACGAAAGCTAGGGTAGCAAATGGG` |
| ASV3481 | 429 | `TGGGGAATTTTGGACAATGGGGGCAACCCTGATCCAGCAACGCCGCGTGAATGAAGAAGGCCTTAGGGTTGTAAAGTTCTTTTATCTGAGAGCAAAGTCGACGTGTTAATAGCATGTTGAGATGAGAGTATCAGAGGAATAAGCACCGGCTAACTACGTGCCAGCAGCCGCGGTAATACGTAGGGTGCGAGCGTTAATCGGAATTACTGGGCGTAAAGCGTGCGCAGGCGGATATGTAAGTTAGATGTGAAATACCCGAGCTCAACTTGGGAATGGCATTTAAGACTGCATGTCTAGAGTTTGTCAGAGGGGGGTAGAATTCCAAGTGTAGCAGTGAAATGCGTAGAGATTTGGAGGAATACCGGTGGCGAAGGCGGCCCCCTGGGATAGAACTGACGCTCATGCACGAAAGCGTGGGTAGCAAACAGG` |
| ASV2637 | 407 | `TGGGGAATTTTCCGCAATGGGCGAAAGCCTGACGGAGCAATACCGCGTGAGGGAGGAAGGCTCTTGGGTCGTAAACCTCTTTTCTCAGGGAAGAACACAATGACGGTACCTGAGGAATAAGCATCGGCTAACTCTGTGCCAGCAGCCGCGGTAATACAGAGGATGCAAGCGTTATCCGGAATGATTGGGCGTAAAGCGTCCGTAGGTGGTTTGTCAAGTCTGTGGTTAAAGAGCGAAGCTCAACTTCGTAAAGGCCATGGAAACTGACAGACTAGAGTGAAGTAGGGGTAGAGGGAATTCCCAGTGTAGCGGTGAAATGCGTAGAGATTGGGAAGAACACCGGTGGCGAAAGCGCTCTACTGGACTTATACTGACACTGAGGGACGAAAGCTAGGGGAGCGAAAGGG` |
| ASV1569 | 429 | `TCGAGAATAATTCACAATGGGGGCAACCCTGATGGTGCAACGCCGCGTGGAGGATGAAGGTCTTCGGATTGTAAACTCCTGTCATCCGGGAGCAAGACCTGACGGTTAACAGCCGGCAGGGTTGATAGTACCGGAAGAGGAAGGGACGGCTAACTTCGTGCCAGCAGCCGCGGTAATACGAAGGTCCCGAGCGTTGTTCGGAATCACTGGGCGTAAAGGGAGCGTAGGCGGCGTGGTAAGTCAGATGTGAAATCCCGGGGCTCAACCCCGGAACTGCATCCGATACTGCCGTGCTAGAGGATTGGAGAGGTAGCTGGAATTCACGGTGTAGCAGTGAAATGCGTGGATATCGTGAGGAACACTCGTGGCGAAAGCGAGCTACTGGACAACTCCTGACGCTGAGGCTCGAAGGCCAGGGTAGCGAAAGGG` |
| ASV2063 | 404 | `TGGGGAATCATAGACAATGGGGGAAACCCTGATCTTGCCATGCCGCGTGGACGATGAAGGCCTTAGGGTTGTAAAGTCCTTTCACCGGTGAAGATAATGACGGTAGCCGGAGAAGAAGCCCCGGCTAACTCCGTGCCAGCAGCCGCGGTAATACGGAGGGGGCTAGCGTTGTTCGGAATTACTGGGCGTAAAGCGCACGTAGGCCGGCTGTTAAGTCGGGGGTGAAATCCTGGGGCTCAACCCCAGAACTGCCCTCGATACTGGCAGCCTAGAGGACGGAAGAGGCTAGTGGAATGGCGAGTGTAGAGGTGAAATTCGTAGATATTCGCCGGAACACCAGTGGCGAAGGCGACTAGCTGGTCCGTTTCTGACGCTGAGGTGCGAAAGCGTGGGGAGCAAACAGG` |
| ASV3295 | 387 | `GCGAGAAACCTTTGCAATGTGCGCAAGCACGACAAGGTTAATCCGAGTGTTTCCTGCTAAAGGAATCTTTTGTTAGTTCTAAAACCACTGATGAATAAGGGGTGGGCAAGTTCTGGTGTCAGCCGCCGCGGTAAAACCAGCACCTCAAGTGGTCAGGAGGATTATTGGGCCTAAAGCATCCGTAGCCGGTTTTGTAAGTTTCCGGTTAAATCCACTAGCTCAACTAATGGGCTGCCGGAAATACTGTAGAACTAGGGAGTGGGAGAGGTAGACGGTACTCGGTAGGAAGGGGTAAAATCCTTTGATCTATTGATGACCACCTGTGGCGAAGGCGGTCTACCAGAACACGTTCGACGGTGAGGGATGAAAGCTGGGGGAGCAAACCGG` |
| ASV4854 | 429 | `TGGGGAATATTGGACAATGGGCGCAAGCCTGATCCAGCCATACCGCGTGGGTGAAGAAGGCCTTCGGGTTGTAAAGCCCTTTTGTTGGGAAAGAAATCCTGTTGATTAATACTCGATAGGGATGACGGTACCCAAAGAATAAGCACCGGCTAACTTCGTGCCAGCAGCCGCGGTAATACGAAGGGTGCAAGCGTTACTCGGAATTACTGGGCGTAAAGCGTGCGTAGGTGGTTGTTTAAGTCCGTTGTGAAAGCCCTGGGCTCAACCTGGGAATTGCAGTGGATACTGGGCAACTAGAGTGTGATAGAGGGTGGCGGAATTCCCGGTGTAGCAGTGAAATGCGTAGATATCGGGAGGAACATCCGTGGCGAAGGCGGCCACCTGGGTCAGCACTGACACTGAGGCACGAAAGCGTGGGGAGCAAACAGG` |
| ASV5394 | 424 | `TGAGGAATATTGGTCAATGGACGCAAGTCTGAACCAGCCAAGTCGCGTGAAGGAAGAAGGTTCTATGGATCGTAAACTTCTTTTGTAAGGGGATAAAAAACATCACGAGTGATGTCTTGTAGGTACCTTACGAATAAGGATCGGCTAACTCCGTGCCAGCAGCCGCGGTAATACGGAGGATCCAAGCGTTATCCGGATTTATTGGGTTTAAAGGGTGCGTAGGCGGGATTTTAAGTCAGTGGTGAAAGTTTGCGGCTCAACCGTAAAATTGCCATTGAAACTGAAGTTCTTGAGTACAAATGAGGTAGGCGGAATGTGTTGTGTAGCGGTGAAATGCTTAGATATAACACAGAACACCAATTGCGAAGGCAGCTTACTGGGATGTAACTGACGCTGAGGCACGAAAGCGTGGGGATCGAACAGG` |
| ASV4351 | 424 | `TGAGGAATATTGGTCAATGGACGGAAGTCTGAACCAGCCAAGTAGCGTGCAGGACGACGGCCCTATGGGTTGTAAACTGCTTTTATGTGGGGATAAAGTTCGGGACGTGTCCTGATTTGCAGGTACCACATGAATAAGGACCGGCTAATTCCGTGCCAGCAGCCGCGGTAATACGGAAGGTCCGGGCGTTATCCGGATTTATTGGGTTTAAAGGGAGCGTAGGCTGTCTGGTAAGCGTGTTGTGAAATGTAGGCGCTCAACGTCTGAATTGCAGCGCGAACTACCGGACTTGAGTGCGCAGGAAGTAGGCGGAATTTGTGGTGTAGCGGTGAAATGCTTAGATATCACGAAGAACTCCGATTGCGAAGGCAGCTTACTGTAGCGTTACTGACGCTGAAGTTCGAAAGCGCGGGTATCGAACAGG` |
| ASV1291 | 429 | `TGGGGAATTTTGGACAATGGGCGCAAGCCTGATCCAGCAATGCCGCGTGCAGGAAGAAGGCCTTCGGGTTGTAAACTGCTTTTGTACGGAGCGAAAAGGCTCTCTTTAATACAGAGGGTTCATGACGGTACCGTAAGAATAAGCACCGGCTAACTACGTGCCAGCAGCCGCGGTAATACGTAGGGTGCGAGCGTTAATCGGAATTACTGGGCGTAAAGCGTGCGCAGGCGGTTTTGTAAGACAGGCGTGAAATCCCCGGGCTCAACCTGGGAATGGCGCTTGTGACTGCAAAGCTGGAGTGCGGCAGAGGGGGATGGAATTCCGCGTGTAGCAGTGAAATGCGTAGATATGCGGAGGAACACCGATGGCGAAGGCAATCCCCTGGGCCTGCACTGACGCTCATGCACGAAAGCGTGGGGAGCAAACAGG` |
| ASV3521 | 429 | `TGGGGAATATTGCACAATGGAGGAAACTCTGATGCAGCGACGCCGCGTGGGTGATGAAGGCCTTCGGGTTGTAAAGCTCTGTCGGCTGGGAAAAAGGTAGTCGTGCTAATACCACGATTGCTTGATGGTACCAGCAAAGGAAGCACCGGCTAACCCTGTGCCAGCAGCCGCGGTAATACAGGGGGTGCTAACGTTGTTCGGAATTACTGGGCGTAAAGCGCGCGCAGGCGGCTATACAAGTCAGATGTGAAATCTCGGGGCTTAACTCCGAAAGTGCATTTGAAACTGTATGGCTAGAGTACAGTAGAGGTTGGTGGAATTCCCAGTGTAGAGGTGAAATTCGTAGATATTGGGAGGAACACCGGTGGCGAAGGCGGCCAACTGGGCTGTAACTGACGCTGAGGCGCGAAAGCGTGGGGAGCAAACAGG` |
| ASV4202 | 404 | `TGGGGAATATTGCACAATGGGCGAAAGCCTGATGCAGCAACGCCGCGTGAGTGATGAAGGCCTTCGGGTTGTAAAGCTCTGTCTTCTGGGACGATAATGACGGTACCAGAGGAGGAAGCCACGGCTAACTACGTGCCAGCAGCCGCGGTAATACGTAGGTGGCAAGCGTTGTCCGGATTTACTGGGCGTAAAGAATGCGTAGGCGGATACTTAAGTCAGATGTGAAATACCCGAGCTCAACTTGGGTGCTGCATTTGAAACTGGGTATCTAGAGTGCGGGAGAGGTAAGTGGAATTCCTAGTGTAGCGGTGAAATGCGTAGAGATTAGGAAGAACACCAGTGGCGAAGGCGACTTACTGGACCGTAACTGACGCTGAGGCATGAAAGCGTGGGGAGCAAACAGG` |
| ASV500 | 404 | `TGGGGAATATTGGACAATGGGCGAAAGCCTGATCCAGCAATGCCGCGTGAGTGATGAAGGCCTTAGGGTTGTAAAGCTCTTTTACCAGGGATGATAATGACAGTACCTGGAGAATAAGCTCCGGCTAACTCCGTGCCAGCAGCCGCGGTAATACGGAGGGAGCTAGCGTTGTTCGGAATTACTGGGCGTAAAGCGTACGTAGGCGGTTATTCAAGTCAGAGGTGAAAGCCTGGAGCTCAACTCCAGAACTGCCTTTGAAACTAGATAGCTAGAATCTTGGAGAGGTTAGTGGAATTCCGAGTGTAGAGGTGAAATTCGTAGATATTCGGAAGAACACCAGTGGCGAAGGCGACTAACTGGACAAGTATTGACGCTGAGGTACGAAAGCGTGGGGAGCAAACAGG` |
| ASV3987 | 429 | `TGGGGAATCTTGTGCAATGGGCGAAAGCCTGACACAGCCATGCCGCGTGTGTGAAGAAGGCCTTCGGGTTGTAAAGCACTTTCAGTCGTAAAGAAAAGCTTCGGGCTAATACCTCGGAGTCTTGACGTTAGCGACAGAAGAAGCACCGGCTAACTCCGTGCCAGCAGCCGCGGTAATACGGAGGGTGCAAACGTTAATCGGAATTACTGGGCGTAAAGCGCGCGTAGGCGGTCTGCTAAGTGGGATGTGAAAGCCCCGGGCTTAACCTGGGAACTGCATTCCAAACTGACAGGCTAGAGTCTGGTAGAGGGAGGTGGAATTTCCTGTGTAGCGGTGAAATGCGTAGATATAGGAAGGAACACCAGTGGCGAAGGCGGCCTCCTGGACCAAGACTGACGCTGAGGTGCGAAAGCGTGGGGAGCAAACAGG` |
| ASV588 | 430 | `TGGGGAATATTGCGCAATGGGCGAAAGCCTGACGCAGCAACGCCGCGTGAGCGATGAAGGCCTTCGGGTTGTAAAGCTCTTTCGGTTGGGAAGAAGGGTGTGAGGGCGAACAGTCCTTACATTTGATGGTACCAAAAGAAGAAGCACCGGCTAACTTCGTGCCAGCAGCCGCGGTAATACGAAGGGTGCAAGCGTTGTTCGGAATTACTGGGCGTAAAGGGTTCGTAGGCGGGAGTTTAAGTCAAGTGTGAAATCCCCAGGCTCAACCTGGGACGTGCACCTGAAACTGGATTTCTTGAGTACTAGAGAGGGTGGTGGAATTGCTGGTGTAGGAGTGACATCCGTAGAGATCAGCAGGAACACCGGAGGCGAAGGCGACCACCTGGCTAGACACTGACGCTGAGGAACGAAAGCGTGGGGAGCAAACAGG` |
| ASV3512 | 429 | `TGGGGAATTTTGGACAATGGGGGCAACCCTGATCCAGCAATGCCGCGTGTGTGAAGAAGGCCTTCGGGTTGTAAAGCACTTTTGGCGGGAACGAAACGGTTTTCTCTAATACAGGGAGCTAATGACGGTACCCGCAGAATAAGCACCGGCTAACTACGTGCCAGCAGCCGCGGTAATACGTAGGGTGCAAGCGTTAATCGGAATTACTGGGCGTAAAGCGTGCGCAGGCGGTTTTTTAAGACAGATGTGAAATGCCCGGGCTTAACCTGGGAACTGCATTTGTGACTGGAAGGCTAGAATCTGGCAGAGGGGGGTAGAATTCCACGTGTAGCAGTGAAATGCGTAGAGATGTGGAGGAACACCGATGGCGAAGGCAGCCCCCTGGGCTAAGATTGACGCTCATGCACGAAAGCGTGGGGAGCAAACAGG` |
| ASV2333 | 404 | `TGAGGAATCTTCCGCAATGGGCGAAAGCCTGACGGAGCGACACCGCGTGGAGGATGACGCGTTTACTCGTGTAAACTCCTTTTCTGTGGGAAGATAATGACGGTACCACAGGAATAAGGGGCGGCAAACTACGTGCCAGCAGCCGCGGTAATACGTAGGCCCCAAGCGTTATCCGGAATTACTGGGTGTAAAGCGTCTGTAGGCGGGAAAATAAGTCTTCCATGAAAGACCGGAGCTCAACTCCGTGTTTGTGGGAGATACTGTTTTTCTAGAATCGGGGAGAGGGAAGCGGAATGGTATGAGTAGGGGTGCAATCCGTTGATACATATCAGAACACCAAAAGCGAAGGCAGCTTCCTGGAACCGCATTGACGCTGAGAGACGAAAGCGTGGGGAGCGAAAAGG` |
| ASV4601 | 429 | `TGGGGAATTTTGGACAATGGGGGCAACCCTGATCCAGCAATGCCGCGTGAGTGAAGAAGGCCTTCGGGTTGTAAAGCTCTTTTGTCAGGGAAGAAATCCTTAGTGCTAATATCGCTGAGGGATGACGGTACCTGAAGAATAAGCACCGGCTAACTACGTGCCAGCAGCCGCGGTAATACGTAGGGTGCAAGCGTTAATCGGAATTACTGGGCGTAAAGCGTGCGCAGGCGGCTTCGCAAGTCAGATGTGAAATCCCCGAGCTCAACTTGGGAACTGCATTTGAAACTACGAAGCTAGAGTGTGTCAGAGGGAGGTAGAATTCCACGTGTAGCAGTGAAATGCGTAGAGATGTGGAGGAATACCAGTGGCGAAGGCGGCCTCCTGGGATAACACTGACGCTCATGTACGAAAGCGTGGGGAGCGAACAGG` |
| ASV4695 | 423 | `TAGGGAATATTGGGCAATGGTCGGAAGACTGACCCAGCCATGCCGCGTGCAGGATGAAGGCGCTATGCGTTGTAAACTGCTTTTGTCAGGGAAGAAAACAGCTATGCGTAGCAATTTGACGGTACCTGAAGAATAAGCATCGGCTAACTCCGTGCCAGCAGCCGCGGTAATACGGAGGATGCAAGCGTTGTCCGGATTTATTGGGTTTAAAGGGTGCGTAGGCGGTCTTATAAGTCAGTGGTTAAATAACTTTGCTTAACAAGGGAAATGCCATTGATACTGTTCGACTTGAATACTGACTAGGTAGGCGGAATTGGTAGTGTAGCGGTGAAATGCTTAGATATTACCAGGAACACCGATAGCGAAGGCAGCTTACTGGACAGTTATTGACGCTGAGGCACGAAAGTGCGGGTATCAAACAGG` |
| ASV3034 | 407 | `TGGGGAATTTTCCGCAATGGGCGCAAGCCTGACGGAGCAATACCGCGTGAGGGAGGAAGGCTCTTGGGTCGTAAACCTCTTTTCTCAGGGAAGAACACAATGACGGTACCTGAGGAATCAGCATCGGCTAACTCCGTGCCAGCAGCCGCGGTAATACGGAGGATGCAAGCGTTATCCGGAATGATTGGGCGTAAAGCGTCCGTAGGTGGTTTTGTAAGTCTGTGGTTAAAGCGCGAAGCTTAACTTCGTAAGGGCCATGGAAACTACATAACTTGAGTAAAGTAGGGGTAGAGGGAATTCCCAGTGTAGCGGTGAAATGCGTAGAGATTGGGAAGAACACCGGTGGCGAAAGCGCTCTACTGGACTTAAACTGACACTGAGGGACGAAAGCTAGGGGAGCGAAAGGG` |
| ASV2165 | 424 | `TGAGGAATATTGGTCAATGGGCGGAAGCCTGAACCAGCCAAGTAGCGTGCAGGATGACGGCCCTATGGGTTGTAAACTGCTTTTATACAGGAATAAAGTTAGCCACGTGTGGTTATTTGTAGGTACTGTATGAATAAGGACCGGCTAATTCCGTGCCAGCAGCCGCGGTAATACGGAAGGTCCAGGCGTTATCCGGATTTATTGGGTTTAAAGGGAGCGTAGGCCGTCTGTTAAGCGTGTTGTGAAATGTAGATGCTCAACATCTGAATTGCAGCGCGAACTGGCAGACTTGAGTGTGCGCAACGTAGGCGGAATTCGTGGTGTAGCGGTGAAATGCTTAGATATCACGAAGAACTCCGATTGCGAAGGCAGCTTACGGGAGCACAACTGACGCTGAAGCTCGAAAGTGCGGGTATCGAACAGG` |
| ASV2901 | 404 | `TAGGGAATATTGGACAATGGGGGCAACCCTGATCCAGCAATGCCGCGTGAGTGATGAAGGCCTTAGGGTTGTAAAGCTCTTTCGCCGGGGACGATAATGACGGTACCCGGAGAAGAAGCCTCGGCTAACTCCGTGCCAGCAGCCGCGGTAAGACGGAGGAGGCTAGCGTTGTTCGGAATTACTGGGCGTAAAGGGCGCGTAGGCGGCCTGTTAAGTCGGACGTGAAAGCCCGGGGCTCAACCCCGGAGTTGCGTTCGGGACTGGCGGGCTTGAGGATGGAAGAGGATGGTGGAATTCCCAGTGTAGAGGTGAAATTCGTAGATATTGGGAAGAACACCGGTGGCGAAGGCGGCCATCTGGTCCATACCTGACGCTGAGGCGCGAAAGCGTGGGGAGCAAACAGG` |
| ASV5451 | 404 | `TGGGGAATATTGGACAATGGGCGCAAGCCTGATCCAGCAATACCGCGTGGGTGATGAAGGTCTTCGGATTGTAAAGCCCTTTTAGTGGAGAAGATAATGACGGTATCCACAGAAAAAGCACCGGCAAACTTCGTGCCAGCAGCCGCGGTAATACGAAGGGTGCTAGCGTTGTTCGGAATCACTGGGCGTAAAGGGTACGTAGGCGGCTTGGTAAGTCGATTGTGAAAGCCCCGAGCTCAACTCGGGAACTGCAACCGATACTGCTTGGCTTGAATTTGGTAGGGGATGGCGGAATTCCTAGTGTAGGGGTGAAATCCGTAGAGATTAGGAAGAACACCAGTGGCGAAGGCGGCCATCTATGCCAATATTGACGCTAAGGTACGAAAGCGTGGGGAGCAAACAGG` |
| ASV1592 | 404 | `TTAGGAATATTGGACAATGGGCGCAAGCCTGATCCAGCCATGCCGCGTGAGTGATGAAGGCCTTCGGGTTGTAAAACTCTTTAGCCGATGATTATAATGACTGTAGTCGGAAAATAAGCCCCGGCTAACTTCGTGCCAGCAGCCGCGGTAATACGAAGGGGGCGAGCGTTATTCGGAATCACTGGGCGTAAAGCGTGCGTAGGCGGTCTTGTAAGTTAGGAGTGAAAGCCCAGGGCTTAACCTTGGAATTGCTCTTAAAACTGCAAGGCTCGAATCTCGGAGAGGATAGCGGAATTTCCAGTGTAGCAGTGAAATGCGTAGATATTGGAAGGAACACCAGTGGCGTAAGCGGCTATCTGGACGAGTATTGACGCTGAGGCACGAAAGCGTGGGGATCAAACAGG` |
| ASV2636 | 429 | `TGGGGAATATTGGACAATGGGGGCAACCCTGATCCAGCCATGCCGCGTGTGTGAAGAAGGCCTTCGGGTTGTAAAGCACTTTAAGGGGGGAGGAAGGTTCGCTGGCTAATATCCAGCGGAATTGACGTTACCCCCAGAATAAGCACCGGCTAACTCCGTGCCAGCAGCCGCGGTAATACGGAGGGTGCAAGCGTTAATCGGAATTACTGGGCGTAAAGCGCGCGTAGGCGGCTTGATAAGTCGGATGTGAAATCCCTGGGCTCAACCTGGGAACTGCATTCGATACTGTCTGGCTAGAGTGTGGGAGAGGGTGGTGGAATTCCCGGTGTAGCGGTGAAATGCGTAGATATCGGGAGGAACATCAGTGGCGAAGGCGACCGCCTGGCCCAACACTGACGCTGAGGTGCGAAAGCGTGGGTAGCAAACAGG` |
| ASV4998 | 406 | `TGGGGAATATTGCGCAATGGGCGAAAGCCTGACGCAGCAACGCCGCGTGCGGGATGAAGGCCTTCGGGTTGTAAACCGCTTTCAGTGGGGACGAAAACAGACGGTACCCACAGAAGAAGCCCCGGCCAACTACGTGCCAGCAGCCGCGGTAACACGTAGGGGGCGAGCGTTGTCCGGATTTATTGGGCGTAAAGAGCTCGTAGGCGGCTCGGTAAGTGAGGTGTGAAAACTCCAGGCTCAACCTGGAGACGCCACCTCATACTGCCGTGGCTAGAGTCCGGTAGGGGAGCATGGAATTCCTGGTGTAGCGGTGAAATGCGCAGATATCAGGAGGAACACCAGTGGCGAAGGCGGTGCTCTGGGCCGGAACTGACGCTGAGGAGCGAAAGCGTGGGGAGCAAACAGG` |
| ASV1997 | 429 | `TGGGGAATTTTGGACAATGGGCGAAAGCCTGATCCAGCAATGCCGCGTGCAGGATGAAGGCCTTAGGGTTGTAAACTGCTTTTGTACGGAACGAAACGGTCTCTCTTAATACGGGGGGCTAATGACGGTACCGTAAGAATAAGCACCGGCTAACTACGTGCCAGCAGCCGCGGTAATACGTAGGGTGCGAGCGTTAATCGGAATTACTGGGCGTAAAGCGTGCGCAGGCGGTTATATAAGACAGATGTGAAATCCCCGGGCTCAACCTGGGACCTGCATTTGTGACTGTATAGCTAGAGTACGGTAGAGGGGGATGGAATTCCGCGTGTAGCAGTGAAATGCGTAGATATGCGGAGGAACACCGATGGCGAAGGCAATCCCCTGGACCTGTACTGACGCTCATGCACGAAAGCGTGGGGAGCAAACAGG` |
| ASV3485 | 406 | `TGGGGAATTTTCCGCAATGGGCGAAAGCCTGACGGAGCAACGCCGCGTGAGGGAGGAAGGCCTGTGGGTTGTAAACCTCTTTTCTCAAGGAAGAAGTGCTGACGGTACTTGAGGAATCAGCATCGGCTAACTCCGTGCCAGCAGCCGCGGTAAGACGGAGGATGCAAGCGTTATCCGGAATTATTGGGCGTAAAGCGTCCGTAGGCGGTTTCAAAAGTCTGTTGTTAAAGCTCACAGCTCAACTGTGAATCGGCAATGGAAACTGTGAGACTAGAGAGTGGTAGGGGTAGAGGGAATTCCTAGTGTAGCGGTGAAATGCGTAGATATTAGGAAGAACACCAGTGGCGAAGGCGCTCTACTGGGCCATTACTGACGCTGATGGACGAAAGCTAGGGGAGCGAAAGGG` |
| ASV3449 | 424 | `TAAGGAATATTGGTCAATGGACGCAAGTCTGAACCAGCCATGCCGCGTGGAGGATGAAGGTCCTCTGGATTGTAAACTTCTTTTATTTGGGAAGAAATCCAAGGTTTCTACTTTGGTTGACGGTACCAGATGAATAAGCACCGGCTAACTCCGTGCCAGCAGCCGCGGTAATACGGAGGGTGCAAGCGTTATCCGGATTCACTGGGTTTAAAGGGTGCGTAGGCGGGCAGGTAAGTCAGTGGTGAAATCCCCGAGCTTAACTTGGGAACTGCCGTTGATACTATCTGTCTTGAATACTGTGGAGGTAAGCGGAATATGTCATGTAGCGGTGAAATGCTTAGATATGACATAGAACACCTATTGCGAAGGCAGCTTGCTACACAGTCATTGACGCTGAGGCACGAAAGCGTGGGGATCAAACAGG` |
| ASV4744 | 388 | `GCGCGAAAACTTTACAATGCACGAAAGTGTGATAAGGGAATCCCAAGTGCTTTGTCTTCGGATAAAGCTTTTGCCAAGTCTAAACAGCTTGGAGAATAAGTGATGGGTAAGACGGGTGCCAGCCGCCGCGGTAACACCTGCGTCACAAGTGGCGATCACAATTATTGGGTCTAAAGCGTTCGTAGCCGGTTTAATAAGTCTTTTGTGAAATTGTTGGTCTTAAGCTAACGGCGTGCAAAAGAAACTATCAAACTAGAGACAGGGAGGGGGCAATGGTATTTCAGGGGGAGCGGTAAAATGTTAGAATCCCTGGAGGACCACCAGTGGCGAAGGCGATTGCCTAGAACTGGTCTGACGGTGAGGAACGAAGGCTAGAGGAGCAAAACGG` |
| ASV863 | 429 | `TGGGGAATTTTGGACAATGGGCGAAAGCCTGATCCAGCCATGCCGCGTGCAGGATGAAGGCCTTCGGGTTGTAAACTGCTTTTGTACGGAACGAAAAGCTCTGGACTAATACTCTGGGGTCATGACGGTACCGTAAGAATAAGCACCGGCTAACTACGTGCCAGCAGCCGCGGTAATACGTAGGGTGCAAGCGTTAATCGGAATTACTGGGCGTAAAGCGTGCGCAGGCGGTTATATAAGACAGATGTGAAATCCCCGGGCTCAACCTGGGAACTGCATTTGTGACTGTATAGCTAGAGTACGGCAGAGGGGGATGGAATTCCGCGTGTAGCAGTGAAATGCGTAGATATGCGGAGGAACACCGATGGCGAAGGCAATCCCCTGGGCCTGTACTGACGCTCATGCACGAAAGCGTGGGGAGCAAACAGG` |
| ASV3174 | 410 | `TTTGGAATATTGGACAATGGACGAAAGTCTGATCCAGCGACGCCGCGTGCACGATGAAGGCCTTCGGGTCGTAAAGTGCTTTTAGCAGGGAAAAATTTCTGATTGTACCTGCAGAATAAGAGGTTGCTAACTCTGTGCCAGCAGCAGCGGTAATACAGAGACCTCAAGCGTTATCCGGAATCATTGGGCGTAAAGCGTGCCGATAGGTGGTTTATTTAGTCTTGGGTAAAAGCCAGAAGCTCAACTTCTGAGTTTGCTTAAGATACTAATAGACTAGAGGGGCAAAGAGGTGCTTGGAACGAACGGTGTAGTAGTGAAATGCGTTGATATCGTTCGGAACACCGAAAGCGAAGGCAGAGCACTGGTTGCCACCTGACACTGCTAGGACGAAAGCGTGGGGAGCGAATGGG` |
| ASV4868 | 423 | `TGAGGAATATTGGTCAATGGTCGGAAGACTGAACCAGCCATGCCGCGTGAAGGTCAGTGCCCTATGGGCGTTAAACTTCTTTTGTGTGAGAGCAATAAGGACCTCGTGAGGTCCGATGAGAGTATCACACGAATAAGCATCGGCTAACTCCGTGCCAGCAGCCGCGGTAATACGGGGGATGCGAGCGTTATCCGGATTTATTGGGTTTAAAGGGTGCGTAGGCGGCCTGTTAAGTCAGCGGTGAAATATCCTCGCTCAACGAGGAGACTGCCGTTGATACTGGTGGGCTTGAATGCGGATGCCGTTGGAGGAATGTGTGGTGTAGCGGTGAAATGCATAGATATCACACAGAACACCGATTGCGAAGGCATCTGACGAATCCGCGATTGACGCTGATGCACGAAAGCGTGGGTATCAAACAGG` |
| ASV3399 | 429 | `TGGGGAATATTGCGCAATGGAGGAAACTCTGACGCAGCGACGCCGCGTGGGTGATGAAGGCCTTCGGGTTGTAAAGCCCTGTCGGCTGGGAAAAAGGTAGTCGTGCTAATACCACGATTGCTTGATGGTACCAGCAAAGGAAGCACCGGCTAACCCTGTGCCAGCAGCCGCGGTAATACAGGGGGTGCAAGCGTTGTTCGGAATTACTGGGCGTAAAGGGCGCGTAGGCGGCCAGATAAGTCAGATGTGAAATCCCGGGGCTCAACTCCGGACGTGCATTTGAAACTATTTGGCTAGAGTACAGTAGAGGTTGGTGGAATTCCCAGTGTAGAGGTGAAATTCGTAGATATTGGGAGGAACACCGGTGGCGAAGGCGGCCAACTGGGCTGTAACTGACGCTGAGGCGCGAAAGCGTGGGTAGCAAACAGG` |
| ASV1609 | 424 | `TGAGGAATATTGGACAATGGGCGCAAGCCTGATCCAGCCATGCCGCGTGCAGGAAGACGCATCTATGGTGTGTAAACTGCTTTTGTACGGGAAGAAACACTCCTTCGTGAAGGAGCTTGACGGTACCGTAAGAATAAGGATCGGCTAACTCCGTGCCAGCAGCCGCGGTAATACGGAGGATCCAAGCGTTATCCGGAATCATTGGGTTTAAAGGGTCCGTAGGCGGTTTAATAAGTCAGTGGTGAAATCTGGTCGCTCAACGATCAAACGGCCATTGATACTGTTAGACTTGAATTACTAGGAAGTAACTAGAATATGTAGTGTAGCGGTGAAATGCTTAGATATTACATGGAATACCAATTGCGAAGGCAGGTTACTACTAGTGGATTGACGCTGATGGACGAAAGCGTGGGGAGCGAACAGG` |
| ASV2845 | 429 | `TAGGGAATATTGCACAATGGGCGAAAGCCTGATGCAGCAACGCCGCGTGAGTGATGAAGGTTTTCGGATCGTAAAGCTCTGTCGTAAGGGAAAAAACGGGCGTAGGCTAATACCCTTGTCTTTGATTGTACCTTACAAGAAAGCACCGGCTAACTTCGTGCCAGCAGCCGCGGTAATACGAGGGGTGCAAGCGTTGTTCGGAATTACTGGGCGTAAAGCGTGTGTAGGTGGTCATGTAAGTCAGATGTGAAAGCCTGGGGCTCAACCCCAGAAGTGCATCTGAAACTATTTGACTTGAGTATGGGAGAGGGTAGCGGAATTCCTGGTGTAGTGGTAAAATACGTAGATATCAGGAGGAACATCGGTGGCGAAGGCGGCTACCTGGCCCAATACTGACACTGAGACACGAAAGCATGGGGATCAAACAGG` |
| ASV331 | 424 | `TAAGGAATATTGGTCAATGGAGGCAACTCTGAACCAGCCATGCCGCGTGCAGGATGACGGCCCTATGGGTTGTAAACTGCTTTTATACCAGAGAAAACCCGAGTACGTGTACTCGGTTGATAGTATGGTAAGAATAAGCATCGGCTAACTTCGTGCCAGCAGCCGCGGTAAGACGAAGGATGCAAGCGTTATCCGGATTCATTGGGTTTAAAGGGTGCGTAGGCGGACCTGTAAGTCAGTGGTGAAATCTCTTTGCTTAACAAAGAAACTGCCATTGATACTGCAAGTCTAGAGTATAGATGACGTTGGCGGAATATGACATGTAGTGGTGAAATACTTAGATATGTCATAGAACACCGATTGCGAAGGCAGCTAACGAAACTATAACTGACGCTGAGGCACGAAAGTGCGGGGATCAAACAGG` |
| ASV551 | 429 | `TCGAGAATCATTCACAATGGGCGAAAGCCTGATGGTGTGACGCCGCGTGGAGGATGACGGTCTTCGGATTGTAAACTCCTGTCATGTGAGAGTAAGGTTGGAGTAGTAACTGACTGCCAACTTGAGAGTATCACAAGAGGAAGAGGCGGCTAACTCTGTGCCAGCAGCCGCGGTAATACAGAGGCCTCAAGCGTTGTTCGGAATCACTGGGCGTAAAGGGTGCGTAGGCGGCGCGGAAAGTCAGGGGTGAAATCCCGGAGCTCAACTCCGGAACTGCCTTTGATACTCCCGCGCTTGAGAACTGGAGGGGACATCGGAATTCACGGTGGAGCAGTGAAATGCGTGGATATCGTGAGGAACACTAGTGGCGAAGGCGGATGTCTGGACAGTATCTGACGCTGAGGCACGAAGGCCAGGGGAGCGAACGGG` |
| ASV2444 | 404 | `TGGGGAATATTGGACAATGGGCGAAAGCCTGATCCAGCAATACCGCGTGAGTGATGAAGGCCTTAGGGTTGTAAAGCTCTTTTAGTAGGGAAGATAATGACGGTACCTACAGAAAAAGCCCCGGCTAACTCCGTGCCAGCAGCCGCGGTAAGACGGAGGGGGCTAGCGTTGTTCGGAATTACTGGGCGTAAAGAGCGCGTAGGCGGTTTAGTAAGTTGGAAGTGAAAGCCCGAGGCTTAACCTCGGAACTGCTTTCAAAACTGCTAGACTTGAGTATGGTAGAGGGTGATAGAATTCCTAGTGTAGAGGTGAAATTCTTAGATATTAGGAGGAATACCGGTGGCGAAGGCGATCACCTGGGCCATTACTGACGCTGAGGTGCGAAAGCGTGGGGAGCAAACAGG` |
| ASV1827 | 404 | `TGGGGAATCTTGGACAATGGGGGCAACCCTGATCCAGCGATGCCGCGTGAATGAAGAAGGCCTTAGGGTTGTAAAGTTCTTTCACCCGTGACGATAATGACGGTAGCGGGAGAAGAAGCCCCGGCTAACTCCGTGCCAGCAGCCGCGGTAAGACGGAGGGGGCTAGCGTTGTTCGGAATGACTGGGCGTAAAGGGCGTGTAGGCTGTTGATCAAGTCAAGCGTGAAAGCCTCGGGCTTAACCCGAGAATGGCGTTTGATACTGGTTGACTTGAGGACGAGAGAGGAAAGTGGAATTCCGAGTGTAGAGGTGAAATTCGTAGATATTCGGAGGAACATCAGAGGCGAAGGCGACTTTCTGGCTCGTATCTGACGCTGAGGCGCGAAAGCGTGGGGAGCAAACAGG` |
| ASV4091 | 431 | `TGGGGAATATTGGACAATGGGCGCAAGCCTGATCCAGCAATACCGCGTGTGTGAAGAAGGCCTTCGGGTTGTAAAGCACTTTAGGTGGGGAGGACGATGATGAAGGTTAATACCCTTTGTTATTGACGTTACCCACAGAATAAGCACCGGCTAACTCCGTGCCAGCAGCCGCGGTAATACGGAGGGTGCAAGCGTTAATCGGAATTACTGGGCGTAAAGGGCGCGTAGGCGGTCATTTAAGTCAGATGTGAAATCCCCGGGCTCAACCTGGGAATTGCATTTGATACTGGGTGACTTGGAATGTGGTAGAGGGTAGCGGAATTTCTGGTGTAGCGGTGAAATGCGTAGATATCAGAAGGAACATCAATGGCGAAGGCAGCTACCTGGGCCAACATTGACGCTGAGGCGCGAAAGCGTGGGGAGCAAACAGG` |
| ASV925 | 404 | `TGGGGAATCTTAGACAATGGGCGCAAGCCTGATCTAGCCATGCCGCGTGAGCGATGAAGGCCTTAGGGTTGTAAAGCTCTTTCAGTGGGGAAGATAATGACTGTACCCACAGAAGAAGCCCCGGCTAACTCCGTGCCAGCAGCCGCGGTAATACGGAGGGGGCTAGCGTTGTTCGGAATTACTGGGCGTAAAGCGCACGTAGGCGGACTGGAAAGTCAGGGGTGAAATCCCGGGGCTCAACCCCGGAACTGCCTTTGAAACTCCCGGTCTTGAGGTCGAGAGAGGTGAGTGGAATTCCGAGTGTAGAGGTGAAATTCGTAGATATTCGGAGGAACACCAGTGGCGAAGGCGGCTCACTGGCTCGATACTGACGCTGAGGTGCGAAAGCGTGGGGAGCAAACAGG` |
| ASV2505 | 430 | `TAGGGAATCTTCCGCAATGGACGAAAGTCTGACGGAGCAACGCCGCGTGAGTGATGAAGGCTTTCGGGTCGTAAAACTCTGTTGTTAGGGAAGAACAAGTGCTAGTTGAATAAGCTGGCACCTTGACGGTACCTAACCAGAAAGCCACGGCTAACTACGTGCCAGCAGCCGCGGTAATACGTAGGTGGCAAGCGTTATCCGGAATTATTGGGCGTAAAGCGCGCGCAGGTGGTTTCTTAAGTCTGATGTGAAAGCCCACGGCTCAACCGTGGAGGGTCATTGGAAACTGGGAGACTTGAGTGCAGAAGAGGAAAGTGGAATTCCATGTGTAGCGGTGAAATGCGTAGAGATATGGAGGAACACCAGTGGCGAAGGCGACTTTCTGGTCTGTAACTGACACTGAGGCGCGAAAGCGTGGGGAGCAAACAGG` |
| ASV3780 | 424 | `TGAGGAATATTGGTCAATGGGCGTAAGCCTGAACCAGCCAAGTAGCGTGCAGGATGACGGCCCTATGGGTTGTAAACTGCTTTTATGCGGGGATAAAGTGTGGGACGTGTCCCATATTGCAGGTACCGCATGAATAAGGACCGGCTAATTCCGTGCCAGCAGCCGCGGTAATACGGAAGGTCCGGGCGTTATCCGGATTTATTGGGTTTAAAGGGAGCGTAGGCCGTCGTTTAAGCGTGTTGTGAAATGCTGCGGCTCAACCGTGGCACTGCAGCGCGAACTGGATGACTTGAGTTCGCGGGAGGTAGGCGGAATTCGTGGTGTAGCGGTGAAATGCTTAGATATCACGAAGAACTCCGATTGCGAAGGCAGCTTACCGTAGCGTTACTGACGCTGAAGCTCGAAAGTGCGGGTATCGAACAGG` |
| ASV5556 | 407 | `TGGGGAATTTTCCGCAATGGGCGAAAGCCTGACGGAGCAACGCCGCGTGAGGGAGGAAGGCTCTTGGGTTGTAAACCTCTTTTCACAGGGAAGAAGAAAGTGACGGTACCTGAGGAATAAGCATCGGCTAACTCCGTGCCAGCAGCCGCGGTAATACGGAGGATGCAAGCGTTATCCGGAATCATTGGGCGTAAAGCGTCCGCAGGTGGTTATACAAGTCTGTTGTCAAAGACAGTAGCTCAACTACTGAAAGGCAGAAGAAACTGAATAACTAGAGTACGGTAGGGGTAGCAGGAATTCCCAGTGTAGCGGTGAAATGCGTAGAGATTGGGAAGAACATCGGTGGCGAAGGCGTGCTACTGGGCCGAAACTGACACTGAGGGACGAAAGCTAGGGGAGCGAAAGGG` |
| ASV2647 | 406 | `TAGGGAATATTGGACAATGGGGGCAACCCTGATCCAGCGACGCCGCGTGAGCGATGAAGGCCTTCGGGTCGTAAAGCTCTGTTGTAAGGGAAAAAGTTCTGATTGTACCTTACGAGAAAGGATCGGCTAACTTCGTGCCAGCAGCCGCGGTAAGACGAGGGATCCTAGCGTTGTTCGGAATTATTGGGCGTAAAGCGGGTGCAGATGGCCTTGTAAGTCGAGTGTGAAAGCCTGGGGCTTAACCCCAGAAGTGCATTCGATACTGCGAGGCTTGAGTGTTGGAGAGGCTATTAGAATTCCTGGTGTAGTGGTGAAATACGTAGATATCAGGAGGAATACCGGAGGCGAAGGCGGATAGCTGGCCAAACACTGACATTCAGACCCGAAAGCGTGGGTATCAAACAGG` |
| ASV4887 | 429 | `TGGGGAATATTGGACAATGGGGGCAACCCTGATCCAGCGATGCCGCGTGTGTGAAGAAGGCCTGCGGGTTGTAAAGCACTTTAGTGGGGGAGGAAAGTGTTAACGCTAATAACGTTAACAATTGACGTTACCCCAAGAATAAGCACCGGCTAACTCTGTGCCAGCAGCCGCGGTAATACAGAGGGTGCAAGCGTTAATCGGAGTTACTGGGCGTAAAGGGTGCGTAGGAGGCTATGTAAGTTGGATGTGAAATACTCGGGCTTAACCTGGGGGCTGCATTCAATACTGCATAGCTAGAGTAGGGTAGAGGGAAGTGGAATTTCCGGTGTAGCGGTGAAATGCGTAGATATCGGAAGGAACACCAGTGGCGAAGGCGGCTTCCTGGACCTATACTGACTCTGAAGCACGAAAGCGTGGGGATCAAACAGG` |
| ASV4700 | 426 | `TCGAGAATCTTCCGCAATGGGCGAAAGCCTGACGGAGCGACACCGCGTGCAGGATGAAGGCCTTCGGGTTGTAAACTGCTGTCAGGTTCTAGGAATGATTGGATGTGAACAACATCCAAGGTGACCAAGACCAAAGGAAGCAACGGCTAACTCTGTGCCAGCAGCCGCGGTAAGACAGAGGTTGCAAACGTTGTTCGGAATTACTGGGCATAAAGCGCACGTAGGCGGTTCGTTAAGTCATGTGTGAAATCCCCCGGCTCAACCGGGGAATTGCGCCTGATACTGGCGAGCTAGAGATCGATAGGGGTGAGCGGAACTCCAGGTGGAGCGGTGAAATGCGTAGATATCTGGAGGAACACCGGTGGCGAAAGCGGCTCACTGGATCGAAACTGACGCTGAGGTGCGAAAGCTAGGGTAGCAAACGGG` |
| ASV3564 | 405 | `TGGGGAATATTGGACAATGGGCGCAAGCCTGATCCAGCCATGCCGCGTGAGTGATGAAGGCCCTAGGGTTGTAAAGCTCTTTCGACGGGGACGATAATGACGGTACCCGTAGAAGAAGCCCCGGCTAACTTCGTGCCAGCAGCCGCGGTAATACGAAGGGGGCTAGCGTTGTTCGGAATCACTGGGCGTAAAGCGCACGTAGGCGGATCGTTAAGTCGAGGGTGAAATCCCAGGGCTCAACCCTGGAACTGCCTTCGATACTGGCGATCTTCGAGTCCGGGAGAGGTGAGTGGAACTCCGAGTGTAGAGGTGAAATTCGTAGATATTCGGAAGAACACCAGTGGCGAAGGCGGCTCACTGGCCCGGTACTGACGCTGAGGTGCGAAAGCGTGGGGAGCAAACAGG` |
| ASV3042 | 424 | `TGAGGAATATTGGACAATGGGCGCAAGCCTGATCCAGCCATGCCGCGTGCAGGATGAAGCATCTATGGTGTGTAAACTGCTTTTATACAGGAAGAAACCCTCCCACGTGTGGGAGATTGACGGTACTGTAGGAATAAGGATCGGCTAACTCCGTGCCAGCAGCCGCGGTAATACGGAGGATCCAAGCGTTATCCGGAATCATTGGGTTTAAAGGGTCCGTAGGCGGCCTTATAAGTCAGTGGTGAAATCTCCCGGCTCAACCGGGAAATGGCCATTGATACTGTAGGGCTTGAATTATTAGGAAGTAACTAGAATATGTAGTGTAGCGGTGAAATGCTTAGAGATTACATGGAATACCAATTGCGAAGGCAGGTTACTACTAATGGATTGACGCTGATGGACGAAAGCGTGGGTAGCGAACAGG` |
| ASV850 | 404 | `TGAGGAATTTTCCGCAATGGGCTCAAGCCTGACGGAGCAATACCGCGTGAAGGATGAAGGCCTGCGGGTTGTAAACTTCTTTTCTTAGAGAAGATAATGACGGTATCTGAGGAATAAGCATCGGCTAACCCTGTGCCAGCAGCCGCGGTAATACAGGGGATGCAAGCGTTATCCGGAATGATTGGGCGTAAAGCGTCTGTAGGTGGTTTTGAAAGTCGATTGTCAAAAACCAAGGCTTAACCTTGGGCCGGCAATGGAAACTTCAAGACTAGAGTATGGTAGAGGTAGAGGGAATTACCGGTGTAACGGTGAAATGTGCAGATATCGGTAAGAACACCAAAGGCGAAAGCACTCTACTGGGCCAAAACTGACACTCAGAGACGAAAGCTAGGGGAGCGAATAGG` |
| ASV152 | 429 | `TGGGGAATTTTGGACAATGGGCGAAAGCCTGATCCAGCAATGCCGCGTGCAGGAAGAAGGCCTTCGGGTTGTAAACTGCTTTTGTACGGAACGAAACGGTTCGCTTTAATACAGTGGGCTAATGACGGTACCGTAAGAATAAGCACCGGCTAACTACGTGCCAGCAGCCGCGGTAATACGTAGGGTGCGAGCGTTAATCGGAATTACTGGGCGTAAAGCGTGCGCAGGCGGTTATATAAGACAGTTGTGAAATCCCCGGGCTCAACCTGGGAATTGCATCTGTGACTGTATAGCTAGAGTACGGTAGAGGGGGATGGAATTCCGCGTGTAGCAGTGAAATGCGTAGATATGCGGAGGAACACCGATGGCGAAGGCAATCCCCTGGACCTGTACTGACGCTCATGCACGAAAGCGTGGGGAGCAAACAGG` |
| ASV2887 | 428 | `TCGAGAATTTTTCTCAATGGGGGAAACCCTGAAGGAGCGACGCCGCGTGGGGGATGAATGGCTTCGGCCCGTAAACCCCTGTCATTTGCGATCAAACCTTACGACCTAACACGTCGTGAGCTGATAGTAGCGGAAGAGGAAGGGACGGCTAACTCTGTGCCAGCAGCCGCGGTAATACAGAGGTCCCAAGCGTTGTTCGGATTCACTGGGCGTAAAGGGTGCGTAGGCGGTCGGGTAAGTCTGACGTGAAATCTTCAAGCTCAACTTGGAAACTGCGTCGGATACTATTCGGCTAGAGGAATGGAGGGGAGACTGGAATACTTGGTGTAGCAGTGAAATGCGTAGATATCAAGTGGAACACCAGTGGCGAAGGCGAGTCTCTGGACATTTCCTGACGCTGAGGCACGAAAGCCAGGGGAGCAAACGGG` |
| ASV2276 | 404 | `TGGGGAATATTGGACAATGGGCGCAAGCCTGATCCAGCAATGCCGCGTGTGTGAAGAAGGTCTTCGGATTGTAAAGCACTTTCGGCGGGGACGATGATGACGGTACCCGCAGAAGAAGCCCCGGCTAACTTCGTGCCAGCAGCCGCGGTAATACGAAGGGGGCTAGCGTTGCTCGGAATGACTGGGCGTAAAGGGTGCGTAGGCGGCGATGTAAGTCAGACGTGAAATTCCTGGGCTTAACCTGGGGACTGCGTTTGAGACTGTGTTGCTTGAGGGTGGAAGAGGCTCGTGGAATTCCCAGTGTAGAGGTGAAATTCGTAGATATTGGGAAGAACACCGGTGGCGAAGGCGGCGAGCTGGTCCATTACTGACGCTGAGGCACGATAGCGTGGGGAGCAAACAGG` |
| ASV1218 | 404 | `TGGGGAATATTGGACAATGGGGGCAACCCTGATCCAGCAATGCCGCGTGAGTGAAGAAGGCCTTAGGGTTGTAAAGCTCTTTTTTCAGGGAAGATAATGACGGTACCTGAGGAATAAGTCCCGGCTAACTCCGTGCCAGCAGCCGCGGTAATACGGAGGGGGCAAGCGTTGTTCGGAATTACTGGGCGTAAAGGGCGTGTAGGCGGCTTTATAAGTCAGACGTGAAAGTCCTGGGCTTAACCTAGGGATTGCGTTTGAGACTGTGAGGCTAGAGTGCGAGAGAGGAGAGTAGAATTCCAAGTGTAGAGGTGAAATTCGTAGATATTTGGAGGAATACCGGTGGCGAAGGCGGCTCTCTGGATCGTAACTGACGCTGAGGCGCGAAAGCGTGGGGAGCAAACAGG` |
| ASV416 | 424 | `TAGGGAATATTGGGCAATGGGCGAGAGCCTGACCCAGCCATGCCGCGTGCAGGAAGACGGCCCTCTGGGTTGTAAACTGCTTTTGTAGGGGAAGAACGGCAGGGGTGCGCCCTTGTGTGACGGTACTCTACGAATAAGCACCGGCTAACTCCGTGCCAGCAGCCGCGGTAATACGGAGGGTGCAAGCGTTGTCCGGATTTATTGGGTTTAAAGGGTGCGTAGGTGGGTTGGTAAGTCTGGTTTGAAAGTTGGTCGCTTAACGATCAAGGGTGGCTGGATACTGCTGATCTTGAATGTTCTTGAGGTAGCCGGAATGGGTTGTGTAGCGGTGAAATGCATAGAGATGACCCGGAACACCGATTGCGAAGGCAGGCTACTGAGGAATGATTGACACTGAGGCACGAGAGCATGGGGAGCAAACAGG` |
| ASV5243 | 429 | `TGGGGAATTTTGGACAATGGGCGAAAGCCTGATCCAGCCATGCCGCGTGAGTGAAGAAGGCCTTCGGGTTGTAAAGCTCTTTCAGCCGGAAAGAAATCGCGCAGGCTAATACTCTGCGTGGATGACGGTACCGGAAGAAGAAGCACCGGCTAACTACGTGCCAGCAGCCGCGGTAATACGTAGGGTGCGAGCGTTAATCGGAATTACTGGGCGTAAAGCGTGCGCAGGCGGTTATATAAGACAGATGTGAAATCCCCGGGCTCAACCTGGGAACTGCGTTTGTGACTGTATGACTAGAGTACGGCAGAGGGAGGTGGAATTCCGCGTGTAGCAGTGAAATGCGTAGATATGCGGAGGAACACCGATGGCGAAGGCAGCCTCCTGGGCCAGTACTGACGCTCATGCACGAAAGCGTGGGGAGCAAACAGG` |
| ASV1897 | 429 | `TGGGGAATTTTGGACAATGGGCGAAAGCCTGATCCAGCAATGCCGCGTGTAGGATGAAGGCCCTCGGGTTGTAAACTACTTTTGTACGGAACGAAAAGACTCTTTCTAATAAAGAGGGTCCATGACGGTACCGTAAGAATAAGCACCGGCTAACTACGTGCCAGCAGCCGCGGTAATACGTAGGGTGCAAGCGTTAATCGGAATTACTGGGCGTAAAGCGTGCGCAGGCGGTTATGTAAGACAGAGGTGAAATCCCCGGGCTCAACCTGGGAACGGCCTTTGTGACTGCATAGCTAGAGTACGGTAGAGGGGGATGGAATTCCGCGTGTAGCAGTGAAATGCGTAGATATGCGGAGGAACACCGATGGCGAAGGCAATCCCCTGGACCTGTACTGACGCTCATGCACGAAAGCGTGGGGAGCAAACAGG` |
| ASV812 | 424 | `TGAGGAATATTGGTCAATGGGCGCAAGCCTGAACCAGCCATGCCGCGTGCAGGATGACGCATCTATGGTGTGTAAACTGCTTTTGCACAGGAAGAAACACTCCCTCGTGAGGGAGCTTGACGGTACTGTAAGAATAAGGATCGGCTAACTCCGTGCCAGCAGCCGCGGTAATACGGAGGATCCAAGCGTTATCCGGAATCATTGGGTTTAAAGGGTCCGTAGGCGGTTTGATAAGTCAGTGGTGAAATCCCATCGCTCAACGATGGAACGGCCATTGATACTGTCAGACTTGAATTACTAGGAAGTAACTAGAATATGTAGTGTAGCGGTGAAATGCTTAGATATTACATGGAATACCAATTGCGAAGGCAGGTTACTACTAGTTTATTGACGCTGATGGACGAAAGCGTGGGGAGCGAACAGG` |
| ASV4191 | 410 | `TTTGGAATTTTGGACAATGGGCGAAAGCCTGATCCAGCGACGCCGCGTGGACGATGAAGGCCTTCGGGTCGTAAAGTCCTTTTGCTCGGGAACAATTTTTGAGCGTACCGAGAGAATAAGAGGTTGCTAACTCTGTGCCAGCAGCAGCGGTAATACAGAGACCTCAAGCGTTATCCGGAATCATTGGGCGTAAAGCGTACCGATAGGTGGTTTTACAAGTCAGAAGTGAAATCTTAAAGCTTAACTTTGAGACTGTCTTTTGAAACTGTAAAACTAGAGGGGCAAAGAGGAAGCTGGAACAAACGGTGTAGTAGTGAAATGCGTTGATATCGTTTGGAACACCAATAGCGTAGGCAAGCTTCTGGGTGCCACCTGACACTGCTAGGACGAGAGCGTGGGTAGCGAATGGG` |
| ASV1078 | 404 | `TGGGGAATCTTGGACAATGGGCGCAAGCCTGATCCAGCCATGCCGCGTGAGTGAAGAAGGCCTTCGGGTTGTAAAGCTCTTTTGCCAGGGACGATAATGACGGTACCTGGAGAATAAGCCCCGGCAAACTTCGTGCCAGCAGCCGCGGTAATACGAAGGGGGCTAGCGTTGTTCGGAATTACTGGGCGTAAAGCGCACGTAGGCTGATAGTTAAGTGAGGGGTGAAATCCCGAGGCTCAACCTCGGAACTGCCTTTCATACTGGCTGTCTCGAGTCCGGAAGAGGTTAGTGGAATTCCTAGTGTAGAGGTGAAATTCGTAGATATTAGGAAGAACACCAGTGGCGAAGGCGGCTAACTGGTCCGGTACTGACGCTGAGGTGCGAAAGCGTGGGGAGCAAACAGG` |
| ASV1555 | 429 | `TGGGGAATATTGGACAATGGGCGCAAGCCTGATCCAGCCATGCCGCGTGGGTGAAGAAGGCCTTCGGGTTGTAAAGCCCTTTTGTCCGGAAAGAAAAGCACTGGGTTAATACCTCGGTGTTCTGACGGTACCGGAAGAATAAGCACCGGCTAACTTCGTGCCAGCAGCCGCGGTAATACGAAGGGTGCAAGCGTTACTCGGAATTACTGGGCGTAAAGCGTGCGTAGGTGGTTCGTTAAGTCTGATGTGAAAGCCCTGGGCTCAACCTGGGAATTGCATTGGATACTGGCGGGCTAGAGTGCGGTAGAGGATGGCGGAATTCCCGGTGTAGCAGTGAAATGCGTAGAGATCGGGAGGAACATCTGTGGCGAAGGCGGCCATCTGGACCAGCACTGACACTGAGGCACGAAAGCGTGGGGAGCAAACAGG` |
| ASV215 | 429 | `TCGAGAATAATTCACAATGGGGGCAACCCTGATGGTGCAACGCCGCGTGGAGGATGAAGGTCTTCGGATTGTAAACTCCTGTCATCCGGGAGCAAGACCTGACGGTGAATAGCCGGCAGGGTTGATAGTACCGGAAGAGGAAGGGACGGCTAACTTCGTGCCAGCAGCCGCGGTAATACGAAGGTCCCGAGCGTTGTTCGGAATCACTGGGCGTAAAGGGAGCGTAGGCGGTGCGGTAAGTCAGATGTGAAATCCCGGGGCTCAACCCCGGAACTGCATCCGATACTGCCGTGCTTGAGTGATGGAGAGGTAGCTGGAATTTACGGTGTAGCAGTGAAATGCGTGGATATCGTAAGGAACACTCGTGGCGAAAGCGAGCTACTGGACATTTACTGACGCTGAGGCTCGAAGGCCAGGGTAGCGAAAGGG` |
| ASV1439 | 429 | `TGGGGAATATTGGACAATGGGCGCAAGCCTGATCCAGCCATGCCGCGTGTGTGAAGAAGGCCTTCGGGTTGTAAAGCACTTTTGTTGGGGAAGAAAAGCATTCGGTTAATACCCGGGTGTTCTGACGGTACCCAAAGAATAAGCACCGGCTAACTTCGTGCCAGCAGCCGCGGTAATACGAAGGGTGCAAGCGTTACTCGGAATTACTGGGCGTAAAGCGTGCGTAGGTGGTTCGTTAAGTCTGTCGTGAAAGCCCCGGGCTCAACCTGGGAATGGCGATGGATACTGGCGGGCTAGAGTGCGGTAGAGGAGAGTGGAATTCCCGGTGTAGCAGTGAAATGCGTAGAGATCGGGAGGAACATCAGTTGCGAAGGCGGCTCTCTGGACCAGCACTGACACTGAGGCACGAAAGCGTGGGGAGCAAACAGG` |
| ASV684 | 421 | `TGGGGAATCTTGCGCAATGGCCGAGAGGCTGACGCAGCGACGCCGCGTGGGGGATGAAGCATTTCGGTGTGTAAACCCCTGTTGCTCGGGACGAACGATCCTTTTCGAGGGATGTGACGGTACCGAGTGAGGAAGCACCGGCTAACTCCGTGCCAGCAGCCGCGGTAATACGGAGGGTGCGAGCGTTGTCCGGAATCACTGGGCGTAAAGGGCGCGTAGGTGGTTGGATGCGCCGGTGGTGAAAGCGCAGGGCTCAACCCTGCGTCGGCCATCGGGACGGTCCGACTGGAGCACTGTAGAGGCAGGTGGAATTCCGGGTGTAGCGGTGGAATGCGTAGAGATCCGGAAGAACACCGGTGGCGAAGGCGGCCTGCTGGGCAGTTGCTGACACTGAGGCGCGACAGCGTGGGGAGCAAACAGG` |
| ASV2822 | 404 | `TGGGGAATATTGGACAATGGGGGCAACCCTGATCCAGCGATGCCGCGTGAGTGATGAAGGCCTTCGGGTTGTAAAGCTCTTTTGTGGGGGACGATGATGACGGTACCCCACGAATAAGTCCCGGCTAACTTCGTGCCAGCAGCCGCGGTAATACGAAGGGGGCTAGCGTTGTTCGAAATGACTGGGCGTAAAGGGTGCGTAGGCGGTTTGATAAGTTTGGTGTGAAAGTCCTGGGCTCAACCTGGGGATTGCATTGGATACTGTTGGACTAGAGATCGAGAGAGGAAAATGGAATTGCGAGTGTAGAGGTGAAATTCGTAGATATTCGCAAGAACACCGGTGGCGAAGGCGGTTTTCTGGCTCGATACTGACGCTAAGGCACGAAAGCGTGGGGAGCAAACAGG` |
| ASV4891 | 423 | `TGAGGAATATTGGTCAATGGGCGAGAGCCTGAACCAGCCAAGTCGCGTGAGGGAAGACGGTCTTAAAGATTGTAAACCTCTTTTGCCGGGGAGCAAAGGATGCCACGCGTGGCGTTTCGAGAGTACCCGGAGAAAAAGCATCGGCTAACTCCGTGCCAGCAGCCGCGGTAATACGGAGGATGCGAGCGTTATCCGGATTTATTGGGTTTAAAGGGTGCGTAGGCGGGCCGGTAAGTCAGCGGTAAAATAGCGGGGCTCAACCCCGTCGAGCCATTGAAACTGCCGGTCTTGAGTGGGCGAGAAGTGCGCGGAATGCGTGGTGTAGCGGTGAAATGCATAGATATCACGCAGAACCCCGATTGCGAAGGCAGCGCACCGGCGCCCTACTGACGCTGAAGCACGAAAGCGTGGGGATCGAACAGG` |
| ASV4568 | 428 | `TCGCGAATTTTTCACAATGGGCGCAAGCCTGATGAAGCCACGCCGCGTGCGGGATGAAGGCCCTCTGGGTTGTAAACCGCTGTCGAGGGAGGGGAACCGCTGTGCGTGAATAGCGTACAGTCTGACTGCTCTCTGAAGGAAGCCCCGGCAAAACATGTGCCAGCAGCCGCGGTAATACATGTGGGGCAAGCGTTGTTCGGAATTACTGGGCATAAAGGGTGCGTAGGTGGTTTGTTAAGTCAGGGGTGAAATCCCGCGGCCCAACCGTGGAACTGCCTTTGATACTGGCGAACTTGAGTGCGTGAGGGGTAGGGGGAACGAGCAGTGTAGCGGTGAAATGCGTAGATATTGCTCGGAAGGCCAGAGCCGAAGGGGCCCTACTGGTGCGTAACTGACACTGAGGCACGAAAGCGTGGGGAGCAAACAGG` |
| ASV3669 | 404 | `TCGGGAATTTTGGGCAATGGGCGAAAGCCTGACCCAGCAACGCCGCGTGAAGGATGAAGTATTTCGGTATGTAAACTTCGCAAGAATAGGAAGAATAAATGACGGTACTATTTGTAAGGTCCGGCTAACTACGTGCCAGCAGCCGCGGTAATACGTAGGGACCAAGCGTTGTTCGGATTTACTGGGCGTAAAGGGCGCGTAGGCGGCGTGGTAAGTCACTTGTGAAATCTCTGAGCTCAACTCAGAACGGTCAAGTGATACTGCTATGCTAGAGTGTAGGAAGGGCAATCGGAATTCTTGGTGTAGCGGTGAAATGCGTAGATATCAAGAGGAATATCAGAGGCGAAGGCGGGTTGCTAGACTAACACTGACGCTGAGGCGCGAAAGCTAGGGTAGCAAACGGG` |
| ASV4034 | 404 | `TGGGGAATATTGGACAATGGGCGCAAGCCTGATCCAGCCATGCCGCGTGAGTGATGAAGGCCTTAGGGTTGTAAAGCTCTTTTGTCCGGGACGATAATGACGGTACCGGAAGAATAAGCCCCGGCTAACTTCGTGCCAGCAGCCGCGGTAATACGAAGGGGGCTAGCGTTGCTCGGAATCACTGGGCGTAAAGGGCGCGTAGGCGGCCATTCAAGTCGGGGGTGAAAGCCTGTGGCTCAACCACAGAATTGCCTTCGATACTGTTTGGCTTGAGTTTGGTAGAGGTTGGTGGAACTGCGAGTGTAGAGGTGAAATTCGTAGATATTCGCAAGAACACCAGTGGCGAAGGCGGCCAACTGGACCAATACTGACGCTGAGGCGCGAAAGCGTGGGGAGCAAACAGG` |
| ASV3175 | 424 | `TAAGGAATATTGGACAATGCCCGCAAGGGTGATCCAGCCATGCCGCGTGCAGGAAGAAGGCCCTATGGGTCGTAAACTGCTTTTGAACAAGAGAAAACCCCGATACGTGTATTGGGCTGATAGTATTGTTAGAATAAGCATCGGCTAACTTCGTGCCAGCAGCCGCGGTAAGACGAAGGATGCAAGCGTTATCCGGATTCATTGGGTTTAAAGGGTGCGTAGGCGGCCTTATAAGTCAGTGGTGAAATCTCCTCGCTTAACGAGGAAACTGCCATTGATACTGTAGGGCTTGAGTACAGATGCTGTGGGCGGAATATGACATGTAGTGGTGAAATACATAGAGATGTCATAGAACACCGATTGCGAAGGCAGCTCACAAAACTGTAACTGACGCTGAGGCACGAAAGTGCGGGGATCAAACAGG` |
| ASV3039 | 429 | `TGGGGAATTTTGGACAATGGGCGCAAGCCTGATCCAGCCATGCCGCGTGTGTGAAGAAGGCCTTCGGGTTGTAAAGCACTTTCGGACGGAACGAAATCGCGCGGTTGAATATTCCGCGTGGATGACGGTACCGTAAGAAGAAGCACCGGCTAACTACGTGCCAGCAGCCGCGGTAATACGTAGGGTGCGAGCGTTAATCGGAATTACTGGGCGTAAAGGGTGCGCAGGCGGCCTCGCAAGTCAGACGTGAAATCCCCGGGCTTAACTTGGGAATGGCGTTTGAAACTACGGGGCTGGAGTGTGGCAGAGGGAGGTGGAATTCCACGTGTAGCGGTGAAATGCGTAGAGATGTGGAGGAACACCGATGGCGAAGGCAGCCTCCTGGGCCAACACTGACGCTCATGCACGAAAGCGTGGGGAGCAAACAGG` |
| ASV4482 | 423 | `TAGGGAATATTGGTCAATGGGCGCAAGCCTGAACCAGCCATGCCGCGTGCAGGATGAAGGCCCTCTGGGTTGTAAACTGCTTTTATATGGAAATAAAAAGCCCTTGCGAGGGTCATTGAAGGTACCATAAGAATAAGCACCGGCTAACTCCGTGCCAGCAGCCGCGGTAATACGGAGGGTGCAAGCGTTATCCGGATTTACTGGGTTTAAAGGGTGCGTAGGCGGCTTTTTAAGTCAGTGGTGAAAGCCTAGTGCTCAACACTAGAAATGCCACTGAAACTGAAGAGCTCGAGTCAAGGAGAGGTAAGTAGAATTTATGGTGTAGCAGTGAAATGCTTAGATACCATAAGGAATACCGACAGCGAAGGCAGCTTACTGGCCTTGTACTGACGCTGAGGCACGAAAGCGTGGGGAGCAAACAGG` |
| ASV2910 | 404 | `TGGGGAATATTGGACAATGGGCGAAAGCCTGATCCAGCAATGCCGCGTGAGTGATGAAGGCCTTAGGGTTGTAAAGCTCTTTTACCCGGGATGATAATGACAGTACCGGGAGAATAAGCTCCGGCTAACTCCGTGCCAGCAGCCGCGGTAATACGGAGGGAGCTAGCGTTATTCGGAATTACTGGGCGTAAAGCGCACGTAGGCGGCTTTGTAAGTAAGAGGTGAAAGCCTGGTGCTCAACACCAGAACTGCCTTTTAGACTGCATCGCTTGAATCCAGGAGAGGTGAGTGGAATTCCGAGTGTAGAGGTGAAATTCGTAGATATTCGGAAGAACACCAGTGGCGAAGGCGGCTCACTGGACTGGTATTGACGCTGAGGTGCGAAAGCGTGGGGAGCAAACAGG` |
| ASV5251 | 424 | `TGAGGAATATTGGTCAATGGACGGAAGTCTGAACCAGCCATGCCGCGTGCAGGAAGAATGTGCTATGCATTGTAAACTGCTTTTGTACGAGGGTAAACTTAGATACGTGTATCTAACTGAAAGTATCGTACGAATAAGGGTCGGCTAACTCCGTGCCAGCAGCCGCGGTAATACGGAGGACCCAAGCGTTATCCGGATTTATTGGGTTTAAAGGGTGCGTAGGCGGTTTTATAAGTTAGAGGTGAAAGCTCGACGCTCAACGTCGAAATTGCCTCTAATACTGTAAGGCTAGAGTATAGTTGCGGAAGGCGGAATGTGTGGTGTAGCGGTGAAATGCTTAGATATCACACAGAACACCGATTGCGAAGGCAGCTTTCCAAGCTATCACTGACGCTGAGGCACGAAAGCGTGGGGAGCGAACAGG` |
| ASV5037 | 407 | `TGGGGAATTTTCCGCAATGGGCGAAAGCCTGACGGAGCAACGCCGCGTGAGGGAGGAAGGTCCTTGGATCGTAAACCTCTTTTCTCAAGGAAGAAAAAAATGACGGTACTTGAGGAATCAGCCTCGGCTAACTCCGTGCCAGCAGCCGCGGTAATACGGGGGAGGCAAGCGTTATCCGGAATTATTGGGCGTAAAGCGTCCGCAGGTGGTCATCCAAGTCTGCCGTTAAAGCGTGGGGCTTAACTCCATAAAGGCGGTGGAAACTGGAAGACTAGAGAGCAGTAGGGGTAGCAGGAATTCCCAGTGTAGCGGTGAAATGCGTAGAGATTGGGAAGAACATCGGTGGCGAAAGCGTGCTACTGGGCTGTATCTGACACTCAGGGACGAAAGCTAGGGTAGCGAAAGGG` |
| ASV4588 | 408 | `TCGAGAATCTTCCGCAATGGACGAAAGTCTGACGGAGCGACGCCGCGTGGTGGATGAAGTCCCTCGGGACGTAAACACCTTTTATGGAGGAGAAAGTTTATTGATGTTACTCCATGAATAAGGGGCTCCCAACTCTGTGCCAGCAGGAGCGGTAATACAGAGGCCCCAAGCATTATCCGGATTTACTGGGCGTAAAGGGTGCGTAGGCGGTTGTATTAGTCGGGTGTTAAATCCTGGAGCTCAACTCCAGAATCGCATTCGAAACGGTACAACTAGAATAAGTCAGAGGCAAGCAGAACTCACGGTGTAGGGGTGAAATCCGTTGATATCGTGGGGAATACCAAATGCGAAGGCAGCTTGCTGGGACTTTATTGACGCTGAGGCACGAAAGCGTGGGTAGCGAAGCGG` |
| ASV1837 | 429 | `TGGGGAATTTTGGACAATGGGGGAAACCCTGATCCAGCCATTCCGCGTGTGCGATGAAGGCCTTCGGGTTGTAAAGCACTTTTGGCAGGAAAGAAATCGCATCAATTAATACTTGGTGTGGATGACGGTACCTGCAGAATAAGCACCGGCTAACTACGTGCCAGCAGCCGCGGTAATACGTAGGGTGCAAGCGTTAATCGGAATTACTGGGCGTAAAGCGTGCGCAGGCGGTTCGGAAAGAGAGGTGTGAAATCCCAGGGCTCAACCTTGGAACTGCACTTTTAACTACCGAGCTAGAGTATGTCAGAGGGGGGTGGAATTCCGCGTGTAGCAGTGAAATGCGTAGATATGCGGAGGAACACCGATGGCGAAGGCAGCCCCCTGGGATAATACTGACGCTCATGCACGAAAGCGTGGGGAGCAAACAGG` |
| ASV949 | 404 | `TGGGGAATATTGGACAATGGGCGAAAGCCTGATCCAGCAATGCCGCGTGAGTGAAGAAGGCCTTCGGGTTGTAAAGCTCTTTTACCCGGGAAGATAATGACCGTACCGGGAGAATAAGCCCCGGCTAACTCCGTGCCAGCAGCCGCGGTAATACGGAGGGGGCTAGCGTTGTTCGGAATCACTGGGCGTAAAGCGTACGTAGGCGGCCATTCAAGTCAGAGGTGAAATCCCAGGGCTCAACCCTGGAACTGCCTTTGAAACTAGGTGGCTGGAACACGGGAGAGGTGAGTGGAATTCCGAGTGTAGAGGTGAAATTCGTAGATATTCGGAAGAACACCAGTGGCGAAGGCGGCTCACTGGACCGTTGTTGACGCTGAGGTACGAAAGCGTGGGGAGCAAACAGG` |
| ASV279 | 429 | `TGGGGAATATTGGACAATGGGCGCAAGCCTGATCCAGCCATGCCGCGTGTGTGAAGAAGGCCTTCGGGTTGTAAAGCACTTTTGTCCGGAAAGAAATCCCAAAGGTTAATACCTTTTGGGGATGACGGTACCGGAAGAATAAGCACCGGCTAACTTCGTGCCAGCAGCCGCGGTAATACGAAGGGTGCAAGCGTTACTCGGAATTACTGGGCGTAAAGCGTGCGTAGGCGGTTTGTTAAGTCTGATGTGAAATCCCCGGGCTCAACCTGGGACCTGCATTGGATACTGGCGAGCTAGAGTACGGTAGAGGGTGGTGGAATTCCCGGTGTAGCAGTGAAATGCGTAGAGATCGGGAGGAACACTCGTGGCGAAGGCGGCCACCTGGACCAGTACTGACGCTGAGGCACGAAAGCGTGGGGAGCAAACAGG` |
| ASV333 | 429 | `TGGGGAATTTTGGACAATGGGGGAAACCCTGATCCAGCCATTCCGCGTGTGCGATGAAGGCCTTCGGGTTGTAAAGCACTTTTGGCAGGAAAGAAATCGCACTGGTTAATAACCAGTGTGGATGACGGTACCTGCAGAATAAGCACCGGCTAACTACGTGCCAGCAGCCGCGGTAATACGTAGGGTGCAAGCGTTAATCGGAATTACTGGGCGTAAAGCGTGCGCAGGCGGTTCGGAAAGAAGGATGTGAAATCCCAGGGCTTAACCTTGGAACTGCATTCTTAACTACCGGGCTAGAGTATGTCAGAGGGGGGTGGAATTCCGCGTGTAGCAGTGAAATGCGTAGATATGCGGAGGAACACCGATGGCGAAGGCAGCCCCCTGGGATAATACTGACGCTCATGCACGAAAGCGTGGGGAGCAAACAGG` |
| ASV4722 | 404 | `TGGGGAATATTGGACAATGGGCGAAAGCCTGATCCAGCGACGCCGCGTGAGTGATGAAGGCCTTCGGGTTGTAAAGCTCTTTTAATAGGGAAGATAATGACGGTACCTATAGAAAAAGCCCCGGCAAACTTCGTGCCAGCAGCCGCGGTAATACGAAGGGGGCTAGCGTTACTCGGAATTACTGGGCGTAAAGCGTGCGTAGGCGGCTTGGTAAGTTAGAAGTGAAAGCCTGGGGCTCAACCTCAGAATTGCTTTTAAAACTGCCTGGCTAGAGTATCAGAGAGGATAGCGGAATTCCTAGTGTAGAGGTGAAATTCGTAGATATTAGGAGGAACACCGGAGGCGAAAGCGGTTATCTGGCTGAAAACTGACGCTGTTGCACGAAAGCGTGGGGAGCAAACAGG` |
| ASV2182 | 387 | `GCGAGAAAACTTTGCAATGTGCGAAAGCACGACAAGGTTAATCCGAGTGATTTCTGCTAAAGAAATCTTTTGTCAGTCCTAGAAACACTGGTGAATAAGGGGTGGGCAAGTTCTGGTGTCAGCCGCCGCGGTAAAACCAGCACCTCAAGTGGTCAGGATGATTATTGGGCCTAAAGCATCCGTAGCCTGCTATGTAAGTTCTCGGTTAAATCCATACGCTTAACGTATGGGCTGCCGGCAATACTGCATAGCTAGGGAGTGGGAGAGGTAGACGGTACTCGGTAGGAAGGGGTAAAATCCTTTGATCTATTGATGACCACCTGTGGCGAAGGCGGTCTACCAGAACACATCCGACGGTGAGGGATGAAAGCTGGGGGAGCAAACCGG` |
| ASV2919 | 413 | `TGGAGAATATGTGGGCAATGAGCGCAAGCTTAACCCCGAAAAGTCTTGTGGGGGAGGAAGGGCCTGAATCTGTAAACCCCTTTCGATTGACTAAAATAAATGATATGAGTCAAAGGCAAGATAGTCCTGACTAAAGGTCGTGCCAGCAGTCGCGGTAAGACGGATGGGGCTAGCGTTATTGGTCTTTACTGGGCGTAAAGGGTATGTAGGTGGTTTATCACTTGTTTTGTGAAATGTCTAGGTCTAACTTTTTCATATTGCATTTTAAAAGGATAAAACTGGAGTTTAATAGAAGTTTGCGGAATTCCTAAGGAAGGGGTAATATCCATCGAACTAAGGGGGAATATCAAAGGCGAAAGCAGCATTCTAAATTAATCTGACACTGAGGTACTAAAGCGTGGGGAGCAAACAGG` |
| ASV4324 | 429 | `TGGGGAATATTGGACAATGGGGGCAACCCTGATCCAGCGACGCCGCGTGTGTGAAGAAGGCCTGCGGGTTGTAAAGCACTTTTAGTGGGGACAAAAAGCTACGGATTAATACCCTGTGGTCTTGATTTAACCCAAAGAAAAAGCACCGGCTAACTCTGTGCCAGCAGCCGCGGTAATACAGAGGGTGCAAGCGTTAATCGGAATTACTGGGCGTAAAGCGTGCGCAGTCGGTTATTTAAGTCGGATGTGAAATCCCCGGGCTCAACCTGGGAATTGCATTCGATACTGGATAGCTAGAGTTCGGCAGAGGGAAGTGGAATTTCAGGTGTAGCGGTGAAATGCGTAGATATCTGAAGGAACATCAGTGGCGAAGGCGACTTCCTGGACCAGAACTGACGATCAGGCACGAAAGCGTGGGGAGCAAACAGG` |
| ASV2433 | 409 | `TGGGGAATATTGCGCAATGGGCGAAAGCCTGACGCAGCGACGCCGCGTGAGGGATGAAGGCCTTCGGGTTGTAAACCTCTTTCAGCAGGGACGAAGCGAAAGTGACTGTACCTGCAGAAGAAGCACCGGCTAACTACGTGCCAGCAGCCGCGGTAATACGTAGGGTGCAAGCGTTGTCCGGATTTATTGGGCGTAAAGAGCTCGTAGGCGGCTCGTCGCGTCGGATGTGAAAACTCGGGGCTTAACCCCGAGCCTGCATTCGATACGGGCGAGCTAGAATTCGGTAGGGGAGACTGGAATTCCTGGTGTAGCGGTGAAATGCGCAGATATCAGGAGGAACACCGATGGCGAAGGCAGGTCTCTGGGCCGATATTGACGCTGAGGAGCGAAAGCGTGGGGAGCGAACAGG` |
| ASV4980 | 424 | `TAAGGAATATTGGTCAATGGACGCAAGTCTGAACCAGCCATGCCGCGTGGAGGATGAAGGTCCTCTGGATTGTAAACTTCTTTTATATGGGACGAAAAAGGATCTTTCCAGATCAACTGACGGTACCATATGAATAAGCACCGGCTAACTCCGTGCCAGCAGCCGCGGTAATACGGAGGGTGCAAGCGTTATCCGGATTCACTGGGTTTAAAGGGAGCGTAGGTGGGTTGGTAAGTCAGTGGTGAAATCCCCGAGCTTAACTTGGGAACTGCCATTGATACTATCAGTCTTGAATACTGTGGAGGTTAGCGGAATATGTCATGTAGCGGTGAAATGCTTAGATATGACATAGAACACCAATTGCGAAGGCAGCTGGCTACACAATTATTGACACTGAGGCTCGAAAGCGTGGGGATCAAACAGG` |
| ASV3439 | 429 | `TGGGGAATATTGGACAATGGGCGCAAGCCTGATCCAGCCATACCGCGTGGGTGAAGAAGGCCTTCGGGTTGTAAAGCCCTTTTGTTGGGAAAGAAAAGCATTCGGTTAATACCCGGGTGTTCTGACGGTACCCAAAGAATAAGCACCGGCTAACTTCGTGCCAGCAGCCGCGGTAATACGAAGGGTGCAAGCGTTACTCGGAATTACTGGGCGTAAAGCGTGCGTAGGTGGTTGTTTAAGTCCGTTGTGAAAGCCCTGGGCTCAACCTGGGAATTGCAGTGGATACTGGGCAACTAGAGTGTGGTAGAGGATAGTGGAATTTCCGGTGTAGCAGTGAAATGCGTAGAGATCGGAAGGAACATCTGTGGCGAAGGCGACTATCTGGGCCAACACTGACACTGAGGCACGAAAGCGTGGGGAGCAAACAGG` |
| ASV1868 | 405 | `TGGGGAATCTTGCGCAATGGGCGAAAGCCTGACGCAGCAACGCCGCGTGTGGGATGACGGCTTTCGGGTTGTAAACCACTTTCAGCGGGAACGAAATTGACGGTACCCGCAGAAGAAGCACCGGCCAACTACGTGCCAGCAGCCGCGGTAATACGTAGGGTGCAAGCGTTGTCCGGATTTATTGGGCGTAAAGAGCTCGTAGGCGGCTTTGTAAGTTGGGTGTGAAAACTCCAGGCTTAACCTGGAGACGCCATCCGATACTGCAATGGCTAGAGTCTGGTAGGGGATCACGGAATTCCTGGTGTAGCGGTGAAATGCGCAGATATCAGGAGGAACACCGGTGGCGAAGGCGGTGATCTGGGCCAGTACTGACGCTGAGGAGCGAAAGCGTGGGGAGCAAACAGG` |
| ASV0 | 408 | `TGGGGAATTTTCCGCAATGGGCGAAAGCCTGACGGAGCAATACCGCGTGAGGGAAGACTGCCTATGGGTTGTAAACCTCTTTTTTCAGGGAGGAACAAAATGACGTGTACCTGAAGAATAAGCATCGGCTAACTCCGTGCCAGCAGCCGCGGTAAGACGGAGGATGCAAGTGTTATCCGGAATCACTGGGCGTAAAGCGTCTGTAGGTGGTTTAATAAGTCAACTGTTAAATCTTGAGGCTCAACTTCAAAATCGCAGTTGAAACTATTAGACTAGAGTATAGTAGAGGTAAAGGGAATTTCCAGTGGAGCGGTGAAATGCGTAGATATTGGAAAGAACACCGATGGCGAAAGCACTTTACTGGGCTATTACTAACACTCAGAGACGAAAGCTAGGGTAGCAAATGGG` |
| ASV2960 | 424 | `TGAGGAATATTGGACAATGGGCGCAAGCCTGATCCAGCCATGCCGCGTGCAGGATGACGGTCCTATGGATTGTAAACTGCTTTTGTACGAGAAGAAACACCCCGACGTGTCGGGACTTGACGGTATCGTAAGAATAAGGATCGGCTAACTCCGTGCCAGCAGCCGCGGTAATACGGAGGATCCAAGCGTTATCCGGAATCATTGGGTTTAAAGGGTCCGTAGGCGGTTTAGTAAGTCAGTGGTGAAAGCCCATCGCTCAACGGTGGAACGGCCATTGATACTGCTAAACTTGAATTATTAGGAAGTAACTAGAATATGTAGTGTAGCGGTGAAATGCTTAGAGATTACATGGAATACCAATTGCGAAGGCAGGTTACTACTAATGGATTGACGCTGATGGACGAAAGCGTGGGTAGCGAACAGG` |
| ASV4339 | 429 | `TGGGGAATTTTTCGCAATGGGCGCAAGCCTGACGAAGCAACGCCGCGTGGAGGATGAAGGTCTTCGGATTGTAAACTCCTGTCGACCGGGACGAATGCACTCGACCTAATACGTCGGAATGTTGACGGTACCGGTGGAGGAAGCCACGGCTAACTCTGTGCCAGCAGCCGCGGTAATACAGAGGTGGCAAGCGTTGTTCGGAATTACTGGGCGTAAAGGGCGCGTAGGCGGCCTTGTAAGTCTCGTGTGAAAGCCCTCGGCTTAACTGAGGATCTGCACGGGATACTGCAAGGCTCGAGTTCGGGAGAGGAAAGCGGAATTCCGGGTGTAGCGGTGAAATGCGTAGATATCCGGAGGAACACCAGTGGCGAAGGCGGCTTTCTGGACCGACACTGACGCTGAGGCGCGAAAGCTAGGGGAGCAAACGGG` |
| ASV3400 | 408 | `TCGAGAATCTTCCGCAATGGACGAAAGTCTGACGGAGCGACGCCGCGTGGTAGATGAAGTCCTTAGGGACGTAAATACCTTTTATGAAGGAAGAAGTTTATTGACATTACTTCATGAATAAGGGGCTCCCAATTCTGTGCCAGCAGGAGCGGTAATACAGAAGCCTCGAGCGTTACCCGGATTTACTGGGCGTAAAGGATGTGTAGGTGGTGTGATTAGTCTTGCGTTAAATCTTTGGGCTCAACCCGAAACTCGCGTGAGAAACGGTCATACTAGAGGAAGTGAGAGGTGTGTGGAACTCAAGGTGTAGGGGTGAAATCCGTTGATATCTTGGGGAACACCAAAAGCGAAGGCAACACACTGGCACTTTCCTGACACTGAAACATGAAAGCGTGGGTAGCGAATCGG` |
| ASV4947 | 405 | `TGGGGAATTTTCCGCAATGGGCGAAAGCCTGACGGAGCAATACCGCGTGAGGGAGGAAGGCTCTTGGGTTGTAAACCTCTTTTCTCAGGGAAGAAAAATGACGGTACCTGAGGAATCAGCATCGGCTAACTCCGTGCCAGCAGCCGCGGTAATACGGAGGATGCAAGCGTTATCCGGAATTATTGGGCGTAAAGCGTCTGTAGGTGGATTAGCAAGTCTGCTGTCAAAGACCGTGGCTCAACCATGGAAAGGCGGTGGAAACTGCAAGTCTAGAGTGAGGTAGGGGCAGAGGGAATTCCCAGTGTAGCGGTGAAATGCGTAGAGATTGGGAAGAACACCGGTGGCGAAAGCGCTCTGCTGGGCCAGAACTGACACTAAGGGACGAAAGCTAGGGGAGCGAATGGG` |
| ASV2579 | 430 | `TGGGGAATTTTGGACAATGGGCGAAAGCCTGATCCAGCCATGCCGCGTGCAGGAAGAAGGCCTTCGGGTTGTAAACTGCTTTAGTTCGGGAAAAAGGGGTGCGAGGTTAATACCCTCGTGCTTTGATGGTACCGGAAGAATAAGCACCGGCTAACTACGTGCCAGCAGCCGCGGTAATACGTAGGGTGCGAGCGTTAATCGGAATTACTGGGCGTAAAGCGTGCGCAGGCGGTCTTGTAAGACAGTTGTGAAAGCCCCGGGCTTAACCTGGGAACTGCAATTGTGACTGCAAGGCTAGAGTGTGTCAGAGGGGGGTGGAATTCCACGTGTAGCAGTGAAATGCGTAGAGATGTGGAGGAACACCGATGGCGAAGGCAGCCCCCTGGGATAACACTGACGCTCATGCACGAAAGCGTGGGGAGCAAACAGG` |
| ASV5513 | 429 | `TGGGGAATATTGGACAATGGGCGAAAGCCTGATCCAGCAATACCGCGTGTGTGAAGAAGGCCTGAGGGTTGTAAAGCACTTTCGTTTGGGAGGAAGAGATTTGTTTTAATAGAGCAAATTGAAGACGTTACCAAAATAAGAAGCACCGGCTAACTCTGTGCCAGCAGCCGCGGTAATACAGAGGGTGCGAGCGTTAATCGGAATTACTGGGCGTAAAGGGTGCGTAGGTTGTTCTGTAAGTCAGATGTGAAAACCCGGGGCTTAACTCCGGAAGGTCGTTTGATACTGCAGGGCTAGAGTTTTGAAGGGGACAGTGGAATTTCCGGTGTAGCGGTGAAATGCGTAGATATCGGAAGGAACACCAGAGGCGAAGGCGACTGTCTGGTCAAAAACTGACACTGAGGCACGATAGCGTGGGTAGCAAACAGG` |
| ASV5642 | 415 | `TCGAGAATCTTTCACAATGGGGGAAACCCTGATGGAGCGACGCCGCGTGGAGGATAAGGTCTTCGGATTGTAAACTCCTGTCATGCGAGAACAAAGCCCGCAAGGGCCTGATAGTATCGCAAGAGGAAGAGACGGCTAACTCTGTGCCAGCAGCCGCGGTAATACAGAGGTCTCAAGCGTTGTTCGGAATCACTGGGCGTAAAGGGTGCGTAGGCGGCGGGGAAAGTCAGATGTGAAATCCGGGGGCTCAACCTCCGAATTGCATCCGATACTCCCTCGCTAGAGAGTTGTAGAGGAGCTTGGAATTCTCGGTGTAGCAGTGAAATGCGTAGATATCGAGAAGAACGCTCGCAGCGAAAGCGAAGCTCTGGGCAATTTCTGACGCTGATGCACGAAGGCCAGGGGAGCAAACGGG` |
| ASV110 | 403 | `TGGGGAATTTTGCGCAATGGAGGAAACTCTGACGCAGCAACGCCGCGTGCGGGATGAAGGCCTTCGGGTTGTAAACCGCTTTCAGCAGGGAAGAATTTGACGGTACCTGCAGAAGAAGCTCCGGCTAACTACGTGCCAGCAGCCGCGGTAATACGTAGGGAGCAAGCGTTATCCGGATTCATTGGGCGTAAAGCGCGCGTAGGCGGCTTGTTAAGCGGAATCTCTAATCTCGGGGCTCAACCCCGAACCGGATTCTGAACTGGCAGGCTCGAGTTTGGTAGAGGAAGATGGAATTCCCAGTGTAGCGGTGAAATGCGCAGATATTGGGAAGAACACCGATTGCGAAGGCAGTCTTCTGGGCCAAAACTGACGCTGAGGTGCGAAAGCTAGGGGAGCGAACAGG` |
| ASV457 | 429 | `TGGGGAATATTGGACAATGGGCGCAAGCCTGATCCAGCCATGCCGCGTGGGTGAAGAAGGCCTTCGGGTTGTAAAGCCCTTTTGTCCGGAAAGAAAAGCACGGGATTAATACCCTCGTGTGATGACGGTACCGGAAGAATAAGCACCGGCTAACTTCGTGCCAGCAGCCGCGGTAATACGAAGGGTGCAAGCGTTACTCGGAATTACTGGGCGTAAAGCGTGCGTAGGTGGTTCGTTAAGTCTGTCGTGAAATCCCCGGGCTCAACCTGGGAATGGCGATGGATACTGGCGAGCTAGAGTGAGGTAGAGGAGAGTGGAATTCCCGGTGTAGCAGTGAAATGCGTAGAGATCGGGAGGAACATCAGTTGCGAAGGCGGCTCTCTGGACCATCACTGACACTGAGGCACGAAAGCGTGGGGAGCAAACAGG` |
| ASV5334 | 388 | `GCGCGAAACCTCCGCAATGTGAGAAATCGCGACGGGGGGATCCCAAGTGCCATTCTTAACGGGATGGCTTTTCATTAGTGTAAAGAGCTTTTGGAATAAGAGCTGGGCAAGACCGGTGCCAGCCGCCGCGGTAACACCGGCAGCTCTAGTGGTAGCAGTTTTTATTGGGCCTAAAGCGTCCGTAGCCGGTTTATTAAGTCTCTGGTGAAATCCTGTAGCTTAACTGTGGGAATTGCTGGAGATACTAGTAGACTTGAGATCGGGAGAGGTTAGAGGTACTCCCAGGGTAGAGGTGAAATTCTGTAATCCTGGGAGGACCGCCTGTGGCGAAGGCGTCTAGCTGGAACGATTCTGACGGTGAGGGACGAAAGCTAGGGGCGCGAACCGG` |
| ASV4691 | 430 | `TGGGGAATATTGCGCAATGGGCGAAAGCCTGACGCAGCGACGCCGCGTGAGGGATGAAGGTTCTCGGATCGTAAACCTCTGTCAGGGGGGAAGAAACATCCTCGTGTGAATAATGCGAGGACTTGACGGTACCCCCAAAGGAAGCACCGGCTAACTCCGTGCCAGCAGCCGCGGTAATACGGAGGGTGCAAGCGTTAATCGGAATCACTGGGCGTAAAGCGCACGTAGGCGGCCCGTTAAGTCAGAGGTGAAATCCCACAGCCCAACTGTGGAACTGCCTTTGATACTGGCGGGCTTGAGTACCGGAGAGGGTGGCGGAATTCCAGGTGTAGGAGTGAAATCCGTAGATATCTGGAGGAACACCGGTGGCGAAGGCGGCCACCTGGACGGTAACTGACGCTGAGGTGCGAAAGCGTGGGTAGCAAACAGG` |
| ASV458 | 404 | `TGGGGAATATTGGACAATGGGCGAAAGCCTGATCCAGCCATGCCGCGTGAATGATGAAGGCCTTAGGGTTGTAAAGTTCTTTTGACGGGGAAGATAATGACGGTACCCGTAGAATAAGCTCCGGCTAACTTCGTGCCAGCAGCCGCGGTAATACGAAGGGAGCTAGCGTTGTTCGGAATTACTGGGCGTAAAGGGCGCGTAGGCGGCTTAACAAGTCAGTGGTGAAATCCCGGGGCTCAACCTCGGAATTGCCTTTGAAACTGTTAAGCTAGAGTTTTGTAGGGGATAGTGGAATTTCTAGTGTAGGGGTGAAATCCGTAGATATTAGAAGGAACACCAGTGGCGAAGGCGGCTATCTGGACAAATACTGACGCTGAGGCGCGAAAGTGTGGGGAGCAAACAGG` |
| ASV3013 | 404 | `TGGGGAATATTGGGCAATGGGCGAAAGCCTGACCCAGCTATGCCGCGTGAGTGATGAAGGCCTTAGGGTTGTAAAGCTCTTTTAAGTGGGAAGATAATGACGGTACCACTAGAATAAGCCCCGGCTAACTTCGTGCCAGCAGCCGCGGTAATACGAAGGGGGCAAGCGTTGCTCGGATTTATTGGGCGTAAAGGGTGTGTAGGTGGTTTGATAAGTTAGGTGTGAAAGCCCTGGGCTTAACCCGGGAATTGCACTTAAAACTATCAGACTTGAAGATCACAGAGGTAAGCAGAATTTCCAGTGTAGAGGTGAAATTCGTAGATATTGGAAAGAATACCAACGGCGTAGGCAGCTTACTGGGTGATACTTGACACTGAGGCACGAAAGCGTGGGGAGCAAACAGG` |
| ASV3120 | 429 | `TGGGGAATATTGGACAATGGGCGCAAGCCTGATCCAGCCATGCCGCGTGTGTGAAGAAGGCCTTCGGGTTGTAAAGCACTTTAAGCGAGGAGGAAGGTCTTTGGACTAATATTTCAAAGAATTGACGTTACTCGCAGAATAAGCACCGGCTAACTCTGTGCCAGCAGCCGCGGTAATACAGAGGGTGCAAGCGTTAATCGGAATTACTGGGCGTAAAGCGCGCGTAGGCGGTATGTTAAGTCGGATGTGAAATCCCTGGGCTCAACCTAGGCATTGCATCCGATACTGGCACACTAGAGTGTGGGAGAGGAAGGTAGAATTCCAGGTGTAGCGGTGAAATGCGTAGAGATCTGGAGGAATACCGATGGCGAAGGCAGCCTTCTGGCCTGACACTGACGCTGAGGTGCGAAAGCATGGGGAGCAAACAGG` |
| ASV5329 | 424 | `TGAGGAATATTGGACAATGGGTGCAAGCCTGATCCAGCCATCCCGCGTGTAGGAAGAAGGCCCTATGGGTTGTAAACTACTTTTCTGCAGGAAGAAACCTACGTACGCGTACGTAGCTGACGGTACTGCAGGAATAAGCACCGGCTAACTCCGTGCCAGCAGCCGCGGTAATACGGAGGGTGCAAGCGTTATCCGGATTTATTGGGTTTAAAGGGTCCGTAGGCGGTAAATTAAGTCAGTGGTGAAATCCTACAGCTTAACTGTAGAATTGCCATTGATACTGGTTTACTTGAGTAAGGTTGAAGTAGCTAGAATGTGTAGTGTAGCGGTGAAATGCATAGATATTACACAGAATACCGATAGCGAAGGCAGGTTACTAAGCCTTTACTGACGCTGATGGACGAAAGCGTGGGGAGCAAACAGG` |
| ASV1575 | 422 | `TGAGGAATTTTGCGCAATGGACGAAAGTCTGACGCAGCGACGCCGCGTGTGGGATGAAGGGGCTACGCCCTGTAAACCACTGTTAGATGTGAAAAAAAGATGGCTTTGCCGTCCGGGATTGTAACATCAAAGAAAGGACCGGCTAACTACGTGCCAGCAGCCGCGGTAATACGTAGGGTCCGAGCGTTGTCCGGAATTACTGGGTATAAAGGGTGCGTAGGCGGGCTTTTACGTCAAGGGTTAAAGACTTCAGCTCAACTGAGGGAGTGCCTTTGAAACGGAAAGTCTTGAATGTAAGAGAGGTAGGTAGAATTCCTGGTGTAGCGGTGAAATGCGTAGATATCAGGAGGAATACCAGTGGCGAAGGCGGCCTACTGGCTTATTATTGACGCTGAGGCACGAAAGCGTGGGTAGCGAACAGG` |
| ASV3276 | 405 | `TGGGGAATATTGGGCAATGGATGAAAGTCTGACCCAGCGACGCCGCGTGAATGAAGAAGGTCTTCGGATTGTAAAGTTCTTTTATACGGGAAGAGAAGAGGACGGTACCGTATGAATAAGTCCCGGCTAACTACGTGCCAGCAGCCGCGGTAATACGTAGGGGACGAGCGTTGTCCGGAATTACTGGGCGTAAAGGGCATGTAGGCTGTTTGGCAAGTCAGTAGTAAAAGAGTGAGGCTTAACCTTATTAAGCTATTGAGACTGTCAGACTGGAGTACTGAAGAGGCAAGGGGAATTCCCGGTGTAGCGGTGAAATGCGTAGATATCGGGAAGAACACCGGTGGCGAAGGCGACTTGCTGGTCAGTAACTGACGCTGAGGTGCGAAAGCCAGGGTAGCAAACGGG` |
| ASV295 | 424 | `TGAGGAATATTGGTCAATGGACGCAAGTCTGAACCAGCCATGCCGCGTGCAGGATGACGGCTCTATGAGTTGTAAACTGCTTTTATACGAGGGTAAAATCATCTACGTGTAGGTGATTGAAAGTATCGTACGAATAAGGATCGGCTAACTCCGTGCCAGCAGCCGCGGTAATACGGAGGATCCAAGCGTTATCCGGATTTATTGGGTTTAAAGGGTGCGTAGGCGGTTTAATAAGTTAGAGGTGAAATATTAGGGCTTAACCCTGAAACTGCCTCTAATACTGTTAGACTAGAGAATAGTTGCGGTAGGCGGAATGTATGGTGTAGCGGTGAAATGCTTAGAGATCATACAGAACACCGATTGCGAAGGCAGCTTACCAAACTATATCTGACGTTGAGGCACGAAAGCGTGGGGAGCAAACAGG` |
| ASV2230 | 425 | `TGAGGAATATTGGACAATGGGCGCAAGCCTGATCCAGCCATGCCGCGTGCAGGAAGACGGTCCTATGGATTGTAAACTGCTTTTATTCGAGAATAAACCCCCTGTCGTGCATAGGGGCTGAAGGTACCGAATGAATAAGCACCGGCTAACTCCGTGCCAGCAGCCGCGGTAATACGGAGGGTGCAAGCGTTATCCGGAATCATTGGGTTTAAAGGGTCCGCAGGCGGACATATAAGTCAGTGGTGAAAGCCCGTTGCTTAACAACGGAACTGCCATTGATACTGTATGTCTTGAATGGGGTCGAAGTGGGCGGAATATGTCATGTAGCGGTGAAATGCTTAGATATGACATAGAACACCGATAGCGAAGGCAGCTCACTAGGCCTTTATTGACGCTCATGGACGAAAGCGTGGGGAGCAAACAGG` |
| ASV2823 | 424 | `TAAGGAATATTGGTCAATGGGCGCAAGCCTGAACCAGCCATGCCGCGTGCAGGAAGAAGGCTCTATGGGTTGTAAACTGCTTTTTTAAGGGAATAAACCTGATTACGTGTAATCAGCTGAATGTACCTTGCGAATAAGGATCGGCTAACTCCGTGCCAGCAGCCGCGGTAATACGGAGGATCCAAGCGTTATCCGGATTTATTGGGTTTAAAGGGTGCGTAGGCGGCTATATAAGTCAGAGGTGAAAACTTCGAGCTCAACTGGAAGCTTGCCTTTGATACTGTATGGCTTGAATACAGATGAAGTAGGCGGAATGTGGCAAGTAGCGGTGAAATGCATAGATATGCCACAGAACACCAATTGCGAAGGCAGCTTACTAAACTGTTATTGACGCTGATGCACGAAAGCGTGGGGATCAAACAGG` |
| ASV3886 | 428 | `TAGGGAATTTTCGGCAATGGGGGAAACCCTGACCGAGCAACGCCGCGTGAGTGAAGAAGGCCTTCGGGTTGTAAAGCTCTGTTGTAAGGGAAGAACAAGTATGAGAGGGAATGCTCGTACTATGACGGTACCTTACCAGAAAGCCACGGCTAACTACGTGCCAGCAGCCGCGGTAATACGTAGGTGGCAAGCGTTATCCGGAATTATTGGGCGTAAAGGGAGCGCAGGCGGTTTATTAAGTTTATGGTGAAAGTTCGGGGCTCAACCCCGTGACGCCATAGAAACTGGTAGACTTGAGTGCAGGAGAGGTAAGTGGAATTCCATGTGTAGCGGTAAAATGCGTAGATATATGGAGGAACACCAGTGGCGAAGGCGGCTTACTGGCCTGTAACTGACGCTGAGGCTCGAAAGCGTGGGGAGCAAATAGG` |
| ASV1470 | 429 | `TGGGGAATTTTGGACAATGGGCGCAAGCCTGATCCAGCAATGCCGCGTGCAGGATGAAGGCCTTCGGGTTGTAAACTGCTTTTGTACGGAGCGAAACGGTCTGCCCTAATACGGTGGGCTAATGACGGTACCGTAAGAATAAGCACCGGCTAACTACGTGCCAGCAGCCGCGGTAATACGTAGGGTGCGAGCGTTAATCGGAATTACTGGGCGTAAAGCGTGCGCAGGCGGTGATGTAAGACAGATGTGAAATCCCCGGGCTCAACCTGGGACCTGCATTTGTGACTGCATCGCTAGAGTACGGTAGAGGGGGATGGAATTCCGCGTGTAGCAGTGAAATGCGTAGATATGCGGAGGAACACCGATGGCGAAGGCAATCCCCTGGACCTGTACTGACGCTCATGCACGAAAGCGTGGGGAGCAAACAGG` |
| ASV4287 | 405 | `TAAAGAATCTTCCACAATGGACGAAAGTCTGATGGAGCGACACCGCGTGCAGGATGAAGGCCTTTGGGTCGTAAACTGCTTTTTTGGGGGAATAATTCTGAATGTACCCCAAGAATAAGCACCGGCAAATCACGTGCCAGCAGCCGCGGTAATACGTGAGGTGCAAACGTTACCCGGAATTATTGGGCGTAAAGCGTCCGCAGGTGGCGTAGTAAGTAGGGTATTAAATCTTGAGGCTCAACTTCAAGGCTGTGCTCTAAACTACTATGCTAGAGAAATGGAGAGGTGAGTGGAATTCTACATGTAGGGGTAAAATCCGTAGATATGTAGAGGAACACCAAAAGCGAAGGCAGCTCACTGGCCATTTTCTGACACTCATGGACGAAAGCGTGGGGAGCGAACGGG` |
| ASV2281 | 423 | `TAGGGAATATTGGACAATGGGTGTAAGCCTGATCCAGCCATGCCGCGTGCAGGAAGAAGGCCTTCTGGGTTGTAAACTGCTTTTGCCGGGGGATAAAATGGCCATGCGTGGCAAATTGAAGGTACCCGGTGAATAAGCCACGGCTAACTACGTGCCAGCAGCCGCGGTAATACGTAGGTGGCAAGCGTTGTCCGGATTTATTGGGTTTAAAGGGTGCGTAGGCGGTCCCATAAGTCAGTGGTGAAATACGGCAGCTCAACTGTCGAGGTGCCATTGATACTGCGGGACTTGAGTACAGACGAGGTAGGCGGAATTGACGGTGTAGCGGTGAAATGCTTAGATATCGTCAAGAACACCGATAGCGAAGGCAGCTTACTAGGCTGTAACTGACGCTGAGGCACGAAGGTGCGGGGATCAAACAGG` |
| ASV2531 | 424 | `TAAGGAATATTGGTCAATGGACGGAAGTCTGAACCAGCCATGCCGCGTGGAGGATGAAGGTCCTCTGGATTGTAAACTTCTTTTCTCTGGGGCGAAAAAAGGGAATTCTTTCTCTCTTGACGGTACCAGAGGAATAAGCACCGGCTAACTCCGTGCCAGCAGCCGCGGTAATACGGAGGGTGCAAGCGTTATCCGGATTCACTGGGTTTAAAGGGAGCGCAGGCGGGTCGGTAAGTCAGTGGTGAAAGCCCCGTGCTTAACATGGGAACTGCCATTGATACTATCGGTCTTGAATGTCGTTGAGGTTTGCGGAATGGGTCATGTAGCGGTGAAATGCATAGATATGACCCGGAACACCTATTGCGAAGGCAGCAGGCTAAGCGAATATTGACGCTGAGGCTCGAAAGCGTGGGGATCAAACAGG` |
| ASV2137 | 404 | `TGGGGAATATTGGACAATGGGGGAAACCCTGATCCAGCAATGCCGCGTGAGTGATGAAGGCCTTCGGGTTGTAAAACTCTTTTGTCGGGGACGATAATGACGGTACCCGACGAATAAGCTCCGGCTAACTTCGTGCCAGCAGCCGCGGTAATACGAAGGGAGCTAGCGTTGTTCGGAATTATTGGGCGTAAAGGGCGCGTAGGCGGTGTTGTAAGTCAGTTGTGAAAGCCCCGGGCTTAACCTGGGAATGGCAATTGAAACTGCAACGCTAGAGATCGAGAGGGGAAAGTGGAATTGCGAGTGTAGAGGTGAAATTCGTAGATATTCGCAAGAACACCAATGGCGAAGGCAGCTTTCTGGCTCGATACTGACGCTAAGGCGCGAAAGCGTGGGGAGCAAACAGG` |
| ASV5582 | 403 | `TGGGGAATATTGCGCAATGGGGGAAACCCTGACGCAGCGACGCCGCGTGAGCGACGAAGTCTTTCGGGATGTAAAGCTCTGTCAAGAGGAACGATTATGACGGTACCTCTAGAGGAAGCCCCGGCTAACTACGTGCCAGCAGCCGCGGTAATACGTAGGGGGCGAGCGTTATCCGGATTTATTGGGCATAAAGAGCGTGTAGGCGGTATTTTAAGTCAGATGTTAAATGCTTCAGCTTAACTGAAGTTCGCGTTTGAAACTGAATTACTAGAGATCAGGAGAGGGAAGCGGAACTCACGGTGTAGCGGTGAAATGCGTAGATATCGTGAGGAACACCAGTTGCGAAGGCGGCTTCCTGGACTGAATCTGACGCTGAGACGCGAAAGCGTGGGGAGCAAACGGG` |
| ASV5405 | 404 | `TAAGGAATCTTCCACAATGGGCGAAAGCCTGATGGAGCGACGCCGCGTGAAGGATGAAGGTCGAAAGGTTGTAAACTTCTTTTCTGAGGGAGCATAATGAGAGTACTTCAGGAATAAGGGACGGCAAATCACGTGCCAGCAGCCGCGGTAATACGTGAGTCCCAAGCATTGCCCGGAATCACTGGGTGTAAAGAGTTTGTAGGTGGTTTGATAAGTAGAGTGTGAAAGACCGAAGCTTAACTTCGTGTTTGTACTCTAAACTATCTCACTAGAATCAGGAAGAGGTAAGCGGAATTTTAAGTGTAGGGGTGCAATCCGTAGATACTTAAAAGAACACCAAAAGCGAAGGCAGCTTACTGGTACTGTATTGACACTGAGAAACGAAAGCGTGGGGAGCAAAAAGG` |
| ASV2377 | 404 | `TAGGGAATATTGGACAATGGGCGAAAGCCTGATCCAGCCATGCCGCGTGAGTGAAGAAGGCCTTAGGGTTGTAAAGCTCTTTTGGCGGGGAAGATAATGACGGTACCCGCAGAATAAGCTCCGGCTAACTTCGTGCCAGCAGCCGCGGTAATACGAAGGGGGCTAGCGTTGTTCGGAATCACTGGGCGTAAAGCGCACGCAGGCGGATTGATAAGTCGGGGGTGAAATCCCGGGGCTCAACCTCGGAATTGCCTTCGATACTGTCTGTCTTGAGTCCGGGAGAGGTGAGTGGAATTCCTAGTGTAGAGGTGAAATTCGTAGATATTAGGAAGAACACCAGTGGCGAAGGCGGCTCACTGGCCCGGTACTGACGCTCATGTGCGAAAGCGTGGGGAGCAAACAGG` |
| ASV652 | 429 | `TGAGGAATATTGCACAATGGGCGAAAGCCTGATGCAGCGACGCCGCGTGGAGGATGACGGTCTTCGGATTGTAAACTCCTGTTAAGTGAAAAGAAAGAACCGCTTTTAATAGAGGCGGGTGATGACGTTATCACTAGAGAAAGCACCGGCTAACTTCGTGCCAGCAGCCGCGGTAATACGAGGGGTGCAAGCGTTATTCGGAATGACTGGGCGTAAAGGGTGCGTAGACGGCACGTTAAGTCTTTTGTTAAATACCTCAGCCTAACTGGGGATCAGCAGGGGAAACTGGCGAGCTTGAGATTGGAAGAGAGAAGTGGAATTCTCGGAGTAGCGGTAAAATGCGTAGATCTCGAGAGGAACACCAATGGCGAAGGCAGCTTCTTGGTCCAAATCTGACGTTGAGGCACGAAAGCGTGGGGAGCAAACAGG` |
| ASV587 | 429 | `TAGGGAATCTTCCGCAATGGGCGAAAGCCTGACGGAGCAACGCCGCGTGAGTGATGAAGGTCTTCGGATCGTAAAACTCTGTTATTAGGGAAGAACAAATGTGTAAGTAACTATGCACGTCTTGACGGTACCTAATCAGAAAGCCACGGCTAACTACGTGCCAGCAGCCGCGGTAATACGTAGGTGGCAAGCGTTATCCGGAATTATTGGGCGTAAAGCGCGCGTAGGCGGTTTTTTAAGTCTGATGTGAAAGCCCACGGCTCAACCGTGGAGGGTCATTGGAAACTGGAAAACTTGAGTGCAGAAGAGGAAAGTGGAATTCCATGTGTAGCGGTGAAATGCGCAGAGATATGGAGGAACACCAGTGGCGAAGGCGACTTTCTGGTCTGTAACTGACGCTGATGTGCGAAAGCGTGGGGATCAAACAGG` |
| ASV2886 | 405 | `TAGGGAATTTTCCACAATGGACGAAAGTCTGATGGAGCAACGCCGCGTGCAGGATGAAGGCTCTTGGGTCGTAAACTGCTTTTCTCTGTGAGGAATTTGACAGTAGCAGAGGAATAAGGACCGGCTAACTACGTGCCAGCAGCCGCGGTCATACGTAGGGTCCAAGCGTTATCCGGAATTACTGGGCGTAAAGAGTTGCGTAGGTGGCAGAGTAAGCAAACAGTGAAAGCGTGGGGCTCAACCTCATATACATTGTTTGAACTGCTCAGCTAGAAGATGAGAGAGGTACCTGGAATTCCTAGTGTAGGAGTGAAATCCGTAGATATTAGGAGGAACACCGATGGCGTAGGCAGGGTACTGGCTCATTCTTGACACTAAGGCACGAAAGCGTGGGGAGCGAACTGG` |
| ASV5585 | 408 | `CCGAGAATATTCGACAATGGGCGAAAGCCTGATCGAGCGACGCCGCGTGCAGGATGAAATCCTTCGGGATGTAAACTGCTTTTATAGGGGAAGAACGTAAGTGACTGTACCCTACGAATAAGGACCTGCAAACCTCGTGCCAGCAGCCGCGGTAATACGAGGGGTCCAAGCGTTGTCCGGATTAACTGGGCGTAAAGGGTGTGTAGGGGGTCGTGTTAGTCCTGCTTTAAATCTCTGAGCTTAACTCAGAGGCTGGGTGGGAAACGGCACGGCTGGAGGGTGCGAGAGGCGAGTGGAACTCACGGTGTAGGGGTGAAATCCGTTGATATCGTGGGGAACACCAAAAGCGAAGGCAACTCGCTGGCGCATACCTGACCCTGAAACACGAAAGCGTGGGGATCAAAAAGG` |
| ASV1170 | 429 | `TGGGGAATTTTGGACAATGGGCGAAAGCCTGATCCAGCAATGCCGCGTGCAGGACGAAGGCCTTCGGGTTGTAAACTGCTTTTGTACGGAACGAAACGGCCTCTTTTAATACAGGGGGCTAATGACGGTACCGTAAGAATAAGCACCGGCTAACTACGTGCCAGCAGCCGCGGTAATACGTAGGGTGCAAGCGTTAATCGGAATTACTGGGCGTAAAGCGTGCGCAGGCGGTTATATAAGACAGATGTGAAATCCCCGGGCTCAACCTGGGACCTGCATTTGTGACTGTATAGCTAGAGTACGGTAGAGGGGGATGGAATTCCGCGTGTAGCAGTGAAATGCGTAGATATGCGGAGGAACACCGATGGCGAAGGCAATCCCCTGGACCTGTACTGACGCTCATGCACGAAAGCGTGGGGAGCAAACAGG` |
| ASV598 | 430 | `TGGGGAATATTGGACAATGGGGGCAACCCTGATCCAGCAATGCCGCGTGTGTGAAGAAGGCCTGCGGGTTGTAAAGCACTTTTGTCCGGGAAGAAAGGCCTTGACCTAATACGTTGGGGAGATGACGGTACCGGGAGAATAAGCACCGGCTAACTTCGTGCCAGCAGCCGCGGTAATACGAAGGGTGCAAGCGTTAATCGGAATTACTGGGCGTAAAGGGTGCGTAGACGGCCTGATAAGTCGACTGTGAAAGTCCTGGGCTTAACCTGGGGACTGCAGTCGATACTGTGAGGCTTGGAGTTTGGGAGAGGGTGGTGGAATTCCCGGTGTAGCGGTGAAATGCGTAGAGATCGGGAGGAACATCCGTGGCGAAGGCGGCCACCTGGCCTGAAACTGACGTTGAGGCACGAAAGCGTGGGGAGCAAACAGG` |
| ASV3332 | 387 | `GCGCGAAACCTTTACACTACACGCAAGTGTGATAAGGGGATCCCAAGTGCTTCCTGCATAAGGAGGCTTTTGCCAAGTGTAAATAACTTGGCGAATAAGTGGTGGGCAAGACTGGTGCCAGCCGCCGCGGTAACCCCAGCGCCACTAGTCGGAATCGCATTTATTGGGTCTAAAGCGTTCGTAGCCGGCCAGGCAAATCTCTTGTGAAATCGTTGGGCTCAACTCAACGTCGAGCATGAGACACTACCAGGCTTGGGACTGGGAGGGGCTTGAGGTATGCTTGGGGGAGCAGTAAAATGCTATAATCCCTTGTAGACCACCTGTGGCGAAGGCGTCAAGCCAGAACAGATCCGACGGTGAGGAACGAAAGCTAGGGGAGCAATCCGG` |
| ASV91 | 429 | `TCGAGAATAATTCACAATGGGGGCAACCCTGATGGTGCAACGCCGCGTGGAGGATGACGGTCTTCGGATTGTAAACTCCTGTCATCTGGGAGTAAGGCATGGTGGTGAATAGCCAACATGATTGATAGTACCAGAAGAGGAAGGGACGGCTAACTTCGTGCCAGCAGCCGCGGTAATACGAAGGTCCCAAGCGTTGTTCGGAATCACTGGGCGTAAAGGGAGCGTAGGCGGCGTGGTAAGTCAGATGTGAAATCCCGGGGCTCAACCCCGGAACTGCATCCGATACTGCCATGCTAGAGGATTGGAGAGGTAGCTGGAATTCTTGGTGTAGCAGTGAAATGCGTAGAGATCAAGAGGAACACTCGTGGCGAAAGCGAGCTACTGGACAATTCCTGACGCTGAGGCTCGAAGGCTAGGGTAGCGAAAGGG` |
| ASV4881 | 430 | `TGGGGAATATTGGACAATGGGGGAAACCCTGATCCAGCAACGCCGCGTGAGGGAAGAAGGCCTTCGGGTTGTAAACCTCTGTCGAAGGGGACGAAACCTATCATGGCTAATATCCATGGTAATTGACGGTACCCTTAGAGGAAGCACCGGCTAACTCCGTGCCAGCAGCCGCGGTAATACGGAGGGTGCAAGCGTTGTTCGGAATTACTGGGCGTAAAGCGTGTCTAGGTGGATTTGTAAGTCAGGCGTGAAATCCCCCGGCTCAACCGGGGAGGGTCGCTTGATACTGCATATCTTGAGTTTCGGAGAGGTGGGCAGAATTCCCAGTGTAGAGGTGAAATTCGTAGAGATTGGGAGGAATACCGGAGACGAAGGTGGCTCACTAGCCGAATACTGACACTCAAGCACGAAAGCGTGGGTAGCAAACAGG` |
| ASV2343 | 429 | `TGGGGAATTTTGGACAATGGGCGCAAGCCTGATCCAGCCATGCCGCGTGCGGGAAGAAGGCCTTCGGGTTGTAAACCGCTTTTGTCAGGGAAGAAACGCTCTGAGCTAATACCTCGGAGTAATGACGGTACCTGAAGAATAAGCACCGGCTAACTACGTGCCAGCAGCCGCGGTAATACGTAGGGTGCAAGCGTTAATCGGAATTACTGGGCGTAAAGCGTGCGCAGGCGGTGATGCAAGACAGATGTGAAATCCCCGGGCTTAACCTGGGAACTGCATTTGTGACTGCATCGCTGGAGTGCGGCAGAGGGGGATGGAATTCCGCGTGTAGCAGTGAAATGCGTAGATATGCGGAGGAACACCGATGGCGAAGGCAATCCCCTGGGCCTGCACTGACGCTCATGCACGAAAGCGTGGGGAGCAAACAGG` |
| ASV868 | 429 | `TGGGGAATTTTGGACAATGGGGGCAACCCTGATCCAGCAATGCCGCGTGAGTGAAGAAGGCCTTCGGGTTGTAAAGCTCTTTTGTTAGGGAAGAAAAGGTCATCGATAATACCGGTGGCTGATGACGGTACCTAAAGAATAAGCACCGGCTAACTACGTGCCAGCAGCCGCGGTAATACGTAGGGTGCAAGCGTTAATCGGAATTACTGGGCGTAAAGCGTGCGCAGGCGGCCTCGCAAGTCAGATGTGAAATCCCCGAGCTTAACTTGGGAACTGCATTTGAAACTACGGGGCTAGAGTGTGTCAGAGGGAGGTAGAATTCCACGTGTAGCAGTGAAATGCGTAGAGATGTGGAGGAATACCAGTGGCGAAGGCGGCCTCCTGGGATAACACTGACGCTCATGTACGAAAGCGTGGGGAGCAAACAGG` |
| ASV2312 | 407 | `TGGGGAATTTTCCGCAATGGGCGAAAGCCTGACGGAGCAATACCGCGTGAGGGAAGAAGGCTCTTGGGTTGTAAACCTCTTTTCTGAGGGAAGAAAAAAATGACGGTACCTCAGGAATCAGCATCGGCTAACTCCGTGCCAGCAGCCGCGGTAATACGGAGGATGCAAGCGTTATCCGGAATGATTGGGCGTAAAGCGTCCGCAGGTGGCAATTCAAGTTTGCGGTTAAAGGCTCTGGCTCAACCAGGGACAGGCCGTGAAAACTGAATAGCTAGAGTATGGTAGGGGCAGAGGGAATTCCCAGTGTAGCGGTGAAATGCGTAGAGATTGGGAAGAACACCGGTGGCGAAAGCGCTCTGCTAGGCCAAAACTGACACTGAGGGACGAAAGCTAGGGGAGCGAATGGG` |
| ASV5117 | 404 | `TGGGGAATATTGGACAATGGGCGAAAGCCTGATCCAGCCATGCCGCGTGGATGATGAAGGCCTTAGGGTTGTAAAATCCTTTCACCGGGGACGATAATGACGGTACCCGGAGAAGAAGCCCCGGCTAACTTCGTGCCAGCAGCCGCGGTAATACGAAGGGGGCTAGCGTTGCTCGGAATTACTGGGCGTAAAGCGCACGTAGGCGGATCGGTCAGTCAGGGGTGAAATCCCAGAGCTCAACTCTGGAACTGCCTTTGATACTGCCGGTCTTGACCAGAGGAGAGGCGAGTGGAATTCCGAGTGTAGAGGTGAAATTCGTAGATATTCGGAGGAACACCAGTGGCGAAGGCGACTCGCTGGACTCTTGGTGACGTTGAGGTGCGAAAGCGTGGGGAGCAAACAGG` |
| ASV1858 | 429 | `TGGGGAATTTTGGACAATGGGCGAAAGCCTGATCCAGCAATGCCGCGTGCAGGATGAAGGCCTTCGGGTTGTAAACTGCTTTTGTACGGAACGAAACGGCCTTTTCTAATAAAGAGGGCTAATGACGGTACCGTAAGAATAAGCACCGGCTAACTACGTGCCAGCAGCCGCGGTAATACGTAGGGTGCAAGCGTTAATCGGAATTACTGGGCGTAAAGCGTGCGCAGGCGGTTATGTAAGACAGTTGTGAAATCCCCGGGCTCAACCTGGGAATTGCATCTGTGACTGCATAGCTAGAGTACGGTAGAGGGGGATGGAATTCCGCGTGTAGCAGTGAAATGCGTAGATATGCGGAGGAACACCGATGGCGAAGGCAATCCCCTGGACCTGTACTGACGCTCATGCACGAAAGCGTGGGGAGCAAACAGG` |
| ASV867 | 429 | `TGGGGAATTTTGGACAATGGGGGAAACCCTGATCCAGCCATCCCGCGTGTGCGATGAAGGCCTTCGGGTTGTAAAGCACTTTTGGCAGGAAAGAAACGTCATGGGATAATACCCTGTGAAACTGACGGTACCTGCAGAATAAGCACCGGCTAACTACGTGCCAGCAGCCGCGGTAATACGTAGGGTGCAAGCGTTAATCGGAATTACTGGGCGTAAAGCGTGCGCAGGCGGTTCGGAAAGAGAGATGTGAAATCCCAGGGCTTAACCTTGGAACTGCATTTTTAACTACCGAGCTAGAGTGTGTCAGAGGGAGGTGGAATTCCGCGTGTAGCAGTGAAATGCGTAGATATGCGGAGGAACACCGATGGCGAAGGCAGCCTCCTGGGATAACACTGACGCTCATGCACGAAAGCGTGGGGAGCAAACAGG` |
| ASV768 | 404 | `TGGGGAATATTGGACAATGGGGGAAACCCTGATCCAGCAATGCCGCGTGAGTGAAGAAGGCCTTCGGGTTGTAAAGCTCTTTTGTCCGTGACGATAATGACGGTAGCGGACGAATAAGCACCGGCTAACTTCGTGCCAGCAGCCGCGGTAATACGAAGGGTGCTAGCGTTGTTCGGAATGACTGGGCGTAAAGGGCGTGTAGGCGGTCTTATGCGTCAGATGTGAAAGTCCTGGGCTTAACCTGGGGACTGCATTTGATACGGTAAGACTAGAGGTTATGAGAGGAGAGTGGAATTGCTCATGTAGAAGTGAAATTCGTAGATATGAGCAGGAACATCAGTGGCGAAGGCGACTCTCTGGTATTAACCTGACGCTAAGGCGCGAAAGCGTGGGGAGCAAACAGG` |
| ASV5554 | 428 | `TGGGGAATCTTGGTCAATGGGCGAAAGCCTGAACCAGCGACGCCGCGTGAGTGATGAAGGCCTTCGGGTTGTAAAGCTCTGTGGGGCGAGACGAAAGCCTACGTTCTAACAGGACGTAGTTTGACGGTATCGCCTTAGCAAGCACCGGCTAACTCTGTGCCAGCAGCCGCGGTAAGACAGAGGGTGCAAACGTTGCTCGGAATCACTGGGCGTAAAGGGCGTGTAGGCGGGAAGGAAAGTCAGATGTGAAATCCCTGGGCTCAACCCAGGAACTGCATTTGAAACTCCCTTTCTTGAGTGTCGGAGAGGAAAGCGGAATTCCTGGTGTAGAGGTGAAATTCGTAGATATCAGGAGGAACACCGGTGGCGAAGGCGGCTTTCTGGACGACAACTGACGCTGAGACGCGAAAGCGTGGGGAGCAAACAGG` |
| ASV1269 | 404 | `TGGGGAATATTGGACAATGGGCGAAAGCCTGATCCAGCAATGCCGCGTGAGTGATGAAGGCCTTAGGGTTGTAAAGCTCTTTTACCCGGGATGATAATGACAGTACCGGGAGAATAAGCCCCGGCTAACTCCGTGCCAGCAGCCGCGGTAATACGGAGGGGGCTAGCGTTGTTCGGAATTACTGGGCGTAAAGCGCGCGTAGGCGGCTTTGTAAGTTAGAGGTGAAAGCCCGGAGCTCAACTCCGGAACTGCCTTTAAGACTGCATCGCTAGAATTGTGGAGAGGTAAGTGGAATTCCGAGTGTAGAGGTGAAATTCGTAGATATTCGGAAGAACACCAGTGGCGAAGGCGACTTACTGGACACATATTGACGCTGAGGTGCGAAAGCGTGGGGAGCAAACAGG` |
| ASV5496 | 407 | `TAGGGAATTTTGCGCAATGGACGAAAGTCTGACGCAGCAATGCCGCGTGTAGGAAGAAGCATCTTGGTGTGTAAACTACTGTCGGCGGGGAAAAACACAATGATTGTACCCGCAAAGGAAGCATCGGCTAACTACGTGCCAGCAGCCGCGGTAAGACGTAGGATGCAAGCGTTGTCCGGATTTATTGGGCGTAAAGAGCTCGTAGGTGGCAATTTAAGTCTAATGTTAAATAATAGAGCTTAACTCTATACCTGCATTGGATACTGAAATGCTTGAGTTAGGTAGGGGTCAGAGGAATTCCTGGTGTAGCGGTGAAATGCGTAGATATCAGGAGGAACACCGGTGGCGAAGGCGTCTGACTGGGCCTATACTGACACTGAGGAACGAAAGCTAGGGGAGCAAAAGGG` |
| ASV2530 | 404 | `TGGGGAATATTGGACAATGGGGGAAACCCTGATCCAGCAATGCCGCGTGAGTGAAGAAGGCCTTAGGGTTGTAAAGCTCTTTTACCCAGGAAGATAATGACTGTACTGGGAGAATAAGCTCCGGCTAACTCCGTGCCAGCAGCCGCGGTAATACGGAGGGAGCTAGCGTTGTTCGGAATTACTGGGCGTAAAGCGCACGTAGGTGGTTATATAAGTCAGAGGTGAAAGCCTGGAGCTTAACTCCAGAATTGCCTTTGAGACTGTATAGCTAGAGTGTAGGAGAGGTAAGTGGAATTCCTAGTGTAGAGGTGAAATTCGTAGATATTAGGAAGAACACCAGTGGCGAAGGCGGCTTACTGGACTACTACTGACACTGAGGTGCGAAAGCGTGGGGAGCAAACAGG` |
| ASV5131 | 407 | `TCGAGAATTTTTCACAATGGGGGCAACCCTGATGGAGCGACGCCGCGTGGAGGATGAAGGCCTTCGGGTTGTAAACTCCTGTCATGCGGGAACAAGCAAGTGATAGTACCGCAAGAGGAAGAGACGGCTAACTCTGTGCCAGCAGCCGCGGTAATACAGAGGTCTCAAGCGTTGTTCGGATTCATTGGGCGTAAAGGGTGCGTAGGTGGTGACGCAAGTCTGGTGTGAAATCTCGGAGCTTAACTCCGAAATTGCACCGGATACTGCGTAACTCGAGGACTGTAGAGGAGATCGGAATTCACGGTGTAGCAGTGAAATGCGTAGATATCGTGAGGAAGACCAGTTGCGAAGGCGGATCTCTGGGCAGTTCCTGACACTGAGGCACGAAGGCCAGGGGAGCAAACGGG` |
| ASV237 | 407 | `TGGGGAATTTTCCGCAATGGGCGAAAGCCTGACGGAGCAATACCGCGTGAGGGATGAAGGCCTATGGGTTGTAAACCTCTTTTTTCAGGGAGGAATAAATGACGTGTACCTGAAGAATAAGCATCGGCTAACTCCGTGCCAGCAGCCGCGGTAAGACGGGGGATGCAAGTGTTATCCGGAATCACTGGGCGTAAAGCGTCTGTAGGTGGTTAAATAAGTCAACTGTTAAATCTTGAGGCTCAACCTCAAAATCGCAGTCGAAACTGTTTGACTAGAGTATAGTAGGGGTAAAGGGAATTTCCAGTGGAGCGGTGAAATGCGTAGATATTGGAAAGAACACCGATGGCGAAGGCACTTTACTGGGCTATTACTAACACTGAGAGACGAAAGCTAGGGTAGCAAATGGG` |
| ASV2691 | 428 | `TCGGGAATCTTCCGCAATGGGCGCAAGCCTGACGGAGCGACGCCGCGTGAGCGATGAAGGCCTTCGGGTTGTAAAGCTCTGTGGGGAGGGAAGAATAAGTTCAGGCCAATACCCTGAATGATGACGGTACCTCCTTAGCAAGCACCGGCTAACTCTGTGCCAGCAGCCGCGGTAAGACAGAGGGTGCAAACGTTGTTCGGATTTACTGGGCGTAAAGCGCGTGTAGGCGGCTTTGCAAGTCGGATGTGAAAGCCCACGGCTCAACCGTGGAAGTGCATCCGAAACTGCATGGCTGGAGTCCCGGAGAGGAAGGTGGAATGCTTGGTGTAGAGGTGAAATTCGTAGATATCAAGCAGAACACCTGCGGCGAAGGCGGCCTTCTGGACGGTGACTGACGCTGAGACGCGAAAGCGTGGGGAGCAAACAGG` |
| ASV3513 | 424 | `TGAGGAATATTGCGCAATGGAGGCAACTCTGACGCAGCCATGCCGCGTGCAGGAAGAAGGCGCTACGCGTTGTAAACTGCTTTTAACCGGGAAGAAACCCCAGGACGTGTCCTGGGTTGACGGTACCGGAGGAATAAGCACCGGCTAACTTCGTGCCAGCAGCCGCGGTAATACGAAGGGTGCAAGCGTTATCCGGATTCATTGGGTTTAAAGGGTGCGTAGGTGGGGCGATAAGTCAGTGGTGAAATCCTGCGGCTTAACCGTAGAATTGCCATTGATACTGTCGTTCTTGAGTACGCTTGACGTGGGCGGAATGTGCCGTGTAGCGGTGAAATGCTTAGATATGGCACAGAACACCGATAGTGAAGACAGCTCACGAAGGCGAAACTGACACTGAGGCACGAAAGCGTGGGGATCGAACAGG` |
| ASV5123 | 421 | `TAGGGAATTTTGCGCAATGGGGGAAACCCTGACGCAGCAACGCCATGTGAGGGATGAAGCATTTAGGTGTGTAAACCTCTGTCAGCAGGGAATAAATGTCCGTTTACGGAAGTTGAATGTACCTGCAAAGGAAGCACCGGCTAACTTCGTGCCAGCAGCCGCGGTAATACGAGGGGTGCAAGTGTTGTTCGGAATCACTGGGCGTAAAGGGAGCGTAGGCGGACCTTTAAGTCAGATGTTTAATTTCAGGGCCCAACCCTGAATTTGCGTTTGAAACTGGAGGTCTTGAATATATGTAGAGGTGAATGGAATTCCCGGTGTAGCGGTGGAATGCGTAGATATCGGGAGGAACACCAGTTGCGAAGGCGATTCACTGGACTAATATTGACGCTGAGGCTCGAAAGCGTGGGGAGCGAACAGG` |
| ASV2084 | 407 | `TCGAGAATTTTTCACAATGGGGGAAACCCTGATGGAGCGACGCCGCGTGGAGGACGAAGGTCTTCGGATTGTAAACTCCTGTCATGCGGGAACAAGAAAGTGATAGTACCGCAAGAGGAAGAGACGGCTAACTCTGTGCCAGCAGCCGCGGTAATACAGAGGTCTCAAGCGTTGTTCGGATTCATTGGGCGTAAAGGGTGCGCAGGCGGCGTGGAAAGTTGAGTGTGAAATCCTGGGGCTTAACCTCAGAACTGCACTCAATACTCCCATGCTAGAGGAATGTAGAGGAGAGTGGAATTCACGGTGTAGCAGTGAAATGCGTAGATATCGTGAGGAAGACCAGTTGCGAAGGCGACTCTCTGGGCATTTCCTGACGCTCATGCACGAAGGCCAGGGGAGCAAACGGG` |
| ASV3300 | 429 | `TGGGGAATTTTGGACAATGGGCGAAAGCCTGATCCAGCAATGCCGCGTGTGTGAAGAAGGCCTTCGGGTTGTAAAGCACTTTTGTCAGGAACGAAACGGCGCTCGCTAATATCGAGCGCTAATGACGGTACCTGAAGAATAAGCACCGGCTAACTACGTGCCAGCAGCCGCGGTAATACGTAGGGTGCAAGCGTTAATCGGAATTACTGGGCGTAAAGCGTGCGCAGGCGGTTATGCAAGACAGATGTGAAATCCCCGGGCTTAACCTGGGCACTGCATTTGTGACTGCATGGCTAGAGTGTGTCAGAGGGGGGTAGAATTCCACGTGTAGCAGTGAAATGCGTAGAGATGTGGAGGAACACCGATGGCGAAGGCAGCCCCCTGGGATAACACTGACGCTCATGCACGAAAGCGTGGGGAGCAAACAGG` |
| ASV4180 | 423 | `TAGGGAATATTGGACAATGGGTGAGAGCCTGATCCAGCCATGCCGCGTGCAGGAAGAAGGCGTTATGCGTTGTAAACTGCTTTAGCCAGGGGATAAAACGGCTCTGCGGAGCTAATTGAAGGTACCTGGATAATAAGCCACGGCTAACTACGTGCCAGCAGCCGCGGTAATACGTAGGTAGCAAGCGTTGTCCGGATTTATTGGGTTTAAAGGGTGCGTAGGCGGCCCTGTAAGTCAGTGCTGAAATATCCCGGCTTAACCGGGAGGGTGGCATTGATACTGCAGGGCTTGAGTACAGATGAAGTAGGCGGAATTGACGGTGTAGCGGTGAAATGCATAGATATCGTCAAGAACACCGATAGCGAAGGCAGCTTACTAAGCTGTAACTGACGCTGAGGCACGAAAGTGTGGGGATCAAACAGG` |
| ASV428 | 407 | `TGGGGAATATTGCGCAATGGGGGAAACCCTGACGCAGCAATACCGCGTGAGTGAAGAAGGTTTTCGGATCGTAAAGCTCTGTTATTGGGGAAGAAAAAAATGACGGTACCCAATGAGGAAGTCCCGGCTAACTACGTGCCAGCAGCCGCGGTAATACGTAGGGGACAAGCGTTGTCCGGAATCACTGGGCGTAAAGGGCGCGTAGGCGGTTATTTAAGTCAGATGTGAAAGGTTACAGCTCAACTGTAAGATGGCATTTGAAACTGAATAACTTGAGTATTGGAGAGGCAAGTGGAATTCCTAGTGTAGCGGTGAAATGCGTAGATATTAGGAGGAACACCGGTGGCGAAGGCGGCTTGCTGGACAAATACTGACGCTGAGGTGCGAAAGCGTGGGGAGCAAACAGG` |
| ASV86 | 404 | `TGGGGAATATTGGACAATGGGCGAAAGCCTGATCCAGCAATGCCGCGTGAGTGATGAAGGCCTTAGGGTTGTAAAGCTCTTTTACCAGGGATGATAATGACAGTACCTGGAGAATAAGCTCCGGCTAACTCCGTGCCAGCAGCCGCGGTAATACGGAGGGAGCTAGCGTTGTTCGGAATTACTGGGCGTAAAGCGTACGTAGGCGGTGATTCAAGTCAGAGGTGAAAGCCTGGAGCTCAACTCCAGAACTGCCTTTGAAACTAGATCGCTAGAATCACGGAGAGGTGAGTGGAATTCCGAGTGTAGAGGTGAAATTCGTAGATATTCGGAAGAACACCAGTGGCGAAGGCGGCTCACTGGACGTGTATTGACGCTGAGGTACGAAAGCGTGGGGAGCAAACAGG` |
| ASV807 | 424 | `TGAGGAATATTGGACAATGGGCGCAAGCCTGATCCAGCCATGCCGCGTGCAGGAAGACGGTCCTATGGATTGTAAACTGCTTTTATACAGGAAGAAACACTCGGACGTGTCCGAACTTGACGGTACTGTATGAATAAGCATCGGCTAACTCCGTGCCAGCAGCCGCGGTAATACGGAGGATGCAAGCGTTATCCGGAATCATTGGGTTTAAAGGGTCCGTAGGCGGTCATATAAGTCAGTGGTGAAAGCCCATCGCTCAACGATGGAACGGCCATTGATACTGTTTGACTTGAATTATTAGGAAGTAACTAGAATATGTAGTGTAGCGGTGAAATGCTTAGAGATTACATGGAATACCAATTGCGAAGGCAGGTTACTACTAATATATTGACGCTGATGGACGAAAGCGTGGGGAGCGAACAGG` |
| ASV4423 | 404 | `TGGGGAATATTGGACAATGGGCGCAAGCCTGATCCAGCCATGCCGCGTGAGTGACGAAGGCCTTAGGGTTGTAAAGCTCTTTTGGCGGGGAAGATAATGACGGTACCCGCAGAATAAGCTCCGGCTAACTTCGTGCCAGCAGCCGCGGTAATACGAAGGGAGCTAGCGTTGTTCGGAATCACTGGGCGTAAAGCGCACGTAGGCGGATTTGCCAGTCAGGGGTGAAATCCCGGGGCTCAACCTCGGAACTGCCTTTGATACAGCAAGTCTTGAGTTCGAGAGAGGTGGGTGGAATTCCTAGTGTAGAGGTGAAATTCGTAGATATTAGGAAGAACACCGGTGGCGAAGGCGGCCCACTGGCTCGACACTGACGCTGAGGTGCGAAAGCGTGGGGAGCAAACAGG` |
| ASV75 | 429 | `TGGGGAATTTTGGACAATGGGCGCAAGCCTGATCCAGCAATGCCGCGTGCAGGATGAAGGCCTTCGGGTTGTAAACTGCTTTTGTACGGAACGAAACGGTCTCTTCTAATAAAGGAGGCTAATGACGGTACCGTAAGAATAAGCACCGGCTAACTACGTGCCAGCAGCCGCGGTAATACGTAGGGTGCGAGCGTTAATCGGAATTACTGGGCGTAAAGCGTGCGCAGGCGGTTATATAAGACAGATGTGAAATCCCCGGGCTCAACCTGGGACCTGCATTTGTGACTGTATAGCTAGAGTACGGTAGAGGGGGATGGAATTCCGCGTGTAGCAGTGAAATGCGTAGATATGCGGAGGAACACCGATGGCGAAGGCAATCCCCTGGACCTGTACTGACGCTCATGCACGAAAGCGTGGGGAGCAAACAGG` |
| ASV5535 | 428 | `TCGAGAATTTTTCACAATGGGGGAAACCCTGATGGAGCGACGCCGCGTGGGGGATGAAGGGCTTCGGTCCGTAAACCCCTGTCATTGGCGAACAAGGGTTGCGTACTAATACGACGTAACTTGATAGTAGCCGAAGAGGAAGGGACGGCTAACTCTGTGCCAGCAGCCGCGGTAATACAGAGGTCCCGAGCGTTGTTCGGATTCACTGGGCGTAAAGGGTGCGTAGGTGGTTGGGTAAGTTTGAGGTGAAAGCTCCGAGCCTAACTCGGAAAATGCCTTGAAGACTATCTGACTAGAGGGTTGGAGGGGAGACTGGAATTCTCGGTGTAGCAGTGAAATGCGTAGATATCGAGAGGAACACCAGTGGCGAAGGCGAGTCTCTGGACAACACCTGACACTGAGGCACGAAAGCCAAGGGAGCAAACGGG` |
| ASV5579 | 405 | `TGAGGAATTTTCCACAATGGACGAAAGTCTGATGGAGCAATACCGCGTGCAGGATGAAGGCCTTCGGGTCGTAAACTGCTTTTTATAAGGAGCAACTATGAGTTTACTTATAGAATAAGTCACGGCTAATCACGTGCCAGCAGCCGCGGTAATACGTGAGTGGCAAGCGTTATCCGGAATCATTGGGCGTAAAGCGTCAGCAGGGGGGCCTTCAAGTGAAATGTAAAATCCTTCGACCCAATCGAAGAGCTGTATTTCATACTGTTGGCCTAGAAGACAGAAGAGGTAAATAGAATTTCCGGAGTAGGGGTAATATCCGTAGATACCGGAAGGAATATCAATAGCGAAGGCAATTTACTGGTCTGTTCTTGACCCTCAGTGACGAAAGCGTGGGTAGCAAACAGG` |
| ASV4008 | 411 | `TTTGGAATTTTGGACAATGGGCGAAAGCCTGATCCAGCGACGCCGCGTGGACGATGAAGGCCTTCGGGTTGTAAAGTCCTTTTGCTCGGGAACAATATTTTGAGCGTACCGAGAGAATAAGAGGTTGCTAACTCTGTGCCAGCAGCAGCGGTAATACAGAGACCTCAAGCGTTATCCGGAATCATTGGGCGTAAAGCGTACCGATAGGTGGTTTTACAAGTCAGAAGTGAAATCTTAAGGCTCAACCTTGAGACTGTCTTTTGAAACTGTAAAACTAGAGGGGCAAAGAGGAAGCTGGAACGAACGGTGTAGTAGTGAAATGCGTTGATATCGTTCGGAACACCAATAGCGTAGGCAGGCTTCTGGGTGCCACCTGACACTGCTAGGACGAAAGCGTGGGGAGCGAATGGG` |
| ASV4135 | 429 | `TGGGGAATATTGCGCAATGGAGGAAACTCTGACGCAGCGACGCCGCGTGGGTGATGAAGGCCTTCGGGTTGTAAAGCCCTGTCGGCTGGGAAAAAGGTAGTCGTGCTAACACCACGATTGCTTGATGGTACCAGCAAAGGAAGCACCGGCTAACTCTGTGCCAGCAGCCGCGGTAATACAGAGGGTGCGAGCGTTGTTCGGAATTACTGGGCGTAAAGGGCGCGTAGGCGGTTTAACAAGTCTGTTGTGAAATCCCGGGGCTTAACCTCGGAAGTGCAGTGGAAACTGTTAGACTAGAGTGCAGTAGAGGTTGGTGGAATACCCAGTGTAGAGGTGAAATTCGTAGATATTGGGTGGAACACCGGTGGCGAAGGCGGCCAACTGGGCTGCAACTGACGCTGAGGCGCGAAAGCGTGGGGATCAAACAGG` |
| ASV1232 | 404 | `TGGGGAATCTTGCGCAATGGGCGAAAGCCTGACGCAGCCATGCCGCGTGAATGATGAAGGTCTTAGGATTGTAAAATTCTTTCACCGGGGACGATAATGACGGTACCCGGAGAAGAAGCCCCGGCTAACTTCGTGCCAGCAGCCGCGGTAATACGAAGGGGGCTAGCGTTGCTCGGAATTACTGGGCGTAAAGGGAGCGTAGGCGGACATTTAAGTCAGGGGTGAAATCCCGGGGCTCAACCTCGGAATTGCCTTTGATACTGGGTGTCTTGAGTATGAGAGAGGTGTGTGGAACTCCGAGTGTAGAGGTGAAATTCGTAGATATTCGGAAGAACACCAGTGGCGAAGGCGACACACTGGCTCATTACTGACGCTGAGGCTCGAAAGCGTGGGGAGCAAACAGG` |
| ASV496 | 429 | `TCGAGAATCATTCACAATGGGCGAAAGCCTGATGGTGTGACGCCGCGTGGAGGATGAAGGTCTTCGGATTGTAAACTCCTGTCATGTGGGGGTAAGGCTTGGCGTTTAACTGGCGTCAAGATTGATAGTACCACAAGAGGAAGGGGCGGCTAACTCTGTGCCAGCAGCCGCGGTAATACAGAGGCCCCAAGCGTTGTTCGGAATCACTGGGCGTAAAGGGTGCGTAGGCGGCGGTGTAAGTCAGATGTGAAATCCCGGGGCTCAACCTCGGAACTGCATCCGATACTGCATTGCTTGAGTACTGGAGGGGAGTCTGGAATTCACGGTGGAGCAGTGAAATGCGTGGATATCGTGAGGAACACTAGTGGCGAAGGCGAGACTCTGGACAGTAACTGACGCTGAGGCACGAAGGCCAGGGGAGCAAACGGG` |
| ASV5712 | 404 | `TGGGGAATATTGGACAATGGGCGAAAGCCTGATCCAGCCATGCCGCGTGTGTGATGAAGGCCTTAGGGTTGTAAAGCACTTTCACCGATGAAGATAATGACGGTAGTCGGAGAAGAAGCCCCGGCTAACTTCGTGCCAGCAGCCGCGGTAATACGAAGGGGGCTAGCGTTGTTCGGAATCACTGGGCGTAAAGCGCACGTAGGCGGATTGTTAAGTGAGGGGTGAAATCCCGGGGCTCAACCCCGGAACTGCCTTTCATACTGGCAATCTTGAGTTCGAGAGAGGTAAGTGGAATTCCGAGTGTAGAGGTGAAATTCGTAGATATTCGGAGGAACACCAGTGGCGAAGGCGGCTTACTGGATCGATACTGACGCTGAGGTGCGAAAGCGTGGGGAGCAAACAGG` |
| ASV1730 | 429 | `TGGGGAATATTGGACAATGGGCGCAAGCCTGATCCAGCCATGCCGCGTGTGTGAAGAAGGCCTTCGGGTTGTAAAGCACTTTTGTTGGGGAAGAAAAGCTATCGGTTAATACCCGGTGGTGATGACGGTACCCAAAGAATAAGCACCGGCTAACTTCGTGCCAGCAGCCGCGGTAATACGAAGGGTGCAAGCGTTACTCGGAATTACTGGGCGTAAAGCGTGCGTAGGTGGTTTGTTAAGTCAGATGTGAAAGCCCCGGGCTCAACCTGGGAATTGCATTTGATACTGGCTAACTAGAGTGAGGTAGAGGAGAGTGGAATTCCCGGTGTAGCAGTGAAATGCGTAGAGATCGGGAGGAACATCAGTTGCGAAGGCGGCTCTCTGGACCATCACTGACACTGAGGCACGAAAGCGTGGGGAGCAAACAGG` |
| ASV2420 | 404 | `TGGGGAATATTGGACAATGGGCGCAAGCCTGATCCAGCCATGCCGCGTGAGTGATGAAGGCCCTAGGGTTGTAAAGCTCTTTTACCTGGGAAGATAATGACTGTACCAGGAGAATAAGCCCCGGCTAACTTCGTGCCAGCAGCCGCGGTAATACGAAGGGGGCTAGCGTTGTTCGGAATCACTGGGCGTAAAGCGCACGTAGGCGGATTCTTAAGTCAGAGGTGAAATCCTGAGGCTCAACCTCAGAACTGCCTTTGATACTGGGAATCTTGAGTTCGGAAGAGGTAAGTGGAACTGCGAGTGTAGAGGTGAAATTCGTAGATATTCGCAAGAACACCAGTGGCGAAGGCGGCTTACTGGTCCGATACTGACGCTGAGGTGCGAAAGCGTGGGGAGCAAACAGG` |
| ASV2561 | 404 | `TGGGGAATATTGGACAATGGGCGAAAGCCTGATCCAGCAATGCCGCGTGAGTGATGAAGGCCTTCGGGTTGTAAAGCTCTTTTGGTGGGGAAAATAATGATGGTACCCACAGAATAAGCTCCGGCTAACTTCGTGCCAGCAGCCGCGGTAATACGAAGGGAGCTAGCGTTGTTCGGAATTACTGGGCGTAAAGAGTACGTAGGCTGTTTTTTAAGTTAGGTGTGAAAGCCCCGGGCTCAACCCGGGAATTGCACTTAAAACTGAAAAGCTAGAGATTGGGAGAGGAAAGTGGAATTACTAGTGTAGAGGTGAAATTCGTAGATATTAGTAAGAACACCAGTGGCGAAGGCGACTTTCTGGACCAATACTGACGCTGAGGTACGAAAGCGTGGGGAGCAAACAGG` |
| ASV2325 | 404 | `TGGGGAATATTGGACAATGGGGGCAACCCTGATCCAGCCATGCCGCGTGCGTGAAGAAGGCCTTAGGGTTGTAAAGCGCTTTTGCTAGGGATGATAATGACCGTACCTAGAGAATAAGCCCCGGCTAACTCCGTGCCAGCAGCCGCGGTAAGACGGAGGGGGCTAGCGTTGTTCGGATTTACTGGGCGTAAAGGGCGCGTAGGCGGCCATGTGTGTCAGAGGTGAAATCCCAAGGCTTAACCTTGGAATTGCTTTTGAAACTGCATGGCTTGAGTGATAGAGAGGCAAGTGGAATTCCGAGTGTAGCAGTGAAATGCGTAGATATTCGGAGGAACATCGGTGGCGAAGGCGACTTGCTGGCTATCAACTGACGCTGAGGCGCGAAAGCGTGGGGAGCAAACAGG` |
| ASV3851 | 429 | `TGGGGAATATTGGACAATGGGGGCAACCCTGATCCAGCCATGCCGCGTGTGTGAAGAAGGCCCTTGGGTTGTAAAGCACTTTCAGTGGGGAGAAAAGTCTGGAACCTAATACGTTTTGGAATTGATGTTACCCACAGAAGAAGCACCGGCTAACTCCGTGCCAGCAGCCGCGGTAATACGGAGGGTGCAAGCGTTAATCGGAATTACTGGGCGTAAAGCGCACGTAGGTGGTTTACTAAGCCAGCTGTGAAATCCCCGGGCTCAACCTGGGCACTGCAGTTGGAACTGGCAAGCTAGAGTAGGGTAGAGGGGTGTGGAATTCCAGGTGTAGCGGTGAAATGCGTAGATATCTGGAGGAACATCAGTGGCGAAGGCGACACCCTGGACTCATACTGACACTGAGGTGCGAAAGCGTGGGGAGCAAACAGG` |
| ASV1287 | 403 | `TGGGGAATATTGCACAATGGACGAAAGTCTGATGCAGCGACGCCGCGTGAGGGAAGAAGGCCTTCGGGTTGTAAACCTCTGTCTTTGGGGACGATAATGACGGTACCCAAGGAGGAAGCACCGGCTAACTACGTGCCAGCAGCCGCGGTAAAACGTAGGGTGCAAGCGTTGTCCGGAATTACTGGGTGTAAAGGGAGCGCAGGCGGATCTATAAGTTGGATGTTTAATCTACGGGCTCAACCCGTATCAGCATTCAAAACTGTGGATCTTGAGTGGTGCAGAGGTAGGCGGAATTCCCGGTGTAGCGGTGGAATGCGTAGATATCGGGAGGAACACCAGTGGCGAAGGCGGCCTACTGGGCACTAACTGACGCTGAGGCTCGAAAGCATGGGTAGCAAACAGG` |
| ASV5442 | 404 | `TGGGGAATATTGGACAATGGGCGCAAGCCTGATCCAGCAATGCCGCGTGGATGAAGAAGGTCTTCGGATTGTAAAGTCCTTTCGGCGGGGACGATGATGACGGTACCCGCAGAAGAAGCCCCGGCTAACTTCGTGCCAGCAGCCGCGGTAATACGAAGGGGGCTAGCGTTGCTCGGAATGACTGGGCGTAAAGGGCGCGTAGGCGGGTGGCTTAGTCAGGCGTGAAATTCCCGGGCTTAACCTGGGGGCTGCGCTTGAGACGGGCTGCCTTGAGTGGGGAAGAGGGTTGTGGAATTCCCAGTGTAGAGGTGAAATTCGTAGATATTGGGAAGAACACCGGTGGCGAAGGCGGCAACCTGGTCCTTGACTGACGCTGAGGCGCGAAAGCGTGGGGAGCAAACAGG` |
| ASV3857 | 408 | `TCGAGAATCTTCCGCAATGGACGCAAGTCTGACGGAGCGATGTCGCGTGATTGATGAAGTCTTTCGGGATGTAAAGATCTTTTCTCAAGGAGGAAGTTTATTGACGTTACTTGAGGAATAAGGGGTTGCTAAACTCGTGCCAGCAGCAGCGGTAATACGAGTGCCCCAAGCGTTATCCGGAATTATTGGGCGTAAAGGGTCCGCAGGCGGTTTTATTAGTCTTGTGTGAAATCTCCCGGCTTAACTGGGAAAACGCATAGGAAACGGTAAAACTAGAGGATGTGAGAGGTATGTGGAACTCATGGTGTAGGGGTGAAATCCGTTGATATCATGGGGAACACCAAATGCGAAGGCAACATACTGGCACATTCCTGACGCTCAGGGACGAAAGCACGGGTAGCGAATGGG` |
| ASV1312 | 430 | `TGGGGAATATTGGACAATGGGCGAAAGCCTGATCCAGCCATGCCGCGTGTGTGAAGAAGGCCTTTTGGTTGTAAAGCACTTTAAGCGGGGAGGAGGGCTCTGTGGTTAATACCCATAGAAGTCGGACGTTACCCGCAGAATAAGCACCGGCTAACTCTGTGCCAGCAGCCGCGGTAATACAGAGGGTGCGAGCGTTAATCGGAATTACTGGGCGTAAAGCGCGCGTAGGCGGCTTGTTAAGTCGGATGTGAAATCCCTGGGCTCAACCTAGGCATTGCATTCGATACTGGCAAGCTAGAGTATGGGAGAGGAAGGTAGAATTCCAGGTGTAGCGGTGAAATGCGTAGAGATCTGGAGGAATACCGATGGCGAAGGCAGCCTTCTGGCCTAATACTGACGCTGAGGTGCGAAAGCATGGGGAGCAAACAGG` |
| ASV3122 | 429 | `TGGGGAATATTGCGCAATGGGCGAAAGCCTGACGCAGCGACGCCGCGTGAGGGATGAAGGTTTTCGGATCGTAAACCTCTGTCAGAAGGGAAGAAACTACGTTGTGCTAATCAGCAGCGTACTGACGGTACCTTCAAAGGAAGCACCGGCTAACTCCGTGCCAGCAGCCGCGGTAATACGGAGGGTGCAAGCGTTAATCGGAATTACTGGGCGTAAAGCGCACGTAGGCTGTAGTGTAAGTCAGGGGTGAAATCCCACGGCTCAACCGTGGAACTGCCTTTGATACTGCACAACTTGAATCCGGGAGAGGGTGGCGGAATTCCAGGTGTAGGAGTGAAATCCGTAGATATCTGGAGGAACATCAGTGGCGAAGGCGGCCACCTGGACCGGTATTGACGCTGAGGTGCGAAAGCGTGGGGAGCAAACAGG` |
| ASV471 | 429 | `TGGGGAATTTTGGACAATGGGGGCAACCCTGATCCAGCCATGCCGCGTGCAGGATGAAGGCCTTCGGGTTGTAAACTGCTTTTGTTAGGAACGAAAAGCCATCTAATAACACTAGGTGGTGCTGACGGTACCTAAAGAATAAGCACCGGCTAACTACGTGCCAGCAGCCGCGGTAATACGTAGGGTGCAAGCGTTAATCGGAATTACTGGGCGTAAAGCGTGCGCAGGCGGTTCGTTAAGTCAGATGTGAAATCCCCGGGCTCAACCTGGGAACTGCATTTGAAACTGGCGAGCTAGAGTGTGTCAGAGGGGGGTAGAATTCCACGTGTAGCAGTGAAATGCGTAGAGATGTGGAGGAATACCAATGGCGAAGGCAACCCCCTGGGATAACACTGACGCTCATGTACGAAAGCGTGGGGAGCAAACAGG` |
| ASV930 | 430 | `TGGGGAATCTTGCACAATGGGCGCAAGCCTGATGCAGCAACGCCGCGTGAGTGAAGACGGTCCTTGGATTGTAAAGCTCTTTCAGTTAGGGAGAAGGGCCTTTAGGTTAATACCCTAAGGGTTTGACGGTACTAGCAGAAGCAGCACCGGCTAATTTCGTGCCAGCAGCCGCGGTAATACGAAAGGTGCAAGCGTTGTTCGGAATGACTGGGCGTAAAGCGCACGTAGGTGGGTTAGCAAGTCGGATGTGAAAGCCCTGGGCTCAACCTGGGAATGGCACCCGAAACTGCTGATCTTGAATGCTCGAGAGGTTGGTAGAATTCCTGGTGTAGGAGTGAAATCCGTAGATATCAGGAGGAACATCGGCGGCGAAGGCGGCCAACTGGCGGTGCATTGACACTGAGGTGCGAAAGCGTGGGGAGCAAACAGG` |
| ASV5232 | 430 | `TCGAGAGGCTTCGGCAATGGGGGAAACCCTGACCGAGCGACGCCGCGTGGGGGATGAAGGCCTTTGGGTTGTAAACCCCTGTCAAGCGGGACGAAACACTCTGAGGTGATAAGCCGAGGGGTTTGACGGTACCGCCAGAGGAAGCTCCGGCTAACTCCGTGCCAGCAGCCGCGGTAATACGGGGGGAGCAAGCGTTGTTCGGAATTACTGGGCGTAAAGGGCGTGTAGGCGGCCTTGTAAGTCGGGGGTGAAATCCACTGGCTCAACCAGTGACGTGCCTTCGAAACTGCTTGGCTTGAGTATGGCAGAGGAGAGCGGAATTCCAGGTGTAGCGGTGAAATGCGTAGATATCTGGAAGAACACCGGTGGCGTAGGCGGCTCTCTGGGCCAATACTGACGCTGATGCGCGAAAGCCAGGGGAGCAAACGGG` |
| ASV3928 | 404 | `TGGGGAATATTGGGCAATGGGCGAAAGCCTGACCCAGCTATGCCGCGTGAGTGATGAAGGCCTTCGGGTTGTAAAGCTCTTTTAGGTGGGACGATGATGACGGTACCACCAGAAAAAGCCCCGGCTAACTTCGTGCCAGCAGCCGCGGTAATACGAAGGGGGCAAGCGTTGCTCGGATTTATTGGGCGTAAAGGGTGTGTAGGTGGCTCTACAAGTTAGATGTGAAAGCCCCGGGCTTAACCCGGGAACTGCATTTAAAACTGTAGAGCTTGAAGATCACAGAGGTAAGCAGAATTTCCAGTGTAGAGGTGAAATTCGTAGATATTGGAAAGAATACCAACGGCGTAGGCAGCTTACTGGGTGACTCTTGACACTGAGGCACGAAAGCGTGGGGAGCAAACAGG` |
| ASV225 | 404 | `TGGGGAATCTTAGACAATGGGGGGAACCCTGATCTAGCCATGCCGCGTGATCGATGAAGGCCTTAGGGTTGTAAAGATCTTTCAGATGGGAAGATAATGACGGTACCATCAGAAGAAGCCCCGGCTAACTCCGTGCCAGCAGCCGCGGTAATACGGAGGGGGCTAGCGTTATTCGGAATTACTGGGCGTAAAGCGCACGTAGGCGGACTGGAAAGTCAGAGGTGAAATCCCAGGGCTCAACCTTGGAACGGCCTTTGAAACTCCCAGTCTTGAGATCGTGAGAGGTGAGTGGAATTCCGAGTGTAGAGGTGAAATTCGTAGATATTCGGAGGAACACCAGTGGCGAAGGCGGCTCACTGGCACGATACTGACGCTGAGGTGCGAAAGCGTGGGGAGCAAACAGG` |
| ASV90 | 429 | `TGGGGAATTTTGGACAATGGACGCAAGTCTGATCCAGCCATTCCGCGTGCAGGATGAAGGCCCTCGGGTTGTAAACTGCTTTTGTACGGAACGAAAAGGTCTCTCTTAATACGGGGGGCTCATGACGGTACCGTAAGAATAAGCACCGGCTAACTACGTGCCAGCAGCCGCGGTAATACGTAGGGTGCAAGCGTTAATCGGAATTACTGGGCGTAAAGCGTGCGCAGGCGGTTATATAAGACAGATGTGAAATCCCCGGGCTCAACCTGGGAACTGCATTTGTGACTGTATAGCTAGAGTACGGTAGAGGGGGATGGAATTCCGCGTGTAGCAGTGAAATGCGTAGATATGCGGAGGAACACCGATGGCGAAGGCAATCCCCTGGACCTGTACTGACGCTCATGCACGAAAGCGTGGGGAGCAAACAGG` |
| ASV1423 | 429 | `TGGGGAATCTTGGACAATGGGCGCAAGCCTGATCCAGCAATGCCGCGTGCGTGAAGAAGGCCTTCGGGTTGTAAAGCGCTTTTGTCAGGGAGCAAATTCTTGTGGCTAATACCCACCGGGGATGAGAGTACCTGAAGAATAAGGACCGGCTAACTACGTGCCAGCAGCCGCGGTAATACGTAGGGTCCAAGCGTTAATCGGAATTACTGGGCGTAAAGCGTCCGCAGGTGGTTTGTTAAGCACGATGTGAAATCCCCGAGCTCAACTTGGGAATTGCATTGTGAACTGGCTAACTAGAGTATGGCAGAGGGGGGTGGAATTCCGCGTGTAGCAGTGAAATGCGTAGAGATGCGGAGGAACACCGATGGCGAAGGCAACCCCCTGGGCTAATACTGACACTCATGGACGAAAGCGTGGGGAGCAAACAGG` |
| ASV3661 | 406 | `TGGGGAATATTGCACAATGGGCGGAAGCCTGATGCAGCGACGCCGCGTGAGCGAAGAAGGTTTTCGGATCGTAAAGCTCTGTCCTTGGGGAAGAAAAAAATGACGGTACCCAAGGAGGAAGCCCCGGCTAACTACGTGCCAGCAGCCGCGGTAATACGTAGGGGGCTAGCGTTGTCCGGATTTACTGGGCGTAAAGGGTGAGTAGGCGGTAACGTATGTCAGATGTTAAATCCATGGGCTTAACCCATGTCAGCATTTGAAACTGCGTAACTTGAGTGCAGGAGAGGTAAGTGGAATTCCTAGTGTAGCGGTGAAATGCGTAGATATTAGGAGGAACACCAGTGGCGAAGGCGACTTACTGGACTGTAACTGACGCTGAGACACGAAAGCGTGGGGAGCGAACAGG` |
| ASV1369 | 404 | `TGGGGAATATTGCACAATGGGGGAAACCCTGATGCAGCGACGCCGCGTGAGTGAAGAAGTATTTCGGTATGTAAAGCTCTATCAGCAGGGAAGATAATGACGGTACCTGACTAAGAAGCCCCGGCTAACTACGTGCCAGCAGCCGCGGTAATACGTAGGGGGCAAGCGTTATCCGGATTTACTGGGTGTAAAGGGAGCGTAGGCGGTTCGGTAAGTCTGGTGTGAAAGCCCGGGGCTCAACCCCGGGACTGCATTGGAAACTATCGGACTTGAGTGTCGGAGAGGTAAGTGGAATTCCTAGTGTAGCGGTGAAATGCGTAGATATTAGGAGGAACACCAGTGGCGAAGGCGACTTACTGGACGATAACTGACGCTGAGGCTCGAAAGCGTGGGGAGCAAACAGG` |
| ASV808 | 404 | `TGGGGAATCTTAGACAATGGGCGCAAGCCTGATCTAGCGATGCCGCGTGAGTGACGAAGGCCTTAGGGTCGTAAAGCTCTTTCGCCAGGGATGATAATGACAGTACCTGGTAAAGAAACCCCGGCTAACTCCGTGCCAGCAGCCGCGGTAATACGGAGGGGGTTAGCGTTGTTCGGAATTACTGGGCGTAAAGCGCACGTAGGCGGATTGATAAGTTGGGGGTGAAATCCCAGGGCTCAACCCTGGAACTGCCTCCAAAACTATTTGTCTTGAGTTCGAGAGAGGTGAGTGGAATTCCGAGTGTAGAGGTGAAATTCGTAGATATTCGGAGGAACACCAGTGGCGAAGGCGGCTCACTGGCTCGATACTGACGCTGAGGTGCGAAAGCGTGGGGAGCAAACAGG` |
| ASV724 | 404 | `TGGGGAATATTGGACAATGGGCGAAAGCCTGATCCAGCAATGCCGCGTGAGTGATGAAGGCCTTAGGGTTGTAAAGCTCTTTTACCAGGGATGATAATGACAGTACCTGGAGAATAAGCTCCGGCTAACTCCGTGCCAGCAGCCGCGGTAATACGGAGGGAGCTAGCGTTGTTCGGAATTACTGGGCGTAAAGCGTACGTAGGCGGTTATTCAAGTCAGAGGTGAAAGCCTGGAGCTCAACTCCAGAACTGCCTTTGAAACTAGATAGCTAGAATCATGGAGAGGTGAGTGGAATTCCGAGTGTAGAGGTGAAATTCGTAGATATTCGGAAGAACACCAGTGGCGAAGGCGGCTCACTGGACATGTATTGACGCTGAGGTACGAAAGCGTGGGGAGCAAACAGG` |
| ASV1333 | 429 | `TGGGGAATTTTGGACAATGGGCGCAAGCCTGATCCAGCAATGCCGCGTGCAGGATGAAGGCCTTCGGGTTGTAAACTGCTTTTGTACGGAGCGAAACGGTTCTTTCTAATACAGAGAGCTAATGACGGTACCGTAAGAATAAGCACCGGCTAACTACGTGCCAGCAGCCGCGGTAATACGTAGGGTGCAAGCGTTAATCGGAATTACTGGGCGTAAAGCGTGCGCAGGCGGTTGTGTAAGACAGATGTGAAATCCCCGGGCTTAACCTGGGACCTGCATTTGTGACTGCACGGCTAGAGTACGGTAGAGGGGGATGGAATTCCGCGTGTAGCAGTGAAATGCGTAGATATGCGGAGGAACACCGATGGCGAAGGCAATCCCCTGGACCTGTACTGACGCTCATGCACGAAAGCGTGGGGAGCAAACAGG` |
| ASV1708 | 405 | `TGGGGAATCTTGCGCAATGGACGAAAGTCTGACGCAGCCATGCCGCGTGAATGATGAAGGTCTTAGGATTGTAAAGTTCTTTAGCCTGTGACGATGATGACGGTAACAGGAGAATAAGCCCCGGCTAACTTCGTGCCAGCAGCCGCGGTAATACGAAGGGGGCTAGCGTTGTTCGGAATTACTGGGCGTAAAGCGCGCGTAGGCGGTTTTACAAGCCAGAGGTGAAATGCCTGAGCTCAACTTAGGAATTGCCTTTGGAACTGTATGGCTTTGAGGACGAGAGAGGTGAGTGGAATTCCCAGTGTAGAGGTGAAATTCGTAGATATTGGGAAGAACACCGGTGGCGAAGGCGGCTCACTGGCTCGTTACTGACGCTGTAGCGCGAAAGCGTGGGGATCAAACAGG` |
| ASV5443 | 428 | `TGGGGAATATTGGACAATGGGCGCAAGCCTGATCCAGCCACGCCGCGTGAGTGATGAAGGCCTTCGGGTCGTAAAGCTCTGTGGGGAGAGACGAACCGCCGGTGGTTAATACCCATCGGCATGACGGTATCTCCTTAGCAAGCACCGGCTAACTTCGTGCCAGCAGCCGCGGTAATACGAAGGGTGCAAACGTTGCTCGGAATCATTGGGCGTAAAGCGCACGTAGGCGGCTCCAAAAGTCGGATGTGAAATCCCTCGGCTCAACCGAGGACGTGCATCCGAAACTGCGGAGCTTGAGTATGGAAGAGGGTCGCGGAATCCCCGGTGTAGAGGTGAAATTCGTAGATATCGGGGGGAACACCAGTGGCGAAGGCGGCGACCTGGGCCAATACTGACGCTGAGGTGCGAAAGCGTGGGGAGCAAACAGG` |
| ASV1875 | 429 | `TGGGGAATTTTGGACAATGGGCGCAAGCCTGATCCAGCCATGCCGCGTGTCTGAAGAAGGCCTTCGGGTTGTAAAGGACTTTTGTCCGGGAGCAAATGCCTGGTGTTAATAGCATCGGGAGCTGAGAGTACCGGAAGAATAAGCACCGGCTAACTACGTGCCAGCAGCCGCGGTAATACGTAGGGTGCAAGCGTTAATCGGAATTACTGGGCGTAAAGCGTGCGCAGGCGGTTTTGTAAGCCAGATGTGAAAGCCCCGGGCTCAACCTGGGAACTGCGTTTGGAACTACAAGACTAGAGTGTGTCAGAGGGGGGTGGAATTCCGCGTGTAGCAGTGAAATGCGTAGAGATGCGGAGGAACACCGATGGCGAAGGCAGCCCCCTGGGATAACACTGACGCTCATGCACGAAAGCGTGGGGAGCAAACAGG` |
| ASV4483 | 406 | `TGGGGAATATTGGGCAATGGAGGCAACTCTGACCCAGCAACGCCGCGTGAATGATGAAGGTCTTCGGATTGTAAAGTTCTGTTGAGAGGGACGAACGAATGACGGTACCTCTTTAGAAAGCCACGGCTAACTACGTGCCAGCAGCCGCGGTAATACGTAGGTGGCAAGCGTTGTCCGGAATGACTGGGCGTAAAGGGAGCGTAGGCGGTCTGTCAAGTTAGGAGTGAAATCCCATGGCTTAACCATGGAACTGCTTTTAAAACTGATGGACTTGAGTGATGGAGAGGAAAGTGGAATTCCTAGTGTAGCGGTGGAATGCGTAGATATTAGGAGGAACACCAGTGGCGAAGGCGACTTTCTGGACATTAACTGACGCTGAGGCTCGAAAGCGTGGGGAGCAAACAGG` |
| ASV1456 | 404 | `TGGGGAATATTGGGCAATGGGCGAAAGCCTGACCCAGCCATGCCGCGTGTGTGATGAAGGCCTTAGGGTTGTAAAGCACTTTCGCTGGTGAAGATAATGACGGTAACCAGATAAGAAGCCCCGGCTAACCTCGTGCCAGCAGCCGCGGTAATACGAGGGGGGCTAGCGTTGTTCGGAATTACTGGGCGTAAAGCGCGTGTAGGCGGATATACAAGCTAGTGGTGAAATCCCAGGGCTCAACCCTGGAACTGCCTTTAGAACTGTATATCTGGAGTATTGGAGGGGAGTGCGGAATTTCTAATGTAGAGGTGAAATTCGTAGATATTAGAAGGAACACCGGTGGCGAAGGCGGCACTCTGGACAATTACTGACGCTGAGACGCGAAAGCGTGGGGAGCAAACAGG` |
| ASV5494 | 405 | `TAAGGAATCTTTCGCAATGGGGGCAACCCTGACGAAGCAACACCGCGTGGAGGATGAAGGCGTTTTCGTCGTAAACTCCTTTTCTGAAGGAATAATATTGAAGGTACTTCAGGAATAAGCATCGGCTAACTACGTGCCAGCAGCCGCGGTAAGACGTAGGATGCAAGCGTTATCCGGAATTATTGGGCGTAAAGCGTTCGTAGGTGGTTTGTTAAGTAGGATGTTAAATCTTTGAGCTCAACTCAAAATTGGTTTCCTAAACTAGCAGACTAGAGACAGGTAGAGGCAAGTAGAATTCTGTATGTAGGGGTAATATCCGTAGATATACAGAGGAATACCAAAAGCGAAGGCAGCTTGCTGGACCTGTTCTGACACTCAAGAACGAAAGCGTGGGGAGCAAACGGG` |
| ASV3254 | 407 | `TGGGGAATATTGCACAATGGGCGCAAGCCTGATGCAGCGACGCCGCGTGAAGGATGAAGGTCTTCGGATCGTAAACTTCTATCAGCAGGGAAGAATACCATGACGGTACCTGACTAAGAAGCCCCGGCTAACTACGTGCCAGCAGCCGCGGTAATACGTAGGGGGCAAGCGTTATCCGGAATTACTGGGTGTAAAGGGTGCGTAGGTGGCGATTTAAGTCAGATGTGAAAACTCAGGGCTCAACCTTGAGACTGCATCTGAAACTGAGTTGCTAGAGTGCAGGAGAGGAAAGCGGAATTCCGAGTGTAGCGGTGAAATGCGTAGATATTCGGAGGAACACCAGTAGCGAAGGCGGCTTTCTGGACTGTAACTGACACTGAGGCACGAAAGCGTGGGGAGCGAACAGG` |
| ASV755 | 429 | `TGGGGAATATTGGACAATGGGCGAAAGCCTGATCCAGCCATGCCGCGTGTGTGAAGAAGGTCTTCGGATTGTAAAGCACTTTAAGTTGGGAGGAAGGGCAGTTACCTAATACGTGATTGTCTTGACGTTACCGACAGAATAAGCACCGGCTAACTCTGTGCCAGCAGCCGCGGTAATACAGAGGGTGCAAGCGTTAATCGGAATTACTGGGCGTAAAGCGCGCGTAGGTGGTTTGTTAAGTTGAATGTGAAATCCCCGGGCTCAACCTGGGAACTGCATCCAAAACTGGCAAGCTAGAGTATGGTAGAGGGTAGTGGAATTTCCTGTGTAGCGGTGAAATGCGTAGATATAGGAAGGAACACCAGTGGCGAAGGCGACTACCTGGACTGATACTGACACTGAGGTGCGAAAGCGTGGGGAGCAAACAGG` |
| ASV2993 | 407 | `TGGGGAATATTGCACAATGGGCGCAAGCCTGATGCAGCGACGCCGCGTGAAGGATGAAGGTTTTCGGATCGTAAACTTCTATCAGCAGGGAGGAACCAAATGACGGTACCTGACTAAGAAGCCCCGGCTAACTACGTGCCAGCAGCCGCGGTAATACGTAGGGGGCAAGCGTTATCCGGATTTACTGGGTGTAAAGGGTGCGTAGGTGGTAATGTAAGTCAGATGTGAAACCTTGGGGCTCAACCTCAAGCATGCATCTGAAACTGCGTAACTAGAGTGCAGGAGAGGAAAGCGGAATTCCGAGTGTAGCGGTGAAATGCGTAGATATTCGGAGGAACACCAGTAGCGAAGGCGGCTTTCTGGACTGTAACTGACACTGAGGCACGAAAGCGTGGGGAGCGAACAGG` |
| ASV3087 | 423 | `TAGGGAATATTGGTCAATGGGTGAGAGCCTGAACCAGCCATGCCGCGTGCAGGAAGACGGCCTTATGGGTTGTAAACTGCTTTTATCAGGGGCTAAAAGGCTTATGCGTAAGCGATTGAAAGTACCTGATGAATAAGCCACGGCTAACTACGTGCCAGCAGCCGCGGTAATACGTAGGTGGCAAGCGTTGTCCGGATTTATTGGGTTTAAAGGGTGCGTAGGCGGCCCTGTAAGTCAGTGCTGAAATATCCCGGCTTAACCGGGAGGGTGGCATTGATACTGCGGGGCTTGAGTACAGATGAAGTCGGCGGAATTGACGGTGTAGCGGTGAAATGCATAGATATCGTCAAGAACACCGATAGCGAAGGCAGCTGACTAAGTTGTAACTGACGCTGAGGCACGAAAGTGTGGGGATCAAACAGG` |
| ASV4257 | 404 | `TGAGGAATCTTCCACAATGGGCGAAAGCCTGATGGAGCGACACCGCGTGAAGGATGAAGCCTTTGTTGGTGTAAACTTCTTTTTTCTCGGAAGATAATGACGGTACGAGAAGAATAAGGGGCGGCAAACTTCGTGCCAGCAGCCGCGGTAATACGAAGGCCCCAAGCGTTATCCGGAATTACTGGGCGTAAAGCGTCTGTAGGTGGTTTTTTAAGTCTTTCCGTTAAAATCCAGGGCTCAACCCTGGACTGCGGGAGAAACTGGAGAACTCGAGTGTGGGAGGGGCTAGCAGAACGGTAAGAGTAGGGGTGCAATCCGTTGATACTTACCAGAATACCAAAAGCGAAGGCAGCTAGCTGGAACACTACTGACACTGAGAGACGAAAGCGTGGGGAGCAAAAGGG` |
| ASV2176 | 405 | `TGAGGAATCTTCCACAATGGACGAAAGTCTGATGGAGCAATGCCGCGTGCAGGATGACGGCCTTCGGGTTGTAAACTGCTTTTATATGGGAAGATTATGACGGTACCATATGAATAAGGACCGGCTAACTACGTGCCAGCAGCCGCGGTAATACGTAGGGTCCAAGCGTTATCCGGAATCACTGGGCGTAAAGCGTTTGTAGGCGGAAATCTAAGTGAGATGCGAAATCTTGTGGCTCAACCATATAGACGGTATCTCATACTGGGTTTCTAGAAGATAGCAGAGGTAAGTGGAATTAGTAGTGTAGCGGTAAAATGCGTAGATATTACTAGGAACACCAATGGCGAAGGCAGCTTACTGGGCTATATTTGACGCTGAGAAACGAAAGCGTGGGGAGCGAACAGG` |
| ASV4013 | 423 | `TAGGGAATATTGGGCAATGGTCGGCAGACTGACCCAGCCATGCCGCGTGCAGGAAGAAGGCGTTCTGCGTCGTAAACTGCTTTTGTTTGGGATGAAAAAGGTCTTGCGAGACAAATTGACAGTACCAAACGAATAAGCCACGGCTAACTACGTGCCAGCAGCCGCGGTAATACGTAGGTGGCAAGCGTTGTCCGGATTTATTGGGTTTAAAGGGTGCGTAGGTGGTTATTTAAGTCAGTGGTGAAAGACGGTCGCTCAACGATTGCAGTGCCATTGATACTGAATAACTTGAGTAGAGATGAGGTGGGCGGAATTGATAGTGTAGCGGTGAAATGCATAGATATTATCAAGAACACCAATTGCGAAGGCAGCTCACTGGGTTCTAACTGACACTGAGGCACGAAAGTGTGGGGATCAAACAGG` |
| ASV1405 | 429 | `TCGAGAATCATTCACAATGGGGGAAACCCTGATGGTGCAACGCCGCGTGTGGGATGAAGGCCTTAGGGTCGTAAACCACTGTCATGTGAGAGTAAGGCTTATAAGTTAATACCTTGTAAGTTTGATAGTATCACAAGAGGAAGGGACGGCTAACTTCGTGCCAGCAGCCGCGGTAATACGAAGGTCCCGAGCGTTATTCGGAATCACTGGGCGTAAAGGGAGCGTAGGCTGCGCGGTAAGTCAGATGTGAAATCTCAGGGCTCAACCCTGAAACTGCATCCGATACTGCCGCGCTAGAGTAATGGAGGGGTAACTGGAATTCTCAGTGTAGCAGTGAAATGCGTAGATATTGAGAGGAAGACCAACGGCGAAGGCAGGTTACTGGACATTTACTGACGCTGAGGCTCGAAGGCTAGGGGAGCAAAAGGG` |
| ASV5584 | 405 | `TGAGGAATCTTCCGCAATGGGCGAAAGCCTGACGGAGCAACACCGCGTGAAGGATGAAGTGGTTCGCCATGTAAACTTCTTTTCTGAGGGAAGAATTCTGACGGTACCTTAGGAATAAGCACCGGCTAACTCTGTGCCAGCAGCCGCGGTAAGACAGAGGGTGCGAGCGTTATCCGGAATTACTGGGCGTAAAGCGTGTGTAGGCGGAACATAAAGTCGGGGGTAAAATCTCCAAGCTCAACTTGGAATGCGTCTCCGAAACTCATGTTCTGGAAGAACGGAGAGGTAAGCGGAATTCTGTGTGTAGGGGTAAAATCCGTAGATACACGGAGGAACGCCAAAAGCGAAAGCAGCTTACCGGCCGTCTCTTGACGCTGAGACACGAAAGCGTGGGGAGCAAACAGG` |
| ASV4703 | 429 | `TGGGGAATATTGGACAATGGGGGAAACCCTGATCCAGCGATGCCGCGTGTGTGAAGAAGGCCTGAGGGTTGTAAAGCACTTTAGTTGGGGAGGAGAGGCTTAAGTCGAATAGGCTTAAGAGTAGACGTTACCCAAAGAATAAGCACCGGCTAACTCTGTGCCAGCAGCCGCGGTAATACAGAGGGTGCAAGCGTTAATCGGAATGACTGGGCGTAAAGGGCACGTAGGCGGATAGATGTGTCATAGGTGAAATCCCCGGGCTCAACCTGGGAACTGCCTATGAAACTGTGTATCTAGAATATAGGAGAGGGAAGTGGAATTTCCGGTGTAGCGGTGAAATGCGTAGATATCGGAAAGAACACCAGTGGCGAAGGCGACTTCCTGGCCTAATATTGACGCTGAGGTGCGAAAGCGTGGGGAGCAAACGGG` |
| ASV2364 | 430 | `TAGGGAATATTGCGCAATGGAGGAAACTCTGACGCAGCGACGCCGCGTGGGTGATGAAGGTCTTCGGATCGTAAAGCCCTGTCGGATGGGAAGAAAAACACTGAGGGTAATATTCTCAGTGCTTGACGGTACCATTAAAGGAAGCCCCGGCTAACTTCGTGCCAGCAGCCGCGGTAATACGAAGGGGGCAAGCGTTGTTCGGAATCATTGGGCGTAAAGCGCGTGTAGGCGGTCTATTAAGTCGAGTGTGAAATCCCTCGGCTCAACCGAGGAAGTGCATTCGAAACTGGTAGACTAGAAGATGGTAGAGGATGGTGGAATTCCAGGTGTAGAGGTGAAATTCGTAGATATCTGGAGGAATACCGGTGGCGAAGGCGGCCATCTGGGCCATTCTTGACGCTGAGACGCGAAAGCGTGGGTAGCAAACAGG` |
| ASV5413 | 430 | `TGGGGAATCTTGCGCAATGGACGAAAGTCTGACGCAGCCACGCCGCGTGAGTGAAGAAGGCCTTCGGGTTGTAAAGCTCTGTCGTGAGGGACGAACATTATTTGGGTTAACAGCCCAAGTAACTGACGGTACCTCGAAAGGAAGCACCGGCTAACTTCGTGCCAGCAGCCGCGGTAATACGAGGGGTGCAAGCGTTGCTCGGAATCATTGGGCGTAAAGGGTAGGTAGGTGGTCTGATTTGTCAGGGGTGAAAGCCTTGGGCTTAACCTGAGAAGTGCCCCTGAAACGGTCAGACTTGAGTCTTGGAGAGGTAAGTGGAATTCCCGGTGTAGCGGTGAAATGCGTAGAGATCGGGAGGAACACCAGAGGCGAAGGCGGCTTACTGGACAAGCACTGACACTCAACTACGAAAGCGTGGGGAGCAAACAGG` |
| ASV5316 | 404 | `TGGGGAATTTTCCGCAATGGGCGAAAGCCTGACGGAGCAACGCCGCGTGAGGGAGGAAGGTTTTTGGACTGTAAACCTCTTTTCTTAGGGAAGATAATGACGGTACCTAAGGAATCAGCATCGGCTAACTTCGTGCCAGCAGCCGCGGTAATACGAGGGATGCAAGCGTTATCCGGAATTATTGGGCGTAAAGCGTCCGTAGGCGGTCTTATAAGTCTGTTGTTAAATAGTGCAGCTTAACTGCATAGGGGCAATGGAAACTGTGGGACTAGAGTGAGATAGGGGTAAGGGGAATTCCAGGTGTAGCGGTGAAATGCGTAGATATCTGGAAGAACACCAGCGACGAAAGTGCCTTACTGGGTCTTAACTGACGCTGATGGACGAAAGCTAGGGGAGCGAAAGGG` |
| ASV1761 | 411 | `TTTGGAATTTTGGACAATGGGCGAAAGCCTGATCCAGCGACGCCGCGTGGACGATGAAGGCCTTCGGGTCGTAAAGTCCTTTTGCTCGGGAACAATATTTTGAGCGTACCGAGAGAATAAGAGGTTGCTAACTCTGTGCCAGCAGCAGCGGTAATACAGAGACCTCAAGCGTTATCCGGAATCATTGGGCGTAAAGCGTACCGATAGGTGGTTTTACAAGTCAGAAGTGAAATCTCTCAGCTTAACTGGGAGTCTGTCTTTTGAAACTGTAAAACTAGAGGGGCAAAGAGGAAGCTGGAACAAGCGGTGTAGTAGTGAAATGCGTTGATATCGCTTGGAACACCAATAGCGTAGGCAGGCTTCTGGGTGCCACCTGACACTGCTAGGACGAAAGCGTGGGGAGCGAATGGG` |
| ASV4035 | 404 | `TGGGGAATATTGGACAATGGGCGCAAGCCTGATCCAGCCATGCCGCGTGAGTGACGAAGGTCTTCGGATTGTAAAGCTCTTTTGGCGGGGACGATAATGACGGTACCCGCAGAATAAGCCCCGGCTAACTTCGTGCCAGCAGCCGCGGTAATACGAAGGGGGCTAGCGTTGTTCGGAATCACTGGGCGTAAAGCGCACGTAGGCGGATGTGTCAGTCAGGGGTGAAATCCCGGGGCTCAACCTCGGAACTGCCTCTGATACAACACGTCTTGAGTCCGGAAGAGGTGGGTGGAATTCCTAGTGTAGAGGTGAAATTCGTAGATATTAGGAAGAACACCGGTGGCGAAGGCGACCCACTGGTCCGGTACTGACGCTGAGGTGCGAAAGCGTGGGGAGCAAACAGG` |
| ASV3494 | 429 | `TGGGGAATATTGGACAATGGGGGCAACCCTGATCCAGCAATGCCGCGTGTGTGAAGAAGGCCTGAGGGTTGTAAAGCACTTTCAGTGGGGAGGAGGATTGACAGGTTAAGAGCTAGTTAATTGGACGTTACCCACAGAAGAAGCACCGGCTAACTCCGTGCCAGCAGCCGCGGTAATACGGAGGGTGCGAGCGTTAATCGGAATTATTGGGCGTAAAGCGTGCGTAGGTGGTTGGATAAGTTATCTGTGAAATCCCCGGGCTTAACCTGGGCAGGTCAGATAAGACTGTTCGACTCGAGTATAGGAGAGGGTAGTGGAATTTCCGGTGTAGCGGTGAAATGCGTAGAGATCGGAAGGAACACCAGTGGCGAAGGCGGCTACCTGGCCTGATACTGACACTGAGGCACGAAAGCGTGGGGAGCAAACAGG` |
| ASV5026 | 430 | `TGGGGAATCTTCCGCAATGGACGAAAGTCTGACGGAGCAACGCCGCGTGAGTGAAGAAGGTTTTCGGATCGTAAAACTCTGTTTTCAGGGACGAATGTATTTAATGTGAATAATGTTAGATAATGACGGTACCTGAAGAGGAAGCCACGGCTAACTACGTGCCAGCAGCCGCGGTAATACGTAGGTGGCAAGCGTTGTCCGGAATTATTGGGCGTAAAGCGCGCGCAGGCGGAATAATAAGTCTGATGTTAAAGTTCGGGGCTCAACCCCGTGAAGCATTTGGAAACTGTTATTCTTGAGTGCAGGAGAGGAAAGTGGAATTCCTAGTGTAGCGGTGAAATGCGTAGATATTAGGAGGAACACCAGTGGCGAAGGCGACTTTCTGGACTGTGTCTGACGCTGAGGCGCGAAAGCCAGGGTAGTGAACGGG` |
| ASV1039 | 429 | `TGGGGAATTTTGGACAATGGGGGCAACCCTGATCCAGCAATGCCGCGTGAGTGAAGAAGGCCTTCGGGTTGTAAAGCTCTTTTGTTAGGGAAGAAAAGGACTCCAATAATACTGGGGTCTGATGACGGTACCTAAAGAATAAGCACCGGCTAACTACGTGCCAGCAGCCGCGGTAATACGTAGGGTGCAAGCGTTAATCGGAATTACTGGGCGTAAAGCGTGCGCAGGCGGCCTCGCAAGTCAGATGTGAAATCCCCGAGCTCAACTTGGGAACTGCATTTGAAACTACGAGGCTAGAGTGTGTCAGAGGGAGGTAGAATTCCACGTGTAGCAGTGAAATGCGTAGATATGTGGAGGAATACCAGTGGCGAAGGCGGCCTCCTGGGATAACACTGACGCTCATGTACGAAAGCGTGGGGAGCAAACAGG` |
| ASV4740 | 429 | `TGGGGAATTTTGGACAATGGGGGCAACCCTGATCCAGCCATTCCGCGTGAGTGAAGAAGGCCTTCGGGTTGTAAAGCTCTTTCGGTGGGGAAGAAAAGGCGGGCTCTAACAGAGTCTGTTCATGACGGTACCCGAAGAAGAAGCACCGGCTAACTACGTGCCAGCAGCCGCGGTAATACGTAGGGTGCGAGCGTTAATCGGAATTACTGGGCGTAAAGGGTGCGCAGGCGGTTATTTAAGACAGATGTGAAATCCCCGGGCTCAACCTGGGAACTGCGTTTGTGACTGAATGGCTAGAGTGCGGCAGAGGGGGGTGGAATTCCACGTGTAGCAGTGAAATGCGTAGAGATGTGGAGGAACACCGATGGCGAAGGCAGCCCCCTGGGCCAGCACTGACGCTCATGCACGAAAGCGTGGGGAGCAAACAGG` |
| ASV2204 | 424 | `TAAGGAATATTGGTCAATGGAGGCAACTCTGAACCAGCCATGCCGCGTGCAGGAAGACGGCCCTCTGGGTTGTAAACTGCTTTTATTCGGGAATAAACGTACTTACGTGTAAGTATTTGAATGTACCGAAGGAATAAGGATCGGCTAACTCCGTGCCAGCAGCCGCGGTAATACGGAGGATCCAAGCGTTATCCGGATTTATTGGGTTTAAAGGGTGCGTAGGCGGTTTTTTAAGTCAGGGGTGAAAGACGGTAGCTCAACTATCGCAGTGCCCTTGATACTGAAGAGCTTGAATGAACTAGAGGTATGCGGAATGAGACAAGTAGCGGTGAAATGCATAGATATGTCTCAGAACACCGATTGCGAAGGCAGCATACTATGGTTTTATTGACGCTGAGGCACGAAAGCGTGGGGATCAAACAGG` |
| ASV1260 | 429 | `TGGGGAATTTTGGACAATGGGCGCAAGCCTGATCCAGCCATGCCGCGTGAGTGAAGAAGGCCTTCGGGTTGTAAAGCTCTTTTGTCCGGGAGGAAATCCCTGAAGTTAATACCTTCAGGGGATGACAGTACTGGAAGAATAAGCACCGGCTAACTACGTGCCAGCAGCCGCGGTAATACGTAGGGTGCAAGCGTTAATCGGAATTACTGGGCGTAAAGCGTGCGCAGGCGGTTTGATAAGTAAGATGTGAAATCCCCGGGCTTAACCTGGGAACTGCGTTTTAAACTGTCAGACTAGAGTACGTCAGAGGGGGGTAGAATTCCACGTGTAGCAGTGAAATGCGTAGAGATGTGGAGGAATACCGATGGCGAAGGCAGCCCCCTGGGATGATACTGACGCTCATGCACGAAAGCGTGGGGAGCAAACAGG` |
| ASV5186 | 429 | `TGGGGAATATTGCGCAATGGACGAAAGTCTGACGCAGCGACGCCGCGTGAAGGAAGACGGTTTTCGGATTGTAAACTTCTGTTAAGTGGGAAGAAATACTTCTGGTTAATACCCAGAAGGGCTGACGGTACCACTAGAGAAAGCACCGGCAAACTTCGTGCCAGCAGCCGCGGTAATACGAAGGGTGCAAGCGTTATTCGGAGTGACTGGGCGTAAAGAGCGCGTAGGCGGTCTTGTAAGTCATATGTTAAATTCCACAGCTTAACTGTGGGTCAGCATGCGATACTGCAAGGCTAGAGGGTAAGAGAGAGCAGTGGAATTCTCGGAGTAGCGGTAAAATGCGTAGATCTCGAGAGGAACACCGGTAGCGAAGGCGGCTGCTTGGCTTATACCTGACGCTGAGGCGCGAAAGCGTGGGGAGCAAACAGG` |
| ASV505 | 429 | `TCGAGAATCATTCACAATGGGGGCAACCCTGATGGTGCAACGCCGCGTGGAGGATGAAGGTCTTCGGATTGTAAACTCCTGTCATCTAGGAGCAAGACATGGCTGTAAATAGCAGACATGTTTGATAGTACTAGAAGAGGAAGGGACGGCTAACTTCGTGCCAGCAGCCGCGGTAATACGAAGGTCCCGAGCGTTATTCGGAATCACTGGGCGTAAAGGGAGCGTAGGCGGCGTGGAAAGTCAGATGTGAAATCCCGGGGCTCAACCTCGGAACTGCATCCGATACTACCATGCTAGAGTATTGGAGGGGTAACTGGAATTCTCGGTGTAGCAGTGAAATGCGTGGATATCGAGAGGAACACCTGTGGCGAAAGCGAGTTACTGGACAATTACTGACGCTGAGGCTCGAAGGCTAGGGGAGCAAAAGGG` |
| ASV3251 | 429 | `TGGGGAATTTTGGACAATGGGCGAAAGCCTGATCCAGCAATGCCGCGTGTGGGAAGAAGGCCTTCGGGTTGTAAACCACTTTTGTCGGGAACGAAACGGTCTGCACTAATAATGCGGGCTAATGACTGTACCCGAAGAATAAGCACCGGCTAACTACGTGCCAGCAGCCGCGGTAATACGTAGGGTGCAAGCGTTAATCGGAATTACTGGGCGTAAAGCGTGCGCAGGCGGTTATGTAAGTCAGATGTGAAATCCCCGGGCTTAACCTGGGAACTGCATTTGAAACTGCATGGCTTGAGTGCGGTAGAGGGGGGTAGAATTCCACGTGTAGCAGTGAAATGCGTAGAGATGTGGAGGAATACCGATGGCGAAGGCAGCCCCCTGGACCGTAACTGACGCTCATGCACGAAAGCGTGGGGAGCAAACAGG` |
| ASV4316 | 403 | `TGAGGAATCTTCCACAATGGACGAAAGTCTGATGGAGCGACACCGCGTGAAGGATGAAGCCTTTGTTGGTGTAAACTTCTTTTCTCTCCGAAGATAATGACGGTAGGAGAGGAATAAGGGGCGGCAAACTATGTGCCAGCAGCCGCGGTAATACATAGGCCCCAAGCGTTATCCGGAATTACTGGGTGTAAAGCGTCTGTAGGTGGTCTTAACAGTCTCGTGTGAAACTTCGGAGCTCAACTCCGAATTGCACGGGAAACTATAAGACTTGAGTGCGGGAGAGGCAAGCAGAACTGTATGAGTAGGGGTGCAATCCGTTGATACATACAAGAATACCAAAAGCGAAGGCAGCTTGCTGGAACGCCACTGACACTGAGAGACGAAAGCGTGGGGAGCAAAAGGG` |
| ASV1091 | 404 | `TGGGGAATTTTGGACAATGGACGAAAGTCTGATCCAGCAATTCTGCGTGCATGATGAAGGCCTGCGGGTCGTAAAGTGCTTTTTTGAGGGAAAACGACTGATGGTACCTCAAGAATAAGGGACGGCTAACTCTGTGCCAGCAGCCGCGGTAATACAGAGGTCCCAAGCGTTATCCGGATTTACTGGGCGTAAAGAGTCCGTAGCTGGCATTGTAAGTCTTGATTCAAATACTCTGACTCACTCAGAGAAAGGGTCAAGATACTGCAAAGCTCGAGATCATAGGGGGTTAGTGGAATTTCCAGTGGAGGGGTGAAATCCGTTGATATTGGAAGGAATGCCGAAAGCGAAAGCAGCTAACTACTATATTCTGACAGTCAAGGACGAAAGCTTGGGGATCGAACGGG` |
| ASV3862 | 404 | `TGAGGAATATTGGTCAATGGGCGCAAGCCTGATCCAGCAATGCCGCGTGAATGAAGAAGGCCTTAGGGTTGTAAAGTTCTTTTAGTTGGGAAGATAATGACGGTACCAACAGAAAAAGCTCCGGCTAACTTCGTGCCAGCAGCCGCGGTAATACGAAGGGAGCTAGCGTTATTCGGAATTACTGGGCGTAAAGAGCATGTAGGCGGTCTAACAAGTTGGAAGTGAAAGCCCAGGGCTTAACCCTGGAACTGCTTTCAAAACTGTTAAACTAGAGATCAATAGGGGATAGCGGAATTCCTAGTGTAGAGGTGAAATTCTTAGATATTAGGAGGAACACCGGAGGCGAAGGCGGCTATCTGGGTTGAATCTGACGCTGAGATGCGAAAGTGTGGGTAGCAAACAGG` |
| ASV1279 | 424 | `TGAGGAATATTGGTCAATGGGCGATAGCCTGAACCAGCCAAGTAGCGTGAAGGATGAAGGCTCTATGGGTCGTAAACTTCTTTTATATGGGAATAAAGTGAGCCACGTGTGGCTTTTTGTATGTACCATATGAATAAGGACCGGCTAACTCCGTGCCAGCAGCCGCGGTAATACGGAGGGTCCGAGCGTTATCCGGATTTATTGGGTTTAAAGGGAGCGTAGGCGGATTGATAAGTCAGTTGTGAAAGTTTGCGGCTCAACCGTAAAATTGCAGTTGATACTGTCAGTCTTGAGTACAGTAGAGGTAAGCGGAATTCGTGGTGTAGCGGTGAAATGCTTAGATATCACGAAGAACTCCAATTGCGAAGGCAGCTTGCTGGACTGTCACTGACGCTGAGGCTCGAAAGTGTGGGTATCAAACAGG` |
| ASV2789 | 406 | `TGGGGAATATTGGGCAATGGGCGCAAGCCTGACCCAGCAACGCCGCGTGAAGGAAGAAGGCTTTCGGGTTGTAAACTTCTTTTGTCAGGGAACAGTAGAAGAGGGTACCTGACGAATAAGCTCCGGCTAACTACGTGCCAGCAGCCGCGGTAATACGTAGGGAGCGAGCGTTGTCCGGATTTACTGGGTGTAAAGGGCGTGTAGGCGGGATTACAAGTCAGATGTGAAATTCCGGGGCTCAACCCCGGAACTGCATTTGAAACTGTAGTTCTTGAGTACTGGAGAGGCAGGCGGAATTCCTAGTGTAGCGGTGAAATGCGTAGATATTAGGAGGAACACCAGTGGCGAAGGCGGCCTGCTGGACAGCAACTGACGCTGAGGCGCGAAAGCGTGGGGAGCAAACAGG` |
| ASV1415 | 429 | `TGGGGAATCTTGGACAATGGGCGCAAGCCTGATCCAGCAATGCCGCGTGCGTGAAGAAGGCCTTCGGGTTGTAAAGCGCTTTTGTTCGGGAGGAAATCCTGGTGGCTAATATCCACCGGGGATGACAGTACCGGAAGAATAAGGACCGGCTAACTACGTGCCAGCAGCCGCGGTAATACGTAGGGTCCAAGCGTTAATCGGAATTACTGGGCGTAAAGGGTGCGCAGGTGGTTGATTAAGTGTGATGTGAAAGCCCCGGGCTCAACCTGGGAATTGCATTGCAAACTGGTCAACTAGAGTATGGCAGAGGGGGGTGGAATTCCGCGTGTAGCAGTGAAATGCGTAGAGATGCGGAGGAACACCGATGGCGAAGGCAACCCCCTGGGCTAATACTGACACTCATGCACGAAAGCGTGGGGAGCAAACAGG` |
| ASV5715 | 404 | `TGGGGAATATTGGACAATGGGGGAAACCCTGATCCAGCCATGCCGCGTGAGTGATGAAGGCCTTAGGGTTGTAAAGCTCTTTTGTCAGGGAAGATGATGACGGTACCTGAAGAATAAGCACCGGCTAACTTCGTGCCAGCAGCCGCGGTAATACGAAGGGTGCTAGCGTTGTTCGGAATCACTGGGCGTAAAGCGCGCGTAGGCGGCTTTGCAAGTCAGAGGTGAAATCCTGAGGCTTAACCTCAGAATTGCCTTTGAAACTGCAAAGCTGGAGTGTTGGAGGGGTTGGCGGAATTCCGAATGTAGAGGTGAAATTCGTAGATATTCGGAGGAACACCGGTGGCGAAGGCGGCCAACTGGACAACAACTGACGCTGAGGCGCGAAAGCGTGGGGATCAAACAGG` |
| ASV783 | 429 | `TGGGGAATTTTGGACAATGGGCGCAAGCCTGATCCAGCAATGCCGCGTGCAGGATGAAGGCCTTCGGGTTGTAAACTGCTTTTGTACGGAGCGAAACGGCTCATCCTAATAAGGTGGGCTAATGACGGTACCGTAAGAATAAGCACCGGCTAACTACGTGCCAGCAGCCGCGGTAATACGTAGGGTGCGAGCGTTAATCGGAATTACTGGGCGTAAAGCGTGCGCAGGCGGTTATATAAGACAGATGTGAAATCCCCGGGCTCAACCTGGGACCTGCATTTGTGACTGTATAGCTAGAGTACGGTAGAGGGGGATGGAATTCCGCGTGTAGCAGTGAAATGCGTAGATATGCGGAGGAACACCGATGGCGAAGGCAATCCCCTGGACCTGTACTGACGCTCATGCACGAAAGCGTGGGGAGCAAACAGG` |
| ASV1835 | 429 | `TGGGGAATTTTGGACAATGGGGGAAACCCTGATCCAGCCATGCCGCGTGAGTGAAGAAGGCCTTCGGGTTGTAAAGCTCTTTCAGCCGGAACGAAACGATTACGGATAATACCTGTGATTAATGACGGTACCGGAAGAAGAAGCACCGGCTAACTACGTGCCAGCAGCCGCGGTAATACGTAGGGTGCGAGCGTTAATCGGAATTACTGGGCGTAAAGCGTGCGCAGGCGGTTTTGTAAGTCAGATGTGAAATCCCCGGGCTTAACCTGGGAACTGCGTTTGAAACTGCAAGGCTAGAGTGTGGCAGAGGGGGGTGGAATTCCACGTGTAGCAGTGAAATGCGTAGAGATGTGGAGGAACATCAATGGCGAAGGCAGCCCCCTGGGTTAACACTGACGCTCAGGCACGAAAGCGTGGGGAGCAAACAGG` |
| ASV460 | 429 | `TGGGGAATTTTGGACAATGGGCGCAAGCCTGATCCAGCCATGCCGCGTGCGGGAAGAAGGCCTTCGGGTTGTAAACCGCTTTTGTCAGGGAAGAAACGCTCTGGGCTAATACCCCGGGGTAATGACGGTACCTGAAGAATAAGCACCGGCTAACTACGTGCCAGCAGCCGCGGTAATACGTAGGGTGCAAGCGTTAATCGGAATTACTGGGCGTAAAGCGTGCGCAGGCGGTTATGCAAGACAGATGTGAAATCCCCGGGCTTAACCTGGGAACTGCATTTGTGACTGCATAGCTAGAGTGCGGCAGAGGGGGATGGAATTCCGCGTGTAGCAGTGAAATGCGTAGATATGCGGAGGAACACCGATGGCGAAGGCAATCCCCTGGGCCTGCACTGACGCTCATGCACGAAAGCGTGGGGAGCAAACAGG` |
| ASV1526 | 429 | `TGGGGAATTTTGGACAATGGGGGCAACCCTGATCCAGCAATGCCGCGTGCAGGACGAAGGCCTTCGGGTTGTAAACTGCTTTTGTACGGAACGAAACGGTCTTTTCTAATACAAGAGGCTAATGACGGTACCGTAAGAATAAGCACCGGCTAACTACGTGCCAGCAGCCGCGGTAATACGTAGGGTGCGAGCGTTAATCGGAATTACTGGGCGTAAAGCGTGCGCAGGCGGTTATATAAGACAGTTGTGAAATCCCCGGGCTCAACCTGGGAATTGCATCTGTGACTGTATAGCTAGAGTACGGTAGAGGGGGATGGAATTCCGCGTGTAGCAGTGAAATGCGTAGATATGCGGAGGAACACCGATGGCGAAGGCAATCCCCTGGACCTGTACTGACGCTCATGCACGAAAGCGTGGGGAGCAAACAGG` |
| ASV3785 | 410 | `TTTGGAATATTGGACAATGGGCGAAAGCCTGATCCAGCGACGCCGCGTGGACGATGAAGGTCTTCGGATTGTAAAGTCCTTTTGGTAGGGAACAATATATTGAGCGTACCTACAGAATAAGTGGTTGCTAACTCTGTGCCAGCAGCAGCGGTAATACAGAGACCACAAACGTTATCCGGAATCATTGGGCGTAAAGCGTACCGATAGGTGGTTTTGTAAGCCAGAGGTTAAACTTGCGAGCTCAACTCGTAATCTGCCTTTGGAACTATAAAACTAGAGGGGCAAAGAGGAAGCTGGAACGTGCGGTGTAGTAGTGAAATGCGTTGATATCGCACGGAACACCAATAGCGAAGGCGGGCTTCTGGGTGCCACCTGACACTGCTAGGACGAAAGCGTGGGGAGTGAATGGG` |
| ASV931 | 429 | `TGGGGAATATTGGACAATGGGGGGAACCCTGATCCAGCAACGCCGCGTGAATGAAGAAGGCCTTCGGGTTGTAAAGTTCTTTTGTCCGAGAGCAAAGCGTGTGGAGTAATATACCATATGAATGAGAGTATCGGAAGAATAAGCACCGGCTAACTACGTGCCAGCAGCCGCGGTAATACGTAGGGTGCGAGCGTTAATCGGAATTACTGGGCGTAAAGAGTGCGTAGGCGGATATATAAGTTAGATGTGAAATACCTGGGCTTAACCTGGGAGGTGCATTTAAGACTGTATGTCTAGAGTTAGTAAGAGGATGGGAGAATTCCGAGTGTAGCAGTGAAATGCGTAGAGATTCGGAGGAATACCGGTGGCGAAGGCGCCCATCTGGGATATAACTGACGCTGAGGCACGAAAGCGTGGGGAGCAAACAGG` |
| ASV1579 | 404 | `TGAGGAATCTTCCACAATGGGCGAAAGCCTGATGGAGCGACACCGCGTGAAGGATGAAGCCTTTGTTGGTGTAAACTTCTTTTCTCTAGGAAGATAATGACGGTACTAGAGGAATAAGGGGCGGCAAACTTCGTGCCAGCAGCCGCGGTAATACGAAGGCCCCAAGCGTTATCCGGAATTACTGGGCGTAAAGCGTCTGTAGGTGGTTCGGAAAGTCTCAAGTGAAAGGTCAGGGCTCAACCCTGTACTTGCTTGGGAAACTATCGAACTTGAGTGCGGGAGAGGCAAGCAGAACTGTATGAGTAGGGGTGCAATCCGTTGATACATACAAGAATACCAAAAGCGAAGGCAGCTTGCTGGAACGCCACTGACACTGAGAGACGAAAGCGTGGGGAGCAAAAGGG` |
| ASV3977 | 424 | `TGAGGAATATTGGACAATGGGTGCGAGCCTGATCCAGCCATCCCGCGTGAAGGACGACGGCCCTATGGGTTGTAAACTTCTTTTGTATAGGGATAAACCTAGATACGTGTATCTAGCTGAAGGTACTATACGAATAAGCACCGGCTAACTCCGTGCCAGCAGCCGCGGTAATACGGAGGGTGCAAGCGTTATCCGGATTTATTGGGTTTAAAGGGTCCGTAGGCGGATCTGTAAGTCAGTGGTGAAATCTCACAGCTTAACTGTGAAACTGCCATTGATACTGCAGGTCTTGAGTGTTGTTGAAGTAGCTGGAATAAGTAGTGTAGCGGTGAAATGCATAGATATTACTTAGAACACCAATTGCGAAGGCAGGTTACTAAGCAACAACTGACGCTGATGGACGAAAGCGTGGGGAGCGAACAGG` |
| ASV4772 | 429 | `TGGGGAATATTGGACAATGGGCGCAAGCCTGATCCAGCCATACCGCGTGGGTGAAGAAGGCCTTCGGGTTGTAAAGCCCTTTTGTTGGGAAAGAAATCCTATCGGTTAATAACCGGTGGGGATGACGGTACCCAAAGAATAAGCACCGGCTAACTTCGTGCCAGCAGCCGCGGTAATACGAAGGGTGCAAGCGTTACTCGGAATTACTGGGCGTAAAGCGTGCGTAGGTGGTGGTTTAAGTCTGCTGTGAAAGCCCTGGGCTCAACCTGGGAATTGCAGTGGATACTGGGTCACTAGAGTGTGGTAGAGGGATGCGGAATTTCTGGTGTAGCAGTGAAATGCGTAGAGATCAGAAGGAACATCCGTGGCGAAGGCGGCATCCTGGGCCAACACTGACACTGAGGCACGAAAGCGTGGGGAGCAAACAGG` |
| ASV1191 | 407 | `TGGGGAATATTGGACAATGGGCCACAAGCCTGATCCAGCAATTCTGTGTGCACGATGAAGGTCTTCGGATTGTAAAGTGCTTTCAGTTGGGAAGAAGAAAGTGACGGTACCAACAGAAGAAGCGACGGCTAAATACGTGCCAGCAGCCGCGGTAATACGTATGTCGCAAGCGTTATCCGGATTTATTGGGCGTAAAGCGCGTCTAGGCGGAAAAGTAAGTCTGATGTTAAAATGCGGGGCTCAACTCCGTATTGCGTTGGAAACTGCTTTTCTAGAGTACTGGAGAGGTGGGCGGAACTACAAGTGTAGAGGTGAAATTCGTAGATATTTGTAGGAATGCCGATGGAGAAGTCAGCTCACTGGACAGATACTGACGCTAAAGCGCGAAAGCGTGGGGAGCAAACAGG` |
| ASV3006 | 387 | `GCGAGAAACCTTTGCAATGTGCGAAAGCACGACAAGGTTAATCCGAGTGATTCCTGCTAAAGGAATCTTTTGTTAGTCATAAAACCACTGATGAATAAGGGGTGGGCAAGTTCTGGTGTCAGCCGCCGCGGTAATACCAGCACCTCGAGTGGTCAGGAGGTTTATTGGGCCTAAAGCATCCGTAGCCGGCTGTTCAAGTTTCCGGTTAAATCCACACGCTTAACGTATGGGCTGCCGGAAATACTGTTCAGCTAGGGAGTGGGAGAGGTAGACGGTACTCGATAGGTAGGGGTAAAATCCTTTGATCTATTGATGACCACCTGTGGCGAAGGCGGTCTACCAGAACACGTTCGACGGTGAGGGATGAAAGCTGGGGGAGCAAACCGG` |
| ASV683 | 424 | `TAGGGAATATTGGGCAATGGATGCAAGTCTGACCCAGCCATGCCGCGTGCAGGAAGAAGGTCCTCTGGATTGTAAACTGCTTTTTTCGGGGAAGAAGAGGCCTCTTGCGAGGGGTTGTGACGGTACCCGATGAATAAGCCACGGCTAACTACGTGCCAGCAGCCGCGGTAATACGTAGGTGGCAAGCGTTGTCCGGATTTATTGGGTTTAAAGGGTGCGTAGGTGGTTTTATAAGTCAGTGGTGAAAGCTGGTTGCTCAACAATCAAGTTGCCATTGATACTGTTAGACTTGAGAGAAGTGGAGGTTCATGGAATGGATAGTGTAGCGGTGAAATGCATAGATATTATCCAGAACGTCGATTGCGAAGGCAGTGGGCTGTACTTTTTCTGACACTGAGGCACGAAAGCGTGGGGATCAAACAGG` |
| ASV2129 | 424 | `TGAGGAATATTGGACAATGGGCGCAAGCCTGATCCAGCCATGCCGCGTGCAGGAAGAAGCATCTATGGTGTGTAAACTGCTTTTGTACGGGAAGAAACACTCCTACGTGTAGGAACTTGACGGTACCGTAAGAATAAGGATCGGCTAACTCCGTGCCAGCAGCCGCGGTAATACGGAGGATCCAAGCGTTATCCGGAATCATTGGGTTTAAAGGGTCCGTAGGCGGTTTAATAAGTCAGTGGTGAAAGCCCATCGCTCAACGATGGAACGGCCATTGATACTGTTAGACTTGAATTACTAGGAAGTAACTAGAATATGTAGTGTAGCGGTGAAATGCTTAGATATTACATGGAATACCAATTGCGAAGGCAGGTTACTACTAGTGGATTGACGCTGATGGACGAAAGCGTGGGGAGCGAACAGG` |
| ASV2934 | 404 | `TGGGGAATATTGGACAATGGGCGCAAGCCTGATCCAGCCATGCCGCGTGAGTGATGAAGGCCTTAGGGTTGTAAAGCTCTTTTACCAGGGAAGATAATGACTGTACCTGGAGAATAAGCCCCGGCTAACTTCGTGCCAGCAGCCGCGGTAATACGAAGGGGGCTAGCGTTGTTCGGAATCACTGGGCGTAAAGCGCACGTAGGCGGACTCTTAAGTCAGGGGTGAAAGCCTGAGGCTCAACCTCAGAACTGCCTTTGATACTGGGAGTCTTGAGTGTGGTAGAGGTTGGTGGAACTGCGAGTGTAGAGGTGAAATTCGTAGATATTCGCAAGAACACCAGTGGCGAAGGCGGCCAACTGGACCATAACTGACGCTGAGGTGCGAAAGCGTGGGGAGCAAACAGG` |
| ASV4540 | 429 | `TGGGGAATATTGGACAATGGGCGCAAGCCTGATCCAGCAATGCCGCGTGGGTGAAGAAGGCCTTCGGGTTGTAAAGCCCTTTTGTCCGGAACGAAAAGCTGTGGGTTAATACCCTGCAGTGCTGACGGTACCGGAAGAATAAGCACCGGCTAACTTCGTGCCAGCAGCCGCGGTAATACGAAGGGTGCAAGCGTTAATCGGAATTACTGGGCGTAAAGCGTGCGTAGGCGGCCTGTTAAGTCTGCTGTGAAAGCCCCGGGCTCAACCTGGGAACTGCAGTGGATACTGACGGGCTAGAGTGTGATAGAGGATGGTGGAATTCCCGGTGTAGCGGTGAAATGCGTAGAGATCGGGAGGAACATCAGTGGCGAAGGCGGCCATCTGGATCAACACTGACGCTGAGGCACGAAAGCGTGGGGAGCAAACAGG` |
| ASV364 | 407 | `TGGGGAATTTTCCGCAATGGGCGAAAGCCTGACGGAGCAATACCGCGTGAGGGAAGAAGGCTCTTGGGTTGTAAACCTCTTTTCTTAGGGAATAATAAAGTGAAGGTACCTAAGGAATAAGCATCGGCTAACTCCGTGCCAGCAGCCGCGGTAATACGGAGGATGCAAGCGTTATCCGGAATGATTGGGCGTAAAGCGTCCGCAGGTGGAACAACAAGTCTAGTGTCAAAGACCGGGGCTCAACCCCGGGTCGGCGGTAGAAACTGTTGAACTAGAGGGTCGTAGGGGTAGAGGGAATTCCCAGTGTAGCGGTGAAATGCGTAGAGATTGGGAAGAACATCGGTGGCGAAGGCGCTCTACTGGACGGCACCTGACACTCAGGGACGAAAGCTAGGGGAGCGAATGGG` |
| ASV5704 | 405 | `TAGGGAATATTGCGCAATGGGCGAAAGCCTGACGCAGCAATTCCGCGTGGAGGATGACGGATTTCGGTCTGTAAACTCCTGTCAGCAGGGAAGTAAAAGGACGGTACCTGCAGAGGAAGCACCGGCTAACTACGTGCCAGCAGCCGCGGTAATACGTAGGGTGCAAGCGTTGTCCGGAATTATTGGGCGTAAAGCGTCCGTAGGCGGTTCATTAAGTCTGACGTGAAAGGTCGTGGCTCAACCACGGTTATGTGTTGGAAACTAGTGGACTAGAATATGGTAGAGGTTGCTGGAATTCCTGGTGTAGCGGTGAAATGCGTAGATATCAGGAGGAACACCTGTGGCGAAAGCGTGCAACTGGGCCTATATTGACGCTAATGGACGAAAGCCAGGGTAGCGAAAGGG` |
| ASV4481 | 430 | `TGGGGAATCTTGCGCAATGGACGAAAGTCTGACGCAGCCACGCCGCGTGAGTGAAGAAGGCCTTCGGGTTGTAAAGCTCTGTCGGAAGGGACAAAAACCATCTAGGTTAATAGCCTAGATGCTTGATGGTACCTTTAAAGGAAGCACCGGCTAACTTCGTGCCAGCAGCCGCGGTAATACGAAGGGTGCAAGCGTTGCTCGGAATTATTGGGCGTAAAGGGTTGGTAGGTGGTTACGTATGTCTGAGGTGAAAGCCCTGAGCTCAACTCAGGAAGTGCCTTGGAAACGGCGTAACTAGAGTGCTAGAGAGGTTCGTGGAATTCCCAGTGTAGCGGTGAAATGCGTAGAGATTGGGAGGAACACCAGAGGCGAAGGCGGCGAACTGGATAGCAACTGACACTGAACAACGAAAGCGTGGGTAGCAAACAGG` |
| ASV871 | 404 | `TGGGGAATATTGGGCAATGGGCGAAAGCCTGACCCAGCCATGCCGCGTGAGTGATGAAGGCTCTAGGGTTGTAAAGCTCTTTCGCCGATGAAGATAATGACGGTAGTCGGAGAAGAAGCCCCGGCTAACTTCGTGCCAGCAGCCGCGGTAATACGAAGGGGGCTAGCGTTGTTCGGAATTACTGGGCGTAAAGCGAGCGTAGGTCGCTTGGCAAGTCAGTGGTGAAATCCCAGAGCTTAACTCTGGAACTGCCATTGAAACTGCTGGGCTAGAATCTCAGAGGGGATAGCGGAATTCCAAATGTAGGGGTGAAATCCGTAGATATTTGGAGGAACACCGGTGGCGAAGGCGGCTATCTGGATGAGTATTGACACTGAGGCTCGAAAGCGTGGGGATCAAACAGG` |
| ASV2711 | 429 | `TGGGGAATATTGGACAATGGGGGCAACCCTGATCCAGCAATACCGCGTGTGTGAAGAAGGCCTGAGGGTTGTAAAGCACTTTCAGTGGGGAGGAGGTCAATTAGGTTAAGAGCTAGATTGATGGACGTTACCCACAGAAGAAGCACCGGCTAACTCCGTGCCAGCAGCCGCGGTAATACGGAGGGTGCGAGCGTTAATCGGAATTACTGGGCGTAAAGGGTGCGTAGGTGGTCTGATAAGTTATCTGTAAAATCCCTGGGCTCAACCTGGGCAGGTCAGATAATACTGTTAGACTCGAGTATGGGAGAGGGTAGTGGAATTTCCGGTGTAGCGGTGAAATGCGTAGAGATCGGAAGGAACACCAGTGGCGAAGGCGGCTACCTGGCCTAATACTGACACTGAGGCACGAAAGCGTGGGGAGCAAACAGG` |
| ASV2157 | 389 | `TCGGGAAAATTTTGCAATGTGCGCAAGCATGACAGAGCAAGCCAGAGTGCATTTCATTTTATGAAATGCTTTTGTGGCATGTAAACAGTGCCACGAATAAGGACCGGGAGAGACTGGTGCCAGCCGCCGCGGTAATCCCAGCGGTCCAAGTCGCAGCCACATTTGTTGGGTCTAAAACATCCGTAGCTTGCTCTACAAGTTCATTGTGAAATCGGGACTCTCAAGGTTCCGGCGGGCAATGAATACTGTTGAGCTAGAGATCGGGAGATGCAGTGAGTATGAACAGGGAAGCGGTAAAATGCGTTGATCCTTTTCAGACTAACACCAGCGAAGGCACGCTGCAAGAACGAATCTGACAGTGAGGGATGAAGGCTAGGGGCGCAAAGTGG` |
| ASV2360 | 430 | `TGGGGAATATTGGACAATGGGCGAAAGCCTGATCCAGCAATGCCGCGTGTGTGAAGAAGGCCTTCGGGTTGTAAAGCACTTTCAGCGGGGAGGAAGTGGTGCTGCTTAATACGCAGTATTGTTGACGTTACCCGCAGAAGAAGCACCGGCTAACTCTGTGCCAGCAGCCGCGGTAATACAGAGGGTGCAAGCGTTAATCGGAATTACTGGGCGTAAAGGGTGTGTAGGTGGTTCAAGTAAGTCAGGTGTAAAAACCCCGGGCTCAACCTGGGAATGTCGCTTGAAACTGCTTAGCTAGAGTACTATAGAGGATAGTGGAATTTCCAGTGTAGCGGTGAAATGCGTAGATATTGGAAGGAACACCAGTGGCGAAGGCGACTATCTGGATAGATACTGACACTGAAACACGAAAGCATGGGTAGCAAACAGG` |
| ASV3470 | 404 | `TGGGGAATATTGGGCAATGGGGGGAACCCTGACCCAGCGACGCCGCGTGAGTGAAGAAATCCCTCGGGATGTAAAGCTCTGTTGTGTGGGAAGATAATGACGGTACCACACGAGGAAGCCCCGGCAAACTACGTGCCAGCAGCCGCGGTAATACGTAGGGGGCAAGCGTTGTTCGGAATTACTGGGCGTAAAGCGCACGCAGGCGGAATGGTAAGTCAAACGTCAAAGGCAGAGGCTCAACTTCTGTTCCACGTTTGATACTGTCATTCTAGAGTATGTGAGAGGAAGATGGAATTCCCGGTGTAGCGGTGAAATGCGTAGATATCGGGAGGAACACCAGTGGCGAAGGCGATCTTCTGGCACAAAACTGACGCTCATGTGCGAAAGCTAGGGCAGCGAACGGG` |
| ASV4714 | 404 | `TGGGGAATATTGGGCAATGGGCGAAAGCCTGACCCAGCAACGCCGCGTGGAGAATGAAGGCCTTAGGGTTGTAAACTCCTTTTATTAGGGAAGATAGTGACGGTACCTAATGAATAAGCATCGGCTAACTTCGTGCCAGCAGCCGCGGTAATACGAAGGATGCTAGCGTTACTCGGAATTATTGGGCGTAAAGAATGCGTAGGCGGATTGTTAAGTTGATAGTGAAATCCCCAAGCTTAACTTGGGAACTGCTATCAAAACTGATAATCTAGAATGTCGGAGAGAGTAAGGGAATTCTTGGTGTAGGGGTAATATCCGTAGATATCAGGAGGAAGACCGGAAGCGAAAGCGCTTACTTGGACGACTATTGACGCTGAGGCATGAAAGCGTGGGGATCGAACAGG` |
| ASV2275 | 429 | `TGGGGAATTTTGGACAATGGGCGCAAGCCTGATCCAGCCATGCCGCGTGCGGGAAGAAGGCCTTCGGGTTGTAAACCGCTTTTGTCAGGGAAGAAACGCACCGAGCTAATATCTCGGCGTAATGACGGTACCTGAAGAATAAGCACCGGCTAACTACGTGCCAGCAGCCGCGGTAATACGTAGGGTGCAAGCGTTAATCGGAATTACTGGGCGTAAAGCGTGCGCAGGCGGTCCTACAAGACAGATGTGAAATCCCCGGGCTCAACCTGGGAACTGCATTTGTGACTGTAGGGCTGGAGTGCGGCAGAGGGGGATGGAATTCCGCGTGTAGCAGTGAAATGCGTAGATATGCGGAGGAACACCGATGGCGAAGGCAATCCCCTGGGCCTGCACTGACGCTCATGCACGAAAGCGTGGGGAGCAAACAGG` |
| ASV374 | 423 | `TAGGGAATATTGGGCAATGGAGGCAACTCTGACCCAGCCATGCCGCGTGCAGGAAGAAGGCGTTATGCGTTGTAAACTGCTTTTATATAGGAAGAAAGAGTTTCTGAGGAGACAGTTGACGGTACTATATGAATAAGCACCGGCTAACTCCGTGCCAGCAGCCGCGGTAATACGGAGGGTGCAAGCGTTGTCCGGATTTATTGGGTTTAAAGGGTGCGTAGGCGGTTTATTAAGTCAGTGGTGAAAGACGGTCGCTCAACGATTGCAGTGCCATTGATACTGGTAGACTTGAGTGAGTTTGAGGTAGCTGGAATGGATAGTGTAGCGGTGAAATGCATAGATATTATCCAGAACACCAATTGCGTAGGCAAGTTACTAAGACTCAACTGACGCTGAGGCACGAAAGTGTGGGTATCAAACAGG` |
| ASV2179 | 429 | `TGGGGAATTTTGGACAATGGGGGCAACCCTGATCCAGCCATGCCGCGTGCAGGATGAAGGCCTTCGGGTTGTAAACTGCTTTTGTTAGGAACGAAAAGGTAGGCAATAACACTGCTTACTGCTGACGGTACCTAAAGAATAAGCACCGGCTAACTACGTGCCAGCAGCCGCGGTAATACGTAGGGTGCAAGCGTTAATCGGAATTACTGGGCGTAAAGCGTGCGCAGGCGGTTGAGTAAGTTAGATGTGAAATCCCCGGGCTCAACCTGGGAACTGCATTTAAAACTGCTTGGCTAGAGTGTGTCAGAGGGGGGTAGAATTCCACGTGTAGCAGTGAAATGCGTAGAGATGTGGAGGAATACCAATGGCGAAGGCAACCCCCTGGGATAACACTGACGCTCATGTACGAAAGCGTGGGGAGCAAACAGG` |
| ASV5008 | 404 | `TGGGGAATATTGGACAATGGGGGAAACCCTGATCCAGCAATGCCGCGTGAATGATGAAGGCCTTCGGGTTGTAAAGTTCTTTTACCTGAGAAGATAATGACGGTATCAGGGGAAAAAGCACCGGCTAACTTCGTGCCAGCAGCCGCGGTAATACGAAGGGTGCAAGCGTTGTTCGGAATGACTGGGCGTAAAGGGCGCGTAGGCGGTTTGGTACGTTTGATGTGAAAGTCCTGGGCTTAACCTGGGGACGGCATTGGATACGGCCAAGCTAGAGAGCGAGAGAGGGAGACGGAATAGTGCGTGTAGAGGTGAAATTCGTAGATATGCACTAGAACACCGGAGGCGAAGGCGGTTTCCTGGCTCGCAACTGACGCTAAGGCGCGAAAGCGTGGGGAGCAAACAGG` |
| ASV615 | 429 | `TGGGGAATTTTGGACAATGGGCGCAAGCCTGATCCAGCAATGCCGCGTGAGTGAAGAAGGCCTTCGGGTTGTAAAGCTCTTTTGTCAGGGAAGAAACGGTGGGAGCTAATATCTCCTGCTAATGACGGTACCTGAAGAATAAGCACCGGCTAACTACGTGCCAGCAGCCGCGGTAATACGTAGGGTGCAAGCGTTAATCGGAATTACTGGGCGTAAAGCGTGCGCAGGCGGTTTTGTAAGTCTGATGTGAAATCCCCGGGCTCAACCTGGGAATTGCATTGGAGACTGCAAGGCTAGAATCTGGCAGAGGGGGGTAGAATTCCACGTGTAGCAGTGAAATGCGTAGATATGTGGAGGAACACCGATGGCGAAGGCAGCCCCCTGGGTCAAGATTGACGCTCATGCACGAAAGCGTGGGGAGCAAACAGG` |
| ASV3843 | 407 | `TAGGGAATATTGCGCAATGGGGGAAACCCTGACGCAGCAATTCCGCGTGGAGGATGACGGATTTCGGTCTGTAAACTCCTGTCAGCAGGGAAGTGAACAAGGACGGTACCTGCAGAGGAAGCACCGGCTAACTACGTGCCAGCAGCCGCGGTAATACGTAGGGTGCAAGCGTTGTCCGGAATTATTGGGCGTAAAGCGTCCGTAGGCGGTTAATCAAGTCATACGTTAAAGGTACGGGCTCAACCCGTGTATGGTGTATGAAACTGTTTGACTCGAATTAGGTAGAGGTTGCTGGAATTCCTGGTGTAGCGGTGAAATGCGTAGATATCAGGAGGAACACCTGTGGCGAAAGCGTGCAACTGGGCCTTAATTGACGCTAATGGACGAAGGCTAGGGTAGCGAAAGGG` |
| ASV2703 | 424 | `TAAGGAATATTGGTCAATGGAGGCAACTCTGAACCAGCCATGCCGCGTGCAGGATGAAGGCCCTATGGGTCGTAAACTGCTTTTATACCGGAGAAAACCCGAGTACGAGTACTCGGTTGATAGTATGGTAAGAATAAGCATCGGCTAACTTCGTGCCAGCAGCCGCGGTAAGACGAAGGATGCAAGCGTTATCCGGATTCATTGGGTTTAAAGGGTGCGTAGGCGGACTTGTAAGTCAGTGGTGAAATCTCTTTGCTTAACAAAGAAACTGCCATTGATACTGCAGGTCTAGAGTATAGATGACGTTGGCGGAATATGACATGTAGTGGTGAAATACTTAGATATGTCATAGAACACCGATTGCGAAGGCAGCTAACGAAACTATAACTGACGCTGAGGCACGAAAGTGCGGGGATCAAACAGG` |
| ASV4914 | 405 | `TGAGGAATCTTGCACAATGGGCGAAAGCCTGATGCAGCGACGTCGCGTGCGGGAAGAAGGCCTTCGGGTCGTAAACCGCTTTTCTGGGGGACGAGTAAGGACGGTACCTCAGGAATAAGTCCCGGCTAACTACGTGCCAGCAGCCGCGGTAATACGTAGGGGGCAAGCGTTATCCGGAATTACTGGGCGTAAAGGGTGTCTAGGCGGCAAAGCAAGTCTTGCGTGAAAGCTCCCGGCTCAACTGGGAGAGCGCGCAGGAAACTGCTTGGCTAGAGACTCGGAGGGGGGTGTGGAATTCCCGGTGGAGTGGTGAAATGCGTTGAGATCGGGAGGAACACCAGTAGCGAAGGCGGCACCCTGGCCGAGGTCTGACGCTGAAGCACGACAGCGTGGGGAGCAAACGGG` |
| ASV824 | 429 | `TGGGGAATATTGGACAATGGGCGCAAGCCTGATCCAGCGATGCCACGTGTGCGAAGAAGGCCTTAGGGTTGTAAAGCACTTTAGTGAGGGAGGAAGTTAGTCTGGTTAATACCTGGATTGATTGACAGTACCTCAAGAATAAGCACCGGCAAACTCTGTGCCAGCAGCCGCGGTAATACAGAGGGTGCAAGCGTTAATCGGAATTATTGGGCGTAAAGAGCTTGTAGGCGGTTAGATAAGTTAGATGTGAAAACCCTGGGCTCAACCTGGGGCTGGCATTTAATACTGTTTAGCTAGAGTATAGGAGAGGAGAAGGGAATTTCTGGTGTAGCGGTGAAATGCGTAGATATCAGAAGGAACATCAGTGGCGAAGGCGCTTCTCTGGCCTAATACTGACGCTGAGGAGCGAAGGCGTGGGGATCAAACAGG` |
| ASV1296 | 424 | `TAGGGAATATTGGGCAATGGGCGAGAGCCTGACCCAGCCATGCCGCGTGCAGGACGAAGGCCCTCTGGGTTGTAAACTGCTTTTTTGGGGGAAGAATAGCATTCCTGCGGGGGTGTATGACGGTACCCTAAGAATAAGCACCGGCTAACTCCGTGCCAGCAGCCGCGGTAATACGGAGGGTGCAAGCGTTGTCCGGATTTATTGGGTTTAAAGGGTGCGTAGGTGGGTGAGTAAGTCTGGTTTGAAAGTTGGTCGCTTAACGATCAAGGGTGGCTGGATACTGTTCATCTTGAATATTTTGGAGGTAGCTGGAATGGGTTGTGTAGCGGTGAAATGCATAGAGATGACCCGGAACTCCAATTGCGTAGGCAGGCTACTACGAAATGATTGACACTGAGGCACGAGAGCATGGGTAGCAAACAGG` |
| ASV1519 | 429 | `TGGGGAATATTGGACAATGGGCGCAAGCCTGATCCAGCAATGCCGCGTGTGTGAAGAAGGCCTGCGGGTTGTAAAGCACTTTTGTTCGGGAAGAAATCATGCCGACTAATACTTGGTATGGATGACGGTACCGGAAGAATAAGCACCGGCTAACTTCGTGCCAGCAGCCGCGGTAATACGAAGGGTGCAAGCGTTAATCGGAATTACTGGGCGTAAAGGGTGCGTAGGCGGCTTTTTAAGTCGGATGTGAAATCCCCGGGCTTAACCTGGGAATGGCATACGATACTGGGGAGCTAGAGTTTGGGAGAGGGTGGTGGAATTCCCGGTGTAGCGGTGAAATGCGTAGAGATCGGGAGGAACATCAGTGGCGAAGGCGGCCACCTGGCCTAAAACTGACGCTGAGGCACGAAAGCGTGGGGAGCAAACAGG` |
| ASV5653 | 416 | `TTAGGAATATTGTGCAATGGAGGGAACTCTGACACAGCGACGCCGCGTGAGGGATGAAGGTTTTCGGATCGTAAACCTCTGAATTAGGGACGAAAGACGCGTAAGCGGGATGACGGTACCTAAGTAATAGCACCGGCTAACTCCGTGCCAGCAGCCGCGGTAATACGGAGGGTGCAAGCGTTACCCGGAATCACTGGGCGTAAAGGGCGTGTAGGCGGTGGAATAAGTCTGGATTTAAAGACTGAAGCTCAACTTCAGGAATGGTTCGGATACTGTTTCACTAGACAAATGGAGAGGTAATTGGAATTCTTGGTGTAGCGGTGAAATGCGTAGATACCAAGAGGAACACCAATGGCGAAGGCAGGTTACTGGACATTTTGTGACGCTGAGGCGCGAAAGTGTGGGGAGCAAACCGG` |
| ASV4607 | 429 | `TGGGGAATATTGGACAATGGGGGCAACCCTGATCCAGCAATGCCGCGTGTGTGAAGAAGGCCTGCGGGTTGTAAAGCACTTTCAGTGGGGAGGAAATGTTGTAACTGAATAGGTTGCAAAGAAGACGTTACCCACAGAAGAAGCACCGGCTAACTCTGTGCCAGCAGCCGCGGTAATACAGAGGGTGCAAGCGTTAATCGGAATTACTGGGCGTAAAGCGCACGTAGGTGGATAATTAAGTTAGATGTGAAAGACCCGGGCTCAACCTGGGAAGGTCATTTAATACTGATTATCTAGAGTACAGTAGAGGGAAGTGGAATTTCCGGTGTAGCGGTGAAATGCGTAGATATCGGAAGGAACATCAGTGGCGAAGGCGGCTTCCTGGACTGATACTGACACTGAGGTGCGAAAGCGTGGGTAGCGAACAGG` |
| ASV1522 | 425 | `TAAGGAATATTGGACAATGGAGGGAACTCTGATCCAGCCATGCCGCGTGAAGGAAGAAGGCCCTCTGGGTTGTAAACTTCTTTTATATGGGAACAAACCTGTGGATTGATCCGCAGCTGAGGGTACCATAAGAATAAGCACCGGCTAACTCCGTGCCAGCAGCCGCGGTAATACGGAGGGTGCAAGCGTTATCCGGAATCACTGGGTTTAAAGGGTGCGTAGGCGGTCTTATAAGTCAGTCGTGAAATCGCTGGGCTCAACCCATGCAATTGCGATTGATACTGTTTGACTTGAATCAGGTTGAGGTAGGCGGAATGTGGCATGTAGCGGTGAAATGCATAGATATGCCATAGAACACCGATTGCGAAGGCAGCTTGCTGGACCTGTATTGACGCTGAGGCACGAAAGCGTGGGGAGCAAACAGG` |
| ASV166 | 429 | `TGGGGAATTTTGGACAATGGACGCAAGTCTGATCCAGCCATGCCGCGTGCGGGAAGAAGGCCTTCGGGTTGTAAACCGCTTTTGTCAGGGAAGAAATCTTCTGAGTTAATACCTCGGGAGGATGACGGTACCTGAAGAATAAGCACCGGCTAACTACGTGCCAGCAGCCGCGGTAATACGTAGGGTGCAAGCGTTAATCGGAATTACTGGGCGTAAAGCGTGCGCAGGCGGTTATGCAAGACAGGTGTGAAATCCCCGAGCTCAACTTGGGAACTGCACTTGTGACTGCATAGCTAGAGTACGGTAGAGGGGAGTGGAATTCCGCGTGTAGCAGTGAAATGCGTAGATATGCGGAGGAACACCGATGGCGAAGGCAGCTCCCTGGACCTGTACTGACGCTCATGCACGAAAGCGTGGGGAGCAAACAGG` |
| ASV5531 | 430 | `TGGGGAATATTGGGCAATGGGCGAAAGCCTGACCCAGCCACGCCGCGTGAGTGATGAAGGCCTTCGGGTCGTAAAGCTCTGTGGGGAGGGACGAAAAAGTCCGGCCTTAATACCGCCGGGCCCTGACGGTACCTCCTTAGCAAGCACCGGCTAACCCTGTGCCAGCAGCCGCGGTAATACAGGGGGTGCAAACGTTGCTCGGAATTATTGGGCGTAAAGCGCGCGTAGGCGGCTACTTAAGTCGGATGTGAAAGCCCCTGGCTCAACTGGGGAAGTGCACCCGAAACTGAGCAGCTCGAGTACAAAAGAGGGCCGCGGAATTCCCGGTGTAGAGGTGAAATTCGTAGATATCGGGAGGAACACCGGTGGCGAAGGCGGCGGCCTGGGTTGATACTGACGCTGAGGCGCGAAAGCGTGGGGAGCAAACAGG` |
| ASV4595 | 404 | `TGGGGAATATTGGACAATGGGCGCAAGCCTGATCCAGCAATACCGCGTGAATGATGAAGGTCTTCGGATTGTAAAGTTCTTTTAGTGGAGAAGATAATGACGGTATCCACAGAAAAAGCACCGGCTAACTTCGTGCCAGCAGCCGCGGTAATACGAAGGGTGCTAGCGTTGTTCGGAATCATTGGGCGTAAAGCGTATGTAGGCTGTTTAGTAAGTCGGTTGTGAAATCCCGGGGCTCAACCCCGGAATTGCAATCGAAACTGCTTTGCTAGAATTTGGTGGGGGATGATGGAATTCCTAGTGTAGGGGTGAAATCCGTAGATATTAGGAGGAACGCCAGAGGCGAAAGCGATCATCTACGCCAACATTGACGCTGAGATACGAAAGCGTGGGGAGCAAACAGG` |
| ASV2192 | 428 | `TCGGGAATCTTCCGCAATGGGCGAAAGCCTGACGGAGCGACGCCGCGTGAGCGATGAAGGCCTTCGGGTTGTAAAGCTCTGTGGGGAGAGAAGAATAAGTTCAAGCGAATACCTTGAATGATGACGGTATCTCCTTAGCAAGCACCGGCTAACTCTGTGCCAGCAGCCGCGGTAAGACAGAGGGTGCAAACGTTGTTCGGATTTACTGGGCGTAAAGCGCGTGTAGGCGGCTTGGCAAGTCGGATGTGAAAGCCCACGGCTCAACCGTGGAAGCGCATCCGAAACGGCCTCGCTCGAGTCTCGGAGAGGAAGGTGGAATGCTTGGTGTAGAGGTGAAATTCGTAGATATCAAGCAGAACACCTGCGGCGAAGGCGGCCTTCTGGACGATGACTGACGCTGAGACGCGAAAGCGTGGGGAGCAAACAGG` |
| ASV4413 | 429 | `TGGGGAATATTGGACAATGGGCGCAAGCCTGATCCAGCAATGCCGCGTGTGTGAAGAAGGCCTGCGGGTTGTAAAGCACTTTAAGCAGGAAAGAAGGTTTCAGGGCTAATATCCCTGGAAATTGACGTTACCTGCAGAATAAGCACCGGCTAACTCCGTGCCAGCAGCCGCGGTAATACGGAGGGTGCGAGCGTTAATCGGAATTACTGGGCGTAAAGCGCGCGTAGGCGGTTGACTAAGTCTGCTGTGAAAGCCCTGGGCTTAACCTGGGAACGGCAGTGGATACTGGTTGGCTAGAGTATGGTAGAGGAGAGTGGAATTTCCGGTGTAGCAGTGAAATGCGTAGAGATCGGAAGGAACACCAGTGGCGAAGGCGGCTCTCTGGACCAATACTGACGCTGAGGTGCGAAAGCGTGGGGAGCAAACAGG` |
| ASV4872 | 407 | `TGGGGAATATTGGACAATGGACCAAAAGTCTGATCCAGCAATTCTGTGTGCACGATGACGTTTTTCGGAATGTAAAGTGCTTTCAGTTGGGAAGAAAAAAATGACGGTACCAACAGAAGAAGTGACGGCTAAATACGTGCCAGCAGCCGCGGTAATACGTATGTCACAAGCGTTATCCGGATTTATTGGGCGTAAAGCGCGTCTAGGTGGTTATGTAAGTCTGATGTGAAAATGCAGGGCTCAACTCTGTATTGCGTTGGAAACTGCATGACTAGAGTACTGGAGAGGTAAGCGGAACTACAAGTGTAGAGGTGAAATTCGTAGATATTTGTAGGAATGCCGATGGGGAAGCCAGCTTACTGGACAGATACTGACGCTAAAGCGCGAAAGCGTGGGTAGCAAACAGG` |
| ASV5543 | 424 | `TGAGGAATATTGGACAATGGGTGAGAGCCTGATCCAGCCATCCCGCGTGAAGGAAGACGGCCCTACGGGTTGTAAACTTCTTTTATATAGGAAGAAACCTACATACGTGTATGTAGCTGACGGTACTATATGAATAAGCATCGGCTAACTCCGTGCCAGCAGCCGCGGTAATACGGAGGATGCGAGCGTTATCCGGATTTATTGGGTTTAAAGGGTCCGTAGGCGGTAGATTAAGTCAGTGGTGAAATCCTACAGCTCAACTGTAGAATTGCCATTGATACTGGTTTACTTGAGTAAGGTTGAAGTGGCTAGAATGTGTAGTGTAGCGGTGAAATGCATAGATATTACACGGAATACCGATAGCGAAGGCAGGTCACTAAGCCTTTACTGACGCTGAGGGACGAAAGCGTGGGGAGCAAACAGG` |
| ASV4976 | 430 | `TAGGGAATATTGCGCAATGGAGGAAACTCTGACGCAGCGACGCCGCGTGGGTGATGAAGGTCTTCGGATCGTAAAGCCCTGTCGGATGGGAAGAAAAACACTGAGGGTAATATTCTCAGTGCTTGACGGTACCATTAAAGGAAGCCCCGGCTAACTTCGTGCCAGCAGCCGCGGTAATACGAAGGGGGCAAGCGTTGTTCGGAATCATTGGGCGTAAAGCGCGTGTAGGCGGCCTATTAAGTCGAGTGTGAAATCCCTGGGCTCAACCGAGGAAGTGCATTCGAAACTAATGGGCTAGAAGATGGTAGAGGATGGTGGAATTCCAGGTGTAGAGGTGAAATTCGTAGATATCTGGAGGAATACCGGTGGCGAAGGCGGCCATCTGGGCCATTCTTGACGCTGAGACGCGAAAGCGTGGGTAGCAAACAGG` |
| ASV4987 | 416 | `TTAGGAATCTTCCCCAATGGGCGCAAGCCTGAGGGAGCGACGCCGCTTGAAGGACGAAGGTCTTCGGATTGTAAACTTCTGAAACAAGGACGAAATCCTAGAAATAGGTCTGACGGTACTTGTGTAATAGCACCGGCTAACTCCGTGCCAGCAGCCGCGGTAATACGGAGGGTGCAAGCGTTACCCGGAATCACTGGGCGTAAAGGGCGTGTAGGCGGCTTGGTAAGTCTGGATTTAAAGACCGAAGCTCAACTTCGGGAATGGTCTGGATACTGCCATGCTAGACGACTGGAGGGGAGAGTGGAATTCTCGGTGTAGCGGTGGAATGCGTAGATACCGAGAGGAACACCAGTGGCGAAGGCGGCTCTCTGGACAGTGCGTGACGCTGAGGCGCGAAAGTGTGGGGAGCGAACCGG` |
| ASV4418 | 429 | `TGGGGAATTTTGGACAATGGGGGCAACCCTGATCCAGCCATTCCGCGTGAGTGAAGAAGGCCTTCGGGTTGTAAAGCTCTTTTGTCGGGGACGAAACGGTTTCACCTAATACGTGAGACTACTGACGGTACCCGAAGAATAAGCACCGGCTAACTACGTGCCAGCAGCCGCGGTAATACGTAGGGTGCGAGCGTTAATCGGAATTACTGGGCGTAAAGCGTGCGCAGGCGGTTTTGTAAGCCAGATGTGAAATCCCCGGGCTCAACCTGGGAATGGCATTTGGGACTGCAAGGCTAGAGTACGGCAGAGGAGACTAGAATTCCTGGTGTAGCAGTGAAATGCGTAGATATCAGGAGGAATACCGATGGCGAAGGCAGGTCTCTGGGCTGATACTGACGCTCATGCACGAAAGCGTGGGGAGCAAACAGG` |
| ASV3064 | 429 | `TGGGGAATTTTGGACAATGGGCGAAAGCCTGATCCAGCCATGCCGCGTGCAGGATGAAGGCCTTCGGGTTGTAAACTGCTTTTGTACGGAACGAAAAGGTCTTTTCTAATAAAGAAGGCCCATGACGGTACCGTAAGAATAAGCACCGGCTAACTACGTGCCAGCAGCCGCGGTAATACGTAGGGTGCAAGCGTTAATCGGAATTACTGGGCGTAAAGCGTGCGCAGGCGGTTATATAAGACAGATGTGAAATCCCCGGGCTCAACCTGGGAACTGCATTAGTGACTGTATAGCTAGAGTGCGGCAGAGGGGGATGGAATTCCGCGTGTAGCAGTGAAATGCGTAGATATGCGGAGGAACACCGATGGCGAAGGCAATCCCCTGGGCCTGCACTGACGCTCATGCACGAAAGCGTGGGGAGCAAACAGG` |
| ASV3759 | 404 | `TGGGGAATATTGGACAATGGGCGCAAGCCTGATCCAGCAATACCGCGTGAGTGATGAAGGTCTTCGGATTGTAAAGCTCTTTTAGTGGGGAAGATAATGACGGTACCCACAGAAAAAGCACCGGCAAACTTCGTGCCAGCAGCCGCGGTAATACGAAGGGTGCTAGCGTTGTTCGGAATCACTGGGCGTAAAGCGTACGTAGGCGGTTTAGTAAGTCGATTGTGAAAGCCCCAAGCTTAACTTGGGAATTGCAACCGAAACTGCTTTTCTAGAATATGGTGGGGGATAGTGGAATTCCTAGTGTAGGGGTGAAATCCGTAGATATTAGGAGGAATACCGGTGGCGAAAGCGACTATCTATGCCATTATTGACGCTAAGGTACGAAAGCGTGGGGAGCAAACAGG` |
| ASV140 | 424 | `TGAGGAATATTGGTCAATGGGCGCAAGCCTGAACCAGCCATGCCGCGTGCAGGATGACGGTCCTATGGATTGTAAACTGCTTTTGTACAGGAAGAAACACCTCTTCGTGAAGAGACTTGACGGTACTGTAAGAATAAGGATCGGCTAACTCCGTGCCAGCAGCCGCGGTAATACGGAGGATCCAAGCGTTATCCGGAATCATTGGGTTTAAAGGGTCCGTAGGCGGTTTGATAAGTCAGTGGTGAAAGCCCATCGCTCAACGGTGGAACGGCCATTGATACTGTTAGACTTGAATTATTAGGAAGTAACTAGAATATGTAGTGTAGCGGTGAAATGCTTAGAGATTACATGGAATACCAATTGCGAAGGCAGGTTACTACTAATGGATTGACGCTGATGGACGAAAGCGTGGGTAGCGAACAGG` |
| ASV1355 | 408 | `TCGAGAATCTTCCGCAATGGGCGAAAGCCTGACGGAGCGACGCCGCGTGGTGGATGAAGCGCTTAGGCGCGTAAACACCTTTTATGGAGGAGAAAGTTTATTGATGTTACTCCATGAATAAGGGGCTCCCAACTCTGTGCCAGCAGGAGCGGTAATACAGAGGCCCCAAGCATTATCCGGATTTACTGGGCGTAAAGGGTGCGTAGGCGGTGTGATTAGTCGGGTGTTAAATCCTGGGGCTCAACCTCAGAATCGCATTCGAAACGGTCACACTAGAAGAAGTCAGAGGTAAACAGAACTCTCGGTGTAGGGGTGAAATCCGTTGATATCGAGGGGAATACCAAATGCGAAGGCAGTTTACTGGGACTTTCTTGACGCTGAGGCACGAAAGCGTGGGTAGCGAATCGG` |
| ASV3815 | 404 | `TAGGGAATATTGCACAATGGACGAAAGTCTGATGCAGCGACGCCGCGTGAGTGATGAAGGCCTTCGGGTTGTAAAACTCTGTGATAAGGGAAGATGATGACGGTACCTTATCAGAAAAGACCGGCTAACTTCGTGCCAGCAGCCGCGGTAATACGAGGGGTCTAAGCGTTGTTCGGAATCACTGGGCGTAAAGCGGGTGTAGGTGGCTCAGCAAGTTAGGTGTGAAAGCCCCGGGCTCAACCTGGGAAGTGCATCTAAAACTACTGAGCTTGAGTACGGGAGAGGGAAGCAGAATTCCTGGTGTAGTGGTGAAATACGTAGATATCAGGAGGAACACCGGTGGCGAAGGCGGTTTCCTGGCCCAGTACTGACACTCAGACCCGAAAGCGTGGGTAGCAAACAGG` |
| ASV799 | 424 | `TAAGGAATATTGGGCAATGGACGCAAGTCTGACCCAGCCATGCCGCGTGGAGGATGAAGGTCCTCTGGATTGTAAACTTCTTTTTTCTGGGGCGAAAACCGGTTTTTCTAAACCATTTGACAGTACCAGAAGAATAAGCACCGGCTAACTCCGTGCCAGCAGCCGCGGTAATACGGAGGGTGCAAGCGTTATCCGGATTCACTGGGTTTAAAGGGTGCGTAGGTGGGCAGGTAAGTCCGCGGTGAAATCCCCGAGCTTAACTTGGGAACTGCCGTTGATACTATCTGTCTTGAATGTCGTGGAGGTAAGCGGAATATGTCATGTAGCGGTGAAATGCTTAGAGATGACATAGAACACCAATTGCGAAGGCAGCTTGCTACACGAAAATTGACACTGAGGCACGAAAGCGTGGGGAGCAAACAGG` |
| ASV1738 | 429 | `TCGAGAATCATTCACAATGGGCGAAAGCCTGATGGTGTGACGCCGCGTGGAGGATGAAGGTCTTCGGATTGTAAACTCCTGTCATGTGAGAGTAAGGTTGAGGGAGTAACTGCCTGTCAACTTGAGAGTATCACAAGAGGAAGAGGCGGCTAACTCTGTGCCAGCAGCCGCGGTAATACAGAGGCCTCAAGCGTTGTTCGGAATCACTGGGCGTAAAGGGTGCGTAGGCGGCGCGGAAAGTCAGGGGTGAAATCCCGGAGCTCAACTCCGGAACTGCCTTTGATACTCCCGCGCTTGAGAACTGGAGGGGACATCGGAATTCACGGTGGAGCAGTGAAATGCGTGGATATCGTGAGGAACACTAGTGGCGAAGGCGGATGTCTGGACAGTATCTGACGCTGAGGCACGAAGGCCAGGGGAGCGAACGGG` |
| ASV4563 | 430 | `TAGGGAATATTGCACAATGGGCGCAAGCCTGATGCAGCAATGCCGCGTGAGTGAAGAAGGCCTTCGGGTTGTAAAGCTCTTTCGACAGAGAAGAAGGGTATTATGGTGAATAATCATGGTATTTGACGGTACCTGGAGAAGAAGCACCGGCTAACTCCGTGCCAGCAGCCGCGGTAATACGGAGGGTGCAAGCGTTGTTCGGAATCACTGGGCGTAAAGCGCACGTAGGCGGATCTGTTAGCCAATTGTGAAATCCCGGGGCTCAACCCCGGAATGGCAGTTGGAACTGCAGACCTTGAATGCTCAAGAGGTTGGGGGAATTCCTGGTGTAGAAGTGAAATTCGTAGATATCAGGAGGAACACCTGAGGCGAAGGCGCCTGACTGGTGGGCCATTGACGCTGAGGTGCGAAAGCGTGGGTAGCAAACAGG` |
| ASV777 | 424 | `TGAGGAATATTGGACAATGGGCGCAAGCCTGATCCAGCCATGCCGCGTGCAGGATGACGGTCCTATGGATTGTAAACTGCTTTTGCACAGGAAGAAACAACATTACGTGTAATGTCTTGACGGTACTGTGAGAATAAGGATCGGCTAACTCCGTGCCAGCAGCCGCGGTAATACGGAGGATCCAAGCGTTATCCGGAATCATTGGGTTTAAAGGGTCCGTAGGCGGTCTTATAAGTCAGTGGTGAAATCTCCCCGCTCAACGGGGAAACGGCCATTGATACTGTAGGACTTGAATTATTAGGAAGTAACTAGAATATGTAGTGTAGCGGTGAAATGCTTAGAGATTACATGGAATACCAATTGCGAAGGCAGGTTACTACTAATGGATTGACGCTGATGGACGAAAGCGTGGGTAGCGAACAGG` |
| ASV4009 | 404 | `TGAGGAATCTTCCACAATGGGCGAAAGCCTGATGGAGCGACACCGCGTGAAGGATGAAGCCTTTGTTGGTGTAAACTTCTTTTCTCTGGGAAGATAATGACGGTACCAGAGGAATAAGGGGCGGCAAACTTCGTGCCAGCAGCCGCGGTAATACGAAGGCCCCAAGCGTTATCCGGAATTACTGGGTGTAAAGGGTATGTAGGTGGTCTTTTAAGTCTATCTGTGAAACTTCGGGGCTCAACCCCGAATTGCAGTGGAAACTGGAAGACTTGAGTGTGGGAGAGGCAAGCAGAACAGTAAGAGTAGGGGTGCAATCCGTTGATACTTACTAGAATACCAAAAGCGAAGGCAGCTTGCTGGAACACTACTGACACTGAGATACGAAAGCGTGGGGAGCGAAAGGG` |
| ASV2741 | 404 | `TGGGGAATATTGGACAATGGGCGAAAGCCTGATCCAGCAATGCCGCGTGAGTGATGAAGGCCTTAGGGTTGTAAAGCTCTTTTACCCGGGATGATAATGACAGTACCGGGAGAATAAGCTCCGGCTAACTCCGTGCCAGCAGCCGCGGTAATACGGAGGGAGCTAGCGTTGTTCGGAATTACTGGGCGTAAAGCGCGCGTAGGCGGTTTTTTAAGTCAGAGGTGAAAGCCCAGTGCTCAACACTGGAACTGCCTTTGAAACTGGAAAACTTGAATCTTGGAGAGGTCAGTGGAATTCCGAGTGTAGAGGTGAAATTCGTAGATATTCGGAAGAACACCAGTGGCGAAGGCGACTGACTGGACAAGTATTGACGCTGAGGTGCGAAAGCGTGGGGAGCAAACAGG` |
| ASV1725 | 404 | `TGGGGAATCTTGGACAATGGGCGCAAGCCTGATCCAGCCATGCCGCGTGGATGATGAAGGCCTTAGGGTTGTAAAATCCTTTCGTGGGGGACGATAATGACGGTACCCCAAGAAGAAGCTCCGGCTAACTTCGTGCCAGCAGCCGCGGTAATACGAAGGGGGCTAGCGTTGTTCGGATTTACTGGGCGTAAAGCGCGTGTAGGCGGGTCGTTAAGTTGGGGGTGAAATCCCGAGGCTCAACCTCGGAACTGCCTTCAAAACTGGCGACCTGGACTCAAGTAGAGGCAAGTGGAATTGCGAGTGTAGAGGTGAAATTCGTAGATATTCGCAGGAACACCAGTGGCGAAGGCGACTTGCTGGACTTGTAGTGACGCTGAGACGCGAAAGCGTGGGGAGCAAACAGG` |
| ASV1411 | 404 | `TGGGGAATCTTGGACAATGGGCGCAAGCCTGATCCAGCCATGCCGCGTGAGTGACGAAGGCCTTAGGGTTGTAAAGCTCTTTTGCCGGGGACGATAATGACGGTACCCGGAGAATAAGTCCCGGCTAACTTCGTGCCAGCAGCCGCGGTAATACGAAGGGGACTAGCGTTGTTCGGAATTACTGGGCGTAAAGCGCACGTAGGCGGAATTGTCAGTCAGGGGTGAAATCCCGGGGCTCAACCTCGGAACTGCCTTTGATACAGCAATTCTCGAGTCCGATAGAGGTGGGTGGAATTCCTAGTGTAGAGGTGAAATTCGTAGATATTAGGAGGAACACCGGTGGCGAAGGCGGCCCACTGGATCGGTACTGACGCTGAGGTGCGAAAGCGTGGGGAGCAAACAGG` |
| ASV2967 | 429 | `TGGGGAATTTTGGACAATGGGGGCAACCCTGATCCAGCCATGCCGCGTGAGGGAAGAAGGCCTTCGGGTTGTAAACCTCTTTCGGGCGGGACGAAAGCGCCTGGGCGAATAGCCTGGGTGGATGACGGTACCGCAAGAAGAAGCACCGGCTAACTACGTGCCAGCAGCCGCGGTAATACGTAGGGTGCGAGCGTTAATCGGAATTACTGGGCGTAAAGCGTGCGCAGGCGGCTTTGCAAGCCAGACGTGAAAGCCCCGGGCTCAACCTGGGAATGGCGTTTGGGACTGCGAGGCTGGAGTGCGGCAGAGGGAACTGGAATTCCTGGTGTAGCAGTGAAATGCGTAGATATCAGGAGGAACACCGATGGCGAAGGCAGGTTCCTGGGCCAGCACTGACGCTCATGCACGAAAGCGTGGGGAGCAAACAGG` |
| ASV431 | 429 | `TGGGGAATATTGGACAATGGGCGCAAGCCTGATCCAGCCATGCCGCGTGTGTGAAGAAGGTCTTCGGATTGTAAAGCACTTTAAGTTGGGAGGAAGGGTTGTAGATTAATACTCTGCAATTTTGACGTTACCGACAGAATAAGCACCGGCTAACTCTGTGCCAGCAGCCGCGGTAATACAGAGGGTGCAAGCGTTAATCGGAATTACTGGGCGTAAAGCGCGCGTAGGTGGTTATGTAAGTTGGATGTGAAATCCCCGGGCTCAACCTGGGAACTGCATCCAAAACTGCATGGCTAGAGTACGGTAGAGGGTGGTGGAATTTCCTGTGTAGCGGTGAAATGCGTAGATATAGGAAGGAACACCAGTGGCGAAGGCGACCACCTGGACCGATACTGACACTGAGGTGCGAAAGCGTGGGGAGCAAACAGG` |
| ASV125 | 404 | `TGGGGAATCTTAGACAATGGGGGAAACCCTGATCTAGCCATGCCGCGTGATCGATGAAGGCCTTAGGGTTGTAAAGATCTTTCAGTGGGGAAGATAATGACGGTACCCACAGAAGAAGCCCCGGCTAACTCCGTGCCAGCAGCCGCGGTAATACGGAGGGGGCTAGCGTTGTTCGGAATTACTGGGCGTAAAGCGCACGTAGGCGGACCGGAAAGTCAGAGGTGAAATCCCAGGGCTCAACCTTGGAACTGCCTTTGAAACTCCTGGTCTTGAGGTCGAGAGAGGTGAGTGGAATTCCGAGTGTAGAGGTGAAATTCGTAGATATTCGGAGGAACACCAGTGGCGAAGGCGGCTCACTGGCTCGATACTGACGCTGAGGTGCGAAAGCGTGGGGAGCAAACAGG` |
| ASV4728 | 404 | `TAGGGAATATTGGGCAATGGGCGAAAGCCTGACCCAGCCATGCCGCGTGAATGATGAAGGCCTTAGGGTTGTAAAGTTCTTTCGCCGATGAAGATAATGACGGTAGTCGGATAAGAAGCCCCGGCTAACTTCGTGCCAGCAGCCGCGGTAATACGAAGGGGGCAAGCGTTGTTCGGAATTACTGGGCGTAAAGCGCATGTAGGCGGTCCTACAAGCCAGAGGTGAAATCCCGGAGCTCAACTTCGGAATTGCCTTTGGAACTGTAGGACTGGAGTCTCGGAGGGGATGGTGGAATTCCAAGTGTAGAGGTGAAATTCGTAGATATTTGGAGGAACACCGGTGGCGAAGGCGACCATCTGGACGAGTACTGACGCTGAGATGCGAAAGCGTGGGGAGCAAACAGG` |
| ASV527 | 424 | `TAAGGAATATTGGACAATGGTCGAAAGACTGATCCAGCCATGCCGCGTGAAGGATGAAGGCCCTATGGGTTGTAAACTTCTTTTGTACGGGAAAAAACCTCTTTACGTGTAAAGAGCTGATTGTACTGTAAGAATAAGGATCGGCTAACTCCGTGCCAGCAGCCGCGGTAATACGGAGGATCCAAGCGTTATCCGGATTCATTGGGTTTAAAGGGTGCGTAGGCGGAATAATAAGTCAGTGGTGAAATCCTGCAGCTTAACTGCAGAACTGCCATTGATACTGTTAATCTTGAGTATATTTGATGTGGGCGGAATGTGTCATGTAGCGGTGAAATGCTTAGATATGACACAGAACACCGATTGCGAAGGCAGCTCACAAAACTATAACTGACGCTGAGGCACGAAAGCGTGGGTATCAAACAGG` |
| ASV2110 | 404 | `TGGGGAATCTTAGACAATGGGGGCAACCCTGATCTAGCCATGCCGCGTGATCGATGAAGGCCTTAGGGTTGTAAAGATCTTTCAGATGGGAAGATAATGACGGTACCATCAGAAGAAGCCCCGGCTAACTCCGTGCCAGCAGCCGCGGTAATACGGAGGGGGCTAGCGTTATTCGGAATTACTGGGCGTAAAGCGCACGTAGGCGGATGAGAAAGTCAGAGGTGAAATCCCAGGGCTCAACCCTGGAACGGCCTTTGAAACTCCTTGTCTTGAGGTCGTGAGAGGTGAGTGGAATTCCGAGTGTAGAGGTGAAATTCGTAGATATTCGGAGGAACACCAGTGGCGAAGGCGGCTCACTGGCACGATACTGACGCTGAGGTGCGAAAGCGTGGGGAGCAAACAGG` |
| ASV3947 | 420 | `TAAGGAATATTGCTCAATGGGAGAAATCCTGAAGCAGCAACGCCGCGTGGAGGATGAAATACTTTTGTATGTAAACTCCTGTAAGAAGGGAAAAATACACAGTTCTTCTGTGTTGATTGTACCTTCAGAGTAAGCCCCGGCTAACTACGTGCCAGCAGCCGCGGTAATACGTAGGGGGCAAGCGTTGTCCGGATTTACTGGGTGTAAAGGGTGCTCAGGTGGATTTGTAAGTCAGAGGTGAAATCCCAAAGCTTAACTTTGGTGCTGCCTTTGATACTGCAAGTCTTGAGTTTGAGAGAGGATAATGGAATATCTGGTGTAGCAGTGAAATGCGTAGATATCAGATAGAACACCAATGGCGAAGGCAGTTATCTGGCTCAAAACTGACGCTAAAGCACGAAAGTGTGGGGAGCAAACAGG` |
| ASV944 | 404 | `TGGGGAATATTGGACAATGGGGGCAACCCTGATCCAGCCATGCCGCGTGCGTGAAGAAGGCCTTAGGGTTGTAAAGCGCTTTTGTCAGGGAAGATAATGACTGTACCTGAAGAATAAGCCCCGGCTAACTCCGTGCCAGCAGCCGCGGTAAGACGGAGGGGGCTAGCGTTGTTCGGAATTACTGGGCGTAAAGGGCGCGTAGGCGGTTATGTGTGTCAGATGTGAAATCCCAAGGCTCAACCTTGGAACTGCATTTGAAACTGCATGACTAGAGTGAGTAAGAGGCGGACGGAATTCCGAGTGTAGCAGTGAAATGCGCAGATATTCGGAGGAACATCGGTGGCGAAGGCGGTTCGCTGGTACTCTACTGACGCTGAGGCGCGAAAGCGTGGGGAGCAAACAGG` |
| ASV4615 | 404 | `TGGGGAATATTGGACAATGGGGGCAACCCTGATCCAGCAATGCCGCGTGAGTGATGAAGGCCTTCGGGTTGTAAAGCTCTTTTGTTAGGGAAGATAATGACGGTACCTAACGAATAAGCCCCGGCTAACTTCGTGCCAGCAGCCGCGGTAATACGAAGGGGGCTAGCGTTGTTCGGAATGACTGGGCGTAAAGGGAGCGTAGGCGGTATGACACGTCTGATGTGAAAGTCCTGGGCTTAACCTGGGGACTGCATTGGATACGGTCGTACTAGAGGCGTAGAGGGGAAATTGGAATTGCGTGTGTAGAGGTGAAATTCGTAGATATACGCAAGAACACCAATGGCGAAGGCAAATTTCTGGCTACGATCTGACGCTAAGGCTCGAAAGCGTGGGGAGCAAACAGG` |
| ASV3667 | 423 | `TAGGGAATATTGGGCAATGGGCGAAAGCCTGACCCAGCCATGCCGCGTGCCGGAAGAAGGCCCTCTGGGTTGTAAACGGCTTTTGACGGGGAAGAAACGACTCTTGCGAGAGGCATTGACGGTACCCGCAGAATAAGCACCGGCTAACTCCGTGCCAGCAGCCGCGGTAATACGGAGGGTGCAAGCGTTGTCCGGATTTATTGGGTTTAAAGGGTGCGTAGGCGGTCTGGTAAGTCGGCGATGAAAGCCCGGTGCTCAACACCGGAACTGTCGTCGATACTGTCGGACTTGAGTATGGTGGAGGCTGGCGGAATGGGCGGTGTAGCGGTGAAATGCATAGATACCGTCCAGAACCCCGATTGCGAAGGCAGCTGGCTGTACCATAACTGACGCTGAGGCACGAAAGCGTGGGGATCGAACAGG` |
| ASV4392 | 424 | `TGAGGAATATTGGTCAATGGGCGAGAGCCTGAACCAGCCAAGTAGCGTGCAGGATGACGGCCCTATGGGTTGTAAACTGCTTTTATGCGGGGATAAAGTGTACCATGTATGGTGTTTTGCAGGTACCGCATGAATAAGGACCGGCTAATTCCGTGCCAGCAGCCGCGGTAATACGGAAGGTCCGGGCGTTATCCGGATTTATTGGGTTTAAAGGGAGCGTAGGCCGTTTTTTAAGCGTGTTGTGAAATGCCGCGGCTCAACCGTGGCACTGCAGCGCGAACTGGATGACTTGAGTGCGCGGGAGGTAGGCGGAATTCGTGGTGTAGCGGTGAAATGCTTAGATATCACGAAGAACTCCGATTGCGAAGGCAGCTTACCGTAGCGTTACTGACGCTGAAGCTCGAAAGTGCGGGTATCGAACAGG` |
| ASV5524 | 405 | `TGGGGAATATTGGACAATGGGGGCAACCCTGATCCAGCCATGCCGCGTGAATGATGAAGGCCTTCGGGTTGTAAAGTTCTTTTGGTGGGGACGATGATGACGGTACCCACAGAATAAGCTCCGGCTAACTTCGTGCCAGCAGCCGCGGTAATACGAAGGGAGCTAGCGTTGTTCGGAATTACTGGGCGTAAAGGGCGCGTAGGCGGTTAAACAAGTCAGGCGTGAAAGCCCCAAGCTCAACTTGGGAACTGCGCTTGAGACTGTTTTTCTAAGAGTACGGGAGAGGTGAGTGGAATTTCCAGTGTAGCAGTGAAATGCGTAGATATTGGAAGGAACACCAGTGGCGAAGGCGGCTCACTGGACCGTGACTGACGCTGAGGCGCGAAAGCGTGGGGAGCAAACAGG` |
| ASV5696 | 429 | `TGGGGAATTTTGGACAATGGGCGAAAGCCTGATCCAGCCATGCCGCGTGAGTGAAGAAGGCCTTCGGGTTGTAAAGCTCTTTCGGCGGGGAAGAAATCGCACTTTCTAATACAGGGTGTGGATGACGGTACCCGAATAAGAAGCACCGGCTAACTACGTGCCAGCAGCCGCGGTAATACGTAGGGTGCGAGCGTTAATCGGAATTACTGGGCGTAAAGCGTGCGCAGGCGGCTATACAAGACAGGCGTGAAATCCCCGGGCTTAACCTGGGAACTGCGTTTGTGACTGTATGGCTAGAGTACGGCAGAGGGGGGTGGAATTCCACGTGTAGCAGTGAAATGCGTAGAGATGTGGAGGAACACCGATGGCGAAGGCAGCCCCCTGGGCCAGTACTGACGCTCATGCACGAAAGCGTGGGGAGCAAACAGG` |
| ASV4962 | 408 | `TGGGGAATATTGGGCAATGGGCGAAAGCCTTACCCAGCAATGCCGCGTGAGTGAAGAAGGTCTTCGGATTGTAAAGCTCTTTGATTGGGGACGAACAAAATGACGGTACCCAAAGAACAAGCCCCGGCTAACTACGTGCCAGCAGCCGCGGTAATACGTAGGGGGCAAGCGTTGTCCGGAATGACTGGGCGTAAAGGGTGTGTAGGCGGGCCTGTAAGTTGGATGTGTAATACCCAGAGCTTAACTCGGGTGCTGCATCTGAAACTACAAGTCTTGAGTGTCGGAGAGGTAAGTGGAATTCCTAGTGTAGCGGTGGAATGCGTAGATATTAGGAGGAACATCAGTGGCGAAGGCGACTTACTGGACGATAACTGACGCTGAGGCACGAAAGCGTGGGGAGCAAACAGG` |
| ASV3211 | 404 | `TGGGGAATCTTGGACAATGGGCGCAAGCCTGATCCAGCCATGCCGCGTGAGTGACGAAGGCCTTAGGGTTGTAAAGCTCTTTTGCCGGGGACGATAATGACGGTACCCGGAGAATAAGTCCCGGCTAACTTCGTGCCAGCAGCCGCGGTAATACGAAGGGGACTAGCGTTGTTCGGAATCACTGGGCGTAAAGCGCACGTAGGCGGATGTGTCAGTCAGGGGTGAAATCCCGGAGCTCAACTTCGGAACTGCCTTTGATACAGCACGTCTTGAATCCGGTAGAGGTGAGTGGAATTCCTAGTGTAGAGGTGAAATTCGTAGATATTAGGAAGAACACCAGTGGCGAAGGCGGCTCACTGGACCGGTATTGACGCTGAGGTGCGAAAGCGTGGGGAGCAAACAGG` |
| ASV222 | 429 | `TGGGGAATTTTGGACAATGGGCGAAAGCCTGATCCAGCCATTCCGCGTGAGTGAAGAAGGCCTTCGGGTTGTAAAGCTCTTTCGCAAGGGAAGAAAGATTACTCTCTAACATAGAGTGATTATGACGGTACCTTGATAAGAAGCACCGGCTAACTACGTGCCAGCAGCCGCGGTAATACGTAGGGTGCGAGCGTTAATCGGAATTACTGGGCGTAAAGCGTGCGCAGGCGGTTTTGTAAGTCAGATGTGAAATCCCCGAGCTCAACTTGGGAACTGCGTTTGAAACTACAAGACTAGAATATGTCAGAGGGGGGTAGAATTCCACGTGTAGCAGTGAAATGCGTAGAGATGTGGAGGAATACCAATGGCGAAGGCAGCCCCCTGGGATAATATTGACGCTCATGCACGAAAGCGTGGGGAGCAAACAGG` |
| ASV1244 | 429 | `TGGGGAATTTTGGACAATGGGGGCAACCCTGATCCAGCCATGCCGCGTGCGGGAAGAAGGCCTTCGGGTTGTAAACCGCTTTTGTCAGGGAAGAAACGCTCTGAGTTAATACCTCGGGGTAATGACGGTACCTGAAGAATAAGCACCGGCTAACTACGTGCCAGCAGCCGCGGTAATACGTAGGGTGCAAGCGTTAATCGGAATTACTGGGCGTAAAGCGTGCGCAGGCGGTTATGCAAGACAGATGTGAAATCCCCGGGCTCAACCTGGGAACTGCATTTGTGACTGCATAGCTGGAGTGCGGCAGAGGGGGATGGAATTCCGCGTGTAGCAGTGAAATGCGTAGATATGCGGAGGAACACCGATGGCGAAGGCAATCCCCTGGGCCTGCACTGACGCTCATGCACGAAAGCGTGGGGAGCAAACAGG` |
| ASV554 | 429 | `TGGGGAATTTTGGACAATGGGCGCAAGCCTGATCCAGCAATGCCGCGTGAGTGAAGAAGGCCTTCGGGTTGTAAAGCTCTTTTGTCAGGGAAGAAACGGGTTTTTCTAATACAGAGATCTAATGACGGTACCTGAAGAATAAGCACCGGCTAACTACGTGCCAGCAGCCGCGGTAATACGTAGGGTGCAAGCGTTAATCGGAATTACTGGGCGTAAAGCGTGCGCAGGCGGTTTTGTAAGACTGTCGTGAAATCCCCGGGCTCAACCTGGGAATGGCGATGGTGACTGCAAGGCTAGAGTTTGGCAGAGGGGGGTAGAATTCCACGTGTAGCAGTGAAATGCGTAGATATGTGGAGGAACACCGATGGCGAAGGCAGCCCCCTGGGTCAAAACTGACGCTCATGCACGAAAGCGTGGGGAGCAAACAGG` |
| ASV2280 | 429 | `TGGGGAATATTGCACAATGGGCGCAAGCCTGATGCAGCCATGCCGCGTGTATGAAGAAGGCCTTCGGGTTGTAAAGTACTTTCAGTGGGGAGGAAGGCGATGGAGTTAATAGCTCCGTCGATTGACGTTACCCGCAGAAGAAGCACCGGCTAACTCCGTGCCAGCAGCCGCGGTAATACGGAGGGTGCAAGCGTTAATCGGAATTACTGGGCGTAAAGCGCACGCAGGCGGTCTGTCAAGTCGGATGTGAAATCCCCGGGCTCAACCTGGGAACTGCATTCGAAACTGGCAGACTAGAGTCTTGTAGAGGGGGGTAGAATTCCAGGTGTAGCGGTGAAATGCGTAGAGATCTGGAGGAATACCGGTGGCGAAGGCGGCCCCCTGGACAAAGACTGACGCTCAGGTGCGAAAGCGTGGGGAGCAAACAGG` |
| ASV1514 | 423 | `TAGGGAATATTGGTCAATGGGCGAAAGCCTGAACCAGCCATGCCGCGTGCAGGATGAAGGCCCTCAGGGTTGTAAACTGCTTTTGTACGGGAACAAAAAGCTCCTGCGGGAGCCATTGAGGGTACCGTAAGAATAAGCACCGGCTAACTCCGTGCCAGCAGCCGCGGTAATACGGAGGGTGCAAGCGTTATCCGGATTTACTGGGTTTAAAGGGTGCGTAGGCGGCTCTTTAAGTCAGTGGTGAAAGCCTGGCGCTCAACGCCAGAAATGCCATTGATACTGTGGAGCTTGAGTCAAGAAGAGGTAGGCAGAATTCATGGTGTAGCGGTGAAATGCATAGATATCATGAGGAATTCCAACAGCGAAGGCAGCTTACTGGTCTTTGACTGACGCTGAGGCACGAAAGCGTGGGGAGCAAACAGG` |
| ASV3369 | 429 | `TGGGGAATTTTGGACAATGGGCGCAAGCCTGATCCAGCCATTCCGCGTGAGTGAAGAAGGCCTTCGGGTTGTAAAGCTCTTTCGCAAGGGAAGAAACGTTATGGGCGAATAACTCATGATAATGACGGTACCTTGATAAGAAGCACCGGCTAACTACGTGCCAGCAGCCGCGGTAATACGTAGGGTGCGAGCGTTAATCGGAATTACTGGGCGTAAAGCGTGCGCAGGCTGTTTTGTAAGTCAGATGTGAAATCCCTGAGCTCAACTTAGGAACTGCGTTTGAAACTACAAGACTAGAATATGTCAGAGGGGGGTAGAATTCCACGTGTAGCAGTGAAATGCGTAGAGATGTGGAGGAATACCAATGGCGAAGGCAGCCCCCTGGGATAATATTGACGCTCATGCACGAAAGCGTGGGGAGCAAACAGG` |
| ASV4239 | 404 | `TGAGGAATCTTGGACAATGGGGGAAACCCTGATCCAGCCATGCCGCGTGAGTGATGAAGGCCTTCGGGTTGTAAAGCTCTTTCAGATGTGAAGATGATGACGGTAGCATCAGAAGAAGCTCCGGCTAACTCCGTGCCAGCAGCCGCGGTAATACGGAGGGAGCGAGCGTTGTTCGGAATTACTGGGCGTAAAGCGTATGTAGGCGGCTTCGTAAGACAGGTGTGAAATCCCAGAGCTTAACTTTGGAACTGCATTTGTGACTGCGAGGCTTGAGTATCGGAGAGGTTGGCGGAATTCCCAGTGTAGAGGTGAAATTCGCAGATATTGGGAGGAACACCGATGGCGTAAGCAGCCAACTGGACGATTACTGACGCTGAGATACGAAAGCGTGGGGAGCAAACAGG` |
| ASV2450 | 404 | `TCGGGAATATTGGACAATGGGCGAAAGCCTGATCCAGCGACGCCGCGTGTGTGATGAAGGCCTTCGGGTTGTAAAGCACTTTTGGCAGGGAAGATAATGACGGTACCTGCAGAATAAGCCCCGGCTAACTTCGTGCCAGCAGCCGCGGTAATACGAAGGGGGCTAGCGTTGTTCGGAATTACTGGGCGTAAAGCGTGTGTAGGCTGTGCTGATAGTTAGGTGTGAAATCCCCGGGCTCAACCCGGGAATTGCACTTAATACTACAGTGCTAGAGTATGGTAGAGGATAGTGGAATACCCAGTGTAGAGGTGAAATTCGTAGATATTGGGTGGAACATCGATGGCGAAGGCAGCTATCTGGGCCATTACTGACGCTGAGACACGAAAGCGTGGGGATCAAACAGG` |
| ASV3166 | 416 | `TTAGGAATCTTCCCCAATGGGCGCAAGCCTGAGGGAGCGACGCCGCTTGAAGGACGAAGGTCTTCGGATTGTAAACTTCTGAAACAAGGACGAAATCCTGGAAACAGGTCTGACGGTACTTGTGTAATAGCACCGGCTAACTCCGTGCCAGCAGCCGCGGTAATACGGAGGGTGCAAGCGTTACCCGGAATCACTGGGCGTAAAGGGCGTGTAGGCGGCTTGGTAAGTCTGGATTTAAAGACCGAAGCTCAACTTCGGGAATGGTCTGGATACTGCCATGCTAGACGACTGGAGGGGAGAGTGGAATTCTCGGTGTAGCGGTGGAATGCGTAGATACCGAGAGGAACACCAGTGGCGAAGGCGGCTCTCTGGACAGTGCGTGACGCTGAGGCGCGAAAGTGTGGGGAGCGAACCGG` |
| ASV296 | 424 | `TGAGGAATATTGGTCAATGGACGAGAGTCTGAACCAGCCAAGTAGCGTGCAGGATGACGGCCCTATGGGTTGTAAACTGCTTTTATTCGGGAATAAAAGAGACTACGTGTAGTCTATTGCATGTACCGAAAGAAAAAGGACCGGCTAATTCCGTGCCAGCAGCCGCGGTAATACGGAAGGTCCAGGCGTTATCCGGATTTATTGGGTTTAAAGGGAGCGTAGGCGGACTTTTAAGTCAGCTGTGAAATACCTCGGCTTAACCGAGGCACTGCAGTTGATACTGGGAGTCTTGAGTGCACATAAGGATAGTGGAATTCGTGGTGTAGCGGTGAAATGCTTAGATATCACGAAGAACTCCGATTGCGAAGGCAGCTATCTGGGGTGTAACTGACGCTGAGGCTCGAAAGTGTGGGTATCAAACAGG` |
| ASV1208 | 404 | `TGGGGAATATTGGACAATGGGCGAAAGCCTGATCCAGCAATGCCGCGTGAGTGAAGAAGGCCTTCGGGTTGTAAAGCTCTTTTACCCGGGAAGATAATGACTGTACCGGGAGAATAAGCCCCGGCTAACTCCGTGCCAGCAGCCGCGGTAATACGGAGGGGGCTAGCGTTGTTCGGAATTACTGGGCGTAAAGCGCACGTAGGCGGCTTTTTAAGTCAGAGGTGAAAGCCCGGGGCTCAACTCCGGAATTGCCTTTGAAACTGGAAAGCTTGAACACGGGAGAGGTGAGTGGAATTCCGAGTGTAGAGGTGAAATTCGTAGATATTCGGAGGAACACCAGTGGCGAAGGCGGCTCACTGGACCGTTGTTGACGCTGAGGTGCGAAAGCGTGGGGAGCAAACAGG` |
| ASV3139 | 429 | `TGGGGAATTTTGGACAATGGGCGAAAGCCTGATCCAGCAATGCCGCGTGTGCGAAGAAGGCCTTCGGGTTGTAAAGCACTTTTGTTTGGGAAGAAAATGTTCGTGTTAATACCATGAGCAGATGACGGTACCAAAAGAATAAGCACCGGCTAACTACGTGCCAGCAGCCGCGGTAATACGTAGGGTGCGAGCGTTAATCGGAATTACTGGGCGTAAAGCGTGCGCAGGCGGCTATGTAAGACAGGTGTGAAATCCCCGAGCTTAACTTGGGAACTGCATTTGTGACTGCATGGCTAGAGTGTGTCAGAGGGGGGTGGAATTCCACGTGTAGCAGTGAAATGCGTAGATATGTGGAGGAACACCAATGGCGAAGGCAGCCCCCTGGGATAACACTGACGCTCATGCACGAAAGCGTGGGGAGCAAACAGG` |
| ASV675 | 429 | `TCGAGAATAATTCACAATGGGGGCAACCCTGATGGTGCAACGCCGCGTGGAGGATGACGGTCTTCGGATTGTAAACTCCTGTCATCTGGGAGTAAGGCATGGTGGTAAATAGCCAACATGATTGATAGTACCAGAAGAGGAAGGGACGGCTAACTTCGTGCCAGCAGCCGCGGTAATACGAAGGTCCCAAGCGTTGTTCGGAATCACTGGGCGTAAAGGGAGCGTAGGCGGCGTGGTAAGTCAGATGTGAAATCCCGGGGCTCAACCCCGGAACTGCATCCGATACTGCCATGCTAGAGGATTGGAGAGGTAGCTGGAATTCTTGGTGTAGCAGTGAAATGCGTAGAGATCAAGAGGAACACTCGTGGCGAAAGCGAGCTACTGGACAATTCCTGACGCTGAGGCTCGAAGGCTAGGGTAGCGAAAGGG` |
| ASV1202 | 409 | `TGGGGAATATTGCACAATGGGCGGAAGCCTGATGCAGCGACGCCGCGTGAGGGATGACGGCCTTCGGGTTGTAAACCTCTTTCAGCAGGGACGAAGCGTAAGTGACGGTACCTGCAGAAGAAGCACCGGCTAACTATGTGCCAGCAGCCGCGGTAATACGTAGGGTGCGAGCGTTGTCCGGAATTACTGGGCGTAAAGAGCTCGTAGGCGGTTTGTCACGTCGTCTGTGAAATCCTAGGGCTTAACCCTGGACGTGCAGGCGATACGGGCTGACTTGAGTACTACAGGGGAGACTGGAATTTCTGGTGTAGCGGTGGAATGCACAGATATCAGGAAGAACACCGATGGCGAAGGCAGGTCTCTGGGTAGTAACTGACGCTGAGGAGCGAAAGCATGGGTAGCGAACAGG` |
| ASV1917 | 404 | `TGGGGAATATTGGACAATGGGCGAAAGCCTGATCCAGCCATGCCGCGTGAATGATGAAGGCCTTAGGGTTGTAAAGTTCTTTCGTTGGTGAAGATAATGACGGTAACCAAAGAAGAAGCCCCGGCTAACTTCGTGCCAGCAGCCGCGGTAATACGAAGGGGGCTAGCGTTGTTCGGAATCACTGGGCGTAAAGCGCGCGTAGGCGGTCATGCAAGTCAGAGGTGAAATGCCGGGGCTTAACCCCGGAACTGCCTTTGAAACTGCATGACTGGAATCTTGGAGGGGGTAGTGGAATTCCTAGTGTAGAGGTGAAATTCGTAGATACTAGGAGGAACACCGGTGGCGAAGGCGGCTACCTGGACAAGTATTGACGCTGAGGCGCGAAAGCGTGGGGAGCAAACAGG` |
| ASV4051 | 404 | `TGGGGAATATTGGACAATGGGCGAAAGCCTGATCCAGCAATACCGCGTGAGTGATGAAGGTCTTCGGATTGTAAAGCTCTTTTAGTAGGGAAGATAATGACGGTACCTACAGAAAAAGCACCGGCAAACTTCGTGCCAGCAGCCGCGGTAATACGAAGGGTGCTAGCGTTGTTCGGAATCATTGGGCGTAAAGCGTACGTAGGCGGCTTAGTAAGTCGATTGTGAAAGCCCAGGGCTTAACCTTGGAATTGCAATCGAAACTGCTTCGCTAGAATTTGGTCGGGGATGGTGGAATTCCTAGTGTAGGGGTGAAATCCGTAGAGATTAGGAGGAATATCGGTGGCGAAAGCGACCATCTAGGCCAACATTGACGCTGAGGTACGAAAGCGTGGGGAGCAAACAGG` |
| ASV2776 | 429 | `TGGGGAATTTTGGACAATGGGCGCAAGCCTGATCCAGCCATGCCGCGTGCGGGAAGAAGGCCTTCGGGTTGTAAACCGCTTTTGTCAGGGAAGAAACGGTTCGAGCTAATACCTTGAGCTAATGACGGTACCTGAAGAATAAGCACCGGCTAACTACGTGCCAGCAGCCGCGGTAATACGTAGGGTGCGAGCGTTAATCGGAATTACTGGGCGTAAAGCGTGCGCAGGCGGCTTTGCAAGACAGATGTGAAATCCCCGGGCTTAACCTGGGAACTGCATTTGTGACTGCAAGGCTGGAGTGTGGCAGAGGGGGATGGAATTCCGCGTGTAGCAGTGAAATGCGTAGATATGCGGAGGAACACCGATGGCGAAGGCAATCCCCTGGGCCTGCACTGACGCTCATGCACGAAAGCGTGGGGAGCAAACAGG` |
| ASV2661 | 404 | `TGGGGAATCTTGCGCAATGGGCGAAAGCCTGACGCAGCCATGCCGCGTGAATGATGAAGGTCTTAGGATTGTAAAATTCTTTTAGCAGGGACGATAATGACGGTACCTGCAGAAAAAGCCCCGGCTAACTTCGTGCCAGCAGCCGCGGTAATACGAAGGGGGCTAGCGTTGCTCGGAATTACTGGGCGTAAAGGGAGCGTAGGCGGGTTATCAAGTTGGAGGTGAAAGCCCAGGGCTCAACCCTGGAATTGCCTTCAAAACTGATAGCCTTGAGTATGGAAGAGGTAAGTGGAACTCCGAGTGTAGAGGTGAAATTCGTAGATATTCGGAAGAACACCAGTGGCGAAGGCGACTTACTGGTCCATGACTGACGCTGAGGCTCGAAAGCGTGGGGAGCAAACAGG` |
| ASV2507 | 387 | `GCGAGAAACCTTTGCAATGTGCGAAAGCACGACAAGGTTAATCCGAGTGTCTTCTGCTAAAGAAGTCTTTTGTTAGTCATAGAACCACTGATGAATAAGGGGTGGGCAAGTTCTGGTGTCAGCCGCCGCGGTAAAACCAGCACCTCGAGTGGTCAGGAGGTTTATTGGGCCTAAAGCATCCGTAGCCGGTTGTTTAAGTTTTCGGTTAAATCTATGCGCTTAACGTATAGGCTGCCGAAAATACTGGACAGCTAGGGAGTGGGAGAGGTACACGGTACTCAATAGGAAGGGGTAAAATCCTTTGATCTGTTGATGACCACCTGTGGCGAAGGCGGTGTACCAGAACACGTTCGACGGTGAGGGATGAAAGCTGGGGGAGCAAACCGG` |
| ASV3071 | 404 | `TGGGGAATATTGGACAATGGGGGAAACCCTGATCCAGCCATGCCGCGTGAGTGATGAAGGCCTTAGGGTTGTAAAGCTCTTTTACCAGGGAAGATGATGACGGTACCTGGAGAATAAGCACCGGCTAACTTCGTGCCAGCAGCCGCGGTAATACGAAGGGTGCTAGCGTTGTTCGGAATCACTGGGCGTAAAGCGTGCGTAGGCGGCTTTGCAAGCCAGAGGTGAAAGCCTGGAGCTTAACTCCAGAATTGCCTTTGGAACTGCATGGCTAGAGTGTCGGAGGGGATAGCGGAATTGCTAATGTAGAGGTGAAATTCGTAGATATTAGCAGGAACACCGGTGGCGAAGGCGGCTATCTGGACGACAACTGACGCTGAGGCACGAAAGCGTGGGGATCAAACAGG` |
| ASV4807 | 421 | `TGAGGAATATTGGTCAATGGGAGCAATCCTGAACCAGCAATGCCGCGTGTGGGATGAAGGGGCTTCGCCTCGTAAACCACTGTCGGACGGGACGAAAAGAGGTAGTAACCTCTGGGACGGTACCGTCAAAGGAAGGGTCGGCTAACTACGTGCCAGCAGCCGCGGTAATACGTAGGACCCAAGCGTTGTCCGGAATCACTGGGTATAAAGGGTGCGTAGGCGGCCAAGTGCGTCAGAGGTGAAATATCCGGGCTTAACCCGGAGGGTGCCTTTGATACGGCTTGGCTTGAGTGCAGGAGAGGATGATGGAATTTCTGGTGTAGCGGTGAAATGCGTAGATATCAGAAGGAACACCGGTGGCGAAGGCGGTCATCTGGCCTGCAACTGACGCTGAGGCACGAAAGCGCGGGGATCAAACAGG` |
| ASV298 | 404 | `TGGGGAATATTGGACAATGGGGGCAACCCTGATCCAGCAATACCGCGTGAGTGAAGAAGGCCTTCGGGTTGTAAAGCTCTTTTACCGGGGAAGATAATGACGGTACCCGGGGAATAAGCTCCGGCTAACTTCGTGCCAGCAGCCGCGGTAATACGAAGGGAGCTAGCGTTGTTCGGAATTACTGGGCGTAAAGGGCGCGTAGGCGGCTCTGTAAGTCAGATGTGAAAGCCCCGGGCTCAACCTGGGAACTGCATTTGATACTGCAAGGCTAGAGATCGAGAGAGGAAAGTGGAATTACGAGTGTAGAAGTGAAATTCGTAGATATTCGTAGGAACACCAGTGGCGAAGGCGACTTTCTGGCTCGATACTGACGCTAAAGCGCGAAAGCGTGGGGAGCAAACAGG` |
| ASV5731 | 428 | `TGGGGAATCTTGCGCAATGGACGAAAGTCTGACGCAGCGACGCCGCGTGGGTGATGAAGGCCTTCGGGTTGTAAAGCCCTGTGGGGAGAGACGAATAAGTGCGGGCTAACATCTTGCATGATGACGGTATCTCCTTAGCAAGCACCGGCTAACTCTGTGCCAGCAGCCGCGGTAAGACAGAGGGTGCAAACGTTGTTCGGAATTACTGGGCGTAAAGCGTGTGTAGGCGGTTCGCAAAGTCGGATGTGAAAGCCCCGGGCTCAACCTGGGAAGTGCACTCGATACTGGCGAACTTGAGTATTGGAGAGGTCGGTGGAATTCTCGGTGGAGAGGTGAAATTCGTAGATATCGAGGGGAACACCAGTGGCGAAGGCGGCCGACTGGACAAATACTGACGCTGAGACACGAAAGCGTGGGGAGCAAACAGG` |
| ASV4210 | 405 | `TGGGGAATATTGGACAATGGGCGAAAGCCTGATCCAGCAATGCCGCGTGAGTGATGAAGGCCCTCTGGGTTGTAAAGCTCTTTTATTAGAGAAGATAATGACGGTATCTAATGAATAAGCACCGACTAACTACGTGCCAGCAGTCGCGGTAATACGTAGGGTGCTAGCGTTACTCGGATTTACTGGGCGTAAAGCACACGTAGGTGGATTTGTAAGACAGAAGTGCAATCCCTAGGCTCAACCTAGGAACTGCTTTTGTAACTGCTTATCTGGAGTGCGGTAGGGGATAATGGAATTTCTAGTGGAGGGGTAAAATCCGTAGATATTAGAAGGAACACCAAAAGCGAAAGCAATTATCTGGGCCGTTACTGACACTGAGGTGTGAAAGCGTGGGGAGCAAACAGG` |
| ASV4580 | 424 | `TGAGGAATATTGGTCAATGGGCGAGAGCCTGAACCAGCCAAGTAGCGTGAAGGATGACTGCCCTATGGGTTGTAAACTTCTTTTATACGGGAATAACACGCGGCACGTGTGCCGTTCTGCATGTACCGTATGAATAAGCATCGGCTAACTCCGTGCCAGCAGCCGCGGTAATACGGAGGATGCGAGCGTTATCCGGATTTATTGGGTTTAAAGGGAGCGTAGACGGGTCCTTAAGTCAGTTGTGAAAGTTTGCGGCTCAACCGTAAAATTGCAGTTGATACTGGGGACCTTGAGTGCGGCAGAGGCAGGCGGAATTCGTGGTGTAGCGGTGAAATGCTTAGATATCACGAAGAACTCCGATTGCGAAGGCAGCTTGCTGGACCGTAACTGACGTTGATGCTCGAAAGTGCGGGTATCAAACAGG` |
| ASV2017 | 429 | `TGGGGAATTTTGGACAATGGGCGCAAGCCTGATCCAGCCATGCCGCGTGCGGGAAGAAGGCCTTCGGGTTGTAAACCGCTTTTGTCAGGGAAGAAATCCTTTGAGTTAATACCTCGGGGGGATGACGGTACCTGAAGAATAAGCACCGGCTAACTACGTGCCAGCAGCCGCGGTAATACGTAGGGTGCAAGCGTTAATCGGAATTACTGGGCGTAAAGCGTGCGCAGGCGGTTTTGTAAGACAGAGGTGAAATCCCCGGGCTTAACCTGGGAACTGCCTTTGTGACTGCAAAGCTTGAGTGCGGCAGAGGGGGATGGAATTCCGCGTGTAGCAGTGAAATGCGTAGATATGCGGAGGAACACCGATGGCGAAGGCAATCCCCTGGGCCTGCACTGACGCTCATGCACGAAAGCGTGGGGAGCAAACAGG` |
| ASV5322 | 404 | `TGGGGAATATTGGACAATGGGGGAAACCCTGATCCAGCGATGCCGCGTGAGTGAAGAAGGCCCTAGGGTTGTAAAGCTCTTTTAGTGGGGAAGATAATGACGGTACCCACAGAAAATGCACCGGCAAACTCTGTGCCAGCAGCCGCGGTAATACAGAGGGTGCAAGCGTTGTTCGGAATTACTGGGCGTAAAGAGCGCGTAGGCGGTTTTATAAGTCAGACGTGAAAGCCCTGGGCTTAACCCAGGAGGTGCGTTTGAAACTGTAGAGCTAGAGGACGAGAGAGGTGAGTGGAATTCCTAGTGTAGAAGTGAAATTCGTAGATATTAGGAGGAACATTAGTGGCGAAGGCGGCTCACTGGCTCGTAACTGACGCTGAGGCGCGAAAGCGTGGGGAGCAAACAGG` |
| ASV180 | 418 | `TGGGGAATTTTCCGCAATGGGCGAAAGCCTGACGGAGCAAGACCGCGTGGGGGAAGAAGGCTCTTGGGTTGTAAACCCCTTTTCTCTGGGAAGAAAGTTGTGAAAGCAGCCTGACGGTACCAGAGGAATCAGCATCGGCTAACTCCGTGCCAGCAGCCGCGGTAAGACGGAGGATGCAAGCGTTATCCGGAATGATTGGGCGTAAAGCGTCCGCAGGTGGCAGTTCAAGTCTGCTGTCAAAGACCGGGGCTTAACCTCGGAAAGGCAGTGGAAACTGAACAGCTAGAGTATGGTAGGGGCAAAGGGAATTCCTGGTGTAGCGGTGAAATGCGTAGAGATCAGGAAGAACATCGGTGGCGAAGGCGCTTTGCTGGACCATAACTGACACTCAGGGACGAAAGCTAGGGGAGCGAATGGG` |
| ASV5564 | 425 | `TGGGGAATATTGCGCAATGGGCGAAAGCCTGACGCAGCCACGCCGCGTGAGCGATGAAGGCCTTCGGGTCGTAAAGCTCTGTGGGGAGAGAAGAATAAAGCCTGTGAAGAGCGGGCCTTGACGGTATCTCCTTAGCAAGCACCGGCTAACTCCGTGCCAGCAGCCGCGGTAATACGGAGGGTGCAAACGTTGCTCGGAATCATTGGGCGTAAAGCGCACGTAGGCGGCGAAGCAAGTCGGGTGTGAAAGCCCTGGGCTCAACCCAGGAAGTGCATTCGAAACTATTTCGCTTGAATCTCGGAGGGGGTCAGAGAATTCCCGGTGTAGAGGTGAAATTCGTAGATATCGGGAGGAATACCAGTGGCGAAGGCGCTGACCTGGACGAAGATTGACGCTGAGGTGCGAAAGCGCGGGGAGCAAACAGG` |
| ASV4157 | 405 | `TGGGGAATTTTCCGCAATGGGCGAAAGCCTGACGGAGCAATACCGCGTGAGGGAGGAAGGCTCTTGGGTTGTAAACCTCAAAACTTAGGGAAGAAGAAAGTGACGGTACCTAATATAAGCATCGGCTAACTCCGTGCCAGCAGCCGCGGTAATACGGAGGATGCAAGCGTTATCCGGAATCATTGGGCGTAAAGAGTCTGTAGGTGGCATTTAAAGTCTGCTGTCAAAGACCGAAGCTCAACTTCGGGCCGGCGGTAGAAACTGAAAAGCTAGAGTATAGTAGGGGTAGAGGGAATTCCTAGTGTAGCGGTGAAATGCGTAGAGATTAGGAAGAACACCAGTGGCGAAGGCGCTCTACTGGGCATATACTGACACTGAGAGACGAAAGCTAGGGTAGCGAAAGGG` |
| ASV208 | 429 | `TGGGGAATTTTGGACAATGGGCGCAAGCCTGATCCAGCAATGCCGCGTGCAGGATGAAGGCCTTCGGGTTGTAAACTGCTTTTGTACGGAACGAAACGGCTCTGGTTAATACCCGGAGCTAATGACGGTACCGTAAGAATAAGCACCGGCTAACTACGTGCCAGCAGCCGCGGTAATACGTAGGGTGCAAGCGTTAATCGGAATTACTGGGCGTAAAGCGTGCGCAGGCGGTTATGTAAGACAGTCGTGAAATCCCCGGGCTCAACCTGGGAACTGCGATTGTGACTGCATAGCTAGAGTACGGTAGAGGGGGGTGGAATTCCGCGTGTAGCAGTGAAATGCGTAGATATGCGGAGGAACACCGATGGCGAAGGCAGCCCCCTGGACCTGTACTGACGCTCATGCACGAAAGCATGGGGAGCAAACAGG` |
| ASV542 | 404 | `TGGGGAATATTGGACAATGGGCGAAAGCCTGATCCAGCAATACCGCGTGAGTGATGAAGGCCTTAGGGTTGTAAAGCTCTTTCAGTTGGAAAGATAATGACGGTACCAACAGAAGAAGCCCCGGCTAACTACGTGCCAGCAGCCGCGGTAAGACGTAGGGGGCTAGCGTTGTTCGGAATTACTGGGCGTAAAGAGCGCGTAGGCGGTCTGTTAAGTTGGAAGTGAAAGCCCGGGGCTTAACCCCGGAATTGCTTTCAAAACTGGCAGACTAGAGTGCGAGAGGGGGTGATGGAATTCCTAGTGTAGAGGTGAAATTCTTAGATATTAGGAGGAACACCGGTGGCGAAGGCGATCACCTGGCTCGATACTGACGCTGAGGCGCGAAAGCGTGGGGAGCAAACAGG` |
| ASV3918 | 429 | `TGGGGAATATTGGACAATGGGGGCAACCCTGATCCAGCAATGCCACGTGTGTGAAGAAGGCCTGAGGGTTGTAAAGCACTTTTGTTGGGGAAGAAGTTGATTAGGTTAAGAGCTAAATCAATTGACATTACCCAAAGAAAAAGCACCGGCTAACTCTGTGCCAGCAGCCGCGGTAATACAGAGGGTGCAAGCGTTAATCGGACTTACTGGGCGTAAAGGGCGCGTAGGTGGAATTTTAAGTCAGGTGTGAAATCCCAGGGCTCAACCTTGGAATTGCACTTGATACTGGAGTTCTGGAGTATGGTAGAGGGAAGGGGAATTTCCGGTGTAGCGGTGAAATGCGTAGATATCGGAAGGAACATCAGTGGCGAAGGCGCCTTCCTGGACCAATACTGACACTGAGGCGCGAAAGCGTGGGTAGCAAACAGG` |
| ASV4269 | 405 | `TAAAGAATCTTTCGCAATGGGGGCAACCCTGACGAAGCAACACCGCGTGGAGGATGAAGGCGTTTTCGTCGTAAACTCCTTTTCTGAGGGAATAACTTTGAAGGTACCTCAGGAATAAGCATCGGCTAACTACGTGCCAGCAGCCGCGGTAAGACGTAGGATGCGAGCGTTATCCGGAATTATTGGGTGTAAAGCGTTTGTAGGCGGTTTATTAAGTTGGGTGTTAAAGCTTTGAGCTTAACTCAAAATTGGTGCCCAAAACTGATAGGCTTGAGACAAGGAAAGGCAGATGGAATTCTGTATGTAGGGGTAATATCCGTAGATATACAGAGGAACACCAAAAGCGAAGGCAGTCTGCTGGCCTTGTTCTGACGCTGAGAAACGAAAGCGTGGGGATCAAACGGG` |
| ASV1381 | 429 | `TGGGGAATTTTGGACAATGGGGGGAACCCTGATCCAGCCATGCCGCGTGTCTGAAGAAGGCCTTCGGGTTGTAAAGGACTTTTGTTCGGGAGCAAAAGCTGCGGCCTAATACGTCGTGGTGATGAGAGTACCGAAAGAATAAGCACCGGCTAACTACGTGCCAGCAGCCGCGGTAATACGTAGGGTGCGAGCGTTAATCAGAATTACTGGGCGTAAAGCGTGCGCAGGCGGTTTATTAAGTTTGATGTGAAATCCCCGGGCTTAACCTGGGAACTGCATTGGATACTGGTAGACTAGAGTGCGTCAGAGGGGGGTAGAATTCCGCGTGTAGCAGTGAAATGCGTAGATATGCGGAGGAATACCGATGGCGAAGGCAGCCCCCTGGGATGACACTGACGCTCATGTACGAAAGCGTGGGTAGCAAACAGG` |
| ASV2524 | 429 | `TGGGGAATATTGGACAATGGGCGAAAGCCTGATCCAGCAATACCGCGTGTGTGAAGAAGGCCTGAGGGTTGTAAAGCACTTTCGTCGAGGAGGAAGTGTGTGTAGTTAATAGCTGCATGCATTGACGTTACTCGAATAAGAAGCACCGGCTAACTCTGTGCCAGCAGCCGCGGTAATACAGAGGGTGCAAGCGTTAATCGGAATTACTGGGCGTAAAGCGTGCGTAGGTGGATGCTTAAGTCAGATGTGAAAGCCCCGGGCTCAACCTGGGAATTGCATCTGATACTGGGTTTCTAGAGTATGGTAGAGGATGGCGGAATTTCCGGTGTAGCGGTGAAATGCGTAGATATCGGAAGGAACACCAGTGGCGAAGGCGGCCGTCTGGGCCAATACTGACACTGAGGCACGAAAGCGTGGGTAGCAAACAGG` |
| ASV2518 | 404 | `TGGGGAATATTGGACAATGGGCGCAAGCCTGATCCAGCCATGCCGCGTGGATGATGAAGGCCTTAGGGTTGTAAAGTCCTTTTAACGGGGAAGATAATGACGGTACCCGTAGAATAAGCCCCGGCTAACTTCGTGCCAGCAGCCGCGGTAATACGAAGGGGGCTAGCGTTGCTCGGAATCACTGGGCGTAAAGCGCACGTAGGCGGCTTTCTAAGTCAGGGGTGAAATCCTGGAGCTCAACTCCAGAACTGCCTTTGATACTGGAGAGCTTGAGTTCGGGAGAGGTGAGTGGAACTGCGAGTGTAGAGGTGAAATTCGTAGATATTCGCAAGAACACCAGTGGCGAAGGCGGCTCACTGGCCCGATACTGACGCTGAGGTGCGAAAGCGTGGGGAGCAAACAGG` |
| ASV5191 | 422 | `TAGGGAATTTTGCGCAATGGGGGAAACCCTGACGCAGCAACGCCATGTGTGGGATGAAGCATTTCGGTGTGTAAACCACTGTCGGCAGGGAATAAAGCCCACATTTATGTGGGGGTGAATGTACCTGCAGAGGAAGCCCCGGCTAACTTCGTGCCAGCAGCCGCGGTAATACGAGGGGGGCAAGTGTTGTTCGGAATCACTGGGCGTAAAGGGAGCGTAGGCGGATGTTTAAGTTGGAGGTTTAAAACCGGGGCCCAACCTCGGAGCGGCCTCCAAAACTGGACGTCTTGAATGGAACAGAGGCTAGTGGAATTGGGGGTGTAGCGGTGGAATGCGTAGAGATCCCCAAGAACACCAATTGCGAAGGCAGCTAGCTGGGTTCACATTGACGCTGAGGCTCGAAAGCATGGGGAGCAAACAGG` |
| ASV4160 | 429 | `TGGGGAATATTGGACAATGGGCGAAAGCCTGATCCAGCGACGCCGCGTGGGTGAAGAAGGCCTGCGGGTTGTAAAGCCCTTTCGGTGGGGATAAAAAGTCTTGGTCTAACACACCAGGGTCTTGATCTAACCCAAGAAAGAAGCACCGGCTAACTCTGTGCCAGCAGCCGCGGTAATACAGAGGGTGCGAGCGTTAATCGGAATTACTGGGCGTAAAGCGTACGTAGGTGGCTTTGCAAGTCGATTGTGAAATCCCCGGGCTTAACCTGGGAACTGCAGTCGAGACTGCTTTGCTTGAGTATGGGAGAGGGAGGTGGAATTTCCGGTGTAGCGGTGAAATGCGTAGATATCGGAAGGAACATCAGTGGCGAAGGCGACCTCCTGGACCAATACTGACACTCAAGTACGAAAGCGTGGGGAGCAAACAGG` |
| ASV1969 | 429 | `TGGGGAATATTGCGCAATGGAGGAAACTCTGACGCAGCCATGCCGCGTGTGTGAAGAAGGCCTTCGGGTTGTAAAGCACTTTCAGCAAGGAGGAAAGGGTGTGCGTTAATAGCGTACATCTGTGACGTTACTTGCAGAAGAAGCACCGGCTAATTCAGTGCCAGCAGCCGCGGTAATACTGAAGGTGCAAGCGTTAATCGGAATTACTGGGCGTAAAGCGCGCGTAGGCGGCTTGTTAAGTCGGATGTGAAAGCCCTGGGCTCAACCTGGGAACTGCACTCGATACTGACAAGCTAGAGTATGGGAGAGGGCTGTGGAATTCCACGTGTAGCGGTGAAATGCGTAGATATGTGGAGGAACATCAGTGGCGAAGGCGGCAGCCTGGCCCAATACTGACGCTGAGGTGCGAAAGCGTGGGGAGCAAACAGG` |
| ASV2966 | 425 | `TAGGGAATATTGGGCAATGGGCGAAAGCCTGACCCAGCCATGCCGCGTGCAGGAAGACGGCCCTCTGGGTTGTAAACTGCTTTTGAATGGGAAGAAAGGTATGCTTGCGAGCGTAATTGACGGTACCATTAGAATAAGCCACGGCTAACTACGTGCCAGCAGCCGCGGTAATACGTAGGTGGCGAGCGTTATCCGGATTTATTGGGTTTAAAGGGTGCGTAGGCGGCTTTTTAAGTCGGTGGTTAAAGGTAGCAGCTCAACTGTTTTACATGCTGCCGATACTGAAGAGCTTGAGTTATTGGAAGGCAGATGGAATTCCTGGTGTAGCGGTGAAATGCATAGATACCAGGAGGAACACCGATTGCGAAGGCATTCTGCTGCAAATATACTGACGCTGAGGCACGAAAGCGTGGGGATCAAACAGG` |
| ASV3818 | 404 | `TGAGGAATATTGGACAATGGGGGAAACCCTGATCCAGCCATGCCGCGTGAGTGAAGAAGGCCTTCGGGTTGTAAAGCTCTTTCAGATGCGAAGATAATGACGGTACCATCAGAAGAAGCCCCGGCTAAATTCGTGCCAGCAGCCGCGGTAATACGAATGGGGCAAGCGTTGTTCGGAATAACTGGGCTTAAAGGGTGCGCAGGCGGTCTTGAAAGTCAGGAGTGAAAGCCTGGGGCTTAACCCCAGAATTGCTCTTGAAACTTCAAGACTAGAATCCGGGAGAGGTTGGGAGAATTCCTAGTGTAGAGGTGAAATTCGCAGATATTAGGAGGAATACCAGTGGCGAAGGCGCCCAACTGGACCGGTATTGACGCTCATGCACGAAAGCGTGGGGAGCAAACAGG` |
| ASV4291 | 403 | `TAATGAATCTTCCGCAATGCACGAAAGTGTGACGGAGCGACGCCGAGTGAAGGATGAAGTTCTTCGGAATGTAAACTTCTAAAACCTGCTAGAAAGTACTGATCGGCAGGCTATAAGTAGCGACTAACTCTGTGCCAGCAGTCGCGGTAATACAGAGGCTACGAGCGTTAGGCGGAATCACTGGGCTTAAAGCGTGTGTAGGTGGATTGGTAAGTATCTTGTGAAATCCCACAGCTCAACTGTGGAACTGCTGGATATACTGCCAGTCTTGAGGCACTCAGGGGCGAGCGGAACAGATGGTGGAGCGGTGAAATGCATAGATATCATCTGGAACGCCAATGGTGAAGACAGCTCGCTGGGGGTGTACTGACACTGAGACACGAAAGCCAGGGGAGCAAACGGG` |
| ASV2310 | 431 | `TGGGGAATATTGGACAATGGGGGCAACCCTGATCCAGCAATGCCGCGTGTGTGAAGAAGGCCTGCGGGTTGTAAAGCACTTTTGTTGGGGAAGAAATCGTAGTTAGTAATATTGATTACGGATGACGGTACCCAAAGAATAAGCACCGGCTAACTTCGTGCCAGCAGCCGCGGTAATACGAAGGGTGCGAGCGTTAATCGGAATTACTGGGCGTAAAGCGTGCGTAGATGGCCTATTAAGTCGGATGTGAAAGCCCCGGGCTCAACCTGGGAACGGCATCCGATACTGATTGGGCTTTGAATCTAGGAGAGGGTGATGGAATTCCCGGTGTAGCGGTGAAATGCGTAGAGATCGGGAGGAACATCTGTGGCGAAGGCGGTCACCTGGCCTAAGATTGACGTTGATGCACGAAAGCGTGGGTAGCAAACAGG` |
| ASV4484 | 429 | `TGGGGAATTTTGGACAATGGGCGAAAGCCTGATCCAGCAATGCCGCGTGCAGGATGAAGGCCTTCGGGTTGTAAACTGCTTTTGTACAGAACGAAACGGCTTCCATTAATACTGGAGGCTAATGACGGTACTGTAAGAATAAGCACCGGCTAACTACGTGCCAGCAGCCGCGGTAATACGTAGGGTGCAAGCGTTAATCGGAATTACTGGGCGTAAAGCGTGCGCAGGCGGTCTTGTAAGACAGGTGTGAAATCCCCGGGCTTAACCTGGGAACTGCACTTGTGACTGCAAGGCTAGAGTACGGTAGAGGGGGGTGGAATTCCGCGTGTAGCAGTGAAATGCGTAGATATGCGGAGGAACACCGATGGCGAAGGCAGCCCCCTGGACCTGTACTGACGCTCATGCACGAAAGCATGGGGAGCAAACAGG` |
| ASV5444 | 404 | `TTAGGAATCTTGCGCAATGGGGGAAACCCTGACGCAGCCATGCCGCGTGAATGATGAAGGTCTTAGGATTGTAAAATTCTTTCAGCGGGGACGATAATGACGGTACCCGCAGAAGAAGCCCCGGCTAACTTCGTGCCAGCAGCCGCGGTAATACGAAGGGGGCTAGCGTTGCTCGGAATTACTGGGCGTAAAGGGCGCGTAGGCGGATGTTTAAGTCAGCGGTGAAAGCCCAGGGCTCAACCTTGGAACTGCCATTGATACTGGACATCTTGAGTACGGGAGAGGTGAGTGGAACACCGAGTGTAGAGGTGAAATTCGTAGATATTCGGTAGAACACCAGTGGCGAAGGCGACTCACTGGCCCGTTACTGACGCTGAGGCGCGAAAGCGTGGGGAGCAAACAGG` |
| ASV1542 | 429 | `TGGGGAATTTTGGACAATGGGCGCAAGCCTGATCCAGCCATTCCGCGTGAGTGAAGAAGGCCTTCGGGTTGTAAAGCTCTTTTGTCAGGGAAGAAAGCTTACCTTCGAATAAAGGGTGAGTATGACGGTATCTGACGAATAAGCACCGGCTAACTACGTGCCAGCAGCCGCGGTAATACGTAGGGTGCAAGCGTTAATCGGAATTACTGGGCGTAAAGCGAGCGCAGGCGGTTTTGTAAGTCAGATGTGAAATCCCCGGGCTTAACCTGGGAACTGCGTTTGAAACTGCAAGGCTAGAGTATGGGAGAGGGGGGTAGAATTCCACGTGTAGCAGTGAAATGCGTAGAGATGTGGAGGAATACCAATGGCGAAGGCAGCCCCCTGGCCTACTACTGACGCTCAGGCTCGAAAGCGTGGGGAGCAAACAGG` |
| ASV214 | 424 | `TGAGGAATATTGGTCAATGGGCGAGAGCCTGAACCAGCCAAGTCGCGTGAAGGATGAAGGATCTATGGTTCGTAAACTTCTTTTACAGGAGAATAAAGTGCAGGACGTGTCCTGTTTTGTATGTACCCTAGGAATAAGGATCGGCTAACTCCGTGCCAGCAGCCGCGGTAATACGGAGGATCCGAGCGTTATCCGGATTTATTGGGTTTAAAGGGTGCGTAGGTGGCTGTTTAAGTCAGCGGTGAAAGTTTGTGGCTCAACCATAAAATTGCCGTTGAAACTGGATAGCTTGAGTATATTTGAGGTAGGCGGAATGCGTGGTGTAGCGGTGAAATGCTTAGATATCACGCAGAACTCCAATTGCGAAGGCAGCTTACTAAACTATAACTGACACTGAAGCACGAAAGCGTGGGTATCAAACAGG` |
| ASV1073 | 428 | `TTTCGAATCATTCACAATGGGCGAAAGCCTGATGGTGCGACGCCGCGTGAGGGATGAAGGTCTTCGGATTGTAAACCTCTGTCACCGGGGAAGAAACGCTTCATTTTAACAGAATGAAGCCTGACTTAACCCGGAGAGGAAGCAGTGGCTAACTCTGTGCCAGCAGCCGCGGTAATACAGAGACTGCAAGCGTTATTCGGATTCACTGGGCGTAAAGGGTGCGCAGGCGGTTGGGTGTGTCAGATGTGAAATCCCGAGGCTCAACCTCGGAACTGCGTCTGAAACTACTCGACTAGAGTACTGGAGAGGGAAACGGAATTCACGGTGTAGCAGTGAAATGCGTAGATATCGTGAGGAACACCAGAGGCGAAGGCGGTTTCCTGGACAGTTACTGACGCTCAGGCACGAAAGCGTGGGGAGCAAAAGGG` |
| ASV1045 | 429 | `TCGAGAATAATTCACAATGGGGGCAACCCTGATGGTGCAACGCCGCGTGGAGGATGACGGTCTTCGGATTGTAAACTCCTGTCATCTGGGAGTAAGGCATGGCTGTGAAAAGCAGACATGATTGATAGTACCAGAAGAGGAAGGGACGGCTAACTTCGTGCCAGCAGCCGCGGTAATACGAAGGTCCCAAGCGTTGTTCGGAATCACTGGGCGTAAAGGGAGCGTAGGCGGCGTGGTAAGTCAGATGTGAAATCCCGGGGCTCAACCCCGGAACTGCATCCGATACTGCCATGCTAGAGGATTGGAGAGGTAGCTGGAATTCTTGGTGTAGCAGTGAAATGCGTAGAGATCAAGAGGAACACTCGTGGCGAAAGCGAGCTACTGGACAATTCCTGACGCTGAGGCTCGAAGGCTAGGGTAGCGAAAGGG` |
| ASV1812 | 429 | `TGGGGAATTTTGGACAATGGGCGAAAGCCTGATCCAGCAATGCCGCGTGCAGGATGAAGGCCTTAGGGTTGTAAACTGCTTTTGTACGGAACGAAACGGCCCCTTCCAATACAGGGGGCTAATGACGGTACCGTAAGAATAAGCACCGGCTAACTACGTGCCAGCAGCCGCGGTAATACGTAGGGTGCGAGCGTTAATCGGAATTACTGGGCGTAAAGCGTGCGCAGGCGGTTATATAAGACAGATGTGAAATCCCCGGGCTCAACCTGGGACCTGCATTTGTGACTGTATAGCTAGAGTACGGTAGAGGGGGATGGAATTCCGCGTGTAGCAGTGAAATGCGTAGATATGCGGAGGAACACCGATGGCGAAGGCAATCCCCTGGACCTGTACTGACGCTCATGCACGAAAGCGTGGGGAGCAAACAGG` |
| ASV945 | 429 | `TCGAGAATAATTCACAATGGGGGCAACCCTGATGGTGCAACGCCGCGTGGAGGATGACGGTCTTCGGATTGTAAACTCCTGTCATCCGGGAGTAAGACCTGGCTGTGAATAGCAGACAGGGTTGATAGTACCGGAAGAGGAAGGGACGGCTAACTTCGTGCCAGCAGCCGCGGTAATACGAAGGTCCCGAGCGTTGTTCGGAATCACTGGGCGTAAAGGGAGCGTAGGCGGCGTGGTAAGTCAGATGTGAAATCCCGGGGCTCAACCCCGGAACTGCATCCGATACTGCCGTGCTAGAGGATTGGAGAGGTAGCTGGAATTCTTGGTGTAGCAGTGAAATGCGTGGATATCAAGAGGAACACTCGTGGCGAAAGCGAGCTACTGGACAACTCCTGACGCTGAGGCTCGAAGGCCAGGGTAGCGAAAGGG` |
| ASV1060 | 429 | `TGGGGAATATTGGACAATGGGCGCAAGCCTGATCCAGCCATGCCGCGTGTGTGAAGAAGGCCCTAGGGTTGTAAAGCACTTTCAGTGGGGAGAAAGGTTTGCCACCTAATACGTGGTGAAATTGATGTTACCCACAGAAGAAGCACCGGCTAACTCCGTGCCAGCAGCCGCGGTAATACGGAGGGTGCAAGCGTTAATCGGAATTACTGGGCGTAAAGCGCATGTAGGTGGTTTGCTAAGCCAGCTGTGAAATCCCCGGGCTCAACCTGGGACTTGCAGTTGGAACTGGTAAGCTAGAGTAGGGTAGAGGGGTGTGGAATTCCAGGTGTAGCGGTGAAATGCGTAGATATCTGGAGGAACATCAGTGGCGAAGGCGACACCCTGGACTCATACTGACACTGAGATGCGAAAGCGTGGGGAGCAAACAGG` |
| ASV3524 | 404 | `TGGGGAATCTTGCACAATGGGGGAAACCCTGATGCAGCGACGCCGCGTGAGTGATGAAGCCCTTCGGGGTGTAAAGCTCTTTCGGCAGGGACGATAATGACGGTACCTGCGGAAGAAGCTGCGGCTAACTACGTGCCAGCAGCCGCGGTAATACGTAGGCAGCAAGCGTTGTTCGGAATTACTGGGCGTAAAGAGTGTGTAGGTGGTGTCGTAAGTTTGGTGTGAAATCTCCCGGCTCAACTGGGAGGGTGCGCCGAAGACTGCGATGCTTGAGGGTGGGAGAGGTAAGCGGAATTCCTGGTGTAGCGGTGAAATGCGTAGATATCAGGAGGAACACCTGCGGTGTAGACGGCTTACTGGACCACACCTGACACTGAGACACGAAAGCGTGGGTAGCAAACAGG` |
| ASV706 | 429 | `TGGGGAATTTTGGACAATGGGCGCAAGCCTGATCCAGCCATGCCGCGTGCGGGAAGAAGGCCTTCGGGTTGTAAACCGCTTTTGTCAGGGAAGAAACGCTCTGGGTTAATACCCTGGGGTAATGACGGTACCTGAAGAATAAGCACCGGCTAACTACGTGCCAGCAGCCGCGGTAATACGTAGGGTGCAAGCGTTAATCGGAATTACTGGGCGTAAAGCGTGCGCAGGCGGTCTTGCAAGACAGATGTGAAATCCCCGGGCTCAACCTGGGAACTGCATTTGTGACTGCAAGGCTGGAGTACGGCAGAGGGGGATGGAATTCCGCGTGTAGCAGTGAAATGCGTAGATATGCGGAGGAACACCGATGGCGAAGGCAATCCCCTGGGCCTGTACTGACGCTCATGCACGAAAGCGTGGGGAGCAAACAGG` |
| ASV2104 | 404 | `TGGGGAATATTGGACAATGGGGGCAACCCTGATCCAGCAATGCCGCGTGAGTGATGAAGGCCTTAGGGTTGTAAAGCTCTTTTACCGGGGACGATGATGACGGTACCCGGAGAATAAGCCCCGGCTAACTTCGTGCCAGCAGCCGCGGTAATACGAAGGGGGCGAGCGTTGTTCGGAGTTACTGGGCGTAAAGGGCGCGTAGGCGGCGTTCCAAGTTAGGCGTGAAAGCCCCGGGCTCAACCTGGGAATTGCGCTTAAGACTGGAATGCTGGAGTGCGGGAGAGGTGAGTGGAATTCCCAGTGTAGAGGTGAAATTCGTAGATATTGGGAAGAACACCAGTGGCGAAGGCGGCTCACTGGCCCGCAACTGACGCTGAGGCGCGAAAGCGTGGGGAGCAAACAGG` |
| ASV4440 | 405 | `TAGGGAATTTTCCACAATGGACGAAAGTCTGATGGAGCAACGCCGCGTGCAGGATGAATGCCTTAGGGTTGTAAACTGCTTTTATCTGTGACGAATATGACGGTAGCAGATGAATAAGGATCGGCTAACTCCGTGCCAGCAGCCGCGGTCATACGGAGGATCCAAGCGTTATCCGGAATTACTGGGCGTAAAGAGTTGCGTAGGTGGCATAGTAAGCGAGTAGTGAAAGCGTTCGGCTCAACCGAATATCCATTACTTGAACTGCTAAGCTAGAAGATGAGAGAGGTCACTGGAATTCCCTGTGTAGGAGTGAAATCCGTAGATATAGGGAGGAACACCGATGGCGTAGGCAGGTGACTGGCTCATTCTTGACACTAAGGCACGAAAGCGTGGGTAGCAAACGGG` |
| ASV4409 | 407 | `TGGGGAATTTTCCGCAATGGGCGAAAGCCTGACGGAGCAATACCGCGTGAGGGAAGAAGGCTCTTGGGTTGTAAACCTCTTTTCTCAGGGAATAAAAAAATGAAGGTACCTGAGGAATAAGCATCGGCTAACTCCGTGCCAGCAGCCGCGGTAATACGGAGGATGCAAGCGTTATCCGGAATGATTGGGCGTAAAGCGTCCGCAGGTGGAACAACAAGTCTAGTGTCAAAGACCGGGGCTCAACCCCGGGTCGGCGGTAGAAACTGTTGAACTAGAGGGTCGTAGGGGTAGAGGGAATTCCCAGTGTAGCGGTGAAATGCGTAGAGATTGGGAAGAACATCGGTGGCGAAGGCGCTCTACTGGACGGCACCTGACACTCAGGGACGAAAGCTAGGGGAGCGAATGGG` |
| ASV2195 | 405 | `TGGGGAATCTTGCGCAATGGGCGAAAGCCTGACGCAGCAACGCCGCGTGCGGGAAGAAGGCCCTCGGGTTGTAAACCGCTTTCAGCAGGAACGAAAATGACGGTACTTGCAGAAGAAGGTGCGGCCAACTACGTGCCAGCAGCCGCGGTGACACGTAGGCACCAAGCGTTGTCCGGATTTATTGGGCGTAAAGAGCTCGTAGGCGGTTCGGTAAGTCGGGTGTGAAAACTTTGGGCTTAACCCAAAGCCTGCATCCGATACTGCTGTGACTAGAGTTCGGTAGGGGAGCGGGGAATTCCTAGTGTAGCGGTGAAATGCGCAGATATTAGGAGGAACACCGGTGGCGAAGGCGCCGCTCTGGGCCGATACTGACGCTGAGGAGCGAAAGCGTGGGTAGCAAACAGG` |
| ASV116 | 429 | `TGGGGAATTTTGGACAATGGGCGCAAGCCTGATCCAGCAATGCCGCGTGCAGGATGAAGGCCTTCGGGTTGTAAACTGCTTTTGTACGGAACGAAACGGTCGCCTCTAATAAAGGTGGCTAATGACGGTACCGTAAGAATAAGCACCGGCTAACTACGTGCCAGCAGCCGCGGTAATACGTAGGGTGCGAGCGTTAATCGGAATTACTGGGCGTAAAGCGTGCGCAGGCGGTTATATAAGACAGATGTGAAATCCCCGGGCTCAACCTGGGACCTGCATTTGTGACTGTATAGCTAGAGTACGGTAGAGGGGGATGGAATTCCGCGTGTAGCAGTGAAATGCGTAGATATGCGGAGGAACACCGATGGCGAAGGCAATCCCCTGGACCTGTACTGACGCTCATGCACGAAAGCGTGGGGAGCAAACAGG` |
| ASV4098 | 416 | `TGGGGAATTTTGGACAATGGGCGAAAGCCTGATCCAGCAATTCTGCGTGAGGGACGAAGTCTTTCGGGATGTAAACCTCTTTTGTTTGGGACGAAAACCCGCAAGGGCCTGACGGTACCAGACGAATAAGCAACGGCTAACTACGTGCCAGCAGCCGCGGTAAGACGTAGGTTGCAAGCGTTATTCGGAATTACTAGGCGTAAAGCGAGTGTAGGCGGGTACTTAAGTCCGTCGTGAAATCTCCCGGCTTAACTGGGAGGGGTCGACAGATACTGGGTATCTTGAGTGGGGTAGGGGGCAGTGGAATTCCCGGTGTAGCGGTGAAATGCGTAGATATCGGGAGGAACACCTATGGCGAAAGCAGCTGCCTGGGCCTTTACTGACGCTAAGACTCGAAAGCTAGGGGAGCAAACAGG` |
| ASV5372 | 424 | `TGGGGAATCTTGCGCAATGGGGGCAACCCTGACGCAGCAATGCCGCGTGGAGGACGAAGGTCTTCGGATTGTAAACTCCTTTTACAGGGGACAAATGCCTTGGTGAAGAGCCAAGGGTGATGGTACTCTGTGAATAAGCCACGGCTAACTTCGTGCCAGCAGCCGCGGTAAGACGAAGGTGGCGAGCGTTACTCGGATTTACTAGGCGTAAAGCGTGGGCAGGCGGTTCGGTCAGTCTGTTGTGAAAGCTCCCGGCTCAACTGGGAGAGGCCAATGGATACTACCGGGCTTGAATGCGGGAGAGGTGACTGGAATTCCTGGTGTAGCGGTGAAATGCGTAGATATCAGGAGGAACACCTATGGCGAAAGCAGGTCACTGGACCGTTATTGACGCTCATCCACGAAAGCTGGGGGATCAAACAGG` |
| ASV1475 | 429 | `TGGGGAATATTGGACAATGGGCGAAAGCCTGATCCAGCGACGCCGCGTGGGTGAAGAAGGCCTGCGGGTTGTAAAGCCCTTTCGGTTGGGAAGAAAAGTGTTGACCTAACACGTCAGCACCTTGACGTAACCAACAAAAGAAGCACCGGCTAACTCTGTGCCAGCAGCCGCGGTAATACAGAGGGTGCGAGCGTTAATCGGAATTACTGGGCGTAAAGCGCACGTAGGCGGCTTGATTAGTCGAGTGTGAAATCCCCGGGCTTAACCTGGGACCTGCATTCGAAACTGTCTTGCTAGAGTATGGGAGAGGGTAGTGGAATTTCAGGTGTAGCGGTGAAATGCGTAGATATCTGAAGGAACATCAGTGGCGAAAGCGACTGCCTGGACCAATACTGACGCTCAGGTGCGAAAGCGTGGGGAGCAAACAGG` |
| ASV2552 | 404 | `TGGGGAATCTTGGACAATGGGCGCAAGCCTGATCCAGCCATGCCGCGTGGATGATGAAGGCCTTCGGGTTGTAAAATCCTTTCGTTGGGGACGATAATGACGGTACCCAAAGAAGAAGCTCCGGCTAACTTCGTGCCAGCAGCCGCGGTAATACGAAGGGGGCTAGCGTTGTTCGGATTTACTGGGCGTAAAGCGCGTGTAGGCGGGTCGTTAAGTCGGAGGTGAAAGCCCAGAGCTCAACTCTGGAATTGCCTTCGATACTGGCGACCTGGACCCATGTAGAGGCAAGTGGAATTGCGAGTGTAGAGGTGAAATTCGTAGATATTCGCAGGAACACCAGTGGCGAAGGCGACTTGCTGGACATGTGGTGACGCTGAGACGCGAAAGCGTGGGGAGCAAACAGG` |
| ASV5412 | 404 | `TGGGGAATATTGGACAATGGGGGCAACCCTGATCCAGCAATGCCGCGTGAGTGATGAAGGCCTTCGGGTTGTAAAGCTCTTTTACCTACGACGATAATGACGGTAGTAGGAGAATAAGCCCCGGCTAACTTCGTGCCAGCAGCCGCGGTAATACGAAGGGGGCTAGCGTTGTTCGGAATTACTGGGCGTAAAGGGCGCGTAGGCGGTCTTATAAGTCTGATGTGAAAGGCCTGGGCTTAACCTGGGAACTGCATTGGATACTGTGAGACTTGAGGGTGTGAGAGGAAAGTGGAATGACGAGTGTAGAGGTGAAATTCGTAGATATTCGTTGGAACACCAGAGGCGAAGGCGGCTTTCTGGCACACTACTGACGCTAAGGCGCGAAAGCGTGGGGAGCAAACAGG` |
| ASV2978 | 421 | `TGGGGAATATTGCGCAATGGCCGAAAGGCTGACGCAGCGACGCCGCGTGGGGGATGAAGCATTTCGGTGTGTAAACCCCTGTTGCCCGGGACGAACAACTGGATTCTTCCAGTCTGACGGTACCGGGTGAGGAAGCACCGGCTAACTCCGTGCCAGCAGCCGCGGTAATACGGAGGGTGCGAGCGTTGTCCGGAATCACTGGGCGTAAAGGGTGAGTAGGCGGTCTGCTAAGCGTGTGGTGAAAGCCCGGGGCTCAACCCCGGGTCTGCCATGCGAACTGGTGGACTTGAGCACTGTAGAGGCAGGTGGAATTCCGGGTGTAGCGGTGGAATGCGTAGAGATCCGGAAGAACACCGGTGGCGAAGGCGGCCTGCTGGGCAGTAGCTGACGCTGAATCACGACAGCGTGGGGAGCAAACAGG` |
| ASV4549 | 404 | `TAGGGAATATTGCGCAATGGGGGAAACCCTGACGCAGCGACGCCGCGTGAGCGATGAAGGCCTTAGGGTTGTAAAGCTCTGTCGTAAGGGAAGATAATGACGGTACCTTACAAGAAAGGGACGGCTAACTTCGTGCCAGCAGCCGCGGTAATACGAAGGTCCCAAGCGTTGTTCGGAATCATTGGGCGTAAAGCGGGTGTAGGCGGTTCTATAAGTCGGATGTGAAATCCCTGGGCTCAACCTAGGAACTGCATTCGATACTGTTTAACTTGAGTACGGGAGAGGGTAGTGGAATTCCTGGTGTAGTGGTGAAATACGTAGATATCAGGAGGAACTCCGGCGGCGAAGGCGGCTGCCTGGCCCAGTACTGACGCTGAGACCCGAAAGCGTGGGGATCAAACAGG` |
| ASV2958 | 430 | `TGGGGAATCTTCCGCAATGGACGAAAGTCTGACGGAGCAACGCCGCGTGAGTGAAGAAGGTTTTCGGATCGTAAAACTCTGTTTTCAGGGACGAATGTGCAGAACGTGAATAATGTTTTGTAATGACGGTACCTGAGTAGAAAGCCACGGCTAACTACGTGCCAGCAGCCGCGGTAATACGTAGGTGGCAAGCGTTGTCCGGAATTATTGGGCGTAAAGCGCGCGCAGGTGGGATATTAAGTCTGGTGTTAAAGTTCGGGGCTCAACCCCGTGAAGCATTTGGAAACTGATATTCTTGAGTGCAGGAGAGGAAAGTGGAATTCCTAGTGTAGCGGTGAAATGCGTAGATATTAGGAGGAACACCAGTGGCGAAGGCGACTTTCTGGACTGTGTCTGACACTGAGGCGCGAAAGCTAGGGTAGTGAACAGG` |
| ASV3350 | 429 | `TGGGGAATTTTGGACAATGGGCGAAAGCCTGATCCAGCAATGCCGCGTGCAGGATGAAGGCCTTCGGGTTGTAAACTGCTTTTGTACGGAACGAAAAAGCTTCTCCTAATACGAGAGGCCCATGACGGTACCGTAAGAATAAGCACCGGCTAACTACGTGCCAGCAGCCGCGGTAATACGTAGGGTGCGAGCGTTAATCGGAATTACTGGGCGTAAAGCGTGCGCAGGCGGTTATGTAAGACAGATGTGAAATCCCCGGGCTCAACCTGGGAACTGCATTTGTGACTGCATGGCTAGAGTACGGTAGAGGGGGATGGAATTCCGCGTGTAGCAGTGAAATGCGTAGATATGCGGAGGAACACCGATGGCGAAGGCAATCCCCTGGACCTGTACTGACGCTCATGCACGAAAGCGTGGGGAGCAAACAGG` |
| ASV1315 | 430 | `TGGGGAATATTGGACAATGGGCGGAAGCCTGATCCAGCAATACCGCGTGTGTGAAGAAGGCCTTCGGGTTGTAAAGCACTTTTGTTAGGGAGGATGGTTATTTGGGCAAATACCCCAAGTATCTGACGTTACCTAAAGAATAAGCACCGGCTAACTCCGTGCCAGCAGCCGCGGTAATACGGAGGGTGCAAGCGTTAATCGGATTTACTGGGCGTAAAGGGTGCGTAGGCGGCCCGTTAAGTCAGATGTGAAAGCCCCGGGCTCAACCTGGGAATTGCATTTGATACTGGCGGGCTAGAATGTGGTAGAGGGTAGCGGAATTTCTGGTGTAGCGGTGAAATGCGTAGATATCAGAAGGAACATCAATGGCGAAGGCAGCTACCTGGGCCAACATTGACGCTGAGGCACGAAAGCGTGGGGAGCAAACAGG` |
| ASV3147 | 405 | `TGGGGAATATTGGACAATGGGGGCAACCCTGATCCAGCCATGCCGCGTGAATGATGAAGGCCTTCGGGTTGTAAAGTTCTTTTGGTAGGGACGATGATGACGGTACCTACAGAATAAGCTCCGGCTAACTTCGTGCCAGCAGCCGCGGTAATACGAAGGGAGCTAGCGTTGTTCGGAATTACTGGGCGTAAAGAGCGCGTAGGCGGTTTAACAAGTCAGGCGTGAAAGCCCTGGGCTCAACCCGGGAATTGCGCTTGAGACTGTTTTTCTAGGAGTACGAGAGAGGTAAGTGGAATTCCCAGTGTAGCAGTGAAATGCGTAGATATTGGGAGGAACACCAGTGGCGAAGGCGGCTTACTGGCTCGTAACTGACGCTAAGGCGCGAAAGCGTGGGGAGCAAACAGG` |
| ASV2865 | 429 | `TGGGGAATTTTGGACAATGGACGCAAGTCTGATCCAGCCATTCCGCGTGCAGGAAGAAGGCCTTCGGGTTGTAAACTGCTTTTGTATGGGGTGAAAAGCGCTATATTAATACCATGGCGTCATGACAGTACCATAAGAATAAGCACCGGCTAACTACGTGCCAGCAGCCGCGGTAATACGTAGGGTGCAAGCGTTAATCGGAATTACTGGGCGTAAAGCGTGCGCAGGCGGCTATGTAAGACAGAGGTGAAATCCCCGGGCTCAACCTGGGAACTGCCTTTGTGACTGCATAGCTGGAGTACGGCAGAGGGGGATGGAATTCCGCGTGTAGCAGTGAAATGCGTAGATATGCGGAGGAACACCGATGGCGAAGGCAATCCCCTGGGCCTGTACTGACGCTCATGCACGAAAGCGTGGGGAGCAAACAGG` |
| ASV3483 | 405 | `TGGGGAATATTGGGCAATGGAGGAAACTCTGACCCAGCAACGCCGCGTGAAGGATGAAGGTTTTCGGATTGTAAACTTCTGTCGTGAGGGAAGAAGAATGACAGTACCTCACAAGAAAGTCCCGGCAAACTACGTGCCAGCAGCCGCGGTAATACGTAGGGGACGAGCGTTGTCCGGAATGACTGGGTGTAAAGGGAGTGTAGGCGGCCTTTTAAGTTATATGTGAAAGCCCACGGCTTAACCGTGGAACTGCATATAAAACTGGAGGGCTGGAGTGCAGGAGAGGTAAGTGGAATTCCTAGTGTAGCGGTGGAATGCGTAGATATTAGGAGGAACACCAGTGGCGAAGGCGACTTACTGGACTGTAACTGACGCTGAGGCTCGAAAGCGTGGGGAGCAAACAGG` |
| ASV2171 | 428 | `TAGGGAATTTTCGGCAATGGGGGAAACCCTGACCGAGCAACGCCGCGTGAGTGAAGAAGGCCTTCGGGTTGTAAAGCTCTGTTGTAAGGGAAGAACGATAGTTAGAGGGAATGCTAGCTATATGACGGTACCTTACCAGAAAGCCACGGCTAACTACGTGCCAGCAGCCGCGGTAATACGTAGGTGGCAAGCGTTATCCGGAATTATTGGGCGTAAAGGGAGCGCAGGCGGTTTATTAAGTTTATGGTGAAAGTTCGGGGCTCAACCCCGTGATGCCATAGAAACTGGTAGACTTGAGTACAGGAGAGGTTAGTGGAATTCCATGTGTAGCGGTAAAATGCGTAGATATATGGAGGAACACCAGTGGCGAAGGCGGCTAACTGGCCTGTAACTGACGCTGAGGCTCGAAAGCGTGGGGAGCAAATAGG` |
| ASV4190 | 404 | `TTAGGAATCTTGGGCAATGGGCGAAAGCCTGACCCAGCCATGCCGCGTGAATGATGAAGGTCCTAGGATTGTAAAATTCTTTCACCGGGGAAGATAATGACGGTACCCGGAGAAGAAGCTCCGGCTAACTTCGTGCCAGCAGCCGCGGTAATACGAAGGGGGCTAGCGTTGCTCGGAATTACTGGGCGTAAAGGGCGCGTAGGCGGACGATTAAGTTGGGGGTGAAATCCCAGGGCTCAACCCTGGAACTGCCTCCAAAACTGGTCGTCTTGAGTACGGAAGAGGTATGTGGAACTCCGAGTGTAGAGGTGAAATTCGTAGATATTCGGAAGAACACCAGTGGCGAAGGCGACATACTGGTCCGTTACTGACGCTGAGGCGCGAAAGCGTGGGGAGCAAACAGG` |
| ASV1688 | 404 | `TGGGGAATATTGGACAATGGGCGAAAGCCTGATCCAGCAATGCCGCGTGAGTGATGAAGGCCTTCGGGTTGTAAAGCTCTTTTGTCCGGGAAGATAATGACTGTACCGGAAGAATAAGCCCCGGCTAACTTCGTGCCAGCAGCCGCGGTAATACGAAGGGGGCTAGCGTTGTTCGGAATCACTGGGCGTAAAGCGTACGTAGGCGGCTATTCAAGTCAGAGGTGAAAGCCCAGGGCTCAACCTTGGAACTGCCTTTGAGACTGGATAGCTAGAGCACGGAAGAGGTAAGTGGAATTCCGAGTGTAGAGGTGAAATTCGTAGATATTCGGAGGAACACCCGTGGCGAAGGCGGCTTACTGGGCCGTTGCTGACGCTGAGGTACGAAAGCGTGGGGATCAAACAGG` |
| ASV5023 | 404 | `TGGGGAATATTGGACAATGGGCGCAAGCCTGATCCAGCCATGCCGCGTGAGTGATGAAGGCCTTAGGGTTGTAAAGCTCTTTCGTCAGGGAAGATAATGACGGTACCTGAAGAAGAAGCCCCGGCTAACTTCGTGCCAGCAGCCGCGGTAATACGAAGGGGGCTAGCGTTGCTCGGAATCACTGGGCGTAAAGCGCACGTAGGCGGCTTTCTAAGTCAGGGGTGAAATCCTGGAGCTCAACTCCAGAACTGCCTTTGATACTGGAAAGCTTGAGTTCGGGAGAGGTGAGTGGAACTGCGAGTGTAGAGGTGAAATTCGTAGATATTCGCAAGAACACCAGTGGCGAAGGCGGCTCACTGGCCCGATACTGACGCTGAGGTGCGAAAGCGTGGGGAGCAAACAGG` |
| ASV3047 | 423 | `TAGGGAATATTGGGCAATGGGCGCAAGCCTGACCCAGCCATGCCGCGTGCAGGAAGAATGCCCTCTGGGTTGTAAACTGCTTTTGGGAGGGGACAAAAAGCCAATGCGTTGGTAATTGAGTGTACCTCCAGAATAAGCACCGGCTAACTCCGTGCCAGCAGCCGCGGTAATACGGAGGGTGCAAGCGTTGTCCGGATTTATTGGGTTTAAAGGGTGCGTAGGCGGCCTTATAAGTCGGAGGTGAAACCCACAGGCTTAACCTGTGGAGTGCCTTCGATACTGTAAGGCTTGGGTTATCTCTAGGCGGGCGGAATGGAAGGTGTAGCGGTGAAATGCATAGATATCTACCAGAACGCCGATAGCGAAGGCAGCTCGCTGGGGATATACCGACGCTGAGGCACGATAGCGTGGGGATCAAACAGG` |
| ASV4934 | 430 | `TGGGGAATATTGGACAATGGGGGCAACCCTGATCCAGCAATGCCGCGTGTGTGAAGAAGGCCTTCGGGTTGTAAAGCACTTTCAGTGGGAAAGATTGATTTTGGTACTAATACTATCGAAGTCTGACGTTACCCAAAGAAGAAGCACCGGCTAACTCCGTGCCAGCAGCCGCGGTAATACGGAGGGTGCAAGCGTTAATCGGAATTACTGGGCGTAAAGCGCATGTAGGCGGCGAATTAAGTCAGATGTGAAAGCCCCGGGCTTAACCTGGGAATTGCATCTGATACTGGTTTGCTAGAGTATGCTAGAGAGAAGCGGAATTCCGGGTGTAGCGGTGAAATGCGTAGATATCCGGAGGAACATCAATGGCGAAGGCAGCTTCTTGGGGCAATACTGACGCTGAGGTGCGAAAGCGTGGGGATCAAACAGG` |
| ASV4712 | 424 | `TGAGGAATATTGGTCAATGGGCGAGAGCCTGAACCAGCCAAGTCGCGTGAAGGATGACTGCCCTATGGGTTGTAAACTTCTTTTATAAGAGAGTAAAAAGAGCTACGTGTAGTTTATTGCAAGTATCTTATGAATAAGCACCGGCTAACTCCGTGCCAGCAGCCGCGGTAATACGGAGGGTGCGAGCGTTATCCGGATTTATTGGGTTTAAAGGGTGCGTAGGCGGTCTTATAAGTCAGCGGTGAAAGTTTTAGGCTCAACCTAAAAATTGCCGTTGAAACTGTTTGACTTGAGTATAGATGAAGTGGGCGGAATTTGTGGTGTAGCGGTGAAATGCATAGATATCACGAGGAACTCCAATTGCGCAGGCAGCTCACTAAGCTATAACTGACGCTGATGCACGAAAGCGTGGGTATCCAACAGG` |
| ASV1478 | 429 | `TGGGGAATATTGGACAATGGGCGAAAGCCTGATCCAGCCATGCCGCGTGTGTGAAGAAGGCCCTTGGGTTGTAAAGCACTTTCAGTGGGGAGAAAGGTCTGTCACCTAATACGTGATGGAATTGATGTTACCCACAGAAGAAGCACCGGCTAACTCCGTGCCAGCAGCCGCGGTAATACGGAGGGTGCAAGCGTTAATCGGAATTACTGGGCGTAAAGCGCACGTAGGTGGTTTGCTAAGCCAGCTGTGAAATCCCCGGGCTCAACCTGGGCACTGCAGTTGGAACTGGTAAGCTAGAGTAGGGTAGAGGGGTGTGGAATTCCAGGTGTAGCGGTGAAATGCGTAGATATCTGGAGGAACATCAGTGGCGAAGGCGACACCCTGGACTCATACTGACACTGAGGTGCGAAAGCGTGGGGAGCAAACAGG` |
| ASV1824 | 429 | `TGGGGAATTTTGGACAATGGGGGCAACCCTGATCCAGCCATTCCGCGTGAGTGAAGAAGGCCTTCGGGTTGTAAAGCTCTTTCGCAAGGGAAGAAACGTTATTAGTGAATAACTAATGATAATGACGGTACCTTGATAAGAAGCACCGGCTAACTACGTGCCAGCAGCCGCGGTAATACGTAGGGTGCGAGCGTTAATCGGAATTACTGGGCGTAAAGCGTGCGCAGGCTGTTTTGTAAGTCAGATGTGAAATCCCCGAGCTCAACTTGGGAACTGCGTTTGAAACTACAAGACTAGAATAGGTCAGAGGGGGGTAGAATTCCACGTGTAGCAGTGAAATGCGTAGAGATGTGGAGGAATATCAATGGCGAAGGCAGCCCCCTGGGATCATATTGACGCTCATGCACGAAAGCGTGGGGAGCAAACAGG` |
| ASV3090 | 404 | `TGGGGAATATTGGACAATGGGCGAAAGCCTGATCCAGCCATGCCGCGTGAGTGATGAAGGCCTTAGGGTTGTAAAGCTCTTTTATCAGGGAAGATAATGACGGTACCTGAAGAATAAGCACCGGCTAACTTCGTGCCAGCAGCCGCGGTAATACGAAGGGTGCTAGCGTTGTTCGGAATTACTGGGCGTAAAGAGTTTGTAGGCGGTCCTACAAGTCAGAGGTGAAATCCCGAAGCTTAACTTCGGAACTGCCTTTGAAACTGTATGACTGGAGTATGTGAGGGGATAGCGGAATTCTTAATGTAGAGGTGAAATTCGTAGATATTAAGAGGAACACCAGTGGCGAAGGCGGCTATCTGGCACATTACTGACGCTGAGAAACGAAAGCGTGGGGATCAAACAGG` |
| ASV477 | 429 | `TGGGGAATTTTGGACAATGGGCGCAAGCCTGATCCAGCAATGCCGCGTGCAGGAAGAAGGCCTTCGGGTTGTAAACTGCTTTTGTACGGAGCGAAAAGGCTCTCCTTAATACGGGGGGCTCATGACGGTACCGTAAGAATAAGCACCGGCTAACTACGTGCCAGCAGCCGCGGTAATACGTAGGGTGCGAGCGTTAATCGGAATTACTGGGCGTAAAGCGTGCGCAGGCGGTTTTGTAAGACAGGCGTGAAATCCCCGGGCTCAACCTGGGAATGGCGCTTGTGACTGCAAAGCTGGAGTGCGGCAGAGGGGGATGGAATTCCGCGTGTAGCAGTGAAATGCGTAGATATGCGGAGGAACACCGATGGCGAAGGCAATCCCCTGGGCCTGCACTGACGCTCATGCACGAAAGCGTGGGGAGCAAACAGG` |
| ASV3784 | 430 | `TGGGGAATCTTGCGCAATGGAGGAAACTCTGACGCAGCGATACCGCGTGAGTGAGGAAGGCCTTCGGGTTGTAAAGCTCTGTTAGGGTAGAAAAATGATTTACATGCGAATAGGATGTAGATTTGATGGTACACCCAAAGAAATCACCGGCTAACTTCGTGCCAGCAGCCGCGGTAATACGAAGGGTGAGAGCGTTGTTCGGATTTATTGGGCGTAAAGCGCGCGCAGGCGGGTGATCAAGTCAGATGTGAAATCTCGGGGCTCAACTCCGAAACAGCGTCTGAAACTAATCACCTTGAATTCTGGCGGGGGAGGGGGAATTTCACATGTAGGGGTAAAATCCGTAGAGATGTGAAGGAACACCAGAGGCGAAGGCGCCCTCCTAGACAGACATTGACGCTGAGGCGCGAAAGCGTGGGTAGCAAACAGG` |
| ASV1779 | 423 | `TAGGGAATATTGGACAATGGGTGCGAGCCTGATCCAGCCATGCCGCGTGCAGGAAGAAGGCCTTCTGGGTTGTAAACTGCTTTTGCCGGGGGATAAAATGGCCATGCGTGGCAAATTGAAGGTACCCGGTGAATAAGCCACGGCTAACTACGTGCCAGCAGCCGCGGTAATACGTAGGTGGCAAGCGTTGTCCGGATTTATTGGGTTTAAAGGGTGCGTAGGCGGTCCCATAAGTCAGTGGTGAAATACGGCAGCTCAACTGTCGAGGTGCCATTGATACTGCGGGACTTGAGTACAGACGAGGTAGGCGGAATTGACGGTGTAGCGGTGAAATGCTTAGATATCGTCAAGAACACCGATAGCGAAGGCAGCTTACTAGGCTGTAACTGACGCTGAGGCACGAAGGTGCGGGGATCAAACAGG` |
| ASV1763 | 429 | `TGGGGAATATTGGACAATGGGCGCAAGCCTGATCCAGCAATGCCGCGTGTGTGAAGAAGGCCTGTGGGTTGTAAAGCACTTTCAGGGGTGAGGAAGTTTAATGTGCTAATAGTATATTAAATTGACGTTAGCCCCAGAAGAAGCACCGGCTAACTCTGTGCCAGCAGCCGCGGTAATACAGAGGGTGCTAGCGTTAATCGGAATTACTGGGCGTAAAGGGTGTGTAGGTGGTATAGTAAGTCAGATGTAAAATCCCCGGGCTTAACCTGGGGCTGTCGTTTGAAACTGCTAAGCTAGAGTATTATAGAGGGTAGTGGAATTTCCGGAGTAGCGGTGAAATGCGTAGATACCGGAAGGAACACCAGTGGCGAAGGCGGCTACCTGGATAATTACTGACACTGAGGCACGAAAGCGTGGGTAGCAAACAGG` |
| ASV4295 | 429 | `TGGGGAATTTTGGACAATGGGGGAAACCCTGATCCAGCAACGCCGCGTGAATGAAGAAGGCCTTAGGGTTGTAAAGTTCTTTTATCTAGGAGCAAATCCTGTGGTTTAATATTCCACGGGGATGAGAGTACTAGAAGAATAAGCACCGGCTAACTACGTGCCAGCAGCCGCGGTAATACGTAGGGTGCGAGCGTTAATCGGAATTACTGGGCGTAAAGCGTGCGCAGGCGGATATGTAAGTTAGATGTGAAATCCCCGAGCTCAACTTGGGAATGGCATTTAAGACTGCATGTCTAGAGTTTGTCAGAGGGGGGTAGAATTCCAAGTGTAGCAGTGAAATGCGTAGAGATTTGGAGGAATACCGGTGGCGAAGGCGGCCCCCTGGGATAGAACTGACGCTCATGCACGAAAGCGTGGGTAGCAAACAGG` |
| ASV337 | 429 | `TGGGGAATTTTGGACAATGGGCGCAAGCCTGATCCAGCAATGCCGCGTGCAGGATGAAGGCCTTCGGGTTGTAAACTGCTTTTGTACGGAGCGAAACGGTTCTTTCTAATACAAAGGGCTAATGACGGTACCGTAAGAATAAGCACCGGCTAACTACGTGCCAGCAGCCGCGGTAATACGTAGGGTGCGAGCGTTAATCGGAATTACTGGGCGTAAAGCGTGCGCAGGCGGTTATATAAGACAGATGTGAAATGCCCGGGCTCAACCTGGGACCTGCATTTGTGACTGTATAGCTAGAGTACGGTAGAGGGGGATGGAATTCCGCGTGTAGCAGTGAAATGCGTAGATATGCGGAGGAACACCGATGGCGAAGGCAATCCCCTGGACCTGTACTGACGCTCATGCACGAAAGCGTGGGGAGCAAACAGG` |
| ASV4139 | 429 | `TGGGGAATTTTGGACAATGGGCGAAAGCCTGATCCAGCCATGCCGCGTGAGTGAAGAAGGCCTTCGGGTTGTAAAGCTCTTTCAGACGGAAAGAAATTGCTCTGGATAATACCTGGAGTAGATGACGGTACCGTAAGAAGAAGCACCGGCTAACTACGTGCCAGCAGCCGCGGTAATACGTAGGGTGCGAGCGTTAATCGGAATTACTGGGCGTAAAGCGTGCGCAGGCGGTTATATAAGACAGATGTGAAATCCCCGGGCTTAACCTGGGAACTGCGTTTGTGACTGTATAACTGGAGTACGGTAGAGGGGGGTGGAATTCCTGGTGTAGCAGTGAAATGCGTAGATATCAGGAGGAACATCGATGGCGAAGGCAGCCCCCTGGACCTGTACTGACGCTCATGCACGAAAGCGTGGGGAGCAAACAGG` |
| ASV4272 | 407 | `TGGGGAATTTTCCGCAATGGGCGAAAGCCTGACGGAGCAATACCGCGTGAGGGAGGAAGGCTCTTGGGTTGTAAACCTCTTTTCTCAGGGAAGAACGCAATGACGGTACCTGAGGAATCAGCATCGGCTAACTCCGTGCCAGCAGCCGCGGTAATACGGAGGATGCAAGCGTTATCCGGAATGATTGGGCGTAAAGGGTTCGCAGGTGGTAATGAAAGTCTGCTGTTAAAAGTCGGGGCTCAACCCCGAACCGGCGGTGGAAACTACAATGCTAGAGTGTGGTAGGGGCAGAGGGAATTCCTGGTGTAGCGGTGAAATGCGTAGAGCTCAGGAAGAACACCGGTGGCGAAAGCGCTCTGCTAGACCAAAACTGACACTGAGGGACGAAAGCTAGGGGAGCGAATGGG` |
| ASV490 | 430 | `TAGGGAATATTGCGCAATGGGGGAAACCCTGACGCAGCAACGCCGCGTGAGTGAGGAAGGTCTTCGGATTGTAAAACTCTTTTGCTAGGGAAAAATGACCTTGTGGCTAATATCCATAAGGAGTGATGGTACCTAAAGAATAAGGACCGGCTAACTTCGTGCCAGCAGCCGCGGTAATACGAAGGGTCCAAGCGTTGTTCGGAATCATTGGGCGTAAAGCGCGCGCAGGCGGGTCAGCAAGTCAGATGTGAAATCTCGAAGCTCAACTTCGAAACTGCGTCTGAAACTGCTAGTCTAGAGTGTCGGAGGGGGTAGGGGAATTTCACGTGTAGGGGTAAAATCCGTAGAGATGTGAAGGAACACCTGGGGCGAAGGCGCCTACCTGGACGACTACTGACGCTGAGGCGCGAAAGCGTGGGGAGCAAACAGG` |
| ASV5218 | 424 | `TGAGGAATATTGGTCAATGGGCGAGAGCCTGAACCAGCCAAGTCGCGTGAAGGATGACTGTCTTATGGATTGTAAACTTCTTTTATTAGGGAATAAAAAAGGGTATGTATACTCTCTTGCAAGTACCTGATGAATAAGCATCGGCTAACTCCGTGCCAGCAGCCGCGGTAATACGGAGGATGCGAGCGTTATCCGGATTTATTGGGTTTAAAGGGTGCGTAGGCGGGATATTAAGTCAGCGGTGAAAGTTTTCGGCTCAACCGAGAAATTGCCGTTGAAACTGTTATTCTTGAGTGTAAATGAGGTTTGCGGAATGCGTGGTGTAGCGGTGAAATGCATAGATATCACGCAGAACTCCGATTGCGAAGGCAGCAAACTAGGATACAACTGACGCTGAGGCACGAAAGCGTGGGGATCAAACAGG` |
| ASV2373 | 429 | `TGGGGAATTTTGGACAATGGGCGCAAGCCTGATCCAGCCATTCCGCGTGAGTGAAGAAGGCCTTCGGGTTGTAAAGCTCTTTCGCAAGGGAAGAAACGATTTAGGTAAACAATCTAAGTTAATGACGGTACCTTGATAAGAAGCACCGGCTAACTACGTGCCAGCAGCCGCGGTAATACGTAGGGTGCGAGCGTTAATCGGAATTACTGGGCGTAAAGCGTGCGCAGGCTGTTTGGCAAGTCAGATGTGAAATCCCCGGGCTCAACCTGGGAACTGCGTTTGAAACTGCCAGACTAGAATCGGTCAGAGGGGGGTAGAATTCCACGTGTAGCAGTGAAATGCGTAGAGATGTGGAGGAATATCAATGGCGAAGGCAGCCCCCTGGGATCAGATTGACGCTCATGCACGAAAGCGTGGGGAGCAAACAGG` |
| ASV4073 | 429 | `TCGAGAATTTTTCACAATGGGGGAAACCCTGATGGAGCGACGCCGCGTGGAGGATGAAGGTTTTCGGATTGTAAACTCCTGTCACCACAGAACAAGCAATGTTATTTAATAAATGACAAAGTTGATAGTATGTGGGGAGGAAGGGACGGCTAACTCTGTGCCAGCAGCCGCGGTAATACAGAGGTCCCAAGCGTTGTTCGGATTCACTGGGCGTAAAGGGTGTGTAGGAGGTTTGATAAGTCTGATGTGAAATCCCGTCGCTCAACGACGGAACTGCATTGGATACTGTCAGGCTAGAGGGTCTGAGGGGGAAGCGGAATTCTTGGTGTAGCGGTGAAATGCGTAGATATCAAGAGGAACACCCGTGGCTAACGCGGCTTCCTGGAAGACTCCTGACTCTGAAACACGAAAGCCAGGGTAGCGAATGGG` |
| ASV5510 | 418 | `TCGAGAATCTTCCGCAATGGGCGAAAGCCTGACGGAGCGACGCCGCGTGAGGGAAGAAGTCCTTCGGGATGTAAACCTCTTTTAACAGGGAAGAATCCGCCGTTTGGCGGATGACGGTACCTGTGGAATAAGGAACTGCTAACTTCGTGCCAGCAGCAGCGGTAATACGAAGGTTCCAAGCGTTATCCGGAGTTACTGGGCGTAAAGGGTGCGTAAGCGGTTTGGTTAGTCTGATGTTAAATCCTGAGGCTTAACTTCAGGACCGCATTGGAAACGGCCAAACTAGATCAAGTCAGAGGCTGACGGAACGTGCGGTGTAGGGGTGAAATCCGTTGATATCGCACGGAACACCAAAGGCGAAGGCAGTCAGCTGGGGCTTTGATGACGCCGAGGCACGAAAGCGTGGGGATCAAAAAGG` |
| ASV4866 | 429 | `TGGGGAATATTGGACAATGGGCGCAAGCCTGATCCAGCAACGCCGCGTGTGTGAAGAAGGCCTTCGGGTTGTAAAGCACTTTCGATGGGGAAGAAAAAGCTGAAGTTAACACCTTTGGAAATTGACGTTACCCATAGAAGAAGCACTGGCTAACTCTGTGCCAGCAGCCGCGGTAATACAGAGAGTGCAAGCGTTAATCGGAATTACTGGGCGTAAAGCGCGCGTAGGCGGATGCTTAAGTCGGATGTGAAATCCCCGAGCTTAACTTGGGAATTGCATTCGATACTGGGTATCTGGAGTATGGTAGAGGGAAGTGGAATTTCTGGTGTAGCGGTGAAATGCGTAGATATCAGAAAGAACATCAGTGGCGAAGGCGACTTCCTGGACCAATACTGACGCTGAGGCGCGAAAGCGTGGGGAGCAAACAGG` |
| ASV4208 | 430 | `TGGGGAATCTTGCGCAATGGACGAAAGTCTGACGCAGCCACGCCGCGTGAGTGAAGAAGGCCTTCGGGTCGTAAAGCTCTGTCGGGCGGGACGAAAATTATTTAGGTTAATAGCCTAGGTAACTGACGGTACCGCCAAAGGAAGCACCGGCTAACTCCGTGCCAGCAGCCGCGGTAATACGGAGGGTGCAAGCGTTGCTCGGAATTATTGGGCGTAAAGGGTAGGTAGGTGGTCTCATTTGTCAGAGGTGAAAGCCTTGGGCTTAACCCAAGAAGTGCCTCTGAAACGGTGAGACTAGAGTTCTGGAGAGGGTCGTGGAATTCCCGGTGTAGCGGTGAAATGCGTAGAGATCGGGAGGAACACCAGAGGCGAAGGCGGCGACCTGGACAGATACTGACACTCAACTACGAAAGCGTGGGTAGCAAACAGG` |
| ASV1785 | 404 | `TGGGGAATATTGGACAATGGGCGAAAGCCTGATCCAGCAATACCGCGTGAATGATGAAGGCCTTAGGGTTGTAAAGTTCTTTTAGTAGGGAAGATAATGACGGTACCTACAGAAAAAGCCCCGGCTAACTCCGTGCCAGCAGCCGCGGTAAGACGGAGGGGGCTAGCGTTGTTCGGAATTACTGGGCGTAAAGAGCGCGTAGGCGGATTAGTAAGTTGGAAGTGAAAGCCCGGAGCTTAACTTCGGAATAGCTTTCAAAACTACTAGTCTTGAGTGTAGTAAGGGATAATGGAATTCCTAGTGTAGAGGTGAAATTCTTAGATATTAGGAGGAACACCGGTGGCGAAAGCGATTATCTAGGCTACAACTGACGCTGAGGCGCGAAAGCGTGGGGAGCAAACAGG` |
| ASV3790 | 404 | `TGGGGAATATTGGACAATGGGCGCAAGCCTGATCCAGCCATGCCGCGTGAGTGAAGAAGGCCTTAGGGTTGTAAAGCTCTTTGGACAGGGACGATAATGACGGTACCTGTAGAACAAGCCCCGGCTAACTCCGTGCCAGCAGCCGCGGTAATACGGAGGGGGCTAGCGTTATTCGGAATTACTGGGCGTAAAGCGCGCGTCGGCGGCTTGCCAAGTCAGGTGTGAAAGCCCGGGGCTTAACCCCGGAATTGCATTTGAAACTGGCAGGCTAGAGTCTGGTAGAGGGTGGTGGAATTCCACGTGTAGAGGTGAAATTCGTAGATATGTGGAGGAACACCGGTGGCGAAGGCGACCACCTGGACCAGTACTGACGCTATAGCGCGAAAGCGTGGGGAGCAAACAGG` |
| ASV5099 | 428 | `TGGGGAATATTGGACAATGGGCGCAAGCCTGATCCAGCAATTCCGCGTGGGTGAAGAAGGCCTTCGGGTTGTAAAGCCCTTTAAGTTGGGACAAAAAAATCGGCGATAACATCGCCGGTCTTGATGGTACCGACAGAATAAGCAACGGCTAACTCTGTGCCAGCAGCCGCGGTAATACAGAGGTTGCAAGCGTTAATCGGATTTACTGGGCGTAAAGCGTGCGTAGGAGGCTTGCTAAGTCGGGCGTGAAATCCCTGGGCTCAACCCGGGAATGGCACTCGATACTGGTGAGCTAGAGTTCGGTAGAGGGCGGCGGAATTCCCGGTGTAGCAGTGAAATGCGTAGATATCGGGAGGAACATCGATGGCGAAGGCAACCGCCTGGGCCTGAACTGACTCTGAGGCACGAAAGCGTGGGGAGCAAACAGG` |
| ASV5053 | 405 | `TGAGGAATCTTCCGCAATGGGCGAAAGCCTGACGGAGCAACACCGCGTGAAGGATGAAGTGGTTCGCCATGTAAACTTCTTTTCTGAGGGAAGAATTCTGACGGTACCTTAGGAATAAGCACCGGCTAACTCTGTGCCAGCAGCCGCGGTAAGACAGAGGGTGCGAGCGTTATCCGGAATTATTGGGCGTAAAGCGTGTGTAGGTGGAATAGTAAGTCGGGGATAAAATCTCTGTGCTCAACGCAGAACCTGTTCCCGATACTGTTATTCTTGAAGTATGCAGAGGCACGTGGAATTCTGTGTGTAGGGGTAAAATCCGTAGATACACGGAGGAACACCAAAAGCGAAAGCAGCGTGCTGGGCATATCTTGACACTGAGACACGAAAGCGTGGGGATCAAACAGG` |
| ASV4501 | 429 | `TGGGGAATTTTGGACAATGGGGGCAACCCTGATCCAGCAACGCCGCGTGAATGAAGAAGGCCTTCGGGTTGTAAAGTTCTTTTGTCAGGGAGCAAAGTCTGAGAGTTAATACCTCTTGGAGCTGAGAGTACCTGAAGAATAAGCACCGGCTAACTACGTGCCAGCAGCCGCGGTAATACGTAGGGTGCGAGCGTTAATCGGAATTACTGGGCGTAAAGCGTGCGCAGGCGGATAGGTAAGTTAGAAGTGAAATCCCCGGGCTCAACCTGGGAACTGCTTTTAAGACTGCCCATCTAGAGTTTGTCAGAGGGGGGTGGAATTCCAAGTGTAGCAGTGAAATGCGTAGAGATTTGGAGGAACACCGATGGCGAAGGCAGCCCCCTGGGATAAAACTGACGCTCATGTACGAAAGCGTGGGTAGCAAACAGG` |
| ASV1168 | 429 | `TGGGGAATTTTGGACAATGGGCGCAAGCCTGATCCAGCAATGCCGCGTGCAGGACGAAGGCCTTCGGGTTGTAAACTGCTTTTGTACAGAGCGAAAAGGTCTTCTCTAATACAGGAGACTGATGACGGTACTGTAAGAATAAGCACCGGCTAACTACGTGCCAGCAGCCGCGGTAATACGTAGGGTGCAAGCGTTAATCGGAATTACTGGGCGTAAAGCGTGCGCAGGCGGTTTTGTAAGACAGAGGTGAAATCCCCGGGCTCAACCTGGGAACTGCCTTTGTGACTGCAAAGCTAGAGTGCGGCAGAGGGGGATGGAATTCCGCGTGTAGCAGTGAAATGCGTAGATATGCGGAGGAACACCAATGGCGAAGGCAATCCCCTGGGCCTGCACTGACGCTCATGCACGAAAGCGTGGGGAGCAAACAGG` |
| ASV5278 | 424 | `TAGGGAATATTGGGCAATGGAGGCAACTCTGACCCAGCCATGCCGCGTGAAGGATGACCCCCCTATGGGGCGTAAACTTCTTTTATCATAGAGTAACCGCAGGGACGTGTCCCTGCCTGCAAGTATATGATGAATAAGCACCGGCTAACTCCGTGCCAGCAGCCGCGGTAATACGGAGGGTGCAAGCGTTATCCGGATTTATTGGGTTTAAAGGGTGCGCAGGCGGATCGATAAGTCAGGGGTGAAATTTTGCAGCTTAACTGCAACACTGCCTTTGATACTGTCGGACTAGAGTGTAATTGACGTTGCTGGAATGTGATGTGTAGCGGTGAAATGCTTAGATATGTCACAGAACGCCGATCGTGAAGACAGGTAACGAAATTAATACTGACGCTCAGGCACGAAAGCGTGGGGATCAAACAGG` |
| ASV5323 | 424 | `TGAGGAATATTGGACAATGGGTGAGAGCCTGATCCAGCCATCCCGCGTGAAGGAATAAGGTCCTATGGATTGTAAACTTCTTTTATACTGGGATAAACCTACCCTCGTGAGGGTAGCTGAAGGTACAGTATGAATAAGCACCGGCTAACTCCGTGCCAGCAGCCGCGGTAATACGGAGGGTGCAAGCGTTATCCGGATTTATTGGGTTTAAAGGGTCCGTAGGCGGGCTTATAAGTCAGCGGTGAAATCTCCTAGCTTAACTAGGAAACTGCCATTGATACTGTAAGCCTTGAGTATATTTGAGGTAGCTGGAATAAGTAGTGTAGCGGTGAAATGCATAGATATTACTTAGAACACCAATTGCGAAGGCAGGTTACCAAGATATAACTGACGCTGATGGACGAAAGCGTGGGGAGCGAACAGG` |
| ASV462 | 404 | `TGGGGAATATTGGGCAATGGACGAAAGTCTGACCCAGCCATGCCGCGTGAATGATGAAGGCCTTAGGGTTGTAAAGTTCTTTCGCCGATGAAGATAATGACGGTAGTCGGAGAAGAAGCCCCGGCTAACTTCGTGCCAGCAGCCGCGGTAATACGAAGGGGGCTAGCGTTGTTCGGAATTACTGGGCGTAAAGCGAGCGTAGGTTGCTCGATAAGTCAGTGGTGAAAGCCCAGAGCTCAACTCTGGAACTGCCATTGAAACTGTCGAGCTGGAATCTCAGAGGGGGTAGCGGAATTCCAAATGTAGGGGTGAAATCCGTAGATATTTGGAGGAACACCGGTGGCGAAGGCGGCTACCTGGATGAGTATTGACACTGAGGCTCGAAAGCGTGGGGATCAAACAGG` |
| ASV2621 | 406 | `TAGGGAATATTGCGCAATGGGGGAAACCCTGACGCAGCGACGCCGCGTGAGTGATGAAGGCCTTCGGGTCGTAAAGCTCTGTTATATGGGAATAAGATCTGAATGTACCATGTGAGAAAGGATCGGCTAACTTCGTGCCAGCAGCCGCGGTAAGACGAGGGATCCTAGCGTTGCTCGGAATCATTGGGCGTAAAGCGGGTGTAGGTGGCTCTATAAGTCAGGTGTGAAAGCCCAGGGCTCAACCCTGGAAGTGCACTTGATACTGTTTAGCTTGAGTGTGGGAGAGGTTACTGGAATACCTGGTGTAGTGGTGAAATACGTAGATATCAGGTGGAACACCGGAGGCGAAGGCGGGTAACTGGCCCAACACTGACACTCAGACCCGAAAGCGTGGGGATCAAACAGG` |
| ASV3584 | 428 | `TCGGGAATCTTCCGCAATGGGCGAAAGCCTGACGGAGCGACGCCGCGTGAGCGATGAAGGCCTTCGGGTTGTAAAGCTCTGTGGGGAGGGAAGAATAAGGGTGAGTTAATAGCTCATCTGATGACGGTACCTCCTTAGCAAGCACCGGCTAACTCTGTGCCAGCAGCCGCGGTAAGACAGAGGGTGCAAACGTTGTTCGGATTTACTGGGCGTAAAGCGCGTGTAGGCGGCCTTGCAAGTCGGATGTGAAAGCCCACGGCTCAACCGTGGAAGTGCATCCGAAACTGCATCGCTCGAGTCTCGGAGAGGAAGGTGGAATGCTTGGTGTAGAGGTGAAATTCGTAGATATCAAGCAGAACACCTGCGGCGAAGGCGGCCTTCTGGACGATGACTGACGCTGAGACGCGAAAGCGTGGGGAGCAAACAGG` |
| ASV5373 | 405 | `TGAGGAATATTGCTCAATGGGCGCAAGCCTGAAGCAGCGACGCCGCGTGGATGAAGAAGGTTTTCGGACTGTAAAATCCTTTTCTGTGTGACGAGAGTGGACGGTAGCACAGGAATAAGTCTCGGCTAACTACGTGCCAGCAGCCGCGGTAAAACGTAGGAGGCAAGCGTTATCCGGAGTTACTGGGCGTAAAGCGCGTGTAGGTGGGTTGGTAAGTTACTTATAAAAGCCCCTGGCTAAACTGGGGGAGGTTGAGTGATACTGCCAGTCTTGAATAGAGGAGAGGTTGACGGAATTCCGCGTGTAGTGGTGAAATGCGTAGAGATGCGGAAGAACACCAGTGGCGAAGGCGGTCAACTGGCCTCATATTGACACTCAGACGCGAAGGCATGGGTAGCGAACGGG` |
| ASV5025 | 405 | `TGGGGAATCTTGCGCAATGGGCGAAAGCCTGACGCAGCAACGCCGCGTGCGGGATGAAGGCCTTCGGGTTGTAAACCGCTTTCAGCAGGAACGAAAATGACGGTACCTGCAGAAGAAGGTCCGGCCAACTACGTGCCAGCAGCCGCGGTAACACGTAGGGGCCAAGCGTTGTCCGGATTTATTGGGCGTAAAGAGCTCGTAGGCGGTTCGGTAAGTCGGGTGTGAAAACTCTGGGCTCAACCCAGAGACGCCACCCGATACTGCTGTGACTAGAGTCCGGTAGGGGAGTGGGGAATTCCTGGTGTAGCGGTGAAATGCACAGATATCAGGAGGAACACCGGTGGCGAAGGCGCCACTCTGGGCCGGTACTGACGCTGAGGAGCGAAAGCGTGGGTAGCAAACAGG` |
| ASV423 | 429 | `TGGGGAATTTTGGACAATGGGCGCAAGCCTGATCCAGCAATGCCGCGTGCAGGATGAAGGCCTTCGGGTTGTAAACTGCTTTTGTACGGAGCGAAACGGCGAGTTCTAATAAGACTCGCTAATGACGGTACCGTAAGAATAAGCACCGGCTAACTACGTGCCAGCAGCCGCGGTAATACGTAGGGTGCAAGCGTTAATCGGAATTACTGGGCGTAAAGCGTGCGCAGGCGGTTATATAAGACAGATGTGAAATCCCCGGGCTCAACCTGGGACCTGCATTTGTGACTGTATGGCTAGAGTACGGTAGAGGGGGATGGAATTCCGCGTGTAGCAGTGAAATGCGTAGATATGCGGAGGAACACCGATGGCGAAGGCAATCCCCTGGACCTGTACTGACGCTCATGCACGAAAGCGTGGGGAGCAAACAGG` |
| ASV1517 | 405 | `TAGGGAATTTTCCACAATGGGCGAAAGCCTGATGGAGCAACGCCGCGTGCAGGATGACGGCCTTAGGGTTGTAAACTGCTTTTCTGTGTGAAGAATATGACGGTAACATAGGAATAAGGATCGGCTAACTACGTGCCAGCAGCCGCGGTCATACGTAGGATCCAAGCGTTATCCGGAATTACTGGGCGTAAAGAGTTGCGTAGGTGGTTTATTAAGTGGAGCGTGAAAGCTTCCCGCTCAACGGGATATCCGCGTTCCAAACTGGTAAACTAGAGTGTGAGAGAGGTAACTGGAATTCCAAGTGTAGGAGTGAAATCCGTAGATATTTGGAGGAACACCGATGGCGTAGGCAGGTTACTGGCTCATTACTGACACTAAGGCACGAAAGCGTGGGGAGCAAACGGG` |
| ASV562 | 404 | `TGGGGAATCTTAGACAATGGGGGAAACCCTGATCTAGCCATGCCGCGTGAGCGATGAAGGCCTTAGGGTTGTAAAGCTCTTTCAGTGGGGAAGATAATGACGGTACCCACAGAAGAAGCCCCGGCTAACTCCGTGCCAGCAGCCGCGGTAATACGGAGGGGGCTAGCGTTGTTCGGAATTACTGGGCGTAAAGCGCACGTAGGCGGATCGGAAAGTTGGGGGTGAAATCCCGGGGCTCAACCCTGGAACTGCCTTCAAAACTCCCGGTCTTGAGGTCGAGAGAGGTGAGTGGAATTCCGAGTGTAGAGGTGAAATTCGTAGATATTCGGAGGAACACCAGTGGCGAAGGCGACTCACTGGCTCGATACTGACGCTGAGGTGCGAAAGCGTGGGGAGCAAACAGG` |
| ASV2363 | 404 | `TGGGGAATCTTGGACAATGGGCGCAAGCCTGATCCAGCCATGCCGCGTGAGTGACGAAGGCCTTAGGGTTGTAAAGCTCTTTTGCCGGGGACGATAATGACGGTACCCGGAGAATAAGTCCCGGCTAACTTCGTGCCAGCAGCCGCGGTAATACGAAGGGGACTAGCGTTGTTCGGAATTACTGGGCGTAAAGCGCACGTAGGCGGATGTGTCAGTCAGAGGTGAAATCCCGGAGCTCAACTTCGGAACTGCCTTTGATACAGCACGTCTTGAATCCGGTAGAGGTGGGTGGAATTCCTAGTGTAGAGGTGAAATTCGTAGATATTAGGAAGAACACCGGTGGCGAAGGCGGCCCACTGGACCGGTATTGACGCTGAGGTGCGAAAGCGTGGGGAGCAAACAGG` |
| ASV4519 | 416 | `TTAGGAATCTTCCCCAATGGGCGAAAGCCTGAGGGAGCGACGCCGCTTGAAGGACGAAGGTCTTCGGATCGTAAACTTCTGAAACAAGGACGAAATCCCTGCAAAGGGTCTGACGGTACTTGTGTAATAGCACCGGCTAACTCCGTGCCAGCAGCCGCGGTAATACGGAGGGTGCAAGCGTTACCCGGAATCACTGGGCGTAAAGGGCGTGTAGGCGGCTTGGTAAGTCTGGATTTAAAGACCGAAGCTCAACTTCGGGAATGGTCTGGATACTGCTTTGCTTGACGACTGGAGGGGAGAGTGGAATTCTCGGTGTAGCGGTGGAATGCGTAGATACCGAGAGGAACACCAGTGGCGAAGGCGGCTCTCTGGACAGTGCGTGACGCTGAGGCGCGAAAGTGTGGGGAGCGAACCGG` |
| ASV5344 | 406 | `GCGAGAATATTCGACAATGGACGAAAGTCTGATCGAGCGACGCCGCGTGTACGATGAAGTTTTTCGGAATGTAAAGTACTTTTATTAGGGAAGAACGTTGACGGTACCTAATGAATAAGGGGTTGCTAACTCTGTGCCAGCAGCAGCGGTAATACAGAGACCCCAAGCGTTATCCGGAATTATTGGGCGTAAAGGGTGAGTAGGTGGTATTGTTAGTCGAGTTTAAAAGATTGCGGGCTCAACCCGTGATACGGACTCGAAACGGCAAAACTAGAATATGTAAGAGGTTAGTGGAATTATATGTGTAGGGGTAAAATCCGTTGATATATATGAGAACACCTAAAGCGAAGGCAGCTAACTGGTACATTATTGACACTGAATCACGAAAGCGTGGGTAGCGATACGG` |
| ASV599 | 429 | `TGGGGAATCTTGGACAATGGGCGAAAGCCTGATCCAGCAATGCCGCGTGCGTGAAGAAGGCCTTCGGGTTGTAAAGCGCTTTTGTCGGGGAGGAAATCCTAGTATCTAATAAATGCTGGGGATGACAGTACCCGAAGAATAAGGACCGGCTAACTACGTGCCAGCAGCCGCGGTAATACGTAGGGTCCAAGCGTTAATCGGAATTACTGGGCGTAAAGCGTCCGCAGGTGGCTTGATAAGATAGAGGTGAAATCCCTGGGCTCAACCTAGGAATTGCCTTTATGACTGTCTCGCTAGAGTATGGGAGAGGGGGGTGGAATTCCACGTGTAGCAGTGAAATGCGTAGAGATGTGGAGGAACACCGATGGCGAAGGCAACCCCCTGGCCTAATACTGACACTCATGGACGAAAGCGTGGGGAGCAAACAGG` |
| ASV4717 | 404 | `TGGGGAATATTGGACAATGGGCGAAAGCCTGATCCAGCAATACCGCGTGAGTGATGAAGGCCTTAGGGTTGTAAAGCTCTTTCAGTAGGGAAGATAATGACGGTACCTACAGAAGAAGCACCGGCTAACTCCGTGCCAGCAGCCGCGGTAATACGGAGGGTGCTAGCGTTGTTCGGAATTACTGGGCGTAAAGAGTGCGTAGGCGGTCTGACAAGTTGGAAGTGAAATCCCGGGGCTTAACCTCGGAATTGCTTTCAAAACTGTTAGACTAGAGTATGGTAGGGGATAGTGGAATTCTTAGTGTAGGGGTGAAATCCGTAGATATTAAGAGGAACACCAGAGGCGAAGGCGACTATCTGGGCCATTACTGACGCTAAGGTACGAAAGCGTGGGGAGCAAACAGG` |
| ASV2825 | 404 | `TCGGGAATCTTGGACAATGGGCGCAAGCCTGATCCAGCCATGCCGCGTGAATGATGAAGGCCTTAGGGTTGTAAAATTCTTTCGCAGGGGACGATAGTGACGGTACCCTGATAAGAAGCTCCGGCTAACTTCGTGCCAGCAGCCGCGGTAATACGAAGGGGGCTAGCGTTGTTCGGAATTACTGGGCGTAAAGCGCGTGTAGGCGGGCCGTTAAGTCGGGGGTGAAAGCCCAGAGCTCAACTCTGGAATTGCCTTCGATACTGGCGGCCTGGACCCAAGTAGAGGCGAGTGGAATTGCGAGTGTAGAGGTGAAATTCGTAGATATTCGCAGGAACACCAGTGGCGAAGGCGACTCGCTGGACTTGTGGTGACGCTGAGACGCGAAAGCGTGGGGAGCAAACAGG` |
| ASV1404 | 407 | `TGGGGAATTTTCCGCAATGGGCGCAAGCCTGACGGAGCAATACCGCGTGAGGGAGGAAGGCTCTTGGGTCGTAAACCTCTTTTCTCAGGGAAGAACAAAATGACGGTACCTGAGGAATAAGCATCGGCTAACTCCGTGCCAGCAGCCGCGGTAATACGGAGGATGCAAGCGTTATCCGGAATGATTGGGCGTAAAGCGTCCGTAGGTGGTTTTGTAAGTCTGTGGTTAAAGCGTGTAGCTCAACTACATAACGGCCATGGAAACTACAAGACTAGAGTGAAGTAGGGGTAGAGGGAATTCCCAGTGTAGCGGTGAAATGCGTAGAGATTGGGAAGAACACCGGTGGCGAAAGCGCTCTACTGGACTTATACTGACACTGAGGGACGAAAGCTAGGGGAGCGAAAGGG` |
| ASV5327 | 404 | `TAGGGAATATTGCGCAATGGGCGAAAGCCTGACGCAGCAATTCCGCGTGGAGGATGACGGATTTCGGTCTGTAAACTCCTGTCAGCAGGGAAGTATCGGACGGTACCTGCAGAGGAAGCACCGGCTAACTACGTGCCAGCAGCCGCGGTAATACGTAGGGTGCAAGCGTTGTCCGGAATTATTGGGCGTAAAGCGTCCGTAGGCGGTTTGGTAAGTCTAATGTAAAAGGTCGTGGCTTAACCACGGTTATGTGTTGGAAACTACTAGACTTGAGTATGGTAGAGGTTGCTGGAATTCCTGGTGTAGCGGTGAAATGCGTAGATATCAGGAGGAACACCTGTGGCGAAAGCGTGCAACTGGGCCTATACTGACGCTAATGGACGAAAGCCAGGGTAGCGAAAGGG` |
| ASV5542 | 429 | `TGGGGAATATTGGACAATGGGGGCAACCCTGATCCAGCCATGCCGTGTGTGTGAAGAAGGCCTTAGGGTTGTAAAGCACTTTTGACGGAGAAGAAGGGATATTGGTTAATACCCGATATTTTTGACATGAACTGTAGAAAAAGCACCGGCTAACTCCGTGCCAGCAGCCGCGGTAATACGGAGGGTGCAAGCGTTAATCGGAATTACTGGGCGTAAAGGGCGTGTAGGCGGTTGTATAAGTTGGATGTGAAATCCCCGAGCTCAACTTGGGAACTGCATTCAAAACTGTACGACTAGAGTACTGTAGAGGATGGTGGAATTTTCTGTGTAGCAGTAAAATGCTTAGAGATTGAAAGGAACATCAGAGGCGAAGGCGGCCATCTGGACATGTACTGACGCTGAGGCGCGAAAGCGTGGGGAGCAAACAGG` |
| ASV5505 | 501 | `CAGCACCATCGTGGGGCCGCCGCTCTTGCTGCTGCTGACCGAGAAGCCCCGGACGAACCGCCCGCTCGCTGCGGCGAGTGCGGCCTTGCTCATCGGCCGGGGGAGAACACGCTATGCAGCCGATGACCCCGCCCGAACCTAAGGAGACGACGACGTGAGCAGAATCCGCAACGCATGGGTCAACCGCTGCCTGCTCTGCCTGCTGCGGCCATTTCACGGGAGCCATGATGCCTGAGTCTGTTTCTGGGGTGGACGCGATCCTTGACGGGCTGCGGGAGGCGACGCGCTGGACATGGCCGACGGTGCTTCACGGCCCGCCGTCCCGCGACCCAGCGACGGACGGGCGAGTCGAGATGGTCCCGCTCGCTGATGCTGAGGCTGCGGTTCGCGCCGCAGCCGACGCCCAACGACTCCCCCGCACGGATGACCGCGCGTACGACCTGCGCCAGCGGCTTAACCGCGCCGTGCGATGGCTGCGCGACGCCGAGGATATCGCTCGCA` |
| ASV922 | 429 | `TGGGGAATATTGGACAATGGGGGCAACCCTGATCCAGCCATGCCGCGTGTGTGAAGAAGGCCTTAGGGTTGTAAAGCACTTTCAGTGGGGAGAAAGGTGATTCACCTAATACGTGAGTCAATTGATGCTACCCACAGAAGAAGCACCGGCTAACTCCGTGCCAGCAGCCGCGGTAATACGGAGGGTGCAAGCGTTAATCGGAATTACTGGGCGTAAAGCGTACGTAGGTGGTTTGTTAAGCCAGCTGTGAAATCCCCGGGCTCAACCTGGGCACTGCAGTTGGAACTGTCAAGCTAGAGTAGGGTAGAGGGGTGTGGAATTCCAGGTGTAGCGGTGAAATGCGTAGATATCTGGAGGAACATCAGTGGCGAAGGCGACACCCTGGACTCATACTGACACTGAGGTACGAAAGCGTGGGGAGCAAACAGG` |
| ASV5685 | 406 | `TCGAGAATATTCGACAATGGGCGAAAGCCTGATCGAGCGACGCCGCGTGCAGGATGAAGTGCTTCGGTATGTAAACTGCTTTTGTGAACCAGAAAGTTTATTGATCGGTTCAAGAATAAGAGGTTGCTAAACTCGTGCCAGCAGCAGCGGTAATACGAGTGCCTCGAGCGTTATCCGGAATCATTGGGCGTAAAGGGTGTGCAGGCGGTCTTATTAGTCTTTTGTTAAATTCTTCGGCTCAACCGGGGAAGTGCAGGGGAAACGGTAAGACTCGAGGATGCGAGAGGTTAACGGAACTCATAGTGTAGCGGTGAAATGCGTTGATATTATGGGGAACACCAAATGCGAAGGCAGTTAACTGGAGCACTCCTGACGTTGAAACACGAAAGCGTGGGTAGCGAATGGG` |
| ASV405 | 429 | `TGGGGAATCTTGGACAATGGGCGAAAGCCTGATCCAGCAATGCCGCGTGCGTGAAGAAGGCCTTCGGGTTGTAAAGCGCTTTTGTCTGGGAGCAAATCCTGGCCTCTAATACAGGTCGGGGATGAGAGTACCAGAAGAATAAGGACCGGCTAACTACGTGCCAGCAGCCGCGGTAATACGTAGGGTCCAAGCGTTAATCGGAATTACTGGGCGTAAAGCGTCCGCAGGTGGCTTGATAAGATAGACGTGAAATCCCTGGGCTCAACCTAGGAATTGCGTTTATGACTGTCTCGCTAGAGTATGGGAGAGGGGGGTGGAATTCCACGTGTAGCAGTGAAATGCGTAGAGATGTGGAGGAACACCGATGGCGAAGGCAACCCCCTGGCCTAATACTGACACTCATGGACGAAAGCGTGGGGAGCAAACAGG` |
| ASV2324 | 404 | `CAGGGAATTTTGCGCAATGGGCGAAAGCCTGACGCAGCAACGCCGCGTGGGGGATGAAGGCTCTTGGGTCGTAAACCCCTTTTGCCGGGGAAGATAATGACGGTACCCGGCGAATAAGCCACGGCTAACTACGTGCCAGCAGCCGCGGTAATACGTAGGTGGCAAGCGTTGTCCGGATTTATTGGGCGTAAAGGGCGCGCAGGCGGTTCGTTAAGTTCGAGGTGAAATCTCCCGGCTCAACTGGGAGGGGTCTTCGGATACTGGCGGACTTGAGGAGAGCAGAGGTGAGTGGAATTCTGGGTGTAGTGGTGAAATGCGTAGATATCCAGAGGAACACCAGTGGCGAAGGCGACTCACTGGGCTCCACCTGACGCTGAGGCGCGAAAGCGTGGGGAGCGAACCGG` |
| ASV5506 | 407 | `CCGAGAATATTGGACAATGGGCGAAAGCCTGATCCAGCGACATTGCGTGGAGGATGAAGACGTTCGCGCCGTAAACTCCTTTTATAGGGAAAGAAGTAATTGACGGTACCCTATGAATAAGCGGGGGCTAATTCTGTGCCAGCAGCAGCGGTAATACAGAACCCGCAAGCGTTATCCGGATTTATTGGGCGTAAAGTGTTCGTAGGCGGTTTGGAAAGTCTGATATCAAATATCCGGGCTCAACCCGGGACGCGTATCGGAAACTTCCAAACTTGAAGGTGCTAGGGGCTATCGGAACGCACGGTGTAGGGGTGAAATCCGTTGATATCGTGCGGAACACCAAAAGCGAAGGCAGATAGCTGGGGCATTCTTGACGCTGAGGAACGAAAGCGTGGGGAGCAAAAAGG` |
| ASV2447 | 429 | `TGGGGAATTTTGGACAATGGGCGCAAGCCTGATCCAGCAATGCCGCGTGCAGGATGAAGGCCTTCGGGTTGTAAACTGCTTTTGTACGGAACGAAAAGGTCCTTTCTAATACAAGGGGCCCATGACGGTACCGTAAGAATAAGCACCGGCTAACTACGTGCCAGCAGCCGCGGTAATACGTAGGGTGCGAGCGTTAATCGGAATTACTGGGCGTAAAGCGTGCGCAGGCGGTTATATAAGACAGTTGTGAAATCCCCGGGCTCAACCTGGGAACTGCATCTGTGACTGTATAGCTAGAGTACGGTAGAGGGGGATGGAATTCCGCGTGTAGCAGTGAAATGCGTAGATATGCGGAGGAACACCGATGGCGAAGGCAATCCCCTGGACCTGTACTGACGCTCATGCACGAAAGCGTGGGGAGCAAACAGG` |
| ASV3063 | 429 | `TGGGGAATTTTGGACAATGGGGGGAACCCTGATCCAGCAATGCCGCGTGAGTGAAGAAGGCCTTCGGGTTGTAAAGCTCTTTTGTTAGGGAAGAAAAGGTCTGGGTTAATACCCTGGGCTGATGACGGTACCTAAAGAATAAGCACCGGCTAACTACGTGCCAGCAGCCGCGGTAATACGTAGGGTGCGAGCGTTAATCGGAATTACTGGGCGTAAAGCGTGCGCAGGCGGTTTAGTAAGTCAGATGTGAAATCCCCGAGCTTAACTTGGGAACTGCATTTGAAACTGCGAGACTAGAGTGTGTCAGAGGGAGGTAGAATTCCACGTGTAGCAGTGAAATGCGTAGATATGTGGAGGAATACCGGTGGCGAAGGCGGCCTCCTGGGATAACACTGACGCTCATGTACGAAAGCGTGGGGAGCAAACAGG` |
| ASV159 | 429 | `TGGGGAATTTTGGACAATGGGGGCAACCCTGATCCAGCAACGCCGCGTGAATGAAGAAGGCCTTAGGGTTGTAAAGTTCTTTTATCTGAGAGCAAAGACGAATGGTTAATAGCTATTTGGGATGAGAGTATCAGAGGAATAAGCACCGGCTAACTACGTGCCAGCAGCCGCGGTAATACGTAGGGTGCAAGCGTTAATCGGAATTACTGGGCGTAAAGCGTGCGCAGGCGGATATGTAAGTTAGATGTGAAATACCCGAGCTCAACTTGGGAATGGCATTTAAGACTGCATGTCTAGAGTTTGTCAGAGGGGGGTAGAATTCCAAGTGTAGCAGTGAAATGCGTAGAGATTTGGAGGAATACCGGTGGCGAAGGCGGCCCCCTGGGATAGAACTGACGCTCATGCACGAAAGCGTGGGTAGCAAACAGG` |
| ASV855 | 429 | `TGGGGAATTTTGGACAATGGGCGCAAGCCTGATCCAGCAATGCCGCGTGAGTGAAGAAGGCCTTCGGGTTGTAAAGCTCTTTTGTCAGGGAAGAAACGGTATCCTCTAATACAGGGTGCTAATGACGGTACCTGAAGAATAAGCACCGGCTAACTACGTGCCAGCAGCCGCGGTAATACGTAGGGTGCAAGCGTTAATCGGAATTACTGGGCGTAAAGCGTGCGCAGGCGGTTATATAAGTCAGATGTGAAATCCCCGGGCTCAACCTGGGAACTGCATTTGAGACTGTATGGCTAGAGTGTGTCAGAGGGGGGTAGAATTCCACGTGTAGCAGTGAAATGCGTAGATATGTGGAGGAATACCGATGGCGAAGGCAGCCCCCTGGGATAACACTGACGCTCATGCACGAAAGCGTGGGGAGCAAACAGG` |
| ASV4688 | 429 | `TGGGGAATTTTGGACAATGGGGGCAACCCTGATCCAGCCATGCCGCGTGCGGGAAGAAGGCCTTCGGGTTGTAAACCGCTTTTGTCCGGAAAGAAAAGGCATCCCCTAATACGGGGTGTCCTTGACGGTACCGGAAGAATAAGCACCGGCTAACTACGTGCCAGCAGCCGCGGTAATACGTAGGGTGCGAGCGTTAATCGGAATTACTGGGCGTAAAGCGTGCGCAGGCGGTTCGCTAAGTCTGATGTGAAAGCCCCGGGCTCAACCTGGGAACTGCATTGGAAACTGGCGAGCTTGAGTGTGGCAGAGGGGGGTGGAATTCCACGTGTAGCAGTGAAATGCGTAGAGATGTGGAGGAACACCGATGGCGAAGGCAGCCCCCTGGGCTAACACTGACGCTCAGGCACGAAAGCGTGGGGAGCAAACAGG` |
| ASV2896 | 404 | `TGGGGAATATTGGACAATGGGCGCAAGCCTGATCCAGCCATGCCGCGTGAGTGATGAAGGCCTTCGGGTTGTAAAGCTCTTTTGGCGGGGACGATAATGACGGTACCCGCAGAATAAGCCCCGGCTAACTTCGTGCCAGCAGCCGCGGTAATACGAAGGGGGCAAGCATTGTTCGGAATTACTGGGCGTAAAGGGTGCGTAGGCGGATTAGACAGTCAGGTGTGAAAGCCCGAGGCTCAACCTCGGAATTGCATTTGATACTACTAGTCTAGAGTATTGGAGAGGAGCGTGGAATTCCCAGTGTAGAGGTGAAATTCGTAGATATTGGGAGGAACACCAGTGGCGTAGGCGACGCTCTGGCCAAATACTGACGCTGAGGCACGAAAGTGTGGGGAGCAAACAGG` |
| ASV2672 | 407 | `TAGGGAATATTGCGCAATGGGGGAAACCCTGACGCAGCGACGCCGCGTGAGTGACGAAGGCCTTAGGGTCGTAAAGCTCTGTTGAATGGGAAGAAACAAATGACGGTACCATTCGAGAAAGGACCGGCTAACTTCGTGCCAGCAGCCGCGGTAATACGAGGGGTCCTAGCGTTGTTCGGAATTATTGGGCGTAAAGGGTGTGTAGGCGGCCTTGTAAGTCAGTTGTGAAAGCCCCGGGCTCAACCCGGGAAGTGCTTCTGATACTGCTTGGCTTGAGTACTGGAGAGGATAGTGGAATTCCAGGTGTAGTGGTGAAATACGTAGATATCTGGAGGAACACCGGTGGCGAAGGCGGCTATCTGGCCATGTACTGACGCTGAAACACGAAAGCGTGGGGAGCAAACAGG` |
| ASV4909 | 430 | `TGGGGAATCTTGCGCAATGGACGAAAGTCTGACGCAGCCACGCCGCGTGAGTGAAGAAGGCCTTCGGGTTGTAAAGCTCTGTCGGGAGGGACGAAAAGCGGGTCGGTGAATAGCCGATTCGATTGACGGTACCTTCAAAGGAAGCACCGGCTAACTCCGTGCCAGCAGCCGCGGTAATACGGAGGGTGCAAGCGTTGCTCGGAATTATTGGGCGTAAAGGGCAGGTAGGTGGTCTCATTTGTCTGGGGTGAAAGCCTTGGGCTTAACTCAAGAAGTGCCCCAGAAACGGTGAGACTGGAGTCCTGGAGAGGTTTGCGGAATTCCCGGTGTAGCGGTGAAATGCGTAGAGATCGGGAGGAACACCAGAGGCGAAGGCGGCAAACTGGACAGGTACTGACACTCAACTGCGAAAGCGTGGGGAGCAAACAGG` |
| ASV781 | 424 | `TGAGGAATATTGGTCAATGGGCGCAAGCCTGAACCAGCCATGCCGCGTGCAGGAAGACGGCTCTATGAGTTGTAAACTGCTTTTATACGAGGGTAAACTTATCTACGTGTAGGTAATTGAAAGTATCGTATGAATAAGGATCGGCTAACTCCGTGCCAGCAGCCGCGGTAATACGGAGGATTCGAGCGTTATCCGGATTTATTGGGTTTAAAGGGTGCGTAGGCGGGAGAGAAAGTTAGTCGTTAAATCGGGGTGCTTAACACTTCAACGGCGATTGATACTATTTTCCTAGAGAACAGTTGCAGTGGGCGGAATGAGTAGTGTAGCGGTGAAATGCTTAGATATTACTCAGAACACCGATTGCGTAGGCAGCTCACTAAGCTGTTTCTGACGCTGAGGCACGAAAGCGTGGGGATCAAACAGG` |
| ASV5493 | 403 | `TGGGGAATACTGGACAATGAGTTCACGCTTGATCCGGCAATGTTGAATGAATGAATCGAAAGTAATGAGCTGTAAAATTTGTAAAGTTGAATAAGTTAAATTGAAAAACTCAATAAAGCCCCGACTAACTTCGTGCCAGCAGTCGCGGTAATACGGAGGGGGCAAGTGTTACTCATCATGACTAGGCGTAAAGGGTGTTAAGATTGCTAACGTTTGTTTTAACTAAAAAATCAATGAAATATTGATTAAGGTTACTTAAACGAGTTAGCTTGGGTCGATTGGAAAATAGAGTTACTAGTTTTTAAGGGGTAGAATCCAACAAGAAGATTAGGAGAAACAAAGGCGCAGGCAACTATTTATAATCTACTAAAATTGATATACAAAAGCGTGGGGAGCAAACAGG` |
| ASV1186 | 429 | `TGGGGAATTTTGGACAATGGGCGCAAGCCTGATCCAGCCATGCCGCGTGTGGGAAGAAGGCCTTCGGGTTGTAAACCACTTTTGTCAGGGAAGAAACGGCTCTTACTAATATTAGGGGCTAATGACGGTACCTGAAGAATAAGCACCGGCTAACTACGTGCCAGCAGCCGCGGTAATACGTAGGGTGCAAGCGTTAATCGGAATTACTGGGCGTAAAGCGTGCGCAGGCGGCTTTGCAAGACAGATGTGAAATCCCCGGGCTCAACCTGGGAACTGCATTTGTGACTGCATGGCTAGAGTACGGTAGAGGGGGATGGAATTCCGCGTGTAGCAGTGAAATGCGTAGATATGCGGAGGAACACCGATGGCGAAGGCAATCCCCTGGACCTGTACTGACGCTCATGCACGAAAGCGTGGGGAGCAAACAGG` |
| ASV5285 | 429 | `TGGGGAATTTTGCGCAATGGGCGAAAGCCTGACGCAGCGACGCCGCGTGAATGAAGAAGGCCTTACGGTCGTAAAGTTCTTTCAGCAGGGAAGAAAGTGTATGTTAGGCAATGATCATATTTTGACGTTACCTGCAGAAGCAGCCCCGGCTAACTCCGTGCCAGCAGCCGCGGTAATACGGAGGGGGCAAGCGTTGTTCGGAGTGACTGGGCGTAAAGCGCACGTAGGCGGTGATGTAAGTCATGTGTCAAAGGCAACAGCTTAACTGTTGTAAGGCCTGTGAAACTACATCACTAGAGTACTGGAGAGGAATGCGGAATATCTGGTGTAGCGGTGGAATGCGTAGATATCAGATGGAACACCTGTTGCGAAGGCAGCATTCTGGTCAGTAACTGACGCTGAGGTGCGAAAGCGTGGGGAGCAAACAGG` |
| ASV5483 | 424 | `TGAGGAATATTGGTCAATGGACGAGAGTCTGAACCAGCCAAGTCGCGTGAAGGAAGAAGGTTCTATGGATTGTAAACTTCTTTTGTAAGGGAATAAAACAGACCACGTGTGGTCTCTTGTATGTACTTTACGAATAAGGATCGGCTAACTCCGTGCCAGCAGCCGCGGTAATACGGAGGATCCGAGCGTTATCCGGATTTATTGGGTTTAAAGGGTGCGTAGGCGGACCTGTAAGTCAGTGGTGAAAGTTTGCGGCTCAACCGTAAAATTGCCATTGAAACTACCGGTCTTGAGTGTAAATGAGGTAGGCGGAATGTGTTGTGTAGCGGTGAAATGCTTAGATATAACACAGAACACCAATTGCGAAGGCAGCTTACTAGGATACAACTGACGCTGAGGCACGAAAGCGTGGGGATCAAACAGG` |
| ASV4394 | 423 | `TAGGGAATATTGGACAATGGGTGCGAGCCTGATCCAGCCATGCCGCGTGCAGGAAGACGGCCTTCTGGGTTGTAAACTGCTTTTGCCAGGGGATAAAAAGTCCCTGCGGGGAAAATTGAAGGTACCTGGTGAATAAGCCACGGCTAACTACGTGCCAGCAGCCGCGGTAATACGTAGGTGGCAAGCGTTGTCCGGATTTATTGGGTTTAAAGGGTGCGTAGGTTGTTTTGTAAGTCAGTGGTGAAATCCGGCAGCTCAACTGTCGATGTGCCATTGATACTGCAGAACTTGAGTACAGACGAGGTAGGCGGAATTGACGGTGTAGCGGTGAAATGCTTAGATATCGTCAAGAACACCGATAGCGAAGGCAGCTTACTAGACTGTAACTGACACTGATGCACGAAAGTGTGGGGATCAAACAGG` |
| ASV4550 | 406 | `TGAGGAATTTTCCGCAATGGGCGACAGCCTGACGGAGCAATGCCGCGTGAAGGATGAAGGCCTATGGGTTGTAAACTTCTTTTCTCAGAGAAGAAGCATTGACGGTATCTGAGGAATAAGCATCGGCTAACTCTGTGCCAGCAGCCGCGGTAAGACAGAGGATGCAAGCGTTATCCGGAATGATTGGGCGTAAAGCGTCTGTAGGTGGCTTAAAAAGTCTCCTGTCAAAGATCAGGGCTTAACCCTGGGCCGGCAGGAGAAACTCTTAGGCTAGAGTTTGGTAGGGGCAGAGGGAATTCCCGGTGGAGCGGTGAAATGCGTAGAGATCGGGAGGAACACCAAAGGCGAAAGCACTCTGCTGGGCCATAACTGACACTGAGAGACGAAAGCGAGGGGAGCAAAAGGG` |
| ASV142 | 407 | `TGGGGAATTTTCCGCAATGGGCGAAAGCCTGACGGAGCAATACCGCGTGAGGGATGACGGCCTATGGGTTGTAAACCTCTTTTTTCAGGGAGGAATCAATGACGTGTACCTGAAGAATAAGCATCGGCTAACTCCGTGCCAGCAGCCGCGGTAAGACGGAGGATGCAAGTGTTATCCGGAATCACTGGGCGTAAAGCGTCTGTAGGTGGTTTAATAAGTCAACTGTTAAATCTTGAGGCTTAACCTCGAAATCGCAGTCGAAACTATTAGACTAGAGTATAGTAGGGGTAAAGGGAATTTCCAGTGGAGCGGTGAAATGCGTAGATATTGGAAAGAACACCGATGGCGAAGGCACTTTACTGGGCTATTACTAACACTGAGAGACGAAAGCTAGGGTAGCAAATGGG` |
| ASV815 | 406 | `TGGGGAATATTGCACAATGGGCGCAAGCCTGATGCAGCAACGCCGCGTGAGCGATGAAGGCTTTCGGGTCGTAAAGCTCTGTCCTTGGGGAAGAAACAAATGACGGTACCCTTGGAGGAAGCCCCGGCTAACTACGTGCCAGCAGCCGCGGTAATACGTAGGGGGCGAGCGTTATCCGGAATTATTGGGCGTAAAGAGTACGTAGGTGGTTTCATAAGCGTAAGGTGAAAGGCAATGGCTCAACCATTGTTAGCCTCGCGAACTGTGAGACTTGAGTGCAGGAGAGGAAAGCGGAATTCCTAGTGTAGCGGTGAAATGCGTAGATATTAGGAGGAACACCAGTGGCGAAGGCGGCTTTCTGGACTGTAACTGACACTGAGGTACGAAAGCGTGGGGAGCAAACAGG` |
| ASV2386 | 429 | `TGGGGAATATTGGACAATGGGCGCAAGCCTGATCCAGCAATGCCGCGTGGGTGAAGAAGGCCTTCGGGTTGTAAAGCCCTTTTGTACGGGAAGAAATCCTGTCGGTTAATACCCGGCGGGGATGACGGTACCGTAAGAATAAGCACCGGCTAACTTCGTGCCAGCAGCCGCGGTAATACGAAGGGTGCAAGCGTTAATCGGAATTACTGGGCGTAAAGCGTGCGTAGGCGGTTGGCTAAGTCAGATGTGAAAGCCCTGGGCTCAACCTGGGAATGGCATTTGAAACTGGCGAGCTAGAGTGTGATAGAGGATGGCGGAATTCCCGGTGTAGCGGTGAAATGCGTAGAGATCGGGAGGAACATCCGTGGCGAAGGCGGCCATCTGGATCAACACTGACGCTGAGGCACGAAAGCGTGGGGAGCAAACAGG` |
| ASV1308 | 423 | `TAGGGAATATTGGTCAATGGGCGGAAGCCTGAACCAGCCATGCCGCGTGCAGGAAGAAGGCCCTCTGGGTTGTAAACTGCTTTTGATAGGGAACAAATGCCACTTGCGAGTGGAGCTGAGTGTACCTATAGAATAAGCACCGGCTAACTCCGTGCCAGCAGCCGCGGTAATACGGAGGGTGCAAGCGTTGTCCGGATTTATTGGGTTTAAAGGGTGCGTAGGCGGCTATTTAAGTCAGTGGTGAAATCCTTCAGCTTAACTGGAGAACTGCCATTGATACTGAATAGCTTGAGTATAGTTGAGGTAGGCGGAATTGACAGTGTAGCGGTGAAATGCTTAGATATTGTCAAGAACACCGATTGCGAAGGCAGCTTACTAAACTATAACTGACGCTGAGGCACGAAAGCGTGGGTATCAAACAGG` |
| ASV448 | 424 | `TGAGGAATATTGGTCAATGGGCGCAAGCCTGAACCAGCCATGCCGCGTGCAGGATGACGGTCCTATGGATTGTAAACTGCTTTTGTACGAGAAGAAACACTCCAACGTGTTGGAGCTTGACGGTATCGTAAGAATAAGGATCGGCTAACTCCGTGCCAGCAGCCGCGGTAATACGGAGGATCCAAGCGTTATCCGGAATCATTGGGTTTAAAGGGTCCGTAGGCGGTTTAGTAAGTCAGTGGTGAAAGCCCATCGCTCAACGGTGGAACGGCCATTGATACTGCTATACTTGAATTATTAGGAAGTAACTAGAATATGTAGTGTAGCGGTGAAATGCTTAGAGATTACATGGAATACCAATTGCGAAGGCAGGTTACTACTAATATATTGACGCTGATGGACGAAAGCGTGGGTAGCGAACAGG` |
| ASV5155 | 423 | `TAGGGAATATTGGGCAATGGAGGCAACTCTGACCCAGCCACGCCGCGTGCAGGAGGAAGGCGCTCTGCGTTGTAAACTGCTTTAGCAGGGGAAGAAAAAACCGATGCGTCGGGAATTGACGGTACCCTGAGAATAAGCACCGGCTAACTACGTGCCAGCAGCCGCGGTAATACGTAGGGTGCAAGCGTTGTCCGGATTTATTGGGTTTAAAGGGTGCGTAGGCGGTCTGTTAAGTCAGTGTTTAAATTTTGCAGCTTAACTGTAGGTATGGCATTGATACTGATGGACTTGAATATTAAGGAGGTAGCTGGAATTTATGGTGTAGCGGTGAAATGCTTAGATACCATAAGGAACACCGATAGCGAAGGCAGGTTACCACTTGATTATTGACGCTGAGGCACGAAAGTGTGGGGATCAAACAGG` |
| ASV5596 | 387 | `GCGCGAAACCTTTTCAATGCGCGCAAGCGTGAGAAGGGAATTCCAAGTGCTCAAGTTTTACTTGGGCTTTTGCCAAGTGTAAAAAGCTTGGCGAATAAGGGGTGGGAAAGACTGGTGCCAGCCGCCGCGGTAACACCAGCGCCTCGAGTGGGGGTCGCGATTATTGGGTCTAAAGCGTCCGTAGCAGGCTTTGTAAATGCTTTGTGAAATTGGCAGGCTTAACCTGCCAGCGTGCAGAGTAGACTGCAGAGCTCGAGACCGGGAGAGGTTAGAAGTATTCCTAGGGTACCGGTAAAATGGGTTAATCCTAGGAGGACTACCTATGGCGAAGGCATCTAACCAGAACGGTTCTGACTGTGAGGGACGAAAGCTAGGGGAGCAAAACGG` |
| ASV52 | 429 | `TGGGGAATTTTGGACAATGGGCGAAAGCCTGATCCAGCAATGCCGCGTGTGGGAAGAAGGCCTTCGGGTTGTAAACCACTTTTGTACGGAACGAAACGGTTCGCTTTAATACAGTGGGCTAATGACGGTACCGTAAGAATAAGCACCGGCTAACTACGTGCCAGCAGCCGCGGTAATACGTAGGGTGCGAGCGTTAATCGGAATTACTGGGCGTAAAGCGTGCGCAGGCGGTTATATAAGACAGTTGTGAAATCCCCGGGCTCAACCTGGGAATTGCATCTGTGACTGTATAGCTAGAGTACGGTAGAGGGGGATGGAATTCCGCGTGTAGCAGTGAAATGCGTAGATATGCGGAGGAACACCGATGGCGAAGGCAATCCCCTGGACCTGTACTGACGCTCATGCACGAAAGCGTGGGGAGCAAACAGG` |
| ASV916 | 404 | `TAAGGAATATTGGACAATGGGGGAAACCCTGATCCAGCCATGCCGCGTGAATGAAGAAGGCCTTAGGGTTGTAAAGTTCTTTTAGCTGTGACGATAATGACGGTAGCAGCAGAAAAAGCTCCGGCTAACTCCGTGCCAGCAGCCGCGGTAATACGGAGGGAGCGAGCGTTGTTCGGAATCACTGGGCGTAAAGCGTGCGTAGGCGGTATGACAAGTCGGAAGTGAAAGCCCGGGGCTCAACCTCGGAATTGCTTTCGAAACTGTCAAACTAGAGTCCTGGAGAGGTAAGCGGAATTCCGAGTGTAGAGGTGAAATTCGTAGATATTCGGAGGAACACCAGTGGCGTAAGCGGCTTACTGGACAGGAACTGACGCTGAGGCACGAAAGCGTGGGGAGCAAACAGG` |
| ASV4177 | 430 | `TGGGGAATCTTGCGCAATGGACGAAAGTCTGACGCAGCCACGCCGCGTGAGTGAAGAAGGCCTTCGGGTCGTAAAGCTCTGTCGGGCGGGACGAACATCCTAGTTGTTAACAGCGACTGGGACTGACGGTACCGCCAAAGGAAGCACCGGCTAACTTCGTGCCAGCAGCCGCGGTAATACGAGGGGTGCGAGCGTTGCTCGGAATTATTGGGCGTAAAGGGTAGGTAGGTGGTCTCATTTGTCTGGGGTGAAAGCCTTGAGCTTAACTCAAGAAGTGCCCCGGAAACGGTGAGACTAGAGTCTTGGAGAGGGTCGTGGAATTCCCGGTGTAGCGGTGAAATGCGTAGAGATCGGGAGGAACACCAGAGGCGAAGGCGGCGACCTGGACAAGTACTGACACTCAACTACGAAAGCGTGGGGAGCAAACAGG` |
| ASV3933 | 424 | `TGAGGAATATTGGTCAATGGGCGCAAGCCTGAACCAGCCATGCCGCGTGAAGGATGAAGGTTCTATGGATTGTAAACTTCTTTTATTTGGGAATAAACCTCGGGACGTGTCCCGAGCTGAAGGTACCAAACGAATAAGCACCGGCTAACTCCGTGCCAGCAGCCGCGGTAATACGGAGGGTGCAAGCGTTATCCGGAATCATTGGGTTTAAAGGGTCCGCAGGCGGATGTATAAGTCAGTGGTGAAAGCCTACAGCTCAACTGTAGAACTGCCATTGATACTGTACGTCTTGAATTCGGTCGAAGTGGGCGGAATATGACATGTAGCGGTGAAATGCTTAGATATGTCATAGAACACCGATAGCGAAGGCAGCTCACTAGGCCTGGATTGACGCTCAGGGACGAAAGCGTGGGGAGCAAACAGG` |
| ASV5222 | 405 | `TAGGGAATATTGCTCAATGGGGGAAACCCTGAAGCAGCAACGCCGCGTGAGTGATGAAGGCCTTCGGGTCGTAAAGCTCTGTCGCAGGGGAAAATTCAAGATGGTACCCTGTAAGAAAGGACCGGCCAACTTCGTGCCAGCAGCCGCGGTAATACGGGGGGTCCTAGCGTTGTTCGGAATCACTGGGCGTAAAGCGTTTGTAGGTGGCTTTGTAAGTTGAATGTGAAAGCCCAGGGCCTAACCCTGGAAGTGCATTCAATACTGCAGAGCTTGAGTATGAGAGAGGAAGGTGGAATACCTGGTGTAGTGGTGAAATACGTAGATATCAGGTGGAACATCGGCGGCGAAAGCGGCCTTCTGGCTTAATACTGACACTGAAAAACGAAAGCGTGGGGATCAAACAGG` |
| ASV4716 | 424 | `TAAGGAATATTGGTCAATGGACGGAAGTCTGAACCAGCCATGCCGCGTGGAGGATGAAGGTCCTCTGGATTGTAAACTTCTTTTATAGGGGGCGAAAAAAGGCCATTCTTGGTCGTCTGACGGTACCCTATGAATAAGCACCGGCTAACTCCGTGCCAGCAGCCGCGGTAATACGGAGGGTGCAAGCGTTATCCGGATTCACTGGGTTTAAAGGGAGCGTAGGCGGGCATGTAAGTCCGTGGTGAAAGCCCTCAGCTTAACTGAGGAACTGCCATGGATACTATATGTCTTGAATGATGTGGAGGTTTGCGGAATAGGTCATGTAGCGGTGAAATGCTTAGATATGACCTAGAACACCTATTGCGAAGGCAGCAGGCTACACATATATTGACGCTGAGGCTCGAAAGCGTGGGGATCAAACAGG` |
| ASV2405 | 424 | `TGAGGAATATTGGTCAATGGACGAGAGTCTGAACCAGCCAAGTCGCGTGAAGGATGACTGCCCTATGGGTTGTAAACTTCTTTTGTACAGGGGTAAAACAGGCTACGTGTAGCTTCTTGCAAGTACTGTACGAATAAGCACCGGCTAACTCCGTGCCAGCAGCCGCGGTAATACGGAGGGTGCGAGCGTTATCCGGATTTATTGGGTTTAAAGGGTGCGTAGGCGGGAAATTAAGTCAGCGGTGAAAGTTTGAGGCTCAACCTTAAAATTGCCGTTGAAACTGGTTTTCTTGAGTATAGATGAAGTAGGCGGAATTTGTGGTGTAGCGGTGAAATGCATAGATATCACGAGGAACTCCGATAGCGCAGGCAGCTTACTAAGGTATAACTGACGCTGATGCACGAAAGCGTGGGTATCAAACAGG` |
| ASV2243 | 404 | `TGGGGAATATTGGACAATGGGCGAAAGCCTGATCCAGCAATACCGCGTGAGTGATGAAGGCCTTCGGGTTGTAAAGCTCTTTTGGTAGGGAAGATGATGACGGTACCTACAGAATAAGCACCGGCTAACTCTGTGCCAGCAGCCGCGGTAATACAGAGGGTGCTAGCGTTGTTCGGAATTACTGGGCGTAAAGGGCGCGTAGGCGGTTTATTAAGTCAAAGGTGAAATCCCCGAGCTCAACTTGGGAACTGCCTTTGATACTGGTAGGCTAGAGATCGGGAGAGGAAAGTGGAATTCCTAGTGTAGAGGTGAAATTCTTAGATATTAGGAGGAACACCAGAGGCGAAGGCGGCTTTCTGGACCGTTTCTGACGCTGAGGCGCGAAGGCGTGGGTAGCGAAAGGG` |
| ASV5273 | 416 | `TGGGGAATTTTGGACAATGGGCGAAAGCCTGATCCAGCAACTCTGCGTGAGGGACGAAGGTCTTCGGATCGTAAACCTCTTTTGCTAGGGGCGAATACCCGCAAGGGTTTGACGGTACCTGGCGAATAAGCCACGGCTAACTATGTGCCAGCAGCCGCGGTAAGACATAGGTGGCAAGCGTTATTCGGAATTACTAGGCGTAAAGCGAGTGTAGGCGGGCGCTTAAGTCCGACGTTAAATCTCCAGGCTTAACTTGGAGCTGTCGTCGGATACTGGGTGTCTCGAGAGGGGTAGGGGGCAGTGGAATTCCCGGTGTAGCGGTGAAATGCGTAGATATCGGGAGGAACACCTATGGCGAAAGCAGCTGCCTGGGCCTTTTCTGACGCTAAGACTCGAAAGCTGGGGGAGCAAACAGG` |
| ASV3560 | 404 | `TGGGGAATCTTGGACAATGGGGGGAACCCTGATCCAGCCATGCCGCGTGAGCGATGAAGGCCTTCGGGTTGTAAAGCTCTTTCGTGGGGGAAGATAATGACTGTACCCCAAGAAGAAGCCCCGGCTAACTCCGTGCCAGCAGCCGCGGTAATACGGAGGGGGCTAGCGTTGTTCGGAATTACTGGGCGTAAAGCGCACGTAGGCGGGGCGGAAAGTCAGAGGTGAAATCCCAGGGCTCAACCCTGGAACTGCCTTTGAAACTCCCGCTCTTGAGGTCGTGAGAGGTGAGTGGAATTCCGAGTGTAGAGGTGAAATTCGTAGATATTCGGAGGAACACCAGTGGCGAAGGCGGCTCACTGGCACGATACTGACGCTGAGGTGCGAAAGCGTGGGGAGCAAACAGG` |
| ASV4089 | 404 | `TGGGGAATATTGGACAATGGGCGAAAGCCTGATCCAGCCATGCCGCGTGAGTGATGAAGGCTTTAGGGTTGTAAAGCTCTTTCGCCGATGAAGATAATGACGGTAGTCGGAGAAGAAGCCCCGGCTAACTTCGTGCCAGCAGCCGCGGTAATACGAAGGGGGCTAGCGTTGTTCGGAATTACTGGGCGTAAAGCGCACGTAGGCTGTGCCGTAAGTCAGGGGTGAAATCCCAGAGCTCAACTCTGGAACTGCCTTTGAAACTACGGTGCTAGAATCTCAGAGGGGGTAGCGGAATTCCAAATGTAGGGGTGAAATCCGTAGATATTTGGAGGAACACCGGTGGCGAAGGCGGCTACCTGGATGAGTATTGACGCTGAGGTGCGAAAGCGTGGGGATCAAACAGG` |
| ASV1536 | 404 | `TGGGGAATCTTGGACAATGGGGGCAACCCTGATCCAGCGACGCCGCGTGGGTGACGAAGGCCTTCGGGTTGTAAAGCCCTTTTTCCGGGGACGATGATGACGGTACCCGGGGAATAAGCACCGGCTAACTCCGTGCCAGCAGCCGCGGTAAGACGGAGGGTGCTAGCGTTGTTCGGATTTACTGGGCGTAAAGGGCGCGTAGGCGGCCTGATCAGTCGGGTGTGAAAGCCCGGGGCTCAACCCCGGAACGGCACCCGAGACGGTCAGGCTGGAGACCGGGAGAGGAGGGTGGAATAGCCAGTGTAGAGGTGAAATTCGTAGATATTGGCTGGAACACCGGTGGCGAAAGCGGCTCTCTGGCCCGGTTCTGACGCTGAGGCGCGAAAGCGTGGGGAGCAAACAGG` |
| ASV57 | 424 | `TAAGGAATATTGGTCAATGGACGCAAGTCTGAACCAGCCATGCCGCGTGCAGGAAGACGGTCCTCTGGATTGTAAACTGCTTTTGTTAGGGAAGAAACCTTCGGATGCGTTCGAAGCTGACGGTACCTAAAGAATAAGGATCGGCTAACTCCGTGCCAGCAGCCGCGGTAATACGGAGGATCCAAGCGTTATCCGGATTTATTGGGTTTAAAGGGTGCGTAGGCGGCGTTGTAAGTCAGTGGTGAAAGTTTGCAGCTTAACTGTAAAATTGCCATTGATACTGCAGTGCTTGAGTACAGATGAGGTGGGCGGAATGTGTCATGTAGCGGTGAAATGCATAGATATGACACAGAACACCGATTGCGAAGGCAGCTCACTAAACTGTAACTGACGCTGAGGCACGAAAGCGTGGGGATCAAACAGG` |
| ASV703 | 407 | `TAGGGAATATTGCACAATGGGGGAAACCCTGATGCAGCGACGCCGCGTGAGTGATGAAGGCCTTCGGGTCGTAAAGCTCTGTCGTATGGGACTAACACAATGAAGGTACCATGCAAGAAAGGATCGGCTAACTTCGTGCCAGCAGCCGCGGTAAGACGAGGGATCCTAGCGTTGTTCGGAATCATTGGGCGTAAAGCGGGTGTAGGCGGTCTTATAAGTCAGGTGTGAAATCCCAGGGCTCAACCCTGGAAGTGCACTTGATACTGCAAGACTTGAGTGTGGTAGAGGCTACTGGAATTCCTGGTGTAGTGGTAAAATACGTAGATATCAGGAGGAATACCGGAGGCGAAGGCGGGTAGCTGGACCAACACTGACGCTGAGACCCGAAAGCGTGGGGATCAAACAGG` |
| ASV2665 | 429 | `TGGGGAATTTTGGACAATGGGCGCAAGCCTGATCCAGCCATTCCGCGTGAGTGAAGAAGGCCTTCGGGTTGTAAAGCTCTTTCGCAAGAGAAGAAAACTTACCCTCTAACATAGGGTGAGGTTGACGGTACCTTGATAAGAAGCACCGGCTAACTACGTGCCAGCAGCCGCGGTAATACGTAGGGTGCGAGCGTTAATCGGAATTACTGGGCGTAAAGCGTGCGCAGGCGGTTTTGAAAGTCAGATGTGAAATCCCCGGGCTCAACCTGGGAACTGCGTTTGAAACTCCAAGACTAGAGTATAGGAGAGGGGGGTAGAATTCCACGTGTAGCAGTGAAATGCGTAGAGATGTGGAGGAATACCAATGGCGAAGGCAGCCCCCTGGCCTAATACTGACGCTCATGCACGAAAGCGTGGGGAGCAAACAGG` |
| ASV4597 | 429 | `TGGGGAATTTTGGACAATGGGCGGAAGCCTGATCCAGCCATGCCGCGTGAGTGAAGAAGGCCTTCGGGTTGTAAAGCTCTTTCGGTGGGGAAGAAATCGCAATGGTTAATACCTATTGTGGATGACGGTACCCGCATAAGAAGCACCGGCTAACTACGTGCCAGCAGCCGCGGTAATACGTAGGGTGCGAGCGTTAATCGGAATTACTGGGCGTAAAGCGTGCGCAGGCGGTTATATAAGTCAGACGTGAAATCCCCGGGCTCAACCTGGGAACTGCGTTTGAGACTGTATGACTAGAGGATGGCAGAGGGGGGTAGAATTCCACGTGTAGCAGTGAAATGCGTAGAGATGTGGAGGAATACCGATGGCGAAGGCAGCCCCCTGGGCTATTACTGACGCTCATGCACGAAAGCGTGGGGAGCAAACAGG` |
| ASV5525 | 429 | `TGGGGAATTTTGGACAATGGGGGCAACCCTGATCCAGCCATGCCGCGTGCGGGAAGAAGGCCTTCGGGTTGTAAACCGCTTTTAGTCGGAAAGAAAAGGCACTCCCTAATACGGGGTGTTCTTGACGGTACCGGCGGAATAAGCACCGGCTAACTACGTGCCAGCAGCCGCGGTAATACGTAGGGTGCGAGCGTTAATCGGAATTACTGGGCGTAAAGCGTGCGCAGGCGGTTCGCTGTGTCCCGTGTGAAAGCCCCGGGCTTAACCTGGGAATTGCATGGGAAACTGGCGGGCTTGAGTGCGGCAGAGGGGGGTGGAATTCCGCGTGTAGCAGTGAAATGCGTAGAGATGCGGAGGAACACCGATGGCGAAGGCAGCCCCCTGGGCCGACACTGACGCTCAGGCACGAAAGCGTGGGGAGCAAACAGG` |
| ASV4565 | 404 | `TGGGGAATATTGGACAATGGGCGAAAGCCTGATCCAGCAATCCTGCGTGAAGGATGAAGCCTTATGGGGTGTAAACTTCTTTTCTGGGGGAAGACGAATGACGGTACCTCAGGAATAAGAGACGGCTAACTACGTGCCAGCAGCCGCGGTAATACGTAGGTCTCGAGCGTTATCCGGAATTACTGGGCGTAAAGCGTCCGCAGCTGGATAGATAAGTCTGTTTTCAAATATCCCGATTCAATCGGGAAAAGGGTATAGATACTGTCTATCTAGAGTGTATTAGGGGTTAATGGAATTTCCGGTGGAGCGGTGAAATGCGTTGATATCGGAAGGAATGCCAAAAGCGAAAGCAGTTAACTATAGTACACTGACAGTCAGGGACGACAGCTTGGGTAGCAAACGGG` |
| ASV1001 | 429 | `TGGGGAATATTGCACAATGGGCGCAAGCCTGATGCAGCCATGCCGCGTGTGTGAAGAAGGCCTTCGGGTTGTAAAGCACTTTCAGCGAGGAGGAAGGGTTTGGTGTTAATAGCCCCATTCATTGACGTTACTCGCAGAAGAAGCACCGGCTAACTCCGTGCCAGCAGCCGCGGTAATACGGAGGGTGCAAGCGTTAATCGGAATTACTGGGCGTAAAGCGCACGCAGGCGGTTTGTTAAGTCAGATGTGAAATCCCCGAGCTTAACTTGGGAACTGCATTTGAAACTGGCAAGCTAGAGTCTTGTAGAGGGGGGTAGAATTCCAGGTGTAGCGGTGAAATGCGTAGAGATCTGGAGGAATACCGGTGGCGAAGGCGGCCCCCTGGACAAAGACTGACGCTCAGGTGCGAAAGCGTGGGGAGCAAACAGG` |
| ASV485 | 404 | `TGGGGAATATTGGACAATGGGCGCAAGCCTGATCCAGCCATGCCGCGTGAGTGATGAAGGCCTTCGGGTTGTAAAGCTCTTTCGCACGCGACGATGATGACGGTAGCGTGAGAAGAAGCCCCGGCTAACTTCGTGCCAGCAGCCGCGGTAATACGAAGGGGGCTAGCGTTGTTCGGAATTACTGGGCGTAAAGGGCGCGTAGGCGGCTTACCAAGTCAGGCGTGAAAGCCCCGGGCTTAACCTGGGAACTGCGCTTGAGACTGATAGGCTTGAGTTTCGGAGAGGTAGGTGGAATTCCCAGTGTAGAGGTGAAATTCGTAGATATTGGGAAGAACACCGGTGGCGAAGGCGGCCTACTGGACGAATACTGACGCTGAGGCGCGAAAGCGTGGGGAGCAAACAGG` |
| ASV3544 | 429 | `TGGGGAATTTTGGACAATGGGCGAAAGCCTGATCCAGCAATGCCGCGTGCAGGAAGAAGGCCTTCGGGTTGTAAACTGCTTTTGTACGGAGCGAAAAGGTCTGCCCTAATACGGTGGGCCCATGACGGTACCGTAAGAATAAGCACCGGCTAACTACGTGCCAGCAGCCGCGGTAATACGTAGGGTGCGAGCGTTAATCGGAATTACTGGGCGTAAAGCGTGCGCAGGCGGTTATATAAGACAGTTGTGAAATCCCCGGGCTCAACCTGGGAATTGCATCTGTGACTGTATAGCTAGAGTACGGTAGAGGGGGATGGAATTCCGCGTGTAGCAGTGAAATGCGTAGATATGCGGAGGAACACCGATGGCGAAGGCAATCCCCTGGACCTGTACTGACGCTCATGCACGAAAGCGTGGGGAGCAAACAGG` |
| ASV1104 | 404 | `TGAGGAATATTGGACAATGGGCGAAAGCCTGATCCAGCCATGCCGCGTGAGTGAAGAAGGCCTTAGGGTTGTAAAGCTCTTTCAATAGTGAAGATAATGACGGTAGCTATAGAAGAAGCCCCGGCTAAATTCGTGCCAGCAGCCGCGGTAATACGAATGGGGCAAGCGTTGTTCGGAATCACTGGGCTTAAAGCGTGCGTAGGTGGATTTGCAAGTCGGGAGTGAAATCCCAGGGCTCAACCCTGGAACTGCTTCCGAAACTGCAAGTCTAGAATCCGGGAGAGGTTAGTAGAATTCCTAGTGTAGAGGTGAAATTCGCAGATATTAGGAGGAATACCAGTGGCGAAGGCGGCTAACTGGACCGGTATTGACACTGAGGCACGAAAGCGTGGGGAGCAAACAGG` |
| ASV1690 | 409 | `TGGGGAATATTGGGCAATGGACGAAAGTCTGACCCAGCGACGCCGCGTGCGGGATGAAGGCCTTCGGGTTGTAAACCGCTTTCAGCAGGGAATAAGCGCAAGTGAAGGTACCTGCAGAAGAAGCACCGGCTAACTATGTGCCAGCAGCCGCGGTAATACATAGGGTGCAAGCGTTGTCCGGAATTATTGGGCGTAAAGAGCTCGTAGGTGGTTTGTTACGTCGGTTGTGAAATTCAGGGGCTCAACCCCTGACTTGCAGCCGATACGGGCAAGCTAGAGTTTGGTAGGGGAGACTGGAATTCCTGGTGTAGCGGTGGAATGCGCAGATATCAGGAGGAACACCGATGGCGAAGGCAGGTCTCTGGGCCAATACTGACACTGAGGAGCGAAAGCGTGGGGAGCGAACAGG` |
| ASV1878 | 409 | `TGGGGAATATTGCACAATGGGCGAAAGCCTGATGCAGCAACGCCGCGTGAGGGATGAAGGCCTTCGGGTTGTAAACCTCTTTTAGCAGGGAAGAAGCGAAAGTGACGGTACCTGCAGAAAAAGCACCGGCTAACTACGTGCCAGCAGCCGCGGTAATACGTAGGGTGCAAGCGTTGTCCGGAATTATTGGGCGTAAAGAGCTCGTAGGCGGTTTGTCGCGTCTGCTGTGAAAATCCAAGGCTCAACCTTGGACCTGCAGTGGGTACGGGCAAGCTAGAGTGCGGTAGGGGAGATGGGAATTCCTGGTGTAGCGGTGGAATGCGCAGATATCAGGAGGAACACCAATGGCGAAGGCACATCTCTGGGCCGTAACTGACGCTGAGGAGCGAAAGCGTGGGGAGCGAACAGG` |
| ASV391 | 404 | `TGGGGAATATTGGACAATGGGCGAAAGCCTGATCCAGCAATGCCGCGTGAGTGAAGAAGGCCTTCGGGTTGTAAAGCTCTTTTACCCGGGAAGATAATGACTGTACCGGGAGAATAAGCCCCGGCTAACTCCGTGCCAGCAGCCGCGGTAATACGGAGGGGGCTAGCGTTGTTCGGAATTACTGGGCGTAAAGCGTACGTAGGCGGCTTTCTAAGTCAGAGGTGAAATCCCACGACTCAATCGTGGAACTGCCTTTGAGACTGGATCGCTTGAAGATGGGAGAGGTAAGTGGAATTCCGAGTGTAGAGGTGAAATTCGTAGATATTCGGAAGAACACCAGTGGCGAAGGCGGCTTACTGGACCATTCTTGACGCTGAGGTACGAAAGCGTGGGGAGCAAACAGG` |
| ASV3259 | 404 | `TGGGGAATATTGGACAATGGGCGCAAGCCTGATCCAGCCATGCCGCGTGAGTGATGAAGGTCTTAGGATTGTAAAGCTCTTTCAGTGGGGACGATAATGACGGTACCCACAGAAGAAGCCCCGGCTAACTTCGTGCCAGCAGCCGCGGTAATACGAAGGGGGCTAGCGTTGTTCGGAATTACTGGGCGTAAAGCGCACGTAGGCGGATATTTAAGTCAGGGGTGAAATCCCGGGGCTCAACCCCGGAACTGCCTTTGATACTGGATATCTTGAGTTCGGAAGAGGTGAGTGGAATTGCGAGTGTAGAGGTGAAATTCGTAGATATTCGCAGGAACACCAGTGGCGAAGGCGGCTCACTGGTCCGATACTGACGCTGAGGTGCGAAAGCGTGGGGAGCAAACAGG` |
| ASV1388 | 404 | `TGGGGAATATTGCACAATGGGGGAAACCCTGATGCAGCAACGCCGCGTGAGTGATGACGGTCTTCGGATTGTAAAGCTCTGTCTTTGGGGACGATAATGACGGTACCCAAGGAGGAAGCCACGGCTAACTACGTGCCAGCAGCCGCGGTAATACGTAGGTGGCAAGCGTTGTCCGGATTTACTGGGCGTAAAGGGAGCGTAGGCGGATTTTTAAGTGGGATGTGAAATACCCGGGCTCAACCTGGGTGCTGCATTCCAAACTGGAAATCTAGAGTGCAGGAGGGGAGAGTGGAATTCCTAGTGTAGCGGTGAAATGCGTAGAGATTAGGAAGAACACCAGTGGCGAAGGCGACTCTCTGGACTGTAACTGACGCTGAGGCTCGAAAGCGTGGGGAGCGAACAGG` |
| ASV1798 | 430 | `TGGGGAATCTTGCACAATGGGCGAAAGCCTGATGCAGCAACGCCATGTGAGTGAAGAAGGCCCTTGGGTTGTAAAGCTCTTTCGGTTAGGAAGAAGGGTGAGATAGTTAATACCTATTTTATCTGACGGTACTAGACTAAGAAGCACTGGCTAATTCCGTGCCAGCAGCCGCGGTAATACGGAAGGTGCAAGCATTGTTCGGATTGACTGGGCGTAAAGAGCGCGTAGGTGGGTTTGTATGTCGGATGTGAAATCCCTAGGCTTAACCTGGGAACTGCATCCGAAACGGCAGATCTTGAATGTTTGAGAGGTTGGCGGAATTCCAGGTGTAGGAGTGAAATCCGTAGATATCTGGAGGAACACCGGAGGCGAAGGCGGCTGACTAACAAAACATTGACACTGAGGCGCGAAAGCGTGGGTAGCAAACAGG` |
| ASV2573 | 429 | `TGGGGAATATTGGACAATGGGGGCAACCCTGATCCAGCCATGCCGCGTGTGTGAAGAAGGCCTTCGGGTTGTAAAGCACTTTAAGTGGGGAGAAAGGCTCCTTGGCTAACATCCAAGGGGATTGATGCTACCCACAGAATAAGCACCGGCTAACTCTGTGCCAGCAGCCGCGGTAATACAGAGGGTGCAAGCGTTAATCGGAATTACTGGGCGTAAAGCGCGCGTAGGCGGCTGCTTAAGTCGGATGTGAAATCCCCGGGCTCAACCTGGGAACTGCATTCGATACTGAGCAGCTAGAGTATGAGAGAGGGAGGTAGAATTCCACGTGTAGCGGTGAAATGCGTAGATATGTGGAGGAATACCGGTGGCGAAGGCGGCCTCCTGGCTTAATACTGACGCTGAGGTGCGAAAGCGTGGGGAGCAAACAGG` |
| ASV5569 | 422 | `TAGGGAATATTGGGCAATGGGCGCAAGCCTGACCCAGCCACGCCGCGTGAGGGACGAAGGTGCACAGCATTGTAAACCTCTTTTCTAAGAGAATAACGGTCCTTTAGAAGGAAGATGCAGGTATCTTAGGAATAAGCACCGGCAAACTACGTGCCAGCAGCCGCGGTAATACGTAGGGTGCAAGCGTTGTCCGGATTTACTGGGTTTAAAGGGTGCGCAGGCGGCTTTGTAAGTCGGAGATGAAATTATGGAGCTTAACTCTGTAGCTGTCTCCGAAACTATGAGGCTAGGGTTAGTTTGAGGTTGCCGGAATGGACGGTGTAGCGGTGAAATGCGTAGAGATCGTCCAGAACACCAATTGCGAAGGCAGGCAACTAAGACTCAACCGACGCTCAGGCACGAAAGTGTGGGTAGCAAACAGG` |
| ASV1435 | 429 | `TGGGGAATTTTGGACAATGGGGGCAACCCTGATCCAGCAATGCCGCGTGAGTGAAGAAGGCCTTCGGGTTGTAAAGCTCTTTTGTTAGGGAAGAAAAGGTTCTCAATAATACTGGGAACTGATGACGGTACCTAAAGAATAAGCACCGGCTAACTACGTGCCAGCAGCCGCGGTAATACGTAGGGTGCAAGCGTTAATCGGAATTACTGGGCGTAAAGCGTGCGCAGGCGGCCTCGCAAGTCAGATGTGAAATCCCCGAGCTCAACTTGGGAACTGCATTTGAAACTACGAGGCTAGAGTGTGTCAGAGGGAGGTAGAATTCCACGTGTAGCAGTGAAATGCGTAGATATGTGGAGGAATACCAGTGGCGAAGGCGGCCTCCTGGGATAACACTGACGCTCATGTACGAAAGCGTGGGGAGCAAACAGG` |
| ASV1687 | 425 | `TAGGGAATATTGGGCAATGGGCGAAAGCCTGACCCAGCCACGCCGCGTGCAGGAAGACGGCCCTCTGGGTTGTAAACTGCTTTTTTGGGGGAAGAAAGAAGCCTATGCGTAGGAAGTTGACGGTACCCTAAGAATAAGCCACGGCTAACTACGTGCCAGCAGCCGCGGTAATACGTAGGTGGCGAGCGTTATCCGGATTTATTGGGTTTAAAGGGAGCGTAGGCGGCTGATTAAGTCAGCGGTGAAAGGTAGCAGCTTAACTGTTTTACATGCCGTTGATACTGGTTAGCTTGAGTTGACAGAAGGCAGATAGAATTCCTGATGTAGCGGTGAAATGCTTAGATATCAGGAGGAATACCGATTGCGAAGGCAGTCTGCTGCAGTCACACTGACGCTGAGGCTCGAAGGTGCGGGGATCAAACAGG` |
| ASV2923 | 428 | `TCGAGAATTTTTCACAATGGGCGAAAGCCTGATGGAGCGACGCCGCGTGGGGGATGAAGGGCTTCGGCTCGTAAACCCCTGTCATTCGAGAACAAACCCTGCGATTAAATAGATCGCGGGCTGATAGTATCGGAAGAGGAAGGGACGGCTAACTCTGTGCCAGCAGCCGCGGTAATACAGAGGTCCCAAGCGTTGTTCGGATTTACTGGGCGTAAAGGGTGCGTAGGCGGCGAGGAAAGTTTGAGGTGAAATCTCCAGGCTCAACTTGGAAAATGCCTTGAAGACTTCCTTGCTCGAGGGTCGTAGAGGAGACTGGAATTCTCGGTGTAGCAGTGAAATGCGTAGATATCGAGAGGAACACCAGTGGCGAAAGCGGGTCTCTGGACGACACCTGACGCTGAGGCACGAAAGCCAGGGGAGCAAACGGG` |
| ASV5287 | 405 | `TAGGGAATTTTCCACAATGGACGAAAGTCTGATGGAGCAACGCCGCGTGCAGGATGAATGCCTTCGGGTTGTAAACTGCTTTTATATGTGACGAATATGACGGTAGCATATGAATAAGGATCGGCTAATTCCGTGCCAGCAGCCGCGGTCATACGGAAGATCCAAGCGTTATCCGGAATTACTGGGCGTAAAGAGTTGCGTAGGTGGCATTGTAAGTTAGTAGTGAAAGCGTGGGGCTCAACCTCATATCCATTACTAAAACTGCAAAGCTAGAGGACGAGAGAGGTAACTAGAATTCCTTGTGTAGGAGTGAAATCCGTAGATATAAGGAGGAATACCGATGGCGTAGGCAGGTTACTGGCTCGTTCCTGACACTAAGGCACGAAAGCGTGGGGAGCAAACGGG` |
| ASV47 | 429 | `TGGGGAATTTTGGACAATGGACGAAAGTCTGATCCAGCCATGCCGCGTGCGGGAAGAAGGCCTTCGGGTTGTAAACCGCTTTTGTCAGGGAAGAAACGGCTCGCTCTAATACAGTGGGCTAATGACGGTACCTGAAGAATAAGCACCGGCTAACTACGTGCCAGCAGCCGCGGTAATACGTAGGGTGCAAGCGTTAATCGGAATTACTGGGCGTAAAGCGTGCGCAGGCGGTTATGCAAGACAGATGTGAAATCCCCGGGCTCAACCTGGGAACTGCATTTGTGACTGCATGGCTAGAGTACGGTAGAGGGGGATGGAATTCCGCGTGTAGCAGTGAAATGCGTAGATATGCGGAGGAACACCGATGGCGAAGGCAATCCCCTGGACCTGTACTGACGCTCATGCACGAAAGCGTGGGGAGCAAACAGG` |
| ASV3108 | 429 | `TGGGGAATTTTGGACAATGGGGGCAACCCTGATCCAGCAACGCCGCGTGAATGAAGAAGGCCTTAGGGTTGTAAAGTTCTTTTATTTGGGAGCAAAGCTTATTTGTGAATAGCAGATGAGGATGAGAGTACCAAAAGAATAAGCACCGGCTAACTACGTGCCAGCAGCCGCGGTAATACGTAGGGTGCGAGCGTTAATCGGAATTATTGGGCGTAAAGGGTGCGTAGGCGGATATATAAGTTAGATGTGAAAAGCCAGGGCTTAACCTTGGTCGTGCATTTAAGACTGTATGTCTAGAGTTTGTAAGAGGGGGGTAGAATTCCAAGTGTAGCAGTGAAATGCGTAGAGATTTGGAGGAATACCGATGGCGAAGGCAGCCCCCTGGTATAGAACTGACGCTGAGGCACGAAAGCGTGGGTAGCAAACAGG` |
| ASV1971 | 429 | `TGGGGAATTTTGGACAATGGGCGCAAGCCTGATCCAGCCATTCCGCGTGAGTGAAGAAGGCCTTCGGGTTGTAAAGCTCTTTCGGCAGGGAAGAAACGGTGCGGGCTAATACCTCGCATTAATGACGGTACCTGAAGAAGAAGCACCGGCTAACTACGTGCCAGCAGCCGCGGTAATACGTAGGGTGCGAGCGTTAATCGGAATTACTGGGCGTAAAGCGTGCGCAGGCGGTTTTGTAAGACAGGTGTGAAATCCCCGGGCTTAACCTGGGAACTGCGCTTGTGACTGCAAGACTCGAGTACGGCAGAGGGGGGTGGAATTCCACGTGTAGCAGTGAAATGCGTAGAGATGTGGAGGAACACCGATGGCGAAGGCAGCCCCCTGGGTCGATACTGACGCTCATGCACGAAAGCGTGGGGAGCAAACAGG` |
| ASV1861 | 387 | `GCGAGAAACCTTTGCAATGTGCGCAAGCACGACAAGGTTAATCCGAGTGTTTCCTGCTAAAGGAATCTTTTGTTAGTTCTAAAACCACTGATGAATAAGGGGTGGGCAAGTTCTGGTGTCAGCCGCCGCGGTAAAACCAGCACCTCAAGTGGTCAGGAGGATTATTGGGCCTAAAGCATCCGTAGCCGGTTCTGTAAGTTTCCGGTTAAATCCACTAGCTCAACTAATGGGCTGCCGGAAATACTGCAGAACTAGGGAGTGGGAGAGGTAGACGGTACTCGGGAGGAAGGGGTAAAATCCTTTGATCTCTTGATGACCACCTGTGGCGAAGGCGGTCTACCAGAACACGTTCGACGGTGAGGGATGAAAGCTGGGGGAGCAAACCGG` |
| ASV1012 | 424 | `TAGGGAATATTGGGCAATGGAGGCAACTCTGACCCAGCCATGCCGCGTGCAGGACGAAGGCTCTTCGAGTCGTAAACTGCTTTTATCGGGGAAGAACGGCTTTCTTGCGAGGAAGTGTGACGGTATCCGATGAATAAGCACCGGCTAACTCCGTGCCAGCAGCCGCGGTAATACGGAGGGTGCAAGCGTTGTCCGGATTTATTGGGTTTAAAGGGTGCGTAGGCGGGTGTTTAAGTCTGGTTTAAAATCAGGTCGCTCAACGATCTAACGTGGCTGGATACTGGACATCTTGAAATTGTTGGCCGCAGCCGGAATGGGTCATGTAGCGGTGAAATGCATAGATATGCCCCGGAACCCCGATTGCGAAGGCAGGCTGCGACGACTGATTTGACGCTGAGGCACGAGAGCATGGGTAGCAAACAGG` |
| ASV2288 | 405 | `TGGGGAATATTGGGCAATGGACGAAAGTCTGACCCAGCAATACCGCGTGAATGATGAAGGCCCTCTGGGTTGTAAAGTTCTTTTGTCAGGGACGATAATGACGGTACCTGAAGAATAAGCACCGACTAACTATGTGCCAGCAGTCGCGGTAATACATAGGGTGCTAGCGTTGCTCGGAATTACTGGGCGTAAAGAACATGTAGGAGGACTTGTAAGTCAAAAGTGCAATTCCTGGGCTTAACCCAGGCGCTGCTTTTGATACTGCTTGTCTAGAGTAAGGTAGAGGATAGCGGAATTTCTAGTGTAGGGGTAAAATCCGTAGATATTAGAAGGAACACCGAAAGCGAAAGCAGCTATCTGGGCCTTTACTGACTCTGAGATGTGAAAGCGTGGGGAGCAAACAGG` |
| ASV1533 | 404 | `TGGGGAATATTGGACAATGGACGCAAGTCTGATCCAGCAATACCACGTGAGTGATGAAGGCCTTCGGGTTGTAAAACTCTTTTAGTAGGGAAGATAATGACGGTACCTACAGAAAAAGCACCGGCTAACTTCGTGCCAGCAGCCGCGGTAATACGAAGGGTGCTAGCGTTGTTCGGAATCATTGGGCGTAAAGCGCACGTAGGCGGCTTAATAAGTCAGCTGTGAAAGCCCAGAGCTTAACTCTGGAATTGCAGTTGAAACTATTTCGCTAGAGTGTGGTGGGGGATAGTGGAATTCCTAGTGTAGGGGTGAAATCCGTAGATATTAGGAGGAACACCGGTGGCGAAAGCGACTATCTACGCCATTACTGACGCTAAGGTGCGAAAGCGTGGGGAGCAAACAGG` |
| ASV5299 | 429 | `TGGGGAATTTTGGACAATGGGGGGAACCCTGATCCAGCAACGCCGCGTGAATGAAGAAGGCCTTAGGGTTGTAAAGTTCTTTTATCTGGGAGCAAAGTCTTGGTGCTAATACCATCAAGATATGAGAGTACCAGAAGAATAAGCACCGGCTAACTACGTGCCAGCAGCCGCGGTAATACGTAGGGTGCGAGCGTTAATCGGAATTACTGGGCGTAAAGGGTGCGCAGGCGGATGATTAAGTTAGAAGTGAAATCCCCGGGCTTAACCTGGGAACTGCTTTTAAGACTGGTCGTCTAGAGTTTGTCAGAGGGGGGTGGAATTCCAAGTGTAGCAGTGAAATGCGTAGAGATTTGGAGGAACACCAATGGCGAAGGCAGCCCCCTGGGATAGAACTGACGCTCAGGCACGAAAGCGTGGGTAGCAAACAGG` |
| ASV1911 | 429 | `TGGGGAATATTGGACAATGGGCGCAAGCCTGATCCAGCGATGCCGCGTGTGTGAAGAAGGCCTGCGGGTTGTAAAGCACTTTCAGTGGGGAGAAAGGACTTGTTCCTAATATGGACAAGGAGAGATGTTACCCACAGAAGAAGCACCGGCTAACTCTGTGCCAGCAGCCGCGGTAATACAGAGGGTGCGAGCGTTAATCGGAATTACTGGGCGTAAAGGGTGCGTAGGTGGTTAAATTAGTTAGGTGTGAAAGTCCTGAGCTCAACTTGGGGATTGCGCCTAATACTGTTTGACTAGAGTATTGTAGAGGAAAGTAGAATTTCCGGTGTAGCGGTGAAATGCGTAGATATCGGAAGGAATATCAGTGGCGAAGGCGACTTTCTGGACAAATACTGACACTGAGGCACGAAAGCATGGGGAGCAAACAGG` |
| ASV3962 | 404 | `TGGGGAATATTGGACAATGGGCGCAAGCCTGATCCAGCAATACCACGTGAATGATGAAGGCCTTCGGGTTGTAAAGTTCTTTTAGTAGGGAAGATAATGACGGTACCTACAGAAAAAGCGCCGGCTAACTTCGTGCCAGCAGCCGCGGTAATACGAAGGGCGCTAGCGTTGTTCGGAATCATTGGGCGTAAAGCGTGCGTAGGCGGCATGATAAGTCAGCTGTGAAAGCCCAGGGCTCAACCTTGGAATTGCAGTTGAAACTATCAAGCTAGAGTATGGCGGGGGATAGTGGAATTCTTAGTGTAGGGGTGAAATCCGTAGATATTAAGAGGAACACCAGTGGCGAAAGCGACTATCTACACCATTACTGACGCTGAGGTACGAAAGCGTGGGGAGCAAACAGG` |
| ASV1164 | 404 | `TGGGGAATCTTGGACAATGGGCGCAAGCCTGATCCAGCCATGCCGCGTGAGTGAAGAAGGCCTTCGGGTTGTAAAGCTCTTTTACCAGGGACGATAATGACGGTACCTGGAGAATAAGCCCCGGCTAACTTCGTGCCAGCAGCCGCGGTAATACGAAGGGGGCTAGCGTTGTTCGGAATTACTGGGCGTAAAGCGCACGTAGGTTGATTGTTAAGTCAGGGGTGAAATCCCAGAGCTCAACTCTGGAACTGCCCTTGATACTGGCAATCTCGAGTCCGGAAGAGGTAAGTGGAATTCCCAGTGTAGAGGTGAAATTCGTAGATATTGGGAAGAACACCAGTGGCGAAGGCGGCTTACTGGTCCGGTACTGACACTGAGGTGCGAAAGCGTGGGGATCAAACAGG` |
| ASV3258 | 407 | `TAGGGAATATTGCACAATGGGGGAAACCCTGATGCAGCGACGCCGCGTGAGTGATGAAGGCCTTCGGGTCGTAAAGCTCTGTCGTATGGGACTAACACAATGAAGGTACCATGCAAGAAAGGATCGGCTAACTTCGTGCCAGCAGCCGCGGTAAGACGAGGGATCCTAGCGTTGTTCGGAATCATTGGGCGTAAAGCGGGTGTAGGCGGTCTTATAAGTCAGGTGTGAAATCCCAGGGCTCAACCCTGGAAGTGCACTTGATACTGTAGGACTTGAGTGTGGTAGAGGTTACTGGAATTCCTGGTGTAGTGGTAAAATACGTAGATATCAGGAGGAATACCGGAGGCGAAGGCGGGTAACTGGGCCAACACTGACGCTGAGATCCGAAAGCGTGGGGATCAAACAGG` |
| ASV5436 | 404 | `TGAGGAATATTGGACAATGGGGGAAACCCTGATCCAGCCATGCCGCGTGAGTGATGAAGGCCTTAGGGTTGTAAAGCTCTTTTAGATGGGAAGATAATGACTGTACCATCAGAATAAGCCCCGGCTAAATTCGTGCCAGCAGCCGCGGTAATACGAATGGGGCAAGCGTTGTTCGGAATCACTGGGCGTAAAGCGTATGTAGGCGGTTCGGCAAGTCAGAAGTGAAATCCCAGAGCTTAACTCTGGAATTGCTTTTGAAACTGCCAAGCTTGAGTACTGGAGAGGTTAGCAGAATTCCGAGTGTAGAGGTGAAATTCGCAGATATTCGGAGGAATACCAGTGGCGTAAGCGGCTAACTGGACAGTAACTGACGCTGAGATACGAAAGCGTGGGGATCAAACAGG` |
| ASV1076 | 407 | `TGGGGAATTTTCCGCAATGGGCGAAAGCCTGACGGAGCAATACCGCGTGAGGGATGAAGGCCTTTGGGTTGTAAACCTCTTTTATCAAGGAGGAAGTTCTGACGTGTACTTGATGAATAAGCATCGGCTAACTCCGTGCCAGCAGCCGCGGTAAGACGGAGGATGCAAGTGTTATCCGGAATCACTGGGCGTAAAGCGTCTGTAGGTGGTTTCAAAAGTCCACTGTTAAAGATTGAAGCTTAACTTCAAAAATGCCGTGGAAACTTTGAAACTTGAGTATGATAGGGGTAGAGGGAATTTCCAGTGGAGCGGTGAAATGCGTAGATATTGGAAAGACCACCAATGGCGAAGGCACTCTACTGGGCCATTACTGACACTGAGAGACGAAAGCTAGGGGAGCAAATGGG` |
| ASV4554 | 430 | `TGGGGAATATTGGACAATGGGGGAAACCCTGATCCAGCGATGCCGCGTGTGTGAAGAAGGCCTGCGGGTTGTAAAGCACTTTTGTTGGGGAAGAGGGTGTTATGTGTTCATAGCACATGGCATTGACGGTACCCAAAGAATAAGCACCGGCTAACTCTGTGCCAGCAGCCGCGGTAATACAGAGGGTGCGAGCGTTAATCGGAATTACTGGGCGTAAAGGGCGCGTAGGCGGAGAAGTATGTCTGATGTGAAAGTCCTGGGCTTAACCTGGGGATGGCATCAGAAACTGCTTTTCTGGAGTGAAGTAGAGGGTGGCGGAATTTCCGGTGTAGCGGTGAAATGCGTAGATATCGGAAAGAACACCAGTGGCGAAGGCGGCCACCTGGACTTTAACTGACGCTGAGGCGCGAAAGCATGGGGAGCGAACAGG` |
| ASV1531 | 429 | `TGGGGAATATTGGACAATGGGCGCAAGCCTGATCCAGCCACGCCGCGTGAGTGATGAAGGCCTTCGGGTTGTAAAGCTCTGTGGGGAGAGGCGAAAACAGTCATCAAGAAACGGGTGATTCTTGACGGTATCTCCTTAGCAAGCACCGGCTAACTCTGTGCCAGCAGCCGCGGTAATACAGAGGGTGCGAACGTTGCTCGGAATTACTGGGCGTAAAGCGCGTGTAGGCGGACCGGTAAGTCAGATGTGAAATCCCTGGGCTCAACCCAGGAACTGCATTTGAAACTGCTGGTCTAGAGTATCGGAGAGGGTGGCGGAATTCCTGGTGTAGAGGTGAAATTCGTAGATATCAGGAGGAACACCGGTGGCGAAGGCGGCCACCTGGACGGTCACTGACGCTGAGACGCGAAAGCGTGGGGAGCAAACAGG` |
| ASV2539 | 407 | `TAGGGAATATTGCACAATGGAGGAAACTCTGATGCAGCGACGCCGCGTGAGTGACGAAGGCCTTCGGGTTGTAAAGCTCTGTTCTCAGGGAAAAAGAAAGTGATTGTACCTGAGAAGAAAGGACCGGCTAACTTCGTGCCAGCAGCCGCGGTAAGACGAGGGGTCCAAGCGTTGTTCGGAATTATTGGGCGTAAAGCGGGTGTAGGTGGCTCTATAAGTCAGGAGTGAAAGCCCCGAGCTTAACTTGGGAAGTGCTTTTGATACTGTAGAGCTTGAATGTGGGAGAGGATCGTAGAATTCCAGGTGTAGTGGTGAAATACGTAGATATCTGGAGGAATACCGGTGGCGAAGGCGGCGATCTGGCCCAACATTGACACTGAGACCCGAAAGTGCGGGGATCAAACAGG` |
| ASV640 | 404 | `TGGGGAATCTTAGACAATGGGCGCAAGCCTGATCTAGCCATGCCGCGTGAGTGATGAAGGCCTTAGGGTCGTAAAGCTCTTTCGCTGGGGAAGATAATGACGGTACCCAGTAAAGAAACCCCGGCTAACTCCGTGCCAGCAGCCGCGGTAATACGGAGGGGGTTAGCGTTGTTCGGAATTACTGGGCGTAAAGCGCGCGTAGGCGGATCAGAAAGTTGGGGGTGAAATCCCAGGGCTCAACCCTGGAATTGCCTTCAAAACTCCTGGTCTTGAGTTCGAGAGAGGTGAGTGGAATTCCGAGTGTAGAGGTGAAATTCGTAGATATTCGGAGGAACACCAGTGGCGAAGGCGGCTCACTGGCTCGATACTGACGCTGAGGTGCGAAAGCGTGGGGAGCAAACAGG` |
| ASV5435 | 429 | `TGGGGAATTTTGGACAATGGGGGCAACCCTGATCCAGCCATGCCGCGTGCGGGAAGAAGGCCTTCGGGTTGTAAACCGCTTTTGTTCGGGAAGAAATCGTCCTCGCTAATACCGGGGGCGGATGACGGTACCGGAAGAATAAGCACCGGCTAACTACGTGCCAGCAGCCGCGGTAATACGTAGGGTGCAAGCGTTAATCGGAATTACTGGGCGTAAAGCGTGCGCAGGCGGTTTCGCAAGTCAGATGTGAAAGCCCCGGGCTTAACCTGGGAACTGCATTTGAAACTACGAGGCTAGAGTGTGTCAGAGGGGGGTAGAATTCCACGTGTAGCAGTGAAATGCGTAGAGATGTGGAGGAATACCGATGGCGAAGGCAGCCCCCTGGGATAACACTGACGCTCATGCACGAAAGCGTGGGGAGCAAACAGG` |
| ASV2146 | 429 | `TGGGGAATTTTGGACAATGGGCGCAAGCCTGATCCAGCAATGCCGCGTGAGTGAAGAAGGCCTTCGGGTTGTAAAGCTCTTTTGTCAGGGAAGAAACGGGTTTTTCTAATACAGAGACCTAATGACGGTACCTGAAGAATAAGCACCGGCTAACTACGTGCCAGCAGCCGCGGTAATACGTAGGGTGCAAGCGTTAATCGGAATTACTGGGCGTAAAGCGTGCGCAGGCGGTTTTGTAAGACTGACGTGAAATCCCCGGGCTCAACCTGGGAATGGCGTTGGTGACTGCAAGGCTAGAGTTTGGCAGAGGGGGGTAGAATTCCACGTGTAGCAGTGAAATGCGTAGATATGTGGAGGAACACCGATGGCGAAGGCAGCCCCCTGGGTCAAAACTGACGCTCATGCACGAAAGCGTGGGGAGCAAACAGG` |
| ASV1558 | 406 | `TGGGGAATTTTCCGCAATGGGCGCAAGCCTGACGGAGCAACGCCGCGTGAGGGAGGAAGGCCTTTGGGTTGTAAACCTCTTTTCTCAAGGAAGAAGATCTGACGGTACTTGAGGAATAAGCATCGGCTAACTCCGTGCCAGCAGCCGCGGTAAGACGGAGGATGCAAGCGTTATCCGGAATTATTGGGCGTAAAGCGTCCGTAGGTGGCAGACCAAGTCTGTTTTTAAATAGTGCAGCTCAACTGCATAAGGGGAATGGAAACTGGTCAGCTAGAGTTCGGTAGGGGCAGAGGGAATTCCCGGTGTAGCGGTGAAATGCGTAGATATCGGGAAGAACACCAGTGGCGAAAGCGCTCTGCTGGGCCTGAACTGACACTGATGGACGAAAGCTAGGGGAGCGAAAGGG` |
| ASV3201 | 404 | `TCGGGAATTTTGGGCAATGGGCGAAAGCCTGACCCAGCAACGCCGCGTGAAGGATGAAGTATTTCGGTATGTAAACTTCGAAAGAATAGGAAGAATCAATGACGGTACTATTTATAAGGTCCGGCTAACTACGTGCCAGCAGCCGCGGTAATACGTAGGGACCAAGCGTTGTTCGGATTTACTGGGCGTAAAGGGCGCGTAGGCGGCGTTGTAAGTCACTTGTGAAATCTCTGAGCTTAACTCAGAACGGCCAAGTGATACTGCAGTGCTAGAGTGTGGAAGGGGCAATCGGAATTCTTGGTGTAGCGGTGAAATGCGTAGATATCAAGAGGAACACCTGAGGTGAAGACGGGTTGCTGGGCCAACACTGACGCTGAGGCGCGAAAGCCAGGGGAGCAAACGGG` |
| ASV1278 | 429 | `TGGGGAATTTTGGACAATGGACGCAAGTCTGATCCAGCCATGCCGCGTGCGGGAAGAAGGCCTTCGGGTTGTAAACCGCTTTTGTCAGGGAAGAAACGAGTTTCTCTAATACAGAGACTTAATGACGGTACCTGAAGAATAAGCACCGGCTAACTACGTGCCAGCAGCCGCGGTAATACGTAGGGTGCAAGCGTTAATCGGAATTACTGGGCGTAAAGCGTGCGCAGGCGGTTATGCAAGACAGAGGTGAAATCCCCGGGCTCAACCTGGGAACTGCCTTTGTGACTGCATAGCTAGAGTACGGCAGAGGGGGATGGAATTCCGCGTGTAGCAGTGAAATGCGTAGATATGCGGAGGAACACCGATGGCGAAGGCAATCCCCTGGGCCTGTACTGACGCTCATGCACGAAAGCGTGGGGAGCAAACAGG` |
| ASV1215 | 423 | `TAGGGAATATTGGACAATGGGTGAGAGCCTGATCCAGCCATGCCGCGTGCAGGAAGAAGGCCTTCTGGGTTGTAAACTGCTTTTGCCGGGGGATAAAAGATCCCTGCGGGGAGCATTGAAGGTACCCGGTGAATAAGCCACGGCTAACTACGTGCCAGCAGCCGCGGTAATACGTAGGTGGCAAGCGTTGTCCGGATTTATTGGGTTTAAAGGGTGCGTAGGCGGTCCTGTAAGTCAGTGGTGAAATACGGCAGCTCAACTGTCGAGGTGCCATTGATACTGCGGGACTTGAGTACAGTCGAGGTAGGCGGAATTGACGGTGTAGCGGTGAAATGCTTAGATATCGTCAAGAACACCGATAGCGAAGGCAGCTTACTAGGCTGTAACTGACGCTGAGGCACGAAGGTGCGGGGATCAAACAGG` |
| ASV5179 | 365 | `CACACTACGGCCCAGCGCGGCCGATGAGGCCCTTGCACTACGCCGTGCGCTCTCGGCCAGGTCGTACTTGGTGGCGCGCGGCGTCTCGCCGCTCAAGATCATGGTGAACTTCGCATCGGCCGCCGATTTCGTGGCAGACAACTCGACGGCCGAGGGCCGGCGCGAGAACCAGCGCGTGGACATTGAGGTGGTCTACATCCCCACCTACTGAGCGATCCGGCATCGCCATTCCTGGCGGTGCTGCAGGTCGCTCCAGCAGCTTCGCATCGTCGGCTCTGACGATGCAGGCCCGGCCGGCTTCCCTTGACAGCATCGCCATTTCTGACGATGCTGGCCAAGTCCCGATTTACTCCAGGAAGAAGGCC` |
| ASV2931 | 404 | `TGGGGAATATTGCACAATGGGCGAAAGCCTGATGCAGCGACGCCGCGTGAGCGAAGAAGTATTTCGGTATGTAAAGCTCTATCAGCAGGGAAGAAAATGACGGTACCTGACTAAGAAGCACCGGCTAAATACGTGCCAGCAGCCGCGGTAATACGTATGGTGCAAGCGTTATCCGGATTTACTGGGTGTAAAGGGAGCGTAGGCGGCAAGGCAAGTCTGATGTGAAAGCCCGGGGCTCAACCCCGGGACTGCATTGGAAACTGCTTGGCTAGAGTGTCGGAGAGGTAAGTGGAATTCCTAGTGTAGCGGTGAAATGCGTAGATATTAGGAGGAACACCAGTGGCGAAGGCGACTTACTGGACGATAACTGACGCTGAGGCTCGAAAGCGTGGGGAGCAAACAGG` |
| ASV2437 | 429 | `TCGAGAATAATTCACAATGGGGGCAACCCTGATGGTGCAACGCCGCGTGGAGGATGACGGTCTTCGGATTGTAAACTCCTGTCATCTGGGAGAAAGACCTGACGGTAAATAGCCGGCAGGGTTGATAGTACCAGAAGAGGAAGGGACGGCTAACTTCGTGCCAGCAGCCGCGGTAATACGAAGGTCCCGAGCGTTGTTCGGAATCACTGGGCGTAAAGGGAGCGTAGGCGGTTTGGTAAGTCAGATGTGAAATCCCGGGGCTCAACCCCGGAACTGCATCCGATACTGCCAGACTTGAGTAATGGAGAGGTAGCTGGAATTATCGGTGTAGCAGTGAAATGCGTAGATATCGATAGGAACACTCGTGGCGAAAGCGAGCTACTGGACATTTACTGACGCTGAGGCTCGAAGGCTAGGGGAGCGAAAGGG` |
| ASV3221 | 387 | `GCGAGAAAACTTTGCAATGTGCGAAAGCACGACAAGGTTAATCCGAGTGGTTTCTGCTAAAGAAACCTTTTGCCAGTCCTAGAAACACTGGTGAATAAGGGGTGGGCAAGTTCTGGTGTCAGCCGCCGCGGTAAAACCAGCACCTCAAGTGGTCAGGATGATTATTGGGCCTAAAGCATCCGTAGCCTGCTTTGTAAGTTCTCGGTTAAATCCATACGCTTAACGTATGGGCTGCCGGCAATACTGCATAGCTAGGGAGTGGGAGAGGTAGACGGTACTCGGTAGGAAGGGGTAAAATCCTTTGATCTATTGATGACCACCTGTGGCGAAGGCGGTCTACCAGAACACGTCCGACGGTGAGGGATGAAAGCTGGGGGAGCAAACCGG` |
| ASV780 | 416 | `TTAGGAATCTTCCCCAATGGGCGAAAGCCTGAGGGAGCGACGCCGCTTGAAGGATGAAGGTTTTCGGATCGTAAACTTCTGAAACAAGGACGAAATCCCCGCGAGGGGTCTGACGGTACTTGTGTAATAGCACCGGCTAACTCCGTGCCAGCAGCCGCGGTAATACGGAGGGTGCAAGCGTTACCCGGAATCACTGGGCGTAAAGGGCGTGTAGGCGGCTTGGTAAGTCTGGATTTAAAGACCGAAGCTCAACTTCGGGAATGGTCTGGATACTGCCTTGCTTGACGACTGGAGGGGAGAGTGGAATTCTCGGTGTAGCGGTGGAATGCGTAGATACCGAGAGGAACACCAGTGGCGAAGGCGGCTCTCTGGACAGTGCGTGACGCTGAGGCGCGAAAGTGTGGGGAGCGAACCGG` |
| ASV1226 | 424 | `TGAGGAATATTGGTCAATGGGCGCAAGCCTGAACCAGCCATGCCGCGTGCAGGAAGACGGTCCTATGGATTGTAAACTGCTTTTATTTGGGAATAAACCTCTTTACGTGTAGAGAGCTGAAGGTACCAAATGAATAAGCACCGGCTAACTCCGTGCCAGCAGCCGCGGTAATACGGAGGGTGCAAGCGTTATCCGGAATCATTGGGTTTAAAGGGTCCGCAGGCGGGAAGATAAGTCAGTGGTGAAATCTCACAGCTCAACTGTGAAACTGCCATTGATACTGTCTTTCTTGAATTCGATCGAAGTGGGCGGAATGTGACATGTAGCGGTGAAATGCTTAGATATGTCACAGAACACCGATAGCGAAGGCAGCTCACTAGGTCTGGATTGACGCTCAGGGACGAAAGCGTGGGGAGCAAACAGG` |
| ASV1161 | 428 | `TCGAGAATTTTTCACAATGGGCGAAAGCCTGATGGAGCGACGCCGCGTGGGGGATGAAGGGCTTCGGCTCGTAAACCCCTGTCATTCGCGAACAAACCCTGTGATCGAATAGATCGTAGGCTGATAGTAGCGGAAGAGGAAGGGACGGCTAACTCTGTGCCAGCAGCCGCGGTAATACAGAGGTCCCAAGCGTTGTTCGGATTCACTGGGCGTAAAGGGTGCGTAGGCGGCAAGGAAAGTTCTAGGTGAAATCTCCAGGCTCAACTTGGAAAATGCCTAGAAAACTTCCTTGCTAGAGAGTTGCAGAGGAGATTGGAATTCTCGGTGTAGCAGTGAAATGCGTAGATATCGAGAGGAACATCAGTGGCGAAAGCGAATCTCTGGGCAACATCTGACGCTGAGGCACGAAAGCCAGGGGAGCAAACGGG` |
| ASV1806 | 429 | `TGGGGAATTTTGGACAATGGGCGAAAGCCTGATCCAGCAATGCCGCGTGCAGGATGAAGGCCTTCGGGTTGTAAACTGCTTTTGTACGGAACGAAAAGGTCTGCCCTAATACGGTGGGCTCATGACGGTACCGTAAGAATAAGCACCGGCTAACTACGTGCCAGCAGCCGCGGTAATACGTAGGGTGCGAGCGTTAATCGGAATTACTGGGCGTAAAGCGTGCGCAGGCGGTTATATAAGACAGATGTGAAATCCCCGGGCTCAACCTGGGACCTGCATTTGTGACTGTATAGCTAGAGTACGGTAGAGGGGGATGGAATTCCGCGTGTAGCAGTGAAATGCGTAGATATGCGGAGGAACACCGATGGCGAAGGCAATCCCCTGGACCTGTACTGACGCTCATGCACGAAAGCGTGGGGAGCAAACAGG` |
| ASV403 | 429 | `TGGGGAATTTTGGACAATGGACGCAAGTCTGATCCAGCCATGCCGCGTGCGGGAAGAAGGCCTTCGGGTTGTAAACCGCTTTTGTCAGGGAAGAAACGGCTCGCTCTAATACAGCGGGCTAATGACGGTACCTGAAGAATAAGCACCGGCTAACTACGTGCCAGCAGCCGCGGTAATACGTAGGGTGCAAGCGTTAATCGGAATTACTGGGCGTAAAGCGTGCGCAGGCGGTTATGCAAGACAGATGTGAAATCCCCGGGCTCAACCTGGGAACTGCATTTGTGACTGCATGGCTAGAGTACGGTAGAGGGGGATGGAATTCCGCGTGTAGCAGTGAAATGCGTAGATATGCGGAGGAACACCGATGGCGAAGGCAATCCCCTGGACCTGTACTGACGCTCATGCACGAAAGCGTGGGGAGCAAACAGG` |
| ASV1601 | 429 | `TGGGGAATATTGGACAATGGGCGCAAGCCTGATCCAGCCATGCCGCGTGGGTGAAGAAGGCCTTCGGGTTGTAAAGCCCTTTTGTCCGGAAAGAAAAGCACTGGATTAATACTTCGGTGTACTGACGGTACCGGAAGAATAAGCACCGGCTAACTTCGTGCCAGCAGCCGCGGTAATACGAAGGGTGCAAGCGTTACTCGGAATTACTGGGCGTAAAGCGTGCGTAGGTGGTTCGTTAAGTCTGATGTGAAAGCCCTGGGCTCAACCTGGGAATTGCATTGGATACTGGCGAGCTAGAGTGCGGTAGAGGATGGCGGAATTCCCGGTGTAGCAGTGAAATGCGTAGAGATCGGGAGGAACATCTGTGGCGAAGGCGGCCATCTGGACCAGCACTGACACTGAGGCACGAAAGCGTGGGGAGCAAACAGG` |
| ASV882 | 429 | `TGGGGAATATTGGACAATGGGCGCAAGCCTGATCCAGCCATGCCGCGTGTGTGAAGAAGGCCTTCGGGTTGTAAAGCACTTTTATCCGGAAAGAAAAGCTTTGGGTTAATACCCCGAAGTCATGACGGTACCGGAAGAATAAGCACCGGCTAACTTCGTGCCAGCAGCCGCGGTAATACGAAGGGTGCAAGCGTTACTCGGAATTACTGGGCGTAAAGCGTGCGTAGGTGGTTCGTTAAGTCTGATGTGAAAGCCCTAGGCTCAACCTGGGAATTGCATTGGATACTGGCGGGCTAGAGTGCGGTAGAGGATAGTGGAATTCCCGGTGTAGCAGTGAAATGCGTAGAGATCGGGAGGAACATCTGTGGCGAAGGCGACTATCTGGGCCAGCACTGACACTGAGGCACGAAAGCGTGGGGAGCAAACAGG` |
| ASV4361 | 422 | `TAGGGAATATTGGGCAATGGATGAAAGTCTGACCCAGCCATTCCGCGTGGAGGAAGAAGGTTTTCGGATTGTAAACTCCTTTTGCACGAGAACAAATACATTTTGCGAAATGGGTTGAGTGTATCGTGAGAATAAGCACCGGCTAACTACGTGCCAGCAGCCGCGGTAATACGTAGGGTGCAAGCGTTGTCCGGATTTATTGGGTTTAAAGGGTACGTAGGCGGGACGATAAGTCAGCGGTGAAAGCTGAAAGCTTAACTTTCAAATTGCCGTTGATACTGTTGTTCTTGAATTATGGAGAGGCACTTGGAATTTGGGATGTAGCGGTGAAATGCATAGATATCCCAAAGAACACCAACAGTGAAGACATGGTGCTGGTCATATATTGACGCTGAGGTACGAAAGCGTGGGTAGCAAACAGG` |
| ASV446 | 404 | `TGGGGAATATTGGACAATGGGGGAAACCCTGATCCAGCAATGCCGCGTGAATGAAGAAGGCCTTAGGGTTGTAAAATTCTTTTGTTCGTGACGATAATGACGGTAGCGGACGAATAAGCCCTGGCTAACTCTGTGCCAGCAGCCGCGGTAATACAGAGAGGGCAAGCGTTGTCCGGAATGACTGGGCGTAAAGCGCGCGCAGGCGGTTTCTTAAGTTGGATGTGAAATCCCCGGGCTTAACCTGGGAACTGCATTCGATACTGGGGAACTAGAGGCCGAGAGAGGAAAGCGGAATGACGAGTGTAGAGGTGAAATTCGTAGATATTCGTCGGAACACCAGAGGCGAAGGCGGCTTTCTGGCTCGGACCTGACGCTCAGGCGCGAAAGCGTGGGGAGCAAACAGG` |
| ASV4963 | 428 | `TGGGGAATATTGGACAATGGGCGCAAGCCTGATCCAGCCATGCCGCGTGAGTGATGAAGGCCTTCGGGTCGTAAAGCTCTGTGGAGGGGGAAGAACACTTGCAGCATCAGATCCTGCAAACTGACGGTACCCCTTTAGCAAGCACCGGCTAACTCCGTGCCAGCAGCCGCGGTAATACGGAGGGTGCAAACGTTGCTCGGAATTATTGGGCGTAAAGCGCACGTAGGCGGACCATCAAGTCGGTTGTGAAATCCCTCGGCTTAACCGAGGACGTGCATTCGAAACTGATGGCCTTGAGTACTAAAGAGGATCGCGGAATTCCCGGTGTAGAGGTGAAATTCGTAGATATCGGGAGGAACACCAGTGGCGAAGGCGGCGATCTGGGTAGATACTGACGCTGAGGTGCGAAAGCGTGGGGAGCAAACAGG` |
| ASV212 | 429 | `TGGGGAATTTTGGACAATGGGCGAAAGCCTGATCCAGCCATGCCGCGTGAGTGAAGAAGGCCTTCGGGTTGTAAAGCTCTTTTGTCCGGGAGGAAATCCTGACCCCTAATACGGGTCGGGGATGACAGTACTGGAAGAATAAGCACCGGCTAACTACGTGCCAGCAGCCGCGGTAATACGTAGGGTGCAAGCGTTAATCGGAATTACTGGGCGTAAAGCGTGCGCAGGCGGTTTTGTAAGCCAGATGTGAAATCCCCGAGCTTAACTTGGGAACTGCGTTTGGAACTGCAAGACTAGAGTGTGTCAGAGGGGGGTAGAATTCCACGTGTAGCAGTGAAATGCGTAGAGATGTGGAGGAATACCGATGGCGAAGGCAGCCCCCTGGGATAACACTGACGCTCATGCACGAAAGCGTGGGGAGCAAACAGG` |
| ASV4368 | 423 | `TAAGGAATATTGGACAATGGACGGAAGTCTGATCCAGCCATCCCGCGTGGAGGAATAAGGCCCTATGGGTTGTAAACTCCTTTAGCAGGGGAATAAAAAGGTCATTTATGATCAATTGAAGGTACCCTGAGAATAAGCACCGGCTAACTCCGTGCCAGCAGCCGCGGTAATACGGAGGGTGCAAGCGTTATCCGGATTTACTGGGTTTAAAGGGTGCGTAGGCGGACTATTAAGTCAAATGTGAAAGCTTGCAGCTCAACTGTAAGATTGCGTTTGATACTGGTAGTCTTGAATATGGTAGAAGTTGGCGGAATAAATCATGTAGCGGTGAAATGCTTAGATATGATTTAGAACACCGATTGCGAAGGCAGCTAGCTATGTCATAATTGACGCTGAGGCACGAAAGCGTGGGGATCAAACAGG` |
| ASV3633 | 429 | `TCGAGAATCATTCACAATGGGGGCAACCCTGATGGTGCAACGCCGCGTGGAGGATGAAGGTCTTCGGATTGTAAACTCCTGTCATTGGAGAGCAAGGCATGGGTGTGAATAGCACACATGATTGATAGTATCCAAAGAGGAAGGGACGGCTAACTTCGTGCCAGCAGCCGCGGTAATACGAAGGTCCCGAGCGTTGTTCGGAATCACTGGGCGTAAAGGGAGCGTAGGCTGCGTGGTAAGTCAGATGTGAAATCCCAGAGCTCAACTCTGGAACTGCATCCGATACTGCCATGCTAGAGTATTGGAGGGGTAACTGGAATTTTCGGTGTAGCAGTGAAATGCGTGGATATCGAAAGGAACACCTGTGGCGAAAGCGAGTTACTGGACAATTACTGACGCTGAGGCTCGAAGGCTAGGGGAGCAAAAGGG` |
| ASV21 | 423 | `TAGGGAATATTGGGCAATGGACGCAAGTCTGACCCAGCCATGCCGCGTGCAGGATGAAGGCCCTCTGGGTTGTAAACTGCTTTTGACAGGGAAGAAATACACCATGCGTGGTGTGCTGACGGTACCTGTAGAATAAGCACCGGCTAACTCCGTGCCAGCAGCCGCGGTAATACGGAGGGTGCAAGCGTTGTCCGGATTTATTGGGTTTAAAGGGTGCGTAGGCGGTTCTGTAAGTCAGTGGTGAAAGCTAGCAGCTTAACTGTTAAACTGCCATTGATACTGCAGAGCTTGAGTTCAGATGAGGTAGGCGGAATTTATAGTGTAGCGGTGAAATGCTTAGATACTATAAAGAACTCCGATAGCGTAGGCAGCTTACTAAACTGATACTGACGCTGAGGCACGAAAGTGTGGGGATCAAACAGG` |
| ASV2237 | 429 | `TGGGGAATATTGGACAATGGGGGCAACCCTGATCCAGCCATGCCGCGTGTGTGAAGAAGGCCTTCGGGTTGTAAAGCACTTTAAGCGGGGAGGAAGGCTCGATGGCTAATATCTATCGGGATTGACGTTACCCGCAGAATAAGCACCGGCAAACTTCGTGCCAGCAGCCGCGGTAATACGAAGGGTGCGAGCGTTAATCGGAATTACTGGGCGTAAAGCGCGCGTAGGTGGTTTGGTAAGCGATATGTGAAATCCCTGGGCTCAACCTGGGAACTGCATGTCGAACTGCCAGGCTAGAGTATGGTAGAGGGGTGTGGAATTTCCTGTGTAGCGGTGAAATGCGTAGATATAGGAAGGAACATCAGTGGCGAAGGCGACACCCTGGACCGATACTGACACTGAGGCGCGAAAGCGTGGGTAGCGAACAGG` |
| ASV122 | 404 | `TGGGGAATATTGGACAATGGGCGCAAGCCTGATCCAGCAATGCCGCGTGTGTGAAGAAGGTCTTCGGATCGTAAAGCACTTTCGACAGGGACGATGATGACGGTACCTGTAGAAGAAGCCCCGGCTAACTTCGTGCCAGCAGCCGCGGTAATACGAAGGGGGCTAGCGTTACTCGGAATTACTGGGCGTAAAGGGCGCGTAGGCGGCTCGGTCAGTCAGGCGTGAAATTCCTGGGCTTAACCTGGGGACTGCGCTTGATACTGTCGAGCTTGAGGCCGAGAGAGGGTGGTGGAATTCCCAGTGTAGAGGTGAAATTCGTAGATATTGGGAAGAACACCGGTGGCGAAGGCGACCACCTGGCTCGGTACTGACGCTGAGGCGCGACAGCGTGGGGAGCAAACAGG` |
| ASV512 | 404 | `TGGGGAATCTTGCACAATGGGCGAAAGCCTGATGCAGCCATGCCGCGTGAATGATGAAGGCCTTAGGGTTGTAAAATTCTTTCGTCAGGGATGATAATGACCGTACCTGAAGAAGAAGCCCCGGCTAACTTCGTGCCAGCAGCCGCGGTAATACGAAGGGGGCTAGCGTTGTTCGGAATTACTGGGCGTAAAGCGCACGTAGGCGGACTTTTAAGTCAGGTGTGAAATCCCGGGGCTCAACCTCGGAACTGCATTTGAAACTGGGAGTCTTGAGACCAGGAGAGGTTAGCGGAATACCGAGTGTAGAGGTGAAATTCGTAGATATTCGGTGGAACACCAGTGGCGAAGGCGGCTAACTGGACTGGTACTGACGCTGAGGTGCGAAAGTGTGGGGAGCAAACAGG` |
| ASV4104 | 424 | `TAGGGAATATTGGTCAATGGAGGGAACTCTGAACCAGCCATGCCGCGTGAACGAAGAAGGCGCTCTGCGTCGTAAAGTTCTTTTAAATGGGACGAAAAAGGGGAACTTGTTCTCAACTGACGGTACCATTGGAATAAGCACCGGCTAACTCTGTGCCAGCAGCCGCGGTAATACAGAGGGTGCAAGCGTTATCCGGAATCACTGGGTTTAAAGGGTGCGTAGGCGGGTTAATAAGTCAGAGGTGAAAGGTAGTCGCTTAACGATTAAATTGCCTTTGATACTGTTAGTCTTGAATCAAGATGAGGTTGGCGGAATGAGACATGTAGCGGTGAAATGCATAGATATGTCTTAGAACACCGATTGCGAAGGCAGCTGACTGGACTTCGATTGACGCTGAGGCACGAAAGCGTGGGGATCAAACAGG` |
| ASV3336 | 404 | `TGGGGAATATTGGACAATGGGCGAAAGCCTGATCCAGCAATGCCGCGTGAGTGAAGAAGGCCTTCGGGTTGTAAAGCTCTTTTACCCGGGATGATAATGACCGTACCGGGAGAATAAGCCCCGGCTAACTCCGTGCCAGCAGCCGCGGTAATACGGAGGGGGCTAGCGTTGTTCGGAATCACTGGGCGTAAAGCGTACGTAGGCGGCTATTCAAGTCAGAGGTGAAAGCCTGGGGCTCAACCCCAGAACTGCCTTTGAAACTAGGTAGCTAGAACACGGGAGAGGTGAGTGGAATTCCGAGTGTAGAGGTGAAATTCGTAGATATTCGGAAGAACACCAGTGGCGAAGGCGGCTCACTGGACCGTTGTTGACGCTGAGGTACGAAAGCGTGGGGAGCAAACAGG` |
| ASV919 | 429 | `TGGGGAATTTTGGACAATGGGCGCAAGCCTGATCCAGCCATGCCGCGTGAGTGAAGAAGGCCTTCGGGTTGTAAAGCTCTTTCGGCGGGGACGAAACGGTTCGTGACAACACCACGGACTAATGACGGTACTCGCAGAAGAAGCACCGGCTAACTACGTGCCAGCAGCCGCGGTAATACGTAGGGTGCAGGCGTTAATCGGAATTACTGGGCGTAAAGCGTGCGCAGGCGGCTCCTTAAGTCAGATGTGAAATCCCCGGGCTTAACCTGGGAACTGCGTTTGAAACTGGGGAGCTGGAGTGTGGAAGAGGGGGGTGGAATTCCAGGTGTAGCAGTGAAATGCGTAGAGATCTGGAGGAACACCGATGGCGAAGGCAGCCCCCTGGGCTAACACTGACGCTCATGCACGAAAGCGTGGGGAGCAAACAGG` |
| ASV1686 | 429 | `TGGGGAATCTTGGACAATGGGCGCAAGCCTGATCCAGCAATGCCGCGTGCGTGAAGAAGGCCTTCGGGTTGTAAAGCGCTTTTGTTCGGGAGGAAATCCTAGTGGCTAATATCCATTGGGGATGACAGTACCGGAAGAATAAGGACCGGCTAACTACGTGCCAGCAGCCGCGGTAATACGTAGGGTCCAAGCGTTAATCGGAATTACTGGGCGTAAAGGGTGCGCAGGTGGTTGATTAAGTGTGATGTGAAAGCCCCGGGCTCAACCTGGGAATTGCATTGCAAACTGGTGAACTAGAGTATGGCAGAGGGGGGTGGAATTCCGCGTGTAGCAGTGAAATGCGTAGAGATGCGGAGGAACACCGATGGCGAAGGCAACCCCCTGGGCTAATACTGACACTCATGCACGAAAGCGTGGGGAGCAAACAGG` |
| ASV1395 | 403 | `TGGGGAATATTGGACAATGGGGGCAACCCTGATCCAGCCATACCGCGTGAGTGCTAAGGCCTTCGGGTTGTAAAACTCTTTTACCCACGACGATGATGACGGTAGTGGGAGAATAAGTCCCGGCAAACTTCGTGCCAGCAGCCGCGGTAATACGAAGGGGACTAGCGTTGTTCGGATTTACTGGGCGTAAAGGGCGCGTAGGCTGTAGAATAAGTCAGGTGTGAAATCCCCGAGCTCAACTTGGGAACTGCATTTGATACTGTTTTACTGGAGTATGGGAGAGGTGAGCGGAATTCCGAGTGTAGAGGTGAAATTCGTAGATATTCGGAAGAACACCGATGGCGAAGGCAGCTCACTGGACCATAACTGACGCTGAGGCGCGAAAGCGTGGGGATCAAACAGG` |
| ASV304 | 429 | `TGGGGAATTTTGGACAATGGGCGCAAGCCTGATCCAGCCATGCCGCGTGTGGGAAGAAGGCCTTCGGGTTGTAAACCACTTTTGTCAGGGAAGAAACGGCTTTTTCTAATAAAGAGAGCTAATGACGGTACCTGAAGAATAAGCACCGGCTAACTACGTGCCAGCAGCCGCGGTAATACGTAGGGTGCGAGCGTTAATCGGAATTACTGGGCGTAAAGCGTGCGCAGGCGGCTTTGCAAGACAGATGTGAAATCCCCGGGCTCAACCTGGGAACTGCATTTGTGACTGCAAGGCTAGAGTACGGTAGAGGGGGATGGAATTCCGCGTGTAGCAGTGAAATGCGTAGATATGCGGAGGAACACCGATGGCGAAGGCAATCCCCTGGACCTGTACTGACGCTCATGCACGAAAGCGTGGGGAGCAAACAGG` |
| ASV1493 | 429 | `TGGGGAATTTTGGACAATGGGGGCAACCCTGATCCAGCCATGCCGCGTGAGTGAAGAAGGCCTTCGGGTTGTAAAGCTCTTTCAGCCGGGAAGAAAACGCATAGGCTAATACCCTGTGTGAATGACGGTACCGGAATAAGAAGCACCGGCTAACTACGTGCCAGCAGCCGCGGTAATACGTAGGGTGCGAGCGTTAATCGGAATTACTGGGCGTAAAGCGTGCGCAGGCGGTTTTGTAAGACAGGCGTGAAATCCCCGGGCTTAACCTGGGAACTGCGTTTGTGACTGCAAGGCTAGAGTACGGCAGAGGGGGGTGGAATTCCACGTGTAGCAGTGAAATGCGTAGAGATGTGGAGGAACACCAATGGCGAAGGCAGCCCCCTGGGTCGATACTGACGCTCATGCACGAAAGCGTGGGTAGCAAACAGG` |
| ASV4277 | 404 | `TGGGGAATATTGGACAATGGGGGAAACCCTGATCCAGCAATGCCGCGTGAGTGATGAAGGCCTTCGGGTTGTAAAGCTCTTTTACCTGTGACGATAATGACGGTAACAGGAGAATAAGCCCCGGCTAACTTCGTGCCAGCAGCCGCGGTAATACGAAGGGGGCTAGCGTTGTTCGGAATTACTGGGCGTAAAGGGCGCGTAGGCGGCTTTTTGCGTGAGATGTGAAAGACCTGGGCTCAACCTGGGAAGTGCATTTCATACGGGAAAGCTAGAGTGCGAGAGGGGAAAGTGGAATGATGCGTGTAGAGGTGAAATTCGTAGATATGCATCGGAACACCAGTGGCGAAGGCGACTTTCTGGCTCGTAACTGACGCTAAGGCGCGAAAGCGTGGGGAGCAAACAGG` |
| ASV650 | 424 | `TAGGGAATATTGGACAATGGGGGCAACCCTGATCCAGCCATGCCGCGTGCAGGAAGAAGGCCCTATGGGTCGTAAATTGCTTTTGTACGGGAAGAAACCTTACCATGCGTGGTAAGTTGACGGTACCGTGAGAATAAGGATCGGCTAACTTCGTGCCAGCAGCCGCGGTAATACGAAGGATCCAAGCGTTGTCCGGATTTACTGGGTTTAAAGGGTGCGTAGGCGGACTTTTAAGTCAGTGGTGAAAGCCTGTAGCTTAACTATAGAATTGCCATTGAAACTGAAAGTCTCGAGTGTGGTTGAGGTAACTGGAATATATCATGTAGCGGTGAAATGCTTAGATATGATATAGAACACCAATTGCGAAGGCAGGTTACTGAGCCATAACTGACGCTGAGGCACGAAAGCGTGGGGATCAAACAGG` |
| ASV5643 | 429 | `TGGGGAATATTGGACAATGGGGGAAACCCTGATCCAGCAATGCCGCGTGTGTGAAGAAGGCCTTCGGGTTGTAAAGCACTTTTAGTCATGAGGAAGAGTTTATGGTTAATACCCATAAACTTTGACGTTAGTGACAGAAAAAGCACCGGCTAACTCTGTGCCAGCAGCCGCGGTAATACAGAGGGTGCAAGCGTTAATCGGAATTACTGGGCGTAAAGCGCGCGTAGGTGGGTAAGCAAGTTGGATGTGAAAGCCCTGGGCTCAACCTGGGAACTGCATTCAAAACTATTTATCTAGAGTACAGGAGAGGGAAGTGGAATTTCCGGTGTAGTGGTGAAATACGTAGATATCGGAAGGAACACCAATGGCGAAGGCAACTTCCTGGACTGATACTGACACTGAGGCGCGAAAGCGTGGGGAGCAAACAGG` |
| ASV2134 | 429 | `TGGGGAATTTTGGACAATGGGGGCAACCCTGATCCAGCCATGCCGCGTGCGGGAAGAAGGCCTTCGGGTTGTAAACCGCTTTTGTCAGGGAAGAAATCCTCCGGGCTAACACCTTGGGGGGATGACGGTACCTGGAGAATAAGCACCGGCTAACTACGTGCCAGCAGCCGCGGTAATACGTAGGGTGCAAGCGTTAATCGGAATTACTGGGCGTAAAGCGTGCGCAGGCGGTTGTGCAAGACAGATGTGAAATCCCCGGGCTTAACCTGGGAACTGCATTTGTGACTGCACGGCTGGAGTGCGGCAGAGGGGGATGGAATTCCGCGTGTAGCAGTGAAATGCGTAGATATGCGGAGGAACACCGATGGCGAAGGCAATCCCCTGGGCCTGCACTGACGCTCATGCACGAAAGCGTGGGGAGCAAACAGG` |
| ASV5007 | 424 | `TGAGGAATATTGGACAATGGGTGAGAGCCTGATCCAGCCATCCCGCGTGAAGGACGACGGCCCTATGGGTTGTAAACTTCTTTTGTATAGGGATAAACCTACTCTCGTGAGAGTAGCTGAAGGTACTATACGAATAAGCACCGGCTAACTCCGTGCCAGCAGCCGCGGTAATACGGAGGGTGCAAGCGTTATCCGGATTTATTGGGTTTAAAGGGTCCGTAGGCTGATTTGTAAGTCAGTGGTGAAATCTCACAGCTCAACTGTGAAACTGCCATTGATACTGCAAGTCTTGAGTGTTGTTGAAGTAGCTGGAATAAGTAGTGTAGCGGTGAAATGCATAGATATTACTTAGAACACCAATTGCGAAGGCAGGTTACTAAGCAACAACTGACGCTGATGGACGAAAGCGTGGGGAGCGAACAGG` |
| ASV4748 | 429 | `TGGGGAATCTTGCGCAATGCGCGAAAGCGTGACGCAGCAACGCCGCGTGCGGGAAGACGGCCTTCGGGTTGTAAACCGCTTTCAGTTGGGACGAAGGTTCGCCGGTGAATAGCCGGCTGGACTGACGGTACCTTCAGAAGAAGCCCCGGCTAACTACGTGCCAGCAGCCGCGGTAATACGTAGGGGGCAAGCGTTGTCCGGAATCATTGGGCGTAAAGCGCGTGTAGGCGGCTCGGTAAGTCTGCTGTGAAAGTCCAGGGCTCAACCCTGGGATGCCGGTGGATACTGTCGAGCTCGAGTCCGGAAGAGGCGAGTGGAATTCCTGGTGTAGCGGTGAAATGCGCAGATATCAGGAGGAACACCAATGGCGAAGGCAGCTCGCTGGGACGTGACTGACGCTGAGACGCGAAAGCGTGGGGAGCAAACAGG` |
| ASV4742 | 423 | `TAAGGAATATTGGTCAATGGACGGAAGTCTGAACCAGCCACGCCGCGTGCAGGAAGAAGGTGCTTCGCATCGTAAACTGCTTTTGTCAGGGAAGAAAAGGCTCTTGCGAGAGGAATTGACGGTACCTGATGAATAAGCATCGGCTAACTACGTGCCAGCAGCCGCGGTAATACGTAGGATGCAAGCGTTATCCGGATTTATTGGGTTTAAAGGGTGCGTAGGCGGCTTTATAAGTCGTGGGTGAAAGTCAGACGCTTAACGTCTGGATTGCCTGCGATACTGTGAGGCTTGGATTCGGAGGAGGTAATTGGAATGTCTGATGTAGCGGTGAAATGCATAGATATCAGATGGAACGCCCATTGCGAAGGCAGGTTACTGTACCGAGATTGACGCTGAGGCACGAAAGCATGGGGAGCAAACAGG` |
| ASV1679 | 405 | `TGGGGAATATTGGACAATGGGCGAAAGCCTGATCCAGCGATTCTGCGTGAAGGATGAAGCCTCGTGGGGTGTAAACTTCTTTTCTGGGAGAAGACGAATGACGGTATCTCAGGAATAAGAGACGGCTAACTACGTGCCAGCAGCCGCGGTAATACGTAGGTCTCGAGCGTTATCCGGAATTACTGGGCGTAAAGCGTCTGTAGCCGGATGTATAAGTCTGTCTTTAAATACTCCGATTCAATCGGAGAAAAGGGATAGATACTGTATATCTAGAGGATTTTAGAGGTTAGTGGAATTTCCGGTGGAGCGGTGAAATGCGTTGATATCGGAAGGAATGCCGAAAGCGAAAGCAGCTAACTATAAAATAACTGACGGTCAGAGACGAAAGCTTGGGGAGCGAACGGG` |
| ASV4121 | 389 | `GCGCGAAAACTCTCCAATGTGCGAAAGCGTGAGAGGGGAATTCACAGTGCTTACGGTTTAGCCGTAGGCTTTTGCCAAGTGTAAATAGCTTGGCGAATAAGTGGTGGGTAAGACTGGTGCCAGCCGCCGCGGTAACACCAGCGCCACGAGTGGCTATCGCTATTATTGGGCCTAAAGCGTCCGTAGTCGGTTCTGTGCATCCTCTGTGAAATCGCAGCGCTCAACGTTGCGGCATGCGGGGGACACGGCAGAGCTTGAGACCGGGAGGCGTGTAGGGTATTCCACGGGGACCGGTAAAATGGTATAATCCGTGGAGGACCACCTGTGGCGAAGGCGCTACACGAGAACGGATCTGACGATGAGGGACGAAAGCCAGGGGAGCAAACCGG` |
| ASV2968 | 404 | `TGGGGAATCTTGCACAATGGAGGAAACTCTGATGCAGCGACGCCGCGTGGACGACGAAGCCCTTCGGGGTGTAAAGTCCTTTCGACCGGAACGATAATGACGGTACCGGTGGAAGAAGCTGCGGCTAACTACGTGCCAGCAGCCGCGGTAATACGTAGGCAGCAAGCGTTGTTCGGAATTACTGGGCGTAAAGAGTTCGTAGGCGGTGCGGCAAGTTCGGTGTGAAATCTCCCGGCTCAACTGGGAGGGTGCGCTAAATACTGCCGTGCTCGAGTATAGGAGAGGTAAGCGGAATTCCTGGTGTAGCGGTGAAATGCGTAGATATCAGGAGGAACACCTGTGGTGTAGACGGCTTACTGGACTATAACTGACGCTGAGGAACGAAAGCGTGGGTAGCAAACAGG` |
| ASV35 | 408 | `TGGGGAATTTTCCGCAATGGGCGAAAGCCTGACGGAGCAATACCGCGTGAGGGATGACGGCCTATGGGTTGTAAACCTCTTTTTTCAGGGAGGAATAAAATGACGTGTACCTGAAGAATAAGCATCGGCTAACTCCGTGCCAGCAGCCGCGGTAAGACGGGGGATGCAAGTGTTATCCGGAATCACTGGGCGTAAAGCGTCTGTAGGTGGTCCAATAAGTCAACTGTTAAATCTTGGGGCTCAACCTCAAAATCGCAGTCGAAACTATTAGACTAGAGTATAGTAGGGGTAAAGGGAATTTCCAGTGGAGCGGTGAAATGCGTAGATATTGGAAAGAACACCGATGGCGAAAGCACTTTACTGGGCTATTACTAACACTCAGAGACGAAAGCTAGGGTAGCAAATGGG` |
| ASV5276 | 430 | `TAGGGAATATTGCGCAATGGAGGAAACTCTGACGCAGCGACGCCGCGTGGGTGATGAAGGCTTTCGGGTCGTAAAGCCCTGTCGGAAGGGAAGAAAAATATCATGGCTAATATCCATGGTACTTGACGGTACCTTCAAAGGAAGCACCGGCTAACTACGTGCCAGCAGCCGCGGTAATACGTAGGGTGCAAGCGTTGTTCGGAATCATTGGGCGTAAAGCGCGTGTAGGCGGTTAGTTAAGTCGAATGTGAAATCCCCCGGCTCAACCGAGGAAGTGCATTCGAAACTAGCTAGCTAGAAGATGGCAGAGGAAGGTGGAATTCCAAGTGTAGAGGTGAAATTCGTAGATATTTGGAGGAATACCGGTGGCGAAGGCGGCCTTCTGGACCATTCTTGACGCTGAGACGCGAAAGCGTGGGGAGCAAACAGG` |
| ASV3797 | 404 | `TGGGGAATATTGGACAATGGGGGAAACCCTGATCCAGCCATGCCGCGTGCGTGAAGAAGGCCTTAGGGTTGTAAAGCGCTTTTGTCAGGGAAGATAATGACTGTACCTGAAGAATAAGCCCCGGCTAACTCCGTGCCAGCAGCCGCGGTAAGACGGAGGGGGCTAGCGTTGTTCGGAATTACTGGGCGTAAAGGGCGCGTAGGCGGCTATGTATGTCGGGGGTGAAATCCCAGAGCTTAACTCTGGAACTGCTCTCGAAACTGCATAGCTAGAGTGAGTAAGAGGCGGACGGAATTCCGAGTGTAGCAGTGAAATGCGTAGATATTCGGAGGAACATCAGTGGCGAAGGCGGTTCGCTGGTACTCAACTGACGCTGAGGCGCGAAAGCGTGGGGAGCAAACAGG` |
| ASV4937 | 404 | `TGGGGAATATTGGACAATGGGCGAAAGCCTGATCCAGCAATGCCGCGTGAGTGATGAAGGCTTTAGGGTTGTAAAACTCTTTCAGCGGGGAAGATAATGACGGTACCCGCAGAAGAAGCACCGGCTAACTCCGTGCCAGCAGCCGCGGTAATACGGAGGGTGCTAGCGTTATTCGGAATTACTGGGCGTAAAGCGTATGTAGGCGGTTTAGTAAGTTGGGAGTGAAATCCCGGGGCTCAACCCCGGAGCTGCTTTCAAAACTGCTAGGCTAGAGTTTGGTAGGGGACAGTGGAATTTCTAGTGTAGAGGTGAAATTCGTAGATATTAGAAAGAACACCAGTGGCGAAGGCGACTGTCTAGGCCAATACTGACGCTGAGATACGAAAGCGTGGGGAGCAAACAGG` |
| ASV5541 | 404 | `TGAGGAATATTGGACAATGGGCGAAAGCCTGATCCAGCCATGCCGCGTGAGTGATGAAGGCCTTCGGGTTGTAAAACTCTTTTAGTGGGGACAATAATGATGGTACCCACAGAAAAAGCACCGGCTAACTTCGTGCCAGCAGCCGCGGTAATACGAAGGGTGCAAGCGTTATTCGGAATCACTGGGCGTAAAGCGAGTGTAGGCGGTTTGTTAAGTCAGGTGTGAAAGCCTGGGGCTCAACCCCAGAATTGCACTTGATACTGGCAAACTAGAGTATGGTAGAGGGTGGTGGAATTCCAAGTGTAGAGGTGAAATTCGTAGATATTTGGAGGAACACCGGTGGCGAAGGCGACCACCTGGACCATTACTGACGCTAAGACTCGAAAGCGTGGGGAGCAAACGGG` |
| ASV3855 | 423 | `TAGGGAATATTGGACAATGGGTGAGAGCCTGATCCAGCCATGCCGCGTGCAGGAAGAAGGCGTTATGCGTTGTAAACTGCTTTTGCCAGGGGATAAAAAGACTCTGCGGAGTTTATTGAAGGTACCTGGTGAATAAGCCACGGCTAACTACGTGCCAGCAGCCGCGGTAATACGTAGGTGGCAAGCGTTGTCCGGATTTATTGGGTTTAAAGGGTGCGTAGGCGGCCCTGTAAGTCAGTGCTGAAATATCCCGGCTTAACCGGGAGGGTGGCATTGATACTGCAGGGCTTGAGTACAGATGAAGTAGGCGGAATTGACGGTGTAGCGGTGAAATGCATAGATATCGTCAAGAACACCGATAGCGAAGGCAGCTTACTAAGCTGTAACTGACGCTGAGGCACGAAAGTGTGGGGATCAAACAGG` |
| ASV995 | 404 | `TGGGGAATATTGGACAATGGGCGCAAGCCTGATCCAGCCATGCCGCGTGAGTGATGAAGGCCTTAGGGTTGTAAAGCTCTTTCGACGGGGACGATGATGACGGTACCCGTAGAAGAAGCCCCGGCTAACTTCGTGCCAGCAGCCGCGGTAATACGAAGGGGGCTAGCGTTGTTCGGAATTACTGGGCGTAAAGCGCACGCAGGCGGTTCGATTAGTCAGAAGTGAAAGCCCCGGGCTCAACCTGGGAACTGCTTTTGATACTGTCGAGCTTGAATCACGGAGAGGGTAGTGGAATTCCGAGTGTAGAGGTGAAATTCGTAGATATTCGGAAGAACACCAGTGGCGAAGGCGACTACCTGGCCGTCGATTGACGCTCATGTGCGAAAGCGTGGGGAGCAAACAGG` |
| ASV3265 | 404 | `TGGGGAATATTGGACAATGGGCGCAAGCCTGATCCAGCCATGCCGCGTGAGTGATGAAGGCCTTAGGGTTGTAAAGCTCTTTTGTCCGGGACGATAATGACGGTACCGGAAGAATAAGCCCCGGCTAACTTCGTGCCAGCAGCCGCGGTAATACGAAGGGGGCTAGCGTTGCTCGGAATCACTGGGCGTAAAGGGCGCGTAGGCGGCCATTCAAGTCGGGGGTGAAAGCCTGTGGCTCAACCACAGAATTGCCTTCGATACTGTTTGGCTTGAGACCGGAAGAGGTAAGTGGAACTGCGAGTGTAGAGGTGAAATTCGTAGATATTCGCAAGAACACCAGTGGCGAAGGCGGCTTACTGGTCCGGTTCTGACGCTGAGGCGCGAAAGCGTGGGGAGCAAACAGG` |
| ASV2734 | 404 | `TAGGGAATATTGGACAATGGAGGAAACTCTGATCCAGCCATGCCGCGTGAATGATGAAGGCCTTCGGGTTGTAAAGTTCTTTAGTCCGGGAAGATAATGACGGTACCGGAAGAATAAGCTCCGGCTAACTCTGTGCCAGCAGCCGCGGTAATACAGAGGGAGCTAGCGTTGTTCGGAATTACTGGGCGTAAAGCGCGCGCAGGCGGCCATTTAAGTCAGATGTGAAAGCCCGGGGCTTAACCTCGGAACTGCATTTGAAACTGAGTGGCTAGAGTATGGTAGGGGAGAGGGGAATTCCTAGTGTAGCGGTGAAATGCGTAGAGATTAGGAGGAACACCAGTGGCGAAGGCGCCTCTCTGGGCCATTACTGACGCTGAGGCGCGAAAGCGTGGGGAGCAAACAGG` |
| ASV2805 | 429 | `TGGGGAATTTTGGACAATGGGCGCAAGCCTGATCCAGCCATGCCGCGTGCGGGAAGAAGGCCTTCGGGTTGTAAACCGCTTTTGTCAGGGAAGAAATCCTCTGGGATAACACCTCGGGGGGATGACGGTACCTGGAGAATAAGCACCGGCTAACTACGTGCCAGCAGCCGCGGTAATACGTAGGGTGCAAGCGTTAATCGGAATTACTGGGCGTAAAGCGTGCGCAGGCGGTTGTGCAAGACAGATGTGAAATCCCCGGGCTTAACCTGGGAACTGCATTTGTGACTGCACGGCTGGAGTGCGGCAGAGGGGGATGGAATTCCGCGTGTAGCAGTGAAATGCGTAGATATGCGGAGGAACACCGATGGCGAAGGCAATCCCCTGGGCCTGCACTGACGCTCATGCACGAAAGCGTGGGGAGCAAACAGG` |
| ASV4787 | 424 | `TAGGGAATATTGGGCAATGGAGGCAACTCTGACCCAGCCATGCCGCGTGAAGGATGAAGGCGCTTTGCGTCGTAAACTTCTTTTGGGTGGGAAGAAAAGCAGACGTTCTCGTTTGTTTGACGGTACCATCAGAATAAGCACCGGCTAACTCTGTGCCAGCAGCCGCGGTAATACAGAGGGTGCAAGCGTTATCCGGATTTACTGGGTTTAAAGGGTGCGTAGGCGGGACTTTAAGTCAGTGGTGAAAGCTCGTCGCTTAACGACGGAATTGCCATTGAAACTGAGGTTCTTGAGATACAGTGAGGTTGGCGGAATGAGACATGTAGCGGTGAAATGCATAGAGATGTCTTAGAACACCAATTGCGAAGGCAGCTGACTGGCTGTTATCTGACGCTGAGGCACGAAAGCGTGGGGATCAAACAGG` |
| ASV56 | 429 | `TGGGGAATTTTGGACAATGGGCGCAAGCCTGATCCAGCAATGCCGCGTGCAGGATGAAGGCCTTCGGGTTGTAAACTGCTTTTGTACGGAGCGAAACGGCCTGCCCTAATACGGCGGGCTAATGACGGTACCGTAAGAATAAGCACCGGCTAACTACGTGCCAGCAGCCGCGGTAATACGTAGGGTGCGAGCGTTAATCGGAATTACTGGGCGTAAAGCGTGCGCAGGCGGTTATATAAGACAGATGTGAAATCCCCGGGCTCAACCTGGGACCTGCATTTGTGACTGTATAGCTAGAGTACGGTAGAGGGGGATGGAATTCCGCGTGTAGCAGTGAAATGCGTAGATATGCGGAGGAACACCGATGGCGAAGGCAATCCCCTGGACCTGTACTGACGCTCATGCACGAAAGCGTGGGGAGCAAACAGG` |
| ASV2720 | 428 | `TGGGGAATTGTTCGCAATGGGCGCAAGCCTGACGACGCAACGCCGCGTGGAGGATGAAGGTCTTCGGATTGTAAACTCCTGTTGATCGGGACGAAAAGCTCCAACCTAATACGTTGGAGACTGACGGTACCGGTTGAGGAAGCCACGGCTAACTCTGTGCCAGCAGCCGCGGTAATACAGAGGTGGCAAGCGTTGTTCGGAATTACTGGGCGTAAAGGGCGCGTAGGCGGCCTCCTAAGTCGAACGTGAAAGCCCCAGGCTTAACCTGGGAACTGCGTTTGATACTGGGAGGCTTGGATTCGGGAGAGGGATGTGGAATTCCAGGTGTAGCGGTGAAATGCGTAGATATCTGGAGGAACACCGGTGGCGAAGGCGGCATCCTGGACCGAGATCGACGCTGAGGCGCGAAAGCTAGGGGAGCAAACGGG` |
| ASV823 | 424 | `TAGGGAATATTGGACAATGGGGGCAACCCTGATCCAGCCATTCCGCGTGCAGGAATAAGGCCCTATGGGTCGTAAACTGCTTTTGTATGGGAAAAAATCATTCTACGTGTAGAGTGTTGATGGTACCATGAGAATAAGGATCGGCTAACTTCGTGCCAGCAGCCGCGGTAATACGAAGGATCCAAGCGTTGTCCGGATTTACTGGGTTTAAAGGGTGCGTAGGCGGGGAATTAAGTCAGTGGTGAAAGCTGGTAGCTTAACTATCAAATTGCCATTGAAACTGATTTTCTCGAGTACGGTTGAGGTAGCTGGAATGTAACATGTAGCGGTGAAATGCTTAGATATGTTACAGAACACCAATTGCGAAGGCAGGCTACTAAACCGTAACTGACGCTGAGGCACGAAAGTGTGGGGATCAAACAGG` |
| ASV2898 | 404 | `TGAGGAATATTGGACAATGGGCGAAAGCCTGATCCAGCCATGCCGCGTGAGTGAAGAAGGCCTTCGGGTTGTAAAGCTCTTTCAGTTGCGAAGATAATGACGGTAGCAACAGAAGAAGCTCCGGCTAACTCCGTGCCAGCAGCCGCGGTAATACGGGGGGAGCGAGCGTTATTCGGAATCACTGGGCGTAAAGCGTGCGCAGGCGGATTGATAAGTCAGAAGTGAAATCCCAGGGCTCAACCCTGGAACTGCTTTTGAAACTATCTTTCTAGAGTATGGTAGAGGTTGGGAGAATTCCTAGTGTAGAGGTGAAATTCGTAGATATTAGGAGGAATACCAGTGGCGTAGGCGCCCAACTGGACCATTACTGACGCTCAGGCACGAAAGCGTGGGGAGCAAACAGG` |
| ASV3688 | 404 | `TGGGGAATATTGGGCAATGGGGGCAACCCTGACCCAGCGACGCCGCGTGAGTGAAGAAATCCCTCGGGATGTAAAGCTCTGTTGTGTGGGAAGATAATGACGGTACCACACGAGGAAGCCCCGGCAAACTACGTGCCAGCAGCCGCGGTAATACGTAGGGGGCAAGCGTTGTCCGGAATTACTGGGCGTAAAGCGCACGCAGGCGGATTAAAAAGTCAGCCGTAAAATGCACGGGCTCAACCTGTGTTAGTCAGCTGATACTATTAGTCTAGAGTATGTGAGAGGGAAGTGGAATTCCCGGTGTAGCGGTGAAATGCGTAGATATCGGGAGGAACACCAGTGGCGAAGGCGGCTTCCTGGCACAAAACTGACGCTCATGTGCGAAAGCTAGGGCAGCGAACGGG` |
| ASV697 | 429 | `TAGGGAATCTTCCACAATGGACGAAAGTCTGATGGAGCAACGCCGCGTGAGTGATGAAGGTTTTCGGATCGTAAAACTCTGTTGTAAGGGAAGAACAGGTGTGAGAGGAAATGCTCATGCTGTGACGGTACCTTGCGAGAAAGCCACGGCTAACTACGTGCCAGCAGCCGCGGTAATACGTAGGTGGCAAGCGTTGTCCGGAATTATTGGGCGTAAAGCGCGCGCAGGCGGCCTTTTAAGTCTGATGTGAAAGCCCCCGGCTCAACCGGGGAGGGTCATTGGAAACTGGAAGGCTTGAGTACAGAAGAGAAGAGTGGAATTCCATGTGTAGCGGTGAAATGCGTAGAGATGTGGAGGAACACCAGTGGCGAAGGCGACTCTTTGGTCTGTAACTGACGCTGAGGCGCGAAAGCGTGGGGAGCAAACAGG` |
| ASV577 | 423 | `TAGGGAATATTGGACAATGGGTGAGAGCCTGATCCAGCCATGCCGCGTGCAGGAAGAAGGCCTTCTGGGTTGTAAACTGCTTTTGCCAGGGGATAAAATTTCCATGCGTGGAGAATTGAAGGTACCTGGTGAATAAGCCACGGCTAACTACGTGCCAGCAGCCGCGGTAATACGTAGGTGGCAAGCGTTGTCCGGATTTATTGGGTTTAAAGGGTGCGTAGGCGGTTCCATAAGTCAGTGGTGAAATACAATAGCTTAACTATTGAGGTGCCATTGATACTGCGGAACTTGAGTACAGACGAGGTAGGCGGAATTGACGGTGTAGCGGTGAAATGCTTAGATATCGTCAAGAACACCGATAGCGAAGGCAGCTTACTAGACTGTAACTGACGCTGAGGCACGAAAGTGTGGGGATCAAACAGG` |
| ASV5561 | 405 | `TCGAGAATATTCGACAATGGGCGAAAGCCTGATCGAGCGACGCCGCGTGTGGGAAGAAGGTCTGCGGATCGTAAACCACTTTTCTCGCTGACGAATTTTGACGGTAAGCGAGGAATAAGGGGCTGCTAACTTCGTGCCAGCAGCAGCGGTAATACGAAGGCCCCGAGCGTTATCCGGAATCATTGGGCGTAAAGCGTGTGTAGGCGGTAGGTATTGTCGGTGGTTAAAGCCCGGGGCCTAACCTCGGGACTGCTTCCGAAACGTACCAACTAGAAGGTGTGAGAGGTGAGCGGAACTCATGGTGTAGGGGTGAAATCCGTTGATATCATGGGGAACACCAAAGGCGAAGGCAGCTCACTGGTGCACTCTTGACGCTGAAATACGAAAGCGTGGGGATCAAAAAGG` |
| ASV5046 | 407 | `TAGGGAATATTGCACAATGGAGGAAACTCTGATGCAGCGACGCCGCGTGAGTGATGAAGGACTTCGGTTCGTAAAGCTCTGTCGCATGGGAATAATAAAGTGAAGGTACCATGCAAGAAAGGATCGGCTAACTTCGTGCCAGCAGCCGCGGTAAGACGAGGGATCCTAGCGTTGTTCGGAATCATTGGGCGTAAAGCGGGTGCAGGTGGCTTTGTAAGTCAGGTGTGAAAGCCCAGGGCTCAACCCTGGAAGTGCATTTGATACTGCGAAGCTTGAGTGCTAGAGAGGTTATTAGAATACATGGTGTAGTGGTGAAATACGTAGATATCATGTGGAATACCAGAGGCGAAGGCGGATAACTGGCTAGACACTGACACTCAGACCCGAAAGCGTGGGGATCAAACAGG` |
| ASV3390 | 404 | `TGGGGAATATTGGGCAATGGGCGCAAGCCTGACCCAGCCATGCCGCGTGTGTGATGAAGGCCTTAGGGTTGTAAAGCACTTTCGTTGGTGACGATAATGACGGTAACCAAAGAAGAAGCCCCGGCTAACTTCGTGCCAGCAGCCGCGGTAATACGAAGGGGGCTAGCGTTGTTCGGAATTACTGGGCGTAAAGAGTTCGTAGGCGGACTTGTAAGTCAGAGGTGAAATCCCAGAGCTTAACTCTGGAACTGCCTTTGAAACTATAAGTCTGGAATCTCAGAGGGGATAGCGGAATTCCAAATGTAGGGGTGAAATCCGTAGATATTTGGAGGAACACCGGTGGCGAAGGCGGCTATCTGGATGAGTATTGACGCTGAGGAACGAAAGCGTGGGGATCAAACAGG` |
| ASV1473 | 429 | `TGGGGAATTTTGGACAATGGACGCAAGTCTGATCCAGCCATGCCGCGTGCGGGAAGAAGGCCTTCGGGTTGTAAACCGCTTTTGTCAGGGAAGAAAAGGATCCTTCTAATACAGGGGTCTCATGACGGTACCTGAAGAATAAGCACCGGCTAACTACGTGCCAGCAGCCGCGGTAATACGTAGGGTGCAAGCGTTAATCGGAATTACTGGGCGTAAAGCGTGCGCAGGCGGTTATGCAAGACAGAGGTGAAATCCCCGGGCTCAACCTGGGAACTGCCTTTGTGACTGCATAGCTAGAGTACGGTAGAGGGGGATGGAATTCCGCGTGTAGCAGTGAAATGCGTAGATATGCGGAGGAACACCGATGGCGAAGGCAATCCCCTGGACCTGTACTGACGCTCATGCACGAAAGCGTGGGGAGCAAACAGG` |
| ASV1692 | 431 | `TGGGGAATCTTGCGCAATGGGCGAAAGCCTGACGCAGCAACGCCGCGTGAGTGATGAAGGCCCTTGGGTTGTAAAGCTCTGTCGGCAGGGAAGATGGGAGATGGTTTTAATACAGCTGTTTCTTGACGGTACCTGCAAAGGAAGCACCGGCTAACTCTGTGCCAGCAGCCGCGGTAATACAGAGGGTGCAAGCGTTGTTCGGAATTATTGGGCGTAAAGCGCGCGTAGGCTGTTTGTTAAGTCTGACTGTGAAATCCCGGGGCTCAACTCCGGAAGTGCATCGGAAACTGGCAAACTAGAGTCACGGAGAGGGTGGTGGAATTCCTGGTGTAGAGGTGAAATTCGTAGATATCAGGAGGAACATCGGTGGCGAAGGCGACCACCTGGACGATGACTGACGCTGAGGTGCGAAAGCGTGGGGAGCAAACAGG` |
| ASV4338 | 429 | `TGGGGAATATTGGACAATGGGCGAAAGCCTGATCCAGCAACGCCGTGTGTGTGAAGAAGGCCTTCGGGTTGTAAAGCACTTTTAGTTGGGAAGAAAAGCTCAAGACTAATACCCTTGAGTGATGACGTTACCAACAGAAAAAGCACTGGCTAACTCTGTGCCAGCAGCCGCGGTAATACAGAGAGTGCGAGCGTTAATCGGAATTACTGGGCGTAAAGCGCACGTAGGTGGAATTTTAAGTCGGATGTGAAATCCCCGGGCTTAACCTGGGAATTGCATTCGATACTGTGATTCTAGAGTATGGTAGAGGGAAGTGGAATTTCCGGTGTAGCGGTGAAATGCGTAGATATCGGAAGGAACATCAGTGGCGAAAGCGGCTTCCTGGACCAATACTGACACTTAGGTGCGAAAGCGTGGGGAGCAAACAGG` |
| ASV343 | 404 | `TGGGGAATCTTAGACAATGGGCGCAAGCCTGATCTAGCCATGCCGCGTGAGCGATGAAGGCCTTAGGGTTGTAAAGCTCTTTCAGTGGGGAAGATAATGACTGTACCCACAGAAGAAGCCCCGGCTAACTCCGTGCCAGCAGCCGCGGTAATACGGAGGGGGCTAGCGTTGTTCGGAATTACTGGGCGTAAAGCGCACGTAGGCGGACTGGAAAGTCAGAGGTGAAATCCCAGGGCTCAACCTTGGAACTGCCTTTGAAACTCCCGGTCTTGAGGTCGAGAGAGGTGAGTGGAATTCCGAGTGTAGAGGTGAAATTCGTAGATATTCGGAGGAACACCAGTGGCGAAGGCGGCTCACTGGCTCGATACTGACGCTGAGGTGCGAAAGCGTGGGGAGCAAACAGG` |
| ASV2151 | 404 | `TGGGGGATATTGCACAATGGGGGAAACCCTGATGCAGCGACGCCGCGTGAGTGAAGAAGTATTTCGGTATGTAAAGCTCTATCAGCAGGAAAGATAATGACGGTACCTGACTAAGAAGCACCGGCTAAATACGTGCCAGCAGCCGCGGTAATACGTATGGTGCAAGCGTTATCCGGATTTACTGGGTGTAAAGGGAGCGCAGACGGCATGGCAAGCCAGATGTGAAAGGTGGGGGCTCAACCCCCAAGCTGCATTTGGAACTGTCAAGCTAGAGTATCGGAGAGGTAAGTGGAATTCCTAGTGTAGCGGTGAAATGCGTAGATATTAGGAGGAACACCAGTGGCGAAGGCGGCTTACTGGACGATAACTGACGTTGAGGCTCGAAAGCGTGGGGAGCAAACAGG` |
| ASV3747 | 404 | `TGGGGAATATTGGACAATGGGCGAAAGCCTGATCCAGCAATGCCGCGTGAGTGAAGAAGGCCTTAGGGTTGTAAAGCTCTTTTAGTAGGGAAGATAATGACGGTACCTACAGAAAAAGCACCGGCTAACTCCGTGCCAGCAGCCGCGGTAATACGGAGGGTGCTAGCGTTGTTCGGAATTACTGGGCGTAAAGCGTACGTAGGCGGCCTGACAAGTTGGAAGTGAAAGCCCAAGGCTCAACCTTGGAATTGCTTTCAAAACTGTTAGGCTAGAGAATGGTAGGGGAAAGTGGAATTCCTAGTGTAGAGGTGAAATTCGTAGATATTAGGAGGAACACCAGAGGCGAAAGCGGCTTTCTGGACCATTTCTGACGCTAAGGTACGAAAGCGTGGGGAGCAAACAGG` |
| ASV168 | 429 | `TGGGGAATTTTGGACAATGGGCGCAAGCCTGATCCAGCAATGCCGCGTGCAGGATGAAGGCCTTCGGGTTGTAAACTGCTTTTGTACGGAACGAAACGGCCTGTCCTAATACGGCGGGTTAATGACGGTACCGTAAGAATAAGCACCGGCTAACTACGTGCCAGCAGCCGCGGTAATACGTAGGGTGCGAGCGTTAATCGGAATTACTGGGCGTAAAGCGTGCGCAGGCGGTTATATAAGACAGATGTGAAATCCCCGGGCTCAACCTGGGACCTGCATTTGTGACTGTATAGCTAGAGTACGGTAGAGGGGGATGGAATTCCGCGTGTAGCAGTGAAATGCGTAGATATGCGGAGGAACACCGATGGCGAAGGCAATCCCCTGGACCTGTACTGACGCTCATGCACGAAAGCGTGGGGAGCAAACAGG` |
| ASV1762 | 405 | `TGAGGAATTTTCCGCAATGGGCGAAAGCCTGACGGAGCAATGCCGCGTGAAGGATGAAGGCCTATGGGTTGTAAACTTCTTTTCTCAGAGAAGAATTTTGACGGTATCTGAGGAATAAGCATCGGCTAACTCTGTGCCAGCAGCCGCGGTAAGACAGAGGATGCAAGCGTTATCCGGAATGATTGGGCGTAAAGCGTCTGTAGGTGGCTTAAAAAGTCTCCTGTCAAAGATCAGGGCTTAACCCTGGGCCGGCAGGAGAAACTCTTAGGCTAGAGTTTGGTAGGGGCAGAGGGAATTCCCGGTGGAGCGGTGAAATGCGTAGAGATCGGGAGGAACACCAAAGGCGAAAGCACTTTGCTGGGCCACAACTGACACTGAGAGACGAAAGCGAGGGGAGCAAAAGGG` |
| ASV4373 | 406 | `TGGGGAATTTTCCGCAATGGGCGAAAGCCTGACGGAGCAACGCCGCGTGAGGGAGGAAGGTCTTTGGATTGTAAACCTCTTTTCTCAAGGAAGAAGTTCTGACGGTACTTGAGGAATCAGCCTCGGCTAACTCCGTGCCAGCAGCCGCGGTAATACGGGGGAGGCAAGCGTTATCCGGAATTATTGGGCGTAAAGCGTCCGCAGGTGGTCAGCCAAGTCTGCCGTCAAATCAGGTTGCTTAACGACCTAAAGGCGGTGGAAACTGGCAGACTAGAGAGCAGTAGGGGTAGCAGGAATTCCCAGTGTAGCGGTGAAATGCGTAGAGATTGGGAAGAACATCGGTGGCGAAAGCGTGCTACTGGGCTGTATCTGACACTCAGGGACGAAAGCTAGGGTAGCGAAAGGG` |
| ASV2122 | 429 | `TGGGGAATATTGGACAATGGGCGAAAGCCTGATCCAGCAATGCCGCGTGTGTGAAGAAGGCCTTCGGGTTGTAAAGCACTTTCAGTAGGGAAGAAAAATTCAGGGTTAATACCTCTGAGTTTTGACGTTACCTACAAAAGAAGCACCGGCTAACTCTGTGCCAGCAGCCGCGGTAATACAGAGGGTGCGAGCGTTAATCGGAATTACTGGGCGTAAAGCGTGCGTAGGCGGATATTTAAGTCGAATGTGAAATCCCCGGGCTTAACCTGGGAACTGCATGCGATACTGAGTATCTGGAGTATGGTAGAGGAAAGTGGAATTCCCGGTGTAGCGGTGAAATGCGTAGATATCGGGAGGAACATCAGTGGCGAAGGCGACTTTCTGGACCAATACTGACGCTGAGGCACGAAAGCGTGGGGAGCAAACAGG` |
| ASV2219 | 404 | `TGGGGAATATTGGACAATGGGCGCAAGCCTGATCCAGCCATGCCGCGTGAACGATGAAGGCCTTAGGGTTGTAAAGTTCTTTTGTCAGGGAAGATAATGACGGTACCTGAAGAATAAGCACCGGCTAACTTCGTGCCAGCAGCCGCGGTAATACGAAGGGTGCTAGCGTTGTTCGGAATTACTGGGCGTAAAGCGCGCGTAGGCGGCTTTGCAAGTCAGAGGTGAAATCCCCAGGCTTAACCTGGGAACTGCCTTTGAAACTGCCAAGCTGGAGTATTGGAGGGGAGCGGGGAATTCCTAATGTAGAGGTGAAATTCGTAGATATTAGGAGGAACACCGGTGGCGAAGGCGCCGCTCTGGACAATCACTGACGCTGAGGCGCGAAAGCGTGGGGATCAAACAGG` |
| ASV417 | 404 | `TGGGGAATATTGGACAATGGGCGAAAGCCTGATCCAGCCATGCCGCGTGAGTGATGAAGGCCTTAGGGTTGTAAAGCTCTTTTACCAGGGAAGATAATGACTGTACCTGGAGAATAAGCCCCGGCTAACTTCGTGCCAGCAGCCGCGGTAATACGAAGGGGGCTAGCGTTGTTCGGAATCACTGGGCGTAAAGCGCACGTAGGCGGACTCTTAAGTCAGAGGTGAAAGCCCAGGGCTCAACCCTGGAATTGCCTTTGATACTGGGAGTCTTGAGTATGGTAGAGGTTGGTGGAACAGCAAGTGTAGAGGTGAAATTCGTAGATATTTGCTAGAACACCGGTGGCGAAGGCGGCCAACTGGACCATAACTGACGCTGAGGTGCGAAAGCGTGGGGAGCAAACAGG` |
| ASV3595 | 404 | `TGGGGAATATTGGACAATGGGCGCAAGCCTGATCCAGCAATACCGCGTGAATGATGAAGGTCTTCGGATTGTAAAGTTCTTTTAGTGAGGAAGATAATGACGGTACTCACAGAAAAAGCACCGGCTAACTTCGTGCCAGCAGCCGCGGTAATACGAAGGGTGCTAGCGTTGTTCGGAATCATTGGGCGTAAAGCGTATGTAGGCTGTTTGGTAAGTCAGTTGTGAAATCCCTGAGCTCAACTTAGGAATTGCAGCTGAAACTGCCTTGCTAGAATTTGGTGGGGGATAGTGGAATTCTTAGTGTAGGGGTGAAATCCGTAGATATTAAGAGGAACACCGGAGGCGAAAGCGACTATCTACACCAATATTGACGCTGAGATACGAAAGCGTGGGGAGCAAACAGG` |
| ASV2319 | 404 | `TTAGGAATATTGGACAATGGGGGCAACCCTGATCCAGCCATGCCGCGTGTGTGACGAAGGCCTTAGGGTTGTAAAGCACTTTCAGCTGTGAAGATAATGACAGTAGCAGCAGAAGAAGCTCCGGCTAAATTCGTGCCAGCAGCCGCGGTAATACGAATGGAGCGAGCGTTGTTCGGAATCACTGGGCGTAAAGGGTGCGCAGGCGGTCATGAAAGTTAGAAGTGAAATCCCAGGGCTCAACCCTGGAATTGCTTTTAAAACTCCATGACTTGAGTACTGGAGAGGTTGGCGGAATTCCGAGTGTAGCAGTGAAATGCGTAGATATTCGGAGGAACACCGATGGCGTAGGCAGCCAACTGGACAGTTACTGACGCTCATGCACGAAAGCGTGGGGATCAAACAGG` |
| ASV2988 | 429 | `TGGGGAATTTTGGACAATGGGCGAAAGCCTGATCCAGCAATGCCGCGTGCAGGATGAAGGCCTTCGGGTTGTAAACTGCTTTTGTACGGAGCGAAACGGCGAGTTTTAATAAAGCTCGCTAATGACGGTACCGTAAGAATAAGCACCGGCTAACTACGTGCCAGCAGCCGCGGTAATACGTAGGGTGCAAGCGTTAATCGGAATTACTGGGCGTAAAGCGTGCGCAGGCGGTTATATAAGACAGATGTGAAATCCCCGGGCTCAACCTGGGACCTGCATTTGTGACTGTATAGCTAGAGTACGGTAGAGGGGGATGGAATTCCGCGTGTAGCAGTGAAATGCGTAGATATGCGGAGGAACACCGATGGCGAAGGCAATCCCCTGGACCTGTACTGACGCTCATGCACGAAAGCGTGGGGAGCAAACAGG` |
| ASV2059 | 429 | `TGGGGAATATTGGACAATGGGCGAAAGCCTGATCCAGCCATGCCGCGTGTGTGAAGAAGGTCTTCGGATTGTAAAGCACTTTAAGTTGGGAGGAAGGGCCATTACCTAATACGTGATGGTTTTGACGTTACCGACAGAATAAGCACCGGCTAACTCTGTGCCAGCAGCCGCGGTAATACAGAGGGTGCAAGCGTTAATCGGAATTACTGGGCGTAAAGCGCGCGTAGGTGGTTTGTTAAGTTGAATGTGAAATCCCCGGGCTCAACCTGGGAACTGCATCCAAAACTGGCAAGCTAGAGTAGGGCAGAGGGTGGTGGAATTTCCTGTGTAGCGGTGAAATGCGTAGATATAGGAAGGAACACCAGTGGCGAAGGCGACCACCTGGGCTCATACTGACACTGAGGTGCGAAAGCGTGGGGAGCAAACAGG` |
| ASV2329 | 404 | `TGGGGAATATTGGACAATGGGCGAAAGCCTGATCCAGCAATGCCGCGTGGGTGAAGAAGGTCTTCGGATTGTAAAGCCCTTTCAGCGGGGAAGATGATGACGGTACCCGCAGAAGAAGCCCCGGCTAACTTCGTGCCAGCAGCCGCGGTAATACGAAGGGGGCTAGCGTTGCTCGGAATTACTGGGCGTAAAGGGCGCGTAGGTGGCTCTGTGAGTCGGACGTGAAATTCCTGGGCTCAACCTGGGGACTGCGTTCGATACGGCAGGGCTAGAGGACGGAAGAGGGTCGTGGAATTCCCAGTGTAGAGGTGAAATTCGTAGATATTGGGAAGAACACCGGTGGCGAAGGCGGCGACCTGGTCCGTTACTGACACTGAGGCGCGACAGCGTGGGGAGCAAACAGG` |
| ASV2145 | 429 | `TGGGGAATTTTGGACAATGGGCGCAAGCCTGATCCAGCCATGCCGCGTGCGGGAAGAAGGCCTTCGGGTTGTAAACCGCTTTTGTCAGGGAAGAAACGGCTCCTACTAATACTGGGGGCTAATGACGGTACCTGAAGAATAAGCACCGGCTAACTACGTGCCAGCAGCCGCGGTAATACGTAGGGTGCAAGCGTTAATCGGAATTACTGGGCGTAAAGCGTGCGCAGGCGGCTTTGCAAGACAGATGTGAAATCCCCGGGCTCAACCTGGGAACTGCATTTGTGACTGCAAGGCTAGAGTACGGTAGAGGGGGATGGAATTCCGCGTGTAGCAGTGAAATGCGTAGATATGCGGAGGAACACCGATGGCGAAGGCAATCCCCTGGACCTGTACTGACGCTCATGCACGAAAGCGTGGGGAGCAAACAGG` |
| ASV4952 | 425 | `TAAGGAATATTGGACAATGGTCGGAAGACTGATCCAGCCATGCCGCGTGAAGGAAGAAGGTCCTCTGGATTGTAAACTTCTTTTGGACGGGAACAAAACCACCCTTGCGAGGGTGTCTGAGGGTACCGTCAGAATAAGCACCGGCTAACTCCGTGCCAGCAGCCGCGGTAATACGGAGGGTGCAAGCGTTATCCGGAATCACTGGGTTTAAAGGGTGCGTAGGCGGTCATATAAGTCAGTCGTGAAATCACTTGGCTCAACCAAAGTAACTGCGATTGATACTGTTTGACTTGAATTAGGTTGAGGTAGGCGGAATGTGGCATGTAGCGGTGAAATGCATAGATATGCCATAGAACACCAATTGCGAAGGCAGCTTGCTGGACCTATATTGACGCTGAGGCACGAAAGCGTGGGGAGCAAACAGG` |
| ASV791 | 404 | `TGGGGAATATTGGACAATGGGCGCAAGCCTGATCCAGCAATGCCGCGTGGGTGAAGAAGGTCTTCGGATCGTAAAGCCCTTTCGACAGGGAAGATGATGACGGTACCTGTAGAAGAAGCCCCGGCTAACTTCGTGCCAGCAGCCGCGGTAATACGAAGGGGGCTAGCGTTACTCGGAATTACTGGGCGTAAAGGGCGCGTAGGCGGCTCGGTCAGTCAGGCGTGAAATTCCTGGGCTTAACCTGGGGACTGCGCTTGATACTGTCGAGCTTGAGGCCGAGAGAGGGTGGTGGAATTCCCAGTGTAGAGGTGAAATTCGTAGATATTGGGAAGAACACCGGTGGCGAAGGCGACCACCTGGCTCGGTACTGACGCTGAGGCGCGACAGCGTGGGGAGCAAACAGG` |
| ASV1782 | 404 | `TGGGGAATCTTGGACAATGGGCGCAAGCCTGATCCAGCCATGCCGCGTGAGCGATGAAGGCCTTCGGGTTGTAAAGCTCTTTCGTGGGGGAAGATAATGACTGTACCCCAAGAAGAAGCCCCGGCTAACTCCGTGCCAGCAGCCGCGGTAATACGGAGGGGGCTAGCGTTGTTCGGAATTACTGGGCGTAAAGCGCACGTAGGCGGACCAGAAAGTCAGGGGTGAAATCCCGGGGCTCAACCCCGGAACTGCCTTTGAAACTTCTGGTCTTGAGGTCGAGAGAGGTGAGTGGAATTCCGAGTGTAGAGGTGAAATTCGTAGATATTCGGAGGAACACCAGTGGCGAAGGCGGCTCACTGGCTCGATACTGACGCTGAGGTGCGAAAGCGTGGGGAGCAAACAGG` |
| ASV1067 | 423 | `TAGGGAATATTGGTCAATGGGCGAGAGCCTGAACCAGCCATGCCGCGTGCAGGAAGAAGGCCCTCTGGGTTGTAAACTGCTTTTGACTAGGGCCAAAATACTCATGCGTGAGGAATTGAGAGTACTAGTAGAATAAGCACCGGCTAACTCCGTGCCAGCAGCCGCGGTAATACGGAGGGTGCAAGCGTTGTCCGGATTTATTGGGTTTAAAGGGTGCGTAGGTGGCTTTTTAAGTCAGTGGTGAAAGCCTGCAGCTTAACTGTAGGACTGCCATTGATACTGAAGAGCTTGAGTACTGATGAGGTAGGCGGAATTGATAGTGTAGCGGTGAAATGCATAGATATTATCAAGAACCCCGATTGCGAAGGCAGCTTACTAAGGAGTTACTGACACTGAGGCACGAAAGCGTGGGGATCAAACAGG` |
| ASV866 | 429 | `TGGGGAATTTTGGACAATGGGCGAAAGCCTGATCCAGCCATGCCGCGTGCAGGATGAAGGCCTTCGGGTTGTAAACTGCTTTTGTACGGAACGAAAAGACTCTTTCTAATAAAGAGGGTCCATGACGGTACCGTAAGAATAAGCACCGGCTAACTACGTGCCAGCAGCCGCGGTAATACGTAGGGTGCAAGCGTTAATCGGAATTACTGGGCGTAAAGCGTGCGCAGGCGGTTATATAAGACAGATGTGAAATCCCCGGGCTCAACCTGGGAACTGCATTTGTGACTGTATAGCTAGAGTACGGCAGAGGGGGATGGAATTCCGCGTGTAGCAGTGAAATGCGTAGATATGCGGAGGAACACCGATGGCGAAGGCAATCCCCTGGGCCTGTACTGACGCTCATGCACGAAAGCGTGGGGAGCAAACAGG` |
| ASV1418 | 428 | `TCGAGAATCTTTCACAATGGGGGCAACCCTGATGGAGCGACGCCGCGTGGAGGATAAGGTCTTCGGATTGTAAACTCCTGTCATGCGCGAACAAGGTTGCAGTGTTAATAGCATTGCAAATTGATGGTACCGCAAGAGGAAGAGACGGCTAACTCTGTGCCAGCAGCCGCGGTAATACAGAGGTCTCAAGCGTTGTTCGGAATCACTGGGCGTAAAGGGTGCGTAGGTGGCGTGGAAAGTCAGATGTGAAAGCCCGGGGCTCAACCTCGGAACTGCATCCGATACTACCATGCTAGAGTATTGAAGGGGAAACTAGAATTCTCGGTGTAGCAGTGAAATGCGTAGATATCGAGAGGAATACCAAAGGCGAAGGCAGGTTTCTTGGCAATTACTGACACTGAGGCACGAAGGCCAGGGTAGCGAACGGG` |
| ASV843 | 404 | `TGGGGAATATTGGGCAATGGAGGAAACTCTGACCCAGCCATGCCGCGTGAATGATGAAGGCCTTAGGGTTGTAAAGTTCTTTCGCCGATGAAGATAATGACGGTAGTCGGAGAAGAAGCCCCGGCTAACTTCGTGCCAGCAGCCGCGGTAATACGAAGGGGGCTAGCGTTGTTCGGAATTACTGGGCGTAAAGCGAGCGTAGGTTGCTCGACAAGTCAGTGGTGAAAGCCCAGAGCTCAACTCTGGAACTGCCATTGAAACTGTCGAGCTGGAATCTCAGAGGGGGTAGCGGAATTCCAAATGTAGGGGTGAAATCCGTAGATATTTGGAGGAACACCGGTGGCGAAGGCGGCTACCTGGATGAGTATTGACACTGAGGCTCGAAAGCGTGGGGATCAAACAGG` |
| ASV1972 | 429 | `TGGGGAATATTGGACAATGGGGGAAACCCTGATCCAGCAATGCCGCGTGTGTGAAGAAGGCCTGAGGGTTGTAAAGCACTTTAGGTAATGAGGAAAGGCCATTTGCAAATAGTGAATGGAATTGACGTTAGTTACAGAATAAGCACCGGCTAACTCTGTGCCAGCAGCCGCGGTAATACAGAGGGTGCAAGCGTTAATCGGAATGACTGGGCGTAAAGCGTGCGTAGGTGGTGACTTAAGTTAGATGTGAAATCCCTGGGCTTAACCTAGGAACTGCATATAATACTGGGACACTAGAGTATGGGAGAGGTAAGTGGAATTTCCGGTGTAGCGGTGAAATGCGTAGATATCGGAAGGAACACCAGTGGCGAAGGCGGCTTACTGGCCTAATACTGACACTGAGGCACGAAAGCGTGGGGAGCAAACAGG` |
| ASV4774 | 404 | `TGGGGAATCTTGCACAATGGAGGAAACTCTGATGCAGCGACGCCGCGTGGACGACGAAGCCCTTCGGGGTGTAAAGTCCTTTCGACCGGAACGATAATGACGGTACCGGTGGAAGAAGCTGCGGCTAACTACGTGCCAGCAGCCGCGGTAATACGTAGGCAGCAAGCGTTGTTCGGAATTACTGGGCGTAAAGAGTTCGTAGGCGGTCTGCCAAGTCTGATGTGAAATCTCCCGGCTTAACTGGGAGGGTGCGTTAGATACTGGCAGGCTTGAGTATAGGATAGGAAAGCGGAATTCCTGGTGTAGCGGTGAAATGCGTAGATATCAGGAGGAACACCTGTGGTGTAGACGGCTTTCTGGACTATAACTGACGCTGAGGAACGAAAGCGTGGGTAGCAAACAGG` |
| ASV1483 | 407 | `TAGGGAATATTGCGCAATGGGGGAAACCCTGACGCAGCGACGCCGCGTGAGTGATGAAGGCCTTCGGGTCGTAAAGCTCTGTCGTATGGGAATAACACAATGAATGTACCATGCAAGAAAGGATCGGCTAACTTCGTGCCAGCAGCCGCGGTAAGACGAGGGATCCTAGCGTTGTTCGGAATCATTGGGCGTAAAGCGGGTGTAGGCGGGCTTCTAAGTCAGATGTGAAATCCCAGGGCTCAACCCTGGAAGTGCATTTGATACTGGAAGTCTTGAGTGTGGTAGAGGTTACTGGAATTGTTGGTGTAGTGGTGAAATACGTAGATATCAACAGGAATACCGGAGGCGAAGGCGAGTAACTGGGCCAACACTGACGCTGAGACCCGAAAGCGTGGGGATCAAACAGG` |
| ASV2623 | 429 | `TGGGGAATTTTGGACAATGGGCGCAAGCCTGATCCAGCAATGCCGCGTGAGTGAAGAAGGCCTTCGGGTTGTAAAGCTCTTTTGTCAGGGAAGAAACGGATTTTTCTAATACAGAGATCTAATGACGGTACCTGAAGAATAAGCACCGGCTAACTACGTGCCAGCAGCCGCGGTAATACGTAGGGTGCAAGCGTTAATCGGAATTACTGGGCGTAAAGCGTGCGCAGGCGGTTTTGTAAGACTGTCGTGAAATCCCCGGGCTCAACCTGGGAATGGCGATGGTGACTGCAAGGCTAGAGTTTGGCAGAGGGGGGTAGAATTCCACGTGTAGCAGTGAAATGCGTAGATATGTGGAGGAACACCGATGGCGAAGGCAGCCCCCTGGGTCAAAACTGACGCTCATGCACGAAAGCGTGGGGAGCAAACAGG` |
| ASV2808 | 403 | `TAGGGAATATTGCGCAATGGGCGAAAGCCTGACGCAGCAATTCCGCGTGGAGGATGACGGATTTCGGTCTGTAAACTCCTGTCAGCAGGGAAGTATTGACGGTACCTGCAGAGGAAGCACCGGCTAACTACGTGCCAGCAGCCGCGGTAATACGTAGGGTGCAAGCGTTGTCCGGAATTATTGGGCGTAAAGCGTCCGTAGGCGGTTAGATAAGTCTGGCGTGAAAGGTCGTGGCTCAACCACGGTTATGTGTTGGAAACTGTTTAACTAGAATATGGTAGAGGTTGCTGGAATTCCTGGTGTAGCGGTGAAATGCGTAGATATCAGGAGGAACACCTGTGGCGAAAGCGTGCAACTGGGCCTATATTGACGCTAATGGACGAAAGCCAGGGTAGCGAAAGGG` |
| ASV604 | 424 | `TGAGGAATATTGGACAATGGGCGCAAGCCTGATCCAGCCATGCCGCGTGCAGGAAGAAGCATCTATGGTGTGTAAACTGCTTTTGTACAGGAAGAAACACTCCCTCGTGAGGGAGCTTGACGGTACTGTAAGAATAAGGATCGGCTAACTCCGTGCCAGCAGCCGCGGTAATACGGAGGATCCAAGCGTTATCCGGAATCATTGGGTTTAAAGGGTCCGTAGGCGGTTTAATAAGTCAGTGGTGAAAGCCCATCGCTCAACGATGGAACGGCCATTGATACTGTTAGACTTGAATTACTAGGAAGTAACTAGAATATGTAGTGTAGCGGTGAAATGCTTAGATATTACATGGAATACCAATTGCGAAGGCAGGTTACTACTAGTGGATTGACGCTGATGGACGAAAGCGTGGGGAGCGAACAGG` |
| ASV2428 | 424 | `TAGGGAATATTGGACAATGGGGGCAACCCTGATCCAGTCATGCCGCGTGCAGGAAGACTGCCCTATGGGTTGTAAACTGCTTTTGTACGGGAAGAAACCCCTCTACGTGTAGAGGGTTGACGGTACCGTGAGAATAAGGATCGGCTAACTTCGTGCCAGCAGCCGCGGTAATACGAAGGATCCAAGCGTTGTCCGGATTTACTGGGTTTAAAGGGTGCGTAGGCGGGCTATTAAGTCAGTGGTGAAAGCCGATCGCTTAACGATCGAACTGCCATTGAAACTGATAGTCTTGAGTATGTTTGAGGTTACTGGAATATAACATGTAGCGGTGAAATGCTTAGATATGTTATAGAACACCAATTGCGAAGGCAGGTAACTAAGTCATAACTGACGCTGAGGCACGAAAGCGTGGGGAGCAAACAGG` |
| ASV3920 | 429 | `TGGGGAATATTGGACAATGGGGGAAACCCTGATCCAGCAATGCCGCGTGTGTGAAGAAGGCCTGAGGGTTGTAAAGCACTTTCAGTGGGGAGGAGGATTATCCGGTTAAGAGCTAGATAATTGGACGTTACCCACAGAAGAAGCACCGGCTAACTCCGTGCCAGCAGCCGCGGTAATACGGAGGGTGCGAGCGTTAATCGGAATTACTGGGCGTAAAGGGTGCGTAGGTGGTTTGATAAGTTATCTGTGAAAGCCCTGGGCTCAACCTAGGAAGGCCAGATAAGACTGTTAGACTCGAGTATGGGAGAGGGTAGTGGAATTTCCGGTGTAGCGGTGAAATGCGTAGAGATCGGAAGGAACACCAGTGGCGAAGGCGGCTACCTGGCCTGATACTGACACTGAAGCACGAAAGCGTGGGGAGCAAACAGG` |
| ASV104 | 429 | `TGGGGAATATTGGACAATGGGCGCAAGCCTGATCCAGCCATGCCGCGTGTGTGAAGAAGGCCTTCGGGTTGTAAAGCACTTTTGTTGGGGAAGAAAAGACCGCGGTTAATAACCGAGGTTCATGACGGTACCCAAAGAATAAGCACCGGCTAACTTCGTGCCAGCAGCCGCGGTAATACGAAGGGTGCAAGCGTTACTCGGAATTACTGGGCGTAAAGCGTGCGTAGGTGGTTCGTTAAGTCTGCCGTGAAAGCCCCGGGCTCAACCTGGGAATGGCGGTGGATACTGGCGGACTAGAGTGCGGTAGAGGGTGGTGGAATTCCCGGTGTAGCAGTGAAATGCGTAGAGATCGGGAGGAACATCTGTGGCGAAGGCGGCCACCTGGACCAGCACTGACACTGAGGCACGAAAGCGTGGGGAGCAAACAGG` |
| ASV1847 | 424 | `TGAGGAATATTGGACAATGGGCGCAAGCCTGATCCAGCCATGCCGCGTGCAGGAAGAAGGTCCTATGGATTGTAAACTGCTTTTATACAGGAAGAAACACCTCTACGTGTAGAGACTTGACGGTACTGTATGAATAAGGACCGGCTAACTCCGTGCCAGCAGCCGCGGTAATACGGAGGGTCCAAGCGTTATCCGGAATCATTGGGTTTAAAGGGTCCGTAGGCGGTTTAATAAGTCAGTGGTGAAAGCCCATCGCTCAACGATGGAACGGCCATTGATACTGTTAAACTTGAATTATTAGGAAGTAACTAGAATATGTAGTGTAGCGGTGAAATGCTTAGAGATTACATGGAATACCAATTGCGAAGGCAGGTTACTACTAATATATTGACGCTGATGGACGAAAGCGTGGGGAGCGAACAGG` |
| ASV2513 | 404 | `TGGGGAATATTGGACAATGGGCGCAAGCCTGATCCAGCCATGCCGCGTGAGTGACGAAGGCCTTAGGGTTGTAAAGCTCTTTTGCCGGGGAAGATAATGACGGTACCCGGAGAATAAGTCCCGGCTAACTTCGTGCCAGCAGCCGCGGTAATACGAAGGGGACTAGCGTTGTTCGGAATTACTGGGCGTAAAGCGCACGTAGGCTGATATGTAAGTCAGGGGTGAAATCCCGGGGCTCAACCTCGGAACTGCCCTTGATACAGCATATCTAGAATCCGGAAGAGGTGAGTGGAATTCCTAGTGTAGAGGTGAAATTCGTAGATATTAGGAAGAACACCGGTGGCGAAGGCGGCTCACTGGTCCGGTATTGACGCTGAGGTGCGAAAGCGTGGGGAGCAAACAGG` |
| ASV1645 | 429 | `TCGAGAATAATTCACAATGGGGGAAACCCTGATGGTGCAACGCCGCGTGGAGGATGAAGGTCTTCGGATTGTAAACTCCTGTCATCGGGGAGTAAGACCTGGGCGTAAATAGCGCACAGGGTTGATAGTACCCGAAGAGGAAGGGACGGCTAACTTCGTGCCAGCAGCCGCGGTAATACGAAGGTCCCAAGCGTTGTTCGGAATCACTGGGCGTAAAGGGAGCGTAGGCGGCGTGGTAAGTCAGATGTGAAATCCCGGGGCTCAACCCCGGAACTGCATCCGATACTGCCGTGCTTGAGGGTTGGAGAGGTAGCTGGAATTCTCGGTGTAGCAGTGAAATGCGTGGATATCGAGAGGAACACTCGTGGCGAAGGCGAGCTACTGGACAACATCTGACGCTGAGGCTCGAAGGCCAGGGGAGCAAAAGGG` |
| ASV3394 | 429 | `TGGGGAATATTGGACAATGGGGGCAACCCTGATCCAGCCATGCCGCGTGTGTGAAGAAGGCCTTAGGGTTGTAAAGCACTTTAAGTTGTGAGGAAGTTGAGTTTGTGAATAATGGATTCAATTGACGTTAGCAACAGAATAAGCACCGGCTAACTCCGTGCCAGCAGCCGCGGTAATACGGAGGGTGCGAGCGTTAATCGGAATTACTGGGCGTAAAGAGCACGTAGGCGGTTTGTTAAGTTGGATGTGAAATCCCAGGGCTCAACCTTGGAACTGCATCCAAAACTGGGAGACTAGAGTAAGGCAGAGGAGAGTGGAATTTCCGGTGTAGCGGTGAAATGCATAGATATCGGAAGGAACATCAGTGGCGAAGGCGACTCTCTGGGCTATTACTGACGCTGAGGTGCGAAAGCGTGGGGATCAAACAGG` |
| ASV4085 | 424 | `TAAGGAATATTGGTCAATGGAGGAAACTCTGAACCAGCCATCCCGCGTGCAGGAATAAGGTCCTATGGATTGTAAACTGCTTTTAGTAGAGAAGAAACGCCTTCATTTATGAGGGTCTGACGGTACCTACAGAATAAGCACCGGCTAACTCCGTGCCAGCAGCCGCGGTAATACGGAGGGTGCGAGCGTTATCCGGAATCACTGGGTTTAAAGGGTGCGTAGGCGGGTTATTAAGTCAGTGGTGAAAGCTGGTCGCTTAACGATCAAACTGCCATTGATACTGATAGTCTTGAATTGGGTTGAGGTTAGCGGAATGTGACATGTAGCGGTGAAATGCGTAGATATGTCATGGAACACCAATTGCGAAGGCAGCTAGCTAGGCCTTGATTGACGCTGAGGCACGAAAGCGTGGGTAGCGAACAGG` |
| ASV557 | 404 | `TGGGGAATCTTAGACAATGGGGGAAACCCTGATCTAGCCATGCCGCGTGATCGATGAAGGCCTTAGGGTTGTAAAGATCTTTCAGATGGGAAGATAATGACGGTACCATCAGAAGAAGCCCCGGCTAACTCCGTGCCAGCAGCCGCGGTAATACGGAGGGGGCTAGCGTTATTCGGAATTACTGGGCGTAAAGCGCACGTAGGCGGATAAGAAAGTCAGAGGTGAAATCCCAGGGCTCAACCTTGGAACTGCCTTTGAAACTCCTTGTCTTGAGGTCGTGAGAGGTGAGTGGAATTCCGAGTGTAGAGGTGAAATTCGTAGATATTCGGAGGAACACCAGTGGCGAAGGCGGCTCACTGGCACGATACTGACGCTGAGGTGCGAAAGCGTGGGGAGCAAACAGG` |
| ASV4491 | 404 | `TGGGGAATATTGGACAATGGGCGAAAGCCTGATCCAGCCATGCCGCGTGAGTGACGAAGGCCTTAGGGTTGTAAAGCTCTTTTGTCAGGGAAGATAATGACGGTACCTGAAGAATAAGACACGGCTAACTTCGTGCCAGCAGCCGCGGTAATACGAAGGTGTCTAGCGTTGTTCGGAATCACTGGGCGTAAAGCGCGCGTAGGCGGCTTTTCAAGTCAGAGGTGAAAGCCTGGAGCTTAACTCCAGAATTGCCTTTGAAACTGTAAAGCTAGAGTATTGGAGAGGAGTGCGGAATACCTAATGTAGCGGTGAAATGCGTAGATATTAGGTGGAACACCGATGGCGAAGGCAGCACTCTGGACAATTACTGACGCTGAGGTGCGAAAGCGTGGGGATCAAACAGG` |
| ASV2491 | 404 | `TGGGGAATCTTGGACAATGGGCGCAAGCCTGATCCAGCCATGCCGCGTGAGTGACGAAGGCCTTAGGGTTGTAAAGCTCTTTTGCCGGGGACGATAATGACGGTACCCGGAGAATAAGTCCCGGCTAACTTCGTGCCAGCAGCCGCGGTAATACGAAGGGGACTAGCGTTGTTCGGAATTACTGGGCGTAAAGCGCACGTAGGCGGATATGCCAGTCAGGGGTGAAATCCCGGGGCTCAACCTCGGAACTGCCTTTGATACAGCATATCTGGAGTCCGATAGAGGTGAGTGGAATTCCTAGTGTAGAGGTGAAATTCGTAGATATTAGGAAGAACACCGGTGGCGAAGGCGGCTCACTGGATCGGTACTGACGCTGAGGTGCGAAAGCGTGGGGAGCAAACAGG` |
| ASV960 | 404 | `TGGGGAATATTGGACAATGGGCGAAAGCCTGATCCAGCAATGCCGCGTGAGTGATGAAGGCCTTAGGGTTGTAAAGCTCTTTTACCCGGGAAGATAATGACTGTACCGGGAGAATAAGCCCCGGCTAACTCCGTGCCAGCAGCCGCGGTAATACGGAGGGGGCTAGCGTTGTTCGGAATTACTGGGCGTAAAGCGCGCGTAGGCGGCTTTGTAAGTTAGAGGTGAAAGCCCGGAGCTCAACTCCGGAACTGCCTTTAAGACTGCATCGCTAGAATCATGGAGAGGTGAGTGGAATTCCGAGTGTAGAGGTGAAATTCGTAGATATTCGGAAGAACACCAGTGGCGAAGGCGACTCACTGGACATGTATTGACGCTGAGGTGCGAAAGCGTGGGGAGCAAACAGG` |
| ASV4655 | 429 | `TCGAGAATCTTCCGCAATGGGCGAAAGCCTGACGGAGCGACGCCGCGTGGAGGACGAAGGCCTTCGGGTTGTAAACTCCTGTCACGAGTTAAGAAGGGCATGCAGCGAACAGCTGTAGGCTTTGACGTAAGCTCGAGAGGAAGCACTGGCTAACTCTGTGCCAGCAGCCGCGGTAATACAGAGAGTGCAAACGTTGTTCGGAATTACTGGGCTTAAAGCGCACGTAGGCGGCATCGTAAGTGTCGGGTGAAATCCCACGGCTCACCCGTGGAACTGCCCGGCAGACTGCGATGCTCGAGTGTGGTAGGGGAGAGCGGAACTCCTGGTGGAGCGGTGAAATGCGTAGATATCAGGAGGAACACCGGTGGCGAAGGCGGCTCTCTGGTCCACAACTGACGCTGAGGTGCGAAAGCTAGGGGAGCAAACGGG` |
| ASV1503 | 404 | `TGGGGAATCTTGGACAATGGGCGCAAGCCTGATCCAGCCATGCCGCGTGAGTGACGAAGGCCTTAGGGTTGTAAAGCTCTTTTGCCGGGGACGATAATGACGGTACCCGGAGAATAAGTCCCGGCTAACTTCGTGCCAGCAGCCGCGGTAATACGAAGGGGACTAGCGTTGTTCGGAATTACTGGGCGTAAAGCGCACGTAGGCGGATTTGTCAGTCAGGGGTGAAATCCCGGGGCTCAACCTCGGAACTGCCTTTGATACAGCAAGTCTTGAGTCCGATAGAGGTGGGTGGAATTCCTAGTGTAGAGGTGAAATTCGTAGATATTAGGAAGAACACCGGTGGCGAAGGCGGCCCACTGGATCGGTACTGACGCTGAGGTGCGAAAGCGTGGGGAGCAAACAGG` |
| ASV929 | 429 | `TGGGGAATTTTGGACAATGGGCGCAAGCCTGATCCAGCCATGCCGCGTGCGGGAAGAAGGCCTTCGGGTTGTAAACCGCTTTTGTCAGGGAAGAAACGCTCTGGGCTAATACCCTGGGGTAATGACGGTACCTGAAGAATAAGCACCGGCTAACTACGTGCCAGCAGCCGCGGTAATACGTAGGGTGCAAGCGTTAATCGGAATTACTGGGCGTAAAGCGTGCGCAGGCGGTTATGCAAGACAGATGTGAAATCCCCGGGCTTAACCTGGGAACTGCATTTGTGACTGCATAGCTAGAGTGCGGCAGAGGGGGATGGAATTCCGCGTGTAGCAGTGAAATGCGTAGATATGCGGAGGAACACCGATGGCGAAGGCAATCCCCTGGGCCTGCACTGACGCTCATGCACGAAAGCGTGGGGAGCAAACAGG` |
| ASV5297 | 404 | `TGGGGAATATTGGACAATGGGGGAAACCCTGATCCAGCAATGCCGCGTGAATGAAGAAGGCCTTCGGGTTGTAAAGTTCTTTCGCCTGTGACGATAATGACGGTAGCAGGAAAAGAAGTCCCGGCTAACTCCGTGCCAGCAGCCGCGGTAATACGGAGGGGACTAGCGTTGCTCGGAATGACTGGGCGTAAAGGGTGCGTAGGCGGTTCTTTAAGTAAGGCGTGAAAGCCCTGGGCTTAACCCAGGAAGTGCGCTTTAAACTGAAGAGCTAGAGTGTGAGAGAGGAAAGTGGAATTCCTAGTGTAGAGGTGAAATTCGCAGATATTAGGAAGAACACCAGAGGCGAAGGCGGCTTTCTGGCTCACAACTGACGCTGAGGCACGAAAGCGTGGGGAGCAAACAGG` |
| ASV2344 | 387 | `GCGAGAAACCTTTGCAATGTGCGAAAGCACGACAAGGTTAATCCGAGTGTCTCCTGCTAAAGGAGTCTTTTGCCGGTCATAGAACCACCGGTGAATAAGGGGTGGGCAAGTTCTGGTGTCAGCCGCCGCGGTAAAACCAGCACCTCGAGTGGTCAGGAGGTTTATTGGGCCTAAAGCATCCGTAGCCGGCTGCTCAAGTTTTCGGTTAAATCTATGCGCTTAACGTATAGGCCGCCGAAAATACTGGGCAGCTAGGGAGTGGGAGAGGTACACGGTACTCAATAGGAAGGGGTAAAATCCTTTGATCTATTGATGACCACCTGTGGCGAAGGCGGTGTACCAGAACACGTTCGACGGTGAGGGATGAAAGCTGGGGGAGCAAACCGG` |
| ASV730 | 421 | `TGGGGAATATTGCGCAATGGCCGAAAGGCTGACGCAGCGACGCCGCGTGGGGGATGAAGCATTTCGGTGTGTAAACCCCTGTTGCTCGGGACGAACAAGCCTTTTCGAGGGCTCTGACGGTACCGAGTGAGGAAGCACCGGCTAACTCCGTGCCAGCAGCCGCGGTAATACGGAGGGTGCGAGCGTTGTCCGGAATCACTGGGCGTAAAGGGCGCGTAGGCGGTCTGCTAAGCGTGTGGTGAAAGCTCGGGGCTCAACCCCGAGTCGGCCATGCGAACTGGTGGACTAGAGCACTGTAGAGGCAGGTGGAATTCCGGGTGTAGCGGTGGAATGCGTAGAGATCCGGAAGAACACCGGTGGCGAAGGCGGCCTGCTGGGCAGTAGCTGACGCTGAGGCGCGATAGCGTGGGGAGCAAACAGG` |
| ASV5238 | 429 | `TGGGGAATATTGGACAATGGGGGCAACCCTGATCCAGCGATGCCGCGTGAGTGAAGAAGGCCCTCGGGTCGTAAAGCTCTTTAGGTTGGGAAGAAGGGTGTTAGGGGATAATCCTAGCACTTTGACGGTACCGACAGAATAAGCACCGGCAAACTCTGTGCCAGCAGCCGCGGTAATACAGAGGGTGCGAGCGTTAATCGGAATTACTGGGCGTAAAGGGCGCGTAGGCGGTCATAAGAGTGTGATGTGAAAGCCCCGGGCTTAACCTGGGAAGCGCATCGCAAACGCTATGACTGGAGTATATGAGAGGGTGGCGGAATTTCCGGTGTAGCGGTGAAATGCGTAGATATCGGAAGGAACGTCGATGGCGAAGGCAGCCACCTGGCATAATACTGACGCTGAAGCGCGAAAGCGTGGGGAGCGAACAGG` |
| ASV3455 | 407 | `TCGAGAATATTCGACAATGGGCGCAAGCCTGATCGAGCGACGCCGCGTGCAGGATGAAGGACTTTGGTCTGTAAACTGCTTTTCATGGGGAAAAAACAAATGATGGTACCCATGGAATAAGGGGTTGCTAACCTCGTGCCAGCAGCCGCGGTAATACGAGGGCCCCGAGCGTTATCCGGATTTATTGGGCGTAAAGAGCGAGTAGGCGGTCTTGTAAGTTTCGTGTCAAATACCGGGGCTCAACCTCGGATCAGCGCGAAAGACTGCGAGACTAGAGGGTGCTAGAGGCTACTGGAACTCACAGTGTAGGGGTGAAATCCGTTGATATTGTGGGGAACACCGAAGGCGAAGGCAGGTAGCTGGGGCATCCCTGACGCTCAATCGCGAAAGCGTGGGGATCAAAAAGG` |
| ASV803 | 429 | `TGGGGAATTTTGGACAATGGACGCAAGTCTGATCCAGCCATGCCGCGTGCGGGAAGAAGGCCTTCGGGTTGTAAACCGCTTTTGTCAGGGAAGAAACGGCTTTGGTTAATACCTGAGGCTAATGACGGTACCTGAAGAATAAGCACCGGCTAACTACGTGCCAGCAGCCGCGGTAATACGTAGGGTGCAAGCGTTAATCGGAATTACTGGGCGTAAAGCGTGCGCAGGCGGTTATGCAAGACAGATGTGAAATCCCCGGGCTCAACCTGGGAACTGCATTTGTGACTGCATGGCTAGAGTACGGTAGAGGGGGATGGAATTCCGCGTGTAGCAGTGAAATGCGTAGATATGCGGAGGAACACCGATGGCGAAGGCAATCCCCTGGACCTGTACTGACGCTCATGCACGAAAGCGTGGGGAGCAAACAGG` |
| ASV579 | 404 | `TGGGGAATATTGGACAATGGGCGAAAGCCTGATCCAGCAATGTCGCGTGAATGATGAAGGCCTTAGGGTTGTAAAGTTCTTTCGGTGGGGAAGATAATGACGGTACCCACATAAGAAGCTCCGGCTAACTCCGTGCCAGCAGCCGCGGTAATACGGAGGGAGCTAGCGTTGTTCGGAATTACTGGGCGTAAAGAGCGCGTAGGCGGCTAACTAAGTTGGGAGTGAAATCCCAGGGCTTAACCCTGGAATGGCTTTCAAAACTGGTTAGCTAGAGGACGATAGAGGATGGTGGAATTCCTAGTGTAGAGGTGAAATTCTTAGATATTAGGAGGAACATCGGAGGCGAAGGCGACCATCTGGGTCGTATCTGACGCTGAGGCGCGAAAGCGTGGGGAGCAAACAGG` |
| ASV2202 | 404 | `TGGGGAATTTTCCGCAATGGGCGAAAGCCTGACGGAGCAATACCGCGTGAGGGACGAAGGCTCTTGGGTCGTAAACCTCTTTTCTCAAGGAAGATAATGACGGTACTTGAGGAATAAGCATCGGCTAACTCTGTGCCAGCAGCCGCGGTAATACAGAGGATGCAAGCGTTATCCGGAATGATTGGGCGTAAAGCGTCCGCAGGTGGTTTTATAAGTCTGCTGTTAAAGCGTCATGCTCAACATGATAAGGGCAGTGGAAACTATGAGACTAGAGTATGTTAGGGGTAGAGGGAATTCCCAGTGTAGCGGTGAAATGCGTAGAGATTGGGAAGAACACCGGTGGCGAAAGCGCTCTGCTAGGACAATACTGACACTGAGGGACGAAAGCTAGGGGAGCGAATGGG` |
| ASV616 | 407 | `TGGGGAATATTGGGCAATGGGCGCAAGCCTGACCCAGCAACGCCGCGTGAAGGAAGAAGGCTTTCGGGTTGTAAACTTCTTTTGGCAGGGACGAAAAAAATGACGGTACCTGCAGAATAAGCTCCGGCTAACTACGTGCCAGCAGCCGCGGTAATACGTAGGGAGCAAGCGTTATCCGGATTTACTGGGTGTAAAGGGCGCGTAGGCGGGATGGCAAGTCAGATGTGAAATGTAGGGGCTCAACCCCTGAACTGCATTTGAAACTGCTGTTCTTGAGTGATGGAGAGGCAAGTGGAATTCCTAGTGTAGCGGTGAAATGCGTAGATATTAGGAGGAACACCAGTGGCGAAGGCGACTTGCTGGACATTAACTGACGCTGAGGCGCGAAAGCGTGGGGAGCAAACAGG` |
| ASV1366 | 429 | `TGGGGAATATTGGACAATGGGCGGAAGCCTGATCCAGCAATGCCGCGTGTGTGAAGAAGGCCTTTGGGTTGTAAAGCACTTTCAGCGGGGAAAAAGAAGCTGCGAGGAATATACGTAGCGGAAGATGTTACCCGCAAAAGAAGCACCGGCTAACTCTGTGCCAGCAGCCGCGGTAATACAGAGGGTGCGAGCGTTAATCGAAATGACTGGGCGTAAAGGGTGCGTAGGTGGTTTATTAAGTTAGGTGTGAAAGTCCTAGGCTCAACCTGGGGATTGCGCCTAATACTGGTGAGCTAGAGTACTGGAGAGGAAAGTGGAATTTCCGGTGTAGCGGTGAAATGCGTAGATATCGGAAGGAACATCAGTGGCGAAGGCGACTTTCTGGCCAGATACTGACACTGAGGCACGAAAGCATGGGGAGCAAACAGG` |
| ASV2487 | 410 | `TGGGGAATATTGCGCAATGGGCGGAAGCCTGACGCAGCGACGCCGCGTGGGGGATGACGGCCTTCGGGTTGTAAACCTCTTTCGGCCCCGACGAAGCGTGAGTGACGGTAGGGGTGGAAGAAGCACCGGCCAACTACGTGCCAGCAGCCGCGGTAACACGTAGGGTGCGAGCGTTGTCCGGAATTATTGGGCGTAAAGAGCTCGTAGGCGGTGTGTCGCGTCGGCCGTGAAAACCTGTGGCTTAACTGTGGGCTTGCGGTCGATACGGGCATCACTGGAGTTCGGCAGGGGAGACTGGAATTCCTGGTGTAGCGGTGAAATGCGCAGATATCAGGAGGAACACCGGTGGCGAAGGCGGGTCTCTGGGCCGATACTGACGCTGAGGAGCGAAAGCGTGGGGAGCGAACAGG` |
| ASV1906 | 429 | `TCGAGAATAATTCACAATGGGGGCAACCCTGATGGTGCAACGCCGCGTGGAGGATGAAGGTCTTCGGATTGTAAACTCCTGTCATCTGGGAGAAAGACCTGGCCGTTAATAGCGGACAGGGTTGATAGTACCAGAAGAGGAAGGGACGGCTAACTTCGTGCCAGCAGCCGCGGTAATACGAAGGTCCCGAGCGTTGTTCGGAATCACTGGGCGTAAAGGGAGCGTAGGCGGTTTGGTAAGTCAGATGTGAAATCCCGGGGCTCAACCCCGGAACTGCATCCGATACTGCCAGACTTGAGTAATGGAGAGGTAGCTGGAATTATCGGTGTAGCAGTGAAATGCGTAGATATCGATAGGAACACTCGTGGCGAAAGCGAGCTACTGGACATTTACTGACGCTGAGGCTCGAAGGCTAGGGGAGCGAAAGGG` |
| ASV1952 | 429 | `TGGGGAATTTTGGACAATGGGGGCAACCCTGATCCAGCAATGCCGCGTGAGTGAAGAAGGCCTTCGGGTTGTAAAGCTCTTTTATTTGGGAAGAAAAGTACTGTGTTAATAGCGCGGTACGCTGACGGTACCAAAGGAATAAGCACCGGCTAACTACGTGCCAGCAGCCGCGGTAATACGTAGGGTGCAAGCGTTAATCGGAATTACTGGGCGTAAAGCGTGCGTAGGCGGTTTAGTAAGACAGGCGTGAAATCCCCGAGCTCAACTTGGGAACTGCGTTTGTTACTGCTAGGCTAGAGTGTGTCAGAGGGGGGTAGAATTCCACGTGTAGCAGTGAAATGCGTAGATATGTGGAGGAATACCGATGGCGAAGGCAGCCCCCTGGGATAACACTGACGCTCAAGTACGAAAGCGTGGGGAGCAAACAGG` |
| ASV4715 | 430 | `TGGGGAATATTGCGCAATGGGCGAAAGCCTGACGCAGCGACGCCGCGTGGGTGATGAAGGCCTTCGGGTCGTAAAGCCCTGTCGGGAGGGACGAAACCTCGGTGACCTAATACGTCATCGACCTGACGGTACCTCCTAAGGAAGCACCGGCTAACTCCGTGCCAGCAGCCGCGGTAATACGGAGGGTGCAAGCGTTGTTCGGAATTACTGGGCGTAAAGCGCGTGTAGGCGGTCGCGTAAGTCTGATGTGAAAGCCCGGGGCTCACCCCCGGAAGTGCATTGGAAACTGCGCGACTAGAGTGTCGGAGAGGAGGGTGGAATTCCTGGTGTAGCGGTGAAATGCGTAGATATCAGGAGGAACACCGGTGGCGAAGGCGGCCCTCTGGACGAATACTGACGCTGAGACGCGAAAGCGTGGGGAGCAAACAGG` |
| ASV4844 | 429 | `TGGGGAATTTTGCGCAATGGGCGAAAGCCTGACGCAGCGACGCCGCGTGTGCGATGAAGGCCTTTGGGTCGTAAAGCACTGTCGAGTGGGAAGAAAGTCTCGGATCTAATACATTCGAGATCTGACGGTACCGCTAAAGGAAGCACCGGCTAACTCTGTGCCAGCAGCCGCGGTAATACAGAGGGTGCAAACGTTGCTCGGAATCATTGGGCGTAAAGCGCGTGTAGGCGGCTTGAAAAGTCGACTGTGAAAGCCCTTGGCTCAACCAAGGAAGTGCAGTCGAAACTCTCGAGCTAGAATACCGAAGAGGGTGGCGGAATGCGCAGTGTAGAGGTGAAATTCGTAGATATTGCGTAGAACACCGGTGGCGAAGGCGGCCACCTGGGAGGTTATTGACGCTGAGACGCGAAAGCGTGGGGAGCAAACAGG` |
| ASV1885 | 429 | `TGGGGAATTTTGGACAATGGGCGCAAGCCTGATCCAGCAATGCCGCGTGCAGGACGAAGGCCTTCGGGTTGTAAACTGCTTTTGTACGGAGCGAAACGGTCTTCTCTAATACAGGGGGCTAATGACGGTACCGTAAGAATAAGCACCGGCTAACTACGTGCCAGCAGCCGCGGTAATACGTAGGGTGCAAGCGTTAATCGGAATTACTGGGCGTAAAGCGTGCGCAGGCGGTTATGTAAGACAGATGTGAAATCCCCGGGCTCAACCTGGGACCTGCATTTGTGACTGCATAGCTAGAGTACGGTAGAGGGGGATGGAATTCCGCGTGTAGCAGTGAAATGCGTAGATATGCGGAGGAACACCGATGGCGAAGGCAATCCCCTGGACCTGTACTGACGCTCATGCACGAAAGCGTGGGGAGCAAACAGG` |
| ASV5657 | 407 | `TAGGGAATATTGGGCAATGGAGGCAACTCTGACCCAGCAACACCGCGTGAAGGAAGAAGGTCTTCGGATTGTAAACATCTGTTGTGAGGGAAAAAGAAAATGATGGTACCTCATAAGAAAGCACCGGCAAACTATGTGCCAGCAGCCGCGGTAATACATAGGGTGCAAGCGTTGTCCGGAATCATTGGGCGTAAAGGGAGTGTAGGTGGCTATGCAAGTTAAATGTGAAATCCTATGGCTTAACCATAGAACTGCATTTAAAACTATGTAGCTTGAGTATGGAAGAGGTAAGTGGAATTCCTAGTGTAGCGGTGGAATGCGTAGATATTAGGAGGAACACCAGAGGCGAAGGCGGCTTACTGGGACATTACTGACACTGAAACTCGAAAGCGTGGGGAGCAAACAGG` |
| ASV2125 | 424 | `TAAGGAATATTGGTCAATGGACGAAAGTCTGAACCAGCTATGCCGCGTGAAGGATTAAGGTCCTCTGGATTGTAAACTTCTTTTATATGGGACGAAAAAAGGTCTTTCTAGATCACTTGACGGTACCATATGAATAAGCACCGGCTAACTCCGTGCCAGCAGCCGCGGTAATACGGAGGGTGCAAGCGTTATCCGGATTCACTGGGTTTAAAGGGTGCGTAGGCGGGCAGGTAAGTCAGTGGTGAAATCCTGGAGCTCAACTCCAGAACTGCCATTGATACTATCTGTCTTGAATATTGTGGAGGTAAGCGGAATATGTCATGTAGCGGTGAAATGCTTAGATATGACATAGAACACCTATTGCGAAGGCAGCTTACTACGCATATATTGACGCTGAGGCACGAAAGCGTGGGGATCAAACAGG` |
| ASV1041 | 429 | `TGGGGAATTTTGGACAATGGGGGCAACCCTGATCCAGCAACGCCGCGTGAATGAAGAAGGCCTTAGGGTTGTAAAGTTCTTTTGTCAGGGAAGAAAGTTTCTGTGCTAATATCATAGAACGATGACGGTACCTGAAGAATAAGCACCGGCTAACTACGTGCCAGCAGCCGCGGTAATACGTAGGGTGCAAGCGTTAATCGGAATTACTGGGCGTAAAGCGTGCGCAGGCGGATATGTAAGTTAGAAGTGAAATCCCCGGGCTCAACCTGGGAACTGCTTTTAAGACTGCGTATCTAGAGTTTGTCAGAGGGGGGTGGAATTCCAAGTGTAGCAGTGAAATGCGTAGAGATTTGGAGGAACACCGATGGCGAAGGCAGCCCCCTGGGATAAAACTGACGCTCATGCACGAAAGCGTGGGTAGCAAACAGG` |
| ASV4164 | 429 | `TGGGGAATTTTGGACAATGGGGGCAACCCTGATCCAGCAATGCCGCGTGAGTGAAGAAGGCCTTCGGGTTGTAAAGCTCTTTTGTCAGGGAAGAAAAGGCACTTTCTAATAAAGAGTGCTGATGACGGTACCTGAAGAATAAGCACCGGCTAACTACGTGCCAGCAGCCGCGGTAATACGTAGGGTGCAAGCGTTAATCGGAATTACTGGGCGTAAAGCGTGCGCAGGCGGTTATATAAGTCTGATGTGAAATCCCCGGGCTCAACCTGGGAACTGCATTGGAGACTGTATGGCTAGAGTGTGTCAGAGGGGGGTAGAATTCCACGTGTAGCAGTGAAATGCGTAGAGATGTGGAGGAATACCGATGGCGAAGGCAGCCCCCTGGGATAACACTGACGCTCATGCACGAAAGCGTGGGGAGCAAACAGG` |
| ASV174 | 404 | `TGGGGAATATTGGACAATGGGCGCAAGCCTGATCCAGCCATGCCGCGTGAGTGATGAAGGTCTTAGGATTGTAAAGCTCTTTCACCGATGAAGATAATGACGGTAGTCGGAGAAGAAGCCCCGGCTAACTTCGTGCCAGCAGCCGCGGTAATACGAAGGGGGCTAGCGTTGTTCGGAATTACTGGGCGTAAAGCGCACGTAGGCGGATATTTAAGTCAGGGGTGAAATCCCGCAGCTCAACTGCGGAACTGCCTTTGATACTGGGTATCTTGAGTATGGAAGAGGTGAGTGGAATTCCGAGTGTAGAGGTGAAATTCGTAGATATTCGGAGGAACACCAGTGGCGAAGGCGGCTCACTGGTCCATTACTGACGCTGAGGTGCGAAAGCGTGGGGAGCAAACAGG` |
| ASV4039 | 429 | `TGGGGAATATTGCGCAATGGAGGAAACTCTGACGCAGCGACGCCGCGTGGGTGATGAAGGCCTTCGGGTTGTAAAGCCCTGTCGGCTGGGAAAAAGGTAGTCGTGCTAATACCACGATTGCTTGATGGTACCAGCAAAGGAAGCACCGGCTAACTCTGTGCCAGCAGCCGCGGTAATACAGAGGGTGCAAGCGTTGTTCGGAATTACTGGGCGTAAAGGGCGCGTAGGCGGCTTGGTAAGTCAGATGTGAAATCCCGGGGCTTAACTCCGGAAGTGCATTTGAAACTGCCAGGCTAGAGTGCAGTAGAGGTTGGTGGAATTCCCAGTGTAGAGGTGAAATTCGTAGATATTGGGAGGAACACCAGTGGCGAAGGCGGCCAACTGGGCTGTAACTGACGCTGAGGCGCGAAAGCGTGGGGAGCAAACAGG` |
| ASV390 | 429 | `TGGGGAATCTTGGACAATGGGCGAAAGCCTGATCCAGCAATGCCGCGTGCGTGAAGAAGGCCTTCGGGTTGTAAAGCGCTTTTGTCGGGGAGGAAATCCTAGTCTCTAATAAAGGCTGGGGATGACAGTACCCGAAGAATAAGGACCGGCTAACTACGTGCCAGCAGCCGCGGTAATACGTAGGGTCCAAGCGTTAATCGGAATTACTGGGCGTAAAGCGTCCGCAGGTGGCTTGATAAGATAGACGTGAAATCCCTGGGCTCAACCTAGGAATTGCGTTTATGACTGTCTCGCTAGAGTATGGGAGAGGGGGGTGGAATTCCACGTGTAGCAGTGAAATGCGTAGAGATGTGGAGGAACACCGATGGCGAAGGCAACCCCCTGGCCTAATACTGACACTCATGGACGAAAGCGTGGGGAGCAAACAGG` |
| ASV1052 | 429 | `TGGGGAATATTGGACAATGGGCGCAAGCCTGATCCAGCCATGCCGCGTGTGTGAAGAAGGCCTTCGGGTTGTAAAGCACTTTTGTCCGGAAAGAAAAGCTTCGGGTTAATACCCCGGGGTTATGACGGTACCGGAAGAATAAGCACCGGCTAACTTCGTGCCAGCAGCCGCGGTAATACGAAGGGTGCAAGCGTTACTCGGAATTACTGGGCGTAAAGCGTGCGTAGGTGGTTTGTTAAGTCTGATGTGAAAGCCCTGGGCTCAACCTGGGAATTGCATTGGATACTGGCAGGCTAGAGTGCGGTAGAGGATGGCGGAATTCCCGGTGTAGCAGTGAAATGCGTAGAGATCGGGAGGAACATCTGTGGCGAAGGCGGCCATCTGGACCAGCACTGACACTGAGGCACGAAAGCGTGGGGAGCAAACAGG` |
| ASV3093 | 424 | `TAAGGAATATTGGTCAATGGACGCAAGTCTGAACCAGCCATGCCGCGTGGAGGATGAAGGTCCTCTGGATTGTAAACTTCTTTTATTTGGGACGAAAAAAGGCTTTTCTAAGTCGTCTGACGGTACCAGATGAATAAGCACCGGCTAACTCCGTGCCAGCAGCCGCGGTAATACGGAGGGTGCAAGCGTTATCCGGATTCACTGGGTTTAAAGGGTGCGTAGGTGGGTTGGTAAGTCAGTGGTGAAATCTCCGAGCTTAACTTGGAAACTGCCATTGATACTATCAGTCTTGAATATTGTGGAGGTTAGCGGAATATGTCATGTAGCGGTGAAATGCTTAGATATGACATAGAACACCAATTGCGAAGGCAGCTGGCTACACATATATTGACACTGAGGCACGAAAGCGTGGGGATCAAACAGG` |
| ASV5178 | 405 | `TTAGGGATCTTGCGCAATGGGCGAAAGCCTGACGCAGCGACGCCGCGTGCGGGAGGACGGCCCTCGGGTCGTAAACCGCTTTTCTAGGGGACGAGGATGGACGGTACTCTAGGAATAAGCATCGGCTAACTCCGTGCCAGCAGCCGCGGTAATACGGGGGATGCGAGCGTTGTCCGGATTCACTGGGCGTAAAGGGCGCGTAGGCGGCTATCACAGTCCGATCTATAAGTGCGGGGCTCAACCTCGTGATGGGTTGGGGACGTGGTAGCTGGGGCGCGGCAGAGGGAGACGGAATGCCGGGTGTAGTGGTGAAATGCGTAGAGATCCGGCGGAACACCGGTGATGAAGATGGTCTCCTGGGCCGAGGGCCGACGCTGAGGCGCGAGAGCGTGGGGAGCGAACCGG` |
| ASV3961 | 429 | `TGGGGAATTTTGGACAATGGGCGCAAGCCTGATCCAGCCATGCCGCGTGCGGGATGAAGGCCTTCGGGTTGTAAACCGCTTTTGTTGGGGAAGAAATACTTCGAGTTAATACCTCGGAAGGATGACGGTACCCAAAGAATAAGCACCGGCTAACTACGTGCCAGCAGCCGCGGTAATACGTAGGGTGCAAGCGTTAATCGGAATTACTGGGCGTAAAGCGTGCGCAGGCGGTCTTGCAAGACCGATGTGAAATCCCCGGGCTTAACCTGGGAACTGCATTGGTGACTGCAAGGCTAGAGTACGGTAGAGGGGGATGGAATTCCGCGTGTAGCAGTGAAATGCGTAGAGATGCGGAGGAACACCGATGGCGAAGGCAATCCCCTGGACCTGTACTGACGCTCATGCACGAAAGCGTGGGGAGCAAACAGG` |
| ASV1673 | 404 | `TGGGGAATATTGGACAATGGGCGAAAGCCTGATCCAGCAATGCCGCGTGAGTGATGAAGGCCTTAGGGTTGTAAAGCTCTTTTACCCGGGATGATAATGACAGTACCGGGAGAATAAGCCCCGGCTAACTCCGTGCCAGCAGCCGCGGTAATACGGAGGGGGCTAGCGTTGTTCGGAATTACTGGGCGTAAAGCGCACGTAGGCGGCTATTCAAGTCAGAGGTGAAAGCCTGGAGCTCAACTCCAGAACTGCCTTTGAAACTAGATAGCTAGAATCATGGAGAGGTGGGTGGAATTCCGAGTGTAGAGGTGAAATTCGTAGATATTCGGAAGAACACCAGTGGCGAAGGCGGCCCACTGGACATGTATTGACGCTGAGGTGCGAAAGCGTGGGGAGCAAACAGG` |
| ASV1169 | 404 | `TGGGGAATCTTGGACAATGGGCGCAAGCCTGATCCAGCCATGCCGCGTGAGTGACGAAGGCCTTAGGGTTGTAAAGCTCTTTTGCCGGGGACGATAATGACGGTACCCGGAGAATAAGTCCCGGCTAACTTCGTGCCAGCAGCCGCGGTAATACGAAGGGGACTAGCGTTGTTCGGAATTACTGGGCGTAAAGCGCACGTAGGCGGATTTGTCAGTCAGGGGTGAAATCCCGGGGCTCAACCTCGGAACTGCCTTTGATACAGCAAGTCTTGAATCCGGTAGAGGTGGGTGGAATTCCTAGTGTAGAGGTGAAATTCGTAGATATTAGGAAGAACACCGGTGGCGAAGGCGGCCCACTGGACCGGTATTGACGCTGAGGTGCGAAAGCGTGGGGAGCAAACAGG` |
| ASV162 | 429 | `TGGGGAATTTTGGACAATGGGCGAAAGCCTGATCCAGCAATGCCGCGTGTGGGAAGAAGGCCTTCGGGTTGTAAACCACTTTTGTACGGAACGAAACGGCCTGCTCCAACACAGCGGGCTAATGACGGTACCGTAAGAATAAGCACCGGCTAACTACGTGCCAGCAGCCGCGGTAATACGTAGGGTGCGAGCGTTAATCGGAATTACTGGGCGTAAAGCGTGCGCAGGCGGTTATATAAGACAGTTGTGAAATCCCCGGGCTCAACCTGGGAATTGCATCTGTGACTGTATAGCTAGAGTACGGTAGAGGGGGATGGAATTCCGCGTGTAGCAGTGAAATGCGTAGATATGCGGAGGAACACCGATGGCGAAGGCAATCCCCTGGACCTGTACTGACGCTCATGCACGAAAGCGTGGGGAGCAAACAGG` |
| ASV5361 | 429 | `TGGGGAATATTGGACAATGGGGGCAACCCTGATCCAGCAATGCCGCGTGTGTGAAGAAGGCCTGAGGGTTGTAAAGCACTTTCGGTAGGGAGGATGTTATGGTGGTTAATAACCATTATGATGGACGTTACCTACATAAGAAGCACCGGCTAACTCCGTGCCAGCAGCCGCGGTAATACGGAGGGTGCGAGCGTTAATCGGAATTACTGGGCGTAAAGGGTGCGTAGGTGGTTATATAAGTTATCTGTGAAATACCTGGGCTCAACCTGGGCAGGTCAGATAAAACTGTGTGACTCGAGTATGGGAGAGGGTAGTGGAATTTCCGGTGTAGCGGTGAAATGCGTAGAGATCGGAAGGAACACCGGTGGCGAAGGCGGCTACCTGGCCTAATACTGACACTGAGGCACGAAAGCGTGGGGAGCAAACAGG` |
| ASV2311 | 429 | `TCGAGAATAATTCACAATGGGGGCAACCCTGATGGTGCAACGCCGCGTGGAGGATGAAGGTCCTTGGATTGTAAACTCCTGTCATCTGGGAGTAAGACCTGGCGATGAATAGTCGACAGGGTTGATAGTACCAGAAGAGGAAGGGACGGCTAACTTCGTGCCAGCAGCCGCGGTAATACGAAGGTCCCAAGCGTTGTTCGGAATCACTGGGCGTAAAGGGAGCGTAGGCGGCGTGGTAAGTCAGATGTGAAATCCCGGGGCTCAACCCCGGAACTGCATCCGATACTGCCATGCTAGAGGATTGGAGAGGTAGCTGGAATTCTTGGTGTAGCAGTGAAATGCGTAGAGATCAAGAGGAACACTCGTGGCGAAAGCGAGCTACTGGACAATATCTGACGCTGAGGCTCGAAGGCTAGGGTAGCGAAAGGG` |
| ASV381 | 429 | `TGGGGAATTTTGGACAATGGGCGCAAGCCTGATCCAGCCATGCCGCGTGTGGGAAGAAGGCCTTCGGGTTGTAAACCACTTTTGTCAGGGAAGAAACGGCTCTTACTAATATTAGGAGCTAATGACGGTACCTGAAGAATAAGCACCGGCTAACTACGTGCCAGCAGCCGCGGTAATACGTAGGGTGCAAGCGTTAATCGGAATTACTGGGCGTAAAGCGTGCGCAGGCGGCTATGCAAGACAGATGTGAAATCCCCGGGCTCAACCTGGGAACTGCATTTGTGACTGCATGGCTAGAGTACGGTAGAGGGGGATGGAATTCCGCGTGTAGCAGTGAAATGCGTAGATATGCGGAGGAACACCGATGGCGAAGGCAATCCCCTGGACCTGTACTGACGCTCATGCACGAAAGCGTGGGGAGCAAACAGG` |
| ASV5571 | 408 | `TCGAGAATATTCGACAATGGACGAAAGTCTGATCGAGCGACGCCGCGTGATGGATGAAGTCCTTCGGGACGTAAACATCTTTTGGGAGGGACGAAGTAATTGACGGTACCTCCAGAATAAGGGGCTCCTAACTCTGTGCCAGCAGGAGCGGTAATACAGAGGCCCCAAGCGTTACCCGGAATCACTGGGCGTAAAGGATGCGTAGGTGGCGTTGTTAGTCCTTCGTTAAATGCTTCCCGCTCAACGGGAAGATCGCGATGGAAACGGCAATGCTCGAGGATGTACGAGGCGTGCGGAACTCATGGTGTAGGGATTAAATCCGTTGATATCATGGGGAACACCGAAAGCGAAGGCAGCACGCTGGGACATTCCTGACACTGAGGCATGAAAGCGTAGGTAGCGAAAAGG` |
| ASV2679 | 407 | `TCGAGAATATTCGACAATGGGCGCAAGCCTGATCGAGCGACGCCGCGTGCAGGATGAAGGACTTTGGTCTGTAAACTGCTTTTCATGGGGAAAAAACAAATGATGGTACCCATGGAATAAGGGGTTGCTAACCTCGTGCCAGCAGCCGCGGTAATACGAGGGCCCCGAGCGTTATCCGGATTTATTGGGCGTAAAGAGCGAGTAGGCGGTCGTATAAGTTTCGTGTCAAATACCGGGGCTCAACCCCGGACTAGCGCGAAATACTGTACGACTAGAGGGTGCTAGAGGCTACTGGAACTCACAGTGTAGGGGTGAAATCCGTTGATATTGTGGGGAACACCGAAGGCGAAGGCAGGTAGCTGGGGCATCCCTGACGCTCAATCGCGAAAGCGTGGGGATCAAAAAGG` |
| ASV5061 | 428 | `TGGGGAATCTTGCGCAATGGGCGAAAGCCTGACGCAGCGACGCCGCGTGAGTGATGAAGGCCTTCGGGTTGTAAAGCTCTGTGGAGAGGGACGAATAAGTGTTGGCTAATATCCAGCATGATGACGGTACCTTTTTAGCAAGCACCGGCTAACTCTGTGCCAGCAGCCGCGGTAAGACAGAGGGTGCAAACGTTGTTCGGAATTACTGGGCGTAAAGCGCGTGTAGGCGGCTTAGCAAGTCGGGTGTGAAAGCCCCGGGCTCAACCTGGGAAGTGCACTCGAAACTGCCGAGCTGGAGTCTCGGAGAGGGAAGCGGAATTCTCGGTGTAGAGGTGAAATTCGTAGATATCGAGAGGAACACCGGTGGCGAAGGCGGCTTCCTGGACGACGACTGACGCTGAGACGCGAAAGCGTGGGGAGCAAACAGG` |
| ASV1922 | 429 | `TCGAGAATAATTCACAATGGGGGCAACCCTGATGGTGCAACGCCGCGTGGAGGATGACGGTCTTCGGATTGTAAACTCCTGTCATCTGGGAGTAAGGCGTAGTTGTTAAAAGCAAATACGATTGATAGTACCAGAAGAGGAAGGGACGGCTAACTTCGTGCCAGCAGCCGCGGTAATACGAAGGTCCCAAGCGTTGTTCGGAATCACTGGGCGTAAAGGGAGCGTAGGCGGCGTGGTAAGTCAGATGTGAAATCCCGGGGCTCAACCCCGGAACTGCATCCGATACTGCCATGCTAGAGGATTGGAGAGGTAGCTGGAATTCTTGGTGTAGCAGTGAAATGCGTAGAGATCAAGAGGAACACTCGTGGCGAAAGCGAGCTACTGGACAATAACTGACGCTGAGGCTCGAAGGCTAGGGTAGCGAAAGGG` |
| ASV450 | 430 | `TGGGGAATATTGCGCAATGGGGGAAACCCTGACGCAGCAACGCCGCGTGAGTGAGGAAGGCCTTCGGGTTGTAAAGCTCTGTCACTGTAGAAAAATGTACATTGGTCTAATAGGCCAGTGTAGTGATGGTATACAGAGAGGAAGCACCGGCTAACTTCGTGCCAGCAGCCGCGGTAATACGAAGGGTGCAAGCGTTGTTCGGATTTATTGGGCGTAAAGCGCGCGCAGGCGGATGATTAAGCCAGATGTGAAATCTCGGGGCTCAACCCCGAAACTGCGTCTGGAACTGGTTATCTAGAGTACTGGAGGGGGAAGGGGAATTTCGCATGTAGGGGTAAAATCCGTAGAGATGCGAAGGAACACCAGCGGGGAAACCGCCTTCCTGGACAGTTACTGACGCTGAGGCGCGAAAGCGTGGGGATCAAACAGG` |
| ASV3715 | 404 | `TGAGGAATATTGGACAATGGAGGAAACTCTGATCCAGCCATGCCGCGTGAGTGAAGAAGGCCTTAGGGTTGTAAAGCTCTTTCGATAGTGAAGATAATGACGGTAGCTATAGAAGAAGCCCCGGCTAAATTCGTGCCAGCAGCCGCGGTAATACGAATGGGGCAAGCGTTGTTCGGAATCACTGGGCTTAAAGCGTGCGTAGGCGGACTTGCAAGTCAGGAGTGAAATCCCAGGGCTCAACCCTGGAATTGCTTTTGAAACTATGAGTCTAGAATCCGGCAGGGGTTGGGAGAATTCCTAGTGTAGAGGTGAAATTCGCAGATATTAGGAGGAATACCAGTGGCGTAGGCGCCCAACTGGACCGGTATTGACGCTGAGGCACGAAAGCGTGGGGAGCAAACAGG` |
| ASV2983 | 424 | `TGAGGAATATTGGTCAATGGACGCAAGTCTGAACCAGCCATGCCGCGTGAAGGATGAAGGCGTTCTGCGTTGTAAACTTCTTTTATCTGGGAAGAAACCATCCTTTTCTAAGGATGCCGACGGTACCAGAGGAATAAGCACCGGCTAACTCCGTGCCAGCAGCCGCGGTAATACGGAGGGTGCAAGCGTTATCCGGATTCACTGGGTTTAAAGGGTGTGTAGGCGGGTATTTAAGTCAGTGGTGAAATCTCCGAGCTCAACTCGGAAACTGCCATTGATACTATTTATCTTGAATATTGTTGAGGTAGGCGGAATATGTCATGTAGCGGTGAAATGCTTAGATATGACATAGAACACCGATTGCGAAGGCAGCTTGCTAAACAATTATTGACGCTGAGGCACGAAAGCGTGGGGATCAAACAGG` |
| ASV1155 | 407 | `TGGGGAATTTTCCGCAATGGGCGAAAGCCTGACGGAGCAATACCGCGTGAGGGATGAAGGCCTATGGGTTGTAAACCTCTTTTTTCAGGGAGGAATAAATGACGTGTACCTGAAGAATAAGCATCGGCTAACTCCGTGCCAGCAGCCGCGGTAAGACGGGGGATGCAAGTGTTATCCGGAATCACTGGGCGTAAAGCGTCTGTAGGTGGTTAAGTAAGTCAACTGTTAAATCTTGAGGCTTAACTTCAAAATCGCAGTCGAAACTGCTTGACTAGAGTATAGTAGGGGTAAAGGGAATTTCCAGTGGAGCGGTGAAATGCGTAGATATTGGAAAGAACACCGATGGCGAAGGCACTTTACTGGGCTATTACTAACACTGAGAGACGAAAGCTAGGGTAGCAAATGGG` |
| ASV637 | 404 | `TGGGGAATATTGGGCAATGGACGCAAGTCTGACCCAGCCATGCCGCGTGAATGATGAAGGCCTTAGGGTTGTAAAGTTCTTTCGCCGATGAAGATAATGACGGTAGTCGGAGAAGAAGCCCCGGCTAACTTCGTGCCAGCAGCCGCGGTAATACGAAGGGGGCTAGCGTTGTTCGGAATTACTGGGCGTAAAGCGAGCGTAGGTCGCTTAGCAAGTCAGTGGTGAAAGCCCAAGGCTCAACCTTGGAACTGCCATTGAAACTGCTAAGCTGGAATCTCAGAGGGGGTAGCGGAATTCCAAATGTAGGGGTGAAATCCGTAGATATTTGGAGGAACACCGGTGGCGAAGGCGGCTACCTGGATGAGTATTGACACTGAGGCTCGAAAGCGTGGGGATCAAACAGG` |
| ASV4282 | 404 | `TGGGGAATATTGGACAATGGGCGAAAGCCTGATCCAGCAATGCCGCGTGAGTGAAGAAGGCCTTCGGGTTGTAAAGCTCTTTTGCCCGAGACGATAATGACGGTATCGGGAGAATAAGCCCCGGCTAACTTCGTGCCAGCAGCCGCGGTAATACGAAGGGGGCTAGCGTTGTTCGGAATCACTGGGCGTAAAGCGCACGTAGGCGGTTTTTCAAGTTAGGGGTGAAATCCTGGGGCTTAACCTCAGAACTGCCCTTAAGACTGGGAGACTTGAATCCAGGAGAGGTTGGTGGAATTCCGAGTGTAGAGGTGAAATTCGTAGATATTCGGAAGAACACCAGTGGCGAAGGCGGCCAACTGGACTGGTATTGACGCTGAGGTGCGAAAGCGTGGGGAGCAAACAGG` |
| ASV4106 | 428 | `TGGGGAATATTGGACAATGGGGGGAACCCTGATCCAGCAATGCCGCGTGTGTGAAGAAGGCCTTAGGGTTGTAAAGCACTTTCAGTTGGGAGGAATATCCTATGGCTAATAACCATAGGTTTGACGCTACCGACAGAAGAAGCACCGGCTAACTCTGTGCCAGCAGCCGCGGTAATACAGAGGGTGCAAGCGTTAATCGGAATTACTGGGCGTAAAGCGCGCGTAGGTGGATAAGTAAGTTGGATGTGAAATCCCCGGGCTCAACCTGGGACTGGCATTCAAAACTGCTTTTCTAGAGTATAGTAGAGGGGAGTGGAATTTCCGGTGTAGCGGTGAAATGCGTAGATATCGGAAGGAACACCAGTGGCGAAGGCGACTCCCTGGACTAATACTGACACTGAGGTGCGAAAGCGTGGGGAGCAAACAGG` |
| ASV250 | 405 | `TGGGGAATATTGGACAATGGGCGAAAGCCTGATCCAGCGATTCTGCGTGAAGGATGAAGCCTTGTGGGGTGTAAACTTCTTTTCTGGGAGAAGACGAATGACGGTATCTCAGGAATAAGAGACGGCTAACTACGTGCCAGCAGCCGCGGTAATACGTAGGTCTCGAGCGTTATCCGGAATTACTGGGCGTAAAGCGTCTGTAGCCGGATGTATAAGTCTGTCTTTAAATACTCTGATTCAATCGGAGAAAAGGGATAGATACTGTATATCTAGAGGATTTTAGGGGTTAGTGGAATTTCCGGTGGAGCGGTGAAATGCGTTGATATCGGAAGGAATGCCGAAAGCGAAAGCAGCTAACTATAAAATATCTGACGGTCAGAGACGACAGCTTGGGGAGCGAACGGG` |
| ASV834 | 429 | `TGGGGAATTTTGGACAATGGGCGAAAGCCTGATCCAGCAATGCCGCGTGCAGGATGAAGGCCTTCGGGTTGTAAACTGCTTTTGTACGGAACGAAACGGCTCTGGTTAATACCTGGGGCTAATGACGGTACCGTAAGAATAAGCACCGGCTAACTACGTGCCAGCAGCCGCGGTAATACGTAGGGTGCAAGCGTTAATCGGAATTACTGGGCGTAAAGCGTGCGCAGGCGGTGATGTAAGACAGTGGTGAAATCCCCGGGCTCAACCTGGGAACTGCCATTGTGACTGCATCGCTAGAGTACGGTAGAGGGGGGTGGAATTCCGCGTGTAGCAGTGAAATGCGTAGATATGCGGAGGAACACCGATGGCGAAGGCAGCCCCCTGGACCTGTACTGACGCTCATGCACGAAAGCATGGGGAGCAAACAGG` |
| ASV2705 | 429 | `TGGGGAATATTGGACAATGGGGGCAACCCTGATCCAGCCATGCCGCGTGTGTGAAGAAGGCCTTCGGGTTGTAAAGCACTTTAAGTGGGGAGAAAGGTTCGCTGTTTAATACGCAGCGGAATTGATGCTACCCACAGAATAAGCACCGGCTAACTCTGTGCCAGCAGCCGCGGTAATACAGAGGGTGCAAGCGTTAATCGGAATTACTGGGCGTAAAGCGCGCGTAGGCGGCTGATTAAGTCGGATGTGAAAGCCCCGGGCTCAACCTGGGAACTGCATTCGATACTGATCGGCTAGAGTATGAGAGAGGGAGGTAGAATTCCACGTGTAGCGGTGAAATGCGTAGATATGTGGAGGAATACCGGTGGCGAAGGCGGCCTCCTGGTTCAATACTGACGCTGAGGTGCGAAAGCGTGGGGAGCAAACAGG` |
| ASV1497 | 429 | `TGGGGAATATTGGACAATGGGGGCAACCCTGATCCAGCAATGCCGCGTGTGTGAAGAAGGCCTGAGGGTTGTAAAGCACTTTAAGCGGGGAGGAAGACGGGTATATTAATACTGTATTCGTTTGACGTTACCCGCAGAATAAGCACCGGCTAACTCTGTGCCAGCAGCCGCGGTAATACAGAGGGTGCGAGCGTTAATCGGAATTACTGGGCGTAAAGGGCGCGTAGGCGGTTTGGTAAGTTAGATGTGAAATCCCTGGGCTTAACCTAGGAATGGCATCTGATACTGCTGAGCTGGAGTATAGGAGAGGGCAGTGGAATTTCCGGTGTAGCGGTGAAATGCGTAGATATCGGAAGGAACACCAGTGGCGAAGGCGACTGTCTGGCCTAATACTGACGCTGAGGCGCGAAAGCGTGGGGAGCAAACAGG` |
| ASV3293 | 429 | `TGGGGAATTTTGCACAATGGGGGAAACCCTGATGCAGCGACGCCGCGTGGGTGATGAAGGCCTTCGGGTTGTAAAGCCCTGTCGGCTGGGAAAAAGGTAGTCGTGCTAATACCACGATTGCTTGATGGTACCAGCAAAGGAAGCACCGGCTAACTCTGTGCCAGCAGCCGCGGTAATACAGAGGGTGCAAGCGTTGTTCGGAATTACTGGGTGTAAAGGGCGCGTAGGCGGCTATTTAAGTCAGATGTGAAAACCCGAAGCTCAACTTCGGACGTGCATTTGAAACTATTTAGCTAGAGTACAGTAGAGGATGGTGGAATTCCCAGTGTAGAGGTGAAATTCGTAGAGATTGGGAGGAACACCGGTGGCGAAGGCGGCCATCTGGGCTGTAACTGACGCTGAGGCGCGAAAGCGTGGGGATCAAACAGG` |
| ASV3771 | 424 | `TAAGGAATATTGGTCAATGGACGCAAGTCTGAACCAGCCATGCCGCGTGCAGGAAGACGGCCCTATGGGTTGTAAACTGCTTTTGTCGGGGAAGAAACCCATCTACGTGTAGATGGCTGACGGTACTCGAAGAATAAGGATCGGCTAACTCCGTGCCAGCAGCCGCGGTAATACGGAGGATCCAAGCGTTATCCGGATTTATTGGGTTTAAAGGGTGCGTAGGCGGCGTTGTAAGTCAGTGGTGAAAGTTTTCAGCTTAACTGGAAAATTGCCATTGATACTGCAGTGCTTGAGTATAGATGAGGTGGGCGGAATGTGTCATGTAGCGGTGAAATGCATAGATATGACACAGAACACCGATTGCGAAGGCAGCTCACTAAACTATAACTGACGCTGAGGCACGAAAGCGTGGGGATCAAACAGG` |
| ASV5605 | 430 | `TAGGGAATATTGCGCAATGGGGGAAACCCTGACGCAGCAACGCCGTGTGAGGGAGGAAGGTCTTCGGATTGTAAACCTCTGTCGAGGGGGAAAAATGGCAGGTGCGTGAATAATGCATCCGCATGATGGTACCCCTGAAGGAAGCACCGGCTAACTTCGTGCCAGCAGCCGCGGTAATACGAAGGGTGCAAGCGTTGTTCGGATTTATTGGGCGTAAAGCGCGCGCAGGCGGAATTGTGTGTCAGATGTGAAATCTCGAAGCTTAACTTCGAACGTGCGTCTGAAACTGCAGTTCTAGAGTGCCGGAGGGGGAAGGGGAATTTCACGTGTAGGGGTAAAATCCGTAGAGATGTGAAGGAACACCGGAGGCGAAGGCGCCTTCCTGGCCGGCCACTGACGCTGAGGCGCGAAAGCGTGGGGATCAAACAGG` |
| ASV3387 | 429 | `TGGGGAATATTGCGCAATGGGCGAAAGCCTGACGCAGCCATGCCGCGTGTGTGAAGAAGGCCTTAGGGTTGTAAAGCACTTTCAGTAGGGAGGAAAGGTGTAAGGTTAATACCCTTGCGCTGTGACGTTACCTACAGAAGAAGCACCGGCTAACTCTGTGCCAGCAGCCGCGGTAATACAGAGGGTGCAAGCGTTAATCGGAATGACTGGGCGTAAAGCGCGCGTAGGTGGTTTGTTAAGTTGGATGTGAAAGCCCCGGGCTCAACCTGGGAATGGCATCCAAAACTGCCAGACTAGAGTATAGTAGAGGGGGGTAGAATTTCCGGTGTAGCGGTGAAATGCGTAGAGATCGGAAGGAATACCAGTGGCGAAGGCGGCCCCCTGGACTGATACTGACACTGAGGTGCGAAAGCGTGGGTAGCAAACAGG` |
| ASV183 | 404 | `TGGGGAATCTTAGACAATGGGGGAAACCCTGATCTAGCCATGCCGCGTGAGCGATGAAGGCCTTAGGGTTGTAAAGCTCTTTCAGTGGGGAAGATAATGACGGTACCCACAGAAGAAGCCCCGGCTAACTCCGTGCCAGCAGCCGCGGTAATACGGAGGGGGCTAGCGTTGTTCGGAATTACTGGGCGTAAAGCGCACGTAGGCGGATTGGAAAGTCAGAGGTGAAATCCCAGGGCTCAACCTTGGAACTGCCTTTGAAACTCCCAGTCTTGAGGTCGAGAGAGGTGAGTGGAATTCCGAGTGTAGAGGTGAAATTCGTAGATATTCGGAGGAACACCAGTGGCGAAGGCGGCTCACTGGCTCGATACTGACGCTGAGGTGCGAAAGCGTGGGGAGCAAACAGG` |
| ASV3109 | 424 | `TGAGGAATATTGGACAATGGGTGCGAGCCTGATCCAGCCATCCCGCGTGAAGGACTAAGGCCCTATGGGTTGTAAACTTCTTTTATACTGGGATAAACCTACTTACGAGTAAGTAGCTGAAGGTACAGTATGAATAAGCACCGGCTAACTCCGTGCCAGCAGCCGCGGTAATACGGAGGGTGCAAGCGTTATCCGGATTTATTGGGTTTAAAGGGTCCGTAGGCGGACTCGTAAGTCAGTGGTGAAATCTCATAGCTTAACTATGAAACTGCCATTGATACTGCGGGTCTTGAGTAAAATAGAAGTGGCTGGAATAAGTAGTGTAGCGGTGAAATGCATAGATATTACTTAGAACACCAATTGCGAAGGCAGGTCACTATGTTTTAACTGACGCTGATGGACGAAAGCGTGGGGAGCGAACAGG` |
| ASV4328 | 428 | `TCGAGAATTTTTCTCAATGGGGGAAACCCTGAAGGAGCGACGCCGCGTGGAGGATGAAGGTCTTCGGATTGTAAACTCCTGTCATTTGGGAACAATTGTCACCGATTAACTGTCGGGGGCTTGATAGTACCAGAAGAGGAAGAGACGGCTAACTCTGTGCCAGCAGCCGCGGTAATACAGAGGTCTCAAGCGTTGTTCGGATTCATTGGGCGTAAAGGGTGCGTAGGTGGCGGGGTAAGTCTGGTGTGAAATCTCGGGGCTCAACCCCGAAACTGCACTGGATACTGCCTTGCTCGAGTACTGGAGAGGAGATTGGAATTTACGGTGTAGCAGTGAAATGCGTAGATATCGTAAGGAAGACCAGTGGCGAAGGCGAATCTCTGGACAGTTACTGACACTGAGGCACGAAGGCCAGGGGAGCAAACGGG` |
| ASV3532 | 429 | `TGGGGAATATTGCGCAATGGACGAAAGTCTGACGCAGCGACGCCGCGTGAGGGATGAAGGCCTTAGGGTCGTAAACCTCTGTCAGGAGGGAAGAACTGTACGGGTGCTAATCAGCCTGTATTTGACGGTACCTCCAAAGGAAGCACCGGCTAACTCCGTGCCAGCAGCCGCGGTAATACGGAGGGTGCAAGCGTTAATCGGAATCACTGGGCGTAAAGCGCGCGTAGGCGGCTTTTTAAGTCAGGGGTGAAAGCCCACGGCTCAACCGTGGAACTGCCTTTGATACTGGAAAGCTTGAGTGTGTGAGAGGATAGTGGAATTCCTGGTGTAGGAGTGAAATCCGTAGAGATCAGGAGGAAGATCAGTGGCGAAGGCGACTATCTGGCACAATACTGACGCTGAGGTGCGAAAGCGTGGGTAGCAAACAGG` |
| ASV1040 | 429 | `TGGGGAATTTTGGACAATGGGGGAAACCCTGATCCAGCCATTCCGCGTGAGTGAAGAAGGCCTTCGGGTTGTAAAGCTCTTTCGGCAGGGAAGAAACGGTGTCCCCTAATACGGGGCGCTAATGACGGTACCTGAAGAAGAAGCACCGGCTAACTACGTGCCAGCAGCCGCGGTAATACGTAGGGTGCGAGCGTTAATCGGAATTACTGGGCGTAAAGCGTGCGCAGGCGGTTTTGTAAGACAGATGTGAAATCCCCGGGCTCAACCTGGGAACTGCGTTTGTGACTGCAAGACTTGAGTATGGCAGAGGGGGGTAGAATTCCACGTGTAGCAGTGAAATGCGTAGAGATGTGGAGGAATACCGATGGCGAAGGCAGCCCCCTGGGTCAATACTGACGCTCATGCACGAAAGCGTGGGGAGCAAACAGG` |
| ASV3959 | 404 | `TGGGGAATATTGGACAATGGGCGCAAGCCTGATCCAGCCATGCCGCGTGAGTGATGAAGGTCTTAGGATTGTAAAGCTCTTTCACCGGGGACGATAATGACGGTACCCGGAGAAGAAGCCCCGGCTAACTTCGTGCCAGCAGCCGCGGTAATACGAAGGGGGCTAGCGTTGTTCGGAATTACTGGGCGTAAAGCGCACGTAGGCGGACATTTAAGTCAGGGGTGAAATCCCGGGGCTCAACCCCGGAACTGCCTTTGATACTGGGTGTCTTGAGTGTGGTAGAGGTGAGTGGAATTGCGAGTGTAGAGGTGAAATTCGTAGATATTCGCAGGAACACCGGTGGCGAAGGCGGCTCACTGGACCACAACTGACGCTGAGGTGCGAAAGCGTGGGGAGCAAACAGG` |
| ASV2131 | 429 | `TGGGGAATTTTGGACAATGGGCGCAAGCCTGATCCAGCAATGCCGCGTGCAGGATGAAGGCCTTCGGGTTGTAAACTGCTTTTGTACGGGAAGAAAAGGCTCTCACTAATACTGGGGGTTGATGACGGTACCGTAAGAATAAGCACCGGCTAACTACGTGCCAGCAGCCGCGGTAATACGTAGGGTGCAAGCGTTAATCGGAATTACTGGGCGTAAAGCGTGCGCAGGCGGTTTTGTAAGACAGACGTGAAAGCCCCGGGCTCAACCTGGGAATGGCGTTTGTGACTGCAAAGCTAGAGTGCGGCAGAGGGGGATGGAATTCCGCGTGTAGCAGTGAAATGCGTAGATATGCGGAGGAACACCAATGGCGAAGGCAATCCCCTGGGCCTGCACTGACGCTCATGCACGAAAGCGTGGGGAGCAAACAGG` |
| ASV1585 | 429 | `TGGGGAATTTTGGACAATGGGCGCAAGCCTGATCCAGCAATGCCGCGTGCAGGATGAAGGCCTTCGGGTTGTAAACTGCTTTTGTACGGAACGAAACGGCCCTTTCTAATACAAGGGGCTAATGACGGTACCGTAAGAATAAGCACCGGCTAACTACGTGCCAGCAGCCGCGGTAATACGTAGGGTGCGAGCGTTAATCGGAATTACTGGGCGTAAAGCGTGCGCAGGCGGTTATGTAAGACAGATGTGAAATCCCCGGGCTTAACCTGGGACCTGCATTTGTGACTGCATAGCTAGAGTACGGTAGAGGGGGATGGAATTCCGCGTGTAGCAGTGAAATGCGTAGATATGCGGAGGAACACCGATGGCGAAGGCAATCCCCTGGACCTGTACTGACGCTCATGCACGAAAGCGTGGGGAGCAAACAGG` |
| ASV4345 | 430 | `TGGGGAATTTTGCACAATGGGCGCAAGCCTGATGCAGCAACGCCGCGTGGGTGAAGAAGGTTTTCGGATTGTAAAGCCCTTTCAGCTGAGGAGAAGGTTTTTTGGATGAATAAGCCAAAAGGTTGACGGTATCAGCAGAAGAAGCACCGGCTAATTCCGTGCCAGCAGCCGCGGTAATACGGAAGGTGCAAGCGTTGTTCGGAATGATTGGGCGTAAAGCGCACGTAGGTGGGTTTGTAAGTTAGATGTGAAATCCCCAGGCTCAACCTGGGAAGGTCATCTAAGACTGCGAATCTTGAATGCTTGAGAGGTTGGTGGAATTCCTGGTGTAGGAGTGAAATCCGTAGATATCAGGAGGAACATCGGAGGCGAAGGCGGCCAACTGGCGAAGCATTGACGCTGAGGTGCGAAAGCGTGGGTAGCAAACAGG` |
| ASV3416 | 424 | `TAAGGAATATTGGTCAATGGTGGGAACACTGAACCAGCCATCCCGCGTGCAGGACGAAGGCCCTCTGGGTTGTAAACTGCTTTTACCAGGAAAGAAACGAGCGGGCTTGCCTGCTCCTGACGGTACCTGGGGAATAAGCACCGGCTAACTCCGTGCCAGCAGCCGCGGTAATACGGAGGGTGCGAGCGTTATCCGGAATCACTGGGTTTAAAGGGTGCGTAGGCGGCTTGATAAGTCAGGGGTGAAAGCTTCCCGCTCAACGGGAGAACTGCCCTTGATACTGTCAGGCTCGAATTGGGTTGAGGCAGGCGGAACGTGGCATGTAGCGGTGAAATGCTTAGATATGCCACAGAACACCGATTGCGAAGGCAGCCTGCCAAGCCTTGATTGACGCTGAGGCACGAAAGCGTGGGGAGCGAACAGG` |
| ASV865 | 429 | `TGGGGAATATTGCACAATGGGCGCAAGCCTGATGCAGCCATGCCGCGTGTGTGAAGAAGGCCTTCGGGTTGTAAAGCACTTTCAGCGAGGAGGAAGGGTTCGGTGTTAATAGCACCGTTCATTGACGTTACTCGCAGAAGAAGCACCGGCTAACTCCGTGCCAGCAGCCGCGGTAATACGGAGGGTGCAAGCGTTAATCGGAATTACTGGGCGTAAAGCGCACGCAGGCGGTTTGTTAAGTCAGATGTGAAATCCCCGAGCTTAACTTGGGAACTGCATTTGAAACTGGCAAGCTAGAGTCTTGTAGAGGGGGGTAGAATTCCAGGTGTAGCGGTGAAATGCGTAGAGATCTGGAGGAATACCGGTGGCGAAGGCGGCCCCCTGGACAAAGACTGACGCTCAGGTGCGAAAGCGTGGGGAGCAAACAGG` |
| ASV2502 | 429 | `TGGGGAATTTTGGACAATGGGGGCAACCCTGATCCAGCCATTCCGCGTGAGTGAAGAAGGCCTTCGGGTTGTAAAGCTCTTTCGCAAGGGAAGAAAACTTATCCTCTAACATAGGATGAGGTTGACGGTACCTTGATAAGAAGCACCGGCTAACTACGTGCCAGCAGCCGCGGTAATACGTAGGGTGCGAGCGTTAATCGGAATTACTGGGCGTAAAGCGTGCGCAGGCTGTTTTGTAAGTCAGATGTGAAATCCCCGAGCTCAACTTGGGAACTGCGTTTGAAACTACAAGACTAGAATATGTCAGAGGGGGGTAGAATTCCACGTGTAGCAGTGAAATGCGTAGAGATGTGGAGGAATACCAATGGCGAAGGCAGCCCCCTGGGATAATATTGACGCTCATGCACGAAAGCGTGGGGAGCAAACAGG` |
| ASV5415 | 430 | `TGGGGAATATTGCGCAATGGGCGAAAGCCTGACGCAGCAACGCCGCGTGAGTGAAGAAGGCCTTCGGGTTGTAAAGCTCTTTCGGCTGGGAAGAAGGGTGTGCGTGTGAATAATGCGTGCATTTGATGGTACCAGAAGAAGAAGCACCGGCTAACTTCGTGCCAGCAGCCGCGGTAATACGAAGGGTGCGAGCGTTGTTCGGAATTATTGGGCGTAAAGGGTTCGTAGGTGGAAAGATAAGTCAGATGTGAAATCCCCGAGCTTAACTTGGGACGTGCATTTGAAACTCTCTTTCTTGAGTGTTGGAGAGGGTAGTGGAATTGCTGGTGTAGGAGTGACATCCGTAGAGATCAGCAGGAACACCGGAGGCGAAAGCGACTGCCTGGCCAAACACTGACACTGAGGAACGAAAGCGTGGGGAGCAAACAGG` |
| ASV1640 | 404 | `TGAGGAATATTGGACAATGGAGGAAACTCTGATCCAGCCATGCCGCGTGAGTGACGAAGGCCTTCGGGTTGTAAAACTCTTTAAGTGGGGAAGATAATGACGGTACCCACAGAATAAGCACCGGCTAACTTCGTGCCAGCAGCCGCGGTAATACGAAGGGTGCTAGCGTTACTCGGAATCACTGGGCGTAAAGCGTGCGTAGGCGGAAATATAAGTCAGAGGTGAAATCCCGGAGCTTAACTTCGGAATTGCCTTTGAAACTGTATTTCTTGAGTATGGTAGAGGATGGTGGAATTCCTAGTGTAGAGGTGAAATTCGTAGATATTAGGAGGAACACCGGTGGCGAAGGCGGCCATCTGGGCCATCACTGACGCTGAGGCACGAAAGCGTGGGTAGCAAACAGG` |
| ASV3613 | 429 | `TGGGGAATTTTGGACAATGGGCGCAAGCCTGATCCAGCAATGCCGCGTGAGTGAAGAAGGCCTTCGGGTTGTAAAGCTCTTTTGTCAGGGAAGAAACGGTGGTGGCTAATACCTACTGCTAATGACGGTACCTGAAGAATAAGCACCGGCTAACTACGTGCCAGCAGCCGCGGTAATACGTAGGGTGCAAGCGTTAATCGGAATTACTGGGCGTAAAGCGTGCGCAGGCGGTTATATAAGTCAGATGTGAAATCCCCGGGCTCAACCTGGGAACTGCATTTGAGACTGTATGGCTAGAGTGTGTCAGAGGGGGGTAGAATTCCACGTGTAGCAGTGAAATGCGTAGATATGTGGAGGAATACCGATGGCGAAGGCAGCCCCCTGGGATAACACTGACGCTCATGCACGAAAGCGTGGGGAGCAAACAGG` |
| ASV3085 | 429 | `TGGGGAATTTTGGACAATGGGCGCAAGCCTGATCCAGCAATGCCGCGTGAGTGAAGAAGGCCCTTGGGTTGTAAAGCTCTTTCGTTTGGGAAGAAGGAAGTGTGGTTAATACCCATACTGACAGACGGTACCAAAATAAGAAGCACCGGCTAACTACGTGCCAGCAGCCGCGGTAATACGTAGGGTGCAAGCGTTAATCGGAATGACTGGGCGTAAAGGGTGTGTAGGCGGTTTATTAAGTCTGATGTGAAAGCCCCGGGCTCAACCTGGGAACTGCGTTGGATACTGGTAAACTAGAGTACGTAAGAGGGGGGTGGAATTCCACGTGTAGCAGTGAAATGCGTAGAGATGTGGAGGAATACCGATGGCGAAGGCAACCCCCTGGGACGATACTGACGCTGAGGCACGAAAGCGTGGGGAGCAAACAGG` |
| ASV219 | 423 | `TAGGGAATATTGGGCAATGGACGAAAGTCTGACCCAGCCATGCCGCGTGCAGGATGAAGGCCCTCTGGGTTGTAAACTGCTTTTGACAGGGAAGAAATAGACCATGCGTGGTGTGCTGACGGTACCTGTAGAATAAGCACCGGCTAACTCCGTGCCAGCAGCCGCGGTAATACGGAGGGTGCAAGCGTTGTCCGGATTTATTGGGTTTAAAGGGTGCGTAGGCGGTTTTGTAAGTCAGTGGTGAAAGCTAGCAGCTTAACTGTTAAACTGCCATTGATACTGCAGAGCTTGAGTTCAGATGAGGTAGGCGGAATTTATAGTGTAGCGGTGAAATGCTTAGATACTATAAAGAACTCCGATAGCGTAGGCAGCTTACTAAACTGATACTGACGCTGAGGCACGAAAGTGTGGGGATCAAACAGG` |
| ASV4757 | 407 | `TTAGGAATCTTGCGCAATGGGGGCAACCCTGACGCAGCGACGCCGCGTGGAGGAAGAAGGTTCTCGGATCGTAAACTCCTTTTATATGGGAAGAATAACTGACGGTACCATATGAATAAGCACCTGCTAACTACGTGCCAGAAGCATCGGTGATGCGTAGGGTGCAAACATTATCCGGATTTATTGGGCGTAAAGAGATGCGTAGGCGTTTTTGTAAGTCTTGTGTCAAAGGCCATTGCTCAACAATGGAAAGGCACTGGATACTGCATGAATAGAGAAAGGAAGAGGTGAGCGGAATTCCCGGTGGAGCGGTGAAATGCGTTGATATCGGGAGGAACACCAGAGGCGAAAGCGGCTCACTAGTCCTTTTCTGACGCTAAGGCTCGAAAGCGTGGGTAGCAAAACGG` |
| ASV4657 | 423 | `TAGGGAATATTGGACAATGGGTGAGAGCCTGATCCAGCCATGCCGCGTGCAGGAAGACGGCCTTCTGGGTTGTAAACTGCTTTTATCTGGGGATAAAAAGGTCTTGCGAGACCAACTGAAGGTACCAGATGAATAAGCCACGGCTAACTACGTGCCAGCAGCCGCGGTAATACGTAGGTGGCAAGCGTTGTCCGGATTTATTGGGTTTAAAGGGTGCGTAGGCGGTCTATTAAGTCAGTGGTGAAATACTTCAGCTCAACTGGAGAGGTGCCATTGATACTGATGGACTTGAGTGCAGTCGAGGTAGGCGGAATGGACGGTGTAGCGGTGAAATGCATAGATATCGTCCAGAACACCGATAGTGAAGACAGCTTACCAGGCTGTAACTGACGCTGAGGCACGAAAGTGTGGGGATCAAACAGG` |
| ASV3723 | 428 | `TCGAGAATTTTTCTCAATGGGGGAAACCCTGAAGGAGCGACGCCGCGTGGAGGATGAAGGTCTTCGGATTGTAAACTCCTGTCATTGGGGAACAAAGCTTCCGGTTAACTGCCGGCGAGTCTGATAGTACCCGAAGAGGAAGAGACGGCTAACTCTGTGCCAGCAGCCGCGGTAATACAGAGGTCTCAAGCGTTGTTCGGATTCATTGGGCGTAAAGGGTGCGTAGGCGGCGAGGTAAGTCGGATGTGAAATCCCGGGGCTCAACCCCGGAACTGCATTCGATACTGCCTTGCTCGAGTACTGGAGAGGAGACTGGAATTTACGGTGTAGCAGTGAAATGCGTAGATATCGTAAGGAAGACCAGTGGCGAAGGCGAGTCTCTGGACAGTTACTGACGCTGAGGCACGAAGGCCAGGGGAGCAAACGGG` |
| ASV510 | 429 | `TGGGGAATTTTGGACAATGGGCGCAAGCCTGATCCAGCAATGCCGCGTGCAGGATGAAGGCCTTCGGGTTGTAAACTGCTTTTGTATGGAACGAAACGGCTCTGGTTAATACCCGGGGCTAATGACGGTACCGTAAGAATAAGCACCGGCTAACTACGTGCCAGCAGCCGCGGTAATACGTAGGGTGCAAGCGTTAATCGGAATTACTGGGCGTAAAGCGTGCGCAGGCGGTTATGTAAGACAGTCGTGAAATCCCCGGGCTCAACCTGGGAACTGCGATTGTGACTGCATAGCTAGAGTACGGTAGAGGGGGGTGGAATTCCGCGTGTAGCAGTGAAATGCGTAGATATGCGGAGGAACACCAATGGCGAAGGCAGCCCCCTGGACCTGTACTGACGCTCATGCACGAAAGCATGGGGAGCAAACAGG` |
| ASV4605 | 424 | `TGGGGAATTTTGGACAATGGGGGCAACCCTGATCCAGCAACGCCGCGTGAAGGATGAAGGTCTTCGGATCGTAAACTTCTTTTGTAGGGGACGAATTCTCTGTTGAATAAACAGAATTGACGGTACTCTACGAATAAGCCACGGCTAACTTCGTGCCAGCAGCCGCGGTAATACGAAGGTGGCAAGCGTTACTCGGAATTACTAGGCGTAAAGCGCAGGTAGGTGGTTAGGTAAGTCTGTAATGAAATCTCCCGGCTTAACTGGGAGGGGTTTATAGATACTGCTTAACTCGAGTGTGGGAGAGGTAACTGGAATTCCTGGTGTAGCGGTGAAATGCGTAGATATCAGGAGGAACACCAATGGCGAAAGCAGGTTACTGGCCCAATACTGACACTGAGCTGCGAAAGCTAGGGGAGCAAACAGG` |
| ASV5430 | 404 | `TTGGGAATCTTGGACAATGGGCGAAAGCCTGATCCAGCCATGCCGCGTGAGTGATGAAGGCCTTAGGGTTGTAAAGCTCTTTTACCCGGGAAGATAATGACGGTACCGGGAGAATAAGCTCCGGCTAACTTCGTGCCAGCAGCCGCGGTAATACGAAGGGGGCTAGCGTTGTTCGGAATTACTGGGCGTAAAGCGTGCGCAGGCGGCTTTTCAAGTCAGGGGTGAAAGCCCAGAGCTCAACTCTGGAATTGCCTTTGAAACTGTCAAGCTCGAGTGCGGGAGAGGTGAGTGGAATTCCCAGTGTAGAGGTGAAATTCGTAGATATTGGGAAGAACACCGGTGGCGAAGGCGGCTCACTGGCCCGTTTCTGACGCTCATGCACGATAGCGTGGGGATCAAACAGG` |
| ASV2411 | 404 | `TGGGGAATATTGCACAATGGGGGAAACCCTGATGCAGCGACGCCGCGTGAAGGAAGAAGTATTTCGGTATGTAAACTTCTATCAGCAGGGAAGATATTGACGGTACCTGACTAAGAAGCCCCGGCTAACTACGTGCCAGCAGCCGCGGTAATACGTAGGGGGCAAGCGTTATCCGGATTTACTGGGTGTAAAGGGAGCGTAGACGGCAGCATAAGTCTGATGTGAAAGCCCAAGGCTCAACCTTGGGACTGCATTGGAAACTATGCGGCTAGAGTGTCGGAGAGGTAAGTGGAATTCCTAGTGTAGCGGTGAAATGCGTAGATATTAGGAGGAACATCAGTGGCGAAGGCGACTTACTGGACGATAACTGACGTTGAGGCTCGAAAGCGTGGGGAGCAAACAGG` |
| ASV1083 | 417 | `TTAGGAATCTTCCACAATGGGGGCAACCCTGATGGAGCGACGCCGCGTGAGGGATGAAGGTTTTCGGATCGTAAACCTCTGAATCTGGGACGAAAGACGCGACGAGCGGGATGACGGTACCAGAGTAATAGCACCGGCTAACTCCGTGCCAGCAGCCGCGGTAATACGGAGGGTGCAAGCGTTACCCGGAATCACTGGGCGTAAAGGGCGTGTAGGCGGGAACATAAGTCTGGCTTTAAAGCCCACGGCTCAACCGTGGAGATGGGCTGGGTACTGTGTTTCTAGACCTCTGGAGAGAGAACTGGAATTCCTGGTGTAGCGGTGGAATGCGTAGATACCAGGAGGAACACCAATGGCGAAGGCAGGTTCTTGGACAGAAGGTGACGCTGAGGCGCGAAAGTGTGGGGAGCGAACCGG` |
| ASV3700 | 429 | `TGGGGAATTTCGGACAATGGGGGCAACCCTGATCCAGCAACGCCGCGTGAATGAAGAAGGCCTTAGGGTTGTAAAGTTCTTTTATCTAGGAGCAAAGCCTGTTGGTGAATAGCTAATGGGGATGAGAGTACTAGAGGAATAAGCACCGGCTAACTACGTGCCAGCAGCCGCGGTAATACGTAGGGTGCGAGCGTTAATCGGAATTACTGGGCGTAAAGCGTGCGCAGGCGGATGTGTAAGTTAGATGTGAAATCCCCGAGCTCAACTTGGGAATGGCATTTAAGACTGCATGTCTAGAGTTTGTCAGAGGGGGGTAGAATTCCAAGTGTAGCAGTGAAATGCGTAGAGATTTGGAGGAATACCGGTGGCGAAGGCGGCCCCCTGGGATAGAACTGACGCTCATGCACGAAAGCGTGGGTAGCAAACAGG` |
| ASV4983 | 404 | `TGGGGAATCTTGGACAATGGGGGCAACCCTGATCCAGCCATGCCGCGTGAGCGATGAAGGCCTTAGGGTTGTAAAGCTCTTTCAGCGGGGAAGATAATGACGGTACCCGCAGAAGAAGCCCCGGCTAACTCCGTGCCAGCAGCCGCGGTAATACGGAGGGGGCTAGCGTTGTTCGGAATTACTGGGCGTAAAGCGCACGTAGGCGGACACTTAAGTCAGGGGTGAAATCCCGGGGCTCAACCCCGGAACTGCCTTTGATACTGGGTGTCTGGAGGTCGAGAGAGGTGAGTGGAATTCCGAGTGTAGAGGTGAAATTCGTAGATATTCGGAGGAACACCAGTGGCGAAGGCGGCTCACTGGCTCGATACTGACGCTGAGGTGCGAAAGCGTGGGGAGCAAACAGG` |
| ASV2959 | 424 | `TGAGGAATATTGGACAATGGGCGCAAGCCTGATCCAGCCATGCCGCGTGCAGGATGAAGCATCTATGGTGTGTAAACTGCTTTTGTACGAGAAGAAACACTCCAACGTGTTGGGGCTTGACGGTATCGTAAGAATAAGGATCGGCTAACTCCGTGCCAGCAGCCGCGGTAATACGGAGGATCCAAGCGTTATCCGGAATCATTGGGTTTAAAGGGTCCGTAGGCGGGCTAGTAAGTCAGTGGTGAAATCTCTCGGCTCAACCGGGAAATGGCCATTGATACTGCTAGTCTTGAATTATTAGGAAGTAACTAGAATATGTAGTGTAGCGGTGAAATGCTTAGAGATTACATGGAATACCAATTGCGAAGGCAGGTTACTACTAATGGATTGACGCTGATGGACGAAAGCGTGGGTAGCGAACAGG` |
| ASV4901 | 429 | `TGGGGAATTTTGGACAATGGGCGAAAGCCTGATCCAGCCATGCCGCGTGAGTGAAGAAGGCCTTCGGGTTGTAAAGCTCTTTCGGACAGAAAGAAATCGCATGAACTAATACTTTGTGCGGATGACGGTACTGTAAGAAGAAGCACCGGCTAACTACGTGCCAGCAGCCGCGGTAATACGTAGGGTGCGAGCGTTAATCGGAATTACTGGGCGTAAAGGGTGCGCAGGCGGTTATATAAGACAGATGTGAAATCCCCGGGCTCAACCTGGGAACTGCGTTTGTGACTGTATGACTAGAGTACGGTAGAGAGGGGTGGAATTCCTGGTGTAGCAGTGAAATGCGTAGATATCAGGAGGAACATCGATGGCGAAGGCAGCCCCTTGGACCTGTACTGACGCTCATGCACGAAAGCGTGGGGAGCAAACAGG` |
| ASV832 | 429 | `TGGGGAATTTTGGACAATGGGCGCAAGCCTGATCCAGCCATGCCGCGTGCGGGATGAAGGCCTTCGGGTTGTAAACCGCTTTTGTCAGGGAAGAAACGCTCCGAGCGAATACCTCGGGGTAATGACGGTACCTGAAGAATAAGCACCGGCTAACTACGTGCCAGCAGCCGCGGTAATACGTAGGGTGCAAGCGTTAATCGGAATTACTGGGCGTAAAGCGTGCGCAGGCGGTCTTGTAAGACAGAGGTGAAATCCCCGGGCTCAACCTGGGAACTGCCTTTGTGACTGCAAGGCTTGAGTGCGGCAGAGGGGGATGGAATTCCGCGTGTAGCAGTGAAATGCGTAGATATGCGGAGGAACACCGATGGCGAAGGCAATCCCCTGGGCCTGCACTGACGCTCATGCACGAAAGCGTGGGGAGCAAACAGG` |
| ASV1883 | 406 | `TGGGGAATTTTCCGCAATGGGCGCAAGCCTGACGGAGCAATACCGCGTGAGGGAAGACGGCTTTTGGGTTGTAAACCTCTTTTATTCGGGAAGAAGATCTGACGGTACCGAATGAATCAGCATCGGCTAACTCCGTGCCAGCAGCCGCGGTAATACGGAGGATGCAAGCGTTATCCGGAATTATTGGGCGTAAAGGGTCCGCAGGTGGTCATTCAAGTCTGCTGTTAAAGCGTGGAGCTCAACTCCATATCGGCAGTGGAAACTGAGTGACTAGAGTTCGGTAGGGGTAGCGGGAATTCCCAGTGTAGCGGTGAAATGCGTAGATATTGGGAAGAACACCGGCGGCGAAGGCGCGCTACTGGGCCGAAACTGACGCTGAGGGACGAAAGCTAGGGGAGCGAAAGGG` |
| ASV5352 | 407 | `TCGAGAATATTCCACAATGGGCGAAAGCCTGATGGAGCGACGCCGCGTGCAGGAAGAAGGCCTTCGGGTTGTAAACTGCGGTAATCCGCCAACAAGCTTCGGCTGAGTGGCGAATGGAAAGAGGTTGGTAACTACGTGCCAGCACCCGCGGTAATACGTAGACCTCGAGCGTTGTCCGGATTTATTGGGCGTAAAGGGTGTGTAGGGGGCTCTGCGCGTCTCATGTTAAATCTTATGGCTTAACCATAGGGCTGCATGGGATACGGCAGTGCTAGAGGGCGCTAGAGGTGAATAGAACGCACGGTGTAGGGGTGAAATCCGTTGATATCGTGCGGAATACCAAAGGCGAAGGCAGTTCACTGGGGTGCACCTGACCCTGAAACACGAAAGCGTGGGGAGCAAAAAGG` |
| ASV151 | 406 | `TGGGGAATATTGGACAATGGGCGAAAGCCTGATCCAGCAATACCGAGTGGGTGATGAAGGCCTTAGGGTTGTAAAGCCCTTTTCAGCAGGGAAGATAATGACGGTACCTGACCAAGAAAGCCCCGGCTAACTCCGTGCCAGCAGCCGCGGTAAGACGGAGGGGGCTAGCGTTGTTCGGAATTACTGGGCGTAAAGAGTGCGTAGGCGGTTTAGTAAGTTGGAAGTGAAAGCCCGAGGCTTAACCTCGGAACTGCTTTCAAAACTACTAATCTAGAGTGTAGTAGGGGATGATGGAATTCCTAGTGTAGAGGTGAAATTCTTAGATATTAGGAGGAACACCGGTGGCGAAGGCGGTCATCTAGGCTACAACTGACGCTGATGCACGAAAGCGTGGGGAGCAAACAGG` |
| ASV4059 | 430 | `TAGGGAATATTGCGCAATGGAGGAAACTCTGACGCAGCGACGCCGCGTGGGTGATGAAGGTCTTCGGATCGTAAAGCCCTGTCGGAAGGGACGAAAAACGATATGGCTAATATCCATATCGCTTGACGGTACCTTTAAAGGAAGCACCGGCTAACTTCGTGCCAGCAGCCGCGGTAATACGAAGGGTGCAAGCGTTGTTCGGAATCATTGGGCGTAAAGCGAGTGTAGGCTGCTAATTAAGTCAGTTGTGAAATCCCTGGGCTCAACCCAGGAAGTGCATCTGAAACTAGTTAGCTAGAAGATGGTAGAGGAAGGTGGAATTCCAAGTGTAGAGGTGAAATTCGTAGATATTTGGAGGAATACCTGAGGCGAAGGCGGCCTTCTGGGCCATTCTTGACGCTGAGACTCGAAAGCGTGGGTAGCAAACAGG` |
| ASV4956 | 429 | `TGGGGAATATTGGACAATGGGCGCAAGCCTGATCCAGCAATGCCGCGTGTGTGAAGAAGGCCTGCGGGTTGTAAAGCACTTTCGCCGGGGAGGAAGGTCTCCAACTTAATACGTTGGGGAATTGACGCTACCCGGATAAGAAGCACCGGCTAACTCCGTGCCAGCAGCCGCGGTAATACGGAGGGTGCAAGCGTTAATCGGAATTACTGGGCGTAAAGCGCGTGTAGGCGGTTGTTTAAGTTGGATGTGAAATCCCCGGGCTTAACCTGGGAACTGCATTCAATACTGGGCAACTGGAGTCTGAGAGAGGAGGGTGGAATTTCCGGTGTAGCAGTGAAATGCGTAGAGATCGGAAGGAACACCAGTGGCGAAGGCGGCCCTCTGGCTCAAGACTGACGCTGAGACGCGAAAGCGTGGGGAGCAAACAGG` |
| ASV3707 | 404 | `TGGGGAATATTGCACAATGGGGGAAACCCTGATGCAGCAACGCCGCGTGAGTGATGACGGTCTTCGGATTGTAAAGCTCTGTCTTTAGGGACGATAATGACGGTACCTAAGGAGGAAGCCACGGCTAACTACGTGCCAGCAGCCGCGGTAATACGTAGGTGGCAAGCGTTGTCCGGATTTACTGGGCGTAAAGGGAGCGTAGGTGGATATTTAAGTGGGGTGTGAAATACCCGGGCTTAACCTGGGTGCTGCATTCCAAACTGGATATCTAGAGTGCAGGAGAGGAAAGGAGAATTCCTAGTGTAGCGGTGAAATGCGTAGAGATTAGGAAGAATACCAGTGGCGAAGGCGCCTTTCTGGACTGTAACTGACACTGAGGCTCGAAAGCGTGGGGAGCAAACAGG` |
| ASV5389 | 404 | `TGGGGAATATTGGACAATGGGGGCAACCCTGATCCAGCAATGCCGCGTGAGTGAAGAAGGCCTTAGGGTTGTAAAGCTCTTTTGGCGGGGAAGATGATGACGGTACCCGCAGAATAAGTCCCGGCTAACTCCGTGCCAGCAGCCGCGGTAATACGGAGGGGGCAAGCGTTGTTCGGAATTACTGGGCGTAAAGGGCGTGTAGGCGGTATATCAAGTCAGGCGTGAAAGCCCCGGGCTCAACCTGGGATGTGCGTTTGATACTGATAGACTGGAGATCGGGAGAGGAAAGCAGAATTTCCAGTGTAGAGGTGAAATTCGTAGATATTGGAAGGAATACCAGTGGCGAAGGCGGCTTTCTGGACCGAATCTGACGCTGAGGCGCGAAAGCGTGGGGAGCAAACAGG` |
| ASV194 | 429 | `TGGGGAATCTTGGACAATGGGCGCAAGCCTGATCCAGCAATGCCGCGTGCGTGAAGAAGGCCTTCGGGTTGTAAAGCGCTTTTGTTCGGGAGGAAATCCTGGTGGTTAATACCTGCCGGGGATGACAGTACCGGAAGAATAAGGACCGGCTAACTACGTGCCAGCAGCCGCGGTAATACGTAGGGTCCAAGCGTTAATCGGAATTACTGGGCGTAAAGGGTGCGCAGGTGGTTGATTAAGTGTGATGTGAAAGCCCCGGGCTCAACCTGGGAATTGCATTGCAAACTGGTCAACTAGAGTATGGCAGAGGGGGGTGGAATTCCGCGTGTAGCAGTGAAATGCGTAGAGATGCGGAGGAACACCGATGGCGAAGGCAACCCCCTGGGCTAATACTGACACTCATGCACGAAAGCGTGGGGAGCAAACAGG` |
| ASV2242 | 430 | `TAGGGAATATTGCGCAATGGGGGAAACCCTGACGCAGCAACGCCGCGTGAGTGAGGAAGGTCTTCGGATTGTAAAACTCTTTTATTTGGGAAAAATGTCCATATGGCTAATATCCATGTGGAGTGATGGTACCAAAGGAATAAGGACCGGCTAACTTCGTGCCAGCAGCCGCGGTAATACGAAGGGTCCAAGCGTTGTTCGGAATCATTGGGCGTAAAGCGCGCGCAGGCGGATCAGTAAGTCAGATGTGAAATTTCGAGGCTCAACCTCGAGACTGCGTCTGAAACTACTAGTCTAGAATGTCGGAGGGGGTAGGGGAATTTCACGTGTAGGGGTAAAATCCGTAGAGATGTGAAGGAACACCGGAGGCGAAGGCGCCTGCCTAGACGACTATTGACGCTGAGGCGCGAAAGCGTGGGGAGCAAACAGG` |
| ASV5058 | 404 | `TAGGGAATCTTGCGCAATGGGGGAAACCCTGACGCAGCAACGCCGCGTGAGTGATGAAGGCTTTCGGGTCGTAAAGCTCTGTCGTATGGGGCAATTATGATGGTACCATACAAGAAAGGACCGGCCAACTTCGTGCCAGCAGCCGCGGTAATACGGGGGGTCCTAGCGTTATTCGGAATCACTGGGCGTAAAGCGCATGTAGGTGGACTTACAAGTCACATGTGAAAGCCCCGGGCTTAACCTGGGAAGGGCATGTGATACTGTTTGTCTTGAGTACTAAAGAGGATAGTGGAATTTCTGGTGTAGTGGTGAAATACGTAGATATCAGAAGGAACATCGGTGGCGAAGGCGGCTATCTGGTTAGATACTGACACTGAGATGCGAAAGCGTGGGGAGCAAACAGG` |
| ASV112 | 429 | `TGGGGAATCTTGGACAATGGGCGAAAGCCTGATCCAGCAATGCCGCGTGCGTGAAGAAGGCCTTCGGGTTGTAAAGCGCTTTTGTCGGGGAGGAAATCCTAGTGGTTAATAACTGCTGGGGATGACAGTACCCGAAGAATAAGGACCGGCTAACTACGTGCCAGCAGCCGCGGTAATACGTAGGGTCCAAGCGTTAATCGGAATTACTGGGCGTAAAGCGTCCGCAGGTGGCTTGATAAGATAGACGTGAAATCCCTGGGCTCAACCTAGGAATTGCGTTTATGACTGTCTCGCTAGAGTATGGGAGAGGGGGGTGGAATTCCGCGTGTAGCAGTGAAATGCGTAGAGATGCGGAGGAACACCGATGGCGAAGGCAACCCCCTGGCCTAATACTGACACTCATGGACGAAAGCGTGGGGAGCAAACAGG` |
| ASV2009 | 404 | `TGGGGAATATTGGACAATGGGCGAAAGCCTGATCCAGCAATGCCGCGTGAGTGATGAAGGCCTTAGGGTTGTAAAGCTCTTTTACTGGGGATGATAATGACAGTACCCAGAGAATAAGCCCCGGCTAACTCCGTGCCAGCAGCCGCGGTAATACGGAGGGGGCTAGCGTTGTTCGGAAATACTGGGCGTAAAGCGCACGTAGGCGGCTCAGCAAGTCAGAGGTGAAATCCCAGGGCTCAACCCTGGAACTGCCTTTGAAACTGCTAGGCTAGAATCATGGAGAGGTCAGTGGAATTCCGAGTGTAGAGGTGAAATTCGTAGATATTCGGAAGAACACCAGTGGCGAAGGCGACTGACTGGACATGTATTGACGCTGAGGTGCGAAAGCGTGGGGAGCAAACAGG` |
| ASV753 | 430 | `TGGGGAATATTGGACAATGGGGGCAACCCTGATCCAGCAATGCCGCGTGTGTGAAGAAGGCCTTCGGGTTGTAAAGCACTTTTGTTCGGGGCGAAATCTTACGAATTAATACCTCGTGAGGATGACGGTACCGAAAGAATAAGCACCGGCTAACTTCGTGCCAGCAGCCGCGGTAATACGAAGGGTGCAAGCGTTAATCGGAATTACTGGGCGTAAAGCGTGCGTAGACGGCGCATTAAGTCAGCTGTGAAACCCCCGGGCTCAACCTGGGACCTGCAGCTGATACTGATGTGCTTGGAGTTTGAGAGAGGGTGGTGGAATTCCTGGTGTAGCGGTGAAATGCGTAGAGATCAGGAGGAACACTAGTGGCGAAGGCGGCCACCTGGCTTAAAACTGACGTTGAGGCACGAAAGCGTGGGGAGCAAACAGG` |
| ASV1935 | 425 | `TAGGGAATATTGGGCAATGGATGCAAGTCTGACCCAGCCATGCCGCGTGCAGGAAGACGGTCCTCTGGATTGTAAACTGCTTTTGACAGGGAAGAAAAAAAGGCATGCGTGCTTTATTGACGGTACCTGCAGAATAAGCCACGGCTAACTACGTGCCAGCAGCCGCGGTAATACGTAGGTGGCAAGCGTTGTCCGGATTTATTGGGTTTAAAGGGTGCGTAGGCGGCTTTATAAGTCAGTGGTAAAAGGTAGCAGCTTAACTGTTTTACACGCCATTGAAACTGTAGAGCTTGAGAGTTTTGGAGGATGATAGAATTTCTGATGTAGCGGTGAAATGCTTAGATATCAGGAGGAATACCAATTGCGAAGGCAGTCATCTGTGAAATTTCTGACGCTGAGGCACGAAAGTGCGGGTATCAAACAGG` |
| ASV3913 | 429 | `TGGGGAATTTTGGACAATGGGGGCAACCCTGATCCAGCCATTCCGCGTGAGTGAAGAAGGCCTTCGGGTTGTAAAGCTCTTTCGGCAGGGAAGAAACGGCGGGTGCTAATACCACCCGTTAATGACGGTACCTGAAGAAGAAGCACCGGCTAACTACGTGCCAGCAGCCGCGGTAATACGTAGGGTGCGAGCGTTAATCGGAATTACTGGGCGTAAAGCGTGCGCAGGCGGTTTTGTAAGACAGATGTGAAATCCCCGGGCTCAACCTGGGAACTGCGTTTGTGACTGCAAGACTCGAGTGCGGCAGAGGGGGGCGGAATTCCACGTGTAGCAGTGAAATGCGTAGAGATGTGGAGGAACACCGATGGCGAAGGCAGCCCCCTGGGTCGACACTGACGCTCATGCACGAAAGCGTGGGGAGCAAACAGG` |
| ASV4186 | 429 | `TGGGGAATATTGGACAATGGGGGCAACCCTGATCCAGCAATGCCGCGTGTGTGAAGAAGGCCTGAGGGTTGTAAAGCACTTTCAGTGGGGAGGAGGATTAGCGGGTTAAGAGCTGGCTAATTGGACGTTACCCACAGAAGAAGCACCGGCTAACTCCGTGCCAGCAGCCGCGGTAATACGGAGGGTGCGAGCGTTAATCGGAATTACTGGGCGTAAAGGGTGCGTAGGTGGTTAAATAAGTTAGATGTAAAATCCCTGGGCTTAACCTGGGAATGAGATTTAATACTGTTTGACTAGAGTATGGGAGAGGGTAGTGGAATTTCCGGTGTAGCGGTGAAATGCGTAGAGATCGGAAGGAACACCAGTGGCGAAGGCGGCTACCTGGCCTAATACTGACACTGAAGCACGAAAGCGTGGGGAGCAAACAGG` |
| ASV5545 | 405 | `TGGGGAATATTGGACAATGGGCGAAAGCCTGATCCAGCCACGCCGCGTGGGTGATGAAGGCCTTCGGGTTGTAAAGCCCTGTCGGGAGGGACGAATACTGACGGTACCTCCCAAGAAAGCACCGGCTAACTCCGTGCCAGCAGCCGCGGTAATACGGAGGGTGCAAGCGTTGCTCGGAATTACTGGGCGTAAAGCGTACGCAGGCGGCCTGATAAGTCGGGTGTGAAAGCCCTGGGCTCAACCCAGGAACTGCATCCGAAACTGCCAGGCTTGAGTTCGGGAGAGGGAAGTGGAATTCCTGGTGTAGAGGTGAAATTCGTTGATATCAGGAGGAACACCGGCGGCGAAAGCGGCTTCCTGGCCCGATACTGACGCTCATGTACGAAAGCGTGGGGAGCAAACAGG` |
| ASV4541 | 404 | `TGGGGAATATTGGACAATGGGCGAAAGCCTGATCCAGCAATGCCGCGTGTGTGAAGAAGGCCTGCGGGTTGTAAAGCACTTTCGGTAGGGAAGATAATGACGGTACCTACAGAAGAAGCACCGGCTAACTCTGTGCCAGCAGCCGCGGTAATACAGAGGGTGCGAGCGTTAATCGGAATTACTGGGCGTAAAGCGCGCGTAGGCGGTTTGACAAGTCGGGTGTGAAAGCCCCAGGCTCAACCTGGGAATTGCATTCGATACTATTTTACTAGAGTCTGGTAGAGGGAAGTGGAATTCTCGGTGTAGCGGTGAAATGCGTAGATATCGGGAGGAACATCAGTGGCGAAGGCGACTTCCTGGACCAAGACTGACGCTGAGGCGCGAAAGCGTGGGGAGCAAACAGG` |
| ASV173 | 404 | `TGGGGAATATTGGACAATGGGCGCAAGCCTGATCCAGCAATGCCGCGTGGGTGATGAAGGCCTTCGGGTTGTAAAGCCCTTTTGGCGGGGACGATGATGACGGTACCCGCCGAATAAGCTCCGGCTAACTTCGTGCCAGCAGCCGCGGTAATACGAAGGGAGCTAGCGTTGTTCGGAATTACTGGGCGTAAAGGGCGCGTAGGCGGTTTCGATAGTCGGGCGTGAAATCCCCAGGCTCAACCTGGGAATTGCGTTCGATACTGCGAGACTTGAGTGAGGGAGAGGATAGTGGAATTCCCAGTGTAGAGGTGAAATTCGTAGATATTGGGAAGAACACCAGTGGCGAAGGCGGCTATCTGGACCTCAACTGACGCTGAGGCGCGAAAGCGTGGGGAGCAAACAGG` |
| ASV2659 | 429 | `TGGGGAATTTTGGACAATGGGGGGAACCCTGATCCAGCAACGCCGCGTGAATGAAGAAGGCCTTAGGGTTGTAAAGTTCTTTTGTCAGGGAGCAAAGTCTAGCAGCTAATATCTGTTGGAGCTGAGAGTACATGAAGAATAAGCACCGGCTAACTACGTGCCAGCAGCCGCGGTAATACGTAGGGTGCGAGCGTTAATCGGAATTACTGGGCGTAAAGCGTGCGCAGGCGGATATGTAAGTTAGGAGTGAAATCCCCGGGCTCAACCTGGGAACTGCTTTTAAGACTGCATATCTAGAGTTTGTCAGAGGGGGGTGGAATTCCAAGTGTAGCAGTGAAATGCGTAGAGATTTGGAGGAACACCGATGGCGAAGGCAGCCCCCTGGGATAGAACTGACGCTCATGTACGAAAGCGTGGGTAGCAAACAGG` |
| ASV1624 | 429 | `TGGGGAATTTTGGACAATGGGCGCAAGCCTGATCCAGCCATGCCGCGTGAGTGAAGAAGGCCTTCGGGTTGTAAAGCTCTTTCGGCCGGGAAAAAATCGCTTGGGTTAATACCCTGAGTGGATGATGGTACCGGAATAAGAAGCACCGGCTAACTACGTGCCAGCAGCCGCGGTAATACGTAGGGTGCGAGCGTTAATCGGAATTACTGGGCGTAAAGCGTGCGCAGGCGGTTTTGTAAGACAGGCGTGAAATCCCCGGGCTCAACCTGGGAACTGCGTTTGTGACTGCAAGGCTAGAGTATGGCAGAGGGGGGTGGAATTCCACGTGTAGCAGTGAAATGCGTAGAGATGTGGAGGAACACCAATGGCGAAGGCAGCCCCCTGGGCCAATACTGACGCTCATGCACGAAAGCGTGGGGAGCAAACAGG` |
| ASV3323 | 429 | `TAGGGAATATTGCGCAATGGGGGAAACCCTGACGCAGCAACGCCGCGTGAGTGAGGAAGGTCTTCGGATTGTAAAACTCTTTTATTTGGGAAAAATGGTCATGGAGCTAATACCCCATGATTTGATGGTACCAAAGGAATAAGGACCGGCTAACTTCGTGCCAGCAGCCGCGGTAATACGAAGGGTCCAAGCGTTGTTCGGAATCATTGGGCGTAAAGCGCGCGCAGGCGGATCAGCAAGTCAGATGTGAAATCTCGAAGCTCAACTTCGAAACTGCGTCTGAAACTGCTAGTCTAGAATGTCGGAGGGGGCAGGGGAATTTCACGTGTAGGGGTAAAATCCGTAGAGATGTGAAGGAACACCGGAGGCGAAGGCGCCTGCCTGGACGACTATTGACGCTGAGGCGCGAAAGCGTGGGGAGCAAACAGG` |
| ASV476 | 424 | `TAGGGAATATTGGGCAATGGAGGCAACTCTGACCCAGCCATGCCGCGTGCAGGACGAAGGCTCTTTGAGTCGTAAACTGCTTTTATCGGGGAAGAACGGCTTTCTTGCGAGGAAGTGTGACGGTATCCGATGAATAAGCACCGGCTAACTCCGTGCCAGCAGCCGCGGTAATACGGAGGGTGCAAGCGTTGTCCGGATTTATTGGGTTTAAAGGGTGCGTAGGCGGGTGTTTAAGTCTGGTTTAAAATCAGGTCGCTCAACGATCTAACGTGGCTGGATACTGGACATCTTGAAATTGTTGGCCGCAGCCGGAATGGGTCATGTAGCGGTGAAATGCATAGATATGCCCCGGAACCCCGATTGCGAAGGCAGGCTGCGACGACTGATTTGACGCTGAGGCACGAGAGCATGGGTAGCAAACAGG` |
| ASV2150 | 404 | `TGGGGAATATTGGACAATGGGCGCAAGCCTGATCCAGCCATGCCGCGTGAGTGATGAAGGCCTTCGGGTTGTAAAGCTCTTTTGGCGGGGACGATGATGACGGTACCCGCAGAATAAGCCCCGGCAAACTTCGTGCCAGCAGCCGCGGTAAGACGAAGGGGGCAAGCGTTGTTCGGAATTACTGGGCGTAAAGGGCGCGTAGGCGGTCCATTAAGTCAGGCGTGAAAGCCCTGGGCTCAACCTGGGAGTGGCGCTTGATACTGGAGGACTAGAGAGTGGGAGAGGTTGGTGGAATTCCCAGTGTAGAGGTGAAATTCGTAGATATTGGGAAGAACACCGGTGGCGAAGGCGGCCAACTGGACCATTTCTGACGCTGAGGCGCGAAAGCGTGGGGAGCAAACAGG` |
| ASV4298 | 424 | `TAAGGAATATTGGACAATGGGCGCAAGCCTGATCCAGCCATGCCGCGTGAAGGATTAAGGTCCTCTGGATTGTAAACTTCTTTTATAAGGGACGAAAAAAGGGCATTCTTGCTCACTTGACGGTACCTTATGAATAAGCACCGGCTAACTCCGTGCCAGCAGCCGCGGTAATACGGAGGGTGCAAGCGTTATCCGGATTCACTGGGTTTAAAGGGTGCGTAGGCGGGTATTTAAGTCAGTGGTGAAATCCTGGAGCTTAACTCCAGAACTGCCATTGATACTATTTACCTTGAATATTGTGGAGGTAAGCGGAATATGTCATGTAGCGGTGAAATGCTTAGATATGACATAGAACACCTATTGCGAAGGCAGCTTACTACGCATATATTGACGCTGAGGCACGAAAGCGTGGGGATCAAACAGG` |
| ASV1722 | 406 | `TTGGGAATCTTGCACAATGGGGGAAACCCTGATGCAGCGACGCCGCGTGGACGATGAAGGCCTTAGGGTTGTAAAGTCCTTTTTTGGGGAAAGACTTAGGACGGTACCCCAAGAATAAGCTCCGGCTAACTACGTGCCAGCAGCCGCGGTAAGACGTAGGGAGCAAGCGTTGTCCGGATTTACTGGGCGTAAAGAGCGCGTAGGCGGCTCGTTAAGTGTGAAGTGAAATCTCCAGTGCTCAACACGGAAACTGCTTTACATACTGGCGAGCTTGAGGAAAGCAGAGGCAACTAGAATTCCTGGTGTAGCGGTGAAATGCGTTGATATCAGGAGGAATACCAATGGCGAAGGCAGGTTGCTGGGCTTTATCTGACGCTGAGGCGCGAAAGCGTGGGTAGCAAACAGG` |
| ASV2071 | 430 | `TGGGGAATATTGGACAATGGGCGAAAGCCTGATCCAGCAATACCGCGTGTGTGAAGAAGGCCTTCGGGTTGTAAAGCACTTTTGTTAGGGAGGATGATTGTTTAAGCGAATACCTTAAGCAGTTGACGTTACCTAAAGAATAAGCACCGGCTAACTCCGTGCCAGCAGCCGCGGTAATACGGAGGGTGCAAGCGTTAATCGGATTTACTGGGCGTAAAGGGTGCGTAGGCGGCCTATTAAGTCAGATGTGAAAGCCCTGGGCTCAACCTGGGAATTGCATTTGAAACTGGTAGGCTAGAATGTGGTAGAGGGTAGCGGAATTTCTGGTGTAGCGGTGAAATGCGTAGATATCAGAAGGAACATCAATGGCGAAGGCAGCTACCTGGGCCAACATTGACGCTGAGGCACGAAAGCGTGGGGAGCAAACAGG` |
| ASV4668 | 408 | `TCGAGAATCTTCCACAATGGACGAAAGTCTGATGGAGCGACGCCGCGTGATCGATGAAGTGCTTCGGTATGTAAAGATCTTTTATGGGGGAAGAAGTTTATTGACGGTACCCCATGAATAAGGGCCTGCAAAACTCGTGCCAGCAGCAGCGGTAATACGAGTGGCCCAAGCGTTATCCGGAATTATTGGGCGTAAAGGATATGTAGGTGGTTGTATTAGTCGAATGTCTAACCTCTGAGCTCAACTTGGAGACAGCATTCGAAACGGTACGGCTAGAGTATGTCAGGGGTAAGCGGAACTCATAGTGTAGGGGTGAAATCCGTTGATATTATGGGGAACACCAAAAGCGAAGGCAGCTTACTGGGACATTACTGACACTGAAATATGAAAGCGTGGGTAGCGAATGGG` |
| ASV3406 | 430 | `TGGGGAATCTTCCGCAATGGACGAAAGTCTGACGGAGCAACGCCGCGTGAGTGAAGAAGGTTTTCGGATCGTAAAACTCTGTTTTCAGGGACGAATGTGCAGATTGTAAATAATGATTTGTAATGACGGTACCTGAGTAGAAAGCCACGGCTAACTACGTGCCAGCAGCCGCGGTAATACGTAGGTGGCAAGCGTTGTCCGGAATTATTGGGCGTAAAGCGCGCGCAGGTGGGATATTAAGTCTGGTGTTAAAGTTCGGGGCTCAACCCCGTGAAGCATTTGGAAACTGATATTCTTGAGTGCAGGAGAGGAAAGTGGAATTCCTAGTGTAGCGGTGAAATGCGTAGATATTAGGAGGAACACCAGTGGCGAAGGCGACTTTCTGGACTGTGTCTGACACTGAGGCGCGAAAGCTAGGGTAGTGAACAGG` |
| ASV5472 | 407 | `TGGGGAATTTTACGCAATGGGCGAAAGCCTGACGTAGCGACACCGCGTGAGCGAAGAAGCCCCTTGGGGTGTAAAGCTCTGTCAGCTGGAACGAAAAAAATGACGGTACCAGCAGAGGAAGCATCGGCTAACTACGTGCCAGCAGCCGCGGTAAGACGTAGGATGCAAGCGTTGTCCGGATTTATTGGGCGTAAAGAGTTCGTAGGTGGTTTGTTAAGTTTGGTGTTAAAGATTGGGGCTCAACCCTGAAACTGCACTGAATACTGGCAGACTCGAGTGTGGTAGAGGCTAGTGGAATTCCCAGTGTAGCGGTGAAATGCGTAGATATTGGGAAGAACACCGGTGGCGTAGGCGACTAGCTGGGCCATAACTGACACTGAGGAACGAAAGCCAGGGGAGCGAATGGG` |
| ASV4830 | 428 | `TCGAGAATTTTTCACAATGGGCGAAAGCCTGATGGAGCGACGCCGCGTGGGGGATGAAGGGCTTCGGCTCGTAAACCCCTGTCATTCGAGAACAAACCCTGCAATTTAAAAGATTGCGGGCTGATAGTATCGGAAGAGGAAGGGACGGCTAACTCTGTGCCAGCAGCCGCGGTAATACAGAGGTCCCAAGCGTTGTTCGGATTTACTGGGCGTAAAGGGTGCGTAGGCGGCGAGGAAAGTTTGAGGTGAAATCTCCAGGCTCAACTTGGAAAATGCCTTGAAGACTTCCTTGCTCGAGGGTCGTAGAGGAGACTGGAATTCTCGGTGTAGCAGTGAAATGCGTAGATATCGAGAGGAACACCAGTGGCGAAAGCGGGTCTCTGGACGACACCTGACGCTGAGGCACGAAAGCCAGGGGAGCAAACGGG` |
| ASV886 | 429 | `TGGGGAATTTTGGACAATGGGCGCAAGCCTGATCCAGCAATGCCGCGTGCAGGATGAAGGCCTTCGGGTTGTAAACTGCTTTTGTACGGAGCGAAAAGGCTCCCTCTAATACAGGGGGCTCATGACGGTACCGTAAGAATAAGCACCGGCTAACTACGTGCCAGCAGCCGCGGTAATACGTAGGGTGCAAGCGTTAATCGGAATTACTGGGCGTAAAGCGTGCGCAGGCGGTTATATAAGACAGATGTGAAATCCCCGGGCTCAACCTGGGACCTGCATTTGTGACTGTATAGCTAGAGTACGGTAGAGGGGGATGGAATTCCGCGTGTAGCAGTGAAATGCGTAGATATGCGGAGGAACACCGATGGCGAAGGCAATCCCCTGGACCTGTACTGACGCTCATGCACGAAAGCGTGGGGAGCAAACAGG` |
| ASV726 | 429 | `TGGGGAATTTTGGACAATGGGCGAAAGCCTGATCCAGCCATTCCGCGTGAGTGAAGAAGGCCTTCGGGTTGTAAAGCTCTTTCGGCAGGGAAGAAACGGTCCTGGATAATACCTTGGGCTAATGACGGTACCTGAAGAAGAAGCACCGGCTAACTACGTGCCAGCAGCCGCGGTAATACGTAGGGTGCGAGCGTTAATCGGAATTACTGGGCGTAAAGCGTGCGCAGGCGGTTTTGTAAGACAGGTGTGAAATCCCCGGGCTTAACCTGGGAACTGCGCTTGTGACTGCAAGACTCGAGTACGGCAGAGGGGGGTAGAATTCCACGTGTAGCAGTGAAATGCGTAGAGATGTGGAGGAATACCGATGGCGAAGGCAGCCCCCTGGGTCGATACTGACGCTCATGCACGAAAGCGTGGGGAGCAAACAGG` |
| ASV170 | 429 | `TGGGGAATTTTGGACAATGGACGCAAGTCTGATCCAGCCATGCCGCGTGCGGGAAGAAGGCCTTCGGGTTGTAAACCGCTTTTGTCAGGGAAGAAATCTTCTGAACTAATACTTTGGGAGGATGACGGTACCTGAAGAATAAGCACCGGCTAACTACGTGCCAGCAGCCGCGGTAATACGTAGGGTGCAAGCGTTAATCGGAATTACTGGGCGTAAAGCGTGCGCAGGCGGTTATGCAAGACAGGTGTGAAATCCCCGAGCTCAACTTGGGAACTGCACTTGTGACTGCATAGCTAGAGTACGGTAGAGGGGAGTGGAATTCCGCGTGTAGCAGTGAAATGCGTAGATATGCGGAGGAACACCGATGGCGAAGGCAGCTCCCTGGACCTGTACTGACGCTCATGCACGAAAGCGTGGGGAGCAAACAGG` |
| ASV4112 | 407 | `TAGGGAATATTGCACAATGGAGGAAACTCTGATGCAGCGACGCCGCGTGAGTGACGAAGGCCTTCGGGTTGTAAAGCTCTGTTCTTAGGGAAAAAGAAAGTGATTGTACCTAAGGAGAAAGGACCGGCTAACTTCGTGCCAGCAGCCGCGGTAAGACGAGGGGTCCAAGCGTTGTTCGGAATTATTGGGCGTAAAGCGGGTGTAGGTGGCTCTATAAGTCAGGAGTGAAATCCCCGAGCTTAACTTGGGAAGTGCTTTTGATACTGCAGAGCTTGAATGTGGGAGAGGATAATAGAATTCCAGGTGTAGTGGTGAAATACGTAGATATCTGGAGGAATACCGGTGGCGAAGGCGGTTATCTGGCCCAACATTGACACTGAGACCCGAAAGCGTGGGGATCAAACAGG` |
| ASV4836 | 403 | `TGGGGAATATTGCGCAATGGGCGAAAGCCTGACGCAGCGACGCCGCGTGAGTGAAGAAGTCTTTCGGGATGTAAAACTCTGTCAGGTGGGAAGATAATGACGGTACCACCAGAGGAAGCCCCGGCTAACTACGTGCCAGCAGCCGCGGTAATACGTAGGGGGCGAGCGTTATCCGGATTCACTGGGCATAAAGCGCGCGTAGGCGGTTTGCTAAGTCAGATGTGAAATGTCTCGGCTCAACCGAGGCCTGCATTTGAAACTGGCGAACTAGAGATAAGGAGAGGAAAGCGGAACTCACGGTGTAGCGGTGAAATGCGTAGATATCGTGAGGAACATCAGTTGCGAAGGCGGCTTTCTGGACTTATTCTGACGCTGAGGCGCGAAAGCGTGGGGAGCAAACGGG` |
| ASV2715 | 428 | `TCGAGAATTTTTCTCAATGGGGGAAACCCTGAAGGAGCGACGCCGCGTGGAGGATGAAGGTCTTCGGATTGTAAACTCCTGTCATTTGGGAACAATTGTTGGCGATTAACTGTCGTCAACTTGATAGTACCAGAAGAGGAAGAGACGGCTAACTCTGTGCCAGCAGCCGCGGTAATACAGAGGTCTCAAGCGTTGTTCGGATTCATTGGGCGTAAAGGGTGCGTAGGTGGCGGGGTAAGTCTGGTGTGAAATCTCGGGGCTCAACCCCGAAACTGCACTGGATACTGCCTTGCTCGAGTACTGGAGAGGAGATTGGAATTTACGGTGTAGCAGTGAAATGCGTAGATATCGTAAGGAAGACCAGTGGCGAAGGCGAATCTCTGGACAGTTACTGACACTGAGGCACGAAGGCCAGGGGAGCAAACGGG` |
| ASV3620 | 424 | `TAAGGAATATTGGACAATGGTGGCAACACTGATCCAGCCATGCCGCGTGAAGGATGAAGGTCCTATGGATTGTAAACTTCTTTTATACGGGAAAAAACCTCTGAACGTGTTCGGAGCTGATGGTACCGTAAGAATAAGGATCGGCTAACTCCGTGCCAGCAGCCGCGGTAATACGGAGGATCCAAGCGTTATCCGGATTCATTGGGTTTAAAGGGTGCGTAGGCGGAATAGTAAGTCAGTGGTGAAAGCCTGCAGCTTAACTGCAGAATTGCCATTGATACTGCTAATCTAGAGTACATTTGATGTGGGCGGAATGTGTCGTGTAGCGGTGAAATGCTTAGATATGACACAGAACACCAATTGCGAAGGCAGCTCACAAAACTGTAACTGACGCTGAGGCACGAAAGCGTGGGGATCAAACAGG` |
| ASV4660 | 429 | `TGGGGAATATTGGACAATGGGCGCAAGCCTGATCCAGCCATGCCGCGTGTGTGAAGAAGGCCTTCGGGTTGTAAAGCACTTTTGTTCGGGAAGAAATCGGGCGGGTTAATACCCAGTCCGGATGACGGTACCGAAAGAATAAGCACCGGCTAACTTCGTGCCAGCAGCCGCGGTAATACGAAGGGTGCAAGCGTTACTCGGAATCACTGGGCGTAAAGCGTGCGTAGGCGGTTCGTTAAGTCTGCTGTGAAAGCCCTGGGCTCAACCTGGGAACTGCAGTGGATACTGGCGGACTAGAGTGTGATAGAGGATGGTGGAATTCCCGGTGTAGCGGTGAAATGCGTAGAGATCGGGAGGAACACCAGTGGCGAAGGCGGCCATCTGGATCAACACTGACGCTGAGGCACGAAAGCGTGGGGAGCAAACAGG` |
| ASV3674 | 429 | `CCGAGAATCTTTCGCAATGGGGGAAACCCTGACGAAGCGACGCCGCGTGCAGGATGAAGGTCTTCGGATTGTAAACTGCTGTCGGCTGGGAAAAAAGATCAAGTATTAACAGTATTTGATTCTGATGGTACCAGCAAAGGAAGCCACGGCTAACTCTGTGCCAGCAGCCGCGGTAATACAGAGGTGGCAAGCGTTGTTCGGATTTATTGGGCGTAAAGGGTCCGTAGGCGGTCTGAAAAGTCGGGTGTGAAAGCCCACAGCTTAACTGTGGAATTGCATTCGAAACTCTCAGACTCGAGTGTGAGAGGGGTAAAGGGAATTCCCGGTGTAAGGGTGAAATCTGCAGAGATCGGGAAGAACACCAGTCGCGAAGGCGCTTTACTGGCTCACAACTGACGCTGAGGGACGAAAGCTAGGGGAGCAAACGGG` |
| ASV5302 | 404 | `TGGGGAATATTGGACAATGGGGGAAACCCTGATCCAGCAATGCCGCGTGAGTGACGAAGGCCTTAGGGTTGTAAAGCTCTTTTACCCGTGAAGATAATGACGGTAGCGGGAGAATAAGCCCTGGCTAACTCTGTGCCAGCAGCCGCGGTAATACAGAGAGGGCAAGCGTTGTCCGGAATGACTGGGCGTAAAGAGCGCGTAGGCGGTTTTTTAAGTTGGATGTGAAATCCCTGGGCTTAACCTAGGAACTGCATTCAATACTGGGAAACTAGAGGATGAGAGAGGAAAGTGGAATGACGAGTGTAGAGGTGAAATTCGTAGATATTCGTCGGAACACCAGAGGCGAAGGCGGCTTTCTGGCTCATACCTGACGCTGAGGCGCGAAAGCGTGGGGAGCAAACAGG` |
| ASV4681 | 389 | `GTAGGAAACTTTTGCAATGCGCGCAAGCGTGACAAAGCAAGCCAGAGTGATTTTCTATAATTGAAAATCTTTTGCGGGATGTAAAAAGTCCCGCGAATAAGGACTGGGCAAGACTGGTGCCAGCCGCCGCGGTAATCCCAGCAGTCCGAGTCGCAGCCACATTTGTTGGGTCTAAAACATCCGTAGCTTGCCTTTCAAGTCTCTTGTGAAATCGGGCCTCTTAAGGGTCCGGCGAGCAAGAGATACTGTTTGGCTAGAGACCGGGAGACGTAAGGAGTACGATAGGAGTAGCGGTTAAATGCGTTGATCCTTATCGGACTAACAATAGCGAAGGCACCTTACGATAACGGTTCTGACAGTGAGGGATGAAGGCTAGGGGCGCAAAGTGG` |
| ASV1636 | 421 | `TAAGGAATATTGCGCAATGGGCGAAAGCCTGACGCAGCGACGCCGCGTGGGGGATGAAGGTCTTCGGATTGTAAACCCCTTTCGGGAGGGAAGATGGAATGGGGTAACCCATTCGGACGGTACCTCCAGAAGCAGCCACGGCTAACTTCGTGCCAGCAGCCGCGGTAATACGAAGGTGGCAAGCGTTATTCGGATTTACTGGGCGTACAGGGAGCGTAGGCGGTTCGGTAAGCCCTCTGTGAAATCTTCAGGCTTAACCTGGAAAAGTCGGAGGGGACTGCTGGGCTAGAGGACGGGAGAGGAGCGCGGAATTCCCGGTGTAGCGGTGAAATGCGTAGAGATCGGGAGGAAGGCCGGTGGCGAAGGCGGCGCTCTGGAACGTTTCTGACGCTGAGGCTCGAAAGCGTGGGGAGCAAACAGG` |
| ASV143 | 429 | `TGGGGAATTTTGGACAATGGACGCAAGTCTGATCCAGCCATGCCGCGTGCGGGAAGAAGGCCTTCGGGTTGTAAACCGCTTTTGTCAGGGAAGAAACGGGCGTCTCTAATACAGATGCCTAATGACGGTACCTGAAGAATAAGCACCGGCTAACTACGTGCCAGCAGCCGCGGTAATACGTAGGGTGCAAGCGTTAATCGGAATTACTGGGCGTAAAGCGTGCGCAGGCGGTTATGCAAGACAGATGTGAAATCCCCGGGCTCAACCTGGGAACTGCATTTGTGACTGCATGGCTAGAGTACGGTAGAGGGGGATGGAATTCCGCGTGTAGCAGTGAAATGCGTAGATATGCGGAGGAACACCGATGGCGAAGGCAATCCCCTGGACCTGTACTGACGCTCATGCACGAAAGCGTGGGGAGCAAACAGG` |
| ASV3140 | 423 | `TAAGGAATATTGGTCAATGGGCGGAAGCCTGAACCAGCCACGCCGCGTGAAGGAAGAAGGTGCTTCGCATCGTAAACTTCTTTTGTCAGGGAAGAAAAAGCTCCTGTGGGAGAAATTGCCGGTACCTGATGAATAAGCATCGGCTAACTACGTGCCAGCAGCCGCGGTAATACGTAGGATGCGAGCGTTATCCGGATTTATTGGGTTTAAAGGGTGCGTAGGCGGCTTCATAAGTCGTGGGTGAAAGTCAAACGCTTAACGTTTGGATTGCCTGCGATACTGTGAGGCTTGGATTCGGCGGAGGTAACTGGAATGGTAGATGTAGCGGTGAAATGCATAGATATCTGCTGGAACGCCAATTGCGAAGGCAAGTTACTGTACCGAGATTGACGCTGAGGCACGAAAGCATGGGGAGCAAACAGG` |
| ASV5367 | 405 | `TAGGGAATTTTCCACAATGGGCGCAAGCCTGATGGAGCAACGCCGCGTGCAGGATGAAGGCCTTCGGGTCGTAAACTGCTTTTTTCTGTGATGAATATGACAGTAACAGACGAATAAGGATCGGCTAACTCCGTGCCAGCAGCCGCGGTCATACGGAGGATCCAAGCGTTATCCGGAATTACTGGGCGTAAAGAGTTGCGTAGGTGGTTTGTTAAGTAAGGCATGAAATCGTGTGGCTCAACCATACGGCTATGCTTTAAACTGGCAGACTTGAAGATGAGAGAGGTTATTGGAATTCCCAGTGTAGGAGTGAAATCCGTAGATATTGGGAGGAACACCGATGGCGTAGGCAGATAACTGGCTCATTCTTGACACTAAGGCACGAAAGCGTGGGTAGCAAACGGG` |
| ASV3499 | 404 | `TGGGGAATATTGGACAATGGGCGCAAGCCTGATCCAGCCATGCCGCGTGGGTGAAGAAGGCCCTAGGGTTGTAAACCCCTTTCAGCGGGGAAGATAATGACGGTACCCGCAGAAGAAGCCCCGGCTAACTCCGTGCCAGCAGCCGCGGTAATACGGAGGGGGCTAGCGTTGTTCGGAATTACTGGGCGTAAAGCGCGCGTAGGCGGACCTGCAAGTCGGGGGTGAAAGCCCGAGGCTCAACCTCGGAACTGCCTTCGATACTGCGGGTCTCGAGTCCGGGAGAGGTGAGTGGAATTCCTAGTGTAGAGGTGAAATTCGTAGATATTAGGAAGAACACCAGTGGCGAAGGCGGCTCACTGGCCCGGTACTGACGCTGAGGCGCGAAAGCGTGGGGAGCAAACAGG` |
| ASV5331 | 423 | `TGGGGAATCTTGCGCAATGGGCGGAAGCCTGACGCAGCAACGCCGCGTGAGGGATGACGGCCTTCGGGTTGTAAACCTCTTTCGCGATCGACGAAGCTCCATCTTTTGGGTGGGGTGACGGTAGGTCGAGAAGAAGCACCGGCCAACTACGTGCCAGCAGCCGCGGTAATACGTAGGGTGCGAGCGTTGTCCGGAATTATTGGGCGTAAAGAGCTCGTAGGCGGTGTGTCGCGTCGGCCGTGAAAACCTGGGGCTTAACTCTGGGCGTGCGGTCGATACGGGCATCACTAGAGTTCGGCAGGGGAGACTGGAATTCCTGGTGTAGCGGTGAAATGCGCAGATATCAGGAGGAACACCGGTGGCGAAGGCGGGTCTCTGGGCCGATACTGACGCTGAGGAGCGAAAGCATGGGTAGCGAACAGG` |
| ASV27 | 429 | `TGGGGAATTTTGGACAATGGGCGAAAGCCTGATCCAGCAATGCCGCGTGCAGGACGAAGGCCTTCGGGTTGTAAACTGCTTTTGTACGGAACGAAACGGTCTCTTTTAATACAGGGGGCTAATGACGGTACCGTAAGAATAAGCACCGGCTAACTACGTGCCAGCAGCCGCGGTAATACGTAGGGTGCAAGCGTTAATCGGAATTACTGGGCGTAAAGCGTGCGCAGGCGGTTATATAAGACAGATGTGAAATCCCCGGGCTCAACCTGGGACCTGCATTTGTGACTGTATAGCTAGAGTACGGTAGAGGGGGATGGAATTCCGCGTGTAGCAGTGAAATGCGTAGATATGCGGAGGAACACCGATGGCGAAGGCAATCCCCTGGACCTGTACTGACGCTCATGCACGAAAGCGTGGGGAGCAAACAGG` |
| ASV148 | 409 | `TGGGGAATATTGCACAATGGGCGGAAGCCTGATGCAGCAACGCCGCGTGCGGGATGACGGCCTTCGGGTTGTAAACCGCTTTCGCCTGTGACGAAGCGTGAGTGACGGTAATGGGTAAAGAAGCACCGGCTAACTACGTGCCAGCAGCCGCGGTGATACGTAGGGTGCGAGCGTTGTCCGGATTTATTGGGCGTAAAGGGCTCGTAGGTGGTTGATCGCGTCGGAAGTGTAATCTTGGGGCTTAACCCTGAGCGTGCTTTCGATACGGGTTGACTTGAGGAAGGTAGGGGAGAATGGAATTCCTGGTGGAGCGGTGGAATGCGCAGATATCAGGAGGAACACCAGTGGCGAAGGCGGTTCTCTGGGCCTTTCCTGACGCTGAGGAGCGAAAGCGTGGGGAGCGAACAGG` |
| ASV533 | 404 | `TGGGGAATATTGGACAATGGGCGCAAGCCTGATCCAGCAATATCACGTGAATGATGAAGGCCTTAGGGTTGTAAAATTCTTTTAGTAGGGAAGATAGTGACGGTACCTACAGAAAAAGCACCGGCTAACTTCGTGCCAGCAGCCGCGGTAATACGAAGGGTGCTAGCGTTGTTCGGAATTATTGGGCGTAAAGCGTGCGTAGGCGGCATAGTAAGTCAGCTGTGAAAGCCCAGGGCTCAACCTTGGAATTGCGGTTGAAACTATTAAGCTAGAGTATGGCGGGGGATAGTGGAATTCTTAGTGTAGGGGTGAAATCCGTAGATATTAAGAGGAACACCAGTGGCGAAAGCGACTATCTACACCATTACTGACGCTGAGGTACGAAAGCGTGGGGAGCAAACAGG` |
| ASV3188 | 404 | `TGGGGAATATTGGACAATGGGGGCAACCCTGATCCAGCAATGCCGCGTGAATGATGAAGGCCTTCGGGTTGTAAAGTTCTTTTGTAGATGACGATAATGACGGTAGTCTACGAATAAGCACCGGCTAACTTCGTGCCAGCAGCCGCGGTAATACGAAGGGTGCTAGCGTTGTTCGGAATTACTGGGCGTAAAGGGTGCGTAGGCGGCCTTATAAGTGAGATGTGAAATACCTGGGCTTAACCTGGGAACTGCATTTCATACTGTAAGGCTAGAGTGGGCGAGAGGAAGATGGAATTGTGCGTGTAGAGGTGAAATTCGTAGATATGCACAAGAACACCAGTGGCGAAGGCGATCTTCTGGTACCTAACTGACGCTAATGCACGAAAGCGTGGGGAGCAAACAGG` |
| ASV993 | 429 | `TGGGGAATATTGGACAATGGGGGCAACCCTGATCCAGCAATGCCGCGTGTGTGAAGAAGGCCTGAGGGTTGTAAAGCACTTTCAGTGGGGAGGAGGATTATTGGGTTAAGAGCTAGATAATTGGACGTTACCCACAGAAGAAGCACCGGCTAACTCCGTGCCAGCAGCCGCGGTAATACGGAGGGTGCGAGCGTTAATCGGAATTATTGGGCGTAAAGGGTGCGTAGGTGGTTAGATAAGTTATCTGTGAAATCCCCGGGCTCAACCTGGGAAGGTCAGATAAGACTGTTTGACTCGAGTATAGGAGAGGGTAGTGGAATTTCCGGTGTAGCGGTGAAATGCGTAGAGATCGGAAGGAACACCAGTGGCGAAGGCGGCTACCTGGCCTGATACTGACACTGAAGCACGAAAGCGTGGGGAGCAAACAGG` |
| ASV4304 | 429 | `TGGGGAATATTGCACAATGGGCGCAAGCCTGATGCAGCCATGCCGCGTGTGTGAAGAAGGCCTTCGGGTTGTAAAGCACTTTCAGCGAGGAGGAAAGGGGATAACTTAATACGTTATTCCTGTGACGTTACTCGCAGAAGAAGCACCGGCTAACTCCGTGCCAGCAGCCGCGGTAATACGGAGGGTGCAAGCGTTAATCGGAATTACTGGGCGTAAAGCGCACGCAGGCGGTTTGTTAAGTCAGATGTGAAATCCCCGAGCTCAACTTGGGAACTGCATTTGAAACTGGCAAGCTAGAGTCTTGTAGAGGGGGGTAGAATTCCAGGTGTAGCGGTGAAATGCGTAGAGATCTGGAGGAATACCGGTGGCGAAGGCGGCCCCCTGGACAAAGACTGACGCTCAGGTGCGAAAGCGTGGGGAGCAAACAGG` |
| ASV1115 | 429 | `TGGGGAATATTGGACAATGGGGGCAACCCTGATCCAGCCATGCCGCGTGTGTGAAGAAGGCCTTCGGGTTGTAAAGCACTTTAGGTCGGGAGGAAGGTTCGCTGGCTAATATCCAGCGGAATTGACGTTACCGACAGAATAAGCACCGGCTAACTCCGTGCCAGCAGCCGCGGTAATACGGAGGGTGCAAGCGTTAATCGGAATTACTGGGCGTAAAGCGCGCGTAGGCGGCTTGGTAAGTCGGATGTGAAAGCCCTGGGCTCAACCTGGGAACTGCATTCGATACTGTCAAGCTAGAGTGTGGGAGAGGGTGGTGGAATTCCCGGTGTAGCGGTGAAATGCGTAGATATCGGGAGGAACATCAGTGGCGAAGGCGACCGCCTGGCCCAACACTGACGCTGAGGTGCGAAAGCGTGGGTAGCAAACAGG` |
| ASV1410 | 429 | `TGGGGAATTTTGGACAATGGGCGCAAGCCTGATCCAGCAATGCCGCGTGCAGGAAGAAGGCCTTCGGGTTGTAAACTGCTTTTGTCAGGGAAGAAATCCTGTGGGTTAATACCTCGTGGGGATGACGGTACCTGAAGAATAAGCACCGGCTAACTACGTGCCAGCAGCCGCGGTAATACGTAGGGTGCGAGCGTTAATCGGAATTACTGGGCGTAAAGCGTGCGCAGGCGGTTGTGCAAGACAGGTGTGAAATCCCCGGGCTTAACCTGGGAACTGCACTTGTGACTGCACGGCTAGAGTACGGTAGAGGGGGATGGAATTCCGCGTGTAGCAGTGAAATGCGTAGATATGCGGAGGAACACCGATGGCGAAGGCAATCCCCTGGACCTGTACTGACGCTCATGCACGAAAGCGTGGGGAGCAAACAGG` |
| ASV722 | 424 | `TAGGGAATATTGGACAATGGGGGCAACCCTGATCCAGCCATGCCGCGTGCAGGAAGACTGCCCTATGGGTTGTAAACTGCTTTTGTACGGGAAGAAACCTCACTACGTGTAGTGAGTTGACGGTACCGTGAGAATAAGGATCGGCTAACTTCGTGCCAGCAGCCGCGGTAATACGAAGGATCCAAGCGTTGTCCGGATTTACTGGGTTTAAAGGGTGCGTAGGCGGGCTATTAAGTCAGTGGTGAAAGCCTACAGCTTAACTGTAGAATTGCCATTGAAACTGATAGTCTTGAGTATGTTTGAGGTTACTGGAATATAACATGTAGCGGTGAAATGCTTAGATATGTTATAGAACACCAATTGCGAAGGCAGGTAACTAAGTCATAACTGACGCTGAGGCACGAAAGCGTGGGGAGCAAACAGG` |
| ASV1303 | 404 | `TGGGGAATATTGCACAATGGAGGAAACTCTGATGCAGCGACGCCGCGTGAGTGAAGAAGTATTTCGGTACGTAAAGCTCTATCAGCAAGGAAGAAAATGACGGTACTTGACTAAGAAGCCCCGGCTAACTACGTGCCAGCAGCCGCGGTAATACGTAGGGGGCAAGCGTTATCCGGATTTACTGGGTGTAAAGGGAGCGTAGACGGCATGGTAAGTCAGATGTGAAAGCCCGGGGCTCAACCCCGGGACTGCATTTGAAACTATCAGGCTAGAGTGCAGGAGAGGTAAGTGGAATTCCTAGTGTAGCGGTGAAATGCGTAGATATTAGGAGGAACACCGGTGGCGAAGGCGACTTACTGGACTGTAACTGACGTTGAGGCTCGAAAGCGTGGGGAGCAAACAGG` |
| ASV581 | 430 | `TGGGGAATTTTGGACAATGGGCGAAAGCCTGATCCAGCCATGCCGCGTGCAGGAAGAAGGCCTTCGGGTTGTAAACTGCTTTAGTTCGGGAAAAAGGGGTGCGGGGTTAATACCCTCGTGCTTTGATGGTACCGGAAGAATAAGCACCGGCTAACTACGTGCCAGCAGCCGCGGTAATACGTAGGGTGCGAGCGTTAATCGGAATTACTGGGCGTAAAGCGTGCGCAGGCGGTCCGTTAAGACAGTTGTGAAATCCCCGGGCTTAACCTGGGAACTGCAATTGTGACTGACGGGCTAGAGTGTGTCAGAGGGGGGTGGAATTCCACGTGTAGCAGTGAAATGCGTAGAGATGTGGAGGAACACCGATGGCGAAGGCAGCCCCCTGGGATAACACTGACGCTCATGCACGAAAGCGTGGGGAGCAAACAGG` |
| ASV1914 | 429 | `TGGGGAATTTTGGACAATGGGCGCAAGCCTGATCCAGCAATGCCGCGTGCAGGATGAAGGCCTTCGGGTTGTAAACTGCTTTTGTACGGAACGAAACGGCCTGTCCTAATACGATGGGCTAATGACGGTACCGTAAGAATAAGCACCGGCTAACTACGTGCCAGCAGCCGCGGTAATACGTAGGGTGCGAGCGTTAATCGGAATTACTGGGCGTAAAGCGTGCGCAGGCGGTTATATAAGACAGATGTGAAATCCCCGGGCTCAACCTGGGACCTGCATTTGTGACTGTATAGCTAGAGTACGGTAGAGGGGGATGGAATTCCGCGTGTAGCAGTGAAATGCGTAGATATGCGGAGGAACACCGATGGCGAAGGCAATCCCCTGGACCTGTACTGACGCTCATGCACGAAAGCGTGGGGAGCAAACAGG` |
| ASV3550 | 429 | `TAGGGAATCTTCGGCAATGGACGAAAGTCTGACCGAGCAACGCCGCGTGAGTGAAGAAGGTTTTCGGATCGTAAAACTCTGTTGTTAGAGAAGAACAAGGATGAGAGTAACTGTTCATCCCTTGACGGTATCTAACCAGAAAGCCACGGCTAACTACGTGCCAGCAGCCGCGGTAATACGTAGGTGGCAAGCGTTGTCCGGATTTATTGGGCGTAAAGCGAGCGCAGGCGGTTTCTTAAGTCTGATGTGAAAGCCCCCGGCTCAACCGGGGAGGGTCATTGGAAACTGGGAGACTTGAGTGCAGAAGAGGAGAGTGGAATTCCATGTGTAGCGGTGAAATGCGTAGATATATGGAGGAACACCAGTGGCGAAGGCGGCTCTCTGGTCTGTAACTGACGCTGAGGCTCGAAAGCGTGGGGAGCAAACAGG` |
| ASV574 | 429 | `TGGGGAATTTTGGACAATGGGCGCAAGCCTGATCCAGCAATGCCGCGTGCAGGATGAAGGCCTTCGGGTTGTAAACTGCTTTTGTACGGAACGAAACGGTCCTTTCTAATACAGGGGGCTAATGACGGTACCGTAAGAATAAGCACCGGCTAACTACGTGCCAGCAGCCGCGGTAATACGTAGGGTGCGAGCGTTAATCGGAATTACTGGGCGTAAAGCGTGCGCAGGCGGTTATGTAAGACAGATGTGAAATCCCCGGGCTCAACCTGGGACCTGCATTTGTGACTGCATAGCTAGAGTACGGTAGAGGGGGATGGAATTCCGCGTGTAGCAGTGAAATGCGTAGATATGCGGAGGAACACCGATGGCGAAGGCAATCCCCTGGACCTGTACTGACGCTCATGCACGAAAGCGTGGGGAGCAAACAGG` |
| ASV4380 | 425 | `TGGGGAATATTGGACAATGGGCGAAAGCCTGATCCAGCAATGCCGCGTGAGTGATGAAGGCCTTCGGGTTGTAAAGCTCTGTCGGAAGGGACGAATGGTGCCGGGTAACTCCGGTAATTGACGGTACCTCCAAAGGAAGCACCGGCTAACTCTGTGCCAGCAGCCGCGGTAATACAGAGGGTGCAAGCGTTGTTCGGAATTACTGGGCGTAAAGCGCGTGTAGGCGGCGTTATAAGTCGGATGTGAAAGCCCTCGGCTCAACCGAGGAAGTGCATCTGATACTGTAGTGCTTGAGTGTCGGAGGGGGTGGCGGAATTCCCGGTGTAGAGGTGAAATTCGTAGATATCGGGAGGAACACCTGTGGCGAAGGCGGCCACCTGGACGACAACTGACGCTGAGACGCGAAAGCGTGGGTAGCAAACAGG` |
| ASV556 | 424 | `TAGGGAATATTGGTCAATGGAGGCAACTCTGAACCAGCCATGCCGCGTGCAGGACTAAGGCCCTATGGGTCGTAAACTGCTTTTGAATGGGAAGAAATGTATCTACGTGTAGATATTTGACGGTACCATTAGAATAAGGATCGGCTAACTTCGTGCCAGCAGCCGCGGTAATACGAAGGATCCAAGCGTTGTCCGGATTTACTGGGTTTAAAGGGTGCGTAGGCGGACTCATAAGTCAGTGGTGAAAGCCTTCAGCTTAACTGAAGAACTGCCATTGAAACTGTGAGTCTCGAGTTCGGTTGAGGTCATTGGAATGTAACATGTAGCGGTGAAATGCATAGATATGTTACAGAACACCAATTGCGAAGGCAGATGACTAAACCGAAACTGACGCTGAGGCACGAAAGCGTGGGGATCAAACAGG` |
| ASV4621 | 408 | `ACGAGAATATTCCGCAATGGGCGAAAGCCTGACGGAGCGACGCCGCGTGATCGATGAAGTGCTTCGGTACGTAAAGATCTTTTATCGGGGACGAAGTTTATTGACGGTACCCGATGAATAAGGGGCTCCTAACTCTGTGCCAGCAGGAGCGGTAATACAGAGGCCCCAAGCATTACCCGGAATCACTGGGCGTAAAGGGTGTCTCGGCGGCCATATTAGTCTCTCGTTAAATGTCCGGGCTCAACCTGGTCACCGCGAGGGAAACGGTATGGCTCGAGGGCGCAAGAGGTGCACGGAACTCATGGTGGAGGGGTGAAATCCGTTGATATCATGGGGAACACCAAAGGCGAAGGCAGTGCACTGGTGCGTTCCTGACGCTCAAACACGAAAGCCAGGGTAGCGAACGGG` |
| ASV5357 | 429 | `TGGGGAATTTTGGACAATGGGGGAAACCCTGATCCAGCAATGCCGCGTGCGGGAAGAAGGCCTTCGGGTTGTAAACCGCTTTTGTTGGGAGCGAAACGGCGACGGTTAATACCTGTTGCTAATGACGGTACCCAAAGAATAAGCACCGGCTAACTACGTGCCAGCAGCCGCGGTAATACGTAGGGTGCAAGCGTTAATCGGAATTACTGGGCGTAAAGCGTGCGCAGGCTGTTGAGTAAGACAGATGTGAAATCCCTGGGCTTAACCTAGGAACTGCATTTGTGACTGCTCAGCTAGAGTGCGGCAGAGGGGGATGGAATTCCGCGTGTAGCAGTGAAATGCGTAGATATGCGGAGGAACACCGATGGCGAAGGCAATCCCCTGGGCCTGCACTGACGCTCATGCACGAAAGCGTGGGGAGCAAACAGG` |
| ASV2693 | 422 | `TGAGGAATTTTGCACAATGGAGGTAACTCTGATGCAGCGACGCCGTGTGTAGGATGAAGGGGCTCTGCCCTGTAAACTACTGTCGGATAGGAATAAAAGTTGGGTTTCCCAACTGGGAATGTACTATCAAAGGAAGGATCGGCTAACTACGTGCCAGCAGCCGCGGTAATACGTAGGATCCGAGCGTTGTCCGGATTTACTGGGTATAAAGGGTGCGTAGGCGGGTTTGTGCTTCAGAGGTTAAAGACCGCAGCTTAACTGCGGGAGTGCCTCTGATAAGGCAAATCTTGGATATGAGAGGGGTAGGTAGAATTCCTGGTGTAGCGGTGAAATGCGTAGATATCAGGAGGAATACCAGTGGCGAAGGCGGCCTACTGGCTCATAATCGACGCTGAGGCACGAAAGCGTGGGGATCAAACAGG` |
| ASV2459 | 424 | `TGAGGAATATTGCGCAATGGAGGCAACTCTGACGCAGCCATGCCGCGTGCAGGAAGAAGGCGCTACGCGTTGTAAACTGCTTTTAACCGGGAAGAAACCCCCAGACGTGTCTGGGGTTGACGGTACCGGAGGAATAAGCACCGGCTAACTTCGTGCCAGCAGCCGCGGTAATACGAAGGGTGCAAGCGTTATCCGGATTCATTGGGTTTAAAGGGTGCGTAGGCGGGGCGTTAAGTCAGTGGTGAAATCCTACAGCTCAACTGTAGACTTGCCATTGATACTGACGCTCTTGAGTGCGCTTGAAGTAGGCGGAATGTGCCGTGTAGCGGTGAAATGCTTAGATATGGCACAGAACACCGATTGCGAAGGCAGCTTACTAAGGCGATACTGACGCTGAGGCACGAAAGCGTGGGGATCGAACAGG` |
| ASV2803 | 404 | `TGGGGAATATTGGACAATGGGCGAAAGCCTGATCCAGCAATACCGCGTGAATGATGAAGGCCTTAGGGTTGTAAAGTTCTTTTAGTAGGGAAGATAATGACGGTACCTACAGAAAAAGCCCCGGCTAACTCCGTGCCAGCAGCCGCGGTAAGACGGAGGGGGCTAGCGTTGTTCGGAATTACTGGGCGTAAAGAGCGCGTAGGCGGATTAGTAAGTTGGAAGTGAAAGCCCAAGGCTTAACCTTGGAATTGCTTTCAAAACTGCTAGTCTAGAGTGAAGTAGGGGGTGATGGAATTCCTAGTGTAGAGGTGAAATTCTTAGATATTAGGAGGAACACCGGTGGCGAAGGCGGTCACCTGGGCTTCAACTGACGCTGAGGCGCGAAAGCGTGGGGAGCAAACAGG` |
| ASV5231 | 408 | `TGAGGAATCTTCCGCAATGGGGGAAACCCTGACGGAGCGACACCGCGTGAAGGATGAAGGTCTATTCGATCGTAAACTTCTGTTCTGAGGGACGAAATTTTTGACGGTACCTCAGGAGAAAGCACCGGCTAATTCTGTGCCAGCAGCCGCGGTAAGACAGAAGGTGCAAGCGTTACTCGGAATTACTGGGCGTAAAGCGTCCGCAGGTGACTGGGGTAGTCTGGTGTTAAAGCACGGAGCTCAACTCCGTGTCCGTGCCGGAAACTCCCCGGATTGAGTCATACAGAGGCATCTGGAATGCTGTGTGTAGGGGTAAAATCCGTTGATCCACAGTAGAACGCCAAAAGCGAAGGCAGGATGCTGGGTATGTACTGACACTCAGGGACGAAAGCGTGGGGAGCAAACCGG` |
| ASV467 | 429 | `TGGGGAATTTTGGACAATGGGCGCAAGCCTGATCCAGCCATGCCGCGTGCGGGAAGAAGGCCTTCGGGTTGTAAACCGCTTTTGTCAGGGAAGAAACGGTCTGGGTTAATACCCTGGGCTAATGACGGTACCTGAAGAATAAGCACCGGCTAACTACGTGCCAGCAGCCGCGGTAATACGTAGGGTGCAAGCGTTAATCGGAATTACTGGGCGTAAAGCGTGCGCAGGCGGTTATGCAAGACAGATGTGAAATCCCCGGGCTCAACCTGGGAACTGCATTTGTGACTGCATAGCTAGAGTACGGTAGAGGGGGATGGAATTCCGCGTGTAGCAGTGAAATGCGTAGATATGCGGAGGAACACCGATGGCGAAGGCAATCCCCTGGACCTGTACTGACGCTCATGCACGAAAGCGTGGGGAGCAAACAGG` |
| ASV3765 | 407 | `TAGGGAATATTGCGCAATGGAGGAAACTCTGACGCAGCGACGCCGCGTGAGTGATGAAGGCCTTCGGGTTGTAAAGCTCTGTCGTACGGGACTAAGAAAGTGAAGGTACCGTGCAAGAAAGGATCGGCTAACTTCGTGCCAGCAGCCGCGGTAAGACGAGGGATCCTAGCGTTGTTCGGAATTATTGGGCGTAAAGAGGGCGTAGGTGGCTTGATAAGTCAGGTGTGAAAGCCCAGGGCTCAACCCTGGAAGTGCATTTGATACTGTTAAGCTTGAGTGCGAGAGAGGTTAGTGGAATTCCTGGTGTAGTGGTGAAATACGTAGATATCAGGAGGAACACCGGAGGCGAAGGCGGCTAACTGGCTCGACACTGACACTGAGGCCCGAAAGCGTGGGGATCAAACAGG` |
| ASV4363 | 428 | `TGGGGAATCTTGCGCAATGGGCGAAAGCCTGACGCAGCGACGCCGCGTGCGGGAAGAAGGCCTTAGGGTTGTAAACCGCTTTCAGGAGGGACGAAGCCACTCGGGTTAATAGCCCAGAGGGTGACGGTACCTCCAGAAGAAGCCCCGGCTAACTACGTGCCAGCAGCCGCGGTAATACGTAGGGGGCAAGCGTTGTCCGGATTCATTGGGCGTAAAGAGCGTGTAGGCGGCCAGGTAGGTGGGTTGTGAAAACTGGAGGCTCAACCTTCAGACGTCGACCCAAACCCCCTGGCTAGAGTCCGGGAGAGGAGAGTGGAATTCCTGGTGTAGCGGTGGAATGCGCAGATATCAGGAAGAACACCCGTGGCGAAAGCGGCTCTCTGGAACGGTACTGACGCTGAGACGCGAAAGCGTGGGGAGCGAACAGG` |
| ASV20 | 404 | `TGGGGAATCTTAGACAATGGGGGAAACCCTGATCTAGCCATGCCGCGTGATCGATGAAGGCCTTAGGGTTGTAAAGATCTTTCAGATGGGAAGATAATGACGGTACCATCAGAAGAAGCCCCGGCTAACTCCGTGCCAGCAGCCGCGGTAATACGGAGGGGGCTAGCGTTATTCGGAATTACTGGGCGTAAAGCGCACGTAGGCGGATTGGAAAGTCAGAGGTGAAATCCCAGGGCTCAACCTTGGAACTGCCTTTGAAACTCCCAGTCTTGAGGTCGAGAGAGGTGAGTGGAATTCCGAGTGTAGAGGTGAAATTCGTAGATATTCGGAGGAACACCAGTGGCGAAGGCGGCTCACTGGCTCGATACTGACGCTGAGGTGCGAAAGCGTGGGGAGCAAACAGG` |
| ASV2244 | 429 | `TGGGGAATATTGCACAATGGGCGCAAGCCTGATGCAGCCATGCCGCGTGTGTGAAGAAGGCCTTCGGGTTGTAAAGCACTTTCAGCGAGGAGGAAGGGTGGTGTGTTAATAGCACACGACATTGACGTTACTCGCAGAAGAAGCACCGGCTAACTCCGTGCCAGCAGCCGCGGTAATACGGAGGGTGCAAGCGTTAATCGGAATTACTGGGCGTAAAGCGCACGCAGGCGGTTTGTTAAGTCAGATGTGAAATCCCCGCGCTTAACGTGGGAACTGCATTTGAAACTGGCAAGCTAGAGTCTTGTAGAGGGGGGTAGAATTCCAGGTGTAGCGGTGAAATGCGTAGAGATCTGGAGGAATACCGGTGGCGAAGGCGGCCCCCTGGACAAAGACTGACGCTCAGGTGCGAAAGCGTGGGGAGCAAACAGG` |
| ASV1003 | 429 | `TGGGGAATTTTGGACAATGGGCGCAAGCCTGATCCAGCAATGCCGCGTGCAGGATGAAGGCCTTCGGGTTGTAAACTGCTTTTGTACGGAGCGAAACGGTCTCTACTAATACTGGGGGCTAATGACGGTACCGTAAGAATAAGCACCGGCTAACTACGTGCCAGCAGCCGCGGTAATACGTAGGGTGCGAGCGTTAATCGGAATTACTGGGCGTAAAGCGTGCGCAGGCGGTTTTGTAAGACAGATGTGAAATCCCCGGGCTCAACCTGGGACCTGCATTTGTGACTGCAAGGCTAGAGTACGGTAGAGGGGGATGGAATTCCGCGTGTAGCAGTGAAATGCGTAGATATGCGGAGGAACACCGATGGCGAAGGCAATCCCCTGGACCTGTACTGACGCTCATGCACGAAAGCGTGGGGAGCAAACAGG` |
| ASV2133 | 404 | `TGGGGAATATTGGACAATGGGCGCAAGCCTGATCCAGCCATGCCGCGTGGATGATGAAGGCCCTAGGGTTGTAAAGTCCTTTCGGCGGGGAAGATAATGACGGTACCCGCAGAAGAAGCCCCGGCTAACTTCGTGCCAGCAGCCGCGGTAATACGAAGGGGGCTAGCGTTGCTCGGAATCACTGGGCGTAAAGCGCACGTAGGCGGCTTTTTAAGTCAGGGGTGAAATCCTGGAGCTCAACTCCAGAACTGCCTTTGATACTGAGAAGCTTGAGTCCGGGAGAGGTGAGTGGAACTGCGAGTGTAGAGGTGAAATTCGTAGATATTCGCAAGAACACCAGTGGCGAAGGCGGCTCACTGGCCCGGTACTGACGCTGAGGTGCGAAAGCGTGGGGAGCAAACAGG` |
| ASV2806 | 429 | `TGGGGAATTTTGGACAATGGACGAAAGTCTGATCCAGCCATTCCGCGTGCAGGATGAAGGCCTTCGGGTTGTAAACTGCTTTTGTACGGAACGAAAAGGTTTTCATTAATACTGAAGATCCATGACGGTACCGTAAGAATAAGCACCGGCTAACTACGTGCCAGCAGCCGCGGTAATACGTAGGGTGCAAGCGTTAATCGGAATTACTGGGCGTAAAGCGTGCGCAGGCGGTTATGTAAGACAGAGGTGAAATCCCCGGGCTCAACCTGGGAACTGCCTTTGTGACTGCATAGCTAGAGTACGGTAGAGGGGGATGGAATTCCGCGTGTAGCAGTGAAATGCGTAGATATGCGGAGGAACACCGATGGCGAAGGCAATCCCCTGGACCTGTACTGACGCTCATGCACGAAAGCGTGGGGAGCAAACAGG` |
| ASV1626 | 424 | `TGAGGAATATTGGTCAATGGGCGCAAGCCTGAACCAGCCATGCCGCGTGCAGGAAGACGGTCCTATGGATTGTAAACTGCTTTTATTTGGGAATAAACTCCGATACGTGTATCGGCCTGAAGGTACCAAATGAATAAGCACCGGCTAACTCCGTGCCAGCAGCCGCGGTAATACGGAGGGTGCAAGCGTTATCCGGAATCATTGGGTTTAAAGGGTCCGCAGGCGGATTTATAAGTCAGTGGTGAAAGCCTGATGCTTAACATCAGAACTGCCATTGATACTGTAAGTCTTGAATTCGATCGAAGTGGGCGGAATATGACATGTAGCGGTGAAATGCTTAGATATGTCATAGAACACCGATAGCGAAGGCAGCTCACTAGGTCTGGATTGACGCTCAGGGACGAAAGCGTGGGGAGCAAACAGG` |
| ASV4397 | 405 | `TGAGGAATATTGGGCAATGGGCGGAAGCCTGACCCAGCAACGCCGCGTGGAGGAAGACGGCCTTCGGGTTGTAAACTCCTTTTACGGGGGAAGAGGAAGGACGGTACCCCGAGAATAAGTCACGGCTAACTACGTGCCAGCAGCCGCGGTAATACGTAGGTGACAAGCGTTATCCGGAATTACTGGGCGTAAAGCGCACGCAGGCGGCGCATTAAGTCTGATGTGAAATCTCCCGGCTTAACTGGGAGGGGTCATTGGAAACTGATGTGCTTGAGGCGGTGAGAGGGGTGTAGAATTCCCGGTGTAGTGGTGGAATGCGTAGATATCGGGAGGAATACCAGTGGCGAAAGCGGCACCCTGGCACTAGCCTGACGCTCATGTGCGAAGGCGTGGGGAGCGAACGGG` |
| ASV179 | 404 | `TGGGGAATCTTAGACAATGGGCGCAAGCCTGATCTAGCCATGCCGCGTGAGCGATGAAGGCCTTAGGGTTGTAAAGCTCTTTCGTGGGGGAAGATAATGACTGTACCCCAAGAAGAAGCCCCGGCTAACTCCGTGCCAGCAGCCGCGGTAATACGGAGGGGGCTAGCGTTGTTCGGAATTACTGGGCGTAAAGCGCACGTAGGCGGACTGGAAAGTCAGAGGTGAAATCCCAGGGCTCAACCTTGGAACTGCCTTTGAAACTCCCGGTCTTGAGGTCGAGAGAGGTGAGTGGAATTCCGAGTGTAGAGGTGAAATTCGTAGATATTCGGAGGAACACCAGTGGCGAAGGCGGCTCACTGGCTCGATACTGACGCTGAGGTGCGAAAGCGTGGGGAGCAAACAGG` |
| ASV1458 | 404 | `TGGGGAATATTGGACAATGGGCGAAAGCCTGATCCAGCAATGCCGCGTGAGTGATGAAGGCCTTAGGGTTGTAAAGCTCTTTTACCTGGGATGATAATGACAGTACCAGGAGAATAAGCCCCGGCTAACTCCGTGCCAGCAGCCGCGGTAATACGGAGGGGGCTAGCGTTGTTCGGAAATACTGGGCGTAAAGCGCACGTAGGCGGCTCAGCAAGTCAGGGGTGAAATCCCGGGGCTCAACCCCGGAACTGCCCTTGAAACTGCTAGGCTAGAATCTTGGAGAGGCGAGTGGAATTCCGAGTGTAGAGGTGAAATTCGTAGATATTCGGAAGAACACCAGTGGCGAAGGCGACTCGCTGGACAAGTATTGACGCTGAGGTGCGAAAGCGTGGGGAGCAAACAGG` |
| ASV3368 | 404 | `TGGGGAATATTGGACAATGGGCGAAAGCCTGATCCAGCAATGCCGCGTGAATGATGAAGGCCTTAGGGTTGTAAAGTTCTTTTAGTGGGGAAGATAATGACGGTACCCACAGAAAAAGCACCGGCTAACTTCGTGCCAGCAGCCGCGGTAATACGAAGGGTGCAAGCGTTGTTCGGAATTACTGGGCGTAAAGCGTGCGTAGGCGGTTTAGTAAGTTGGGAGTGAAATCCCGGAGCTTAACTCCGGAATTGCTTTCAAAACTACTAATCTAGAATATGGCAGGGGACAGTGGAATTCCTAGTGTAGAGGTGAAATTCGTAGATATTAGGAGGAACACCAGTGGCGAAAGCGACTGTCTGGACCATTATTGACGCTGAGGCACGAAAGCGTGGGGAGCAAACAGG` |
| ASV3725 | 404 | `TGGGGAATATTGGACAATGGGCGCAAGCCTGATCCAGCAATACCGCGTGAGTGATGAAGGTCTTCGGATTGTAAAGCTCTTTTAGTGGGGAAGATAATGACGGTACCCACAGAAAAAGCACCGGCAAACTTCGTGCCAGCAGCCGCGGTAATACGAAGGGTGCTAGCGTTGTTCGGAATCATTGGGCGTAAAGCGTACGTAGGCGGTTTAGTAAGTCGATTGTGAAAGCCCCAAGCTCAACTTGGGAATTGCAACCGATACTGCTTTGCTTGAGTTTGGTCGGGGATGGTGGAATTTCTAGTGTAGGGGTGAAATCCGTAGATATTAGAAGGAATATCGGTGGCGAAGGCGACCATCTAGGCCAATACTGACGCTAAGGTACGAAAGCGTGGGGAGCAAACAGG` |
| ASV1530 | 429 | `TAGGGAATATTGCGCAATGGGGGAAACCCTGACGCAGCAACGCCGCGTGAGTGAGGAAGGTCTTCGGATTGTAAAACTCTTTTATTAGGGAAAAATGGTTAGGGAGCTAATACCCCCTGATTTGATGGTACCTAAAGAATAAGCACCGGCTAACTTCGTGCCAGCAGCCGCGGTAATACGAAGGGTGCAAGCGTTGTTCGGATTTATTGGGCGTAAAGCGCGCGCAGGCGGATCAGCAAGTCAGATGTGAAATCTCAGGGCTCAACCCTGAAACTGCGTCTGAAACTGCTAGTCTAGAATGTCGGAGGGGGCAGGGGAATTTCACGTGTAGGGGTAAAATCCGTAGAGATGTGAAGGAACACCGGAGGCGAAGGCGCCTGCCTGGACGACTATTGACGCTGAGGCGCGAAAGCGTGGGGAGCAAACAGG` |
| ASV4776 | 424 | `TGAGGAATATTGGTCAATGGGCGCAAGCCTGAACCAGCCATGCCGCGTGCAGGAAGACGGTCCTATGGATTGTAAACTGCTTTTATTCGGGAATAAACCTCCTTACGTGTAGGGAGCTGAAGGTACCGAATGAATAAGCACCGGCTAACTCCGTGCCAGCAGCCGCGGTAATACGGAGGGTGCAAGCGTTATCCGGAATCATTGGGTTTAAAGGGTCCGCAGGCGGTCTAATAAGTCAGTGGTGAAAGCCTACAGCTCAACTGTAGAACTGCCATTGATACTGTTAGACTTGAATTCGGTCGAAGTGGGCGGAATGTGACATGTAGCGGTGAAATGCTTAGATATGTCACAGAACACCGATAGCGAAGGCAGCTCACTAGGCCTGGATTGACGCTCAGGGACGAAAGCGTGGGGAGCAAACAGG` |
| ASV698 | 429 | `TGGGGAATTTTGGACAATGGACGAAAGTCTGATCCAGCAATGCCGCGTGCAGGACGAAGGCCTTCGGGTTGTAAACTGCTTTTGTACGGAACGAAAAAGTCTTTTTTAATAAAGAGGGCTTATGACGGTACCGTAAGAATAAGCACCGGCTAACTACGTGCCAGCAGCCGCGGTAATACGTAGGGTGCAAGCGTTAATCGGAATTACTGGGCGTAAAGCGTCCGCAGGCGGTTGTGTAAGACAGATGTGAAATCCCCGGGCTCAACCTGGGAACTGCATTTGTGACTGCACGGCTAGAGTACGGCAGAGGGGGATGGAATTCCGCGTGTAGCAGTGAAATGCGTAGATATGCGGAGGAACACCGATGGCGAAGGCAATCCCCTGGGCCTGTACTGACGCTCATGGACGAAAGCGTGGGGAGCAAACAGG` |
| ASV1550 | 424 | `TAAGGAATATTGGACAATGCCCGCAAGGGTGATCCAGCCATGCCGCGTGCAGGAAGAAGGCCCTATGGGTCGTAAACTGCTTTTACACCGGAGAAAACCCTCGTTCGTGAACGAGGCTGATAGTATGGTGAGAATAAGCATCGGCTAACTTCGTGCCAGCAGCCGCGGTAAGACGAAGGATGCAAGCGTTATCCGGATTCATTGGGTTTAAAGGGAGCGTAGGTGGACTTATAAGTCAGTGGTGAAATCTTTGGGCTTAACCCAAAAATTGCCATTGATACTGTAGGTCTTGAGTACAGTTGCTGTGGGCGGAATATGACATGTAGTGGTGAAATACATAGAGATGTCATAGAACACCGATTGCGAAGGCAGCTCACAAAGCTGTAACTGACACTGAGGCTCGAAAGTGCGGGGATCAAACAGG` |
| ASV4384 | 404 | `TGGGGAATATTGGACAATGGGCGAAAGCCTGATCCAGCAATGCCGCGTGAGTGATGAAGGCCTTAGGGTTGTAAAGCTCTTTTACCCGGGAAGATAATGACTGTACCGGGAGAATAAGCCCCGGCTAACTCCGTGCCAGCAGCCGCGGTAATACGGAGGGGGCTAGCGTTGTTCGGAATTACTGGGCGTAAAGCGTACGTAGGCGGCTTGATAAGTTAGAGGTGAAAGCCCGGGGCTCAACTCCGGAATTGCCTTTAAGACTGTCTCGCTTGAACGTCGGAGAGGTGAGTGGAATTCCGAGTGTAGAGGTGAAATTCGTAGATATTCGGAAGAACACCAGTGGCGAAGGCGGCTCACTGGACGACTGTTGACGCTGAGGTACGAAAGCGTGGGGAGCAAACAGG` |
| ASV4487 | 428 | `TCGAGAATTTTTCTCAATGGGCGAAAGCCTGAAGGAGCGACGCCGCGTGGAGGATGAAGGTCTTCGGATTGTAAACTCCTGTCATTAGGGAACAAAACTTACGGTTAACTGCCGTGGAGTCTGATAGTACCTGAAGAGGAAGAGACGGCTAACTCTGTGCCAGCAGCCGCGGTAATACAGAGGTCTCAAGCGTTGTTCGGATTCATTGGGCGTAAAGGGTGCGTAGGCGGCGCGGTAAGTCGGATGTGAAATCCCGGGGCTCAACCCCGGAACTGCATTCGATACTGCCTTGCTAGAGTACTGGAGAGGAGACTGGAATTTACGGTGTAGCAGTGAAATGCGTAGATATCGTAAGGAAGACCAGTGGCGAAGGCGAGTCTCTGGACAGTTACTGACGCTGAGGCACGAAGGCCAGGGGAGCAAACGGG` |
| ASV4119 | 405 | `TTGGGAATCTTGCACAATGGGCGAAAGCCTGATGCAGCAACGCCGCGTGGAGGATGAAGGTCTTCGGATCGTAAACTCCTTTTGCTGAGGAGCACCACCGAGAGTACTCAGCGAATAAGCACCGGCTAACTCCGTGCCAGCAGCCGCGGTAATACGGAGGGTGCAAACGTTATCCGGAATCATTGGGCGTAAAGCGCGTGTAGGTGGTTTGGTCAGTCTACGATTAAATCCCAGGGCTTAACCCTGGATCGGTTGTAGATACTGCCAGACTTGAGGGTAGCAGGGGTACGTGGAACTTTGAGTGTAGGAGTGAAATCCGTAGATATTCAAAGGAACACCAATGGCGAAGGCAGCGTACTGGGCTACACCTGACACTGAGACGCGAAAGCGTGGGGAGCAAACGGG` |
| ASV693 | 404 | `TGGGGAATATTGCACAATGGGGGAAACCCTGATGCAGCAACGCCGCGTGAGTGATGACGGCCTTCGGGTTGTAAAGCTCTGTCTTCAGGGACGATAATGACGGTACCTGAGGAGGAAGCCACGGCTAACTACGTGCCAGCAGCCGCGGTAATACGTAGGTGGCGAGCGTTGTCCGGATTTACTGGGCGTAAAGGGAGCGTAGGCGGATTTTTAAGTGAGATGTGAAATACTCGGGCTTAACCTGAGTGCTGCATTTCAAACTGGAAGTCTAGAGTGCAGGAGAGGAGAAGGGAATTCCTAGTGTAGCGGTGAAATGCGTAGAGATTAGGAAGAACACCAGTGGCGAAGGCGCTTCTCTGGACTGTAACTGACGCTGAGGCTCGAAAGCGTGGGGAGCAAACAGG` |
| ASV607 | 423 | `TAGGGAATATTGGTCAATGGAGGCAACTCTGAACCAGCCATGCCGCGTGTAGGAAGAAGGCGTTCTGCGTTGTAAACTACTTTTGATTGGGAACAAATGACTCTTGCGAGAGTAGCTGAGTGTACCAATAGAATAAGCCACGGCTAACTACGTGCCAGCAGCCGCGGTAATACGTAGGTGGCAAGCGTTGTCCGGATTTATTGGGTTTAAAGGGTGCGTAGGCGGCTTAATAAGTCAGTGGTGAAAGCCGATCGCTTAACGATCGAACTGCCATTGATACTGCTAGGCTTGAGTATAGTTGAGGTAGGCGGAATTGACAGTGTAGCGGTGAAATGCATAGATATTGTCAAGAACACCAATTGCGAAGGCAGCTTACTAAAGTATAACTGACGCTGAGGCACGAAAGTGCGGGGATCAAACAGG` |
| ASV827 | 424 | `TGAGGAATATTGGACAATGGGCGCAAGCCTGATCCAGCCATGCCGCGTGCAGGATGACGGTCCTATGGATTGTAAACTGCTTTTATACGAGAAGAAACACTGCTTCGTGAAGCAGCTTGACGGTATCGTAAGAATAAGGATCGGCTAACTCCGTGCCAGCAGCCGCGGTAATACGGAGGATCCAAGCGTTATCCGGAATCATTGGGTTTAAAGGGTCCGTAGGCGGTTTAATAAGTCAGTGGTGAAAGCCCATCGCTCAACGGTGGAACGGCCATTGATACTGTTAAACTTGAATTATTAGGAAGTAACTAGAATATGTAGTGTAGCGGTGAAATGCTTAGAGATTACATGGAATACCAATTGCGAAGGCAGGTTACTACTAATGGATTGACGCTGATGGACGAAAGCGTGGGTAGCGAACAGG` |
| ASV2346 | 429 | `TGGGGAATTTTGGACAATGGGCGCAAGCCTGATCCAGCCATGCCGCGTGCGGGAAGAAGGCCTTCGGGTTGTAAACCGCTTTTGTCAGGGAAGAAAAGCTCTGGGCTAATACCCTGGGGTGATGACGGTACCTGAAGAATAAGCACCGGCTAACTACGTGCCAGCAGCCGCGGTAATACGTAGGGTGCAAGCGTTAATCGGAATTACTGGGCGTAAAGCGTGCGCAGGCGGTTGTGCAAGACAGATGTGAAATCCCCGGGCTCAACCTGGGAACTGCATTTGTGACTGCACAGCTAGAGTACGGTAGAGGGGGATGGAATTCCGCGTGTAGCAGTGAAATGCGTAGATATGCGGAGGAACACCGATGGCGAAGGCAATCCCCTGGACCTGTACTGACGCTCATGCACGAAAGCGTGGGGAGCAAACAGG` |
| ASV5586 | 407 | `TCGAGAATATTTGACAATGGGCGCAAGCCTGATCAAGCGACGCCGCGTGTAGGATGAAGGCCTTCGGGTCGTAAACTACTTTTCTACGGGAAAAAACAAATGATTGTACTGTAGGAATAAGGGGTGGCTAACTTCGTGCCAGCAGCAGCGGTAATACGAAGGCCCCGAGCGTTATCCGGAATTATTGGGCGTAAAGGATGCGTAGGTGGTTTTGTGCGTCTTTTGTAAAATCTCACTGCTCAACGGTGAGACCGCAGAGGATACGGCAAAACTAGAGGATGCAAGAGGCTTGTGGAATTTACGGTGTAGGGGTGAAATCCGTTGATATCGTAGAGAACACCAAAGGCGAAGGCAGCAAGCTGGTGCATTTCTGACACTGAGGCATGAAAGCGTGGGGAGCAAAAAGG` |
| ASV1507 | 424 | `TAAGGAATATTGGTCAATGGAGGGAACTCTGAACCAGCCACGCCGCGTGGAGGATGAAGGCTCTATGGGTTGTAAACTTCTTTAGTCTGGGACGAAAAAGGGTCATTTATGATCAACTGACGGTACCAGAAGAATAAGCACCGGCTAACTCCGTGCCAGCAGCCGCGGTAATACGGAGGGTGCAAGCGTTATCCGGAATCACTGGGTTTAAAGGGTGCGTAGGCTGTTTTATAAGTCAGCGGTGAAAGGTGTTCGCTTAACGAATGAATTGCCGTTGATACTGTTTAACTAGAATTGGGCTGAGGTAACTAGAATGTAACATGTAGCGGTGAAATGCTTAGATATGTTATAGAACACCGATTGCGAAGGCAGGTTACTGGACCTTGATTGACGCTGAGGCACGAAAGCGTGGGTAGCGAACAGG` |
| ASV1153 | 424 | `TGAGGAATATTGGACAATGGGCGCAAGCCTGATCCAGCCATGCCGCGTGCAGGATGACGGTCCTATGGATTGTAAACTGCTTTTATACGAGAAGAAACACTGATTCGTGAATCAGCTTGACGGTATCGTAAGAATAAGGATCGGCTAACTCCGTGCCAGCAGCCGCGGTAATACGGAGGATCCAAGCGTTATCCGGAATCATTGGGTTTAAAGGGTCCGTAGGCGGTTTAATAAGTCAGTGGTGAAAGCCCATCGCTCAACGGTGGAACGGCCATTGATACTGTTAAACTTGAATTATTAGGAAGTAACTAGAATATGTAGTGTAGCGGTGAAATGCTTAGAGATTACATGGAATACCAATTGCGAAGGCAGGTTACTACTAATGGATTGACGCTGATGGACGAAAGCGTGGGTAGCGAACAGG` |
| ASV3413 | 430 | `TGGGGAATTGTTCGCAATGGGCGAAAGCCTGACGACGCAACGCCGCGTGGAGGATGAAGAATTTCGGTTCGTAAACTCCTTTCGACCGAGATGAATACCCACGGACCTAACACGTCTGTGGGCTGACAGTATCGAGAGAAGAAGCCCCGGCTAACTCTGTGCCAGCAGCCGCGGTAATACAGGGGGGGCAAGCGTTGTTCGGAATTACTGGGCGTAAAGGGTTCGTAGGTGGCCAACTAAGTCAGACGTGAAATCCCTCAGCTTAACTGGGGAACTGCGTCTGATACTGGTTGGCTTGAGTGCAGGAGAGGAACGCAGAATTCCAGGTGTAGCGGTGAAATGCGTAGATATCTGGAGGAATACCGGTGGCGAAGGCGGCGTTCTGGACTGTAACTGACACTGAGGAACGAAAGCCAGGGGAGCAAACGGG` |
| ASV4480 | 428 | `TGGGGAATTTTGCACAATGGACGCAAGTCTGATGCAGCAACGCCGCGTGAGTGAAGAAGGCTTTCGGGTTGTAAAGCTCTGTCATTCGGGACGAAAAAATTTAGGTGCATAGCCTAAGTCTTGACGGTACCGAAGAAGGAAGCACCGGCTAACTCCGTGCCAGCAGCCGCGGTAATACGGAGGGTGCAAGCGTTGTTCGGAATTATTGGGCGTAAAGGGGAGGTAGGTGGTCTCATTTGTCTGGGGTGAAATCCCGGGGCTTAACCCCGGACGTGCCTCAGAAACGGTGAGACTGGAATCTTGGCGAGGGTCGCGGAATTCCCGGTGTAGCGGTGAAATGCGTAGATATCGGGAGGAACACCAGAGGCGAAAGCGGCGGCCTGGACAAGTATTGACACTAAACTCCGAAAGCGTGGGTAGCAAACAGG` |
| ASV5400 | 422 | `TGAGGAATATTGGTCAATGGGCGGAAGCCTGAACCAGCCAAGTCGCGTGAGGGAAGACGGTCCTACGGATTGTAAACCTCTTTTGCCGGGGAGCAAAACGGCCATGTATGGCCGACTGAGAGTACCCGGAGAAAAAGCATCGGCTAACTCCGTGCCAGCAGCCGCGGTAATACGGAGGATGCGAGCGTTATCCGGATTTATTGGGTTTAAAGGGTGCGTAGGCGGATGGTTAAGTCAGCGGTAAAATCGAGGGGCTCAACCCCTTCCGGCCGTTGAAACTGGCTGTCTTGAGTGGGCGAGAAGTATGCGGAATGCGAGGTGTAGCGGTGAAATGCATAGATATCACGCAGAACTCCGATTGCGAAGGCAGCATACCGGCGCCCGACTGACGCTGAGGCACGAAAGCGTGGGGATCAAACAGG` |
| ASV4539 | 424 | `TGGGGAATATTGGGCAATGGGGGAAACCCTGACCCAGCAACGCCGCGTGAGTGATGAAGGCCTTCGGGTCGTAAAGCTCTGTTGAGGGGGACGAAACAACAGCTTAACAAACTGTTCTGACTTAACCCCTTGAGGAAGCACCGGCTAACTCTGTGCCAGCAGCCGCGGTAATACAGAGGGTGCAAGTGTTGTTCGGAATCATTGGGCGTAAAGGGCGCGTAGGTGGTCAGGTCAGTCAGATGTGAAAGCCCTGGGCTTAACCCAGGAACTGCATTTGATACTGCCTGACTTGAGTTCAGTAGAGGGTAGTGGAATTCCCGGTGTAGAGGTGAAATTCGTAGAGATCGGGAGGAACACCAGTGGCGAGAGCGGCTACCTGGGCTGACACTGACACTGAGGCGCGAGAGCGTGGGGAGCAAACAGG` |
| ASV2700 | 387 | `GCGAGAAACCTTTGCAATGTGCGAAAGCACGACAAGGTTAATCCGAGTGTCTCCCGCTAAGGGAGTCTTTTGTCAGTTCTAGAACCACTGGTGAATAAGGGGTGGGCAAGTTCTGGTGTCAGCCGCCGCGGTAAAACCAGCACCTCGAGTGGTCAGGAGGTTTATTGGGCCTAAAGCATCCGTAGCCGGTTGCATAAGTTTTCGGTTAAATCTATGCGCTTAACGTATAGGCCGCCGAAAATACTGTGCAGCTAGGGAGTGGGAGAGGTACACGGTACTCGATAGGAAGGGGTAAAATCCTTTGATCTATTGATGACCACCTGTGGCGAAGGCGGTGTACCAGAACACGTTCGACGGTGAGGGATGAAAGCTGGGGGAGCAAACCGG` |
| ASV3744 | 404 | `TGGGGAATATTGGACAATGGGCGAAAGCCTGATCCAGCAACGCCGCGTGAATGATGAAGGCCTTAGGGTTGTAAAGTTCTTTTAGTGGGGAAGATGATGACTGTACCCACAGAATAAGTCCCGGCTAACTTCGTGCCAGCAGCCGCGGTAATACGAAGGGGGCTAGCGTTACTCGGAATTATTGGGCGTAAAGCGTGCGCAGGCGGCAAAGTAAGTTGGGAGTGAAATCCTAGGGCTCAACCCTAGAACTGCTTCCAATACTGCTTTACTAGAGTGTCGGAGGGGGTGACGGAATTCCTAGTGTAGAGGTGAAATTCGTAGATATTAGGAGGAACACCGGTGGCGAAAGCGGTCACCTGGAAGAACACTGACGCTGTTGCACGAAAGCGTGGGGAGCAAACAGG` |
| ASV5377 | 430 | `TGGGGAATATTGGACAATGGGGGCAACCCTGATCCAGCAATGCCGCGTGTGTGAAGAAGGCCTGCGGGTTGTAAAGCACTTTTGTTCGAGAAGAAATCCTGTTTGTGAATAGCAGATAGGGATGACGGTATCGAAAGAATAAGCACCGGCTAACTTCGTGCCAGCAGCCGCGGTAATACGAAGGGTGCAAGCGTTAATCGGAATTACTGGGCGTAAAGCGTGCGTAGATGGCTTTTTAAGTCGACTGTGAAACCCCTGGGCTCAACCTGGGACGTGCAGTCGATACTGAGAAGCTTGGAGTTTGAGAGAGGGTGGTGGAATTCCTGGTGTAGCGGTGAAATGCGTAGAGATCAGGAGGAACACTAGTGGCGAAGGCGGCCACCTGGCTTAAAACTGACGTTGAGGCACGAAAGCGTGGGGAGCAAACAGG` |
| ASV2323 | 429 | `TGGGGAATTTTGGACAATGGGGGCAACCCTGATCCAGCCATGCCGCGTGTCTGAAGAAGGCCTTCGGGTTGTAAAGGACTTTTGTCAGGGAGCAAATCCTGCTTGTGAATAATGAGTGGGGATGAGAGTACCTGAAGAATAAGCACCGGCTAACTACGTGCCAGCAGCCGCGGTAATACGTAGGGTGCAAGCGTTAATCGGAATTACTGGGCGTAAAGCGTGCGCAGGCGGTTCTGCAAGTCTGATGTGAAAGCCCCGGGCTTAACCTGGGAACGGCATTGGAGACTGCAGAGCTAGAGTACGTCAGAGGGGGGTAGAATTCCACGTGTAGCAGTGAAATGCGTAGAGATGTGGAGGAATACCGATGGCGAAGGCAGCCCCCTGGGATGATACTGACGCTCATGCACGAAAGCGTGGGGAGCAAACAGG` |
| ASV2506 | 429 | `TGGGGAATTTTGGACAATGGGCGCAAGCCTGATCCAGCAATGCCGCGTGCAGGATGAAGGCCTTCGGGTTGTAAACTGCTTTTGTACGGAGCGAAACAGCCCCTTCTAATACAGGGGGTGAATGACGGTACCGTAAGAATAAGCACCGGCTAACTACGTGCCAGCAGCCGCGGTAATACGTAGGGTGCAAGCGTTAATCGGAATTACTGGGCGTAAAGCGTGCGCAGGCGGTCTTGTAAGACAGATGTGAAATCCCCGGGCTTAACCTGGGACCTGCATTTGTGACTGCAAGGCTGGAGTACGGCAGAGGGGGATGGAATTCCGCGTGTAGCAGTGAAATGCGTAGATATGCGGAGGAACACCGATGGCGAAGGCAGTCCCCTGGGCCTGTACTGACGCTCATGCACGAAAGCGTGGGGAGCAAACAGG` |
| ASV2872 | 429 | `TGGGGAATTTTGGACAATGGGGGCAACCCTGATCCAGCAATGCCGCGTGAGTGAAGAAGGCCTTCGGGTTGTAAAGCTCTTTTGTCAGGGAAGAAAAGGTTCTGGATAATACCTGGAACTGATGACGGTACCTGAAGAATAAGCACCGGCTAACTACGTGCCAGCAGCCGCGGTAATACGTAGGGTGCAAGCGTTAATCGGAATTACTGGGCGTAAAGCGTGCGCAGGCGGTTATGTAAGACAGGTGTGAAATCCCCGAGCTCAACTTGGGAACTGCACTTGTGACTGCATGGCTAGAGTGTGTCAGAGGGGGGTAGAATTCCACGTGTAGCAGTGAAATGCGTAGAGATGTGGAGGAATACCGATGGCGAAGGCAGCCCCCTGGGATAACACTGACGCTCATGTACGAAAGCGTGGGGAGCAAACAGG` |
| ASV297 | 404 | `TGGGGAATATTGGACAATGGGGGAAACCCTGATCCAGCAATGCCGCGTGTGTGATGAAGGTCTTCGGGTTGTAAAGCACTTTTGTCAGGGACGATGATGACGGTACCTGACGAATAAGCTCCGGCTAACTTCGTGCCAGCAGCCGCGGTAATACGAGGGGAGCTAGCGTTGTTCGGAATTACTGGGCGTAAAGGGCGTGTAGGCGGTACCATAAGTCAGATGTGAAAGCCCTAGGCTTAACCTAGGAGGTGCATTTGATACTGTGGAACTAGAGATCGAGAGAGGTAAGTGGAATTACGAGTGTAGAGGTGAAATTCGTAGATATTCGTAAGAACACCAGTGGCGAAGGCGACTTACTGGCTCGATACTGACGCTAAGGCGCGAAAGCGTGGGGAGCAAACAGG` |
| ASV2105 | 429 | `TGGGGAATATTGGACAATGGGGGCAACCCTGATCCAGCAATGCCGCGTGTGTGAAGAAGGCCTTCGGGTTGTAAAGCACTTTAGGTTGGGAAGAAGGTGTATTGTTTAAGAGATGATACATTTGACGGTACCGACAGAATAAGCACCGGCTAACTCTGTGCCAGCAGCCGCGGTAATACAGAGGGTGCAAGCGTTAATCGGAGTTACTGGGCGTAAAGGGCGCGTAGGTGGATATTTAAGTTGGATGTGAAATCCCTGGGCTTAACCTAGGAATGGCATACAAAACTGGATATCTGGAGTACTGTAGAGGGTAGTGGAATTTCCGGTGTAGCGGTGAAATGCGTAGAGATCGGAAGGAACACCAGTGGCGAAGGCGGCTACCTGGACAGATACTGACACTGAGGCGCGAAAGCGTGGGGATCAAACAGG` |
| ASV5406 | 404 | `TGGGGAATATTGGACAATGGGGGAAACCCTGATCCAGCGATGCCGCGTGAGTGATGAAGGCCTTAGGGTTGTAAAGCTCTTTCGCCCACGACGATGATGACGGTAGTGGGAGAAGAAGCCCCGGCTAACTCCGTGCCAGCAGCCGCGGTAATACGGAGGGGGCTAGCGTTGTTCGGAATTACTGGGCGTAAAGCGCGCGTAGGCGGCTTAGCAAGTCAGGGGTGAAAGGCCTGGGCTTAACCCGGGAGTGGCCCTTGAGACTGCTAGGCTTGAGAGCGGGAGAGGATGGTGGAATTCCCAGTGTAGAGGTGAAATTCGTAGATATTGGGAAGAACACCGGTTGCGAAGGCGGCCATCTGGCCCGCCACTGACGCTGGAGCGCGAAAGCGTGGGGAGCAAACAGG` |
| ASV5213 | 429 | `TGGGGAATTTTGGACAATGGGGGAAACCCTGATCCAGCAACGCCGCGTGAATGAAGAAGGCCTTAGGGTTGTAAAGTTCTTTTGTCAGAGAGCAAATCCTTGATGCTAATACCATTAAGGGATGAGAGTATCTGAAGAATAAGCACCGGCTAACTACGTGCCAGCAGCCGCGGTAATACGTAGGGTGCGAGCGTTAATCGGAATTACTGGGCGTAAAGGGTGCGCAGGCGGATATTTAAGTTAGATGTGAAATCCCTGAGCTTAACTTGGGAATGGCATTTAAGACTGGATATCTAGAGTTGGTCAGAGGGGGGTGGAATTTCTGGTGTAGCAGTGAAATGCGTAGAGATCAGAAGGAACATCGATGGCGTAGGCAGCCCCCTGGGATTAAACTGACGCTCAGGCACGAAAGCGTGGGTAGCAAACAGG` |
| ASV4499 | 429 | `TGGGGAATTTTGGACAATGGGCGCAAGCCTGATCCAGCCATGCCGCGTGAGTGAAGAAGGCCTTCGGGTTGTAAAGCTCTTTCGGCAGGAACGAAATGGTTCGTGCTAATACCACGGATTGATGACGGTACCTGAAGAAGAAGCACCGGCTAACTACGTGCCAGCAGCCGCGGTAATACGTAGGGTGCAGGCGTTAATCGGAATTACTGGGCGTAAAGCGTGCGCAGGCGGTTTCTTAAGTCAGATGTGAAAGCCCCGGGCTTAACCTGGGAACTGCGTTTGAAACTGGGAGACTTGAGTGTGGCAGAGGGGGGTGGAATTCCACGTGTAGCAGTGAAATGCGTAGATATGTGGAGGAACACCGATGGCGAAGGCAGCCCCCTGGGTTAACACTGACGCTCATGCACGAAAGCGTGGGGAGCAAACAGG` |
| ASV4813 | 405 | `TTAGGAATCTTGGGCAATGGGCGCAAGCCTGACCCAGCAACGCCGCGTGGGTGACGAAGGCCTTCGGGTCGTAAAGCTCTTTTCCCGGGGAAGATAATGACGGTACCTGGGGAATAAGCCCCGGCTAACTACGTGCCAGCAGCCGCGGTAATACGTAGGGGGCGAGCGTTGTCCGGATTTATTGGGCGTAAAACGCTTGTAGGCGGCCACGCCACGTCGGACGTGAAATCTCCCGGCTTAACTGGGAGGGGTCGTGCGATACGGGCGGGCTCGAGGCCGGTAGGGGGAAGCGGAATTCCCGGTGTAGTGGTGGAATGCGTAGATATCGGGAGGAACACCAGAGGCGCAGGCGGCTTCCTGGACCGGAACTGACGCTGAGGAGCGAAAGCGTGGGGAGCGATCCGG` |
| ASV5125 | 417 | `TTAGGAATCTTCCACAATGGGCGCAAGCCTGATGGAGCGACGCCGCGTGAGGGATGACGGTTTTCGGATTGTAAACCTCTGAATCTGGGACGAAAGACGCGACGAGCGGGATGACGGTACCAGAGTAATAGCACCGGCTAACTCCGTGCCAGCAGCCGCGGTAATACGGAGGGTGCAAGCGTTACCCGGAATCACTGGGCGTAAAGGGCGTGTAGGCGGGTCACTCAGTCTGGTTTTAAAGACCGGGGCTCAACCCCGGGGATGGACTGGATACTGGTGACCTTGACCTCTGGAGAGAGAACTGGAATTCCTGGTGTAGCGGTGGAATGCGTAGATACCAGGAGGAACACCAATGGCGAAGGCAGGTTCTTGGACAGAAGGTGACGCTGAGGCGCGAAAGTGTGGGGAGCGAACCGG` |
| ASV406 | 429 | `TGGGGAATTTTGGACAATGGGCGCAAGCCTGATCCAGCAATGCCGCGTGAGTGAAGAAGGCCTTCGGGTTGTAAAGCTCTTTTGTCAGGGAAGAAACGGTTGTGGCTAATATCTGCAACTAATGACGGTACCTGAAGAATAAGCACCGGCTAACTACGTGCCAGCAGCCGCGGTAATACGTAGGGTGCAAGCGTTAATCGGAATTACTGGGCGTAAAGCGTGCGCAGGCGGTTTTGTAAGTCTGTCGTGAAATCCCCGGGCTCAACCTGGGAATGGCGATGGAGACTGCAAGGCTAGAATCTGGCAGAGGGGGGTAGAATTCCACGTGTAGCAGTGAAATGCGTAGAGATGTGGAGGAACACCGATGGCGAAGGCAGCCCCCTGGGTCAAGATTGACGCTCATGCACGAAAGCGTGGGGAGCAAACAGG` |
| ASV2365 | 403 | `TGGGGAATATTGCACAATGGGCGAAAGCCTGATGCAGCAACGCCGCGTGAGCGATGAAGGCCTCCGGGTCGTAAAGCTCTGTCCTCAAGGAAGATAATGACGGTACTTGAGGAGGAAGCCCCGGCTAACTACGTGCCAGCAGCCGCGGTAATACGTAGGGGGCTAGCGTTATCCGGAATTACTGGGCGTAAAGGGTGCGTAGGTGGTTTCTTAAGTCAGAAGTGAAAGGCTACGGCTCAACCGTAGTAAGCTTTTGAAACTAAGAAACTTGAGTGCAGGAGAGGAGAGTAGAATTCCTAGTGTAGCGGTGAAATGCGTAGATATTAGGAGGAATACCAGTTGCGAAGGCGGCTCTCTGGACTGTAACTGACACTGAGGCACGAAAGCGTGGGGAGCAAACAGG` |
| ASV4522 | 404 | `TGGGGAATATTGGACAATGGGGGAAACCCTGATCCAGCGATGCCGCGTGAGTGAAGAAGGCCTTCGGGTTGTAAAGCTCTTTCATCTGTGAAGATAATGACGGTAGCAGAAGAAGAAGCACCGGCAAACTCTGTACCAGCAGCCGCGGTAATACAGAGGGTGCGAGCGTTGTTCGGAATGACTGGGCGTAAAGGGCGCGTAGGTGGCTGATCAAGTCAGGTGTGAAAGCCCCGGGCTTAACCTGGGATGGGCATTTGATACTGGTCGGCTAGAGTACGAAAGAGGAAAGTGGAATTCCTAGTGTAGAGGTGAAATTCGTAGATATTAGGAGGAACATCGGAGGCGAAGGCGGCTTTCTGGTTCGTAACTGACACTGAGGCGCGAAAGTGTGGGGAGCAAACAGG` |
| ASV1362 | 422 | `TAGGGAATATTGGTCAATGGGCGCAAGCCTGAACCAGCCACGCCGCGTGCGGGAGGAAGGCCCTATGGGTCGTAAACCGCTTTTGCCGGGGAAGAAACCTCTCTTTTAGGAGAGCTGACGGTACCCGGAGAATAAGGACCGGCTAACTCCGTGCCAGCAGCCGCGGTAATACGGAGGGTCCGAGCGTTGTCCGGATTTATTGGGTTTAAAGGGTGCGTAGGCGGGCCACTAAGCGGGCGGTGAAAGCGCGCCGCTCAACGGCGCAAGCGCCGCCCGAACTGGTGGCCTGGAGTGCAGACGACGTGGGCGGAATGTGCCGTGTAGCGGTGAAATGCTTAGATATGGCACAGAACGCCGATTGCGAAGGCAGCTCACGAGGGTGCAACTGACGCTGAGGCACGAAAGCGTGGGGATCAAACAGG` |
| ASV2841 | 404 | `TGGGGAATATTGGACAATGGGGGAAACCCTGATCCAGCAATGCCGCGTGGATGATGAAGGCCTTCGGGTTGTAAAGTCCTTTTGGCGGGGACGATGATGACGGTACCCGCCGAATAAGCCCCGGCTAACTTCGTGCCAGCAGCCGCGGTAATACGAAGGGGGCTAGCGTTGTTCGGAATTACTGGGCGTAAAGGGCGCGTAGGCGGCTTCAACAGTCGGGCGTGAAATGCCTGGGCTCAACCCGGGAACTGCGTTCGAAACTGCGAGGCTAGAGTGTGGGAGAGGTTGGTGGAATTCCCAGTGTAGAGGTGAAATTCGTAGATATTGGGAAGAACACCGGTGGCGAAGGCGGCCAACTGGACCACAACTGACGCTGAGGCGCGAAAGCGTGGGGAGCAAACAGG` |
| ASV4044 | 429 | `TGGGGAATTTTGGACAATGGGCGCAAGCCTGATCCAGCCATGCCGCGTGAGTGAAGAAGGCCTTCGGGTTGTAAAGCTCTTTTGTCCGGGAGCAAATCCTACGTCCTAATACGGCGTGGGGATGAGAGTACTGGAAGAATAAGCACCGGCTAACTACGTGCCAGCAGCCGCGGTAATACGTAGGGTGCGAGCGTTAATCGGAATTACTGGGCGTAAAGCGTGCGCAGGTGGTTTTGTAAGCCAGATGTGAAATCCCCGGGCTTAACCTGGGAATGGCATTTGGGACTGCAAGGCTAGAGTGTGGCAGAGGGGGGTGGAATTCCGCGTGTAGCAGTGAAATGCGTAGAGATGCGGAGGAACACCGATGGCGAAGGCAGCCCCCTGGGCTAACACTGACACTCATGCACGAAAGCGTGGGGAGCAAACAGG` |
| ASV4232 | 404 | `TGGGGAATATTGGACAATGGGGGCAACCCTGATCCAGCAATACCACGTGAGTGATGAAGGCCTTCGGGTTGTAAAACTCTTTTAGTAGGGAAGATAATGACGGTACCTACAGAAAAAGCACCGGCTAACTTCGTGCCAGCAGCCGCGGTAATACGAAGGGTGCTAGCGTTGTTCGGAATCATTGGGCGTAAAGCGTGCGTAGGCGGTTTAGTAAGTTGACTGTGAAAGCCCAGGGCTTAACCTTGGAATTGCAGCCAAAACTACTAAACTCGAGTATGGCGGGGGATAGTGGAATTCTTAGTGTAGGGGTGAAATCCGTAGATATTAAGAGGAACACCAGTGGCGAAGGCGACTATCTACACCATCACTGACGCTGAGGCACGAAAGCGTGGGGAGCAAACAGG` |
| ASV5431 | 404 | `TTAGGAATATTGGACAATGGGCGCAAGCCTGATCCAGCCATGCCGCGTGAACGATGAAGGCCTTCGGGTTGTAAAGTTCTTTAGCCGATGATTATAATGACTGTAGTCGGAAAATAAGCCCCGGCTAACTTCGTGCCAGCAGCCGCGGTAATACGAAGGGGGCGAGCGTTATTCGGAATCACTGGGCGTAAAGCGTGCGTAGGCGGCCTCGCAAGTTAGAAGTGAAAGCCCAGGGCTCAACCCTGGAATTGCTTTTAAAACTGCAAGGCTCGAATCTCGGAGAGGATAGCGGAATTTCCAGTGTAGCAGTGAAATGCGTAGATATTGGAAGGAACACCAGTGGCGTAAGCGGCTATCTGGACGAGTATTGACGCTGAGGCACGAAAGCGTGGGGATCAAACAGG` |
| ASV5005 | 404 | `TGGGGAATATTGCACAATGGGGGAAACCCTGATGCAGCAACGCCGCGTGAAGGACGACGTATTTCGGTATGTAAACTTCTATCAGCAAGGAAGATGATGACGGTACTTGACTAAGAAGCCCCGGCTAACTACGTGCCAGCAGCCGCGGTAATACGTAGGGGGCAAGCGTTATCCGGATTTACTGGGTGTAAAGGGAGCGTAGGCGGCCATGCAAGTCAGAAGTGAAAGCCCGGGGCTCAACCCCGGGACTGCTTTTGAAACTGTAAGGCTAGATTGCCGGAGAGGTAAGCGGAATTCCTAGTGTAGCGGTGAAATGCGTAGATATTAGGAGGAACACCAGTGGCGAAGGCGGCTTACTGGACGGTCAATGACGCTGAGGCTCGAAAGCGTGGGGAGCAAACAGG` |
| ASV3326 | 429 | `TCGAGAATAATTCACAATGGGGGCAACCCTGATGGTGCAACGCCGCGTGGAGGATGACGGTCTTCGGATTGTAAACTCCTGTCATCTGGGAGAAAGACCTGGGAGTTAATAGCTCACAGGGTTGATAGTACCAGAAGAGGAAGGGACGGCTAACTTCGTGCCAGCAGCCGCGGTAATACGAAGGTCCCAAGCGTTGTTCGGAATCACTGGGCGTAAAGGGAGCGTAGGCGGCGCGGTAAGTCAGATGTGAAATCCCGGGGCTCAACCCCGGAACTGCATCCGATACTGCCGTGCTAGAGCAATGGAGAGGTAGCTGGAATTATCGGTGTAGCAGTGAAATGCGTAGATATCGATAGGAACACTCGTGGCGAAAGCGAGCTACTGGACATTTGCTGACGCTGAGGCTCGAAGGCTAGGGGAGCGAAAGGG` |
| ASV5497 | 405 | `TGAGGAATATTGGGCAATGGGCGCAAGCCTGACCCAGCAATGCCGCGTGCAGGATGACGCCCTTCGGGGTGTAAACTGCTTTTGTGCGGGAGGAGACAGGACCGTACCGCACGAATCAGTCACGGCTAACTACGTGCCAGCAGCCGCGGTAACACGTAGGTGACGAACGTTATCCGGAATTACTGGGCGTAAAGGGTCCGTAGGCGGGATGGCAAGTCTGGTGTGAAAGCCCTCGGCTCAACTGGGGGAAGCCATTGGAGACTGCCGTTCTTGAATCCTAGCGAGGTCGGTGGAATTCCCGGTGTAGTGGTGGAATGCGTAGAGATCGGGAGGAACCTCGAAAGCGAAGGCAACCGGCTAGCTGCGGATTGACGCTGAGGGACGAGAGCGTGGGGAGCGAACAGG` |
| ASV2296 | 429 | `TGGGGAATTTTGGACAATGGGCGCAAGCCTGATCCAGCCATGCCGCGTGTGTGAAGAAGGCCTTCGGGTTGTAAAGCACTTTCGGCCGGAACGAAATCGCGCGGGTGAACATCCCGCGTGGATGACGGTACCGGAAGAAGAAGCACCGGCTAACTACGTGCCAGCAGCCGCGGTAATACGTAGGGTGCGAGCGTTAATCGGAATTACTGGGCGTAAAGGGTGCGCAGGCGGCCCCGCAAGTCAGGCGTGAAATCCCCGGGCTTAACCTGGGAATGGCGCTTGAAACTACGGGGCTGGAGTATGGCAGAGGGAGGTGGAATTCCACGTGTAGCGGTGAAATGCGTAGAGATGTGGAGGAACACCGATGGCGAAGGCAGCCTCCTGGGCCAATACTGACGCTCATGCACGAAAGCGTGGGGAGCAAACAGG` |
| ASV2461 | 429 | `TGGGGAATTTTGGACAATGGGCGCAAGCCTGATCCAGCCATGCCGCGTGAGTGAAGAAGGCCTTCGGGTTGTAAAGCTCTTTCGGTGAAGACGAAACGGTTCGCGCTAATAATGCGAACTAATGACGGTACTCACAGAAGAAGCACCGGCTAACTACGTGCCAGCAGCCGCGGTAATACGTAGGGTGCAGGCGTTAATCGGAATTACTGGGCGTAAAGCGTGCGCAGGCGGTTTGCTAAGACAGGTGTGAAATCCCCGGGCTTAACCTGGGAATTGCGCTTGTAACTGGCAGGCTAGAGTGTGGCAGAGGGGGGTGGAATTCCACGTGTAGCAGTGAAATGCGTAGATATGTGGAGGAACACCGATGGCGAAGGCAGCCCCCTGGGTTAACACTGACGCTCATGCACGAAAGCGTGGGGAGCAAACAGG` |
| ASV4037 | 404 | `TGGGGAATATTGGGCAATGGGCGAAAGCCTGACCCAGCTATGCCGCGTGAGTGATGAAGGCCTTAGGGTTGTAAAGCTCTTTTAGATGGGAAGATAATGACGGTACCATCAGAAAAAGCCCCGGCTAACTTCGTGCCAGCAGCCGCGGTAATACGAAGGGGGCAAGCGTTGCTCGGATTTATTGGGCGTAAAGGGTGTGTAGGTGGTTTGATAAGTTAGATGTGAAAGCCCGGGGCTTAACCCCGGAACTGCATTTAAAACTGTTGAACTTGAAGATCACAGAGGTAAGCAGAATTTCCAGTGTAGAGGTGAAATTCGTAGATATTGGAAAGAATACCAACGGCGAAGGCAGCTTACTGGGTGACTCTTGACACTGAGGCACGAAAGCGTGGGGAGCAAACAGG` |
| ASV1283 | 429 | `TGGGGAATATTGCACAATGGGCGCAAGCCTGATGCAGCCATGCCGCGTGTGTGAAGAAGGCCTTCGGGTTGTAAAGCACTTTCAGCGAGGAGGAAGGGTTAAGTGTTAATAGCACTGAACATTGACGTTACTCGCAGAAGAAGCACCGGCTAACTCCGTGCCAGCAGCCGCGGTAATACGGAGGGTGCAAGCGTTAATCGGAATTACTGGGCGTAAAGCGCACGCAGGCGGTTTGTTAAGTCAGATGTGAAATCCCCGAGCTTAACTTGGGAACTGCATTTGAAACTGGCAAGCTAGAGTCTTGTAGAGGGGGGTAGAATTCCAGGTGTAGCGGTGAAATGCGTAGAGATCTGGAGGAATACCGGTGGCGAAGGCGGCCCCCTGGACAAAGACTGACGCTCAGGTGCGAAAGCGTGGGGAGCAAACAGG` |
| ASV4612 | 420 | `TAGGGAATTTTGCGCAATGGACGAAAGTCTGACGCAGCAACGCCATGTGTGGGATGAAGCATTTCGGTGTGTAAACCACTGTCAGCAGGGAATAACAACCGCTTCGGCGGCCATGAATGTACCTGCAAAGGAAGCCCCGGCAAACTTCGTGCCAGCAGCCGCGGTAATACGAGGGGGGCAAATGTTGTTCGGAATCACTGGGCGTAAAGGGAGCGTAGGTGGGAGTTTAAGTTGGGTGTTTAATCCCGGGGCCCAACCCCGGATCGGCACCCAAAACTGGGCTTCTTGAATGGGACAGAGGCTGATGGAATTGGGGGTGTAGCGGTGGAATGCGTAGAGATCCCCAAGAACACCAGTTGCGAAGGCGGTCAGCTGGGTCCACATTGACACTGAGGCTCGAAAGCGTGGGGAGCAAACAGG` |
| ASV1970 | 428 | `TTTCGAATCATTCACAATGGGCGAAAGCCTGATGGTGCGACGCCGCGTGAGGGATGAAGGTCTTCGGATTGTAAACCTCTGTCACCGGGGAAGAAACGCTTCAGGTTAATAGTCTGAAGCCTGACTTAACCCGGAGAGGAAGCAGTGGCTAACTCTGTGCCAGCAGCCGCGGTAATACAGAGACTGCAAGCGTTATTCGGATTCACTGGGCGTAAAGGGTGCGCAGGCGGCCATGTGTGTGAGGCGTGAAAGCCCGGGGCTTAACCCCGGAATTGCACCTCAAACTACATGGCTAGAGCATTGGAGAGGGTAGCAGAATTCACGGTGTAGCAGTGAAATGCGTAGATATCGTGAGGAATACCAGAGGCGAAGGCGGCTACCTGGACAATTGCTGACGCTCAGGCACGAAAGCGTGGGGAGCAAAAGGG` |
| ASV5138 | 428 | `TCGAGAATTTTTCTCAATGGGGGAAACCCTGAAGGAGCGACGCCGCGTGGAGGATGAAGGTCTTCGGATTGTAAACTCCTGTCATTAGGGAACAAAGCCTGCGGTTAACTGCCGTTGGGACTGATAGTACCTGAAGAGGAAGAGACGGCTAACTCTGTGCCAGCAGCCGCGGTAATACAGAGGTCTCAAGCGTTGTTCGGATTTATTGGGCGTAAAGGGTGCGTAGGCGGCGGAGTAAGTCAGGTGTGAAATCCCGGGGCTCAACCTCGGAACTGCACTTGATACTGCTTTGCTTGAGAACTGGAGAGGAGACTGGAATTTACGGTGTAGCAGTGAAATGCGTAGATATCGTAAGGAAGGCCAGTGGCGAAGGCGAGTCTCTGGAAAGTATCTGACGCTGAGGCACGAAGGCCAGGGGAGCAAACGGG` |
| ASV2979 | 404 | `TGGGGAATCTTAGACAATGGGCGAAAGCCTGATCTAGCCATGCCGCGTGGGTGATGAAGGCCTTAGGGTCGTAAAGCCCTTTCGCCGGGGAAGATAATGACGGTACCCGGTAAAGAAACCCCGGCTAACTCCGTGCCAGCAGCCGCGGTAATACGGAGGGGGTTAGCGTTGTTCGGAATGACTGGGCGTAAAGCGCGCGTAGGCGGATCGGATAGTTGGGGGTGAAAGCCCAGGGCTCAACCCTGGAATGGCCTTCAAGACTCCCGGTCTGGAGTTCGAGAGAGGTGGGTGGAATTCCGAGTGTAGAGGTGAAATTCGTAGATATTCGGAGGAACACCAGTGGCGAAGGCGGCCCACTGGCTCGACACTGACGCTGAGGCGCGAAAGCGCGGGGAGCAAACAGG` |
| ASV2180 | 429 | `TGGGGAATATTGGACAATGGGGGCAACCCTGATCCAGCCATGCCGCGTGTGTGAAGAAGGCCTTCGGGTTGTAAAGCACTTTAAGCGAGGAGGAAGGTTTACTAGATAATACCTAGTAGAATTGACGTTACTCGCAGAATAAGCACCGGCTAACTCTGTGCCAGCAGCCGCGGTAATACAGAGGGTGCAAGCGTTAATCGGAATTACTGGGCGTAAAGCGCGCGTAGGCGGTTTGTTAAGTCGGATGTGAAATCCCTGGGCTCAACCTAGGCACTGCATCCGATACTGGCTAACTAGAGTGTGGGAGAGGAAGGTAGAATTCCAGGTGTAGCGGTGAAATGCGTAGAGATCTGGAGGAATACCGATGGCGAAGGCAGCCTTCTGGCCTAACACTGACGCTGAGGTGCGAAAGCATGGGGAGCAAACAGG` |
| ASV347 | 429 | `TGGGGAATTTTGGACAATGGGCGCAAGCCTGATCCAGCAATGCCGCGTGAGTGAAGAAGGCCTTCGGGTTGTAAAGCTCTTTTGTCAGGGAAGAAACGGTTGTTCCTAATACGGATGGCTAATGACGGTACCTGAAGAATAAGCACCGGCTAACTACGTGCCAGCAGCCGCGGTAATACGTAGGGTGCAAGCGTTAATCGGAATTACTGGGCGTAAAGCGTGCGCAGGCGGTTATATAAGTCAGATGTGAAATCCCCGGGCTCAACCTGGGAACTGCATTTGAGACTGTATGGCTAGAGTGTGTCAGAGGGGGGTAGAATTCCACGTGTAGCAGTGAAATGCGTAGATATGTGGAGGAATACCGATGGCGAAGGCAGCCCCCTGGGATAACACTGACGCTCATGCACGAAAGCGTGGGGAGCAAACAGG` |
| ASV525 | 404 | `TGGGGAATCTTAGACAATGGGGGAAACCCTGATCTAGCCATGCCGCGTGATCGATGAAGGCCTTAGGGTTGTAAAGATCTTTCAGTGGGGAAGATAATGACGGTACCCACAGAAGAAGCCCCGGCTAACTCCGTGCCAGCAGCCGCGGTAATACGGAGGGGGCTAGCGTTATTCGGAATTACTGGGCGTAAAGCGCACGTAGGCGGACTGGAAAGTCAGAGGTGAAAGCCCAGGGCTCAACCCTGGAACTGCCTTTGAAACTCCCAGTCTTGAGGTCGTGAGAGGTGAGTGGAATTCCGAGTGTAGAGGTGAAATTCGTAGATATTCGGAGGAACACCAGTGGCGAAGGCGGCTCACTGGCACGATACTGACGCTGAGGTGCGAAAGCGTGGGGAGCAAACAGG` |
| ASV3606 | 404 | `TGGGGAATATTGGACAATGGGCGCAAGCCTGATCCAGCAATGCCGCGTGAGTGATGAAGGCCTTAGGGTTGTAAAGCTCTTTCGCACGTGACGATGATGACGGTAACGTGAGAAGAAGCCCCGGCTAACTTCGTGCCAGCAGCCGCGGTAATACGAAGGGGGCTAGCGTTGTTCGGAATTACTGGGCGTAAAGGGCGCGTAGGCGGCCCGATCAGTCAGAAGTGAAAGCCCCGGGCTCAACCTGGGAACTGCTTTTGATACTGTCGGGCTTGAGTTTCGGAGAGGATGGTGGAATTCCCAGTGTAGAGGTGAAATTCGTAGATATTGGGAAGAACACCAGTGGCGAAGGCGGCCATCTGGACGAACACTGACGCTGAGGCGCGAAAGCGTGGGGAGCAAACAGG` |
| ASV39 | 423 | `TAGGGAATATTGGTCAATGGACGGAAGTCTGAACCAGCCATGCCGCGTGCAGGAAGAAGGCGCTCTGCGTTGTAAACTGCTTTAGACTAGGAACAAAACGACTCTGCGGAGTTACTTGAGTGTACTAGTAGAATAAGCCACGGCTAACTACGTGCCAGCAGCCGCGGTAATACGTAGGTGGCAAGCGTTGTCCGGATTTATTGGGTTTAAAGGGTGCGTAGGCGGCTAAATAAGTCAGTGGTGAAATACTCGAGCTTAACTTGAGAACGGCCAATGATACTGTTTAGCTTGAGTACAAACGAAGTGGGCGGAATTGAAAGTGTAGCGGTGAAATGCATAGATATTTTCAAGAACACCGATAGCGAAGGCAGTTCACTAGGATGTAACTGACGCTGAGGCACGAAAGTGTGGGGATCAAACAGG` |
| ASV5707 | 428 | `TCGAGAATTTTTCTCAATGGGGGAAACCCTGAAGGAGCGACGCCGCGTGGAGGATGAAGGTCTTCGGATTGTAAACTCCTGTCATTGGGGAACAAAACTTCCGGTTAACTGCCGGCGAGTCTGATAGTACCCGAAGAGGAAGAGACGGCTAACTCTGTGCCAGCAGCCGCGGTAATACAGAGGTCTCAAGCGTTGTTCGGATTCATTGGGCGTAAAGGGTGCGTAGGCGGCGAGGTAAGTCGGATGTGAAATCCCGGGGCTCAACCCCGGAACTGCATTCGATACTGCCTTGCTCGAGTACTGGAGAGGAGACTGGAATTTACGGTGTAGCAGTGAAATGCGTAGATATCGTAAGGAAGACCAGTGGCGAAGGCGAGTCTCTGGACAGTTACTGACGCTGAGGCACGAAGGCCAGGGGAGCAAACGGG` |
| ASV2912 | 404 | `TGGGGAATATTGGACAATGGGCGCAAGCCTGATCCAGCCATGCCGCGTGAGTGATGAAGGCCTTAGGGTTGTAAAGCTCTTTTGCCGGGGACGATAATGACGGTACCCGGAGAATAAGTCCCGGCTAACTTCGTGCCAGCAGCCGCGGTAATACGAAGGGGACTAGCGTTGTTCGGAATCACTGGGCGTAAAGCGCACGTAGGCGGATCATTTAGTCAGGGGTGAAATCCCGGGGCTCAACCTCGGAACTGCCTTTGATACTGATGGTCTCGAGTCCGATAGAGGTGGGTAGAATTCCTAGTGTAGAGGTGAAATTCGTAGATATTAGGAAGAATACCGGTGGCGAAGGCGGCCCACTGGATCGGTACTGACGCTGAGGTGCGAAAGCGTGGGGAGCAAACAGG` |
| ASV4547 | 404 | `TCGGGAATTTTGGGCAATGGGCGAAAGCCTGACCCAGCAACGCCGCGTGAAGGATGAAGTTTTTCGGAATGTAAACTTCGCAAGAATAGGAAGAATAAATGACGGTACTATTTGTAAGCTCCGGCTAACTACGTGCCAGCAGCCGCGGTAATACGTAGGGAGCAAGCGTTGTTCGGATTTACTGGGCGTAAAGGGTGCGTAGGCGGCGTGATAAGTCAGCTGTGAAATCTCCGAGCTTAACTCGGAACGGTCAGCTGATACTGTCGTGCTAGAGTGCAGGAAGGGCAATCGGAATTCTTGGTGTAGCGGTGAAATGCGTAGATATCAAGAGGAACACCTGAGGCGAAGGCGGGTTGCTAGACTGACACTGACGCTGAGGCACGAAAGCTAGGGGAGCAAACAGG` |
| ASV1031 | 424 | `TGAGGAATATTGGACAATGGGCGCAAGCCTGATCCAGCCATGCCGCGTGCAGGATGACGGTCCTATGGATTGTAAACTGCTTTTGTACGAGAAGAAACACTACTTCGTGAAGTAGCTTGACGGTATCGTAAGAATAAGGATCGGCTAACTCCGTGCCAGCAGCCGCGGTAATACGGAGGATCCAAGCGTTATCCGGAATCATTGGGTTTAAAGGGTCCGTAGGCGGTTTAGTAAGTCAGTGGTGAAAGCCCATCGCTCAACGGTGGAACGGCCATTGATACTGCTAGACTTGAATTATTAGGAAGTAACTAGAATATGTAGTGTAGCGGTGAAATGCTTAGAGATTACATGGAATACCAATTGCGAAGGCAGGTTACTACTAATGGATTGACGCTGATGGACGAAAGCGTGGGTAGCGAACAGG` |
| ASV3163 | 404 | `TGGGGAATATTGGACAATGGGCGAAAGCCTGATCCAGCAATGCCGCGTGAGTGATGAAGGCCTTCGGGTTGTAAAGCTCTTTTACCCGGGAAGATAATGACTGTACCGGGAGAATAAGCCCCGGCTAACTTCGTGCCAGCAGCCGCGGTAATACGAAGGGGGCTAGCGTTGTTCGGAATTACTGGGCGTAAAGCGCACGTAGGCGGCTTCTTAAGTTAGGGGTGAAAGCCCAGGGCTCAACCCTGGAAATGCCCTTAAGACTGGGAAGCTTGAACACGGGAGAGGTGAGTGGAATTCCGAGTGTAGAGGTGAAATTCGTAGATATTCGGAGGAACACCAGTGGCGAAGGCGGCTCACTGGACCGTAGTTGACGCTGAGGTGCGAAAGCGTGGGGAGCAAACAGG` |
| ASV5134 | 409 | `TGGGGAATATTGCACAATGGGCGGAAGCCTGATGCAGCAACGCCGCGTGCGGGATGACGGCCTTCGGGTTGTAAACCGCTTTCAGTAGGGACGAAGCGCAAGTGACGGTACCTACAGAAGAAGCACCGGCTAACTACGTGCCAGCAGCCGCGGTGATACGTAGGGTGCGAGCGTTGTCCGGAATTATTGGGCGTAAAGAGCTTGTAGGCGGTTGGTTGCGTCGTAAGTGAAAACTCAGGGCTTAACCCTGGGCCTGCTTGCGATACGGGCTGACTTGAGGAAGGTAGGGGAGAATGGAATTCCCGGTGGAGCGGTGGAATGCGCAGATATCGGGAGGAACACCAGTGGCGAAGGCGGTTCTCTGGACCTTTCCTGACGCTGAGAAGCGAAAGCGTGGGGAGCAAACAGG` |
| ASV2929 | 429 | `TCGAGAATTTTTCTCAATGGGGGAAACCCTGAAGGAGCGACGCCGCGTGAAGGATGAAGGTCTTCGGATCGTAAACTTCTGTCATTAGAGAACAAAGCCCACCGATTAACTGTCGGGGGGTCTGATAGTATCTGAAGAGGAAGGGACGGCTAACTCTGTGCCAGCAGCCGCGGTAATACAGAGGTCCCAAGCGTTGTTCGGATTTATTGGGCGTAAAGGGTGCGTAGGCGGTCGCCTAAGTCAGGTGTGAAATCTCGGGGCTCAACCTCGAAACTGCACTTGATACTGGGTGGCTAGAGTACTGGAGAGGAGACTGGAATTTACGGTGTAGCAGTGAAATGCGTAGATATCGTAAGGAAGACCAGTGGCGAAGGCGAGTCTCTGGACAGTTACTGACGCTGAGGCACGAAGGCCAGGGGAGCAAACGGG` |
| ASV1949 | 404 | `TGGGGAATATTGGACAATGGGGGCAACCCTGATCCAGCCATGCCGCGTGGATGATGAAGGCCTTAGGGTTGTAAAATCCTTTCACCGGAGAAGATAATGACGGTATCCGGAGAAGAAGCCCCGGCTAACTTCGTGCCAGCAGCCGCGGTAATACGAAGGGGGCTAGCGTTGCTCGGAATTACTGGGCGTAAAGCGCACGTAGGCGGGTCATTAAGTCAGGGGTGAAAGCCCACAGCTCAACTGTGGAACTGCCCTTGATACTGGTGACCTAGACCAAAGTAGAGGCAGGTGGAATTCCGAGTGTAGAGGTGGAATTCGTAGATATTCGGAGGAACACCAGTGGCGAAGGCGGCCTGCTGGACTTTTGGTGACGCTGAGGTGCGAAAGCGTGGGGAGCAAACAGG` |
| ASV4193 | 404 | `TCGGGAATTTTGGGCAATGGGCGAAAGCCTGACCCAGCAACGCCGCGTGAAGGATGAAGTATTTCGGTATGTAAACTTCGTAAGAATAGGAAGAATAAATGACGGTACTATTTGTAAGGTCCGGCTAACTACGTGCCAGCAGCCGCGGTAATACGTAGGGACCAAGCGTTGTTCGGATTTACTGGGCGTAAAGGGCGCGTAGGCGGCGTGGTAAGTCATCTGTGAAATCTCTGAGCTTAACTCGGAACGGTCAGATGATACTGCTGTGCTAGAGTACAGAAGGGGCAATCGGAATTCTTGGTGTAGCGGTGAAATGCGTAGATATCAAGAGGAACACCTGAGGCGAAGGCGGGTTGCTGGGCTGATACTGACGCTGAAGCGCGAAAGCTAGGGTAGCAAACGGG` |
| ASV4337 | 430 | `TGGGGAATTTTGCACAATGGAGGAAACTCTGATGCAGCAACGCCGCGTGAGTGATGAAGGCCTTTGGGTTGTAAAGCTCTTTCGGTTGGGGAGAAGGGTACAGTGGCTAATATCCATTGTATTTGACGGTACCAAAAGAAGAAGCACCTGCTAACTTCGTGCCAGCAGCAGCGGTAATACGAAGGGTGCAAGCGTTGTTCGGAATTACTGGGCGTAAAGGGCGCGTAGGCTGGTTTGTAAGTCTAATGTGAAATCCCCGGGCTTAACCCGGGACGTGCATTAGAAACTGCAGATCTTGAATGCTTAAGAGGGTGGTGGAATTCCAGGAGTAGAAGTGAAATTCGTAGATATCTGGAGGAACACCTGAGGCGAAGGCGGCCACCTGGTAAGACATTGACGCTGAGGCGCGAAAGTGCGGGGAGCAAACAGG` |
| ASV5032 | 407 | `TAGGGAATTTTGCACAATGGACGAAAGTCTGATGCAGCGACGCCGCGTGAGTGATGAAGGCCTTCGGGTTGTAAAGCTCTGTTCTCAGGGAATAAAAAAATGAAGGTACCTGAGGAGAAAGGACCGGCTAACTTCGTGCCAGCAGCCGCGGTAAGACGAGGGGTCCAAGCGTTGTTCGGAATCATTGGGCGTAAAGCGGGTGTAGGTGGATTTGTAAGTCAGGAGTGAAAGCCCCAGGCTTAACCTGGGAAGTGCTTTTGATACTGCAAATCTTGAATGTGGGAGAGGATGGTGGAATTCCTGGTGTAGTGGTGAAATACGTAGATATCAGGAGGAACTCCGGTGGCGAAGGCGGCCATCTGGCCCAACATTGACACTGAGACCCGAAAGCGTGGGGATCAAACAGG` |
| ASV5106 | 404 | `TGGGGAATATTGGACAATGGGCGAAAGCCTGATCCAGCGACGCCGCGTGGATGATGAAGGCCTTAGGGTTGTAAAGTCCTTTCAGTGGGGAAGATAATGACGGTACCCACAGAAGAAGCTCCGGCTAACTTCGTGCCAGCAGCCGCGGTAATACGAAGGGAGCTAGCGTTACTCGGAATTATTGGGCGTAAAGCGTGCGTAGGCGGTTTTGTAAGTTGGAAGTGAAAGCCCAGGGCTCAACCCTGGAATAGCTTTCAAAACTACAAGACTAGAGTATCAGAGAGGATAGTGGAATTCCTAGTGTAGAGGTGAAATTCGTAGATATTAGGAGGAACACCGGTGGCGAAGGCGACTATCTGGCTGAATACTGACGCTGTTGCACGAAAGCGTGGGGAGCAAACAGG` |
| ASV2049 | 404 | `TGGGGAATATTGGACAATGGGCGCAAGCCTGATCCAGCCATGCCGCGTGAATGATGAAGGCCTTAGGGTTGTAAAATTCTTTCAACGGGGACGATAATGACGGTACCCGTAGAAGAAGCCCCGGCTAACTTCGTGCCAGCAGCCGCGGTAATACGAAGGGGGCTAGCGTTGCTCGGAATTACTGGGCGTAAAGAGTACGTAGGCGGACTGATTAGTTGGTGGTGAAATCCCAGGGCTCAACCTTGGAACTGCCATCAATACTGTCAGTCTGGAGCATAGTAGAGGTTAGTGGAATTCCAAGTGTAGAGGTGAAATTCGTAGATATTTGGAGGAACACCAGTGGCGAAGGCGACTGACTGGGCTATTGCTGACGCTGAGGTACGAAAGCGTGGGGAGCAAACAGG` |
| ASV773 | 429 | `TGGGGAATTTTGGACAATGGGGGCAACCCTGATCCAGCCATGCCGCGTGAGTGAAGAAGGCCTTCGGGTTGTAAAGCTCTTTCGGCCGGGAAAAAATCGCATGGGCTAATACCCTGTGTGGATGATGGTACCGGAATAAGAAGCACCGGCTAACTACGTGCCAGCAGCCGCGGTAATACGTAGGGTGCGAGCGTTAATCGGAATTACTGGGCGTAAAGCGTGCGCAGGCGGTTTTTTAAGACAGGCGTGAAATCCCCGGGCTCAACCTGGGAACTGCGCTTGTGACTGGAAGGCTAGAGTACGGCAGAGGGGGGTGGAATTCCACGTGTAGCAGTGAAATGCGTAGAGATGTGGAGGAACACCAATGGCGAAGGCAGCCCCCTGGGTCGATACTGACGCTCATGCACGAAAGCGTGGGGAGCAAACAGG` |
| ASV5029 | 423 | `TAGGGAATATTGGACAATGGGCGAGAGCCTGATCCAGCCATGCCGCGTGCAGGAAGAAGGCCCTCTGGGTTGTAAACTGCTTTTGACAGGGGCCAAAATGGGCATGCGTGCCAACTTGAGAGTACCTGTAGAATAAGCACCGGCTAACTCCGTGCCAGCAGCCGCGGTAATACGGAGGGTGCAAGCGTTGTCCGGATTTATTGGGTTTAAAGGGTGCGTAGGTGGCTTTATAAGTCAGTGGTGAAAGTCTGCAGCTTAACTGTAGAATTGCCATTGATACTGTGGAGCTTGAGTACTGATGAGGTAGGCGGAATTGATAGTGTAGCGGTGAAATGCATAGATATTATCAAGAACACCGATTGCGAAGGCAGCTTACTAAGGAGTTACTGACACTGAGGCACGAAAGCGTGGGGATCAAACAGG` |
| ASV3523 | 429 | `TGGGGAATATTGGACAATGGGCGCAAGCCTGATCCAGCAATGCCGCGTGTGTGAAGAAGGCCTTCGGGTTGTAAAGCACTTTTATCAGGAACGAAAAGCGGAGGGCTAATACCTATTCGTGCTGACGGTACCTGAGGAATAAGCACCGGCTAACTTCGTGCCAGCAGCCGCGGTAATACGAAGGGTGCAAGCGTTAATCGGAATTACTGGGCGTAAAGGGTGCGTAGATGGCCTATTAAGTCGGATGTGAAAGCCCCGGGCTCAACCTGGGAACGGCATCCGATACTGATGGGCTAGAGTGCGGTAGAGGAGAGTGGAATTCCCGGTGTAGCGGTGAAATGCGTAGAGATCGGGAGGAACATTTGTGGCGAAGGCGGCTCTCTGGACCAGCACTGACATTGAGGCACGAAAGCGTGGGGAGCAAACAGG` |
| ASV2284 | 423 | `TAGGGAATATTGGTCAATGGGCGAGAGCCTGAACCAGCCATGCCGCGTGCAGGAAGAAGGCCCTCTGGGTTGTAAACTGCTTTTGACAGGGAACAAAGAGTCCATGCGTGGATTGATGAGTGTACCTGTAGAATAAGCACCGGCTAACTCCGTGCCAGCAGCCGCGGTAATACGGAGGGTGCAAGCGTTGTCCGGATTTATTGGGTTTAAAGGGTGCGTAGGCGGCTTTTTAAGTCAGCGGTGAAATACTTCGGCTCAACCGAGGAACTGCCGTTGATACTGGAAGGCTTGAGTACTGATGAAGTGGGCGGAATTGAAGGTGTAGCGGTGAAATGCATAGATATCTTCAAGAACACCGATTGCGAAGGCAGCTCACTAAGGAGTAACTGACGCTGAGGCACGAAAGCGTGGGTAGCAAACAGG` |
| ASV4131 | 404 | `TGGGGAATATTGGACAATGGACGCAAGTCTGATCCAGCAATACCGCGTGGGTGATGAAGGTCTTCGGATTGTAAAGCCCTTTTAGTGAGGAAGATAATGACGGTACTCACAGAAAAAGCACCGGCAAACTTCGTGCCAGCAGCCGCGGTAATACGAAGGGTGCTAGCGTTGTTCGGAATCACTGGGCGTAAAGCGTACGTAGGTGGTCTTGTAAGTCGATTGTGAAATCCCCAAGCTTAACTTGGGAATTGCAACCGAAACTGCTCGGCTAGAGTGTGGTCGGGGATAGTGAAATTTCTAGTGTAGGGGTGAAATCCGTAGATATTAGAAGGAATACCGGTGGCGAAAGCGACTGTCTAGGCCATTACTGACACTGAGGTACGAAAGCGTGGGGAGCAAACAGG` |
| ASV779 | 429 | `TGGGGAATATTGGACAATGGGCGCAAGCCTGATCCAGCCATGCCGCGTGTGTGAAGAAGGCCTTCGGGTTGTAAAGCACTTTTGTTGGGAAAGAAAAGCAGCCGGTTAATACCCGGTTGTTCTGACGGTACCCAAAGAATAAGCACCGGCTAACTTCGTGCCAGCAGCCGCGGTAATACGAAGGGTGCAAGCGTTACTCGGAATTACTGGGCGTAAAGCGTGCGTAGGTGGTTTGTTAAGTCAGATGTGAAAGCCCCGGGCTCAACCTGGGAATTGCATTTGATACTGGCAGACTAGAGTGCGGTAGAGGAGAGTGGAATTCCCGGTGTAGCAGTGAAATGCGTAGAGATCGGGAGGAACATCAGTTGCGAAGGCGGCTCTCTGGACCAGCACTGACACTGAGGCACGAAAGCGTGGGGAGCAAACAGG` |
| ASV2298 | 404 | `TGGGGAATATTGGACAATGGGCGCAAGCCTGATCCAGCCATGCCGCGTGAGTGACGAAGGTCTTCGGATTGTAAAGCTCTTTTGGCGGGGACGATAATGACGGTACCCGCAGAATAAGTCCCGGCTAACTTCGTGCCAGCAGCCGCGGTAATACGAAGGGGACTAGCGTTGTTCGGAATCACTGGGCGTAAAGCGCACGTAGGCGGATTTGTAAGTCAGGGGTGAAATCCCGGGGCTCAACCTCGGAACTGCCTTTGATACTGCGAATCTCGAGTCCGGAAGAGGTGGGTGGAATTCCTAGTGTAGAGGTGAAATTCGTAGATATTAGGAAGAACACCAGTGGCGAAGGCGGCCCACTGGTCCGGTACTGACGCTGAGGTGCGAAAGCGTGGGGAGCAAACAGG` |
| ASV2790 | 431 | `TGGGGAATATTGCACAATGGGGGAAACCCTGATGCAGCAACGCCGCGTGAGTGAAGAAGGCTCTTGGGTCGTAAAGCTCTTTCGGCTGGGAAGAAGGGTCGTACGGTGAACAATCGTGCGATCTGACGGTACCAGAAGAAGAAGGACCTGCTAACTGCGTGCCAGCAGCAGCGGTAATACGTGGGGTCCGAGCGTTGTTCGGAATTACTGGGCGTAAAGCGCACATAGGTGGGCATAAAAGTCGATTGTGAAATCCCTGGGCTAAACCTGGGAACTGCAGTCGAAACGTTATGTCTTGAGTGCCTGAGAGGGTGGAGGAATTCCTGGTGGAGTAGTGAAATACGTAGAGATCAGGAGGAACACCGGAGGCGAAGGCGTCTGCCTGGCGGTGCACTGACACTAATTGTGCGAAAGCGCGGGGAGCAAACAGG` |
| ASV3724 | 429 | `TGGGGAATTTTGGACAATGGGGGCAACCCTGATCCAGCAACGCCGCGTGAATGAAGAAGGCCTTAGGGTTGTAAAGTTCTTTTATCTGAGAGCAAAGACGACGTGTTAATAGCATGTTGGGATGAGAGTATCAGAGGAATAAGCACCGGCTAACTACGTGCCAGCAGCCGCGGTAATACGTAGGGTGCAAGCGTTAATCGGAATTACTGGGCGTAAAGCGTGCGCAGGCGGATATGTAAGTTAGATGTGAAATACCCGAGCTCAACTTGGGAATGGCATTTAAGACTGCATGTCTAGAGTTTGTCAGAGGGGGGTAGAATTCCAAGTGTAGCAGTGAAATGCGTAGAGATTTGGAGGAATACCGGTGGCGAAGGCGGCCCCCTGGGATAGAACTGACGCTCATGCACGAAAGCGTGGGTAGCAAACAGG` |
| ASV2794 | 430 | `TAGGGAATTTTGCACAATGGGCGCAAGCCTGATGCAGCAACGCCGCGTGAGTGAAGAAGGCTCTTGGGTTGTAAAGCTCTTTCGGCCAAGAAGAAGGGGTAGCCTGCGAACAGTTGGCTATTCTGACGGTACTGGAAGAAGAAGCACCTGCTAACTACGTGCCAGCAGCAGCGGTAATACGTAGGGTGCAAGCGTTGTTCGGAATTACTGGGCGTAAAGCGCGCGTAGGCGGGCTTGTAAGTCGATTGTGAAAGCCCTGGGCTTAACCCGGGAATGGCAGTCGAAACTACAAGTCTTGAGTGCCTGAGAGGATGGCGGAATTCCCGGTGTAGAAGTGAAATTCGTAGATATCGGGAAGAACACCTGAGGCGAAGGCGGCTATCTGGCGGTGCACTGACGCTAAGGCGCGAAAGCGTGGGGAGCAAACAGG` |
| ASV1599 | 404 | `TGAGGAATCTTCCACAATGGGCGCAAGCCTGATGGAGCGACACCGCGTGAAGGATGAAGCGTTTACTCGTGTAAACTTCTTTTCTGTGGGAAGATAATGACGGTACCACAGGAATAAGGGGCGGCAAACTTCGTGCCAGCAGCCGCGGTAATACGAAGGCCCCAAGCGTTATCCGGAATTACTGGGTGTAAAGAGTTCGTAGGTGGAGGAATAAGTCAGTGGTGAAAGGTCTGGGCTCAACCCAGTATTTGCCACCGAAACTATTCCCCTTGAGTGAGGGAGAGGTAAGCAGAACTGTATGAGTAGGGGTGCAATCCGTTGATACATACAAGAATACCAAAAGCGAAGGCAGCTTACTGGAACTCTACTGACACTGAGGAACGAAAGCGTAGGGAGCGAAAAGG` |
| ASV1235 | 424 | `TGAGGAATATTGGTCAATGGACGCAAGTCTGAACCAGCCAAGTCGCGTGAAGGAAGACTGCCCTTAGGGTTGTAAACTTCTTTTACTTGAGAGTAAAGTGTCCCACGAGTGGGATATTGCAAGTATCAAGAGAATAAGCATCGGCTAATTCCGTGCCAGCAGCCGCGGTAATACGGAAGATGCGAGCGTTATCCGGATTTATTGGGTTTAAAGGGTGCGTAGGCGGACCACACAGTCAGCGGTGAAATCTTCCGGCTTAACCGGGAAACTGCCGTTGAAACTAGCGGTCTTGAGTGTAAATGAGGTATGCGGAATGCGTGGTGTAGCGGTGAAATGCATAGATATCACGCAGAACTCCGATTGCGAAGGCAGCATGCTGGGATACAACTGACGCTGAGGCACGAAAGCGTGGGGATCGAACAGG` |
| ASV5363 | 404 | `TGGGGAATATTGGACAATGGGGGAAACCCTGATCCAGCCATGCCGCGTGAGTGATGACGGCCTTAGGGTTGTAAAGCTCTTTTACTCGGGAAGATAATGACTGTACCGAGAGAATAAGCCCCGGCAAACTTCGTGCCAGCAGCCGCGGTGATACGAAGGGGGCTAGCGTTGTTCGGATTTACTGGGCGTAAAGAGCTCGTAGGCGGATTATTAAGTCAGAGGTGAAATCCTGGGGCTCAACCCCAGAATTGCCTTTGATACTGGTAATCTTGAGTGTGATAGAGGTAAGTGGAATTCCTAGTGTAGAGGTGAAATTCGTAGATATTAGGAAGAACACCAGTGGCGAAGGCGGCTTACTGGATCACAACTGACGCTGAGGAGCGAAAGCGTGGGGATCAAACAGG` |
| ASV3380 | 424 | `TAGGGAATATTGGGCAATGGACGAAAGTCTGACCCAGCCACGCCGCGTGAAGGATGACTGCCCTATGGGTTGTAAACTTCTTTTGTATGGGAAGAAACCTCCGAACGTGTTCGGAGCTGACGGTACCATAAGAATAAGCATCGGCTAACTCCGTGCCAGCAGCCGCGGTAATACGGAGGATGCAAGCGTTGTCCGGATTCATTGGGTTTAAAGGGTGCGTAGGCGGAGAATTAAGTCAGTGGTGAAAGCTTACAGCTTAACTGTAAAAATGCCATTGAAACTGATTTTCTTGAGTTCGATTGAGGTAGATGGAATGTATCATGTAGCGGTGAAATGCTTAGATATGATACAGAACACCAATTGCGAAGGCAGTCTACTAAGTCGATACTGACGCTGAGGCACGAAAGCATGGGGAGCAAACAGG` |
| ASV3706 | 424 | `TAGGGAATATTGGTCAATGGGCGCAAGCCTGAACCAGCCATGCCGCGTGCAGGAAGAATGCCCTCTGGGTTGTAAACTGCTTTTGCACAGGAAGAAACACTCCCTCGTGAGGGAGCTTGACGGTACTGTGAGAATAAGGATCGGCTAACTCCGTGCCAGCAGCCGCGGTAATACGGAGGATCCAAGCGTTATCCGGAATCATTGGGTTTAAAGGGTCCGTAGGCGGTTTAGTAAGTCAGTGGTGAAAGCCCATCGCTCAACGGTGGAACGGCCATTGATACTGCTAGACTTGAATTATTAGGAAGTAACTAGAATATGTAGTGTAGCGGTGAAATGCTTAGAGATTACATGGAATACCAATTGCGAAGGCAGGTTACTACTAATGGATTGACGCTGATGGACGAAAGCGTGGGTAGCGAACAGG` |
| ASV4589 | 409 | `TGGGGAATATTGGGCAATGGAGGAAACTCTGACCCAGCGACGCCGCGTGAGGGATGAAGGCCTTCGGGTTGTAAACCTCTTTCAGCAGGGAATAAGCGAAAGTGAAGGTACCTGCAGAAGAAGCACCGGCTAACTATGTGCCAGCAGCCGCGGTAATACATAGGGTGCAAGCGTTGTCCGGAATTATTGGGCGTAAAGAGCTCGTAGGTGGTCGATCACGTCGGATGTGAAATTCTGAGGCTTAACCTCGGACCTGCATTCGATACGGGTTGGCTAGAGTGTGGTAGGGGAGACTGGAATTCCTGGTGTAGCGGTGGAATGCGCAGATATCAGGAGGAACACCGATGGCGAAGGCAGGTCTCTGGGCCATTACTGACACTGAGGAGCGAAAGCGTGGGGAGCGAACAGG` |
| ASV5354 | 424 | `TAGGGAATATTGCGCAATGGACGAAAGTCTGACGCAGCAACTCCACGTGTGGGATGACGCATTTCGGTGTGTAAACCACTGTCGGATGGGAATAATGGTTTGGTGTTGAGCCAAATTTGAATGTACCATCAAAGGAAGCATCGGCTAACTTCGTGCCAGCAGCCGCGGTAATACGAGGGATGCGAGCGTTGTTCGGAATCACTGGGCGTAAAGAGAGCGTAGGCGGGGAAATAAGTTTGGTGTTTAAAGCAAGGGCCTAACCCTTGTGTGGCACTGAATACTGTTTCTCTTGAGTAGGTTAGGGGTTATCGGAATTCCTGGTGTAGCGGTGGAATGCGTAGATATCAGGAAGAACACCAATGGCGAAGGCAGATAACTGGGACCATACTGACGCTGAGGCTCGAAAGTGCGGGGAGCAAACAGG` |
| ASV605 | 404 | `TGGGGAATATTGGACAATGGGCGAAAGCCTGATCCAGCAATGCCGCGTGAGTGATGAAGGCCTTAGGGTTGTAAAGCTCTTTTACCAGGGATGATAATGACAGTACCTGGAGAATAAGCTCCGGCTAACTCCGTGCCAGCAGCCGCGGTAATACGGAGGGAGCTAGCGTTGTTCGGAATCACTGGGCGTAAAGCGTGCGTAGGCGGTGACTCAAGTCAGAGGTGAAAGCCTGGAGCTCAACTCCAGAACTGCCTTTGAAACTAGGTCGCTAGAACATCGGAGAGGTGAGTGGAATTCCGAGTGTAGAGGTGAAATTCGTAGATATTCGGAAGAACACCAGTGGCGAAGGCGGCTCACTGGACGATTGTTGACGCTGAGGCACGAAAGCGTGGGGAGCAAACAGG` |
| ASV5662 | 424 | `TGAGGAATATTGGTCAATGGTCGCAAGACTGAACCAGCCATGCCGCGTGCAGGATGAAGGCCCTCTGGGTTGTAAACTGCTTTTCTCAGGGAATAAACCCTTCTACGTGTAGAAGGCTGAAGGTACCTGAGGAATAAGCACCGGCTAACTCCGTGCCAGCAGCCGCGGTAATACGGAGGGTGCAAGCGTTATCCGGATTTATTGGGTTTAAAGGGTGCGCAGGCGGGGCCTTAAGTCAGTGGTGAAATCTCTTCGCTTAACGAAGAAACTGCCATTGATACTGAGGTTCTTGAATCTAGTTGACGTTAGTGGAATGTGTCATGTAGCGGTGAAATGCTTAGATATGACACAGAACACCGATCGCGAAGGCAGCTAACGAAGCTAGTATTGACGCTCAGGCACGACAGCATGGGGAGCAAACAGG` |
| ASV2801 | 429 | `TGGGGAATTTTGGACAATGGGGGCAACCCTGATCCAGCAATGCCGCGTGTGTGAAGAAGGCCTTCGGGTTGTAAAGCACTTTTGGCGGGAACGAAAAGGTTTTCTCTAATACAGAATGCTAATGACGGTACCCGCAGAATAAGCACCGGCTAACTACGTGCCAGCAGCCGCGGTAATACGTAGGGTGCAAGCGTTAATCGGAATTACTGGGCGTAAAGCGTGCGCAGGCGGTTTTGTAAGACAGATGTGAAATGCCCGGGCTTAACCTGGGAACTGCATTTGTGACTGCAAGGCTAGAATCTGGCAGAGGGGGGTAGAATTCCACGTGTAGCAGTGAAATGCGTAGAGATGTGGAGGAACACCGATGGCGAAGGCAGCCCCCTGGGCTAAGATTGACGCTCATGCACGAAAGCGTGGGGAGCAAACAGG` |
| ASV4651 | 404 | `TAGGGAATATTGGACAATGGGGGCAACCCTGATCCAGCAATGCCGCGTGAGTGATGAAGGCCTTAGGGTTGTAAAGCTCTTTCGCCGGGGACGATAATGACGGTACCCGGAGAAGAAGCCTCGGCTAACTCCGTGCCAGCAGCCGCGGTAAGACGGAGGAGGCTAGCGTTGTTCGGAATTACTGGGCGTAAAGGGCGCGTAGGCGGCCTGTTAAGTCGGACGTGAAAGCCCGGGGCTCAACTCCGGAGTTGCGTTCGGGACTGACGGGCTTGAGGATGGAAGAGGATGGTGGAATTCCCAGTGTAGAGGTGAAATTCGTAGATATTGGGAAGAACACCGGTGGCGAAGGCGGCCATCTGGTCCATACCTGACGCTGAGGCGCGAAAGCGTGGGGAGCAAACAGG` |
| ASV3355 | 424 | `TGAGGAATATTGGTCAATGGACGCAAGTCTGAACCAGCCATGCCGCGTGCAGGAAGACGGTCCTATGGATTGTAAACTGCTTTTGTACGAGAAGAAACACTGCTTCGTGAAGCAGCTTGACGGTATCGTAAGAATAAGGATCGGCTAACTCCGTGCCAGCAGCCGCGGTAATACGGAGGATCCAAGCGTTATCCGGAATCATTGGGTTTAAAGGGTCCGTAGGCGGTCTTATAAGTCAGTGGTGAAATCCCGTCGCTCAACGATGGAACGGCCATTGATACTGTAGGACTTGAATTATTAGGAAGTAACTAGAATATGTAGTGTAGCGGTGAAATGCTTAGAGATTACATGGAATACCAATTGCGAAGGCAGGTTACTACTAATGGATTGACGCTGATGGACGAAAGCGTGGGTAGCGAACAGG` |
| ASV3810 | 404 | `TGGGGAATATTGGACAATGGGCGAAAGCCTGATCCAGCCATGCCGCGTGAATGATGAAGGCCTTAGGGTTGTAAAGTTCTTTCGCCGATGAAGATAATGACGGTAGTCGGAGAAGAAGCCCCGGCTAACTTCGTGCCAGCAGCCGCGGTAATACGAAGGGGGCTAGCGTTGTTCGGAATTACTGGGCGTAAAGCGAGCGTAGGTCGCTGTGTGTGTCAGTGGTGAAAGCCCAAGGCTCAACCTTGGAACTGCCATTGAAACTGCACAGCTGGAATCTCAGAGGGGATAGCGGAATTCCAAATGTAGAGGTGAAATTCGTAGATATTTGGAGGAACACCGGTGGCGAAGGCGGCTATCTGGATGAGTATTGACACTGAGGCTCGAAAGTGTGGGGATCAAACAGG` |
| ASV4362 | 404 | `TGGGGAATATTGGACAATGGGCGAAAGCCTGATCCAGCAATGCCGCGTGAGTGATGAAGGCCTTAGGGTTGTAAAGCTCTTTTACCCGGGATGATAATGACAGTACCGGGAGAATAAGCTCCGGCTAACTCCGTGCCAGCAGCCGCGGTAATACGGAGGGAGCTAGCGTTATTCGGAATTACTGGGCGTAAAGCGCGCGTAGGCGGCTTTTTAAGTCAGAGGTGAAAGCCTGGAGCTCAACTCCAGAACTGCCTTTGAAACTGGAGAGCTAGAATCTTGGAGAGGCGAGTGGAATTCCGAGTGTAGAGGTGAAATTCGTAGATATTCGGAAGAACACCAGTGGCGAAGGCGACTCGCTGGACAAGTATTGACGCTGAGGTGCGAAAGCGTGGGGAGCAAACAGG` |
| ASV2485 | 404 | `TGGGGAATCTTGGACAATGGGCGCAAGCCTGATCCAGCCATGCCGCGTGAGTGAAGAAGGCCTTCGGGTTGTAAAGCTCTTTTGTCAGGGACGATAATGACGGTACCTGAAGAATAAGCCCCGGCAAACTTCGTGCCAGCAGCCGCGGTAATACGAAGGGGGCTAGCGTTGTTCGGAATTACTGGGCGTAAAGCGCACGTAGGCGGCTTGTTAAGTCAGGGGTGAAATCCCGGAGCTCAACTCCGGAACTGCCTTTGATACTGGCAAGCTAGAGTTCGGAAGAGGTTAGTGGAATTCCCAGTGTAGAGGTGAAATTCGTAGATATTGGGAAGAACACCAGTGGCGAAGGCGGCTAACTGGTCCGATACTGACGCTGAGGTGCGAAAGCGTGGGGAGCAAACAGG` |
| ASV1307 | 409 | `TGGGGAATATTGCACAATGGGCGAAAGCCTGATGCAGCAACGCCGCGTGAGGGATGAAGGCCTTCGGGTTGTAAACCTCTTTTAGCAGGGAAGAAGCGAAAGTGACGGTACCTGCAGAAAAAGCACCGGCTAACTACGTGCCAGCAGCCGCGGTAATACGTAGGGTGCAAGCGTTGTCCGGAATTATTGGGCGTAAAGAGCTCGTAGGCGGTTTGTCGCGTCTGCTGTGAAAATCCGAGGCTCAACCTCGGACCTGCAGTGGGTACGGGCAAGCTAGAGTGCGGTAGGGGAGATGGGAATTCCTGGTGTAGCGGTGGAATGCGCAGATATCAGGAGGAACACCAATGGCGAAGGCACATCTCTGGGCCGTAACTGACGCTGAGGAGCGAAAGCGTGGGGAGCGAACAGG` |
| ASV3598 | 430 | `TAGGGAATATTGCGCAATGGGGGAAACCCTGACGCAGCAACGCCGCGTGAGTGAGGAAGGTCTTCGGATTGTAAAACTCTTTTATTTGGGAAAAATGTCTTAGTGGCTAATATCCATTAAGAGTGATGGTACCAAAGGAATAAGGACCGGCTAACTTCGTGCCAGCAGCCGCGGTAATACGAAGGGTCCAAGCGTTGTTCGGAATCATTGGGCGTAAAGCGCGCGCAGGCGGATCAGTAAGTCAGGTGTGAAATCTCGAAGCTCAACTTCGAAACTGCGCCTGAAACTACTAGTCTAGAATGTCGGAGGGGGCAGGGGAATTTCACGTGTAGGGGTAAAATCCGTAGAGATGTGAAGGAACACCGGGGGCGAAGGCGCCTGCCTGGACGACTATTGACGCTGAGGCGCGAAAGCGTGGGGAGCAAACAGG` |
| ASV5487 | 430 | `TGGGGAATCTTGCGCAATGGACGAAAGTCTGACGCAGCGACGCCGCGTGGGTGAAGAAGGCCTTCGGGTTGTAAAGCCCTGTCGAACGGGAAAAATGGCGTGTGCAATAATAAGGCACACGATTGATGGTACCGTTAGAGGAAGCACCGGCTAACTCTGTGCCAGCAGCCGCGGTAATACAGAGGGTGCAAGCGTTGTTCGGAATTATTGGGCGTAAAGGGCAGGTAGGTTGTCTTATAAGTCTGCTGTGAAATCCCCGAGCTTAACTTGGGAAGTGCGGCAGATACTGTAGGACTAGAGTGCTGGAGGGGTACGTGGAATTCCCGGTGTAGCGGTGAAATGCGTAGATATCGGGAGGAACACCAGAGGCGAAGGCGGCGTACTGGACAGCAACTGACACTAAACTGCGAAAGCGTGGGGAGCAAACAGG` |
| ASV1275 | 423 | `TAGGGAATATTGGACAATGGGCGAAAGCCTGATCCAGCCATGCCGCGTGAAGGATGAAGGGCCTCTGGCTTGTAAACTTCTTTTGGCGGGGGATAAACCACCACTACAAGTGGTGCTGAAGGTACCCGCAGAATAAGCACCGGCTAACTCCGTGCCAGCAGCCGCGGTAATACGGAGGGTGCAAGCGTTGTCCGGATTCATTGGGTTTAAAGGGTGCGTAGGCGGTACGCTAAGTCAGTGGTGAAATCCTGGGGCTCAACCCCGGAATTGCCATAGATACTGGCGTGCTTGAGTTCGACCGAGGCGGTTGGAATGCGTGGTGTAGCGGTGAAATGCTTAGATATCACGCGGAACACCGATTGCGAAGGCAGATCGCTAGGTCGCGACTGACGCTGAGGCACGAAAGTGCGGGGATCAAACAGG` |
| ASV1856 | 404 | `TGAGGAATATTGGACAATGGGCGAAAGCCTGATCCAGCCATGCCGCGTGAATGATGAAGGCCTTAGGGTTGTAAAGTTCTTTTAGCTGTGACGATAATGACGGTAGCAGCAGAAAAAGCCCCGGCTAACTCCGTGCCAGCAGCCGCGGTAATACGGAGGGGGCGAGCGTTGTTCGGATTTACTGGGCGTAAAGCGTGCGTAGGTGGTTTCCTAAGTTAGAAGTGAAAGCCCAAGGCTTAACCTTGGAATTGCTTTTAAGACTGGGTCACTAGAATCTGGGAGAGGTGAATGGAATTCCGAGTGTAGAGGTGAAATTCGTAGATATTCGGAGGAACACCAATGGCGAAAGCAGTTCACTGGACCAGTATTGACACTGAGGCACGAAAGCGTGGGTAGCAAACAGG` |
| ASV3778 | 406 | `TGGGGAATTTTCCGCAATGGGCGAGAGCCTGACGGAGCAACGCCGCGTGAGGGAGGAAGGCCTGTGGGTTGTAAACCTCTTTTCTCAAGGAAGAAGTTCTGACGGTACTTGAGGAATCAGCATCGGCTAACTCCGTGCCAGCAGCCGCGGTAAGACGGAGGATGCAAGCGTTATCCGGAATTATTGGGCGTAAAGCGTCCGTAGGCGGTTTTAAAAGTCTGCTGTCAAAGCTCACAGCTCAACTGTGAATGGGCAGTGGAAACTATAAGACTAGAGAGTGGTAGGGGTAGAGGGAATTCCTAGTGTAGCGGTGAAATGCGTAGATATTAGGAAGAACACCAGTGGCGAAGGCGCTCTACTGGGCCATTACTGACGCTGATGGACGAAAGCTAGGGGAGCGAAAGGG` |
| ASV2197 | 429 | `TGGGGAATTTTGGACAATGGGGGCAACCCTGATCCAGCCATGCCGCGTGAGTGAAGAAGGCCTTCGGGTTGTAAAGCTCTTTCAGCCGGGAAGAAAATGCATGGGTTAATACCCTGTGCAGATGACGGTACCGGAATAAGAAGCACCGGCTAACTACGTGCCAGCAGCCGCGGTAATACGTAGGGTGCGAGCGTTAATCGGAATTACTGGGCGTAAAGCGTGCGCAGGCGGTTTTGTAAGACAGGCGTGAAATCCCCGGGCTTAACCTGGGAACTGCGTTTGTGACTGCAAGGCTAGAGTACGGCAGAGGGGGGTGGAATTCCACGTGTAGCAGTGAAATGCGTAGAGATGTGGAGGAACACCAATGGCGAAGGCAGCCCCCTGGGTCGATACTGACGCTCATGCACGAAAGCGTGGGTAGCAAACAGG` |
| ASV2499 | 424 | `TAAGGAATATTGGGCAATGGAGGCAACTCTGACCCAGCCATGCCGCGTGCAGGATGACTGCCCTATGGGTTGTAAACTGCTTTTGTATGGGAGAAAACCCTCGTTTGCGAACGGGGCTGATAGTACCATAAGAATAAGCATCGGCTAACTCCGTGCCAGCAGCCGCGGTAATACGGAGGATGCAAGCGTTATCCGGATTCATTGGGTTTAAAGGGTGCGTAGGTGGTCTTATAAGTCAGTGGTGAAAGCCTACAGCTTAACTGTAGAATTGCCATTGATACTGTAGGACTTGAGTACAGACGACGTTGGCGGAATATGACATGTAGCGGTGAAATGCTTAGATATGTCATAGAACACCGATTGCGAAGGCAGCTGACGAGACTGTAACTGACACTGAGGCACGAAAGTGTGGGGATCAAACAGG` |
| ASV4926 | 429 | `TGGGGAATTTTGGACAATGGGGGCAACCCTGATCCAGCCATGCCGCGTGCGGGAAGAAGGCCTTCGGGTTGTAAACCGCTTTTGTCCGGGAAGAAAAGGCACCCCCTAATACGGGATGCTCTTGACGGTACTGGAAGAATAAGCACCGGCTAACTACGTGCCAGCAGCCGCGGTAATACGTAGGGTGCGAGCGTTAATCGGAATTACTGGGCGTAAAGCGTGCGCAGGCGGTTCGTGATGTCTGATGTGAAAGCCCCGGGCTCAACCTGGGAATTGCATTGGAAACTAGCGGGCTGGAGTGTGGCAGAGGGGGGTGGAATTCCACGTGTAGCAGTGAAATGCGTAGAGATGTGGAGGAACACCGATGGCGAAGGCAGCCCCCTGGGCTAACACTGACGCTCATGCACGAAAGCGTGGGGAGCAAACAGG` |
| ASV2379 | 429 | `TGGGGAATTTTGGACAATGGGGGAAACCCTGATCCAGCCATTCCGCGTGTGCGATGAAGGCCTTCGGGTTGTAAAGCACTTTTGGCAGGAAAGAAATCGCACAAATTAATACTTTGTGTGGATGACGGTACCTGCAGAATAAGCACCGGCTAACTACGTGCCAGCAGCCGCGGTAATACGTAGGGTGCAAGCGTTAATCGGAATTACTGGGCGTAAAGCGTGCGCAGGCGGTTCGGAAAGAGAGGTGTGAAATCCCAGGGCTTAACCTTGGAACTGCACTTTTAACTCCCGAGCTAGAGTATGTCAGAGGGGGGTGGAATTCCGCGTGTAGCAGTGAAATGCGTAGATATGCGGAGGAACACCGATGGCGAAGGCAGCCCCCTGGGATAATACTGACGCTCATGCACGAAAGCGTGGGGAGCAAACAGG` |
| ASV5182 | 406 | `TCAAGAATATTCGACAATGGGCGAAAGCCTGATCGAGCGACGCCGCGTGCAGGAAGAAGCCCCTCGGGGTGTAAACTGCTTTTCTCAGGGAAGAATTTGTGACGGTACCTGAAGAATAAGAGGTTGCTAACTCTGTGCCAGCAGCAGCGGTAATACAGAGACCTCAAGCGTTATCCGGATTCATTGGGCGTAAAGCGTCCGCAGGTGGTTTGATAAGTCAATAGTCAAATCTTGGGGCTTAACTCCGAGGCTGCTATTGATACTGACAAACTAGAGACTGGGAGAGGCAAGCGGAACTATCGGTGTAGTCGTAATAAGCGCTGATATCGATAGGAACACCAAAGGCGAAGGCAGCTTGCTAGAACAGTTCTGACACTCATGGACCAAAGCGTGGGTAGCGAAAGGG` |
| ASV346 | 404 | `TGGGGAATATTGGACAATGGGCGAAAGCCTGATCCAGCAATACCGCGTGAATGATGAAGGCCTTAGGGTTGTAAAGTTCTTTTAGTTGGGAAGATAATGACGGTACCAACAGAAAAAGCCCCGGCTAACTCCGTGCCAGCAGCCGCGGTAAGACGGAGGGGGCTAGCGTTGTTCGGAATTACTGGGCGTAAAGGGCGCGTAGGCGGATTAGTAAGTTGGGAGTGAAAGCCCGGGGCTTAACCTCGGAACTGCTTTCAAAACTGCTAGTCTAGAGTGAAGTAGGGGGTGATGGAATTCCTAGTGTAGAGGTGAAATTCTTAGATATTAGGAGGAACACCGGTGGCGAAGGCGGTCACCTGGACTTCAACTGACGCTGAGGCGCGAAAGCGTGGGGAGCAAACAGG` |
| ASV4407 | 430 | `TGGGGAATATTGGACAATGGGGGCAACCCTGATCCAGCCATGCCGCGTGTGTGAAGAAGGCCTTTTGGTTGTAAAGCACTTTAAGCGGGGAGGAGGGTACTCTGACTAATAATCGGATGTATCGGACGTTACCCGCAGAATAAGCACCGGCTAACTCTGTGCCAGCAGCCGCGGTAATACAGAGGGTGCGAGCGTTAATCGGAATTACTGGGCGTAAAGCGCGCGTAGGCGGTTATTTAAGTCGGATGTGAAATCCCCGAGCTCAACTTGGGAATTGCATTCGATACTGGGTAGCTAGAGTATGGGAGAGGAAGGTAGAATTCCAGGTGTAGCGGTGAAATGCGTAGAGATCTGGAGGAATACCGATGGCGAAGGCAGCCTTCTGGCCTAATACTGACGCTGAGGTGCGAAAGCATGGGGAGCAAACAGG` |
| ASV3902 | 404 | `TGGGGAATATTGGACAATGGGCGAAAGCCTGATCCAGCCATGCCGCGTGAATGATGAAGGCCTTAGGGTTGTAAAGTTCTTTTGTCAGGGAAGATGATGACGGTACCTGAAGAATAAGCACCGGCTAACTTCGTGCCAGCAGCCGCGGTAATACGAAGGGTGCTAGCGTTGTTCGGAATCACTGGGCGTAAAGCGCGCGTAGGCGGCTTTCCAAGTCAGAGGTGAAATCCCACAGCTTAACTGTGGAACTGCCTTTGAAACTGGTAAGCTGGAGTGTCGGAGGGGATAGTGGAATTGCTAATGTAGCGGTGAAATGCGTAGATATTAGCAGGAACACCGGTGGCGAAGGCGGCTATCTGGACGACAACTGACGCTGAGGCGCGAAAGCGTGGGGATCAAACAGG` |
| ASV3693 | 407 | `TAGGGAATATTGCACAATGGAGGAAACTCTGATGCAGCGACGCCGCGTGAGTGATGAAGGCCTTCGGGTCGTAAAGCTCTGTCGCAGGGGAATAACACAATGAATGTACCCTGTAAGAAAGGATCGGCTAACTTCGTGCCAGCAGCCGCGGTAAGACGAGGGATCCTAGCGTTGTTCGGAATTATTGGGCGTAAAGCGGGTGTAGGTGGCTATGTAAGTCAAGTGTGAAAGCCTGGGGCTCAACCCCAGAAGTGCATTTGATACTGCGTAGCTTGAGTGCTAGAGAGGATAGTAGAATTGTTGGTGTAGTGGTGAAATACGTAGATATCAACAGGAATACCGGTGGCGAAGGCGGCTATCTGGCTAGACACTGACACTCAGACCCGAAAGCGTGGGGATCAAACAGG` |
| ASV4174 | 388 | `GCGCGAAAACTTTACACTGCTAGAAATAGCGATAAGGGGACCCCGAGTGCCAGTACCTAGTACTGGCTGTCCCGCTGTCTAAACAACAGTGGTTAGCAAGGGCCGGGCAAGACCGGTGCCAGCCGCCGCGGTAACACCGGCGGCCCGAGTGGTAGCCGTTATTATTGGGTTTAAAGGGTCCGTAGCCGGCCTATTAAGTCTCTTGGGAAATCTGGCGACTTAATCGTCAGGCGTCCAAGAGATACTGATAGGCTTGGGACCGGGAGAGGTGGGAGGTACTCCAGGGGTAGGGGTGAAATCTCGTAATCCTTGGGGGACCACCGATGGCGAAGGCATCCCACCAGAACGGGTCCGACGGTGAGGGACGAAAGCTGGGGGCACGAACCGG` |
| ASV2355 | 429 | `TGGGGAATTTTGGACAATGGGGGGAACCCTGATCCAGCCATGCCGCGTGAGTGAAGAAGGCCTTCGGGTTGTAAAGCTCTTTCGGCCGGGAAGAAATCGTGCAGGCTAATACCCTGTATGGATGACGGTACCGGAATAAGAAGCACCGGCTAACTACGTGCCAGCAGCCGCGGTAATACGTAGGGTGCGAGCGTTAATCGGAATTACTGGGCGTAAAGCGTGCGCAGGCGGTTTTGTAAGACAGGCGTGAAATCCCCGGGCTCAACCTGGGCATTGCGCTAGTGACTGCAAGGCTAGAGTACGGCAGAGGGGGGTAGAATTCCACGTGTAGCAGTGAAATGCGTAGAGATGTGGAGGAATACCGATGGCGAAGGCAGCCCCCTGGGTCGATACTGACGCTCATGCACGAAAGCGTGGGGAGCAAACAGG` |
| ASV2698 | 417 | `TTAGGAATCTTCCACAATGGGCGAAAGCCTGATGGAGCGACGCCGCGTGAGGGATGAAGGTTTTAGGATTGTAAACCTCTGAATTAGGGACGAAAGACGCTTTATGCGGGATGACGGTACCTAAGTAATAGCACCGGCTAACTCCGTGCCAGCAGCCGCGGTAATACGGAGGGTGCAAGCGTTACCCGGAATCACTGGGCGTAAAGGGCGTGTAGGCGGAACGTTAAGTCTGGTTTTAAAGACCGTCGCTCAACGACGGGCATGGATTGGATACTGACGATCTTGACCTCTGGAGAGGTAACTGGAATTTCTGGTGTAGCGGTGGAATGCGTAGATACCAGAAGGAACACCAATGGCGAAGGCAAGTTACTGGACAGAAGGTGACGCTGAGGCGCGAAAGTGTGGGGAGCGAACCGG` |
| ASV966 | 429 | `TGGGGAATTTTGGACAATGGGGGCAACCCTGATCCAGCAACGCCGCGTGAATGAAGAAGGCCTTAGGGTTGTAAAGTTCTTTTACATGGGAGCAAGTTTAGTAGGTTAAGAGCTGATTAAATTGAGAGTACCATGAGAATAAGCACCGGCTAACTACGTGCCAGCAGCCGCGGTAATACGTAGGGTGCGAGCGTTAATCGGAATTACTGGGCGTAAAGGGTGAGTAGGCGGATGTACAAGTTAGATGTGAAATACCTAGGCTTAACCTGGGAGGTGCATTTAAGACTGTATATCTAGAGTCTGTTAGAGGGGGGTAGAATTCCAAGTGTAGCAGTGAAATGCGTAGAGATTTGGAGGAATACCGAAGGCGAAGGCAGCCCCCTGGGATAAGACTGACGCTGAGTCACGAAAGCGTGGGTAGCAAACAGG` |
| ASV2458 | 424 | `TAGGGAATATTGGGCAATGGGCGCAAGCCTGACCCAGCCATGCCGCGTGCAGGACGAAGGCCCTCCGGGTTGTAAACTGCTTTTGCAGGAGAAGAATCATTGGTTTGCGAACCAATCTGACGGTATTCTGCGAATAAGCCACGGCTAACTACGTGCCAGCAGCCGCGGTAATACGTAGGTGGCAAGCGTTGTCCGGATTTATTGGGTTTAAAGGGTGCGTAGGCGGACTGTTAAGTCAGAGGTGAAAGACGGTGGCTCAACCATCGCAGTGCCTTTGATACTGGCAGTCTTGAGTGCAGACGAGGTCTGTGGAATGGATGGTGTAGCGGTGAAATGCTTAGATACCATCCAGAACACCAATTGCGAAGGCAGCAGACTAGGGTGCAACTGACGCTGAGGCACGAAAGTGCGGGGATCAAACAGG` |
| ASV1231 | 424 | `TGAGGAATATTGGTCAATGGGTGCAAGCCTGAACCAGCCATCCCGCGTGAAGGAAGACGGTCCTCTGGATTGTAAACTTCTTTTATATAGGAATAAACCTACATACGTGTATGTAGCTGAAGGTACTATATGAATAAGCACCGGCTAACTCCGTGCCAGCAGCCGCGGTAATACGGAGGGTGCAAGCGTTATCCGGATTTATTGGGTTTAAAGGGTCCGTAGGCGGGCTGATAAGTCAGTGGTGAAATCCTTCAGCTTAACTGGAGAACTGCCATTGATACTGTTAGCCTTGAGTGTTTTTGATGTAGCTGGAATGTGTGGTGTAGCGGTGAAATGCATAGATATCACACAGAACACCAATTGCGAAGGCAGGTTACAAAGCAACAACTGACGCTGATGGACGAAAGCGTGGGTAGCGAACAGG` |
| ASV2297 | 430 | `TGGGGAATCTTGCGCAATGGAGGAAACTCTGACGCAGTGATACCGCGTGAGTGAGGAAGGCCTTCGGGTTGTAAAGCTCTGTCAAAATGGAAAAACGAGACTATGTCGAACAGGCATAGTAAGTGATGGTACATTGAGAGGAATCACCGGCTAACTTCGTGCCAGCAGCCGCGGTAATACGAAGGGTGAGAGCGTTGTTCGGATTTATTGGGCGTAAAGCGCGCGCAGGCGGATCGTCAAGTCAGATGTGAAATCTCGGGGCTCAACTCCGAAACAGCGTCTGAAACTAATGATCTAGAGTCTTGGCAGGGGAGGGGGAATTTCACATGTAGGGGTAAAATCCGTAGAGATGTGAAGGAACACCAGAGGCGAAGGCGCCCTCCTGGACAAGTACTGACGCTGAGGCGCGAAAGCGTGGGGAGCAAACAGG` |
| ASV5184 | 429 | `TCGGGAATCTTGTCCAATGGGGGAAACCCTGAGACAGCAACGCCGCGTGAGGGATGAAGGCCCTAGGGTCGTAAACCTCTGTCAGGAGGGAAGAAAACTTCATTACAAATAGTTCTGAAGTCTGACGGTACCTCCAAAGGAAGCACCGGCTAACTCCGTGCCAGCAGCCGCGGTAATACGGAGGGTGCAAGCGTTGTTCGGAATTATTGGGCGTAAAGCGGGTGCAGGCGGTGAAGTAAGTCAGATGTGAAATCCCAGGGCCCAACCCTGGAACTGCATCCGATACTGCTTCACTAGAGTCTCGGAGAGGAAAGCGGAATTCCCGGTGTAGAGGTGAAATTCGTAGATATCGGGAGGAACACCAGTGGCGAAGGCGGCTTTCTGGACGATGACTGACGCTCAGACCCGAAAGCGTGGGGAGCAAACAGG` |
| ASV1199 | 429 | `TGGGGAATTTTGGACAATGGGCGCAAGCCTGATCCAGCAATGCCGCGTGAGTGAAGAAGGCCTTCGGGTTGTAAAGCTCTTTTGTCAGGGAAGAAACGGGTGGGGCTAATATCCCCATCTAATGACGGTACCTGAAGAATAAGCACCGGCTAACTACGTGCCAGCAGCCGCGGTAATACGTAGGGTGCAAGCGTTAATCGGAATTACTGGGCGTAAAGCGTGCGCAGGCGGTTTTGTAAGTCTGTCGTGAAATCCCCGGGCTCAACCTGGGAATGGCGATGGAGACTGCAAGGCTAGAATCTGGCAGAGGGGGGTAGAATTCCACGTGTAGCAGTGAAATGCGTAGAGATGTGGAGGAACACCGATGGCGAAGGCAGCCCCCTGGGTCAAGATTGACGCTCATGCACGAAAGCGTGGGGAGCAAACAGG` |
| ASV5207 | 430 | `TGGGGAATATTGCGCAATGGACGAAAGTCTGACGCAGCGACGCCGCGTGAGGGATGAAGGCCTTCGGGTTGTAAACCTCTGTCAGGTGGGAAGAACCGCCATGGTAGTAACTGCCCATGGTCTGACGGTACCACCAAAGGAAGCACCGGCTAACTCCGTGCCAGCAGCCGCGGTAATACGGAGGGTGCAAGCGTTAATCGGAATCACTGGGCGTAAAGCGCGCGTAGGCGGCTTTGTAAGTCAGGGGTGAAATCCCACGGCTCAACCGTGGAACTGCCCTTGATACTGCTTAGCTACGAGTTTGTGAGAGGATAGTGGAATTCCAGGTGTAGGAGTGAAATCCGTAGAGATCTGGAGGAACATCAGTGGCGAAGGCGACTATCTGGCACAACACTGACGCTGAGGTGCGAAAGCGTGGGTATCAAACAGG` |
| ASV1733 | 429 | `TGGGGAATATTGGACAATGGGGGCAACCCTGATCCAGCCATGCCGCGTGTGTGAAGAAGGCCTTTTGGTTGTAAAGCACTTTAAGCAGTGAAGAAGACTCCGTGGTTAATACCCACGGACGATGACATTAGCTGCAGAATAAGCACCGGCTAACTCTGTGCCAGCAGCCGCGGTAATACAGAGGGTGCAAGCGTTAATCGGAATTACTGGGCGTAAAGGGAGCGTAGGTGGCTCGATAAGTCAGATGTGAAATCCCCGGGCTCAACCTGGGAACTGCATCTGATACTGTTGAGCTAGAGTATGTGAGAGGAAGGTAGAATTCCAGGTGTAGCGGTGAAATGCGTAGAGATCTGGAGGAATACCGATGGCGAAGGCAGCCTTCTGGCATAATACTGACACTGAGGCTCGAAAGCGTGGGTAGCAAACAGG` |
| ASV4838 | 429 | `TGGGGAATTGTTCACAATGGGCGCAAGCCTGATGACGCAACGCCGCGTGGGGGATGAAGGCCTTCGGGTCGTAAACCCCTGTCGAACGGGACGAAAGACCTTGGATCTAATCAACCAGGGAGTGACGGTACCGTTAAAGGAAGCCACGGCTAACTCTGTGCCAGCAGCCGCGGTAATACAGAGGTGGCAAGCGTTGTTCGGAATTACTGGGCGTAAAGGGCGCGTAGGCGGCCTTCTAAGTCAGACGTGAAATCCCCCGGCTTAACCTGGGAACTGCGTCTGATACTGGGAGGCTCGAGTATGGGAGAGGGATGTAGAATTCCAGGTGTAGCGGTGAAATGCGTAGATATCTGGAGGAATACCGGTGGCGAAGGCGGCATCCTGGACCATTACTGACGCTGAGGCGCGAAAGCTAGGGGAGCAAACGGG` |
| ASV3198 | 421 | `TGGGGAATATTGCGCAATGGACGAAAGTCTGACGCAGCGACGCCGCGTGTGGGATGAAGCTCTTCGGGGTGTAAACCACTGTTGCCCGGGAAGAATCTCCTGATTCATCAGGATTGACGGTACCGGGTGAGGAAGCACCGGCTAACTCTGTGCCAGCAGCCGCGGTAATACAGAGGGTGCGAGCGTTGTCCGGAATCACTGGGCGTAAAGGGCGCGTAGGTGGCTAGGTAAGTGTGCGGTGAAAGCCCGGGGCTCAACCCCGGGTCGGCCGTGCAGACTGCTTGGCTCGAGCATAGTAGAGGCACGTGGAATTCCGGGTGTAGCGGTGGAATGCGTAGAGATCCGGAAGAACACCGGTGGCGAAGGCGGCGTGCTGGGCTATAGCTGACACTGAGGCGCGACAGCGTGGGGAGCAAACAGG` |
| ASV439 | 404 | `TGGGGAATATTGGACAATGGGCGAAAGCCTGATCCAGCAATGCCGCGTGAGTGAAGAAGGCCTTCGGGTTGTAAAGCTCTTTTACCCGGGAAGATAATGACCGTACCGGGAGAATAAGCCCCGGCTAACTCCGTGCCAGCAGCCGCGGTAATACGGAGGGGGCTAGCGTTGTTCGGAATCACTGGGCGTAAAGCGTACGTAGGCGGCCATTCAAGTCAGAGGTGAAAGCCCAGGGCTCAACCCTGGAACTGCCTTTGAAACTAGATGGCTGGAACACGGGAGAGGTGAGTGGAATTCCGAGTGTAGAGGTGAAATTCGTAGATATTCGGAAGAACACCAGTGGCGAAGGCGGCTCACTGGACCGTTGTTGACGCTGAGGTACGAAAGCGTGGGGAGCAAACAGG` |
| ASV2973 | 430 | `TGGGGAATCTTGCACAATGGGCGAAAGCCTGATGCAGCAACGCCGCGTGAGTGATGAAGGTCTTTGGATTGTAAAGCTCTTTCGGCCGGGAAGAAGTGTATGTGAGTTAATACCTCATGTATGTGACGGTACCGGCATAAGAAGCACTGGCTAATTCCGTGCCAGCAGCTGCGGTAATACGGAAGGTGCAAGCGTTGTTCGGAGTGACTGGGCGTAAAGGGCGCGTAGGCTGGAGGGTAAGAAGATTGTGAAACCCCCGGGCTCAACCCGGGACTTGCAGTTTTGACTGCTTTTCTTGAATGTCTGAGAGGTTAGTGGAATTCCTGGTGTAGGAGTGAAATCCGTAGATATCAGGAGGAACACCGGAGGCGAAGGCGGCTGACTGGTGGAGCATTGACGCTGAGGCGCGAAAGCACGGGTAGCAAACAGG` |
| ASV1944 | 404 | `TGGGGAATATTGCACAATGGGCGGAAGCCTGATGCAGCAACGCCGCGTGCGGGATGAAGGCCTTCGGGTTGTAAACCGCTTTCACCAGGGGCGAATGTGACGGTACCTGGAGAAGAAGCACCGGCTAACTACGTGCCAGCAGCCGCGGTGATACGTAGGGTGCGAGCGTTGTCCGGATTTATTGGGCGTAAAGAGCTCGTAGGCGGTTTGTCGCGTCGGAAGTGAAAACACGGGGCTTAACTTCGTGCTTGCTTTCGATACGGGCAGACTTGAGGAAGGTAGGGGAGAACGGAATTCCTGGTGGAGCGGTGGAATGCGCAGATATCAGGAGGAACACCGGTGGCGAAGGCGGTTCTCTGGACCTTTCCTGACGCTGAGGAGCGAAAGCGTGGGGAGCAAACAGG` |
| ASV3762 | 405 | `TAGGGAATATTGGACAATGGACGAAAGTCTGATCCAGCAATACCGCGTGGATGATGAAGGCGTTATGCGTTGTAAAATCCTTTTGTCGGGGAAGATAATGACGGTACCCGAAGAATAAGCACCGACTAACTATGTGCCAGCAGTCGCGGTAATACATAGGGTGCTAGCGTTGCTCGGAATTATTGGGCGTAAAGCATGTGTAGGAGGACTTGAAAGTCAAAAGTGTAATTCCTAGGCTCAACCTAGGCGCTGCTTTTGATACTCCAAGTCTAGAGTGAGGTAGAGGATAGCGGAATTCTTAGTGTAGGGGTAAAATCCGTAGATATTAAGAGGAACACCGAAAGCGAAGGCAGCTATCTGGGCCTTAACTGACTCTGAGACATGAAAGCGTGGGGAGCAAACAGG` |
| ASV3415 | 430 | `TGGGGAATATTGCGCAATGAGCGAAAGCTTGACGCAGCGATGCCGCGTGGAGGATGAAGGTCTTCGGATTGTAAACTTCTGTTGCTCGGGAAGAAAGACTCTAGGCGAATAACCTGAGAGGGATGACGGTACCGAGAAAGAAAGCACCGGCTAACTTCGTGCCAGCAGCCGCGGTAATACGAAGGGTGCGAGCGTTATTCGGAATAACTGGGCGTAAAGAGCGTGTTGGTGGCTTTGTAAGTCGGCTGTGAAAGACCATGACCTAATCGTGGAATAGCGGTCGAGACTGCAGGGCTAGAGGGTGAGAGAGAAGAGTGGAATTCTTGGAGTAGCGGTAATATGCGTAGATCTCAAGAGGAACACCGATGGCGAAGGCAGCTCTTTGGCTCATTCCTGACACTTAAGCGCGAAAGCGTGGGGAGCAAACAGG` |
| ASV5118 | 429 | `TGGGGAATATTGGACAATGGGGGTAACCCTGATCCAGCGATGCCGCGTGAGTGAAGAAGGCCTTCGGGTCGTAAAGCTCTTTAGGTTGGGAAGAAGGACATGAGGGGATAATCCTTGTGTTTTGACGGTACCGACAGAATAAGCACCGGCAAACTCTGTGCCAGCAGCCGCGGTAATACAGAGGGTGCGAGCGTTAATCGGAATTACTGGGCGTAAAGGGCGCGTAGGCGGTGATATGAGTGTGATGTGAAAGCCCCGGGCTTAACCTGGGAAGTGCATCGCAAACGATATGACTGGAGTAGATGAGAGGGTGGCGGAATTTCCGGTGTAGCGGTGAAATGCGTAGAGATCGGAAGGAACGTCAATGGCGAAGGCAGCCACCTGGCATCATACTGACGCTGAGGCGCGAAAGCGTGGGGAGCGAACAGG` |
| ASV3207 | 429 | `TGGGGAATTTTGGACAATGGACGCAAGTCTGATCCAGCCATGCCGCGTGAGTGAAGAAGGCCTTCGGGTTGTAAAGCTCTTTTGTCCGGGAGCAAATCCTGTTCACTAATACTGAATGGGGCTGAGAGTACCGGAAGAATAAGGACCGGCTAACTACGTGCCAGCAGCCGCGGTAATACGTAGGGTCCAAGCGTTAATCGGAATTACTGGGCGTAAAGGGTGCGCAGGTGGTTACGCAAGTCTGATGTGAAAGCCCCGGGCTTAACCTGGGAACTGCATTGGAGACTGCGTGACTAGAGTGTGGCAGAGGGGGGTGGAATTCCGCGTGTAGCAGTGAAATGCGTAGATATGCGGAGGAACACCAATGGCGAAGGCAGCCCCCTGGGTTAACACTGACACTCATGCACGAAAGCGTGGGGAGCAAACAGG` |
| ASV2442 | 404 | `TTAGGAATATTGGACAATGGGGGCAACCCTGATCCAGCCATGCCGCGTGAGTGATGAAGGCCTTCGGGTTGTAAAGCTCTTTAGTCCACGAATATAATGACTGTAGTGGAAGAATAAGCCCCGGCTAACTTCGTGCCAGCAGCCGCGGTAATACGAAGGGGGCGAGCGTTGTTCGGAATCACTGGGCGTAAAGAGTGCGTAGGCGGTTATGAAAGTTAGGAGTGAAAGCCCCAGGCTCAACTTGGGAATTGCTCTTAATACTACATGACTTGAATTCGGGAGAGGATAGCGGAATTGTCAGTGTAGCAGTGAAATGCGTAGATATTGACAGGAACACCAGTGGCGTAAGCGGCTATCTGGACCGACATTGACGCTGAGGCACGAAAGCGTGGGGATCAAACAGG` |
| ASV3138 | 429 | `TGGGGAATATTGGACAATGGGCGCAAGCCTGATCCAGCAATGCCGCGTGTGTGAAGAAGGCCTTAGGGTTGTAAAGCACTTTCAGTGGGGAGGAAGTTTGTGGTGCTAATATCATCATAAATTGACGTTACCCACAGAAGAAGCACCGGCTAACTCTGTGCCAGCAGCCGCGGTAATACAGAGGGTGCGAGCGTTAATCGGAATTACTGGGCGTAAAGCGTGTGTAGGTGGTTAGGTAAGTTAGATGTGAAATCCCCGGGCTCAACCTGGGAATTGCGTTTAAAACTGCTTGGCTAGAGTATTGTAGAGGATAGTGGAATTTCCAGTGTAGCGGTGAAATGCGTAGATATTGGAAGGAACACCAGTGGCGAAGGCGACTATCTGGGCACATACTGACACTGAGACACGAAAGCATGGGGAGCAAACAGG` |
| ASV5639 | 429 | `TGAGGAATCTTGCACAATGGGCGAAAGCCTGATGCAGCGACGCCGCGTGGAGGATGAAGGTTTTCGGATCGTAAACTCCTGTTAAGTGGGAAGAAATGCTTGCTCCTAATATGGGCAAGTAATGACGGTACCATTAGAGAAAGCACCGGCTAACTTCGTGCCAGCAGCCGCGGTAATACGAGGGGTGCGAGCGTTATTCGGAATCATTGGGCGTAAAGGGTGCGTAGACGGCATATTAAGTCAACTGTTAAATTCCCCGGCCTAACCGGGGGCCAGCAGTAGATACTGATGTGCTAGAGGATGGAAGAGAGAAGTGGAATTCTCGGAGTAGCGGTAAAATGCGTAGATCTCGAGAGGAACACCGATGGCGAAGGCAGCTTCTTGGTCCATTCCTGACGTTGAGGCACGAAAGCGTGGGGAGCAAACAGG` |
| ASV4322 | 404 | `TAGGGAATATTGGGCAATGGGCGAAAGCCTGACCCAGCCATGCCGCGTGAATGATGAAGGCCTTAGGGTTGTAAAGTTCTTTCGCCGATGAAGATAATGACGGTAGTCGGATAAGAAGCCCCGGCTAACTTCGTGCCAGCAGCCGCGGTAATACGAAGGGGGCAAGCGTTGTTCGGAATTACTGGGCGTAAAGCGCATGTAGGCGGTCCTACAAGCCAGAGGTGAAATCCCGGAGCTCAACTTCGGAATTGCCTTTGGAACTGTAGGACTGGAGTATCGGAGGGGATGGTGGAATTCCTAGTGTAGAGGTGAAATTCGTAGATATTAGGAGGAACACCGGTGGCGAAGGCGACCATCTGGACGATAACTGACGCTGAGATGCGAAAGCGTGGGGAGCAAACAGG` |
| ASV1309 | 421 | `TGGGGAATCTTGCGCAATGGCCGAGAGGCTGACGCAGCGACGCCGCGTGGGGGATGAAGCATTTCGGTGTGTAAACCCCTGTTGCTCGGGACGAACGACCCTTTTCGAGGGGTGTGACGGTACCGAGTGAGGAAGCACCGGCTAACTCCGTGCCAGCAGCCGCGGTAATACGGAGGGTGCGAGCGTTGTCCGGAATCACTGGGCGTAAAGGGCGCGTAGGTGGTTGGATGCGCCGGTGGTGAAAGCGCAGGGCTCAACCCTGCGTCGGCCATCGGGACGGTCCGACTGGAGCACTGTAGAGGCAGGTGGAATTCCGGGTGTAGCGGTGGAATGCGTAGAGATCCGGAAGAACACCGGTGGCGAAGGCGGCCTGCTGGGCAGTTGCTGACACTGAGGCGCGACAGCGTGGGGAGCAAACAGG` |
| ASV794 | 403 | `TGGGGAATATTGCACAATGGAGGAAACTCTGATGCAGCGACGCCGCGTGAGGGAAGAAGGTCTTCGGATTGTAAACCTCTGTCTTCGGGGACGATAATGACGGTACCCGAGGAGGAAGCACCGGCTAACTACGTGCCAGCAGCCGCGGTAATACGTAGGGTGCTAGCGTTGTCCGGAATTACTGGGTGTAAAGGGAGCGCAGGCGGGAAGATAAGTTGGATGTTTAATCTACGGGCTCAACCCGTATCAGCATTCAAAACTGTTTTTCTTGAGTGGTGCAGAGGTAGGCGGAATTCCCGGTGTAGCGGTGGAATGCGTAGATATCGGGAGGAACACCAGTGGCGAAGGCGGCCTACTGGGCACTAACTGACGCTGAGGCTCGAAAGCATGGGTAGCAAACAGG` |
| ASV3891 | 407 | `TAGGGAATATTGCACAATGGAGGAAACTCTGATGCAGCGACGCCGCGTGAGTGAAGAAGGACTTCGGTTTGTAAAGCTCTGTTCTTAGGGAATAAGACAGTGAATGTACCTAAGGAGAAAGGGACGGCTAACTTCGTGCCAGCAGCCGCGGTAATACGAAGGTCCCAAGCGTTGTTCGGAATCATTGGGCGTAAAGCGGGTGTAGGTGGCTTTATAAGTCAGAGGTGAAAGCCCAGGGCTCAACCCTGGAAGTGCCTTTGAAACTGTAAAGCTTGAATGTGGGAGAGGGTGCTAGAATTCCTGGTGTAGTGGTGAAATACGTAGAGATCAGGAGGAATTCCGGTGGCGAAGGCGGGTGCCTGGCCCAACATTGACACTGAGACCCGAAAGCGTGGGGATCAAACAGG` |
| ASV2270 | 429 | `TGGGGAATCTTGGACAATGGGGGAAACCCTGATCCAGCAACGCCGCGTGAATGAAGAAGGCCCTAGGGTTGTAAAGTTCTTTTGTCTAGGAGCAAAAGTCTACTATAAATAGTAGTAGATGTTGAGAGTACTAGAAGAATAAGCACCGGCTAACTACGTGCCAGCAGCCGCGGTAATACGTAGGGTGCGAGCGTTAATCGGAATTACTGGGCGTAAAGAGTGCGTAGGCGGACATAAAAGTTAGATGTGAAATACCCAAGCTCAACTTGGGAACTGCATTTAATACTCTATGTCTAGAGTTTATCAGAGGGAAGTAGAACTCCAAGTGTAGTAGTGAAATGCGTAGATATTTGGAAGAATACCGATGGCGAAGGCAACTTCCTGGGATAAAACTGACGCTGAGGTACGAAAGCGTGGGGAGCAAACAGG` |
| ASV1179 | 430 | `TAGGGAATATTGCGCAATGGGGGAAACCCTGACGCAGCAACGCCGCGTGAGTGAGGAAGGTCTTCGGATTGTAAAACTCTTTTATTTGGGAAAAATGTCCATATGGCTAATATCCATGTGGAGTGATGGTACCAAAGGAATAAGGACCGGCTAACTTCGTGCCAGCAGCCGCGGTAATACGAAGGGTCCAAGCGTTGTTCGGAATCATTGGGCGTAAAGCGCGCGCAGGCGGATCAGCAAGTCAGATGTGAAATCTCGGAGCTCAACTTCGAAACTGCGTCTGAAACTGCTAGTCTAGAATGTCGGAAGGGGCAGGGGAATTTCACGTGTAGGGGTAAAATCCGTAGAGATGTGAAGGAACACCGGAGGCGAAGGCGCCTGCCTGGACGACTATTGACGCTGAGGCGCGAAAGCGTGGGGAGCAAACAGG` |
| ASV455 | 424 | `TGAGGAATATTGGTCAATGGGCGCAAGCCTGAACCAGCCATGCCGCGTGCAGGATGACGGTCCTATGGATTGTAAACTGCTTTTGTACAGGAAGAAACACTCCCTCGTGAGGGAGCTTGACGGTACTGTAAGAATAAGGATCGGCTAACTCCGTGCCAGCAGCCGCGGTAATACGGAGGATCCAAGCGTTATCCGGAATCATTGGGTTTAAAGGGTCCGTAGGCGGTTTTATAAGTCAGTGGTGAAATCTGGTCGCTCAACGATCAAACGGCCATTGATACTGTAAGACTTGAATTACTTGGAAGTAACTAGAATATGTAGTGTAGCGGTGAAATGCTTAGAGATTACATGGAATACCAATTGCGAAGGCAGGTTACTACAAGTGGATTGACGCTGATGGACGAAAGCGTGGGGAGCGAACAGG` |
| ASV3974 | 404 | `TGGGGAATATTGGACAATGGGCGAAAGCCTGATCCAGCAATACCGCGTGAATGATGAAGGCCTTAGGGTTGTAAAGTTCTTTTAGTAGGGAAGATAATGACGGTACCTACAGAAAAAGCCCCGGCTAACTCCGTGCCAGCAGCCGCGGTAAGACGGAGGGGGCTAGCGTTGTTCGGAATTACTGGGCGTAAAGAGCGCGTAGGCGGTTTGGTAAGTTGGAAGTGAAAGCCCGGGGCTTAACCTCGGAATTGCTTTCAAAACTGCCAATCTAGAGTGTAGTAGGGGATGATGGAATTCCTAGTGTAGAGGTGAAATTCTTAGATATTAGGAGGAACACCGGTGGCGAAGGCGGTCATCTGGGCTACAACTGACGCTAAGGCGCGAAAGCGTGGGGAGCAAACAGG` |
| ASV4551 | 405 | `TGGGGAATATTGCACAATGGACGAAAGTCTGATGCAGCAACGCCGCGTGAAGGATGAAGGTTTTCGGATTGTAAACTTCTTTGATGGGGGACGAATAATGACGGTACCCCAAAAACAAGCCACGGCTAACTACGTGCCAGCAGCCGCGGTAATACGTAGGTGGCGAGCGTTGTCCGGAATTACTGGGTGTAAAGGGCGTGTAGGCGGGAATGCAAGTCAGATGTGAAATACCGGAGCTTAACTCCGGGGCTGCATCTGAAACTGCATTTCTTGAGTGCTGGAGAGGAAAGCGGAATTCCTAGTGTAGCGGTGAAATGCGTAGATATTAGGAGGAACACCAGTGGCGAAGGCGGCTTTCTGGACAGTAACTGACGCTGAGGCGCGAAAGCGTGGGGAGCAAACAGG` |
| ASV2638 | 404 | `TGGGGAATATTGGACAATGGGCGAAAGCCTGATCCAGCAATGCCGCGTGAGTGATGAAGGCCTTAGGGTTGTAAAGCTCTTTTACCCGGGATGATAATGACAGTACCGGGAGAATAAGCCCCGGCTAACTCCGTGCCAGCAGCCGCGGTAATACGGAGGGGGCTAGCGTTGTTCGGAATTACTGGGCGTAAAGCGCACGTAGGCGGCTTTGTAAGTTAGAGGTGAAAGCCCAGGGCTCAACCCTGGAATTGCCTTTAAGACTGCATCGCTTGAACATCGGAGAGGTAAGTGGAATTCCGAGTGTAGAGGTGAAATTCGTAGATATTCGGAAGAACACCAGTGGCGAAGGCGACTTACTGGACGATTGTTGACGCTGAGGTGCGAAAGCGTGGGGAGCAAACAGG` |
| ASV4968 | 405 | `CAGGGAATTTTGCACAATGGGCGAAAGCCTGATGCAGCGACGCCGCGTGAGGGATGAAGGCCTTAGGGTCGTAAACCTCTTTTCTCAGGGACGAGCAAGGACGGTACCTGAGGAATAAGCAACGGCTAACTACGTGCCAGCAGCCGCGGTAATACGTAGGTTGCAAGCGTTGTCCGGAATTACTGGGCGTAAAGGGTCTGCAGGCGGCCTGTTAAGTCTCTTGTGAAATCTCCGGGCTTAACCCGGAGCGGTCGAGGGATACTGGCAGGCTAGAGGCCGTCAGAGGGAAGCGGAATTCCCGGTGTAGTGGTGAAATGCGTAGATATCGGGAGGAACACCAGTGGCGAAAGCGGCTTCCTAGGGCGGCCCTGACGCTCATAGACGAAAGCGTGGGGAGCAAACCGG` |
| ASV4777 | 429 | `TGGGGAATTTTGGACAATGGGGGCAACCCTGATCCAGCCATGCCGCGTGTGTGAAGAAGGCCTTCGGGTTGTAAAGCACTTTCGGCCGGAACGAAATCGCGCGGGCTAACACCCCGTGTGGATGACGGTACCGGAAGAAGAAGCACCGGCTAACTACGTGCCAGCAGCCGCGGTAATACGTAGGGTGCGAGCGTTAATCGGAATTACTGGGCGTAAAGTGTGCGCAGGTGGCGGCGCAAGTCGAGTGTGAAATCCCCGGGCTTAACTTGGGAATTGCGCTCGAAACTACGCGGCTCGAGTGTGGCAGAGGAAGGTGGAATTCCACGTGTAGCGGTGAAATGCGTAGAGATGTGGAGGAACACCGATGGCGAAGGCAGCCTTCTGGGCCAACACTGACACTCATGCACGAAAGCGTGGGGAGCAAACAGG` |
| ASV5197 | 404 | `TGGGGAATATTGGACAATGGGCGCAAGCCTGATCCAGCCATGCCGCGTGAGTGACGAAGGCCTTCGGGTTGTAAAGCTCTTTTGGCGGGGACGATAATGACGGTACCCGCAGAATAAGCCCCGGCTAACTTCGTGCCAGCAGCCGCGGTAATACGAAGGGGGCAAGCATTGTTCGGAATTACTGGGCGTAAAGGGTGCGTAGGCGGATTAGACAGTCAGGTGTGAAAGCCCGGGGCTCAACCTCGGAATTGCATTTGATACTACTAGTCTAGAGTATTGGAGAGGAGCGTGGAATTCCCAGTGTAGAGGTGAAATTCGTAGATATTGGGAGGAACACCAGTGGCGTAGGCGACGCTCTGGCCAAATACTGACGCTGAGGCACGAAAGTGTGGGGAGCAAACAGG` |
| ASV2001 | 423 | `TAGGGAATATTGGACAATGGGTGAGAGCCTGATCCAGCCATGCCGCGTGCAGGAAGAAGGCCTTCTGGGTTGTAAACTGCTTTTGCCGGGGGATAAAAGATCCCTGCGGGGAGAATTGAAGGTACCCGGTGAATAAGCCACGGCTAACTACGTGCCAGCAGCCGCGGTAATACGTAGGTGGCAAGCGTTGTCCGGATTTATTGGGTTTAAAGGGTGCGTAGGCGGTTCTTTAAGTCAGTGGTGAAATACAGCCGCTCAACGGTTGAGGTGCCATTGATACTGAAGAACTTGAGTACAGTCGAGGTAGGCGGAATTGACGGTGTAGCGGTGAAATGCTTAGATATCGTCAAGAACACCGATAGCGAAGGCAGCTTACTAGGCTGTAACTGACGCTGAGGCACGAAGGTGCGGGGATCAAACAGG` |
| ASV2817 | 429 | `TGGGGAATATTGGACAATGGGGGCAACCCTGATCCAGCAATACCGCGTGTGTGAAGAAGGCCTGAGGGTTGTAAAGCACTTTCAGTGGGGAGGAGGTTTGCTAGGTTAAGAGCTAAGTAGATGGACGTTACCCACAGAAGAAGCACCGGCTAACTCCGTGCCAGCAGCCGCGGTAATACGGAGGGTGCAAGCGTTAATCGGAATTACTGGGCGTAAAGGGTGCGTAGGTGGTTTGGTAAGTTATCTGTGAAATCCCCGGGCTCAACCTGGGCAGGTCAGATAATACTGCTGAACTCGAGTATGGGAGAGGGTAGTGGAATTTCCGGTGTAGCGGTGAAATGCGTAGAGATCGGAAGGAACACCAGTGGCGAAGGCGGCTACCTGGCCCAATACTGACACTGAGGCACGAAAGCGTGGGGAGCAAACAGG` |
| ASV981 | 430 | `TGGGGAATATTGCGCAATGGGGGAAACCCTGACGCAGCAACGCCGCGTGAGTGAGGAAGGACTTCGGTCTGTAAAGCTCTGTTAATGAGGAAAAATGGTAGTTGGTCTAATAGGCCAATTATTTGATGGTACTCATAGAGGAAGCACCGGCTAACTTCGTGCCAGCAGCCGCGGTAATACGAAGGGTGCAAGCGTTGTTCGGATTTATTGGGCGTAAAGCGCGCGTAGGCGGACCTGCAAGTCAGATGTGAAATCTCGAGGCTCAACCTCGAAACTGCGTCTGAAACTACAGGTCTAGAATCTCGGAGGGGGAAGGGGAATATCGCATGTAGGGGTAAAATCCGTAGATATGCGATGGAACACCAGAGGCGAAGGCGCCTTCCTGGACGAGTATTGACGCTGAGGCGCGAAAGCGTGGGGATCAAACAGG` |
| ASV2500 | 429 | `TGGGGAATTTTGGACAATGGGCGCAAGCCTGATCCAGCCATGCCGCGTGCGGGAAGAAGGCCTTCGGGTTGTAAACCGCTTTTGTCAGGGAAGAAACGCTCTGGGCTAATACCCCGGGGTAATGACGGTACCTGAAGAATAAGCACCGGCTAACTACGTGCCAGCAGCCGCGGTAATACGTAGGGTGCAAGCGTTAATCGGAATTACTGGGCGTAAAGCGTGCGCAGGCGGTTATGCAAGACAGATGTGAAATCCCCGGGCTCAACCTGGGAACTGCATTTGTGACTGTATAGCTAGAGTACGGTAGAGGGGGATGGAATTCCGCGTGTAGCAGTGAAATGCGTAGATATGCGGAGGAACACCGATGGCGAAGGCAATCCCCTGGACCTGTACTGACGCTCATGCACGAAAGCGTGGGGAGCAAACAGG` |
| ASV5039 | 428 | `TCGAGAATTTTTCTCAATGGGCGAAAGCCTGAAGGAGCGACGCCGCGTGGGGGATGAAGGGCTTCGGCTCGTAAACCCCTGTCATTTGTGAACAAATGGCAATAGTTAATAGCTGTTGCCCTGATAGTAGCAGAAGAGGAAGGGACGGCTAACTCTGTGCCAGCAGCCGCGGTAATACAGAGGTCCCAAGCGTTGTTCGGATTCACTGGGCGTAAAGGGTGCGTAGGCGGTTGAGTAAGTTTGTTGTGAAATCTCCGGGCTTAACCCGGAAAATGCAATGAAAACTGCTTGACTAGAGGATTGTAGGGGAGGCTGGAATTCTCGGTGTAGCAGTGAAATGCGTAGATATCGAGAGGAACACCAGTGGCGAAGGCGAGTCTCTGGACAAATCCTGACGCTGATGCACGAAAGCTGGGGGAGCAAACAGG` |
| ASV4619 | 430 | `TGGGGAATCTTCCGCAATGGGCGAAAGCCTGACGGAGTGACGCCGCGTGGGGGACGAAGGCCTTCGGGTCGTAAACCCCTGTCAGAGGGAAAGATGGGTGTCGGCGTGAACAGCGCCGGCATCTGACGGTACCTTCAGAGGAAGCCCCGGCCAACTCTGTGCCAGCAGCCGCGGTAAGACAGAGGGGGCAAGCGTTGCTCGGAATTACTGGGCGTAAAGGGCGTGTAGGCGGAGCGGCAAGTCGGTCGTGAAAGCCTCCGGCTCAACTGGAGAACGTCGATCGATACTGCCGCCCTTGAGGGTAGCAGAGGAGACTGGAATTCCCGGTGTAGCGGTGAAATGTGTAGAGATCGGGAGGAACACCGGTGGCGAAGGCGGGTCTCTGGGCTACTCCTGACGCTGAGGCGCGAAAGCGTGGGGAGCAAACGGG` |
| ASV1077 | 429 | `TGGGGAATTTTGGACAATGGGCGCAAGCCTGATCCAGCAATGCCGCGTGCAGGATGAAGGCCTTCGGGTTGTAAACTGCTTTTGTACGGAACGAAACGGTCCTTTCTAATACAAGGGGCTAATGACGGTACCGTAAGAATAAGCACCGGCTAACTACGTGCCAGCAGCCGCGGTAATACGTAGGGTGCGAGCGTTAATCGGAATTACTGGGCGTAAAGCGTGCGCAGGCGGTTATGTAAGACAGATGTGAAATCCCCGGGCTCAACCTGGGACCTGCATTTGTGACTGCATAGCTAGAGTACGGTAGAGGGGGATGGAATTCCGCGTGTAGCAGTGAAATGCGTAGATATGCGGAGGAACACCGATGGCGAAGGCAATCCCCTGGACCTGTACTGACGCTCATGCACGAAAGCGTGGGGAGCAAACAGG` |
| ASV2054 | 409 | `TGAGGAATCTTCCGCAATGGGCGAAAGCCTGACGGAGCGACGCCGCGTGGTGGATGAAGGCCTTTACCGGTCGTAAACACCTGTTCTGAGGGACGAAATTTTTGACGGTACCTCAGGAGAAAGTATCGGCAAACCCTGTGCCAGCAGCCGCGGTAATACAGGGGATACAAGCGTTACTCGGAATCACTGGGCGTAAAGCGTCTGTAGGTGCTTAAGAAAGTCTGAGGTAAAAACCCGGGGCTTAACCCCGGGATCGTCTTGGAAACTTCTTGAGTTGAGCAGTGGAGAGGCATCTGGAATGTCGCATGTAGGAGTAAAATCCGTAGATATGCGATGGAACGCCAAAAGCGAAGGCAGGATGCTAGCTACTTGCTGACACTGAGAGACGAAAGCGTGGGGAGCGAAACGG` |
| ASV4796 | 404 | `TCGGGAATTTTGCTCAATGGGGGAAACCCTGAAGCAGCAACGCCGCGTGAGGGATGAAGGCCTTCGGGTTGTAAACCTCTTTTGTCAGGGACGATGATGACGGTACCTGGCGAATAAGCCACGGCTAACTACGTGCCAGCAGCCGCGGTAATACGTAGGTGGCAAGCGTTGTCCGGATTTACTGGGCGTAAAGAGCGCGCAGGCGGTCGGGTAAGTCGAATGTGAAAGCCCCTGGCTCAACCGGGGAGGGTCATTCGATACTGCTCGACTCGAAGGCAGGAGAGGGAAGCGGAATTCCCGGTGTAGTGGTGAAATGCGTAGATATCGGGAGGAACACCAGTGGCGAAGGCGGCTTTCTGGCCTGTTCTTGACGCTGAGGCGCGAAAGCTAGGGGAGCAAACGGG` |
| ASV4025 | 421 | `TGGGGAATATTGCGCAATGGACGAAAGTCTGACGCAGCGACGCCGCGTGTGGGATGAAGCCTTTCGGGGCGTAAACCACTGTTGCCCGGGACGAACCTCTCCTTTCGAGGAGACTGACGGTACCGGGTGAGGAAGCACCGGCTAACTCTGTGCCAGCAGCCGCGGTAATACAGAGGGTGCGAGCGTTGTCCGGAATCACTGGGCGTAAAGGGCGCGTAGGTGGCTTGGTAAGCGTGTGGTGAAAGTCCGGGGCTCAACCCCGGATCTGCCGTGCGAACTGCTGAGCTTGAGCGATGTAGAGGCAGGTGGAATTTCGGGTGTAGCGGTGGAATGCGTAGATATCCGAAAGAACACCAGTGGCGAAGGCGGCCTGCTGGGCATCAGCTGACACTGAGGCGCGACAGCGTGGGGAGCAAACAGG` |
| ASV469 | 429 | `TGGGGAATATTGCACAATGGGGGAAACCCTGATGCAGCCATGCCGCGTGTGTGAAGAAGGCCTTCGGGTTGTAAAGCACTTTCAGCGAGGAGGAAAGGTTGGCGCCTAATACGTGTCAACTGTGACGTTACTCGCAGAAGAAGCACCGGCTAACTCCGTGCCAGCAGCCGCGGTAATACGGAGGGTGCAAGCGTTAATCGGAATTACTGGGCGTAAAGCGCACGCAGGCGGTTGGATAAGTTAGATGTGAAAGCCCCGGGCTCAACCTGGGAATTGCATTTAAAACTGTCCAGCTAGAGTCTTGTAGAGGGGGGTAGAATTCCAGGTGTAGCGGTGAAATGCGTAGAGATCTGGAGGAATACCGGTGGCGAAGGCGGCCCCCTGGACAAAGACTGACGCTCAGGTGCGAAAGCGTGGGGAGCAAACAGG` |
| ASV4851 | 428 | `TTTCGAATCATTCACAATGGGCGCAAGCCTGATGGTGCGACGCCGCGTGAGGGATGAAGGTCTTCGGATTGTAAACCTCTGTCACTGGGGAGGAAACGCTTATGCAAAATATGTATAAGCCTGACTTAACCCGGAGAGGAAGCAGTGGCTAACTCTGTGCCAGCAGCCGCGGTAATACAGAGACTGCAAGCGTTATTCGGATTTACTGGGCGTAAAGGGTGCGCAGGCGGTTCTGTGTGTGATGTGTGAAATCCTGAGGCTCAACCTCAGAATTGCATCTCAAACTACAAAACTAGAGCATTGGAGAGAGTAGCAGAATTCATGGTGTAGCAGTGAAATGCGTAGATATCATGAGGAATACCAGAGGCGAAGGCGGCTACTTGGACAATTGCTGACGCTCAAGCACGAAAGCGTGGGGAGCAAAAGGG` |
| ASV357 | 425 | `TAGGGAATATTGGGCAATGGGCGAAAGCCTGACCCAGCCACGCCGCGTGCAGGAAGACGGCCCTCTGGGTTGTAAACTGCTTTTAACTGGGAAGAAAGCAGCCCTTGCGAGGGAATTTGACGGTACCAGTTGAATAAGCCACGGCTAACTACGTGCCAGCAGCCGCGGTAATACGTAGGTGGCAAGCGTTATCCGGATTTATTGGGTTTAAAGGGTGCGTAGGCGGCTTGATAAGTCAGTGGTTAAAGGTAGCAGCTTAACTGTTTTACATGCCACTGATACTGCCAAGCTTGAGTTGACAGAAGGCAGATAGAATTTCTGGTGTAGCGGTGAAATGCTTAGATACCAGAAGGAATACCGATTGCGAAGGCAGTCTGCTGCAGTCACACTGACGCTGAGGCACGAAAGTGCGGGGATCAAACAGG` |
| ASV504 | 424 | `TTTCGAATCATTCACAATGGGGGCAACCCTGATGGTGCGACGCCGCGTGGAGGATGAAGGTCTTCGGATTGTAAACTCCTGTCAAGGGGGACTAACCACACGGTTCATACCCGTGTCTGCATTAACCCCTAGAGGAAGCAGTGGCTAACTCTGTGCCAGCAGCCGCGGTAATACAGAGACTGCAAGCGTTACTCGGATTCACTGGGCGTAAAGGGAGCGCAGGCGGATTGGTGTGTCAGATGTGAAATCCCGGGGCTCAACTCCGGGGCTGCGTCTGAAACTACCGATCTAGAGATTCGGAGGGGCTAGTGGAATTCCTGGTGGAGCAGTGAAATGCGTAGATATCAGGAGGAACACCAACGGCGAAGGCAGCTAGCTGGACGAAATCTGACGCTCAGGCTCGAAAGTATGGGGAGCAAAAAGG` |
| ASV3241 | 429 | `TGGGGAATATTGGACAATGGGCGACAGCCTGATCCAGCGATGCCGCGTGTGTGAAGAAGGCCTTCGGGTTGTAAAGCACTTTCAGTAGGGAAGAAAGAACGATCATTAATACTGATCGGTGATGACGTTACCTAAAGAAGAAGCACCGGCTAACTCTGTGCCAGCAGCCGCGGTAATACAGAGGGTGCAAGCGTTAATCGGAATTACTGGGCGTAAAGGGCGCGTAGGTGGCTGGGTAAGTTGGATGTGAAATTCCTGGGCTCAACCTGGGGACTGCATTCAAAACTGCTCGGCTAGAGTAGGATAGAGGGAAGTGGAATTTCCGGTGTAGCGGTGAAATGCGTAGATATCGGAAGGAACACCAGTGGCGAAGGCGGCTTCCTGGATCCATACTGACACTGAGGCGCGAAAGCGTGGGGAGCAAACAGG` |
| ASV1377 | 429 | `TGGGGAATTTTGGACAATGGGCGCAAGCCTGATCCAGCCATGCCGCGTGCGGGATGAAGGCCTTCGGGTTGTAAACCGCTTTTGTCAGGGAAGAAACGCTCTGGATTAATACTCCGGGGTAATGACGGTACCTGAAGAATAAGCACCGGCTAACTACGTGCCAGCAGCCGCGGTAATACGTAGGGTGCAAGCGTTAATCGGAATTACTGGGCGTAAAGCGTGCGCAGGCGGTCTTGTAAGACAGAGGTGAAATCCCCGGGCTCAACCTGGGAACTGCCTTTGTGACTGCAAGGCTTGAGTGCGGCAGAGGGGGATGGAATTCCGCGTGTAGCAGTGAAATGCGTAGATATGCGGAGGAACACCGATGGCGAAGGCAATCCCCTGGGCCTGCACTGACGCTCATGCACGAAAGCGTGGGGAGCAAACAGG` |
| ASV1561 | 408 | `TGGGGAATTTTCCGCAATGGGCGAAAGCCTGACGGAGCAATACCGCGTGAGGGAAGACTGCCTATGGGTTGTAAACCTCTTTTTTCAGGGAGGAATCAAATGACGTGTACCTGAAGAATAAGCATCGGCTAACTCCGTGCCAGCAGCCGCGGTAAGACGGAGGATGCAAGTGTTATCCGGAATCACTGGGCGTAAAGCGTCTGTAGGTGGTCCAATAAGTCAACTGTTAAATCTTGAGGCTCAACCTCAAAATCGCAGTCGAAACTATTAGACTAGAGTATAGTAGGGGTAAAGGGAATTTCCAGTGGAGCGGTGAAATGCGTAGATATTGGAAAGAACACCGATGGCGAAAGCACTTTACTGGGCTATTACTAACACTCAGAGACGAAAGCTAGGGTAGCAAATGGG` |
| ASV4669 | 405 | `TGAGGAATCTTGCGCAATGGGCGAAAGCCTGACGCAGCGATGCCGCGTGAGCGAAGAAGGCCTTCGGGTCGTAAAGCTCTTTATCTCAGGGAAGATAATGACGGTACCTGAGGAATAAGCACCGGCTAACTACGTGCCAGCAGCCGCGGTAATACGTAGGGTGCGAGCGTTGTTCGGAATTACTGGGCGTAAAGGGTGCGTAGGCGGATCGACAAGCTAAGGGTGAAAGCCCGGGGCTCAACCTCGGAACGGCTTTTAGAACTATCGATCTAGAGAGGGTCAGGGGTCTGTGGAATTCCTGGTGTAGAGGTGAAATTCGTAGATATCAGGAGGAACACCGGAGGCGAAAGCGGCGGACTGGGGCCACTCTGACGCTGAGGCACGAAAGCGTGGGGAGCAAACAGG` |
| ASV968 | 407 | `TAGGGAATATTGCACAATGGAGGAAACTCTGATGCAGCGACGCCGCGTGAGTGATGAAGGCCTTCGGGTCGTAAAGCTCTGTCGCAGGGGAATAACACAATGAAGGTACCCTGCAAGAAAGGATCGGCTAACTTCGTGCCAGCAGCCGCGGTAAGACGAGGGATCCAAGCGTTGTTCGGAATTATTGGGCGTAAAGCGGGTGTAGGTGGCTATGTAAGTCAGGTGTGAAAGCCCAGGGCTCAACCCTGGAAGTGCATTTGATACTGCGTAGCTTGAGTGTAGGAGAGGATAGTAGAATTCCTGGTGTAGTGGTGAAATACGTAGATATCAGGAGGAATACCGGAGGCGAAGGCGGCTATCTGGCCTAACACTGACACTCAGACCCGAAAGCGTGGGGATCAAACAGG` |
| ASV5060 | 424 | `TGAGGAATATTGGACAATGGGGGCAACCCTGATCCAGCCATGCCGCGTGCAGGAAGACGGCCCTATGGGTTGTAAACTGCTTTTGTACAGGAAGAAACGTTCCCACGTGTGGGGATTTGACGGTACTGTAAGAATAAGGATCGGCTAACTCCGTGCCAGCAGCCGCGGTAATACGGAGGATCCGAGCGTTATCCGGAATCATTGGGTTTAAAGGGTCCGTAGGCGGGGCGGTAAGTCAGTGGTGAAAGTCGGTAGCTCAACTATCGAATTGCCATTGAAACTGCTGTCCTTGAGTTATTGTGAAGTGGTTGGAACATGTAGTGTAGCGGTGAAATGCATAGATATTACATAGAACACCGATTGCGAAGGCAGGTCACTAACAATACACTGACGCTGAGGGACGAAAGCGTGGGTAGCGAACAGG` |
| ASV2368 | 429 | `TGGGGAATTTTGGACAATGGGGGCAACCCTGATCCAGCCATGCCGCGTGCAGGATGAAGGCCTTCGGGTTGTAAACTGCTTTTGTTAGGAACGAAAAGCCATCCAATAACACTGGGTGGTGCTGACGGTACCTAAAGAATAAGCACCGGCTAACTACGTGCCAGCAGCCGCGGTAATACGTAGGGTGCAAGCGTTAATCGGAATTACTGGGCGTAAAGCGTGCGCAGGCGGTTCGTTAAGTTAGATGTGAAATCCCTGGGCTCAACCTAGGAACTGCATTTAAAACTGGCGAGCTAGAGTGTGTCAGAGGGGGGTAGAATTCCACGTGTAGCAGTGAAATGCGTAGAGATGTGGAGGAATACCAATGGCGAAGGCAACCCCCTGGGATAACACTGACGCTCATGTACGAAAGCGTGGGGAGCAAACAGG` |
| ASV1649 | 429 | `TGGGGAATTTTGGACAATGGGCGCAAGCCTGATCCAGCAATGCCGCGTGAGTGAAGAAGGCCTTCGGGTTGTAAAGCTCTTTTGTCAGGGAAGAAAAGGTTGCGGCTAATATCCGCAACTGCTGACGGTACCTGAAGAATAAGCACCGGCTAACTACGTGCCAGCAGCCGCGGTAATACGTAGGGTGCAAGCGTTAATCGGAATTACTGGGCGTAAAGCGTGCGCAGGCGGTTTCGTAAGTCTGTCGTGAAATCCCCGGGCTTAACCTGGGAATGGCGATGGAGACTGCGAGGCTAGAGTTTGGCAGAGGGGGGTAGAATTCCACGTGTAGCAGTGAAATGCGTAGAGATGTGGAGGAACACCGATGGCGAAGGCAGCCCCCTGGGTCAAAACTGACGCTCATGCACGAAAGCGTGGGGAGCAAACAGG` |
| ASV3841 | 408 | `TGGGGAATATTGGGCAATGGAGGCAACTCTTACCCAGCAATGCCGCGTGAATGAAGAAGGCCTTCGGGTCGTAAAGTTCTTTAATTGGGGACGAAGAAAATGACGGTACCCAAGGAATAAGCTCCGGCTAACTACGTGCCAGCAGCCGCGGTAATACGTAGGGAGCAAGCGTTGTCCGGAATGACTGGGTGTAAAGGGTGTGTAGGCGGTTTGATAAGTTAGAAGTGTAATACCTAGTGCTTAACACAGGTGCTGCTTCTAAAACTATTAGGCTTGAGTGCAAGAGAGGATAGTGGAATTCCTAGTGTAGCGGTGGAATGCGTAGATATTAGGAGGAACATCAGTGGCGAAGGCGACTATCTGGCTTGTAACTGACGCTGAGGCACGAAAGTGTGGGGAGCAAACAGG` |
| ASV1023 | 429 | `TGGGGAATTTTGGACAATGGGCGCAAGCCTGATCCAGCAATGCCGCGTGCAGGATGAAGGCCTTCGGGTTGTAAACTGCTTTTGTACGGAGCGAAAAGGCTTCCCCTAATACGGGGGGCTCATGACGGTACCGTAAGAATAAGCACCGGCTAACTACGTGCCAGCAGCCGCGGTAATACGTAGGGTGCAAGCGTTAATCGGAATTACTGGGCGTAAAGCGTGCGCAGGCGGTTTTGTAAGACAGATGTGAAATCCCCGGGCTCAACCTGGGACCTGCATTTGTGACTGCAAGGCTAGAGTACGGTAGAGGGGGATGGAATTCCGCGTGTAGCAGTGAAATGCGTAGATATGCGGAGGAACACCGATGGCGAAGGCAATCCCCTGGACCTGTACTGACGCTCATGCACGAAAGCGTGGGGAGCAAACAGG` |
| ASV356 | 404 | `TGGGGAATCTTGCGCAATGGGCGAAAGCCTGACGCAGCCATGCCGCGTGGATGATGAAGGTCTTAGGATTGTAAAATCCTTTCACCGGTGAAGATAATGACTGTAGCCGGAGAAGAAGCCCCGGCTAACTTCGTGCCAGCAGCCGCGGTAATACGAAGGGGGCTAGCGTTGCTCGGAATTACTGGGCGTAAAGGGAGCGTAGGCGGACATTTAAGTCAGGGGTGAAATCCCAGAGCTCAACTCTGGAACTGCCTTTGATACTGGGTGTCTTGAGTGTGATAGAGGTATGTGGAACTCCGAGTGTAGAGGTGAAATTCGTAGATATTCGGAAGAACACCAGTGGCGAAGGCGACATACTGGATCATTACTGACGCTGAGGCTCGAAAGCGTGGGGAGCAAACAGG` |
| ASV4636 | 406 | `TAAGGAATCTTCCACAATGGGCGAAAGCCTGATGGAGCAACGCCGCGTGCAGGATGAAGGCCCTCGGGTCGTAAACTGCTTTTATGAGTGAAGAATATGACGGTAACTCATGAATAAGGGTCGGCTAACTACGTGCCAGCAGCCGCGGTCATACGTAGGACCCAAGCGTTATCCGGAGTGACTGGGCGTAAAGAGTTGCGTAGGTGGCTTGTAAAGTGAATAGTGAAATCTGGGGGCTCAACCTCACAGGCTATTATTCAAACTCACAAGCTCGAGAATGATAGAGGTAACTGGAATTTCTGAAGTAGGGGTAATATCCGTAGATATCAGAAGGAACACCAATGGCGTAGGCAGGTTACTGGATCATTTCTGACACTGAGGCACGAAAGCGTGGGGAGCGAACCGG` |
| ASV352 | 404 | `TGGGGAATCTTAGACAATGGGGGAAACCCTGATCTAGCCATGCCGCGTGATCGATGAAGGCCTTAGGGTTGTAAAGATCTTTCAGATGGGAAGATAATGACGGTACCATCAGAAGAAGCCCCGGCTAACTCCGTGCCAGCAGCCGCGGTAATACGGAGGGGGCTAGCGTTATTCGGAATTACTGGGCGTAAAGCGCACGTAGGCGGATTGGAAAGTTGGAGGTGAAATCCCAGGGCTCAACCTTGGAACTGCCTTCAAAACTCCCAGTCTTGAGGTCGAGAGAGGTGAGTGGAATTCCGAGTGTAGAGGTGAAATTCGTAGATATTCGGAGGAACACCAGTGGCGAAGGCGGCTCACTGGCTCGATACTGACGCTGAGGTGCGAAAGCGTGGGGAGCAAACAGG` |
| ASV5169 | 430 | `TGGGGAATATTGGACAATGGGCGCAAGCCTGATCCAGCGACGCCGCGTGAGTGATGAAGGCCTTCGGGTTGTAAAGCTCTGTTGAGAGGGAAGAAAACGGGTGGCGCGAATAGCTCCATCCCTTGACGGTACCTCTTTAGAAAGCACCGGCTAACTCTGTGCCAGCAGCCGCGGTAAGACAGAGGGTGCAAACGTTGCTCGGAATTACTGGGCGTAAAGCGCGTGTAGGCGGACCAGCAAGTCAGGCGTGAAATCCCTAGGCTCAACCTAGGAACTGCACCTGAAACTGCTGGTCTGGAGTAGTGGAGAGGAAAGCAGAATTCCTGGTGTAGAGGTGAAATTCGTAGATATCAGGAGGAACACCGGTGGCGAAGGCGGCTTTCTGGACACTTACTGACGCTGAGACGCGAAAGCGTGGGGAGCAAACAGG` |
| ASV4420 | 403 | `TGAGGAATCTTCCACAATGGGCGAAAGCCTGATGGAGCGACACCGCGTGAAGGATGAAGCCTTTGTTGGTGTAAACTTCTTTTCTCTCCGAAGATAATGACGGTAGGAGAGGAATAAGGGGCGGCAAACTATGTGCCAGCAGCCGCGGTAATACATAGGCCCCAAGCGTTATCCGGAATTACTGGGTGTAAAGCGTCTGTAGGTGGTCTTAACAGTCTCGTGTGAAACTTCGGAGCTCAACTCCGAATTGCACGGGAAACTATAAGACTTGAGTGCGGGAGAGGCAAGCAGAACTGTATGAGTAGGGGTGCAATCCGTTGATACATACAAGAATACCAAAAGCGAAGGCAGCTTGCTGGAACGCCACTGACACTGAGAGACGAAAGCGTGGGGAGCAAAAGGG` |
| ASV3846 | 408 | `TGGGGAATATTGGGCAATGGAGGAAACTCTTACCCAGCAATGCCGCGTGAATGAAGAAGGTCTTCGGATTGTAAAGTTCTTTAGTCAGGGACGAAAAAAATGACGGTACCTGAAAAATAAGCTCCGGCTAACTACGTGCCAGCAGCCGCGGTAATACGTAGGGAGCGAGCGTTGTCCGGAATGACTGGGCGTAAAGGGTGTGTAGGCGGTTTGTCAAGTTAGAAGTGTAATACCTAGAGCTTAACTCAGGTGCTGCTTCTAAAACTGATAGACTTGAGTGCAGCAGAGGTAAATGGAATTCCTAGTGTAGCGGTGGAATGCGTAGATATTAGGAGGAACACCAGAGGCGAAGGCGGTTTACTGGGCTGTAACTGACGCTGAGACACGAAAGTGTGGGGAGCAAACAGG` |
| ASV1472 | 407 | `TGGGGAATTTTCCGCAATGGGCGAAAGCCTGACGGAGCAATACCGCGTGAGGGAGGAAGGCTCTTGGGTCGTAAACCTCTTTTGTCAGGGAAGAACACAATGACGGTACCTGACGAATCAGCATCGGCTAACTCCGTGCCAGCAGCCGCGGTAATACGGGGGATGCAAGCGTTATCCGGAATTATTGGGCGTAAAGCGTCCGCAGGTGGTTGACCAAGTCTGCTGTCAAAGCATGGGGCTTAACTCCATATAGGCAGTGGAAACTGGTCGACTAGAGTACGGTAGGGGTCGCGGGAATTCCCAGTGTAGCGGTGAAATGCGTAGATATTGGGAAGAACACCAGTGGCGAAAGCGCGCGGCTGGACCTGTACTGACACTGAGGGACGAAAGCTAGGGGAGCGAAAGGG` |
| ASV739 | 424 | `TGAGGAATATTGGTCAATGGACGCAAGTCTGAACCAGCCATGCCGCGTGAAGGATGAAGGTCCTCTGGATTGTAAACTTCTTTTATATGGGAAGAAATGTGGATTTTCTAATCCACTTGACGGTACCATGTGAATAAGCACCGGCTAACTCCGTGCCAGCAGCCGCGGTAATACGGAGGGTGCAAGCGTTATCCGGATTCACTGGGTTTAAAGGGTGCGTAGGTGGACTTATAAGTCAGTGGTGAAATCCCGAAGCTCAACTTCGGAACTGCCATTGATACTGTTAGTCTTGAATATCCTGGAGGTGGGCGGAATATGTCATGTAGCGGTGAAATGCTTAGATATGACATAGAACACCAATTGCGAAGGCAGCTCACTACGGGATGATTGACACTGAGGCACGAAAGCGTGGGGATCAAACAGG` |
| ASV293 | 429 | `TGGGGAATCTTGGACAATGGGCGAAAGCCTGATCCAGCAATGCCGCGTGCGTGAAGAAGGCCTTCGGGTTGTAAAGCGCTTTTGTCGGGGAGGAAATCCTAGTCTCTAATAAAGGCTGGGGATGACAGTACCCGAAGAATAAGGACCGGCTAACTACGTGCCAGCAGCCGCGGTAATACGTAGGGTCCAAGCGTTAATCGGAATTACTGGGCGTAAAGCGTCCGCAGGTGGCTTGATAAGATAGAGGTGAAATCCCTGGGCTCAACCTAGGAATTGCCTTTATGACTGTCTCGCTAGAGTATGGGAGAGGGGGGTGGAATTCCACGTGTAGCAGTGAAATGCGTAGAGATGTGGAGGAACACCGATGGCGAAGGCAACCCCCTGGCCTAATACTGACACTCATGGACGAAAGCGTGGGGAGCAAACAGG` |
| ASV1364 | 430 | `TAGGGAATATTGCGCAATGGGGGAAACCCTGACGCAGCAACGCCGCGTGAGTGAGGAAGGTCTTCGGATTGTAAAACTCTTTTATCAGGGAAAAATGTCTTGGTGGTTAATACCCATTAGGAGTGATGGTACCTGAGGAATAAGCACCGGCTAACTTCGTGCCAGCAGCCGCGGTAATACGAAGGGTGCAAGCGTTGTTCGGAATCATTGGGCGTAAAGCGCGCGCAGGCGGATCAGCAAGTCAGATGTGAAATCTCGAAGCTCAACTTCGAAACTGCGTCTGAAACTGCTAGTCTAGAGTGTCAGAGGGGGTAGGGGAATTTCACGTGTAGGGGTAAAATCCGTAGAGATGTGAAGGAACACCGGTGGCGAAGGCGCCTACCTGGATGACTACTGACGCTGAGGCGCGAAAGCGTGGGGAGCAAACAGG` |
| ASV1674 | 429 | `TGGGGAATATTGGACAATGGGCGCAAGCCTGATCCAGCAATGCCGCGTGTGTGAAGAAGGCCTTCGGGTTGTAAAGCACTTTTGTCCGGAACGAAAAGCGACAGGTTAATAACCTGGCGTGCTGACGGTACCGGTTGAATAAGCACCGGCTAACTTCGTGCCAGCAGCCGCGGTAATACGAAGGGTGCAAGCGTTAATCGGAATTACTGGGCGTAAAGGGTGCGTAGACGGTTTGTTAAGTCGGATGTGAAAGCCCCGGGCTCAACCTGGGAACTGCATCCGAAACTGGCAGGCTAGAGTGCGATAGAGGATGGTGGAATTCCCGGTGTAGCGGTGAAATGCGTAGAGATCGGGAGGAACATCTGTGGCGAAGGCGGCCATCTGGATCAGCACTGACGTTGAGGCACGAAAGCGTGGGGAGCAAACAGG` |
| ASV3151 | 429 | `TGGGGAATATTGGACAATGGGGGCAACCCTGATCCAGCGATGCCGCGTGAGTGAAGAAGGCCCTCGGGTCGTAAAGCTCTTTAGGTTGGGAAGAAGGACGTTAGGTTAATACCCTGATGTTTTGACGGTACCGACAGAATAAGCACCGGCAAACTCTGTGCCAGCAGCCGCGGTAATACAGAGGGTGCGAGCGTTAATCGGATTTACTGGGCGTAAAGGGCGCGTAGGCGGTAATGTGAGTGTGATGTGAAAGCCCCGGGCTTAACCTGGGAAGTGCATCGCAAACGACATTGCTGGAGTATAAGAGAGGGTGGCGGAATTTCCGGTGTAGCGGTGAAATGCGTAGAGATCGGAAGGAACGTCAATGGCGAAGGCAGCCACCTGGCTTAATACTGACGCTGAGGCGCGAAAGCGTGGGGATCAAACAGG` |
| ASV236 | 429 | `TGGGGAATTTTGGACAATGGGCGAAAGCCTGATCCAGCCATGCCGCGTGCGGGAAGAAGGCCTTCGGGTTGTAAACCGCTTTTGTCAGGGAAGAAATCCTTTGGGTTAATACCTCGGAGGGATGACGGTACCTGAAGAATAAGCACCGGCTAACTACGTGCCAGCAGCCGCGGTAATACGTAGGGTGCGAGCGTTAATCGGAATTACTGGGCGTAAAGCGTGCGCAGGCGGTCTTGCAAGACAGGTGTGAAATCCCCGGGCTTAACCTGGGAACTGCACTTGTGACTGCAAGGCTGGAGTACGGCAGAGGGGGATGGAATTCCGCGTGTAGCAGTGAAATGCGTAGATATGCGGAGGAACACCGATGGCGAAGGCAATCCCCTGGGCCTGTACTGACGCTCATGCACGAAAGCGTGGGGAGCAAACAGG` |
| ASV2948 | 404 | `TGGGGAATATTGGACAATGGGCGAAAGCCTGATCCAGCAATGCCGCGTGAGTGATGAAGGCCTTAGGGTTGTAAAGCTCTTTTACCCGGGATGATAATGACAGTACCGGGAGAATAAGCCCCGGCTAACTCCGTGCCAGCAGCCGCGGTAATACGGAGGGGGCTAGCGTTGTTCGGAATTACTGGGCGTAAAGCGCGCGTAGGCGGTCTTTTAAGTCAGAGGTGAAAGCCTGGAGCTCAACTCCAGAACTGCCTTTGAAACTGGAAGACTTGAATCATGGAGAGGTTAGTGGAATTCCGAGTGTAGAGGTGAAATTCGTAGATATTCGGAAGAACACCAGTGGCGAAGGCGACTAACTGGACATGTATTGACGCTGAGGTGCGAAAGCGTGGGGAGCAAACAGG` |
| ASV1880 | 404 | `TGGGGAATATTGGACAATGGGGGAAACCCTGATCCAGCGATGCCGCGTGAGTGAAGAAGGCCTTCGGGTTGTAAAGCTCTTTTAGTAGGGAAGATGATGACGGTACCTACAGAAAAAGCACCGGCTAACTTCGTGCCAGCAGCCGCGGTAATACGAAGGGTGCAAGCGTTGTTCGGAATTACTGGGCGTAAAGCGTGCGTAGGCGGTTTGTTAAGTCAGACGTGAAAGCCCCGAGCTCAACTTGGGAAATGCGTTTGATACTGGCAGGCTAGAGTATTGGAGAGGAGAGTGGAATTCCTAGTGTAGAGGTGAAATTCGTAGATATTAGGAAGAACACCAGTGGCGAAGGCGACTCTCTGGCCAATTACTGACGCTGAGGTACGAAAGCGTGGGGAGCAAACAGG` |
| ASV4977 | 429 | `TGGGGAATATTGCACAATGGGCGCAAGCCTGATGCAGCCATGCCGCGTGTATGAAGAAGGCCTTAGGGTTGTAAAGTACTTTCAGTCGGGAGGAAGGTGTTGAGTTTAATATGCTCAGCAATTGACGTTACCGACAGAAGAAGCACCGGCTAACTCCGTGCCAGCAGCCGCGGTAATACGGAGGGTGCAAGCGTTAATCGGAATTACTGGGCGTAAAGCGCACGCAGGCGGTTGATTAAGTCAGATGTGAAATCCCCGGGCTCAACCCGGGAATTGCATCTGAGACTGGTCGGCTAGAGTCTTGTAGAGGGGGGTAGAATTCCATGTGTAGCGGTGAAATGCGTAGAGATGTGGAGGAATACCGGTGGCGAAGGCGGCCCCCTGGACAAAGACTGACGCTCAGGTGCGAAAGCGTGGGGAGCAAACAGG` |
| ASV3020 | 428 | `TCGGGAATCTTCCGCAATGGGCGAAAGCCTGACGGAGCGACGCCGCGTGAGTGATGAAGGCCTTCGGGTTGTAAAGCTCTGTGGGGAGGGAAGAATAAGTTCAAGTGAATAGCTTGAATGATGACGGTACCTCCTTAGCAAGCACCGGCTAACTCTGTGCCAGCAGCCGCGGTAAGACAGAGGGTGCAAACGTTGTTCGGATTTACTGGGCGTAAAGCGCGTGTAGGCGGCTTTGCAAGTCGGATGTGAAAGCCCACGGCTCAACCGTGGAAGTGCATCCGAAACTGCATGGCTGGAGTCTCGGAGAGGAAGGTGGAATGCTTGGTGTAGAGGTGAAATTCGTAGATATCAAGCAGAACACCTGCGGCGAAGGCGGCCTTCTGGACGATGACTGACGCTGAGACGCGAAAGCGTGGGGAGCAAACAGG` |
| ASV2024 | 404 | `TGGGGAATATTGCACAATGGAGGAAACTCTGATGCAGCGACGCCGCGTGAGGGAAGAAGGTCTTCGGATTGTAAACCTCTGTCTTTGGGGACGATAATGACGGTACCCAAGGAGGAAGCACCGGCTAACTACGTGCCAGCAGCCGCGGTAAAACGTAGGGTGCAAGCGTTGTCCGGAATTACTGGGTGTAAAGGGAGCGCAGGCGGAAGAGTAAGTTGGAAGTGAAAACTATGGGCTCAACCCATAACCTGCTTTCAAAACTATTTTTCTTGAGTGGTGTAGAGGTAGGCGGAATTCCCGGTGTAGCGGTGGAATGCGTAGATATCGGGAGGAACACCAGTGGCGAAGGCGGCCTACTGGGCACTAACTGACGCTGAGGCTCGAAAGCATGGGTAGCAAACAGG` |
| ASV961 | 404 | `TGGGGAATATTGGACAATGGGGGAAACCCTGATCCAGCAATGCCGCGTGTATGATGAAGGTCTTTGGATTGTAAAGTACTTTTATTGGTGACGATGATGACGGTAACCAATGAATAAGCTCCGGCTAACTTCGTGCCAGCAGCCGCGGTAATACGAAGGGAGCTAGCGTTGTTCGGAATAACTGGGCGTAAAGGGTGCGTAGGTGGGCGTGTGCGTTAGATGTGAAATCCCCGGGCTTAACCTGGGAACTGCATTTAATACGGCACGTCTAGAGTGCGAGAGGGGAAAGCGGAATGGCGAGTGGAGCAGTGAAATGCATTGAGATTCGCTAGAACACCAGAGGCGAAGGCGGCTTTCTGGCTCGCTACTGACACTAAGGCACGAAAGCGTGGGGAGCAAACAGG` |
| ASV4974 | 428 | `TGGGGAATCTTGGACAATGGGGGCAACCCTGATCCAGCAATGCCGCGTGTGTGAAGAAGGCCTTCGGGTTGTAAAGCACTTTCGGTGGGGACGAACATGGTCGGGTGAATATCCCGGCCCTTGACGGTACCCAAAGAAGAAGCACCGGCTAACTCTGTGCCAGCAGCCGCGGTAATACAGAGGGTGCAAGCGTTAATCGGAATTACTGGGCGTAAAGCGTGCGTAGGTGGTTTGATAAGTCAGATGTGAAATCCCTGGGCTCAACCTGGGAACTGCATTTGATACTGTCGAACTCGAGTTCAGTAGAGGGTAGTAGAATTCCCGGTGTAGCGGTGAAATGCGTAGATATCGGGAGGAATACCAGTGGCGAAGGCGGCTACCTGGACTGAAACTGACACTGAGGTACGAAAGCGTGGGTAGCAAACAGG` |
| ASV2026 | 423 | `TAGGGAATATTGGACAATGGGCGAAAGCCTGATCCAGCCATGCCGCGTGAAGGATGAAGGGCCTCTGGCTTGTAAACTTCTTTTGGCGGGGGATAAACCACCACTACAAGTGGTGCTGAAGGTACCCGCAGAATAAGCACCGGCTAACTCCGTGCCAGCAGCCGCGGTAATACGGAGGGTGCAAGCGTTGTCCGGATTTATTGGGTTTAAAGGGTGCGTAGGCGGTACGCTAAGTCAGTGGTGAAATCCTGGGGCTCAACCCCGGAATTGCCATAGATACTGGCGTGCTTGAGTTCGACCGAGGCGGTTGGAATGCGTGGTGTAGCGGTGAAATGCTTAGATATCACGCGGAACACCGATTGCGAAGGCAGATCGCTAGGTCGCGACTGACGCTGAGGCACGAAAGTGCGGGGATCAAACAGG` |
| ASV444 | 428 | `TGGGGAATTTTGGACAATGGGGGCAACCCTGATCCAGCAATGCCGCGTGAGTGAAGAAGGCCTTCGGGTTGTAAAGCTCTTTTGTCAGGGAAGAAACAGCAGCTCTAACACAGTCTGCGAATGACGGTACCTGAAGAATAAGCACCGGCTAACTACGTGCCAGCAGCCGCGGTAATACGTAGGGTGCAAGCGTTAATCGGAATTACTGGGCGTAAAGCGTGCGCAGGCGGTTATACAAGACAGGCGTGAAATCCCCGGGCTTAACCTGGGAATGGCGTCTGTGACTGTATGACTAGAGTGTGTCAGAGGGGGGTAGAATTCCACGTGTAGCAGTGAAATGCGTAGATATGTGGAGGAATACCAATGGCGAAGGCAGCCCCCTGGGATAACACTGACGCTCATGCACGAAAGCGTGGGGAGCAAACAGG` |
| ASV1873 | 429 | `TGGGGAATTTTGGACAATGGACGCAAGTCTGATCCAGCAATGCCGCGTGCAGGACGAAGGCCTTCGGGTTGTAAACTGCTTTTGTACGGAACGAAAAGGTCTGCTTTAATACAGTGGGCTCATGACGGTACCGTAAGAATAAGCACCGGCTAACTACGTGCCAGCAGCCGCGGTAATACGTAGGGTGCGAGCGTTAATCGGAATTACTGGGCGTAAAGCGTGCGCAGGCGGTTATATAAGTCAGATGTGAAATCCCCGGGCTCAACCTGGGACCTGCATTTGAGACTGTATAGCTAGAGTACGGTAGAGGGGGATGGAATTCCGCGTGTAGCAGTGAAATGCGTAGATATGCGGAGGAACACCGATGGCGAAGGCAATCCCCTGGACCTGTACTGACGCTCATGCACGAAAGCGTGGGGAGCAAACAGG` |
| ASV4922 | 428 | `TTTCGAATCATTCACAATGGGCGAAAGCCTGATGGTGCGACGCCGCGTGAGGGATGAAGGTCTTCGGATTGTAAACCTCTGTCACTGGGGAAGAAACGCTAAATCCTAATACGATTTAGCCTGACTTAACCCGGAGAGGAAGCAGTGGCTAACTCTGTGCCAGCAGCCGCGGTAATACAGAGACTGCAAGCGTTATTCGGATTCACTGGGCGTAAAGGGTGCGCAGGCGGCCATGTGTGTCAGATGTGAAATCCCGGAGCTTAACTCCGGAACTGCGTCTGAAACTACATGGCTAGAGCATTGGAGAGGGAAGTGGAATTCACGGTGTAGCAGTGAAATGCGTAGATATCGTGAGGAACACCAGAGGCGAAGGCGACTTCCTGGACAATTGCTGACGCTCAGGCACGAAAGCGTGGGTAGCCAAAGGG` |
| ASV1712 | 429 | `TGGGGAATTTTGGACAATGGACGCAAGTCTGATCCAGCCATGCCGCGTGTGGGAAGAAGGCCTTCGGGTTGTAAACCACTTTTGTCAGGGAAGAAACGGCTCTTACTAATATTAGGAGCTAATGACGGTACCTGAAGAATAAGCACCGGCTAACTACGTGCCAGCAGCCGCGGTAATACGTAGGGTGCAAGCGTTAATCGGAATTACTGGGCGTAAAGCGTGCGCAGGCGGTTATGCAAGACAGAGGTGAAATCCCCGGGCTCAACCTGGGAACTGCCTTTGTGACTGCATAGCTAGAGTACGGTAGAGGGGGATGGAATTCCGCGTGTAGCAGTGAAATGCGTAGATATGCGGAGGAACACCGATGGCGAAGGCAATCCCCTGGACCTGTACTGACGCTCATGCACGAAAGCGTGGGGAGCAAACAGG` |
| ASV4507 | 429 | `TGGGGAATATTGGACAATGGGGGCAACCCTGATCCAGCAATGCCGCGTGTGTGAAGAAGGCCTTCGGGTTGTAAAGCACTTTGGTTGGGGAGGAAGGGGCGTGCGTGAATAACGTAGGTCATTGACGTTACCCAAAGAACAAGCACCGGCTAACTCTGTGCCAGCAGCCGCGGTAATACAGAGGGTGCGAGCGTTAATCGGAATTACTGGGCGTAAAGGGTGCGTAGGTGGTTTTGTAAGTTGTATGTGAAAGCCCTGGGCTTAACCTGGGAACGGCATACAATACTGCGGAGCTAGAGTCGTGTAGAGGGAAGTGGAATTTCCGGTGTAGCGGTGAAATGCGTAGATATCGGAAGGAACATCGGTGGCGAAGGCGGCTTCCTGGACACAGACTGACACTGAGGCACGAAAGCGTGGGTAGCAAACAGG` |
| ASV4918 | 408 | `TGGGGAATTTTCCACAATGGGCGAAAGCCTGATGGAGCAATACCGCGTGGGTGAAGACGGTTCGTGGATTGTAAAGCCCTTTTCTTAAGGAAGAATAAATGACGGTACTTAAGGAATAAGCACCGGCTAACTCTGTGCCAGCAGCCGCGGTAATACAGGGGGTGCAAGCGTTGTCCGGAATGATTGGGCGTAAAGCATCTGTAGGTGGTTCTTAAAGTCAACTGTTAAATCCCAGGGCTCAACCTTGGAAAAGCAGTTGAGTACTTAAGGACTTGAGTGTGGTAGAGGCAGAGGGAATTCCTAGTGGAGCGGTGAAATGCACAGAGATTAGGAAGAACACCAGAGGCGAAAGCGCTCTGCTGGGCCATCCACTGACACTGAGAGATGAAAGTTAGGGGAGCGAAAAGG` |
| ASV5245 | 431 | `TGGGGAATATTGCACAATGGGGGAAACCCTGATGCAGCAACGCCGCGTGAGTGAAGAAGGCTCTTGGGTCGTAAAGCTCTTTCGGCTGGAAAGAAGGGTTTCAAGTCTAATAAGCTTGGAATTTGACGGTACCAGAAGAAGAAGGACCTGCTAATTGCGTGCCAGCAGCAGCGGTAATACGTGAGGTCCGAGCGTTGTTCGGAATTACTGGGCGTAAAGCGCTCATAGGCGGGTAAGAAAGTCGATTGTGAAATCCCTGGGCTAAACCTAGGAACTGCAGTCGAAACGTTTTATCTTGAGTGCCTGAGAGGGTGGGGGAATTCCTGGTGGAGTAGTGAAATACGTAGAGATCAGGAGGAACACCGGAGGCGAAGGCGCCTACCTGGCGGTGCACTGACGCTAATTGAGCGAAAGCGCGGGGATCAAACAGG` |
| ASV990 | 429 | `TGGGGAATTTTGGACAATGGGCGAAAGCCTGATCCAGCAATGCCGCGTGCAGGATGAAGGCCTTCGGGTTGTAAACTGCTTTTGTACGGAACGAAAAGCTTTGGGTTAATACCCTGGAGTCATGACGGTACCGTAAGAATAAGCACCGGCTAACTACGTGCCAGCAGCCGCGGTAATACGTAGGGTGCAAGCGTTAATCGGAATTACTGGGCGTAAAGCGTGCGCAGGCGGTTTTGTAAGACAGAGGTGAAATCCCCGGGCTCAACCTGGGAACTGCCTTTGTGACTGCAAGGCTAGAGTACGGCAGAGGGGGGTGGAATTCCGCGTGTAGCAGTGAAATGCGTAGATATGCGGAGGAACACCGATGGCGAAGGCAACCCCCTGGGCCTGTACTGACGCTCATGCACGAAAGCGTGGGGAGCAAACAGG` |
| ASV3179 | 406 | `TAGGGAATTTTCCACAATGGACGAAAGTCTGATGGAGCAACGCCGCGTGCAGGATGACGGCCTTCGGGTTGTAAACTGCTTTTGTGAGTGAAGATTTAAGACAGTAACTCAAGAATAAGGGACGGCTAACTACGTGCCAGCAGCCGCGGTCATACGTAGGTCCCAAGCGTTATCCGGAATCACTGGGCGTAAAGAGTTGCGTAGGTGGCTTATTAAGCAGCGTGTGAAAGCGTACGGCTCAACCGTATACACATACACTGAACTGGTAAGCTTGAGGATGAGAGAGGTTATTGGAATTCCAAGTGTAGGAGTGAAATCCGTAGATATTTGGAGGAACACCGATGGCGTAGGCAGATAACTGGCTCATTCCTGACACTAAGGCACGAAAGCGTGGGGAGCAAACGGG` |
| ASV4736 | 406 | `TGGGGAATATTGGACAATGGGCGCAAGCCTGATCCAGCAATGCCGCGTGAGTGAAGAAGGCCTTAGGGTTGTAAAGCTCTTTCGGCGGGGAGGATGATGACGCTACCCGCAGAAGAAGCCCCGGCTAACTCCGTGCCAGCAGCCGCGGTAATACGGAGGGGGCTAGCGTTGTTCGGAATCACTGGGCGTAAAGGGCGCGTAGGCGGCTCTGTCAGTCAGGCGTGAAAGGCCCGGGCTCAACCTGGGGACGGCGCTTGATACTGCGGGGCTTTCGAGTCCGGGAGAGGATGGTGGAATTCCCAGTGTAGAGGTGAAATTCGTAGATATTGGGAAGAACACCGGTGGCGAAGGCGGCCATCTGGTCCGGTACTGACGCTGAGGCGCGAAAGCGTGGGGAGCAAACAGG` |
| ASV466 | 404 | `TGGGGAATCTTAGACAATGGGGGAAACCCTGATCTAGCCATGCCGCGTGAGCGATGAAGGCCTTAGGGTTGTAAAGCTCTTTCAGTGGGGAAGATAATGACGGTACCCACAGAAGAAGCCCCGGCTAACTCCGTGCCAGCAGCCGCGGTAATACGGAGGGGGCTAGCGTTGTTCGGAATTACTGGGCGTAAAGCGCACGTAGGCGGATTGGAAAGTCAGAGGTGAAATCCCAGGGCTCAACCTTGGAACTGCCTTTGAAACTCCCAGTCTTGAGATCGAGAGAGGTGAGTGGAATTCCGAGTGTAGAGGTGAAATTCGTAGATATTCGGAGGAACACCAGTGGCGAAGGCGGCTCACTGGCTCGATACTGACGCTGAGGTGCGAAAGCGTGGGGAGCAAACAGG` |
| ASV4960 | 430 | `TGGGGAATATTGGACAATGGGGGAAACCCTGATCCAGCAACGCCGCGTGGGTGATGAAGGCCTTCGGGTCGTAAAGCCCTGTCAACAGGGAAGAAACTTCAGTGGTCTAATAGGCCATTGGATTGACGGTACCTGTGGAGGAAGCACCGGCTAACTACGTGCCAGCAGCCGCGGTAAAACGTAGGGTGCTAGCGTTGTTCGGAATTACTGGGCGTAAAGCGCGTGCAGGTGGTCTTTTAAGTCCCATGTGAAAGCCCTCGGCTTAACTGAGGAACGGCATGAGAAACTGAAAGACTCGAGTTCGGCAGAAGCAGGCGGAATTCCTAGTGTAGAGGTGAAATTCGTAGATATTAGGAGGAACACCGGTGGCGAAAGCGGCTTGTTAGACCGATACTGACACTCATACGCGACAGCGCGGGGAGCAAACAGG` |
| ASV11 | 404 | `TGGGGAATATTGGACAATGGGCGAAAGCCTGATCCAGCAATGCCGCGTGAGTGATGAAGGCCTTAGGGTTGTAAAGCTCTTTTACTGGGGATGATAATGACAGTACCCAGAGAATAAGCTCCGGCTAACTCCGTGCCAGCAGCCGCGGTAATACGGAGGGAGCTAGCGTTGTTCGGAATTACTGGGCGTAAAGCGCACGTAGGCGGCTTTTTAAGTCAGGGGTGAAATCCCGGGGCTCAACCCCGGAACTGCCCTTGAAACTGGGAAGCTAGAATCTTGGAGAGGCGAGTGGAATTCCGAGTGTAGAGGTGAAATTCGTAGATATTCGGAAGAACACCAGTGGCGAAGGCGACTCGCTGGACAAGTATTGACGCTGAGGTGCGAAAGCGTGGGGAGCAAACAGG` |
| ASV190 | 429 | `TGGGGAATTTTGGACAATGGGCGCAAGCCTGATCCAGCAATGCCGCGTGCAGGATGAAGGCCTTCGGGTTGTAAACTGCTTTTGTACGGAACGAAACGGCCCTTTCTAATATAAGGGGCTAATGACGGTACCGTAAGAATAAGCACCGGCTAACTACGTGCCAGCAGCCGCGGTAATACGTAGGGTGCGAGCGTTAATCGGAATTACTGGGCGTAAAGCGTGCGCAGGCGGTTATATAAGACAGATGTGAAATCCCCGGGCTCAACCTGGGACCTGCATTTGTGACTGTATGGCTAGAGTACGGTAGAGGGGGATGGAATTCCGCGTGTAGCAGTGAAATGCGTAGATATGCGGAGGAACACCGATGGCGAAGGCAATCCCCTGGACCTGTACTGACGCTCATGCACGAAAGCGTGGGGAGCAAACAGG` |
| ASV2403 | 404 | `TGGGGAATATTGGACAATGGGGGCAACCCTGATCCAGCCATGCCGCGTGCGTGAAGAAGGCCTTAGGGTTGTAAAGCGCTTTTGCTAGGGATGATAATGACCGTACCTAGAGAATAAGCCCCGGCTAACTCCGTGCCAGCAGCCGCGGTAAGACGGAGGGGGCTAGCGTTGTTCGGATTTACTGGGCGTAAAGGGCGCGTAGGCGGCCATGTGTGTCAGGGGTGAAATCCCGGGGCTTAACCTCGGAACTGCTCCTGAAACTGCATGGCTTGAGTGATAGAGAGGCAAGTGGAATTCCGAGTGTAGCAGTGAAATGCGTAGATATTCGGAGGAACATCGGTGGCGAAGGCGACTTGCTGGCTATCAACTGACGCTGAGGCGCGAAAGCGTGGGGAGCAAACAGG` |
| ASV844 | 411 | `TGGGGAATATTGCACAATGGGCGCAAGCCTGATGCAGCGACGCCGCGTGGGGGATGACGGCCTTCGGGTTGTAAACTCCTTTCGCTAGGGACGAAGCTTTTTGTGACGGTACCTAGATAAGAAGCACCGGCTAACTACGTGCCAGCAGCCGCGGTAATACGTAGGGTGCGAGCGTTGTCCGGAATTACTGGGCGTAAAGGGCTCGTAGGTGGTTTGTCGCGTCGTCTGTGAAATTCCGGGGCTTAACTCCGGGCGTGCAGGCGATACGGGCATAACTTGAGTACTGTAGGGGTAACTGGAATTCCTGGTGTAGCGGTGAAATGCGCAGATATCAGGAGGAACACCGATGGCGAAGGCAGGTTACTGGGCAGTTACTGACGCTGAGGAGCGAAAGCATGGGTAGCGAACAGG` |
| ASV4713 | 404 | `TGGGGAATATTGGACAATGGGCGAAAGCCTGATCCAGCAATGCCGCGTGAGTGATGAAGGCCTTAGGGTTGTAAAGCTCTTTTACCCGGGATGATAATGACAGTACCGGGAGAATAAGCCCCGGCTAACTCCGTGCCAGCAGCCGCGGTAATACGGAGGGGGCTAGCGTTGTTCGGAATTACTGGGCGTAAAGCGTACGTAGGCGGCTTCGCAAGTCAGAGGTGAAAGCCCGGGGCTCAACTCCGGAACTGCCTTTGAGACTGCGTCGCTTGATCACGGGAGAGGTGAGTGGAATTCCGAGTGTAGAGGTGAAATTCGTAGATATTCGGAAGAACACCAGTGGCGAAGGCGGCTCACTGGACCGTAGATGACGCTGAGGTACGAAAGCGTGGGGAGCAAACAGG` |
| ASV2019 | 429 | `TGGGGAATTTTGGACAATGGGCGAAAGCCTGATCCAGCAATGCCGCGTGCAGGATGAAGGCCTTCGGGTTGTAAACTGCTTTTGTACGGAACGAAAAGCTTCGGGTTAATACCCTGGAGTCATGACGGTACCGTAAGAATAAGCACCGGCTAACTACGTGCCAGCAGCCGCGGTAATACGTAGGGTGCAAGCGTTAATCGGAATTACTGGGCGTAAAGCGTGCGCAGGCGGTTTTGTAAGACAGAGGTGAAATCCCCGGGCTCAACCTGGGAACTGCCTTTGTGACTGCAAGGCTAGAGTACGGCAGAGGGGGGTGGAATTCCGCGTGTAGCAGTGAAATGCGTAGATATGCGGAGGAACACCGATGGCGAAGGCAACCCCCTGGGCCTGTACTGACGCTCATGCACGAAAGCGTGGGGAGCAAACAGG` |
| ASV2109 | 404 | `TGGGGAATATTGGACAATGGGCGAAAGCCTGATCCAGCAATGCCGCGTGAGTGATGAAGGCCTTAGGGTTGTAAAGCTCTTTTACCAGGGATGATAATGACAGTACCTGGAGAATAAGCTCCGGCTAACTCCGTGCCAGCAGCCGCGGTAATACGGAGGGAGCTAGCGTTGTTCGGAATTACTGGGCGTAAAGCGCGCGTAGGCCGCGATTCAAGTCAGAGGTGAAAGCCCGGGGCTCAACCCCGGAACTGCCTTTGAAACTAGATTGCTAGAATCTTGGAGAGGTCAGTGGAATTCCGAGTGTAGAGGTGAAATTCGTAGATATTCGGAAGAACACCAGTGGCGAAGGCGACTGACTGGACAAGTATTGACGCTGAGGTGCGAAAGCGTGGGGAGCAAACAGG` |
| ASV2871 | 404 | `TGGGGGATATTGCACAATGGAGGAAACTCTGATGCAGCAACGCCGCGTGAGGGAAGAAGGTTTTCGGATTGTAAACCTCTGTCCTTGGTGAAGATAATGACGGTAGCCAAGGAGGAAGCTCCGGCTAACTACGTGCCAGCAGCCGCGGTAATACGTAGGGAGCAAGCGTTGTCCGGATTTACTGGGTGTAAAGGGTGCGTAGGCGGCTCTGCAAGTCAGGCGTGAAATATATGGGCTTAACCCATAGACTGCGTTTGAAACTGTGGAGCTTGAGTGAAGTAGAGGTAGGCGGAATTCCCGGTGTAGCGGTGAAATGCGTAGAGATCGGGAGGAACACCAGTGGCGAAGGCGGCCTACTGGGCTTTAACTGACGCTGAGGCACGAAAGCATGGGTAGCAAACAGG` |
| ASV691 | 429 | `TGGGGAATTTTGGACAATGGGCGCAAGCCTGATCCAGCAATGCCGCGTGCAGGAAGAAGGCCTTCGGGTTGTAAACTGCTTTTGTCAGGGAAGAAATCCTCTGGGTTAATACCTCGGGGGGATGACGGTACCTGAAGAATAAGCACCGGCTAACTACGTGCCAGCAGCCGCGGTAATACGTAGGGTGCGAGCGTTAATCGGAATTACTGGGCGTAAAGCGTGCGCAGGCGGTTGTGCAAGACAGGTGTGAAATCCCCGGGCTTAACCTGGGAACTGCACTTGTGACTGCACAGCTAGAGTACGGTAGAGGGGGATGGAATTCCGCGTGTAGCAGTGAAATGCGTAGATATGCGGAGGAACACCGATGGCGAAGGCAATCCCCTGGACCTGTACTGACGCTCATGCACGAAAGCGTGGGGAGCAAACAGG` |
| ASV1392 | 404 | `TGGGGAATATTGGACAATGGGCGCAAGCCTGATCCAGCAATACCACGTGAGTGATGAAGGCCTTCGGGTTGTAAAACTCTTTTAGTGGGGAAGATAATGACTGTACCCACAGAAAAAGCACCGGCTAACTTCGTGCCAGCAGCCGCGGTAATACGAAGGGTGCTAGCGTTGTTCGGAATCATTGGGCGTAAAGCGTGCGTAGGCGGCTTGACAAGTCAGCTGTGAAAGCCCAGGGCTCAACCTTGGAATTGCAGTTGAAACTGTTTCGCTAGAGTGTGAAGAGGGATAGTGGAATTCTTAGTGTAGGGGTGAAATCCGTAGATATTAAGAGGAACACCGGTGGCGAAAGCGACTATCTATGTCATTACTGACGCTGTTGCACGAAAGCGTGGGGAGCAAACAGG` |
| ASV344 | 404 | `TGGGGAATATTGGACAATGGGCGCAAGCCTGATCCAGCCATGCCGCGTGAGTGATGAAGGCCTTAGGGTTGTAAAGCTCTTTCAGTGGGGACGATAATGACGGTACCCACAGAAGAAGCCCCGGCTAACTTCGTGCCAGCAGCCGCGGTAATACGAAGGGGGCTAGCGTTGTTCGGATTTACTGGGCGTAAAGCGCACGTAGGCGGATCGTTAAGTCGGGGGTGAAATCCTGGAGCTCAACTCCAGAACTGCCTTCGATACTGGCGATCTTGAGTCCGGAAGAGGTGAGTGGAACTCCTAGTGTAGAGGTGGAATTCGTAGATATTAGGAAGAACACCAGTGGCGAAGGCGGCTCACTGGTCCGGTACTGACGCTGAGGTGCGAAAGCGTGGGGAGCAAACAGG` |
| ASV4689 | 429 | `TGGGGAATTTTGGACAATGGGGGCAACCCTGATCCAGCAATGCCGCGTGAGTGAAGAAGGCCTTCGGGTTGTAAAGCTCTTTTGTCAGGGAAGAAAAGGACATCAATAATACTGGTGTCTGATGACGGTACCTGAAGAATAAGCACCGGCTAACTACGTGCCAGCAGCCGCGGTAATACGTAGGGTGCAAGCGTTAATCGGAATTACTGGGCGTAAAGCGTGCGCAGGCGGCTTCGCAAGTCAGATGTGAAATCCCCGAGCTCAACTTGGGAACTGCATTTGAAACTACGAAGCTAGAGTGTGTCAGAGGGAGGTAGAATTCCACGTGTAGCAGTGAAATGCGTAGAGATGTGGAGGAATACCAGTGGCGAAGGCGGCCTCCTGGGATAACACTGACGCTCATGTACGAAAGCGTGGGGAGCAAACAGG` |
| ASV1442 | 404 | `TGGGGAATCTTGCACAATGGAGGAAACTCTGATGCAGCGACGCCGCGTGAACGATGAAGCCCTTCGGGGTGTAAAGTTCTTTCGCCAGGAACGATAATGACGGTACCTGGAGAAGAAGCTGCGGCTAACTACGTGCCAGCAGCCGCGGTAATACGTAGGCAGCAAGCGTTGTTCGGAATTATTGGGCGTAAAGAGTATGTAGGTGGTGTCGTAAGTTTGGTGTGAAATCTCCTGGCTTAACTGGGAGGGTGCGCCGAAGACTGCGATGCTTGAGTATGGGAGAGGGAATCGGAATTCCTGGTGTAGCGGTGAAATGCGTAGATATCAGGAGGAACACCTGTGGTGTAGACGGGTTCCTGGACCATTACTGACACTGAGATACGAAAGCGTGGGTAGCAAACAGG` |
| ASV2522 | 405 | `TGGGGAATCTTGCGCAATGGGCGAAAGCCTGACGCAGCAACGCCGCGTGCGGGATGAAGGCCCTCTGGGCTGTAAACCGCTTTCAGCAGGAACGAAAATGACGGTACCTGCAGAAGAAGGAGCGGCCAACTATGTGCCAGCAGCCGCGGTGATACATAGGCTTCAAGCGTTGTCCGGATTTATTGGGCGTAAAGAGTTCGTAGGCGGTCGAGTAAGTCGGGTGTGAAAATTCTGGGCTCAACCCAGAAACGCCACCCGATACTGCTTAACTTGAGTTCGATAGGGGAGTGGGGAATTCCTAGTGTAGCGGTGAAATGCGCAGATATTAGGAGGAACACCGGTGGCGAAGGCGCCACTCTGGATCGAAACTGACGCTGAGGAACGAAAGCATGGGTAGCAAACAGG` |
| ASV2754 | 404 | `TGGGGAATCTTAGACAATGGGCGCAAGCCTGATCTAGCCATGCCGCGTGGACGATGAAGGCCTTAGGGTTGTAAAGTCCTTTCAGCTGGGAAGATAATGACGGTACCAGCAGAAGAAGCCCCGGCTAACTCCGTGCCAGCAGCCGCGGTAATACGGAGGGGGCTAGCGTTGTTCGGAATTACTGGGCGTAAAGCGCGCGTAGGCGGACTGGAAAGTTGGGGGTGAAATCCCAGGGCTCAACCCTGGAACTGCCTTCAAAACTATCAGTCTGGAGTTCGAGAGAGGTGAGTGGAATTCCGAGTGTAGAGGTGAAATTCGTAGATATTCGGAGGAACACCAGTGGCGAAGGCGGCTCACTGGCTCGATACTGACGCTGAGGTGCGAAAGCGTGGGGAGCAAACAGG` |
| ASV3652 | 424 | `TGAGGAATATTGGACAATGGGCGCAAGCCTGATCCAGCCATGCCGCGTGCAGGAAGACGGTCCTATGGATTGTAAACTGCTTTTGCACAGGAAGAAACAACGCTACGTGTAGCGTCTTGACGGTACTGTGAGAATAAGGATCGGCTAACTCCGTGCCAGCAGCCGCGGTAATACGGAGGATCCAAGCGTTATCCGGAATCATTGGGTTTAAAGGGTCCGTAGGCGGTTTAATAAGTCAGTGGTGAAATCCCATCGCTCAACGGTGGAACGGCCATTGATACTGTTAAACTTGAATTATAAGGAAGTAACTAGAATATGTAGTGTAGCGGTGAAATGCTTAGAGATTACATGGAATACCAATTGCGAAGGCAGGTTACTACTTATATATTGACGCTGATGGACGAAAGCGTGGGTAGCGAACAGG` |
| ASV2014 | 409 | `TGAGGAATTTTCTGCAATGGGCGAAAGCCTGACAGAGCAATACCGCGTGAGGGAAGAAGGCTTACTGAGTTGTAAACCTCTGTTACCTAAGGAGGAAGATCTGACGTTACTTAGGAGACGAAGCATCGGCTAACTCCGTGCCAGCAGCCGCGGTAAGACGGAGGATGCAAGTGTTATCCGGAATTACTGGGCGTAAAGCGTCTGCAGGTGGTTTCTTAAGTCCACTGTTAAACCTTGAGGCTCAACCTCAAATCAGCATTGGAAACTGGGAAACTTGAGTATAGTAGGGGTAGAGGGAATTTCCAGTGGAGCGGTGAAATGCGTAGATATTGGAAAGAACACCGATGGCGAAGGCACTCTACTGGGCTATTACTGACACTCAGAGACGAAAGCTAGGGGAGCAAATGGG` |
| ASV5252 | 424 | `TTGGGAATCTTGCGCAATGGGGGAAACCCTGACGCAGCGACGCCGCGTGAGCGAAGAAGGCCTTCGGGTTGTAAAGCTCTGTCAGAAGGGAAGAACGCATAGATGATGAGTTTATGTTGACGGTACCTTCAGAGGAAGCACCGGCTAACTCTGTGCCAGCAGCCGCGGTAATACAGAGGGTGCAAGCGTTGTTCGGAATCACTGGGCGTAAAGAGTTCGTAGGTGGACTTATTAGTACAGGGTTAAATACCGGGGCCTAACCCCGGACCTGCTCTGTAAACGGTAAGTCTAGAGTACTGAAGAGGATAGCGGAATTCCTGGTGTAGAGGTGAAATTCGTAGATATCAGGAGGAACATCTGAGGCGAAGGCGGCTATCTGGTCAGGCACTGACACTAAAGAACGAAAGCATGGGGATCAAACAGG` |
| ASV2657 | 429 | `TCGAGAATAATTCACAATGGGGGCAACCCTGATGGTGCAACGCCGCGTGGAGGATGAAGGTCTTCGGATTGTAAACTCCTGTCATCTGGGAGTAAGACCTGGCTGTGAAAAGCAGACAGGGTTGATAGTACCAGAAGAGGAAGGGACGGCTAACTTCGTGCCAGCAGCCGCGGTAATACGAAGGTCCCGAGCGTTGTTCGGAATCACTGGGCGTAAAGGGAGCGTAGGCGGCGCGGTAAGTCAGATGTGAAATCCCGGGGCTCAACCCCGGAACTGCATCCGATACTGCCGTGCTAGAGGATTGGAGAGGTAGCTGGAATTCTTGGTGTAGCAGTGAAATGCGTGGAGATCAAGAGGAACACTCGTGGCGAAAGCGAGCTACTGGACAATAACTGACGCTGAGGCTCGAAGGCCAGGGTAGCGAAAGGG` |
| ASV164 | 404 | `TGGGGAATATTGGACAATGGGCGAAAGCCTGATCCAGCAATGCCGCGTGAGTGATGAAGGCCTTAGGGTTGTAAAGCTCTTTTACCAGGGATGATAATGACAGTACCTGGAGAATAAGCTCCGGCTAACTCCGTGCCAGCAGCCGCGGTAATACGGAGGGAGCTAGCGTTGTTCGGAATTACTGGGCGTAAAGAGTACGTAGGCGGTTATTCAAGTCAGAGGTGAAAGCCTGGAGCTCAACTCCAGAACTGCCTTTGAAACTAGATAGCTAGAATCTTGGAGAGGTGAGTGGAATTCCGAGTGTAGAGGTGAAATTCGTAGATATTCGGAAGAACACCAGTGGCGAAGGCGACTCACTGGACAAGTATTGACGCTGAGGTACGAAAGCGTGGGGAGCAAACAGG` |
| ASV1160 | 429 | `TGGGGAATTTTGGACAATGGGCGAAAGCCTGATCCAGCCATTCCGCGTGAGTGAAGAAGGCCTTCGGGTTGTAAAGCTCTTTCGCAAGGGAAGAAAGATTATCCTCTAACATAGGATGATTATGACGGTACCTTGATAAGAAGCACCGGCTAACTACGTGCCAGCAGCCGCGGTAATACGTAGGGTGCGAGCGTTAATCGGAATTACTGGGCGTAAAGCGTGCGCAGGCGGTTTTGTAAGTCAGATGTGAAATCCCCGAGCTCAACTTGGGAACTGCGTTTGAAACTACAAGACTAGAATATGTCAGAGGGGGGTAGAATTCCACGTGTAGCAGTGAAATGCGTAGAGATGTGGAGGAATACCAATGGCGAAGGCAGCCCCCTGGGATAATATTGACGCTCATGCACGAAAGCGTGGGGAGCAAACAGG` |
| ASV3935 | 430 | `TGGGGAATATTGGACAATGGGCGAAAGCCTGATCCAGCAATGCCGCGTGTGTGAAGAAGGCCTTCGGGTTGTAAAGCACTTTCAGTGAGGAGGAAAAGTCTAGGGTTAATACCTCTAGGCATTGACGTTACTCACAGAAGAAGCACCGGCTAACTCTGTGCCAGCAGCCGCGGTAATACAGAGGGTGCAAGCGTTAATCGGAATTACTGGGCGTAAAGCGTGCGTAGGCGGATATTTAAGTCGAATGTGAAAGCCCTGGGCTTAACCTGGGAACTGCATTCGATACTGGGTATTCTTGAGTATAGTAGAGGAAAGTGGAATTCTCGGTGTAGCGGTGAAATGCGTAGATATCGGGAGGAACATCAATGGCGAAGGCAACTTTCTGGACTAATACTGACGCTGAGGCACGAAAGCGTGGGGAGCAAACAGG` |
| ASV689 | 429 | `TGGGGAATTTTGGACAATGGGCGCAAGCCTGATCCAGCAATGCCGCGTGCAGGAAGAAGGCCTTCGGGTTGTAAACTGCTTTTGTACGGAGCGAAAAGGTACTTTCTAATAAAGAGTGCTGATGACGGTACCGTAAGAATAAGCACCGGCTAACTACGTGCCAGCAGCCGCGGTAATACGTAGGGTGCGAGCGTTAATCGGAATTACTGGGCGTAAAGCGTGCGCAGGCGGTTTTGTAAGACAGGCGTGAAATCCCCGGGCTCAACCTGGGAATGGCGCTTGTGACTGCAAAGCTAGAGTGCGGCAGAGGGGGATGGAATTTTGCGTGTAGCAGTGAAATGCGTAGATATGCAAAGGAACACCGATGGCGAAGGCAATCCCCTGGGCCTGCACTGACGCTCATGCACGAAAGCGTGGGGAGCAAACAGG` |
| ASV4063 | 404 | `TGGGGAATATTGGACAATGGGGGCAACCCTGATCCAGCCATGCCGCGTGAGTGATGAAGGCCTTAGGGTTGTAAAGCTCTTTTACCTGGGAAGATAATGACTGTACCAGGAGAATAAGCCCCGGCTAACTTCGTGCCAGCAGCCGCGGTAATACGAAGGGGGCTAGCGTTGTTCGGAATCACTGGGCGTAAAGCGCATGTAGGCGGGCTGTTAAGTCAGGGGTGAAATCCCAGAGCTCAACTCTGGAACTGCCTTTGATACTGATGGTCTTGAGTGTGGAAGAGGTTGGTGGAACTGCAAGTGTAGAGGTGAAATTCGTAGATATTTGCAAGAACACCAGTGGCGAAGGCGGCCAACTGGTCCATTACTGACGCTGAGATGCGAAAGCGTGGGGAGCAAACAGG` |
| ASV4077 | 429 | `TGGGGAATTTTGGACAATGGGCGCAAGCCTGATCCAGCCATGCCGCGTGAGTGAAGAAGGCCTTCGGGTTGTAAAGCTCTTTCGGTGGGGAAGAAATGGTTCGTGCCAATACCACGAACTGATGACGGTACCCGAAGAAGAAGCACCGGCTAACTACGTGCCAGCAGCCGCGGTAATACGTAGGGTGCAGGCGTTAATCGGAATTACTGGGCGTAAAGCGTACGCAGGCGGTTTGCTAAGACAGGTGTGAAATCCCCGGGCTTAACCTGGGAACTGCGCTTGTTACTGGCAAGCTAGAGTGTGGCAGAGGGGGGTAGAATTCCAGGTGTAGCAGTGAAATGCGTAGATATCTGGAGGAATACCGATGGCGAAGGCAGCCCCCTGGGTTAACACTGACGCTCATGTACGAAAGCGTGGGGAGCAAACAGG` |
| ASV3169 | 419 | `TAAGGAATATTGCGCAATGGGCGACAGCCTGACGCAGCGACGCCGCGTGGGGGATGAAGGTCTTCGGATTGTAAACCCCTTTCGGCAGGGAAGATGGAACGGGTAACCGTTCGGACGGTACCTGCAGAAGCAGCCACGGCTAACTTCGTGCCAGCAGCCGCGGTAATACGAAGGTGGCAAGCGTTGTTCGGATTTACTGGGCGTACAGGGAGCGTAGGCGGTTGGGTAAGCCCTCCGTGAAATCTCCGGGCCTAACCCGGAAAGTGCGGAGGGGACTGCTCGGCTAGAGGATGGGAGAGGAGCGCGGAATTCCCGGTGTAGCGGTGAAATGCGTAGAGATCGGGAGGAAGGCCGGTGGCGAAGGCGGCGCTCTGGAACATTTCTGACGCTGAGGCTCGAAAGCGTGGGGAGCAAACAGG` |
| ASV3794 | 424 | `TAAGGAATATTGGGCAATGGACGAAAGTCTGACCCAGCCATGCCGCGTGAAGGATTAAGGTCCTCTGGATTGTAAACTTCTTTTCTCTGGGGCGAAAACCACCCTTTCTAGGGTATTTGACAGTACCAGAGGAATAAGCACCGGCTAACTCCGTGCCAGCAGCCGCGGTAATACGGAGGGTGCAAGCGTTATCCGGATTCACTGGGTTTAAAGGGTGCGTAGGTGGGTCTGTAAGTCAGTGGTGAAATCCCCGAGCTTAACTTGGGAACTGCCGTTGATACTATAGATCTTGAATATCGTGGAGGTAAGCGGAATATGTCATGTAGCGGTGAAATGCTTAGATATGACATAGAACACCAATTGCGAAGGCAGCTTACTACACGATCATTGACACTGAGGCACGAAAGCGTGGGGAGCAAACAGG` |
| ASV2300 | 424 | `TGAGGAATATTGGACAATGGGCGCAAGCCTGATCCAGCCATGCCGCGTGCAGGAAGACGCATCTATGGTGTGTAAACTGCTTTTGTACGGGAAGAAACACTCCTTCGTGAAGGAGCTTGACGGTACCGTAAGAATAAGGATCGGCTAACTCCGTGCCAGCAGCCGCGGTAATACGGAGGATCCAAGCGTTATCCGGAATCATTGGGTTTAAAGGGTCCGTAGGCGGTTTAGTAAGTCAGTGGTGAAAGCCCATCGCTCAACGATGGAACGGCCATTGATACTGCTAGACTTGAATTACAAGGAAGTAACTAGAATATGTAGTGTAGCGGTGAAATGCTTAGATATTACATGGAATACCAATTGCGAAGGCAGGTTACTACTTGTGTATTGACGCTGATGGACGAAAGCGTGGGGAGCGAACAGG` |
| ASV4939 | 428 | `TCGCGAATTTTTCACAATGGGCGCAAGCCTGATGGAGCCATGCCGCGTGCGGGATGAAGGCCCTCTGGGTTGTAAACCGCTGTCGAGTGGGGGGAACCGCGTCCAGTGAATAGCTGGACGTCTGACTGCCCGCTGAAGGAAGCCCCGGCAAAACATGTGCCAGCAGCCGCGGTAATACATGTGGGGCAAGCGTTGTTCGGAATTACTGGGCATAAAGGGTGCGTAGGCGGTATGATAAGTCAGTGGTGAAATCCTGCGGCCCAACCGTAGTACTGCCATTGATACTGTCGTGCTTGAGTGCGTGAGGGGTGACTGGAACGGGTAGTGTAGCGGTGAAATGCGTAGATATTACCCGGAACACCAACGGCGAAGGCAGGTCACTGGCACGCAACTGACGCTGAGGCACGAAAGCGTGGGGAGCAAACAGG` |
| ASV541 | 404 | `TGGGGAATATTGGACAATGGGCGAAAGCCTGATCCAGCAATGCCGCGTGAGTGATGAAGGCCTTAGGGTTGTAAAGCTCTTTTACCCGGGATGATAATGACAGTACCGGGAGAATAAGCTCCGGCTAACTTCGTGCCAGCAGCCGCGGTAATACGAGGGGAGCTAGCGTTGTTCGGAATTACTGGGCGTAAAGCGCGCGTAGGCGGTCTTTTAAGTCAGAGGTGAAAGCCTGGTGCTCAACACCAGAACTGCCTTTGAAACTGAAAAACTTGAATCATGGAGAGGTCAGTGGAATTCCGAGTGTAGAGGTGAAATTCGTAGATATTCGGAAGAACACCAGTGGCGAAGGCGACTGACTGGACATGTATTGACGCTGAGGTGCGAAAGCGTGGGGAGCAAACAGG` |
| ASV1525 | 404 | `TGGGGAATCTTAGACAATGGGGGCAACCCTGATCTAGCCATGCCGCGTGATCGATGAAGGCCTTAGGGTTGTAAAGATCTTTCAGATGGGAAGATAATGACGGTACCATCAGAAGAAGCCCCGGCTAACTCCGTGCCAGCAGCCGCGGTAATACGGAGGGGGCTAGCGTTATTCGGAATTACTGGGCGTAAAGCGCACGTAGGCGGACTGGAAAGTCAGGGGTGAAATCCCGGGGCTCAACCCCGGAACGGCCTTTGAAACTCCCCGTCTTGAGATCGTGAGAGGTGAGTGGAATTCCGAGTGTAGAGGTGAAATTCGTAGATATTCGGAGGAACACCAGTGGCGAAGGCGGCTCACTGGCACGATACTGACGCTGAGGTGCGAAAGCGTGGGGAGCAAACAGG` |
| ASV393 | 404 | `TGGGGAATATTGGACAATGGGCGAAAGCCTGATCCAGCGACGCCGCGTGAGTGAAGAAGGCCTTCGGGTTGTAAAGCTCTTTTAGTGGAGAAGATAATGACGGTATCCACAGAAAAAGCCCCGGCTAACTTCGTGCCAGCAGCCGCGGTAATACGAAGGGGGCTAGCGTTACTCGGAATTATTGGGCGTAAAGCGTGCGTAGGCGGTTATGTAAGTTGGGAGTGAAAGCCCAGGGCTCAACCTTGGAATTGCTTTCAAAACTACGTAACTTGAGTATTGGAGAGGATAGTGGAATTCCTAGTGTAGAGGTGAAATTCGTAGATATTAGGAGGAACACCAGATGCGAAAGCGGCTATCTGGCCAAATACTGACGCTGTTGCACGAAAGCGTGGGGAGCAAACAGG` |
| ASV2991 | 407 | `TGGGGAATTTTCCGCAATGGGCGAAAGCCTGACGGAGCAATACCGCGTGAGGGAAGAAGGCTCTTGGGTTGTAAACCTCTTTTCTGAGGGAAGAAAAAAATGACGGTACCTCAGGAATCAGCATCGGCTAACTCCGTGCCAGCAGCCGCGGTAATACGGAGGATGCAAGCGTTATCCGGAATGATTGGGCGTAAAGCGTCCGCAGGTGGCAATTCAAGTTTGCGGTTAAAGGCTCTGGCTCAACCAGAGACAGGCCGTGAAAACTGAATAGCTAGAGTATGGTAGGGGCAGAGGGAATTCCCAGTGTAGCGGTGAAATGCGTAGAGATTGGGAAGAACACCGGTGGCGAAAGCGCTCTGCTAGGCCAAAACTGACACTGAGGGACGAAAGCTAGGGGAGCGAATGGG` |
| ASV5356 | 429 | `TGGGGAATTTTGGACAATGGGCGCAAGCCTGATCCAGCCATGCCGCGTGAGTGAAGAAGGCCTTCGGGTTGTAAAGCTCTTTCGGCGGGAACGAAATGGTTCGTGCTAATACCACGGATTGATGACGGTACCTGAAGAAGAAGCACCGGCTAACTACGTGCCAGCAGCCGCGGTAATACGTAGGGTGCGGGCGTTAATCGGAATTACTGGGCGTAAAGCGTGCGCAGGCGGTTTCTTAAGTCAGATGTGAAAGCCCCGGGCTTAACCTGGGAACTGCGTTTGAAACTGAGAGACTCGAGTGTGGCAGAGGGGGGTGGAATTCCACGTGTAGCAGTGAAATGCGTAGATATGTGGAGGAACACCGATGGCGAAGGCAGCCCCCTGGGTTAACACTGACGCTCATGCACGAAAGCGTGGGGAGCAAACAGG` |
| ASV4756 | 428 | `TCGCGAATTTTTCACAATGGACGCAAGTCTGATGAAGCCACGCCGCGTGCGGGATGAAGGCCCTTTGGGTTGTAAACCGCTGTCGAGAGAGGGGAACCGCTGCAAGTGAATAGCTTGCAGTCTGACTGCTTTCTGAAGGAAGCCCCGGCAAAACATGTGCCAGCAGCCGCGGTAATACATGTGGGGCAAGCGTTGTTCGGAATTACTGGGCATAAAGGGTGCGTAGGTGGTTTGTTAAGTCAGGGGTGAAATCCCGCGGCCCAACCGTGGAACTGCCTTTGATACTGGCGAACTTGAGTGCGTGAGGGGTAGGAGGAACGAGCAGTGTAGCGGTGAAATGCGTAGATATTGCTCGGAAGGCCAGAGCCGAAGGGGTCCTACTGGTACGTAACTGACACTGAGGCACGAAAGCGTGGGGAGCAAACAGG` |
| ASV3951 | 404 | `TGGGGAATCTTGCGCAATGGGGGCAACCCTGACGCAGCCATACCGCGTGAGTGATGAAGGTCTTCGGATTGTAAAGCTCTTTCGGCGGGGACGACGATGACGGTACCCGCAAAAGAAGTCACGGCTAACTTCGTGCCAGCAGCCGCGGTAATACGAAGGTGGCGAATGTTGCTCGGATTCACTGGGCGTAAAGCGTCTGTAGGTGGCTTGTTAAGTTAGAGGTGAAAGCCCCGCGCTCAACGTGGGAACTGCCTTTAAAACTGGCGAGCTTGAGAGTATCAGAGGAAGGCGGAATAACTGGTGTAGAGGTAAAATTCATAGATATCAGTTGGAACATCCATGGCGAAGGCAGCTTTCTGGGATATTTCTGACACTGAGAGACGACAGCGTGGGGAGCAAACAGG` |
| ASV1510 | 430 | `TGGGGAATATTGGACAATGGGCGAAAGCCTGATCCAGCAATGCCGCGTGTGTGAAGAAGGCCTTCGGGTTGTAAAGCACTTTCAGCGGGGAGGAAGTGGTGCTGCTTAATACGCAGTACTGTTGACGTTACCCGCAGAAGAAGCACCGGCTAACTCTGTGCCAGCAGCCGCGGTAATACAGAGGGTGCAAGCGTTAATCGGAATTACTGGGCGTAAAGGGTGTGTAGGTGGTTCAAGTAAGTCAGGTGTAAAAACCCCGGGCTCAACCTGGGAATGTCGCTTGAAACTGCTTAGCTAGAGTACTATAGAGGATAGTGGAATTTCCAGTGTAGCGGTGAAATGCGTAGATATTGGAAGGAACACCAGTGGCGAAGGCGACTATCTGGATAGATACTGACACTGAAACACGAAAGCATGGGTAGCAAACAGG` |
| ASV857 | 429 | `TGGGGAATTTTGGACAATGGGGGCAACCCTGATCCAGCCATTCCGCGTGAGTGAAGAAGGCCTTCGGGTTGTAAAGCTCTTTTGTCGGGGACGAAACGGTTAAAGCTAATAACTTTGACTACTGACGGTACCCGAAGAATAAGCACCGGCTAACTACGTGCCAGCAGCCGCGGTAATACGTAGGGTGCAAGCGTTAATCGGAATTACTGGGCGTAAAGCGTGCGCAGGCGGTTTTGTAAGCCAGATGTGAAATCCCCGGGCTCAACCTGGGAATGGCATTTGGGACTGCAAGGCTAGAGTATGGCAGAGGAGACTAGAATTCCTGGTGTAGCAGTGAAATGCGTAGATATCAGGAGGAATACCGATGGCGAAGGCAGGTCTCTGGGTCAATACTGACGCTCATGCACGAAAGCGTGGGGAGCAAACAGG` |
| ASV5460 | 404 | `TGGGGAATATTGGACAATGGGCGAAAGCCTGATCCAGCGACGCCGCGTGGGTGATGAAGGCCTTCGGGTTGTAAAGCCCTTTCGTCAGGGAAGATAATGACGGTACCTGAAGAAGAAGCTCCGGCTAACTTCGTGCCAGCAGCCGCGGTAATACGAAGGGAGCTAGCGTTACTCGGAATTATTGGGTGTAAAGCGTGCGTAGGCGGTTTTATAAGTCAGAAGTGAAAGCCTGGGGCTCAACCCTAGAATTGCTTTTGAAACTATAAAACTAGAGTGTCAGAGGGGATAGCGGAATTCCTAGTGTAGAGGTGAAATTCGTAGATATTAGGAGGAACACCGGTGGCGAAGGCGGCTATCTGGCTGACAACTGACGCTGTTGCACGAAAGCGTGGGGAGCAAACAGG` |
| ASV271 | 429 | `TGGGGAATTTTGGACAATGGACGCAAGTCTGATCCAGCCATTCCGCGTGCAGGATGAAGGCCCTCGGGTTGTAAACTGCTTTTGTACGGAACGAAAAGGTCTCTATTAATACTAGGGGCTCATGACGGTACCGTAAGAATAAGCACCGGCTAACTACGTGCCAGCAGCCGCGGTAATACGTAGGGTGCAAGCGTTAATCGGAATTACTGGGCGTAAAGCGTGCGCAGGCGGTTATGTAAGACAGAGGTGAAATCCCCGGGCTCAACCTGGGAACTGCCTTTGTGACTGCATAGCTAGAGTACGGTAGAGGGGGATGGAATTCCGCGTGTAGCAGTGAAATGCGTAGATATGCGGAGGAACACCGATGGCGAAGGCAATCCCCTGGACCTGTACTGACGCTCATGCACGAAAGCGTGGGGAGCAAACAGG` |
| ASV4886 | 429 | `TGGGGAATATTGGACAATGGGGGCAACCCTGATCCAGCGATGCCGCGTGTGTGAAGAAGGCCTGAGGGTTGTAAAGCACTTTAGTTGGGGAGGAAAGGCTCTTGCCGAATAGGTAAGGGAGAAGACGTTACCCAAAGAATAAGCACCGGCTAACTCTGTGCCAGCAGCCGCGGTAATACAGAGGGTGCAAGCGTTAATCGGAATGACTGGGCGTAAAGGGCACGTAGGCGGATAGATGTGTCATAGGTGAAATCCCCGGGCTCAACCTGGGAACTGCCTGTGAAACTGTGTATCTAGAATAGAGTAGAGGTAAGTGGAATTTCCGGTGTAGCGGTGAAATGCGTAGATATCGGAAAGAACACCAGTGGCGAAGGCGACTTACTGGACTCATATTGACGCTGAGGTGCGAAAGCGTGGGGAGCAAACGGG` |
| ASV4197 | 406 | `TAGGGAATATTGCACAATGGAGGAAACTCTGATGCAGCGACGCCGCGTGAGTGATGAAGGCCTTTGGGTTGTAAAGCTCTGTTCTGTGGGAATAAGTTCTGAATGTACCACAGGAGAAAGGACCGGCTAACTTCGTGCCAGCAGCCGCGGTAAGACGAGGGGTCCTAGCGTTGTTCGGAATTATTGGGCGTAAAGCGAGTGTAGGTGGATCGAAAAGTTAGGAGTGAAAGCCCTGGGCTTAACCTGGGAAGTGCTTTTAAAACTGCCGATCTTGAGTGTGGGAGAGGATAGTAGAATTCCTGGTGTAGTGGTGAAATACGTAGATATCAGGAGGAATTTCGGTGGGGAAGCCGGCTATCTGGCCCAACACTGACACTCAGACTCGAAAGCGTGGGGATCAAACAGG` |
| ASV4630 | 428 | `TGGGGAATCTTGCGCAATGGGCGGAAGCCTGACGCAGCGACGCCGCGTGCGGGAAGAAGGCCTTCGGGTTGTAAACCGCTTTCAGAAGGGACGAAGCCACTCGGGTGAATAGCCCAGAGGGTGACGGTACCTTCAGAAGAAGCCCCGGCTAACTACGTGCCAGCAGCCGCGGTAATACGTAGGGGGCCAGCGTTGTCCGGATTCATTGGGCGTAAAGCGCGTGTAGGCGGCCAGGTAGGTCCGATGTGAAATCTGGAGGCTCAACCTCCAGCGGTCATTGGAAACCATCTGGCTAGAGTCCGGAAGAGGAGAGTGGAATTCCTGGTGTAGCGGTGAAATGCGCAGATATCAGGAAGAACACCGATGGCGAAGGCAGCTCTCTGGGACGGTACTGACGCTGAGACGCGAAAGCGTGGGGAGCGAACAGG` |
| ASV5618 | 429 | `TGGGGAATATTGGACAATGGGGGCAACCCTGATCCAGCAATGCCGCGTGTGTGAAGAAGGCCTTCGGGTTGTAAAGCACTTTAGACTAGGAAGAATGTATGGTGTTTAAGAGATGCCATATTTGACTGTACTAGTAGAATAAGCACCGGCTAACTCTGTGCCAGCAGCCGCGGTAATACAGAGGGTGCAAGCGTTAATCGGAGTTACTGGGCGTAAAGGGCGCGTAGGCGGTTAGTTAAGTGAGATGTGAAATCCCTGGGCTTAACCTAGGAACTGCATCTTAAACTGGCTAGCTGGAGTACAGTAGAGGGTAGCGGAATTTCCGGTGTAGCGGTGAAATGCGTAGAGATCGGAAGGAACACCAGTGGCGAAGGCGGCTATCTGGACTGATACTGACGCTGAGGCGCGAGAGCGTGGGGAGCAAACAGG` |
| ASV3528 | 430 | `TGGGGAATATTGCGCAATGGGCGAAAGCCTGACGCAGCGACGCCGCGTGGGTGATGAAGGCCTTCGGGTCGTAAAGCCCTTTCGAGGGGAAAGAAAAAGCTGGGTTCAAATAGGGCCCAGCCCTGACGGTACCCCTAGAAGAAGCACCGGCTAACTCCGTGCCAGCAGCCGCGGTAATACGGAGGGTGCGAGCGTTGTTCGGAATCACTGGGCGTAAAGCGCGTGTAGGCGGCTTGATAAGTCTGGTGTGAAAGCCCGGGGCTCACCCCCGGAAGTGCACTGGATACTGTTGAGCTCGAGTCCCGGAGAGGAGAGTGGAATTCCTGGTGTAGCGGTGAAATGCGTAGATATCAGGAGGAACACCGGTGGCGAAGGCGGCTCTCTGGACGGTGACTGACGCTGAGACGCGAAAGCGTGGGGAGCAAACAGG` |
| ASV3805 | 416 | `TTAGGAATCTTCCCCAATGGGCGCAAGCCTGAGGGAGCGACGCCGCGTGCGGGATGAAGGTTTTCGGATCGTAAACCGCTGAAACAAGGACGAAATCCCTGTAAAGGGTCTGACGGTACTTGTGTAATAGCACCGGCTAACTCCGTGCCAGCAGCCGCGGTAATACGGAGGGTGCAAGCGTTACCCGGAATCACTGGGCGTAAAGGGCATGTAGGCGGTGACTTAAGTCTGGATTTAAAGACCGAAGCTCAACTTCGGGCATGGTCTGGATACTGGGTTGCTGGACGACTGGAGGGGAGAGTGGAATTCTCGGTGTAGCGGTGGAATGCGTAGATACCGAGAGGAACACCAGTGGCGAAGGCGGCTCTCTGGACAGTGCGTGACGCTGAGGTGCGAAAGTGTGGGGAGCAAACCGG` |
| ASV5547 | 424 | `TAAGGAATATTGGTCAATGGACGAAAGTCTGAACCAGCCATGCCGCGTGCAGGATGAAGGTCCTCTGGATCGTAAACTGCTTTTATCTATGAAGAAATGTCCCGTTTCTACGGGATTTGACGGTAATAGAGGAATAAGCACCGGCTAACTCCGTGCCAGCAGCCGCGGTAATACGGAGGGTGCAAGCGTTATCCGGATTTACTGGGTTTAAAGGGTGCGTAGGTGGATATTTAAGTCAGTGGTGAAATCCCCGAGCTTAACTCGGGAACTGCCATTGATACTATTTATCTTGAATACGGTTGAGGTCGGCGGAATGTGTCATGTAGCGGTGAAATGCTTAGATATGACACAGAACACCTATTGCGAAGGCAGCTGGCTAAACCGTGATTGACACTCAGGCACGAAAGCGTGGGGAGCAAACAGG` |
| ASV4625 | 407 | `TAGGGAATATTGCGCAATGGGGGAAACCCTGACGCAGCGACGCCGCGTGAGTGATGAAGGCCTTAGGGTCGTAAAGCTCTGTCGTATGGGACTAACAAAATGAATGTACCATGCAAGAAAGGATCGGCTAACTTCGTGCCAGCAGCCGCGGTAAGACGAGGGATCCTAGCGTTGTTCGGAATTATTGGGCGTAAAGAGGGCGTAGGTGGCTTGATAAGTCAGATGTGAAAGCCCAGGGCTTAACCCTGGAAGTGCATTTGATACTGTTAAGCTTGAGTGCGAGAGAGGTTAGTAGAATTCCTGGTGTAGTGGTGAAATACGTAGATATCAGGAGGAATACCGGAGGCGAAGGCGGCTAACTGGCTCGACACTGACACTGAGGCCCGAAAGTGTGGGGATCAAACAGG` |
| ASV54 | 404 | `TGGGGAATATTGGACAATGGGCGAAAGCCTGATCCAGCAATGCCGCGTGAGTGATGAAGGCCTTAGGGTTGTAAAGCTCTTTTACCAGGGATGATAATGACAGTACCTGGAGAATAAGCTCCGGCTAACTCCGTGCCAGCAGCCGCGGTAATACGGAGGGAGCTAGCGTTGTTCGGAATTACTGGGCGTAAAGCGTACGTAGGCGGTTATTCAAGTCAGAGGTGAAAGCCTGGAGCTCAACTCCAGAACTGCCTTTGAAACTAGATAGCTAGAATCACGGAGAGGTGAGTGGAATTCCGAGTGTAGAGGTGAAATTCGTAGATATTCGGAAGAACACCAGTGGCGAAGGCGGCTCACTGGACGTGTATTGACGCTGAGGTACGAAAGCGTGGGGAGCAAACAGG` |
| ASV4804 | 407 | `CTGGGAATTTTCCGCAATGGACGAAAGTCTGACGGAGCAACGCCGCGTGAAGGATGAAGGTCTTTAGGGATTGTAAACTTCTTTTATGTAGGACGAGAAAGGACGGTACTACATGAATAAGTCCCGGCTAACTACGTGCCAGCAGCCGCGGTAATACGTAGGGGGCAAGCGTTGTTCGGAATTACTGGGCATAAAGGGTGCGCAGGCGGCTTATTAAGCAAGAGGTGAAATGTCAAGGCTCAACCATGGCACTGCCTTTTGAACTAATAATCTGGAGTACAGGAGGGGAGAGCAGAATTTCCGGTGTAGGGGTGGAATCTATAGATATCGGAAGGAATACCGTTCGCGAAGGCGGCTCTCTGGTCTGTTACTGACGCTCATGCACGAAAGCGTGGGGAGCAAACAGG` |
| ASV3103 | 407 | `TGGGGAATTTTCCGCAATGGGCGCAAGCCTGACGGAGCAATACCGCGTGAGGGAGGAAGGCTCTTGGGTCGTAAACCTCTTTTCTCAGGGAAGAACAAAATGACGGTACCTGAGGAATAAGCATCGGCTAACTCTGTGCCAGCAGCCGCGGTAATACAGAGGATGCAAGCGTTATCCGGAATGATTGGGCGTAAAGCGTCCGTAGGTGGTTTTGTAAGTCTGTGGTTAAAGCGTGTAGCTCAACTACATAACGGCCATGGAAACTACAAGACTAGAGTTAAGTAGGGGTAGAGGGAATTCCCAGTGTAGCGGTGAAATGCGTAGAGATTGGGAAGAACACCGGTGGCGAAAGCGCTCTACTGGACTTATACTGACACTGAGGGACGAAAGCTAGGGGAGCGAAAGGG` |
| ASV851 | 429 | `TGGGGAATCTTGGACAATGGGCGAAAGCCTGATCCAGCAATGCCGCGTGCGTGAAGAAGGCCTTCGGGTTGTAAAGCGCTTTTGTCGGGGAGGAAATCCTAACTTCTAATATAAGTTGGGGATGACAGTACCCGAAGAATAAGGACCGGCTAACTACGTGCCAGCAGCCGCGGTAATACGTAGGGTCCAAGCGTTAATCGGAATTACTGGGCGTAAAGCGTCCGCAGGTGGCTTGATAAGATAGACGTGAAATCCCTGGGCTCAACCTAGGAATTGCGTTTATGACTGTCTCGCTAGAGTATGGGAGAGGGGGGTGGAATTCCGCGTGTAGCAGTGAAATGCGTAGAGATGCGGAGGAACACCGATGGCGAAGGCAACCCCCTGGCCTAATACTGACACTCATGGACGAAAGCGTGGGGAGCAAACAGG` |
| ASV5211 | 405 | `TGGGGAATTTTGCACAATGGACGAAAGTCTGATGCAGCGATTCCGCGTGGAGGATGACGCATTACGGTGTGTAAACTCCTTTTTTGGTGGAAGACGAATGACGGTACACCAAGAATAAGGGACGGCTAACTACGTGCCAGCAGCCGCGGTAATACGTAGGTCCCAAGCGTTATCCGGATTTACTGGGCGTAAAGTGTCCGTAGCCTGATATACAAGTCAGTTTTCAAATATCTCGACTCAATCGAGAAAAGGGAATAGATACTGTATATCTCGAGATTATTGGAGGTTAGTGGAATTTCCGGTGGAGCGGTGAAATGCGTTGATATCGGAAAGAACGCCGAAAGCGAAAGCAGCTAACTATGATAATTCTGACGGTGAGGGACGAAAGCTTGGGTAGCAAACGGG` |
| ASV584 | 404 | `TTAGGAATATTGGACAATGGGGGAAACCCTGATCCAGCCATGCCGCGTGTGTGATGAAGGCCCTAGGGTTGTAAAGCACTTTCGTTGGGGATGATAATGACCGTACCCAAAGAAGAAGCCACGGCTAAATTCGTGCCAGCAGCCGCGGTAATACGAATGTGGCAAGCGTTGTTCGGAATCACTGGGCGTAAAGAATGCGTAGGCGGCTTAACAAGTCGGGAGTGAAATCCCAGGGCTCAACCCTGGAACTGCTCTCGAAACTGTTTTGCTAGAGTCCCGGAGGGGTTAGCGGAATTCCGAGTGTAGAGGTGAAATTCGCAGATATTCGGAGGAACACCAGTGGCGAAGGCGGCTAACTGGACGGGAACTGACGCTGAGGCATGAAAGTGTGGGGATCAAACAGG` |
| ASV1907 | 406 | `TGGGGAATATTGCACAATGGGCGAAAGCCTGATGCAGCAACGCCGCGTGAGCGATGAAGGCCTTTGGGTCGTAAAGCTCTGTCCTAGGGGAAGAAAAAAATGACGGTACCCTTGGAGGAAGCCCCGGCTAACTACGTGCCAGCAGCCGCGGTAATACGTAGGGGGCGAGCGTTATCCGGAATTATTGGGCGTAAAGAGTGCGTAGGTGGTCTCGTAAGCGCGGGGTGAAAGGCAATGGCTCAACCATTGTAAGCCTTGCGAACTGCGAGACTTGAGTGCGGGAGAGGAAAGTGGAATTCCTAGTGTAGCGGTGAAATGCGTAGATATTAGGAGGAACACCAGTGGCGAAGGCGACTTTCTGGACCGTAACTGACACTGAGGCACGAAAGCGTGGGGAGCAAACAGG` |
| ASV4175 | 424 | `TGGGGAATATTGGTCAATGGGCGAAAGCCTGAACCAGCAATGCCGCGTGAGTGATGAAGGCCTTCGGGTTGTAAAGCTCTGTCAGAGGGAACGAAAAAACTGCGTAACGAGCAGCCTTGACGGTACCCTCAAAGGAAGCACCGGCTAACTCTGTGCCAGCAGCCGCGGTAATACAGAGGGTGCAAGCGTTGTTCGGAATTACTGGGCGTAAAGCGCGTGTAGGCGGCTTATTAAGTCAGATGTGAAATCCCTCGGCTCAACTGAGGAACTGCATCTGATACTGCTAAGCTTGAGTGTCGGAGGGGATGGTAGAATTCCTGGTGTAGAGGTGAAATTCGTAGATATCAGGAGGAATACCTGTGGCGAAGGCGACCATCTGGAAGACAACTGACGCTGAGACGCGAAAGCGTGGGTAGCAAACAGG` |
| ASV1289 | 429 | `TGGGGAATTTTGGACAATGGGCGAAAGCCTGATCCAGCAATGCCGCGTGCAGGATGAAGGCCTTCGGGTTGTAAACTGCTTTTGTACGGAACGAAAAGCTTCTGGTTAATACCTGGGAGTCATGACGGTACCGTAAGAATAAGCACCGGCTAACTACGTGCCAGCAGCCGCGGTAATACGTAGGGTGCAAGCGTTAATCGGAATTACTGGGCGTAAAGCGTGCGCAGGCGGTCTTGTAAGACAGAGGTGAAATCCCCGGGCTCAACCTGGGAACTGCCTTTGTGACTGCAAGGCTGGAGTGCGGCAGAGGGGGATGGAATTCCGCGTGTAGCAGTGAAATGCGTAGATATGCGGAGGAACACCGATGGCGAAGGCAATCCCCTGGGCCTGCACTGACGCTCATGCACGAAAGCGTGGGGAGCAAACAGG` |
| ASV2971 | 406 | `TGGGGAATTTTCCGCAATGGGCGCAAGCCTGACGGAGCAATACCGCGTGAGGGAGGAAGGCTCTTGGGTCGTAAACCTCTTTTCTCTGGGAAGAATAACTGACGGTACCAGAGGAATCAGCATCGGCTAACTCCGTGCCAGCAGCCGCGGTAATACGGGGGATGCAAGCGTTATCCGGAATTATTGGGCGTAAAGCGTCCGCAGGTGGCCAATCAAGTCTGTTGTCAAAGCGCGGAGCTCAACTCCGTACAGGCGATGGAAACTGGTTAGCTAGAGTTAGGTAGGGGTAAGAGGAATTCCCAGTGTAGCGGTGAAATGCGTAGATATTGGGAAGAACACCGGCGGCGAAAGCGTCTTACTGGGCCTAAACTGACACTGAGGGACGAAAGCTAGGGGAGCGAAAGGG` |
| ASV80 | 429 | `TGGGGAATATTGGACAATGGGGGGAACCCTGATCCAGCAATGCCGCGTGTGTGAAGAAGGCCTGAGGGTTGTAAAGCACTTTCAGTGGGGAGGAGGGTACTTAGGTTAAGAGCTGGAGTATTGGACGTTACCCACAGAAGAAGCACCGGCTAACTCCGTGCCAGCAGCCGCGGTAATACGGAGGGTGCGAGCGTTAATCGGAATTACTGGGCGTAAAGGGTGCGTAGGTGGTTTATTAAGTTAGCTGTGAAATTCCTGGGCTTAACCTGGGCAGGTCAGCTAAGACTGGTAGACTCGAGTATGGGAGAGGGTAGTGGAATTTCCGGTGTAGCGGTGAAATGCGTAGAGATCGGAAGGAACACCAGTGGCGAAGGCGGCTACCTGGCCTAATACTGACACTGAGGCACGAAAGCGTGGGGAGCAAACAGG` |
| ASV1378 | 429 | `TGGGGAATTTTGGACAATGGGCGAAAGCCTGATCCAGCCATGCCGCGTGCGGGAAGAAGGCCTTAGGGTTGTAAACCGCTTTTGTCAGGGAAGAAACGCTTCGGAATAATACTCTGAGGTAATGACGGTACCTGAAGAATAAGCACCGGCTAACTACGTGCCAGCAGCCGCGGTAATACGTAGGGTGCAAGCGTTAATCGGAATTACTGGGCGTAAAGCGTGCGCAGGCGGCTTTGCAAGACAGGTGTGAAATCCCCGGGCTCAACCTGGGAACTGCATTTGTGACTGCAAGGCTAGAGTACGGTAGAGGGGGATGGAATTCCGCGTGTAGCAGTGAAATGCGTAGATATGCGGAGGAACACCGATGGCGAAGGCAATCCCCTGGACCTGTACTGACGCTCATGCACGAAAGCGTGGGGAGCAAACAGG` |
| ASV2669 | 429 | `TGGGGAATTTTGGACAATGGGGGCAACCCTGATCCAGCCATGCCGCGTGCGGGAAGAAGGCCTTCGGGTTGTAAACCGCTTTTGTCAGGGAAGAAATGCTCCGCGCCAATACCGCGGGGTGATGACGGTACCTGAAGAATAAGCACCGGCTAACTACGTGCCAGCAGCCGCGGTAATACGTAGGGTGCAAGCGTTAATCGGAATTACTGGGCGTAAAGCGTGCGTAGGCGGTTGCGCAAGACAGGTGTGAAATCCCCGGGCTTAACCTGGGCACTGCACTTGTGACTGTGCGGCTGGAGTACGGCAGAGGGGGATGGAATTCCGCGTGTAGCAGTGAAATGCGTAGATATGCGGAGGAACACCGATGGCGAAGGCAGTCCCCTGGGCCTGTACTGACGCTCATGCACGAAAGCGTGGGGAGCAAACAGG` |
| ASV1853 | 429 | `TGGGGAATATTGGACAATGGGGGGAACCCTGATCCAGCAATGCCGCGTGTGTGAAGAAGGCCTGAGGGTTGTAAAGCACTTTCAGTGGGGAGGAGAGTTGGCAGGTTAAGAGCTAGCTGATGAGACGTTACCCACAGAAGAAGCACCGGCTAACTCCGTGCCAGCAGCCGCGGTAATACGGAGGGTGCAAGCGTTAATCGGAATTACTGGGCGTAAAGGGTGCGTAGGTGGTATGGTAAGTTATCTGTGAAATCCCCGGGCTCAACCTGGGCAGGTCAGATAAAACTGCAGTACTCGAGTATGGGAGAGGGTAGTGGAATTTCCGGTGTAGCGGTGAAATGCGTAGAGATCGGAAGGAACACCAGTGGCGAAGGCGGCTACCTGGCCTAATACTGACACTGAGGCACGAAAGCGTGGGGAGCAAACAGG` |
| ASV4114 | 404 | `TGGGGGATATTGCACAATGGGCGAAAGCCTGATGCAGCAACGCCGCGTGAGGGAAGACGGTTTTCGGATTGTAAACCTCTGTCCTTAGTGACGATAATGACGGTAGCTAAGGAGGAAGCCACGGCTAACTACGTGCCAGCAGCCGCGGTAATACGTAGGTGGCAAGCGTTGTCCGGAATTACTGGGTGTAAAGGGAGCGTAGGCGGGAAGGCAAGTCGGATGTGAAAACTATGGGCTCAACCCATAGCCTGCATTCGAAACTGTTTTTCTTGAGTGAAGTAGAGGTTGGCGGAATTCCCGGTGTAGCGGTGAAATGCGTAGATATCGGGAGGAACACCAGTGGCGAAGGCGGCCAACTGGGCTTTTACTGACGCTGAGGCTCGAAAGCATGGGGAGCAAACAGG` |
| ASV2495 | 429 | `TGGGGAATATTGGACAATGGGCGCAAGCCTGATCCAGCAATGCCGCGTGTGTGAAGAAGGCCTTCGGGTTGTAAAGCACTTTTAGTGGGGACAAAAAAGGCACGGGTAATATTCGTGCAAATTGATGGTACCCACAGAAAAAGCACCGGCTAACTCTGTGCCAGCAGCCGCGGTAATACAGAGGGTGCGAGCGTTAATCGGAATTACTGGGCGTAAAGCGCACGTAGGCGGTTTGTTAAGTCAGATGTGAAAGCCCCGGGCTTAACCTGGGAATGGCATTTGATACTGGCGAGCTAGAGTTTGGTAGAGGAAGGCGGAATTCCCGGTGTAGCGGTGAAATGCGTAGATATCGGGAGGAACACCAGTGGCGAAGGCGGCCTTCTGGACTAAAACTGACGCTGAGGTGCGAAAGCGTGGGGAGCAAACAGG` |
| ASV1622 | 404 | `TGGGGAATCTTAGACAATGGGCGCAAGCCTGATCTAGCCATGCCGCGTGAGCGATGAAGGCCTTAGGGTTGTAAAGCTCTTTCAGTGGGGAAGATAATGACTGTACCCACAGAAGAAGCCCCGGCTAACTCCGTGCCAGCAGCCGCGGTAATACGGAGGGGGCTAGCGTTGTTCGGAATTACTGGGCGTAAAGCGCACGTAGGCGGACCGGAAAGTCAGAGGTGAAATCCCAGAGCTCAACTTTGGAACTGCCTTTGAAACTCCTGGTCTTGAGGTCGAGAGAGGTGAGTGGAATTCCGAGTGTAGAGGTGAAATTCGTAGATATTCGGAGGAACACCAGTGGCGAAGGCGGCTCACTGGCTCGATACTGACGCTGAGGTGCGAAAGCGTGGGGAGCAAACAGG` |
| ASV4454 | 404 | `TGGGGAATATTGGGCAATGGACGAAAGTCTGACCCAGCAACGCCGCGTGGGGGATGAAGGCCTTAGGGTTGTAAACTCCTTTTATTAGGGAAGATAATGACTGTACCTAATGAATAAGCATCGGCTAACTTCGTGCCAGCAGCCGCGGTAATACGAAGGATGCTAGCGTTACTCGGAATTACTGGGCGTAAAGAATGCGTAGGCGGGCTTTTAAGTTGATAGTGCAATCCCCAAGCTTAACTTAGGAATTGCTATCAAAACTGATTGCCTAGAATATGAAAGAGAGTAAGGGAATTCCTGGTGTAGGGGTAAAATCCGTAGATATCAGGAGGAAGACCGGAAGCGAAAGCGCTTACTTGGTTCATTATTGACGCTGAGGCATGAGAGCGTGGGGATCGAACAGG` |
| ASV1929 | 424 | `TAAGGAATATTGGACAATGGAGGGAACTCTGATCCAGCCATGCCGCGTGAAGGATGAAGGCGCTCAGCGTTGTAAACTTCTTTTGGACGGGAACAAAAGCAATCATTTATGATTGTCTGAGGGTACCGTCAGAATAAGCACCGGCTAACTCCGTGCCAGCAGCCGCGGTAATACGGAGGGTGCAAGCGTTATCCGGAATCACTGGGTTTAAAGGGTGCGTAGGCGGTCCGATAAGTCAGTCGTGAAATCGTGTGGCTTAACCATGCAACTGCGATTGATACTGTTGGACTTGAATCAGGTTGAGGTAGGCGGAATGTGACATGTAGCGGTGAAATGCATAGATATGTCATAGAACACCGATTGCGAAGGCAGCTTACTGGGCCTGAATTGACGCTGAGGCACGAAAGCGTGGGGATCAAACAGG` |
| ASV3824 | 429 | `TGGGGAATATTGGACAATGGGCGCAAGCCTGATCCAGCAATGCCGCGTGTGTGAAGAAGGCCTCAGGGTTGTAAAGCACTTTCAGTGGGGAGAAATAGGCTTTTGCTAATATCAGAAGCTGATGATGTTACCCACAGAAGAAGCACCGGCTAACTCTGTGCCAGCAGCCGCGGTAATACAGAGGGTGCGAGCGTTAATCGGAATTACTGGGCGTAAAGGGTGCGTAGGCGGTTTTTTAAGTTAGATGTGAAATTCCTGGGCTTAACCTGGGAACTGCGTCTGATACTGGGAAGCTAGAGTATTGTAGAGGAAAGTGGAATTTCCGGTGTAGCGGTGAAATGCGTAGATATCGGAAGGAACATCAATGGCGAAGGCAACTTTCTGGACAAATACTGACGCTGAGGCACGAAAGCATGGGGAGCAAACAGG` |
| ASV741 | 430 | `TGGGGAATATTGGACAATGGGCGGAAGCCTGATCCAGCAATACCGCGTGTGTGAAGAAGGCCTTCGGGTTGTAAAGCACTTTTGTTAGGGAGGATGGTTATTTGGGCTAATATCCCAAGTAGCTGACGTTACCTAAAGAATAAGCACCGGCTAACTCCGTGCCAGCAGCCGCGGTAATACGGAGGGTGCAAGCGTTAATCGGATTTACTGGGCGTAAAGGGTGCGTAGGCGGCCTATTAAGTCAGATGTGAAAGCCCTGGGCTCAACCTGGGAATTGCATTTGAAACTGGTAGGCTAGAATGTGGTAGAGGGTAGCGGAATTTCTGGTGTAGCGGTGAAATGCGTAGATATCAGAAGGAACATCAATGGCGAAGGCAGCTACCTGGGCCAACATTGACGCTGAGGCACGAAAGCGTGGGGAGCAAACAGG` |
| ASV2334 | 429 | `TCGAGAATAATTCACAATGGGGGCAACCCTGATGGTGCAACGCCGCGTGGAGGATGACGGTCTTCGGATTGTAAACTCCTGTCATCCGGGAGAAAGACCTGGCCGTTAATAGCGGACAGGGTTGATAGTACCGGAAGAGGAAGGGACGGCTAACTTCGTGCCAGCAGCCGCGGTAATACGAAGGTCCCAAGCGTTGTTCGGAATCACTGGGCGTAAAGGGAGCGTAGGCGGCGCGGTAAGTCAGATGTGAAATCCCGGGGCTCAACCCCGGAACTGCATCCGATACTGCCGTGCTAGAGCAATGGAGAGGTAGCTGGAATTATCGGTGTAGCAGTGAAATGCGTAGATATCGATAGGAACACTCGTGGCGAAAGCGAGCTACTGGACATTTGCTGACGCTGAGGCTCGAAGGCTAGGGGAGCGAAAGGG` |
| ASV25 | 429 | `TGGGGAATTTTGGACAATGGACGCAAGTCTGATCCAGCCATGCCGCGTGCGGGAAGAAGGCCTTCGGGTTGTAAACCGCTTTTGTCAGGGAAGAAACGGCTCGCTCTAATACAGTGGGCTAATGACGGTACCTGAAGAATAAGCACCGGCTAACTACGTGCCAGCAGCCGCGGTAATACGTAGGGTGCAAGCGTTAATCGGAATTACTGGGCGTAAAGCGTGCGCAGGCGGTTATGCAAGACAGATGTGAAATCCCCGGGCTCAACCTGGGAACTGCATTTGTGACTGCATGGCTAGAGTACGGTAGAGGGGGATGGAATTCCGCGTGTAGCAGTGAAATGCGTAGATATGCGGAGGAACACCGATGGCGAAGGCAATCCCCTGGACCTGTACTGACGCTCATGCACGAAAGCGTGGGGAGCAAACAGG` |
| ASV3630 | 409 | `TGGGGAATATTGCACAATGGGCGAAAGCCTGATGCAGCAACGCCGCGTGAGGGACGAAGGCCTTCGGGTTGTAAACCTCTTTTAGCAGGGAAGAAGCGAAAGTGACGGTACCTGCAGAAAAAGCACCGGCTAACTACGTGCCAGCAGCCGCGGTAATACGTAGGGTGCAAGCGTTGTCCGGAATTATTGGGCGTAAAGAGCTCGTAGGCGGTTTGTCACGTCTGCTGTGAAATTTCGAGGCTCAACCTCGAACTTGCAGTGGGTACGGGCAGGCTAGAGTGCGGTAGGGGAGATGGGAATTCCTGGTGTAGCGGTGGAATGCGCAGATATCAGGAGGAACACCAATGGCGAAGGCACATCTCTGGGCCGTAACTGACGCTGAGGAGCGAAAGCGTGGGGAGCGAACAGG` |
| ASV30 | 404 | `TGGGGAATCTTAGACAATGGGGGAAACCCTGATCTAGCCATGCCGCGTGATCGATGAAGGCCTTAGGGTTGTAAAGATCTTTCAGTGGGGAAGATAATGACTGTACCCACAGAAGAAGCCCCGGCTAACTCCGTGCCAGCAGCCGCGGTAATACGGAGGGGGCTAGCGTTGTTCGGAATTACTGGGCGTAAAGCGCACGTAGGCGGATCAGAAAGTCAGAGGTGAAATCCCAGGGCTCAACCTTGGAACTGCCTTTGAAACTCCTGATCTTGAGGTCGAGAGAGGTGAGTGGAATTCCGAGTGTAGAGGTGAAATTCGTAGATATTCGGAGGAACACCAGTGGCGAAGGCGGCTCACTGGCTCGATACTGACGCTGAGGTGCGAAAGCGTGGGGAGCAAACAGG` |
| ASV2200 | 429 | `TGGGGAATTTTGGACAATGGGCGCAAGCCTGATCCAGCAATGCCGCGTGCAGGACGAAGGCCTTCGGGTTGTAAACTGCTTTTGTACGGAACGAAACGGTCCTTTCTAATACAGAGGGCTAATGACGGTACCGTAAGAATAAGCACCGGCTAACTACGTGCCAGCAGCCGCGGTAATACGTAGGGTGCAAGCGTTAATCGGAATTACTGGGCGTAAAGCGTGCGCAGGCGGTCTTTTAAGACAGATGTGAAATCCCCGGGCTTAACCTGGGAACCGCATTTGTGACTGAAAGGCTAGAGTACGGCAGAGGGGGATGGAATTCCGCGTGTAGCAGTGAAATGCGTAGATATGCGGAGGAACACCGATGGCGAAGGCAGTCCCCTGGGCCTGTACTGACGCTCATGCACGAAAGCGTGGGGAGCAAACAGG` |
| ASV4370 | 388 | `TCGAGAAACTTCTGCAATACGCGCAAGCGTGACAGAGCAAGCCAGAGTGCTTTTCCGTATTGGAAAAGCTTTTGTGATATGTAAAAAGTATCACGAATAAGGACTGGGCAAGACTGGTGCCAGCCGCCGCGGTAATCCCAGCAGTCCAAGTCGCAATCACTTTTATTGGGTCTAAAACATCCGTAGCTTGTTCTGCAAGTCTCTTGTGAAATCTGGTCTCTTAAGGGCCAAAGTGCGAGAGATACTACAGGACTAGAGACCGGGAGAGGTAAAGAGTACAAATGGTGTACCGGTAAAATGGGTTGATCCTATTTGGACTATCAACAGCGAAGGCACTTTACCAGAACGGATCTGACAGTGAGGGATGAAGGCTAGGGGCGCAAAACGG` |
| ASV1768 | 429 | `TGGGGAATTTTGGACAATGGGCGCAAGCCTGATCCAGCAATGCCGCGTGAGTGAAGAAGGCCTTCGGGTTGTAAAGCTCTTTTGTCAGGGAAGAAACGGGCCTTTCTAATACAGGGGCCTAATGACGGTACCTGAAGAATAAGCACCGGCTAACTACGTGCCAGCAGCCGCGGTAATACGTAGGGTGCAAGCGTTAATCGGAATTACTGGGCGTAAAGCGTGCGCAGGCGGTTTTGTAAGTCTGTTGTGAAATCCCCGGGCTCAACCTGGGAATGGCAATGGAGACTGCAAGGCTAGAGTTTGGCAGAGGGGGGTAGAATTCCACGTGTAGCAGTGAAATGCGTAGATATGTGGAGGAACACCGATGGCGAAGGCAGCCCCCTGGGTCAAAACTGACGCTCATGCACGAAAGCGTGGGGAGCAAACAGG` |
| ASV2947 | 424 | `TGAGGAATATTGGACAATGGGCGCAAGCCTGATCCAGCCATGCCGCGTGCAGGATGACGGTCCTATGGATTGTAAACTGCTTTTATACGAGAAGAAACACTCCTTCGTGAAGGAATTTGACGGTATCGTAAGAATAAGGATCGGCTAACTCCGTGCCAGCAGCCGCGGTAATACGGAGGATCCAAGCGTTATCCGGAATCATTGGGTTTAAAGGGTCCGTAGGCGGTCTTGTAAGTCAGTGGTGAAAGCCCATCGCTCAACGGTGGAACGGCCATTGATACTGCAGGACTTGAATTACTAGGAAGTAACTAGAATATGTAGTGTAGCGGTGAAATGCTTAGAGATTACATGGAATACCAATTGCGAAGGCAGGTTACTACTAGTGGATTGACGCTGATGGACGAAAGCGTGGGTAGCGAACAGG` |
| ASV1999 | 403 | `TGGGGAATATTGCACAATGGGCGAAAGCCTGATGCAGCAACGCCGCGTGAGCGATGAAGGCCTTCGGGTCGTAAAGCTCTGTCCTCAAGGAAGATAATGACGGTACTTGAGGAGGAAGCCCCGGCTAACTACGTGCCAGCAGCCGCGGTAATACGTAGGGGGCTAGCGTTATCCGGAATTACTGGGCGTAAAGGGTGCGTAGGTGGTTTCTTAAGTCAGAAGTGAAAGGCTACGGCTCAACCGTAGTAAGCTTTTGAAACTAAGAAACTTGAGTGCAGGAGAGGAGAGTAGAATTCCTAGTGTAGCGGTGAAATGCGTAGATATTAGGAGGAATACCAGTTGCGAAGGCGGCTCTCTGGACTGTAACTGACACTGAGGCACGAAAGCGTGGGGAGCAAACAGG` |
| ASV5092 | 423 | `TAGGGAATATTGGGCAATGGAAGCAATTCTGACCCAGCCATGCCGCGTGCAGGATGAAGGCGCTAAGCGTTGTAAACTGCTTTTGTCAAGGAAGAAAAAGAGAGTGCGCTCTTCATTGACGGTACTTGATGAATAAGCATCGGCTAACTCCGTGCCAGCAGCCGCGGTAATACGGAGGATGCAAGCGTTGTCCGGATTTATTGGGTTTAAAGGGTGCGTAGGTGGTTTTGTAAGTCAGTGGTAAAATTAAGCAGCTCAACTGTTTGTGGGCCATTGATACTGCAAGACTTGAGACTTGTGGGGGTAAGCGGAATTGGTAGTGTAGCGGTGAAATGCATAGATACTACCAGGAATATCGATAGCGAAGGCAGCTTACTACGGAAGGACTGACACTGAGGCACGAAAGTGCGGGGATCAAACAGG` |
| ASV3143 | 404 | `TGGGGAATCTTGGACAATGGGCGCAAGCCTGATCCAGCCATGCCGCGTGAATGATGAAGGCCTTAGGGTTGTAAAGTTCTTTCGCCGGGGACGATAATGACGGTACCCGGAGAAGAAGCCCCGGCTAACTTCGTGCCAGCAGCCGCGGTAATACGAAGGGGGCTAGCGTTGTTCGGAATTACTGGGCGTAAAGCGCATGTAGGCGGACCATCAAGTTGGAGGTGAAAGCCCAAGGCTCAACCTTGGAATTGCCTTCAAAACTGGTGGTCTGGAGCATATTAGAGGCAGGTGGAATTCCGAGTGGAGAGGTGAAATTCGTAGATATTCGGAGGAACACCGGTGGCGAAGGCGACCTGCTGGGATATTGCTGACGCTGAGATGCGAAAGCGTGGGGAGCAAACAGG` |
| ASV4216 | 431 | `TAGGGAATCTTCCGCAATGGGCGCAAGCCTGACGGAGCAATGCCGCGTGAACGATGAAGGCCTTCGGGTCGTAAAGTTCTGTTGTGGGGGAAGAAATCGCGTCACATTAATACGGTGACGTGTTTGACGGTACTCCACGAGGAAGCCCCGGCTAATTACGTGCCAGCAGCCGCGGTAATACGTAAGGGGCAAGCGTTGTCCGGAATCACTGGGCGTAAAGCGCACGTAGGCGGTTTAGCAAGTTGGGGGTGTAAGCTCTAGGCTTAACCTAGAAATTGCCTCCAAAACTACTTCACTTGAGTATTAGAGGGGAAGCTGGAATTCACGGTGTAGCGGTGAAATGCGTAGATATCGTGGAGAACACCCGTGGCGAAGGCGGGCTTCTGGCTAATTACTGACGCTGAGGTGCGAAAGCTAGGGGAGCGAACGGG` |
| ASV4899 | 429 | `TGGGGAATATTGGACAATGGGGGCAACCCTGATCCAGCGATGCCGCGTGGGTGAAGAAGGCCTTCGGGTTGTAAAGCCCTTTAGGCCGGGAAGAAGTGTAGTAGTTTAAGAGATAATTACATTGACGGTACCGGCAGAATAAGCACCGGCAAACTCTGTGCCAGCAGCCGCGGTAATACAGAGGGTGCGAGCGTTAATCGGAATTACTGGGCGTAAAGGGCGCGTAGGCGGTTAAGTGAGTGTGATGTGAAAGCCCTGGGCTTAACCTAGGAAGTGCATCGCAAACGACTTAACTGGAGTATATGAGAGGGTGGTGGAATTTCCGGTGTAGCGGTGAAATGCGTAGATATCGGAAGGAACGTCAATGGCGAAGGCAGCCACCTGGCATAATACTGACGCTGAGGCGCGAAAGCGTGGGGATCGAACAGG` |
| ASV1591 | 404 | `TAGGGAATCTTGCGCAATGGGCGAAAGCCTGACGCAGCCATGCCGCGTGAATGATGAAGGTCTTAGGATTGTAAAATTCTTTCACCGGGGAAGATAATGACGGTACCCGGAGAAGAAGCCCCGGCTAACTTCGTGCCAGCAGCCGCGGTAATACGAAGGGGGCTAGCGTTGCTCGGAATTACTGGGCGTAAAGGGCGCGTAGGCGGATAGTTTAGTCAGAGGTGAAAGCCCAGGGCTCAACCTTGGAACTGCCTTTGATACTGGCTATCTTGAGTTCGGGAGAGGTGAGTGGAATGCCGAGTGTAGAGGTGAAATTCGTAGATATTCGGCGGAACACCAGTGGCGAAGGCGACTCACTGGCCCGATACTGACGCTGAGGCGCGAAAGCGTGGGGAGCAAACAGG` |
| ASV1851 | 405 | `TGGGGGATATTGCACAATGGAGGAAACTCTGATGCAGCGACGCCGCGTGTAGGAAGACGGTCCTATGGATTGTAAACTACTGTCTTCGGGGACGATAATGACGGTACCCGAAGAGGAAGCTCCGGCTAACTACGTGCCAGCAGCCGCGGTAATACGTAGGGAGCGAGCGTTGTCCGGAATTACTGGGTGTAAAGGGAGCGTAGGCGGGGAGGCAAGTTGGATGTGAAAACTATCGGCTCAACCGGTAGATTGCATTCAAAACTGTTTCTCTTGAGTGAAGTAGAGGCAGGCGGAATTCCTAGTGTAGCGGTGAAATGCGTAGATATTAGGAGGAACACCAGTGGCGAAGGCGGCCTGCTGGGCTTTTACTGACGCTGAGGCTCGAAAGTGTGGGTAGCAAACAGG` |
| ASV3452 | 404 | `TGGGGAATATTGGACAATGGGCGCAAGCCTGATCCAGCAATGCCGCGTGGATGAAGAAGGTCTTCGGATTGTAAAGTCCTTTCGGCGGGGACGATGATGACGGTACCCGCAGAAGAAGCCCCGGCTAACTTCGTGCCAGCAGCCGCGGTAATACGAAGGGGGCTAGCGTTGCTCGGAATTACTGGGCGTAAAGGGCGCGTAGGCGGCGGCATCAGTCAGGCGTGAAAGCCCCGGGCTCAACCTGGGAACTGCGCTTGAGACGGTGTTGCTTGAGGACGGGAGAGGGTGGTGGAATTCCCAGTGTAGAGGTGAAATTCGTAGATATTGGGAAGAACACCGGTGGCGAAGGCGGCCACCTGGCCCGTTACTGACGCTGAGGCGCGAAAGCGTGGGGAGCAAACAGG` |
| ASV3850 | 406 | `CGAGGAATCTTGGGCAATGGGCGAAAGCCTGACCCAGCGACGCCGCGTGAGGGATGAAGGCCTTCGGGTCGTAAACCTCTTTTGTGAGGGAAGAAGTTCTGACGGTACCTCACGAATAAGCCACGGCTAACTACGTGCCAGCAGCCGCGGTAATACGTAGGTGGCAAGCGTTGTCCGGATTTACTGGGCGTAAAGGGCTTGTAGGCGGTCCGTTAAGTCCGGTGTGAAATCTCCCGGCTCAACTGGGAGGGGCCATCGGATACTGGCGGACTAGAGGTGGGCAGAGGAAAGCAGAATTCCCGGTGTAGCGGTGAAATGCGTAGATATCGGGAGGAATACCAGTGGCGAAGGCGGCTTTCTGGGCCTATCCTGACGCTGAGGAGCGAAAGCGTGGGGAGCAAACCGG` |
| ASV3144 | 429 | `TGGGGAATTTTGGACAATGGGGGCAACCCTGATCCAGCAACGCCGCGTGAATGAAGAAGGCCCTTGGGTTGTAAAGTTCTTTTATAACTGGGAAGAAGTTCATGTTTAAGAGATGTGAATGGAGATAGTAGGTTATGAATAAGCACCGGCTAACTACGTGCCAGCAGCCGCGGTAATACGTAGGGTGCGAGCGTTAATCGGAATTATTGGGCGTAAAGGGTGAGTAGGCGGTTAGGTAAGTTAATAGTGAAAGCATTGGGCTCAACCTGATAAGTGCTATTAAAACTGCTTAACTAGAGTTAGTTAGAGGATTGTGGAATTCCAAGTGTAGTAGTGAAATGCGTAGATATTTGGAGGAACCTTAATGGCGAAGGCAGCAATCTGGGATGAGACTGACGCTGAATCACGAAAGCGTGGGGAGCAAACAGG` |
| ASV4747 | 429 | `TGGGGAATATTGGACAATGGGCGCAAGCCTGATCCAGCCACGCCGCGTGAGTGATGAAGGCCTTCGGGTCGTAAAGCTCTGTGGGGAGAGACGAACCGCCAGGGGTTAATAACCGCTTGGCATGACGGTATCTCCTTAGCAAGCACCGGCTAACTTCGTGCCAGCAGCCGCGGTAATACGAAGGGTGCAAACGTTGCTCGGAATTATTGGGCGTAAAGCGCACGTAGGCGGTCTATCAAGTCGGATGTGAAATCCCTCGGCTCAACCAAGGAAGTGCATTCGAAACTGACAGACTTGAGTACTTAAGAGGATCGCGGAATTCCCGGTGTAGAGGTGAAATTCGTAGATATCGGGAGGAACACCAGTGGCGAAGGCGGCGATCTGGGAAGATACTGACGCTGAGGTGCGAAAGCGTGGGGAGCAAACAGG` |
| ASV231 | 429 | `TGGGGAATCTTGGACAATGGGCGCAAGCCTGATCCAGCAATGCCGCGTGCGTGAAGAAGGCCTTCGGGTTGTAAAGCGCTTTTGTTCGGGAGGAAATCCTAGTGGCTAATATCCATTGGGGATGACAGTACCGGAAGAATAAGGACCGGCTAACTACGTGCCAGCAGCCGCGGTAATACGTAGGGTCCAAGCGTTAATCGGAATTACTGGGCGTAAAGGGTGCGCAGGTGGTTGATTAAGTGTGATGTGAAAGCCCCGGGCTCAACCTGGGAATTGCATTGCAAACTGGTCAACTAGAGTATGGCAGAGGGGGGTGGAATTCCGCGTGTAGCAGTGAAATGCGTAGAGATGCGGAGGAACACCGATGGCGAAGGCAACCCCCTGGGCTAATACTGACACTCATGCACGAAAGCGTGGGGAGCAAACAGG` |
| ASV860 | 429 | `TGGGGAATATTGCGCAATGGACGAAAGTCTGACGCAGCGACGCCGCGTGAGGGATGAAGGTCTTCGGATCGTAAACCTCTGTCAGAGGGGAAGAAACTACTTGGTGCTAATCAGCCAGGTACTGACGGTACCCTCAAAGGAAGCACCGGCTAACTCCGTGCCAGCAGCCGCGGTAATACGGAGGGTGCAAGCGTTAATCGGAATCACTGGGCGTAAAGCGCTCGTAGGCTGTTTGTTAAGTCAGAGGTGAAATCCCACGGCTCAACCGTGGAACTGCCTTTGATACTGGCAGACTAGAATCCGGGAGAGGGTGGTGGAATTCCAGGTGTAGGAGTGAAATCCGTAGATATCTGGAGGAACATCAGTGGCGAAGGCGACCACCTGGACCGGTATTGACGCTGAGGAGCGAAAGCGTGGGGAGCAAACAGG` |
| ASV2508 | 404 | `TTAGGAATATTGGACAATGGGCGAAAGCCTGATCCAGCCATGCCGCGTGAATGATGAAGGCCTTAGGGTTGTAAAGTTCTTTTAGGGGTGAAGATAATGACGGTAGCCCCAGAAAAAGCTCCGGCTAACTCCGTGCCAGCAGCCGCGGTAATACGGAGGGAGCGAGCGTTGTTCGGAATCATTGGGCGTAAAGGGTGCGTAGGCGGTTCGACAAGTCAGAAGTGAAATCCCTGGGCTTAACCCAGGAACTGCTTTTGATACTGTCGGTCTAGAGTATGGGAGAGGTTGGCGGAATTCCGAGTGTAGAGGTGAAATTCGTAGATATTCGGAAGAACACCAGTGGCGTAGGCGGCCAACTGGACCATTACTGACGCTGAGGCACGAAAGCGTGGGGAGCAAACAGG` |
| ASV2597 | 409 | `TGGGGAATATTGCGCAATGGGCGAAAGCCTGACGCAGCAACGCCGCGTGGGGGATGAAGGCCTTCGGGTTGTAAACCTCTTTCAGCAGGGAAGAAGCGAAAGTGACGGTACCTGCAGAAGAAGCACCGGCTAACTACGTGCCAGCAGCCGCGGTAATACGTAGGGTGCAAGCGTTGTCCGGATTTATTGGGCGTAAAGAGCTCGTAGGCGGTTAGTCACGTCGGATGTGAAAACCCGGAGCTTAACTCCGGGCCTGCATTCGATACGGGCTGACTAGAGTGTTGTAGGGGAGACTGGAACTCCTGGTGTAGCGGTGAAATGCGCAGATATCAGGAAGAACACCGGTGGCGAAGGCGGGTCTCTGGGCAACTACTGACGCTGAGGAGCGAAAGCGTGGGGAGCAAACAGG` |
| ASV1120 | 429 | `TGAGGAATATTGCACAATGGGCGAAAGCCTGATGCAGCGACGTCGCGTGGAGGATGAAGGTCTTCGGATTGTAAACTTCTGTTAAGTGGGAAGAAAGAATTGTCTTTAATAGAGGCAAAAGATGACAGTACCATTAGAGAAAGCACCGGCTAACTTCGTGCCAGCAGCCGCGGTAATACGAAGGGTGCAAACGTTATTCGGAATCACTGGGCGTAAAGGGTGTGTAGACGGCTTGTCAAGTCGATTGTTAAATTTTCCAGCTTAACTGGAACCCGGCAGTCGAAACTAGCAAGCTAGAGAATGAAAGAGAGAAGCGGAATTCTCGGAGTAGCGGTTAAATGCGTAGATCTCGAGAGGAACACCGGTGGCGAAGGCGGCTTCTTGGTTCATTTCTGACGTTGAAACACGAAAGCGTGGGTAGCAAACAGG` |
| ASV701 | 403 | `TGGGGAATATTGCACAATGGGCGAAAGCCTGATGCAGCAACGCCGCGTGAGCGATGAAGGCCTTCGGGTCGTAAAGCTCTGTCCTCAAGGAAGATAATGACGGTACTTGAGGAGGAAGCCCCGGCTAACTACGTGCCAGCAGCCGCGGTAATACGTAGGGGGCTAGCGTTATCCGGATTTACTGGGCGTAAAGGGTGCGTAGGTGGTTTCTTAAGTCAGGAGTGAAAGGCTACGGCTCAACCGTAGTAAGCTCTTGAAACTGGGAAACTTGAGTGCAGGAGAGGAAAGTGGAATTCCTAGTGTAGCGGTGAAATGCGTAGATATTAGGAGGAACACCAGTAGCGAAGGCGGCTTTCTGGACTGTAACTGACACTGAGGCACGAAAGCGTGGGGAGCAAACAGG` |
| ASV2670 | 405 | `TAGGGAATTTTCCACAATGGGCGAAAGCCTGATGGAGCAATACCGCGTGCAGGATGAAGGCCTTCGGGTCGTAAACTGCTTTTATGTGCGAAGAATTTGACGGTAACACATGAATAAGGATCGGCTAACTACGTGCCAGCAGCCGCGGTCATACGTAGGATCCAAGCGTTATCCGGAATTACTGGGCGTAAAGAGTTGCGTAGGTGGCATTGTAAGTGGAGCGTGAAAGCGTGCGGCTCAACCGCATTACTGCGTTCCAAACTGCAAAGCTTGAGTATGAGAGGGGTAGATGGAATTTCTAGTGTAGGAGTGAAATCCGTAGATATTAGAAGGAACACCGATAGCGTAGGCAGTCTACTGGCTCATTACTGACACTAAGGCACGAAAGCGTGGGGAGCAAACAGG` |
| ASV107 | 429 | `TGGGGAATTTTGGACAATGGGCGCAAGCCTGATCCAGCCATGCCGCGTGCGGGAAGAAGGCCTTCGGGTTGTAAACCGCTTTTGTCAGGGAAGAAACGGCTTTCTCTAATACAGAGGGCTAATGACGGTACCTGAAGAATAAGCACCGGCTAACTACGTGCCAGCAGCCGCGGTAATACGTAGGGTGCAAGCGTTAATCGGAATTACTGGGCGTAAAGCGTGCGCAGGCGGTTATGCAAGACAGAGGTGAAATCCCCGGGCTCAACCTGGGAACTGCCTTTGTGACTGCATAGCTAGAGTACGGTAGAGGGGGATGGAATTCCGCGTGTAGCAGTGAAATGCGTAGATATGCGGAGGAACACCGATGGCGAAGGCAATCCCCTGGACCTGTACTGACGCTCATGCACGAAAGCGTGGGGAGCAAACAGG` |
| ASV3772 | 429 | `TGGGGAATATTGGACAATGGGGGAAACCCTGATCCAGCAATGCCACGTGTGTGAAGAAGGCCTGAGGGTTGTAAAGCACTTTTGTCAGTGAGGAAGCTTGTTAGGTTAAGAGCTAGACAAGTTGACGTTAGCTGAAGAAAAAGCACCGGCTAACTCTGTGCCAGCAGCCGCGGTAATACAGAGGGTGCAAGCGTTAATCGGAATTACTGGGCGTAAAGCGCACGTAGGTGGATATTTAAGTCAGATGTGAAAACCCCGGGCTCAACCTGGGAATGGCATTTGATACTGGGTATCTAGAGTATGATAGAGGGTAGAGGAATTTCTGGTGTAGCGGTGAAATGCGTAGATATCAGAAGGAACATCAGTGGCGAAGGCGTCTACCTGGATTAATACTGACACTGAGGAGCGAAAGCATGGGTAGCAAACAGG` |
| ASV4107 | 430 | `TGGGGAATATTGGACAATGGGGGCAACCCTGATCCAGCGATGCCACGTGTGTGAAGAAGGCCTTAGGGTTGTAAAGCACTTTTGTTAGTGAGGAAGGTTGTTACTGCGAATAGTGGTAATGATTGACGTTAGCTAAAGAAAAAGCACCGGCTAACTCTGTGCCAGCAGCCGCGGTAATACAGAGGGTGCAAGCGTTAATCGGAATTACTGGGCGTAAAGCGCTCGTAGGTGGATCATTAAGTCAGTCGTAAAATACCTGGGCTCAACCTGGGAGGGTCGATTGAAACTGGTGATCTAGAGTATGGGAGAGGGTAGAGGAATTTCTGGTGTAGCGGTGAAATGCGTAGATATCAGAAGGAACATCAGTGGCGAAGGCGTCTACCTGGCCTAATACTGACACTGAGGAGCGAAAGCGTGGGGAGCAAACAGG` |
| ASV1613 | 407 | `TTAGGAATCTTGCGCAATGGGGGAAACCCTGACGCAGCAACGCCGCGTGAGTGAAGAAGGCCTTCGGGTTGTAAAGCTCTTTCGACAGGGAAGAAAAAAATGACGGTACCTGTATAAGAAGCACCGGCTAACTCTGTGCCAGCAGCCGCGGTAATACAGAGGGTGCAAGCGTTATTCGGAATTACTGGGCGTAAAGCGCGTGTAGGCTGTTTTGTAAGTCAGTTGTGAAAGCCCTTGGCTCAACCAAGGAAGTGCAACTGATACTGCTAAACTAGAGTATGAGAGGGGAGAACGGAATTCCTGGTGTAGAGGTAAAATTCGTAGATATCAGGAGGAACAACAGTGGCGAAGGCGGTTCTCTGGATCATTACTGACGCTGATACGCGAAAGCGTGGGGAGCAAACAGG` |
| ASV5240 | 430 | `TGGGGAATCTTGCGCAATGGACGAAAGTCTGACGCAGCCACGCCGCGTGAGTGAAGAAGGCCTTCGGGTTGTAAAGCTCTGTCGCCTGGGACGAACATCCCTATAGTTAACAGCTGTAGGGACTGACGGTACCGGGAGAGGAAGCACCGGCTAACTCTGTGCCAGCAGCCGCGGTAATACAGAGGGTGCAAGCGTTGCTCGGAATCATTGGGCGTAAAGGGCAGGTAGGTGGTCTCATTTGTCTGGGGTGAAAGCCTTGGGCTTAACCCAAGAAGTGCCTCGGAAACGGTGAGACTAGAGTACTGGAGAGGGTCGTGGAATTCCCGGTGTAGCGGTGAAATGCGTAGAGATCGGGAGGAACACCAGCGGCGAAGGCGGCGACCTGGACAGTTACTGACACTCAACTGCGAAAGCGTGGGGAGCAAACAGG` |
| ASV2250 | 429 | `TGGGGAATTTTGGACAATGGGGGCAACCCTGATCCAGCCATGCCGCGTGTGTGAAGAAGGCCTTCGGGTTGTAAAGCACTTTCGGCCGGAACGAAATCGCCTGCGTTAATAGCGCAGGTGGATGACGGTACCGGAAGAAGAAGCACCGGCTAACTACGTGCCAGCAGCCGCGGTAATACGTAGGGTGCGAGCGTTAATCGGAATTACTGGGCGTAAAGGGTGCGCAGGCGGCGTCGCAAGTCAGGCGTGAAATCCCCGGGCTTAACCTGGGAATGGCACTTGAAACTACGATGCTGGAGTATGGCAGAGGGAGGTGGAATTCCACGTGTAGCGGTGAAATGCGTAGAGATGTGGAGGAACACCGATGGCGAAGGCAGCCTCCTGGGCCAATACTGACGCTCATGCACGAAAGCGTGGGGAGCAAACAGG` |
| ASV1884 | 429 | `TGGGGAATTTTGGACAATGGGCGAAAGCCTGATCCAGCAATGCCGCGTGCAGGACGAAGGCCTTCGGGTTGTAAACTGCTTTTGTACGGAGCGAAAGGGCTCTTCCTAATACGAGGGGTCTATGACGGTACCGTAAGAATAAGCACCGGCTAACTACGTGCCAGCAGCCGCGGTAATACGTAGGGTGCAAGCGTTAATCGGAATTACTGGGCGTAAAGCGTCCGCAGGCGGTTCTATAAGACAGATGTGAAATCCCCGGGCTCAACCTGGGAACTGCATTTGTGACTGTAGAGCTAGAGTACGGCAGAGGGGGATGGAATTCCGCGTGTAGCAGTGAAATGCGTAGATATGCGGAGGAACACCGATGGCGAAGGCAATCCCCTGGGCCTGTACTGACGCTCATGGACGAAAGCGTGGGGAGCAAACAGG` |
| ASV4516 | 404 | `TGAGGAATCTTCCGCAATGGACGAAAGTCTGACGGAGCGACACCGCGTGGAGGATGAAGGTTTTGTTATTGTAAACTCCTTTTCTCAGGGACGATAATGACGGTACCTGAGGAATAAGGGACGGCAAACTATGTGCCAGCAGCCGCGGTAATACATAGGTCCCAAGCGTTATCCGGAATTACTGGGCGTAAAGCGTCTGCAGGTGGAAAAGTGTGTGAGATGTGAAAGACCGGGGCTCAACCCCGCGTTTGTATCTCAAACTGCTTTTCTCGAGTGAGCAAGAGGTATGCGGAATTTATGGAGTAGGAGTGCAATCCGTTGACACCATAAAGAACACCAAAAGCGAAGGCAGCATACTGGGGCTCTACTGACACTCAGAGACGAAAGCGTGGGGAGCGAAAGGG` |
| ASV5537 | 430 | `TGGGGAATATTGCACAATGGGCGCAAGCCTGATGCAGCAACGCCGCGTGAGTGAAGAAGGCCTTCGGGTTGTAAAGCTCTTTCGATTGGGAAGAAGGGTGATTGCACAAATAGTGCCGTCATTTGACTGTACCAGAATAAGAAGCACCGGCTAACTCCGTGCCAGCAGCCGCGGTAATACGGGGGGTGCAAGCGTTGTTCGGAATTACTGGGCGTAAAGCGCACGTAGGCGGATTCGTAAGTCAATTGTGAAAGCCCTGGGCTTAACCTGGGAATGGCAGTTGAAACTGCGGACCTTGAATACTTGAGAGGATGACGGAATTCCTGGTGTAGAAGTGAAATTCGTAGATATCAGGAGGAACACCCGAGGCGAAGGCGGTTATCTGGCAAGCTATTGACGCTGAGGTGCGAAAGCGTGGGGAGCAAACAGG` |
| ASV907 | 428 | `TGGGGAATTTTGGACAATGGGGGCAACCCTGATCCAGCAATGCCGCGTGAGTGAAGAAGGCCTTCGGGTTGTAAAGCTCTTTTGTCAGGGAAGAAACAGCAGCTCTAACACAGTTTGCGAATGACGGTACCTGAAGAATAAGCACCGGCTAACTACGTGCCAGCAGCCGCGGTAATACGTAGGGTGCAAGCGTTAATCGGAATTACTGGGCGTAAAGCGTGCGCAGGCGGTTATGTAAGACAGGCGTGAAATCCCCGGGCTTAACCTGGGAATTGCGCTTGTGACTGCATAGCTAGAGTATGTCAGAGGGGGGTAGAATTCCACGTGTAGCAGTGAAATGCGTAGAGATGTGGAGGAATACCAATGGCGAAGGCAGCCCCCTGGGATAATACTGACGCTCATGCACGAAAGCGTGGGGAGCAAACAGG` |
| ASV2044 | 404 | `TTGGGAATCTTGGACAATGGGGGAAACCCTGATCCAGCCATGCCGCGTGAGTGATGAAGGCCTTCGGGTTGTAAAACTCTTTCGACGGGGACGATAATGACGGTACCCGTAGAAGAAGCTCCGGCTAACTTCGTGCCAGCAGCCGCGGTAATACGAAGGGGGCTAGCGTTGTTCGGAATTACTGGGCGTAAAGCGCGCGCAGGCGGTCTTCCAAGTCAGTGGTGAAAGCCCGGAGCTCAACTCCGGAACTGCCATTGAAACTGTTAGACTTGAGGACGAGAGAGGTGAGTGGAATTCCCAGTGTAGAGGTGAAATTCGTAGATATTGGGAAGAACACCGGTGGCGAAGGCGGCTCACTGGCTCGTATCTGACGCTCAGGCGCGATAGCGTGGGGATCAAACAGG` |
| ASV1548 | 405 | `TGGGGAATCTTGCACAATGGGCGAAAGCCTGATGCAGCAACGCCGCGTGCGGGAAGAAGGCCCTAGGGTTGTAAACCGCTTTCAGTAGGGAAGAAAATGACGGTACCTACAGAAGAAGGTGCGGCCAACTACGTGCCAGCAGCCGCGGTGACACGTAGGCACCAAGCGTTATCCGGATTTATTGGGCGTAAAGAGCTCGTAGGCGGTTTGGTAAGTCGGGTGTGAAAACTCTGGGCTCAACCCAGAGACGCCACCCGATACTGCTATGACTAGAGTACGGTAGGGGAGCGGGGAATTCATGGTGTAGCGGTGAAATGCGCAGATATCATGAGGAACACCAGCGGCGAAGGCGCCGCTCTGGGCCGTAACTGACGCTGAGGAGCGAAAGCATGGGTAGCAAACAGG` |
| ASV3117 | 429 | `TGGGGAATTTTGGACAATGGGCGGAAGCCTGATCCAGCAATGCCGCGTGTGCGAAGAAGGCCTTCGGGTTGTAAAGCACTTTTATCAGAGAAAAAAAGTCTTATGTAAATAATGTAGGATGATGATGGTATCTGAAGAATAAGCACCGGCTAACTACGTGCCAGCAGCCGCGGTAATACGTAGGGTGCGAGCGTTAATCGGAATTATTGGGCGTAAAGCGTGCGCAGGCGGTTATATAAGATAGATGTGAAATCCTTGGGCTTAACCTGAGAACTGCATATATGACTGTATAACTAGAGTGTATCAGAGGGGGGTAGAATTCCGCGTGTAGCAGTGAAATGCGTAGAGATGCGGAGGAATACCGATGGCGAAGGCAGCCTCCTGGGAAGACACTGACGCTCATGCACGAAAGCGTGGGGAGCAAACAGG` |
| ASV4870 | 430 | `TGGGGAATCTTGCACAATGGGCGAAAGCCTGATGCAGCAACGCCGCGTGAGTGAAGAAGGCCCTTGGGTCGTAAAGCTCTTTAGGCTGGGGAGAAGGGTCTTGGTGTTAATACCATCAGGATTTGACGGTACCAGCAGAATAAGCACTGGCTAATTCCGTGCCAGCAGCCGCGGTAATACGGAAGGTGCAAGCGTTGTTCGGATTGACTGGGCGTAAAGAGCACGTAGGCGGTCTTTTAAGTCGATTGTGAAAGCCCTGGGCTTAACCTAGGAATAGCAGTCGAAACTGAGAGTCTTGAATACCCGAGAGGTTAGTGGAATTCCTGGTGTAGGAGTGAAATCCGTAGATATCAGGAGGAACACCGGAGGCGAAGGCGGCTAACTGGTGGTGTATTGACGCTGAGGTGCGAAAGCGTGGGTAGCAAACAGG` |
| ASV2615 | 429 | `TAGGGAATATTGCGCAATGGGGGAAACCCTGACGCAGCAACGCCGCGTGAGTGAGGAAGGTCTTCGGATTGTAAAACTCTTTTGTTAGGGAAAAATGGTCACGTAACTAATAGCGCGTGATTTGATGGTACCTAAAGAATAAGCACCGGCTAACTTCGTGCCAGCAGCCGCGGTAATACGAAGGGTGCAAGCGTTATTCGGAATCATTGGGCGTAAAGCGCGCGCAGGCGGATCAGCAAGTCAGATGTGAAATCTCGAAGCTCAACTTCGAAACTGCGTCTGAAACTGCTAGTCTAGAGTGTCGGAGGGGGTAGGGGAATTTCACGTGTAGGGGTAAAATCCGTAGAGATGTGAAGGAACACCGGGGGCGAAGGCGCCTACCTGGACGACTACTGACGCTGAGGCGCGAAAGCGTGGGGAGCAAACAGG` |
| ASV3421 | 404 | `TAAGGAATATTGGACAATGGGGGAAACCCTGATCCAGCCATGCCGCGTGAGTGACGAAGGCCTTAGGGTTGTAAAGCTCTTTTGGCGGGGAAGATAATGACTGTACCCGCAGAATAAGCCCCGGCTAAATTCGTGCCAGCAGCCGCGGTAATACGAATGGGGCAAGCGTTGTTCGGAATCACTGGGCGTAAAGCGTGCGTAGGCGGTTTTGCAAGTCAGAAGTGAAAGCCCAAGGCTCAACCTTGGAATTGCTTTTGAAACTGCATTACTTGAATCCCGGAGAGGTTAGTAGAATTCCGAGTGTAGAGGTGAAATTCGCAGATATTCGGAGGAATACCAGTGGCGTAAGCGGCTAACTGGACGGGCATTGACGCTGAGGTACGAAAGCGTGGGGATCAAACAGG` |
| ASV732 | 404 | `TGGGGAATATTGGACAATGGGGGAAACCCTGATCCAGCAATGCCGCGTGAGTGATGAAGGCCTTAGGGTTGTAAAGCTCTTTTCTTAAGGAAGATAATGACGGTACTTAAGGAATAAGCCCCGGCTAACTTCGTGCCAGCAGCCGCGGTAATACGAAGGGGGCAAGCGTTGTTCGGAATAACTGGGCGTAAAGGGCGCGTAGGCGGTGAAACATGTCACATGTGAAAGACCTGGGCTTAACCTGGGAATAGCATGTGAAACGGTATTACTAGAGACCGAGAGGGGAAAAGGGAATTGTGCGTGTAGAGGTGAAATTCGTAGATATGCACAGGAACACCGGTGGCGAAGGCGCTTTTCTGGCTCGGATCTGACGCTAAGGCGCGAAAGCGTGGGGAGCAAACAGG` |
| ASV4258 | 430 | `TGGGGAATATTGCGCAATGGGCGAAAGCCTGACGCAGCAACGCCGCGTGAGTGATGAAGGCCTTCGGGTTGTAAAGCTCTTTCGGCCGGGAAGAAGGGTGTGCGGGTGAATAATCCGTTCATTTGATGGTACCGGAAGAAGAAGCACCGGCTAACTTCGTGCCAGCAGCCGCGGTAATACGAAGGGTGCGAGCGTTGTTCGGACTTACTGGGCGTAAAGGGTTCGTAGGCGGGAGCCTAAGTAGCGTGTGAAATCCCCGGGCTCAACCCGGGACGTGCACGCTAAACTGGGCTTCTTGAGTATTGGAGAGGGTCATGGAATTGCTGGTGTAGGAGTGACATCCGTAGAGATCAGCAGGAACACCGGAGGCGTAAGCGATGACCTGGCCAAATACTGACGCTGAGGAACGAAAGCGTGGGGAGCAAACAGG` |
| ASV4984 | 430 | `TAGGGAATATTGCGCAATGGGGGAAACCCTGACGCAGCAACGCCGCGTGAGTGAGGAAGGTCTTCGGATTGTAAAACTCTTTTGCTAGGGAAAAATGACTTTTTGGCTAATATCCAAGAAGAGTGATGGTACCTAAAGAATAAGGACCGGCTAACTTCGTGCCAGCAGCCGCGGTAATACGAAGGGTCCAAGCGTTGTTCGGAATCATTGGGCGTAAAGCGAGCGCAGGCGGATCAGCAAGTCAGATGTGAAATCTCGAAGCTCAACTTCGAAACTGCGTCTGAAACTGCTAGTCTAGAATGTCGGAGGGGATAGGGGAATTTCACGTGTAGGGGTAAAATCCGTAGAGATGTGAAGGAACACCGGAGGCGAAGGCGCCTATCTGGACGACTATTGACGCTGAGGCTCGAAAGCGTGGGGAGCAAACAGG` |
| ASV4330 | 404 | `TGGGGAATATTGGACAATGGGGGAAACCCTGATCCAGCAATGCCGCGTGAGTGATGAAGGCCTTAGGGTTGTAAAGCTCTTTCGACGGGGACGATGATGACGGTACCCGTAGAAGAAGCCCCGGCTAACTTCGTGCCAGCAGCCGCGGTAATACGAAGGGGGCTAGCGTTGTTCGGAATTACTGGGCGTAAAGGGCGCGTAGGCGGTCTTGTAAGTCAGATGTGAAAGCCCCGGGCTCAACCCGGGAATAGCATTTGATACTGCGAGGCTTGAGTCCGGGAGAGGATGGCGGAATTCCCAGTGTAGAGGTGAAATTCGTAGATATTGGGAAGAACACCGGTGGCGAAGGCGGCCATCTGGACCGGTACTGACGCTGAGGCGCGAAAGCGTGGGGAGCAAACAGG` |
| ASV1447 | 404 | `TGAGGAATATTGGACAATGGGCGCAAGCCTGATCCAGCCATGCCGCGTGAGTGATGAAGGCCCTAGGGTTGTAAAACTCTTTTAGTGGGGAAAATAATGATGGTACCCACAGAAAAAGCTCCGGCTAACTTCGTGCCAGCAGCCGCGGTAATACGAAGGGAGCTAGCGTTACTCGGAATAACTGGGCGTAAAGCGCATGTAGGCGGAAATACAAGTCAGAGGTGAAAGCCCTGGGCTCAACCCAGGAATTGCCTTTGAAACTGTATTTCTTGAGTATGACAGAGGATGGTGGAATTTTCAGTGTAGAGGTGAAATTCGTAGATATTGAAAAGAACACCGGTGGCGAAGGCGACCATCTGGGTCATAACTGACGCTGAGATGCGAAAGCGTGGGGAGCGAACGGG` |
| ASV1184 | 409 | `TGAGGAATTTTCTGCAATGGGCGAAAGCCTGACAGAGCAATACCGCGTGGGGGATGAAGACTTACTGAGTTGTAAACCTCGGTGCCTTAAGGAAGAAGATCTGACGGTACTTAAGGTGGAAAGCATCGGCTAACTCCGTGCCAGCAGCCGCGGTAAGACGGGGGATGCAAGTGTTATCCGGATTTACTGGGCGTAAAGCGTCTGCAGGTGGTTTCTTAAGTCTACTGTTAAATCTTGAGGCTCAACCTCAAATCTGCAGTAGAAACTAGGAGACTTGAGTATAGTAGGGGTAGAGGGAATTTCCAGTGGAGCGGTGAAATGCGTAGATATTGGAAAGAACACCGATGGCGAAGGCACTCTACTGGGCTATTACTGACACTCAGAGACGAAAGCTAGGGGAGCAAATGGG` |
| ASV5369 | 405 | `TGGGGAATCTTGGACAATGAGTGAAAACTTGATCCAGCGATATCACATGGGTGAAGAAGGCCATTTGGTCGTAAAACTCTTTCGTTAACGAAGATAGTGACAGTAGTTAAAGTAAGAAGTCCTGGCAAATCCTGTGCCAGCAGCCGCGGTAATACAGGAGGGGCTAGCGTTATTCGGATTTAATGGGCGTAAAGGGTGCGTAGGCGATAAATTTGATTAAATATGAAATCCCAAAGCTTAACTTTGGAAGAGTGTTTAAAATCGGTTTATTAGAGTTAGATAGAGGTTTGTAGAATTCTTAAAGTAAAGGTAATATTTTTCGATATTAGGAGGAATACTAGTTGCGAAGGCGGCATTCTGGATCTATACTGACGCTGAGGCACGAAAGCGTGGGGAGCAAACAGG` |
| ASV3172 | 424 | `TAGGGAATATTGGGCAATGGAGGCAACTCTGACCCAGCCATGCCGCGTGCAGGAAGACGGTCCTCTGGATTGTAAACTGCTTTTGAATAGGAAGAATGTGTGGGATGCGTCCCATAATGACGGTACTATTAGAATAAGCCACGGCTAACTACGTGCCAGCAGCCGCGGTAATACGTAGGTGGCAAGCGTTATCCGGATTTATTGGGTTTAAAGGGTGCGTAGGCGGCTTGTTAAGTCGATGGTGAAAGGTAGCAGCTTAACTGTATACATGCTGTCGATACTGGCGAGCTTGAGTAATTTGGAGGCAAGTAGAATTTCGGGTGTAGCGGTGAAATGCATAGATACCCGGAGGAATACCGATTGCGAAGGCAGCTTGCTACGAATTTACTGACGCTGATGCACGAAAGCGTGGGGATCAAACAGG` |
| ASV5682 | 404 | `TTGGGAATATTGCGCAATGGGGGAAACCCTGACGCAGCGACGCCGCGTGCGGGATGAAGGTCTTCGGATCGTAAACCGCTTTTCTAAAGGACGATAATGACGGTACTTTAGGAATAAGCCCCGGCTAACTCCGTGCCAGCAGCCGCGGTAAGACGGAGGGGGCAAGCGTTGTCCGGAATTACTGGGCGTAAAGCGCTCGTAGGCGGTTTGTTACGGTAGTGGTGAAAGCTCCTGGCTCAACTGGGAGAGGTCCACTACAACGGGCAGACTCGAGGTATGTAGAGGCAAATGGAATTCCTGGTGTAGCGGTGAAATGCGTAGAGATCAGGAGGAACACCAATGGCGAAGGCAGTTTGCTGGGCATATTCTGACGCTGAGGAGCGAAAGCTAGGGGAGCAAACGGG` |
| ASV1777 | 407 | `TAGGGAATATTGCACAATGGACGAAAGTCTGATGCAGCGACGCCGCGTGAGTGATGAAGGCCTTCGGGTCGTAAAGCTCTGTCGTATGGGACTAACACAATGAAGGTACCATGCAAGAAAGGATCGGCTAACTTCGTGCCAGCAGCCGCGGTAAGACGAGGGATCCTAGCGTTGTTCGGAATCATTGGGCGTAAAGCGGGTGTAGGCGGCTTTATAAGTCAGGTGTGAAAGCCCGGGGCTCAACCCCGGAAGTGCACTTGATACTGTTTAGCTTGAGTGTGGTAGAGGCTACTGGAATTTCTGGTGTAGTGGTGAAATACGTAGATATCAGAAGGAATACCGGAGGCGAAGGCGGGTAGCTGGGCCAACACTGACGCTGAGACCCGAAAGCGTGGGGATCAAACAGG` |
| ASV3748 | 430 | `TGGGGAATATTGCGCAATGGGCGAAAGCCTGACGCAGCAACGCCGTGTGAGTGATGAAGGCCTTCGGGTCGTAAAGCTCTGTCGGCAGGAACGATGGGTGAACGTATTAATACAACGTTTATTTGACGGTACCTGCAAAGGAAGCACCGGCTAACTCTGTGCCAGCAGCCGCGGTAAGACAGAGGGTGCAAGCGTTGTTCGGAATCATTGGGCGTAAAGCGCGCGTAGGCGGCTCATTAAGTTGAATGTGAAATCCCCGGGCTCAACTTGGGAAGTGCATTCAAAACTGGTGAGCTAGAGTCCCGGAGAGGGTGGTGGAATTCCCAGTGTAGAAGTGAAATTCGTAGATATTGGGAGGAACATCGGTGGCGAAGGCGACCACCTGGACGGAGACTGACGCTGAGGCGCGAAAGCGTGGGTAGCAAACAGG` |
| ASV1426 | 429 | `TGGGGAATTTTGGACAATGGACGCAAGTCTGATCCAGCCATGCCGCGTGCGGGAAGAAGGCCTTCGGGTTGTAAACCGCTTTTGTCAGGGAAGAAAAGGGTTTCTCTAATACAGAGGCCTAATGACGGTACCTGAAGAATAAGCACCGGCTAACTACGTGCCAGCAGCCGCGGTAATACGTAGGGTGCAAGCGTTAATCGGAATTACTGGGCGTAAAGCGTGCGCAGGCGGTTATGCAAGACAGAGGTGAAATCCCCGGGCTCAACCTGGGAACTGCCTTTGTGACTGCATGGCTAGAGTACGGTAGAGGGGGATGGAATTCCGCGTGTAGCAGTGAAATGCGTAGATATGCGGAGGAACACCGATGGCGAAGGCAATCCCCTGGACCTGTACTGACGCTCATGCACGAAAGCGTGGGGAGCAAACAGG` |
| ASV432 | 404 | `TGGGGAATATTGGACAATGGGCGAAAGCCTGATCCAGCAATGCCGCGTGAGTGATGAAGGCCTTAGGGTTGTAAAGCTCTTTTACCAGGGATGATAATGACAGTACCTGGAGAATAAGCTCCGGCTAACTCCGTGCCAGCAGCCGCGGTAATACGGAGGGAGCTAGCGTTGTTCGGAATTACTGGGCGTAAAGCGTGCGTAGGCGGTTATTCAAGTCAGAGGTGAAAGCCTGGAGCTCAACTCCAGAACGGCCTTTGAAACTAGGTAACTAGAATCTTGGAGAGGTGAGTGGAATTCCGAGTGTAGAGGTGAAATTCGTAGATATTCGGAAGAACACCAGTGGCGAAGGCGACTCACTGGACAAGTATTGACGCTGAGGTACGAAAGCGTGGGGAGCAAACAGG` |
| ASV4303 | 404 | `TTGGGAATCTTGGACAATGGGGGAAACCCTGATCCAGCCATGCCGCGTGAGTGATGAAGGCCTTCGGGTTGTAAAACTCTTTCGACGGGGACGATAATGACGGTACCCGTAGAAGAAGCTCCGGCTAACTTCGTGCCAGCAGCCGCGGTAATACGAAGGGGGCTAGCGTTGTTCGGAATTACTGGGCGTAAAGCGCGCGCAGGCGGCTATCCAAGTCAGTGGTGAAAGCCCGGAGCTCAACTCCGGAACTGCCATTGAAACTGTTTAGCTTGAGGACGAGAGAGGTGAGTGGAATTCCCAGTGTAGAGGTGAAATTCGTAGATATTGGGAAGAACACCGGTGGCGAAGGCGGCTCACTGGCTCGTATCTGACGCTCAGGCGCGATAGCGTGGGGATCAAACAGG` |
| ASV589 | 429 | `TGGGGAATATTGGACAATGGGCGCAAGCCTGATCCAGCCATGCCGCGTGTGTGAAGAAGGCCTTCGGGTTGTAAAGCACTTTTGTCCGGAAAGAAAAGCTTCGGGTTAATACCCCGGGGTTCTGACGGTACCGGAAGAATAAGCACCGGCTAACTTCGTGCCAGCAGCCGCGGTAATACGAAGGGTGCAAGCGTTACTCGGAATTACTGGGCGTAAAGCGTGCGTAGGTGGTTTGTTAAGTCTGATGTGAAAGCCCTGGGCTCAACCTGGGAATTGCATTGGATACTGGCAGGCTAGAGTGCGGTAGAGGATGGCGGAATTCCCGGTGTAGCAGTGAAATGCGTAGAGATCGGGAGGAACATCTGTGGCGAAGGCGGCCATCTGGACCAGCACTGACACTGAGGCACGAAAGCGTGGGGAGCAAACAGG` |
| ASV5730 | 405 | `TGGGGAATATTGCGCAATGGGGGAAACCCTGACGCAGCCACGCCGCGTGGGTGATGAAGGCCTTCGGGTTGTAAAGCCCTGTCGAGGGGGAAGAAGTATGACGGTACCCCTCAAGAAAGCACCGGCTAACCCTGTGCCAGCAGCCGCGGTAATACAGGGGGTGCAAGCGTTGCTCGGAATCACTGGGCGTAAAGCGTGCGCAGGCGGACCGTTAAGTCAGGCGTGAAAGCCCTGGGCTCAACCCAGGAATTGCGCTTGATACTGGCGGTCTTGAGTACGGGAGAGGTAAGCGGAATTCCTGGTGTAGAGGTGAAATTCGTAGATATCAGGAGGAACACCGGTGGCGAAAGCGGCTTACTGGCCCGATACTGACGCTCATGCACGAAAGCGTGGGGAGCAAACAGG` |
| ASV5116 | 424 | `TAGGGAATATTGGGCAATGGGCGAGAGCCTGACCCAGCCATGCCGCGTGCAGGATGAAGGCCCTCTGGGTTGTAAACTGCTTTTTTGGGGGAAGAACAGCAGGGGTGCGCCCCTGTATGACGGTACCCTAAGAATAAGCACCGGCTAACTCCGTGCCAGCAGCCGCGGTAATACGGAGGGTGCAAGCGTTGTCCGGATTTATTGGGTTTAAAGGGTGCGTAGGTGGTTTGATAAGTCTGGTTTGAAAGTTGGTCGCTTAACGATCAAGGGTGGCTGGATACTGTCGGACTTGAATGTTTTGGAGGTAGCTGGAATGGGTTGTGTAGCGGTGAAATGCATAGAGATGACCCGGAACTCCAATTGCGTAGGCAGGCTACTACGAAATGATTGACACTGAGGCACGAGAGCATGGGTAGCAAACAGG` |
| ASV5593 | 429 | `TCGAGAATCTTTCGCAATGGGCGCAAGCCTGACGAAGCGACGCCGTGTGAGCGAAGAAGGCCTTCGGGTTGTAAAGCTCTTTCGCTAGGGAACAAGAAAGACAGGATAATACCTTGTCGATTTGATGGTACTTGGTAAAGAAGCACCGGCTAACTCCGTGCCAGCAGCTGCGGTAATACGGAGGGTGCAAGCATTGATCGGAATTACTGGGCGTAAAGGGCGCGTAGGCGGCTGGGTAAGTCAGATGTGAAATTCCGGGGCTCAACCCCGGAGCTGCATTTGAAACTGCTTAGCTAGAGTGCAGTTAGGGAAAACGGAATTCCACGTGTAGCGGTGAAATGCGTAGATATGTGGAAGAACACCGGTGGTGAAGACGGTTTTCTGGGCTGTAACTGACGCTGAGGCGCGAAAGCATGGGGAGCAAACAGG` |
| ASV511 | 429 | `TGGGGAATTTTGGACAATGGGCGAAAGCCTGATCCAGCCATGCCGCGTGCAGGATGAAGGCCTTCGGGTTGTAAACTGCTTTTGTACGGAACGAAAAGACTCCTTCTAATAAAGGGGGTCCATGACGGTACCGTAAGAATAAGCACCGGCTAACTACGTGCCAGCAGCCGCGGTAATACGTAGGGTGCAAGCGTTAATCGGAATTACTGGGCGTAAAGCGTGCGCAGGCGGTTATGTAAGACAGATGTGAAATCCCCGGGCTCAACCTGGGAACTGCATTTGTGACTGCATAGCTAGAGTACGGTAGAGGGGGATGGAATTCCGCGTGTAGCAGTGAAATGCGTAGATATGCGGAGGAACACCGATGGCGAAGGCAATCCCCTGGACCTGTACTGACGCTCATGCACGAAAGCGTGGGGAGCAAACAGG` |
| ASV2859 | 427 | `TCGAGAATTTTTCTCAATGGGGGAAACCCTGAAGGAGCGACGCCGCGTGGAGGATGAAGGTCTTCGGATTGTAAACTCCTGTCATTTGGGAACAACTATGCGGTTTAACTGACTGCAAAACGATAGTACCAGAAGAGGAAGGGACGGCTAACTCTGTGCCAGCAGCCGCGGTAATACAGAGGTCTCAACCGTTGTTCGGATTCATTGGGCGTAAAGGGTGCGTAGGCGGTCGCGTAAGTCAGATGTGAAATCCCGGGGCTCAACCCCGGAACTGCATTTGATACTGCGTGGCTAGGATACTGGAGAGGAGACTGGAATTTACGGTGTAGCAGTGAAATGCGTAGATATCGTAAGGAAGACCAGTGGCGAAGGCGAGTCTCTGGACAGTTATCGACGCTGAGGCACGAAGGCTAGGGGAGCAAACGGG` |
| ASV4075 | 430 | `TGGGGAATTTTGGGCAATGGGCGAAAGCCTGACCCAGCCACGCCGCGTGGGTGAAGAAGGCCTTCGGGTCGTAAAGCCCTGTTGTGGGGGGCGAAGGTTCCGGCGTCAAATAGACGTCGGGATTGACGGTACTCCACGAGGAAGGACTGGCCAACTCCGTGCCAGCAGCCGCGGTAAAACGGAGAGTCCAAGCGTTGTCCGGAATTACTGGGCATAAAGGGCGCGTAGGCGGCCTTGTATGTCGGTGGTGAAATCCCCTTGCTTAACAAGGGAACGGCCGCCGAAACTACAGGGCTAGAGGACGGGAGAGGAAGGCGGAATTCCCGGTGTAGCGGTGAAATGCTAAGATATCGGGAGGAACACCTACGGCGAAAGCAGCCTTCTAGAACGTCCCTGACGCTGAGGCGCGAAAGCGTGGGGAGCAAACAGG` |
| ASV1515 | 429 | `TGGGGAATTTTGGACAATGGGGGCAACCCTGATCCAGCAATGCCGCGTGAGTGAAGAAGGCCTTCGGGTTGTAAAGCTCTTTTGTCAGGGAAGAAACGGTAGTAGCTAATATCTATTACTAATGACGGTACCTGAAGAATAAGCACCGGCTAACTACGTGCCAGCAGCCGCGGTAATACGTAGGGTGCAAGCGTTAATCGGAATTACTGGGCGTAAAGCGTGCGCAGGCGGTTGTGTAAGTCAGATGTGAAATCCCCGGGCTCAACCTGGGAATTGCATTTGAGACTGCATGGCTAGAGTGTGTCAGAGGGGGGTAGAATTCCACGTGTAGCAGTGAAATGCGTAGATATGTGGAGGAATACCGATGGCGAAGGCAGCCCCCTGGGATAACACTGACGCTCATGCACGAAAGCGTGGGGAGCAAACAGG` |
| ASV3529 | 405 | `TAGGGAATTTTCCACAATGGGCGAAAGCCTGATGGAGCAATGCCGCGTGCAGGATGAATGCCTTCGGGTTGTAAACTGCTTTTATGTGCGAAGAATTTGACGGTAACACATGAATAAGGGTCGGCTAACTACGTGCCAGCAGCCGCGGTCATACGTAGGACCCAAGCGTTATCCGGAATTACTGGGCGTAAAGAGTTGCGTAGGTGGCATTATAAGTGGAGCGTGAAAGCGTGCGGCTCAACCGCATTATTGCGTTCCAAACTGTAAAGCTTGAGTATGAGAGAGGTAGATGGAATTTCTAGTGTAGGAGTGAAATCCGTAGATATTAGAAGGAACACCGATGGCGTAGGCAGTCTACTGGCTCATTACTGACACTAAGGCACGAAAGCGTGGGGAGCAAACAGG` |
| ASV2290 | 404 | `TGGGGAATATTGGACAATGGGGGAAACCCTGATCCAGCAATGCCGCGTGAGTGAAGAAGGCCTTCGGGTTGTAAAACTCTTTTGTTAGGGACGATAATGACGGTACCTAACGAATAAGCACCGGCTAACTTCGTGCCAGCAGCCGCGGTAATACGAAGGGTGCTAGCGTTGTTCGGAATGACTGGGCGTAAAGGGTGTGTAGGCGGTTAGACACGTCAGATGTGAAATCCCCGGGCTTAACCTGGGAATGGCATTTGATACGGTCTAGCTAGAGATTGGTAGAGGGTAGTGGAATTGCGAGTGTAGAGGTGAAATTCGTAGAGATTCGCAGGAACACCGGTGGCGAAGGCGGCTACCTGGGCCAATACTGACGCTGAGGCACGAAAGCGTGGGGAGCAAACAGG` |
| ASV2174 | 429 | `TGGGGAATTTTGGACAATGGGGGCAACCCTGATCCAGCAACGCCGCGTGAATGAAGAAGGCCTTAGGGTTGTAAAGTTCTTTTATTTGGGAGCAAAGCTGATGTGTGAATAGCATATTAGTATGAGAGTACCAAAGGAATAAGCACCGGCTAACTACGTGCCAGCAGCCGCGGTAATACGTAGGGTGCGAGCGTTAATCGGAATTATTGGGCGTAAAGGGTGCGTAGGCGGATATATAAGTTAGATGTGAAAAGCCAGGGCTTAACCTTGGTCGTGCATTTAAGACTGTATGTCTAGAGTTTGTAAGAGGGGGGTAGAATTCCAAGTGTAGCAGTGAAATGCGTAGAGATTTGGAGGAATACCGATGGCGAAGGCAGCCCCCTGGTATAGAACTGACGCTGAGGCACGAAAGCGTGGGTAGCAAACAGG` |
| ASV2478 | 421 | `TAGGGAATTTTGCGCAATGGGGGAAACCCTGACGCAGCAACGCCATGTGAGGGATGAAGCATTTAGGTGTGTAAACCTCTGTCAGCAGGGAATAAATGTCCGTTTACGGAAGTTGAATGTACCTGCAAAGGAAGCACCGGCTAACTTCGTGCCAGCAGCCGCGGTAATACGAGGGGTGCAAGTGTTGTTCGGAATCACTGGGCGTAAAGGGAGCGTAGGCGGACTTTTAAGTCAGATGTTTAATTTCAGGGCCCAACCCTGAATTTGCGTTTGAAACTGGAAGTCTTGAATATATGTAGAGGTGAATGGAATTCCCGGTGTAGCGGTGGAATGCGTAGATATCGGGAGGAACACCAGTTGCGAAGGCGATTCACTGGACTAATATTGACGCTGAGGCTCGAAAGCGTGGGGAGCGAACAGG` |
| ASV245 | 408 | `TGGGGAATTTTCCGCAATGGGCGAAAGCCTGACGGAGCAATACCGCGTGAGGGATGACGGCCTATGGGTTGTAAACCTCTTTTTTCAGGGAGGAATCAAATGACGTGTACCTGAAGAATAAGCATCGGCTAACTCCGTGCCAGCAGCCGCGGTAAGACGGAGGATGCAAGTGTTATCCGGAATCACTGGGCGTAAAGCGTCTGTAGGTGGTCCAATAAGTCAACTGTTAAATCTTGGGGCTCAACCCCAAAATCGCAGTCGAAACTATTAGACTAGAGTATAGTAGGGGTAAAGGGAATTTCCAGTGGAGCGGTGAAATGCGTAGATATTGGAAAGAACACCGATGGCGAAGGCACTTTACTGGGCTATTACTAACACTCAGAGACGAAAGCTAGGGTAGCAAATGGG` |
| ASV4378 | 404 | `TGGGGAATATTGGACAATGGGCGCAAGCCTGATCCAGCAATTCCGCGTGGGTGAAGAAGGTCTTCGGATTGTAAAGCCCTTTCGGCGGGGACGATGATGACGGTACCCGCAGAAGAAGCCCCGGCTAACTTCGTGCCAGCAGCCGCGGTAATACGAAGGGGGCTAGCGTTGCTCGGAATTACTGGGCGTAAAGGGCGCGTAGGCGGCGCATTTGGTCAGGCGTGAAATTCCCGGGCTCAACCTGGGGACTGCGCTTGAGACGGGTGTGCTGGAGGACGGAAGAGGCTCGCGGAATTCCCAGTGTAGAGGTGAAATTCGTAGATATTGGGAAGAACACCGGTGGCGAAGGCGGCGAGCTGGTCCGTTACTGACGCTGAGGCGCGACAGCGTGGGGAGCAAACAGG` |
| ASV487 | 429 | `TGGGGAATTTTGGACAATGGGCGCAAGCCTGATCCAGCAATGCCGCGTGCAGGATGAAGGCCTTCGGGTTGTAAACTGCTTTTGTACGGAACGAAACGGCTTCTTCTAATAAAGGGAGCTAATGACGGTACCGTAAGAATAAGCACCGGCTAACTACGTGCCAGCAGCCGCGGTAATACGTAGGGTGCGAGCGTTAATCGGAATTACTGGGCGTAAAGCGTGCGCAGGCGGTTATATAAGACAGATGTGAAATCCCCGGGCTCAACCTGGGACCTGCATTTGTGACTGTATAGCTAGAGTACGGTAGAGGGGGATGGAATTCCGCGTGTAGCAGTGAAATGCGTAGATATGCGGAGGAACACCGATGGCGAAGGCAATCCCCTGGACCTGTACTGACGCTCATGCACGAAAGCGTGGGGAGCAAACAGG` |
| ASV3717 | 429 | `TGGGGAATTTTGGACAATGGGGGAAACCCTGATCCAGCCATTCCGCGTGTGTGAAGAAGGCCTTCGGGTTGTAAAGCACTTTTGTCAGGAAAGAAATCGCTTAGGTTAATACCCTGAGTGGATGACGGTACCTGAAGAATAAGCACCGGCTAACTACGTGCCAGCAGCCGCGGTAATACGTAGGGTGCAAGCGTTAATCGGAATTACTGGGCGTAAAGCGTGCGCAGGCGGTTTGGAAAGAAAGATGTGAAATCCCAGAGCTCAACTTTGGAACTGCATTTTTAACTGCCAGACTAGAGTATGTCAGAGGGGGGTAGAATTCCGCGTGTAGCAGTGAAATGCGTAGATATGCGGAGGAATACCGATGGCGAAGGCAGCCCCCTGGGATAATACTGACGCTCATGCACGAAAGCGTGGGGAGCAAACAGG` |
| ASV3199 | 429 | `TAGGGAATATTGGACAATGGGCGCAAGCCTGATCCAGCCATGCCGCGTGAGTGAAGAAGGCCCTCGGGTTGTAAAGCTCTTTTGTCCGGAAAGAAAAGCATCCAGTTAATACCTGGGTGTCCTGACGGTACCGGAAGAATAAGCACCGGCTAACTTCGTGCCAGCAGCCGCGGTAATACGAAGGGTGCAAGCGTTACTCGGAATTACTGGGCGTAAAGCGTGCGTAGGTGGTTTGTTAAGTCTGATGTGAAAGCCCTGGGCTCAACCTGGGAATGGCATTGGATACTGGCAGGCTAGAGTGCGGTAGAGGGTAGTGGAATTCCCGGTGTAGCAGTGAAATGCGTAGAGATCGGGAGGAACATCTGTGGCGAAGGCGACTACCTGGACCAGCACTGACACTGAGGCACGAAAGCGTGGGGAGCAAACAGG` |
| ASV3626 | 423 | `TAGGGAATATTGGGCAATGGGCGCAAGCCTGACCCAGCCATGCCGCGTGCAGGAAGAAGGCGTTATGCGTTGTAAACTGCTTTTATACGGGAAGAAAACGGCCATGCGTGGCAAACTGACGGTACCGTATGAATAAGCACCGGCTAACTCCGTGCCAGCAGCCGCGGTAATACGGAGGGTGCAAGCGTTGTCCGGATTTATTGGGTTTAAAGGGTGCGTAGGCGGCTTTTTAAGTCAGCGGTGAAAGACTGTCGCTCAACGACGGAACTGCCGTTGATACTGATTAGCTTGAGTTTGATTGAGGTAGCCGGAATGGATAGTGTAGCGGTGAAATGCATAGATATTATCCAGAACGCCAATAGCGTAGGCAGGTTACTAAGTCAATACTGACGCTGAGGCACGAGAGTGCGGGGATCAAACAGG` |
| ASV2086 | 404 | `TGGGGAATATTGGACAATGGGGGAAACCCTGATCCAGCAATGCCGCGTGAGTGATGAAGGCCTTCGGGTTGTAAAACTCTTTTGTCAGGGACGATGATGACGGTACCTGACGAATAAGCTCCGGCTAACTTCGTGCCAGCAGCCGCGGTAATACGAAGGGAGCTAGCGTTGTTCGGAATTACTGGGCGTAAAGGGCGTGTAGGCGGTATCATAAGTCAGATGTGAAAGACCTGGGCTTAACCTAGGAGGTGCATTTGATACTGTGAAACTAGAGATCGAGAGAGGAAAGCGGAATTACGAGTGTAGAGGTGAAATTCGTAGATATTCGTAAGAACACCAGTGGCGAAGGCGGCTTTCTGGCTCGATACTGACGCTAAGGCGCGAAAGCGTGGGGAGCAAACAGG` |
| ASV246 | 424 | `TGAGGAATATTGGACAATGGGTGGAAACCTGATCCAGCCATGCCGCGTGCAGGAAGACGGCCCTATGGGTTGTAAACTGCTTTTGTATGGGAGCAATAAGGTCTACGAGTAGACTGATGAGAGTACCATACGAATAAGCATCGGCTAACTCCGTGCCAGCAGCCGCGGTAATACGGAGGATGCGAGCGTTATCCGGATTTATTGGGTTTAAAGGGTGCGTAGGCTGTTCGATAAGTCAGCGGTGAAATACATGTGCTCAACACATGAACTGCCGTTGATACTGTCGAGCTAGAATGGGGATGCTGTTGGCGGAATGTGTAGTGTAGCGGTGAAATGCATAGATATTACACAGAACACCAATTGCGAAGGCAGCTGACAAATCCCTAATTGACGCTGAGGCACGAAAGTGTGGGGATCAAACAGG` |
| ASV785 | 430 | `TGGGGAATATTGCGCAATGGGGGAAACCCTGACGCAGCAACGCCGCGTGAGTGAGGAAGGACTTCGGTCTGTAAAGCTCTGTTAATGTGGAAAAATGGTAGTTGGTCTAATAGGCCGATTATTTGATGGTACACATAGAGGAAGCACCGGCTAACTTCGTGCCAGCAGCCGCGGTAATACGAAGGGTGCAAGCGTTGTTCGGATTTATTGGGCGTAAAGCGCGCGTAGGCGGACCTGCAAGTCAGATGTGAAATCTCGGGGCTCAACCCCGAAACTGCGTCTGAAACTACAGGTCTAGAATCTTGGAGAGGGAAGGGGAATTTCGCATGTAGGGGTAAAATCCGTAGATATGCGAAGGAACACCAGGGGCGAAGGCGCCTTCCTGGACAAGTATTGACGCTGAGGCGCGAAAGCGTGGGGATCAAACAGG` |
| ASV2707 | 424 | `TAGGGAATTTTGCGCAATGGGGGAAACCCTGACGCAGCAACGCCATGTGTGGGATGAAGCATTTCGGTGTGTAAACCACTGTCAGCAGGGAATAAACGGAGGTCCTCGGGACCTCCTTGAATGTACCTGCAAAGGAAGCGCCGGCTAACTTCGTGCCAGCAGCCGCGGTAATACGAGGGGCGCAAGTGTTGTTCGGAATCACTGGGCGTAAAGGGAGCGTAGGCGGGCTTTTAAGTTAGGTGTTTAATACCGGGGCCCAACCCCGGACCGGCACCTAAAACTGGGAGTCTTGAATAGTGGAGAGGTCGGTGGAATTGGGGGTGTAGCGGTGGAATGCGTAGAGATCCCCAAGAACACCAGTTGCGAAGGCGGCCGACTGGACACATATTGACGCTGAGGCTCGAAAGCATGGGGAGCAAACAGG` |
| ASV5225 | 424 | `TAAGGAATATTGGTCAATGGGCGAAAGCCTGAACCAGCCATGCCGCGTGTAGTATGAAGGCCCTCTGGGTTGTAAGCTACTTTTGAGTGGGAAGAAACCCTTGGATTCATCCGAGGCTGACGGTACCACTAGAATAAGCACCGGCTAACTCCGTGCCAGCAGCCGCGGTAATACGGAGGGTGCAAGCGTTATCCGGAATCACTGGGTTTAAAGGGTGCGTAGGCGGTCTGATAAGTCAGTATTGAAAGGCTGGAGCTTAACTCCGGGACTGGTATTGATACTGTCAGACTTGAATCAGGTTGAGGTAGGCGGAATGTGACATGTAGCGGTGAAATGCTTAGATATGTCATAGAACACCGATTGCGTAGGCAGCCTACTGGCCCTCGATTGACGCTGAGGCACGAAAGCGTGGGGAGCAAACAGG` |
| ASV1372 | 424 | `TGAGGAATATTGGTCAATGGGCGCAAGCCTGAACCAGCCATGCCGCGTGCAGGATGACGGTCCTATGGATTGTAAACTGCTTTTGTACAGGAAGAAACACTCCCTCGTGAGGGAGCTTGACGGTACTGTAAGAATAAGGATCGGCTAACTCCGTGCCAGCAGCCGCGGTAATACGGAGGATCCAAGCGTTATCCGGAATCATTGGGTTTAAAGGGTCCGTAGGCGGGTTTATAAGTCAGTGGTGAAAGCCCATCGCTCAACGATGGAACGGCCATTGATACTGTAGATCTTGAATTATTAGGAAGTAACTAGAATATGTAGTGTAGCGGTGAAATGCTTAGAGATTACATGGAATACCAATTGCGAAGGCAGGTTACTACTAATGGATTGACGCTGATGGACGAAAGCGTGGGTAGCGAACAGG` |
| ASV5370 | 430 | `TGGGGAATTTTGCGCAATGGGCGAAAGCCTGACGCAGTAACGCCGCGTGGAGGATGAAGGCCTTCGGGTTGTAAACTCCTGTCAGAAGGAACGACGGAGAAGGGGTGAACAATCTCTTCTTCCAGACGGTACCTTCGAAGGAAGCCCCGGCTAATTCCGTGCCAGCAGCCGCGGTAATACGGAAGGGGCGAGCGTTGTTCGGAATCATTGGGCGTAAAGGGCGCGTAGGTGGTTTTGTGTGTCTATCGTGAAAGCACGCGGCTCAACCGCGTTCCGTCGGTAGAAACTGCAGAACTAGAGGCTGGGAGGGGCTGGTGGAATTCCCTGTGTAGCGGTGAAATGCGTAGAGATGGGGGAGAACACTCGTGGCGAAGGCGGCCAGCTAGACCAGTTCTGACACTGAGGCGCGATAGCGTGGGGAGCAAACAGG` |
| ASV516 | 429 | `TGGGGAATATTGGACAATGGGCGCAAGCCTGATCCAGCCATGCCGCGTGTGTGAAGAAGGCCTTCGGGTTGTAAAGCACTTTTGTTGGGGAAGAAAAGCCGTCGGTTAATACCCGGTGGTGATGACGGTACCCAAAGAATAAGCACCGGCTAACTTCGTGCCAGCAGCCGCGGTAATACGAAGGGTGCAAGCGTTACTCGGAATTACTGGGCGTAAAGCGTGCGTAGGTGGTTTGTTAAGTCAGATGTGAAAGCCCCGGGCTCAACCTGGGAATTGCATTTGATACTGGCTAACTAGAGTGAGGTAGAGGAGAGTGGAATTCCCGGTGTAGCAGTGAAATGCGTAGAGATCGGGAGGAACATCAGTTGCGAAGGCGGCTCTCTGGACCATCACTGACACTGAGGCACGAAAGCGTGGGGAGCAAACAGG` |
| ASV4786 | 406 | `TAGGGAATTTTCCACAATGGGCGAAAGCCTGATGGAGCAACGCCGCGTGCAGGATGAAGGCTTTCGGGTCGTAAACTGCTTTTATAAGTGAAGATTATGACGGTAACTTATGAATAAGGACCGGCTAACTCCGTGCCAGCAGCCGCGGTCATACGGAGGGTCCAAGCGTTATCCGGAATTACTGGGCGTAAAGAGTTGCGTAGGCGGTTAAGTAAGCGAATAGTGAAACTGTGTGGCTCAACCATGCACCCATTGTTCGAACTGCTTTGACTAGAGAATGGAAGAGGTTGCTAGAATTCCTAGTGTAGGGGTGAAATCCGTAGATATTAGGAGGAATACCGATGGCGTAGGCAGGCAACTGGTCCATTTCTGACGCTAAGGCACGAAAGCGTGGGGAGCGATCGGG` |
| ASV498 | 404 | `TGGGGAATATTGGACAATGGGGGCAACCCTGATCCAGCAATGCCGCGTGAGTGATGAAGGCCTTCGGGTTGTAAAGCTCTTTTGTTAGGGAAGATAATGACGGTACCTAACGAATAAGCTCCGGCTAACTTCGTGCCAGCAGCCGCGGTAATACGAAGGGAGCTAGCGTTGTTCGGAATTACTGGGCGTAAAGGGCGCGTAGGCGGTTATCTAAGTCAGATGTGAAAGCCTTGGGCTTAACCTAAGAATTGCATTTGATACTAGATAACTAGAGATCGAGAGAGGAAAGTGGAATTACGAGTGTAGAGGTGAAATTCGTAGATATTCGTAAGAACACCAGTGGCGAAGGCGGCTTTCTGGCTCGATACTGACGCTAAGGCGCGAAAGCGTGGGGAGCAAACAGG` |
| ASV1136 | 429 | `TGGGGAATTTTGGACAATGGGCGCAAGCCTGATCCAGCCATGCCGCGTGCGGGAAGAAGGCCTTCGGGTTGTAAACCGCTTTTGTCAGGGAAGAAACGGCCTGGGTTAATACCTTGGGCTAATGACGGTACCTGAAGAATAAGCACCGGCTAACTACGTGCCAGCAGCCGCGGTAATACGTAGGGTGCAAGCGTTAATCGGAATTACTGGGCGTAAAGCGTGCGCAGGCGGCCATGCAAGACAGATGTGAAATCCCCGGGCTCAACCTGGGAACTGCATTTGTGACTGCATGGCTCGAGTACGGCAGAGGGGGATGGAATTCCGCGTGTAGCAGTGAAATGCGTAGATATGCGGAGGAACACCGATGGCGAAGGCAATCCCCTGGGCCTGTACTGACGCTCATGCACGAAAGCGTGGGGAGCAAACAGG` |
| ASV3069 | 429 | `TGGGGAATTTTGGACAATGGGCGAAAGCCTGATCCAGCAATGCCGCGTGCAGGATGAAGGCCTTCGGGTTGTAAACTGCTTTTGTACGGAACGAAAAGCTCTGGGCTAATACCCCGGAGTCATGACGGTACCGTAAGAATAAGCACCGGCTAACTACGTGCCAGCAGCCGCGGTAATACGTAGGGTGCAAGCGTTAATCGGAATTACTGGGCGTAAAGCGTGCGCAGGCGGTTTTGTAAGACAGTGGTGAAATCCCCGGGCTCAACCTGGGAACTGCCATTGTGACTGCAAAGCTAGAGTACGGTAGAGGGGGATGGAATTCCGCGTGTAGCAGTGAAATGCGTAGATATGCGGAGGAACACCGATGGCGAAGGCAATCCCCTGGACCTGTACTGACGCTCATGCACGAAAGCGTGGGGAGCAAACAGG` |
| ASV4133 | 429 | `TGGGGAATATTGGACAATGGGGGCAACCCTGATCCAGCCATGCCGCGTGTGTGAAGAAGGCCTTCGGGTTGTAAAGCACTTTAAGTGGGGAGGAAGGGGTGATTCCTAATACGAATCATCATTGACGCTACCCACAGAATAAGCACCGGCTAACTCTGTGCCAGCAGCCGCGGTAATACAGAGGGTGCAAGCGTTAATCGGAATTACTGGGCGTAAAGCGCGCGTAGGCGGCTGATTAAGTCGGGTGTGAAAGCCCCGGGCTCAACCTGGGAACTGCATTCGATACTGATCAGCTAGAGTATGAGAGAGGGAGGTAGAATTCCACGTGTAGCGGTGAAATGCGTAGATATGTGGAGGAATACCGGTGGCGAAGGCGGCCTCCTGGTTCAATACTGACGCTGAGGTGCGAAAGCGTGGGGAGCAAACAGG` |
| ASV4101 | 423 | `TGAGGAATATTGGTCAATGGGCGGGAGCCTGAACCAGCCAAGCCGCGTGGAGGAAGGAGGTACAGAGTATCGTAAACTCCTTTTGTCAGGGGATAAAAGGTCCCACGCGTGGGACTCTGAAGGTACCTGAAGAAAAAGCATCGGCTAACTCCGTGCCAGCAGCCGCGGTAATACGGAGGATGCGAGCGTTATCCGGATTTATTGGGTTTAAAGGGTGCGTAGGCCGTTCCTTAAGTCAGCGGTGAAAGCCCGGGGCTCAACCCCGGCGAGCCGTTGAAACTGGGGAGCTGGAGATGAAGAAAGGTATGCGGAATTCGCGGTGTAGCGGTGAAATGCTTAGATATCGCGAAGAACTCCGATTGCGAAGGCAGCATACCGGCTTCTTTCTGACGCTGAGGCACGAAAGCGTGGGGATCGAACAGG` |
| ASV1570 | 388 | `TCGGGAAAATTTTGCAATACACGCAAGTGTGACAGAGCAAGCCGAAGTGCTGTTCTTTTTTGAACAGCTTTTTTGAGATGTAAAAAGTCTCTAGAATAAGGACCGGGCAAGACTGGTGCCAGCCGCCGCGGTAATCCCAGCGGTCCAAGTCGCAGCCACATTTGTTGGGTCTAAAACATCCGTAGCTTGCTTTTCAAGTCTTTTGTGAAATCGGGACTCTTAAGGTTCCGGCGTGCAAAAGATACTGTTAAGCTAGAGACCGGGAGGCGTAAGAAGTACGTATAGGGTAGCGGTAAAATGCGTTGATCCTATGTGGACTCACAGTCGCGAAGGCGTCTTACGAGAACGGTTCTGACAGTGAGGGATGAAGGCTAGGGGCGCAAAACGG` |
| ASV1496 | 429 | `TGGGGAATTTTGGACAATGGGCGCAAGCCTGATCCAGCCATTCCGCGTGAGTGAAGAAGGCCTTCGGGTTGTAAAGCTCTTTCGGCAGGGAAGAAACGGTTTGGGCGAATACCCCAAGCTAATGACGGTACCTGAAGAAGAAGCACCGGCTAACTACGTGCCAGCAGCCGCGGTAATACGTAGGGTGCGAGCGTTAATCGGAATTACTGGGCGTAAAGCGTGCGCAGGCGGTTTTGTAAGACAGATGTGAAATCCCCGGGCTTAACCTGGGAACTGCGTTTGTGACTGCAAGACTCGAGTACGGCAGAGGGGGGTGGAATTCCACGTGTAGCAGTGAAATGCGTAGAGATGTGGAGGAACACCGATGGCGAAGGCAGCCCCCTGGGTCGATACTGACGCTCATGCACGAAAGCGTGGGGAGCAAACAGG` |
| ASV4435 | 430 | `TGGGGGATCTTGCGCAATGGGCGAAAGCCTGACGCAGCGACGCCGCGTGGGGGATGACGGCCTTCGGGTTGTAAACCCCTGTCGGGTGGAACGAACAAGCCCGGTGCGAATACCACCGGGCCCTGACGGTACCACCGAAGGAAGCCCCGGCTAACTACGTGCCAGCAGCCGCGGTAATACGTAGGGGGCGAGCGTTGTTCGGATTTACTGGGCGTAAAGAGCGCGTAGGCGGCCGGATAAGTCCGTTGTGAAATCCCCCGGCTCAACCGGGGACCCGCTTCGGATACTGTCCGGCTTGAGTCGCGGAGAGGAGAGCGGAATTCCCAGTGTAGCGGTGAAATGCGTAGATATTGGGAGGAACACCGGTGGCGAAGGCGGCTCTCTGGACGCGTACTGACGCTGAGGCGCGAAAGCTAGGGGAGCAAACGGG` |
| ASV5459 | 404 | `TAGGGAATCTTGGACAATGGGCGCAAGCCTGATCCAGCCATGCCGCGTGAGTGACGAAGGCCTTAGGGTTGTAAAACTCTTTCGCACGTGACGATAATGACGGTAACGTGAGAAGAAGCTCCGGCTAACTTCGTGCCAGCAGCCGCGGTAATACGAAGGGGGCTAGCGTTGTTCGGATTTACTGGGCGTAAAGCGCGTGTAGGCGGGCCGTTAAGATGGGGGTGAAAGCCCGGAGCTCAACTCCGGAACTGCCTTCATGACTGGCGGCCTGGACTCAAGGAGAGGCAAGTGGAATTGCGAGTGTAGAGGTGAAATTCGTAGATATTCGCAGGAACACCAGTGGCGAAGGCGACTTGCTGGACTTGTAGTGACGCTGAGACGCGAAAGCGTGGGGAGCAAACAGG` |
| ASV2468 | 404 | `TGAGGAATCTTCCACAATGGGCGAAAGCCTGATGGAGCGACACCGCGTGAAGGATGAAGCCTTTGTTGGTGTAAACTTCTTTTCTCTTGGAAGATAATGACGGTACAAGAGGAATAAGGGGCGGCAAACTTCGTGCCAGCAGCCGCGGTAATACGAAGGCCCCAAGCGTTATCCGGAATTACTGGGCGTAAAGCGTCTGTAGGTGGTCTGGAAAGTCTCAAGTGAAAGGTCAGGGCTCAACCCTGTACTTGCTTGGGAAACTATCAGACTAGAGTGCGGGAGAGGCAAGCAGAACTGTATGAGTAGGGGTGCAATCCGTTGATACATACAAGAATACCAAAAGCGAAGGCAGCTTGCTGGAACGCCACTGACACTGAGAGACGAAAGCGTGGGGAGCAAAAGGG` |
| ASV5529 | 430 | `TGGGGAATCTTGCGCAATGGGCGAAAGCCTGACGCAGCAATGCCGCGTGAGCGAGGAAGGTCTTCGGATTGTAAAGCTCTGTCAGAATGGAAAAATGGTAGATGGTCTAATAGGCCATTTAATTGATGGTACATTCAGAGGAAGCACCGGCTAACTTCGTGCCAGCAGCCGCGGTAATACGAAGGGTGCAAGCGTTGTTCGGATTTATTGGGCGTAAAGCGCGCGCAGGCGGATCTTTAAGTCAGATGTGAAATCTCGGGGCTCAACCCCGAAACTGCGTCTGAAACTGGGGATCTAGAATGGCGTAGGGGGAAGGGGAATTTCACATGTAGGGGTAAAATCCGTAGAGATGTGAAGGAACACCAGAGGCGAAGGCGCCTTCCTGGACGCATATTGACGCTGAGGCGCGAAAGCGTGGGGAGCAAACAGG` |
| ASV750 | 424 | `TAGGGAATATTGGGCAATGGATGCAAGTCTGACCCAGCCATGCCGCGTGCAGGAAGAAGGTCCTCTGGATTGTAAACTGCTTTTGTCGGGGAAGAATAGGTCTCTTGCGAGGGATTGTGACGGTACCCGATGAATAAGCCACGGCTAACTACGTGCCAGCAGCCGCGGTAATACGTAGGTGGCAAGCGTTGTCCGGATTTATTGGGTTTAAAGGGTGCGTAGGTGGTTTGCTAAGTCAGTGGTGAAAGCTGGTTGCTCAACAATCAAGTTGCCATTGATACTGTCAGACTTGAGAGAAGTGGAGGCTCATGGAATGGATGGTGTAGCGGTGAAATGCATAGATATCATCCAGAACGTCAATTGCGAAGGCAGTGGGCTGTACTTTTTCTGACACTGAGGCACGAAAGCGTGGGGATCAAACAGG` |
| ASV1089 | 408 | `TGGGGAATTTTCCGCAATGGGCGAAAGCCTGACGGAGCAATACCGCGTGAGGGATGACGGCCTATGGGTTGTAAACCTCTTTTTTCAGGGAGGAATAAAATGACGTGTACCTGAAGAATAAGCATCGGCTAACTCCGTGCCAGCAGCCGCGGTAAGACGGAGGATGCAAGTGTTATCCGGAATCACTGGGCGTAAAGCGTCTGTAGGTGGTCCAATAAGTCAACTGTTAAATCTTGAGGCTCAACCTCAAAATCGCAGTCGAAACTATTAGACTAGAGTATAGTAGGGGTAAAGGGAATTTCCAGTGGAGCGGTGAAATGCGTAGATATTGGAAAGAACACCGATGGCGAAAGCACTTTACTGGGCTATTACTAACACTCAGAGACGAAAGCTAGGGTAGCAAATGGG` |
| ASV2472 | 429 | `TAGGGAATATTGCGCAATGGGGGAAACCCTGACGCAGCAACGCCGCGTGAGTGAGGAAGGTCTTCGGATTGTAAAACTCTGTTCTTAGGGAAAAATGGTCATGGAGCTAATACCCCATGATTTGATGGTACCTAAGTAGAAAGCACCGGCTAACTTCGTGCCAGCAGCCGCGGTAATACGAAGGGTGCAAGCGTTGTTCGGAATCATTGGGCGTAAAGCGCGCGCAGGCGGATCAGCAAGTCAGATGTGAAATCCCGGGGCTCAACCTCGGAACTGCGTCTGAAACTGCTAGTCTAGAATGTCGGAGGGGGCAGGGGAATTTCACGTGTAGGGGTAAAATCCGTAGAGATGTGAAGGAACACCGGGGGCGAAGGCGCCTGCCTGGACGACTATTGACGCTGAGGCGCGAAAGCGTGGGGAGCAAACAGG` |
| ASV3721 | 429 | `TGGGGAATTTTGGACAATGGGCGCAAGCCTGATCCAGCCATTCCGCGTGAGTGAAGAAGGCCTTCGGGTTGTAAAGCTCTTTCGGCAGGGAAGAAACGGTTGTGGTTAATACCCATGGCTAATGACGGTACCTGAAGAAGAAGCACCGGCTAACTACGTGCCAGCAGCCGCGGTAATACGTAGGGTGCGAGCGTTAATCGGAATTACTGGGCGTAAAGCGTGCGCAGGCGGCTCTATAAGACAGATGTGAAATCCCCGGGCTTAACCTGGGAACTGCGTTTGTGACTGTAGGGCTCGAGTGCGGCAGAGGGGGGTGGAATTCCACGTGTAGCAGTGAAATGCGTAGAGATGTGGAGGAACACCGATGGCGAAGGCAGCCCCCTGGGTCGACACTGACGCTCATGCACGAAAGCGTGGGGAGCAAACAGG` |
| ASV3252 | 429 | `TGGGGAATATTGCACAATGGGGGAAACCCTGATGCAGCCATGCCGCGTGTGTGAAGAAGGCCTTCGGGTTGTAAAGCACTTTCAGTAGGGAGGAAAGGGTGAGTCTTAATACGGCTCATCTGTGACGTTACCTACAGAAGAAGGACCGGCTAACTCCGTGCCAGCAGCCGCGGTAATACGGAGGGTCCGAGCGTTAATCGGAATTACTGGGCGTAAAGCGTGCGCAGGCGGTTTGTTAAGCGAGATGTGAAAGCCCTGGGCTCAACCTAGGAATCGCATTTCGAACTGGCGAACTAGAGTCTTGTAGAGGGGGGTAGAATTCCAGGTGTAGCGGTGAAATGCGTAGAGATCTGGAGGAATACCGGTGGCGAAGGCGGCCCCCTGGACAAAGACTGACGCTCATGCACGAAAGCGTGGGGAGCAAACAGG` |
| ASV1290 | 404 | `TGGGGAATCTTAGACAATGGGCGCAAGCCTGATCTAGCCATGCCGCGTGAGTGATGAAGGCCTTAGGGTCGTAAAGCTCTTTCGCTGGGGAAGATAATGACGGTACCCAGTAAAGAAACCCCGGCTAACTCCGTGCCAGCAGCCGCGGTAATACGGAGGGGGTTAGCGTTGTTCGGAATTACTGGGCGTAAAGCGCGCGTAGGCGGATCGGAAAGTTGGGGGTGAAATCCCGGGGCTCAACCCCGGAATTGCCTTCAAAACTCCTGGTCTTGAGTTCGAGAGAGGTGAGTGGAATTCCGAGTGTAGAGGTGAAATTCGTAGATATTCGGAGGAACACCAGTGGCGAAGGCGGCTCACTGGCTCGATACTGACGCTGAGGTGCGAAAGCGTGGGGAGCAAACAGG` |
| ASV513 | 407 | `TGGGGAATTTTCCGCAATGGGCGAAAGCCTGACGGAGCAATCTCGCGTGAGGGATGACTGCCTATGGGTTGTAAACCTCTTTTTTCAGGGAGGAATCAATGACGTGTACCTGAAGAATAAGCATCGGCTAACTCCGTGCCAGCAGCCGCGGTAAGACGGAGGATGCAAGTGTTATCCGGAATCACTGGGCGTAAAGCGTCTGTAGGTGGTTTGATAAGTCAACTGTTAAATCTTGAAGCTCAACTTCAAAATCGCAGTCGAAACTATCAGACTAGAGTATAGTAGGGGTAAAGGGAATTTCCAGTGGAGCGGTGAAATGCGTAGAGATTGGAAAGAACACCGATGGCGAAGGCACTTTACTGGGCTATTACTGACACTCAGAGACGAAAGCTAGGGTAGCAAATGGG` |
| ASV4654 | 429 | `TGGGGAATATTGGACAATGGGGGAAACCCTGATCCAGCAATGCCGCGTGTGTGAAGAAGGCCTGCGGGTTGTAAAGCACTTTCAGTGGGGAGGAAAGGTCGTAGTTGAATAGGCTACGAAGAAGACGATACCCACAGAAGAAGCACCGGCTAACTCTGTGCCAGCAGCCGCGGTAATACAGAGGGTGCAAGCGTTAATCGGAATTACTGGGCGTAAAGCGCACGTAGGTGGATAATTAAGTTAGATGTGAAAGCCCTGGGCTCAACCTGGGAACGGCATTTAATACTGGTTGACTAGAGTATAGTAGAGGGAAGTGGAATTTCCGGTGTAGCGGTGAAATGCGTAGATATCGGAAGGAACATCAGTGGCGAAGGCGGCTTCCTGGACTAATACTGACACTGAGGTGCGAAAGCGTGGGTAGCAAACAGG` |
| ASV4171 | 428 | `TGGGGAATCTTGCGCAATGGGCGAAAGCCTGACGCAGCGACGCCGCGTGGGTGATGAAGGCCTTCGGGTCGTAAAGCTCTGTGGGGAGAGACGAATAAGGCACGGCGAATACCCGTGGTGATGACGGTATCTCCTTAGCAAGCACCGGCTAACTCTGTGCCAGCAGCCGCGGTAAGACAGAGGGTGCAAACGTTGTTCGGAATTACTGGGCGTAAAGCGTGTGTAGGCGGCCATGTAAGTTGGATGTGAAAGCCCGGGGCTCAACCTCGGAAGTGCATTCAATACTGCGTGGCTTGAGTATCGGAGAGGTTGGTGGAATTCTCGGTGTAGAGGTGAAATTCGTAGATATCGAGAGGAACACCGGTGGCGAAGGCGGCCAACTGGACGAATACTGACGCTGAGACACGAAAGCGTGGGGAGCAAACAGG` |
| ASV5647 | 428 | `TGGGGAATCTTGGTCAATGGGCGAAAGCCTGAACCAGCGACGCCGCGTGAGTGATGAAGGCCTTCGGGTTGTAAAGCTCTGTGGGGCGAGACGAAAGCCTACGTTCTAACAGGACGTAGTTTGACGGTATCGCCTTAGCAAGCACCGGCTAACTCTGTGCCAGCAGCCGCGGTAAGACAGAGGGTGCAAACGTTGCTCGGAATCACTGGGCGTAAAGGGCGTGTAGGCGGGAGAGAAAGTCGGGCGTGAAATCCCTGGGCTCAACCCAGGAACTGCACTCGAAACTTCTTTTCTTGAGTGCCGGAGAGGAAAGCGGAATACCTGGTGTAGAGGTGAAATTCGTAGATATCAGGTGGAACACCAGTGGCGAAGGCGGCTTTCTGGACGGTAACTGACGCTGAGACGCGAAAGCGTGGGGAGCAAACAGG` |
| ASV1071 | 429 | `TGGGGAATTTTGGACAATGGGCGAAAGCCTGATCCAGCAATGCCGCGTGCAGGAAGAAGGCCTTCGGGTTGTAAACTGCTTTTGTACGGAACGAAACGGCTTTTTCTAATAAAGAAGGCTAATGACGGTACCGTAAGAATAAGCACCGGCTAACTACGTGCCAGCAGCCGCGGTAATACGTAGGGTGCGAGCGTTAATCGGAATTACTGGGCGTAAAGCGTGCGCAGGCGGTTATGCAAGACAGTTGTGAAATCCCCGGGCTTAACCTGGGAATTGCATCTGTGACTGCATAGCTAGAGTACGGTAGAGGGGGATGGAATTCCGCGTGTAGCAGTGAAATGCGTAGATATGCGGAGGAACACCGATGGCGAAGGCAATCCCCTGGACCTGTACTGACGCTCATGCACGAAAGCGTGGGGAGCAAACAGG` |
| ASV608 | 406 | `TAGGGAATATTGCGCAATGGGGGAAACCCTGACGCAGCGACGCCGCGTGAGTGATGAAGGCCTTCGGGTCGTAAAGCTCTGTTGCAAGGGAAAAATTAATGATTGTACCTTGCGAGAAAGGATCGGCTAACTTCGTGCCAGCAGCCGCGGTAAGACGAGGGATCCTAGCGTTGTTCGGAATTATTGGGCGTAAAGCGGGTGTAGGTGGCTTTATAAGTCAGATGTGAAAGCCCAGGGCTTAACCCTGGAAGGGCATTTGATACTATTTAGCTTGAGTGTTGGAGAGGTTACTAGAATTCCTGGTGTAGTGGTGAAATACGTAGATATCAGGAGGAATACCGGAGGCGAAGGCGGGTAACTGGCCAAACACTGACACTGAGACCCGAAAGCGTGGGGATCAAACAGG` |
| ASV3338 | 409 | `TGGGGAATATTGCACAATGGGCGCAAGCCTGATGCAGCAACGCCGCGTGAGGGATGACGGCCTTCGGGTTGTAAACCTCTTTTAGTAGGGAAGAAGCGAAAGTGACGGTACCTGCAGAAAAAGCACCGGCTAACTACGTGCCAGCAGCCGCGGTAATACGTAGGGTGCAAGCGTTGTCCGGAATTATTGGGCGTAAAGAGCTCGTAGGCGGTTTGTCGCGTCTGCTGTGAAATCCCGAGGCTCAACCTCGGGCCTGCAGTGGGTACGGGCAAACTAGAGTGTGGTAGGGGAGATTGGAACTCCTGGTGTAGCGGTGGAATGCGCAGATATCAGGAAGAACACCGATGGCGAAGGCAGATCTCTGGGCCATTACTGACGCTGAGGAGCGAAAGCGTGGGGAGCGAACAGG` |
| ASV4795 | 429 | `TGGGGAATATTGCACAATGGGCGCAAGCCTGATGCAGCGACGCCGCGTGGAGGATGAAGGTTTTCGGATTGTAAACTCCTGTTAAGTGGGAAAAAAGACCTGTACCTAATACGTACGGGGGATGATGGTACCACTAGAGAAAGCACCGGCAAACTTCGTGCCAGCAGCCGCGGTAATACGAAGGGTGCGAGCGTTATTCGGAATCACTGGGTGTAAAGAGCGTGTAGGCGGTTTCTTAAGTCATTCGTTAAAGAGCTCGGCTTAACCGAGCATATGCGGGCGAAACTGGGAAACTAGAGTGCAGAAGAGAAGAGTGGAATTCTCGGAGTAGCGGTAAAATGCGTAGATCTCGAGAGGAACACCGGTTGCGAAGGCGGCTCTTTGGTCTGTAACTGACGCTAAAGTGCGAAAGCGTGGGGAGCAAACAGG` |
| ASV928 | 429 | `TGGGGAATATTGGACAATGGGCGAAAGCCTGATCCAGCCATGCCGCGTGTGTGAAGAAGGCCTTCGGGTTGTAAAGCACTTTAAGCGAGGAGGAATGGCTCTGGGTTAATACCCTTGAGCAGTGACGTTACTCGCAGAATAAGCACCGGCTAACTCTGTGCCAGCAGCCGCGGTAATACAGAGGGTGCAAGCGTTAATCGGAATTACTGGGCGTAAAGCGCGCGTAGGCGGTTACGTAAGTTGGATGTGAAATCCCCGGGCTCAACCTGGGAATTGCATTCAAAACTGCGTGGCTAGAGTGTGGGAGAGGAAGGTAGAATTCCAGGTGTAGCGGTGAAATGCGTAGATATCTGGAGGAATACCGATGGCGAAGGCAGCCTTCTGGCCTAACACTGACGCTGAGGTGCGAAAGCATGGGGAGCAAACAGG` |
| ASV2383 | 430 | `TGGGGAATTTTGGACAATGGGGGCAACCCTGATCCAGCCATGCCGCGTGCGGGAAGAAGGCCTTCGGGTTGTAAACCGCTTTAGTCCGGGAAAAATGTCAGCTAGGTTAATACCTTGGCTGGTTGATGGTACTGGAAGAATAAGCACCGGCTAACTACGTGCCAGCAGCCGCGGTAATACGTAGGGTGCAAGCGTTAATCGGAATTACTGGGCGTAAAGCGTGCGCAGGCGGTTTCATAAGACAGATGTGAAAGCCCCGGGCTTAACCTGGGAATGGCATTTGTGACTGTGAGGCTAGAGTGTGTCAGAGGGGGGTGGAATTCCGCGTGTAGCAGTGAAATGCGTAGATATGCGGAGGAACACCGATGGCGAAGGCAGCCCCCTGGGATAACACTGACGCTCATGCACGAAAGCGTGGGGAGCAAACAGG` |
| ASV326 | 425 | `TAGGGAATATTGGGCAATGGAGGCAACTCTGACCCAGCCATGCCGCGTGCAGGAAGACGGTCCTCTGGATTGTAAACTGCTTTTGAATGGGAAGAATGTGCTCCTTGCGAGGGGTAATGACGGTACCATTAGAATAAGCCACGGCTAACTACGTGCCAGCAGCCGCGGTAATACGTAGGTGGCGAGCGTTATCCGGATTTATTGGGTTTAAAGGGTGCGTAGGCGGCTTAATAAGTCGGTGGTTAAAAGTAGCAGCTTAACTGTTTTACATGCCATCGATACTGTTGAGCTTGAGATATTTGGAGGCAGCTAGAATTTCCGGTGTAGCGGTGAAATGCATAGATACCGGAAGGAATACCAATTGCGAAGGCAGGTTGCTACGAATAAACTGACGCTGATGCACGAAAGCGTGGGGATCAAACAGG` |
| ASV1247 | 424 | `TGAGGAATATTGGACAATGGGTTAGCGCCTGATCCAGCCATCCCGCGTGAAGGACGACTGCCCTATGGGTTGTAAACTTCTTTTGTACAGGGATAAACCTATCTACGTGTAGATAGCTGAAGGTACTGTACGAATAAGCACCGGCTAACTCCGTGCCAGCAGCCGCGGTAATACGGAGGGTGCAAGCGTTATCCGGATTTATTGGGTTTAAAGGGTCCGTAGGCGGGCTCGTAAGTCAGTGGTGAAATCTCATAGCTTAACTATGAAACTGCCATTGATACTGCGGGTCTTGAGTAAAGTAGAAGTGGCTGGAATAAGTAGTGTAGCGGTGAAATGCATAGATATTACTTAGAACACCAATTGCGAAGGCAGGTCACTATGTTTTAACTGACGCTGATGGACGAAAGCGTGGGGAGCGAACAGG` |
| ASV3593 | 423 | `TAGGGAATATTGGTCAATGGGCGAGAGCCTGAACCAGCCATGCCGCGTGCAGGAAGAAGGCCCTCTGGGTTGTAAACTGCTTTTGACCAGGGCCAAAATACTCATGCGTGAGGAATTGAGAGTACTGGTAGAATAAGCACCGGCTAACTCCGTGCCAGCAGCCGCGGTAATACGGAGGGTGCAAGCGTTGTCCGGATTTATTGGGTTTAAAGGGTGCGTAGGTGGCTTTTTAAGTCAGTGGTGAAAGCCTGCAGCTTAACTGTAGGACTGCCATTGATACTGAAGAGCTTGAGTACTGATGAGGTAGGCGGAATTGATAGTGTAGCGGTGAAATGCATAGATATTATCAAGAACCCCGATTGCGAAGGCAGCTTACTAAGGAGTTACTGACACTGAGGCACGAAAGCGTGGGGATCAAACAGG` |
| ASV3712 | 429 | `TGGGGAATTTTGGACAATGGGGGCAACCCTGATCCAGCCATGCCGCGTGCGGGAAGAAGGCCTTCGGGTTGTAAACCGCTTTTGTCAGGGAAGAAACGCGCTGAGCTAATACCTCGGCGTAATGACGGTACCTGAAGAATAAGCACCGGCTAACTACGTGCCAGCAGCCGCGGTAATACGTAGGGTGCAAGCGTTAATCGGAATTACTGGGCGTAAAGCGTGCGCAGGCGGCTTTGCAAGACAGATGTGAAATCCCCGGGCTCAACCTGGGAACTGCATTTGTGACTGCAAAGCTAGAGTACGGCAGAGGGGGATGGAATTCCGCGTGTAGCAGTGAAATGCGTAGATATGCGGAGGAACACCGATGGCGAAGGCAGTCCCCTGGGCCTGTACTGACGCTCATGCACGAAAGCGTGGGGAGCAAACAGG` |
| ASV3806 | 407 | `TAGGGAATATTGCGCAATGGAGGAAACTCTGACGCAGCGACGCCGCGTGAGTGATGAAGGCCTTCGGGTTGTAAAGCTCTTTTGCAAGGGAATAAGAAAGTGAATGTACTTTGCGAATAAGGATCGGCTAACTTCGTGCCAGCAGCCGCGGTAAGACGAGGGATCCTAGCGTTGTTCGGAATTATTGGGCGTAAAGCGGGTGCAGGTGGCTCGTTAAGTCAGGTGTGAAATCCCAGGGCTCAACCCTGGACGTGCATTTGATACTGACTAGCTTGAGTGTGGGAGAGGCTACTAGAATTCTTGGTGTAGTGGTGAAATACGTAGATATCAAGAGGAATACCGGTGGCGAAGGCGGGTAGCTGGCCCAACACTGACACTCAGACCCGAAAGCGTGGGGATCAAACAGG` |
| ASV5084 | 406 | `TGAGGAATCTTCCGCAATGGGCGAAAGCCTGACGGAGCAACACCGCGTGAAGGATGAAGGGCCTCGGTCCGTAAACTTCTTTTCTGAGGGAAGAATTCTGACGGTACCTTAGGAATAAGCACCGGCTAACTCTGTGCCAGCAGCCGCGGTAAGACAGAGGGTGCGAGCGTTATCCGGAATCATTGGGCGTAAAGCGTATGTAGGCGGAGACGCAAGTCGGGGGTTAAATCTCCAGGCTCAACCTGGAATCTGTTCCCGATACTGCGTTTCTTTGAAGCATTCAGAAGCATGTGGAATTCTGTGTGTAGGGGTAAAATCCGTAGATACACGGAGGAATGCCAAAAGCGAAAGCAGCATGCTAGGAATGTCTTGACGCTGAGATACGAAAGCGTGGGGATCAAACAGG` |
| ASV977 | 429 | `TGGGGAATTTTGGACAATGGGCGCAAGCCTGATCCAGCCATGCCGCGTGCGGGAAGAAGGCCTTCGGGTTGTAAACCGCTTTTGTCAGGGAAGAAATCCCTTGGGATAATACCTCGGGGGGATGACGGTACCTGAAGAATAAGCACCGGCTAACTACGTGCCAGCAGCCGCGGTAATACGTAGGGTGCAAGCGTTAATCGGAATTACTGGGCGTAAAGCGTGCGCAGGCGGTTGTGCAAGACAGATGTGAAATCCCCGGGCTCAACCTGGGAACTGCATTTGTGACTGCACGGCTAGAGTACGGTAGAGGGGGATGGAATTCCGCGTGTAGCAGTGAAATGCGTAGATATGCGGAGGAACACCGATGGCGAAGGCAATCCCCTGGACCTGTACTGACGCTCATGCACGAAAGCGTGGGGAGCAAACAGG` |
| ASV544 | 424 | `TAGGGAATATTGGGCAATGGCCGCAAGGCTGACCCAGCCATGCCGCGTGCAGGAAGAAGGCCCTCCGGGTCGTAAACTGCTTTTGCGGGGGAAGAGCACCCGCGCCGAGGCGCGGGCCGACGGTACCCCGCGAATAAGCCACGGCTAACTACGTGCCAGCAGCCGCGGTAATACGTAGGTGGCGAGCGTTGTCCGGATTTATTGGGTTTAAAGGGTGCGCAGGCGGGCCGCCGAGTCAGGGGTGAAAGACGGTGGCTCAACCATCGCAGTGCCCCTGATACTGGCGGCCTTGAGTGCGGACGAGGCCTGCGGAATGGGCGGTGTAGCGGTGAAATGCTCAGATACCGCCCGGAACACCGATTGCGAAGGCAGCAGGCCAGGGCGCCACTGACGCTGAGGCACGAAAGTGCGGGGATCAAACAGG` |
| ASV3407 | 429 | `TCGAGAATAATTCACAATGGGGGCAACCCTGATGGTGCAACGCCGCGTGGAGGATGAAGGTCCTCGGATTGTAAACTCCTGTCATCCGGGAGTAAGACCTGGCCGTTAATAGCGGACAGGGTTGATAGTACCGGAAGAGGAAGGGACGGCTAACTTCGTGCCAGCAGCCGCGGTAATACGAAGGTCCCGAGCGTTGTTCGGAATCACTGGGCGTAAAGGGAGCGTAGGCGGCGTGGCAAGTCAGACGTGAAATCCCGGGGCTCAACCCCGGAACCGCGTCCGATACTGCCATGCTCGAGCATTGGAGAGGCAGCTGGAATTCTTGGTGTAGCAGTGAAATGCGTGGATATCAAGAGGAACACTCGTGGCGAAAGCGAGCTGCTGGACAATTGCTGACGCTGAGGCTCGAAGGCCGGGGGAGCGAAAGGG` |
| ASV1764 | 404 | `TGAGGAATCTTCCGCAATGGGCGAAAGCCTGACGGAGCGACACCGCGTGGAGGATGACGCGTTTACTCGTGTAAACTCCTTTTCTCAGGGAAGATAATGACGGTACCTGAGGAATAAGGGGCGGCAAACTACGTGCCAGCAGCCGCGGTAATACGTAGGCCCCAAGCGTTATCCGGAATTACTGGGTGTAAAGCGTCTGTAGGCGGGAAAACAAGTCTTCCATGAAAGACCGGAGCTCAACTCCGTGTTTGTGGGAGATACTATTTTTCTAGAATCGGGGAGAGGGAAGCGGAATGGTATGAGTAGGGGTGCAATCCGTTGATACATATCAGAACACCAAAAGCGAAGGCAGCTTCCTGGAACCGCATTGACGCTGAGAGACGAAAGCGTGGGGAGCGAAAAGG` |
| ASV1138 | 429 | `TGGGGAATTTTGGACAATGGGGGCAACCCTGATCCAGCAATGCCGCGTGAGTGAAGAAGGCCTTCGGGTTGTAAAGCTCTTTTGTCAGGGAAGAAACGGGGTTGGCTAATATCTGATCCTAATGACGGTACCTGAAGAATAAGCACCGGCTAACTACGTGCCAGCAGCCGCGGTAATACGTAGGGTGCAAGCGTTAATCGGAATTACTGGGCGTAAAGCGTGCGCAGGCGGTTTTGTAAGACTGTCGTGAAATCCCCGGGCTCAACCTGGGAATGGCGATGGTGACTGCAAGGCTAGAGTTTGGCAGAGGGGGGTAGAATTCCACGTGTAGCAGTGAAATGCGTAGATATGTGGAGGAACACCGATGGCGAAGGCAGCCCCCTGGGTCAAAACTGACGCTCATGCACGAAAGCGTGGGGAGCAAACAGG` |
| ASV1265 | 424 | `TGAGGAATATTGGTCAATGGGCGCAAGCCTGAACCAGCCATGCCGCGTGCAGGATGACGGTCCTATGGATTGTAAACTGCTTTTGTACAGGAAGAAACACTCCCTCGTGAGGGAGCTTGACGGTACTGTAAGAATAAGGATCGGCTAACTCCGTGCCAGCAGCCGCGGTAATACGGAGGATCCAAGCGTTATCCGGAATCATTGGGTTTAAAGGGTCCGTAGGCGGTTTAATAAGTCAGTGGTGAAATCTGGTCGCTCAACGATCAAACGGCCATTGATACTGTTGGACTTGAATTACTTGGAAGTAACTAGAATATGTAGTGTAGCGGTGAAATGCTTAGAGATTACATGGAATACCAATTGCGAAGGCAGGTTACTACAAGTATATTGACGCTGATGGACGAAAGCGTGGGGAGCGAACAGG` |
| ASV2351 | 407 | `TTAGGAATTTTGCGCAATGGGGGAAACCCTGACGCAGCAACGCCGCGTGGAGGATGACGGCCTTCGGGTTGTAAACTCCTTTTAGCAGGGAAGAAATAAATGACGGTATCTGCAGAAAAAGCACCGGCTAACTCTGTGCCAGCAGCCGCGGTAATACAGAGGGTGCAAGCGTTATTCGGAATCACTGGGCGTAAAGCGCTCGTAGGCTGTTAATTAAGTCAGATGTGAAATCCCTGGGCTCAATCCAGGACGTGCATCTGATACTGATTGACTAGAGTGCAGGAGAGGAAAGCGGAATTCCAGGTGTAGAGGTGAAATTCGTAGATATCTGGAGGAACAACAGAGGCGAAGGCGGCTTTCTGGACTGTCACTGACGCTGAGGAGCGAAAGCGTGGGGAGCAAACAGG` |
| ASV3273 | 429 | `TGGGGAATATTGGACAATGGGCGAAAGCCTGATCCAGCGACGCCGCGTGGGTGAAGAAGGCCTGCGGGTTGTAAAGCCCTTTCGGTGGGGAAGAAAAGCCTCGACCTAACACGTCGGGGTATTGACGTAACCCATTAAAGAAGCACCGGCTAACTCTGTGCCAGCAGCCGCGGTAATACAGAGGGTGCGAGCGTTAATCGGAATTACTGGGCGTAAAGCGCGCGTAGGCGGTTTTGTAAGTCGGGTGTGAAATCCCCAAGCTCAACTTGGGAACTGCATTCGAGACTGCATTGCTAGAGTATGGGAGAGGGAAGTGGAATTTCCGGTGTAGCGGTGAAATGCGTAGATATCGGAAGGAACATCAGTGGCGAAAGCGACTTCCTGGACCAATACTGACGCTCAAGTGCGAAAGCGTGGGGAGCAAACAGG` |
| ASV1974 | 404 | `TGGGGAATATTGGACAATGGGGGCAACCCTGATCCAGCAATGCCGCGTGGGTGAAGAAGGTCTTCGGATTGTAAAGCCCTTTCGACGGGGACGATGATGACGGTACCCGTAGAAGAAGCCCCGGCTAACTTCGTGCCAGCAGCCGCGGTAATACGAAGGGGGCTAGCGTTGTTCGGAATGACTGGGCGTAAAGGGCGCGTAGGCGGCTTTGTAAGTCAGGCGTGAAATTCCTGGGCTTAACCTGGGGGCTGCGTCTGAGACTGCGGAGCTAGAGGCCGAGAGAGGGTGGTGGAATTCCCAGTGTAGAGGTGAAATTCGTAGATATTGGGAAGAACACCGGTGGCGAAGGCGGCCACCTGGCTCGGAACTGACGCTGAGGCGCGATAGCGTGGGGAGCAAACAGG` |
| ASV962 | 404 | `TGGGGAATCTTGGACAATGGGCGCAAGCCTGATCCAGCCATGCCGCGTGAGTGAAGAAGGCCTTCGGGTTGTAAAGCTCTTTTGTCAGGGACGATAATGACGGTACCTGAAGAATAAGCCCCGGCAAACTTCGTGCCAGCAGCCGCGGTAATACGAAGGGGGCTAGCGTTGTTCGGAATTACTGGGCGTAAAGCGCACGTAGGCTGACATTTAAGTCAGGGGTGAAATCCCAAGGCTCAACCTTGGAACTGCCTTTGATACTGGGTGTCTCGAGTCCGGAAGAGGTTAGTGGAATTCCCAGTGTAGAGGTGAAATTCGTAGATATTGGGAAGAACACCAGTGGCGAAGGCGGCTAACTGGTCCGGTACTGACGCTGAGGTGCGAAAGCGTGGGGAGCAAACAGG` |
| ASV1406 | 429 | `TGGGGAATATTGGACAATGGGCGCAAGCCTGATCCAGCAATGCCGCGTGTGTGAAGAAGGCCTTAGGGTTGTAAAGCACTTTCAGTTGGGAAGAATGCCTTATGGCTAATATCCATGAGGCTTGACGCTACCAACAGAAGAAGCACCGGCTAACTCTGTGCCAGCAGCCGCGGTAATACAGAGGGTGCGAGCGTTAATCGGAATTACTGGGCGTAAAGAGTGCGTAGGCGGTTATATAAGTTAGATGTGAAAGCCCCAGGCTTAACCTGGGAACTGCGTTTAATACTGTGTAGCTAGAGTACTGAAGAGGTTAGTGGAATTTCCAGTGTAGCGGTGAAATGCGTAGATATTGGAAGGAACACCAGTGGCGAAGGCGACTAACTGGGCAGATACTGACGCTGAGGCACGAAAGCGTGGGGAGCAAACAGG` |
| ASV2058 | 429 | `TGGGGAATATTGGACAATGGGGGCAACCCTGATCCAGCCATGCCGCGTGTGTGAAGAAGGCCTTCGGGTTGTAAAGCACTTTTAATAGAGAAGAAGTGATATTGGCTAATACCTGATATTATTGACGTTATTTATAGAAAAAGCACCGGCTAACTCCGTGCCAGCAGCCGCGGTAATACGGAGGGTGCTAGCGTTAATCGGAATTACTGGGCGTAAAGGGCATGTAGGCGGATGAGTAAGTTGGATGTGAAATCCCTGGGCTTAACCTAGGAACTGCATACAAAACTGCTTATCTAGAATGTAGTAGAGGATGATGGAATTTTCTGTGTAGTAGTAAAATGCTTAGAGATAGAAAGGAACACCAGAGGCGAAGGCGATCGTCTGGGCTACAATTGACGCTGAGGTGCGAAAGCGTGGGGAGCAAACAGG` |
| ASV1128 | 424 | `TGAGGAATATTGGACAATGGGCGCAAGCCTGATCCAGCCATGCCGCGTGCAGGAAGAAGGTCCTATGGATTGTAAACTGCTTTTATACAGGAAGAAACACTCCCTCGTGAGGGAACTTGACGGTACTGTATGAATAAGGACCGGCTAACTCCGTGCCAGCAGCCGCGGTAATACGGAGGGTCCAAGCGTTATCCGGAATCATTGGGTTTAAAGGGTCCGTAGGCGGCCAGATAAGTCAGTGGTGAAAGCCCATCGCTCAACGATGGAACGGCCATTGATACTGTTTGGCTTGAATTATTAGGAAGTAACTAGAATATGTAGTGTAGCGGTGAAATGCTTAGAGATTACATGGAATACCAATTGCGAAGGCAGGTTACTACTAATATATTGACGCTGATGGACGAAAGCGTGGGGAGCGAACAGG` |
| ASV1901 | 404 | `TCGGGAATTTTGGGCAATGGGCGAAAGCCTGACCCAGCAACGCCGCGTGAAGGATGAAGTATTTCGGTATGTAAACTTCGAAAGAATAGGAAGAATAAATGACGGTACTATTTATAAGGTCCGGCTAACTACGTGCCAGCAGCCGCGGTAATACGTAGGGACCAAGCGTTGTTCGGATTTACTGGGCGTAAAGGGCGCGTAGGCGGCTTGCCAAGTCACTTGTGAAATCTCTGGGCTTAACCCAGAACGGCCAAGTGAAACTGGCATGCTAGAGTGCAGAAGGGGCAATCGGAATTCTTGGTGTAGCGGTGAAATGCGTAGATATCAAGAGGAACACCTGAGGTGAAGACGGGTTGCTGGGCTGACACTGACGCTGAGGCGCGAAAGCCAGGGGAGCAAACGGG` |
| ASV1528 | 430 | `TGGGGAATCTTGGTCAATGGGCGAAAGCCTGAACCAGCGATGCCGCGTGAGTGATGAAGGCCTTCGGGTTGTAAAGCTCTGTGGGGAGAGAAGAAAACATTCTGGTCAAATAGGCCAGGATCTTGACGGTATCTCCTTAGCAAGCACCGGCTAACTCTGTGCCAGCAGCCGCGGTAATACAGAGGGTGCAAACGTTGCTCGGAATTACTGGGCGTAAAGCGCGTGTAGGCGGACTGATAAGTCAGATGTGAAATCCCTGGGCTCAACCTAGGAACTGCATTTGAAACTGTCTGTCTTGAGTATCGGAGAGGATTGCGGAATTCCTGGTGTAGAGGTGAAATTCGTAGATATCAGGAGGAACACCGGTGGCGAAGGCGGCAATCTGGACGATTACTGACGCTGAGACGCGAAAGCGTGGGGAGCAAACAGG` |
| ASV5495 | 404 | `TGGGGAATAATGGACAATGCGCGCAAGCGTGATCCTGCTACGCCGCGTGAGTGAAGAAGGCTTTAGAGTTGTAAAACTCTTTTAGTGGGAAAGATAATGACGGTACCCACAGAAAAAGTCCCGGCTAACTCCGTGCCAGCTGCCGCGGTGACACGGAGGGGGCAAGCGTTACTCGGCATTACTGGGCGTAAAGAGTGTGTAGGCGGTTTGGTAAGTTGAAAGTGAAATCCCAGGGCTCAACCTTGGAACTGCTTTCAAAACTGCTAGACTAGAGTTGGATAGAGGGTGATGGAATTTCTAGTGTAAAGGTGGAATTTGTAGATATTAGAAGGAACACCAGAGGCGAAGGCGATCTCCTGGGTCTATACTGACGCTGAGACACGAAGGCATGGGGAGCAAACAGG` |
| ASV4583 | 429 | `TGGGGAATCTTGCGCAATGGACGGAAGTCTGACGCAGCCACGCCGTGTGAGTGAAGAAGGCCTTCGGGTTGTAAAGCTCTGTTGAACGGGACGAAGGGAGTGGGGTGAATAGCCTAGCTTTTTGACGGTACCGTTAGAGGAAGCACCGGCTAACCTCGTGCCAGCAGCCGCGGTAATACGAGGGGTGCAAGCGTTGTTCGGAATTACTGGGCGTAAAGGGCAAGTAGGTGGTCTCGTTTGTCTAGGGTGAAATCCTCGGGCTTAACCCGAGAAGTGCCCTGGAAACGGCGAGACTAGAGTTCTGGAGAGGGTCGTGGAATTCCCGGTGTAGCGGTGAAATGCGTAGAGATCGGGAGGAACACCAGAGGCGAAAGCGGCGACCTAGACAGATACTGACACTCAATTGCGAAAGCGTGGGGAGCAAACAGG` |
| ASV3121 | 421 | `TGGGGAATCTTGCGCAATGGCCGAGAGGCTGACGCAGCGACGCCGCGTGGGGGATGAAGCATTTCGGTGTGTAAACCCCTGTTGCTCGGGACGAACGACGTCTTTCGAGGCGTGTGACGGTACCGAGTGAGGAAGCACCGGCTAACTCCGTGCCAGCAGCCGCGGTAATACGGAGGGTGCGAGCGTTGTCCGGAATCACTGGGCGTAAAGGGCGCGTAGGTGGTTGGATGCGCCGGTGGTGAAAGCGCAGGGCTCAACCCTGCGTCGGCCATCGGGACGGTCCGACTGGAGCACTGTAGAGGCAGGTGGAATTCCGGGTGTAGCGGTGGAATGCGTAGAGATCCGGAAGAACACCGGTGGCGAAGGCGGCCTGCTGGGCAGTTGCTGACACTGAGGCGCGACAGCGTGGGGAGCAAACAGG` |
| ASV4343 | 424 | `TAGGGAATATTGGGCAATGGATGCAAGTCTGACCCAGCCATGCCGCGTGCCGGATGAAGGCCCTCAGGGTTGTAAACGGCTTTTATTCGGGAAGAAGAGCAGGGATGCGTCCTTGTGTGACGGTACCGAATGAATAAGCACCGGCTAACTCCGTGCCAGCAGCCGCGGTAATACGGAGGGTGCGAGCGTTGTCCGGATTTATTGGGTTTAAAGGGTGCGTAGGTGGCTTTATAAGTCAGTGGTGAAATACAGCCGCTCAACGGTTGAGGTGCCATTGATACTGTAGAGCTTGAAATAATTGGAGGCTGCCGGAATGGATGGTGTAGCGGTGAAATGCATAGATATCATCCAGAACACCGATTGCGAAGGCAGGTGGCTACGATTTATTTGACACTGAGGCACGAAAGCATGGGGAGCAAACAGG` |
| ASV3465 | 422 | `TAAGGAATATTGGTCAATGGGCGCAAGCCTGAACCAGCCATGCCGCGTGCAGGAAGAAGGTCCTCTGGATTGTAAACTGCTTTTGTCTGGGAATAATACTTGACTATACGTTAAGTTGAAGGTACCAGAAGAATAAGCCACGGCTAACTACGTGCCAGCAGCCGCGGTAATACGTAGGTGGCAAGCGTTGTCCGGATTTATTGGGTTTAAAGGGTGCGTAGGCGGTTTTATAAGTCAGTGGTCAAATCGTGTGGCTTAACCATACTTCGCCATTGATACTGTAAGACTTGAATATGTGCGAGGATAGTGGAATACGTAGTGTAGCGGTGAAATGCATAGATATTACGTAGAACACCGATTGCGAAGGCAGCTATCTGGTACATGATTGACGCTGAGGCACGAAAGCATGGGGAGCAAACAGG` |
| ASV4045 | 429 | `TGGGGAATATTGGACAATGGGCGGAAGCCTGATCCAGCAATGCCGCGTGTGTGAAGAAGGCCTTAGGGTTGTAAAGCACTTTCAGTGGGGAGGAAGTTGTGAGTGATAATACCATTTGCAATTGACGTTACCCACAGAAGAAGCACCGGCTAACTCTGTGCCAGCAGCCGCGGTAATACAGAGGGTGCTAGCGTTAATCGGAATTACTGGGCGTAAAGGGTGTGTAGGTGGTTGGGTAAGTTAGATGTGAAATCCCCGGGCTTAACCTGGGAACTGCGTTTAAAACTGCTTGGCTAGAGTATTGCAGAGGATAGTGGAATTTCCAGTGTAGCGGTGAAATGCGTAGATATTGGAAGGAACACCAGTGGCGAAGGCGACTATCTGGGCAAATACTGACACTGAGACACGAAAGCATGGGGAGCAAACAGG` |
| ASV5508 | 385 | `TTAGGAATAATCTGCAATGTACGAAAGTACGACAGTTTGAGCCAGAGTGCTTCTCAATGAGAAGCTTTTGCCAAATGTAAAAAGTTTGGTGAATAAGGACTGGGTAAGATGAGTGTTAGTCGTCGCGGTAATACTCGCGGTCCAAGTCGCAGCCATATTTATTGGGTCTAAAACATCCGTAGCTTGCTCTATAAGTTTCCTGTGAAATTCCAGGTCTCAAACCTGGAGCGCGCGGGAAGTACTGCAGAGCTAGAGACCGGGAGGCGTAAAGAGTACGGTTAAAGTAACAGTAAAATGTGTTAATCTAAGCCGGACTAACAAGAGCGAAGGCACTTTACGAGAACGGTTCTGACAGTGAGGGATGAAGGCTAGGGGCGCAAAACGG` |
| ASV3308 | 423 | `TAGGGAATATTGGGCAATGGGCGGAAGCCTGACCCAGCCATGCCGCGTGCAGGAAGAAGGTCCTCTGGATTGTAAACTGCTTTTGTCGAGGACGAAAAAGGTGCTGCGGCACAAATTGACGGTATTCGACGAATAAGCCACGGCTAACTACGTGCCAGCAGCCGCGGTAATACGTAGGTGGCAAGCGTTGTCCGGATTTATTGGGTTTAAAGGGTGCGTAGGTGGCTTGGTAAGTCAGCGGTGAAAGACGGTCGCTCAACGATTGCAGTGCCGTTGATACTGCCGAGCTTGAGTAAAGATGAGGTAGGCGGAACGGATAGTGTAGCGGTGAAATGCATAGATATTATCCAGAACACCGATTGCGAAGGCAGCTTACTGGACTTTAACTGACACTGAGGCACGAGAGCGTGGGGAGCAAACAGG` |
| ASV5398 | 429 | `TGGGGAATATTGGACAATGGGCGCAAGCCTGATCCAGCAATGCCGCGTGTGTGAAGAAGGCTTTCGGGTTGTAAAGCACTTTCGGTGGGGAGAAAGGGTCCGTTCCTAATATGAACGGAAGATGATGTTACCCACAGAAGAAGCACCGGCTAACTCTGTGCCAGCAGCCGCGGTAATACAGAGGGTGCGAGCGTTAATCGGAATTACTGGGCGTAAAGGGTGCGTAGGTGGTTGCATCAGTTAGGTGTGAAAGTCCTGGGCTTAACCTGGGGATTGCGCCTAATACTGTGTAGCTAGAGTACTGTAGAGGAAAGTGGAATTTCCGGTGTAGCGGTGAAATGCGTAGATATCGGAAGGAACATCAGTGGCGAAGGCGACTTTCTGGACAGCTACTGACACTGAGGCACGAAAGCGTGGGGAGCAAACAGG` |
| ASV680 | 429 | `TGGGGAATATTGGACAATGGGCGCAAGCCTGATCCAGCAATGCCGCGTGTGTGAAGAAGGCCTTCGGGTTGTAAAGCACTTTTATCGGGAGCGAAAAGCGGACGGTGAATACCCGTCCGTGCTGACGGTACCCGAGGAATAAGCACCGGCTAACTTCGTGCCAGCAGCCGCGGTAATACGAAGGGTGCAAGCGTTAATCGGAATTACTGGGCGTAAAGCGTGCGTAGGCGGTTCGCTAAGTCTGCCGTGAAAGCCCCGGGCTCAACCTGGGAATTGCGGTGGATACTGGCGAGCTGGAGTGTGGTAGAGGAGGGTGGAATTCCCGGTGTAGCGGTGAAATGCGTAGAGATCGGGAGGAACATCAGTGGCGAAGGCGGCCCTCTGGACCAACACTGACGCTGAGGCACGAAAGCGTGGGGAGCAAACAGG` |
| ASV2030 | 407 | `TGGGGAATATTGGGCAATGGGCGAAAGCCTGACCCAGCAACGCCGCGTGAAGGAAGAAGGCTTTCGGGTTGTAAACTTCTTTTACCAGGGATGAAAAAAATGACAGTACCTGGAGAAAAAGCTCCGGCTAACTACGTGCCAGCAGCCGCGGTAATACGTAGGGAGCAAGCGTTGTCCGGATTTACTGGGTGTAAAGGGCGTGTAGGCGGAGTAATAAGTTAGATGTGAAATCTGGTGGCTCAACCACCAAACTGCATTTAAAACTGTTACCCTTGAGTGATGGAGAGGCAGGCGGAATTCCTAGTGTAGCGGTGAAATGCGTAGATATTAGGAGGAACACCAGTGGCGAAGGCGGCCTGCTGGACATTAACTGACGCTGAGGCGCGAAAGCGTGGGGAGCAAACAGG` |
| ASV2610 | 386 | `TCGCGAAAACTTCACACTGGGGGTAACCCCGATGAGGGAATTCCTAGTGCTAGTACAAAGTATTAGCTTTTCTTCAGTATAGATAGCTGGAGGAATAAGGGCTGGGTAAGACGGGTGCCAGCCGCCGCGGTAATACCCGCAGCCCAAGTGGTGGTCGATATTATTGAGTCTAAAACGTTCGTAGCCGGTCTGATAAATCCCTGGGTAAATCGGTGGGCTTAACCCACCGACTTCCGGGGAGACTGTCAGGCTTGGGACCGGGAGAGGCTAGAGGTACTTTTGGGGTAGGGGTAAAATCCTGTAATCCTAAAAGGACCACCGGTGGCGAAGGCGTCTAGCTAGAACGGATCCGACGGTGAGGAACGAAGCCCTGGGGCGCAAACGGG` |
| ASV4259 | 404 | `TGGGGAATATTGGACAATGGGCGAAAGCCTGATCCAGCAACGCCGCGTGAGTGATGAAGGCCTTCGGGTTGTAAAGCTCTTTTGATAGGGAAGATAATGACTGTACCTATAGAATAAGTCCCGGCTAACTTCGTGCCAGCAGCCGCGGTAATACGAAGGGGACTAGCGTTGTTCGGAATGACTGGGCGTAAAGGGCGCGTAGGCGGTAAAGTAAGTGATGCGTGAAAGTCCTGGGCTTAACCTAGGGAGTGCGTCTCATACTGCTTTGCTAGAGGACAGGAGGGGGTGGTAGAATTCTCAGTGTAGAGGTGAAATTCGTAGATATTGAGAGGAATACCAGAGGCGAAGGCGACCACCTGGTCTGTCACTGACGCTGAGGCGCGAAAGCGTGGGGAGCAAACAGG` |
| ASV5036 | 424 | `TAAGGAATATTGGTCAATGGGCGCAAGCCTGAACCAGCCATGCCGCGTGCAGGAAGACGGCCCTATGGGTTGTAAACTGCTTTTGTACCGGAATAAACCTTCTCTTGCGAGAGAAGCTGAACGTACGGTAAGAATAAGGATCGGCTAACTCCGTGCCAGCAGCCGCGGTAATACGGAGGATCCAAGCGTTATCCGGATTTATTGGGTTTAAAGGGTGCGTAGGCGGCTTTTTAAGTCAGGGGTGAAAACTTGCGGCTCAACCGTAAGCTTGCCTTTGATACTGAGGAGCTTGAATACACTTGAGGTAGGCGGAATGTGACAAGTAGCGGTGAAATGCATAGATATGTCACAGAACACCAATTGCGAAGGCAGCTTACTAAAGTGTGATTGACGCTGAGGCACGAAAGCGTGGGGATCAAACAGG` |
| ASV3811 | 429 | `TGAGGAATATTGCACAATGGGCGAAAGCCTGATGCAGCGACGCCGCGTGGAGGATGAAGGTTTTCGGATTGTAAACTCCTGTTAAGTGAGAAAAAAGACTTGCCTTTAATAGGGGCGAGGGATGATTGTATCACTAAAGAAAGCACCGGCTAACTTCGTGCCAGCAGCCGCGGTAATACGAGGGGTGCAAACGTTATTCGGAATCATTGGGCGTAAAGGGTACGTAGACGGCGAAATAAGTCAGTTATTAAATCTTCCAGCTCAACTGGAAGCTTGTGGCTGAAACTGATTTGCTTGAGGATGGAAGAGAGAAGCGGAATTCTCGGAGTAGCGGTAAAATGCGTAGATCTCGAGAGGAACACCGATGGCGAAGGCAGCTTCTTGGTCCATTTCTGACGTTGAGGTACGAAAGCGTGGGGAGCAAACAGG` |
| ASV885 | 404 | `TGGGGAATCATAGACAATGGGGGAAACCCTGATCTTGCCATGCCGCGTGGACGATGAAGGCCTTAGGGTTGTAAAGTCCTTTCACCGGTGAAGATAATGACGGTAGCCGGAGAAGAAGCCCCGGCTAACTCCGTGCCAGCAGCCGCGGTAATACGGAGGGGGCTAGCGTTGTTCGGAATTACTGGGCGTAAAGCGCACGTAGGCCGACTGTTAAGTCGGGGGTGAAATCCTGGGGCTCAACCCCAGAACTGCCCTCGATACTGGCAGTCTAGAGGACGGAAGAGGCTAGTGGAATGGCGAGTGTAGAGGTGAAATTCGTAGATATTCGCCGGAACACCAGTGGCGAAGGCGACTAGCTGGTCCGTTTCTGACGCTGAGGTGCGAAAGCGTGGGGAGCAAACAGG` |
| ASV1903 | 429 | `TGGGGAATTTTGGACAATGGACGAAAGTCTGATCCAGCCATTCCGCGTGCAGGATGAAGGCCCTCGGGTTGTAAACTGCTTTTGTACGGAACGAAAAGGTCTCTATTAATACTAGGGGCTCATGACGGTACCGTAAGAATAAGCACCGGCTAACTACGTGCCAGCAGCCGCGGTAATACGTAGGGTGCAAGCGTTAATCGGAATTACTGGGCGTAAAGCGTGCGCAGGCGGTTATATAAGACAGATGTGAAATCCCCGGGCTCAACCTGGGAACTGCATTTGTGACTGTATAGCTAGAGTACGGTAGAGGGGGATGGAATTCCGCGTGTAGCAGTGAAATGCGTAGATATGCGGAGGAACACCGATGGCGAAGGCAATCCCCTGGACCTGTACTGACGCTCATGCACGAAAGCGTGGGGAGCAAACAGG` |
| ASV3116 | 430 | `TGGGGAATATTGCACAATGGGCGAAAGCCTGATGCAGCGACGTCGCGTGGAGGATGAAGGTTTTCGGATTGTAAACTCCTGTTAAACGGAAAGAAAGAGGTAAGGCTAATAACCTTATCATGATGACGTTACCGTTAAAGAAAGCACCGGCTAACTTCGTGCCAGCAGCCGCGGTAATACGAAGGGTGCGAGCGTTATTCGGAATAACTGGGCGTAAAGGGTGCGTAGGCGGTATTTTAAGTCGACGGTTAAAGACTATGGCTTAACCATAGAAATGCCGGTGATACTGAAATGCTAGAGGGTAGTAGAGAGGAGTGGAATTCTCGGAGTAGCGGTAAAATGCGTAGATCTCGAGAGGAACACCGATGGCGAAGGCAGCTCCTTGGGCTACAACTGACGCTGAGGCACGAAAGCGTGGGGAGCAAACAGG` |
| ASV1330 | 424 | `TGAGGAATATTGGACAATGGGCGCAAGCCTGATCCAGCCATGCCGCGTGCAGGATGACGGTCCTATGGATTGTAAACTGCTTTTGTACAGGAAGAAACAACGCTACGTGTAGCGTCTTGACGGTACTGTGAGAATAAGGATCGGCTAACTCCGTGCCAGCAGCCGCGGTAATACGGAGGATCCAAGCGTTATCCGGAATCATTGGGTTTAAAGGGTCCGTAGGCGGTCTTATAAGTCAGTGGTGAAATCTCCCCGCTCAACGGGGAAACGGCCATTGATACTGTAGGACTTGAATTATTAGGAAGTAACTAGAATATGTAGTGTAGCGGTGAAATGCTTAGAGATTACATGGAATACCAATTGCGAAGGCAGGTTACTACTAATGGATTGACGCTGATGGACGAAAGCGTGGGTAGCGAACAGG` |
| ASV2786 | 429 | `TGGGGAATTTTGGACAATGGGCGCAAGCCTGATCCAGCCATGCCGCGTGAGTGAAGAAGGCCTTCGGGTTGTAAAGCTCTTTCGGTGAAGACGAAACGGTTCGCGTTAATAGCGCGGACTAATGACGGTACTCACAGAAGAAGCACCGGCTAACTACGTGCCAGCAGCCGCGGTAATACGTAGGGTGCAGGCGTTAATCGGAATTACTGGGCGTAAAGCGTGCGCAGGCGGTTTGCTAAGACAGGTGTGAAATCCCCGGGCTTAACCTGGGAATTGCGCTTGTAACTGGCAAGCTAGAGTGTGGCAGAGGGGGGTGGAATTCCACGTGTAGCAGTGAAATGCGTAGATATGTGGAGGAACACCGATGGCGAAGGCAGCCCCCTGGGTTAACACTGACGCTCAGGCACGAAAGCGTGGGGAGCAAACAGG` |
| ASV339 | 429 | `TGGGGAATTTTGGACAATGGGCGCAAGCCTGATCCAGCAATGCCGCGTGCAGGATGAAGGCCTTCGGGTTGTAAACTGCTTTTGTACGGAGCGAAAAGGCTCTCTCTAATAAAGGGGGCTCATGACGGTACCGTAAGAATAAGCACCGGCTAACTACGTGCCAGCAGCCGCGGTAATACGTAGGGTGCAAGCGTTAATCGGAATTACTGGGCGTAAAGCGTGCGCAGGCGGTTATGTAAGACAGATGTGAAATCCCCGGGCTCAACCTGGGACCTGCATTTGTGACTGCATAGCTAGAGTACGGCAGAGGGGGATGGAATTCCGCGTGTAGCAGTGAAATGCGTAGATATGCGGAGGAACACCGATGGCGAAGGCAATCCCCTGGGCCTGTACTGACGCTCATGCACGAAAGCGTGGGGAGCAAACAGG` |
| ASV1086 | 404 | `TGGGGAATATTGGACAATGGGCGCAAGCCTGATCCAGCAATTCCGCGTGGGTGAAGAAGGTCTTCGGATTGTAAAGCCCTTTCGTTGGGGACGATGATGACGGTACCCAAAGAAGAAGCCCCGGCTAACTTCGTGCCAGCAGCCGCGGTAATACGAAGGGGGCTAGCGTTGCTCGGAATGACTGGGCGTAAAGGGCGCGTAGGCGGCACAACTAGTCAGGCGTGAAATTCCTGGGCTTAACCTGGGGGCTGCGTTTGATACGGTTGAGCTAGAGGATGGAAGAGGCTCGTGGAATTCCCAGTGTAGAGGTGAAATTCGTAGATATTGGGAAGAACACCGGTGGCGAAGGCGGCGAGCTGGTCCATTACTGACGCTGAGGCGCGAAAGCGTGGGGAGCAAACAGG` |
| ASV4431 | 407 | `TGGGGAATTTTCCGCAATGGGCGAAAGCCTGACGGAGCAATACCGCGTGAGGGAGGAAGGCTCTTGGGTTGTAAACCTCTTTTCTCAAGGAAGAAAAAAATGACGGTACTTGAGGAATCAGCATCGGCTAACTCCGTGCCAGCAGCCGCGGTAATACGGAGGATGCAAGCGTTATCCGGAATCATTGGGCGTAAAGCGTCCGCAGGTGGCATTTCAAGTCTGCTGTCAAAGACAGAAGCTCAACTTCTGAGCGGCAGTGGAAACTGAGAAGCTAGAGTACGGTAGGGGCAGAGGGAATTTCCAGTGTAGCGGTGAAATGCGTAGAGATTGGAAAGAACACCAGTGGCGAAGGCGCTCTGCTGGGCCTGTACTGACACTGAGGGACGAAAGCTAGGGGAGCGAAAGGG` |
| ASV1444 | 404 | `TGGGGAATCTTGCGCAATGGGCGAAAGCCTGACGCAGCCATGCCGCGTGAATGATGAAGGTCTTAGGATTGTAAAATTCTTTCACCGGGGACGATAATGACGGTACCCGGAGAAGAAGCCCCGGCTAACTTCGTGCCAGCAGCCGCGGTAATACGAAGGGGGCTAGCGTTGCTCGGAATTACTGGGCGTAAAGGGCGCGTAGGCGGATCGTTAAGTCAGAGGTGAAATCCCGGAGCTCAACTTCGGAACTGCCTTTGATACTGGCGATCTTGAGTGTGAGAGAGGTATGTGGAACTCCGAGTGTAGAGGTGAAATTCGTAGATATTCGGAAGAACACCAGTGGCGAAGGCGACATACTGGCTCATTACTGACGCTGAGGCGCGAAAGCGTGGGGAGCAAACAGG` |
| ASV336 | 429 | `TGGGGAATTTTGGACAATGGGCGCAAGCCTGATCCAGCAATGCCGCGTGCAGGATGAAGGCCTTCGGGTTGTAAACTGCTTTTGTACGGAACGAAACGGTCTACCCTAATACGGTGGGCTAATGACGGTACCGTAAGAATAAGCACCGGCTAACTACGTGCCAGCAGCCGCGGTAATACGTAGGGTGCGAGCGTTAATCGGAATTACTGGGCGTAAAGCGTGCGCAGGCGGTTATATAAGACAGATGTGAAATCCCCGGGCTCAACCTGGGACCTGCATTTGTGACTGTATAGCTAGAGTACGGTAGAGGGGGATGGAATTCCGCGTGTAGCAGTGAAATGCGTAGATATGCGGAGGAACACCGATGGCGAAGGCAATCCCCTGGACCTGTACTGACGCTCATGCACGAAAGCGTGGGGAGCAAACAGG` |
| ASV4672 | 424 | `TAAGGAATATTGGACAATGGACGCAAGTCTGATCCAGCCATGCCGCGTGAAGGATTAAGGTCCTCTGGATTGTAAACTTCTTTTATTTGGGAAGAAAAAGGGGAAATCTTTCCCCCTTGACGGTACCAAACGAATAAGCACCGGCTAACTCCGTGCCAGCAGCCGCGGTAATACGGAGGGTGCAAGCGTTATCCGGATTTACTGGGTTTAAAGGGTGCGTAGGCGGGTAAATAAGTCAGTGGTGAAATCCTTGAGCTTAACTCGAGAACTGCCATTGATACTATTTGTCTTGAATATTGTGGAGGTAAGCGGAATATGTCATGTAGCGGTGAAATGCTTAGATATGACATAGAACACCTATTGCGAAGGCAGCTTACTACGCATATATTGACGCTGAGGCACGAAAGCGTGGGGATCAAACAGG` |
| ASV1839 | 406 | `TGAGGAATATTCTGCAATGGGCGAAAGCCTGACAGAGCAATACCGCGTGAAGGATGAAAGCCTATTGGTTGTAAACTTCTTTTCTCAAAGACGAAGATCTGACATTATTTGAGGAATAAGCATCGGCTAACCCTGTGCCAGCAGCCGCGGTAATACAGGGGATGCAAGTGTTATCCGGAATCATTGGGCGTAAAGCGTTTGTAGGTGGATATTTAAGTCTTCTGTTAAATCTCTAAGCTTAACTTAGAACAGGCAAAAGATACTGAATATCTTGAGTATGGTAGAGGTAAAGGGAATTTTCAGTGGAGCGGTGAAATGCGTAGATATTGAAAAGAACACCAATAGCGAAAGCACTTTACTGGGCCATCACTGACACTCAGAAACGAAAGCTGGGGGAGCAAACAGG` |
| ASV2440 | 429 | `TGGGGAATTTTGGACAATGGGCGCAAGCCTGATCCAGCCATGCCGCGTGCGGGAAGAAGGCCTTCGGGTTGTAAACCGCTTTTGTCAGGGAAGAAACGGCTCTTACTAATATTAGGAGCTAATGACGGTACCTGAAGAATAAGCACCGGCTAACTACGTGCCAGCAGCCGCGGTAATACGTAGGGTGCAAGCGTTAATCGGAATTACTGGGCGTAAAGCGTGCGCAGGCGGCTTTGCAAGACAGATGTGAAATCCCCGGGCTCAACCTGGGAACTGCATTTGTGACTGCAAGGCTAGAGTACGGTAGAGGGGGATGGAATTCCGCGTGTAGCAGTGAAATGCGTAGATATGCGGAGGAACACCGATGGCGAAGGCAATCCCCTGGACCTGTACTGACGCTCATGCACGAAAGCGTGGGGAGCAAACAGG` |
| ASV2205 | 404 | `TGAGGAATATTGGACAATGGGCGAAAGCCTGATCCAGCCATGCCGCGTGAGTGATGAAGGCCTTAGGGTTGTAAAGCTCTTTTGGTGGGGAAGATAATGACTGTACCCACAGAATAAGCCCCGGCTAAATTCGTGCCAGCAGCCGCGGTAATACGAATGGGGCAAGCGTTGTTCGGAATCACTGGGCGTAAAGCGTGCGTAGGCGGTTTAACAAGTCAGAAGTGAAAGCCCAGGGCTCAACCCTGGAACCGCTTTTGAAACTGTTAGACTAGTGTCCCGGAGAGGTTGGCGGAATTCCCAGTGTAGAGGTGAAATTCGCAGATATTGGGAGGAACACCAGTGGCGCAAGCGGCCAACTGGACGGGTACAGACGCTGAGGCACGAAAGCGTGGGGATCAAACAGG` |
| ASV2748 | 404 | `TAGGGAATATTGCACAATGGACGAAAGTCTGATGCAGCGACGCCGCGTGAGTGATGAAGGCCTTCGGGTCGTAAAACTCTGTGATAAGGGAAGATAATGACGGTACCTTATCAGAAAAGACCGGCTAACTTCGTGCCAGCAGCCGCGGTAATACGAGGGGTCTAAGCGTTGTTCGGAATCACTGGGCGTAAAGCGGGTGTAGGTGGCTATGCAAGTTGGATGTGAAAGCCCCGGGCTCAACCCGGGAAGTGCATCCAAAACTACGTAGCTTGAGTGCGGGAGAGAAAGGCAGAATTCCTGGTGTAGTGGTGAAATACGTAGATATCAGGAGGAACTCCGGTGGCGAAGGCGGCCTTTTGGCCCAGCACTGACACTCAGACCCGAAAGCGTGGGTAGCAAACAGG` |
| ASV2944 | 424 | `TAAGGAATATTGGACAATGCCCGCAAGGGTGATCCAGCCATGCCGCGTGCAGGAAGAAGGCCCTATGGGTCGTAAACTGCTTTTACACCGGAGAAAACCCCCTTACGTGTAAGGGGCTGATAGTATGGTGAGAATAAGCATCGGCTAACTTCGTGCCAGCAGCCGCGGTAAGACGAAGGATGCAAGCGTTATCCGGATTCATTGGGTTTAAAGGGAGCGTAGGTGGACTTATAAGTCAGTGGTGAAATCTTTGGGCTTAACCCAAAAATTGCCATTGATACTGTAGGTCTTGAGTACAGTTGCTGTGGGCGGAATATGACATGTAGTGGTGAAATACATAGAGATGTCATAGAACACCGATTGCGAAGGCAGCTCACAAAGCTGTAACTGACACTGAGGCTCGAAAGTGCGGGGATCAAACAGG` |
| ASV3305 | 429 | `TGGGGAATTTTGGACAATGGGCGAAAGCCTGATCCAGCAATGCCGCGTGCAGGATGAAGGCCTTCGGGTTGTAAACTGCTTTTGTACGGAACGAAAAGGCGCCTACTAATACTGGGTGTTGATGACGGTACCGTAAGAATAAGCACCGGCTAACTACGTGCCAGCAGCCGCGGTAATACGTAGGGTGCGAGCGTTAATCGGAATTACTGGGCGTAAAGCGTGCGCAGGCGGTGTTGTAAGACAGTTGTGAAATCCCCGGGCTCAACCTGGGAACTGCATCTGTGACTGCAATGCTGGAGTGCGGCAGAGGGGGATGGAATTCCGCGTGTAGCAGTGAAATGCGTAGATATGCGGAGGAACACCGATGGCGAAGGCAATCCCCTGGGCCTGCACTGACGCTCATGCACGAAAGCGTGGGGAGCAAACAGG` |
| ASV3043 | 404 | `TGGGGAATATTGGACAATGGGCGCAAGCCTGATCCAGCAATGCCGCGTGAATGATGAAGGCCTTAGGGTTGTAAAGTTCTTTCAGTGGGGAAGATAGTGACGGTACCCACAGAAGAAGCTCCGGCTAACTCCGTGCCAGCAGCCGCGGTAATACGGAGGGAGCTAGCGTTTTTCGGATTTACTGGGCGTAAAGCGCGTGTAGGCGGTTAATTAAGTTGGAAGTGAAAGCCCAGGGCTCAACCCTGGAACTGCTTCCAAAACTGGTTAACTAGAGAATGGTAGAGGATGGTGGAATTCCTAGTGTAGAGGTGAAATTCTTAGATATTAGGAGGAACATCGATGGCGAAGGCAACCATCTGGACCATTTCTGACGCTGAGATGCGAAAGCGTGGGGAGCAAACAGG` |
| ASV172 | 404 | `TGGGGAATCTTAGACAATGGGGGCAACCCTGATCTAGCCATGCCGCGTGATCGATGAAGGCCTTAGGGTTGTAAAGATCTTTCAGATGGGAAGATAATGACGGTACCATCAGAAGAAGCCCCGGCTAACTCCGTGCCAGCAGCCGCGGTAATACGGAGGGGGCTAGCGTTATTCGGAATTACTGGGCGTAAAGCGCACGTAGGCGGATCGGAAAGTCAGAGGTGAAATCCCAGGGCTCAACCCTGGAACGGCCTTTGAAACTCCTGGTCTTGAGGTCGTGAGAGGTGAGTGGAATTCCGAGTGTAGAGGTGAAATTCGTAGATATTCGGAGGAACACCAGTGGCGAAGGCGGCTCACTGGCACGATACTGACGCTGAGGTGCGAAAGCGTGGGGAGCAAACAGG` |
| ASV5399 | 424 | `TGGGGAATATTGGACAATGGGCGCAAGCCTGATCCAGCAATGCCGCGTGTGTGAAGAAGGCCCTAGGGTTGTAAAGCACTTTCGGTTGGGAGGAAAACACCCAGTGAACCTGGGAATTGACGTAACCAACAGAAGAAGCACCGGCTAACTCTGTGCCAGCAGCCGCGGTAATACAGAGGGTGCAAGCGTTAATCGGAATTACTGGGCGTAAAGCGCGTGTAGGTGGATAAATCAGTCGGATGTGAAATCCCCGGGCTTAACCTGGGAACTGCATTCGATACTACTTATCTAGAGTATGGGAGAGGGAAGTGGAATTTCCGGTGTAGCGGTGAAATGCGTAGATATCGGAAAGAACACCAGTGGCGAAGGCGGCTTCCTGGCCTAATACTGACACTGAGACGCGAAAGCGTGGGGAGCAAACAGG` |
| ASV3301 | 404 | `TGGGGAATATTGGACAATGGGCGCAAGCCTGATCCAGCCATGCCGCGTGAGTGACGAAGGTCTTCGGATTGTAAAGCTCTTTTGGCGGGGACGATAATGACGGTACCCGCAGAATAAGCCCCGGCTAACTTCGTGCCAGCAGCCGCGGTAATACGAAGGGGGCTAGCGTTGTTCGGAATTACTGGGCGTAAAGCGCACGTAGGCGGATTTGTAAGTCAGGGGTGAAATCCCGGGGCTCAACCTCGGAACTGCCTTTGATACTGCGAATCTCGAGACCGGAAGAGGTGAGTGGAATTCCTAGTGTAGAGGTGAAATTCGTAGATATTAGGAAGAACACCAGTGGCGAAGGCGGCTCACTGGTCCGGTTCTGACGCTGAGGTGCGAAAGCGTGGGGAGCAAACAGG` |
| ASV1846 | 404 | `TAGGGAATATTGGACAATGGGGGCAACCCTGATCCAGCAATGCCGCGTGAGTGATGAAGGCCTTAGGGTTGTAAAGCTCTTTTACCCGAGATGATAATGACAGTATCGGGAGAATAAGCTCCGGCTAACTCCGTGCCAGCAGCCGCGGTAATACGGAGGGAGCTAGCGTTGTTCGGAATTACTGGGCGTAAAGCGCACGTAGGCGGCGATTTAAGTCAGAGGTGAAAGCCCGGGGCTCAACCCCGGAACTGCCTTTGAGACTGGATTGCTAGAATCTTGGAGAGGCGGGTGGAATTCCGAGTGTAGAGGTGAAATTCGTAGATATTCGGAAGAACACCAGTGGCGAAGGCGGCCCGCTGGACAAGTATTGACGCTGAGGTGCGAAAGCGTGGGGAGCAAACAGG` |
| ASV4307 | 409 | `TGGGGAATATTGCACAATGGGCGAAAGCCTGATGCAGCAACGCCGCGTGCGGGATGACGGCCTTCGGGTTGTAAACCGCTTTCAGCTCCGACGAAGCTAACGTGACGGTAGGAGCAGAAGAAGGACCGGCCAACTACGTGCCAGCAGCCGCGGTGATACGTAGGGTCCGAGCGTTGTCCGGAATTATTGGGCGTAAAGGGCTTGTAGGCGGTTTGTCGCGTCGGAAGTGAAAACTCAGGGCTTAACCCTGAGCCTGCTTCCGATACGGGCAAACTAGAGGAATGCAGGGGAGAATGGAATTCCTGGTGGAGCGGTGGAATGCGCAGATATCAGGAGGAACACCAGTGGCGAAGGCGGTTCTCTGGGCATTTCCTGACGCTGAGAAGCGAAAGCGTGGGGAGCAAACAGG` |
| ASV1332 | 424 | `TGAGGAATATTGGTCAATGGACGCAAGTCTGAACCAGCCATGCCGCGTGCAGGATGACGGCTCTATGAGTTGTAAACTGCTTTTGTATTAGAGTAAATTCACCTACGTGTAGGTGATTGAAAGTATAGTACGAATAAGGATCGGCTAACTCCGTGCCAGCAGCCGCGGTAATACGGAGGATCCAAGCGTTATCCGGATTTATTGGGTTTAAAGGGTGCGTAGGCGGTTTGATAAGTTAGAGGTGAAATATTAGGGCTTAACCCTGAAACTGCCTCTAATACTGTTGAACTAGAGAATAGTTGCGGTAGGCGGAATGTATGGTGTAGCGGTGAAATGCTTAGAGATCATACAGAACACCGATTGCGAAGGCAGCTTACCAAACTATATCTGACGTTGAGGCACGAAAGCGTGGGTAGCAAACAGG` |
| ASV4865 | 429 | `TGGGGAATATTGGACAATGGGGGCAACCCTGATCCAGCAATGCCGCGTGTGTGAAGAAGGCCTGCGGGTTGTAAAGCACTTTCAGTAGGGAAGAAAAGCCTGGGGCTAATACCCCCGGGTCTTGACGTTACCTAAAGAAGAAGCACCGGCTAACTCCGTGCCAGCAGCCGCGGTAATACGGAGGGTGCGAGCGTTAATCGGAATTACTGGGCGTAAAGCGCACGTAGGCGGTCGGATAAGTCGGATGTGAAAGCCCTGGGCTTAACCTGGGAACTGCATTCGATACTGTCTGGCTAGAGTATGGGAGAGGGAAGCGGAATTTCCGGTGTAGCGGTGAAATGCGTAGATATCGGAAGGAACATCAATGGCGAAGGCAGCTTCCTGGCCCAATACTGACGCTCAGGTGCGAAAGCGTGGGGATCAAACAGG` |
| ASV2350 | 429 | `TGAGGAATATTGGACAATGGGCGAAAGCCTGATCCAGCAATGCCGCGTGTGTGAAGAAGGCCTTCGGGTTGTAAAGCACTTTCAGTAGGGAAGAAGGATTAATGGCTAATACCCATTGATATTGACATTACCTACAGAAGAAGCACCGGCTAACTCTGTGCCAGCAGCCGCGGTAATACAGAGGGTGCAAGCGTTAATCGGAATTACTGGGCGTAAAGCGTACGTAGGCGGTTATTTAAGTCGGATGTGAAATCCCCGGGCTCAACCTGGGAATTGCATCCGATACTGAATAACTAGAGTATGGTAGAGGAAAGTGGAATTTCCGGTGTAGCGGTGAAATGCGTAGATATCGGAAGGAACACCAGTGGCGAAGGCGGCTTTCTGGACCAATACTGACGCTGAGGTACGAAAGCGTGGGGAGCAAACAGG` |
| ASV4900 | 404 | `TGGGGAGTATTGGACAATGGGGGAAACCCTGATCCAGCCATGCCGCGTGAGTGATGAAGGCCTTCGGGTTGTAAAGCTCTTTTAGCGGGGAAGATAATGACGGTACCCGCAGAATAAGCCCCGGCTAACTTCGTGCCAGCAGCCGCGGTAAGACGAAGGGGGCTAGCGTTGTTCGGAATTACTGGGCGTAAAGGGCGCGCAGGCGGCTCTACAAGTCAGGCGTTAAAGACCCAGGCTCAACCTGGGGAATGCGCTTGATACTGTGGGGCTTGAGATCGGAAGAGGAGAGTGGAATTCCCAGTGTAGAGGTGAAATTCGTAGATATTGGGAAGAACACCAGTGGCGAAGGCGGCTCTCTGGTCCGATTCTGACGCTGAGGCGCGAAAGCGTGGGGAGCAAACAGG` |
| ASV5433 | 429 | `TGGGGAATATTGGACAATGGGGGGAACCCTGATCCAGCAATGCCGCGTGTGTGAAGAAGGCCTGAGGGTTGTAAAGCACTTTCAGTGGGGAGGAGGATTGTTTGGTTAAGAGCTGAGTAATTGGACGTTACCCACAGAAGAAGCACCGGCTAACTCCGTGCCAGCAGCCGCGGTAATACGGAGGGTGCAAGCGTTAATCGGAATTACTGGGCGTAAAGGGTGCGTAGGTGGTATGGTAAGTTATCTGTGAAATCCCCGGGCTTAACCTGGGCAGGTCAGATAAGACTGCGATACTTGAGTGAGGGAGAGGGTAGTGGAATTTCCGGTGTAGCGGTGAAATGCGTAGAGATCGGAAGGAACACCAGTGGCGAAGGCGGCTACCTGGCCTAACACTGACACTGAAGCACGAAAGCGTGGGGAGCAAACAGG` |
| ASV4985 | 404 | `TGGGGAATATTGGACAATGGGCGCAAGCCTGATCCAGCCATGCCGCGTGAGTGATGAAGGCCTTCGGGTTGTAAAGCTCTTTCGCCGGGGAAGATAATGACGGTACCCGGAGAAGAAGCCTCGGCTAACTCCGTGCCAGCAGCCGCGGTAAGACGGAGGAGGCTAGCGTTGTTCGGAATTACTGGGCGTAAAGCGCACGTAGGCGGCCCGACAAGTCGGATGTGAAAGCCCGGGGCTTAACCCCGGAACAGCATCCGAAACTAGCGGGCTCGAGACCGGGAGAGGAGAGTGGAATTCCCAGTGTAGAGGTGAAATTCGTAGATATTGGGAAGAACACCTGTGGCGAAGGCGGCTCTCTGGTCCGGTACTGACGCTCAAGTGCGAAAGCGTGGGGAGCAAACAGG` |
| ASV5664 | 424 | `TAAGGAATATTGGGCAATGGAGGCAACTCTGACCCAGCCATGCCGCGTGCAGGATGAAGGTCCTCTGGATTGTAAACTGCTTTTATTGGGGAATAAACCTAGGTATTTATACTTAGCTGAAGGTACCCAAGGAATAAGCACCGGCTAACTCCGTGCCAGCAGCCGCGGTAATACGGAGGGTGCAAGCGTTATCCGGATTTACTGGGTTTAAAGGGTGCGTAGGCGGATCTTTAAGTCAGAGGTGAAAGCCCGAAGCTCAACTTCGGAACTGCCTTTGATACTATTGATCTTGAATTCGGTTGAGGTGGCTGGAATGTGTCATGTAGCGGTGAAATGCTTAGATATGACACAGAACACCAATTGTGAAGACAGGTTGCTAAACCGACATTGACGCTGAGGCACGAAAGCGTGGGGATCAAACAGG` |
| ASV2816 | 429 | `TGGGGAATATTGGACAATGGGGGCAACCCTGATCCAGCGACGCCGCGTGTGTGAAGAAGGCCTGCGGGTTGTAAAGCACTTTTAGTGGGGAGGAAATGCTCAGACCTAACACGTCTGGGAGCTGACTTAACCCAAAGAAAAAGCACCGGCTAACTCTGTGCCAGCAGCCGCGGTAATACAGAGGGTGCGAGCGTTAATCGGAATTACTGGGCGTAAAGCGTGCGTAGACGGTTTTGTAAGTCGGATGTGAAATCCCCGGGCTCAACCTGGGAATTGCATTCGAAACTGCCAAGCTAGGGTACGGTAGAGGGAAGCGGAATTTCCGGTGTAGCGGTGAAATGCGTAGATATCGGAAGGAACATCAGTGGCGAAAGCGGCTTCCTGGACCAGTACCGACGTTCAGGCACGAAAGCGTGGGGAGCAAACAGG` |
| ASV1063 | 429 | `TGGGGAATTTTGGACAATGGGCGCAAGCCTGATCCAGCAATGCCGCGTGAGTGAAGAAGGCCTTCGGGTTGTAAAGCTCTTTTGTCAGGGAAGAAAAGGCTACGGATAATACCTGTGGCTCATGACGGTACCTGAAGAATAAGCACCGGCTAACTACGTGCCAGCAGCCGCGGTAATACGTAGGGTGCAAGCGTTAATCGGAATTACTGGGCGTAAAGCGTGCGCAGGCGGTTTTGTAAGACTGTCGTGAAATCCCCGGGCTCAACCTGGGAATGGCGATGGTGACTGCAAGGCTAGAGTTTGGCAGAGGGGGGTAGAATTCCACGTGTAGCAGTGAAATGCGTAGATATGTGGAGGAACACCGATGGCGAAGGCAGCCCCCTGGGTCAAAACTGACGCTCATGCACGAAAGCGTGGGGAGCAAACAGG` |
| ASV3190 | 423 | `TAGGGAATATTGGACAATGGGTGAGAGCCTGATCCAGCCATGCCGCGTGCAGGAAGACGGCCTTCTGGGTTGTAAACTGCTTTTATCTGGGGATAAAATTCCCTTGCGAGGGAGACTGAAGGTACCAGATGAATAAGCCACGGCTAACTACGTGCCAGCAGCCGCGGTAATACGTAGGTGGCAAGCGTTGTCCGGATTTATTGGGTTTAAAGGGTGCGTAGGCGGCTAAATAAGTCAGTGGTGAAATACTCGAGCTTAACTTGAGAACGGCCAATGATACTGTTTAGCTTGAGTACAAACGAAGTGGGCGGAATTGAAAGTGTAGCGGTGAAATGCATAGATATTTTCAAGAACACCGATAGCGAAGGCAGTTCACTAGGATGTAACTGACGCTGAGGCACGAAAGTGTGGGGATCAAACAGG` |
| ASV3200 | 408 | `TGGGGAATTTTTGGCAATGGGCGAAAGCCTGACCGAGCCATACCGCGTGAAGGAAGAAGGCCCTTGGGTCGTAAACTTCTGTGGAGTTTTACTAAAAAAATGAAGAAAACTCTAGAAAGGAGCTGCAAACTCTGTGCCAGCAGCAGCGGTGATACAGAGGCTCCAAGCGTTATCCGGTTTCACTGGGCGTAAAGCGTTCCGATAGGTTGTTTAATGTGTCAAATGTCAAATACCGGAGCTCAACTCCGCGTCTGCATTCGAAACTATTAGACTAGAGGTGCAAAGAAGTAGCTGGAACTGTGTAAGTAGGGGTAAAATCCGTTGATATGCATAGGAACACCAAAAGCGAAAGCAGGCTACTAAGTGCATCCTGACACTTCTAGGACGAAAGCGTGGGTAGCGAATAGG` |
| ASV3930 | 429 | `TGGGGAATTTTGGACAATGGGCGCAAGCCTGATCCAGCCATTCCGCGTGAGTGAAGAAGGCCTTCGGGTTGTAAAGCTCTTTTGTCAGGGAGGAAATCCCTAAGGATAATACCCTTGGGGGATGACAGTACCTGAAGAATAAGCACCGGCTAACTACGTGCCAGCAGCCGCGGTAATACGTAGGGTGCAAGCGTTAATCGGAATTACTGGGCGTAAAGCGTGCGCAGGCGGTTTCGTAAGCTTGATGTGAAATCCCCGGGCTCAACCTGGGAACTGCATTGAGGACTGCGAGGCTAGAATACGGCAGAGGGGGGTGGAATTCCACGTGTAGCAGTGAAATGCGTAGAGATGTGGAGGAACACCGATGGCGAAGGCAGCCCCCTGGGCCTGTATTGACGCTCATGCACGAAAGCGTGGGGAGCAAACAGG` |
| ASV5439 | 404 | `TAGGGAATATTGCACAATGGACGAAAGTCTGATGCAGCGACGCCGCGTGAGTGATGAAGGCCTTCGGGTCGTAAAACTCTGTGATAAGGGAAGATGATGACGGTACCTTATCAGAAAAGACCGGCTAACTTCGTGCCAGCAGCCGCGGTAATACGAGGGGTCTAAGCGTTGTTCGGAATCACTGGGCGTAAAGCGGGTGTAGGTGGCTATGCAAGTTGGATGTGAAAGCCCCGGGCTCAACCCGGGAAGTGCATCCAAAACTACGTAGCTTGAGTGCGGGAGAGAAAGGCAGAATTCCTGGTGTAGTGGTGAAATACGTAGATATCAGGAGGAACTCCGGTGGCGAAGGCGGCCTTTTGGCCCAGCACTGACACTCAGACCCGAAAGCGTGGGTAGCAAACAGG` |
| ASV5071 | 407 | `TAGGGAATATTGCACAATGGAGGAAACTCTGATGCAGCGACGCCGCGTGAGTGACGAAGGCCTTCGGGTTGTAAAGCTCTGTTCTCAGGGAAAAAGCAAGTGATTGTACCTGAGAAGAAAGGACCGGCTAACTTCGTGCCAGCAGCCGCGGTAAGACGAGGGGTCCAAGCGTTGTTCGGAATCATTGGGCGTAAAGCGGGTGTAGGCGGCTCTATAAGTCAGAAGTGAAAGCCCCGAGCTTAACTTGGGAAGTGCTTTTGATACTGCAGAGCTTGAATGTGGGAGAGGGTAGTGGAATTCCAGGTGTAGTGGTGAAATACGTAGATATCTGGAGGAACACCGGTGGCGAAGGCGGCTACCTGGCCCAACATTGACGCTGAGACCCGAAAGCATGGGGATCAAACAGG` |
| ASV790 | 429 | `TGGGGAATTTTGGACAATGGGGGCAACCCTGATCCAGCAATGCCGCGTGTGTGAAGAAGGCCTTCGGGTTGTAAAGCACTTTTGGCGGGAACGAAACGGGTGTTTCTAATACAGACGCCTAATGACGGTACCCGCAGAATAAGCACCGGCTAACTACGTGCCAGCAGCCGCGGTAATACGTAGGGTGCAAGCGTTAATCGGAATTACTGGGCGTAAAGCGTGCGCAGGCGGTTTTGTAAGTCTGTCGTGAAATCCCCGGGCTCAACCTGGGAATGGCGATGGAGACTGCAAGGCTGGAATCTGGCAGAGGGGGGTAGAATTCCACGTGTAGCAGTGAAATGCGTAGAGATGTGGAGGAACACCGATGGCGAAGGCAGCCCCCTGGGCTAAGATTGACGCTCATGCACGAAAGCGTGGGGAGCAAACAGG` |
| ASV3319 | 424 | `TGAGGAATATTGGTCAATGGAGGCAACTCTGAACCAGCCATGCCGCGTGAAGGATGAAGGTCCTCTGGATTGTAAACTTCTTTTATAGAAGAAGAAACCCCATTATTCTTAGTGGGTTGACGGTACTCTATGAATAAGCACCGGCTAACTCCGTGCCAGCAGCCGCGGTAATACGGAGGGTGCAAGCGTTATCCGGATTCACTGGGTTTAAAGGGAGCGTAGGTGGGTTGGTAAGTCAGTGGTGAAATCCCCGAGCTTAACTTGGGAACTGCCATTGATACTACTGATCTTGAATACTGTGGAGGTAAGCGGAATATGTCATGTAGCGGTGAAATGCTTAGATATGACATAGAACACCGATAGCGAAGGCAGCTTACTACGCAGTTATTGACACTGAGGCTCGAAAGCGTGGGGATCAAACAGG` |
| ASV3275 | 405 | `TAGGGAATTTTCCACAATGGGGGCAACCCTGATGGAGCAACGCCGCGTGCAGGATGAAGGCCCTCGGGTCGTAAACTGCTTTTGTCTGTGACGAATATGACGGTAGCAGACGAATAAGGATCGGCTAACTACGTGCCAGCAGCCGCGGTCATACGTAGGATCCAAGCGTTATCCGGATTTACTGGGCGTAAAGAGTTGCGTAGGTGGCAGAGTAAGTGAATAGTGAAATCGTGCGGCTCAACCGTATACCCATTATTTAAACTGCTCAGCTTGAAGATGAGAGAGGTCACTGGAATTCCTTGTGTAGGAGTGAAATCCGTAGATATAAGGAGGAACACCGATGGCGTAGGCAGGTGACTGGCTCATTCTTGACACTAAGGCACGAAAGCGTGGGGAGCAAACGGG` |
| ASV1135 | 429 | `TAGGGAATATTGCGCAATGGGGGAAACCCTGACGCAGCAACGCCGCGTGAGTGAGGAAGGCCTTCGGGTCGTAAAGCTCTGTTCTTAGGGAAAAATGGTTAGGGAGCTAATACCCCCTGATTTGATGGTACCTAAGGAGAAAGGACCGGCTAACTTCGTGCCAGCAGCCGCGGTAATACGAAGGGTCCAAGCGTTGTTCGGATTTATTGGGCGTAAAGCGCGCGCAGGTGGATCGGTAAGTCAGATGTGAAATCTCAGGGCTCAACCCTGAAACTGCGTCTGAAACTGCCAGTCTAGAGTGTTGGAGGGGGTAGGGGAATTTCACGTGTAGGGGTAAAATCCGTAGAGATGTGAAGGAACACCGGAGGCGAAGGCGCCTACCTGGACAACTACTGACACTGAGGCGCGAAAGCGTGGGGAGCAAACAGG` |
| ASV5404 | 418 | `TGGGGAATATTGGACAATGGGCGAAAGCCTGATCCAGCAATACCGCGTGTGTGAAGAAGGCCTGCGGGTTGTAAAGCACTTTAGTGGGGGAAGAAGTACACGCAAGTGTATTGACGGTACCCCAAGAATAAGCACCGGCTAACTCTGTGCCAGCAGCCGCGGTAATACAGAGGGTGCGAGCGTTAATCGGAATGACTGGGCGTAAAGGGCGCGTAGGCGGTGATGTGTGTTAGATGTGAAAGCCCCGGGCTTAACCCGGGAATTGCATTTAAAACTGCATTGCTAGAGTGAAGTAGAGGTGAGTGGAATTTCCAGAGTAGCGGTGAAATGCGTAGATACTGGAAGGAACACCGGTGGCGAAGGCGGCTCACTGGACTTCAACTGACGCTGAGGCGCGAAAGCGTGGGGAGCAAACAGG` |
| ASV5121 | 430 | `TCGAGAATCTTTCGCAATGGGGGCAACCCTGACGAAGCGACGCCGCGTGGAGGATGAAGGTCTTCGGATTGTAAACTCCTGTCAGCTGGGACGAATAAGCTGCATTTGAATAGAATGCGGCCTTGACGGTACCAGCAGAGGAAGCCACGGCTAACTCTGTGCCAGCAGCCGCGGTAATACAGAGGTGGCAAGCGTTGTTCGGATTTATTGGGCGTAAAGGGTCCGTAGGCGGTTTGGAAAGTCGGATGTGAAATCCCACAGCTTAACTGTGGAACTGCATTCGAAACTTCCGAACTAGAGTGTGAGAGGGGTAAAGGGAATTCCCGGTGTAAGGGTGAAATCTGCAGAGATCGGGAAGAACACCAGTCGCGAAGGCGCTTTACTGGCTCATTACTGACGCTCAGGGACGAAAGCTAGGGGAGCAAACGGG` |
| ASV373 | 429 | `TAGGGAATCTTCGGCAATGGGCGAAAGCCTGACCGAGCAACGCCGCGTGAATGATGAAGGCCTTCGGGTTGTAAAATTCTGTTATAAGGGAAGAACGACTTTAGTAGGAAATGGCTAGAGTGTGACGGTACCTTATGAGAAAGCCACGGCTAACTACGTGCCAGCAGCCGCGGTAATACGTAGGTGGCGAGCGTTATCCGGAATTATTGGGCGTAAAGAGCGCGCAGGTGGTTGATTAAGTCTGATGTGAAAGCCCACGGCTTAACCGTGGAGGGTCATTGGAAACTGGTCGACTTGAGTGCAGAAGAGGGAAGTGGAATTCCATGTGTAGCGGTGAAATGCGTAGAGATATGGAGGAACACCAGTGGCGAAGGCGGCTTCCTGGTCTGTAACTGACACTGAGGCGCGAAAGCGTGGGGAGCAAACAGG` |
| ASV2453 | 429 | `TGGGGAATATTGGACAATGGGGGCAACCCTGATCCAGCAACGCCGCGTGAATGAAGAAGGCCTTAGGGTTGTAAAGTTCTTTTGTCCGAGAGCAAAACTTTGGGGGTAATATACTTAAAGGTTGAGAGTATCGGAAGAATAAGCACCGGCTAACTACGTGCCAGCAGCCGCGGTAATACGTAGGGTGCAAGCGTTAATCGGAATTACTGGGCGTAAAGAGTGCGTAGGCGGATGTAAAAGTTAGATGTGAAATACCTGGGCTTAACCTAGGAGGTGCATTTAAGACTGTATGTCTAGAGTTAGTAAGAGGATGGGAGAATTCCGAGTGTAGCAGTGAAATGCGTAGAGATTCGGAGGAATACCGGTGGCGAAGGCGCCCATCTGGGATATAACTGACGCTGAGGCACGAAAGCGTGGGGAGCAAACAGG` |
| ASV4179 | 404 | `TGGGGAATATTGGACAATGGGCGAAAGCCTGATCCAGCAATGCCGCGTGAGTGAAGAAGGCCTTCGGGTTGTAAAGCTCTTTTGCCCGGGAAGATAATGACTGTACCGGGAGAATAAGCCCCGGCTAACTCCGTGCCAGCAGCCGCGGTAATACGGAGGGGGCTAGCGTTGTTCGGAATTACTGGGCGTAAAGCGCACGTAGGCGGCTGTTCAAGTCAGAGGTGAAAGCCCGGGGCTCAACTCCGGAATTGCCTTTGAAACTGGACGGCTAGAACACGGGAGAGGTGAGTGGAATTCCGAGTGGAGAGGTGAAATTCGTAGATATTCGGAGGAACACCAGTGGCGAAGGCGGCTCACTGGACCGTTGTTGACGCTGAGGTGCGAAAGCGTGGGGAGCAAACAGG` |
| ASV2819 | 404 | `TGGGGAATATTGGACAATGGGGGAAACCCTGATCCAGCAATGCCGCGTGAGTGATGAAGGCCTTAGGGTTGTAAAGCTCTTTTACTAGGGATGATAATGACAGTACCTAGAGAATAAGCCCCGGCTAACTCCGTGCCAGCAGCCGCGGTAATACGGAGGGGGCTAGCGTTGTTCGGAATTACTGGGCGTAAAGAGTACGTAGGCGGTTATTCAAGTCAGAGGTGAAAGCCTGGAGCTCAACTCCAGAACTGCCTTTGAAACTAGATAGCTAGAATCATGGAGAGGTTAGTGGAATTCCGAGTGTAGAGGTGAAATTCGTAGATATTCGGAAGAACACCAGTGGCGAAGGCGACTAACTGGACATGTATTGACGCTGAGGTACGAAAGCGTGGGGAGCAAACAGG` |
| ASV260 | 404 | `TTAGGAATATTGGACAATGGGGGAAACCCTGATCCAGCCATGCCGCGTGTGTGAAGAAGGCCTTAGGGTTGTAAAGCACTTTCGGCTGTGAAGATAATGACAGTAGCAGAAGAAGAAGCTCCGGCTAAATTCGTGCCAGCAGCCGCGGTAATACGAATGGAGCGAGCGTTGTTCGGAATCACTGGGCGTAAAGCGTGCGCAGGCGGTTATGAAAGTCAGAAGTGAAAGCCCAGGGCTTAACCCTGGAATTGCTTTTGAAACTCCATGACTAGAGTACTGGAGGGGTTGGCGGAATTTCGAGTGTAGCAGTGAAATGCGTAGATATTCGAAGGAACACCGATGGCGTAGGCAGCCAACTGGACAGTAACTGACGCTCATGCACGAAAGCGTGGGGATCAAACAGG` |
| ASV3279 | 429 | `TGGGGAATATTGGACAATGGGGGAAACCCTGATCCAGCGACGCCGCGTGTGTGAAGAAGGCCTGCGGGTTGTAAAGCACTTTTAGTGGGGAGGAAATGCTCAGACCTAACACGTCTGGGAGCTGACTTAACCCAAAGAAAAAGCACCGGCTAACTCTGTGCCAGCAGCCGCGGTAATACAGAGGGTGCAAGCGTTAATCGGAATTACTGGGCGTAAAGCGTGCGTAGACGGTTCTGTAAGTCGGATGTGAAATCCCCGGGCTCAACCTGGGAATTGCATTCGAAACTGCAGAGCTAGGGTACGGTAGAGGGAAGCGGAATTTCCGGTGTAGCGGTGAAATGCGTAGATATCGGAAGGAACATCAGTGGCGAAAGCGGCTTCCTGGACCAGTACCGACGTTCAGGCACGAAAGCGTGGGGAGCAAACAGG` |
| ASV2452 | 423 | `TAGGGAATATTGGGCAATGGTCGAAAGACTGACCCAGCCATGCCGCGTGCAGGATGAAGGCGCTATGCGTTGTAAACTGCTTTTGTTAGGGAAGAAAATGGCTATGCGTAGCAACTTGACGGTACCTAAAGAATAAGCATCGGCTAACTCCGTGCCAGCAGCCGCGGTAATACGGAGGATGCAAGCGTTGTCCGGATTTATTGGGTTTAAAGGGTGCGTAGGCTGTTTTATAAGTCAGTGGTTAAATTATCTTGCTTAACAAGAGACCTGCCATTGATACTGTAAAACTTGAATACTGACTAGGTAGGCGGAATTGGTAGTGTAGCGGTGAAATGCTTAGATATTACCAGGAACACCGATAGCGAAAGCAGCTTACTGGACAGTTATTGACGCTGAGGCACGAAAGTGCGGGTATCAAACAGG` |
| ASV5411 | 428 | `TTTCGAATCATTCACAATGGGCGAAAGCCTGATGGTGCGACGCCGCGTGGGGGACGAAGGTCTTCGGATTGTAAACCCCTGTCACCGGGGACAAAACGTCGTCTGCTAATACCAGCCGACCTGATTTAACCCGGAGAGGAAGCAGTGGCTAACTCTGTGCCAGCAGCCGCGGTAATACAGAGACTGCAAGCGTTGTTCGGATTCACTGGGCGTAAAGGGTGCGCAGGCGGCCGGGTGTGTCGGGTGTGAAAGCTCGAGGCTTAACCTCGAAACTGCGCCCGAAACTATCCGGCTAGAGTACTGGAGAGGGAAGCGGAATTCACGGTGTAGCAGTGAAATGCGTAGATATCGTGAGGAACACCAATGGCGAAGGCAGCTTCCTGGACAGTTACTGACGCTCAGGCACGAAAGCGTGGGGAGCAAAAGGG` |
| ASV256 | 404 | `TGGGGAATATTGGACAATGGGCGAAAGCCTGATCCAGCAATACCGCGTGAATGATGAAGGCCTTAGGGTTGTAAAGTTCTTTTAGTTGGAAAGATAATGACGGTACCAACAGAAAAAGCCCCGGCTAACTCCGTGCCAGCAGCCGCGGTAAGACGGAGGGGGCTAGCGTTGTTCGGAATTACTGGGCGTAAAGGGCGCGTAGGCGGATTAGTAAGTTGGGAGTGAAAGCCCGGGGCTTAACCTCGGAACTGCTTTCAAAACTGCTAGTCTTGAGTGAAGTAGGGGATGATGGAATTCCTAGTGTAGAGGTGAAATTCTTAGATATTAGGAGGAACACCGGTGGCGAAGGCGGTCATCTGGACTTCAACTGACGCTGAGGCGCGAAAGCGTGGGGAGCAAACAGG` |
| ASV4821 | 429 | `TGGGGAATATTGGACAATGGGCGAAAGCCTGATCCAGCGATGCCGCGTGTGTGAAGAAGGCCTTAGGGTTGTAAAGCACTTTTGTTGGAGACGAAAAAAAGTAGGCTAATACCCTGCTTATTGACTTAACCCAAAGAATAAGCACCGGCTAACTCTGTGCCAGCAGCCGCGGTAAGACAGAGGGTGCAAGCGTTAATCGGAATTACTGGGCGTAAAGGGCTTGTAGGCGGTTTGCTAAGTCGAGTGTTAAAACCCCGGGCTTAACCTGGGAACGTCATTCGATACTGGTAGACTTCGAGTTGAGTAGAGGTAAGTGGAATTTCCGGTGTAGCGGTGAAATGCGTAGATATCGGAAGGAACATCAGTGGCGAAGGCGGCTTACTGGACTCAAACTGACGCTGAGGAGCGAAAGCGTGGGGAGCAAACAGG` |
| ASV1731 | 429 | `TGGGGAATATTGGACAATGGGCGCAAGCCTGATCCAGCCATGCCGCGTGTGTGAAGAAGGCCTTCGGGTTGTAAAGCACTTTTGTTGGGGAAGAAAAGCATTCGGTTAATACCCGGGTGTTCTGACGGTACCCAAAGAATAAGCACCGGCTAACTTCGTGCCAGCAGCCGCGGTAATACGAAGGGTGCAAGCGTTACTCGGAATTACTGGGCGTAAAGCGTGCGTAGGTGGTTCGTTAAGTCTGCCGTGAAAGCCCCGGGCTCAACCTGGGAATTGCGGTGGATACTGGCGGGCTAGAGTGCGGTAGAGGAGAGTGGAATTCCCGGTGTAGCAGTGAAATGCGTAGAGATCGGGAGGAACATCAGTTGCGAAGGCGGCTCTCTGGACCAGCACTGACACTGAGGCACGAAAGCGTGGGGAGCAAACAGG` |
| ASV1963 | 404 | `TGGGGAATCTTAGACAATGGGCGCAAGCCTGATCTAGCCATGCCGCGTGATCGATGAAGGCCTTAGGGTTGTAAAGATCTTTCAGTGGGGAAGATAATGACTGTACCCACAGAAGAAGCCCCGGCTAACTCCGTGCCAGCAGCCGCGGTAATACGGAGGGGGCTAGCGTTGTTCGGAATTACTGGGCGTAAAGCGCACGTAGGCGGACCAGAAAGTCAGAGGTGAAATCCCAGGGCTCAACCTTGGAACTGCCTTTGAAACTCCTGGTCTTGAGTTCGAGAGAGGTGAGTGGAATTCCGAGTGTAGAGGTGAAATTCGTAGATATTCGGAGGAACACCAGTGGCGAAGGCGGCTCACTGGCTCGATACTGACGCTGAGGTGCGAAAGCGTGGGGAGCAAACAGG` |
| ASV4770 | 429 | `TGGGGAATATTGGACAATGGGGGAAACCCTGATCCAGCAATACCGCGTGTGTGAAGAAGGCCTTCGGGTTGTAAAGCACTTTAGGTTGGGAAGAAGGTATGTAGTTTAAGAGATGACATATTTGACTGTACCAACAGAATAAGCACCGGCTAACTCTGTGCCAGCAGCCGCGGTAATACAGAGGGTGCAAGCGTTAATCGGAGTTACTGGGCGTAAAGGGCGCGTAGGTGGTTGTATAAGTGAGATGTGAAAGCCCTGGGCTTAACCTAGGAACTGCATCTTATACTGTGCAACTGGAGTACTGTAGAGGGTAGTGGAATTTCCGGTGTAGCGGTGAAATGCGTAGAGATCGGAAGGAACACCAGTGGCGAAGGCGGCTACCTGGACAGATACTGACACTGAGGCGCGAGAGCGTGGGGAGCAAACAGG` |
| ASV3432 | 404 | `TGGGGAATCTTGGACAATGGGCGCAAGCCTGATCCAGCCATGCCGCGTGAGTGATGAAGGCCTTAGGGTTGTAAAGCTCTTTTGGCGGGGACGATAATGACGGTACCCGCAGAATAAGTCCCGGCTAACTTCGTGCCAGCAGCCGCGGTAATACGAAGGGGACTAGCGTTGTTCGGAATCACTGGGCGTAAAGCGCACGTAGGCGGATTTGTAAGTCAGGGGTGAAATCCCGGGGCTCAACCTCGGAACTGCCTTTGATACTGCGGATCTCGAGTCCGATAGAGGTGGGTGGAATTCCTAGTGTAGAGGTGAAATTCGTAGATATTAGGAAGAACACCGGTGGCGAAGGCGGCCCACTGGATCGGTACTGACGCTGAGGTGCGAAAGCGTGGGGAGCAAACAGG` |
| ASV5199 | 424 | `TGAGGAATATTGGACAATGGATGGAAATCTGATCCAGCCATGCCGCGTGCAGGAAGACGGCCCTATGGGTTGTAAACTGCTTTTGTATGGGAGCAATAAGATCTACGTGTAGATTGATGAGAGTACCATACGAATAAGCACCGGCTAACTCCGTGCCAGCAGCCGCGGTAATACGGAGGGTGCAAGCGTTATCCGGATTTATTGGGTTTAAAGGGTGCGTAGGCGGTTTGATAAGTCAGCGGTGAAATATTTCAGCTTAACTGGAAGGGTGCCGTTGATACTGTCGAGCTTGAATTTAGTTGCTGTGGGTGGAATGTGTGGTGTAGCGGTGAAATGCATAGATATCACACAGAATATCGATTGCGAAGGCAGCTCACAAAGCTAATATTGACGCTGATGCACGAAAGTGTGGGGATCAAACAGG` |
| ASV3968 | 430 | `TGGGGAATTTTGGACAATGGGGGCAACCCTGATCCAGCCATGCCGCGTGCGGGAAGAAGGCCTTCGGGTTGTAAACCGCTTTAGTCCGGGAAAAATGTCACTTTCGTTAATACCGAAGGTGGTTGATGGTACCGGAAGAATAAGGACCGGCTAACTACGTGCCAGCAGCCGCGGTAATACGTAGGGTCCAAGCGTTAATCGGAATTACTGGGCGTAAAGCGTGCGCAGGCGGTTTTGTAAGACAGAGGTGAAATCCCCGAGCTTAACTTGGGAACGGCCTTTGTGACTGCAAAGCTAGAGTGTGTCAGAGGGGGGTGGAATTCCACGTGTAGCAGTGAAATGCGTAGAGATGTGGAGGAACACCGATGGCGAAGGCAACCCCCTGGGATAACACTGACGCTCATGCACGAAAGCGTGGGGAGCAAACAGG` |
| ASV5454 | 428 | `TCGAGAATCTTTCACAATGGGGGCAACCCTGATGGAGCGACGCCGCGTGGAGGATAAGGTCTTCGGATTGTAAACTCCTGTCATGCGCGAACAAGGTTGCAGAGTTAATAGCTTTGCAAATTGATGGTACCGCAAGAGGAAGAGACGGCTAACTCTGTGCCAGCAGCCGCGGTAATACAGAGGTCTCAAGCGTTGTTCGGAATCACTGGGCGTAAAGGGTGCGTAGGTGGCGTGGAAAGTCAGATGTGAAAGCCCGGGGCTCAACCTCGGAATTGCATCCGATACTCCCATGCTAGAGAACTGGAGAGGAATGTAGAATTCTCGGTGTAGCAGTGAAATGCGTAGATATCGAGAGGAATACCAACGGCGAAGGCAGCATTCTGGACAGTTTCTGACACTGAGGCACGAAGGCCAGGGGAGCAAACGGG` |
| ASV114 | 404 | `TGGGGAATATTGGACAATGGGCGAAAGCCTGATCCAGCAATACCGCGTGAGTGATGAAGGCCTTAGGGTTGTAAAGCTCTTTCAGTTGGAAAGATAATGACGGTACCAACAGAAGAAGCCCCGGCTAACTACGTGCCAGCAGCCGCGGTAAGACGTAGGGGGCTAGCGTTGTTCGGAATTACTGGGCGTAAAGAGCGCGTAGGCGGTCTGTTAAGTTGGAAGTGAAAGCCCGGGGCTTAACCCCGGAATTGCTTTCAAAACTGGCAAGCTAGAGTGCGAGAGGGGGTGATGGAATTCCTAGTGTAGAGGTGAAATTCTTAGATATTAGGAGGAACACCGGTGGCGAAGGCGATCACCTGGCTCGATACTGACGCTGAGGCGCGAAAGCGTGGGGAGCAAACAGG` |
| ASV2159 | 407 | `TGGGGAATTTTCCGCAATGGGCGAAAGCCTGACGGAGCAATACCGCGTGAGGGAAGAAGGCTCTTGGGTTGTAAACCTCTTTTCTTGGGGAAGAAAAAAATGACGGTACCCAAGGAATCAGCATCGGCTAACTCCGTGCCAGCAGCCGCGGTAATACGGAGGATGCAAGCGTTATCCGGAATGATTGGGCGTAAAGCGTCCGCAGGTGGCATTTCAAGTTTGCGGTTAAAGGTTCTGGCTCAACCAGAGACAAGCCGTGAAAACTGAGAAGCTAGAGTATGGTAGGGGCAGAGGGAATTCCCAGTGTAGCGGTGAAATGCGTAGAGATTGGGAAGAACACCGGTGGCGAAAGCGCTCTGCTAGGCCAAAACTGACACTGAGGGACGAAAGCTAGGGGAGCGAATGGG` |
| ASV3731 | 406 | `TTAGGAATCTTGCACAATGGGCGAAAGCCTGATGCAGCGACGCCGCGTGAAGGATGAAGGTTCTCGGATCGTAAACTTCTTTTAAGTGGGAAGAAATTTGACGGTACCACTTGAATAAGCCCCGGCTAACTACGTGCCAGCAGCCGCGGTAATACGTAGGGGGCGAGCGTTGTCCGAAGTTACTGGGCGTAAAGCGCGCGTAGGCGGTTTCTTAAGTCTGGGGTGAAAGGTTCAACGCTCAACGTGAACAGTGCCTTGGATACTGGGAGACTTGAGTTAGGGAGAGGGTAGTAGAATTCCTGGTGTAGCGGTGAAATGCGTAGATATCAGGAGGAATACCAATGGCGAAGGCAGCTACCTGGCCTATAACTGACGCTGAGGTGCGAAAGCGTGGGGAGCAAACGGG` |
| ASV433 | 429 | `TGGGGAATATTGGACAATGGGGGAAACCCTGATCCAGCAATGCCGCGTGTGTGAAGAAGGCCTGAGGGTTGTAAAGCACTTTCAGTGGGGAGGAGGATTTCCGGGTTAAGAGCTAGGGGATTGGACGTTACCCACAGAAGAAGCACCGGCTAACTCCGTGCCAGCAGCCGCGGTAATACGGAGGGTGCAAGCGTTAATCGGAATTACTGGGCGTAAAGGGTGCGTAGGTGGTTTGATAAGTTATGTGTAAAATCCCTGGGCTCAACCTGGGCAGGTCATATAAAACTGTTAGACTCGAGTATGGGAGAGGGTAGTGGAATTTCCGGTGTAGCGGTGAAATGCGTAGAGATCGGAAGGAACACCAGTGGCGAAGGCGGCTACCTGGCCTAATACTGACACTGAGGCACGAAAGCGTGGGGAGCAAACAGG` |
| ASV5043 | 429 | `TGGGGAATATTGGACAATGGGCGCAAGCCTGATCCAGCAATGCCGCGTGTGTGAAGAAGGCCTTCGGGTTGTAAAGCACTTTTAGTGGGGACAAAAAAGGTACGGGTAATATTCGTGCAAATTGATGGTACCCACAGAAAAAGCACCGGCTAACTCTGTGCCAGCAGCCGCGGTAATACAGAGGGTGCGAGCGTTAATCGGAATTACTGGGCGTAAAGCGCACGTAGGTGGTTTGTTAAGTCAGATGTGAAAGCCCCGGGCTTAACCTGGGAATGGCATTTGATACTGGCGAGCTAGAGTTTGGTAGAGGAAGGCGGAATTCCCGGTGTAGCGGTGAAATGCGTAGATATCGGGAGGAACACCAGTGGCGAAGGCGGCCTTCTGGACTAAAACTGACACTGAGGTGCGAAAGCGTGGGGAGCAAACAGG` |
| ASV105 | 423 | `TAGGGAATATTGGTCAATGGGCGCAAGCCTGAACCAGCCATGCCGCGTGCAGGAAGAATGCCCTCTGGGTTGTAAACTGCTTTTGACAGGGAAGAAATCACTTATGCGTAAGTGTCTGACGGTACCTGTAGAATAAGCACCGGCTAACTCCGTGCCAGCAGCCGCGGTAATACGGAGGGTGCAAGCGTTATCCGGATTTATTGGGTTTAAAGGGTGCGTAGGCGGTAGAGTAAGTCAGTGGTGAAAGCCTGTTGCCTAACAACAGAACTGCCATTGATACTGCTTTACTTGAGTATAGATGAGGTAGGCGGAATTAATAGTGTAGCGGTGAAATGCTTAGATATTATTAAGAACTCCGATAGCGAAGGCAGCTTACTAAACTATAACTGACGCTGAGGCACGAAAGTGTGGGGATCAAACAGG` |
| ASV1008 | 404 | `TGGGGAATCTTGCGCAATGGGCGAAAGCCTGACGCAGCCATGCCGCGTGAATGATGAAGGTCTTAGGATTGTAAAATTCTTTCACCGGGGACGATAATGACGGTACCCGGAGAAGAAGCCCCGGCTAACTTCGTGCCAGCAGCCGCGGTAATACGAAGGGGGCTAGCGTTGCTCGGAATTACTGGGCGTAAAGGGCGCGTAGGCGGACATTTAAGTCAGGGGTGAAATCCCAGAGCTCAACTCTGGAACTGCCTTTGATACTGGGTGTCTTGAGTGTGAGAGAGGTATGTGGAACTCCGAGTGTAGAGGTGAAATTCGTAGATATTCGGAAGAACACCAGTGGCGAAGGCGACATACTGGCTCATTACTGACGCTGAGGCGCGAAAGCGTGGGGAGCAAACAGG` |
| ASV1655 | 424 | `TGAGGAATATTGGTCAATGGGCGTAAGCCTGAACCAGCCAAGTAGCGTGAAGGACGAAGGCCCTATGGGTCGTAAACTTCTTTTATAGGGGAATAAAGTGGGGAACGTGTTCCCCTTTGTATGTACCCTACGAATAAGGATCGGCTAACTCCGTGCCAGCAGCCGCGGTAATACGGAGGATCCGAGCGTTATCCGGATTTATTGGGTTTAAAGGGAGCGTAGGTGGATATATAAGTCAGTTGTGAAAGTTTACGGCTCAACCGTGAAATTGCAGTTGATACTGTGTATCTTGAGTGTACAAGAGGTGGGCGGAATTCGTGGTGTAGCGGTGAAATGCTTAGATATCACGAAGAACTCCGATTGCGAAGGCAGCTCACTGGGGTACAACTGACACTGAGGCTCGAAAGTGTGGGTATCAAACAGG` |
| ASV5139 | 429 | `TCGAGAATCATTCACAATGGGGGAAACCCTGATGGTGCAACGCCGCGTGGAGGATGAAGGCCCTAGGGTCGTAAACTCCTGTCATAGCAGAGTAAGACATATTAGTTAATAGCTAGTATGTTTGATAGTATGCTAAGAGGAAGGGACGGCTAACTTCGTGCCAGCAGCCGCGGTAATACGAAGGTCCCGAGCGTTATTCGGAATCACTGGGCGTAAAGGGAGCGTAGGCTGCGCGGTAAGTCAGATGTGAAATCTGGGGGCTCAACCCCCAAACTGCATTCGATACTGCCGTGCTAGAGTAATGGAGGGGTAACTGGAATTCTCAGTGTAGCAGTGAAATGCGTAGATATTGAGAGGAAGACCAATGGCGAAAGCAGGTTACTGGACATTTACTGACGCTGAGGCTCGAAGGCTAGGGGAGCAAAAGGG` |
| ASV2006 | 406 | `TTGGGAATCTTGCACAATGGGGGAAACCCTGATGCAGCGACGCCGCGTGGACGATGAAGGGTCTAGGCTCGTAAAGTCCTTTTTCAGGGAAAGACTTAGGACGGTACCCTGAGAATAAGCACCGGCTAACTACGTGCCAGCAGCCGCGGTAAGACGTAGGGTGCAAGCGTTGTCCGGATTTACTGGGCGTAAAGAGCTCGTAGGCGGTCTGTTAAGTGAGAAGTGAAATCCCCAGCGCTCAACGCGGGAACTGCTTTTCATACTGGCAGACTTGAGGAATGCAGAGGTTAGTAGAATTCCTGGTGTACCGGTGACATGGGTTGATATCAGGAGGAATACCAATGGCGAAGGCAGCTAACTGGGCATTATCTGACGCTGAGGAGCGAAAGCGTGGGTAGCAAACAGG` |
| ASV5374 | 404 | `TGAGGAATATTGGACAATGGGGGAAACCCTGATCCAGCCATGCCGCGTGAGTGATGAAGGCCTTCGGGTTGTAAAGCTCTTTCAGATGCGAAGATAATGACGGTAACATCAGAAGAAGCCCCGGCTAAATTCGTGCCAGCAGCCGCGGTAATACGAATGGGGCAAGCGTTGTTCGGAATCACTGGGCTTAAAGCGTGCGCAGGCGGCCAAGCAAGTCAGAAGTGAAATCCCAGGGCTTAACCCTGGAATTGCTTTTGAAACTGCATGGCTAGAATATGGGAGAGGTTAGCAGAATTCCGAGTGTAGAGGTGAAATTCGCAGATATTCGGAGGAATACCAGTGGCGAAGGCGGCTAACTGGACCATTATTGACGCTCAGGCACGAAAGCGTGGGGATCAAACAGG` |
| ASV575 | 429 | `TCGAGAATAATTCACAATGGGGGCAACCCTGATGGTGCAACGCCGCGTGGAGGATGACGGTCTTCGGATTGTAAACTCCTGTCATCTGGGAGTAAGGCATGGTCGTGAATAGCGGACATGATTGATAGTACCAGAAGAGGAAGGGACGGCTAACTTCGTGCCAGCAGCCGCGGTAATACGAAGGTCCCAAGCGTTGTTCGGAATCACTGGGCGTAAAGGGAGCGTAGGCGGCGTGGTAAGTCAGATGTGAAATCCCGGGGCTCAACCCCGGAACTGCATCCGATACTGCCATGCTAGAGGATTGGAGAGGTAGCTGGAATTCTTGGTGTAGCAGTGAAATGCGTAGAGATCAAGAGGAACACTCGTGGCGAAAGCGAGCTACTGGACAATTCCTGACGCTGAGGCTCGAAGGCTAGGGTAGCGAAAGGG` |
| ASV1518 | 429 | `TGGGGAATTTTGGACAATGGGCGCAAGCCTGATCCAGCAATGCCGCGTGCAGGATGAAGGCCTTCGGGTTGTAAACTGCTTTTGTACGGAACGAAACGGCTCTGGTTAATACCCGGGGCTAATGACGGTACCGTAAGAATAAGCACCGGCTAACTACGTGCCAGCAGCCGCGGTAATACGTAGGGTGCGAGCGTTAATCGGAATTACTGGGCGTAAAGCGTGCGCAGGTGGTTATGTAAGACAGATGTGAAATCCCCGGGCTCAACCTGGGACCTGCATTTGTGACTGCATGGCTAGAGTACGGCAGAGGGGGATGGAATTCCGCGTGTAGCAGTGAAATGCGTAGATATGCGGAGGAACACCGATGGCGAAGGCAGTCCCCTGGGCCTGTACTGACACTCATGCACGAAAGCGTGGGGAGCAAACAGG` |
| ASV3397 | 425 | `TAAGGAATATTGGACAATGGGCGGAAGCCTGATCCAGCCATGCCGCGTGAAGGATGAAGGCGCTCAGCGTTGTAAACTTCTTTTGGATGGGAACAATAACTCCGACTTGTTGGAGCTTGAGGGTACCATCAGAATAAGCACCGGCTAACTCCGTGCCAGCAGCCGCGGTAATACGGAGGGTGCAAGCGTTATCCGGAATCACTGGGTTTAAAGGGTGCGTAGGCGGTCTAATAAGTCAGTCGTGAAATCGCTGGGCTCAACCCATGCAATTGCGATTGATACTGTTAGACTTGAATTGGGTTGAGGCTAGCGGAATGCGACATGTAGCGGTGAAATGCTTAGATATGTCGCAGAACACCGATTGCGAAGGCAGCTAGCTGGGCCTTTATTGACGCTGAGGCACGAAAGCGTGGGGAGCAAACAGG` |
| ASV1890 | 424 | `TAGGGAATATTGGGCAATGGGCGAAAGCCTGACCCAGCCATGCCGCGTGAAGGAATGAAGGCGTTATGCGTTGTAAACTTCTTTTATATGGGAAGAAAAAGCTCATGCGTGAGAAATTGACGGTACCATATGAATAAGCATCGGCTAACTCCGTGCCAGCAGCCGCGGTAATACGGAGGATGCAAGCGTTGTCCGGATTTATTGGGTTTAAAGGGTGCGTAGGTGGTTCTATAAGTCCGTGGTAAAATCCTGCTGCTTAACAGTAGACTTGCCATTGATACTGTAGAACTTGAGTACTGAAGAGGTAGGCGGAATTGGTGGTGTAGCGGTGAAATGCATAGATACCACCAGGAACTCCGATTGCGAAGGCAGCTTACTGGTCAGTTACTGACACTGAGGCACGAAAGCGTGGGGATCAAACAGG` |
| ASV4069 | 407 | `TGGGGAATTTTCCACAATGGGCGAAAGCCTGATGGAGCAATACCGCGTGAGGGATGAAGGATCGTGGTCCGTAAACCTCTTTTCTTAACGAAGAAGTTCTGACGGTAGTTAAGGAATAAGCACCGGCTAACTCCGTGCCAGCAGCCGCGGTAATACGGGGGGTGCAAGCGTTGTCCGGAATGATTGGGCGTAAAGCATCTGTAGGTGGTTTTTAAAGTCGACTGTTAAAGCCCAGGGCTCAACTTTGGAAAAGCGGTCGAGTACTTAAGAACTTGAGTTTGGTAGGGGTAGAGGGAATTCCTTGTGGAGCGGTGAAATGCACAGAGATAAGGAAGAACACCAATGGCGAAAGCACTCTACTGGGCCAAGACTGACACTGAGAGATGAAAGTCGGGGGAGCGAAAAGG` |
| ASV5132 | 375 | `TCGGGAATTTTGGTCAATGGACGAAAGTCTGAACCAGCGACGCCGCGTGAAGGATGACGGCCTTCGGGTTGTAAACTTCTGAAAATAGCACCGGCTAACTACGTGCCAGCAGCAGCGGTCATACGTAGGGTGCAAGCGTTGTCCGGATTTATTGGGCGTAAAGAGTTCTGTAGGCGGTTTTGTAAGTCAAGGGTTAAATCTTCCAGCTTAACTGGAAAACTGCTTTTGATACTGCATGACTAGAACCAGTTAGGGGACAGCGGAACTTATGGTGGAGCAGTGAAATGCGTTGATATCATAAGGAACACCAAGGGGGAAGCCAGCTGTCTGGGACTGTGTTGACGCTGAGAGACGAAAGCGTGGGGAGCAAAACGG` |
| ASV891 | 404 | `TGGGGAATATTGGACAATGGGCGAAAGCCTGATCCAGCAATACCGCGTGAATGATGAAGGCCTTAGGGTTGTAAAGTTCTTTTAATAGGGAAGATAATGACGGTACCTATAGAAAAAGCCCCGGCTAACTCCGTGCCAGCAGCCGCGGTAAGACGGAGGGGGCTAGCGTTGTTCGGAATTACTGGGCGTAAAGGGCGCGTAGGCGGATTAGTAAGTTGGGAGTGAAAGCCCGGGGCTTAACCTCGGAACTGCTTTCAAAACTGCTAGTCTAGAGTGAAGTAGGGGGTGATGGAATTCCTAGTGTAGAGGTGAAATTCTTAGATATTAGGAGGAACACCGGTGGCGAAGGCGGTCACCTGGACTTCAACTGACGCTGAGGCGCGAAAGCGTGGGGAGCAAACAGG` |
| ASV522 | 424 | `TGAGGAATATTGGTCAATGGACGCAAGTCTGAACCAGCCATGCCGCGTGAAGGATGAAGGCGTTCTGCGTTGTAAACTTCTTTTATCTGGGAAGAAACCTACCTTTTCTAAGGTAGCCGACGGTACCAGAGGAATAAGCACCGGCTAACTCCGTGCCAGCAGCCGCGGTAATACGGAGGGTGCAAGCGTTATCCGGATTTACTGGGTTTAAAGGGTGTGTAGGCGGGTATTTAAGTCAGTGGTGAAATCTCCGAGCTCAACTCGGAAACTGCCATTGATACTATTTATCTTGAATATTGTTGAGGTAGGCGGAATATGTCATGTAGCGGTGAAATGCTTAGATATGACATAGAACACCGATTGCGAAGGCAGCTTGCTAAACAATTATTGACGCTGAGGCACGAAAGCGTGGGGATCAAACAGG` |
| ASV2414 | 429 | `TGGGGAATATTGGACAATGGGGGCAACCCTGATCCAGCCATGCCGCGTGTGTGAAGAAGGCCTTCGGGTTGTAAAGCACTTTTATCCGGAACGAAAAGCTGTCGGTTAATAACCGGCAGTGCTGACGGTACCGGAAGAATAAGCACCGGCTAACTTCGTGCCAGCAGCCGCGGTAATACGAAGGGTGCAAGCGTTACTCGGAATTACTGGGCGTAAAGCGTATGTAGGCGGTTTGCTAAGTCTGATGTGAAAGCCCCGGGCTCAACCTGGGAATGGCATTGGATACTGGTGAGCTAGAGTACGGTAGAGGAGAGTGGAATTCCCGGTGTAGCAGTGAAATGCGTAGAGATCGGGAGGAACATCAATGGCGAAGGCAACTCTCTGGACCAGTACTGACGCTGAGATACGAAAGCGTGGGGAGCAAACAGG` |
| ASV5676 | 405 | `TGAGGAATATTGGTCAATGGGCGCAAGCCTGAACCAGCAACGCCGCGTGAAGGATGAAGGCCTTCGGGTTGTAAACTTCTTTTGCAGGGGACAAGAAAGGATGGTACTTTGCGAATAAGTCACGGCTAACTACGTGCCAGCAGCCGCGGTAATACGTAGGTGGCAAGCGTTATCCGGAGTTACTGGGCGTAAAGCGCACGCAGGCGGTTAGATAAGTTTGATGTAAAATCTTCTGGCTTAACTGGGAGAAGTCGTTGAATACTGTCTGACTCGAGGCAATGAGAGGAGTGCAGAATTCCTGGTGTAATGGTAAAATATGTAGAGATCAGGAGGAATATCTGAGGCGAAAGCGGCACTCTGGCATTAGCCTGACGCTCAGGTGCGAGAGCGTGGGTAGCGAACGGG` |
| ASV2807 | 419 | `TGAGGAATATTGCGCAATGGGCGAAAGCCTGACGCAGCGACGCCGCGTGGGGGATGAAGGTCTTCGGATTGTAAACCCCTTTCGGGAGGGAAGATGGGACGGGCAACCGTTCGGACGGTACCTCCAGAAGCAGCCACGGCTAACTTCGTGCCAGCAGCCGCGGTAATACGAAGGTGGCAAGCGTTGTTCGGATTTACTGGGCGTACAGGGAGCGTAGGCGGTTTGGTAAGCCCTCCGGGAAATCTTCGGGCTTAACCCGAAAAGGTCGGAGGGGACTGCCAGGCTAGAGGACGGGAGAGGAGCGCGGAATTCCCGGTGTAGCGGTGAAATGCGTAGAGATCGGGAGGAAGGCCGGTGGCGAAGGCGGCGCTCTGGAACGTTCCTGACGCTGAGGCTCGAAAGCGTGGGGAGCAAACAGG` |
| ASV5638 | 429 | `TGGGGAATATTGGACAATGGGGGCAACCCTGATCCAGCAATACCGCGTGTGTGAAGAAGGCCTGCGGGTTGTAAAGCACTTTCAGTGGGGAGGAAAGGTTAACTCGGATAATGGGTTGACTGTGACGTTACCCACAGAAGAAGCACCGGCTAACTCTGTGCCAGCAGCCGCGGTAATACAGAGGGTGCAAGCGTTAATCGGAATTACTGGGCGTAAAGGGTGCGTAGGTGGTTAAGTAAGTCAGGTGTGAAATCCCAGGGCTCAACCTTGGAATTGCGCTTGAAACTGCATAGCTAGAGTCCTGTAGAGGGTAGTGGAATTTCCAGTGTAGCGGTGAAATGCGTAGATATTGGAAGGAACATCAGTGGCGAAGGCGACTACCTGGACAGAGACTGACACTGAGGCACGAAAGCGTGGGGAGCAAACAGG` |
| ASV602 | 429 | `TGGGGAATTTTGGACAATGGGCGCAAGCCTGATCCAGCAATGCCGCGTGCAGGATGAAGGCCTTCGGGTTGTAAACTGCTTTTGTACGGAACGAAACGGCGAGCTCTAATAAAGTTTGCTAATGACGGTACCGTAAGAATAAGCACCGGCTAACTACGTGCCAGCAGCCGCGGTAATACGTAGGGTGCGAGCGTTAATCGGAATTACTGGGCGTAAAGCGTGCGCAGGCGGTTATATAAGACAGATGTGAAATCCCCGGGCTCAACCTGGGACCTGCATTTGTGACTGTATAGCTAGAGTACGGTAGAGGGGGATGGAATTCCGCGTGTAGCAGTGAAATGCGTAGATATGCGGAGGAACACCGATGGCGAAGGCAATCCCCTGGACCTGTACTGACGCTCATGCACGAAAGCGTGGGGAGCAAACAGG` |
| ASV4070 | 404 | `TGGGGAATATTGGACAATGGGGGAAACCCTGATCCAGCAATGCCGCGTGGGTGATGAAGGTCTTCGGATTGTAAAGCCCTTTTGGCAGGGACGATGATGACGGTACCTGCAGAATAAGCTCCGGCTAACTTCGTGCCAGCAGCCGCGGTAATACGAAGGGAGCAAGCGTTGTTCGGATTTACTGGGCGTAAAGGATGCGTAGGTGGTTTATTAAGTTGGAAGTGAAAGCCCCAGGCTTAACTTGGGAATTGCTTTCAAAACTGGTAGACTTGAGTATGGAAGAGGTTGGTGGAATTCCCAGTGTAGAGGTGAAATTCGTAGATATTGGGAAGAACATCCGTGGCGAAGGCGGCCAACTGGTCCATTACTGACACTGAGGCATGACAGCGTGGGGAGCAAACAGG` |
| ASV430 | 430 | `TGGGGAATATTGGACAATGGGGGCAACCCTGATCCAGCAATGCCGCGTGTGTGAAGAAGGCCTTCGGGTTGTAAAGCACTTTTGTTCGGGACGAAATCTTACGAATTAATACCTCGTGAGGATGACGGTACCGAAAGAATAAGCACCGGCTAACTTCGTGCCAGCAGCCGCGGTAATACGAAGGGTGCAAGCGTTAATCGGAATTACTGGGCGTAAAGCGTGCGTAGACGGCACACTAAGTCAGCTGTGAAACCCCCGGGCTCAACCTGGGACCTGCAGCTGATACTGGTGAGCTTGGAGTTTGAGAGAGGGTGGTGGAATTCCTGGTGTAGCGGTGAAATGCGTAGAGATCAGGAGGAACACTAGTGGCGAAGGCGGCCACCTGGCTTAAAACTGACGTTGAGGCACGAAAGCGTGGGGAGCAAACAGG` |
| ASV3551 | 404 | `TGGGGAATATTGGACAATGGGCGCAAGCCTGATCCAGCAATGCCGCGTGAGTGATGAAGGCCTTAGGGTTGTAAAGCTCTTTTACCCGGGAAGATAATGACTGTACCGGGAGAATAAGCCCCGGCTAACTCCGTGCCAGCAGCCGCGGTAATACGGAGGGGGCTAGCGTTGTTCGGAATTACTGGGCGTAAAGCGCACGTAGGCGGTTGTTCAAGTCAGAGGTGAAAGCCCGGAGCTCAACTTCGGAATTGCCTTTGAAACTAGACAACTTGAACGTCGGAGAGGTGAGTGGAATTCCGAGTGTAGAGGTGAAATTCGTAGATATTCGGAAGAACACCAGTGGCGAAGGCGGCTCACTGGACGACTGTTGACGCTGAGGTGCGAAAGCGTGGGGAGCAAACAGG` |
| ASV5612 | 406 | `TGAGGAATCTTCCGCAATGGGCGAAAGCCTGACGGAGCGACACCGCGTGGAGGATGAAGGCGTTTTCGTTGTAAACTCCTTTTCTGAGGGAATAACTGATGACGGTACCTCAGGAATAAGCACCGGCTAACTACGTGCCAGCAGCCGCGGTAATACGTAGGGTGCAAGCGTTATCCGGAATCATTGGGCGTAAAGCGTTCGTAGGCGTCCTTTTAAGTCATTTGTTAAATCCATCGGCTCAACCGATGACCGGCAAATGAAACTATCAGGATAGAGAATGGCAGAGGCAACTGGAATTCCGCATGTAGGGGTAAAATCCGTTGATATGCGGAGGAACACCAAAAGCGAAGGCAGGTTGCTGGGCCATTTCTGACGCTGAGGAACGAAAGCGTGGGGAGCAAACAGG` |
| ASV3088 | 404 | `TGGGGAATATTGGACAATGGGCGCAAGCCTGATCCAGCAATACCGCGTGAGTGATGAAGGTCTTCGGATTGTAAAGCTCTTTTAGTGGGGAAGATAATGACGGTACCCACAGAAAAAGCACCGGCAAACTTCGTGCCAGCAGCCGCGGTAATACGAAGGGTGCTAGCGTTGTTCGGAATTACTGGGCGTAAAGCGTACGTAGGCGGCTTAGTAAGTCGATTGTGAAAGCCCCAGGCTCAACTTGGGAACTGCAATCGATACTGCTTCGCTCGAGTTTGGTGGGGGATAGTGGAATTCCTAGTGTAGGGGTGAAATCCGTAGATATTAGGAGGAACACCGGTGGCGAAAGCGACTATCTACGCCAATACTGACGCTAAGGTACGAAAGCGTGGGGAGCAAACAGG` |
| ASV1284 | 404 | `TGGGGAATATTGGACAATGGGCGCAAGCCTGATCCAGCCATGCCGCGTGGATGAAGAAGGCCCTAGGGTTGTAAAGTCCTTTCGGTGGTGAAGATAATGACGGTAACCACAGAAGAAGCCCCGGCTAATTTCGTGCCAGCAGCCGCGGTAATACGAAAGGGGCTAGCGTTGTTCGGAATTACTGGGCGTAAAGCGCACGTAGGCGGCCTTGTAAGATGGGGGTGAAATCCCCGGGCTCAACCCGGGAACTGCCTTCATGACTGCAAAGCTAGAGGATGCGAGAGGTTAGCGGAATTCCGAGTGTAGAGGTGAAATTCGTAGATATTCGGAAGAACACCAGTGGCGAAGGCGGCTAACTGGCGCATTTCTGACGCTGAGGTGCGAAAGCGTGGGGAGCAAACAGG` |
| ASV254 | 404 | `TGGGGAATATTGGACAATGGGCGAAAGCCTGATCCAGCAATGCCGCGTGAGTGATGAAGGCCTTAGGGTTGTAAAGCTCTTTTACCAGGGATGATAATGACAGTACCTGGAGAATAAGCTCCGGCTAACTCCGTGCCAGCAGCCGCGGTAATACGGAGGGAGCTAGCGTTGTTCGGAATTACTGGGCGTAAAGAGTACGTAGGCGGTGATTCAAGTCAGAGGTGAAAGCCTGGAGCTCAACTCCAGAACTGCCTTTGAAACTAGATCGCTAGAATCATGGAGAGGTTAGTGGAATTCCGAGTGTAGAGGTGAAATTCGTAGATATTCGGAAGAACACCAGTGGCGAAGGCGACTAACTGGACATGTATTGACGCTGAGGTACGAAAGCGTGGGGAGCAAACAGG` |
| ASV3359 | 404 | `TGGGGAATATTGGACAATGGGGGAAACCCTGATCCAGCAATGCCGCGTGTATGATGAAGGTCTTAGGATTGTAAAGTACTTTTATTGGTGACGATGATGACGGTAACCAATGAATAAGCTCCGGCTAACTTCGTGCCAGCAGCCGCGGTAATACGAAGGGAGCTAGCGTTGTTCGGAATAACTGGGCGTAAAGGGTGCGTAGGTGGGTGTGTGCGTTAGATGTGAAATCCCCGGGCTTAACCTGGGAACTGCATTTAATACGGCACGTCTAGAGTGCGAGAGGGGAAAGCGGAATGGCGAGTGGAGCAGTGAAATGCATTGAGATTCGCTAGAACACCAGAGGCGAAGGCGGCTTTCTGGCTCGCTACTGACACTAAGGCACGAAAGCGTGGGGAGCAAACAGG` |
| ASV5576 | 425 | `TAGGGAATATTGGGCAATGGGCGCAAGCCTGACCCAGCCACGCCGCGTGCAGGAAGAAGGCGCTCTGCGTTGTAAACTGCTTTTGTCCGGGAAGAATAACTTGTACGTGTACAGGGAATGACGGTACCGGGGGAATAAGCACCGGCTAACTCCGTGCCAGCAGCCGCGGTAATACGGAGGGTGCAAGCGTTGTCCGGATTTATTGGGTTTAAAGGGTGCGTAGGCGGCGCCTTAAGTCCGGGGTGAAAGTTCGTAGCTCAACTACGAAAGGGCCTTGGATACTGAGGGGCTAGAGTACAGGCGAGGCTGGTGGAACGGGTGGTGTAGCGGTGAAATGCATAGATACCATCCAGAACACCGATTGCGAAGGCAGCCAGCTAGACTGTCACTGACGCTGAGGCACGAAAGCGCGGGGAGCAAACAGG` |
| ASV2779 | 429 | `TCGAGAATAATTCACAATGGGGGCAACCCTGATGGTGCAACGCCGCGTGGAGGATGAAGGTCTTCGGATTGTAAACTCCTGTCATCCGGGAGAAAAACCTGACGGTCAATAGCCGGCAGGGTTGATAGTACCGGAAGAGGAAGGGACGGCTAACTTCGTGCCAGCAGCCGCGGTAATACGAAGGTCCCAAGCGTTGTTCGGAATCACTGGGCGTAAAGGGTGCGTAGGCGGCATGGTAAGTCAGATGTGAAATCCCGGGGCTCAACCCCGGAACTGCATCCGATACTGCCATGCTAGAGGACTGGAGAGGTGACTGGAATTCTCGGTGTAGCAGTGAAATGCGTGGAGATCGAGAGGAACACTTATGGCGAAGGCAAGTCACTGGACAGTATCTGACGCTGAGGCACGAAGGCTAGGGGAGCGAAAGGG` |
| ASV5293 | 404 | `TGGGGAATATTGGACAATGGGCGAAAGCCTGATCCAGCAATGCCGCGTGAGTGATGAAGGCCCTAGGGTTGTAAAACTCTTTCAGTAGGGAAGATAATGACGGTACCTACAGAAGAAGCACCGGCTAACTTCGTGCCAGCAGCCGCGGTAATACGAAGGGTGCTAGCGTTGTTCGGAATTACTGGGCGTAAAGCGCGCGTAGGCGGCCTCGCAAGTTGGAAGTGAAATCCCAGGGCTTAACCCTGGAATTGCTTTCAAAACTGCAAGGCTAGAGAATGGTAGGGGAAAGTGGAATTCCTAGTGTAGAGGTGAAATTCGTAGATATTAGGAGGAACACCAGAGGCGAAGGCGGCTTTCTGGGCCATTTCTGACGCTAAGGCGCGAAAGCGTGGGGAGCAAACAGG` |
| ASV3370 | 430 | `TAGGGAATATTGCGCAATGGGGGAAACCCTGACGCAGCAACGCCGCGTGAGTGAGGAAGGTCTTCGGATTGTAAAACTCTTTTGCTAGGGAAAAATGACTTTGTGGCTAATATCCATAGAGAGTGATGGTACCTAAAGAATAAGGACCGGCTAACTTCGTGCCAGCAGCCGCGGTAATACGAAGGGTCCAAGCGTTGTTCGGAATCATTGGGCGTAAAGCGCGCGCAGGCGGATCAGTAAGTCAGGTGTGAAATCTCGAAGCTCAACTTCGAAACTGCGCCTGAAACTACTAGTCTAGAGTGTCGGAGGGGGTAGGGGAATTTCACGTGTAGGGGTAAAATCCGTAGAGATGTGAAGGAACACCGGGGGCGAAGGCGCCTACCTGGACGACTACTGACGCTGAGGCGCGAAAGCGTGGGGAGCAAACAGG` |
| ASV4529 | 429 | `TCGAGAATCTTTCGCAATGGGCGCAAGCCTGACGAAGCGACGCCGTGTGAGCGAAGAAGGCCTTCGGGTTGTAAAGCTCTTTCGCTAGGGAACAAGAAAGACGGAACAATACTCCGTCGATTTGATGGTACTTGGTAAAGAAGCACCGGCTAACTCCGTGCCAGCAGCTGCGGTAATACGGAGGGTGCAAGCATTGATCGGAATTACTGGGCGTAAAGGGCGCGCAGGCGGTCAGGAAAGTCAGATGTGAAATTCCGGGGCTCAACCCCGGAGCTGCATTTGAAACTTCCTATCTAGAGTTCAGTTAGGGAAAACGGAATTCCACGTGTAGCGGTGAAATGCGTAGATATGTGGAAGAACACCGGTGGTGAAGACGGTTTTCTGGGCTGATACTGACGCTGAGGCGCGAAAGCATGGGGAGCAAACAGG` |
| ASV3561 | 423 | `TAGGGAATATTGGTCAATGGGCGAGAGCCTGAACCAGCCATGCCGCGTGCAGGATGAAGGCCTTCTGGGTTGTAAACTGCTTTTGATCGGGAACAAAAAGACCATGCGTGGTAAATTGAGTGTACCGATAGAATAAGCACCGGCTAACTCCGTGCCAGCAGCCGCGGTAATACGGAGGGTGCAAGCGTTGTCCGGATTTATTGGGTTTAAAGGGTGCGTAGGCGGCCTTATAAGTCAGTGGTGAAATACGACAGCTCAACTGTCGAACTGCCATTGATACTGTAGGGCTTGAGTACAGACGAGGTAGGCGGAATTGATAGTGTAGCGGTGAAATGCATAGATACTATCAAGAACACCGATTGCGAAGGCAGCTTACTAGGCTGTAACTGACGCTGAGGCACGAAAGCGTGGGGATCAAACAGG` |
| ASV3911 | 430 | `TGGGGAATATTGGACAATGGGCGCAAGCCTGATCCAGCGACGCCGCGTGAGTGATGAAGGCCTTCGGGTTGTAAAGCTCTGTTGAGGGGAAAGAAAACGGTCAGCGCGAATAGCTCTGACCCTTGACGGTACCCCTTTAGAAAGCACCGGCTAACTCTGTGCCAGCAGCCGCGGTAAGACAGAGGGTGCAAACGTTGCTCGGAATTACTGGGCGTAAAGCGCGTGTAGGCGGGCCAACAAGTCGGACGTGAAAGCCCTGAGCTCAACTCAGGAACTGCATCCGAAACTGTTGGCCTGGAGTGGTGGAGAGGGAAGCAGAATTCCTGGTGTAGAGGTGAAATTCGTAGATATCAGGAGGAACACCGGTGGCGAAGGCGGCTTCCTGGACACTTACTGACGCTGAGACGCGAAAGCGTGGGGAGCAAACAGG` |
| ASV979 | 429 | `TGGGGAATTTTGGACAATGGGGGCAACCCTGATCCAGCAATGCCGCGTGAGTGAAGAAGGCCTTCGGGTTGTAAAGCTCTTTTGTCAGGGAAGAAAAGGTCGCCTCTAATACAGGCGGCTCATGACGGTACCTGAAGAATAAGCACCGGCTAACTACGTGCCAGCAGCCGCGGTAATACGTAGGGTGCAAGCGTTAATCGGAATTACTGGGCGTAAAGCGTGCGCAGGCGGTTATGTAAGTCAGATGTGAAATCCCCGGGCTCAACCTGGGAACTGCATTTGAGACTGCATGGCTAGAGTGTGTCAGAGGGGGGTAGAATTCCACGTGTAGCAGTGAAATGCGTAGATATGTGGAGGAATACCGATGGCGAAGGCAGCCCCCTGGGATAACACTGACGCTCATGCACGAAAGCGTGGGGAGCAAACAGG` |
| ASV2234 | 422 | `TAGGGAATTTTGCGCAATGGAGGAAACTCTGACGCAGCAACGCCATGTGTGGGATGAAGCATTTCGGTGTGTAAACCACTGTCAGCAGGGAATAAATAGTCTCTTCGGAGGCGGTTGAATGTACCTGCAAAGGAAGCCCCGGCTAACTTCGTGCCAGCAGCCGCGGTAATACGAGGGGGGCAAATGTTGTTCGGAATCACTGGGCGTAAAGGGAGCGTAGGCGGGCTTTTAAGTTAGGTGTTTAATCCCGGGGCCCAACCCCGGACAGGCACCTAAAACTGGGAGTCTTGAATAGTGGAGAGGACGGCGGAATTGGGGGTGTAGCGGTGGAATGCGTAGAGATCCCCAAGAACACCAGTTGCGAAGGCGGCCGTCTGGACACATATTGACGCTGAGGCTCGAAAGCATGGGGAGCAAACAGG` |
| ASV1097 | 404 | `TGGGGAATCTTAGACAATGGGGGCAACCCTGATCTAGCCATGCCGCGTGAGTGAAGAAGGCCTTAGGGTTGTAAAGCTCTTTCACCTGTGAAGATGATGACAGTAGCAGGATAAGAAGGACCGGCTAACTCCGTGCCAGCAGCCGCGGTAAGACGGAGGGTCCTAGCGTTGTTCGGAATAACTGGGCGTAAAGGGTACGTAGGCGGTTTGGTCAGTTAGATGTGAAAGCCCAGGGCTCAACCTTGGAACTGCATTTAATACTGCCAGACTTGAGATCGGAAGAGGAAAGCGGAATTCCTAGTGTAGAGGTGAAATTCGTAGATATTAGGAGGAACATCAGAGGCGAAGGCGGCTTTCTGGTCCGATACTGACGCTGAGGTACGAAAGCGTGGGGAGCAAACAGG` |
| ASV85 | 429 | `TGGGGAATATTGGACAATGGGCGCAAGCCTGATCCAGCCATGCCGCGTGTGTGAAGAAGGCCTTCGGGTTGTAAAGCACTTTTGTTGGGAAAGAAAAGCGGTCGGTTAATACCCGGTCGTTCTGACGGTACCCAAAGAATAAGCACCGGCTAACTTCGTGCCAGCAGCCGCGGTAATACGAAGGGTGCAAGCGTTACTCGGAATTACTGGGCGTAAAGCGTGCGTAGGTGGTTTGTTAAGTCAGATGTGAAAGCCCCGGGCTCAACCTGGGAATTGCATTTGATACTGGCAGACTAGAGTGCGGTAGAGGAGAGTGGAATTCCCGGTGTAGCAGTGAAATGCGTAGAGATCGGGAGGAACATCAGTTGCGAAGGCGGCTCTCTGGACCAGCACTGACACTGAGGCACGAAAGCGTGGGGAGCAAACAGG` |
| ASV1757 | 404 | `TGGGGAATCTTAGACAATGGGCGCAAGCCTGATCTAGCCATGCCGCGTGATCGATGAAGGCCTTAGGGTTGTAAAGATCTTTCAGCTGGGAAGATAATGACTGTACCAGCAGAAGAAGCCCCGGCTAACTCCGTGCCAGCAGCCGCGGTAATACGGAGGGGGCTAGCGTTGTTCGGAATTACTGGGCGTAAAGCGCACGTAGGCGGACCAGAAAGTTGGAGGTGAAATCCCAGGGCTCAACCTTGGAACTGCCTTCAAAACTCCTGGTCTTGAGTTCGAGAGAGGTGAGTGGAATTCCGAGTGTAGAGGTGAAATTCGTAGATATTCGGAGGAACACCAGTGGCGAAGGCGGCTCACTGGCTCGATACTGACGCTGAGGTGCGAAAGCGTGGGGAGCAAACAGG` |
| ASV681 | 430 | `TAGGGAATATTGCGCAATGGGGGAAACCCTGACGCAGCAACGCCGCGTGAGTGAGGAAGGTCTTCGGATTGTAAAACTCTTTTGCTAGGGAAAAATGACTTAACGGCTAATATCCGTTAGGAGTGATGGTACCTAAAGAATAAGGACCGGCTAACTTCGTGCCAGCAGCCGCGGTAATACGAAGGGTCCAAGCGTTGTTCGGAATCATTGGGCGTAAAGCGCGCGCAGGCGGATCAGCAAGTCAGATGTGAAATCTCGAAGCTCAACTTCGAAACTGCGTCTGAAACTGCTAGTCTAGAGTGTCGGAGGGGGTAGGGGAATTTCACGTGTAGGGGTAAAATCCGTAGAGATGTGAAGGAACACCGGGGGCGAAGGCGCCTACCTGGACGACTACTGACGCTGAGGCGCGAAAGCGTGGGGAGCAAACAGG` |
| ASV4080 | 429 | `TGGGGAATTTTGGACAATGGGCGCAAGCCTGATCCAGCCATTCCGCGTGAGTGAAGAAGGCCTTCGGGTTGTAAAGCTCTTTCGCAAGGGAAGAAACGATACCGACGAATAATCGGTGTTAATGACGGTACCTTGATAAGAAGCACCGGCTAACTACGTGCCAGCAGCCGCGGTAATACGTAGGGTGCGAGCGTTAATCGGAATTACTGGGCGTAAAGCGTGCGCAGGCGGTTTTGTAAGTCAGATGTGAAATCCCCGGGCTCAACCTGGGAACTGCGTTTGAAACTACAAGGCTAGAGTATGGTAGAGGGGGGTAGAATTCCACGTGTAGCAGTGAAATGCGTAGAGATGTGGAGGAATACCAATGGCGAAGGCAGCCCCCTGGACTAATACTGACGCTCATGCACGAAAGCGTGGGGAGCAAACAGG` |
| ASV1490 | 429 | `TGGGGAATTTTGGACAATGGGCGCAAGCCTGATCCAGCAATGCCGCGTGCAGGATGAAGGCCTTCGGGTTGTAAACTGCTTTTGTACGGAACGAAAAGGTTCTCTCTAATACAGGGGGCCCATGACGGTACCGTAAGAATAAGCACCGGCTAACTACGTGCCAGCAGCCGCGGTAATACGTAGGGTGCAAGCGTTAATCGGAATTACTGGGCGTAAAGCGTGCGCAGGCGGTTATGTAAGTCAGATGTGAAATCCCCGGGCTCAACCTGGGACCTGCATTTGAGACTGCATAGCTGGAGTGCGGCAGAGGGGGATGGAATTCCGCGTGTAGCAGTGAAATGCGTAGATATGCGGAGGAACACCGATGGCGAAGGCAATCCCCTGGGCCTGCACTGACGCTCATGCACGAAAGCGTGGGGAGCAAACAGG` |
| ASV2874 | 404 | `TAAGGAATATTGGACAATGGGCGAAAGCCTGATCCAGCCATGCCGCGTGAGTGATGAAGGCCTTAGGGTTGTAAAGCTCTTTTAGATGGGAAGATAATGACTGTACCATCAGAATAAGCCCCGGCTAAATTCGTGCCAGCAGCCGCGGTAATACGAATGGGGCAAGCGTTGTTCGGAATCACTGGGCGTAAAGCGTGTGTAGGCGGTTTATCAAGTCAGAAGTGAAAGCCCAGGGCTCAACCCTGGAATTGCTTTTGAAACTGATAGGCTAGAGTACTGGAGAGGTTAGCAGAATTCCGAGTGTAGAGGTGAAATTCGCAGATATTCGGAGGAATACCAGTGGCGCAAGCGGCTAACTGGACAGTAACTGACGCTGAGACACGAAAGCGTGGGGATCAAACAGG` |
| ASV4834 | 407 | `TTAGGAATTTTGCGCAATGGGCGAAAGCCTGACGCAGCAACGCCGCGTGAGTGATGAAGGCCTTCGGGTTGTAAAGCTCTTTTAGTTGGGAAAAACACAATGATGGTACCAACAGAAACAGCACCGGCTAACTCTGTGCCAGCAGCCGCGGTAATACAGAGGGTGCAAGCGTTATTCGGAATTATTGGGCATAAAGCGCATGTAGGTGGTTTGGTAAGTTTGATGTGAAAGCCCCGGACTCAATTCGGGAAGTGCATTGAATACTGCCAGGCTAGAGTATGGGAGAGGTGAGCGGAATTCCAGGTGTAGAGGTGAAATTCGTAGATATCTGGAGGAACAACAGAGGCGAAGGCGGCTCACTGGACCATTACTGACACTGAGATGCGAAAGCGTGGGGAGCAAACAGG` |
| ASV2050 | 428 | `TTTCGAATCATTCACAATGGGCGAAAGCCTGATGGTGCGACGCCGCGTGAGGGATGAAGGTCTTCGGATTGTAAACCTCTGTCACCGGGGACGAAACTCTTTGAGTTAATAGTTCAAAGATTGACTTAACCCGGAGAGGAAGCAGTGGCTAACTCTGTGCCAGCAGCCGCGGTAATACAGAGACTGCAAGCGTTATTCGGATTTACTGGGCGTAAAGGGTGCGCAGGCGGTTATGTGTGTGATGTGTGAAATCCCATGGCTCAACCTTGGAATTGCACTTCAAACTACATAACTAGAGCATTGGAGAGGGTAGCAGAATTCATGGTGTAGCAGTGAAATGCGTAGATATCATGAGGAATACCAGAGGCGAAGGCGGCTACCTGGACAATTGCTGACGCTCAGGCACTAAAGCGTGGGGAGCAAAAGGG` |
| ASV4064 | 429 | `TCGGGAATATTGGACAATGGGGGCAACCCTGATCCAGCCATGCCGCGTGTGTGAAGAAGGCCTTCGGGTTGTAAAGCACTTTAAGCAGGGAGGAAGGGCGTGAGGATAATACCCTTATGCATTGACGTTACCTGCAGAATAAGCACCGGCTAACTCCGTGCCAGCAGCCGCGGTAATACGGAGGGTGCAAGCGTTAATCGGAATTACTGGGCGTAAAGCGCGCGTAGGTGGTTTGTTAAGTGTGATGTGAAAGCCCAGGGCTTAACCTTGGAACTGCATCACATACTGGCAGGCTAGAGTACGGTAGAGGGGGGTAGAATTCCACGTGTAGCGGTGAAATGCGTAGAGATGTGGAGGAATACCGGTGGCGAAGGCGGCCCCCTGGATCGATACTGACGCTGAGGTGCGAAAGCGTGGGGAGCAAACAGG` |
| ASV590 | 429 | `TGGGGAATATTGGACAATGGGGGCAACCCTGATCCAGCAATGCCATGTGTGTGAAGAAGGCCTTAGGGTTGTAAAGCACTTTAGTGAGGGGGGAAGGCTGTTAGGTTAATACCCTGATGGTTTGACAGTACCTCAAGAATAAGCACCGGCAAACTCTGTGCCAGCAGCCGCGGTAATACAGAGGGTGCGAGCGTTAATCGGAATGACTGGGCGTAAAGGGCGAGTAGGCGGTTGTGTAAGTTAGATGTGAAAGACCTGGGCTTAACCTGGGAGGTGCATTTAATACTGCGCAACTGGAGTATTGTAGAGGTAACGGGAATTTCCGGTGTAGCAGTGAAATGCGTAGATATCGGAAGGAACACCAATGGCGAAGGCAAGTTACTGGACAAATACTGACGCTGAGGCGCGAAGGCGTGGGGAGCAAACAGG` |
| ASV4629 | 429 | `TGGGGAATATTGGACAATGGGCGCAAGCCTGATCCAGCAATGCCGCGTGGGTGAAGAAGGCCTTCGGGTTGTAAAGCCCTTTTGTACGGGAAGAAATCCTGTCGGTTAATACCCGGCGGGGATGACGGTACCGTAAGAATAAGCACCGGCTAACTTCGTGCCAGCAGCCGCGGTAATACGAAGGGTGCAAGCGTTAATCGGAATTACTGGGCGTAAAGCGTGCGTAGGCGGTTCGTTAAGTCTGTCGTGAAAGCCCTGGGCTCAACCTGGGAATGGCGATGGATACTGGCGAGCTAGAGTGTGATAGAGGATGGCGGAATTCCCGGTGTAGCGGTGAAATGCGTAGAGATCGGGAGGAACATCCGTGGCGAAGGCGGCCATCTGGATCAACACTGACGCTGAGGCACGAAAGCGTGGGGAGCAAACAGG` |
| ASV5690 | 406 | `TGGGGAATATTCCGCAATGGGCGAAAGCCTGACGGAGCGACGCCGCGTGGGGGATGAAGGCCTTCGGGTCGTAAACCCCTTTCGGCGGGGAAAATAATGATGGTACCCGCAGAAGCAGCCCCGGCTAATCTTACGTGCCAGCAGCCGCGGTAATACGTAAGGGGCAAGCGTTGTTCGGAATTACTGGGTGTAAAGGGTCCGTAGGCGGTGGCGTGCGTCACGCGTGAAATCCGACGGCTTAACCGTCGAACGGCGCGTGATACGGCGCTACTCGAGTTTCGGAGAGGCTAGTGGAATTCCCGGTGTAGCGGTGAAATGCGTAGAGATCGGGAGGAACGCCGATGGCGAAGGCAGCTGGCTGGCCGGATACTGACGCTGAGGGACGAAGGCGTGGGGAGCAAACAGG` |
| ASV2369 | 404 | `TGGGGAATATTGGACAATGGGCGAAAGCCTGATCCAGCCATGCCGCGTGAGTGATGAAGGCCTTAGGGTTGTAAAGCTCTTTTATCAGGGAAGATAATGACGGTACCTGAAGAATAAGCACCGGCTAACTTCGTGCCAGCAGCCGCGGTAATACGAAGGGTGCTAGCGTTGTTCGGAATTACTGGGCGTAAAGAGTTTGTAGGCGGCTATACAAGTCAGAGGTGAAATCCCGAAGCTTAACTTCGGAACTGCCTTTGAAACTGTATAGCTGGAGTATGTGAGGGGATAGCGGAATTCTTAATGTAGAGGTGAAATTCGTAGATATTAAGAGGAACACCAGTGGCGAAGGCGGCTATCTGGCACATTACTGACGCTGAGAAACGAAAGCGTGGGGATCAAACAGG` |
| ASV2178 | 424 | `TAAGGAATATTGGTCAATGGACGCAAGTCTGAACCAGCCATGCCGCGTGGAGGATGAAGGTCCTCTGGATTGTAAACTTCTTTTCTCTGGGGCGAAAACCTCCCATTCTTGGGAGCTTGACAGTACCAGAGGAATAAGCACCGGCTAACTCCGTGCCAGCAGCCGCGGTAATACGGAGGGTGCAAGCGTTATCCGGATTCACTGGGTTTAAAGGGTGCGTAGGCGGGCAGGTAAGTCAGTGGTGAAATCTCTGAGCTTAACTCAGAAACTGCCGTTGATACTATCTGTCTTGAATACTGTGGAGGTAAGCGGAATATGTCATGTAGCGGTGAAATGCTTAGATATGACATGGAACACCTATTGCGAAGGCAGCTTGCTACACAGTAATTGACGCTGAGGCACGAAAGCGTGGGGATCAAACAGG` |
| ASV4661 | 425 | `TAGGGAATATTGGGCAATGGAGGCAACTCTGACCCAGCCATGCCGCGTGCAGGAAGACGGTCCTCTGGATTGTAAACTGCTTTTGAATGGGAAGAATGTGCTCCTTGCGAGGGGTAATGACGGTACCATTAGAATAAGCCACGGCTAACTACGTGCCAGCAGCCGCGGTAATACGTAGGTGGCGAGCGTTATCCGGATTTATTGGGTTTAAAGGGTGCGTAGGCGGCTTAATAAGTCGGTGGTTAAAGGTAGCAGCTCAACTGTTTTACATGCCATCGATACTGTTTAGCTTGAGATATATGGAGGCAGCTAGAATTCCCGGTGTAGCGGTGAAATGCATAGATACCGGGAGGAATACCGATTGCGAAGGCATGTTGCTACGTATATTCTGACGCTGATGCACGAAAGCGTGGGGATCAAACAGG` |
| ASV5680 | 411 | `TCGAGGATCTTCGGCAATGGGCGCAAGCCTGACCGAGCGATGCCGCGTGGGCGATGAAGGCCTTCGGGTTGTAAAGCCCTGTCGAGAGGGAGAAATGCGAAAGCTTGATCTATCTCTGGAGGAAGGACGGGCTAAGTTCGTGCCAGCAGCCGCGGTAAGACGAACCGTCCAAACGTTGTTCGGAATCACTGGGCTTAAAGGGCGCGTAGGCGGGCTGCCAAGTCCGGGGTGAAATCCTCCAGCTTAACTGGAGAACTGCCTCGGATACTGGAAGTCTCGAGGGGGGTAGGGGCATGCGGAACCGTGGGTGGAGCGGTGAAATGCGTTGATATCCATGGGAACTCCGGTGGCGAAGGCGGCATGCTGGACCTTTTCTGACGCTGAGGCGCGAAAGCCAGGGGAGCAAACGGG` |
| ASV2295 | 430 | `TCGAGGATCATGCACAATGGGGGAAACCCTGATGCTGCGACGCCGCGTGGAGGATGAAGGTCTTCGGATTGTAAACTCCTGTCATCAATGAGTATGCCAAGAACGGTTAATACCCTATCTTGGTGACAGTAATTGAAGAGGAAGGGACGGCTAACTTCGTGCCAGCAGCCGCGGTAATACGAAGGTCCCAAGCGTTGTTCGGAATCACTGGGCGTAAAGGGTGCGTAGGCGGCGCGGAAAGTCAGATGTGAAAGCCCCGGGCTCAACCCGGGAATTGCATCCGATACTCCCGCGCTAGAGGATTGGAGAGGAGTCTGGAATTCACGGTGTAGCAGTGAAATGCGTAGATATCGTGAGGAACACTCGTGGCGAAGGCGAGACTCTGGACAATTCCTGACGCTGAGGCACGAAGGCCGGGGGAGCAAACAGG` |
| ASV653 | 406 | `TGGGGAATTTTCCGCAATGGGCGAAAGCCTGACGGAGCAATCTCGCGTGAGGGATGACTGCCTATGGGTTGTAAACCTCTTTTTTCAGGGAGGAATCAATGACGTTACCTGAAGAATAAGCATCGGCTAACTCCGTGCCAGCAGCCGCGGTAAGACGGAGGATGCAAGTGTTATCCGGAATCACTGGGCGTAAAGAGTCTGTAGGTGGTTTAATAAGTCAACTGTTAAATCTTGAAGCTCAACTTCAAAATCGCAGTCGAAACTATTAGACTAGAGTATAGTAGGGGTAAAGGGAATTTCCAGTGGAGCGGTGAAATGCGTAGAGATTGGAAAGAACACCGATGGCGAAGGCACTTTACTGGGCTATTACTGACACTCAGAGACGAAAGCTAGGGTAGCAAATGGG` |
| ASV606 | 404 | `TGGGGAATATTGGACAATGGGGGAAACCCTGATCCAGCAATGCCGCGTGAATGATGAAGGCCTTCGGGTTGTAAAGTTCTTTTACCCGAGACGATAATGACGGTATCGGGAGAATAAGCACCGGCTAACTTCGTGCCAGCAGCCGCGGTAATACGAAGGGTGCAAGCGTTGTTCGGAATGACTGGGCGTAAAGGGCGCGTAGGCGGTTCGGTACGTTTGATGTGAAAGCCCCGGGCTCAACCTGGGAATTGCATCGAATACGGCCGAGCTTGAGTGCGAGAGAGGAAAATGGAATAGTGCGTGTAGAGGTGAAATTCGTAGATATGCACTAGAACACCAGAGGCGAAGGCGATTTTCTGGCTCGTAACTGACGCTGAGGCGCGAAAGCGTGGGGAGCAAACAGG` |
| ASV3533 | 430 | `TGGGGAATATTGGACAATGGGGGCAACCCTGATCCAGCGATGCCGCGTGTGTGAAGAAGGCCTGCGGGTTGTAAAGCACTTTTATTGGGGAAGAGGGTTTTGTAGTTGAATAGGCTATGGAGCTGACGGTACCTGAGGAATAAGCACCGGCTAACTCTGTGCCAGCAGCCGCGGTAATACAGAGGGTGCGAGCGTTAATCGGAATTACTGGGCGTAAAGGGCGTGTAGGCGGGAGAATAAGTCAGATGTGAAAGTCCTGGGCTCAACCTGGGGATTGCATATGAAACTGTTTTTCTTGAGTGAAGTAGAGGGTGGCGGAATTTCCGGTGTAGCGGTGAAATGCGTAGATATCGGAAGGAACACCAGTGGCGAAGGCGGCCACCTGGACTTTAACTGACGCTGAGGCGCGAAAGCGTGGGGAGCAAACAGG` |
| ASV4667 | 429 | `TTTGGAATATTCCACAATGGGCGAAAGCCTGATGGAGCGACACCGCGTGAAGGATGAAGGTCTTCGGATTGTAAACTTCTTTAGACCTAGATGAAGACTCAGGGGTGAACAATCCCTGAGTTTGACCGTATAGGTAGAATAAGCCACGGCTAACTCTGTGCCAGCAGCCGCGGTAATACAGAGGTGGCGAACGTTGTCCGGATTTATTGGGTGTAAAGGGCATGTAGGTGGTCTTGTAAGTCAAAGGTGAAATGGCTCGGCTCAACCGAGTCATTGCCTTTGAAACTGCCGGACTCGAGTACGGAAGAGGAGAGCGGAATTCCCAGTGTAGCGGTGAAATGCGTAGATATTGGGAGGAACACCGGTGGCGAAAGCGGCTCTCTGGTCCGAAACTGACACTGAGATGCGAAAGCCAGGGGAGCAAACGGG` |
| ASV3896 | 390 | `TTGCGAAAACTCCACACTGCGCGAAAGCGTGATGGGGGGATTCCGAGTGTCGATGCTTTTACGTGTCGACTTTTCTTTAGAGTAAATATCTAGAGGAATAAGTGGTGGGCAAGACCGGTGCCAGCCGCCGCGGTAACCCCGGCGCCACAAGTGGGAACCACGTTTATTGGGTCTAAAGCATCCGTAGCTGGCTTTGTAAATGTCCTGTGAAATCGTTGCGCTCAACGCAACGGCGGGCAGGGCAGACTGCATTGCTAGGGACCAGGAGGGGTAGGGAGTATTCCCGGGGGAGCGGTAAAATGTTATAATCCCGGGAGGACTACCGATGGCGAAGGCACTCTACCAGAATGGATCCGACGGTGAGGGATGAAAGCTAGGGGAGCGATCCGG` |
| ASV187 | 429 | `TGGGGAATTTTGGACAATGGGCGAAAGCCTGATCCAGCCATTCCGCGTGAGTGAAGAAGGCCTTCGGGTTGTAAAGCTCTTTCGCAAGGGAAGAAAACTTAATCTCTAATATAGATTGAGGTTGACGGTACCTTGATAAGAAGCACCGGCTAACTACGTGCCAGCAGCCGCGGTAATACGTAGGGTGCGAGCGTTAATCGGAATTACTGGGCGTAAAGCGTGCGCAGGCTGTTTGGCAAGTCAGATGTGAAATCCCCGAGCTCAACTTGGGAACTGCGTTTGAAACTGCCAGACTAGAGTAGGTCAGAGGGGGGTAGAATTCCACGTGTAGCAGTGAAATGCGTAGAGATGTGGAGGAATATCAATGGCGAAGGCAGCCCCCTGGGATCATACTGACGCTCATGCACGAAAGCGTGGGGAGCAAACAGG` |
| ASV2632 | 429 | `TGGGGAATATTGGACAATGGGCGCAAGCCTGATCCAGCCATGCCGCGTGTGTGAAGAAGGCCCTCGGGTTGTAAAGCACTTTTGTCCGGAAAGAAAAGCACGGGGTTAATACCCACGTGTGATGACGGTACCGGAAGAATAAGCACCGGCTAACTTCGTGCCAGCAGCCGCGGTAATACGAAGGGTGCAAGCGTTACTCGGAATTACTGGGCGTAAAGCGTGCGTAGGTGGTGCGTTAAGTCTGTCGTGAAAGCCCCGGGCTCAACCTGGGAATGGCGATGGATACTGGCGTGCTAGAGTGAGGTAGAGGAGAGTGGAATTCCCGGTGTAGCAGTGAAATGCGTAGAGATCGGGAGGAACATCAGTTGCGAAGGCGGCTCTCTGGACCATCACTGACACTGAGGCACGAAAGCGTGGGGAGCAAACAGG` |
| ASV2681 | 404 | `TGGGGAATATTGGACAATGGGGGCAACCCTGATCCAGCAATGCCGCGTGAGTGATGAAGGCCTTCGGGTTGTAAAGCTCTTTTGCCAGGGACGATGATGACGGTACCTGGAGAATAAGCCCCGGCTAACTTCGTGCCAGCAGCCGCGGTAATACGAAGGGGGCAAGCGTTGTTCGGAATTACTGGGCGTAAAGGGCGCGTAGGCGGCTTATCAAGTCAGGCGTGAAATTCCCGGGCTCAACCTGGGGGCTGCGCTTGATACTGATGAGCTTGAATGCGGGAGAGGATAGTGGAATTCCCAGTGTAGAGGTGAAATTCGTAGATATTGGGAAGAACACCGGTGGCGAAGGCGGCTATCTGGCCCGTAATTGACGCTGAGGCGCGAAAGCGTGGGGAGCAAACAGG` |
| ASV3157 | 404 | `TGGGGAATTTTCCGCAATGGGCGAAAGCCTGACGGAGCAATACCGCGTGAGGGACGAAGGCTTTTGGGTCGTAAACCTCTTTTCTCAAGGAAGATAATGACGGTACTTGAGGAATAAGCATCGGCTAACTCTGTGCCAGCAGCCGCGGTAATACAGAGGATGCAAGCGTTATCCGGAATGATTGGGCGTAAAGCGTCCGCAGGTGGTTTGATAAGTCTGTTGTTAAAGCGTTAGGCTCAACCTAATAAGGGCAATGGAAACTATTAGACTAGAGTACGTTCGGGGCAGAGGGAATTCCCAGTGTAGCGGTGAAATGCGTAGAGATTGGGAAGAACACCGGTGGCGAAAGCGCTCTGCTAGGCCTGTACTGACACTGAGGGACGAAAGCTAGGGGAGCGAATGGG` |
| ASV3637 | 429 | `TCGAGAATAATTCACAATGGGGGCAACCCTGATGGTGCAACGCCGCGTGGAGGATGACGGTCTTCGGATTGTAAACTCCTGTCATCTGGGAGTAAGACCTGGGAGTGAATAGCTCACAGGGTTGATAGTACCAGAAGAGGAAGGGACGGCTAACTTCGTGCCAGCAGCCGCGGTAATACGAAGGTCCCAAGCGTTGTTCGGAATCACTGGGCGTAAAGGGAGCGTAGGCGGTGTGGTAAGTCAGATGTGAAATCCCGGGGCTCAACCCCGGAACTGCATCCGATACTGCCATACTAGAGGATTGGAGAGGTAGCTGGAATTCTTGGTGTAGCAGTGAAATGCGTAGAGATCAAGAGGAACACTCGTGGCGAAAGCGAGCTACTGGACAATTCCTGACGCTGAGGCTCGAAGGCTAGGGGAGCGAAAGGG` |
| ASV2581 | 404 | `TGGGGAATATTGGACAATGGGCGAAAGCCTGATCCAGCAATGCCGCGTGAGTGATGAAGGCCTTAGGGTTGTAAAGCTCTTTTACCAGGGATGATAATGACAGTACCTGGAGAATAAGCTCCGGCTAACTCCGTGCCAGCAGCCGCGGTAATACGGAGGGAGCTAGCGTTGTTCGGAATTACTGGGCGTAAAGCGCGCGTAGGCGGTTACTCAAGTCAGAGGTGAAAGCCCGGGGCTCAACCCCGGAACTGCCTTTGAAACTAGGTGACTAGAATCTTGGAGAGGTCAGTGGAATTCCGAGTGTAGAGGTGAAATTCGTAGATATTCGGAAGAACACCAGTGGCGAAGGCGACTGACTGGACAAGTATTGACGCTGAGGTGCGAAAGCGTGGGGAGCAAACAGG` |
| ASV754 | 429 | `TGGGGAATTTTGGACAATGGGGGCAACCCTGATCCAGCAATGCCGCGTGAGTGAAGAAGGCCTTCGGGTTGTAAAGCTCTTTTGTTAGGGAAGAAAAGGTTTTGGATAATACCCAGAACTGATGACGGTACCTAAAGAATAAGCACCGGCTAACTACGTGCCAGCAGCCGCGGTAATACGTAGGGTGCAAGCGTTAATCGGAATTACTGGGCGTAAAGCGTGCGCAGGCGGCCTATTAAGTCAGATGTGAAATCCCCGAGCTCAACTTGGGAACTGCATTTGAAACTGATAGGCTAGAGTGTGTCAGAGGGAGGTAGAATTCCACGTGTAGCAGTGAAATGCGTAGAGATGTGGAGGAATACCAGTGGCGAAGGCGGCCTCCTGGGATAACACTGACGCTCATGTACGAAAGCGTGGGGAGCAAACAGG` |
| ASV1891 | 429 | `TGGGGAATATTGGACAATGGGGGCAACCCTGATCCAGCCATGCCGCGTGTGTGAAGAAGGCCTTCGGGTTGTAAAGCACTTTAAGTTGGGAGGAAAGGGTGCTAGCTAATATCTAGTATCTATGACGTTACCGACAGAATAAGCACCGGCTAACTCCGTGCCAGCAGCCGCGGTAATACGGAGGGTGCAAGCGTTAATCGGAATTACTGGGCGTAAAGCGCGCGTAGGCGGCTTGGTAAGCTAGATGTGAAATCCCTGGGCTCAACCTGGGAACTGCATTTAGAACTGCTAGGCTAGAGTGCAGAAGAGGAGTGTGGAATTTCCGGTGTAGCGGTGAAATGCGTAGAGATCGGAAGGAACATCAGTGGCGAAGGCGACACTCTGGTCTGACACTGACGCTGAGGTGCGAAAGCGTGGGTAGCAAACAGG` |
| ASV1487 | 406 | `TGGGGAATATTGGACAATGGGCGAAAGCCTGATCCAGCAATACCGAGTGGGTGATGAAGGCCTTAGGGTTGTAAAGCCCTTTTCAGCAGGGAAGATAATGACGGTACCTGACCAAGAAAGCCCCGGCTAACTCCGTGCCAGCAGCCGCGGTAAGACGGAGGGGGCTAGCGTTGTTCGGAATTACTGGGCGTAAAGAGTGCGTAGGCGGTTTAGTAAGTTGGAAGTGAAAGCCCGAGGCTTAACCTCGGAACTGCTTTCAAAACTACTAATCTAGAGTGTAGTAGGGGATGATGGAATTCCTAGTGTAGAGGTGAAATTCTTAGATATTAGGAGGAACACCGGTGGCGAAGGCGGTCATCTGGGCTACAACTGACGCTGATGCACGAAAGCGTGGGGAGCAAACAGG` |
| ASV5546 | 429 | `TGGGGAATATTGCACAATGGGGGGAACCCTGATGCAGCCATGCCGCGTGTATGAAGAAGGCCTTCGGGTTGTAAAGTACTTTCGGTAATGAGGAAGGTGGTGTATCGAATAGGTGCATCAATTGACGTTAATTACAGAAGAAGCACCGGCTAACTCCGTGCCAGCAGCCGCGGTAATACGGAGGGTGCGAGCGTTAATCGGAATGACTGGGCGTAAAGGGCATGTAGGCGGATAATTAAGTTAGGTGTGAAAGCCCCGGGCTCAACCTGGGAACTGCACTTAAAACTGGTTATCTGGAGTATTGTAGAGGAAGGTAGAATTCCACGTGTAGCGGTGAAATGCGTAGAGATGTGGAGGAATACCGGTGGCGAAGGCGGCCTTCTGGACAGATACTGACGCTGAGATGCGAAAGCGTGGGGAGCAAACAGG` |
| ASV3033 | 424 | `TGAGGAATATTGGACAATGGGCGCAAGCCTGATCCAGCCATGCCGCGTGCAGGAAGAAGCATCTATGGTGTGTAAACTGCTTTTGTACAGGAAGAAACACTCCCTCGTGAGGGAGCTTGACGGTACTGTAAGAATAAGGATCGGCTAACTCCGTGCCAGCAGCCGCGGTAATACGGAGGATCCAAGCGTTATCCGGAATCATTGGGTTTAAAGGGTCCGTAGGCGGTTTAATAAGTCAGTGGTGAAATCTGGTCGCTCAACGATCAAACGGCCATTGATACTGTTAGACTTGAATTACTAGGAAGTAACTAGAATATGTAGTGTAGCGGTGAAATGCTTAGATATTACATGGAATACCAATTGCGAAGGCAGGTTACTACTAGTGGATTGACGCTGATGGACGAAAGCGTGGGGAGCGAACAGG` |
| ASV2477 | 430 | `TGGGGAATCTTGCACAATGGGCGCAAGCCTGATGCAGCAACGCCGCGTGAGTGAAGAAGGTCTTTGGATTGTAAAGCTCTTTCGGCTAGGAAGAAGGGTTTTAAGGTAAATAATCTTAGAATTTGACGGTACTAGAAGAAGAAGCACCGGCTAACTCCGTGCCAGCAGCCGCGGTAATACGGAGGGTGCAAGCGTTGTTCGGATTGATTGGGCGTAAAGCGCACGTAGGCGGGTATATAAGTCGAATGTGAAAGCCCCGGGCTTAACCTGGGAATTGCATCCGAAACTATATATCTTGAATACTCGAGAGGTTGGTGGAATTCTTGGTGTAGGAGTGAAATCCGTAGATATCAAGAGGAATATCGGAGGCGAAGGCGGCCGACTGGCGATGTATTGACGCTGAGGTGCGAAAGCGTGGGGAGCAAACAGG` |
| ASV1547 | 404 | `TGGGGAATATTGGACAATGGGCGAAAGCCTGATCCAGCCATGCCGCGTGAGTGATGAAGGCCTTAGGGTTGTAAAGCTCTTTTACCAGGGATGATAATGACAGTACCTGGAGAATAAGCTCCGGCTAACTCCGTGCCAGCAGCCGCGGTAATACGGAGGGAGCTAGCGTTGTTCGGAATTACTGGGCGTAAAGAGTACGTAGGCGGTTTTTTAAGTCAGAGGTGAAATCCCGGAGCTCAACTCCGGAACTGCCTTTGAAACTGGAAAGCTAGAATCATGGAGAGGTTAGTGGAATTCCGAGTGTAGAGGTGAAATTCGTAGATATTCGGAAGAACACCAGTGGCGAAGGCGACTGACTGGACATGTATTGACGCTGAGGTACGAAAGCGTGGGGAGCAAACAGG` |
| ASV2245 | 429 | `TGGGGAATTTTGGACAATGGGCGAAAGCCTGATCCAGCAATGCCGCGTGTGGGAAGAAGGCCTTCGGGTTGTAAACCACTTTTGTACGGAACGAAACGGTCCGCTTTAATACAGCGGGCTAATGACGGTACCGTAAGAATAAGCACCGGCTAACTACGTGCCAGCAGCCGCGGTAATACGTAGGGTGCGAGCGTTAATCGGAATTACTGGGCGTAAAGCGTGCGCAGGCGGTTATGTAAGACAGTTGTGAAATCCCCGGGCTCAACCTGGGAATTGCATCTGTGACTGCATAGCTAGAGTACGGTAGAGGGGGATGGAATTCCGCGTGTAGCAGTGAAATGCGTAGATATGCGGAGGAACACCGATGGCGAAGGCAATCCCCTGGACCTGTACTGACGCTCATGCACGAAAGCGTGGGGAGCAAACAGG` |
| ASV2875 | 420 | `TAGGGAATTTTGCGCAATGGGGGAAACCCTGACGCAGCAACGCCATGTGTGGGATGAAGCATTTCGGTGCGTAAACCACTGTCAGCAGGGAATAAAAGACTCGCAAGGGTATTTGAATGTACCTGCAAAGGAAGCGCCGGCTAACTTCGTGCCAGCAGCCGCGGTAATACGAGGGGCGCAAGTGTTGTTCGGAATCACTGGGCGTAAAGGGAGCGTAGGCGGGCTTTTAAGTTAGGTGTTTAATCCCGGGGCCCAACCCCGGACCGGCACCTAAAACTGGGAGTCTTGAATAGTGGAGAGGTCGGTGGAATTGGGGGTGTAGCGGTGGAATGCGTAGAGATCCCCAAGAACACCAGTTGCGAAGGCGGCCGACTGGACACATATTGACGCTGAGGCTCGAAAGCATGGGGAGCAAACAGG` |
| ASV5176 | 404 | `TAAGGAATCTTCCGCAATGGGCGAAAGCCTGACGGAGCGACACCGCGTGAAGGATGAAGGCTCTCGGGTTGTAAACTTCTTTTCTGGGGGAGCATTATGAGAGTACCCCAGGAATAAGCATCGGCTAACTACGTGCCAGCAGCCGCGGTAATACGTAGGATGCAAGCGTTATCCGGAATCATTGGGCGTAAAGCGTCCGCAGGTGGAACAGTGTGTCGAGCGATAAATACCAGTGCCTACCATTGGGAACTCGCTCGAAACTGCTGATCTAGAGGATGGGAGGGGCAGACGGAATTCCCGGTGTAGGGGTAAAATCCGTAGATATCGGGAAGAACACCAAAAGCGAAGGCAGTCTGCTGGAACATTCCTGACACTCATGGACGAAAGCGTGGGGAGCGAAAAGG` |
| ASV3396 | 404 | `TGGGGAATATTGGACAATGGGCGAAAGCCTGATCCAGCAATGCCGCGTGAGTGATGAAGGCCTTAGGGTTGTAAAGCTCTTTCAGCAGGGAAGATAATGACGGTACCTGCAGAAGAAGCCCCGGCTAACTCCGTGCCAGCAGCCGCGGTAATACGGAGGGGGCTAGCGTTGTTCGGAATTACTGGGCGTAAAGAGCGCGTAGGCGGTTTGACAAGTTGGAAGTGAAAGCCCAGGGCTTAACCCTGGAATTGCTTTCAAAACTGTCTGACTAGAGAGTGGTAGAGGATGGCGGAATTCCTAGTGTAGAGGTGAAATTCGTAGATATTAGGAGGAACACCGGTGGCGAAGGCGGCCATCTGGGCCATTTCTGACGCTGAGGCGCGAAAGCGTGGGGAGCAAACAGG` |
| ASV3781 | 408 | `TTAGGAATCTTCCGCAATGGGCGCAAGCCTGACGGAGCGACACCGCGTGAAGGATGAAGCCATTTTTTGGTGTAAACTTCTGTTGTGAGGGACGAAATTTTTGACGGTACCTCACGAGAAAGCACCGGCTAATTCTGTGCCAGCAGCCGCGGTAAGACAGAAGGTGCAAGCGTTACTCGGAATTACTGGGCGTAAAGCGTCTGTAGGTGTCCTTCCACGTCTGGTCATAAAATGCGGGGCTCAACCCCGCATCCTGGCTGGAAACGAGAAGGATCGAGTCATTCAGAGGCATCTGGAATGTCGTGTGTAGGGGTAAAATCCGTAGATACACGATGGAACGCCGAAAGCGAAAGCAGGATGCTGGGAATGTACTGACACTCAGAGACGAAAGCGTGGGGAGCAAAGGGG` |
| ASV2942 | 409 | `TGGGGAATATTGCACAATGGGCGCAAGCCTGATGCAGCGACGCCGCGTGAGGGATGACGGCCTTCGGGTTGTAAACCTCTGTTAGCATCGAAGAAGCGAAAGTGACGGTAGGTGCAGAGAAAGCGCCGGCTAACTACGTGCCAGCAGCCGCGGTAATACGTAGGGCGCGAGCGTTGTCCGGAATTATTGGGCGTAAAGAGCTTGTAGGCGGTTGGTCGCGTCTGCTGTGAAAGGCTGGGGCTTAACCCTGGTTTTGCAGTGGGTACGGGCTAACTAGAGTGCAGTAGGGGAGACTGGAATTCCTGGTGTAGCGGTGGAATGCGCAGATATCAGGAGGAACACCGATGGCGAAGGCAGGTCTCTGGGCTGTAACTGACGCTGAGAAGCGAAAGCATGGGGAGCGAACAGG` |
| ASV4567 | 387 | `GCGCGAAACCTTTTCAATGCACGCAAGTGTGAGAAGGGAATTCCAAGTGCTTAAGTTTAACTTAGGCTTTTGCCAAGTGTAAAAAGCTTGGCGAATAAGGGGTGGGTAAGACTGGTGCCAGCCGCCGCGGTAACACCAGCGCCTCGAGTGGGGATCGCGATTATTGGGTCTAAAGCGTCCGTAGCAGGCTTTGTAAATGCTTTGTGAAATCGGCAGGCATAACCTGTCGTCGTGCAGAGTAGACTGCAGAGCTCGAGACCGGGAGAGGTTGGCAGTATTCCTAGGGTACCGGTAAAATGGGTTAATCCTGGGAGGACTACCGATGGCGAAGGCAGCCAACCAGAACGGTCCTGACTGTGAGGGACGAAAGCTAGGGGAGCAAAACGG` |
| ASV3012 | 424 | `TGAGGAATATTGGTCAATGGACGAAAGTCTGAACCAGCCATGCCGCGTGCAGGAAGACGGCTCTATGAGTTGTAAACTGCTTTTGTACGAGGGTAAACTTGGATACGTGTATCCAACTGAAAGTATCGTACGAATAAGGATCGGCTAACTCCGTGCCAGCAGCCGCGGTAATACGGAGGATCCGAGCGTTATCCGGATTTATTGGGTTTAAAGGGTGCGTAGGCGGTTGTATAAGTTAGAGGTGAAATTCACGGGCTCAACCCGTGCCATGCCTCTAATACTGTATGACTAGAGTTTAGATGCAGAGGGCGGAATGTGTGGTGTAGCGGTGAAATGCTTAGAGATCACACAGAACACCGATTGCGAAGGCAGCTCTCTAATCTACAACTGACGCTGAGGCACGAAAGCGTGGGTAGCGAACAGG` |
| ASV4110 | 429 | `TGGGGAATATTGGACAATGGGGGCAACCCTGATCCAGCGATGCCGCGTGTGTGAAGAAGGCCTGCGGGTTGTAAAGCACTTTCAGTGGTGAGGAAAGGTTGGAGTTTAAGAGATACCAACTGTGACGTTAACCACAGAAGAAGCACCGGCTAACTCTGTGCCAGCAGCCGCGGTAATACAGAGGGTGCAAGCGTTAATCGGACTTACTGGGCGTAAAGGGTGCGTAGGTGGAGATTTAAGTTGAGTGTGAAATATCTGGGCTTAACCTGGAGGGTGCATTCAAAACTGAGTTTCTAGAGTATGGTAGAGGGAAGTGGAATTTCCGGTGTAGCGGTGAAATGCGTAGATATCGGAAGGAACACCAGTGGCGAAGGCGACTTTCTGGACCAATACTGACACTGAGGCACGAAAGCGTGGGTAGCAAACAGG` |
| ASV771 | 429 | `TGGGGAATTTTGGACAATGGGCGCAAGCCTGATCCAGCCATTCCGCGTGAGTGAAGAAGGCCTTCGGGTTGTAAAGCTCTTTCGCAAGGGAAGAAAACTTACTTTTTAATAAAGAGTGAGGTTGACGGTACCTTGATAAGAAGCACCGGCTAACTACGTGCCAGCAGCCGCGGTAATACGTAGGGTGCGAGCGTTAATCGGAATTACTGGGCGTAAAGCGTGCGCAGGCGGTTTTGTAAGTCAGATGTGAAATCCCCGAGCTCAACTTGGGAACTGCGTTTGAAACTACAAGACTAGAATATGTCAGAGGGGGGTAGAATTCCACGTGTAGCAGTGAAATGCGTAGAGATGTGGAGGAATACCAATGGCGAAGGCAGCCCCCTGGGATAATATTGACGCTCATGCACGAAAGCGTGGGGAGCAAACAGG` |
| ASV2321 | 406 | `TGGGGAATATTGCACAATGGGCGCAAGCCTGATGCAGCAACGCCGCGTGAGTGATGAAGGCCTTTGGGTCGTAAAACTCTGTCCTAGGGGAAGAATAAAATGACGGTACCCTGGGAGGAAGCCCCGGCAAACTACGTGCCAGCAGCCGCGGTAATACGTAGGGGGCAAGCGTTATCCGGAATCACTGGGCGTAAAGGGTGCGTAGGCGGTCCTTCAAGCCAGAGGTAAAAGGCATTGGCTTAACCAATGTAAGCCTTTGGAACTGTGGGACTTGAGTGTAGGAGAGGTAAGTGGAATTCCTAGTGTAGCGGTGAAATGCGTAGATATTAGGAGGAACACCAGTGGCGAAGGCGACTTACTGGACTATAACTGACGCTGAGGCACGAAAGCGTGGGGAGCAAACAGG` |
| ASV3316 | 404 | `TGGGGAATCTTGCGCAATGGGCGAAAGCCTGACGCAGCAACGCCGCGTGCGGGATGAAGGCCTTCGGGTTGTAAACCGCTTTCAGCAGGGACGAAAATGACGGTACCTGCAGAAGAAGCCCCGGCTAACTACGTGCCAGCAGCCGCGGTAATACGTAGGGGGCAAGCGTTGTCCGGATTTATTGGGCGTAAAGAGCTCGTAGGCGGTTCCGTAAGTCGGGTGTGAAAAATCTGGGCTCAACCCAGTGGAGCACCCGATACTGCGGTGACTTGAGTCCGGTAGGGGAGTGTGGAATTCCTGGTGTAGCGGTGAAATGCGCAGATATCAGGAGGAACACCGACGGCGAAGGCAGCACTCTGGGCCGGAACTGACGCTGAGGAGCGAAAGCGTGGGGAGCGAACAGG` |
| ASV3990 | 428 | `TTTCGAATCATTCACAATGGGCGAAAGCCTGATGGTGCGACGCCGCGTGAGGGATGAAGGTCTTCGGATTGTAAACCTCTGTCACCGGGGAAGAAACGTTTTGAGTTAATAGTTCAAAGCCTGACTTAACCCGGAGAGGAAGCAGTGGCTAACTCTGTGCCAGCAGCCGCGGTAATACAGAGACTGCAAGCGTTATTCGGATTCACTGGGCGTAAAGGGTGCGCAGGCGGCCGTGTGTGTCAGGTGTGAAATCCCGGGGCTTAACTCCGGAACTGCGCCTGAAACTACATGGCTAGAGTATTGGAGAGGGTAACGGAATTCACGGTGTAGCAGTGAAATGCGTAGATATCGTGAGGAACACCAGAGGCGAAGGCGGTTACCTGGACAATTACTGACGCTCAGGCACGAAAGCATGGGGAGCAAAAGGG` |
| ASV5307 | 405 | `TGGGGAATCTTGCTCAATGGGCGAAAGCCTGAAGCAGCAACGCCGCGTGCGGGATGACGGCCCTCGGGTTGTAAACCGCTTTCAGCAGGAACGAATCTGACGGTACCTGCAGAAGAAGGTCCGGCCAACTACGTGCCAGCAGCCGCGGTAACACGTAGGGACCAAGCGTTGTCCGGATTTATTGGGCGTAAAGAGCTCGTAGGCGGTTCAGTCAGTCGGGTGTGAAAACTTCGGGCTTAACTCGAAGCATGCATCCGATACTGCTGTGACTTGAGTCCGGTAGGGGAGTGGGGAATTCCTGGTGTAGCGGTGAAATGCGCAGATATCAGGAGGAACACCGGTGGCGAAGGCGCCACTCTGGGCCGGTACTGACGCTGAGGAGCGAAAGCGTGGGTAGCAAACAGG` |
| ASV5180 | 429 | `TCGAGGATCTTCCGCAATGGGGGAAACCCTGACGGAGCGACGCCGCGTGCGGGATGAAGGCCCTCGGGTTGTAAACCGCTGTCACACGTTAAGAAAAAACCCTGGCTAATACCCGGTGTCTTGACAAAGGCGTGGGAGGAAGTCACGGCTAACTTCGTGCCAGCAGCTGCGGTAAGACGAAGGTGACAAGCGTTGCTCGGAATTACTGGGCATAAAGCGCGCGTAGGTGGCTGGGTAAGCGGGAAATGAAATCCCATGGCTCACCCATGGAACTGTTTCTCGAACTGCCCTGCTTGAGGACGGTAGGGGAAAGTGGAATTCCTGGTGTAGCGGTGAAATGCGTAGATATCAGGAGGAACACCGGTGGCGAAAGCGACTTTCTGGACCGTTTCCTGACACTGAGGCGCGAAAGCTAGGGGAGCAAACAGG` |
| ASV1699 | 424 | `TAGGGAATATTGGTCAATGGAGGCAACTCTGAACCAGCCATGCCGCGTGCAGGACTAAGGCCCTATGGGTCGTAAACTGCTTTTGAATGGGAAGAAATGTATCTACGTGTAGATATTTGACGGTACCATTAGAATAAGGATCGGCTAACTTCGTGCCAGCAGCCGCGGTAATACGAAGGATCCAAGCGTTGTCCGGATTTACTGGGTTTAAAGGGTGCGTAGGCGGACTTATAAGTCAGTGGTGAAAGCCTTCAGCTTAACTGAAGAACTGCCATTGAAACTGTAAGTCTCGAGTTCGGTTGAGGTCATTGGAATGTAACATGTAGCGGTGAAATGCATAGATATGTTACAGAACACCAATTGCGAAGGCAGATGACTAAACCGAAACTGACGCTGAGGCACGAAAGCGTGGGGATCAAACAGG` |
| ASV2388 | 429 | `TGGGGAATTTTGGACAATGGGGGCAACCCTGATCCAGCCATGCCGCGTGTGTGAAGAAGGCCTTCGGGTTGTAAAGCACTTTCGGCCGGAACGAAATCGCGCGGATTAATAACCCGCGTGGATGACGGTACCGGAAGAAGAAGCACCGGCTAACTACGTGCCAGCAGCCGCGGTAATACGTAGGGTGCGAGCGTTAATCGGAATTACTGGGCGTAAAGGGTGCGCAGGCGGTTCTGCAAGTCAGGCGTGAAATCCCCGGGCTTAACCTGGGAATGGCGCTTGAAACTACGGGACTGGAGTATGGCAGAGGGAGGTGGAATTCCACGTGTAGCGGTGAAATGCGTAGAGATGTGGAGGAACACCGATGGCGAAGGCAGCCTCCTGGGCCAATACTGACGCTCATGCACGAAAGCGTGGGGAGCAAACAGG` |
| ASV905 | 404 | `TCGGGAATTTTGGGCAATGGGCGAAAGCCTGACCCAGCAACGCCGCGTGAAGGATGAAGTATTTCGGTATGTAAACTTCGAAAGAATAGGAAGAATAAATGACGGTACTATTTATAAGGTCCGGCTAACTACGTGCCAGCAGCCGCGGTAATACGTAGGGACCAAGCGTTGTTCGGATTTACTGGGCGTAAAGGGCGCGTAGGCGGCATGACAAGTCACTTGTGAAATCTCCGGGCTTAACTCGGAACGGCCAAGTGATACTGTCACGCTAGAGTATTGGAGGGGCAATCGGAATTCTTGGTGTAGCGGTGAAATGCGTAGATATCAAGAGGAACACCTGAGGTGAAGACGGGTTGCTGGCCAAATACTGACGCTGAGGCGCGAAAGCCAGGGGAGCAAACGGG` |
| ASV1119 | 424 | `TGAGGAATATTGGACAATGGGTGAGAGCCTGATCCAGCCATCCCGCGTGAAGGACGACGGCCCTATGGGTTGTAAACTTCTTTTGTATAGGGATAAACCTACTCTCGTGAGAGTAGCTGAAGGTACTATACGAATAAGCACCGGCTAACTCCGTGCCAGCAGCCGCGGTAATACGGAGGGTGCAAGCGTTATCCGGATTTATTGGGTTTAAAGGGTCCGTAGGCGGGCTTGTAAGTCAGTGGTGAAATCTCATAGCTTAACTATGAAACTGCCATTGATACTGCAGGTCTTGAGTAAAGTAGAAGTGGCTGGAATAAGTAGTGTAGCGGTGAAATGCATAGATATTACTTAGAACACCAATTGCGAAGGCAGGTCACTATGTTTTAACTGACGCTGATGGACGAAAGCGTGGGGAGCGAACAGG` |
| ASV2261 | 429 | `TGGGGAATTTTGGACAATGGGGGCAACCCTGATCCAGCCATTCCGCGTGAGTGAAGAAGGCCTTCGGGTTGTAAAGCTCTTTCGGACGGAACGAAATGGTTACGGTCAATACCCGTGACTGATGACGGTACTGTAAGAAGAAGCACCGGCTAACTACGTGCCAGCAGCCGCGGTAATACGTAGGGTGCGAGCGTTAATCGGAATTACTGGGCGTAAAGCGTGCGCAGGCGGCGGCTCAAGTCAGGTGTGAAATCCCCGGGCTTAACCTGGGAATTGCGCTTGAAACTAGGTGGCTAGAGTGTGGCAGAGGGGGGTGGAATTCCACGTGTAGCAGTGAAATGCGTAGAGATGTGGAGGAACACCGATGGCGAAGGCAGCCCCCTGGGTCAACACTGACGCTCAGGCACGAAAGCGTGGGGAGCAAACAGG` |
| ASV714 | 429 | `TGGGGAATTTTGGACAATGGACGAAAGTCTGATCCAGCCATGCCGCGTGCGGGAAGAAGGCCTTCGGGTTGTAAACCGCTTTTGTCAGGGAAGAAATCCTTTGAGCTAATACCTCGGGGGGATGACGGTACCTGAAGAATAAGCACCGGCTAACTACGTGCCAGCAGCCGCGGTAATACGTAGGGTGCAAGCGTTAATCGGAATTACTGGGCGTAAAGCGTGCGCAGGCGGTTATGTAAGACAGAGGTGAAATCCCCGGGCTCAACCTGGGAACTGCCTTTGTGACTGCATAGCTTGAGTGCGGCAGAGGGGGATGGAATTCCGCGTGTAGCAGTGAAATGCGTAGATATGCGGAGGAACACCGATGGCGAAGGCAATCCCCTGGGCCTGCACTGACGCTCATGCACGAAAGCGTGGGGAGCAAACAGG` |
| ASV4835 | 404 | `TGGGGAATATTGGACAATGGGCGAAAGCCTGATCCAGCCATGCCGCGTGAATGATGAAGGCCTTAGGGTTGTAAAATTCTTTTGGCGGGGACGATAATGACGGTACCCGCAGAATAAGCTCCGGCTAACTTCGTGCCAGCAGCCGCGGTAATACGAAGGGAGCTAGCGTTGTTCGGAATCACTGGGCGTAAAGCGCGCGCAGGCGGTGGTGCAAGTCAGAGGTGAAATCCCGGAGCTTAACTCCGGAACTGCCTTTGAAACTGCATCGCTAGAATAAGAAAGAGGGTGGCGGAATTCCGAATGTAGCGGTGAAATGCGTAGATATTCGGAGGAACACCGATGGCGAAGGCAGCCACCTGGTTCTTTATTGACGCTGAGGCGCGAAAGCGTGGGGATCAAACAGG` |
| ASV4246 | 429 | `TGGGGAATTTTGGACAATGGGGGCAACCCTGATCCAGCAATGCCGCGTGTGTGAAGAAGGCCTTCGGGTTGTAAAGCACTTTTGTCCGGGAAGAAAACGTCTGTGCTAATACCACGGATGGATGACGGTACCGGAAGAATAAGGACCGGCTAACTACGTGCCAGCAGCCGCGGTAATACGTAGGGTCCAAGCGTTAATCGGAATTACTGGGCGTAAAGCGTGCGCAGGCGGTTGTGTAAGACAGATGTGAAATCCCCGGGCTTAACCTGGGAACTGCATTTGTGACTGCACGGCTAGAGTGTGATAGAGGGGGGTAGAATTCCACGTGTAGCAGTGAAATGCGTAGATATGTGGAGGAATACCAATGGCGAAGGCAGCCCCCTGGATCAATACTGACGCTCATGCACGAAAGCGTGGGGAGCAAACAGG` |
| ASV4302 | 430 | `TGGGGAATATTGCACAATGGGCGAAAGCCTGATGCAGCAACGCCGCGTGGGTGATGAAGGTTCTTGGATTGTAAAGCCCTTTCGGCTAGGAAGAAGGGCGTTATAGTTAATACCTATAGCGTTTGACGGTACTAGAAGAAGAAGCACCGGCTAACTCCGTGCCAGCAGCCGCGGTAATACGGAGGGTGCAAGCGTTGTTCGGATTGATTGGGCGTAAAGCGCACGTAGGCGGGTTTGTAAGTCGGATGTGAAAGCCCAGGGCTTAACCTTGGAATTGCATCCGAAACTATAAATCTTGAATGCCCGAGGGGTTGGTGGAATTCCTGGTGTAGGAGTGAAATCCGTAGATATCAGGAGGAACACCGGAGGCGAAGGCGACCAACTGGTGGTGCATTGACGCTGAGGTGCGAAAGCGTGGGGAGCAAACAGG` |
| ASV3991 | 430 | `TCGGGAATCTTGGGCAATGGGCGCAAGCCTGACCCAGCGACGCCGCGTGAGGGATGAAGGCCTTCGGGTCGTAAACCTCTGTCGGGAGGGAAGAATGGCGGTAGGGCGAATAGTCCTGCCGTTTGACGGTACCTCCAAAGGAAGCGCCGGCTAACTCCGTGCCAGCAGCCGCGGTAATACGGAGGGCGCAAGCGTTGTTCGGAATCACTGGGCGTAAAGCGCGTGTAGGCGGCCGGGCAAGTCGGATGTGAAATCCCGAGGCTCAACCTCGGAACTGCACTCGAAACTGCTCGGCTCGAGTCCCGGAGAGGATGGCGGAATTCCCGGTGTAGGGGTGAAATCCGTAGATATCGGGAGGAACACCAGTGGCGAAGGCGGCCATCTGGACGGTGACTGACGCTGAGACGCGAAAGCGTGGGGAGCAAACAGG` |
| ASV1116 | 407 | `TGGGGAATTTTCCGCAATGGGCGAAAGCCTGACGGAGCAATACCGCGTGAGGGAAGAAGGCTCTTGGGTTGTAAACCTCTTTTCTTAGGGAAGAAAAAAATGACGGTACCTAAGGAATCAGCATCGGCTAACTCCGTGCCAGCAGCCGCGGTAATACGGAGGATGCAAGCGTTATCCGGAATGATTGGGCGTAAAGCGTCCGCAGGTGGCAATTCAAGTTTGCGGTTAAAGGTTCTGGCTCAACCAGAGACAAGCCGTGAAAACTGAATAGCTAGAGTATGGTAGGGGCAGAGGGAATTCCCAGTGTAGCGGTGAAATGCGTAGAGATTGGGAAGAACACCGGTGGCGAAAGCGCTCTGCTAGGCCAAAACTGACACTGAGGGACGAAAGCTAGGGGAGCGAATGGG` |
| ASV936 | 424 | `TGAGGAATATTGGACAATGGGCGCAAGCCTGATCCAGCCATGCCGCGTGCAGGAAGACGCATCTATGGTGTGTAAACTGCTTTTGTACGGGAAGAAACACTCCTACGTGTAGGAGCTTGACGGTACCGTAAGAATAAGGATCGGCTAACTCCGTGCCAGCAGCCGCGGTAATACGGAGGATCCAAGCGTTATCCGGAATCATTGGGTTTAAAGGGTCCGTAGGCGGTTTGATAAGTCAGTGGTGAAAGCCCATCGCTCAACGATGGAACGGCCATTGATACTGTCAGACTTGAATTACTAGGAAGTAACTAGAATATGTAGTGTAGCGGTGAAATGCTTAGATATTACATGGAATACCAATTGCGAAGGCAGGTTACTACTAGTGGATTGACGCTGATGGACGAAAGCGTGGGGAGCGAACAGG` |
| ASV2515 | 404 | `TGGGGAATATTGGACAATGGGCGCAAGCCTGATCCAGCCATGCCGCGTGAGTGATGAAGGTCTTAGGATTGTAAAGCTCTTTCACCGGAGAAGATAATGACGGTATCCGGAGAAGAAGCCCCGGCTAACTTCGTGCCAGCAGCCGCGGTAATACGAAGGGGGCTAGCGTTGTTCGGAATTACTGGGCGTAAAGCGCACGTAGGCGGATATTTAAGTCAGGGGTGAAATCCCGCAGCTCAACTGCGGAACTGCCTTTGATACTGGGTATCTTGAGTATGGAAGAGGTAAGTGGAATTCCGAGTGTAGAGGTGAAATTCGTAGATATTCGGAGGAACACCAGTGGCGAAGGCGGCTTACTGGTCCATTACTGACGCTGAGGTGCGAAAGCGTGGGGAGCAAACAGG` |
| ASV5362 | 404 | `TAGGGAATATTGGACAATGGGCGAAAGCCTGATCCAGCAATGCCGCGTGAGCGATGAAGGCCTTAGGGTTGTAAAGCTCTTTTACCCGAGATGATAATGACAGTATCGGGAGAATAAGCCCCGGCTAACTCCGTGCCAGCAGCCGCGGTAATACGGAGGGGGCTAGCGTTGTTCGGAATTACTGGGCGTAAAGCGCACGTAGGCGGCTATTCAAGTCAGAGGTGAAAGCCCGGGGCTCAACCCCGGAATTGCCTTTGAAACTAGATAGCTAGAATCACGGAGAGGTAAGTGGAATTCCGAGTGTAGAGGTGAAATTCGTAGATATTCGGAAGAACACCAGTGGCGAAGGCGGCTTACTGGACGTGAATTGACGCTGAGGTGCGAAAGCGTGGGGAGCAAACAGG` |
| ASV2160 | 424 | `TAAGGAATATTGGACAATGGGCGCAAGCCTGATCCAGCCATGCCGCGTGCAGGATTAAGGCCCTATGGGTTGTAAACTGCTTTTGGCGGGGAAAAAACCCTCTCACGTGTGAGAGGCTGATGGTACCCGCAGAATAAGGATCGGCTAACTCCGTGCCAGCAGCCGCGGTAATACGGAGGATCCAAGCGTTATCCGGATTTATTGGGTTTAAAGGGTGCGTAGGCGGATTAGTAAGTCAGTGGTGAAAGCCTGCAGCTTAACTGTAGAATTGCCATTGATACTGCTAGTCTTGAATGTAGTTGAGGTGGGCGGAATATGACATGTAGCGGTGAAATGCTTAGATATGTCATAGAACACCGATTGCGAAGGCAGCTCACTAAGCTATGATTGACGCTGAGGCACGAAAGCGTGGGGAGCAAACAGG` |
| ASV1450 | 406 | `TAAGGAATCTTCCACAATGGGCGAAAGCCTGATGGAGCAACGCCGCGTGCAGGATGAAGGCCTTCGGGTCGTAAACTGCTTTTATGAGTGAAGAATATGACGGTAACTCATGAATAAGGGTCGGCTAACTACGTGCCAGCAGCCGCGGTCATACGTAGGACCCAAGCGTTATCCGGAGTGACTGGGCGTAAAGAGTTGCGTAGGTGGTCAGTAAAGCAAATGGTGAAATTTGGCGGCTCAACCGTACAAACTATTATTTGAACTCACTGACTCGAGAGTAGCAGAGGTAACTGGAATTTCTGAAGTAGGGGTAATATCCGTAGATATCAGAAGGAACACCAATGGCGTAGGCAGGTTACTGGGCTATTTCTGACACTGAGGCACGAAAGCGTGGGGAGCGAACCGG` |
| ASV2436 | 429 | `TGGGGAATATTGGACAATGGGCGCAAGCCTGATCCAGCCATACCGCGTGGGTGAAGAAGGCCTTCGGGTTGTAAAGCCCTTTTGTTGGGAAAGAAAAGCAGTCGGTTAATACCCGGTTGTTCTGACGGTACCCAAAGAATAAGCACCGGCTAACTTCGTGCCAGCAGCCGCGGTAATACGAAGGGTGCAAGCGTTACTCGGAATTACTGGGCGTAAAGCGTGCGTAGGTGGTTGTTTAAGTCTGTTGTGAAAGCCCTGGGCTCAACCTGGGAATTGCAGTGGATACTGGGCGACTAGAGTGTGGTAGAGGGTAGTGGAATTCCCGGTGTAGCAGTGAAATGCGTAGAGATCGGGAGGAACATCCATGGCGAAGGCAGCTACCTGGACCAACACTGACACTGAGGCACGAAAGCGTGGGGAGCAAACAGG` |
| ASV1671 | 429 | `TGGGGAATTTTGGACAATGGGGGCAACCCTGATCCAGCAATGCCGCGTGAGTGAAGAAGGCCTTCGGGTTGTAAAGCTCTTTTGTCAGGGAAGAAACGGATTGGTCTAATACACTAATCTAATGACGGTACCTGAAGAATAAGCACCGGCTAACTACGTGCCAGCAGCCGCGGTAATACGTAGGGTGCAAGCGTTAATCGGAATTACTGGGCGTAAAGCGTGCGCAGGCGGTTATGTAAGACAGATGTGAAATGCCCGGGCTTAACCTGGGAACTGCATTTGTGACTGCATGGCTAGAGTGTGTCAGAGGGGGGTAGAATTCCACGTGTAGCAGTGAAATGCGTAGATATGTGGAGGAACACCGATGGCGAAGGCAGCCCCCTGGGATAACACTGACGCTCATGCACGAAAGCGTGGGGAGCAAACAGG` |
| ASV3388 | 407 | `CCGAGAATATTCGACAATGGGCGAAAGCCTGATCGAGCGATACCGCGTGGTGGATGAAGTGCTTCGGCACGTAAACACCTTTTATGAGGGAGGAAGTTATTGACGTTACCTCATGAATAAGGGGCTCCTAACTCTGTGCCAGCAGGAGCGGTAATACAGAGGCCCCAAGCATTACCCGGAATCACTGGGCGTAAAGGGTGCGTAGGTGGTCGTATTAGTCGTTCGTTAAAACCCAGGGCTTAACCCTGGGACTGCGAGCGAAACGGTACGACTCGAGAGAGTGAGAGGTGAATGGAACTCATGGTGTAGGGGTGAAATCCGTTGATATCATGGGGAACACCGAAAGCGAAGGCAATTCACTGGCACTTTTCTGACACTCAAGCACGAAAGCGTGGGTAGCGAACTGG` |
| ASV2614 | 429 | `TCGAGAATAATTCACAATGGGGGCAACCCTGATGGTGCAACGCCGCGTGGAGGATGACGGTCTTCGGATTGTAAACTCCTGTCATCCGGGAGAAAAACCTGGCCGTTAATAGCGGACAGGGTTGATAGTACCGGAAGAGGAAGGGACGGCTAACTTCGTGCCAGCAGCCGCGGTAATACGAAGGTCTCAAGCGTTGTTCGGAATCACTGGGCGTAAAGGGAGCGTAGGCGGCGCGGTAAGTCAGATGTGAAATCCCGGGGCTCAACCCCGGAACTGCATCCGATACTGCCGTGCTTGAGCAATGGAGAGGTAGCTGGAATTCTCGGTGTAGCAGTGAAATGCGTAGATATCGAGAGGAACACTCGTGGCGAAAGCGAGCTACTGGACATTTGCTGACGCTGAGGCTCGAAGGCTAGGGGAGCGAAAGGG` |
| ASV1386 | 424 | `TAAGGAATATTGGGCAATGGACGAAAGTCTGACCCAGCCATGCCGCGTGAAGGATTAAGGTCCTCTGGATTGTAAACTTCTTTTCTCTGGGGCGAAAACCAGCTATTCTTAGCTATTTGACAGTACCAGAGGAATAAGCACCGGCTAACTCCGTGCCAGCAGCCGCGGTAATACGGAGGGTGCAAGCGTTATCCGGATTCACTGGGTTTAAAGGGTGCGTAGGTGGGCTAGTAAGTCCGTGGTGAAATCCCCGAGCTTAACTTGGGAACTGCCATTGATACTATTAGTCTTGAATATCGTGGAGGTAAGCGGAATATGTCATGTAGCGGTGAAATGCTTAGATATGACATAGAACACCAATTGCGAAGGCAGCTTGCTACACGGTCATTGACACTGAGGCACGAAAGCGTGGGGAGCAAACAGG` |
| ASV348 | 429 | `TGGGGAATTTTGGACAATGGGCGAAAGCCTGATCCAGCCATGCCGCGTGCGGGAAGAAGGCCTTCGGGTTGTAAACCGCTTTTGTCAGGGAAGAAACGCTTCGGAATAATACTCTGAGGTAATGACGGTACCTGAAGAATAAGCACCGGCTAACTACGTGCCAGCAGCCGCGGTAATACGTAGGGTGCAAGCGTTAATCGGAATTACTGGGCGTAAAGCGTGCGCAGGCGGCTTTGCAAGACAGATGTGAAATCCCCGGGCTCAACCTGGGAACTGCATTTGTGACTGCAAGGCTAGAGTACGGTAGAGGGGGATGGAATTCCGCGTGTAGCAGTGAAATGCGTAGATATGCGGAGGAACACCGATGGCGAAGGCAATCCCCTGGACCTGTACTGACGCTCATGCACGAAAGCGTGGGGAGCAAACAGG` |
| ASV3328 | 408 | `TCGAGAATCTTCCGCAATGGGCGAAAGCCTGACGGAGCGACGCCGCGTGGTGGATGAAGCACTTTGGTGCGTAAACACCTTTTATGAGGGAGGAAGTTTATTGACGTTACCTCATGAATAAGGGGCTCCCAACTCTGTGCCAGCAGGAGCGGTAATACAGAGGCCCCAAGCATTATCCGGATTCACTGGGCGTAAAGGGTGCGTAGGCGGTGTTATTAGTCGGGTGTTAAATCCTGGGGCTCAACCTCAGAATCGCATTCGAAACGGTAACACTAGAAGAAGTCAGAGGTGAACAGAACTCTCGGTGTAGGGGTGAAATCCGTTGATATCGAGGGGAATACCAAATGCGAAGGCAGTTCACTGGGGCTTTCTTGACGCTGAGGCACGAAAGCGTGGGTAGCGAATGGG` |
| ASV1132 | 407 | `TGGGGAATATTGCGCAATGGGGGAAACCCTGACGCAGCAACGCCGCGTGATTGAAGAAGGCCTTCGGGTTGTAAAGATCTTTAATTGGGGACGAAACAAATGACGGTACCCAAAGAATAAGCTCCGGCTAACTACGTGCCAGCAGCCGCGGTAATACGTAGGGAGCAAGCGTTATCCGGATTTACTGGGTGTAAAGGGCGAGTAGGCGGGCTGGCAAGTTGGGAGTGAAATCCGGGGGCTCAACCCCCGAACTGCTTTCAAAACTGCTGGTCTTGAGTGATGGAGAGGCAGGCGGAATTCCCAGTGTAGCGGTGAAATGCGTAGATATTGGGAGGAACACCAGTGGCGAAGGCGGCCTGCTGGACATTAACTGACGCTGAGGCGCGAAAGCGTGGGGAGCAAACAGG` |
| ASV752 | 423 | `TAGGGAATATTGGGCAATGGAGGCAACTCTGACCCAGCCATGCCGCGTGCAGGAAGAAGGCGTTATGCGTTGTAAACTGCTTTTATATAGGAAGAAATAGACCTTGCGAGGTAATTTGACGGTACTATATGAATAAGCACCGGCTAACTCCGTGCCAGCAGCCGCGGTAATACGGAGGGTGCAAGCGTTGTCCGGATTTATTGGGTTTAAAGGGTGCGTAGGCGGTTTATTAAGTCAGTGGTGAAAGACGGTCGCTCAACGATTGCAGTGCCATTGATACTGGTAGACTTGAGTGGGTTTGAGGTAGCTGGAATGGATAGTGTAGCGGTGAAATGCATAGATATTATCCAGAACACCAATTGCGTAGGCAAGTTACTAAGACTCAACTGACGCTGAGGCACGAAAGTGTGGGTATCAAACAGG` |
| ASV4057 | 430 | `TAGGGAATATTGCGCAATGGGGGAAACCCTGACGCAGCAACGCCGCGTGAGTGAGGAAGGTCTTAGGATTGTAAAACTCTTTTATTTGGGAAAAATGTCTCAGTGGCTAATATCTATTGAGAGTGATGGTACCAAAGGAATAAGGACCGGCTAACTTCGTGCCAGCAGCCGCGGTAATACGAAGGGTCCAAGCGTTGTTCGGAATCATTGGGCGTAAAGCGCGCGCAGGCGGATCAGTAAGTCAGGTGTGAAATCTCGAGGCTCAACCTCGAAATTGCGCCTGAAACTACTAGTCTAGAGTGTCGGAGGGGGCAGGGGAATTTCACGTGTAGGGGTAAAATCCGTAGAGATGTGAAGGAACACCGGAGGCGAAGGCGCCTGCCTGGACGACTACTGACGCTGAGGCGCGAAAGCGTGGGGAGCAAACAGG` |
| ASV4676 | 404 | `TAGGGAATATTGGACAATGGACGCAAGTCTGATCCAGCTATGCCGCGTGAATGATGAAGGCCTTAGGGTTGTAAAGTTCTTTTAGCGAAGAAAATAATGATGGTACTCGCAGAAAAAGCCCCGGCTAACTTCGTGCCAGCAGCCGCGGTAATACGAAGGGGGCTAGCGTTGTTCGGAATCACTGGGCGTAAAGCGTACGTAGGCGGTTTGCAAAGTCAGATGTGAAATCCTGGGGCTTAACCCTAGAATTGCATTTGAAACTGGCAAGCTAGAGACCGGTAGGGGATAGTGGAATTCCTAGTGTAGAGGTGAAATTCGTAGATATTAGGAGGAACACCAGTGGCGCAAGCGACTATCTGGGCCGGTACTGACGCTAAGGTACGAAAGCGTGGGTAGCAAACAGG` |
| ASV1552 | 429 | `TGGGGAATTTTGGACAATGGGCGCAAGCCTGATCCAGCAATGCCGCGTGCAGGATGAAGGCCTTCGGGTTGTAAACTGCTTTTGTACGGAGCGAAACGGCTTCCACTAATACTGGGGGCTAATGACGGTACCGTAAGAATAAGCACCGGCTAACTACGTGCCAGCAGCCGCGGTAATACGTAGGGTGCGAGCGTTAATCGGAATTACTGGGCGTAAAGCGTGCGCAGGCGGTTATGTAAGACAGGTGTGAAATCCCCGGGCTTAACCTGGGAACTGCATTTGTGACTGCATAGCTAGAGTACGGTAGAGGGGGATGGAATTCCGCGTGTAGCAGTGAAATGCGTAGATATGCGGAGGAACACCGATGGCGAAGGCAATCCCCTGGACCTGTACTGACGCTCATGCACGAAAGCGTGGGGAGCAAACAGG` |
| ASV3060 | 424 | `TGAGGAATATTGGTCAATGGGCGCAAGCCTGAACCAGCCATGCCGCGTGAAGGATGAAGGTTCTATGGATTGTAAACTTCTTTTATTTGGGAATAAACCTCTTTACGTGTAGAGAGCTGAAGGTACCAAATGAATAAGCACCGGCTAACTCCGTGCCAGCAGCCGCGGTAATACGGAGGGTGCAAGCGTTATCCGGAATCATTGGGTTTAAAGGGTCCGCAGGCGGGCTTATAAGTCAGTGGTGAAAGCCTACAGCTTAACTGTAGAACTGCCATTGATACTGTAAGTCTTGAATGTGGTCGAAGTGGGCGGAATATGACATGTAGCGGTGAAATGCATAGATATGTCATAGAACACCGATAGCGAAGGCAGCTCACTAGGCCATTATTGACGCTCATGGACGAAAGCGTGGGGAGCAAACAGG` |
| ASV3356 | 404 | `TGGGGAATTTTCCGCAATGGGCGCAAGCCTGACGGAGCAATACCGCGTGCGGGAGGAAGCTTTTTGGAGTGTAAACCGCTTTTCTCAGGGAAGATGATGACGGTACCTGAGGAATCAGCATCGGCTAACTTCGTGCCAGCAGCCGCGGTAATACGAGGGATGCAAGCGTTATCCGGAATTATTGGGCGTAAAGCGTTCGTAGGTGGCTAAGTTAGTCTGTTGTTAAAACGTGGAGCTTAACTCCATATCGGCAATGGAAACTGCTTGGCTAGAGTATGGTAGGGGTAAGAGGAATTCCCAGTGTAGCGGTGAAATGCGTAGATATTGGGAAGAACACCAGCGGCGAAAGCGTCTTACTGGGCCATTACTGACACTGAGGGACGAAAGCTAGGGGAGCGAAAGGG` |
| ASV4415 | 406 | `TGGGGAATATTGCACAATGGGCGAAAGCCTGATGCAGCAACGCCGCGTGAGCGATGAAGGCCTTTGGGTCGTAAAGCTCTGTCCTAGGGGAAGAAAAAAATGACGGTACCCTTGGAGGAAGCCCCGGCTAACTACGTGCCAGCAGCCGCGGTAATACGTAGGGGGCAAGCGTTATCCGGAATTATTGGGCGTAAAGAGTGCGTAGGTGGTTTTGTAAGCGCGGGGTGAAAGGCAATAGCTCAACTATTGTAAGCCCTGCGAACTGTGAGACTTGAGTGCAGGAGAGGAAAGTGGAATTCCTAGTGTAGCGGTGAAATGCGTAGATATTAGGAGGAACACCAGTGGCGAAGGCGACTTTCTGGACTGTAACTGACACTGAGGCACGAAAGCGTGGGGAGCAAACAGG` |
| ASV5637 | 404 | `TGGGGAATATTGGACAATGGGCGAAAGCCTGATCCAGCCATGCCGCGTGAGTGATGACGGCCTTCGGGTTGTAAAGCTCTTTTGGGTGGGACGATGATGACGGTACCACCAGAATAAGATCCGGCTAACTTCGTGCCAGCAGCCGCGGTAATACGAAGGGATCTAGCGTTGTTCGGATTTACTGGGCGTAAAGGGCGCGTAGGCGGTCTAGCAAGTAAGGTGTGAAAGCCCTGAGCTTAACTCGGGAACTGCACTTTAAACTACTAGGCTAGAGTATGGGAGAGGTAAGTGGAATTTCCAGTGTAGAGGTAAAATTCGTAGATATTGGAAAGAACACCAGTGGCGAAGGCGACTTACTGGACCATTACTGACGCTGAGGCGCGAAAGCGTGGGGAGCAAACAGG` |
| ASV4941 | 404 | `TGGGGAATATTGGACAATGGGCGCAAGCCTGATCCAGCCATGCCGCGTGAGTGATGAAGGTCTTAGGATTGTAAAGCTCTTTCAGTGGGGACGATAATGACGGTACCCACAGAAGAAGCCCCGGCTAACTTCGTGCCAGCAGCCGCGGTAATACGAAGGGGGCTAGCGTTGTTCGGAATTACTGGGCGTAAAGCGCACGTAGGCGGATATTTAAGTCAGGGGTGAAATCCCGGGGCTCAACCCCGGAACTGCCTTTGATACTGGGTATCTTGAGTTCGGAAGAGGTAAGTGGAATTGCGAGTGTAGAGGTGAAATTCGTAGATATTCGCAGGAACACCAGTGGCGAAGGCGGCTTACTGGTCCGATACTGACGCTGAGGTGCGAAAGCGTGGGGAGCAAACAGG` |
| ASV5678 | 428 | `TGGGGAGTATTGCGCAATGGGCGAAAGCCTGACGCAGCAACGCCGCGTGAGTGAAGAAGGCCTTCGGGTTGTAAAGCTCTGTCGGCAGGGACGAAACCTTGTGAGTTAACAGCTTACAAGCTGACGGTACCTGCAAAGGAAGCACCGGCTAACTCTGTGCCAGCAGCCGCGGTAATACAGAGGGTGCAAGCGTTGTTCGGAATTATTGGGCGTAAAGGGGAGGCAGGTGGTCTTATTTGTCTAGGGTGAAATCCCGGGGCTTAACCCCGGACGTGCCTTGGAAACGGTAAGACTAGAGTTCTAGAGAGGCTAGCGGAATTCCCGGTGTAGCGGTGAAATGCGTAGAGATCGGGAGGAACACCAGAGGCGTAAGCGGCTAGCTGGATAGATACTGACACTCATCTCCGAAAGCGTGGGGAGCAAACAGG` |
| ASV2974 | 430 | `TAGGGAATATTGCGCAATGGGGGAAACCCTGACGCAGCAACTCCGCGTGAGTGAGGAAGGCCTTCGGGTTGTAAAGCTCTGTTATTAGGGAAAAATGATCATGTGGGTAATATTCATGTGATGTGATGGTACCTGATGAGGAAGCACCGGCTAACTTCGTGCCAGCAGCCGCGGTAATACGAAGGGTGCAAGCGTTGTTCGGAATTATTGGGCGTAAAGCGCGCGCAGGCGGATAAGCAAGTCAGATGTGAAATCTCGGGGCTCAACCTCGAAACTGCGTCTGAAACTGTTTGTCTAGAGTACTGTAGGGGACAGGGGAATTTCACGTGTAGGGGTAAAATCCGTAGAGATGTGAAGGAACACCGGAGGCGAAGGCGCCTGTCTGGCCAGTAACTGACGCTGAGGCGCGAAAGCGTGGGGAGCAAACAGG` |
| ASV209 | 428 | `TCGAGAATCTTTCACAATGGGGGCAACCCTGATGGAGCGACGCCGCGTGGAGGATAAGGTCTTCGGATTGTAAACTCCTGTCATGCGCGAACAAGGTTGCATGGTTAATAACTGTGCAAATTGATGGTAGCGCAAGAGGAAGAGACGGCTAACTCTGTGCCAGCAGCCGCGGTAATACAGAGGTCTCAAGCGTTGTTCGGAATCACTGGGCGTAAAGGGTGCGTAGGTGGCATGGTAAGTCAGATGTGAAAGCCCAGGGCTCAACCTTGGAATTGCATCCGATACTGCTGTGCTAGAGTACTGGAGGGGTGACTAGAATTCTCGGTGTAGCAGTGAAATGCGTAGATATCGAGAGGAATACCAAAGGCGTAGGCAGGTCACTGGACAGTTACTGACACTGAGGCACGAAGGCCAGGGGAGCAAACGGG` |
| ASV1931 | 424 | `TGAGGAATATTGGTCAATGGTCGCAAGACTGAACCAGCCATGCCGCGTGCAGGAAGACGGTCCTATGGATTGTAAACTGCTTTTATACAGGAAGAAACCCTCCCACGTGTGGGAGCTTGACGGTACTGTAGGAATAAGGATCGGCTAACTCCGTGCCAGCAGCCGCGGTAATACGGAGGATCCAAGCGTTATCCGGAATCATTGGGTTTAAAGGGTCCGTAGGCGGTCTTATAAGTCAGTGGTGAAAGCCCATCGCTCAACGATGGAACTGCCATTGATACTGTAAGACTTGAATGCTTAGGAAGTAACTAGAATATGTAGTGTAGCGGTGAAATGCTTAGATATTACATGGAATACCAATTGCGAAGGCAGGTTACTACTAAGTGATTGACGCTGATGGACGAAAGCGTGGGGAGCGAACAGG` |
| ASV2158 | 406 | `TGGGGAATATTGCGCAATGGGGGAAACCCTGACGCAGCAACGCCGCGTGAAGGATGAAGGCTTTCGGGTTGTAAACTTCTTTAGTCGGGGAAGAATAAATGACGGTACCCGAAGAATAAGCCACGGCTAACTACGTGCCAGCAGCCGCGGTAATACGTAGGTGGCAAGCGTTGTCCGGAATTACTGGGTGTAAAGGGCGTGTAGGCGGGTTATTAAGTCAGATGTGAAATTCCAGGGCTCAACCCTGGCGCTGCATCTGAAACTGATGATCTTGAGTACTGGAGAGGGAAGCGGAATTCCTAGTGTAGCGGTGAAATGCGTAGATATTAGGAGGAACACCAGTGGCGAAGGCGGCTTTCTGGACAGTAACTGACGCTGAGGCGCGAAAGCGTGGGGAGCGAACAGG` |
| ASV3776 | 404 | `TGGGGAATATTGGACAATGGGCGAAAGCCTGATCCAGCCATGCCGCGTGAGTGATGAAGGCCTTAGGGTTGTAAAGCTCTTTTGTCAGGGAAGATAATGACGGTACCTGAAGAATAAGCACCGGCTAACTTCGTGCCAGCAGCCGCGGTAATACGAAGGGTGCTAGCGTTGTTCGGAATTACTGGGCGTAAAGCGCGCGTAGGCTGTACTACAAGTCAGGGGTGAAATCCCGGGGCTCAACCTCGGAATTGCCTTTGAAACTGTAGTACTGGAATCTCAGAGGGGATAGCGGAATTCCAAATGTAGGGGTGAAATCCGTAGATATTTGGAGGAACACCGGTGGCGAAGGCGGCTATCTGGATGAGTATTGACGCTGAGGCGCGAAAGCGTGGGGATCAAACAGG` |
| ASV3682 | 429 | `TGGGGAATTTTGGACAATGGGCGCAAGCCTGATCCAGCCATGCCGCGTGAGTGAAGAAGGCCTTCGGGTTGTAAAGCTCTTTCGGCCGGGAAGAAATCGTATGGGTTAATACCCTGTGCGGATGACGGTACCGGCATAAGAAGCACCGGCTAACTACGTGCCAGCAGCCGCGGTAATACGTAGGGTGCGAGCGTTAATCGGAATTACTGGGCGTAAAGCGTGCGCAGGCGGTTTTTTAAGACAGGCGTGAAATCCCCGGGCTCAACCTGGGAACTGCGCTTGTGACTGGAAGACTAGAGTATGGCAGAGGGGGGTGGAATTCCACGTGTAGCAGTGAAATGCGTAGAGATGTGGAGGAACACCGATGGCGAAGGCAGCCCCCTGGGCCAATACTGACGCTCATGCACGAAAGCGTGGGTAGCAAACAGG` |
| ASV2039 | 424 | `TGAGGAATATTGGTCAATGGGCGCAAGCCTGAACCAGCCATGCCGCGTGCAGGATGACGGTCCTATGGATTGTAAACTGCTTTTGTACAGGAAGAAACACTCCTACGTGTAGGAACTTGACGGTACTGTAAGAATAAGGATCGGCTAACTCCGTGCCAGCAGCCGCGGTAATACGGAGGATCCAAGCGTTATCCGGAATCATTGGGTTTAAAGGGTCCGTAGGCGGGCTAATAAGTCAGTGGTGAAAGCCCATCGCTCAACGATGGAACGGCCATTGATACTGTTAGTCTTGAATTATTAGGAAGTAACTAGAATATGTAGTGTAGCGGTGAAATGCTTAGAGATTACATGGAATACCAATTGCGAAGGCAGGTTACTACTAATGGATTGACGCTGATGGACGAAAGCGTGGGTAGCGAACAGG` |
| ASV4560 | 404 | `TAAGGAATATTGGACAATGGGGGCAACCCTGATCCAGCCATGCCGCGTGAGTGATGAAGGCCTTCGGGTTGTAAAGCTCTTTTAGATGTGACGATGATGACGGTAGCATCAGAAAAAGCTCCGGCTAACTTCGTGCCAGCAGCCGCGGTAATACGAAGGGAGCGAGCGTTGTTCGGAATCACTGGGCTTAAAGCGTACGTAGGCGGATATACAAGTCAGGAGTGAAATCCCAGGGCTTAACCCTGGAATTGCTTTTGAAACTGTAAATCTGGAATCCGGTAGAGGTTGGCGGAACTCCGAGTGTAGAGGTGAAATTCGCAGATATTCGGAAGAACACCAGTGGCGAAGGCGGCCAACTGGACCGGTATTGACGCTGAGGTACGAAAGCGTGGGTAGCAAACAGG` |
| ASV37 | 429 | `TGGGGAATTTTGGACAATGGGCGAAAGCCTGATCCAGCAATGCCGCGTGCAGGATGAAGGCCTTCGGGTTGTAAACTGCTTTTGTACGGAACGAAACGGTCTCTCCTAATACGAGGGGCTAATGACGGTACCGTAAGAATAAGCACCGGCTAACTACGTGCCAGCAGCCGCGGTAATACGTAGGGTGCAAGCGTTAATCGGAATTACTGGGCGTAAAGCGTGCGCAGGCGGTTATATAAGACAGATGTGAAATCCCCGGGCTCAACCTGGGACCTGCATTTGTGACTGTATAGCTAGAGTACGGTAGAGGGGGATGGAATTCCGCGTGTAGCAGTGAAATGCGTAGATATGCGGAGGAACACCGATGGCGAAGGCAATCCCCTGGACCTGTACTGACGCTCATGCACGAAAGCGTGGGGAGCAAACAGG` |
| ASV3056 | 429 | `TGGGGAATTTTGGACAATGGACGAAAGTCTGATCCAGCAATGCCGCGTGCAGGACGAAGGCCTTCGGGTTGTAAACTGCTTTTGTACGGAGCGAAAAGGTTCATTTTAATAAAGTGGGCTCATGACGGTACCGTAAGAATAAGCACCGGCTAACTACGTGCCAGCAGCCGCGGTAATACGTAGGGTGCAAGCGTTAATCGGAATTACTGGGCGTAAAGCGTCCGCAGGCGGTTCTATAAGACAGATGTGAAATCCCTGGGCTCAACCTAGGAACTGCATTTGTGACTGTAGAGCTAGAGTACGGCAGAGGGGGATGGAATTCCGCGTGTAGCAGTGAAATGCGTAGATATGCGGAGGAACACCGATGGCGAAGGCAATCCCCTGGGCCTGTACTGACGCTCATGGACGAAAGCGTGGGGAGCAAACAGG` |
| ASV3926 | 430 | `TGGGGAATTTTGCACAATGGGGGAAACCCTGATGCAGCAACGCCGCGTGAGTGAAGAAGGCCCTTGGGTTGTAAAGCTCTTTCGACTGGGAAGCAGGGTAGCGATGTAAATAATGTCGTTATTTGACGGTACCAGTATAAGAAGCACTGGCTAATTCCGTGCCAGCAGCCGCGGTAATACGGAAGGTGCAAGCGTTGTTCGGATTGACTGGGCGTAAAGCGCGCGCAGGCTGGTTTGCAAGTCGAATGTGAAAGCCCCGGGCTTAACCTGGGAATTGCATTCGAAACTGTAAATCTTGAATGTTTGAGAGGTTGGTAGAATTCCAGGTGTAGAAGTGAAATTCGTAGAGATCTGGAGGAATACCGGAGGCGAAGGCGGCCAACTGGCAAAACATTGACGCTGAGGCGCGAAAGCGTGGGGAGCAAACAGG` |
| ASV4863 | 404 | `TGGGGAATATTGGGCAATGGGCGCAAGCCTGACCCAGCCATGCCGCGTGAGTGATGAAGGCCTTAGGGTTGTAAAGCTCTTTTAGCAGGGACGATGATGACGGTACCTGCAGAATAAGCTCCGGCTAACTTCGTGCCAGCAGCCGCGGTAATACGAAGGGAGCTAGCGTTGTTCGGAATCACTGGGCGTAAAGCGCGCGTAGGCGGCGTCTCAAGTCAGGGGTGAAATGCCAAGGCTTAACCTTGGAACTGCCCTTGAAACTAGGACGCTGGAGTACTGGAGGGGATGGCGGAATTCCTAATGTAGCGGTGAAATGCGTAGATATTAGGAGGAACACCAGCGGCGAAGGCGGCCATCTGGACAGTAACTGACGCTGAGGCGCGAAAGCGTGGGGATCAAACAGG` |
| ASV4448 | 429 | `TGGGGAATATTGGACAATGGGGGCAACCCTGATCCAGCAATGCCGCGTGTGTGAAGAAGGCCTGCGGGTTGTAAAGCACTTTCAGTAGAGAAGAAAAGCCTAAGGTTAATAACCTTGAGTCTTGACGTAACCTACAGAAGAAGCACCGGCTAACTCCGTGCCAGCAGCCGCGGTAATACGGAGGGTGCAAGCGTTAATCGGAATTACTGGGCGTAAAGCGCACGTAGGTGGTTTTGTAAGTCGGATGTGAAAGCCCTGGGCTCAACCTGGGAATTGCATTCGATACTGCTTAACTAGAGTATGGTAGAGGGAAGTGGAATTTCCGGTGTAGCGGTGAAATGCGTAGATATCGGAAGGAACACCAGTGGCGAAGGCGACTTCCTGGGCCAATACTGACACTGAGGTGCGAAAGCGTGGGTAGCAAACAGG` |
| ASV4154 | 429 | `TGGGGAATTTTGGACAATGGGCGCAAGCCTGATCCAGCCATGCCGCGTGAGTGAAGAAGGCCTTCGGGTTGTAAAGCTCTTTCGGCCGGGACGAAATCGCATGGGTGAATATCCCGTGTGGATGACGGTACCGGAATAAGAAGCACCGGCTAACTACGTGCCAGCAGCCGCGGTAATACGTAGGGTGCGAGCGTTAATCGGAATTACTGGGCGTAAAGCGTGCGCAGGCGGTTTCGTAAGCCAGATGTGAAATCCCCGGGCTTAACCTGGGAACTGCATTTGGGACTGCGAGGCTAGAGTACGGCAGAGGGAGGTGGAATTCCACGTGTAGCAGTGAAATGCGTAGATATGTGGAGGAACACCGATGGCGAAGGCAGCCTCCTGGGCTAGTACTGACGCTCATGCACGAAAGCGTGGGGAGCAAACAGG` |
| ASV158 | 404 | `TGGGGAATATTGGGCAATGGACGAAAGTCTGACCCAGCCATGCCGCGTGAATGATGAAGGCTTTAGGGTTGTAAAGTTCTTTCGCCGATGAAGATAATGACGGTAGTCGGAGAAGAAGCCCCGGCTAACTTCGTGCCAGCAGCCGCGGTAATACGAAGGGGGCTAGCGTTGTTCGGAATTACTGGGCGTAAAGCGAGCGTAGGTTGCTCGACAAGTCAGTGGTGAAAGCCCAGAGCTCAACTCTGGAACTGCCATTGAAACTGTCGAGCTGGAATCTCAGAGGGGGTAGCGGAATTCCAAATGTAGGGGTGAAATCCGTAGATATTTGGAGGAACACCGGTGGCGAAGGCGGCTACCTGGATGAGTATTGACACTGAGGCTCGAAAGCGTGGGGATCAAACAGG` |
| ASV5041 | 404 | `TGGGGAATATTGGACAATGGGGGAAACCCTGATCCAGCAATGCCGCGTGAATGATGAAGGCCTTCGGGTTGTAAAGTTCTTTTATAGATGACGATAATGACGGTAGTCTATGAATAAGCACCGGCTAACTTCGTGCCAGCAGCCGCGGTAATACGAAGGGTGCTAGCGTTGTTCGGAATTACTGGGCGTAAAGGGTGCGTAGGCGGCCTTATAAGTGAGATGTGAAATACCTGGGCTTAACCTGGGAACGGCATTTTATACTGTAAGGCTAGAGGAAGTGAGAGGAAGATGGAATTGTGCGTGTAGAGGTGAAATTCGTAGATATGCACAAGAACACCAATGGCGAAGGCAGTCTTCTGGCACTTTACTGACGCTAATGCACGAAAGCGTGGGGAGCAAACAGG` |
| ASV2996 | 430 | `TGGGGAATATTGGACAATGGGGGCAACCCTGATCCAGCAATGCCGCGTGTGTGAAGAAGGCCTGCGGGTTGTAAAGCACTTTCGTTGGGGAGGAAGGGTTTTTGGTGAATAGCCAGGAATTCAGACGCTACCCAAAAAAGCAGCACCGGCTAACTCTGTGCCAGCAGCCGCGGTAATACAGAGGGTGCAAGCGTTAATCGGAATTATTGGGCGTAAAGGGCGCGTAGGTGGTTAATTAAGTTGACTGTGAAAGCCCCGGGCTTAACCTGGGACGGTCAGTCAATACTGGTTAACTTAGAGTACAGTAGAGGGTAGTGGAATTTCCAGTGTAGCGGTGAAATGCGTAGATATTGGAAGGAACACCAGTGGCGAAGGCGGCTACCTGGACTGATACTGACACTGAGGCGCGAAAGCGTGGGTAGCGAACAGG` |
| ASV2455 | 404 | `TAAGGAATATTGGACAATGGGCGAAAGCCTGATCCAGCCATGCCGCGTGAGTGAAGAAGGCCTTAGGGTTGTAAAGCTCTTTCAATAGTGAAGATAATGACGGTAGCTATAGAAGAAGCCCCGGCTAAATTCGTGCCAGCAGCCGCGGTAATACGAATGGGGCAAGCGTTGTTCGGAATCACTGGGCTTAAAGCGTGCGCAGGCGGATATGAAAGTCAGAAGTGAAATCCTTGGGCTCAACCCAAGAATTGCTTTTGAAACTTCATATCTTGAATCCGGTAGAGGTTGGGAGAATTCCTAGTGTAGAGGTGAAATTCGCAGATATTAGGAGGAATACCAGTGGCGTAGGCGCCCAACTGGACCGGTATTGACGCTCAGGCACGAAAGCGTGGGGAGCAAACAGG` |
| ASV2851 | 429 | `TGGGGAATATTGGACAATGGGGGCAACCCTGATCCAGCCATGCCGCGTGTGTGAAGAAGGCCTTCGGGTTGTAAAGCACTTTCGGTCAGAAAGAAGGGTGTGTAGTTAATAACTGCGCATCTTGACGGTACTGACATAAGAAGCACCGGCTAACTCCGTGCCAGCAGCCGCGGTAATACGGAGGGTGCAAGCGTTAATCGGAATTACTGGGCGTAAAGCGCGCGTAGGCGGTTTGTTAAGTCGGATGTGAAAGCCCCGGGCTCAACCTGGGAACTGCATACGATACTGGCAAGCTAGAGTGCAATAGAGGGGGGTAGAATTCCAGGTGTAGCGGTGAAATGCGTAGATATCTGGAGGAATACCGGTGGCGAAGGCGGCCCCCTGGATTGACACTGACGCTGAGGTGCGAAAGCGTGGGTAGCAAACAGG` |
| ASV2985 | 429 | `TAGGGAATATTGGACAATGGGCGCAAGCCTGATCCAGCCATGCCGCGTGAGTGAAGAAGGCCTTCGGGTTGTAAAGCTCTTTTGTCCGGAAAGAAAAGCTTCGGGTTAATACCCTGGAGTCCTGACGGTACCGGAAGAATAAGCACCGGCTAACTTCGTGCCAGCAGCCGCGGTAATACGAAGGGTGCAAGCGTTACTCGGAATTACTGGGCGTAAAGCGTGCGTAGGTGGTTTGTTAAGTCTGATGTGAAAGCCCTGGGCTCAACCTGGGAATGGCATTGGATACTGGCAGGCTAGAGTGCGGTAGAGGGTAGTGGAATTCCCGGTGTAGCAGTGAAATGCGTAGAGATCGGGAGGAACATCTGTGGCGAAGGCGACTACCTGGACCAGCACTGACACTGAGGCACGAAAGCGTGGGGAGCAAACAGG` |
| ASV3021 | 424 | `TAAGGAATATTGGACAATGCCCGCAAGGGTGATCCAGCCATGCCGCGTGCAGGAAGACGGCCCTATGGGTTGTAAACTGCTTTTGCACCAGAGAAAACCTCCCTTCGTGAAGGGAGCTGATAGTATGGTGAGAATAAGCATCGGCTAACTTCGTGCCAGCAGCCGCGGTAAGACGAAGGATGCAAGCGTTATCCGGATTCATTGGGTTTAAAGGGAGCGTAGGTGGTCTTATAAGTCAGTGGTGAAATCTCCTTGCTTAACAAGGAAATTGCCATTGATACTGTAGGACTTGAGTACAGTTGCTGTGGGCGGAATATGACATGTAGTGGTGAAATACATAGAGATGTCATAGAACACCGATTGCGAAGGCAGCTCACAAAACTGTAACTGACACTGAGGCTCGAAAGTGCGGGGATCAAACAGG` |
| ASV5424 | 404 | `TGGGGAATATTGGACAATGGGCGCAAGCCTGATCCAGCCATGCCGCGTGAGTGATGAAGGCCTTAGGGTTGTAAAGCTCTTTCAGCTGGGAAGATAATGACGGTACCAGCAGAAGAAGCCCCGGCTAACTTCGTGCCAGCAGCCGCGGTAATACGAAGGGGGCTAGCGTTGTTCGGAATCACTGGGCGTAAAGCGCACGTAGGCGGATCGATAAGTCAGGGGTGAAATCCCGAGGCTCAACCTCGGAACTGCCTTTGATACTGTCGACCTTGAGTCCGGGAGAGGTGAGTGGAACTCCGAGTGTAGAGGTGAAATTCGTAGATATTCGGAAGAACACCAGTGGCGAAGGCGGCTCACTGGCCCGGTACTGACGCTGAGGTGCGAAAGCGTGGGGAGCAAACAGG` |
| ASV4183 | 429 | `TGGGGAATTTTGGACAATGGGGGCAACCCTGATCCAGCCATTCCGCGTGAGTGAAGAAGGCCTTCGGGTTGTAAAGCTCTTTCGCAGGGGAAGAAACGATGCAGACGAATAATCTGCGTTAATGACGGTACCTTGATAAGAAGCACCGGCTAACTACGTGCCAGCAGCCGCGGTAATACGTAGGGTGCGAGCGTTAATCGGAATTACTGGGCGTAAAGCGTGCGCAGGCGGTTAGATAAGTCAGATGTGAAATCCCCGGGCTCAACCTGGGAACTGCGTTTGAAACTATCTAGCTAGAGTATGGGAGAGGGAGGTAGAATTCCACGTGTAGCAGTGAAATGCGTAGAGATGTGGAGGAATACCAATGGCGAAGGCAGCCTCCTGGCTTAATACTGACGCTCATGCACGAAAGCGTGGGGAGCAAACAGG` |
| ASV92 | 404 | `TGGGGAATATTGGACAATGGGGGCAACCCTGATCCAGCCATGCCGCGTGCGTGAAGAAGGCCTTAGGGTTGTAAAGCGCTTTTGTCAGGGAAGATAATGACTGTACCTGAAGAATAAGCCCCGGCTAACTCCGTGCCAGCAGCCGCGGTAAGACGGAGGGGGCTAGCGTTGTTCGGAATTACTGGGCGTAAAGGGCGCGTAGGCTGCCATGTGTGTCAGGTGTGAAATCCCAAGGCTTAACCTTGGAACTGCATCTGAAACTGCATGGCTTGAGTGAGTAAGAGGCGGACGGAATTCCGAGTGTAGCAGTGAAATGCGTAGATATTCGGAGGAACATCGGTGGCGAAGGCGGTTCGCTGGTACTCTACTGACGCTGAGGCGCGAAAGCGTGGGGAGCAAACAGG` |
| ASV682 | 429 | `TGGGGAATTTTGGACAATGGGCGCAAGCCTGATCCAGCCATGCCGCGTGCGGGAAGAAGGCCTTCGGGTTGTAAACCGCTTTTGTCAGGGAAGAAACGGCTTTTTCTAATAAAGAGAGCTAATGACGGTACCTGAAGAATAAGCACCGGCTAACTACGTGCCAGCAGCCGCGGTAATACGTAGGGTGCAAGCGTTAATCGGAATTACTGGGCGTAAAGCGTGCGCAGGCGGTTATGCAAGACAGAGGTGAAATCCCCGGGCTCAACCTGGGAACTGCCTTTGTGACTGCATAGCTAGAGTACGGTAGAGGGGGATGGAATTCCGCGTGTAGCAGTGAAATGCGTAGATATGCGGAGGAACACCGATGGCGAAGGCAATCCCCTGGACCTGTACTGACGCTCATGCACGAAAGCGTGGGGAGCAAACAGG` |
| ASV4235 | 409 | `TTTGGAATTTTGGACAATGGGCGAAAGCCTGATCCAGCGACGCCGCGTGGACGACGAAGGCCTTCGGGTTGTAAAGTCCTTTTGGCAGGGAACAATTTTGAGTGTACCTGCAGAATAAGAGGTTGCTAACTCTGTGCCAGCAGCAGCGGTAATACAGAGACCTCAAGCGTTATCCGGAATCATTGGGCGTAAAGCGTACCGACAGGTGGTTTAAAAAGTCAGAAGTGAAATCTCTCAGCTTAACTGAGAGTCTGTCTTTTGAAACTTTTAAACTAGAGGGACAAAGAGGAAGCTGGAACCAGTGGTGTAGTAGTGAAATGCGTTGATATCACTGGGAACACCAATAGCGAAGGCAGGCTTCTGGGTGTCACCTGACACTGCTAGGACGAAAGCGTGGGGAGCGAATGGG` |
| ASV3552 | 429 | `TGGGGAATTTTGGACAATGGGGGCAACCCTGATCCAGCCATTCCGCGTGAGTGAAGAAGGCCTTCGGGTTGTAAAGCTCTTTTGTCCGGAACGAAACGGTGAGCGTGAATATCGCTCGCTACTGACGGTACCGGAAGAATAAGCACCGGCTAACTACGTGCCAGCAGCCGCGGTAATACGTAGGGTGCGAGCGTTAATCGGAATTACTGGGCGTAAAGCGTGCGCAGGCGGTTTTGTAAGCCAGATGTGAAATCCCCGGGCTTAACCTGGGAATGGCATTTGGGACTGCAAGGCTGGAGTTTGGCAGAGGAGACTGGAATTCCCGGTGTAGCAGTGAAATGCGTAGATATCGGGAGGAATACCGATGGCGAAGGCAGGTCTCTGGGCTATAACTGACGCTCATGCACGAAAGCGTGGGGAGCAAACAGG` |
| ASV3895 | 388 | `TCGGGAAAATTCTGCAATGCGCGAAAGCGTGACAGAGCAAGCCAAAGTGATTTTCCGTTTTGGAAAATCTTTTATGCAATGTAAAAAGTCGCATGAATAAGGACTGGGCAAGACTGGTGCCAGCCGCCGCGGTAATCCCAGCAGTCCAAGTCGCAGCCACATTTATTGGGTCTAAAACATCCGTAGCTAGCATAGTAAGTCTCTTGTAAAATTTGGCTTCTTAAGAGTCGAGCTGCAAGAGATACTGTTTTGCTTGAGACCGGGAGGCGTAAGAAGTACGATAGGGGTAGCGGTAAAATGCGTTGATCCTTTTCGGACTAACAATCGCGAAGGCATCTTACGAGAACGGTTCTGACAGTGAGGGATGAAGGCTAGGGGCGCAAAGTGG` |
| ASV5168 | 429 | `TGGGGAATATTGGACAATGGGGGAAACCCTGATCCAGCAATACCGCGTGTGTGAAGAAGGCCTGCGGGTTGTAAAGCACTTTTGTCGGGGAGGAAAAGTTAAAAGCTAATACCTTTTAACCATGACGCTACCCGAAGAAAAAGCACCGGCTAACTCTGTGCCAGCAGCCGCGGTAATACAGAGGGTGCAAGCGTTAATCGGAATTACTGGGCGTAAAGGGCCTGTAGGTGGTTTCTTAAGTTAGATGTGAAAGCCCTGGGCTCAACCTGGGAACTGCATCTAAAACTGGGGAACTAGAGTATGGTAGAGGGAAGTGGAATTTCTAGTGTAGCGGTGAAATGCGTAGATATTAGAAAGAACACCAGTGGCGAAGGCGACTTCCTGGACCAATACTGACACTGAGGGGCGAAAGCGTGGGGAGCAAACAGG` |
| ASV3343 | 423 | `TAGGGAATATTGGGCAATGGAGGCAACTCTGACCCAGCCATGCCGCGTGAAGGATGAAGGCGTTCTGCGTTGTAAACTTCTTTTATATGGGAAGAAAAGCCTCTTGCGAGAGGGATTGACGGTACCATATGAATAAGCACCGGCTAACTCCGTGCCAGCAGCCGCGGTAATACGGAGGGTGCAAGCGTTGTCCGGATTTATTGGGTTTAAAGGGTGCGTAGGCGGCTTAATAAGTCAGTGGTGAAAGACTGTGGCTCAACCATAGAATTGCCATTGATACTGTTAAGCTTGAGTATAGTTGAGGTAGGCGGAATTGACGGTGTAGCGGTGAAATGCATAGATATCGTCAAGAACACCGATTGCGTAGGCAGCTTACTAAGCTATAACTGACGCTGAGGCACGAAAGTGTGGGGATCAAACAGG` |
| ASV281 | 404 | `TGGGGAATCTTAGACAATGGGGGAAACCCTGATCTAGCCATGCCGCGTGAGCGATGAAGGCCTTAGGGTTGTAAAGCTCTTTCGGGGGGGAAGATAATGACTGTACCCCCAGAAGAAGCCCCGGCTAACTCCGTGCCAGCAGCCGCGGTAATACGGAGGGGGCTAGCGTTGTTCGGAATTACTGGGCGTAAAGCGCACGTAGGCGGACGAGAAAGTCAGAGGTGAAATCCCGGGGCTCAACCCCGGAACGGCCTTTGAAACTCCTTGTCTTGAGGTCGTGAGAGGTGAGTGGAATTCCGAGTGTAGAGGTGAAATTCGTAGATATTCGGAGGAACACCAGTGGCGAAGGCGGCTCACTGGCACGATACTGACGCTGAGGTGCGAAAGCGTGGGGAGCAAACAGG` |
| ASV1397 | 404 | `TGGGGAATATTGGACAATGGGCGCAAGCCTGATCCAGCAATGCCGCGTGGGTGAAGAAGGTCTTCGGATTGTAAAGCCCTTTCGGCGGGGACGATGATGACGGTACCCGCAGAAGAAGCCCCGGCTAACTTCGTGCCAGCAGCCGCGGTAATACGAAGGGGGCTAGCGTTGCTCGGAATTACTGGGCGTAAAGGGCGCGTAGGCGGCACCATCAGTCAGGCGTGAAATTCCCGGGCTTAACCTGGGGACTGCGCTTGATACAGTGGAGCTAGAGGACGGAAGAGGCTCGTGGAATTCCCAGTGTAGAGGTGAAATTCGTAGATATTGGGAAGAACACCGGTGGCGAAGGCGGCGAGCTGGTCCGTTACTGACGCTGAGGCGCGACAGCGTGGGGAGCAAACAGG` |
| ASV4577 | 407 | `TGGGGAATATTGGGCAATGGGGGAAACCCTGACCCAGCAACGCCGCGTGAGGGAAGAAGGTTTTCGGATTGTAAACCTCTGTCCTAAGGGAAGAAAAAAATGACGGTACCTTAGGAGGAAGCCCCGGCTAACTACGTGCCAGCAGCCGCGGTAATACGTAGGGGGCAAGCGTTGTCCGGAATTATTGGGCGTAAAGGGTGCGTAGGCGGCTAGGCAAGTCAGATGTGAAATACCAGGGCTTAACTCTGGAGGTGCATTTGAAACTTCCTGGCTAGAGTGCAGGAGAGGGAAGCGGAATTCCTAGTGTAGCGGTGAAATGCATAGATATTAGGAGGAACACCAGTGGCGAAGGCGGCTTTCTGGACTGTAACTGACGCTGAGGCACGAAAGCGTGGGGAGCAAACAGG` |
| ASV138 | 429 | `TGGGGAATTTTGGACAATGGACGCAAGTCTGATCCAGCCATGCCGCGTGCGGGAAGAAGGCCTTCGGGTTGTAAACCGCTTTTGTCAGGGAAGAAATCTTCTAAGTTAATACCTTGGGAGGATGACGGTACCTGAAGAATAAGCACCGGCTAACTACGTGCCAGCAGCCGCGGTAATACGTAGGGTGCAAGCGTTAATCGGAATTACTGGGCGTAAAGCGTGCGCAGGCGGTTATGCAAGACAGGTGTGAAATCCCCGAGCTCAACTTGGGAACTGCACTTGTGACTGCATAGCTAGAGTACGGTAGAGGGGAGTGGAATTCCGCGTGTAGCAGTGAAATGCGTAGATATGCGGAGGAACACCGATGGCGAAGGCAGCTCCCTGGACCTGTACTGACGCTCATGCACGAAAGCGTGGGGAGCAAACAGG` |
| ASV507 | 421 | `TAAGGAATATTGCGCAATGGGCGAAAGCCTGACGCAGCGACGCCGCGTGGGGGATGAAGGTCTTCGGATTGTAAACCCCTTTCGGGAGGGAAGATGGAATGGGGTAACCCATTCGGACGGTACCTCCAGAAGCAGCCACGGCTAACTTCGTGCCAGCAGCCGCGGTAATACGAAGGTGGCAAGCGTTATTCGGATTTACTGGGCGTACAGGGAGCGTAGGCGGTTCGGTAAGCCCTTTGTGAAATCTCCAGGCTTAACCTGGAACAGTCGGAGGGGACTGCCGGGCTAGAGGACGGGAGAGGAGCGCGGAATTCCCGGTGTAGCGGTGAAATGCGTAGAGATCGGGAGGAAGGCCGGTGGCGAAGGCGGCGCTCTGGAACGTTTCTGACGCTGAGGCTCGAAAGCGTGGGGAGCAAACAGG` |
| ASV1894 | 404 | `TGGGGAATCTTGGACAATGGGGGCAACCCTGATCCAGCCATGCCGCGTGAGCGATGAAGGCCTTCGGGTTGTAAAGCTCTTTCGTGGGGGAAGATAATGACTGTACCCCAAGAAGAAGCCCCGGCTAACTCCGTGCCAGCAGCCGCGGTAATACGGAGGGGGCTAGCGTTGTTCGGAATTACTGGGCGTAAAGCGCACGTAGGCGGGCTGGACAGTCAGGGGTGAAATCCCGGGGCTCAACCCCGGAACTGCCTTTGAAACTCCCAGTCTTGAGGTCGCGAGAGGTGAGTGGAATTCCGAGTGTAGAGGTGAAATTCGTAGATATTCGGAGGAACACCAGTGGCGAAGGCGGCTCACTGGCGCGACACTGACGCTGAGGTGCGAAAGCGTGGGGAGCAAACAGG` |
| ASV5158 | 429 | `TGGGGAATTTTGGACAATGGGGGAAACCCTGATCCAGCAACGCCGCGTGAATGAAGAAGGCTTTCGGGTTGTAAAGTTCTTTTGTCTGGAAACAAAAGTTACAGAGTAATATACTGTAATGCTGAGGGTACCAGAAGAATAAGCACCGGCTAACTACGTGCCAGCAGCCGCGGTAATACGTAGGGTGCAAGCGTTAATCGGAATTATTGGGCGTAAAGAGAGCGTAGGCTGGTGTATAAGTTAGATGTGAAATACCTGAGCTTAACTTGGGAACGGCATTTAAAACTGTACGCCTAGAGTTTGTCAGAGGGAGGTAGAATTCCAAGTGTAGCAGTGAAATGCGTAGAGATTTGGAGGAATATCAGTGGCGAAGGCGGCCTCCTGGGACATGACTGACGCTGAGGTTCGAAAGCGTGGGTAGCAAACAGG` |
| ASV4723 | 404 | `TGAGGAATATTGGACAATGGGCGCAAGCCTGATCCAGCCATGCCGCGTGAGTGAAGAAGGCCTTCGGGTTGTAAAGCTCTTTCAGGTGCGACGATAATGACGGTAACACCAGAAGAAGCCCCGGCTAAATTCGTGCCAGCAGCCGCGGTAATACGAATGGGGCAAGCGTTGTTCGGAATCACTGGGCTTAAAGCGTGCGTAGGCGGACCAACAAGTCAGAAGTGAAATCCCTGGGCTCAACCCAGGAATTGCTTTTGAAACTGTCGGTCTAGAATCCCGGAGAGGTTAGGAGAATTCCGAGTGTAGAGGTGAAATTCGCAGATATTCGGAGGAATACCAGTGGCGTAGGCGCCTAACTGGACGGGCATTGACGCTGAGGCACGAAAGCGTGGGTAGCAAACAGG` |
| ASV5342 | 424 | `TGAGGAATATTGGTCAATGGGCGGAAGCCTGAACCAGCCAAGTAGCGTGAGGGACGAAGGCCCTACGGGTTGTAAACCTCTTTTATGCGGGGATAAAGTGGGGGACGTGTCCCCTATTGCAGGTACCGCATGAATAAGGACCGGCTAATTCCGTGCCAGCAGCCGCGGTAATACGGAAGGTCCGGGCGTTATCCGGATTCATTGGGTTTAAAGGGAGCGTAGGCCGGCCCTTAAGCGTGCCGTGAAATGCCGTGGCTCAACCATGGCACTGCGGCGCGAACTGGGGGTCTTGAGTGCGCGGAAGGCAGGCGGAATTCGTGGTGTAGCGGTGAAATGCTTAGATATCACGAAGAACCCCGATTGCGAAGGCAGCCTGCCGCAGCGCAACTGACGCTGATGCTCGAAAGCGCGGGTATCGAACAGG` |
| ASV848 | 429 | `TGGGGAATTTTGGACAATGGGCGCAAGCCTGATCCAGCCATTCCGCGTGCAGGATGAAGGCCCTCGGGTTGTAAACTGCTTTTGTACGGAACGAAAAGGCCTTTTCTAATACAAGGGGCTCATGACGGTACCGTAAGAATAAGCACCGGCTAACTACGTGCCAGCAGCCGCGGTAATACGTAGGGTGCAAGCGTTAATCGGAATTACTGGGCGTAAAGCGTGCGCAGGCGGTGATGTAAGACAGTTGTGAAATCCCCGGGCTCAACCTGGGAACTGCATCTGTGACTGCATCGCTGGAGTGCGGCAGAGGGGGATGGAATTCCGCGTGTAGCAGTGAAATGCGTAGATATGCGGAGGAACACCGATGGCGAAGGCAATCCCCTGGGCCTGCACTGACGCTCATGCACGAAAGCGTGGGGAGCAAACAGG` |
| ASV436 | 429 | `TGGGGAATTTTGGACAATGGGCGCAAGCCTGATCCAGCCATGCCGCGTGCGGGAAGAAGGCCTTCGGGTTGTAAACCGCTTTTGTCAGGGAAGAAACGCTCCGGGCTAATACCTTGGAGTACTGACGGTACCTGAAGAATAAGCACCGGCTAACTACGTGCCAGCAGCCGCGGTAATACGTAGGGTGCAAGCGTTAATCGGAATTACTGGGCGTAAAGCGTGCGCAGGCGGTCCTACAAGACAGATGTGAAATCCCCGGGCTCAACCTGGGAACTGCATTTGTGACTGTAGGGCTGGAGTGCGGCAGAGGGGGATGGAATTCCGCGTGTAGCAGTGAAATGCGTAGATATGCGGAGGAACACCGATGGCGAAGGCAATCCCCTGGGCCTGCACTGACGCTCATGCACGAAAGCGTGGGGAGCAAACAGG` |
| ASV1230 | 429 | `TGGGGAATATTGGACAATGGGCGCAAGCCTGATCCAGCAATACCGCGTGTGTGAAGAAGGCCTTAGGGTTGTAAAGCACTTTCAGGGGCGAAGAAGGCCTATGCAATAATACTGTATAGGATTGACGTTAGCTCCAGAAGAAGCACCGGCTAACTCTGTGCCAGCAGCCGCGGTAATACAGAGGGTGCGAGCGTTAATCGGAATTACTGGGCGTAAAGGGTGCGTAGGTGGTTAGATAAGTTGGGCGTGAAATCCCCGGGCTCAACCTGGGAACTGCGTTCAAAACTATTTGGCTAGAGTATGGTAGAGGTAAGTGGAATTTCCAGTGTAGCGGTGAAATGCGTAGATATTGGAAGGAACATCAGTGGCGAAGGCGACTTACTGGACTGATACTGACACTGAGGCACGAAAGCGTGGGGAGCAAACAGG` |
| ASV4824 | 410 | `TTTGGAATTTTGGACAATGGACGAAAGTCTGATCCAGCGACGCCGCGTGCACGAAGAAGGTCTTCGGATCGTAAAGTGCTTTTGCAGGGGACGAATTTTTGACTGTACCCTGAGAATAAGAGGTTGCTAACTCTGTGCCAGCAGCAGCGGTAATACAGAGACCTCAAGCGTTATCCGGAATCATTGGGCGTAAAGCGTACCGATAGGTGGTTGAAATAGTCTTGGGTAAAAGCCGGAAGCTCAACTTCCGCGTTTGCTTAAGATACTTTTCGACTAGAGGGGCAAAGAGGTGCTTGGAACCAACGGTGTAGTAGTGAAATGCGTTGATATCGTTGGGAACACCGAAAGCGAAGGCAGAGCACTGGGTGCCACCTGACACTGCTAGGACGAAAGCGTGGGGAGCGAATGGG` |
| ASV5736 | 408 | `TCGAGAATCTTCCGCAATGGGCGAAAGCCTGACGGAGCGACGCCGCGTGGTGGATGAAGCGCTTAGGCGCGTAAACACCTTTTATGGAGGAGAAAGTTTATTGATGTTACTCCATGAATAAGGGGCTCCCAACTCTGTGCCAGCAGGAGCGGTAATACAGAGGCCCCAAGCATTATCCGGATTTACTGGGCGTAAAGGGTGCGTAGGCGGTGTTATTAGTCGGGTGTTAAATCCTGGGGCTCAACCTCAGAATCGCATTCGAAACGGTAACACTAGAAGAAGTCAGAGGTGAACAGAACTCTCGGTGTAGGGGTGAAATCCGTTGATATCGAGGGGAATACCAAATGCGAAGGCAGTTCACTGGGGCTTTCTTGACGCTGAGGCACGAAAGCGTGGGTAGCGAATCGG` |
| ASV720 | 424 | `TGAGGAATATTGGTCAATGGACGCAAGTCTGAACCAGCCATGCCGCGTGCAGGATGACGGTCCTATGGATTGTAAACTGCTTTTGTACGAGAAGAAACACTCCGACGTGTCGGAGCTTGACGGTATCGTAAGAATAAGGATCGGCTAACTCCGTGCCAGCAGCCGCGGTAATACGGAGGATCCAAGCGTTATCCGGAATCATTGGGTTTAAAGGGTCCGTAGGCGGTTTAGTAAGTCAGTGGTGAAAGCCCATCGCTCAACGGTGGAACGGCCATTGATACTGCTAAACTTGAATTATTAGGAAGTAACTAGAATATGTAGTGTAGCGGTGAAATGCTTAGAGATTACATGGAATACCAATTGCGAAGGCAGGTTACTACTAATGGATTGACGCTGATGGACGAAAGCGTGGGTAGCGAACAGG` |
| ASV5599 | 409 | `TGGGGAATTTTCGGCAATGGGGGCAACCCTGACCGAGCCATGCCGCGTGAAGGATGAAGGCCCTTTGGGTTGTAAACTTCTGTGGAGATCTACTAAATAAATGAAGAGATCTCTAGAAAGAGGTTGCTAACTCTGTGCCAGCAGCAGCGGTAATACAGAGACCTCAAGCGTTATCCGGAATCATTGGGCGTAAAGCGTTCCGATCGGTTGTTTAGTGTGTCTGAAGTGAAAGACCGGAGCTCAACTCCGTGTTTGCTTTAGAAACTGCTAGACTAGAGTAGTAAAGAGGTGGCTAGAACTCTGCAAGTAGCGGTGAAATGCGTTGATATGCAGGGGAATACCGAAAGCGAAGGCAGGCCACTGGGTACTTACTGACACTGCTAGGACGAAAGCGTGGGGAGCGAATAGG` |
| ASV4221 | 406 | `TGGGGAATTTTCCGCAATGGGCGAAAGCCTGACGGAGCAATACCGCGTGAGGGATGAAAGCCTGTGGGTTGTAAACCTCTTTTCTCAAGGAAGAAGTTCTGACGGTACTTGAGGAATAAGCATCGGCTAACTCTGTGCCAGCAGCCGCGGTAATACAGAGGATGCAAGCGTTATCCGGAATCACTGGGCATAAAGCGTCTGTAGGTTGCGAATTAAGTCTGTTGTTAAATACTGGGGCCCAACCCTGGAACAGCAATGGAAACTAATACGCTTGAGTGTGGTAGAGGTAGAGGGAATTCCTGGTGTAGCGGTGAAATGCGTAGATATTAGGAAGAACACCAATGGCGAAAGCACTCTACTGGGCCATAACTGACACTGAGAGACGACAGCTAGGGGAGCAAATGGG` |
| ASV710 | 403 | `TGGGGAATATTGCACAATGGAGGAAACTCTGATGCAGCGACGCCGCGTGAGGGAAGAAGGTATTCGTATTGTAAACCTCTGTCTTCGGGGACGATAATGACGGTACCCGAGGAGGAAGCACCGGCTAACTACGTGCCAGCAGCCGCGGTAAAACGTAGGGTGCAAGCGTTGTCCGGAATTACTGGGTGTAAAGGGAGCGCAGGCGGTTCTTTAAGTTGGATGTTTAATCTACGGGCTCAACCCGTATCAGCATTCAAAACTGAAGAACTTGAGTGAAGTAGAGGTAGGCGGAATTTCCGGTGTAGCGGTGGAATGCGTAGATATCGGAAGGAACACCAGTGGCGAAGGCGGCCTACTGGGCTTTTACTGACGCTGAGGCTCGAAAGCATGGGTAGCAAACAGG` |
| ASV5378 | 405 | `TAGGGAATTTTCCACAATGGACGAAAGTCTGATGGAGCAACGCCGCGTGCAGGATGAAGGCTTTCGGGTCGTAAACTGCTTTTGTTTGTGAAGATTATGACTGTAACATTCGAATAAGGACCTGCTAACTACGTGCCAGCAGCCGCGGTCATACGTGGGGTCCAAGCGTTATCCGGAATTACTGGGCGTAAAGAGTTGCGTAGGTGGCAGAGTAAGTTGGTAGTGAAATCGTGTGGCTCAACCATACACACATTATCAAAACTGCTCAGCTAGAGGATAAGAGAGGTAGATGGAATTTCTAGTGTAGGAGTGAAATCCGTAGATATTAGAAAGAACACCGATGGCGTAGGCAGTCTACTGGCTTATTCCTGACACTAAGGCACGAAAGCGTGGGGAGCGAACTGG` |
| ASV2123 | 429 | `TGGGGAATATTGGACAATGGGGGCAACCCTGATCCAGCCATGCCGCGTGTGTGAAGAAGGCCTTTTGGTTGTAAAGCACTTTAAGCGAGGAGGAGGCTTTGTTGGTTAATACCTTTCAAAGTGGACGTTACTCGCAGAATAAGCACCGGCTAACTCTGTGCCAGCAGCCGCGGTAATACAGAGGGTGCGAGCGTTAATCGGAATTACTGGGCGTAAAGCGCGCGTAGGCGGTTTGATAAGTTGGATGTGAAATCCCCGGGCTTAACCTGGGCACTGCATTCAAAACTGTCAGGCTAGAGTATGGGAGAGGATGGTAGAATTCCAGGTGTAGCGGTGAAATGCGTAGAGATCTGGAGGAATACCGATGGCGAAGGCAGCCATCTGGCCTAATACTGACGCTGAGGTGCGAAAGCATGGGGAGCAAACAGG` |
| ASV4961 | 404 | `TGGGGAATATTGGACAATGGGCGCAAGCCTGATCCAGCAATACCGCGTGAGTGATGAAGGTCTTCGGATTGTAAAGCTCTTTTAGTGGGGAAGATAATGACGGTACCCACAGAAAAAGCACCGGCAAACTTCGTGCCAGCAGCCGCGGTAATACGAAGGGTGCTAGCGTTGTTCGGAATTATTGGGCGTAAAGGGTGTGTAGGCGGCTTAGTAAGTCAGTTGTGAAATCCCCGAGCTCAACTTGGGAATTGCGACCGAAACTGCTTCGCTTGAGTTTGGTGGGGGATAGTGGAATTCCTAGTGTAGGGGTGAAATCCGTAGATATTAGGAGGAACACCGGTGGCGAAAGCGACTATCTACGCCAATACTGACGCTAAGACACGAAAGCGTGGGGAGCAAACAGG` |
| ASV2251 | 424 | `TGAGGAATATTGGACAATGGGTGAGAGCCTGATCCAGCCATCCCGCGTGAAGGACGACGGCCCTATGGGTTGTAAACTTCTTTTGTATAGGGATAAACCTACTCTCGTGAGAGTAGCTGAAGGTACTATACGAATAAGCACCGGCTAACTCCGTGCCAGCAGCCGCGGTAATACGGAGGGTGCAAGCGTTATCCGGATTTATTGGGTTTAAAGGGTCCGTAGGCGGATCTGTAAGTCAGTGGTGAAATCTCATAGCTTAACTATGAAACTGCCATTGATACTGCAGGTCTTGAGTAAAGTAGAAGTGGCTGGAATAAGTAGTGTAGCGGTGAAATGCATAGATATTACTTAGAACACCAATTGCGAAGGCAGGTCACTATGTTTTAACTGACGCTGATGGACGAAAGCGTGGGGAGCGAACAGG` |
| ASV3609 | 404 | `TGGGGAATATTGCACAATGGGGGAAACCCTGATGCAGCAACGCCGCGTGAAGGACGAAGGTCTTCGGATTGTAAACTTCTTTTCTAAGGGACGATAATGACGGTACCTTAGGAATAAGCCACGGCTAACTACGTGCCAGCAGCCGCGGTAATACGTAGGTGGCAAGCGTTATCCGGATTTACTGGGTGTAAAGGGCGTGTAGGCGGGACTGCAAGTCAGATGTGAAATTTATGGGCTCAACCCATAACCTGCATTTGAAACTGTGGTTCTTGAGTGATGGAGAGGCAGGCGGAATTCCCGGTGTAGCGGTGAAATGCGTAGATATCGGGAGGAACACCAGTGGCGAAGGCGGCCTGCTGGACATTAACTGACGCTGAGGCGCGAAAGCGTGGGGAGCAAACAGG` |
| ASV2814 | 429 | `TGGGGAATTTTGGACAATGGGGGCAACCCTGATCCAGCCATTCCGCGTGAGTGAAGAAGGCCTTCGGGTTGTAAAGCTCTTTCGGCAGGAACGAAACGGTGTCCCCTAATACGGGATGCTAATGACGGTACCTGAAGAAGAAGCACCGGCTAACTACGTGCCAGCAGCCGCGGTAATACGTAGGGTGCGAGCGTTAATCGGAATTACTGGGCGTAAAGCGTGCGCAGGCGGTTCTATAAGACAGATGTGAAATCCCCGGGCTTAACCTGGGAACTGCGTTTGTGACTGTAGAACTAGAGTGCGGCAGAGGGGGGTGGAATTCCACGTGTAGCAGTGAAATGCGTAGAGATGTGGAGGAACACCGATGGCGAAGGCAGCCCCCTGGGTCGACACTGACGCTCATGCACGAAAGCGTGGGGAGCAAACAGG` |
| ASV2004 | 429 | `TGGGGAATTTTGGACAATGGGCGCAAGCCTGATCCAGCAATGCCGCGTGCAGGAAGAAGGCCTTCGGGTTGTAAACTGCTTTTGTACGGAGCGAAAAGGTCTTCTCTAATACAGGGGGCTGATGACGGTACCGTAAGAATAAGCACCGGCTAACTACGTGCCAGCAGCCGCGGTAATACGTAGGGTGCGAGCGTTAATCGGAATTACTGGGCGTAAAGCGTGCGCAGGCGGTTTTGTAAGACAGGCGTGAAATCCCCGGGCTCAACCTGGGAATGGCGCTTGTGACTGCAAAGCTGGAGTGCGGCAGAGGGGGATGGAATTCCGCGTGTAGCAGTGAAATGCGTAGATATGCGGAGGAACACCGATGGCGAAGGCAATCCCCTGGGCCTGCACTGACGCTCATGCACGAAAGCGTGGGGAGCAAACAGG` |
| ASV5565 | 431 | `TGGGGAATATTGCGCAATGGACGAAAGTCTGACGCAGCGACGCCGCGTGTGGGAGGAAGCTCTTCGGGGTGTAAACCACTGTCAGGAGGGAAGAAAGGCGCTGACTCGAACAGGGTCTAGCGATTGACGGTACCTCCAAAGGAAGCGCCGGCTAACTCCGTGCCAGCAGCCGCGGTAAGACGGAGGGCGCAAGCGTTGTTCGGAATTACTGGGCGTAAAGCGCGGGCAGGCGGTCTGTTAAGTCGTTCGTGAAAATCCGGGGCTCAACCCCGGAAATGCATACGATACTGGCAGGCTGGAGACTGGTAGAGGCTAGCGGAATTCCTGGTGTAGCGGTGGAATGCGTAGATATCAGGAAGAACACCTGTGGCGAAGGCGGCTAGCTGGGCCAGTTCTGACGCTCATCCGCGAAAGCGTGGGGAGCAAACAGG` |
| ASV4205 | 429 | `TCAGGAATATTGGGCAATGGGCGAAAGCCTGACCCAGCAACGCCGCGTGAAGGATGAAGGCCTTCGGGTCGTAAACTTCTGTCGAGAGGGAAGAATGCGTACGGGTGAATAATCCGTGCGTTTGACGGTACCTCCAAAGGAAGCACCGGCTAACTCCGTGCCAGCAGCCGCGGTAATACGGAGGGTGCAAGCGTTGTTCGGATTCATTGGGCGTAAAGCGAGTGTAGGCGGTTTGACAAGTCGGATGTGAAATCCCGGGGCCCAACCCCGGAACTGCATCCGATACTGTCTGGCTAGAGTCCCGGAGAGGGTGGCGGAATTCCCGGTGTAGGGGTGAAATCCGTAGATATCGGGAGGAACACCAGTGGCGAAGGCGGCCACCTGGACGGTGACTGACGCTGAGACTCGAAAGCGTGGGTAGCAAACAGG` |
| ASV5714 | 404 | `TGGGGAATATTGGACAATGGGCGCAAGCCTGATCCAGCCATGCCGCGTGAGTGATGAAGGCCCTAGGGTTGTAAAGCTCTTTTGTACGGGAAGATAATGACGGTACCGTAAGAATAAGCCCCGGCTAACTTCGTGCCAGCAGCCGCGGTAATACGAAGGGGGCTAGCGTTGCTCGGAATCACTGGGCGTAAAGGGTGCGTAGGCGGGTTTTTAAGTCAGAGGTGAAATCCTGGAGCTCAACTCCAGAACTGCCTTTGATACTGGGAATCTTGAGTATGGAAGAGGTGAGTGGAACTGCGAGTGTAGAGGTGAAATTCGTAGATATTCGCAAGAACACCAGTGGCGAAGGCGGCTCACTGGTCCATAACTGACGCTGAGGCACGAAAGCGTGGGGAGCAAACAGG` |
| ASV521 | 406 | `TAGGGAATATTGCGCAATGGGGGAAACCCTGACGCAGCGACGCCGCGTGAGTGATGAAGGCCTTCGGGTCGTAAAGCTCTGTTATATGGGAATAAGATCTGAATGTACCATGTGAGAAAGGATCGGCTAACTTCGTGCCAGCAGCCGCGGTAAGACGAGGGATCCTAGCGTTGCTCGGAATCATTGGGCGTAAAGCGGGTGTAGGTGGCTTTTTAAGTCAGGTGTGAAAGCCCAGGGCTCAACCCTGGAAGTGCATTTGATACTGGAAAGCTTGAGTGTGGGAGAGGCTACTGGAATACCTAGTGTAGTGGTGAAATACGTAGATATTAGGTGGAACACCGGAGGCGAAGGCGGGTAGCTGGCCCAACACTGACACTCAGACCCGAAAGCGTGGGGATCAAACAGG` |
| ASV5609 | 404 | `TGGGGAATATTGGACAATGGGGGAAACCCTGATCCAGCGATGCCGCGTGAGTGACGAAGGCCTTAGGGTTGTAAAACTCTTTCACCTGTGAAGATAATGACGGTAGCAGGAGAAGAAGCACCGGCAAACTCTGTGCCAGCAGCCGCGGTAATACAGAGGGTGCTAGCGTTGTTCGGAATGACTGGGCGTAAAGAGCTCGTAGGCGGTCATATTAGTCGAGCGTGAAATCCCTGGGCTTAACCCAGGAACTGCGTTCGAAACTGTATGACTTGAGTTCAGGAGAGGAAAGCGGAATTCCTAGTGTAGAGGTGAAATTCGTAGATATTAGGAGGAACATCAGAGGCGAAGGCGGCTTTCTGGACTGATACTGACGCTGAGGAGCGAAAGCGTGGGGAGCAAACAGG` |
| ASV3768 | 407 | `TAGGGAATATTGCACAATGGAGGAAACTCTGATGCAGCGACGCCGCGTGAGTGATGAAGGACTTCGGTTCGTAAAGCTCTGTCGTATGGGAATAAGAAAGTGAATGTACCATGCAAGAAAGGATCGGCTAACTTCGTGCCAGCAGCCGCGGTAAGACGAGGGATCCTAGCGTTGTTCGGAATCATTGGGCGTAAAGCGGATGTAGGTGGCTTTGTAAGTCGGGTGTGAAAGCCCAGGGCTCAACCCTGGAAGTGCATTCGATACTGCGAAGCTTGAATGTCGGAGAGGTTATTAGAATACCTGGTGTAGTGGTGAAATACGTAGATATCAGGTGGAATACCGGAGGCGAAGGCGGATAACCAGCCGAACATTGACACTCAGATCCGAAAGTGTGGGGATCAAACAGG` |
| ASV4832 | 404 | `TGGGGAATATTGGACAATGGGCGCAAGCCTGATCCAGCAATACCGCGTGAGTGATGAAGGCTTTCGGGTTGTAAAACTCTTTTAGTAGGGAAGATAATGACGTTACCTACAGAAAAAGCACCGGCTAACTTCGTGCCAGCAGCCGCGGTAATACGAAGGGTGCTAGCGTTGTTCGGAATTATTGGGCGTAAAGCGTGCGTAGGCGGCTTGGTAAGTCAATTGTGAAAGCCCAGAGCTCAACTTTGGAATTGCAGTTGAAACTATCAGACTAGAGTATGGCGGGGGATAGTGGAATTCTTAGTGTAGGGGTGAAATCCGTAGATATTAAGAGGAACACCAGTGGCGAAGGCGACTATCTATACCATTACTGACGCTGAGGTACGAAAGCGTGGGGAGCAAACAGG` |
| ASV1443 | 429 | `TGGGGAATTTTGGACAATGGGCGCAAGCCTGATCCAGCAATGCCGCGTGAGTGAAGAAGGCCTTCGGGTTGTAAAGCTCTTTTGTCAGGGAAGAAACGGTGGGGGCTAATATCCTCCGCTAATGACGGTACCTGAAGAATAAGCACCGGCTAACTACGTGCCAGCAGCCGCGGTAATACGTAGGGTGCAAGCGTTAATCGGAATTACTGGGCGTAAAGCGTGCGCAGGCGGTTTTGTAAGTCTGTCGTGAAAGCCCCGGGCTCAACCTGGGAATTGCGATGGAGACTGCAAGGCTTGAATCTGGCAGAGGGGGGTAGAATTCCACGTGTAGCAGTGAAATGCGTAGAGATGTGGAGGAACACCGATGGCGAAGGCAGCCCCCTGGGTCAAGATTGACGCTCATGCACGAAAGCGTGGGGAGCAAACAGG` |
| ASV751 | 408 | `TGGGGAATTTTCCGCAATGGGCGAAAGCCTGACGGAGCAATACCGCGTGAGGGATGACGGCCTATGGGTTGTAAACCTCTTTTTTCAGGGAGGAATCAAATGACGTGTACCTGAAGAATAAGCATCGGCTAACTCCGTGCCAGCAGCCGCGGTAAGACGGAGGATGCAAGTGTTATCCGGAATCACTGGGCGTAAAGCGTCTGTAGGTGGTCCAATAAGTCAACTGTTAAATCTTGAGGCTCAACCTCAAAATCGCAGTCGAAACTATTAGACTAGAGTATAGTAGAGGTAAAGGGAATTTCCAGTGGAGCGGTGAAATGCGTAGATATTGGAAAGAACACCGATGGCGAAAGCACTTTACTGGGCTATTACTAACACTCAGAGACGAAAGCTAGGGTAGCAAATGGG` |
| ASV747 | 424 | `TAGGGAATATTGGTCAATGGACGCAAGTCTGAACCAGCCATGCCGCGTGAAGGATGAAGGCCCTATGGGTCGTAAACTTCTTTTATTAGGGAAGAAAAAATGGGATGCGTCCTATACTGACGGTACTTAATGAATAAGGATCGGCTAACTTCGTGCCAGCAGCCGCGGTAAGACGAAGGATCCAAGCGTTGTCCGGATTTACTGGGTTTAAAGGGTGCGTAGGCGGAAAATTAAGTCAGTGGTGAAAGCCCGCAGCTCAACTGTGGAACTGCCATTGAAACTGGTTTTCTTGAATATAGCTGAGGCAGATGGAATATAACATGTAGCGGTGAAATGCTTAGATATGTTATAGAACACCGATTGCGAAGGCAGTCTGCTAAACTATTATTGACGCTGAGGCACGAAAGCGTGGGGAGCGAACAGG` |
| ASV3733 | 406 | `TCGAGAATATTCCACAATGGACGCAAGTCTGATGGAGCGACGCCGCGTGCAGGATGAAGGCCTTCGGGTCGTAAACTGCGGTAGCTAGGGAAGAACTTTGTGACGGTACCTAGCGGAAAGAGGCGGGTAACTACGTGCCAGCACCAGCGGTAATACGTAGGCCTCGAGCGTTATCCGGATTTATTGGGCGTAAAGAGCATGTAGGAGGCTTCGCAAGTCTTTGGTTAAAGACCACCGCCTAACGGTGGGAGTGCCGAGGAAACTGCGAAGCTGGAGGAGGTTAGAGGTGCATAGAACGCGCGGTGTAGGGGTGAAATCCGTTGATATCGCGCGGAATACCAAAGGCGAAGGCAGTGCACTGGGACCTTCCTGACTCTGAGATGCGAAAGCGTGGGGAGCAAAAAGG` |
| ASV4149 | 404 | `TGGGGAATCTTGCGCAATGGGGGCAACCCTGACGCAGCTATACCGCGTGTGCGATGAAGGCCTTCGGGTTGTAAAGCACTTTTGGCGGGGACGACGATGACGGTACCCGCAGAATAAGTCACGGCTAACTTCGTGCCAGCAGCCGCGGTAATACGAAGGTGGCGAATGATGCTCGGATTCACTGGGCGTAAAGCGTTGGTAGGTGGTTATGTAAGTTAGAGGTGAAAGCCCCGTGCTCAACATGGGAATTGCCTTTAAAACTGCAAAACTAGAATATGGAAGAGGATGGTAGAATTGCTGGTGTAGAGGTAAAATTCGTAGATATCAGCAGGAATATCCATGGCGAAGGCAGCTATCTGGTCCATTATTGACACTGAGCAACGAAAGCGTGGGGAGCAAACAGG` |
| ASV443 | 409 | `TGGGGAATATTGGGCAATGGAGGAAACTCTGACCCAGCGACGCCGCGTGCGGGATGAAGGCCTTCGGGTTGTAAACCGCTTTCAGCAGGGAAGAAGCGAAAGTGACGGTACCTGAAGAAGAAGCACCGGCTAACTATGTGCCAGCAGCCGCGGTAATACATAGGGTGCAAGCGTTGTCCGGAATTATTGGGCGTAAAGAGCTCGTAGGTGGTTCGTCACGTCGGATGTGAAAATCTGGGGCTTAACCCCAGACCTGCATTCGATACGGGCGAGCTTGAGTATGGTAGGGGAGTCTGGAATTCCTGGTGTAGCGGTGGAATGCGCAGATATCAGGAGGAACACCAATGGCGAAGGCAGGACTCTGGGCCATTACTGACACTGAGGAGCGAAAGCGTGGGGAGCGAACAGG` |
| ASV4342 | 407 | `TAGGGAATATTGCACAATGGAGGAAACTCTGATGCAGCGACGCCGCGTGAGTGATGAAGGACTTCGGTTTGTAAAGCTCTGTTGTCAGGGAATAAAAAAATGAATGTACCTGACGAGAAAGGACCGGCTAACTTCGTGCCAGCAGCCGCGGTAATACGAAGGGTCCAAGCGTTGTTCGGAATCATTGGGCGTAAAGCGAGTGCGGGTGGCTTCTTAAGTCAGGAGTGAAAGCCCAGAGCTTAACTCTGGAAGTGCTTTTGATACTGGGAAGCTTGAATGTGGTAGAGGGTGCTAGAATACCTAATGTAGTGGTGAAATACGTAGATATTAGGTGGAACACCGGTGGCGAAGGCGGGCACCTGGACCAACATTGACACTTAGACTCGAAAGCGTGGGGATCAAACAGG` |
| ASV98 | 404 | `TGGGGAATATTGGACAATGGGCGAAAGCCTGATCCAGCAATGCCGCGTGAGTGATGAAGGCCTTAGGGTTGTAAAGCTCTTTTACCAGGGATGATAATGACAGTACCTGGAGAATAAGCTCCGGCTAACTCCGTGCCAGCAGCCGCGGTAATACGGAGGGAGCTAGCGTTGTTCGGAATTACTGGGCGTAAAGCGCACGTAGGCGGCTATTCAAGTCAGAGGTGAAAGCCCGGGGCTCAACCCCGGAACTGCCTTTGAAACTAGATAGCTTGAATCTTGGAGAGGCGAGTGGAATTCCGAGTGTAGAGGTGAAATTCGTAGATATTCGGAAGAACACCAGTGGCGAAGGCGACTCGCTGGACAAGTATTGACGCTGAGGTGCGAAAGCGTGGGGAGCAAACAGG` |
| ASV1656 | 429 | `TGGGGAATATTGGACAATGGGGGCAACCCTGATCCAGCAATGCCGCGTGTGTGAAGAAGGCCTGAGGGTTGTAAAGCACTTTCAGTGGGGAGGAGGGTTGACAGGTTAAGAGCTGGTCAATTGGACGTTACCCACAGAAGAAGCACCGGCTAACTCCGTGCCAGCAGCCGCGGTAATACGGAGGGTGCGAGCGTTAATCGGAATTACTGGGCGTAAAGGGTGCGTAGGTGGTTTGATAAGTTATCTGTAAAAGCCCTGGGCTCAACCTGGGAAGGCCAGATAAAACTGTTGAACTGGAGTATGGGAGAGGGTAGTGGAATTTCCGGTGTAGCGGTGAAATGCGTAGAGATCGGAAGGAACACCAGTGGCGAAGGCGGCTACCTGGCCTGATACTGACACTGAAGCACGAAAGCGTGGGGAGCAAACAGG` |
| ASV441 | 429 | `TCGAGAATCATTCACAATGGGGGCAACCCTGATGGTGCAACGCCGCGTGGAGGATGAAGGTCTTCGGATTGTAAACTCCTGTCATCTAGGAGCAAGACATGGCTGTGAATAGCGGACATGTTTGATAGTACTAGAAGAGGAAGGGACGGCTAACTTCGTGCCAGCAGCCGCGGTAATACGAAGGTCCCGAGCGTTATTCGGAATCACTGGGCGTAAAGGGAGCGTAGGCGGCGTGGAAAGTCAGATGTGAAATCCCGGGGCTCAACCTCGGAACTGCATCCGATACTACCATGCTAGAGTATTGGAGGGGTAACTGGAATTCTCGGTGTAGCAGTGAAATGCGTGGATATCGAGAGGAACACCTGTGGCGAAAGCGAGTTACTGGACAATTACTGACGCTGAGGCTCGAAGGCTAGGGGAGCAAAAGGG` |
| ASV1222 | 429 | `TGGGGAATATTGGACAATGGGCGAAAGCCTGATCCAGCAATGCCGCGTGTGTGAAGAAGGCCTTAGGGTTGTAAAGCACTTTCAGTGGGGAGGAAGGGATTGATGCTAATATCATCAGTCATTGACGTTACCCACAGAAGAAGCACCGGCTAACTCTGTGCCAGCAGCCGCGGTAATACAGAGGGTGCGAGCGTTAATCGGAATTACTGGGCGTAAAGCGTGTGTAGGTGGTTAGATAAGTTAGATGTGAAATCCCTGGGCTTAACCTGGGCACTGCGTTTAAGACTGTTTAGCTAGAGTACTGTAGAGGATAGTGGAATTTCCAGTGTAGCGGTGAAATGCGTAGATATTGGAAGGAACACCAGTGGCGAAGGCGACTATCTGGACAGATACTGACACTGAGACACGAAAGCGTGGGGAGCAAACAGG` |
| ASV227 | 387 | `TCGCGAAAACTTCACAATGGGGGCAACCCCGATGAGGGAATTCCTAGTGCTAGCACTTTTGTGTTAGCTTTTCTTTAGCGTAGATAACTAGAGGAATAAGGGCTGGGTAAGACGGGTGCCAGCCGCCGCGGTAATACCTGCAGCCCAAGTGGTGGTCGATTTTATTGAGTCTAAAACGTTCGTAGCCGGTTTAATAAATCCTTGGGTAAATCGGGAAGCTTAACTTTCCGAATTCCGAGGAGACTGTTAAACTTGGGACCGGGAGAGGCTAGAGGTACTTCTGGGGTAGGGGTAAAATCCTGTAATCCTAGAAGGACCACCGGTGGCGAAGGCGTCTAGCTAGAACGGATCCGACGGTGAGGGACGAAGCCCTGGGTCGCAAACGGG` |
| ASV1094 | 429 | `TGGGGAATTTTGGACAATGGGCGCAAGCCTGATCCAGCAATGCCGCGTGCAGGAAGAAGGCCTTCGGGTTGTAAACTGCTTTTGTACGGAACGAAACGGTCTGAGTTAATACCTCGGGCTAATGACGGTACCGTAAGAATAAGCACCGGCTAACTACGTGCCAGCAGCCGCGGTAATACGTAGGGTGCAAGCGTTAATCGGAATTACTGGGCGTAAAGCGTGCGCAGGCGGTTTTGTAAGACAGTCGTGAAATCCCCGGGCTCAACCTGGGAATTGCGATTGTGACTGCAAAGCTGGAGTGCGGCAGAGGGGGATGGAATTCCGCGTGTAGCAGTGAAATGCGTAGATATGCGGAGGAACACCGATGGCGAAGGCAATCCCCTGGGCCTGCACTGACGCTCATGCACGAAAGCGTGGGGAGCAAACAGG` |
| ASV1527 | 429 | `TGGGGAATTTTGGACAATGGGCGCAAGCCTGATCCAGCAATGCCGCGTGCAGGATGAAGGCCTTCGGGTTGTAAACTGCTTTTGTACGGAGCGAAAAGGCTTCTCCTAATACGGGGGGCTCATGACGGTACCGTAAGAATAAGCACCGGCTAACTACGTGCCAGCAGCCGCGGTAATACGTAGGGTGCAAGCGTTAATCGGAATTACTGGGCGTAAAGCGTGCGCAGGCGGTTTTGTAAGACAGATGTGAAATCCCCGGGCTCAACCTGGGACCTGCATTTGTGACTGCAAGGCTAGAGTACGGTAGAGGGGGATGGAATTCCGCGTGTAGCAGTGAAATGCGTAGATATGCGGAGGAACACCGATGGCGAAGGCAATCCCCTGGACCTGTACTGACGCTCATGCACGAAAGCGTGGGGAGCAAACAGG` |
| ASV5136 | 429 | `TGGGGAATATTGGACAATGGGCGCAAGCCTGATCCAGCAATGCCGCGTGGGTGAAGAAGGCCTTCGGGTTGTAAAGCCCTTTTGTCCGGAACGAAAAGCTGTGGGCTAATACCTCATAGTGCTGACGGTACCGGAAGAATAAGCACCGGCTAACTTCGTGCCAGCAGCCGCGGTAATACGAAGGGTGCAAGCGTTAATCGGAATTACTGGGCGTAAAGCGTGCGTAGGCGGCCTGTTAAGTCTGCTGTGAAAGCCTCGGGCTCAACCTGGGAACTGCAGTGGATACTGATGGGCTAGAGTGTGATAGAGGATGGTGGAATTCCCGGTGTAGCGGTGAAATGCGTAGAGATCGGGAGGAACATCAGTGGCGAAGGCGGCCATCTGGATCAACACTGACGCTGAGGCACGAAAGCGTGGGGAGCAAACAGG` |
| ASV437 | 404 | `TGGGGAATATTGGACAATGGGCGCAAGCCTGATCCAGCCATGCCGCGTGAGTGACGAAGGCCTTAGGGTTGTAAAGCTCTTTTAACGGGGAAGATAATGACGGTACCCGTAGAATAAGCTCCGGCTAACTTCGTGCCAGCAGCCGCGGTAATACGAAGGGGGCTAGCGTTGTTCGGAATTACTGGGCGTAAAGAGTTCGTAGGCGGTTTATTAAGCTAGATGTGAAATCCCTGGGCTTAACTCAGGAACTGCATTTAGAACTGGTAAGCTAGAGAGTGTTAGAGGTTGGTAGAATTCCTAGTGTAGGGGTGAAATCCGTAGATATTAGGAGGAATACCGATGGCGAAGGCAGCCAACTGGGACATTTCTGACGCTGAGGAACGAAAGCGTGGGTAGCAAACAGG` |
| ASV4809 | 424 | `TAGGGAATATTGGGCAATGGATGAAAGTCTGACCCAGCCATGCCGCGTGCAGGAAGAAGGTCCTCTGGATTGTAAACTGCTTTTATTCGGGGAGAAAAAGCCCATGCGTGGGAAATTGACGGTACCGAATGAATAAGCCACGGCTAACTACGTGCCAGCAGCCGCGGTAATACGTAGGTGGCAAGCGTTGTCCGGATTTATTGGGTTTAAAGGGTGCGTAGGCGGCTTGATAAGTCAGTGGTTAAAGGTAGCAGCTTAACTGTTTTACATGCCATTGATACTGTTGAGCTTGAGAATACTGGAGGATTTTAGAATTTCTGATGTAGCGGTGAAATGCTTAGATATCAGGAGGAATACCAATTGCGAAGGCAGAGATCTGTGGTAATTCTGACGCTGAGGCACGAGAGTATGGGGATCAAACAGG` |
| ASV4538 | 405 | `TGAGGAATATTGCGCAATGGGCGAAAGCCTGACGCAGCGACGCCGCGTGGAGGATGAAGGATTTCGGTCTGTAAACTCCTGTCAGTAGGGAAGAGAATGGACGGTACCTACTGAGGAAGCTTCGGCTAACTACGTGCCAGCAGCCGCGGTAAGACGTAGGAAGCAAGCGTTGTCCGGAATTACTGGGCGTAAAGAGCTCGTAGGCGGGATCGTAAGTCTTAGAGTAAATCTCACGGCTCAACCGTGGGGCCTTCTGAGATACTGCGATTCTTGAGGATGTCAGAGGAAAGTGGAATTCCCGGTGTAGCGGTGAAATGCGTAGATATCGGGAGGAACACCAGTGGCGAAGGCGACTTTCTGGGACATTCCTGACGCTGAGGAGCGAAAGCGTGGGGAGCAAACGGG` |
| ASV2963 | 428 | `TGGGGAATATTGGACAATGGGCGCAAGCCTGATCCAGCCACGCCGCGTGAGTGATGAAGGCCTTCGGGTCGTAAAGCTCTGTGGGGAGAGACGAACCGCTCTTAGTTCATAGCATAGAGCATGACGGTATCTCCTTAGCAAGCACCGGCTAACTTCGTGCCAGCAGCCGCGGTAATACGAAGGGTGCAAACGTTGCTCGGAATCATTGGGCGTAAAGCGCACGTAGGCTGTCTCACAAGTCGGATGTGAAATCCCTCGGCTCAACCAAGGAAGTGCATTCGAAACTGTGAGACTTGAGTACCAAAGAGGATCGTAGAATTCCCGGTGTAGAGGTGAAATTCGTAGATATCGGGAGGAATACCAGTGGCGAAGGCGGCGATCTGGGTGGATACTGACGCTGAGGTGCGAAAGCGTGGGGAGCAAACAGG` |
| ASV2595 | 404 | `TGGGGAATCTTAGACAATGGGCGCAAGCCTGATCTAGCCATGCCGCGTGAGCGATGAAGGCCTTAGGGTTGTAAAGCTCTTTCGTGGGGGAAGATAATGACGGTACCCCAAGAAGAAGCCCCGGCTAACTCCGTGCCAGCAGCCGCGGTAATACGGAGGGGGCTAGCGTTGTTCGGAATTACTGGGCGTAAAGCGCACGTAGGCGGGCCGGAAAGTCAGAGGTGAAATCCCGGGGCTCAACCCCGGAACGGCCTTTGAAACTCCTGGTCTTGAGGTCGAGAGAGGTGAGTGGAATTCCGAGTGTAGAGGTGAAATTCGTAGATATTCGGAGGAACACCAGTGGCGAAGGCGGCTCACTGGCTCGATACTGACGCTGAGGTGCGAAAGCGTGGGGAGCAAACAGG` |
| ASV2999 | 406 | `TAGGGAATTTTCCACAATGGGCGAAAGCCTGATGGAGCAACGCCGCGTGCAGGATGACGGCCTTCGGGTTGTAAACTGCTTTTGTGAGTGAAGATTTAAGACAGTAACTCAAGAATAAGGGACGGCTAACTACGTGCCAGCAGCCGCGGTCATACGTAGGTCCCAAGCGTTATCCGGAATCACTGGGCGTAAAGAGTTGCGTAGGTGGCTTATTAAGCAGCGTGTGAAAGCGTACGGCTCAACCGTATACACATACACTGAACTGGTAAGCTTGAGGATGAGAGAGGTTATTGGAATTCCAAGTGTAGGAGTGAAATCCGTAGATATTTGGAGGAACACCGATGGCGTAGGCAGATAACTGGCTCATTCCTGACACTAAGGCACGAAAGCGTGGGGAGCAAACGGG` |
| ASV1238 | 424 | `TAAGGAATATTGGTCAATGGGCGCAAGCCTGAACCAGCCATGCCGCGTGCAGGAAGACGGCCCTATGGGTTGTAAACTGCTTTTGAACAAGAGAAAACCTCGGTACGTGTACCGAGCTGATAGTATTGTTAGAATAAGCATCGGCTAACTTCGTGCCAGCAGCCGCGGTAAGACGAAGGATGCAAGCGTTATCCGGATTCATTGGGTTTAAAGGGTGCGTAGGCGGACCGATAAGTCAGTGGTGAAATCTCTCAGCTTAACTGAGAAACTGCCATTGATACTGTTGGTCTAGAGTATAGATGATGTGGGCGGAATATGACATGTAGTGGTGAAATACTTAGATATGTCATAGAACACCGATTGCGAAGGCAGCTCACAAAACTATAACTGACGCTGAGGCACGAAAGTGCGGGGATCAAACAGG` |
| ASV2796 | 424 | `TGGGGAATTTTGGACAATGGGGGCAACCCTGATCCAGCAACGCCGCGTGAGGGATGAAGGCTTTCGGGTCGTAAACCTCTTTTATAGGGGACGAATCGCTCCGTGAATAACGGAGTTTGACGGTACCCTATGAATAAGCCACGGCTAACTACGTGCCAGCAGCCGCGGTAATACGTAGGTGGCAAGCGTTACTCGGAATTACTAGGCGTAAAGCGCAGGTAGGCGGTTCGGCAAGTCCGTAATGAAATCTCCCGGCTTAACTGGGAGGGGTTTATGGATACTGCCGGGCTCGAGTGTGGTAGAGGTAACTGGAATTCCCGGTGTAGCGGTGAAATGCGTAGAGATCGGGAGGAACACCTATGGCGAAAGCAGGTTACTGGGCCAATACTGACGCTGAGCTGCGAAAGCTAGGGGAGCAAACAGG` |
| ASV1259 | 429 | `TGGGGAATATTGGACAATGGGCGCAAGCCTGATCCAGCCATGCCGCGTGTGTGAAGAAGGCCTTCGGGTTGTAAAGCACTTTTGTCCGGAAAGAAAAGCATCGGGTTAATACCCTGGTGTTCTGACGGTACCGGAAGAATAAGCACCGGCTAACTTCGTGCCAGCAGCCGCGGTAATACGAAGGGTGCAAGCGTTACTCGGAATTACTGGGCGTAAAGCGTGCGTAGGTGGTTCGTTAAGTCTGATGTGAAAGCCCTGGGCTCAACCTGGGAATTGCATTGGATACTGGCGAGCTAGAGTGCGGTAGAGGATGGCGGAATTCCCGGTGTAGCAGTGAAATGCGTAGAGATCGGGAGGAACATCTGTGGCGAAGGCGGCCATCTGGACCAGCACTGACACTGAGGCACGAAAGCGTGGGGAGCAAACAGG` |
| ASV2189 | 405 | `CAGGGAATCTTGGGCAATGGGCGAAAGCCTGACCCAGCGACGCCGCGTGGAGGAAGAAGGCCTTCGGGTTGTAAACTCCTTTTCTAGGGGAAGAGTAAGGACGGTACCTTGGGAATAAGCCCCGGCTAACTACGTGCCAGCAGCCGCGGTAATACGTAGGGGGCAAGCGTTACCCGGATTTACTGGGCGTAAAGGGTGCGTAGGCGGCTGGGTAAGTCTTCTGTGAAAGCTCCTGGCTTAACTGGGAGAGGTCAGAGGATACTACTCGGCTTGAGGGCAGTAGAGGAAGGTGGAATTCCCGGTGTAGCGGTGAAATGCGTAGATATCGGGAGGAACACCAGTGGCGAAGGCGGCCTTCTGGGCTGTACCTGACGCTGAGGCACGAGAGCGTGGGGAGCGAACTGG` |
| ASV418 | 429 | `TGGGGAATTTTGGACAATGGGCGAAAGCCTGATCCAGCCATTCCGCGTGCAGGATGAAGGCCTTCGGGTTGTAAACTGCTTTTGTACGGAACGAAACGGTCTTTTCTAATACAAGAGGCTAATGACGGTACCGTAAGAATAAGCACCGGCTAACTACGTGCCAGCAGCCGCGGTAATACGTAGGGTGCAAGCGTTAATCGGAATTACTGGGCGTAAAGCGTGCGCAGGCGGTTATGTAAGACAGATGTGAAATCCCCGGGCTCAACCTGGGAACTGCATTTGTGACTGCATAGCTAGAGTACGGCAGAGGGGGATGGAATTCCGCGTGTAGCAGTGAAATGCGTAGATATGCGGAGGAACACCGATGGCGAAGGCAATCCCCTGGGCCTGTACTGACGCTCATGCACGAAAGCGTGGGGAGCAAACAGG` |
| ASV3685 | 429 | `TGGGGAATTTTGGACAATGGGGGCAACCCTGATCCAGCAATGCCGCGTGAGTGAAGAAGGCCTTCGGGTTGTAAAGCTCTTTTGTTAGGGAAGAAAAGTCATTGAATAATACTCGATGATGCTGACGGTACCTAAAGAATAAGCACCGGCTAACTACGTGCCAGCAGCCGCGGTAATACGTAGGGTGCAAGCGTTAATCGGAATTACTGGGCGTAAAGCGTGCGCAGGCGGTTGTGTAAGACAGGTGTGAAATCCCCGAGCTTAACTTGGGAACTGCACTTGTGACTGCACGGCTAGAGTGTGTCAGAGGGGGGTAGAATTCCACGTGTAGCAGTGAAATGCGTAGAGATGTGGAGGAATACCGATGGCGAAGGCAGCCCCCTGGGATAACACTGACGCTCATGTACGAAAGCGTGGGGAGCAAACAGG` |
| ASV1367 | 429 | `TGGGGAATATTGGACAATGGGCGCAAGCCTGATCCAGCCATGCCGCGTGTGTGAAGAAGGCCTTAGGGTTGTAAAGCACTTTCAGTGGGGAGAAAGGTGATTCACCTAATACGTGAGTTAATTGATGTTACCCACAGAAGAAGCACCGGCTAACTCCGTGCCAGCAGCCGCGGTAATACGGAGGGTGCAAGCGTTAATCGGAATTACTGGGCGTAAAGCGCACGTAGGTGGTTTGTTAAGCCAGCTGTGAAATCCCCGGGCTCAACCTGGGCACTGCAGTTGGAACTGGCAAGCTAGAGTAGGGTAGAGGGGTGTGGAATTCCAGGTGTAGCGGTGAAATGCGTAGATATCTGGAGGAACATCAGTGGCGAAGGCGACACCCTGGACTCATACTGACACTGAGGTGCGAAAGCGTGGGGAGCAAACAGG` |
| ASV5332 | 404 | `TGGGGAATATTGGACAATGGGGGAAACCCTGATCCAGCAATGCCGCGTGTATGATGAAGGTCTTCGGATTGTAAAGTACTTTTATAGATGACGATGATGACGGTAATCTATGAATAAGCTCCGGCTAACTTCGTGCCAGCAGCCGCGGTAATACGAAGGGAGCTAGCGTTGTTCGGAATAACTGGGCGTAAAGGGCGCGTAGGTGGAAATATGCGTGAGATGTGAAATCCCCGGGCTCAACCTGGGAACGGCATTTCATACGGTATTTCTGGAGTGCGAGAGGGGAAAGCGGAATGGCGAGTGGAGCAGTGAAATGCATTGAGATTCGCTAGAACACCAGAGGCGAAGGCGGCTTTCTGGCTCGCAACTGACACTAAGGCGCGAAAGCGTGGGGAGCAAACAGG` |
| ASV4012 | 429 | `TGGGGAATATTGGACAATGGGGGCAACCCTGATCCAGCAATGCCACGTGTGTGAAGAAGGCCTGAGGGTTGTAAAGCACTTTTGTGGGGGAAGAAGTGGAGTAGGTTAAGAGCTAGCTCCATTGACATTACCCCAAGAAAAAGCACCGGCTAACTCTGTGCCAGCAGCCGCGGTAATACAGAGGGTGCAAGCGTTAATCGGACTTACTGGGCGTAAAGGGCGCGTAGGTGGATAGTTAAGTCAGGTGTGAAATCCCAGGGCTCAACCTTGGAATGGCACTTGATACTGGCTATCTAGAGTATGGTAGAGGGAAGGGGAATTTCCGGTGTAGCGGTGAAATGCGTAGATATCGGAAGGAACATCAGTGGCGAAGGCGCCTTCCTGGACCAATACTGACACTGAGGCGCGAAAGCGTGGGTAGCAAACAGG` |
| ASV1984 | 407 | `TGGGGAATTTTCCGCAATGGGCGCAAGCCTGACGGAGCAATACCGCGTGAGGGACGAAGGTTTTTGGATTGTAAACCTCTTTTCTCTGGGAAGAACACAATGACGGTACCAGAGGAATCAGCATCGGCTAACTCCGTGCCAGCAGCCGCGGTAATACGGAGGATGCAAGCGTTATCCGGAATTATTGGGCGTAAAGCGTCCGCAGGTGGCGATACAAGTCTGCTGTTAAAGCGCGAGGCTTAACTTCGTATAGGCAGTGGAAACTGTATAGCTAGAGTACGGTAGGGGTAACGGGAATTCCCAGTGTAGCGGTGAAATGCGTAGATATTGGGAAGAACACCAGCGGCGAAGGCGCGTTACTGGGCCTGTACTGACACTGAGGGACGAAAGCTAGGGGAGCGAAAGGG` |
| ASV5641 | 407 | `TAGGGAATATTGGACAATGGGGGAAACCCTGATCCAGCCATGCCGCGTGTGTGATGAAGGCCTTCGGGTTGTAAAGCACTTTTAGTTGGGAAGAAAACAATGACGGTACCAACAGAAAAAGCACCGGCTAACTCTGTGCCAGCAGCCGCGGTAATACAGAGGGTGCAAGCGTTAATCGGATTTACTGGGCGTAAAGAGCACGTAGGTGGTTTTTTAAGTCTGTCATGAAATCCCCGGGCTCAACCTGGGAACTGTGATAGAAACTGGAAAGCTAGAGTATGGGAGAGGAGAGTAGAATTTCTGGTGTAGCGGTGAAATGCGTAGATATCAGGAAGAATGACAGTGGCGAAGGCGACTCTCTGGTCCAAGACTGACACTGAGGTGCGAAAGCGTGGGGAGCAAAAGGG` |
| ASV2824 | 429 | `TGGGGAATTTTGGACAATGGGCGCAAGCCTGATCCAGCCATGCCGCGTGTGTGAAGAAGGCCTTCGGGTTGTAAAGCACTTTCGGCCGGAACGAAATCGCCTTTGTTAATAGCGAGGGTGGATGACGGTACCGGAAGAAGAAGCACCGGCTAACTACGTGCCAGCAGCCGCGGTAATACGTAGGGTGCGAGCGTTAATCGGAATTACTGGGCGTAAAGGGTGCGCAGGCGGCCCCGCAAGTCAGGCGTGAAATCCCCGGGCTTAACCTGGGAATGGCGCTTGAAACTACGGGGCTGGAGTATGGCAGAGGGAGGTGGAATTCCACGTGTAGCGGTGAAATGCGTAGAGATGTGGAGGAACACCGATGGCGAAGGCAGCCTCCTGGGCCAATACTGACGCTCATGCACGAAAGCGTGGGGAGCAAACAGG` |
| ASV4885 | 404 | `TGGGGAATATTGGACAATGGGGGCAACCCTGATCCAGCCATGCCGCGTGAGTGATGAAGGCCTTCGGGTTGTAAAGCTCTTTCGCCCACGACGATAATGACGGTAGTGGGAGAAGAAGCCTCGGCTAACTCCGTGCCAGCAGCCGCGGTAAGACGGAGGAGGCTAGCGTTGTTCGGAATTACTGGGCGTAAAGCGCACGTAGGCTGTTTTGTAAGTTGGGGGTGAAATCCCGGGGCTTAACCCCGGAATGGCCTCCAAGACTGTGAGACTCGAGGATGGTAGAGGAGAGCGGAATTCCCAGTGTAGAGGTGAAATTCGTAGATATTGGGAAGAACACCAGTGGCGAAGGCGGCTCTCTGGACCATACCTGACGCTGAGGTGCGAAAGCGTGGGGAGCAAACAGG` |
| ASV523 | 404 | `TGGGGAATATTGGACAATGGGCGCAAGCCTGATCCAGCAATGCCGCGTGGGTGATGAAGGCCTTCGGGTTGTAAAGCCCTTTTGGCGGGGACGATGATGACGGTACCCGCCGAATAAGCTCCGGCTAACTTCGTGCCAGCAGCCGCGGTAATACGAAGGGAGCTAGCGTTGTTCGGAATTACTGGGCGTAAAGGGCGCGTAGGCGGTTTCGATAGTCGGGCGTGAAATCCCCAGGCTCAACTTGGGAATTGCGTTCGATACTGCGAGACTTGAGTGAGGGAGAGGATAGTGGAATTCCCAGTGTAGAGGTGAAATTCGTAGATATTGGGAAGAACACCAGTGGCGAAGGCGGCTATCTGGACCTCAACTGACGCTGAGGCGCGAAAGCGTGGGGAGCAAACAGG` |
| ASV3779 | 404 | `TGGGGAATATTGGGCAATGGGCGAAAGCCTGACCCAGCAATGCCGCGTGGGTGAAGAAGGCCCTCGGGTCGTAAAGCCCTTTCGGCGGGGACGATGGTGACGGTACCCGCAGAAGAAGCCCCGGCCAACTTCGTGCCAGCAGCCGCGGTAATACGAAGGGGGCTAGCGTTGCTCGGAATCACTGGGCGTAAAGGGCGCGTAGGCGGCCGCGCCAGTCGGGCGTGAAATCCCCGGGCTCAACCCTGGGACTGCGCCCGAGACGGCGGGGCTGGAGGACGGGAGAGGGCCGCGGAATTCCCAGTGTAGAGGTGAAATTCGTAGATATTGGGAAGAACACCGGTGGCGAAGGCGGCGGCCTGGCCCGTGCCTGACGCTGAGGCGCGACAGCGTGGGGAGCAAACAGG` |
| ASV2318 | 424 | `TGAGGAATATTGGTCAATGGGCGCAAGCCTGAACCAGCCATGCCGCGTGAAGGATGAAGGTTCTATGGATTGTAAACTTCTTTTATTTGGGAATAAACCTCTTTACGTGTAGAGAGCTGAAGGTACCAAACGAATAAGCACCGGCTAACTCCGTGCCAGCAGCCGCGGTAATACGGAGGGTGCAAGCGTTATCCGGAATCATTGGGTTTAAAGGGTCCGCAGGCGGACGTATAAGTCAGTGGTGAAAGCCTACAGCTCAACTGTAGAACTGCCATTGATACTGTACGTCTTGAATTCGGTCGAAGTGGGCGGAATATGACATGTAGCGGTGAAATGCTTAGATATGTCCTAGAACACCGATAGCGAAGGCAGCTCACTAGGCCTGGATTGACGCTCAGGGACGAAAGCGTGGGGAGCAAACAGG` |
| ASV963 | 408 | `TGGGGAATTTTCCGCAATGGGCGCAAGCCTGACGGAGCAATACCGCGTGAGGGATGAAGGCCTATGGGTTGTAAACCTCTTTTTTCAGGGAGGAACAAAATGACGTGTACCTGAAGAATAAGCATCGGCTAACTCCGTGCCAGCAGCCGCGGTAAGACGGGGGATGCAAGTGTTATCCGGAATCACTGGGCGTAAAGCGTCTGTAGGTGGTTAAGTAAGTCAACTGTTAAATCTTGAGGCTTAACCTCAAAATCGCAGTCGAAACTGCTTGACTAGAGTATAGTAGGGGTAAAGGGAATTTCCAGTGGAGCGGTGAAATGCGTAGATATTGGAAAGAACACCGATGGCGAAGGCACTTTACTGGGCTATTACTAACACTGAGAGACGAAAGCTAGGGTAGCAAATGGG` |
| ASV3282 | 404 | `TAGGGAATATTGGACAATGGGGGAAACCCTGATCCAGCAATGCCGCGTGAGTGATGAAGGCCTTAGGGTTGTAAAGCTCTTTCGCCCACGACGATGATGACGGTAGTGGGAGAAGAAGCCCCGGCTAACTTCGTGCCAGCAGCCGCGGTAATACGAAGGGGGCTAGCGTTGTTCGGAATTACTGGGCGTAAAGCGTACGCAGGCGGCGCATTAAGTCAGGGGTGAAAGGCCTGGGCTCAACCCAGGAACTGCCTTTGAGACTGATGCGCTCGAGTCCGGAAGAGGGTAGTGGAATTCCCAGTGTAGAGGTGAAATTCGTAGATATTGGGAAGAACACCGGTGGCGAAGGCGGCTACCTGGTCCGGTACTGACGCTCAAGTACGAAAGCGTGGGGAGCAAACAGG` |
| ASV1848 | 384 | `GCGCGCAAACTTTACAATGCGAGCAATCGTGATAAGGAAACCCTGAGTGCCTGTCGATGCAGGCTGTTCATATATTTAAATTGTATGTGAAGAAAGGGCAGGGCAAGACCGGTGCCAGCCGCCGCGGTAATACCGGCTGCTCGAGTGATGGCCACTATTACTGGGTTTAAAGCGTCCGTAGCTTGATTGTTAGGTCCCTTGGGAAATCTTCACGCTCAACGTGAAGGCGTCTAAGAGATACCGGCAATCTTGGAACTGGGAGAGGTAAGCCGTACTTCGGGGGTAGGAGTGAAATCTTGTAATCCTCGAGGGACGACCTATGGCGAAGGCAGCTTACCAGAACAGCTTCGACAGTGAGGGACGAAAGCTGGGGGAGCAAACGGG` |
| ASV3205 | 404 | `TGGGGAATATTGGACAATGGGCGAAAGCCTGATCCAGCCATGCCGCGTGTGTGATGAAGGCCTTAGGGTTGTAAAGCACTTTTGTCAGGGAAGATAATGACGGTACCTGAAGAATAAGCACCGGCTAACTTCGTGCCAGCAGCCGCGGTAATACGAAGGGTGCTAGCGTTGTTCGGAATCACTGGGCGTAAAGCGCGCGTAGGCGGCGTAGCAAGTCAGAGGTGAAAGCCTGGAGCTCAACTCCAGAATTGCCTTTGAAACTGCTATGCTTGAGTGTTGGAGGGGATAGCGGAATTGCTAATGTAGAGGTGAAATTCGTAGATATTAGCAGGAACACCGGTGGCGAAGGCGGCTATCTGGACAACTACTGACGCTGAGGCGCGAAAGCGTGGGGATCAAACAGG` |
| ASV1195 | 429 | `TGGGGAATATTGGACAATGGGCGCAAGCCTGATCCAGCCATGCCGCGTGTGTGAAGAAGGCCTTAGGGTTGTAAAGCACTTTCAGTGGGGAGAAAGGTGGTTCACCTAATACGTGAGCTAATTGATGTTACCCACAGAAGAAGCACCGGCTAACTCCGTGCCAGCAGCCGCGGTAATACGGAGGGTGCAAGCGTTAATCGGAATTACTGGGCGTAAAGCGCACGTAGGTGGTTTGTTAAGCCAGCTGTGAAATCCCCGGGCTCAACCTGGGCACTGCAGTTGGAACTGGCAAGCTAGAGTAGGGTAGAGGGGTGTGGAATTCCAGGTGTAGCGGTGAAATGCGTAGATATCTGGAGGAACATCAGTGGCGAAGGCGACACCCTGGACTCATACTGACACTGAGGTGCGAAAGCGTGGGGAGCAAACAGG` |
| ASV3662 | 430 | `TGGGGAATCTTGCGCAATGGACGAAAGTCTGACGCAGCCACGCCGCGTGAGTGAAGAAGGCCTTCGGGTTGTAAAGCTCTGTCGAGCGGGACGAACAATTCTAGGGTGAATAGCCTTAGGATCTGACGGTACCGCTAAAGGAAGCACCGGCTAACTCCGTGCCAGCAGCCGCGGTAATACGGAGGGTGCAAGCGTTGCTCGGAATTATTGGGCGTAAAGGGTAGGTAGGTGGTCTCATTTGTCTGGGGTGAAATCCTTGAGCTTAACTCAAGAAGTGCCCCAGAAACGGTGAGACTAGAGTCCTGGAGAGGGTCGTGGAATTCCCGGTGTAGCGGTGAAATGCGTAGAGATCGGGAGGAACACCAGAGGCGAAGGCGGCGACCTGGACAGGCACTGACACTCAACTACGAAAGCGTGGGGAGCAAACAGG` |
| ASV3526 | 404 | `TGAGGAATATTGAGCAATGGGCGCAAGCCTGACTCAGCAACGCCGCGTGGATGACGAAGGCCTTCGGGTTGTAAAATCCTTTTCTGGGGGAAGATAATGACGGTACCCCAGGAATAAGTTCCGGCTAACTACGTGCCAGCAGCCGCGGTAAAACGTAGGGGACGAGCGTTATCCGGATTTACTGGGCGTAAAGCGCATGTAGGTGGCGCGGAAAGTTGGACGTGAAAGCTCCCGGCTTAACTGGGAGAGGTCGTACAAAACTACCGAGCTTGAGGATGGAAGAGGAGAGCGGAATTCCCGGTGTAGTGGTGGAATGCGTAGATATCGGGAGGAACACCAGTGGCGAAAGCGGCTCTCTGGTTCAACCCTGACACTGAGATGCGAAAGCGTGGGGAGCGAACGGG` |
| ASV3068 | 429 | `TGGGGAATTTTGGACAATGGGGGCAACCCTGATCCAGCAATGCCGCGTGAGTGAAGAAGGCCTTCGGGTTGTAAAGCTCTTTTGTCAGGGAAGAAACGGTTGGGGCTAATATCCCCGGCTAATGACGGTACCTGAAGAATAAGCACCGGCTAACTACGTGCCAGCAGCCGCGGTAATACGTAGGGTGCAAGCGTTAATCGGAATTACTGGGCGTAAAGCGTGCGCAGGCGGTTTTGTAAGTCTGTCGTGAAATCCCCGGGCTCAACCTGGGAATTGCGATGGAGACTGCAAGGCTAGAGTTTGGCAGAGGGGGGTAGAATTCCACGTGTAGCAGTGAAATGCGTAGAGATGTGGAGGAACACCGATGGCGAAGGCAGCCCCCTGGGTCAAAACTGACGCTCATGCACGAAAGCGTGGGGAGCAAACAGG` |
| ASV876 | 404 | `TGGGGAATCTTAGACAATGGGGGCAACCCTGATCTAGCCATGCCGCGTGGATGATGAAGGCCTTAGGGTCGTAAAGTCCTTTCGCTGGGGAAGATAATGACGGTACCCAGTAAAGAAACCCCGGCTAACTCCGTGCCAGCAGCCGCGGTAATACGGAGGGGGTTAGCGTTGTTCGGAATTACTGGGCGTAAAGCGCGCGTAGGCGGATCAGAAAGTTGGGGGTGAAATCCCGGGGCTCAACCCCGGAATTGCCTTCAAAACTCCTGGTCTTGAGTTCGAGAGAGGTGAGTGGAATTCCGAGTGTAGAGGTGAAATTCGTAGATATTCGGAGGAACACCAGTGGCGAAGGCGGCTCACTGGCTCGATACTGACGCTGAGGTGCGAAAGCGTGGGGAGCAAACAGG` |
| ASV2813 | 406 | `TTAGGAATCTTGCACAATGGGCGAAAGCCTGATGCAGCGACGCCGCGTGAAGGACGAAGGTTCTCGGATCGTAAACTTCTTTTAAGTGGGAAGAAATTTGACGGTACCACTTGAATAAGCCCCGGCTAACTACGTGCCAGCAGCCGCGGTAATACGTAGGGGGCGAGCGTTGTCCGAAGTTACTGGGCGTAAAGCGCGCGTAGGCGGTTTCTTAAGTCTGGGGTGAAAGGTTCAACGCTCAACGTGAACAGTGCCTTGGATACTGGGAGACTTGAGTTAGGGAGAGGGTAGTAGAATTCCTGGTGTAGCGGTGAAATGCGTAGATATCAGGAGGAATACCAATGGCGAAGGCAGCTACCTGGCCTATAACTGACGCTGAGGTGCGAAAGCGTGGGGAGCAAACGGG` |
| ASV3878 | 424 | `TGAGGAATATTGGGCAATGGACGCAAGTCTGACCCAGCCATGCCGCGTGCAGGAAGAAGCATCTATGGTGTGTAAACTGCTTTTGTACAGGAAGAAATGCTTTTACGTGTAGAAGTTTGACGGTACTGTAAGAATAAGGATCGGCTAACTCCGTGCCAGCAGCCGCGGTAATACGGAGGATCCAAGCGTTATCCGGAATCATTGGGTTTAAAGGGTCCGTAGGCGGCTTTATAAGTCAGTGGTGAAATCCAATCGCTCAACGATTGAACTGCCATTGATACTGTAGGGCTTGAATGGCTATGAAGTAACTAGAATATGTAGTGTAGCGGTGAAATGCTTAGATATTACATGGAATACCAATTGCGAAGGCAGGTTACTAATAGTTTATTGACGCTGATGGACGAAAGCGTGGGGAGCGAACAGG` |
| ASV3101 | 429 | `TGGGGAATTTTGGACAATGGGGGCAACCCTGATCCAGCCATGCCGCGTGCGGGAAGAAGGCCTTCGGGTTGTAAACCGCTTTTGTCAGGGAAGAAACGCTCCGGGCTAATACCTCGGGGTAATGACGGTACCTGAAGAATAAGCACCGGCTAACTACGTGCCAGCAGCCGCGGTAATACGTAGGGTGCAAGCGTTAATCGGAATTACTGGGCGTAAAGCGTGCGCAGGTGGTTGTGCAAGACAGATGTGAAATCCCCGGGCTTAACCTGGGAACTGCATTTGTGACTGCATGGCTAGAGTGCGGCAGAGGGGGATGGAATTCCGCGTGTAGCAGTGAAATGCGTAGATATGCGGAGGAACACCGATGGCGAAGGCAATCCCCTGGGCCTGCACTGACACTCATGCACGAAAGCGTGGGGAGCAAACAGG` |
| ASV1669 | 429 | `TGGGGAATTTTGGACAATGGGCGCAAGCCTGATCCAGCCATTCCGCGTGAGTGAAGAAGGCCTTCGGGTTGTAAAGCTCTTTCGGCAGGGAAGAAACGGTTTCGGCTAATACCCGGAGCTAATGACGGTACCTGAAGAAGAAGCACCGGCTAACTACGTGCCAGCAGCCGCGGTAATACGTAGGGTGCGAGCGTTAATCGGAATTACTGGGCGTAAAGCGTGCGCAGGCGGTTTTGTAAGACAGATGTGAAATCCCCGGGCTTAACCTGGGAACTGCGTTTGTGACTGCAAGACTCGAGTACGGCAGAGGGGGGTGGAATTCCACGTGTAGCAGTGAAATGCGTAGAGATGTGGAGGAACACCGATGGCGAAGGCAGCCCCCTGGGTCGATACTGACGCTCATGCACGAAAGCGTGGGGAGCAAACAGG` |
| ASV2183 | 429 | `TGGGGAATTTTGGACAATGGGCGAAAGCCTGATCCAGCAATGCCGCGTGTGTGAAGAAGGCCTTCGGGTTGTAAAGCACTTTTGTCAGGAACGAAACGGCGCTTGCTAATATCGAGCGCTAATGACGGTACCTGAAGAATAAGCACCGGCTAACTACGTGCCAGCAGCCGCGGTAATACGTAGGGTGCAAGCGTTAATCGGAATTACTGGGCGTAAAGCGTGCGCAGGCGGTTATGCAAGACAGATGTGAAATCCCCGGGCTTAACCTGGGCACTGCATTTGTGACTGCATGGCTAGAGTGTGTCAGAGGGGGGTAGAATTCCACGTGTAGCAGTGAAATGCGTAGAGATGTGGAGGAACACCGATGGCGAAGGCAGCCCCCTGGGATAACACTGACGCTCATGCACGAAAGCGTGGGGAGCAAACAGG` |
| ASV3948 | 389 | `TCGGGAGAATTTTGCAATACACGAAAGTGTGACAGAGCAAGCCAGAGTGCTTTTCTATAATTGAAAAGCTTTTGCGGGATGCAAAAAGTCCCGCGAATAAGGACTGGGCAAGACTGGTGCCAGCCGCCGCGGTAATCCCAGCAGTCCAAGTCGCAGCCACATTTGTTGGGTCTAAAATATCCGTAGCTTGCCTTTCAAGTCTCTTGTGAAATCAGGACTCTTAAAGTTCTGGCGTGCAAGAGATACTGTTTGGCTTGAGACCGGGAGACGCAAGAAGTACATTTAGGGTAGCGGTAAAATGTGTTGATCCTATATGGACTAACAATAGCGAAGGCATCTTGCGAGAACGGTTCTGACAGTGAGGGATGAAGGCTAGGGGCGCAAAGTGG` |
| ASV4099 | 404 | `TGGGGAATATTGGACAATGGGGGAAACCCTGATCCAGCAATGCCGCGTGAGTGATGAAGGCCTTCGGGTTGTAAAGCTCTTTTACCTGCGATGATAATGACAGTAGCAGGAGAATAAGCCCCGGCTAACTTCGTGCCAGCAGCCGCGGTAATACGAAGGGGGCTAGCGTTGTTCGGAATTACTGGGCGTAAAGGGCGCGTAGGCGGCGCTGTAAGTGAGATGTGAAAGACCTGGGCTTAACCTGGGAAGTGCATTTCAGACTGCAGTGCTTGAGTGCGAGAGAGGAAAGTGGAATGACGCATGTAGAGGTGAAATTCGTAGATATGCGTTGGAACACCAATGGCGAAGGCAACTTTCTGGCTCGCAACTGACGCTAAGGCGCGAAAGCGTGGGGAGCAAACAGG` |
| ASV1237 | 429 | `TGGGGAATTTTGGACAATGGACGCAAGTCTGATCCAGCCATGCCGCGTGCGGGAAGAAGGCCTTCGGGTTGTAAACCGCTTTTGTCAGGGAAGAAACGGCTTTGGTTAATACCTGAGGCTAATGACGGTACCTGAAGAATAAGCACCGGCTAACTACGTGCCAGCAGCCGCGGTAATACGTAGGGTGCAAGCGTTAATCGGAATTACTGGGCGTAAAGCGTGCGCAGGCGGTTATGCAAGACAGAGGTGAAATCCCCGGGCTCAACCTGGGAACTGCCTTTGTGACTGCATAGCTAGAGTACGGTAGAGGGGGATGGAATTCCGCGTGTAGCAGTGAAATGCGTAGATATGCGGAGGAACACCGATGGCGAAGGCAATCCCCTGGACCTGTACTGACGCTCATGCACGAAAGCGTGGGGAGCAAACAGG` |
| ASV1118 | 424 | `TGAGGAATATTGGTCAATGGGCGCAAGCCTGAACCAGCCATGCCGCGTGCAGGATGAAGCATCTATGGTGTGTAAACTGCTTTTGTACGAGAAGAAACACTCCCTCGTGAGGGAGCTTGACGGTATCGTAAGAATAAGGACCGGCTAACTCCGTGCCAGCAGCCGCGGTAATACGGAGGGTCCAAGCGTTATCCGGAATCATTGGGTTTAAAGGGTTCGTAGGCGGTTAAATAAGTCAGTGGTGAAATCTGGTCGCTCAACGATCAAACGGCCATTGATACTGTTTAACTTGAATTTTTGTGAAGTAACTAGAATATGTAGTGTAGCGGTGAAATGCTTAGATATTACATGGAATACCAATTGCGAAGGCAGGTTACTAACAAACGATTGACGCTGATGAACGAAAGCGTGGGTAGCGAACAGG` |
| ASV1896 | 404 | `TGGGGAATATTGCACAATGGACGAAAGTCTGATGCAGCAACGCCGCGTGGAGGATGACACATTTCGGTGCGTAAACTCCTTTTATATGAGAAGATAATGACGGTATTATATGAATAAGCACCGGCTAACTCCGTGCCAGCAGCCGCGGTAATACGGGGGGTGCAAGCGTTACTCGGAATCACTGGGCGTAAAGAGCATGTAGGCGGATTAATAAGTTTGAAGTGAAATCCTATAGCTTAACTATAGAACTGCTTTGAAAACTGTTAATCTAGAATGTGGGAGAGGTAGATGGAATTTCTGGTGTAGGGGTAAAATCCGTAGAGATCAGAAGGAATACCGATTGCGAAGGCGATCTACTGGAACATTATTGACGCTGAGATGCGAAAGCGTGGGGAGCAAACAGG` |
| ASV4495 | 404 | `TGGGGAATATTGGACAATGGGGGAAACCCTGATCCAGCCATGCCGCGTGAGTGATGAAGGCCTTAGGGTTGTAAAGCTCTTTTGCCAGGGAAGATGATGACGGTACCTGGAGAATAAGCACCGGCTAACTTCGTGCCAGCAGCCGCGGTAATACGAAGGGTGCTAGCGTTGTTCGGAATCACTGGGCGTAAAGCGCGCGTAGGCGGCTTGTCAAGTCAGAGGTGAAATGCCGGGGCTCAACCCCGGAACTGCCTTTGAAACTGACAAGCTAGAGACTCGGAGGGGATAGCGGAATACCTAATGTAGCGGTGAAATGCGTAGATATTAGGTGGAACACCGGTGGCGAAGGCGGCTATCTGGACGAGTACTGACGCTGAGGCGCGAAAGCGTGGGGATCAAACAGG` |
| ASV4072 | 424 | `TGAGGAATATTGGACAATGGGTGAGAGCCTGATCCAGCCATCCCGCGTGAAGGAATAAGGTCCTATGGATTGTAAACTTCTTTTATACTGGGATAAACCTACCCTCGTGAGGGTAGCTGAAGGTACAGTATGAATAAGCACCGGCTAACTCCGTGCCAGCAGCCGCGGTAATACGGAGGGTGCGAGCGTTATCCGGATTTATTGGGTTTAAAGGGTCCGTAGGCGGGCTTATAAGTCAGCGGTGAAATCTCCTAGCTCAACTAGGAAACTGCCATTGATACTGTAAGCCTTGAGTATATTTGAGGTAGCTGGAATAAGTAGTGTAGCGGTGAAATGCATAGATATTACTTAGAACACCAATTGCGAAGGCAGGTTACCAAGATATAACTGACGCTGATGGACGAAAGCGTGGGGAGCGAACAGG` |
| ASV284 | 404 | `TGGGGAATATTGGACAATGGGCGCAAGCCTGATCCAGCAATGCCGCGTGGATGAAGAAGGTCTTCGGATTGTAAAGTCCTTTCGACGGGGACGATGATGACGGTACCCGTAGAAGAAGCCCCGGCTAACTTCGTGCCAGCAGCCGCGGTAATACGAAGGGGGCTAGCGTTACTCGGAATTACTGGGCGTAAAGGGCGCGTAGGCGGCGTTTCAAGTTGGACGTGAAATTCCTGGGCTCAACCTGGGGACTGCGTTCAAGACTGTGATGCTAGAGGATGGAAGAGGGTCGTGGAATTCCCAGTGTAGAGGTGAAATTCGTAGATATTGGGAAGAACACCGGTGGCGAAGGCGGCGACCTGGTCCATTACTGACGCTGAGGCGCGACAGCGTGGGGAGCAAACAGG` |
| ASV4765 | 429 | `TGGGGAATTTTGGACAATGGGGGCAACCCTGATCCAGCCATTCCGCGTGAGTGAAGAAGGCCTTCGGGTTGTAAAGCTCTTTCGGCTGGGAAGAAATCGCATGAGTTAATACCTTGTGCGGATGACGGTACCGGAAGAAGAAGCACCGGCTAACTACGTGCCAGCAGCCGCGGTAATACGTAGGGTGCGAGCGTTAATCGGAATTACTGGGCGTAAAGCGTGCGCAGGCGGTTAGGTAAGACAGATGTGAAATCCCCGGGCTTAACCTGGGAACTGCGTTTGTGACTGCCTAACTAGAGTGCGGCAGAGGGGGGTGGAATTCCACGTGTAGCAGTGAAATGCGTAGATATGTGGAGGAACATCGATGGCGAAGGCAGCCCCCTGGGTCGACACTGACGCTCATGCACGAAAGCGTGGGGAGCAAACAGG` |
| ASV3017 | 428 | `TGGGGAATTGTTCACAATGGGCGCAAGCCTGATGACGCAACGCCGCGTGGAGGATGAAGGTCTTCGGATTGTAAACTCCTGTTGCTCGGGACGAAAAGCTCTGACCTAACACGTCGGAGTCTGACGGTACCGAGTGAGGAAGCCCCGGCTAACTCTGTGCCAGCAGCCGCGGTAATACAGAGGGGGCAAGCGTTGTTCGGAATTACTGGGCGTAAAGGGCGCGTAGGCGGCCCTCTAAGTCAGACGTGAAATCCCCGGGCTCAACCCGGGAACTGCGTCTGATACTGGAGGGCTTGAATCCGGGAGAGGGATGCGGAATTCCAGGTGTAGCGGTGAAATGCGTAGATATCTGGAGGAACACCGGTGGCGAAGGCGGCATCCTGGACCGGTATTGACGCTGAGGCGCGAAAGCCAGGGGAGCAAACGGG` |
| ASV1363 | 429 | `TGGGGAATATTGGACAATGGGGGAAACCCTGATCCAGCAATGCCGCGTGTGTGAAGAAGGCCTGAGGGTTGTAAAGCACTTTCAGTGGGGAGGAGGATTTCCGGGTTAAGAGCTAGGGAATTGGACGTTACCCACAGAAGAAGCACCGGCTAACTCCGTGCCAGCAGCCGCGGTAATACGGAGGGTGCAAGCGTTAATCGGAATTACTGGGCGTAAAGGGTGCGTAGGTGGTTTGATAAGTTATGTGTAAAATCCCTGGGCTCAACCTGGGCAGGTCATATAAAACTGTTAGACTCGAGTATGGGAGAGGGTAGTGGAATTTCCGGTGTAGCGGTGAAATGCGTAGAGATCGGAAGGAACACCAGTGGCGAAGGCGGCTACCTGGCCTAATACTGACACTGAGGCACGAAAGCGTGGGGAGCAAACAGG` |
| ASV4211 | 388 | `GGGGGAAACTTCTGCAATGCGCGAAAGCGTGACAGAGCAAGCCAAAGTGCTTTCATATAATTTGAAAGCTTTTGTGCGATGTAAAAAGTCGCACGAATAAGGACCGGGCAAGACTGGTGCCAGCCGCCGCGGTAATACCAGCGGTCCAAGTCGCAATCAGCATTATTGGGTCTAAAACATCCGTAGCTTGCTTAGTAAGTCTCTCGTGAAATCCGGCTTCTTAAGAGTCGAAGTGCGAGAGATACTGCTTTGCTAGAGACCGGGAGACGTAAAGAGTACGATTAGAGTAGCGGTTAAATGCGTTGATCCTTTTCGGACTATCAACAGCGAAGGCACTTTACGAGAACGGATCTGACAGTGAGGGATGAAGGCTAGGGGCGCAAAACGG` |
| ASV1580 | 404 | `TGGGGAATCTTGGACAATGGGGGAAACCCTGATCCAGCCATGCCGCGTGAATGAAGAAGGCCTTAGGGTTGTAAAGTTCTTTCAGTGGGGATGATAATGACCGTACCCACAGAAGAAGCTCCGGCTAACTCCGTGCCAGCAGCCGCGGTAAGACGGAGGGAGCTAGCGTTGTTCGGAATTACTGGGCGTAAAGGGCGCGTAGGCGGTTCTATAAGTCAGAGGTGAAATTCCCGGGCTCAACCTGGGCGCTGCCTTTGATACTGTAGGACTAGAGATTGAAAGAGGTAGGTAGAATTCCTAGTGTAGAGGTGAAATTCGTAGATATTAGGAGGAATACCGGTGGCGAAGGCGGCCTACTGGTTCATATCTGACGCTGAGGCGCGAAAGCGTGGGGATCAAACAGG` |
| ASV3374 | 429 | `TGGGGAATTTTGGACAATGGGGGCAACCCTGATCCAGCCATGCCGCGTGAGTGAAGAAGGCCTTCGGGTTGTAAAGCTCTTTCGGCCGGGAAGAAATCGCACAGGTTAATACCTTGTGTGGATGACGGTACCGGAATAAGAAGCACCGGCTAACTACGTGCCAGCAGCCGCGGTAATACGTAGGGTGCGAGCGTTAATCGGAATTACTGGGCGTAAAGCGTGCGCAGGCGGTTTTGTAAGACAGATGTGAAATCCCCGGGCTCAACCTGGGAACTGCATTTGTGACTGCAAGGCTAGAGTATGGCAGAGGGGGGTGGAATTCCACGTGTAGCAGTGAAATGCGTAGAGATGTGGAGGAACACCGATGGCGAAGGCAGCCCCCTGGGCCAATACTGACGCTCATGCACGAAAGCGTGGGGAGCAAACAGG` |
| ASV3204 | 424 | `TAAGGAATATTGGACAATGGACGCAAGTCTGATCCAGCCATGCCGCGTGAAGGATTAAGGTCCTCTGGATTGTAAACTTCTTTTATAGGGGACGAAAAAAGGGCATTCTTGCTCACTTGACGGTACCCTATGAATAAGCACCGGCTAACTCCGTGCCAGCAGCCGCGGTAATACGGAGGGTGCAAGCGTTATCCGGATTCACTGGGTTTAAAGGGTGCGTAGGCGGGCAGGTAAGTCAGTGGTGAAATCCTAGAGCTCAACTCTAGAACTGCCATTGATACTATCTGTCTTGAATATTGTGGAGGTAAGCGGAATATGTCATGTAGCGGTGAAATGCTTAGATATGACATAGAACACCTATTGCGAAGGCAGCTTACTACGCATATATTGACGCTGAGGCACGAAAGCGTGGGGATCAAACAGG` |
| ASV4022 | 407 | `TCGAGAATCTTCCGCAATGGACGCAAGTCTGACGGAGCGATACAGCGTGATGGACGAAGTGCTTCGGTACGTAAACATCTTTTATGTGGGACGAAATTATTGACGGTACCACATGAATAAGGGGCTCCTAACTCTGTGCCAGCAGGAGCGGTAATACAGAGGCCCCAAGCATTATCCGGAATCACTGGGCGTAAAGGGTGTCAAGGCGGTTATGTTAGTCGTTCGTTAAATCTGGGGGCTCAACCTCCAGCCTGCGAGCGAAACGGCATAACTTGAGGTCGGGAGAGGTGCGTGGAACTCATGGTGTAGGGGTGAAATCCGTTGATATCATGGGGAACACCAAAGGCGAAGGCAGCGCACTGGCCCGAATCTGACGCTCACACACGAAAGCCAGGGTAGCGAATGGG` |
| ASV4497 | 404 | `TAGGGAATATTGCACAATGGAGGAAACTCTGATGCAGCGACGCCGCGTGAGTGATGAAGGCCTTCGGGTTGTAAAACTCTGTGATATGGGAAGATGATGACGGTACCATATCAGAAAGGACCGGCTAACTTCGTGCCAGCAGCCGCGGTAATACGAGGGGTCCAAGCGTTGTTCGGAATCACTGGGCTTAAAGCGGGTGTAGGTGGCCAAGCAAGTTAGATGTGAAAGCCCCGGGCTCAACCTGGGAAGTGCATCTAAAACTACATGGCTTGAGTAAGGGAGAGAAAGGTAGAATTCCAGGTGTAGTGGTGAAATACGTAGATATCTGGAGGAACACCGGTGGCGAAGGCGGCCTTTTGGCCCATTACTGACACTCAGACCCGAAAGCGTGGGTAGCAAACAGG` |
| ASV4948 | 429 | `TGGGGAATATTGGACAATGGGGGCAACCCTGATCCAGCCATGCCGCGTGTGTGAAGAAGGCCTTCGGGTTGTAAAGCACTTTAAGTTGGGAGGAAGGCCTTAAGGTTAATACCTTTGAGGATTGACGTTACCAACAGAATAAGCACCGGCTAACTCCGTGCCAGCAGCCGCGGTAATACGGAGGGTGCAAGCGTTAATCGGAATTACTGGGCGTAAAGCGCGCGTAGGCGGCTTCTTAAGCTAGATGTGAAATCCCCGGGCTTAACCTGGGAACTGCATTTAGAACTGAGTTGCTAGAGTATGGGAGAGGGGTGTGGAATTTCCGGTGTAGCGGTGAAATGCGTAGAGATCGGAAGGAACATCAGTGGCGAAGGCGACACTCTGGCCCAATACTGACGCTGAGGTGCGAAAGCGTGGGGATCAAACAGG` |
| ASV3869 | 429 | `TGGGGAATCTTGTGCAATGGGCGAAAGCCTGACACAGCCATGCCGCGTGTGTGAAGAAGGCCTTCGGGTTGTAAAGCACTTTCAGTCGTAAAGAAAAGCTTCGGGATAATACCCCGAGGTCTTGACGTTAGCGACAGAAGAAGCACCGGCTAACTCCGTGCCAGCAGCCGCGGTAATACGGAGGGTGCAAACGTTAATCGGAATTACTGGGCGTAAAGCGCGCGTAGGCGGTCTGCTAAGTGGGATGTGAAAGCCCCGGGCTTAACCTGGGAACTGCATTCCAAACTGACAGGCTAGAGTCTGGTAGAGGGAGGTGGAATTTCCTGTGTAGCGGTGAAATGCGTAGATATAGGAAGGAACACCAGTGGCGAAGGCGGCCTCCTGGACCAAGACTGACGCTGAGGTGCGAAAGCGTGGGGAGCAAACAGG` |
| ASV3964 | 429 | `TGGGGAATTTTGGACAATGGGCGCAAGCCTGATCCAGCCATGCCGCGTGTGTGAAGAAGGCCTTCGGGTTGTAAAGCACTTTCGGACGGAACGAAATCGCGCGGGCGAATATCCCGCGTGGATGACGGTACCGTAAGAAGAAGCACCGGCTAACTACGTGCCAGCAGCCGCGGTAATACGTAGGGTGCGAGCGTTAATCGGAATTACTGGGCGTAAAGCGTGCGCAGGTGGCGTCGCAAGTCAGGCGTGAAATCCCCGGGCTTAACCTGGGAATTGCGCTTGAAACTACGATGCTGGAGTGCGGCAGAGGGAGGTGGAATTCCACGTGTAGCGGTGAAATGCGTAGAGATGTGGAGGAACACCGATGGCGAAGGCAGCCTCCTGGGTCGACACTGACGCTCATGCACGAAAGCGTGGGGAGCAAACAGG` |
| ASV82 | 429 | `TCGAGAATAATTCACAATGGGGGCAACCCTGATGGTGCAACGCCGCGTGGAGGATGACGGTCTTCGGATTGTAAACTCCTGTCATCTGGGAGTAAGACCTGGTCGTTAATAGCGGACAGGGTTGATAGTACCAGAAGAGGAAGGGACGGCTAACTTCGTGCCAGCAGCCGCGGTAATACGAAGGTCCCGAGCGTTGTTCGGAATCACTGGGCGTAAAGGGAGCGTAGGCGGCGTGGTAAGTCAGATGTGAAATCCCGGGGCTCAACCCCGGAACTGCATCCGATACTGCCGTGCTAGAGGAATGGAGAGGTAGCTGGAATTCTTGGTGTAGCAGTGAAATGCGTGGATATCAAGAGGAACACTCGTGGCGAAAGCGAGCTACTGGACATTTTCTGACGCTGAGGCTCGAAGGCTAGGGTAGCGAAAGGG` |
| ASV1830 | 406 | `TGGGGAATATTGCACAATGGGCGAAAGCCTGATGCAGCAACGCCGCGTGAGCGATGAAGGCCTTTGGGTCGTAAAGCTCTGTCCTAGGGGAAGAAAAAAATGACGGTACCCTTGGAGGAAGCCCCGGCTAACTACGTGCCAGCAGCCGCGGTAATACGTAGGGGGCAAGCGTTATCCGGAATTATTGGGCGTAAAGAGTGCGTAGGTGGTTTTGTAAGCGCGGGGTGAAAGGCAATAGCTCAACTATTGTAAGCCCTGCGAACTGTAAGACTTGAGTGCAGGAGAGGAAAGTGGAATTCCTAGTGTAGCGGTGAAATGCGTAGATATTAGGAGGAACACCAGTGGCGAAGGCGACTTTCTGGACTGTAACTGACACTGAGGCACGAAAGCGTGGGGAGCAAACAGG` |
| ASV5491 | 386 | `GCGCGAAAACTGCACAATGTGGGAAACCACGATGCGGGAAACCCAAGTGCCAGCACTATGTGTTGGCTGTCCTCCAGTGTTCGTAACTGGGGAAGAAAGAGCTGGGTAAGACGGGTGCCAGCCGCCGCGGTAATACCCGCAGCTCAAGTGGTGGTCATTATTATTGGGCCTAAAGCGTCCGTAGCTGGCTTGTTAGATGCCTGGGTAAATCGATGCGCTCAACGTATCGACTTCCGGGTAGACCGGCAAGCTTGGGACCGGGAGAGGCGTGAGCTACTCGAGGGGTAGGGGTAAAATCCTGTAATCCTTCGGGGAGGACCAGTGGCGAAGGCGTCACGCTAGAACGGCTCCGACAGTGAGGGACGAAGCCCTGGGGCGCAAACCGG` |
| ASV888 | 418 | `TGGGGAATTTTCCGCAATGGGCGAAAGCCTGACGGAGCAAGACCGCGTGGGGGAAGAAGGCTCTTGGGTTGTAAACCCCTTTTCTCTGGGAAGAAAGTTGTGAAAGCAACCTGACGGTACCAGAGGAATCAGCATCGGCTAACTCCGTGCCAGCAGCCGCGGTAAGACGGAGGATGCAAGCGTTATCCGGAATGATTGGGCGTAAAGCGTCCGCAGGTGGCAGTTCAAGTCTGCTGTCAAAGACCGGGGCTTAACTTCGGAAAGGCAGTGGAAACTGAACAGCTAGAGTATGGTAGGGGCAGAGGGAATTCCTGGTGTAGCGGTGAAATGCGTAGAGATCAGGAAGAACATCGGTGGCGAAGGCGCTCTGCTGGACCATAACTGACACTCAGGGACGAAAGCTAGGGGAGCGAATGGG` |
| ASV74 | 429 | `TGGGGAATTTTGGACAATGGGCGCAAGCCTGATCCAGCAATGCCGCGTGCAGGAAGAAGGCCTTCGGGTTGTAAACTGCTTTTGTACGGAGCGAAAAGGCTCTCTCTAATACAGGGGGCTCATGACGGTACCGTAAGAATAAGCACCGGCTAACTACGTGCCAGCAGCCGCGGTAATACGTAGGGTGCAAGCGTTAATCGGAATTACTGGGCGTAAAGCGTGCGCAGGCGGTTTTGTAAGACAGGCGTGAAATCCCCGGGCTCAACCTGGGAATGGCGCTTGTGACTGCAAGGCTGGAGTGCGGCAGAGGGGGATGGAATTCCGCGTGTAGCAGTGAAATGCGTAGATATGCGGAGGAACACCGATGGCGAAGGCAATCCCCTGGGCCTGCACTGACGCTCATGCACGAAAGCGTGGGGAGCAAACAGG` |
| ASV1803 | 430 | `TGGGGAATTTTGGACAATGGGGGAAACCCTGATCCAGCCATGCCGCGTGCAGGAAGAAGGCCTTCGGGTTGTAAACTGCTTTAGTCTAGGAAAAAGGGGTGCACGGTTAATACCCGTGTGCTTTGATGGTACTGGAAGAATAAGCACCGGCTAACTACGTGCCAGCAGCCGCGGTAATACGTAGGGTGCAAGCGTTAATCGGAATTACTGGGCGTAAAGCGTGCGCAGGCGGTCTTGTAAGACAGTCGTGAAATCCCTGGGCTTAACCTAGGAACTGCGATTGTGACTGCAAGGCTAGAGTGTGTCAGAGGGGGGTGGAATTCCACGTGTAGCAGTGAAATGCGTAGAGATGTGGAGGAACACCGATGGCGAAGGCAGCCCCCTGGGATAACACTGACGCTCATGCACGAAAGCGTGGGGAGCAAACAGG` |
| ASV5629 | 424 | `TGAGGAATATTGGTCAATGGACGGAAGTCTGAACCAGCCAAGTAGCGTGCAGGATGACGGCCCTATGGGTTGTAAACTGCTTTTGTATGGGGATAAAGTTAGGGACGTGTCCCTATTTGCAGGTACCATACGAATAAGGACCGGCTAATTCCGTGCCAGCAGCCGCGGTAATACGGAAGGTCCAGGCGTTATCCGGATTTATTGGGTTTAAAGGGAGCGTAGGCTGGAGATTAAGTGTGTTGTGAAATGTAGACGCTCAACGTCTGAATTGCAGCGCATACTGGTTTCCTTGAGTACGCACAACGTTGGCGGAATTCGTCGTGTAGCGGTGAAATGCTTAGATATGACGAAGAACTCCGATTGCGAAGGCAGCTGACGGGAGCGCAACTGACGCTTAAGCTCGAAGGTGCGGGTATCGAACAGG` |
| ASV4816 | 405 | `TGAGGAATATTGCGCAATGGGCGAAAGCCTGACGCAGCGACGCCGCGTGGAGGATGACGGCCTTCGGGTTGTAAACTCCTGTCAGTGGGGAAGAGATAGGACGGTACCCACTGAGGAAGCTTCGGCTAACTACGTGCCAGCAGCCGCGGTAAGACGTAGGGAGCAAGCGTTGTCCGGAATTACTGGGCGTAAAGAGCTCGTAGGCGGGAGCGTAAGTCTGAAAGGAAATCTCAAGGCTCAACTTTGGGGCTTTTTCGGATACTGCGTTTCTTGAGGTTATCAGAGGAAAGTAGAATTCCCGGTGTAGCGGTGAAATGCGTAGATATCGGGAGGAATACCAGTGGCGAAGGCGACTTTCTGGGATACACCTGACGCTGAGGAGCGAAAGCGTGGGGAGCAAACGGG` |
| ASV603 | 428 | `TCGAGAATCTTTCACAATGGGGGCAACCCTGATGGAGCGACGCCGCGTGGAGGATAAGGTCTTCGGATTGTAAACTCCTGTCATGCGCGAACAAGGTTGCAGGGATAATACCTTTGCAAATTGATGGTAGCGCAAGAGGAAGAGACGGCTAACTCTGTGCCAGCAGCCGCGGTAATACAGAGGTCTCAAGCGTTGTTCGGAATCACTGGGCGTAAAGGGTGCGTAGGTGGCATGGTAAGTCAGATGTGAAAGCCCAGGGCTCAACCTTGGAATTGCATCCGATACTGCTGTGCTAGAGTACTGGAGGGGTGACTAGAATTCTCGGTGTAGCAGTGAAATGCGTAGATATCGAGAGGAATACCAAAGGCGTAGGCAGGTCACTGGACAGTTACTGACACTGAGGCACGAAGGCCAGGGGAGCAAACGGG` |
| ASV4606 | 429 | `TGGGGAATTTTGGACAATGGGGGCAACCCTGATCCAGCCATGCCGCGTGAGGGAAGAAGGCCTTCGGGTTGTAAACCTCTTTCGGGCGGGACGAAACGGGGCGCACGAATAGTGCGTCTTACTGACGGTACCGCAAGAAGAAGCACCGGCTAACTACGTGCCAGCAGCCGCGGTAATACGTAGGGTGCGAGCGTTAATCGGAATTACTGGGCGTAAAGCGTGCGCAGGTGGCTGTGCAAGCCAGGCGTGAAATCCCCGGGCTCAACCTGGGAATGGCGCTTGGGACTGCACGGCTAGAGTGCGGCAGAGGGAGCTGGAATTCCTGGTGTAGCAGTGAAATGCGTAGAGATCAGGAGGAACACCGATGGCGAAGGCAGGCTCCTGGGCCAGCACTGACACTCATGCACGAAAGCGTGGGGAGCAAACAGG` |
| ASV2128 | 429 | `TGGGGAATTTTGGACAATGGACGCAAGTCTGATCCAGCAATGCCGCGTGCAGGACGAAGGCCTTCGGGTTGTAAACTGCTTTTGTACGGAACGAAACGGTGAGCTTTAATACAGTTTGCTAATGACGGTACCGTAAGAATAAGCACCGGCTAACTACGTGCCAGCAGCCGCGGTAATACGTAGGGTGCGAGCGTTAATCGGAATTACTGGGCGTAAAGCGTGCGCAGGCGGTTATATAAGACAGATGTGAAATCCCTGGGCTCAACCTAGGACCTGCATCTGTGACTGTATAGCTAGAGTACGGTAGAGGGGGATGGAATTCCGCGTGTAGCAGTGAAATGCGTAGATATGCGGAGGAACACCGATGGCGAAGGCAATCCCCTGGACCTGTACTGACGCTCATGCACGAAAGCGTGGGGAGCAAACAGG` |
| ASV5521 | 403 | `TGGGGAATATTGGGCAATGGACGAAAGTCTGACCCAGCCATGCCGCGTGTGTGATAAGGCCTTAGGGTTGTAAAACACTTTCGTTGGTGAAGATAATGACAGTAGCCAAAGAAGAAGCCCCGGCTAACTTCGTGCCAGCAGCCGCGGTAATACGAAGGGGGCTAGCGTTGTTCGGAATCACTGGGCGTAAAGGGCGCGTAGGTGGTTTAGTAAGTCAGAGGTGAAAGCCTGGAGCTTAACTCCAGAACTGCCTTTGAAACTGCTAAGCTGGAGTCTCGGAGAGGATAGTGGAATTCCTAGTGTAGAGGTGAAATTCGTAGATACTAGGAGGAACACCAGTGGCGAAGGCGACTATCTGGACGAGTACTGACACTGAGGCGCGAAAGCGTGGGTAGCAAACAGG` |
| ASV3828 | 404 | `TGGGGAATATTGGACAATGGGGGCAACCCTGATCCAGCGATGCCGCGTGAGTGATGAAGGCCTTCGGGTTGTAAAGCTCTTTTGTGGGGGACGATGATGACGGTACCCCACGAATAAGCCCCGGCTAACTTCGTGCCAGCAGCCGCGGTAATACGAAGGGGGCTAGCGTTGTTCGGAATGACTGGGCGTAAAGGGCGCGTAGGCGGTTTAGTAAGTTTGGTGTGAAAGTCCTGGGCTCAACCTGGGGATTGCATTGGAAACTGCTAGACTAGAGATCGAGAGAGGAAAATGGAATTGCGAGTGTAGAGGTGAAATTCGTAGATATTCGCAAGAACACCGGTGGCGAAGGCGGTTTTCTGGCTCGATACTGACGCTAAGGCGCGAAAGCGTGGGGAGCAAACAGG` |
| ASV133 | 404 | `TGGGGAATCTTGGACAATGGGCGCAAGCCTGATCCAGCCATGCCGCGTGGATGATGAAGGCCTTAGGGTTGTAAAATCCTTTCAACGGGGACGATAATGACGGTACCCGTAGAAGAAGCCCCGGCTAACTTCGTGCCAGCAGCCGCGGTAATACGAAGGGGGCTAGCGTTGCTCGGAATTACTGGGCGTAAAGCGCACGTAGGCGGATATTTAAGTCGGAGGTGAAAGCCCAGGGCTCAACCCTGGAACTGCCCTCGATACTGGGTATCTGGACCAAAGTAGAGGTAAGTGGAATTCCGAGTGTAGAGGTGAAATTCGTAGATATTCGGAGGAACACCAGTGGCGAAGGCGACTTACTGGACTTTTGGTGACGCTGAGGTGCGAAAGCGTGGGGAGCAAACAGG` |
| ASV4355 | 426 | `TTGGGAATCTTGCGCAATGGGGGCAACCCTGACGCAGCGACGCCGCGTGAGCGAAGAAGGCCTTCGGGTTGTAAAGCTCTGTCAGAAGGGAAGAACGCATAGGATTTATAATCCAGTGTTGACGGTACCTTCAGAGGAAGCACCGGCTAACTCTGTGCCAGCAGCCGCGGTAATACAGAGGGTGCAAGCGTTGTTCGGAATCACTGGGCGTAAAGAGTTCGTAGGTGGACTTATTAGTATGGGGTTAAATACCGGAGCCTAACTCCGGATCTGCTTCGTAAACGGTAAGTCTAGAGTACTAGAGAGGATAGCGGAATTCCTGGTGTAGAGGTGAAATTCGTAGATATCAGGAGGAACATCTGAGGCGAAGGCGGCTATCTGGCTAGGCACTGACACTAAAGAACGAAAGCATGGGGATCAAACAGG` |
| ASV5654 | 430 | `TGGGGAATTTTGCACAATGGGGGAAACCCTGATGCAGCAACGCCGCGTGAGTGAAGAAGGCCTTCGGGTTGTAAAGCTCTTTAGACAGGAAAGAGGGGAGGCTAGGTAAACAATCTAGTCTTCTGACGGTACCTGTAGAATAAGCACCGGCTAACTCCGTGCCAGCAGCCGCGGTAATACGGAGGGTGCAAGCGTTGTTCGGAATTATTGGGCGTAAAGCGCACGTAGGCGGGGTCATATGTCGATTGTGAAAGCCCCAGGCTCAACCTGGGAACTGCAGCCGAAACTGTGACTCTTGAATATCTGAGAGGGTGGGGGAATTGCTGGTGGAGTAGTGAAATACGTAGAGATCAGCAGGAACACCTGAGGCGAAGGCGCCTGCCTGGCAGAATATTGACGCTGAGGTGCGAAAGCGTGGGGAGCAAACAGG` |
| ASV1105 | 425 | `TGGGGAATATTGCGCAATGGGCGAAAGCCTGACGCAGCCACGCCGCGTGAGCGATGAAGGCCTTCGGGTCGTAAAGCTCTGTGGGGAGAGAAGAATAAAGCCTGTGAAGAGCGGGCCTTGACGGTATCTCCTTAGCAAGCACCGGCTAACTCCGTGCCAGCAGCCGCGGTAATACGGAGGGTGCAAACGTTGCTCGGAATCATTGGGCGTAAAGCGCACGTAGGCGGCGAAGCAAGTCGGGTGTGAAAGCCCTGGGCTCAACCCAGGAAGTGCATTCGAAACTATTTCGCTTGAATCTCGGAGGGGGCCAGAGAATTCCCGGTGTAGAGGTGAAATTCGTAGATATCGGGAGGAATACCAGTGGCGAAGGCGCTGGCCTGGACGAAGATTGACGCTGAGGTGCGAAAGCGCGGGGAGCAAACAGG` |
| ASV3263 | 406 | `TGAGGAATTTTCCGCAATGGGCGAAGGCCTGACGGAGCAATGCCGCGTGGAGGATGACAGCCCATGGGTCGTAAACTCCTTTTCTTGGAGAAGAAATTTTGACGGTATCTGAGGAATAAGCATCGGCTAACTCCGTGCCAGCAGCCGCGGTAATACGGAGGATGCAAGCGTTGTCCGGAATCATTGGGCGTAAAGGGTCCTGAGGTGGTTTGCTCAGTCCACTGTGAAAGTTCAGGGCTTACCCTTGAAAATGCGGTGGAAACTAGTAGGCTAGAGTACGGTGGGGGCAGAGGGAATTCCCGATGTAGCGGTGAAATGCATAGATATCGGGAAGAACACCGATGGCGAAGGCACTCTGCTGGGCCGACACTGACACTGAAAGACGAAAGCCAGGGGAGCGAATGGG` |
| ASV358 | 404 | `TGGGGAATCTTGGACAATGGGCGCAAGCCTGATCCAGCCATGCCGCGTGAGTGAAGAAGGCCTTCGGGTTGTAAAGCTCTTTTACCAGGGACGATAATGACGGTACCTGGAGAATAAGCCCCGGCTAACTTCGTGCCAGCAGCCGCGGTAATACGAAGGGGGCTAGCGTTGTTCGGAATTACTGGGCGTAAAGCGCACGTAGGCTGACATTTAAGTGAGGGGTGAAATCCCGAGGCTCAACCTCGGAACTGCCTTTCATACTGGGTGTCTCGAGTCCGGAAGAGGTTAGTGGAATTCCCAGTGTAGAGGTGAAATTCGTAGATATTGGGAAGAACACCAGTGGCGAAGGCGGCTAACTGGTCCGGTACTGACGCTGAGGTGCGAAAGCGTGGGGAGCAAACAGG` |
| ASV820 | 429 | `TGGGGAATTTTGGACAATGGGCGCAAGCCTGATCCAGCCATGCCGCGTGCAGGATGAAGGCCTTCGGGTTGTAAACTGCTTTTGTACGGAACGAAAAGACTCCTTCTAATAAAGGGGGTCCATGACGGTACCGTAAGAATAAGCACCGGCTAACTACGTGCCAGCAGCCGCGGTAATACGTAGGGTGCGAGCGTTAATCGGAATTACTGGGCGTAAAGCGTGCGCAGGCGGTTATATAAGACAGATGTGAAATCCCCGGGCTCAACCTGGGAACTGCATTTGTGACTGTATAGCTAGAGTACGGCAGAGGGGGATGGAATTCCGCGTGTAGCAGTGAAATGCGTAGATATGCGGAGGAACACCGATGGCGAAGGCAATCCCCTGGGCCTGTACTGACGCTCATGCACGAAAGCGTGGGGAGCAAACAGG` |
| ASV62 | 404 | `TGGGGAATCTTGCGCAATGGGCGAAAGCCTGACGCAGCCATGCCGCGTGTATGATGAAGGTCTTAGGATTGTAAAATACTTTCACCGGTGAAGATAATGACTGTAGCCGGAGAAGAAGCCCCGGCTAACTTCGTGCCAGCAGCCGCGGTAATACGAAGGGGGCTAGCGTTGCTCGGAATTACTGGGCGTAAAGGGAGCGTAGGCGGACATTTAAGTCAGAGGTGAAATCCCGGAGCTTAACTTCGGAACTGCCTTTGATACTGGATGTCTTGAGTGTGAGAGAGGTATGTGGAACTCCGAGTGTAGAGGTGAAATTCGTAGATATTCGGAAGAACACCAGTGGCGAAGGCGACATACTGGCTCATTACTGACGCTGAGGCTCGAAAGCGTGGGGAGCAAACAGG` |
| ASV3298 | 424 | `TGAGGAATATTGGACAATGGGCGCAAGCCTGATCCAGCCATGCCGCGTGCAGGATGACGGTCCTATGGATTGTAAACTGCTTTTGTACAGGAAGAAACACCTCTACGTGTAGAGATTTGACGGTACTGTAAGAATAAGGATCGGCTAACTCCGTGCCAGCAGCCGCGGTAATACGGAGGATCCAAGCGTTATCCGGAATCATTGGGTTTAAAGGGTCCGTAGGCGGTTTAGTAAGTCAGTGGTGAAAGCCCATCGCTCAACGGTGGAACGGCCATTGATACTGCTAGACTTGAATTATTAGGAAGTAACTAGAATATGTAGTATAACAATAAAATGCTTAGAGATTACATGGAATACCAATTGCGAAGGCAGGTTACTACTAATTGATTGACGCTGATGGACGAAAGCGTGGGTAGCGAACAGG` |
| ASV1676 | 421 | `TGAGGAATATTGCGCAATGGACGAAAGTCTGACGCAGCAACGCCGCGTGCGGGAAGAAGGGGCTATGCCTCGTAAACCGCTTTAGCATGGGAAGAAAAGTAGCTTAGGGCTATGAGACTGTACCATGAGAATAAGCATCGGCTAACTACGTGCCAGCAGCCGCGGTAATACGTAGGATGCAAGCGTTGTCCGGATTTACTGGGTGTAAAGGGTGCGCAGGCGGGTCTGTAAGTCAGAGGTGAAAGGCTGGAGCTTAACTCCAGAACTGCCTTTGATACTGCGGATCTTGAGTATGGAAGAGGCAGATGGAATATGTGGTGTAGCAGTGAAATGCGTAGATATCACATAGAACACCAGTGGCGAAGGCGGTCTGCTGGTCCATTACTGACGCTCAGGCACGAAAGTGTGGGTAGCAAACAGG` |
| ASV3366 | 429 | `TGGGGAATTTTGGACAATGGGCGAAAGCCTGATCCAGCCATGCCGCGTGAGTGAAGAAGGCCTTCGGGTTGTAAAGCTCTTTCAGACGGAAAGAAATCTTTCGGGTGAATATCCCGGAAGGATGACGGTACCGTAAGAAGAAGCACCGGCTAACTACGTGCCAGCAGCCGCGGTAATACGTAGGGTGCGAGCGTTAATCGGAATTACTGGGCGTAAAGCGTGCGCAGGCGGTTGCATAAGACAGATGTGAAATCCCCGGGCTCAACCTGGGAACTGCGTTTGTGACTGTGTAACTAGAGTACGGTAGAGGGGGGTGGAATTCCTGGTGTAGCAGTGAAATGCGTAGATATCAGGAGGAACATCGATGGCGAAGGCAGCCCCCTGGACCTGTACTGACGCTCATGCACGAAAGCGTGGGGAGCAAACAGG` |
| ASV4450 | 405 | `TAGGGAATCTTGCACAATGAGGGAAACCTTGATGCAGCGACGCCGCGTGCGGGACGAAGGCCCTAGGGTTGTAAACCGCTTTCAGTAGGGACGAAAGTGACGGTACCTACAGAAGAAGCCCCGGCCAAATATGTGCCAGCAGCCGCGGTGATACATATGGGGCAAGCGTTGTCCGGATTTACTGGGCGTAAAGAGCTCGTAGGCGGTTTACCAAGTCGGAAGTGAAATCTCCATGCTCAACATGGAGCGGCCTTTCAATACTGGTATGACTAGAGTATGGTAGAGGAGTGTGGAATTTCCGGTGTAGCGGTGAAATGCGCAGATATCGGAAGGAACACCTATGGCGAAGGCAGCACTCTGGGCCAAAACTGACGCTGAGGAGCGAAAGCGTGGGGAGCGAACAGG` |
| ASV3191 | 404 | `TGGGGAATATTGGACAATGGGGGCAACCCTGATCCAGCCATGCCGCGTGAGTGAAGAAGGCCTTCGGGTTGTAAAGCTCTTTCGACGGGGACGATGATGACGGTACCCGTATAAGAAGCCCCGGCTAACTTCGTGCCAGCAGCCGCGGTAATACGAAGGGGGCTAGCGTTGTTCGGAATTACTGGGCGTAAAGCGCGCGTAGGCGGTGATCCATGTCAGAGGTGAAATCCCGGAGCTCAACTCCGGAACTGCCTTTGAAACGGGATCGCTAGAGTCCGAGAGAGGATGGCGGAATTCCTAGTGTAGAGGTGAAATTCGTAGATATTAGGAAGAACACCGGTGGCGAAGGCGGCCATCTGGCTCGGTACTGACGCTCAGGCGCGAAAGCGTGGGGATCAAACAGG` |
| ASV5346 | 429 | `TGGGGAATATTGCACAATGGGCGCAAGCCTGATGCAGCGACGCCGCGTGGAGGAGGAAGGTCTTCGGATTGTAAACTCCTGTTGAGTGGGAAGAAATGACGGTCTCTAATACAGATCGTAGCTGACGGTACCACTCGAGGAAGCACCGGCAAACTTCGTGCCAGCAGCCGCGGTAATACGAAGGGTGCGAGCGTTATTCGGAGTTATTGGGCGTAAAGAGCGCGTAGGCGGTTTGAAAAGTCAGTTGTTAAAGAGCCCGGCTTAACCGGGTAGAGGCAATTGAAACTGACAGACTAGAGGGTAAGAGAGAGAAGCGGAATTCTCGGAGTAGCGGTAAAATGCGTAGATCTCGAGAGGAACACCGGTGGCGAAGGCGGCTTCTTAGCTTACTACTGACGCTAAAGTGCGAAAGCGTGGGGAGCAAACAGG` |
| ASV5021 | 429 | `TGGGGAATTTTGGACAATGGGCGAAAGCCTGATCCAGCCATGCCGCGTGAGTGAAGAAGGCCTTCGGGTTGTAAAGCTCTTTCAGCCGGGAAGAAAATGTACGGGCTAATACCCAGTGCAGATGACGGTACCGGAAGAAGAAGCACCGGCTAACTACGTGCCAGCAGCCGCGGTAATACGTAGGGTGCGAGCGTTAATCGGAATTACTGGGCGTAAAGCGTGCGCAGGCGGTTGTGTAAGACAGATGTGAAATCCCCGGGCTCAACCTGGGAACTGCGTTTGTGACTGCACGACTAGAATACGGCAGAGGGGGGTGGAATTCCACGTGTAGCAGTGAAATGCGTAGATATGTGGAGGAACACCGATGGCGAAGGCAGCCCCCTGGGCCTGTATTGACGCTCATGCACGAAAGCGTGGGGAGCAAACAGG` |
| ASV2162 | 429 | `TGGGGAATATTGGACAATGGGCGCAAGCCTGATCCAGCAATGCCGCGTGTGTGAAGAAGGCCTTCGGGTTGTAAAGCACTTTTATCAGGAACGAAAAGCGTTGGATTAATACTCTGACGTGCTGACGGTACCTGAGGAATAAGCACCGGCTAACTTCGTGCCAGCAGCCGCGGTAATACGAAGGGTGCAAGCGTTAATCGGAATTACTGGGCGTAAAGCGTGCGTAGATGGTTTGTTAAGTCGAATGTGAAAGCCCCGGGCTCAACCTGGGAACTGCATCCGAAACTGGCAGACTAGAGTGTGGTAGAGGATGGCGGAATTCCCGGTGTAGCGGTGAAATGCGTAGAGATCGGGAGGAACATCCGTGGCGAAGGCGGCCATCTGGACCAACACTGACGTTGAGGCACGAAAGCGTGGGGAGCAAACAGG` |
| ASV5027 | 404 | `TGAGGAATATTGGACAATGGGGGCAACCCTGATCCAGCAATGCCGCGTGAGTGATGAAGGCCTTCGGGTTGTAAAGCTCTTTTGTTAGGGAAGATAATGACGGTACCTAACGAATAAGCACCGGCTAACTTCGTGCCAGCAGCCGCGGTAATACGAAGGGTGCTAGCGTTGTTCGGAATGACTGGGCGTAAAGGGAGCGTAGGTGGCTGTGTACGTTTGATGTGAAAGTCCTGGGCTTAACCTGGGGACTGCATTGAATACGGCATAGCTTGAGGCATAAAGGGGAAGATGGAATTGTGTGTGTAGAGGTGAAATTCGTAGATATACACAAGAACACCAGTGGCGAAGGCGATCTTCTGGTTATGTACTGACACTAAGGCTCGAAAGCGTGGGGAGCAAACAGG` |
| ASV1422 | 429 | `TGGGGAATATTGCACAATGGGCGCAAGCCTGATGCAGCCATGCCGCGTGTGTGAAGAAGGCCTTCGGGTTGTAAAGCACTTTCAGCGAGGAGGAAGGCGGTAAGGTTAATAACCTTATCGATTGACGTTACTCGCAGAAGAAGCACCGGCTAACTCCGTGCCAGCAGCCGCGGTAATACGGAGGGTGCAAGCGTTAATCGGAATGACTGGGCGTAAAGCGCACGCAGGCGGTCTGTTAAGTTGGATGTGAAATCCCCGGGCTTAACCTGGGAACTGCATTCAAAACTGACAGGCTAGAGTCTTGTAGAGGGGGGTAGAATTCCAGGTGTAGCGGTGAAATGCGTAGAGATCTGGAGGAATACCGGTGGCGAAGGCGGCCCCCTGGACAAAGACTGACGCTCAGGTGCGAAAGCGTGGGGAGCAAACAGG` |
| ASV4200 | 404 | `TAACGAATCTTCCGCAATGGGCGAAAGCCTGACGGAGCAATGCCGCGTGTAGGATGAAGCATCTCTGGTGTGTAAACTACTGTCAGGGGGCGTAACCAATGATTGCCCCCAGAGGAAGTGGCGACTAACTCTGTGCCAGCAGTCGCGGTAATACAGAGGCCACAAGCGTTAGTCGGAATCACTGGGCTTAAAGGGTGCGTAGGCGGGTCCGCAAGCGTTCTGTGAAATCCCACGGCTCAACCGTGGAACAGCAGGGCGAACTGCGGGTCTTGAGGCAAGTAGGGGTCGCGAGAACTGTAGGTGGACCGGTGAAATGGGTAGATATCTACAGGAATGCCGATGGTGAAGACGCGCGACTGGGCTTGTCCTGACGCTGAGGCACGAAAGCGTGGGGAGCAAACAGG` |
| ASV317 | 424 | `TAAGGAATATTGGACAATGCCCGCAAGGGTGATCCAGCCATGCCGCGTGCAGGAAGAAGGCGCTATGCGTCGTAAACTGCTTTTACACCAGAGAAAACCCTCCTACGTGTAGGAGGCTGATAGTACGGTGAGAATAAGCATCGGCTAACTTCGTGCCAGCAGCCGCGGTAAGACGAAGGATGCAAGCGTTATCCGGATTCATTGGGTTTAAAGGGAGCGTAGGCGGCCTTATAAGTCAGTGGTGAAATCTCCTCGCTTAACGAGGAAATTGCCATTGATACTGTAGGGCTTGAGTACAGTTGCCGTTGGCGGAATATGACATGTAGTGGTGAAATACATAGAGATGTCATAGAACACCGATTGCGAAGGCAGCTAACGAAGCTGTAACTGACGCTGAGGCTCGAAAGTGCGGGGATCAAACAGG` |
| ASV4486 | 424 | `TGGGGAATATTGGACAATGGGCGCAAGCCTGATCCAGCAATTCCGCGTGTGTGAAGAAGGCCTTCGGGTTGTAAAGCACTTTAAGCAGGGAAGAAAAAGCTTGGCCACACCAAGCCTTGACGGTACCTGAAGAATAAGCACCGGCTAACTCTGTGCCAGCAGCCGCGGTAATACAGAGGGTGCGAGCGTTAATCGGATTTACTGGGCGTAAAGGGTGCGTAGGTGGTTAGATAAGTCGGATGTGAAATCCTCGGGCTCAACCTGAGAACTGCATTCGATACTGTTTGACTAGAGATCGGTAGAGGGTGGCGGAATTCCCGGTGTAGCAGTGAAATGCGTAGATATCGGGAGGAACATCAATGGCGAAGGCAGCCACCTGGGCCTGATCTGACACTGAGGCACGAAAGCGTGGGTAGCAAACAGG` |
| ASV4036 | 404 | `TGGGGAATATTGGACAATGGGCGAAAGCCTGATCCAGCCATGCCGCGTGAATGATGAAGGCCTTAGGGTTGTAAAGTTCTTTTGCCAGGGAAGATAATGACGGTACCTGGAGAATAAGCACCGGCTAACTTCGTGCCAGCAGCCGCGGTAATACGAAGGGTGCTAGCGTTGTTCGGAATTACTGGGCGTAAAGCGCGCGTAGGCGGCTATACAAGTCAGGGGTGAAATCCCACGGCTCAACCGTGGAACTGCCTTTGAAACTGTGTAGCTGGAGTGTGTGAGGGGATGGCGGAATTCCTAATGTAGAGGTGAAATTCGTAGATATTAGGAGGAACACCGGTGGCGAAGGCGGCCATCTGGCACACAACTGACGCTGAGGCGCGAAAGCGTGGGGATCAAACAGG` |
| ASV921 | 404 | `TGGGGAATATTGGACAATGGGGGAAACCCTGATCCAGCAATGCCGCGTGAGTGATGAAGGCCTTCGGGTTGTAAAGCTCTTTTGTCAGGGACGATGATGACGGTACCTGACGAATAAGCTCCGGCTAACTTCGTGCCAGCAGCCGCGGTAATACGAGGGGAGCTAGCGTTGTTCGGAATTACTGGGCGTAAAGGGCGTGTAGGCGGTACCATAAGTCAGATGTGAAAGCCCTGGGCTTAACCTAGGATGTGCATTTGATACTGTGGAACTAGAGATCGAGAGAGGAAAGTGGAATTACGAGTGTAGAGGTGAAATTCGTAGATATTCGTAAGAACACCAGTGGCGAAGGCGACTTTCTGGCTCGATACTGACGCTAAGGCGCGAAAGCGTGGGGAGCAAACAGG` |
| ASV2826 | 429 | `TGGGGAATTTTGGACAATGGGCGCAAGCCTGATCCAGCAATGCCGCGTGCAGGACGAAGGCCTTCGGGTTGTAAACTGCTTTTGTACGGAGCGAAACGGGATGTATTAATACTATGTCCTAATGACGGTACCGTAAGAATAAGCACCGGCTAACTACGTGCCAGCAGCCGCGGTAATACGTAGGGTGCGAGCGTTAATCGGAATTACTGGGCGTAAAGCGTGCGCAGGCGGTTATATAAGACAGATGTGAAATCCCCGGGCTCAACCTGGGAACTGCATTTGTGACTGTATAGCTAGAGTACGGTAGAGGGGGATGGAATTCCGCGTGTAGCAGTGAAATGCGTAGATATGCGGAGGAACACCGATGGCGAAGGCAATCCCCTGGACCTGTACTGACGCTCATGCACGAAAGCGTGGGGAGCAAACAGG` |
| ASV106 | 424 | `TAAGGAATATTGGTCAATGGACGAAAGTCTGAACCAGCCATGCCGCGTGAAGGATGACGGCCCTCTGGGTTGTAAACTTCTTTTATATGGGACGAAAAAAGGGAATTCTTTCTCACTTGACGGTACCATAGGAATAAGCACCGGCTAACTCCGTGCCAGCAGCCGCGGTAATACGGAGGGTGCAAGCGTTATCCGGATTCACTGGGTTTAAAGGGTGCGTAGGTGGGTAGGTAAGTCAGTGGTGAAATCTCCGAGCTTAACTCGGAAACTGCCATTGATACTATTTATCTTGAATTTTTTGGAGGTGAGCGGAATATGTCATGTAGCGGTGAAATGCTTAGATATGACATAGAACACCAATTGCGAAGGCAGCTCACTACGAAATTATTGACACTGAGGCACGAAAGCGTGGGGATCAAACAGG` |
| ASV1343 | 429 | `TGGGGAATTTTGGACAATGGGGGAAACCCTGATCCAGCCATTCCGCGTGAGTGAAGAAGGCCTTCGGGTTGTAAAGCTCTTTCGGCAGGGAAGAAACGGTTACTCCTAATACGAGTGGCTAATGACGGTACCTGAAGAAGAAGCACCGGCTAACTACGTGCCAGCAGCCGCGGTAATACGTAGGGTGCGAGCGTTAATCGGAATTACTGGGCGTAAAGCGTGCGCAGGCGGTTTTGTAAGACAGATGTGAAATCCCCGGGCTCAACCTGGGAACTGCGTTTGTGACTGCAAGACTTGAGTATGGCAGAGGGGGGTAGAATTCCACGTGTAGCAGTGAAATGCGTAGAGATGTGGAGGAATACCGATGGCGAAGGCAGCCCCCTGGGTCAATACTGACGCTCATGCACGAAAGCGTGGGGAGCAAACAGG` |
| ASV5301 | 407 | `TGGGGAATTTTCCGCAATGGGCGAAAGCCTGACGGAGCAAGACCGCGTGAGGGAGGAAGGCTCTTGGGTTGTAAACCTCTTTTCTTGGGGAAGAACAAAATGACGGTACCCAAGGAATCAGCATCGGCTAACTCCGTGCCAGCAGCCGCGGTAATACGGAGGATGCAAGCGTTATCCGGAATTATTGGGCGTAAAGCGTCCGCAGGTGGCATTTCAAGTCTGCTGTCAAAGACCGGGGCTTAACCCTGGAAAGGCAGTGGAAACTGAAGCGCTAGAGTGTCGTAGGGGTAGAGGGAATTCCTGGTGTAGCGGTGAAATGCGTAGAGATCAGGAAGAACATCGGTGGCGAAAGCGCTCTACTGGACGACAACTGACACTCAGGGACGAAAGCTAGGGGAGCGAATGGG` |
| ASV2425 | 424 | `TAAGGAATATTGGTCAATGGGGGCAACCCTGAACCAGCCATGCCGCGTGCAGGAAGACGGACCTATGGTTTGTAAACTGCTTTTGTACGAGAGAAAACCCCTTTACGTGTAGAGGGTTGATAGTATCGTAAGAATAAGGATCGGCTAACTTCGTGCCAGCAGCCGCGGTAATACGAAGGATCCAAGCGTTATCCGGATTCATTGGGTTTAAAGGGTGCGTAGGCGGACTTATAAGTCAGCGGTGAAATCTCTCGGCTTAACCGAGAAACTGCCGTTGATACTGTAGGTCTAGAGTACAGATGATGTTGGCGGAATATGACATGTAGCGGTGAAATGCTTAGATATGTCATAGAACACCGATTGCGAAGGCAGCTGACAAAACTGTAACTGACGCTGAGGCACGAAAGTGCGGGGATCAAACAGG` |
| ASV3656 | 405 | `TGGGGAATCTTGCACAATGGACGAAAGTCTGATGCAGCGATTCCGCGTGGAGGATGAAGCATCATGGTGTGTAAACTCCTTTTCTGGTAGAAGACGAATGACGGTATACCAGGAATAAGGGACGGCTAACTACGTGCCAGCAGCCGCGGTAATACGTAGGTCCCAAGCGTTATCCGGAGTTACTGGGCGTAAAGTGTCCGTAGCCTGATATTTAAGTCCATTTTCAAATATCTCAGCTTAACTGAGAGAAGGGAATAGATACTGGATATCTGGAGGTCTTTGGAGGTTAGTGGAATTTCCGGTGGAGCGGTGAAATGTGTTGATATCGGAAAGAATGCCGAAAGCGAAAGCAACTAACTACAAAGAACCTGACGGTGATGGACGAAAGTTTGGGGAGCAAACGGG` |
| ASV5358 | 429 | `TGGGGAATTTTGGACAATGGGGGAAACCCTGATCCAGCCATGCCGCGTGTCTGAAGAAGGCCTTCGGGTTGTAAAGGACTTTTGTTAGGGAGGAAATCTCACGTGCTAATATCATGTGAGGATGACAGTACCTAAAGAATAAGCACCGGCTAACTACGTGCCAGCAGCCGCGGTAATACGTAGGGTGCAAGCGTTAATCGGAATTACTGGGCGTAAAGCGAGCGCAGGCGGTTTGTTAAGTCAGATGTGAAATCCCCGAGCTCAACTTGGGAATTGCGTTTGAAACTGGCAAGCTAGAGTGTGTCAGAGGGGGGTAGAATTCCACGTGTAGCGGTAAAATGCGTAGAGATGTGGAGGAATACCGATGGCGAAGGCAGCCCCCTGGGATAACACTGACGCTCATGCTCGAAAGCGTGGGTAGCAAACAGG` |
| ASV2791 | 423 | `TAGGGAATATTGGGCAATGGGCGCAAGCCTGACCCAGCCATGCCGCGTGCAGGAAGAATGCCCTCTGGGTTGTAAACTGCTTTTGGGAGGGGACAAAAAGCAGATGCGTCTGTAATTGAGTGTACCTCCAGAATAAGCACCGGCTAACTCCGTGCCAGCAGCCGCGGTAATACGGAGGGTGCAAGCGTTGTCCGGATTTATTGGGTTTAAAGGGTGCGTAGGCGGCTTGATAAGTCGGGGGTGAAACCCACAGGCTTAACCTGTGGAGTGCCCTCGATACTGTCGAGCTTGGGTTATCTCTAGGCTGGCGGAATGGAAGGTGTAGCGGTGAAATGCATAGATATCTACCAGAACACCGATAGCGAAGGCAGCTTGCTGGGGATATACCGACGCTGAGGCACGATAGCGTGGGGATCAAACAGG` |
| ASV2863 | 403 | `TGAGGAATCTTCCACAATGGGCGAAAGCCTGATGGAGCGACACCGCGTGAAGGATGAAGCCTTTGTTGGTGTAAACTTCTTTTCTCTCCGAAGATAATGACGGTAGGAGAGGAATAAGGGGCGGCAAACTTCGTGCCAGCAGCCGCGGTAATACGAAGGCCCCAAGCGTTATCCGGAATTACTGGGTGTAAAGCGTCTGTAGGTGGTTTTAAAGGTTTCGTGTGAAACTTCGGGGCTCAACCCCGAATTGCACGGAAAACCATAAAACTTGAGTGTGGGAGAGGCAAGCAGAATAATAAGAGTAGGGGTGCAATCCGTTGATACTTATTAGAATACCAAAAGCGAAGGCAGCTTGCTGGAACATTACTGACACTGAGAGACGAAAGCGTGGGGAGCAAAAGGG` |
| ASV3430 | 405 | `TCGAGAATATTCGACAATGGGCGAAAGCCTGATCGAGCGACGCCGCGTGGAGGAAGAAGGTCTTCGGATTGTAAACTCCTTTTCCATTCTAGAAAGTTATTGATCAGAGTGGGAATAAGACGCGGCAAACTTCGTGCCAGCAGCAGCGGTAATACGAAGGCGTCAAGCGTTATCCGGATTTATTGGGCGTAAAGAGCGAGTAGGTGGTTTGGTGCGTCTGTCGTTAAATGTCTCCGCTCAACGGGGTCACCGCGATGGAAACGGCCGAACTAGAGGGTGCTAGAGGCAACTGGAACGCACGGTGTAGGGGTGAAATCCGTTGATATCGTGCGGAACACCGAAGGCGAAGGCATTTTGCTAGGGCACTCCTGACACTCAATCGCGAAAGCGTGGGGAGCAAAAAGG` |
| ASV3036 | 429 | `TGGGGAATTTTGGACAATGGGGGCAACCCTGATCCAGCAATGCCGCGTGAGTGAAGAAGGCCTTCGGGTTGTAAAGCTCTTTTGTTAGGGAAGAAAAGGTTCGCAATAATACTGTGGACTGATGACGGTACCTAAAGAATAAGCACCGGCTAACTACGTGCCAGCAGCCGCGGTAATACGTAGGGTGCAAGCGTTAATCGGAATTACTGGGCGTAAAGCGTGCGCAGGCGGCCTCGCAAGTCAGATGTGAAATCCCCGAGCTCAACTTGGGAACTGCATTTGAAACTACGAGGCTAGAGTGTGTCAGAGGGAGGTAGAATTCCACGTGTAGCAGTGAAATGCGTAGATATGTGGAGGAATACCAGTGGCGAAGGCGGCCTCCTGGGATAACACTGACGCTCATGTACGAAAGCGTGGGGAGCAAACAGG` |
| ASV2635 | 429 | `TGGGGAATTTTGGACAATGGGCGAAAGCCTGATCCAGCCATGCCGCGTGCGGGAAGAAGGCCTTCGGGTTGTAAACCGCTTTTGTCAGGGAAGAAATCCTTTGAGTTAATACCTCGGGGGGATGACGGTACCTGAAGAATAAGCACCGGCTAACTACGTGCCAGCAGCCGCGGTAATACGTAGGGTGCGAGCGTTAATCGGAATTACTGGGCGTAAAGCGTGCGCAGGCGGTTATGTAAGACAGGTGTGAAATCCCCGGGCTTAACCTGGGAACTGCACTTGTGACTGCATAGCTTGAGTGCGGCAGAGGGGGATGGAATTCCGCGTGTAGCAGTGAAATGCGTAGATATGCGGAGGAACACCGATGGCGAAGGCAATCCCCTGGGCCTGCACTGACGCTCATGCACGAAAGCGTGGGGAGCAAACAGG` |
| ASV852 | 404 | `TGGGGAATATTGGACAATGGGCGAAAGCCTGATCCAGCAATGCCGCGTGAGTGATGAAGGCCTTAGGGTTGTAAAGCTCTTTTACCCGGGATGATAATGACAGTACCGGGAGAATAAGCTCCGGCTAACTTCGTGCCAGCAGCCGCGGTAATACGAGGGGAGCTAGCGTTGTTCGGAATTACTGGGCGTAAAGCGCACGTAGGCGGTTTTTTAAGTCAGAGGTGAAAGCCTGGTGCTCAACACCAGAACTGCCTTTGAAACTGGAAAACTTGAATCTTGGAGAGGTCAGTGGAATTCCGAGTGTAGAGGTGAAATTCGTAGATATTCGGAAGAACACCAGTGGCGAAGGCGACTGACTGGACAAGTATTGACGCTGAGGTGCGAAAGCGTGGGGAGCAAACAGG` |
| ASV3433 | 428 | `TCGAGAATCTTTCACAATGGGGGCAACCCTGATGGAGCGACGCCGCGTGGAGGATAAGGTCTTCGGATTGTAAACTCCTGTCATGTGAGAACAAGGTTGCAGGGTTAACACCTTTGCAAATTGATGGTACCACAAGAGGAAGAGACGGCTAACTCTGTGCCAGCAGCCGCGGTAATACAGAGGTCTCAAGCGTTGTTCGGAATCACTGGGCGTAAAGGGTGCGTAGGTGGCGTGGTAAGTCAGATGTGAAAGCCCGGGGCTCAACCTCGGAATTGCATCCGATACTGCCATGCTAGAGTACTGAAGAGGTGACTAGAATTCTCGGTGTAGCAGTGAAATGCGTAGATATCGAGAGGAATACCAACGGCGAAGGCAGGTCACTGGGCAGTTACTGACACTGAGGCACGAAGGCCAGGGGAGCAAACGGG` |
| ASV4369 | 429 | `TGGGGAATATTGGACAATGGGGGGAACCCTGATCCAGCAATACCGCGTGTGTGAAGAAGGCCTTCGGGTTGTAAAGCACTTTAGTTGGGGAAGAAGATAATTTGTTTAAGAGATGAGTTATTTGACGGTACCCAAAGAATAAGCACCGGCTAACTCTGTGCCAGCAGCCGCGGTAATACAGAGGGTGCAAGCGTTAATCGGAATTACTGGGCGTAAAGGGCGCGTAGGCGGGTCATTAAGTTAGATGTGAAATCCCTGGGCTTAACCTAGGAACTGCAACTAATACTGGTGATCTAGAGTGCAGTAGAGGGTAGTGGAATTTCCGGTGTAGCGGTGAAATGCGTAGATATCGGAAGGAACACCAGTGGCGAAGGCGGCTACCTGGACTGACACTGACGCTGAGGCGCGAAAGCGTGGGGAGCAAACAGG` |
| ASV1477 | 429 | `TGGGGAATATTGGACAATGGGGGCAACCCTGATCCAGCAATGCCGCGTGTGTGAAGAAGGCCTGCGGGTTGTAAAGCACTTTTATCCGGAACGAAAAGCATTTGGTTAATAACTGGATGTGCTGACGGTACCGGAAGAATAAGCACCGGCTAACTTCGTGCCAGCAGCCGCGGTAATACGAAGGGTGCAAGCGTTAATCGGAATTATTGGGCGTAAAGCGTGCGTAGGTGGTTTTTTAAGTCTTTTGTGAAAGCCCTGGGCTTAACCTGGGAACTGCAGGAGAAACTGGAAGGCTAGAGTCGGTTAGAGGGTTGCGGAATTCCCGGTGTAGCGGTGAAATGCGTAGAGATCGGGAGGAACATCAATGGCGAAGGCAGCAACCTGGAACCAGACTGACGCTGAGGCACGAAAGCGTGGGTAGCAAACAGG` |
| ASV378 | 404 | `TGGGGAATATTGGACAATGGGCGAAAGCCTGATCCAGCAATACCGCGTGAATGATGAAGGTCTTAGGGTTGTAAAGTTCTTTTAGTTGGAAAGATAATGACGGTACCAACAGAAAAAGCCCCGGCTAACTCCGTGCCAGCAGCCGCGGTAAGACGGAGGGGGCTAGCGTTGTTCGGAATTACTGGGCGTAAAGGGCGCGTAGGCGGATTAGTAAGTTGGGAGTGAAAGCCCGGGGCTTAACCTCGGAACTGCTTTCAAAACTGCTAGTCTTGAGTGAAGTAGGAGATGATGGAATTCCTAGTGTAGAGGTGAAATTCTTAGATATTAGGAGGAACACCGGTGGCGAAAGCGGTCATCTGGACTTCAACTGACGCTGAGGCGCGAAAGCGTGGGGAGCAAACAGG` |
| ASV235 | 424 | `TAAGGAATATTGGTCAATGGAGGCAACTCTGAACCAGCCATGCCGCGTGCAGGAAGACAGCCCTATGGGTCGTAAACTGCTTTTATACGGGAATAAACCTTGATTCGTGAATCAAGCTGAATGTACCGTAAGAATAAGGATCGGCTAACTCCGTGCCAGCAGCCGCGGTAATACGGAGGATCCAAGCGTTATCCGGATTTATTGGGTTTAAAGGGAGCGTAGGCGGCCTATTAAGTCAGGGGTGAAAGACGGTGGCTCAACCATCGCAGTGCCTTTGATACTGATGGGCTTGAATATAGTTGAGGTAGGCGGAATGTGACAAGTAGCGGTGAAATGCATAGATATGTCACAGAACACCGATTGCGAAGGCAGCTTACTAAGCTATTATTGACGCTGAGGCTCGAAAGCGTGGGGATCAAACAGG` |
| ASV2617 | 429 | `TGGGGAATTTTGGACAATGGGCGAAAGCCTGATCCAGCAATGCCGCGTGCAGGATGAAGGCCTTCGGGTTGTAAACTGCTTTTGTACGGAGCGAAAAAGTCTTCTCTAATACAGAAGGCTCATGACGGTACCGTAAGAATAAGCACCGGCTAACTACGTGCCAGCAGCCGCGGTAATACGTAGGGTGCAAGCGTTAATCGGAATTACTGGGCGTAAAGCGTGCGCAGGCGGTTATATAAGTCAGATGTGAAATCCCCGGGCTCAACCTGGGACCTGCATTTGAGACTGTATAGCTAGAGTACGGCAGAGGGGGATGGAATTCCGCGTGTAGCAGTGAAATGCGTAGATATGCGGAGGAACACCGATGGCGAAGGCAATCCCCTGGGCCTGTACTGACGCTCATGCACGAAAGCGTGGGGAGCAAACAGG` |
| ASV4383 | 423 | `TGAGGAATATTGGTCAATGGGCGGGAGCCTGAACCAGCCAAGCCGCGTGGAGGAAGGAGGTACAGAGTATCGTAAACTCCTTTTGTCAGGGGATAAAAGGTCCCACGTGTGGGACTCTGAAGGTACCTGAAGAAAAAGCATCGGCTAACTCCGTGCCAGCAGCCGCGGTAATACGGAGGATGCGAGCGTTATCCGGATTTATTGGGTTTAAAGGGTGCGTAGGCCGTTCCTTAAGTCAGCGGTGAAAGCCCGGGGCTCAACCCCGGCGAGCCGTTGAAACTGGGGAGCTGGAGAGGAAGAGAGGCATGCGGAATTCGCGGTGTAGCGGTGAAATGCTTAGATATCGCGAAGAACTCCGATTGCGAAGGCAGCATGCCGGCTTCTGTCTGACGCTGAGGCACGAAAGCGTGGGGATCGAACAGG` |
| ASV55 | 429 | `TGGGGAATTTTGGACAATGGACGCAAGTCTGATCCAGCCATGCCGCGTGCGGGAAGAAGGCCTTCGGGTTGTAAACCGCTTTTGTCAGGGAAGAAACGGGTTTCTCTAATACAGAGACCTAATGACGGTACCTGAAGAATAAGCACCGGCTAACTACGTGCCAGCAGCCGCGGTAATACGTAGGGTGCAAGCGTTAATCGGAATTACTGGGCGTAAAGCGTGCGCAGGCGGTTATGCAAGACAGAGGTGAAATCCCCGGGCTCAACCTGGGAACTGCCTTTGTGACTGCATAGCTAGAGTACGGTAGAGGGGGATGGAATTCCGCGTGTAGCAGTGAAATGCGTAGATATGCGGAGGAACACCGATGGCGAAGGCAATCCCCTGGACCTGTACTGACGCTCATGCACGAAAGCGTGGGGAGCAAACAGG` |
| ASV1800 | 429 | `TGGGGAATATTGCACAATGGGCGCAAGCCTGATGCAGCCATGCCGCGTGTGTGAAGAAGGCCTTAGGGTTGTAAAGCACTTTCAGCGAGGAGGAAGGCGTTGTAGTTAATAGCTGCAGCGATTGACGTTACTCGCAGAAGAAGCACCGGCTAACTCCGTGCCAGCAGCCGCGGTAATACGGAGGGTGCAAGCGTTAATCGGAATTACTGGGCGTAAAGCGCACGCAGGCGGTTTGTTAAGTCAGATGTGAAATCCCCGAGCTTAACTTGGGAACTGCATTTGAAACTGGCAAGCTAGAGTCTTGTAGAGGGGGGTAGAATTCCAGGTGTAGCGGTGAAATGCGTAGAGATCTGGAGGAATACCGGTGGCGAAGGCGGCCCCCTGGACAAAGACTGACGCTCAGGTGCGAAAGCGTGGGGAGCAAACAGG` |
| ASV4942 | 407 | `TGGGGAATATTGGACAATGGACCAAAAGTCTGATCCAGCAATTCTGTGTGCACGATGACGGTCTTAGGATTGTAAAGTGCTTTCAATCGGGAAAAAGAAAGTGATGGTACCGATAGAAGAAGCGACGGCTAAATACGTGCCAGCAGCCGCGGTAATACGTATGTCGCAAGCGTTATCCGGATTTATTGGGCGTAAAGCGCGTCTAGGCGGTCTGGTAAGTCTGATGTGGAAATGCGGGGCTCAACTCCGTATTGCGTTGGAAACTGCCAGACTAGAGTACTGGAGAGGTGGGCGGAACTACAAGTGTAGAGGTGAAATTCGTAGATATTTGTAGGAATGCCGATAGAGAAGTCAGCTCACTGGACAGATACTGACGCTGAAGCGCGAAAGCATGGGGAGCAAACAGG` |
| ASV1930 | 404 | `TGGGGAATCTTAGACAATGGGCGCAAGCCTGATCTAGCCATGCCGCGTGAGTGATGAAGGCCTTAGGGTCGTAAAGCTCTTTCGCTGGGGAAGATAATGACGGTACCCAGTAAAGAAACCCCGGCTAACTCCGTGCCAGCAGCCGCGGTAATACGGAGGGGGTTAGCGTTGTTCGGAATTACTGGGCGTAAAGCGCGCGTAGGCGGATCAGAAAGTAGGGGGTGAAATCCCAGGGCTCAACCCTGGAACTGCCTTCTAAACTCCTGGTCTTGAGTTCGAGAGAGGTGAGTGGAATTCCGAGTGTAGAAGTGAAATTCGTAGATATTCGGAGGAACACCAGTGGCGAAGGCGACTCACTGGCTCGATACTGACGCTGAGGTGCGAAAGCGTGGGGAGCAAACAGG` |
| ASV5351 | 411 | `TTTGGAATTTTGGACAATGGGCGAAAGCCTGATCCAGCGACGCCGCGTGGACGATGAAGGTCTTCGGATTGTAAAGTCCTTTTGCAAGGGAAGAATATTTTGACGGTACCTTGCGAATAAGAGGTTGCTAACTCTGTGCCAGCAGCAGCGGTAATACAGAGACCTCAAGCGTTATCCGGAATTATTGGGCGTAAAGCGTACCGACAGGTGGTTTTGTTAGCCAGTGGTGAAATCTCCGAGCTCAACTCGGAATCTGTCCACTGGAACTGCAAAACTTGAGGGGCAAAGAGGAAGCTAGAACGAACGGTGTAGTAGTGAAATGCGTTGATATCGTTCGGAATACCGATAGCGAAGGCAGGCTTCTGGGTGCCACCTGACACTGCTAGGACGAAAGCGTGGGGAGCGAATGGG` |
| ASV3588 | 429 | `TGGGGAATATTGCACAATGGGCGCAAGCCTGATGCAGCCATGCCGCGTGTGTGAAGAAGGCCTTCGGGTTGTAAAGCACTTTCAGCGAGGAGGAAGGCCAATAACTTAATACGTTGTTGGATTGACGTTACTCGCAGAAGAAGCACCGGCTAACTCCGTGCCAGCAGCCGCGGTAATACGGAGGGTGCAAGCGTTAATCGGAATTACTGGGCGTAAAGCGCACGCATGCGGTTTGTTAAGTCAGATGTGAAATCCCCGCGCTTAACGTGGGAACTGCATTTGAAACTGGCAAGCTAGAGTCTTGTAGAGGGGGGTAGAATTCCAGGTGTAGCGGTGAAATGCGTAGAGATCTGGAGGAATACCGGTGGCGAAGGCGGCCCCCTGGACAAAGACTGACGCTCAGGTGCGAAAGCGTGGGGAGCAAACAGG` |
| ASV4561 | 429 | `TGGGGAATTTTGGACAATGGGGGCAACCCTGATCCAGCAATGCCGCGTGCGGGAAGAAGGCCTTCGGGTTGTAAACCGCTTTTGTCAGGAACGAAAAGGCTTTCGCTAATACCGAAGGCTCATGACGGTACCTGAAGAATAAGCACCGGCTAACTACGTGCCAGCAGCCGCGGTAATACGTAGGGTGCGAGCGTTAATCGGAATTACTGGGCGTAAAGCGTGCGCAGGCGGTTGTGTAAGACAAGTGTGAAATCCCCGGGCTTAACCTGGGAACTGCATTTGTGACTGCACGGCTAGAGTGCGGCAGAGGGGGATGGAATTCCGCGTGTAGCAGTGAAATGCGTAGATATGCGGAGGAACACCAATGGCGAAGGCAATCCCCTGGGCCTGCACTGACGCTCATGCACGAAAGCGTGGGGAGCAAACAGG` |
| ASV2332 | 424 | `TGAGGAATATTGGACAATGGGCGCAAGCCTGATCCAGCCATGCCGCGTGCAGGACGACGGTCCTATGGATTGTAAACTGCTTTTGCACAGGAAGAAACAACATTACGTGTAATGTCTTGACGGTACTGTGAGAATAAGGATCGGCTAACTCCGTGCCAGCAGCCGCGGTAATACGGAGGATCCAAGCGTTATCCGGAATCATTGGGTTTAAAGGGTCCGTAGGCGGTTTAGTAAGTCAGTGGTGAAAGCCCATCGCTCAACGGTGGAACGGCCATTGATACTGCTAGACTTGAATTATTAGGAAGTAACTAGAATATGTAGTGTAGCGGTGAAATGCTTAGAGATTACATGGAATACCAATTGCGAAGGCAGGTTACTACTAATGGATTGACGCTGATGGACGAAAGCGTGGGTAGCGAACAGG` |
| ASV4719 | 424 | `TAAGGAATATTGGACAATGCCCGCAAGGGTGATCCAGCCATGCCGCGTGCAGGAAGACGGCCCTATGGGTTGTAAACTGCTTTTATACCGGAGAAAACCTCCCCTCGTGAGGGGAGCTGATAGTATGGTAAGAATAAGCATCGGCTAACTTCGTGCCAGCAGCCGCGGTAAGACGAAGGATGCAAGCGTTATCCGGATTCATTGGGTTTAAAGGGAGCGTAGGCGGCCTTATAAGTCAGTGGTGAAATCTTTGGGCTTAACCCAAAAATTGCCATTGATACTGTAGGGCTTGAGTACAGTTGCTGTGGGCGGAATATGACATGTAGTGGTGAAATACATAGAGATGTCATAGAACACCGATTGCGAAGGCAGCTCACAAAACTGTAACTGACGCTGAGGCTCGAAAGTGCGGGGATCAAACAGG` |
| ASV2939 | 423 | `TAGGGAATATTGGTCAATGGATGGAAGTCTGAACCAGCCATGCCGCGTGCAGGAAGAAGGCGTTATGCGTTGTAAACTGCTTTTGCCAGGGGATAAAATGAGCGTGCGCGCTTAATTGAAGGTACCTGGTGAATAAGCCACGGCTAACTACGTGCCAGCAGCCGCGGTAATACGTAGGTGGCAAGCGTTGTCCGGATTTATTGGGTTTAAAGGGTGCGTAGGTGGCCCTGTAAGTCAGTGGTGAAATATCTCAGCTTAACTGAGAGGGTGCCATTGATACTGCAGGGCTTGAGTACAGATGAGGTAGGCGGAATTGACGGTGTAGCGGTGAAATGCATAGATATCGTCAAGAACACCGATAGCGAAGGCAGCTTACCAAGCTGTAACTGACACTGAGGCACGAAAGTGTGGGGATCAAACAGG` |
| ASV2567 | 429 | `TGGGGAATTTTGGACAATGGGCGCAAGCCTGATCCAGCCATTCCGCGTGAGTGAAGAAGGCCTTCGGGTTGTAAAGCTCTTTCGCAAGGGAAGAAAGATTACTCTCTAACATAGAGTGATTATGACGGTACCTTGATAAGAAGCACCGGCTAACTACGTGCCAGCAGCCGCGGTAATACGTAGGGTGCGAGCGTTAATCGGAATTACTGGGCGTAAAGCGTGCGCAGGCGGTTTTGTAAGTCAGATGTGAAATCCCCGAGCTCAACTTGGGAACTGCGTTTGAAACTACAAGACTAGAATATGTCAGAGGGGGGTAGAATTCCACGTGTAGCAGTGAAATGCGTAGAGATGTGGAGGAATACCAATGGCGAAGGCAGCCCCCTGGGATAATATTGACGCTCATGCACGAAAGCGTGGGGAGCAAACAGG` |
| ASV4321 | 404 | `TGGGGAATATTGGACAATGGGCGCAAGCCTGATCCAGCAATGCCGCGTGGATGAAGAAGGTTTTCGGATTGTAAAGTCCTTTCGGCGGGGACGATGATGACGGTACCCGCAGAAGAAGCCCCGGCTAACTTCGTGCCAGCAGCCGCGGTAATACGAAGGGGGCTAGCGTTGCTCGGAATGACTGGGCGTAAAGGGCGCGTAGGCGGTATGGACAGTCAGGTGTGAAATTCCCGGGCTTAACCTGGGGACTGCATTTGATACGTTCGAACTAGAGTGTGGAAGAGGGTCGTGGAATTCCCAGTGTAGAGGTGAAATTCGTAGATATTGGGAAGAACACCGGTGGCGAAGGCGGCGACCTGGTCCATTACTGACGCTGAGGCGCGAAAGCGTGGGGAGCAAACAGG` |
| ASV3692 | 405 | `TGGGGAATATTGGACAATGGGCGCAAGCCTGATCCAGCAATACCGCGTGAGTGATGAAGGTCTTCGGATTGTAAAGCTCTTTTAGTGGGGAAGATAATGACGGTACCCACAGAAAAAGCACCGGCAAACTTCGTGCCAGCAGCCGCGGTAATACGAAGGGTGCTAGCGTTGTTCGGAATTACTGGGCGTAAAGCGTACGTAGGCGGCTTAGTAAGTCGATTGTGAAAGCCCAGGGCTTAACCCTGTGAATTGCAATTGAAACTGCTTTGCTTGAGTTTGGTGGGGGATGGCGGAATTCCTAGTGTAGGGGTGAAATCCGTAGATATTAGGAGGAATACCGGTGGCGAAGGCGGCCATCTACGCCAATACTGACGCTAAGGTACGAAAGCGTGGGGAGCAAACAGG` |
| ASV4785 | 428 | `TGGGGAATCTTGCGCAATGGGCGAAAGCCTGACGCAGCGATGCCGCGTGAGCGATGAAGGCCTTTGGGTTGTAAAGCTCTGTGGATGGAGACGAATAAGTGTCGGTTAATACCCGGCGCGATGACGGTATCCATTTAGCAAGCACCGGCTAACTCTGTGCCAGCAGCCGCGGTAATACAGAGGGTGCAAACGTTGTTCGGAATTACTGGGCGTAAAGCGCGTGTAGGCGGCACTGTAGGTCGGATGTGAAATCCCGCGGCTCAACCGTGGAAGTGCATTCGAAACCGCAGAGCTAGAGTATCGGAGAGGAAAGTGGAATGCTTGGTGTAGAGGTGAAATTCGTAGATATCAAGCGGAACACCGGTGGCGAAGGCGGCTTTCTGGACGATTACTGACGCTGAGACGCGAAAGCGTGGGTAGCAAACAGG` |
| ASV5448 | 424 | `TGAGGAATATTGGTCAATGGGCGCAAGCCTGAACCAGCCATGCCGCGTGCAGGAATAAGGTCCTATGGATTGTAAACTGCTTTTGTGCAGGAAGAAATGCTTTTACGCGTAGAAGTTTGACGGTACTGTAAGAATAAGGATCGGCTAACTCCGTGCCAGCAGCCGCGGTAATACGGAGGATCCAAGCGTTATCCGGAATCATTGGGTTTAAAGGGTCTGTAGGCGGTTTAGTAAGTCAGTGGTGAAATCCGGTCGCTTAACGATTGAACTGCCATTGATACTGCGGGACTTGAATAATTAGGAAGTAACTAGAATATGTAGTGTAGCGGTGAAATGCTTAGATATTACATGGAATACCAATTGCGAAGGCAGGTTACTACTAATTTATTGACGCTGATGGACGAAAGCGTGGGTAGCGAACAGG` |
| ASV2233 | 429 | `TGGGGAATTTTGGACAATGGGCGCAAGCCTGATCCAGCAATGCCGCGTGCAGGATGAAGGCCTTCGGGTTGTAAACTGCTTTTGTACGGAGCGAAAAGGCTCTCTCTAATACAGGGGGCTCATGACGGTACCGTAAGAATAAGCACCGGCTAACTACGTGCCAGCAGCCGCGGTAATACGTAGGGTGCGAGCGTTAATCGGAATTACTGGGCGTAAAGCGTGCGCAGGCGGTCTTGTAAGACAGATGTGAAATCCCCGGGCTCAACCTGGGACCTGCATTTGTGACTGCAAGGCTAGAGTACGGCAGAGGGGGATGGAATTCCGCGTGTAGCAGTGAAATGCGTAGATATGCGGAGGAACACCGATGGCGAAGGCAATCCCCTGGGCCTGTACTGACGCTCATGCACGAAAGCGTGGGGAGCAAACAGG` |
| ASV635 | 429 | `TGGGGAATTTTGGACAATGGACGCAAGTCTGATCCAGCCATTCCGCGTGCAGGATGAAGGCCCTCGGGTTGTAAACTGCTTTTGTACGGAACGAAAAGGTTTCCATTAATACTGGGAGCTCATGACGGTACCGTAAGAATAAGCACCGGCTAACTACGTGCCAGCAGCCGCGGTAATACGTAGGGTGCAAGCGTTAATCGGAATTACTGGGCGTAAAGCGTGCGCAGGCGGTTATGTAAGACAGAGGTGAAATCCCCGGGCTCAACCTGGGAACTGCCTTTGTGACTGCATAGCTAGAGTACGGTAGAGGGGGATGGAATTCCGCGTGTAGCAGTGAAATGCGTAGATATGCGGAGGAACACCGATGGCGAAGGCAATCCCCTGGACCTGTACTGACGCTCATGCACGAAAGCGTGGGGAGCAAACAGG` |
| ASV2571 | 428 | `TTTCGAATCATTCACAATGGGCGAAAGCCTGATGGTGCGACGCCGCGTGAGGGATGAAGGTCTTCGGATTGTAAACCTCTGTCACTGGGGAAGAAACGCTAAGTCCTAATACGACTTAGCCTGACTTAACCCGGAGAGGAAGCAGTGGCTAACTCTGTGCCAGCAGCCGCGGTAATACAGAGACTGCAAGCGTTATTCGGATTCACTGGGCGTAAAGGGTGCGCAGGTGGCCAAGTGTGTCAGGTGTGAAATCCCGGGGCTTAACTCCGGAACTGCGCCTGAAACTGCTTGGCTAGAGCATTGGAGAGGGAAGTGGAATTCACGGTGTAGCAGTGAAATGCGTAGATATCGTGAGGAACACCAGAGGCGAAGGCGACTTCCTGGACAATTGCTGACACTCAGGCACGAAAGCGTGGGTAGCCAAAGGG` |
| ASV1909 | 424 | `TGAGGAATATTGGACAATGGGCGCAAGCCTGATCCAGCCATGCCGCGTGCAGGATGACGGTCCTATGGATTGTAAACTGCTTTTGCACAGGAAGAAACAACTCTACGTGTAGAGTCTTGACGGTACTGTGAGAATAAGGATCGGCTAACTCCGTGCCAGCAGCCGCGGTAATACGGAGGATCCAAGCGTTATCCGGAATCATTGGGTTTAAAGGGTCCGTAGGCGGTCTTATAAGTCAGTGGTGAAATCTCCCCGCTCAACGGGGAAACGGCCATTGATACTGTAGGACTTGAATTATTAGGAAGTAACTAGAATATGTAGTGTAGCGGTGAAATGCTTAGAGATTACATGGAATACCAATTGCGAAGGCAGGTTACTACTAATGGATTGACGCTGATGGACGAAAGCGTGGGTAGCGAACAGG` |
| ASV3473 | 428 | `TAGGGAGTTTTCGGCAATGGGGGAAACCCTGACCGAGCAACGCCGCGTGAGTGATGAAGGTCTTCGGATTGTAAAGCTCTGTTATAGAGGATAAATGACGGACCGAGGAAATGCGGTCCGTTTGATAGTACTTTATCAGAAAGCCACGACTAACTACGTGCCAGCAGTCGCGGTAATACGTAGGTGGCAAGCGTTATCCGGAATTATTGGGCGTAAAGAGTGAGCAGGCGGCTGTTTAAGTTTGAGGTTAAATCTATGGGCTCAACCCATATAAGCCTTGAAAACTGGACGGCTAGAGTACGAGAGAGGTAAACGGAACTTCATGTGTAGCGGTGAAATGCGTAGATATATGAAAGAACACCAGTGGCGAAGGCGGTTTACTAGCTCGATACTGACGCTCAGTCACGAAAGCGTGGGTAGCAAATAGG` |
| ASV252 | 429 | `TGGGGAATCTTGGACAATGGGCGAAAGCCTGATCCAGCAATGCCGCGTGCGTGAAGAAGGCCTTCGGGTTGTAAAGCGCTTTTGTCGGGGAGGAAATCCTAGTGGCTAATAACCGCTGGGGATGACAGTACCCGAAGAATAAGGACCGGCTAACTACGTGCCAGCAGCCGCGGTAATACGTAGGGTCCAAGCGTTAATCGGAATTACTGGGCGTAAAGCGTCCGCAGGTGGCTTGATAAGATAGACGTGAAATCCCTGGGCTCAACCTAGGAATTGCGTTTATGACTGTCTCGCTAGAGTATGGGAGAGGGGGGTGGAATTCCACGTGTAGCAGTGAAATGCGTAGAGATGTGGAGGAACACCGATGGCGAAGGCAACCCCCTGGCCTAATACTGACACTCATGGACGAAAGCGTGGGGAGCAAACAGG` |
| ASV5695 | 403 | `TGGGGAATCTTGGACAATGGAGGCAACTCTGATCCAGCCATGCCGCGTGGGTGATAAGGTCTTCGGATTGTAAAGCCCTTTCGGCGGGGACGATAATGACGGTACCCGCAGAAGAAGCTCCGGCTAACTTCGTGCCAGCAGCCGCGGTAATACGAAGGGGGCTAGCGTTGTTCGGAATTACTGGGCGTAAAGCGTGCGCAGGCGGTTTGTTAAGTCAGAGGTGAAATCCCAGAGCTCAACTCTGGAATGGCCTTTGAGACTGGCAGACTGGAGTGCGGAAGAGGTGAGTGGAATTCCCAGTGTAGAGGTGAAATTCGTAGATATTGGGAAGAACACCGGTGGCGAAGGCGGCTCACTGGTCCGCAACTGACGCTCATGCACGACAGCGTGGGGAGCAAACAGG` |
| ASV1618 | 404 | `TGGGGAATATTGGACAATGGGCGAAAGCCTGATCCAGCAATGCCGCGTGAGTGATGAAGGCCTTAGGGTTGTAAAGCTCTTTTACCAGGGATGATAATGACAGTACCTGGAGAATAAGCTCCGGCTAACTCCGTGCCAGCAGCCGCGGTAATACGGAGGGAGCTAGCGTTGTTCGGAATTACTGGGCGTAAAGCGCACGTAGGCGGCTACTCAAGTCAGAGGTGAAAGCCCAGGGCTCAACCCTGGAACTGCCTTTGAAACTAGGTAGCTAGAATCTTGGAGAGGTCAGTGGAATTCCGAGTGTAGAGGTGAAATTCGTAGATATTCGGAAGAACACCAGTGGCGAAGGCGACTGACTGGACAAGTATTGACGCTGAGGTGCGAAAGCGTGGGGAGCAAACAGG` |
| ASV1034 | 429 | `TGGGGAATATTGCACAATGGGCGCAAGCCTGATGCAGCCATGCCGCGTGTGTGAAGAAGGCCTTCGGGTTGTAAAGCACTTTCAGCGAGGAGGAAGGCAGTCGTGTTAATAGCACGGTTGATTGACGTTACTCGCAGAAGAAGCACCGGCTAACTCCGTGCCAGCAGCCGCGGTAATACGGAGGGTGCAAGCGTTAATCGGAATTACTGGGCGTAAAGCGCACGCAGGCGGTTTGTTAAGTCAGATGTGAAATCCCCGCGCTTAACGTGGGAACTGCATTTGAAACTGGCAAGCTAGAGTCTTGTAGAGGGGGGTAGAATTCCAGGTGTAGCGGTGAAATGCGTAGAGATCTGGAGGAATACCGGTGGCGAAGGCGGCCCCCTGGACAAAGACTGACGCTCAGGTGCGAAAGCGTGGGGAGCAAACAGG` |
| ASV4590 | 404 | `TGGGGAATATTGGACAATGGGGGCAACCCTGATCCAGCAATGCCGCGTGAGTGATGAAGGCCTTCGGGTTGTAAAGCTCTTTTGTCTGTGATGATAATGACAGTAACAGACGAATAAGCCCCGGCTAACTTCGTGCCAGCAGCCGCGGTAATACGAAGGGGGCTAGCGTTGTTCGGAATAACTGGGCGTAAAGGGCGCGTAGGCGGTTAGATACGTCTGATGTGAAAGTCCTGGGCTTAACCTAGGGACTGCATTGGATACGGTTTAGCTTGAGTGCGAGAGGGGAAAATGGAATTGCGCGTGTAGAGGTGAAATTCGTAGAGATGCGCAAGAACACCGGTGGCGAAGGCGATTTTCTGGCTCGCAACTGACGCTGAGGCGCGAAAGCGTGGGGAGCAAACAGG` |
| ASV1912 | 429 | `TGGGGAATTTTGGACAATGGGGGCAACCCTGATCCAGCAATGCCGCGTGTGTGAAGAAGGCCTTCGGGTTGTAAAGCACTTTTGTCAGGAACGAAACGGTCTGGGCTAATATCCTGGGCTAATGACGGTACCTGAAGAATAAGCACCGGCTAACTACGTGCCAGCAGCCGCGGTAATACGTAGGGTGCAAGCGTTAATCGGAATTACTGGGCGTAAAGCGTGCGCAGGCGGTTATGTAAGACAGATGTGAAATGCCCGGGCTTAACCTGGGGACTGCATTTGTGACTGCATGGCTAGAATCTGGCAGAGGGGGGTAGAATTCCACGTGTAGCAGTGAAATGCGTAGAGATGTGGAGGAACACCGATGGCGAAGGCAGCCCCCTGGGCTAAGATTGACGCTCATGCACGAAAGCGTGGGGAGCAAACAGG` |
| ASV1589 | 428 | `TCGAGAATCTTTCACAATGGGGGCAACCCTGATGGAGCGACGCCGCGTGGAGGATAAGGTCTTCGGATTGTAAACTCCTGTCATGTGTGAACAAGGTTGCGGGGCTAATACCTTTGCAAATTGATGGTAACACAAGAGGAAGAGACGGCTAACTCTGTGCCAGCAGCCGCGGTAATACAGAGGTCTCAAGCGTTGTTCGGAATCACTGGGCGTAAAGGGTGCGTAGGTGGCGTGGTAAGTCAGATGTGAAAGCCCGGGGCTCAACCTCGGAATTGCATCCGATACTGCCATGCTTGAGTACTGAAGAGGTGACTAGAATTCTCGGTGTAGCAGTGAAATGCGTAGATATCGAGAGGAATACCAAAGGCGAAGGCAGGTCACTGGGCAGTTACTGACACTGAGGCACGAAGGCCAGGGGAGCGAACGGG` |
| ASV2498 | 429 | `TGGGGAATTTTGGACAATGGGGGCAACCCTGATCCAGCAATGCCGCGTGAGTGAAGAAGGCCTTCGGGTTGTAAAGCTCTTTTGTTGGGGAAGAAATCCTTGGTGCTAATATCGCCAAGGGATGACGGTACCTAAAGAATAAGCACCGGCTAACTACGTGCCAGCAGCCGCGGTAATACGTAGGGTGCAAGCGTTAATCGGAATTACTGGGCGTAAAGCGTGCGCAGGCGGCTGTGCAAGTCAGACGTGAAATCCCCGAGCTTAACTTGGGAACTGCGTTTGAAACTACACAGCTAGAATATGTCAGAGGGAGGTAGAATTCCACGTGTAGCAGTGAAATGCGTAGAGATGTGGAGGAATACCGGTGGCGAAGGCGGCCTCCTGGGATAATATTGACGCTCATGTACGAAAGCGTGGGGAGCAAACAGG` |
| ASV1705 | 429 | `TGGGGAATATTGGACAATGGGGGCAACCCTGATCCAGCCATGCCGCGTGTGTGAAGAAGGCCCTTGGGTTGTAAAGCACTTTCAGTGGGGAGAAAAGTCTGTTACTTAATACGTGATGGAATTGATGTTACCCACAGAAGAAGCACCGGCTAACTCCGTGCCAGCAGCCGCGGTAATACGGAGGGTGCAAGCGTTAATCGGAATTACTGGGCGTAAAGCGCACGTAGGTGGTTTACTAAGCCAGCTGTGAAATCCCCGGGCTCAACCTGGGCACTGCAGTTGGAACTGGTAAGCTAGAGTAGGGTAGAGGGGTGTGGAATTCCAGGTGTAGCGGTGAAATGCGTAGATATCTGGAGGAACATCAGTGGCGAAGGCGACACCCTGGACTCATACTGACACTGAGGTGCGAAAGCGTGGGGAGCAAACAGG` |
| ASV3982 | 429 | `TGGGGAATATTGGACAATGGGGGGAACCCTGATCCAGCAATGCCGCGTGTGTGAAGAAGGCCTGAGGGTTGTAAAGCACTTTCAGTGGGGAGGAGGTTACGTAGGTTAAGAGCTAGCGTAATGGACGTTACCCACAGAAGAAGCACCGGCTAACTCCGTGCCAGCAGCCGCGGTAATACGGGGGGTGCAAGCGTTAATCGGAATTACTGGGCGTAAAGAGTGCGTAGGTGGTTTGATAAGTTACATGTAAAATCCCTGGGCTCAACCTGGGCAGGCCATGTAAGACTGTTAGACTCGAGTATGGGAGAGGGTAGTGGAATTTCCGGTGTAGCGGTGAAATGCGTAGAGATCGGAAGGAACACCAGTGGCGAAGGCGGCTACCTGGCCTAATACTGACACTGAGGCACGAAAGCGTGGGGAGCAAACAGG` |
| ASV1344 | 431 | `TAGGGAATTTTCCACAATGGGCGAAAGCCTGATGGAGCAATCCTGCGTGAATGATGAAGGTCTTATATAGATTGTAAAATTCTTTTATTGGAGACGAATAGCAAGAAAAGGAAATGTTTTTTGTTTGACTGTATCCTTTGAATAAGTATCGGCTAACTATGTGCCAGCAGCCGCGGTAATACATAGGATGCAAGCGTTATCCGGATTTACTGGGTGTAAAGCGAGCGCGGGCGGATTTGCAAGTCTAGTGTTAAATGCAATTGCTCAACAATTGTATGCATTGGAAACTGCAAGTCTAGAGTATAGTAGGGAGTTTTGGAACTCCATGTGGAGCGGTGGAATGCGTAGATATATGGAAGAACACCAGTGGCGAAGGCGAAAACTTAGGCTATTACTGACGCTTAGGCTCGAAAGTGTGGGGAGCAAATAGG` |
| ASV923 | 404 | `TGGGGAATCTTAGACAATGGGGGAAACCCTGATCTAGCCATGCCGCGTGATCGATGAAGGCCTTAGGGTTGTAAAGATCTTTCAGTTGGGAAGATAATGACGGTACCAACAGAAGAAGCCCCGGCTAACTCCGTGCCAGCAGCCGCGGTAATACGGAGGGGGCTAGCGTTATTCGGAATTACTGGGCGTAAAGCGCACGTAGGCGGATTAGAAAGTCAGAGGTGAAATCCCAGGGCTCAACCTTGGAACTGCCTTTGAAACTCCTAGTCTTGAGGTCGAGAGAGGTGAGTGGAATTCCGAGTGTAGAGGTGAAATTCGTAGATATTCGGAGGAACACCAGTGGCGAAGGCGGCTCACTGGCTCGATACTGACGCTGAGGTGCGAAAGCGTGGGGAGCAAACAGG` |
| ASV5062 | 430 | `TGGGGAATCTTGCACAATGGACGAAAGTCTGATGCAGCAACGCCGCGTGAGTGAGGAAGGGTTTCGGCCTGTAAAGCTCTGTCAGGGTGGAAAAATGATAAGTGGTCTAATAGGCCATTTAAGTGATGGTACACCCAAAGGAAGCACCGGCTAACTTCGTGCCAGCAGCCGCGGTAATACGAAGGGTGCAAGCGTTGTTCGGAATTATTGGGCGTAAAGCGCGCGCAGGCGGATCAGCAAGTCAGATGTGAAATCTCGGGGCTTAACCCCGAAACTGCGTCTGAAACTACTAATCTAGAATATCGGAGAGGAAGATGGAATTTCGCATGTAGGGGTAAAATCCGTAGAGATGCGAAGGAACACCAGAGGCGAAAGCGATTTTCTGGACGATTATTGACGCTGAGGCGCGAAAGCGTGGGGAGCAAACAGG` |
| ASV1379 | 404 | `TGGGGAATATTGGACAATGGGCGAAAGCCTGATCCAGCAATGCCGCGTGAGTGAAGAAGGCCTTCGGGTTGTAAAGCTCTTTTACCCGGGAAGATAATGACCGTACCGGGAGAATAAGCCCCGGCTAACTCCGTGCCAGCAGCCGCGGTAATACGGAGGGGGCTAGCGTTGTTCGGAATCACTGGGCGTAAAGCGTGCGTAGGCGGCCATTCAAGTCAGAGGTGAAAGCCCAGGGCTCAACCCTGGAACTGCCTTTGAAACTAGATGGCTGGAACACGGGAGAGGTGAGTGGAATTCCGAGTGTAGAGGTGAAATTCGTAGATATTCGGAAGAACACCAGTGGCGAAGGCGGCTCACTGGACCGTTGTTGACGCTGAGGCACGAAAGCGTGGGGAGCAAACAGG` |
| ASV641 | 429 | `TGGGGAATTTTGGACAATGGACGAAAGTCTGATCCAGCCATGCCGCGTGAGTGAAGAAGGCCTTCGGGTTGTAAAGCTCTTTTGTCCGGGAGCAAATCCTGTTCACTAATACTGAGCGGGGCTGAGAGTACCGGAAGAATAAGCACCGGCTAACTACGTGCCAGCAGCCGCGGTAATACGTAGGGTGCGAGCGTTAATCGGAATTACTGGGCGTAAAGGGTGCGCAGGTGGTTACGTAAGTCTGATGTGAAAGCCCCGGGCTCAACCTGGGAACTGCATTGGAGACTGCGTGGCTAGAGTGTGGCAGAGGGGGGTGGAATTCCGCGTGTAGCAGTGAAATGCGTAGAGATGCGGAGGAACACCAATGGCGAAGGCAGCCCCCTGGGTTAACACTGACACTCATGCACGAAAGCGTGGGGAGCAAACAGG` |
| ASV5371 | 430 | `TCGAGAGGCTTCGGCAATGGGGGAAACCCTGACCGAGCGACGCCGCGTGGAGGATGAAGGCCCCTGGGTTGTAAACTCCTTTTGCTGGGGAAGAAACACGATCAGGTGAACAATCTGGTCGTTTGACGGTACCCGGCGAATAAGCTCCGGCTAACTCCGTGCCAGCAGCCGCGGTAACACGGGGGGAGCAAGCGTTGTTCGGAATCACTGGGCGTAAAGGGCGTGTAGGCGGTCAGGTAAGTGGGATGTGAAATCCCTCGGCTCACCCGAGGACCTGCATCCCAAACTGCTTGGCTTGAGTATGGGAGAGGATGAGGGAATTCCTGGTGTAGCGGTGAAATGCGTAGATATCAGGAGGAACACCGGTGGCGAAAGCGCTCATCTGGCCCAATACTGACGCTGAGGCGCGAAAGCTAGGGGAGCAAACGGG` |
| ASV5047 | 429 | `TGGGGAATTTTGGACAATGGGGGCAACCCTGATCCAGCAACGCCGCGTGAATGAAGAAGGCCTTAGGGTTGTAAAGTTCTTTTATCTGGGGACAAAAGTTAATGGTTAATACCTATTAATGCTGAGGGTACCAGAAGAATAAGCACCGGCTAACTACGTGCCAGCAGCCGCGGTAATACGTAGGGTGCGAGCGTTAATCGGAATTACTGGGCGTAAAGGGTGCGTAGGCGGATATATAAGTTAGATGTGAAATCCCCGAGCTTAACTTGGGAATGGCATTTAAGACTGTATGTCTAGAGTCTGTAAGAGGGGGGTAGAATTCCGAGTGTAGCAGTGAAATGCGTAGAGATTCGGAGGAACACCAGTGGCGAAGGCGGCCCCCTGGTATAAGACTGACGCTGAGGCACGAAAGCGTGGGTAGCAAACAGG` |
| ASV4521 | 429 | `TGGGGAATATTGGACAATGGGGGAAACCCTGATCCAGCAATACCGCGTGTGTGAAGAAGGCCTATGGGTTGTAAAGCACTTTCAGTGGGGAGGAAAGTATTATGGTTAATACCCATGATAATTGACGCTACCCACAGAAGAAGCACCGGCTAACTCCGTGCCAGCAGCCGCGGTAATACGGAGGGTGCAAGCGTTAATCGGAATTACTGGGCGTAAAGAGTGCGTAGGTGGTTTAGTGTGTTAGATGTGAAAGCCATGGGCTCAACCTGTGAATTGCATTTAAAACTGCTAGGCTAGAGTGTTGTAGAGGGCGGCGGAATTTCCAGTGTAGCGGTGAAATGCGTAGATATTGGAAGGAACACCAGTGGCGAAGGCGGCCGCCTGGACAAACACTGACACTGAGGCACGAAAGCGTGGGTAGCGAACAGG` |
| ASV4622 | 405 | `TGAGGAATTTTCCACAATGGACGAAAGTCTGATGGAGCAATACCGCGTGCAGGATGAAGGCCTTCGGGTCGTAAACTGCTTTTTATAAGGAGCAACTATGAGTTTACTTATAGAATAAGTCACGGCTAATCACGTGCCAGCAGCCGCGGTAATACGTGAGTGGCAAGCGTTATCCGGAATCATTGGGCGTAAAGCGTCAGCAGGGGGGCCTTCAAGTGAAATGTAAAATCCTTCGACCCAATCGAAGATCTGTATTTCATACTGTTGGCCTAGAAGACAGGAGAGGTAAATAGAATTTCCGGAGTAGGGGTAATATCCGTAGATACCGGAAGGAATATCAATAGCGAAGGCAATTTACTGGTCTGTTCTTGACCCTCAGTGACGAAAGCGTGGGTAGCAAACAGG` |
| ASV4679 | 424 | `TAGGGAATATTGGGCAATGGAGGCAACTCTGACCCAGCCATGCCGCGTGAACGATGAAGGCGCTTTGCGTCGTAAAGTTCTTTTGGGTGGGAAGAAAAGCAGACGTTCTCGTTTGTTTGACGGTACCATCAGAATAAGCACCGGCTAACTCCGTGCCAGCAGCCGCGGTAATACGGAGGGTGCAAGCGTTATCCGGATTTACTGGGTTTAAAGGGTGCGTAGGCGGGATTTTAAGTCAGTGGTGAAAGCTCGTCGCTTAACGACGGAATTGCCATTGAAACTGAAGTTCTTGAGATACAGTGAGGTTGGCGGAATGAGACATGTAGCGGTGAAATGCATAGAGATGTCTTAGAACACCAATTGCGAAGGCAGCTGACTGGCTGTTATCTGACGCTGAGGCACGAAAGCGTGGGGATCAAACAGG` |
| ASV3206 | 429 | `TGGGGAATTTTGGACAATGGGCGAAAGCCTGATCCAGCAATGCCGCGTGCGGGAAGAAGGCCTTCGGGTTGTAAACCGCTTTTGTCAGGGAAGAAACGCTCTGAGCTAATACCTCGGGGTAATGACGGTACCTGAAGAATAAGCACCGGCTAACTACGTGCCAGCAGCCGCGGTAATACGTAGGGTGCGAGCGTTAATCGGAATTACTGGGCGTAAAGCGTGCGCAGGCGGTTATGCAAGACAGATGTGAAATCCCCGGGCTCAACCTGGGAACTGCATTTGTGACTGCATGGCTAGAGTACGGTAGAGGGGGATGGAATTCCGCGTGTAGCAGTGAAATGCGTAGATATGCGGAGGAACACCGATGGCGAAGGCAATCCCCTGGACCTGTACTGACGCTCATGCACGAAAGCGTGGGGAGCAAACAGG` |
| ASV2953 | 424 | `TGAGGAATATTGGTCAATGGACGCAAGTCTGAACCAGCCATGCCGCGTGCAGGATGACGGTCCTATGGATTGTAAACTGCTTTTGTACGAGAAGAAACAACATTACGTGTAATGTCTTGACGGTATCGTAAGAATAAGGATCGGCTAACTCCGTGCCAGCAGCCGCGGTAATACGGAGGATCCAAGCGTTATCCGGAATCATTGGGTTTAAAGGGTCCGTAGGCGGTTTAGTAAGTCAGTGGTGAAAGCCCATCGCTCAACGGTGGAACGGCCATTGATACTGCTAGACTTGAATTATTAGGAAGTAACTAGAATATGTAGTGTAGCGGTGAAATGCTTAGAGATTACATGGAATACCAATTGCGAAGGCAGGTTACTACTAATTGATTGACGCTGATGGACGAAAGCGTGGGTAGCGAACAGG` |
| ASV2843 | 422 | `TAGGGAATCTTCCACAATGGACGAAAGTCTGATGGAGCAACGCCGCGTGAGTGATGAAGGTCTTCGGATTGTAAAGCTCTGTTACCAGGGAAGAAGAGATAGCATGCGTGCTATTGTGACGGTACCTGGGAAGAAAGCCCCGGCTAACTACGTGCCAGCAGCCGCGGTAATACGTAGGGGGCAAGCGTTGTCCGGAATTATTGGGCGTAAAGCGCGTGCAGGCGGTTTTATAAGCGAGACGTATAAGTGCAGGGCTCAACCTTGTGAGGCGTTTCGAACTGTAAGACTTGAGTGCGAGAGAGGAAAGTGGAATTCCACGTGTAGCGGTGAAATGCGTAGAGATGTGGAGGAACACCAGTGGCGAAAGCGACTTTCTGGCTCGTAACTGACGCTGAGGCGCGAAAGCGTGGGGAGCAAACAGG` |
| ASV1717 | 404 | `TAAGGAATATTGGACAATGGGGGAAACCCTGATCCAGCCATGCCGCGTGAGTGACGAAGGCCTTAGGGTTGTAAAGCTCTTTTGGTGGGGAAGATAATGACTGTACCCACAGAATAAGCCCCGGCTAAATTCGTGCCAGCAGCCGCGGTAATACGAATGGGGCAAGCGTTGTTCGGAATCACTGGGCGTAAAGCGTGCGTAGGCGGTTTTGCAAGTCAGAAGTGAAAGCCCAGGGCTCAACCTTGGAATTGCTTTTGAAACTGCATAACTTGAATCCCGGAGAGGTTAGTAGAATTCCGAGTGTAGAGGTGAAATTCGCAGATATTCGGAGGAATACCAGTGGCGTAAGCGGCTAACTGGACGGGCATTGACGCTGAGGTACGAAAGCGTGGGGATCAAACAGG` |
| ASV2070 | 430 | `TGGGGAATATTGGACAATGGGGGGAACCCTGATCCAGCCATGCCGCGTGTGTGAAGAAGGCCTTTTGGTTGTAAAGCACTTTAAGCGAGGAGGAGGCTACTCTAGTTAATACCTAGGGATAGTGGACGTTACTCGCAGAATAAGCACCGGCTAACTCTGTGCCAGCAGCCGCGGTAATACAGAGGGTGCAAGCGTTAATCGGATTTACTGGGCGTAAAGCGCGCGTAGGTGGCCAATTAAGTCAAATGTGAAATCCCCGAGCTTAACTTGGGAATTGCATTCGATACTGGTTGGCTAGAGTATGGGAGAGGATGGTAGAATTCCAGGTGTAGCGGTGAAATGCGTAGAGATCTGGAGGAATACCGATGGCGAAGGCAGCCATCTGGCCTAATACTGACACTGAGGTGCGAAAGCATGGGGAGCAAACAGG` |
| ASV5154 | 429 | `TGGGGAATATTGGACAATGGGGGCAACCCTGATCCAGCGATGCCGCGTGTGTGAAGAAGGCCTGCGGGTTGTAAAGCACTTTCAGTTGGGAAGAAGTAATTTATCCTAATAGGGTAATGCGTTGACATTACCAACAGAAGAAGCACCGGCTAACTCTGTGCCAGCAGCCGCGGTAATACAGAGGGTGCAAGCGTTAATCGGAATTACTGGGCGTAAAGCGCACGTAGGTGGGTTGGTAAGTTGCTGGTGAAAAGCCTGGGCTTAACCTGGGTCGGTCCTGCAAAACTACCAGCCTAGAGTGTATTAGAGGGAAGTGGAATTTCCGGTGTAGCAGTGAAATGCGTAGATATCGGAAGGAACACCAGTGGCGAAGGCGACTTCCTGGGATATAACTGACACTGAGGTGCGACAGCGTGGGGAGCAAACAGG` |
| ASV4229 | 404 | `TGGGGAATATTGGACAATGGGCGCAAGCCTGATCCAGCCATGCCGCGTGAATGATGAAGGCCTTAGGGTTGTAAAGTTCTTTCGCTATCGAGGATAATGACTGTAGATAGATAAGAAGCCCCGGCTAACTTCGTGCCAGCAGCCGCGGTAATACGAAGGGGGCTAGCGTTGTTCGGAATCACTGGGCGTAAAGCGCGCGTAGGCGGCTATACAAGTCAGGGGTGAAATCCCAGAGCTCAACTCTGGAACTGCCTTTGAAACTGTATAGCTGGAGTGTTGGAGGGGATAGCGGAATTCCTAGTGTAGAGGTGAAATTCGTAGATACTAGGAGGAACACCGGTGGCGAAGGCGGCTATCTGGACAACAACTGACGCTGAGGCGCGAAAGCGTGGGTATCAAACAGG` |
| ASV3354 | 405 | `TAGGGAATCTTCCACAATGGGCGAAAGCCTGATGGAGCAACGCCGCGTGCAGGATGAAGGCCTTAGGGTTGTAAACTGCTTTTATATGTGAAGATTTAGACGGTAACATATGAATAAGGATCGGCTAACTCCGTGCCAGCAGCCGCGGTCATACGGAGGATCCAAGCGTTATCCGGAATTACTGGGCGTAAAGAGTTGCGTAGGTGGCATAGTAAGCAGGTAGTAAAATCGTGTGGCTCAACCATACTTATATTATCTGAACTGCTAAGCTAGAGTATATGAGAGGTAGCTGGAATTCCTAGTGTAGGAGTGAAATCCGTAGATATTAGGAGGAACACCGATGGCGTAGGCAGGCTACTGGCATATTACTGACACTAAGGCACGAAAGCGTGGGGAGCGAACGGG` |
| ASV822 | 404 | `TGGGGAGTATTGGACAATGGGGGAAACCCTGATCCAGCAATGCCGCGTGGGTGATGAAGGTCTTCGGATTGTAAAGCCCTTTTGGCAGGGACGATGATGACGGTACCTGCAGAATAAGCTCCGGCTAACTTCGTGCCAGCAGCCGCGGTAATACGAAGGGAGCAAGCGTTGTTCGGATTTACTGGGCGTAAAGGATGCGTAGGTGGTTTGTTAAGTTGGAAGTGAAAGCCCCGGGCTTAACCCGGGAATTGCTTTCAAGACTGGCAGACTAGAGTGTGGAAGAGGTTGGTGGAATTCCCAGTGTAGAGGTGAAATTCGTAGATATTGGGAAGAACATCCGTGGCGAAGGCGGCCAACTGGTCCACAACTGACACTGAGGCATGACAGCGTGGGGAGCAAACAGG` |
| ASV3887 | 423 | `TGAGGAATATTGGTCAATGGTCGAGAGACTGAACCAGCCAAGTCGCGTGAGGGAAGAATGGTCCATGGCCTGTAAACCTCTTTTGACGGGGACGAATAAAGGGAACGCGTTCCCTCTTGCCGGTACCCGTCGAATAAGCATCGGCTAACTCCGTGCCAGCAGCCGCGGTAACACGGGGGATGCGAGCGTTATCCGGATTTATTGGGTTTAAAGGGCGCGTAGGCGGGGGATCAAGTCAGCGGTAAAAGCCTGCAGCTTAACTGTAGCACGCCGTTGAAACTGGTTCTCTAGAGTTCGGTCAAGGGAGGCGGAACAGGTGAAGTAGCGGTGAAATGCATAGATATCACCTGGAACCCCGATAGCGAAGGCAGCTTCCCGGCCCGATACTGACGCTGAAGCGCGAAAGCGTGGGTAGCGAACAGG` |
| ASV3418 | 404 | `TGGGGAATATTGGACAATGGGGGCAACCCTGATCCAGCAATGCCGCGTGAGTGATGAAGGCCTTCGGGTTGTAAAGCTCTTTTATCAGGGACGATGATGACGGTACCTGATGAATAAGCTCCGGCTAACTTCGTGCCAGCAGCCGCGGTAATACGAAGGGAGCTAGCGTTGTTCGGAATTACTGGGCGTAAAGGGCGCGTAGGCGGTTTAGTAAGTCAGATGTGAAAGCCCCGGGCTTAACCTGGGATGTGCATTTGATACTGCTAGACTAGAGATCGAAAGAGGAGAGTGGAATTGCGAGTGTAGAGGTGAAATTCGTAGATATTCGCAGGAACACCAGTGGCGAAGGCGACTCTCTGGTTCGATACTGACGCTAAGGCGCGAAAGCGTGGGGAGCAAACAGG` |
| ASV5604 | 404 | `TGAGGAATCTTCCGCAATGGGCGAAAGCCTGACGGAGCGACACCGCGTGAAGGATGAAGCCTTTATTGGTGTAAACTTCTTTTCTGTGGGAAGATGATGACGGTACCACAGGAATAAGGGGCGGCAAACTATGTGCCAGCAGCCGCGGTAATACATAGGCCCCGAGCGTTATCCGGAATTACTGGGCGTAAAGCGTTCGTAGGCGGAGGGACAAGTCAATCGTGAAAGGTCTGGGCTCAACCCGGCACTTGCGATAGAAACTGTCCCCCTTGAATCGGGGAGAGGTAAGCAGAACTGTACGAGTAGGGGTGCAATCCGTTGATACGTACAAGAATACCAAAAGCGAAGGCAGCTTACTGGAACCGCATTGACGCTGAGGAACGAAAGCGTGGGGAGCGAAAAGG` |
| ASV2855 | 429 | `TGGGGAATTTTGGACAATGGGGGCAACCCTGATCCAGCCATTCCGCGTGAGTGAAGAAGGCCTTCGGGTTGTAAAGCTCTTTCAGCAGGAACGAAACGGTGGCCCCTAATACGGGCTACTAATGACGGTACCTGAAGAAGAAGCACCGGCTAACTACGTGCCAGCAGCCGCGGTAATACGTAGGGTGCGAGCGTTAATCGGAATTACTGGGCGTAAAGCGTGCGCAGGCGGTTCCATAAGACAGATGTGAAATCCCCGGGCTTAACCTGGGAACTGCGTTTGTGACTGTGGGACTCGAGTGTGGCAGAGGGGGGTGGAATTCCACGTGTAGCAGTGAAATGCGTAGAGATGTGGAGGAACACCGATGGCGAAGGCAGCCCCCTGGGTCAACACTGACGCTCATGCACGAAAGCGTGGGGAGCAAACAGG` |
| ASV3288 | 424 | `TGAGGAATATTGGTCAATGGGCGAGAGCCTGAACCAGCCAAGTCGCGTGAAGGAAGAATGTATTATGTATTGTAAACTTCTTTTATCGGGGAATAAAAAAGGGCTCGTGAGCTCTCTTGCAAGTACCCGAAGAATAAGGATCGGCTAACTCCGTGCCAGCAGCCGCGGTAATACGGAGGATCCGAGCGTTATCCGGATTTATTGGGTTTAAAGGGTGCGTAGGCGGCTTGTTAAGTCAGTGGTGAAAGTCCAAGGCTCAACCTTGGAATTGCCGTTGAAACTGGCAGGCTTGAGTACAGTAGAGGTAGGCGGAATTCGTGGTGTAGCGGTGAAATGCATAGATATCACGAAGAACTCCAATTGCGAAGGCAGCTTACCGGAGTGTAACTGACGCTGAGGCACGAAAGTGTGGGGATCAAACAGG` |
| ASV1246 | 423 | `TAGGGAATATTGGACAATGGGTGCGAGCCTGATCCAGCCATGCCGCGTGCAGGAAGAAGGCCTTCTGGGTTGTAAACTGCTTTTATTTGGGGATAAAAAGCTCTTGCGAGAGACATTGAAGGTACCAGATGAATAAGCCACGGCTAACTACGTGCCAGCAGCCGCGGTAATACGTAGGTGGCAAGCGTTGTCCGGATTTATTGGGTTTAAAGGGTGCGTAGGCGGTCTATTAAGTCAGTGGTGAAATACGATAGCTTAACTATCGAGGTGCCATTGATACTGATGGACTTGAGTACAGTCGAGGTAGGCGGAATGGACGGTGTAGCGGTGAAATGCATAGATATCGTCCAGAACACCGATAGTGAAGACAGCTTACCAGACTGTAACTGACGCTGAGGCACGAAAGTGTGGGGATCAAACAGG` |
| ASV2347 | 404 | `TGAGGAATATTGGACAATGGGCGAAAGCCTGATCCAGCAATACCACGTGAATGATGAAGGCCTTAGGGTTGTAAAGTTCTTTTAATAGGGAAGATAATGACGGTACCTATAGAAAAAGCCCCGGCTAACTCCGTGCCAGCAGCCGCGGTAAGACGGAGGGGGCTAGCGTTGTTCGGAATTACTGGGCGTAAAGAGCGCGTAGGCGGATTAATAAGTTGGAAGTGAAAGCCCGGGGCTTAACCTCGGAACAGCTTTCAAAACTGTTAATCTTGAGTGTAGTAGGGGATGATGGAATTCCTAGTGTAGAGGTGAAATTCTTAGATATTAGGAGGAACACCGGTGGCGAAAGCGGTCATCTGGGCTACAACTGACGCTGAGGCGCGAAAGCGTGGGGAGCAAACAGG` |
| ASV3813 | 424 | `TAAGGAATATTGGTCAATGGACGGAAGTCTGAACCAGCCATGCCGCGTGAAGGATGAAGGCCCTCTGGGTTGTAAACTTCTTTTATGGGGGACGAAAAAAGGCCAATCATGGTCACTTGACGGTACCCTAGGAATAAGCACCGGCTAACTCCGTGCCAGCAGCCGCGGTAATACGGAGGGTGCGAGCGTTATCCGGATTCACTGGGTTTAAAGGGTGCGTAGGCGGGATTGTAAGTCAGTGGTGAAAGCCTTCAGCTTAACTGAAGAACTGCCATTGATACTGCGATTCTTGAATACTGTGGAGGTATGCGGAATAGGTCATGTAGCGGTGAAATGCATAGATATGACCTAGAACACCTATTGCGAAGGCAGCATACTACGCAGCGATTGACGCTGAGGCACGAAAGCGTGGGGAGCAAACAGG` |
| ASV184 | 404 | `TGGGGAATATTGGGCAATGGGCGAAAGCCTGACCCAGCCATGCCGCGTGAGTGATGAAGGCCTTAGGGTTGTAAAGCTCTTTCAGGGGGGAAGATAATGACGGTACCCTCAGAAGAAGCTCCGGCTAACTTCGTGCCAGCAGCCGCGGTAATACGAAGGGAGCAAGCGTTACTCGGATTTACTGGGCGTAAAGGGTGCGTAGGCGGCCTGATTAGTCAGATGTGAAAGCCCAGGGCTTAACCCTGGAACTGCATTTGATACTGTCAGGCTTGAGTGTAAGAGAGGTAAGTGGAATTCCCAGTGTAGCAGTGAAATGCGTAGATATTGGGAGGAACATCAGTGGCGAAGGCGACTTACTGGCTTACAACTGACGCTGAGGCACGAAAGCGTGGGGAGCAAACAGG` |
| ASV3215 | 429 | `TGGGGAATATTGGACAATGGGGGAAACCCTGATCCAGCAATACCGCGTGTGGGAAGAAGGCCTTTGGGTTGTAAACCACTGTTATAGGGGAAAAAGGTAGCAAGGTGAACAATCTTGTTGCTTGATGGTACCCTAAGAGGAAGCACCGGCTAACTCCGTGCCAGCAGCCGCGGTAATACGGAGGGTGCAAGCGTTGTTCGGATTTACTGGGCGTAAAGTGTGTCTAGGTGGAATTGTAAGTCAGGTGTGAAAGACCGAGGCTCAACCTCGGAATTGCGCCTGAAACTACTTTTCTTGAGTAATGGAGGGGTGAGTGGAATTCCATGTGTAGCAGTGAAATGCGTAGATATGTGGAAGAACACCGGTGGCGTAGGCGACTCACTGGCCATTTACTGACACTGAAGCACGAAAGCGTGGGGAGCAAACAGG` |
| ASV3186 | 404 | `TGGGGAATATTGGACAATGGGGGCAACCCTGATCCAGCCATGCCGCGTGCGTGAAGAAGGCCTTAGGGTTGTAAAGCGCTTTTGTCAGGGAAGATAATGACTGTACCTGAAGAATAAGCCCCGGCTAACTCCGTGCCAGCAGCCGCGGTAAGACGGAGGGGGCTAGCGTTGTTCGGAATTACTGGGCGTAAAGGGCGCGTAGGCTGTTATGTGTGTCAGATGTGAAATCCCAAGGCTTAACCTTGGAACTGCGTTTGAAACTGCATGACTAGAGTGAGTAAGAGGCGGACGGAATTCCGAGTGTAGCAGTGAAATGCGCAGATATTCGGAGGAACATCGGTGGCGAAGGCGGTTCGCTGGTACTCTACTGACGCTGAGGCGCGAAAGCGTGGGGAGCAAACAGG` |
| ASV4925 | 407 | `TAGGGAATATTGCACAATGGGCGAAAGCCTGATGCAGCGACGCCGCGTGAGTGATGAAGGCCTTCGGGTTGTAAAGCTCTGTTGTCAGGGAATAAAAAAATGAATGTACCTGACGAGAAAGGACCGGCTAACTTCGTGCCAGCAGCCGCGGTAATACGAAGGGTCCTAGCGTTGTTCGGAATCATTGGGCGTAAAGCGAGTGCGGGTGGCTCTCTAAGTCAGGAGTGAAAGCCCAGAGCTTAACTCTGGAAGTGCTTTTGATACTGGAGAGCTTGAATGTGGGAGAGGGTGCTAGAATACCTAATGTAGTGGTGAAATACGTAGATATTAGGTGGAACACCGGTGGCGAAGGCGGGCACCTGGCCCAACATTGACACTTAGACTCGAAAGCGTGGGGATCAAACAGG` |
| ASV5390 | 404 | `TGGGGAATATTGGACAATGGGGGCAACCCTGATCCAGCCATGCCGCGTGAGTGAAGAAGGCCTTCGGGTTGTAAAGCTCTTTTGGCGGGGACGATGATGACGGTACCCGCAGAATAAGCCCCGGCTAACTTCGTGCCAGCAGCCGCGGTAAGACGAAGGGGGCGAGCGTTGTTCGGAATCACTGGGCGTAAAGGGCGCGTAGGCGGCGCGATAAGTCAGGCGTGAAAGCCCCAGGCTCAACTTGGGAAATGCGCTTGGAACTGTCGTGCTAGAGAGCGGGAGAGGATGGTGGAATTCCCAGTGTAGAGGTGAAATTCGTAGATATTGGGAAGAACACCGGTGGCGAAGGCGGCCATCTGGACCGTTTCTGACGCTGAGGCGCGAAAGCGTGGGGAGCAAACAGG` |
| ASV3182 | 429 | `TGGGGAATATTGGACAATGGGGGAAACCCTGATCCAGCGACGCCGCGTGGGTGAAGAAGGCCTGCGGGTTGTAAAGCCCTTTCGGTAGGGAAGAAATCTCTTGCTCTAACAAAGCAGGAGTTTGACTTAACCTACATAAGAAGCACCGGCTAACTCTGTGCCAGCAGCCGCGGTAATACAGAGGGTGCGAGCGTTAATCGGAATTACTGGGCGTAAAGCGCACGTAGTCGGCTTGCCAAGTCGGGTGTGAAATCCCCGGGCTCAACCTGGGAACTGCATCCGAAACTGGCTTGCTAGAGTATGGGAGAGGGAGGCGGAATTTCTGGTGTAGCAGTGAAATGCGTAGATATCAGAAGGAACATCAGTGGCGAAGGCGGCCTCCTGGACCAATACTGACGATCAGGTGCGAAAGCGTGGGGAGCAAACAGG` |
| ASV5668 | 430 | `TAGGGAATTTTGCACAATGGGCGCAAGCCTGATGCAGCAATGCCGCGTGAGTGAAGAAGGCCCTTGGGTTGTAAAGCTCTTTCGGCTGGGAAGAAGGGTGACATTGTGAACAATTATGTCATTTGACGGTACCAGAAGAAGCAGCACCTGCTAACTTCGTGCCAGCAGCAGCGGTAATACGAAGGGTGCAAGCGTTGTTCGGAATTACTGGGCGTAAAGGGCGCGTAGGCGGGTTTATGTGTCGATTGTGAAATCCCTGGGCTTAACCCGGGAATGGCAGTCGAAACTGTAAATCTTGAATGCTTGAGAGGGTGGTGGAATTCCAGGTGTAGAAGTGAAATTCGTAGATATCTGGAGGAACACCGGCGGCGAAGGCGGCCACCTGGCAAGACATTGACGCTGAGGCGCGAAAGTGCGGGGAGCAAACAGG` |
| ASV3146 | 430 | `TGGGGAATCTTGCACAATGGGCGAAAGCCTGATGCAGCAACGCCATGTGAGTGAAGAAGGCCCTTGGGTTGTAAAGCTCTTTCGGTTAGGAAGAAGGGTGGAGTAGTTAATACCTATTTTATCTGACGGTACTAGACTAAGAAGCACTGGCTAATTCCGTGCCAGCAGCCGCGGTAATACGGAAGGTGCAAGCATTGTTCGGATTGACTGGGCGTAAAGAGCGCGTAGGTGGGTTTGTATGTCGGATGTGAAATCCCTAGGCTTAACCTGGGAACTGCATCCGAAACGGCAGATCTTGAATGTTTGAGAGGTTGGCGGAATTCCAGGTGTAGGAGTGAAATCCGTAGATATCTGGAGGAACACCGGAGGCGAAGGCGGCTGACTAACAAAACATTGACACTGAGGTGCGAAAGCGTGGGTAGCAAACAGG` |
| ASV1987 | 429 | `TGGGGAATATTGGACAATGGGGGAAACCCTGATCCAGCAATGCCGCGTGTGTGAAGAAGGCCTGAGGGTTGTAAAGCACTTTCAGTGGGGAGGAGGATTGATAGGTTAAGAGCTAATTAATTGGACGTTACCCACAGAAGAAGCACCGGCTAACTCCGTGCCAGCAGCCGCGGTAATACGGAGGGTGCAAGCGTTAATCGGAATTACTGGGCGTAAAGGGTATGTAGGTGGTTGAATAAGTTGTCTGTGAAAGCCCTGGGCTCAACCTAGGAAGGTCAGACAATACTGTTTAACTCGAGTATGGGAGAGGGTAGTGGAATTTCCGGTGTAGCGGTGAAATGCGTAGAGATCGGAAGGAACACCAGTGGCGAAGGCGGCTACCTGGCCCAATACTGACACTGAAATACGAAAGCGTGGGGAGCAAACAGG` |
| ASV5611 | 430 | `TGGGGAATCTTGCGCAATGGACGAAAGTCTGACGCAGCCACGCCGCGTGAGTGAAGAAGGCCTTCGGGTTGTAAAGCTCTGTCAAGTGGGACAAAAACCATGTGAGTTAATAGCTCATGTGCTTGATGGTACCACTGGAGGAAGCACCGGCTAACTTCGTGCCAGCAGCCGCGGTAATACGAAGGGTGCAAGCGTTGCTCGGAATTATTGGGCGTAAAGGGCAGGTAGGTGGTCTCGTTTGTCTGGAGTGAAATCCCTGGGCTCAACCCAGGAAGTGCTTTGGAAACGGCGAGACTAGAGTTCTAGAGGGGTCCGTGGAATTCTCGGTGTAGCGGTGAAATGCGTAGAGATCGAGAGGAACACCAGAGGCGAAGGCGGCGGACTGGATAGATACTGACACTAAACTGCGAAAGCGTGGGGAGCAAACAGG` |
| ASV2359 | 406 | `TGGGGAATATTGGACAATGGGCCACAAGCCTGATCCAGCAATTCTGTGTGCACGATGAAGGTCTTCGGATTGTAAAGTGCTTTCAGTAGGGAAGAATAAATGACGGTACCTACAGAAGAAGCGACGGCTAAATATGTGCCAGCAGCCGCGGTAATACATATGTCGCAAGCGTTATCCGGATTTATTGGGCGTAAAGCGCGTCTAGGCGGTTTACTAAGTCTGATGTGAAAATGCGGGGCTCAACTCCGTATTGCGTTGGAAACTGGTAGACTAGAGTACTGGAGAGGTGGGCGGAACTACAAGTGTAGAGGTGGAATTCGTAGATATTTGTAGGAATGCCGATGGAGAAGTCAGCTCACTGGACAGATACTGACGCTAAAGCGCGAAAGCGTGGGGAGCAAACAGG` |
| ASV2989 | 429 | `TGGGGAATTTTGGACAATGGGCGAAAGCCTGATCCAGCAATGCCGCGTGCAGGATGAAGGCCTTCGGGTTGTAAACTGCTTTTGTACGGAGCGAAACGGTCTTCTTTAATACAGAGGGCTAATGACGGTACCGTAAGAATAAGCACCGGCTAACTACGTGCCAGCAGCCGCGGTAATACGTAGGGTGCAAGCGTTAATCGGAATTACTGGGCGTAAAGCGTGCGCAGGCGGTTATATAAGACAGTTGTGAAATCCCTGGGCTCAACCTAGGAACTGCATCTGTGACTGTATAGCTAGAGTACGGTAGAGGGGGATGGAATTCCGCGTGTAGCAGTGAAATGCGTAGATATGCGGAGGAACACCGATGGCGAAGGCAATCCCCTGGACCTGTACTGACGCTCATGCACGAAAGCGTGGGGAGCAAACAGG` |
| ASV1771 | 429 | `TGGGGAATTTTGGACAATGGGCGCAAGCCTGATCCAGCCATTCCGCGTGAGTGAAGAAGGCCTTCGGGTTGTAAAGCTCTTTCGCAAGGGAAGAAAACTTAACCTCTAACATAGGTTGAGGCTGACGGTACCTTGATAAGAAGCACCGGCTAACTACGTGCCAGCAGCCGCGGTAATACGTAGGGTGCGAGCGTTAATCGGAATTACTGGGCGTAAAGCGTGCGCAGGCGGCCTGATAAGTCAGATGTGAAATCCCCGAGCTCAACTTGGGAACTGCGTTTGAAACTATCAAGCTAGAATATGTCAGAGGGGGGTAGAATTCCACGTGTAGCAGTGAAATGCGTAGAGATGTGGAGGAATACCAATGGCGAAGGCAGCCCCCTGGGATAATATTGACGCTCATGCACGAAAGCGTGGGGAGCAAACAGG` |
| ASV2733 | 404 | `TGGGGAATCTTAGACAATGGGGGAAACCCTGATCTAGCCATGCCGCGTGGACGATGAAGGCCTTAGGGTTGTAAAGTCCTTTCAGCTGGGAAGATAATGACGGTACCAGCAGAAGAAGCCCCGGCTAACTCCGTGCCAGCAGCCGCGGTAATACGGAGGGGGCTAGCGTTGTTCGGAATTACTGGGCGTAAAGCGCACGTAGGCGGACTGGAAAGTTGGAGGTGAAATCCCAGGGCTCAACCTTGGAACTGCCTTCAAAACTCCCAGTCTAGAGTTCGAGAGAGGTGAGTGGAATTCCGAGTGTAGAGGTGAAATTCGTAGATATTCGGAGGAACACCAGTGGCGAAGGCGGCTCACTGGCTCGATACTGACGCTGAGGTGCGAAAGCGTGGGGAGCAAACAGG` |
| ASV648 | 404 | `TGGGGAATATTGGACAATGGGCGAAAGCCTGATCCAGCCATGCCGCGTGAGTGATGAAGGCCTTAGGGTTGTAAAGCTCTTTTGTCCGGGAAGATAATGACGGTACCGGAAGAATAAGCCCCGGCTAACCTCGTGCCAGCAGCCGCGGTAATACGAGGGGGGCTAGCGTTGTTCGGAATCACTGGGCGTAAAGCGCACGTAGGCGGCTTGTTAAGTCAGGGGTGAAATGCCAAGGCTCAACCTTGGAACTGCCTTTGATACTGGCAAGCTAGAGTGTGGTAGAGGTTGGTGGAACTCCGAGTGTAGAGGTGAAATTCGTAGATATTCGGAAGAACACCAGTGGCGAAGGCGGCCAACTGGACCATTACTGACGCTGAGGTGCGAAAGCGTGGGGAGCAAACAGG` |
| ASV535 | 429 | `TCGAGAATCATTCACAATGGGGGCAACCCTGATGGTGCAACGCCGCGTGGAGGATGAAGGTCTTCGGATTGTAAACTCCTGTCATCTAGGAGCAAGACATGGTGGTAAATAGCCAACATGTTTGATAGTACTAGAAGAGGAAGGGACGGCTAACTTCGTGCCAGCAGCCGCGGTAATACGAAGGTCCCGAGCGTTATTCGGAATCACTGGGCGTAAAGGGAGCGTAGGCGGCGTGGAAAGTCAGATGTGAAATCCCGGGGCTCAACCTCGGAACTGCATCCGATACTACCATGCTAGAGTATTGGAGGGGTAACTGGAATTCTCGGTGTAGCAGTGAAATGCGTGGATATCGAGAGGAACACCTGTGGCGAAAGCGAGTTACTGGACAATTACTGACGCTGAGGCTCGAAGGCTAGGGGAGCAAAAGGG` |
| ASV1521 | 429 | `TGGGGAATCTTGGACAATGGGCGAAAGCCTGATCCAGCAATGCCGCGTGCGTGAAGAAGGCCTTCGGGTTGTAAAGCGCTTTTGTCGGGGAGGAAATCCTAGTGCCTAATAAGTGCTGGGGATGACAGTACCCGAAGAATAAGGACCGGCTAACTACGTGCCAGCAGCCGCGGTAATACGTAGGGTCCAAGCGTTAATCGGAATTACTGGGCGTAAAGCGTCCGCAGGTGGCTTGATAAGATAGACGTGAAATCCCTGGGCTCAACCTAGGAATTGCGTTTATGACTGTCTCGCTAGAGTATGGGAGAGGGGGGTGGAATTCCACGTGTAGCAGTGAAATGCGTAGAGATGTGGAGGAACACCGATGGCGAAGGCAACCCCCTGGCCTAATACTGACACTCATGGACGAAAGCGTGGGGAGCAAACAGG` |
| ASV1532 | 421 | `TGGGGAATATTGCGCAATGGCCGAAAGGCTGACGCAGCGACGCCGCGTGGGGGATGAAGCATTTCGGTGTGTAAACCCCTGTTGCTCGGGACGAACAACTGGTTTCGACCAGTCTGACGGTACCGGGTGAGGAAGCACCGGCTAACTCCGTGCCAGCAGCCGCGGTAATACGGAGGGTGCGAGCGTTGTCCGGAATCACTGGGCGTAAAGGGCGCGTAGGCGGTCTGTTAAGCGTGCGGTGAAAGCCTGGGGCTCAACCCCAGGTCTGCCGTGCGAACTGGTGGACTTGAGCACTGTAGAGGCAGGTGGAATTCCGGGTGTAGCGGTGGAATGCGTAGAGATCCGGAAGAACACCGGTGGCGAAGGCGGCCTGCTGGGCAGTTGCTGACGCTGAGGCGCGACAGCGTGGGGAGCAAACAGG` |
| ASV3863 | 404 | `TGGGGAATATTGCACAATGGGGGAAACCCTGATGCAGCAACGCCGCGTGAGTGATGAAGGCCTTCGGGTTGTAAAGCTCTGTCTTTGGGGACGATAATGACGGTACCCAAGGAGGAAGCCACGGCTAACTACGTGCCAGCAGCCGCGGTAATACGTAGGTGGCAAGCGTTGTCCGGATTTACTGGGCGTAAAGGGAGCGTAGGTGGATTTTTAAGTGGGATGTGAAATACCCGGGCTCAACTTGGGTGCTGCATTCCAAACTGGAAATCTAGAGTGCAGGAGAGGAAAGTGGAATTCCTAGTGTAGCGGTGAAATGCGTAGAGATTAGGAAGAACACCAGTGGCGAAGGCGACTTTCTGGACTGTAACTGACACTGAGGCTCGAAAGCGTGGGGAGCAAACAGG` |
| ASV1112 | 423 | `TAGGGAATATTGGACAATGGGCGCAAGCCTGATCCAGCCATGCCGCGTGCAGGAAGAAGGCCCTCTGGGTTGTAAACTGCTTTTGATAGGGAACAAACGACCCATGCGTGGGGCATTGAGTGTACCTATAGAATAAGCACCGGCTAACTCCGTGCCAGCAGCCGCGGTAATACGGAGGGTGCAAGCGTTGTCCGGATTTATTGGGTTTAAAGGGTGCGTAGGCGGCTCTATAAGTCAGTGGTGAAATACTTGAGCTTAACTTAAGAACTGCCATTGATACTGTAGAGCTTGAGTACTGACGAGGTAGGCGGAATTGACAGTGTAGCGGTGAAATGCATAGATATTGTCAAGAACACCGATAGCGAAGGCAGCTTACTAGAGAGTAACTGACGCTGAGGCACGAAAGCGTGGGGATCAAACAGG` |
| ASV4733 | 426 | `TCGGGAATTTTGGGCAATGGGCGAAAGCCTGACCCAGCAACGCCGCGTGAAGGATGAAGTCTTTCGGGATGTAAACTTCGGAAGGATGGGAAGAATAAATTTAGGTTAATATCTTAGAAGATGACGGTACCATCTTTAAGCCCCGGCTAACTACGTGCCAGCAGCCGCGGTAATACGTAGGGGGCAAGCGTTGTTCGGATTTACTGGGCGTAAAGGGCGCGTAGGCGGCGTGATAAGTCACTTGTGAAATCTCCGAGCTCAACTCGGAACGGTCAAGTGATACTGTTGTGCTAGAGTGCGGAAGGGGCTACTGGAATTCTCGGTGTAGCGGTGAAATGCGTAGATATCGAGAGGAACACCTGAGGCGAAGGCGGGTAGCTGGGCCGACACTGACGCTGAGGCGCGAAAGCTAGGGGAGCGAACGGG` |
| ASV1255 | 407 | `TGGGGAATATTGGACAATGGACCAAAAGTCTGATCCAGCAATTCTGTGTGCACGATGAAGGTCTTCGGATTGTAAAGTGCTTTCAGTTGGGAAGAAGTCAGTGACGGTACCAACAGAAGAAGCGACGGCTAAATATGTGCCAGCAGCCGCGGTAATACATATGTCGCAAGCGTTATCCGGATTTATTGGGCGTAAAGCGCGTCTAGGCGGTTTACTAAGTCTGATGTGAAAATGCGGGGCTCAACCCCGTATTGCGTTGGAAACTGGTAGACTAGAGTACTGGAGAGGTGGGCGGAACTACAAGTGTAGAGGTGGAATTCGTAGATATTTGTAGGAATGCCGATAGAGAAGTCAGCTCACTGGACAGATACTGACGCTAAAGCGCGAAAGCGTGGGGAGCAAACAGG` |
| ASV415 | 404 | `TGGGGAATCTTAGACAATGGGGGAAACCCTGATCTAGCCATGCCGCGTGATCGATGAAGGCCTTAGGGTTGTAAAGATCTTTCAGGTGGGAAGATAATGACTGTACCACCAGAAGAAGCCCCGGCTAACTCCGTGCCAGCAGCCGCGGTAATACGGAGGGGGCTAGCGTTATTCGGAATTACTGGGCGTAAAGCGCACGTAGGCGGACTGAAAAGTCAGAGGTGAAATCCCAGGGCTCAACCTTGGAACTGCCTTTGAAACTCTCAGTCTTGAGATCGAGAGAGGTGAGTGGAATTCCGAGTGTAGAGGTGAAATTCGTAGATATTCGGAGGAACACCAGTGGCGAAGGCGGCTCACTGGCTCGATACTGACGCTGAGGTGCGAAAGCGTGGGGAGCAAACAGG` |
| ASV4462 | 429 | `TGGGGAATATTGGACAATGGGGGCAACCCTGATCCAGCAATGCCACGTGTGCGAAGAAGGCCTTCGGGTTGTAAAGCACTTTAGTGAGGGAGGAAGGTTGTAGTGTTAATACCACTATGATTTGACAGTACCTCAAGAATAAGCACCGGCAAACTCTGTGCCAGCAGCCGCGGTAATACAGAGGGTGCAAGCGTTAATCGGAATTACTGGGCGTAAAGGGCTTGTAGGCGGCTAATTAAGTTAGATGTGAAATACCTGAGCTCAACTTGGGAATGGCATTTAATACTGGTTGGCTAGAGTATAGTAGAGGAAAGGGGAATTTCCGGTGTAGCGGTGAAATGCGTAGATATCGGAAAGAACATCAGTGGCGAAGGCGCCTTTCTGGACTAATACTGACGCTGAGGAGCGAAGGCGTGGGGAGCAAACAGG` |
| ASV3708 | 429 | `TGGGGAATTTTGGACAATGGACGAAAGTCTGATCCAGCCATGCCGCGTGCGGGAAGAAGGCCTTCGGGTTGTAAACCGCTTTTGTCAGGGAAGAAATCCCTTGGGATAATACCTCGGGGGGATGACGGTACCTGAAGAATAAGCACCGGCTAACTACGTGCCAGCAGCCGCGGTAATACGTAGGGTGCAAGCGTTAATCGGAATTACTGGGCGTAAAGCGTGCGCAGGCGGTTATATAAGACAGAGGTGAAATCCCCGGGCTCAACCTGGGAACTGCCTTTGTGACTGTATAGCTTGAGTGCGGCAGAGGGGGATGGAATTCCGCGTGTAGCAGTGAAATGCGTAGATATGCGGAGGAACACCGATGGCGAAGGCAATCCCCTGGGCCTGCACTGACGCTCATGCACGAAAGCGTGGGGAGCAAACAGG` |
| ASV4895 | 388 | `GCGCGCAACTTCCGCAATGCACGAAAGTGTGACGGGGGTAGTCCGAGTGATTCTCGAAAGGGAATCTTTTGGCCAGTCTGAATAACTGGCAGAATAAGTGGTGGGTAAATCCTGTGCCAGCCGCCGCGGTAATACAGGTAGCACGAGTGGTGTCCACGAATATTGAGCCTAAAGCGCCCGTAGCCGGTTCAGTAAATCTTCTGTGAAATCTACCAACTTAATTGGTTTAGGCTTGCAGGAGAAACTGCTGAGCTAGGGAGTGGAGGAGGTGTGAGGTACTGTAGGGGGAATGGTAAAATGTTGTAATCCTTACAGGACCACCAGTGGCGAAAGCGTCACACCAAGACACGTCCGACGGTGAGGGGCGAAGGCTAGGGGAGCAAACCGG` |
| ASV1568 | 404 | `TTGGGAATCTTGGACAATGGGCGCAAGCCTGATCCAGCCATGCCGCGTGAGTGATGAAGGCCTTAGGGTTGTAAAGCTCTTTTACCCGGGAAGATAATGACTGTACCGGGAGAATAAGCTCCGGCTAACTTCGTGCCAGCAGCCGCGGTAATACGAAGGGGGCTAGCGTTGTTCGGAATTACTGGGCGTAAAGCGCGCGCAGGCGGCTTGGTAAGTTGAGGGTGAAAGCCCAGAGCTCAACTCTGGAACTGCCTTCAAAACTATCAAGCTTGAGTACGAGAGAGGTGAGTGGAATTCCCAGTGTAGAGGTGAAATTCGTAGATATTGGGAAGAACACCGGTGGCGAAGGCGGCTCACTGGCTCGTTACTGACGCTCAGGCGCGACAGCGTGGGGATCAAACAGG` |
| ASV1465 | 424 | `TGAGGAATATTGGTCAATGGGCGCAAGCCTGAACCAGCCATGCCGCGTGCAGGATGACGGTCCTATGGATTGTAAACTGCTTTTGTACAGGAAGAAACACTGGTTCGTGAACCAGCTTGACGGTACTGTAAGAATAAGGATCGGCTAACTCCGTGCCAGCAGCCGCGGTAATACGGAGGATCCAAGCGTTATCCGGAATCATTGGGTTTAAAGGGTCCGTAGGCGGTCAGATAAGTCAGTGGTGAAAGCCCATCGCTCAACGGTGGAACGGCCATTGATACTGTCTGACTTGAATTATTAGGAAGTAACTAGAATATGTAGTGTAGCGGTGAAATGCTTAGAGATTACATGGAATACCAATTGCGAAGGCAGGTTACTACTAATGGATTGACGCTGATGGACGAAAGCGTGGGTAGCGAACAGG` |
| ASV5422 | 429 | `TAGGGAATCTTCCGCAATGGACGAAAGTCTGACGGAGCAACGCCGCGTGAGTGATGAAGGTCTTCGGATCGTAAAACTCTGTTGTTAGGGAAGAACAAGTACTAGAGTAACTGCTAGTACCTTGACGGTACCTAACCAGAAAGCCCCGGCTAACTACGTGCCAGCAGCCGCGGTAATACGTAGGGGGCAAGCGTTGTCCGGAATTATTGGGCGTAAAGCGCACGTAGGCGGTCCTTTAAGTCTGATGTGAAAGCCCACGGCTTAACCGTGGAGGGTCATTGGAAACTGGAGGACTTGAGTGCAGAAGAGGAGAGTGGAATTCCACGTGGAGCGGTGAAATGCGTAGAGATGTGGAGGAACACCAGTGGCGAAGGCGACTCTCTGGTCTGTAACTGACGCTGAGGTGCGAAAGCGTGGGTAGCGAACAGG` |
| ASV3736 | 407 | `TTAGGAATTTTGCGCAATGGACGAAAGTCTGACGCAGCGATGCCGCGTGAGGGATGAAGGCCTTCGGGTTGTAAACCTCTTTTAGTTGGAGCGAAAAAAATGACGGTACCAACAGAATAAGCACCGGCTAACCCTGTGCCAGCAGCCGCGGTAATACAGGGGGTGCAAGCGTTACTCGGAATTATTGGGCGTAAAGCGCGTGTAGGTGGTTTTTTAAGTCTGGTGTGAAAGCCCTGGTCTCAAGCCAGGAAGTGCATTGGATACTGAAAGACTAGAGTGCAGGAGAGAGTGGCGGAATTCCCGGTGTAGAGGTGAAATTCGTAGATATCGGGAGGAACAACAGAGGCGAAGGCGGCCACTTGGACTGTTACTGACACTTAGACGCAAAAGCATGGGGAGCAAACAGG` |
| ASV5649 | 424 | `TAAGGAATATTGGACAATGGGCGCAAGCCTGATCCAGCTATGCCGCGTGAAGGATGACGGCCCTATGGGTTGTAAACTTCTTTTAGCAGGGAAAAAACCCCTCGATGCGTTGAGGGCTGATGGTACCTGCAGAATAAGGATCGGCTAACTCCGTGCCAGCAGCCGCGGTAATACGGAGGATCCAAGCGTTATCCGGATTTATTGGGTTTAAAGGGTGCGTAGGCGGATAAATAAGTCAGTGGTGAAAGCCTGCAGCTTAACTGTAGAATTGCCATTGATACTGTTTATCTTGAATGTAGTTGAGGTGGGCGGAATATGACATGTAGTGGTGAAATACTTAGATATGTCATAGAACACCGATTGCGAAGGCAGCTCACTAAGCTATAATTGACGCTGAGGCACGAAAGCGTGGGGAGCAAACAGG` |
| ASV2574 | 407 | `TAGGGAATCTTGCGCAATGGGCGAAAGCCTGACGCAGCAATACCGTGTGCGGGAAGACGCTCTGTGGAGTGTAAACCGCTGTCAGATGGGACGAAATAAATGACTGTACCATCAAAGGAAGCACCGGCTAACTCTGTGCCAGCAGCCGCGGTAATACAGAGGGTGCAAGCGTTGTCCGGATTTATTGGGCGTAAAGCGTCTGTAGGCGGTCTGCCAAGTCCGAAGTGAAATAATGGGGCTTAACTCCATATCCGCTTTGGAAACTGGCAGACTAGAGACTGTTAGAGGTGAATGGAATTTCCAGTGTAGCGGTGAAATGCGTAGATATTGGAAGGAACACCAGAGGCGAAGGCGGTTCACTGGGACTGTTCTGACGCTGAGAGACGAAAGCTAGGGGAGCGAAAGGG` |
| ASV3556 | 424 | `TGAGGAATATTGGACAATGGGCGCAAGCCTGATCCAGCCATGCCGCGTGCAGGATGACGCATCTATGGTGTGTAAACTGCTTTTACACAGGAAGAAACATCCCGACGTGTCGGGACTTGACGGTACTGTGAGAATAAGGATCGGCTAACTCCGTGCCAGCAGCCGCGGTAATACGGAGGATCCAAGCGTTATCCGGAATCATTGGGTTTAAAGGGTCCGTAGGCGGTTTATTAAGTCAGTGGTGAAAGCCCATCGCTCAACGGTGGAACGGCCATTGATACTGATAGACTTGAATTACTAGGAAGTAACTAGAATATGTAGTGTAGCGGTGAAATGCTTAGAGATTACATGGAATACCAATTGCGAAAGCAGGTTACTACTAGTTTATTGACGCTGATGGACGAAAGCGTGGGTAGCGAACAGG` |
| ASV5054 | 404 | `TGGGGAATATTGGACAATGGGGGAAACCCTGATCCAGCCATGCCGCGTGAATGATGAAGGCCTTAGGGTTGTAAAGTTCTTTTGTCAGGGAAGATGATGACGGTACCTGAAGAATAAGCACCGGCTAACTTCGTGCCAGCAGCCGCGGTAATACGAAGGGTGCTAGCGTTGTTCGGAATTACTGGGCGTAAAGCGTGCGTAGGTGGCTTAACAAGTCAGGGGTGAAATCCCACAGCTCAACTGTGGAACTGCCTCTGAAACTGTTAAGCTAGAGTCTTGGAGGGGATAGCGGAATTCCTAATGTAGAGGTGAAATTCGTAGATATTAGGAGGAACACCGGTGGCGAAGGCGGCTATCTGGACAAGCACTGACACTCTGGCACGAAAGCGCGGGGATCAAACAGG` |
| ASV4780 | 404 | `TGGGGAATATTGGACAATGGGGGAAACCCTGATCCAGCCATGCCGCGTGAGTGATGAAGGCCTTAGGGTTGTAAAGCTCTTTTACCAGGGAAGATGATGACGGTACCTGGAGAATAAGCACCGGCTAACTTCGTGCCAGCAGCCGCGGTAATACGAAGGGTGCTAGCGTTGTTCGGAATCACTGGGCGTAAAGCGTGCGTAGGCGGCTTTGCAAGTCAGGGGTGAAATCCCACAGCTCAACTGTGGAATTGCCTTTGAAACTGCATGGCTGGAGTGTCGGAGGGGATAGCGGAATTCCTAATGTAGTGGTGAAATACGTAGATATTAGGAGGAACACCGGTGGCGAAGGCGGCTATCTGGACGACAACTGACGCTGAGGCACGAAAGCGTGGGGATCAAACAGG` |
| ASV3260 | 429 | `TGGGGAATTTTGGACAATGGGGGCAACCCTGATCCAGCAATGCCGCGTGTGTGAAGAAGGCCTTCGGGTTGTAAAGCACTTTTGGCGGGAACGAAACGGCGTGGGTTAATACCTCGCGCTAATGACGGTACCCGCAGAATAAGCACCGGCTAACTACGTGCCAGCAGCCGCGGTAATACGTAGGGTGCAAGCGTTAATCGGAATTACTGGGCGTAAAGCGTGCGCAGGCGGTTGTATAAGACAGATGTGAAATCCCCGGGCTCAACCTGGGAACTGCATTTGTGACTGTACGGCTAGAGTGTGTCAGAGGGGGGTAGAATTCCACGTGTAGCAGTGAAATGCGTAGAGATGTGGAGGAATACCGATGGCGAAGGCAGCCCCCTGGGATAACACTGACGCTCATGCACGAAAGCGTGGGGAGCAAACAGG` |
| ASV468 | 429 | `TGGGGAATTTTGGACAATGGGCGCAAGCCTGATCCAGCAATGCCGCGTGCAGGATGAAGGCCTTCGGGTTGTAAACTGCTTTTGTACGGAACGAAACGGTCCTTTCTAATATAAGGGGCTAATGACGGTACCGTAAGAATAAGCACCGGCTAACTACGTGCCAGCAGCCGCGGTAATACGTAGGGTGCGAGCGTTAATCGGAATTACTGGGCGTAAAGCGTGCGCAGGCGGTTATGTAAGACAGATGTGAAATCCCCGGGCTCAACCTGGGACCTGCATTTGTGACTGCATAGCTAGAGTACGGTAGAGGGGGATGGAATTCCGCGTGTAGCAGTGAAATGCGTAGATATGCGGAGGAACACCGATGGCGAAGGCAATCCCCTGGACCTGTACTGACGCTCATGCACGAAAGCGTGGGGAGCAAACAGG` |
| ASV3753 | 429 | `TGGGGAATATTGCACAATGGGGGAAACCCTGATGCAGCGACGCCGCGTGGGTGATGAAGGCCTTCGGGTTGTAAAGCCCTGTCGGCTGGGAACAAGGTAGACGTGCTCATACCACGTTTGCTTGAGTGTACCAGCAAAGGAAGCACCGGCTAACCCTGTGCCAGCAGCCGCGGTAATACAGGGGGTGCTAGCGTTGTTCGGAATTACTGGGCGTAAAGGGCGCGTAGGCGGTCTTGTAAGTCAGGAGTGAAATCCCGGAGCTTAACTTCGGAATTGCTTTTGAAACTACAAGGCTAGAGTATGTTAGAGGAAATCGGAATTCCTAGTGTAGAAGTGAAATTCGTAGATATTAGGAAGAACACCGGTGGCGAAGGCGGATTTCTGGGACATAACTGACGCTGAGGCGCGAAAGCGTGGGGATCAAACAGG` |
| ASV413 | 404 | `TGGGGAATATTGGACAATGGGGGCAACCCTGATCCAGCCATGCCGCGTGAGTGAAGAAGGCCTTCGGGTTGTAAAGCTCTTTCGACGGGGACGATGATGACGGTACCCGTATAAGAAGCCCCGGCTAACTTCGTGCCAGCAGCCGCGGTAATACGAAGGGGGCTAGCGTTGTTCGGAATTACTGGGCGTAAAGCGCGCGTAGGCGGTGATCCATGTCAGAGGTGAAATCCCGGAGCTCAACTCCGGAACTGCCTTTGAAACGGGATCGCTAGAATCCGAGAGAGGATGGCGGAATTCCTAGTGTAGAGGTGAAATTCGTAGATATTAGGAAGAACACCGGTGGCGAAGGCGGCCATCTGGCTCGGTATTGACGCTCAGGCGCGAAAGCGTGGGGATCAAACAGG` |
| ASV2418 | 429 | `TGGGGAATTTTGGACAATGGGGGAAACCCTGATCCAGCCATTCCGCGTGAGTGAAGAAGGCCTTCGGGTTGTAAAGCTCTTTCGCAAGGGAAGAAACGTTACCGGTGAATATCCGGTGATAATGACGGTACCTTGATAAGAAGCACCGGCTAACTACGTGCCAGCAGCCGCGGTAATACGTAGGGTGCAAGCGTTAATCGGAATTACTGGGCGTAAAGCGTGCGCAGGCTGTTTTGTAAGTCAGATGTGAAATCCCCGAGCTCAACTTGGGAACTGCGTTTGAAACTACAAGACTAGAATATGTCAGAGGGGGGTAGAATTCCACGTGTAGCAGTGAAATGCGTAGAGATGTGGAGGAATACCAATGGCGAAGGCAGCCCCCTGGGATAATATTGACGCTCATGCACGAAAGCGTGGGGAGCAAACAGG` |
| ASV332 | 429 | `TGGGGAATATTGGACAATGGGGGAAACCCTGATCCAGCCATGCCGCGTGTGTGAAGAAGGCCTTAGGGTTGTAAAGCACTTTCAGTGGGGAGAAAGGTTTGTCACCTAATACGTGATGAAATTGATGTTACCCACAGAAGAAGCACCGGCTAACTCCGTGCCAGCAGCCGCGGTAATACGGAGGGTGCAAGCGTTAATCGGAATTACTGGGCGTAAAGCGCACGTAGGTGGTTCGCTAAGTCAGCTGTGAAATCCCCGGGCTCAACCTGGGACTTGCAGTTGAAACTGGTGAGCTAGAGTAGGGTAGAGGGGTGTGGAATTCCAGGTGTAGCGGTGAAATGCGTAGATATCTGGAGGAACATCAGTGGCGAAGGCGACACCCTGGACTCATACTGACACTGAGGTGCGAAAGCGTGGGGAGCAAACAGG` |
| ASV3105 | 423 | `TAGGGAATATTGGTCAATGGGCGGAAGCCTGAACCAGCCATGCCGCGTGCAGGAAGAAGGCCCTCTGGGTTGTAAACTGCTTTTGACAGGGAACAAGTCGGCCATGCGTGGCTAAGTGAGTGTACCTGTAGAATAAGCCACGGCTAACTACGTGCCAGCAGCCGCGGTAATACGTAGGTGGCAAGCGTTGTCCGGATTTATTGGGTTTAAAGGGTGCGTAGGCGGCCATGTAAGTCAGTTGTGAAATACCCCGGCTCAACCGGGGGGGTGCGATTGATACTGCGTGGCTTGAGTGGCAGCGAGGTTGGCGGAATTGACGGTGTAGCGGTGAAATGCATAGATATCGTCAAGAACACCGATAGCGAAGGCAGCCAACTAGGGGCCAACTGACGCTGAGGCACGAAAGTGCGGGGATCAAACAGG` |
| ASV3026 | 424 | `TGAGGAATATTGGTCAATGGACGCAAGTCTGAACCAGCCATGCCGCGTGCAGGATGACGGCTCTATGAGTTGTAAACTGCTTTTGTACTAGGGTAAACTCATCTACGTGTAGGTGATTGAAAGTATAGTACGAATAAGGATCGGCTAACTCCGTGCCAGCAGCCGCGGTAATACGGAGGATCCAAGCGTTATCCGGATTTATTGGGTTTAAAGGGTGCGTAGGCGGTTTTGTAAGTTAGAGGTGAAATTCCAAGGCTCAACCTTGGCACTGCCTCTAAAACTGTGAAGCTAGAGAATAGTTGCTGTGGGCGGAATGTGTAGTGTAGCGGTGAAATGCTTAGATATTACACAGAACACCGATTGCGAAGGCAGCTCACAAAGCTATATCTGACGCTGAGGCACGAAAGCGTGGGTAGCGAACAGG` |
| ASV946 | 429 | `TGGGGAATTTTGGACAATGGGGGCAACCCTGATCCAGCCATTCCGCGTGAGTGAAGAAGGCCTTCGGGTTGTAAAGCTCTTTTGTCGGGGAAGAAACGGTTTTGGTTAATAACCAAGACAACTGACGGTACCCGAAGAATAAGCACCGGCTAACTACGTGCCAGCAGCCGCGGTAATACGTAGGGTGCGAGCGTTAATCGGAATTACTGGGCGTAAAGCGTGCGCAGGCGGTTTTGTAAGCCAGATGTGAAATCCCCGGGCTCAACCTGGGAATGGCATTTGGGACTGCAAGGCTAGAGTATGGCAGAGGAGACTAGAATTCCTGGTGTAGCAGTGAAATGCGTAGATATCAGGAGGAATACCGATGGCGAAGGCAGGTCTCTGGGTCAATACTGACGCTCATGCACGAAAGCGTGGGGAGCAAACAGG` |
| ASV1831 | 404 | `TGAGGAATATTGGACAATGGGCGCAAGCCTGATCCAGCCATGCCGCGTGAGTGAAGAAGGCCTTAGGGTTGTAAAGCTCTTTCAAACGTGAAGATAATGACGGTAGCGTTAGAAGAAGCCCCGGCTAAATTCGTGCCAGCAGCCGCGGTAATACGAATGGGGCAAGCGTTGTTCGGAATCACTGGGCTTAAAGCGTGCGTAGGCGGACATGAAAGTCAGAAGTGAAATCCCAGAGCTTAACTCTGGAACTGCTTTTGAAACTCCATGTCTAGAATCCGGGAGGGGTTAGTGGAATTCCTAGTGTAGAGGTGAAATTCGCAGATATTAGGAGGAACACCAGTGGCGAAGGCGACTAACTGGACCGGTATTGACGCTGAGGTACGAAAGCGTGGGTAGCAAACAGG` |
| ASV3115 | 429 | `TGGGGAATATTGCGCAATGGGCGAAAGCCTGACGCAGCGACGCCGCGTGAGGGATGAAGGTTCTTGGATTGTAAACCTCTGTTAAGTGAGAAGAAAATACTATTTTTAATAGAAATAGGAAATGACGGTATCATTAGAGAAAGCACCGGCTAACTTCGTGCCAGCAGCCGCGGTAATACGAGGGGTGCAAGCGTTATTCGGAATTATTGGGCGTAAAGGGTGTGTAGACGGTATATTAAGTCAATTGCTAAAACCTCTGGCCTAACTGGAGTTCTGCAATAGAAACTGGTGTGCTAGAGTATGAAAGAGAGAAGTGGAATTCTCGGAGTAGCGGTGGAATGCGTAGATCTCGAGAGGAACACCGATGGCGAAAGCAGCTTCTTGGTTCATTACTGACGTTGAAACACGAAAGCGTGGGGAGCAAACAGG` |
| ASV1107 | 405 | `TGGGGGATATTGCACAATGGAGGAAACTCTGATGCAGCGACGCCGCGTGTAGGAAGACGGTCTTATGGATTGTAAACTACTGTCTTTGGGGACGATAATGACGGTACCCAAAGAGGAAGCTCCGGCTAACTACGTGCCAGCAGCCGCGGTAATACGTAGGGAGCGAGCGTTGTCCGGAATTACTGGGTGTAAAGGGAGCGTAGGCGGGGAGGCAAGTTGGATGTGAAAACTATCGGCTCAACCGATAGATTGCATTCAAAACTGCTTCTCTTGAGTGAAGTAGAGGCAGGCGGAATTCCTAGTGTAGCGGTGAAATGCGTAGATATTAGGAGGAACACCAGTGGCGAAGGCGGCCTGCTGGGCTTTTACTGACGCTGAGGCTCGAAAGTGTGGGTAGCAAACAGG` |
| ASV1229 | 430 | `TGGGGAATATTGGACAATGGGGGGAACCCTGATCCAGCCATGCCGCGTGTGTGAAGAAGGCCTTTTGGTTGTAAAGCACTTTAAGCGGGGAGGAGGCTCTTCTAGTTAATACCTAGGATGAGTGGACGTTACCCGCAGAATAAGCACCGGCTAACTCTGTGCCAGCAGCCGCGGTAATACAGAGGGTGCGAGCGTTAATCGGATTTACTGGGCGTAAAGCGTACGTAGGCGGCTTTTTAAGTCGGATGTGAAATCCCTGAGCTTAACTTAGGAATTGCATTCGATACTGGGAAGCTAGAGTATGGGAGAGGATGGTAGAATTCCAGGTGTAGCGGTGAAATGCGTAGAGATCTGGAGGAATACCGATGGCGAAGGCAGCCATCTGGCCTAATACTGACGCTGAGGTACGAAAGCATGGGGAGCAAACAGG` |
| ASV1520 | 405 | `TAGGGAATTTTCCACAATGGGCGAAAGCCTGATGGAGCAACGCCGCGTGCAGGATGAAGGCTTTCGGGTCGTAAACTGCTTTTATGTGCGAAGAATATGACGGTAACACATGAATAAGGGTCGGCTAACTACGTGCCAGCAGCCGCGGTCATACGTAGGACCCAAGCGTTATCCGGAATTACTGGGCGTAAAGAGTTGCGTAGGTGGCATTATAAGTGGAGCGTGAAAGCGTGCGGCTCAACCGCATTACTGCGTTCCAAACTGTAAAGCTTGAGTATGATAGGGGTAACTGGAATTTCTAGTGTAGGAGTGAAATCCGTAGATATTAGAAGGAACACCGATAGCGTAGGCAGGTTACTGGATCATTACTGACACTAAGGCACGAAAGCGTGGGGAGCAAACGGG` |
| ASV4771 | 428 | `TGGGGAATTGTTCACAATGGGCGCAAGCCTGATGACGCAACGCCGCGTGGAGGATGAAGGTCTTCGGATTGTAAACTCCTGTTGCCCGGGACGAATGGCTCCAACCTAATACGTTGGAGTGTGACGGTACCGGGTGAGGAAGCCCCGGCTAACTCTGTGCCAGCAGCCGCGGTAATACAGAGGGGGCAAGCGTTGTTCGGAATTACTGGGCGTAAAGGGCGCGTAGGCGGCCTTCTAAGTCAGACGTGAAATCCCCGGGCTCAACCCGGGAACTGCGTCTGATACTGGAAGGCTTGAATCCGGGAGAGGGATGTGGAATTCCAGGTGTAGCGGTGAAATGCGTAGATATCTGGAGGAACACCGGTGGCGAAGGCGGCATCCTGGACCGGTATTGACGCTGAGGCGCGAAAGCCAGGGGAGCAAACGGG` |
| ASV1741 | 430 | `TGGGGAATTTTGCACAATGGAGGAAACTCTGATGCAGCAACGCCGCGTGAGTGATGAAGGCCTTTGGGTTGTAAAGCTCTTTCGGTTGGGGAGAAGGGTTTATTGGCTAATATCCAATTAATTTGACGGTACCAAAAGAAGAAGCACCTGCTAACTTCGTGCCAGCAGCAGCGGTAATACGAAGGGTGCAAGCGTTGTTCGGAATTACTGGGCGTAAAGGGCGCGTAGGTTGGTTTGTAAGTCTAATGTGAAATCCCCGGGCTTAACCCGGGACGTGCATTGGAAACTGCAGATCTTGAATGCTTAAGAGGGTGGTGGAATTCCAGGAGTAGAAGTGAAATTCGTAGATATCTGGAGGAACACCTGAGGCGAAGGCGGCCACCTGGTAAGACATTGACGCTGAGGCGCGAAAGTGCGGGGAGCAAACAGG` |
| ASV4706 | 404 | `TGGGGAATCTTGGACAATGGGCGCAAGCCTGATCCAGCCATGCCGCGTGGATGACGAAGGCCTTCGGGTTGTAAAGTCCTTTCGCCCGGGACGATAATGACGGTACCGGGAGAAGAAGCCCCGGCTAACTTCGTGCCAGCAGCCGCGGTAATACGAAGGGGGCTAGCGTTGCTCGGATTTACTGGGCGTAAAGCGCGCGTAGGCGGATCTTTAAGTTAGAGGTGAAATCCCGGGGCTCAACCTCGGAACTGCCTTTAATACTGGGGATCTGGAGCAGAGTAGAGGTAAGTGGAATTCCGAGTGTAGAGGTGAAATTCGTAGATATTCGGAGGAACACCAGTGGCGAAGGCGACTTACTGGACTCTTGCTGACGCTGAGGCGCGAAAGCGTGGGGAGCAAACAGG` |
| ASV5204 | 408 | `TGAGGAATCTTCCGCAATGGGCGAAAGCCTGACGGAGCGACACCGCGTGAAGGATGAAACCCCTTTGGGGCGTAAACTTCTGTTCTGAGGGACGAAATTTTTGACGGTACCTCAGGAGAAAGCACCGGCTAATTCTGTGCCAGCAGCCGCGGTAAGACAGAAGGTGCAAGCGTTACTCGGAATTACTGGGCGTAAAGCGTCCGCAGGTGTCTTGTTACGTCTTGTGTTAAAGCACGGAGCCCAACTCCGTGATCGCACTTGAAACGGACGAGATCGAGTCATTCAGAGGTATCTGGAATGTCGTGTGTAGGGGTAAAATCCGTTGATACACGATGGAACGCCAAAAGCGAAGGCAGGATACTGGGAATGTACTGACACTCATGGACGAAAGCGTGGGGAGCAAACCGG` |
| ASV4470 | 404 | `TGGGGAATATTGGACAATGGGCGAAAGCCTGATCCAGCAATGCCGCGTGAGTGATGAAGGCCTTAGGGTTGTAAAGCTCTTTTACCCGAGATGATAATGACAGTATCGGGAGAATAAGCTCCGGCTAACTCCGTGCCAGCAGCCGCGGTAATACGGAGGGAGCTAGCGTTGTTCGGAATTACTGGGCGTAAAGCGCACGTAGGCGGTTTTTCAAGTCAGGGGTGAAAGCCCGGAGCTCAACTCCGGAACTGCCTCTGAAACTGGATGACTTGAATCTTGGAGAGGTCAGTGGAATTCCGAGTGTAGAGGTGAAATTCGTAGATATTCGGAAGAACACCAGTGGCGAAGGCGACTGACTGGACAAGTATTGACGCTGAGGTGCGAAAGCGTGGGGAGCAAACAGG` |
| ASV1815 | 429 | `TGGGGAATTTTGGACAATGGACGCAAGTCTGATCCAGCCATGCCGCGTGCGGGAAGAAGGCCTTCGGGTTGTAAACCGCTTTTGTCAGGGAAGAAATCTTCTGGACTAATACTCTGGGAGGATGACGGTACCTGAAGAATAAGCACCGGCTAACTACGTGCCAGCAGCCGCGGTAATACGTAGGGTGCAAGCGTTAATCGGAATTACTGGGCGTAAAGCGTGCGCAGGCGGTTATGCAAGACAGGTGTGAAATCCCCGAGCTCAACTTGGGAACTGCATTTGTGACTGCATAGCTAGAGTACGGTAGAGGGGAGTGGAATTCCGCGTGTAGCAGTGAAATGCGTAGATATGCGGAGGAACACCGATGGCGAAGGCAGCTCCCTGGACCTGTACTGACGCTCATGCACGAAAGCGTGGGGAGCAAACAGG` |
| ASV53 | 429 | `TGGGGAATATTGGACAATGGGCGCAAGCCTGATCCAGCCATGCCGCGTGTGTGAAGAAGGCCTTCGGGTTGTAAAGCACTTTTGTTGGGGAAGAAAAGACCGTGGTTAATAACCGTGGTTCATGACGGTACCCAAAGAATAAGCACCGGCTAACTTCGTGCCAGCAGCCGCGGTAATACGAAGGGTGCAAGCGTTACTCGGAATTACTGGGCGTAAAGCGTGCGTAGGCGGTTTGTTAAGTCTGTCGTGAAAGCCCCGGGCTCAACCTGGGAATGGCGGTGGATACTGGCAGGCTAGAGTACGGTAGAGGATGGTGGAATTCCCGGTGTAGCAGTGAAATGCGTAGAGATCGGGAGGAACATCTATGGCGAAGGCAGCCATCTGGACCAGTACTGACGCTGAGGCACGAAAGCGTGGGGAGCAAACAGG` |
| ASV2041 | 429 | `TGGGGAATTTTGGACAATGGGCGCAAGCCTGATCCAGCCATGCCGCGTGCAGGATGAAGGCCTTCGGGTTGTAAACTGCTTTTGTACGGAACGAAAAAGTTCTCTCTAATACAGGGGGCGAATGACGGTACCGTAAGAATAAGCACCGGCTAACTACGTGCCAGCAGCCGCGGTAATACGTAGGGTGCGAGCGTTAATCGGAATTACTGGGCGTAAAGCGTGCGCAGGCGGTTATATAAGACAGATGTGAAATCCCCGGGCTCAACCTGGGAACTGCATTAGTGACTGTATAGCTAGAGTGCGGCAGAGGGGGATGGAATTCCGCGTGTAGCAGTGAAATGCGTAGATATGCGGAGGAACACCGATGGCGAAGGCAATCCCCTGGGCCTGCACTGACGCTCATGCACGAAAGCGTGGGGAGCAAACAGG` |
| ASV5148 | 430 | `TGGGGAATCTTGCGCAATGGGGGCAACCCTGACGCAGCAACGCCGCGTGAGCGATGAAGGTCTTCGGATCGTAAAGCTCTGTCGGCAGGAACGACGGGTGGCTTGGCCAATATCCAAGTCGCTTGACGGTACCTGCAAAGGAAGCACCGGCTAACTCTGTGCCAGCAGCCGCGGTAAGACAGAGGGTGCAAGCGTTGTTCGGAATCATTGGGCGTAAAGGGCGCGTAGGTGGCTATGTAAGTCAGATGTGAAATCCTAGAGCTCAACTCTAGAAGTGCATCTGATACTGCATAGCTTGAGTCTCAGAGAGGATGGAGGAATTCCCAGTGTAGAGGTGAAATTCGTAGATATTGGGAGGAACATCGGTGGCGAAGGCGTCCATCTGGATGAAGACTGACACTGAGGCGCGAAAGCGTGGGGAGCAAACAGG` |
| ASV2008 | 407 | `TAGGGAATATTGCACAATGGAGGAAACTCTGATGCAGCGACGCCGCGTGAGTGATGAAGGCCTTCGGGTCGTAAAGCTCTGTCGTATGGGACTAACAAAATGAAGGTACCATGCAAGAAAGGATCGGCTAACTTCGTGCCAGCAGCCGCGGTAAGACGAGGGATCCTAGCGTTGTTCGGAATTATTGGGCGTAAAGCGGGTGTAGGTGGACTTATAAGTCAGGTGTGAAATCCCAGGGCTCAACCCTGGAAGTGCACTTGATACTGCAAGTCTTGAGTGTGGTAGAGGCTATTAGAATTCTTGGTGTAGTGGTGAAATACGTAGATATCAAGAGGAATACCGGAGGCGAAGGCGGATAGCTGGGCCAACACTGACACTGAGACCCGAAAGCGTGGGGATCAAACAGG` |
| ASV1654 | 424 | `TGAGGAATATTGGACAATGGGCGCAAGCCTGATCCAGCCATGCCGCGTGCAGGAAGACGGTCCTATGGATTGTAAACTGCTTTTATACAGGAAGAAACACCTCTACGTGTAGAGACTTGACGGTACTGTATGAATAAGCATCGGCTAACTCCGTGCCAGCAGCCGCGGTAATACGGAGGATGCAAGCGTTATCCGGAATCATTGGGTTTAAAGGGTCCGTAGGCGGTTTAATAAGTCAGTGGTGAAAGCCCATCGCTCAACGATGGAACGGCCATTGATACTGTTAAACTTGAATTATTAGGAAGTAACTAGAATATGTAGTGTAGCGGTGAAATGCTTAGAGATTACATGGAATACCAATTGCGAAGGCAGGTTACTACTAATATATTGACGCTGATGGACGAAAGCGTGGGGAGCGAACAGG` |
| ASV1841 | 404 | `TGGGGAATATTGGGCAATGGGCGAAAGCCTGACCCAGCCATGCCGCGTGAGTGATGAAGGCCTTAGGGTTGTAAAGCTCTTTCAGGGGGGAAGATAATGACGGTACCCCCAGAAGAAGCTCCGGCTAACTTCGTGCCAGCAGCCGCGGTAATACGAAGGGAGCAAGCGTTACTCGGATTTACTGGGCGTAAAGGGTGCGTAGGCGGCCTGATTAGTCAGATGTGAAAGCCCAGGGCTTAACCCTGGAACTGCATTTGATACTGTCAGGCTTGAGTATAAGAGAGGTAAGTGGAATTCCCAGTGTAGCAGTGAAATGCGTAGATATTGGGAGGAACATCAGTGGCGAAGGCGACTTACTGGCTTATAACTGACGCTGAGGCACGAAAGCGTGGGGAGCAAACAGG` |
| ASV3899 | 407 | `TAGGGAATATTGCACAATGGACGAAAGTCTGATGCAGCGACGCCGCGTGAGTGATGAAGGCCTTCGGGTCGTAAAGCTCTGTCGTATGGGACTAACACAATGAATGTACCATGCAAGAAAGGATCGGCTAACTTCGTGCCAGCAGCCGCGGTAAGACGAGGGATCCTAGCGTTGTTCGGAATTATTGGGCGTAAAGCGGGTGTAGGCGGCTTTATAAGTCAGGTGTGAAAGCCCGGGGCTCAACCCCGGAAGTGCACTTGATACTGTAAAGCTTGAATGTGGTAGAGGTTACTGGAATTGTTGGTGTAGTGGTGAAATACGTAGATATCAACAGGAATACCGGAGGCGAAGGCGGGTAACTGGGCCAACATTGACGCTGAGACCCGAAAGCGTGGGGATCAAACAGG` |
| ASV3125 | 415 | `TGGGGAATTTTGGACAATGGGCGAAAGCCTGATCCAGCAACTCTGCGTGAGGGACGAAGGCCTTCGGGTCGTAAACCTCTTTTACATGGGAAGAATACCAGCAATGGTTCGACGGTACCATGTGAATAAGCCACGGCTAACTATGTGCCAGCAGCCGCGGTAAGACATAGGTGGCAAGCGTTATTCGGAATTACTAGGCGTAAAGCGAGTGTAGGCGGACGATTAAGTTCGATGTCAAATCTCCAGGCTTAACTTGGAGTCGCATCGAATACTGGTCGCCTCGAGTGGGGTAGGGGGCAGCGGAATTCCCGGTGTAGCGGTGAAATGCGTAGATATCGGGAGGAACACCTATGGCGAAAGCAGCTGCCTGGGCCTTTACTGACGCTAAGACTCGAAAGCTGGGGGAGCAAACAGG` |
| ASV797 | 424 | `TGAGGAATATTGGTCAATGGGCGCAAGCCTGAACCAGCCATGCCGCGTGCAGGATGACGCATCTATGGTGTGTAAACTGCTTTTGCACAGGAAGAAACACTCCCTCGTGAGGGAGCTTGACGGTACTGTGAGAATAAGGATGGGCTAACTCCGTGCCAGCAGCCGCGGTAATACGGAGGATCCAAGCGTTATCCGGAATCATTGGGTTTAAAGGGTCCGTAGGCGGTTTATTAAGTCAGTGGTGAAAGCCCATCGCTCAACGGTGGAACGGCCATTGATACTGCTAGACTTGAATTATTAGGAAGTAACTAGAATATGTAGTGTAGCGGTGAAATGCTTAGAGATTACATGGAATACCAATTGCGAAGGCAGGTTACTACTAATGGATTGACGCTGATGGACGAAAGCGTGGGTAGCGAACAGG` |
| ASV5456 | 429 | `TGGGGAATTTTGGACAATGGGGGAAACCCTGATCCAGCCATGCCGCGTGTGTGAAGAAGGCCTTCGGGTTGTAAAGCACTTTCGGACGGAACGAAATTGCGCGGGCGAATATCCCGCGTAGATGACGGTACCGTAAGAAGAAGCACCGGCTAACTACGTGCCAGCAGCCGCGGTAATACGTAGGGTGCAAGCGTTAATCGGAATTACTGGGCGTAAAGCGTGCGCAGGCGGCGTCGCAAGTCAGGCGTGAAATCCCCGAGCTCAACTTGGGAATTGCGCTTGAAACTACGGCGCTGGAGTGTGGCAGAGGGAGGTGGAATTCCACGTGTAGCGGTGAAATGCGTAGATATGTGGAGGAACACCGATGGCGAAGGCAGCCTCCTGGGCCAACACTGACGCTCATGCACGAAAGCGTGGGGAGCAAACAGG` |
| ASV818 | 429 | `TGGGGAATTTTGGACAATGGGGGCAACCCTGATCCAGCCATGCCGCGTGCGGGAAGAAGGCCTTCGGGTTGTAAACCGCTTTTGTCAGGGAAGAAACGCTCTGAGTTAATACCTCGGGGTAATGACGGTACCTGAAGAATAAGCACCGGCTAACTACGTGCCAGCAGCCGCGGTAATACGTAGGGTGCAAGCGTTAATCGGAATTACTGGGCGTAAAGCGTGCGCAGGCGGTTATGCAAGACAGATGTGAAATCCCCGGGCTCAACCTGGGAACTGCATTTGTGACTGCATAGCTGGAGTGCGGCAGAGGGGGATGGAATTCCGCGTGTAGCAGTGAAATGCGTAGATATGCGGAGGAACACCAATGGCGAAGGCAATCCCCTGGGCCTGCACTGACGCTCATGCACGAAAGCGTGGGGAGCAAACAGG` |
| ASV2378 | 428 | `TGGGGAATCTTGCGCAATGGGCGAAAGCCTGACGCAGCGACGCCGCGTGGGGGAAGAAGGCCTTCGGGTTGTAAACCTCTTTCAGCAGGGACGAAGCCACTCGGGTGAATAGCCCAGAGGGTGACGGTACCTGCAGAAGAAGCCCCGGCTAACTACGTGCCAGCAGCCGCGGTAATACGTAGGGGGCAAGCGTTGTCCGGATTTATTGGGCGTAAAGAGCGTGTAGGCGGCCAGACAGGTCCGTTGTGAAAACTAGAGGCTTAACCTCTAGACGTCGATGGAAACCGTCTGGCTAGAGTCCGGAAGAGGAGAATGGAATTCCTGGTGTAGCGGTGAAATGCGCAGATATCAGGAAGAACACCCGTGGCTAAGGCGGTTCTCTAGTACGGTACTGACGCTGAGACGCGAAAGCGTGGGGAGCGAACAGG` |
| ASV3187 | 429 | `TGGGGAATATTGGACAATGGGGGCAACCCTGATCCAGCCATGCCGCGTGTGTGAAGAAGGCCTTCGGGTTGTAAAGCACTTTAAGCGAGGAGGAAGGTCTTTGAACTAATATTTCAAAGAATTGACGTTACTCGCAGAATAAGCACCGGCTAACTCTGTGCCAGCAGCCGCGGTAATACAGAGGGTGCAAGCGTTAATCGGAATTACTGGGCGTAAAGCGCGCGTAGGCGGTATGTTAAGTCGGATGTGAAATCCCTGGGCTCAACCTAGGCATTGCATCCGATACTGGCACACTAGAGTGTGGGAGAGGAAGGTAGAATTCCAGGTGTAGCGGTGAAATGCGTAGAGATCTGGAGGAATACCGATGGCGAAGGCAGCCTTCTGGCCTAACACTGACGCTGAGGTGCGAAAGCATGGGGAGCAAACAGG` |
| ASV559 | 404 | `TGGGGAATATTGGACAATGGGCGCAAGCCTGATCCAGCCATGCCGCGTGAGTGATGAAGGCCTTCGGGTTGTAAAGCTCTTTTGTCCGGGAAGATAATGACTGTACCGGAAGAATAAGCCCCGGCAAACTTCGTGCCAGCAGCCGCGGTAATACGAAGGGGGCTAGCGTTGTTCGGATTTACTGGGCGTAAAGCGCACGTAGGCGGCTTGTTAAGTCAGAGGTGAAATCCTGGAGCTCAACTCCAGAACTGCCTTTGATACTGGCAAGCTCGAGTATAGGAGAGGTTGGTGGAACTGCGAGTGTAGAGGTGAAATTCGTAGATATTCGCAAGAACACCAGTGGCGAAGGCGGCCAACTGGCCTATAACTGACGCTGAGGTGCGAAAGCGTGGGGAGCAAACAGG` |
| ASV1981 | 404 | `TGGGGAATATTGGACAATGGGCGAAAGCCTGATCCAGCCATGCCGCGTGAGTGATGAAGGCCTTCGGGTCGTAAAGCTCTTTTGTTGGGGAAAATAATGATGGTACCCAAAGAATAAGCACCGGCTAACTTCGTGCCAGCAGCCGCGGTAATACGAAGGGTGCTAGCGTTGTTCGGAATTACTGGGCGTAAAGGGTTCGTAGGCGGTTATCTAAGTTAGGGGTGAAATCCCGGGGCTCAACCTCGGAATTGCCTTTAAGACTGGATAACTTGAGTTTGGGAGAGGTAAGTGGAATTCCTAGTGTAGAGGTGAAATTCGTAGATATTAGGAGGAACATCAGTGGCGAAGGCGACTTACTGGACCAATACTGACGCTGAGGAACGAAAGTGTGGGGAGCAAACAGG` |
| ASV5315 | 428 | `TGGGGAATCTTGCGCAATGGGCGAAAGCCTGACGCAGCGACGCCGCGTGAGCGATGAAGGCCTTCGGGTTGTAAAGCTCTGTGGGGAGGAAAGAATAAGGTTTGGCTAATATCCAAGCTGATGACGGTACCTCCTTAGCAAGCACCGGCTAACTCTGTGCCAGCAGCCGCGGTAAGACAGAGGGTGCAAACGTTGTTCGGAATTACTGGGCGTAAAGCGCGTGTAGGCGGCCCTGTAAGTCGGGTGTGAAATCCCACGGCTCAACCGTGGAAGTGCACTCGAAACTACAGGGCTAGAGTCCTGGAGAGGAAGGTGGAATGCTTGGTGTAGAGGTGAAATTCGTAGATATCAAGCGGAACACCGGTGGCGAAGGCGGCCTTCTGGACAGTGACTGACGCTGAGACGCGAAAGCGTGGGGAGCAAACAGG` |
| ASV5086 | 428 | `TCGAGGATCTTCCGCAATGGACGAAAGTCTGACGGAGCGACGCCGCGTGGAGGACGAAGGCCTTCGGGTTGTAAACTCCTGTCAACGGTTATGAAAGTCCAGGCGTTAATAATGCTTGGTATGACGTAAGCCGTGGAGGAAGCACCGGCTAACTCTGTGCCAGCAGCCGCGGTAATACAGAGGGTGCAAACGTTGTTCGGAATTACTGGGCATAAAGCGCATGTAGGCGGCTTCGTAAGTGTCGGATGAAATCCCACGGCTTACCCGTGGAACTGTTCGGCATACTGCGAAGCTAGAGGATGGTAGAGGAGAGCGGAACTCTTGGTGGAGCGGTGAAATGCGTAGATATCAAGAGGAACACCGGTGGCGAAGGCGGCTCTCTGGTCCATTACTGACGCTGAGGTGCGAAAGCTAGGGGAGCAAACGGG` |
| ASV2588 | 429 | `TGGGGAATTTTGGACAATGGGGGCAACCCTGATCCAGCCATGCCGCGTGTGTGAAGAAGGCCTTCGGGTTGTAAAGCACTTTCGGCCGGAACGAAATCGCATGGGCTATTACCCCGTGTGGATGACGGTACCGGAAGAAGAAGCACCGGCTAACTACGTGCCAGCAGCCGCGGTAATACGTAGGGTGCGAGCGTTAATCGGAATTACTGGGCGTAAAGGGTGCGCAGGCGGTTGCGCAAGTCAGGCGTGAAATCCCCGGGCTTAACCTGGGAATGGCGCTTGAAACTACGTGACTGGAGTATGGCAGAGGGAGGTGGAATTCCACGTGTAGCGGTGAAATGCGTAGAGATGTGGAGGAACACCGATGGCGAAGGCAGCCTCCTGGGCCAATACTGACGCTCATGCACGAAAGCGTGGGGAGCAAACAGG` |
| ASV29 | 429 | `TGGGGAATTTTGGACAATGGGCGAAAGCCTGATCCAGCAATGCCGCGTGCAGGATGAAGGCCTTCGGGTTGTAAACTGCTTTTGTACGGAGCGAAAAGGTTTTCTCTAATACAGGGAACTGATGACGGTACCGTAAGAATAAGCACCGGCTAACTACGTGCCAGCAGCCGCGGTAATACGTAGGGTGCAAGCGTTAATCGGAATTACTGGGCGTAAAGCGTGCGCAGGCGGTTTTGTAAGACAGGCGTGAAATCCCCGGGCTCAACCTGGGAACTGCGTTTGTGACTGCAAGGCTGGAGTGCGGCAGAGGGGGATGGAATTCCGCGTGTAGCAGTGAAATGCGTAGATATGCGGAGGAACACCGATGGCGAAGGCAATCCCCTGGGCCTGCACTGACGCTCATGCACGAAAGCGTGGGGAGCAAACAGG` |
| ASV1084 | 429 | `TGGGGAATTTTGGACAATGGGCGAAAGCCTGATCCAGCAATGCCGCGTGCAGGAAGAAGGCCTTCGGGTTGTAAACTGCTTTTGTACGGAACGAAACGGTTTGCTTTAATACAGTGGGCTAATGACGGTACCGTAAGAATAAGCACCGGCTAACTACGTGCCAGCAGCCGCGGTAATACGTAGGGTGCGAGCGTTAATCGGAATTACTGGGCGTAAAGCGTGCGCAGGCGGTTATATAAGACAGTTGTGAAATCCCCGGGCTCAACCTGGGAATTGCATTTGTGACTGTATAGCTAGAGTACGGTAGAGGGGGATGGAATTCCGCGTGTAGCAGTGAAATGCGTAGATATGCGGAGGAACACCGATGGCGAAGGCAATCCCCTGGACCTGTACTGACGCTCATGCACGAAAGCGTGGGGAGCAAACAGG` |
| ASV182 | 425 | `TAGGGAATATTGGGCAATGGGCGGAAGCCTGACCCAGCCACGCCGCGTGCAGGAAGACGGCCCTCTGGGTTGTAAACTGCTTTTAACTGGGAAGAAAGTAGCTCATGCGTGAGAATTTGACGGTACCAGTTGAATAAGCCACGGCTAACTACGTGCCAGCAGCCGCGGTAATACGTAGGTGGCGAGCGTTATCCGGATTTATTGGGTTTAAAGGGAGCGTAGGCGGCTGATTAAGTCAGCGGTGAAAGGTAGCAGCTTAACTGTTTTACATGCCGTTGATACTGGTTAGCTTGAGTTGACAGAAGGCAGATAGAATTCCTGATGTAGCGGTGAAATGCTTAGATATCAGGAGGAATACCGATTGCGAAGGCAGTCTGCTGCAGTCACACTGACGCTGAGGCTCGAAGGTGCGGGGATCAAACAGG` |
| ASV1998 | 404 | `TGGGGAATATTGGACAATGGGGGAAACCCTGATCCAGCAATGCCGCGTGAATGACGAAGGCCTTAGGGTTGTAAAGTTCTTTCACCTGTGAAGATAATGACGGTAGCAGGAGAAGAAGCCCCGGCTAACTCCGTGCCAGCAGCCGCGGTAAGACGGAGGGGGCTAGCGTTGTTCGGAATGACTGGGCGTAAAGGGCGCGTAGGCGGTTTTTTAAGTGAGGCGTGAAAGCCCTGGGCTTAACCCAGGAGGTGCGTTTCAGACTGGAAGACTTGAGTGCGAGAGAGGAAAGTGGAATTTCTAGTGTAGAGGTGAAATTCGTAGATATTAGAAAGAACACCCGTGGCGAAGGCGGCTTTCTGGCTCGCAACTGACGCTGAGGCGCGAAAGCGTGGGGAGCAAACAGG` |
| ASV4849 | 429 | `TGGGGAATTTTGGACAATGGACGCAAGTCTGATCCAGCCATGCCGCGTGAGTGAAGAAGGCCTTCGGGTTGTAAAGCTCTTTTGTCAGGGAGCAAATCCTGCCTGCTAATACTAGGTGGGGATGAGAGTACCTGAAGAATAAGCACCGGCTAACTACGTGCCAGCAGCCGCGGTAATACGTAGGGTGCAAGCGTTAATCGGAATTACTGGGCGTAAAGCGTGCGCAGGTGGTTACGCAAGTCTGATGTGAAATCCCCGGGCTCAACCTGGGAACTGCATTGGAGACTGCGTGGCTAGAGTGTGGCAGAGGGGGGTGGAATTCCGCGTGTAGCAGTGAAATGCGTAGATATGCGGAGGAACACCAATGGCGAAGGCAGCCCCCTGGGTTAACACTGACACTCATGCACGAAAGCGTGGGGAGCAAACAGG` |
| ASV5242 | 405 | `TAGGGAATTTTCCACAATGGGCGAAAGCCTGATGGAGCAACGCCGCGTGCAGGATGAATGCCTTCGGGTTGTAAACTGCTTTTATATGTGAAGAATCTGACGGTAACATATGAATAAGGGTCGGCTAACTACGTGCCAGCAGCCGCGGTCATACGTAGGACCCAAGCGTTATCCGGAATTACTGGGCGTAAAGAGTTGCGTAGGTGGATTGTTAAGTGGAGCGTGAAAGCGTGCGGCTCAACCGCATTACTGCGTTCCAAACTGGCAAACTAGAGTGCGAGAGAGGTAGATGGAATTTCCAGTGTAGGAGTGAAATCCGTAGATATTGGAAGGAACACCGATGGCGTAGGCAGTCTACTGGCTCGTTACTGACACTAAGGCACGAAAGCGTGGGGAGCAAACGGG` |
| ASV5200 | 388 | `TCGGGAAAATTTTGCAATGCGCGAAAGCGTGACAGAGCAAGCCAGAGTGATATTCCATTTGGAATATCTTTTGTGGAATGTAGAAAGTTCCACGAATAAGGACCGGGAGAGACTGGTGCCAGCCGCCGCGGTAATCCCAGCGGTCCAAGTCGCAGCCACATTTGTTGGGTCTAAAACATCCGTAGCTTGTCTTACAAGTCTCTTGTGAAATCGGGCATCTTAAGTGTCCGGCGGGCAAGAGATACTGTTTGACTCGAGACCGGGAGGTGCAGAGAGTACTTTGGGGGTAGCGGTTAAATGTGTTGATCCTCAAAGGACTAACAGTCGCGAAGGCGCTCTGCAAGAACGGATCTGACAGTGAGGGATGAAGGCTAGGGGCGCAAAGTGG` |
| ASV2356 | 429 | `TGGGGAATTTTGGACAATGGGCGCAAGCCTGATCCAGCAACGCCGCGTGAGTGAAGAAGGCCTTCGGGTTGTAAAGCTCTTTTGTTAGGGAAGAAATCTCCTAGGTTAATACCTTGGGAGGATGACGGTACCTAAAGAATAAGCACCGGCTAACTACGTGCCAGCAGCCGCGGTAATACGTAGGGTGCAAGCGTTAATCGGAATTACTGGGCGTAAAGCGTGCGCAGGCGGTTGTGTAAGACAGGTGTGAAATCCCAGGGCTTAACCTTGGAATTGCACTTGTGACTGCACGGCTAGAGTGTGTCAGAGGGGGGTAGAATTCCACGTGTAGCAGTGAAATGCGTAGAGATGTGGAGGAATACCGATGGCGAAGGCAGCCCCCTGGGACAATACTGACGCTCATGCACGAAAGCGTGGGGAGCAAACAGG` |
| ASV4213 | 409 | `TCGAGAATCTTCCGCAATGGGCGAAAGCCTGACGGAGCGACACCGCGTGATGGATGAAGACCCTCTGGGTCGTAAACATCTTTTATAGAGGAGAAAGTTTATTGATAGTACTCTACGAATAAGGGGTTGCTAAACTCGTGCCAGCAGCAGCGGTAATACGAGTGCCCCGAGCGTTATCCGGAATCATTGGGCGTAAAGGGTGTGTAGGTGGCAGTGTTAGTCGCTTGTTAAAACCCGGAGCTTAACTCCGGACGTGCGAGCGAAACGGCACAGCTAGAGGATGGCAGGGATGTATGGAACTCATGGAGTAGGGGTGAAATCCGTTGATATCATGGGGAACACCAAAAGCGAAGGCAGTACATTGGGCCACTCCTGACACTGAGACACGAAAGCGTGGGTAGCGAATGGG` |
| ASV3699 | 404 | `TGGGGAATATTGGACAATGGGGGAAACCCTGATCCAGCCATGCCGCGTGAGTGATGAAGGCCTTAGGGTTGTAAAGCTCTTTTGTCAGGGAAGATGATGACGGTACCTGAAGAATAAGCACCGGCTAACTTCGTGCCAGCAGCCGCGGTAATACGAAGGGTGCTAGCGTTGTTCGGAATCACTGGGCGTAAAGCGCGCGTAGGCGGCTATTCAAGTCAGGGGTGAAATCCCACGGCTCAACCGTGGAATTGCCCTTGAAACTGGGTAGCTGGAGTATTGGAGGGGATAGCGGAATTGCTAATGTAGAGGTGAAATTCGTAGATATTAGCAGGAACACCGGTGGCGAAGGCGGCTATCTGGACAATTACTGACGCTGAAGTGCGAAAGCGTGGGGATCAAACAGG` |
| ASV3954 | 408 | `TGAGGAATCTTCCGCAATGGGCGAAAGCCTGACGGAGCGACACCGCGTGAAGGATGAAGGGATTTTTTCCCGTAAACTTCTGTTCTGAGGGACGAAATTTTTGACGGTACCTCAGGAGAAAGCATCGGCTAACCCTGTGCCAGCAGCCGCGGTAAGACAGGGGATGCAAGCGTTACTCGGAATTACTGGGCGTAAAGCGTCTGCAGGTGTCTTGCCACGTTTGGTGTCAAATGATGCGGCTCAACCGCGTCAACGTGCCGAAAACGAGCGAGATCGAGTCATTCAGAGGTACCTGGAATGTCGTGTGTAGGGGTAAAATCCGTAGATCCACGATGGAACGCCAAAAGCGAAGGCAGGGTACTGGGAATGCACTGACACTCAGAGACGAAAGCGTGGGGAGCAAAGGGG` |
| ASV315 | 429 | `TCGAGAATAATTCACAATGGGGGAAACCCTGATGGTGCAACGCCGCGTGGAGGATGAAGGTCTTCGGATTGTAAACTCCTGTCATCGGGGAGTAAGACCTGGGCGTGAATAGCGCACAGGGTTGATAGTACCCGAAGAGGAAGGGACGGCTAACTTCGTGCCAGCAGCCGCGGTAATACGAAGGTCCCAAGCGTTGTTCGGAATCACTGGGCGTAAAGGGAGCGTAGGCGGCGTGGTAAGTCAGATGTGAAATCCCGGGGCTCAACCCCGGAACTGCATCCGATACTGCCGTGCTTGAGGGTTGGAGAGGTAGCTGGAATTATCGGTGTAGCAGTGAAATGCGTGGATATCGATAGGAACACTCGTGGCGAAGGCGAGCTACTGGACAACATCTGACGCTGAGGCTCGAAGGCTAGGGGAGCAAAAGGG` |
| ASV4156 | 428 | `TCGAGAATCTTTCACAATGGGGGCAACCCTGATGGAGCGACGCCGCGTGGAGGATAAGGTCTTCGGATTGTAAACTCCTGTCATGCGAGAACAAGGTTGCAAGGTTAACACCTTTGCAAATTGATGGTACCGCAAGAGGAAGAGACGGCTAACTCTGTGCCAGCAGCCGCGGTAATACAGAGGTCTCAAGCGTTGTTCGGAATCACTGGGCGTAAAGGGTGCGTAGGTGGCGTGGTAAGTCAGATGTGAAAGCCCGGGGCTCAACCTCGGAATTGCATCCGATACTACCACGCTAGAGTACTGAAGAGGTGACTAGAATTCTAGGTGTAGCAGTGAAATGCGTAGATATCTAGAGGAATACCAACGGCGAAGGCAGGTCACTGGGCAGTTACTGACACTGAGGCACGAAGGCCAGGGGAGCAAACGGG` |
| ASV1870 | 404 | `TGGGGAATATTGGACAATGGGGGAAACCCTGATCCAGCCATGCCGCGTGTGTGATGAAGGCCTTAGGGTTGTAAAGCACTTTTGTCCGGGAAGATAATGACTGTACCGGAAGAATAAGCCCCGGCTAACTTCGTGCCAGCAGCCGCGGTAATACGAAGGGGGCTAGCGTTGTTCGGAATCACTGGGCGTAAAGCGCACGTAGGCGGATTATTAAGTCGGAGGTGAAATCCCAGGGCTCAACCTTGGAACTGCCTTCGATACTGGTAATCTCGAGTTTGGAAGAGGTTGGTGGAACTGCGAGTGTAGAGGTGAAATTCGTAGATATTCGCAAGAACACCGGTGGCGAAGGCGGCCAACTGGTCCAATACTGACGCTGAGGTGCGAAAGCGTGGGGAGCAAACAGG` |
| ASV2613 | 429 | `TGGGGAATATTGGACAATGGGGGCAACCCTGATCCAGCCATGCCGCGTGTGTGAAGAAGGCCTTCGGGTTGTAAAGCACTTTAAGTGGGGAGAAAGGCTCGGCAGTTAACACCTGTCGGGATTGATGCTACCCACAGAATAAGCACCGGCTAACTCTGTGCCAGCAGCCGCGGTAATACAGAGGGTGCAAGCGTTAATCGGAATTACTGGGCGTAAAGCGCGCGTAGGCGGCTGATTAAGTCGGATGTGAAATCCCCGGGCTCAACCTGGGAACTGCATTCGATACTGATCGGCTAGAGTATGAGAGAGGGAGGTAGAATTCCACGTGTAGCGGTGAAATGCGTAGATATGTGGAGGAATACCGGTGGCGAAGGCGGCCTCCTGGCTTAATACTGACGCTGAGGTGCGAAAGCGTGGGGAGCAAACAGG` |
| ASV5244 | 430 | `TAGGGAATATTGCGCAATGGGGGAAACCCTGACGCAGCAACGCCGCGTGAGTGAGGAAGGTCTTCGGATTGTAAAACTCTTTTCTTAGAGAAAAATGTCCCCATAGCTAATATCTATGGGGAGTGATGGTATCTAAGGAATAAGCACCGGCTAACTTCGTGCCAGCAGCCGCGGTAATACGAAGGGTGCAAGCGTTGTTCGGATTTATTGGGCGTAAAGCGCGCGCAGGCGGGTCGGCAAGTCAGATGTGAAATCTCGGAGCTCAACTCCGAAATTGCGTCTGAAACTACCAGTCTAGAATGTCGGAGGGGGCAGGGGAATTTCACGTGTAGGGGTAAAATCCGTAGAGATGTGAAGGAACACCGGAGGCGAAGGCGCCTGCCTGGACGACTATTGACGCTGAGGCGCGAAAGCGTGGGGAGCAAACAGG` |
| ASV1162 | 429 | `TGGGGAATTTTGGACAATGGGCGCAAGCCTGATCCAGCAATGCCGCGTGCAGGAAGAAGGCCTTCGGGTTGTAAACTGCTTTTGTCAGGGAAGAAATCTTCTGGGTTAATACCTCGGGAGGATGACGGTACCTGAAGAATAAGCACCGGCTAACTACGTGCCAGCAGCCGCGGTAATACGTAGGGTGCGAGCGTTAATCGGAATTACTGGGCGTAAAGCGTGCGCAGGCGGCTTTGCAAGACAGAGGTGAAATCCCCGGGCTCAACCTGGGAACTGCCTTTGTGACTGCAAGGCTAGAGTACGGCAGAGGGGGATGGAATTCCGCGTGTAGCAGTGAAATGCGTAGATATGCGGAGGAACACCAATGGCGAAGGCAATCCCCTGGGCCTGTACTGACGCTCATGCACGAAAGCGTGGGGAGCAAACAGG` |
| ASV5534 | 407 | `TTAGGAATTTTGCGCAATGGGCGAAAGCCTGACGCAGCAACGCCGCGTGAGGGATGAAGGTCTTCGGATCGTAAACCTCTTTTAGCAGGGAAGAAACAAATGACGGTACCTGCAGAAAAAGCACCGGCTAACTCTGTGCCAGCAGCCGCGGTAATACAGAGGGTGCAAGCGTTATTCGGAATTACTGGGCGTAAAGAGCGCGTAGGCTGTTTGATAAGTTTGATGTGAAAGCCCTGGACTCAATTCAGGAAGTGCATCGAATACTGTCAGACTAGAGTATAGGAGGGGAGAACGGAATTCCTGGTGTAGAGGTGAAATTCGTAGATATCAGGAGGAACAACAGTGGCGAAGGCGGTTCTCTGGACTATAACTGACGCTGAGGCGCGAAAGTGTGGGGAGCAAACAGG` |
| ASV3510 | 409 | `TGGGGAATATTGCACAATGGGCGCAAGCCTGATGCAGCGACGCCGCGTGAGGGATGACGGCCTTCGGGTTGTAAACCTCTTTCAGTACAGACGAAGCGCAAGTGACGGTATGTACAGAAGAAGCACCGGCCAACTACGTGCCAGCAGCCGCGGTAATACGTAGGGTGCGAGCGTTGTCCGGAATTACTGGGCGTAAAGAGCTCGTAGGTGGTTTGTCGCGTTGTTCGTGAAAACCTGAGGCTTAACCTCGGGCGTGCGGGCGATACGGGCAGACTGGAGTACTGCAGGGGAGACTGGAATTCCTGGTGTAGCGGTGGAATGCGCAGATATCAGGAGGAACACCGGTGGCGAAGGCGGGTCTCTGGGCAGTAACTGACGCTGAGGAGCGAAAGCGTGGGGAGCGAACAGG` |
| ASV2894 | 429 | `TGGGGAATCTTGGACAATGGGCGAAAGCCTGATCCAGCAATGCCGCGTGCGTGAAGAAGGCCTTCGGGTTGTAAAGCGCTTTTGTCGGGGAGGAAATCCTAACTTCTAATAAAGGTTGGGGATGACAGTACCCGAAGAATAAGGACCGGCTAACTACGTGCCAGCAGCCGCGGTAATACGTAGGGTCCAAGCGTTAATCGGAATTACTGGGCGTAAAGCGTCCGCAGGTGGCTTGATAAGATAGACGTGAAATCCCTGGGCTCAACCTAGGAATTGCGTTTATGACTGTCTCGCTAGAGTATGGGAGAGGGGGGTGGAATTCCACGTGTAGCAGTGAAATGCGTAGAGATGTGGAGGAACACCGATGGCGAAGGCAACCCCCTGGCCTAATACTGACACTCATGGACGAAAGCGTGGGGAGCAAACAGG` |
| ASV5520 | 428 | `TAGGGAATCTTCCGCAATGGGCGAAAGCCTGACGGAGCAATGCCGCGTGAGTGATGAAGGTTTTCGGATCGTAAAGCTCTGTTGCCAGGGAAGAACGCTTGGGAGAGTAACTGCTCTCAAGGTGACGGTACCTGAGAAGAAAGCCCCGGCTAACTACGTGCCAGCAGCCGCGGTAATACGTAGGGGGCAAGCGTTGTCCGGAATTATTGGGCGTAAAGCGCGCGCAGGCGGTCATTTAAGTCTGGTGTTTAATCCCGGGGCTCAACCCCGGATCGCACTGGAAACTGGGTGACTTGAGTGCAGAAGAGGAGAGTGGAATTCCACGTGTAGCGGTGAAATGCGTAGATATGTGGAGGAACACCAGTGGCGAAGGCGACTCTCTGGGCTGTAACTGACGCTGAGGCGCGAAAGCGTGGGGAGCAAACAGG` |
| ASV4176 | 429 | `TGGGGAATATTGGACAATGGGGGGAACCCTGATCCAGCAATGCCGCGTGTGTGAAGAAGGCCTGAGGGTTGTAAAGCACTTTCAGTAGGGAGGAGGGTTTAGAGGTTAAGAGCTACTAGACTGGACGTTACCTACAGAAGAAGCACCGGCTAACTCCGTGCCAGCAGCCGCGGTAATACGGAGGGTGCGAGCGTTAATCGGAATTACTGGGCGTAAAGGGTGCGTAGGTGGTTAGCTAAGTTATCTGTAAAATCCCTGGGCTTAACCTGGGCAGGTCAGATGATACTGGTTGACTCGAGTATGGGAGAGGGTAGTGGAATTTCCGGTGTAGCGGTGAAATGCGTAGAGATCGGAAGGAACACCAGTGGCGAAGGCGGCTACCTGGCCTAATACTGACACTGAGGCACGAAAGCGTGGGGAGCAAACAGG` |
| ASV2562 | 424 | `TAAGGAATATTGGACAATGCCCGCAAGGGTGATCCAGCCATGCCGCGTGCAGGAAGACGGCCCTATGGGTTGTAAACTGCTTTTACACCGGAGAAAACCTCCCCTCGTGAGGGGAGCTGATAGTATGGTGAGAATAAGCATCGGCTAACTTCGTGCCAGCAGCCGCGGTAAGACGAAGGATGCAAGCGTTATCCGGATTCATTGGGTTTAAAGGGAGCGTAGGTGGACTGATAAGTCAGTGGTGAAATCTCCGAGCTTAACTCGGAAATTGCCATTGATACTGTTGGTCTTGAGTATAGTTGCCGTTGGCGGAATATGACATGTAGTGGTGAAATACATAGAGATGTCATAGAACACCGATTGCGAAGGCAGCTGACGAAACTATAACTGACACTGAGGCTCGAAAGTGTGGGGATCAAACAGG` |
| ASV4618 | 427 | `TGGGGAATCTTCCGCAATGGGCGAAAGCCTGACGGAGCAACGCCGCGTGAATGAAGAAGGTCTTCGGATCGTAAAATTCTGTCTTTAGGGACGAATAAGGTAGGCGAATAGTCTACTGTATGACGGTACCTGAGGAGGAAGCTCCGGCTAACTACGTGCCAGCAGCCGCGGTAATACGTAGGGAGCGAGCGTTGTCCGGATTTACTGGGCGTAAAGGGCGCGTAGGCGGGTGTTTAAGTCAGATGTGAAATCTCCGGGCTCAACTCGGAGCCTGCATTTGAAACTGGATACCTTGAGGGCAGGAGAGGAAAGTGGAATTCCTAGTGTAGCGGTGAAATGCGTAGATATTAGGAGGAACACCAGTGGCGAAGGCGACTTTCTGGACTGTACCTGACGCTGAGGCGCGAAAGCGTGGGGAGCGAACGGG` |
| ASV3870 | 425 | `TAAGGAATATTGGACAATGGGCGGAAGCCTGATCCAGCCATGCCGCGTGAAGGACGAAGGCGCTCAGCGTTGTAAACTTCTTTTGGGTGGGAACAATAACCGAGACTTGTCTTGGCTTGAGGGTACCACCAGAATAAGCACCGGCTAACTCCGTGCCAGCAGCCGCGGTAATACGGAGGGTGCAAGCGTTATCCGGAATCACTGGGTTTAAAGGGTGCGTAGGCGGTCTAATAAGTCAGTCGTGAAATCGCTGGGCTCAACCCATGCAATTGCGATTGATACTGCTAGACTTGAATGGGGTTGAGGCTGGCGGAATGCGACATGTAGCGGTGAAATGCTTAGATATGTCGCAGAACACCGATTGCGAAGGCAGCCAGCTGGGCCTTTATTGACGCTGAGGCACGAAAGCGTGGGGAGCAAACAGG` |
| ASV64 | 429 | `TGGGGAATATTGGACAATGGGCGCAAGCCTGATCCAGCCATGCCGCGTGTGTGAAGAAGGCCTTCGGGTTGTAAAGCACTTTTGTCCGGAAAGAAATCCTTCAGGTTAATACTCTGGAGGGATGACGGTACCGGAAGAATAAGCACCGGCTAACTTCGTGCCAGCAGCCGCGGTAATACGAAGGGTGCAAGCGTTACTCGGAATTACTGGGCGTAAAGCGTGCGTAGGCGGTTCGTTAAGTCTGACGTGAAATCCCCGGGCTCAACCTGGGACGTGCGTTGGAAACTGGCGATCTAGAGTACGGTAGAGGGTGGTGGAATTCCCGGTGTAGCAGTGAAATGCGTAGAGATCGGGAGGAACACTCGTGGCGAAGGCGGCCACCTGGACCAGTACTGACGCTGAGGCACGAAAGCGTGGGGAGCAAACAGG` |
| ASV4726 | 429 | `TGGGGAATTTTGGACAATGGGCGCAAGCCTGATCCAGCCATGCCGCGTGAGTGAAGAAGGCCTTCGGGTTGTAAAGCTCTTTTGTTTGGGAGCAAAAGCTTTGGGCTAATACCCTGAGGCATTGAGAGTACCAAAAGAATAAGCACCGGCTAACTACGTGCCAGCAGCCGCGGTAATACGTAGGGTGCGAGCGTTAATCGGAATTACTGGGCGTAAAGCGTGCGCAGGCGGATTTTTAAGTCAGACGTGAAATCCCCGAGCTTAACTTGGGAACTGCGTTTGAAACTGGGGATCTAGAGTACGTCAGAGGGGGGTGGAATTCCACGTGTAGCAGTGAAATGCGTAGAGATGTGGAGGAACACCGATGGCGAAGGCAGCCCCCTGGGATGATACTGACGCTCATGCACGAAAGCGTGGGGAGCAAACAGG` |
| ASV2544 | 429 | `TCGAGAATCTTTCACAATGGGGGAAACCCTGATGGAGCGACGCCGCGTGGAGGATGAAGGTCTTCGGATTGTAAACTCCTGTCATGAAAGAACAAGTGATGCGGGTAAACAGCCCGCAAAGTTGATAGTATTTCAAGAGGAAGGGACGGCTAACTTCGTGCCAGCAGCCGCGGTAATACGAAGGTCCCAAGCGTTGTTCGGAATCACTGGGCGTAAAGAGTGCGTAGGCGGCGCGGAAAGTCAGATGTGAAATCCCGGGGCTCAACCTCGGAACGGCATCCGATACTCCCGCGCTTGAGGACTGGAGGGGAGTCTGGAATTCACGGTGTAGCAGTGAAATGCGTAGATATCGTGAGGAACACTAGTGGCGAAGGCGAGACTCTGGACAGTTCCTGACGCTGAGGCACGAAGGCCAGGGGAGCGAAGGGG` |
| ASV2639 | 429 | `TGGGGAATTTTGGACAATGGGCGCAAGCCTGATCCAGCCATTCCGCGTGCAGGACGAAGGCCTTCGGGTTGTAAACTGCTTTTGTACGGAACGAAAAAGCCTTGGCTAATACCCAGGGTCCATGACGGTACCGTAAGAATAAGCACCGGCTAACTACGTGCCAGCAGCCGCGGTAATACGTAGGGTGCAAGCGTTAATCGGAATTACTGGGCGTAAAGCGTGCGCAGGCGGTTATATAAGACAAATGTGAAATCCCCGGGCTCAACCTGGGAACTGCATATGTGACTGTATAGCTAGAGTGCGGCAGAGGGGGATGGAATTCCGCGTGTAGCAGTGAAATGCGTAGATATGCGGAGGAACACCGATGGCGAAGGCAATCCCCTGGGCCTGCACTGACGCTCATGCACGAAAGCGTGGGGAGCAAACAGG` |
| ASV2305 | 429 | `TGGGGAATTTTGGACAATGGGGGCAACCCTGATCCAGCAATGCCGCGTGTGTGAAGAAGGCCTGCGGGTTGTAAAGCACTTTAGGTGGGGAGGAAAGCTACTGTTTTAATAAAGCAGTAGGGTGACGTTACCCACAGAATAAGCACCGGCTAACTCTGTGCCAGCAGCCGCGGTAATACAGAGGGTGCAAGCGTTAATCGGAATTACTGGGCGTAAAGCGCACGTAGGTGGTTTAGTAAGTTTGATGTGAAAGCCCTGGGCTTAACCTGGGAATTGCGTTGAAAACTGCTGAACTTGAGTCGGATAGAGGGCAGTGGAATTTCCGGTGTAGCGGTGAAATGCGTAGAGATCGGAAAGAACATCGGTGGCGAAGGCGGCTGCCTGGGTCCAGACTGACACTGAGGTGCGAAAGCGTGGGGAGCAAACAGG` |
| ASV4674 | 428 | `TGGGGAATCTTGCGCAATGGGCGAAAGCCTGACGCAGCGACGCCGCGTGGGTGATGAAGGCCTTCGGGTTGTAAAGCTCTGTGGGGAGAGACGAATAAGTCTTGGCTAATATCCACGATGATGACGGTATCTCCTTAGCAAGCACCGGCTAACTCTGTGCCAGCAGCCGCGGTAAGACAGAGGGTGCAAACGTTGTTCGGAATTACTGGGCGTAAAGCGCATGTAGGTGGTCGAACAGGTCGGATGTGAAAGCCCTCAGCTCAACTGAGGAAGTGCATTCGAAACCGCTCGACTCGAGTCCCGGAGAGGAAGGTAGAATTCTCGGTGTAGAGGTGAAATTCGTAGATATCGAGAGGAACACCGGTGGCGAAGGCGGCCTTCTGGACGAGGACTGACACTGAGATGCGAAAGCATGGGGAGCAAACAGG` |
| ASV911 | 404 | `TGGGGAATCTTGGACAATGGGGGCAACCCTGATCCAGCGATGCCGCGTGAATGAAGAAGGCCTTAGGGTTGTAAAGTTCTTTCACCCGTGACGATAATGACGGTAGCGGGAGAAGAAGCCCCGGCTAACTCCGTGCCAGCAGCCGCGGTAAGACGGAGGGGGCTAGCGTTGTTCGAAATGACTGGGCGTAAAGGGCGTGTAGGCTGTTGATCAAGTCAAGCGTGAAAGCCTCGGGCTTAACCCGAGAACTGCGTTTGATACTGGTTGACTTGAGGACGAGAGAGGAAAGTGGAATTCCGAGTGTAGAGGTGAAATTCGTAGATATTCGGAGGAACATCAGAGGCGAAGGCGGCTTTCTGGCTCGTATCTGACGCTGAGGCGCGAAAGCGTGGGGAGCAAACAGG` |
| ASV3428 | 404 | `GTGGGAATATTGGACAATGGGCGAAAGCCTGATCCAGCAATACCGCGTGAACGATGAAGGCCTTCGGGTTGTAAAGTTCTTTTAATAGGGAAGATAATGACGGTACCTATAGAATAAGCACCGGCAAACTTCGTGCCAGCAGCCGCGGTAATACGAAGGGTGCTAGCGTTGTTCGGAATTATTGGGCGTAAAGGGTGTGTAGGTGGTTTCGTAAGTTAGTTGTGAAATCCCGGGGCTCAACCTCGGAATTGCGGTTAAAACTACGGAACTAGAGTATGATAAGCGATGGCAGAATTTCTAGTGTAGGGGTAAAATCCGCAGATATTAGAAGGAATACCGGTGGTGTAGACGGCTATCTTGGTCATTACTGACACTAAGACACGAAAGTGTGGGGAGCAAACAGG` |
| ASV2143 | 408 | `TAGGGAATATTGCACAATGGAGGAAACTCTGATGCAGCGACGCCGCGTGAGTGATGAAGGCCTTCGGGTTGTAAAGCTCTGTTCTCAGGGAAAAAGACAAGTGATTGTACCTGAGGAGAAAGGACCGGCTAACTTCGTGCCAGCAGCCGCGGTAAGACGAGGGGTCCAAGCGTTGTTCGGAATTATTGGGCGTAAAGCGGGTGTAGGTGGATCTATAAGTCAGGAGTGAAAGCCCCGAGCTTAACTTGGGAAGTGCTTTTGATACTGTAGATCTTGAATGTGGGAGAGGATCGTGGAATTCCAGGTGTAGTGGTGAAATACGTAGATATCTGGAGGAACACCGGTGGCGAAGGCGGCGATCTGGCCCAACATTGACACTGAGACCCGAAAGTGCGGGGATCAAACAGG` |
| ASV3266 | 423 | `TGAGGAATATTGGTCAATGGGCGGGAGCCTGAACCAGCCAAGCCGCGTGGAGGAAGAAGGTACAGAGTATCGTAAACTCCTTTTGTCAGGGGATAAAAGATCCCACGCGTGGGATTCTGAAGGTACCTGAAGAAAAAGCATCGGCTAACTCCGTGCCAGCAGCCGCGGTAATACGGAGGATGCGAGCGTTATCCGGATTTATTGGGTTTAAAGGGTGCGTAGGCCGTTCCTTAAGTCAGCGGTGAAAGCCCGGGGCTCAACCCCGGCGAGCCGTTGAAACTGGGGAGCTGGAGAGGAAGAAAGGTATGCGGAATTCGCGGTGTAGCGGTGAAATGCTTAGATATCGCGAAGAACTCCGATTGCGAAGGCAGCATACCGGCTTCTGTCTGACGCTGAGGCACGAAAGCGTGGGGATCGAACAGG` |
| ASV1977 | 425 | `TAGGGAATATTGGGCAATGGAGGCAACTCTGACCCAGCCATGCCGCGTGCAGGAAGACGGTCCTCTGGATTGTAAACTGCTTTTGAATGGGAAGAATGTGATGCTTGCGAGCGTTAATGACGGTACCATTAGAATAAGCCACGGCTAACTACGTGCCAGCAGCCGCGGTAATACGTAGGTGGCGAGCGTTATCCGGATTTATTGGGTTTAAAGGGTGCGTAGGCGGCTTAATAAGTCGGTGGTTAAAAGTAGCAGCTTAACTGTTTTACATGCCATCGATACTGTTGAGCTTGAGATATTTGGAGGCAGCTAGAATTTCCGGTGTAGCGGTGAAATGCATAGATACCGGAAGGAATACCAATTGCGAAGGCAGGTTGCTACGAATAAACTGACGCTGATGCACGAAAGCGTGGGGATCAAACAGG` |
| ASV1213 | 404 | `TGGGGAATATTGGACAATGGGGGAAACCCTGATCCAGCAATGCCGCGTGAGTGATGAAGGCCTTCGGGTTGTAAAGCTCTTTTGTCAGGGACGATGATGACGGTACCTGACGAATAAGCTCCGGCTAACTTCGTGCCAGCAGCCGCGGTAATACGAGGGGAGCTAGCGTTGTTCGGAATTACTGGGCGTAAAGGGCGTGTAGGCGGTACCATAAGTCAGATGTGAAAGCCCTAGGCTTAACCTAGGAGGTGCATTTGATACTGTGGAACTAGAGATCGAGAGAGGAAAGTGGAATTACGAGTGTAGAGGTGAAATTCGTAGATATTGGTAAGAACACCAGTGGCGAAGGCGACTTTCTGGCTCGATACTGACGCTAAGGCGCGAAAGCGTGGGGAGCAAACAGG` |
| ASV629 | 404 | `TGGGGAATCTTAGACAATGGGGGGAACCCTGATCTAGCCATGCCGCGTGATCGATGAAGGCCTTAGGGTTGTAAAGATCTTTCAGATGGGAAGATAATGACGGTACCATCAGAAGAAGCCCCGGCTAACTCCGTGCCAGCAGCCGCGGTAATACGGAGGGGGCTAGCGTTATTCGGAATTACTGGGCGTAAAGCGCACGTAGGCGGATTGGAAAGTCAGAGGTGAAATCCCAGGGCTCAACCTTGGAACTGCCTTTGAAACTCCCAGTCTTGAGGTCGTGAGAGGTGAGTGGAATTCCGAGTGTAGAGGTGAAATTCGTAGATATTCGGAGGAACACCAGTGGCGAAGGCGGCTCACTGGCACGATACTGACGCTGAGGTGCGAAAGCGTGGGGAGCAAACAGG` |
| ASV1022 | 424 | `TGAGGAATATTGGTCAATGGGCGAGAGCCTGAACCAGCCAAGTAGCGTGAAGGACGAAGGCCCTATGGGTCGTAAACTTCTTTTATAGGGGAATAAAGTGGGGAACGTGTTCCCCTTTGTATGTACCCTACGAATAAGGATCGGCTAACTCCGTGCCAGCAGCCGCGGTAATACGGAGGATCCGAGCGTTATCCGGATTTATTGGGTTTAAAGGGAGCGTAGGTGGATATATAAGTCAGTTGTGAAAGTTTACGGCTCAACCGTGAAATTGCAGTTGATACTGTGTATCTTGAGTGTACAAGAGGTGGGCGGAATTCGTGGTGTAGCGGTGAAATGCTTAGATATCACGAAGAACTCCGATTGCGAAGGCAGCTCACTGGGGTACAACTGACACTGAGGCTCGAAAGTGTGGGTATCAAACAGG` |
| ASV5353 | 381 | `CTTTTATGCAGGACAGAGGAAGCGCTCCATGCACACTGGCTCCCGGAGGCGGGCCCTGCATCCACCGGCCTCCAGCATGCATACGCGCTGTGAGCGCACGGTGCATGGCTGCCAGTGCTTGCTCTGCCCGGTACGCAGATGCATGCAGCGCTCTGAGTGCGGGGCGCCATCTTGCTGGCAGTCCTGGACCGCCGCTGCTGTCTGGGAGAGTGGTGGTCCACCAGATGAGCTCCTGTGCCGCCATGGCACAAGCGTCGGCAGCGCCAGCACAGGCAGCCCAACGAATGGGGTGGGCCAGTCTTGTGGCGGAAGCTGCCTGTGAAAACCCCCGGACACCACCTCCTCGCAGGGCTCGGGTAAGGGCCTGACACCAATTTATCC` |
| ASV1314 | 421 | `TGGGGAATATTGCGCAATGGACGAAAGTCTGACGCAGCGACGCCGCGTGTAGGATGACGCCTTTCGGGGTGTAAACTACTGTTGCCCGGGACGAAACTCCTTTTTCGAGAGGACTGACGGTACCGGGTGAGGAAGCACCGGCTAACTCTGTGCCAGCAGCCGCGGTAATACAGAGGGTGCGAGCGTTGTCCGGAATCACTGGGCGTAAAGGGTGCGTAGGTGGCTTGGTAAGCGTGTGGTGAAAGTCCGGGGCTCAACCCCGGATCTGCCGTGCGAACTGCCGAGCTCGAGCATTGTAGAGGCAGGTGGAATTTCGGGTGTAGCGGTGGAATGCGTAGAGATCCGAAAGAACACCAGTGGCGAAGGCGGCCTGCTGGGCAATTGCTGACACTGAGGCACGACAGCGTGGGGAGCAAACAGG` |
| ASV68 | 404 | `TGGGGAATATTGGACAATGGGCGAAAGCCTGATCCAGCAATGCCGCGTGAGTGATGAAGGCCTTAGGGTTGTAAAGCTCTTTTACCCGAGATGATAATGACAGTATCGGGAGAATAAGCTCCGGCTAACTCCGTGCCAGCAGCCGCGGTAATACGGAGGGAGCTAGCGTTGTTCGGAATTACTGGGCGTAAAGCGCACGTAGGCGGCTTTTTAAGTCAGGGGTGAAATCCCGGGGCTCAACCCCGGAACTGCCCTTGAAACTGGGAAGCTAGAATCTTGGAGAGGCGAGTGGAATTCCGAGTGTAGAGGTGAAATTCGTAGATATTCGGAAGAACACCAGTGGCGAAGGCGACTCGCTGGACAAGTATTGACGCTGAGGTGCGAAAGCGTGGGGAGCAAACAGG` |
| ASV4388 | 429 | `TGAGGAATATTGCACAATGGGCGAAAGCCTGATGCAGCGACGCCGCGTGGAGGATGACGGCCTTAGGGTTGTAAACTCCTGTTAAGTGGGATAAAATCCTTGCCTCTAACACAGGTAAGGGATGATAGTACCATTAGAGAAAGCACCGGCTAACTTCGTGCCAGCAGCCGCGGTAATACGAGGGGTGCAAGCGTTATTCGGATTTATTGGGCGTAAAGGGTGCGTAGGTGGTATGCTAAGTCTGTTGTTAAATTTTCCGGCCTAACCGGAAGCCTGCAATGGAAACTGGTGTACTTGAGGATGGAAGAGGGAAGTGGAATTCTCGGAGTAGCGGTAAAATGCGTGGATCTCGAGAGGAACACCGATGGCGAAGGCAGCTTCCTGGTCCATATCTGACACTGAGGCACGAAAGCGTGGGGAGCAAACAGG` |
| ASV3578 | 407 | `TAGGGAATATTGCACAATGGGGAAAACCCTGATGCAGCGACGCCGCGTGAGTGATGAAGGCCTTCGGGTCGTAAAGCTCTGTCGTATGGGAATAACACAATGAATGTACCATGCAAGAAAGGATCGGCTAACTTCGTGCCAGCAGCCGCGGTAAGACGAGGGATCCTAGCGTTGTTCGGAATTATTGGGCGTAAAGCGGGTGTAGGTGGGCTTATAAGTCAGGTGTGAAATCCCAGGGCTCAACCCTGGAAGTGCATTTGATACTGTAAGCCTTGAATGTGGTAGAGGTTACTAGAATTCCTGGTGTAGTGGTGAAATACGTAGATATCAGGAGGAATACCAGAGGCGAAGGCGGGTAACTGGACCAACATTGACACTGAGACCCGAAAGCGTGGGGATCAAACAGG` |
| ASV4927 | 406 | `TAGGGAATCTTGCACAATGGGGGAAACCCTGATGCAGCGACGCCGCGTGAGTGACGACGGACTTCGGTCTGTAAAGCTCTGTTGCAGGGGAATAAGTACTGAAGGTACCCTGCGAGGAAGGGTCGGCTAACTTCGTGCCAGCAGCCGCGGTAAGACGAGGGACCCTAGCGTTGTTCGGAATCATTGGGCGTAAAGCGGGTGTAGGCGACTATTTAAGTCGAATGTGAAAGCCTTGGGCTCAACCCAAGAAGTGCGTTCGATACTGTTTAGTTTGAGTGTTGGAGAGGCTACCGGAATTCATGGTGTAGTGGTGAAATACGTAGATATCATGAGGAACATCGGAGGCGAAGGCGGGTAGCTGGCCAAACACTGACGCTGAGACCCGAAAGCGTGGGGATCAAACAGG` |
| ASV3769 | 404 | `TGGGGAATATTGGACAATGGGGGAAACCCTGATCCAGCAATGCCGCGTGAGTGATGAAGGCCTTCGGGTTGTAAAGCTCTTTTGTTAGGGACGATGATGACGGTACCTAACGAATAAGCTCCGGCTAACTTCGTGCCAGCAGCCGCGGTAATACGAAGGGAGCTAGCGTTGTTCGGAATTACTGGGCGTAAAGGGCGCGTAGGCGGCTTGATAAGTTTGATGTGAAAGCCCTGGGCTTAACCTAGGAATAGCATTGAATACTGTCAGGCTAGAGGCCGAGAGAGGGAAATGGAATTGCGAGTGTAGAGGTGAAATTCGTAGATATTCGCAAGAACACCAGTGGCGAAGGCGATTTCCTGGCTCGGTACTGACGCTAAGGCGCGAAAGCGTGGGGAGCAAACAGG` |
| ASV4704 | 407 | `TAGGGAATATTGCACAATGGAGGAAACTCTGATGCAGCGACGCCGCGTGAGTGATGAAGGCCTTCGGGTCGTAAAGCTCTGTCGTATGGGAATAAAAAAATGAATGTACCATGCAAGAAAGGATCGGCTAACTTCGTGCCAGCAGCCGCGGTAAGACGAGGGATCCTAGCGTTGTTCGGAATCATTGGGCGTAAAGCGGATGCAGGTGGCTCCATAAGTCAAGTGTGAAAGCCCAGGGCTTAACCCTGGAAGTGCATTTGAAACTGCGGAGCTTGAGTGCCGGAGAGGTCGCTAGAATTCCTGGTGTAGTGGTGAAATACGTAGATATCAGGAGGAACATCGGAGGCGAAGGCGGGTGACCAGCCGGACACTGACACTCATATCCGAAAGTGTGGGGATCAAACAGG` |
| ASV2304 | 422 | `TAAGGAATATTGGTCAATGGTCGGAAGACTGAACCAGCCATGCCGCGTGCAGGAAGAAGGGCTTATGCCTGTAAACTGCTTTTAACAGGGAATAAAACACTCCTGCGGGAGGAACTGAAGGTACCTGTTGAATAAGCATCGGCTAACTACGTGCCAGCAGCCGCGGTAATACGTAGGATGCAAGCGTTGTCCGGATTTATTGGGTTTAAAGGGTGCGTAGGTGGCCCGGTAAGTCAGAGGTGAAAGTCGATTGCTCAACGATCGAACTGCCTTTGATACTGCCGGACTTGAATCAGATTGAGGTTGGCGGAATTGGTGGTGTAGCGGTGAAATGCATAGATACCATCAGGAACTCCGATTGCGAAGGCAGCCAACTAAGTCTGTATTGACACTGAGGCACGAAAGCGTGGGGATCAAACAGG` |
| ASV499 | 429 | `TGGGGAATTTTGGACAATGGGCGAAAGCCTGATCCAGCAATGCCGCGTGCAGGACGAAGGCCTTCGGGTTGTAAACTGCTTTTGTACGGAGCGAAAAGGCTTCCACTAATACTGGGGGCTCATGACGGTACCGTAAGAATAAGCACCGGCTAACTACGTGCCAGCAGCCGCGGTAATACGTAGGGTGCAAGCGTTAATCGGAATTACTGGGCGTAAAGCGTCCGCAGGCGGTTCTATAAGACAGATGTGAAATCCCCGGGCTCAACCTGGGAACTGCATTTGTGACTGTAGAGCTAGAGTACGGCAGAGGGGGATGGAATTCCGCGTGTAGCAGTGAAATGCGTAGATATGCGGAGGAACACCGATGGCGAAGGCAATCCCCTGGGCCTGTACTGACGCTCATGGACGAAAGCGTGGGGAGCAAACAGG` |
| ASV2099 | 424 | `TAAGGAATATTGGTCAATGGAGGCAACTCTGAACCAGCCATGCCGCGTGCAGGAAGACTGCCTTATGGGTTGTAAACTGCTTTTATACGGGAATAAACCTTGATTCGTGAATCAAGCTGAATGTACCGTAAGAATAAGGATCGGCTAACTCCGTGCCAGCAGCCGCGGTAATACGGAGGATCCAAGCGTTATCCGGATTTATTGGGTTTAAAGGGAGCGTAGGCGGCCTATTAAGTCAGGGGTGAAAGACGGCAGCTTAACTGTCGCAGTGCCTTTGATACTGATAGGCTTGAATGTAGTTGAGGTAGGCGGAATGTGACAAGTAGCGGTGAAATGCATAGATATGTCACAGAACACCGATTGCGAAGGCAGCTTACTAAGCTATAATTGACGCTGAGGCTCGAAAGCGTGGGGATCAAACAGG` |
| ASV61 | 424 | `TGAGGAATATTGGACAATGGGCGCAAGCCTGATCCAGCCATGCCGCGTGCAGGATGACGGTCCTATGGATTGTAAACTGCTTTTGTACAGGAAGAAACACTCCCTCGTGAGGGAGCTTGACGGTACTGTAAGAATAAGGATCGGCTAACTCCGTGCCAGCAGCCGCGGTAATACGGAGGATCCAAGCGTTATCCGGAATCATTGGGTTTAAAGGGTCCGTAGGCGGTTTAGTAAGTCAGTGGTGAAAGCCCATCGCTCAACGGTGGAACGGCCATTGATACTGCTAGACTTGAATTATTAGGAAGTAACTAGAATATGTAGTGTAGCGGTGAAATGCTTAGAGATTACATGGAATACCAATTGCGAAGGCAGGTTACTACTAATGGATTGACGCTGATGGACGAAAGCGTGGGTAGCGAACAGG` |
| ASV262 | 429 | `TGGGGAATTTTGGACAATGGGGGCAACCCTGATCCAGCAATGCCGCGTGTGTGAAGAAGGCCTTCGGGTTGTAAAGCACTTTTGGCGGGAACGAAACGGTGGTCTCTAATACAGGCTGCTAATGACGGTACCCGCAGAATAAGCACCGGCTAACTACGTGCCAGCAGCCGCGGTAATACGTAGGGTGCAAGCGTTAATCGGAATTACTGGGCGTAAAGCGTGCGCAGGCGGTTTTGTAAGTCTGTCGTGAAATCCCCGGGCTCAACCTGGGAATGGCGATGGAGACTGCAAGGCTGGAATCTGGCAGAGGGGGGTAGAATTCCACGTGTAGCAGTGAAATGCGTAGAGATGTGGAGGAACACCGATGGCGAAGGCAGCCCCCTGGGCTAAGATTGACGCTCATGCACGAAAGCGTGGGGAGCAAACAGG` |
| ASV3457 | 430 | `TAGGGAATATTGCACAATGGGGGAAACCCTGATGCAGCAACGCCGCGTGAGTGAAGAAGGCCCTTGGGTTGTAAAGCTCTTTAGCGTGGGAAGATGGGAGGTATGGTGAATAATCATATCTTTTGACGGTACCACGAGAATAAGCACCGGCTAATTTCGTGCCAGCAGCCGCGGTAATACGAAAGGTGCAAGCGTTACTCGGAATCACTGGGCGTAAAGCGCACGTAGGCGGACTTACAGGTCAATTGTGAAATACCTGGGCTCAACCCGGGAACTGCAGTTGAAACCGTTTGTCTTGAATATCTGAGAGGGTGGGGGAATACCTGGTGGAGAAGTGAAATTCGTAGAGATCAGGTGGAACACCGGAGGCGAAGGCGCCTGCCTGGCAGTATATTGACGCTGAGGTGCGAAAGTGCGGGGAGCAAACAGG` |
| ASV5004 | 405 | `TAGGGAATTTTGCGCAATGGGCGAAAGCCTGACGCAGCAACGCCGCGTGAATGATGAAGCCCTTCGGGGTGTAAAGTTCTGTCAGTAGGGACGAATCTTGACGGTACCTACAGAGGAAGCACCGGCTAACTCCGTGCCAGCAGCCGCGGTAATACGGAGGGTGCAAGCGTTGTCCGGAATCATTGGGCGTAAAGAGTTCGTAGGCGGTATGTAAAGTCCGGTGTTAAATCCCGAAGCTCAACTTCGGCATGGCACTGGATACTTGCAAACTAGAATGCGGTAGAGGTAAAGGGAATTCCTGGTGTAGCGGTGAAATGCGTAGATATCAGGAGGAACATCGGTGGCGAAAGCGCTTTACCGGGCCGTTATTGACGCTGAGGAACGAAAGCCGGGGTAGCAAATGGG` |
| ASV2142 | 404 | `TGGGGAATATTGGACAATGGGCGAAAGCCTGATCCAGCAATGCCGCGTGAGTGATGAAGGCCTTAGGGTTGTAAAGCTCTTTTACCAGGGATGATAATGACAGTACCTGGAGAATAAGCTCCGGCTAACTCCGTGCCAGCAGCCGCGGTAATACGGAGGGAGCTAGCGTTGTTCGGAATTACTGGGCGTAAAGCGCACGTAGGCGGCGATTCAAGTCAGAGGTGAAAGCCCGGGGCTCAACCCCGGAACTGCCTTTGAAACTAGATTGCTAGAATCCTGGAGAGGTTAGTGGAATTCCGAGTGTAGAGGTGAAATTCGTAGATATTCGGAAGAACACCAGTGGCGAAGGCGACTAACTGGACAGGTATTGACGCTGAGGTGCGAAAGCGTGGGGAGCAAACAGG` |
| ASV3004 | 404 | `TGGGGAATATTGGACAATGGGCGCAAGCCTGATCCAGCCATGCCGCGTGTGTGATGAAGGCCTTAGGGTTGTAAAGCACTTTCACCGGAGAAGATAATGACGGTATCCGGAGAAGAAGCCCCGGCTAACTTCGTGCCAGCAGCCGCGGTAATACGAAGGGGGCTAGCGTTGTTCGGAATTACTGGGCGTAAAGCGCACGTAGGCGGATCGATCAGTCAGGGGTGAAATCCCGCAGCTCAACTGCGGAACTGCCTTTGATACTGTCGGTCTAGAGTATGGAAGAGGTGAGTGGAATTCCGAGTGTAGAGGTGAAATTCGTAGATATTCGGAGGAACACCAGTGGCGAAGGCGGCTCACTGGTCCATTACTGACGCTGAGGTGCGAAAGCGTGGGGAGCAAACAGG` |
| ASV1653 | 404 | `TGGGGAATATTGGACAATGGGCGCAAGCCTGATCCAGCAATGCCGCGTGAGTGAAGAAGGCCTTAGGGTTGTAAAGCTCTTTTAGTAGGGAAGATAATGACGTTACCTACAGAAAAAGCTCCGGCTAACTCCGTGCCAGCAGCCGCGGTAATACGGAGGGAGCTAGCGTTGTTCGGAATGACTGGGCGTAAAGAGCGCGTAGGCGGTATAGTAAGTTGAGGGTGAAATCCCAGGGCTTAACCCTGGAACTGCTTTCAAAACTGCTATACTTGAGATCGATAGGGGATGGTGGAATTCCTAGTGTAGAGGTGAAATTCTTAGATATTAGGAGGAACACCGGTGGCGAAGGCGACCATCTGGGTCGATACTGACGCTGAGGCGCGAAAGCGTGGGGAGCAAACAGG` |
| ASV1336 | 429 | `TAGGGAATATTGCGCAATGGGGGAAACCCTGACGCAGCAACGCCGCGTGAGTGAGGAAGGTCTTCGGATTGTAAAACTCTTTTATTAGGGAAAAATGCTGATGGAGCTAATACCCCATTAGTTGATGGTACCTAAGGAATAAGCACCGGCTAACTTCGTGCCAGCAGCCGCGGTAATACGAAGGGTGCAAGCGTTGTTCGGATTTATTGGGCGTAAAGCGCGCGCAGGCGGATCAGCAAGTCAGATGTGAAATCTCAGGGCTCAACCCTGAAACTGCGTCTGAAACTGCTAGTCTAGAATATTGGAGGGGGCAGGGGAATTTCACGTGTAGGGGTAAAATCCGTAGAGATGTGAAGGAACACCGGAGGCGAAGGCGCCTGCCTGGACAATACTTGACGCTGAGGCGCGAAAGCGTGGGGAGCAAACAGG` |
| ASV2209 | 404 | `TGGGGAATATTGGACAATGGGCGAAAGCCTGATCCAGCCATGCCGCGTGAGTGATGAAGGCCTTAGGGTTGTAAAGCTCTTTCGGTGGTGACGATAATGACGGTAACCACATAAGAAGCCCCGGCTAACTTCGTGCCAGCAGCCGCGGTAATACGAAGGGGGCTAGCGTTGTTCGGAATAACTGGGCATAAAGCGCACGTAGGCGGATTTGCAAGTCAGGGGTGAAATCCCGGGGCTCAACCTCGGAACTGCCTTTGAAACTGCGAATCTGGAATCTCAGAGGGGATAGCGGAATTCCAAATGTAGGGGTGAAATCCGTAGATATTTGGAGGAACACCGGTGGCGAAGGCGGCTATCTGGATGAGTATTGACGCTGAGGTGCGAAAGCGTGGGGATCAAACAGG` |
| ASV2005 | 404 | `TGGGGAATATTGGACAATGGGCGCAAGCCTGATCCAGCAATGCCGCGTGTGTGAAGAAGGTCTTCGGATTGTAAAGCACTTTCGACAGGGACGATGATGACGGTACCTGTAGAAGAAGCCCCGGCTAACTTCGTGCCAGCAGCCGCGGTAATACGAAGGGGGCTAGCGTTGCTCGGAATTACTGGGCGTAAAGGGCGCGTAGGCGGCTCTGTTAGTCAGGCGTGAAATTCCTGGGCTCAACCTGGGGACTGCGCTTGATACGGCGGGGCTAGAATGTGGAAGAGGGTCGTGGAATTCCCAGTGTAGAGGTGAAATTCGTAGATATTGGGAAGAACACCGGTGGCGAAGGCGGCGACCTGGTCCATTATTGACGCTGAGGCGCGACAGCGTGGGGAGCAAACAGG` |
| ASV3734 | 386 | `GCGAGAAACCTTAGCAATGTGCGAAAGCACGACTAGGTTAATCCGAGTGATTTCTGCTGAAGAAATCTTTTGTCAGTTTTAAATCTACTGGTGAATAAGGGGTGGGCAAGTCTGGTGTCAGCCGCCGCGGTAATACCAGCACCTCGAGTGGTTAGGAGGTTTATTGGGCCTAAAGCATCCGTAGCCGGCTACGCAAGTCTCCGGTTAAATCCATCTGCTTAACTGATGGGCTGCCGGAGATACTACATAGCTAGGGAGCGGGAGAGGTAGACGGTATTCGGTGGGTAGGGGTAAAATCCTCTGATCCATTGAGGACCACCAGTGGCGAAGGCGGTCTACCAGAACGCGTTCGACGGTGAGGGATGAAAGCTGGGGGAGCAAACCGG` |
| ASV1840 | 424 | `TAAGGAATATTGGTCAATGGACGCAAGTCTGAACCAGCCATGCCGCGTGAAGGATGACGGCCCTATGGGTTGTAAACTTCTTTTGTACGGGAATAAACCCTTCTACGTGTAGAAGGCTGAATGTACCGTAAGAATAAGGATCGGCTAACTCCGTGCCAGCAGCCGCGGTAATACGGAGGATCCAAGCGTTATCCGGATTCATTGGGTTTAAAGGGTGCGTAGGCGGATTTGTAAGTCAGTGGTGAAAGCCTGCAGCTCAACTGCAGAACTGCCATTGATACTGCAAGTCTTGAGTACACTTGACGTGGGCGGAATGTGTCATGTAGCGGTGAAATGCTTAGATATGACACAGAACACCGATTGCGAAGGCAGCTCACGAAATTGTAACTGACGCTGAGGCACGACAGCGTGGGGATCAAACAGG` |
| ASV2950 | 429 | `TGGGGAATTTTGGACAATGGGGGCAACCCTGATCCAGCAACGCCGCGTGAATGAAGAAGGCCTTAGGGTTGTAAAGTTCTTTTATTTGGGAGCAAAGGTTATTGTTGAATAGACGATGACTATGAGAGTACCAAAAGAATAAGCACCGGCTAACTACGTGCCAGCAGCCGCGGTAATACGTAGGGTGCGAGCGTTAATCGGAATTATTGGGCGTAAAGGGTGCGTAGGCGGATGTATAAGTTAGATGTGAAAAGCCAGGGCTTAACCTTGGTCGTGCATTTAAGACTGTATATCTAGAGTTTGTAAGAGGGGGGTAGAATTCCAAGTGTAGCAGTGAAATGCGTAGAGATTTGGAGGAATACCGATGGCGAAGGCAGCCCCCTGGTATAGAACTGACGCTGAGGCACGAAAGCGTGGGTAGCAAACAGG` |
| ASV3381 | 404 | `TGGGGAATATTGGACAATGGGCGAAAGCCTGATCCAGCCATGCCGCGTGTGTGATGAAGGCCTTAGGGTTGTAAAGCACTTTCGACGGGGACGATAATGACGGTACCCGTAGAAGAAGCCCCGGCTAACTTCGTGCCAGCAGCCGCGGTAATACGAAGGGGGCTAGCGTTGCTCGGAATTACTGGGCGTAAAGGGCGCGTAGGCGGCCATTCAAGTCGGGGGTGAAATCCCAGGGCTCAACCCTGGAATTGCCTTCGATACTGTTTGGCTCGAGAGTGGAAGAGGTAAGTGGAACTGCGAGTGTAGAGGTGAAATTCGTAGATATTCGCAAGAACACCAGTGGCGAAGGCGGCTTACTGGTCCATTTCTGACGCTGAGGCGCGAAAGCGTGGGGAGCAAACAGG` |
| ASV2387 | 430 | `TGGGGAATATTGCGCAATGGGGGAAACCCTGACGCAGCAACGCCGCGTGAGTGAGGAAGGACTTCGGTCTGTAAAGCTCTGTTAATGTGGAAAAATGGCATCGGGTCTAATAGGCCTGGTGTTTGATGGTACACATAGAGGAAGCACCGGCTAACTTCGTGCCAGCAGCCGCGGTAATACGAAGGGTGCAAGCGTTGTTCGGATTTATTGGGCGTAAAGCGCGCGTAGGCGGACCTGCAAGTCAGATGTGAAATCTCGGGGCTCAACCCCGAAACTGCGTCTGAAACTACAGGTCTAGAATCTCGGAGGGGGAAGGGGAATATCGCATGTAGGGGTAAAATCCGTAGATATGCGATGGAACACCAGAGGCGAAGGCGCCTTCCTGGACGAGTATTGACGCTGAGGCGCGAAAGCGTGGGGATCAAACAGG` |
| ASV3634 | 424 | `TGAGGAATATTGGACAATGGGCGCAAGCCTGATCCAGCCATGCCGCGTGCAGGAAGACGGTCCTATGGATTGTAAACTGCTTTTGTACAGGAAGAAACACTCCTACGTGTAGGAACTTGACGGTACTGTAAGAATAAGGATCGGCTAACTCCGTGCCAGCAGCCGCGGTAATACGGAGGATCCAAGCGTTATCCGGAATCATTGGGTTTAAAGGGTCCGTAGGCGGTCTAATAAGTCAGTGGTGAAAGCCCATCGCTCAACGATGGAACGGCCATTGATACTGTTAGACTTGAATTATAAGGAAGTAACTAGAATATGTAGTGTAGCGGTGAAATGCTTAGATATTACATGGAATACCAATTGCGAAGGCAGGTTACTACTTATGGATTGACGCTGATGGACGAAAGCGTGGGGAGCGAACAGG` |
| ASV89 | 429 | `TGGGGAATTTTGGACAATGGACGAAAGTCTGATCCAGCCATGCCGCGTGCGGGAAGAAGGCCTTCGGGTTGTAAACCGCTTTTGTCAGGGAAGAAACGGGTTTCTCTAATACAGAGACCTAATGACGGTACCTGAAGAATAAGCACCGGCTAACTACGTGCCAGCAGCCGCGGTAATACGTAGGGTGCAAGCGTTAATCGGAATTACTGGGCGTAAAGCGTGCGCAGGCGGTTATGCAAGACAGATGTGAAATCCCCGGGCTCAACCTGGGAACTGCATTTGTGACTGCATGGCTAGAGTACGGTAGAGGGGGATGGAATTCCGCGTGTAGCAGTGAAATGCGTAGATATGCGGAGGAACACCGATGGCGAAGGCAATCCCCTGGACCTGTACTGACGCTCATGCACGAAAGCGTGGGGAGCAAACAGG` |
| ASV5202 | 403 | `TGGGGAATATTGCGCAATGGGGGAAACCCTGACGCAGCGACGCCGCGTGAGTGAAGAAGTCTTTCGGGATGTAAAACTCTGTTAGGAGGGACGATTATGACGGTACCTCCAGAGAAAGCCCCGGCTAACTACGTGCCAGCAGCCGCGGTAATACGTAGGGGGCGAGCGTTATCCGGATTTACTGGGCATAAAGCGCGCGTAGGCGGTTTTTTAAGTCAGTTGTTAAATCCCTTGGCTCAACCAAGGTTCGCAATTGATACTGAAGAACTAGAGATCAGGAGAGGGAAGCGGAACTCACGGTGTAGCGGTGAAATGCGTAGATATCGTGAGGAACATCAGTTGCGAAGGCGGCTTCCTGGACTGCATCTGACGCTGAGGCGCGAAAGCGTGGGGAGCAAACAGG` |
| ASV2914 | 429 | `TGGGGAATTTTGGACAATGGGGGCAACCCTGATCCAGCCATTCCGCGTGAGTGAAGAAGGCCTTCGGGTTGTAAAGCTCTTTTGTCCGGAGCGAAACGGTGCTGGTGAATATCCAGCGCTACTGACGGTACCGGAAGAATAAGCACCGGCTAACTACGTGCCAGCAGCCGCGGTAATACGTAGGGTGCGAGCGTTAATCGGAATTACTGGGCGTAAAGCGTGCGCAGGCGGTTTTGTAAGCCAGTCGTGAAATCCCCGGGCTCAACCTGGGAATGGCGATTGGGACTGCAAGGCTTGAGTATGGCAGAGGAGACTGGAATTCCTGGTGTAGCAGTGAAATGCGTAGATATCAGGAGGAATACCGATGGCGAAGGCAGGTCTCTGGGCTGATACTGACGCTCATGCACGAAAGCGTGGGGAGCAAACAGG` |
| ASV3792 | 430 | `TGGGGAATCTTGCGCAATGGACGAAAGTCTGACGCAGCCACGCCGCGTGAGTGAAGAAGGCCTTCGGGTTGTAAAGCTCTGTCACCTGGGACGAAAAATCTTACGGTTAATAGCCGTAGGACTTGACGGTACCAGGGGAGGAAGCACCGGCTAACTTCGTGCCAGCAGCCGCGGTAATACGAAGGGTGCAAGCGTTGCTCGGAATTATTGGGCGTAAAGGGTTGGTAGGTGGTCTTGTTTGTCTAAGGTGAAATCCCACGGCTTAACTGTGGAAGTGCCTTAGAAACGGCAAGACTAGAGTCCTAGAGGGGTTTGTGGAATTCCCGGTGTAGCGGTGAAATGCGTAGAGATCGGGAGGAACACCAGCGGCGAAGGCGGCAAACTGGATAGGAACTGACACTCAACAACGAAAGCGTGGGGATCAAACAGG` |
| ASV1144 | 404 | `TGGGGAATCTTAGACAATGGGGGAAACCCTGATCTAGCCATGCCGCGTGAGCGATGAAGGCCTTAGGGTTGTAAAGCTCTTTCAGTGGGGAAGATAATGACGGTACCCACAGAAGAAGCCCCGGCTAACTCCGTGCCAGCAGCCGCGGTAATACGGAGGGGGCTAGCGTTGTTCGGAATTACTGGGCGTAAAGCGCACGTAGGCGGACTGGAAAGTCAGGGGTGAAATCCCGGGGCTCAACCCCGGAACTGCCTTTGAAACTCCCGGTCTTGAGGTCGTGAGAGGTGAGTGGAATTCCGAGTGTAGAGGTGAAATTCGTAGATATTCGGAGGAACACCAGTGGCGAAGGCGGCTCACTGGCACGATACTGACGCTGAGGTGCGAAAGCGTGGGGAGCAAACAGG` |
| ASV1605 | 430 | `TGGGGAATATTGGACAATGGGGGGAACCCTGATCCAGCCATGCCGCGTGTGTGAAGAAGGCCTTTTGGTTGTAAAGCACTTTAAGCGAGGAGGAGGAGCTCTTGGTTAATACCCAAGATGCTTGGACGTTACTCGCAGAATAAGCACCGGCTAACTCTGTGCCAGCAGCCGCGGTAATACAGAGGGTGCAAGCGTTAATCGGATTTACTGGGCGTAAAGCGCGCGTAGGTGGCCAATTAAGTCAAATGTGAAATCCCCGAGCTTAACTTGGGAATTGCATTCGATACTGGTTGGCTAGAGTATGGGAGAGGATGGTAGAATTCCAGGTGTAGCGGTGAAATGCGTAGAGATCTGGAGGAATACCGATGGCGAAGGCAGCCATCTGGCCTAATACTGACACTGAGGTGCGAAAGCATGGGGAGCAAACAGG` |
| ASV2395 | 404 | `TGGGGAATATTGGACAATGGGCGAAAGCCTGATCCAGCAATGCCGCGTGAGTGATGAAGGCCTTAGGGTTGTAAAGCTCTTTTACCCGGGAAGATAATGACTGTACCGGGAGAATAAGCCCCGGCTAACTCCGTGCCAGCAGCCGCGGTAATACGGAGGGGGCTAGCGTTATTCGGAATTACTGGGCGTAAAGCGCACGTAGGCGGCTATTCAAGTCAGAGGTGAAAGCCCGGAGCTCAACTTCGGAACTGCCTTTGAAACTAGATAGCTTGAACATCGGAGAGGTAAGTGGAATTCCGAGTGTAGAGGTGAAATTCGTAGATATTCGGAAGAACACCAGTGGCGAAGGCGGCTTACTGGACGATTGTTGACGCTGAGGTGCGAAAGCGTGGGGAGCAAACAGG` |
| ASV3267 | 404 | `TGGGGAATATTGGGCAATGGGGGCAACCCTGACCCAGCGACGCCGCGTGAGTGAAGAAATCCCTCGGGATGTAAAGCTCTGTTGTGTGGGAAGATAATGACGGTACCACACGAGGAAGCCCCGGCAAACTACGTGCCAGCAGCCGCGGTAATACGTAGGGGGCAAGCGTTGTTCGGAATTACTGGGCGTAAAGCGCACGCAGGCGGAATGGTAAGTCAAACGTCAAAGGCAGAGGCTCAACTTCTGTTCCACGTTTGATACTGTCATTCTAGAGTATGTGAGAGGAAGATGGAATTCCCGGTGTAGCGGTGAAATGCGTAGATATCGGGAGGAACACCAGTGGCGAAGGCGATCTTCTGGCACAAAACTGACGCTCATGTGCGAAAGCTAGGGCAGCGAACGGG` |
| ASV3501 | 404 | `TGGGGAATCTTGGACAATGGGGGGAACCCTGATCCAGCCATGCCGCGTGAGCGATGAAGGCCTTCGGGTTGTAAAGCTCTTTCGTGGGGGAAGATAATGACTGTACCCCAAGAAGAAGCCCCGGCTAACTCCGTGCCAGCAGCCGCGGTAATACGGAGGGGGCTAGCGTTGTTCGGAATTACTGGGCGTAAAGCGCACGTAGGCGGGACGGAAAGTCAGAGGTGAAATCCCAGGGCTCAACCCTGGAACTGCCTTTGAAACTTCCGTTCTTGAGGTCGAGAGAGGTGGGTGGAATTCCGAGTGTAGAGGTGAAATTCGTAGATATTCGGAGGAACACCAGTGGCGAAGGCGGCCCACTGGCTCGATACTGACGCTGAGGTGCGAAAGCGTGGGGAGCAAACAGG` |
| ASV2880 | 429 | `TGGGGAATTTTGGACAATGGGGGAAACCCTGATCCAGCAACACCGCGTGAATGAAGAAGGCCCTAGGGTTGTAAAGTTCTTTTATCTAGGAGCAAAAGCTTTTTGTTAATAGCAGAGAGTTATGAGAGTACTAGAAGAATAAGCACCGGCTAACTACGTGCCAGCAGCCGCGGTAATACGTAGGGTGCGAGCGTTAATCGGAATTACTGGGCGTAAAGGGTGCGTAGGCGGATATGAAAGTTAGATGTGAAATACCTAAGCTTAACTTGGGAACTGCATTTAATACTGCGTGTCTAGAGTTTATTAGAGGGGGGTAGAATTCCAAGTGTAGTAGTGAAATGCGTAGATATTTGGAGGAATACCGATGGCGAAGGCAGCCCCCTGGGATAAAACTGACGCTGATGCACGAAAGCGTGGGGAGCAAACAGG` |
| ASV70 | 424 | `TGAGGAATATTGGACAATGGGCGCAAGCCTGATCCAGCCATGCCGCGTGCAGGAAGACGGTCCTATGGATTGTAAACTGCTTTTGTACAGGAAGAAACCCTCGCTCGTGAGCGAGATTGACGGTACTGTAAGAATAAGGATCGGCTAACTCCGTGCCAGCAGCCGCGGTAATACGGAGGATCCAAGCGTTATCCGGAATCATTGGGTTTAAAGGGTCCGTAGGCGGTTTGTTAAGTCAGTGGTGAAAGCCCATCGCTCAACGGTGGAACGGCCATTGATACTGACAGACTTGAATTGTTGGGAAGTAACTAGAATATGTAGTGTAGCGGTGAAATGCTTAGATATTACATGGAATACCAATTGCGAAGGCAGGTTACTATCAACATATTGACGCTGATGGACGAAAGCGTGGGTAGCGAACAGG` |
| ASV1396 | 407 | `TAGGGAATTTTCCGCAATGGGCGAAAGCCTGACGGAGCAATACCGCGTGAGGGATGAATGCCTATGGGTTGTAAACCTCTTTTTTCAGGGAGGAATAAATGACGTGTACCTGAAGAATAAGCATCGGCTAACTCCGTGCCAGCAGCCGCGGTAAGACGGAGGATGCAAGTGTTATCCGGAATCACTGGGCGTAAAGCGTCTGTAGGTGGCTTAATAAGTCAACTGTTAAATCTTGAAGCTCAACTTCAAAATCGCAGTCGAAACTATTAGACTAGAGTATAGTAGAGGTAAAGGGAATTTCCAGTGGAGCGGTGAAATGCGTAGAGATTGGAAAGAACACCGATGGCGAAGGCACTTTACTGGGCTATTACTGACACTCAGAGACGAAAGCTAGGGTAGCAAATGGG` |
| ASV3284 | 404 | `TGAGGAATATTGGACAATGGGGGAAACCCTGATCCAGCCATGCCGCGTGAGTGATGAAGGCCTTAGGGTTGTAAAACTCTTTAAGTGGGGAAAATAATGATTGTACCCACAGAATAAGCCACGGCAAACTTCGTGCCAGCAGCCGCGGTAATACGAAGGTGGCTAGCGTTACTCGGAATTACTGGGCGTAAAGCGAGTGCAGGTGGATTTACAAGTCAGTGGTGAAAGCCCAGAGCTTAACTCTGGAATTGCCTTTGAAACTGTAAATCTTGAGGTCGGTAGGGGATAGTGGAATTCTTAGTGTAGAGGTGAAATTCGTAGATATTAAGAGGAACACCGGTGGCGAAGGCGACTGTCTGGACCGATTCTGACACTCAGACTCGAAAGCGTGGGGAGCAAACAGG` |
| ASV3335 | 407 | `TGGGGAATTTTCCGCAATGGGCGCAAGCCTGACGGAGCAAGACCGCGTGAGGGAAGAAGGTCTGTGGATTGTAAACCTCTTTTGATAGGGAAGAAGACAGTGACGGTACCTATCGAATCAGCCTCGGCTAACTCCGTGCCAGCAGCCGCGGTAATACGGAGGAGGCAAGCGTTATCCGGAATTATTGGGCGTAAAGCGTCCGTAGGTGGGTTACCAAGTCAGTTGTTAAATAGTGAAGCTTAACTTCATAGAGGCAATTGAAACTGGTAGTCTAGAGTGCGGTAGGGGTAGAGGGAATTTCCAGTGTAGCGGTGAAATGCGTAGATATTGGAAAGAACACCGGTGGCGAAAGCGCTCTACTGGGCCTGCACTGACACTGAGGGACGAAAGCTAGGGGAGCGAAAGGG` |
| ASV3558 | 425 | `TGGGGAATATTGGTCAATGGGCGCAAGCCTGAACCAGCAATGCCGCGTGAGTGATGAAGGCCTTCGGGTTGTAAAGCTCTGTCGGGAGGGACGAAAAAACTCGGGTAATGCCGAGGCTTGACGGTACCTCCAAAGGAAGCACCGGCTAACTCTGTGCCAGCAGCCGCGGTAATACAGAGGGTGCAAGCGTTGTTCGGAATTACTGGGCGTAAAGCGCGTGTAGGCTGCAATGTCAGTCGGATGTGAAAGCCCTTGGCTCAACCAAGGAAGTGCATCCGATACTGCATCGCTTGAGTGTCGGAGGGGATGGCGGAATTCCCGGTGTAGAGGTGAAATTCGTAGATATCGGGAGGAACACCTGTGGCGAAGGCGGCCATCTGGACGATAACTGACGCTGAGACGCGAAAGCGTGGGTAGCAAACAGG` |
| ASV4281 | 429 | `TGGGGAATATTGGACAATGGGGGCAACCCTGATCCAGCAATACCGCGTGTGTGAAGAAGGCCTTCGGGTTGTAAAGCACTTTGGGCGGGGAAGAAGGTATTCTGTTTAAGAGATGGGATATTTGACGGTACCCGCAGAACAAGCACCGGCTAACTCTGTGCCAGCAGCCGCGGTAATACAGAGGGTGCGAGCGTTAATCGGAATTACTGGGCGTAAAGGGCGCGTAGGCGGATGTGCAAGTGAGATGTGAAATACCTGGGCTTAACCTAGGAACTGCATCTTAAACTGCATATCTGGAGTACTGTAGAGGATAGTGGAATTTCCGGTGTAGCGGTGAAATGCGTAGAGATCGGAAGGAACATCAGTGGCGAAGGCGACTGTCTGGACAGATACTGACGCTGAGGCGCGAAAGCGTGGGGAGCAAACAGG` |
| ASV4134 | 404 | `TTAGGAATATTGGACAATGGGCGCAAGCCTGATCCAGCCATGCCGCGTGAGTGATGAAGGCCTTCGGGTTGTAAAGCTCTTTAGTCCACGAATATAATGACTGTAGTGGAAGAATAAGCCCCGGCTAACTTCGTGCCAGCAGCCGCGGTAATACGAAGGGGGCGAGCGTTATTCGGAATCACTGGGCGTAAAGCGTGCGTAGGCGGTTGTGAAAGTTAGGAGTGAAAGCCCCAGGCTCAACTTGGGAATTGCTCTTAATACTCCACAACTCGAATTTGGGAGAGGATAGCGGAATTGTCAGTGTAGCAGTGAAATGCGTAGATATTGACAGGAACACCGGTGGCGTAAGCGGCTATCTGGACCAACATTGACGCTGAGGCACGAAAGCGTGGGGATCAAACAGG` |
| ASV4379 | 423 | `TAGGGAATATTGGGCAATGGAGGCAACTCTGACCCAGCCATGCCGCGTGAAGGAAGAAGGCGTTATGCGTTGTAAACTTCTTTTATATGGGAAGAAACGACCCTTGCGAGGGGCATTGACGGTACCATATGAATAAGCACCGGCTAACTCCGTGCCAGCAGCCGCGGTAATACGGAGGGTGCAAGCGTTGTCCGGATTTATTGGGTTTAAAGGGTGCGTAGGCGGTTCTTTAAGTCAGCGGTGAAAGTCCAGAGCTCAACTCTGGGATTGCCATTGATACTGAAGAACTTGAGTATAGTTGAGGTAGGCGGAATTGACAGTGTAGCGGTGAAATGCATAGATATTGTCAAGAACACCGATTGCGTAGGCAGCTTACTAAGCTATAACTGACGCTGAGGCACGAAAGTGTGGGGATCAAACAGG` |
| ASV3827 | 404 | `TGGGGAATCTTGCGCAATGGGCGAAAGCCTGACGCAGCAACGCCGCGTGCGGGATGAAGGCCTTCGGGTTGTAAACCGCTTTCAGCAGGGACGAAAATGACGGTACCTGCAGAAGAAGCCCCGGCTAACTACGTGCCAGCAGCCGCGGTAATACGTAGGGGGCAAGCGTTGTCCGGATTTATTGGGCGTAAAGAGCTCGTAGGCGGTTCCGTAAGTCGAGTGTGAAAAATCTGGGCTCAACCCAGTGGAGCACTCGATACTGCGGTGACTAGAGTACGGTAGAGGAGTGTGGAATTCCTGGTGTAGCGGTGAAATGCGCAGATATCAGGAGGAACACCAACGGCGAAGGCAGCACTCTGGGCCGGTACTGACGCTGAGGAGCGAAAGCGTGGGGAGCAAACAGG` |
| ASV2608 | 424 | `TGAGGAATATTGGACAATGGGTGAGAGCCTGATCCAGCCATCCCGCGTGGAGGAAGACGGTCCTATGGATTGTAAACTCCTTTTATATAGGAATAAACCTACGGACGTGTCCGTAGCTGAAGGTACTATATGAATAAGCACCGGCTAACTCCGTGCCAGCAGCCGCGGTAATACGGAGGGTGCAAGCGTTATCCGGATTTATTGGGTTTAAAGGGTCCGTAGGCGGCTCATTAAGTCAGTGGTGAAAGCCTATCGCTTAACGATAGAACTGCCATTGATACTGATGAGCTTGAGTGTGGTTGAAGTAGCTGGAATATGTAGTGTAGCGGTGAAATGCTTAGATATTACATAGAACACCAATTGCGAAGGCAGGTTACTAAGCCACAACTGACGCTGATGGACGAAAGCGTGGGGAGCGAACAGG` |
| ASV475 | 429 | `TGGGGAATTTTGGACAATGGGCGAAAGCCTGATCCAGCAATGCCGCGTGCAGGATGAAGGCCTTCGGGTTGTAAACTGCTTTTGTACGGAACGAAACGGTGGACTTTAATAAAGTTTGCTAATGACGGTACCGTAAGAATAAGCACCGGCTAACTACGTGCCAGCAGCCGCGGTAATACGTAGGGTGCGAGCGTTAATCGGAATTACTGGGCGTAAAGCGTGCGCAGGCGGTTATATAAGACAGATGTGAAATCCCCGGGCTCAACCTGGGACCTGCATTTGTGACTGTATAGCTAGAGTACGGTAGAGGGGGATGGAATTCCGCGTGTAGCAGTGAAATGCGTAGATATGCGGAGGAACACCGATGGCGAAGGCAATCCCCTGGACCTGTACTGACGCTCATGCACGAAAGCGTGGGGAGCAAACAGG` |
| ASV875 | 404 | `TGGGGAATATTGGACAATGGGGGCAACCCTGATCCAGCCATGCCGCGTGAGTGAAGAAGGCCTTCGGGTTGTAAAGCTCTTTCGACGGGGACGATGATGACGGTACCCGTATAAGAAGCCCCGGCTAACTTCGTGCCAGCAGCCGCGGTAATACGAAGGGGGCTAGCGTTGTTCGGAATTACTGGGCGTAAAGCGCGCGTAGGCGGTGCATCATGTCAGAGGTGAAATCCCGGAGCTCAACTCCGGAACTGCCTTTGAAACGGGTGCGCTAGAATCCGAGAGAGGATGGCGGAATTCCTAGTGTAGAGGTGAAATTCGTAGATATTAGGAAGAACACCGGTGGCGAAGGCGGCCATCTGGCTCGGTATTGACGCTCAGGCGCGAAAGCGTGGGGATCAAACAGG` |
| ASV3894 | 430 | `TGGGGAGTCTTGCGCAATGGACGAAAGTCTGACGCAGCCACGCCGCGTGAGTGAAGAAGGCCTTCGGGTTGTAAAGCTCTGTCAAGCGGGACGAACATCCTTTGGGTTAATAGCCCAGAGGACTGACGGTACCGCTGAAGGAAGCACCGGCTAACTTCGTGCCAGCAGCCGCGGTAATACGAAGGGTGCAAGCGTTGCTCGGAATTATTGGGCGTAAAGGGTAGGTAGGTGGTCTCATTTGTCTGGGGTGAAAGCCTTGGGCTTAACCCAAGAAGTGCCCCGGAAACGGTGAGACTAGAGTCCTAGAGAGGGTCGTGGAATTCCCGGTGTAGCGGTGAAATGCGTAGAGATCGGGAGGAACACCAGAGGCGAAAGCGGCGACCTGGATAGGTACTGACACTCAACTACGAAAGCGTGGGGAGCAAACAGG` |
| ASV5165 | 424 | `TGAGGAATATTGGTCAATGGACGCAAGTCTGAACCAGCCATGCCGCGTGCAGGACGAAGGCGCTATGCGTTGTAAACTGCTTTTATTTGGGAAGAAACCCCCCGATGCGTTGGGGGCTGACGGTACCAAATGAATAAGGACCGGCTAACTCCGTGCCAGCAGCCGCGGTAATACGGAGGGTCCAAGCGTTATCCGGATTTATTGGGTTTAAAGGGTTCGTAGGCGGGAAAGTAAGTCAGTGGTGAAATCCTACAGCTTAACTGTAGAACTGCCGTTGATACTGCTTTTCTTGAGTCTAGTTGAGGTGGGCGGAATATGTCATGTAGCGGTGAAATGCATAGATATGACATAGAACACCAATTGCGAAGGCAGCTCACTAAACTAGTACTGACGCTGAGGAACGAAAGCGTGGGTAGCGAACAGG` |
| ASV2916 | 429 | `TGGGGAATATTGGACAATGGGCGGAAGCCTGATCCAGCAATGCCGCGTGTGTGAAGAAGGCCTTAGGGTTGTAAAGCACTTTCAGTGGGGAGGAAGTTGTGAGTGATAATACCATTTGCAATTGACGTTACCCACAGAAGAAGCACCGGCTAACTCTGTGCCAGCAGCCGCGGTAATACAGAGGGTGCTAGCGTTAATCGGAATTACTGGGCGTAAAGGGTGTGTAGGTGGTTAGGTAAGTTAGATGTGAAATCCCCGGGCTTAACCTGGGAACTGCGTTTAAAACTGCTTGGCTAGAGTATTGCAGAGGATAGTGGAATTTCCAGTGTAGCGGTGAAATGCGTAGATATTGGAAGGAACACCAGTGGCGAAGGCGACTATCTGGGCAAATACTGACACTGAGACACGAAAGCATGGGGAGCAAACAGG` |
| ASV1433 | 429 | `TGGGGAATATTGGACAATGGGGGAAACCCTGATCCAGCAATGCCGCGTGTGTGAAGAAGGCCTGAGGGTTGTAAAGCACTTTCAGTGGGGAGGAGTCTTATTAGGTTAAGAGCTAAATAAGAGGACGTTACCCACATAAGAAGCACCGGCTAACTCCGTGCCAGCAGCCGCGGTAATACGGAGGGTGCGAGCGTTAATCGGAATTACTGGGCGTAAAGGGTGCGTAGGTGGTTTGATAAGTTATCTGTGAAAGCCCTGGGCTCAACCTAGGAAGGCCAGATAAGACTGTTAGACTCGAGTATGGGAGAGGGTAGTGGAATTTCCGGTGTAGCGGTGAAATGCGTAGAGATCGGAAGGAACACCAGTGGCGAAGGCGGCTACCTGGCCTAATACTGACACTAAAGCACGAAAGCGTGGGGAGCAAACAGG` |
| ASV4916 | 404 | `TGGGGAATATTGGACAATGGGGGCAACCCTGATCCAGCAATGCCGCGTGGGTGAAGAAGGTCTTCGGATTGTAAAGCCCTTTCGGCGGGGAAGATTGTGACGGTACCCGCAGAAGAAGCCCCGGCTAACTTCGTGCCAGCAGCCGCGGTAATACGAAGGGGGCTAGCGTTGCTCGGAATTACTGGGCGTAAAGGGCGCGTAGGCGGCTCTATCAGTCAGGCGTGAAATTCCTGGGCTTAACCTGGGGGCTGCGCTTGATACAGTGGGGCTTGAGGGTGGAAGAGGGTCGCGGAATTCCCAGTGTAGAGGTGAAATTCGTAGATATTGGGAAGAACACCGGTGGCGAAGGCGGCGACCTGGTCCATTACTGACGCTGAGGCGCGACAGCGTGGGGAGCAAACAGG` |
| ASV1254 | 404 | `TCGGGAATTTTGGGCAATGGGCGAAAGCCTGACCCAGCAACGCCGCGTGAAGGATGAAGTATTTCGGTATGTAAACTTCGAAAGAATGGGAAGAATCAATGACGGTACCATTTATAAGGTCCGGCTAACTACGTGCCAGCAGCCGCGGTAATACGTAGGGACCAAGCGTTGTTCGGATTTACTGGGCGTAAAGGGCGCGTAGGCGGCTTTGTAAGTCACTTGTGAAATCTCCGGGCTTAACTCGGAACGGCCAAGTGATACTGCAAAGCTAGAGTGCAGAAGGGGCAATCGGAATTCTTGGTGTAGCGGTGAAATGCGTAGATATCAAGAGGAACACCTGAGGTGAAGACGGGTTGCTGGGCTGACACTGACGCTGAGGCGCGAAAGCCAGGGGAGCAAACGGG` |
| ASV2737 | 404 | `CAGGGAATTTTGCGCAATGGGCGAAAGCCTGACGCAGCAACGCCGCGTGGGGGAAGAAGGCTCTTGGGTCGTAAACCCCTTTTGCCGGGGAAGATAATGACGGTACCCGGCGAATAAGCCACGGCTAACTACGTGCCAGCAGCCGCGGTAATACGTAGGTGGCAAGCGTTGTCCGGATTTATTGGGCGTAAAGGGCGCGCAGGCGGTTCGTTAAGTTCGAGGTGAAATCTCCCGGCTCAACTGGGAGGGGTCTTCGGATACTGGCGGACTTGAGGAGAGCAGAGGTGAGCGGAATTCTCGGTGTAGTGGTGAAATGCGTAGATATCGAGAGGAACACCAGAGGCGAAGGCGGCTCACTGGGCTCCACCTGACGCTGAGGCGCGAAAGCGTGGGGAGCGAACCGG` |
| ASV3410 | 421 | `TGAGGAATATTGCGCAATGGACGAAAGTCTGACGCAGCAATGCCGCGTGCGGGAAGAAGGTGCTATGCATCGTAAACCGCTGTAGCGTGGGGCAAAAGGCACCTTATGGGTGCGAGATGGTACCACGAAAGTAAGCATCGGCTAACTACGTGCCAGCAGCCGCGGTAATACGTAGGATGCAAGCGTTGTCCGGATTTACTGGGTGTAAAGGGTGCGCAGGCGGGACTGTAAGTCAGAGGTGAAAGCCCGGAGCTTAACTCCGGAAATGCCTTTGATACTGCGGTTCTTGAATATGATAGAGGCTCATGGAATATGTGGTGTAGCGGTGAAATGCGTAGATATCACATAGAACACCAGTGGCGAAGGCGGTGAGCTGGGTCATTATTGACGCTCATGCACGAAAGTGTGGGTAGCAAACAGG` |
| ASV5147 | 451 | `GCCGGACCCTACCTGCCCAAGGACTTCCTTTTCGGGGTGTTCCCGTCGGTCAACCGAACGGACGTGAAGAATCCCGATATGTGGTTCGGCCTTTCCATCGACTCCCATGCCGATGCCCGGCAGGTACGGCTGATCTACTACAATTCGAAGCGGTCAGAGGGGAAGCAGGGCGGCAGGGACGAAACCCGCCTTACCAATTTCGGCGGGGGCAATTCTGCCTTGCTTGACCCGGACAGCACCGGGGCCTTGACCGTCTTCGCCTTTCCGCTCGGGCACGACGGGCAGGCGACAGAGTGCCGAGTCTGGGTGTGCAGGCACGCGACCGAGGAAGACCTGATTGAGGACCGTGTCGGACCGGTGGAACCGGGCCGCTCAGTCACTTGGACGCCCGCCCGCCCCGGCCTGTTTCCACCCCATGCGGCGGTCAGGGCAAGCTGTTGGCTTCAGCCTT` |
| ASV4503 | 429 | `TGGGGAATATTGCACAATGGAGGAAACTCTGATGCAGCGACGCCGCGTGGGTGATGAAGGCCTTCGGGTTGTAAAGCCCTGTCGGCTGGGAACAAGTTAGTCGTGCTCATACCACGATTGATTGAGTGTACCAGCAAAGGAAGCACCGGCTAACCCTGTGCCAGCAGCCGCGGTAATACAGGGGGTGCTAGCGTTGTTCGGAATTACTGGGCGTAAAGGGCGCGTAGGCGGTCTGATAAGTCAGGAGTGAAATCCCGGAGCTCAACTTCGGAATTGCTTTTGAAACTATTAGGCTAGAGTATGTTAGAGGAAATCGGAATTCCTAGTGTAGAAGTGAAATTCGTAGATATTAGGAAGAACACCGGTGGCGAAGGCGGATTTCTGGGACATAACTGACGCTGAGGCGCGAAAGCGTGGGGATCAAACAGG` |
| ASV2986 | 429 | `TGGGGAATTTTGGACAATGGGCGCAAGCCTGATCCAGCCATTCCGCGTGAGTGAAGAAGGCCTTCGGGTTGTAAAGCTCTTTCGCAAGGGAAGAAAACTTAGTCTCTAACATAGACTGAGGCTGACGGTACCTTGATAAGAAGCACCGGCTAACTACGTGCCAGCAGCCGCGGTAATACGTAGGGTGCGAGCGTTAATCGGAATTACTGGGCGTAAAGCGTGCGCAGGCGGCCTGATAAGTCAGATGTGAAATCCCCGAGCTCAACTTGGGAACTGCGTTTGAAACTATCAAGCTAGAATATGTCAGAGGGGGGTAGAATTCCACGTGTAGCAGTGAAATGCGTAGAGATGTGGAGGAATACCAATGGCGAAGGCAGCCCCCTGGGATAATATTGACGCTCATGCACGAAAGCGTGGGGAGCAAACAGG` |
| ASV908 | 429 | `TGGGGAATTTTGGACAATGGGGGAAACCCTGATCCAGCCATTCCGCGTGAGTGAAGAAGGCCTTCGGGTTGTAAAGCTCTTTCAGCAGGAACGAAACGGTAGGCTTTAATAAAGTTTGCTAATGACGGTACCTGAAGAAGAAGCACCGGCTAACTACGTGCCAGCAGCCGCGGTAATACGTAGGGTGCGAGCGTTAATCGGAATTACTGGGCGTAAAGCGTGCGCAGGCGGTTTTGTAAGACAGATGTGAAATCCCCGGGCTTAACCTGGGAACTGCGTTTGTGACTGCAAGACTAGAGTGTGGCAGAGGGGGGTGGAATTCCACGTGTAGCAGTGAAATGCGTAGAGATGTGGAGGAACACCGATGGCGAAGGCAGCCCCCTGGGTCAACACTGACGCTCATGCACGAAAGCGTGGGGAGCAAACAGG` |
| ASV5608 | 388 | `TCGGGAAACTTCTGCAATGCGCGAAAGCGTGACAGAGCAAGCCAGAGTGTTTCTCTATAATTGAGAAACTTTTGTGATATGCAAAAAGTATCACGAATAAGGACTGGGCAAGACTGGTGCCAGCCGCCGCGGTAATCCCAGCAGTCCAAGTCGCAATCACTTTTATTGGGTCTAAAACATCCGTAGCTTGCTTTACAAGTCTCTTGTGAAATCCGGCCTCTTAAGGGTCGAAGTGCGAGAGATACTGTATGGCTAGAGACCGGGAGAGGTAGGGAGTACAGATGGGGTAGCGGTAAAATGTGTTGATCCTATCTGGACTAACAACAGCGAAGGCACCCTACTAGAACGGATCTGACAGTGAGGGATGAAGGCTAGGGGCGCAAAGTGG` |
| ASV5441 | 405 | `TGGGGAATATTGGACAATGGGCGAAAGCCTGATCCAGCCATGCCGCGTGAGTGATGAAGGCCTTAGGGTTGTAAAGCTCTTTCAGCGGGGAAGATAATGACGGTACCCGCAGAAGAAGCCCCGGCTAACTTCGTGCCAGCAGCCGCGGTAATACGAAGGGGGCTAGCGTTGTTCGGAATCACTGGGCGTAAAGCGCGTGTAGGCGGATCGTTAAGTCGGGGGTGAAATCCTGAGGCTCAACCTCAGAACTGCCTTCGATACTGGCGATCTTTGAGTCCGGGAGAGGTGAGTGGAACTCCTAGTGTAGAGGTGAAATTCGTAGATATTAGGAAGAACACCAGTGGCGAAGGCGGCTCACTGGCCCGGTACTGACGCTGATATGCGAAAGCGTGGGGAGCAAACAGG` |
| ASV497 | 407 | `TGGGGAATTTTCCGCAATGGGCGAAAGCCTGACGGAGCAATACCGCGTGAGGGATGACGGCCTATGGGTTGTAAACCTCTTTTTTCAGGGAGGAATAAATGACGTGTACCTGAAGAATAAGCATCGGCTAACTCCGTGCCAGCAGCCGCGGTAAGACGGAGGATGCAAGTGTTATCCGGAATCACTGGGCGTAAAGCGTCTGTAGGTTGTTTAATAAGTCAACTGTTAAATCTTGAGGCTTAACCTCGAAATCGCAGTCGAAACTATTAGACTAGAGTATAGTAGGGGTAAAGGGAATTTCCAGTGGAGCGGTGAAATGCGTAGATATTGGAAAGAACACCAATGGCGAAGGCACTTTACTGGGCTATTACTAACACTGAGAGACGAAAGCTAGGGTAGCAAATGGG` |
| ASV2117 | 429 | `TGGGGAATTTTGGACAATGGGCGCAAGCCTGATCCAGCCATGCCGCGTGTGTGAAGAAGGCCTTCGGGTTGTAAAGCACTTTCGGCCGGAACGAAATCGCCTTTGTTAATAGCGAGGGTGGATGACGGTACCGGAAGAAGAAGCACCGGCTAACTACGTGCCAGCAGCCGCGGTAATACGTAGGGTGCGAGCGTTAATCGGAATTACTGGGCGTAAAGGGTGCGCAGGCGGTTGCGCAAGTCAGGCGTGAAATCCCCGGGCTTAACCTGGGAATGGCGCTTGAAACTACGTGACTGGAGTATGGCAGAGGGAGGTGGAATTCCACGTGTAGCGGTGAAATGCGTAGAGATGTGGAGGAACACCGATGGCGAAGGCAGCCTCCTGGGCCAATACTGACGCTCATGCACGAAAGCGTGGGGAGCAAACAGG` |
| ASV756 | 429 | `TGGGGAATATTGGACAATGGGGGAAACCCTGATCCAGCAATGCCGCGTGTGTGAAGAAGGCCTGCGGGTTGTAAAGCACTTTCAGTAGAGAAGAAATGCCTGGAGCTAACATCTCCGGGTTTTGACGTAACCTACAGAAGAAGCACCGGCTAACTCCGTGCCAGCAGCCGCGGTAATACGGAGGGTGCGAGCGTTAATCGGAATTACTGGGCGTAAAGCGCGCGTAGGCGGTTTGGTCAGTCGATTGTGAAAGCCCTGGGCTTAACCTGGGAATTGCAGTCGATACTGCCTGACTAGAATGTGGAAGAGGGAAGTGGAATTTCCGGTGTAGCGGTGAAATGCGTAGATATCGGAAGGAACACCAGTGGCGAAGGCGGCTTCCTGGTCCAACATTGACGCTGAGGTGCGAAAGCGTGGGGAGCGAACAGG` |
| ASV4557 | 404 | `TGGGGAATATTGGACAATGGGGGCAACCCTGATCCAGCAATGCCGCGTGAGTGAAGAAGGCCTTCGGGTTGTAAAACTCTTTTGTTAGGGACGATGATGACGGTACCTAACGAATAAGCACCGGCTAACTTCGTGCCAGCAGCCGCGGTAATACGAAGGGTGCTAGCGTTGTTCGGAATGACTGGGCGTAAAGGGTGTGTAGGCGGTTAGACACGTCAGATGTGAAATCCCCGGGCTTAACCTGGGAATGGCATTTGATACGGTCTAGCTAGAGATTGGTAGAGGGTAGTGGAATTGCGAGTGTAGAGGTGAAATTCGTAGAGATTCGCAGGAACACCGGTGGCGAAGGCGGCTACCTGGGCCAATACTGACGCTGAGGCACGAAAGCGTGGGGAGCAAACAGG` |
| ASV4230 | 430 | `TGGGGAATATTGCACAATGGGGGAAACCCTGATGCAGCAACGCCGCGTGAGTGATGAAGGCCTTCGGGTTGTAAAGCTCTTTCGGCCGGGAAGAAGGGTGTTGGTGGTAATATCGCCAACATTTGATGGTACCGGAAGAAGAAGCACCGGCTAACTTCGTGCCAGCAGCCGCGGTAATACGAAGGGTGCAAGCGTTGTTCGGAATTATTGGGCGTAAAGGGTTCGTAGGTTGGAAGGTAAGTCGAGTGTGAAATCCCTGAGCTTAACTCAGGACGTGCACCCGAAACTGCTTTTCTTGAATGTTAGAGAGGGTCGTAGAATTGCTGGTGTAGGAGTGAAATCCGTAGATATCAGCAGGAATACCAGAGGCGAAGGCGGCGACCTGGCTAAACATTGACACTGAGGAACGAAAGCGTGGGGAGCAAACAGG` |
| ASV1915 | 430 | `TGGGGAATATTGGACAATGGGCGCAAGCCTGATCCAGCAATACCGCGTGTGTGAAGAAGGCCTTCGGGTTGTAAAGCACTTTAGTCAGGGAGGATGGTAATCATTGCTAATATCAATGGTTGCTGACGTTACCTGAAGAATAAGCACCGGCTAACTCCGTGCCAGCAGCCGCGGTAATACGGGGGGTGCAAGCGTTAATCGGAATTACTGGGCGTAAAGGGTGCGTAGGCGGTCTTTTAAGTCAGATGTGAAATCCCTGGGCTCAACCTAGGAATGGCATTTGATACTGTAAGACTAGAATGTGGTAGAGGATAGCGGAATTTCTGGTGTAGCGGTGAAATGCGTAGATATCAGAAGGAACACCAATGGCGAAGGCAGCTATCTGGGCCAACATTGACGCTGAGGCACGAAAGCGTGGGGAGCAAACAGG` |
| ASV1106 | 404 | `TGGGGAATATTGGACAATGGGCGCAAGCCTGATCCAGCCATGCCGCGTGAGTGATGAAGGCCTTCGGGTTGTAAAGCTCTTTTATCCGGGAAGATAATGACTGTACCGGAAGAATAAGCCCCGGCAAACTTCGTGCCAGCAGCCGCGGTAATACGAAGGGGGCTAGCGTTGTTCGGATTTACTGGGCGTAAAGCGCACGTAGGCGGCTTGTTAAGTCAGGGGTGAAATCCTGGAGCTCAACTCCAGAACTGCCCTTGATACTGGCAAGCTCGAGTATGGTAGAGGTTGGTGGAACTGCGAGTGTAGAGGTGAAATTCGTAGATATTCGCAAGAACACCAGTGGCGAAGGCGGCCAACTGGACCATTACTGACGCTGAGGTGCGAAAGCGTGGGGAGCAAACAGG` |
| ASV3690 | 403 | `TGGGGAATATTGGACAATGGGGGAAACCCTGATCCAGCCATGCCGCGTGAGTGATAAGGCCTTAGGGTTGTAAAGCTCTTTCGCGGGCGAAAATAATGATGGTAACCCGAGAAGAAGCCCCGGCTAACTTCGTGCCAGCAGCCGCGGTAATACGAAGGGGGCTAGCGTTGTTCGGAATCACTGGGCGTAAAGCGCGCGCAGGCGGCTTCCCAAGTCAGGGGTGAAATCCCGGGGCTTAACCCCGGAACTGCCTTTGAAACTGGGAGGCTGGAGACTCGGAGAGGATAGTGGAATTCCAAGTGTAGAGGTGAAATTCGTAGATACTTGGAGGAACACCGATGGCGAAGGCAGCTATCTGGACGAGCACTGACGCTGAGGCGCGAAAGCGTGGGGATCAAACAGG` |
| ASV5135 | 405 | `TTGGGAATCTTGCGCAATGGGGGAAACCCTGACGCAGCGACGTCGCGTGGACGATGAAGGCTCTAGGGTTGTAAAGTCCTTTTTTCAGGAAAGACTTAGGACGGTACCTGAAGAATAAGCACCGGCTAACTACGTGCCAGCAGCCGCGGTAAGACGTAGGGTGCAAGCGTTGTCCGGATTTACTGGGCGTAAAGAGCTCGTAGGCGGTCCGTTAAGTGTGAAGTGAAATCTCCCTGCTCAACAGGGAAACTGCTTTACATACTGGCGGACTAGAGGGATGCAGAGGTAACTGGAATTCCTGGTGTAGCGGTGAAATGCGTTGATATCAGGAGGAACACCAATGGCGAAGGCAGGTTACTGGGCATCATCTGACGCTGAGGAGCGAAAGCGTGGGTAGCAAACAGG` |
| ASV2526 | 417 | `TGGGGAATATTGGACAATGAGCGAAAGCTTGATCCAGCAATACCGCGTGAGTGAAGACATTTTTTAGGTGTAAAACTCTTTCGGTAGTGAAAATATTGATTGTAACTACAAAAGAAGTCCCGGCTAATTCCGTGCCAGCAGCCGCGGTAATACGGGAGGGACGAGTGTTATTCGGAATGACTGGGCGTAAAGAGCGTGTAGGTTGTTTAGTTTTATGATTTAATTTTATAAAGGTCAAACAGGAGGGCCTAACCCTCTACAGCCCCTATAATATTACTAAACTAAGAGTACTTGTCAGGACATTAGAACTTTTGCTGTAGCTTTAATATGCGTAAAAAGCAAGAAGAATGCCGCCACGAAGGTAGATGTCTGGCTTGTTACTGACACTGAGGCGCGAAAGCGTGGGGAGCAAAGCGG` |
| ASV2103 | 423 | `TAGGGAATATTGGTCAATGGGTGAGAGCCTGAACCAGCCATGCCGCGTGCAGGAAGAAGGCCTTCTGGGTTGTAAACTGCTTTTGCCAGGGGATAAAATAGCTTTGCGAAGCAAATTGAAGGTACCTGGTGAATAAGCCACGGCTAACTACGTGCCAGCAGCCGCGGTAATACGTAGGTGGCAAGCGTTGTCCGGATTTATTGGGTTTAAAGGGTGCGTAGGCGGTTCTATAAGTCAGTGGTGAAATACAGCCGCTTAACGGTTGAGGTGCCATTGATACTGCAGGACTTGAGTACAGACGAGGTAGGCGGAATTGACGGTGTAGCGGTGAAATGCTTAGATATCGTCAAGAACACCGATAGCGAAGGCAGCTTACTAGACTGTAACTGACGCTGAGGCACGAAAGTGTGGGGATCAAACAGG` |
| ASV2474 | 404 | `TGGGGAATATTGGACAATGGGGGAAACCCTGATCCAGCAATGCCGCGTGAATGAAGAAGGCCTTAGGGTTGTAAAGTTCTTTCACCTGTGAAGATAATGACGGTAGCAGGAGAAGAAGCCCCGGCTAACTCCGTGCCAGCAGCCGCGGTAAGACGGAGGGGGCTAGCGTTGTTCGGAATGACTGGGCGTAAAGGGCGCGTAGGCGGTTTTTTAAGTGAGGCGTGAAAGCCCTGGGCTTAACCCAGGAGGTGCGTTTCAGACTGGAAGACTTGAGTGCGAGAGAGGAAAGTGGAATTTCTAGTGTAGAGGTGAAATTCGTAGATATTAGAAAGAACACCCGTGGCGAAGGCGGCTTTCTGGCTCGCAACTGACGCTGAGGCGCGAAAGCGTGGGGAGCAAACAGG` |
| ASV3181 | 407 | `TGGGGAATATTGGGCAATGGGCGAAAGCCTGACCCAGCAACGCCGCGTGAGGGAAGAAGGTCTTCGGATTGTAAACCTTTGTCCTTGGGGACGAAAAAAATGACGGTACCCAAGGAGGAAGCTCCGGCTAACTACGTGCCAGCAGCCGCGGTAATACGTAGGGAGCAAGCGTTATCCGGAATTATTGGGCGTAAAGGGTGCGTAGGTGGTCATGCATGTCAGATGTGAAAGACTCGGGCTTAACCCGAGGGTTGCATTTGAAACTGTGTGACTTGAGTGCAGGAGAGGTAAGTGGAATTCCTAGTGTAGCGGTGAAATGCGTAGATATTAGGAGGAACACCAGTGGCGAAGGCGACTTACTGGACTGTAACTGACACTGAGGCACGAAAGCGTGGGGAGCAAACAGG` |
| ASV355 | 429 | `TGGGGAATTTTGGACAATGGACGCAAGTCTGATCCAGCAATGCCGCGTGCAGGACGAAGGCCTTCGGGTTGTAAACTGCTTTTGTACGGAACGAAACGGTGAGCTTTAATACAGTTTGCTAATGACGGTACCGTAAGAATAAGCACCGGCTAACTACGTGCCAGCAGCCGCGGTAATACGTAGGGTGCGAGCGTTAATCGGAATTACTGGGCGTAAAGCGTGCGCAGGCGGTTATATAAGTCAGATGTGAAATCCCCGGGCTCAACCTGGGACCTGCATTTGAGACTGTATAGCTAGAGTACGGTAGAGGGGGATGGAATTCCGCGTGTAGCAGTGAAATGCGTAGATATGCGGAGGAACACCGATGGCGAAGGCAATCCCCTGGACCTGTACTGACGCTCATGCACGAAAGCGTGGGGAGCAAACAGG` |
| ASV1918 | 404 | `TGGGGAATATTGGACAATGGGCGCAAGCCTGATCCAGCCATGCCGCGTGAGTGATGAAGGCCTTAGGGTTGTAAAGCTCTTTTGCCAGGGAAGATAATGACTGTACCTGGAGAATAAGCCCCGGCTAACTTCGTGCCAGCAGCCGCGGTAATACGAAGGGGGCTAGCGTTGTTCGGAATCACTGGGCGTAAAGCGCACGTAGGCGGACTCTTAAGTCAGGGGTGAAAGCCTGAGGCTCAACCTCAGAACTGCCTTTGATACTGGGAGTCTTGAGTGTGGTAGAGGTCGGTGGAATAGCAAGTGTAGAGGTGAAATTCGTAGATATTTGCTAGAACACCAGTGGCGAAGGCGGCCGACTGGACCACAACTGACGCTGAGGTGCGAAAGCGTGGGGAGCAAACAGG` |
| ASV838 | 429 | `TGGGGAATTTTGGACAATGGGCGAAAGCCTGATCCAGCAATACCGCGTGCAGGACGAAGGCCTTCGGGTTGTAAACTGCTTTTGTACGGAACGAAACGGTTCAGATTAATACTCTGAACTAATGACGGTACCGTAAGAATAAGCACCGGCTAACTACGTGCCAGCAGCCGCGGTAATACGTAGGGTGCAAGCGTTAATCGGAATTACTGGGCGTAAAGCGTGCGCAGGCGGTTATGTAAGACAGGCGTGAAATCCCCGAGCTCAACTTGGGAATTGCGTTTGTGACTGCATAGCTAGAGTGCGGCAGAGGGGGATGGAATTCCGCGTGTAGCAGTGAAATGCGTAGATATGCGGAGGAACACCGATGGCGAAGGCAATCCCCTGGGCCTGCACTGACGCTCATGCACGAAAGCGTGGGGAGCAAACAGG` |
| ASV903 | 429 | `TGGGGAATATTGGACAATGGGGGCAACCCTGATCCAGCAATGCCGCGTGTGTGAAGAAGGCCTTAGGGTTGTAAAGCACTTTCAGTGGGGAGGAAGGAGTGTGTGCTAATACCACATACTATTGACGTTACCCACAGAAGAAGCACCGGCTAACTCTGTGCCAGCAGCCGCGGTAATACAGAGGGTGCAAGCGTTAATCGGAATTACTGGGCGTAAAGAGTGCGTAGGTGGTTAGGTAAGTTAGATGTGAAATCCCTGGGCTCAACCTAGGAACTGCGTTTAAAACTGCTTAGCTAGAGTATTGTAGAGGATAGTGGAATTTCCAGTGTAGCGGTGAAATGCGTAGATATTGGAAGGAACACCAGTGGCGAAGGCGACTATCTGGGCAAATACTGACACTGAGGCACGAGAGCATGGGGAGCAAACAGG` |
| ASV3353 | 404 | `TGGGGAATATTGGACAATGGGGGCAACCCTGATCCAGCCATGCCGCGTGAGTGAAGAAGGTCTTAGGATTGTAAAGCTCTTTTATCGGGGATGATAATGACCGTACCCGAGGAATAAGCCCCGGCTAACTCCGTGCCAGCAGCCGCGGTAAGACGGAGGGGGCTAGCGTTGTTCGGAATTACTGGGCGTAAAGGGCGCGTAGGCGGTTTTGTGTGTCAGAGGTGAAATCCCCGAGCTTAACTTGGGAATTGCCTTTGAAACTGCAGGACTTGAGATATGGAGAGGCAAGCGGAATTCCGAGTGTAGCAGTGAAATGCGTAGATATTCGGAGGAACACCGGTGGCGAAGGCGGCTTGCTGGTCATATACTGACGCTGAGGCGCGAAAGCACGGGGAGCAAACAGG` |
| ASV45 | 424 | `TGAGGAATATTGGACAATGGGCGCAAGCCTGATCCAGCCATGCCGCGTGCAGGAAGACGGTCCTATGGATTGTAAACTGCTTTTATACAGGAAGAAACACTCCGACGTGTCGGAGCTTGACGGTACTGTAGGAATAAGGATCGGCTAACTCCGTGCCAGCAGCCGCGGTAATACGGAGGATCCAAGCGTTATCCGGAATCATTGGGTTTAAAGGGTCCGTAGGCGGTTTAATAAGTCAGTGGTGAAATCTGGTCGCTCAACGATCAAACGGCCATTGATACTGTTAGACTTGAATTATAAGGAAGTAACTAGAATATGTAGTGTAGCGGTGAAATGCTTAGATATTACATGGAATACCAATTGCGAAGGCAGGTTACTACTTATGGATTGACGCTGATGGACGAAAGCGTGGGGAGCGAACAGG` |
| ASV456 | 429 | `TGGGGAATTTTGGACAATGGGGGAAACCCTGATCCAGCCATCCCGCGTGTGCGATGAAGGCCTTCGGGTTGTAAAGCACTTTTGGCAGGAAAGAAACGGCGCGTGCTAATACCATGCGCAACTGACGGTACCTGCAGAATAAGCACCGGCTAACTACGTGCCAGCAGCCGCGGTAATACGTAGGGTGCAAGCGTTAATCGGAATTACTGGGCGTAAAGCGTGCGCAGGCGGTTCGGAAAGAAAGATGTGAAATCCCAGGGCTCAACCTTGGAACTGCATTTTTAACTACCGGACTAGAGTGTGTCAGAGGGAGGTGGAATTCCACGTGTAGCAGTGAAATGCGTAGATATGTGGAGGAACACCGATGGCGAAGGCAGCCTCCTGGGATAACACTGACGCTCATGCACGAAAGCGTGGGGAGCAAACAGG` |
| ASV5052 | 429 | `TGGGGAATTTTGGACAATGGGGGCAACCCTGATCCAGCCATGCCGCGTGAGTGAAGAAGGCCTTCGGGTTGTAAAGCTCTTTCGGACGGAACGAAATGGTTACGGTCAATACCCGTGACTGATGACGGTACCGTAAGAAGAAGCACCGGCTAACTACGTGCCAGCAGCCGCGGTAATACGTAGGGTGCGAGCGTTAATCGGAATTACTGGGCGTAAAGCGTGCGCAGGCGGTTTCGTAAGCTGGAGGTGAAATCCCCGGGCTTAACCTGGGAATGGCCTTCAGGACTGCGAGGCTAGAGTGCGGCAGAGGGAGGTGGAATTCCACGTGTAGCAGTGAAATGCGTAGATATGTGGAGGAACACCGATGGCGAAGGCAGCCTCCTGGGCCAGCACTGACGCTCATGCACGAAAGCGTGGGGAGCAAACAGG` |
| ASV1171 | 423 | `TAGGGAATATTGGGCAATGGAGGCAACTCTGACCCAGCCATGCCGCGTGCAGGAAGAAGGCGTTATGCGTTGTAAACTGCTTTTATATAGGAAGAAAGAGTCTTTGCGAAGAAAGTTGACGGTACTATATGAATAAGCACCGGCTAACTCCGTGCCAGCAGCCGCGGTAATACGGAGGGTGCAAGCGTTGTCCGGATTTATTGGGTTTAAAGGGTGCGTAGGCGGTTTATTAAGTCAGTGGTGAAAGACGGTCGCTCAACGATTGCAGTGCCATTGATACTGGTAGACTTGAGTGAGATTGAGGTAGCTGGAATGGATAGTGTAGCGGTGAAATGCATAGATATTATCCAGAACACCAATTGCGTAGGCAAGTTACTAAGTCTCAACTGACGCTGATGCACGAAAGTGTGGGTATCAAACAGG` |
| ASV5262 | 404 | `TGGGGAATATTGGACAATGGGGGAAACCCTGATCCAGCCATGCCGCGTGAGTGATGAAGGCCTTAGGGTTGTAAAGCTCTTTTACCAGGGAAGATGATGACGGTACCTGGAGAATAAGCACCGGCTAACTTCGTGCCAGCAGCCGCGGTAATACGAAGGGTGCTAGCGTTGTTCGGAATCACTGGGCGTAAAGCGTGCGTAGGCGGCTTTTTAAGTCAGAGGTGAAATGCCAAGGCTCAACCTTGGAACTGCCTTTGAAACTGAAAAGCTAGAGTGTCGGAGGGGTCAGGGGAATTCCTAATGTAGCGGTGAAATGCGTAGATATTAGGAGGAACACCGGTGGCGAAGGCGCCTGACTGGACGACAACTGACGCTGAGGCACGAAAGCGTGGGGATCAAACAGG` |
| ASV3345 | 404 | `TGGGGAATCTTAGACAATGGGGGAAACCCTGATCTAGCCATGCCGCGTGGATGATGAAGGCCTTAGGGTCGTAAAGTCCTTTCGCCGGGGAAGATAATGACGGTACCCGGTAAAGAAGCCCCGGCTAACTCCGTGCCAGCAGCCGCGGTAATACGGAGGGGGCTAGCGTTGTTCGGATTGACTGGGCGTAAAGCGCGCGTAGGCGGATCATCAAGTCGGGGGTGAAATCCCGGGGCTCAACCCTGGAATGGCCTTCGAGACTGCTGGTCTTGAGATCGAGAGAGGTGGGTGGAATTCCGAGTGTAGAGGTGAAATTCGTAGATATTCGGAGGAACACCAGTGGCGAAGGCGGCCCACTGGCTCGACACTGACGCTGAGGCGCGAAAGCGTGGGGAGCAAACAGG` |
| ASV1796 | 429 | `TGGGGAATATTGGACAATGGGCGCAAGCCTGATCCAGCCATGCCGCGTGTGTGAAGAAGGCCTTCGGGTTGTAAAGCACTTTTGTTGGGGAAGAAAAGGCCACGGTTAATAACCGTGGTTCATGACGGTACCCAAAGAATAAGCACCGGCTAACTTCGTGCCAGCAGCCGCGGTAATACGAAGGGTGCAAGCGTTACTCGGAATTACTGGGCGTAAAGCGTGCGTAGGCGGTTTGTTAAGTCTGTCGTGAAAGCCCCGGGCTCAACCTGGGAATGGCGGTGGATACTGGCAGGCTAGAGTACGGTAGAGGATGGTGGAATTCCCGGTGTAGCAGTGAAATGCGTAGAGATCGGGAGGAACATCTATGGCGAAGGCAGCCATCTGGACCAGTACTGACGCTGAGGCACGAAAGCGTGGGGAGCAAACAGG` |
| ASV4352 | 408 | `TGAGGAATCTTCCGCAATGGGCGAAAGCCTGACGGAGCGACACCGCGTGGAGGATGAAGCCCTATTCGGGTGTAAACTCCTGTTCTGAGGGACGAAATTTTTGACGGTACCTCAGGAGAAAGCATCGGCAAACCCTGTGCCAGCAGCCGCGGTAAGACAGGGGATGCAAGCGTTACTCGGAATTACTGGGCGTAAAGCGTCTGCAGGTGTTCTGCTGCGTCTGGTGTCAAATGGCGACGCTCAACGTCGTCATCGTGCCGGAAACGAGCAGAATCGAGTTACCCAGAGGCACCTGGAATGTCGTGTGTAGGGGTAAAATCCGTTGATCCACGATGGAACGCCGAAAGCGAAGGCAGGGTGCTGGGGGTGTACTGACACTCAGAGACGAAAGCGTGGGGAGCAAAGGGG` |
| ASV111 | 429 | `TGGGGAATTTTGGACAATGGGCGCAAGCCTGATCCAGCAATGCCGCGTGAGTGAAGAAGGCCTTCGGGTTGTAAAGCTCTTTTGTCAGGGAAGAAACGGTGAGTCCTAATACGGCTTGCTAATGACGGTACCTGAAGAATAAGCACCGGCTAACTACGTGCCAGCAGCCGCGGTAATACGTAGGGTGCAAGCGTTAATCGGAATTACTGGGCGTAAAGCGTGCGCAGGCGGTTATATAAGTCAGATGTGAAATCCCCGGGCTCAACCTGGGAACTGCATTTGAGACTGTATGGCTAGAGTGTGTCAGAGGGGGGTAGAATTCCACGTGTAGCAGTGAAATGCGTAGATATGTGGAGGAATACCGATGGCGAAGGCAGCCCCCTGGGATAACACTGACGCTCATGCACGAAAGCGTGGGGAGCAAACAGG` |
| ASV4853 | 425 | `TGGGGAATATTGGACAATGGGCGAAAGCCTGATCCAGCAATGCCGCGTGAGTGATGAAGGCCCTCGGGTTGTAAAGCTCTGTCGGCAGGGACGAAAAAGTCCGGGTAACGCCGGAACTTGACGGTACCTGCAAAGGAAGCACCGGCTAACTCTGTGCCAGCAGCCGCGGTAATACAGAGGGTGCAAGCGTTGTTCGGAATTACTGGGCGTAAAGCGCGTGTAGGCTGCGATATGAGTCGGATGTGAAAGCCCTCAGCTTAACTGAGGAAGTGCATCCGATACTGTATCGCTCGAGTGTCGGAGGGGGTGGCGGAATTCCCGGTGTAGAGGTGAAATTCGTAGATATCGGGAGGAACACCTGTGGCGAAGGCGGCCACCTGGACGACGACTGACGCTGAGACGCGAAAGCGTGGGTAGCAAACAGG` |
| ASV4698 | 429 | `TGGGGAATATTGCACAATGGGCGCAAGCCTGATGCAGCCATGCCGCGTGTATGAAGAAGGCCTTCGGGTTGTAAAGTACTTTCAGCGGGGAGGAAGGGAGTAAAGTTAATACCTTTACTCATTGACGTTACCCGCAGAAGAAGCACCGGCTAACTCCGTGCCAGCAGCCGCGGTAATACGGAGGGTGCAAGCGTTAATCGGAATTACTGGGCGTAAAGCGCACGCAGGCGGTTTGTTAAGTCAGATGTGAAATCCCCGGGCTCAACCTGGGAACTGCATCTGATACTGGCAAGCTTGAGTCTCGTAGAGGGGGGTAGAATTCCAGGTGTAGCGGTGAAATGCGTAGAGATCTGGAGGAATACCGGTGGCGAAGGCGGCCCCCTGGACGAAGACTGACGCTCAGGTGCGAAAGCGTGGGGAGCAAACAGG` |
| ASV4074 | 408 | `TTCGGAATATTCCGCAATGGGCGAAAGCCTGACGGAGCGACGCCGCGTGCACGAAGACGGCCTTCGGGTTGTAAAGTGCTTTTAGTAGGGAACAATTTTGAGCGTACCTACAGAATAAGAGGTTGCTAACTCTGTGCCAGCAGCAGCGGTAATACAGAGACCTCAAACGTTATCCGGAATCATTGGGCGTAAAGCGTACCGATAGGTGGTTTTGTAAGTCAAAAGTTAAAGCCTGGGGCTCACCCCCAGAATCGCTTTTGAAACTCCAAGACTAGAGGGGCAAAGAGGAAGCTGGAACAAACGGTGTAGCAGTGAAATGCGTTGATATCGTTTGGAACACCGAAAGCGAAGGCGGGCTTCTGGGTGCCACCTGACACTGCTAGGACGAAAGCGTGGGGAGCGAATGGG` |
| ASV3884 | 424 | `TAGGGAATATTGGGCAATGGAAGGAATTCTGACCCAGCCATGCCGCGTGAACGAAGAAGGCGCTCTGCGTCGTAAAGTTCTTTTAGATGGGGCGAAGATGTGGTGTTTACACCATAGTGACGGTACCATCGGAATAAGCACCGGCTAACTCTGTGCCAGCAGCCGCGGTAATACAGAGGGTGCAAGCGTTATCCGGAATCACTGGGTTTAAAGGGTGCGTAGGCTGTGGGATAAGTCAGAGGTGAAATGTGTTCGCTTAACGAATAAATTGCCTTTGATACTGTTTCACTTGAAAGACGATGAGGTTGGCGGAATGTGACATGTAGCGGTGAAATGCTTAGATATGTCATAGAACACCGATTGCGAAGGCAGCTGACTGGTCGAGCTTTGACGCTGAGGCACGAAAGCGTGGGGATCAAACAGG` |
| ASV4284 | 407 | `CTAGGAATTATGCACAATGGGGGAAACCCTGATGCTGCGACGTTGCGTGGAGGATGAAGATTCTAGGATTGTAAACTCCTTTTACTGATGAACAAGATCTGAGTGTAATCAGTGAATAAGTGCCTGCAAACCACGTGCCAGAAGCATCGGTAATACGTGGGGCACAAGCGTTATCCGGATTTACTGGGCGTAAAGAGATGCGTAGGCGTCCTAAAAAGTTTTATGTTAAATCCTAAAGCTCAACTTTAGAACTACATAAAAAACTCTTAGGATTGAGTCAGGTAGAGAGAAGAAGAATTCCTAGTGGAGCGGTTAAATGCGTTGATATTAGGAGGAACACCGAATGCGAAGGCATCTTCTTGGACCTGTACTGACGCTAAGGCTCGAAAGCGTGGGGAGCAAAACGG` |
| ASV1805 | 429 | `TGGGGAATATTGGACAATGGGGGCAACCCTGATCCAGCAATGCCGCGTGTGTGAAGAAGGCCTGAGGGTTGTAAAGCACTTTCAGTGGGGAGGAGGATTGATAGGTTAAGAGCTGATCAATTGGACGTTACCCACAGAAGAAGCACCGGCTAACTCCGTGCCAGCAGCCGCGGTAATACGGAGGGTGCAAGCGTTAATCGGAATTACTGGGCGTAAAGGGTGCGTAGGTGGTTTATTAAGTTATCTGTGAAATTCCTGGGCTCAACCTGGGCAGGTCAGATAAGACTGGTAGACTCGAGTATGGGAGAGGGCAGTGGAATTTCCGGTGTAGCGGTGAAATGCGTAGAGATCGGAAGGAACACCAGTGGCGAAGGCGGCTACCTGGCCTAATACTGACACTGAGGCACGAAAGCGTGGGGAGCAAACAGG` |
| ASV3823 | 429 | `TGGGGAATATTGGACAATGGGCGCAAGCCTGATCCAGCCATGCCGCGTGTGTGAAGAAGGCCTTCGGGTTGTAAAGCACTTTTGTTGGGAAAGAAAAGCTATCGGCTAATACCCGGTGGTACTGACGGTACCCAAAGAATAAGCACCGGCTAACTTCGTGCCAGCAGCCGCGGTAATACGAAGGGTGCAAGCGTTACTCGGAATTACTGGGCGTAAAGCGTGCGTAGGTGGTTTGTTAAGTCAGATGTGAAAGCCCCGGGCTCAACCTGGGAATTGCATTTGATACTGGCAGACTAGAGTGCGGTAGAGGAGAGTGGAATTCCCGGTGTAGCAGTGAAATGCGTAGAGATCGGGAGGAACATCAGTTGCGAAGGCGGCTCTCTGGACCAGCACTGACACTGAGGCACGAAAGCGTGGGGAGCAAACAGG` |
| ASV1137 | 404 | `TGGGGAATATTGGACAATGGGCGCAAGCCTGATCCAGCCATGCCGCGTGAGTGACGAAGGCCTTAGGGTTGTAAAGCTCTTTTAACGGGGAAGATAATGACGGTACCCGTAGAATAAGCTCCGGCTAACTTCGTGCCAGCAGCCGCGGTAATACGAAGGGGGCTAGCGTTGTTCGGAATTACTGGGCGTAAAGAGTTCGTAGGCGGTTTATTAAGCTAGATGTGAAATCCCCGGGCTTAACTCGGGAACTGCATTTAGAACTGGTAAGCTAGAGAGTGTTAGAGGTTGGTAGAATTCCTAGTGTAGGGGTGAAATCCGTAGATATTAGGAGGAATACCGATGGCGAAGGCAGCCAACTGGGACATTTCTGACGCTGAGGAACGAAAGCGTGGGTAGCAAACAGG` |
| ASV1672 | 430 | `TAGGGAATATTGCGCAATGGGGGAAACCCTGACGCAGCAACGCCGCGTGAGTGAGGAAGGTCTTCGGATTGTAAAACTCTTTTGCTAGGGAAAAATGACCAAGTGGCTAATATCCATTTGGAGTGATGGTACCTAAAGAATAAGGACCGGCTAACTTCGTGCCAGCAGCCGCGGTAATACGAAGGGTCCAAGCGTTGTTCGGAATCATTGGGCGTAAAGCGAGCGCAGGCGGATCAGCAAGTCAGATGTGAAATCTCGAAGCTCAACTTCGAAACTGCGTCTGAAACTGCTAGTCTAGAATGTCGGAGGGGATAGGGGAATTTCACGTGTAGGGGTAAAATCCGTAGAGATGTGAAGGAACACCGGAGGCGAAGGCGCCTATCTGGACGACTATTGACGCTGAGGCTCGAAAGCGTGGGGAGCAAACAGG` |
| ASV193 | 429 | `TGGGGAATCTTGGACAATGGGCGAAAGCCTGATCCAGCAATGCCGCGTGCGTGAAGAAGGCCTTCGGGTTGTAAAGCGCTTTTGTCGGGGAGGAAATCCTAACGGTTAATACCTGTTGGGGATGACAGTACCCGAAGAATAAGCACCGGCTAACTACGTGCCAGCAGCCGCGGTAATACGTAGGGTGCAAGCGTTAATCGGAATTACTGGGCGTAAAGCGTCCGCAGGTGGCTTGATAAGATAGACGTGAAATCCCTGGGCTCAACCTAGGAATTGCGTATATGACTGTCTCGCTAGAGTATGGGAGAGGGGGGTGGAATTCCGCGTGTAGCAGTGAAATGCGTAGAGATGCGGAGGAACACCGATGGCGAAGGCAACCCCCTGGCCTAATACTGACACTCATGGACGAAAGCGTGGGGAGCAAACAGG` |
| ASV2976 | 404 | `TGGGGAATATTGGACAATGGGGGCAACCCTGATCCAGCAATGCCGCGTGAATGATGAAGGCCTTAGGGTTGTAAAGTTCTTTTATTGATGACGATAATGACGGTAATCAATGAATAAGCACCGGCTAACTTCGTGCCAGCAGCCGCGGTAATACGAAGGGTGCTAGCGTTGTTCGGAATGACTGGGCGTAAAGGGTGCGTAGGCGGCCAGAAACGTCTGATGTGAAATCCCTGGGCTTAACCTAGGAACTGCATTGGATACGGTCTGGCTAGAGTCCGCGAGAGGAAGATGGAATTGTGTGTGTAGAGGTGAAATTCGTAGATATGCACAAGAACACCGGTGGCGAAGGCGATCTTCTGGAGCGGTACTGACGCTAAGGCACGAAAGCGTGGGGAGCAAACAGG` |
| ASV2262 | 409 | `TGGGGAATATTGCACAATGGGCGCAAGCCTGATGCAGCAACGCCGCGTGAGGGACGACGGCCTTCGGGTTGTAAACCTCTTTTAGCAGGGAAGAAGCGAAAGTGACGGTACCTGCAGAAAAAGCACCGGCTAACTACGTGCCAGCAGCCGCGGTAATACGTAGGGTGCAAGCGTTGTCCGGAATTATTGGGCGTAAAGAGCTCGTAGGCGGTTTGTCACGTCTGCTGTGAAAACGCGAGGCTCAACCTCGCGCCTGCAGTGGGTACGGGCAAACTAGAGTGCGGTAGGGGAGATTGGAATTCCTGGTGTAGCGGTGGAATGCGCAGATATCAGGAGGAACACCAATGGCGAAGGCAGATCTCTGGGCCGTAACTGACGCTGAGGAGCGAAAGCGTGGGGAGCGAACAGG` |
| ASV4800 | 429 | `TGGGGAATATTGGACAATGGGGGTAACCCTGATCCAGCAATGCCGCGTGTGTGAAGAAGGCCTGCGGGTTGTAAAGCACTTTCGTTTGCAAGGAAGGTCATTGTGTTAATAGCGCAGTGAATTGACGTAAGCAAAAGAAGAAGCACCGGCTAACTCTGTGCCAGCAGCCGCGGTAATACAGAGGGTGCAAGCGTTAATCGGAATTACTGGGCGTAAAGAGTGCGTAGGTGGTTAATTAAGTTGAGTGTGAAAGCCCAGGGCTTAACCTTGGAACGGCATTTAAAACTGATTAGCTAGAGTTCAGGAGAGGGAAGTGGAATTTCCGGTGTAGCGGTGAAATGCGTAGATATCGGAAGGAACATCAGTGGCGAAGGCGACTTTCTGGAATGATACTGACACTGAGGTACGACAGCGTGGGTAGCGAACAGG` |
| ASV3602 | 404 | `TGGGGAATATTGGACAATGGGCGAAAGCCTGATCCAGCCATGCCGCGTGAGTGATGAAGGCCTTAGGGTTGTAAAGCTCTTTTGTCCGGGAAGATAATGACTGTACCGGAAGAATAAGCCCCGGCTAACTTCGTGCCAGCAGCCGCGGTAATACGAAGGGGGCTAGCGTTGCTCGGAATCACTGGGCGTAAAGCGCACGTAGGCGGATTTCTAAGTCGGAGGTGAAAGCCCAGGGCTCAACCCTGGAATTGCCTTCGATACTGGAAGTCTCGAGTTCGGAAGAGGTTGGTGGAACTGCGAGTGTAGAGGTGAAATTCGTAGATATTCGCAAGAACACCAGTGGCGAAGGCGGCCAACTGGTCCGATACTGACGCTGAGGTGCGAAAGCGTGGGGAGCAAACAGG` |
| ASV5196 | 404 | `TGAGGAATATTGCACAATGGGGGAAACCCTGATGCAGCCATGCCGCGTGAGTGAAGAAGGCCTTAGGGTTGTAAAGCTCTTTCAATCGTGAAGATAATGACGGTAGCGATAGAAGAAGCCCCGGCTAAATTCGTGCCAGCAGCCGCGGTAATACGAATGGGGCAAGCGTTGTTCGGAATCACTGGGCTTAAAGCGTGCGCAGGCGGCTATGAAAGTCAGGAGTGAAATCCCAGGGCTCAACCCTGGAATTGCTTTTGAAACTCCATAGCTAGAATCCCGGAGAGGTAAGGAGAATTCCGAGTGTAGAGGTGAAATTCGCAGATATTCGGAGGAATACCAGTGGCGAAGGCGCCTTACTGGACGGGCATTGACGCTCAGGCACGAAAGCGTGGGGAGCAAACAGG` |
| ASV4611 | 430 | `TAGGGAATATTGCGCAATGGGGGAAACCCTGACGCAGCAACGCCGCGTGAGTGAGGAAGGTCTTCGGATTGTAAAACTCTGTTCTTAGGGAAAAATGTTTTTATCGTTAATACCGGTAAGAAGTGATGGTACCTAAGTAGAAAGCACCGGCTAACTTCGTGCCAGCAGCCGCGGTAATACGAAGGGTGCAAGCGTTGTTCGGAATCATTGGGCGTAAAGCGCGCGCAGGCGGATCAGCAAGTCAGATGTGAAATCTCGAAGCTCAACTTCGAAACTGCGTCTGAAACTGCTAGTCTAGAATGTCGGAGGGGGCAGGGGAATTTCACGTGTAGGGGTAAAATCCGTAGAGATGTGAAGGAACACCGGGGGCGAAGGCGCCTGCCTGGACGACTATTGACGCTGAGGCGCGAAAGCGTGGGGAGCAAACAGG` |
| ASV5320 | 405 | `TTAGGAATCTTGCGCAATGGGCGAAAGCCTGACGCAGCAACGCCGCGTGTGGGATGAAGGCTTTCGGGTTGTAAACCACTTTCAGCAGGAACGAAATTGACGGTACCTGCAGAAGAAGCTCCGGCCAACTACGTGCCAGCAGCCGCGGTAATACGTAGGGAGCAAGCGTTGTCCGGATTTATTGGGCGTAAAGAGCTCGTAGGCGGTTAAGTAAGTCAGGTGTGAAACCTCCAGGCTCAACCTGGAGACGCCACTTGATACTGCTTTGACTTGAGTTCGGTAGGGGTAAGCGGAATTCCTGGTGTAGCGGTGAAATGCGCAGATATCAGGAGGAACACCGACGGCGAAGGCAGCTTACTGGGCCGATACTGACGCTGAGGAGCGAAAGCGTGGGGAGCGAACAGG` |
| ASV4690 | 424 | `TGAGGAATATTGGACAATGGGCGCAAGCCTGATCCAGCCATGCCGCGTGCAGGATGAAGCATCTATGGTGTGTAAACTGCTTTTATACGGGAAGAAACCCTGGCTCGTGAGCCAGACTGACGGTACCGTAGGAATAAGGATCGGCTAACTCCGTGCCAGCAGCCGCGGTAATACGGAGGATCCAAGCGTTATCCGGAATCATTGGGTTTAAAGGGTCCGTAGGCGGCCCTGTAAGTCAGTGGTGAAATCTCCCGGCTCAACCGGGAAATTGCCATTGATACTGCAGGGCTTGAATTATTGGGAAGTAACTAGAATATGTAGTGTAGCGGTGAAATGCTTAGATATTACATGGAATACCGATTGCGAAGGCAGGTTACTACCAATATATTGACGCTGATGGACGAAAGCGTGGGTAGCGAACAGG` |
| ASV1428 | 429 | `TGGGGAATTTTGGACAATGGGCGAAAGCCTGATCCAGCAATGCCGCGTGCAGGACGAAGGCCTTCGGGTTGTAAACTGCTTTTGTACGGAGCGAAACGGTCTTCTCTAATACAGGGGGCTAATGACGGTACCGTAAGAATAAGCACCGGCTAACTACGTGCCAGCAGCCGCGGTAATACGTAGGGTGCAAGCGTTAATCGGAATTACTGGGCGTAAAGCGTGCGCAGGCGGTTATATAAGACAGATGTGAAATCCCCGGGCTCAACCTGGGACCTGCATTTGTGACTGTATAGCTAGAGTACGGTAGAGGGGGATGGAATTCCGCGTGTAGCAGTGAAATGCGTAGATATGCGGAGGAACACCGATGGCGAAGGCAATCCCCTGGACCTGTACTGACGCTCATGCACGAAAGCGTGGGGAGCAAACAGG` |
| ASV2649 | 429 | `TGGGGAATTTTGGACAATGGGCGCAAGCCTGATCCAGCAATGCCGCGTGAGTGAAGAAGGCCTTCGGGTTGTAAAGCTCTTTTGTCAGGGAAGAAACGGTCTACTCTAATACAGTGGGCTAATGACGGTACCTGAAGAATAAGCACCGGCTAACTACGTGCCAGCAGCCGCGGTAATACGTAGGGTGCAAGCGTTAATCGGAATTACTGGGCGTAAAGCGTGCGCAGGCGGTTATATAAGTCAGATGTGAAATCCCCGGGCTCAACCTGGGAACTGCATTTGAGACTGTATGGCTAGAGTGTGTCAGAGGGGGGTAGAATTCCACGTGTAGCAGTGAAATGCGTAGATATGTGGAGGAATACCGATGGCGAAGGCAGCCCCCTGGGATAACACTGACGCTCATGCACGAAAGCGTGGGGAGCAAACAGG` |
| ASV2645 | 429 | `TGGGGAATTTTGGACAATGGGCGAAAGCCTGATCCAGCAATGCCGCGTGCAGGATGAAGGCCTTCGGGTTGTAAACTGCTTTTGTACGGAGCGAAAAAGCTTCTTCTAATACAGGAGGCTCATGACGGTACCGTAAGAATAAGCACCGGCTAACTACGTGCCAGCAGCCGCGGTAATACGTAGGGTGCAAGCGTTAATCGGAATTACTGGGCGTAAAGCGTGCGCAGGCGGTAATGTAAGACAGAGGTGAAATCCCCGGGCTCAACCTGGGAACTGCCTTTGTGACTGCATTGCTAGAGTACGGTAGAGGGGGGTGGAATTCCGCATGTAGCAGTGAAATGCGTAGATATGCGGAGGAACACCGATGGCGAAGGCAGCCCCCTGGACCTGTACTGACGCTCATGCACGAAAGCATGGGGAGCAAACAGG` |
| ASV4587 | 429 | `TGGGGAATATTGGACAATGGGGGCAACCCTGATCCAGCAATGCCGCGTGTGTGAAGAAGGCCTTCGGGTTGTAAAGCACTTTAGTTGGGGAGGAAGGTGCGTATTTTAAGAGATAATGCATTTGACTGTACCCAAAGAATAAGCACCGGCTAACTCTGTGCCAGCAGCCGCGGTAATACAGAGGGTGCGAGCGTTAATCGGAATTACTGGGCGTAAAGGGCGCGTAGGCGGTTATATAAGTTAGATGTGAAATCCCTGGGCTTAACCTAGGAACTGCATCTAAGACTGTATGACTAGAGTGCAGTAGAGGATAGTGGAATTTCCGGTGTAGCGGTGAAATGCGTAGATATCGGAAGGAACACCAGTGGCGAAGGCGGCTATCTGGACTGACACTGACGCTGAGGCGCGAAAGCGTGGGTAGCAAACAGG` |
| ASV2811 | 404 | `TGGGGAATATTGGACAATGGGCGCAAGCCTGATCCAGCCATGCCGCGTGAGTGACGAAGGCCTTAGGGTTGTAAAGCTCTTTTGTCGGGGACGATAATGACGGTACCCGAAGAATAAGCTCCGGCTAACTTCGTGCCAGCAGCCGCGGTAATACGAAGGGAGCTAGCGTTGTTCGGAATCACTGGGCGTAAAGCGCACGTAGGCGGATTTGTAAGTCAGGGGTGAAATCCCGGGGCTCAACCTCGGAACTGCCTTTGATACTGCGAGTCTAGAGTCCGGAAGAGGTGGGTGGAATTCCTAGTGTAGAGGTGAAATTCATAGATATTAGGAAGAACACCGGTGGCGAAGGCGGCCCACTGGTCCGGTACTGACGCTGAGGTGCGAAAGCGTGGGGAGCAAACAGG` |
| ASV3255 | 429 | `TGGGGAATATTGGACAATGGGGGCAACCCTGATCCAGCCATGCCGCGTGTGTGAAGAAGGCCTTCGGGTTGTAAAGCACTTTCGGTCAGAAAGAAGGGTGTGTAGTTAATAACTGCATATCTTGACGGTACTGACATAAGAAGCACCGGCTAACTCCGTGCCAGCAGCCGCGGTAATACGGAGGGTGCAAGCGTTAATCGGAATTACTGGGCGTAAAGCGCGCGTAGGCGGTTTGTTAAGTCGGATGTGAAAGCCCCGGGCTCAACCTGGGAACTGCATACGATACTGGCAAGCTAGAGTGCAATAGAGGGGGGTAGAATTCCAGGTGTAGCGGTGAAATGCGTAGATATCTGGAGGAATACCGGTGGCGAAGGCGGCCCCCTGGATTGACACTGACGCTGAGGTGCGAAAGCGTGGGTAGCAAACAGG` |
| ASV3722 | 430 | `TAGGGAATATTGCGCAATGGGGGAAACCCTGACGCAGCAACGCCGCGTGAGTGAGGAAGGTCTTCGGATTGTAAAACTCTTTTATTTGGGAAAAATGACCATATGGCTAATATCCATGTGGAGTGATGGTACCAAAGGAATAAGCACCGGCTAACTTCGTGCCAGCAGCCGCGGTAATACGAAGGGTGCAAGCGTTGTTCGGAATCATTGGGCGTAAAGCGCGCGCAGGCGGATTCGTAAGTCAGATGTGAAATCTCGAGGCTCAACCTCGAAACTGCGTCTGAAACTACAAGTCTAGAATGTCGGAGGGGGCAGGGGAATTTCACGTGTAGGGGTAAAATCCGTAGAGATGTGAAGGAACACCGGAGGCGAAGGCGCCTGCCTGGACGACTATTGACGCTGAGGCGCGAAAGCGTGGGGAGCAAACAGG` |
| ASV5492 | 409 | `TCGAGGATCTTCGGCAATGGGCGCAAGCCTGACCGAGCGACGCCGCGTGAGCGATGAAGGCCTTCGGGTCGTAAAGCTCTGTCGGGGGGGAGAAAGGGCAACTTGATCGACCCCCGGAGGAAGCACGGGCTAAGTTCGTGCCAGCAGCCGCGGTAAGACGAACCGTGCGAACGTTATTCGGAATCACTGGGCTTAAAGGGTGCGTAGGCGGGTTCACAAGTCTGGGGTGAAATCCCACGGCCCAACCGTGGAAGTGCCTCAGATACTGTGGGCCTCGAGGGGGATAGGGGCATGCGGAACTCAGGGTGGAGCGGTGAAATGCGTAGATATCCTGAGGAACACCGGTGGCGAAAGCGGCGTGCTAGGTCCCTTCTGACGCTGAGGCACGAAAGCCAGGGGAGCAAACGGG` |
| ASV1695 | 423 | `TAGGGAATATTGGGCAATGGGCGAAAGCCTGACCCAGCCATGCCGCGTGCAGGAAGAAGGCGTTCTGCGTTGTAAACTGCTTTTTTACAAGAAGAAAAGACTCATGCGTGAGGCATTGACGGTATTGTAAGAATAAGCACCGGCTAACTCCGTGCCAGCAGCCGCGGTAATACGGAGGGTGCAAGCGTTGTCCGGATTTATTGGGTTTAAAGGGTGCGTAGGTGGTTTATTAAGTCAGTGGTGAAAGACGGTCGCTTAACGATTGCAGTGCCATTGAAACTGATAGACTTGAGTTTCGTTAAGGTAGGTGGAATTGATAGTGTAGCGGTGAAATGCATAGATATTATCAAGAACTCCAATTGCGTAGGCAACTTACTAGTCGAAAACTGACACTGATGCACGAAAGTGTGGGTATCAAACAGG` |
| ASV4799 | 430 | `TGGGGAATCTTGCACAATGGACGAAAGTCTGATGCAGCAACGCCGCGTGAGTGAAGAAGGTTTTTGGATTGTAAAGCTCTTTCGACTGGGGAGAAGGGGAATTTGGCTAATATCCAAGTTTTTTGACGGTACCAGGAGAAGAAGCACCGGCTAATTCCGTGCCAGCAGCCGCGGTAATACGGAAGGTGCAAGCGTTGTTCGGATTGATTGGGCGTAAAGGGCACGTAGGTGGGTTTGTAAGTCTGTTGTGAAATCCCAGGGCTCAACCTTGGCACAGCAACGGAAACTGCAAGTCTTGAATGCTTGAGGGGTTAGTGGAATTCCAGGTGTAGGAGTGAAATCCGTAGATATCTGGAGGAACACCGGAGGCGAAGGCGGCTAACTGGCGATGCATTGACACTGAGGTGCGAAAGCGTGGGGAGCAAACAGG` |
| ASV1852 | 428 | `TTTCGAATCATTCACAATGGGCGAAAGCCTGATGGTGCGACGCCGCGTGAGGGATGAAGGCCTTCGGGTTGTAAACCTCTGTCACCGGGGAAGAAACGCTTCAGGTTAATAGTCTGAAGCCTGACTTAACCCGGAGAGGAAGCAGTGGCTAACTCTGTGCCAGCAGCCGCGGTAATACAGAGACTGCAAGCGTTATTCGGATTCACTGGGCGTAAAGGGTGCGCAGGCGGCCAAGTGTGTGAGGCGTGAAAGCCCGGGGCTTAACCCCGGAATTGCACCTCAAACTACATGGCTAGAGCATTGGAGAGGGTAGCAGAATTCACGGTGTAGCAGTGAAATGCGTAGATATCGTGAGGAATACCAGAGGCGAAGGCGGCTACCTGGACAATTGCTGACGCTCAGGCACGAAAGCGTGGGGAGCAAAAGGG` |
| ASV4093 | 404 | `TGGGGAATATTGGACAATGGGGGAAACCCTGATCCAGCCATGCCGCGTGAGTGATGAAGGCCTTAGGGTTGTAAAGCTCTTTTACCAGGGAAGATGATGACGGTACCTGGAGAATAAGCACCGGCTAACTTCGTGCCAGCAGCCGCGGTAATACGAAGGGTGCTAGCGTTGTTCGGAATCACTGGGCGTAAAGCGTGCGTAGGCGGCTTTGCAAGTCAGGGGTGAAATCCCACAGCTCAACTGTGGAATTGCCTCTGAAACTGCATCGCTGGAGTGTCGGAGGGGATAGCGGAATTCCTAATGTAGTGGTGAAATACGTAGATATTAGGAGGAACACCGGTGGCGAAGGCGGCTATCTGGACGACAACTGACGCTGAGGCACGAAAGCGTGGGGATCAAACAGG` |
| ASV5517 | 430 | `TGGGGAATCTTGCGCAATGGACGAAAGTCTGACGCAGCCACGCCGCGTGAGTGAAGAAGGCCTTCGGGTTGTAAAGCTCTGTCGGAGGGGACAAAAACGTTTAGGGTTAATAGCCCTAGATCTTGATGGTACCCTTAAAGGAAGCACCGGCTAACTTCGTGCCAGCAGCCGCGGTAATACGAAGGGTGCAAGCGTTGCTCGGAATTATTGGGCGTAAAGGGTAGGTAGGTGGTTACGTATGTCTGAGGTGAAAGCCCTGAGCTTAACTTAGGAAGTGCCTTGGAAACGGCGTAACTAGAGTACTGGAGAGGTTCGTGGAATTCCTAGTGTAGCGGTGAAATGCGTAGATATTAGGAGGAACACCTGAGGCGAAGGCGGCGAACTGGACAGTAACTGACACTCAACTACGAAAGCGTGGGTAGCAAACAGG` |
| ASV813 | 423 | `TAGGGAATATTGGGCAATGGAAGGAATTCTGACCCAGCCATGCCGCGTGCAGGATGACGGCGCTATGCGTTGTAAACTGCTTTTGTCAGGGAAGAATTACACTTTGCGAAGTGTGTTGACGGTACCTGAAGAATAAGCATCGGCTAACTCCGTGCCAGCAGCCGCGGTAATACGGAGGATGCAAGCGTTGTCCGGATTTATTGGGTTTAAAGGGTGCGTAGGCTGTTTTATAAGTCCGTGGTCAAATTTCTTAGCTTAACTAAGACTGTGCCATTGATACTGTAAAACTAGAGTATTGTTTAGGTAGATGGAATTGGTAGTGTAGCGGTGAAATGCATAGATATTACCAAGAACACCGATAGCGAAGGCAATTTACTGAGCAATAACTGACGCTGAGGCACGAAAGTGTGGGTATCAAACAGG` |
| ASV78 | 429 | `TGGGGAATTTTGGACAATGGGCGCAAGCCTGATCCAGCAATGCCGCGTGCAGGATGAAGGCCTTCGGGTTGTAAACTGCTTTTGTACGGAACGAAACGGCCTGCCCTAATACGGTGGGCTAATGACGGTACCGTAAGAATAAGCACCGGCTAACTACGTGCCAGCAGCCGCGGTAATACGTAGGGTGCGAGCGTTAATCGGAATTACTGGGCGTAAAGCGTGCGCAGGCGGTTATATAAGACAGATGTGAAATCCCCGGGCTCAACCTGGGACCTGCATTTGTGACTGTATAGCTAGAGTACGGTAGAGGGGGATGGAATTCCGCGTGTAGCAGTGAAATGCGTAGATATGCGGAGGAACACCGATGGCGAAGGCAATCCCCTGGACCTGTACTGACGCTCATGCACGAAAGCGTGGGGAGCAAACAGG` |
| ASV1323 | 404 | `TGGGGAATCTTAGACAATGGGGGAAACCCTGATCTAGCCATGCCGCGTGATCGATGAAGGCCTTAGGGTTGTAAAGATCTTTCAGGTGGGAAGATAATGACTGTACCACCAGAAGAAGCCCCGGCTAACTCCGTGCCAGCAGCCGCGGTAATACGGAGGGGGCTAGCGTTATTCGGAATTACTGGGCGTAAAGCGCACGTAGGCGGACCAGAAAGTCAGAGGTGAAAGCCCGGGGCTCAACCCCGGAACTGCCTTTGAAACTCCTGGTCTTGAGGTCGAGAGAGGTGAGTGGAATTCCGAGTGTAGAGGTGAAATTCGTAGATATTCGGAGGAACACCAGTGGCGAAGGCGGCTCACTGGCTCGATACTGACGCTGAGGTGCGAAAGCGTGGGGAGCAAACAGG` |
| ASV1541 | 406 | `TGGGGAATTTTCCGCAATGGGCGCAAGCCTGACGGAGCAATACCGCGTGAGGGAGGAAGGCTCTTGGGTCGTAAACCTCTTTTCTCTGGGAAGAATAACTGACGGTACCAGAGGAATCAGCATCGGCTAACTCCGTGCCAGCAGCCGCGGTAATACGGGGGATGCAAGCGTTATCCGGAATTATTGGGCGTAAAGCGTCCGCAGGTGGCTAATCAAGTCTGTTGTCAAAGCGCGGAGCTCAACTCCGTACAGGCGATGGAAACTGATTGGCTAGAGTTAGGTAGGGGTAAGAGGAATTCCCAGTGTAGCGGTGAAATGCGTAGATATTGGGAAGAACACCGGCGGCGAAAGCGTCTTACTGGGCCTAAACTGACACTGAGGGACGAAAGCTAGGGGAGCGAAAGGG` |
| ASV4760 | 429 | `TCGAGAATAATTCACAATGGGGGCAACCCTGATGGTGCAACGCCGCGTGGAGGATGAAGGTCTTCGGATCGTAAACTCCTGTCATCTGGGAGCAAGACCTGGCGGTCAATAGCCGACAGGGTTGATAGTACCAGAAGAGGAAGGGACGGCTAACTTCGTGCCAGCAGCCGCGGTAATACGAAGGTCCCAAGCGTTGTTCGGAATCACTGGGCGTAAAGGGAGCGTAGGCGGCGTGGTAAGTCAGATGTGAAATCCCAAGGCTCAACCTTGGAACTGCATCCGATACTGCCATGCTAGAGGGTTGGAGAGGTAACTGGAATTCTCGGTGTAGCAGTGAAATGCGTAGATATCGAGAGGAACACTCGTGGCGAAAGCGAGTTACTGGACAACACCTGACGCTGAGGCTCGAAGGCTAGGGGAGCGAAAGGG` |
| ASV4042 | 429 | `TGGGGAATTTTGGACAATGGGCGCAAGCCTGATCCAGCAATGCCGCGTGAGTGAAGAAGGCCTTCGGGTTGTAAAGCTCTTTTGTCAGGGAAGAAACGGCTCCAGCTAATATCTGGGGCTAATGACGGTACCTGAAGAATAAGCACCGGCTAACTACGTGCCAGCAGCCGCGGTAATACGTAGGGTGCAAGCGTTAATCGGAATTACTGGGCGTAAAGCGTGCGCAGGCGGTTTTGTAAGTCTGATGTGAAATCCCCGGGCTCAACCTGGGAATTGCATTGGAGACTGCAAGGCTAGAATCTGGCAGAGGGGGGTAGAATTCCACGTGTAGCAGTGAAATGCGTAGAGATGTGGAGGAACACCGATGGCGAAGGCAGCCCCCTGGGTCAAGATTGACGCTCATGCACGAAAGCGTGGGGAGCAAACAGG` |
| ASV1239 | 429 | `TGGGGAATTTTGGACAATGGGCGAAAGCCTGATCCAGCAATGCCGCGTGAGTGAAGAAGGCCTTCGGGTTGTAAAGCTCTTTTGTCAGGGAAGAAACGGTGAGGGCTAATATCTCTTGCTAATGACGGTACCTGAAGAATAAGCACCGGCTAACTACGTGCCAGCAGCCGCGGTAATACGTAGGGTGCAAGCGTTAATCGGAATTACTGGGCGTAAAGCGTGCGCAGGCGGTTTTGTAAGTCTGTCGTGAAATCCCCGGGCTCAACCTGGGAATTGCGATGGAGACTGCAAGGCTAGAATCTGGCAGAGGGGGGTAGAATTCCACGTGTAGCAGTGAAATGCGTAGAGATGTGGAGGAACACCGATGGCGAAGGCAGCCCCCTGGGTCAAGATTGACGCTCATGCACGAAAGCGTGGGGAGCAAACAGG` |
| ASV2021 | 407 | `TGGGGAATATTGGGCAATGGGCGAAAGCCTGACCCAGCAACGCCGCGTGAAGGAAGAAGGCTTTCGGGTTGTAAACTTCTTTTATGTGGGACGAAACAAATGACGGTACCGCATGAATAAGCCACGGCTAACTACGTGCCAGCAGCCGCGGTAATACGTAGGTGGCAAGCGTTATCCGGATTTACTGGGTGTAAAGGGCGTGTAGGCGGGACTGTAAGTCAGGCGTGAAAACTGGTGGCTCAACCACCAGCTTGCGTTTGAAACTGCGGTTCTTGAGTGTCGGAGAGGCAATCGGAATTCCGTGTGTAGCGGTGAAATGCGTAGATATACGGAGGAACACCAGTGGCGAAGGCGGATTGCTGGACGATAACTGACGCTGAGGCGCGAAAGCGTGGGGAGCAAACAGG` |
| ASV1778 | 429 | `TGGGGAATTTTGGACAATGGGCGCAAGCCTGATCCAGCCATGCCGCGTGAGTGAAGAAGGCCTTCGGGTTGTAAAGCTCTTTCGGCGGGGAAGAAATCGCATGGGTTAATACCCTGTGTGGATGACGGTACCCGAATAAGAAGCACCGGCTAACTACGTGCCAGCAGCCGCGGTAATACGTAGGGTGCGAGCGTTAATCGGAATTACTGGGCGTAAAGCGTGCGCAGGCGGTTTTGTAAGTCAGATGTGAAATCCCCGGGCTCAACCTGGGAACTGCATTTGAGACTGCAAGGCTAGAGTGTGGCAGAGGGGGGTGGAATTCCACGTGTAGCAGTGAAATGCGTAGAGATGTGGAGGAACACCGATGGCGAAGGCAACCCCCTGGGCCAATACTGACGCTCATGCACGAAAGCGTGGGGAGCAAACAGG` |
| ASV5324 | 430 | `TGGGGAATATTGGACAATGGGCGCAAGCCTGATCCAGCAATGCCGCGTGTGTGAAGAAGGCCTGCGGGTTGTAAAGCACTTTTGTCCGGAACGAAAAGTGGCGGGATAATACCCTGTCATGCTGACGGTACCGGAAGAATAAGCACCGGCTAACTTCGTGCCAGCAGCCGCGGTAATACGAAGGGTGCAAGCGTTAATCGGAATTACTGGGCGTAAAGGGAGCGTAGGCGGCTCTTTAAGTCGGATGTGAAATCCCTGGGCTCAACCTGGGAACTGCATCCGATACTGGAGAGCTTGGAGTTTGAGAGAGGATGGTGGAATTCCTGGTGTAGCGGTGAAATGCGTAGAGATCAGGAGGAACACTAGTGGCGAAGGCGGCCATCTGGCTCAAAACTGACGCTGAGGCTCGAAAGCGTGGGGAGCAAACAGG` |
| ASV3480 | 404 | `TGGGGAATATTGGACAATGGGCGCAAGCCTGATCCAGCTATGCCGCGTGGATGAAGAAGGCCTTCGGGTTGTAAAGTCCTTTCGTTGGTGACGATAATGACGGTAACCAAAGAAGAAGCCCCGGCTAATTTCGTGCCAGCAGCCGCGGTAATACGAAAGGGGCTAGCGTTGTTCGGAATTACTGGGCGTAAAGCGCACGTAGGCGGCCTTGTAAGATGGGGGTGAAATCCCTGGGCTCAACCCAGGAACTGCCTTCATGACTGCAAAGCTCGAGGATGTGAGAGGTTGGCGGAATTCCGAGTGTAGAGGTGAAATTCGTAGATATTCGGAAGAACACCAGTGGCGAAGGCGGCCAACTGGCACATTTCTGACGCTGAGGTGCGAAAGCGTGGGGAGCAAACAGG` |
| ASV1590 | 424 | `TAAGGAATATTGGGCAATGGAGGCAACTCTGACCCAGCCATCCCGCGTGAAGGATTAAGGCCCTATGGGTTGTAAACTTCTTTTGTTTGGGAAGAAATCTGGACATTTATGTGCAGTTGACGGTACCTTACGAATAAGCACCGGCTAACTCCGTGCCAGCAGCCGCGGTAATACGGAGGGTGCAAGCGTTATCCGGAATCACTGGGTTTAAAGGGTGCGTAGGCGGTTTGATAAGTCAGAGGTGAAAGCTAGCAGCTTAACTGTTAAATTGCCTTTGATACTGTCAGACTTGAATCAAGTTGAGGTAGGCGGAATGTGACATGTAGCGGTGAAATGCATAGATATGTCATAGAACACCAATTGCGTAGGCAGCTTGCTGGGCTTGAATTGACGCTGAGGCACGAAAGCGTGGGGATCAAACAGG` |
| ASV3789 | 404 | `TGGGGAATATTGGGCAATGGAGGAAACTCTGACCCAGCCATGCCGCGTGAGTGATGAAGGCCTTAGGGTTGTAAAGCTCTTTTACCAGGGACGATGATGACGGTACCTGGAGAATAAGCACCGGCTAACTTCGTGCCAGCAGCCGCGGTAATACGAAGGGTGCTAGCGTTGTTCGGAATCACTGGGCGTAAAGCGTGCGCAGGCGGCTTTTTAAGCCAGAGGTGAAATGCCAGGGCTCAACCTTGGAACTGCCTTTGGAACTGGAAAGCTTGAGTACTGGAGAGGTTTGGGGAATTTCTAATGTAGCGGTGAAATGCGTAGATATTAGAAGGAACACCAATGGCGAAGGCACCAAACTGGACAGTTACTGACGCTCAGGCACGAAAGCGTGGGGATCAAACAGG` |
| ASV1872 | 424 | `TGAGGAATATTGGACAATGGGCGCAAGCCTGATCCAGCCATGCCGCGTGCAGGATGACGGTCCTATGGATTGTAAACTGCTTTTATACAGGAAGAAACCCTGGTTCGTGAACCAGCTTGACGGTACTGTAAGAATAAGGATCGGCTAACTCCGTGCCAGCAGCCGCGGTAATACGGAGGATCCAAGCGTTATCCGGAATCATTGGGTTTAAAGGGTCCGTAGGCGGTTTAATAAGTCAGTGGTGAAAGCCCATCGCTCAACGGTGGAACGGCCATTGATACTGTTAAACTTGAATTATTAGGAAGTAACTAGAATATGTAGTGTAGCGGTGAAATGCTTAGAGATTACATGGAATACCAATTGCGAAGGCAGGTTACTACTAATTGATTGACGCTGATGGACGAAAGCGTGGGTAGCGAACAGG` |
| ASV2235 | 404 | `TGGGGAATATTGGACAATGGGGGAAACCCTGATCCAGCAATGCCGCGTGAGTGATGAAGGCCTTCGGGTTGTAAAGCTCTTTTGTCAGGGACGATGATGACGGTACCTGACGAATAAGCTCCGGCTAACTTCGTGCCAGCAGCCGCGGTAATACGAGGGGAGCTAGCGTTGTTCGGAATTACTGGGCGTAAAGGGCGTGTAGGCGGTACCATAAGTCAGATGTGAAAGCCCTAGGCTTAACCTAGGAGGTGCATTTGATACTGTGGAACTAGAGATCGAGAGAGGTAAGTGGAATTACGAGTGTAGAGGTGAAATTCGTAGATATTCGTAAGAACACCAGTGGCGAAGGCGACTTACTGGCTCGATACTGACGCTAAGGCGCGAAAGCGTGGGGAGCAAACAGG` |
| ASV5697 | 425 | `TGGGGAATATTGGTCAATGGGCGAAAGCCTGAACCAGCAATGCCGCGTGAGTGATGAAGGCCTTCGGGTTGTAAAGCTCTGTCGGGAGGGACGAAAAAAGCCGGGTAACTCCGGATCTTGACGGTACCTCCAAAGGAAGCACCGGCTAACTCTGTGCCAGCAGCCGCGGTAATACAGAGGGTGCAAGCGTTGTTCGGAATTACTGGGCGTAAAGCGCGTGTAGGCTGCAACGTAAGTCGGATGTGAAAGCCCTTGGCTTAACCAAGGAAGTGCATCTGATACTGCGTCGCTTGAGTGTCGGAGGGGATGGCGGAATTCCCGGTGTAGAGGTGAAATTCGTAGATATCGGGAGGAACACCTGTGGCGAAGGCGGCCATCTGGACGATTACTGACGCTGAGACGCGAAAGCGTGGGTAGCAAACAGG` |
| ASV1015 | 421 | `TAAGGAATATTGCGCAATGGGCGAAAGCCTGACGCAGCGACGCCGCGTGGGGGATGAAGGTCTTCGGATTGTAAACCCCTTTCGGGAGGGAAGATGGAGTGGGGTAACCCATTCGGACGGTACCTCCAGAAGCAGCCACGGCTAACTTCGTGCCAGCAGCCGCGGTAATACGAAGGTGGCAAGCGTTATTCGGATTTACTGGGCGTACAGGGAGCGTAGGCGGTTCGGTAAGCCCTCTGTGAAATCTTCAGGCTTAACCTGGAAAAGTCGGAGGGGACTGCTGGGCTAGAGGACGGGAGAGGAGCGCGGAATTCCCGGTGTAGCGGTGAAATGCGTAGAGATCGGGAGGAAGGCCGGTGGCGAAGGCGGCGCTCTGGAACGTTTCTGACGCTGAGGCTCGAAAGCGTGGGGAGCAAACAGG` |
| ASV2828 | 404 | `TGGGGAATCTTGCGCAATGGGCGAAAGCCTGACGCAGCCATGCCGCGTGAATGATGAAGGTCTTAGGATTGTAAAATTCTTTCACCGGGGACGATAATGACGGTACCCGGAGAAGAAGCCCCGGCTAACTTCGTGCCAGCAGCCGCGGTAATACGAAGGGGGCTAGCGTTGCTCGGAATTACTGGGCGTAAAGGGAGCGTAGGCGGATAGTTAAGTCAGAGGTGAAAGCCCAGGGCTCAACCTTGGAATTGCCTTTGATACTGGCTATCTTGAGTATGGGAGAGGTAAGCGGAACTCCGAGTGTAGAGGTGAAATTCGTAGATATTCGGAAGAACACCAGTGGCGAAGGCGGCTTACTGGCCCATTACTGACGCTGAGGCTCGAAAGCGTGGGGAGCAAACAGG` |
| ASV5391 | 404 | `TGGGGAATATTGGACAATGGGCGCAAGCCTGATCCAGCCATGCCGCGTGAGTGATGACGGCCTTAGGGTTGTAAAGCTCTTTTATCCGGGACGATAATGACGGTACCGGAAGAATAAGCTCCGGCTAACTTCGTGCCAGCAGCCGCGGTAATACGAAGGGAGCTAGCGTTGCTCGGATTTACTGGGCGTAAAGCGCACGTAGGCGGCTTGATAAGTCGGAGGTGAAATCCTGGGGCTCAACCCCAGAACGGCCTTCGATACTGTTGAGCTTGAGTATGAGAGAGGTAAGTGGAACTCCAAGTGTAGAGGTGAAATTCGTAGATATTTGGAAGAACACCAGTGGCGAAGGCGGCTTACTGGCTCATTACTGACGCTGAGGTGCGAAAGCGTGGGGATCAAACAGG` |
| ASV3689 | 429 | `TGGGGAATTTTGGACAATGGGGGCAACCCTGATCCAGCAATGCCGCGTGAGTGAAGAAGGCCTTCGGGTTGTAAAGCTCTTTTGTCAGGGAAGAAACGGTATGGGTTAATACCCTGTGCTAATGACGGTACCTGAAGAATAAGCACCGGCTAACTACGTGCCAGCAGCCGCGGTAATACGTAGGGTGCAAGCGTTAATCGGAATTACTGGGCGTAAAGCGTGCGCAGGCGGTTTGATAAGTCAGATGTGAAATCCCCGAGCTTAACTTGGGAACTGCATTTGAAACTGTTGAACTAGAGTGTGTCAGAGGGAGGTAGAATTCCACGTGTAGCAGTGAAATGCGTAGAGATGTGGAGGAATACCGGTGGCGAAGGCGGCCTCCTGGGATAACACTGACGCTCATGTACGAAAGCGTGGGGAGCAAACAGG` |
[truncated: 530,551 more chars]
